# Supplementary figures and images for: The Systemin Signaling Cascade As Derived from Time Course Analyses of the Systemin-responsive Phosphoproteome
Source: Mol Cell Proteomics. 2019 May 28;18(8):1526–42. doi: 10.1074/mcp.RA119.001367 (PMC6683004; doi:10.1074/mcp.RA119.001367)

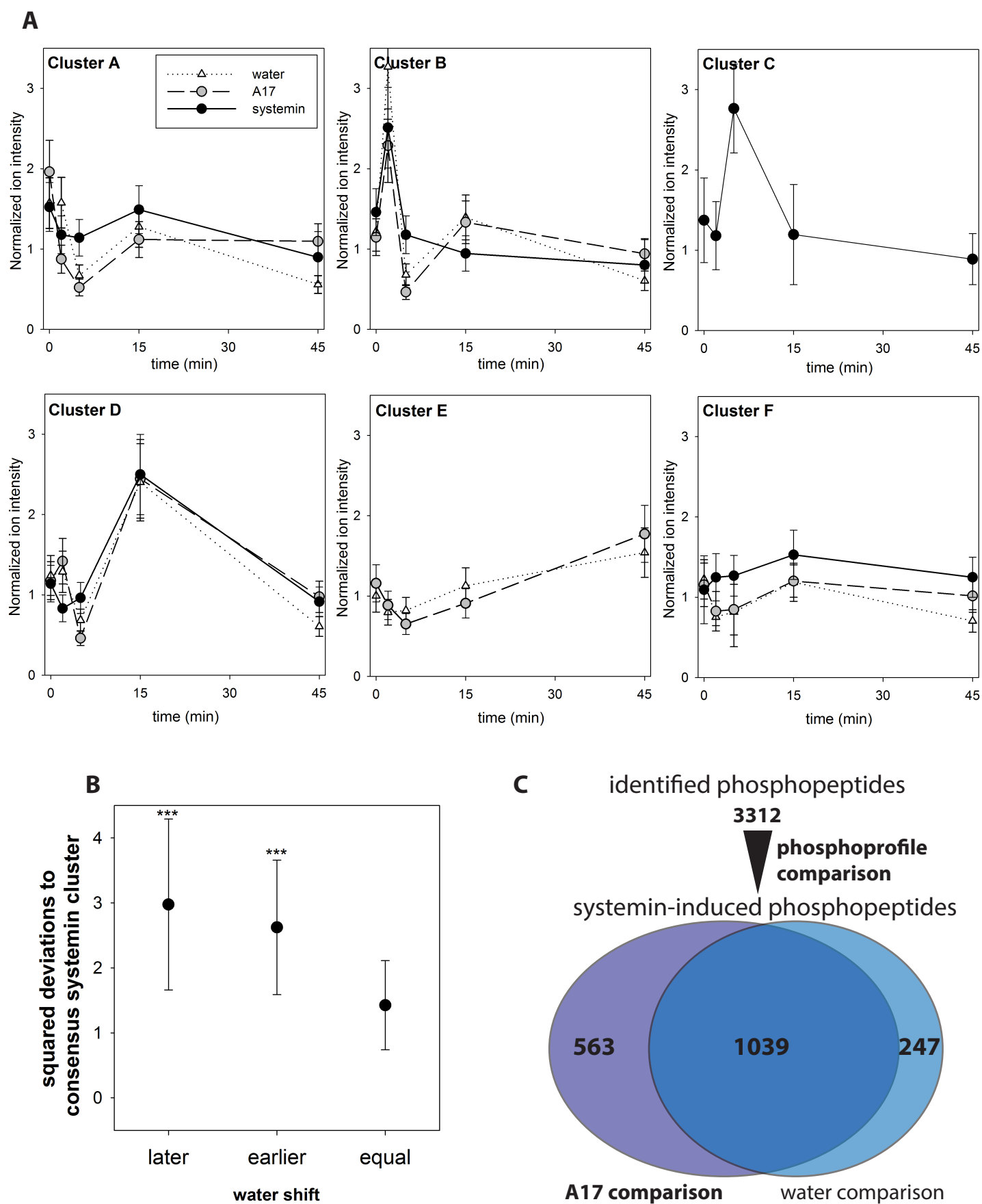

Supplementary Figure S1

Supplement: Supplementary Figure 1 [file 143488_2_supp_334091_ps5gg2.pdf]

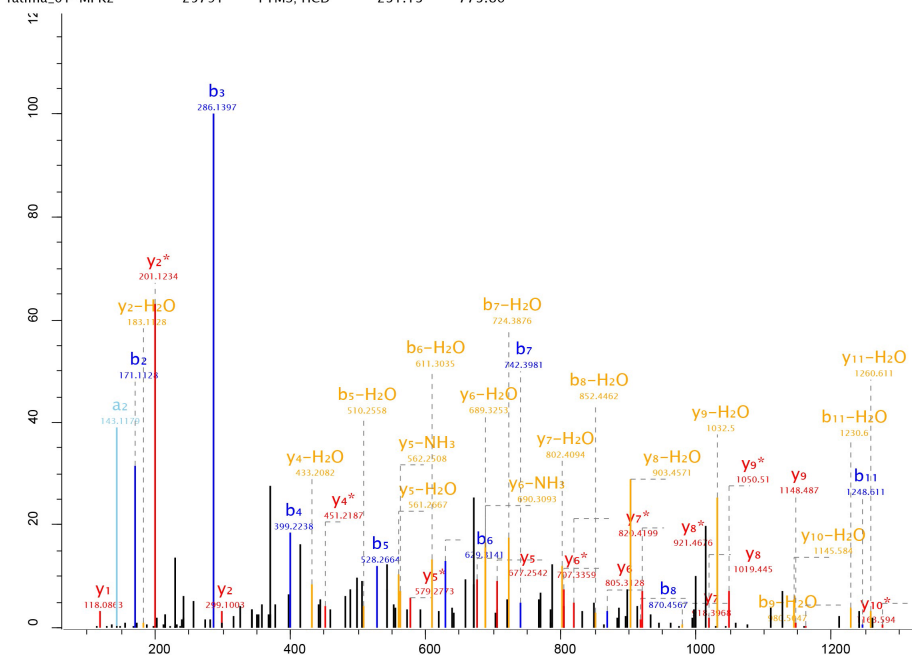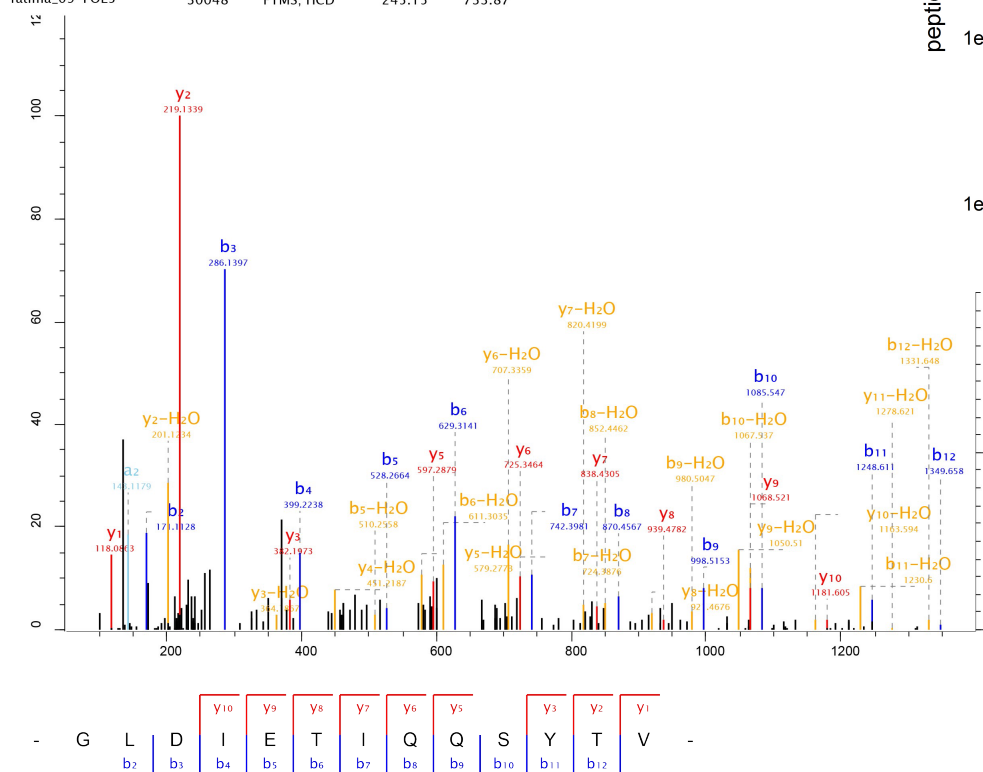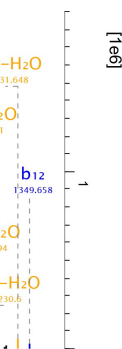

Supplementary Figure S3

Supplement: Supplementary Figure 3 [file 143488_2_supp_334092_ps5gg2.pdf]

**A** Respiratory-Burst Oxidase

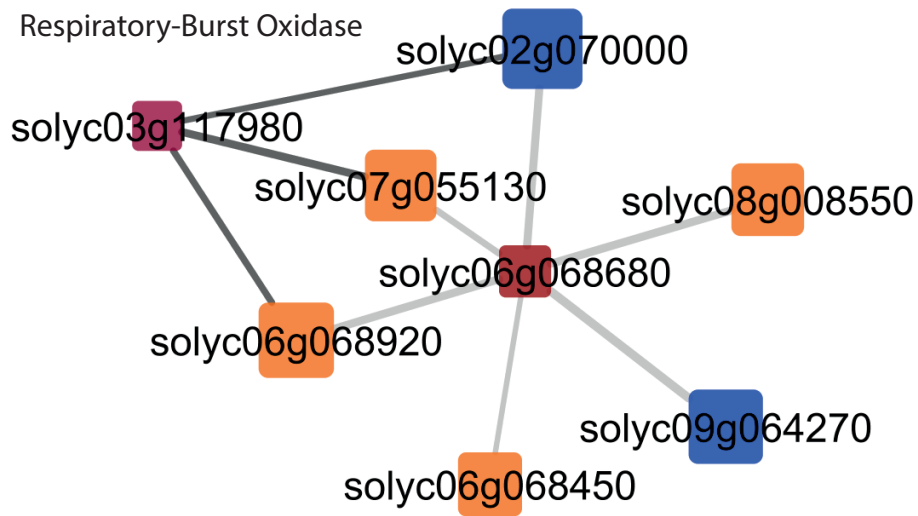

**B** Cellulose Synthase Like

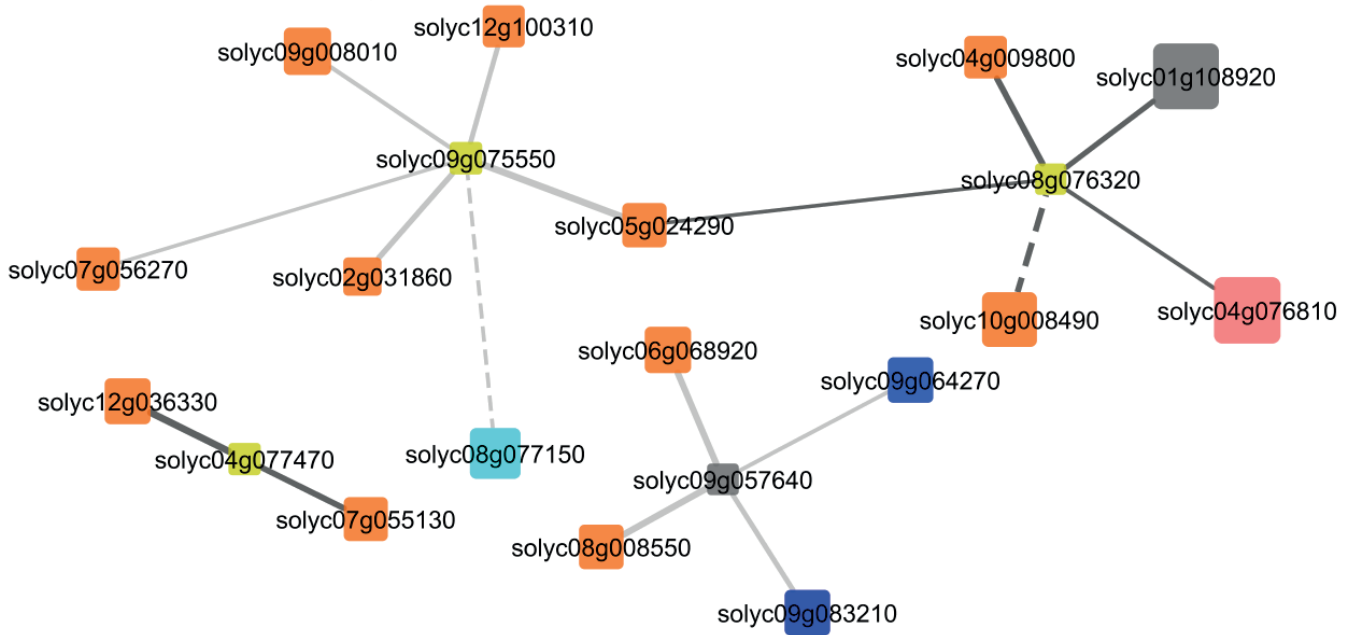

Supplementary Figure S4

Supplement: Supplementary Figure 4 [file 143488_2_supp_334093_ps5gg2.pdf]
