# Supplementary material for: The Systemin Signaling Cascade As Derived from Time Course Analyses of the Systemin-responsive Phosphoproteome
Source: Mol Cell Proteomics. 2019 May 28;18(8):1526–42. doi: 10.1074/mcp.RA119.001367 (PMC6683004; doi:10.1074/mcp.RA119.001367)

Supplementary Figure 2: Representative annotated spectra of identified phosphopeptides under systemin,A17 and water treatment as exported from MaxQuant.

Raw file	Scan	Method	Score	m/z
sys_00_1	12829	FTMS; HCD	62.94	769.86

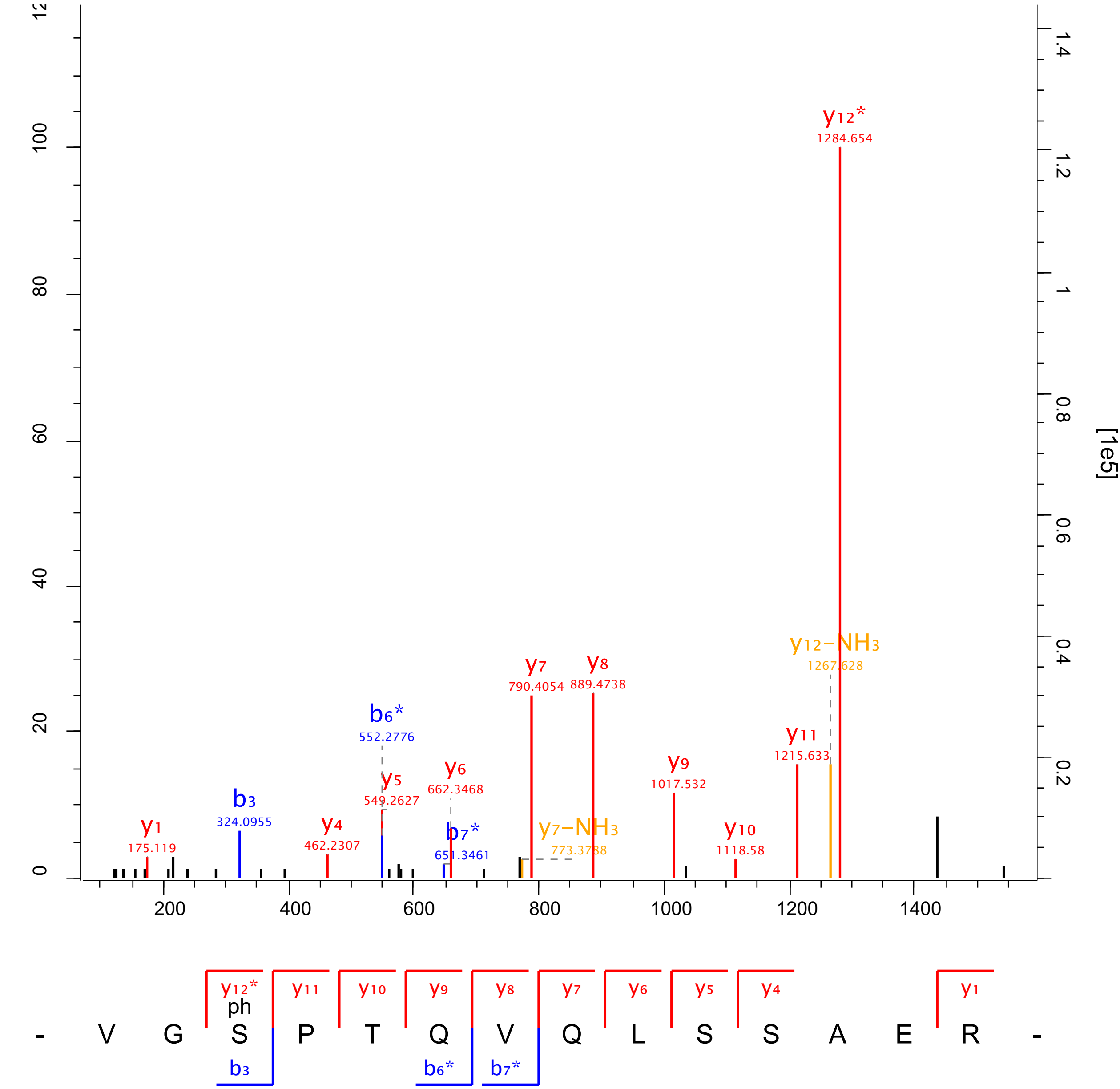

Raw file	Scan	Method	Score	m/z
sys_00_1	1551	FTMS; HCD	62.46	480.19

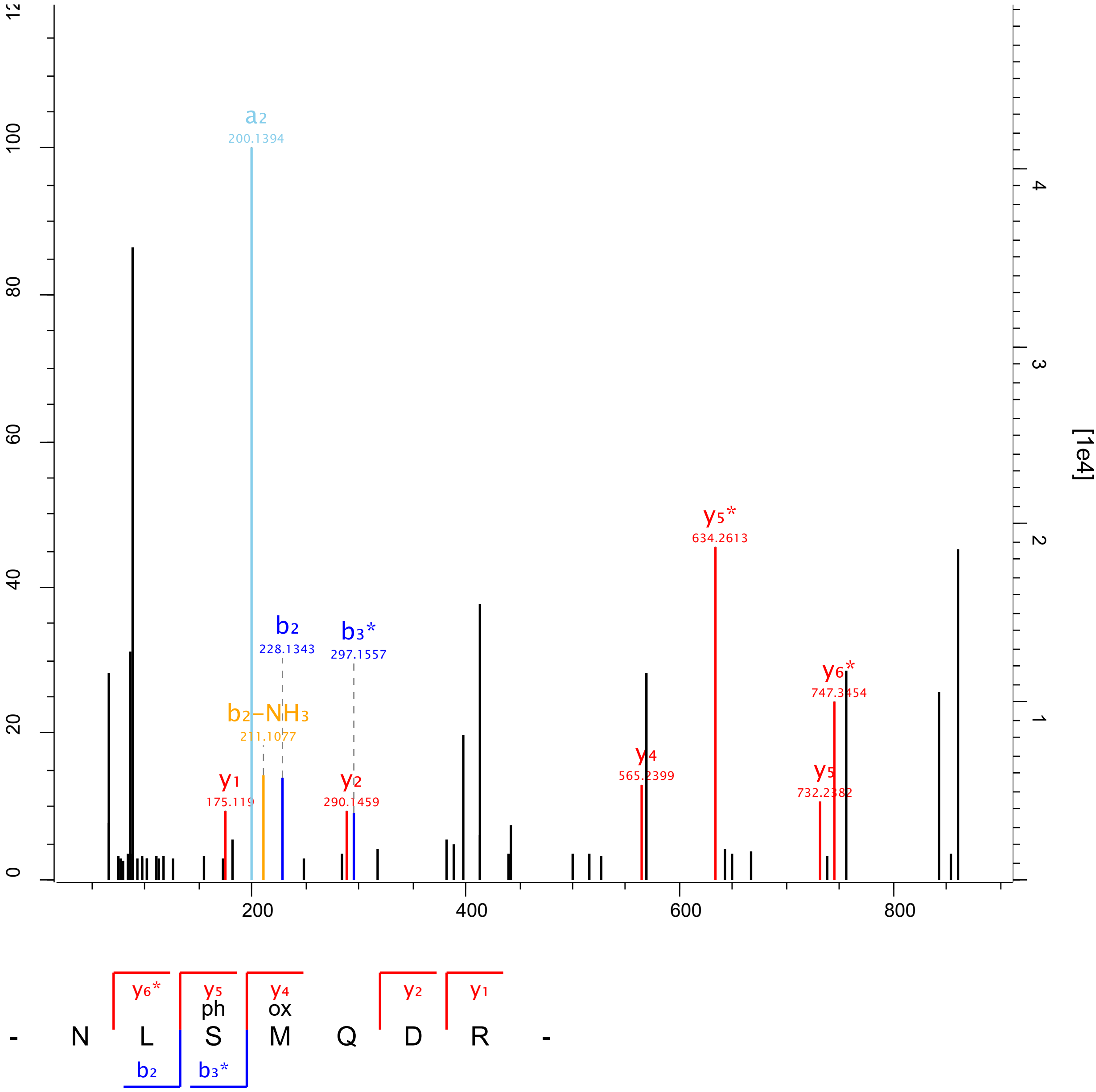

Raw file	Scan	Method	Score	m/z
sys_00_1	1755	FTMS; HCD	50.08	721.8

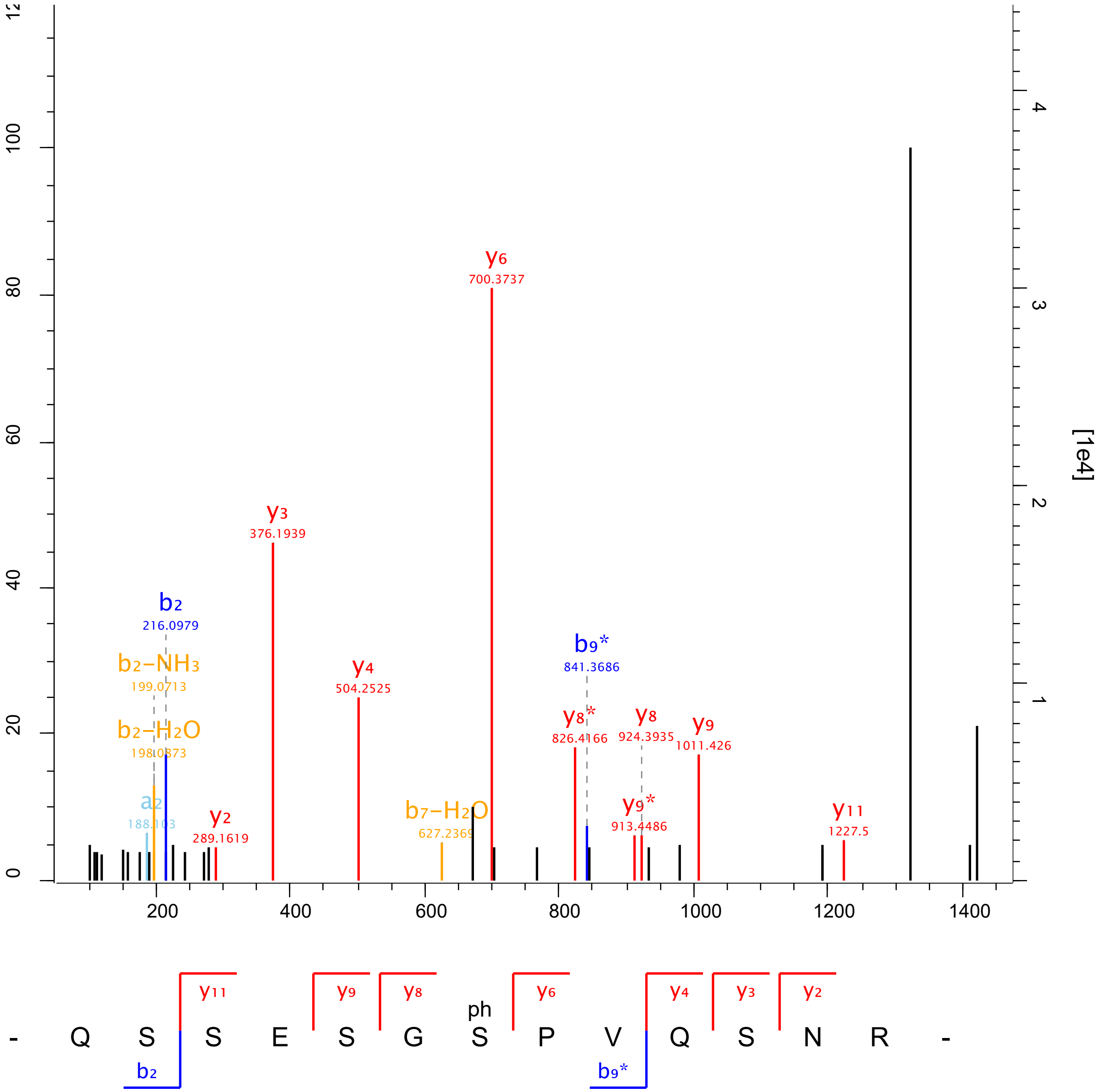

Raw file	Scan	Method	Score	m/z
sys_00_1	2700	FTMS; HCD	51.28	506.73

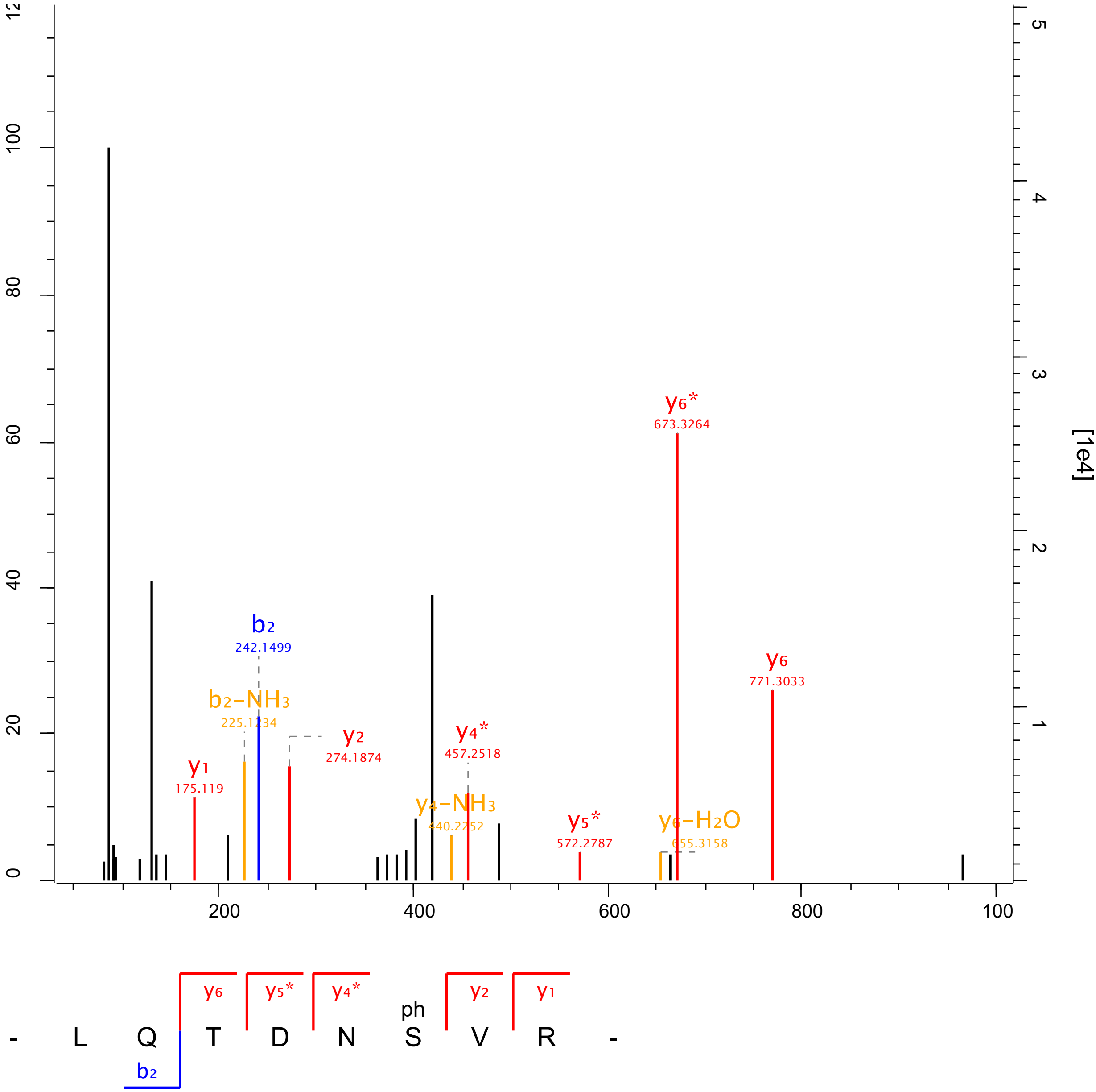

Raw file	Scan	Method	Score	m/z
sys_00_1	2897	FTMS; HCD	46.8	680.74

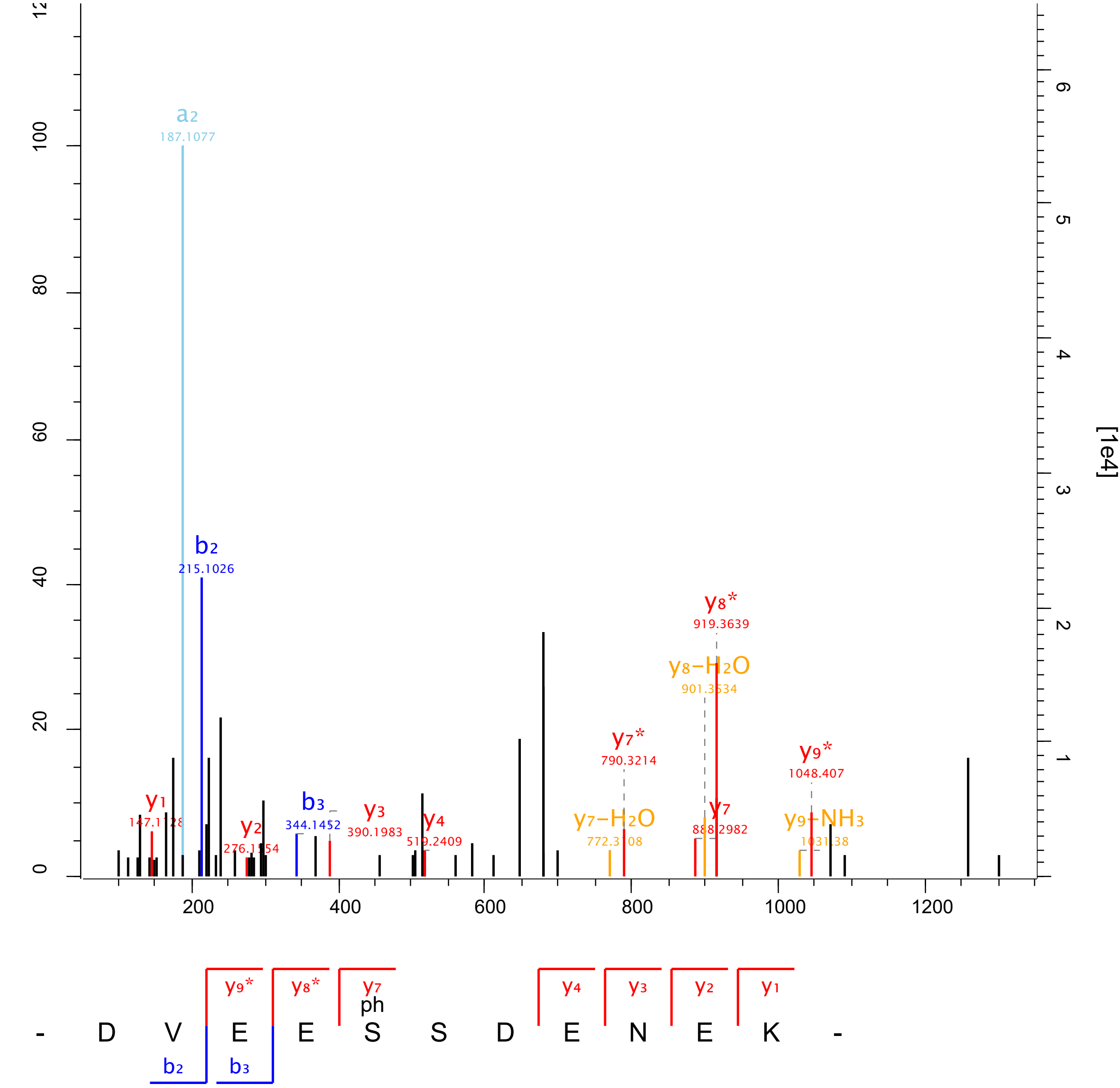

Raw file	Scan	Method	Score	m/z
sys_00_1	3740	FTMS; HCD	139.31	486.7

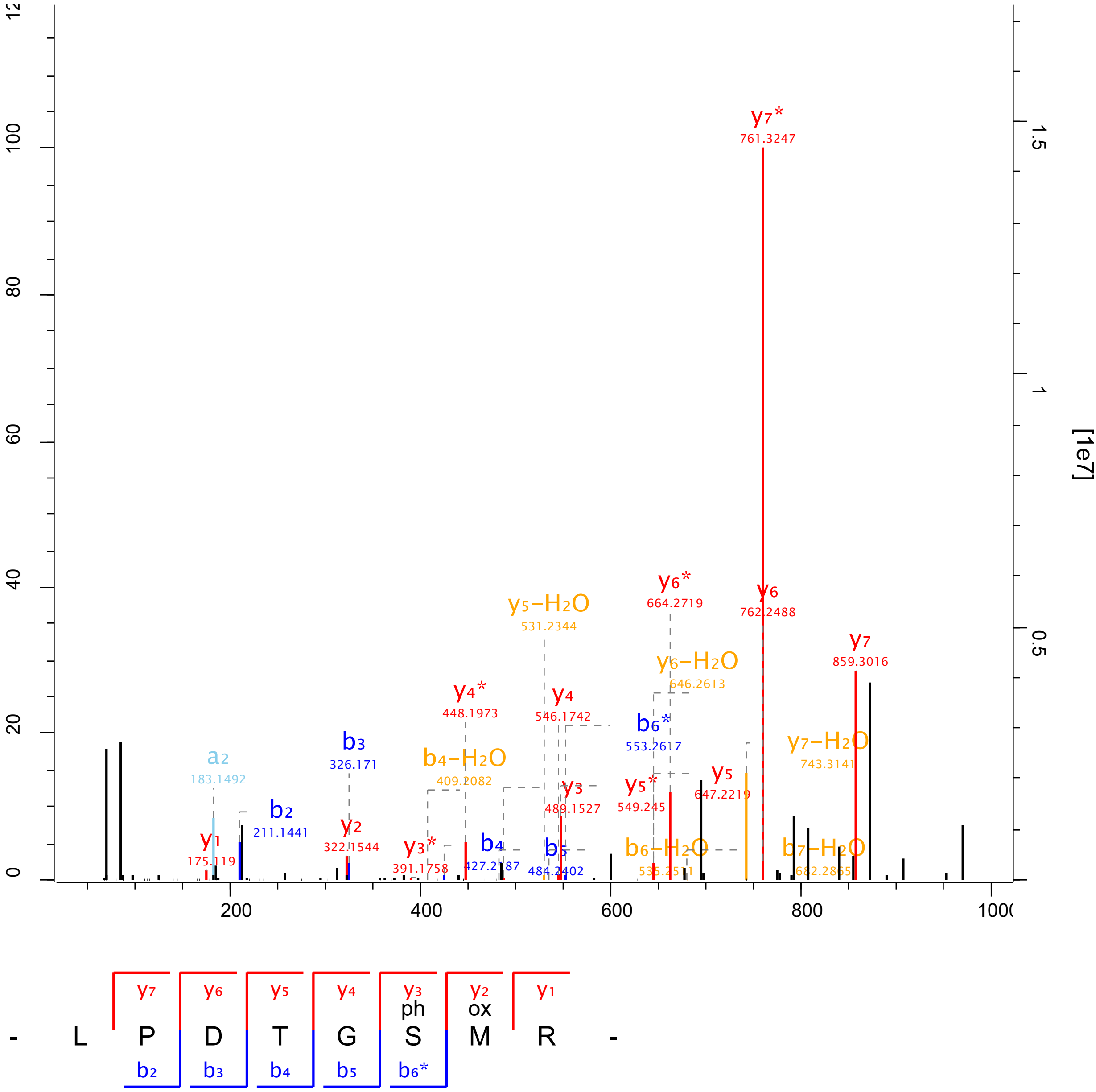

Raw file	Scan	Method	Score	m/z
sys_00_1	3784	FTMS; HCD	54.09	616.7

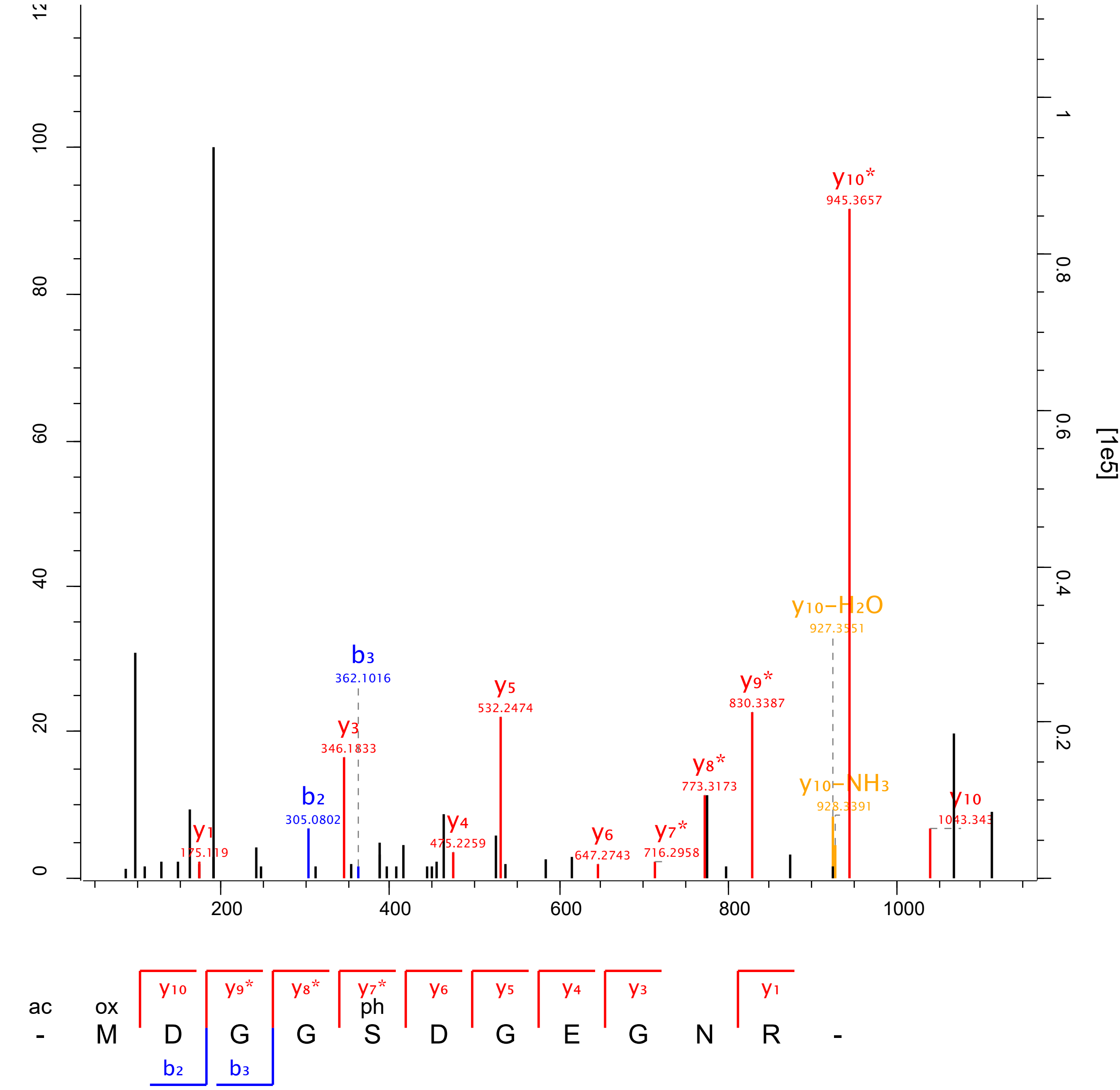

Raw file	Scan	Method	Score	m/z
sys_00_1	4842	FTMS; HCD	68.66	421.7

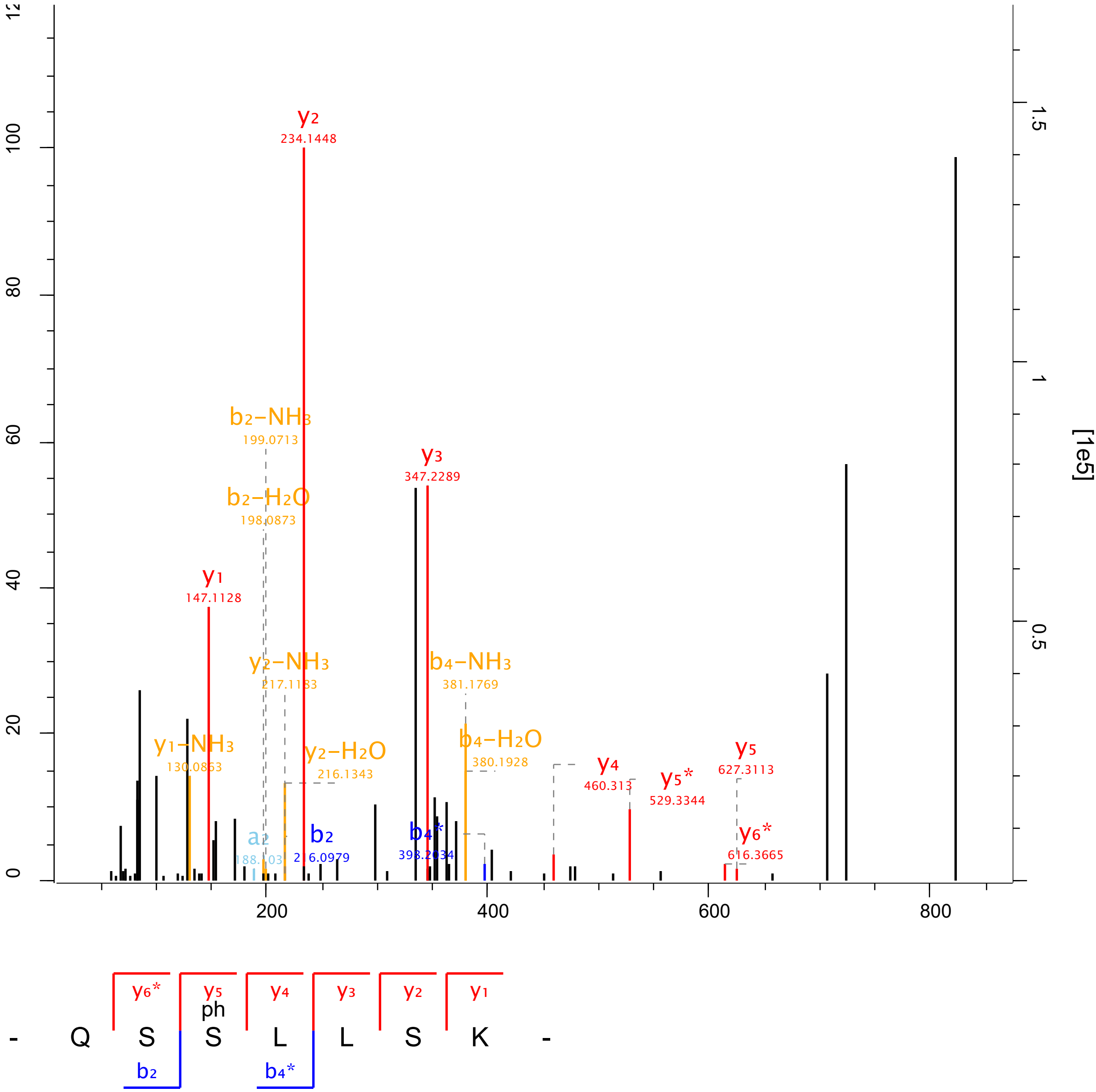

Raw file	Scan	Method	Score	m/z
sys_00_1	5069	FTMS; HCD	51.29	522.73

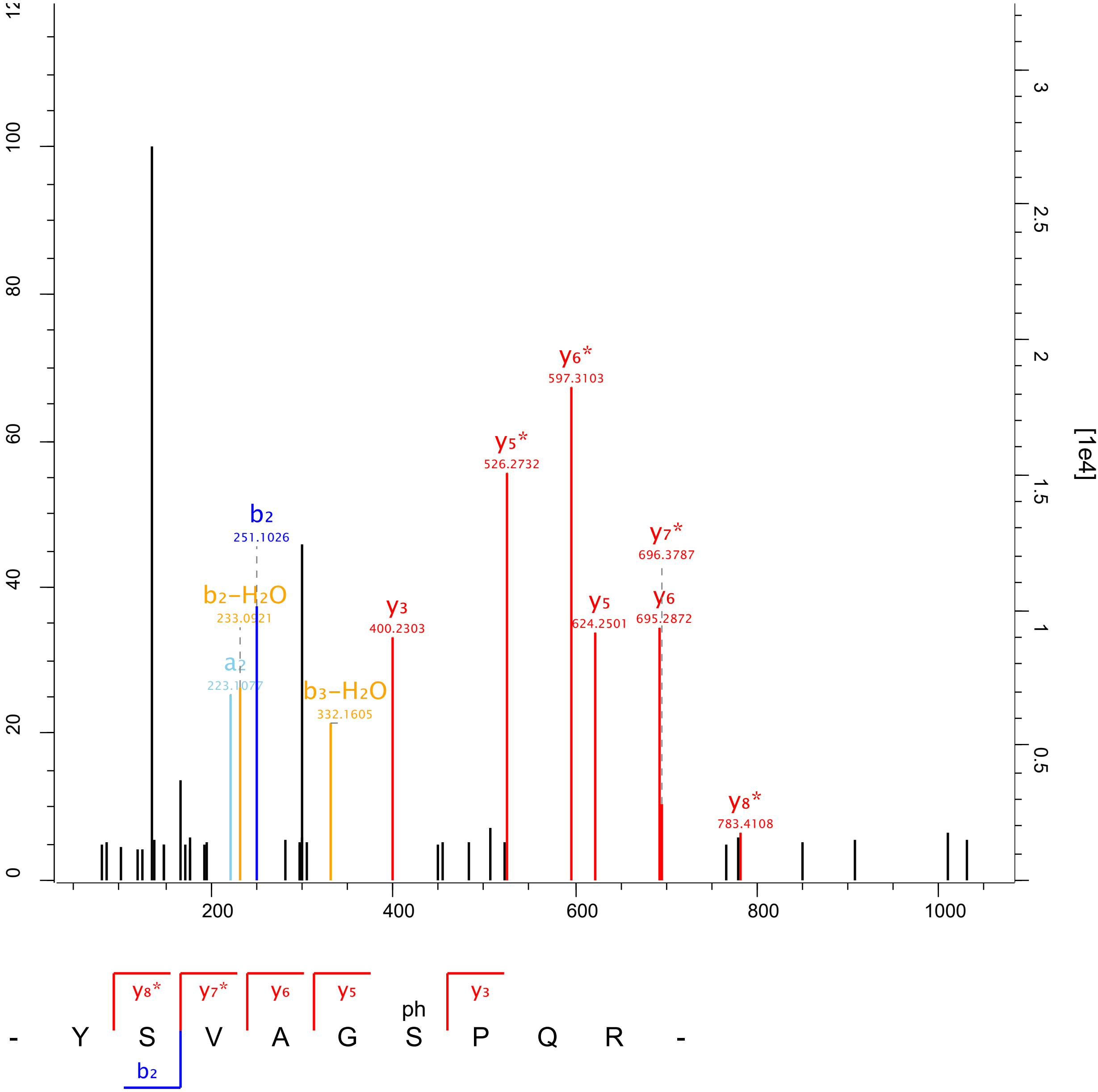

Raw file	Scan	Method	Score	m/z
sys_00_1	6749	FTMS; HCD	114.86	498.23

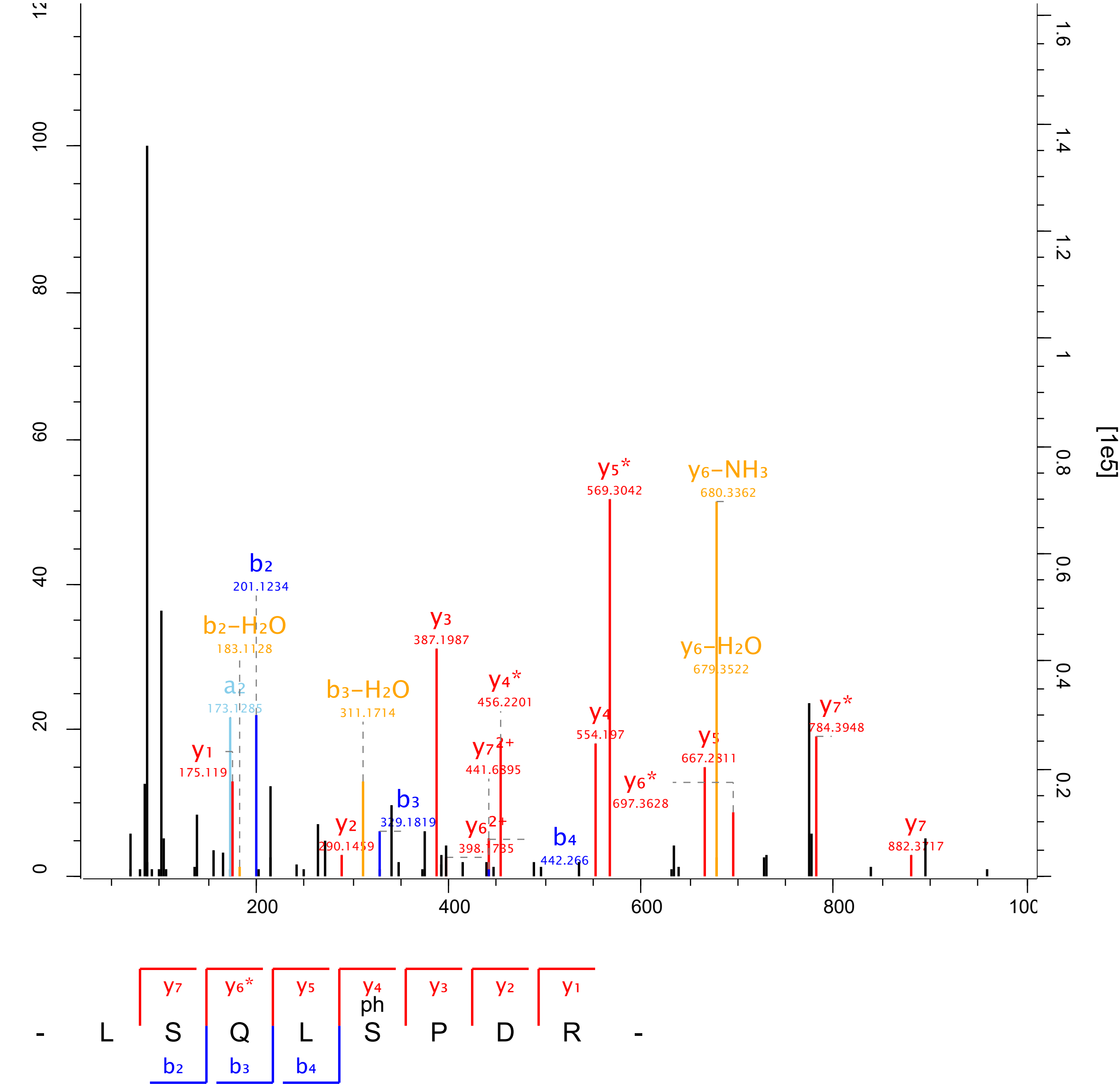

Raw file	Scan	Method	Score	m/z
sys_00_1	7359	FTMS; HCD	115.57	430.7

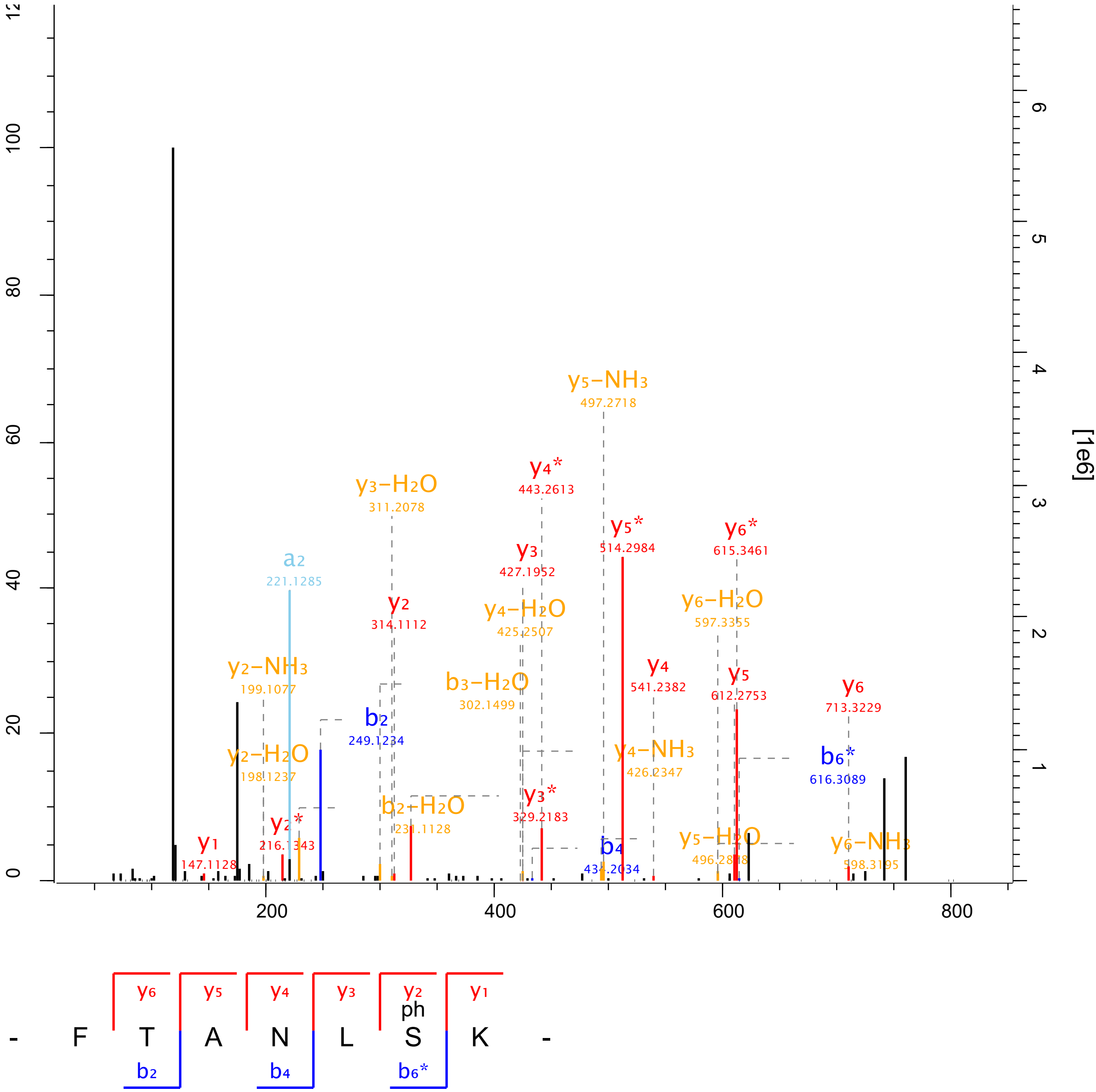

Raw file	Scan	Method	Score	m/z
sys_00_1	7613	FTMS; HCD	81.63	650.75

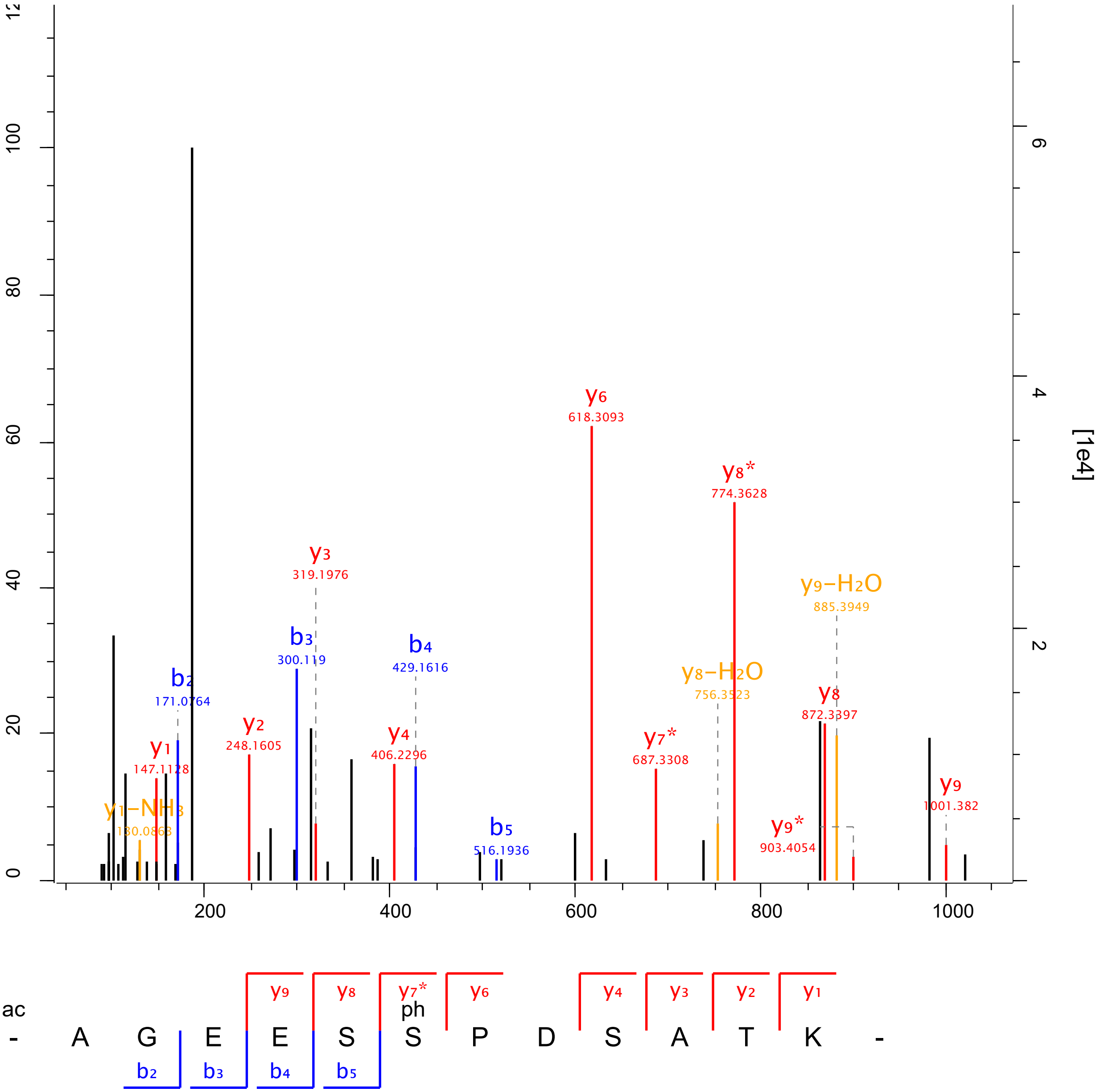

Raw file	Scan	Method	Score	m/z
sys_00_1	8284	FTMS; HCD	97.69	601.75

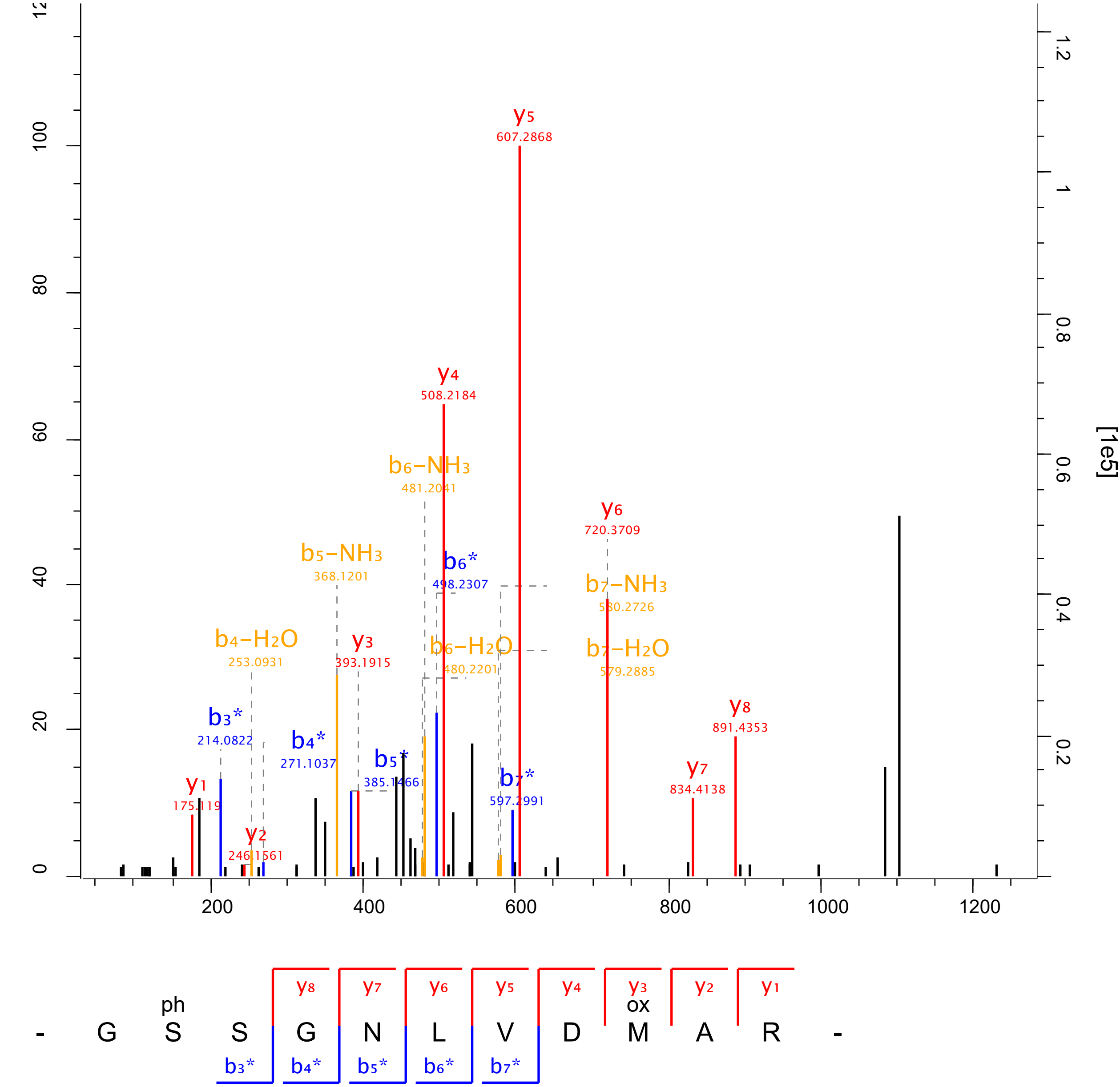

Raw file	Scan	Method	Score	m/z
sys_00_1	8470	FTMS; HCD	83.18	628.76

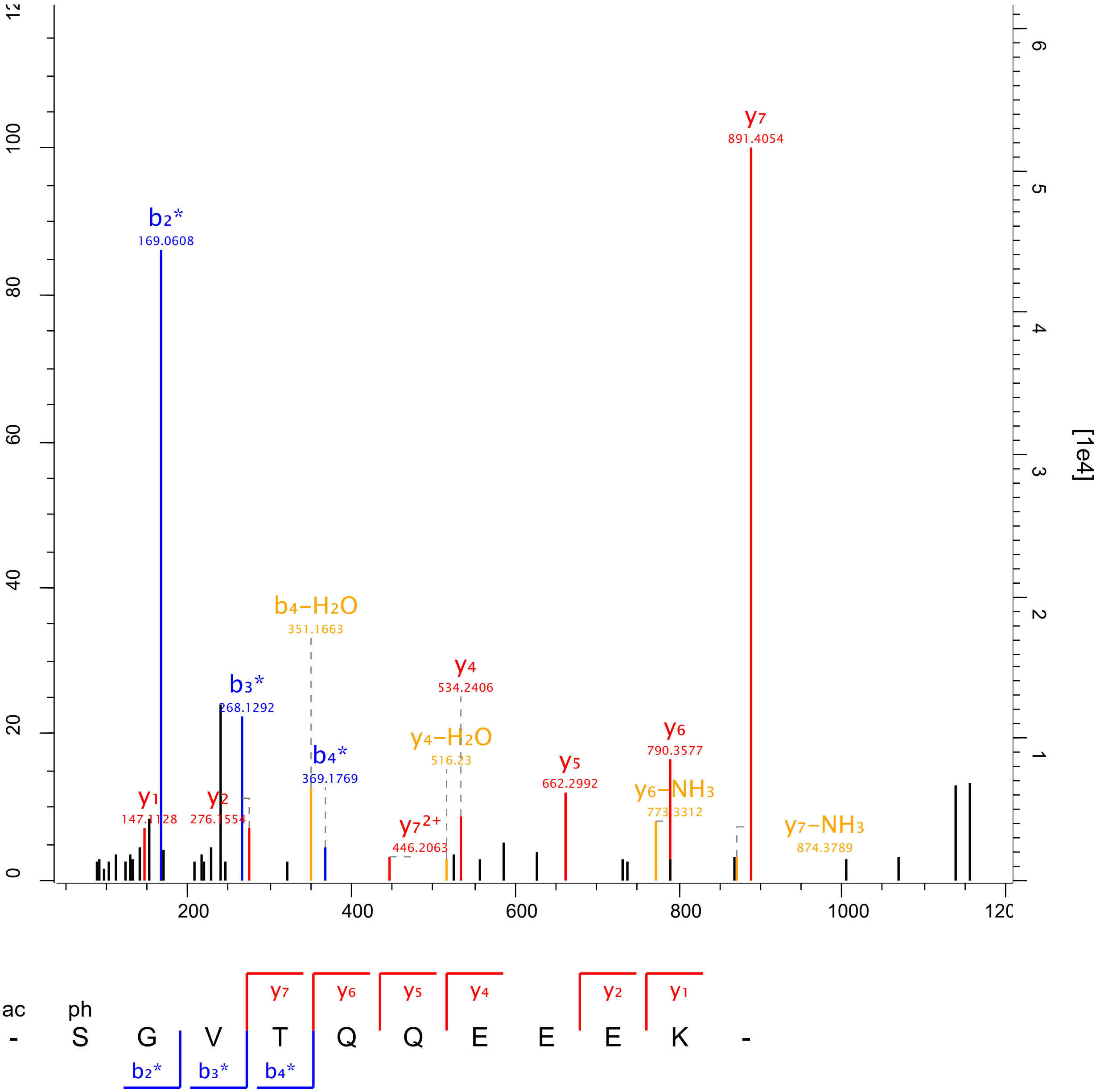

Raw file	Scan	Method	Score	m/z
sys_00_2	10058	FTMS; HCD	67.11	483.18

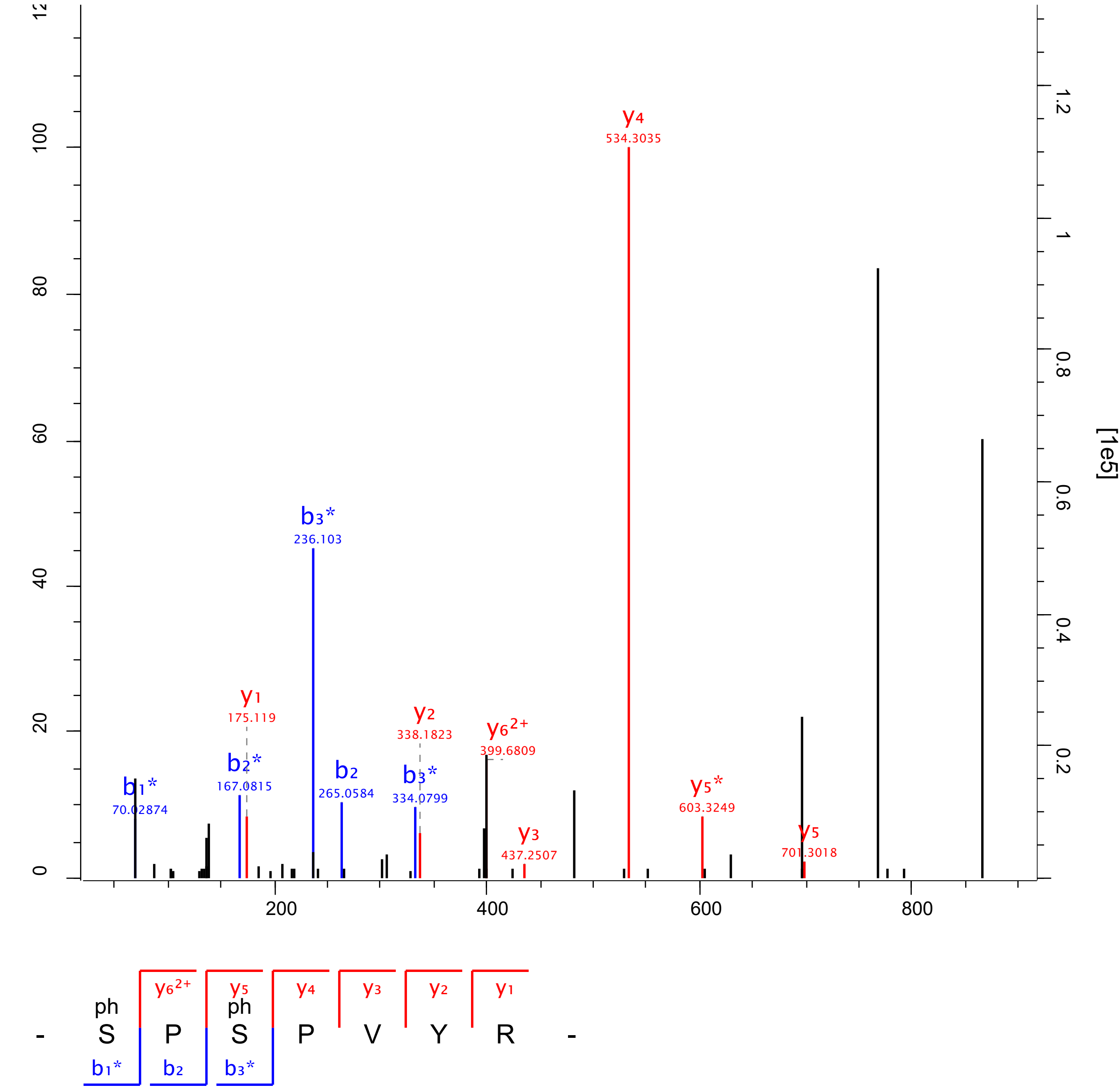

Raw file	Scan	Method	Score	m/z
sys_00_2	10457	FTMS; HCD	44.25	644.27

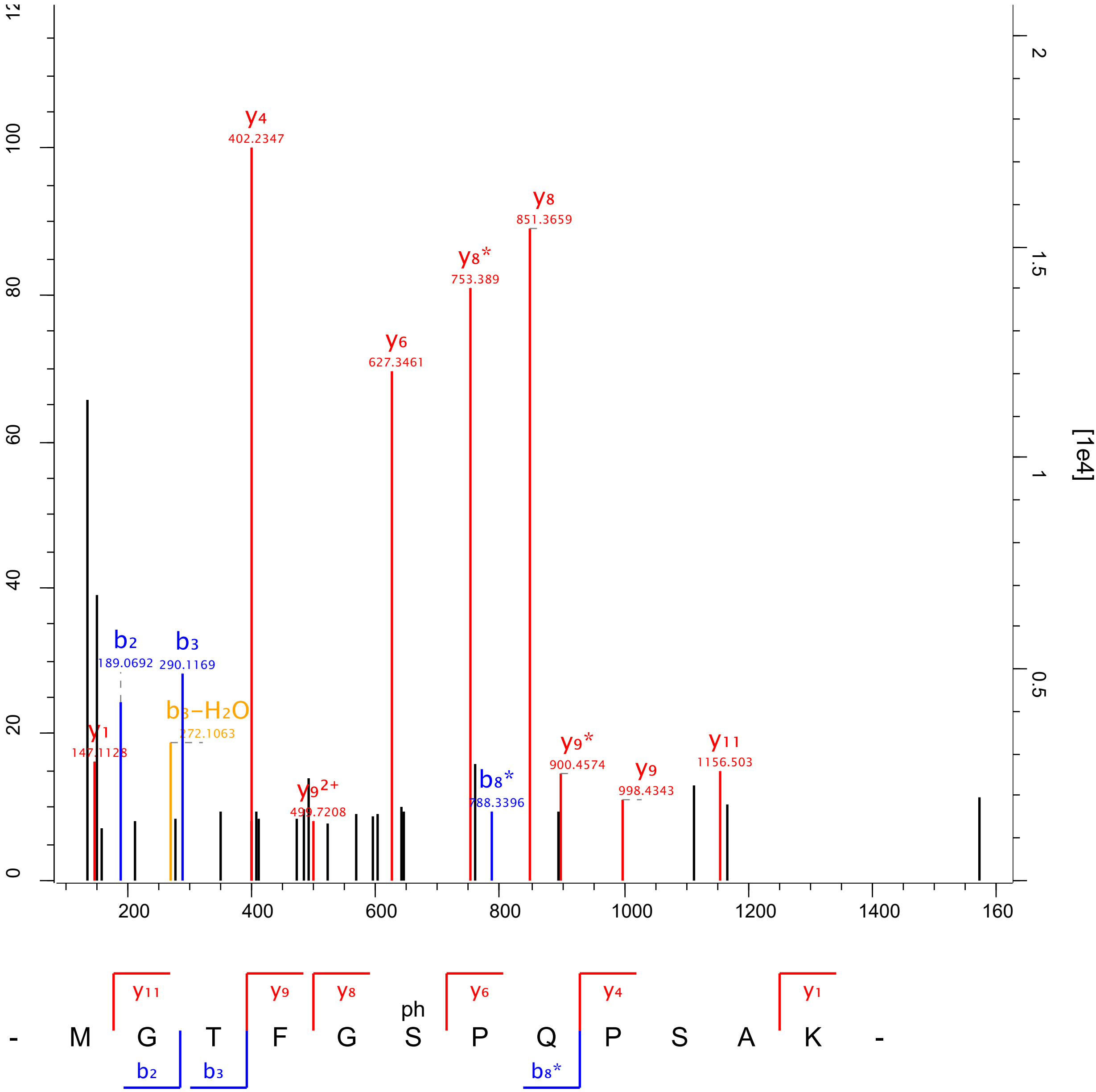

Raw file	Scan	Method	Score	m/z
sys_00_2	10468	FTMS; HCD	54.16	490.71

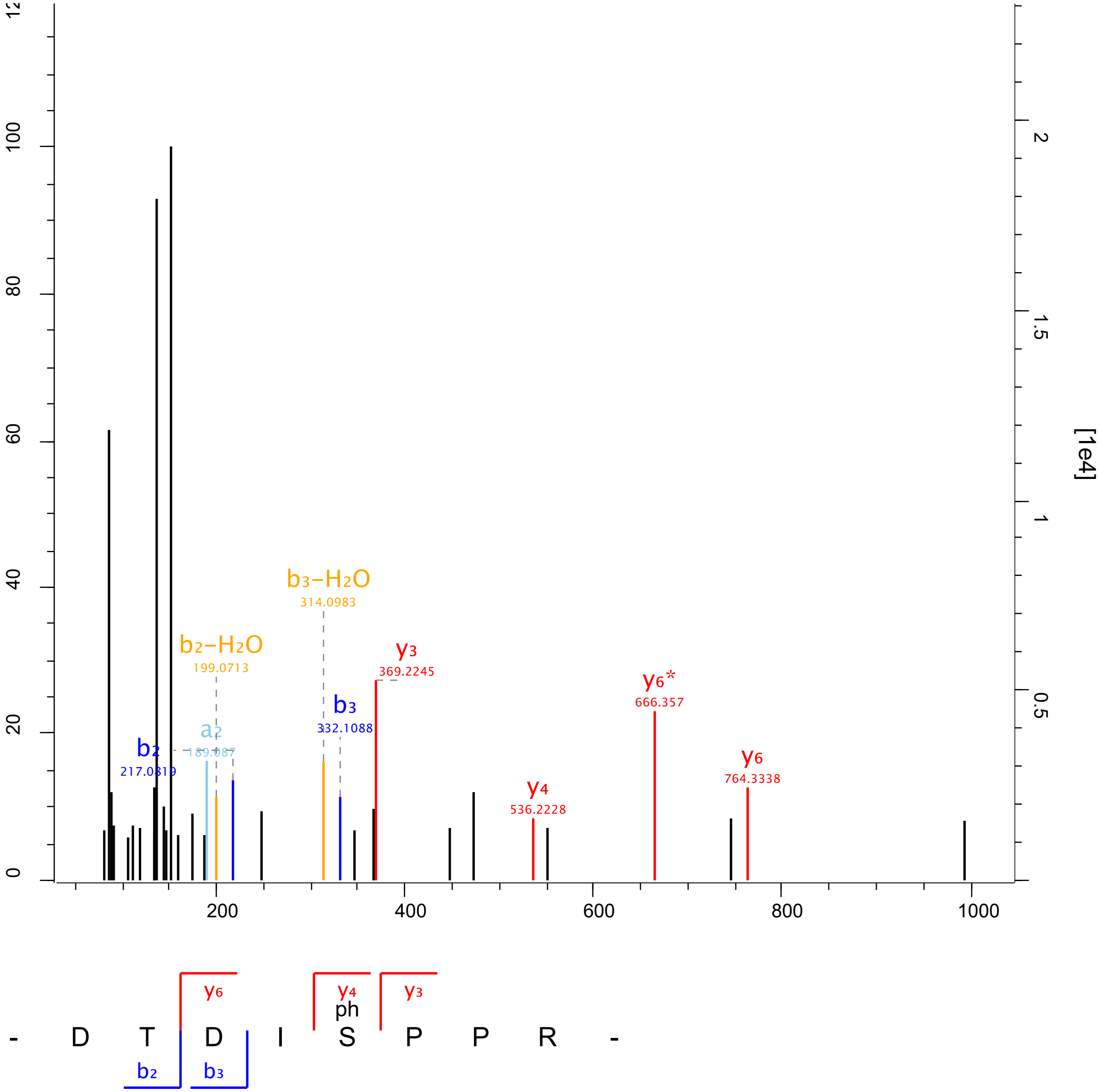

Raw file	Scan	Method	Score	m/z
sys_00_2	10876	FTMS; HCD	147.62	456.73

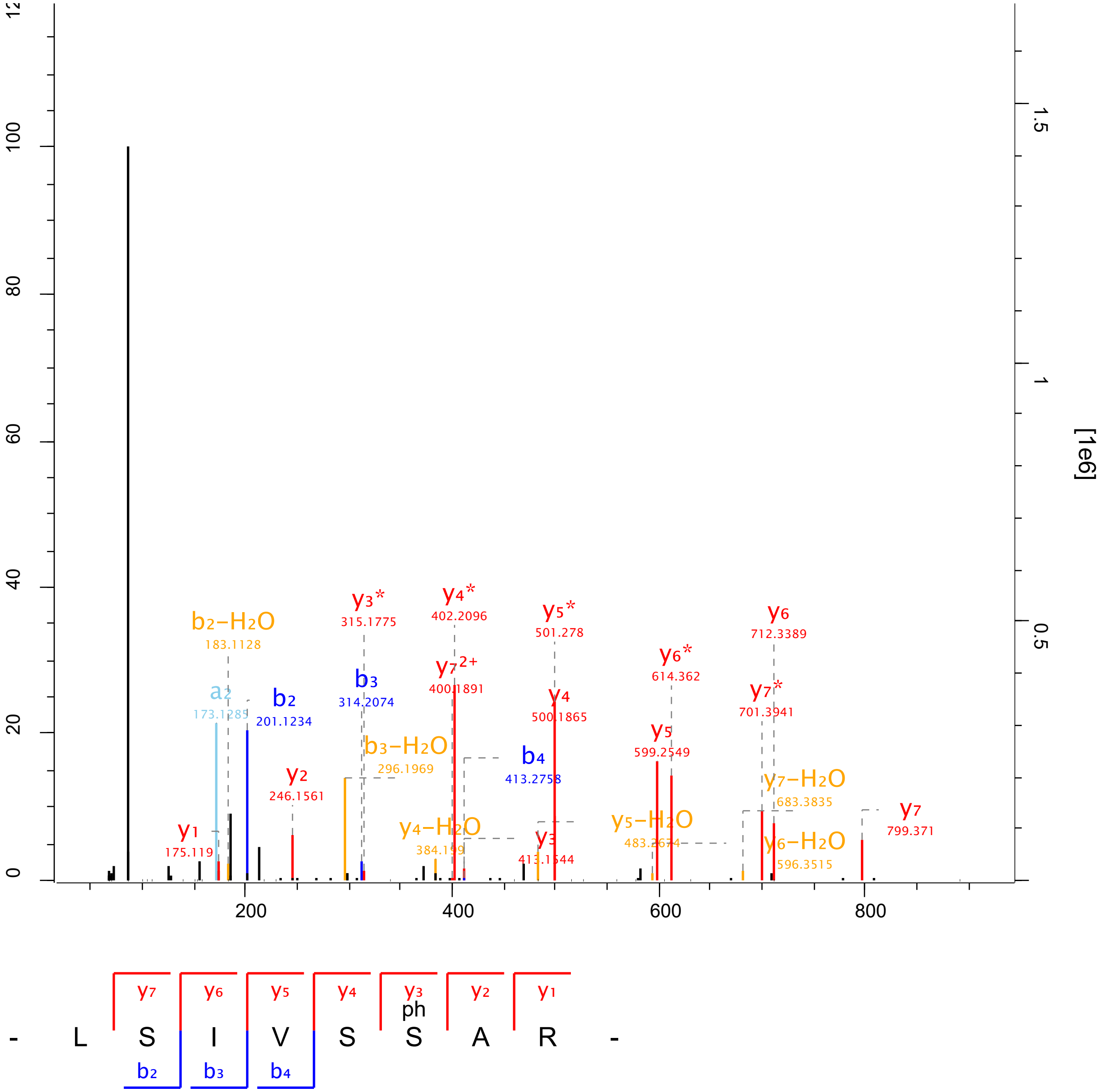

Raw file	Scan	Method	Score	m/z
sys_00_2	11570	FTMS; HCD	109.04	657.3

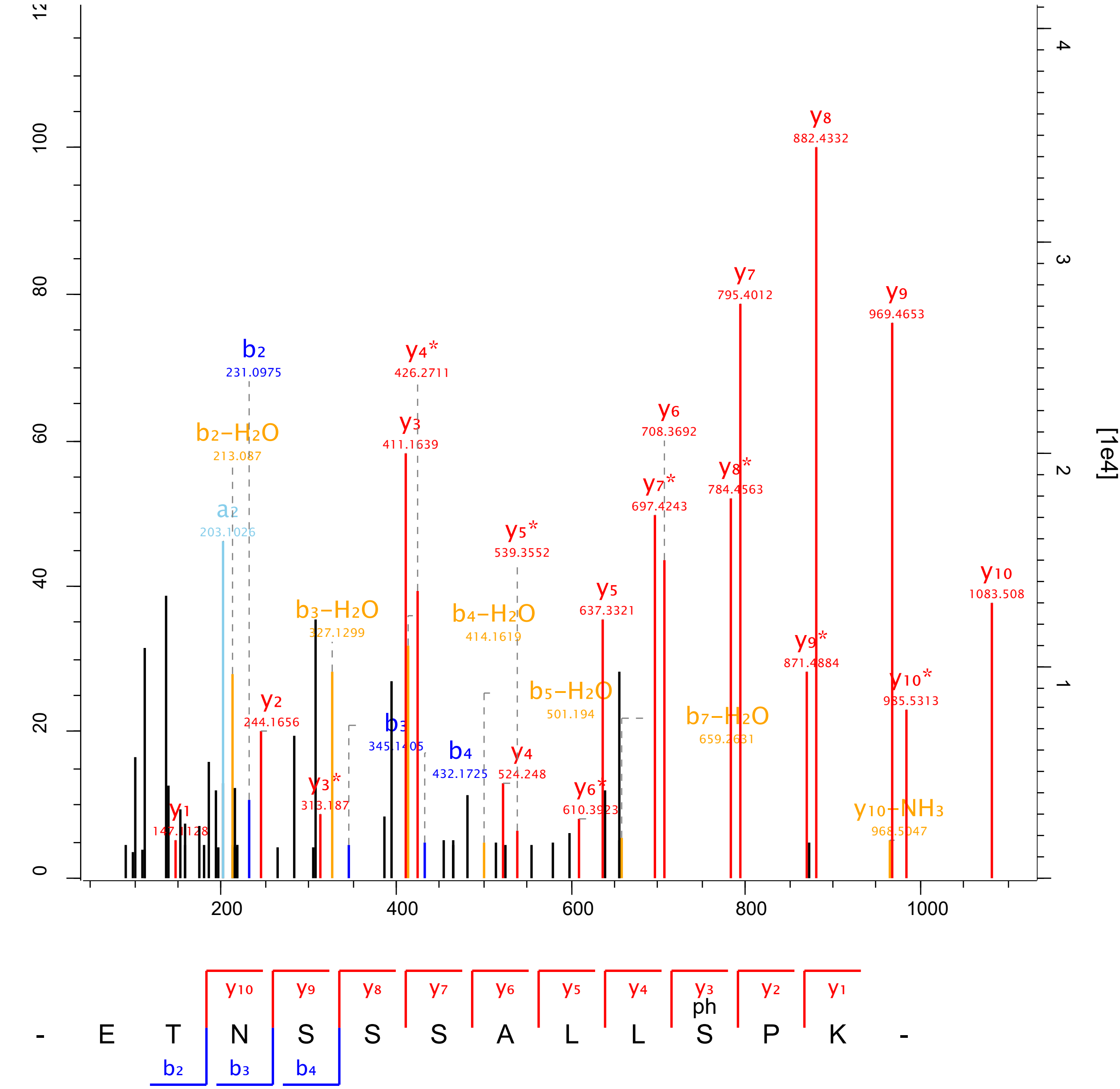

Raw file	Scan	Method	Score	m/z
sys_00_2	13502	FTMS; HCD	40.43	582.23

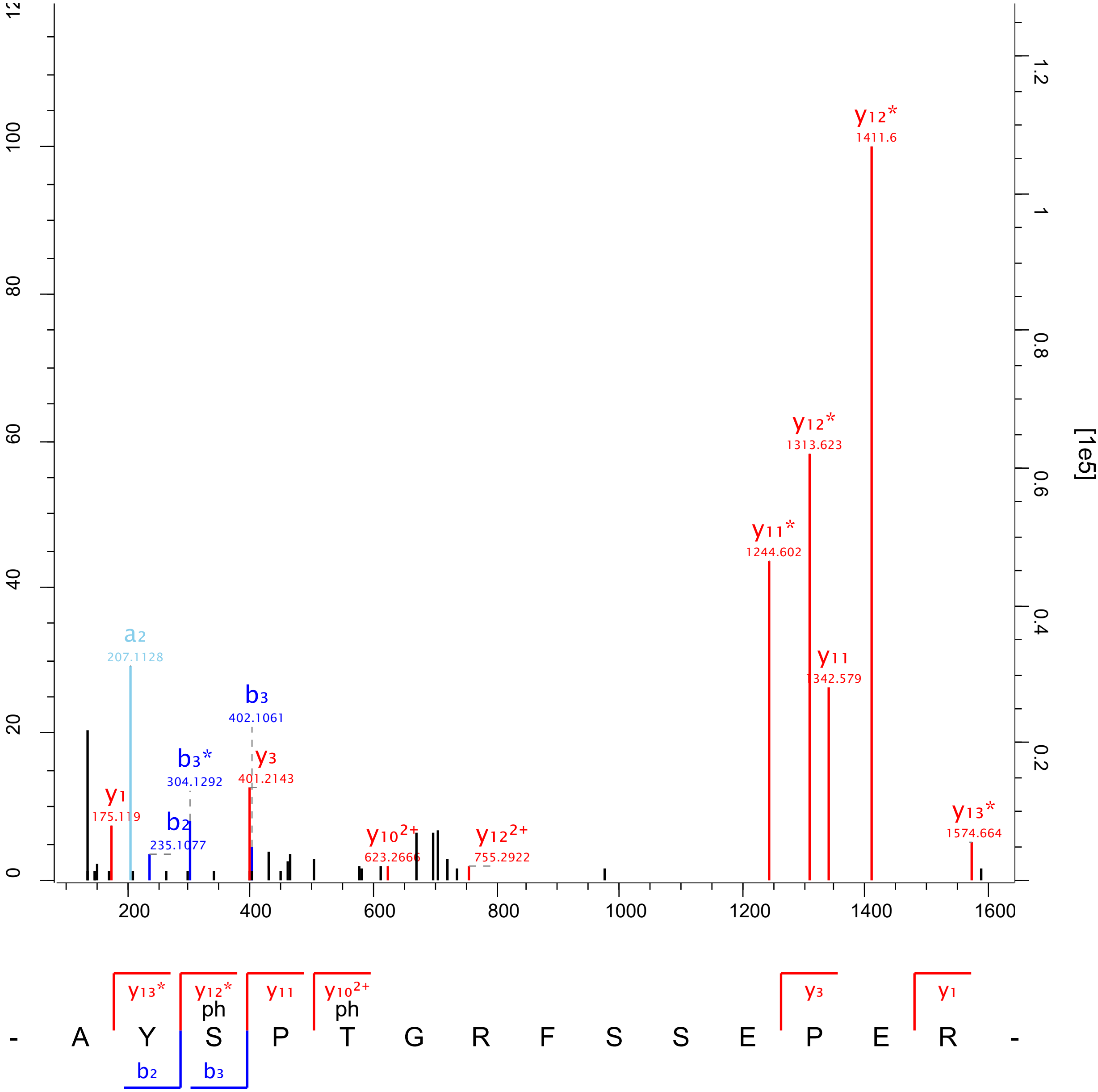

Raw file	Scan	Method	Score	m/z
sys_00_2	14148	FTMS; HCD	69.72	514.24

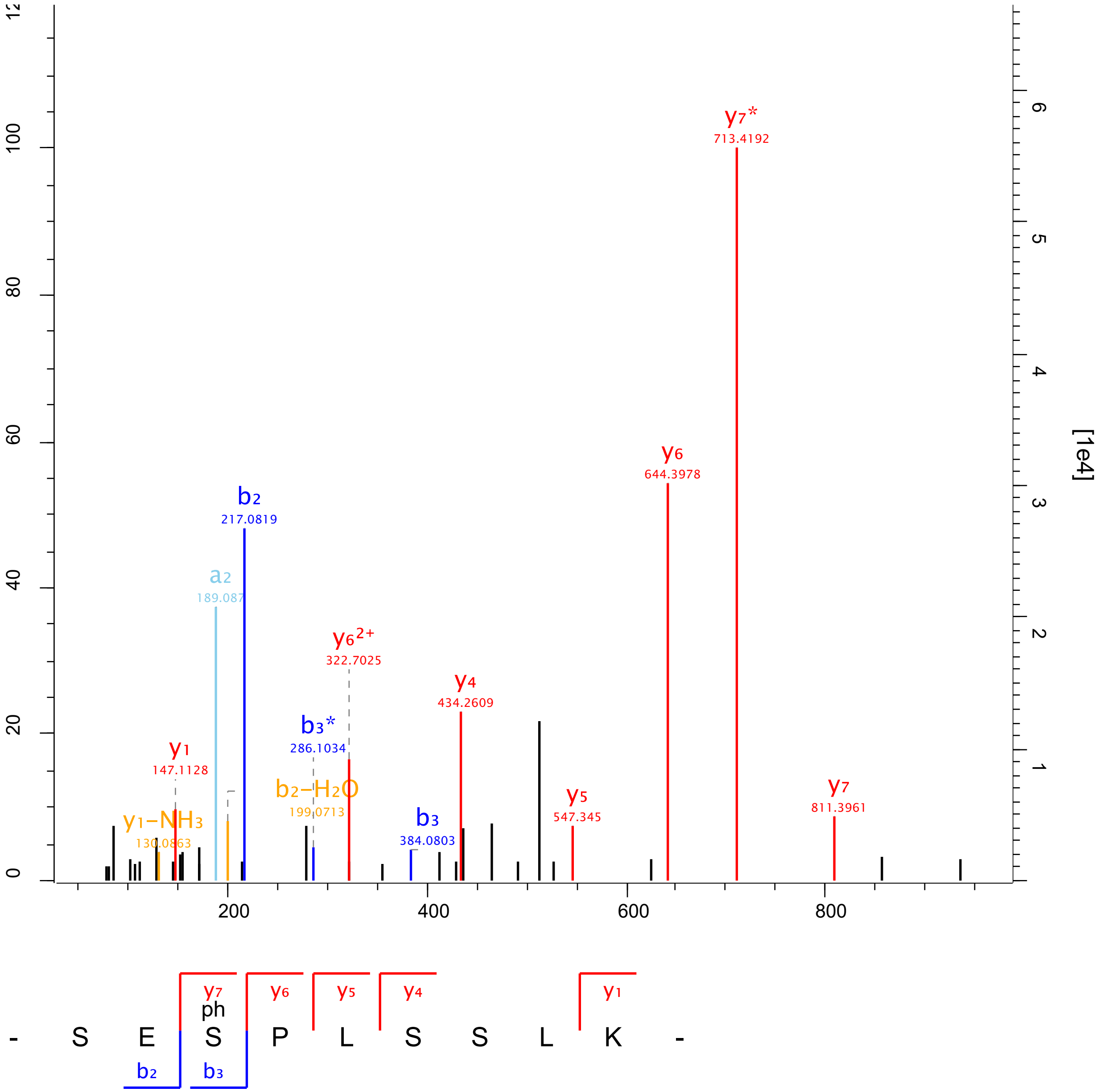

Raw file	Scan	Method	Score	m/z
sys_00_2	14643	FTMS; HCD	54.28	759.83

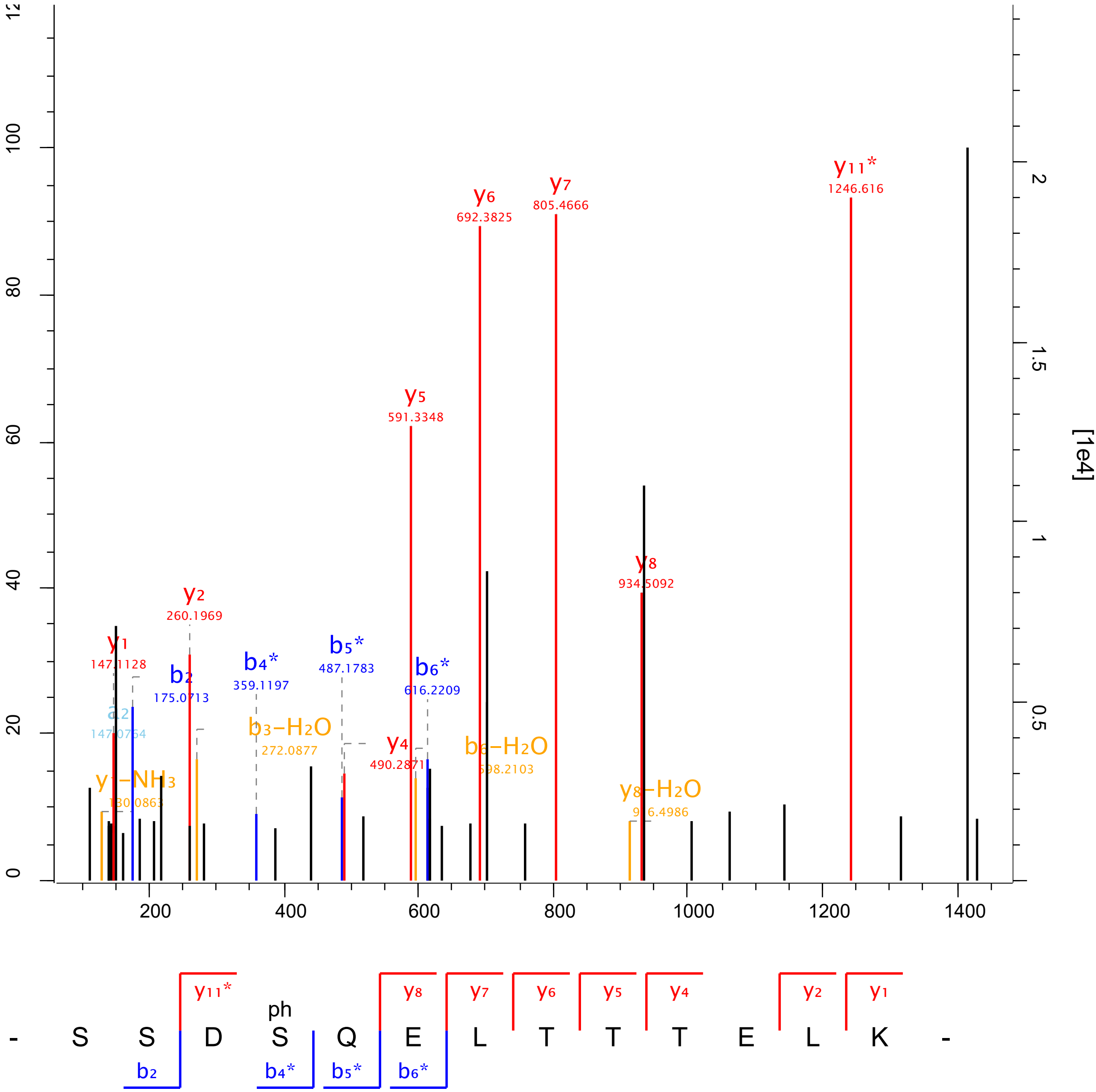

Raw file	Scan	Method	Score	m/z
sys_00_2	14960	FTMS; HCD	305.22	756.29

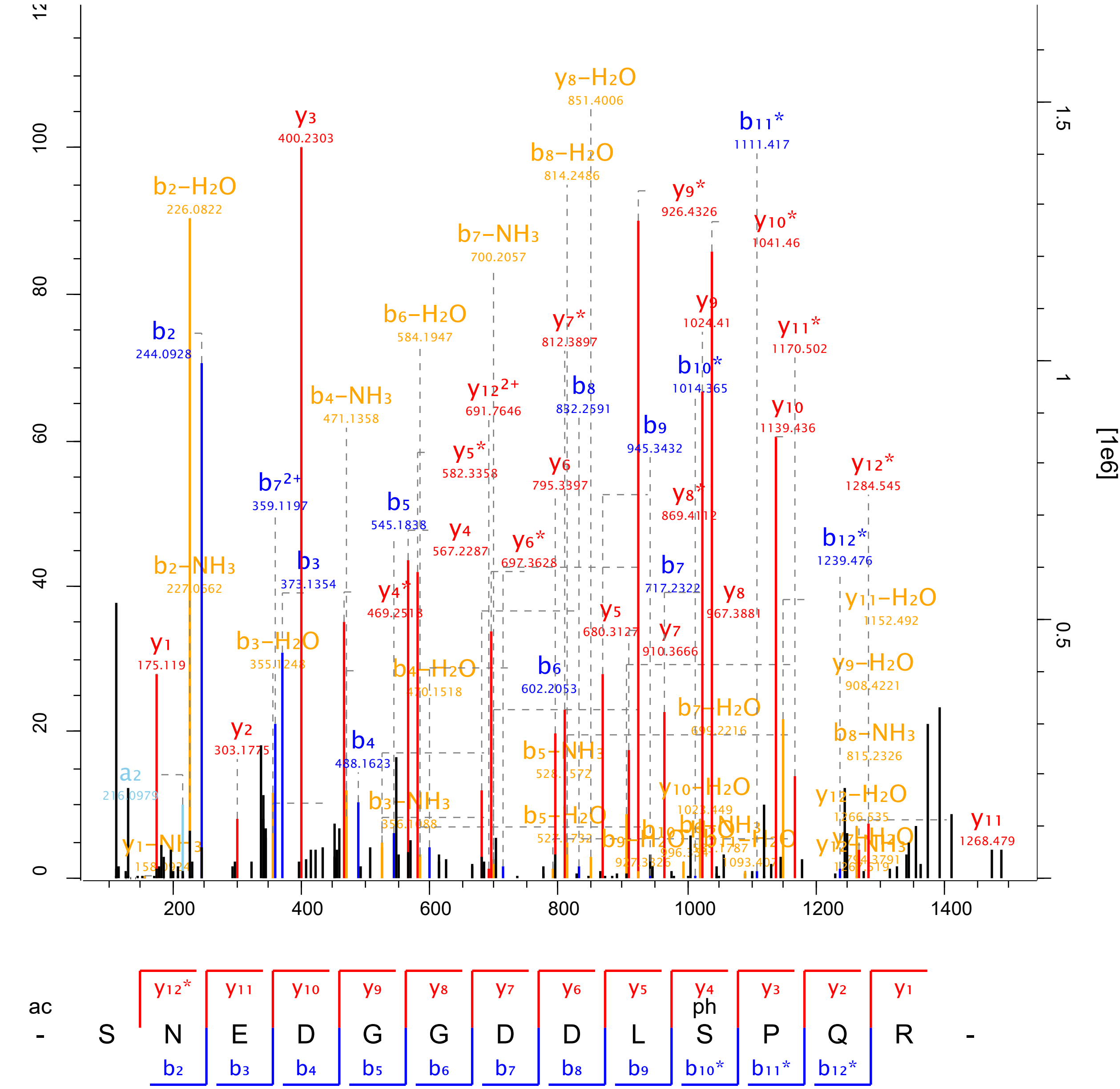

Raw file	Scan	Method	Score	m/z
sys_00_2	1497	FTMS; HCD	43.77	685.93

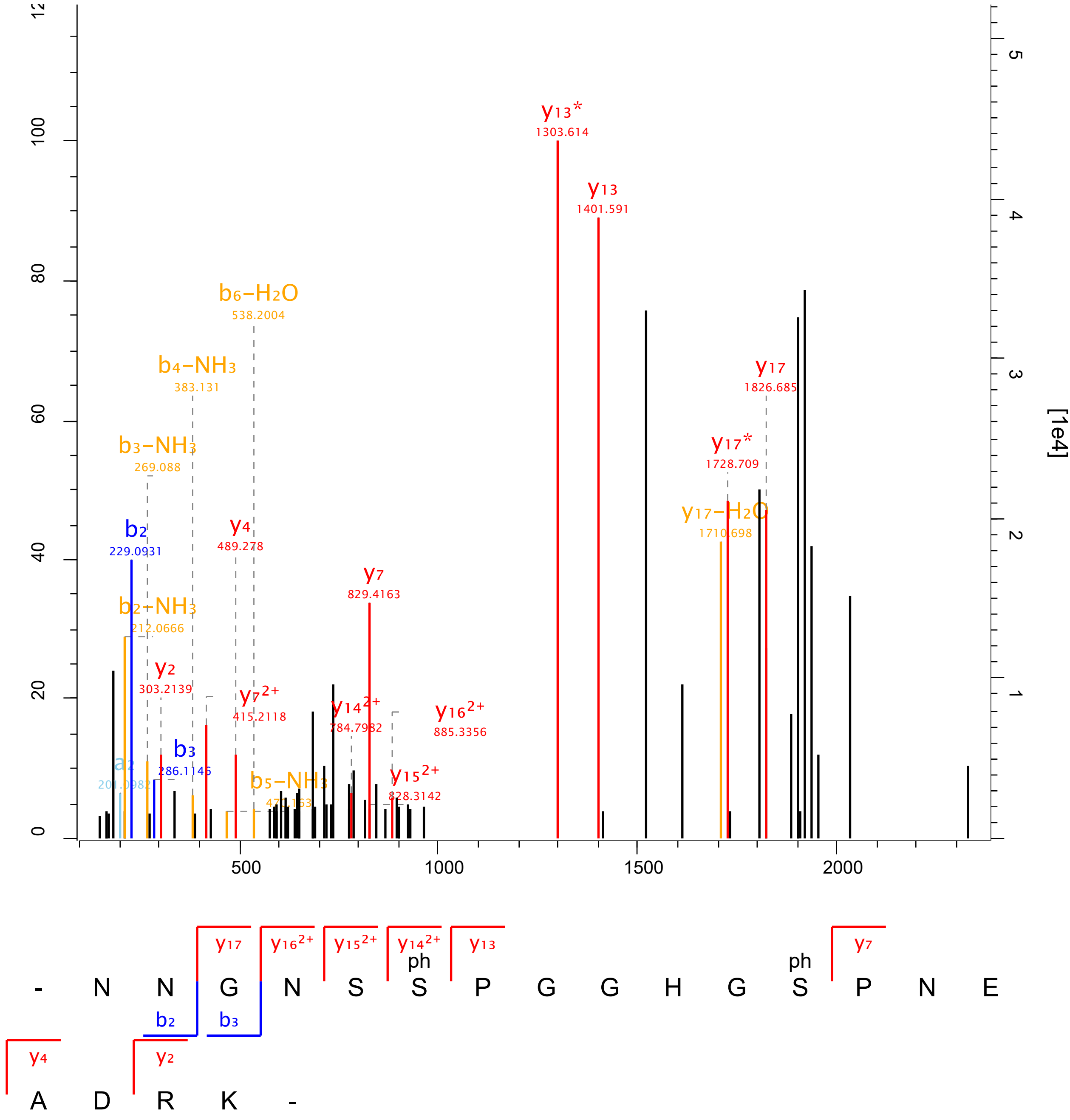

Raw file	Scan	Method	Score	m/z
sys_00_2	15999	FTMS; HCD	85.55	603.75

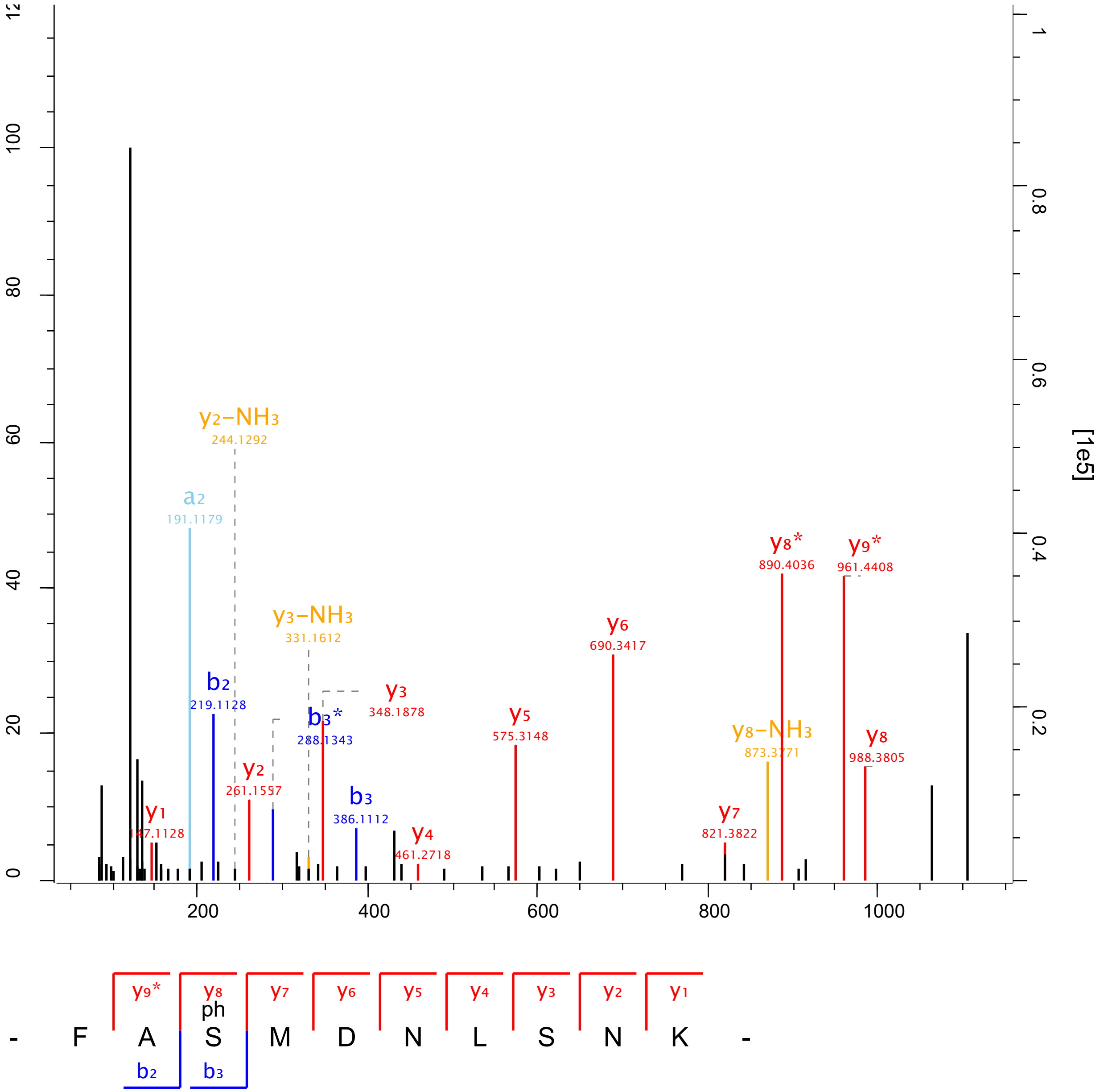

Raw file	Scan	Method	Score	m/z
sys_00_2	16007	FTMS; HCD	117.86	553.24

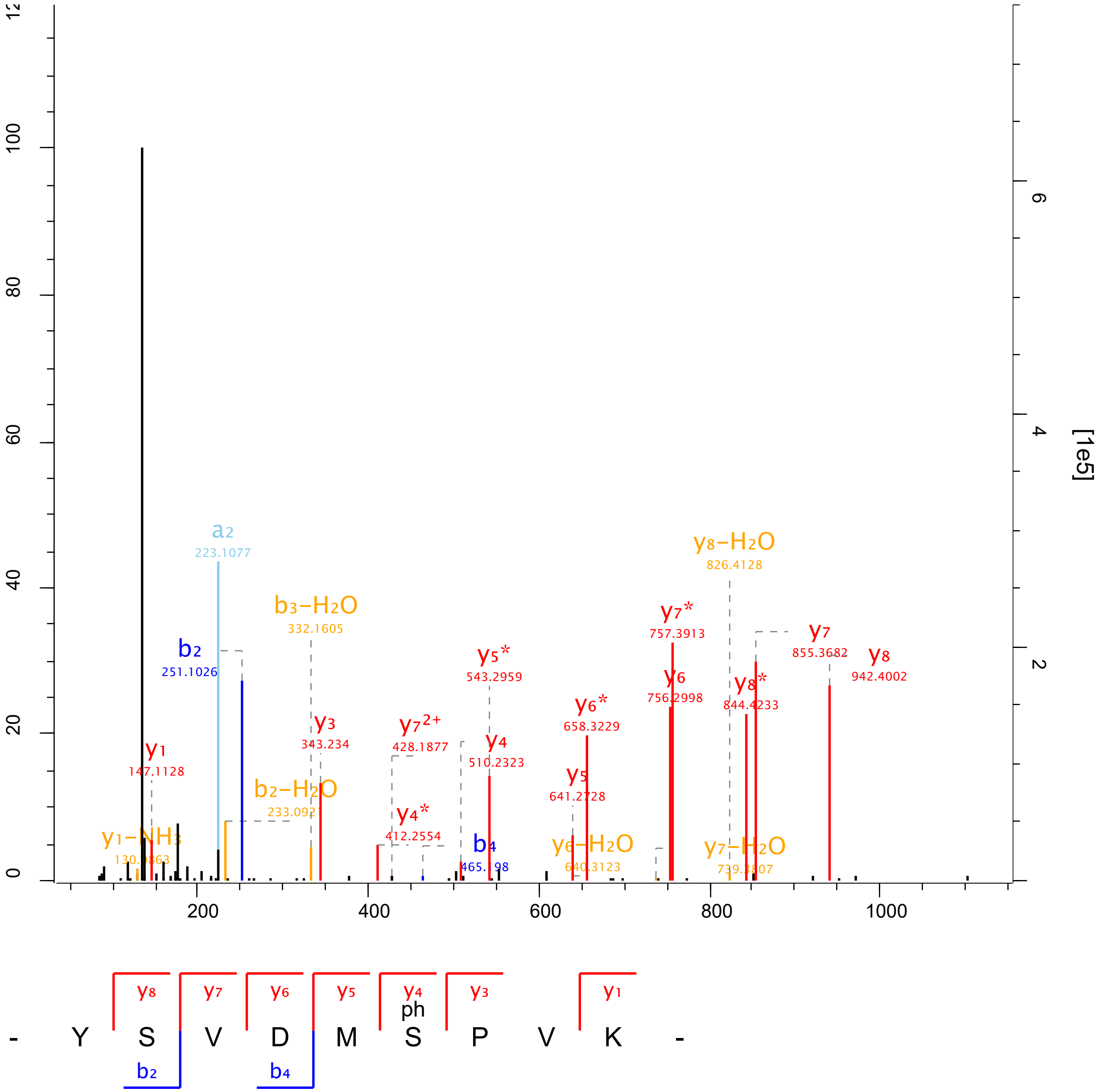

Raw file	Scan	Method	Score	m/z
sys_00_2	17622	FTMS; HCD	177.42	649.25

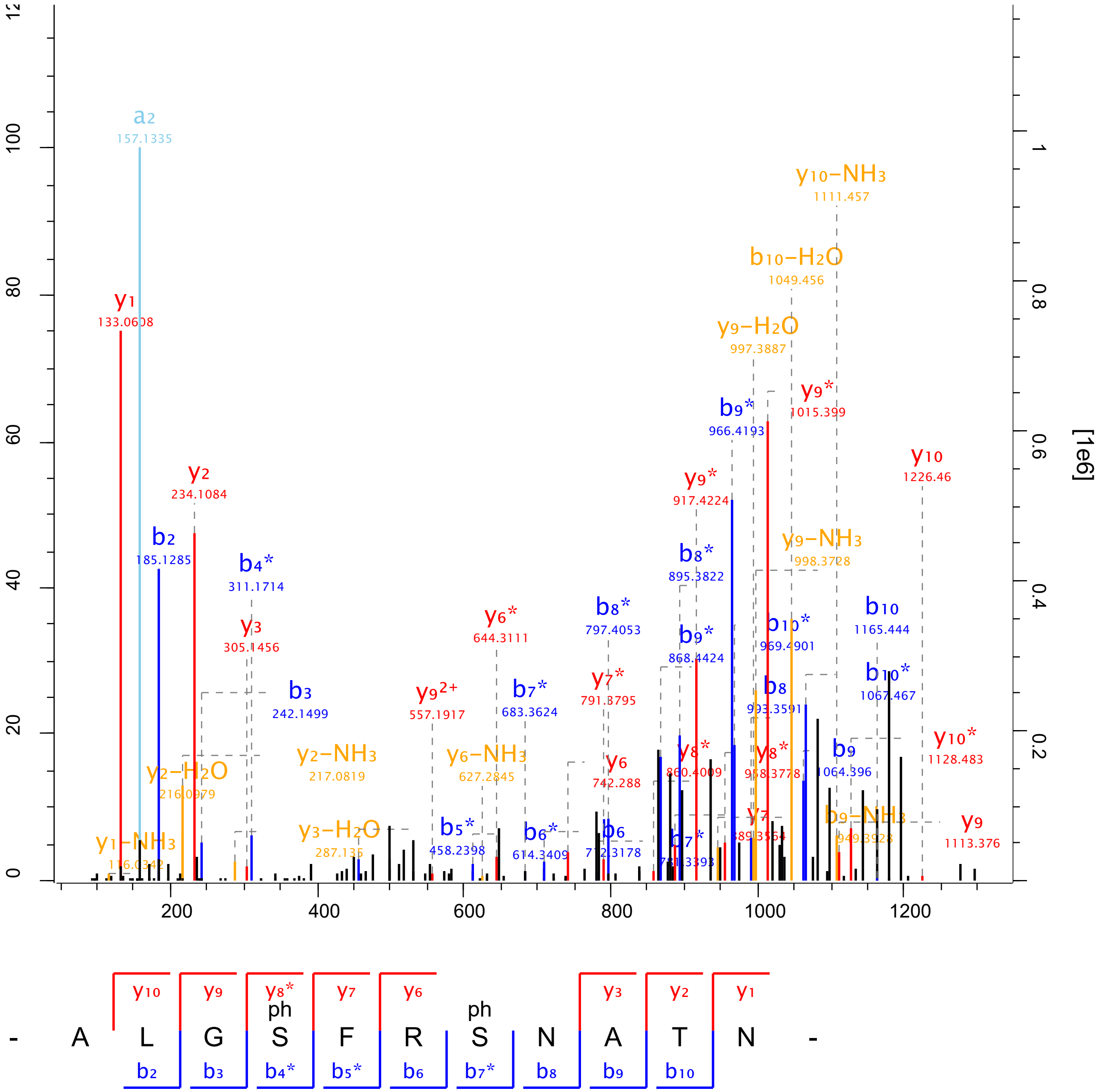

Raw file	Scan	Method	Score	m/z
sys_00_2	19780	FTMS; HCD	46.89	751.84

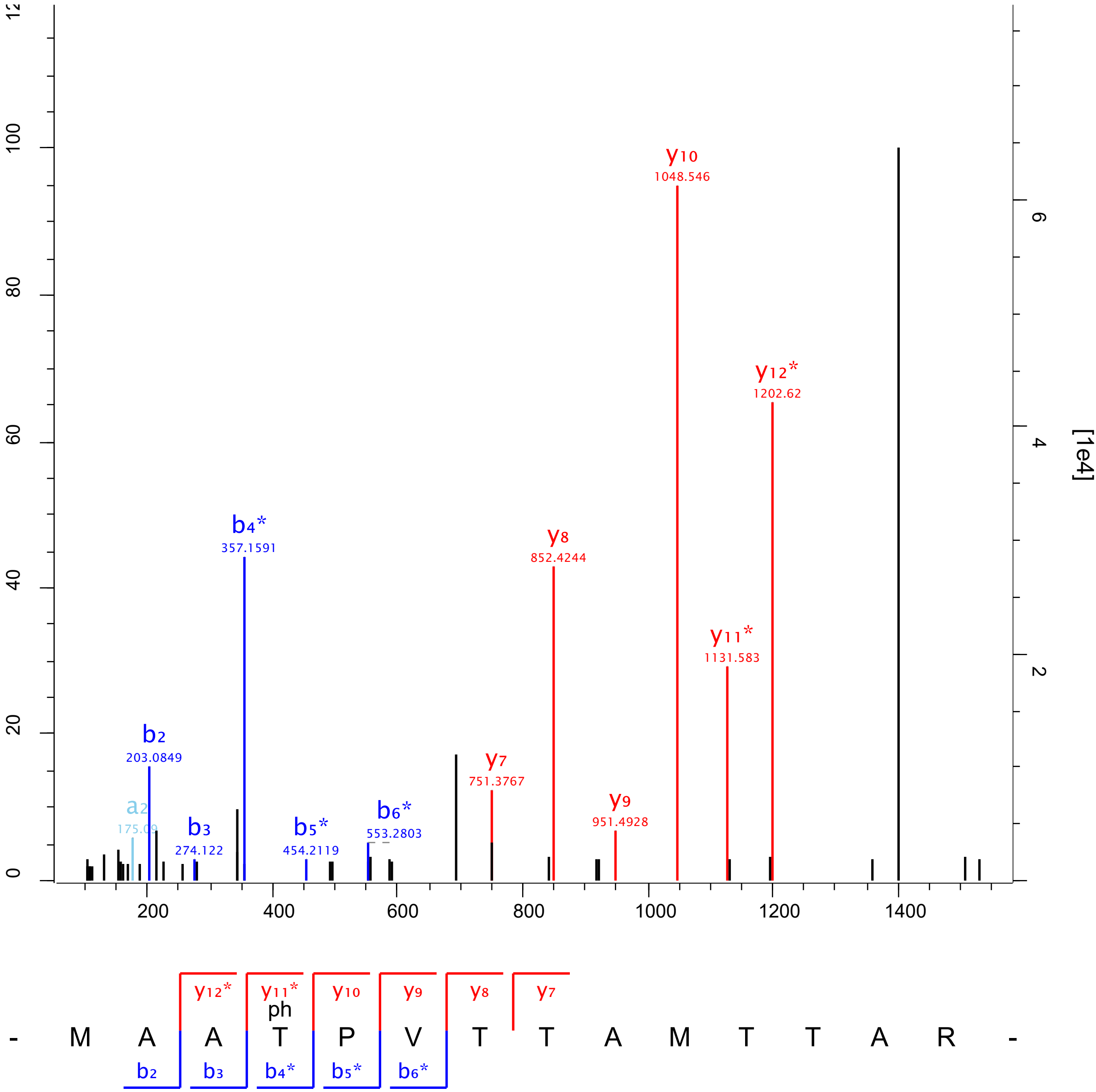

Raw file	Scan	Method	Score	m/z
sys_00_2	20133	FTMS; HCD	210.64	969.92

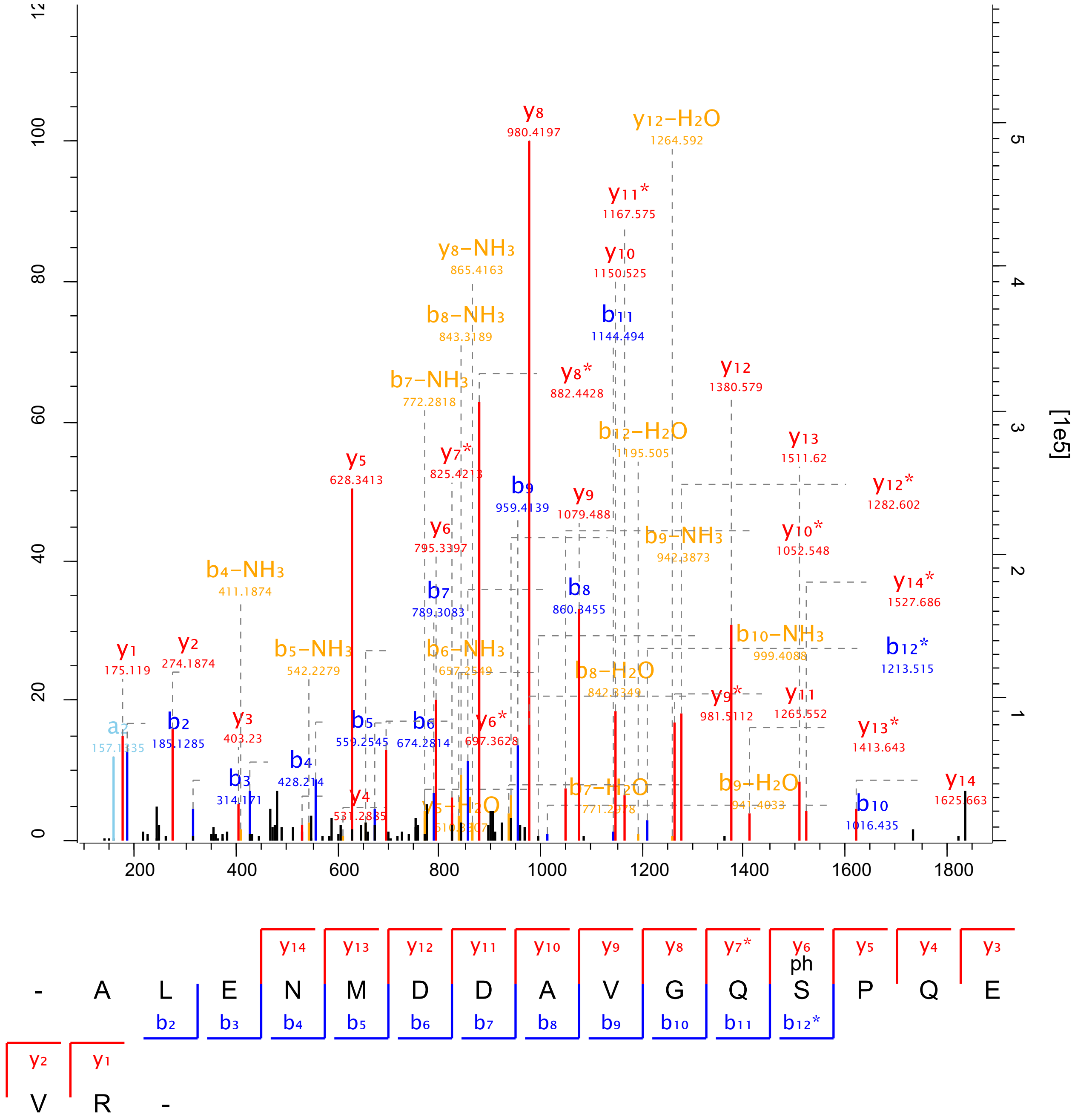

Raw file	Scan	Method	Score	m/z
sys_00_2	21664	FTMS; HCD	201.29	887.88

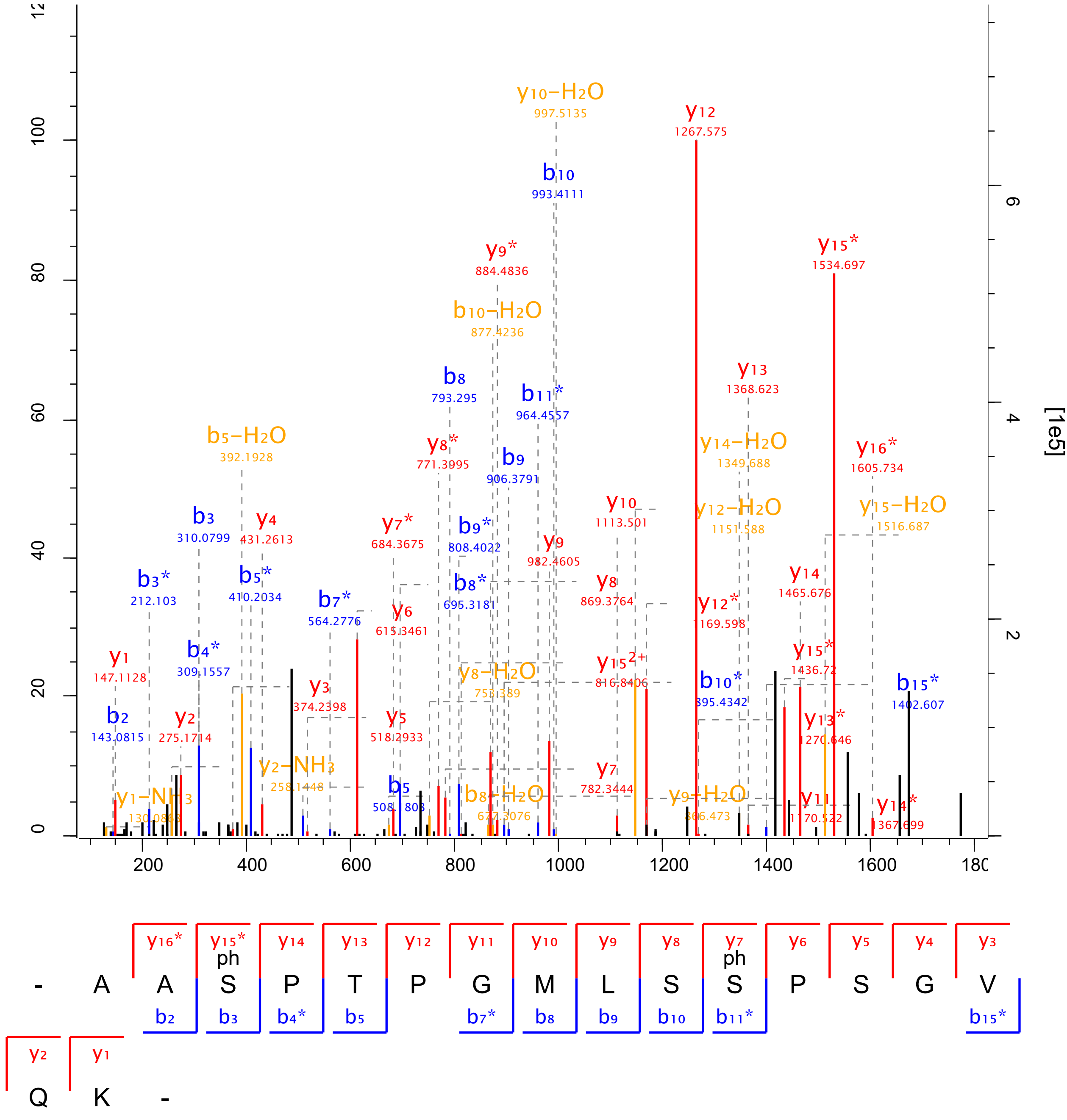

Raw file	Scan	Method	Score	m/z
sys_00_2	22688	FTMS; HCD	241.79	718.86

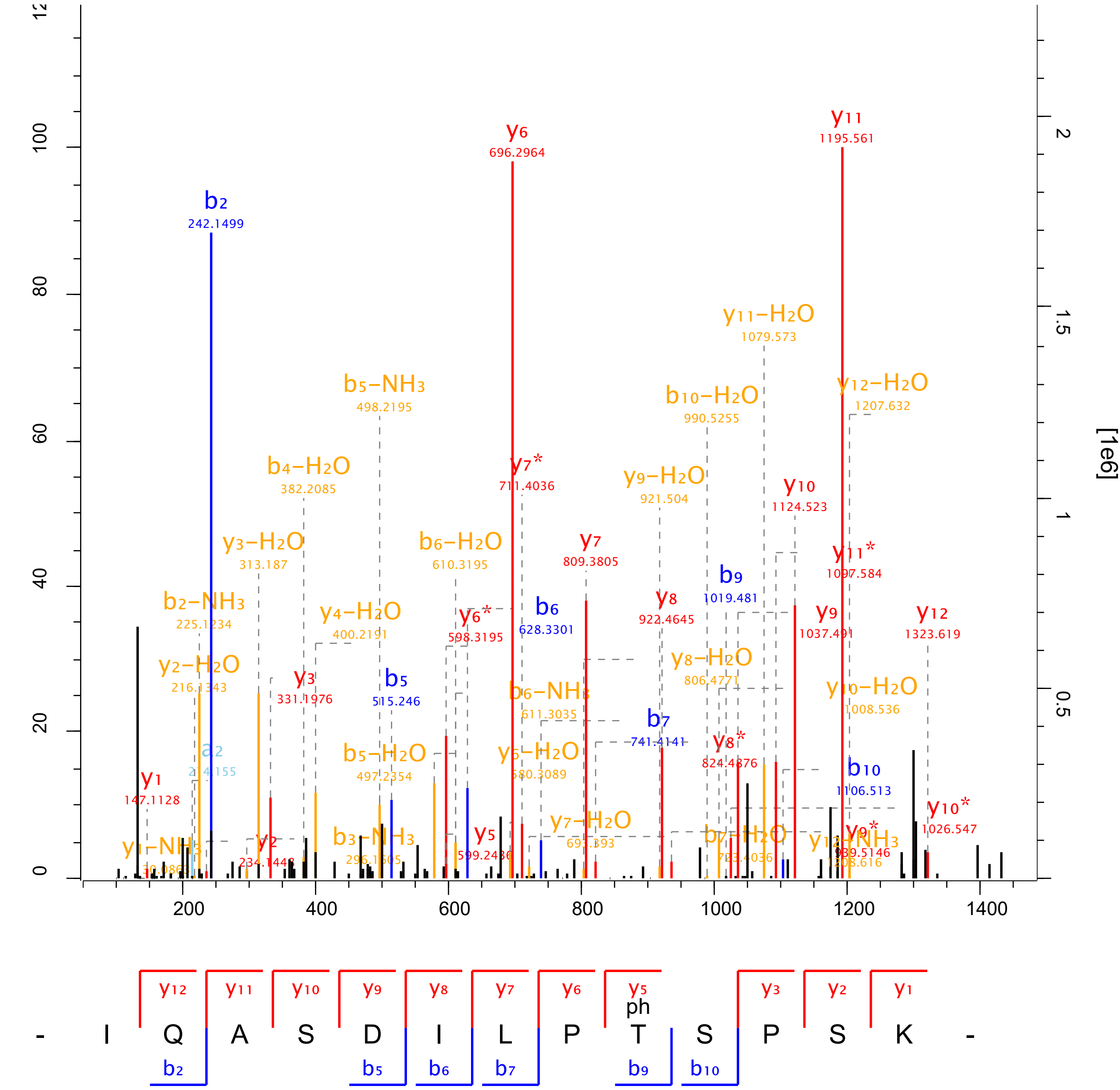

Raw file	Scan	Method	Score	m/z
sys_00_2	23125	FTMS; HCD	134.68	643.29

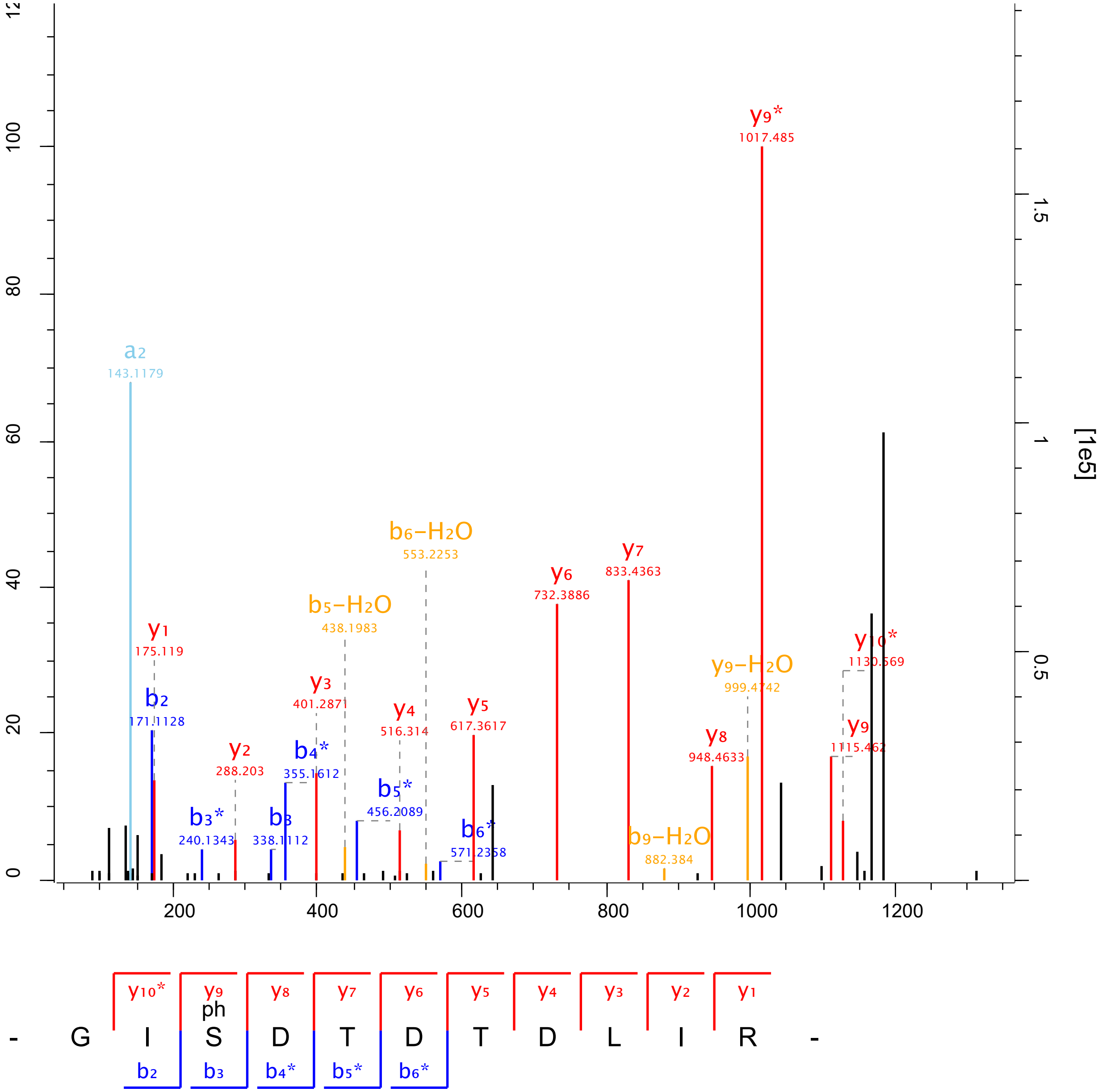

Raw file	Scan	Method	Score	m/z
sys_00_2	25449	FTMS; HCD	167.63	886.36

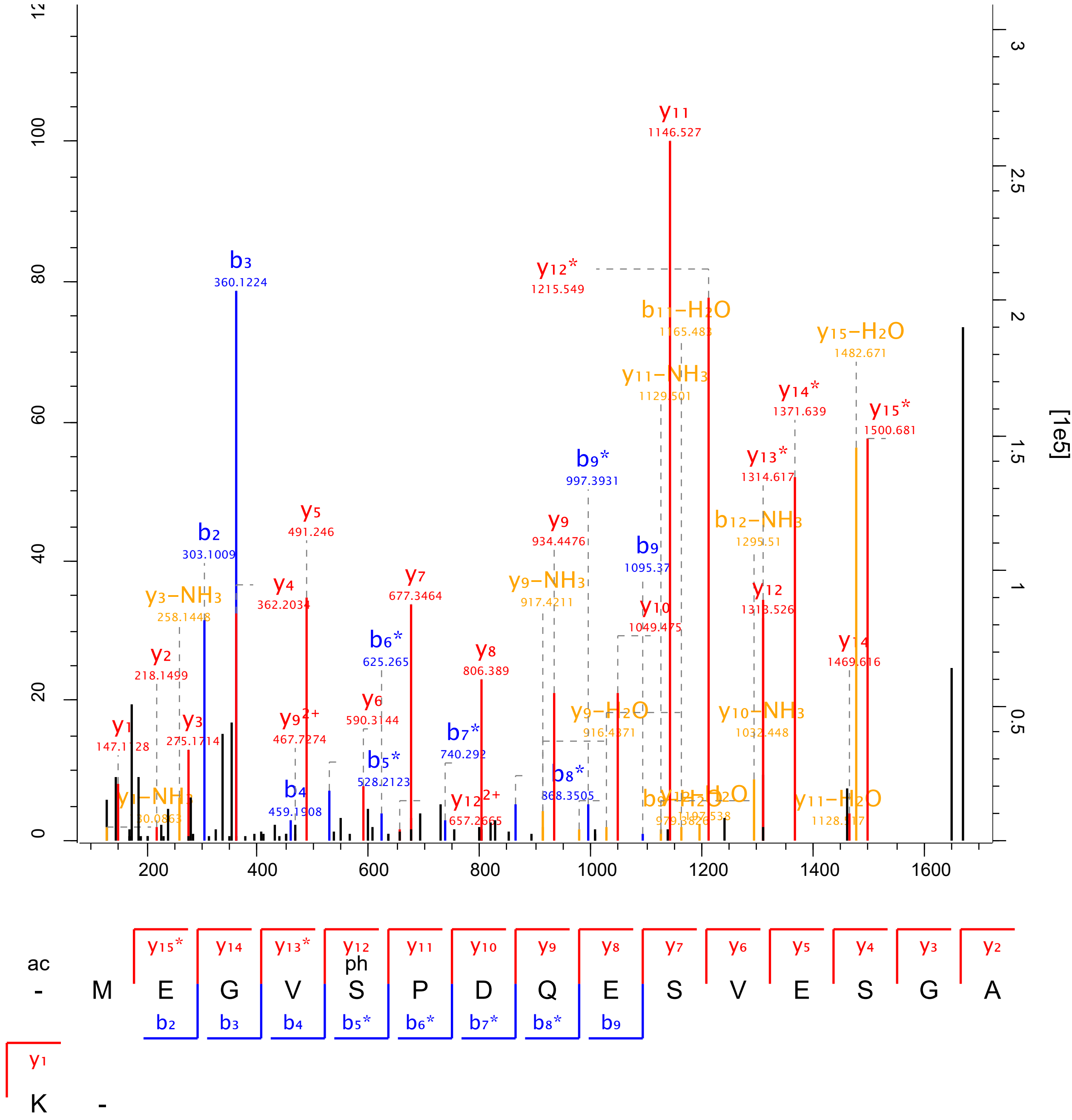

Raw file	Scan	Method	Score	m/z
sys_00_2	28719	FTMS; HCD	135.21	930.38

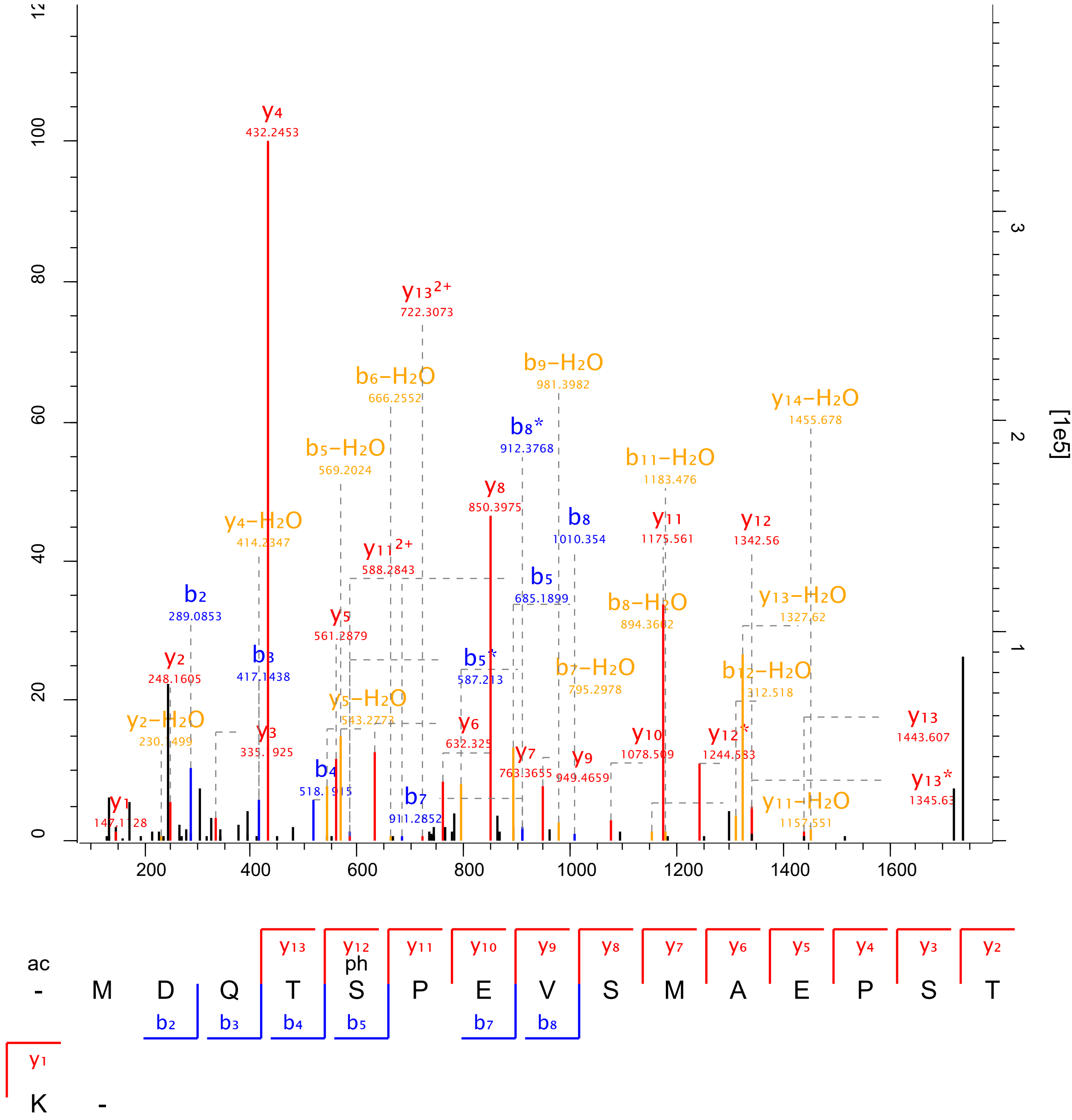

Raw file	Scan	Method	Score	m/z
sys_00_2	30261	FTMS; HCD	131.79	800.34

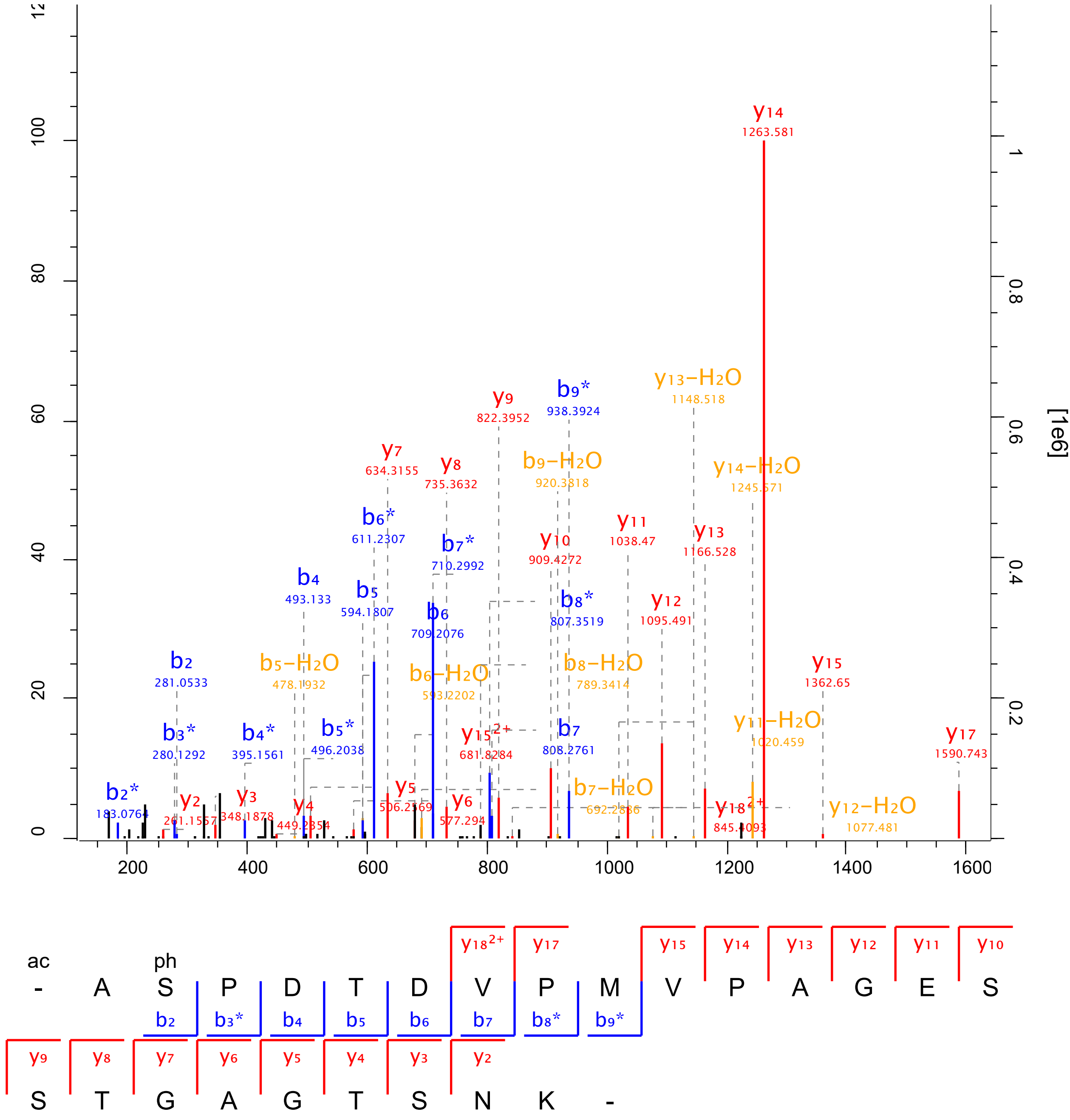

Raw file	Scan	Method	Score	m/z
sys_00_2	3484	FTMS; HCD	121.25	980.36

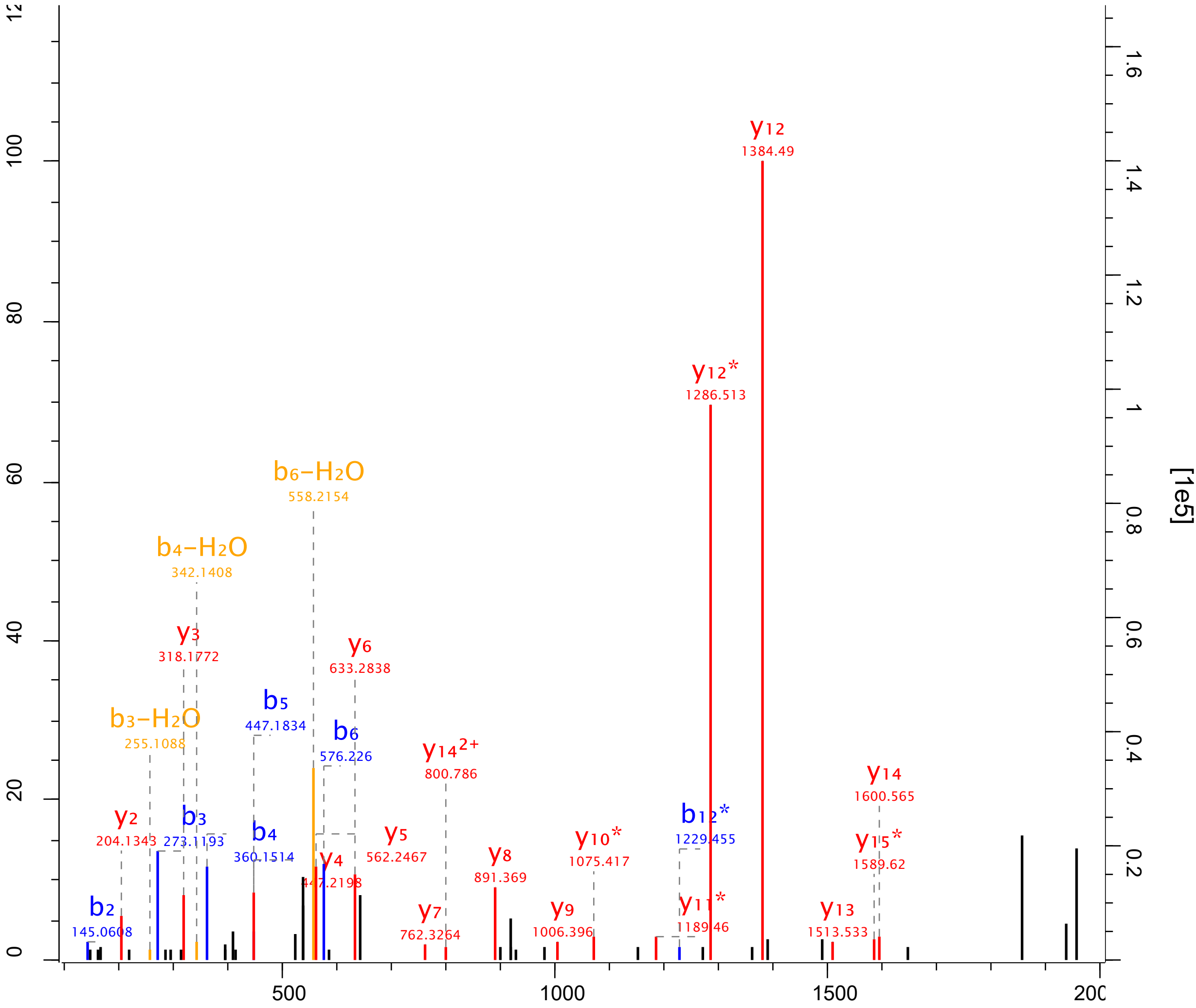

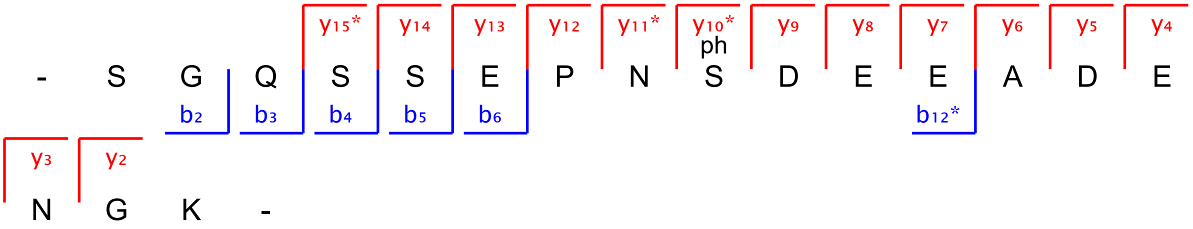

Raw file	Scan	Method	Score	m/z
sys_00_2	36121	FTMS; HCD	76.07	666.27

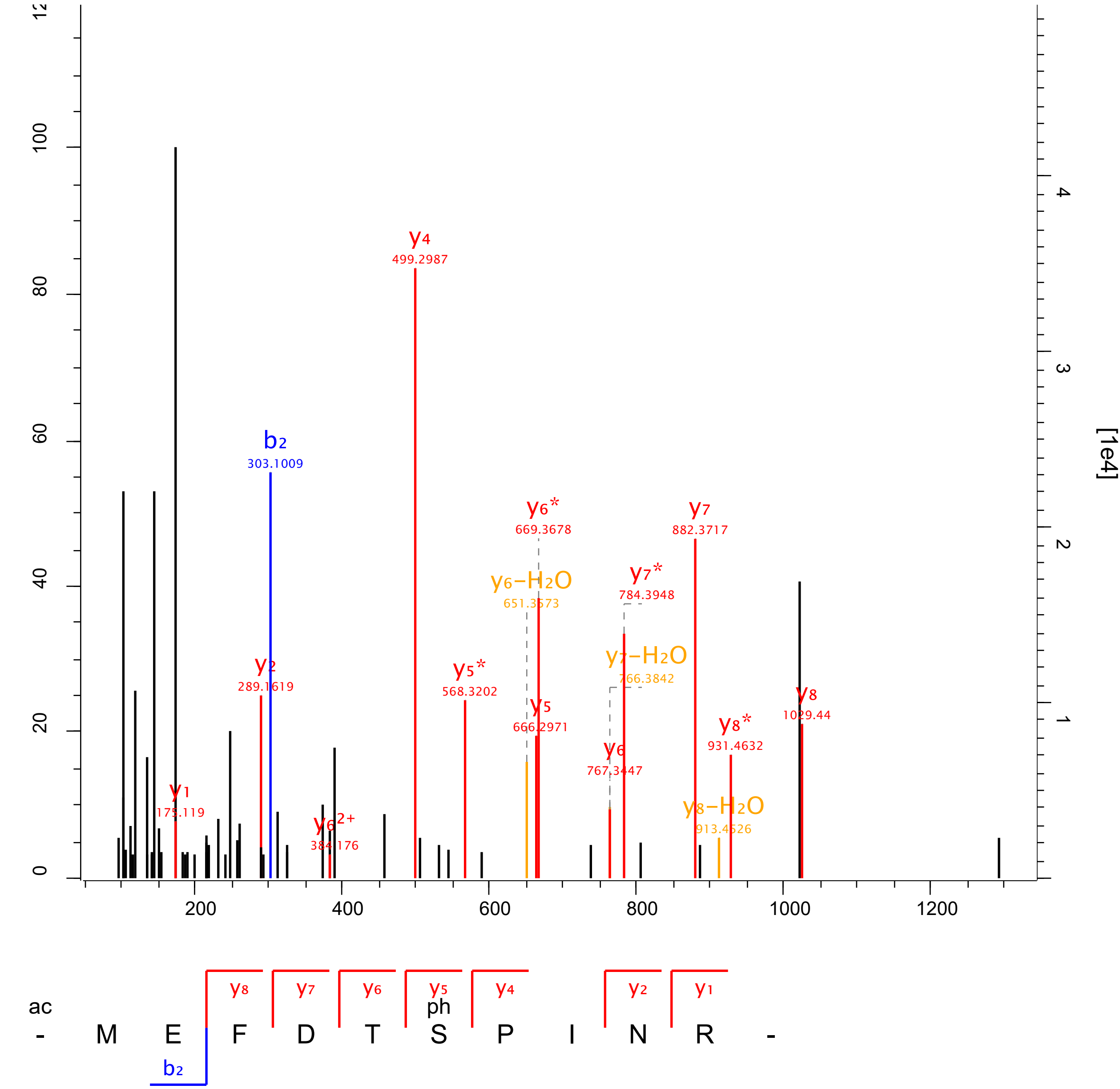

Raw file	Scan	Method	Score	m/z
sys_00_2	3993	FTMS; HCD	50.11	456.22

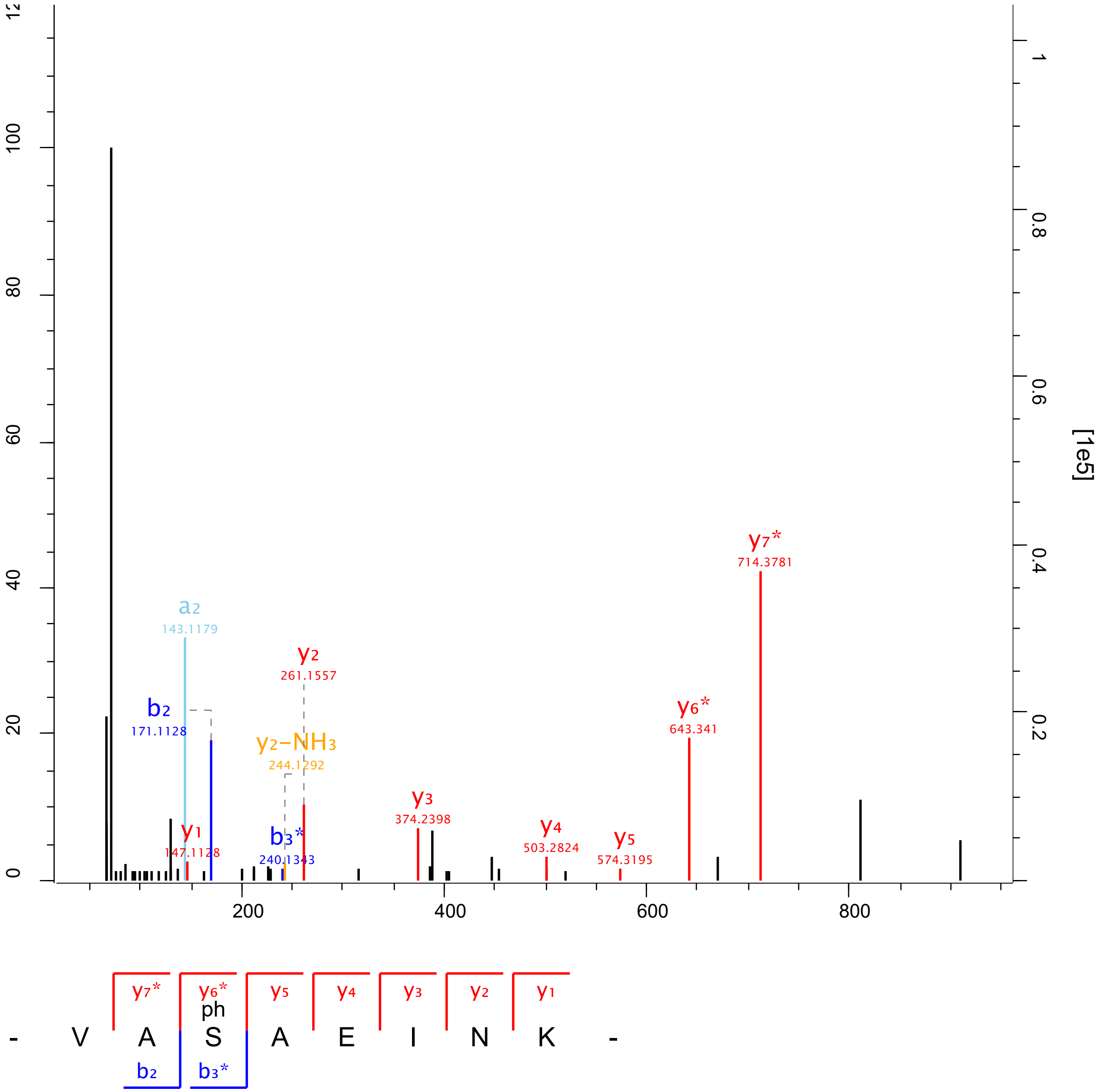

Raw file	Scan	Method	Score	m/z
sys_00_2	4203	FTMS; HCD	110.97	677.78

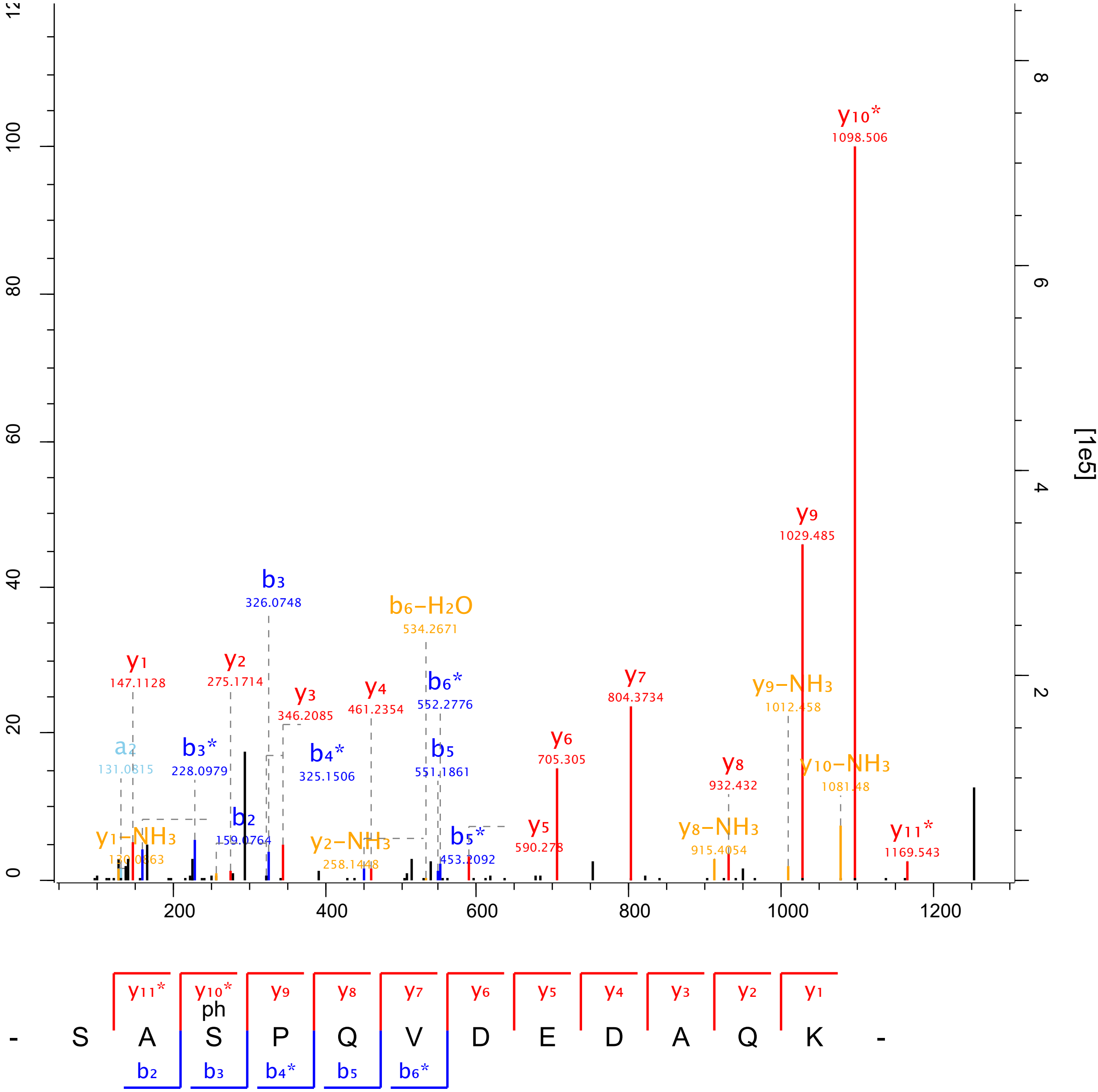

Raw file	Scan	Method	Score	m/z
sys_00_2	4253	FTMS; HCD	62.46	413.17

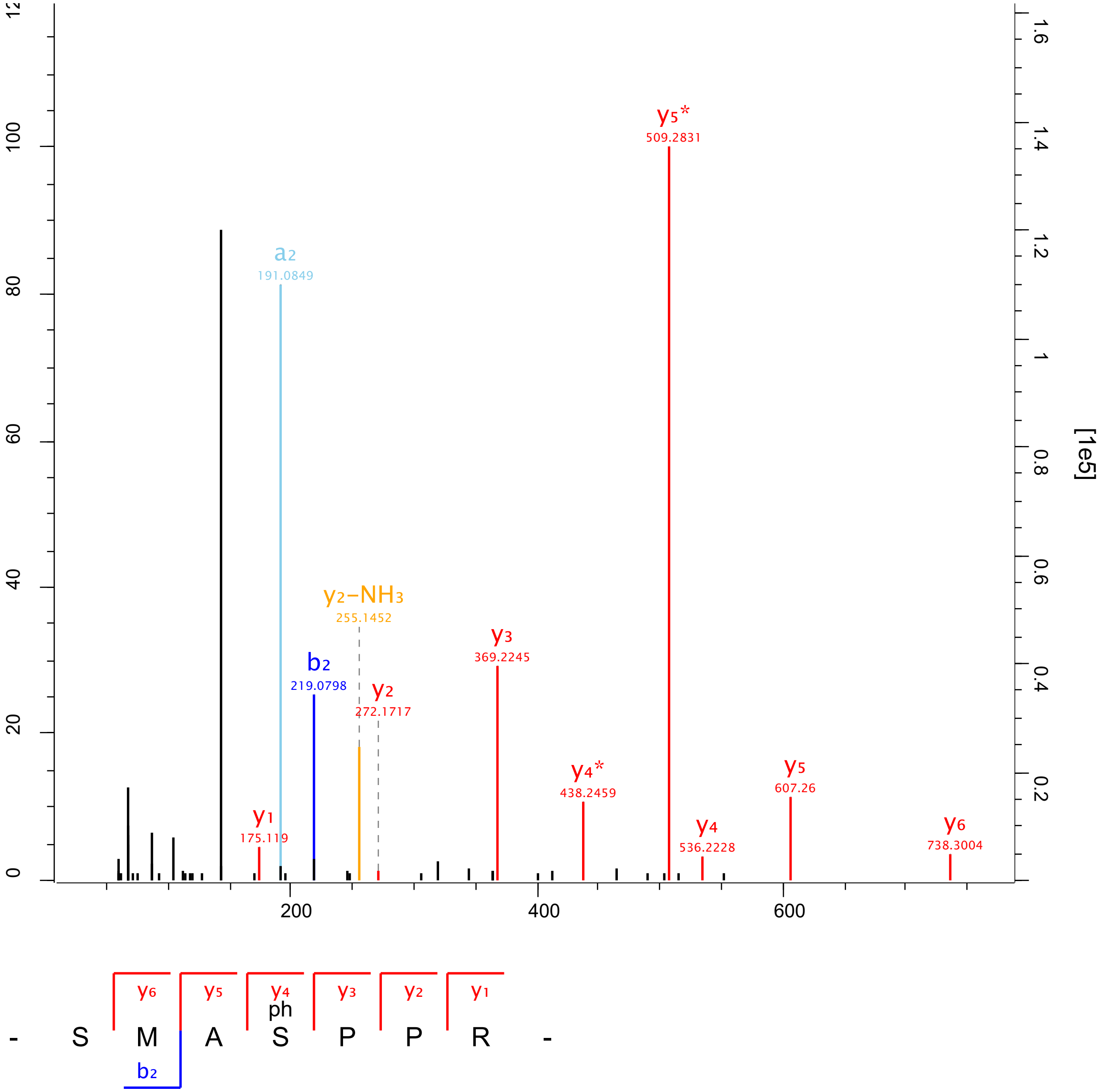

Raw file	Scan	Method	Score	m/z
sys_00_2	4275	FTMS; HCD	113.22	492.72

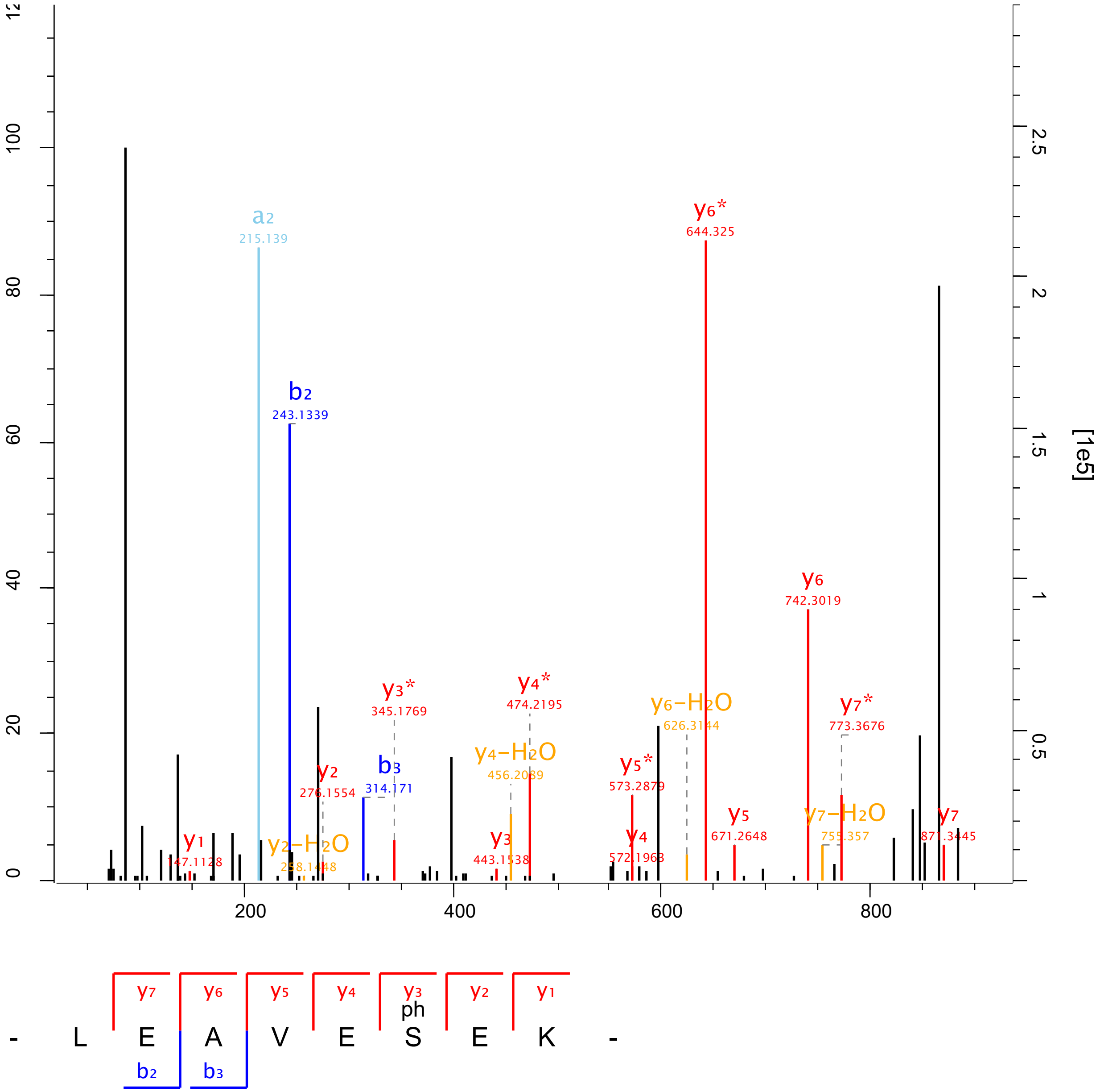

Raw file	Scan	Method	Score	m/z
sys_00_2	4437	FTMS; HCD	42.71	980.37

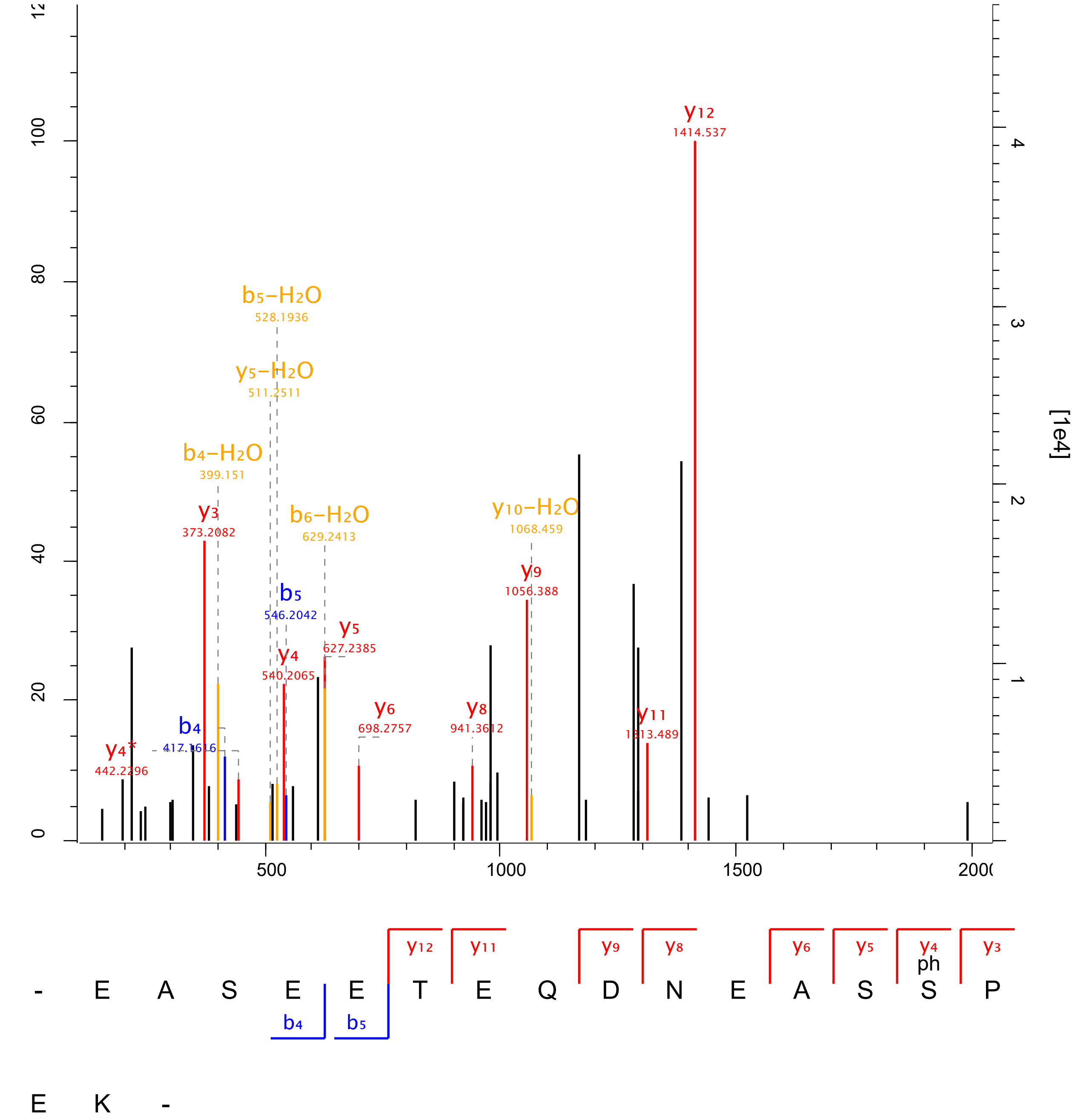

Raw file	Scan	Method	Score	m/z
sys_00_2	44682	FTMS; HCD	72.36	861.88

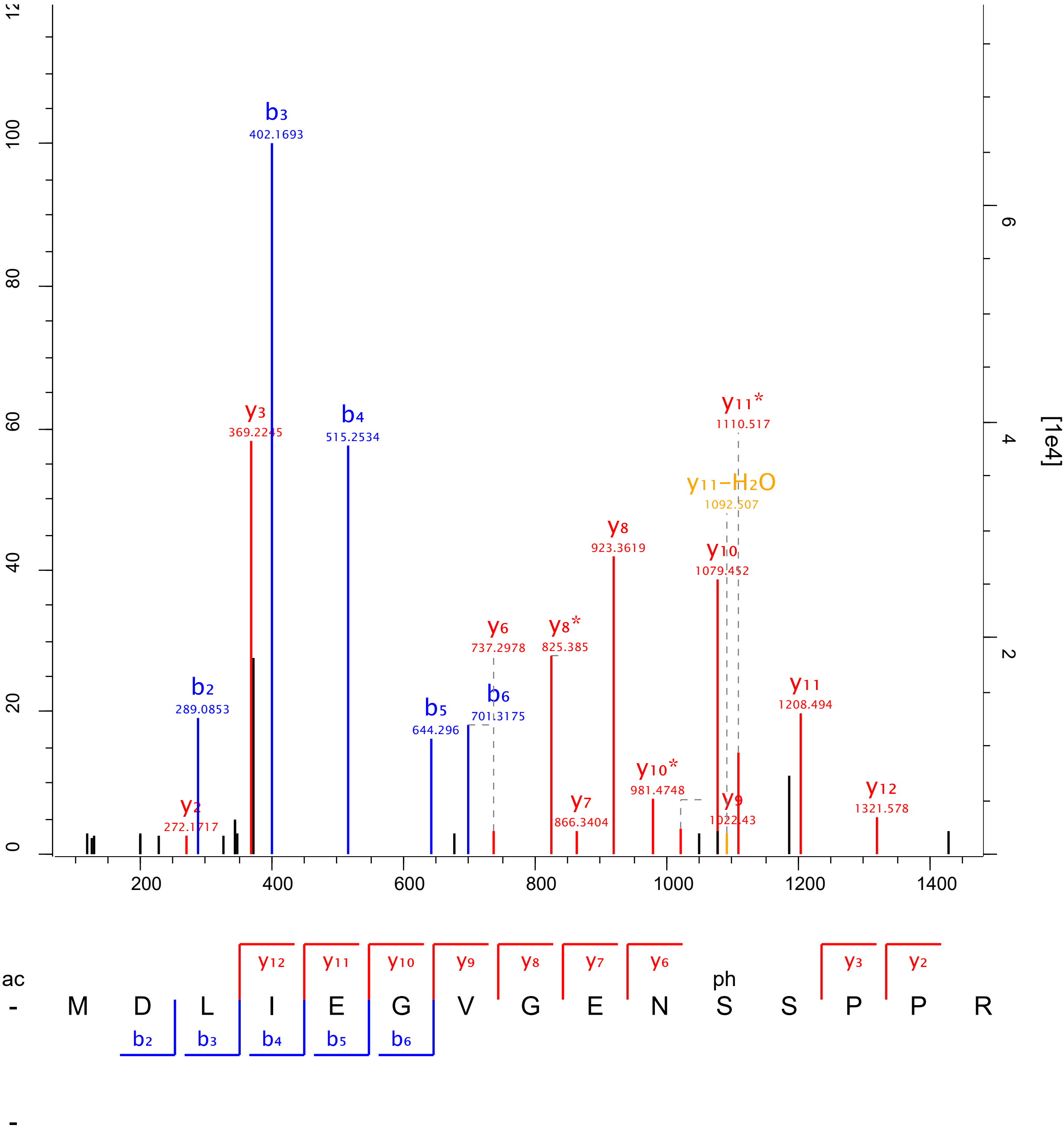

Raw file	Scan	Method	Score	m/z
sys_00_2	4939	FTMS; HCD	72.7	541.25

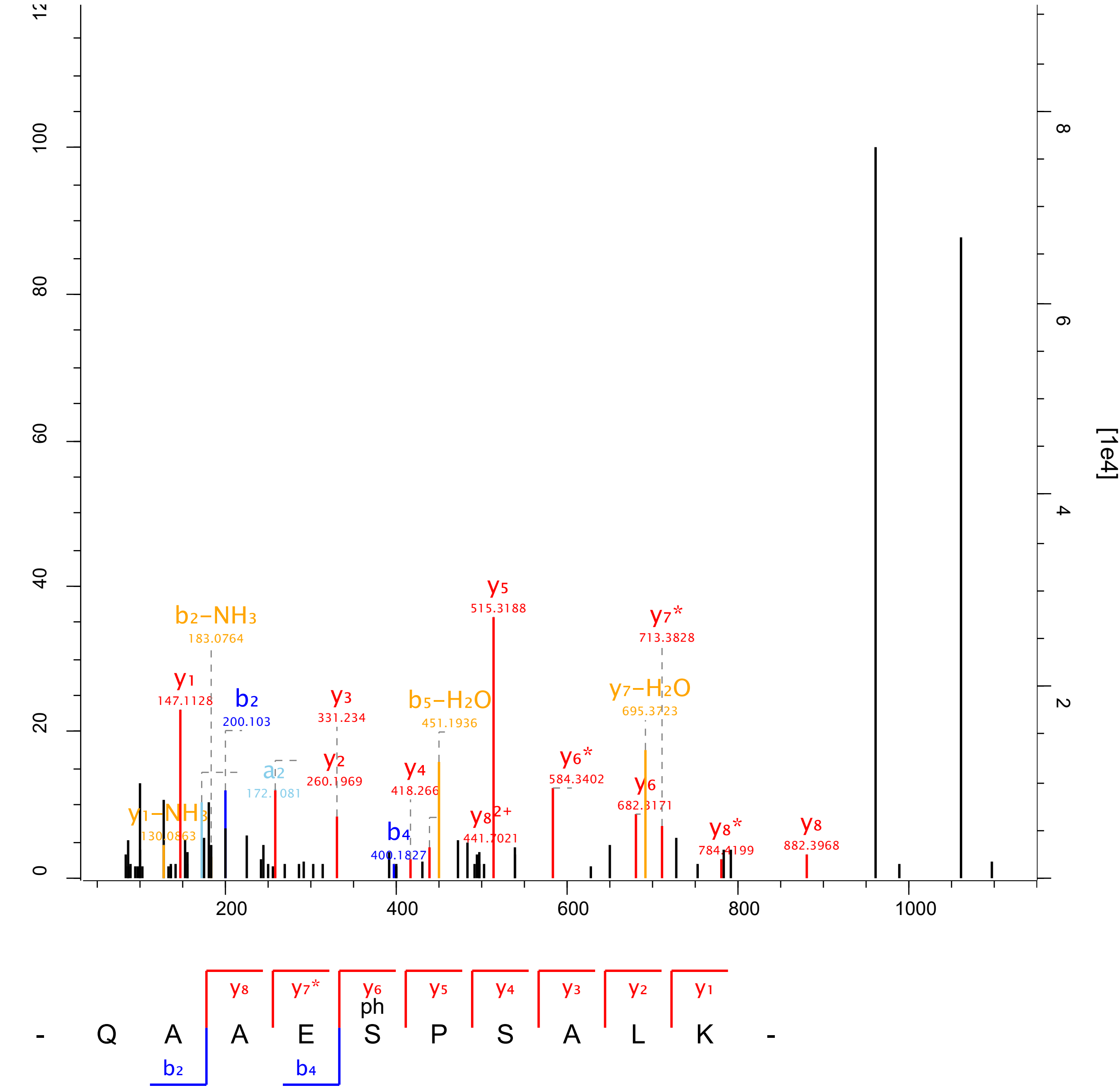

Raw file	Scan	Method	Score	m/z
sys_00_2	5489	FTMS; HCD	77.66	529.72

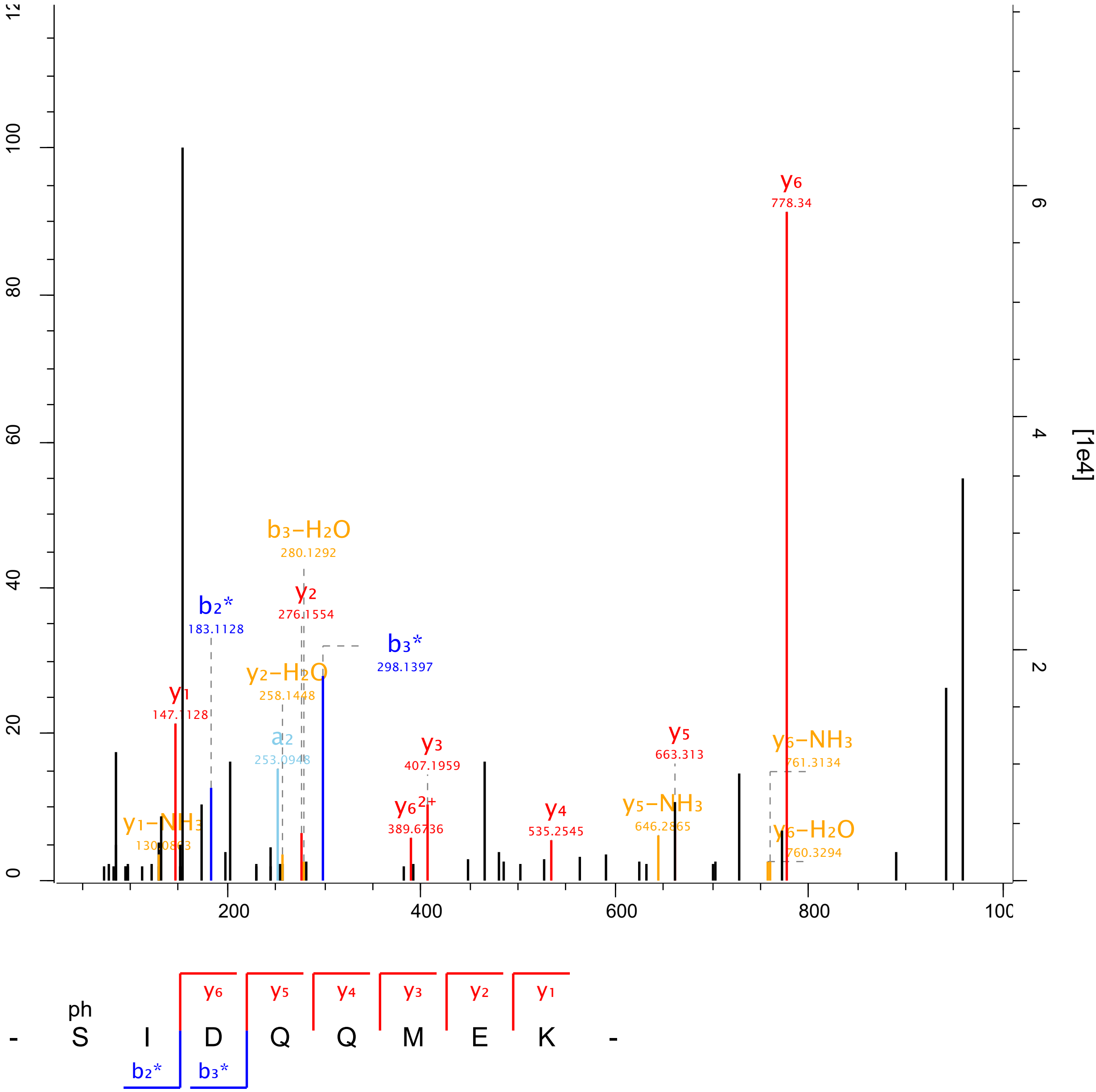

Raw file	Scan	Method	Score	m/z
sys_00_2	5669	FTMS; HCD	55.31	556.24

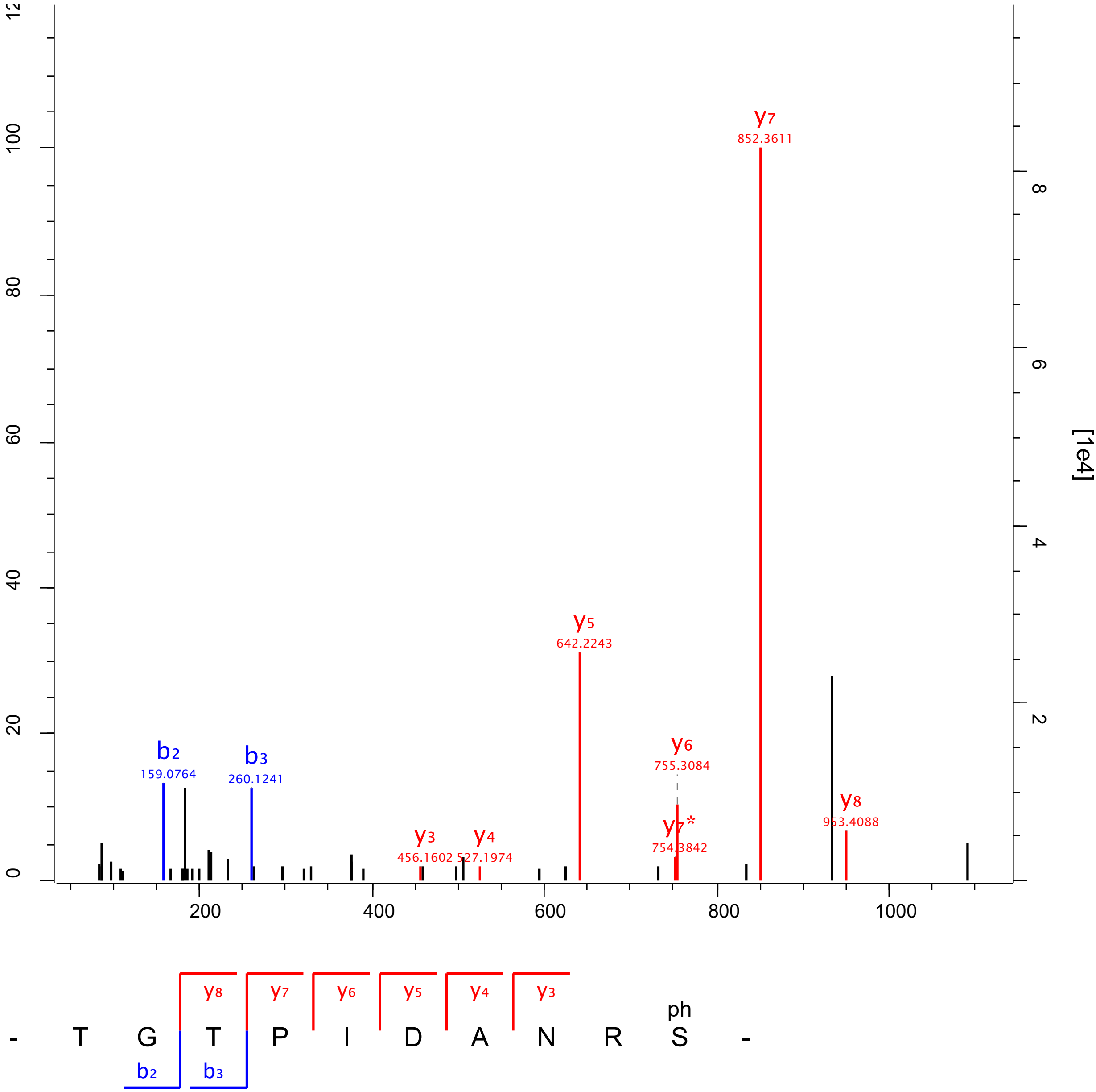

Raw file	Scan	Method	Score	m/z
sys_00_2	5851	FTMS; HCD	99.97	469.72

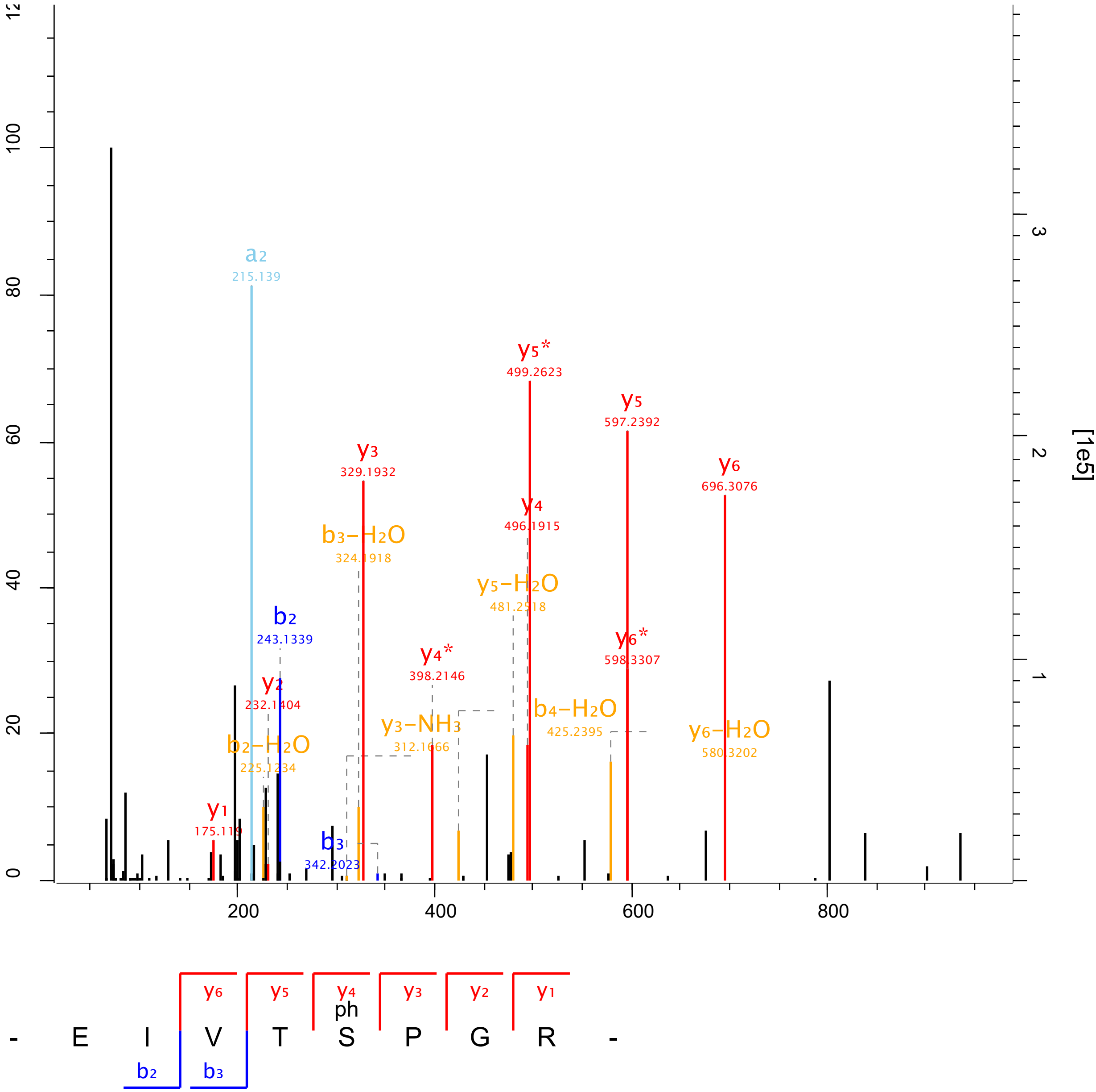

Raw file	Scan	Method	Score	m/z
sys_00_2	6408	FTMS; HCD	91.09	419.2

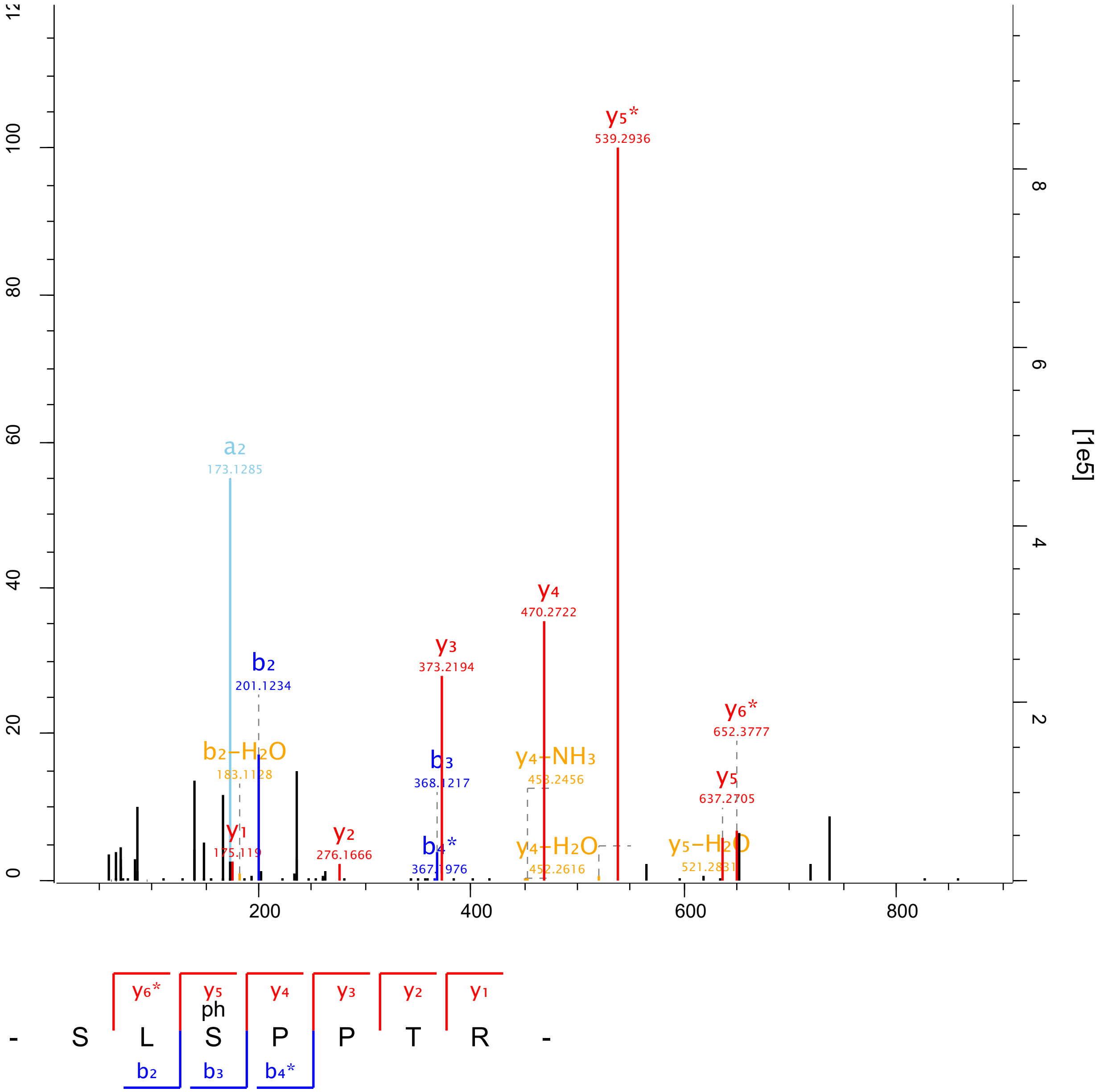

Raw file	Scan	Method	Score	m/z
sys_00_2	6656	FTMS; HCD	42.84	513.23

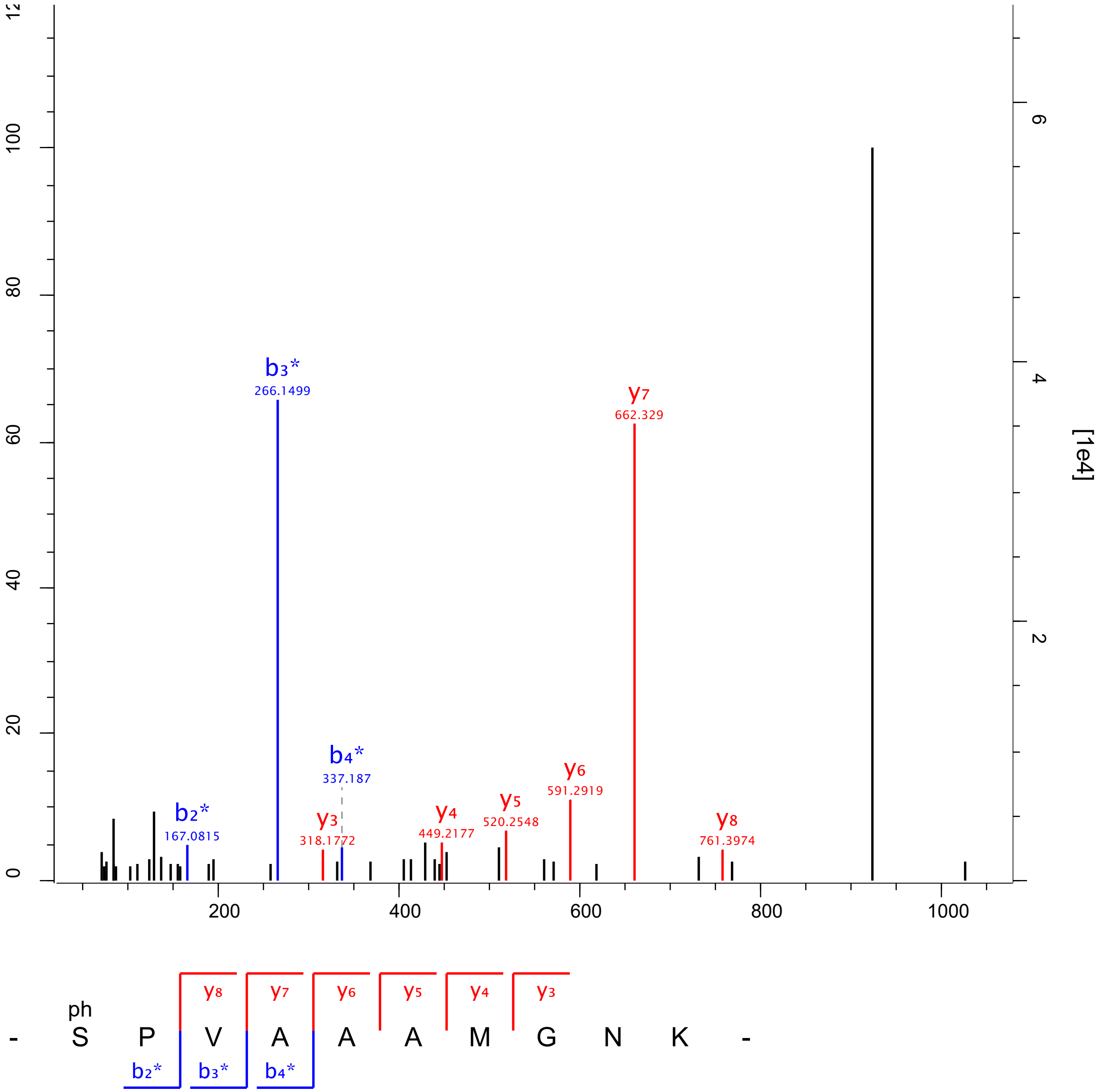

Raw file	Scan	Method	Score	m/z
sys_00_2	6933	FTMS; HCD	78.1	429.18

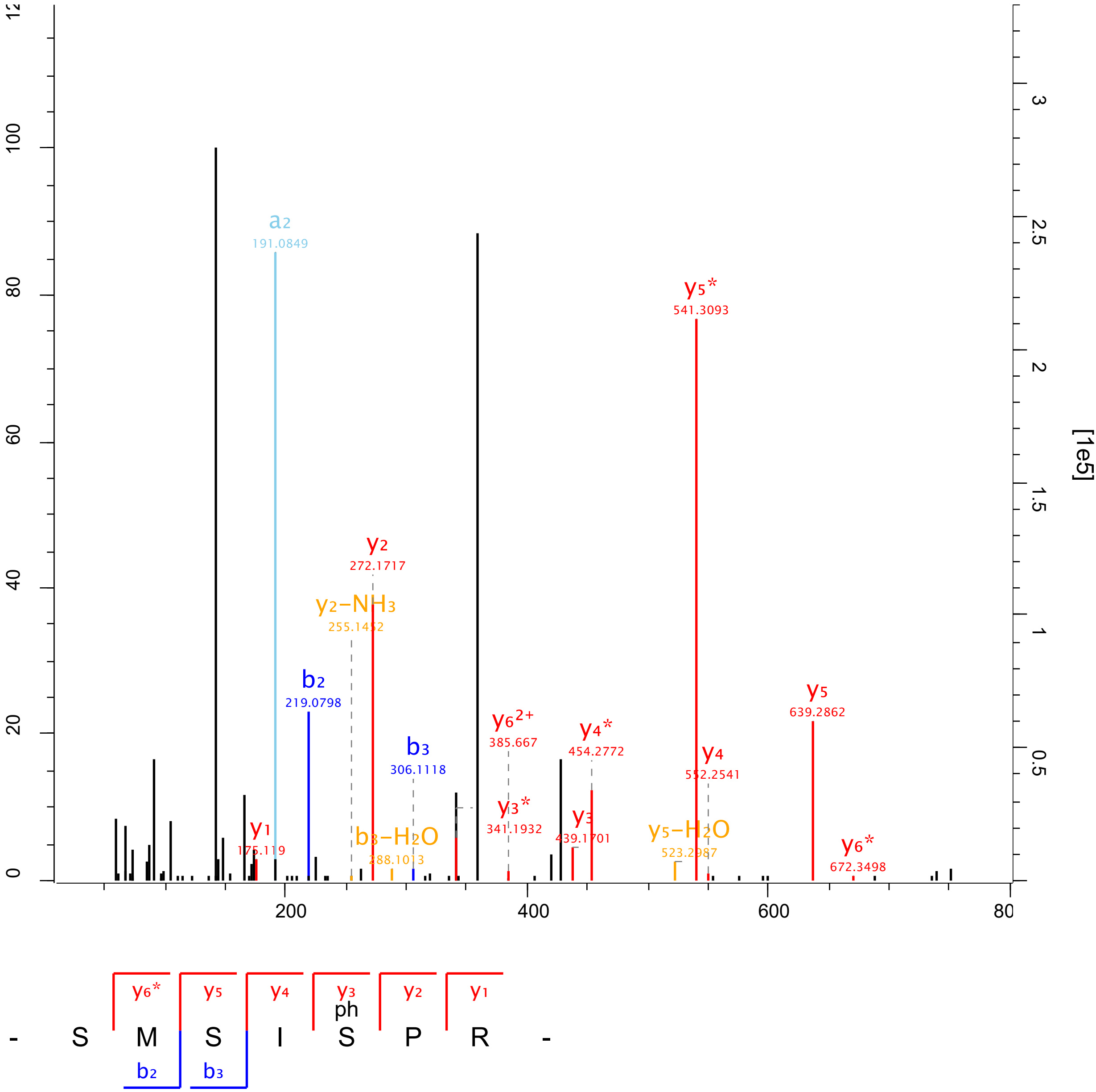

Raw file	Scan	Method	Score	m/z
sys_00_2	7343	FTMS; HCD	91.31	586.23

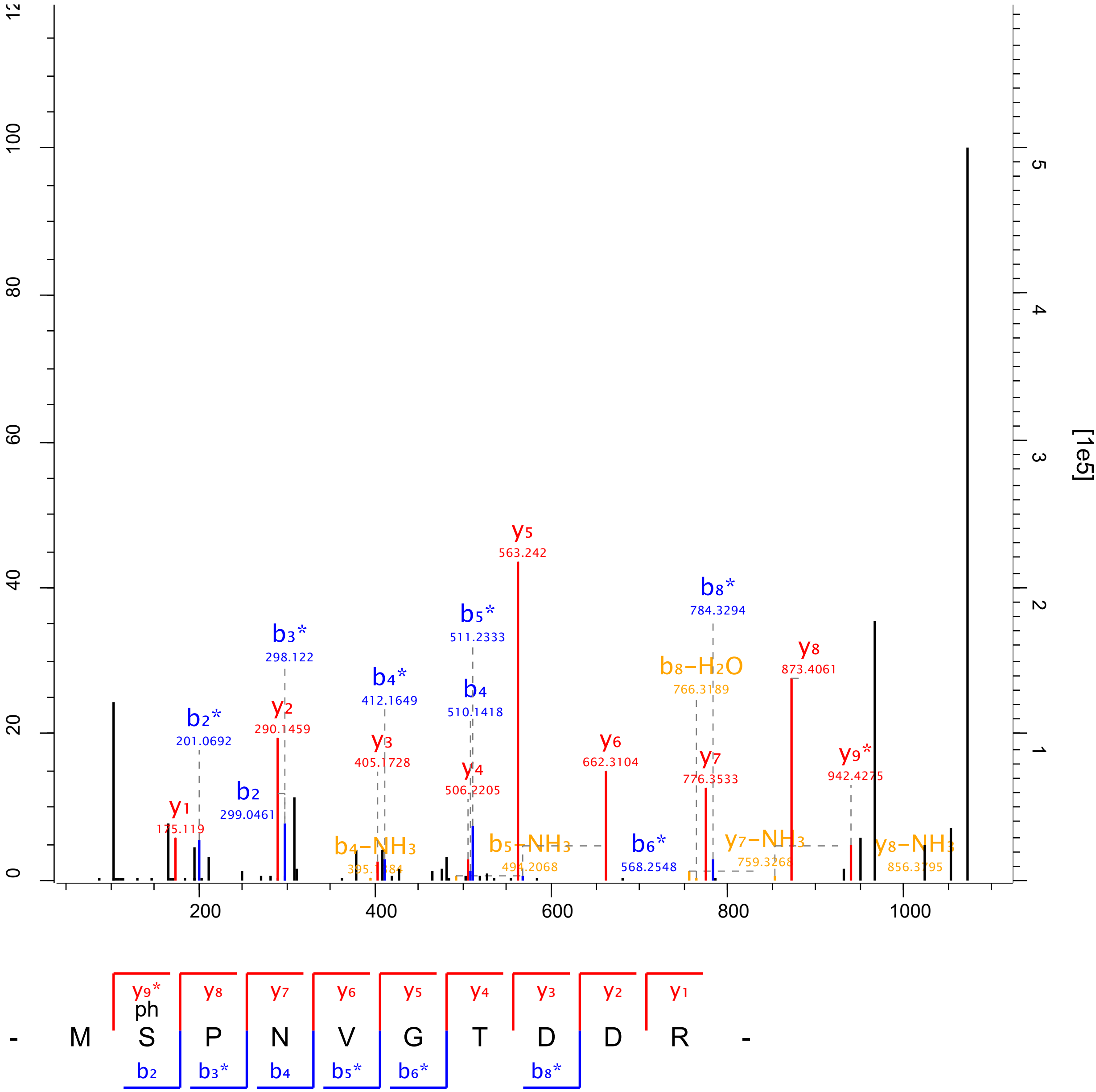

Raw file	Scan	Method	Score	m/z
sys_00_2	7606	FTMS; HCD	62.86	728.32

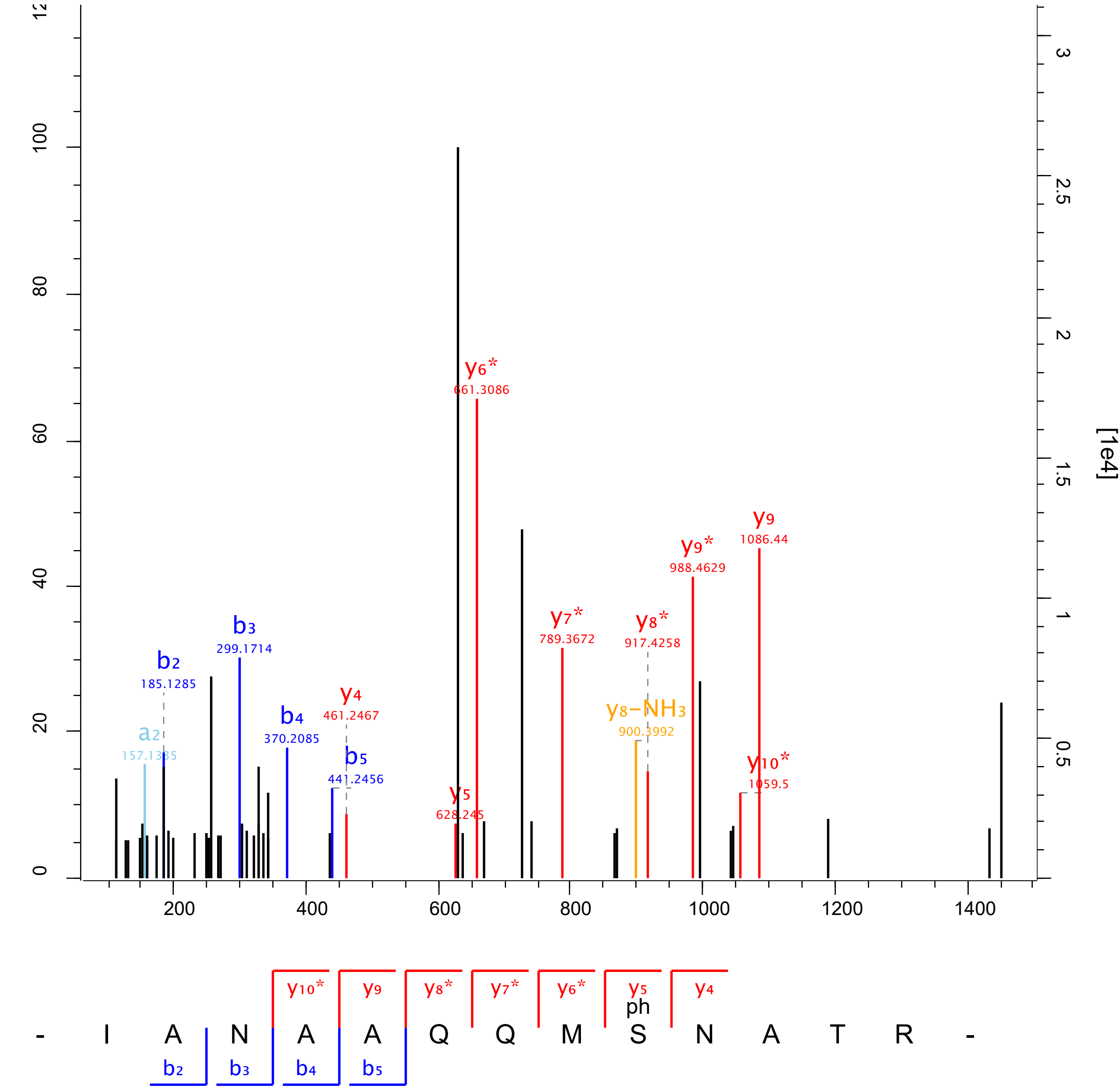

Raw file	Scan	Method	Score	m/z
sys_00_2	8276	FTMS; HCD	126.09	626.76

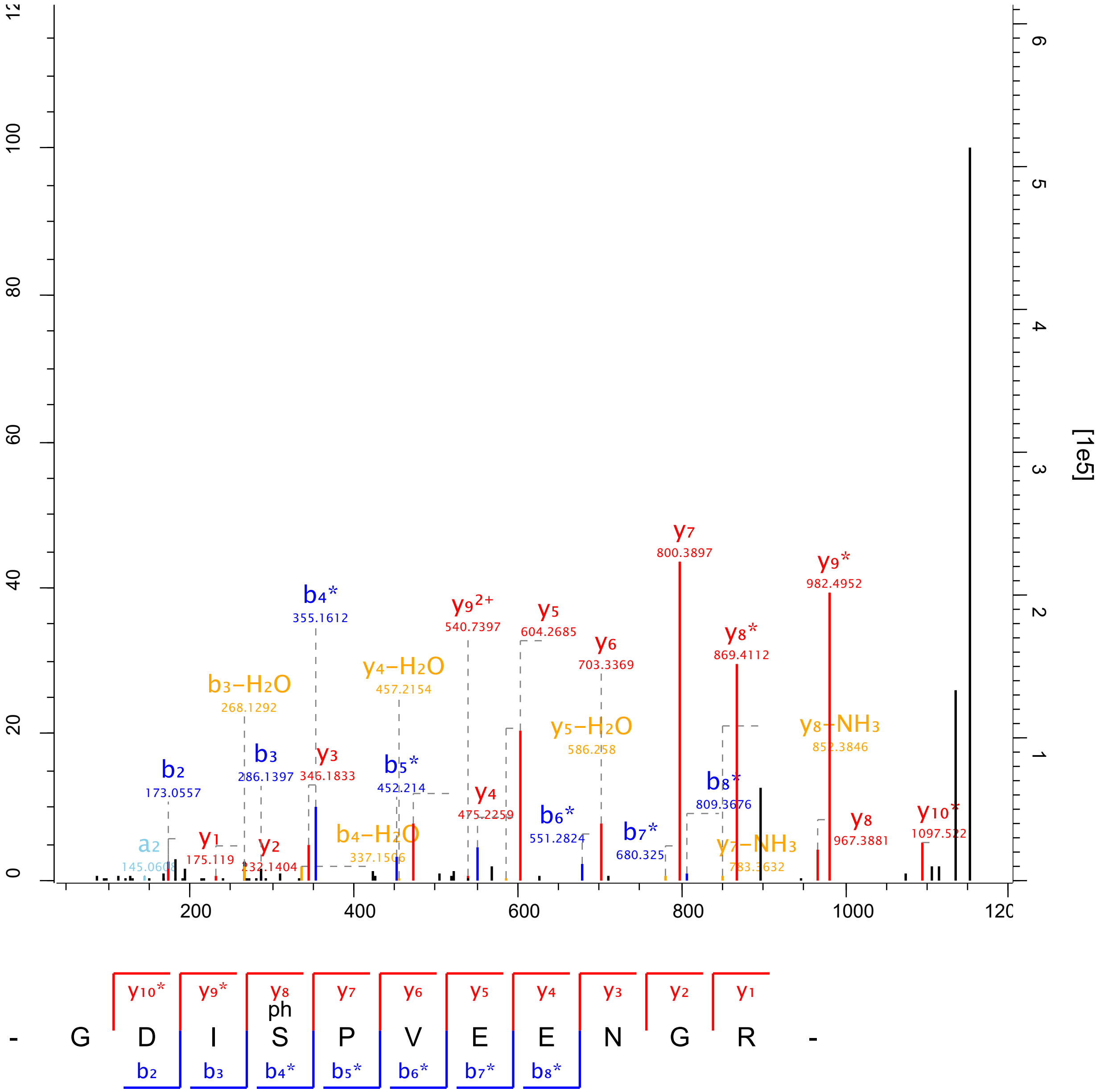

Raw file	Scan	Method	Score	m/z
sys_00_2	8553	FTMS; HCD	139.7	561.27

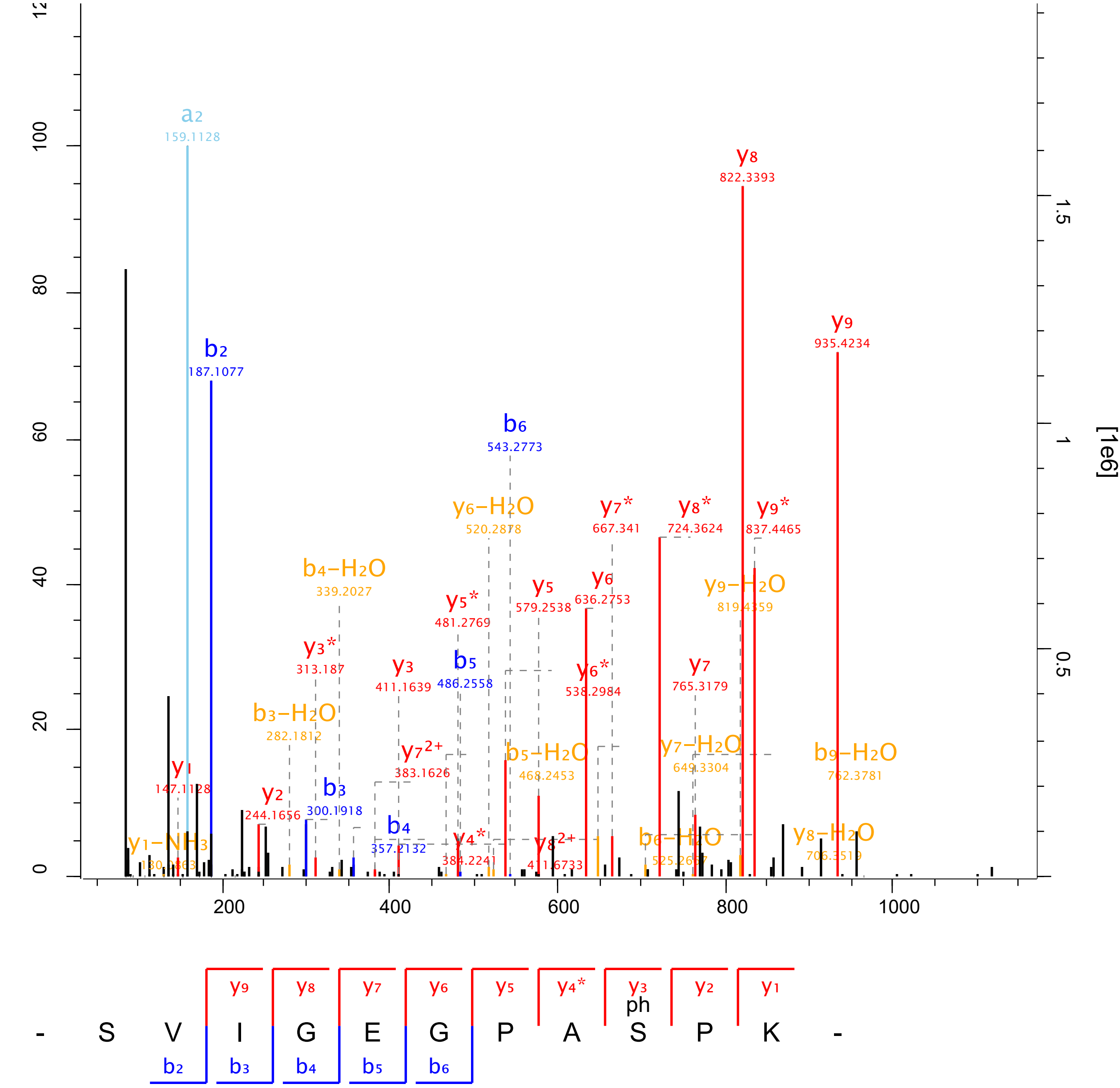

Raw file	Scan	Method	Score	m/z
sys_00_2	8753	FTMS; HCD	148.18	562.76

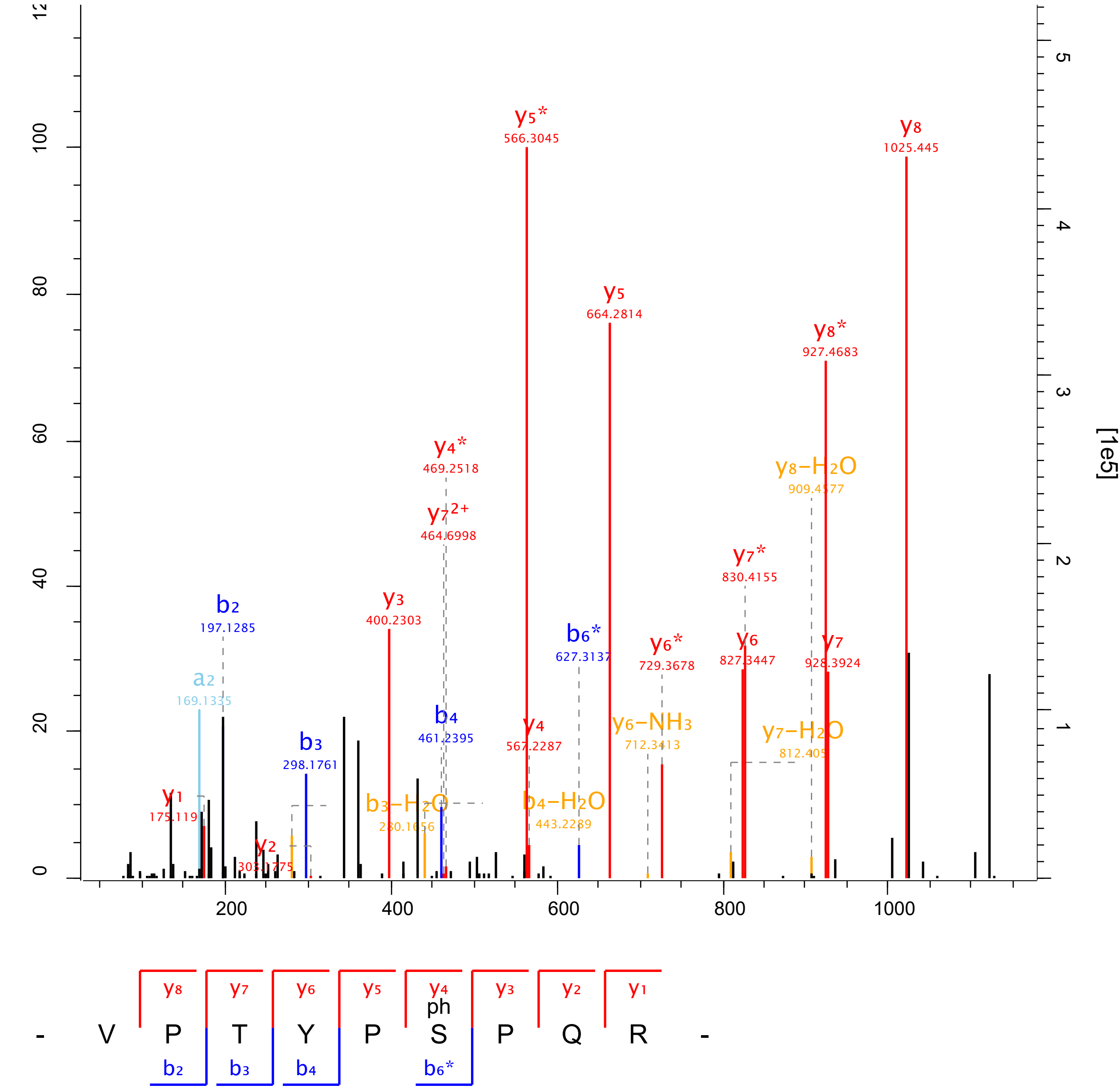

Raw file	Scan	Method	Score	m/z
sys_00_2	8885	FTMS; HCD	40.94	651.26

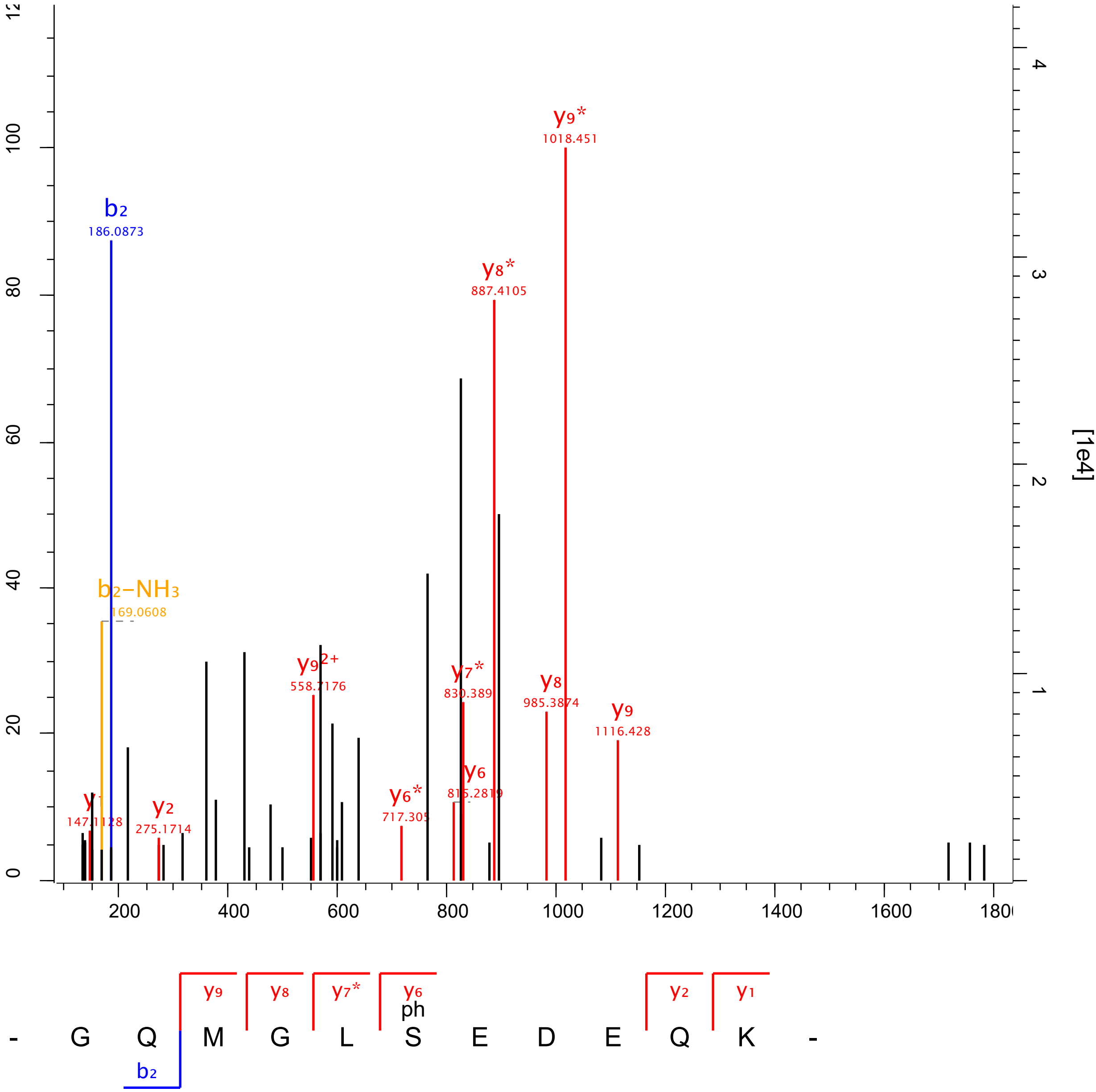

Raw file	Scan	Method	Score	m/z
sys_00_2	9070	FTMS; HCD	179.67	570.77

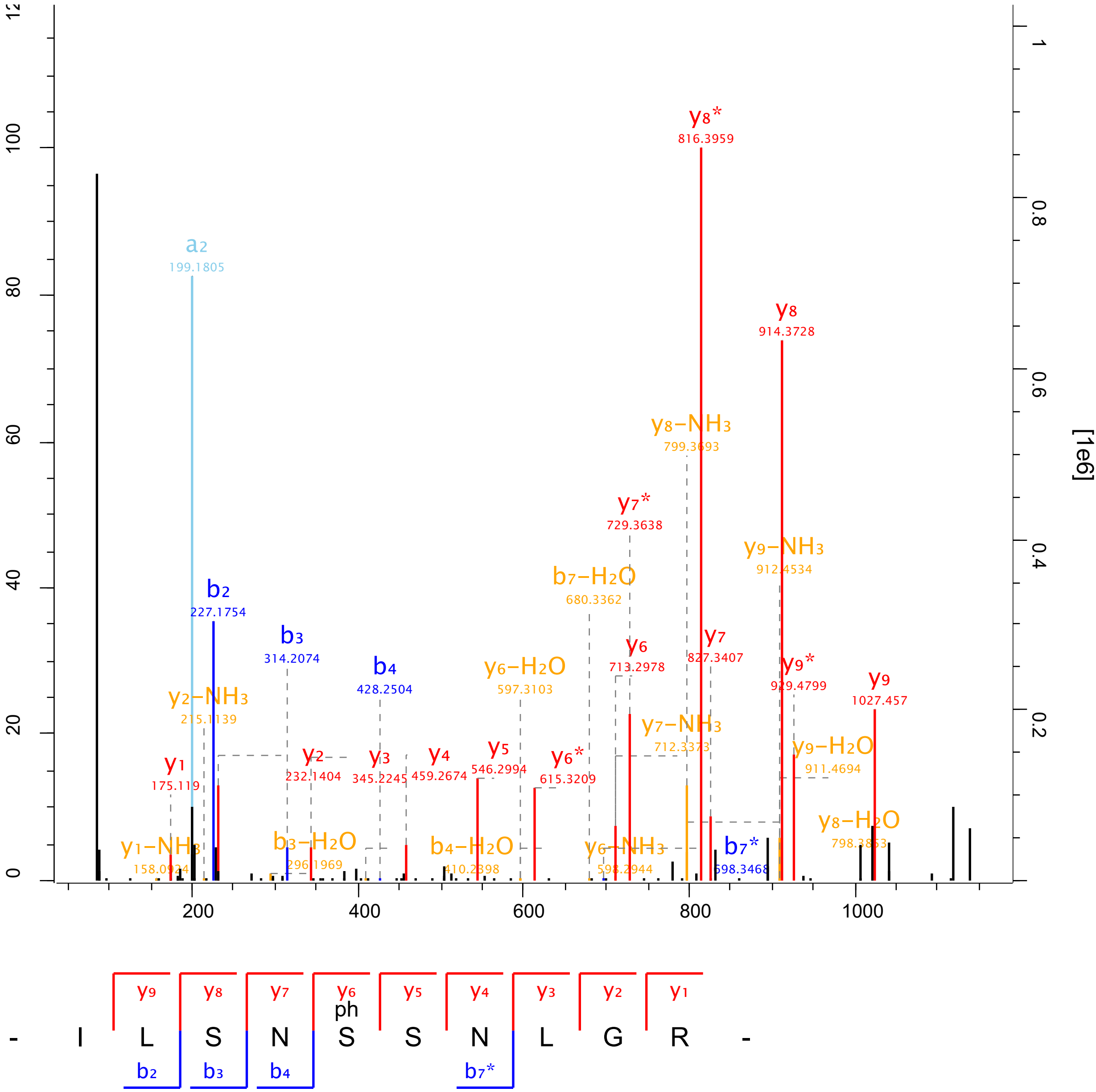

Raw file	Scan	Method	Score	m/z
sys_00_2	9125	FTMS; HCD	72.61	591.28

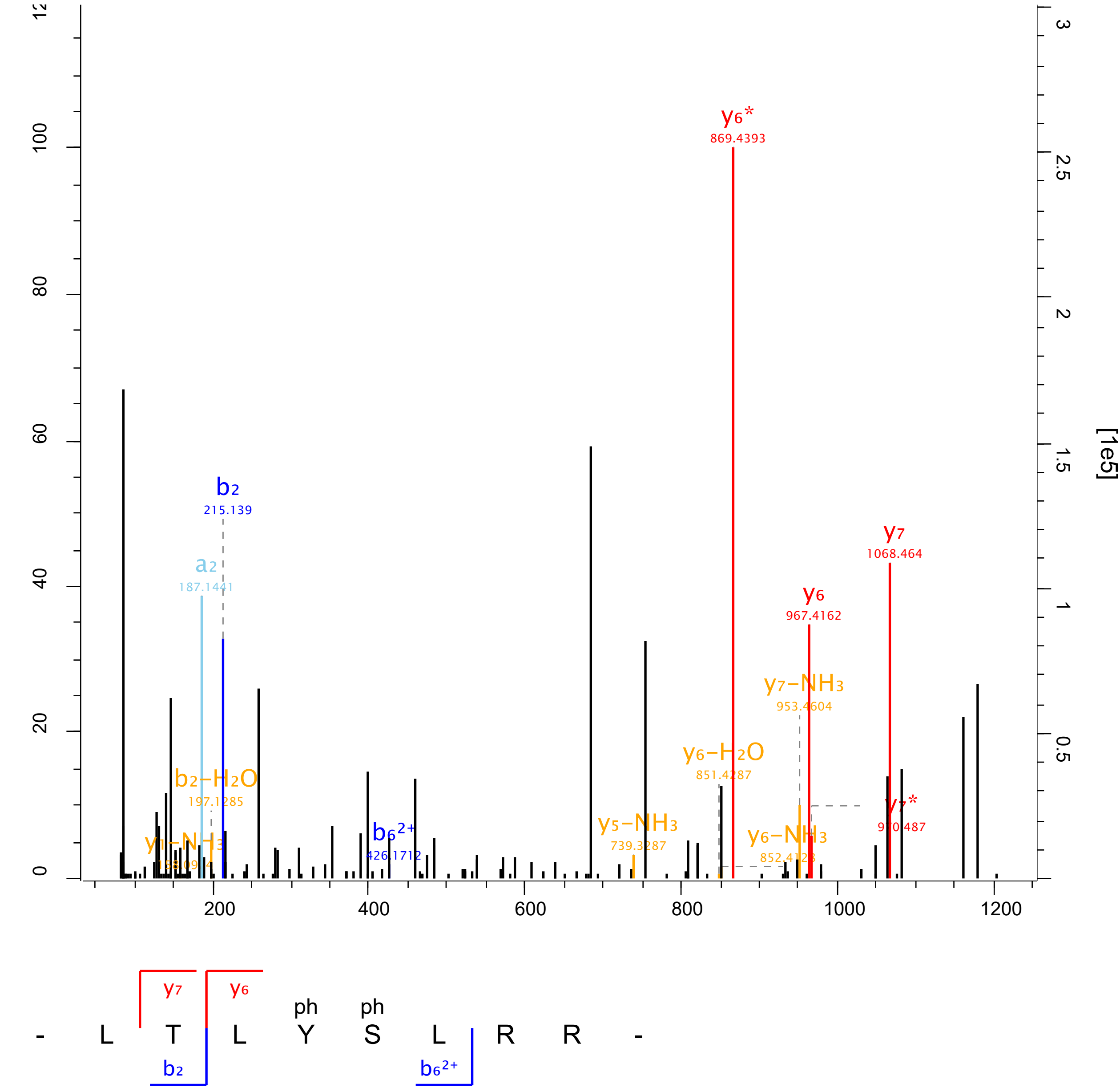

Raw file	Scan	Method	Score	m/z
sys_00_2	9319	FTMS; HCD	118.28	476.71

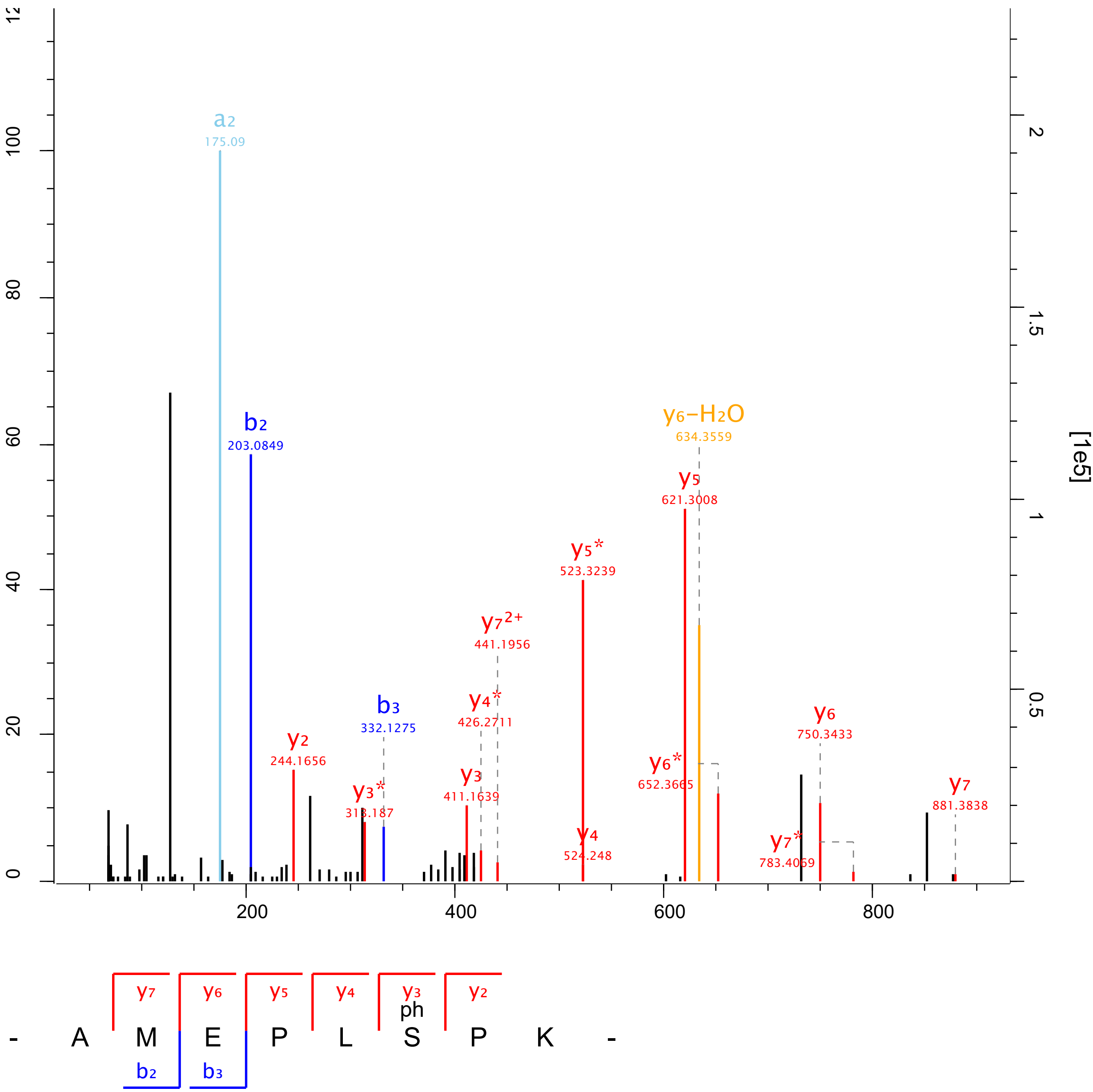

Raw file	Scan	Method	Score	m/z
sys_00_2	9677	FTMS; HCD	137.58	609.27

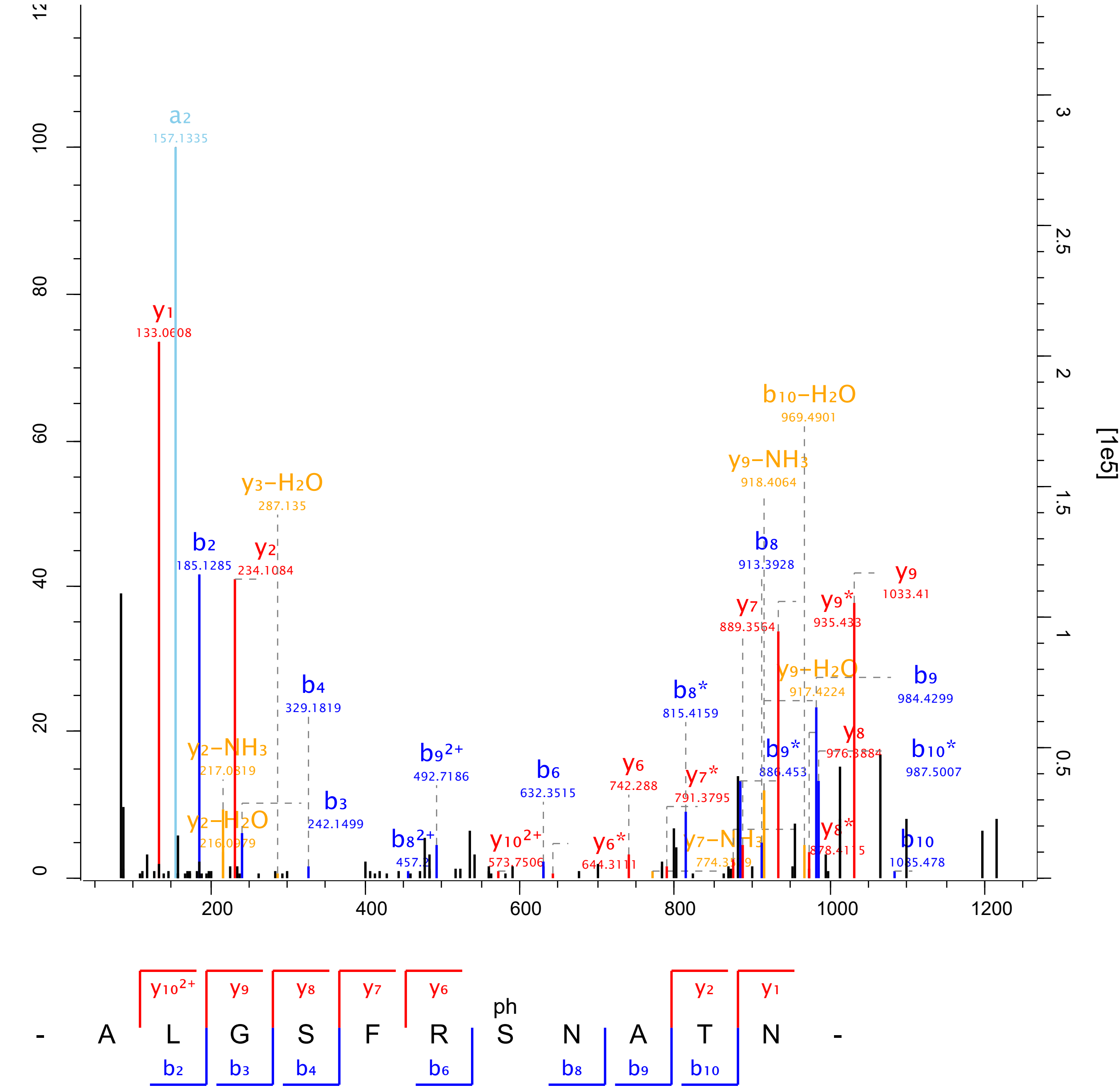

Raw file	Scan	Method	Score	m/z
sys_00_2	9738	FTMS; HCD	94.77	524.22

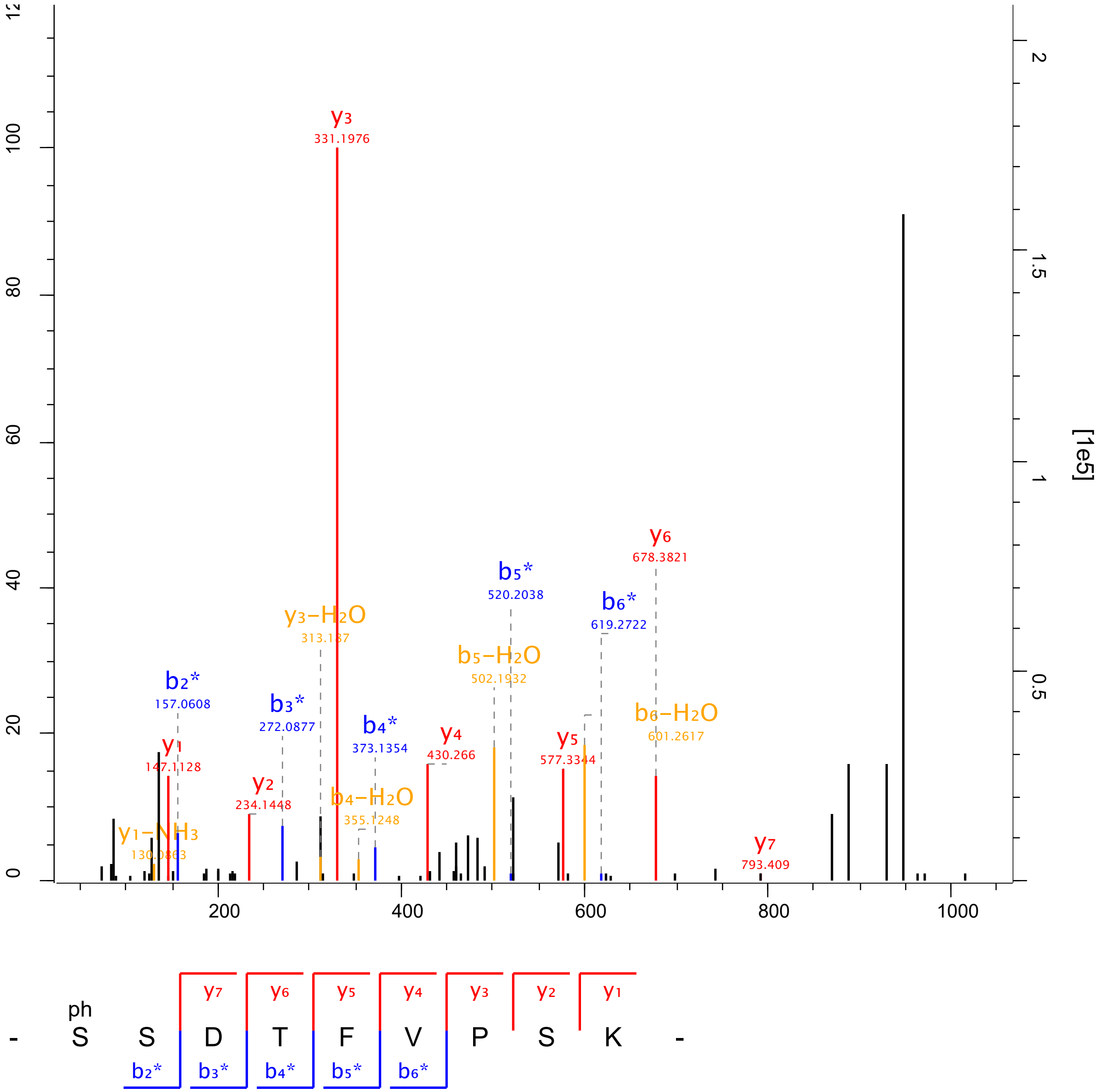

Raw file	Scan	Method	Score	m/z
sys_00_2	9959	FTMS; HCD	79.2	544.73

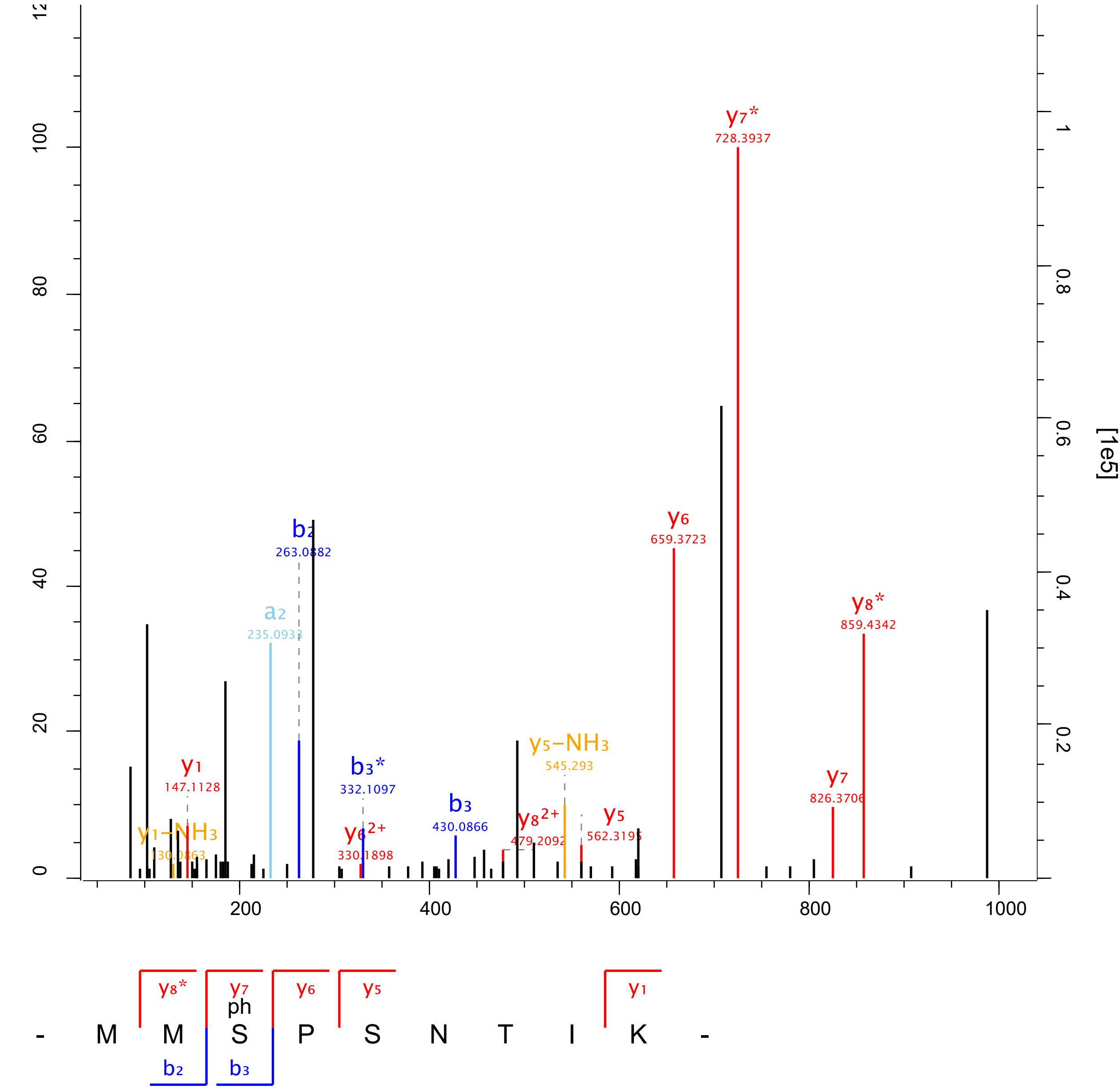

Raw file	Scan	Method	Score	m/z
sys_00_3short	10195	FTMS; HCD	66.27	511.26

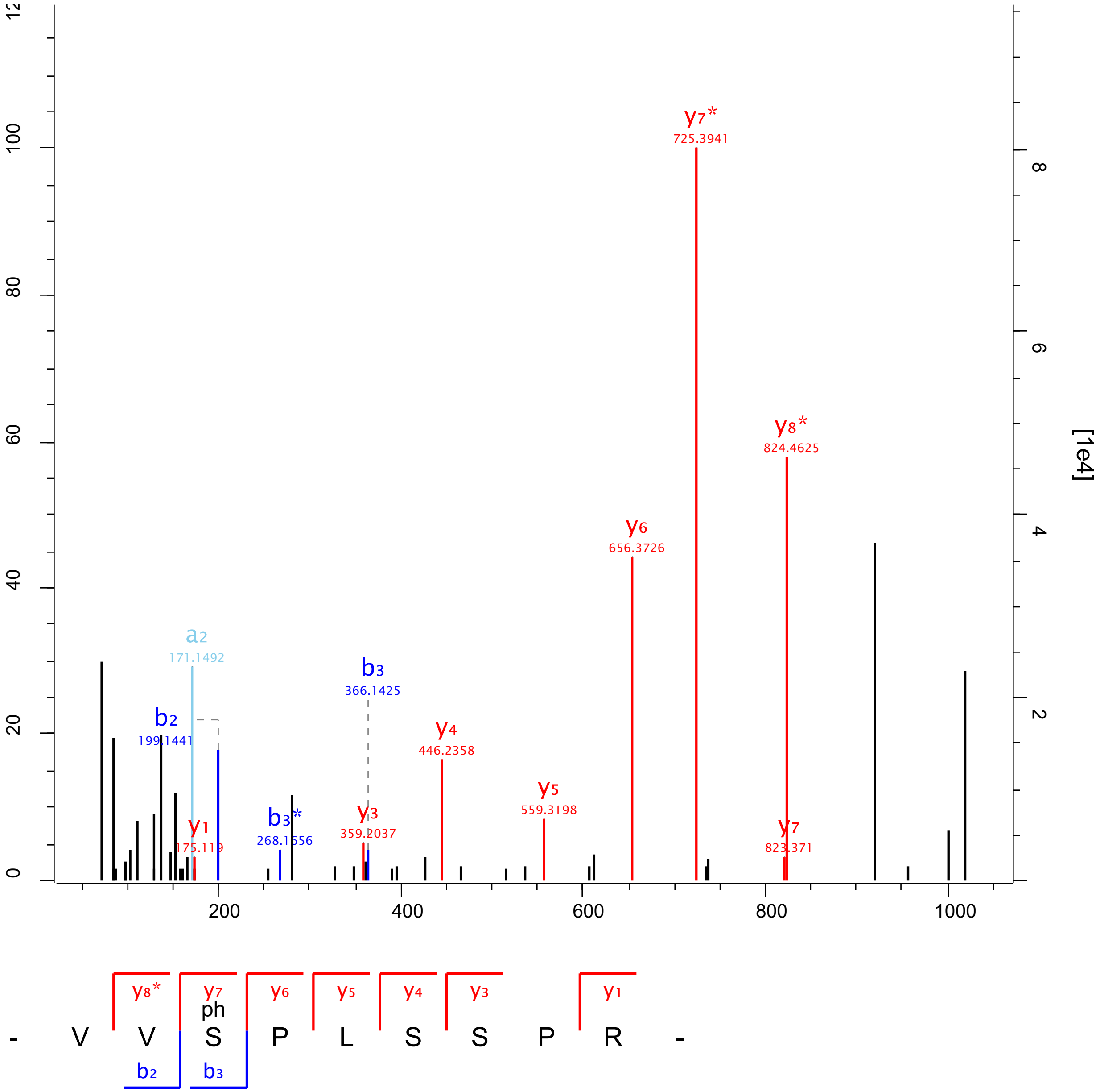

Raw file	Scan	Method	Score	m/z
sys_00_3short	10211	FTMS; HCD	84.05	495.75

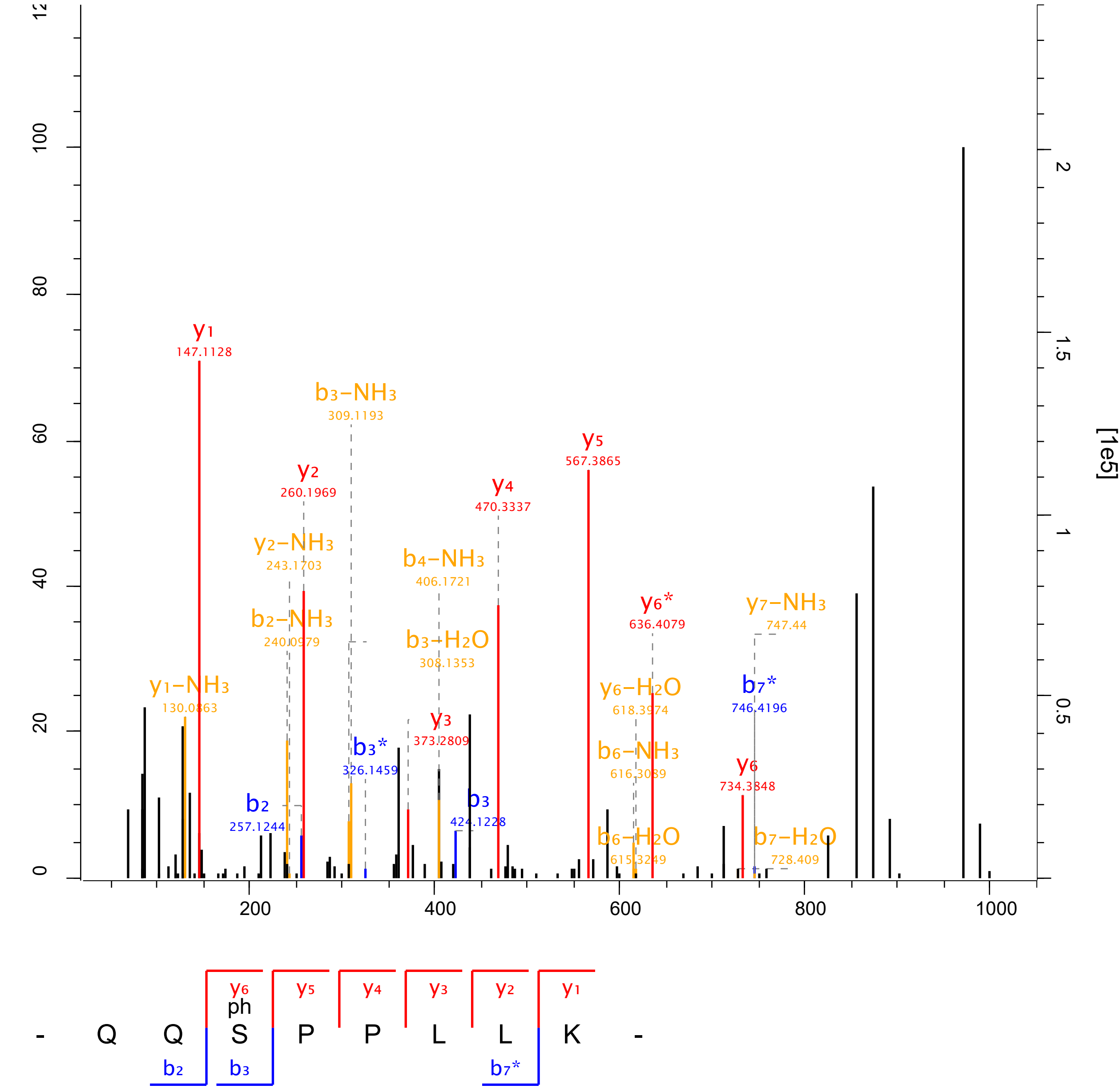

Raw file	Scan	Method	Score	m/z
sys_00_3short	10304	FTMS; HCD	55.01	714.8

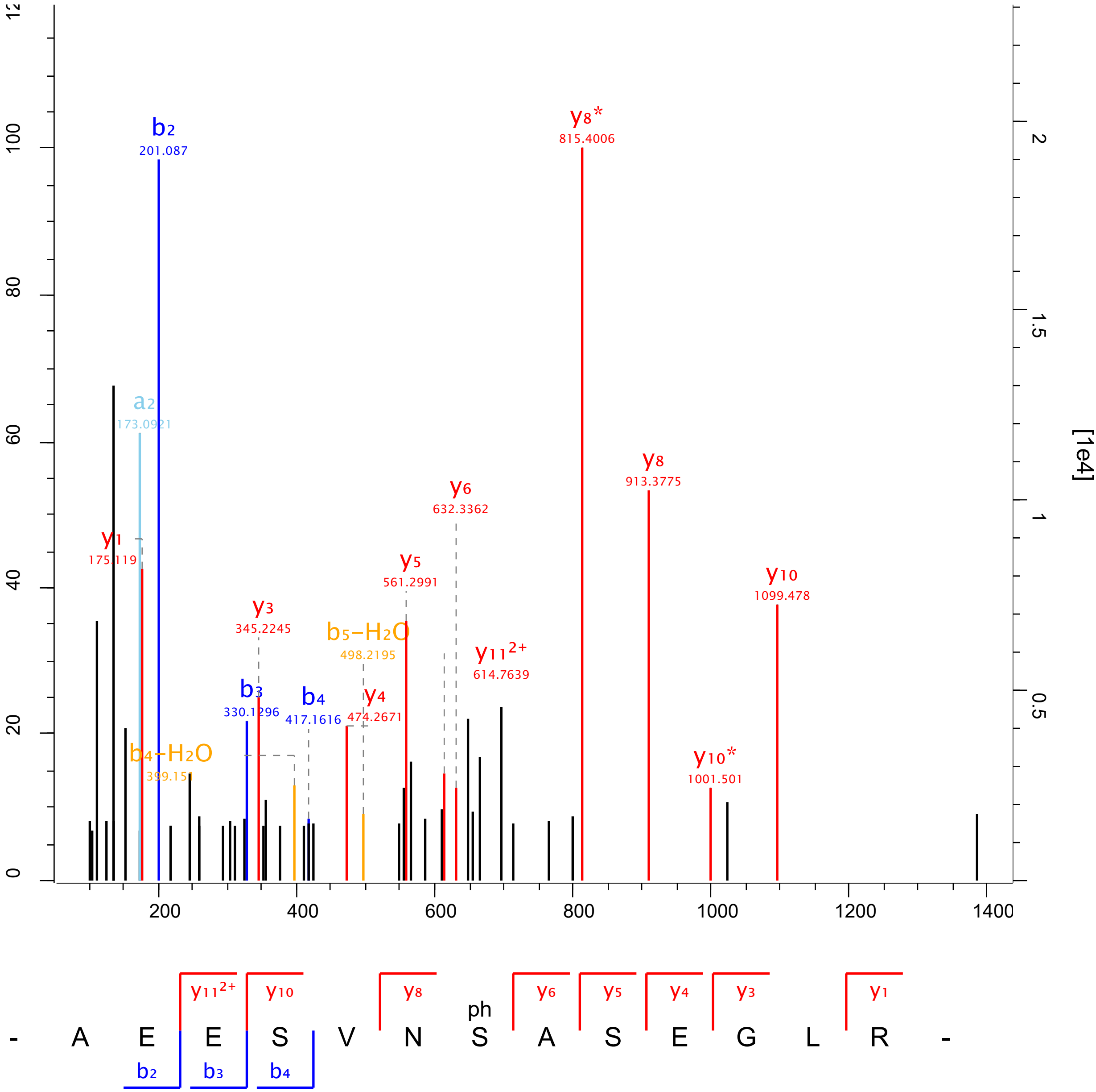

Raw file	Scan	Method	Score	m/z
sys_00_3short	10520	FTMS; HCD	62.47	478.71

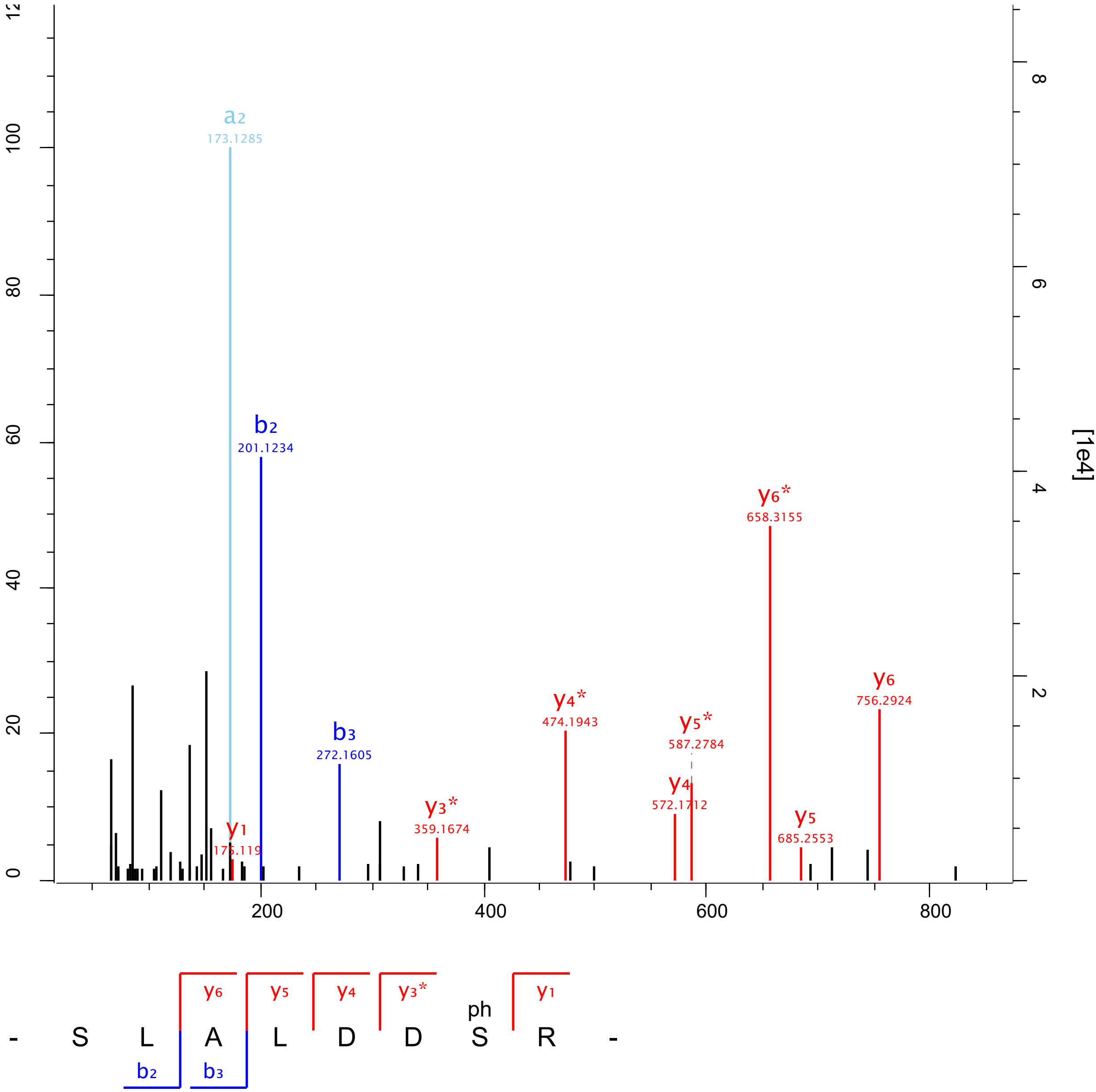

Raw file	Scan	Method	Score	m/z
sys_00_3short	10603	FTMS; HCD	53.26	712.33

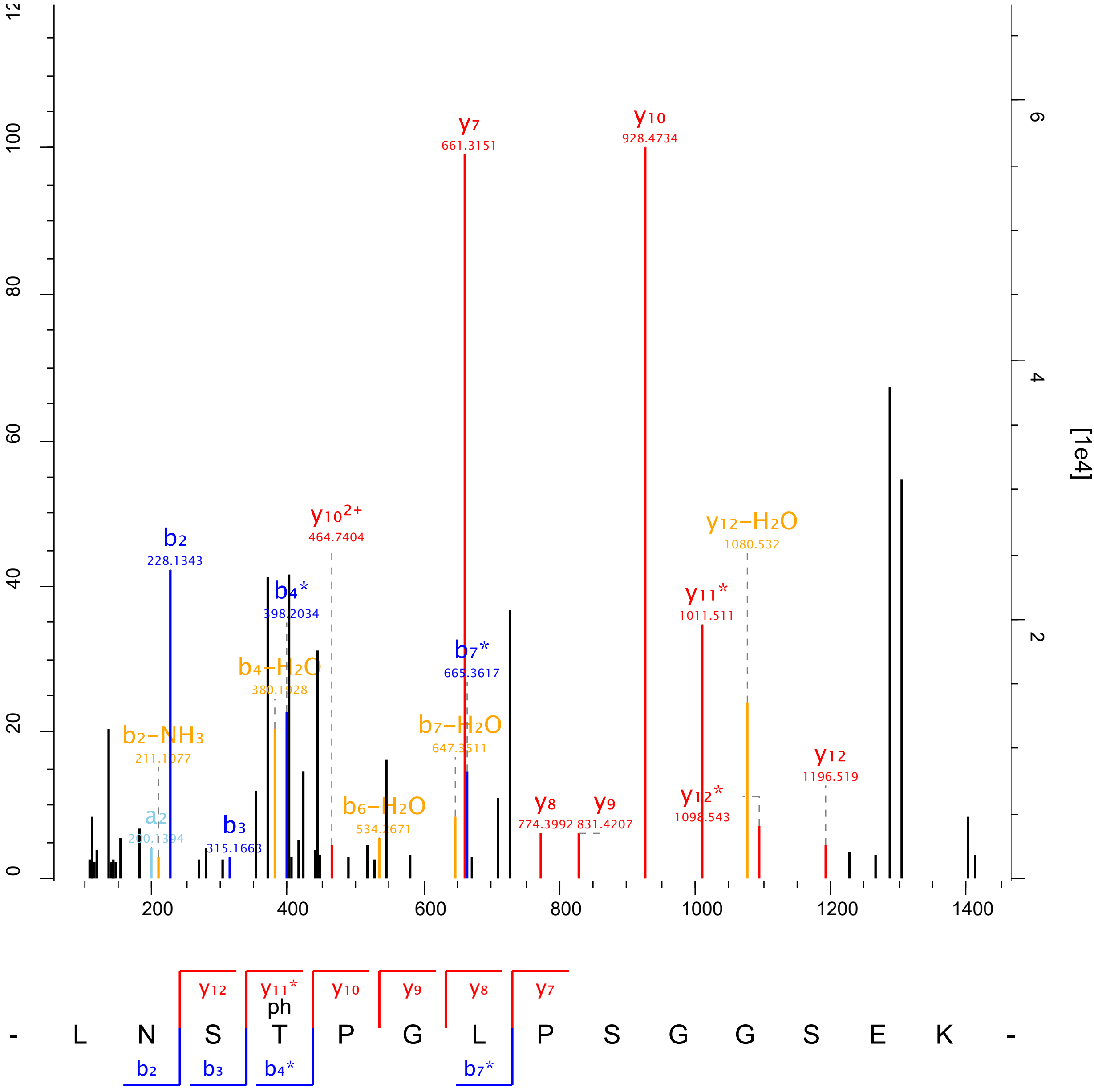

Raw file	Scan	Method	Score	m/z
sys_00_3short	10989	FTMS; HCD	58.08	698.82

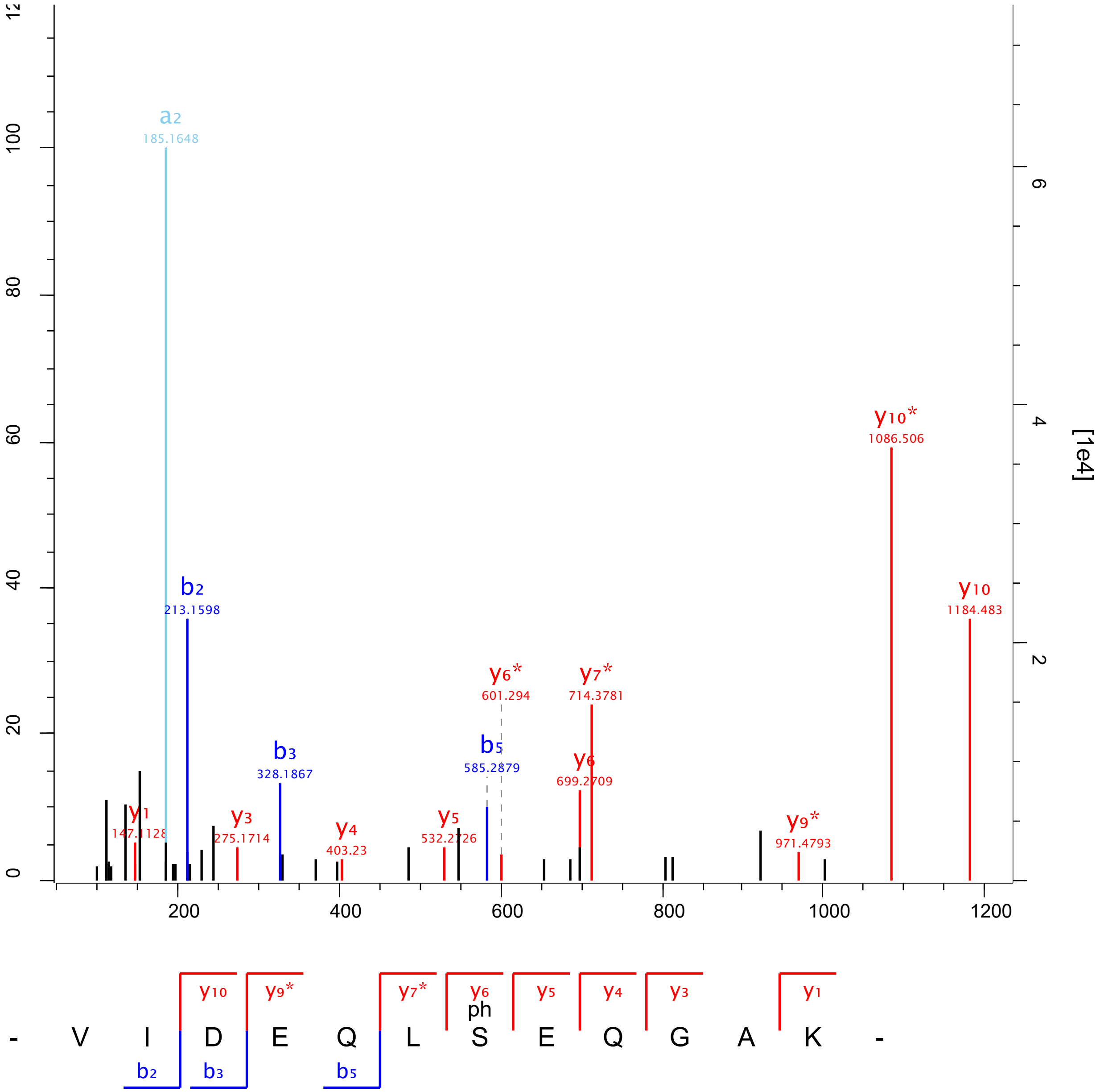

Raw file	Scan	Method	Score	m/z
sys_00_3short	11016	FTMS; HCD	147.7	662.31

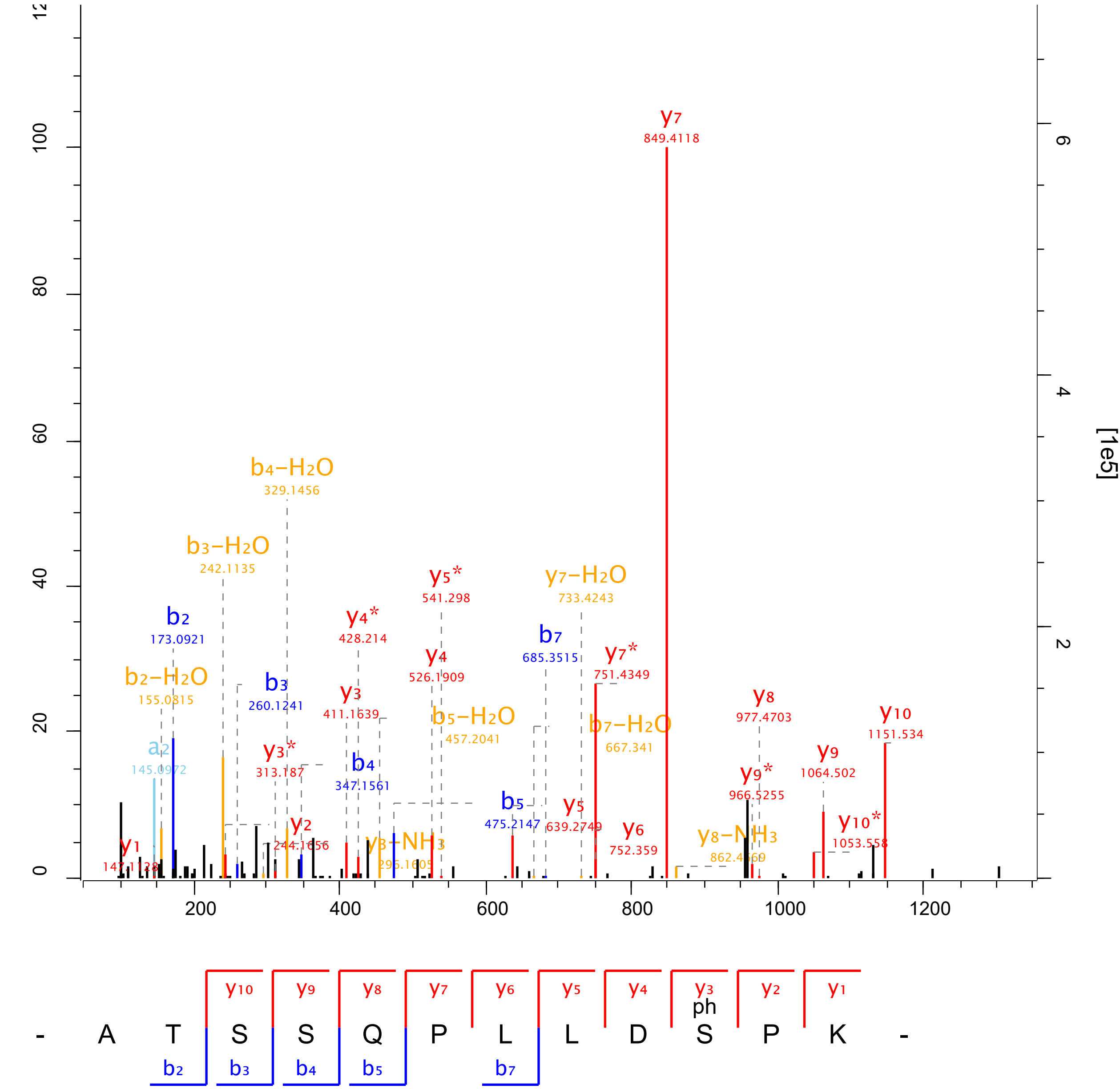

Raw file	Scan	Method	Score	m/z
sys_00_3short	11136	FTMS; HCD	42.34	433.7

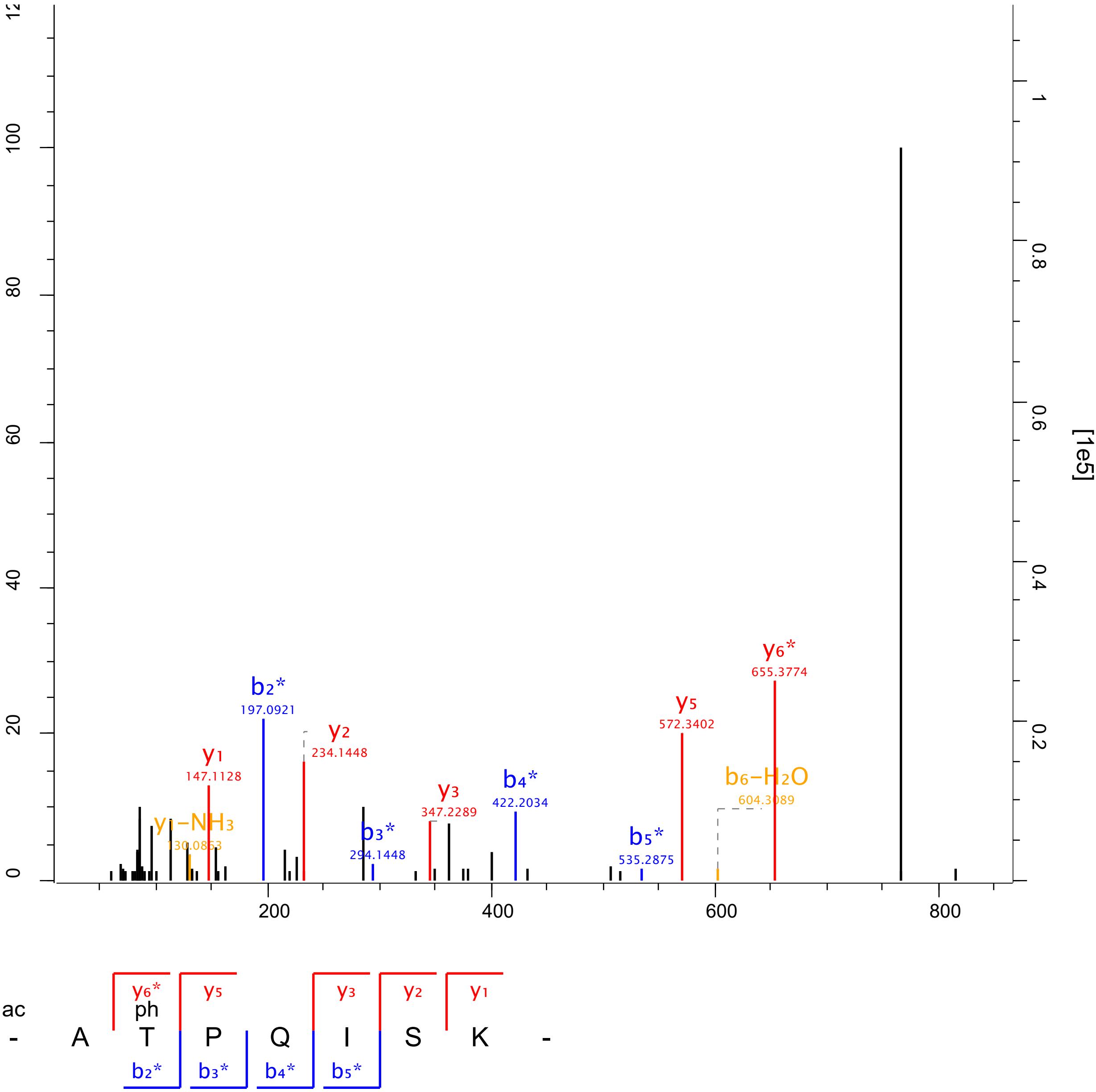

Raw file	Scan	Method	Score	m/z
sys_00_3short	11182	FTMS; HCD	95.42	510.72

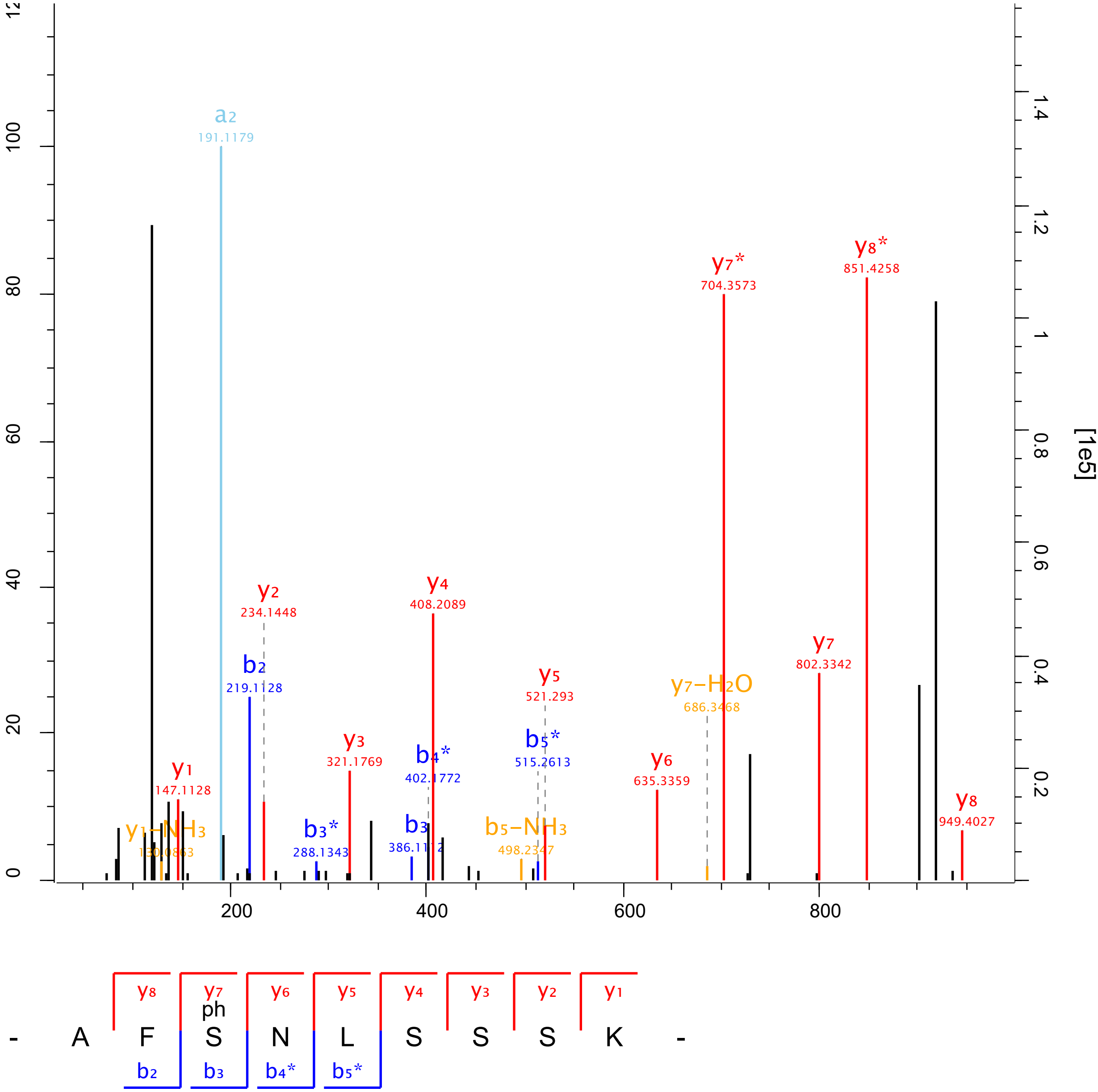

Raw file	Scan	Method	Score	m/z
sys_00_3short	11403	FTMS; HCD	125.97	452.71

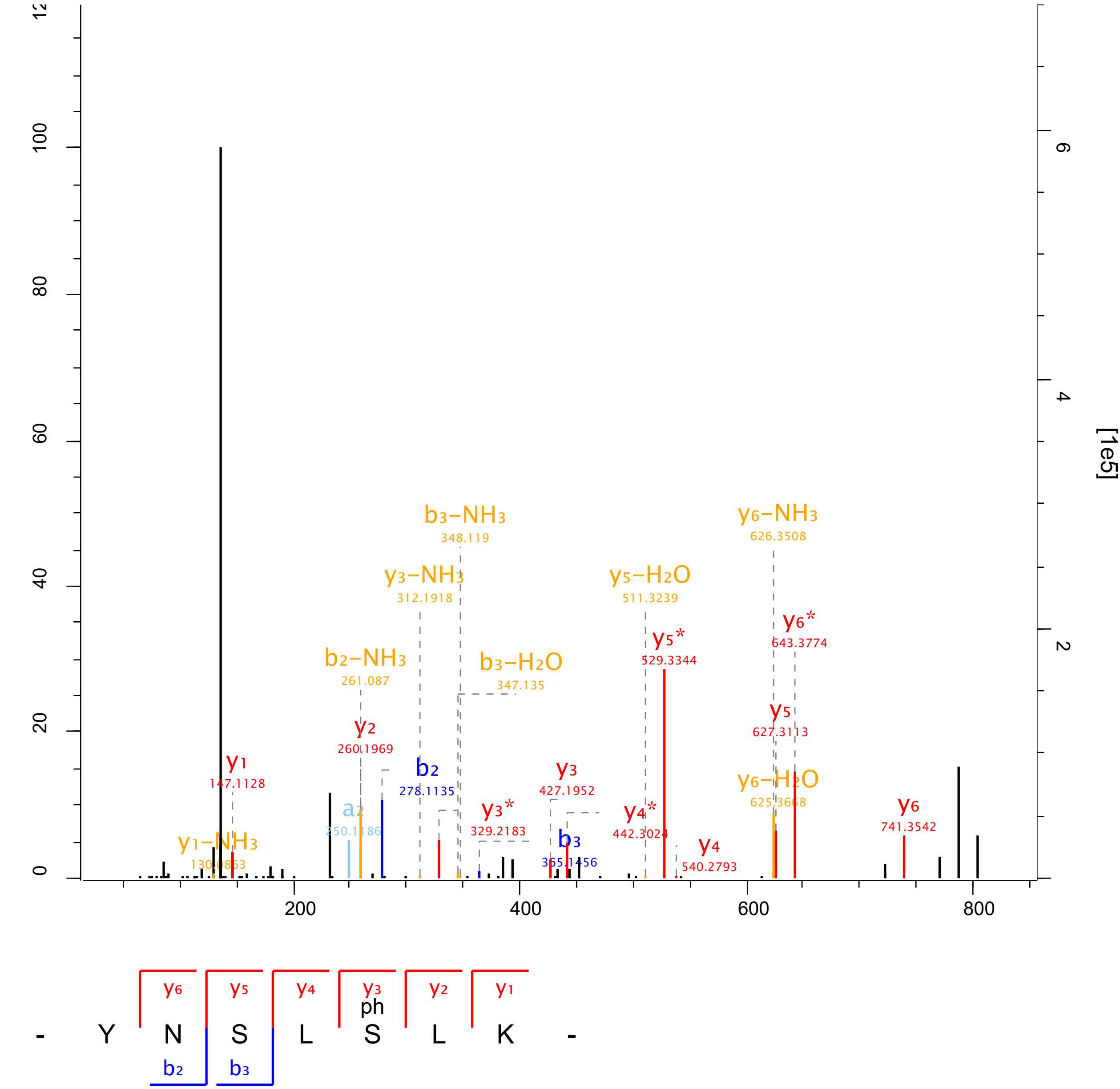

Raw file	Scan	Method	Score	m/z
sys_00_3short	11629	FTMS; HCD	52.58	630.73

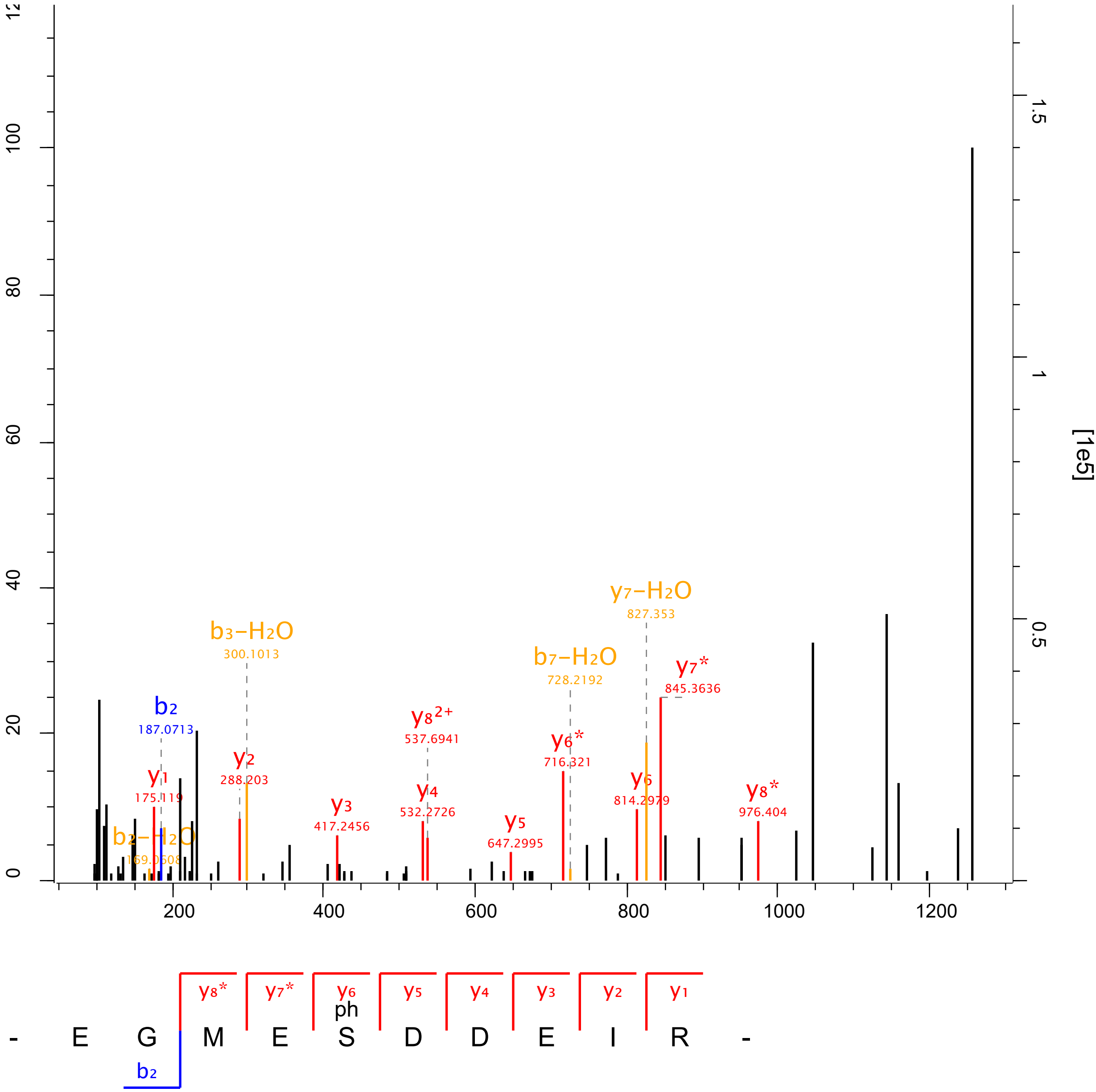

Raw file	Scan	Method	Score	m/z
sys_00_3short	12013	FTMS; HCD	116.24	568.23

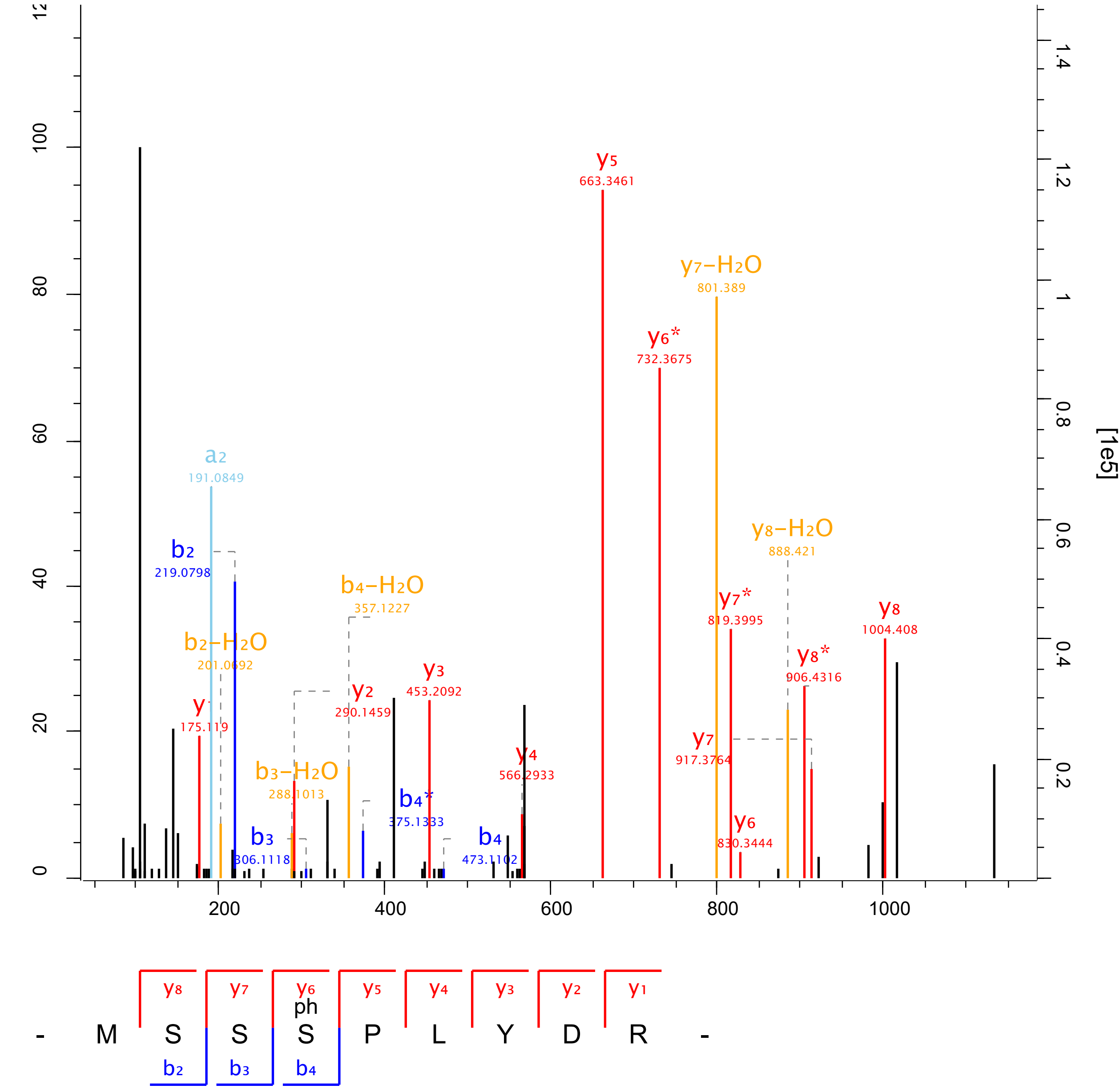

Raw file	Scan	Method	Score	m/z
sys_00_3short	12110	FTMS; HCD	136.39	686.31

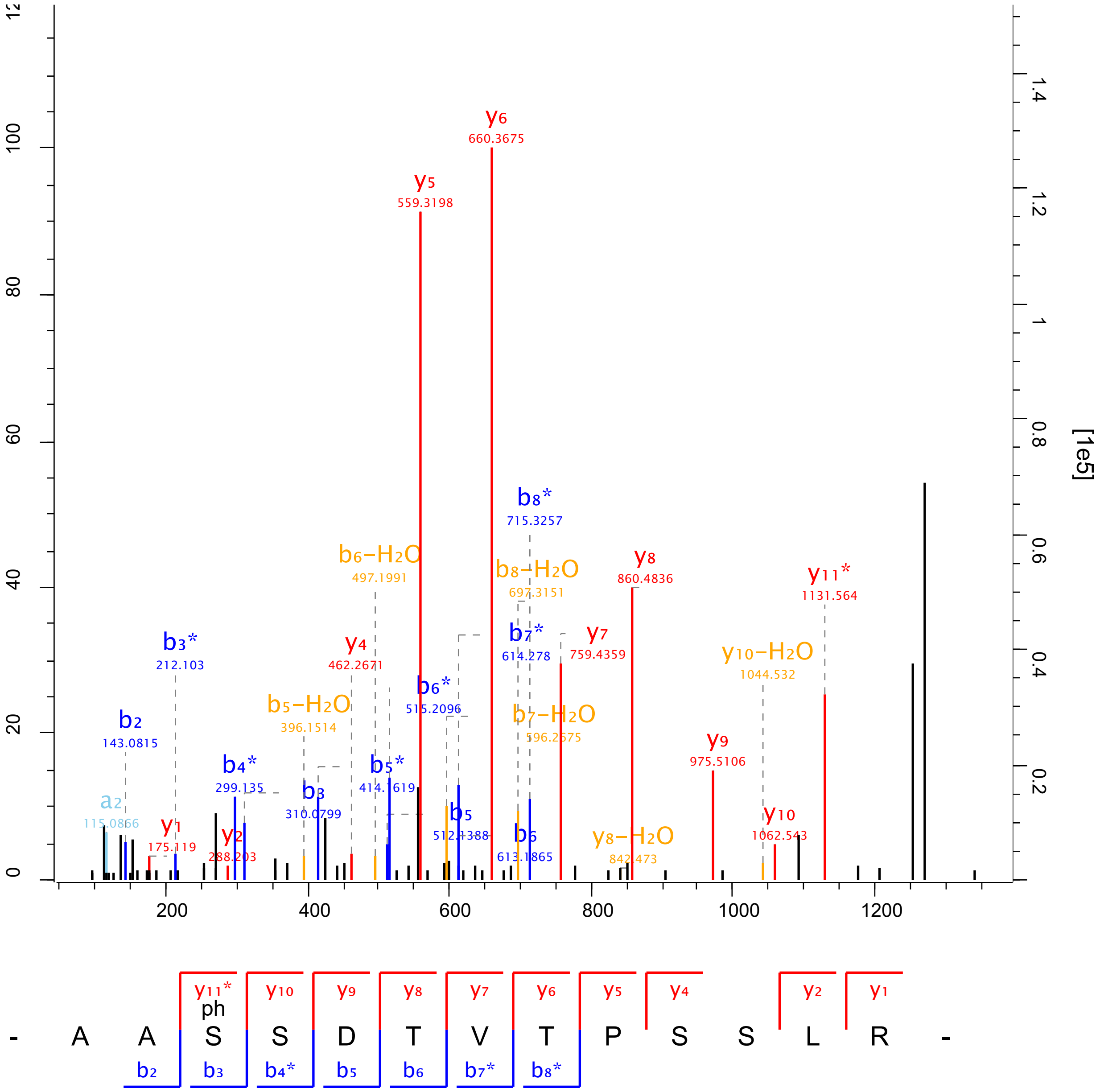

Raw file	Scan	Method	Score	m/z
sys_00_3short	12113	FTMS; HCD	146.16	515.24

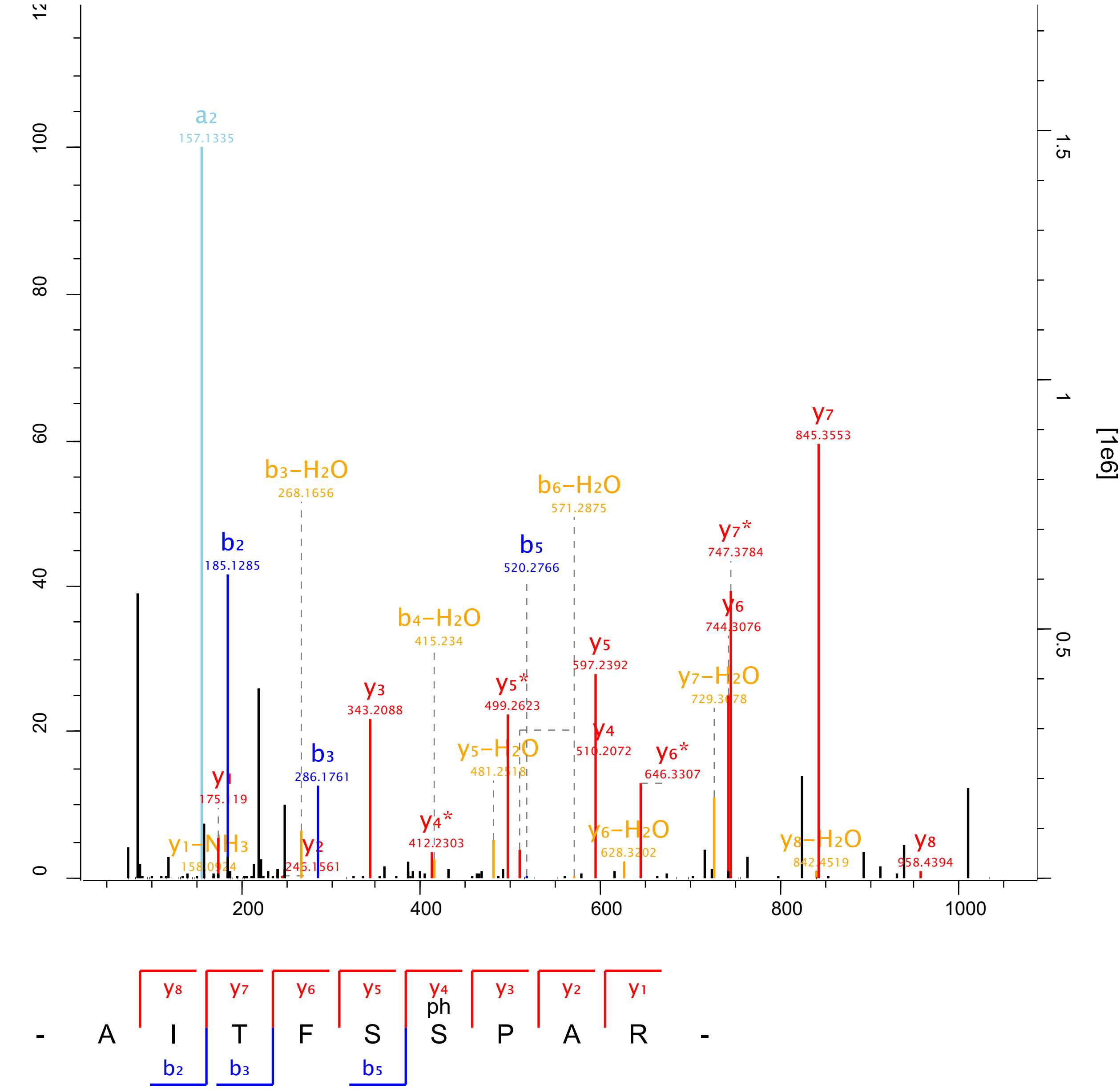

Raw file	Scan	Method	Score	m/z
sys_00_3short	12650	FTMS; HCD	148.62	445.19

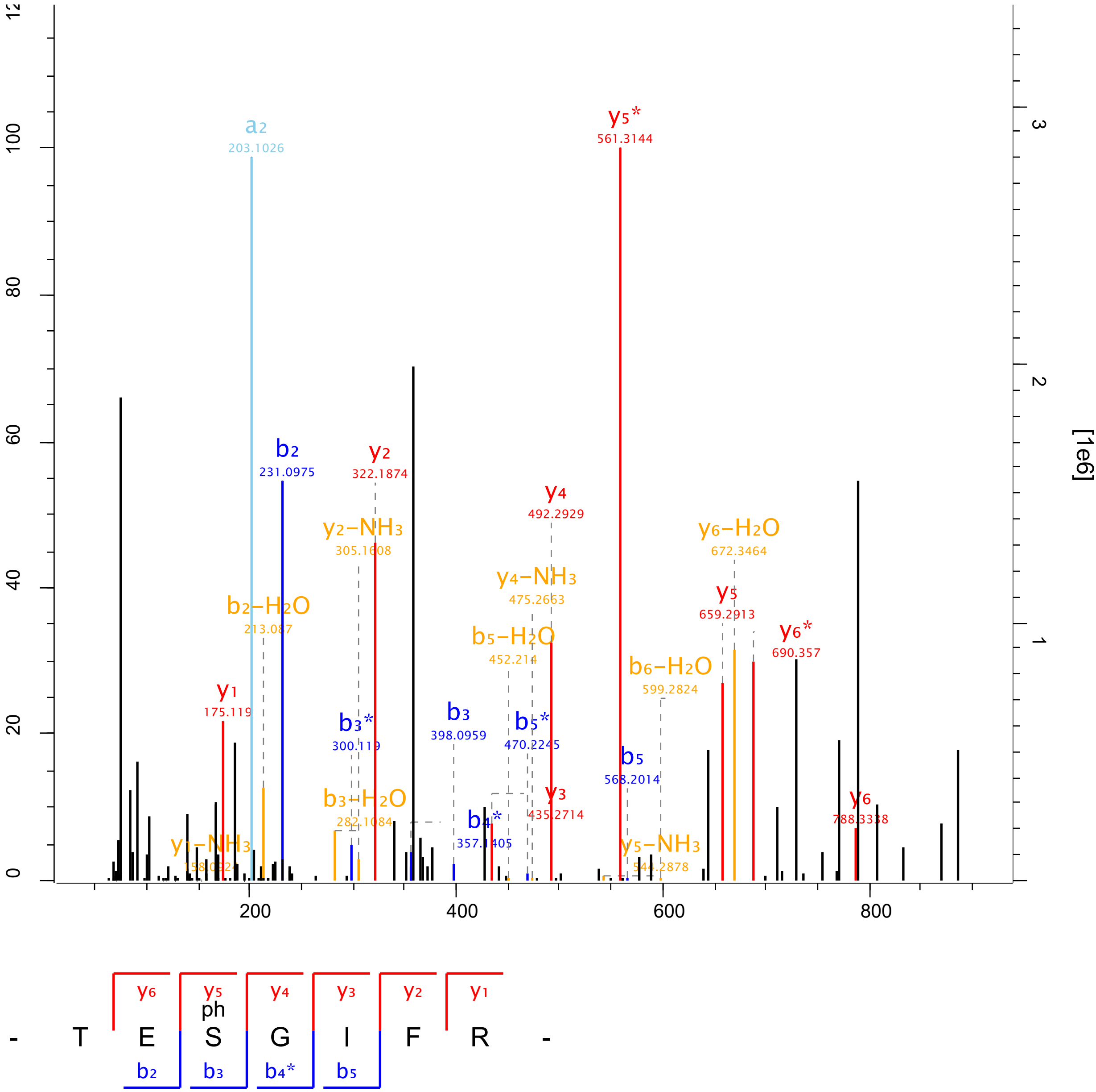

Raw file	Scan	Method	Score	m/z
sys_00_3short	12797	FTMS; HCD	178.51	598.28

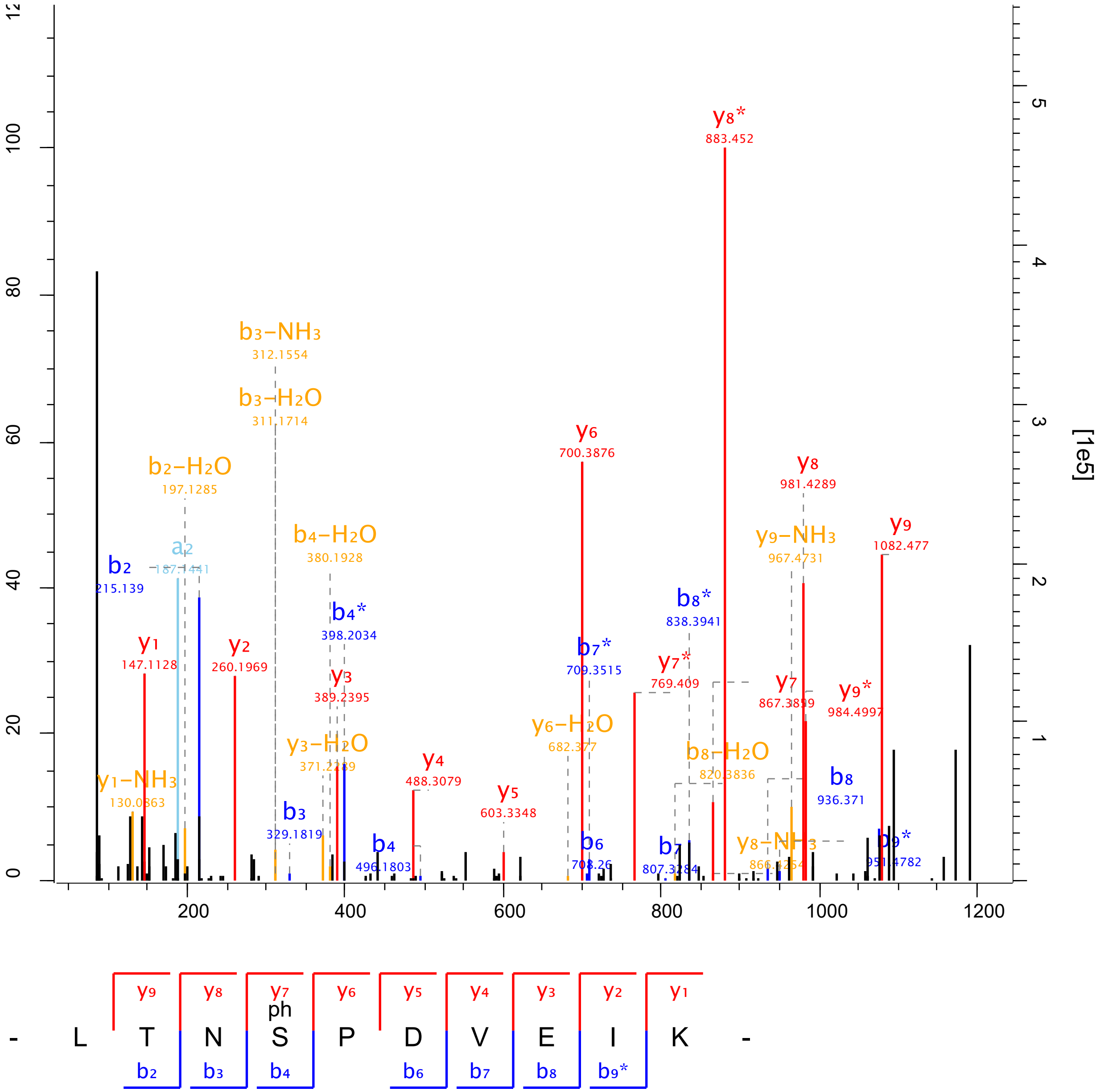

Raw file	Scan	Method	Score	m/z
sys_00_3short	12943	FTMS; HCD	123.75	562.75

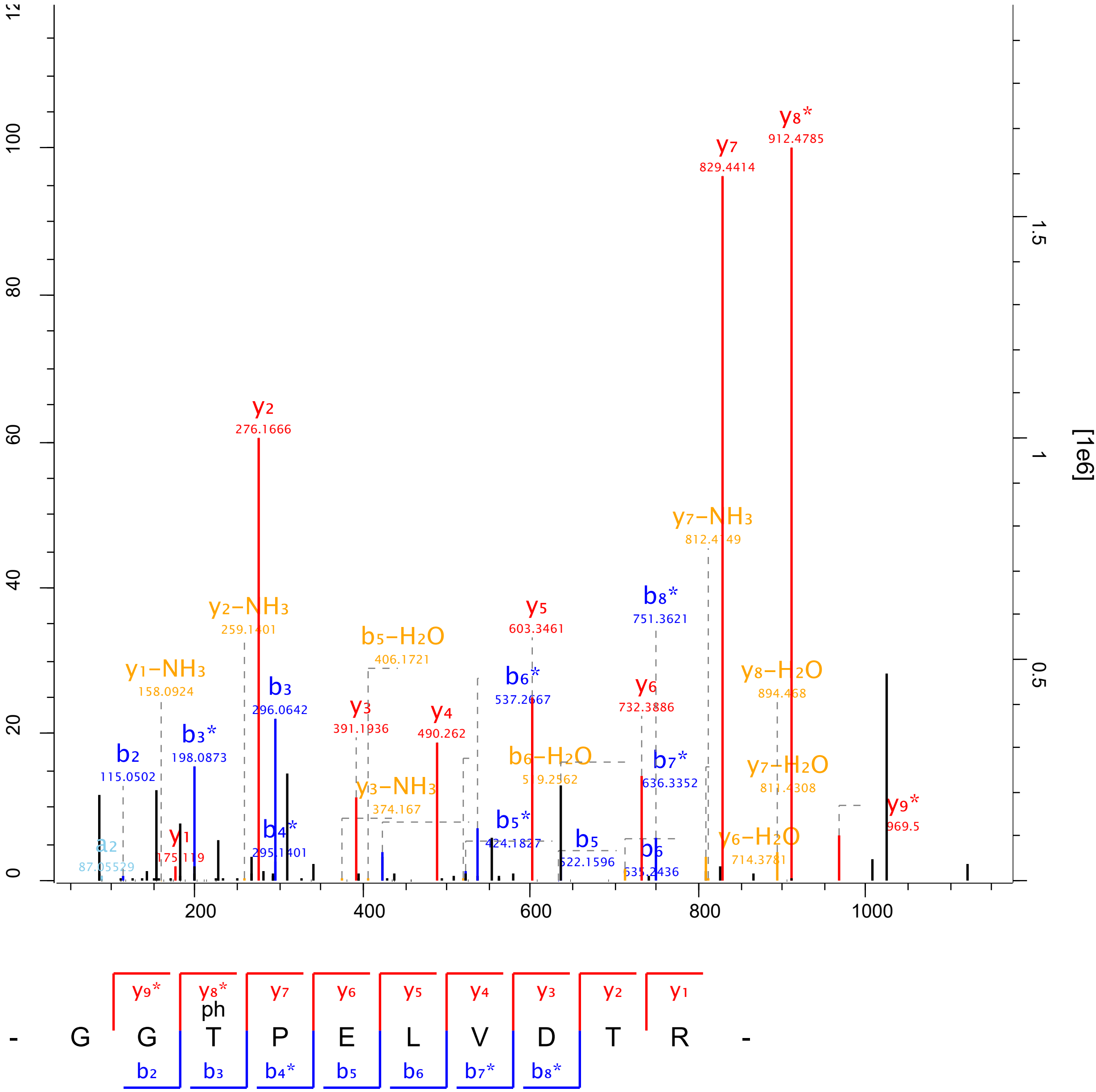

Raw file	Scan	Method	Score	m/z
sys_00_3short	12999	FTMS; HCD	111.63	524.23

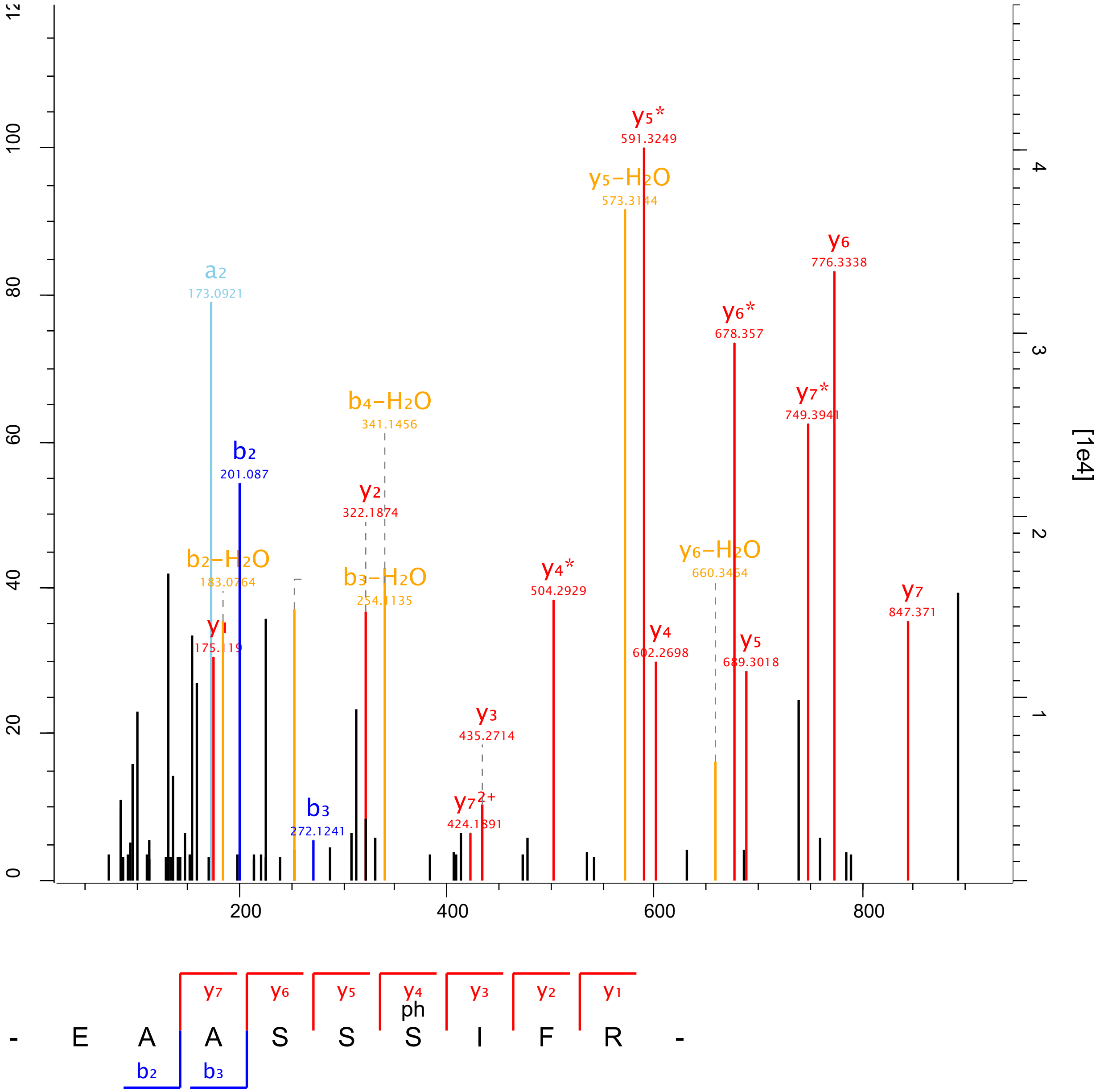

Raw file	Scan	Method	Score	m/z
sys_00_3short	13034	FTMS; HCD	51.73	560.23

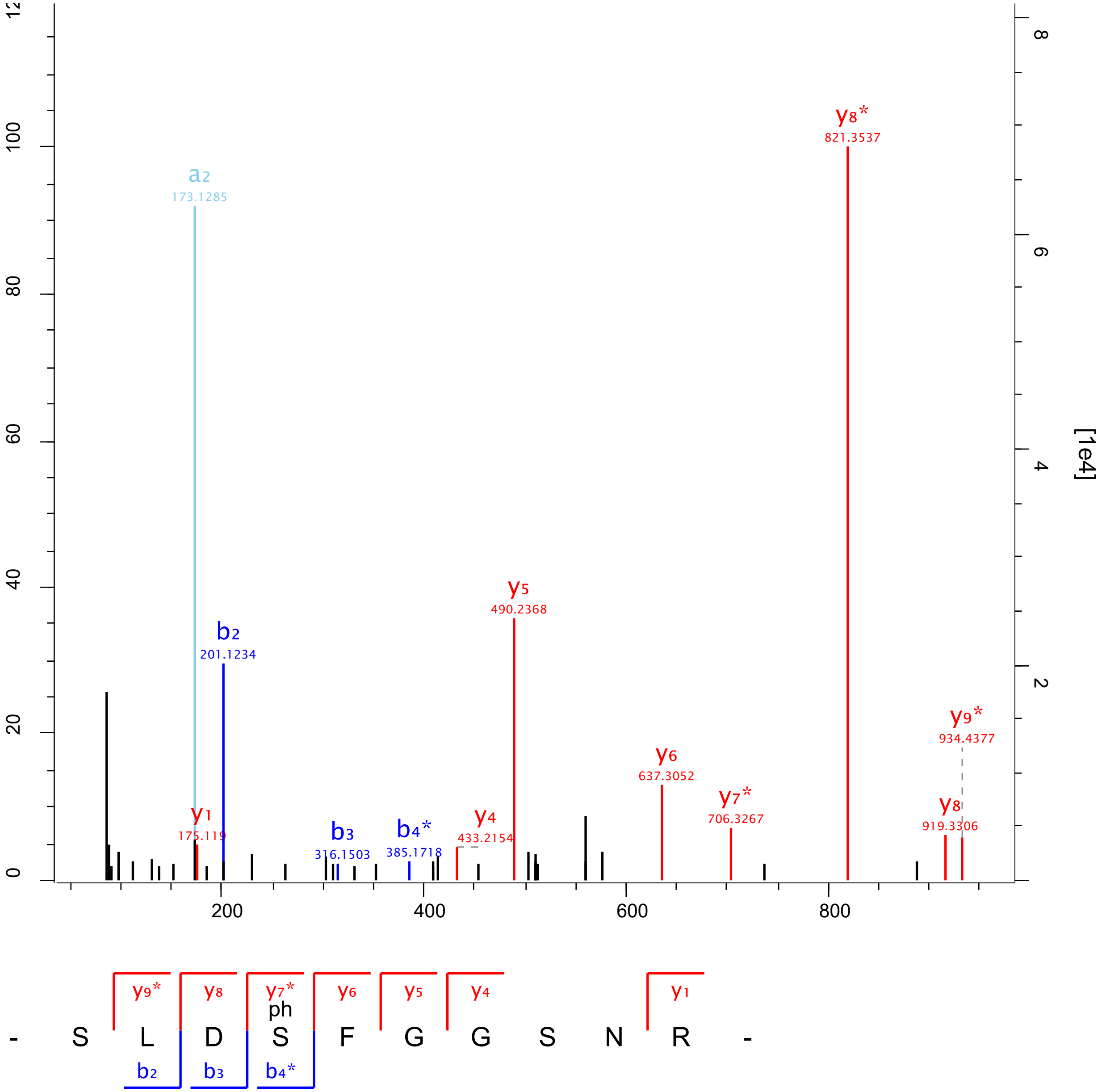

Raw file	Scan	Method	Score	m/z
sys_00_3short	13244	FTMS; HCD	55.26	542.25

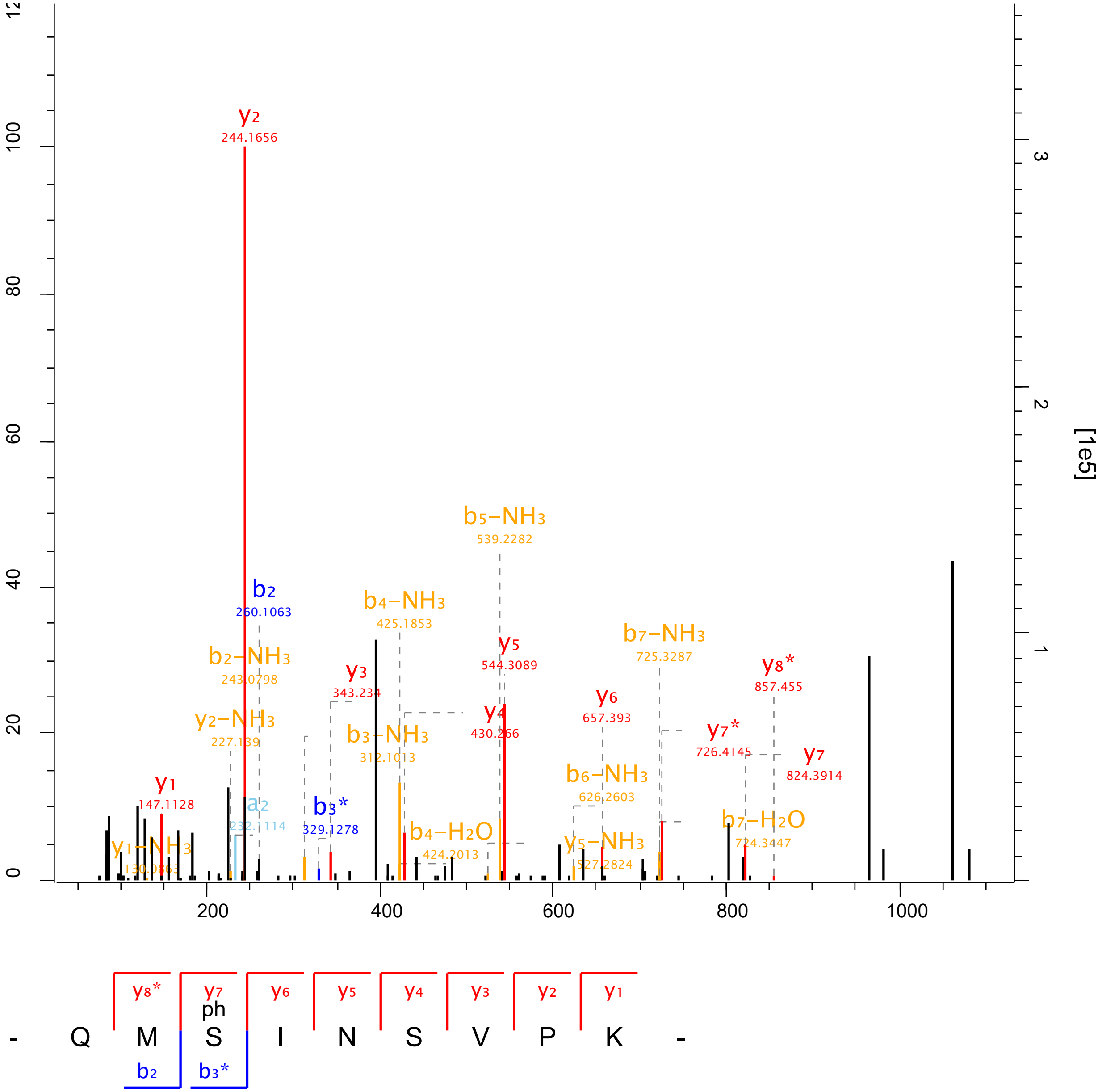

Raw file	Scan	Method	Score	m/z
sys_00_3short	13707	FTMS; HCD	61.24	564.27

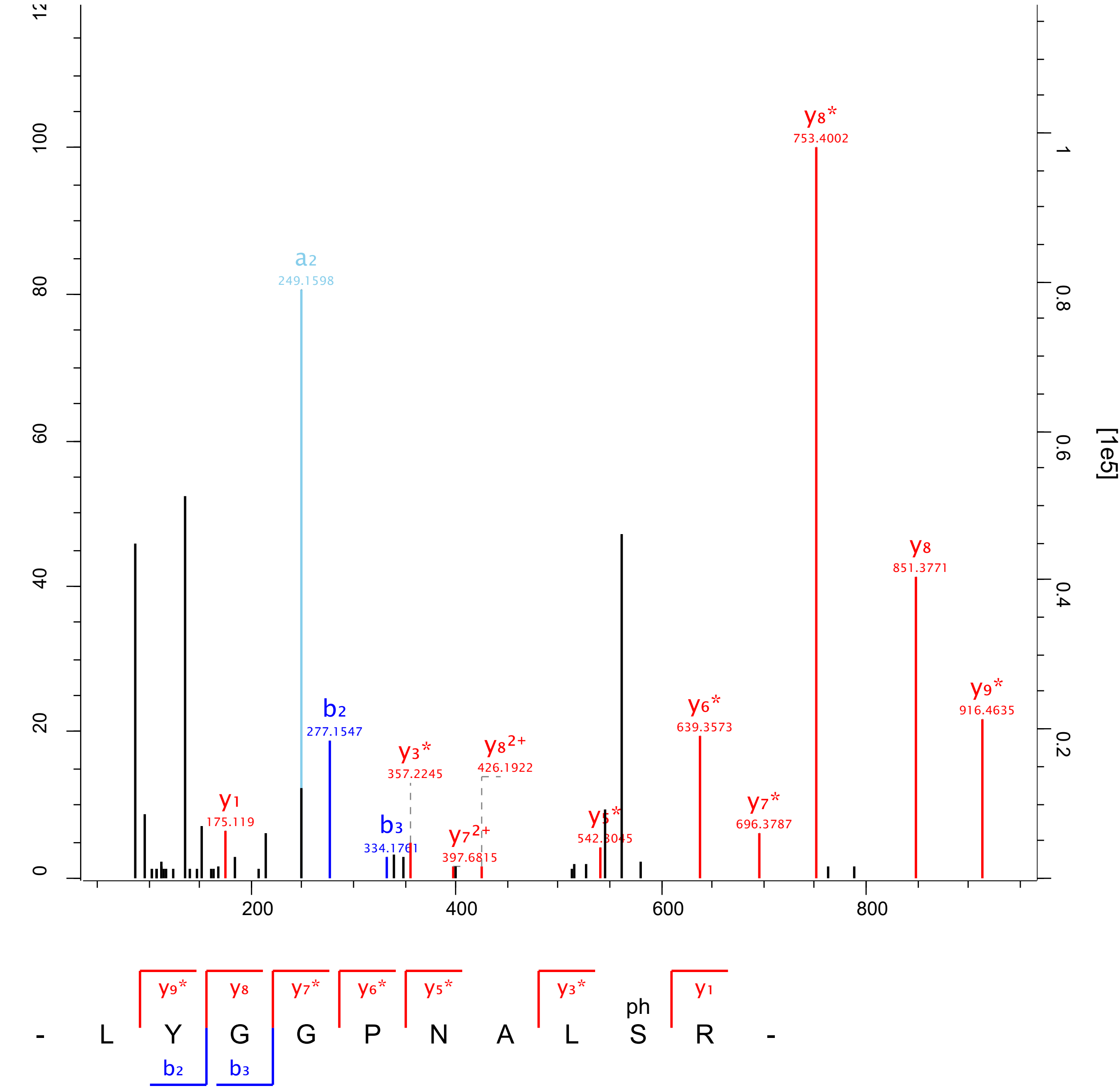

Raw file	Scan	Method	Score	m/z
sys_00_3short	13717	FTMS; HCD	148.78	550.76

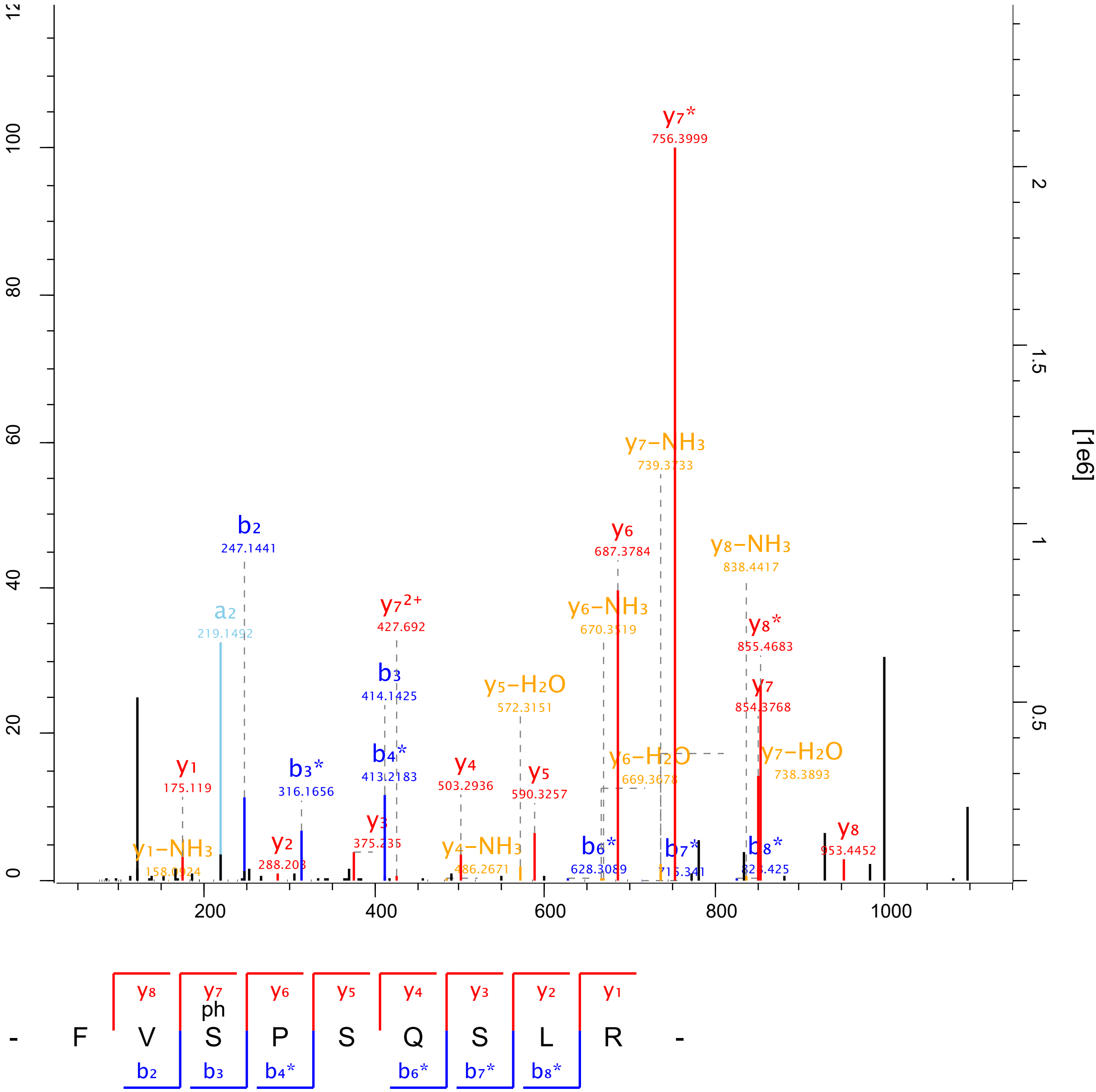

Raw file	Scan	Method	Score	m/z
sys_00_3short	13838	FTMS; HCD	67.52	541.75

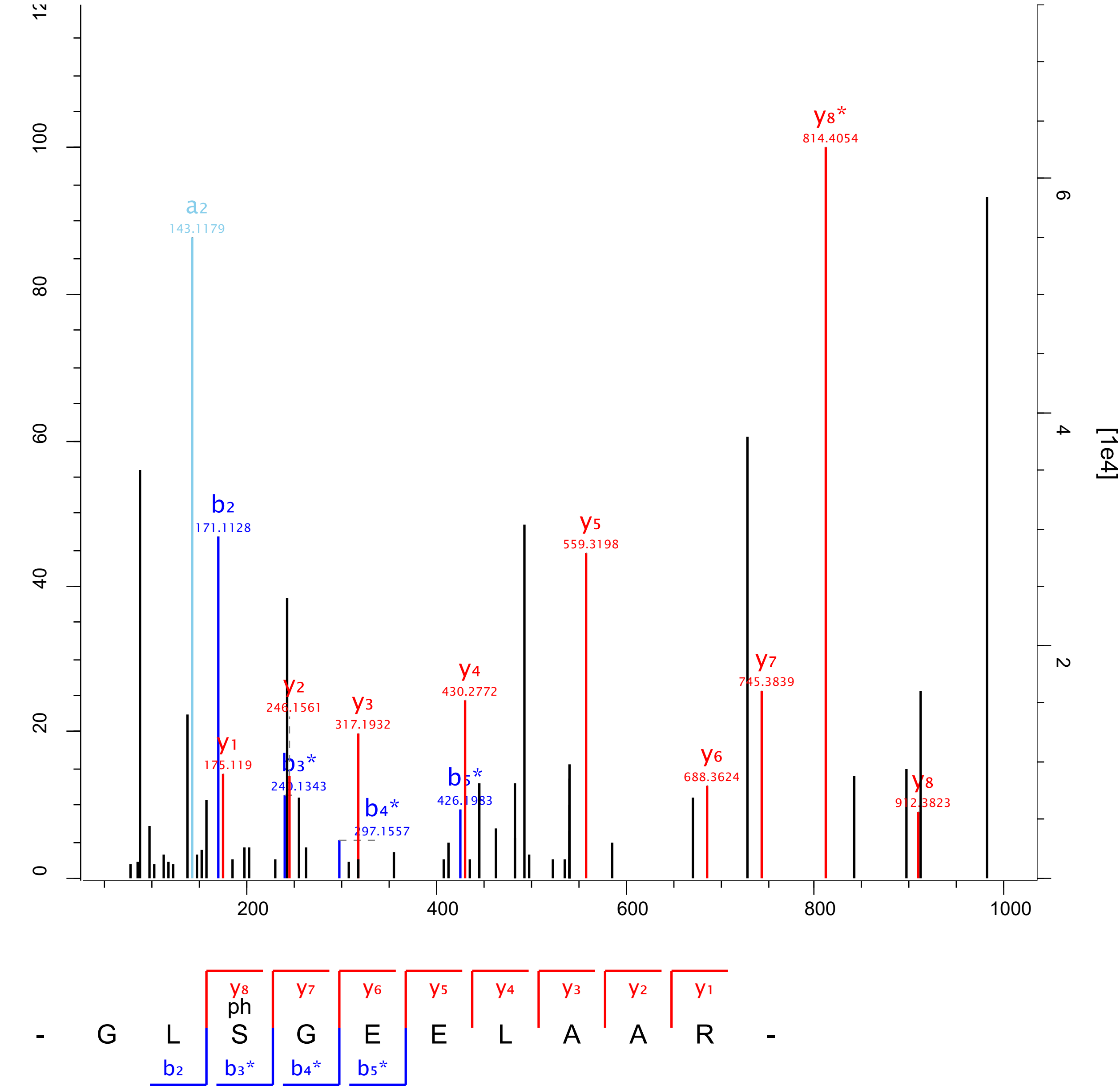

Raw file	Scan	Method	Score	m/z
sys_00_3short	13862	FTMS; HCD	84.51	758.31

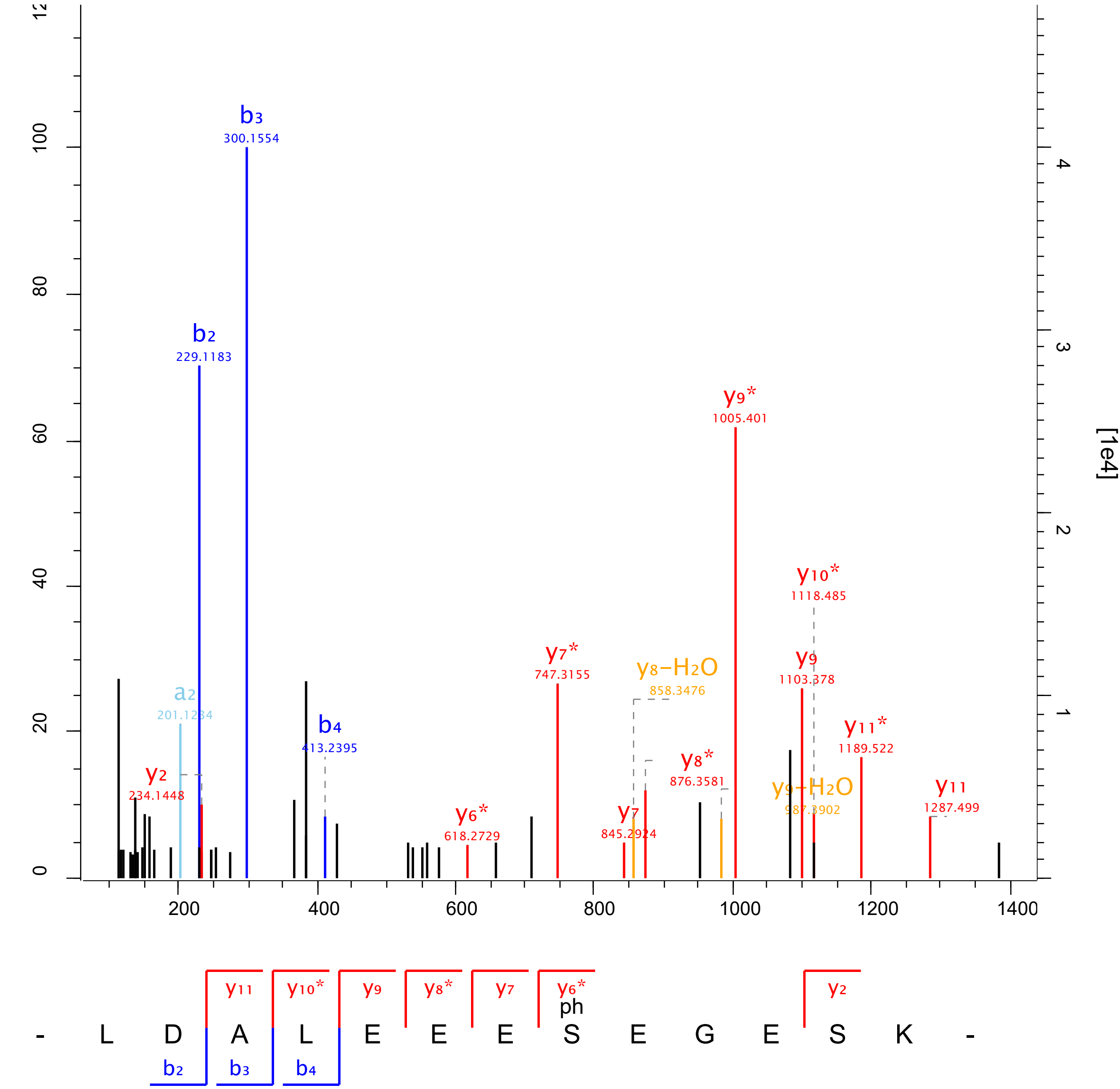

Raw file	Scan	Method	Score	m/z
sys_00_3short	14037	FTMS; HCD	89.51	561.79

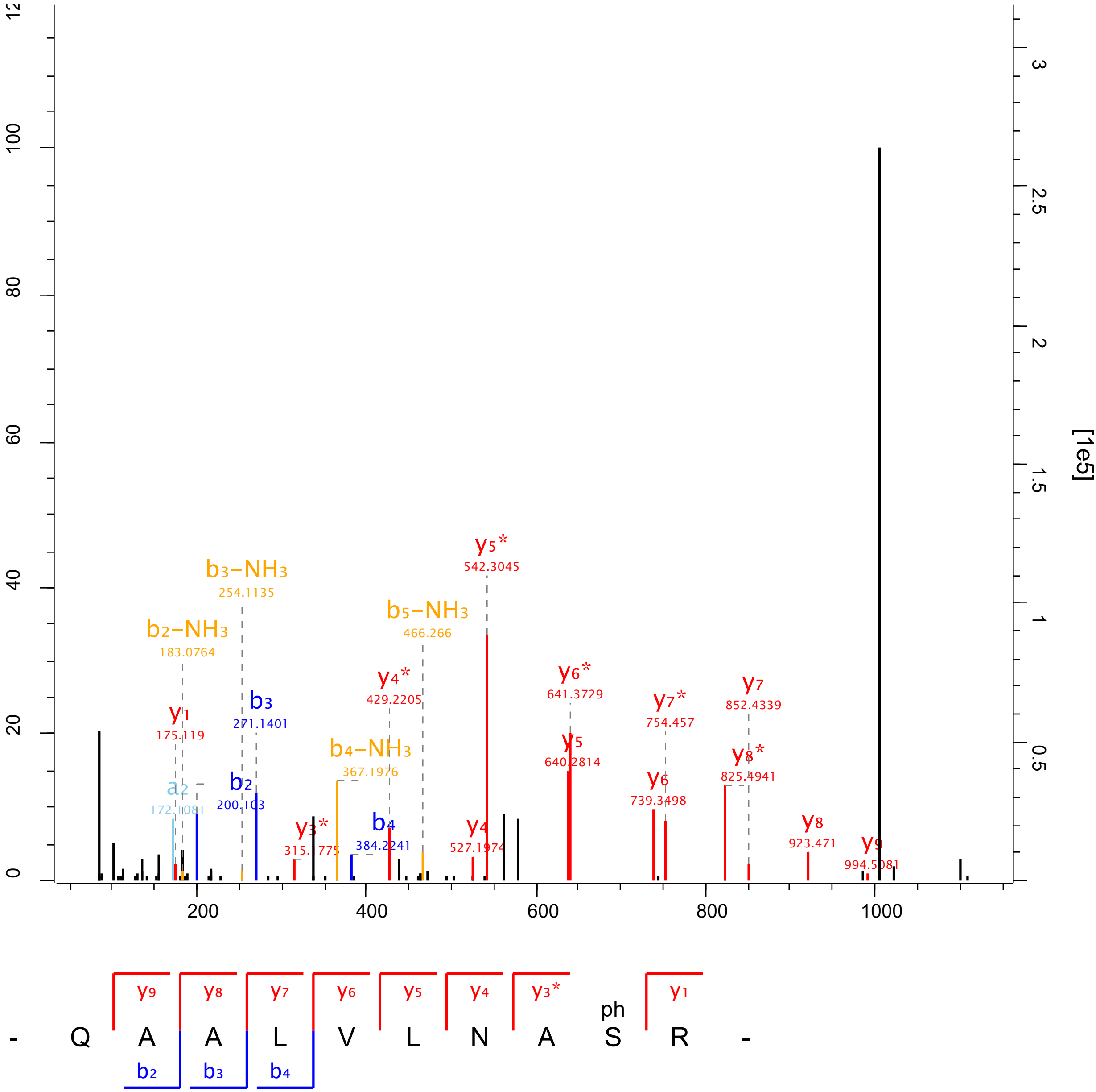

Raw file	Scan	Method	Score	m/z
sys_00_3short	14152	FTMS; HCD	82.35	695.3

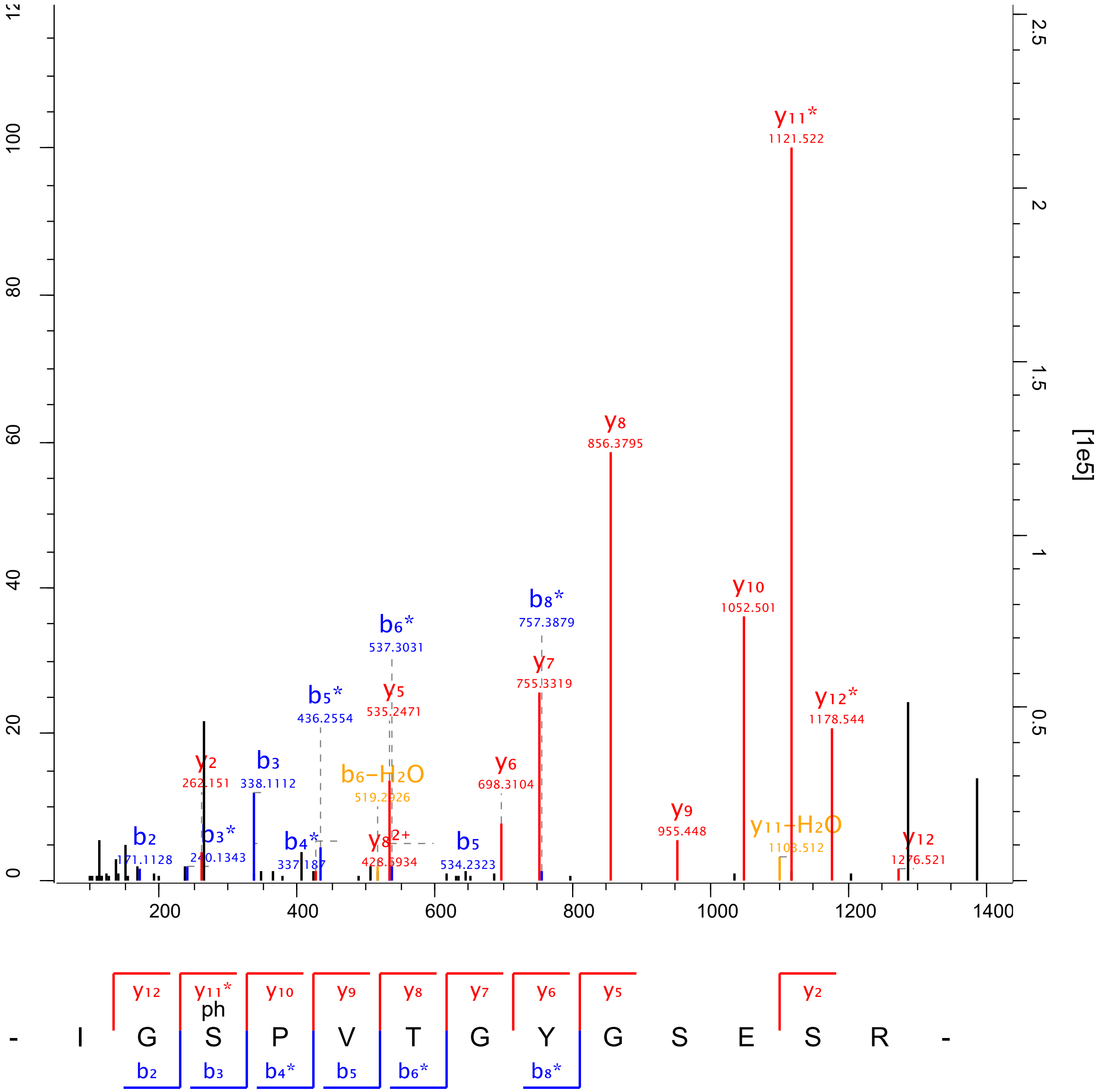

Raw file	Scan	Method	Score	m/z
sys_00_3short	14176	FTMS; HCD	200.58	769.79

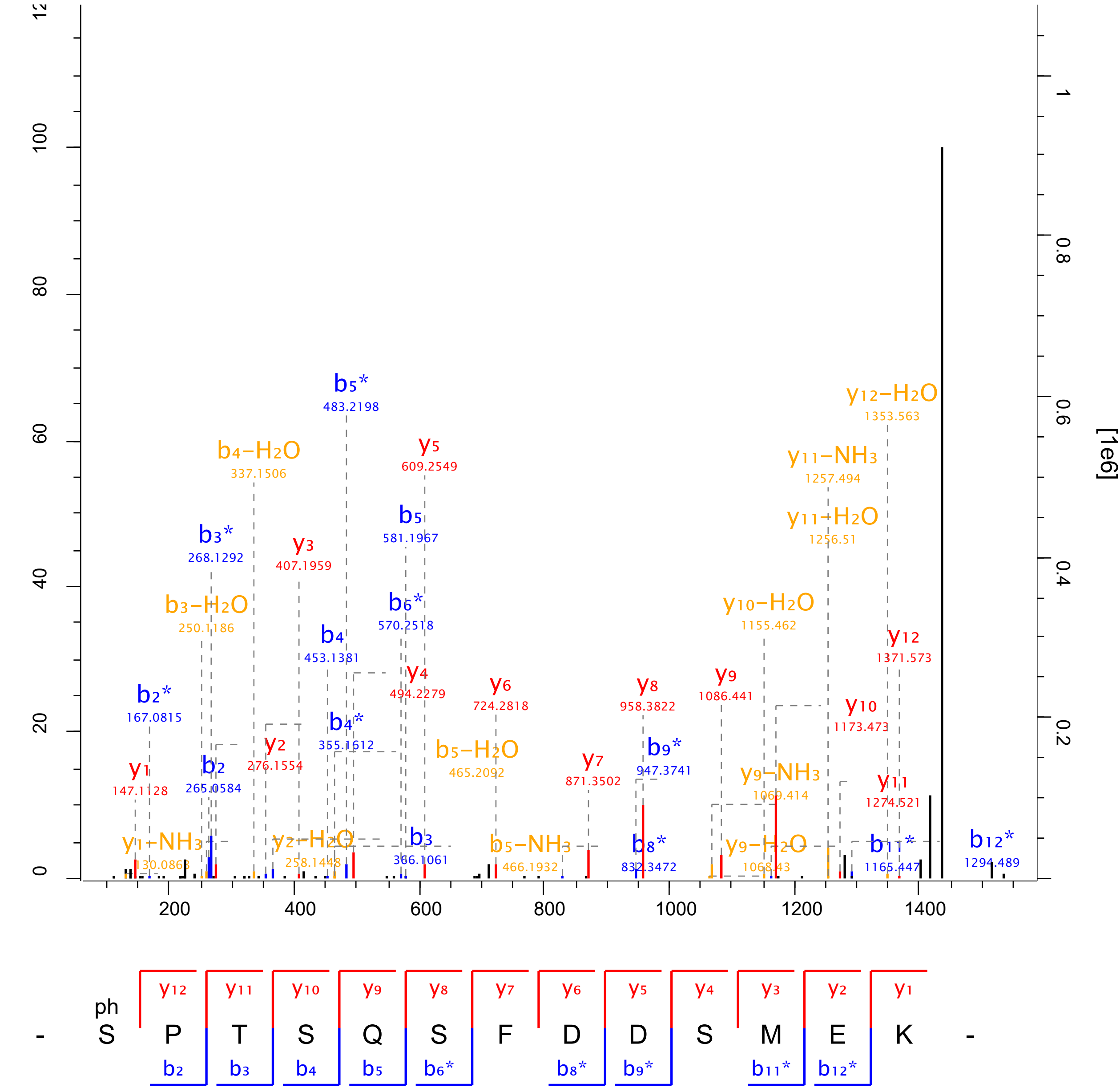

Raw file	Scan	Method	Score	m/z
sys_00_3short	14218	FTMS; HCD	108.72	528.73

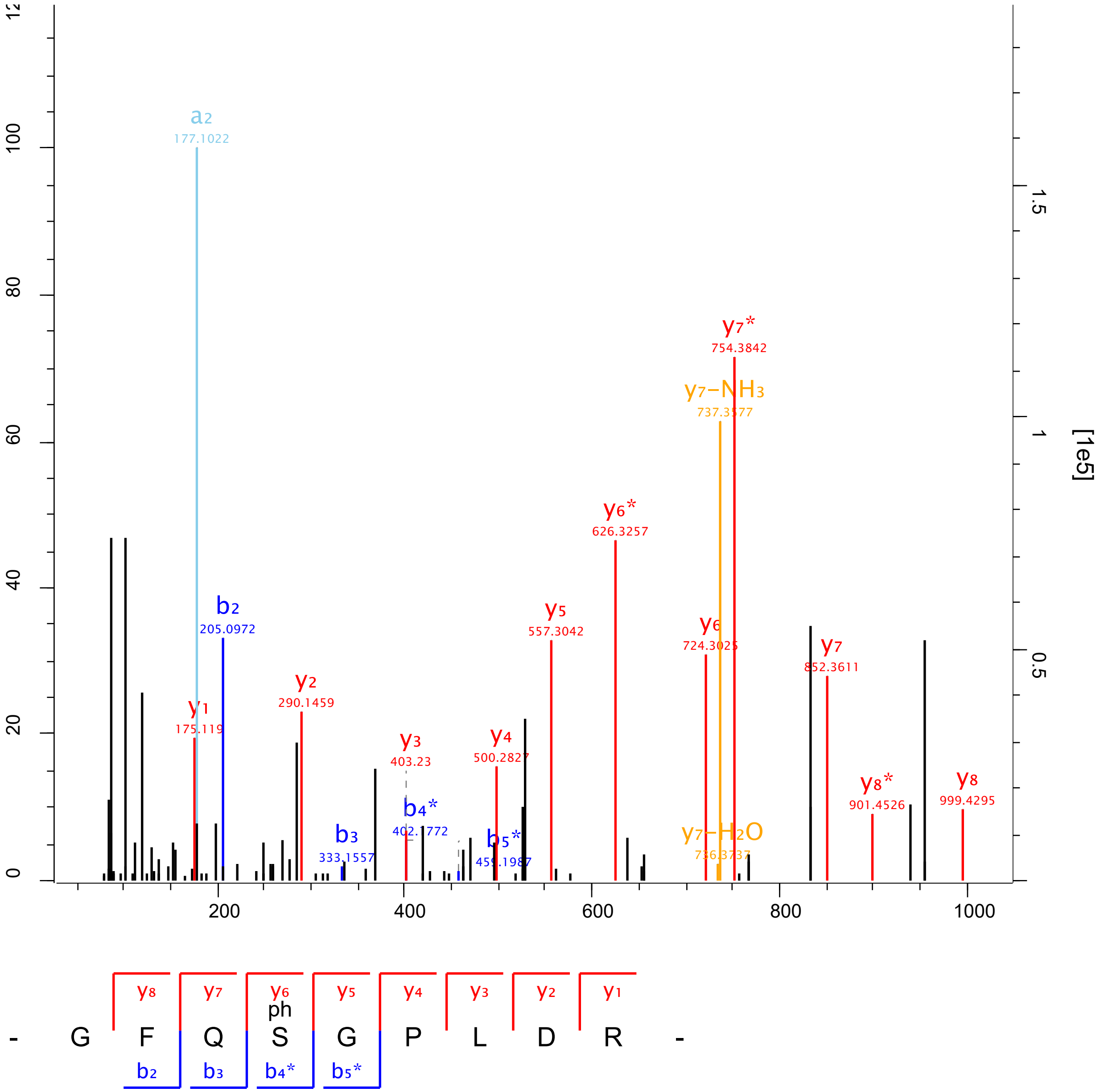

Raw file	Scan	Method	Score	m/z
sys_00_3short	14426	FTMS; HCD	98.05	512.73

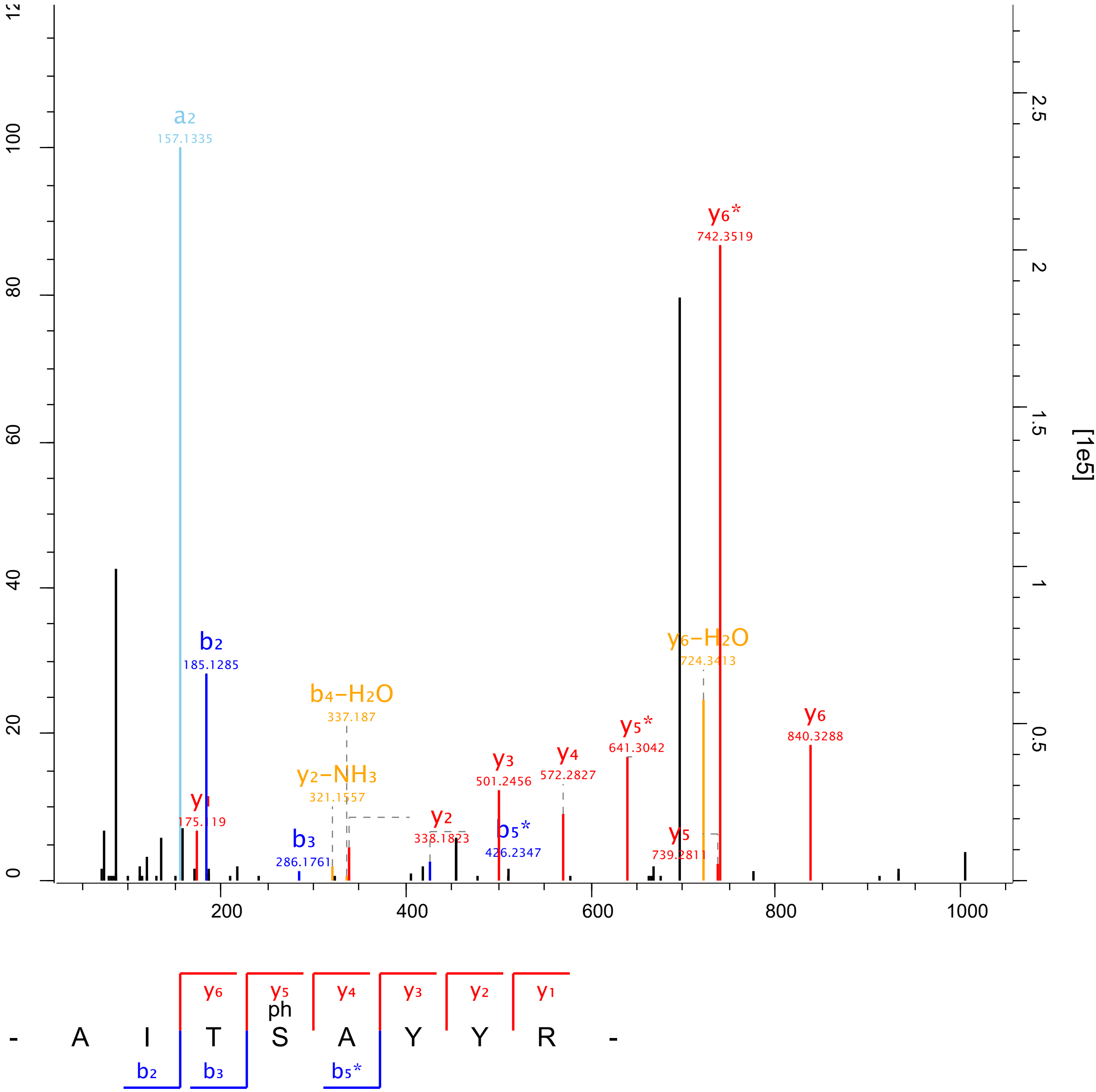

Raw file	Scan	Method	Score	m/z
sys_00_3short	14444	FTMS; HCD	117.18	802.32

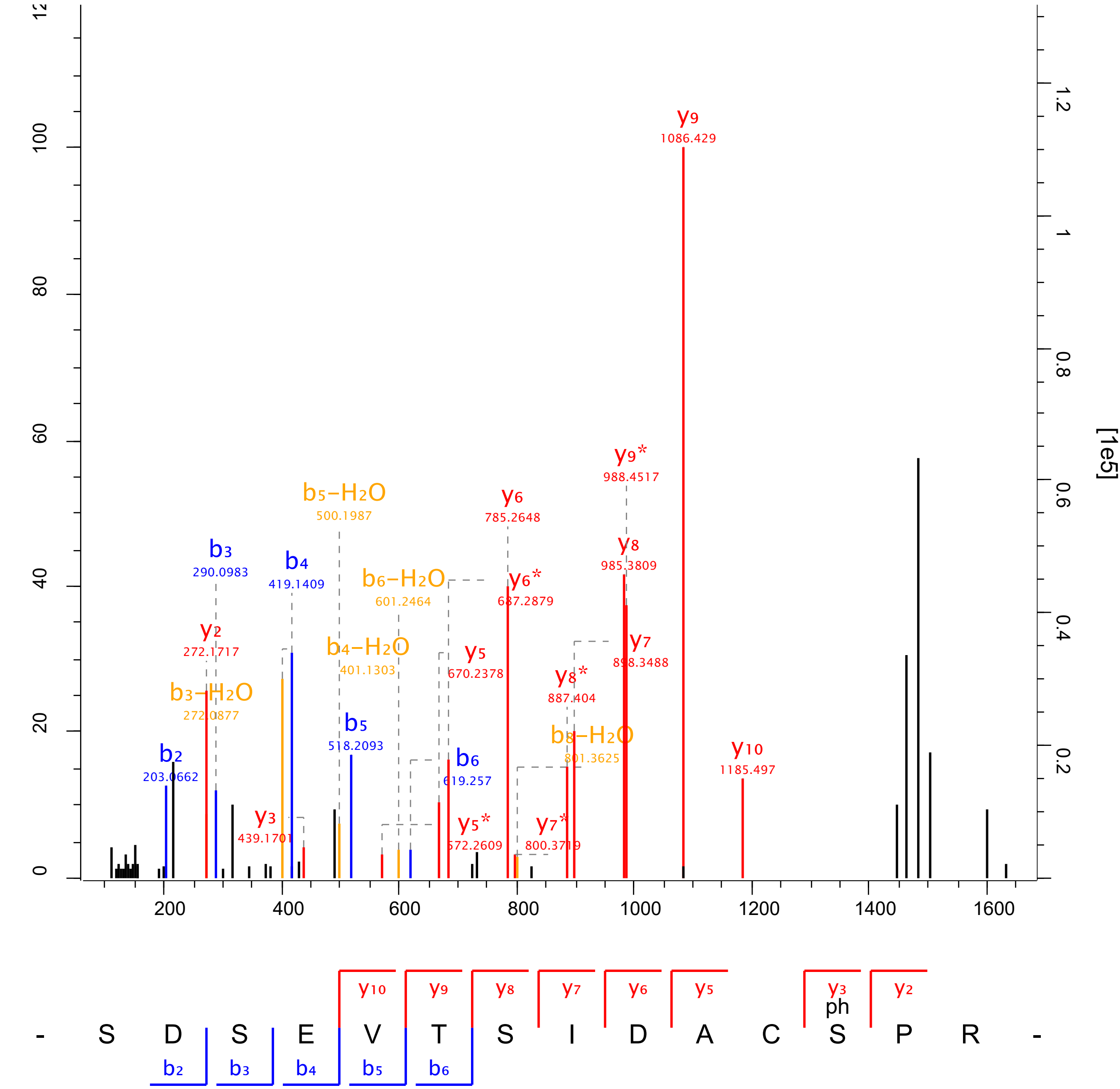

Raw file	Scan	Method	Score	m/z
sys_00_3short	14450	FTMS; HCD	82.07	563.76

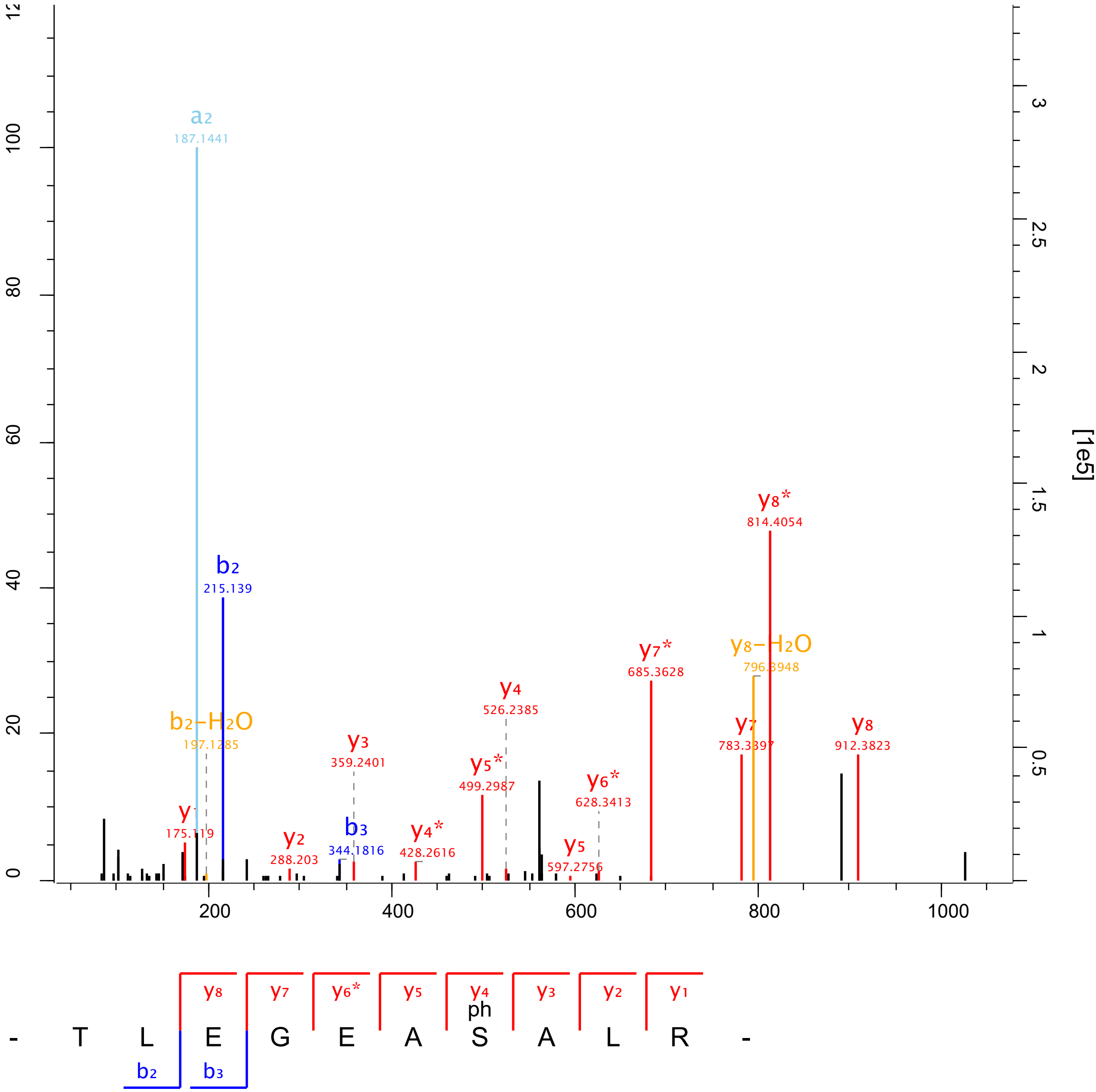

Raw file	Scan	Method	Score	m/z
sys_00_3short	14488	FTMS; HCD	45.89	506.21

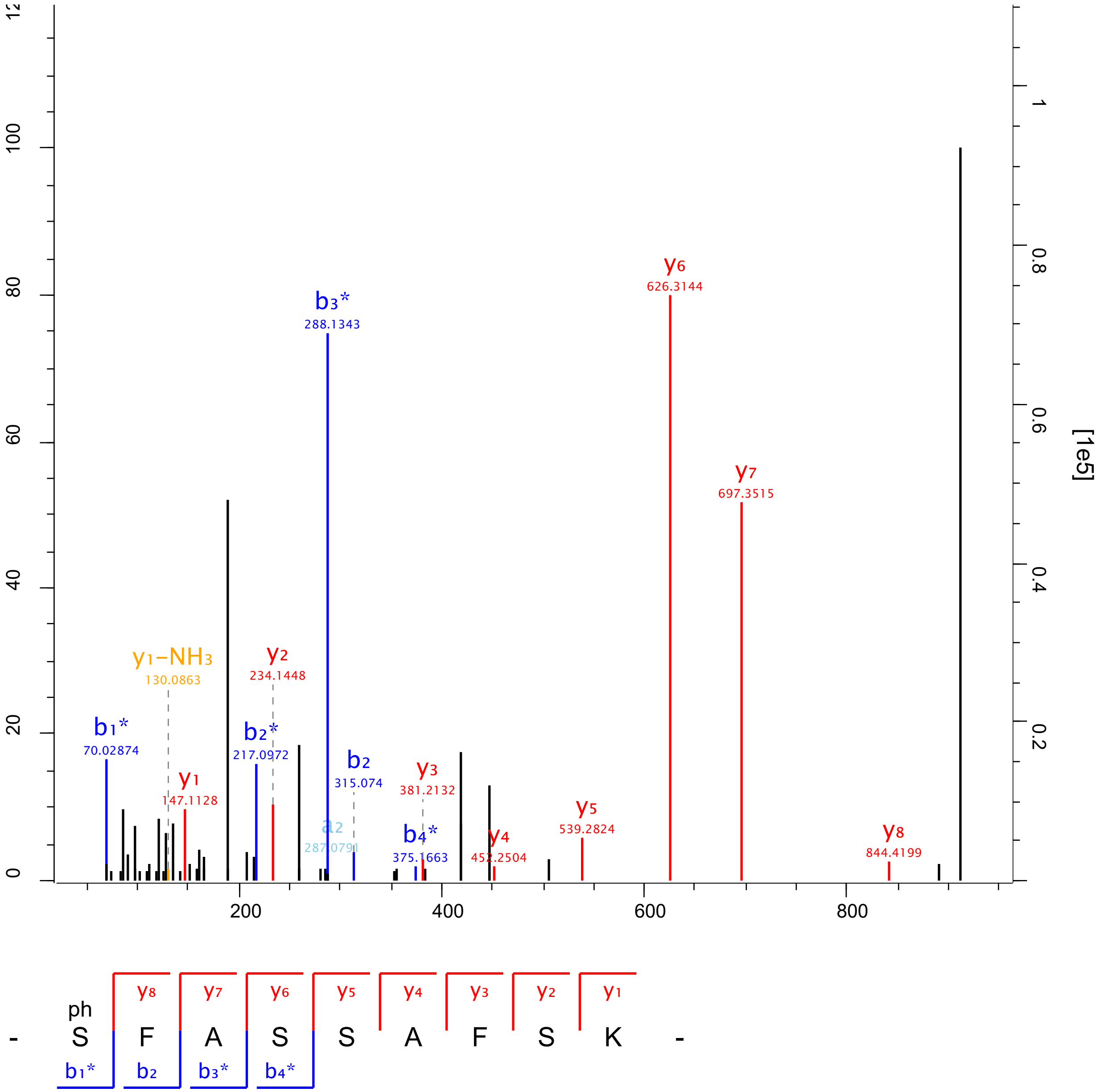

Raw file	Scan	Method	Score	m/z
sys_00_3short	14628	FTMS; HCD	185.96	511.23

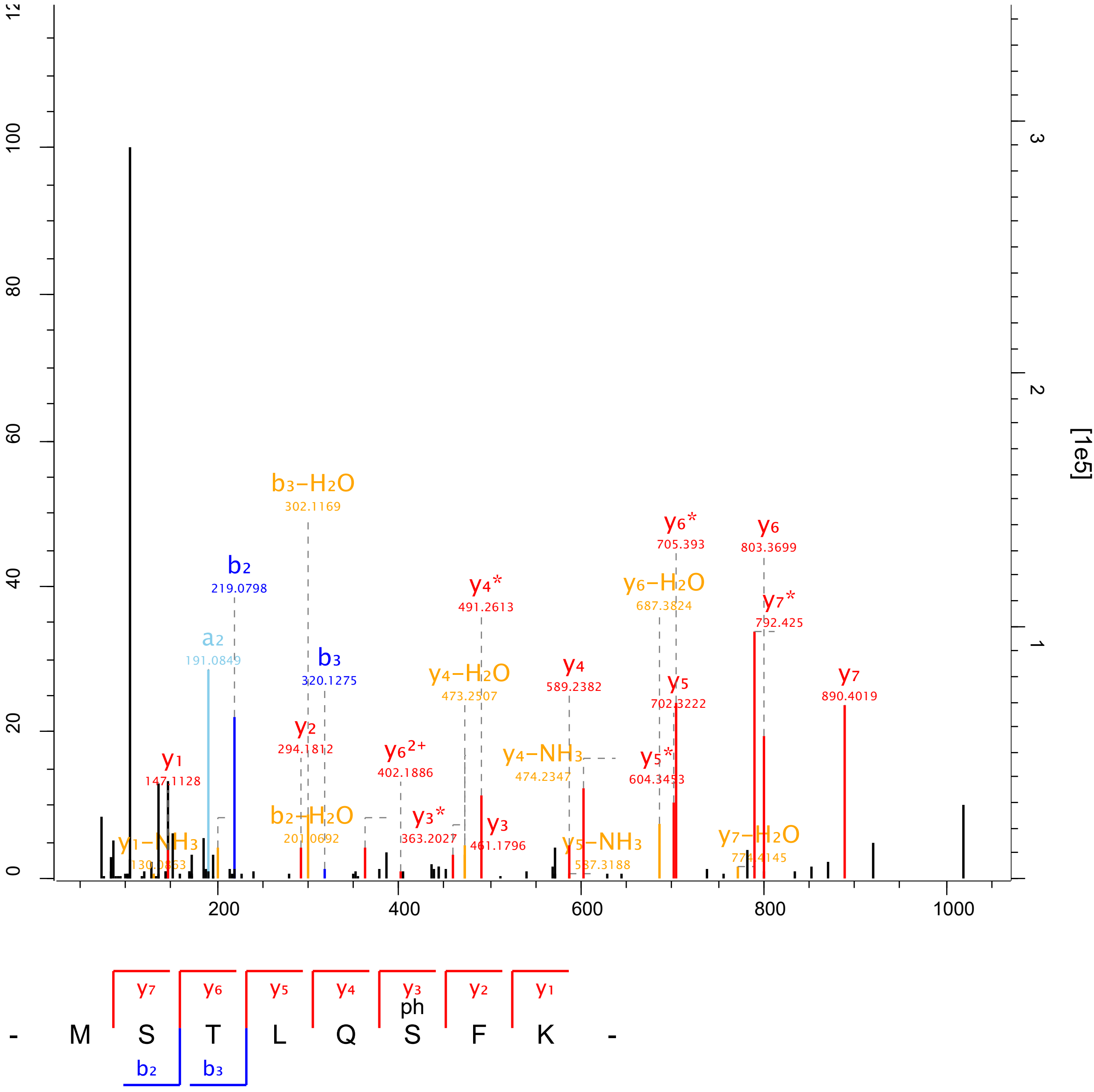

Raw file	Scan	Method	Score	m/z
sys_00_3short	14744	FTMS; HCD	96.46	562.26

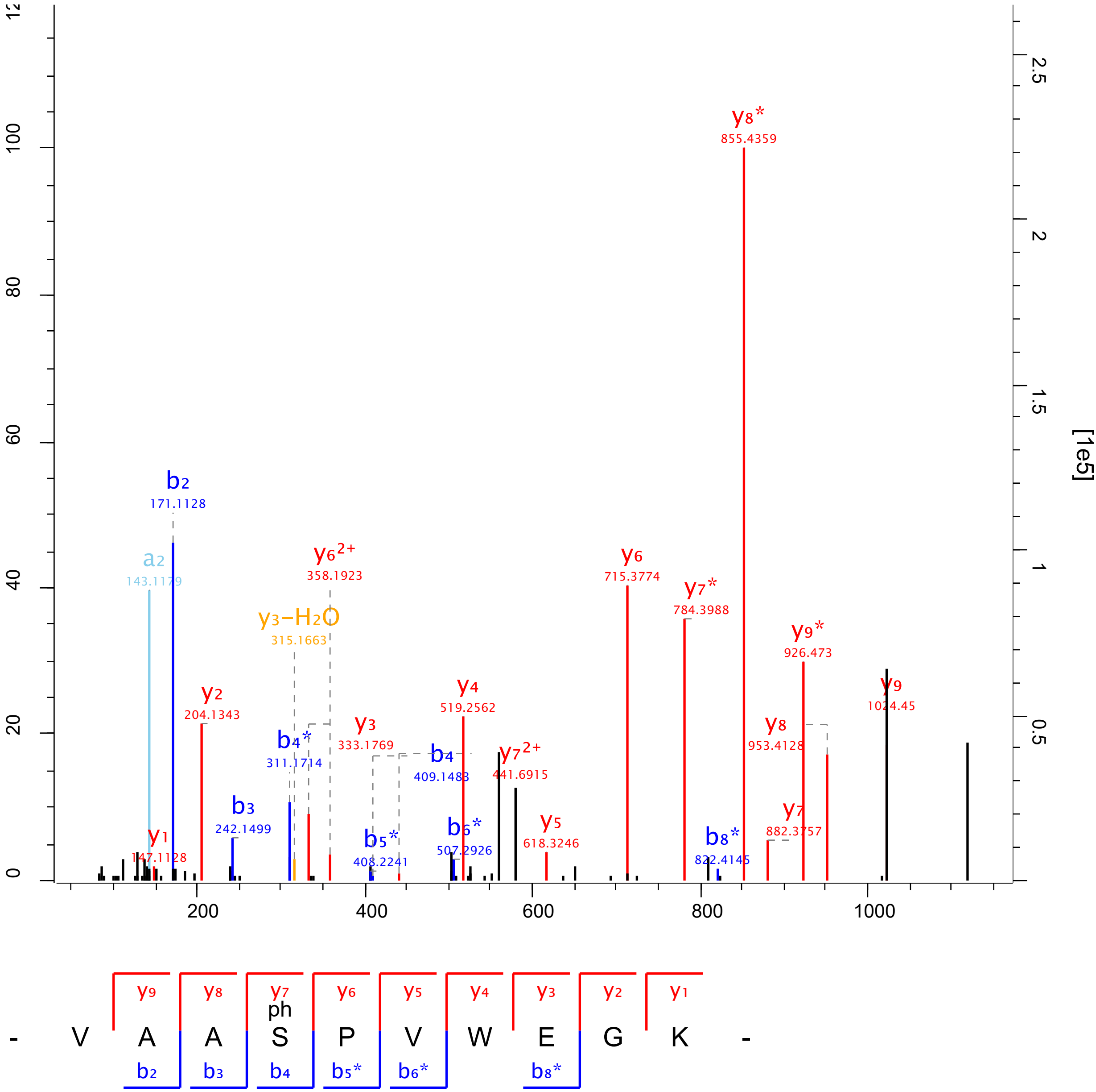

Raw file	Scan	Method	Score	m/z
sys_00_3short	15024	FTMS; HCD	117.93	568.75

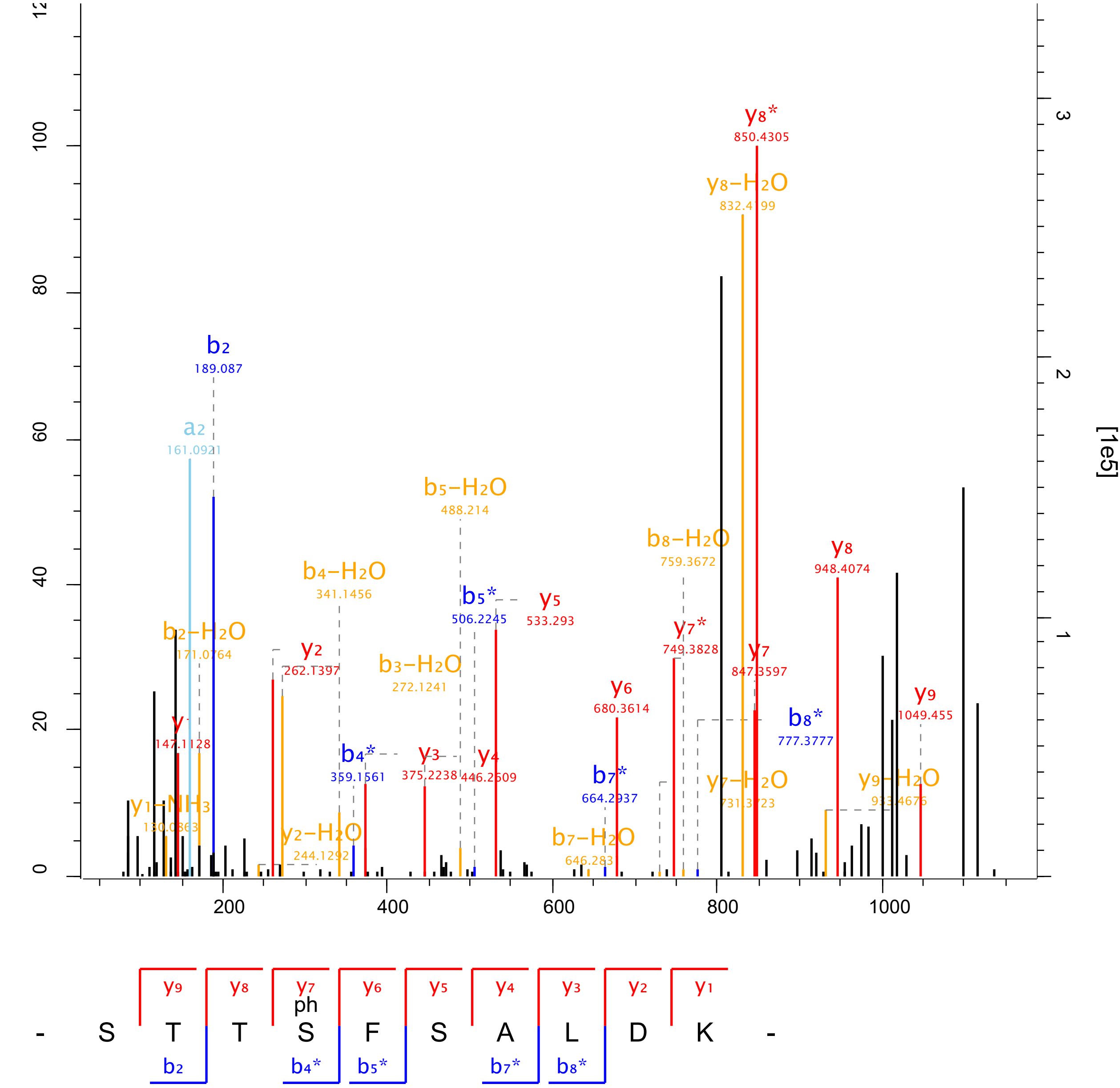

Raw file	Scan	Method	Score	m/z
sys_00_3short	15426	FTMS; HCD	44.85	482.73

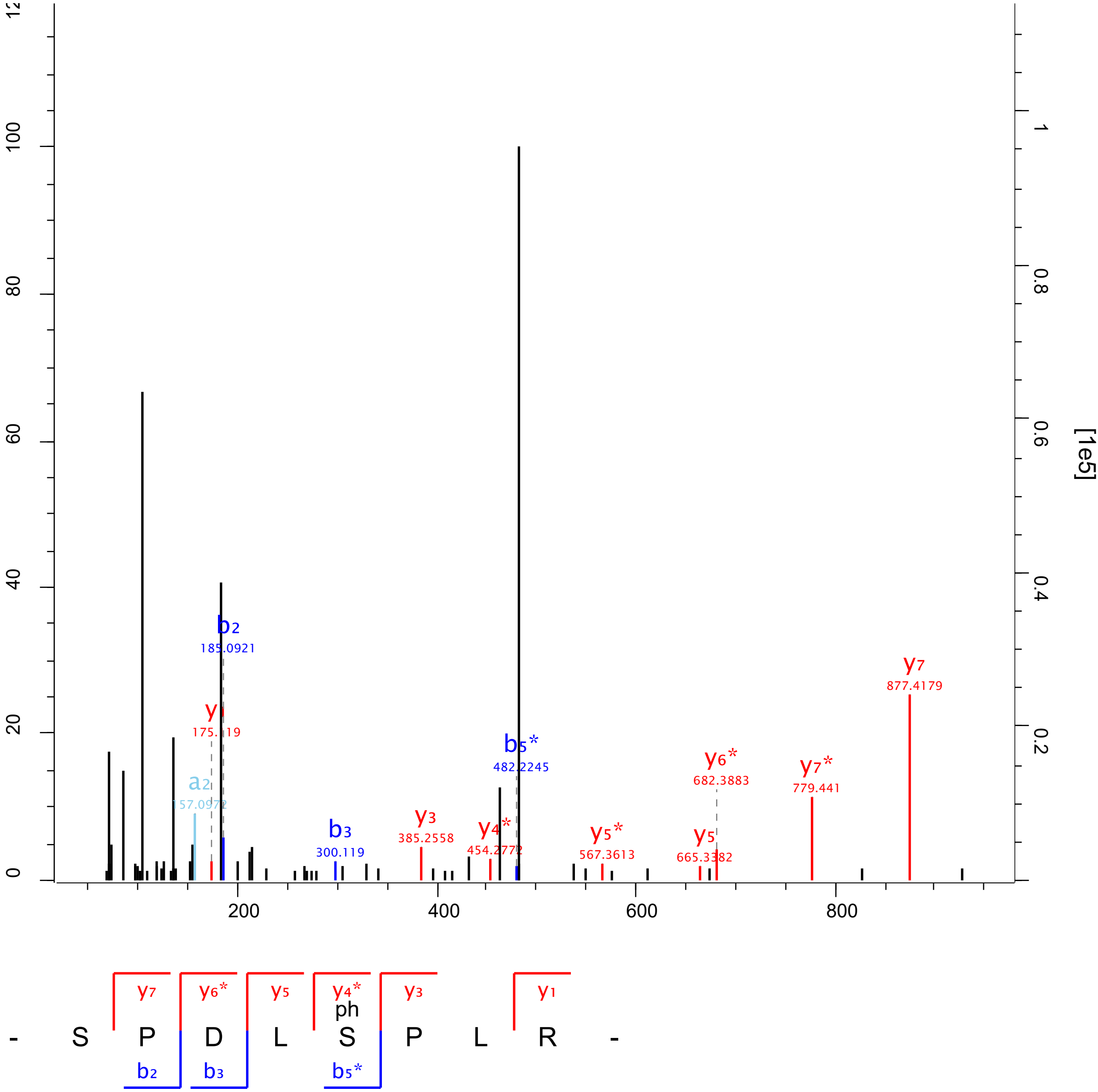

Raw file	Scan	Method	Score	m/z
sys_00_3short	15684	FTMS; HCD	85.81	697.78

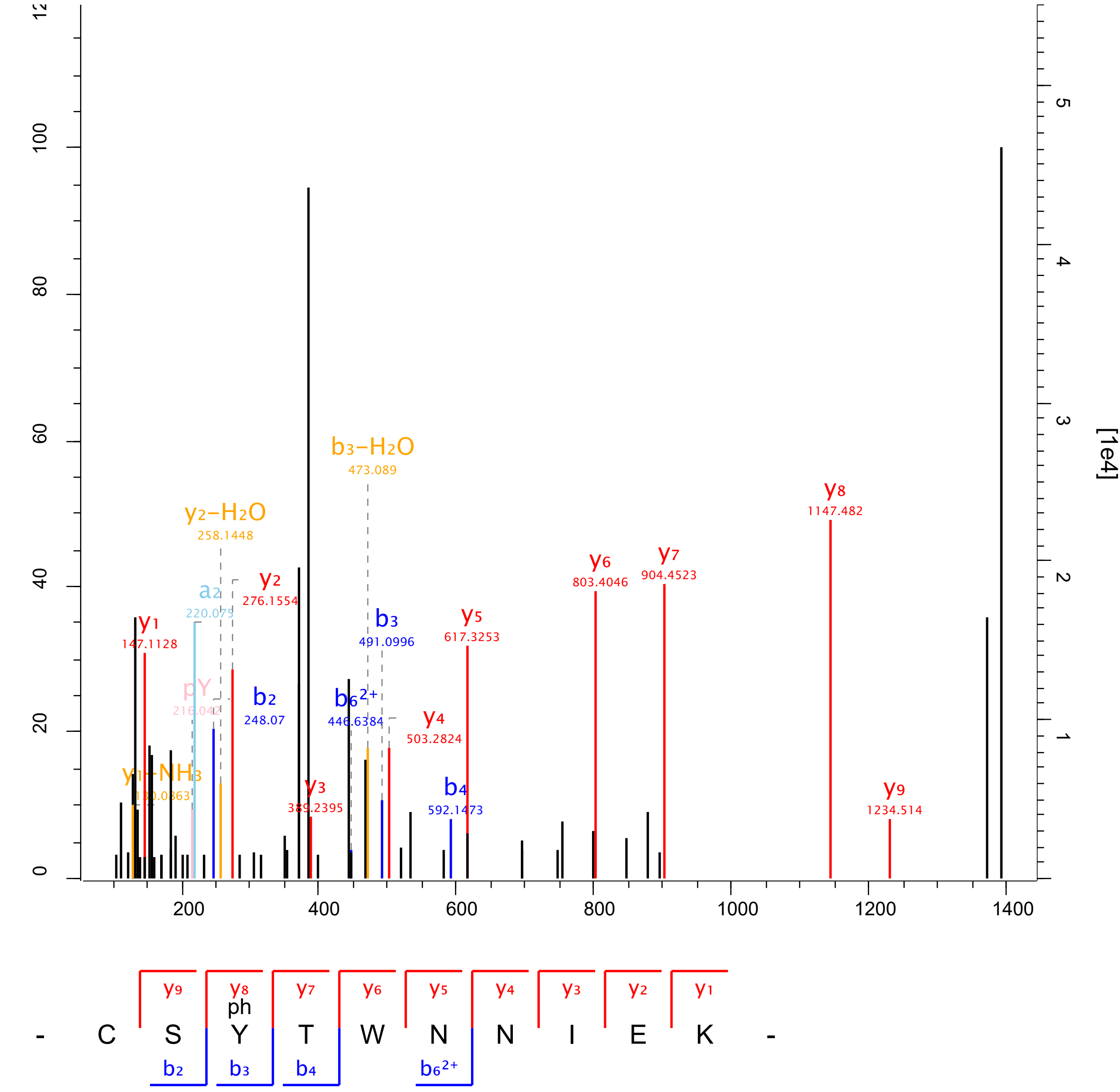

Raw file	Scan	Method	Score	m/z
sys_00_3short	15821	FTMS; HCD	105.03	613.29

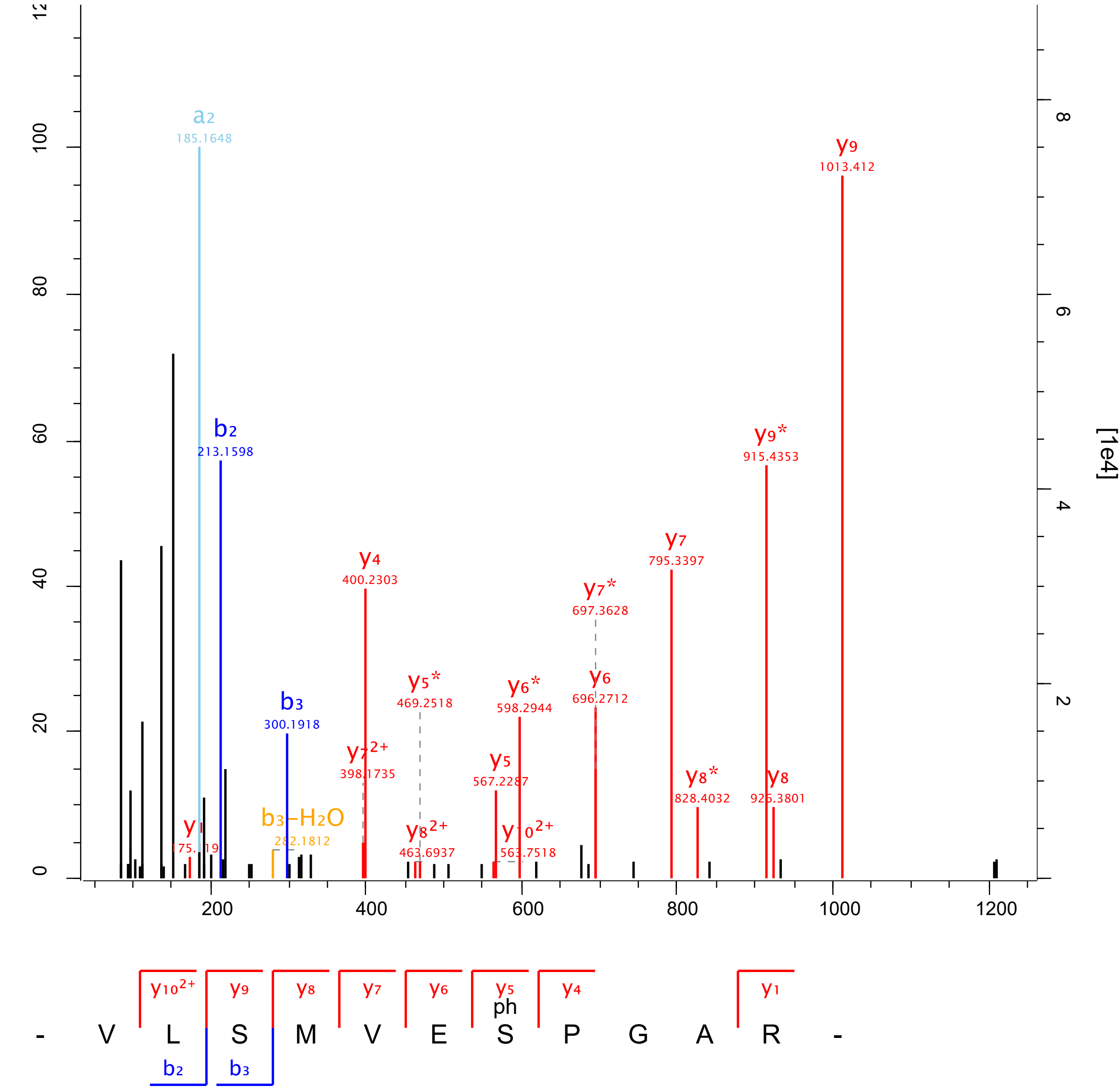

Raw file	Scan	Method	Score	m/z
sys_00_3short	15846	FTMS; HCD	163.99	700.81

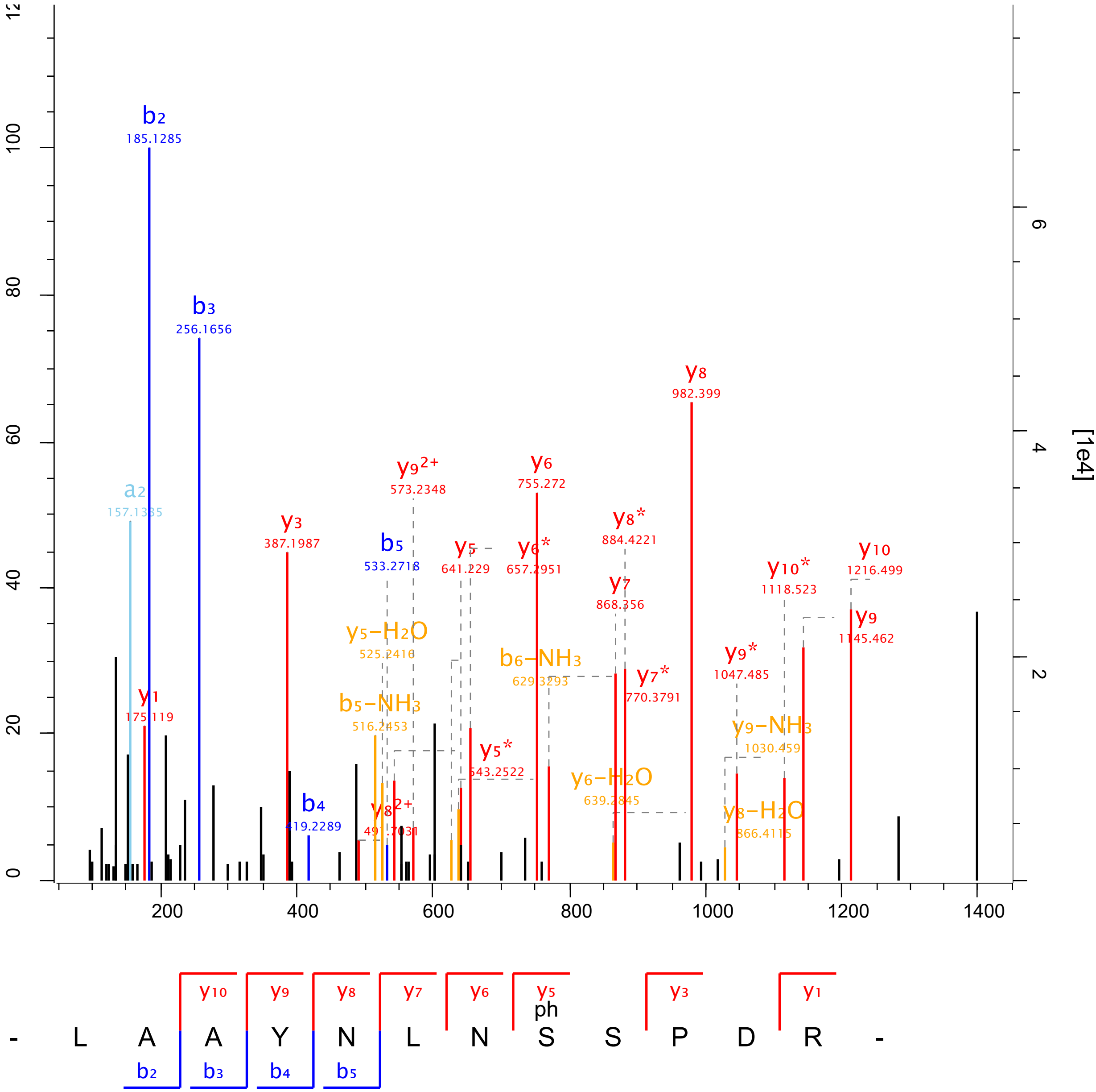

Raw file	Scan	Method	Score	m/z
sys_00_3short	15878	FTMS; HCD	46.84	513.25

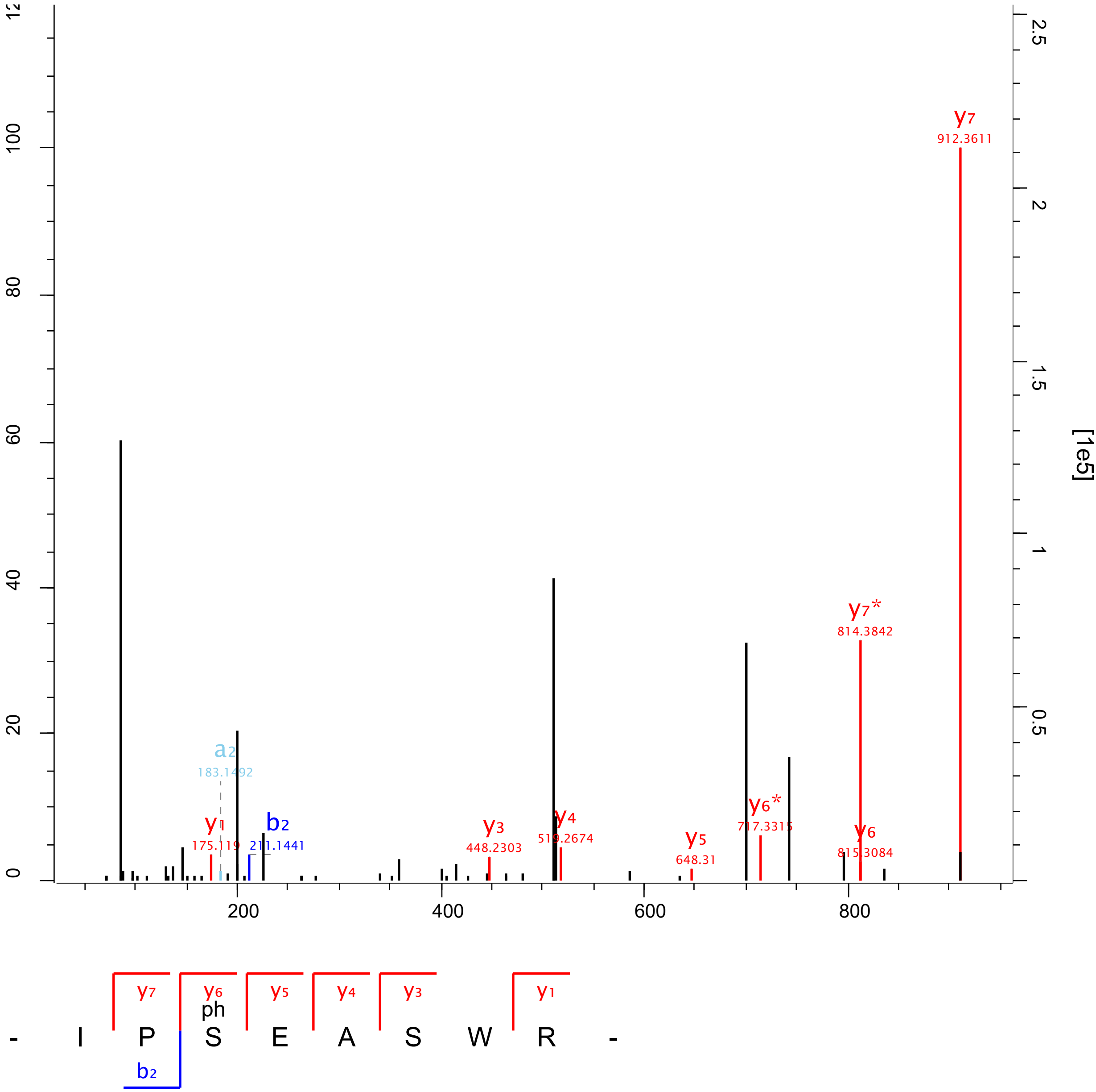

Raw file	Scan	Method	Score	m/z
sys_00_3short	15891	FTMS; HCD	69.72	483.73

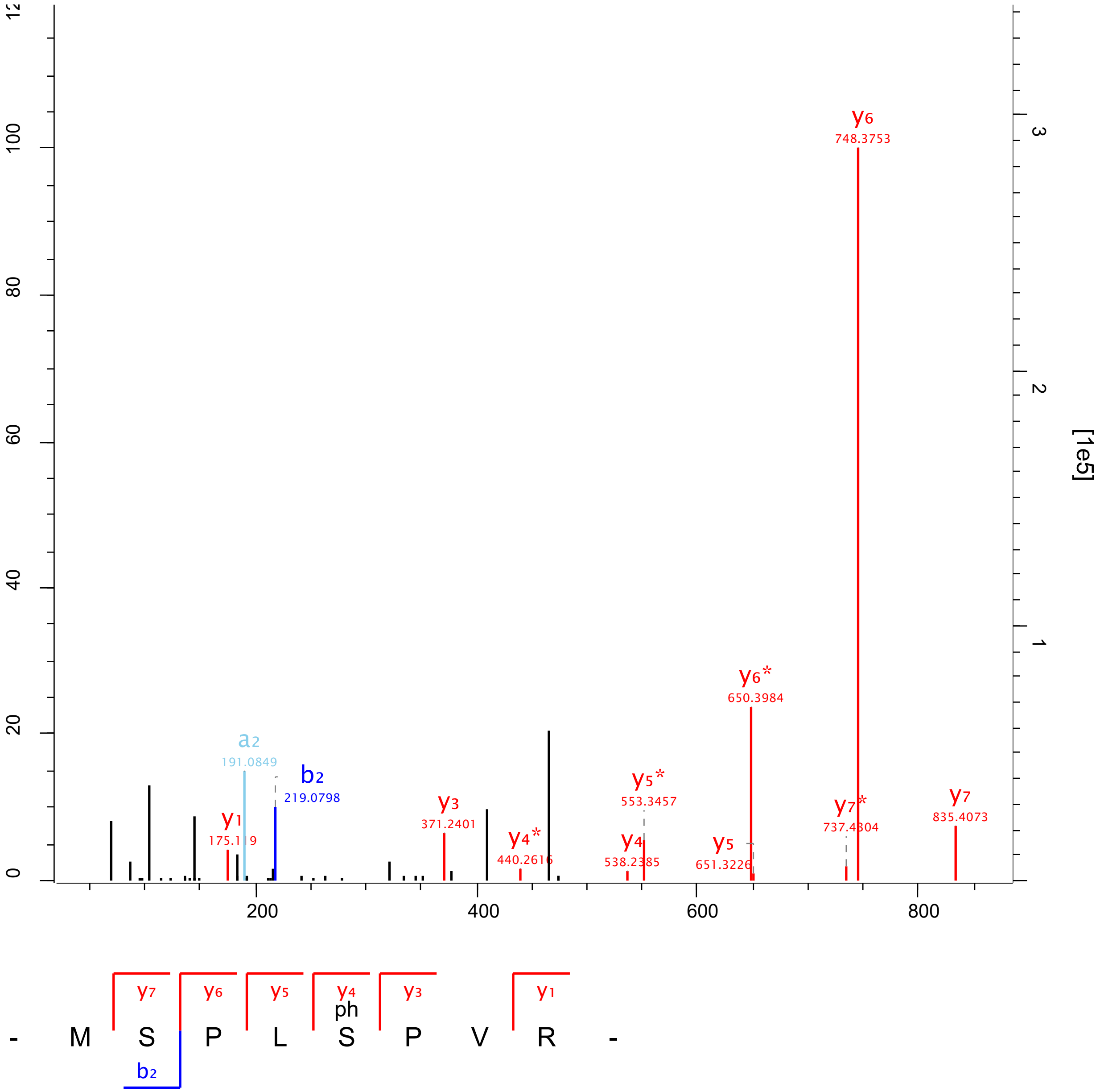

Raw file	Scan	Method	Score	m/z
sys_00_3short	15895	FTMS; HCD	117.7	620.77

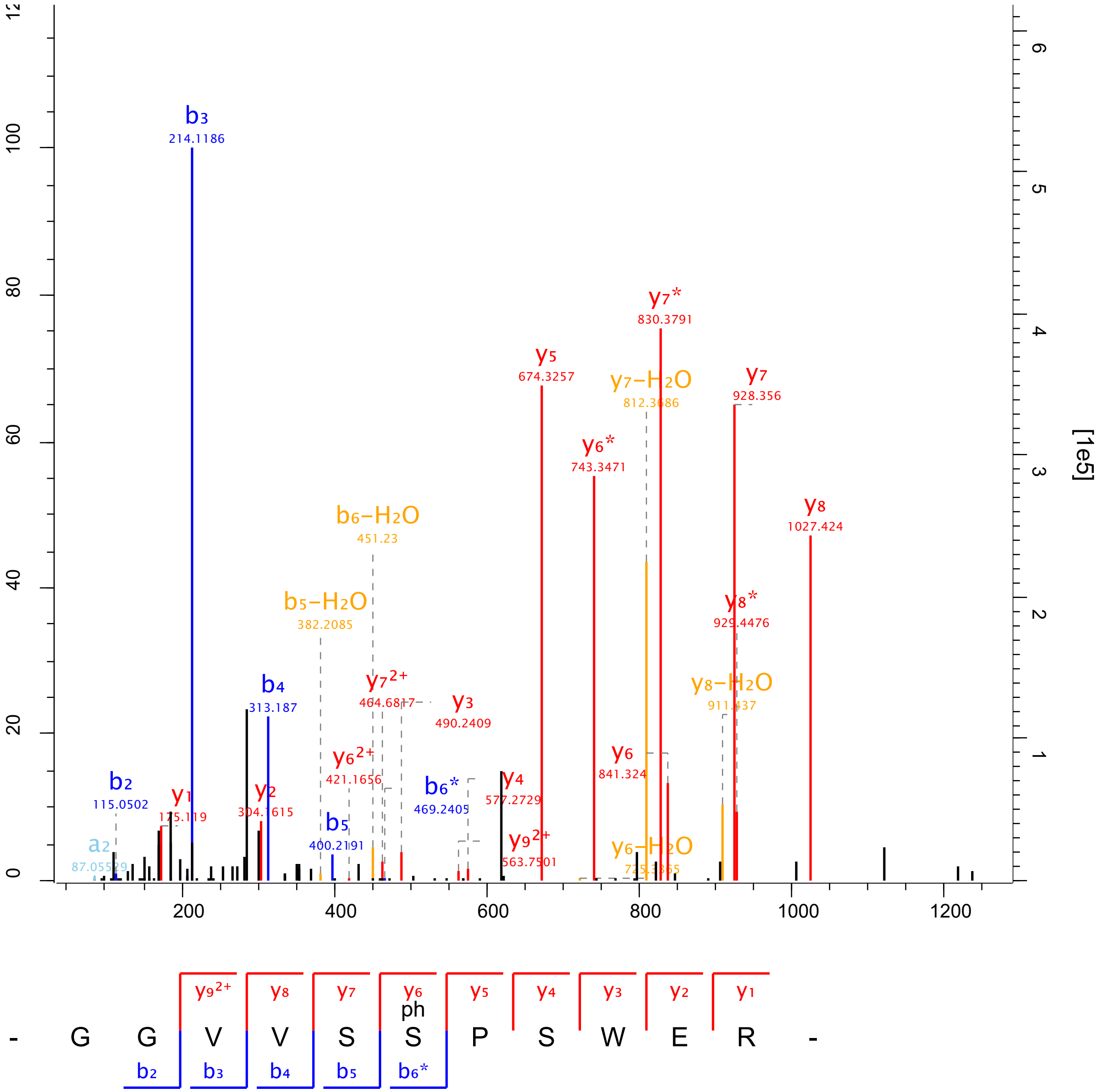

Raw file	Scan	Method	Score	m/z
sys_00_3short	16065	FTMS; HCD	128.59	761.84

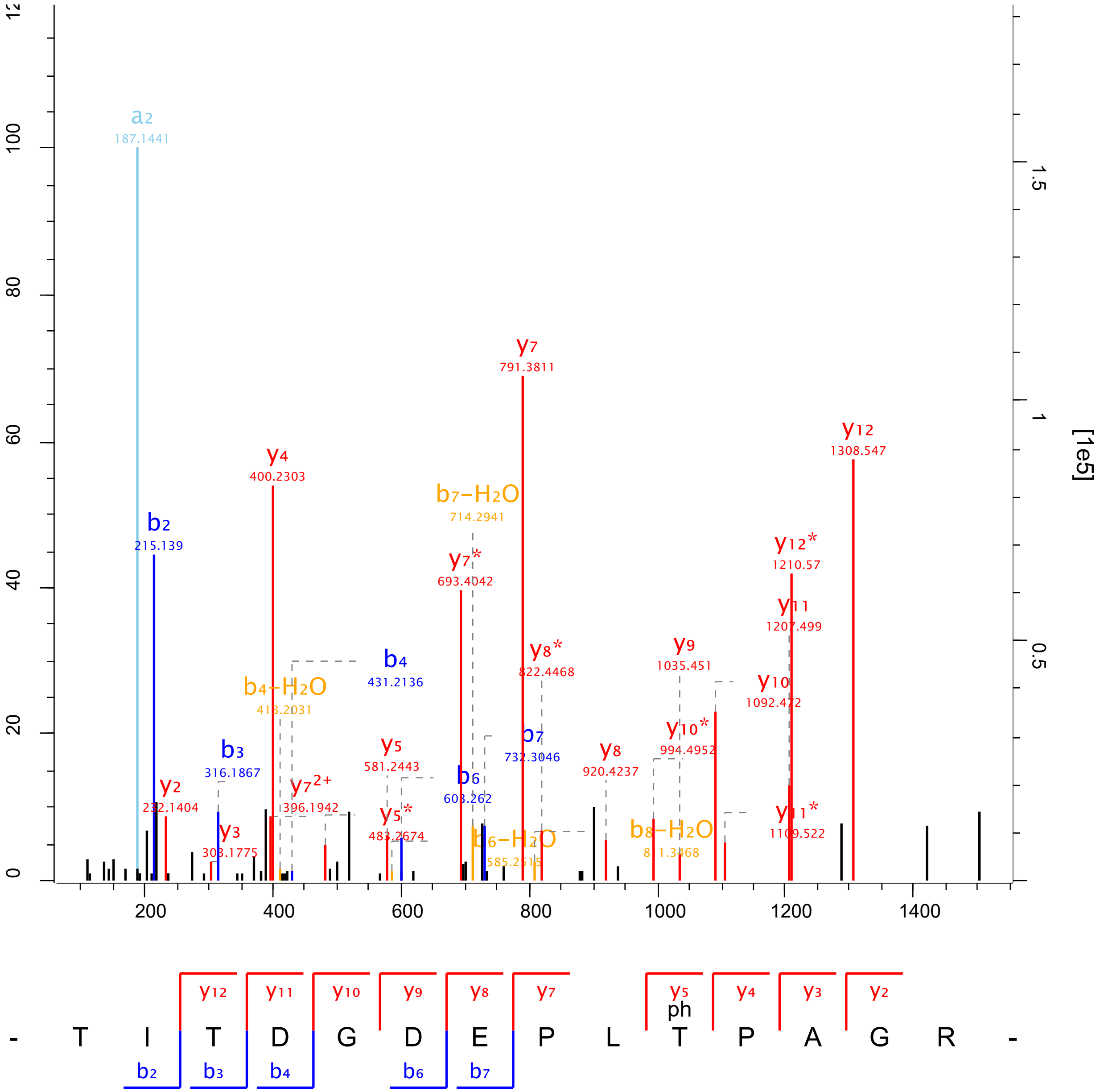

Raw file	Scan	Method	Score	m/z
sys_00_3short	16229	FTMS; HCD	47.5	934.43

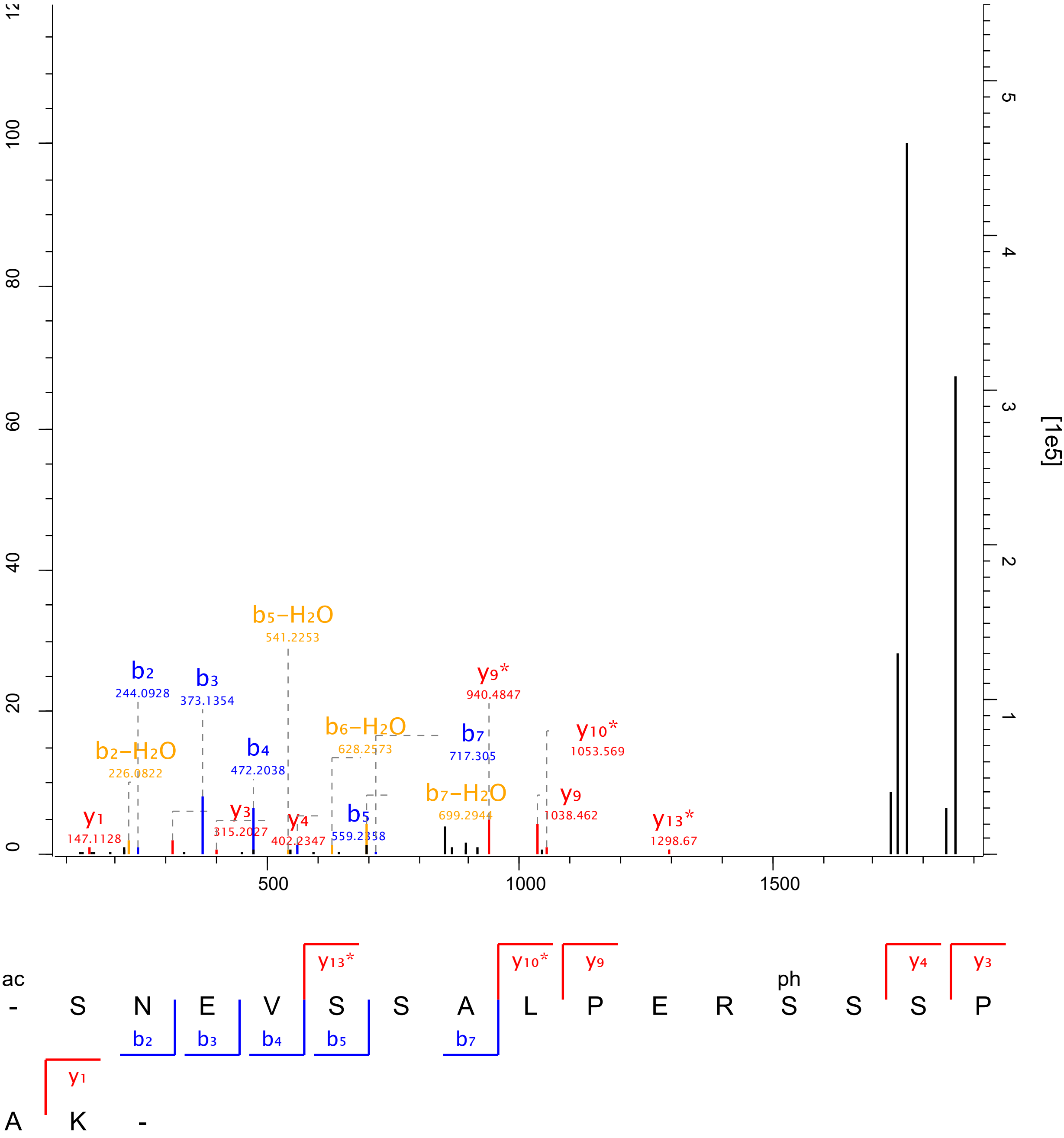

Raw file	Scan	Method	Score	m/z
sys_00_3short	16276	FTMS; HCD	71.88	692.28

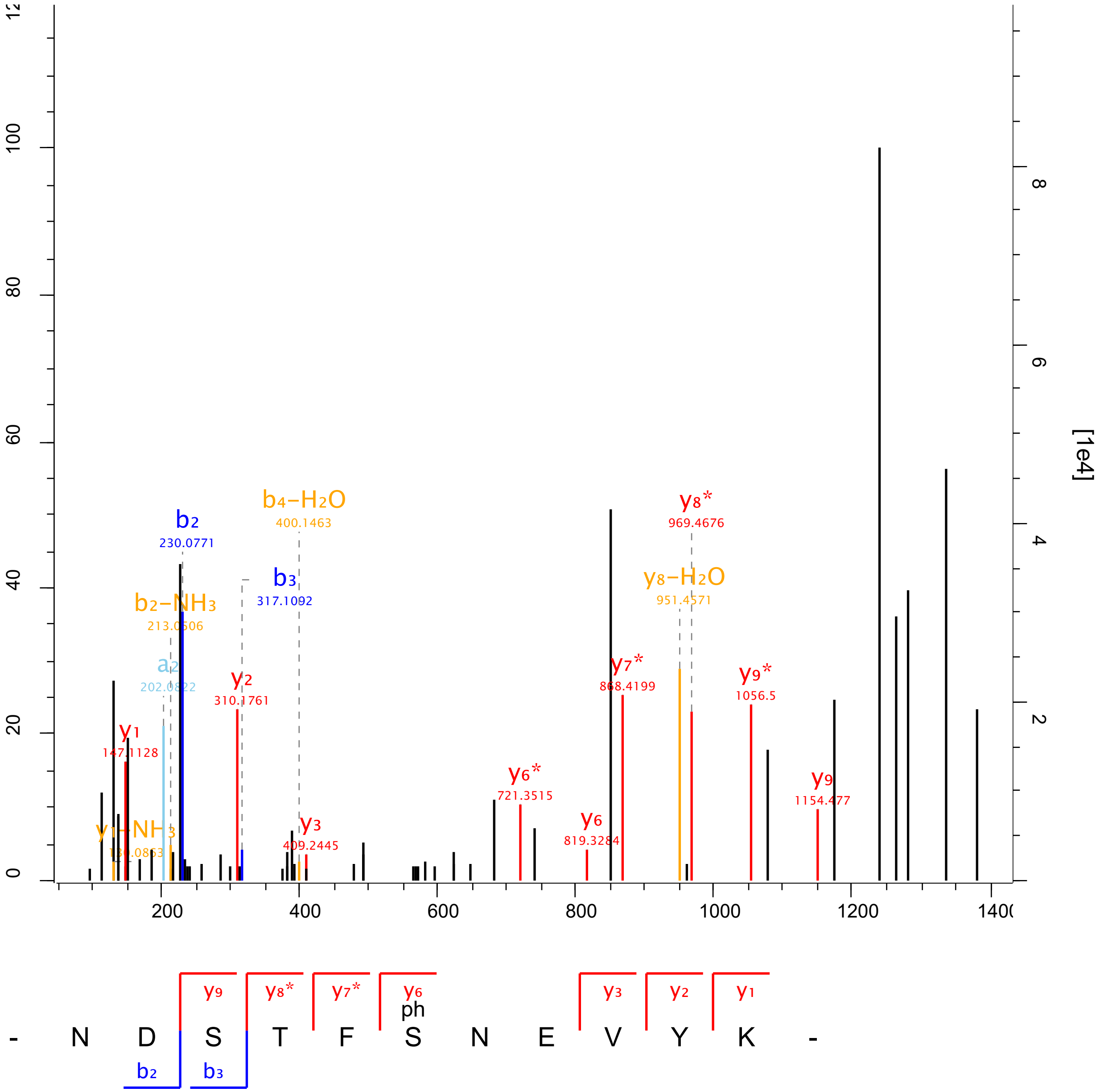

Raw file	Scan	Method	Score	m/z
sys_00_3short	16361	FTMS; HCD	47.6	452.18

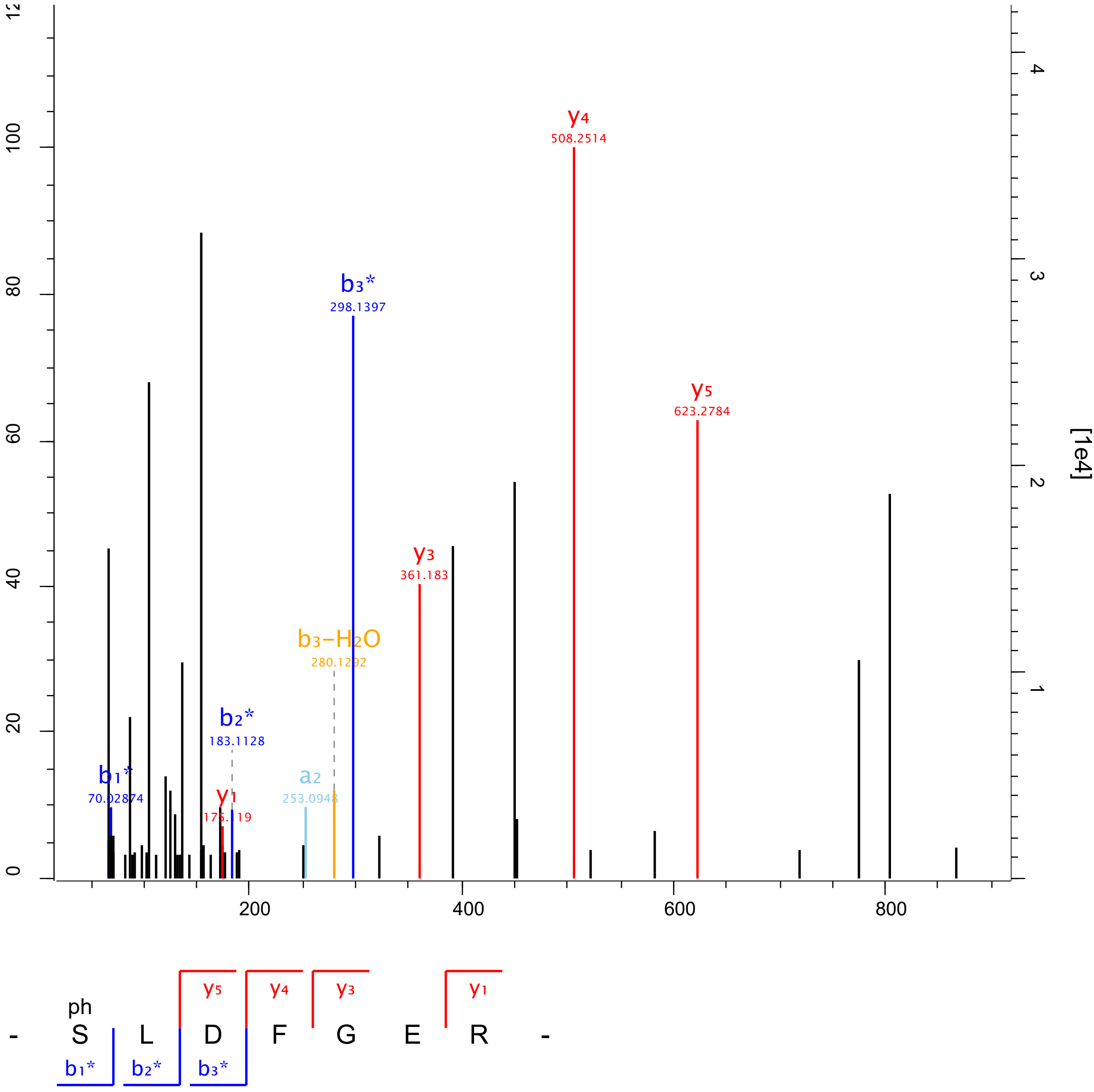

Raw file	Scan	Method	Score	m/z
sys_00_3short	16439	FTMS; HCD	52.58	602.74

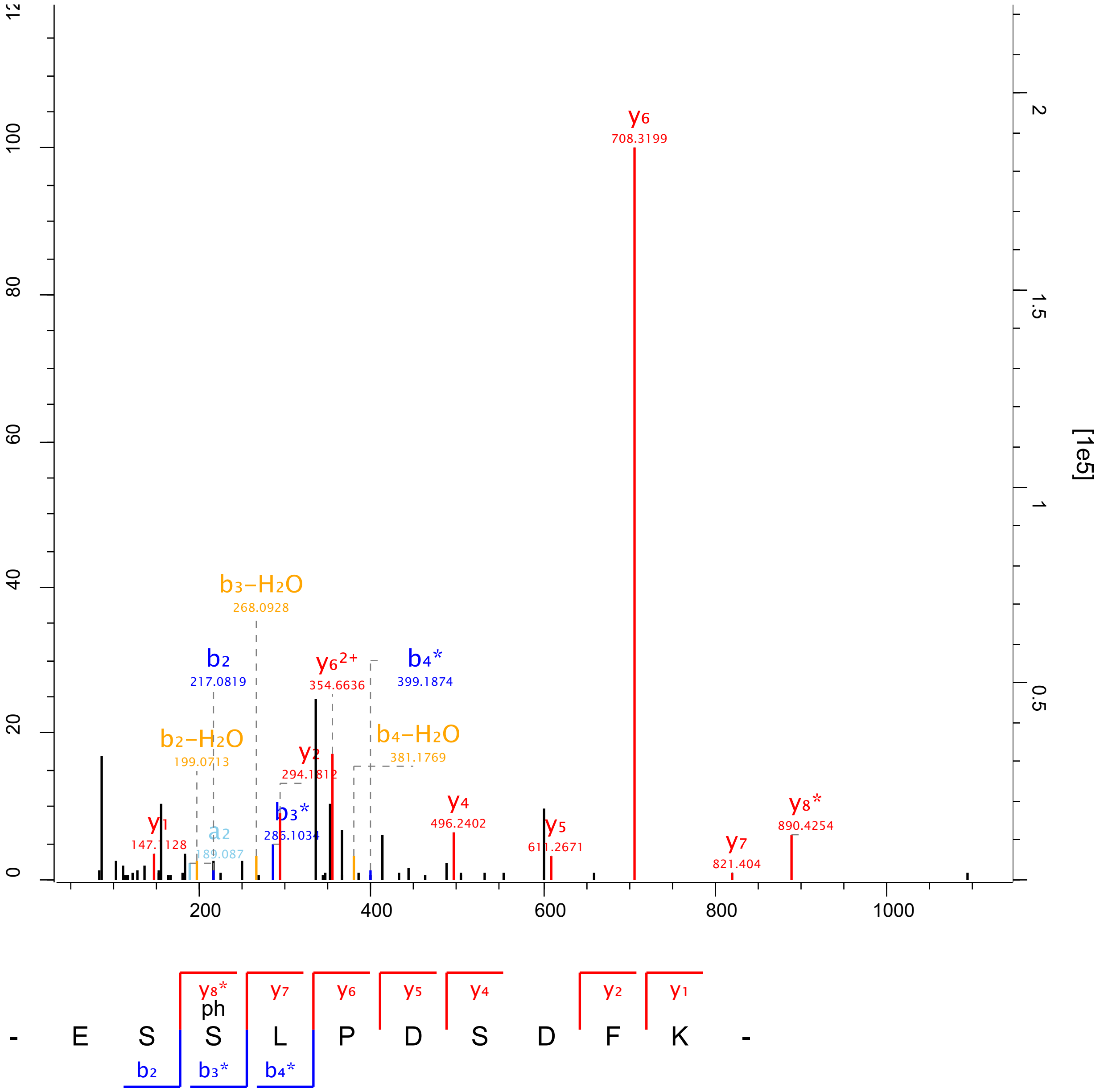

Raw file	Scan	Method	Score	m/z
sys_00_3short	16535	FTMS; HCD	218.83	750.33

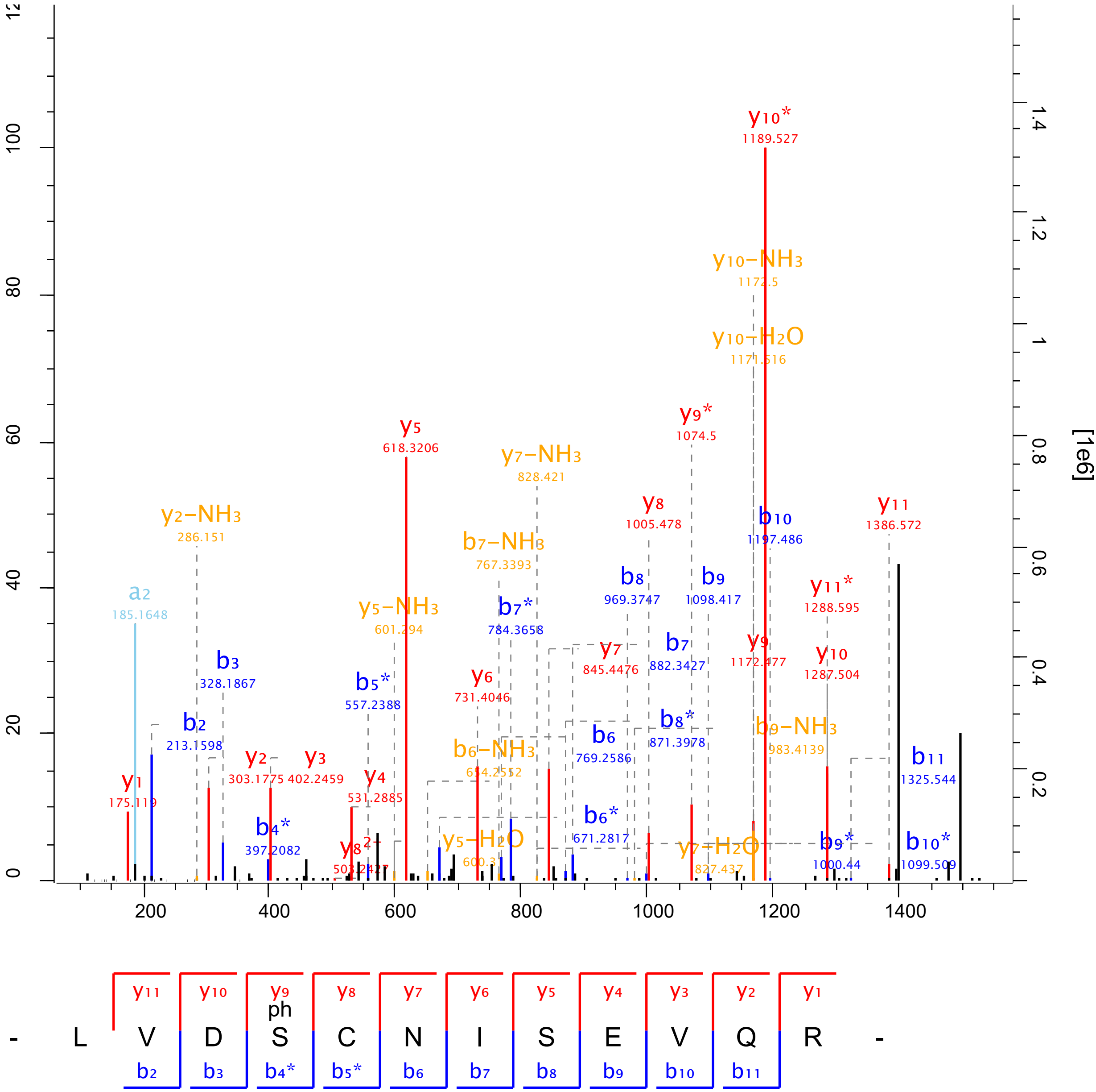

Raw file	Scan	Method	Score	m/z
sys_00_3short	16599	FTMS; HCD	70.2	702.83

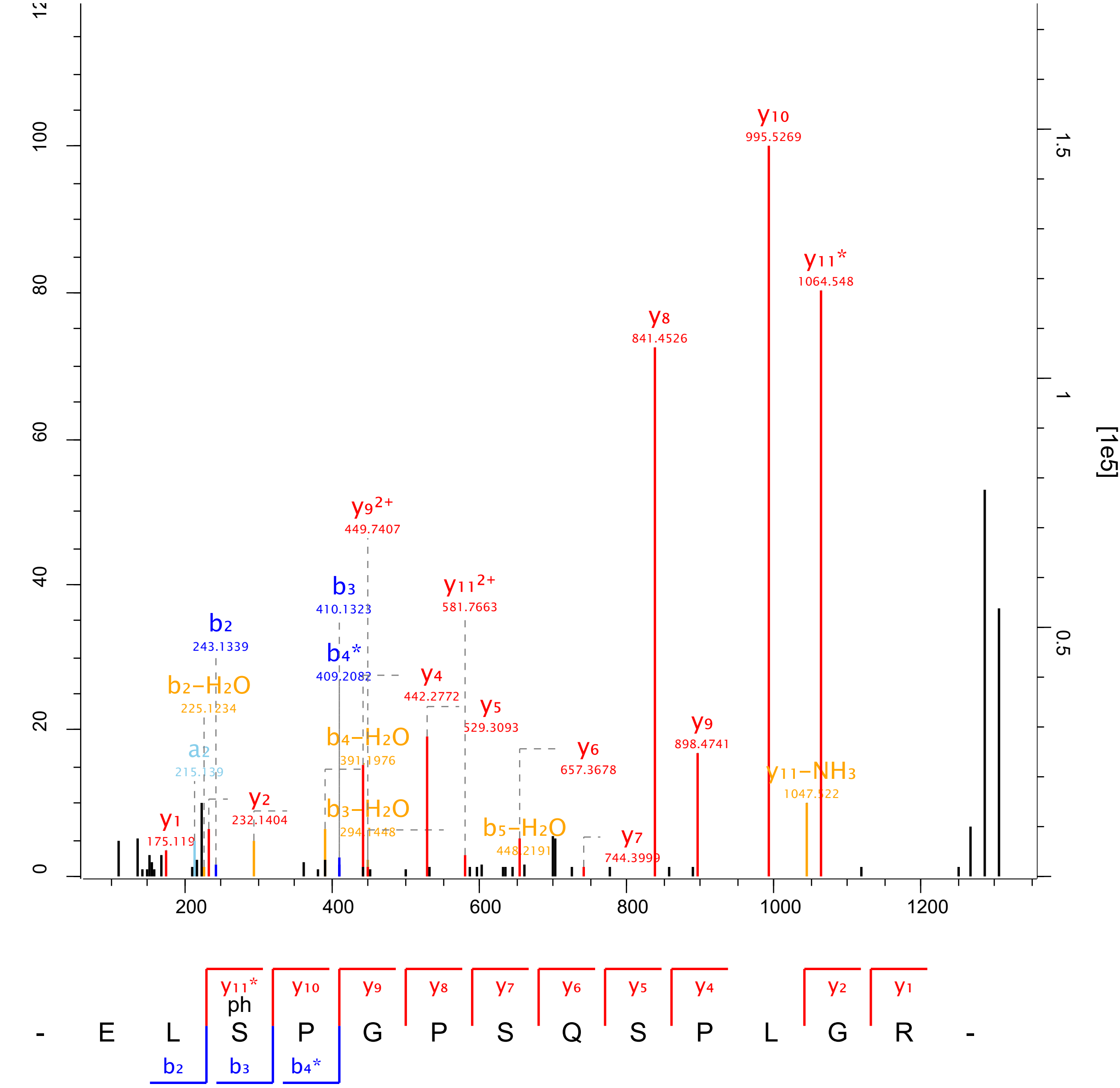

Raw file	Scan	Method	Score	m/z
sys_00_3short	16873	FTMS; HCD	75.55	748.36

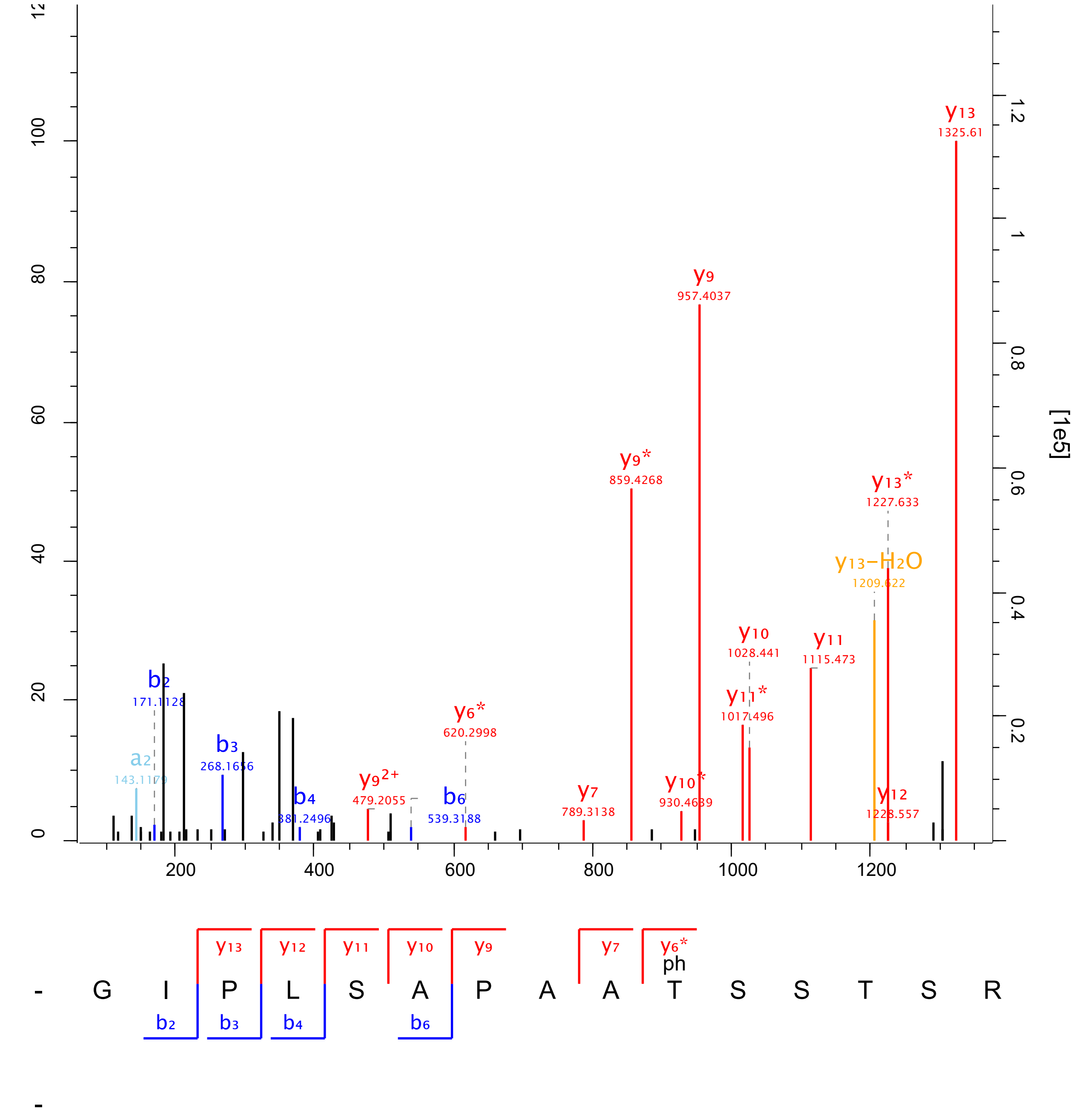

Raw file	Scan	Method	Score	m/z
sys_00_3short	16971	FTMS; HCD	79.49	740.33

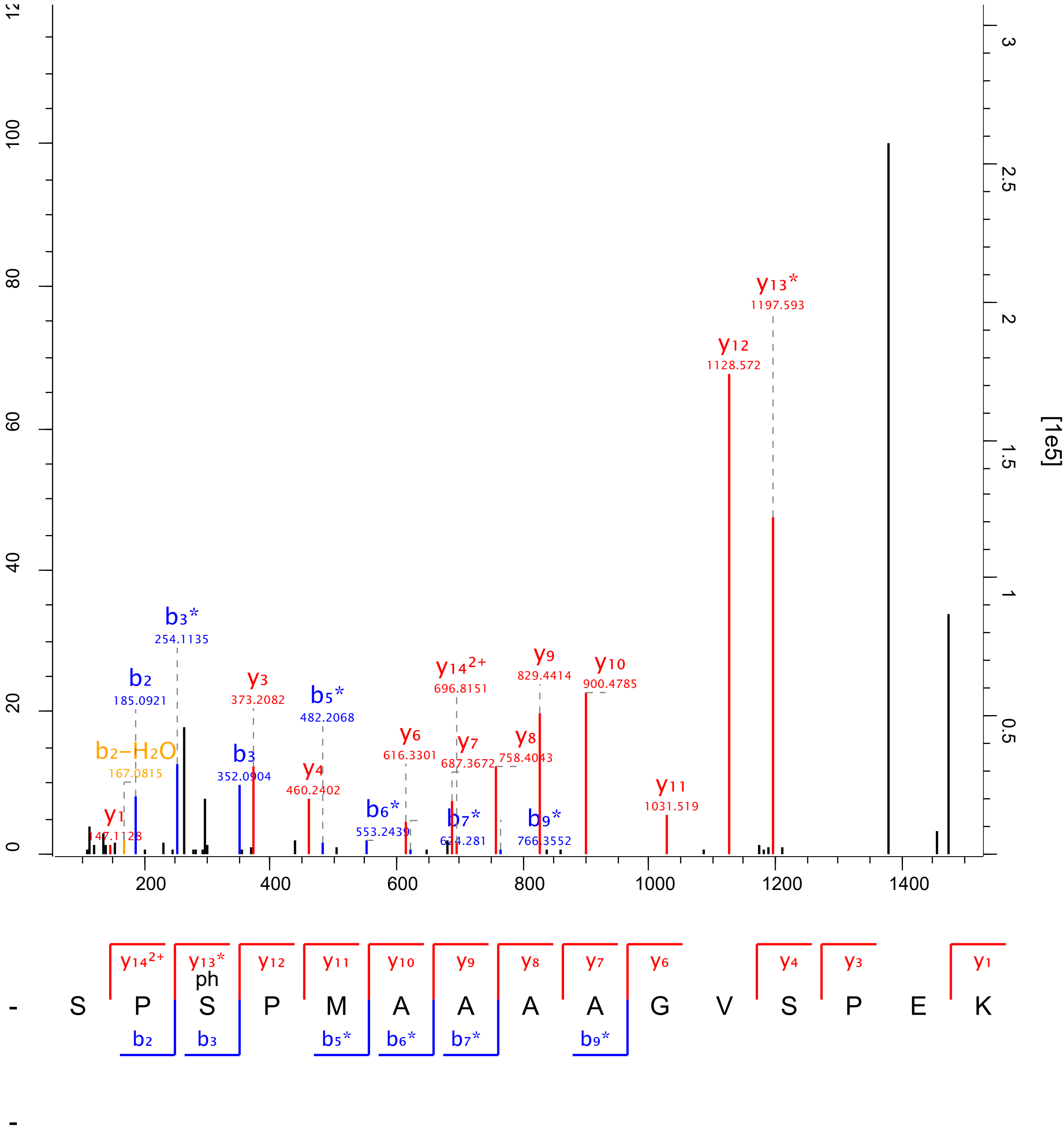

Raw file	Scan	Method	Score	m/z
sys_00_3short	17196	FTMS; HCD	85.84	606.77

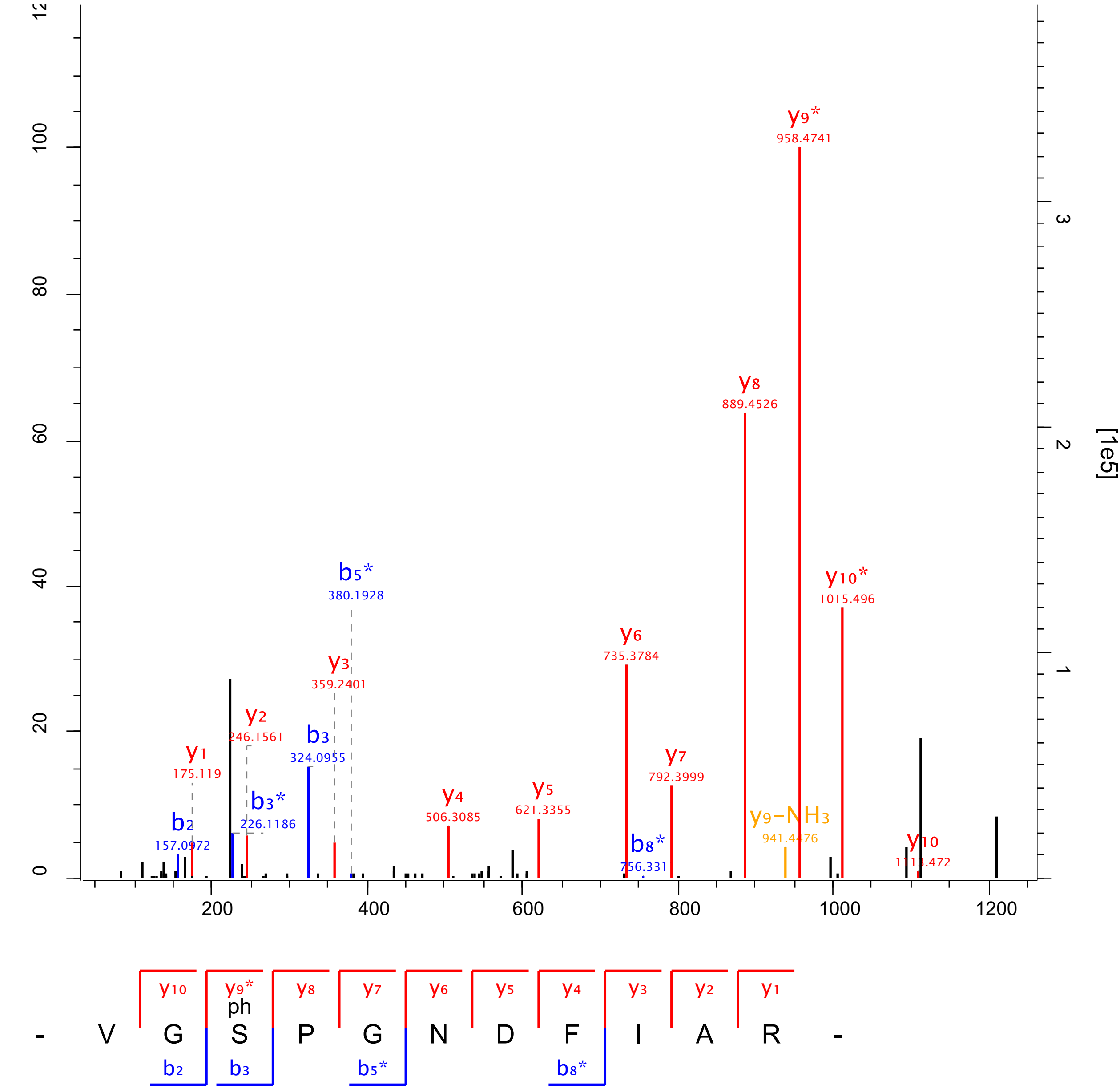

Raw file	Scan	Method	Score	m/z
sys_00_3short	17246	FTMS; HCD	40.95	887.41

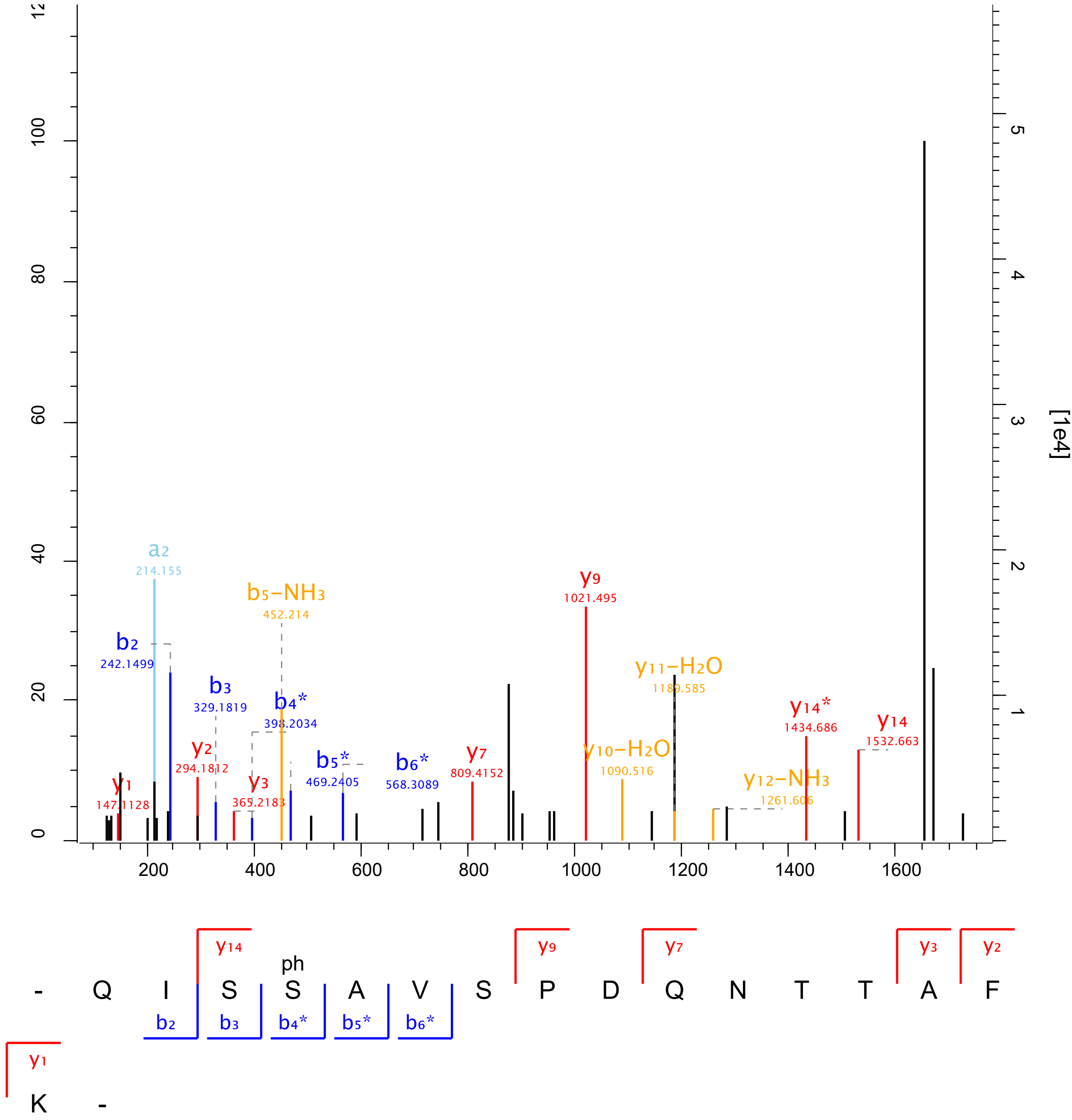

Raw file	Scan	Method	Score	m/z
sys_00_3short	17323	FTMS; HCD	51.45	573.74

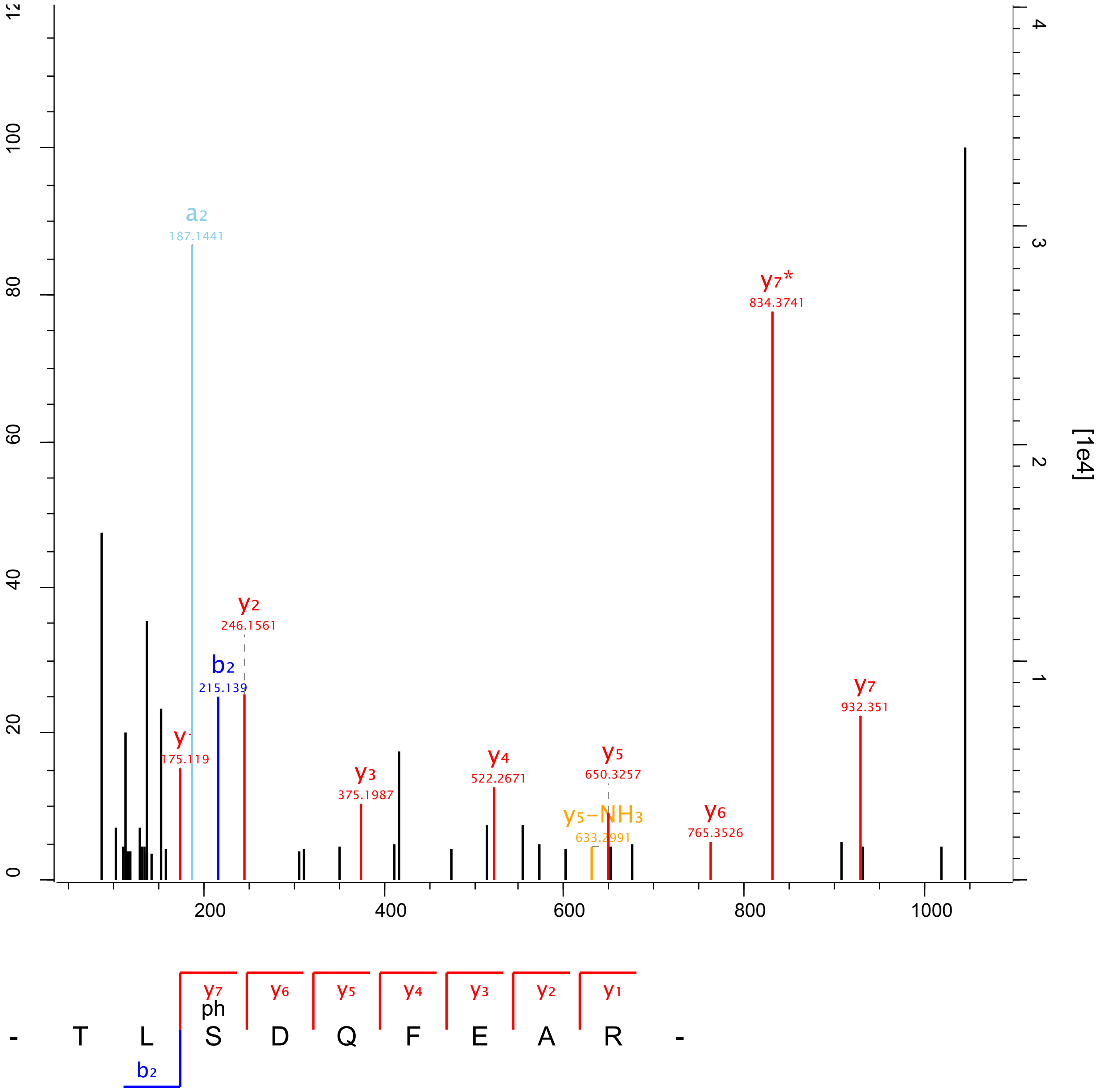

Raw file	Scan	Method	Score	m/z
sys_00_3short	17561	FTMS; HCD	98.59	578.76

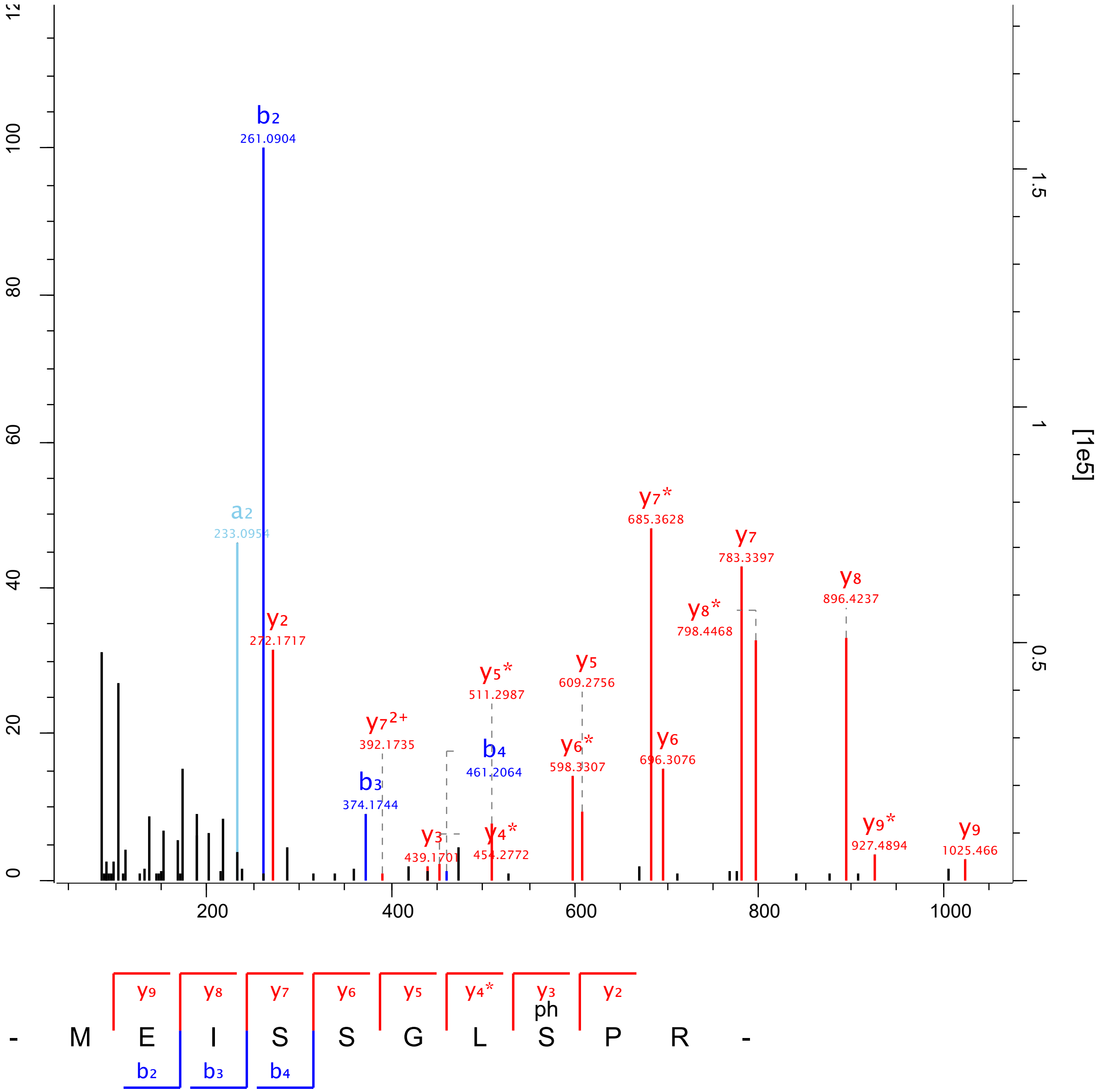

Raw file	Scan	Method	Score	m/z
sys_00_3short	17610	FTMS; HCD	55.72	651.77

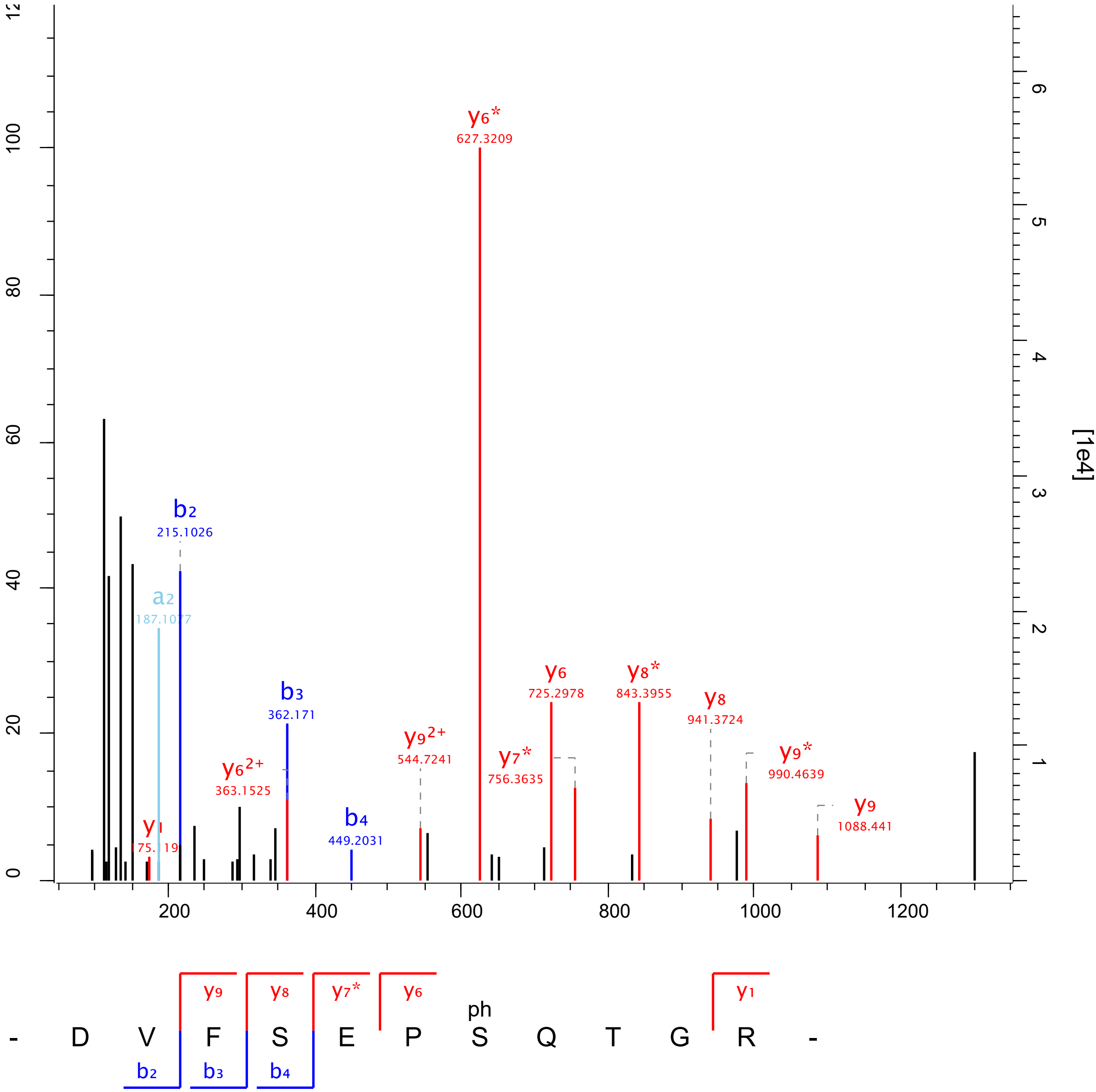

Raw file	Scan	Method	Score	m/z
sys_00_3short	17639	FTMS; HCD	157.98	838.85

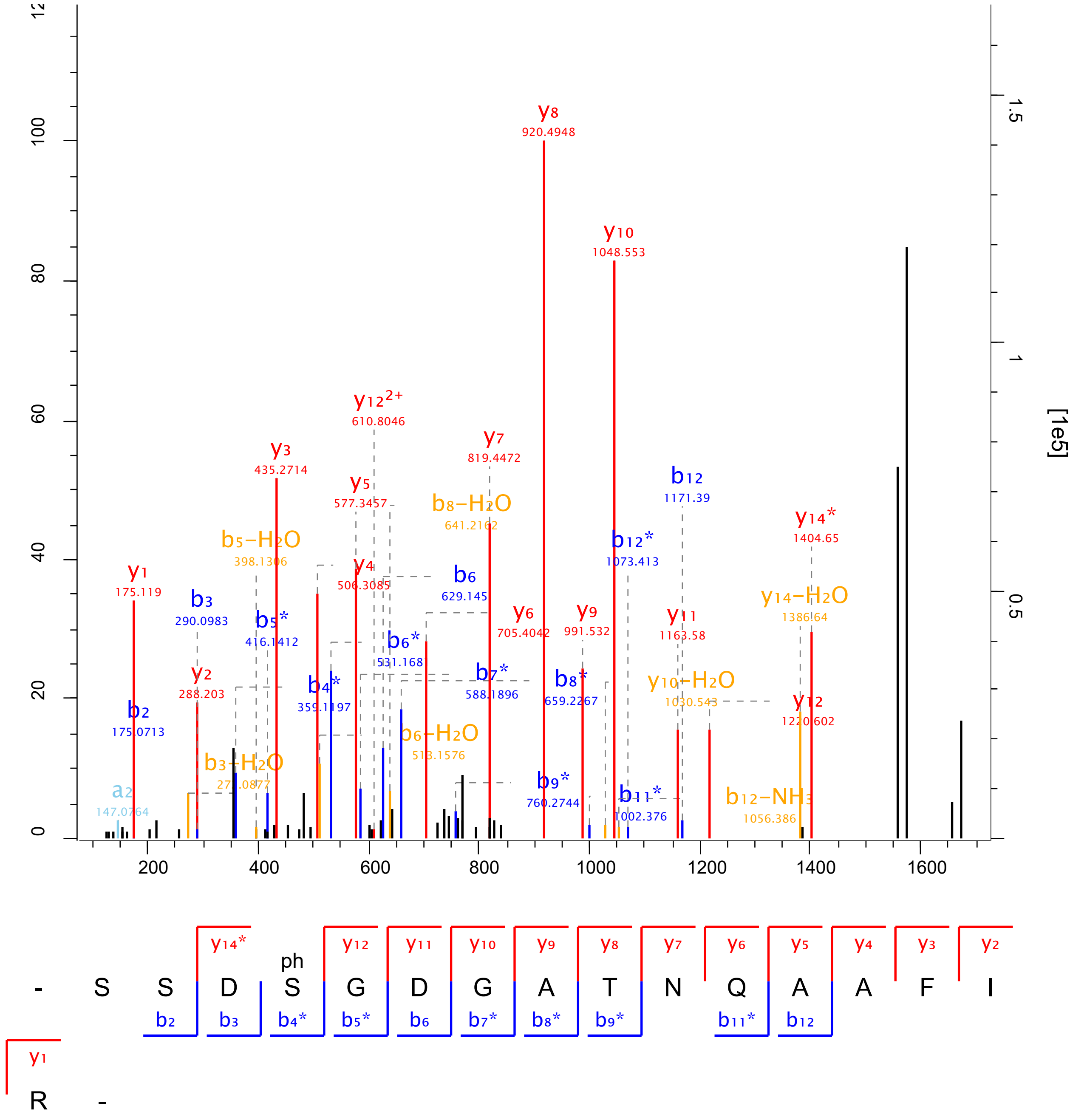

Raw file	Scan	Method	Score	m/z
sys_00_3short	17705	FTMS; HCD	70.03	515.22

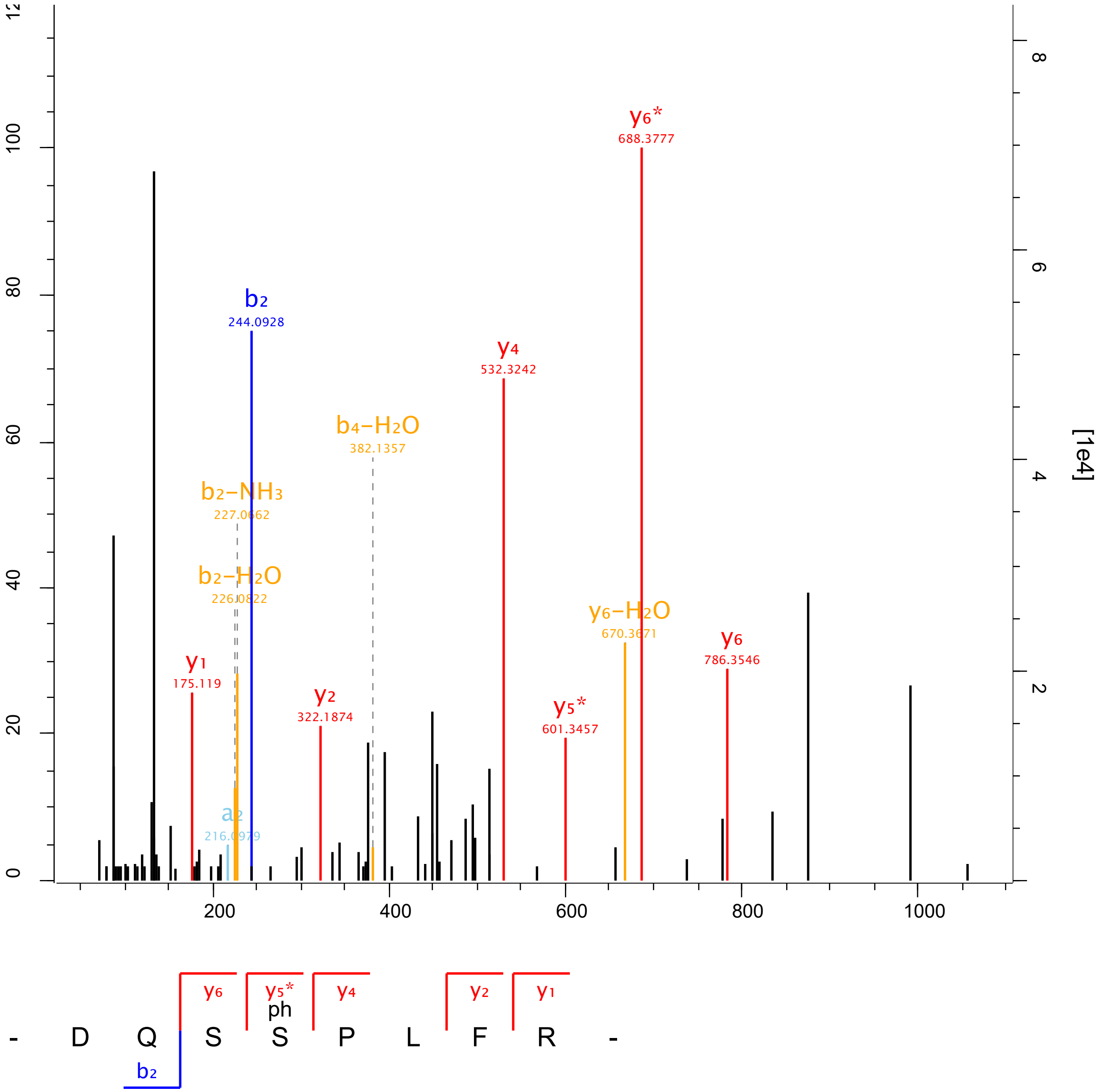

Raw file	Scan	Method	Score	m/z
sys_00_3short	17742	FTMS; HCD	143.25	572.76

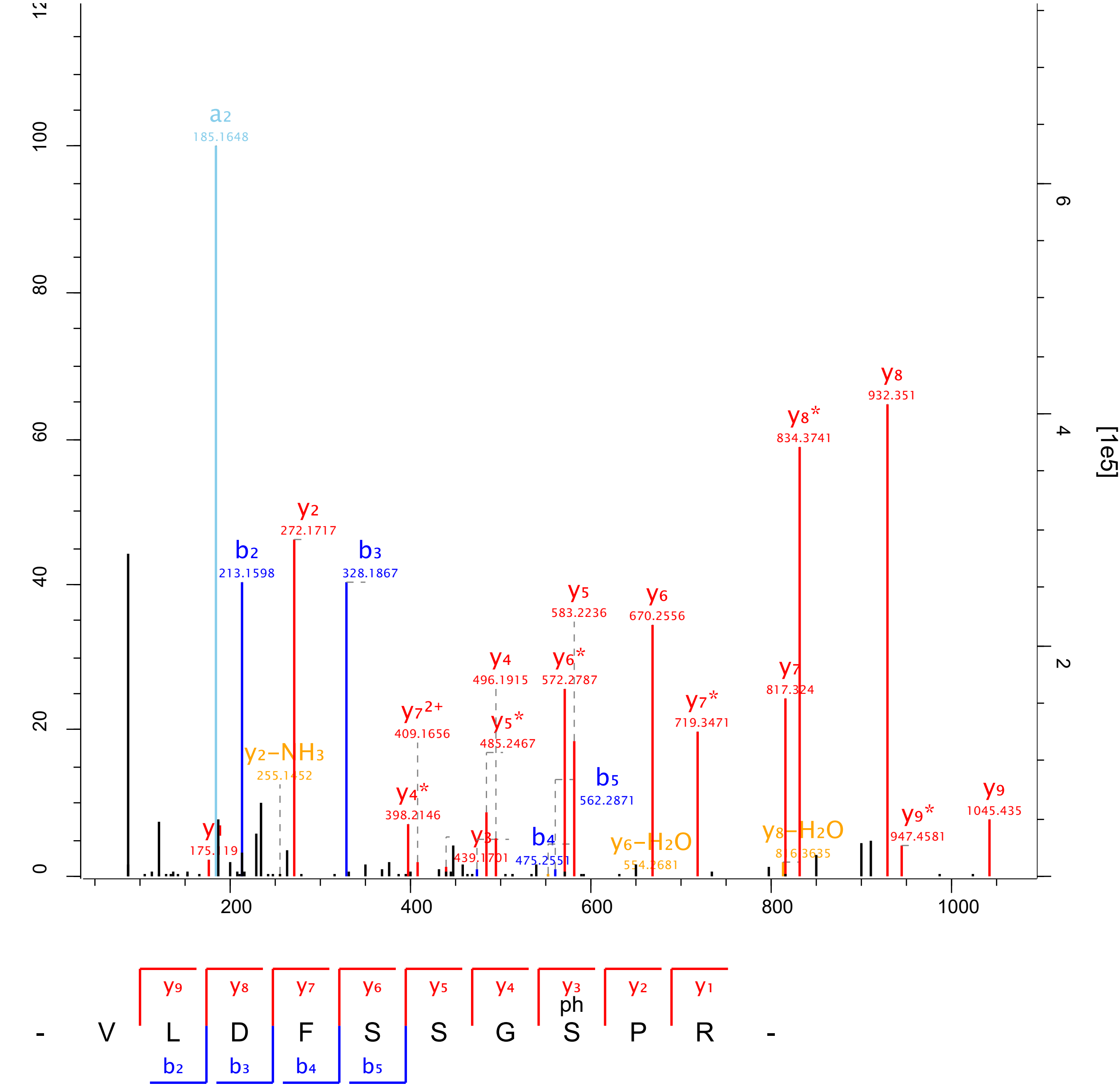

Raw file	Scan	Method	Score	m/z
sys_00_3short	17750	FTMS; HCD	73.93	648.3

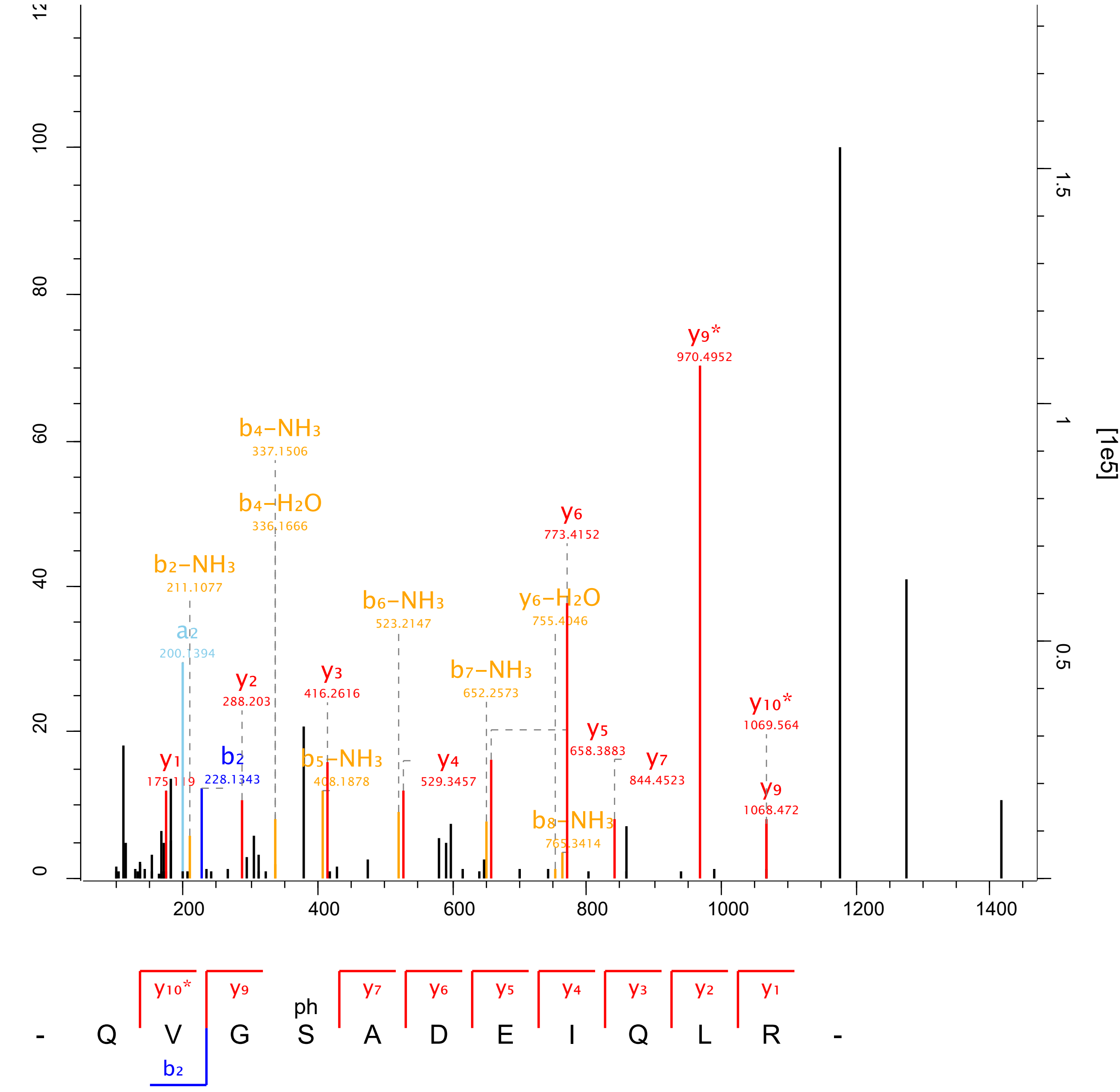

Raw file	Scan	Method	Score	m/z
sys_00_3short	17870	FTMS; HCD	84.36	525.78

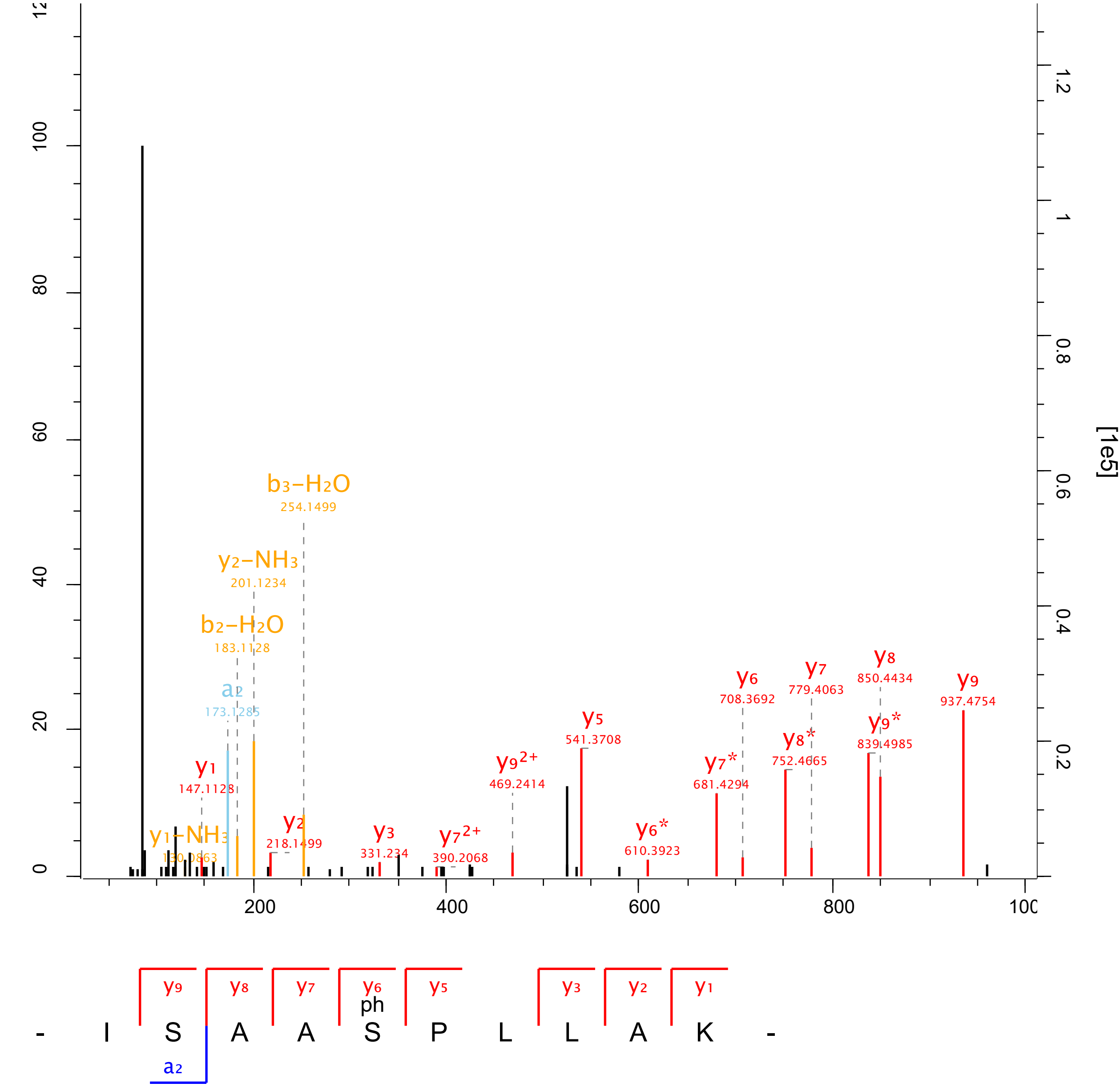

Raw file	Scan	Method	Score	m/z
sys_00_3short	17947	FTMS; HCD	130.15	607.74

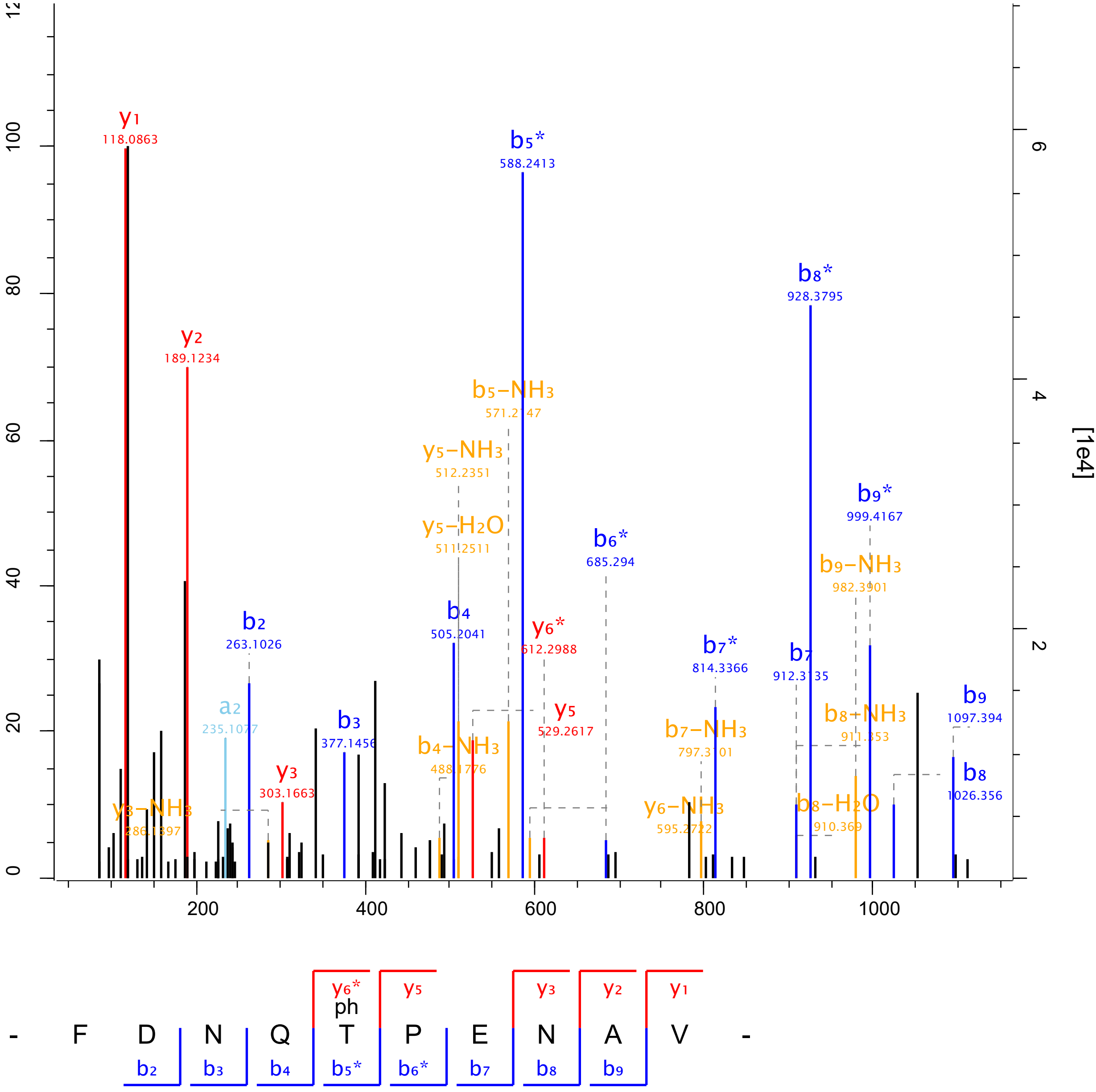

Raw file	Scan	Method	Score	m/z
sys_00_3short	18233	FTMS; HCD	60.16	581.3

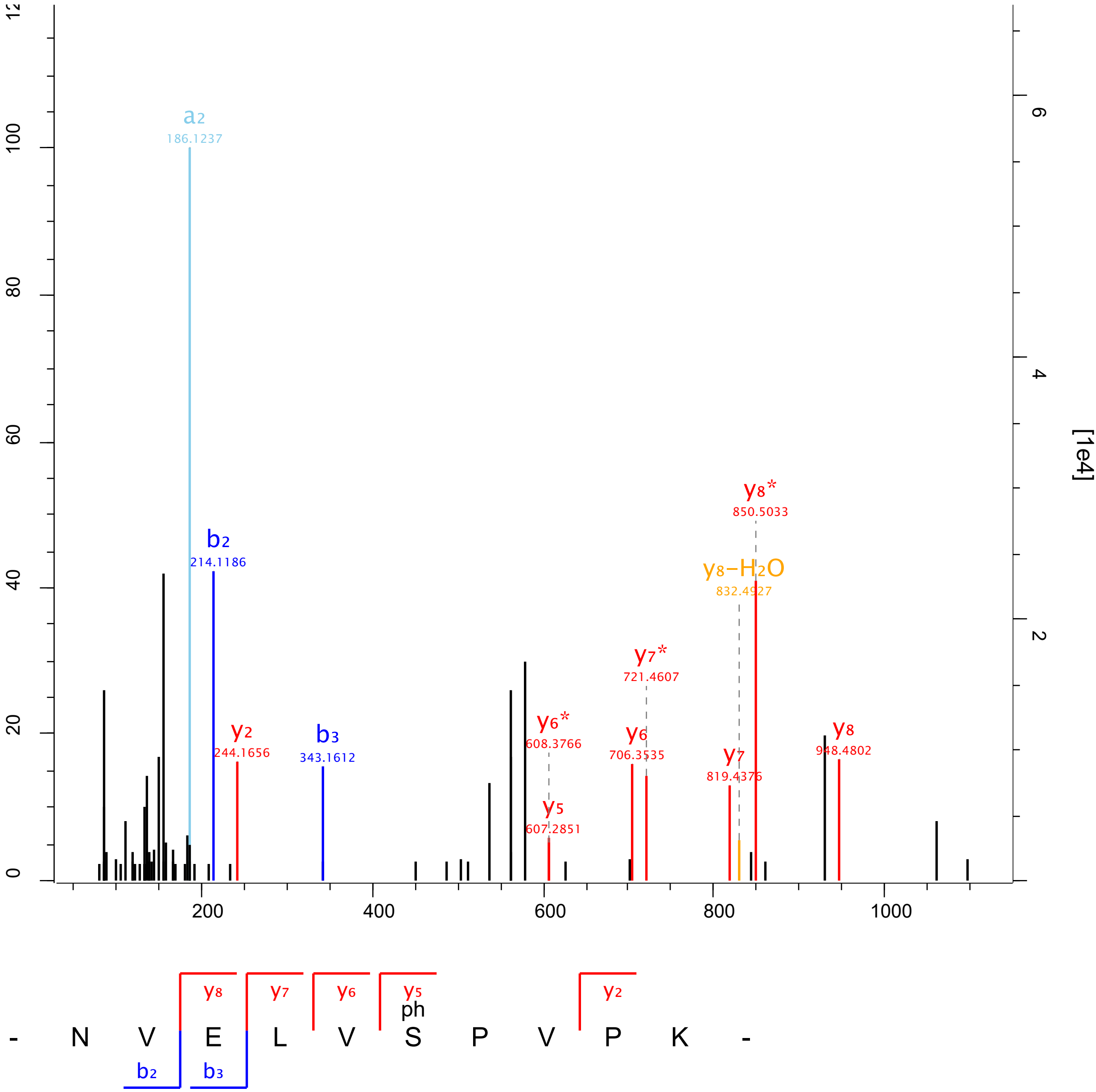

Raw file	Scan	Method	Score	m/z
sys_00_3short	18297	FTMS; HCD	150.11	657.79

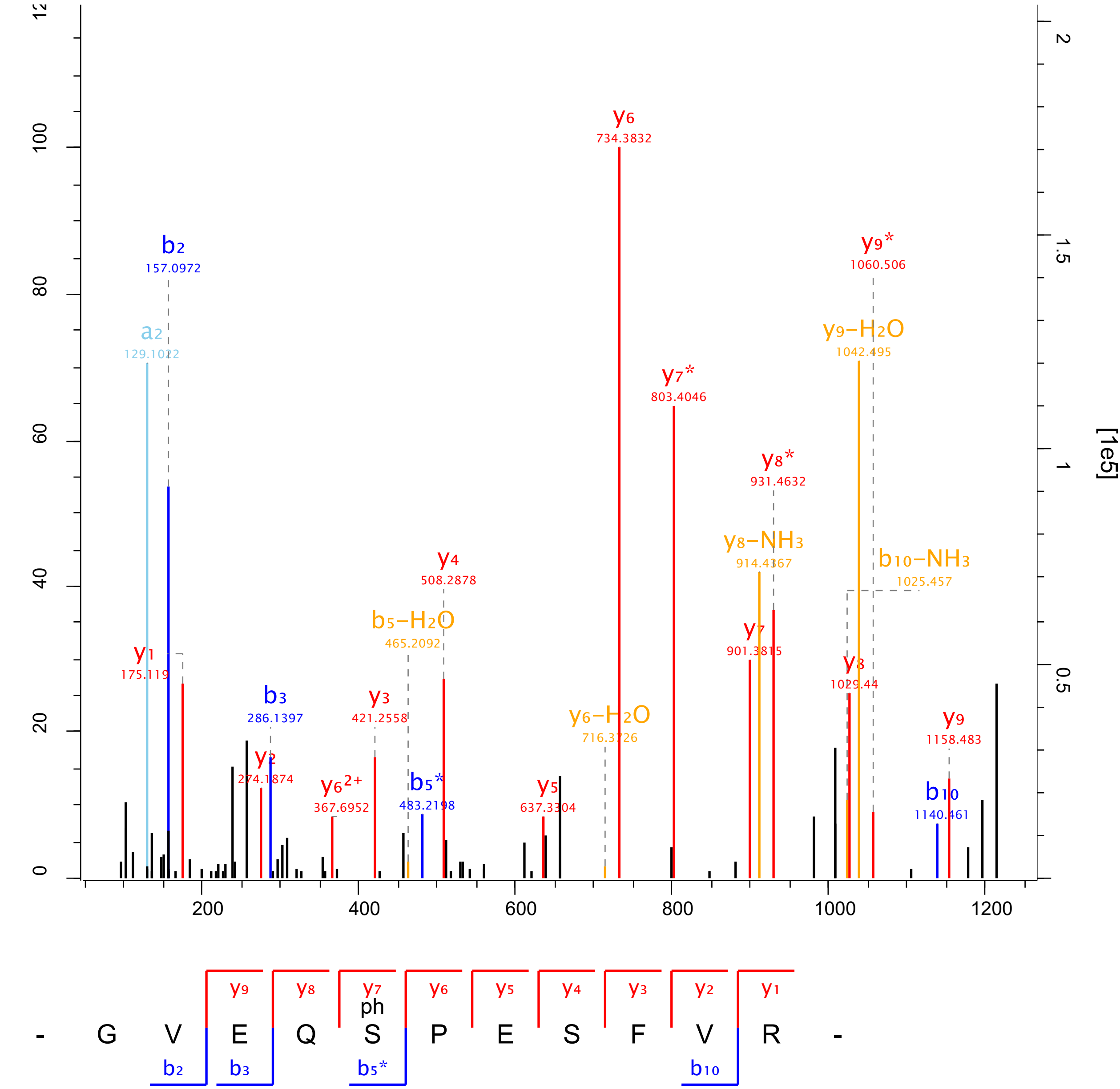

Raw file	Scan	Method	Score	m/z
sys_00_3short	18580	FTMS; HCD	49.33	562.76

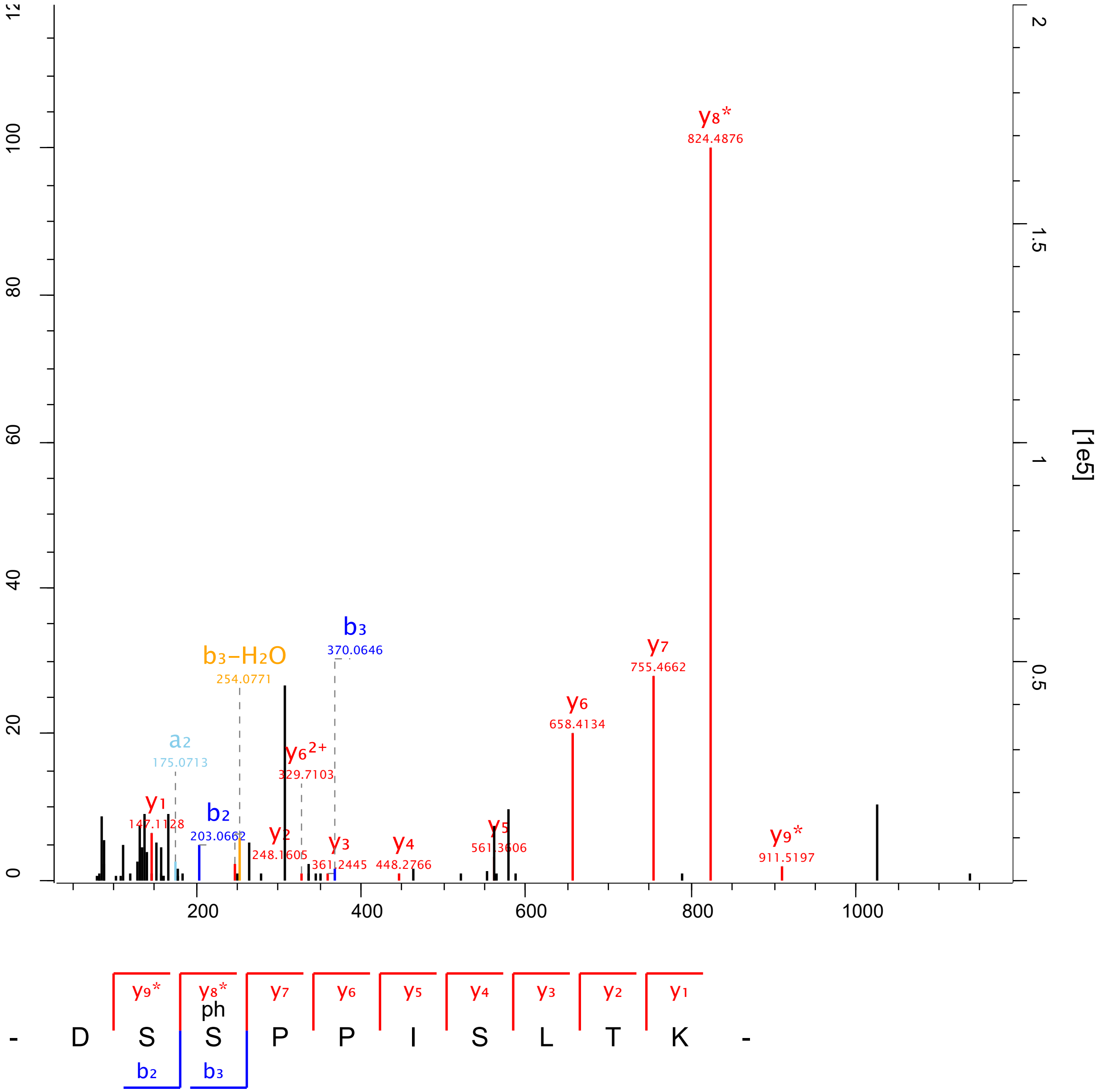

Raw file	Scan	Method	Score	m/z
sys_00_3short	18685	FTMS; HCD	98.57	775.36

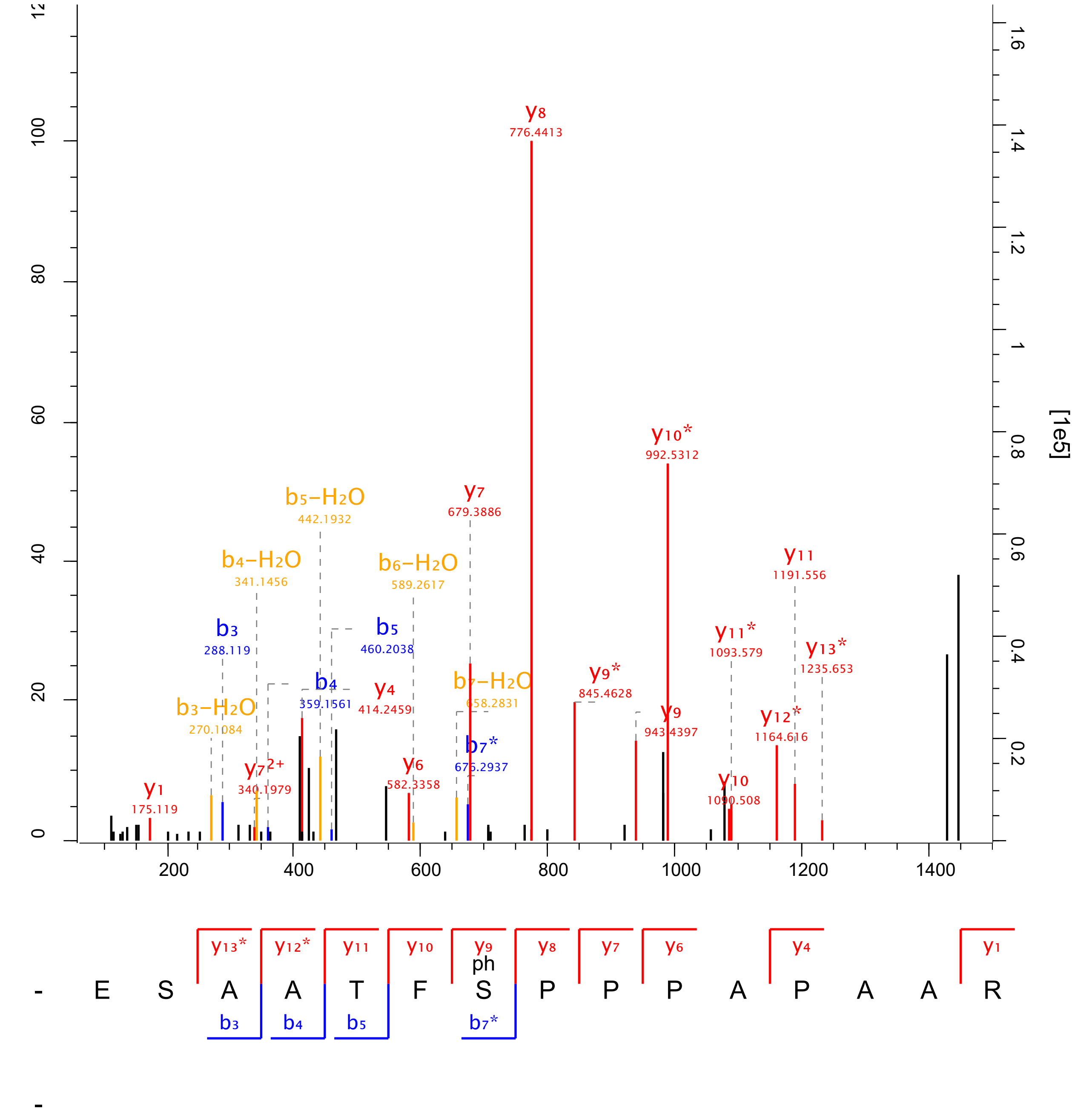

Raw file	Scan	Method	Score	m/z
sys_00_3short	18691	FTMS; HCD	59.71	602.77

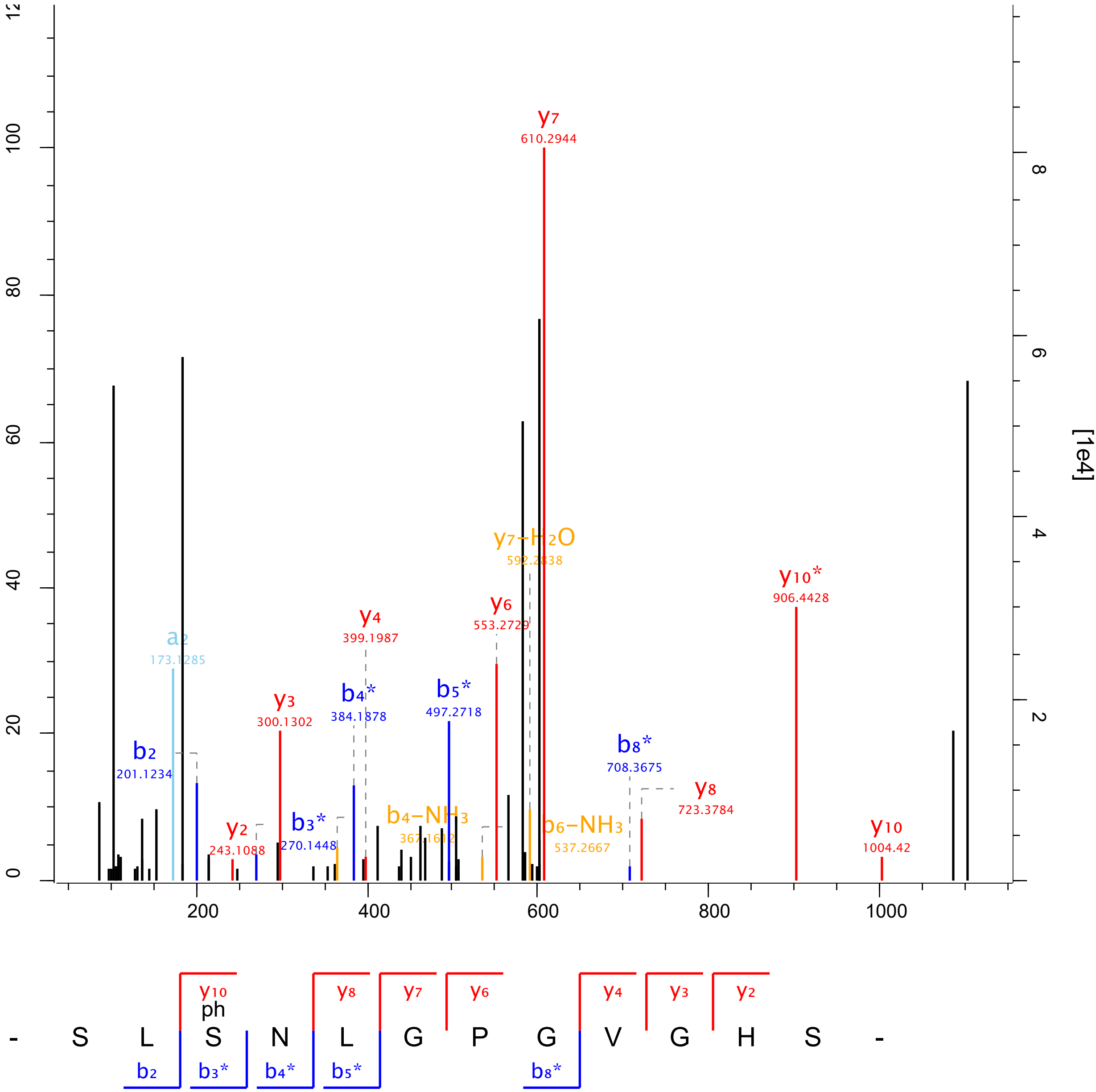

Raw file	Scan	Method	Score	m/z
sys_00_3short	1871	FTMS; HCD	70.81	562.75

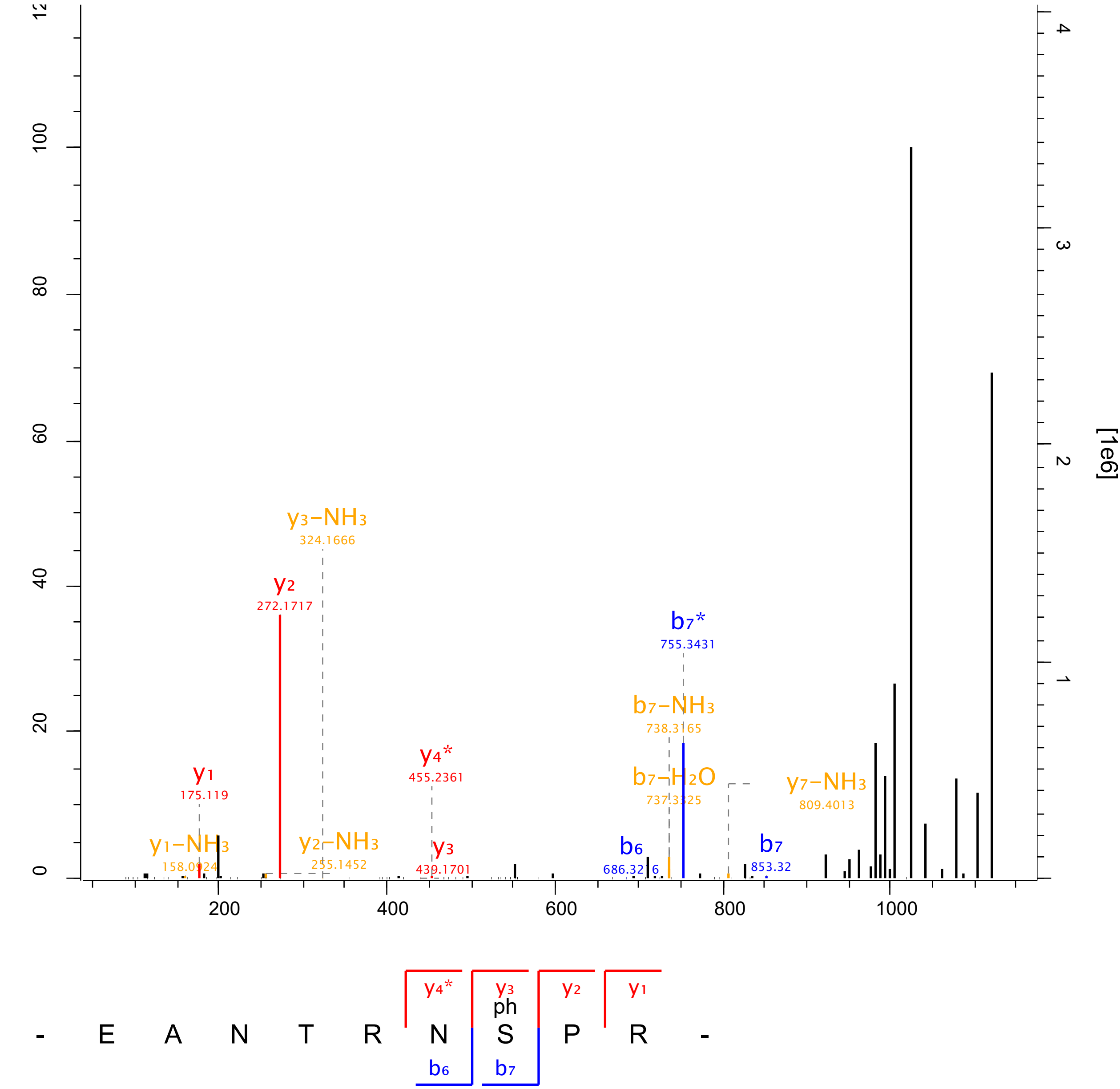

Raw file	Scan	Method	Score	m/z
sys_00_3short	19002	FTMS; HCD	62.09	772.87

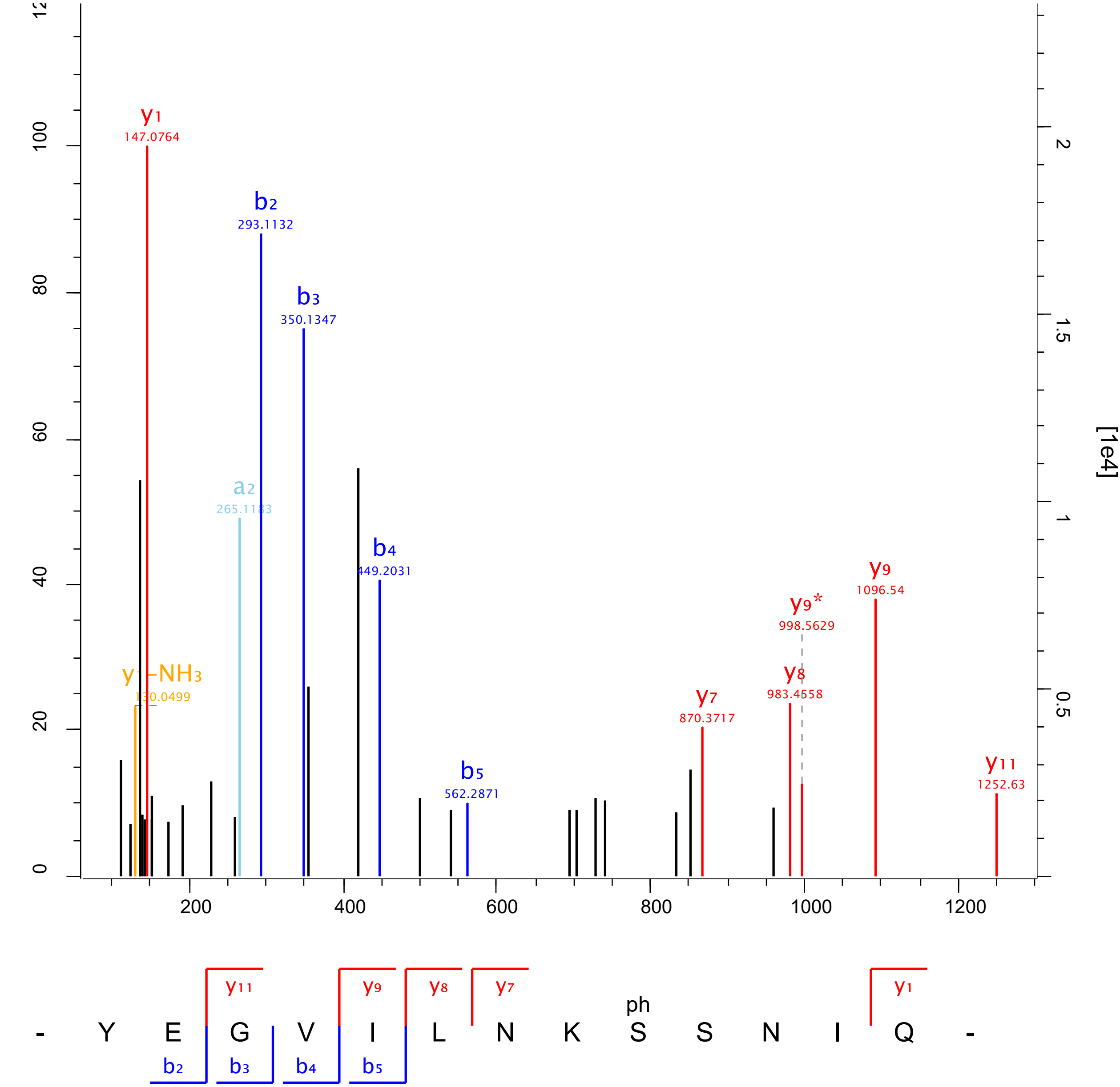

Raw file	Scan	Method	Score	m/z
sys_00_3short	19224	FTMS; HCD	54.26	516.75

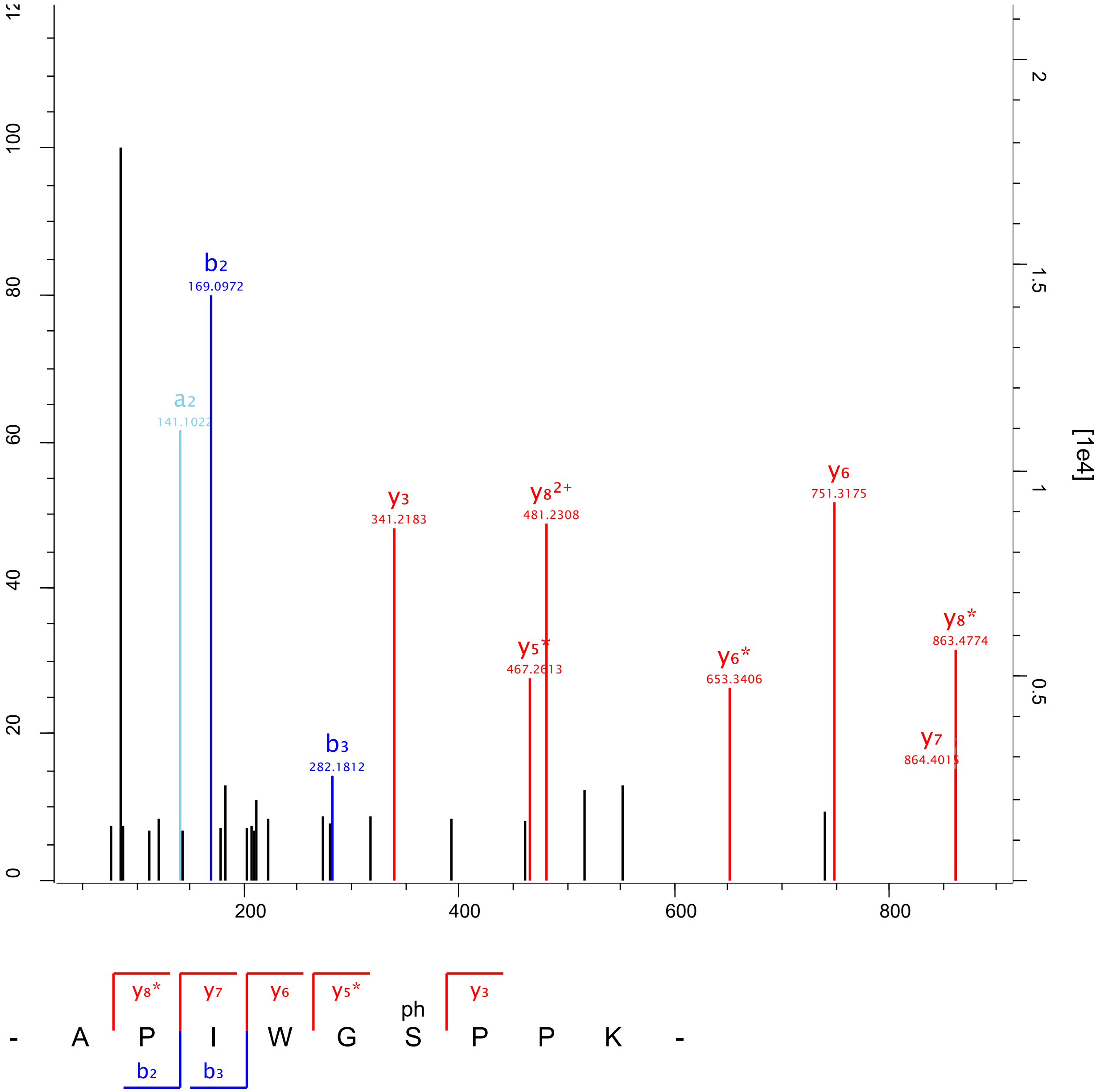

Raw file	Scan	Method	Score	m/z
sys_00_3short	19356	FTMS; HCD	149.42	805.36

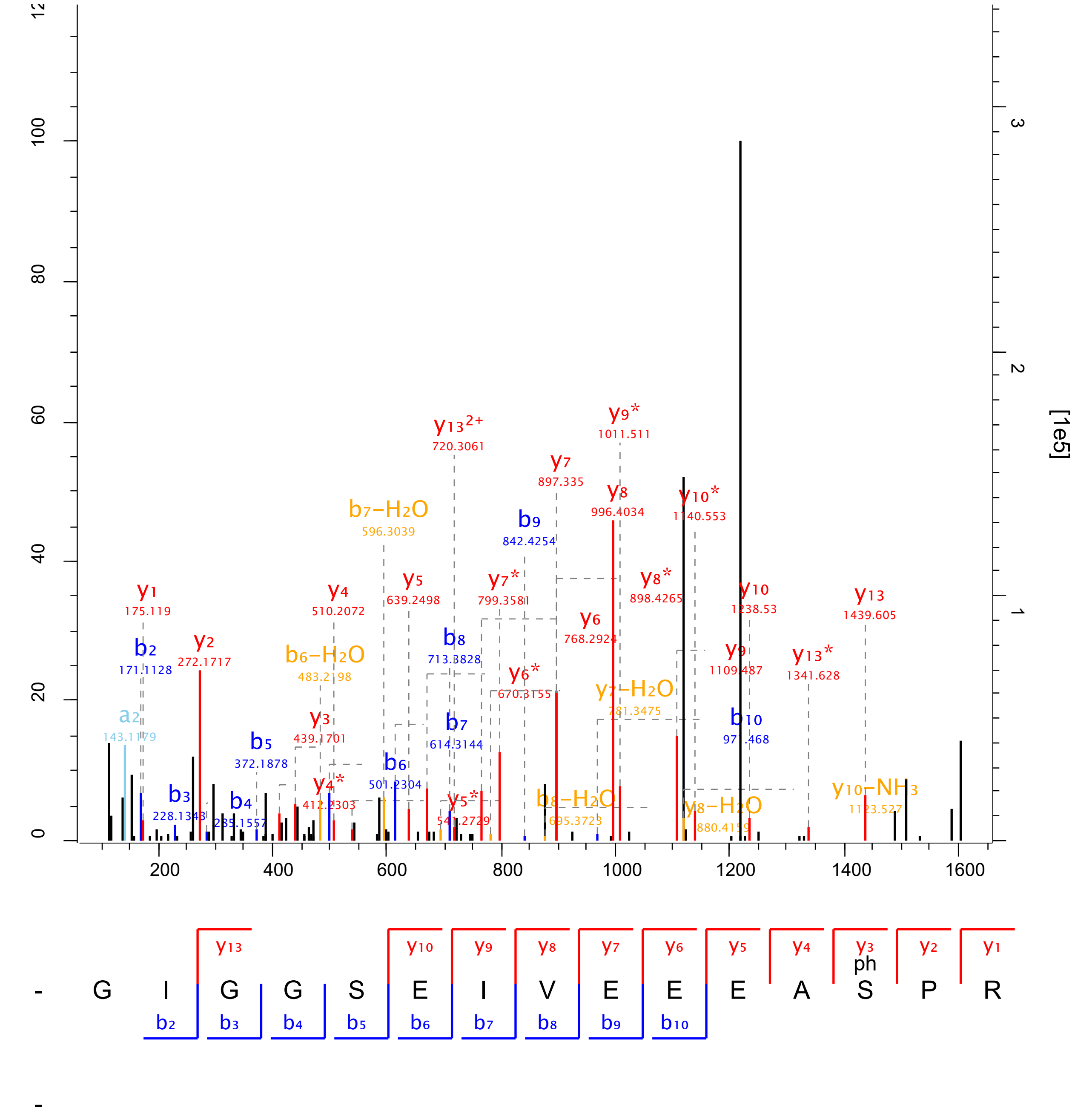

Raw file	Scan	Method	Score	m/z
sys_00_3short	19433	FTMS; HCD	75.06	966.46

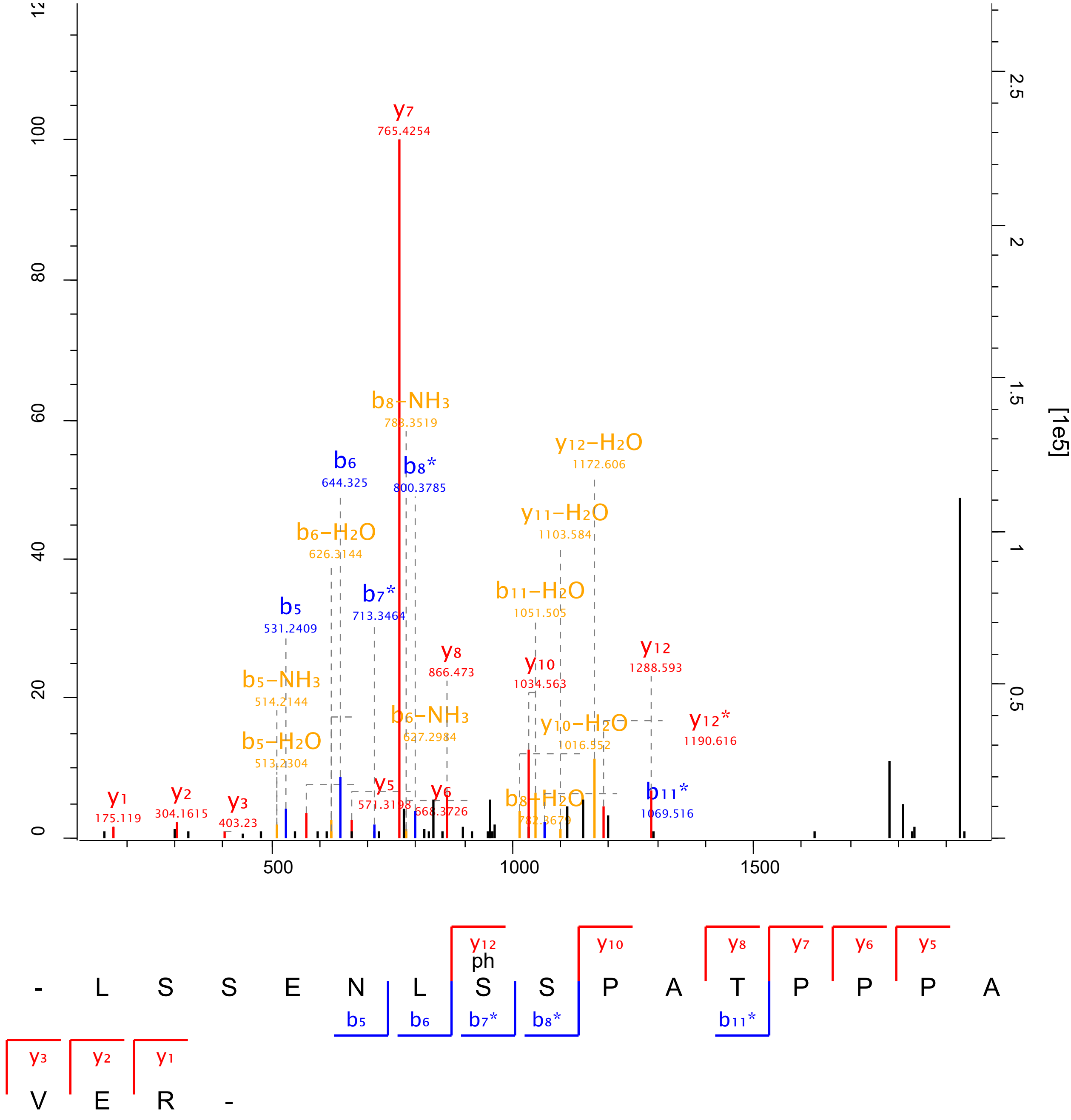

Raw file	Scan	Method	Score	m/z
sys_00_3short	19525	FTMS; HCD	74.84	439.72

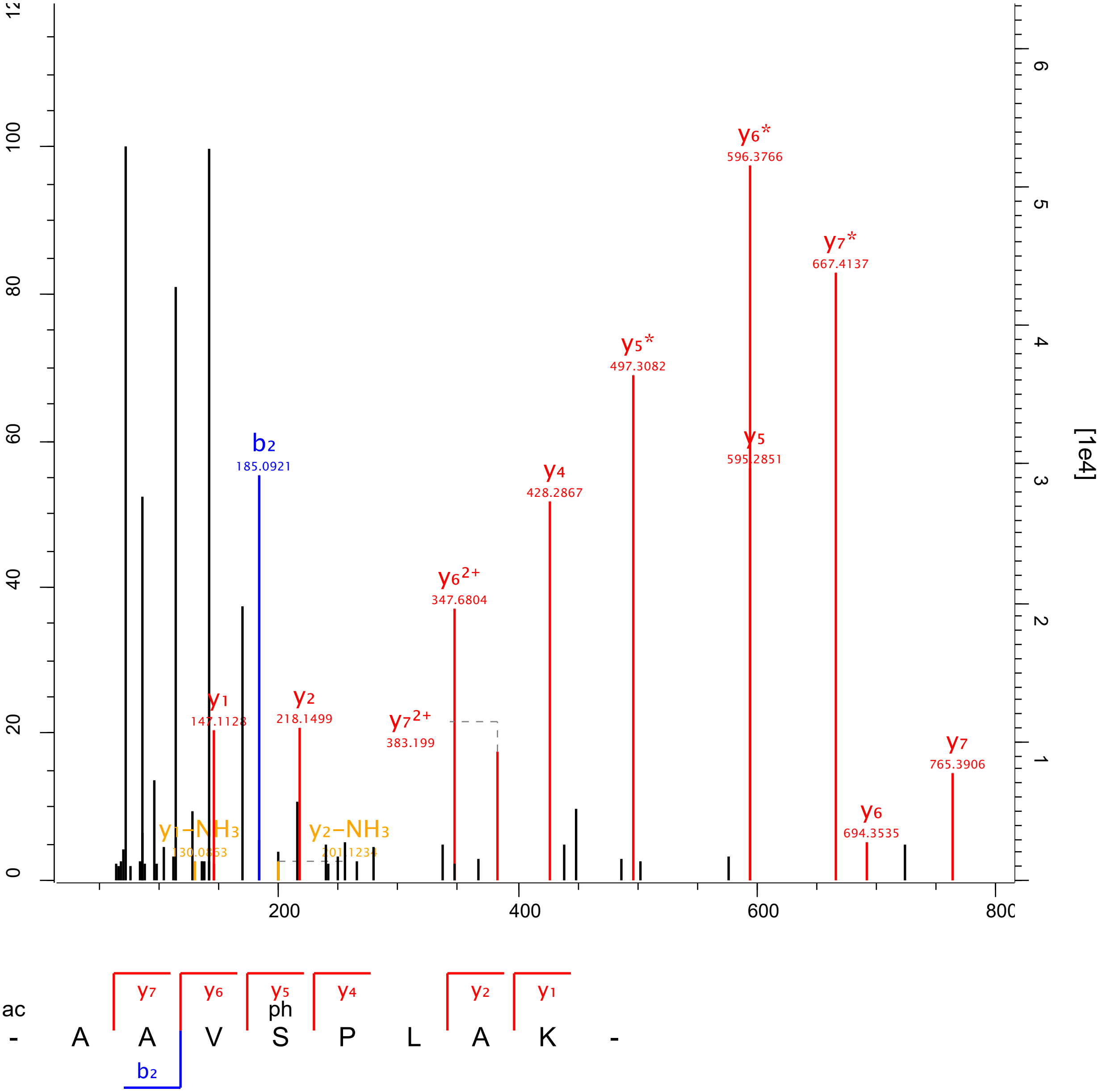

Raw file	Scan	Method	Score	m/z
sys_00_3short	19584	FTMS; HCD	82.64	470.23

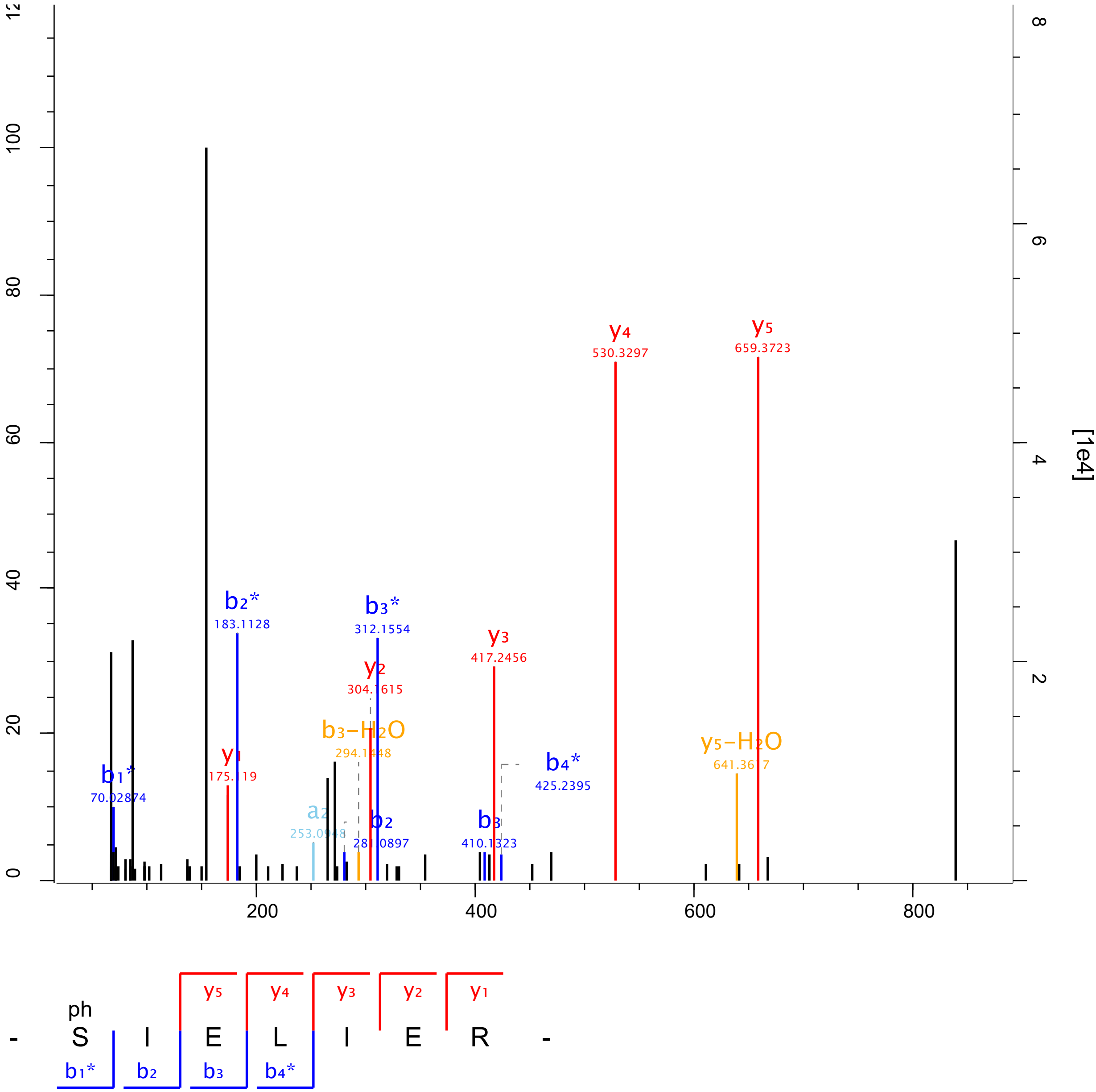

Raw file	Scan	Method	Score	m/z
sys_00_3short	19632	FTMS; HCD	125.98	734.28

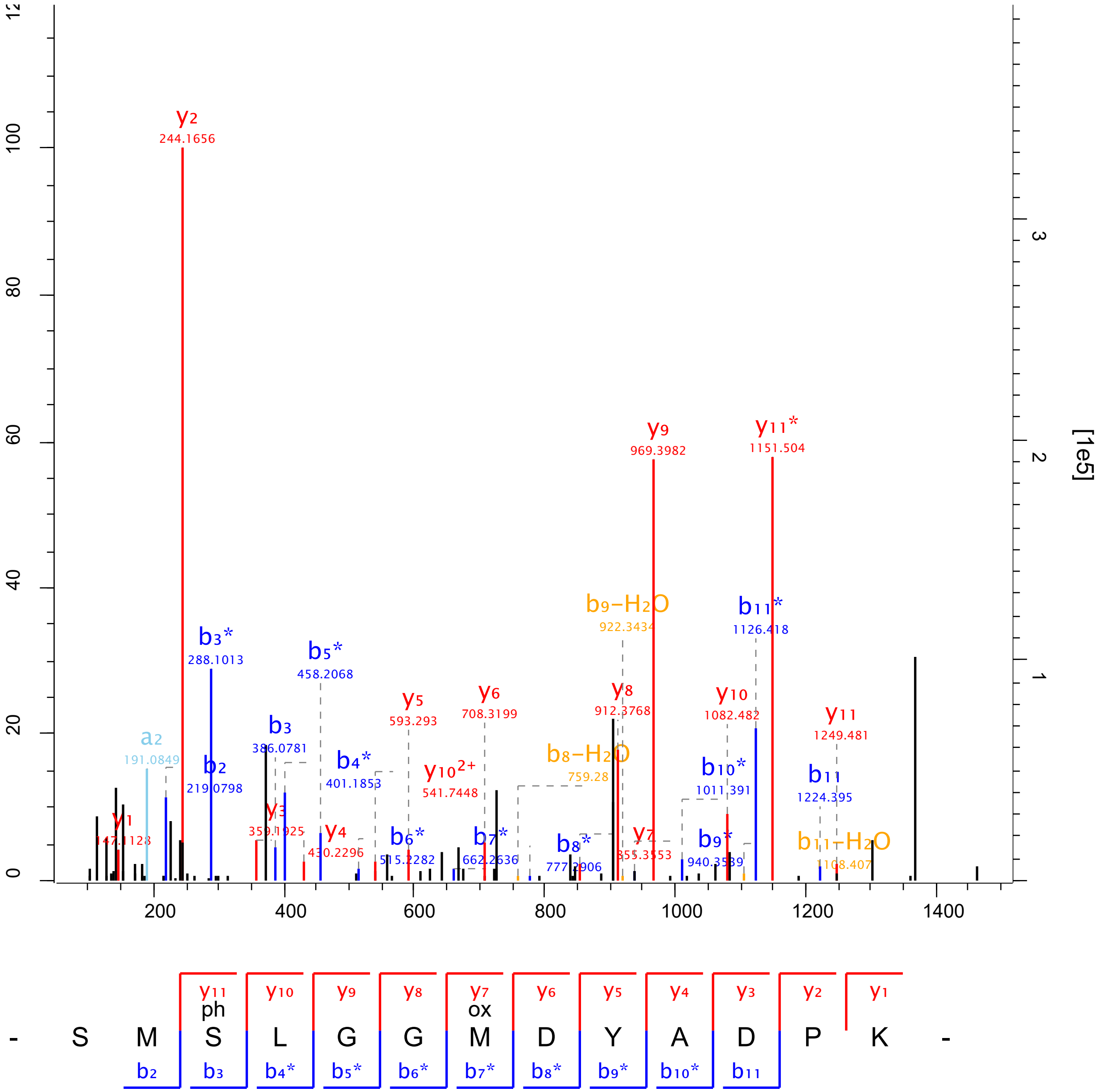

Raw file	Scan	Method	Score	m/z
sys_00_3short	19679	FTMS; HCD	99.41	758.34

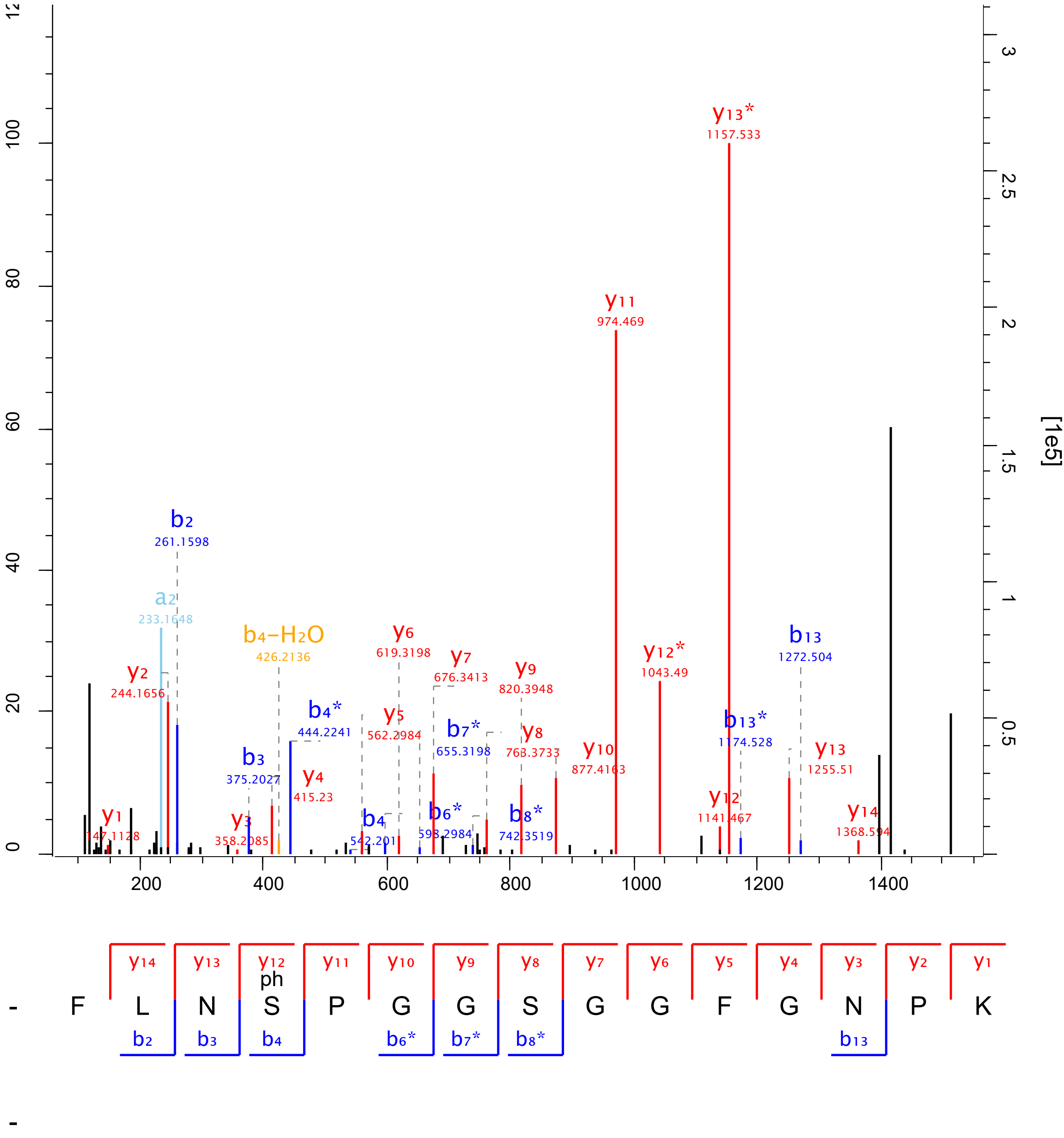

Raw file	Scan	Method	Score	m/z
sys_00_3short	19768	FTMS; HCD	79.84	729.32

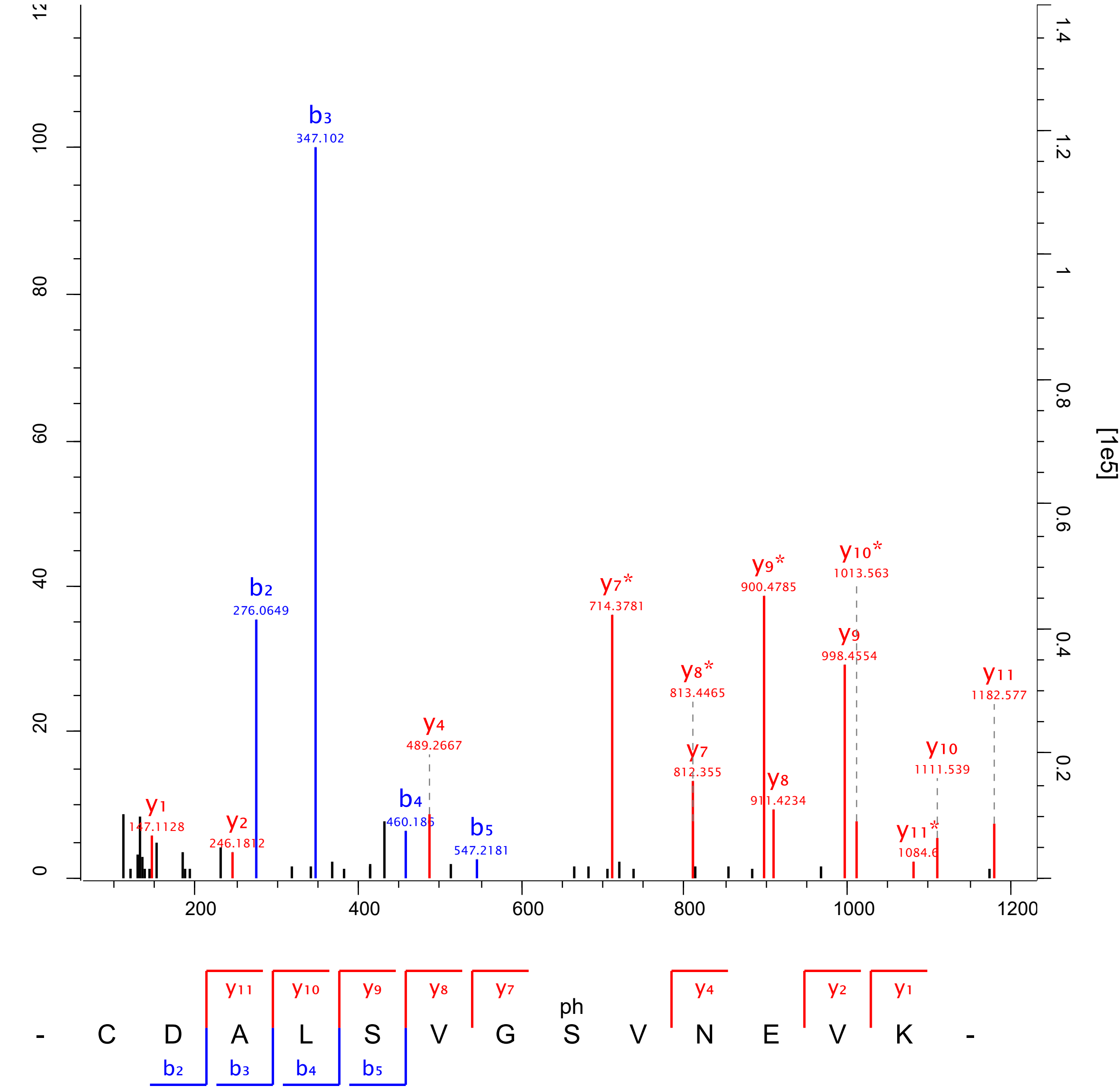

Raw file	Scan	Method	Score	m/z
sys_00_3short	19769	FTMS; HCD	90.61	533.77

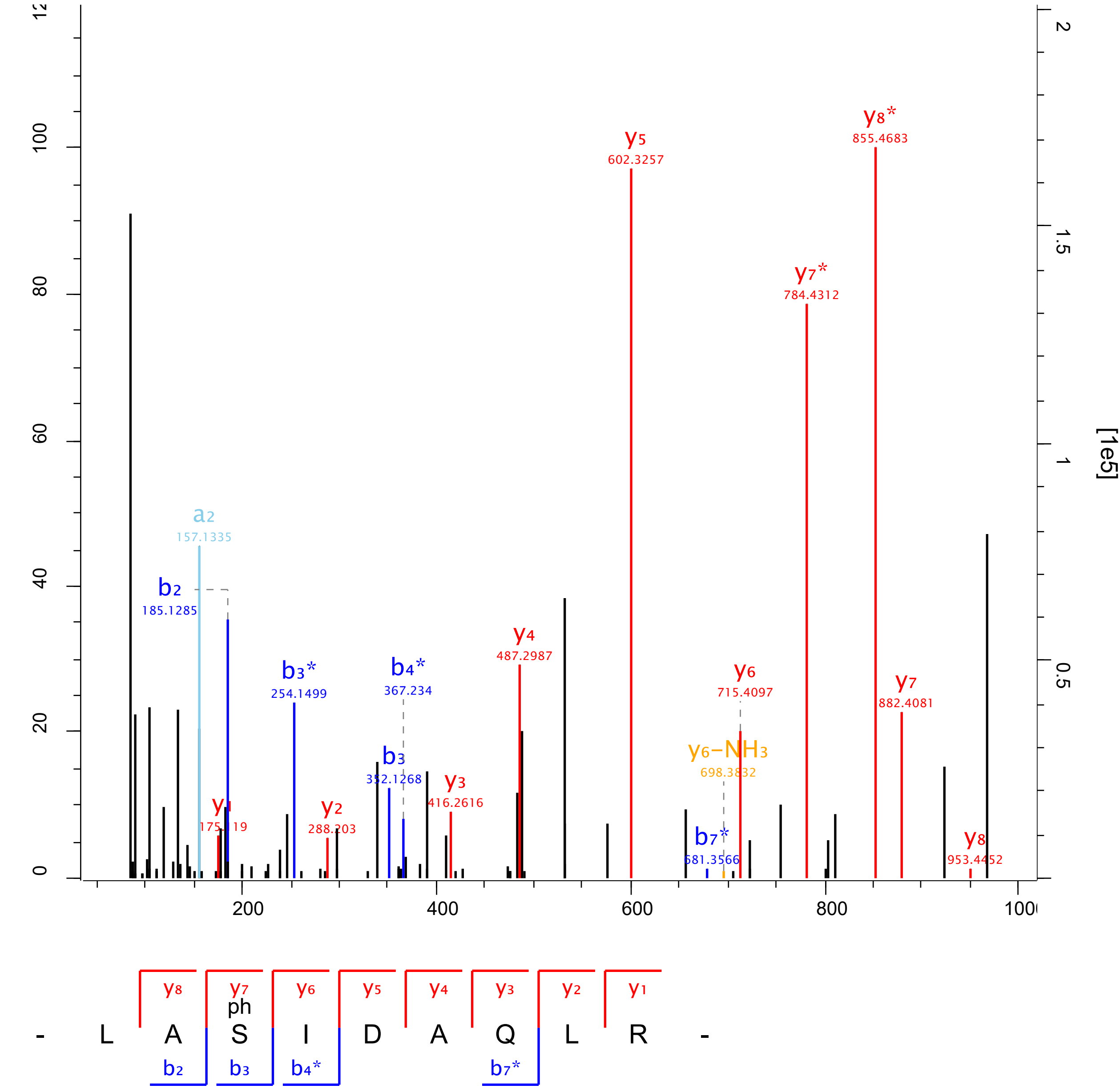

Raw file	Scan	Method	Score	m/z
sys_00_3short	19783	FTMS; HCD	85.52	698.79

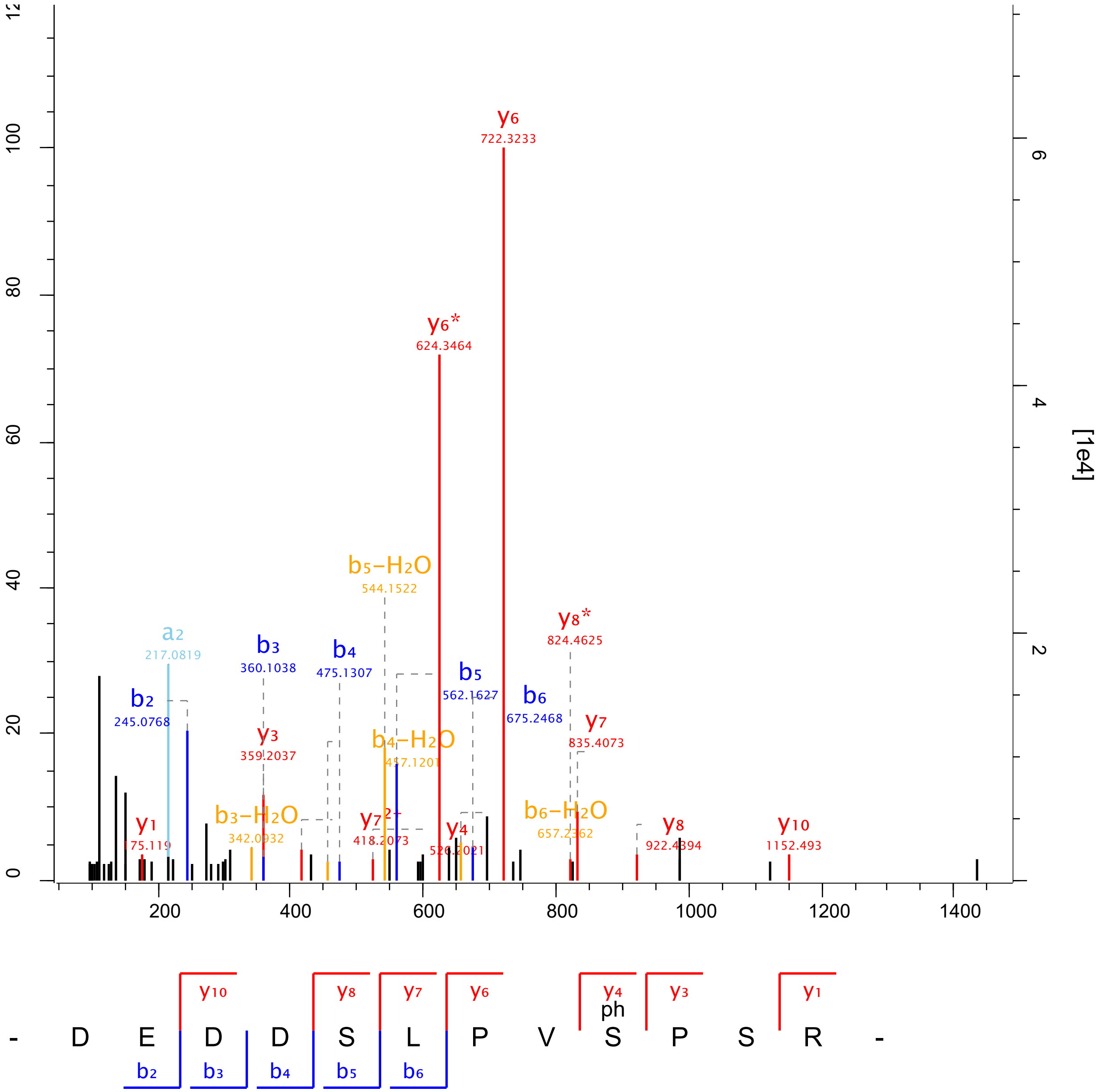

Raw file	Scan	Method	Score	m/z
sys_00_3short	19824	FTMS; HCD	93.24	706.31

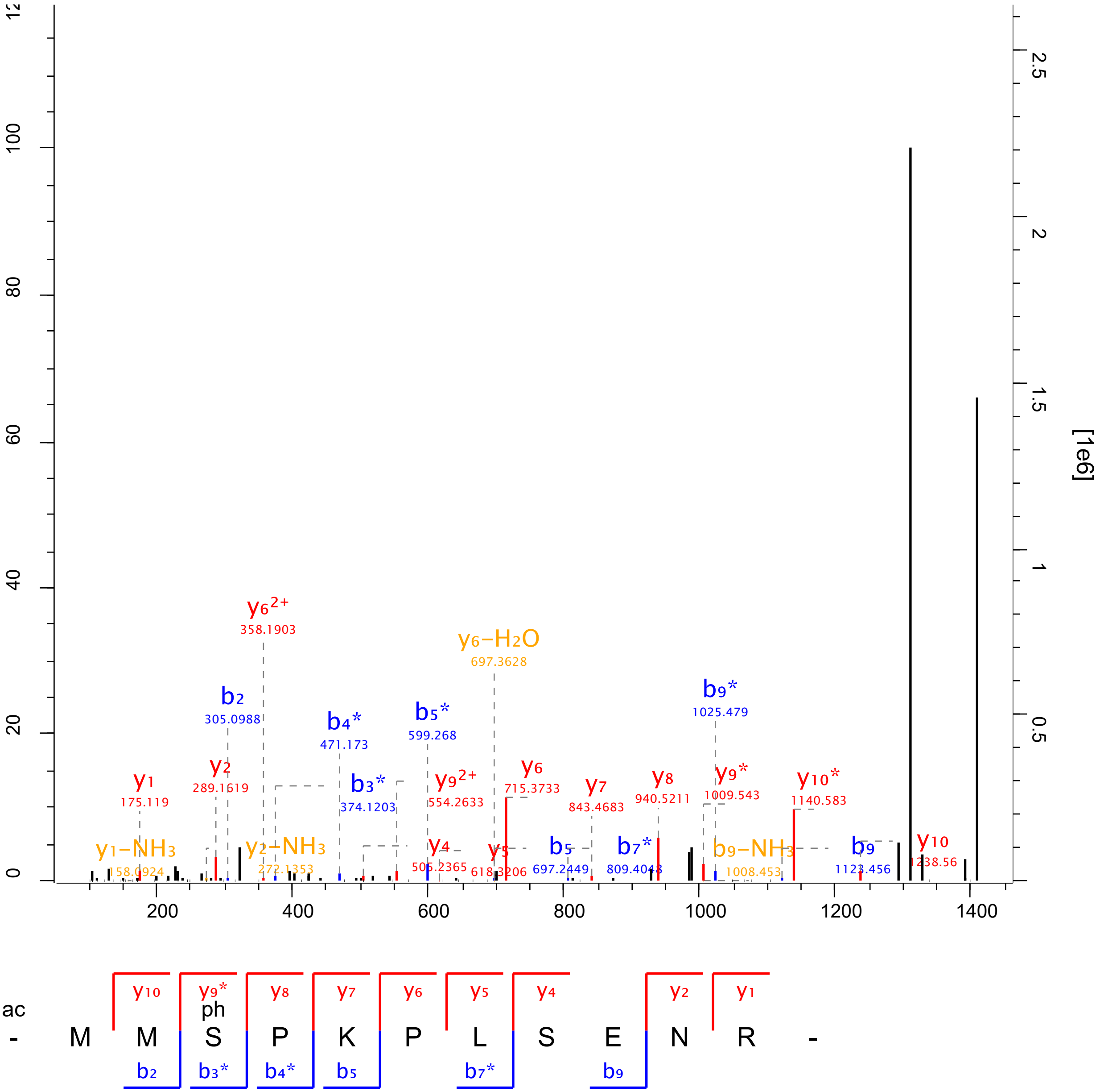

Raw file	Scan	Method	Score	m/z
sys_00_3short	19831	FTMS; HCD	97.16	475.76

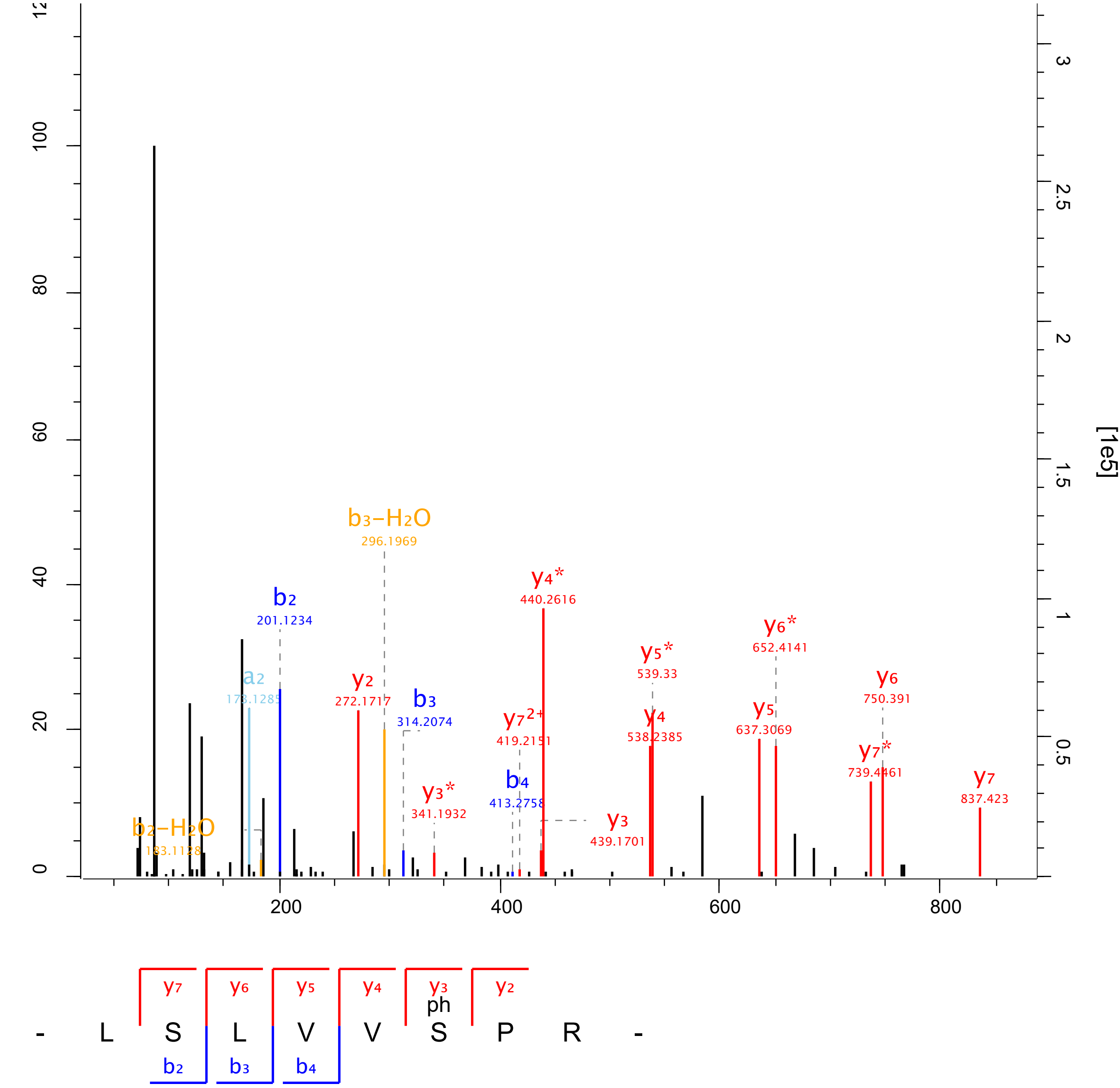

Raw file	Scan	Method	Score	m/z
sys_00_3short	20043	FTMS; HCD	82.01	853.86

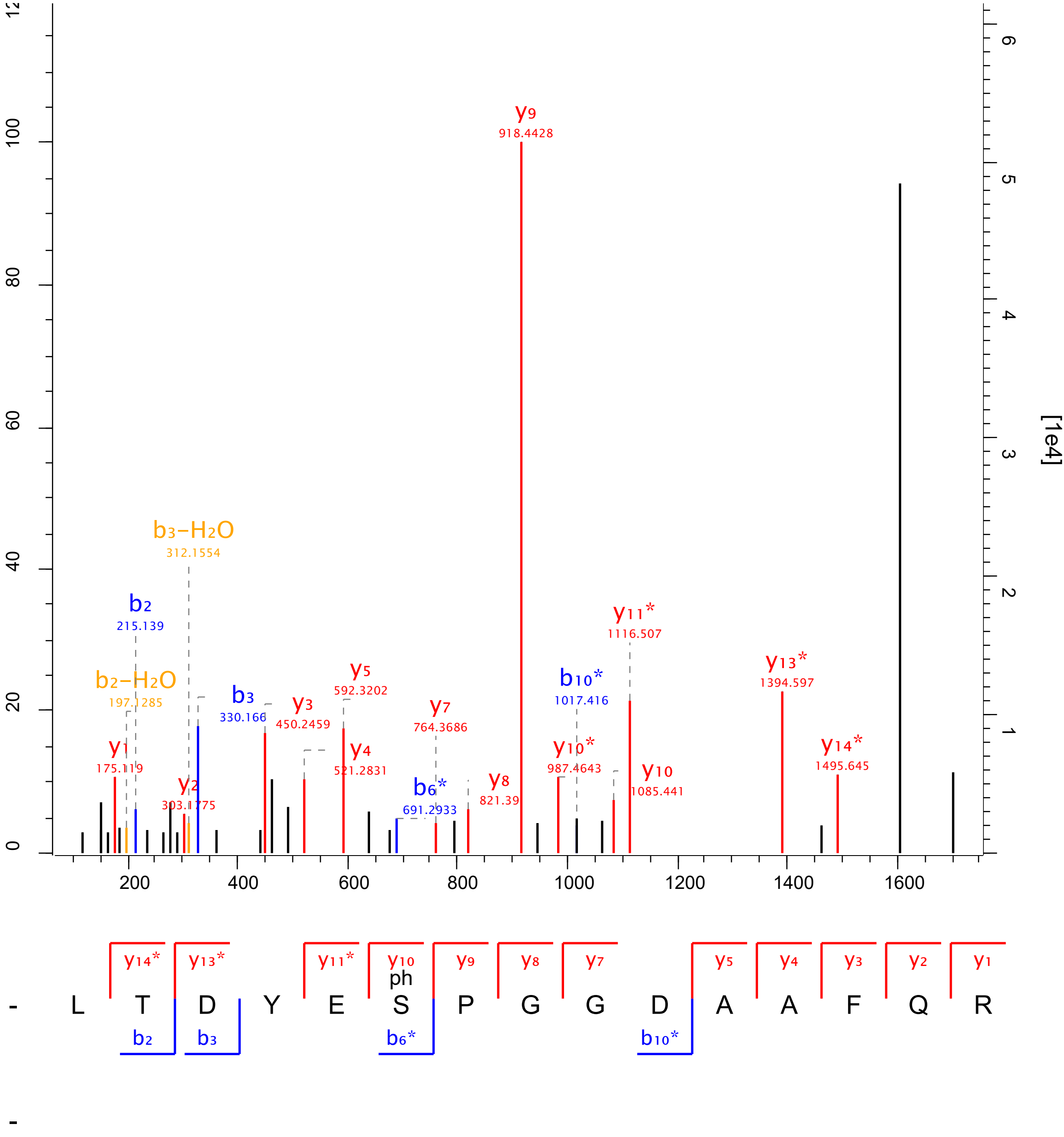

Raw file	Scan	Method	Score	m/z
sys_00_3short	20186	FTMS; HCD	178.71	807.36

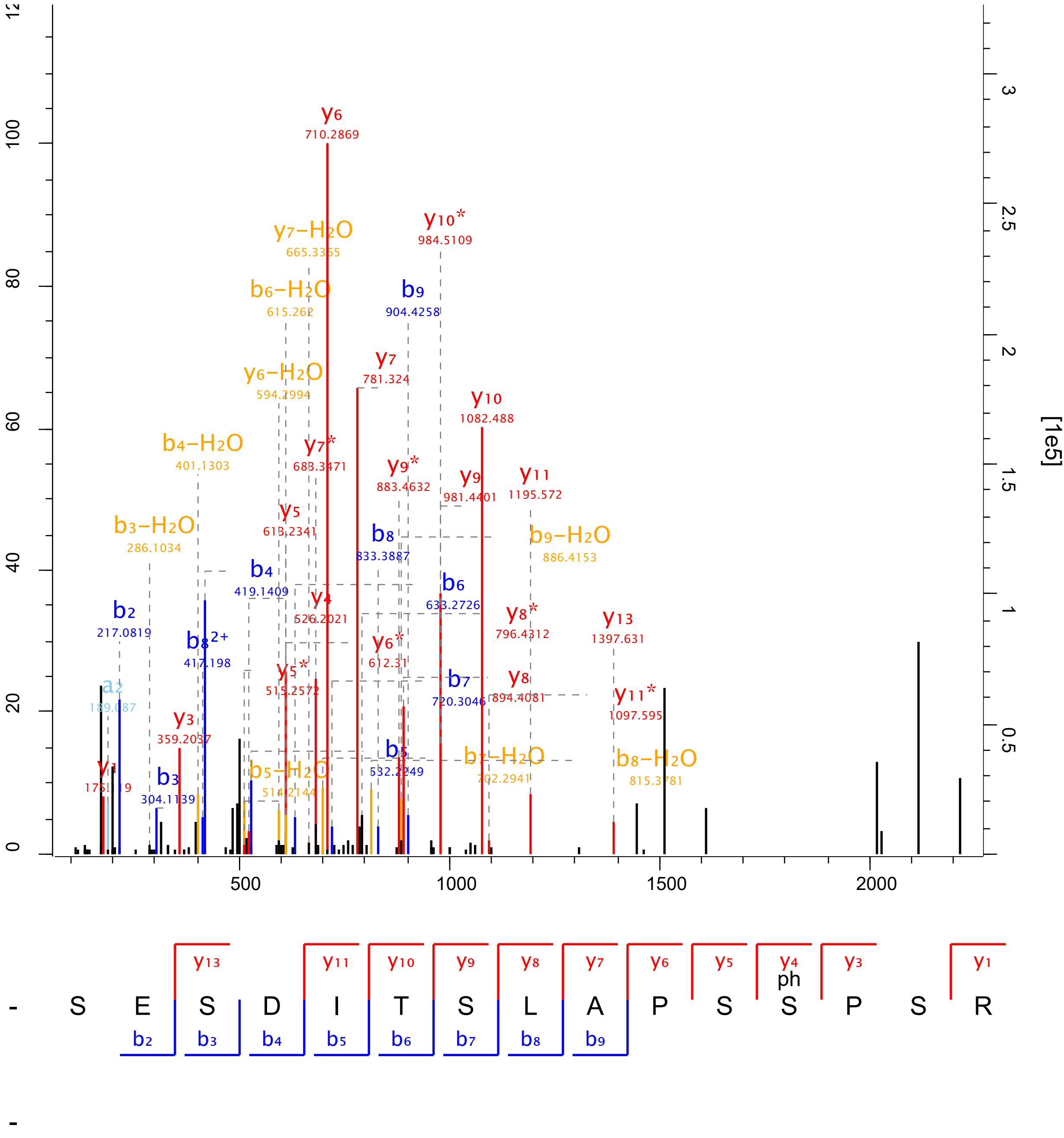

Raw file	Scan	Method	Score	m/z
sys_00_3short	20462	FTMS; HCD	72.9	646.24

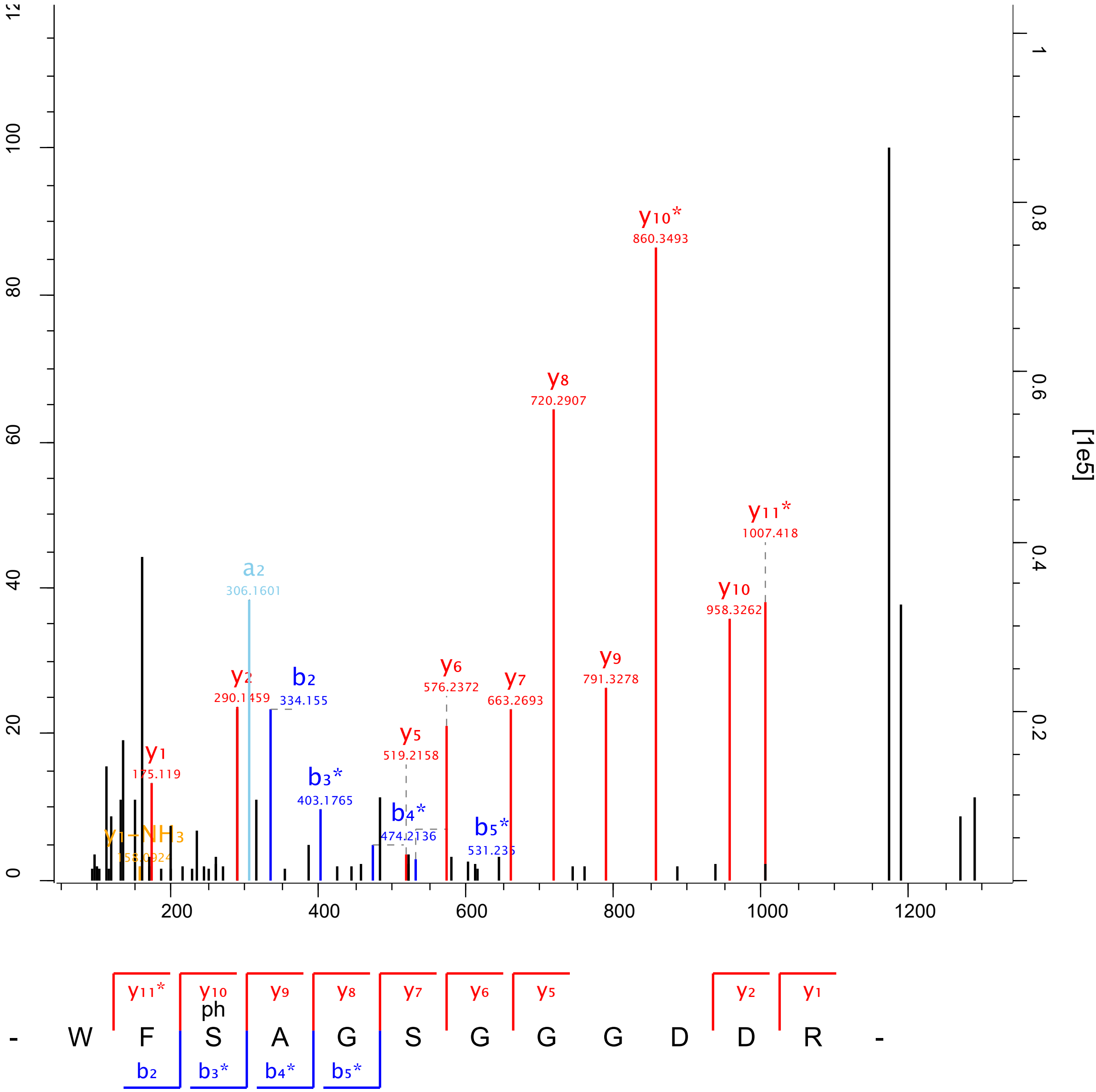

Raw file	Scan	Method	Score	m/z
sys_00_3short	20627	FTMS; HCD	66.27	529.24

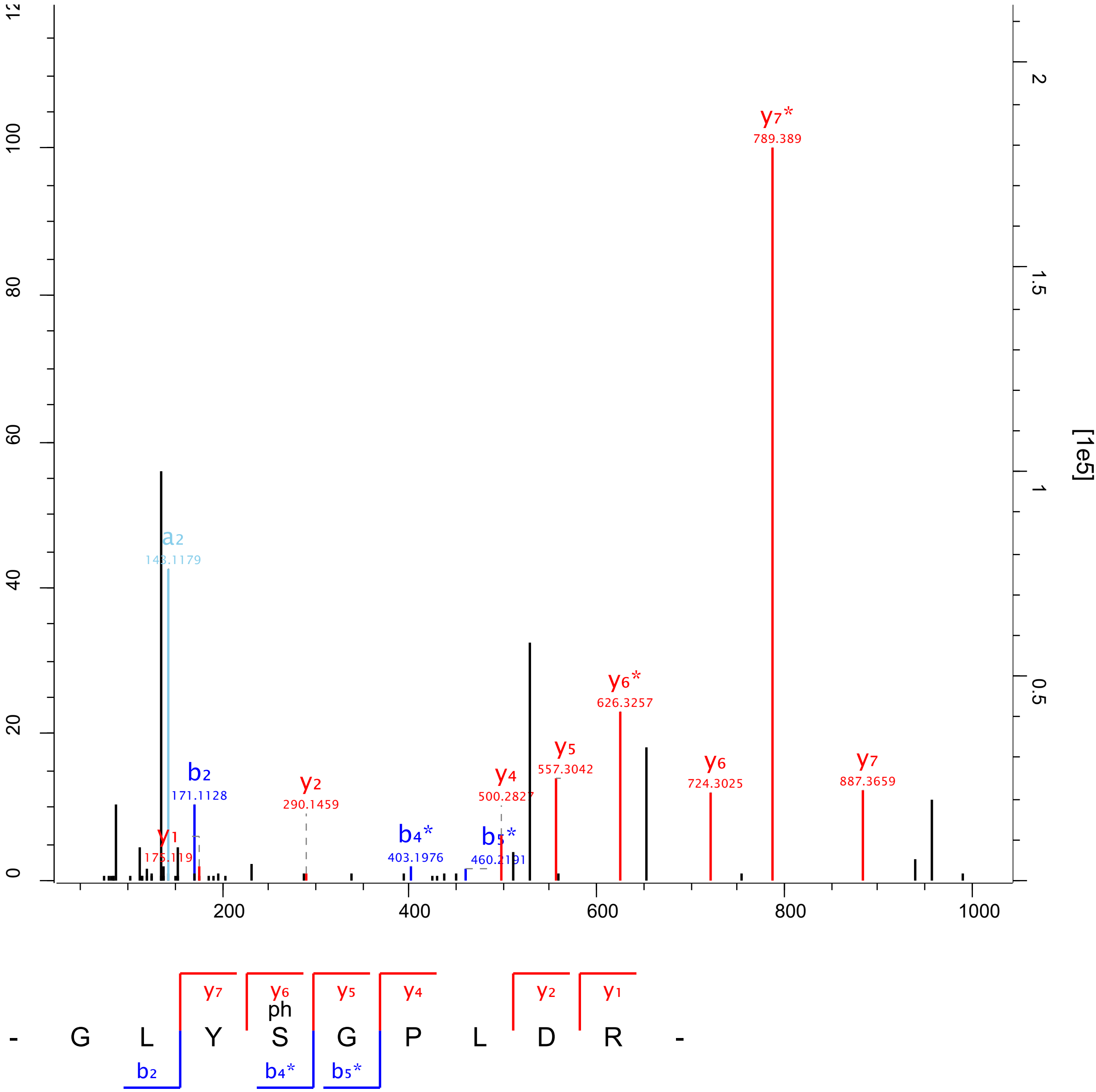

Raw file	Scan	Method	Score	m/z
sys_00_3short	20689	FTMS; HCD	70.47	675.29

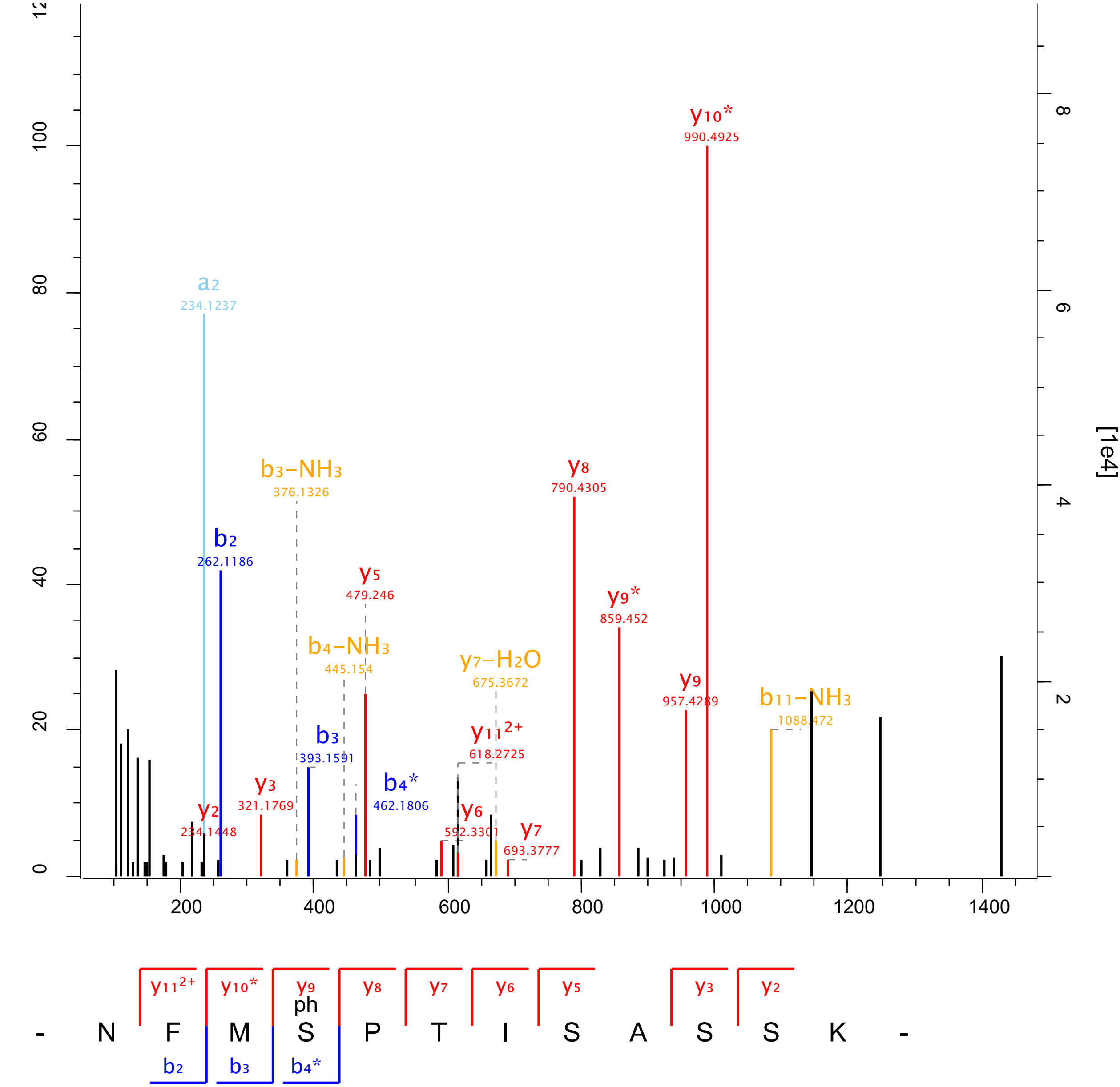

Raw file	Scan	Method	Score	m/z
sys_00_3short	20716	FTMS; HCD	131.26	709.3

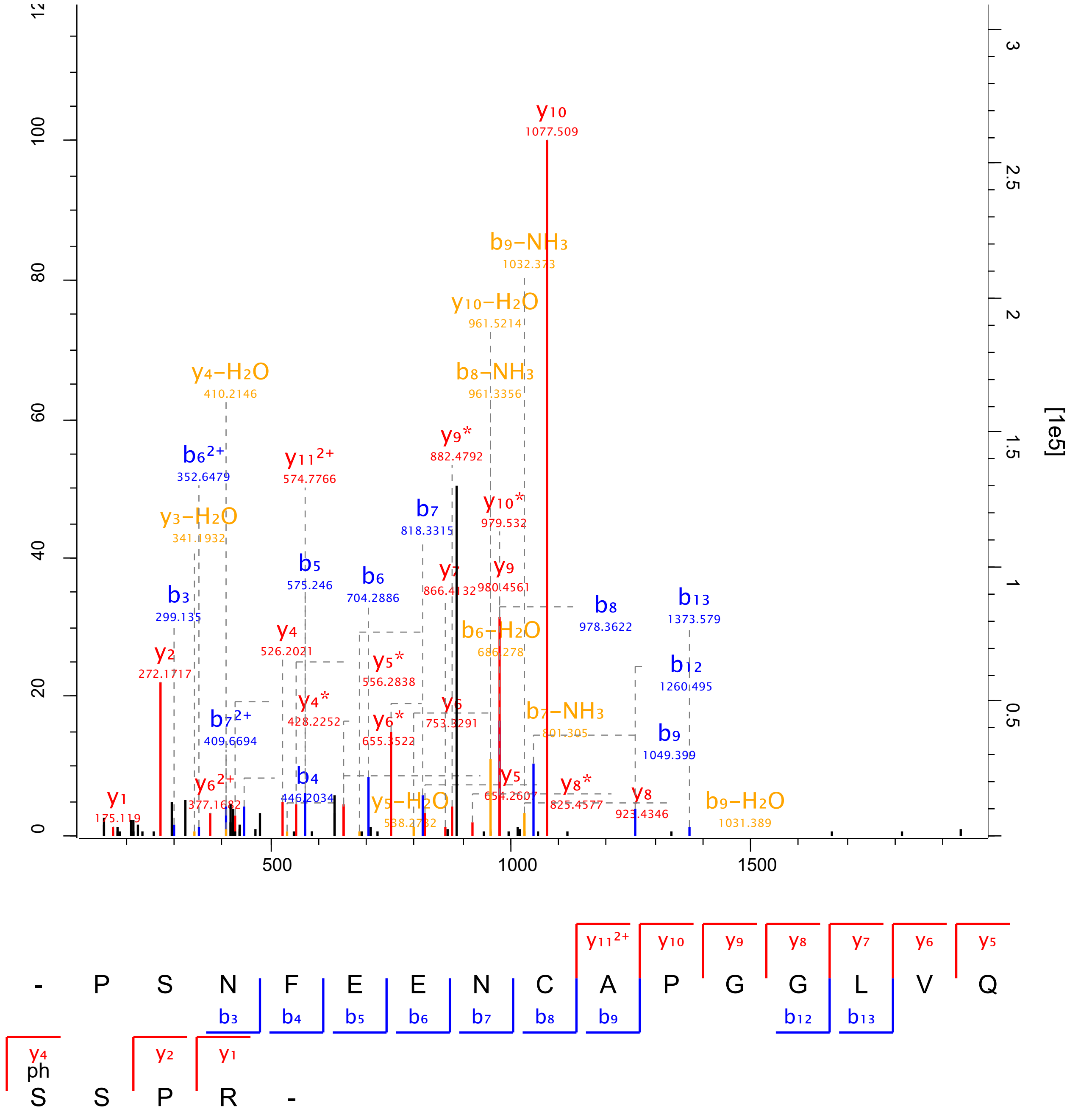

Raw file	Scan	Method	Score	m/z
sys_00_3short	20790	FTMS; HCD	80.24	572.76

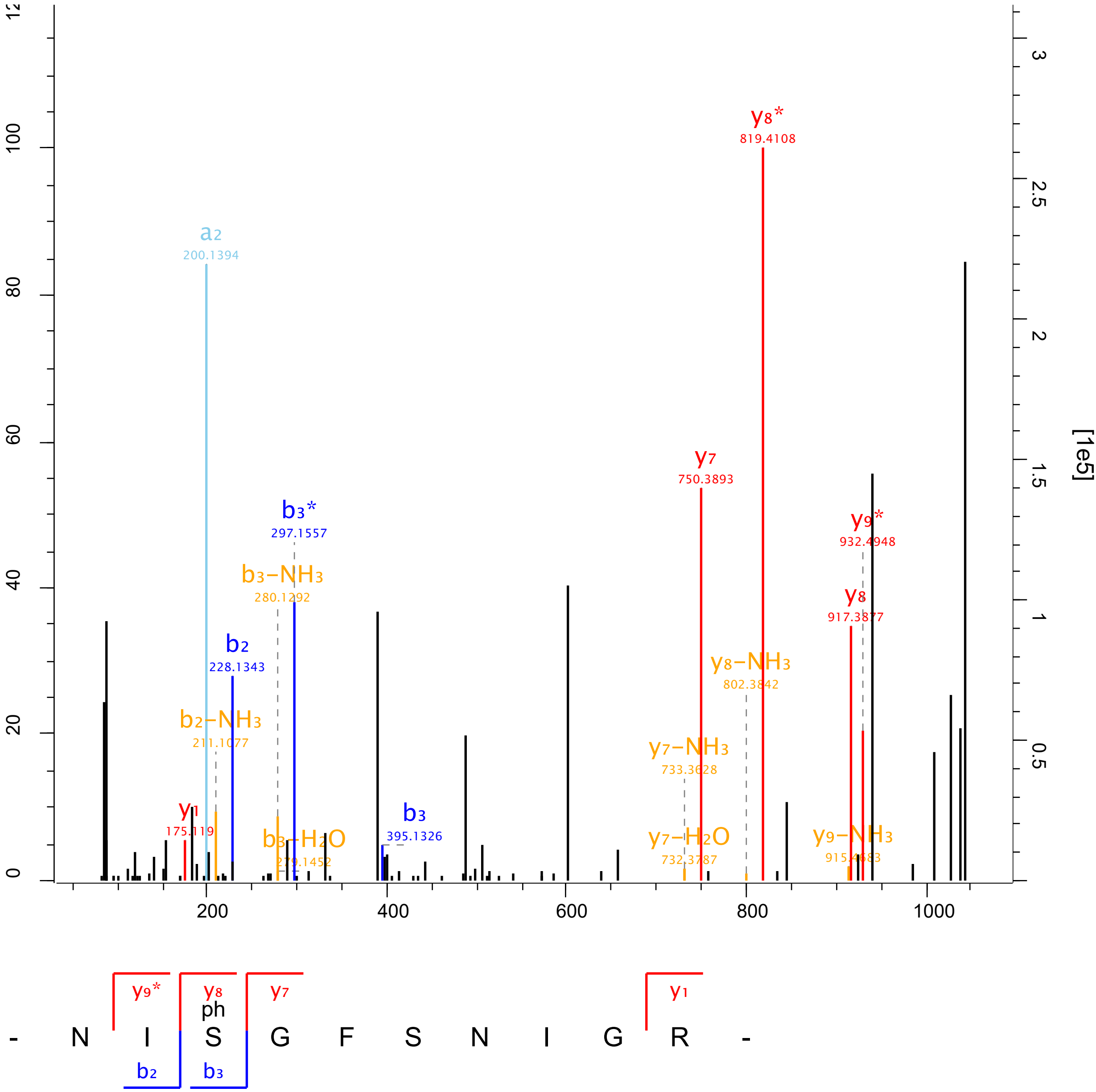

Raw file	Scan	Method	Score	m/z
sys_00_3short	20825	FTMS; HCD	123.14	754.8

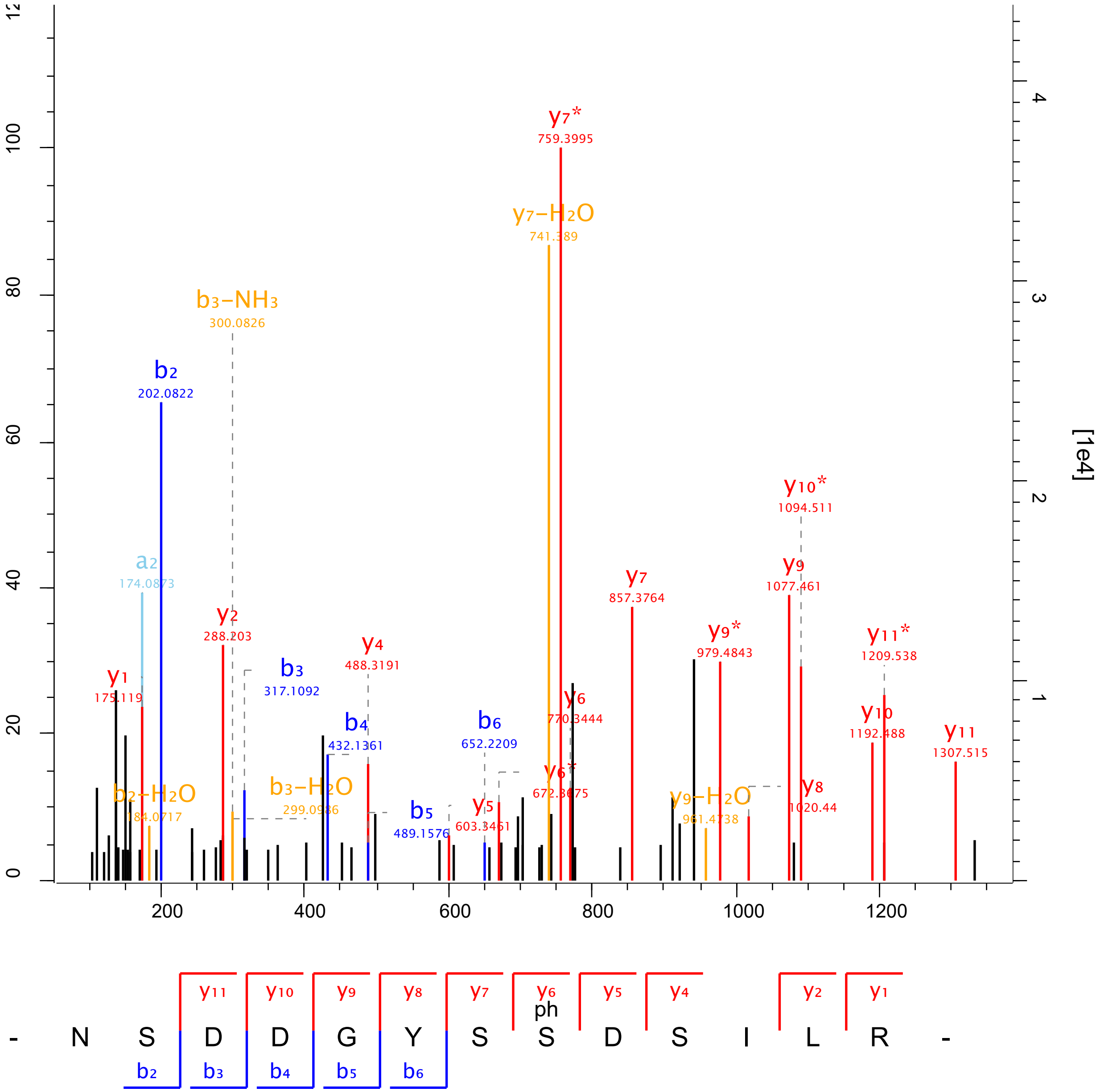

Raw file	Scan	Method	Score	m/z
sys_00_3short	21140	FTMS; HCD	71.15	543.27

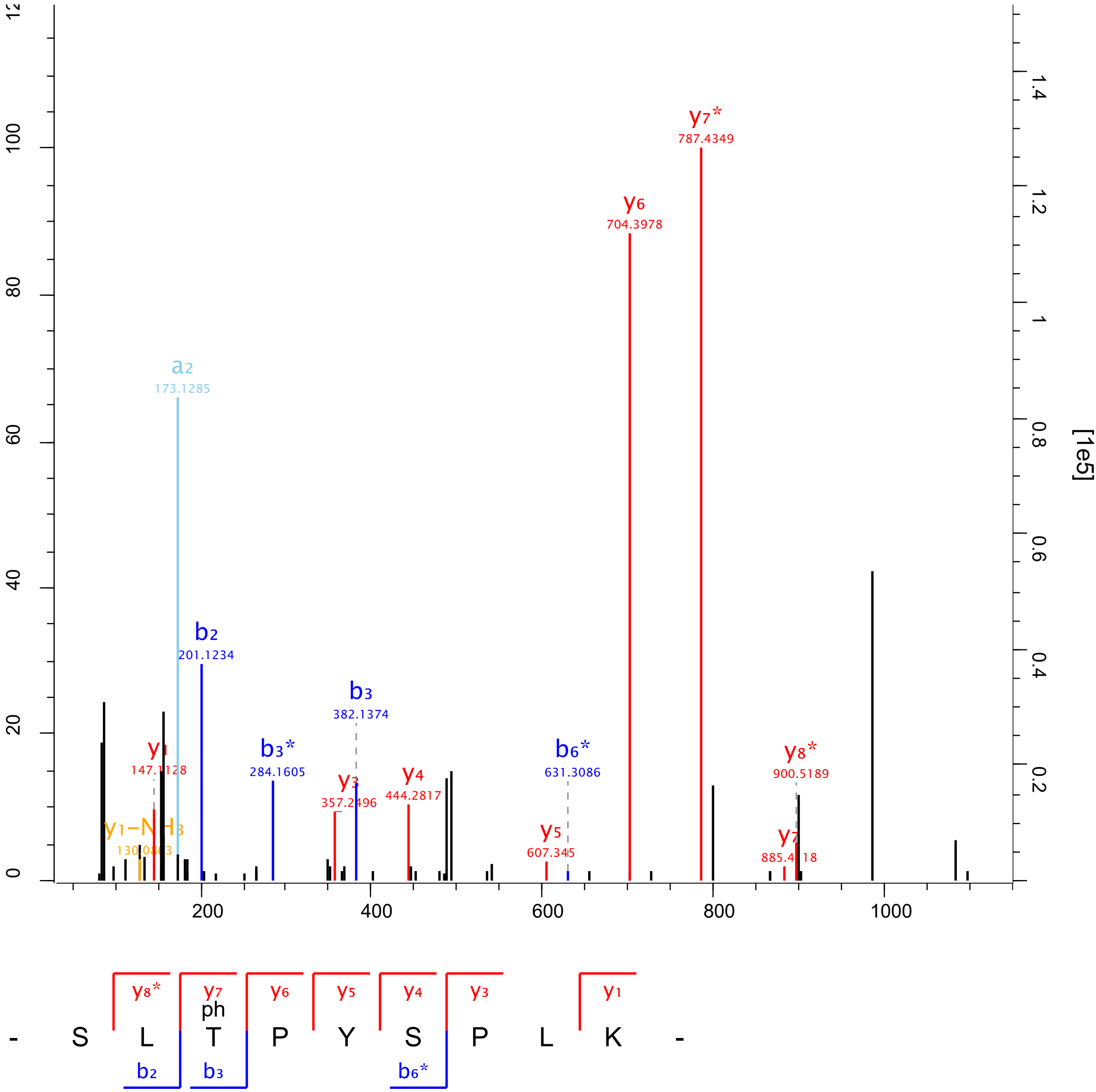

Raw file	Scan	Method	Score	m/z
sys_00_3short	21501	FTMS; HCD	109.72	869.86

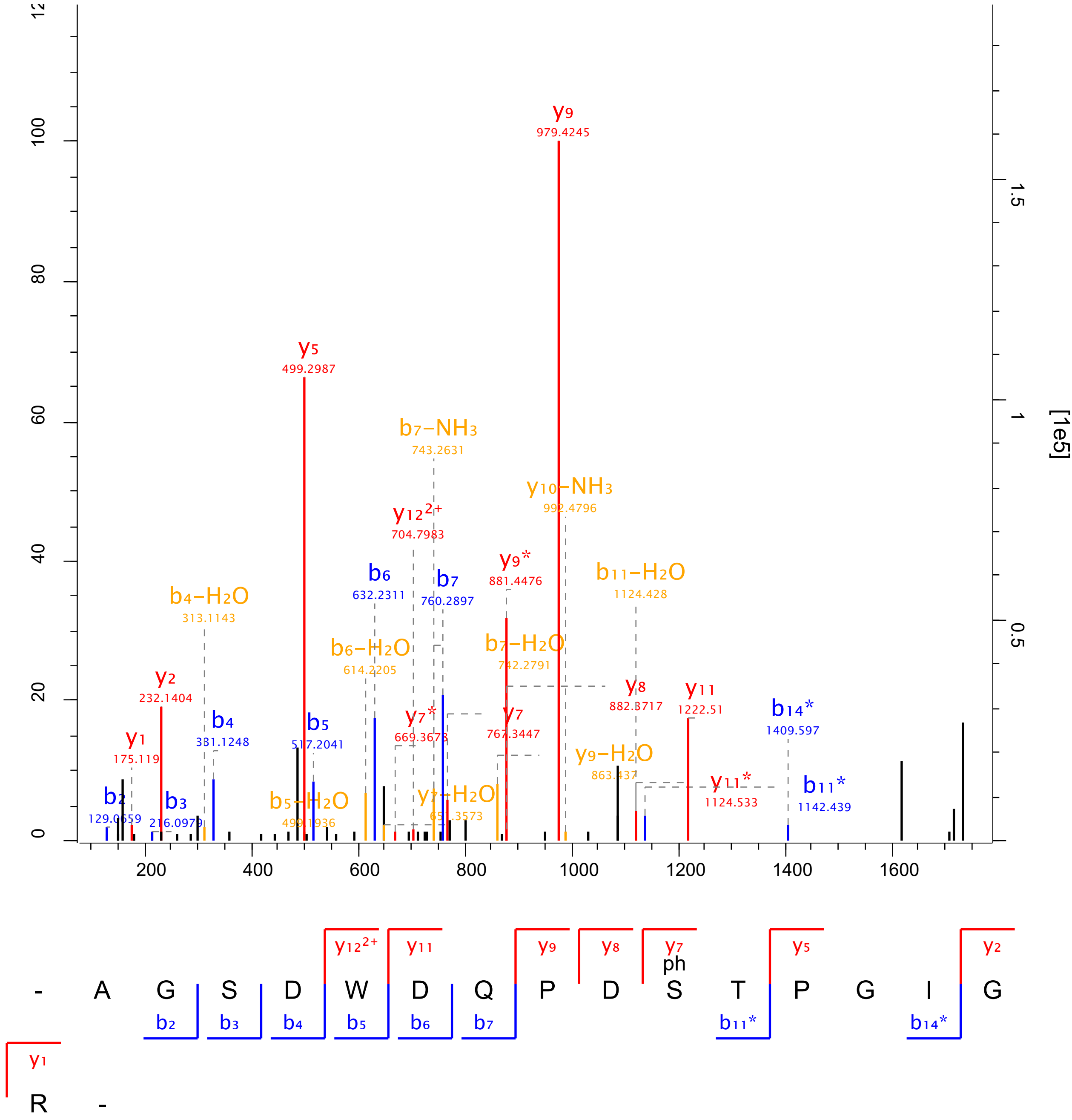

Raw file	Scan	Method	Score	m/z
sys_00_3short	21749	FTMS; HCD	49.59	732.32

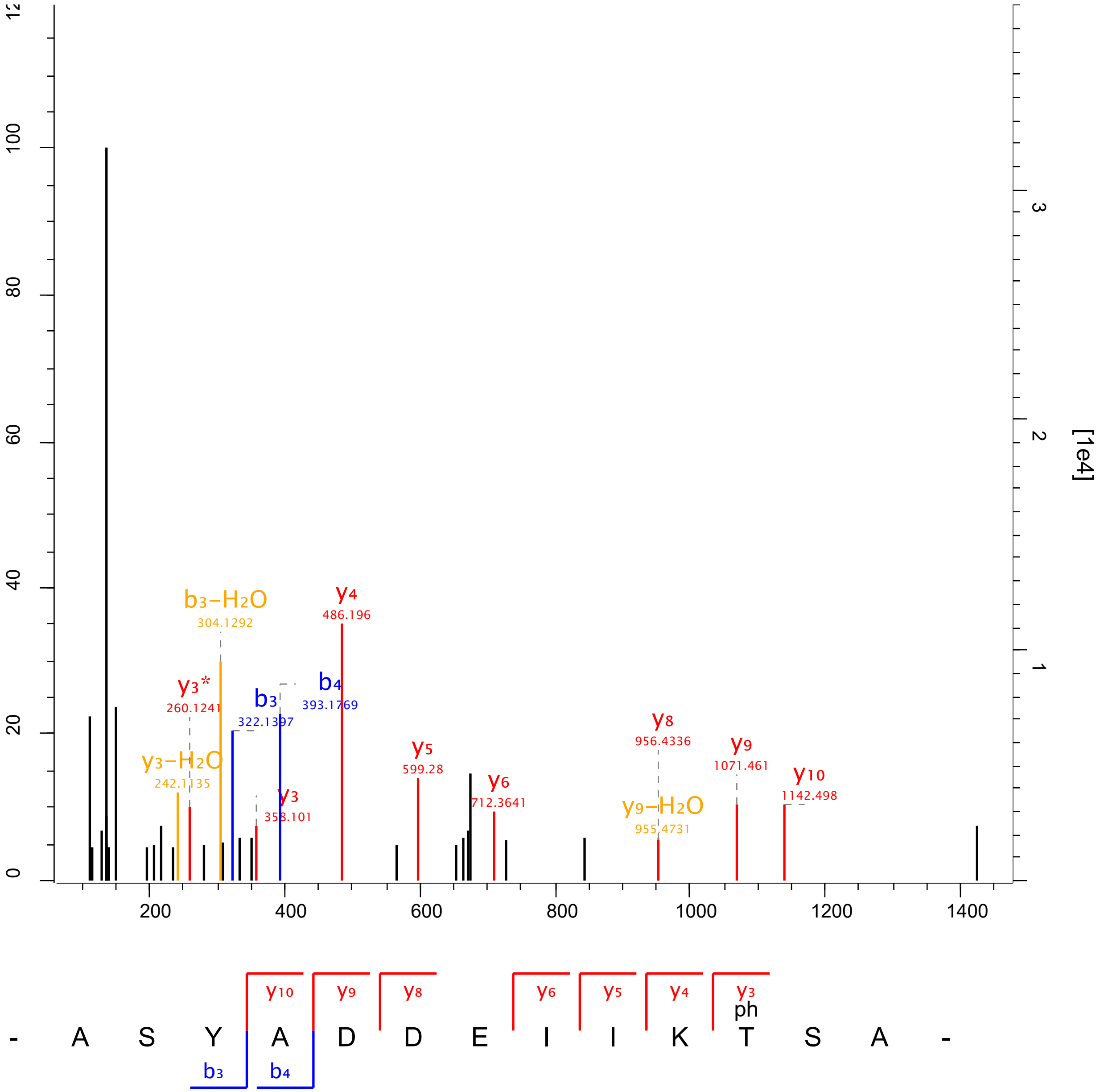

Raw file	Scan	Method	Score	m/z
sys_00_3short	21801	FTMS; HCD	73.44	481.26

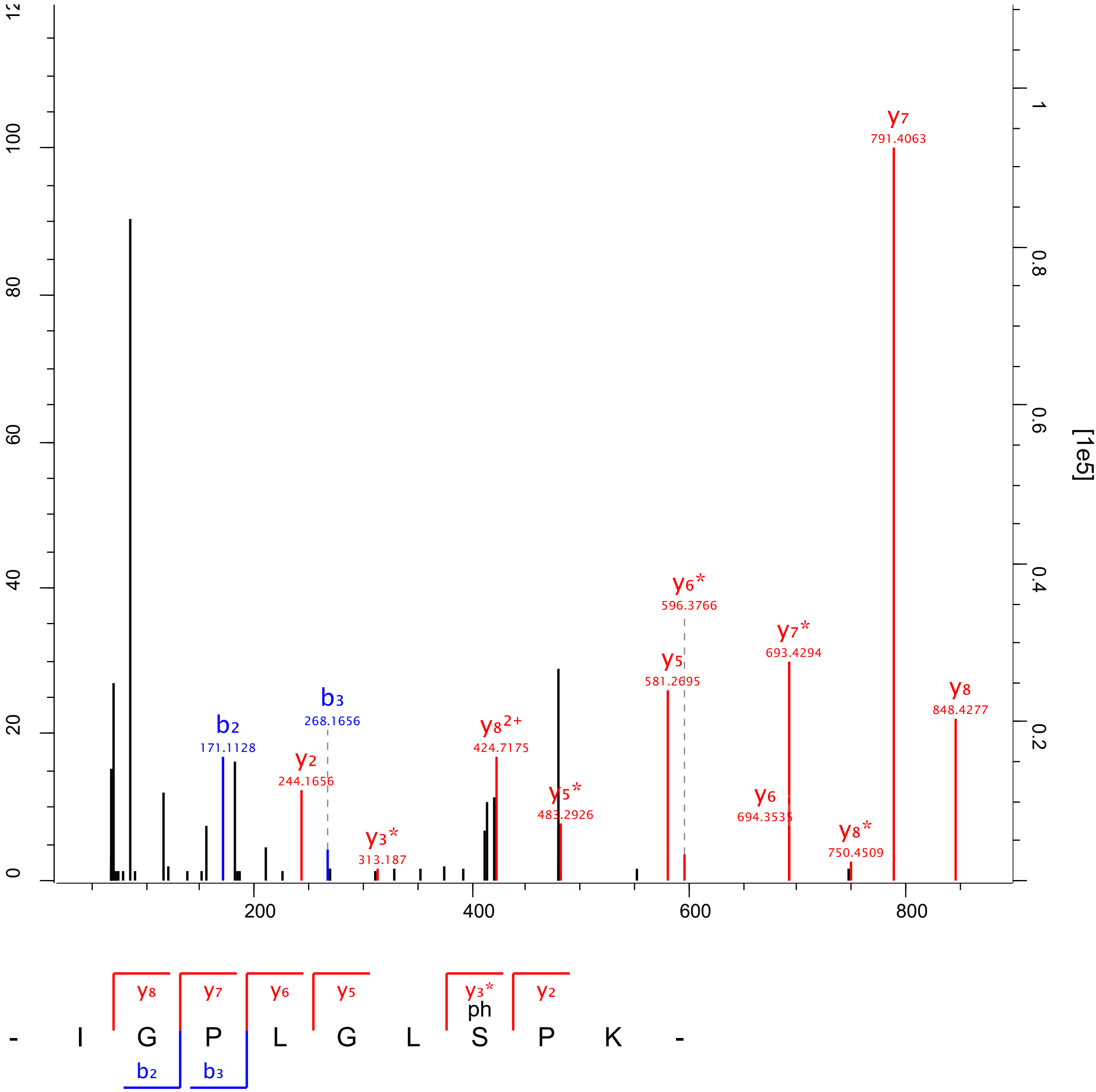

Raw file	Scan	Method	Score	m/z
sys_00_3short	21929	FTMS; HCD	203.2	691.79

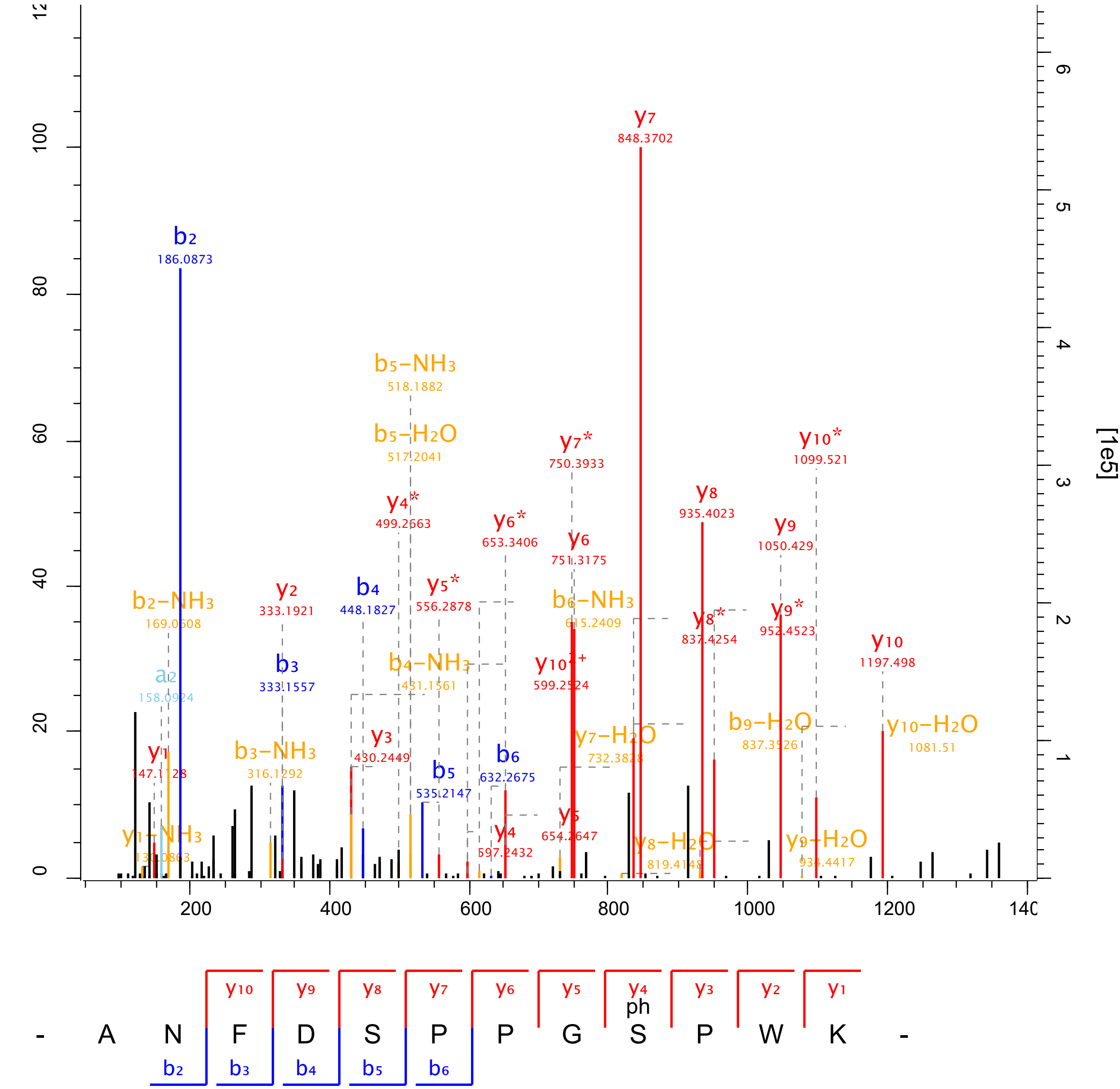

Raw file	Scan	Method	Score	m/z
sys_00_3short	21997	FTMS; HCD	42.31	667.3

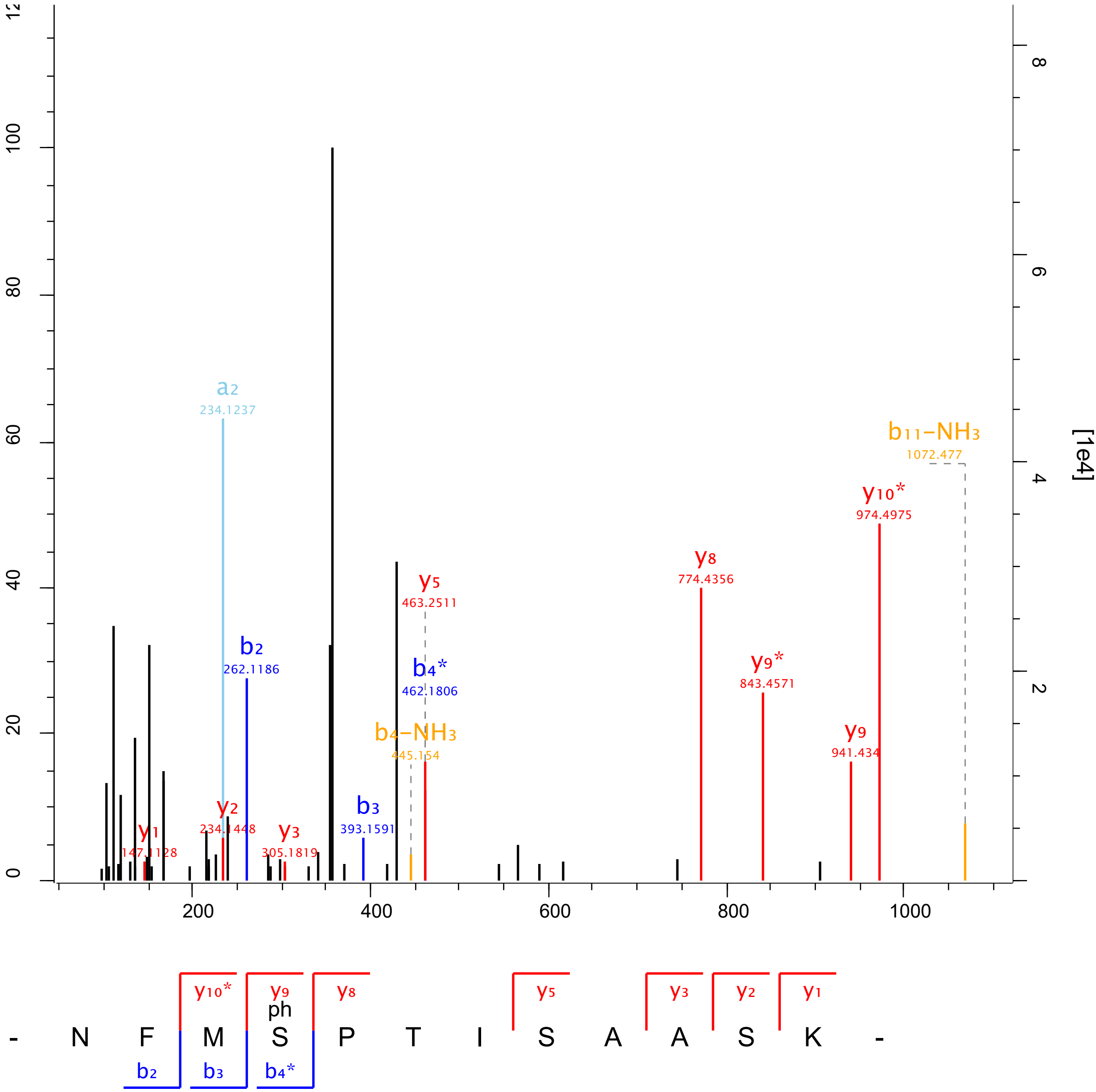

Raw file	Scan	Method	Score	m/z
sys_00_3short	22149	FTMS; HCD	63.69	732.82

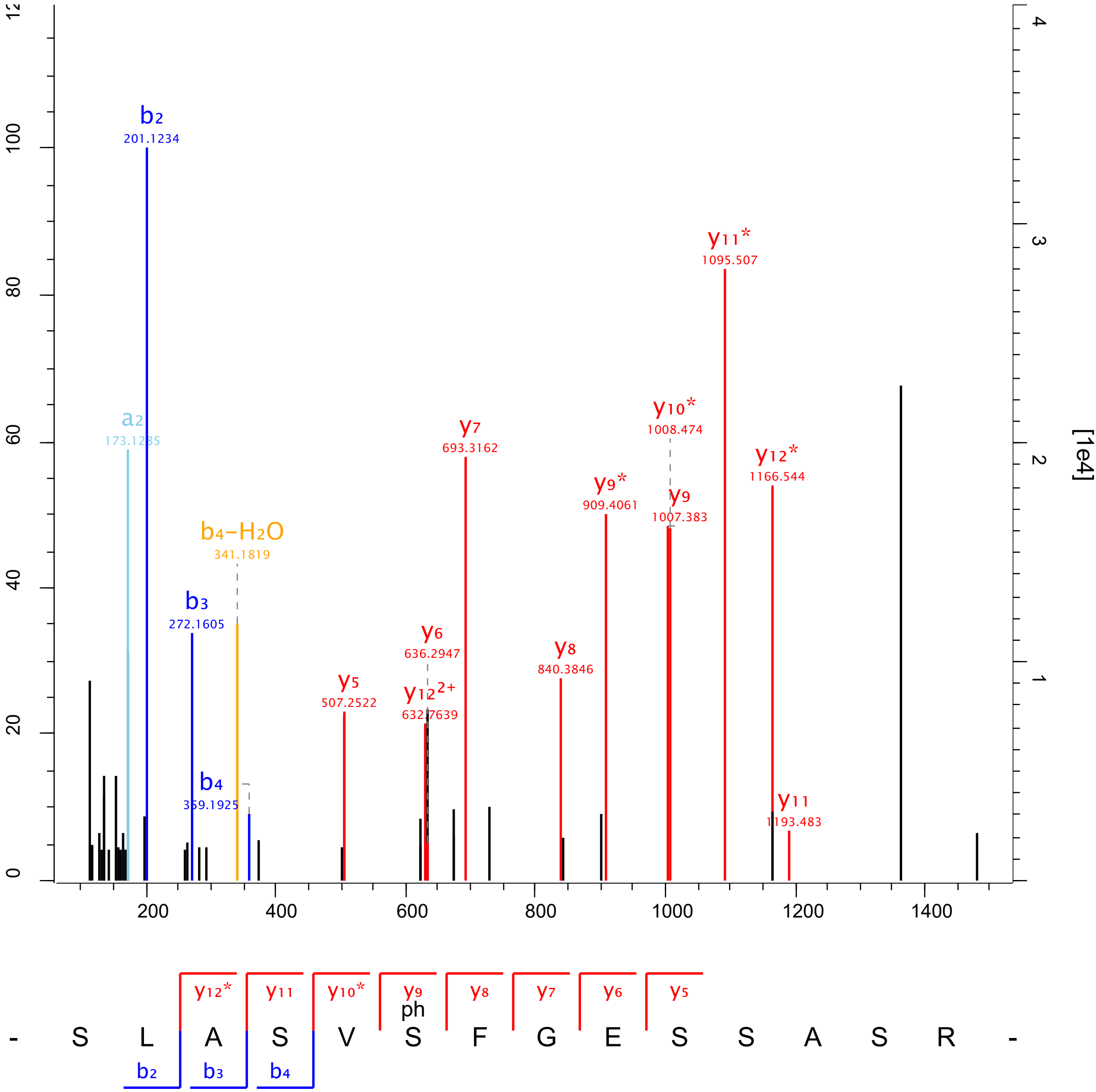

Raw file	Scan	Method	Score	m/z
sys_00_3short	22190	FTMS; HCD	83.75	767.3

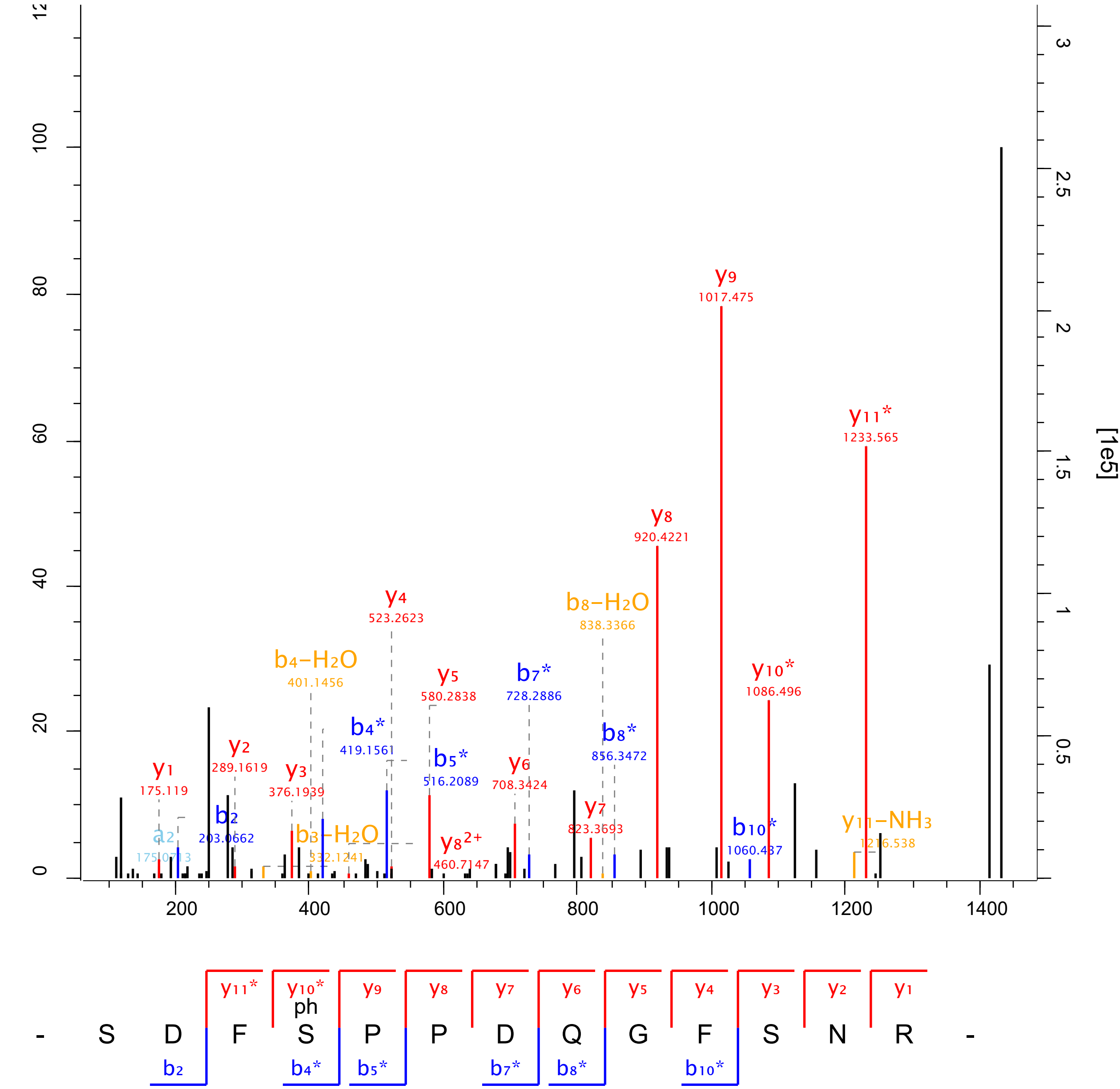

Raw file	Scan	Method	Score	m/z
sys_00_3short	22242	FTMS; HCD	117.37	819.87

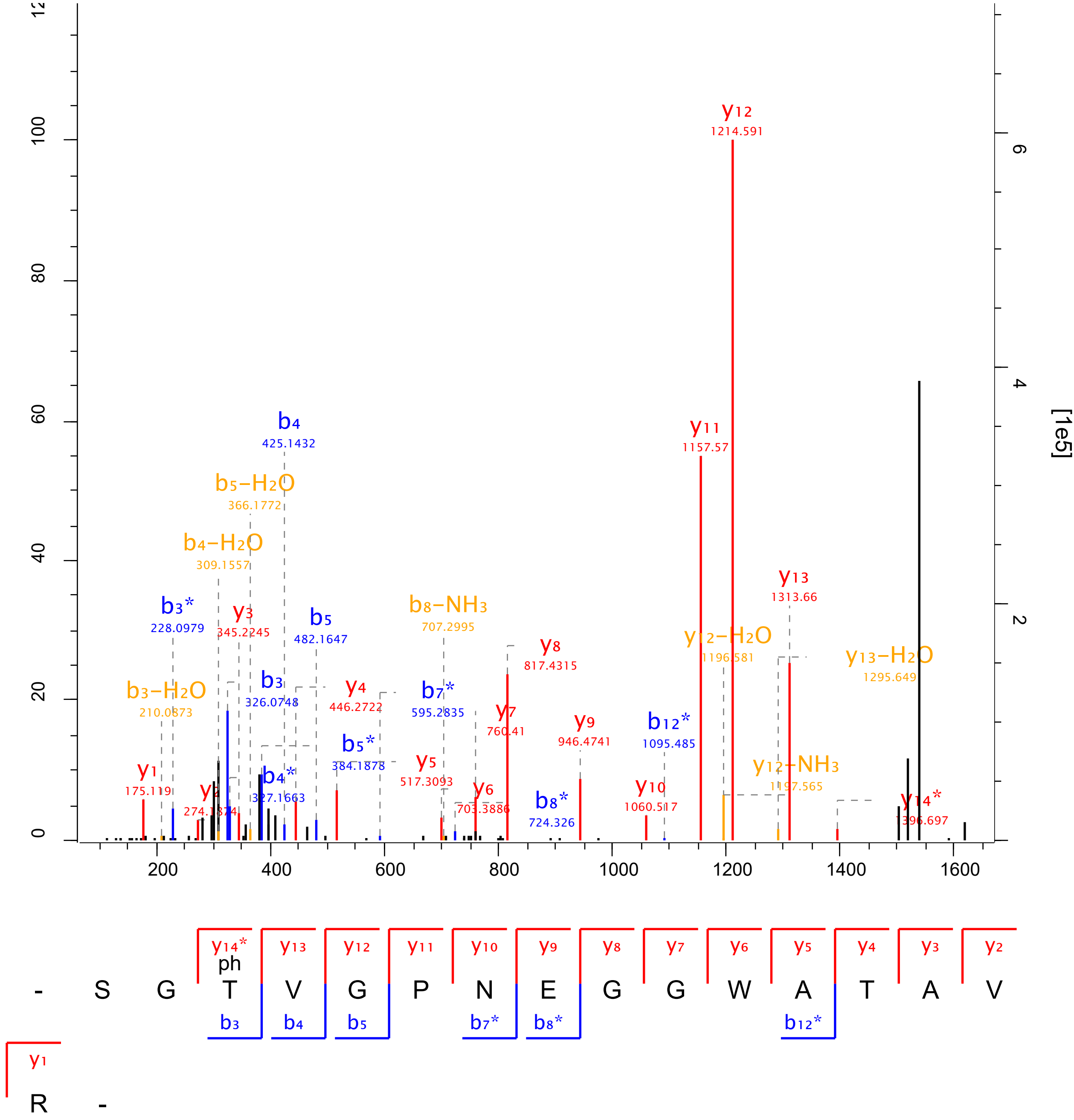

Raw file	Scan	Method	Score	m/z
sys_00_3short	22337	FTMS; HCD	40.28	799.87

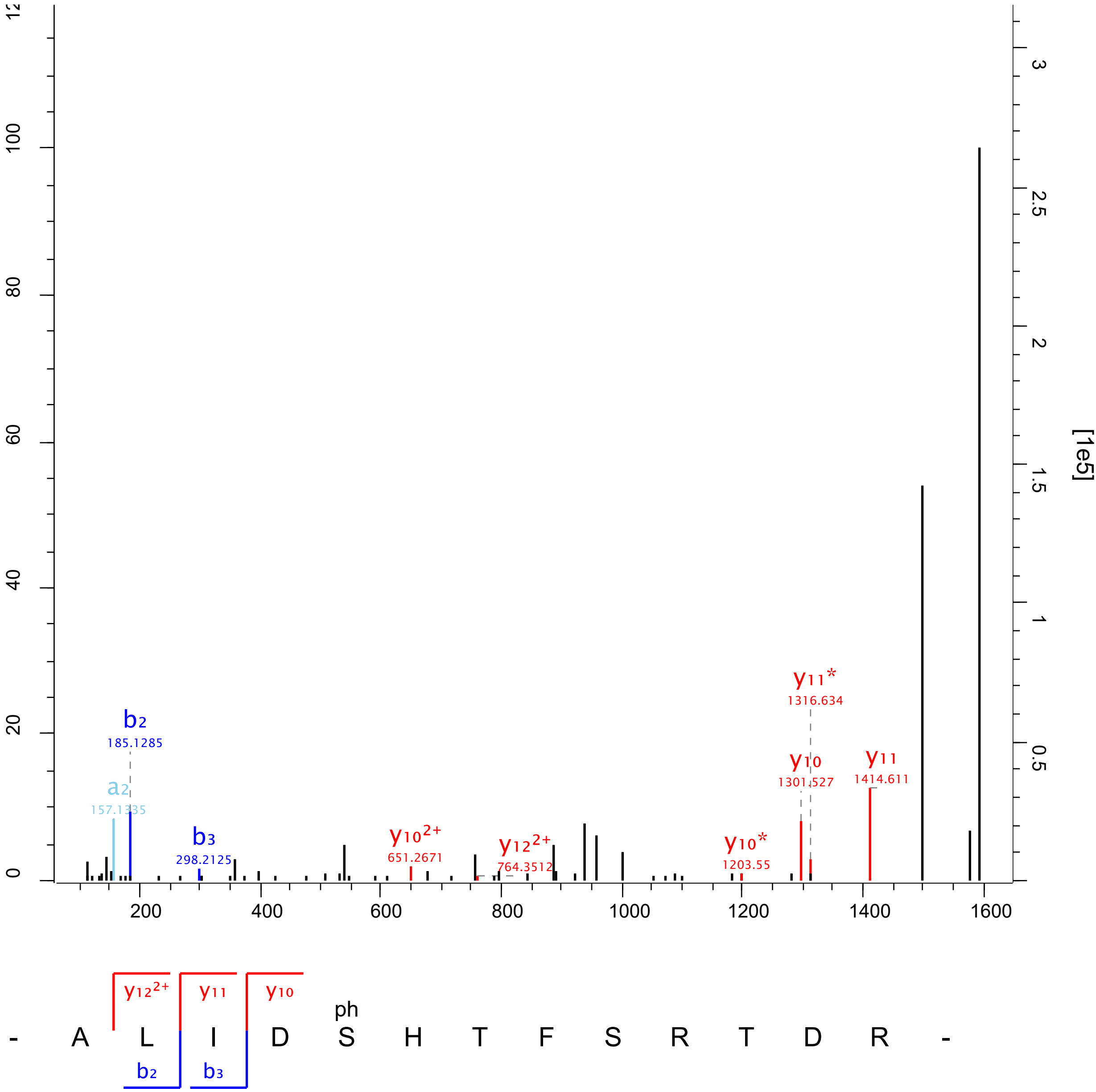

Raw file	Scan	Method	Score	m/z
sys_00_3short	22454	FTMS; HCD	225.29	782.89

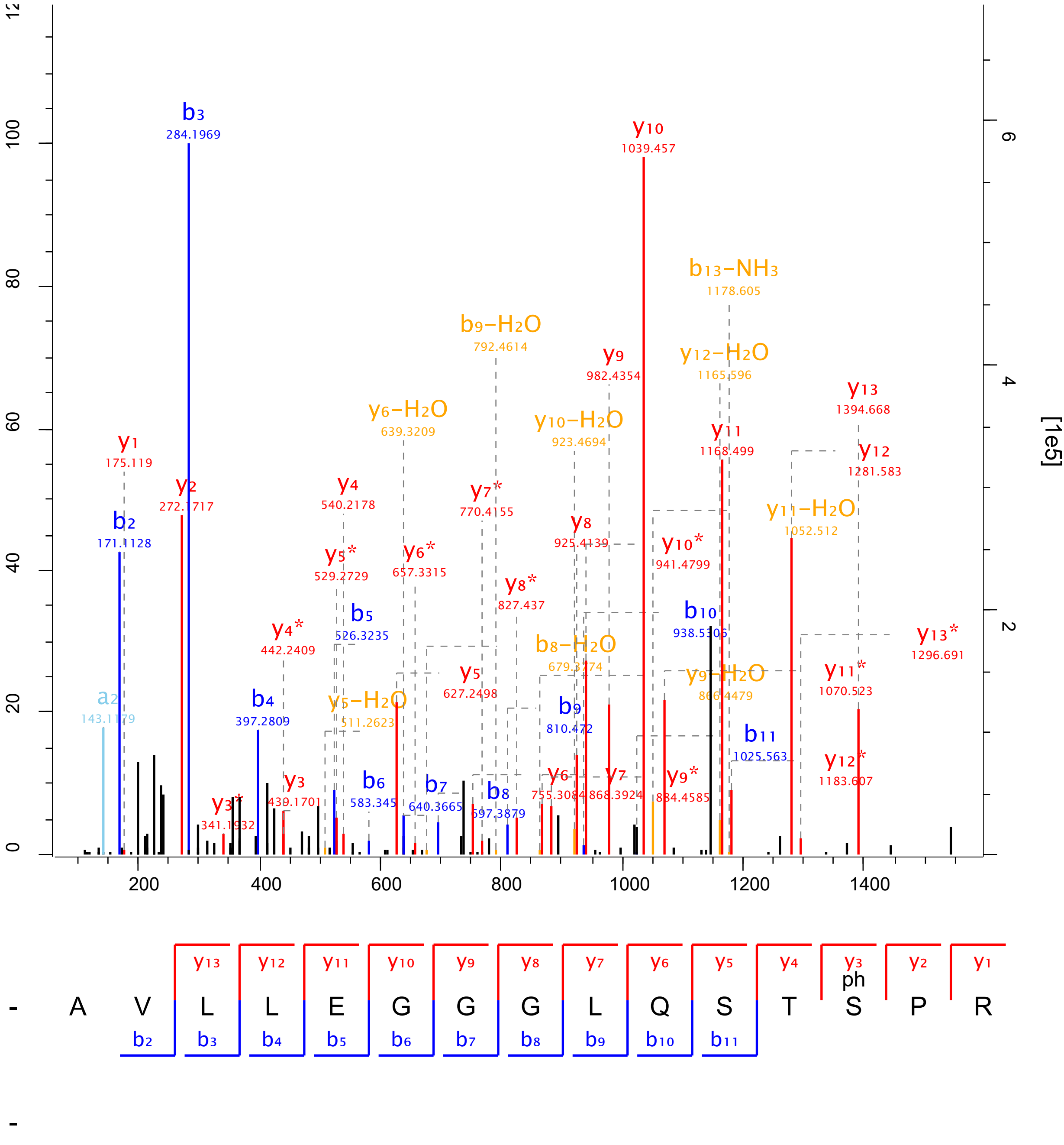

Raw file	Scan	Method	Score	m/z
sys_00_3short	22513	FTMS; HCD	65.42	795.33

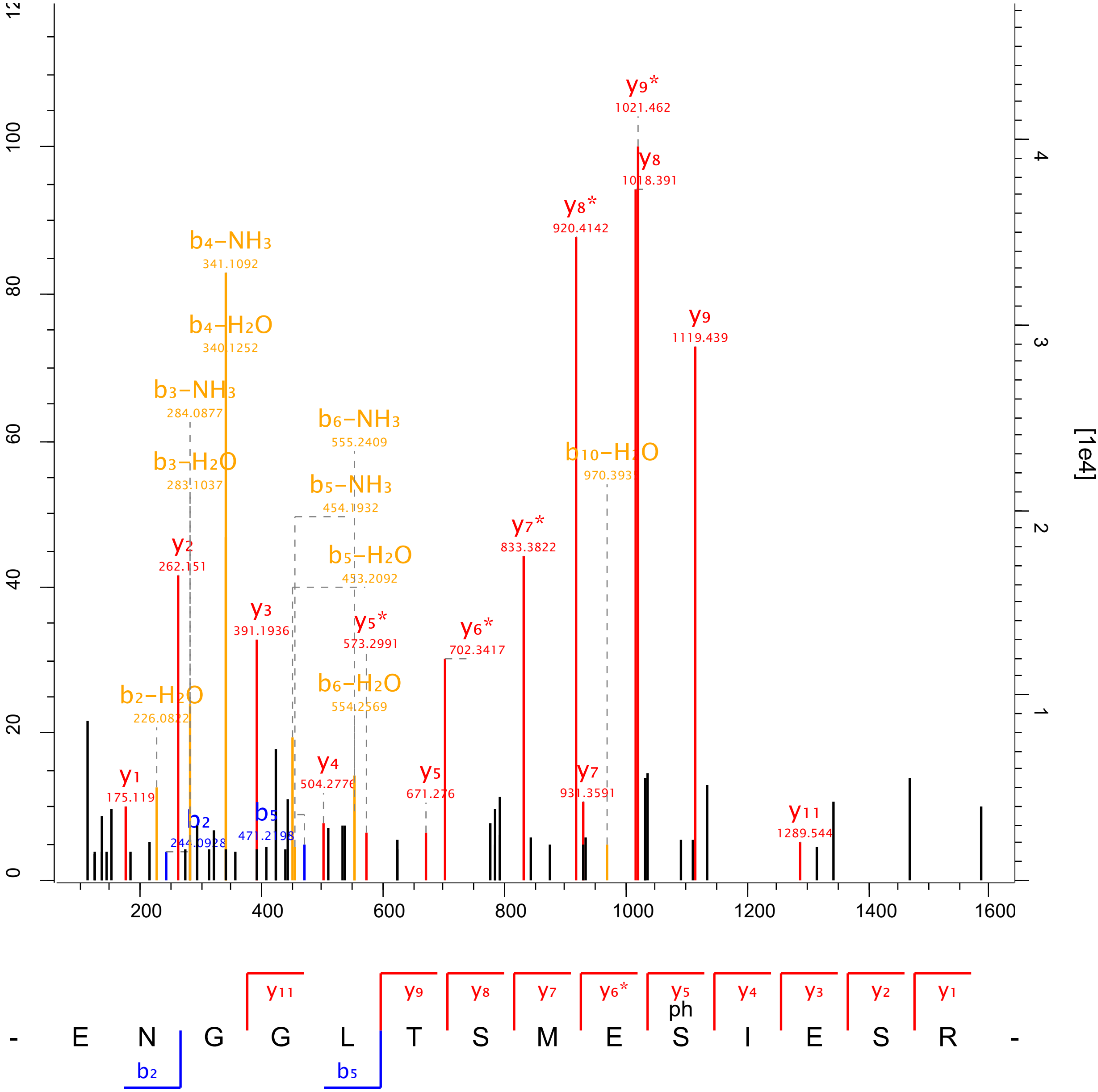

Raw file	Scan	Method	Score	m/z
sys_00_3short	22647	FTMS; HCD	159.18	715.3

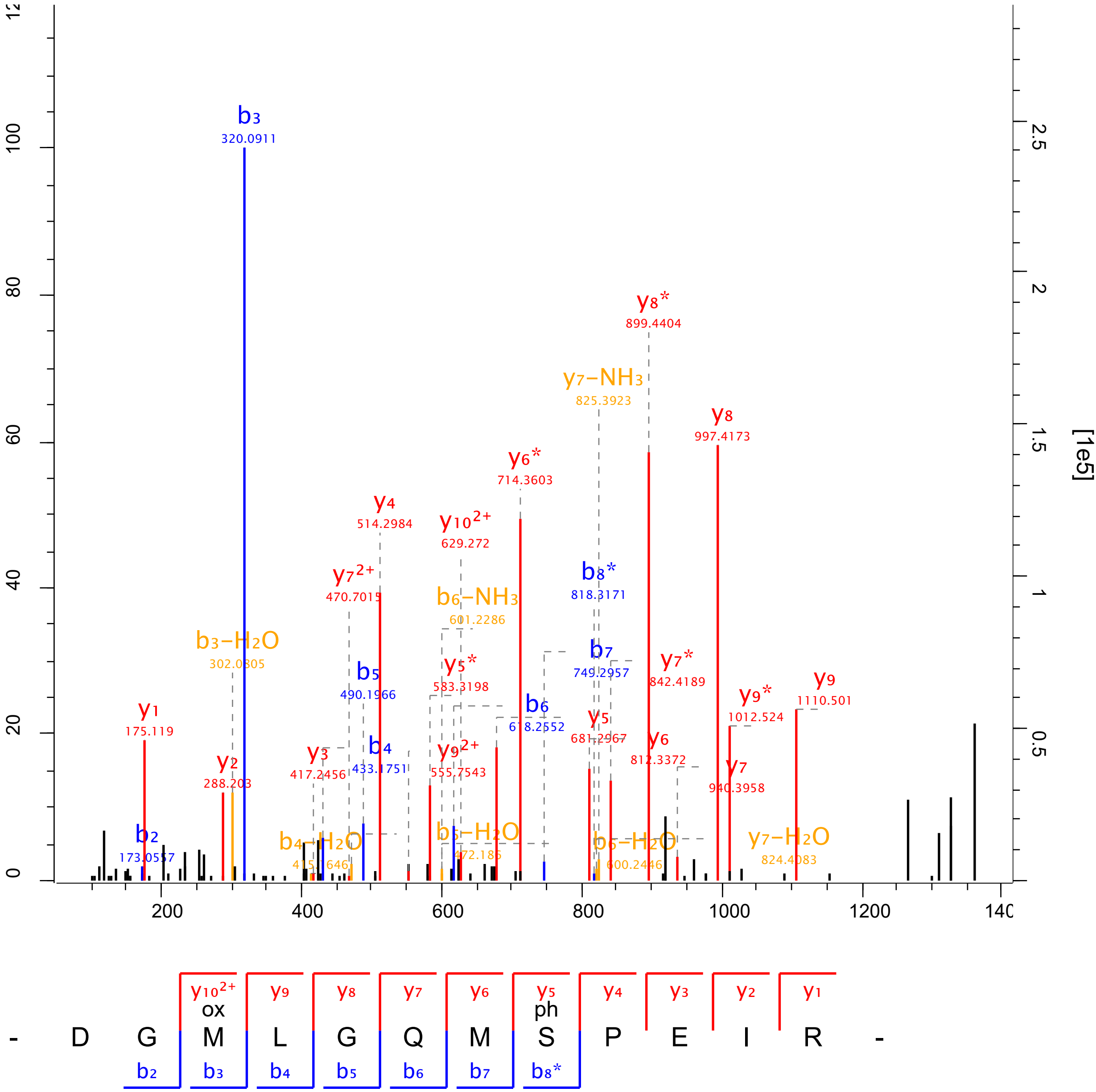

Raw file	Scan	Method	Score	m/z
sys_00_3short	22870	FTMS; HCD	159.64	713.83

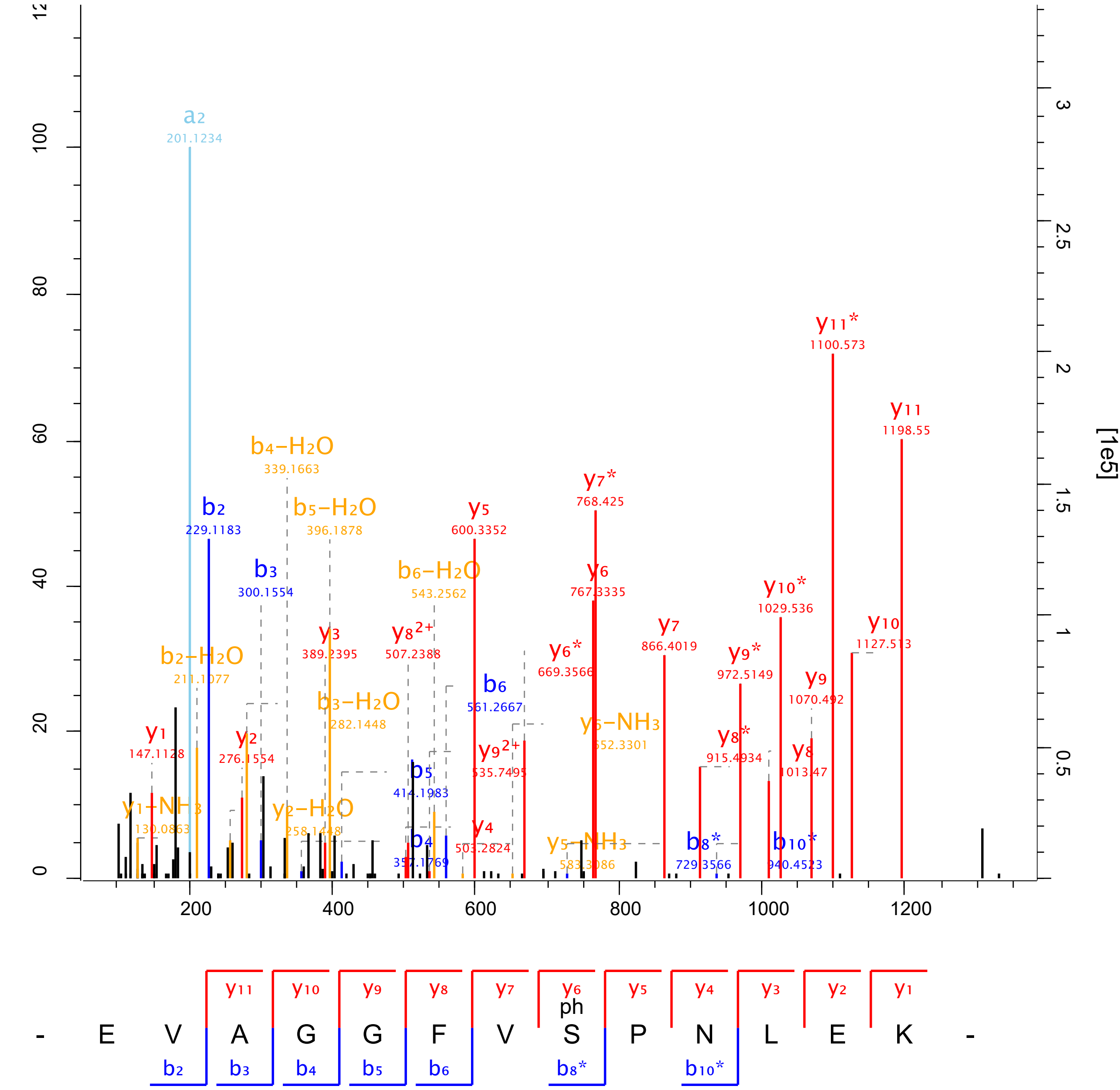

Raw file	Scan	Method	Score	m/z
sys_00_3short	23033	FTMS; HCD	119.18	938.37

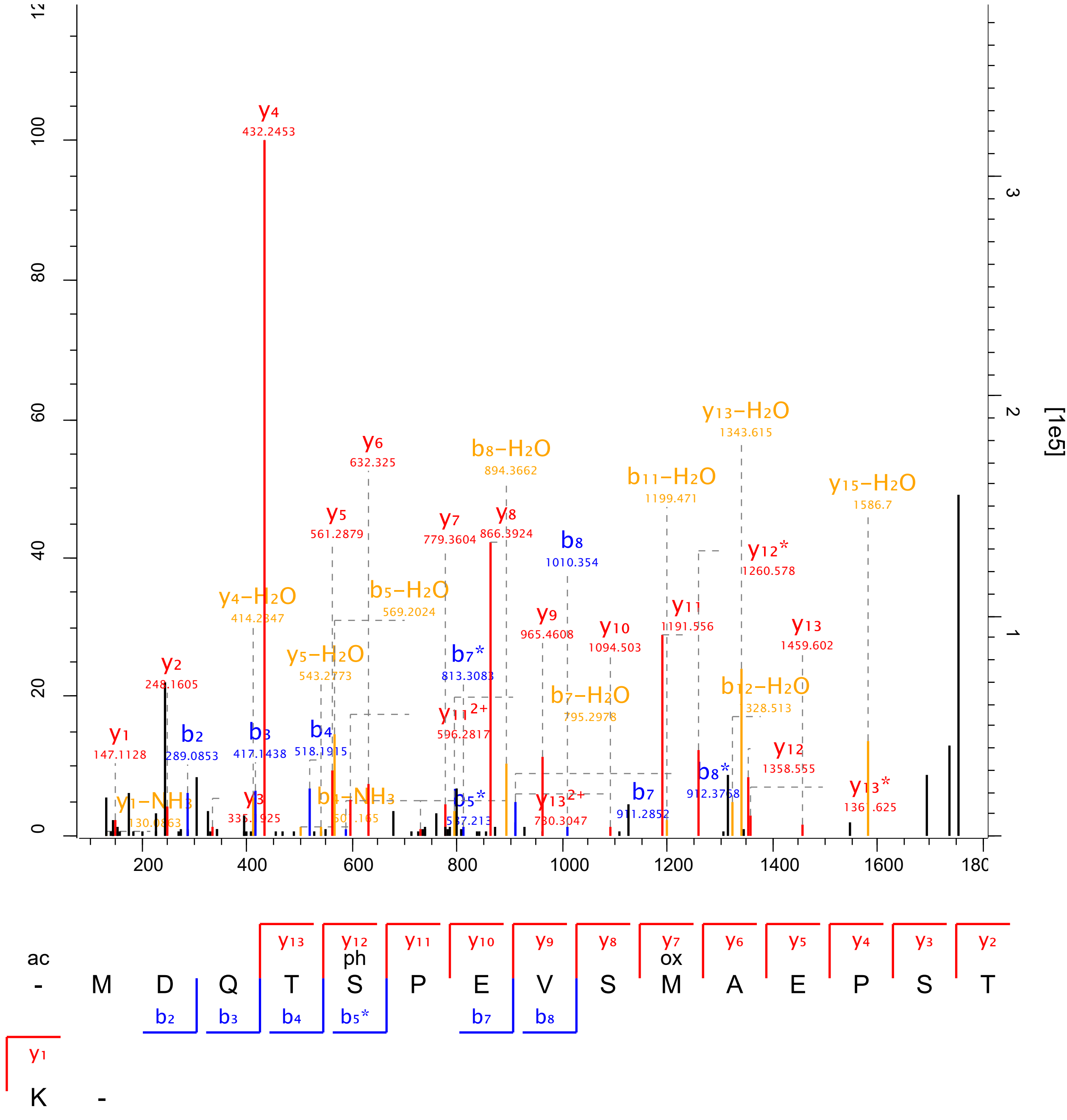

Raw file	Scan	Method	Score	m/z
sys_00_3short	23098	FTMS; HCD	43.76	700.81

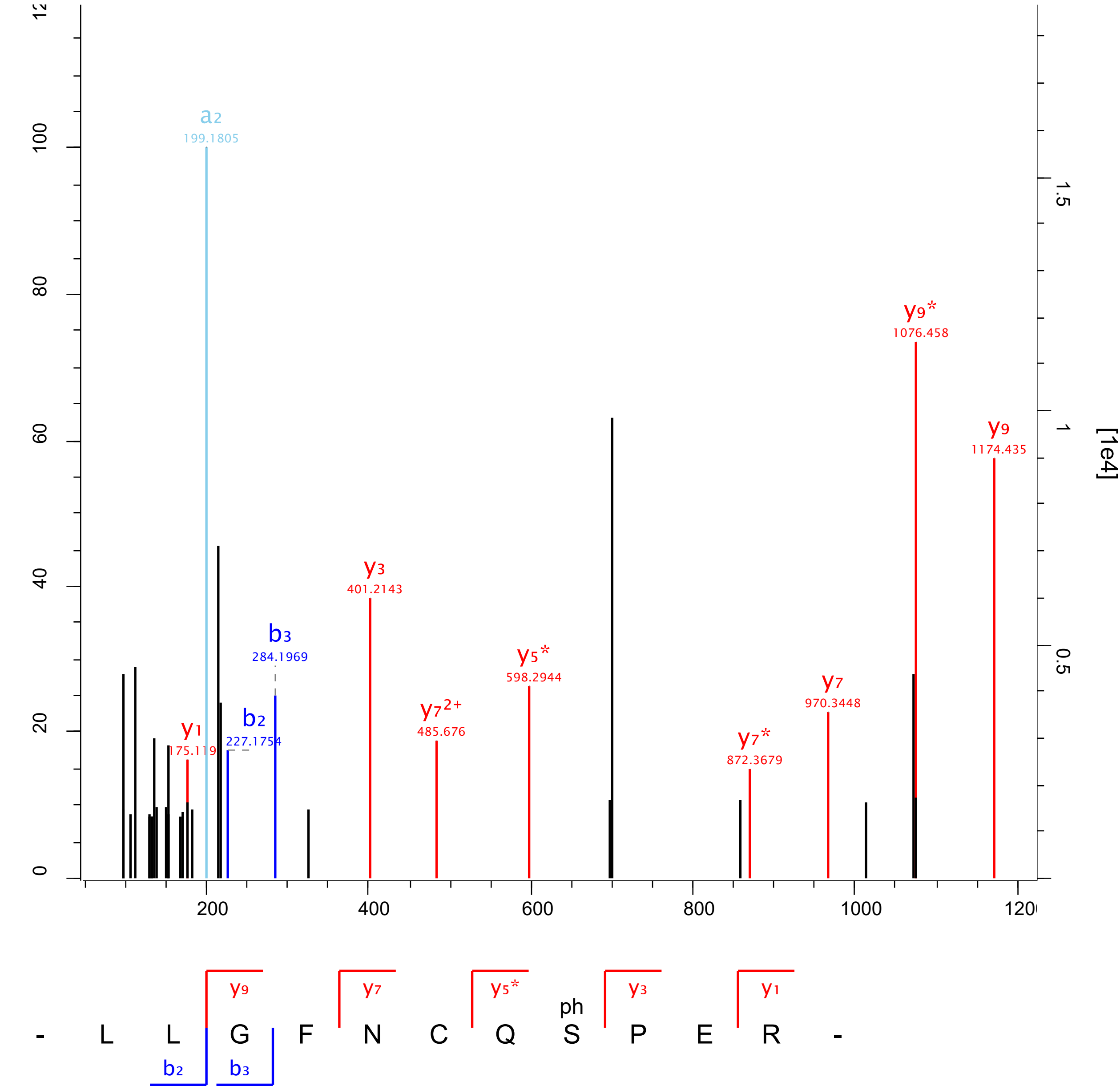

Raw file	Scan	Method	Score	m/z
sys_00_3short	23160	FTMS; HCD	69.92	598.3

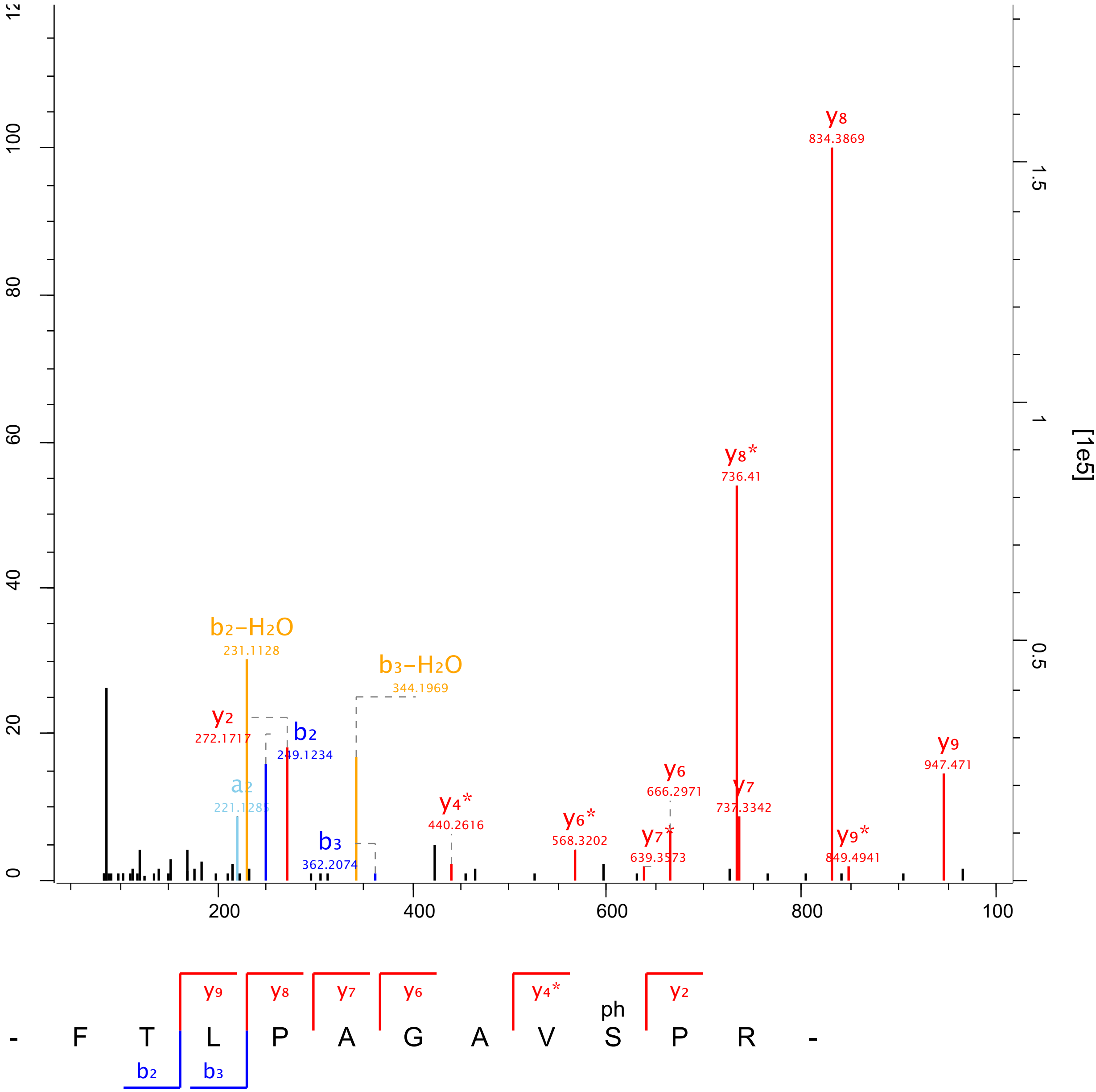

Raw file	Scan	Method	Score	m/z
sys_00_3short	24112	FTMS; HCD	147.12	794.37

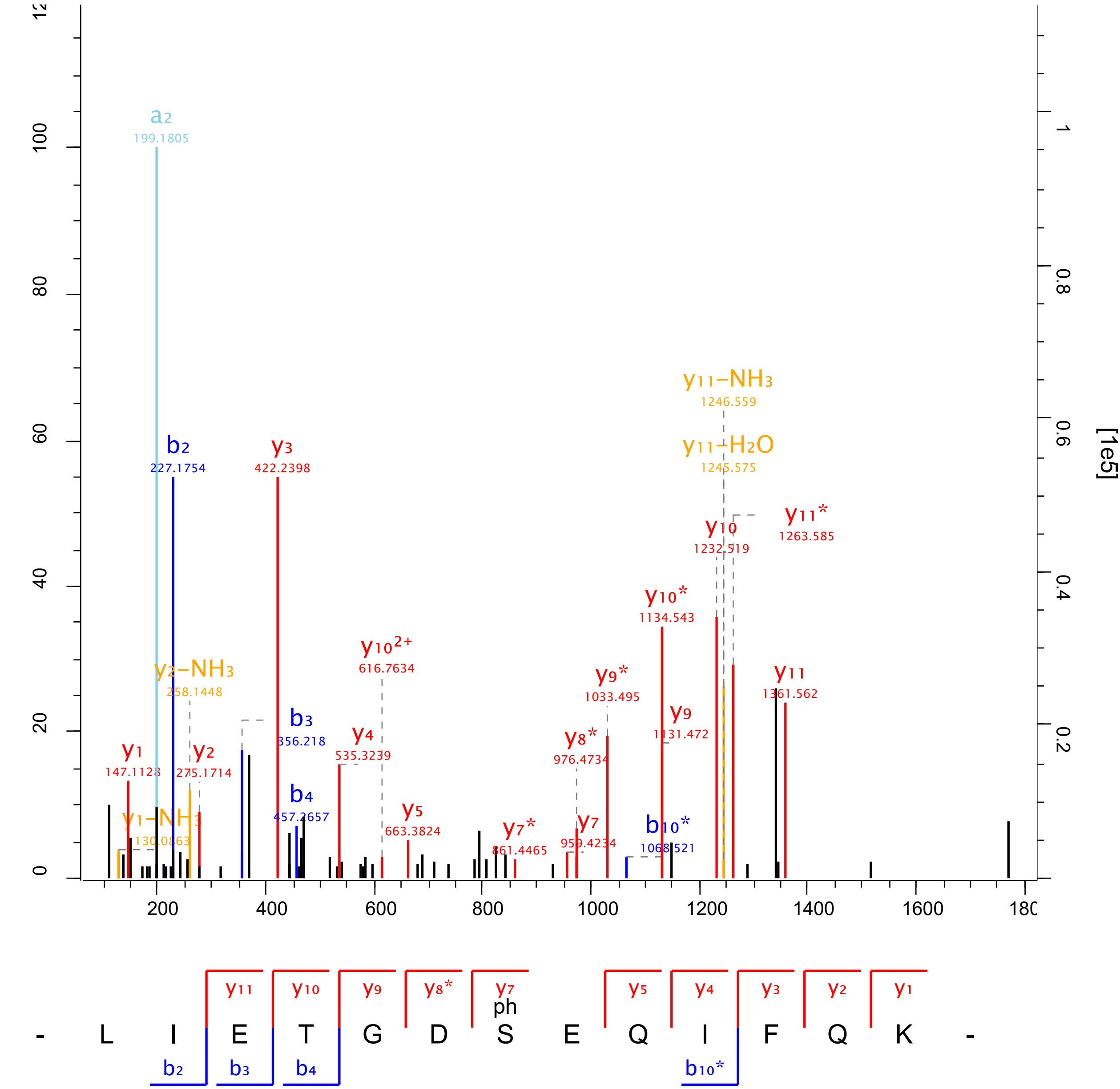

Raw file	Scan	Method	Score	m/z
sys_00_3short	24350	FTMS; HCD	142.45	1049.93

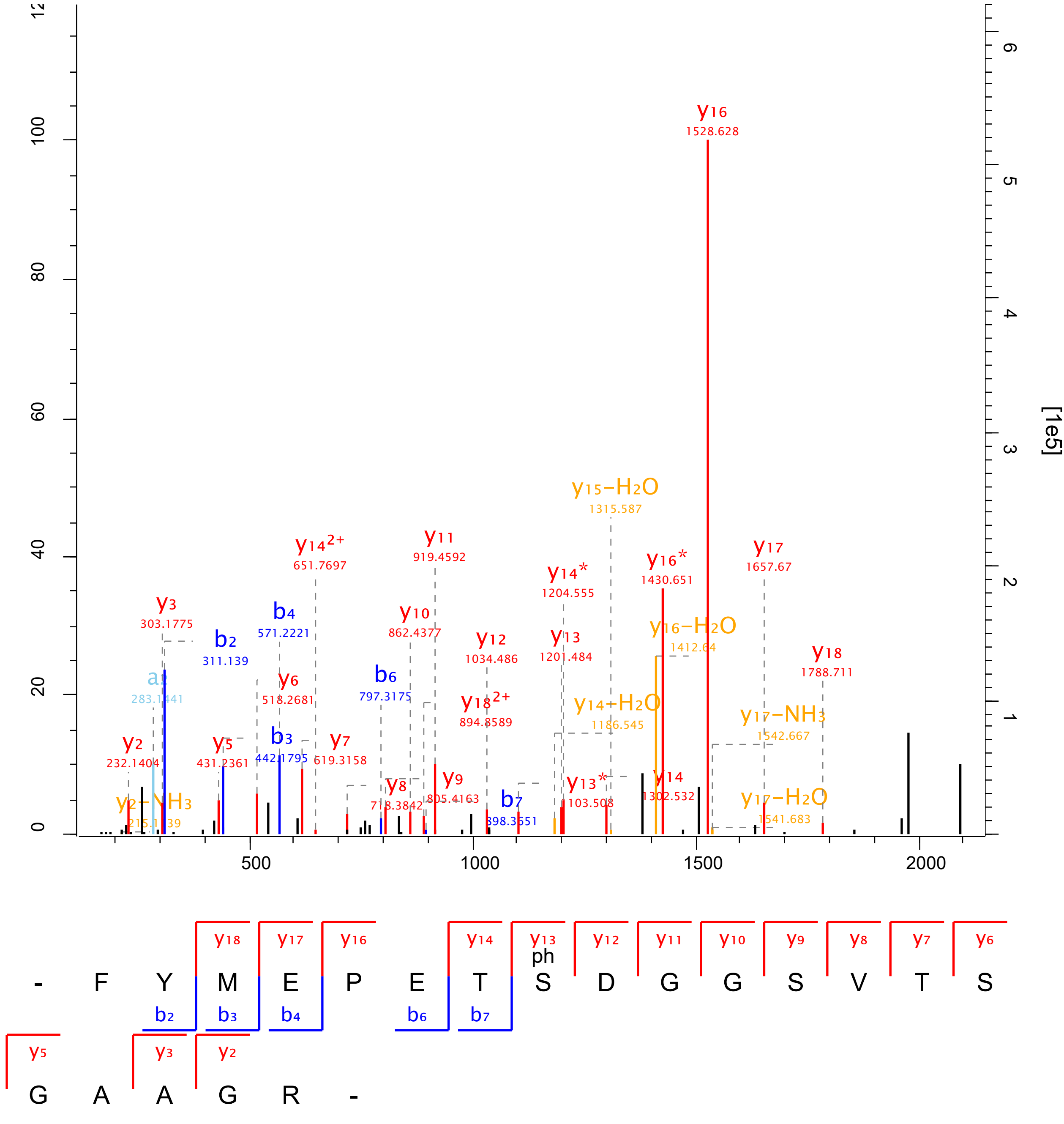

Raw file	Scan	Method	Score	m/z
sys_00_3short	24723	FTMS; HCD	180.27	706.34

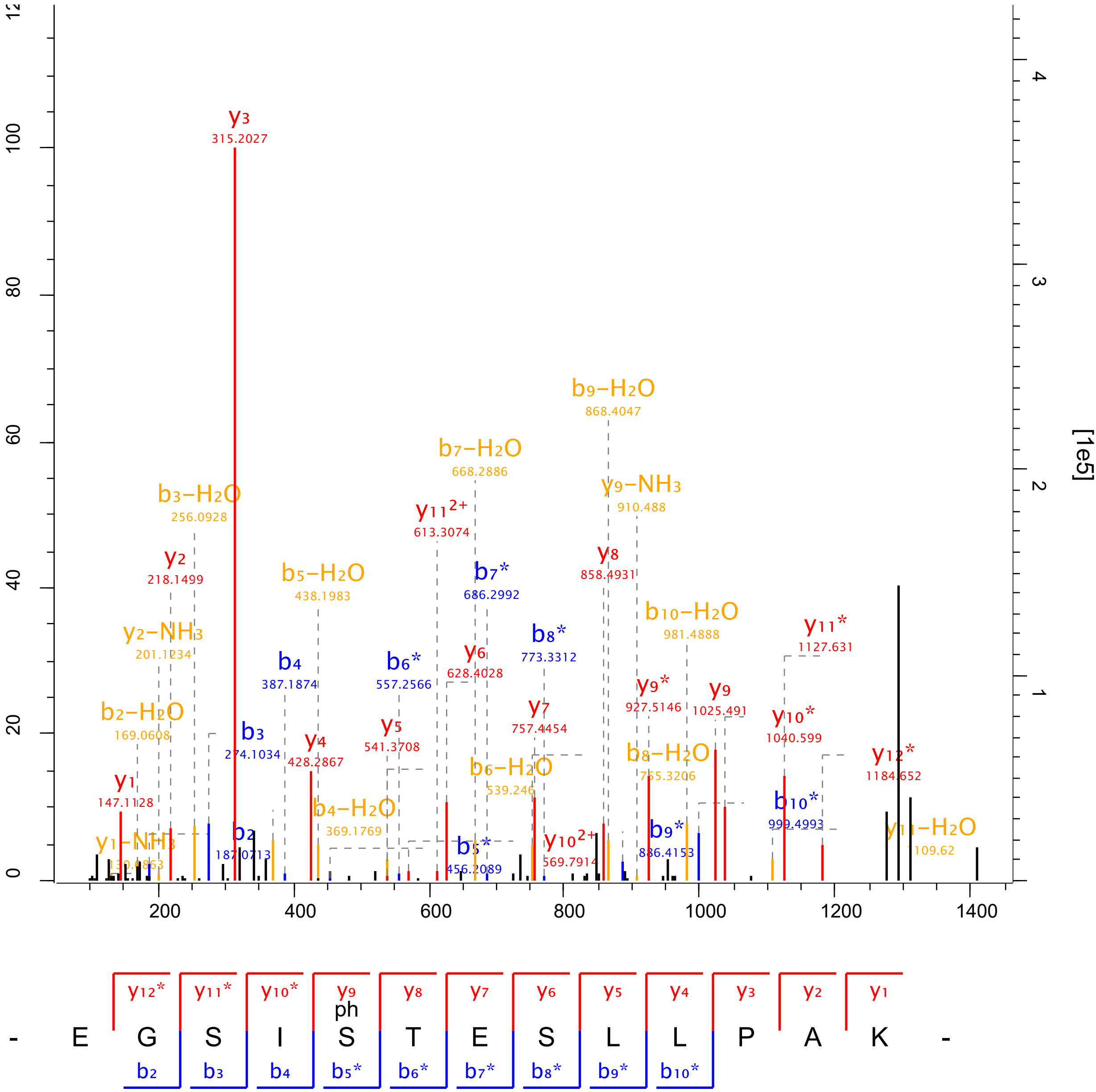

Raw file	Scan	Method	Score	m/z
sys_00_3short	24865	FTMS; HCD	151.44	736.83

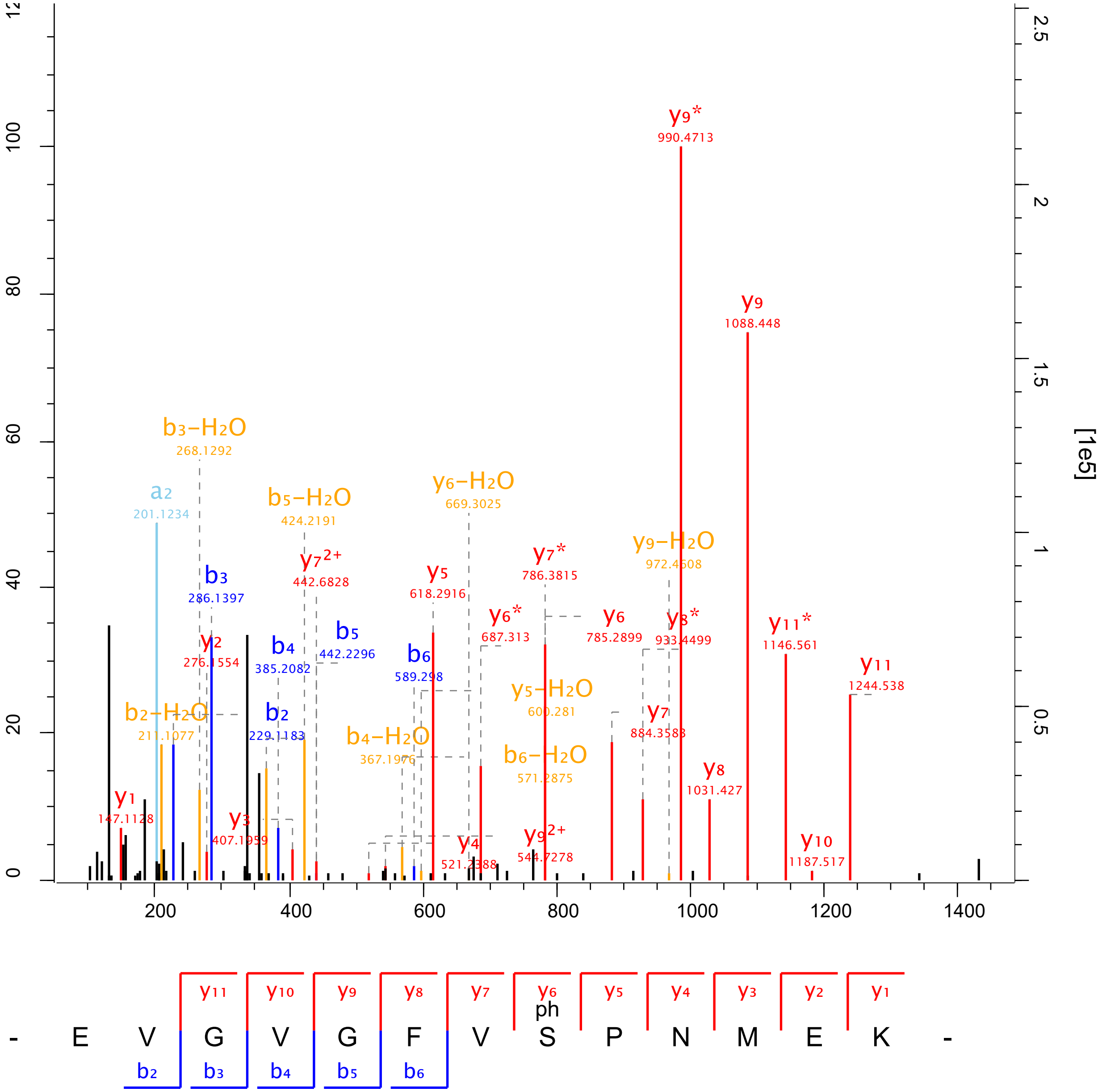

Raw file	Scan	Method	Score	m/z
sys_00_3short	24868	FTMS; HCD	116.86	683.32

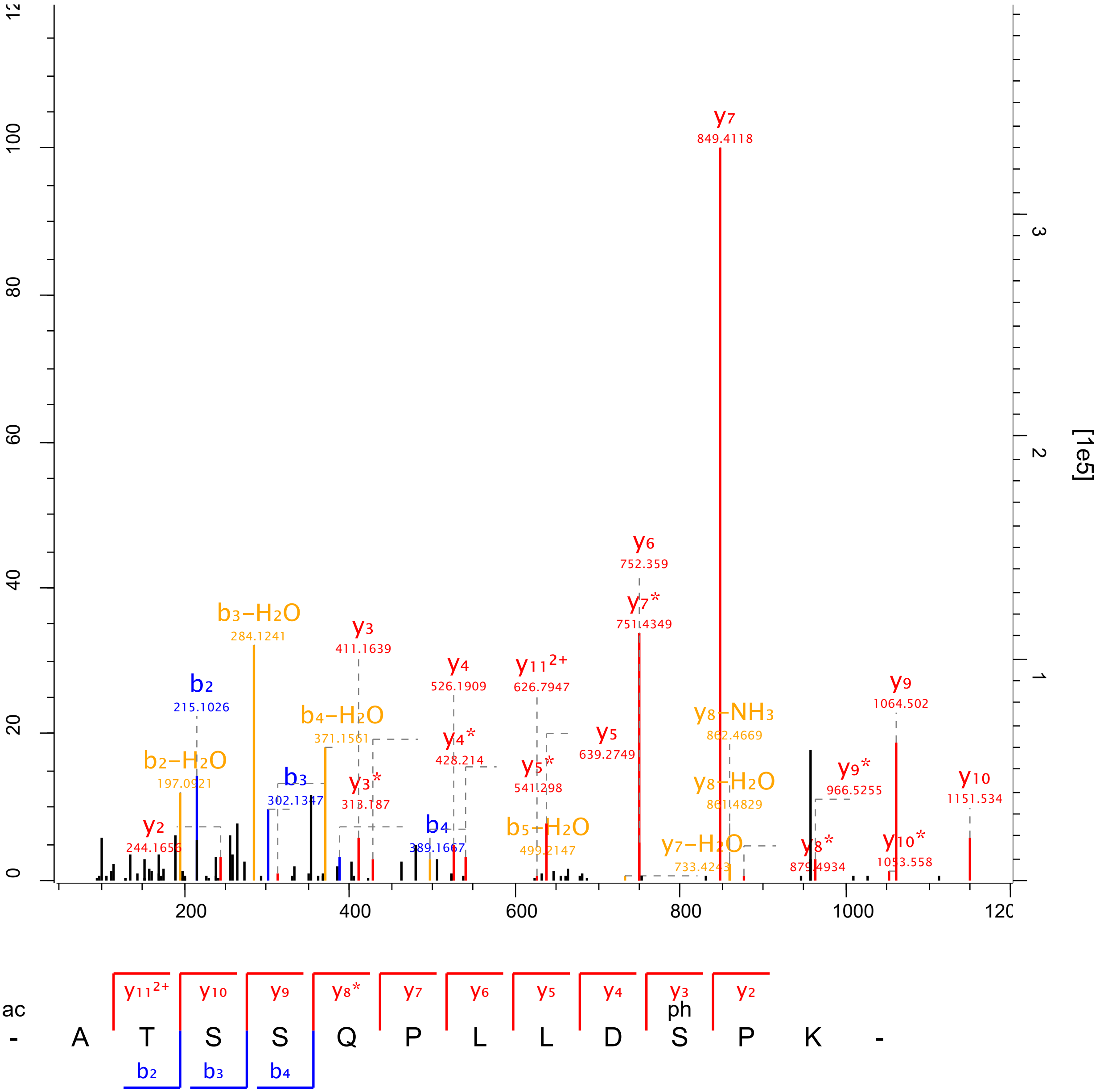

Raw file	Scan	Method	Score	m/z
sys_00_3short	24908	FTMS; HCD	58.98	831.88

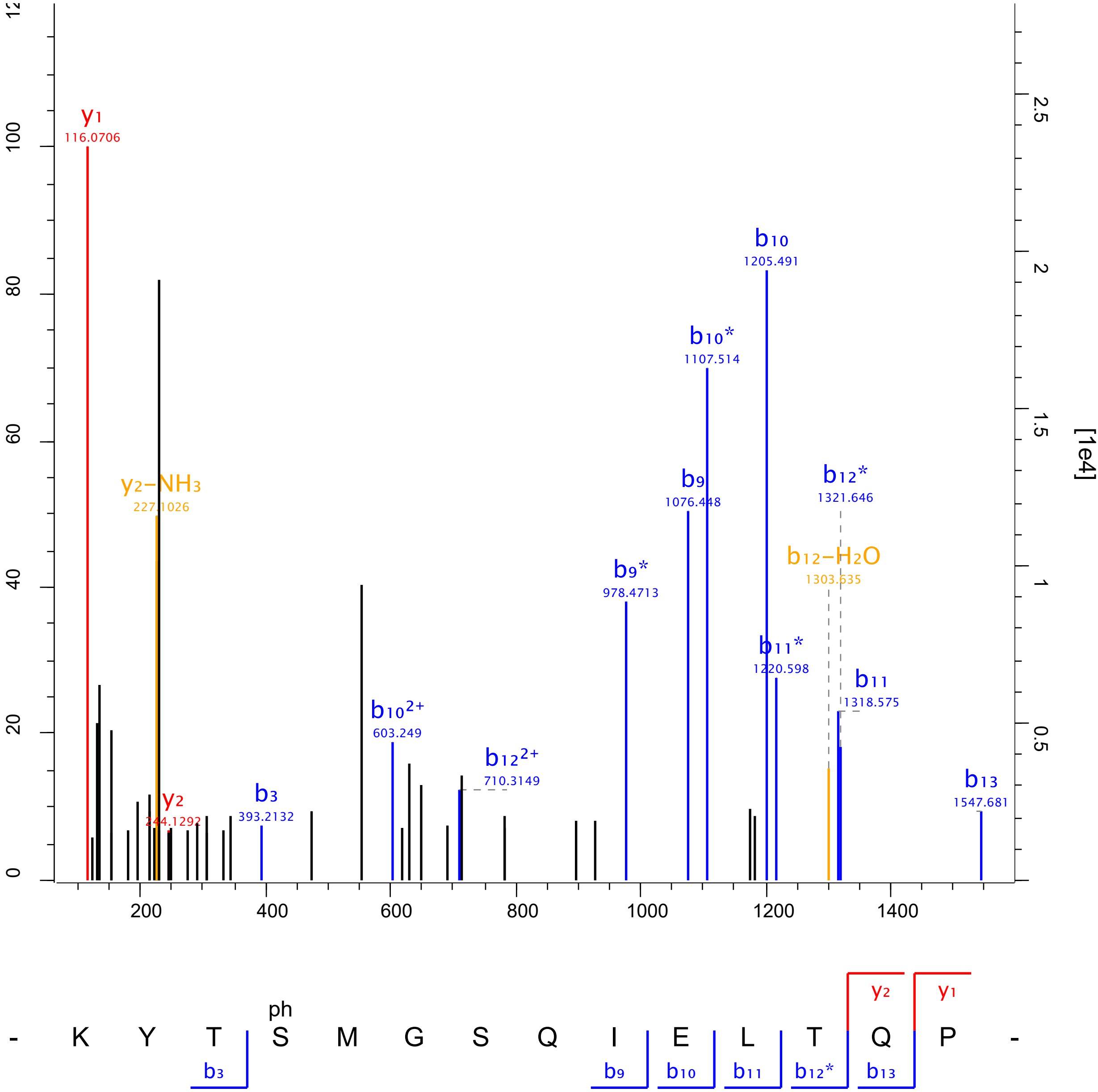

Raw file	Scan	Method	Score	m/z
sys_00_3short	25096	FTMS; HCD	160.78	710.33

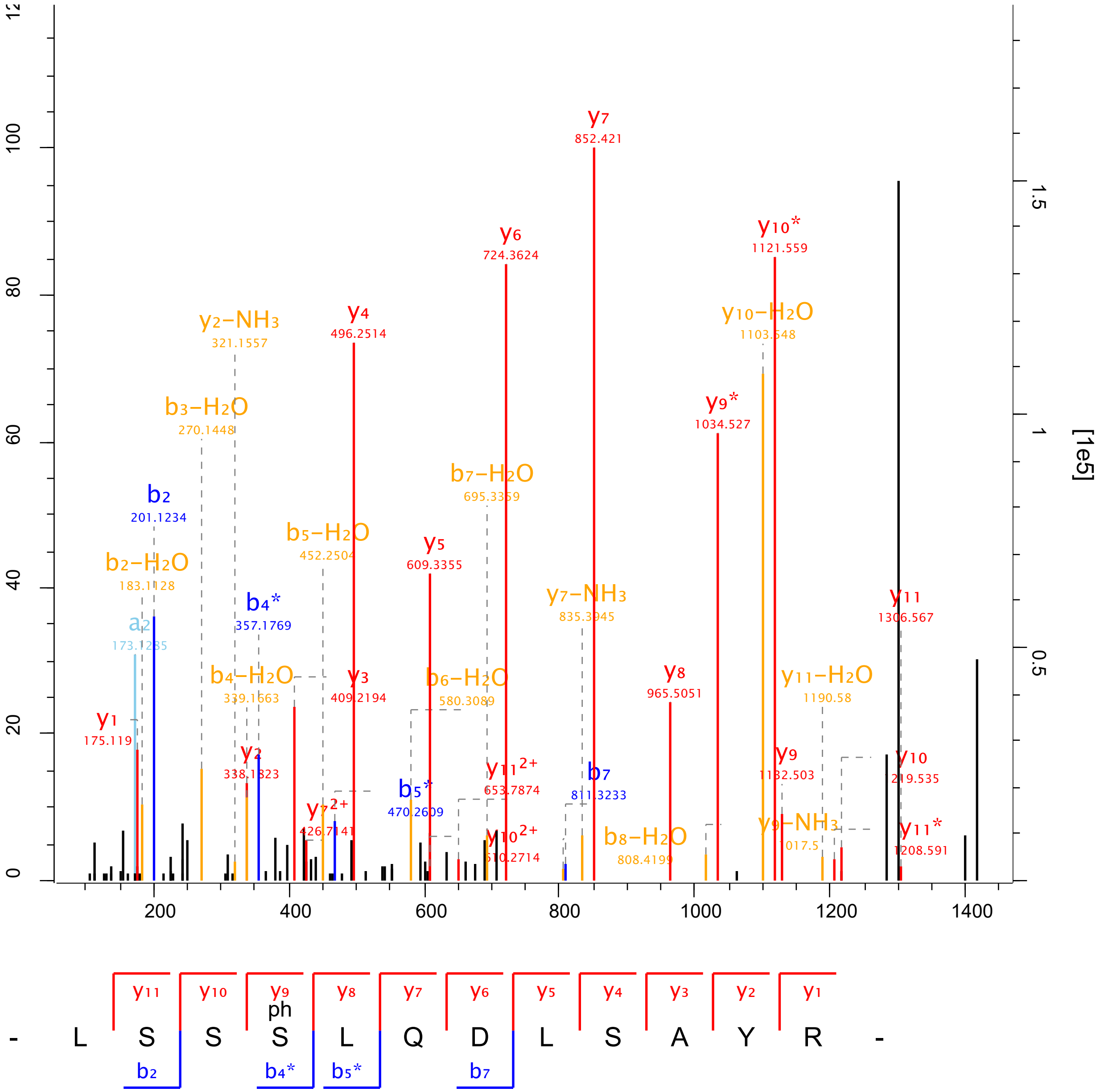

Raw file	Scan	Method	Score	m/z
sys_00_3short	25587	FTMS; HCD	218.11	799.9

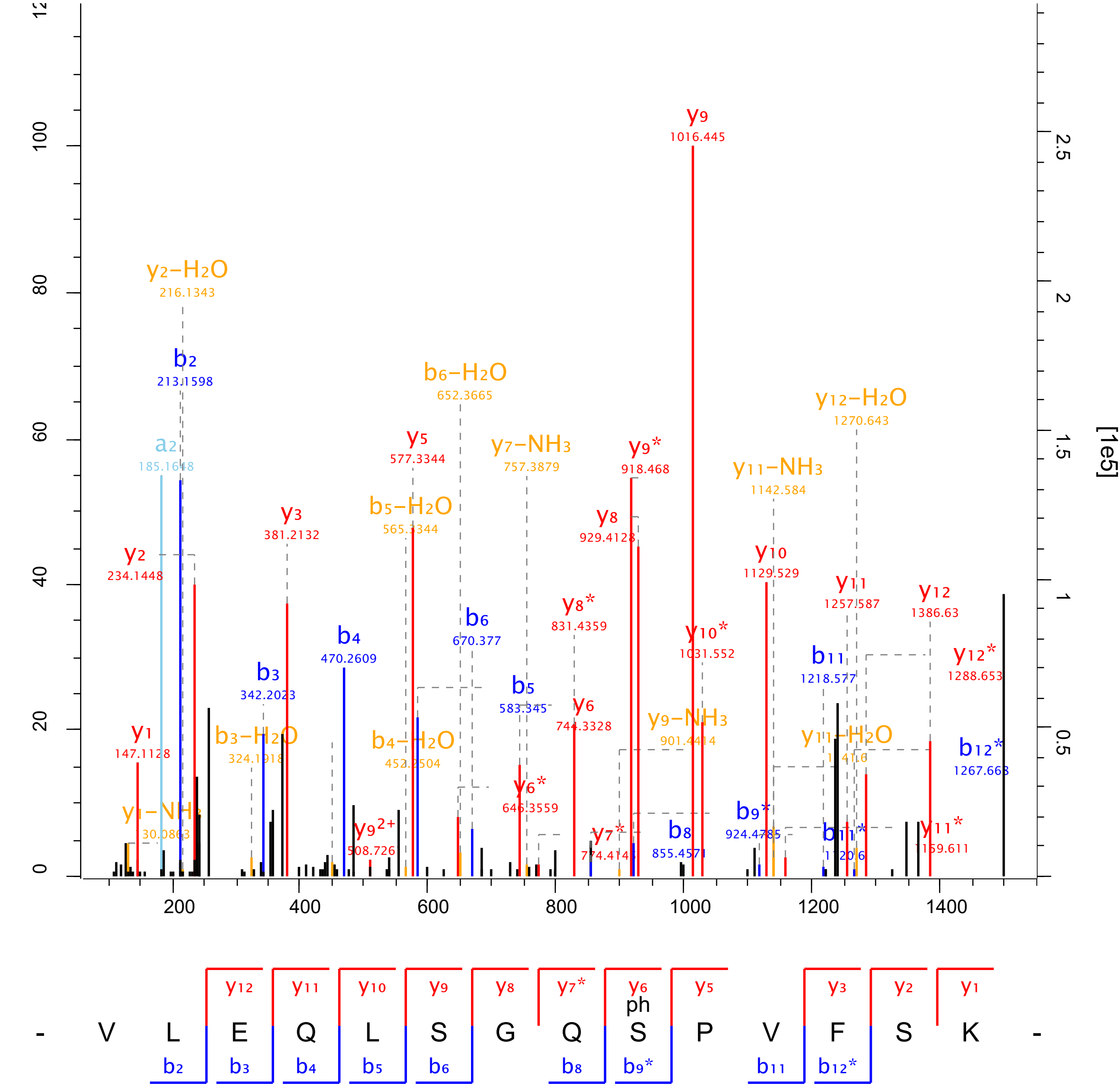

Raw file	Scan	Method	Score	m/z
sys_00_3short	25716	FTMS; HCD	124.25	795.35

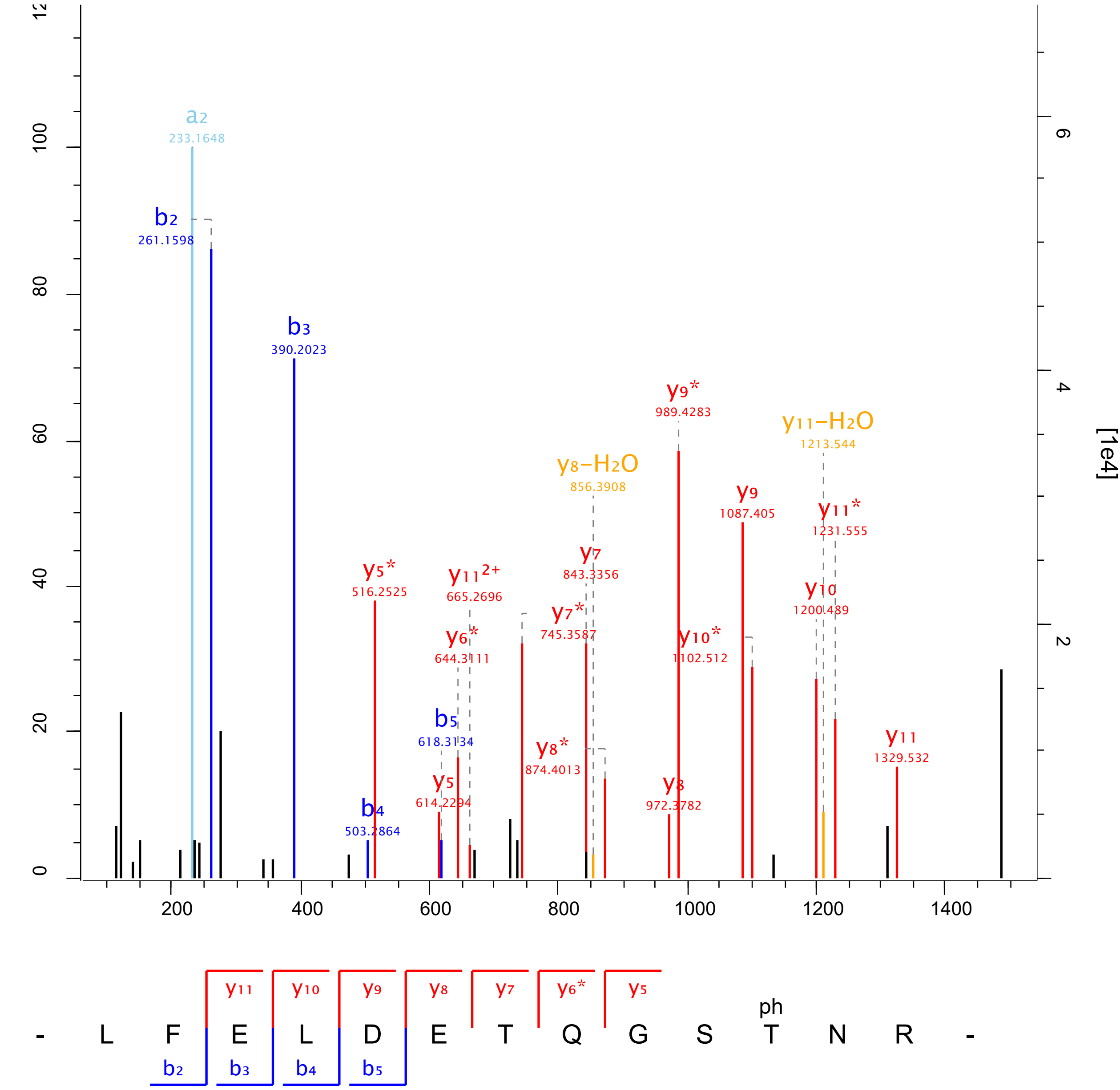

Raw file	Scan	Method	Score	m/z
sys_00_3short	25762	FTMS; HCD	71.88	588.81

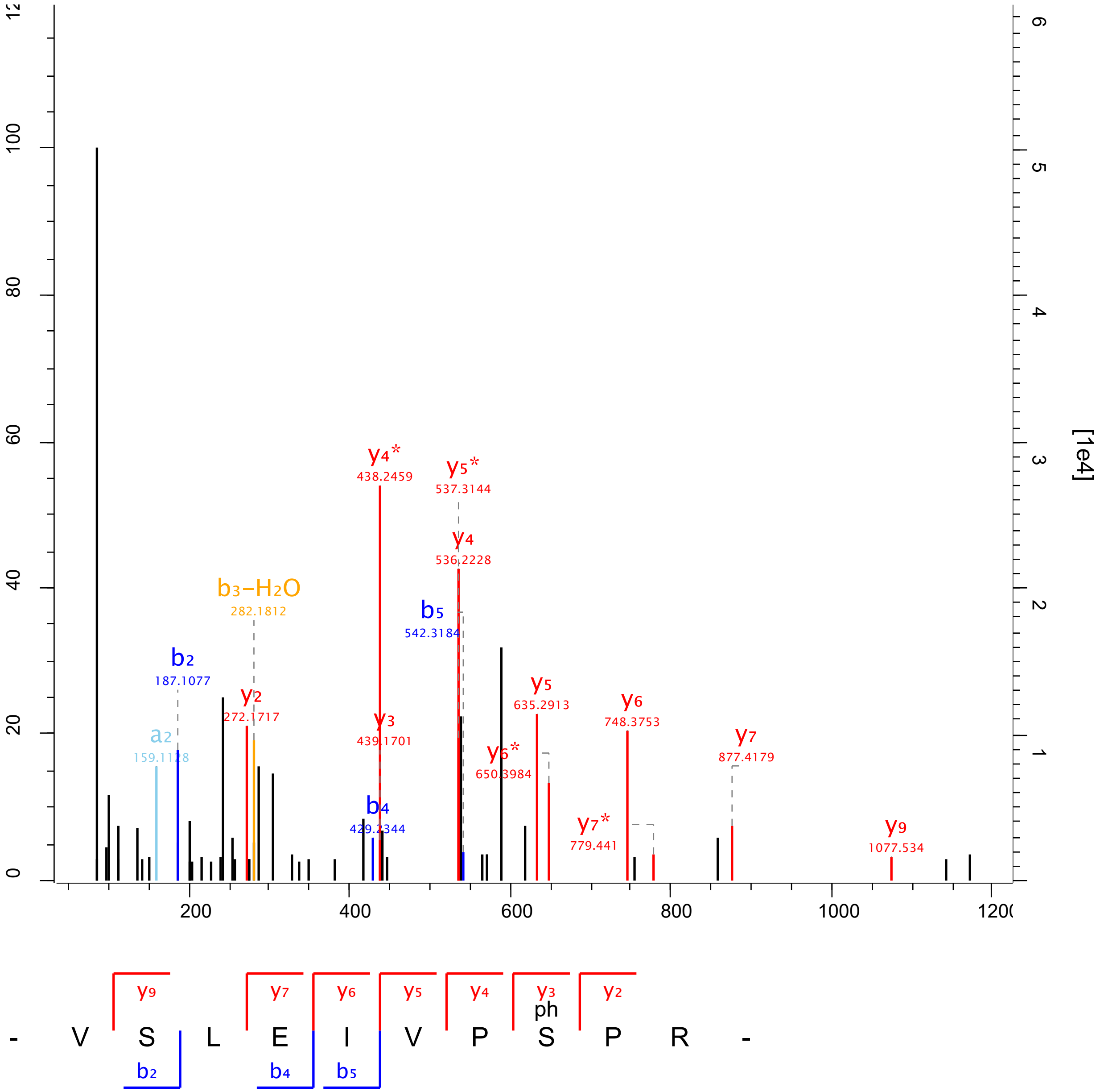

Raw file	Scan	Method	Score	m/z
sys_00_3short	25778	FTMS; HCD	161.21	598.3

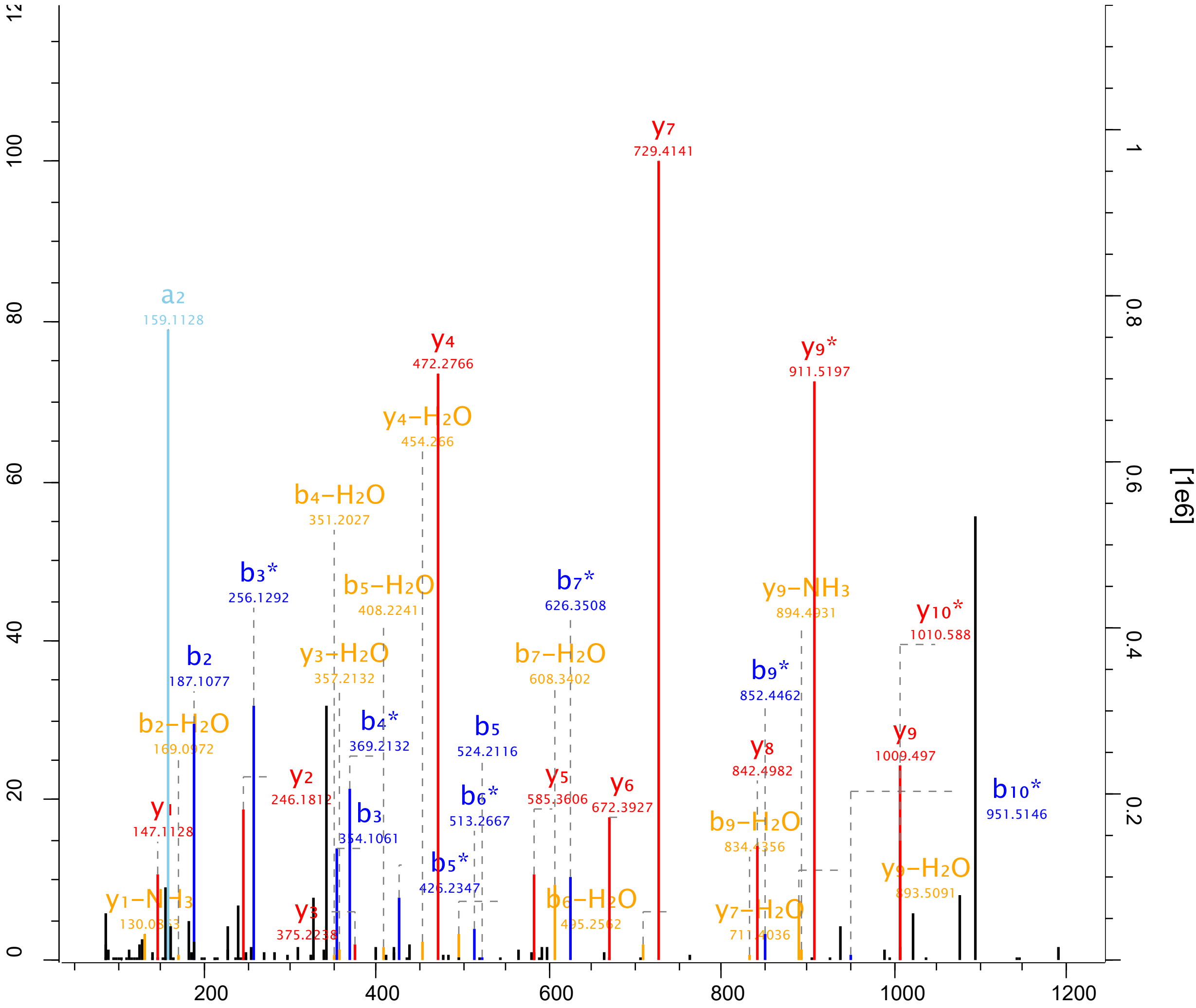

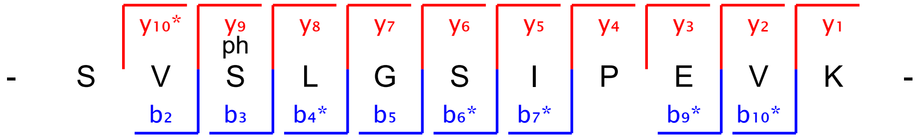

Raw file	Scan	Method	Score	m/z
sys_00_3short	25897	FTMS; HCD	73.69	729.69

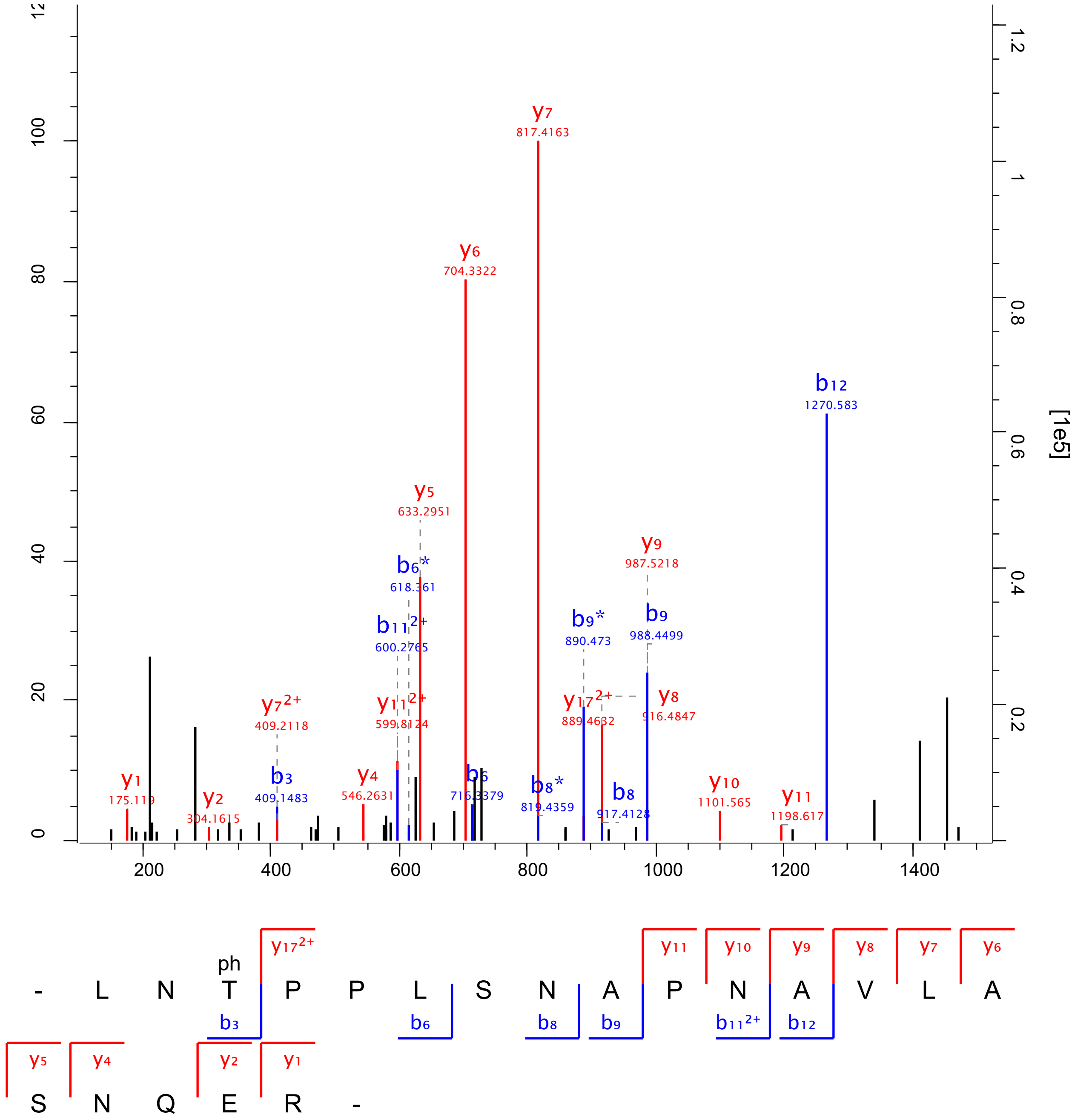

Raw file	Scan	Method	Score	m/z
sys_00_3short	25963	FTMS; HCD	109.44	639.75

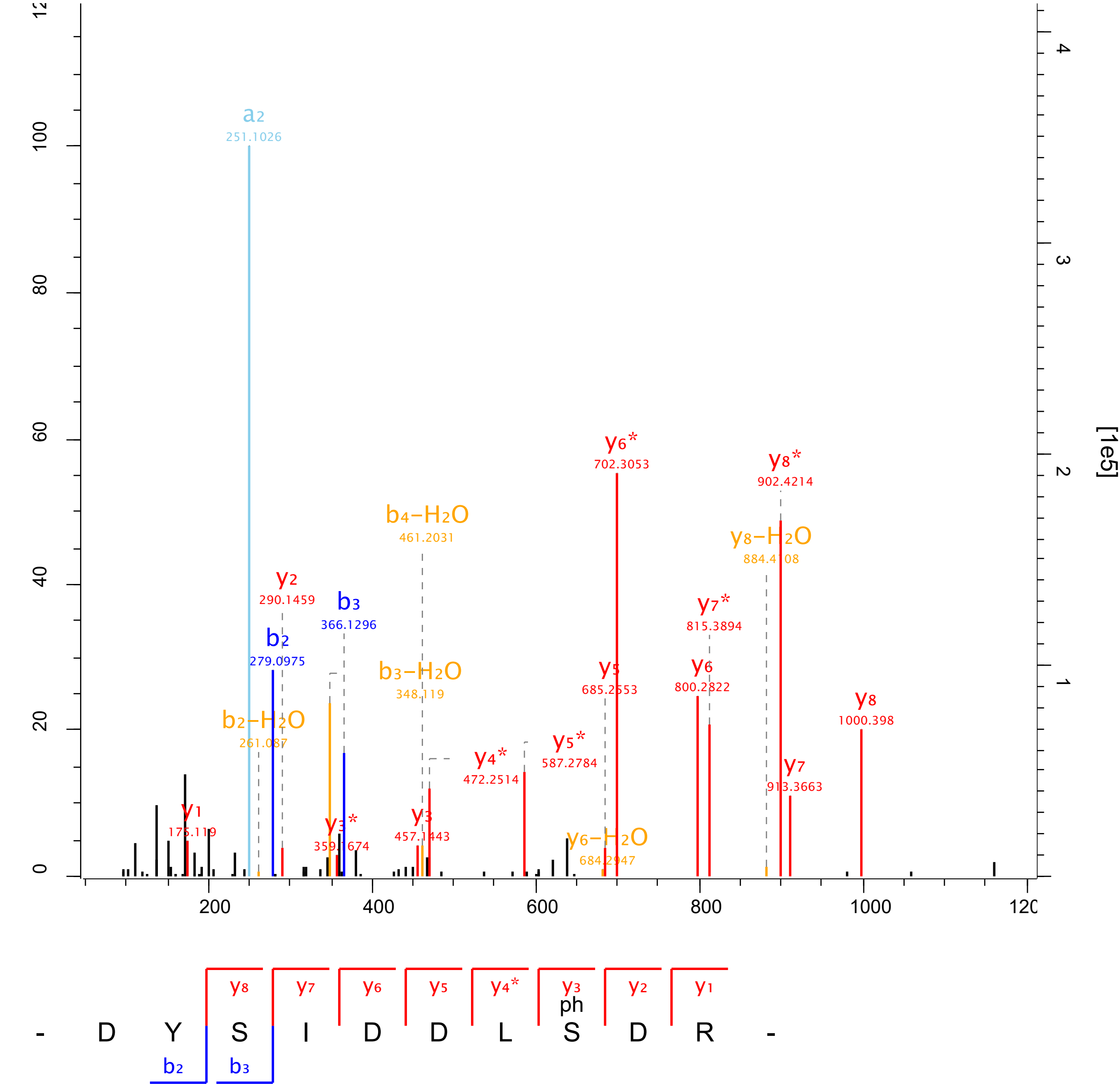

Raw file	Scan	Method	Score	m/z
sys_00_3short	25996	FTMS; HCD	58.32	601.28

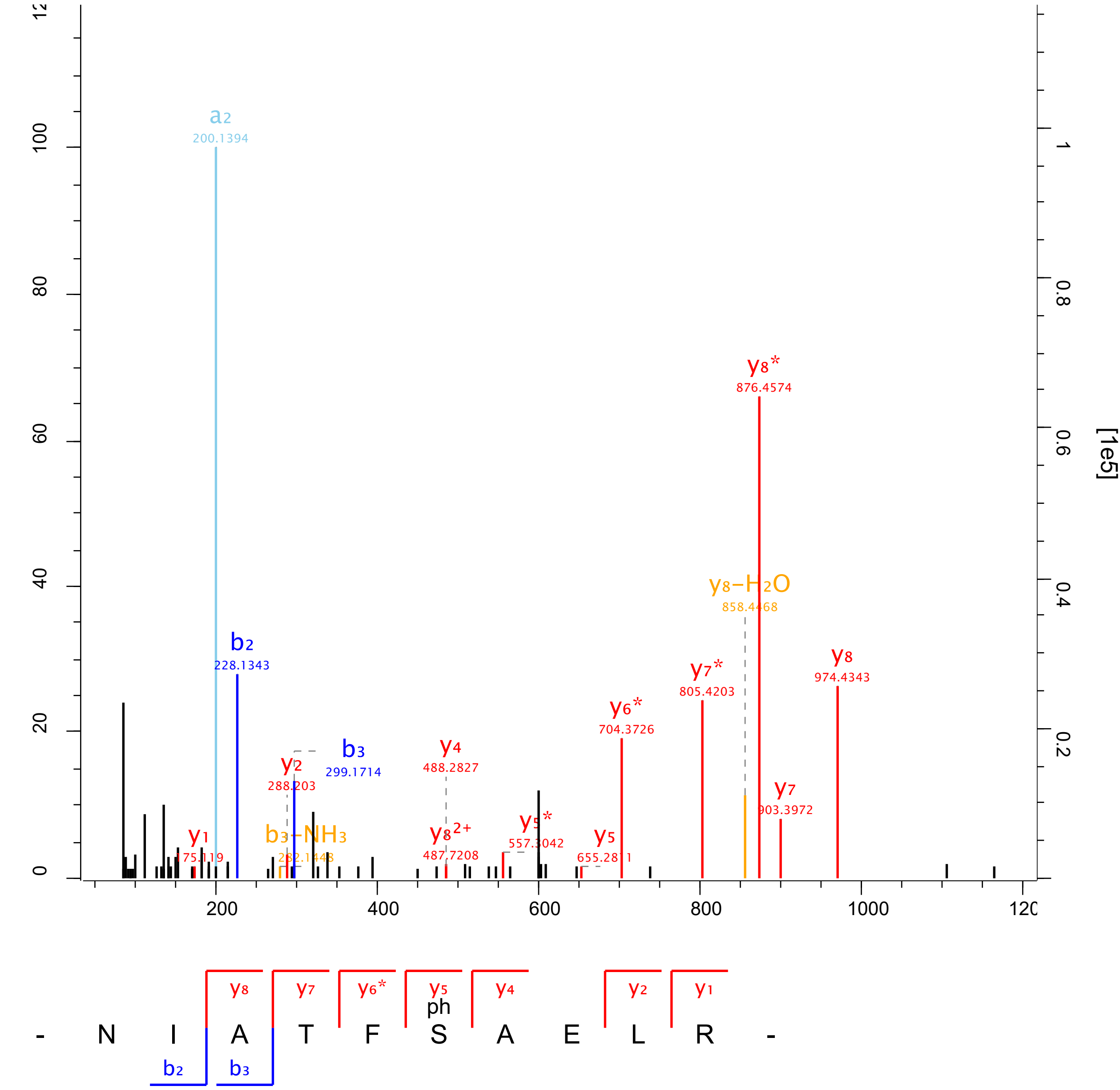

Raw file	Scan	Method	Score	m/z
sys_00_3short	26045	FTMS; HCD	45.22	744.82

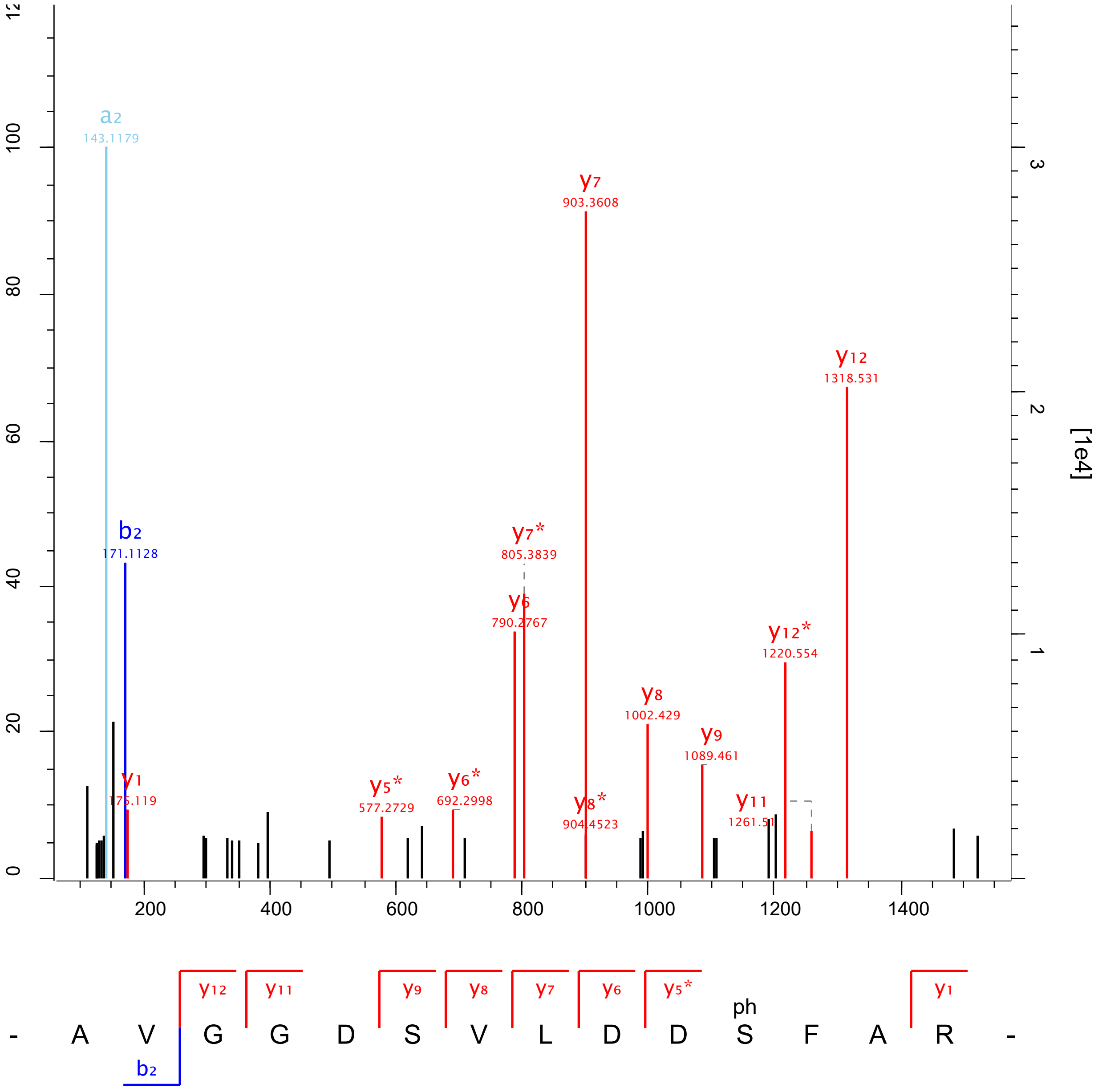

Raw file	Scan	Method	Score	m/z
sys_00_3short	26120	FTMS; HCD	69.33	776.84

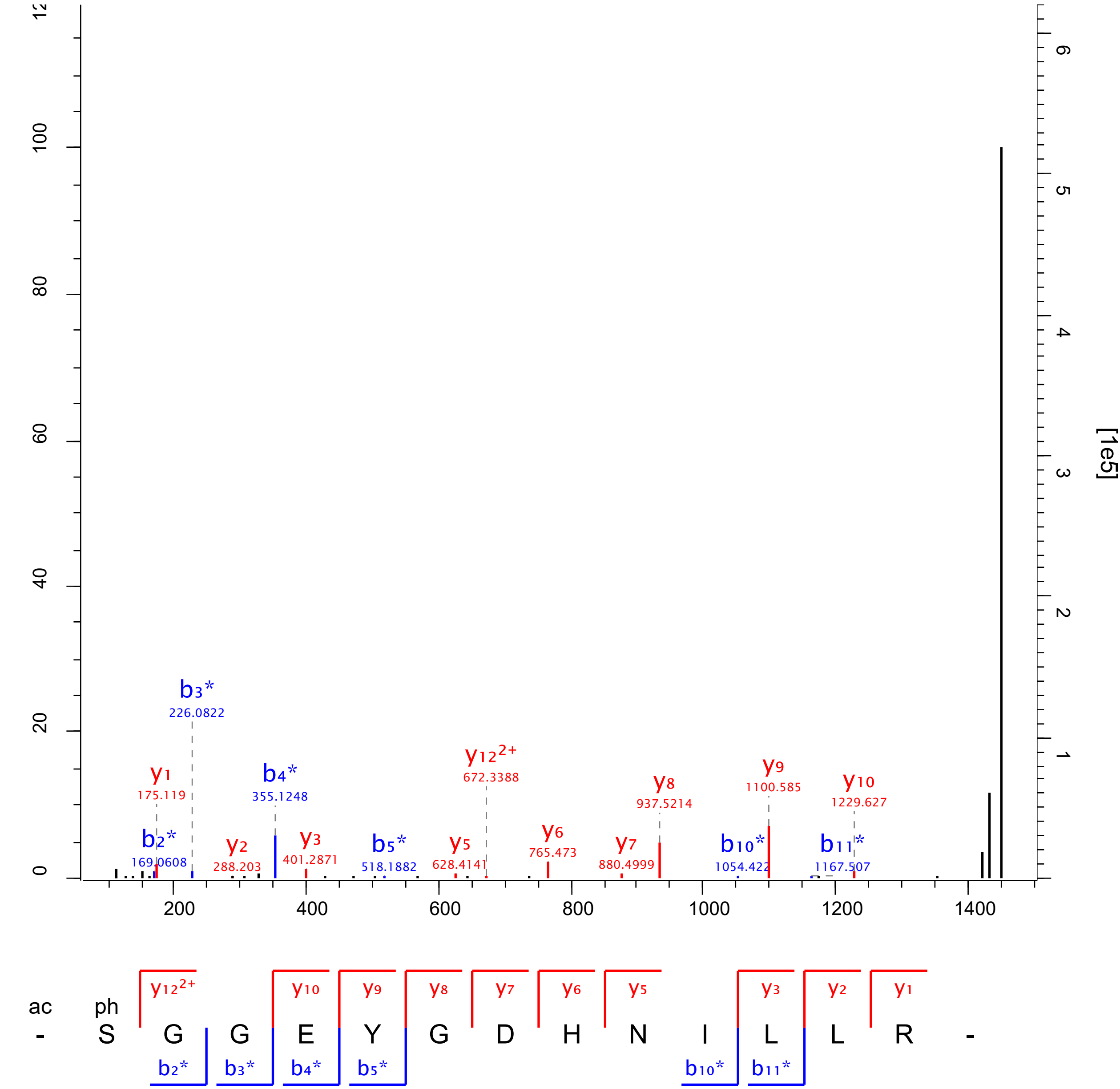

Raw file	Scan	Method	Score	m/z
sys_00_3short	26443	FTMS; HCD	110.12	726.28

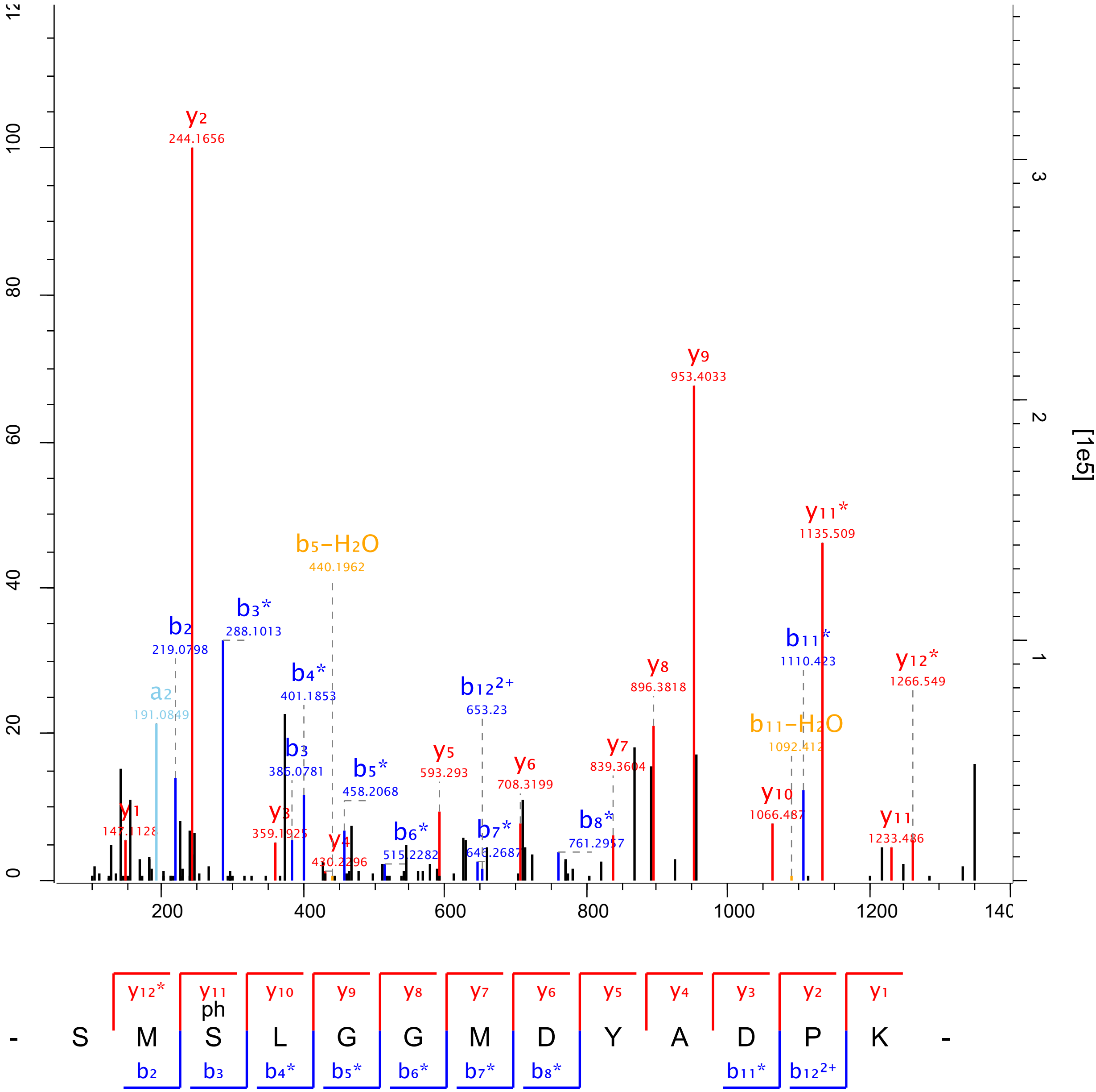

Supplement: Supplementary Figure S2-1 [file 143488_2_supp_334104_ps5hgb.pdf]
