# Supplementary material for: The Systemin Signaling Cascade As Derived from Time Course Analyses of the Systemin-responsive Phosphoproteome
Source: Mol Cell Proteomics. 2019 May 28;18(8):1526–42. doi: 10.1074/mcp.RA119.001367 (PMC6683004; doi:10.1074/mcp.RA119.001367)

Supplementary Figure 2: Representative annotated spectra of identified phosphopeptides under systemin,A17 and water treatment as exported from MaxQuant.

Raw file	Scan	Method	Score	m/z
sys_00_3short	26476	FTMS; HCD	108.63	895.82

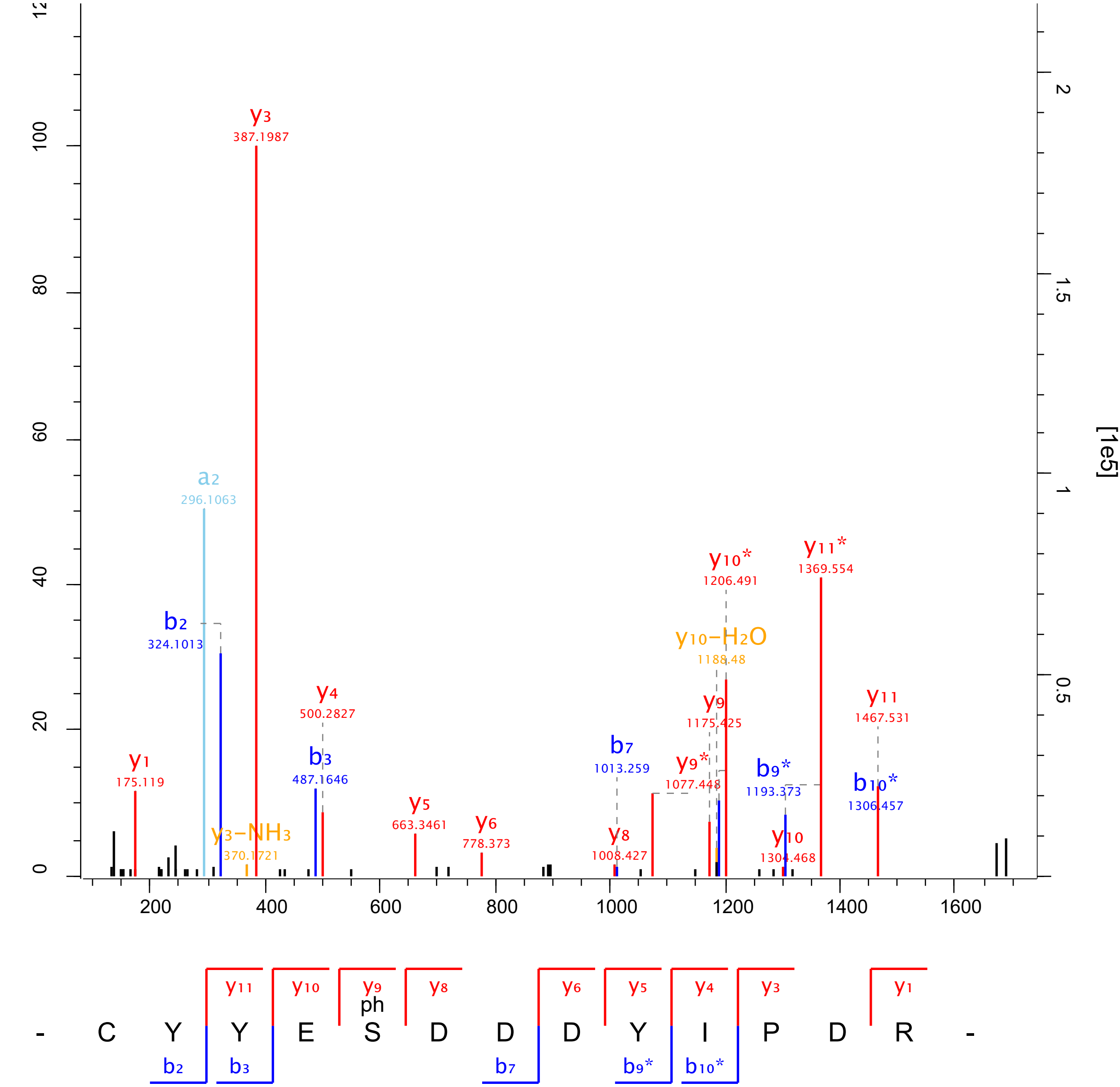

Raw file	Scan	Method	Score	m/z
sys_00_3short	26954	FTMS; HCD	168.84	833.41

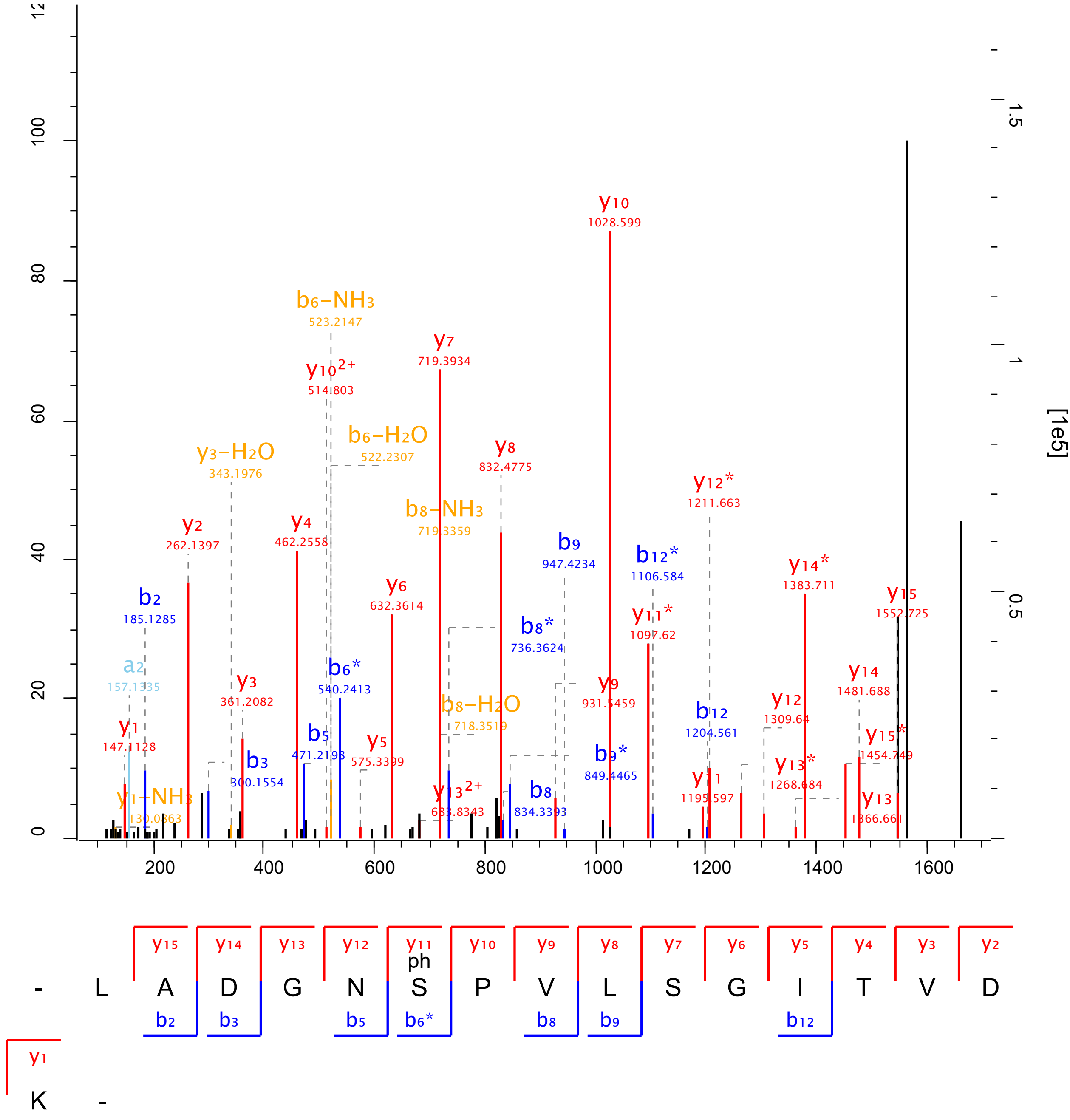

Raw file	Scan	Method	Score	m/z
sys_00_3short	27519	FTMS; HCD	64.52	621.29

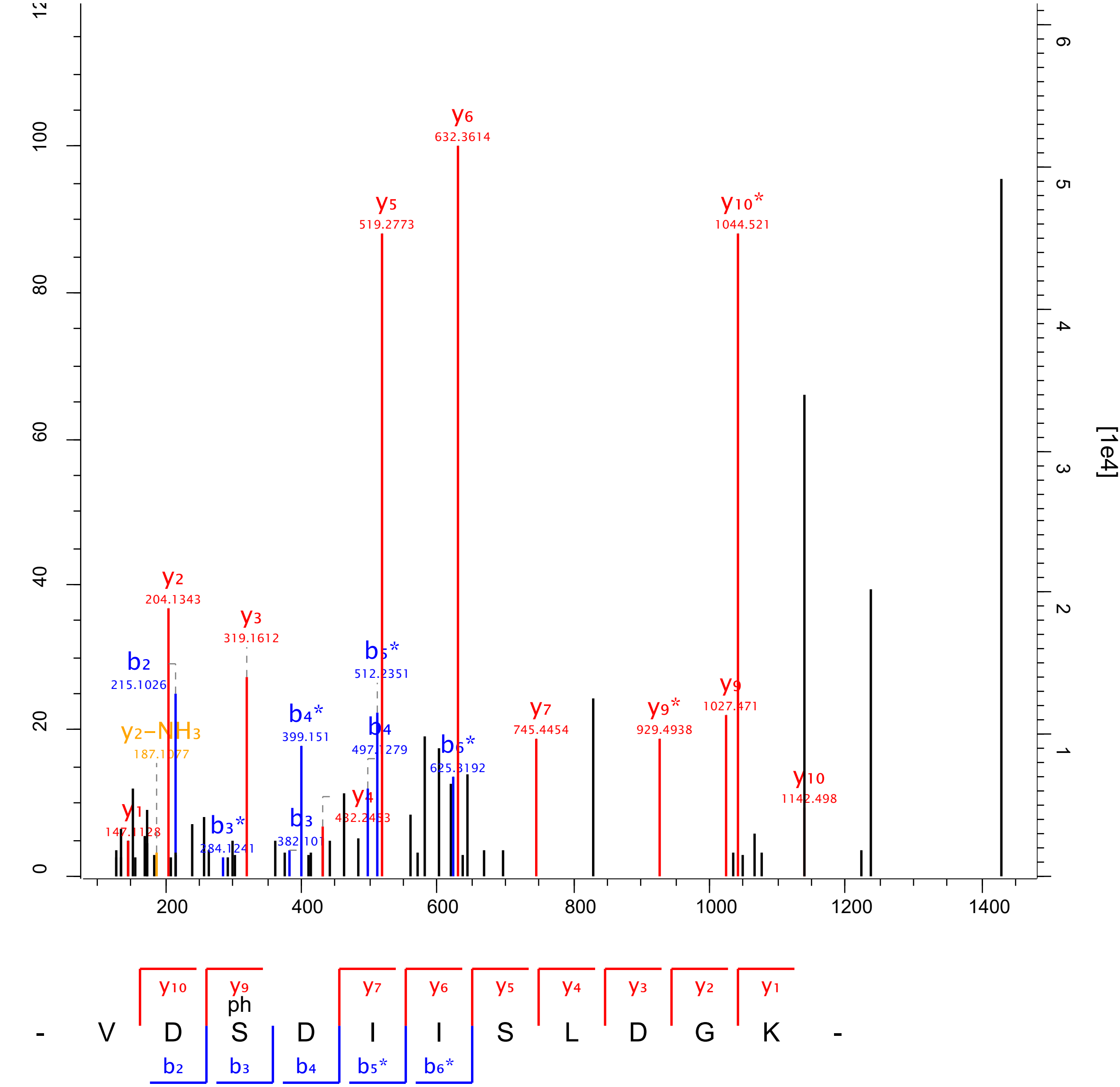

Raw file	Scan	Method	Score	m/z
sys_00_3short	27536	FTMS; HCD	62.09	618.78

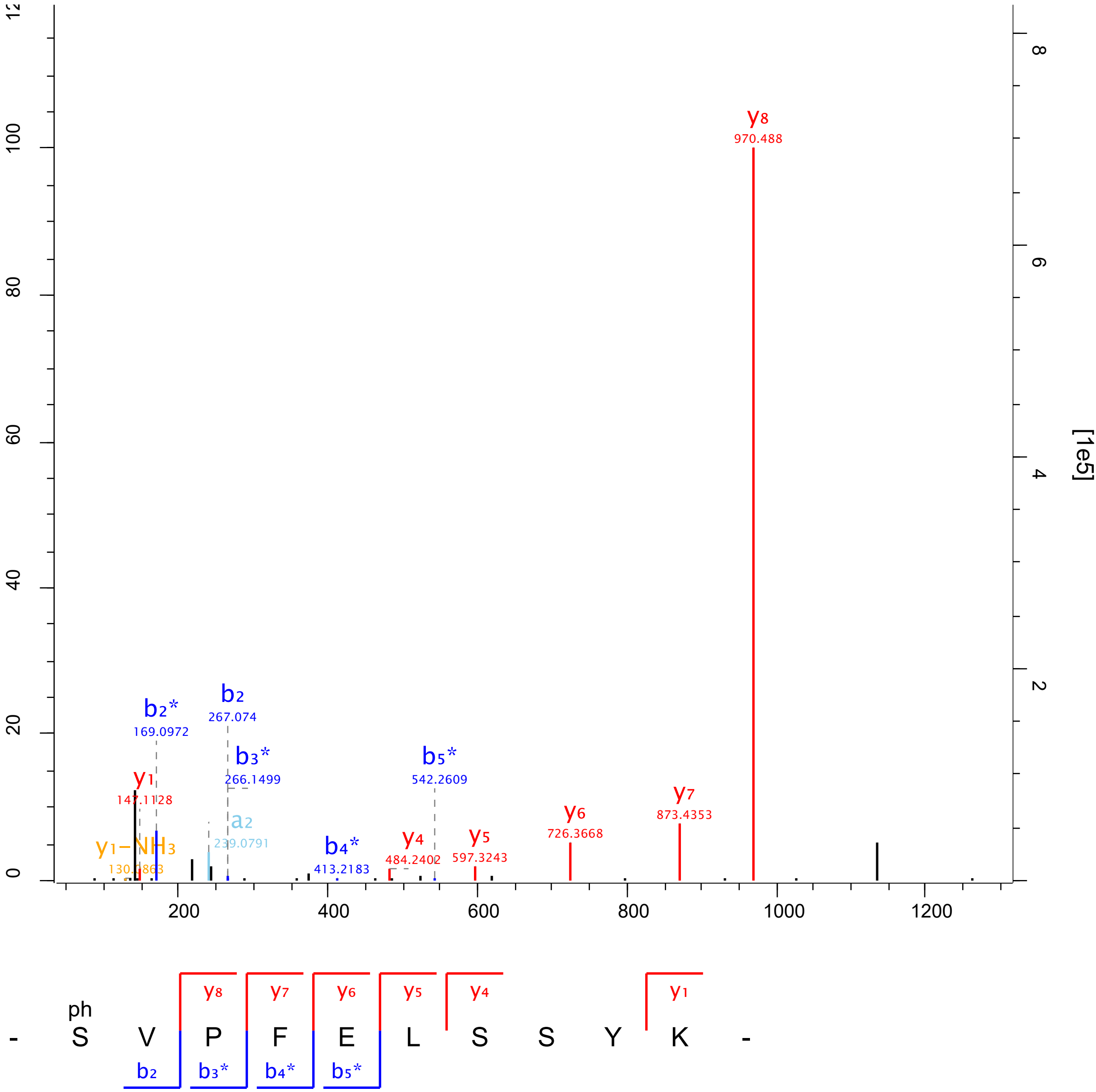

Raw file	Scan	Method	Score	m/z
sys_00_3short	2774	FTMS; HCD	143.7	398.2

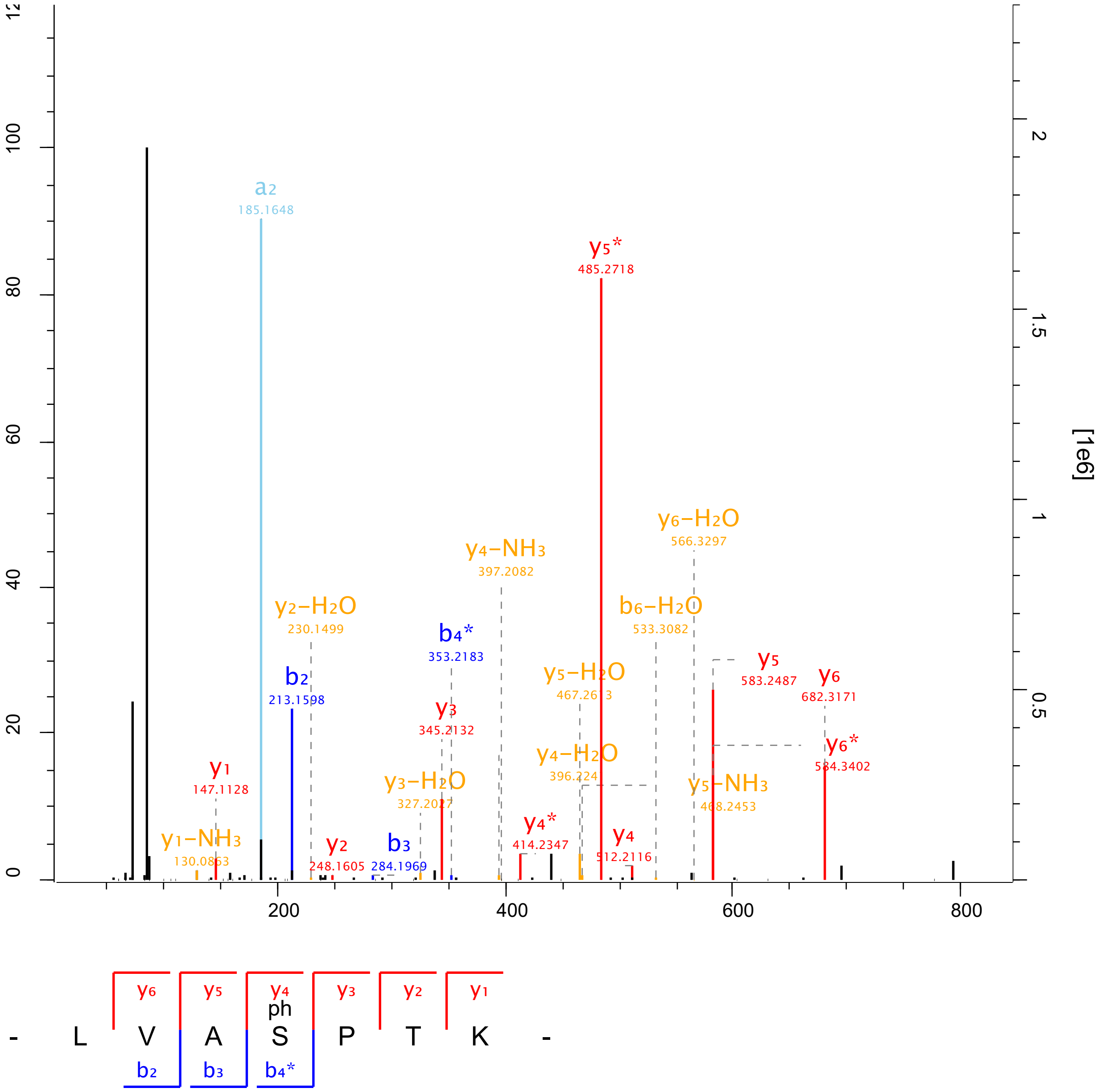

Raw file	Scan	Method	Score	m/z
sys_00_3short	27750	FTMS; HCD	49.12	739.68

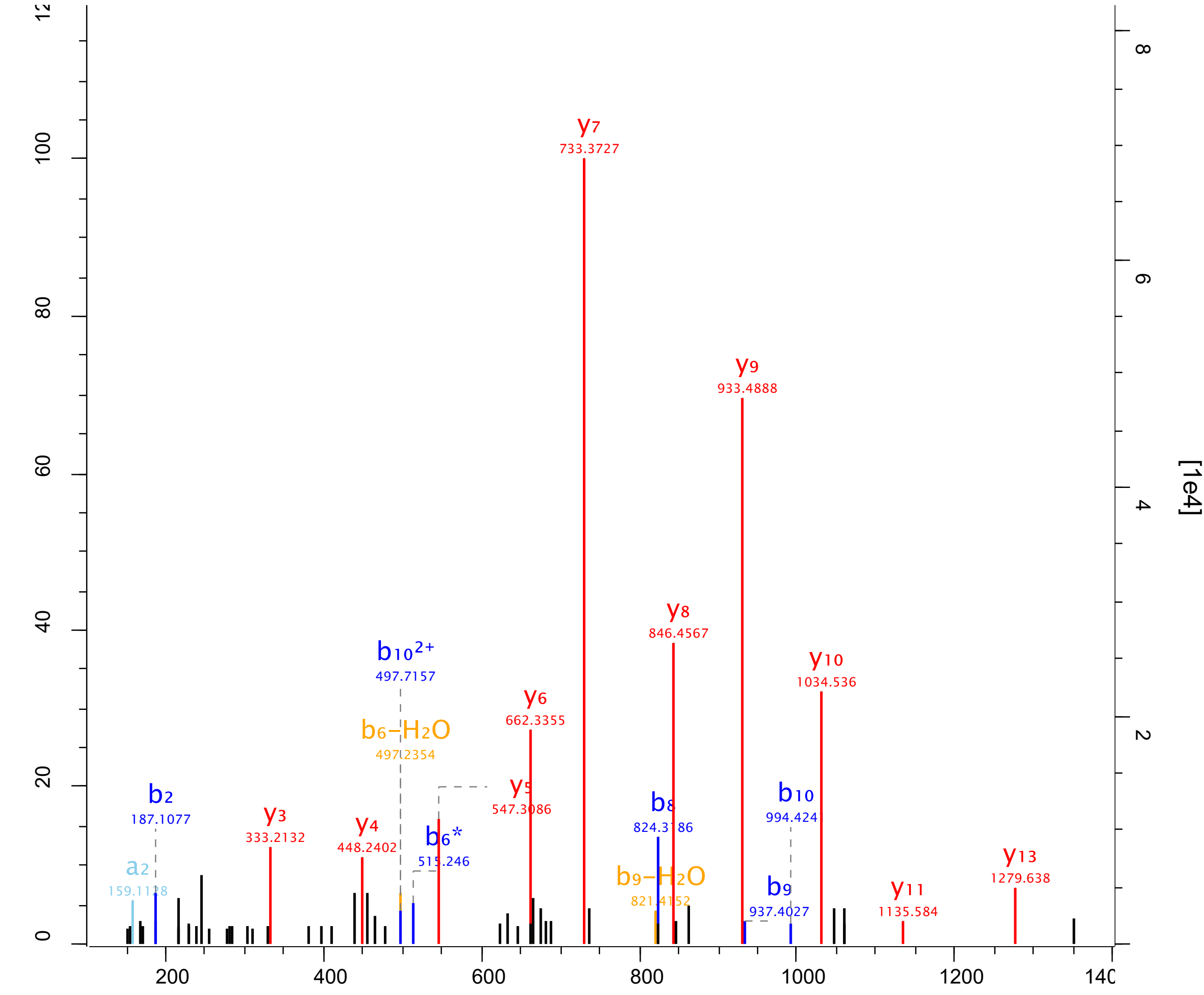

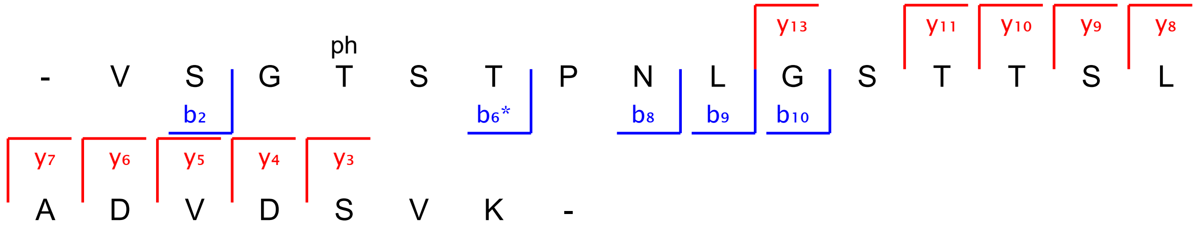

Raw file	Scan	Method	Score	m/z
sys_00_3short	28318	FTMS; HCD	72.2	527.25

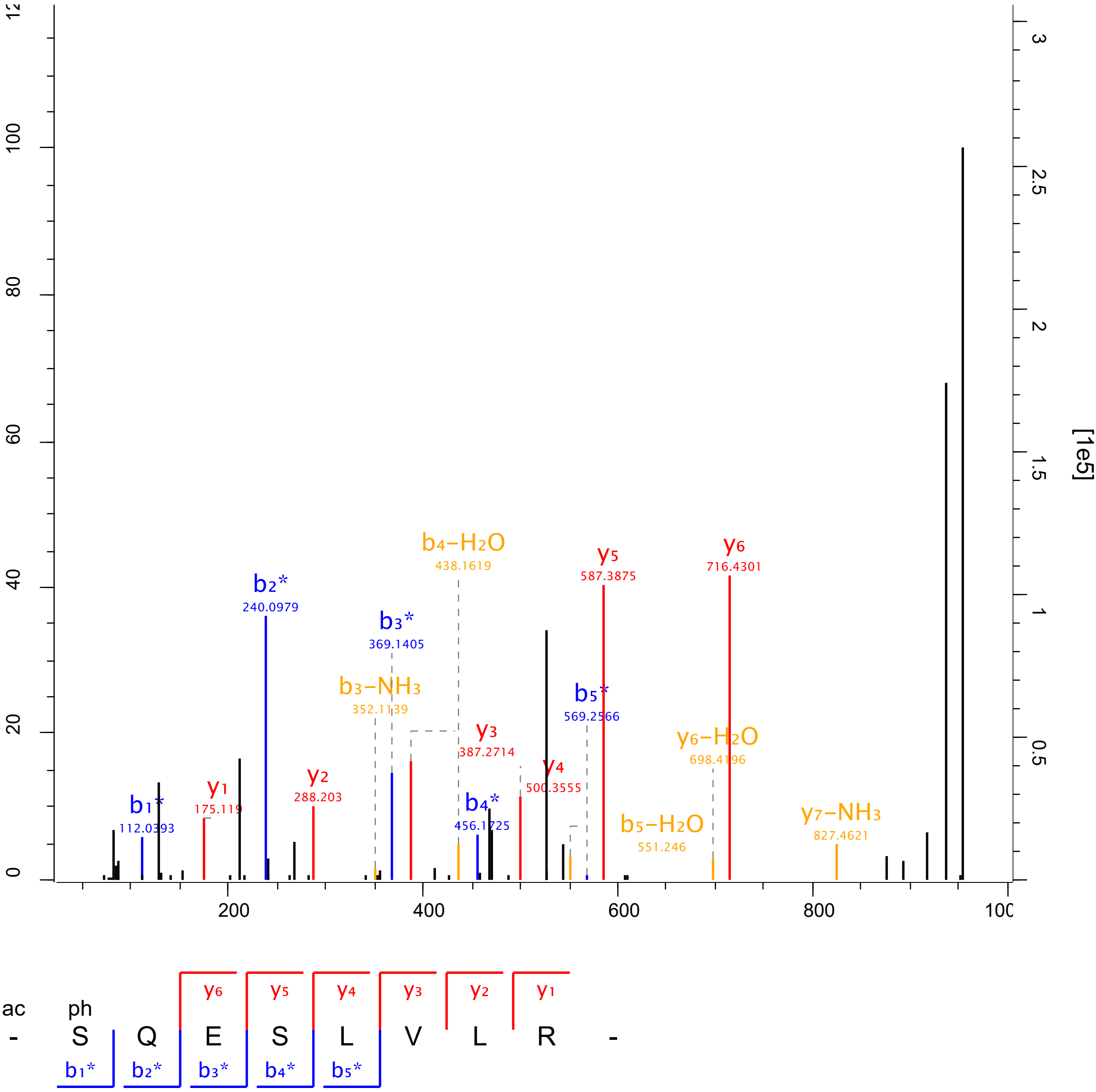

Raw file	Scan	Method	Score	m/z
sys_00_3short	28403	FTMS; HCD	45.58	1151.47

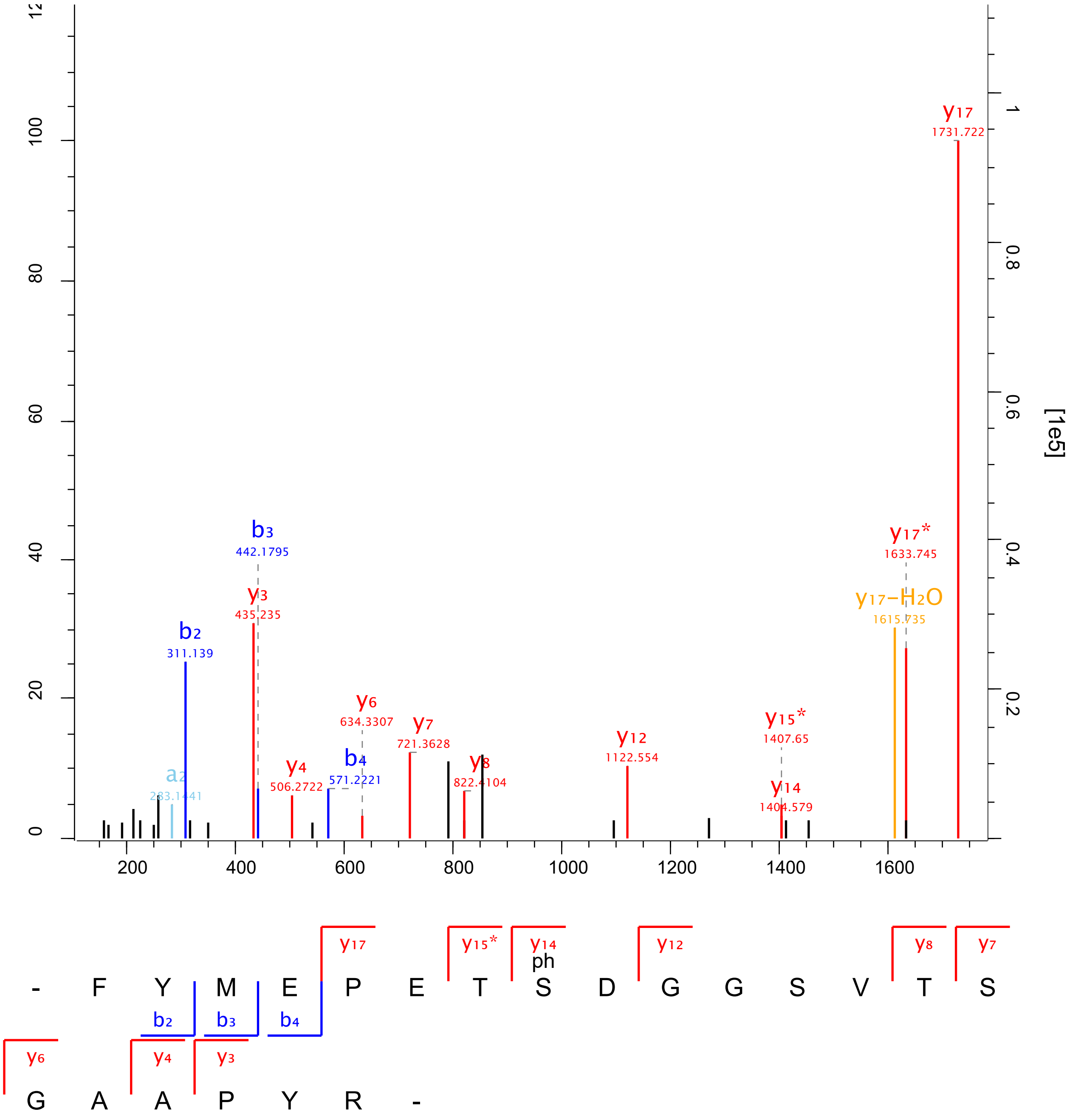

Raw file	Scan	Method	Score	m/z
sys_00_3short	28802	FTMS; HCD	136.6	649.8

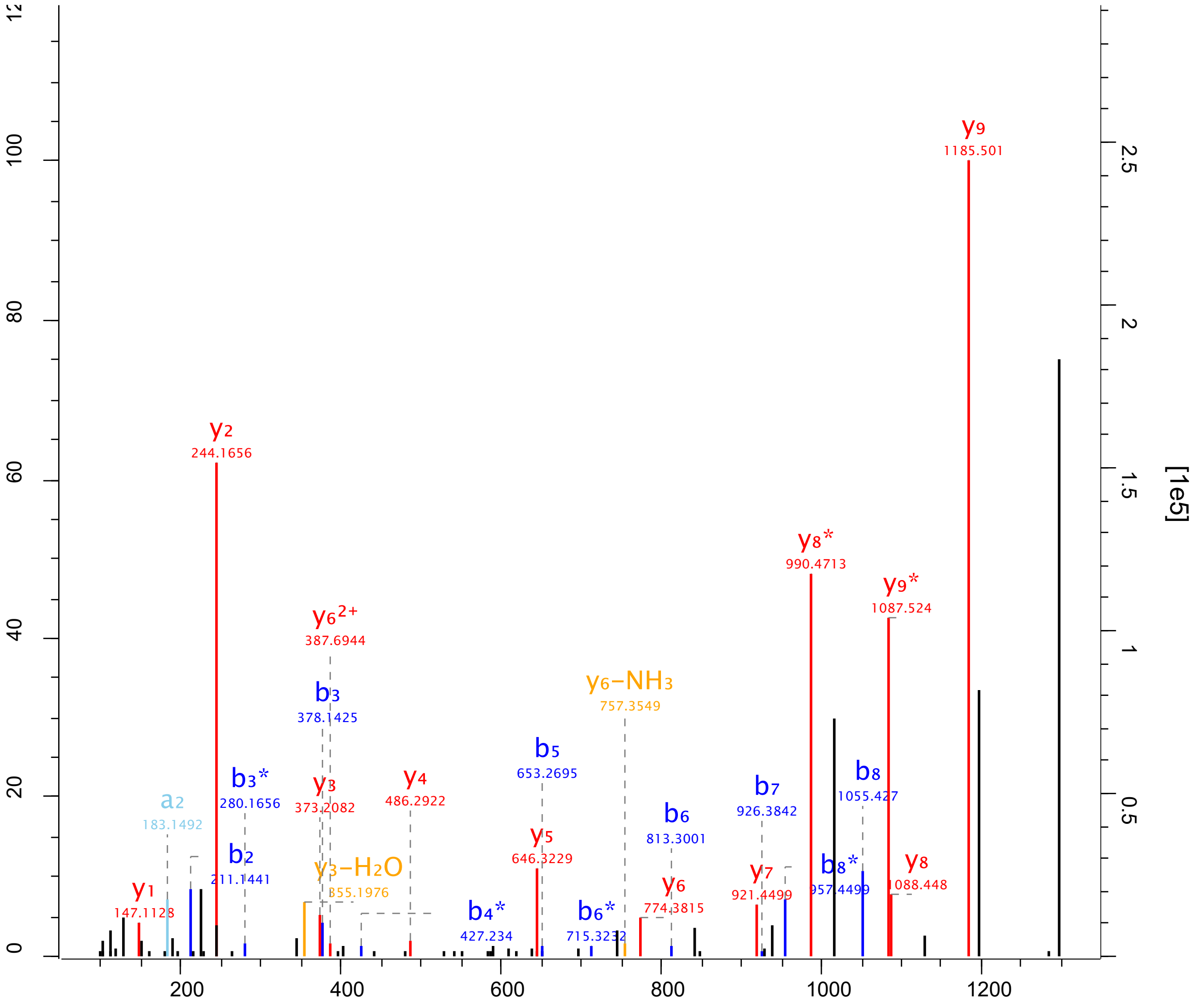

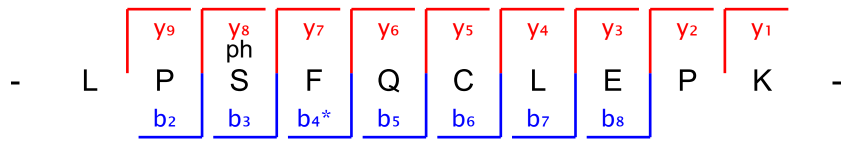

Raw file	Scan	Method	Score	m/z
sys_00_3short	29095	FTMS; HCD	186.94	736.3

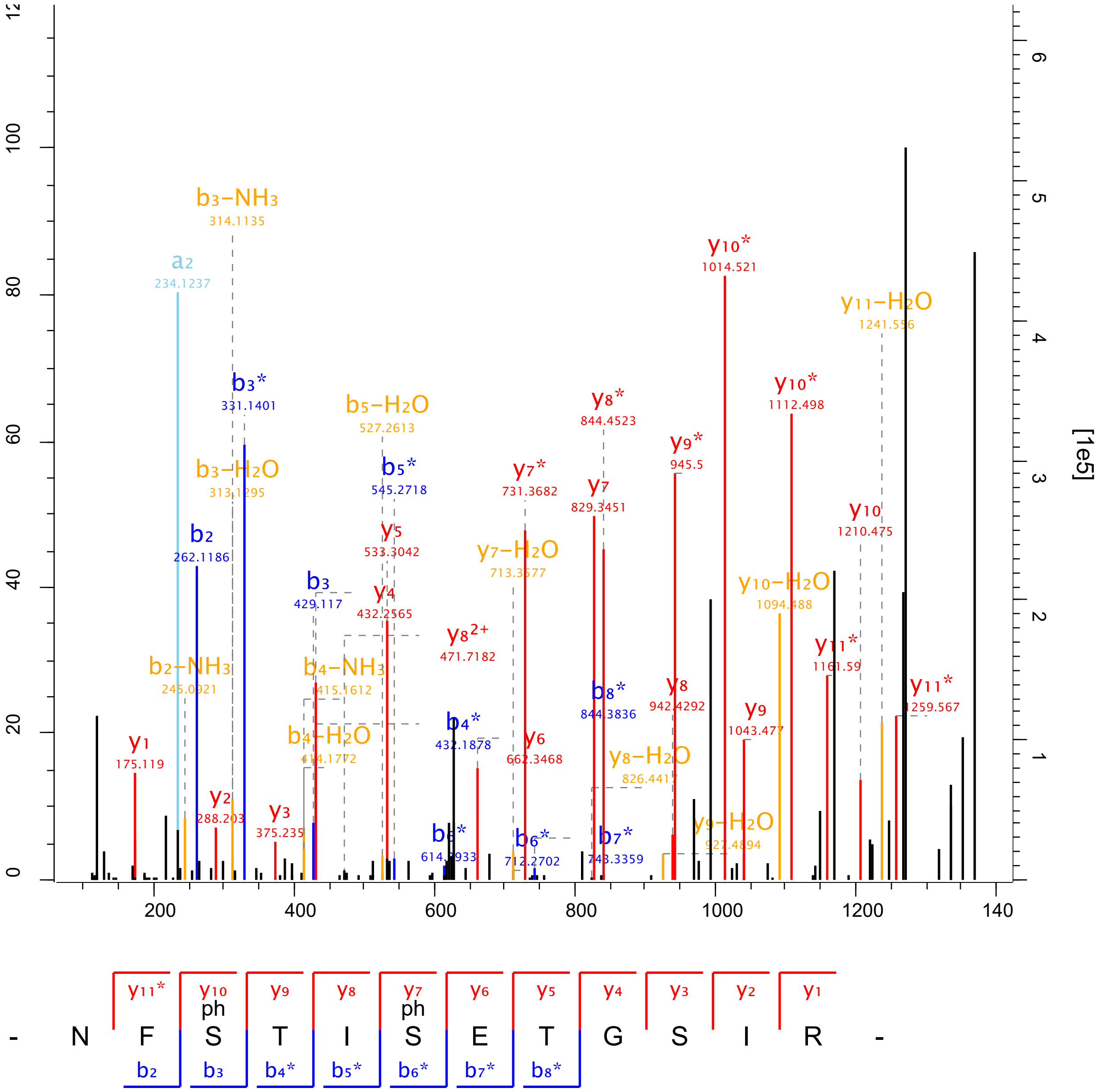

Raw file	Scan	Method	Score	m/z
sys_00_3short	29176	FTMS; HCD	143.28	750.87

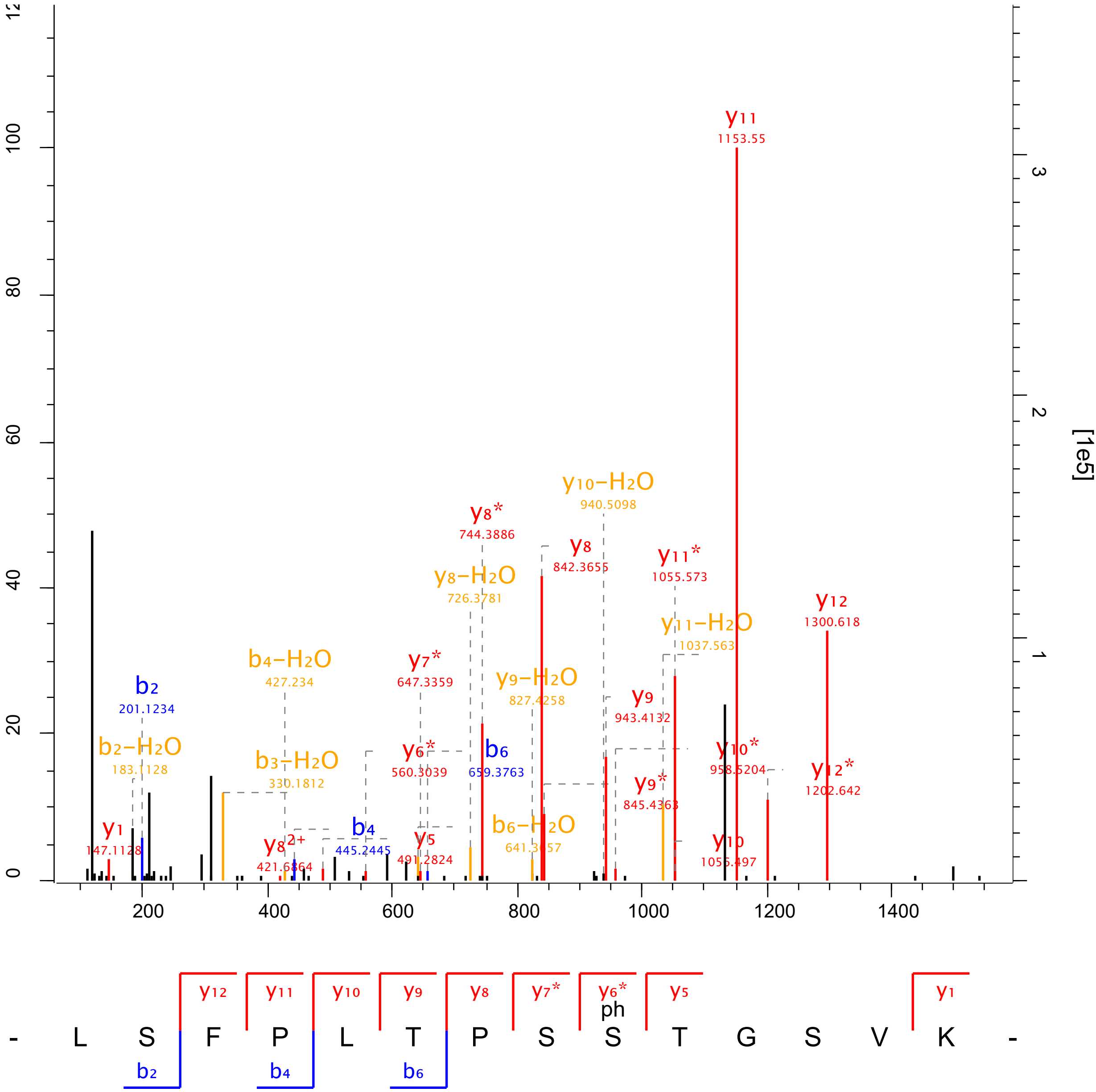

Raw file	Scan	Method	Score	m/z
sys_00_3short	2951	FTMS; HCD	119.39	522.22

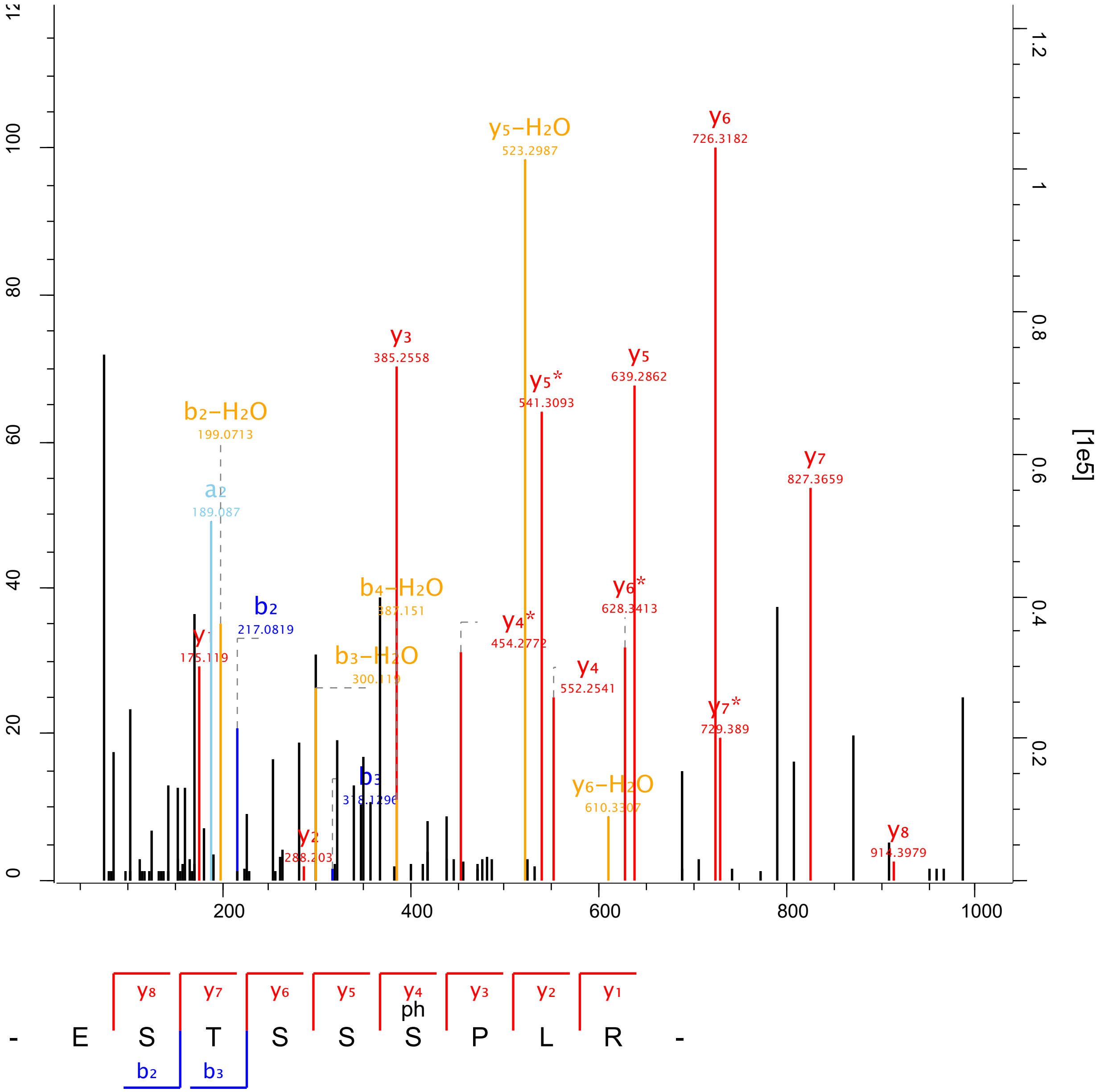

Raw file	Scan	Method	Score	m/z
sys_00_3short	29759	FTMS; HCD	68.48	692.33

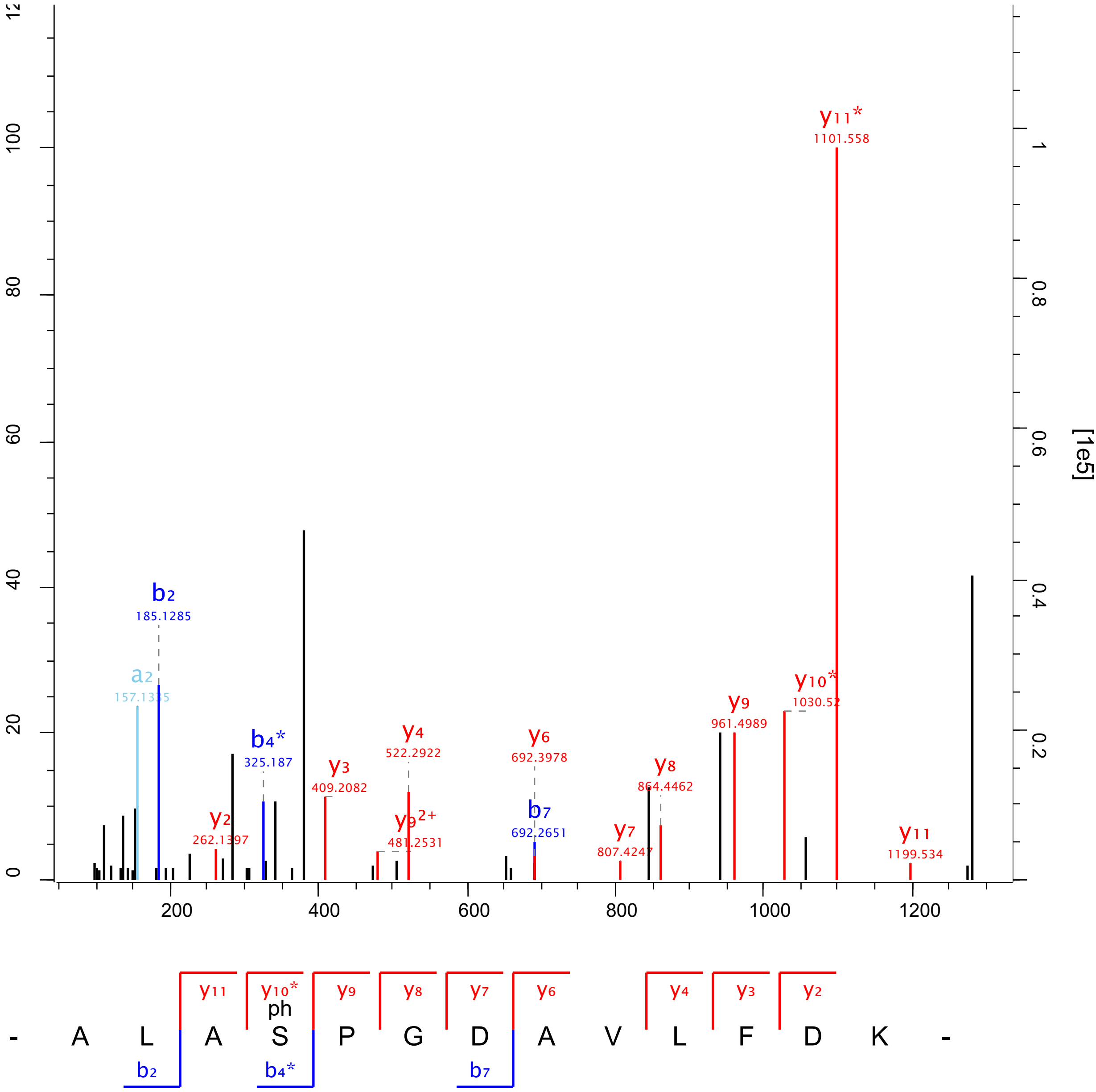

Raw file	Scan	Method	Score	m/z
sys_00_3short	29829	FTMS; HCD	62.2	760.83

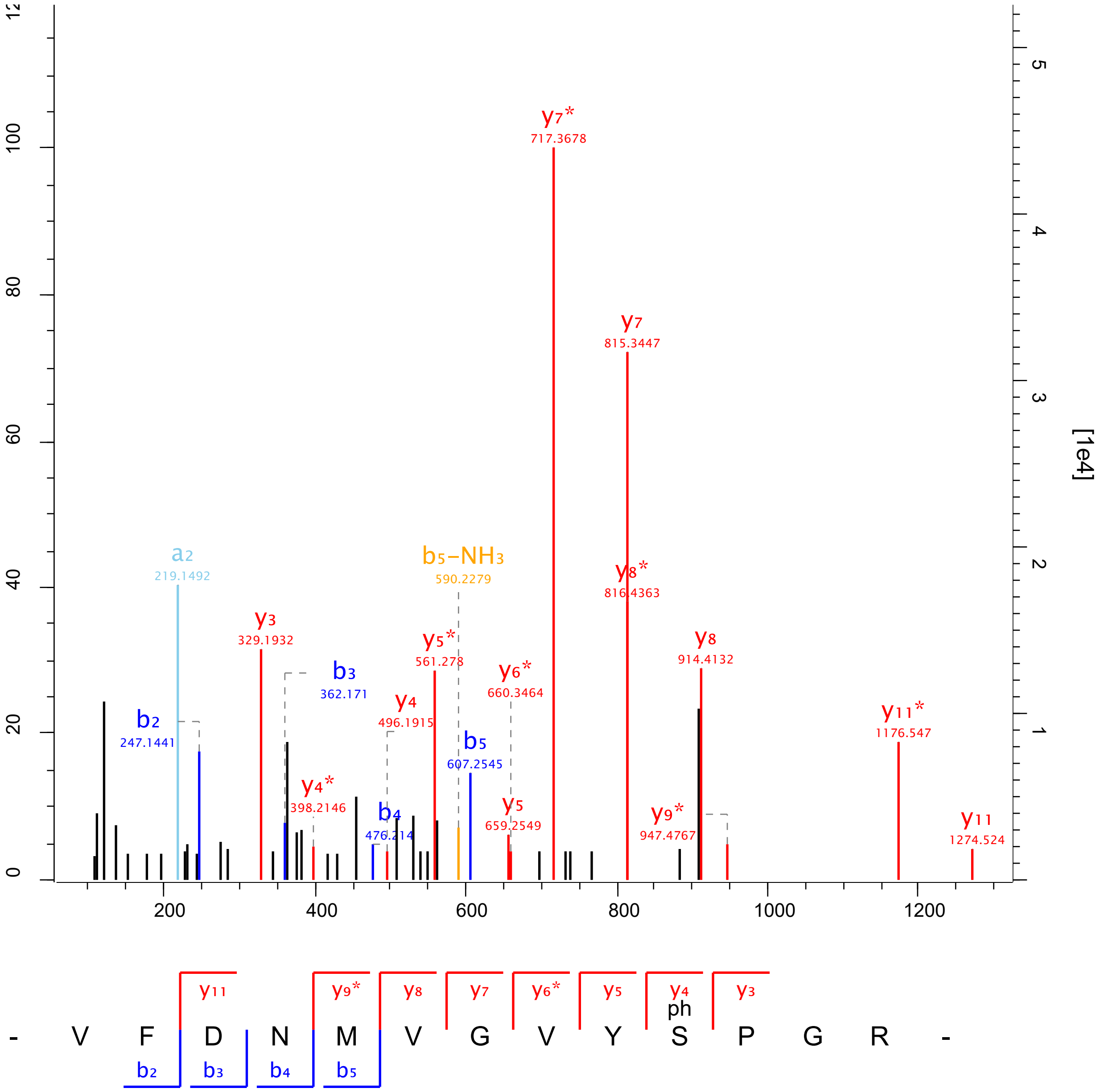

Raw file	Scan	Method	Score	m/z
sys_00_3short	29941	FTMS; HCD	113.38	573.73

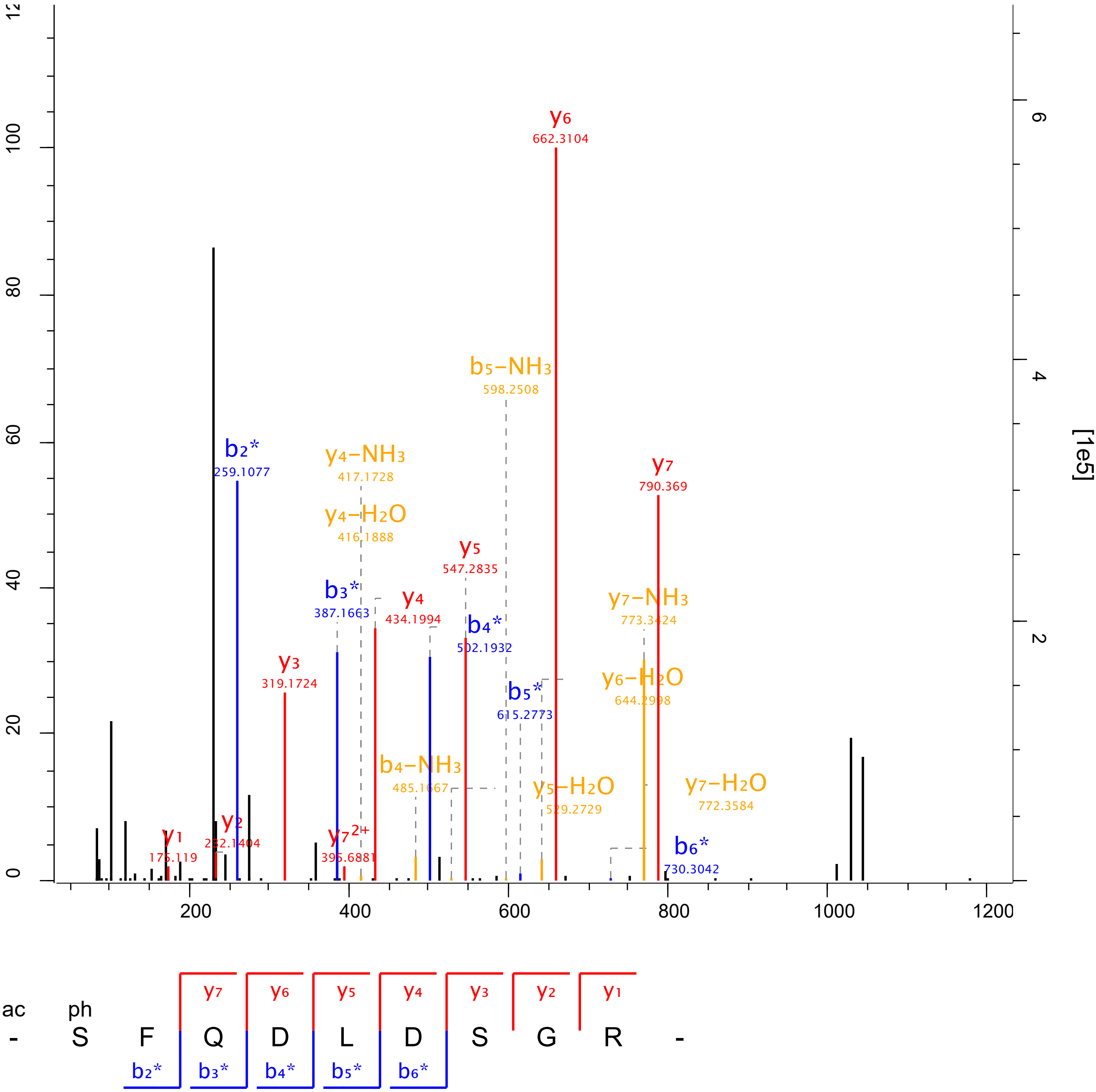

Raw file	Scan	Method	Score	m/z
sys_00_3short	30029	FTMS; HCD	80.31	590.81

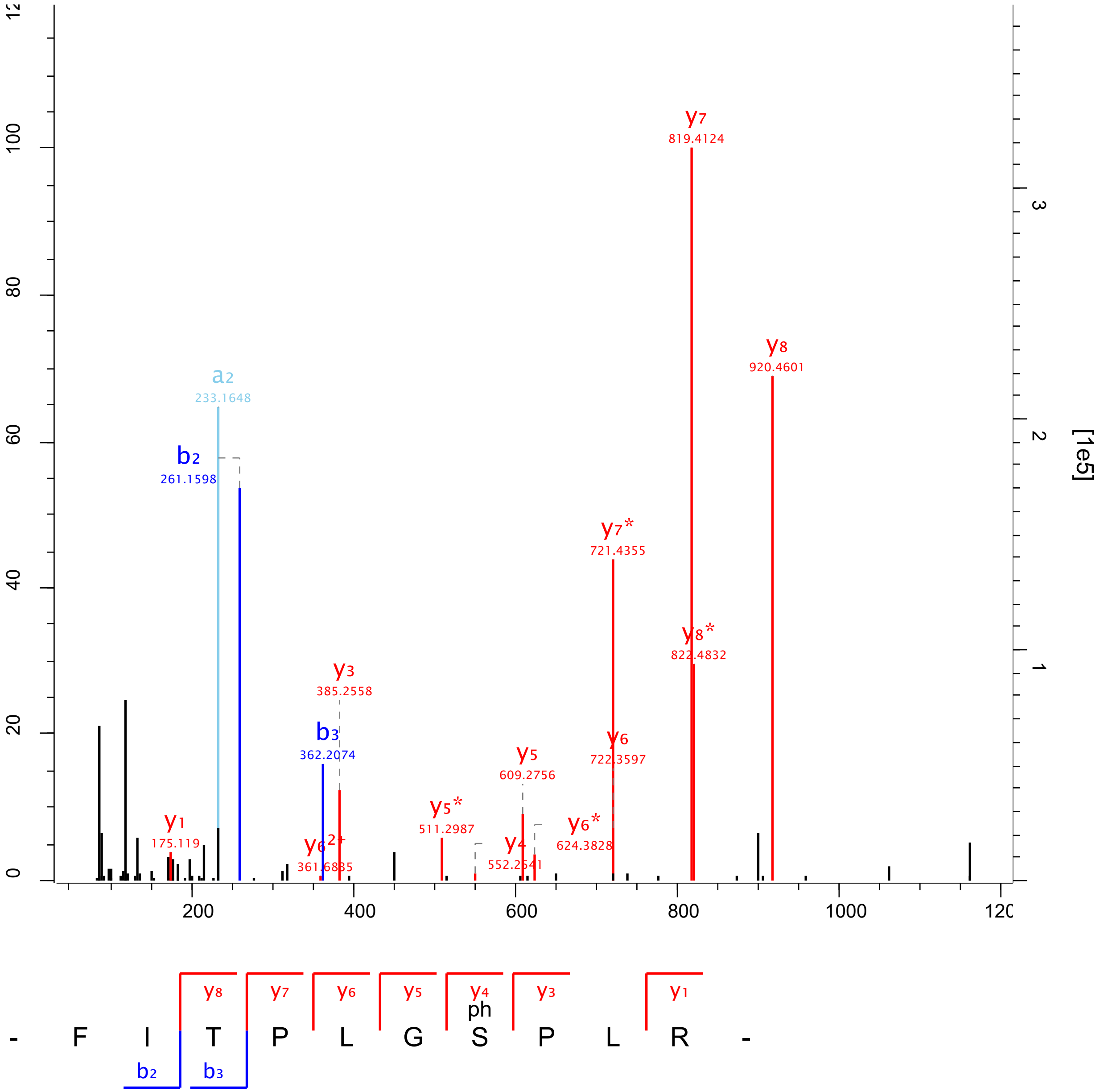

Raw file	Scan	Method	Score	m/z
sys_00_3short	30170	FTMS; HCD	141.55	707.3

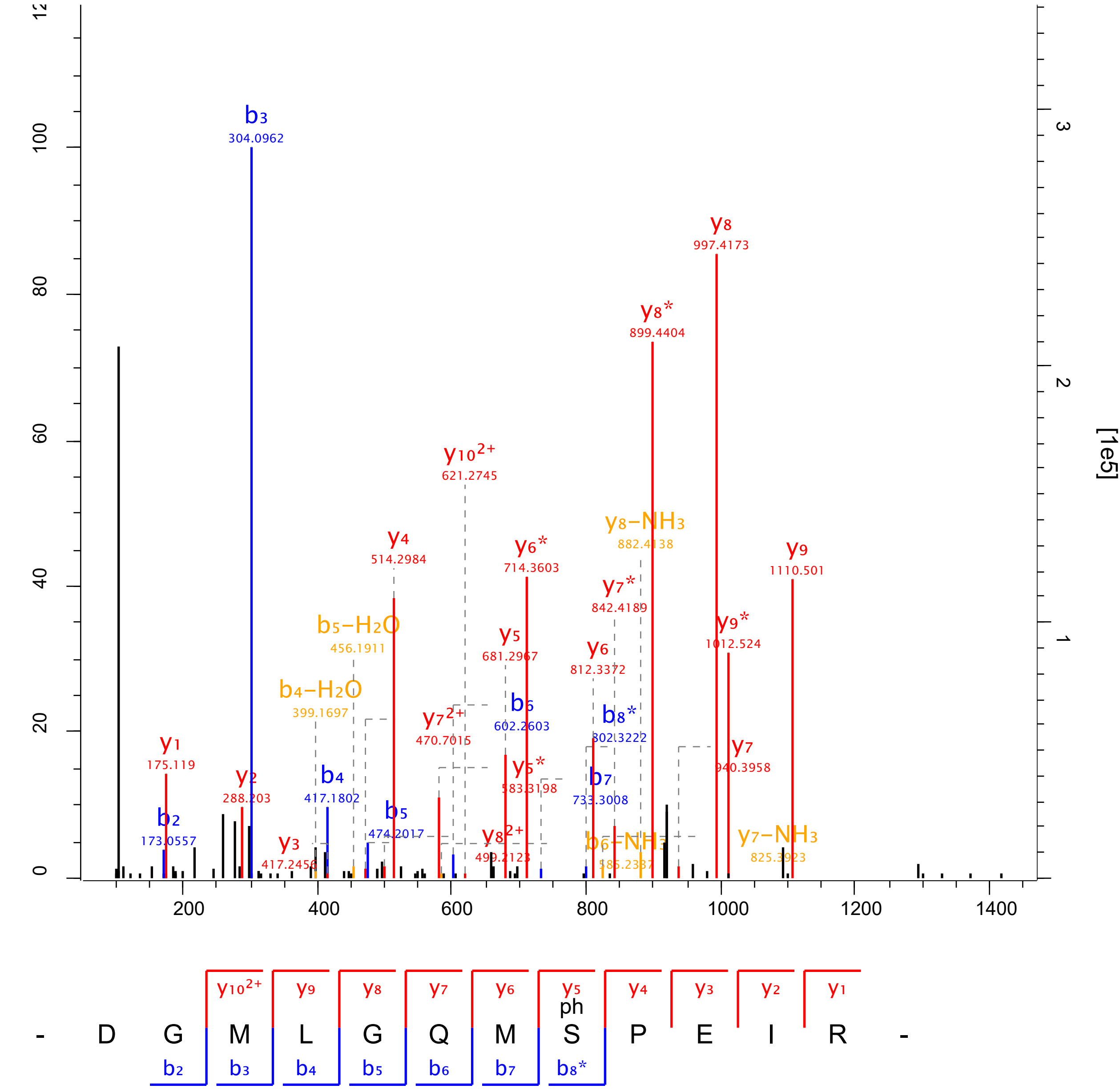

Raw file	Scan	Method	Score	m/z
sys_00_3short	30458	FTMS; HCD	48.05	842.89

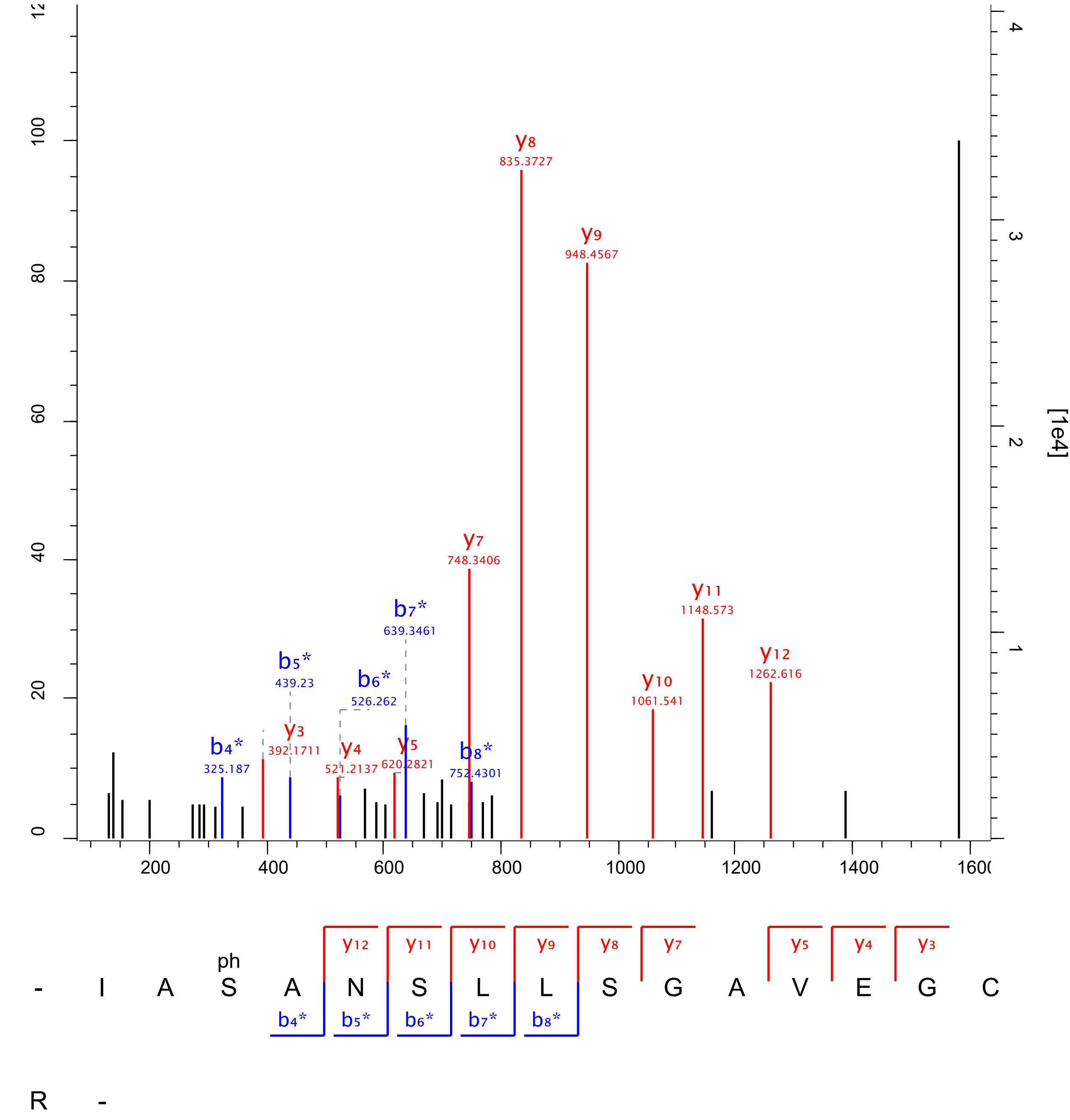

Raw file	Scan	Method	Score	m/z
sys_00_3short	30506	FTMS; HCD	96.14	865.36

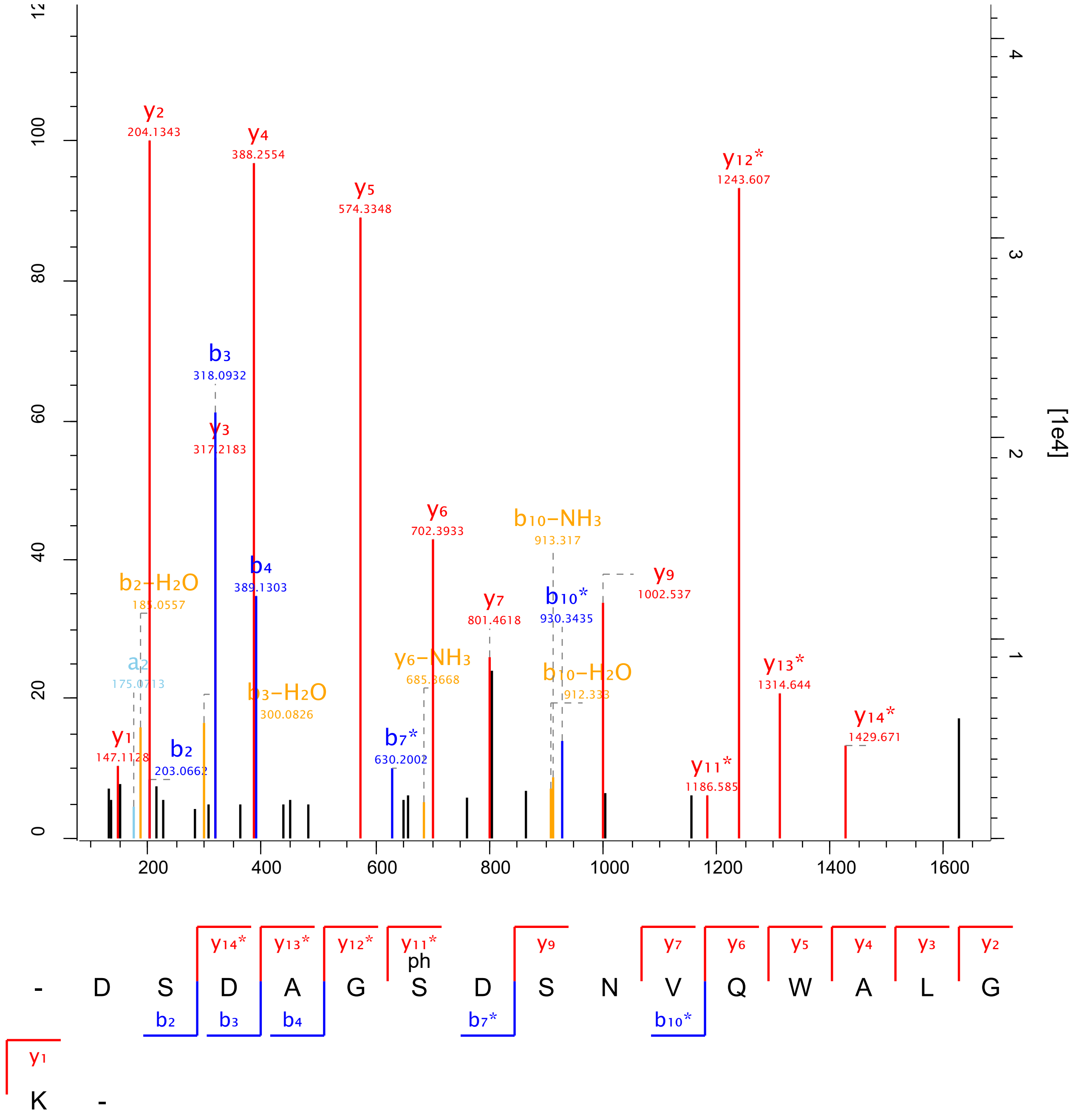

Raw file	Scan	Method	Score	m/z
sys_00_3short	30548	FTMS; HCD	106.57	769.9

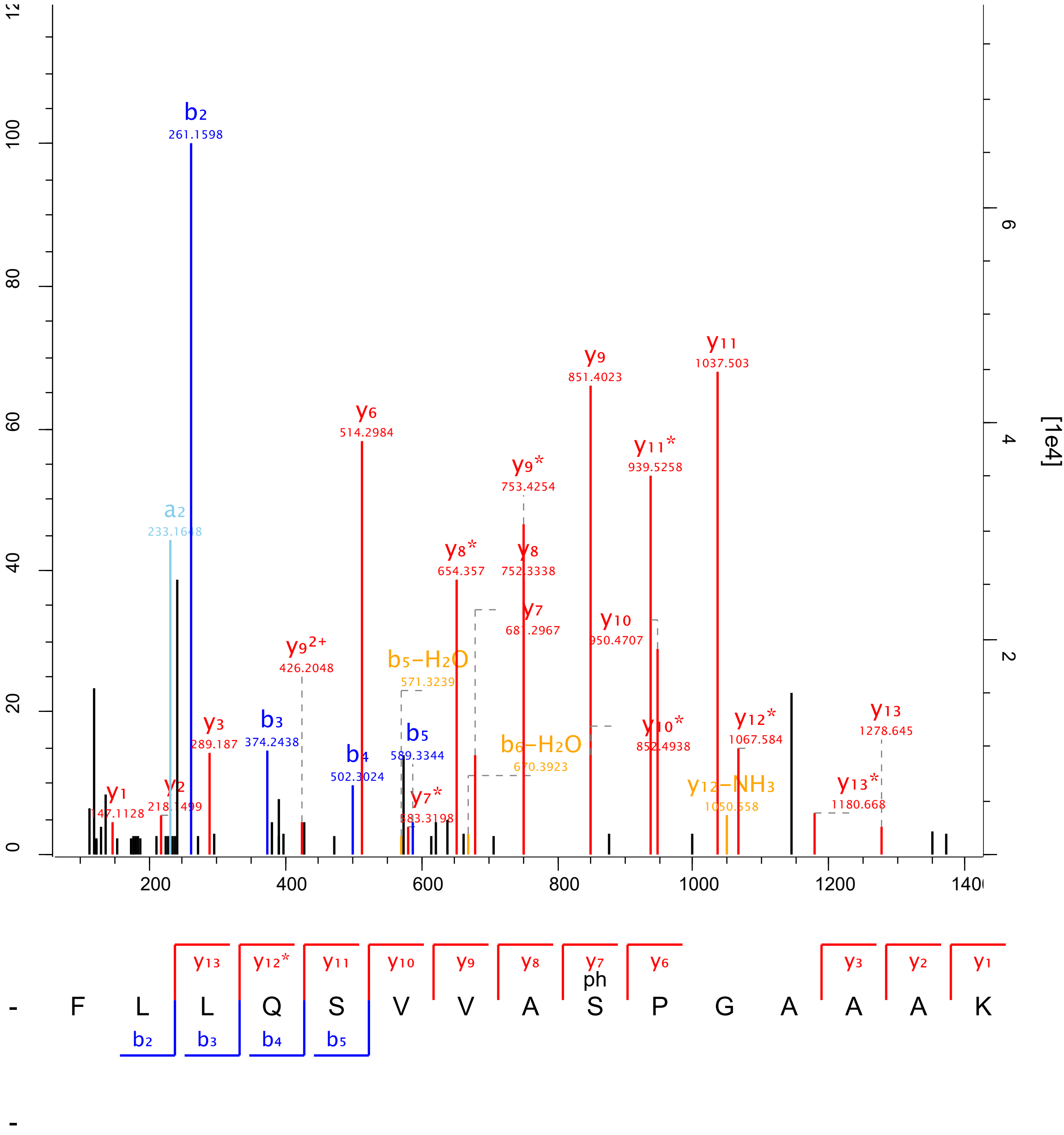

Raw file	Scan	Method	Score	m/z
sys_00_3short	30791	FTMS; HCD	222.81	637.79

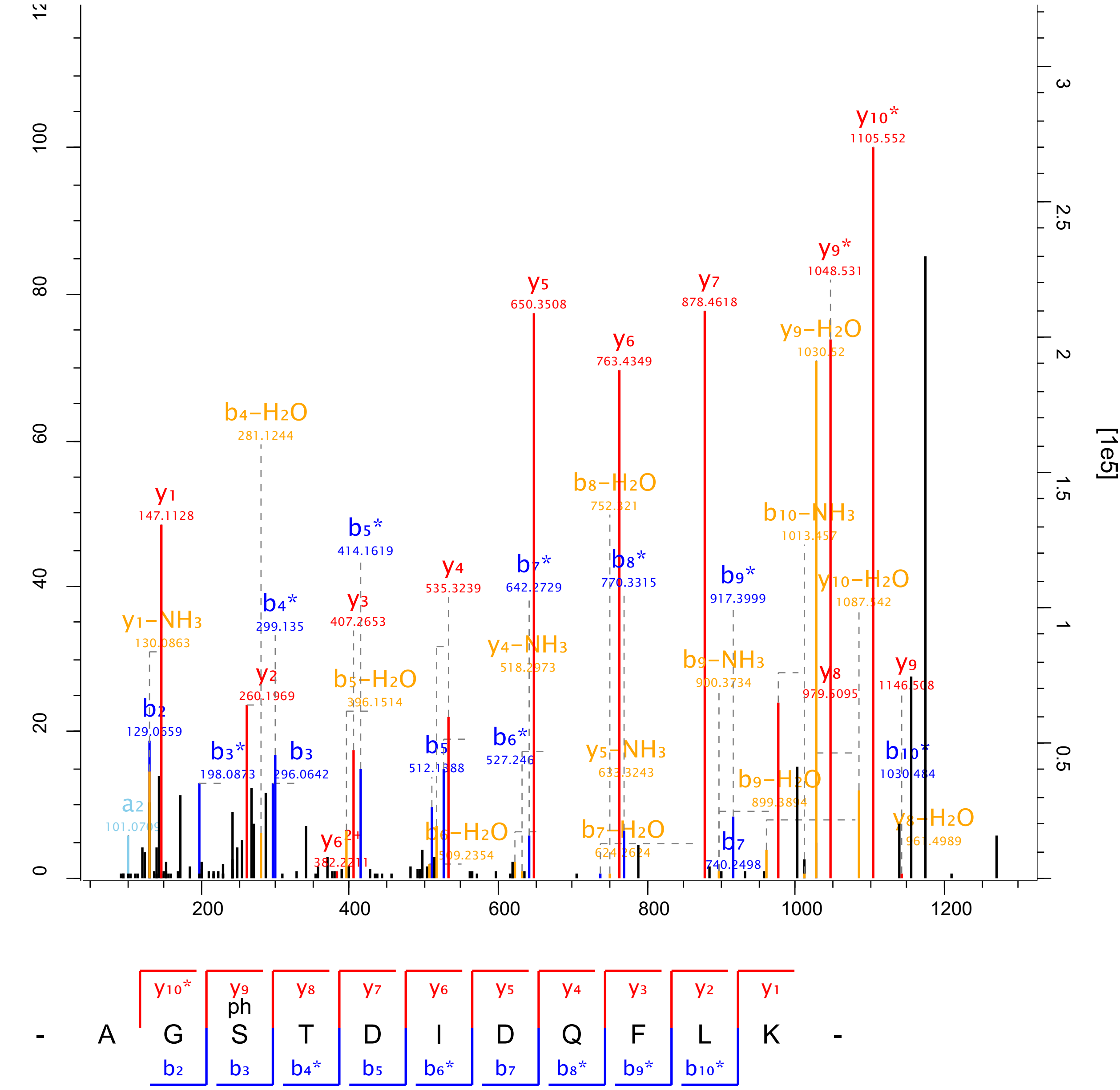

Raw file	Scan	Method	Score	m/z
sys_00_3short	30987	FTMS; HCD	126.71	721.33

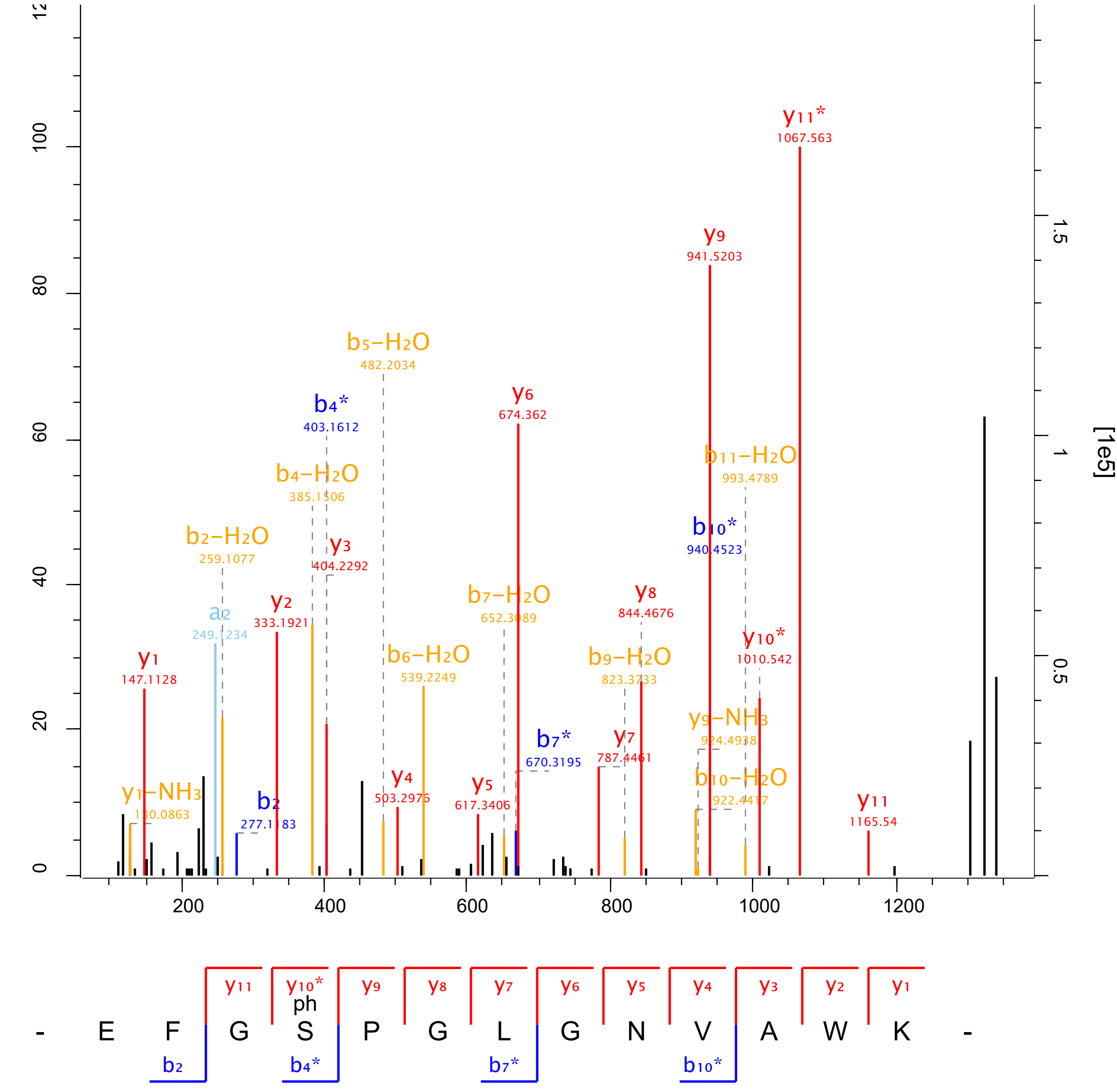

Raw file	Scan	Method	Score	m/z
sys_00_3short	31060	FTMS; HCD	80.75	626.81

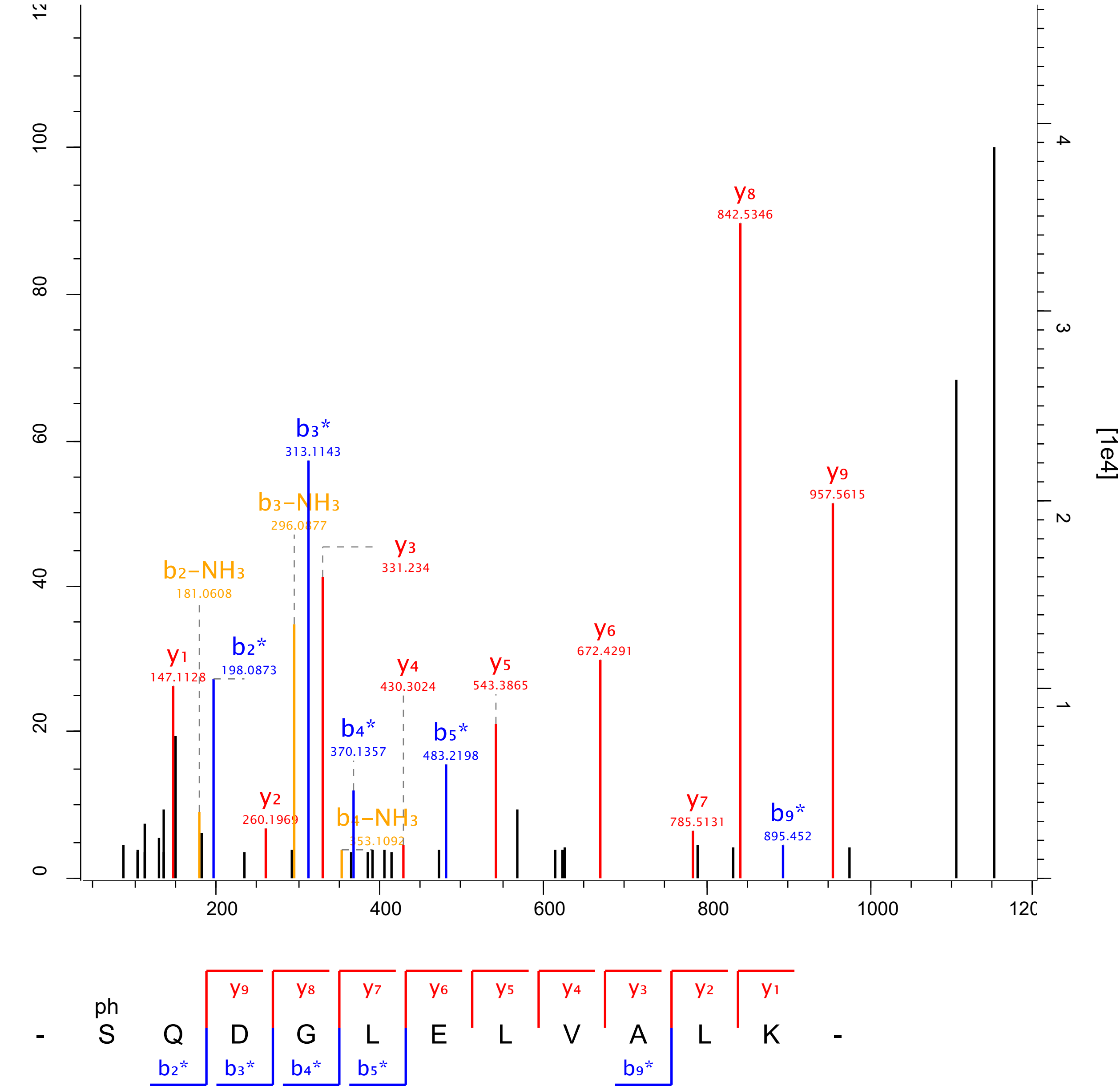

Raw file	Scan	Method	Score	m/z
sys_00_3short	31127	FTMS; HCD	206.62	837.33

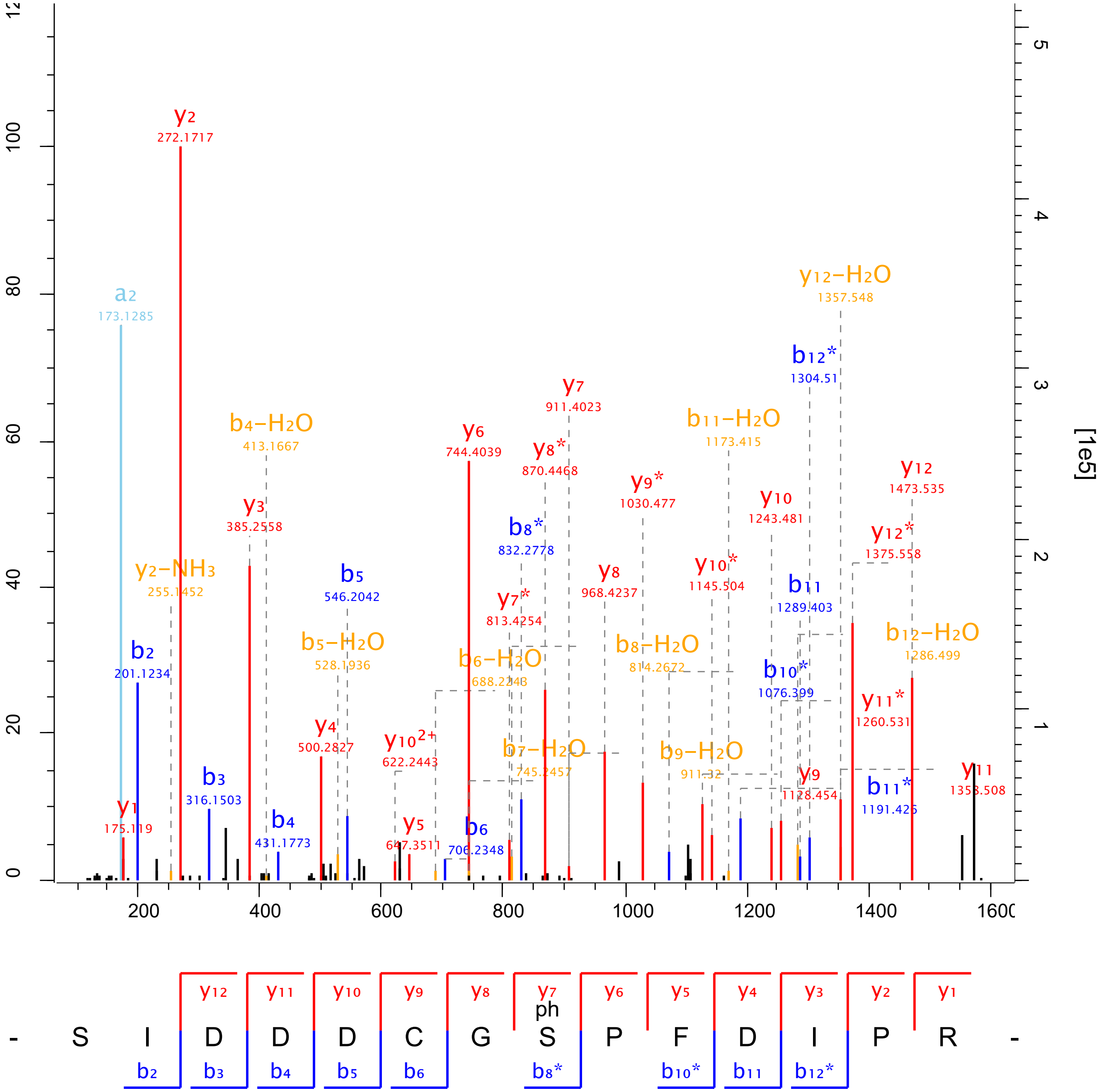

Raw file	Scan	Method	Score	m/z
sys_00_3short	31164	FTMS; HCD	272.72	844.83

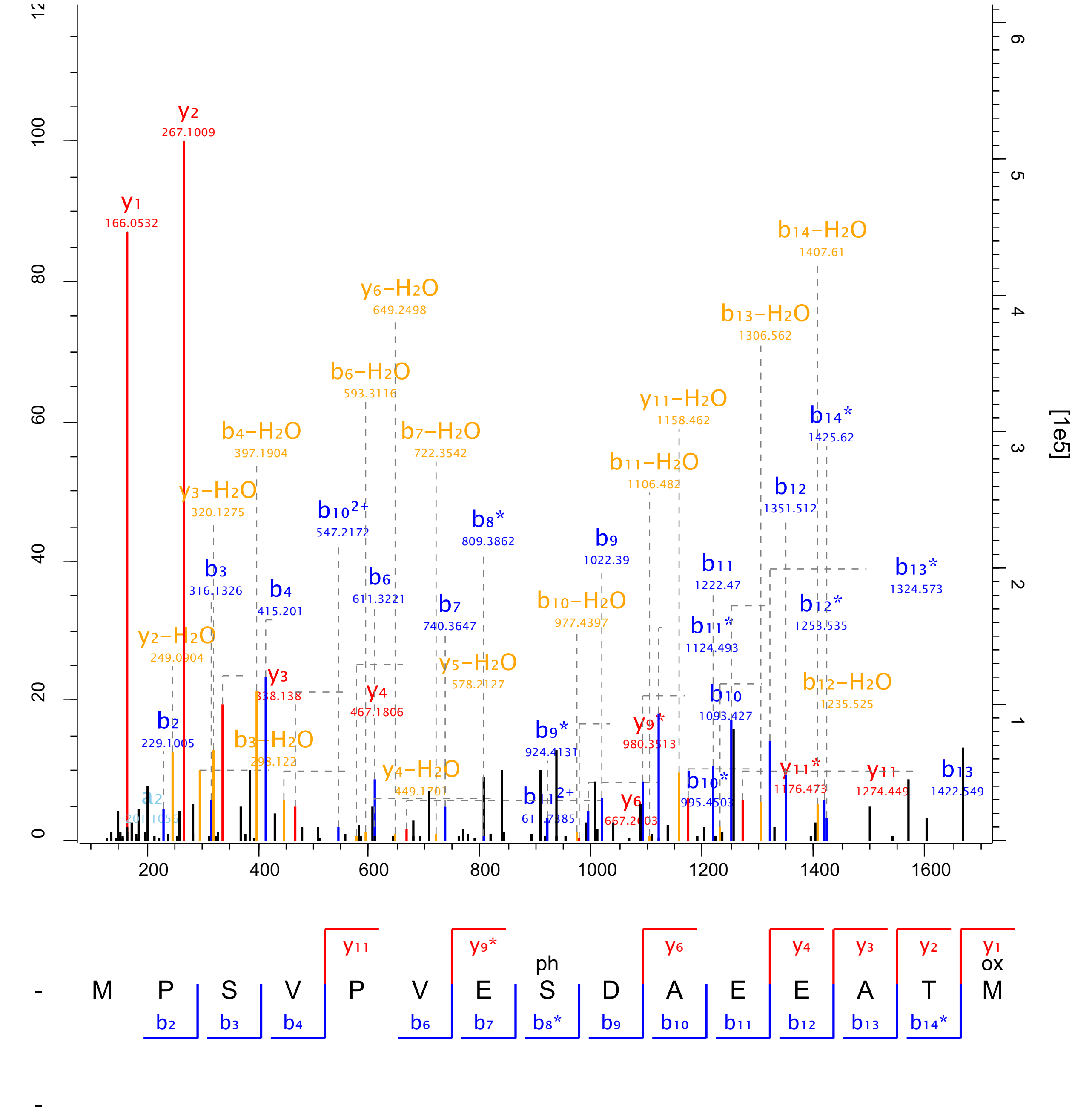

Raw file	Scan	Method	Score	m/z
sys_00_3short	31198	FTMS; HCD	67.65	816.37

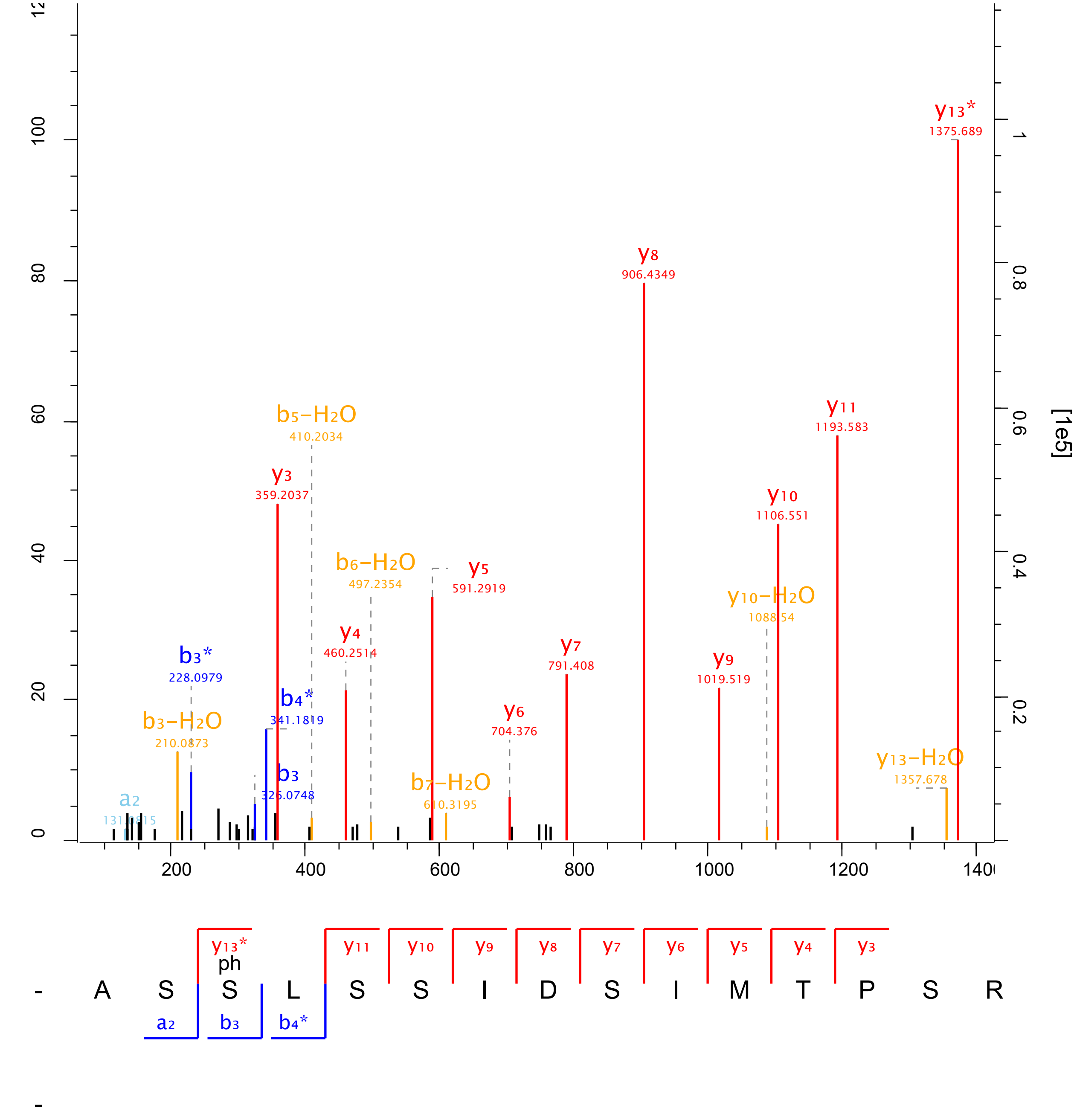

Raw file	Scan	Method	Score	m/z
sys_00_3short	31306	FTMS; HCD	54.34	617.77

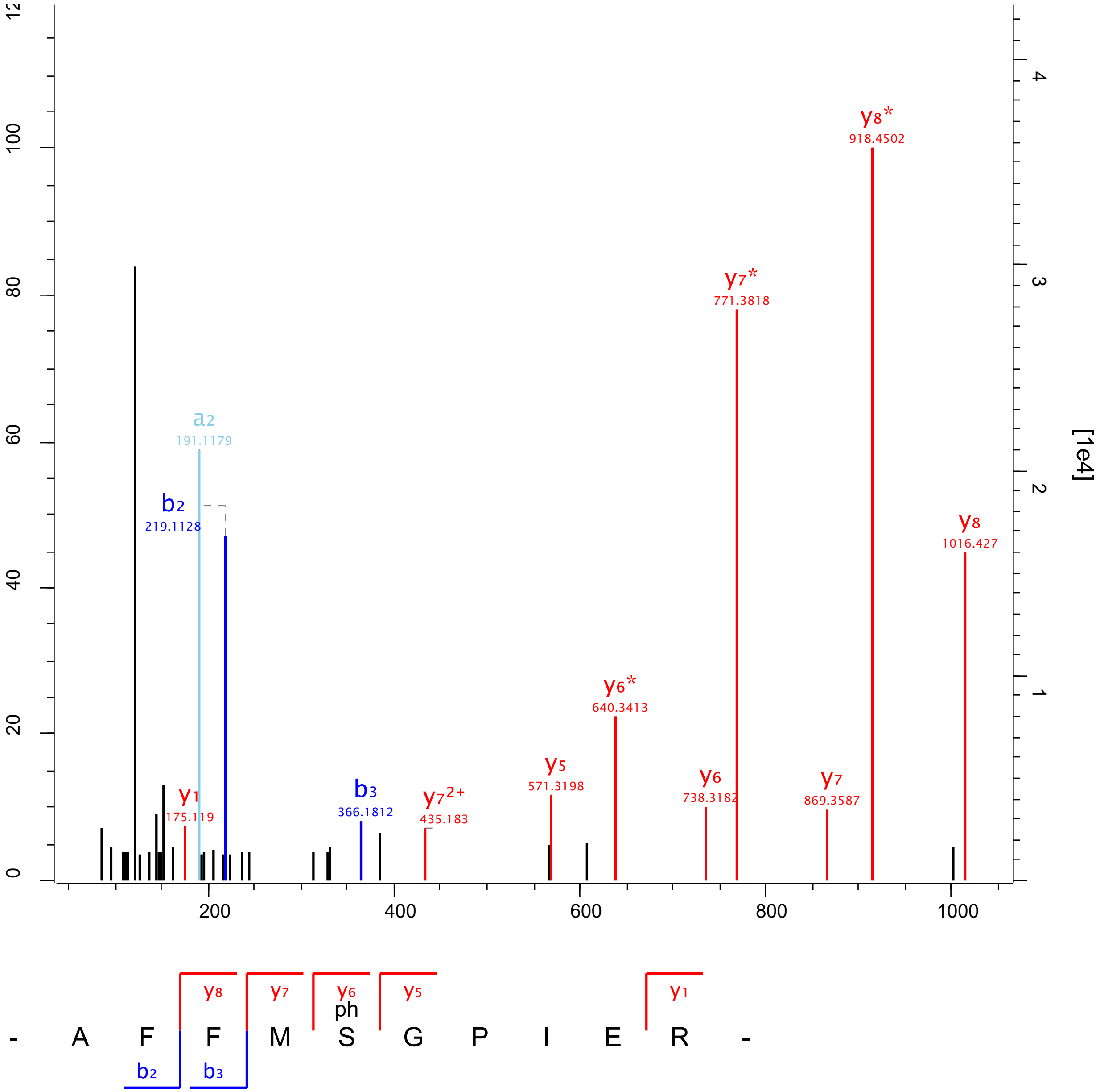

Raw file	Scan	Method	Score	m/z
sys_00_3short	31579	FTMS; HCD	82.01	787.35

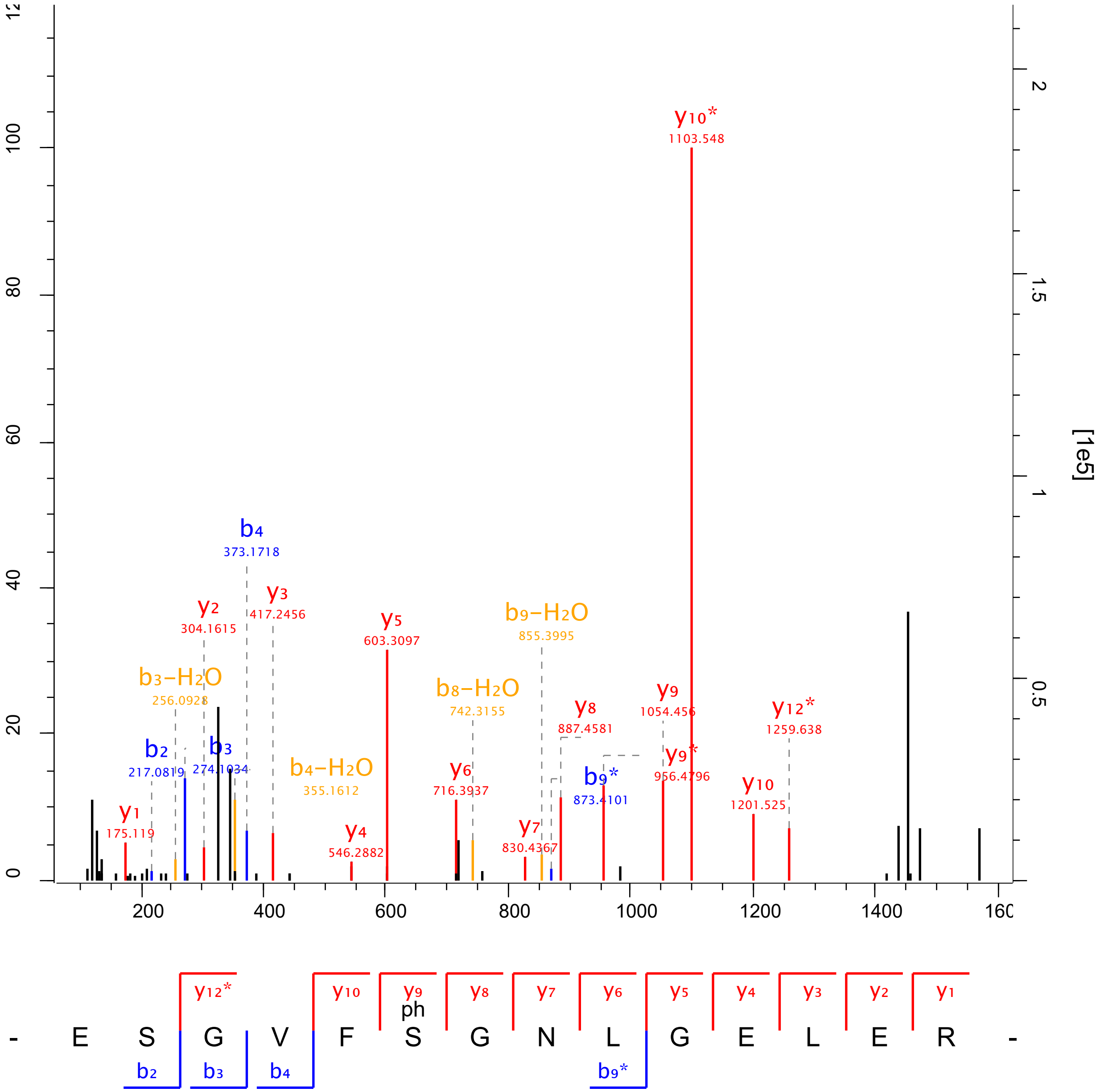

Raw file	Scan	Method	Score	m/z
sys_00_3short	31724	FTMS; HCD	78.19	700.32

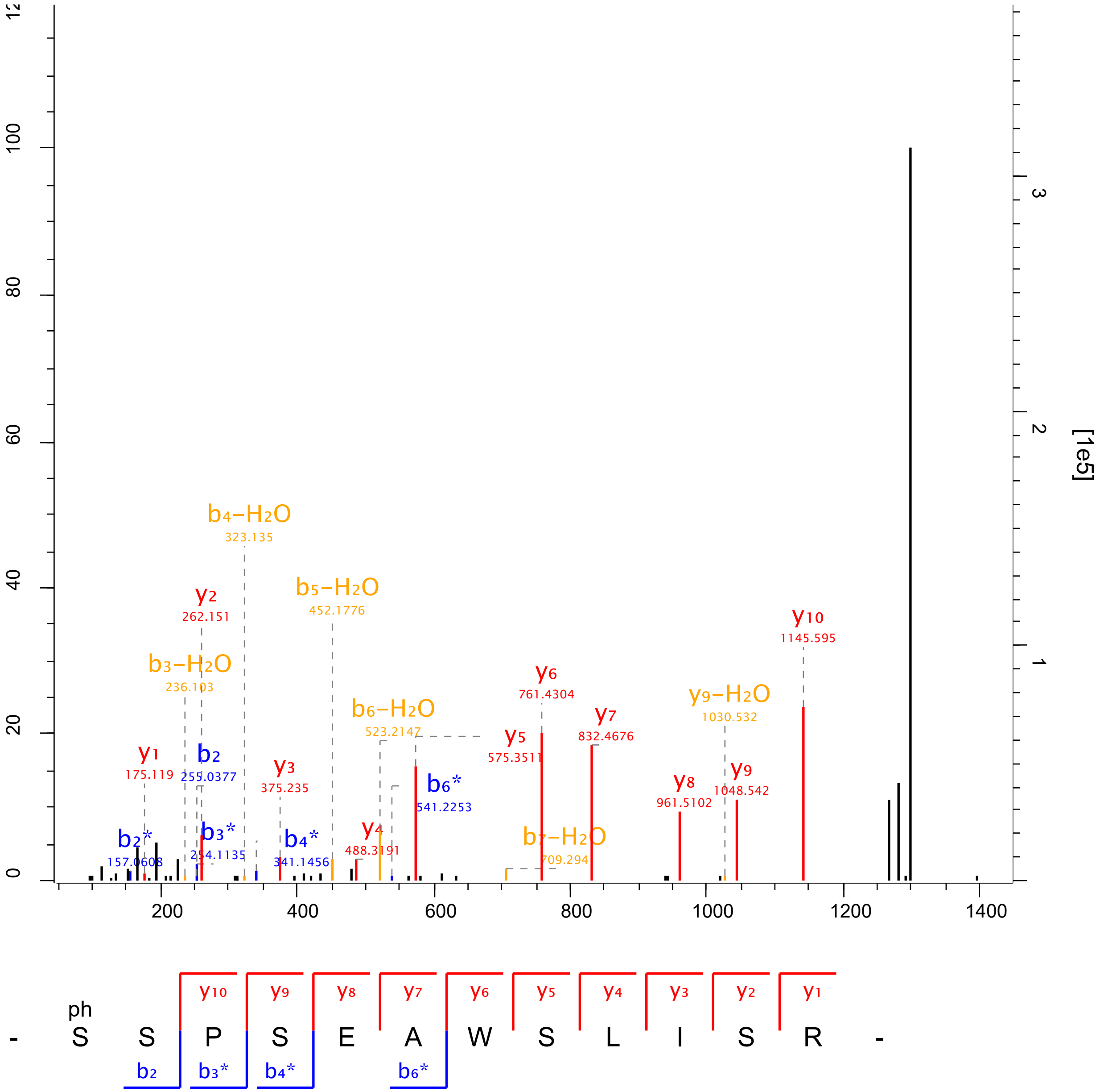

Raw file	Scan	Method	Score	m/z
sys_00_3short	31753	FTMS; HCD	332.56	868.69

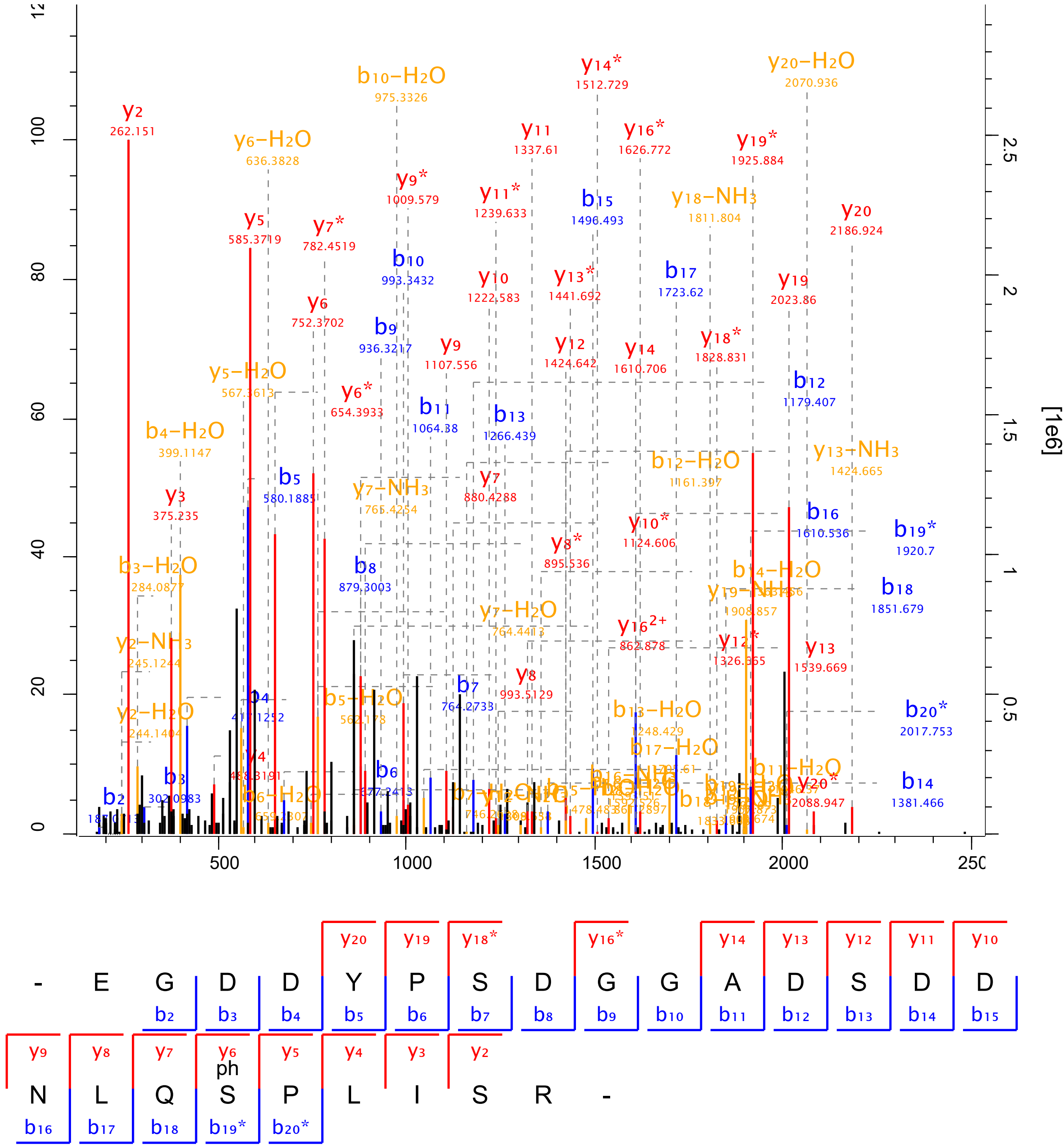

Raw file	Scan	Method	Score	m/z
sys_00_3short	32041	FTMS; HCD	65.18	599.75

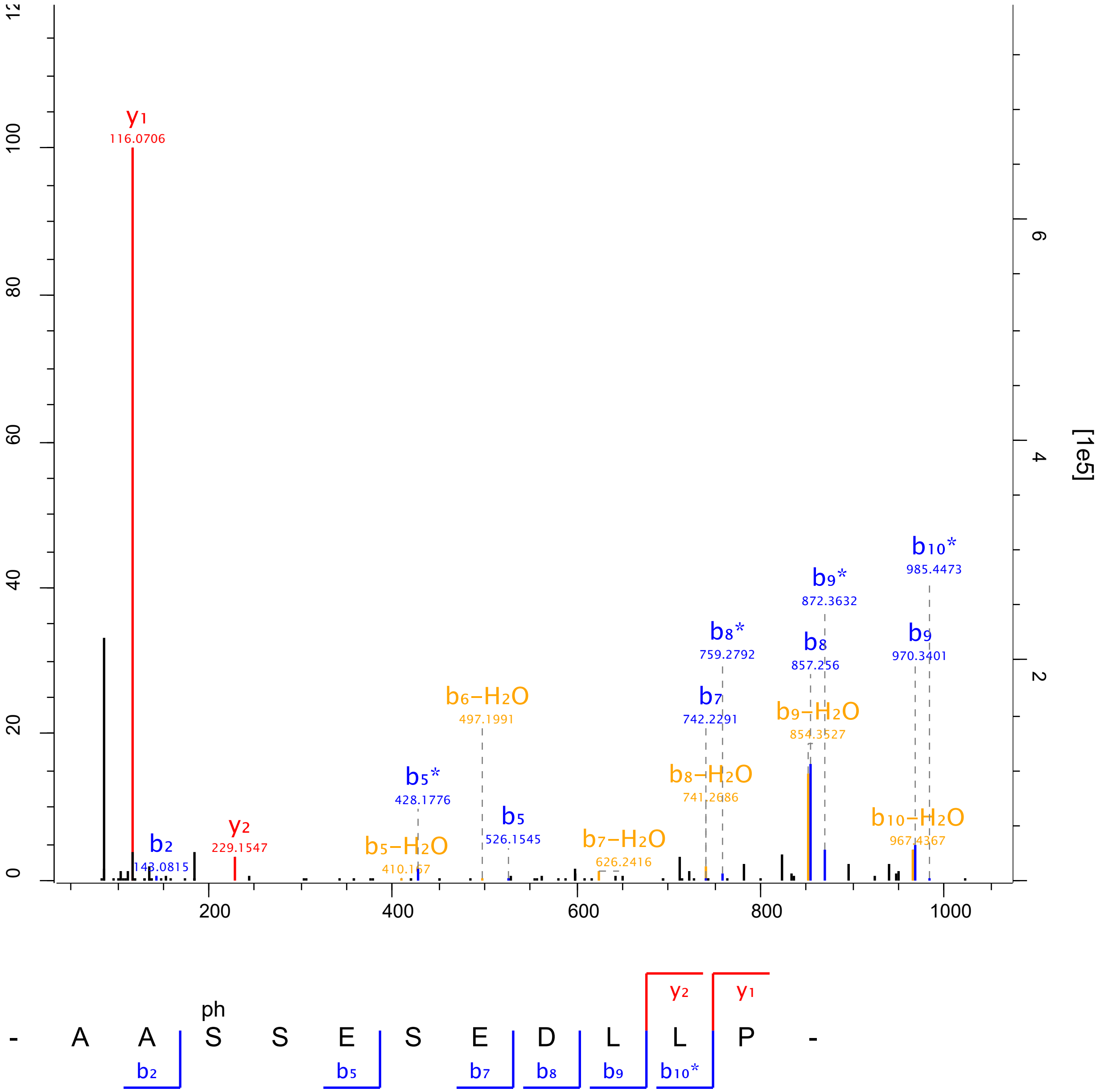

Raw file	Scan	Method	Score	m/z
sys_00_3short	3291	FTMS; HCD	72.29	577.23

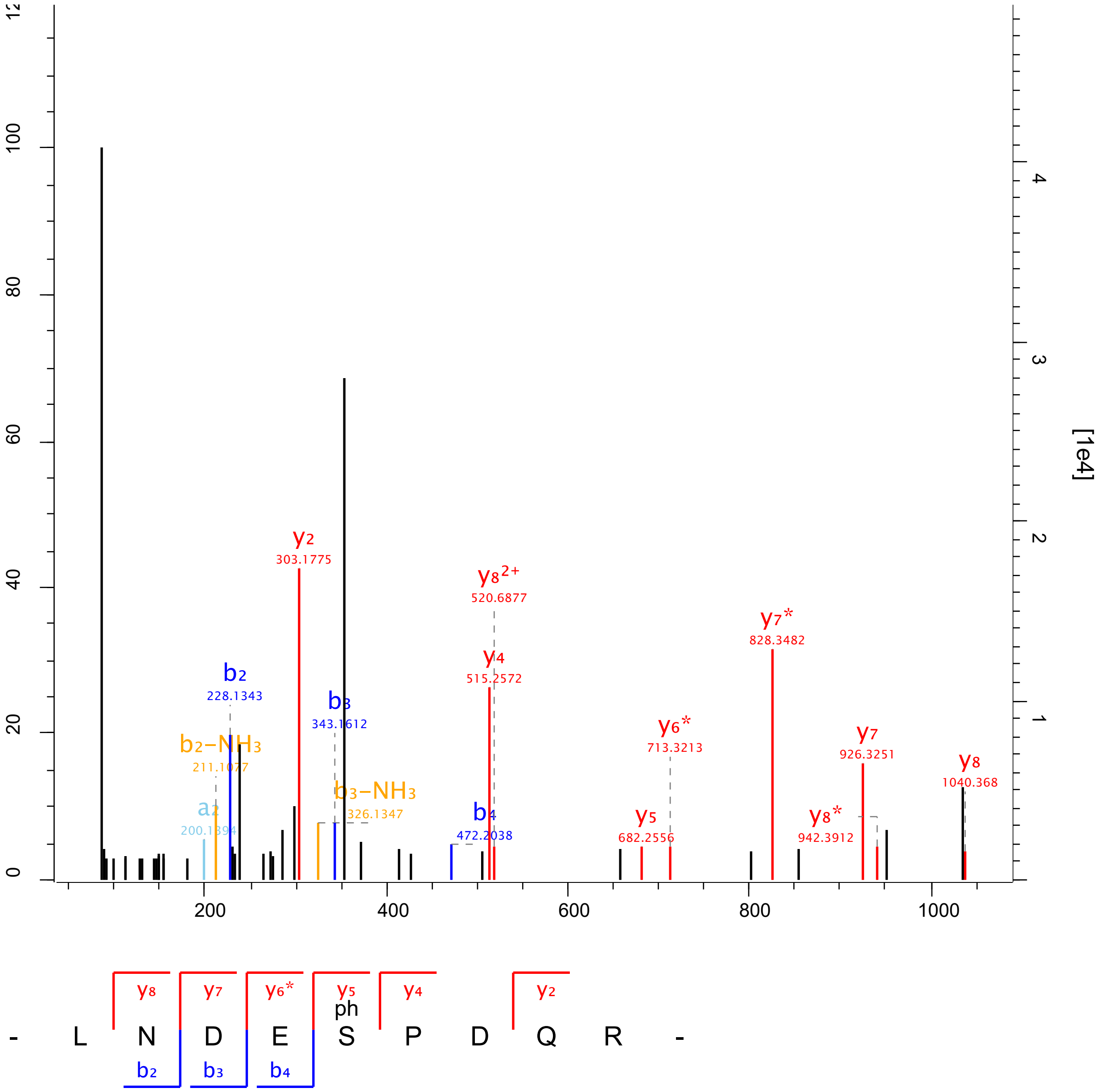

Raw file	Scan	Method	Score	m/z
sys_00_3short	33357	FTMS; HCD	53.24	651.29

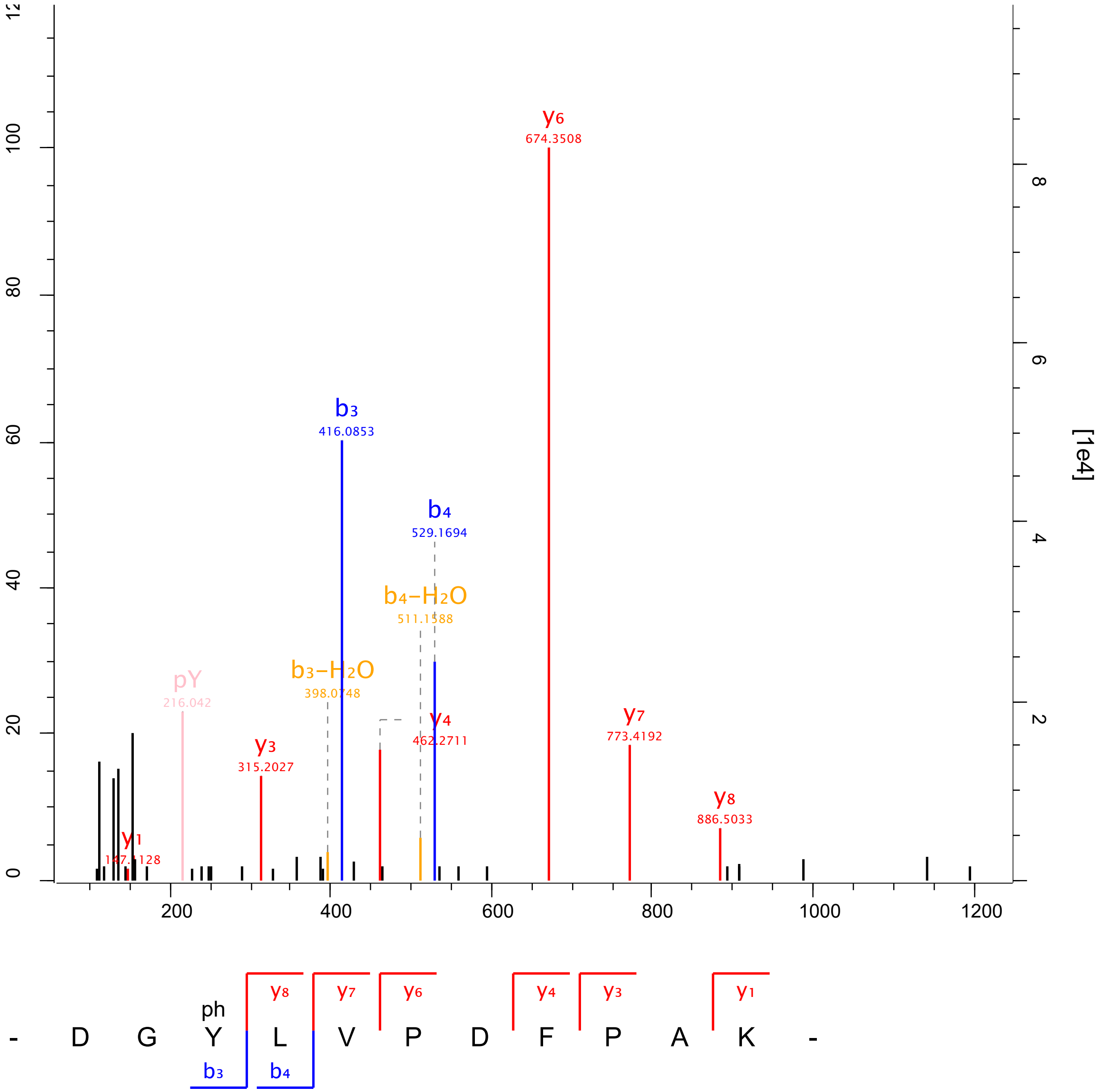

Raw file	Scan	Method	Score	m/z
sys_00_3short	33404	FTMS; HCD	130.89	997.38

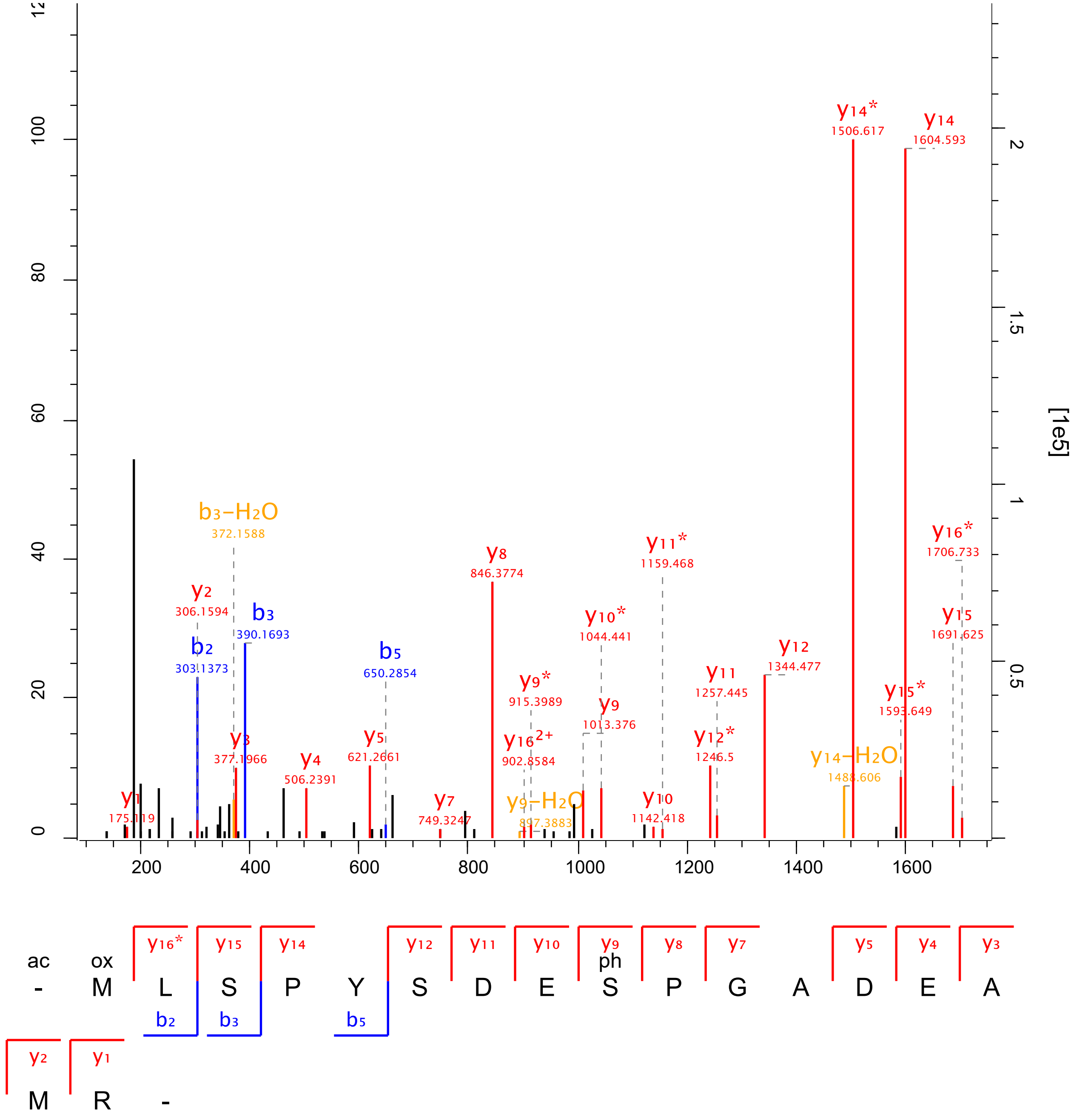

Raw file	Scan	Method	Score	m/z
sys_00_3short	33606	FTMS; HCD	42.78	607.28

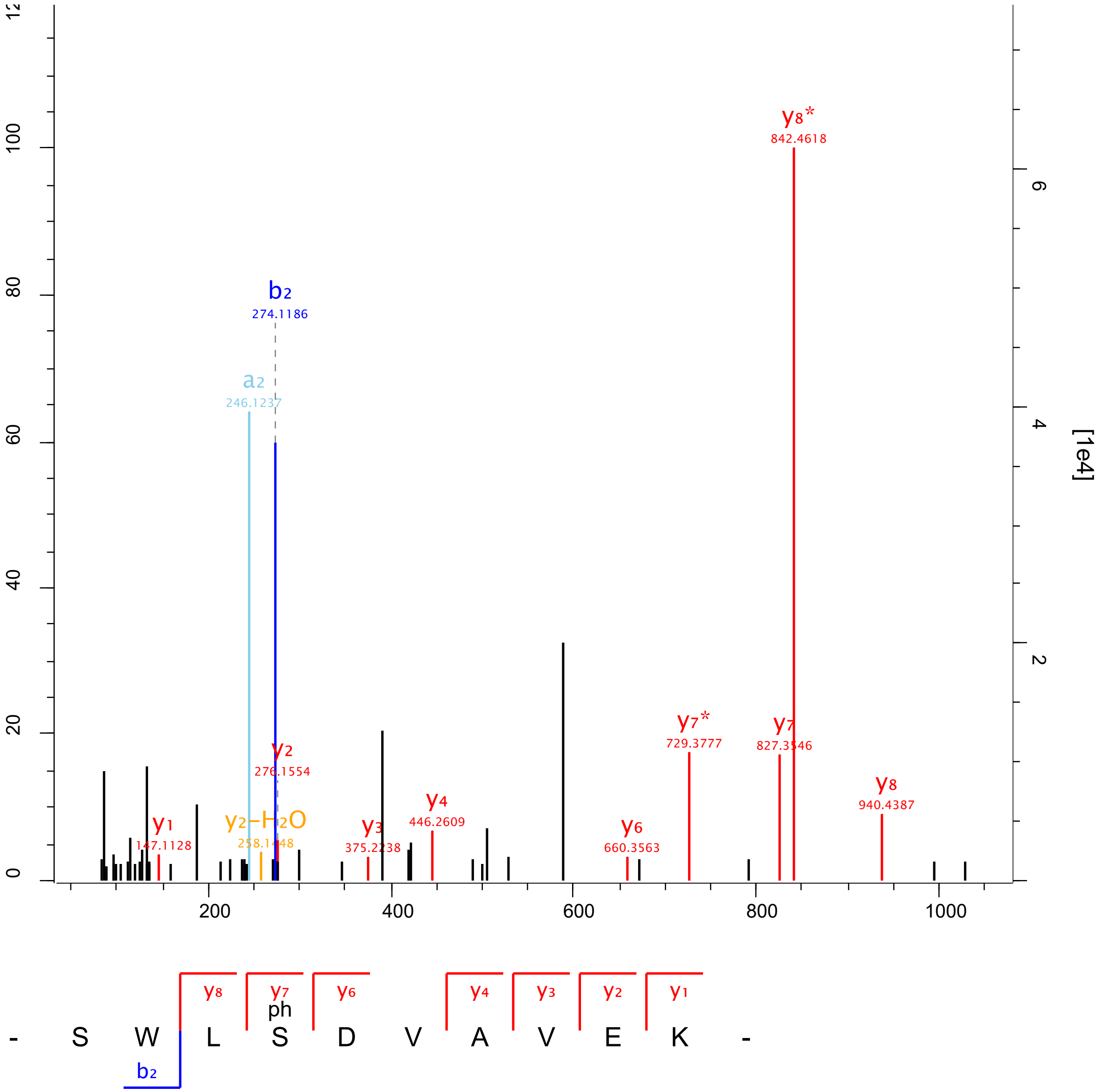

Raw file	Scan	Method	Score	m/z
sys_00_3short	33950	FTMS; HCD	57.29	796.36

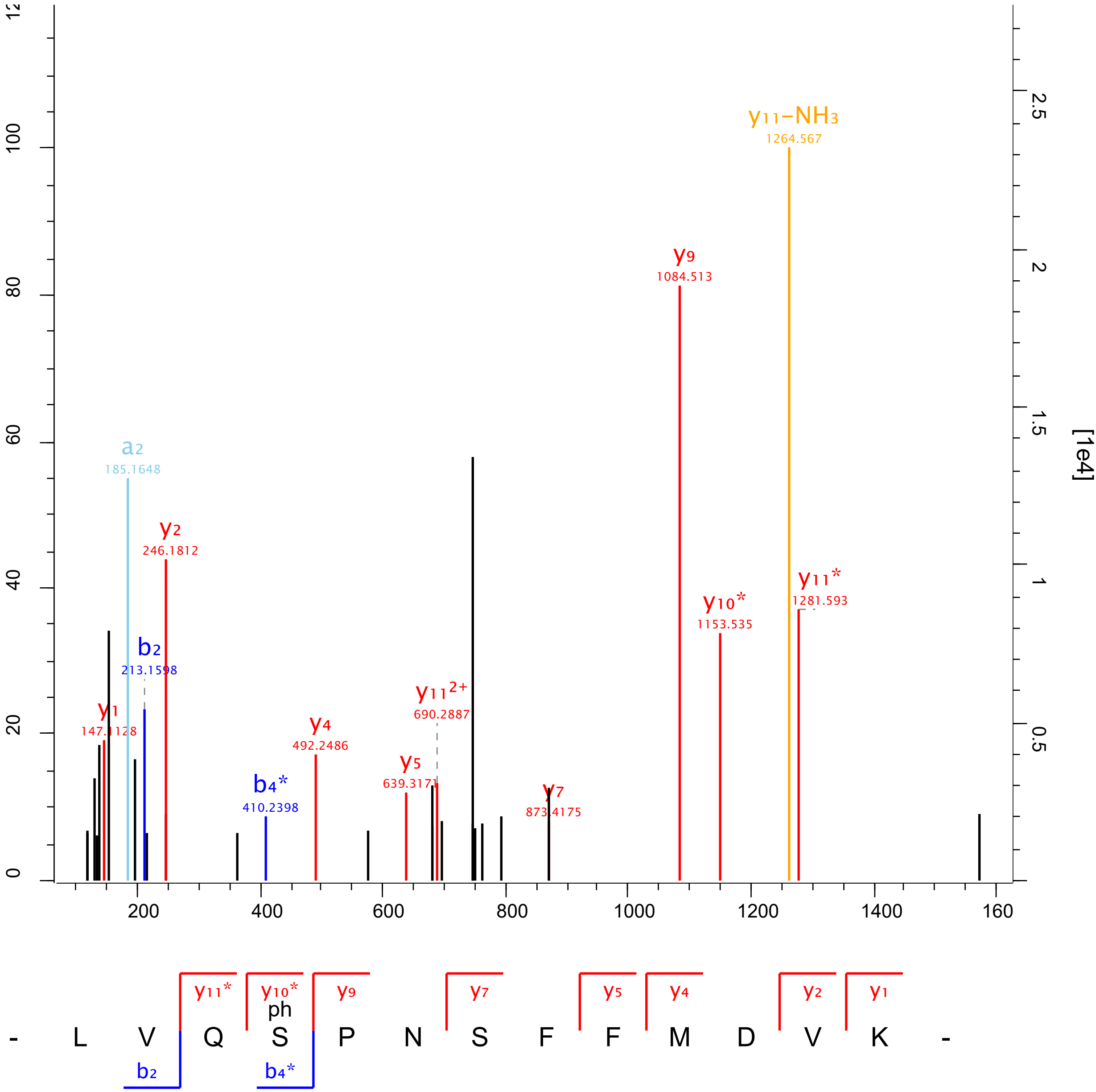

Raw file	Scan	Method	Score	m/z
sys_00_3short	34236	FTMS; HCD	135.91	599.76

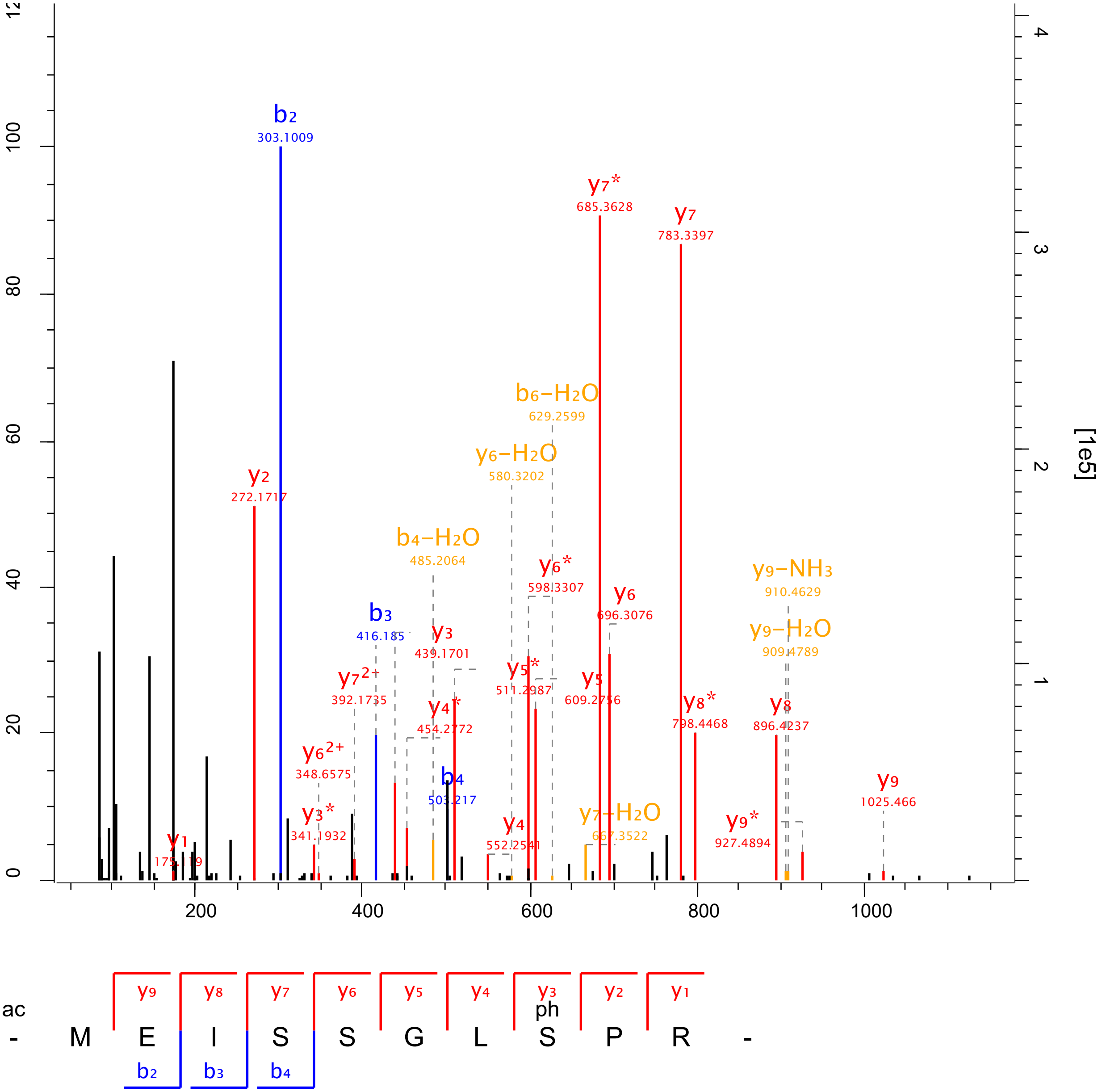

Raw file	Scan	Method	Score	m/z
sys_00_3short	3469	FTMS; HCD	83.2	526.74

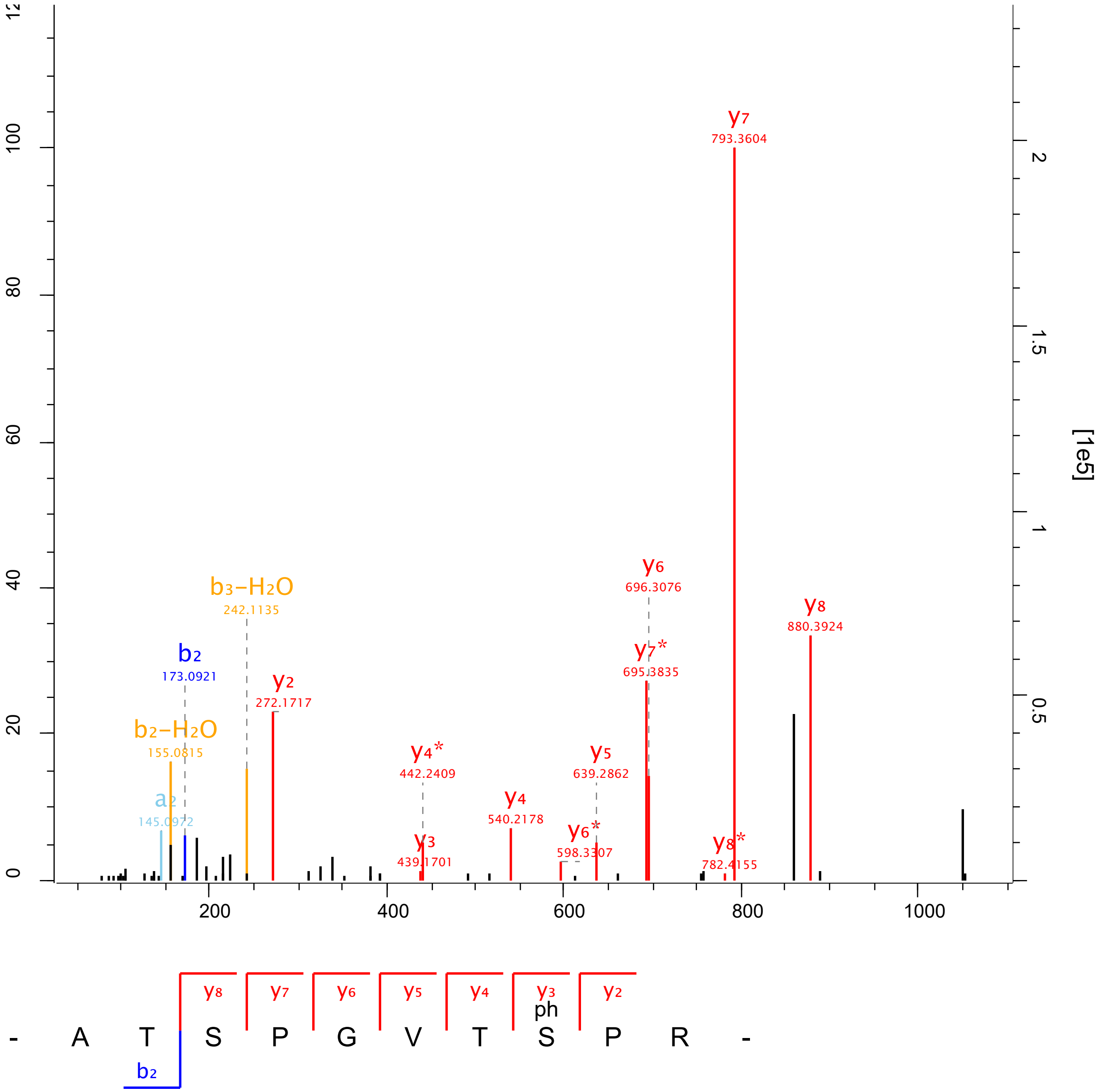

Raw file	Scan	Method	Score	m/z
sys_00_3short	34878	FTMS; HCD	219.95	836.83

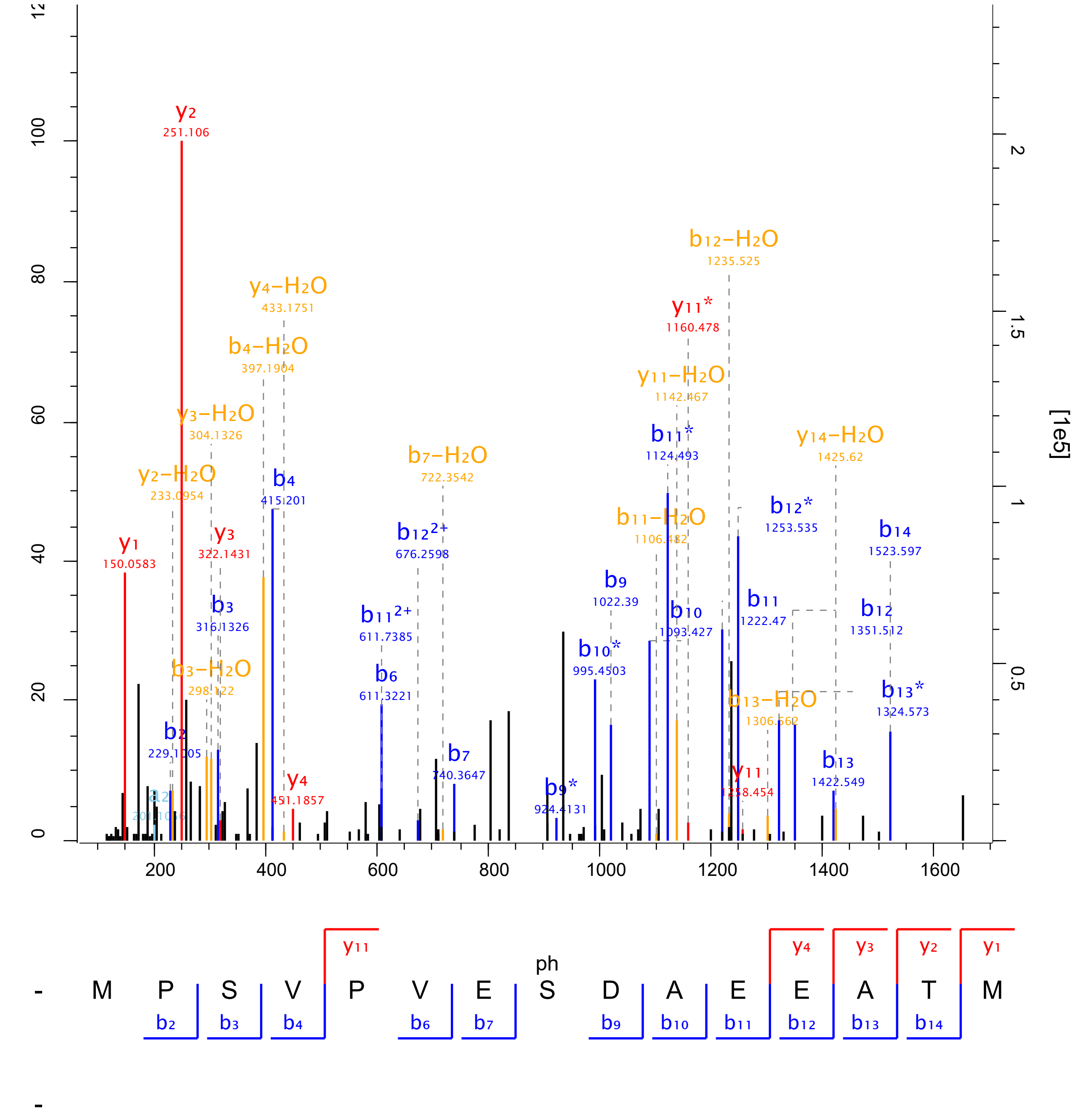

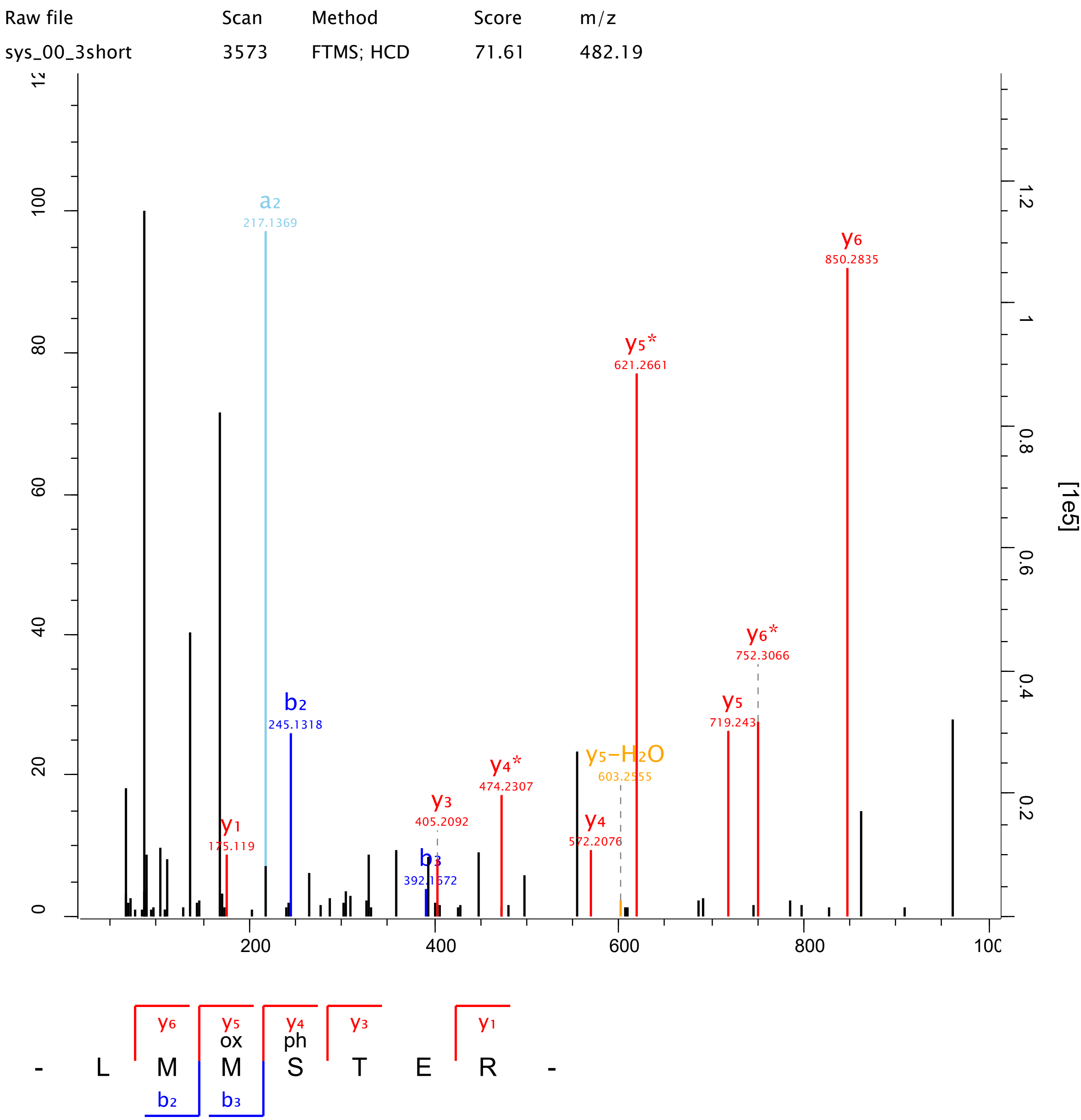
Raw file
Scan
Method
Score
m/z
sys_00_3short
3573
FTMS; HCD
71.61
482.19
a2
217.1369
y6
850.2835
y5*
621.2661
y6*
752.3066
b2
245.1318
y5
719.243
y5−H2O
603.2555
y4*
474.2307
y3
405.2092
y4
572.2076
y1
175.119
b3
392.1572
[1e5]
- L M M S T E R -
ox
ph

Raw file	Scan	Method	Score	m/z
sys_00_3short	3584	FTMS; HCD	68.52	457.21

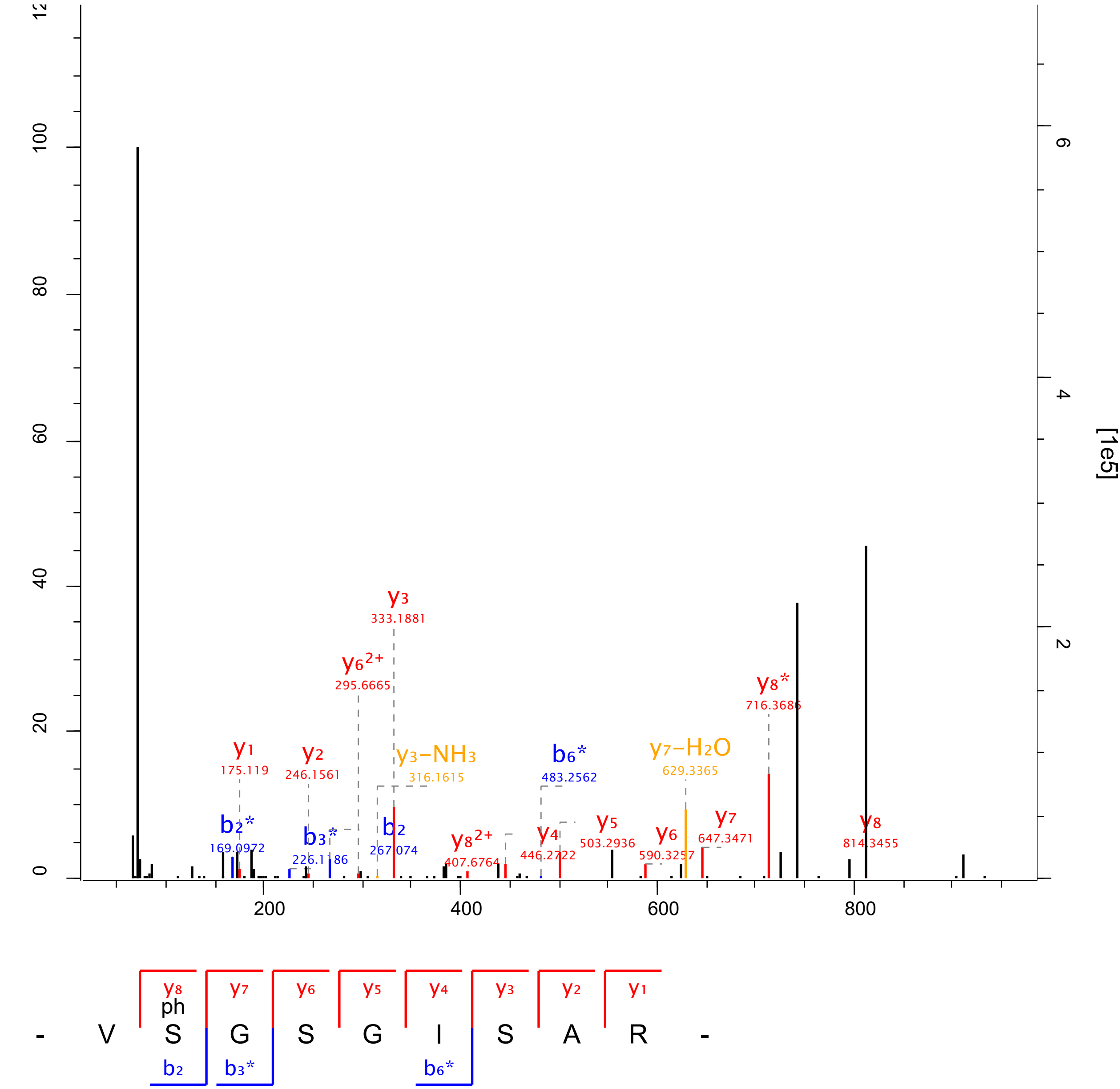

Raw file	Scan	Method	Score	m/z
sys_00_3short	3758	FTMS; HCD	119.51	636.76

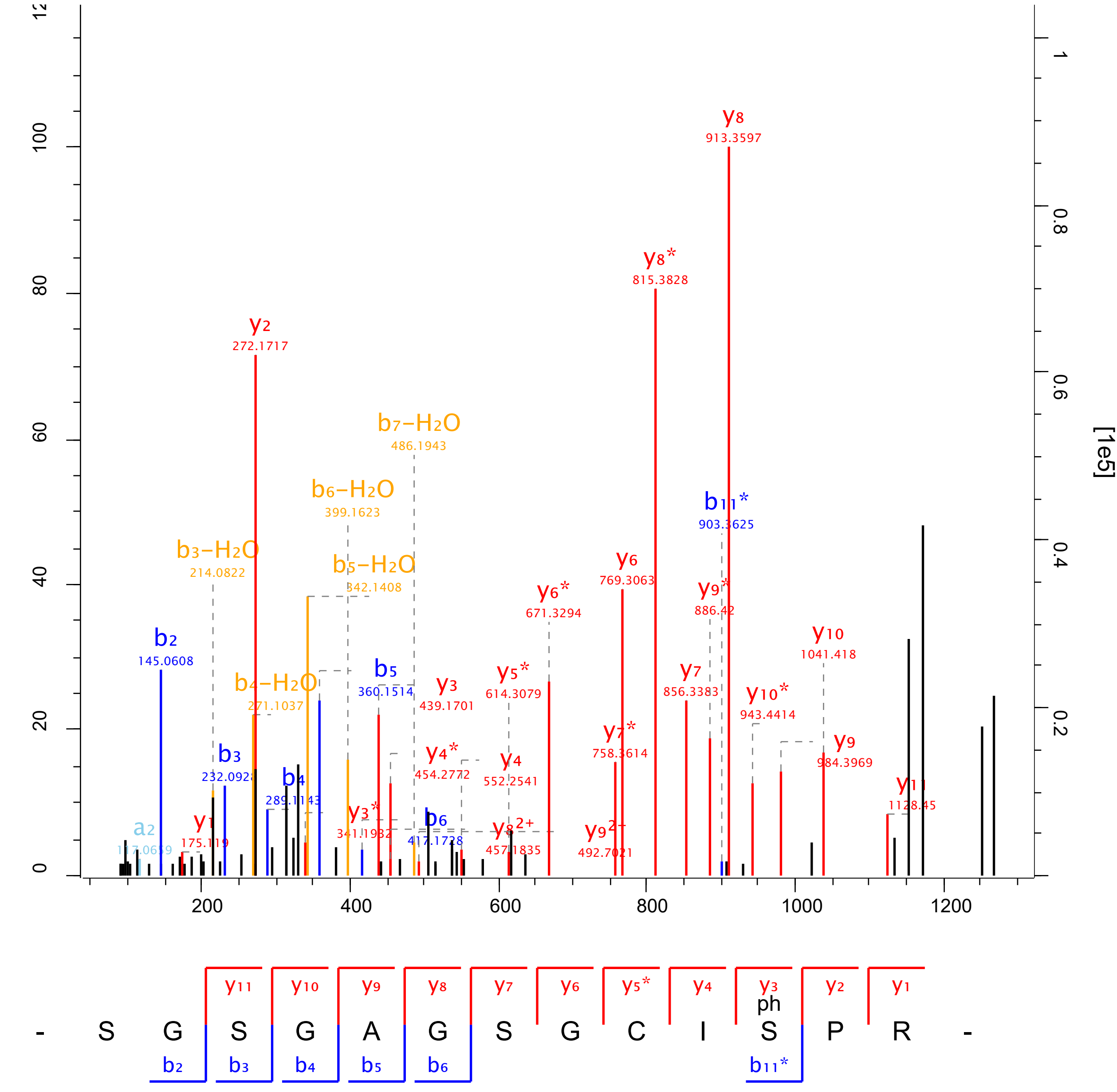

Raw file	Scan	Method	Score	m/z
sys_00_3short	4082	FTMS; HCD	107.06	536.25

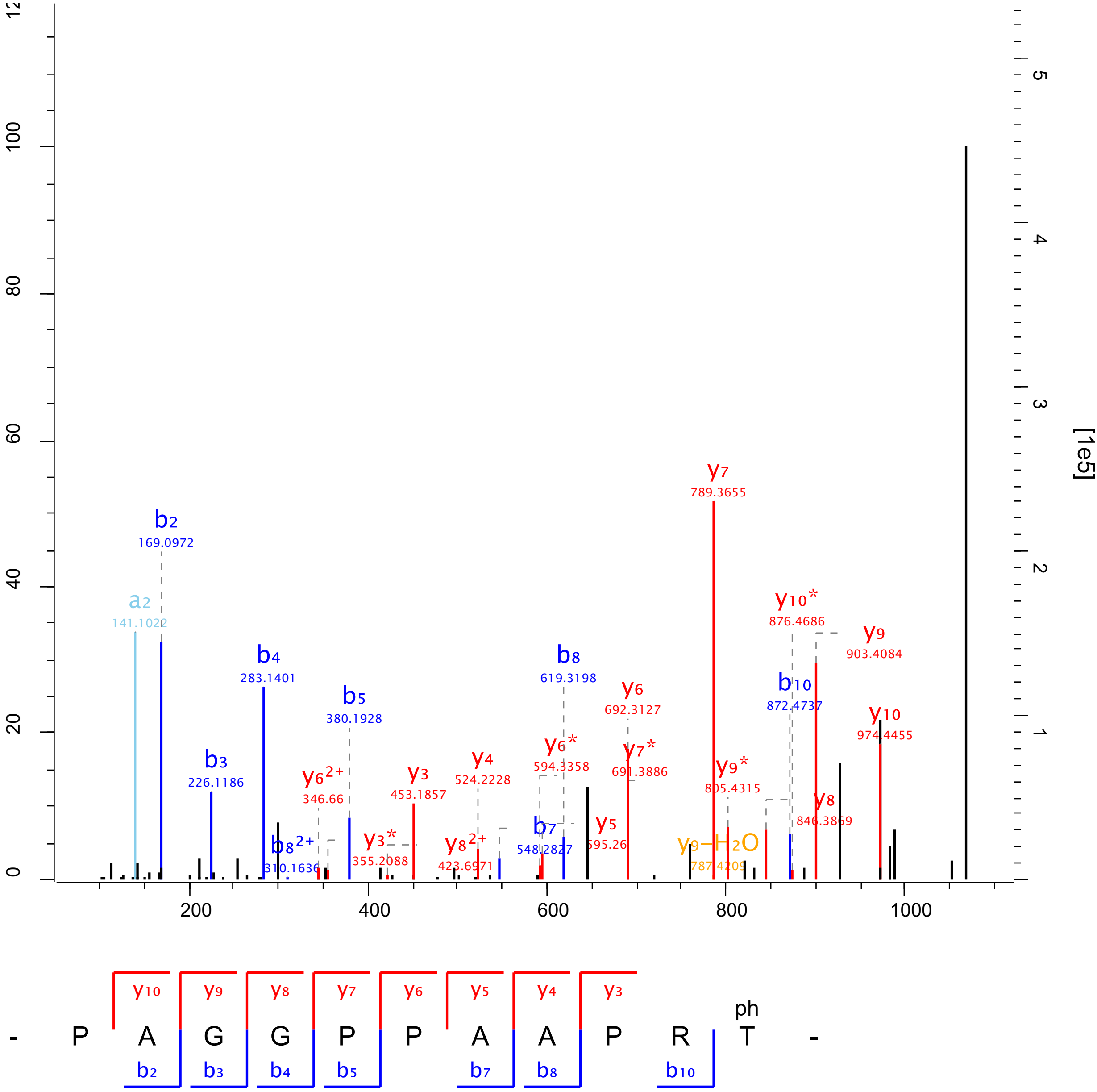

Raw file	Scan	Method	Score	m/z
sys_00_3short	4262	FTMS; HCD	48.57	742.78

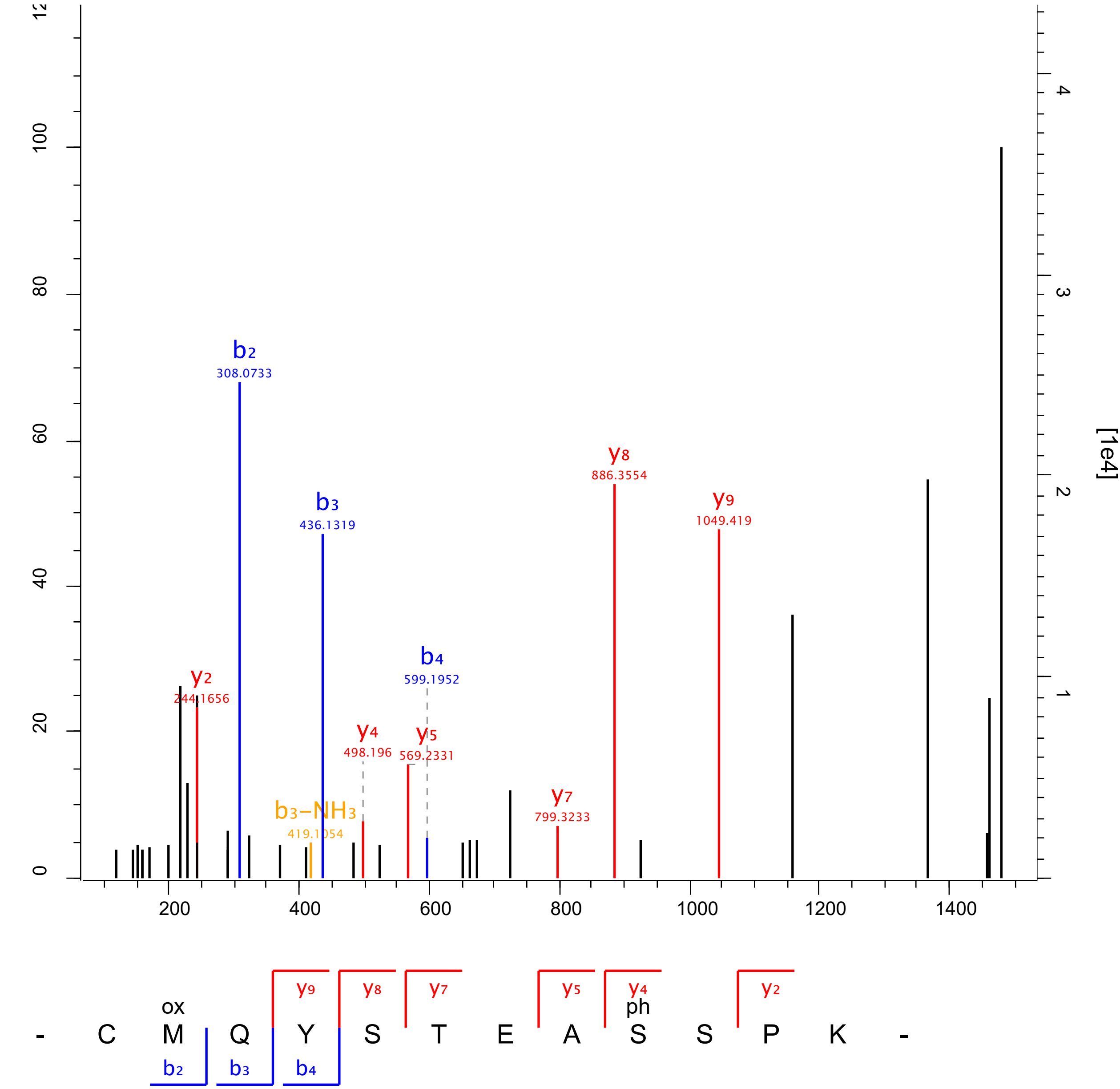

Raw file	Scan	Method	Score	m/z
sys_00_3short	4338	FTMS; HCD	60.16	614.75

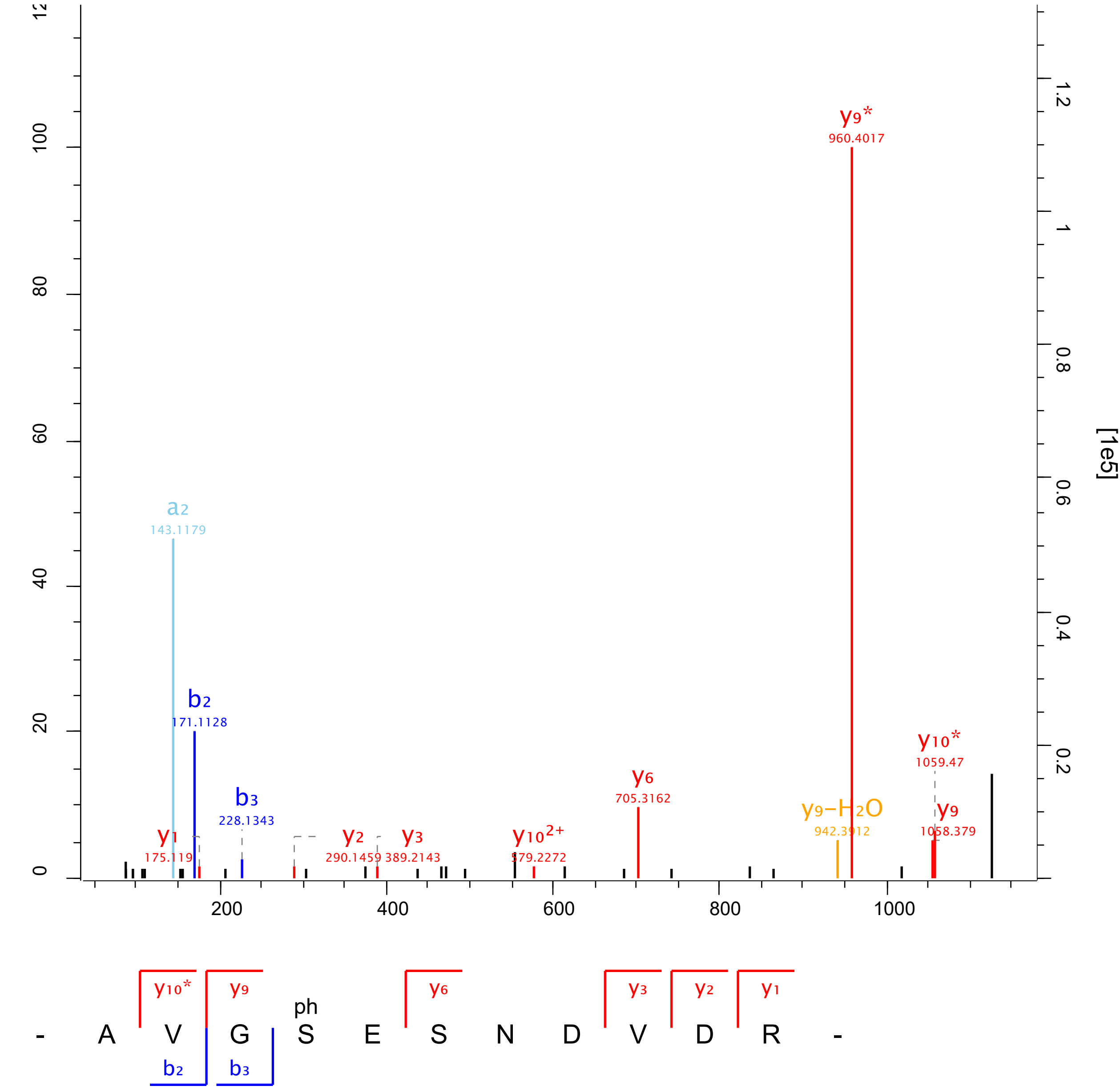

Raw file	Scan	Method	Score	m/z
sys_00_3short	4837	FTMS; HCD	234.28	665.26

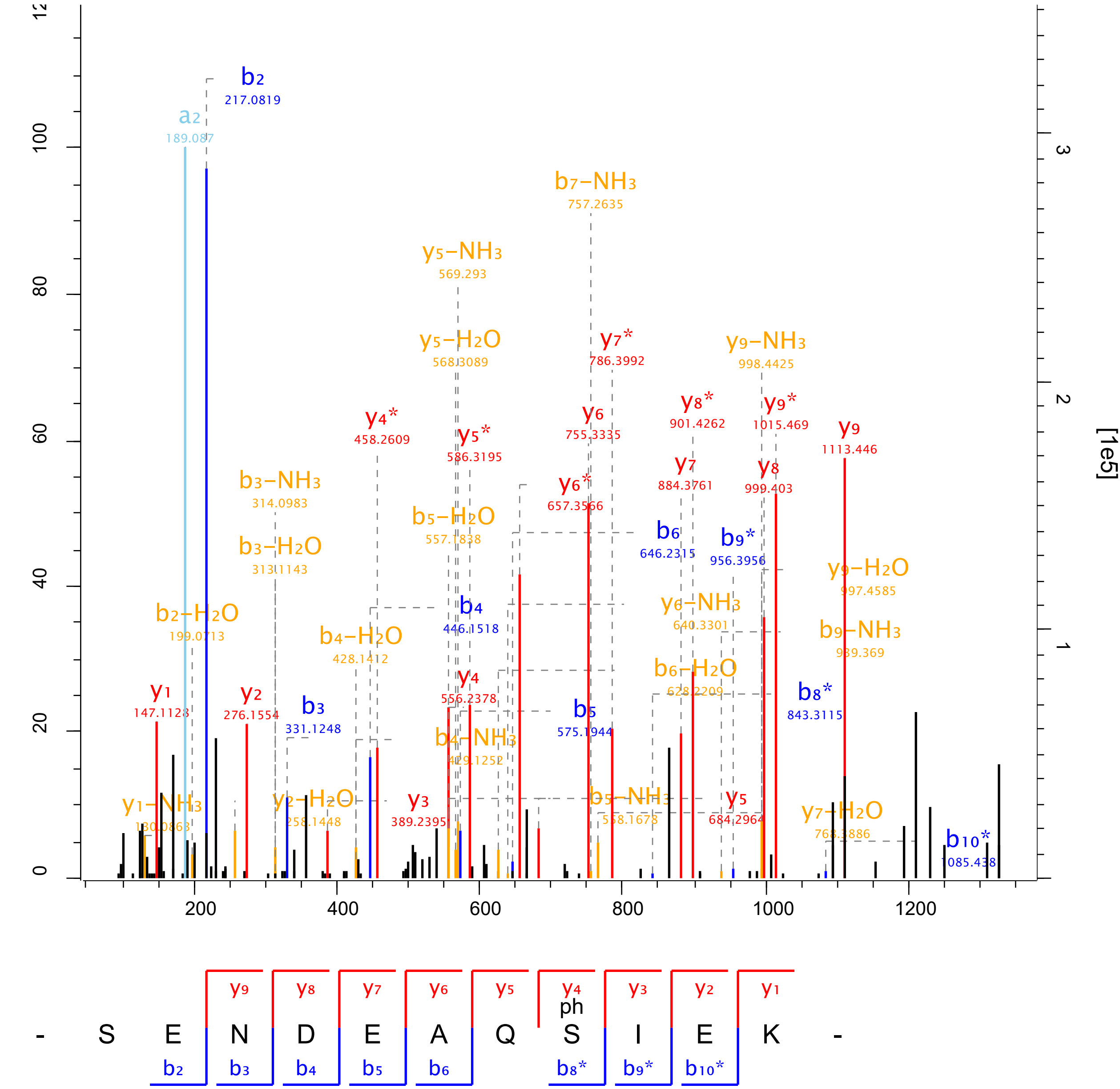

Raw file	Scan	Method	Score	m/z
sys_00_3short	4863	FTMS; HCD	83.86	492.23

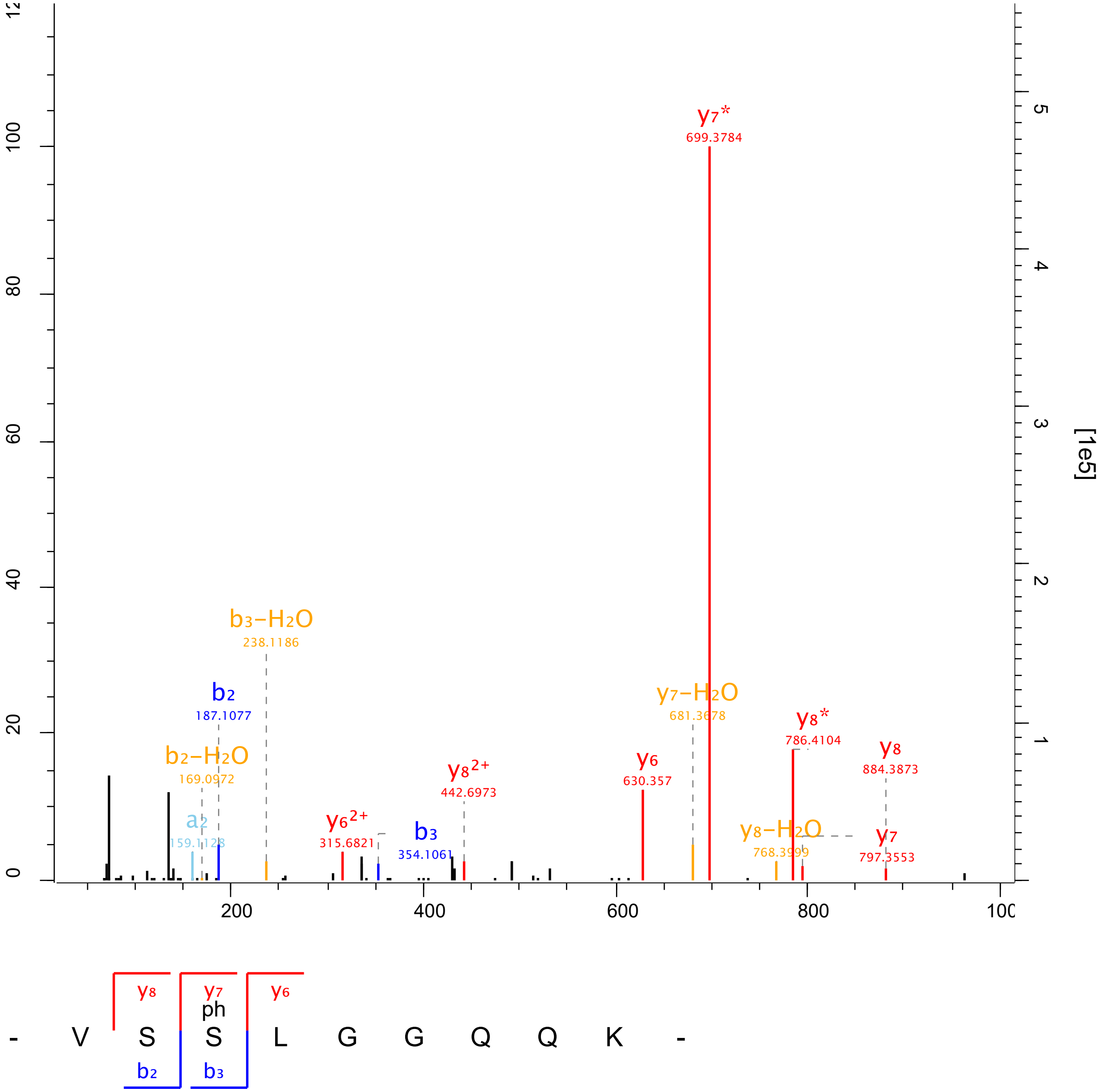

Raw file	Scan	Method	Score	m/z
sys_00_3short	5304	FTMS; HCD	187.51	529.23

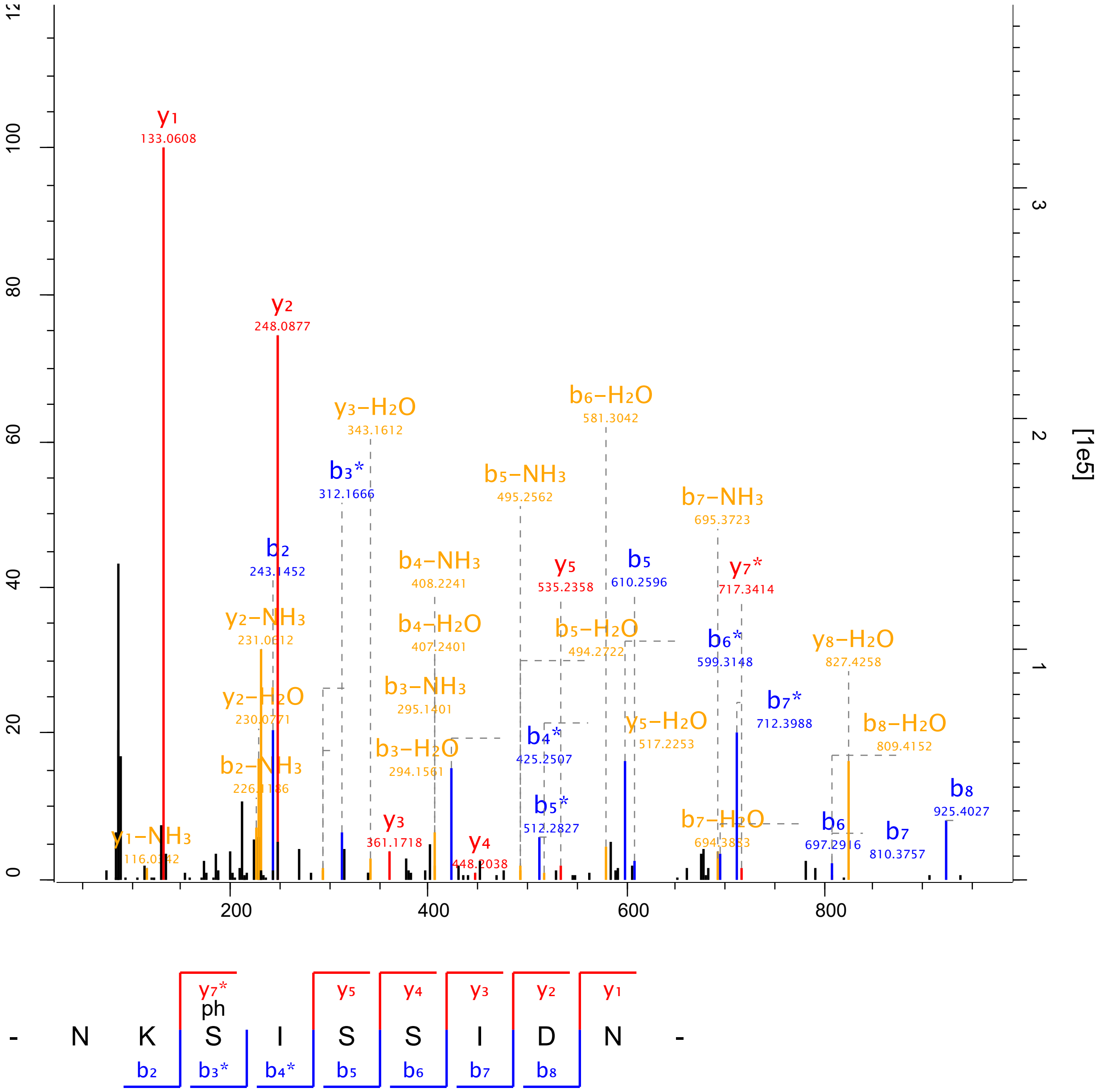

Raw file	Scan	Method	Score	m/z
sys_00_3short	5515	FTMS; HCD	113.74	437.2

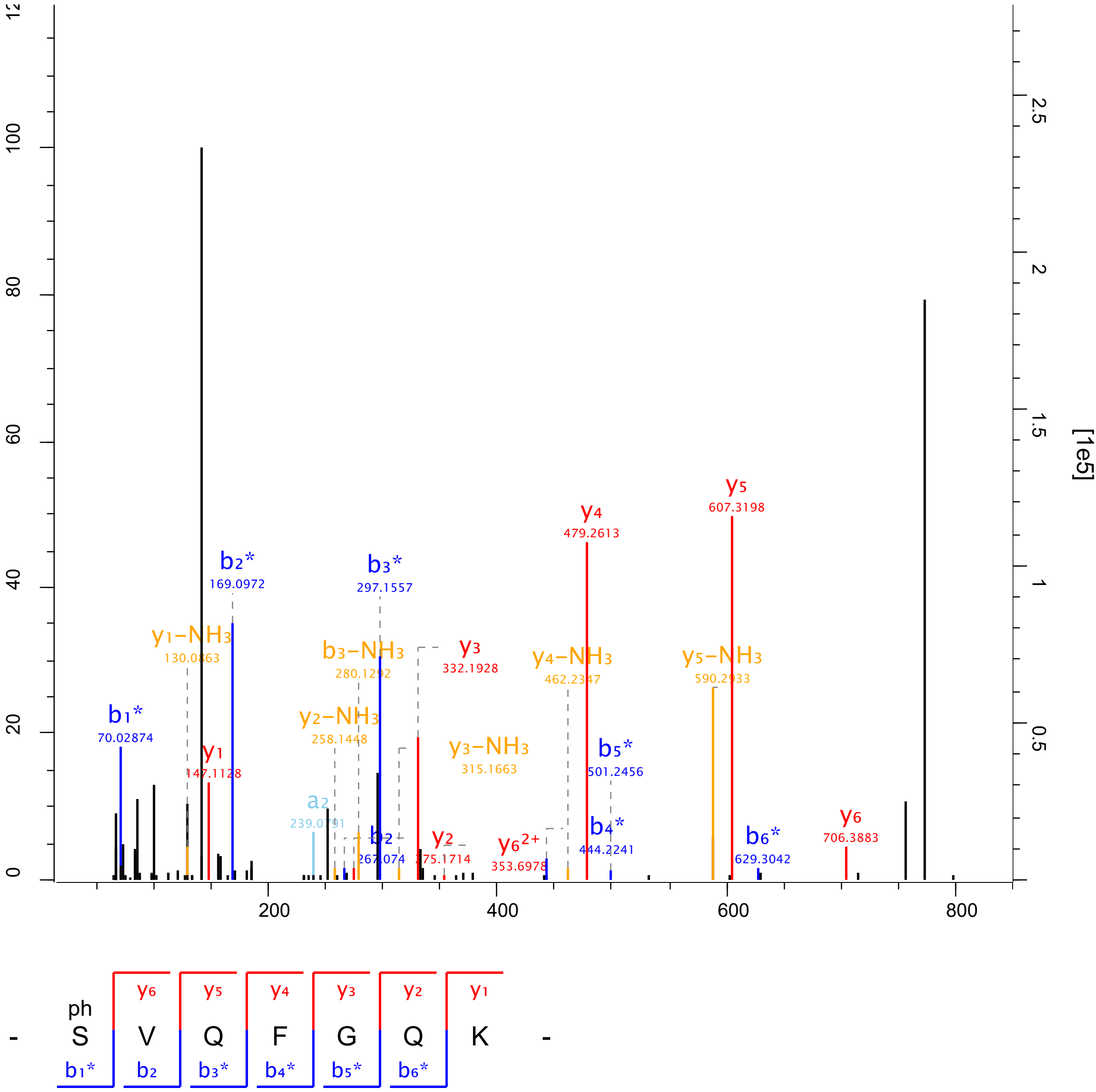

Raw file	Scan	Method	Score	m/z
sys_00_3short	5564	FTMS; HCD	119.25	620.22

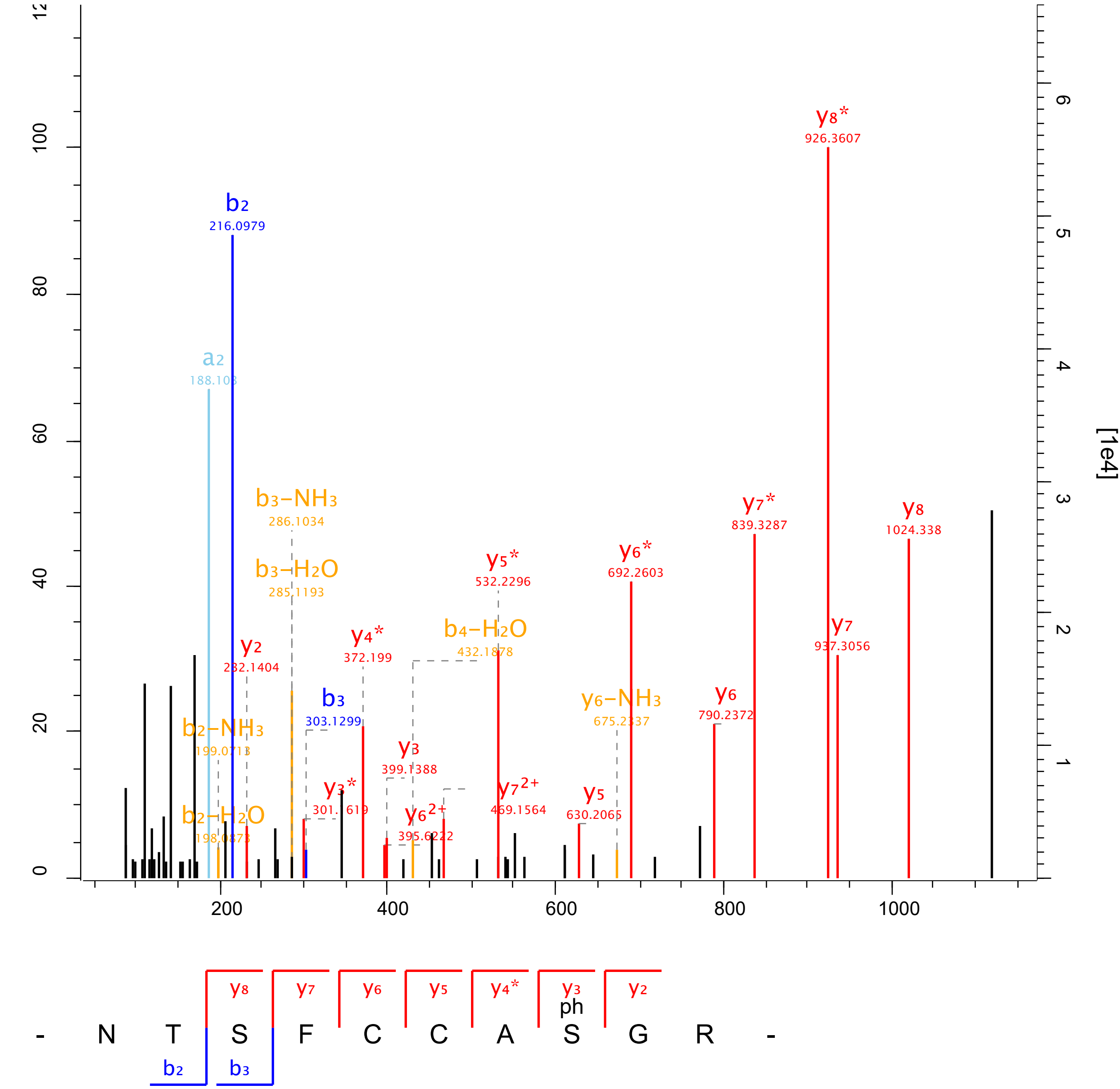

Raw file	Scan	Method	Score	m/z
sys_00_3short	5781	FTMS; HCD	71.88	584.28

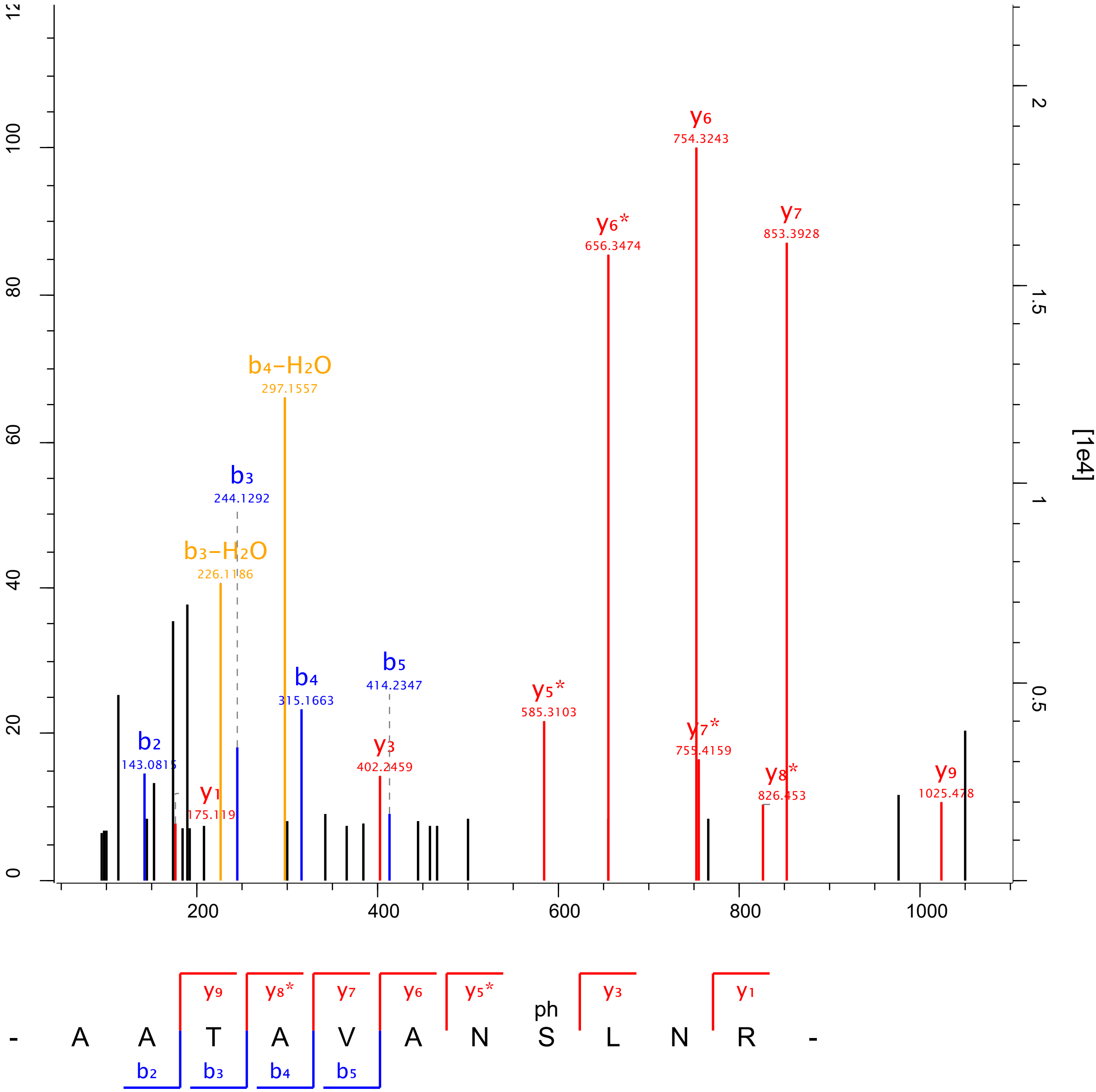

Raw file	Scan	Method	Score	m/z
sys_00_3short	5903	FTMS; HCD	64.45	805.75

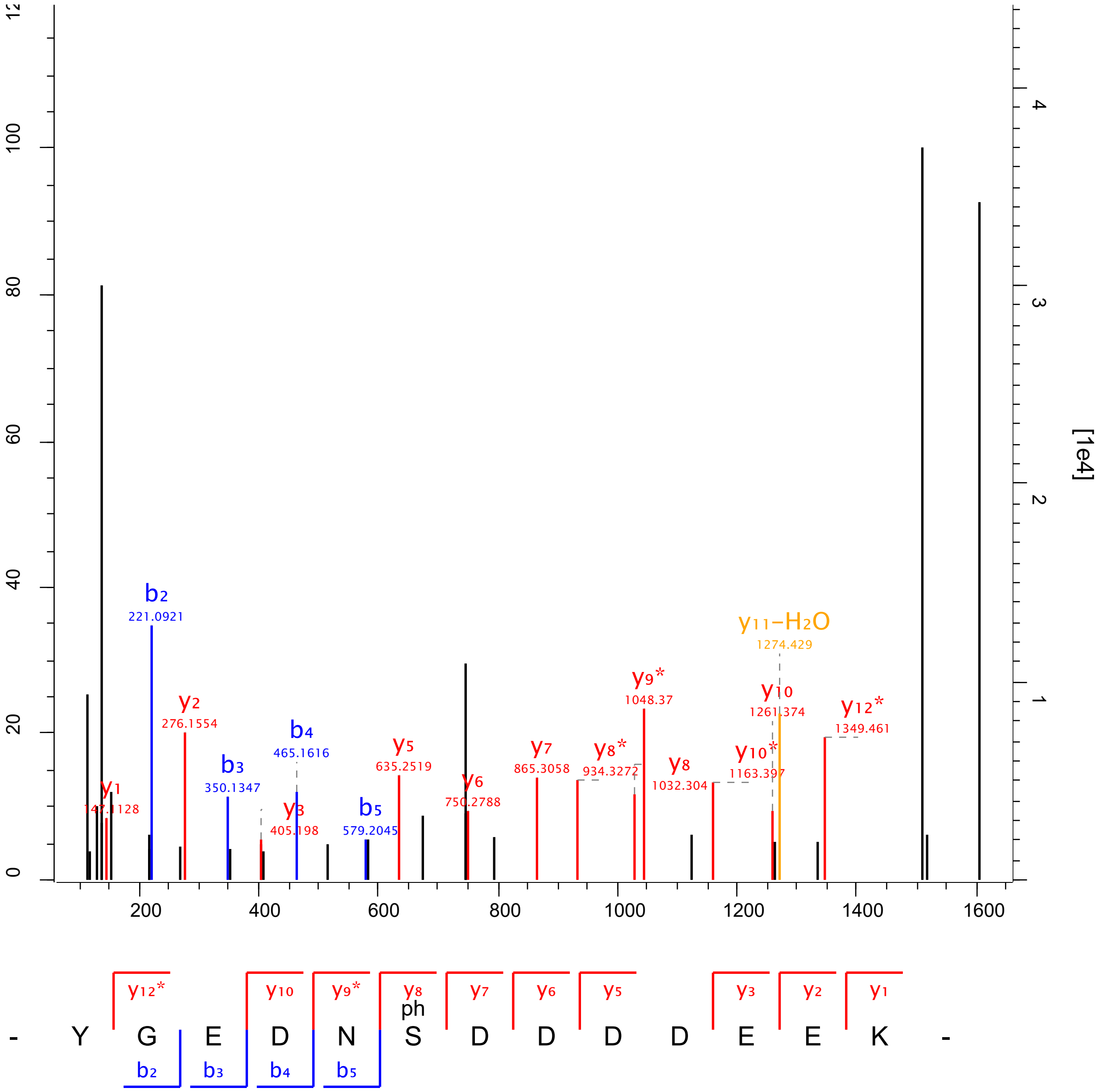

Raw file	Scan	Method	Score	m/z
sys_00_3short	6034	FTMS; HCD	130.05	851.82

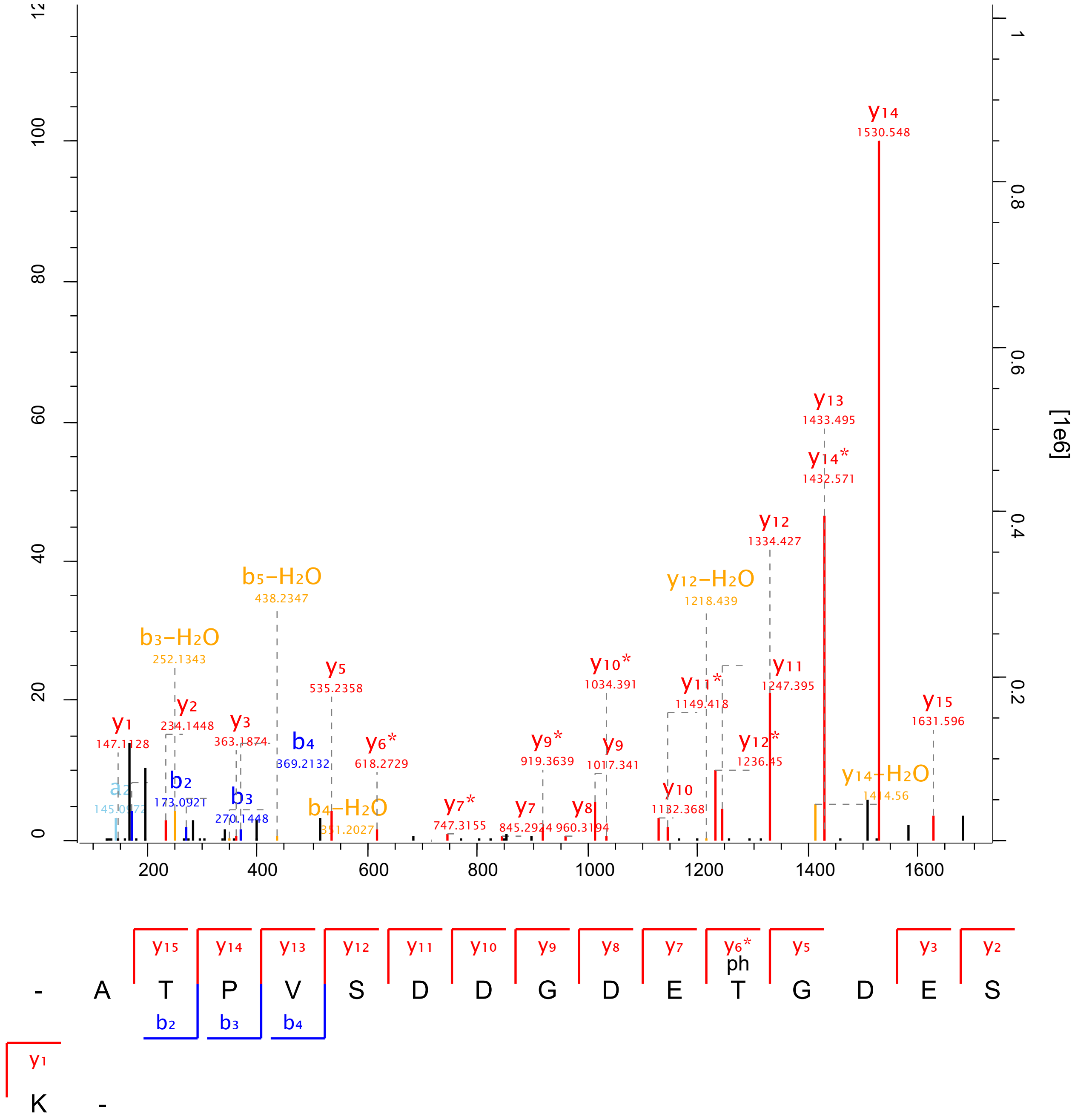

Raw file	Scan	Method	Score	m/z
sys_00_3short	6557	FTMS; HCD	66.71	619.75

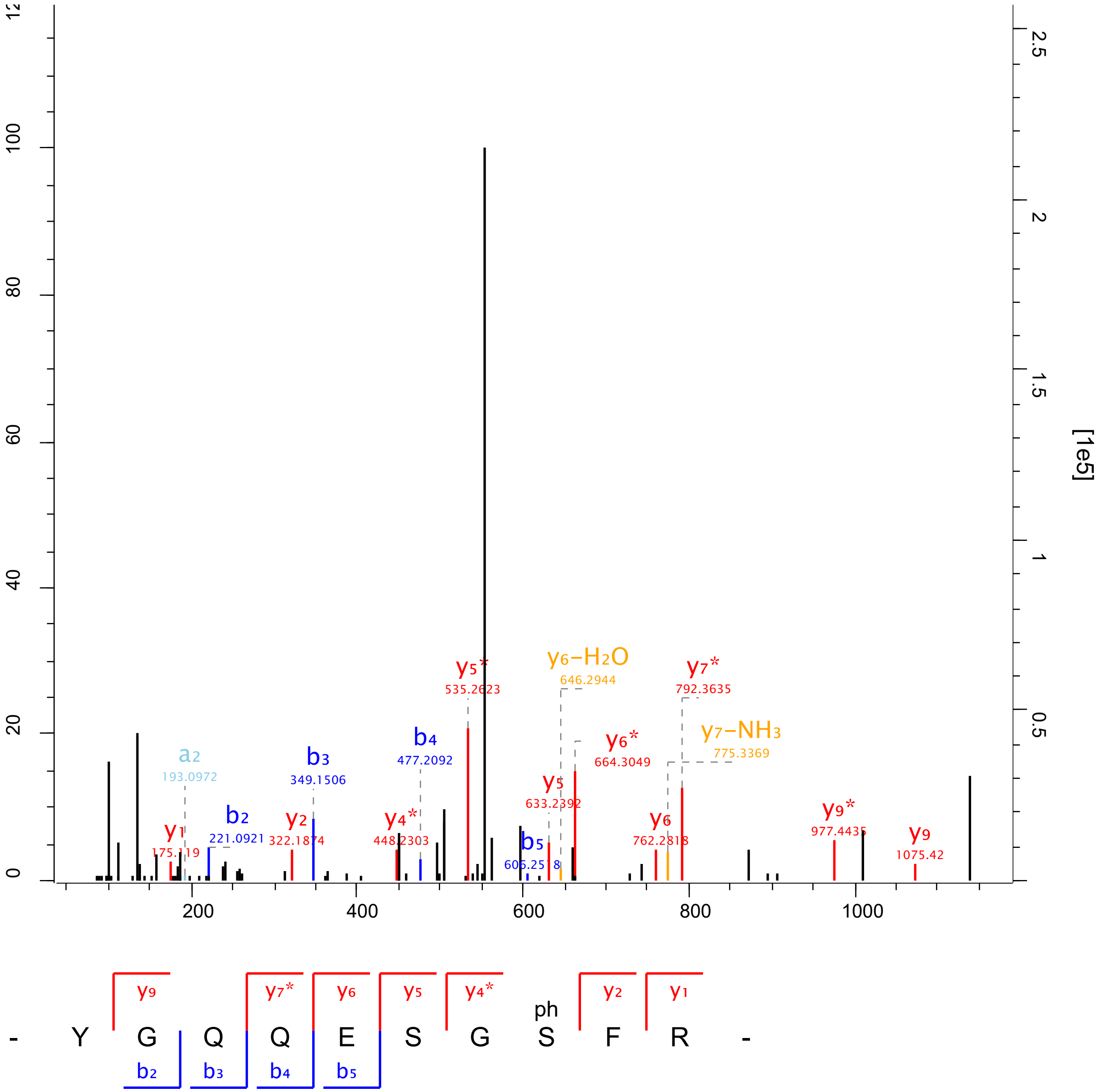

Raw file	Scan	Method	Score	m/z
sys_00_3short	6563	FTMS; HCD	57.17	473.71

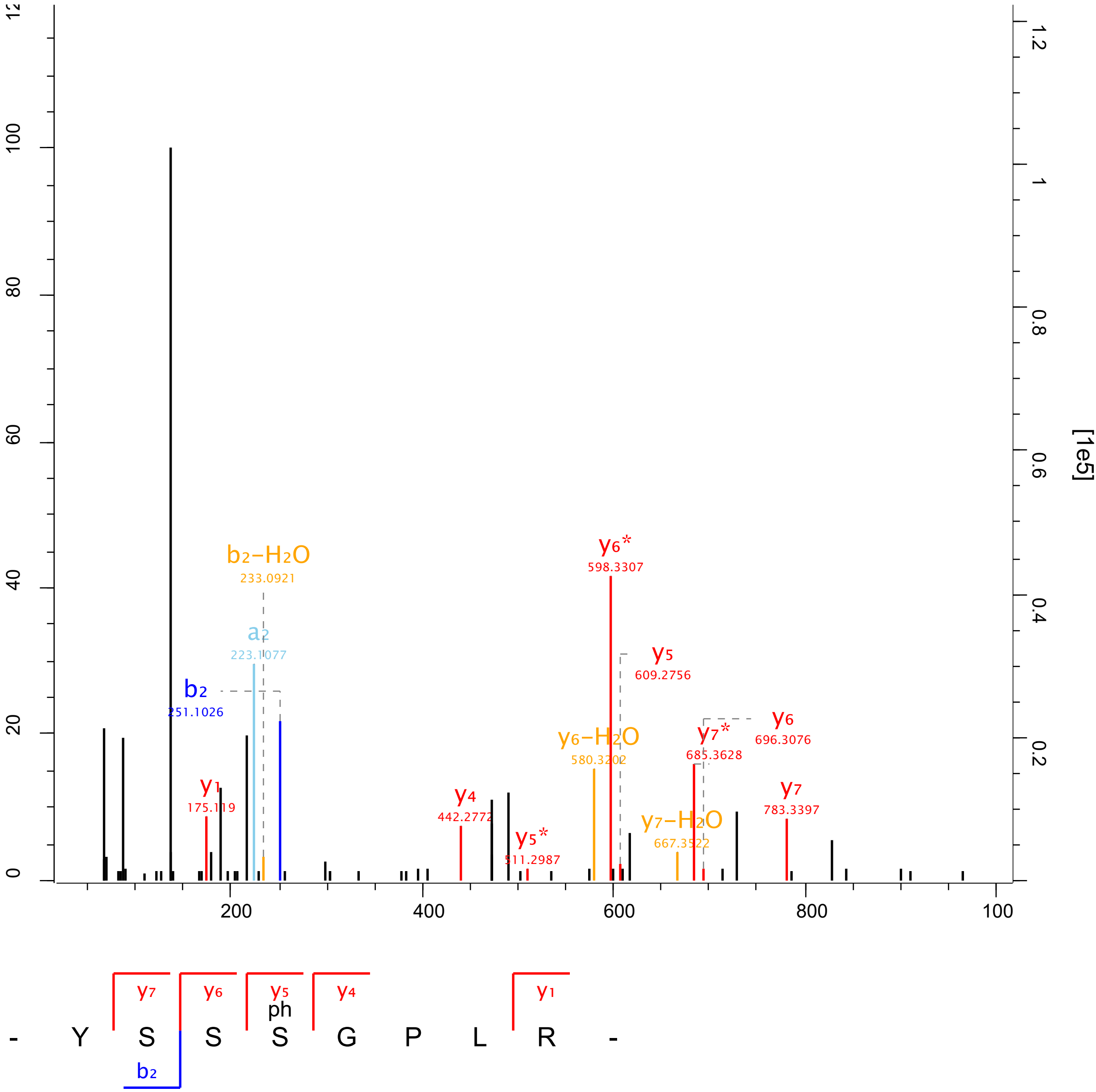

Raw file	Scan	Method	Score	m/z
sys_00_3short	6651	FTMS; HCD	98.17	540.76

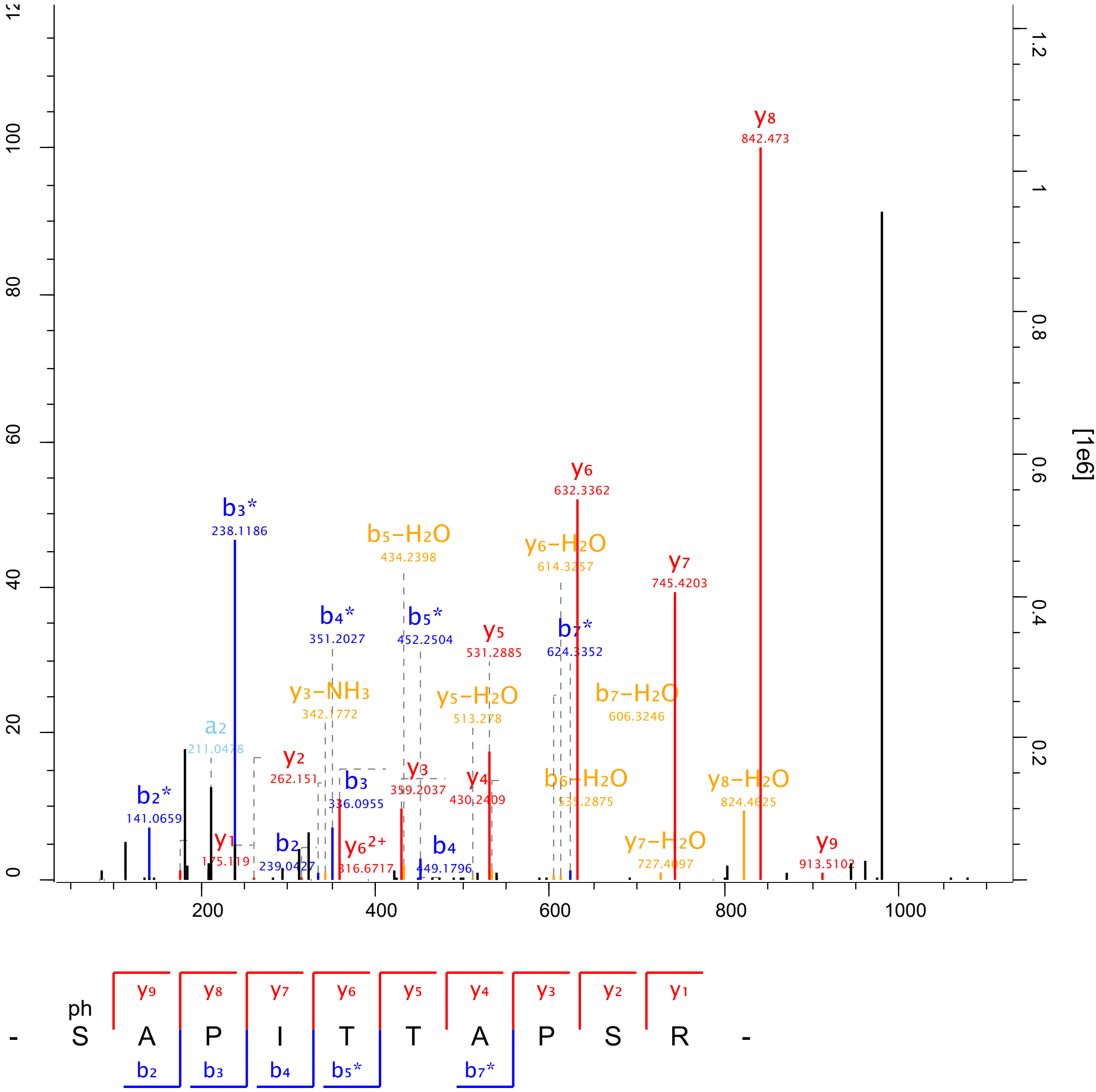

Raw file	Scan	Method	Score	m/z
sys_00_3short	6827	FTMS; HCD	113.99	510.75

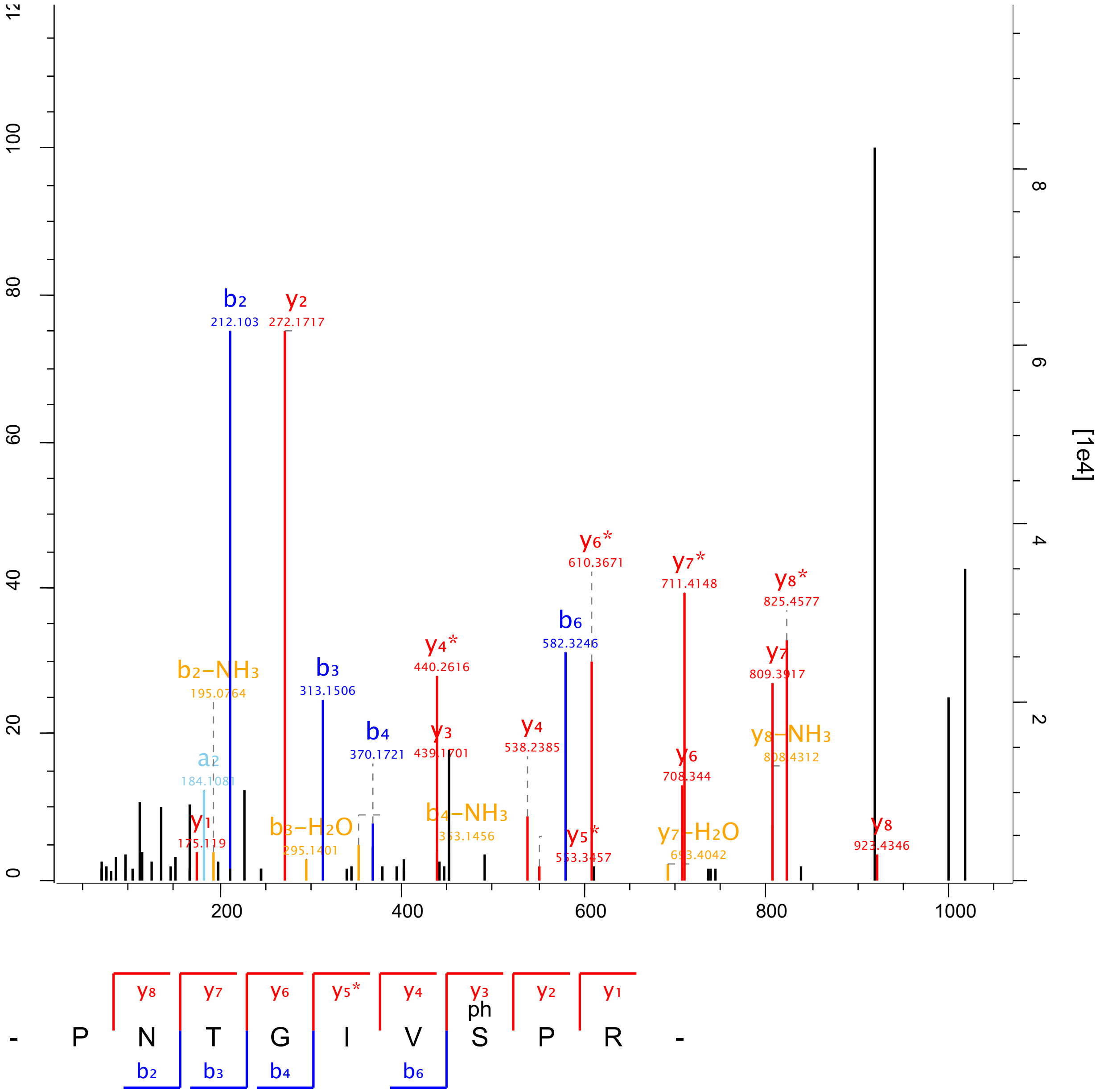

Raw file	Scan	Method	Score	m/z
sys_00_3short	6940	FTMS; HCD	146.59	634.28

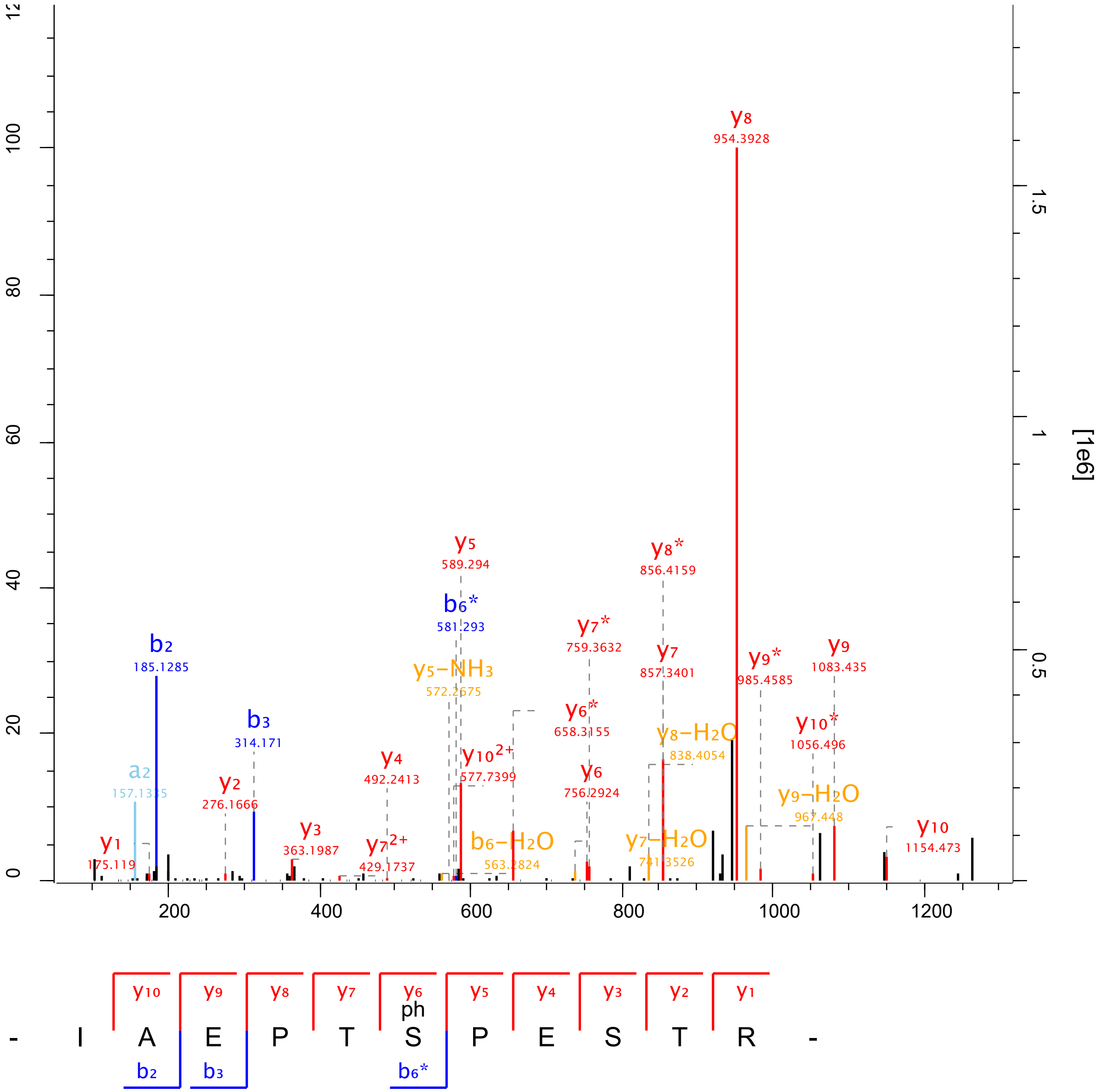

Raw file	Scan	Method	Score	m/z
sys_00_3short	7401	FTMS; HCD	54.9	737.29

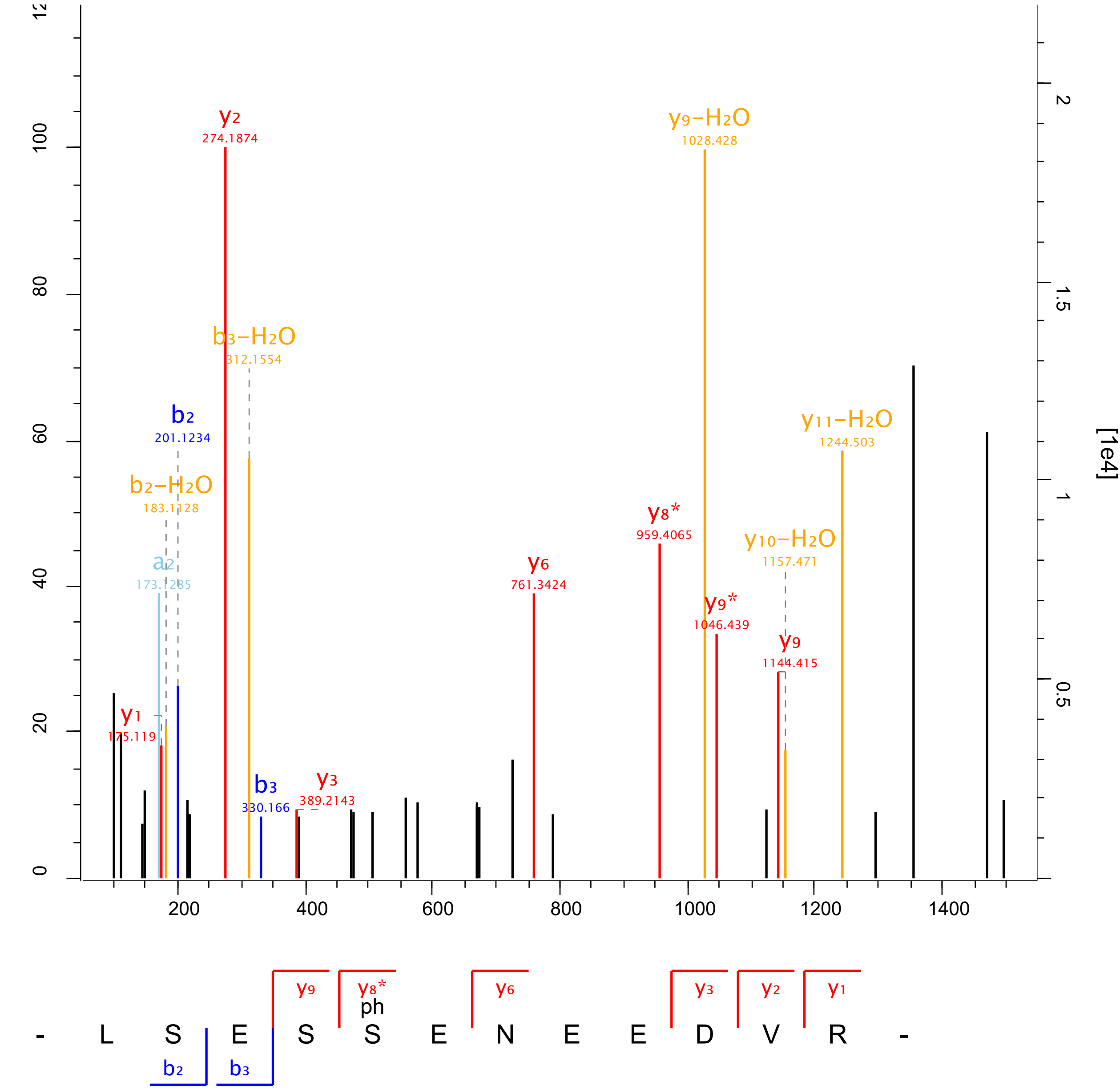

Raw file	Scan	Method	Score	m/z
sys_00_3short	7417	FTMS; HCD	252.66	694.3

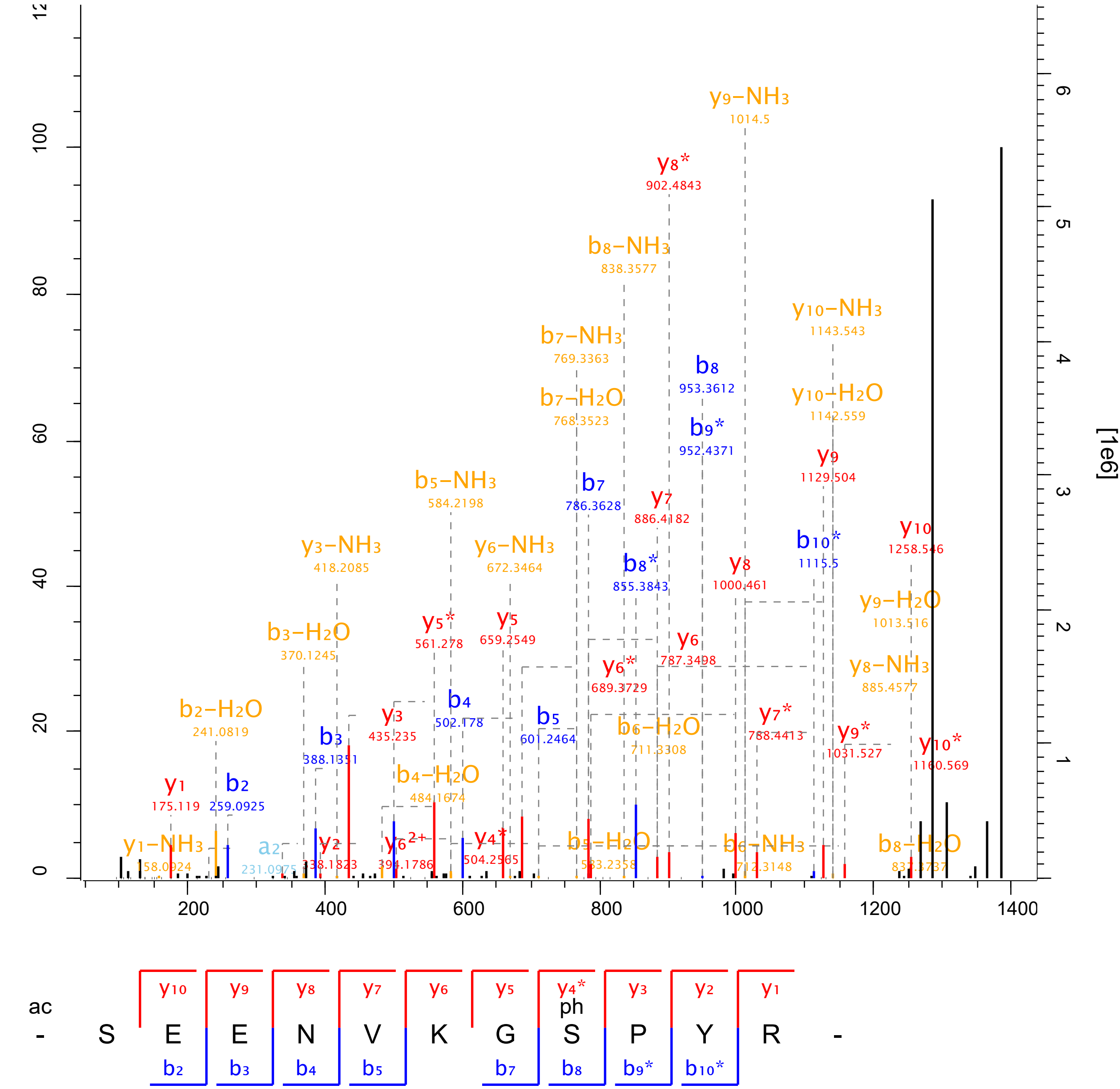

Raw file	Scan	Method	Score	m/z
sys_00_3short	7649	FTMS; HCD	248.75	680.29

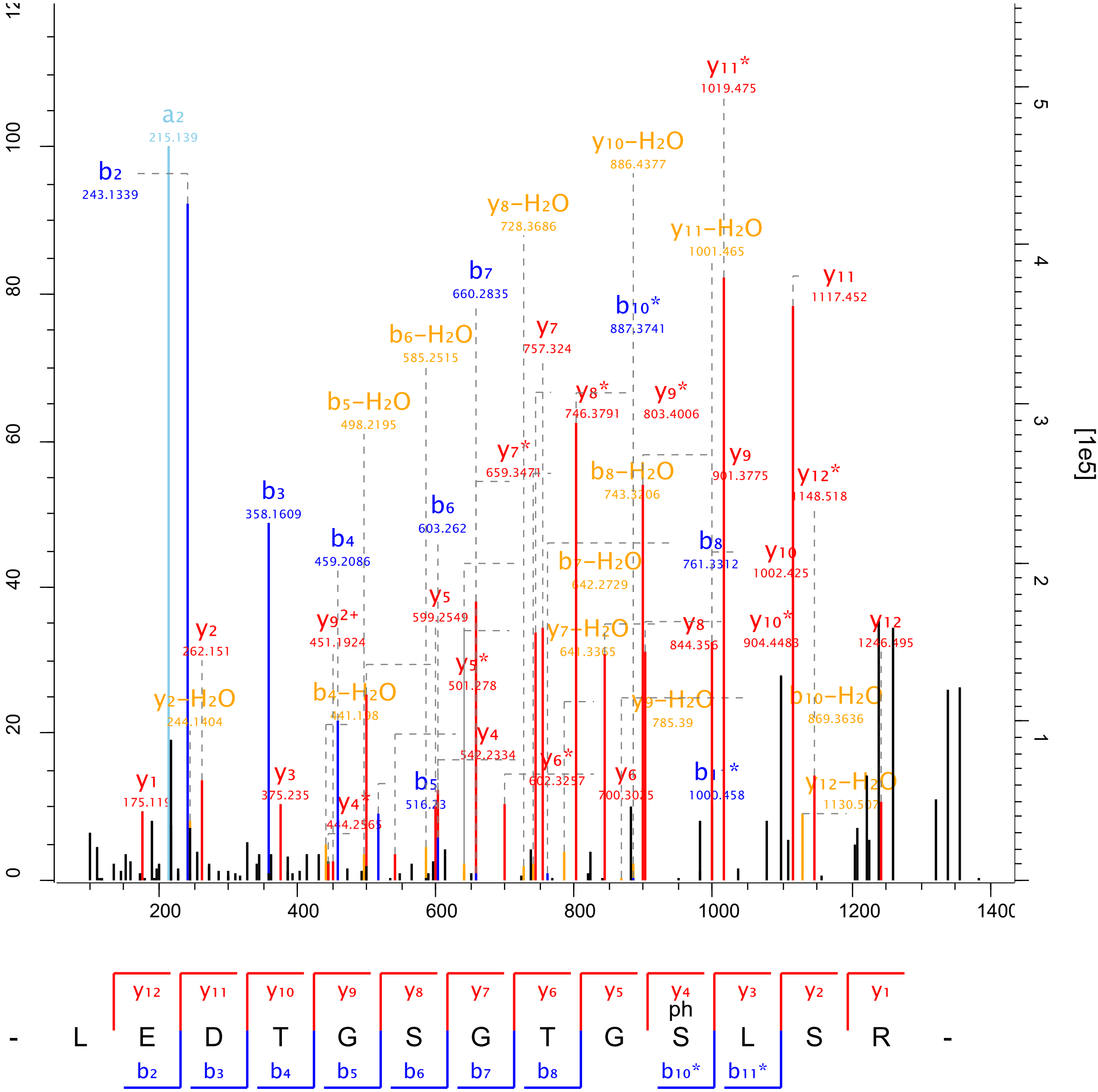

Raw file	Scan	Method	Score	m/z
sys_00_3short	7859	FTMS; HCD	152.04	474.2

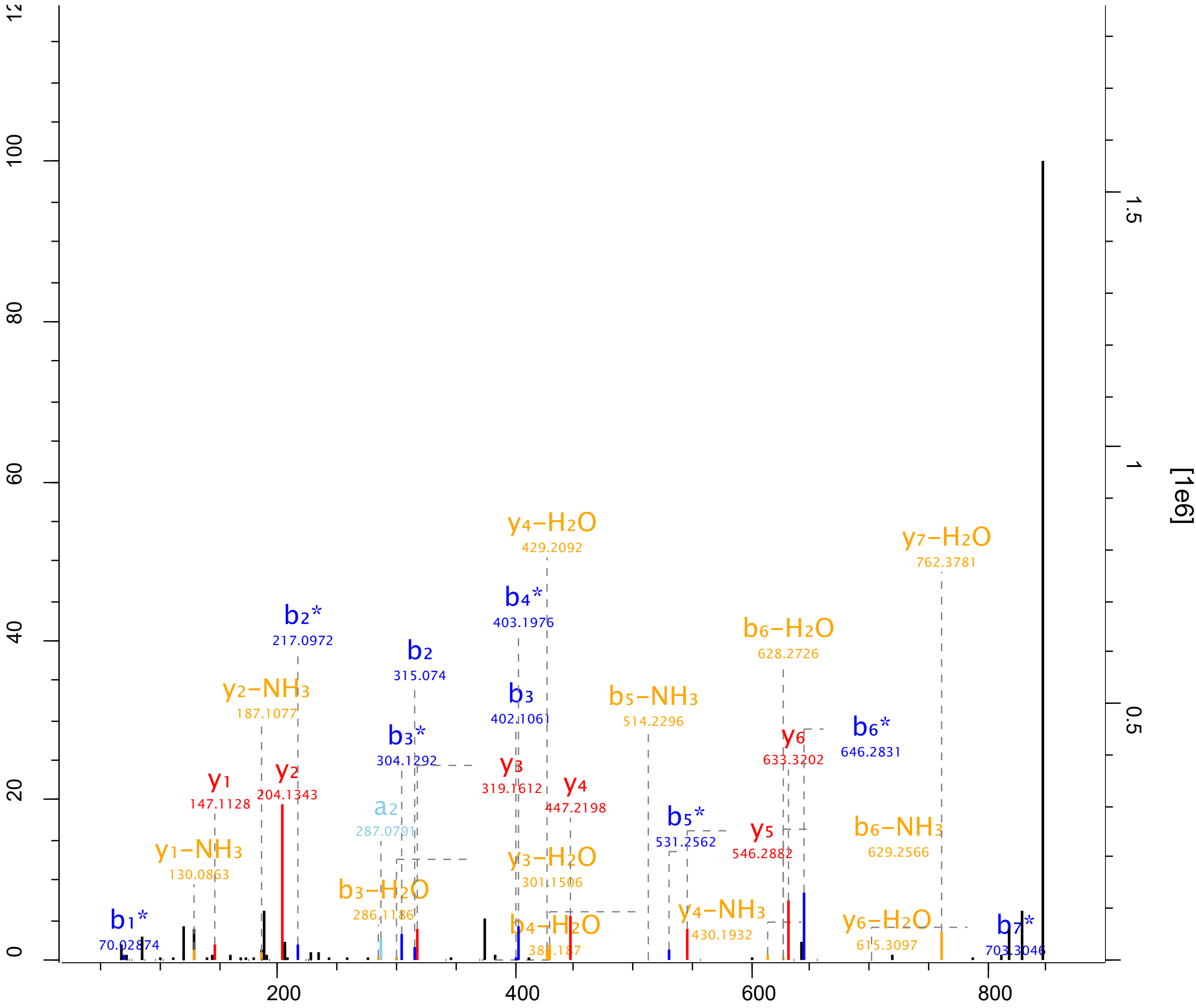

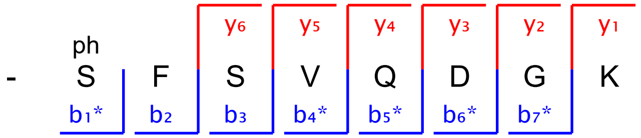

Raw file	Scan	Method	Score	m/z
sys_00_3short	7980	FTMS; HCD	99.14	410.21

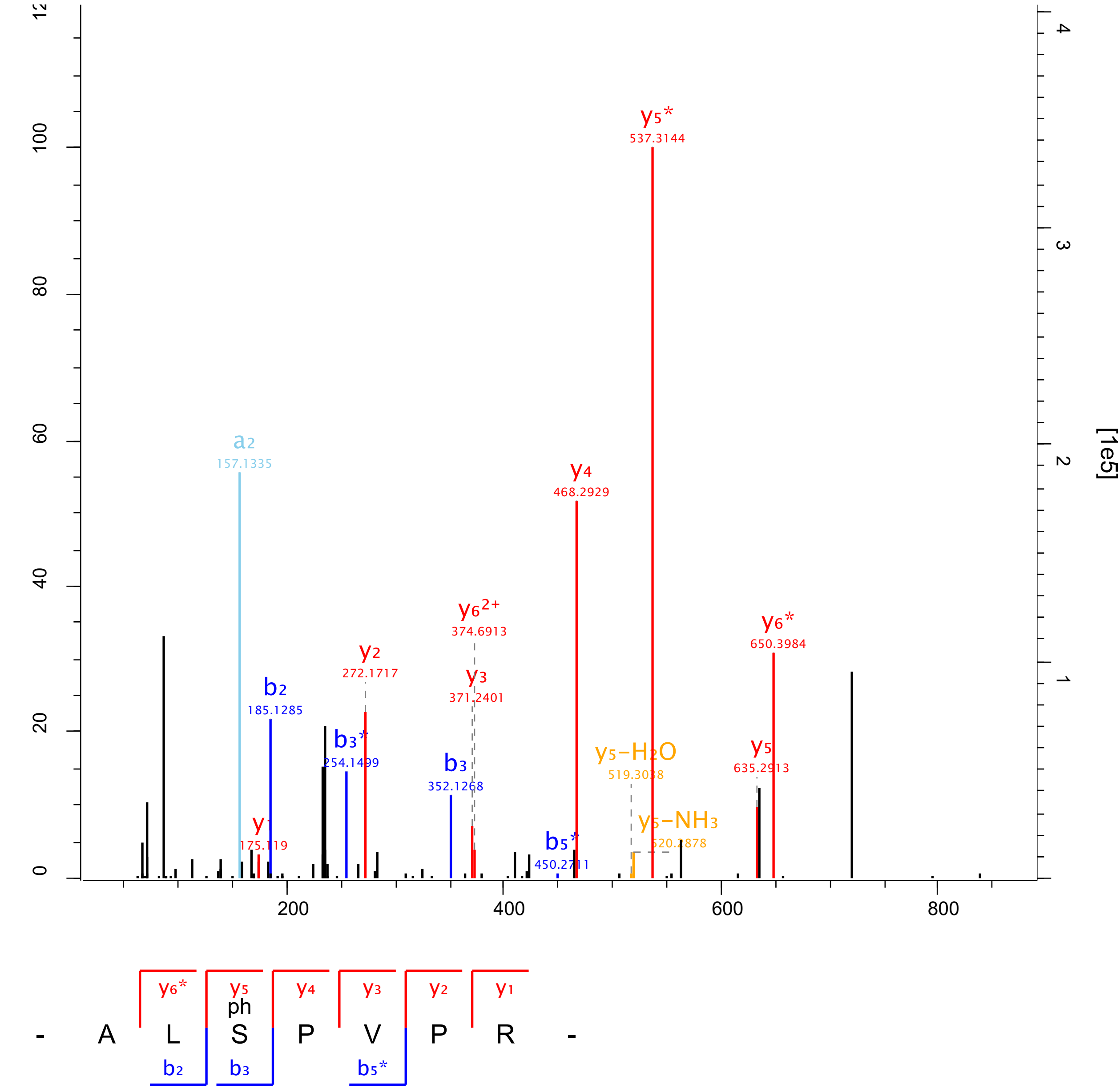

Raw file	Scan	Method	Score	m/z
sys_00_3short	8115	FTMS; HCD	79.66	477.71

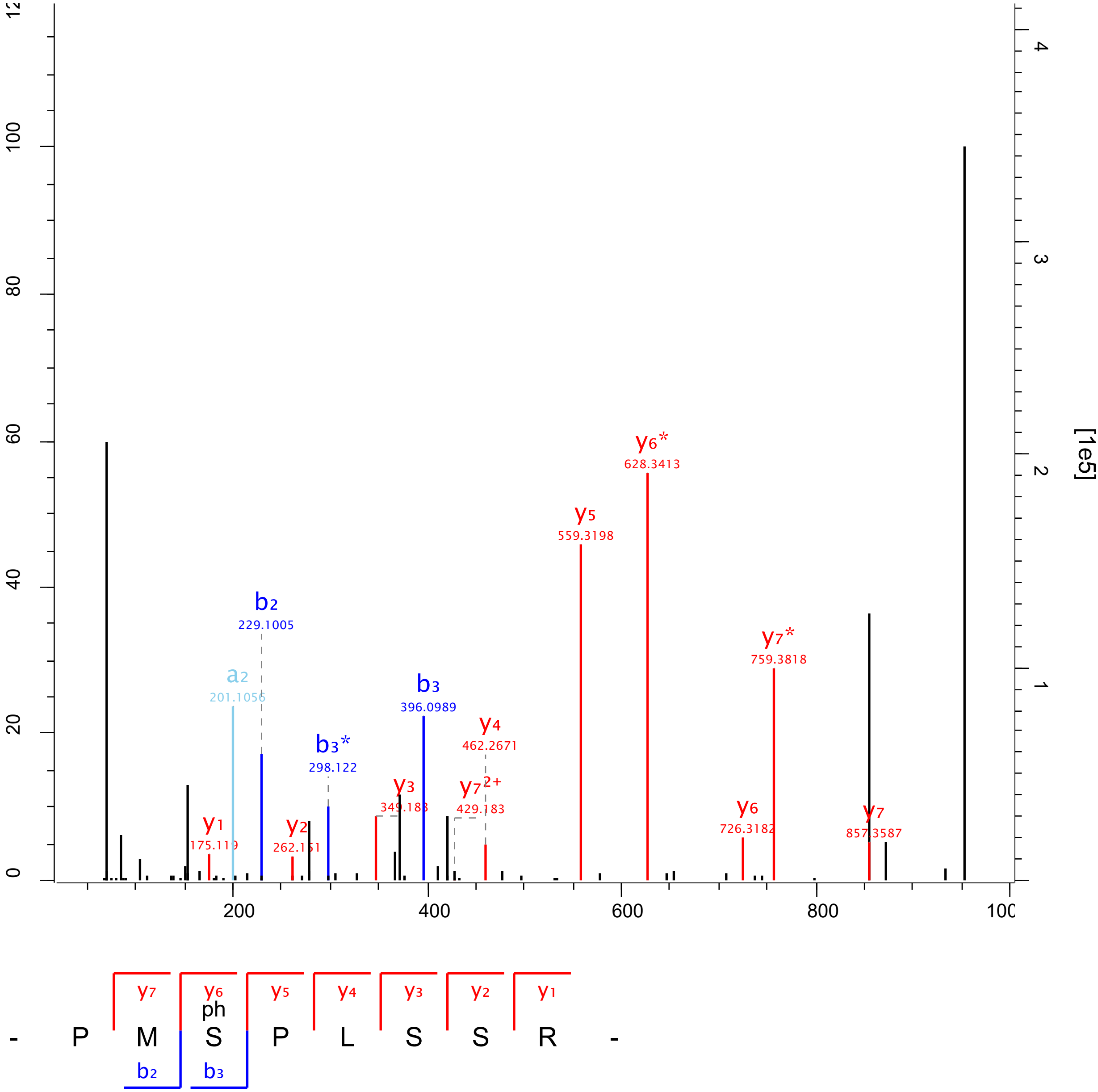

Raw file	Scan	Method	Score	m/z
sys_00_3short	8275	FTMS; HCD	79.09	616.79

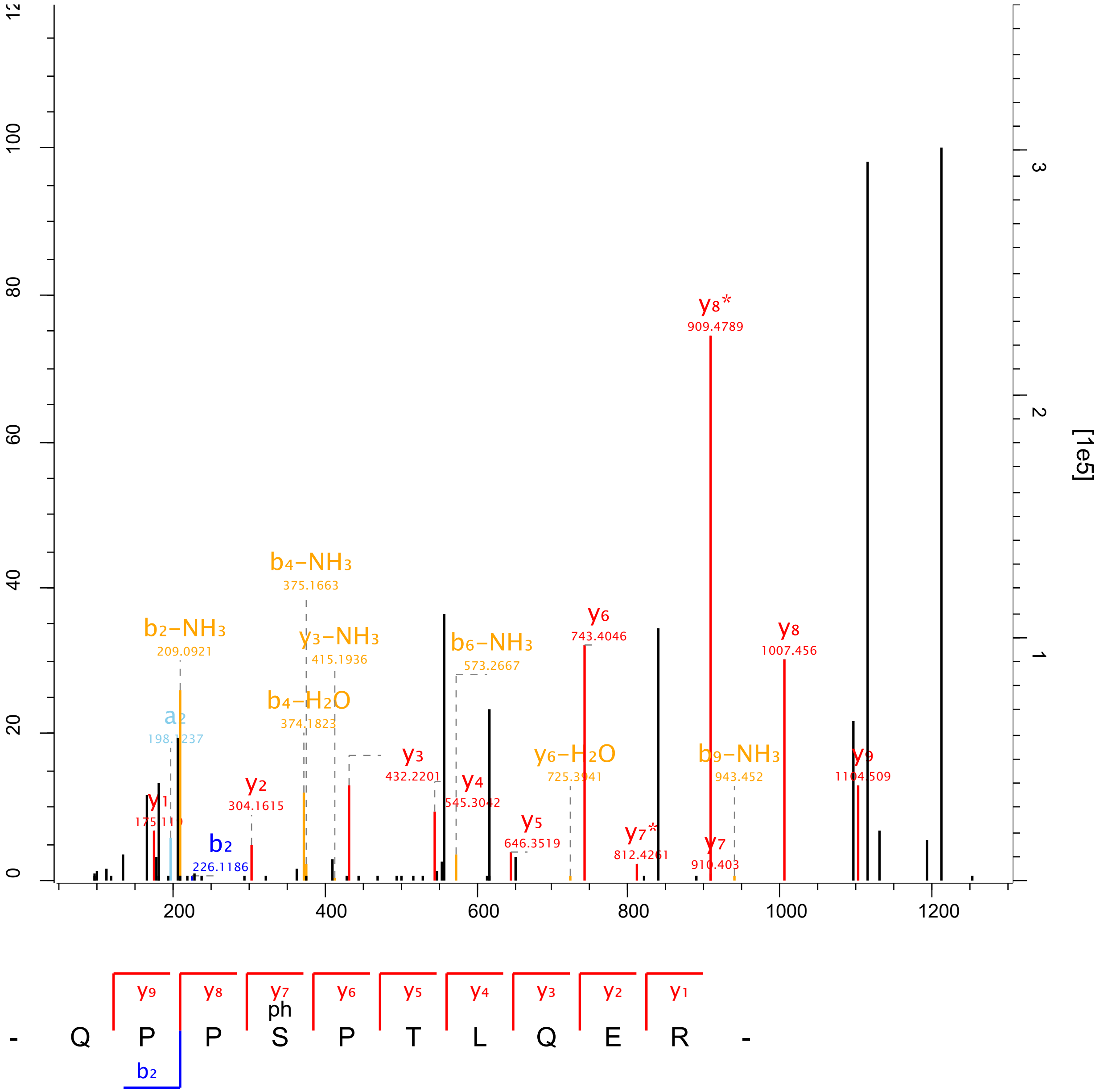

Raw file	Scan	Method	Score	m/z
sys_00_3short	8459	FTMS; HCD	147.33	521.73

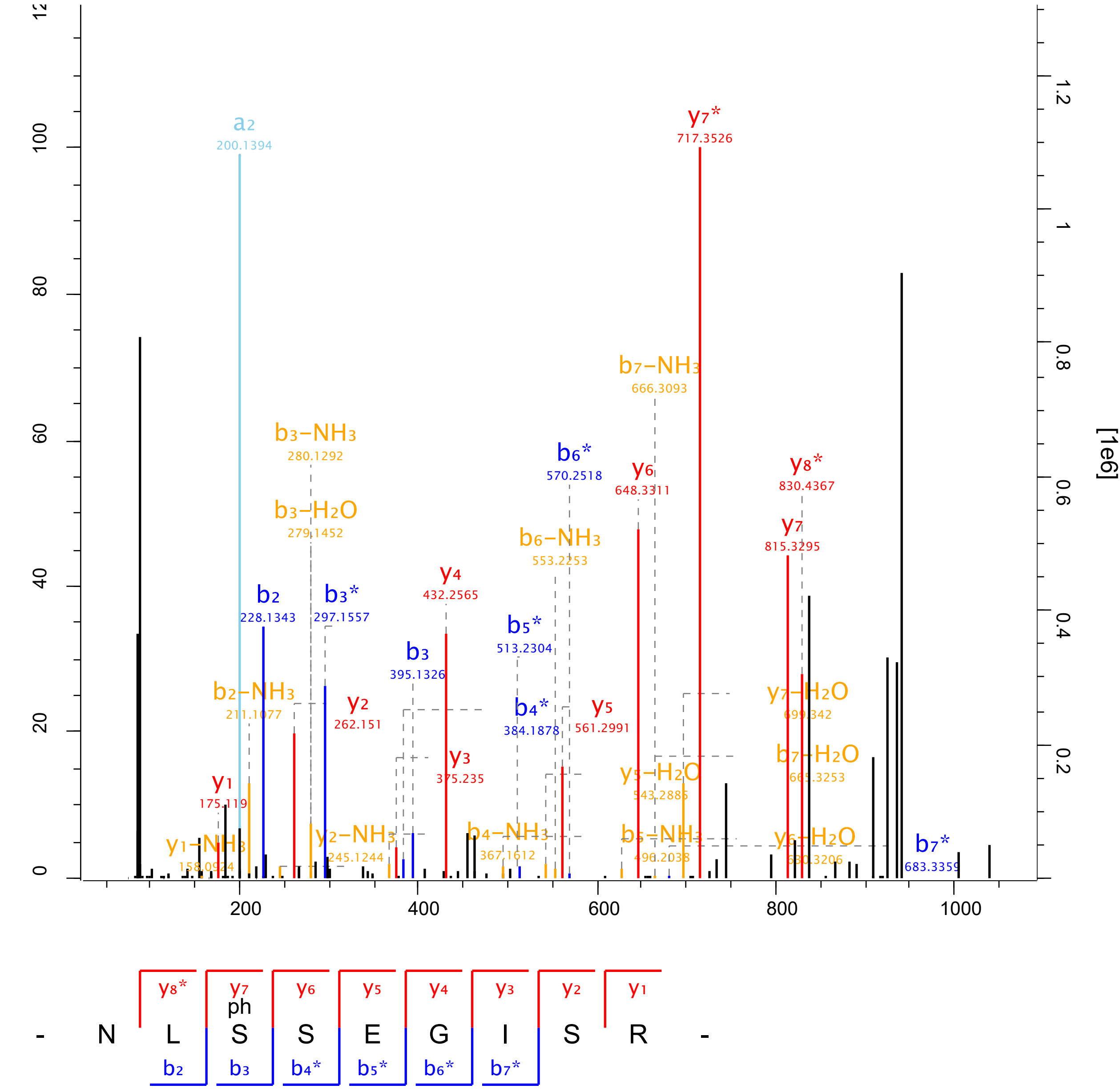

Raw file	Scan	Method	Score	m/z
sys_00_3short	8496	FTMS; HCD	100.69	469.72

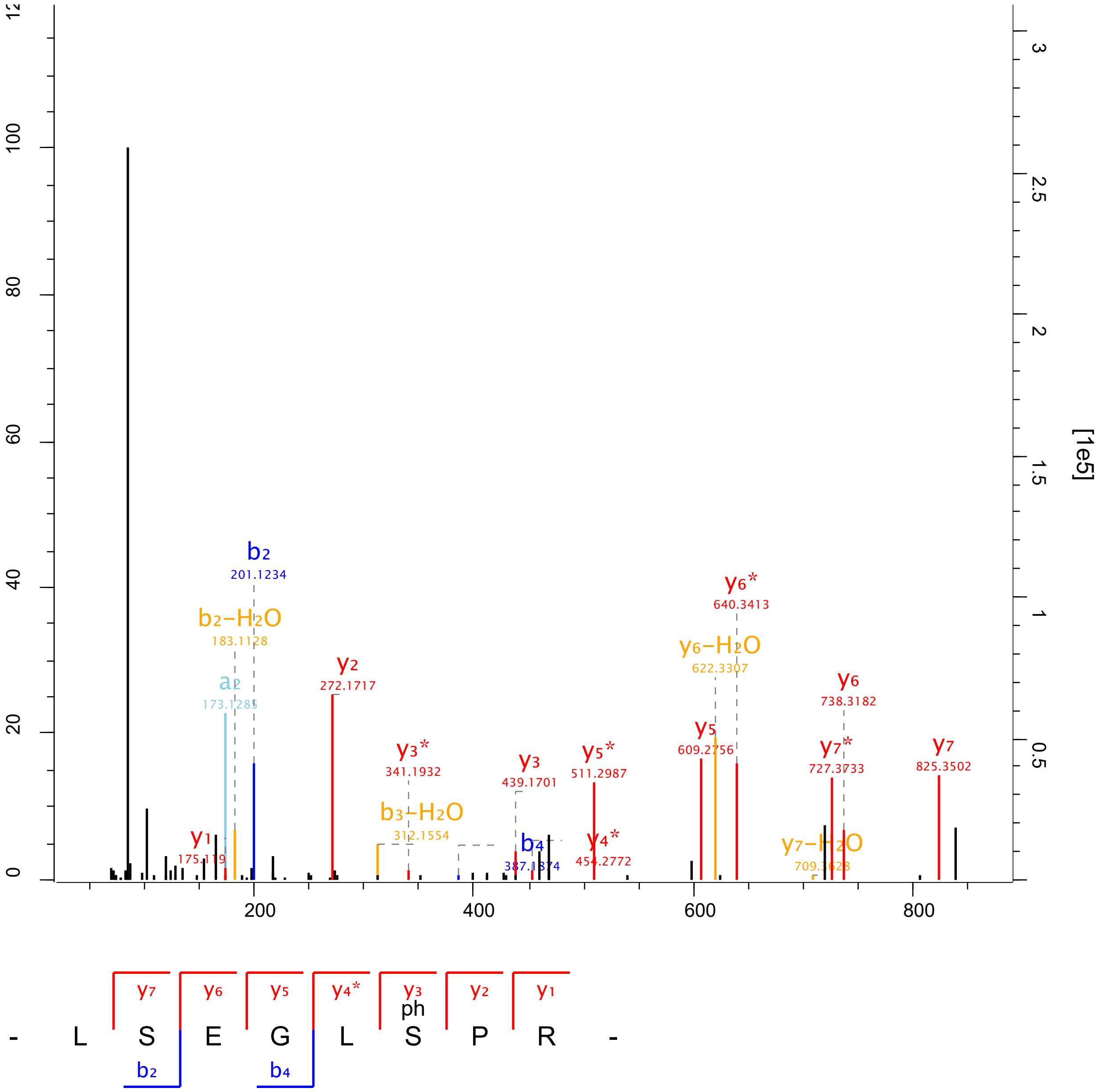

Raw file	Scan	Method	Score	m/z
sys_00_3short	8704	FTMS; HCD	46.13	536.29

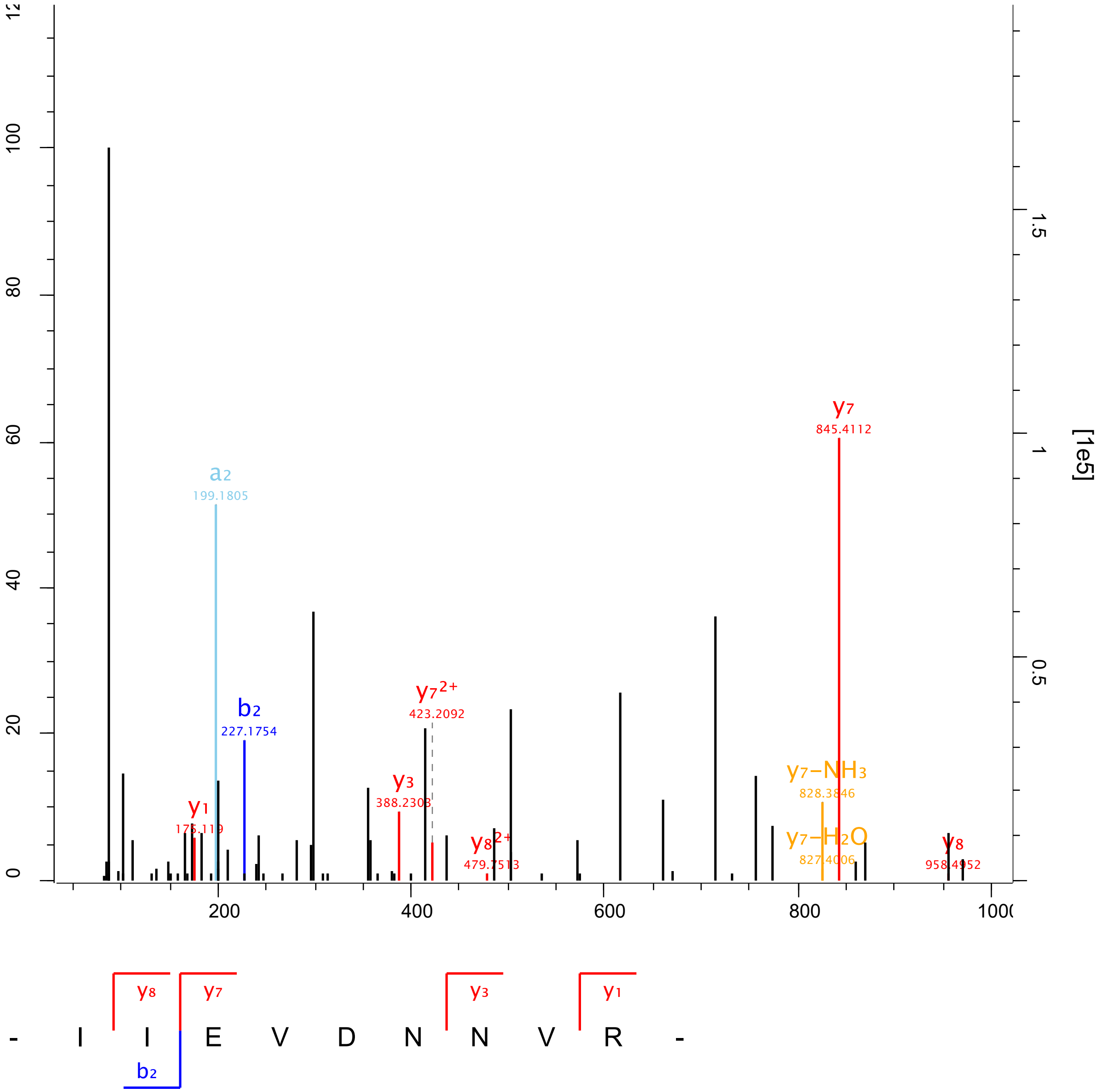

Raw file	Scan	Method	Score	m/z
sys_00_3short	8721	FTMS; HCD	116.55	496.69

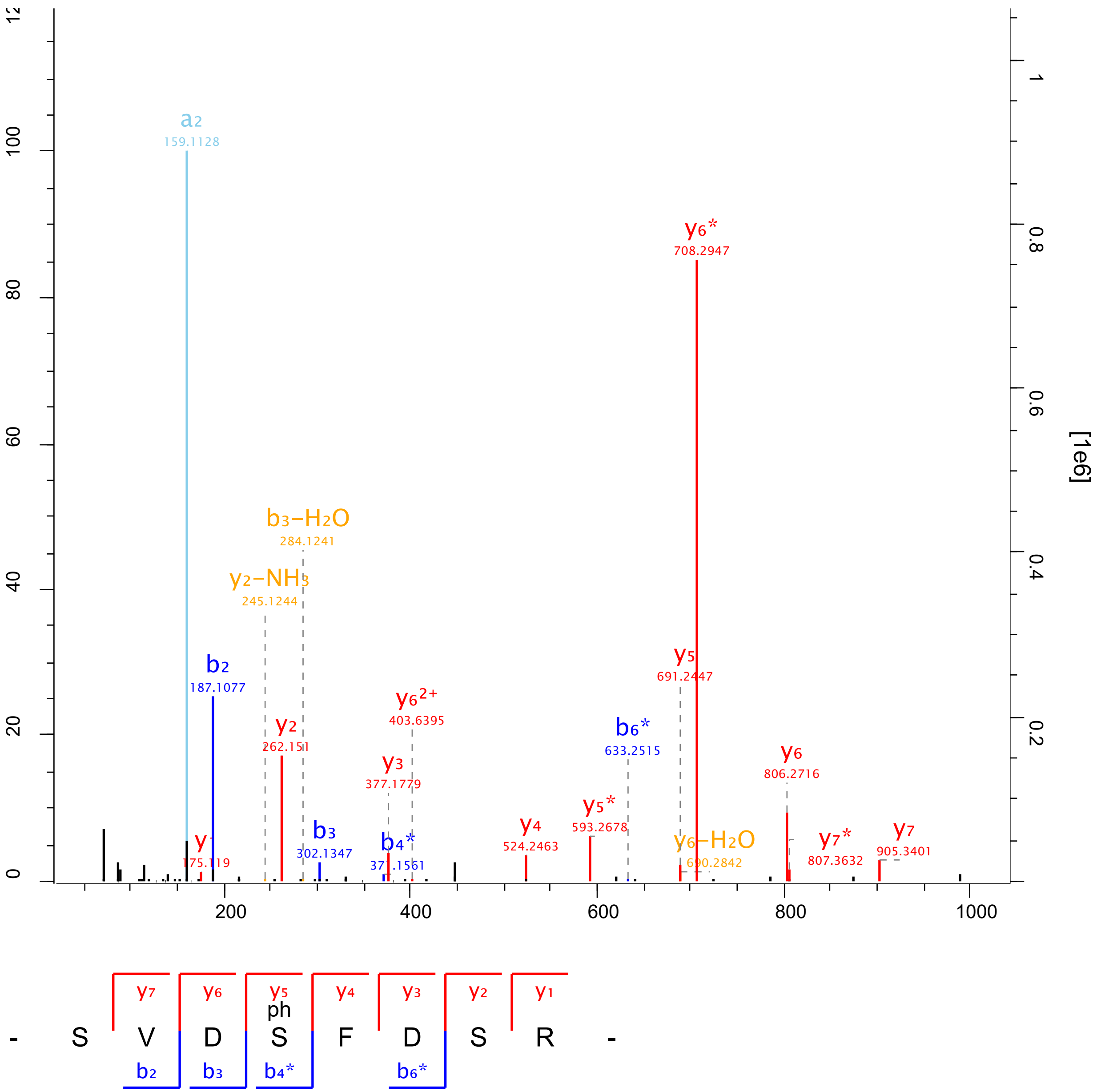

Raw file	Scan	Method	Score	m/z
sys_00_3short	8767	FTMS; HCD	74.99	604.24

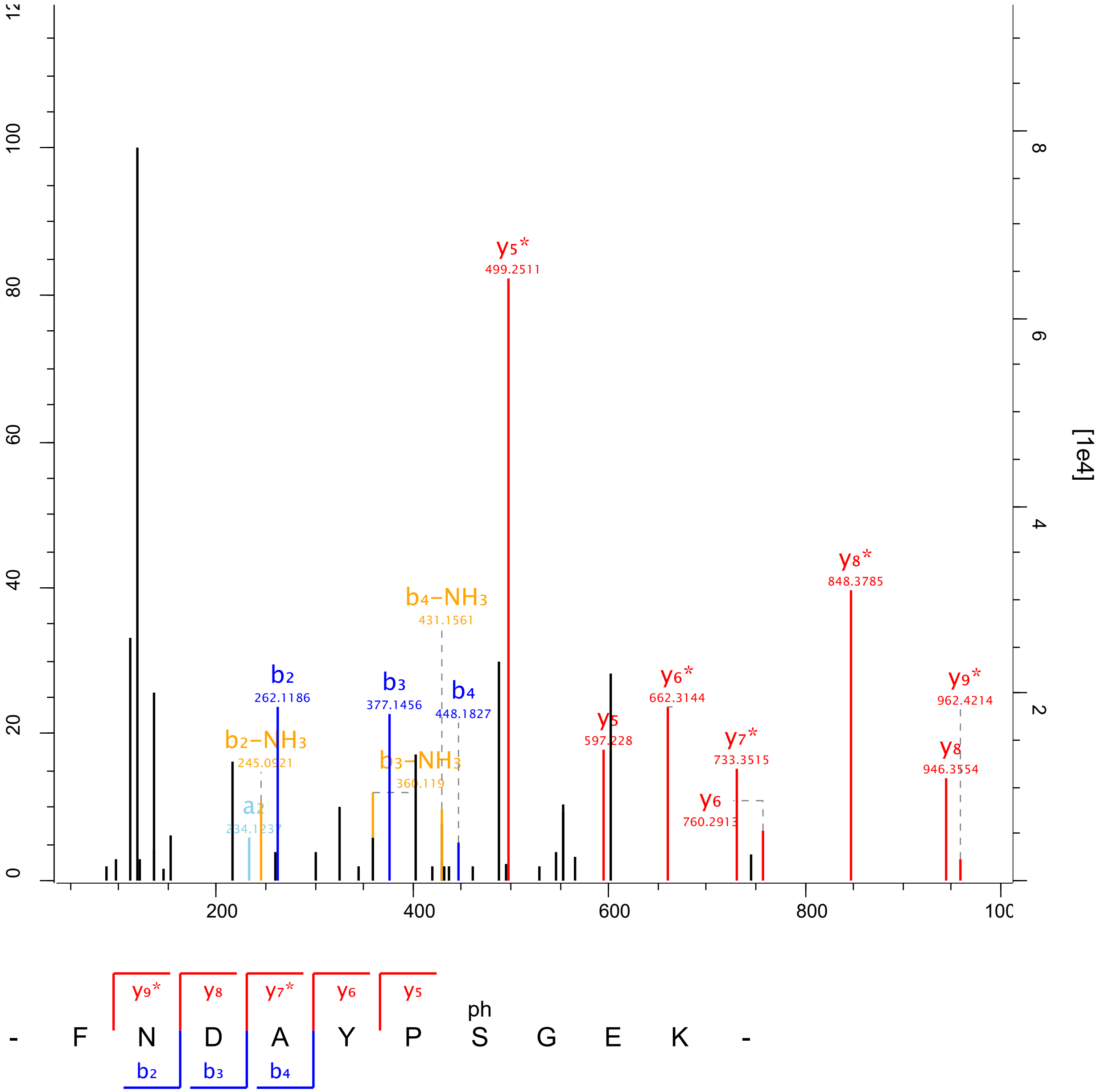

Raw file	Scan	Method	Score	m/z
sys_00_3short	8811	FTMS; HCD	68.66	474.19

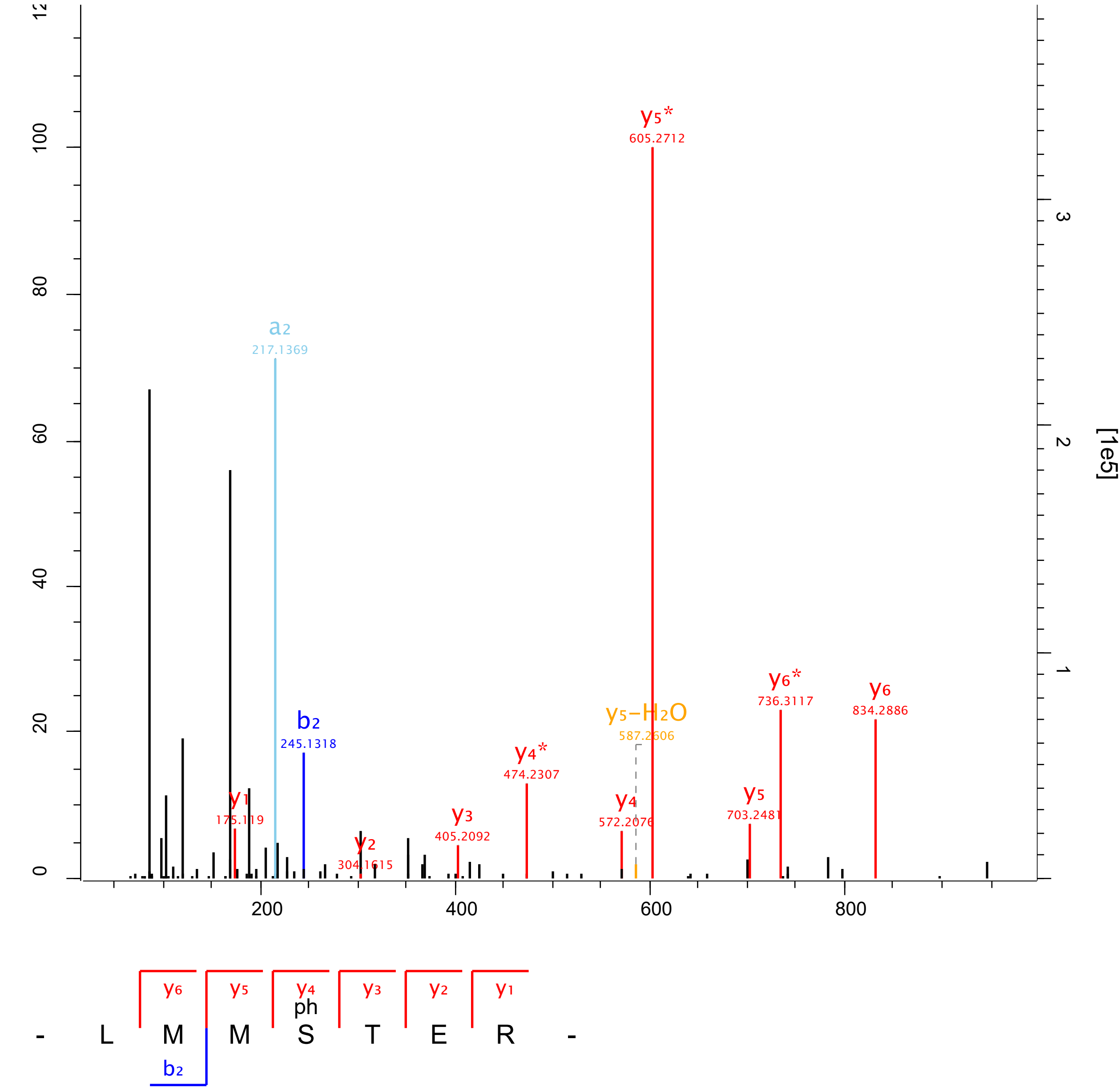

Raw file	Scan	Method	Score	m/z
sys_00_3short	8857	FTMS; HCD	154.1	578.74

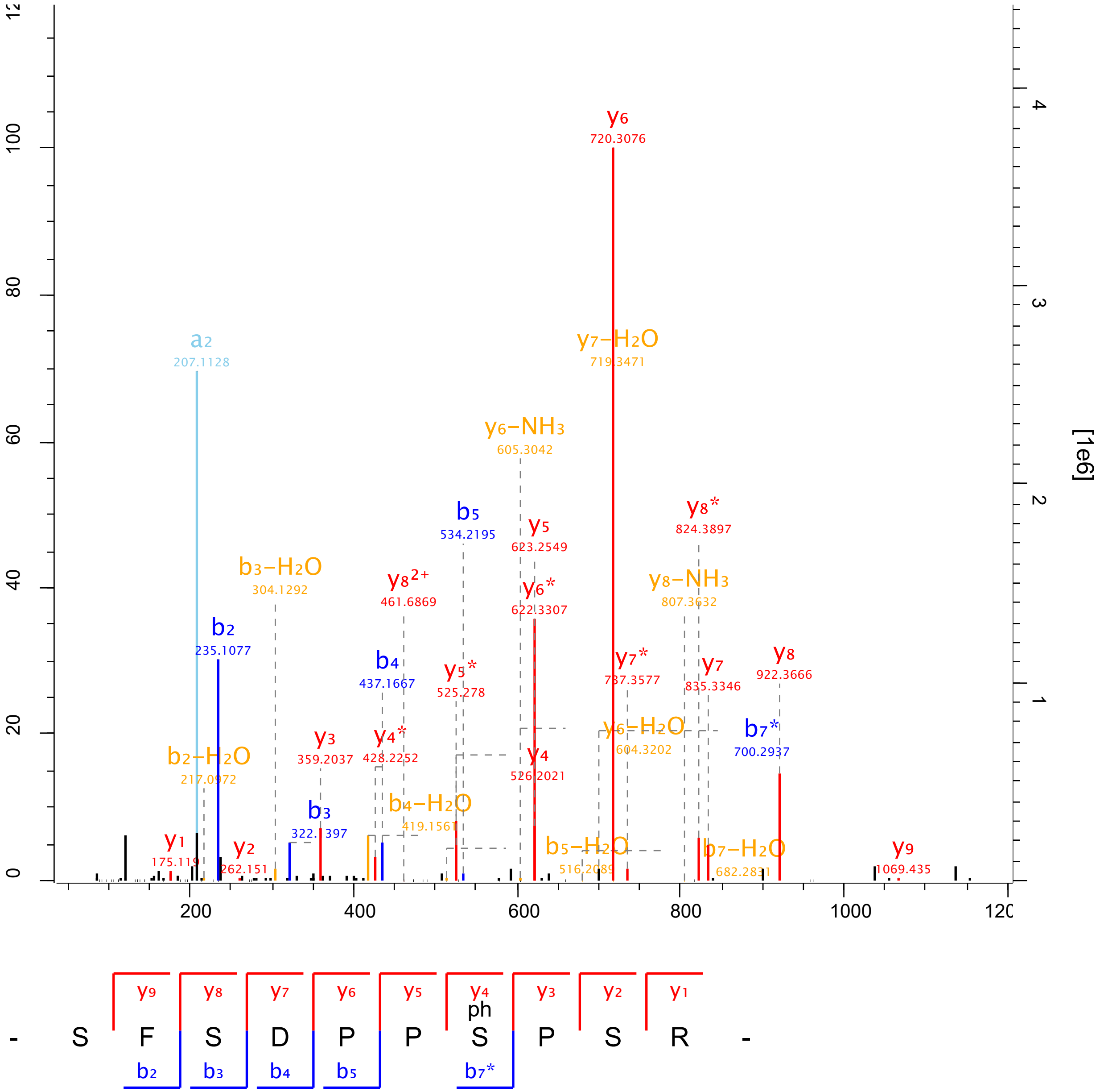

Raw file	Scan	Method	Score	m/z
sys_00_3short	8892	FTMS; HCD	99.5	627.24

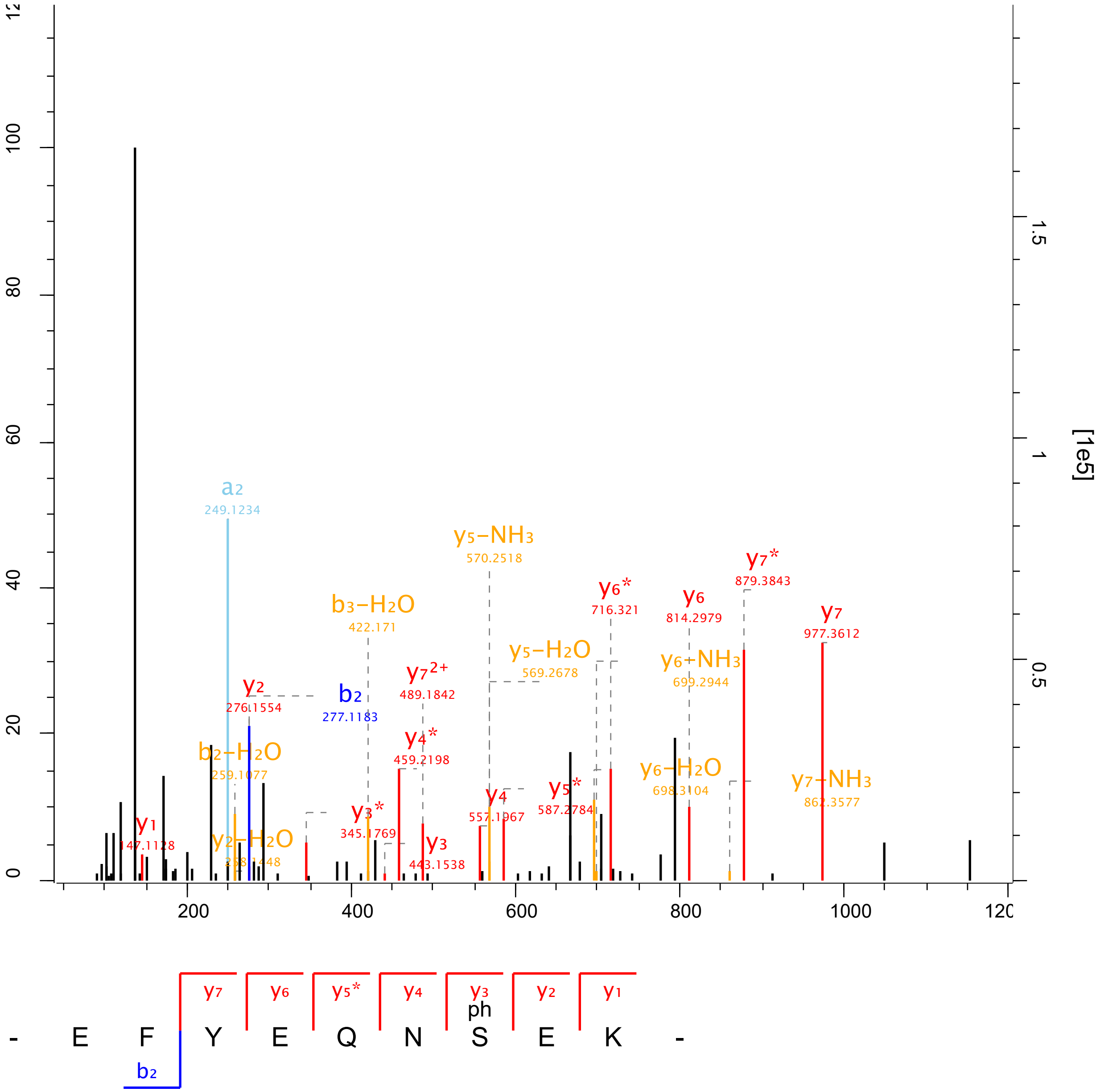

Raw file	Scan	Method	Score	m/z
sys_00_3short	8986	FTMS; HCD	175.08	637.78

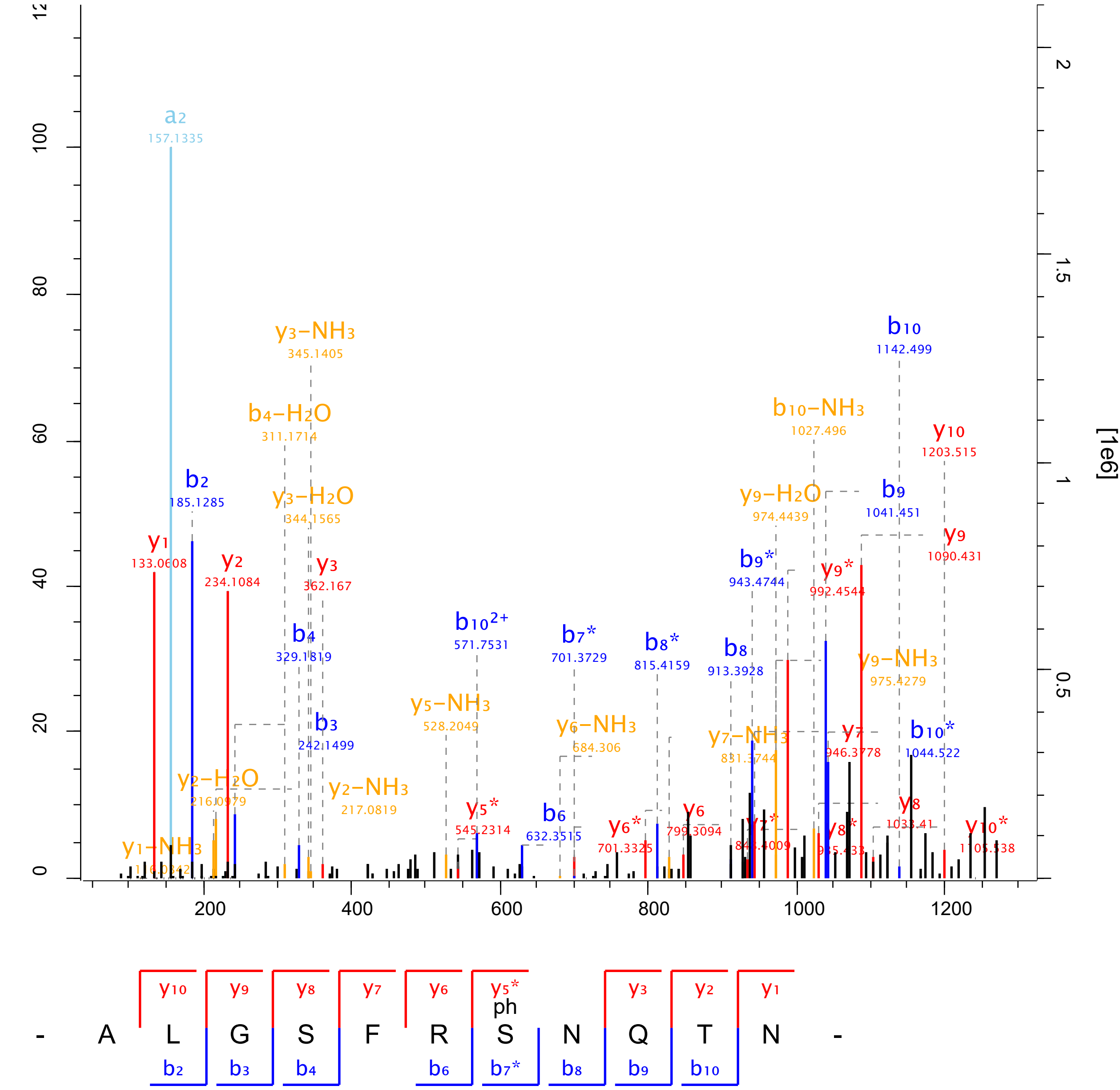

Raw file	Scan	Method	Score	m/z
sys_00_3short	9228	FTMS; HCD	105.58	620.3

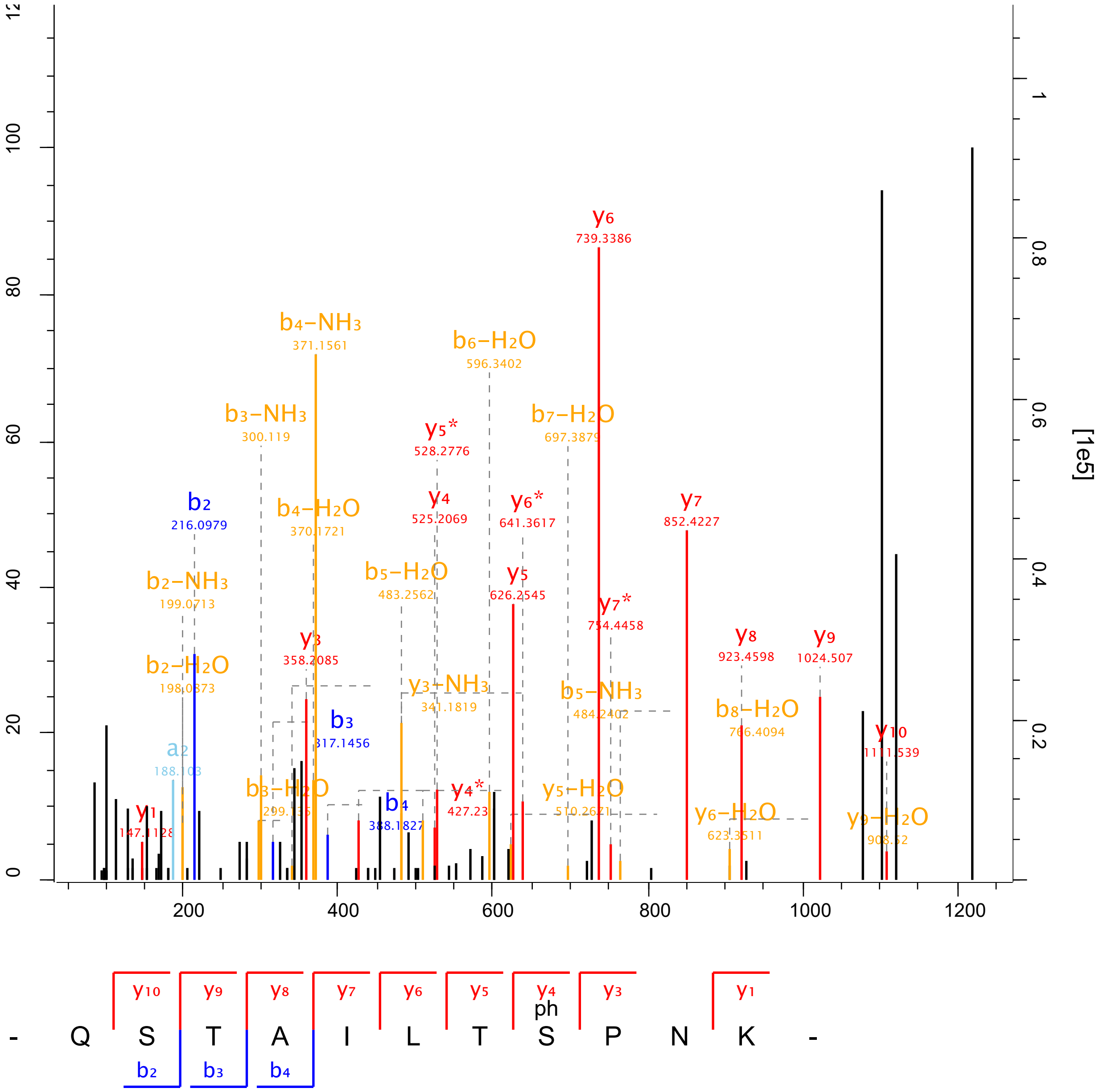

Raw file	Scan	Method	Score	m/z
sys_00_3short	9266	FTMS; HCD	80.23	398.19

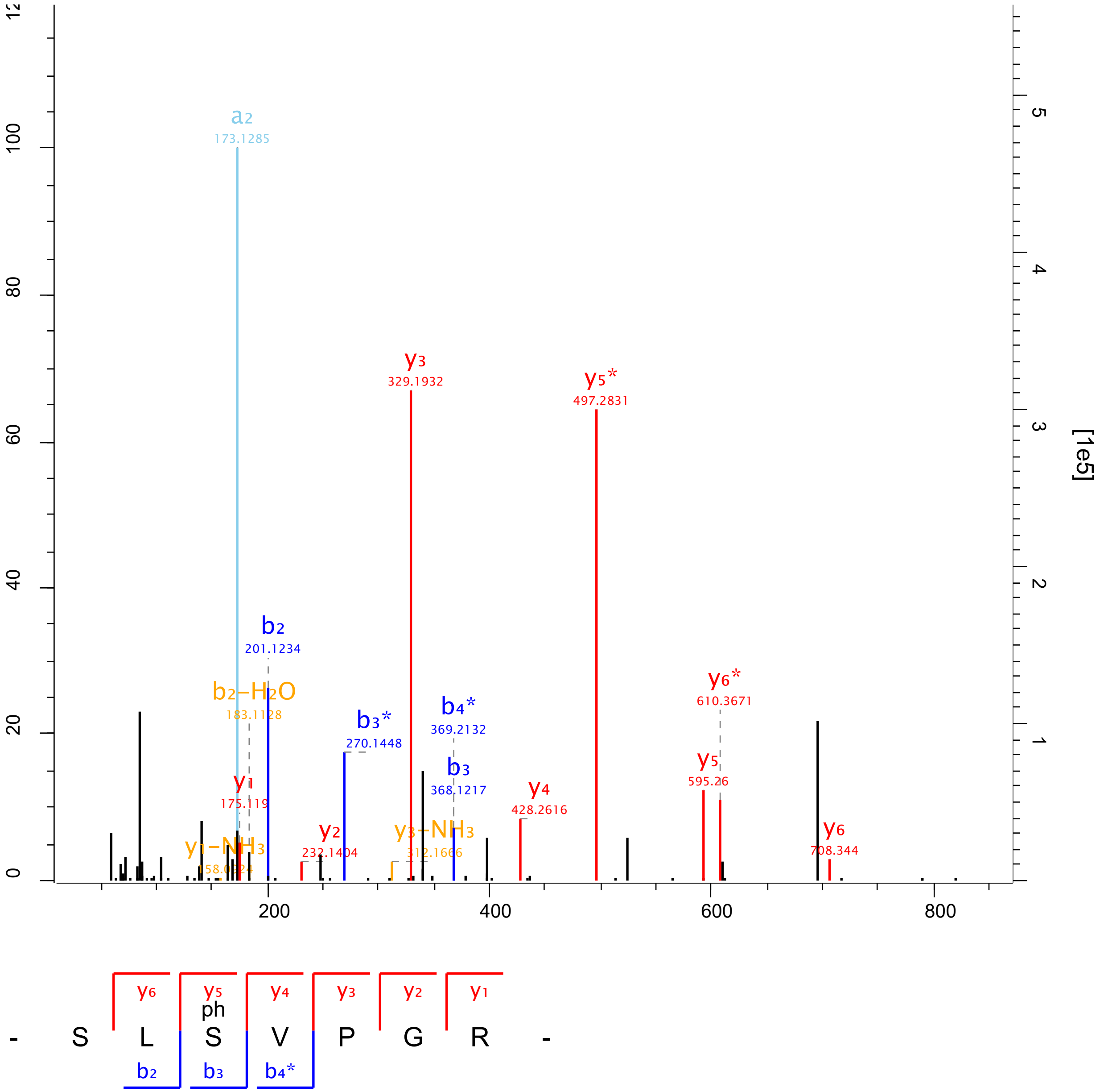

Raw file	Scan	Method	Score	m/z
sys_00_3short	9342	FTMS; HCD	98.9	538.76

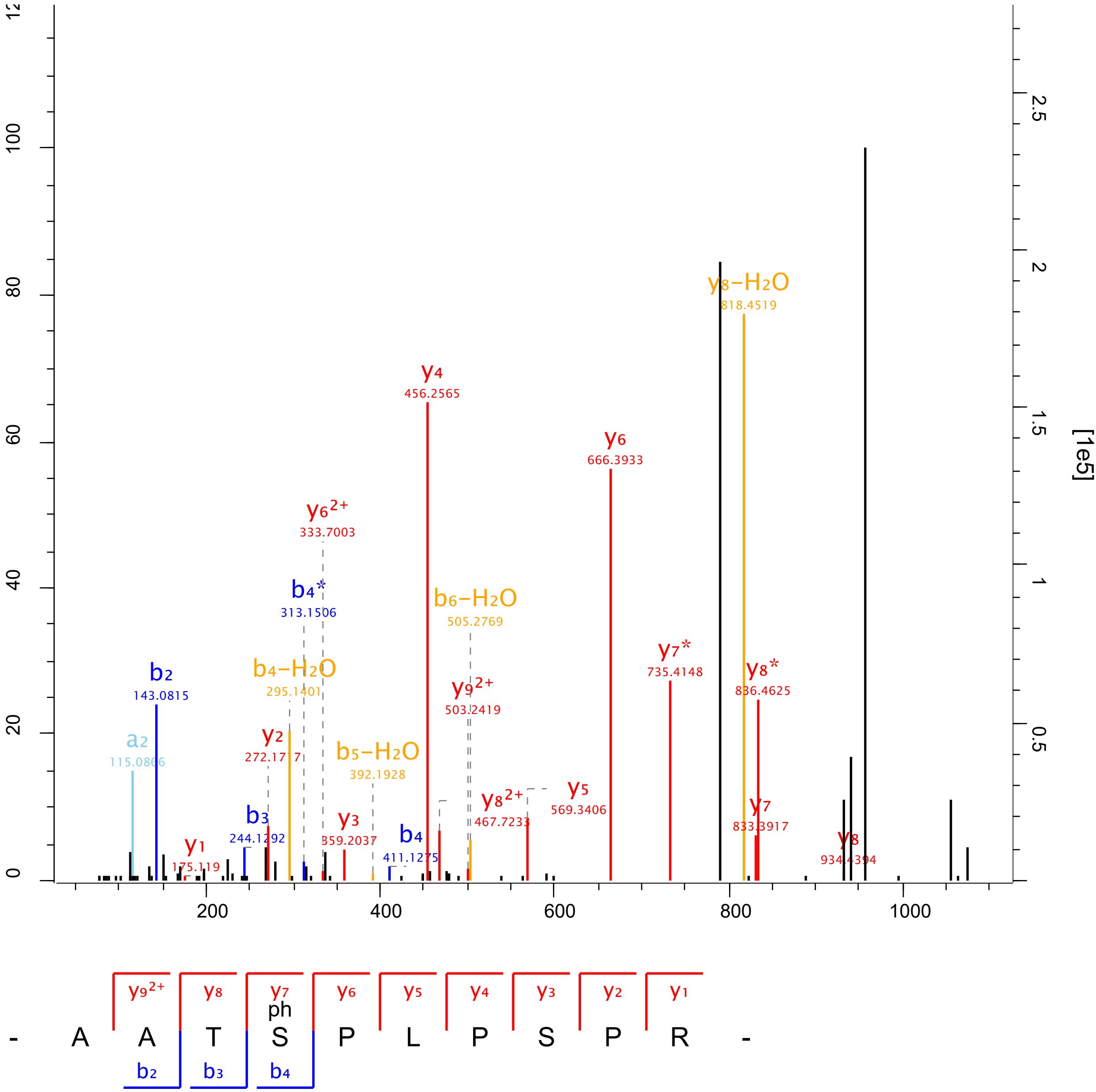

Raw file	Scan	Method	Score	m/z
sys_00_3short	9602	FTMS; HCD	41.4	406.7

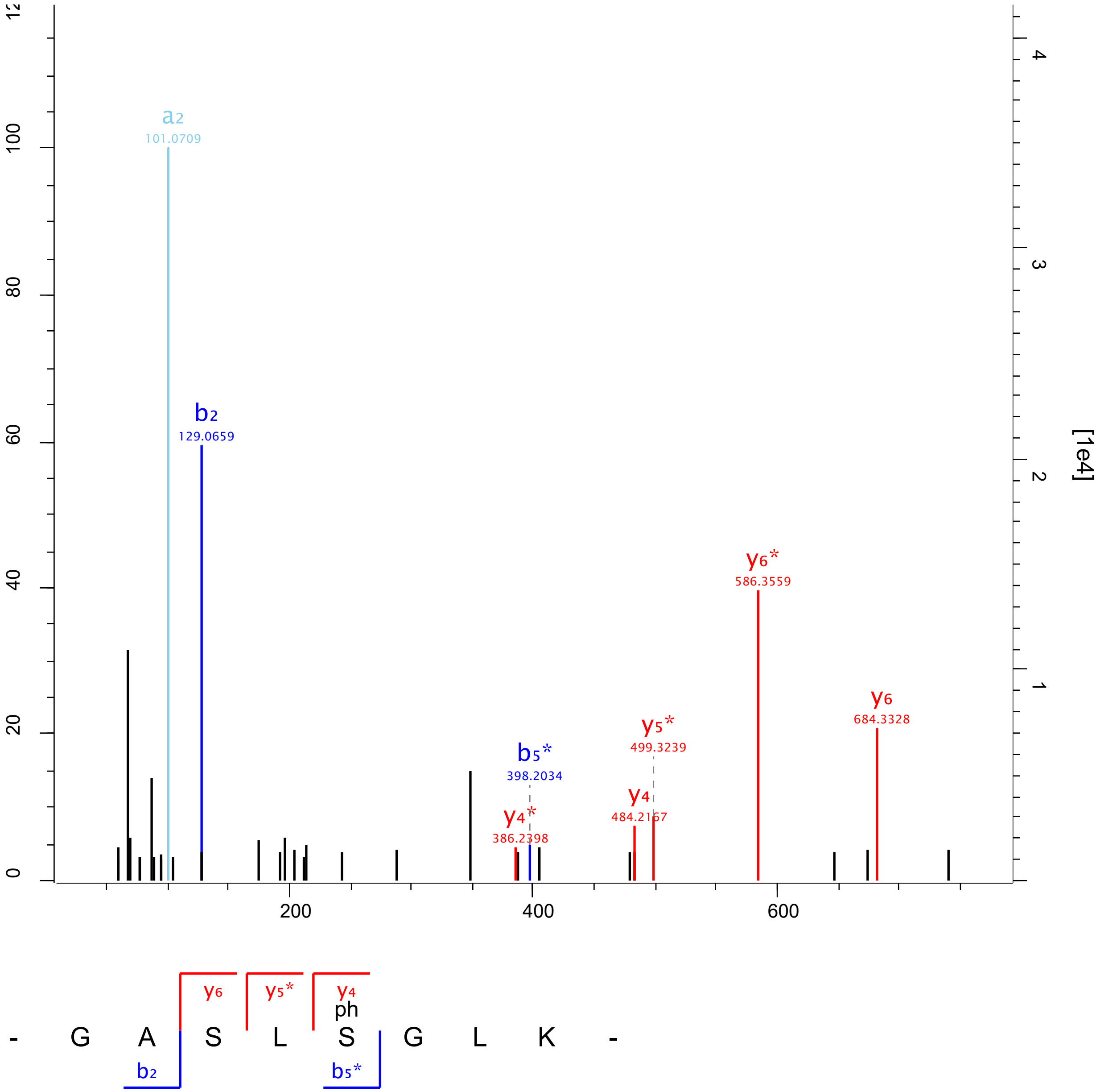

Raw file	Scan	Method	Score	m/z
sys_00_3short	9657	FTMS; HCD	119.04	676.8

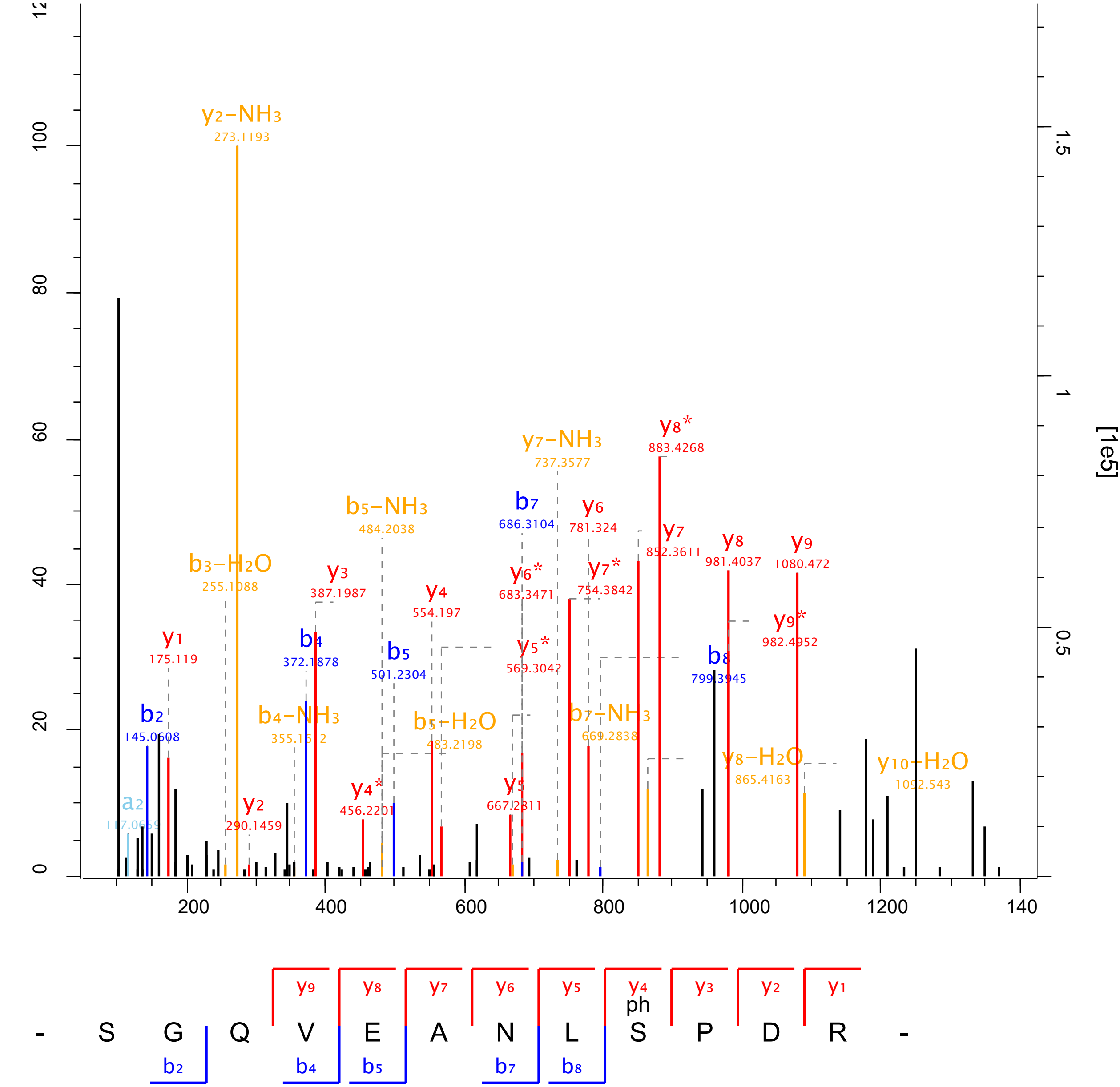

Raw file	Scan	Method	Score	m/z
sys_00_3short	9951	FTMS; HCD	142.12	577.77

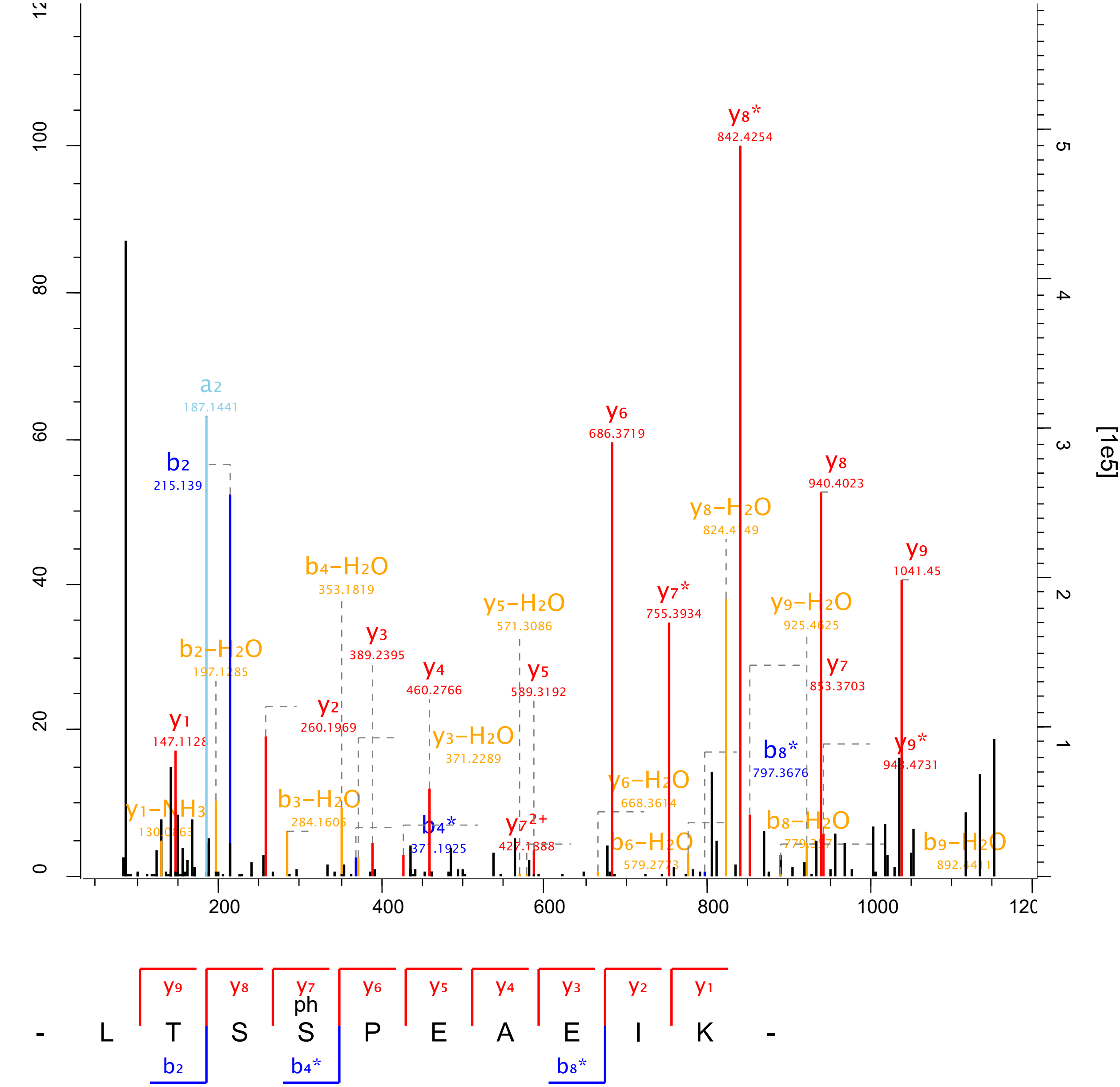

Raw file	Scan	Method	Score	m/z
sys_02_2	10092	FTMS; HCD	50.9	502.24

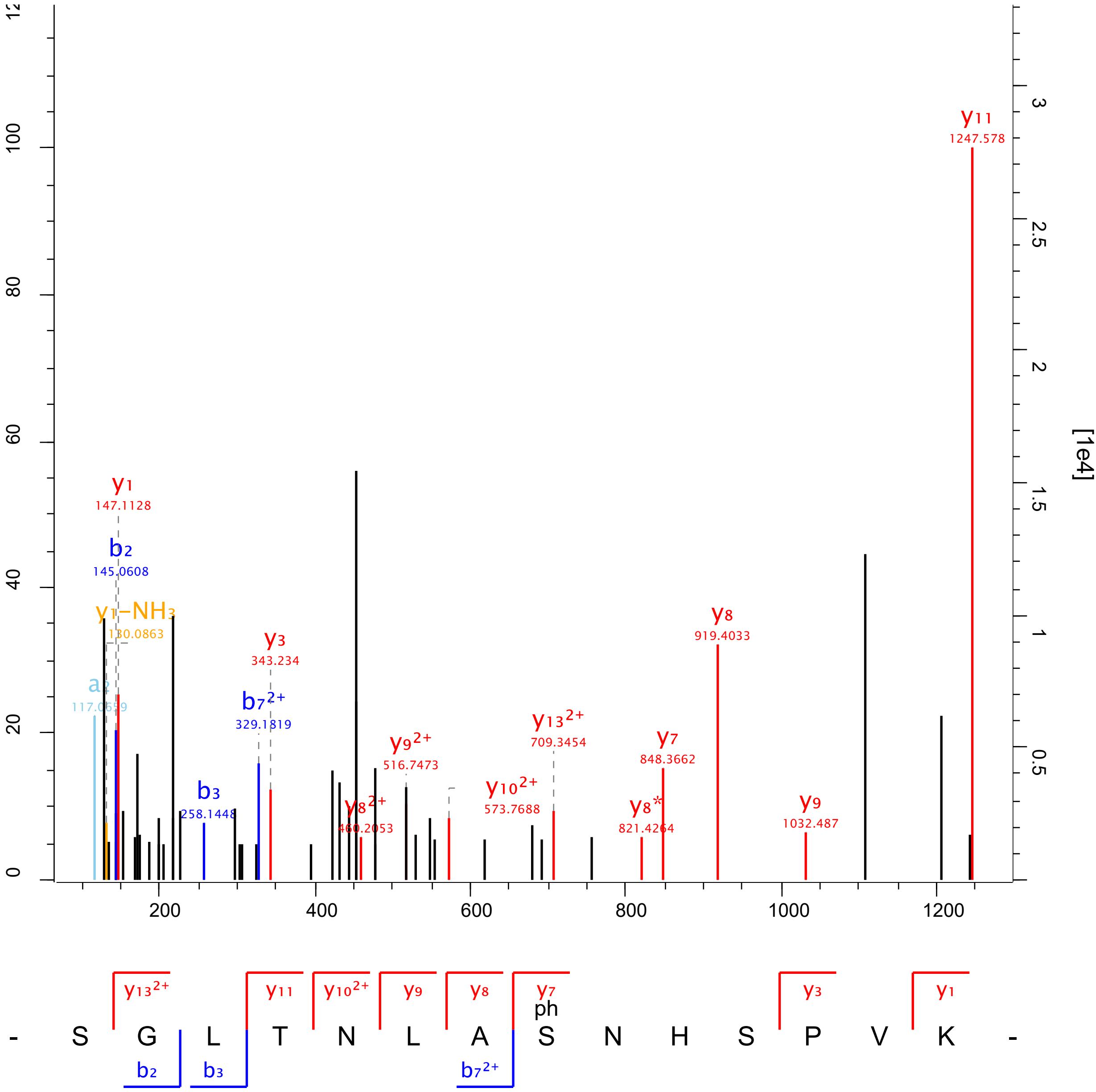

Raw file	Scan	Method	Score	m/z
sys_02_2	10197	FTMS; HCD	42.32	616.29

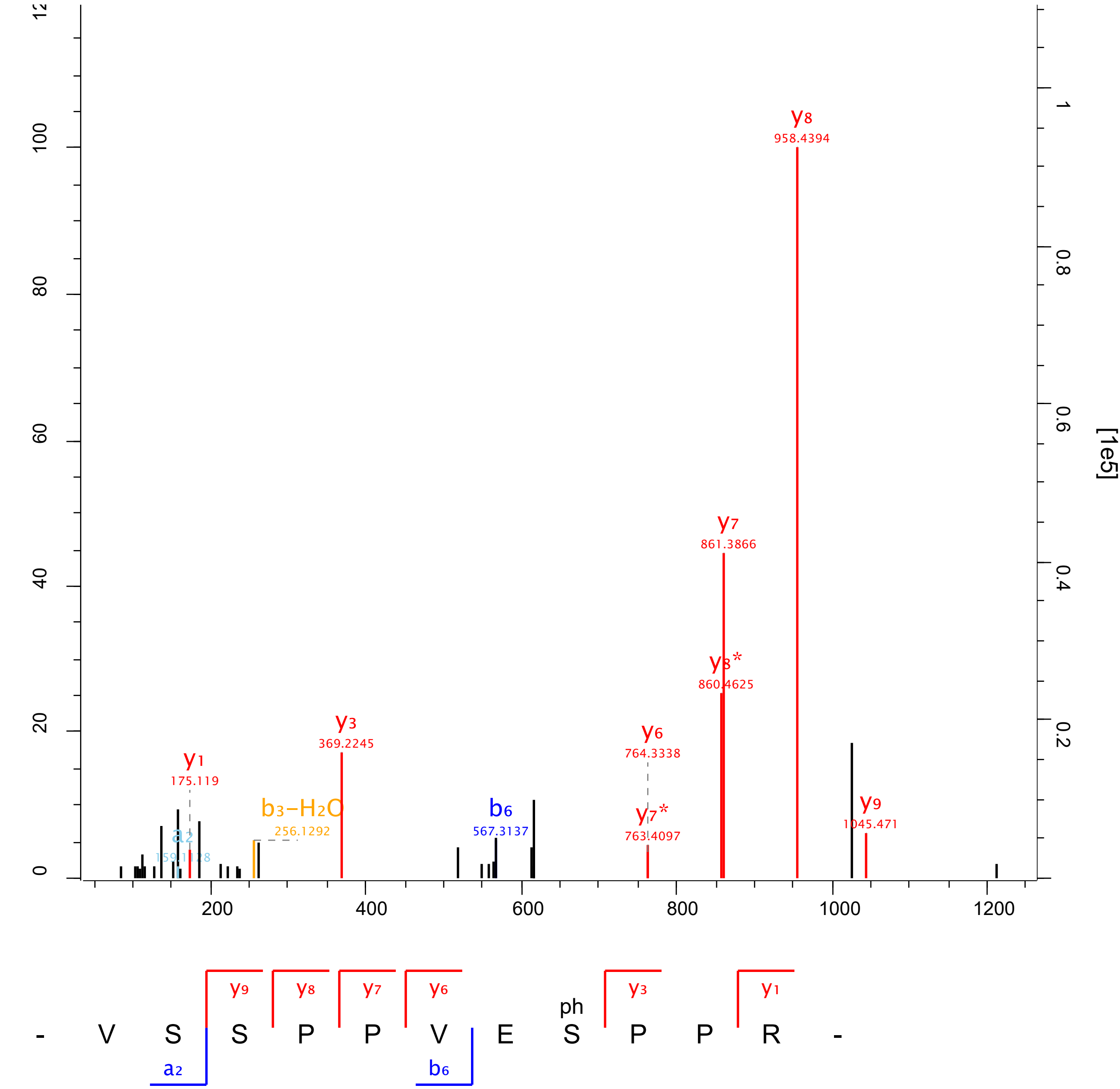

Raw file	Scan	Method	Score	m/z
sys_02_2	10215	FTMS; HCD	94.12	560.78

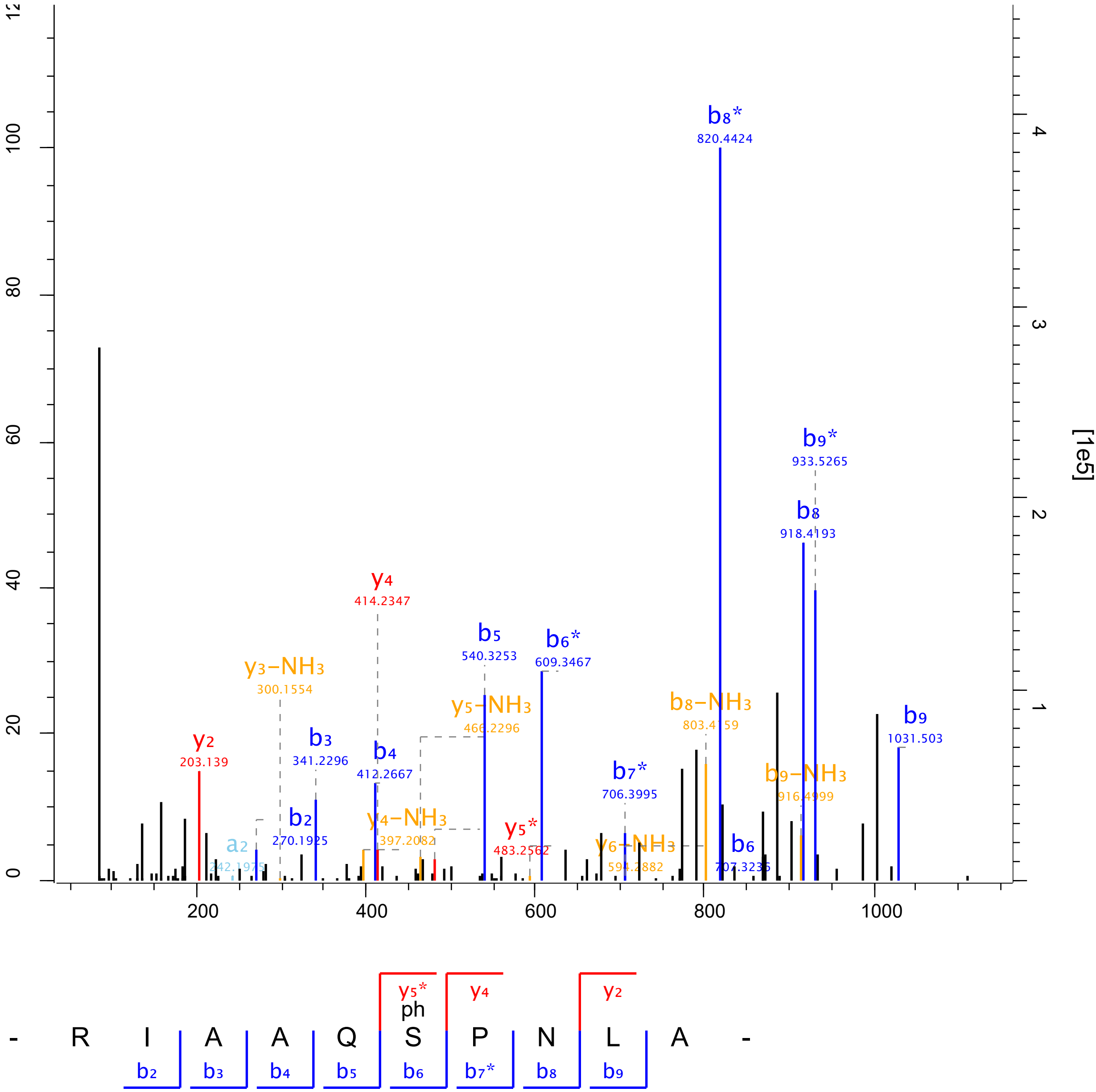

Raw file	Scan	Method	Score	m/z
sys_02_2	10248	FTMS; HCD	130.56	870.38

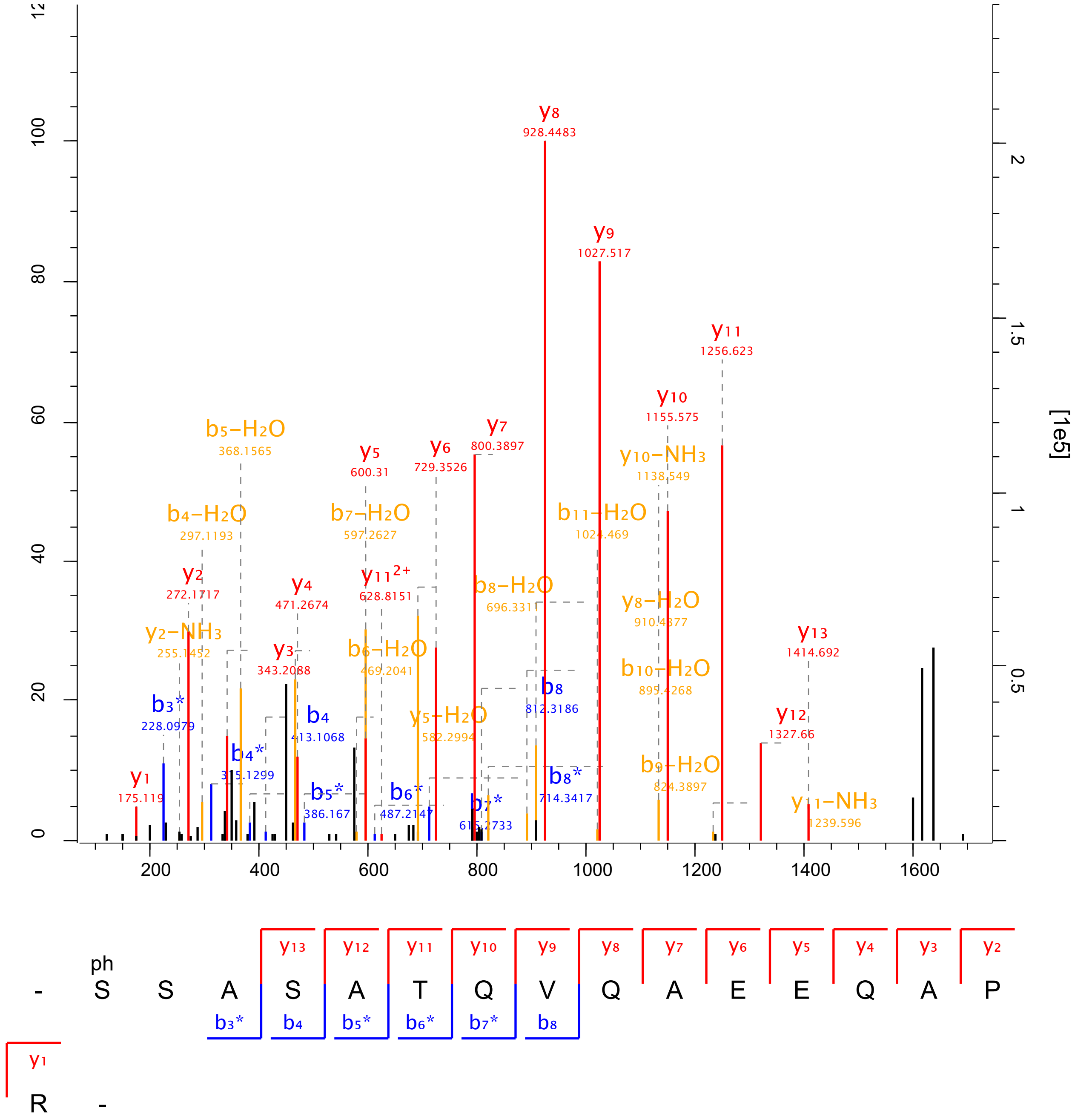

Raw file	Scan	Method	Score	m/z
sys_02_2	10362	FTMS; HCD	47.77	393.86

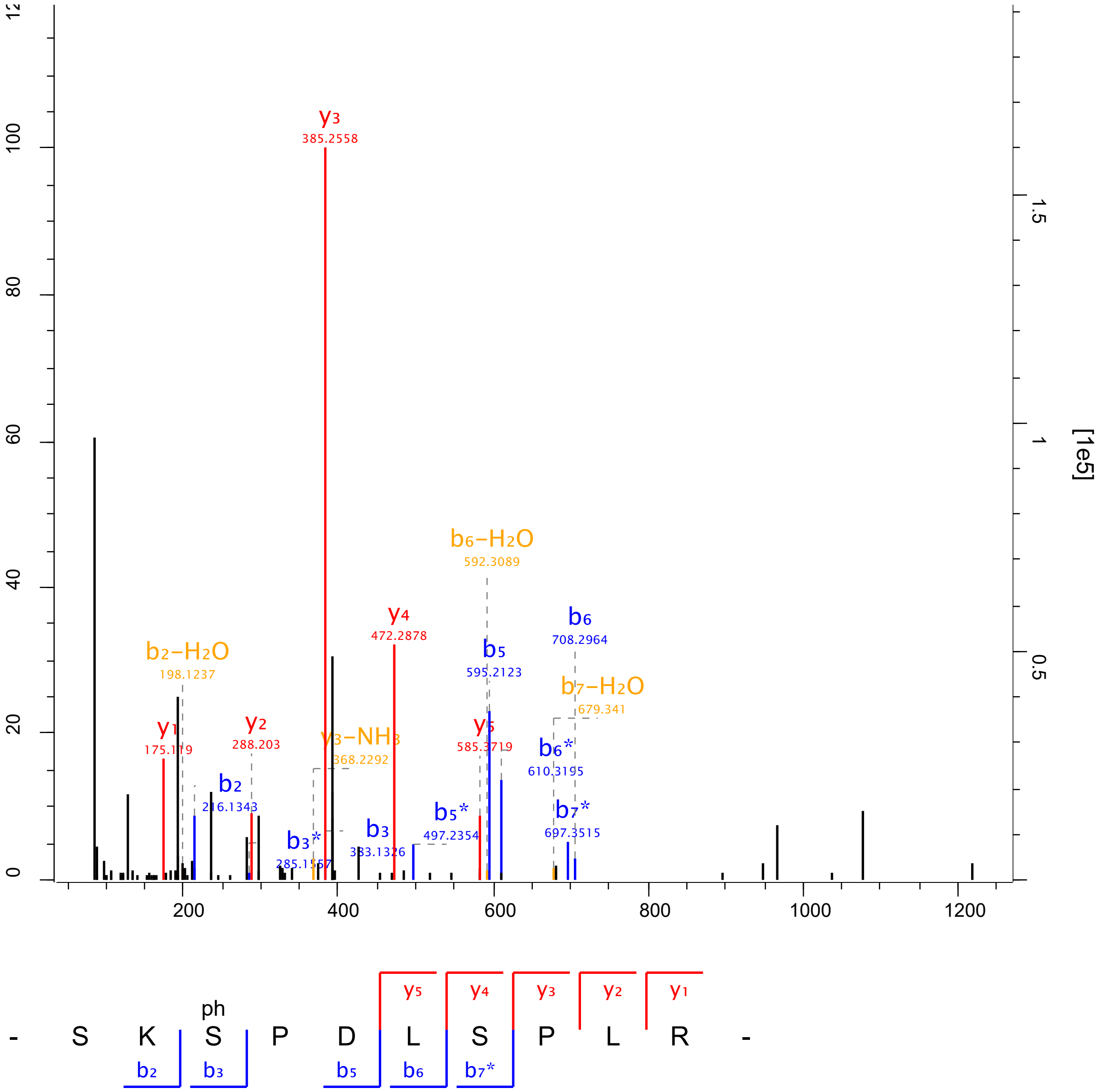

Raw file	Scan	Method	Score	m/z
sys_02_2	10407	FTMS; HCD	248.95	706.31

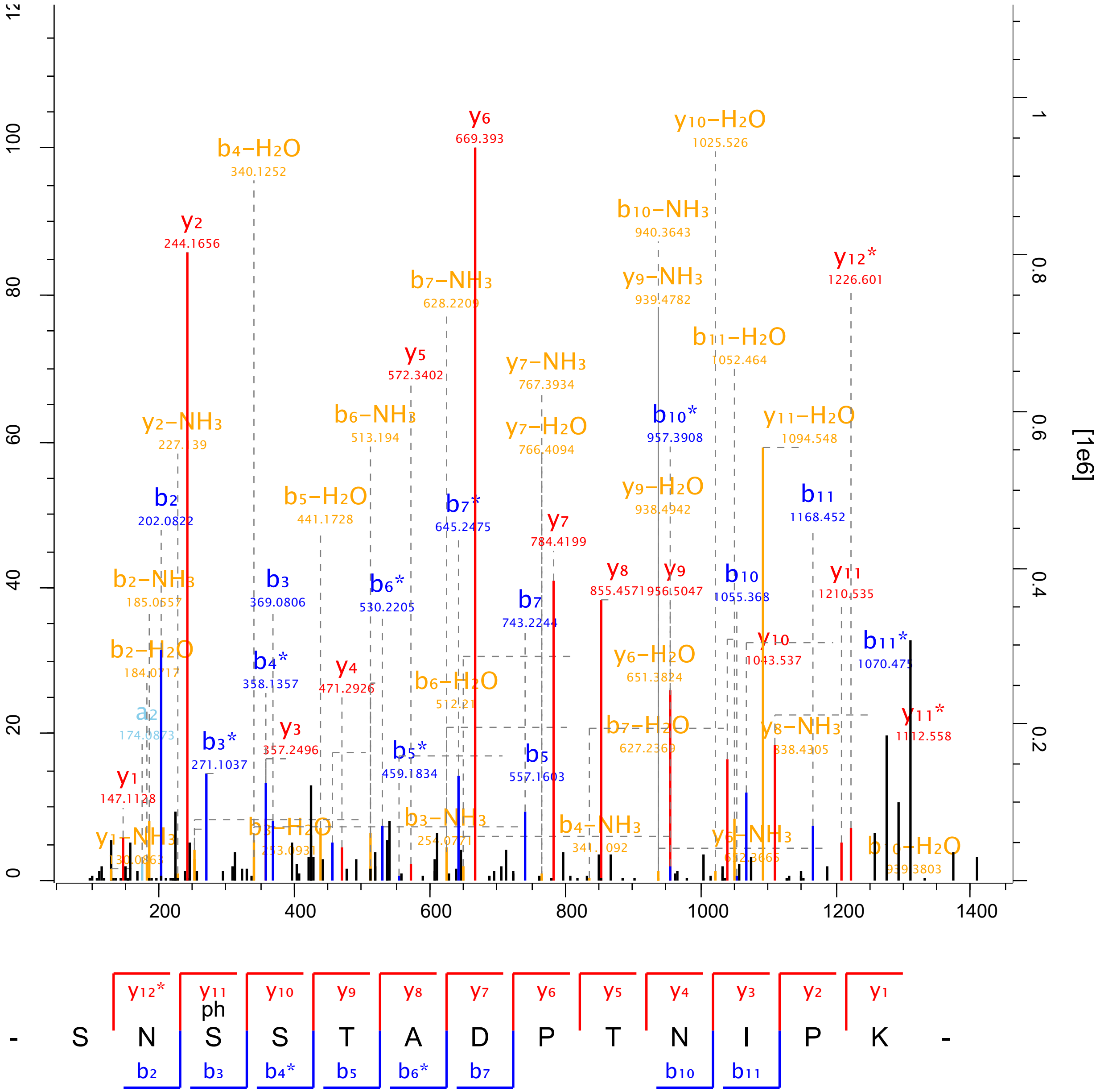

Raw file	Scan	Method	Score	m/z
sys_02_2	10429	FTMS; HCD	142.08	517.72

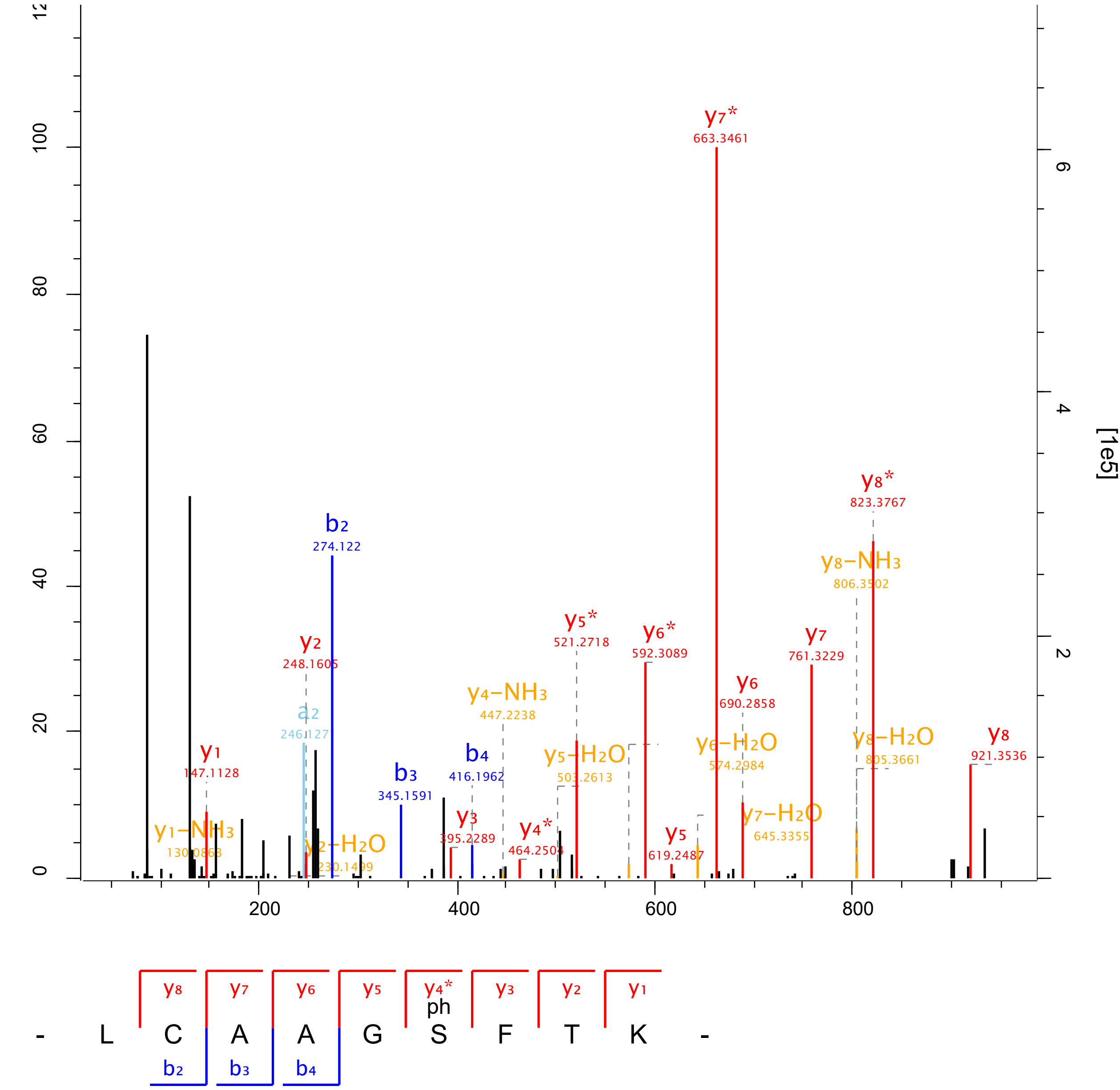

Raw file	Scan	Method	Score	m/z
sys_02_2	10449	FTMS; HCD	70.26	463.22

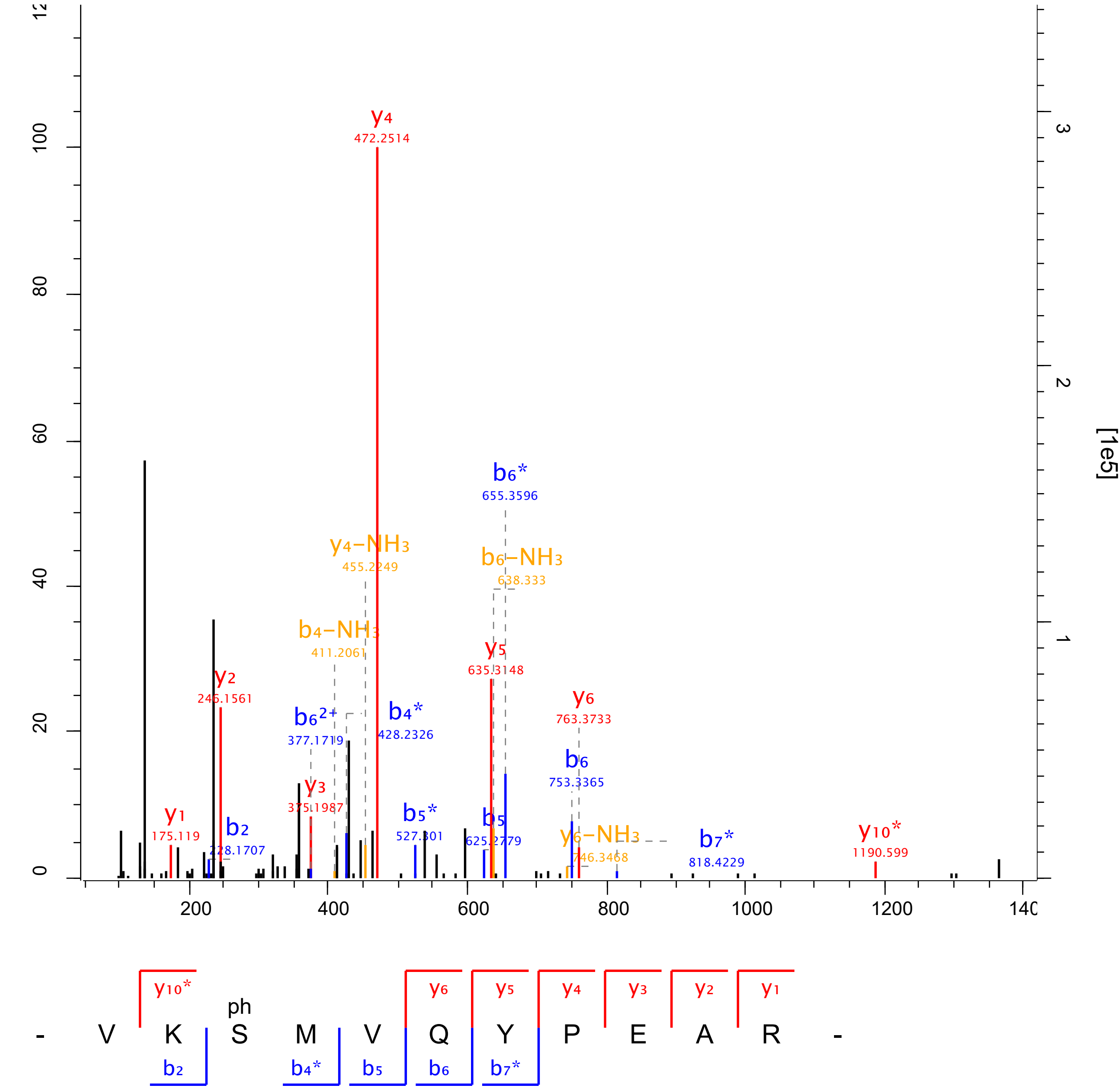

Raw file	Scan	Method	Score	m/z
sys_02_2	10482	FTMS; HCD	190.24	724.28

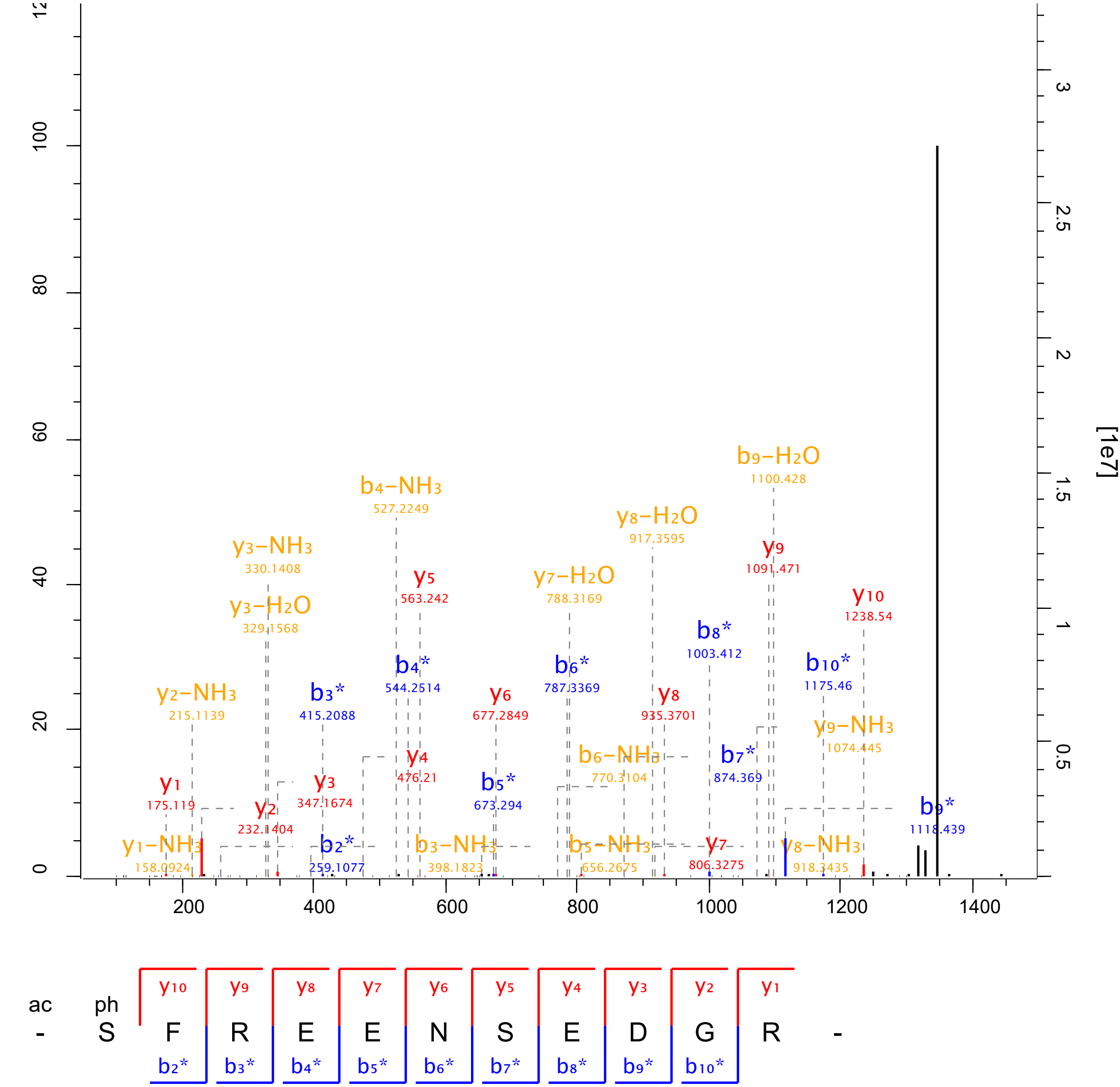

Raw file	Scan	Method	Score	m/z
sys_02_2	10839	FTMS; HCD	190.35	675.94

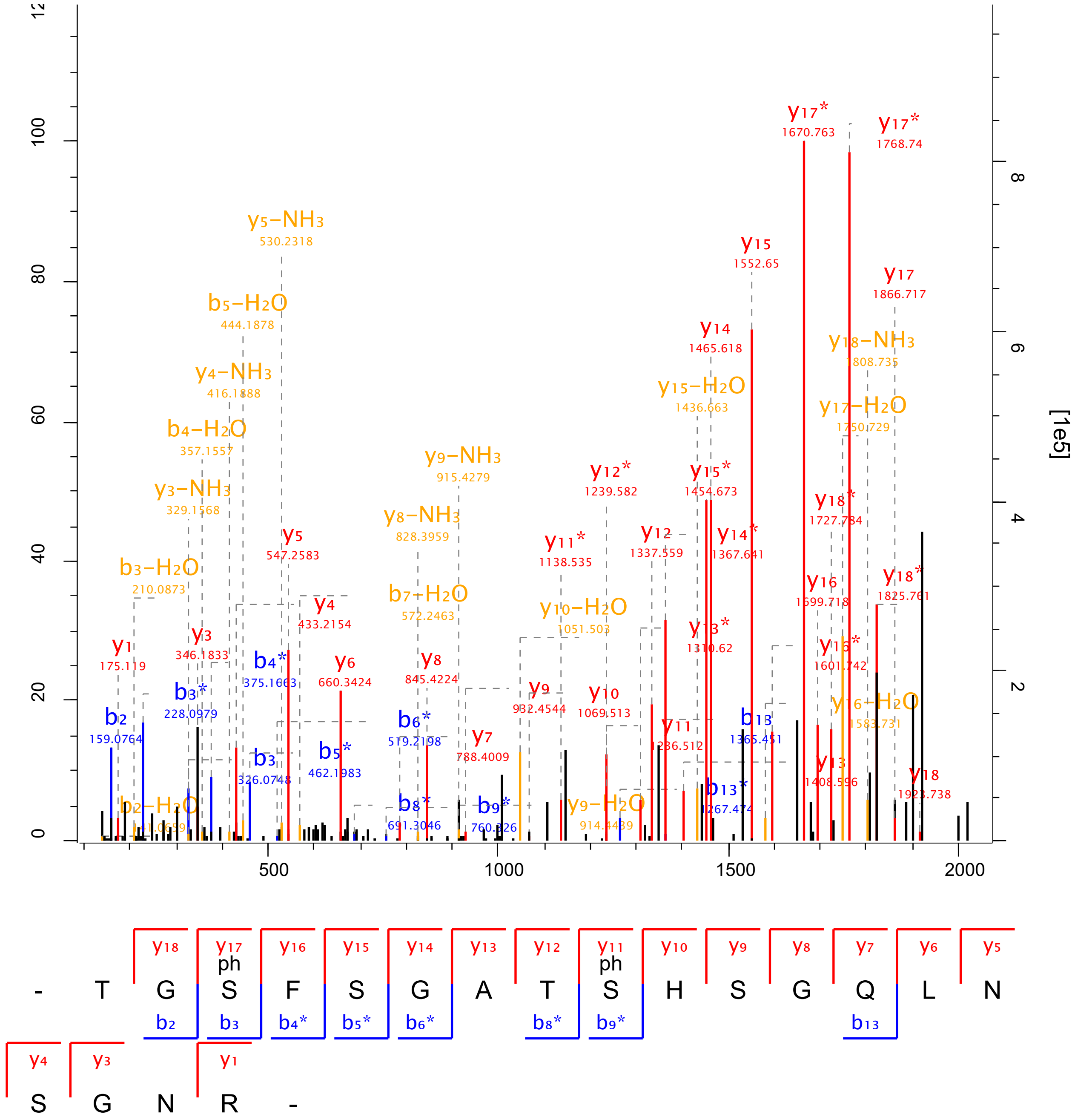

Raw file	Scan	Method	Score	m/z
sys_02_2	10970	FTMS; HCD	230.41	440.72

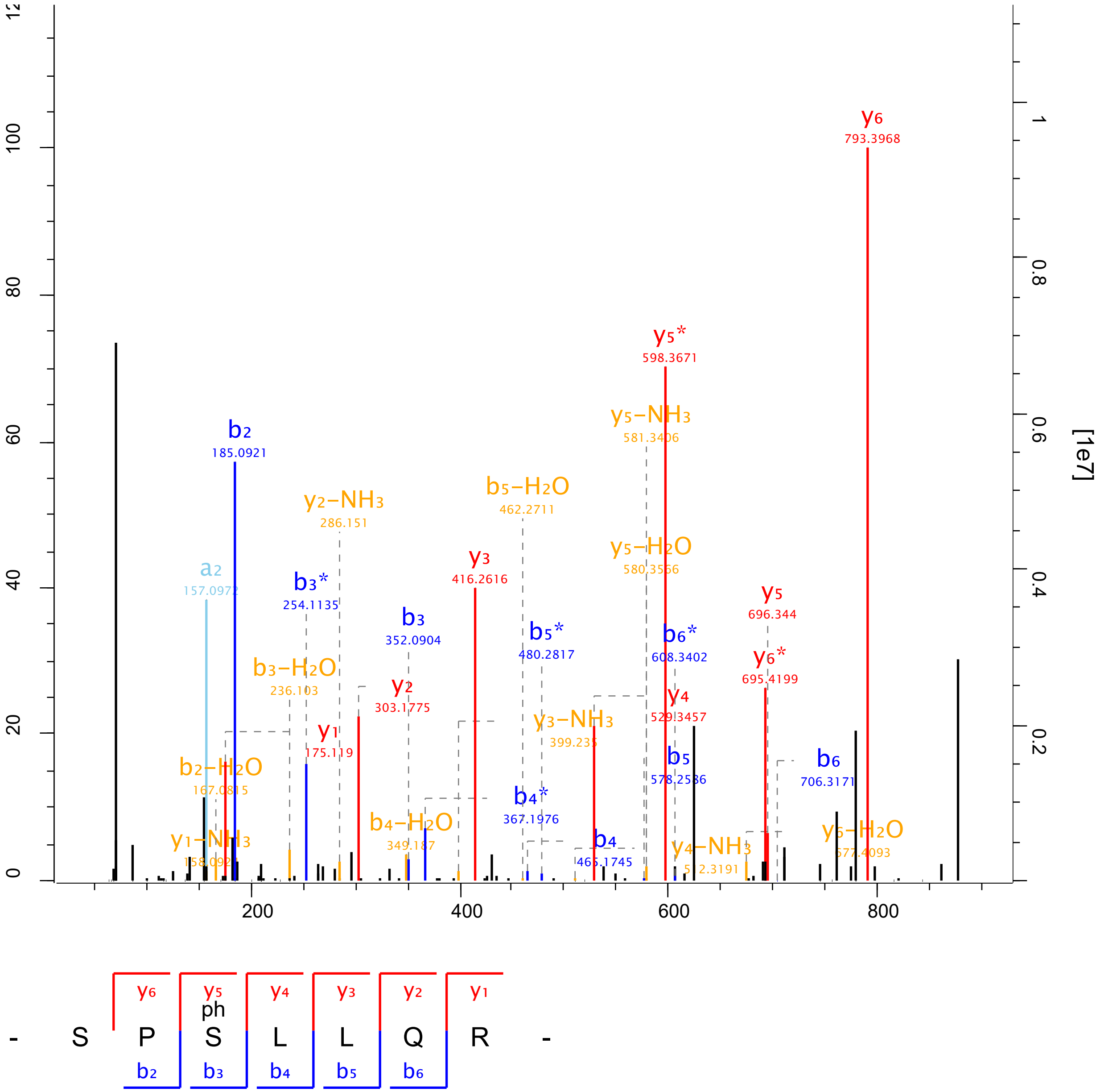

Raw file	Scan	Method	Score	m/z
sys_02_2	10983	FTMS; HCD	47.62	576.75

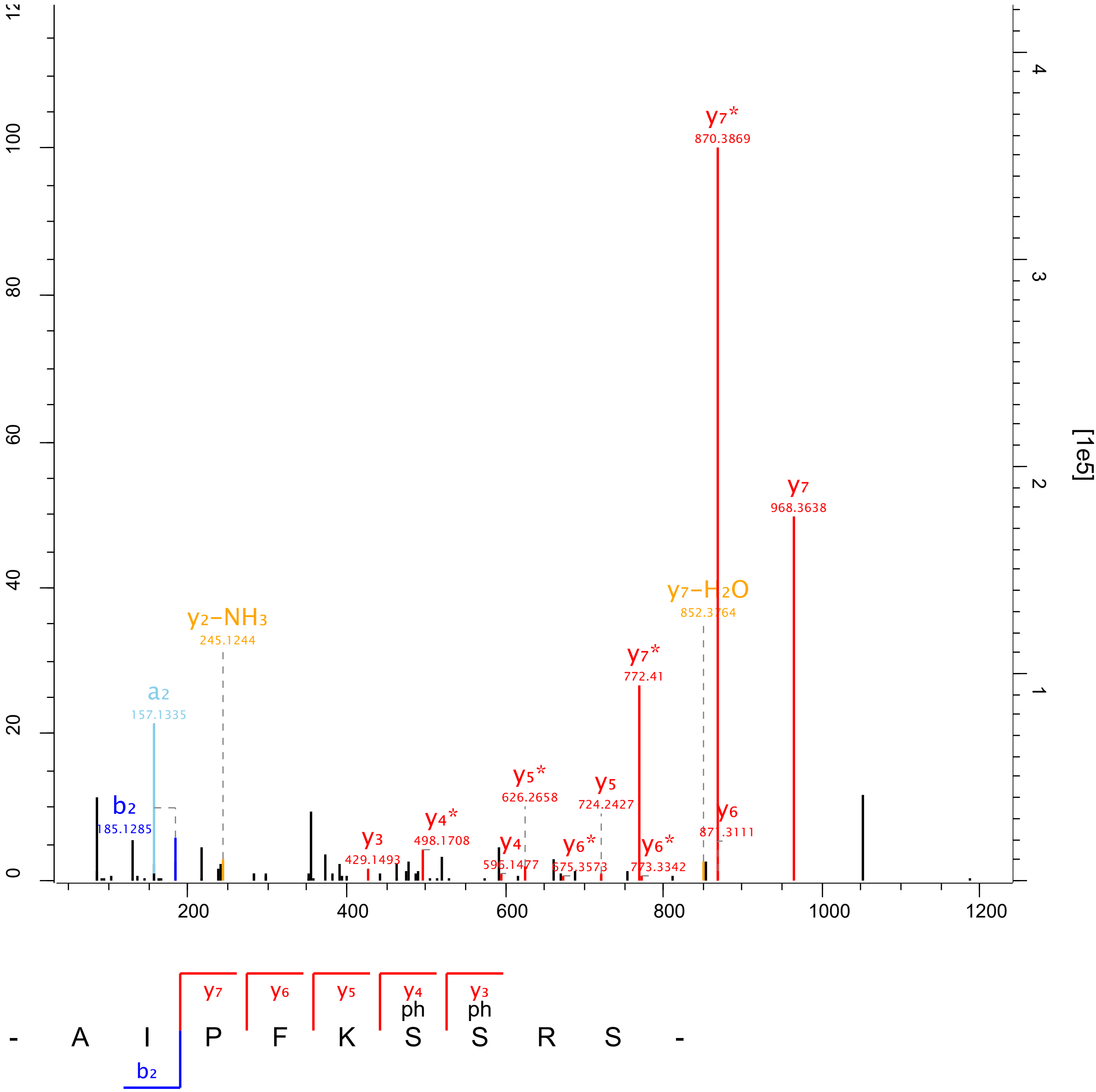

Raw file	Scan	Method	Score	m/z
sys_02_2	11014	FTMS; HCD	84.61	412.21

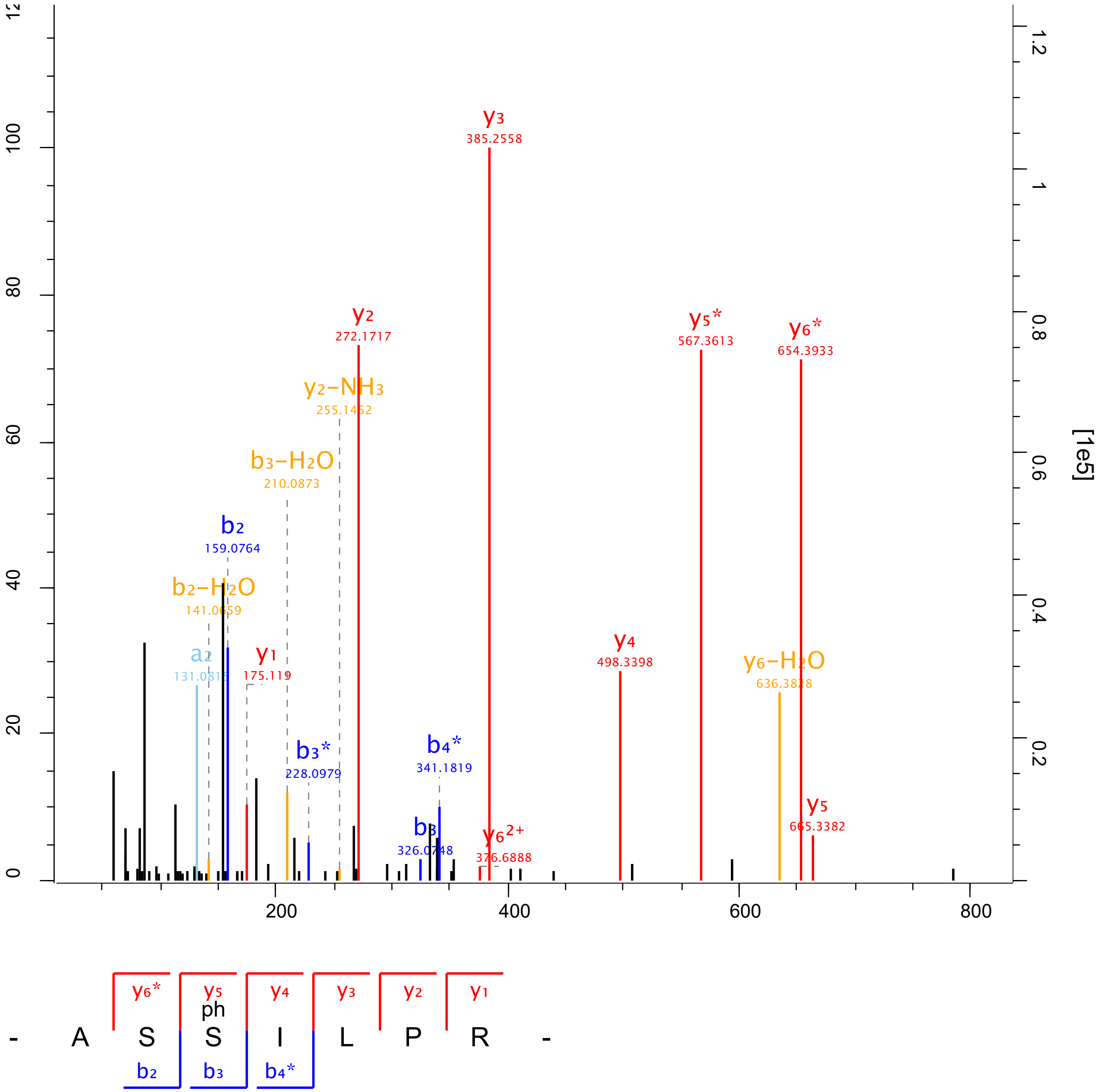

Raw file	Scan	Method	Score	m/z
sys_02_2	11367	FTMS; HCD	196.37	678.3

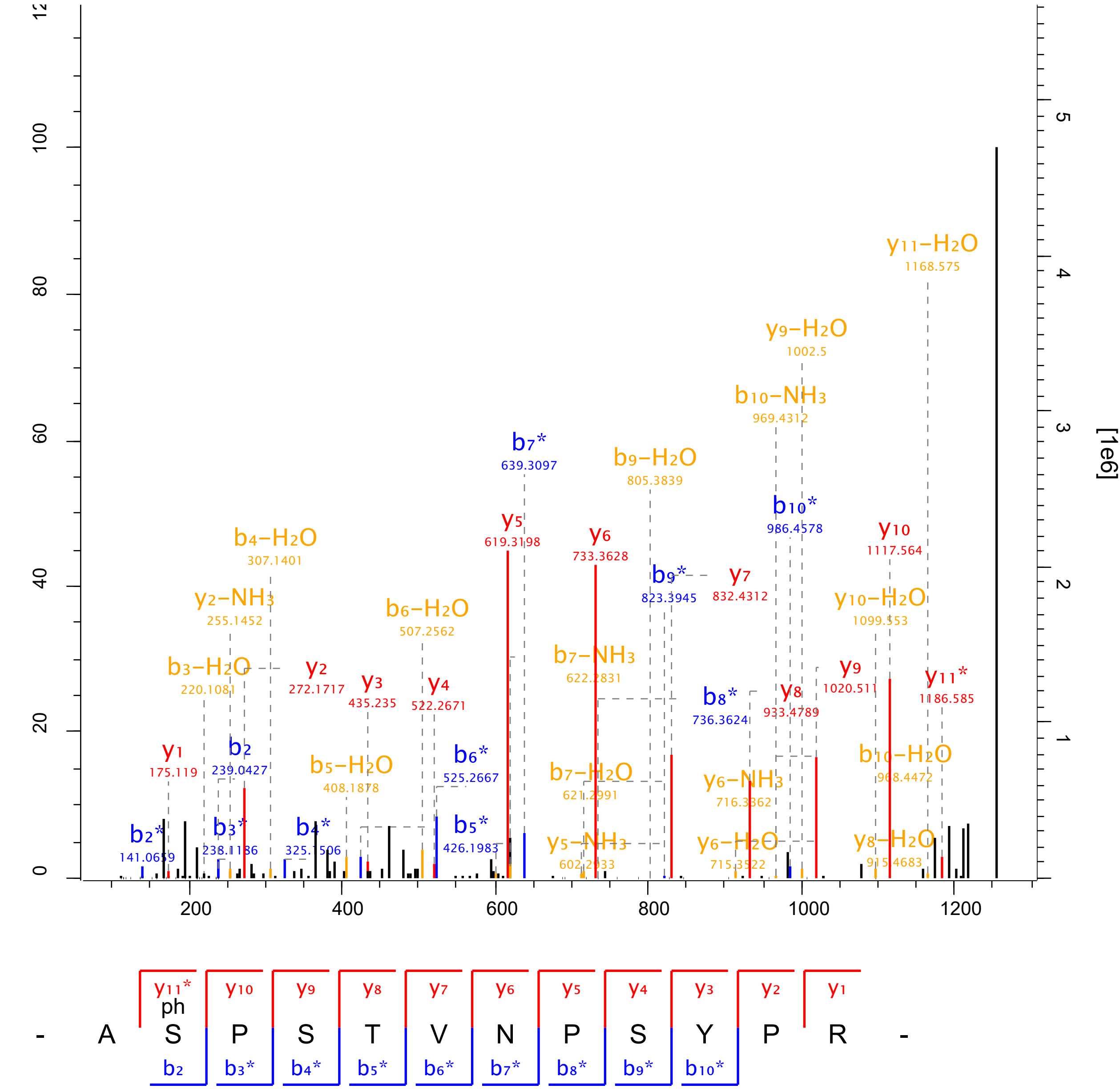

Raw file	Scan	Method	Score	m/z
sys_02_2	11426	FTMS; HCD	46.89	496.25

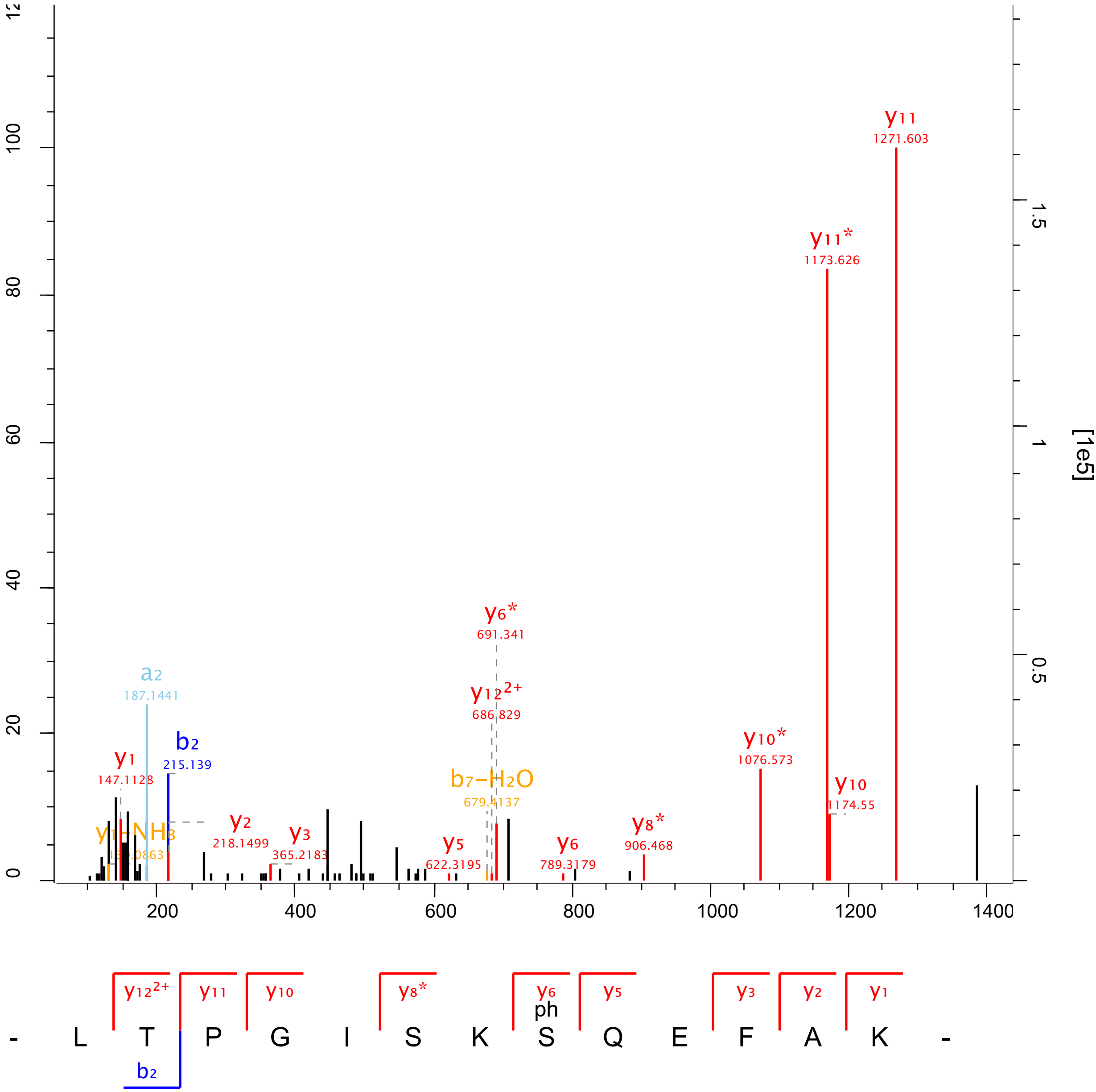

Raw file	Scan	Method	Score	m/z
sys_02_2	11489	FTMS; HCD	99.34	627.6

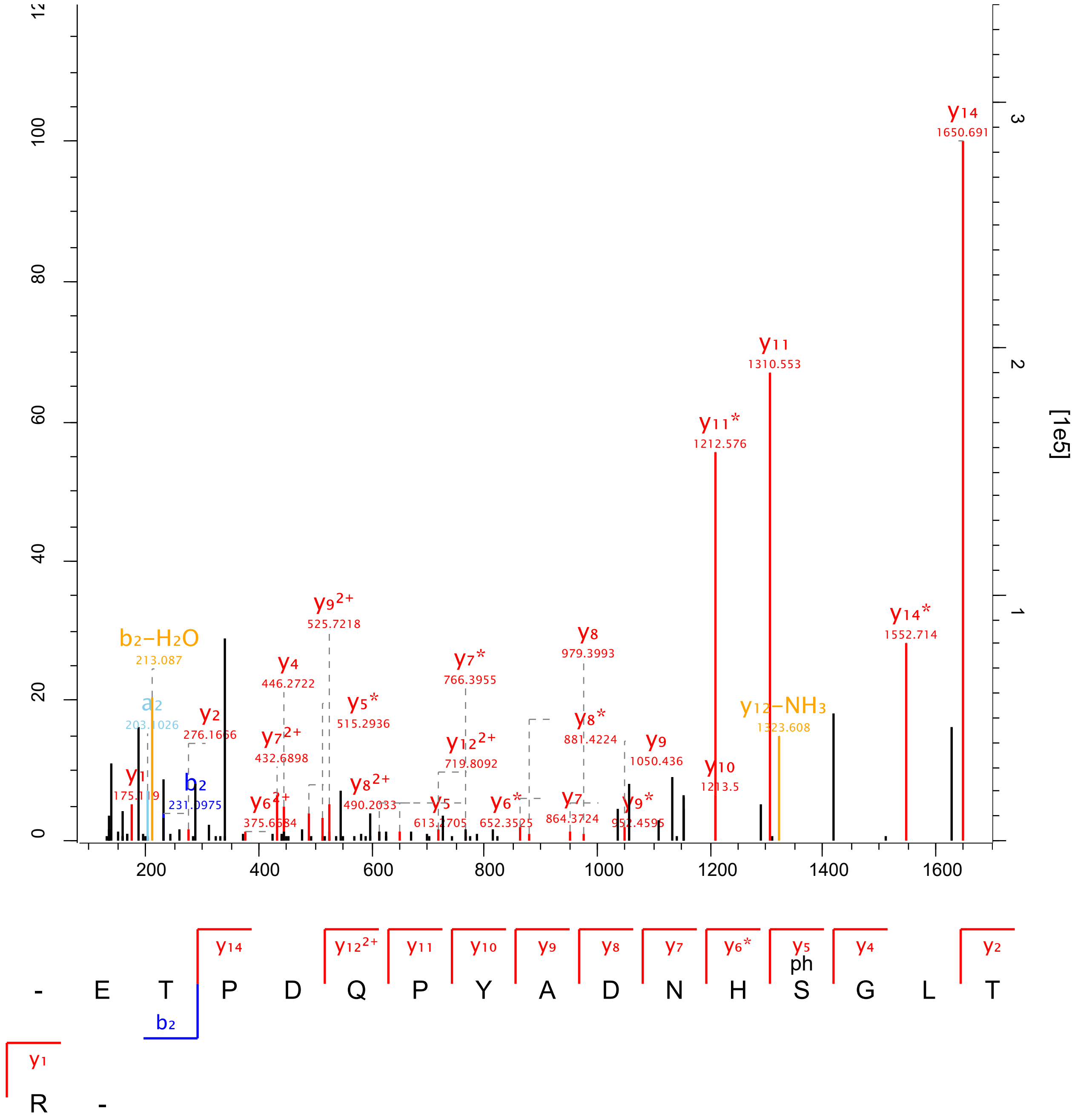

Raw file	Scan	Method	Score	m/z
sys_02_2	11522	FTMS; HCD	51.52	540.94

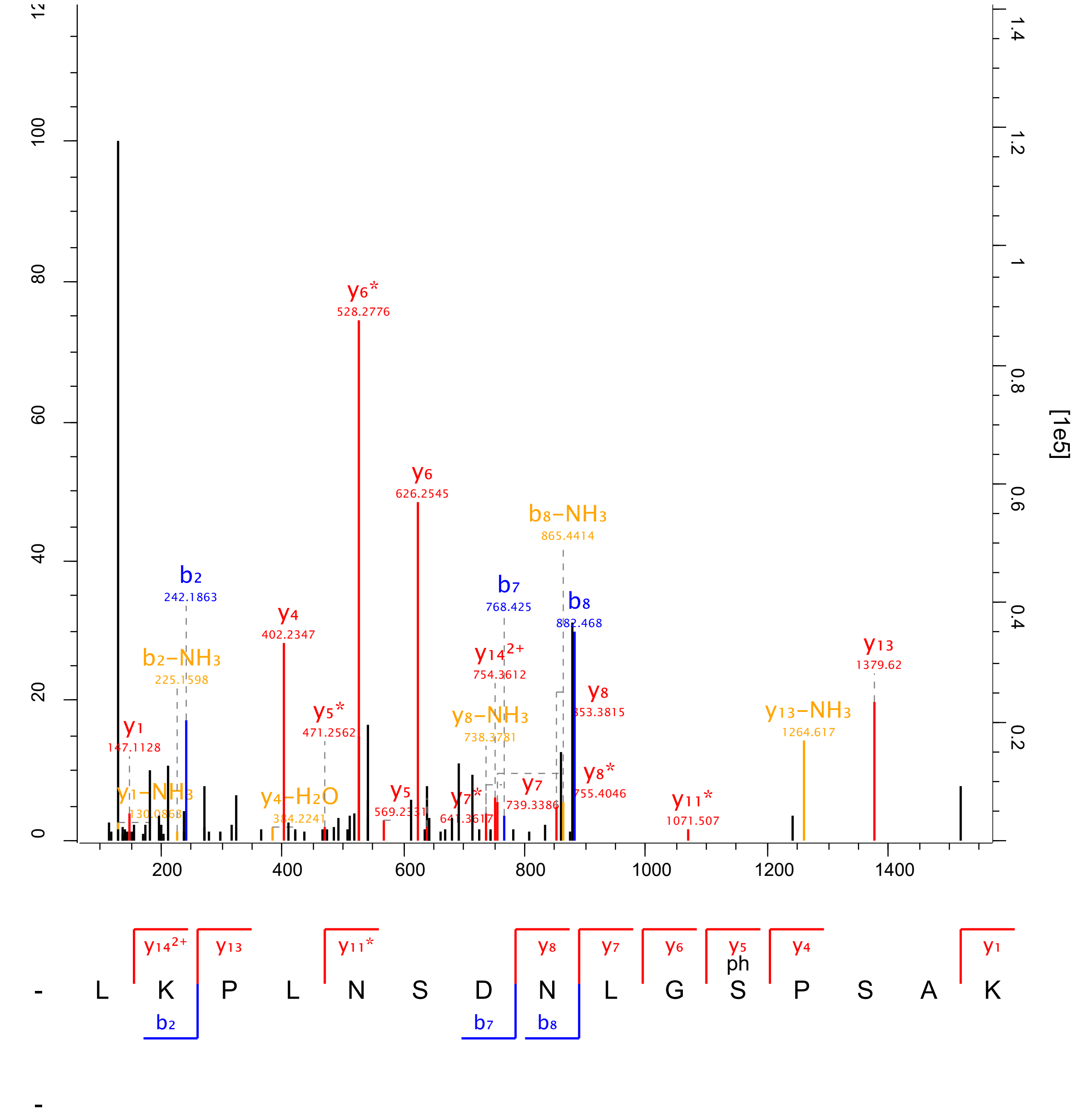

Raw file	Scan	Method	Score	m/z
sys_02_2	11567	FTMS; HCD	72.89	625.3

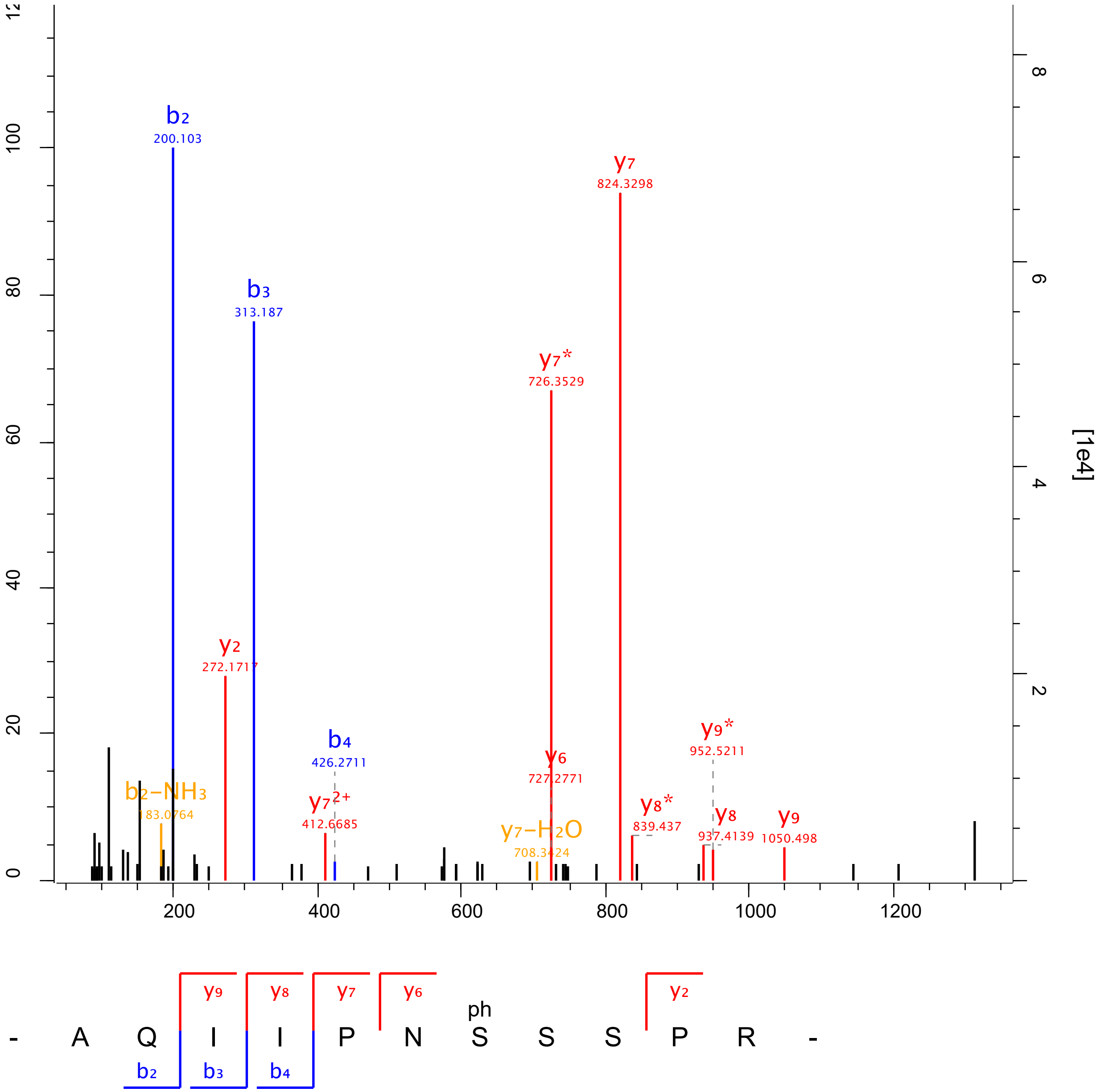

Raw file	Scan	Method	Score	m/z
sys_02_2	11568	FTMS; HCD	52.81	614.96

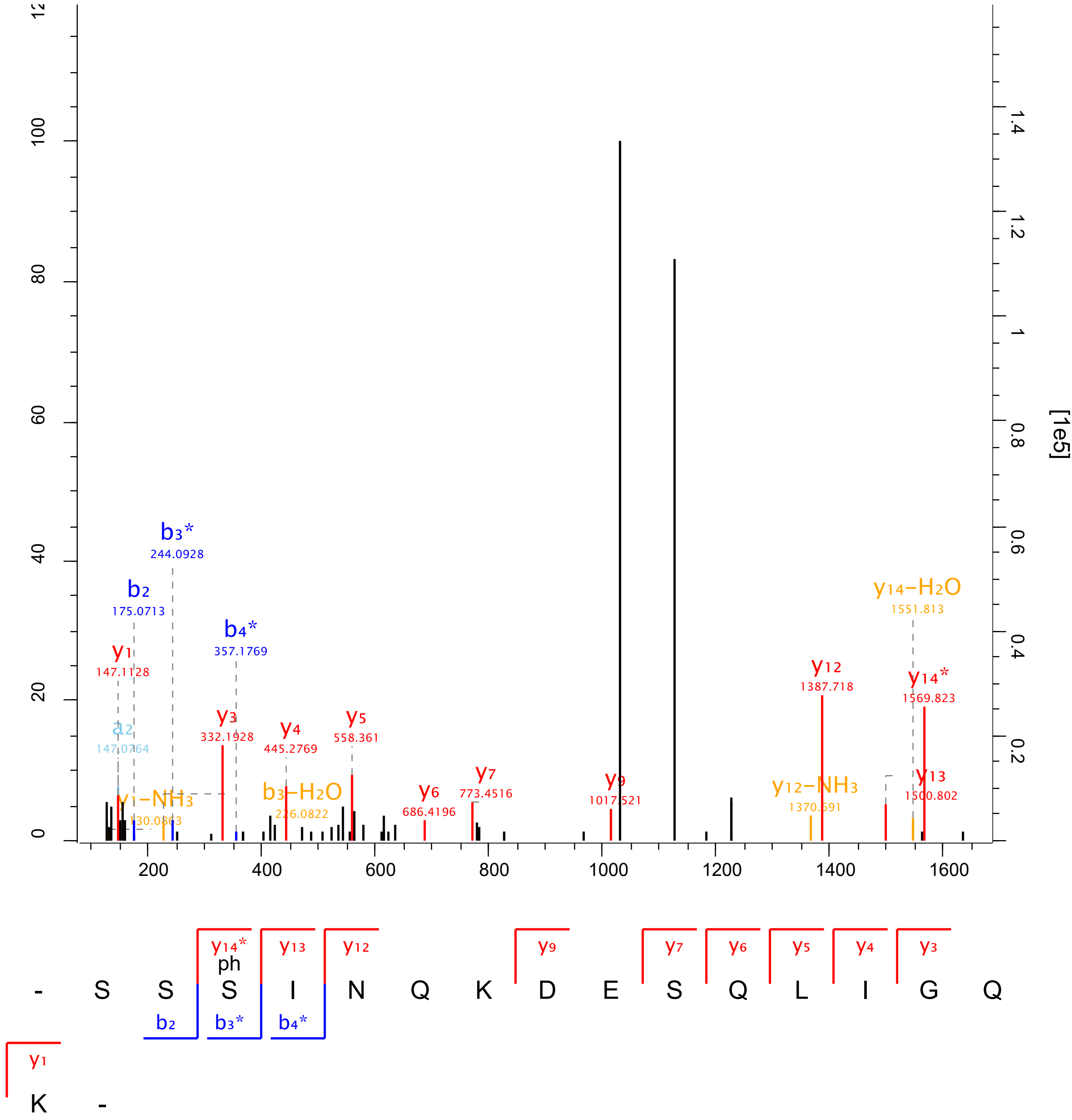

Raw file	Scan	Method	Score	m/z
sys_02_2	11613	FTMS; HCD	44.25	835.35

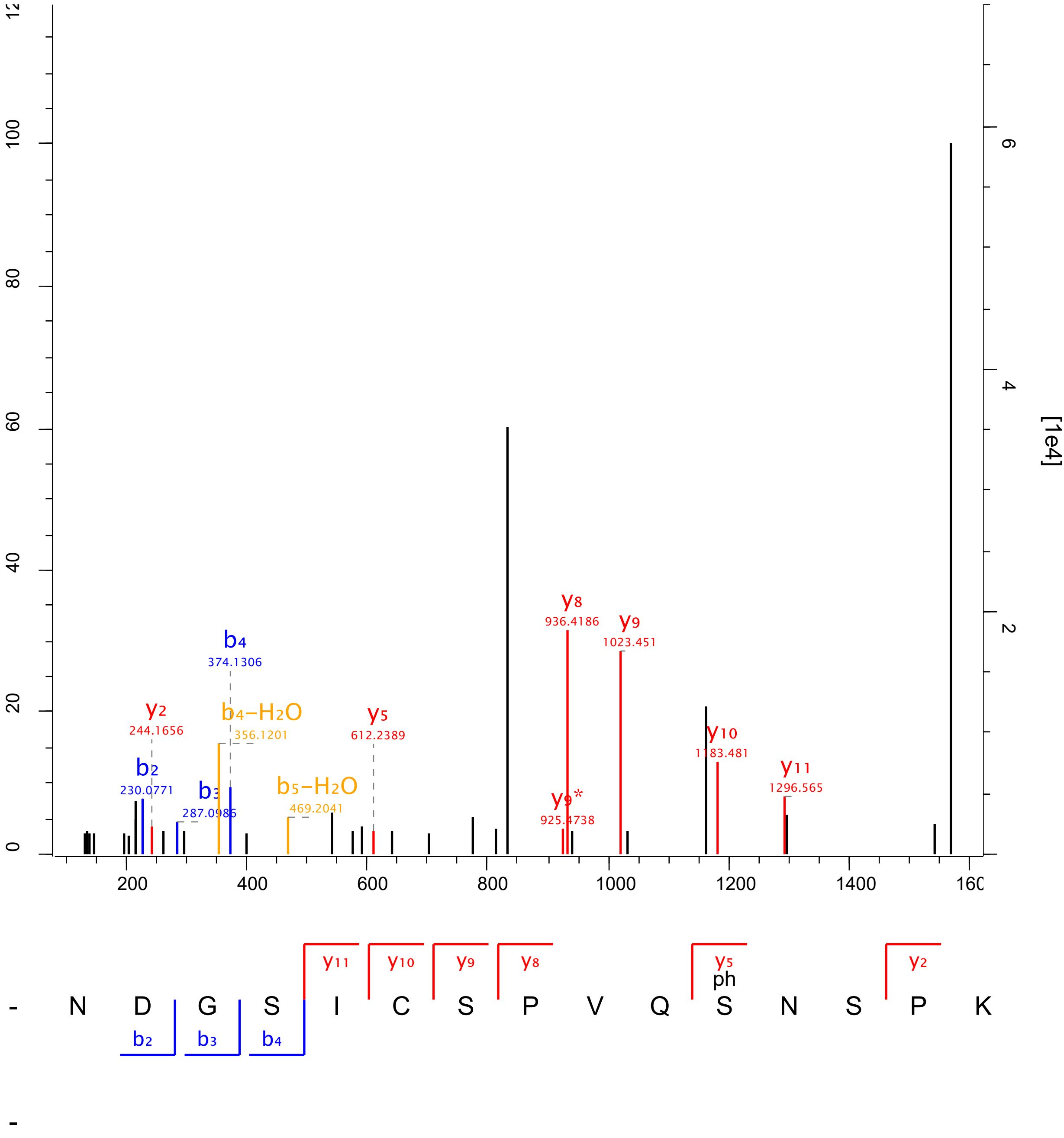

Raw file	Scan	Method	Score	m/z
sys_02_2	11616	FTMS; HCD	77.42	665.79

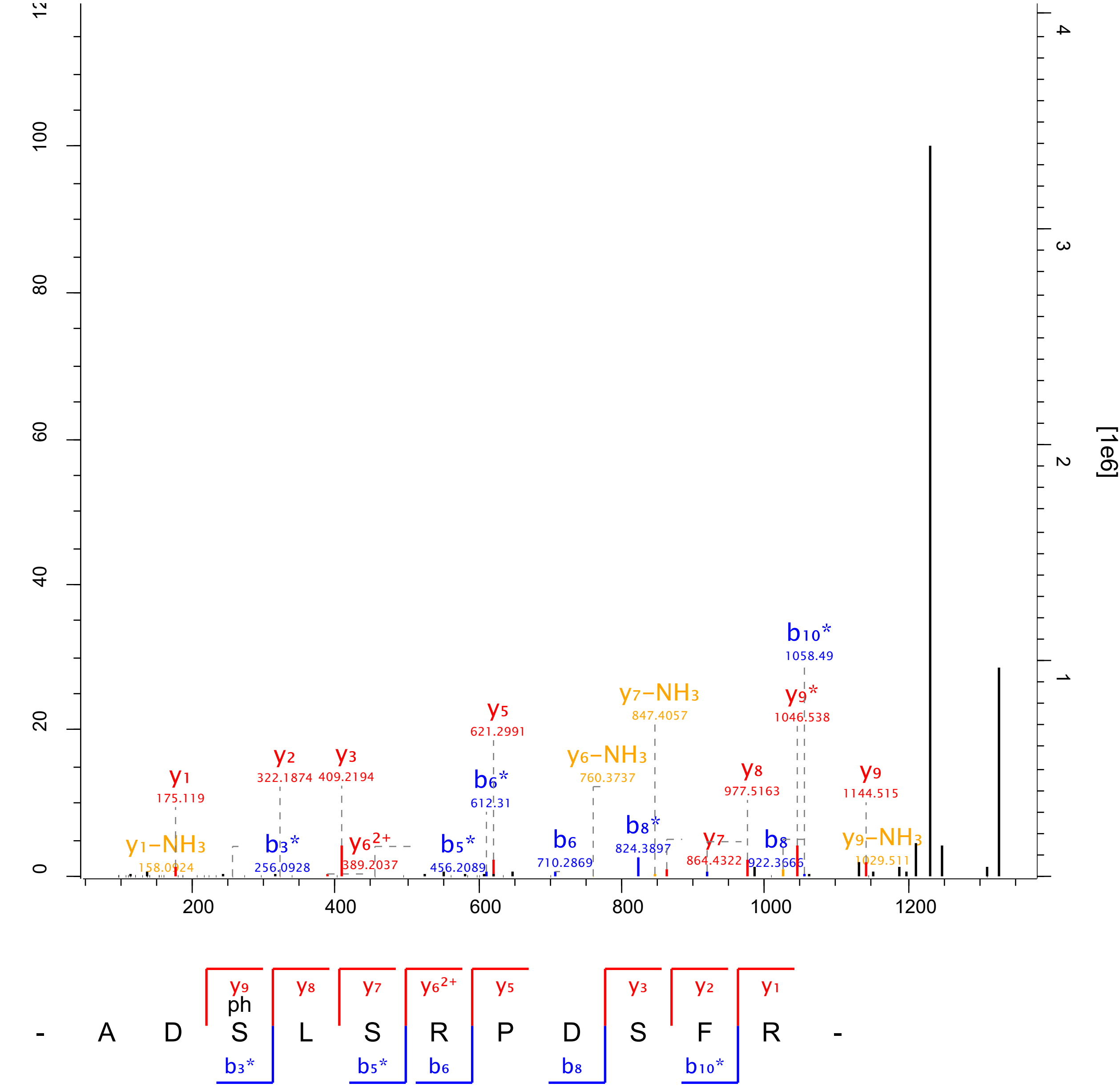

Raw file	Scan	Method	Score	m/z
sys_02_2	11700	FTMS; HCD	56.96	536.26

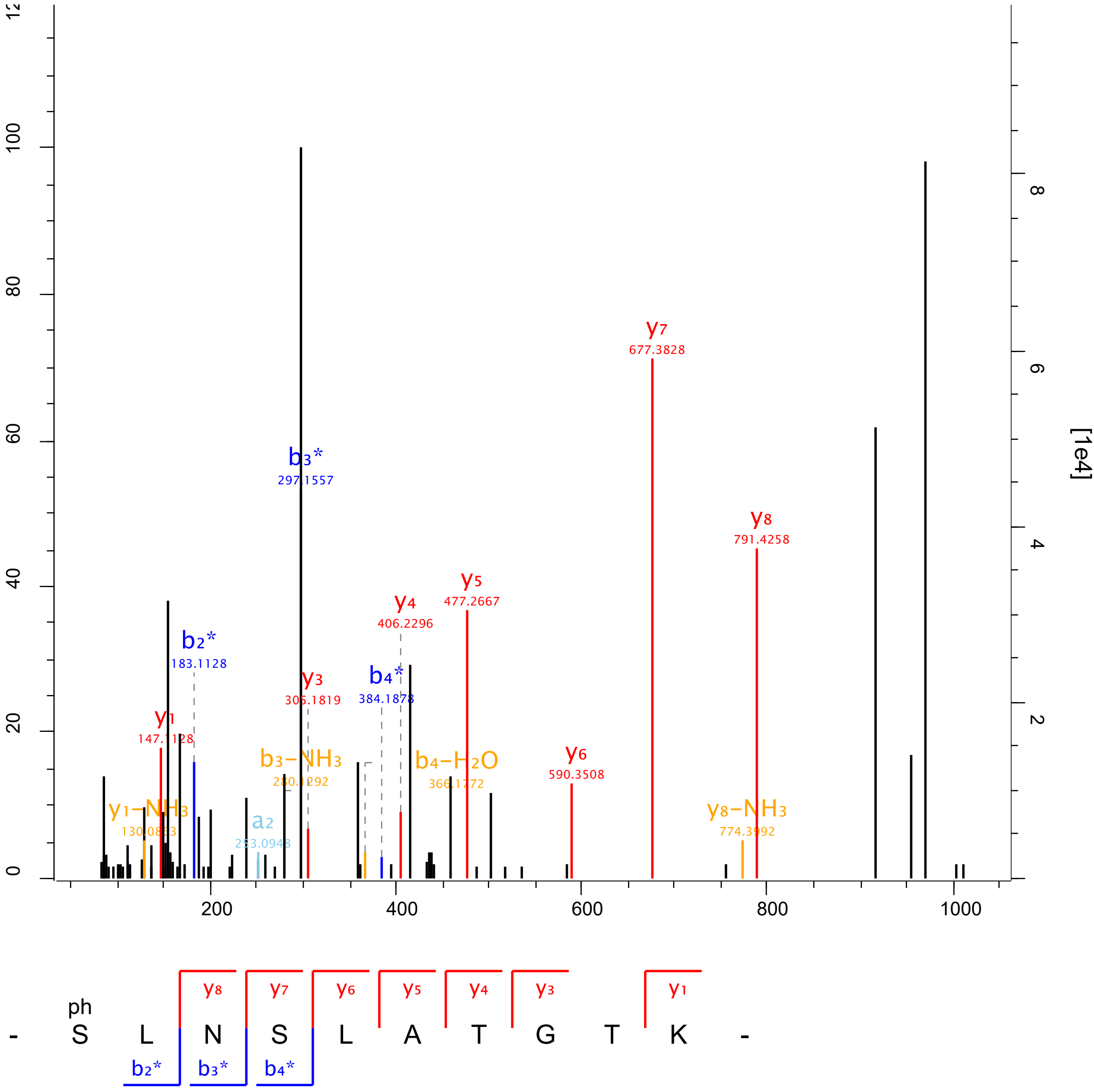

Raw file	Scan	Method	Score	m/z
sys_02_2	11732	FTMS; HCD	42.31	546.25

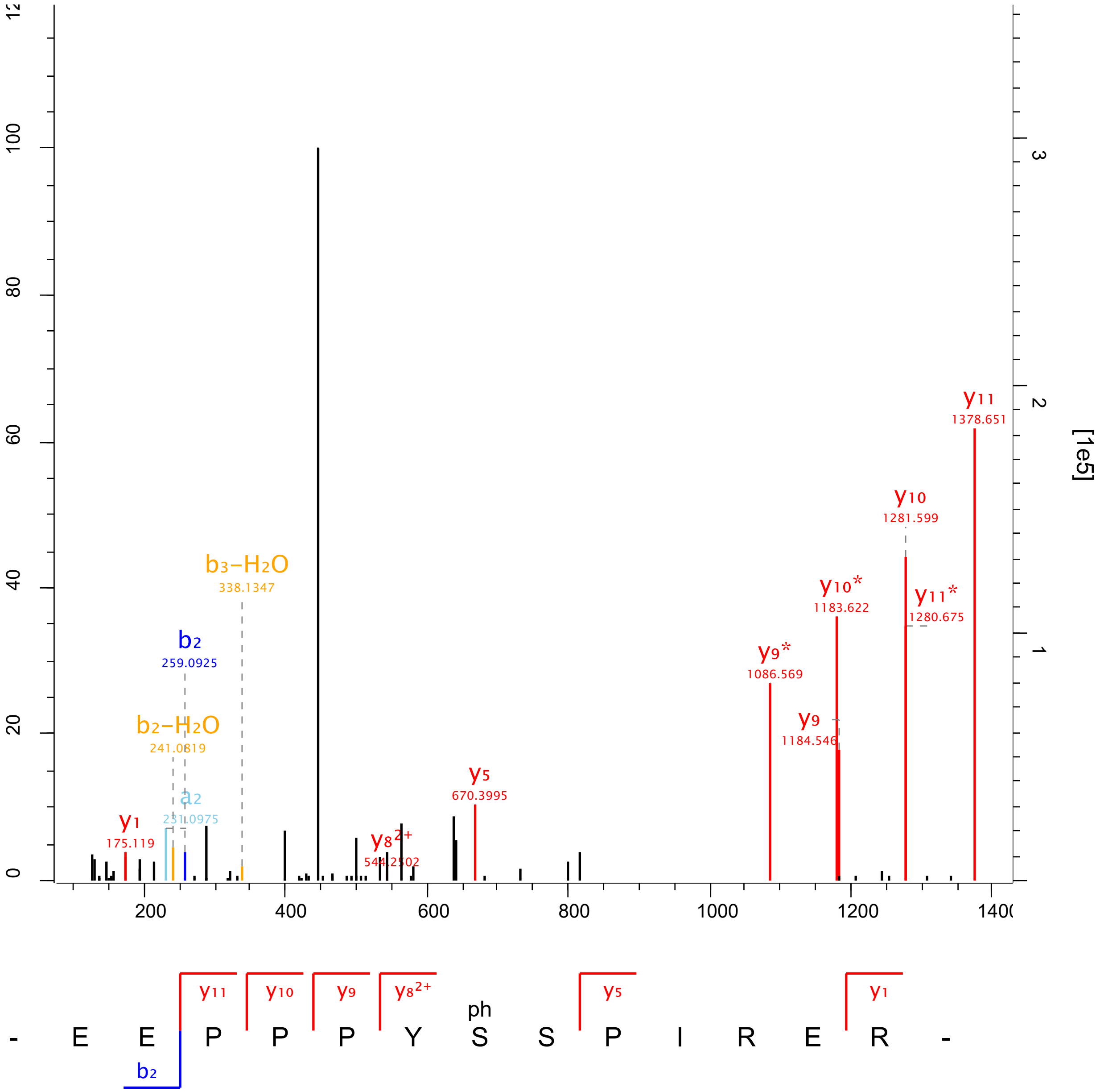

Raw file	Scan	Method	Score	m/z
sys_02_2	11778	FTMS; HCD	138.91	672.29

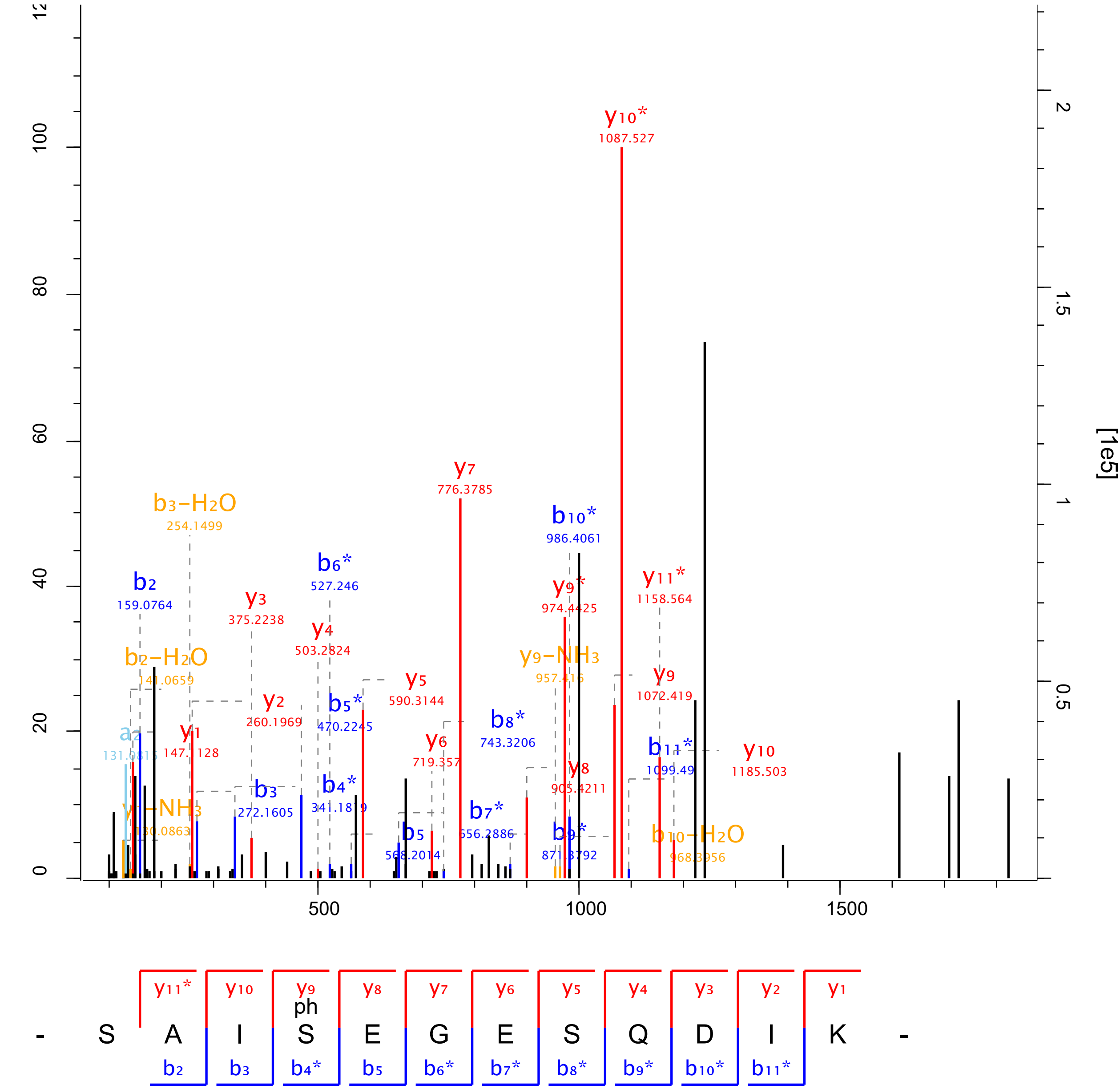

Raw file	Scan	Method	Score	m/z
sys_02_2	11962	FTMS; HCD	56.72	535.25

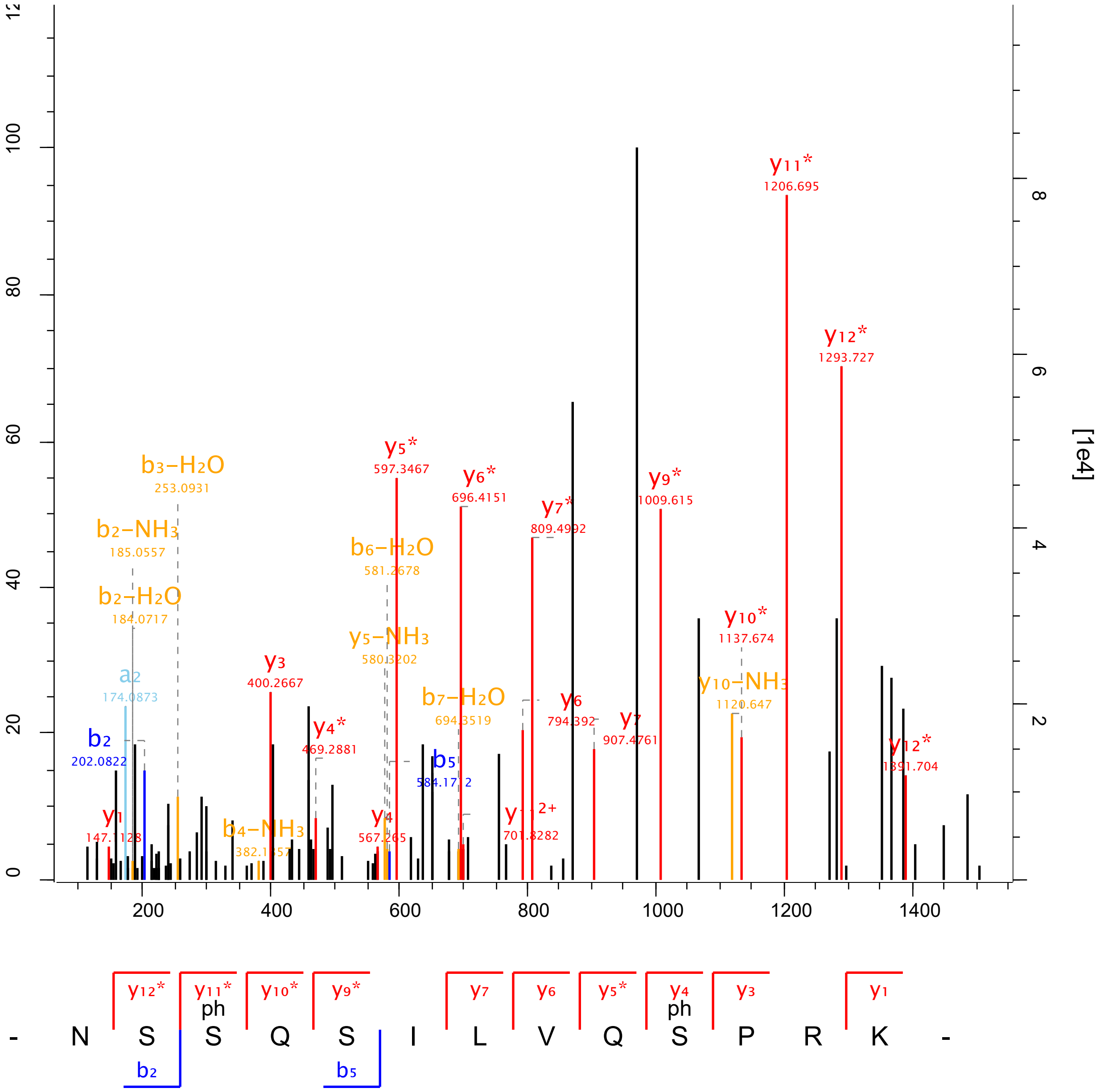

Raw file	Scan	Method	Score	m/z
sys_02_2	12020	FTMS; HCD	108.55	690.98

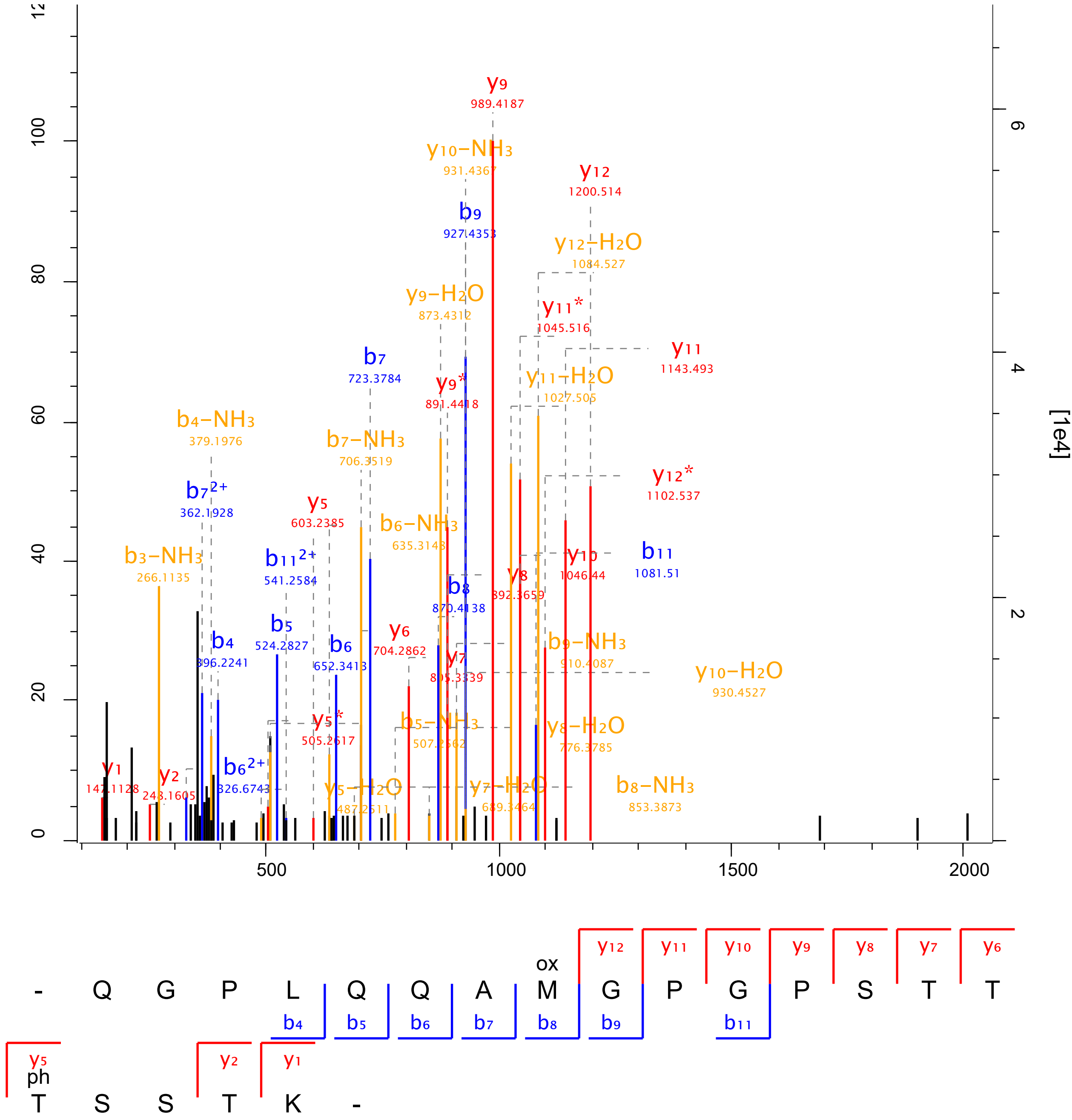

Raw file	Scan	Method	Score	m/z
sys_02_2	12390	FTMS; HCD	42.63	512.72

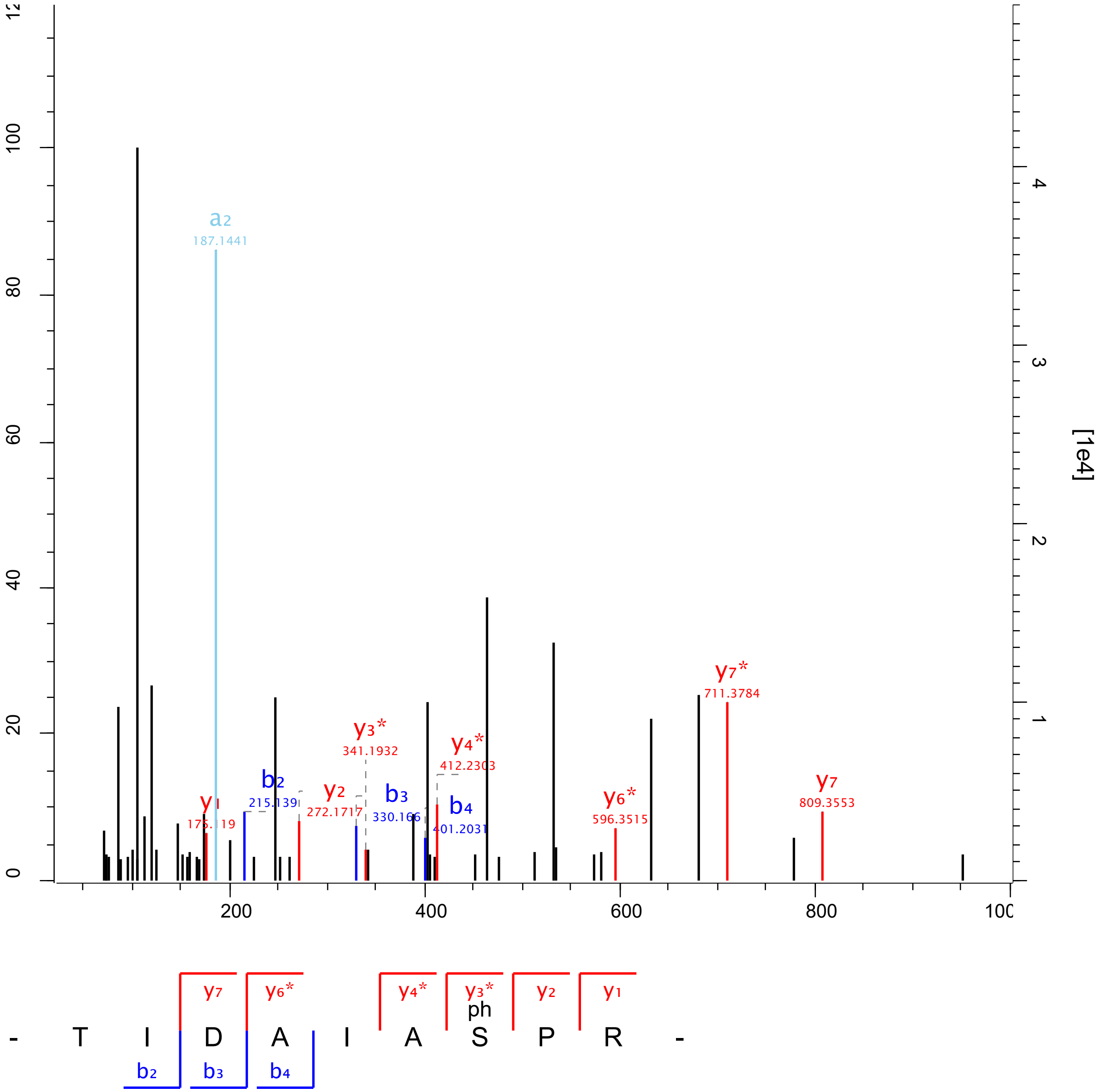

Raw file	Scan	Method	Score	m/z
sys_02_2	12400	FTMS; HCD	72.47	466.56

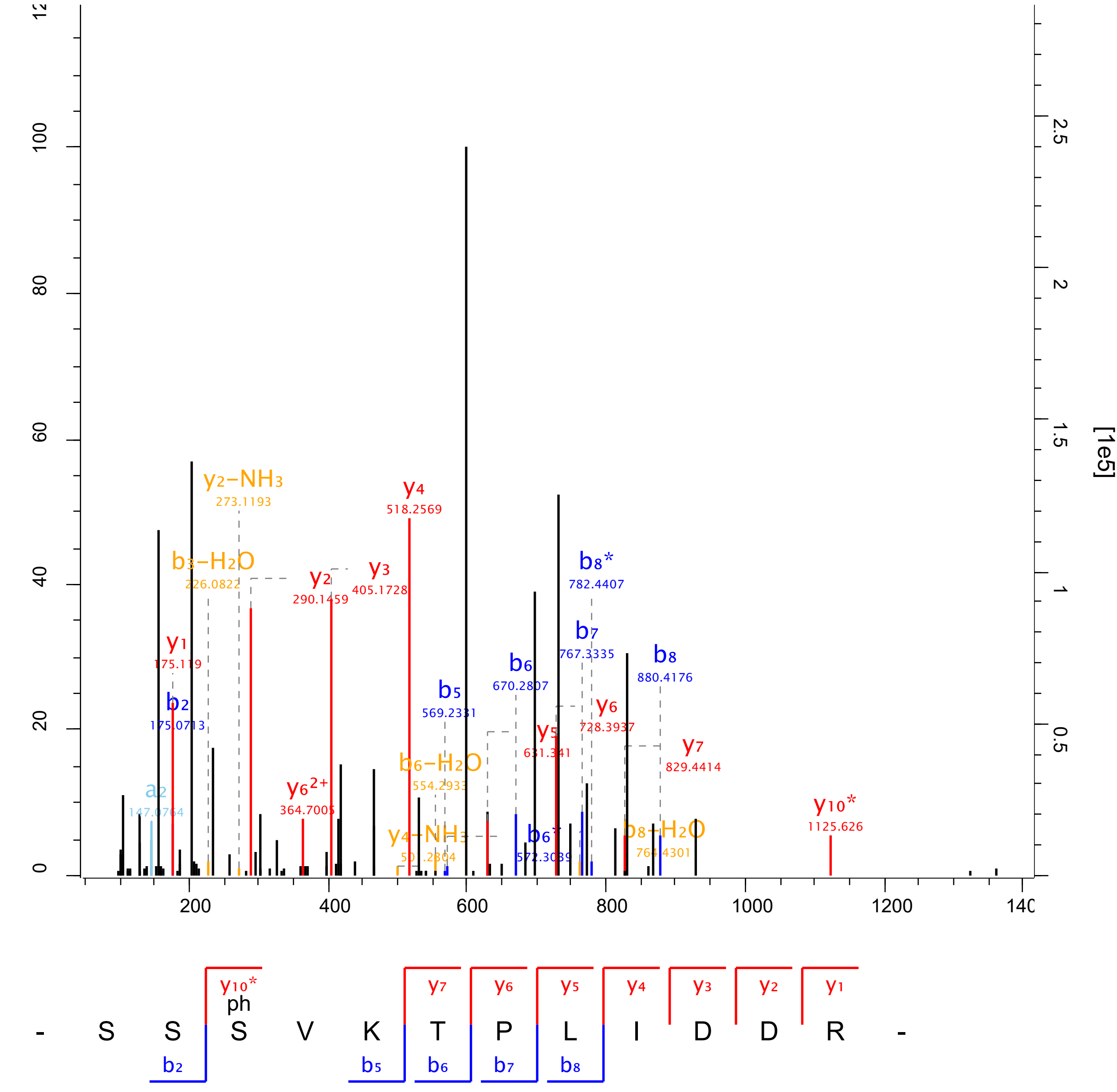

Raw file	Scan	Method	Score	m/z
sys_02_2	12582	FTMS; HCD	73.5	424.2

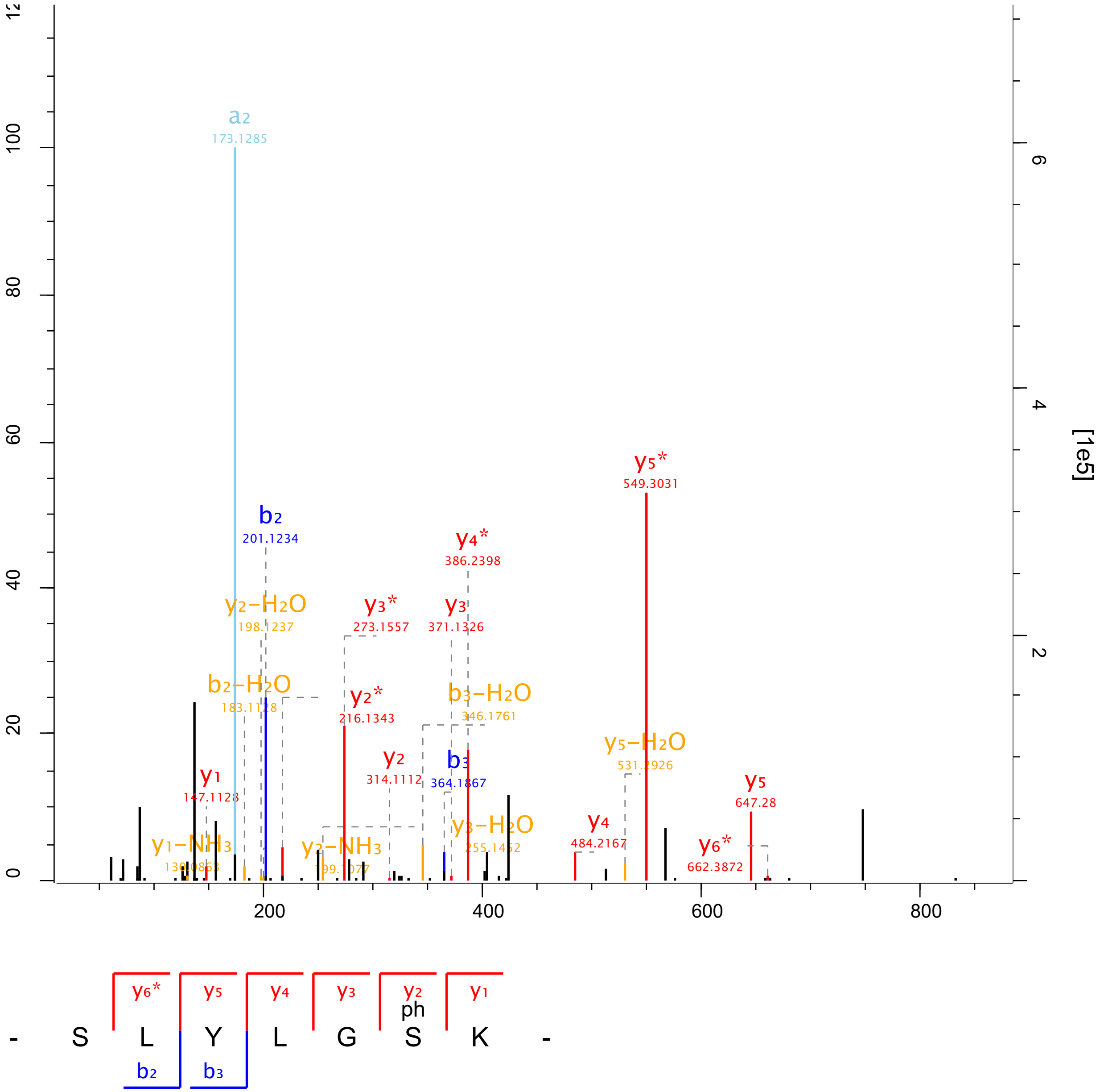

Raw file	Scan	Method	Score	m/z
sys_02_2	12986	FTMS; HCD	149.21	713.78

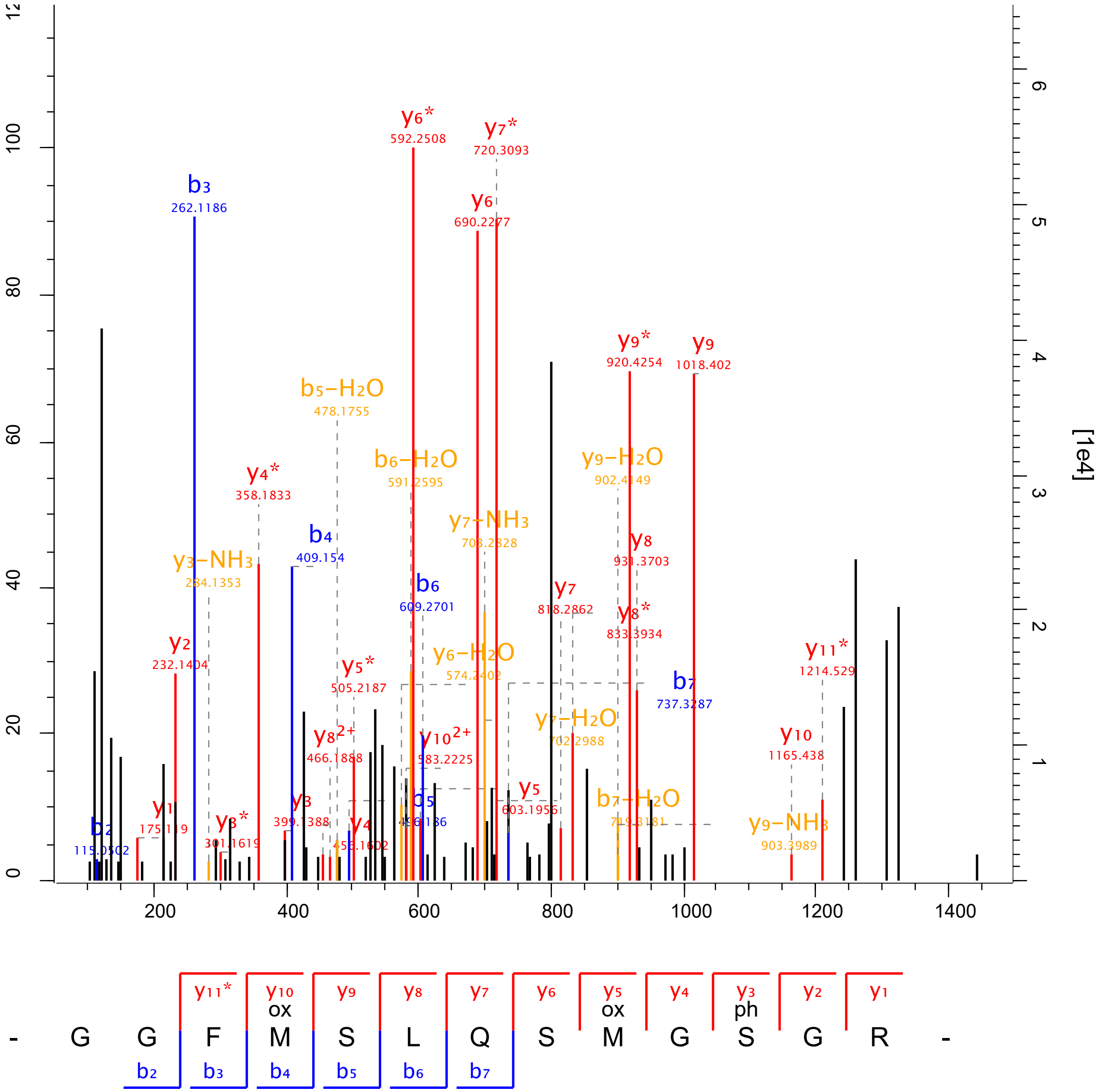

Raw file	Scan	Method	Score	m/z
sys_02_2	13005	FTMS; HCD	47.62	563.76

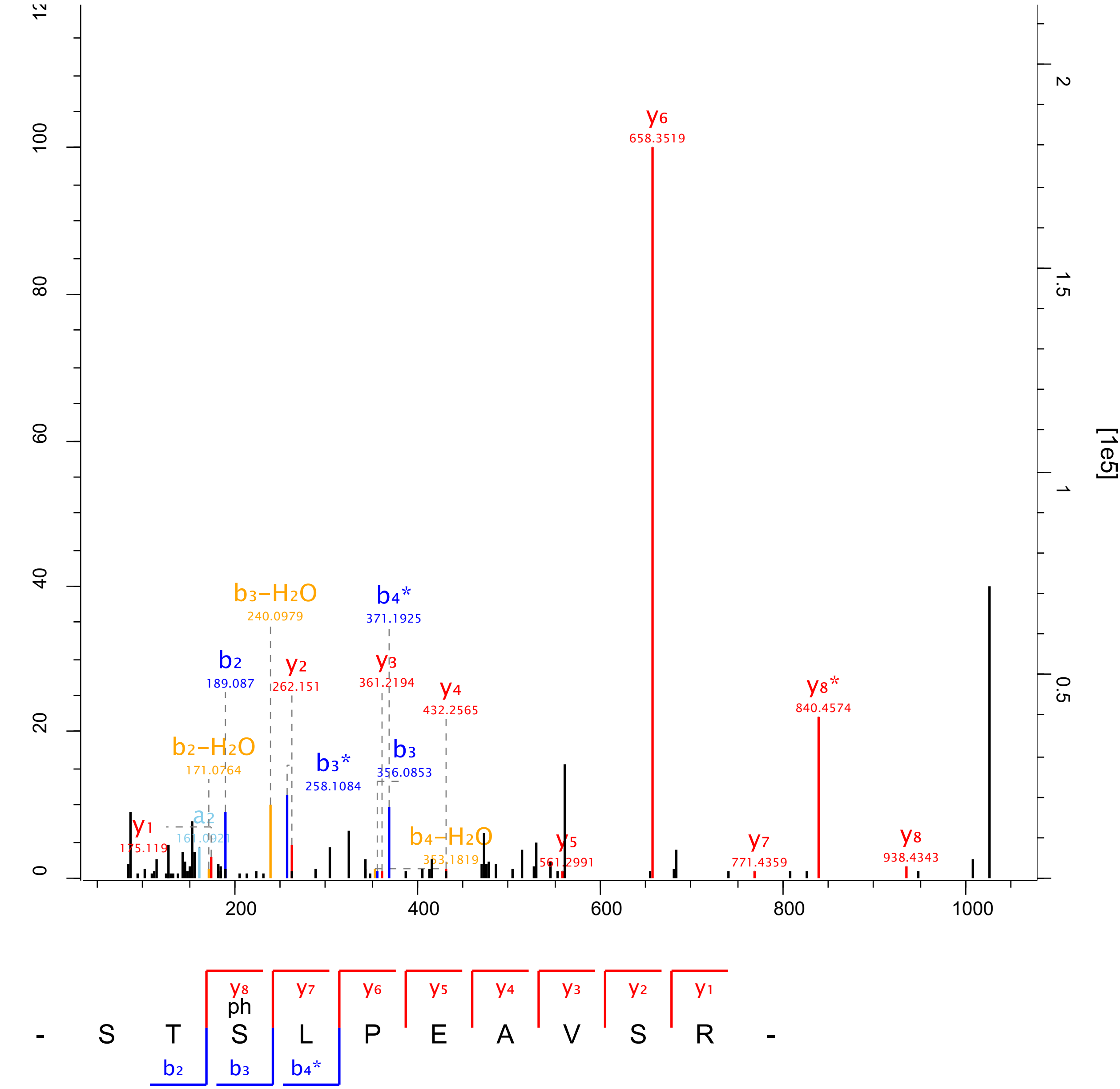

Raw file	Scan	Method	Score	m/z
sys_02_2	13044	FTMS; HCD	155.8	816.84

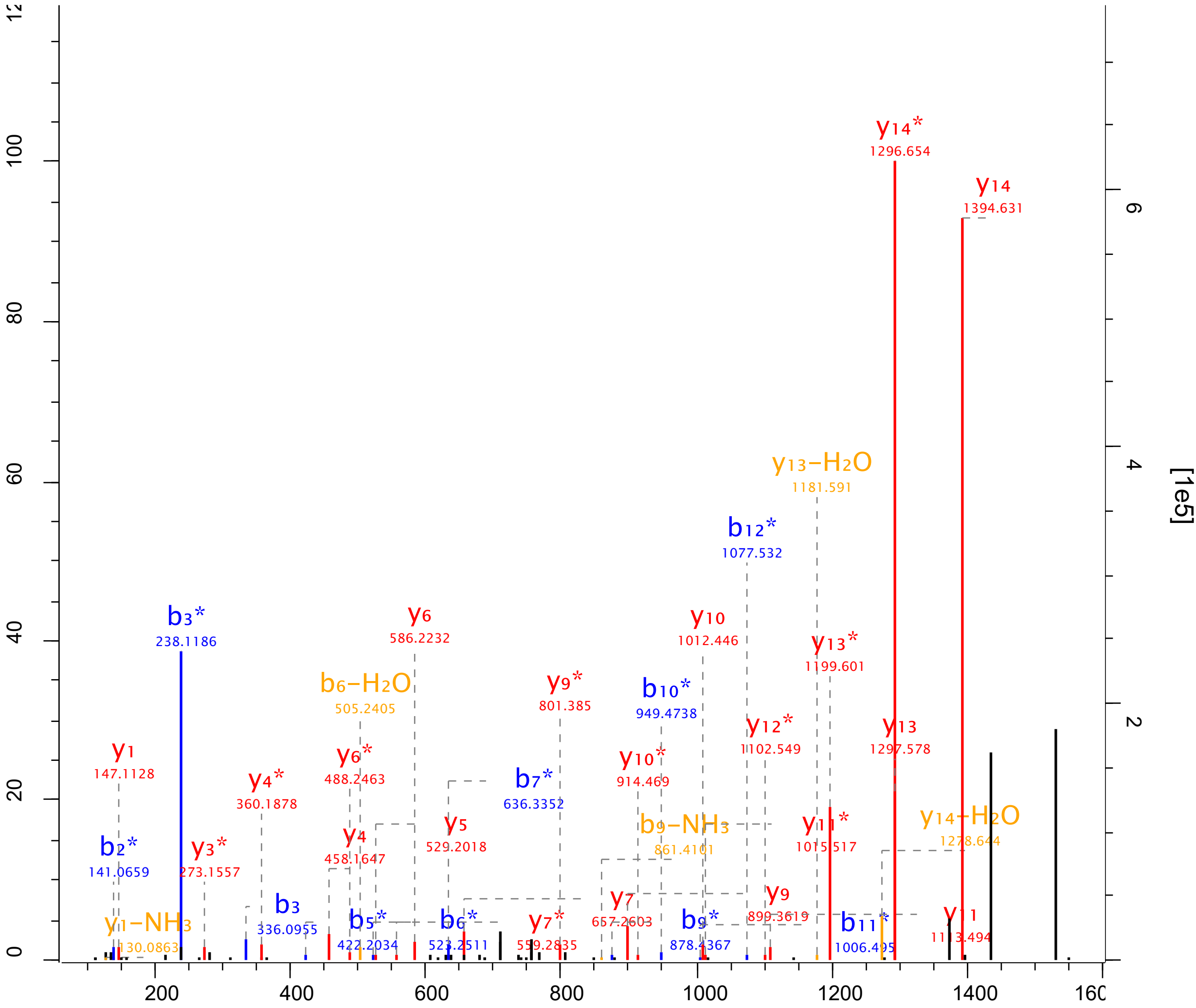

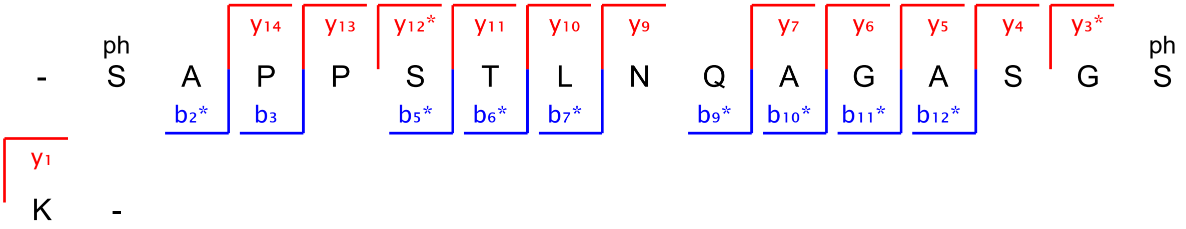

Raw file	Scan	Method	Score	m/z
sys_02_2	13135	FTMS; HCD	42.07	737.3

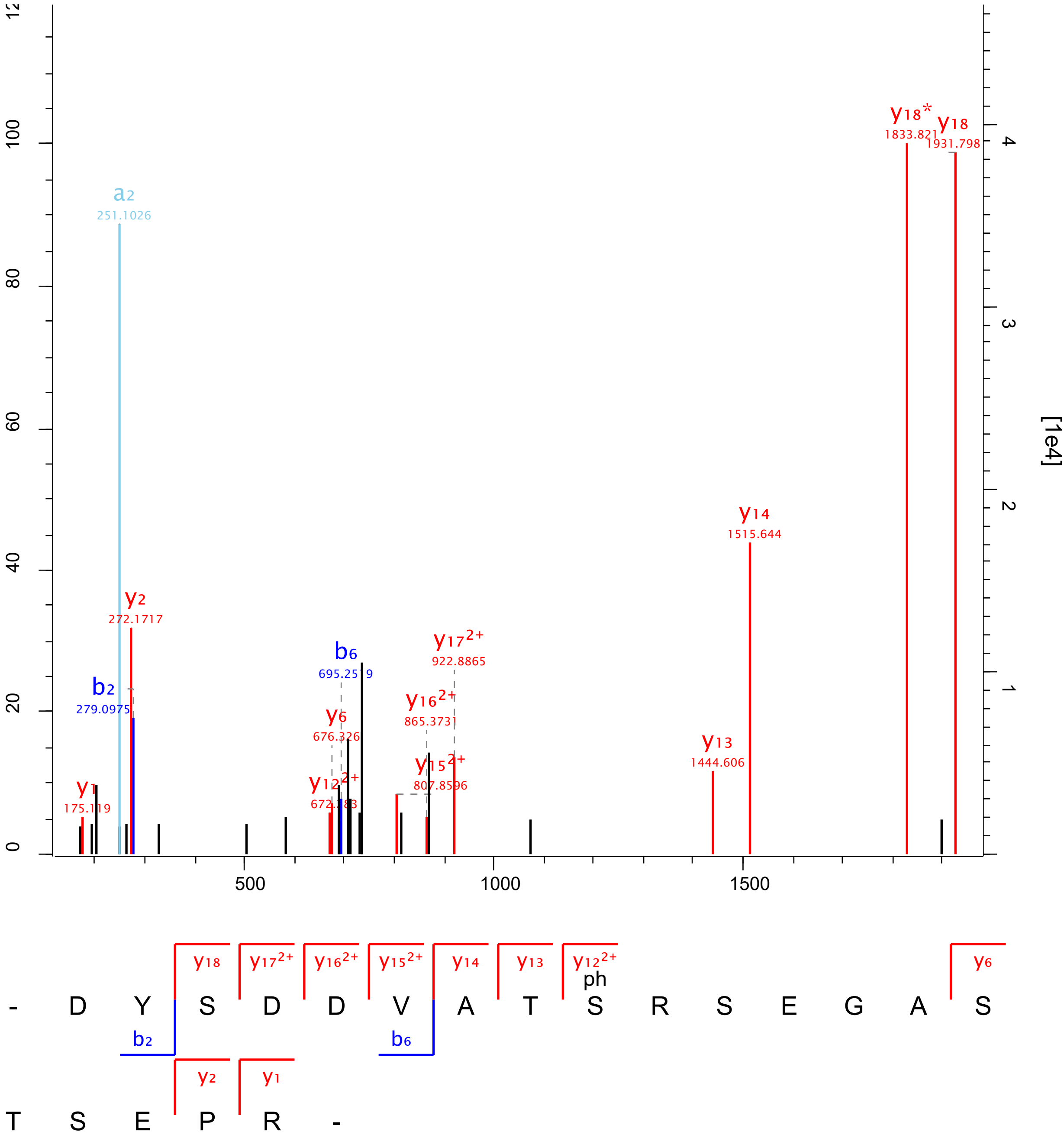

Raw file	Scan	Method	Score	m/z
sys_02_2	13167	FTMS; HCD	139.49	564.27

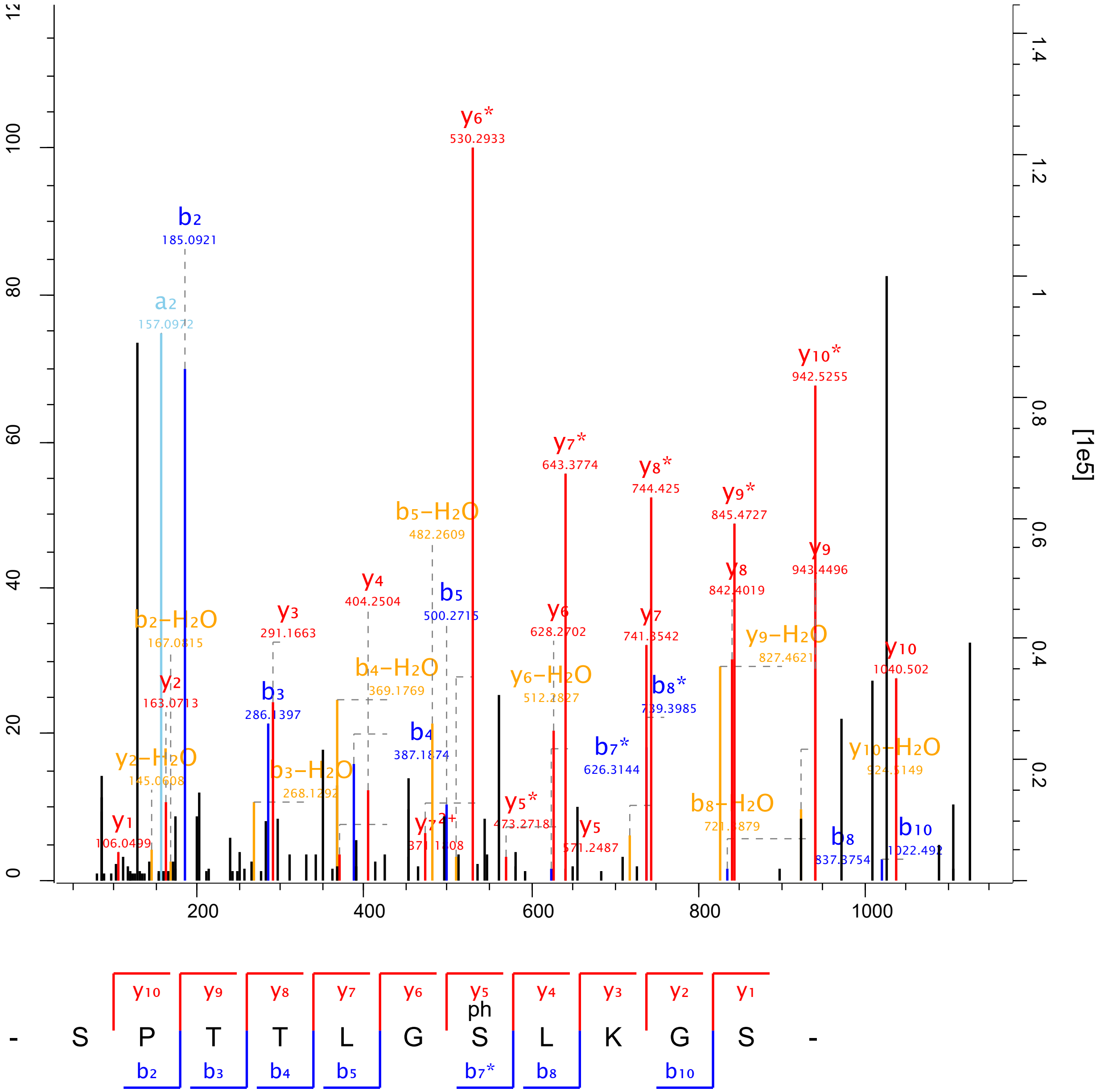

Raw file	Scan	Method	Score	m/z
sys_02_2	13302	FTMS; HCD	94.96	538.94

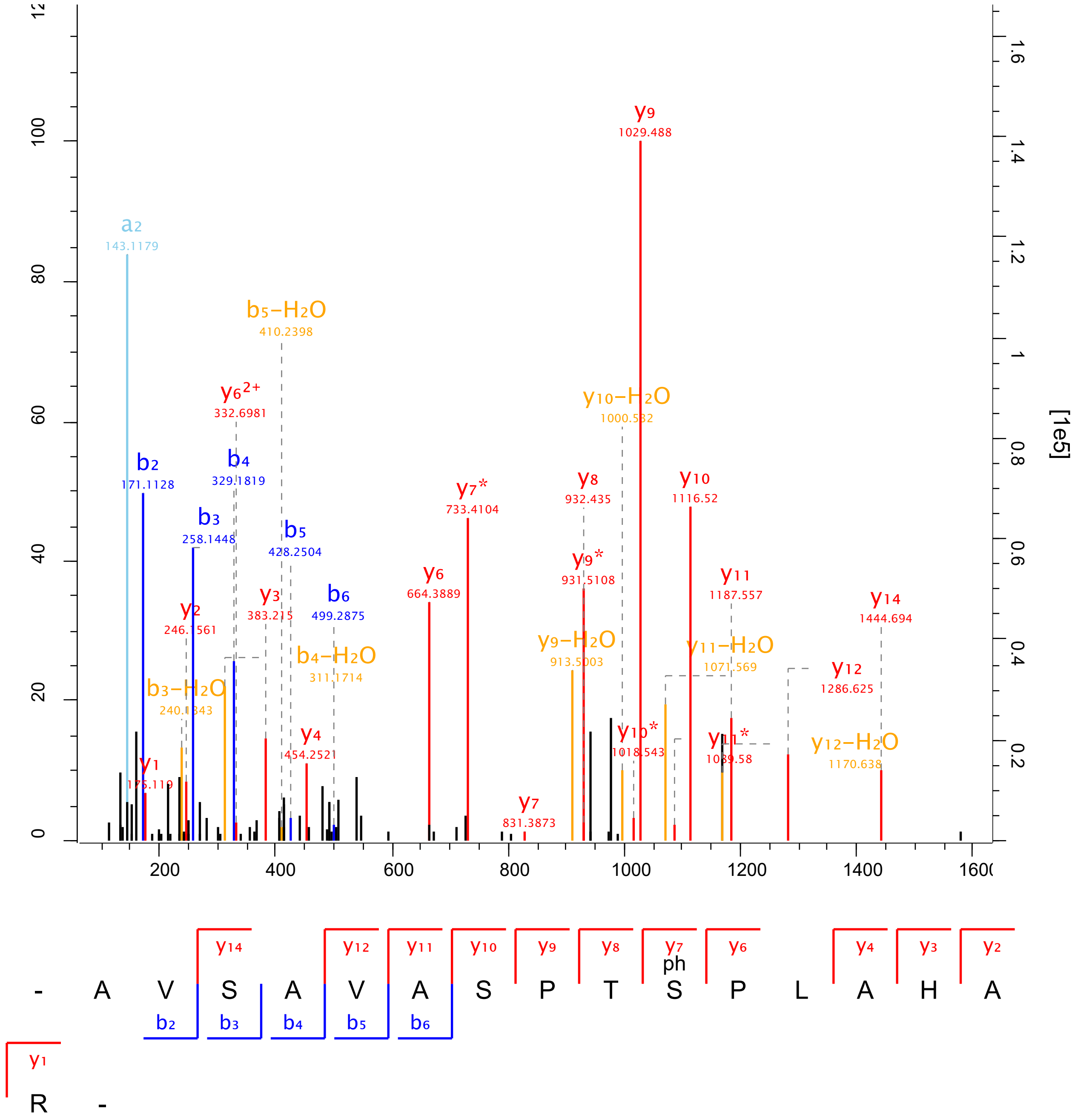

Raw file	Scan	Method	Score	m/z
sys_02_2	13492	FTMS; HCD	120.56	563.93

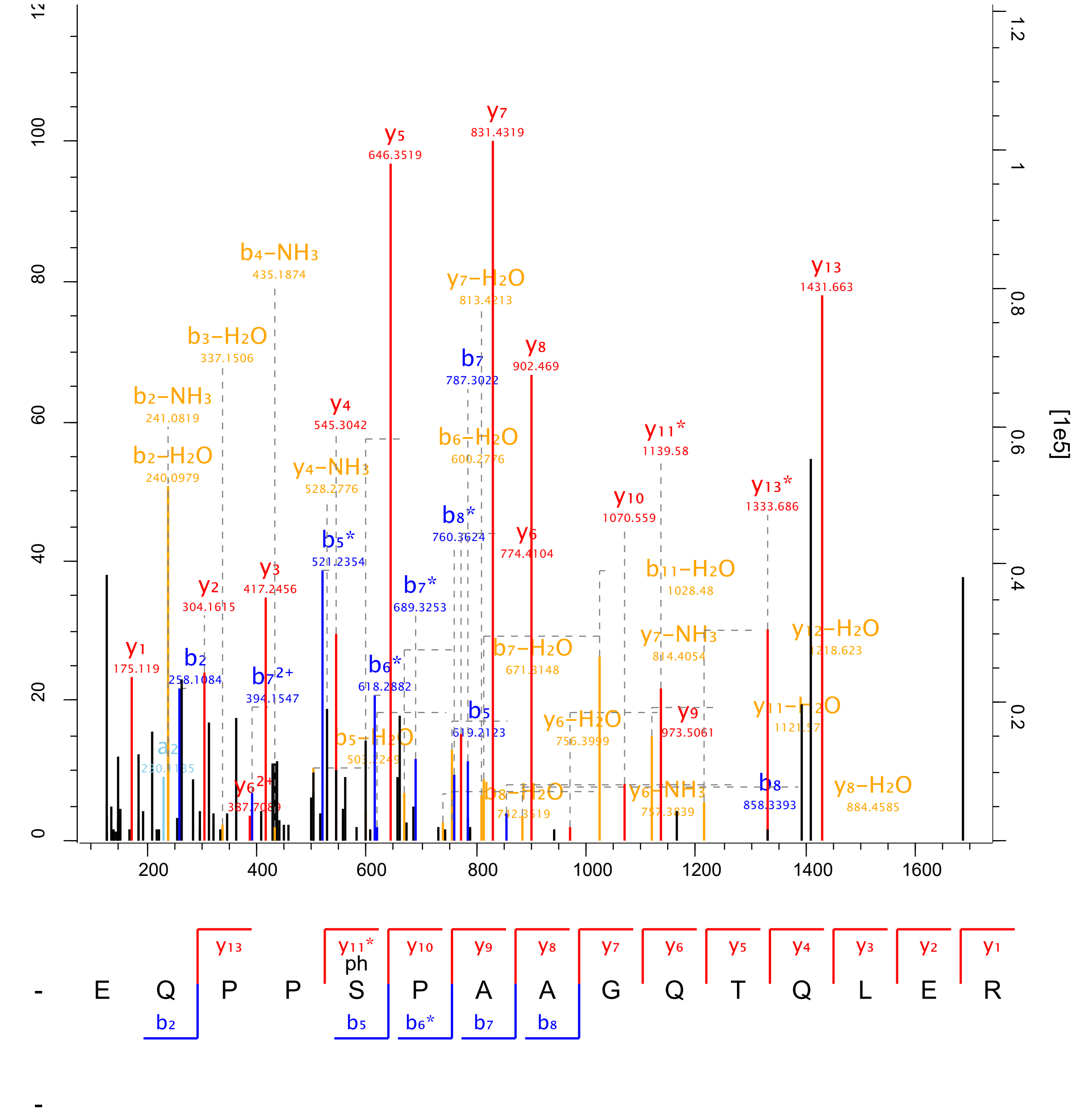

Raw file	Scan	Method	Score	m/z
sys_02_2	13547	FTMS; HCD	113.47	774.83

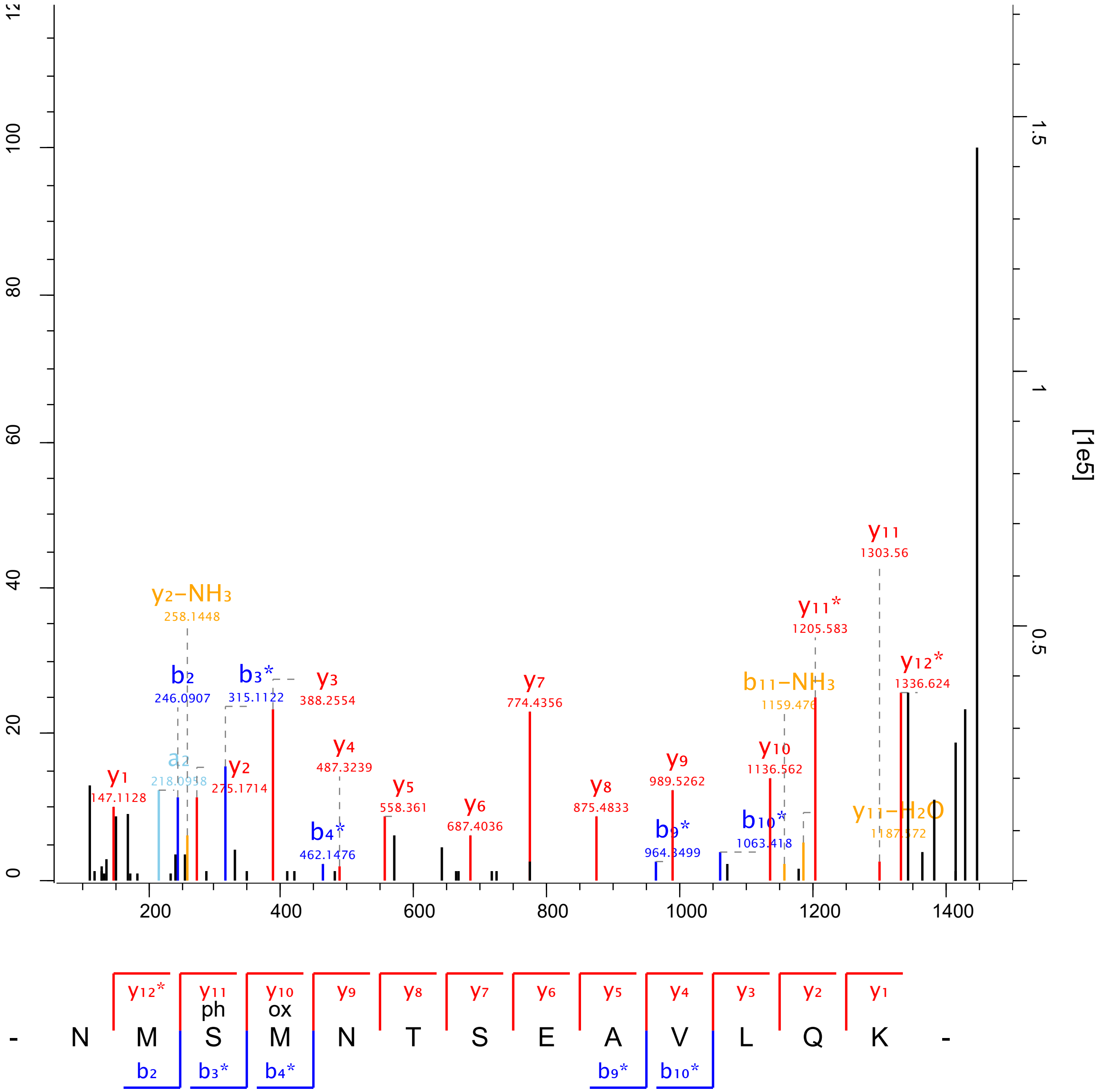

Raw file	Scan	Method	Score	m/z
sys_02_2	13627	FTMS; HCD	75.82	573.78

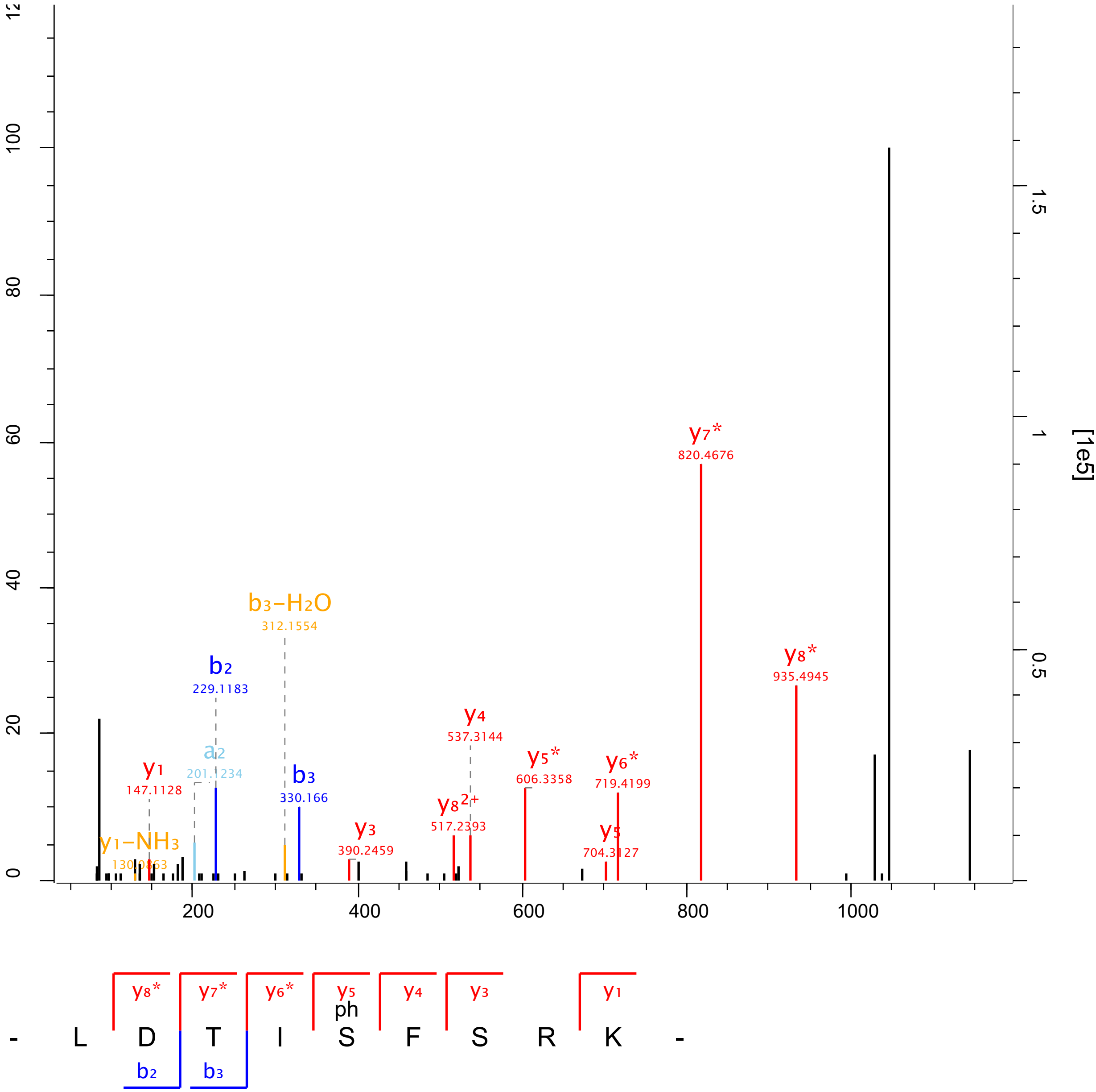

Raw file	Scan	Method	Score	m/z
sys_02_2	13694	FTMS; HCD	289.87	698.33

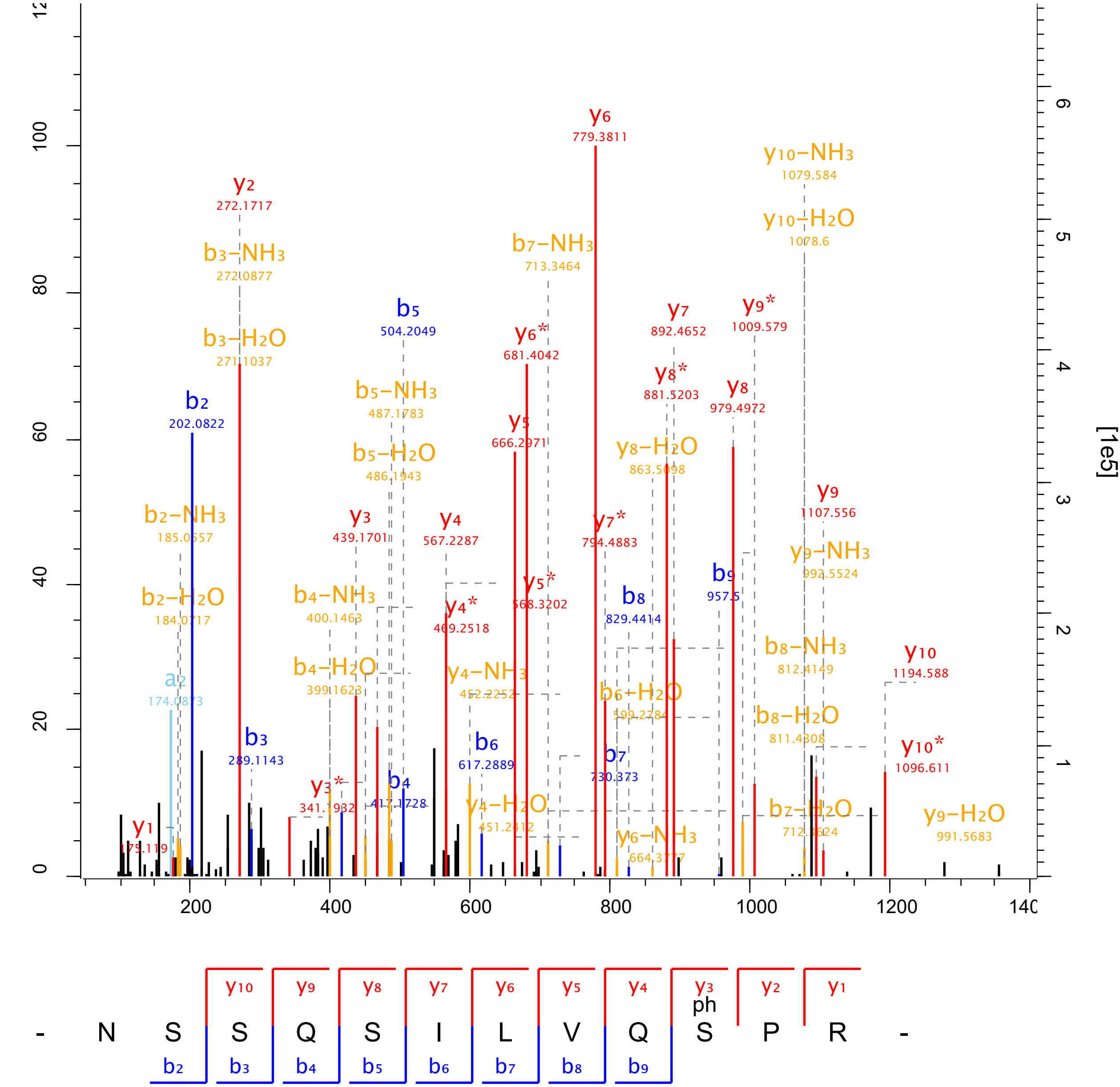

Raw file	Scan	Method	Score	m/z
sys_02_2	13814	FTMS; HCD	129.89	498.75

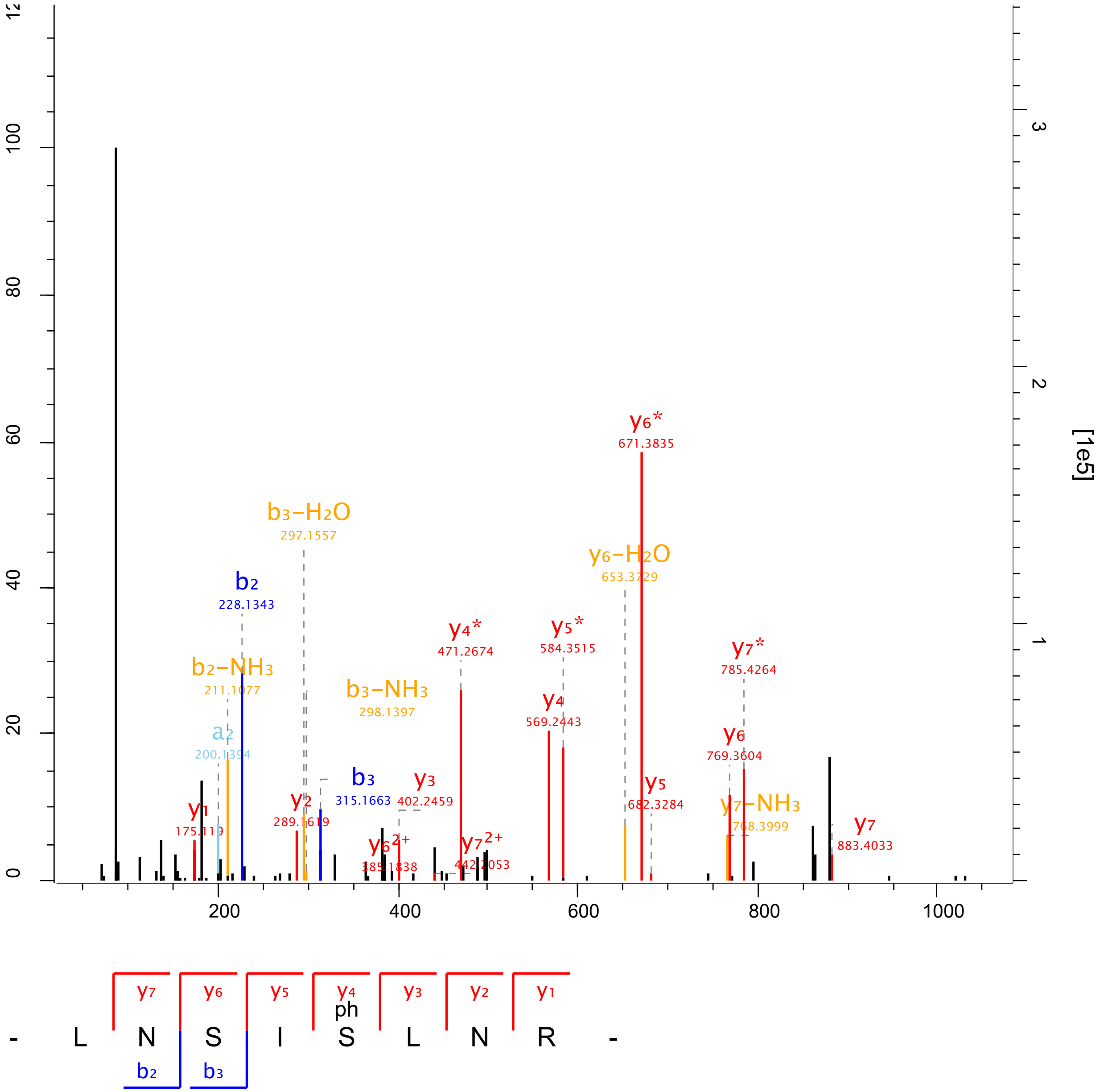

Raw file	Scan	Method	Score	m/z
sys_02_2	13826	FTMS; HCD	204.32	549.76

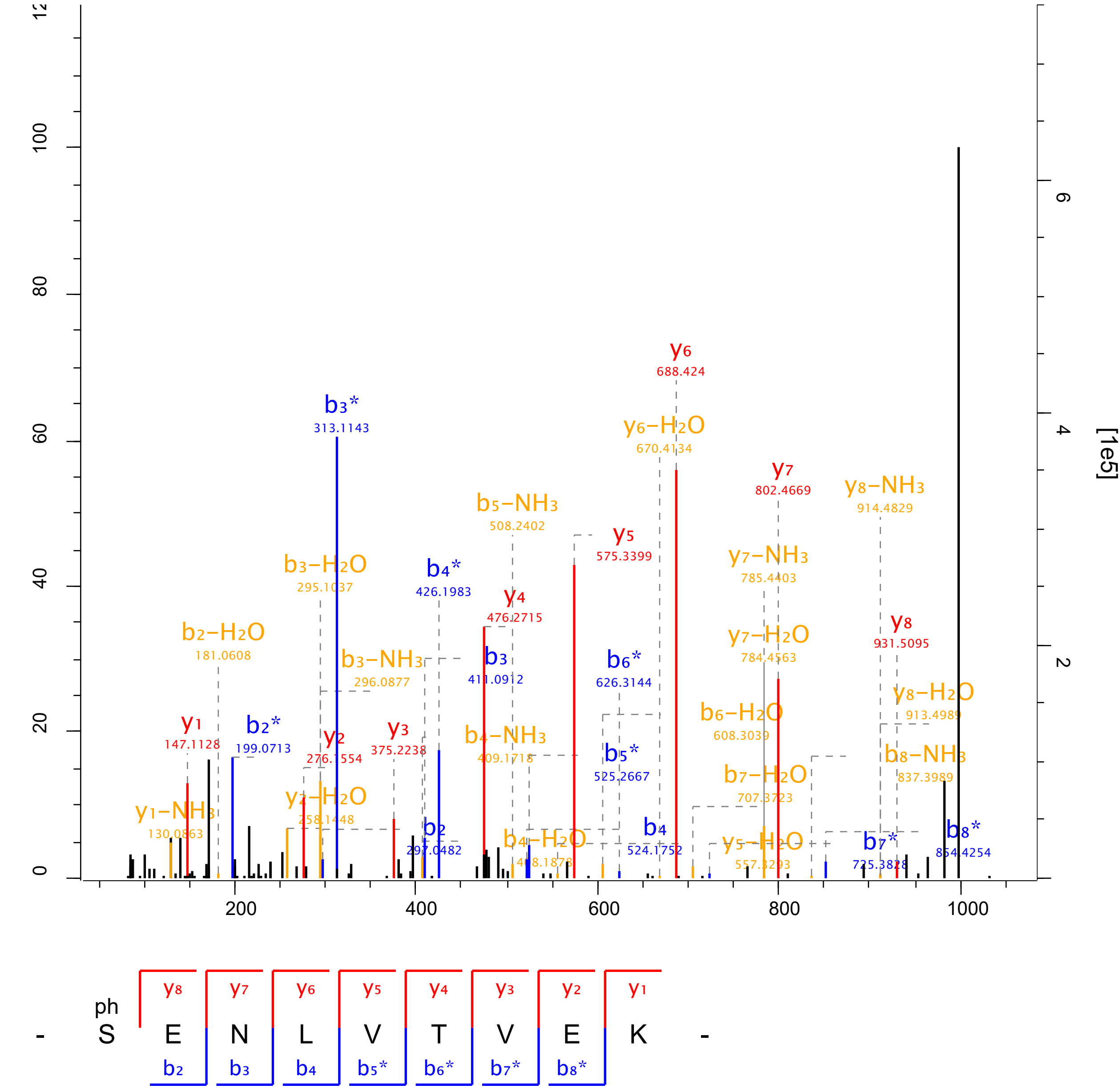

Raw file	Scan	Method	Score	m/z
sys_02_2	13833	FTMS; HCD	148.15	745.33

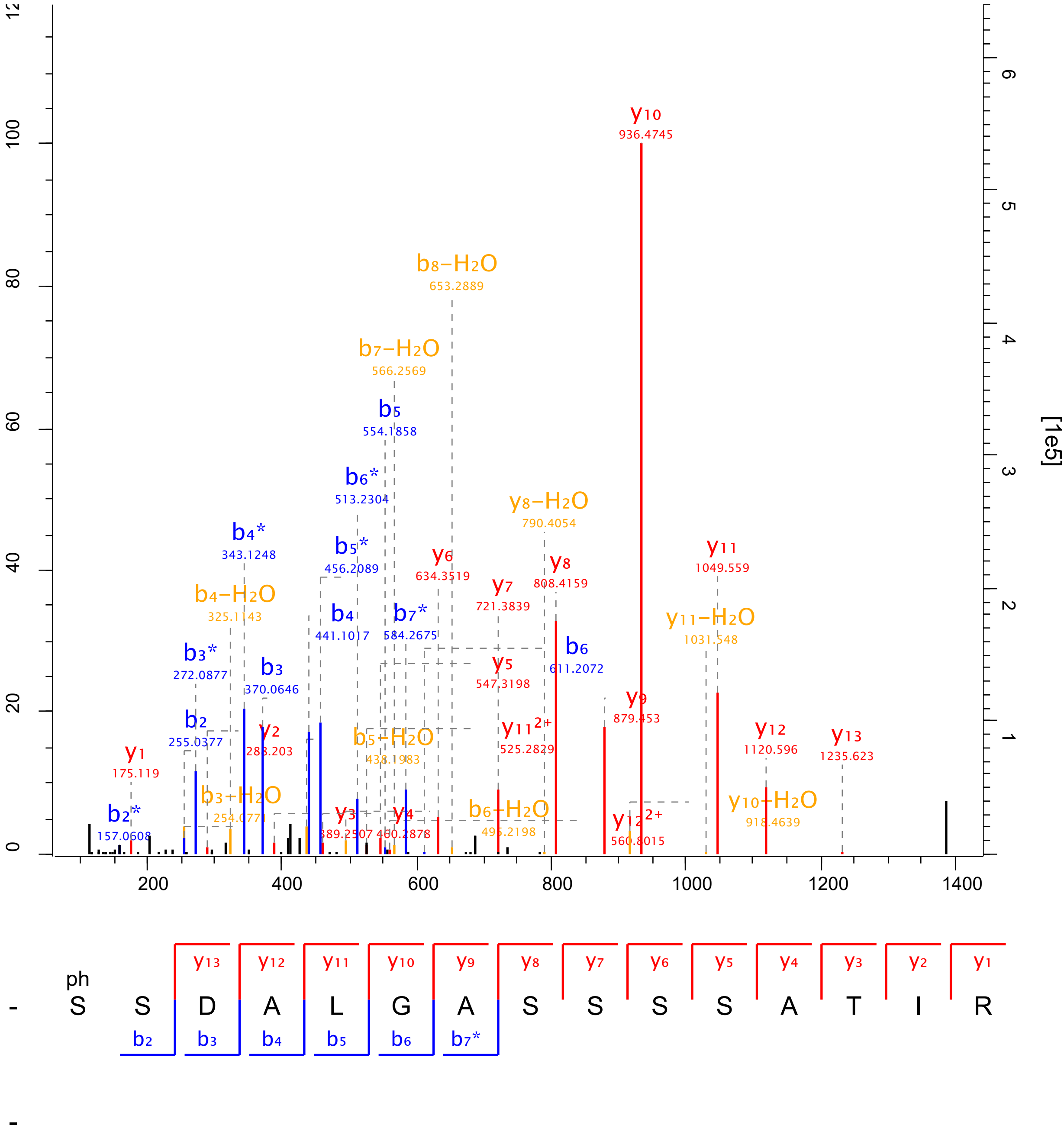

Raw file	Scan	Method	Score	m/z
sys_02_2	14029	FTMS; HCD	50.19	468.25

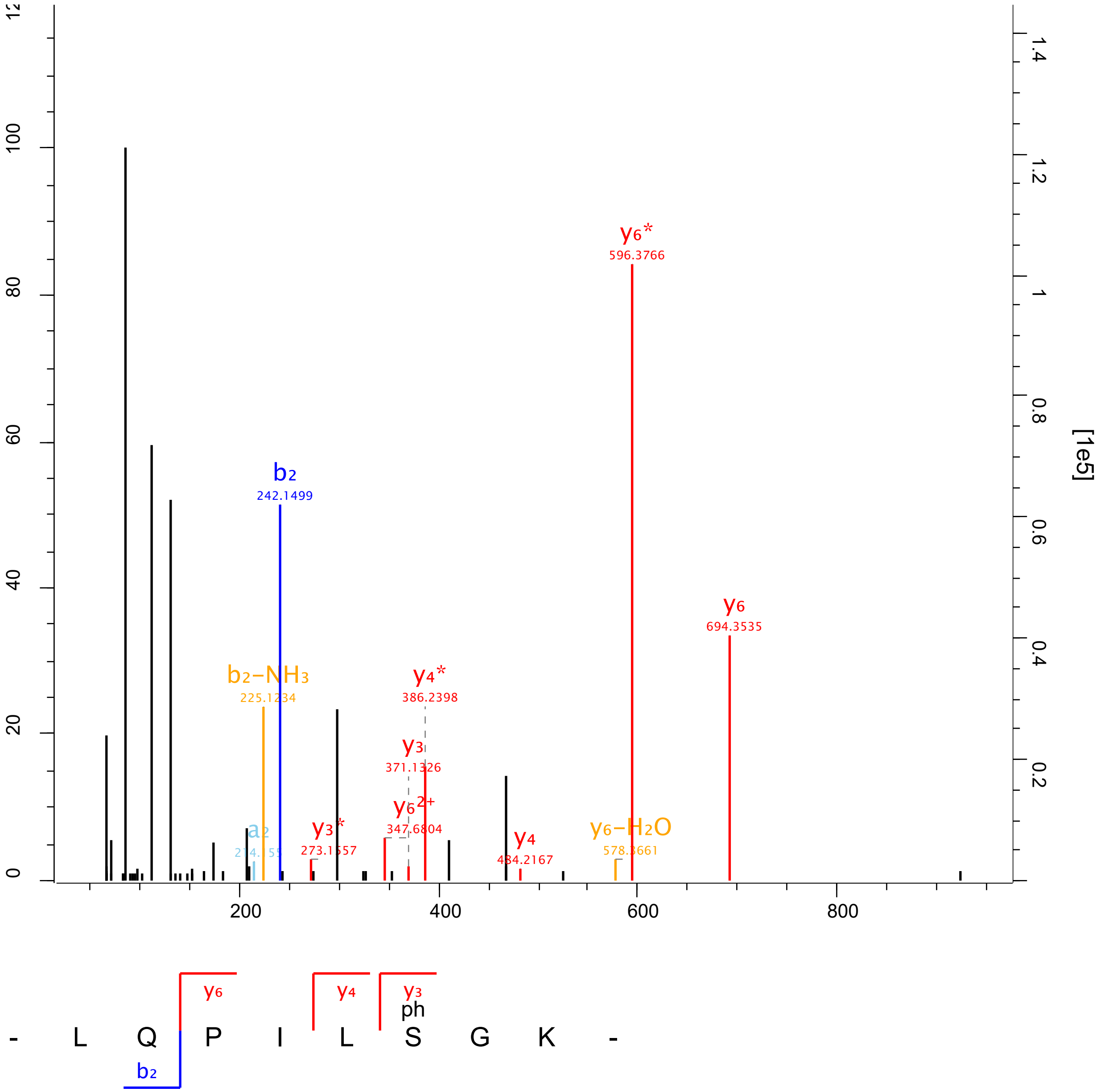

Raw file	Scan	Method	Score	m/z
sys_02_2	14242	FTMS; HCD	136.68	554.22

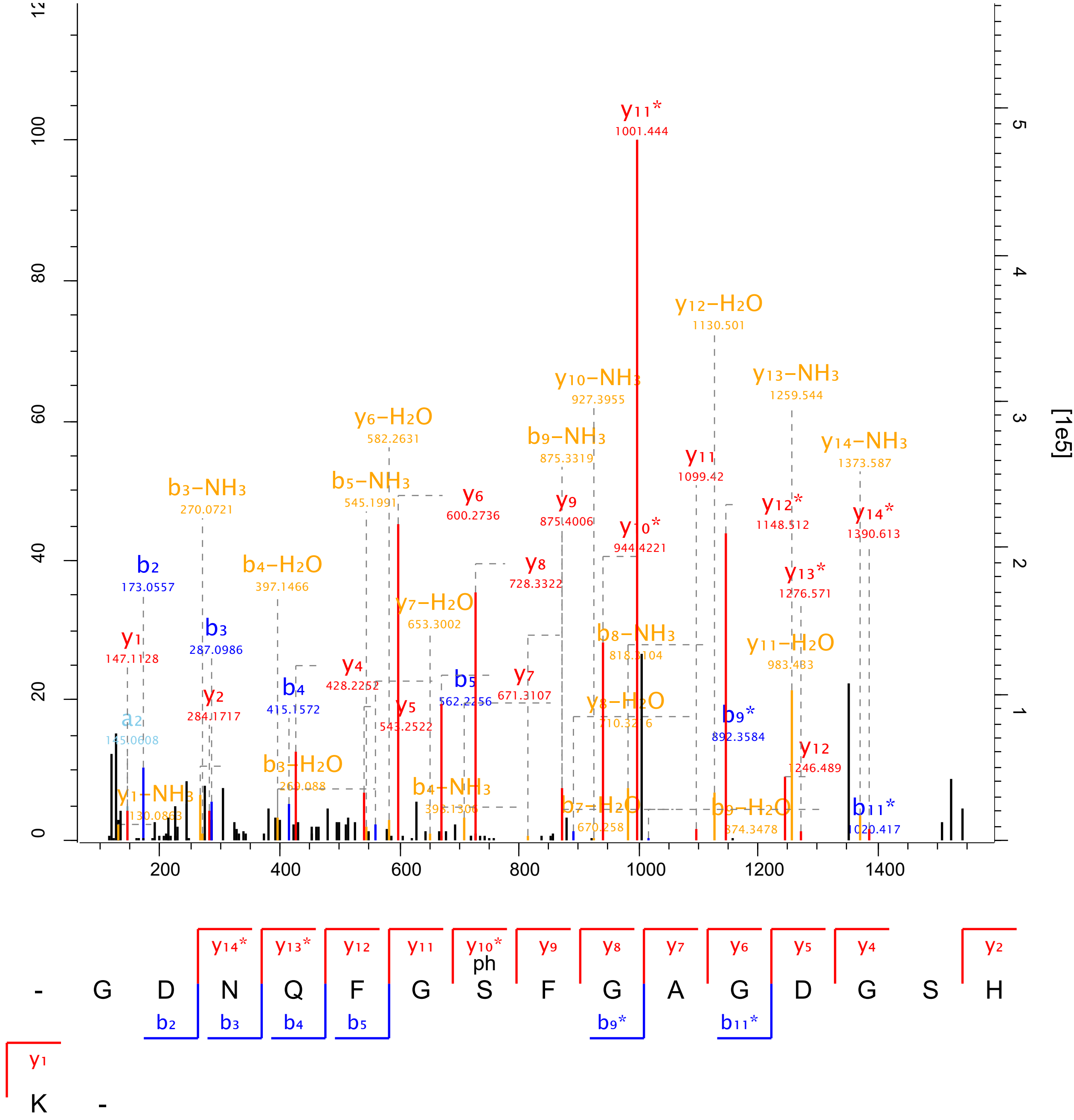

Raw file	Scan	Method	Score	m/z
sys_02_2	14254	FTMS; HCD	111.79	580.74

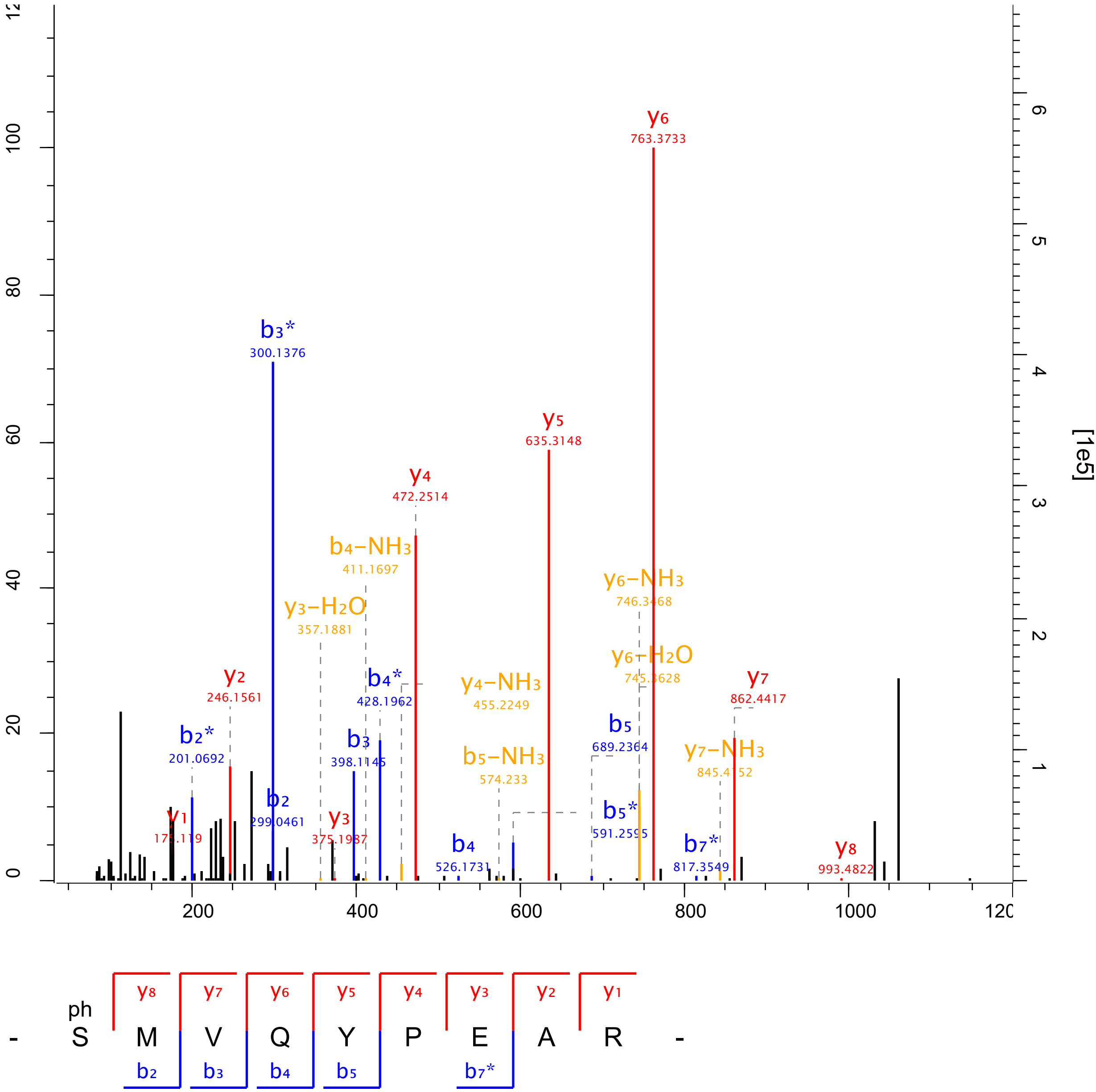

Raw file	Scan	Method	Score	m/z
sys_02_2	14356	FTMS; HCD	69.35	562.75

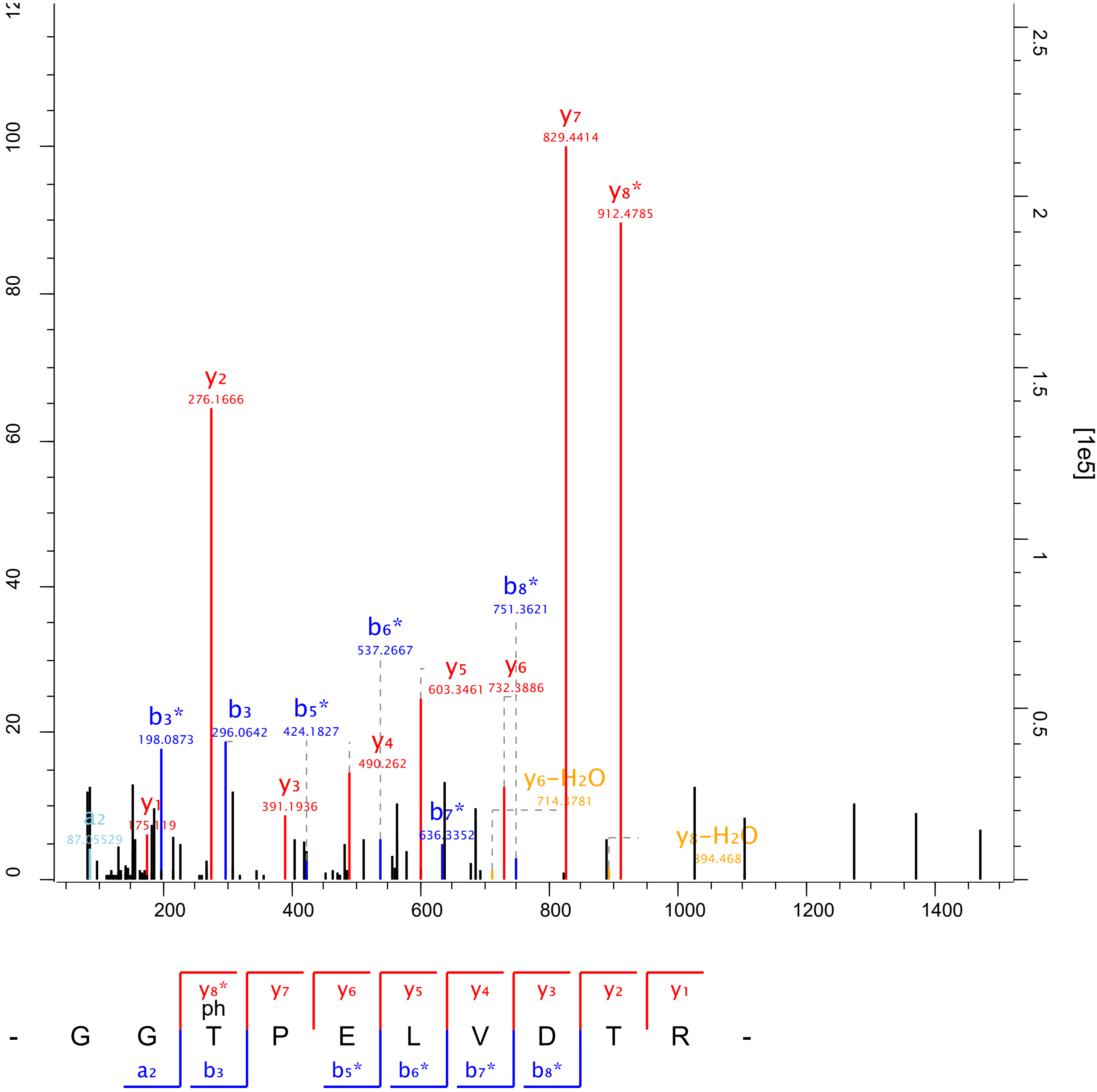

Raw file	Scan	Method	Score	m/z
sys_02_2	14417	FTMS; HCD	111.22	760.8

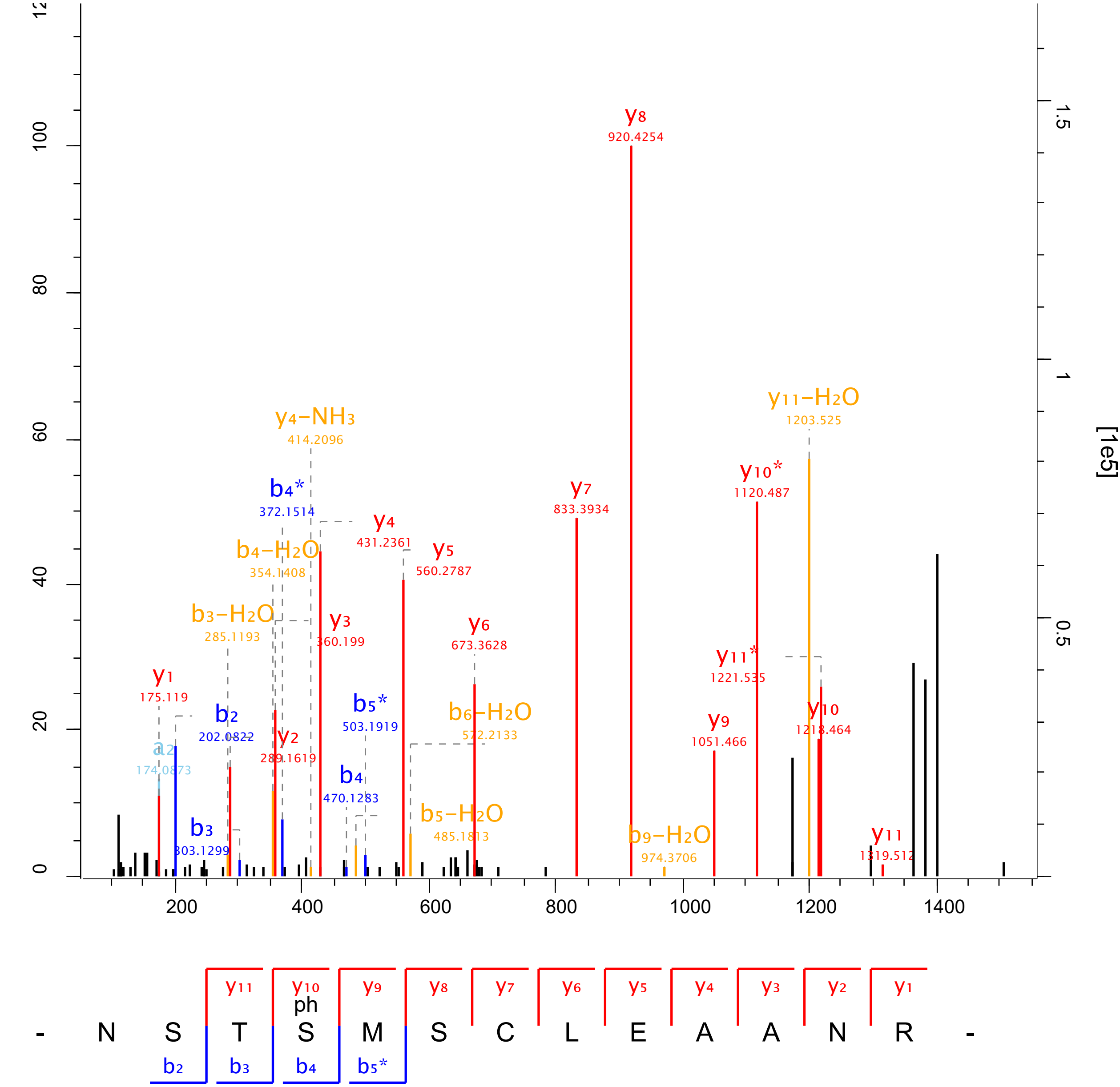

Raw file	Scan	Method	Score	m/z
sys_02_2	1461	FTMS; HCD	48.53	440.86

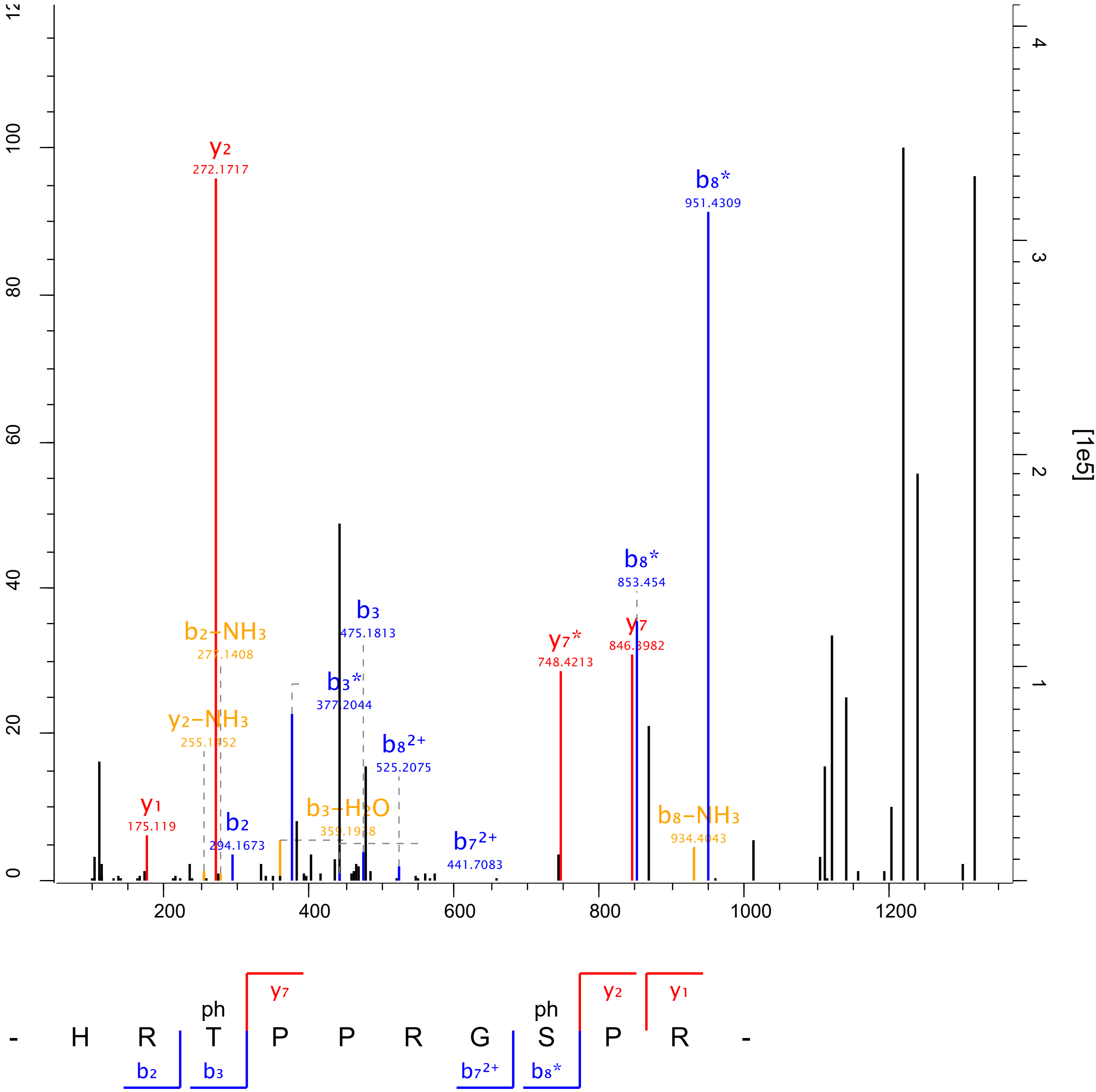

Raw file	Scan	Method	Score	m/z
sys_02_2	14752	FTMS; HCD	58.32	601.26

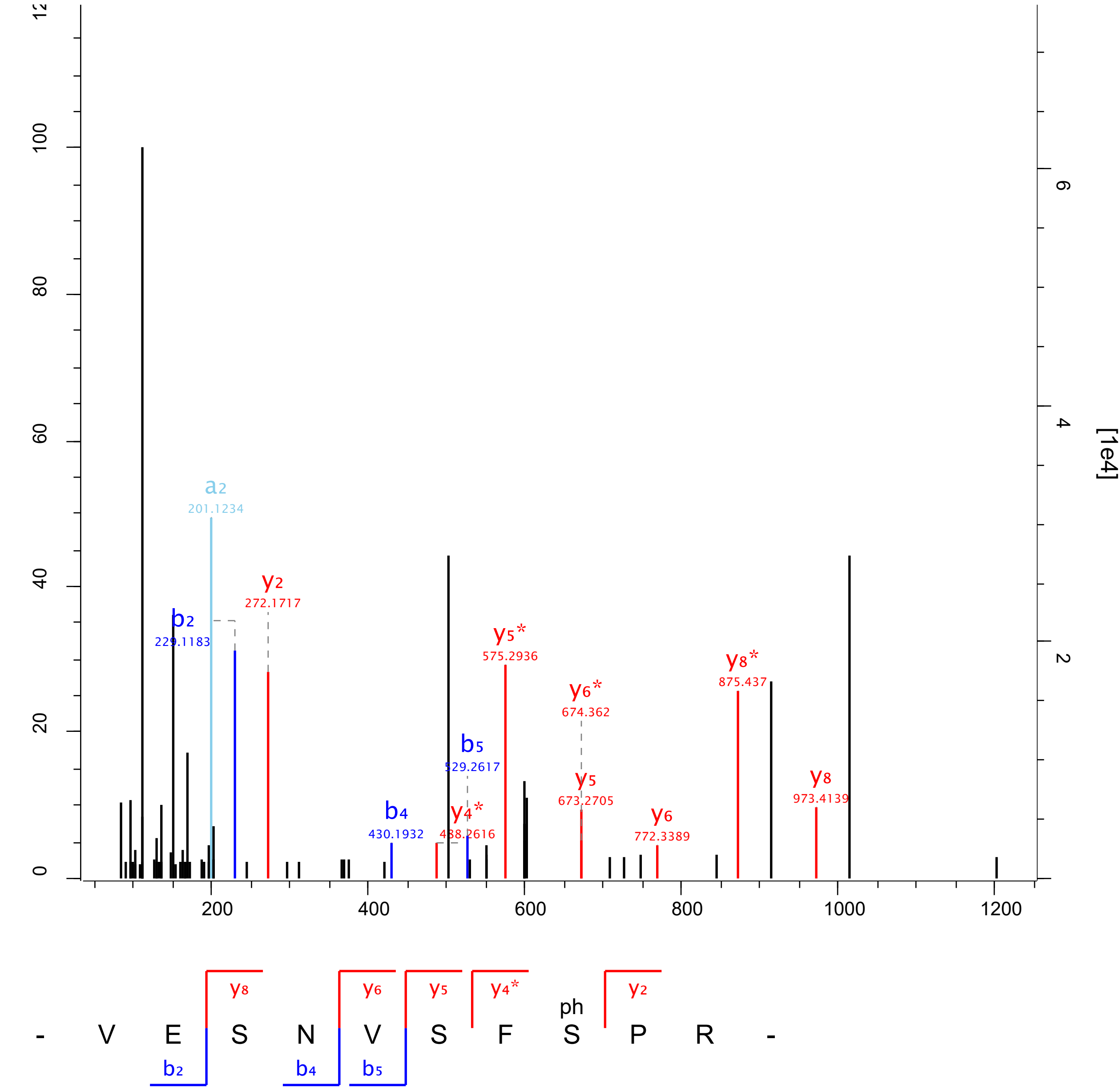

Raw file	Scan	Method	Score	m/z
sys_02_2	1498	FTMS; HCD	43.03	427.16

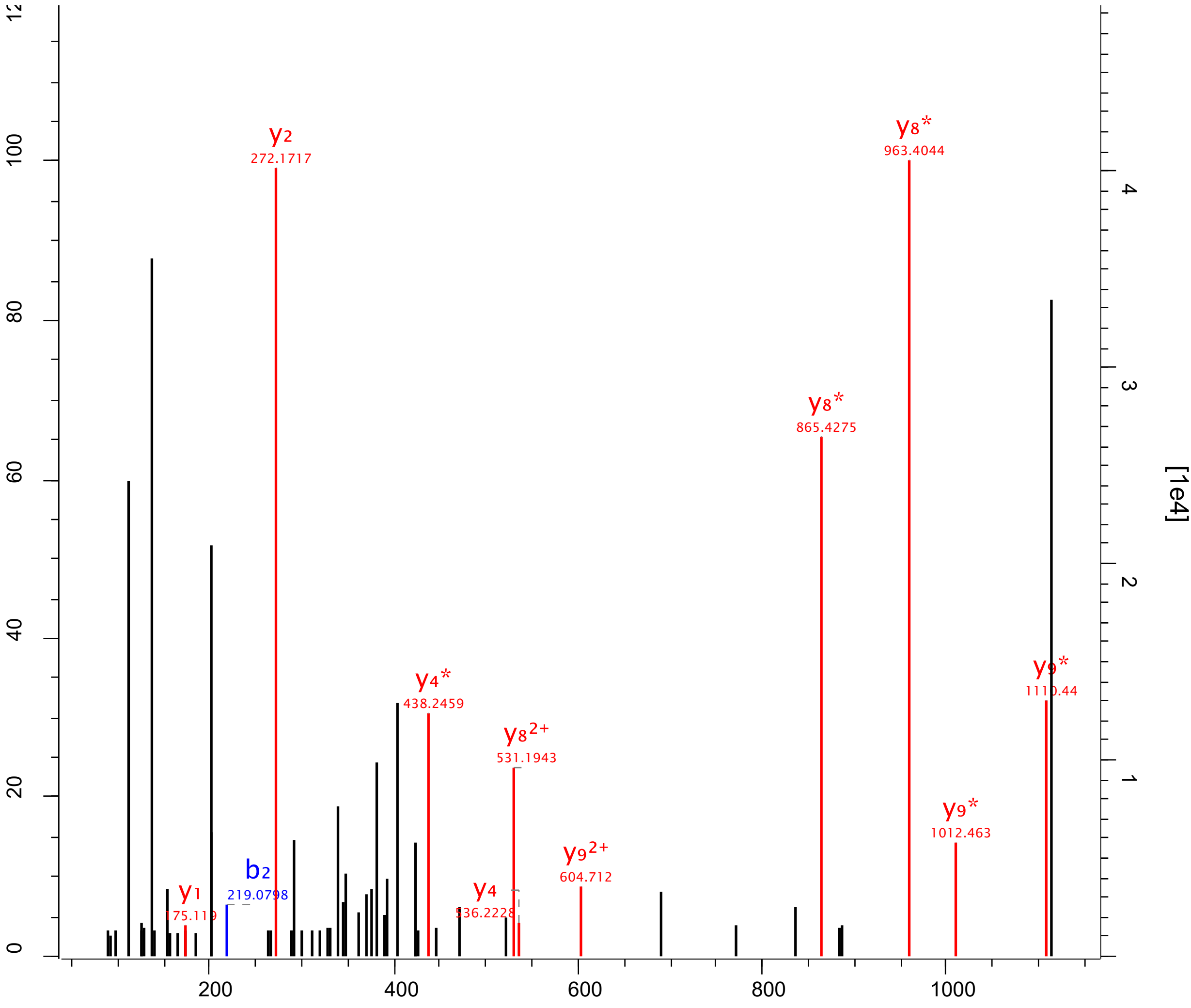

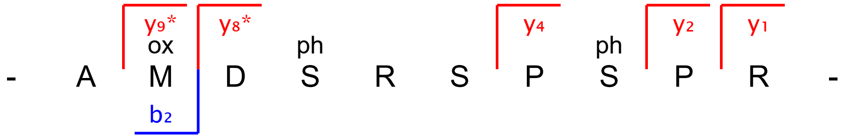

Raw file	Scan	Method	Score	m/z
sys_02_2	15114	FTMS; HCD	74.12	685.67

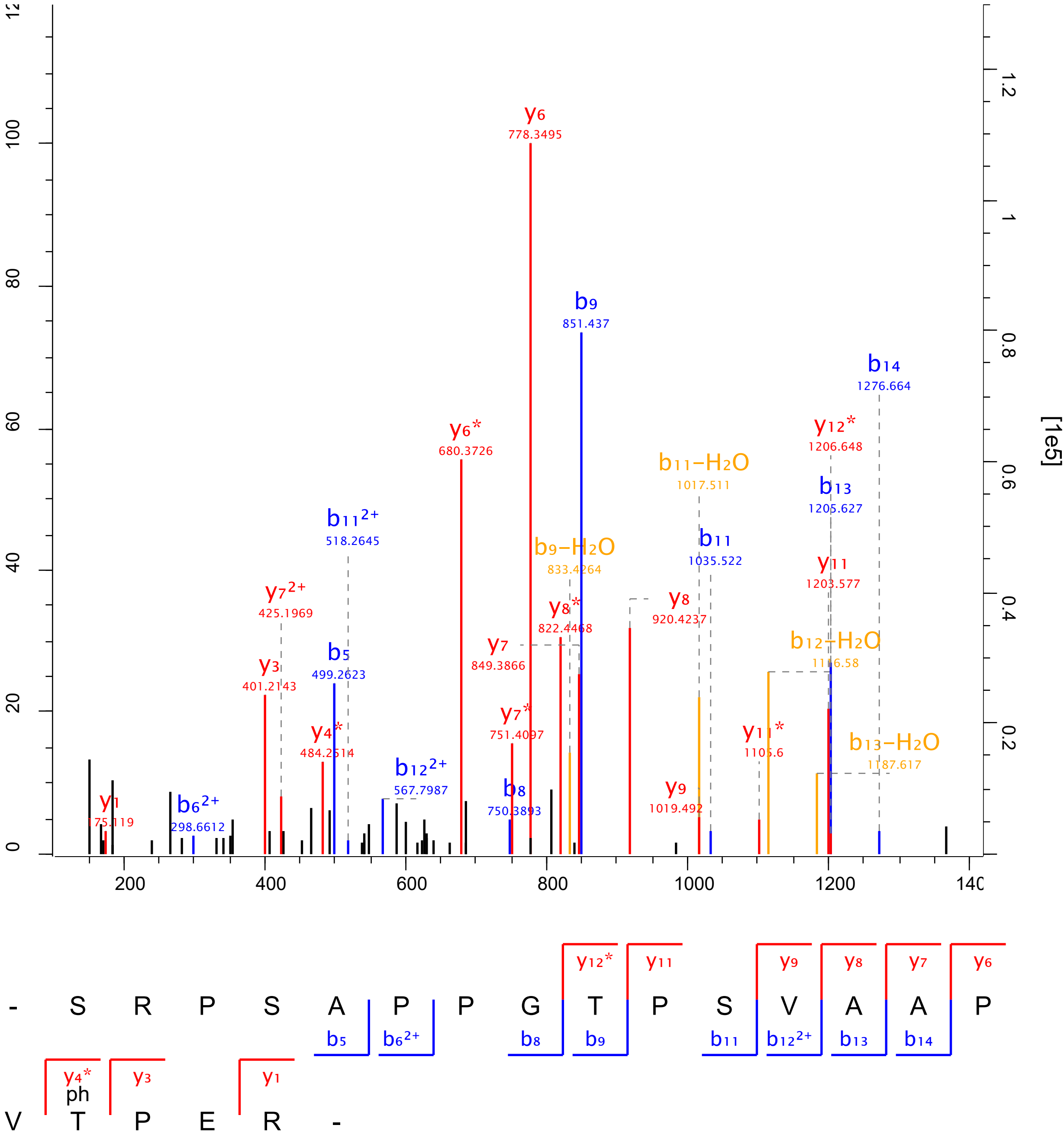

Raw file	Scan	Method	Score	m/z
sys_02_2	15423	FTMS; HCD	160.27	584.95

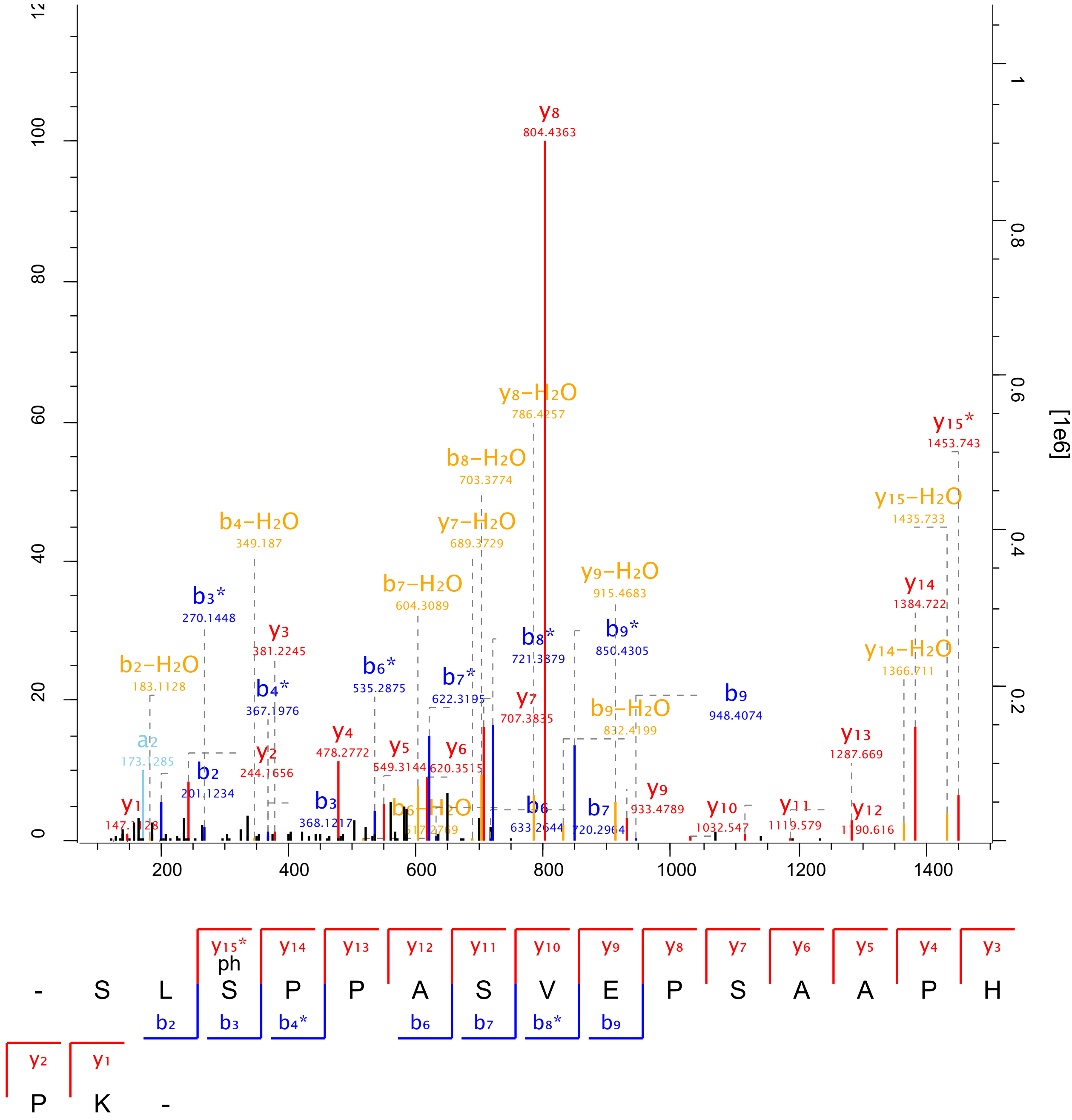

Raw file	Scan	Method	Score	m/z
sys_02_2	1550	FTMS; HCD	68.41	462.23

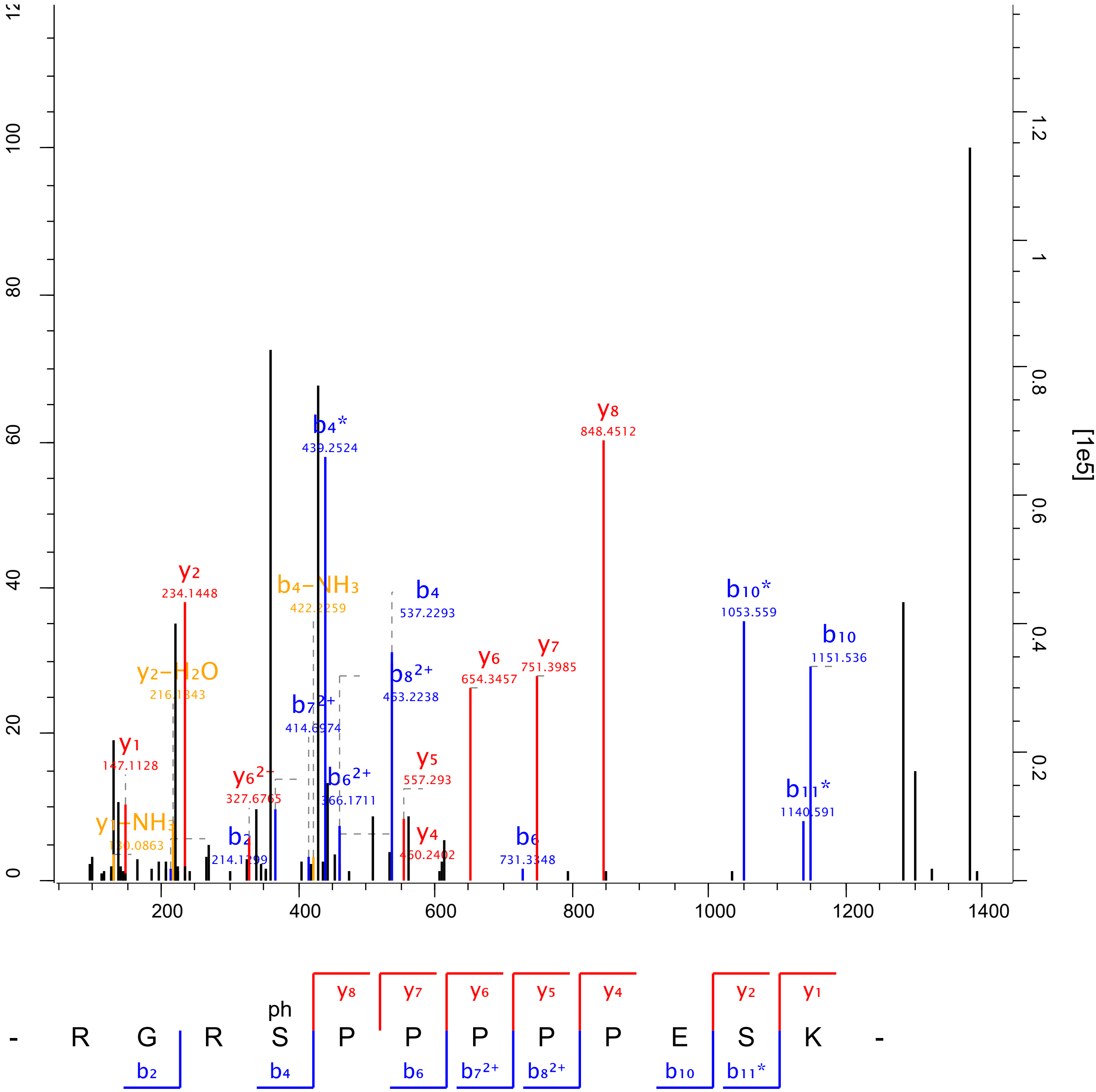

Raw file	Scan	Method	Score	m/z
sys_02_2	15620	FTMS; HCD	69.48	530.24

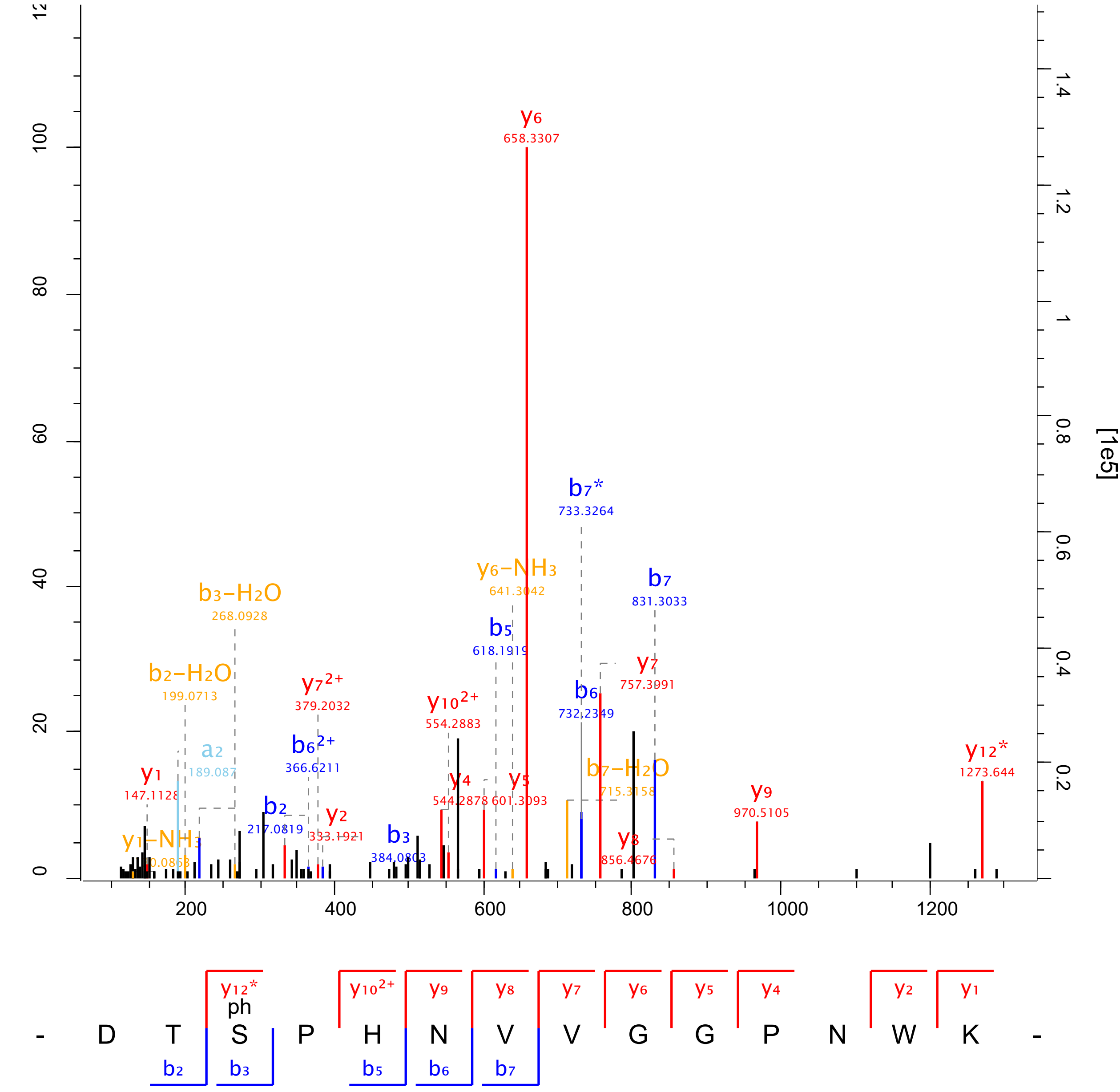

Raw file	Scan	Method	Score	m/z
sys_02_2	15637	FTMS; HCD	80.86	790.81

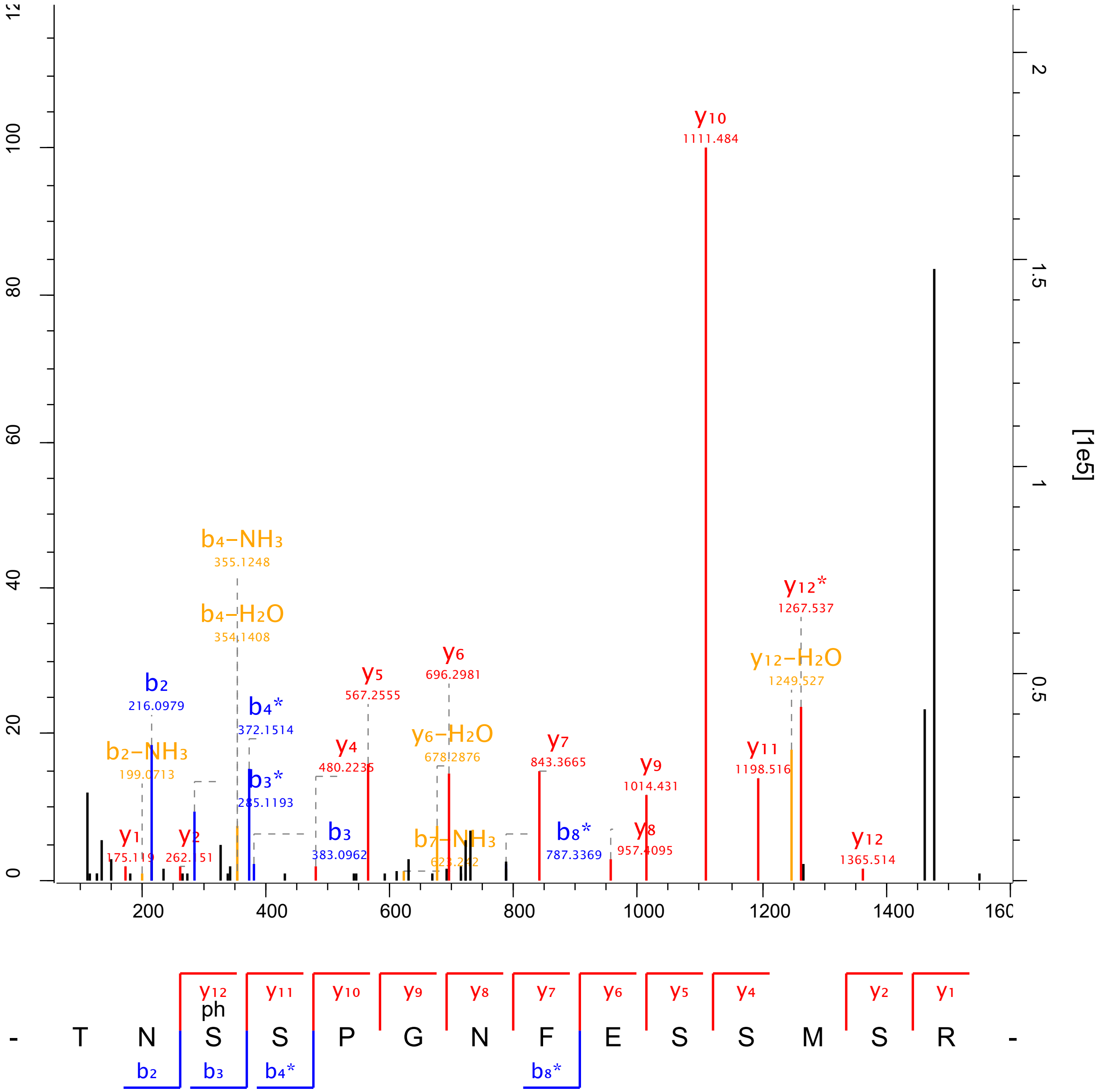

Raw file	Scan	Method	Score	m/z
sys_02_2	15819	FTMS; HCD	46.41	533.2

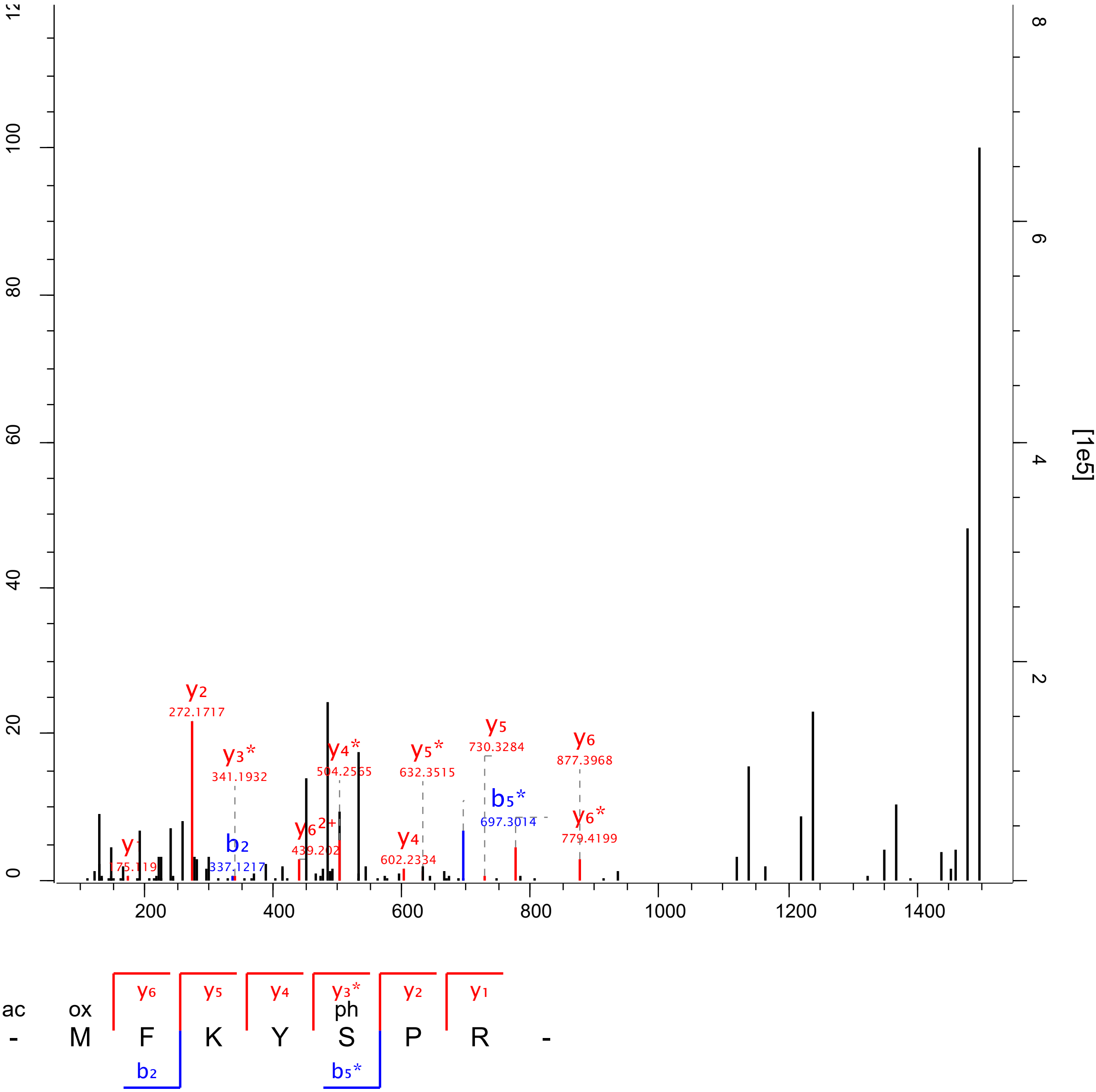

Raw file	Scan	Method	Score	m/z
sys_02_2	15980	FTMS; HCD	66.02	675.28

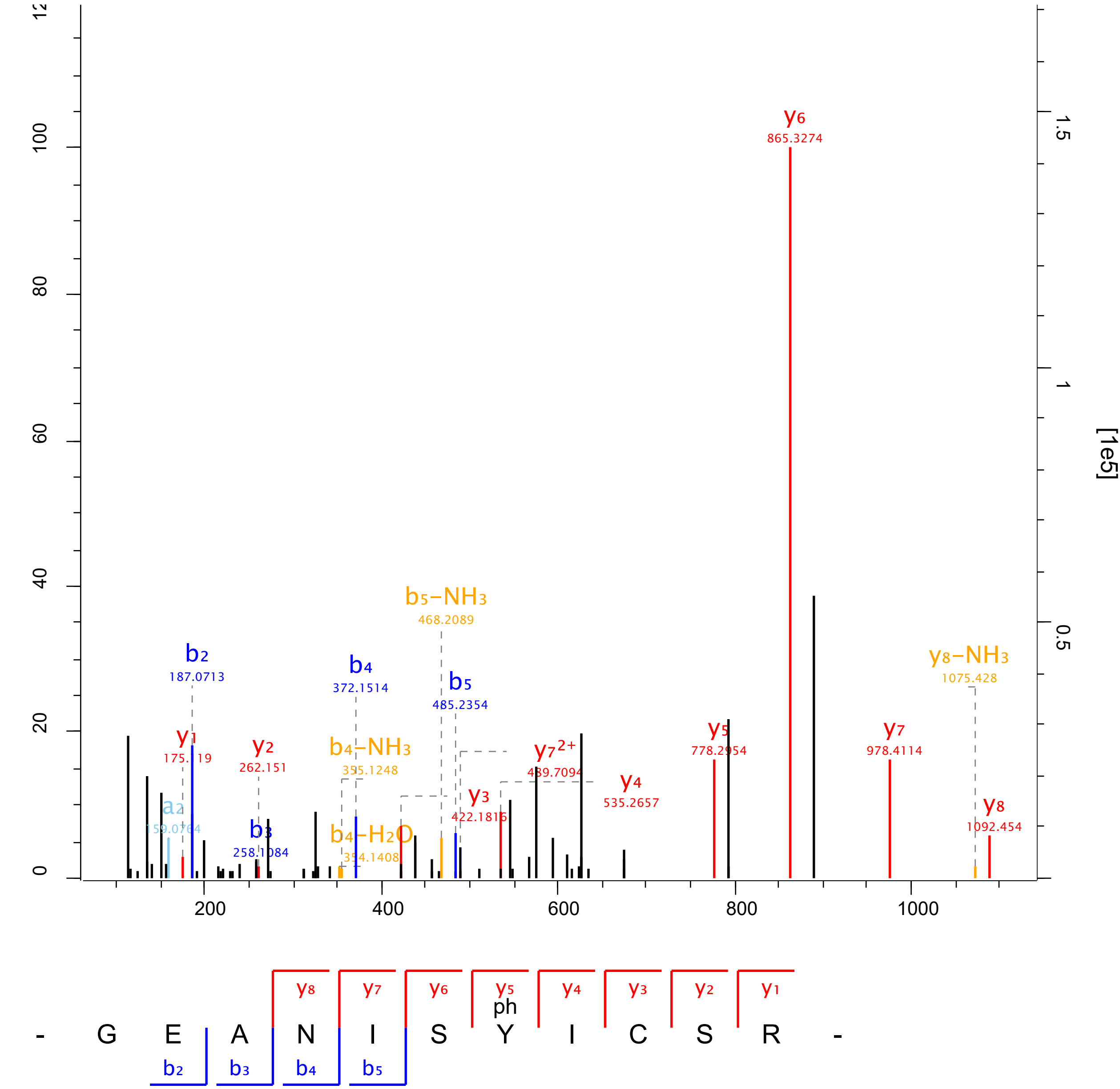

Raw file	Scan	Method	Score	m/z
sys_02_2	16047	FTMS; HCD	135.23	620.79

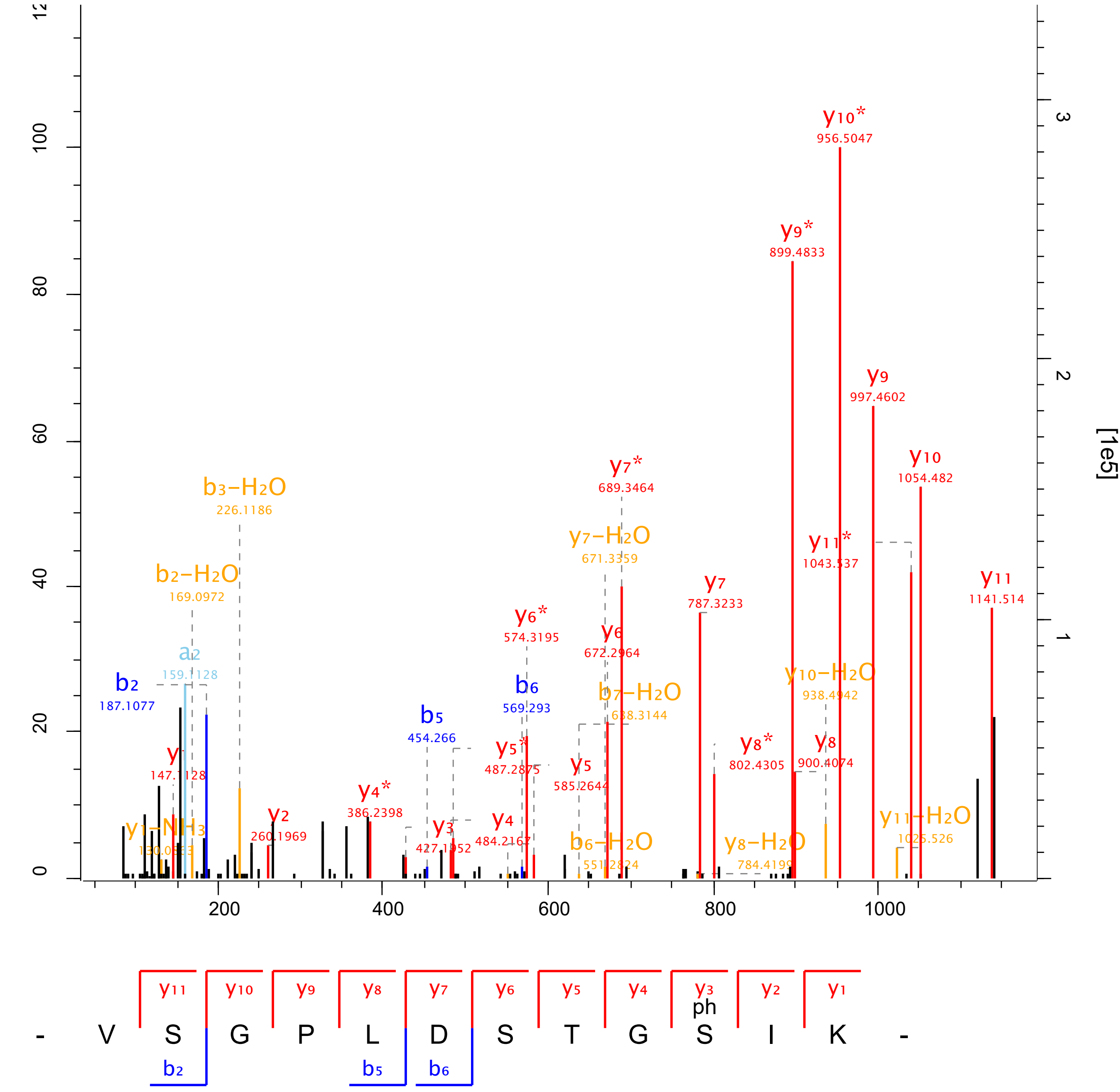

Raw file	Scan	Method	Score	m/z
sys_02_2	16060	FTMS; HCD	98.06	507.59

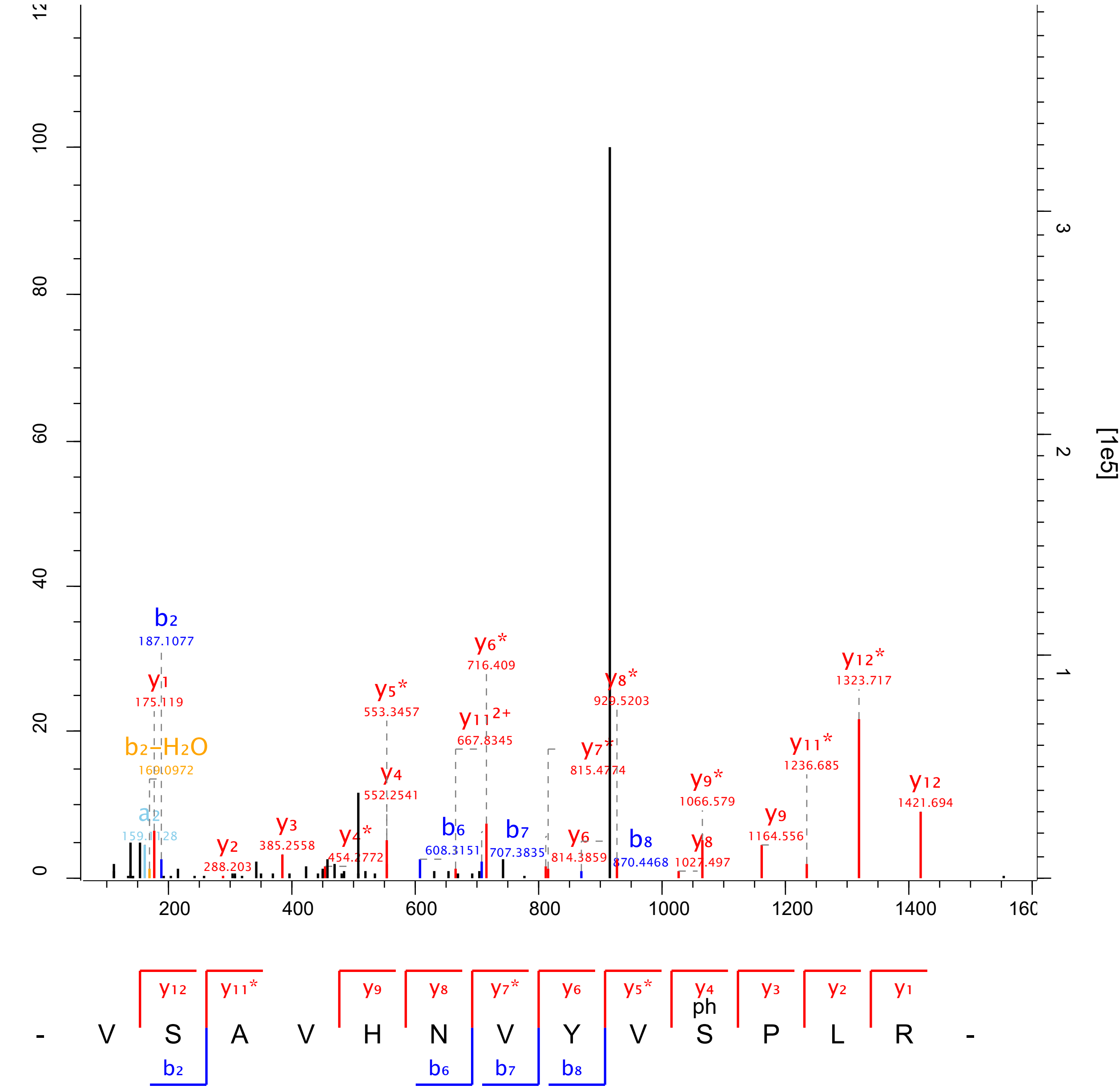

Raw file	Scan	Method	Score	m/z
sys_02_2	16108	FTMS; HCD	257.58	557.27

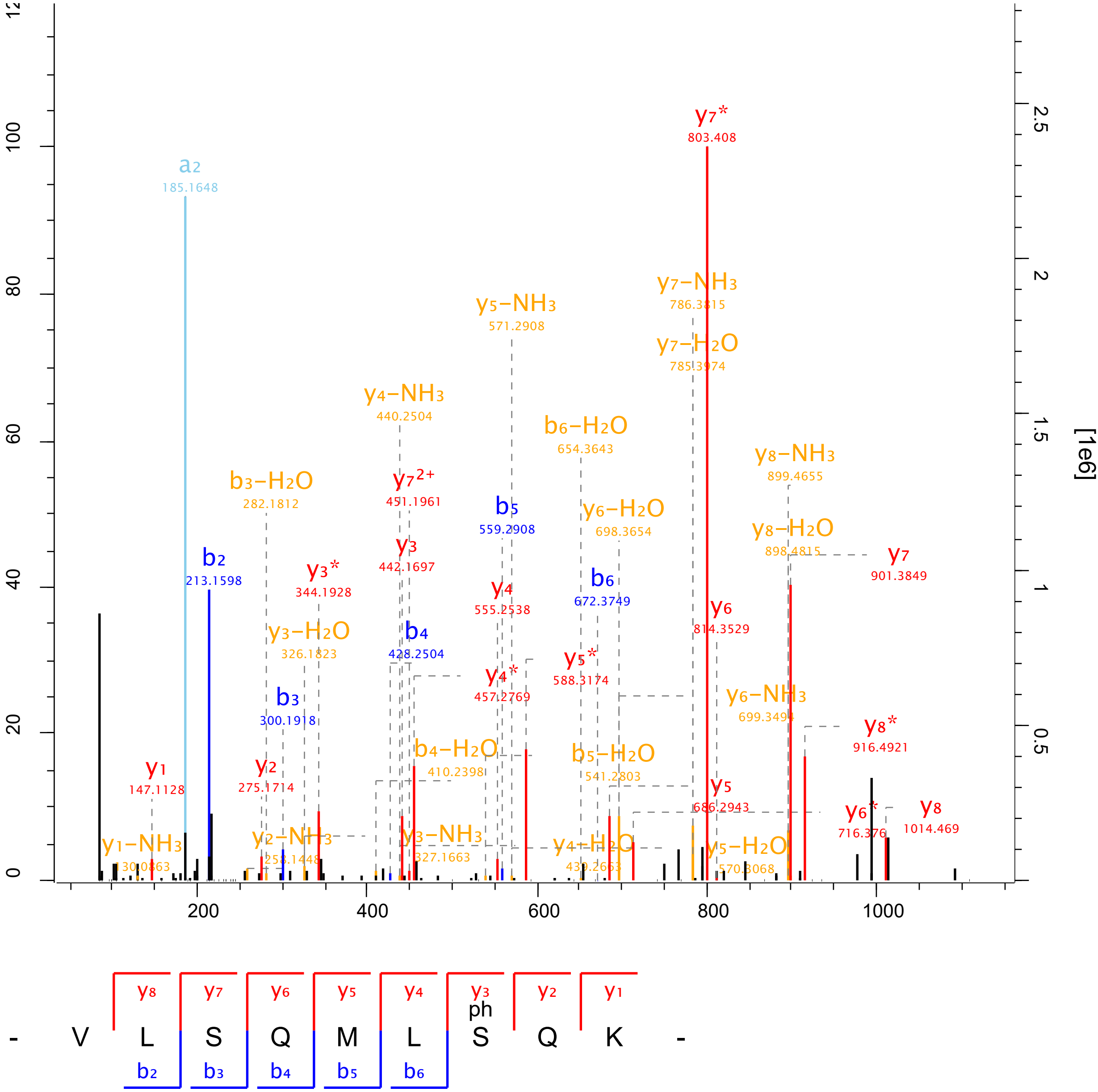

Raw file	Scan	Method	Score	m/z
sys_02_2	16141	FTMS; HCD	98.94	540.76

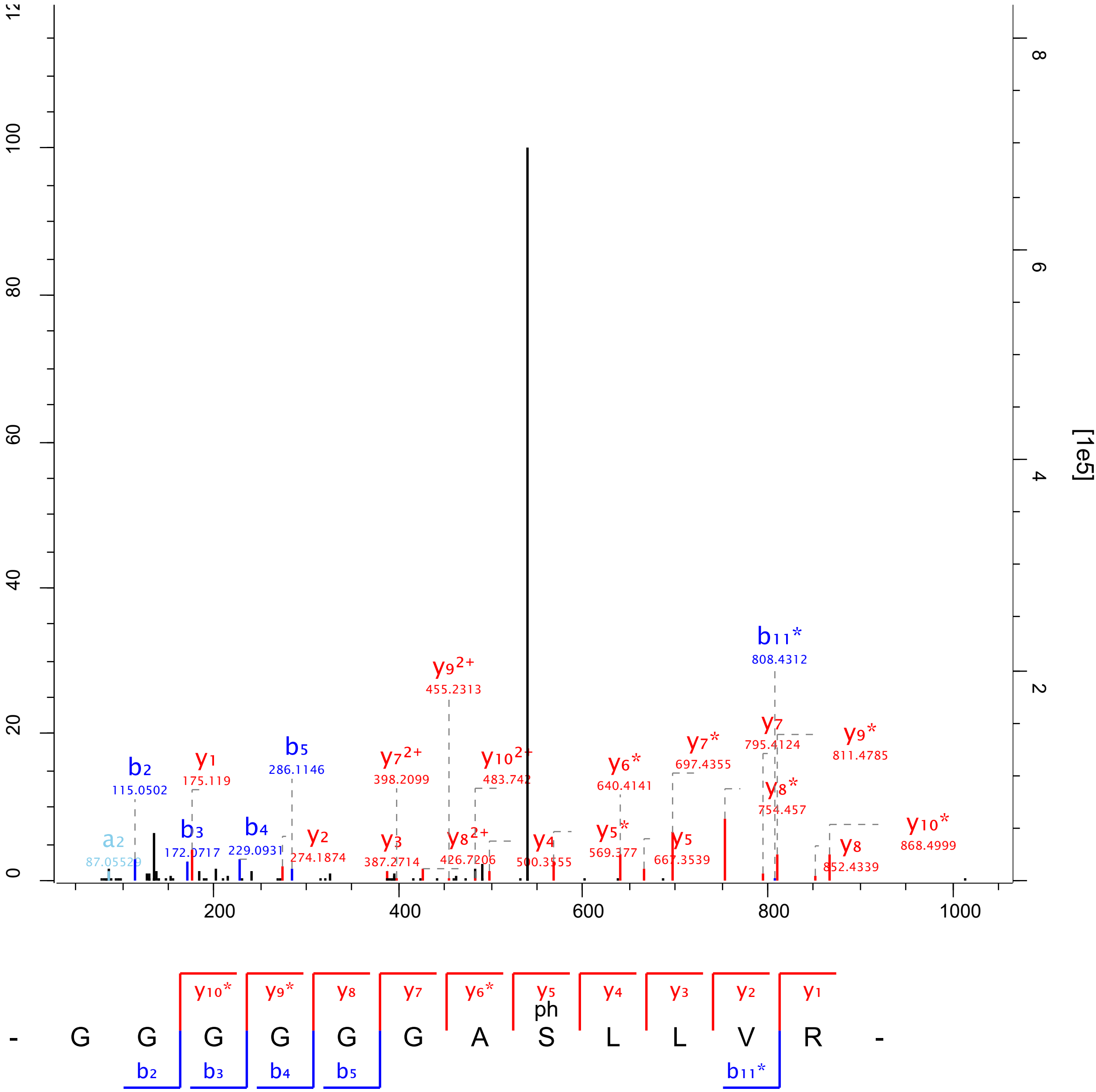

Raw file	Scan	Method	Score	m/z
sys_02_2	16209	FTMS; HCD	57.11	416.55

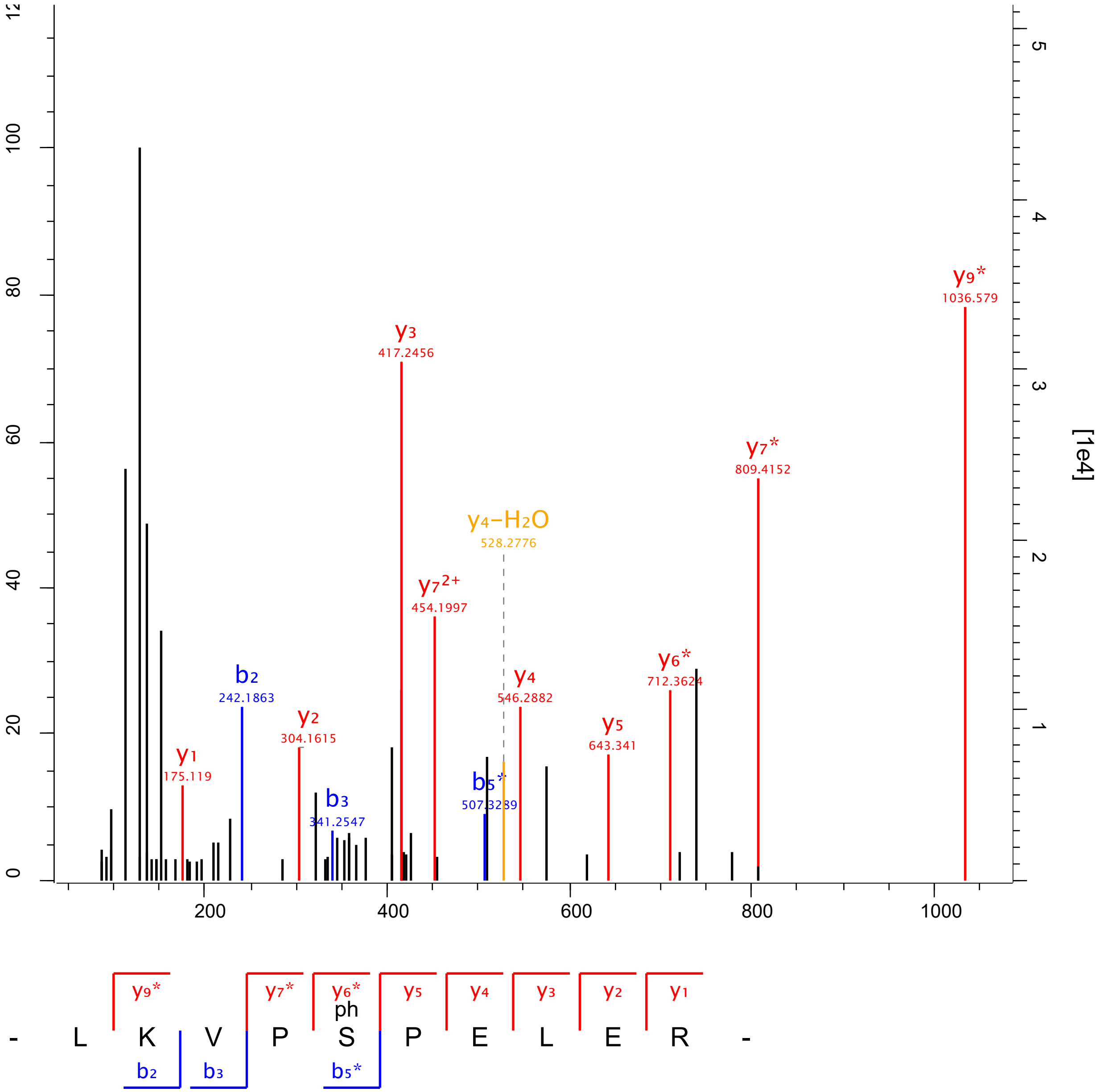

Raw file	Scan	Method	Score	m/z
sys_02_2	1630	FTMS; HCD	41.68	450.22

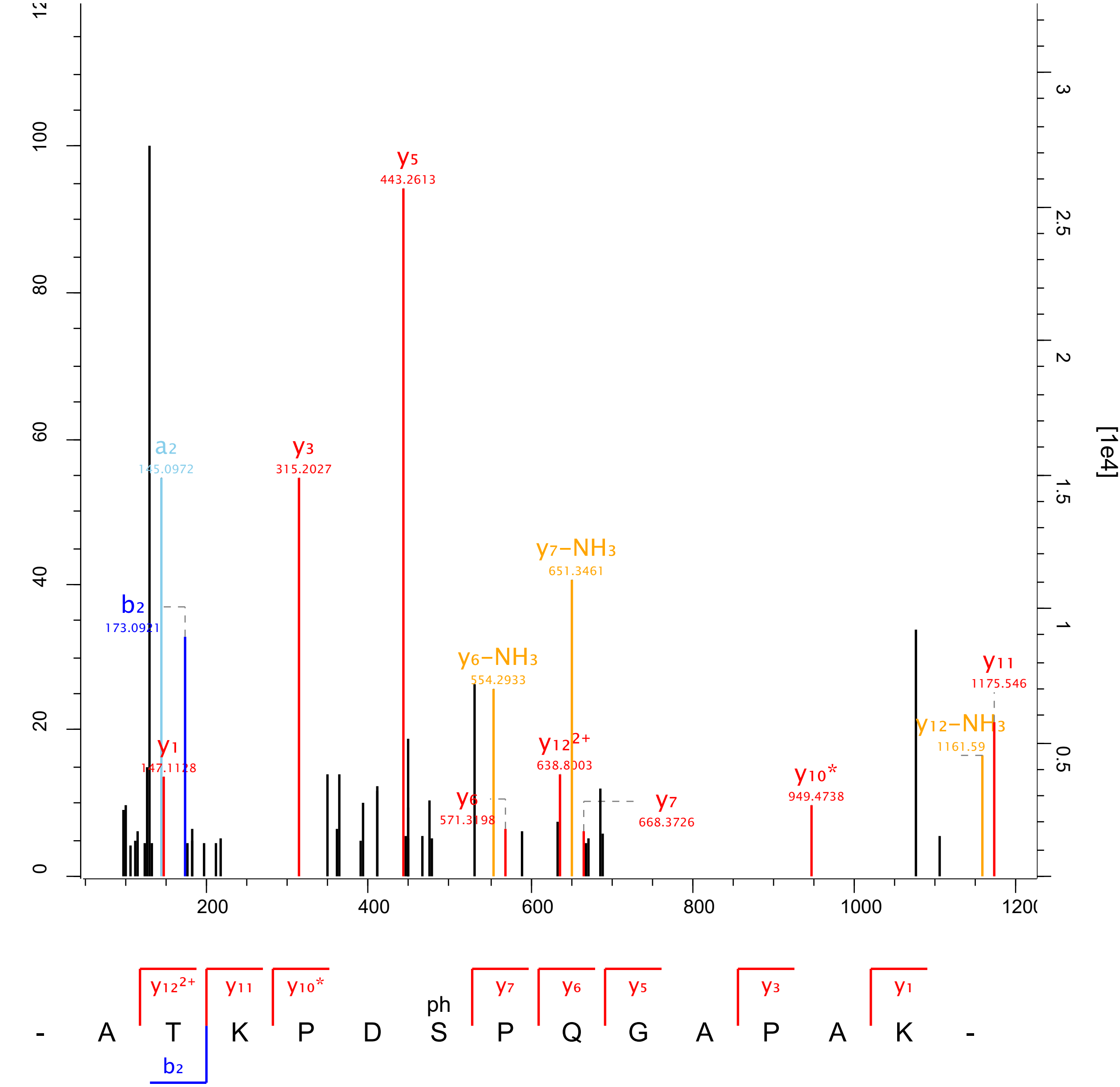

Raw file	Scan	Method	Score	m/z
sys_02_2	16381	FTMS; HCD	43.3	421.88

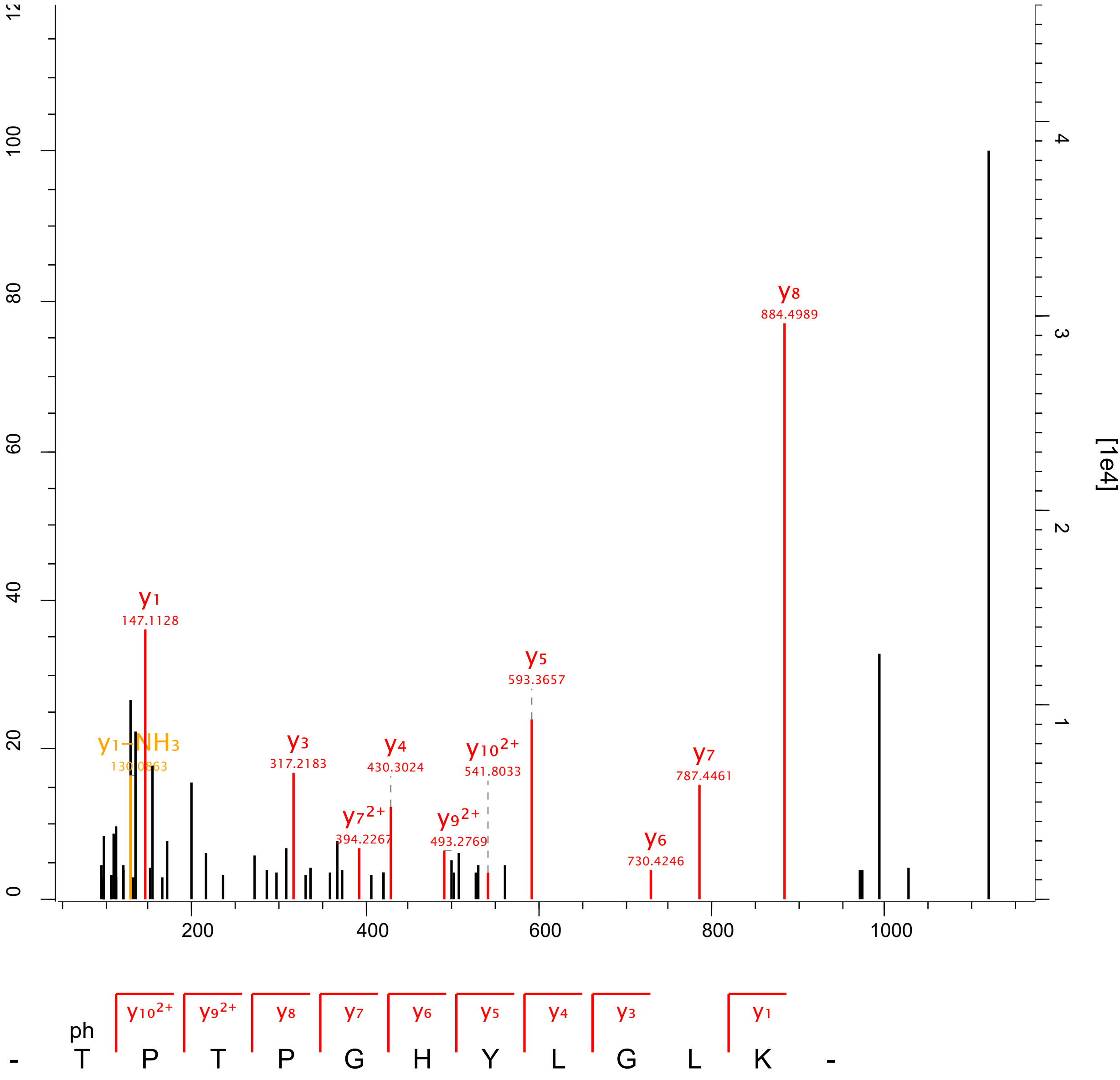

Raw file	Scan	Method	Score	m/z
sys_02_2	16438	FTMS; HCD	81.66	727.82

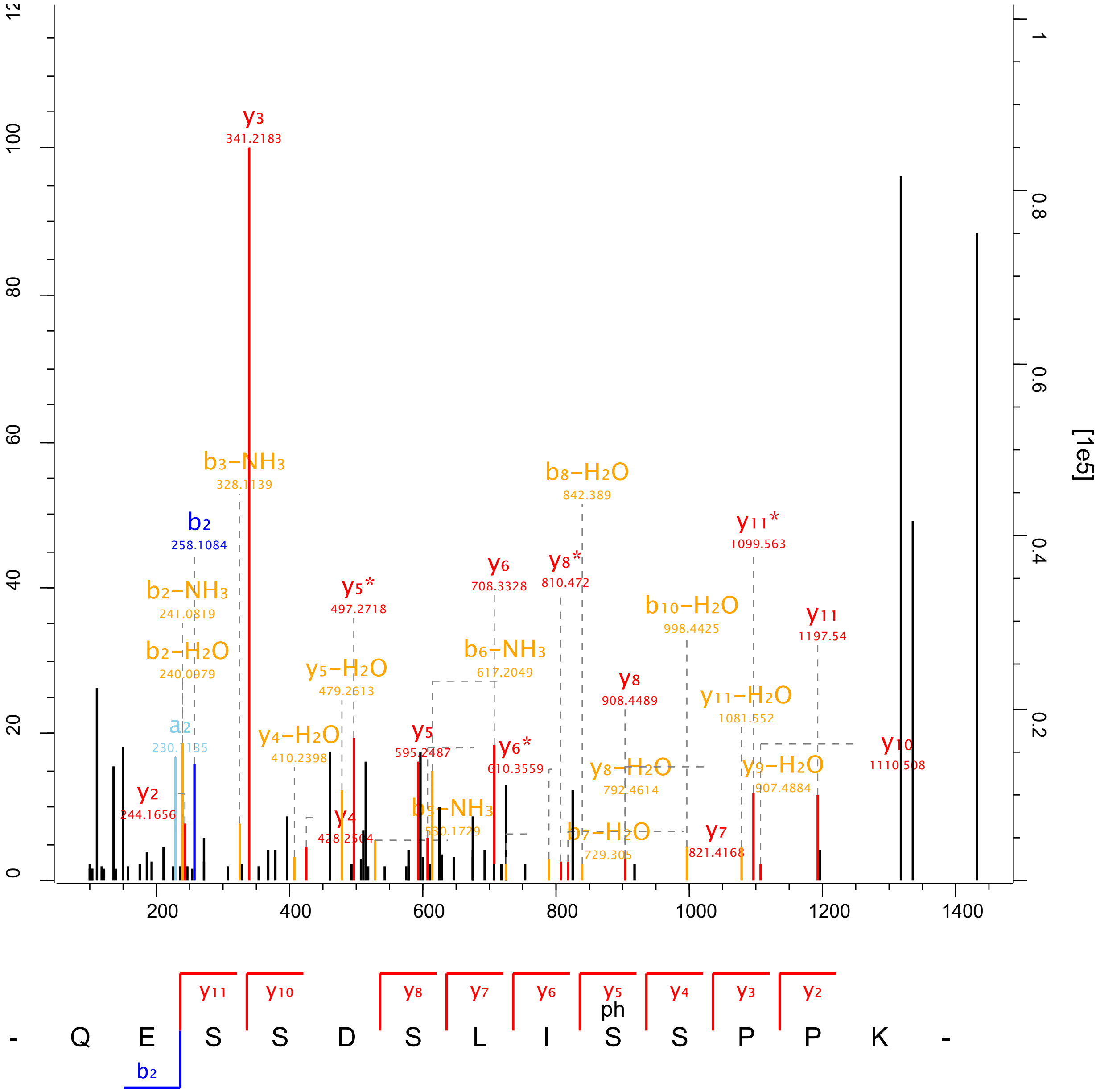

Raw file	Scan	Method	Score	m/z
sys_02_2	16870	FTMS; HCD	102.65	672.79

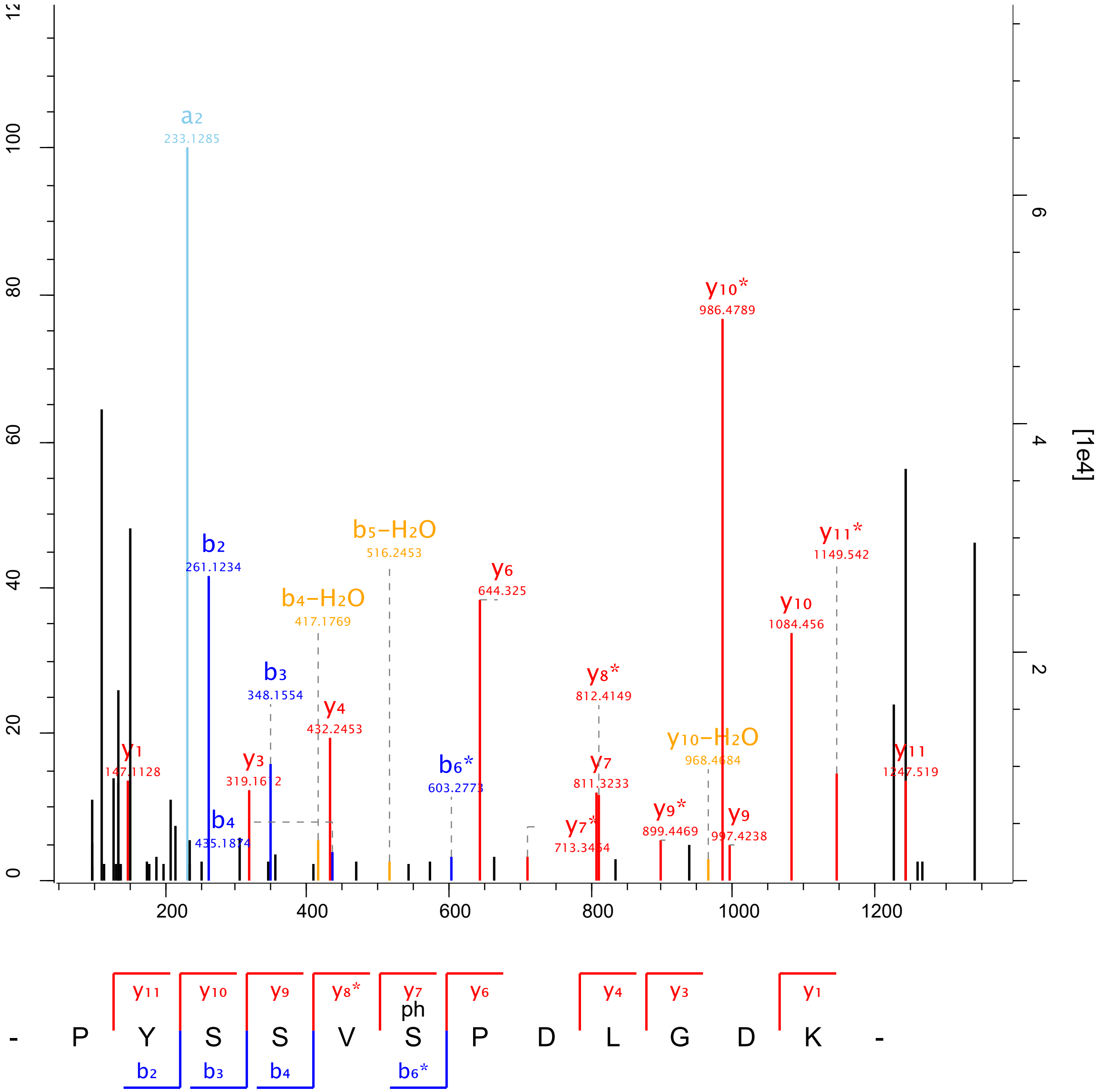

Raw file	Scan	Method	Score	m/z
sys_02_2	16985	FTMS; HCD	60.8	689.31

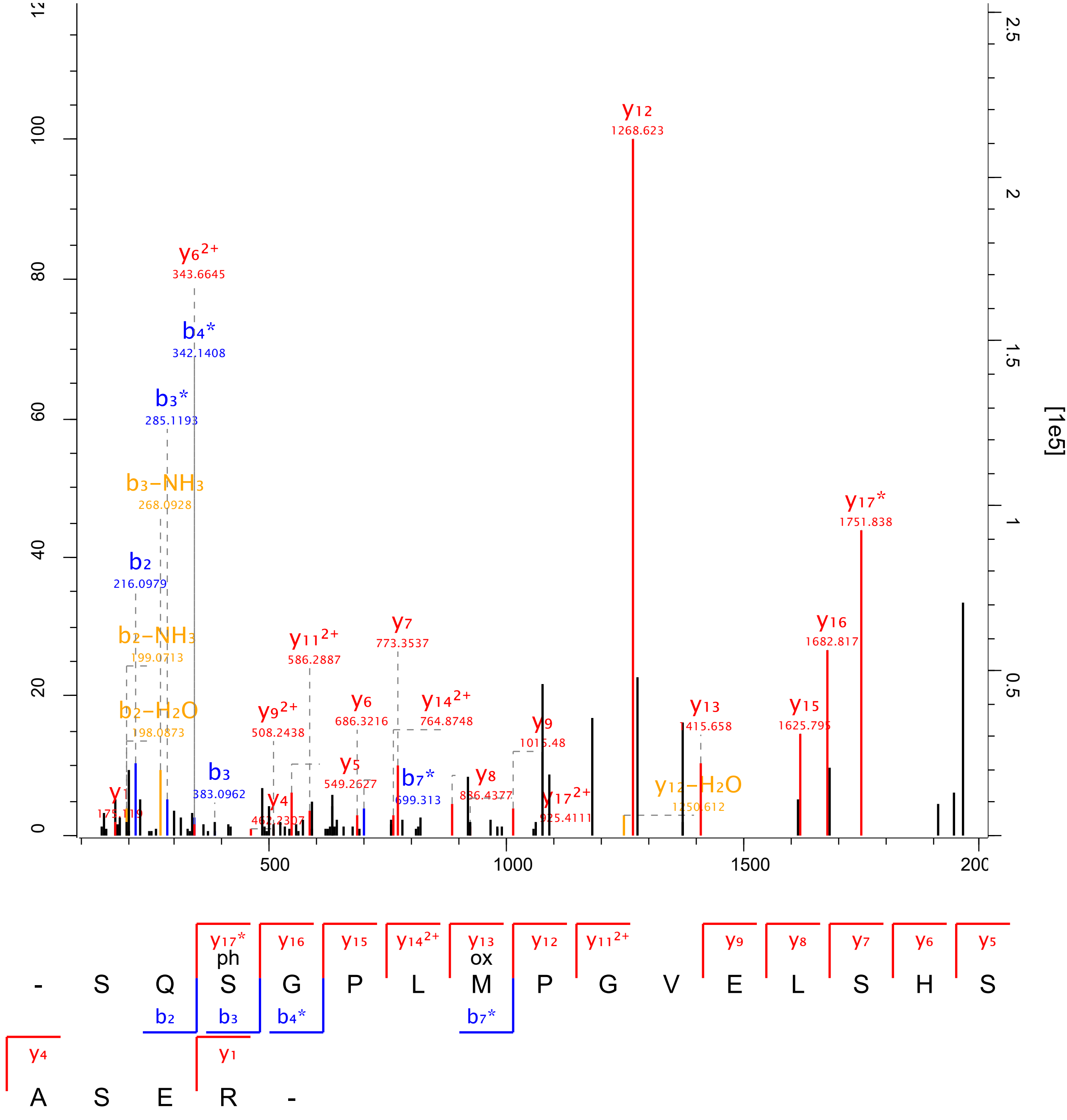

Raw file	Scan	Method	Score	m/z
sys_02_2	17105	FTMS; HCD	80.36	712

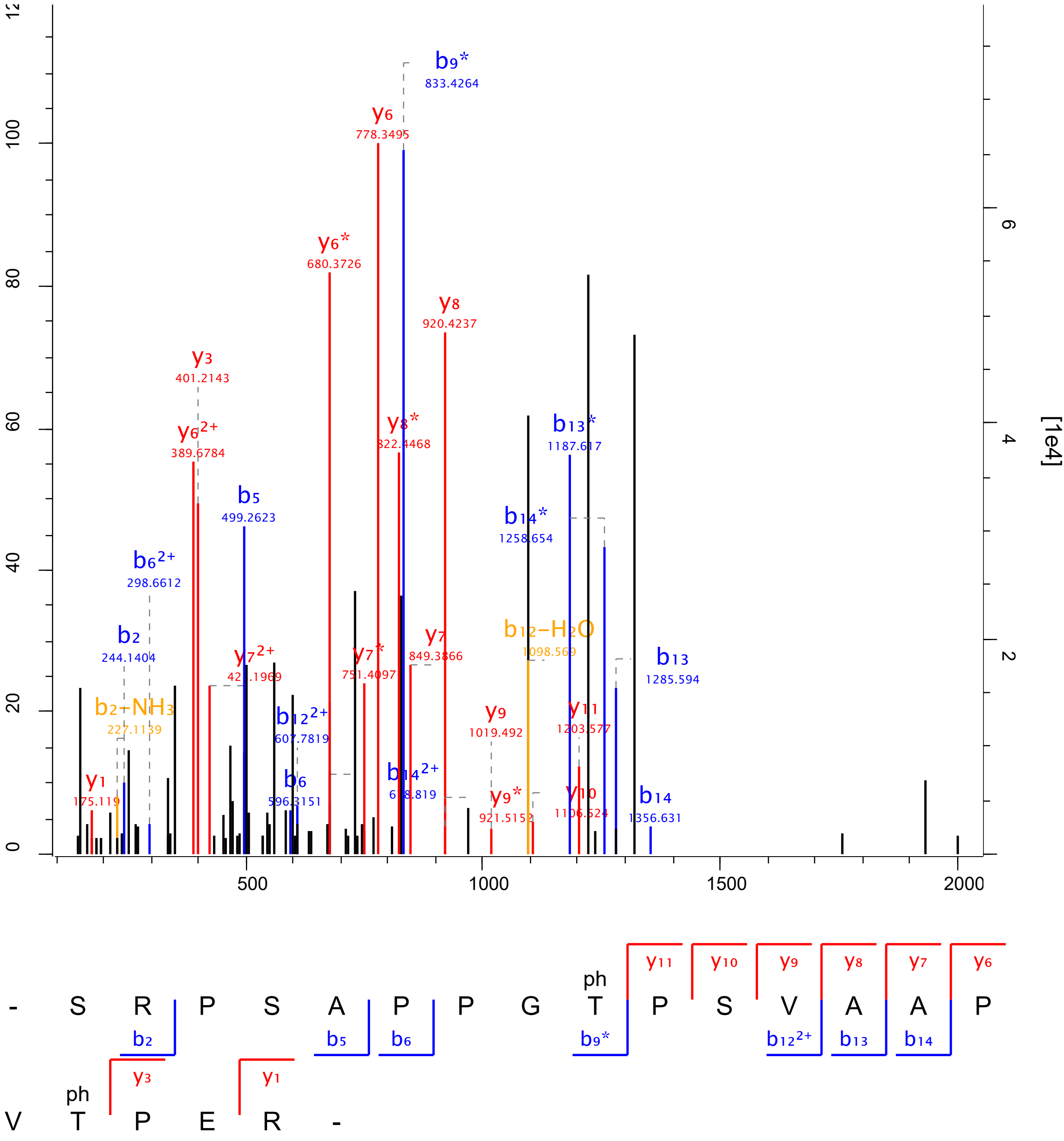

Raw file	Scan	Method	Score	m/z
sys_02_2	17108	FTMS; HCD	144.2	574.26

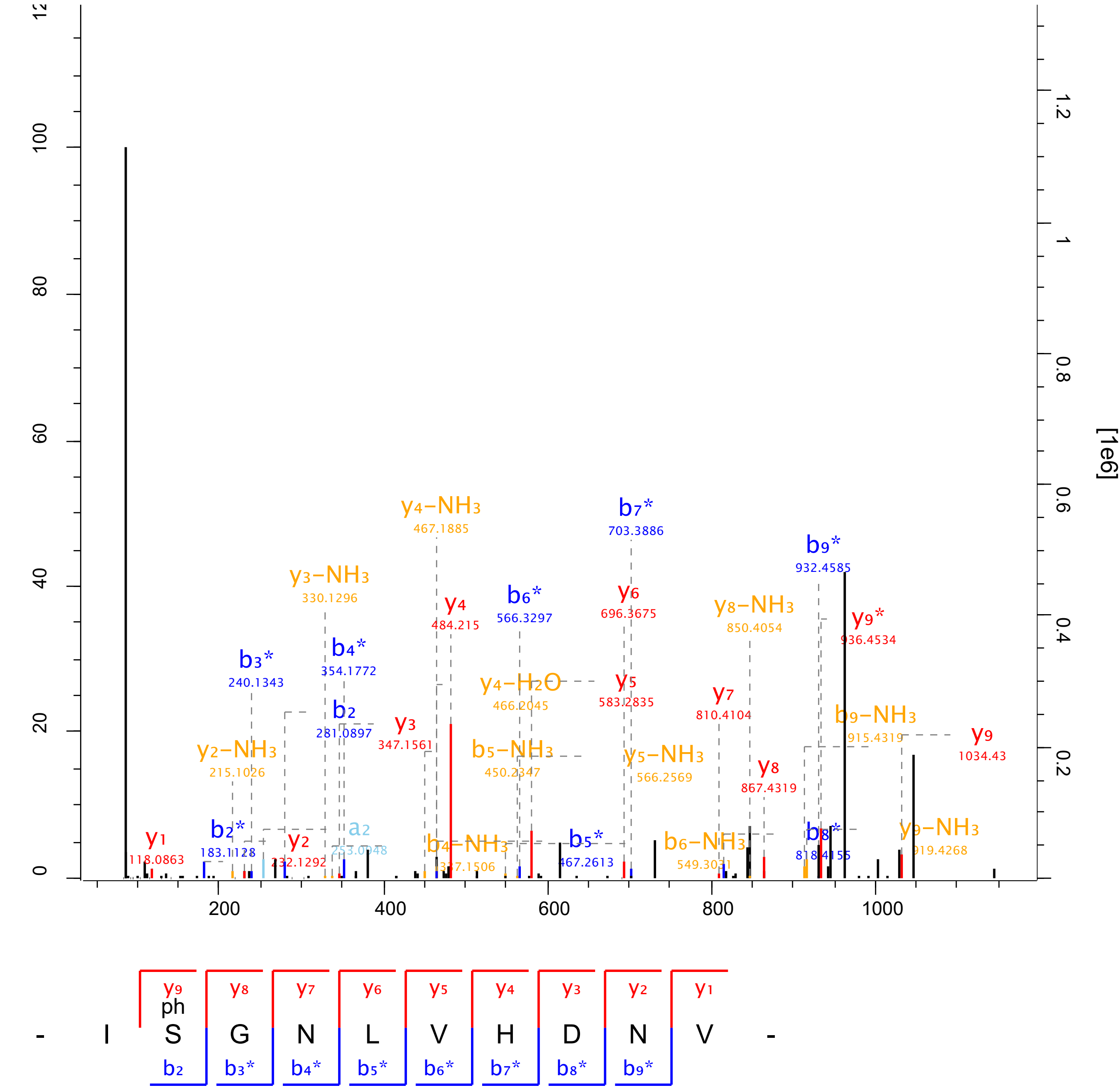

Raw file	Scan	Method	Score	m/z
sys_02_2	17225	FTMS; HCD	73.5	635.26

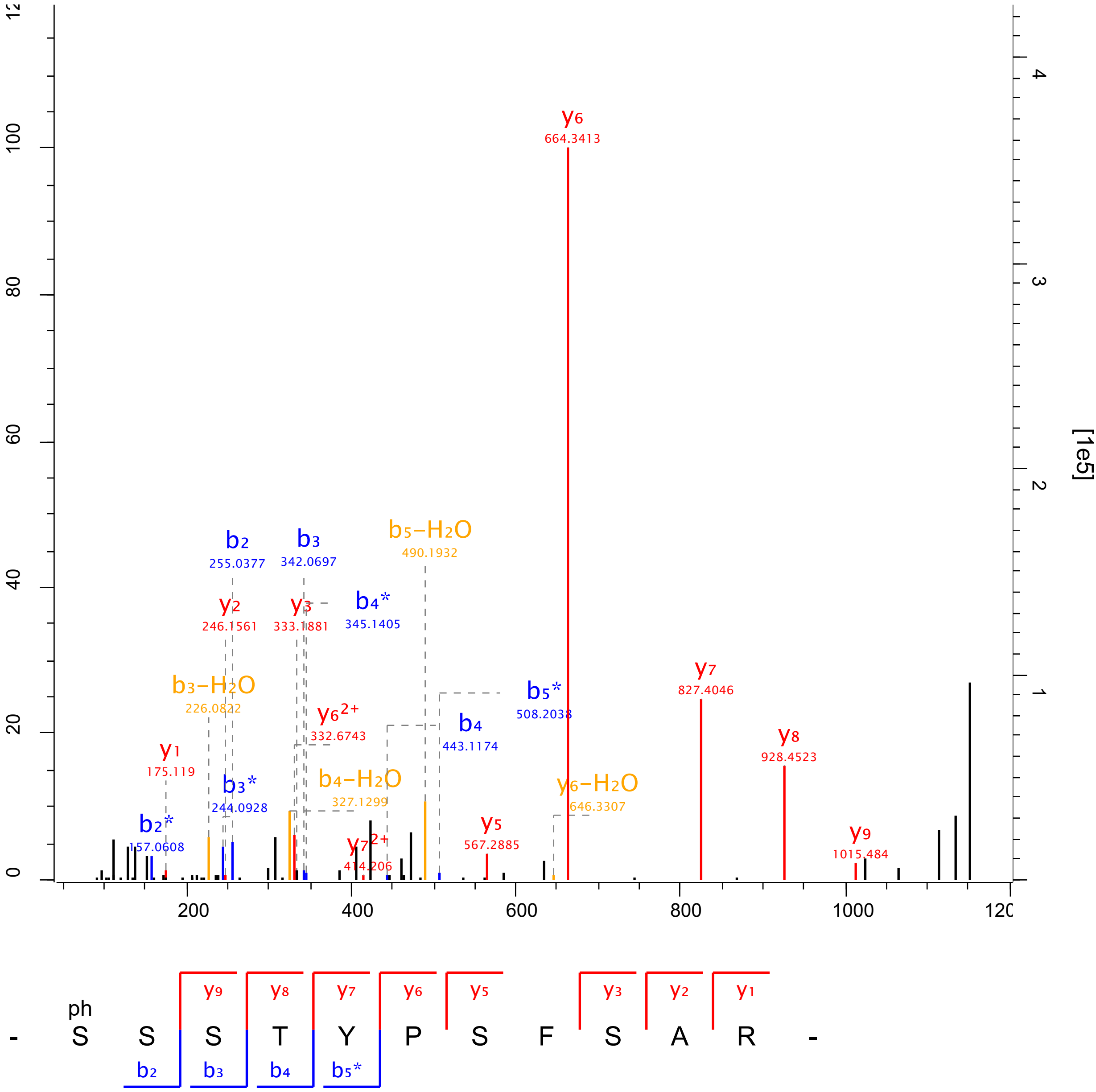

Raw file	Scan	Method	Score	m/z
sys_02_2	17233	FTMS; HCD	137.64	913.9

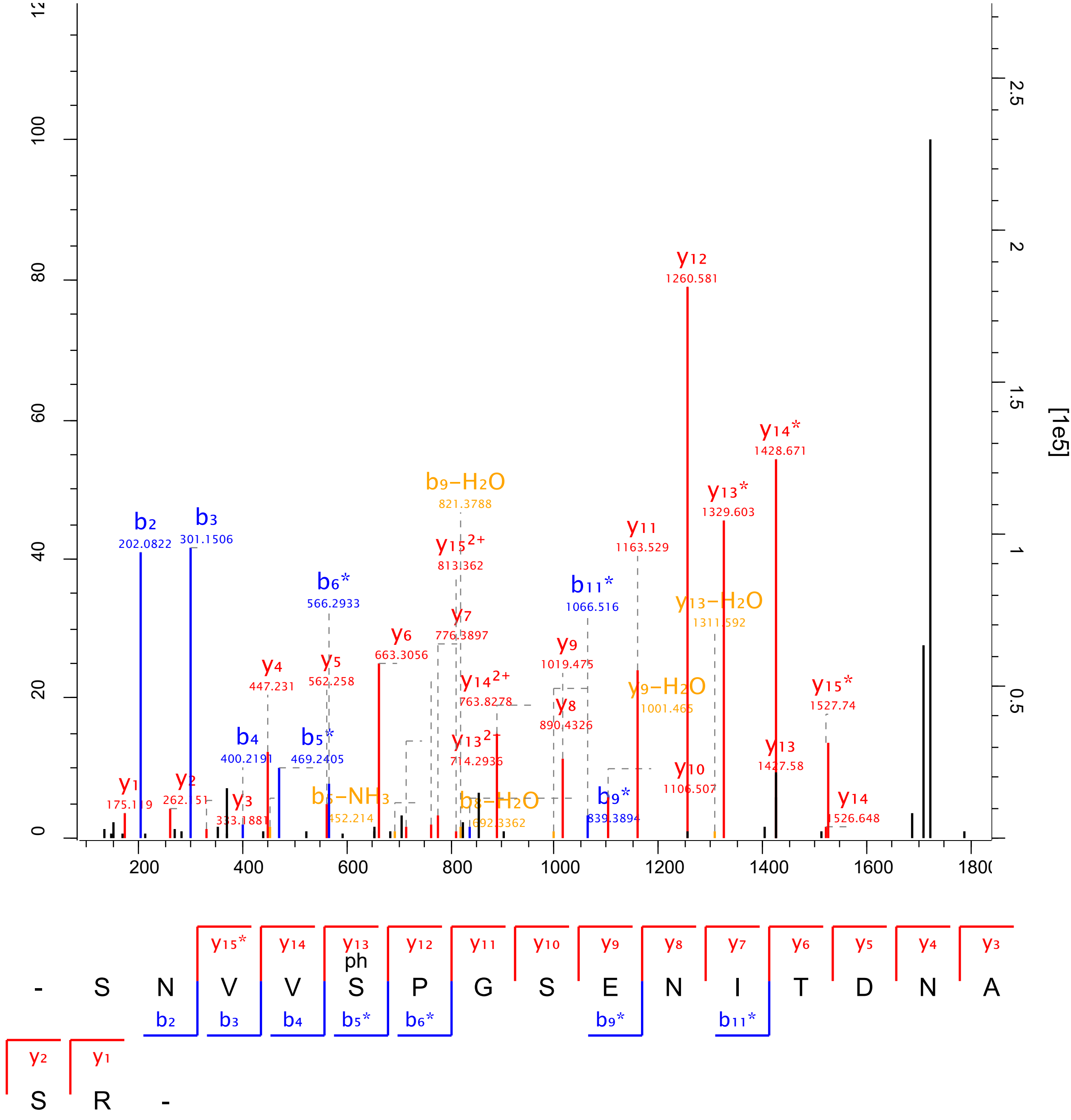

Raw file	Scan	Method	Score	m/z
sys_02_2	17287	FTMS; HCD	260.97	865.85

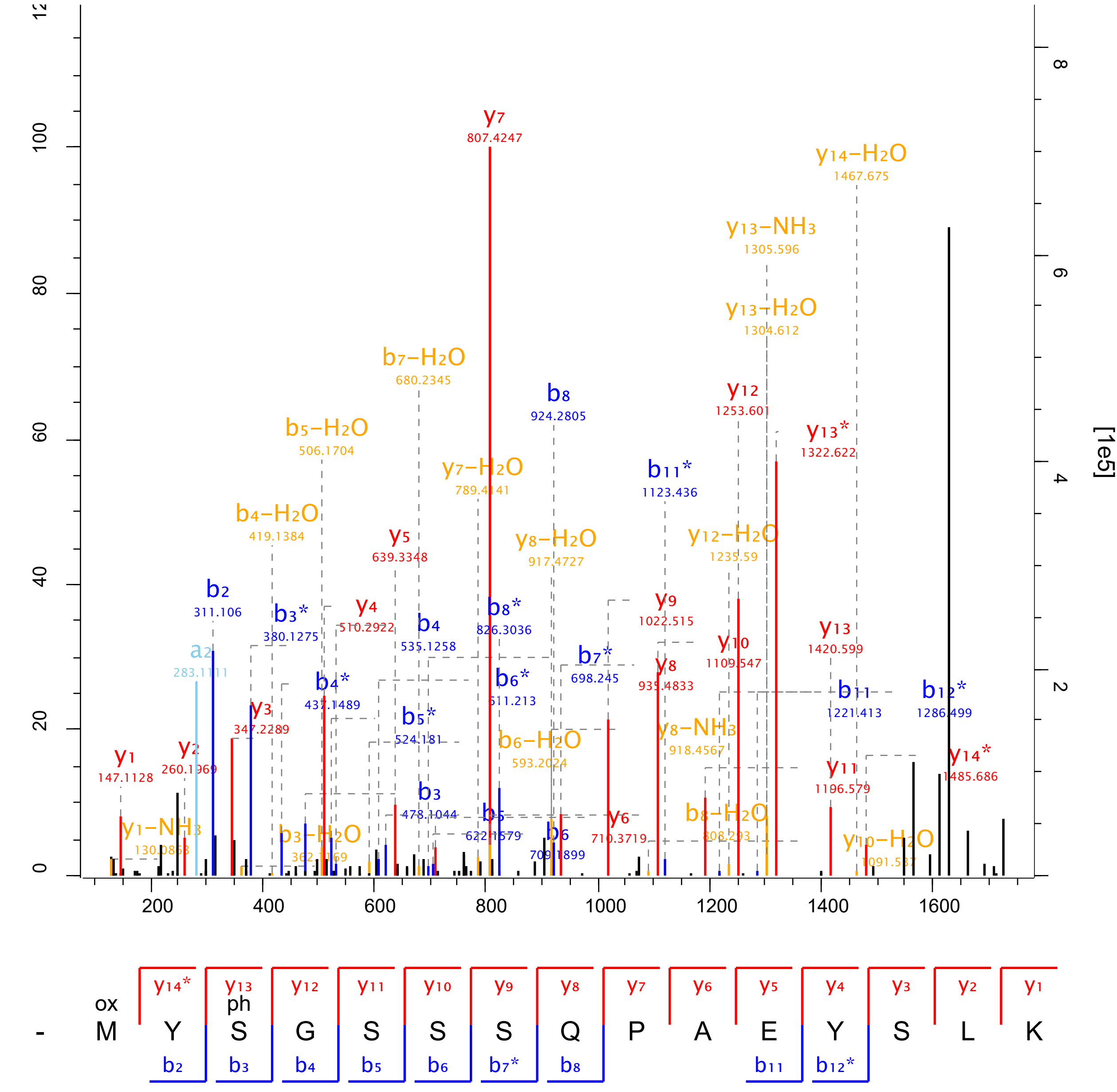

Raw file	Scan	Method	Score	m/z
sys_02_2	17409	FTMS; HCD	42.52	631.83

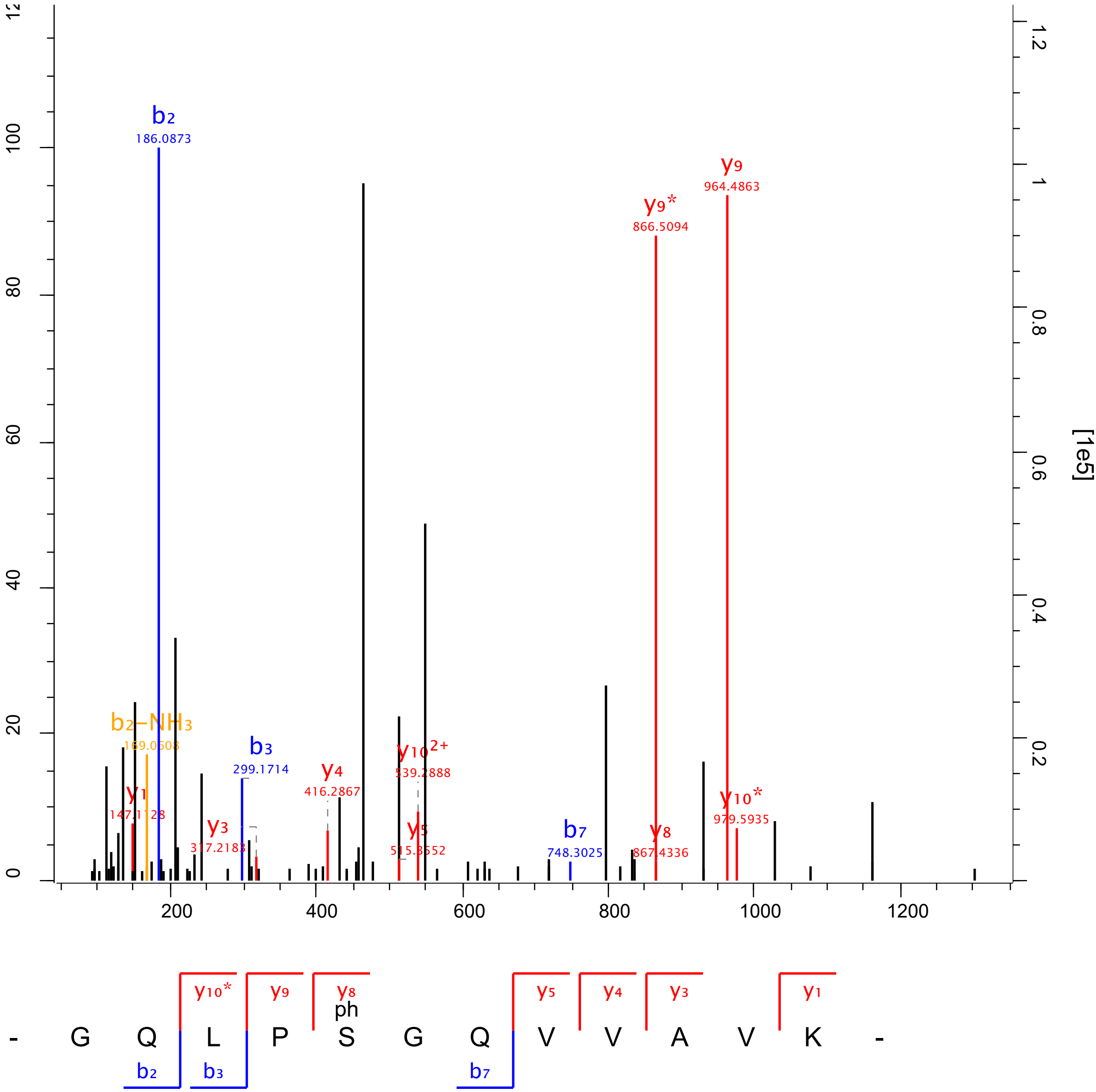

Raw file	Scan	Method	Score	m/z
sys_02_2	17513	FTMS; HCD	171.08	607.78

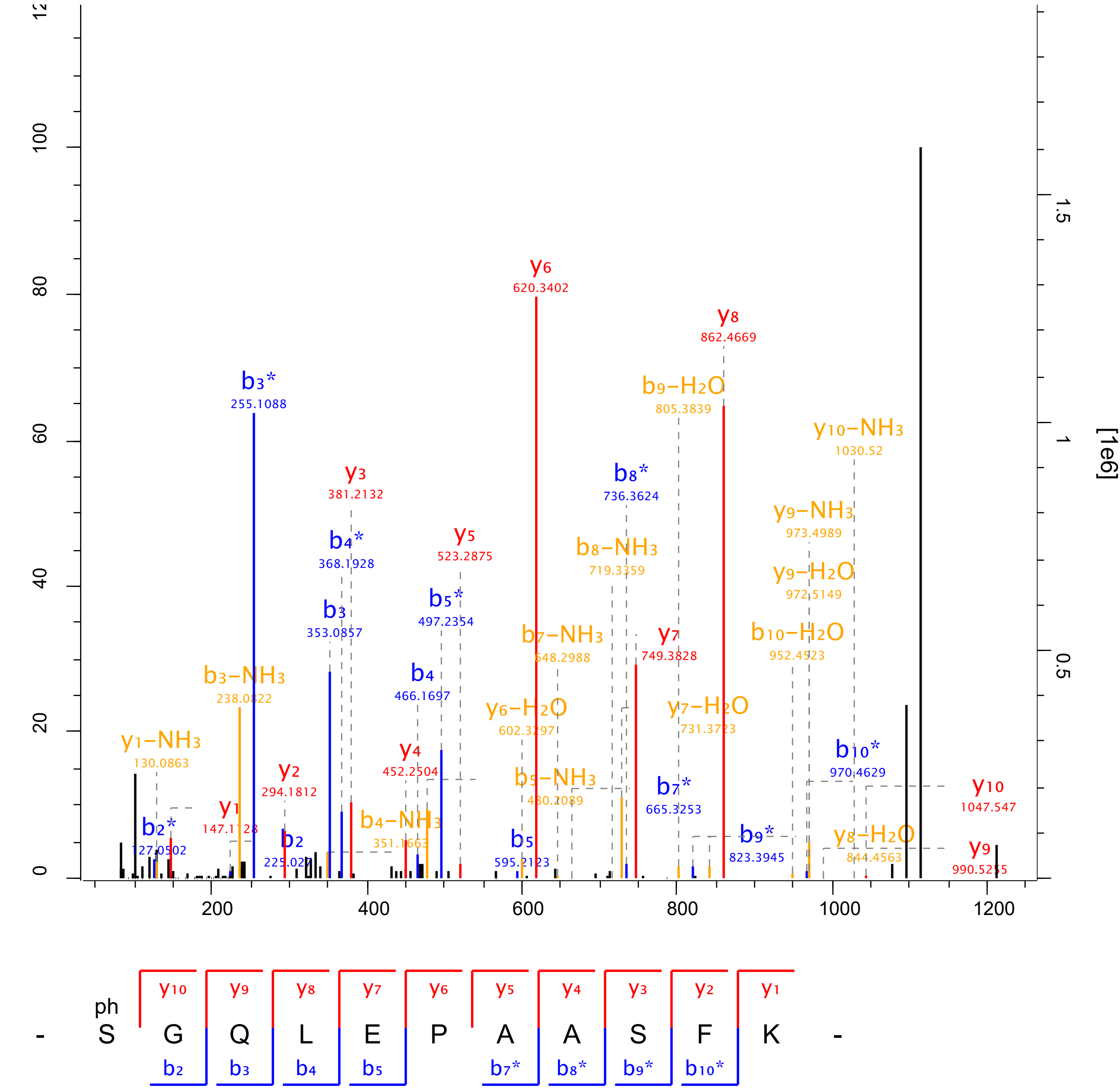

Raw file	Scan	Method	Score	m/z
sys_02_2	17756	FTMS; HCD	318.08	923.42

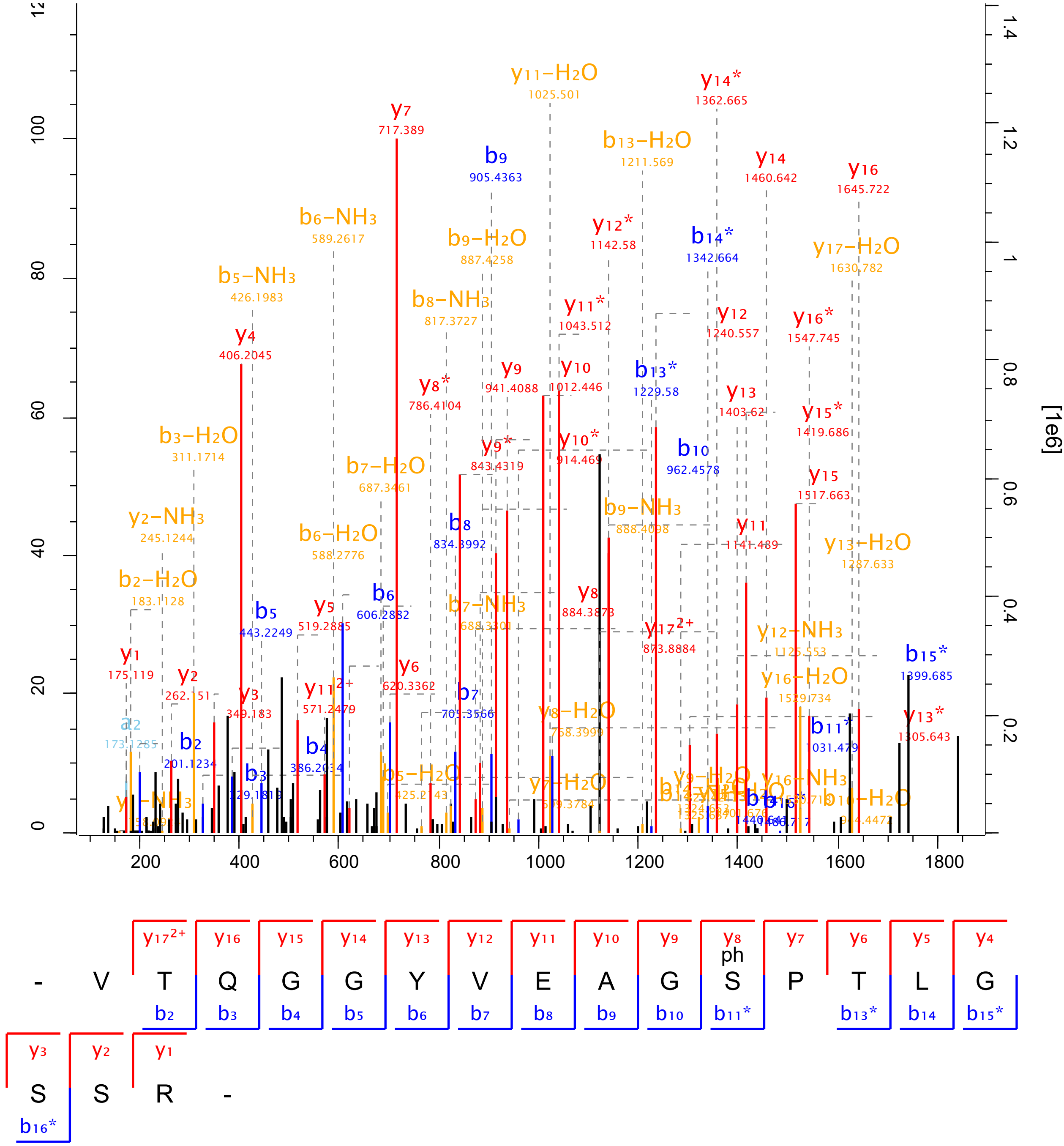

Raw file	Scan	Method	Score	m/z
sys_02_2	18161	FTMS; HCD	45.35	799.38

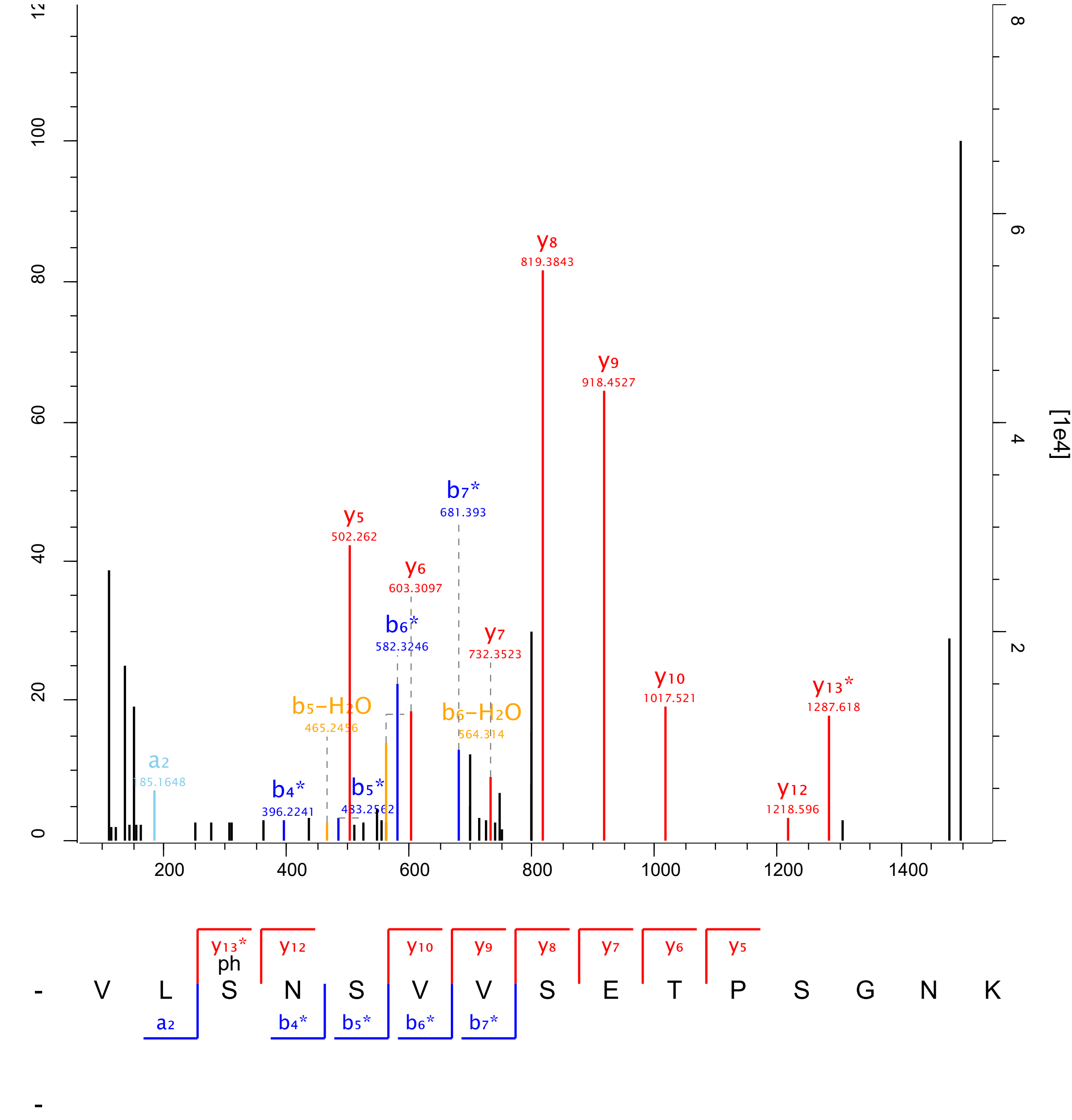

Raw file	Scan	Method	Score	m/z
sys_02_2	18166	FTMS; HCD	97.9	528.6

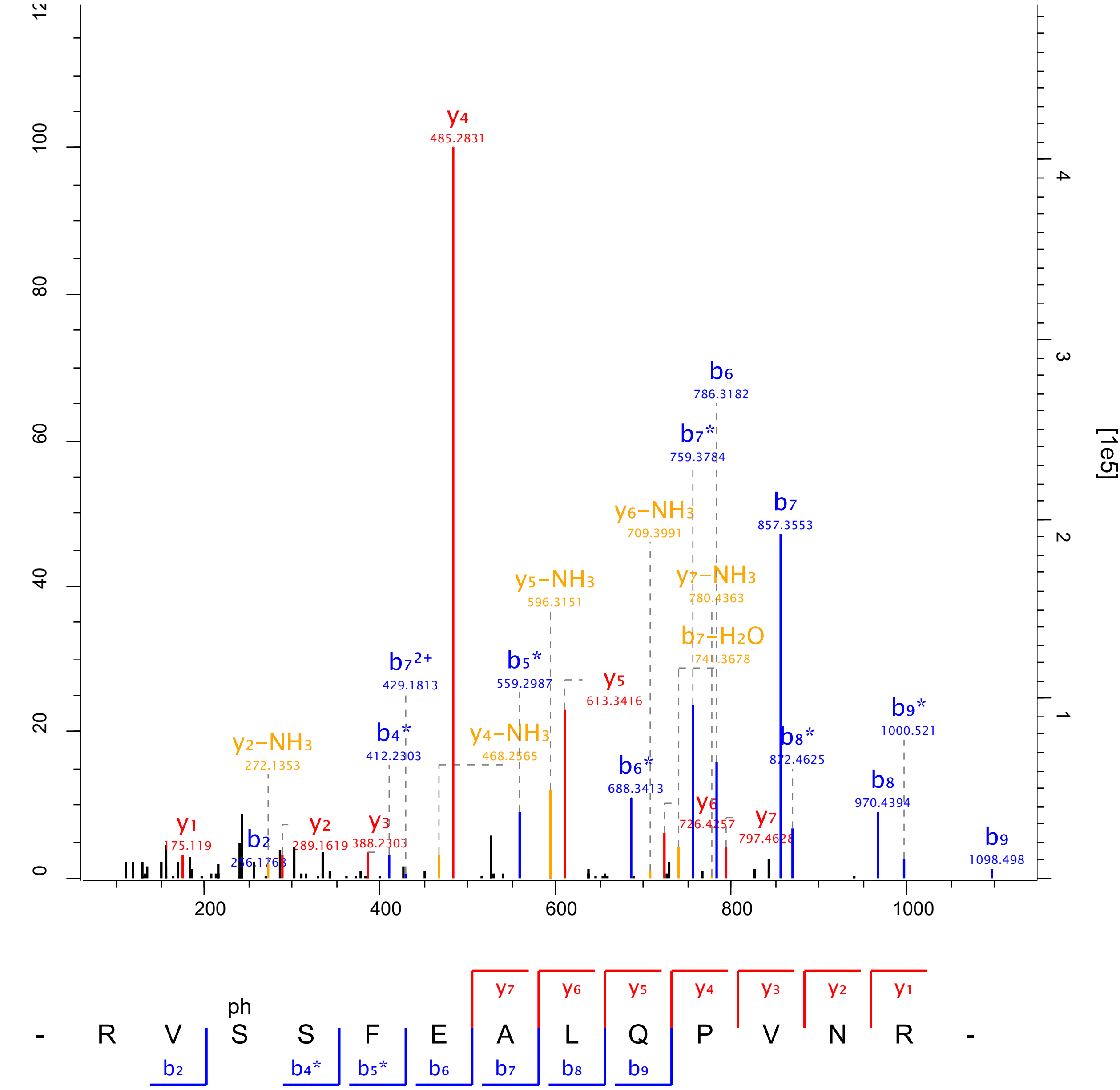

Raw file	Scan	Method	Score	m/z
sys_02_2	18195	FTMS; HCD	218.98	927.35

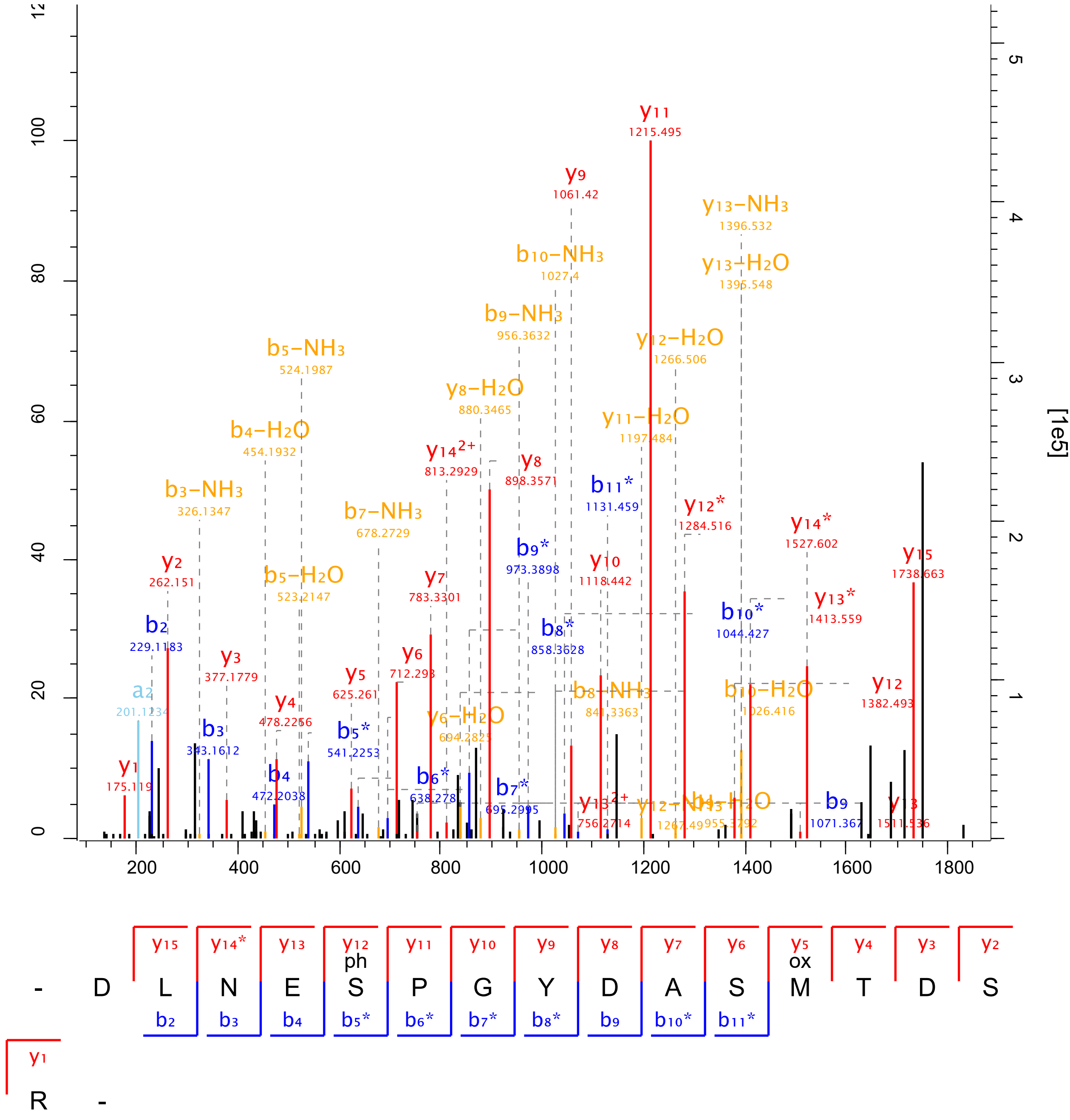

Raw file	Scan	Method	Score	m/z
sys_02_2	18388	FTMS; HCD	60.52	685.32

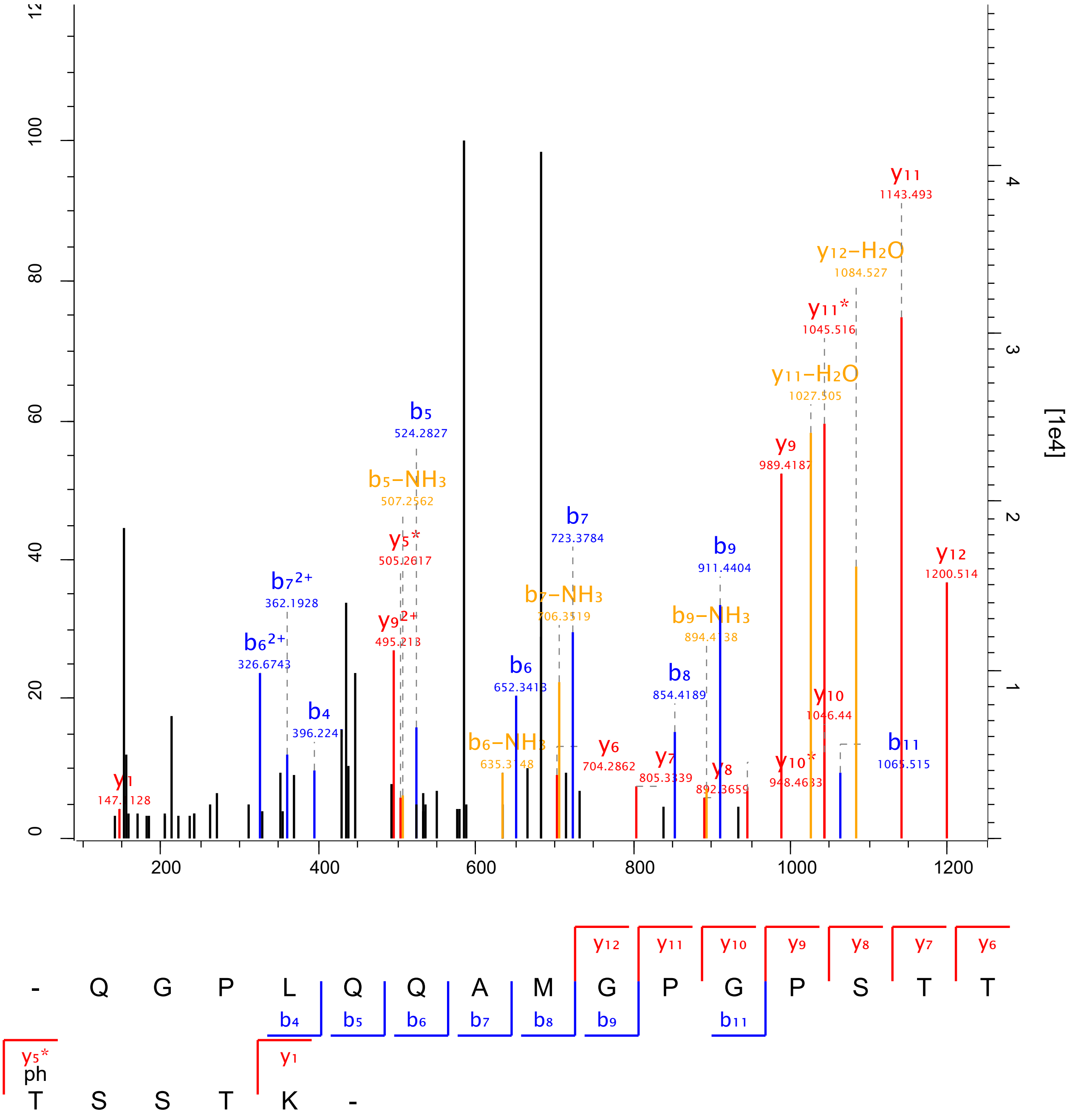

Raw file	Scan	Method	Score	m/z
sys_02_2	18424	FTMS; HCD	124.08	585.6

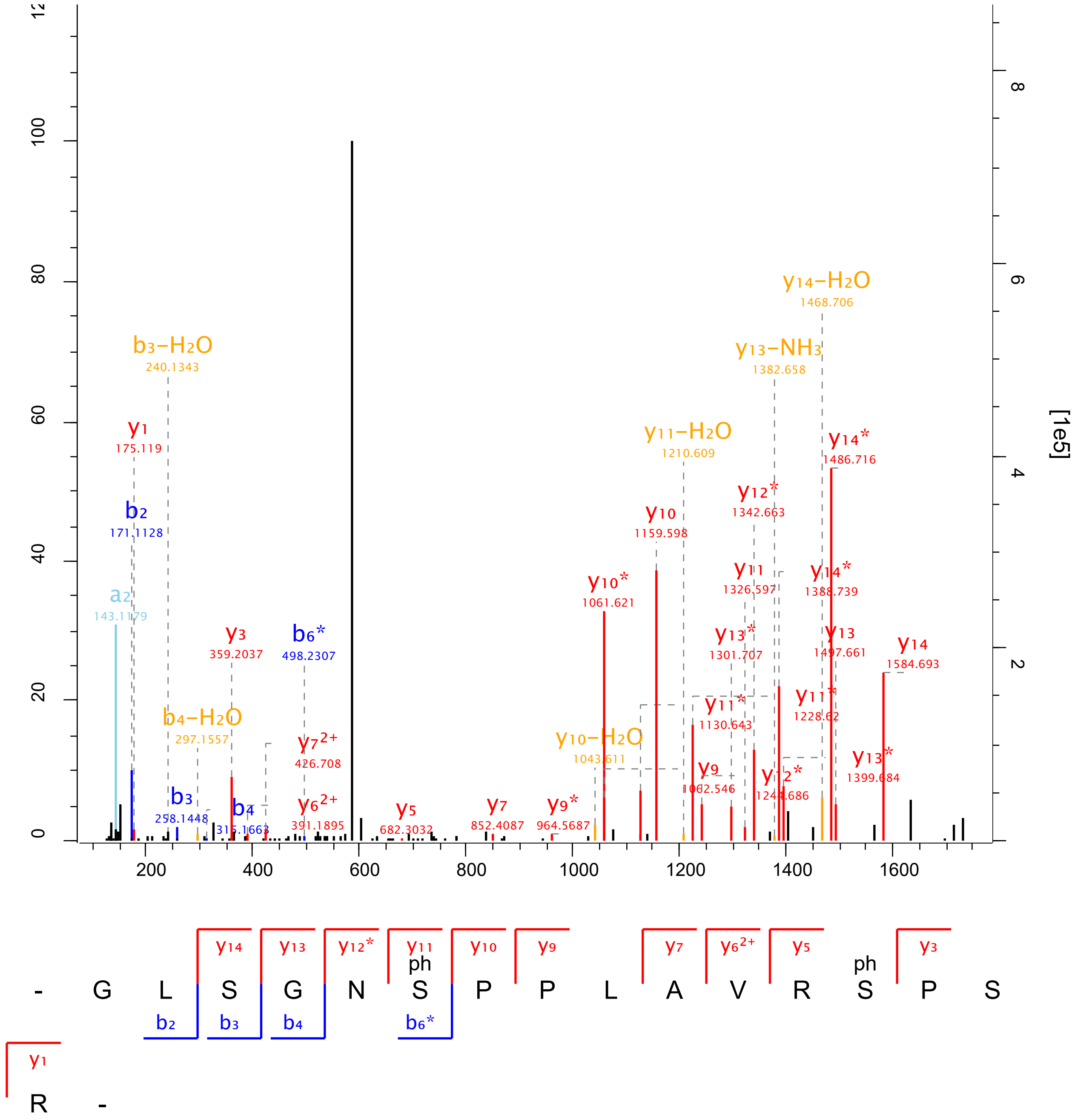

Raw file	Scan	Method	Score	m/z
sys_02_2	18544	FTMS; HCD	140.53	605.25

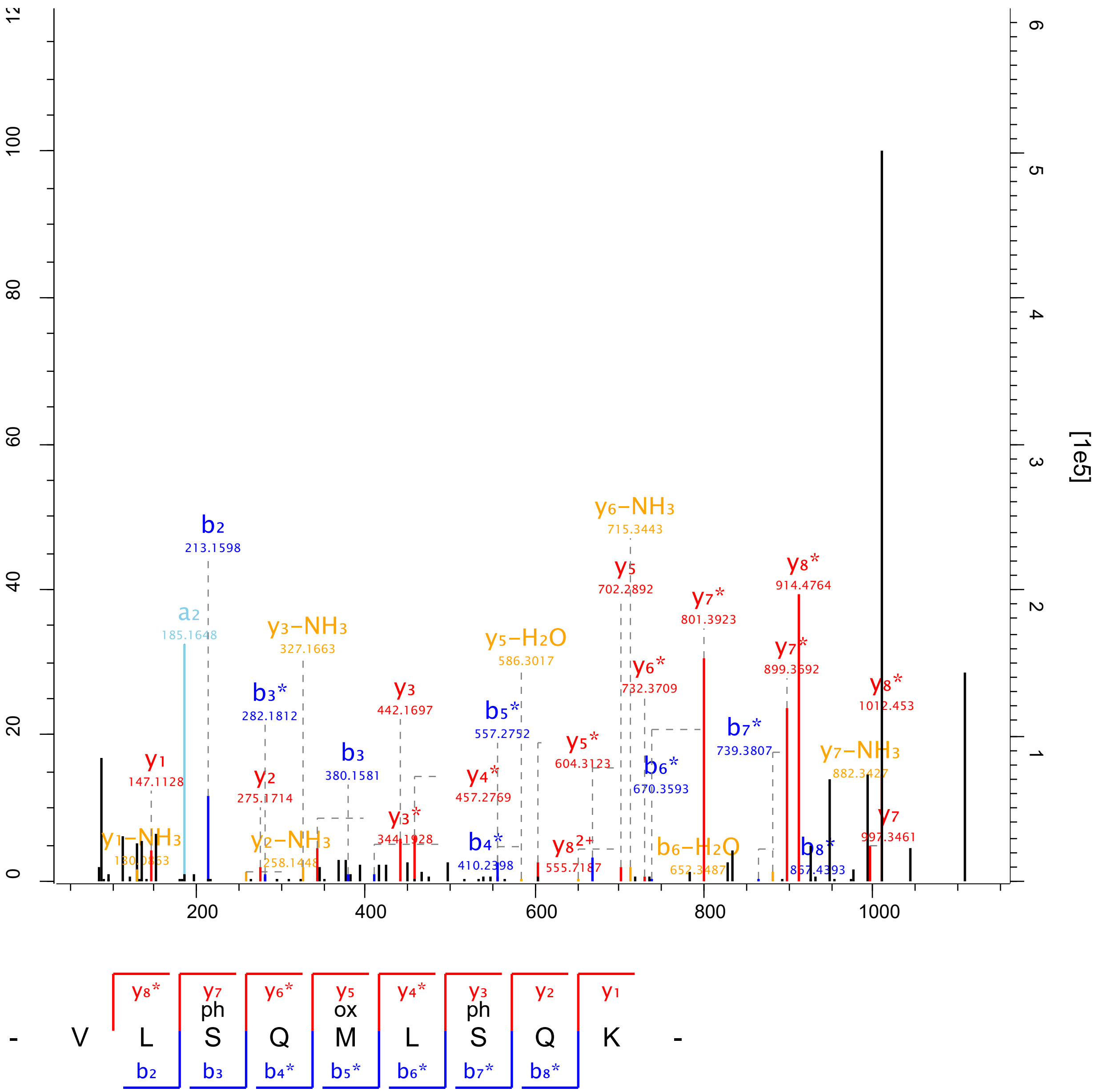

Raw file	Scan	Method	Score	m/z
sys_02_2	18579	FTMS; HCD	56.29	593.75

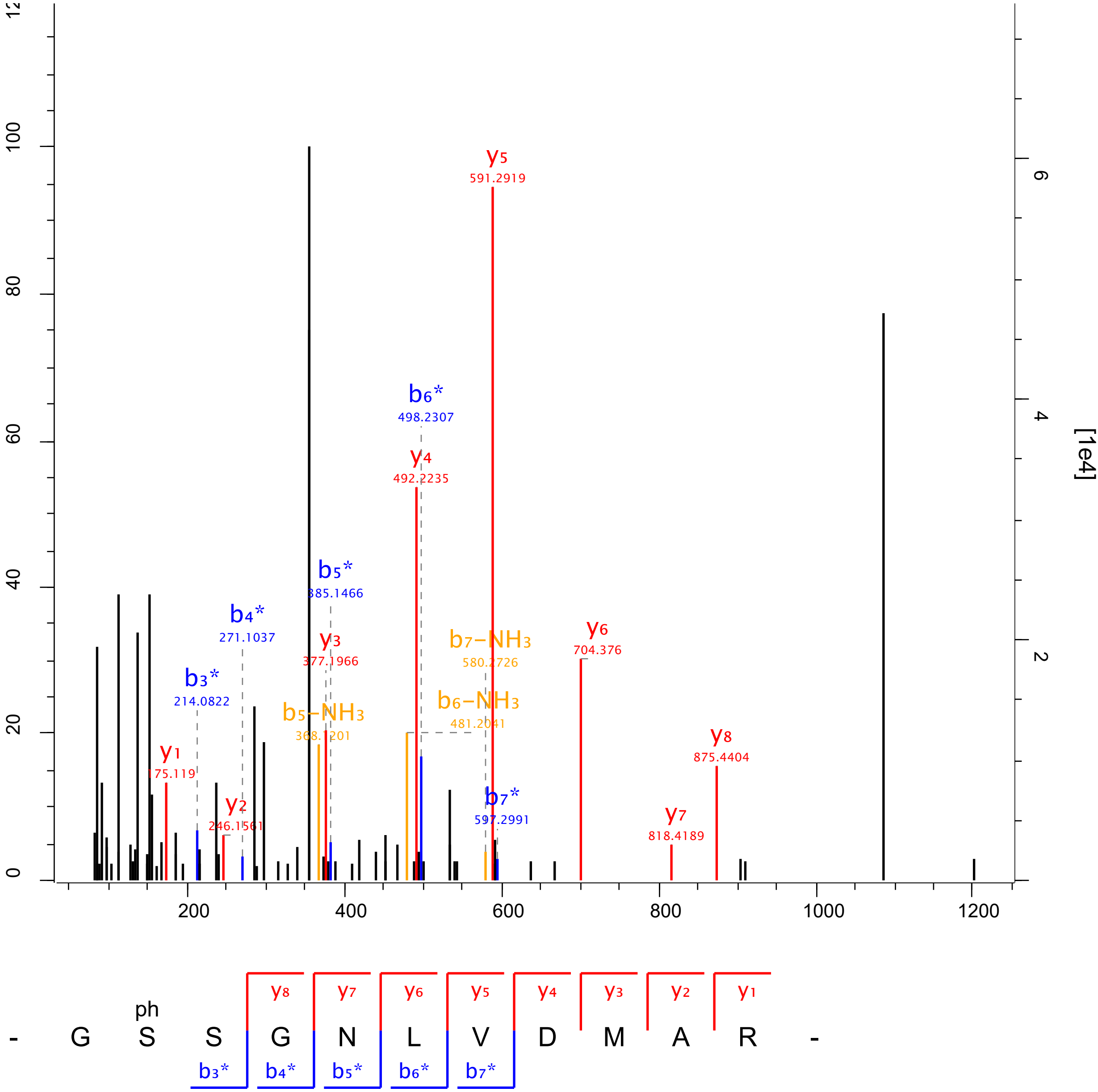

Raw file	Scan	Method	Score	m/z
sys_02_2	18807	FTMS; HCD	128.54	567.76

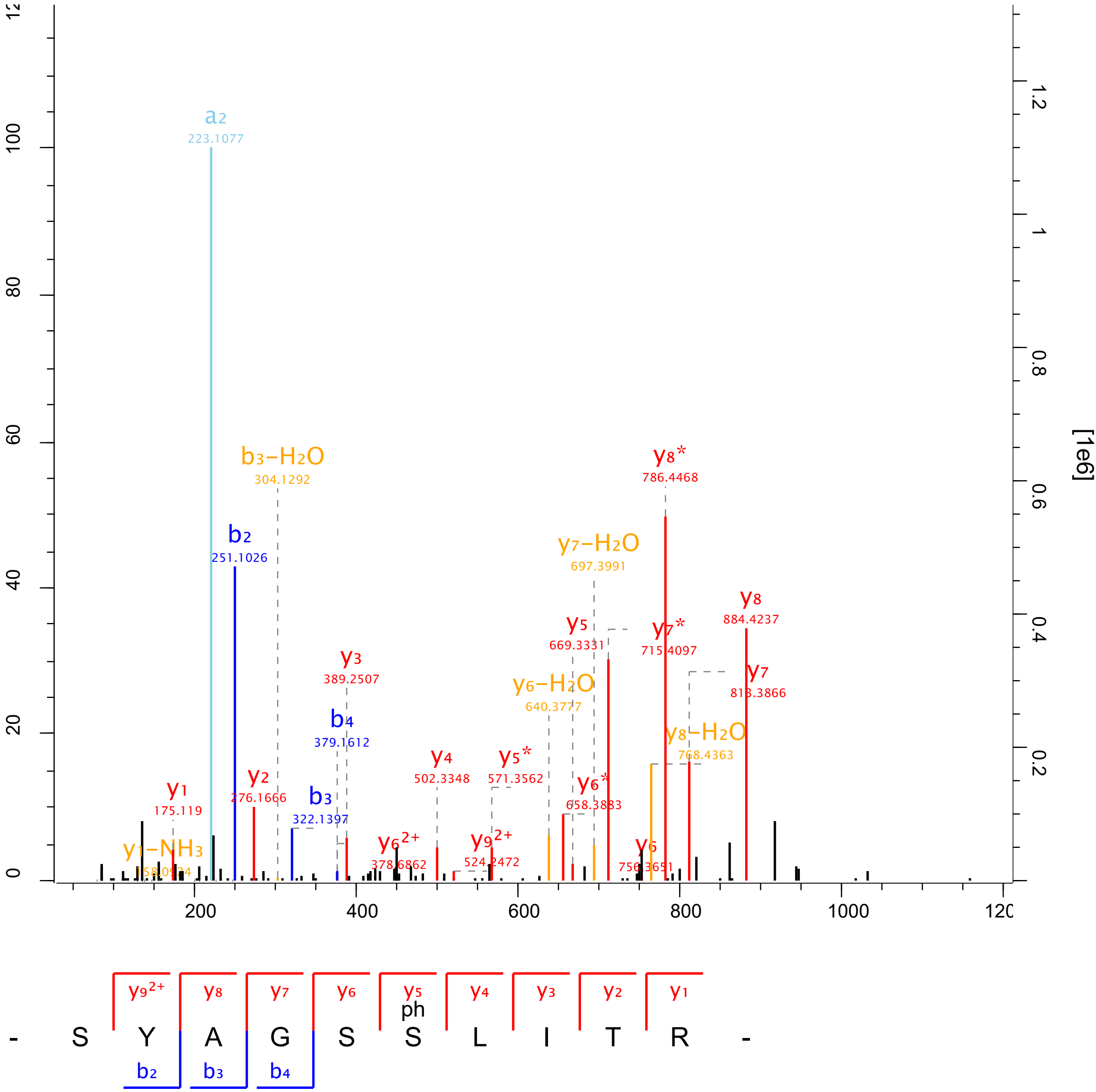

Raw file	Scan	Method	Score	m/z
sys_02_2	1884	FTMS; HCD	42.96	615.27

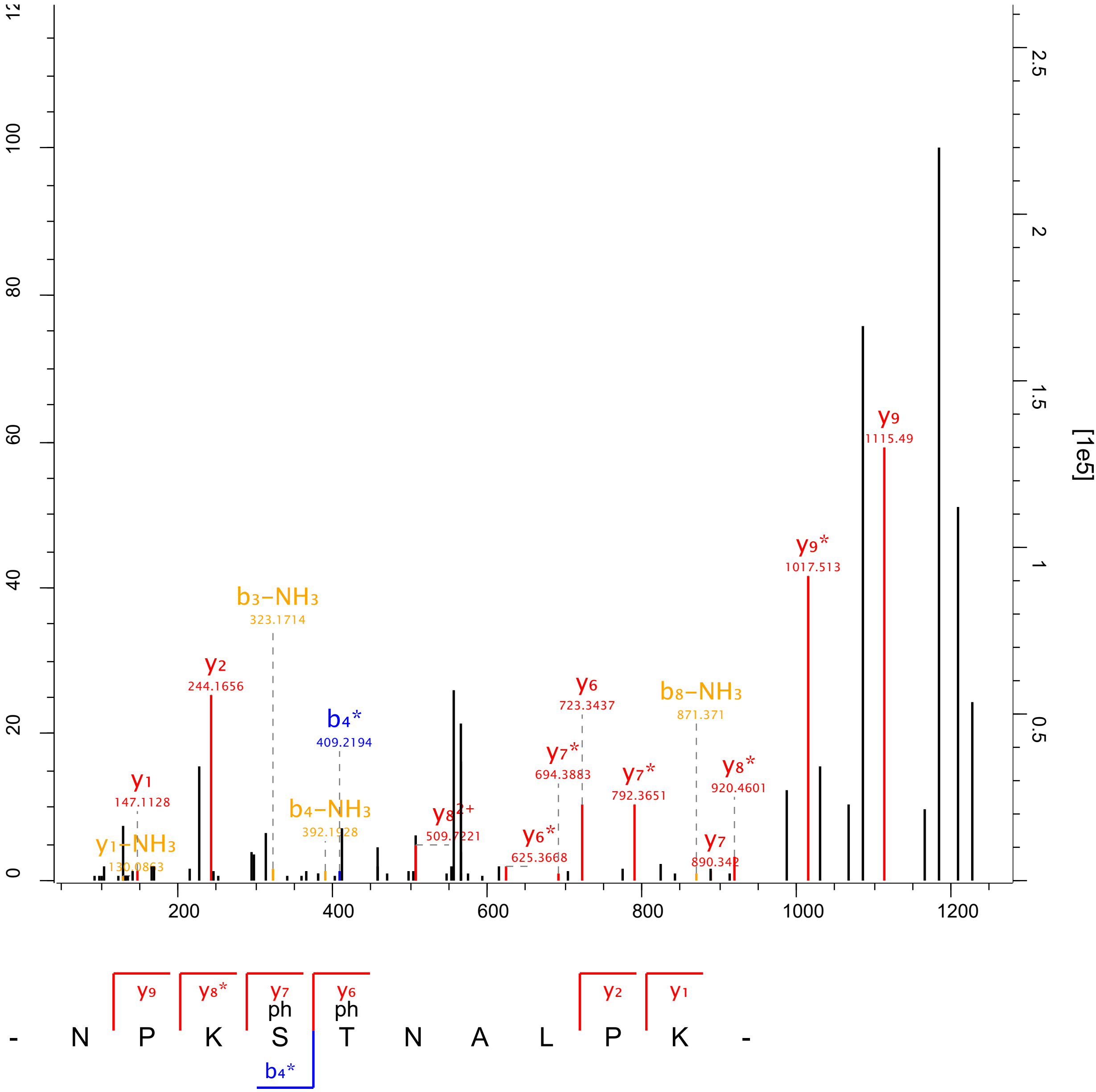

Raw file	Scan	Method	Score	m/z
sys_02_2	1898	FTMS; HCD	125.47	427.53

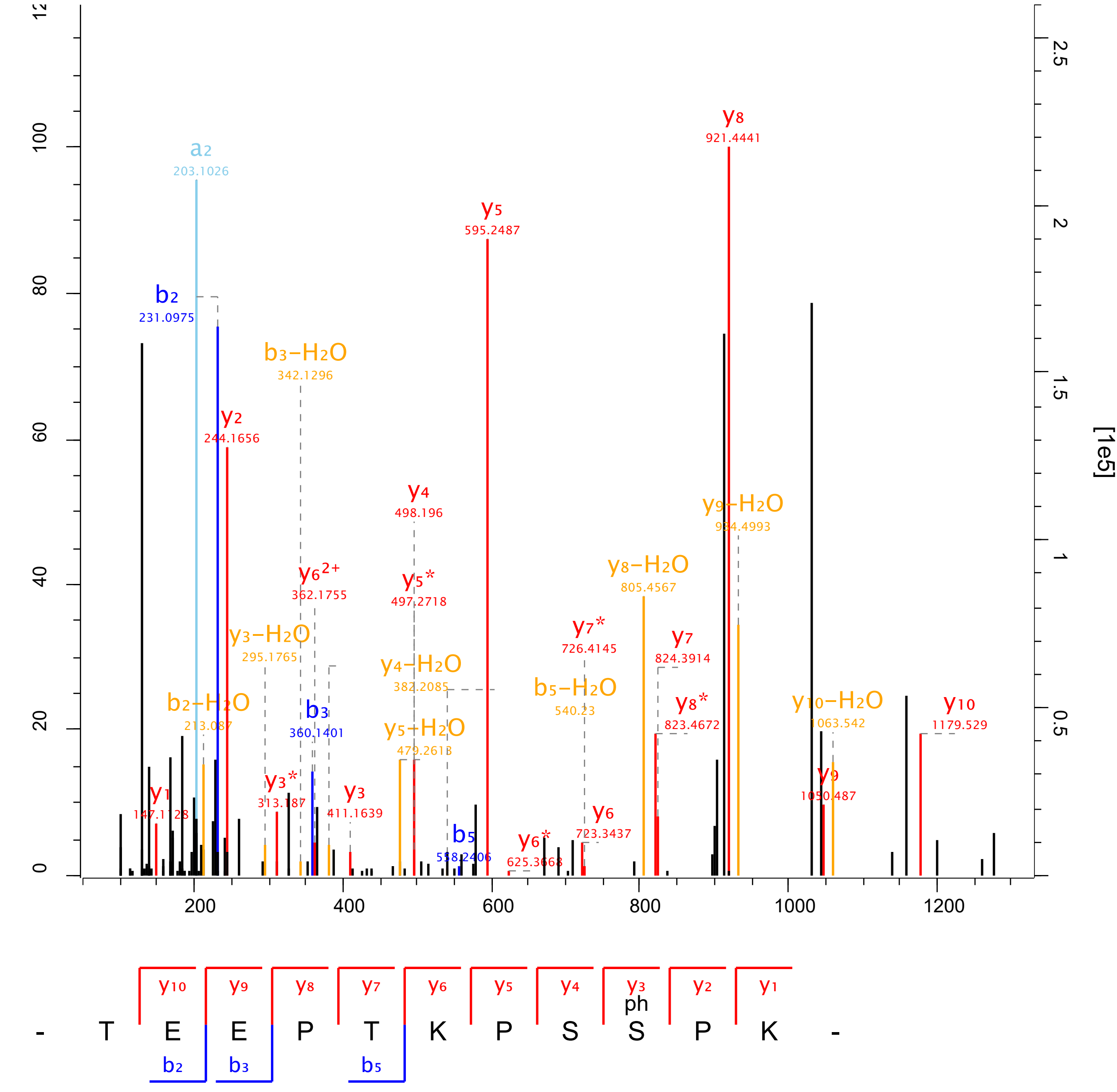

Raw file	Scan	Method	Score	m/z
sys_02_2	19480	FTMS; HCD	155.8	574.77

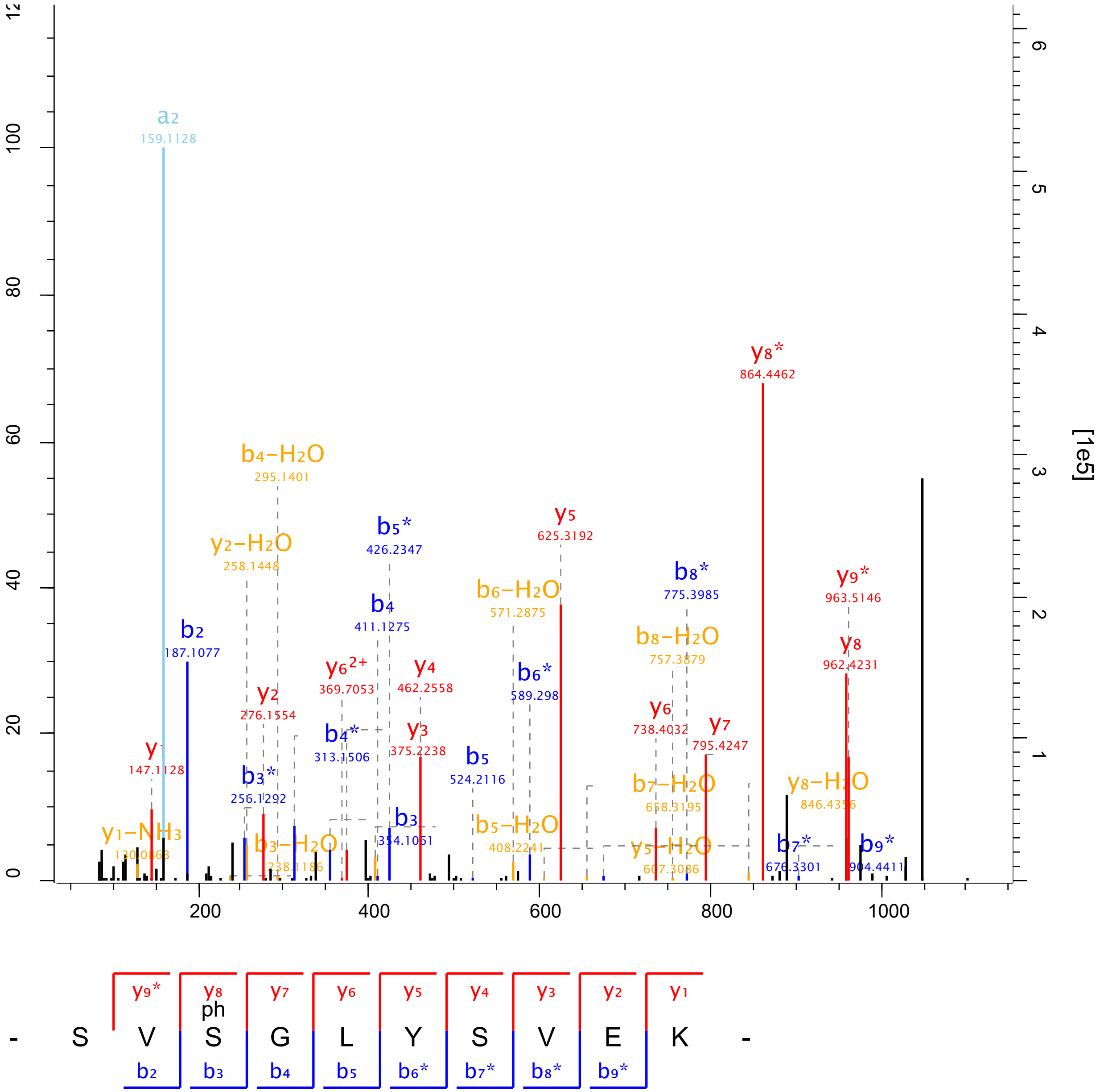

Raw file	Scan	Method	Score	m/z
sys_02_2	19756	FTMS; HCD	47.51	698.79

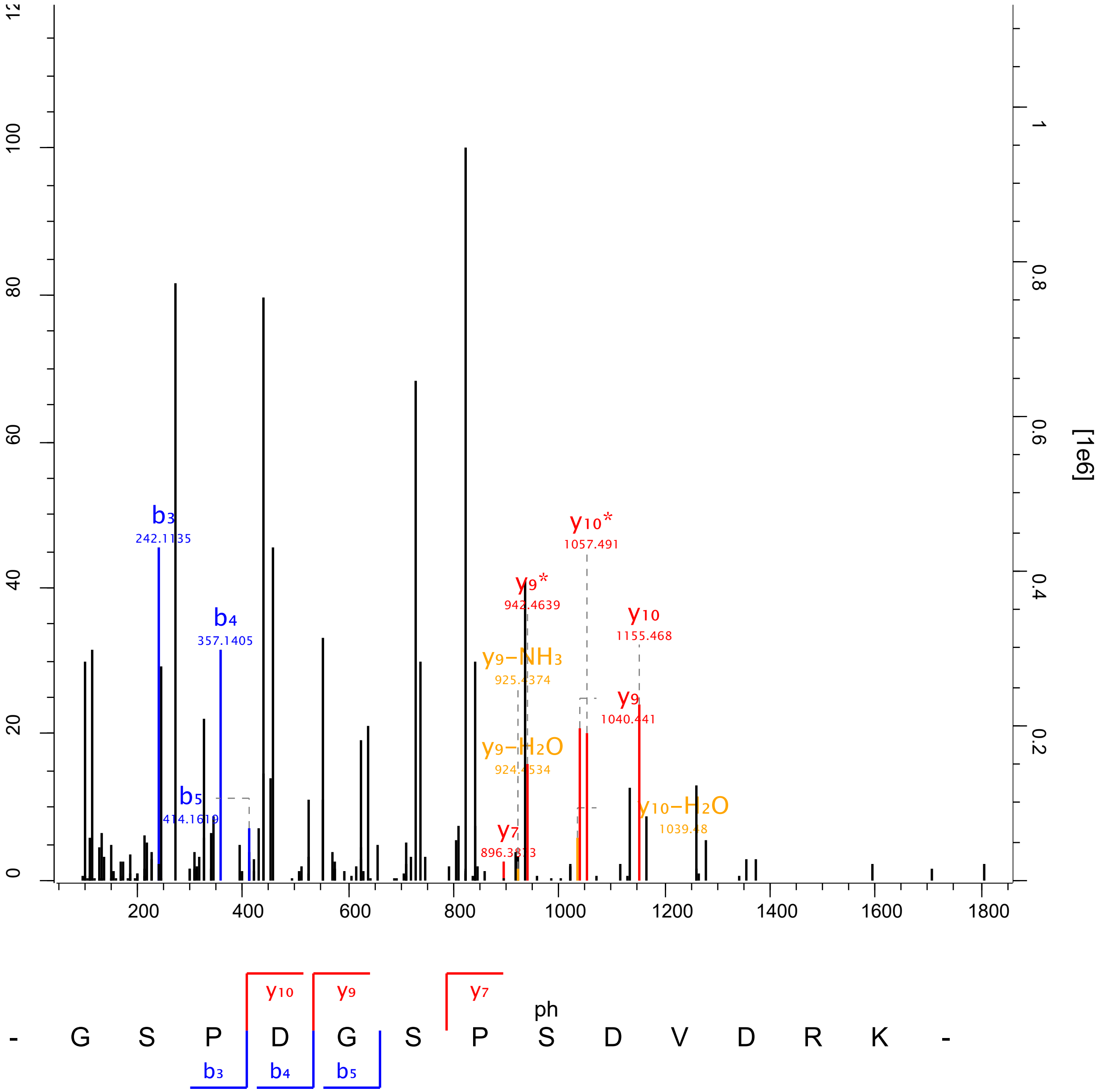

Raw file	Scan	Method	Score	m/z
sys_02_2	19846	FTMS; HCD	62.34	534.75

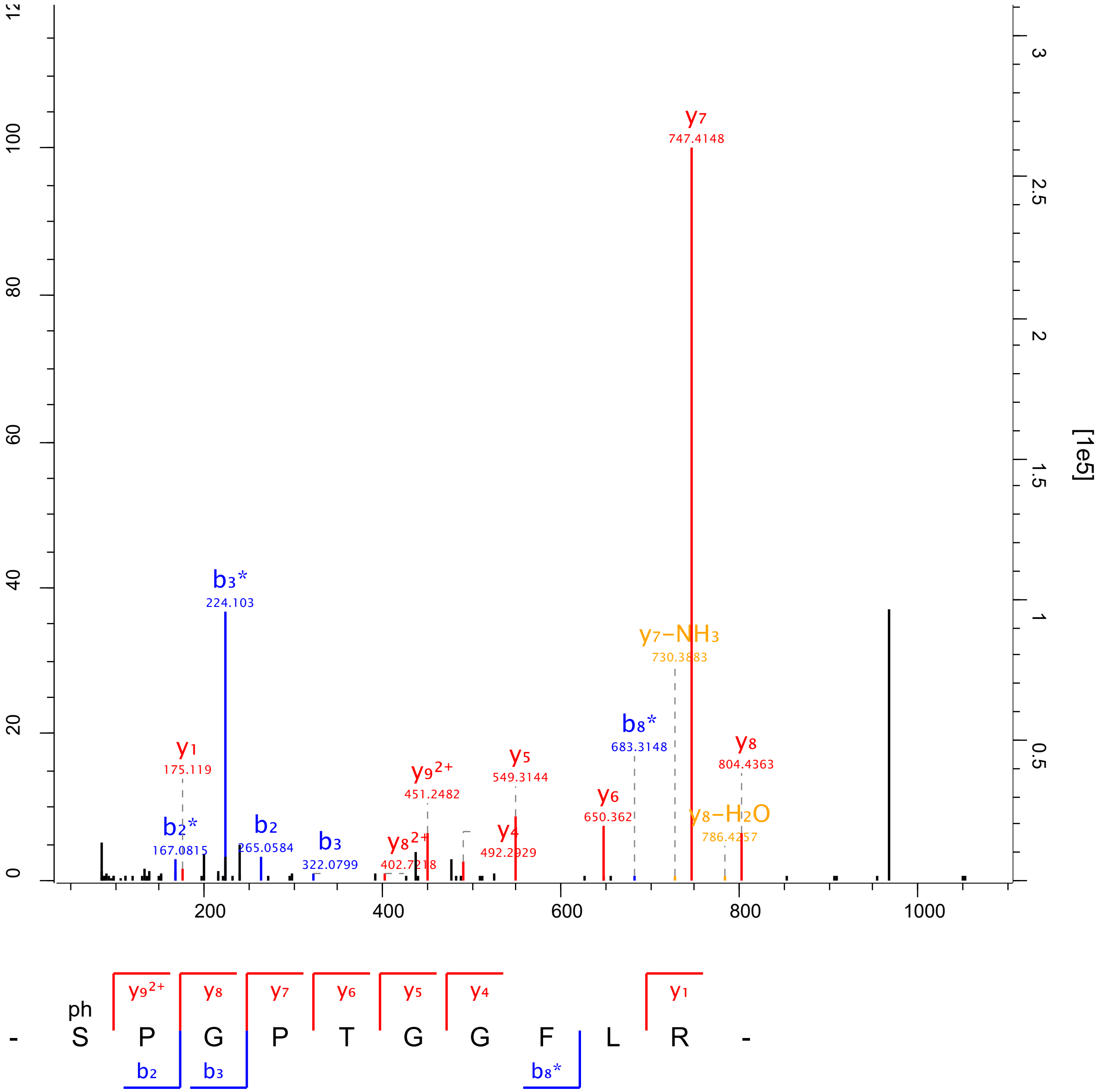

Raw file	Scan	Method	Score	m/z
sys_02_2	20188	FTMS; HCD	72.72	946.42

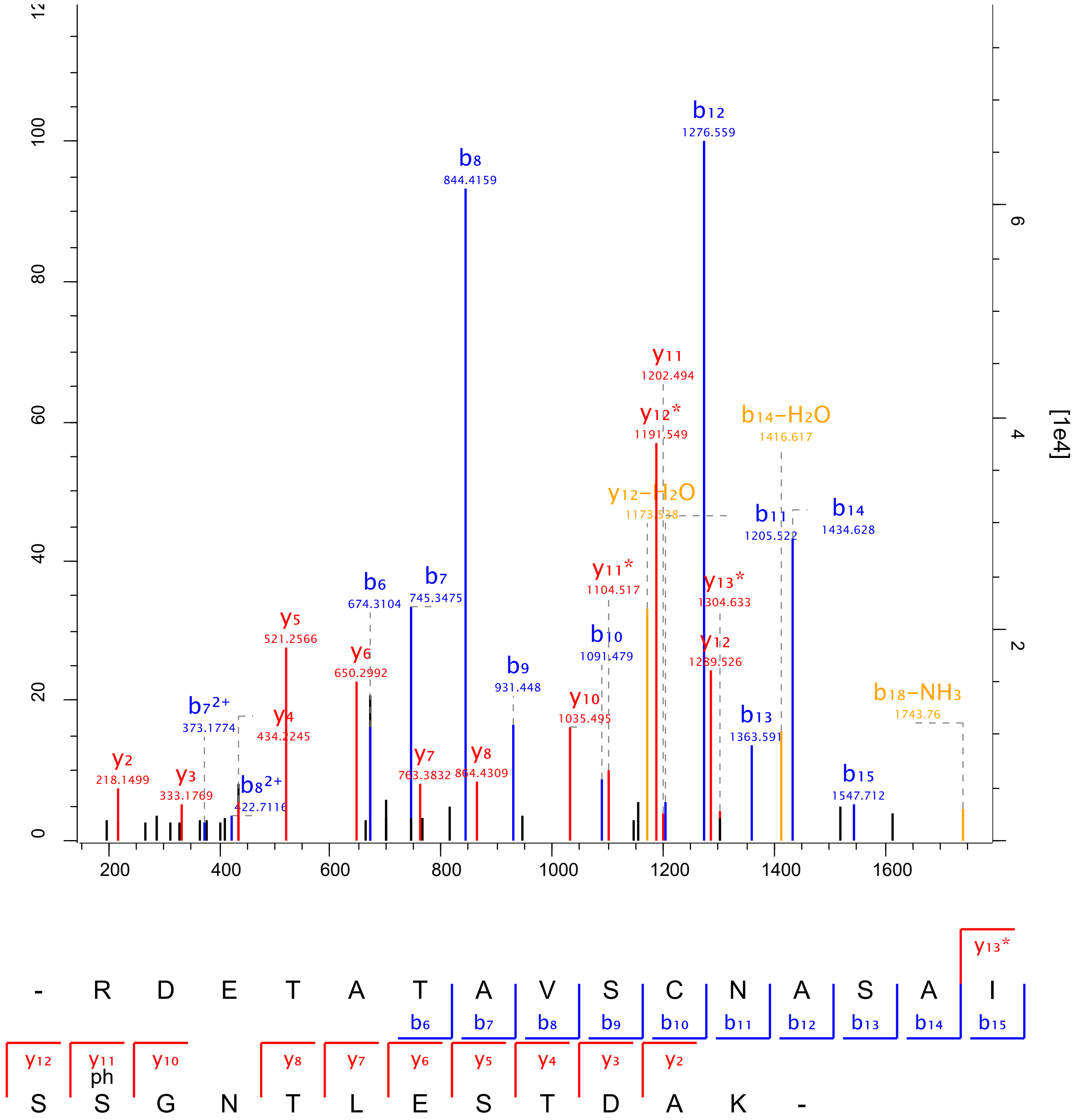

Raw file	Scan	Method	Score	m/z
sys_02_2	20583	FTMS; HCD	141.31	862.86

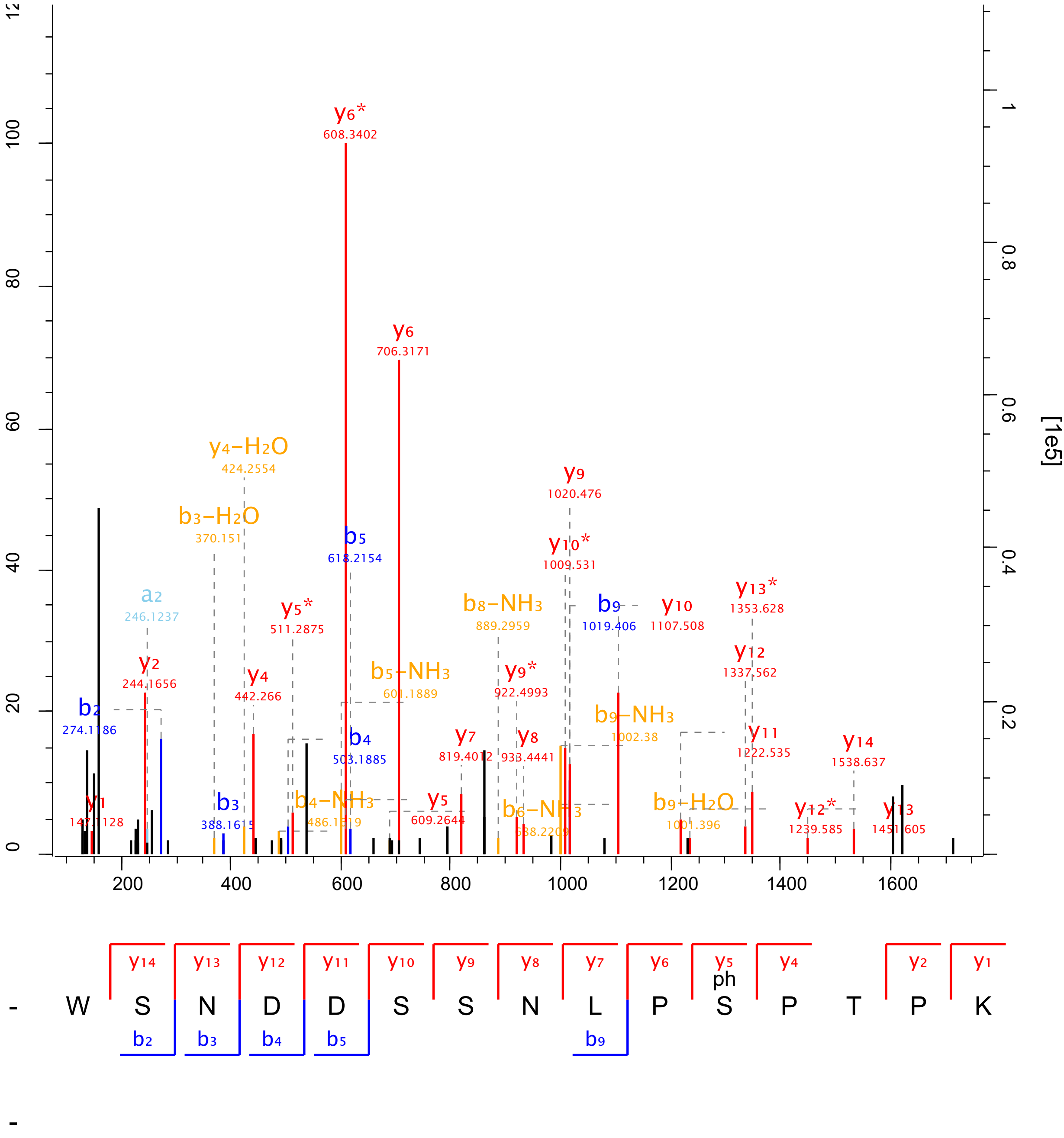

Raw file	Scan	Method	Score	m/z
sys_02_2	2095	FTMS; HCD	45.28	597.78

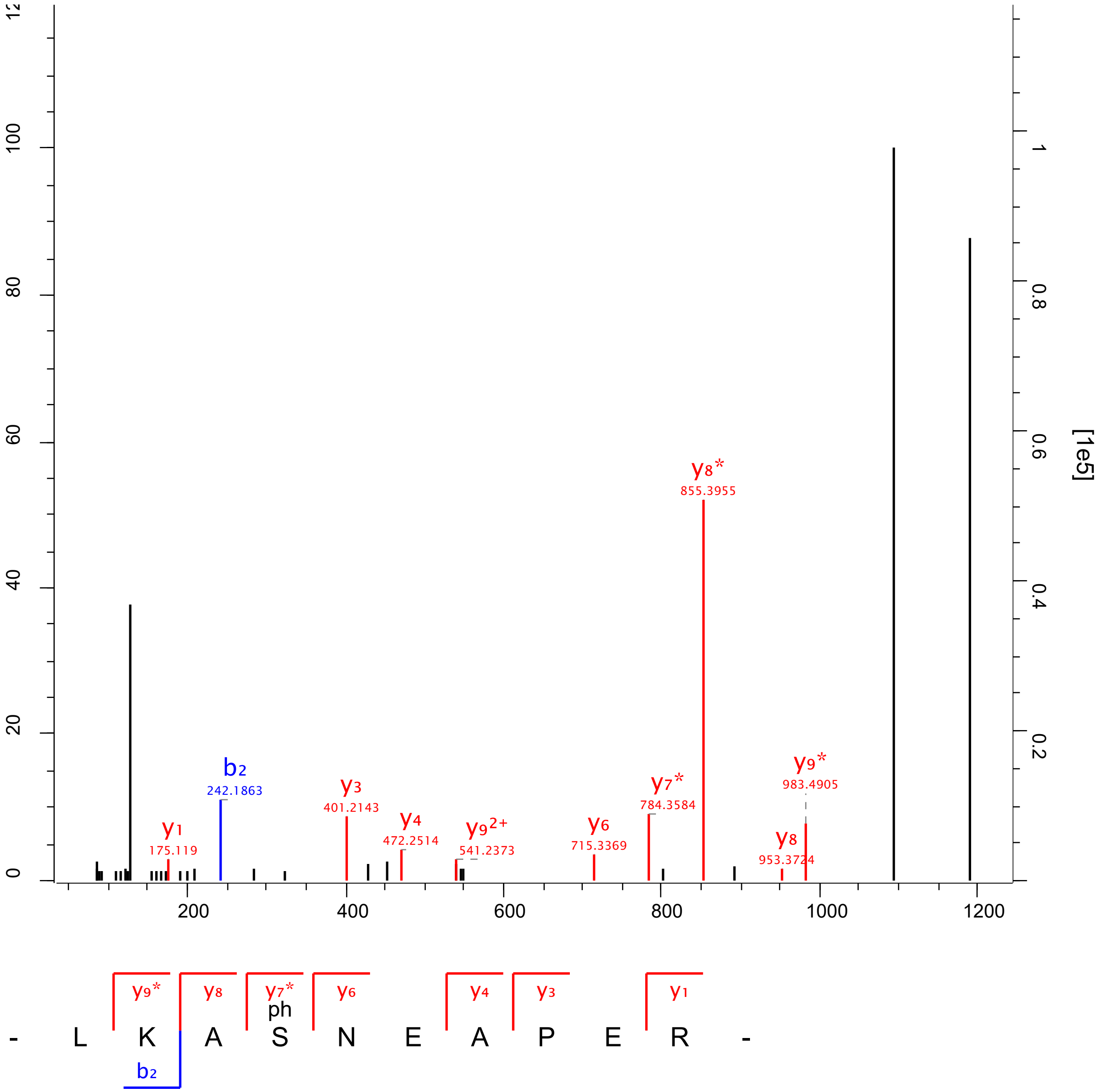

Raw file	Scan	Method	Score	m/z
sys_02_2	21265	FTMS; HCD	44.93	637.62

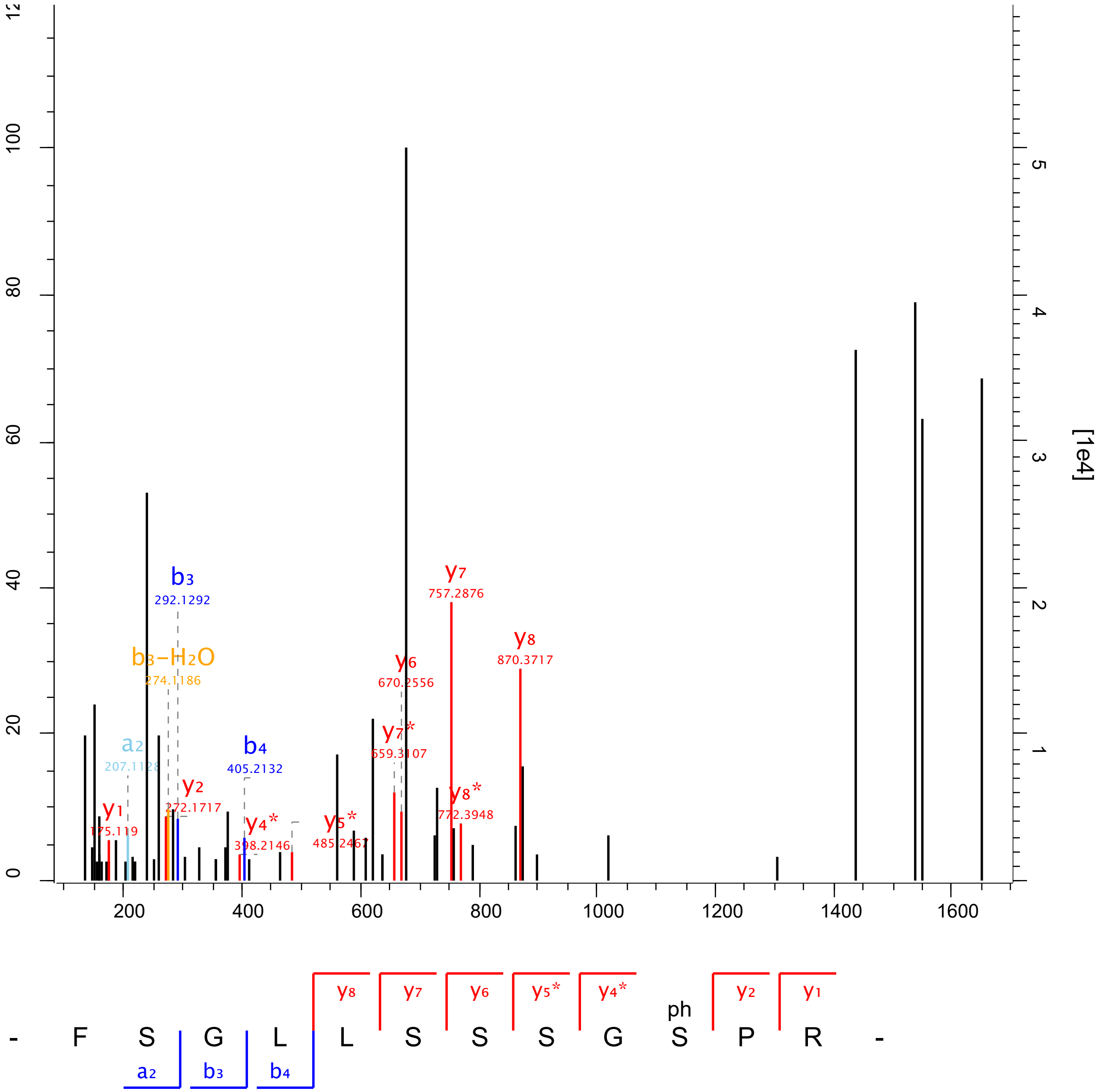

Raw file	Scan	Method	Score	m/z
sys_02_2	2137	FTMS; HCD	128.21	535.27

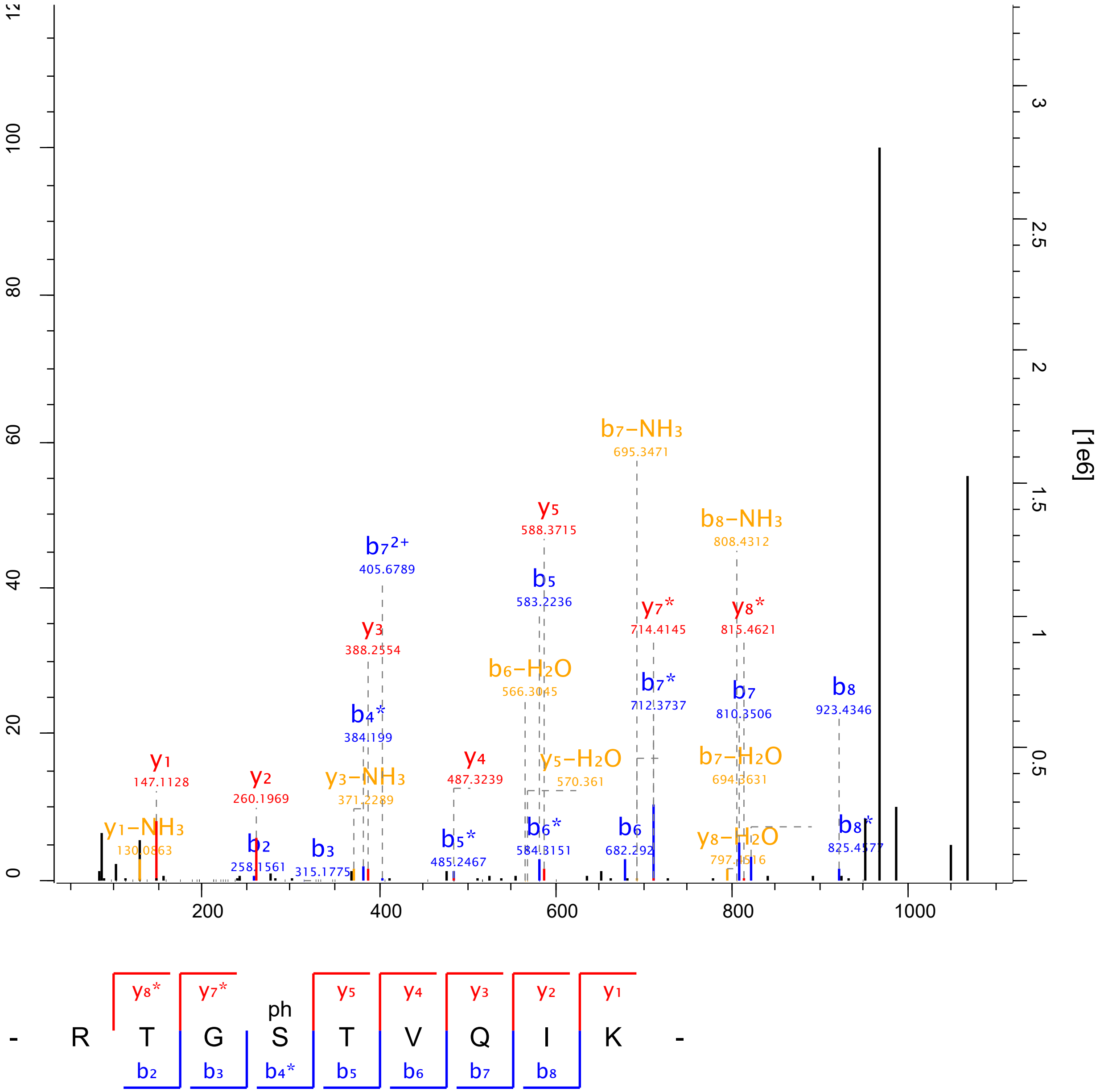

Raw file	Scan	Method	Score	m/z
sys_02_2	2159	FTMS; HCD	40.5	340.84

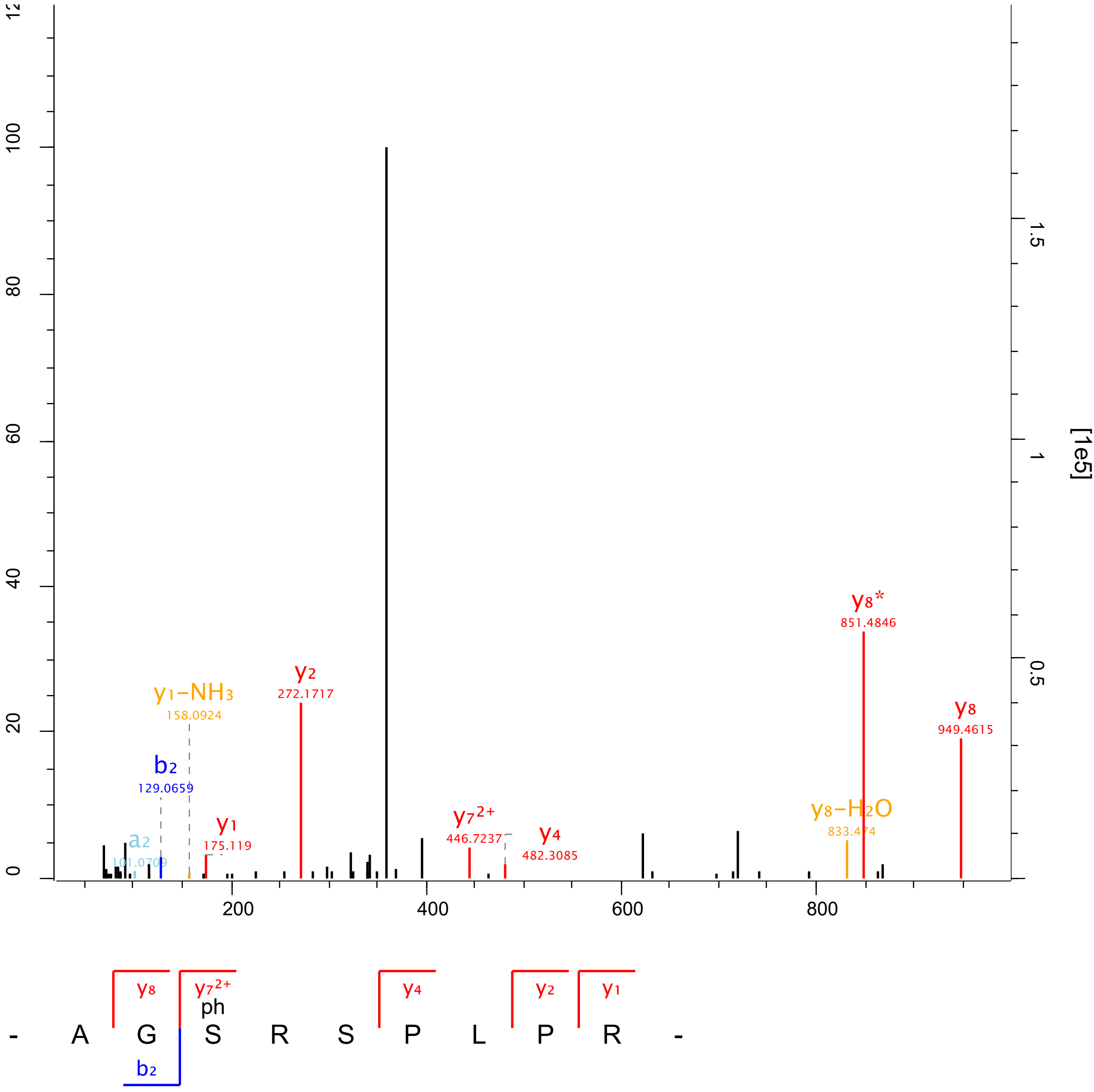

Raw file	Scan	Method	Score	m/z
sys_02_2	21604	FTMS; HCD	108.56	551.75

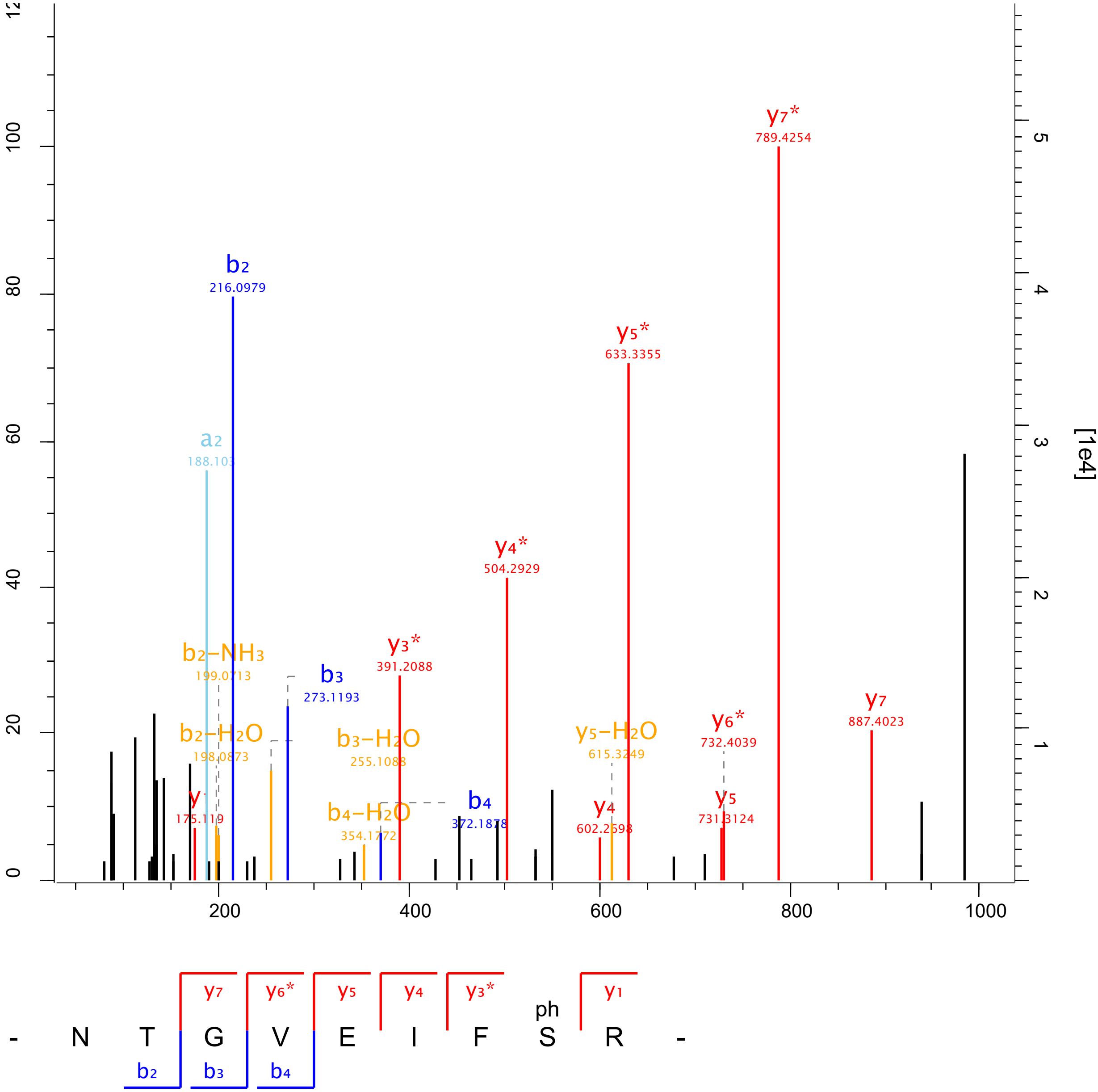

Raw file	Scan	Method	Score	m/z
sys_02_2	21758	FTMS; HCD	54.34	538.76

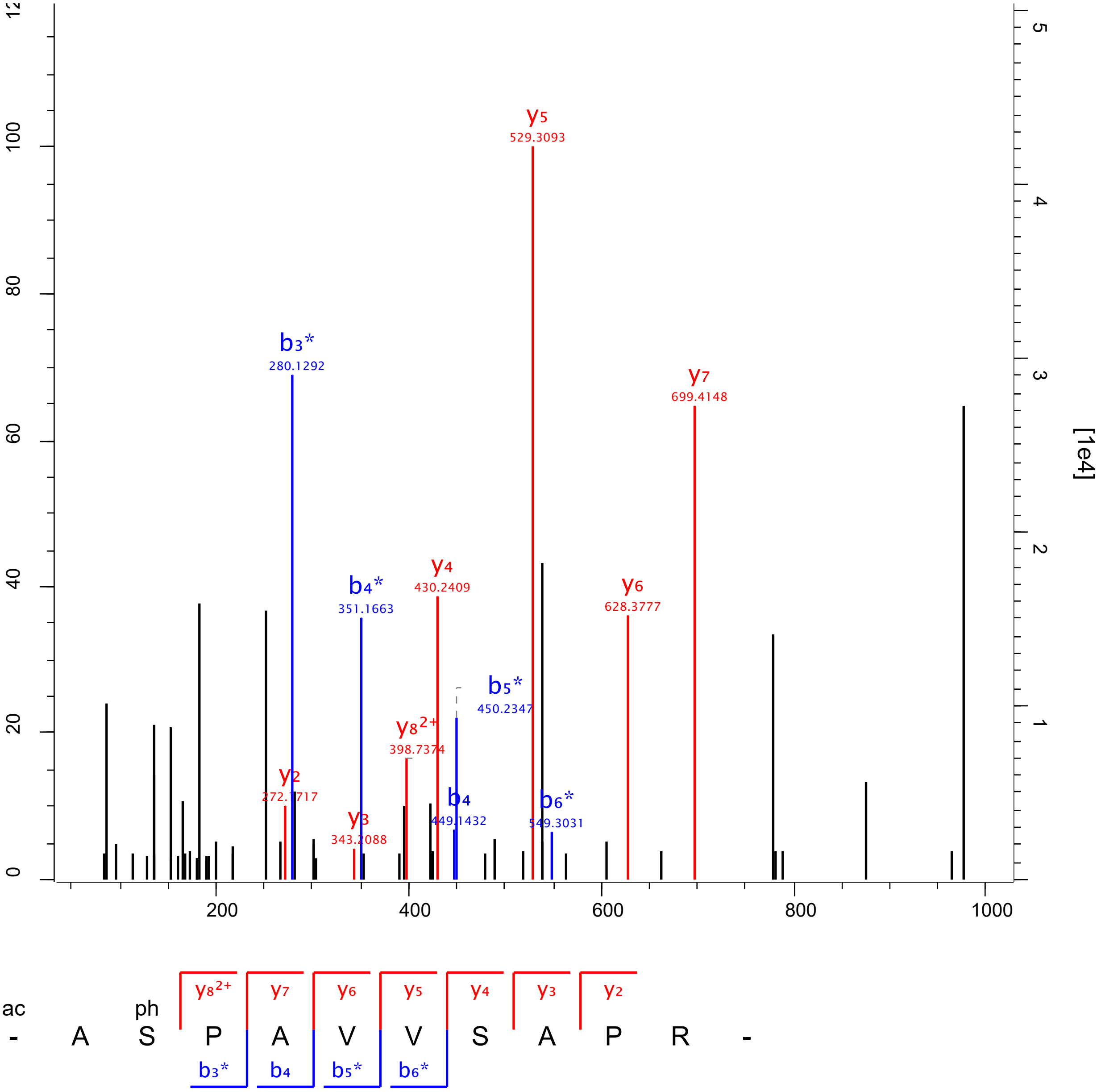

Raw file	Scan	Method	Score	m/z
sys_02_2	21784	FTMS; HCD	54.22	850.4

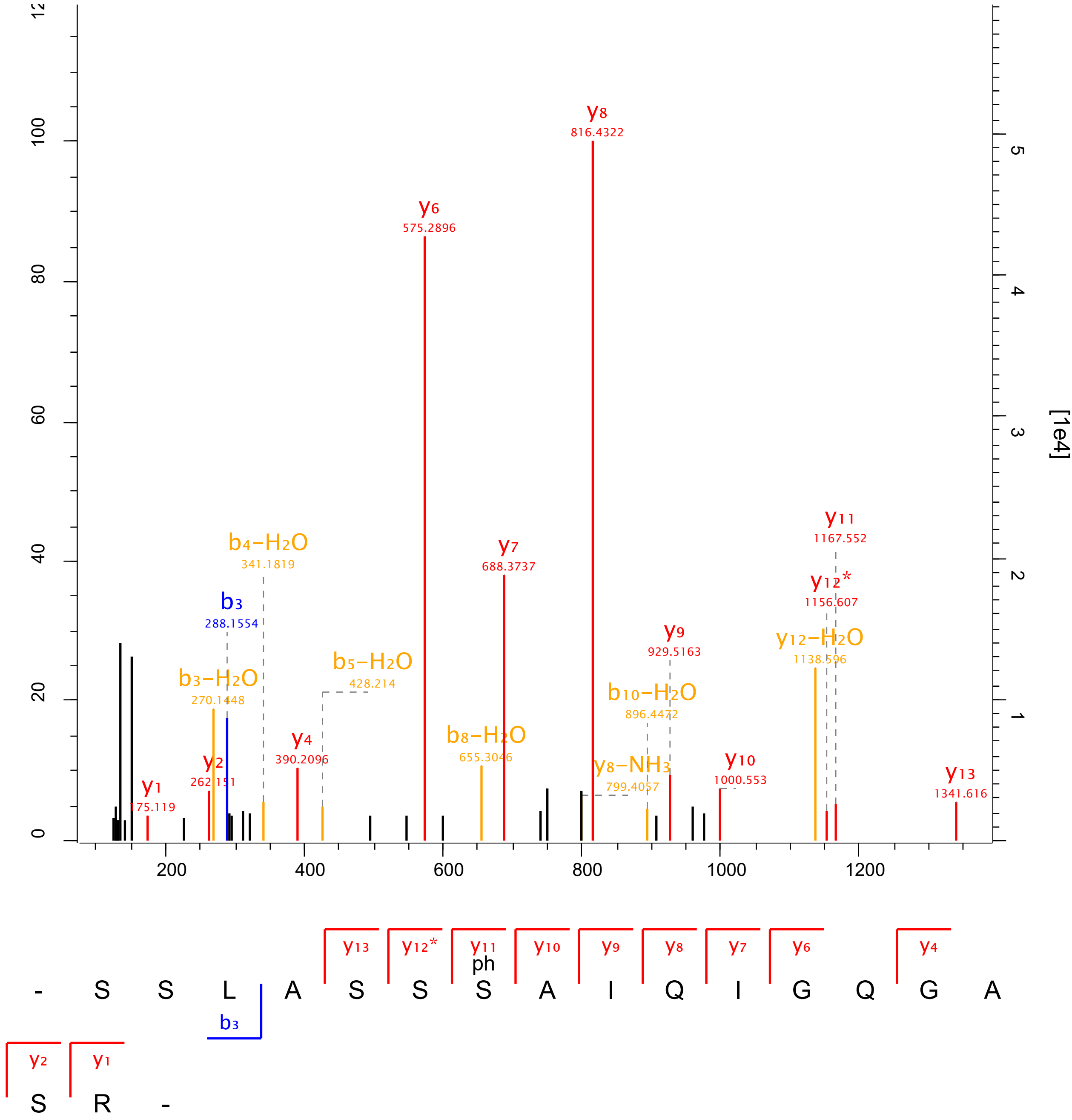

Raw file	Scan	Method	Score	m/z
sys_02_2	21948	FTMS; HCD	82.88	587.63

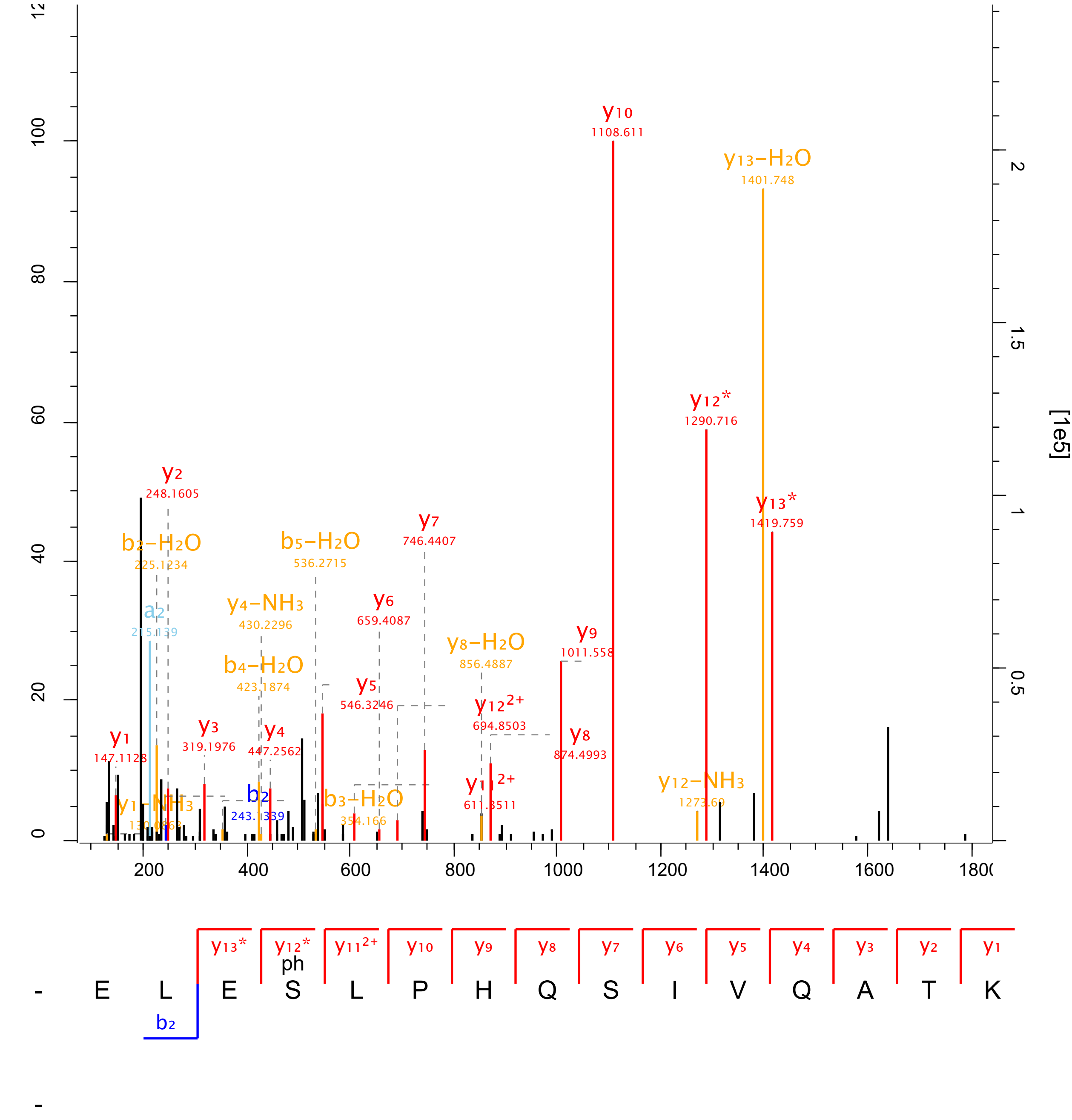

Raw file	Scan	Method	Score	m/z
sys_02_2	22037	FTMS; HCD	48.62	720.79

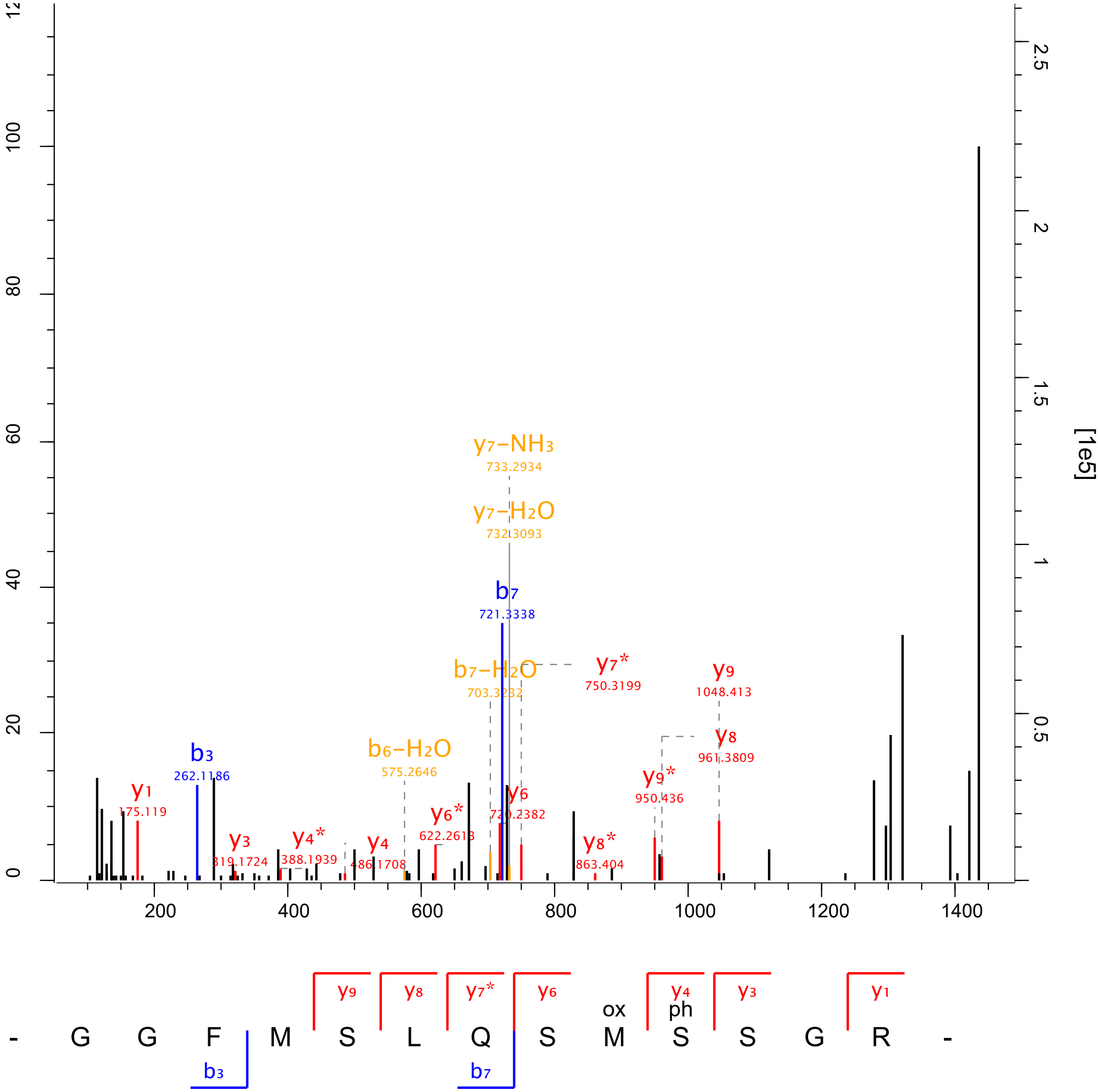

Raw file	Scan	Method	Score	m/z
sys_02_2	2210	FTMS; HCD	112.7	543.72

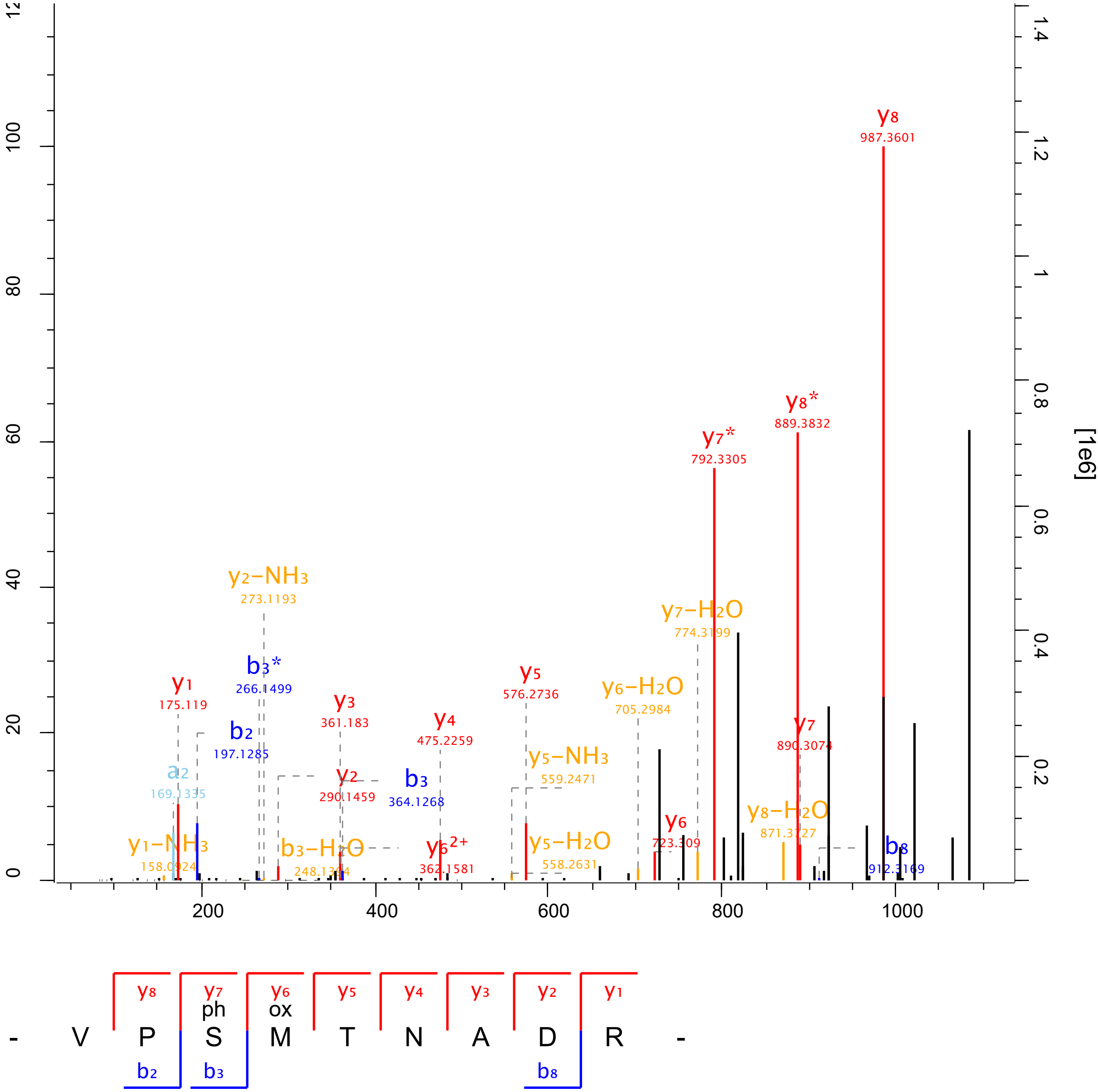

Raw file	Scan	Method	Score	m/z
sys_02_2	22151	FTMS; HCD	42.71	705.78

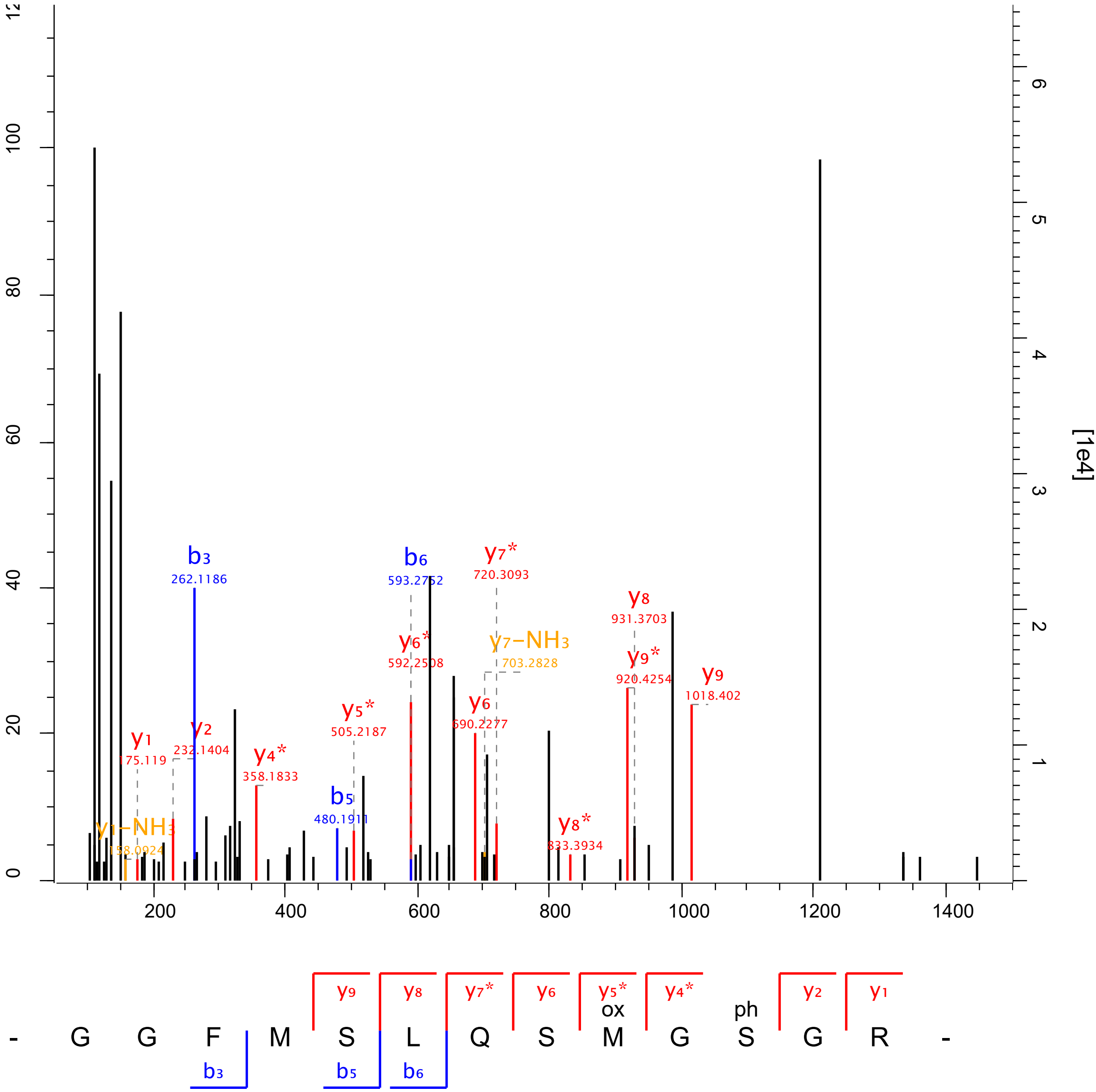

Raw file	Scan	Method	Score	m/z
sys_02_2	22821	FTMS; HCD	49.66	683.98

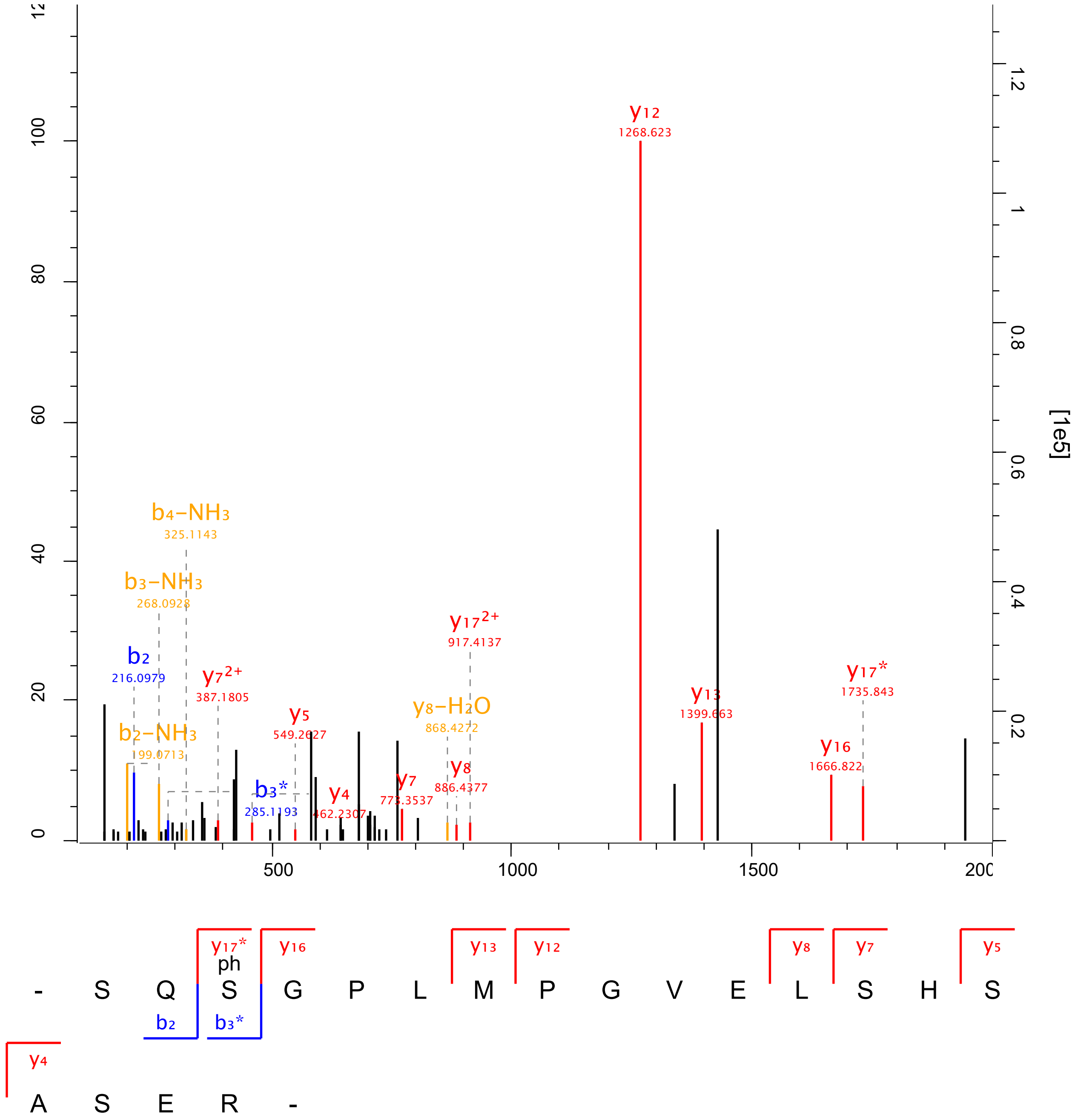

Raw file	Scan	Method	Score	m/z
sys_02_2	22888	FTMS; HCD	81.48	664.95

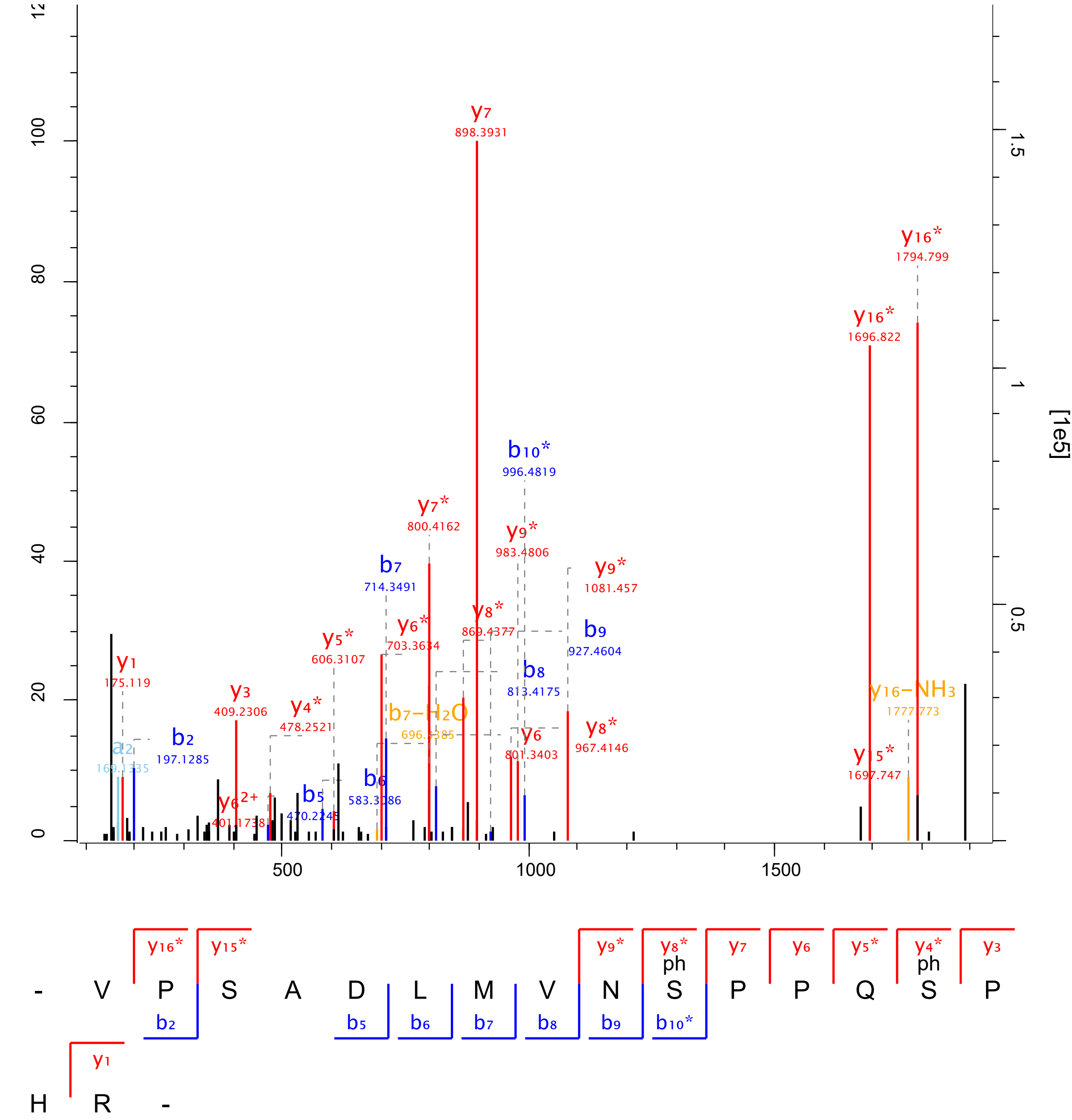

Raw file	Scan	Method	Score	m/z
sys_02_2	22939	FTMS; HCD	56.08	653.98

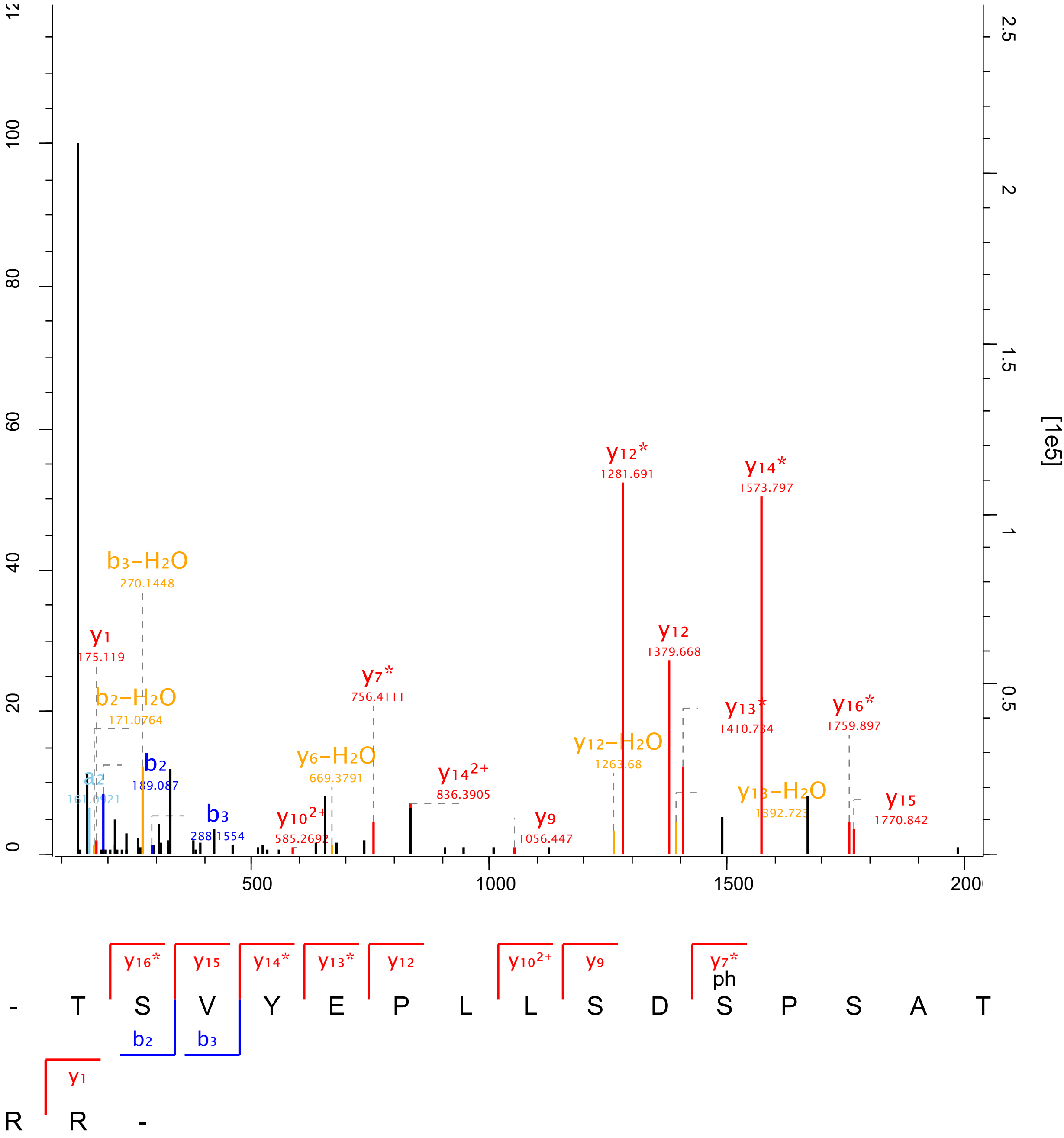

Raw file	Scan	Method	Score	m/z
sys_02_2	23023	FTMS; HCD	138.75	762.36

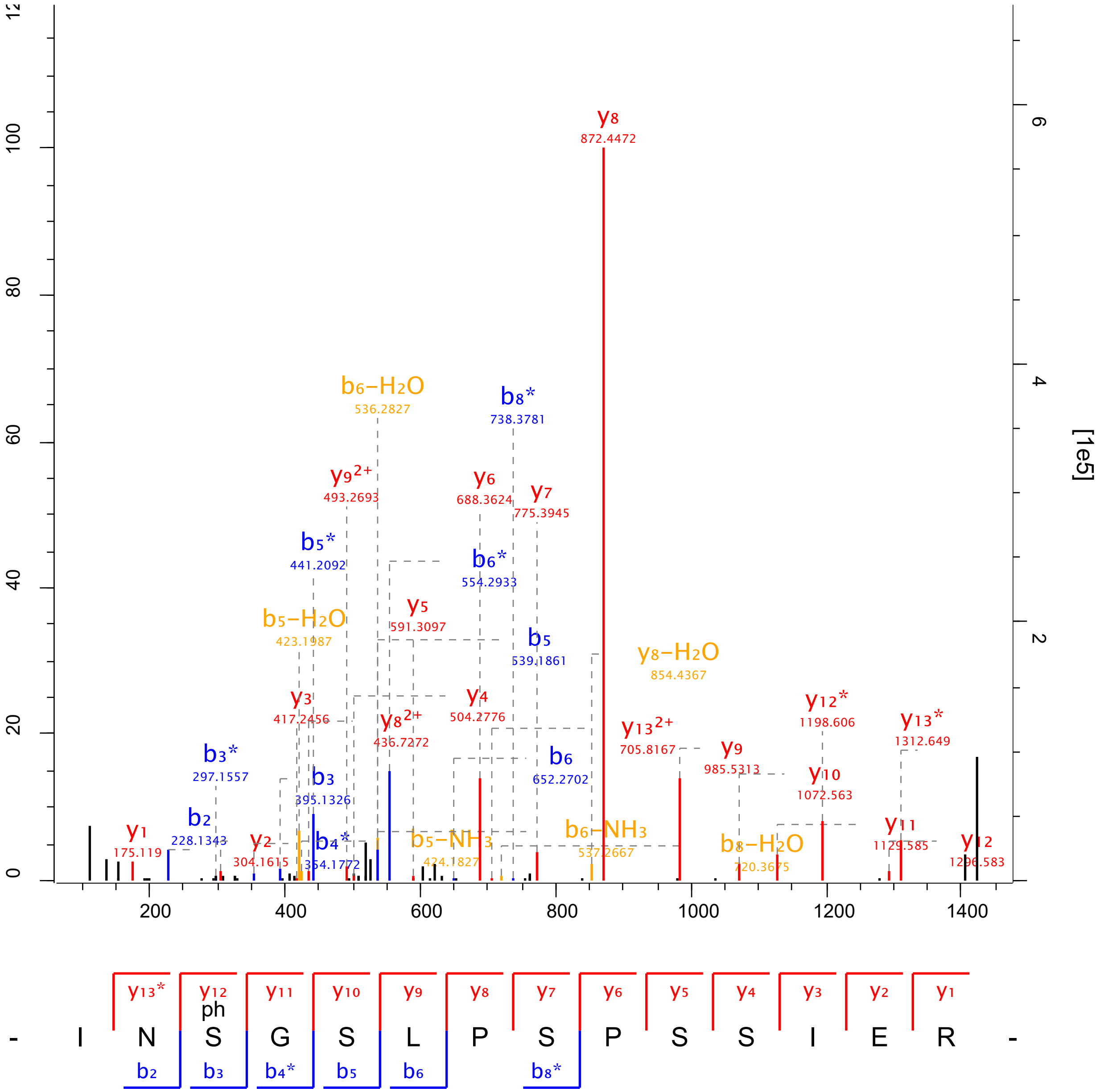

Supplement: Supplementary Figure S2-2 [file 143488_2_supp_334105_ps5hgs.pdf]
