# Supplementary material for: The Systemin Signaling Cascade As Derived from Time Course Analyses of the Systemin-responsive Phosphoproteome
Source: Mol Cell Proteomics. 2019 May 28;18(8):1526–42. doi: 10.1074/mcp.RA119.001367 (PMC6683004; doi:10.1074/mcp.RA119.001367)

Supplementary Figure 2: Representative annotated spectra of identified phosphopeptides under systemin,A17 and water treatment as exported from MaxQuant.

Raw file	Scan	Method	Score	m/z
sys_02_2	23101	FTMS; HCD	75.56	786.84

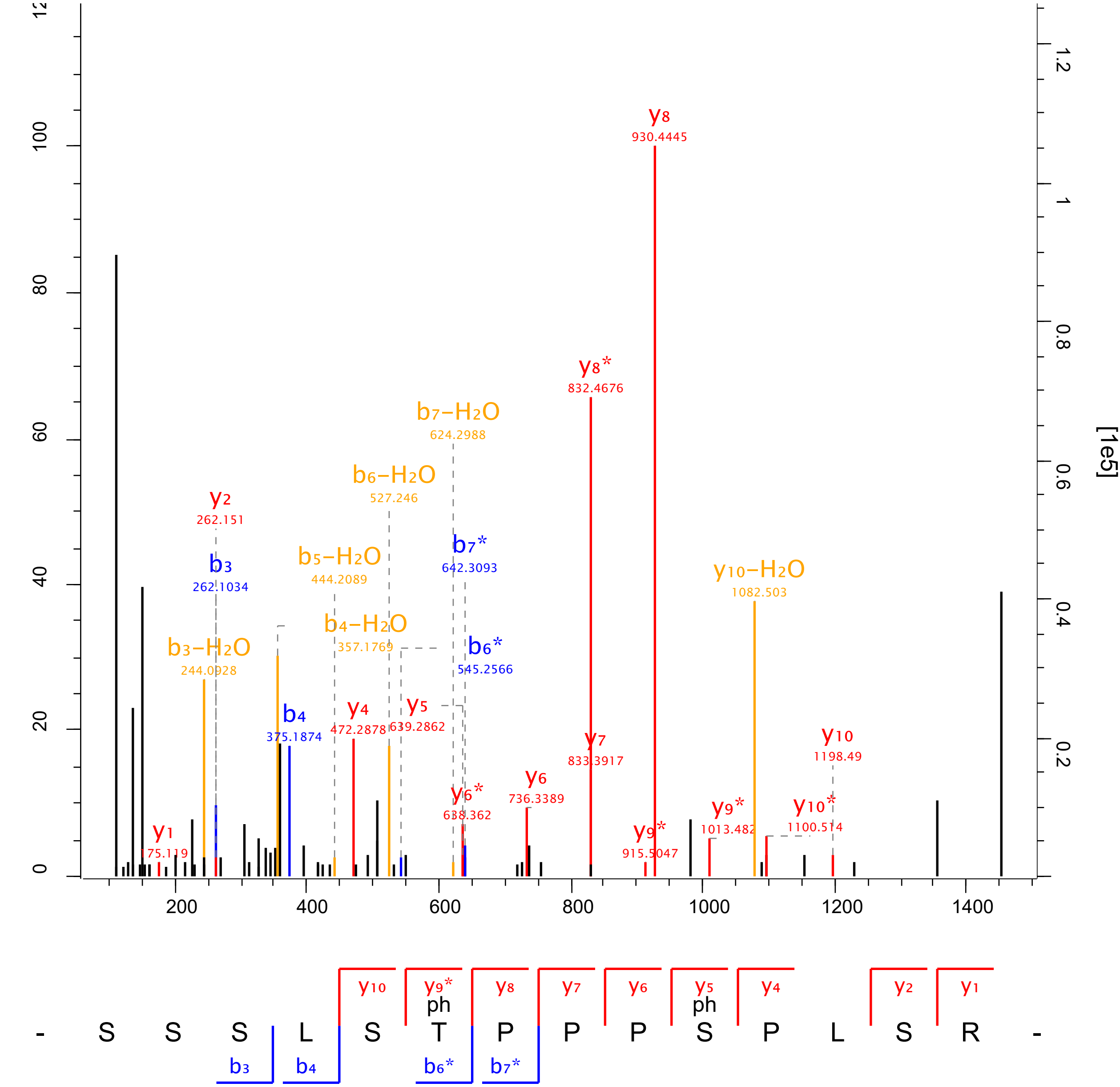

Raw file	Scan	Method	Score	m/z
sys_02_2	23556	FTMS; HCD	124.32	933.06

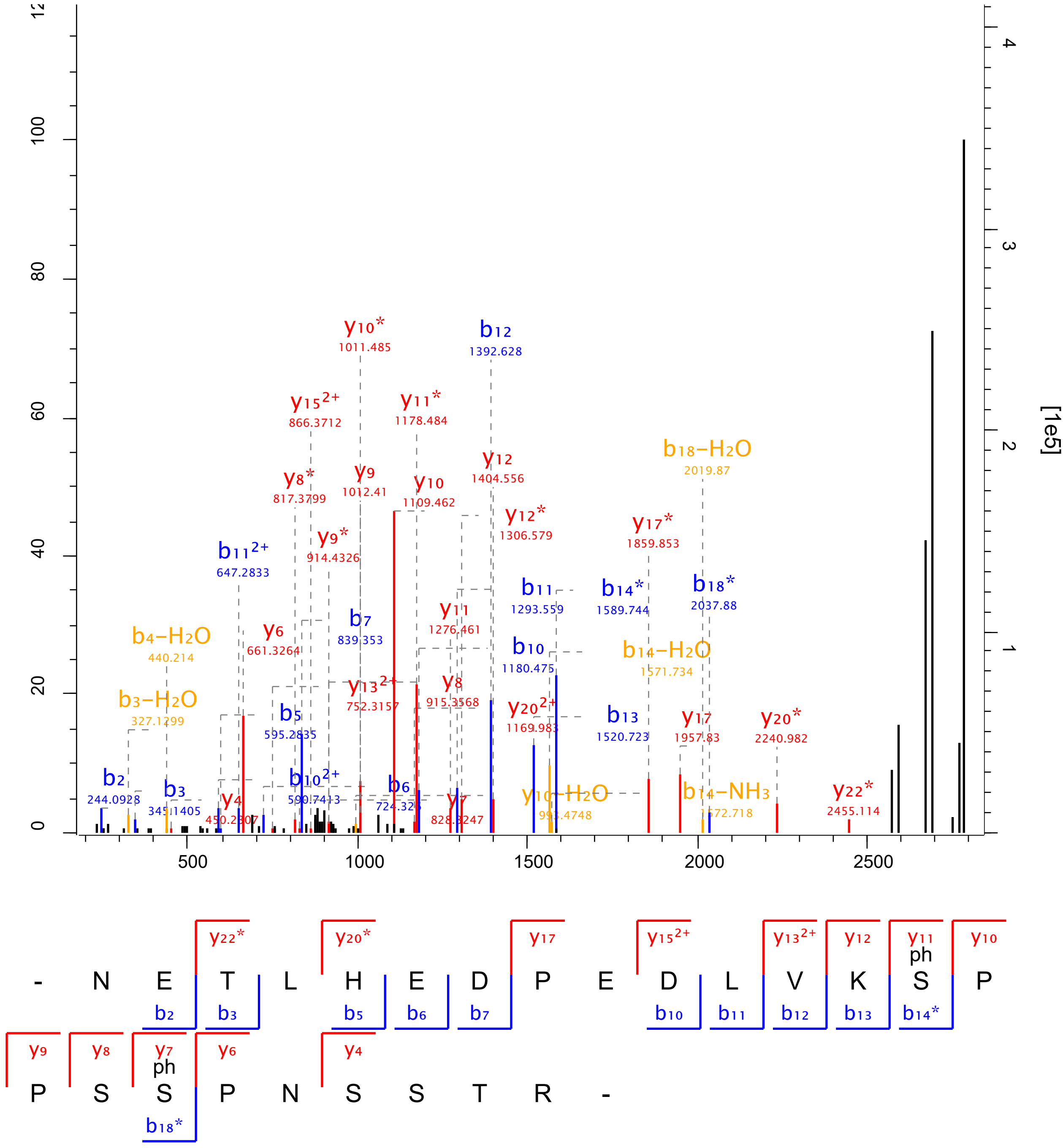

Raw file	Scan	Method	Score	m/z
sys_02_2	23588	FTMS; HCD	117.7	636.31

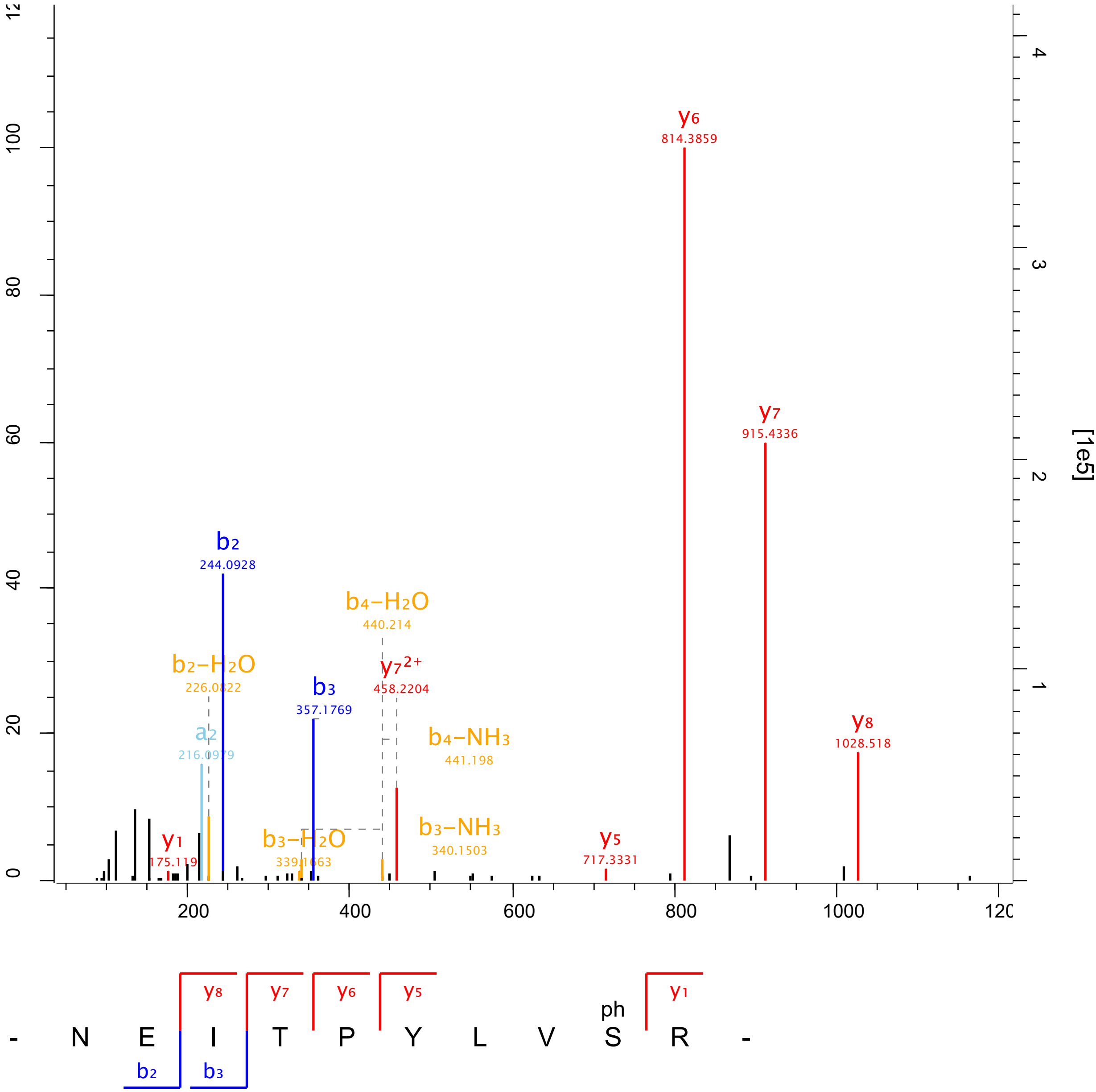

Raw file	Scan	Method	Score	m/z
sys_02_2	23839	FTMS; HCD	89.66	939.41

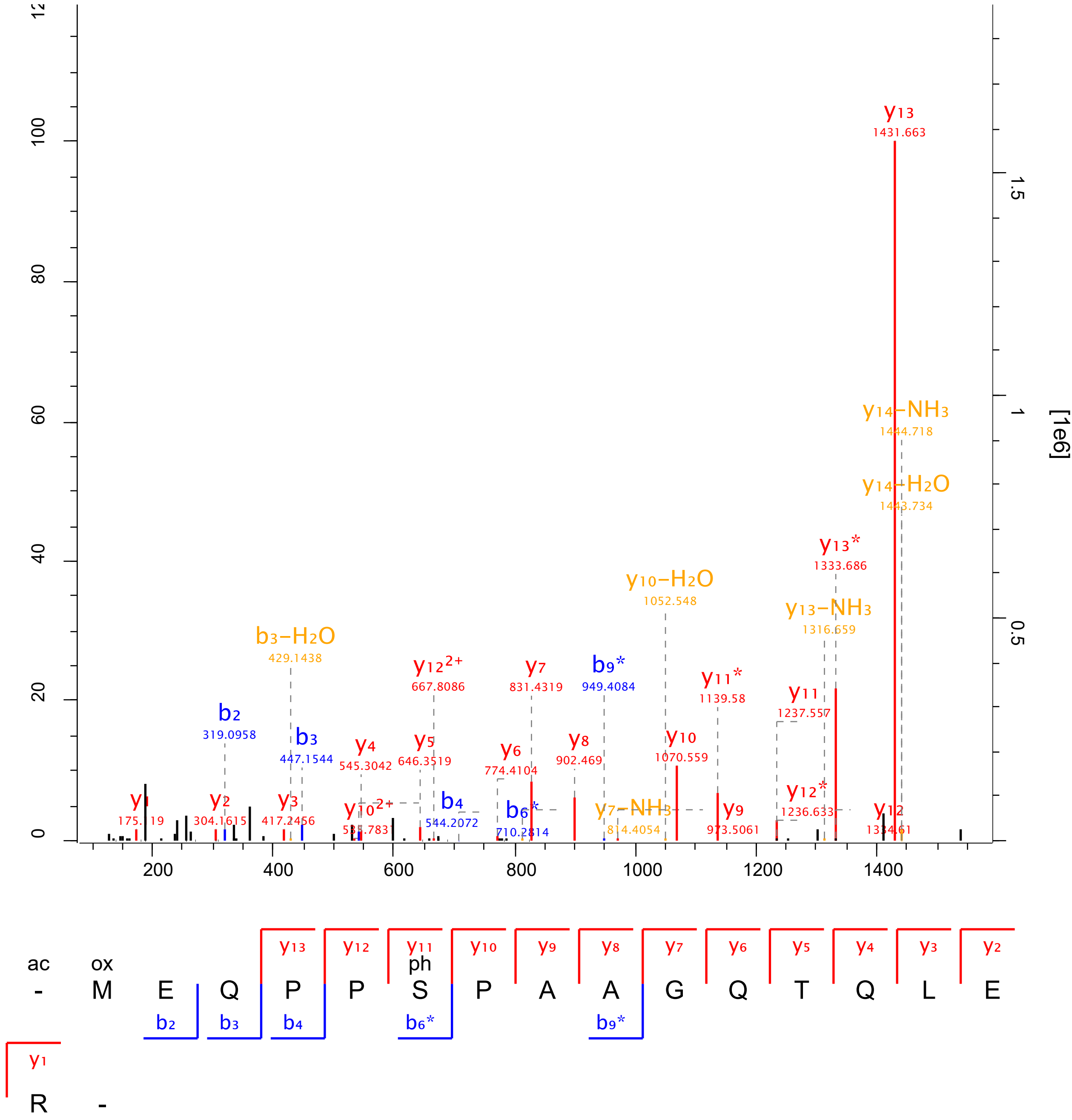

Raw file	Scan	Method	Score	m/z
sys_02_2	23919	FTMS; HCD	158.25	537.77

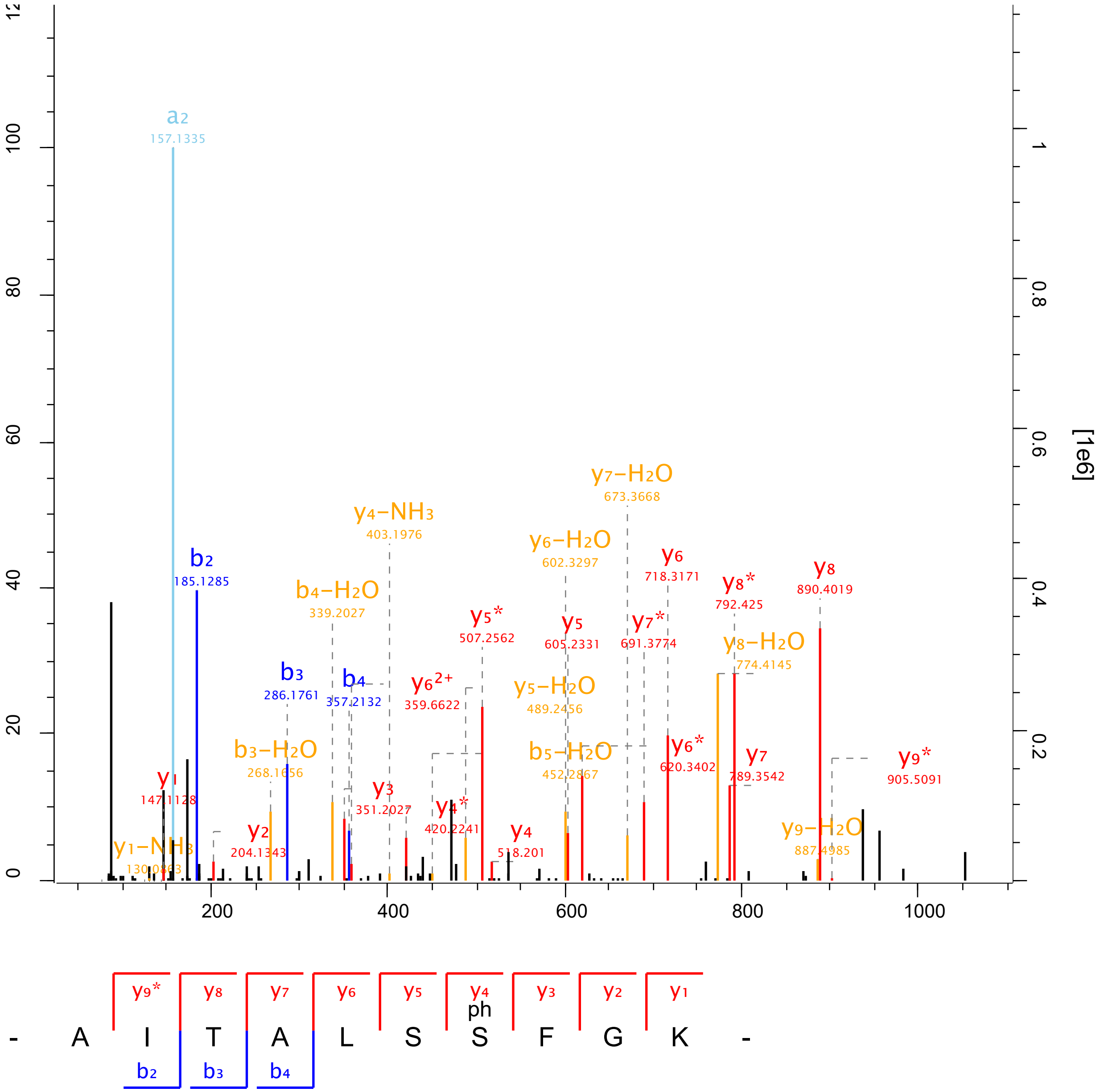

Raw file	Scan	Method	Score	m/z
sys_02_2	24054	FTMS; HCD	229.51	920.39

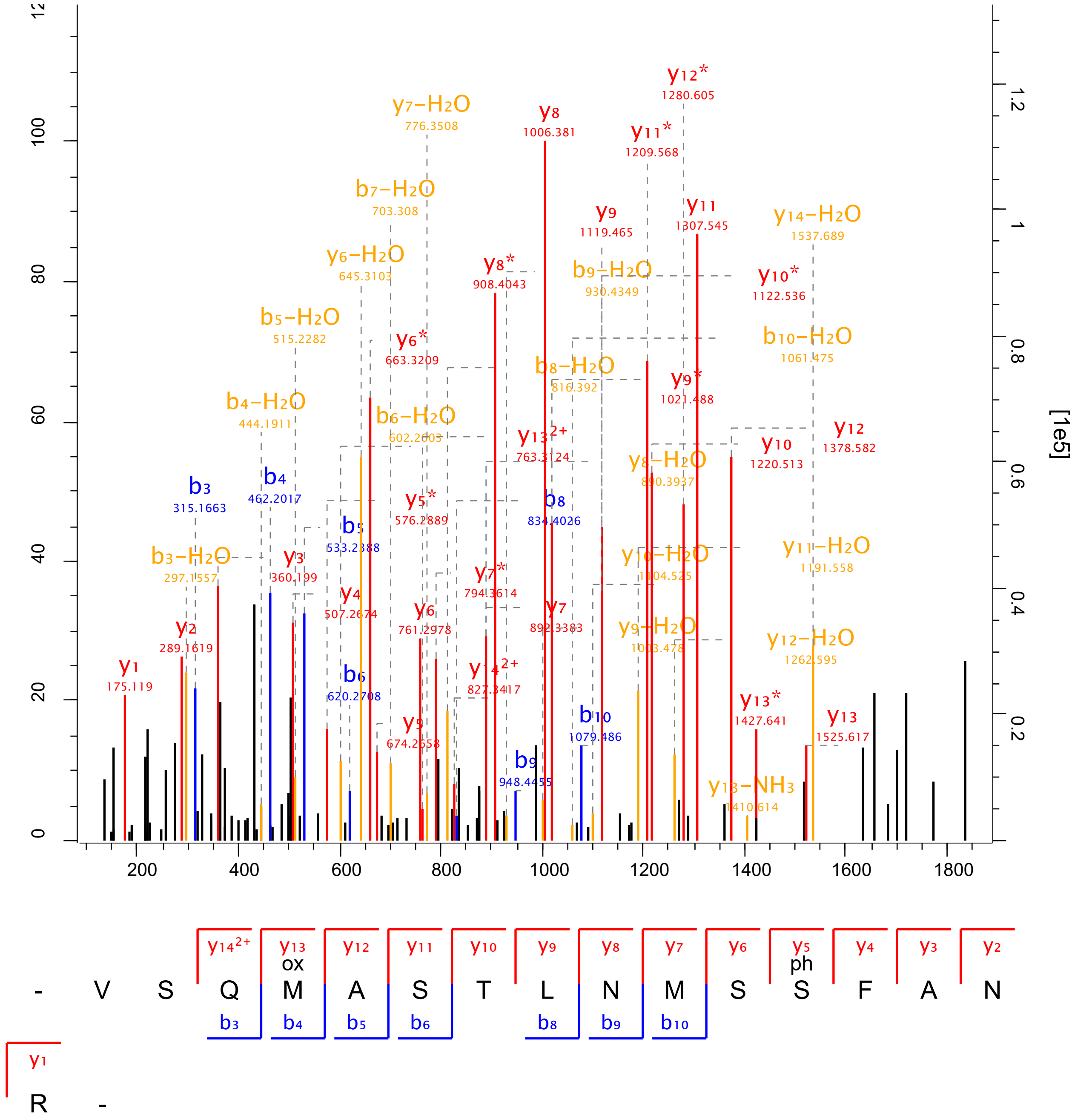

Raw file	Scan	Method	Score	m/z
sys_02_2	24114	FTMS; HCD	44.34	790.72

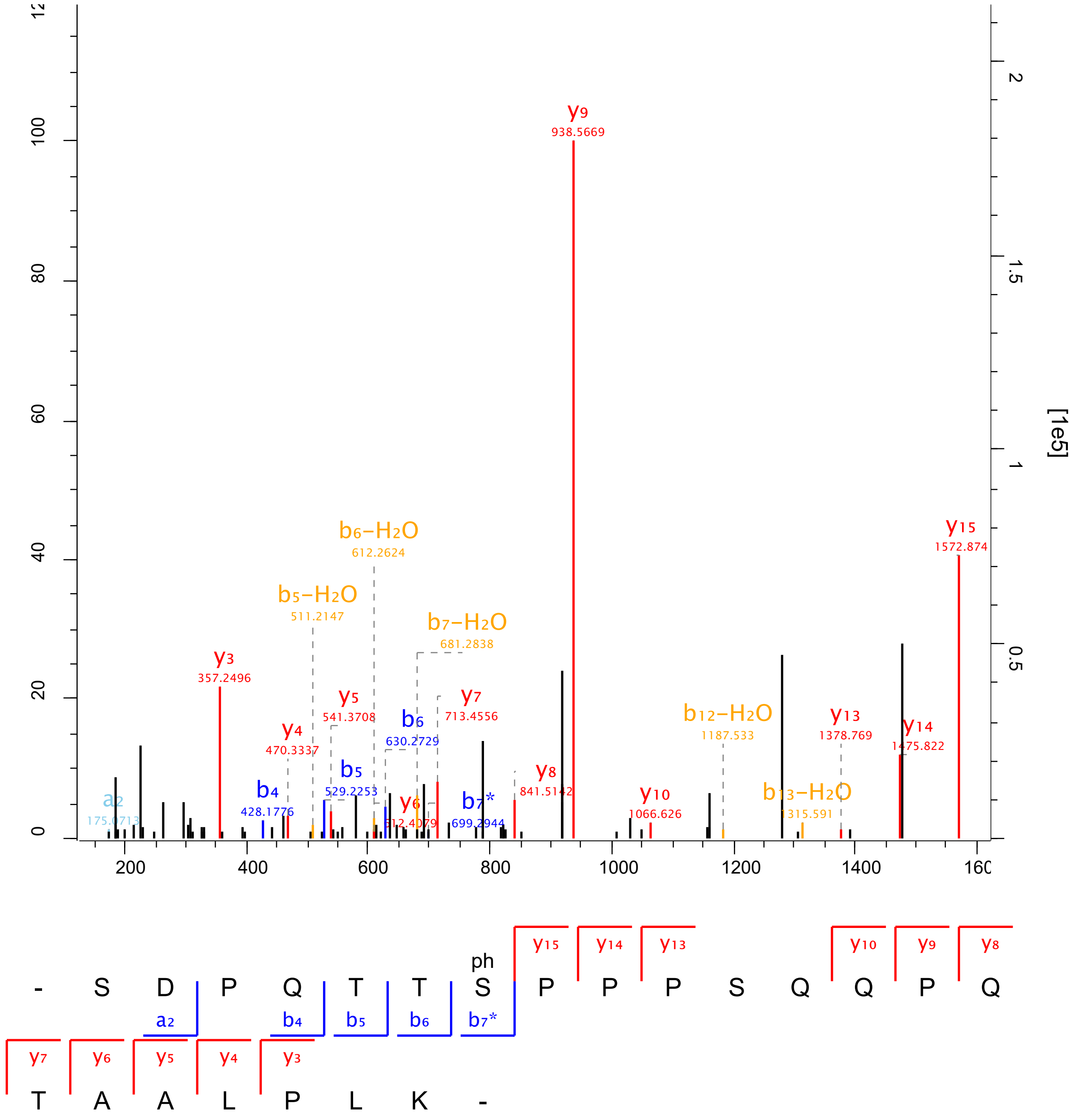

Raw file	Scan	Method	Score	m/z
sys_02_2	24136	FTMS; HCD	67.4	880.9

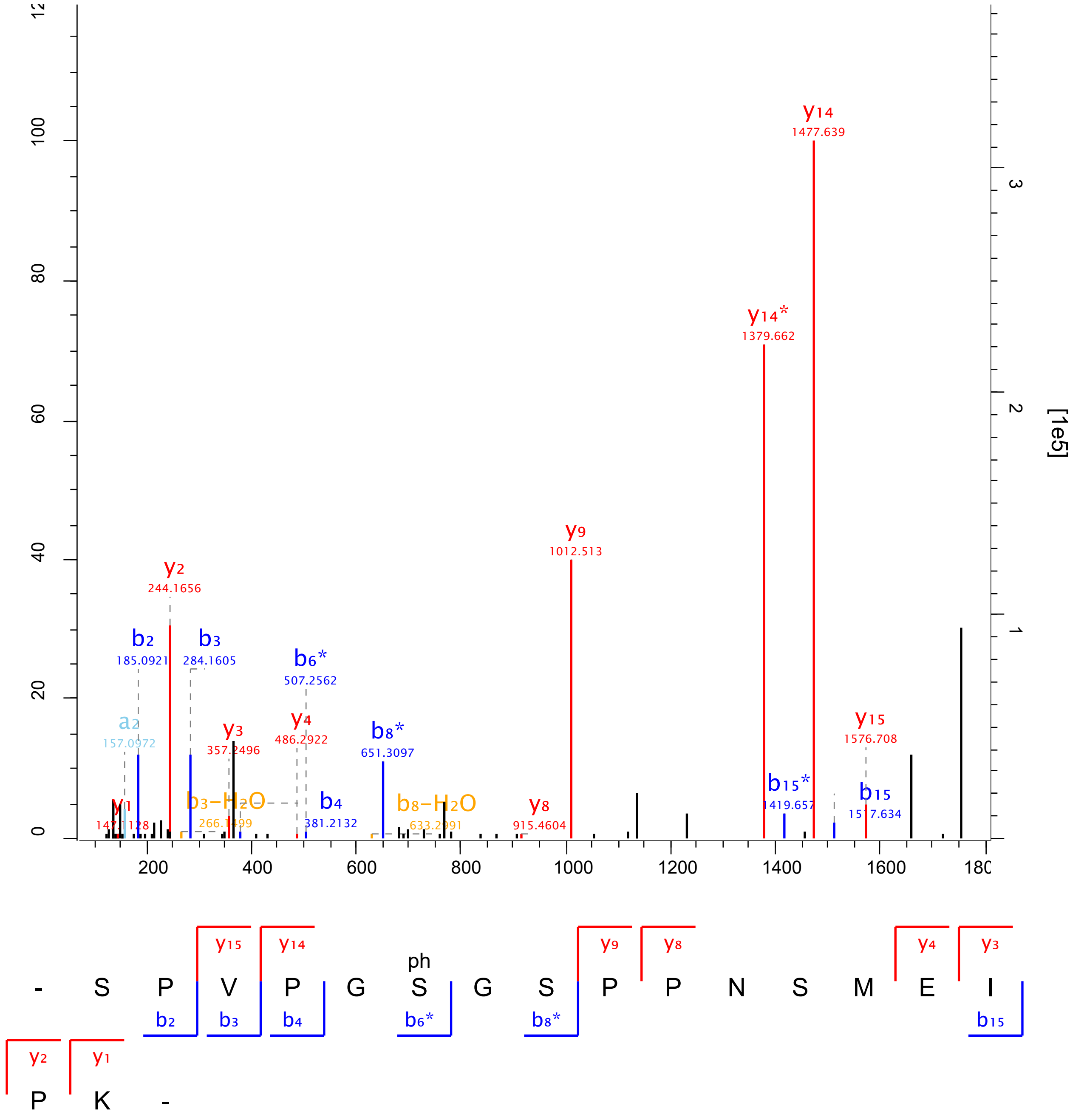

Raw file	Scan	Method	Score	m/z
sys_02_2	24208	FTMS; HCD	45.28	572.76

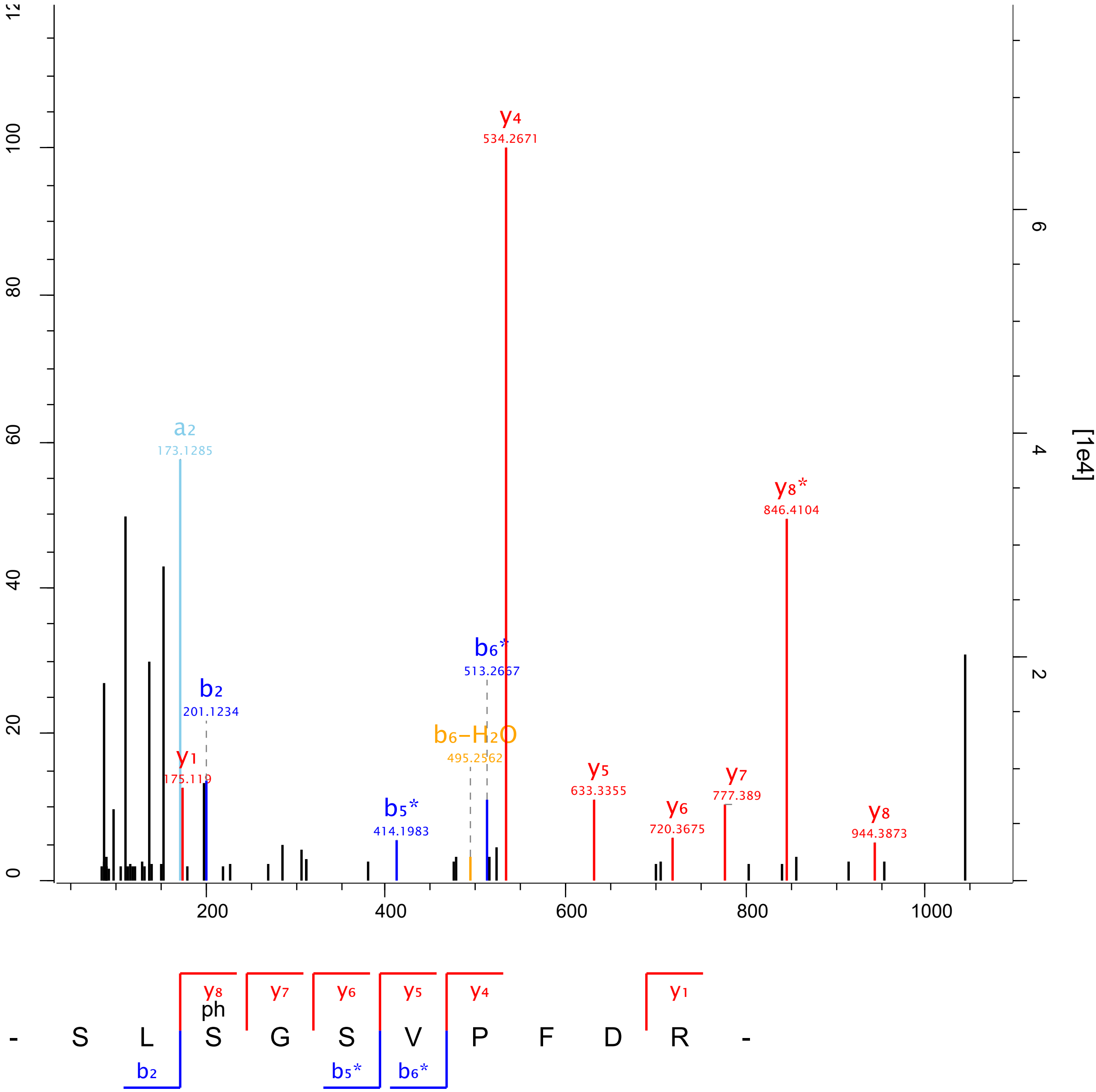

Raw file	Scan	Method	Score	m/z
sys_02_2	2445	FTMS; HCD	42.34	525.68

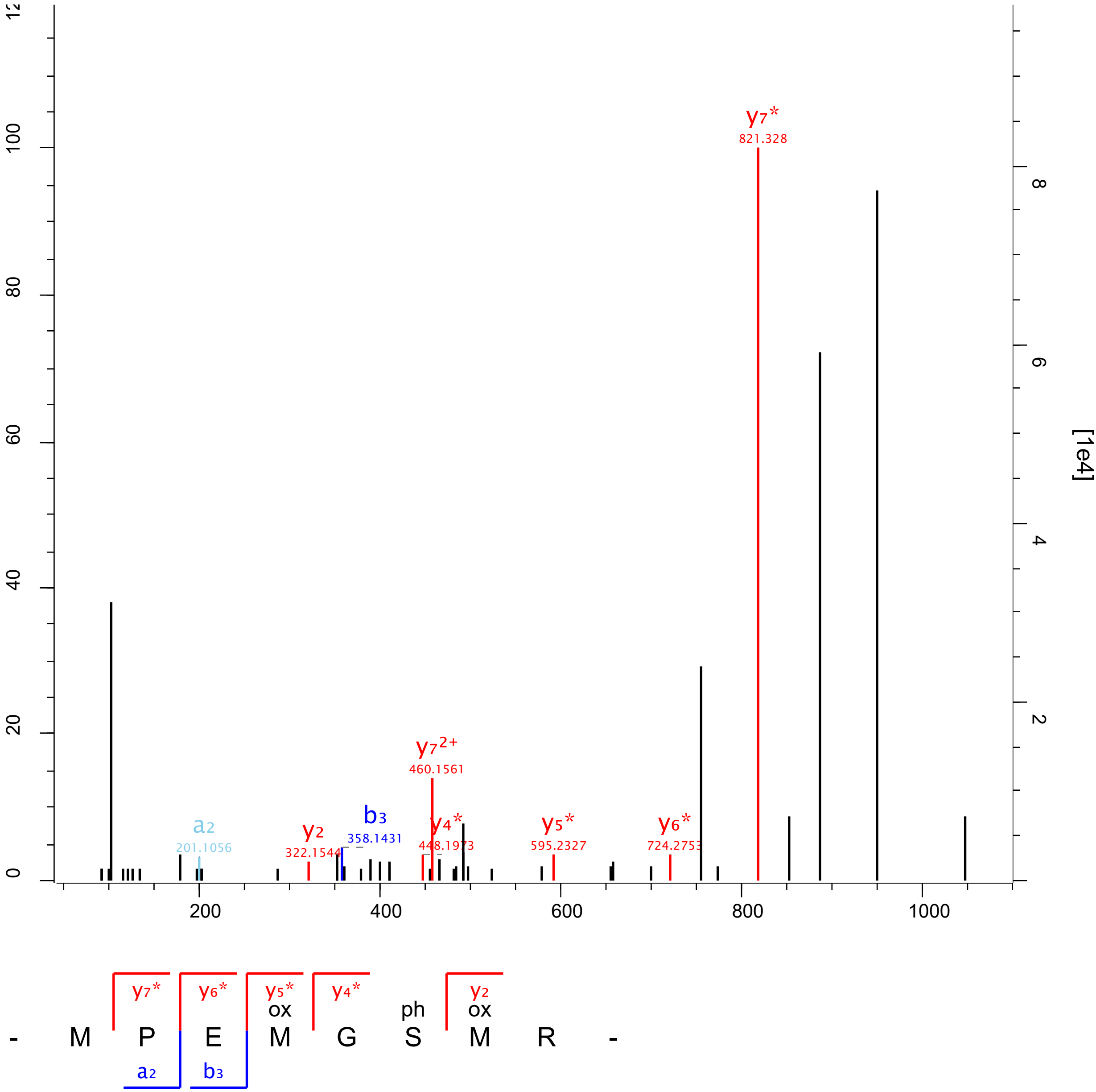

Raw file	Scan	Method	Score	m/z
sys_02_2	24655	FTMS; HCD	115.42	499.26

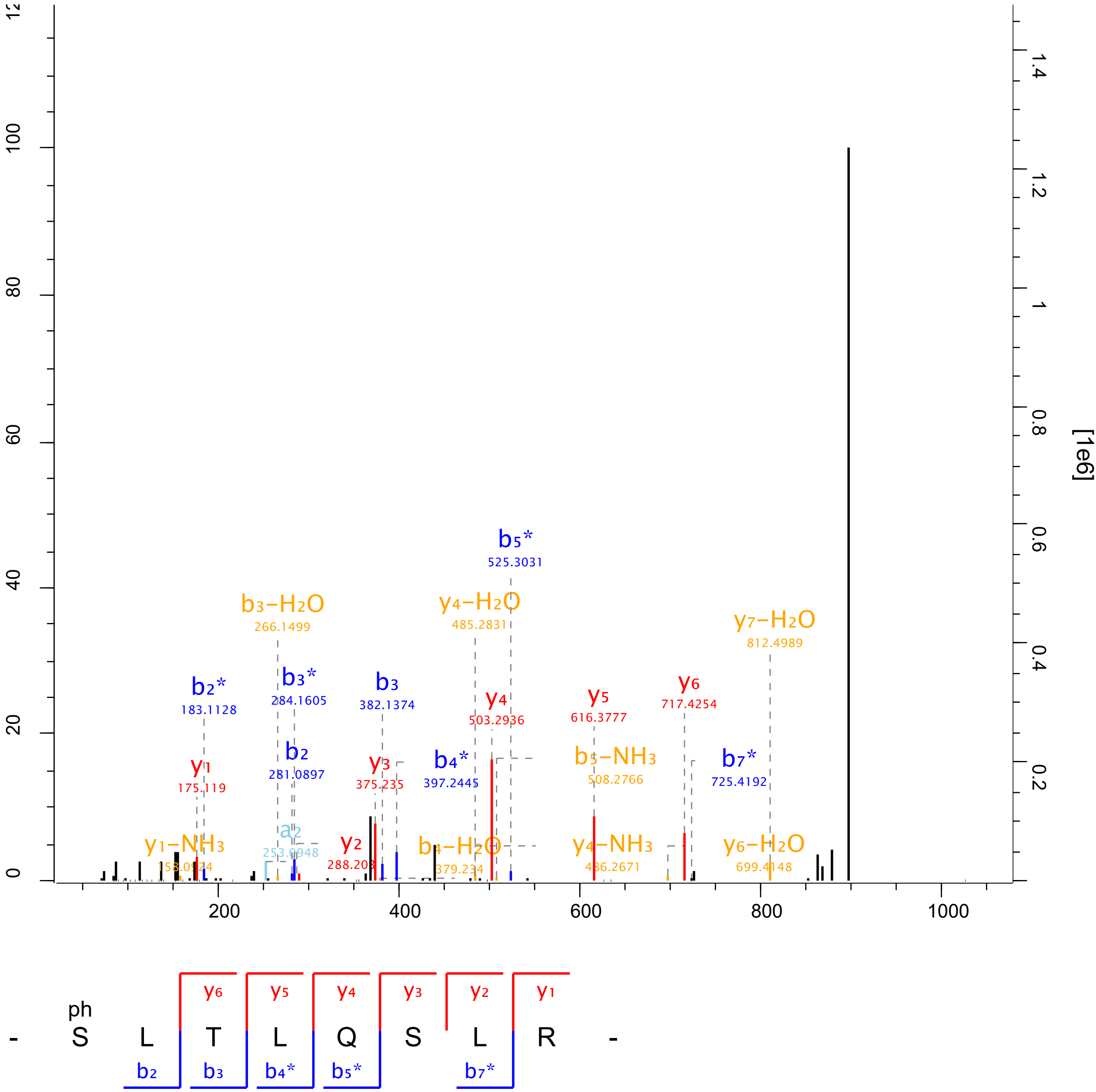

Raw file	Scan	Method	Score	m/z
sys_02_2	25351	FTMS; HCD	127.2	1027.12

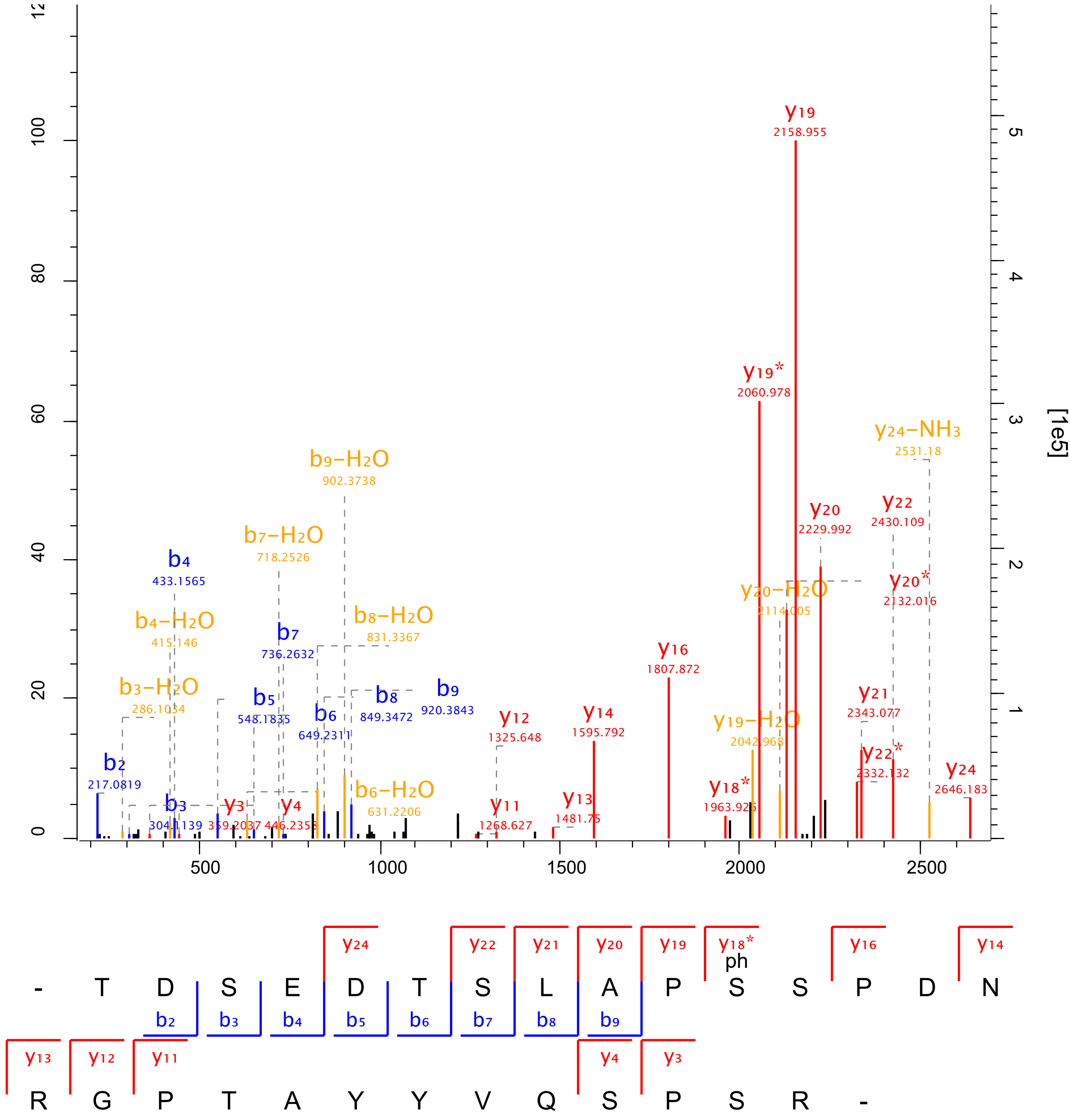

Raw file	Scan	Method	Score	m/z
sys_02_2	25751	FTMS; HCD	199.09	782.87

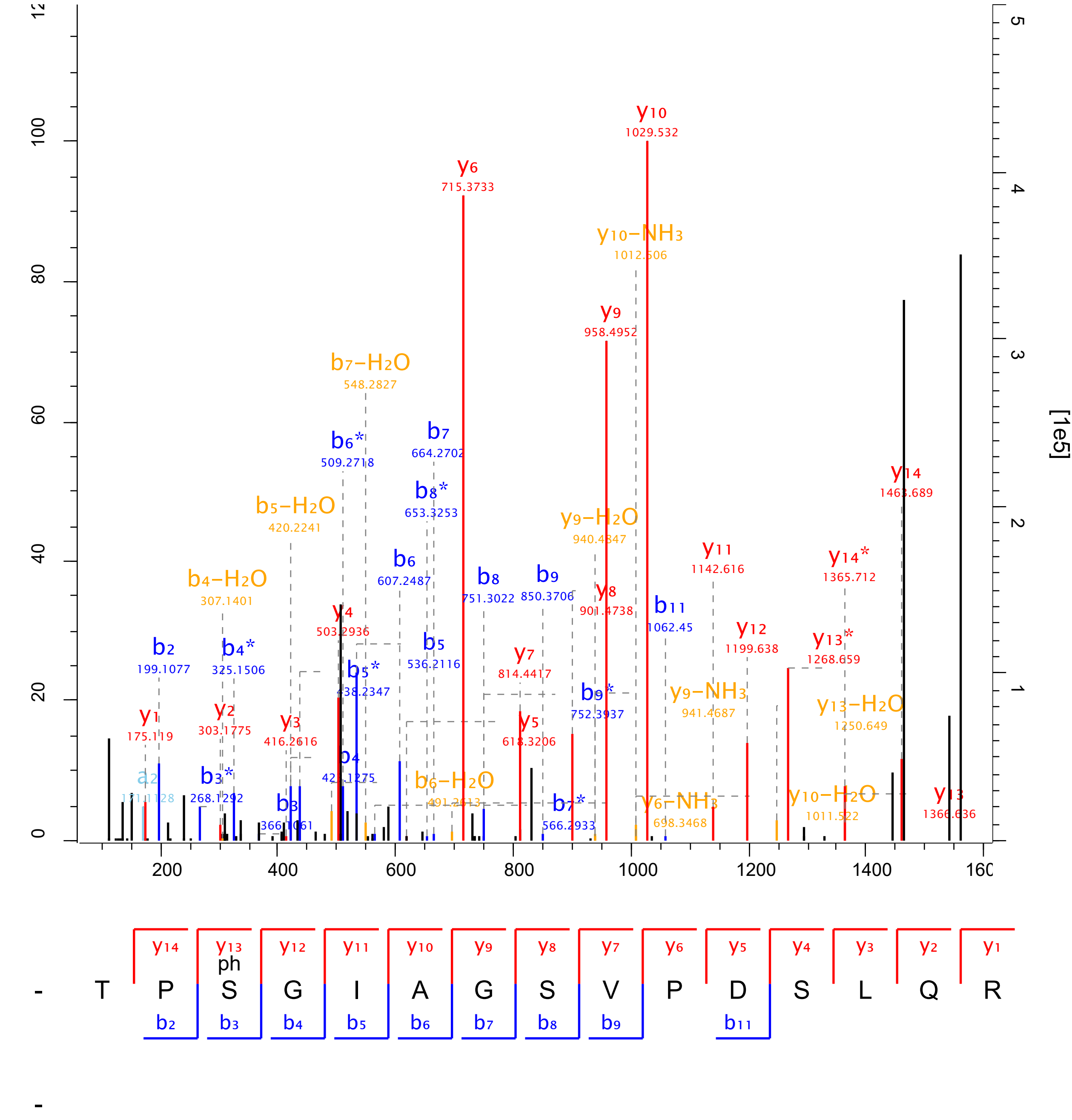

Raw file	Scan	Method	Score	m/z
sys_02_2	25862	FTMS; HCD	219.23	682.35

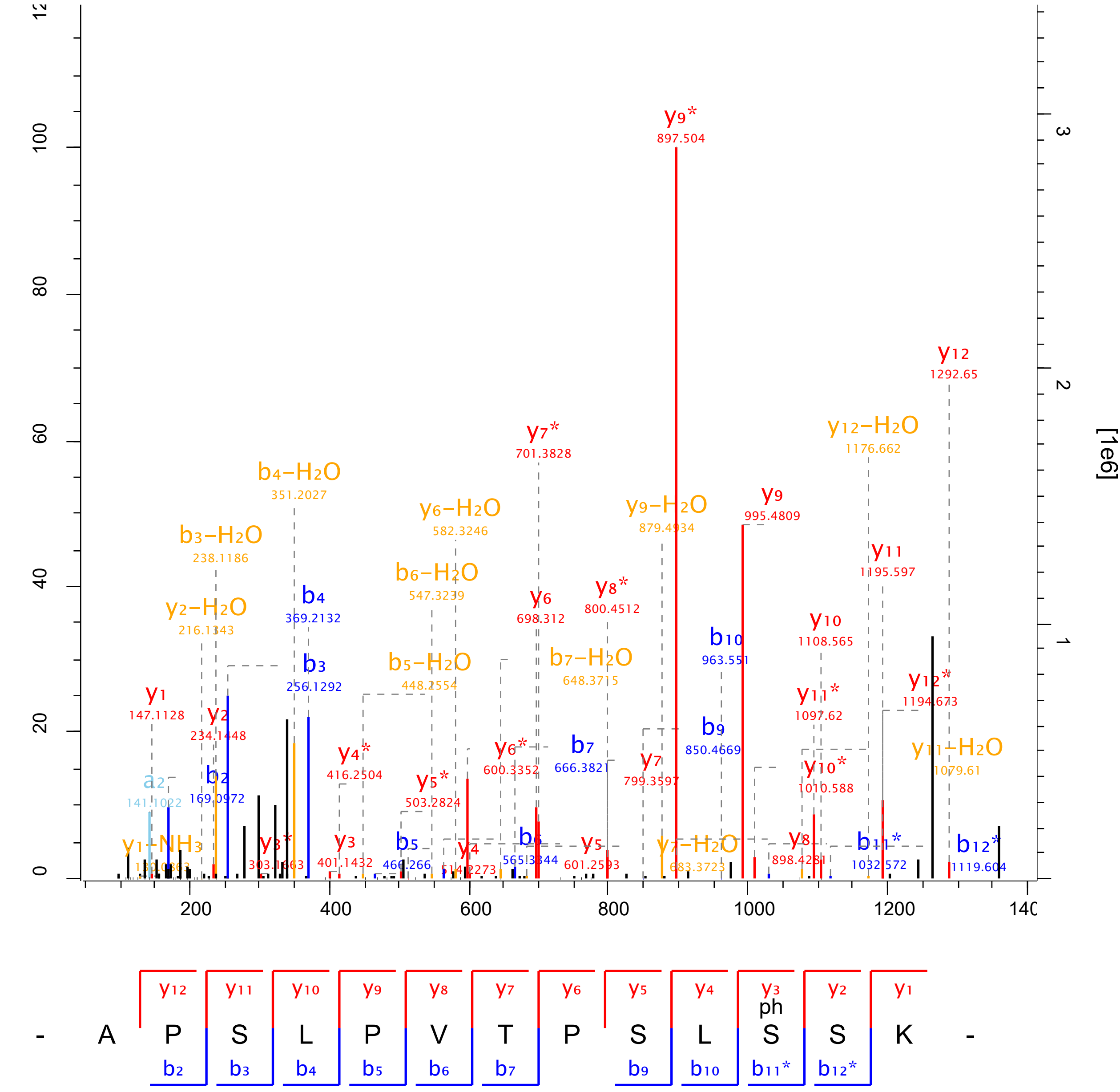

Raw file	Scan	Method	Score	m/z
sys_02_2	2598	FTMS; HCD	119.54	625.8

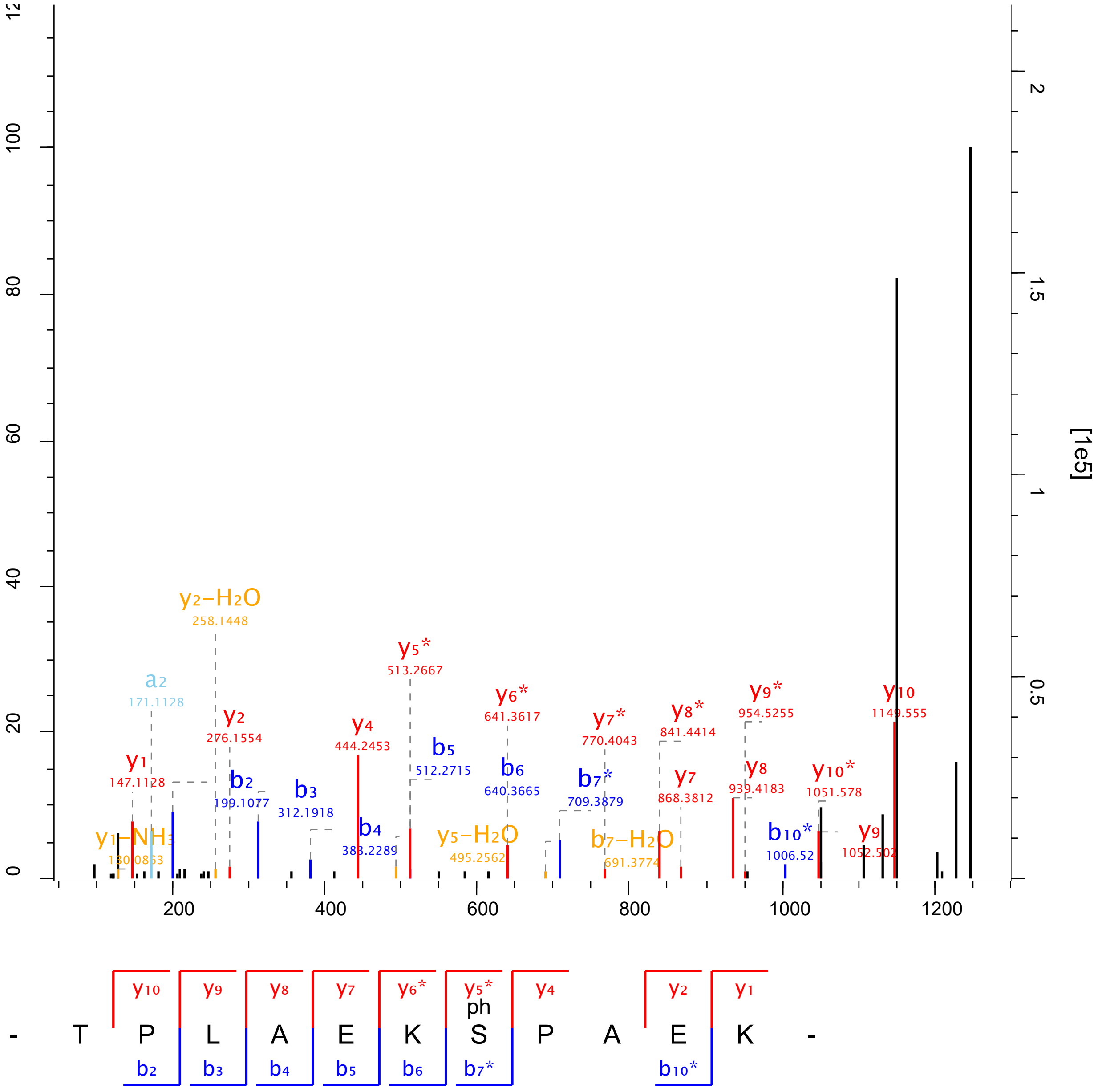

Raw file	Scan	Method	Score	m/z
sys_02_2	26009	FTMS; HCD	55.26	550.77

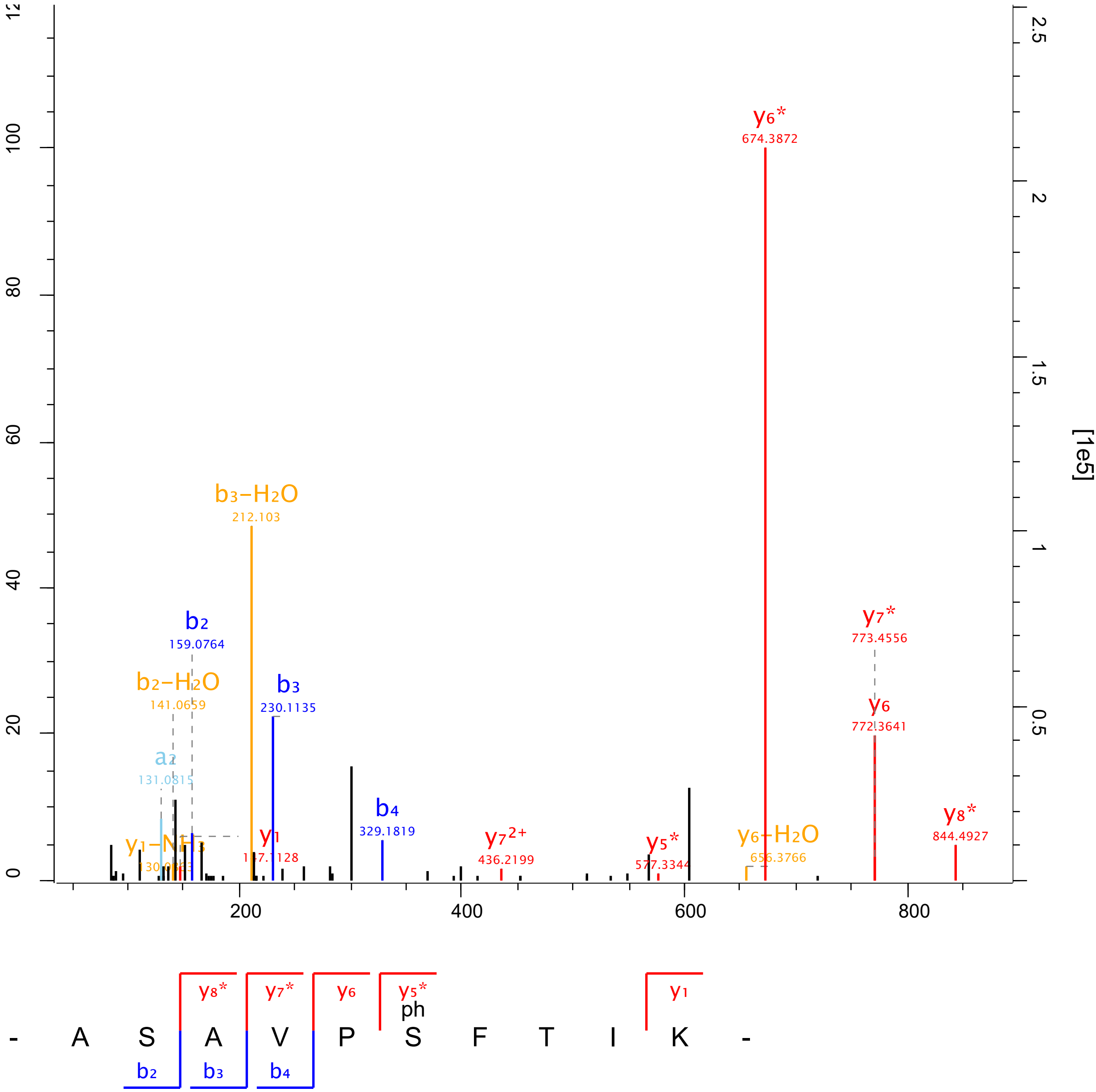

Raw file	Scan	Method	Score	m/z
sys_02_2	2624	FTMS; HCD	43.9	399.17

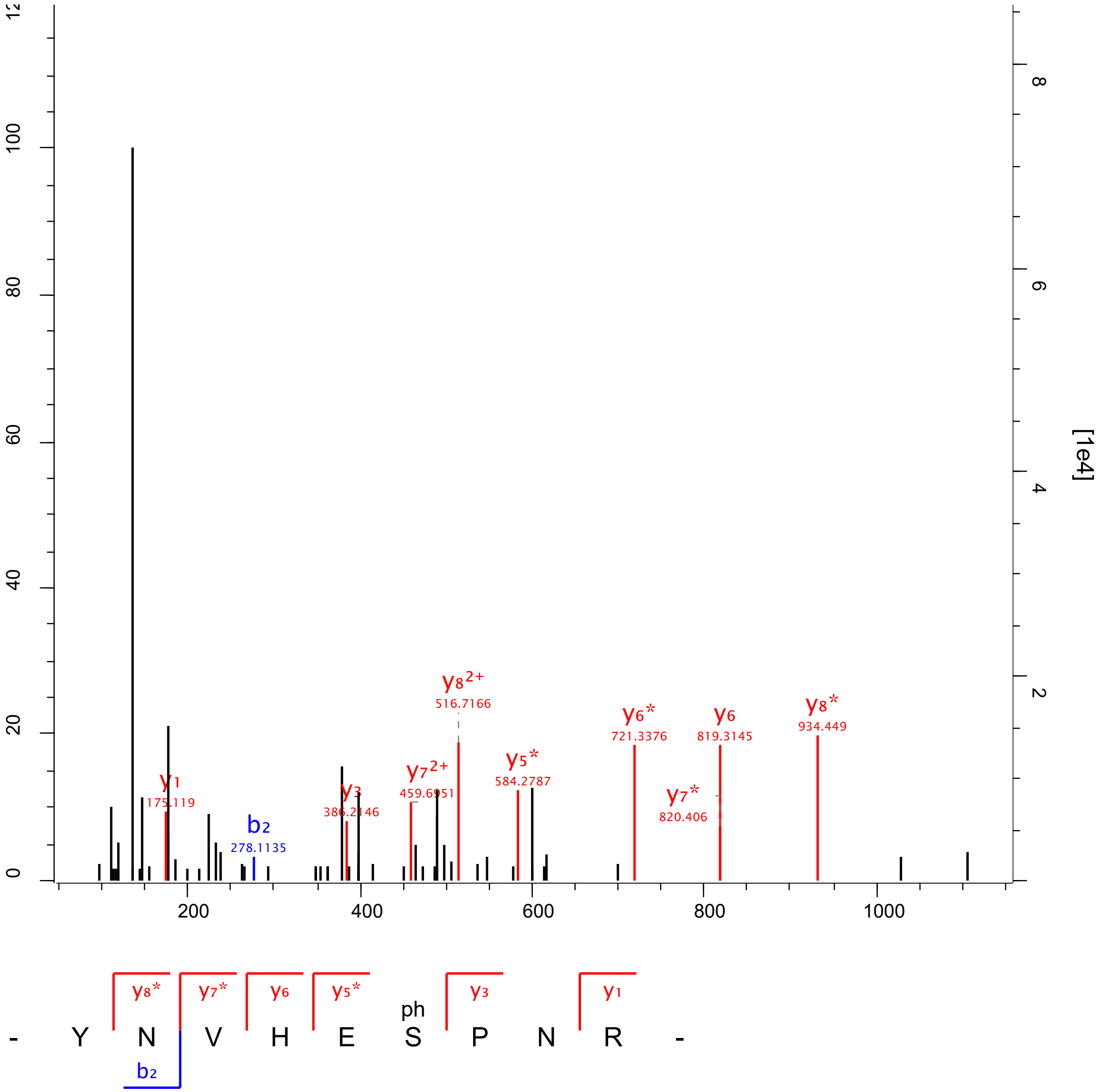

Raw file	Scan	Method	Score	m/z
sys_02_2	26446	FTMS; HCD	188.15	590.75

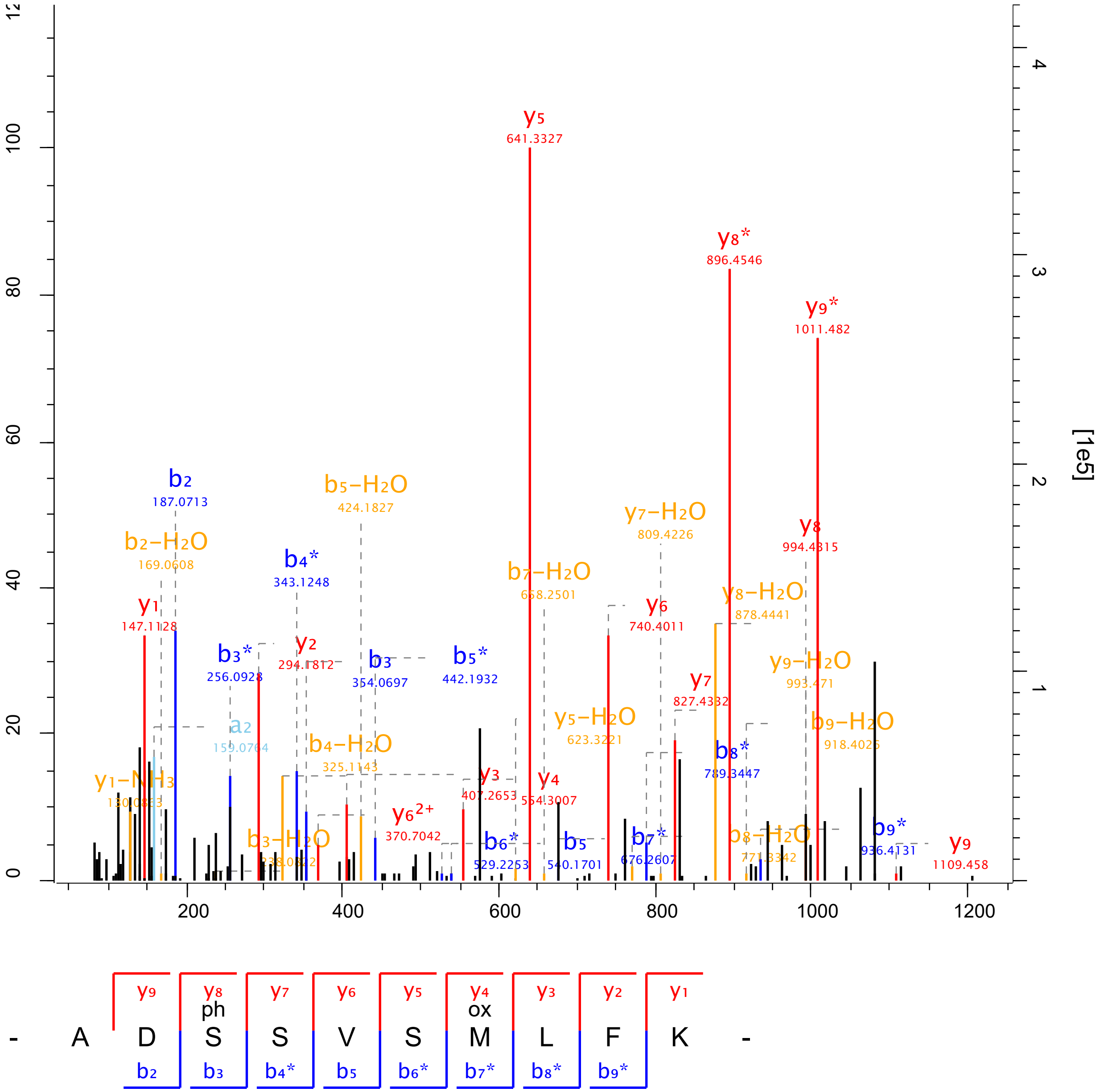

Raw file	Scan	Method	Score	m/z
sys_02_2	2645	FTMS; HCD	66.27	395.86

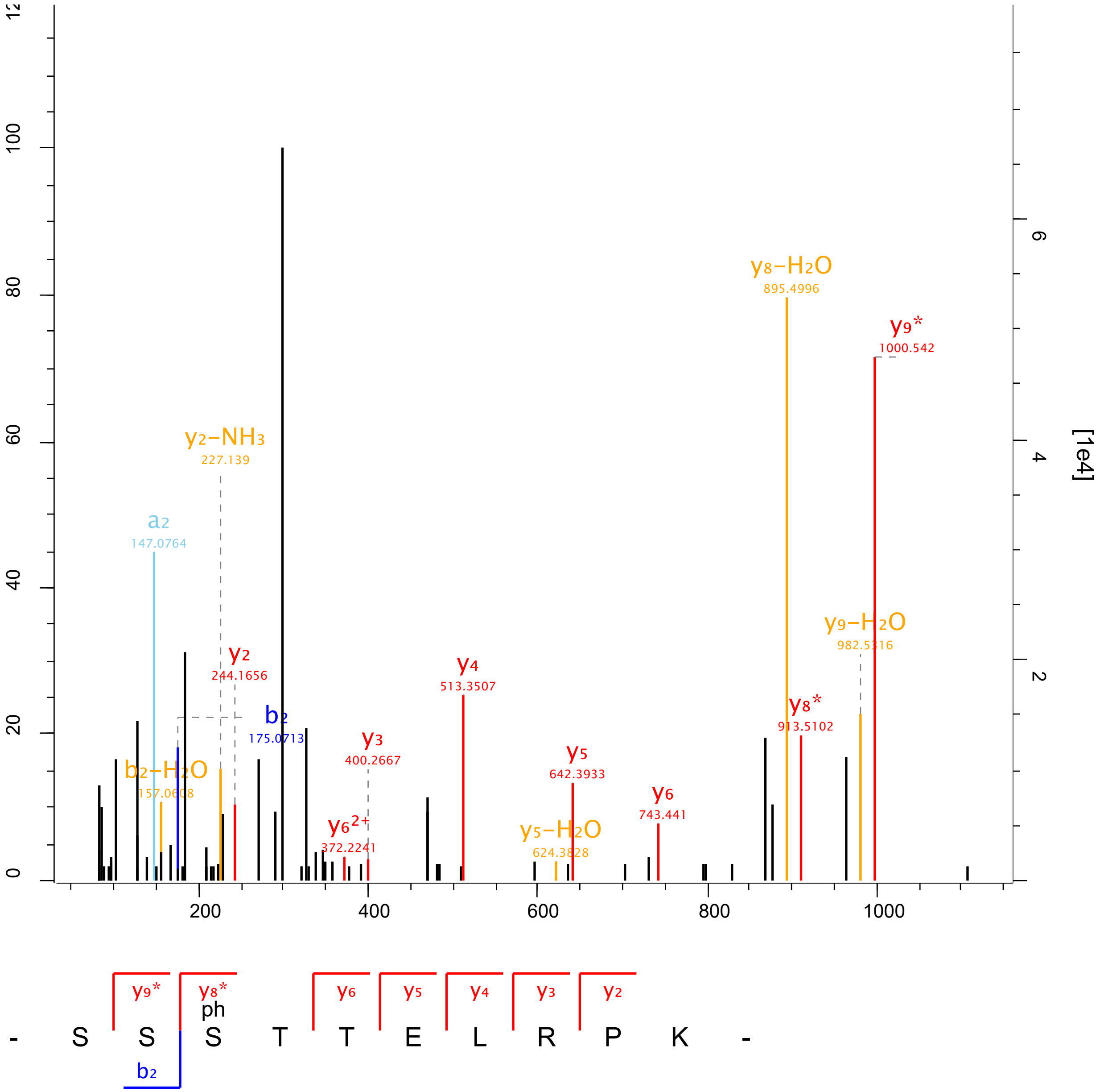

Raw file	Scan	Method	Score	m/z
sys_02_2	26563	FTMS; HCD	95.12	722.96

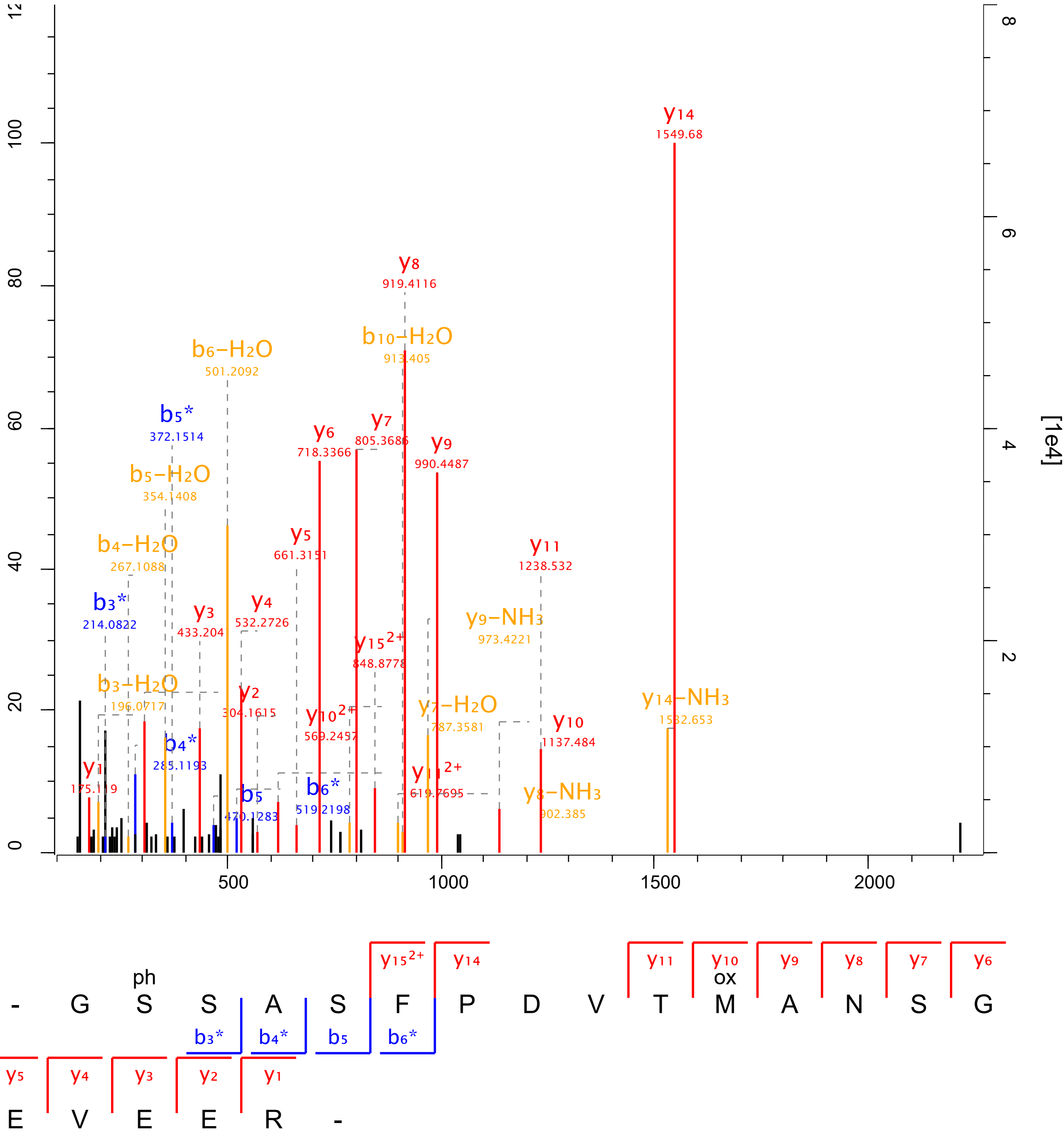

Raw file	Scan	Method	Score	m/z
sys_02_2	27519	FTMS; HCD	42.07	811.39

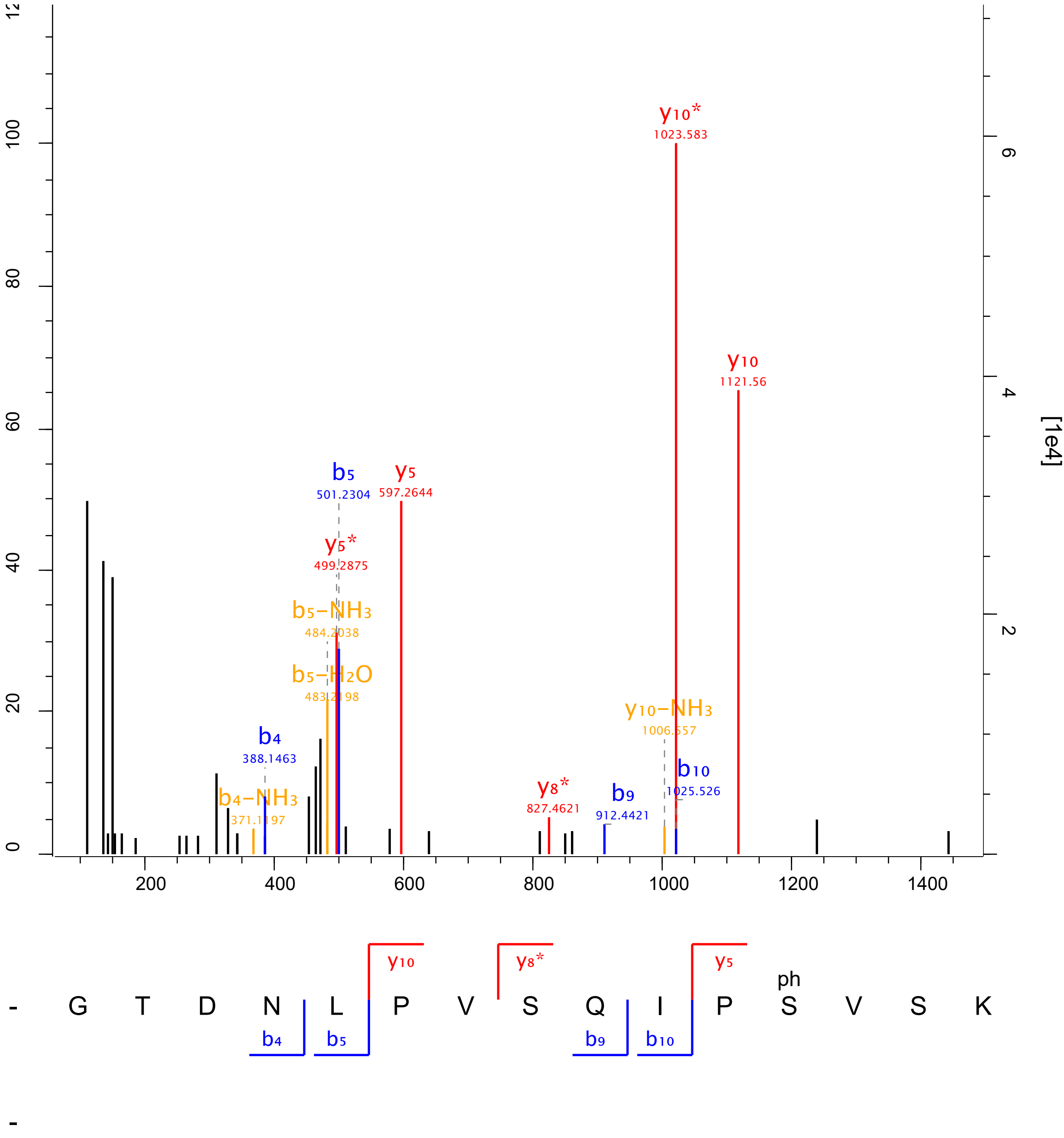

Raw file	Scan	Method	Score	m/z
sys_02_2	27621	FTMS; HCD	61.13	741.32

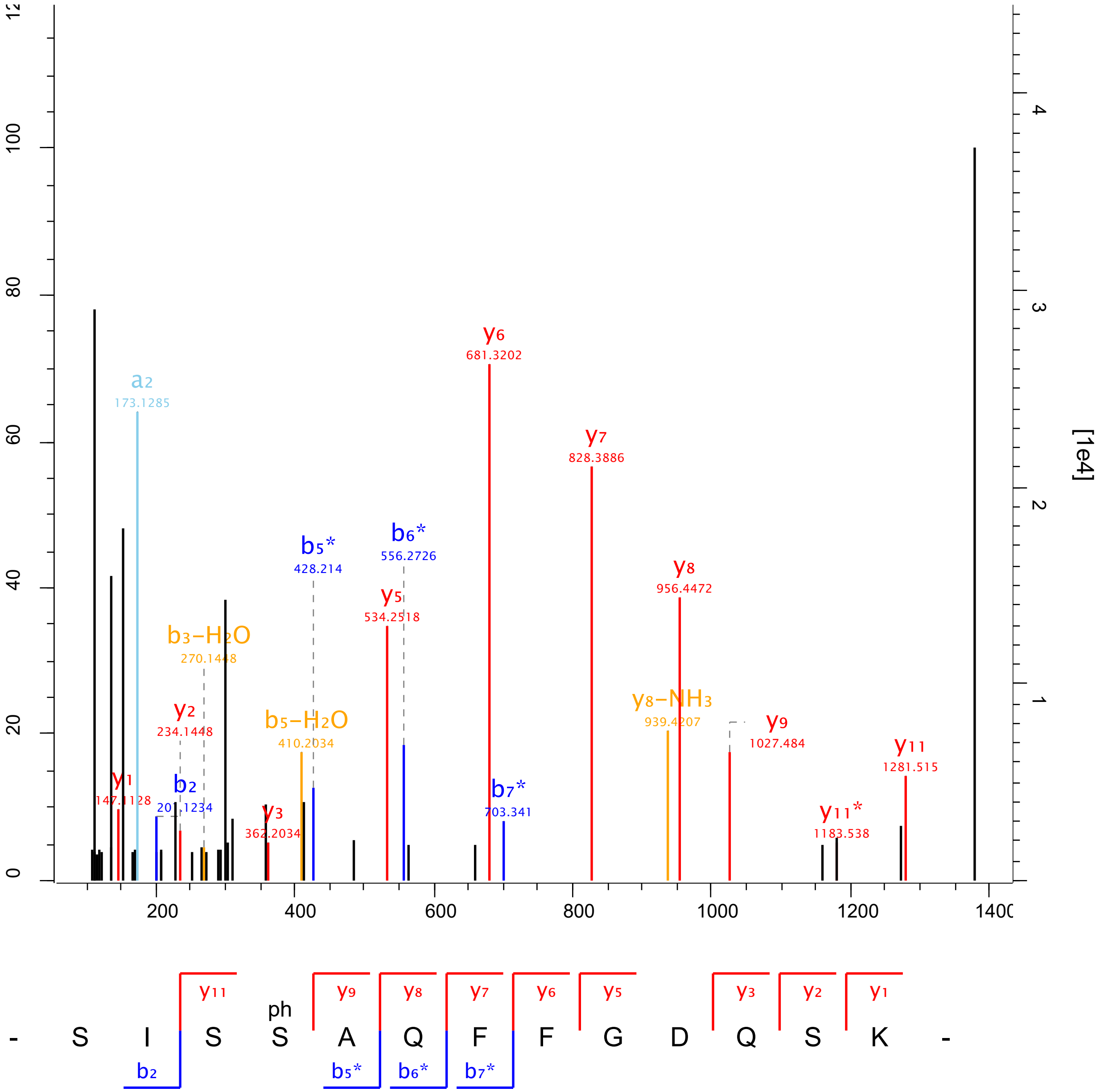

Raw file	Scan	Method	Score	m/z
sys_02_2	2792	FTMS; HCD	75.97	517.87

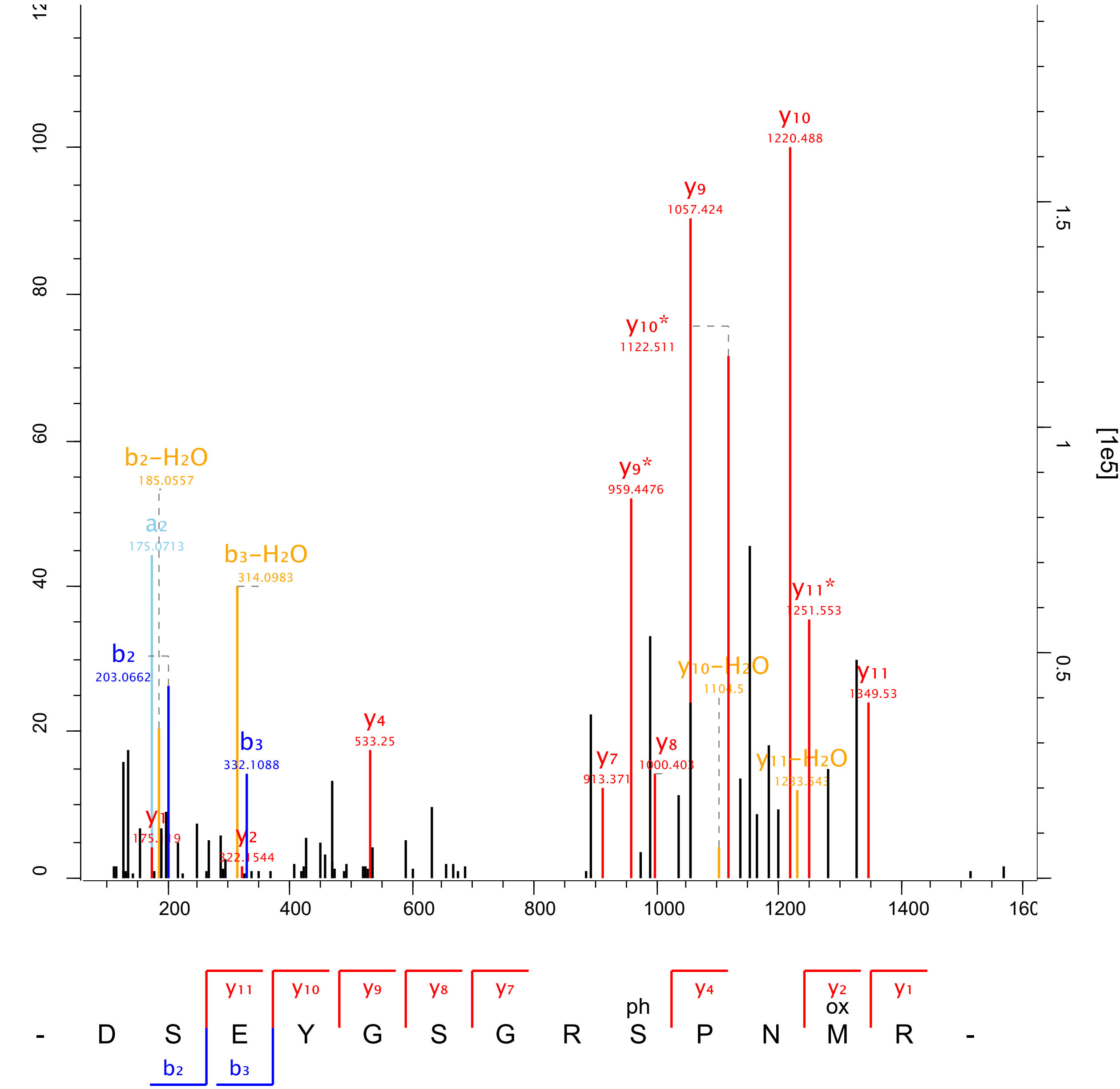

Raw file	Scan	Method	Score	m/z
sys_02_2	2798	FTMS; HCD	70.44	588.79

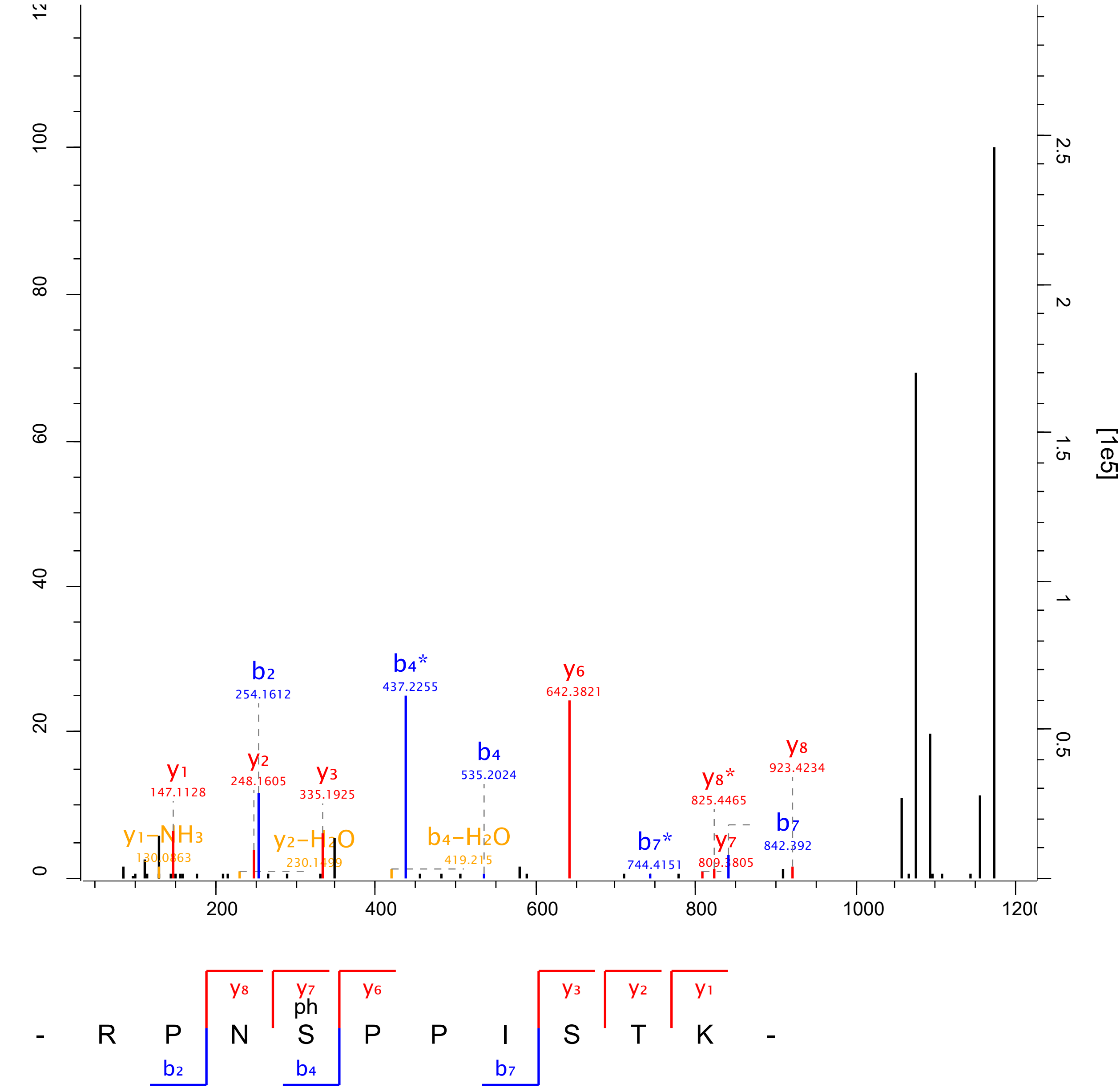

Raw file	Scan	Method	Score	m/z
sys_02_2	2803	FTMS; HCD	116.51	670.3

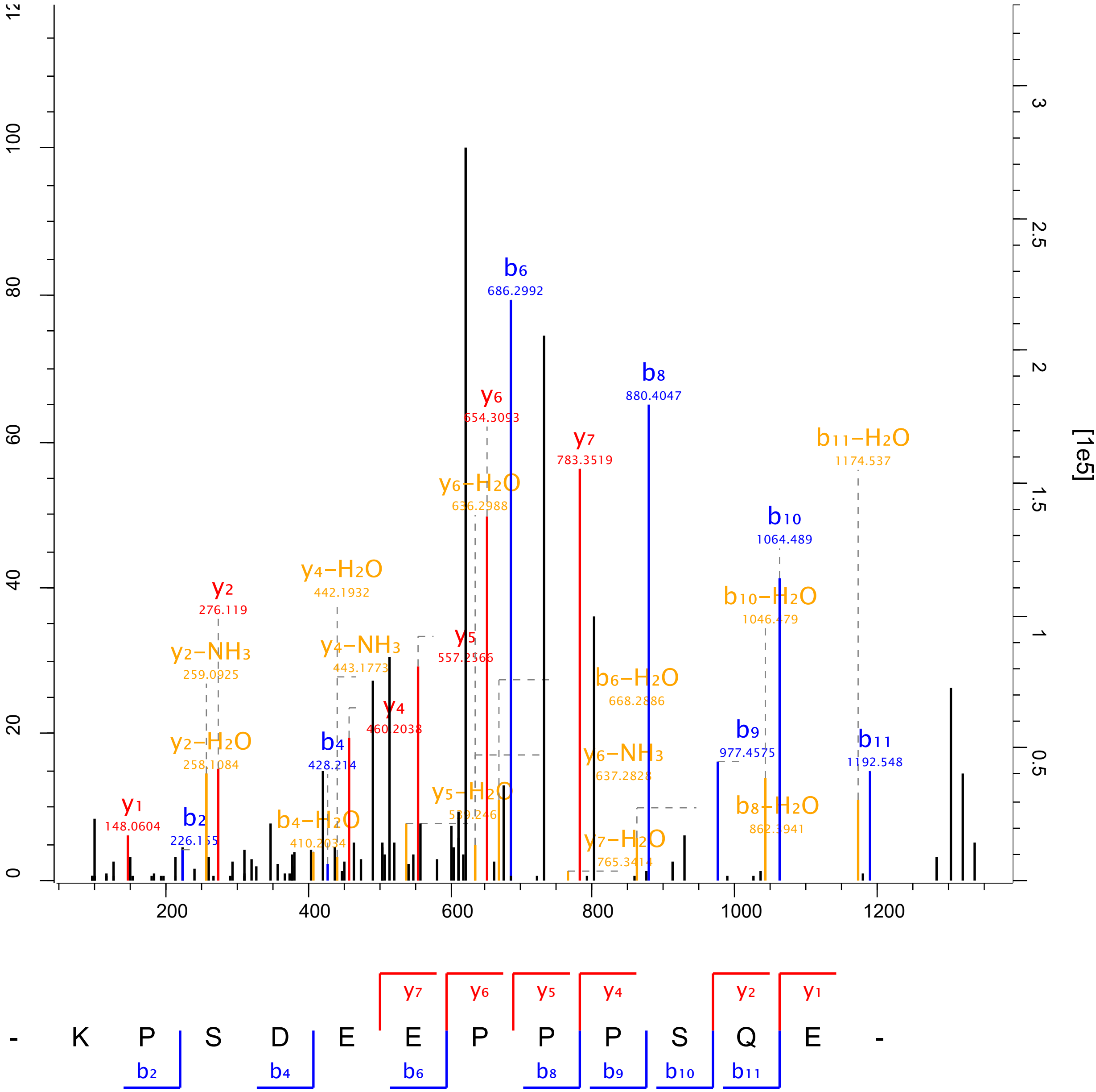

Raw file	Scan	Method	Score	m/z
sys_02_2	2844	FTMS; HCD	43.03	439.53

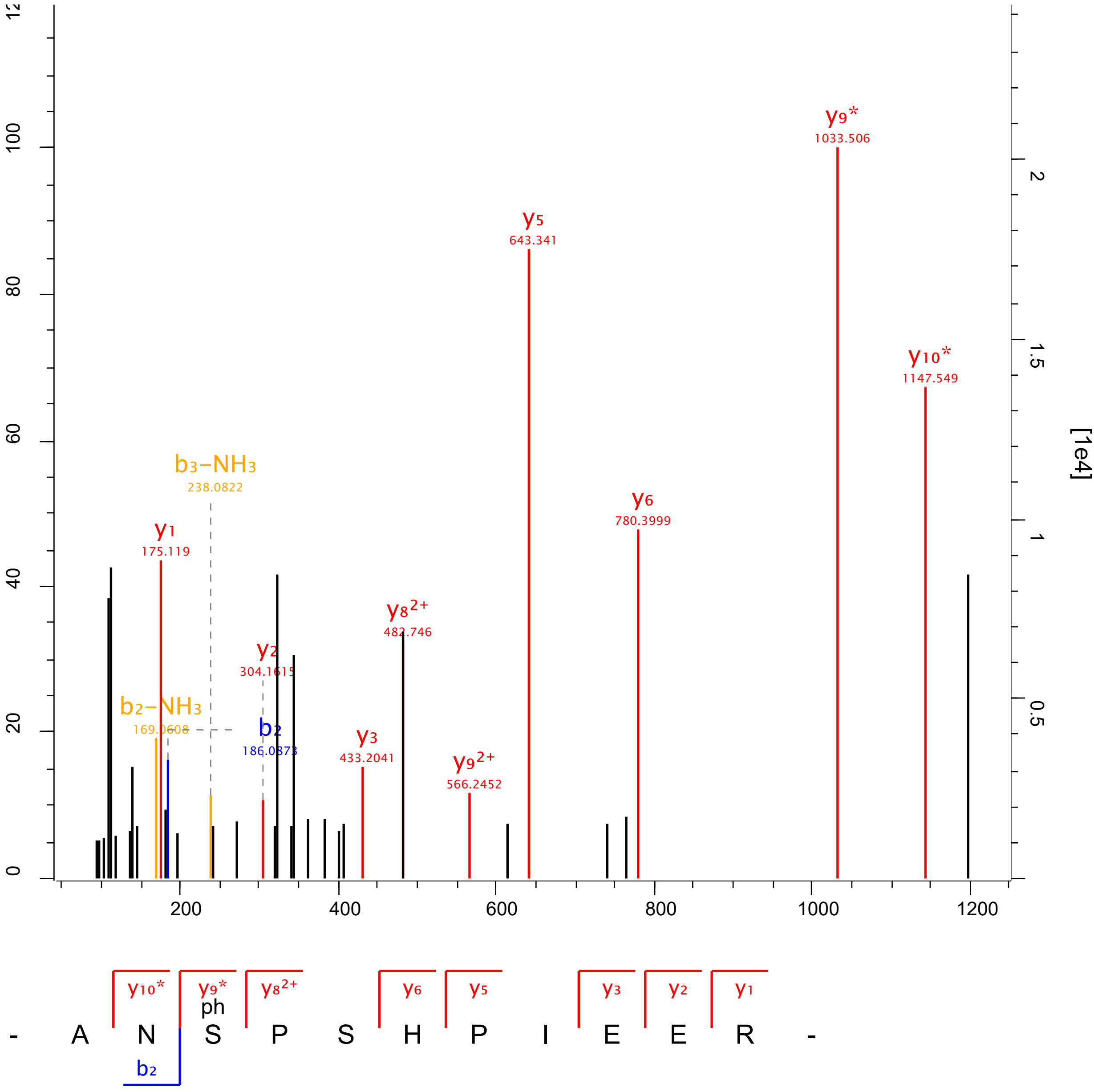

Raw file	Scan	Method	Score	m/z
sys_02_2	28733	FTMS; HCD	46.35	525.78

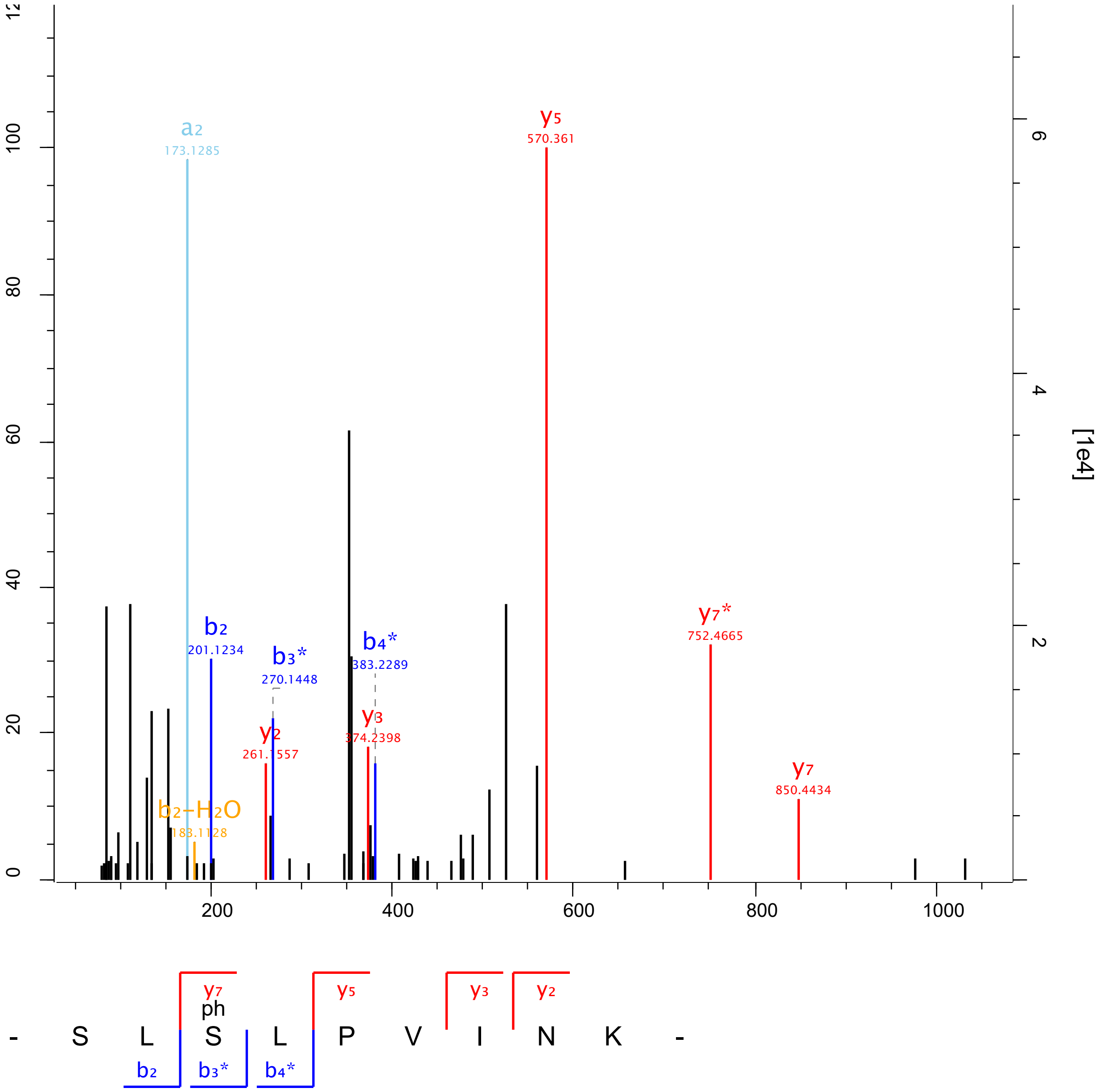

Raw file	Scan	Method	Score	m/z
sys_02_2	2886	FTMS; HCD	62.47	474.24

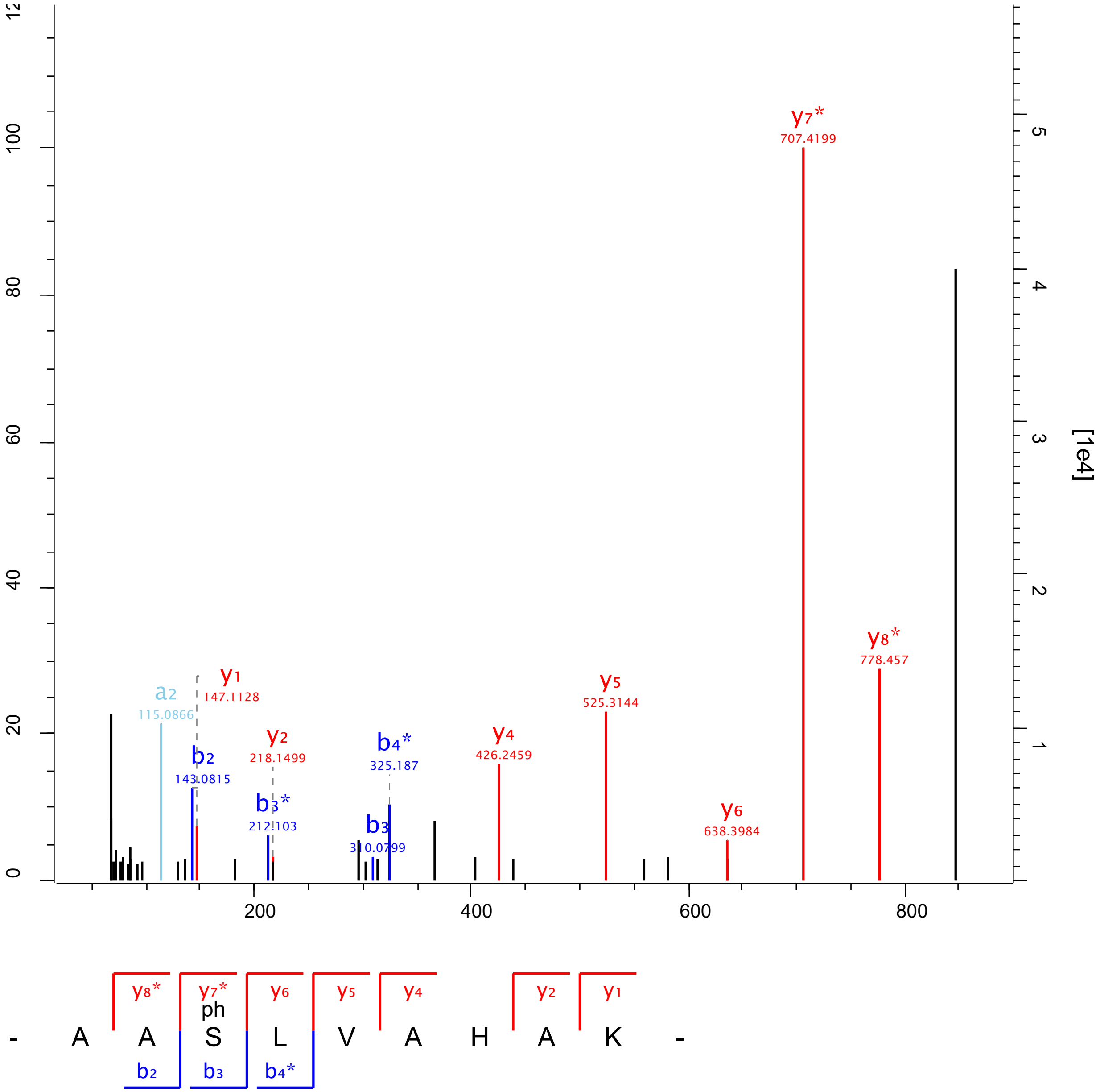

Raw file	Scan	Method	Score	m/z
sys_02_2	2949	FTMS; HCD	148.62	596.26

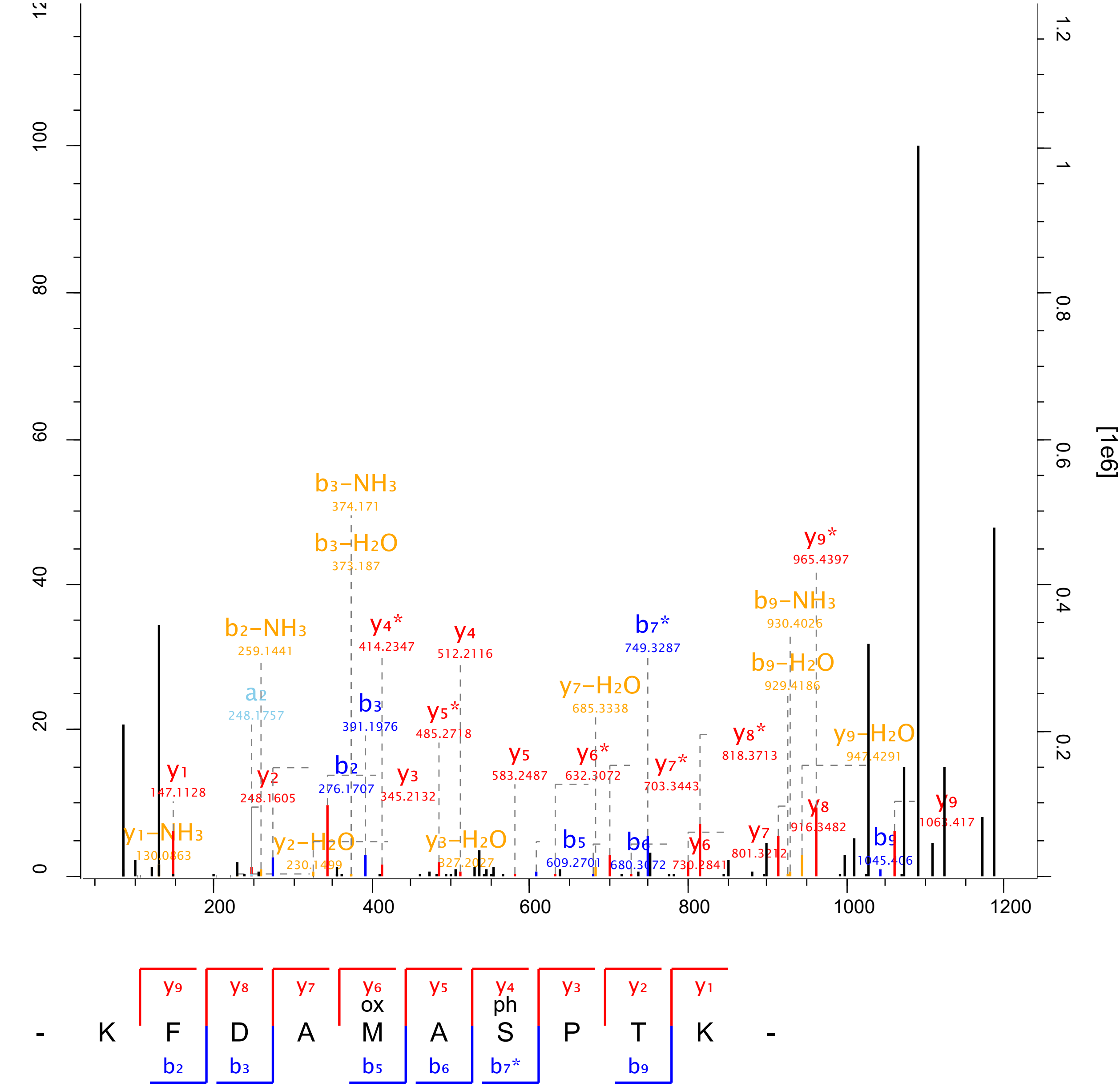

Raw file	Scan	Method	Score	m/z
sys_02_2	3026	FTMS; HCD	95.42	566.74

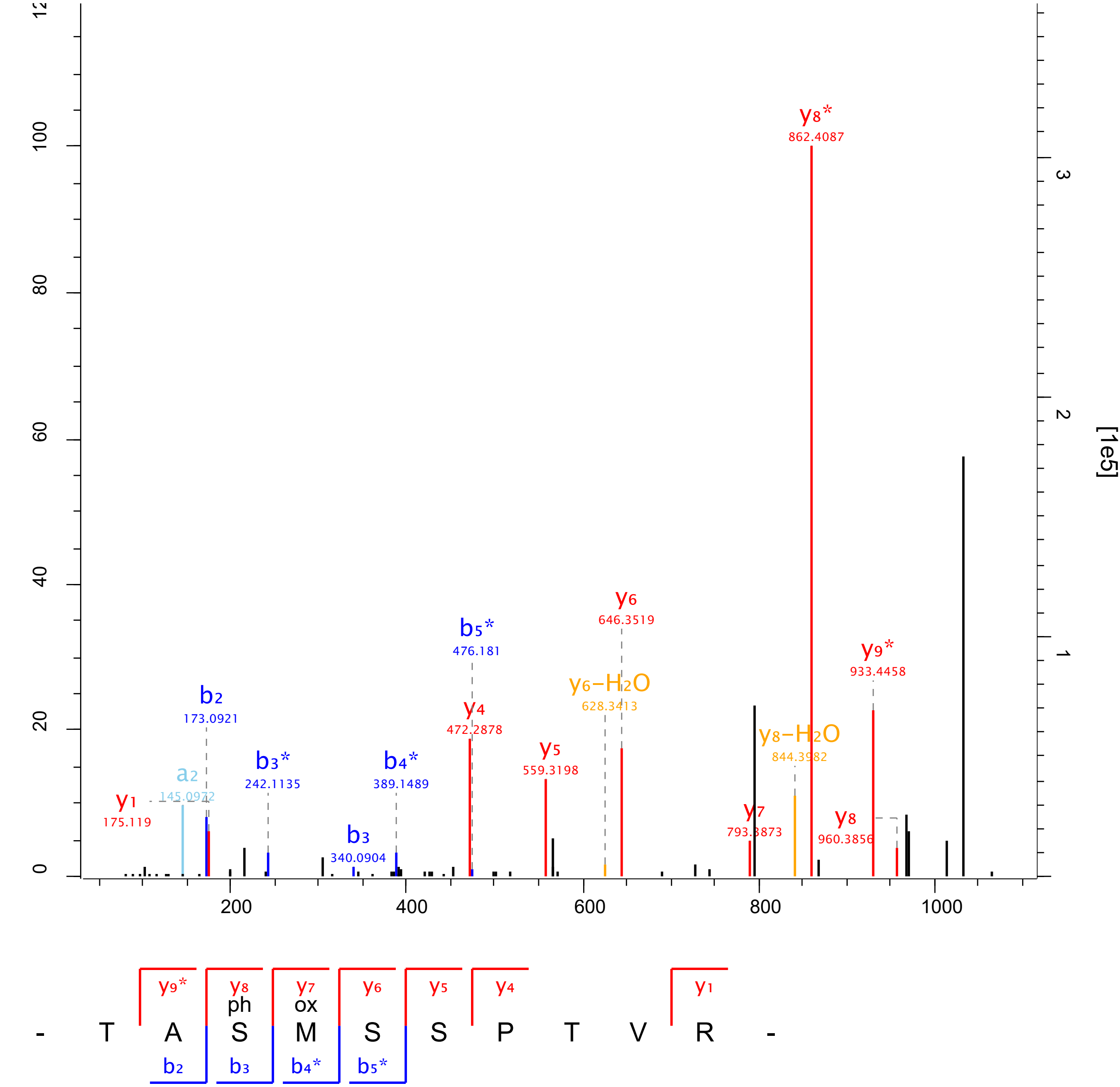

Raw file	Scan	Method	Score	m/z
sys_02_2	3061	FTMS; HCD	97.45	631.58

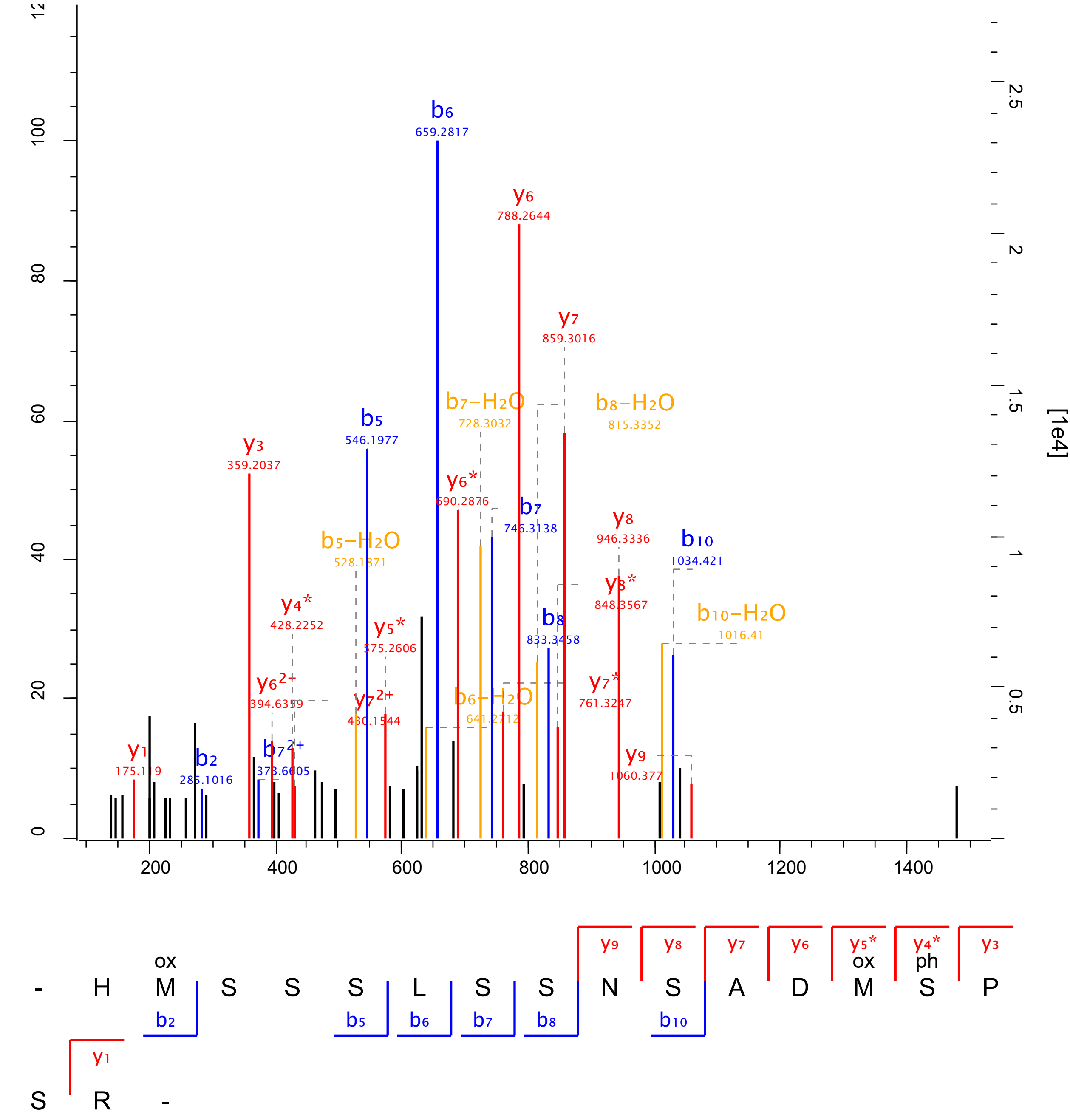

Raw file	Scan	Method	Score	m/z
sys_02_2	3115	FTMS; HCD	58.17	571.74

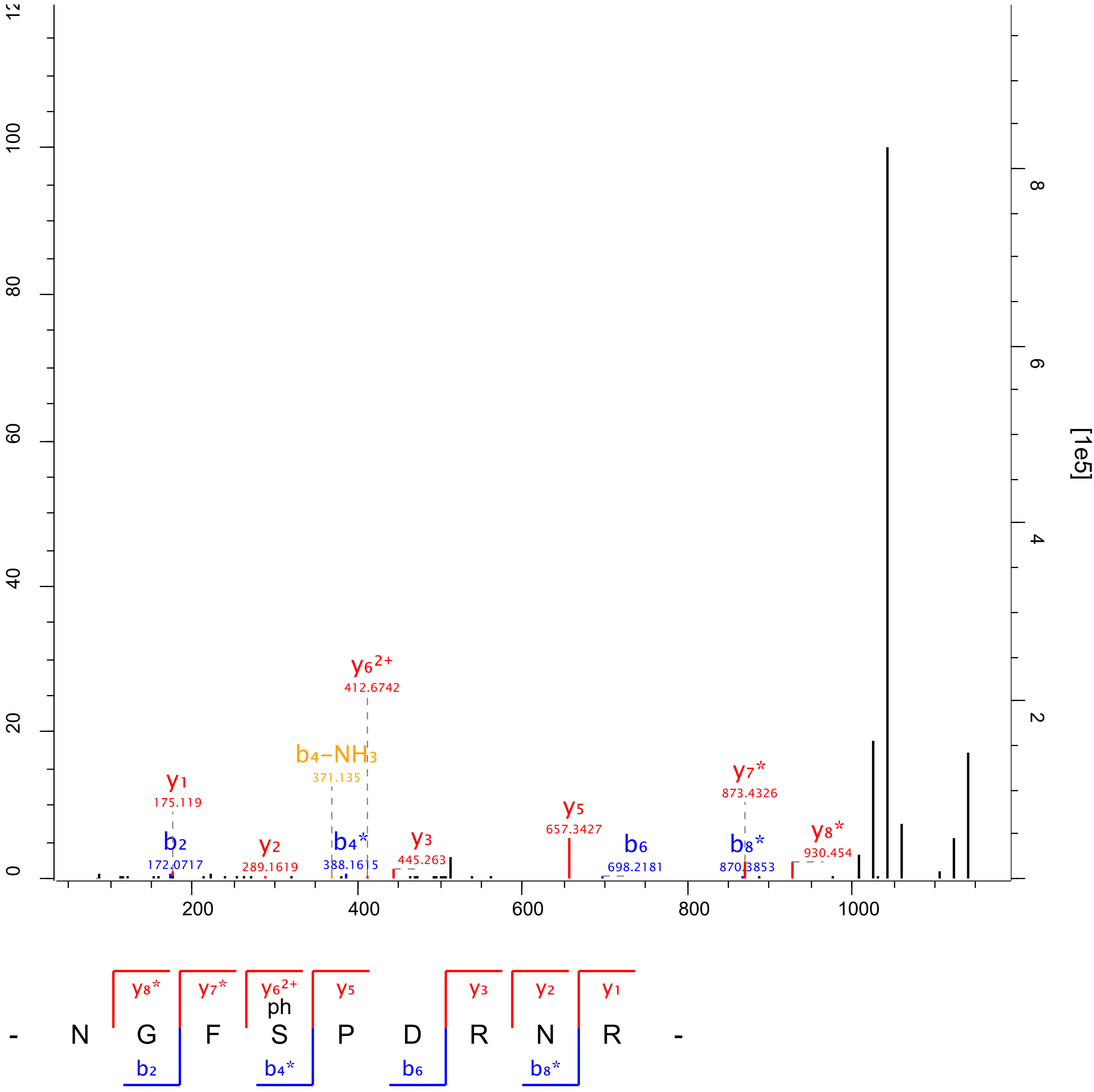

Raw file	Scan	Method	Score	m/z
sys_02_2	3227	FTMS; HCD	50.39	533.21

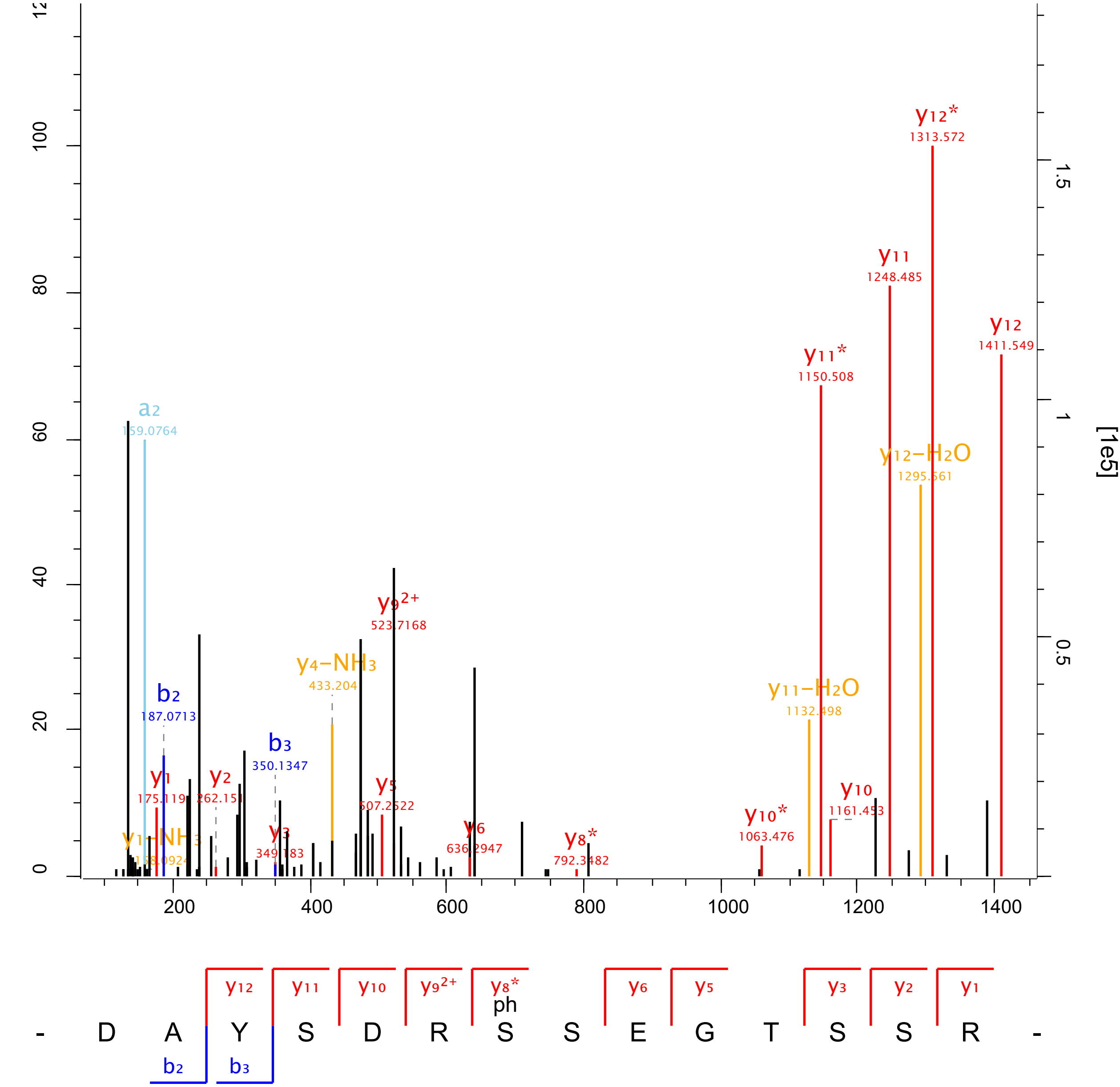

Raw file	Scan	Method	Score	m/z
sys_02_2	3258	FTMS; HCD	42.34	563.24

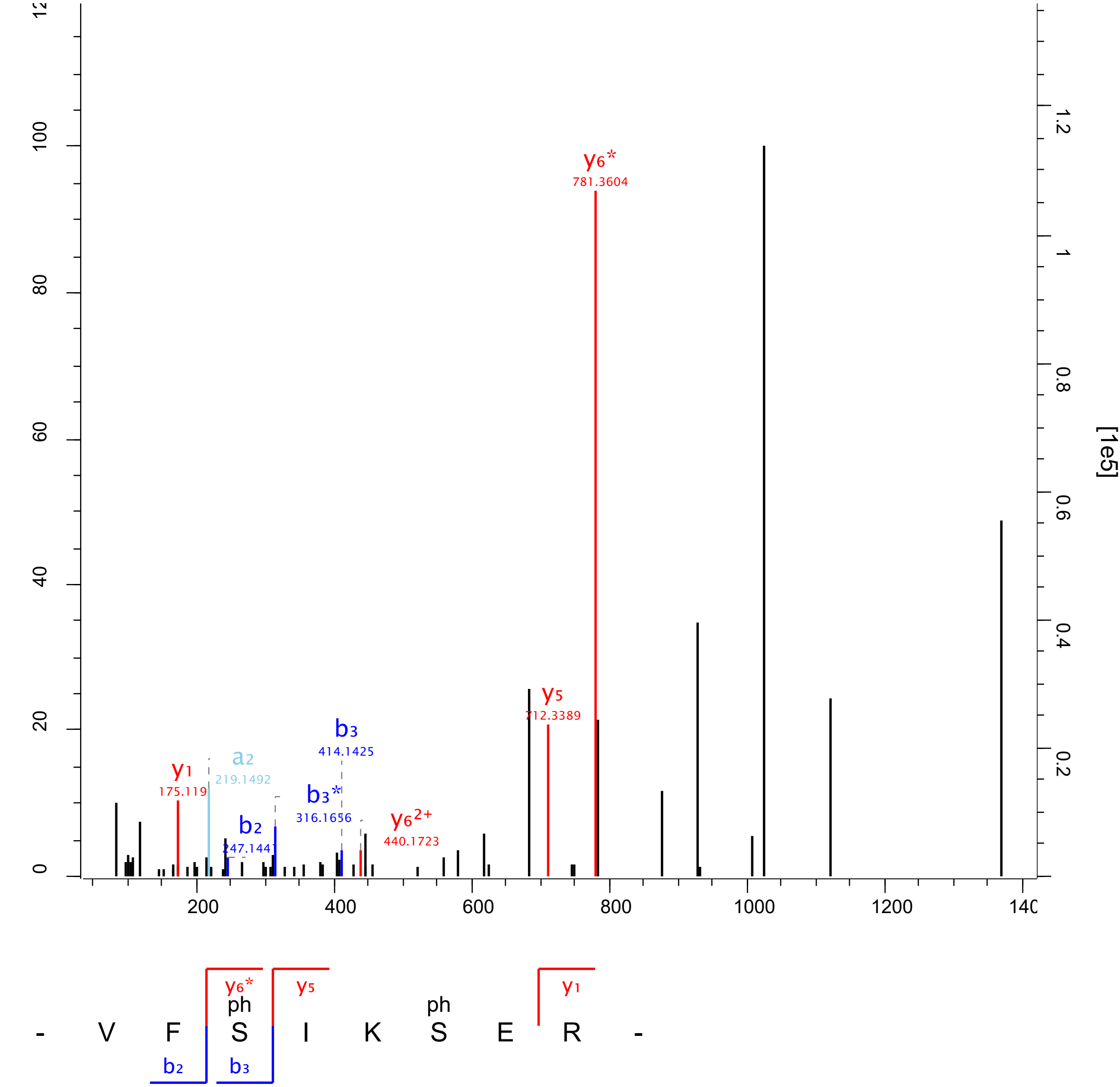

Raw file	Scan	Method	Score	m/z
sys_02_2	32914	FTMS; HCD	230.82	850.36

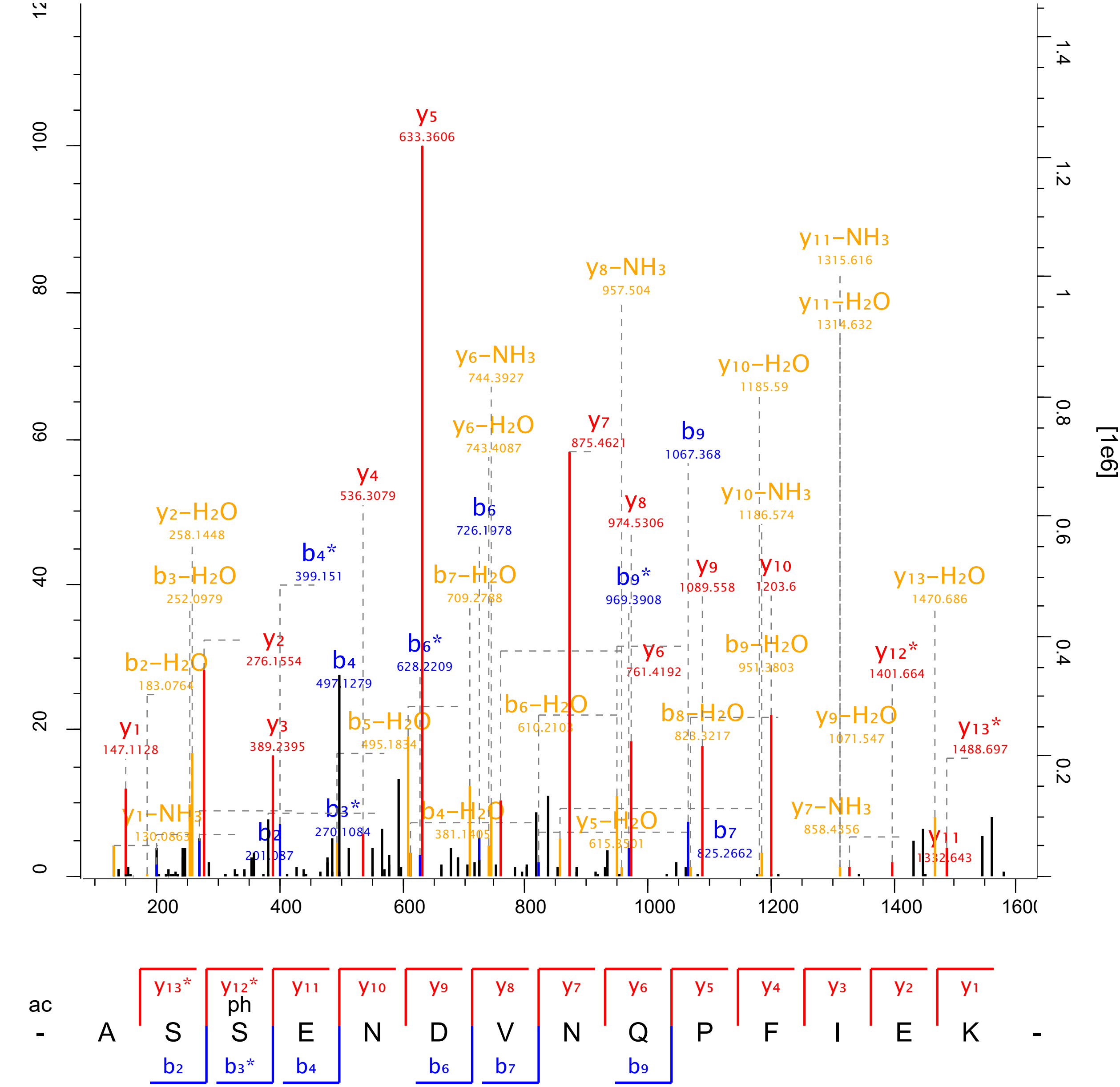

Raw file	Scan	Method	Score	m/z
sys_02_2	3307	FTMS; HCD	107.99	554.25

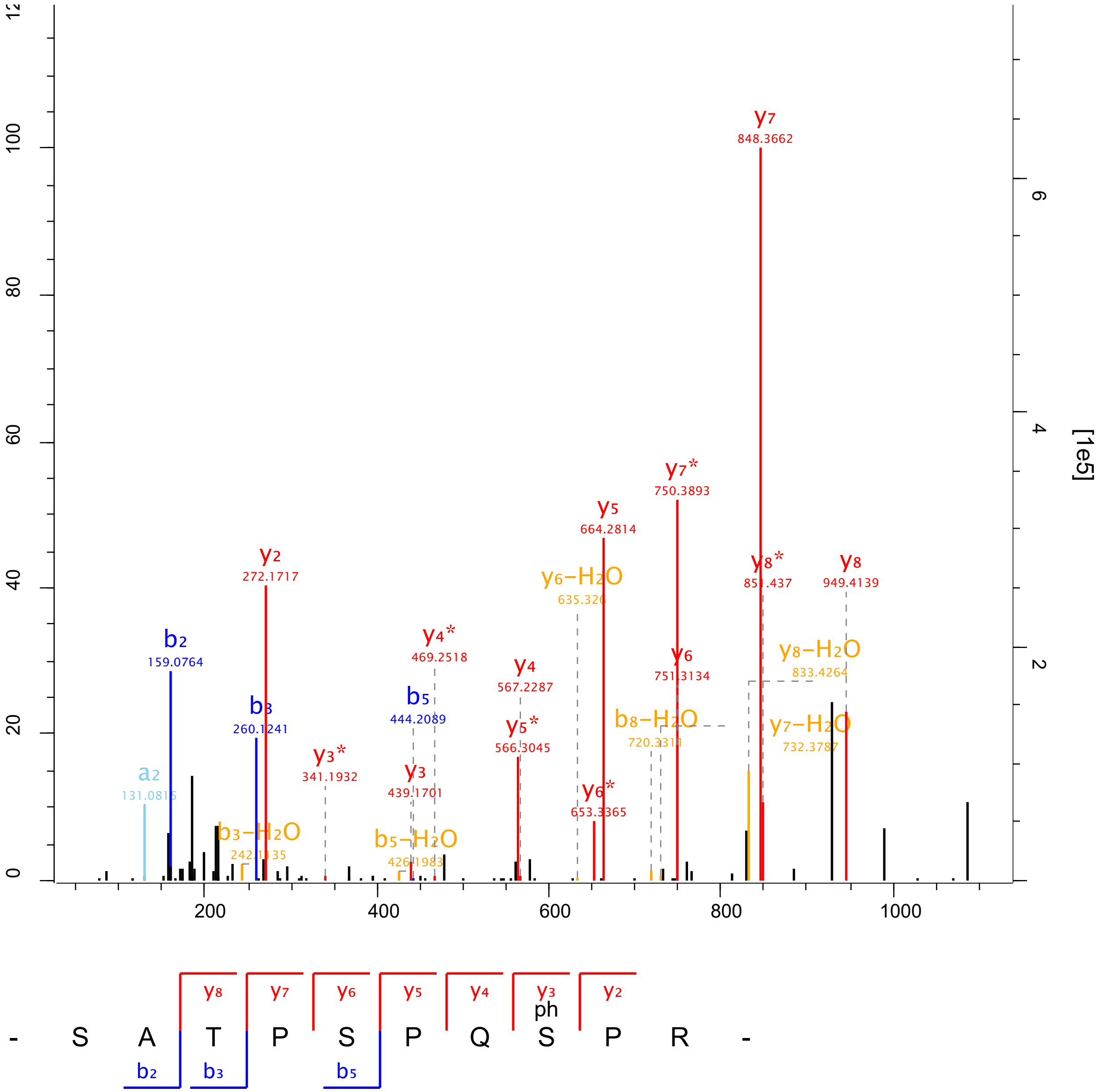

Raw file	Scan	Method	Score	m/z
sys_02_2	3320	FTMS; HCD	60.49	431.87

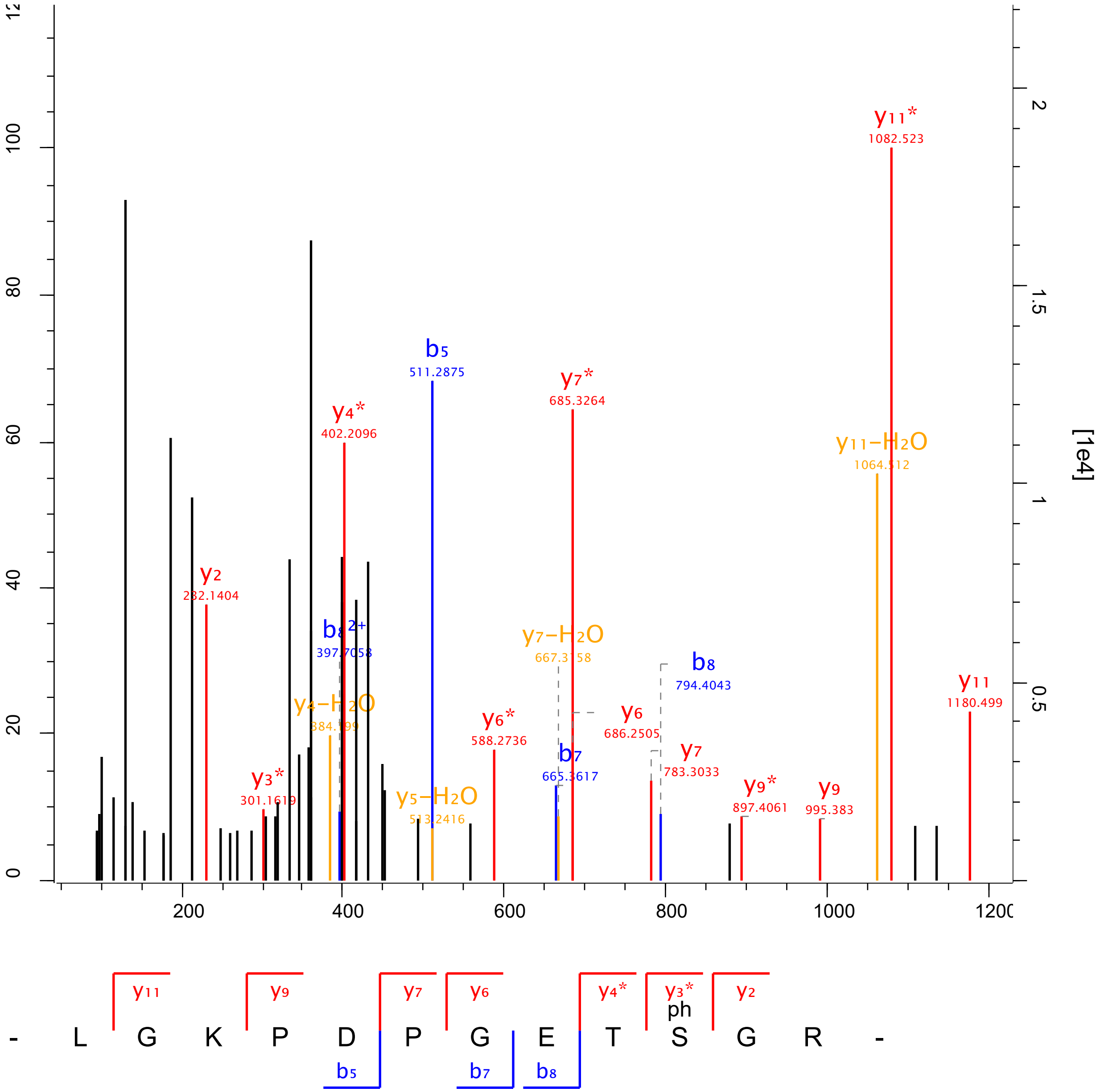

Raw file	Scan	Method	Score	m/z
sys_02_2	3426	FTMS; HCD	240.42	587.58

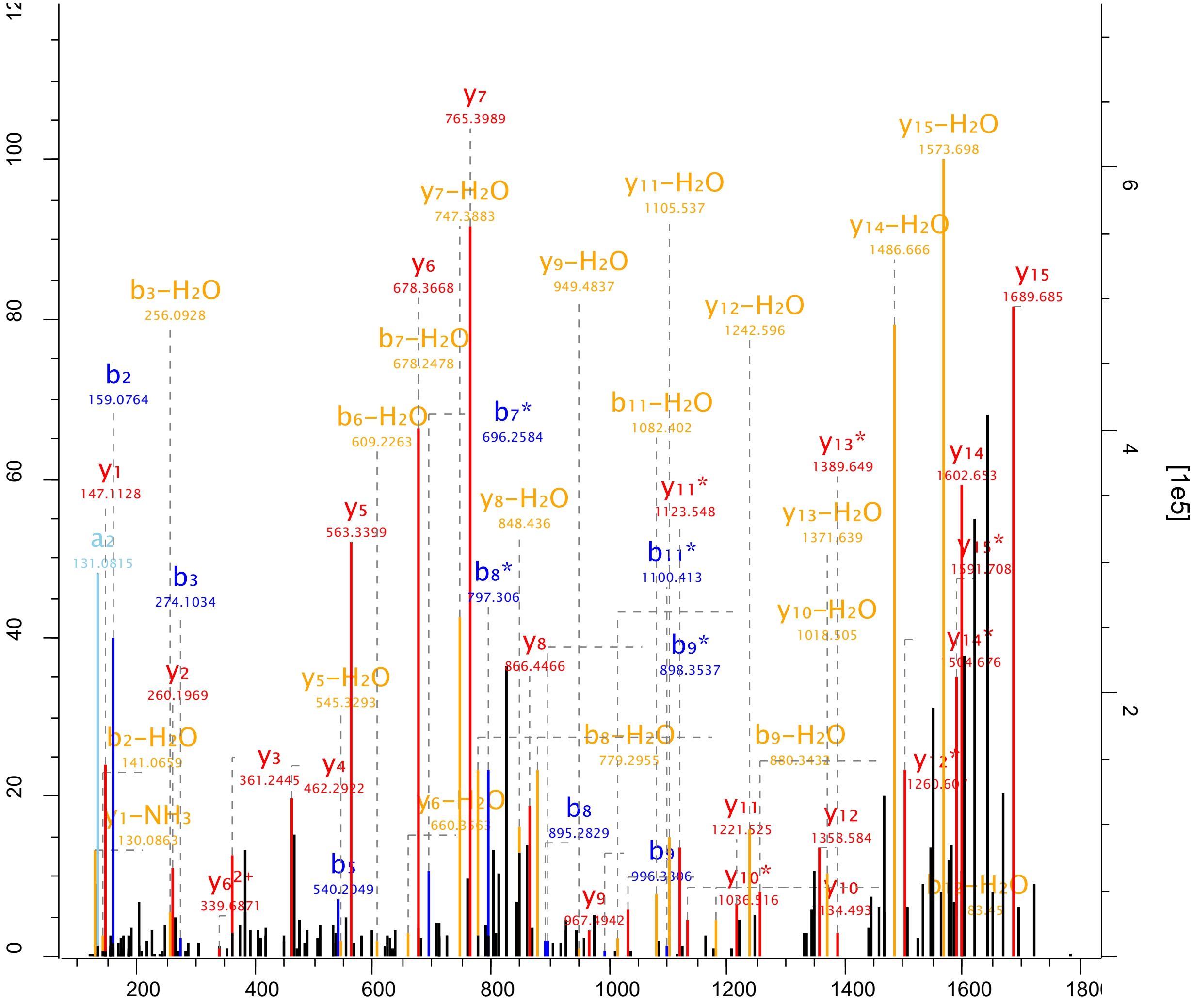

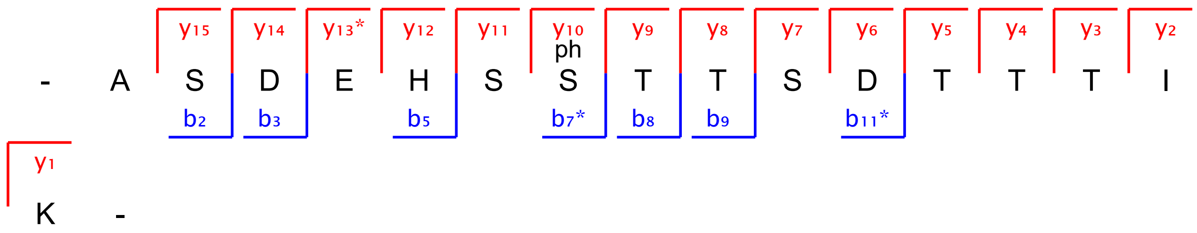

Raw file	Scan	Method	Score	m/z
sys_02_2	3437	FTMS; HCD	88.07	851.83

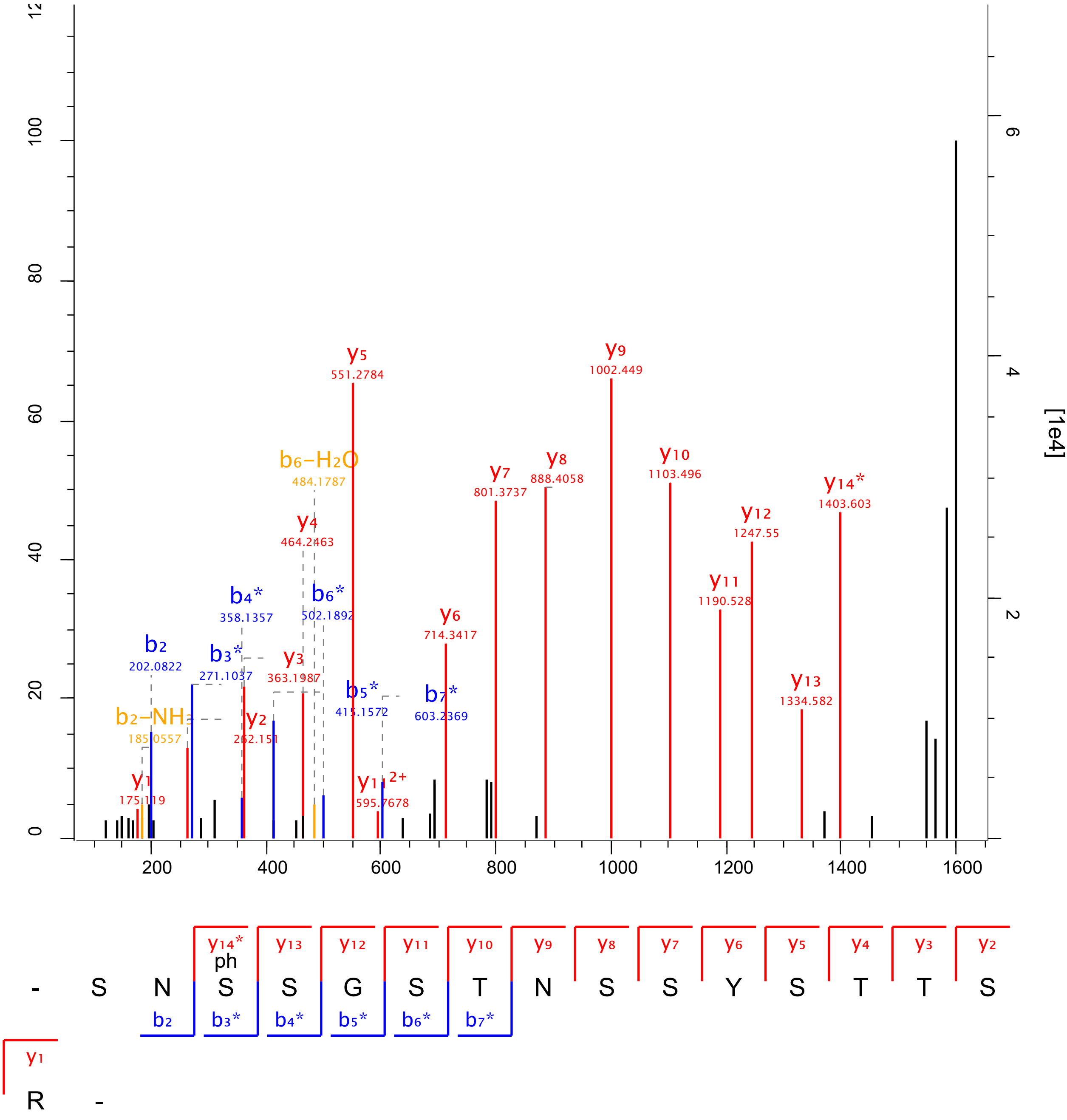

Raw file	Scan	Method	Score	m/z
sys_02_2	3460	FTMS; HCD	168	611.77

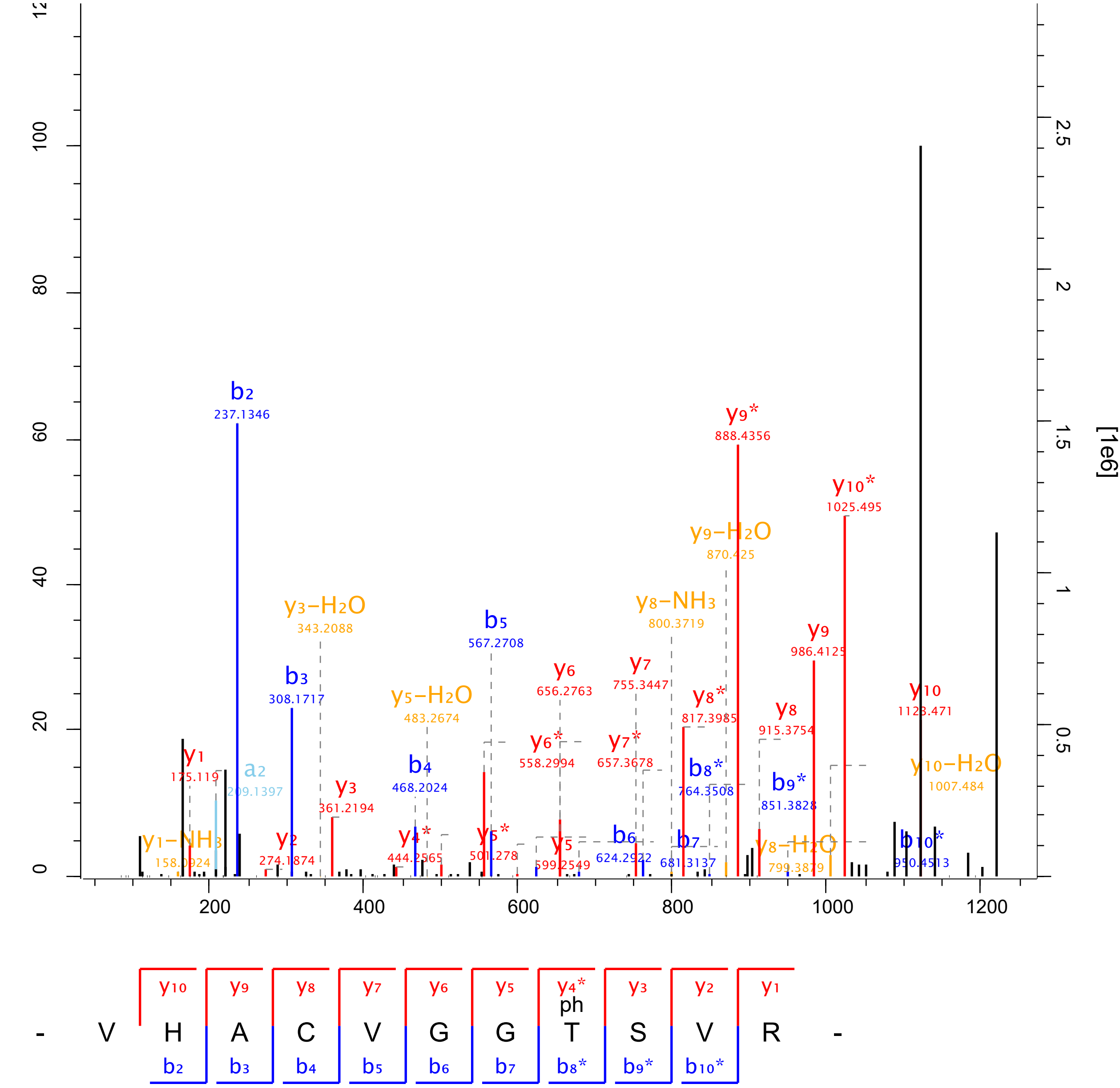

Raw file	Scan	Method	Score	m/z
sys_02_2	3518	FTMS; HCD	80.24	649.61

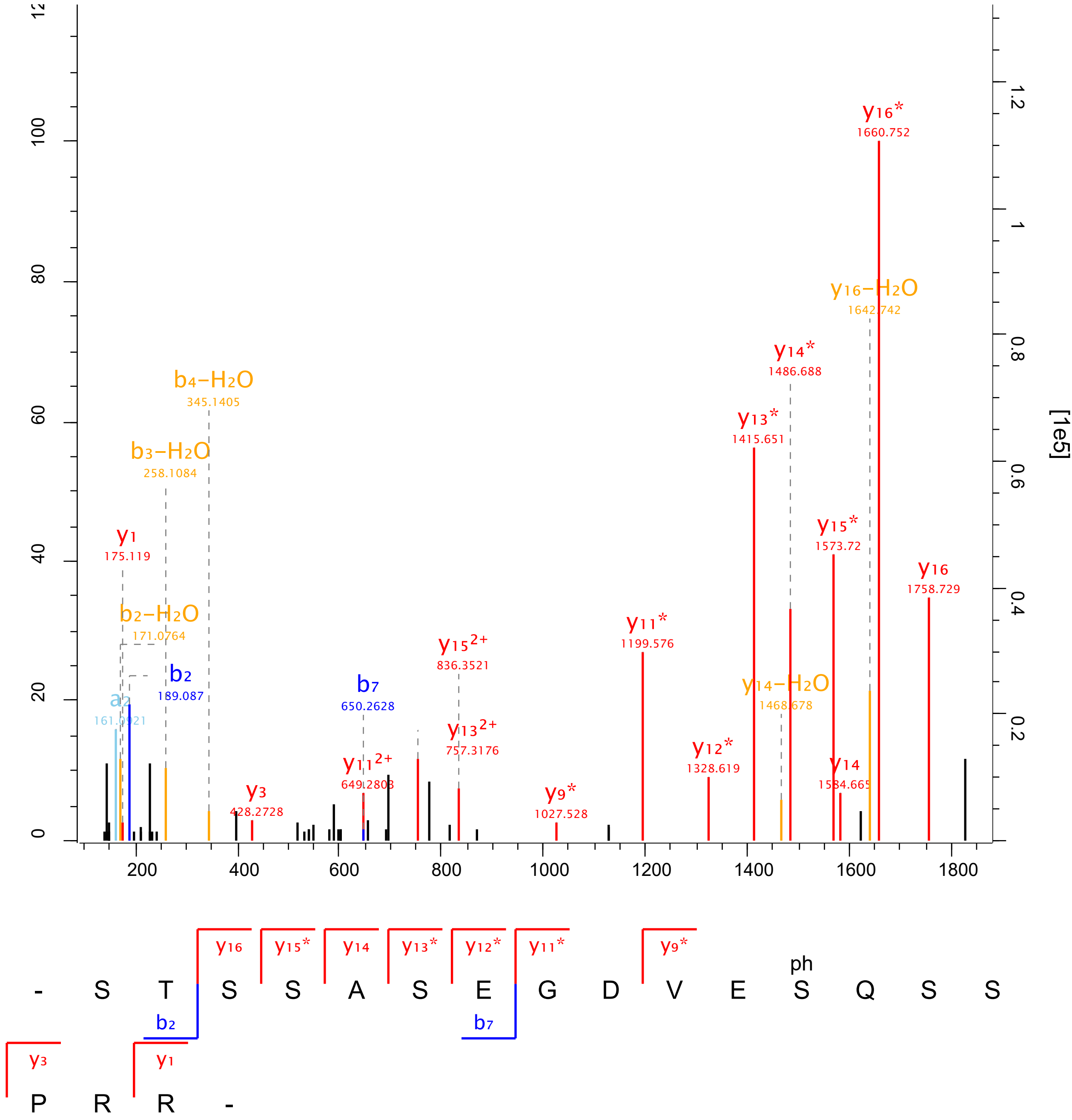

Raw file	Scan	Method	Score	m/z
sys_02_2	3556	FTMS; HCD	89.47	605.59

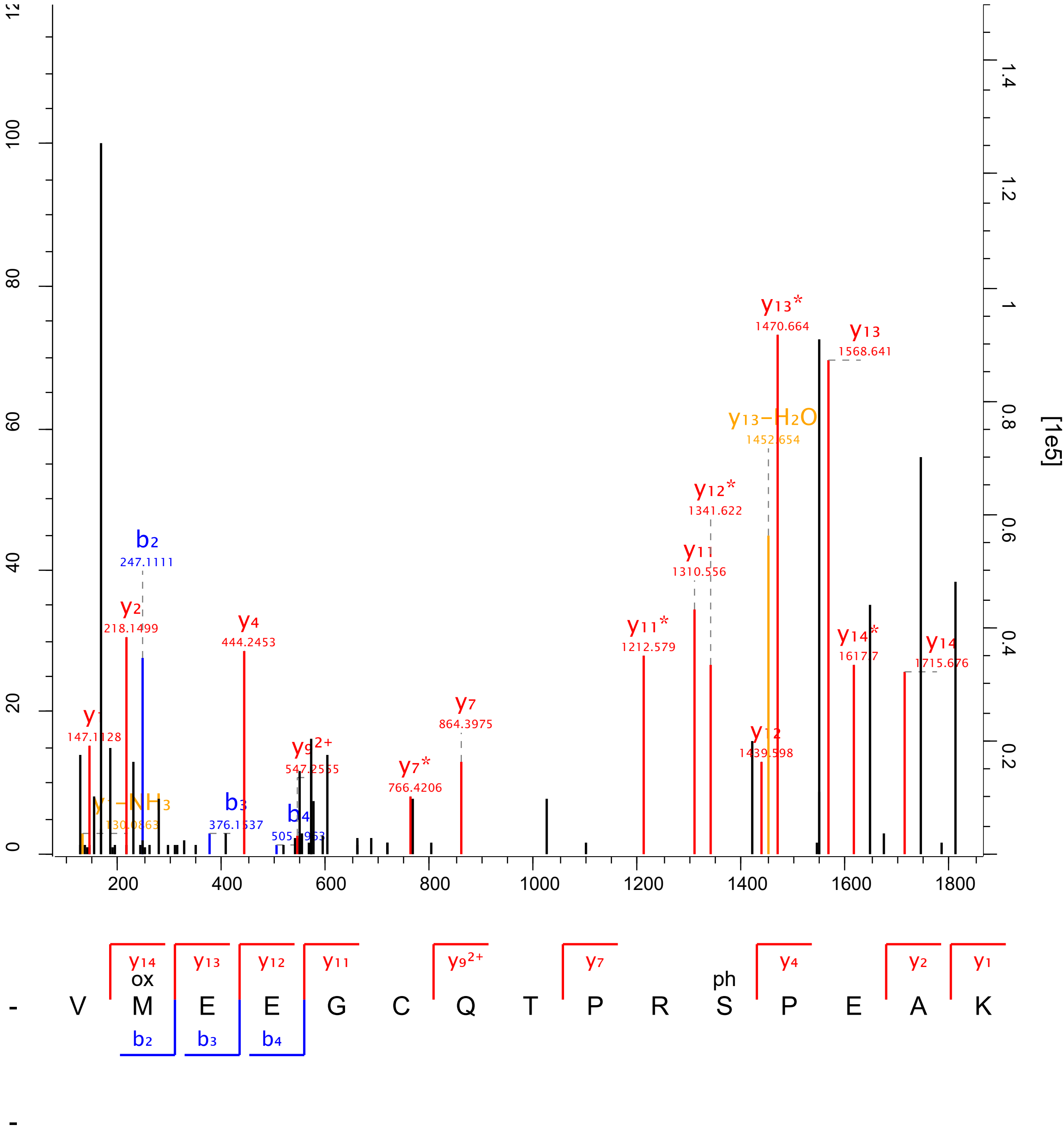

Raw file	Scan	Method	Score	m/z
sys_02_2	3754	FTMS; HCD	62.86	526.2

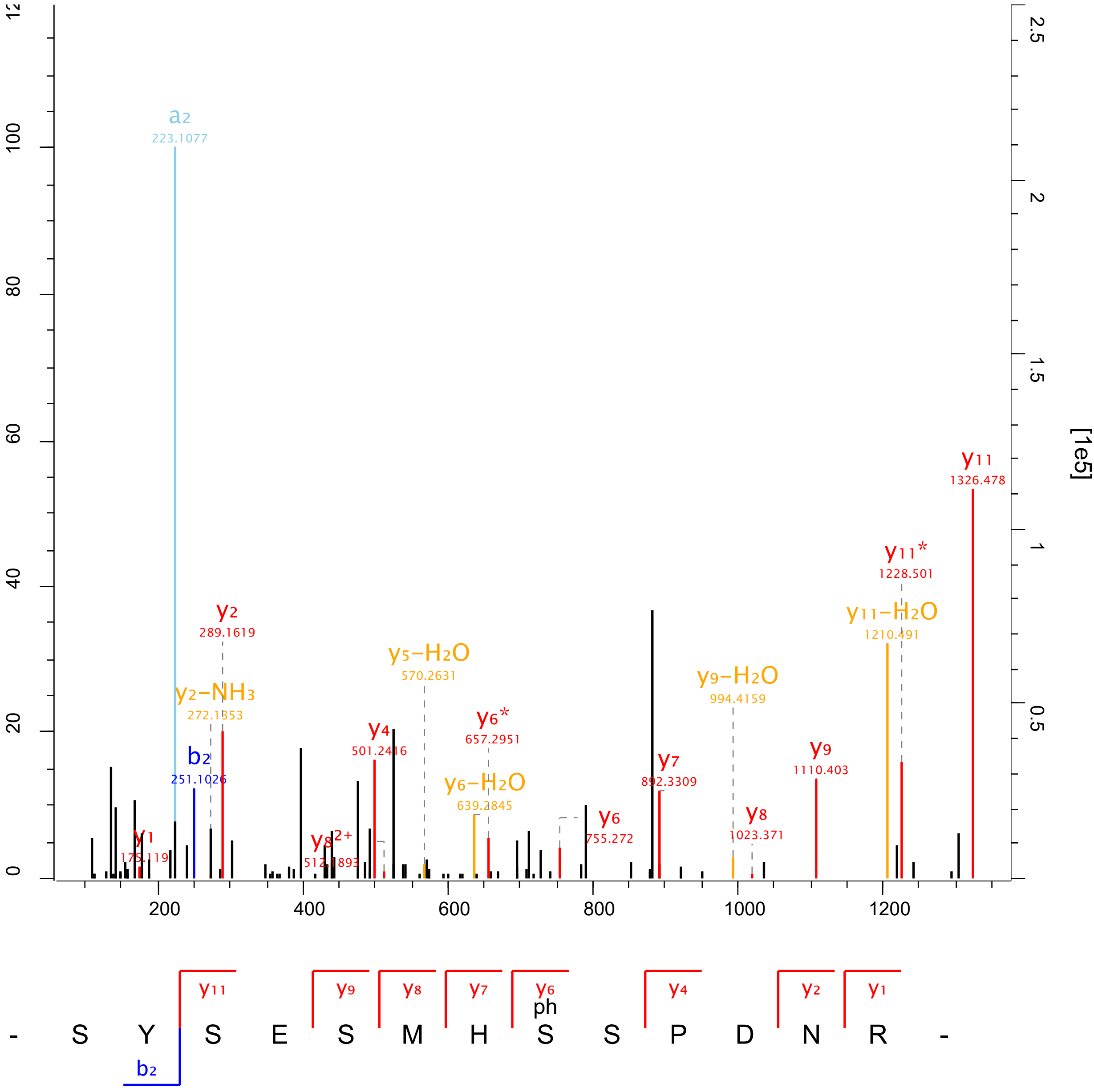

Raw file	Scan	Method	Score	m/z
sys_02_2	3791	FTMS; HCD	168.43	586.27

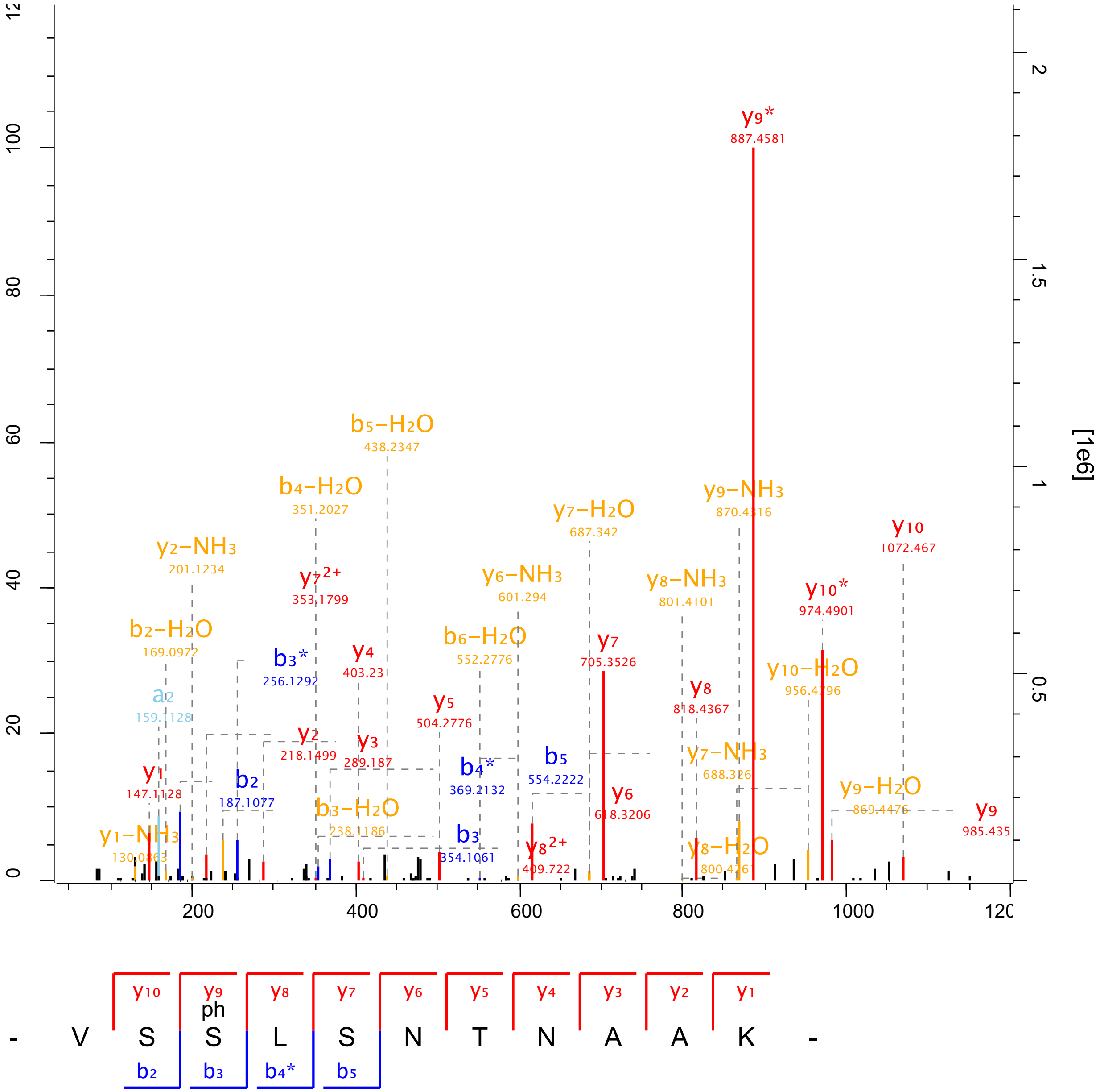

Raw file	Scan	Method	Score	m/z
sys_02_2	3795	FTMS; HCD	58.89	465.89

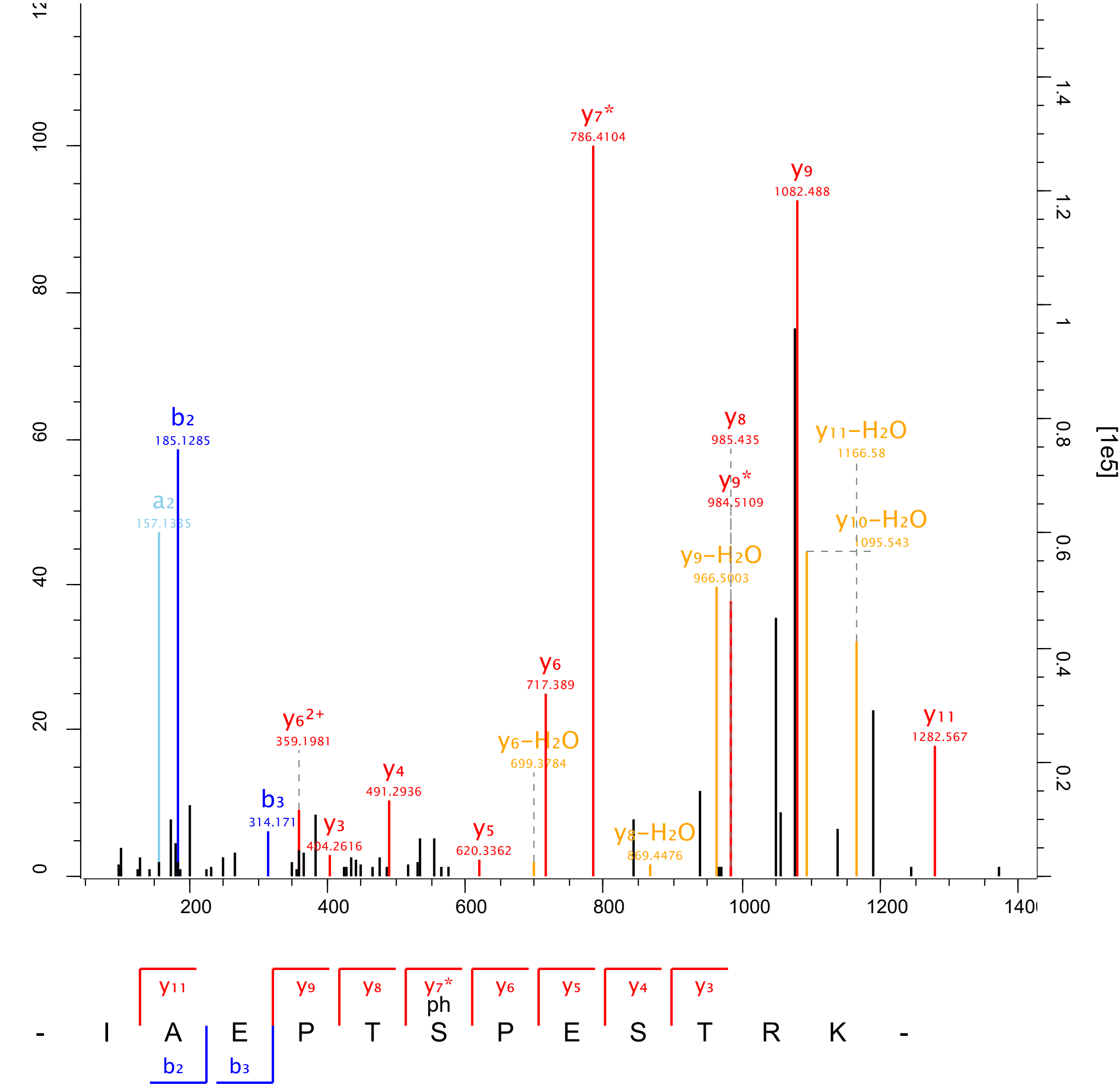

Raw file	Scan	Method	Score	m/z
sys_02_2	38641	FTMS; HCD	206.49	1030.96

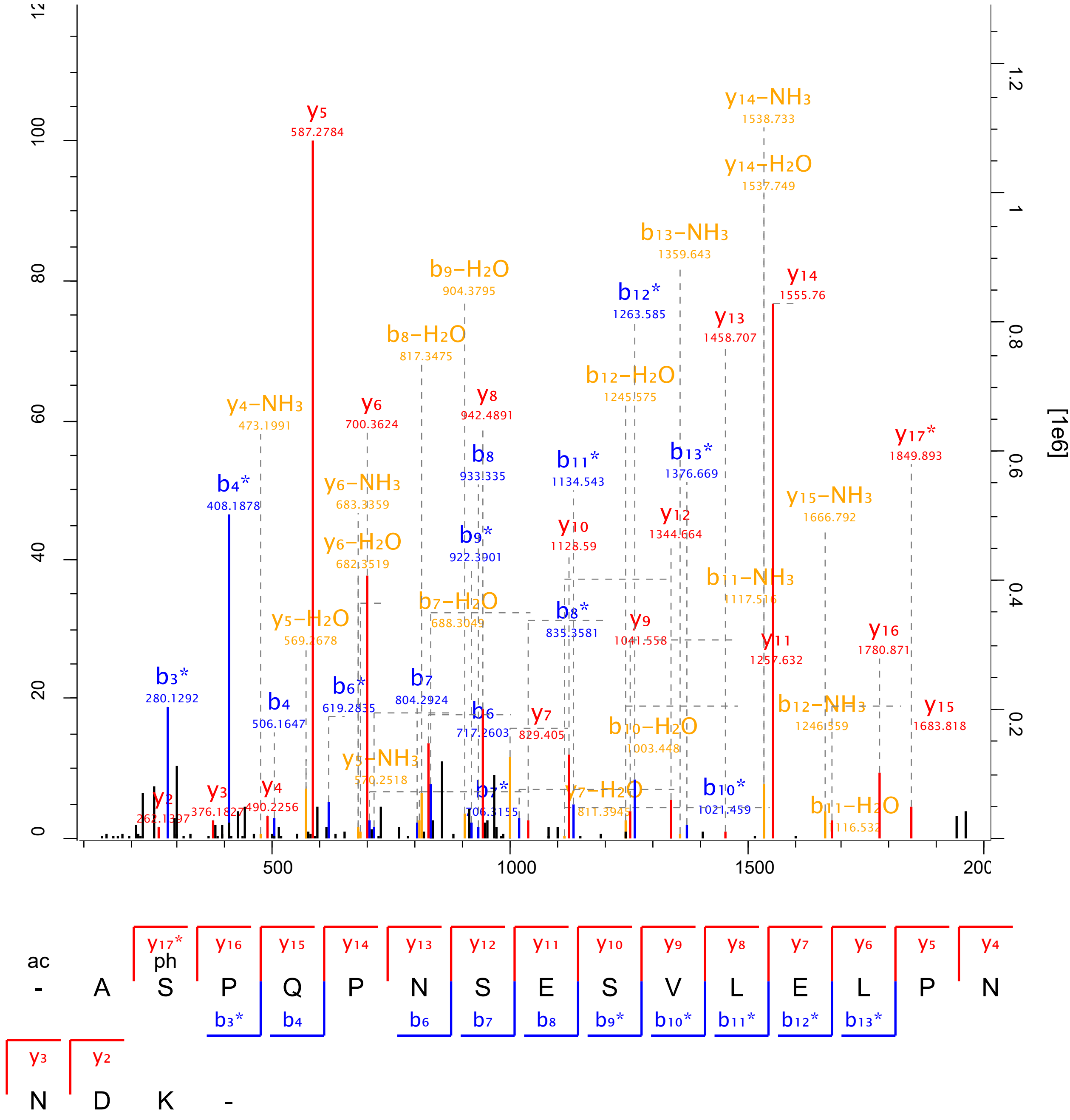

Raw file	Scan	Method	Score	m/z
sys_02_2	3950	FTMS; HCD	40.48	615.27

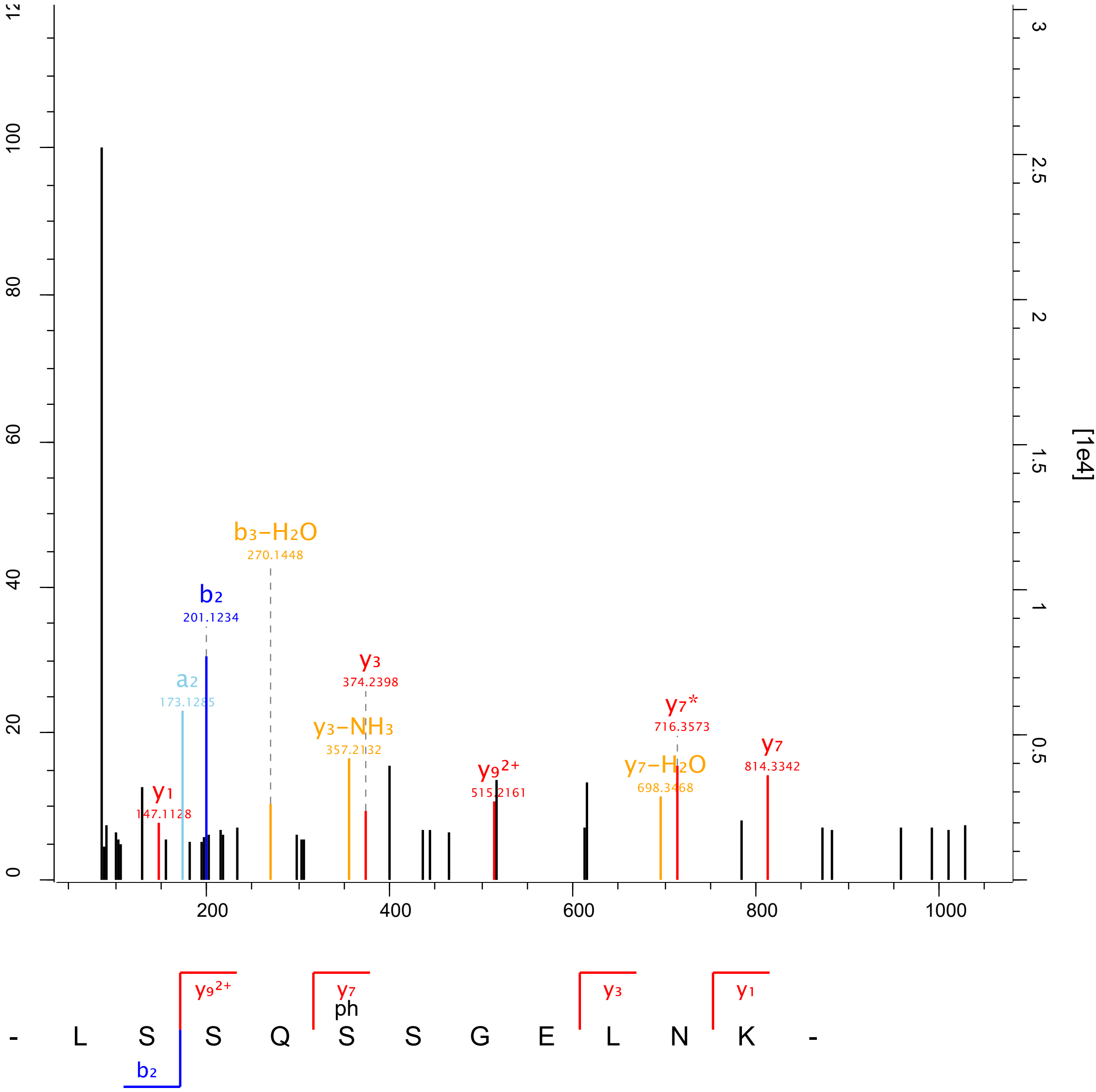

Raw file	Scan	Method	Score	m/z
sys_02_2	3953	FTMS; HCD	124.6	402.19

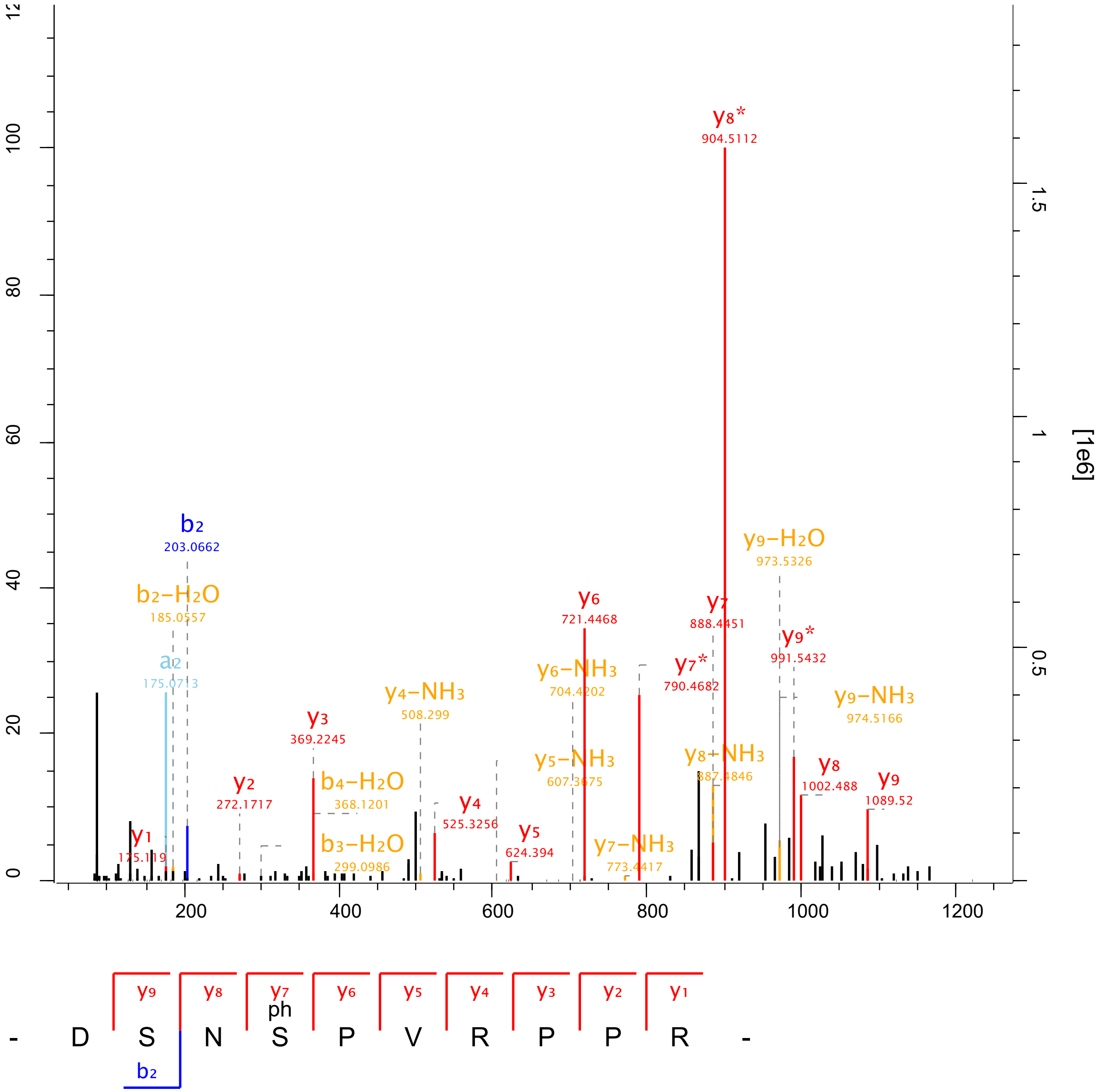

Raw file	Scan	Method	Score	m/z
sys_02_2	4013	FTMS; HCD	70.2	738.79

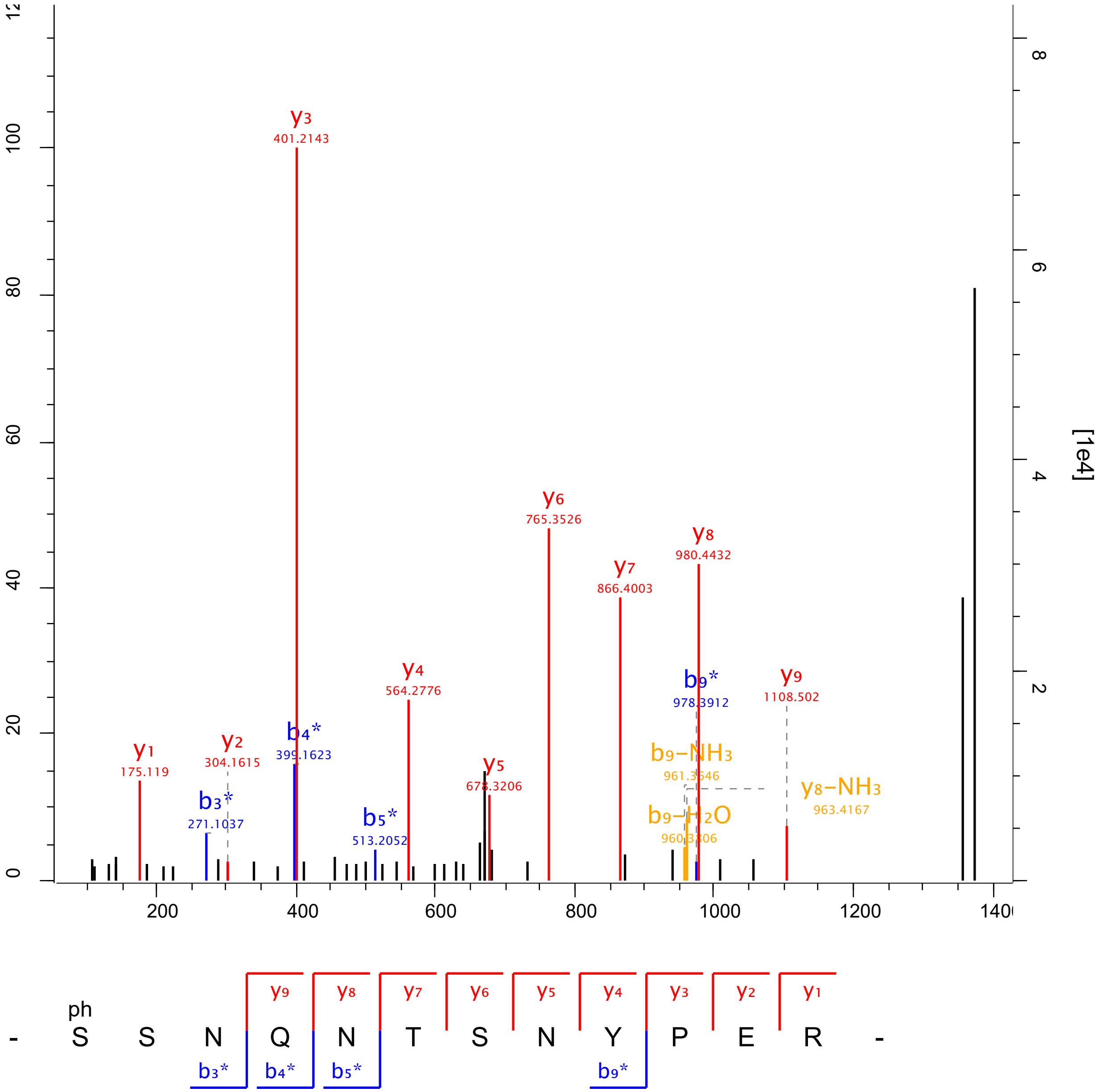

Raw file	Scan	Method	Score	m/z
sys_02_2	4025	FTMS; HCD	149.83	546.78

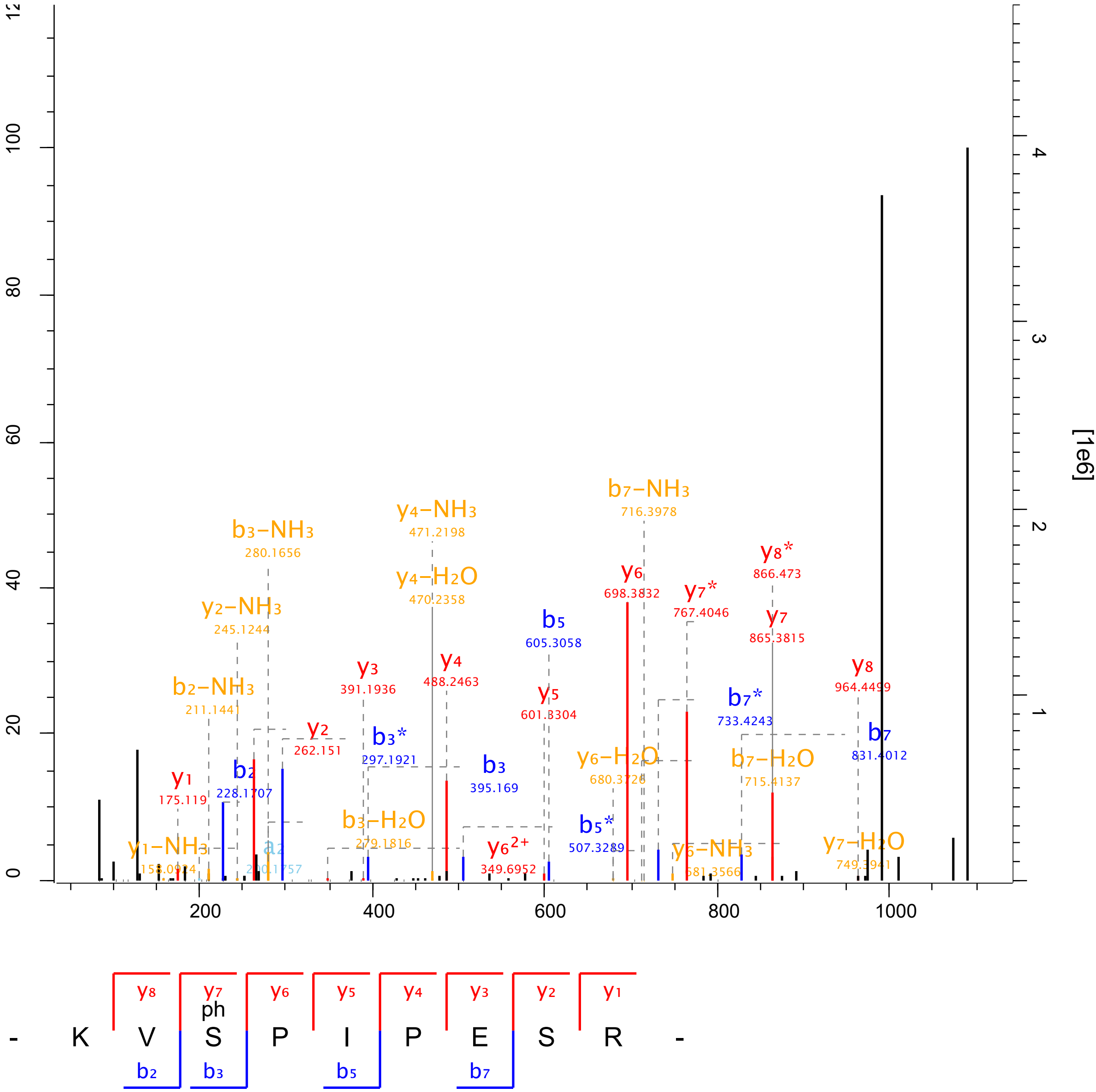

Raw file	Scan	Method	Score	m/z
sys_02_2	4034	FTMS; HCD	90.56	464.88

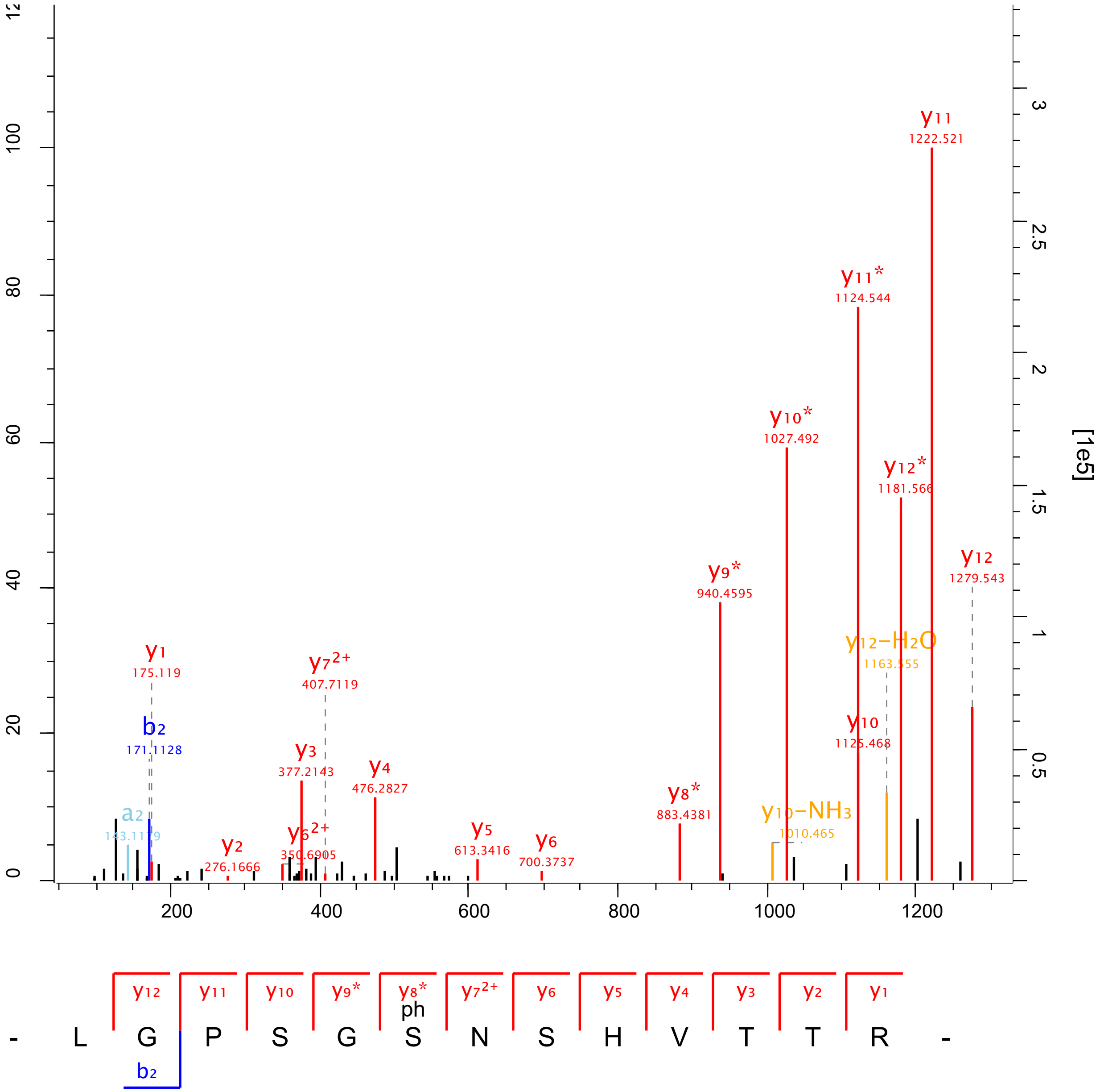

Raw file	Scan	Method	Score	m/z
sys_02_2	4061	FTMS; HCD	68	569.26

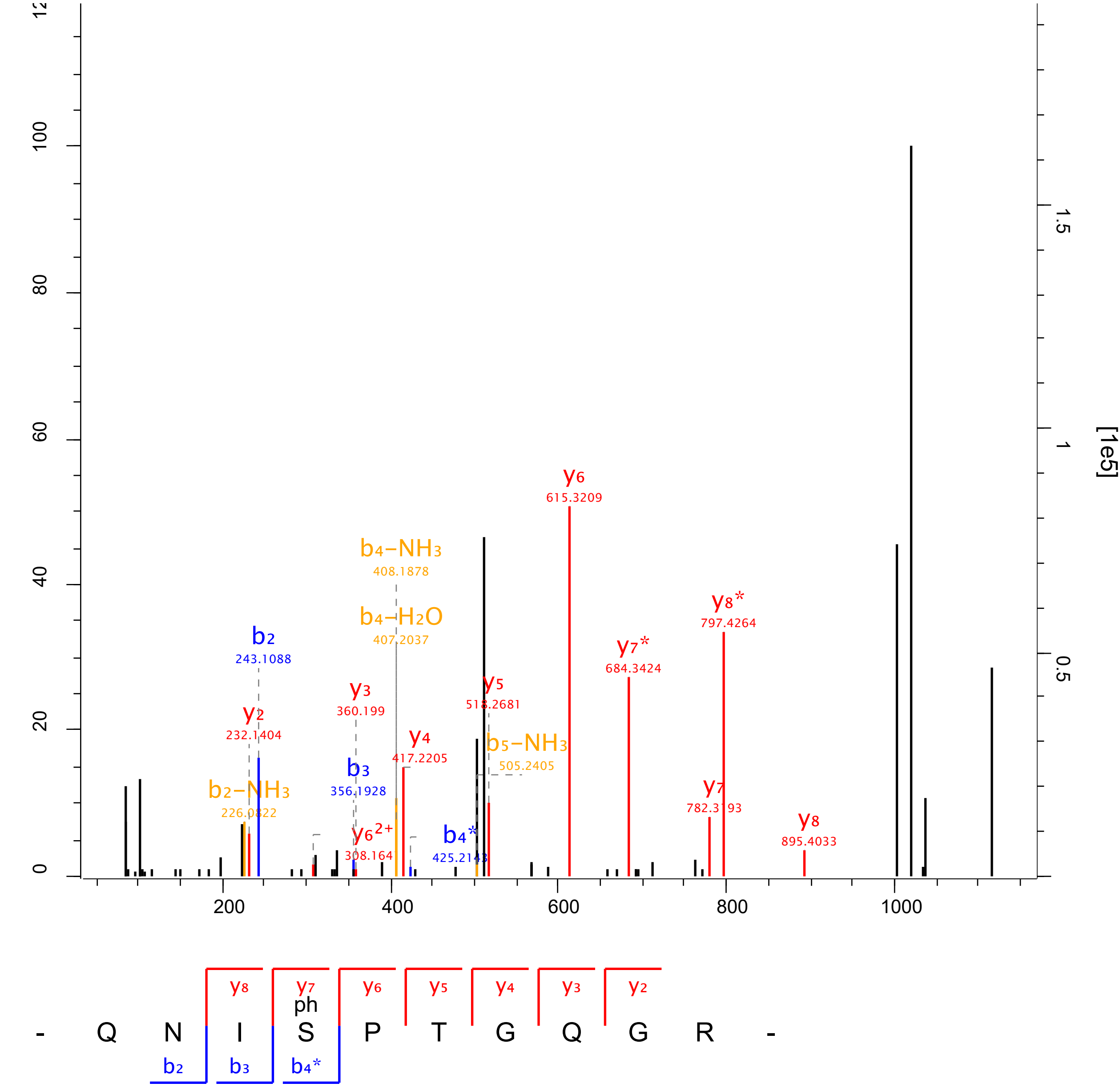

Raw file	Scan	Method	Score	m/z
sys_02_2	4063	FTMS; HCD	49.59	654.29

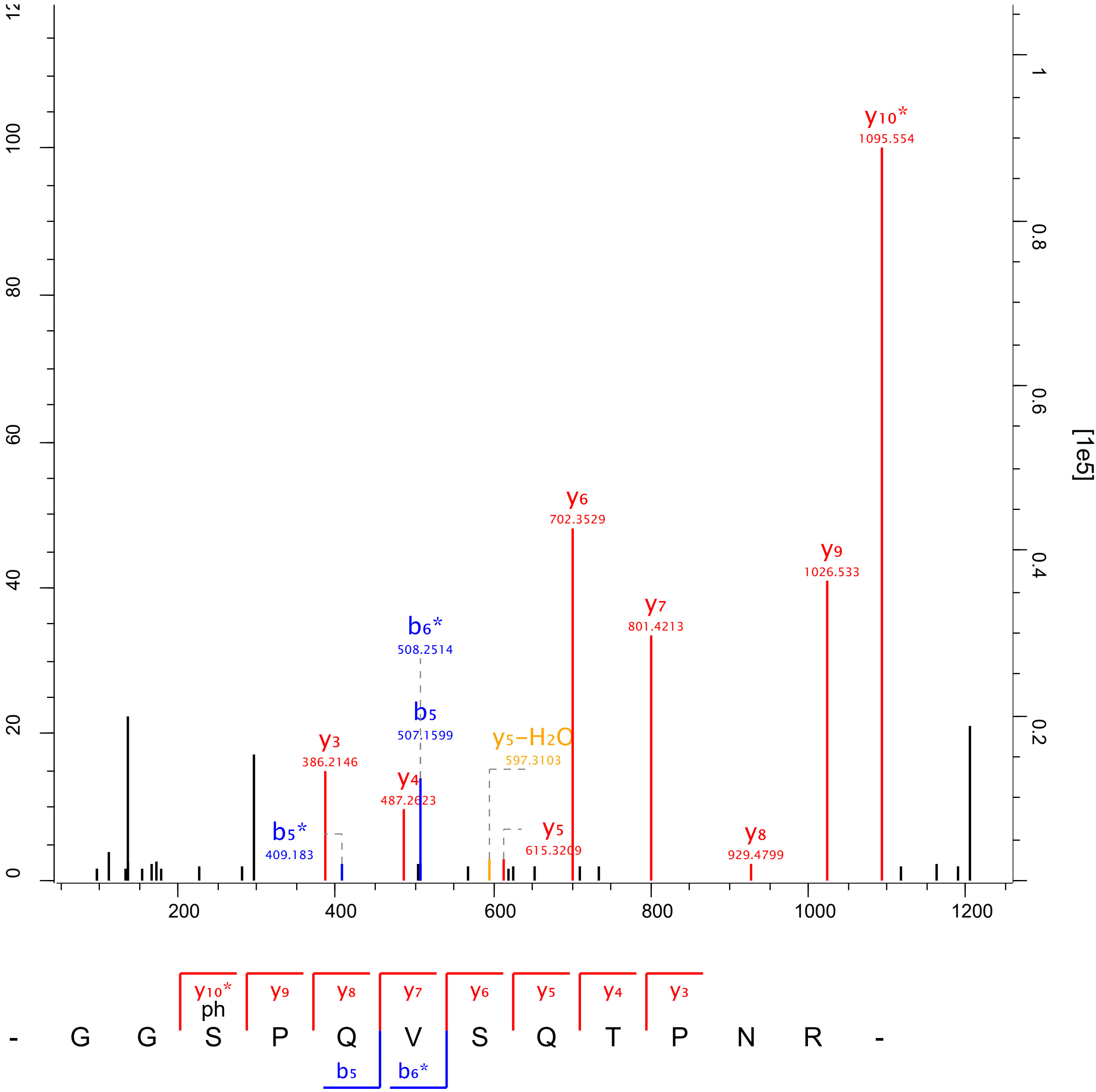

Raw file	Scan	Method	Score	m/z
sys_02_2	4082	FTMS; HCD	52.86	381.49

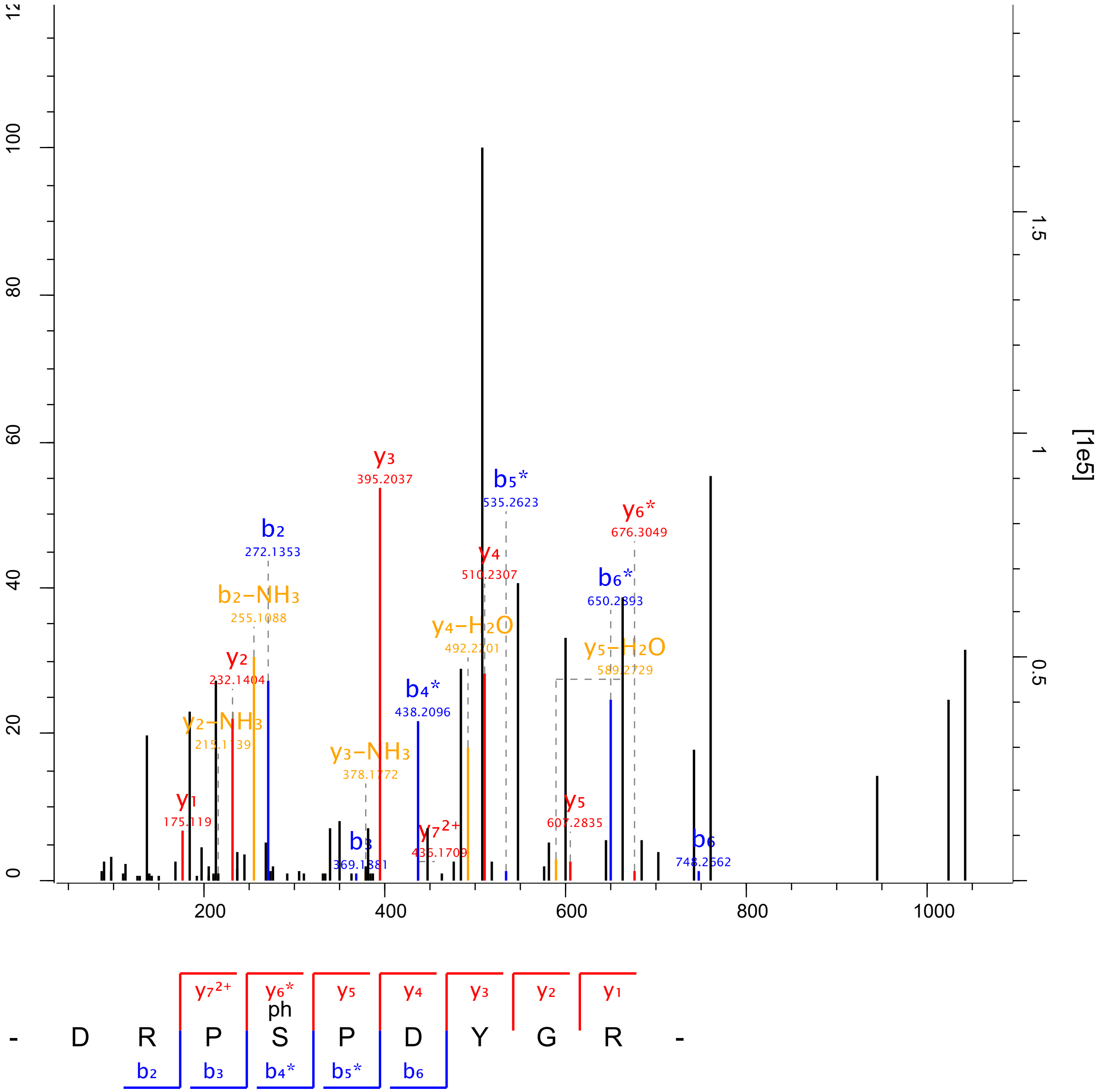

Raw file	Scan	Method	Score	m/z
sys_02_2	4184	FTMS; HCD	133.3	693.64

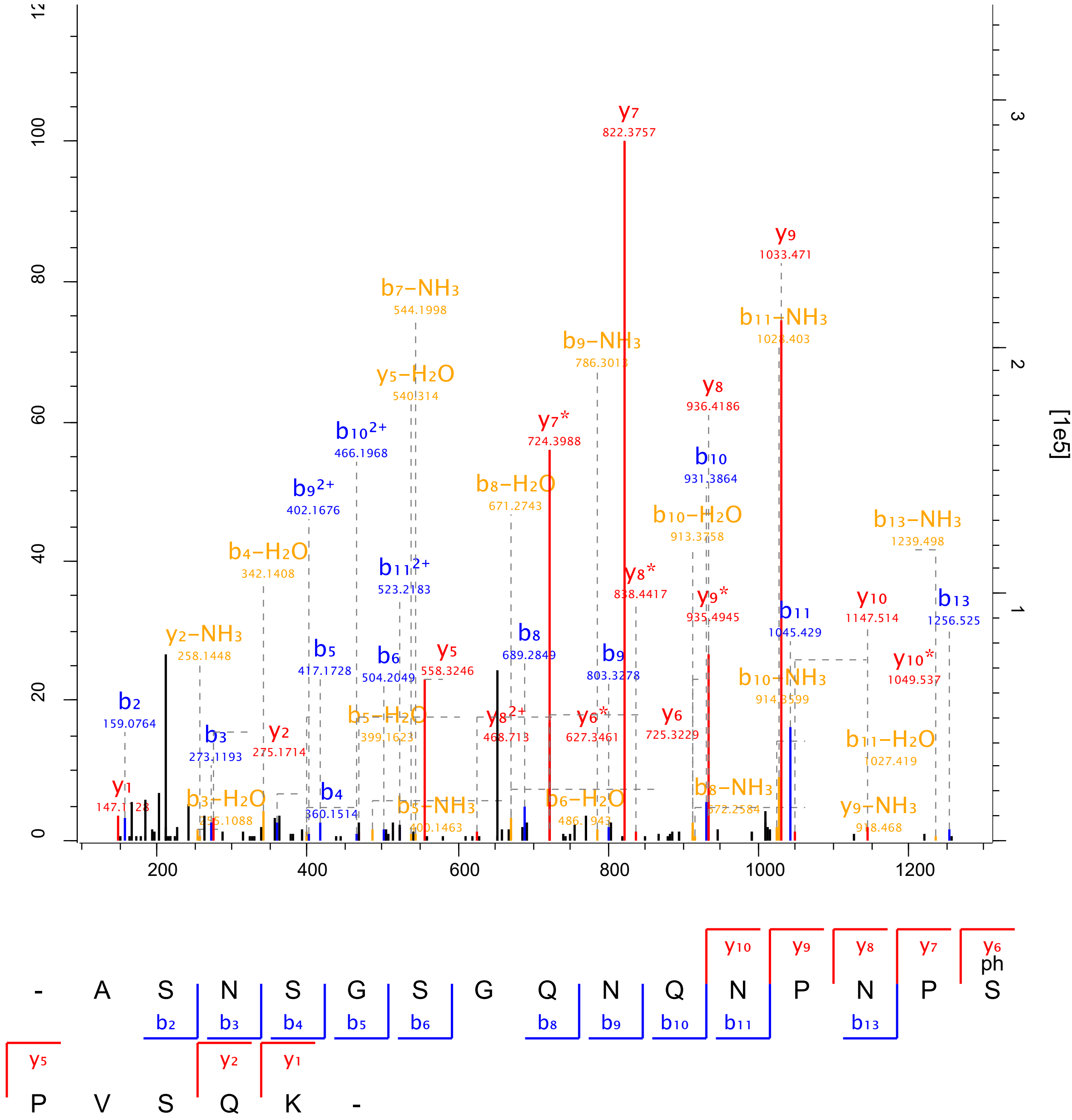

Raw file	Scan	Method	Score	m/z
sys_02_2	4203	FTMS; HCD	81.57	566.24

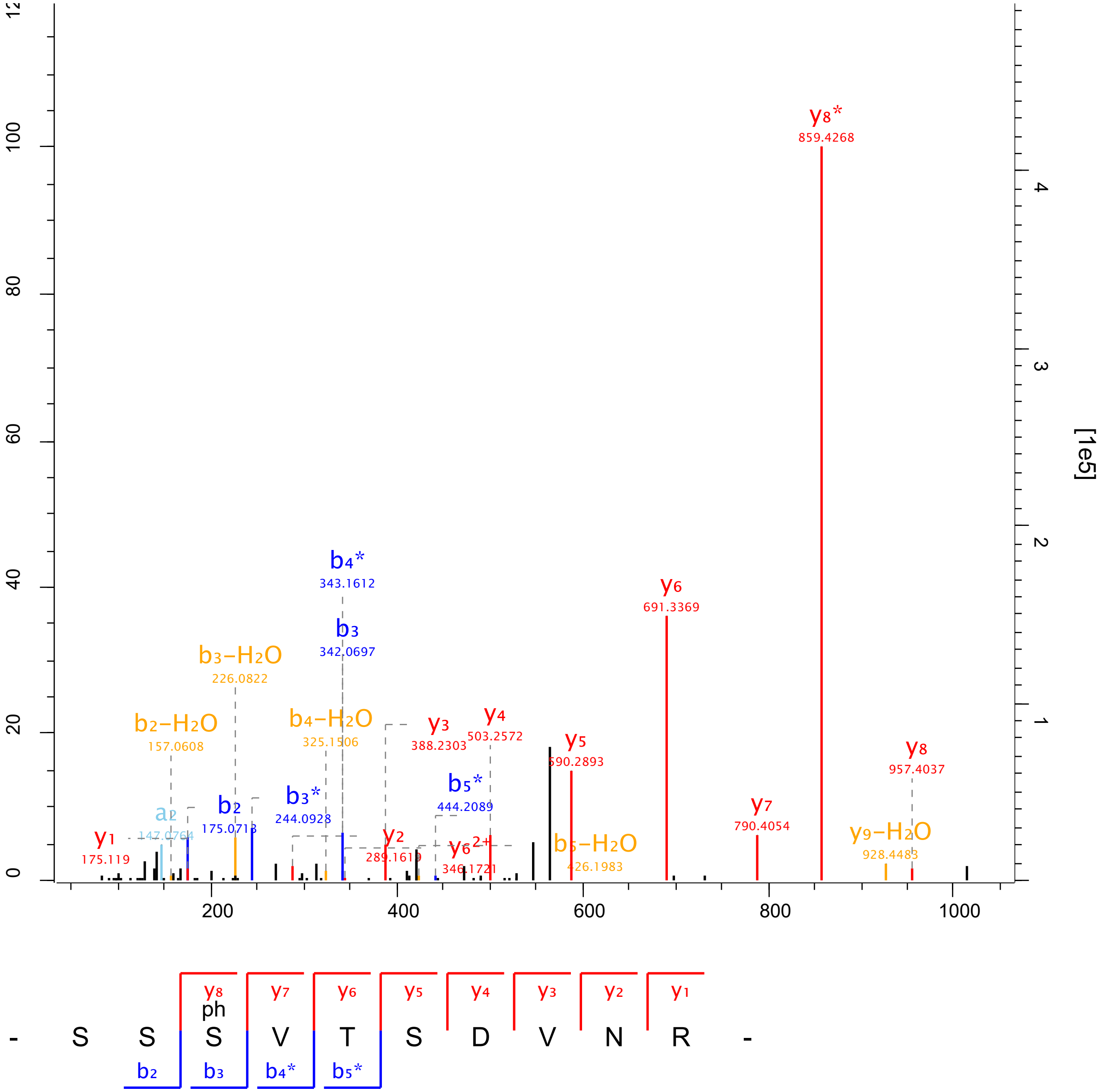

Raw file	Scan	Method	Score	m/z
sys_02_2	4414	FTMS; HCD	112.02	476.23

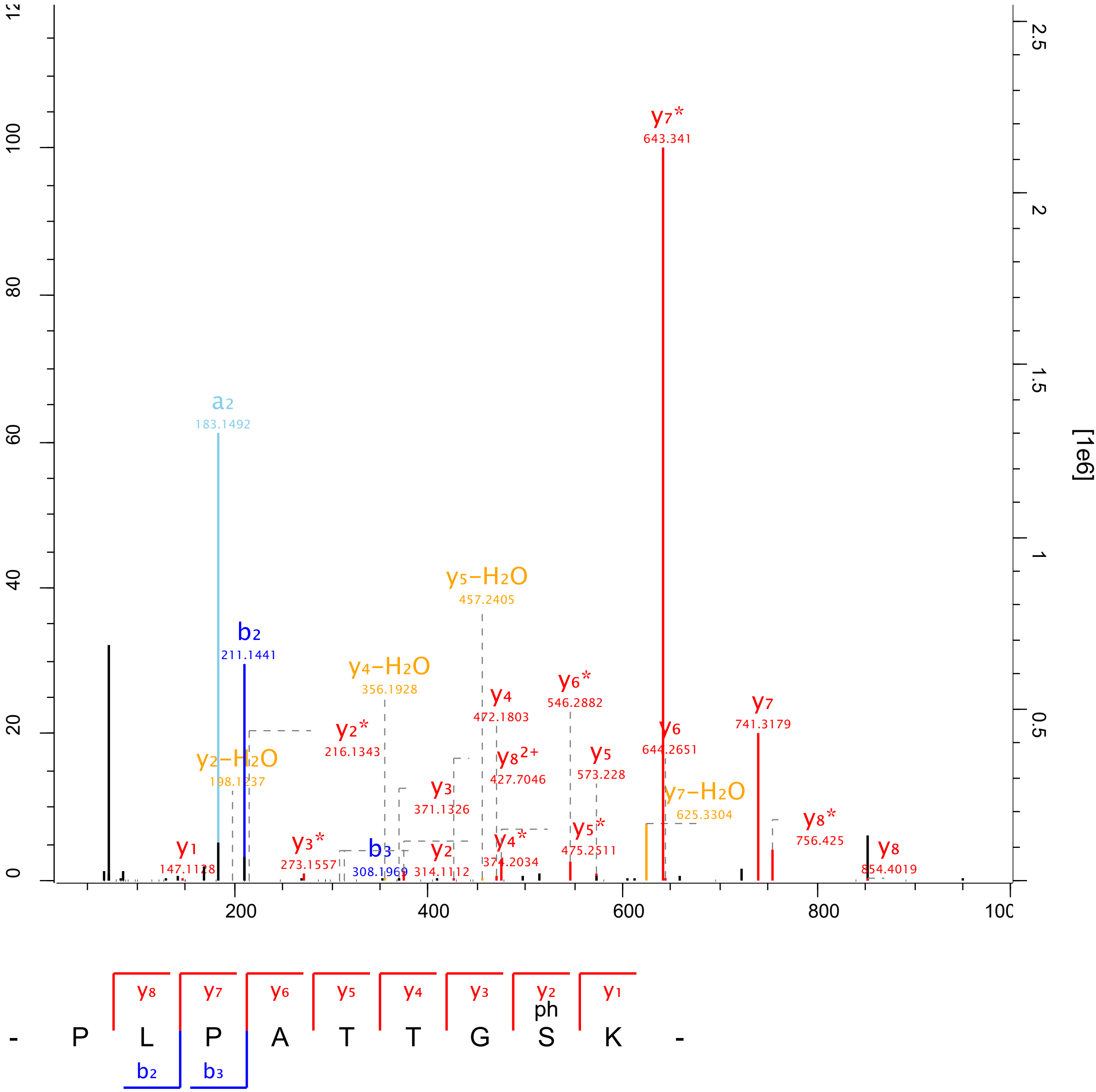

Raw file	Scan	Method	Score	m/z
sys_02_2	4429	FTMS; HCD	101.3	531.74

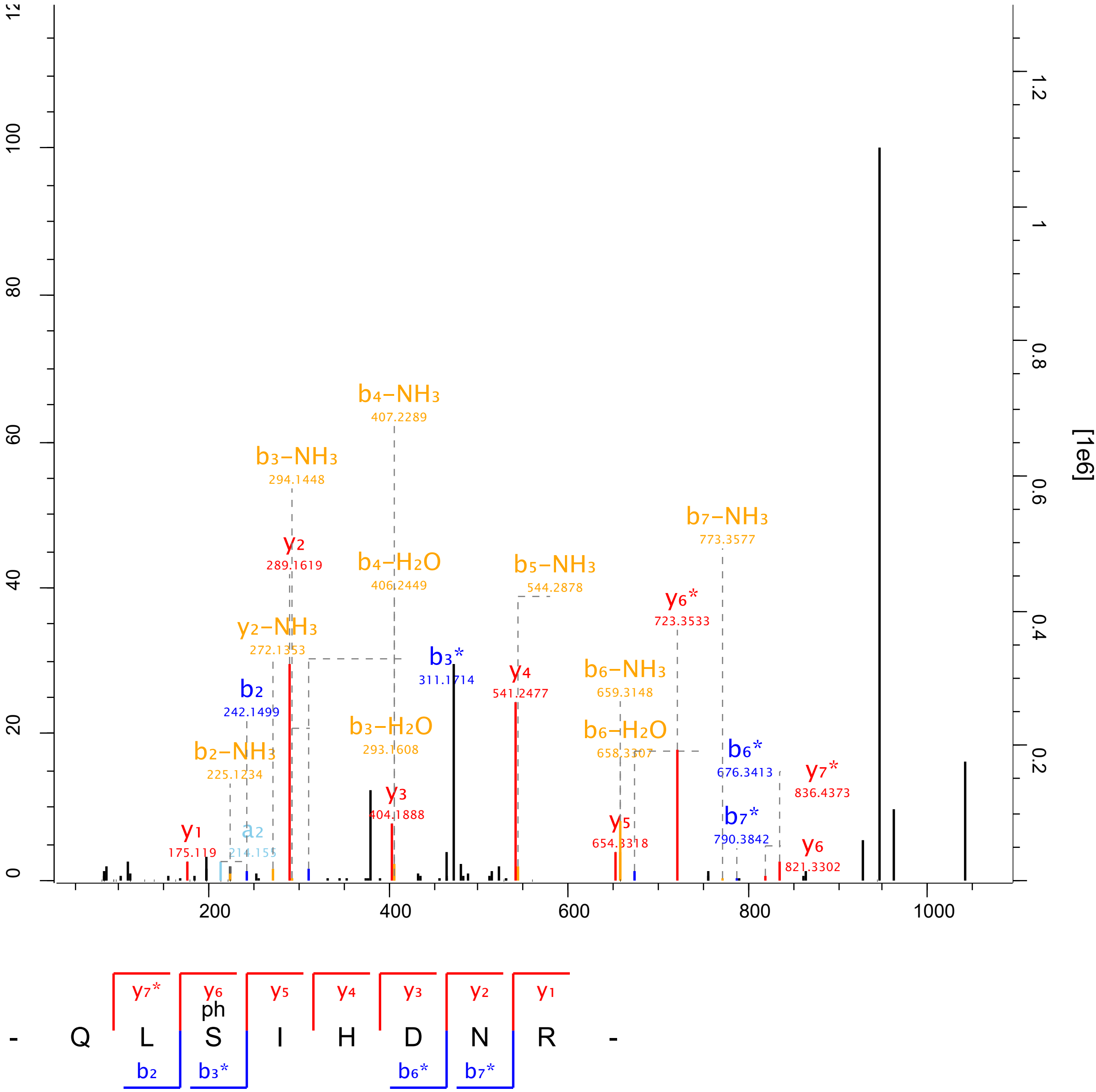

Raw file	Scan	Method	Score	m/z
sys_02_2	4609	FTMS; HCD	67.22	702.32

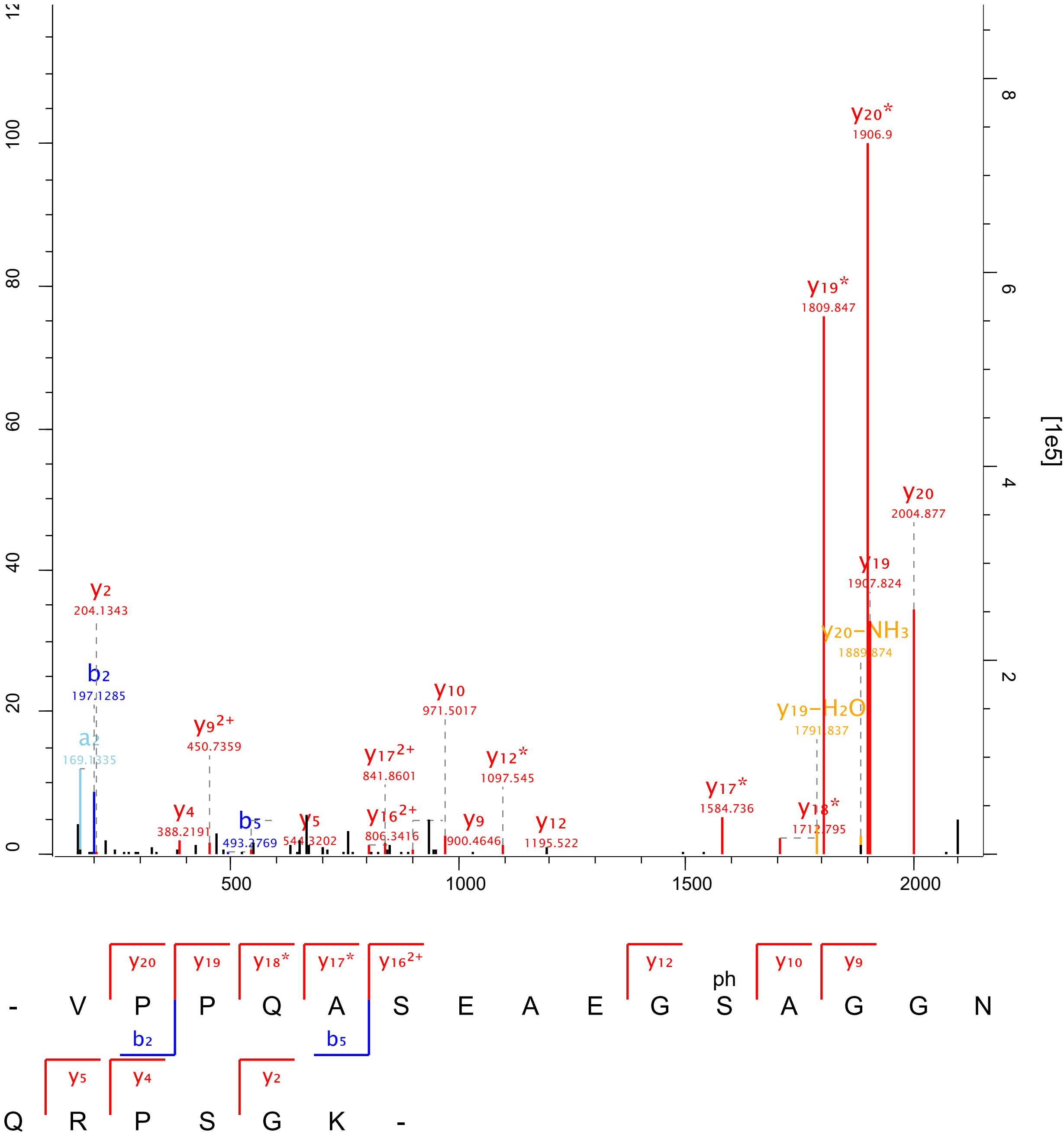

Raw file	Scan	Method	Score	m/z
sys_02_2	4627	FTMS; HCD	41.54	469.55

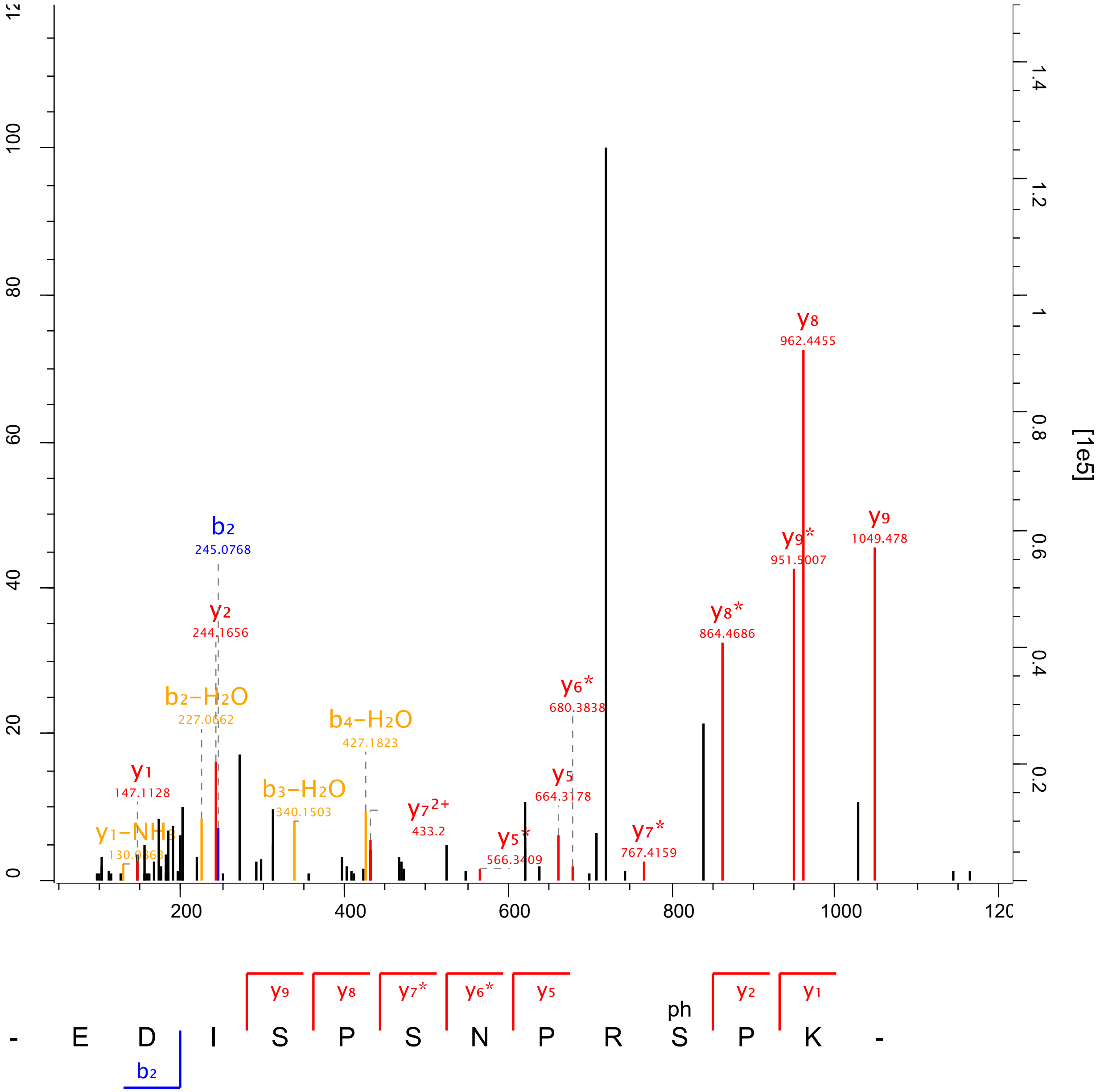

Raw file	Scan	Method	Score	m/z
sys_02_2	4675	FTMS; HCD	76.07	605.78

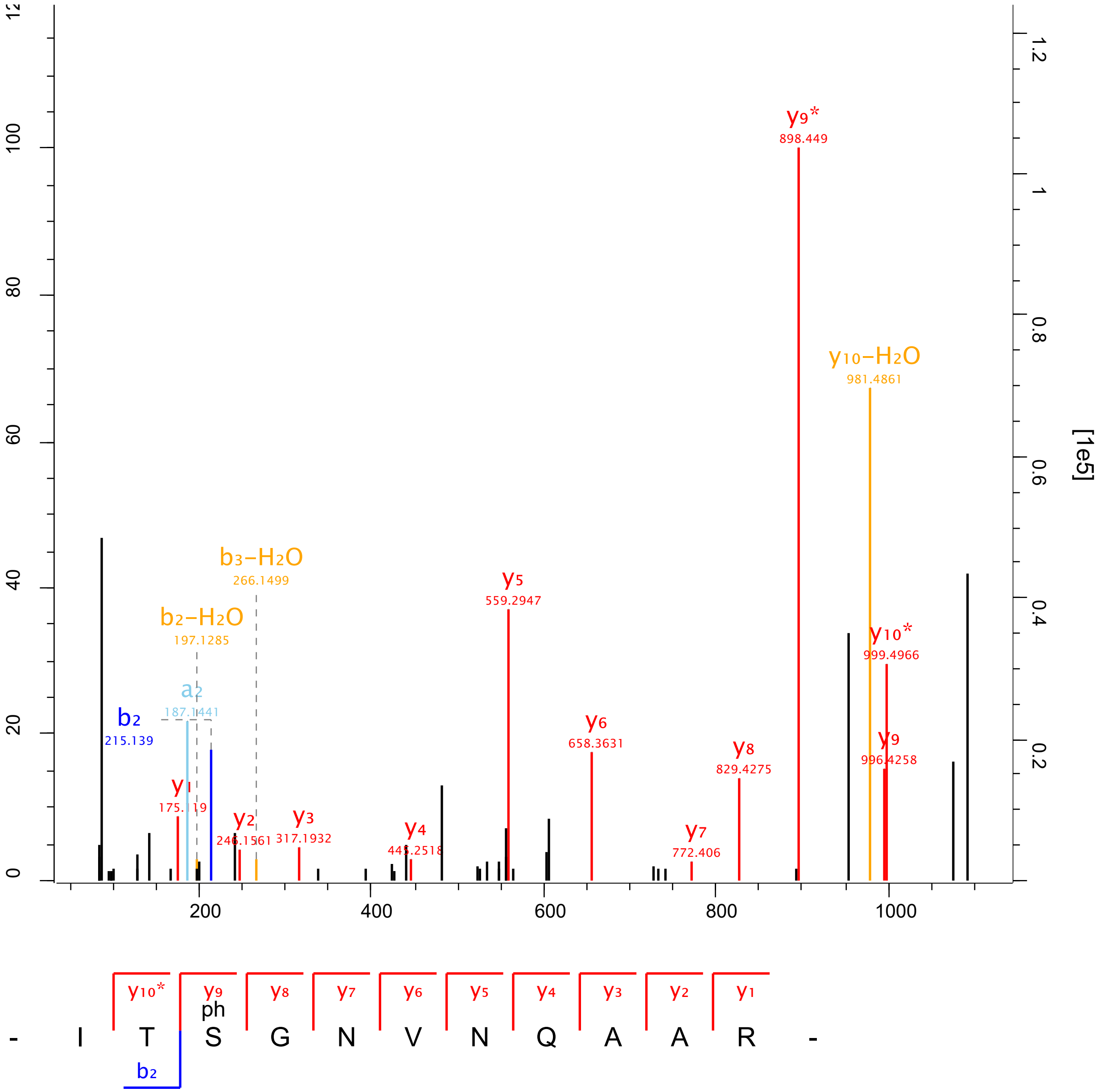

Raw file	Scan	Method	Score	m/z
sys_02_2	4786	FTMS; HCD	41.09	469.23

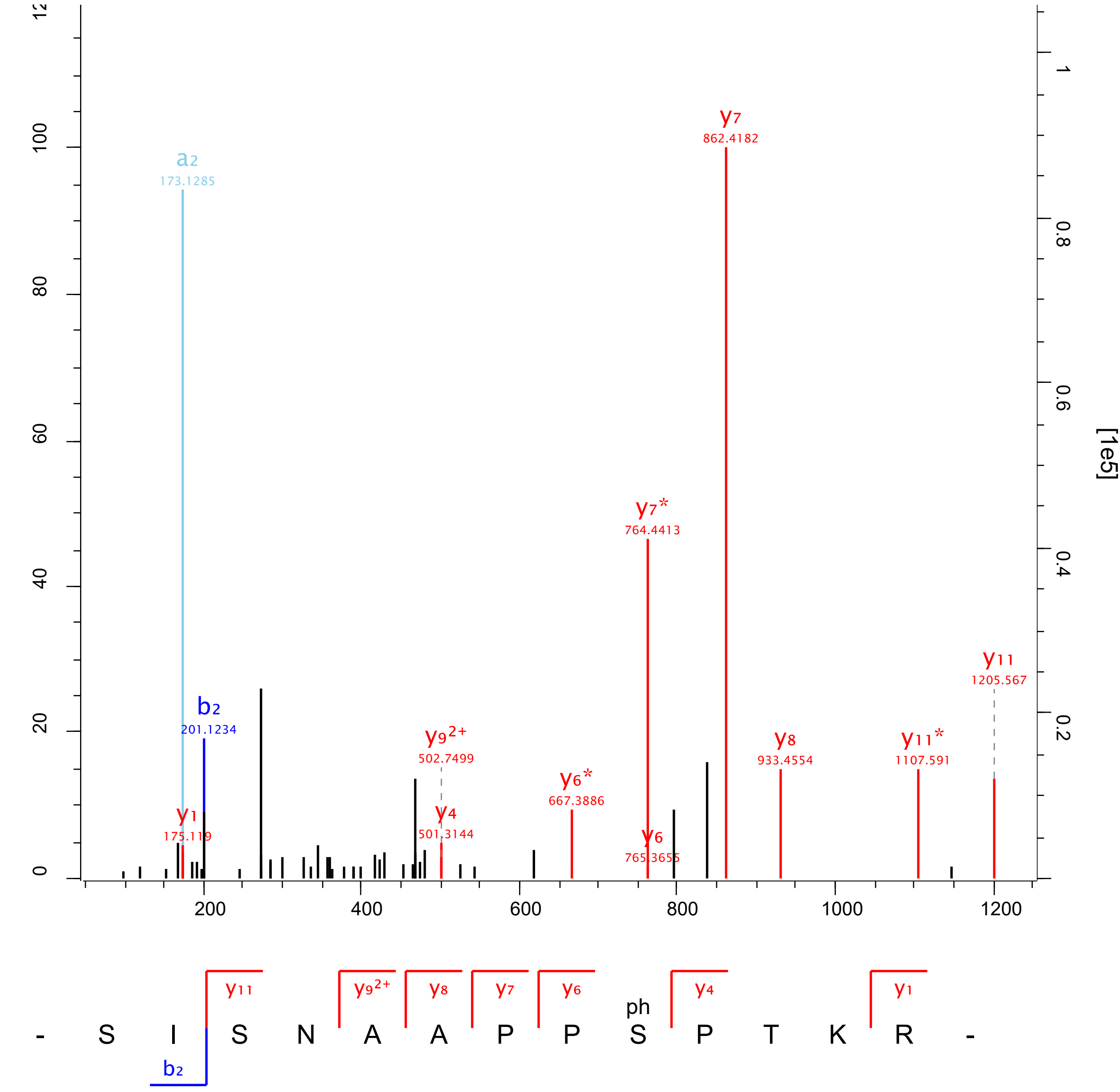

Raw file	Scan	Method	Score	m/z
sys_02_2	4860	FTMS; HCD	50.09	617.79

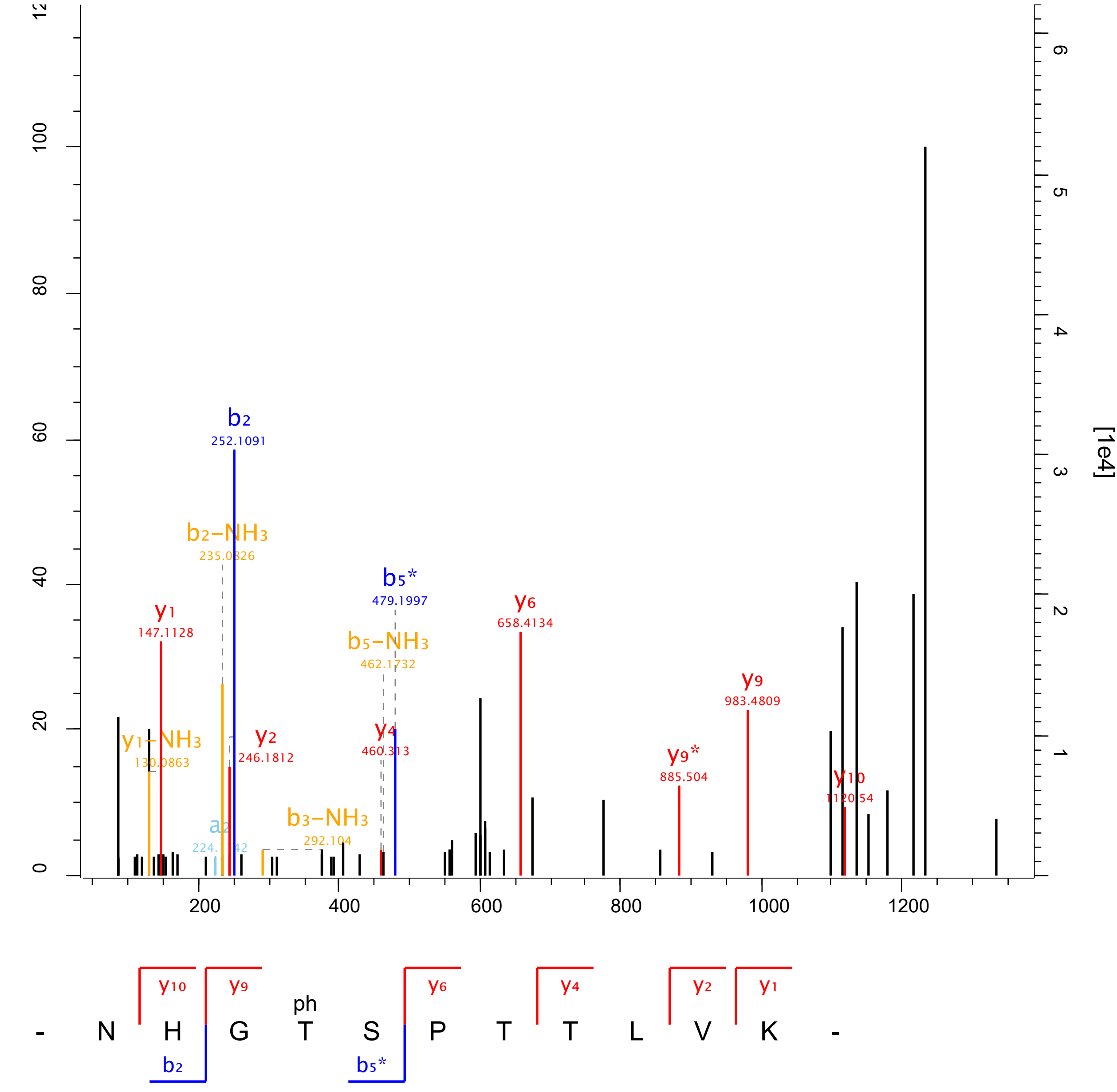

Raw file	Scan	Method	Score	m/z
sys_02_2	4927	FTMS; HCD	64.64	471.21

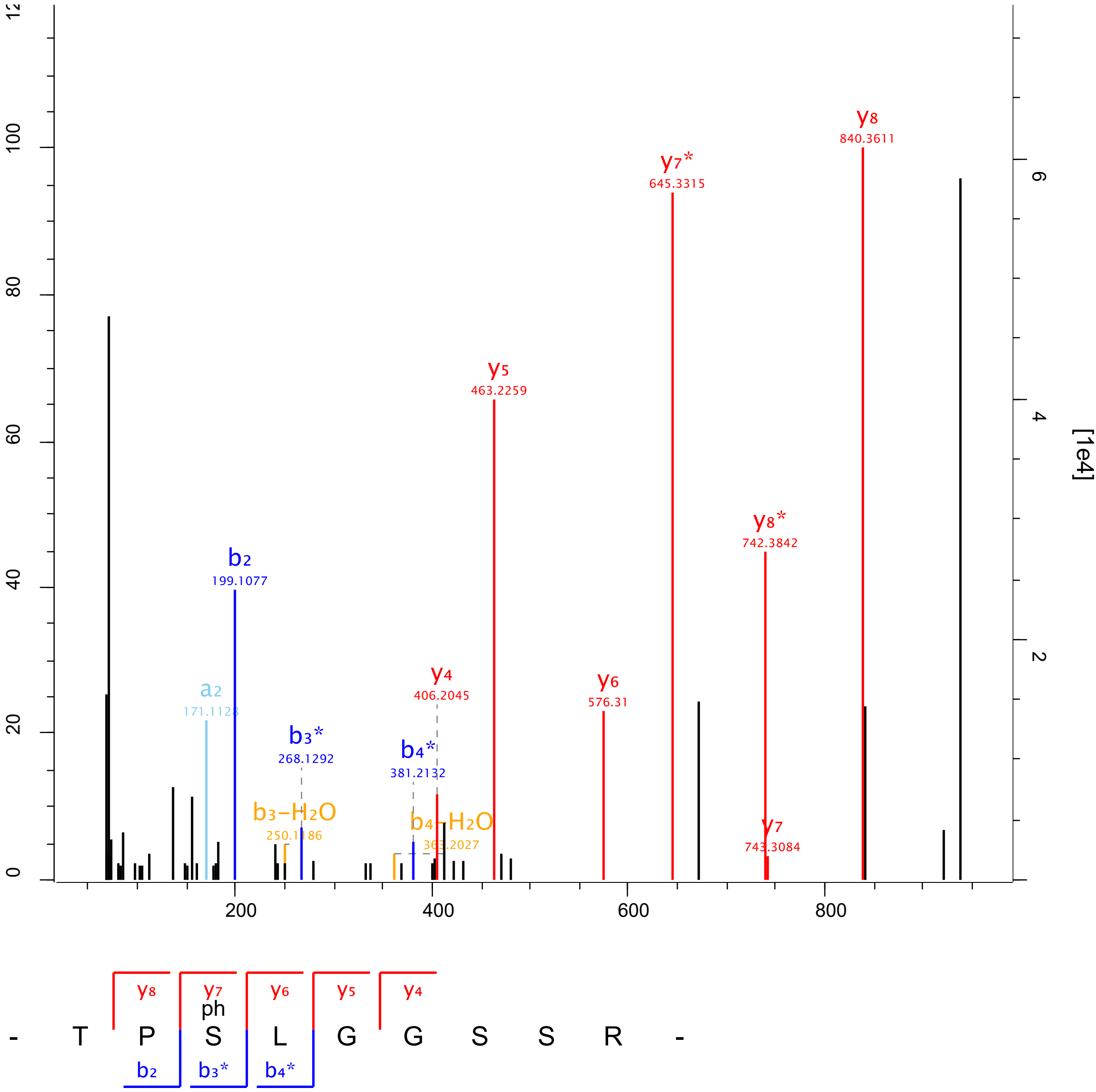

Raw file	Scan	Method	Score	m/z
sys_02_2	4994	FTMS; HCD	95.85	627.28

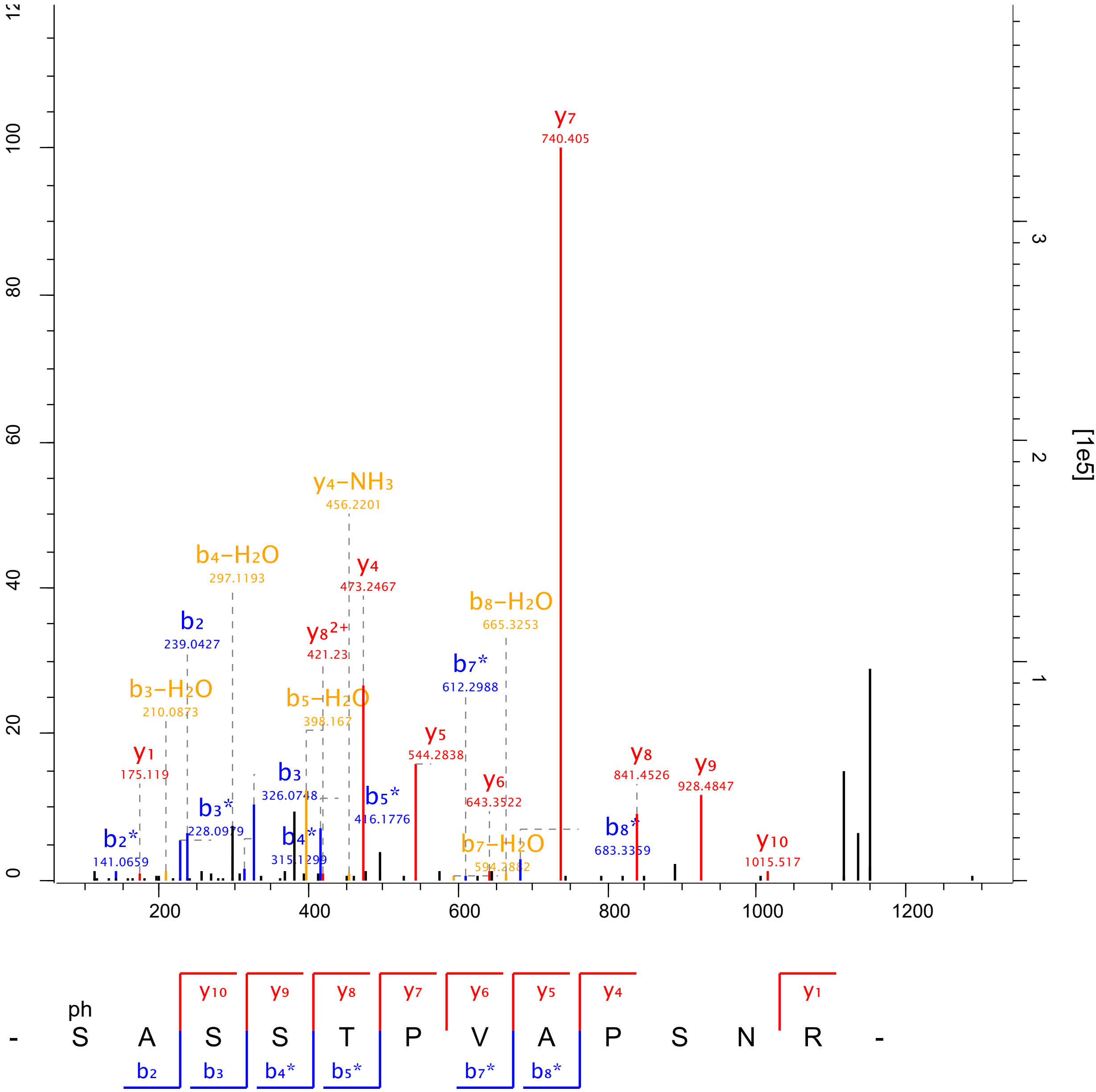

Raw file	Scan	Method	Score	m/z
sys_02_2	5046	FTMS; HCD	155.49	879.86

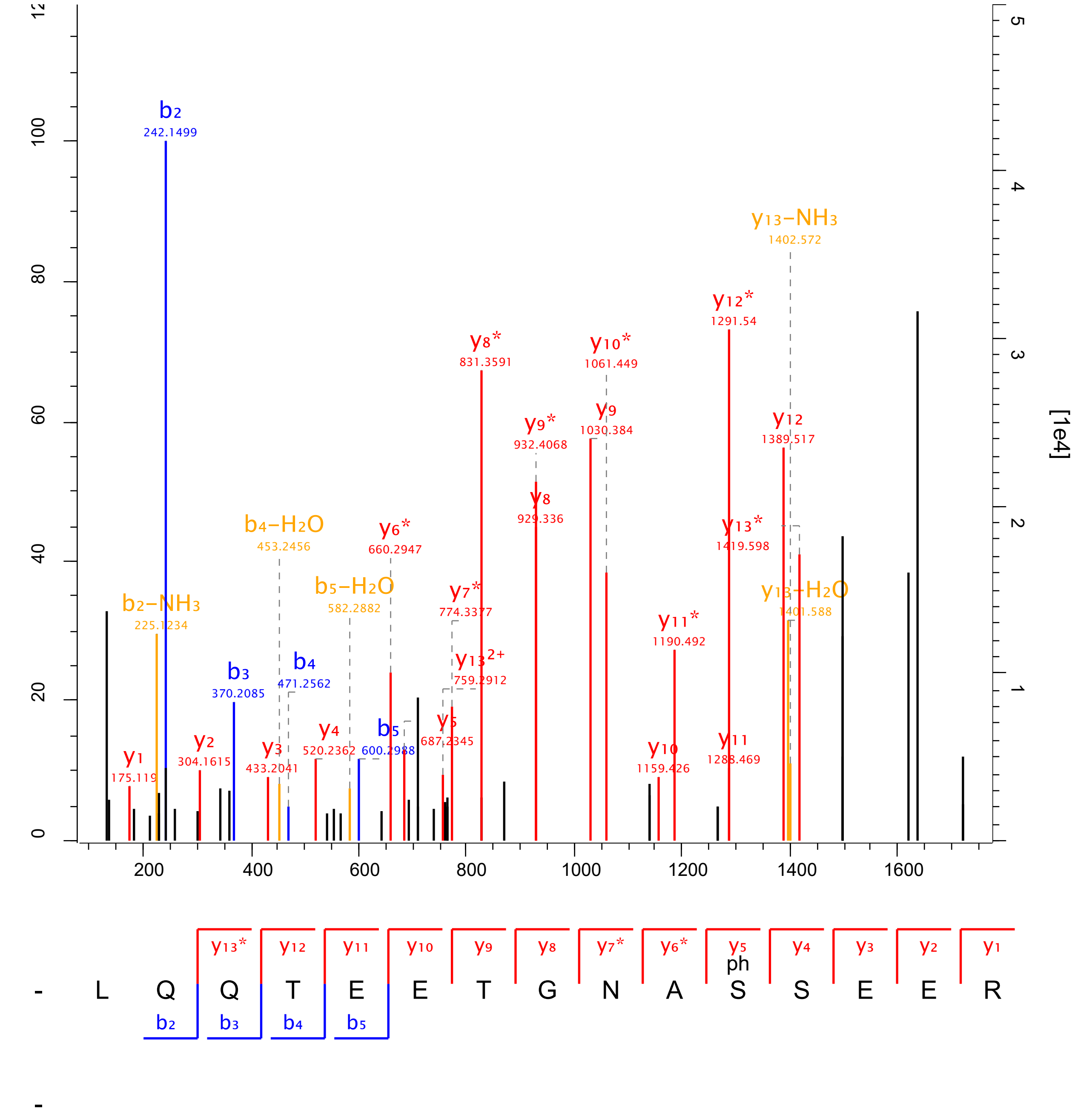

Raw file	Scan	Method	Score	m/z
sys_02_2	5120	FTMS; HCD	43.21	600.59

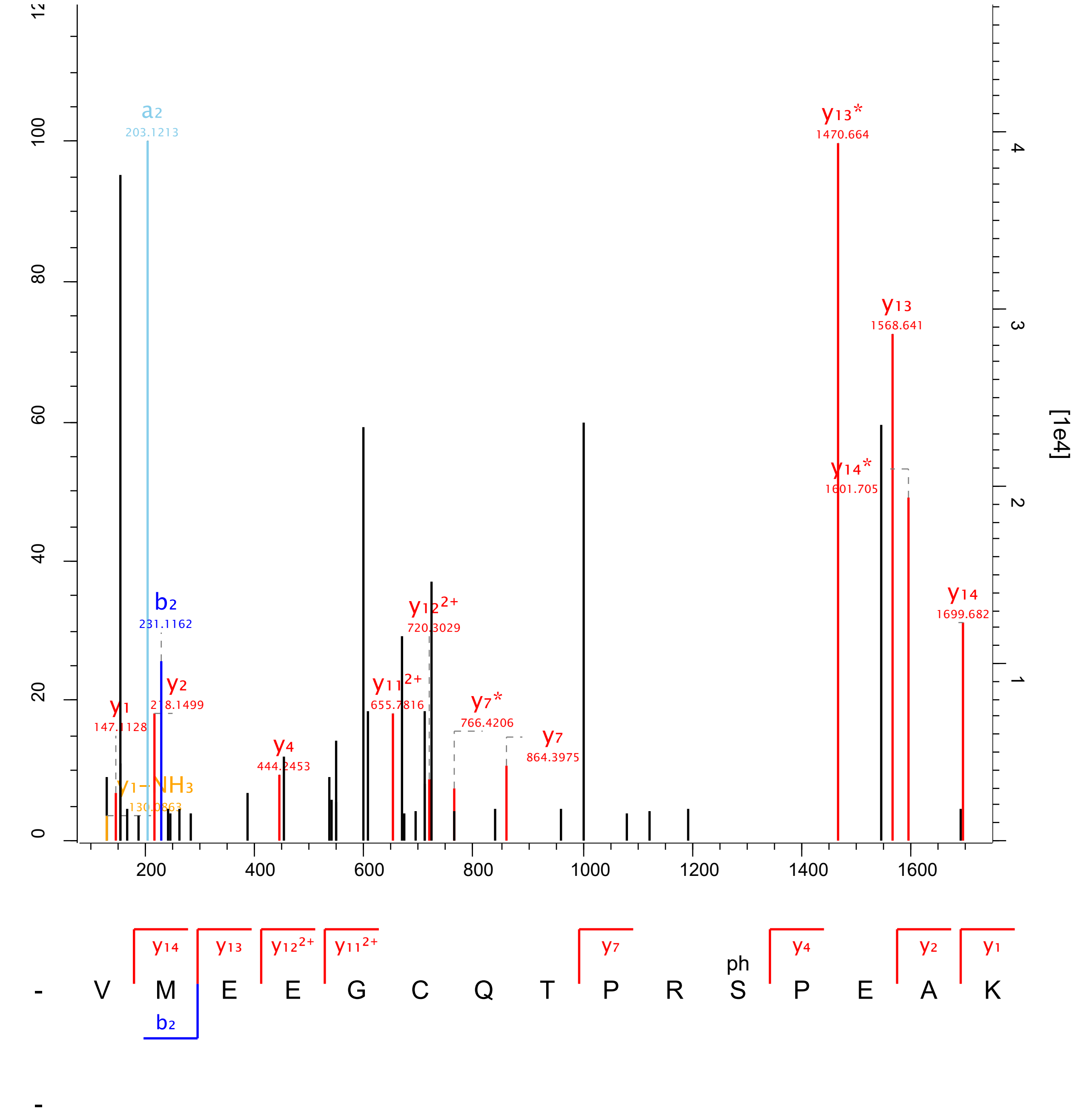

Raw file	Scan	Method	Score	m/z
sys_02_2	5166	FTMS; HCD	77.64	569.25

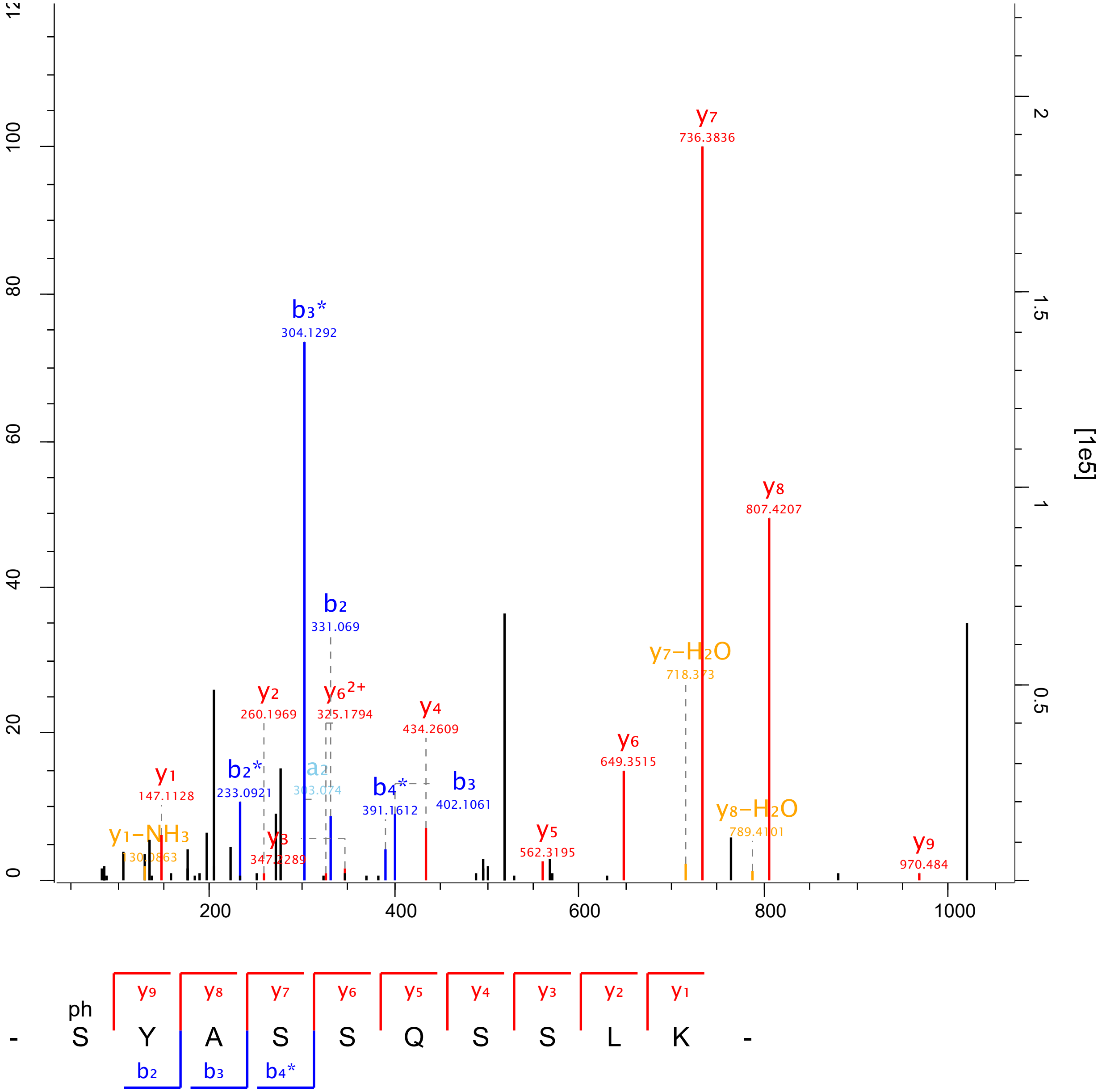

Raw file	Scan	Method	Score	m/z
sys_02_2	5203	FTMS; HCD	71.98	659.29

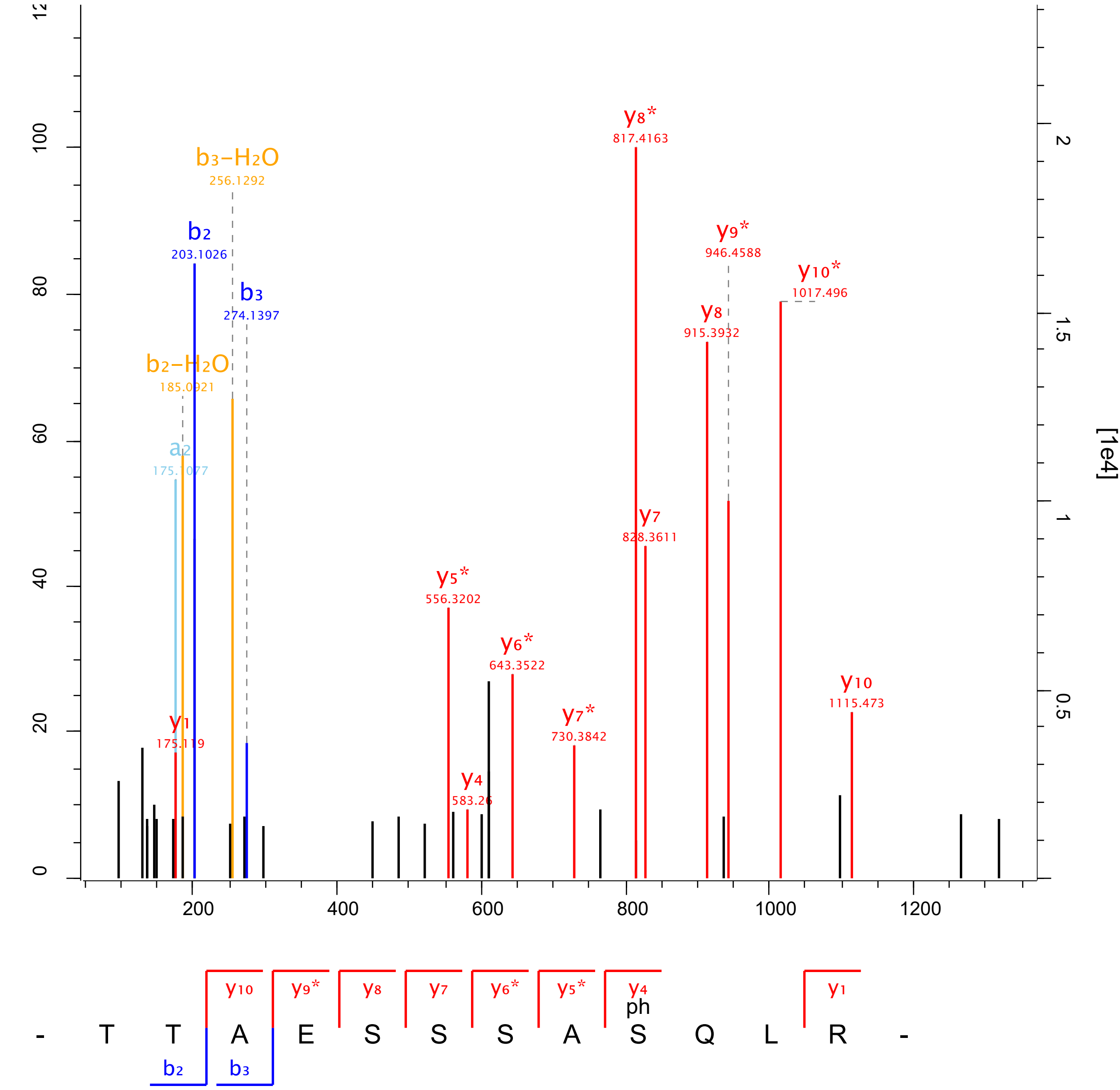

Raw file	Scan	Method	Score	m/z
sys_02_2	5210	FTMS; HCD	40.28	628.79

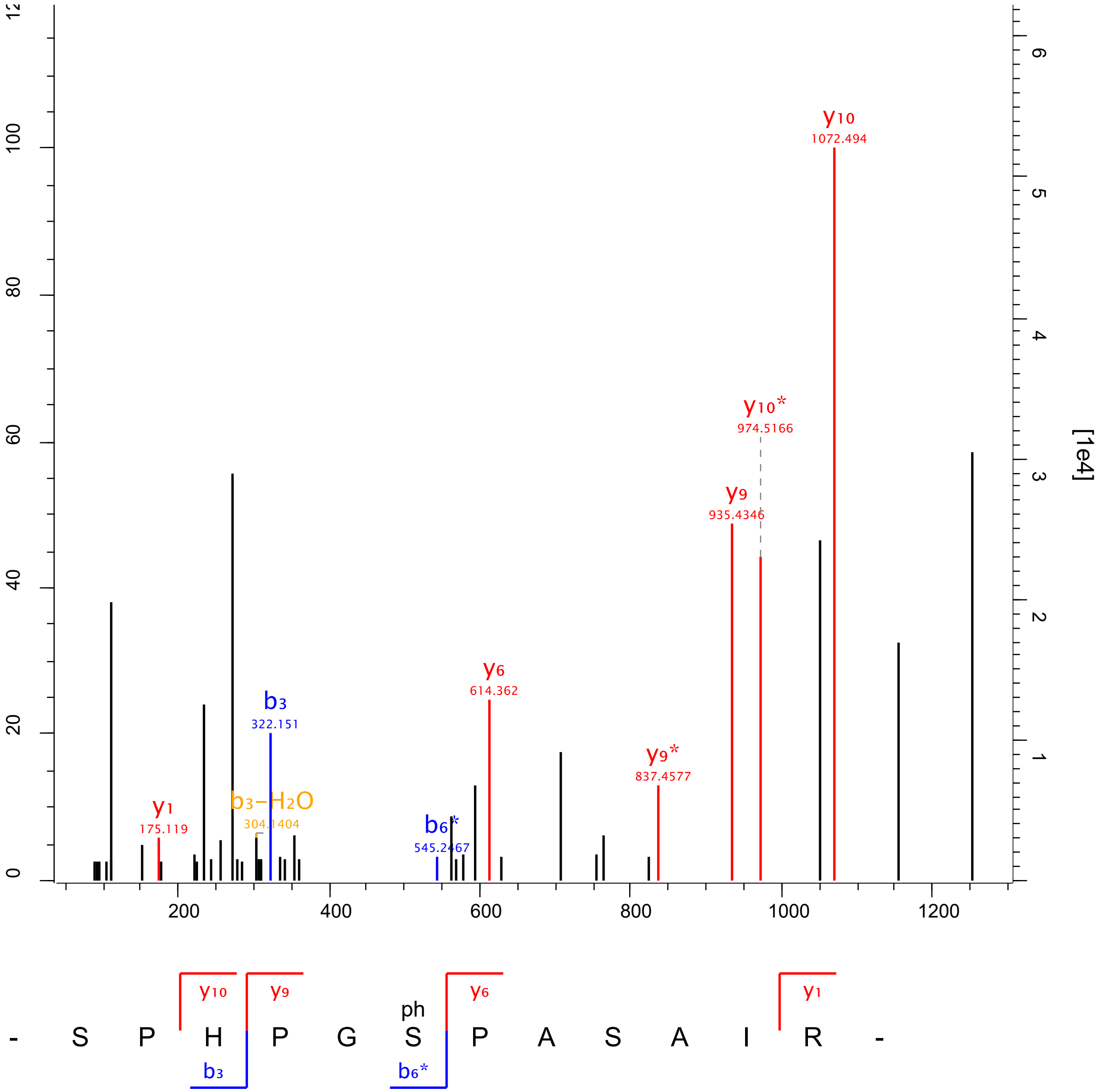

Raw file	Scan	Method	Score	m/z
sys_02_2	5409	FTMS; HCD	143.57	834.34

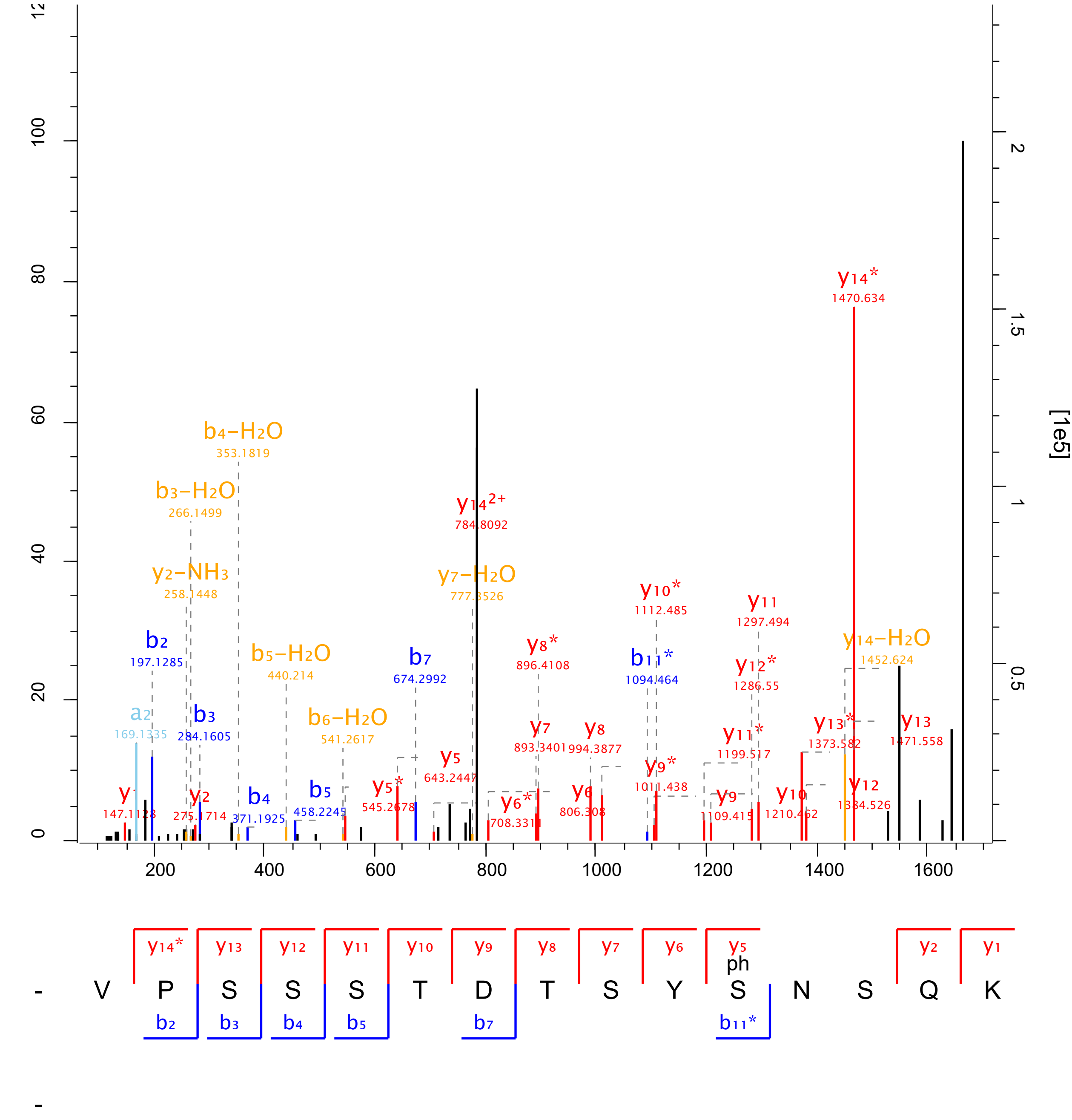

Raw file	Scan	Method	Score	m/z
sys_02_2	5443	FTMS; HCD	52.25	588.22

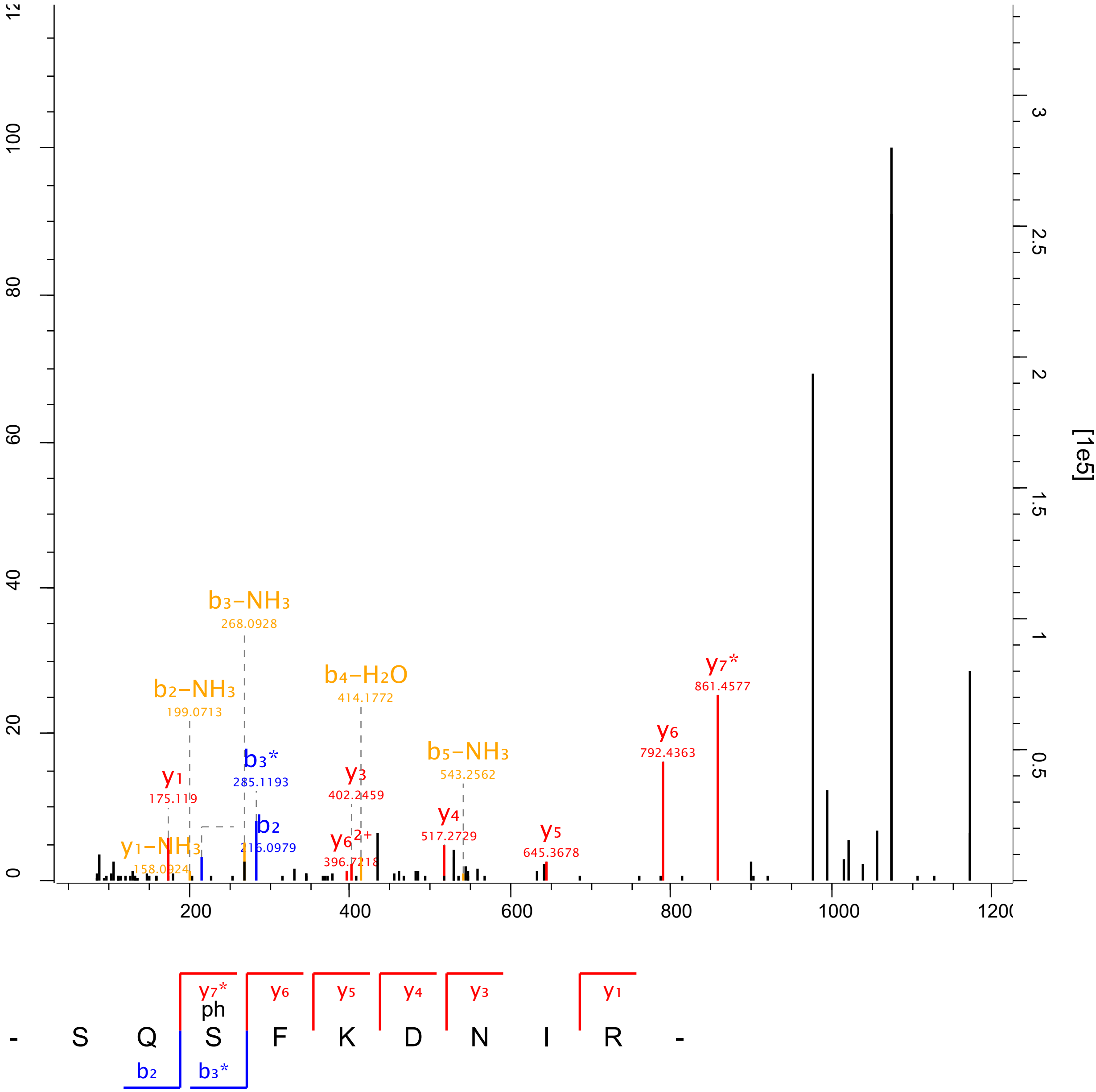

Raw file	Scan	Method	Score	m/z
sys_02_2	5469	FTMS; HCD	211.9	713.33

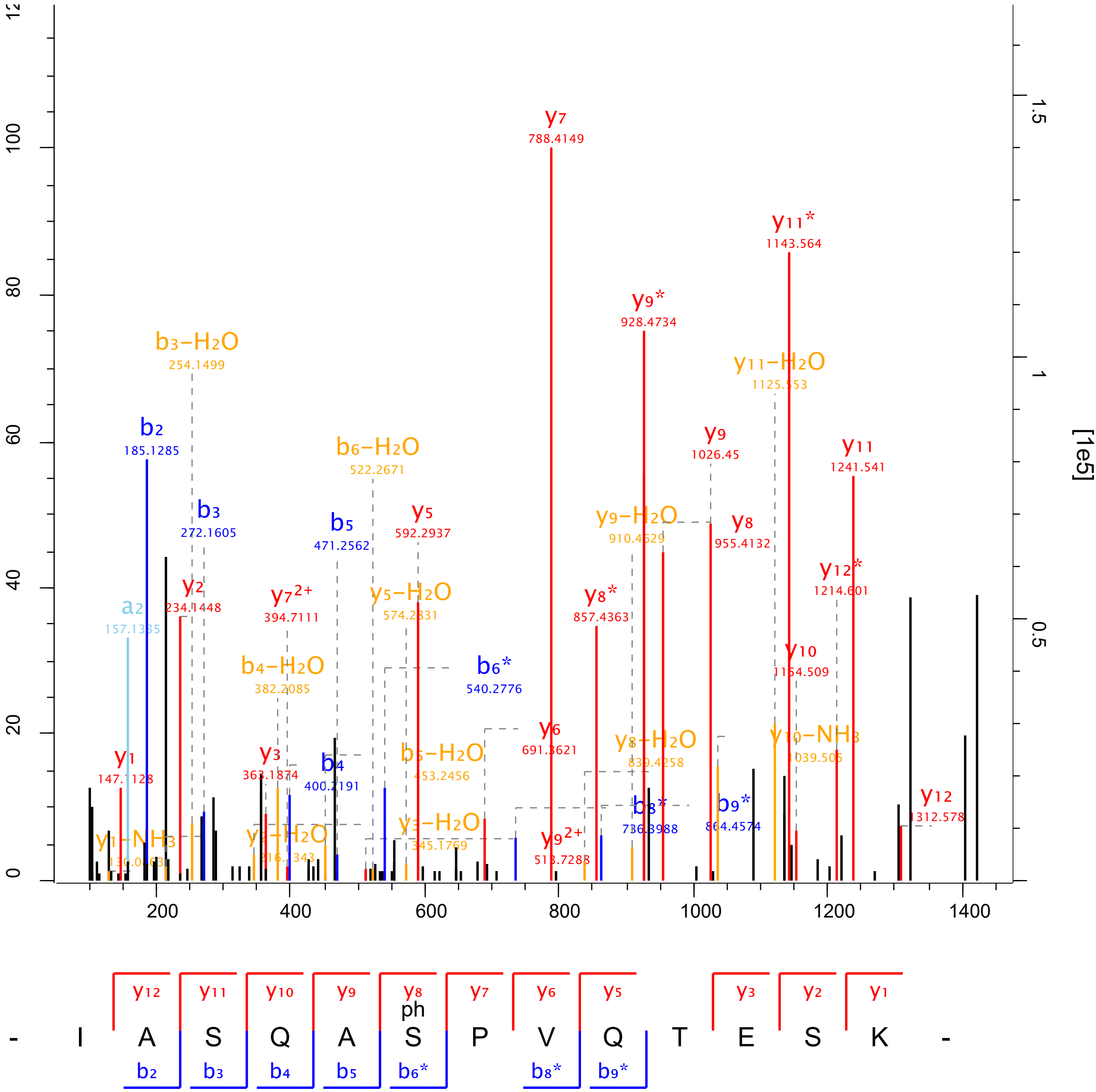

Raw file	Scan	Method	Score	m/z
sys_02_2	5475	FTMS; HCD	58.32	400.86

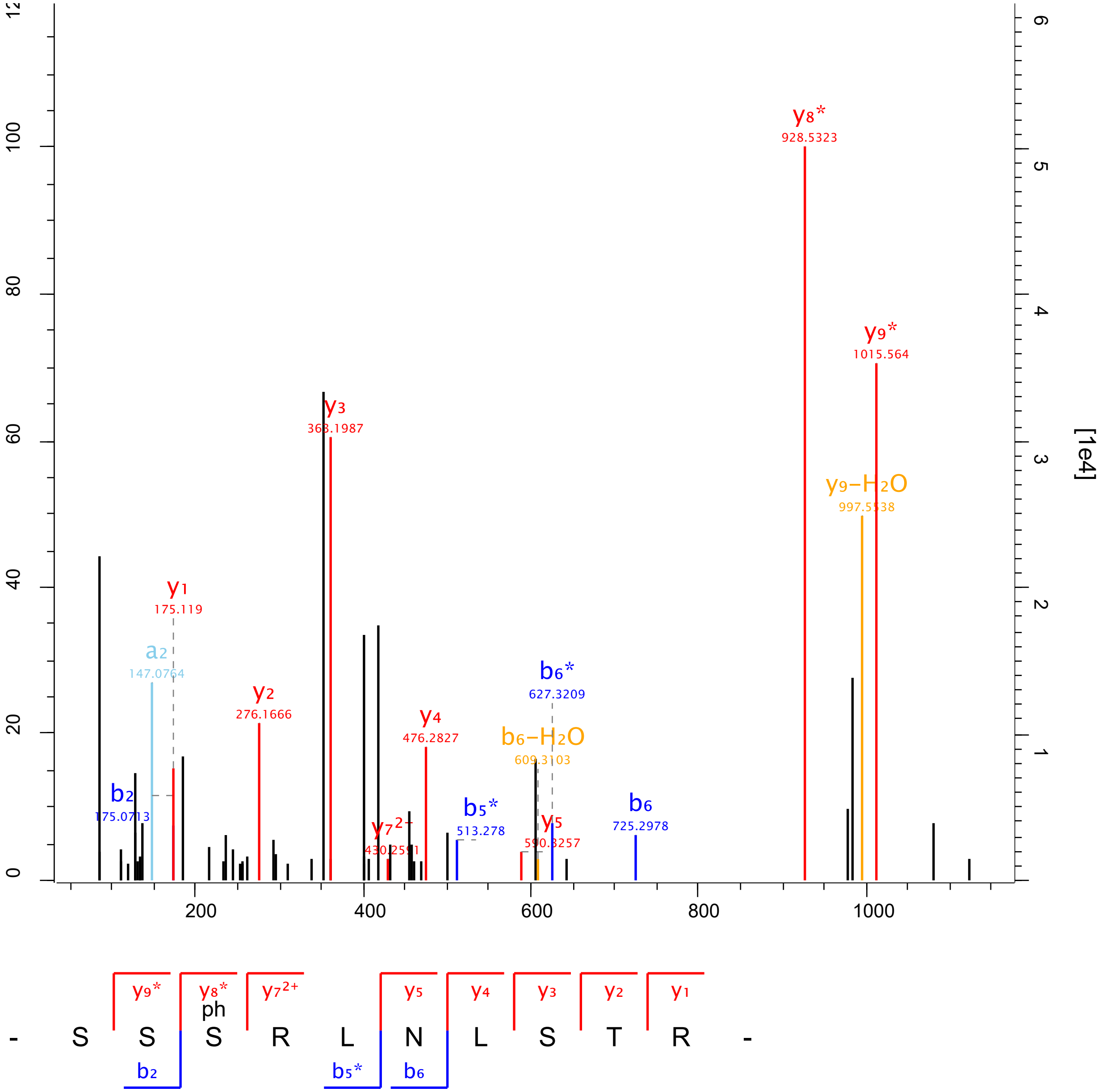

Raw file	Scan	Method	Score	m/z
sys_02_2	5551	FTMS; HCD	206.96	695.65

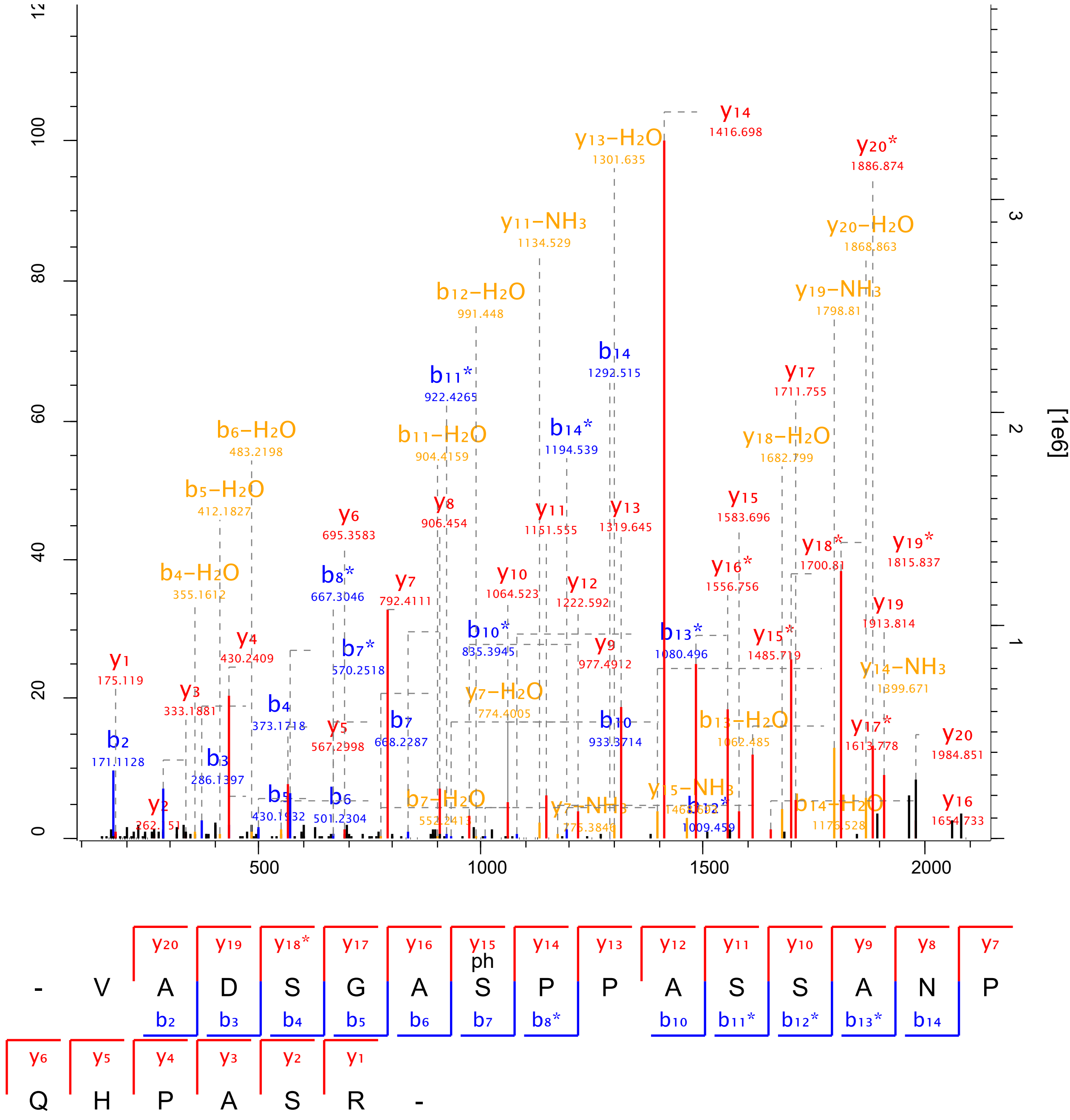

Raw file	Scan	Method	Score	m/z
sys_02_2	5622	FTMS; HCD	102.65	672.78

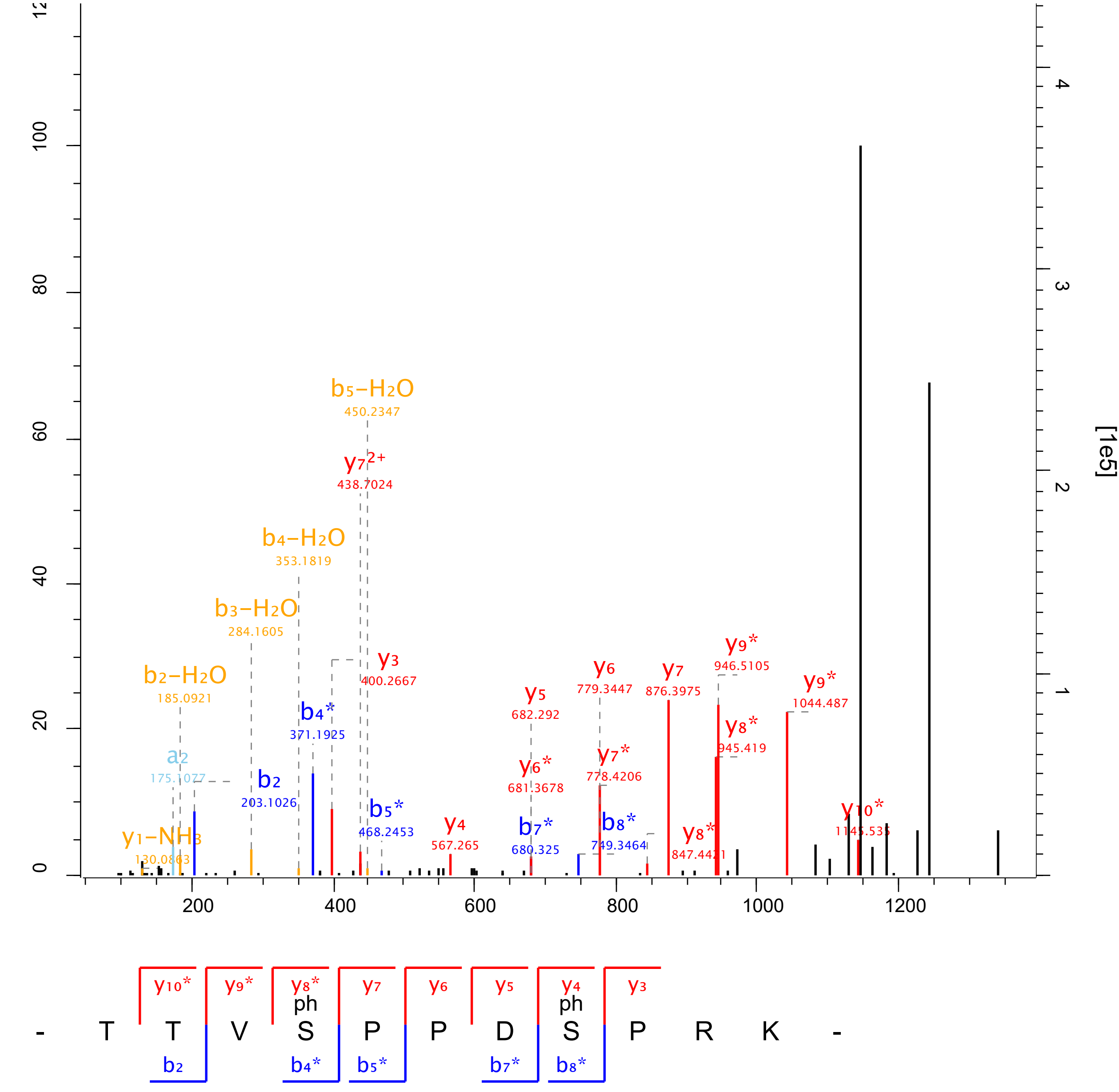

Raw file	Scan	Method	Score	m/z
sys_02_2	5766	FTMS; HCD	149.86	523.89

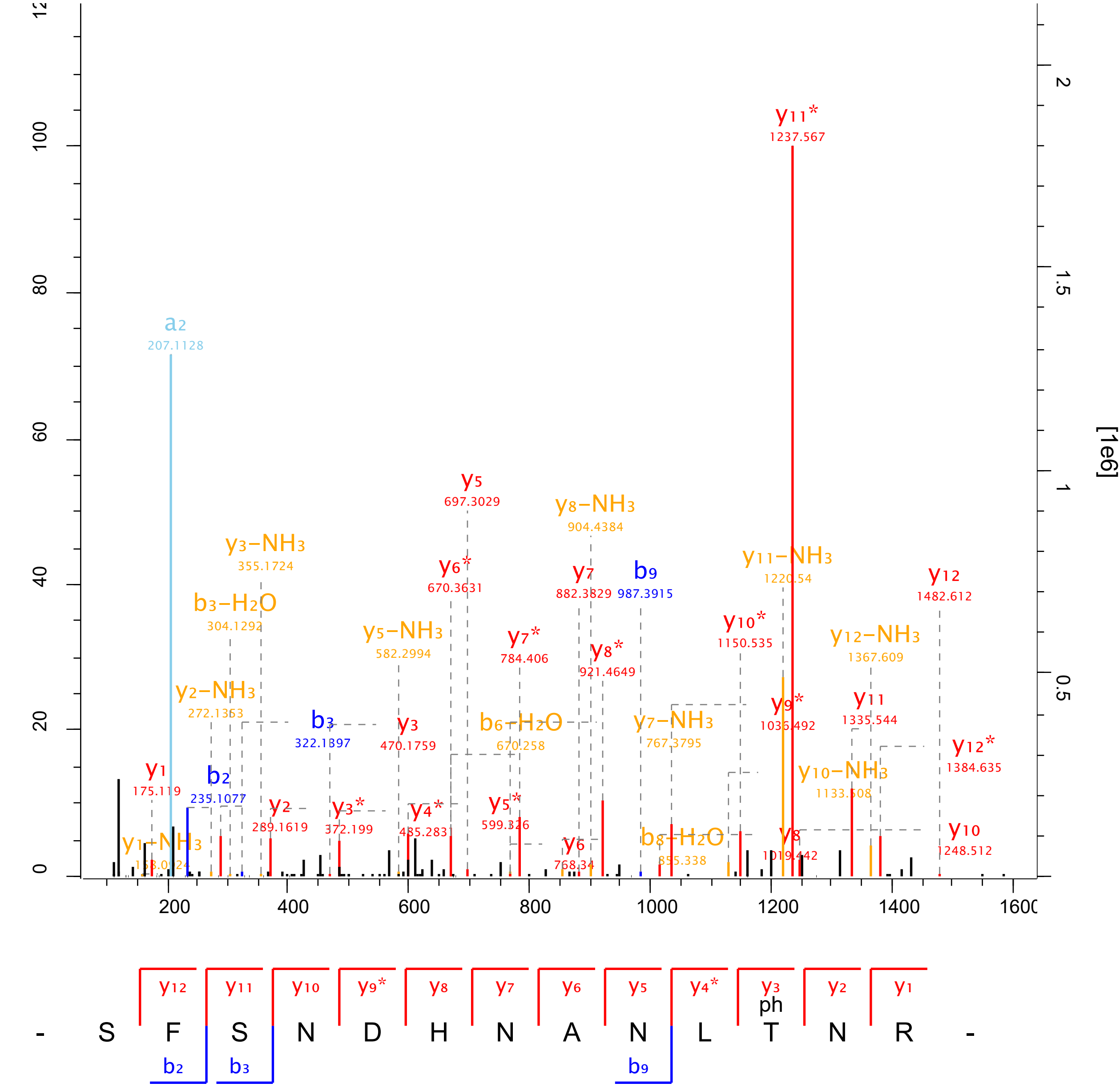

Raw file	Scan	Method	Score	m/z
sys_02_2	5815	FTMS; HCD	74.96	457.22

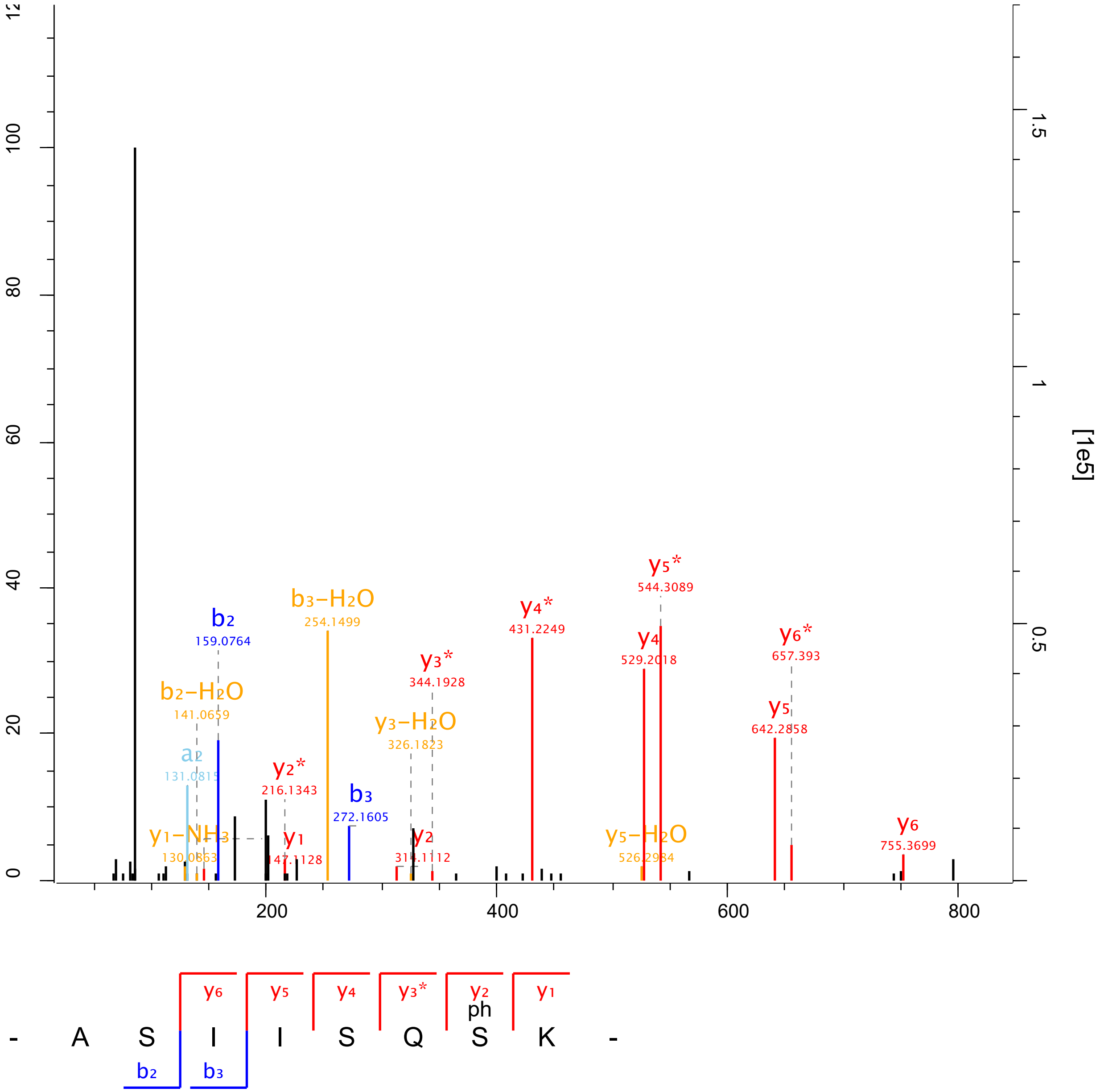

Raw file	Scan	Method	Score	m/z
sys_02_2	5846	FTMS; HCD	66	506.74

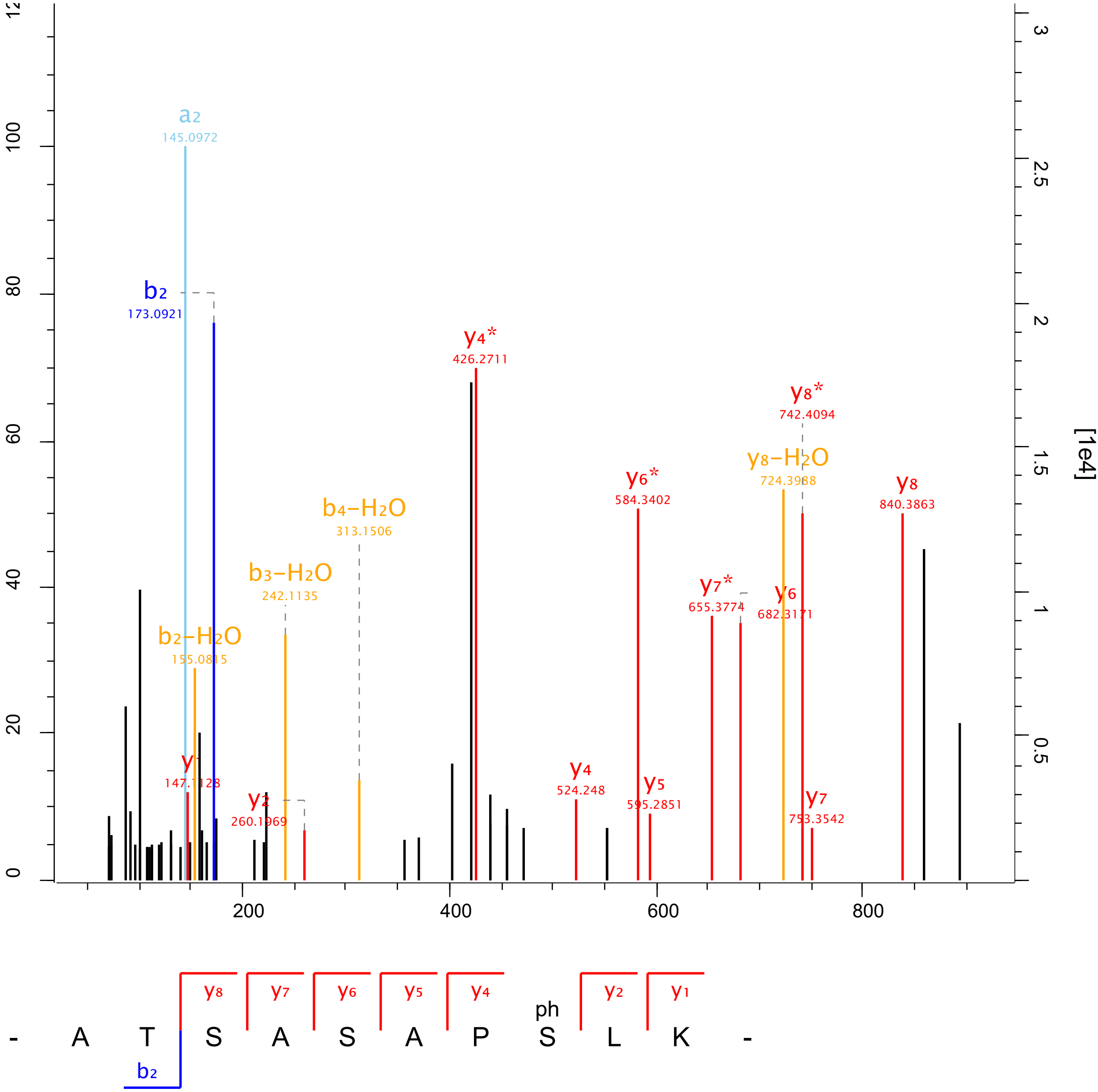

Raw file	Scan	Method	Score	m/z
sys_02_2	5899	FTMS; HCD	54.39	468.55

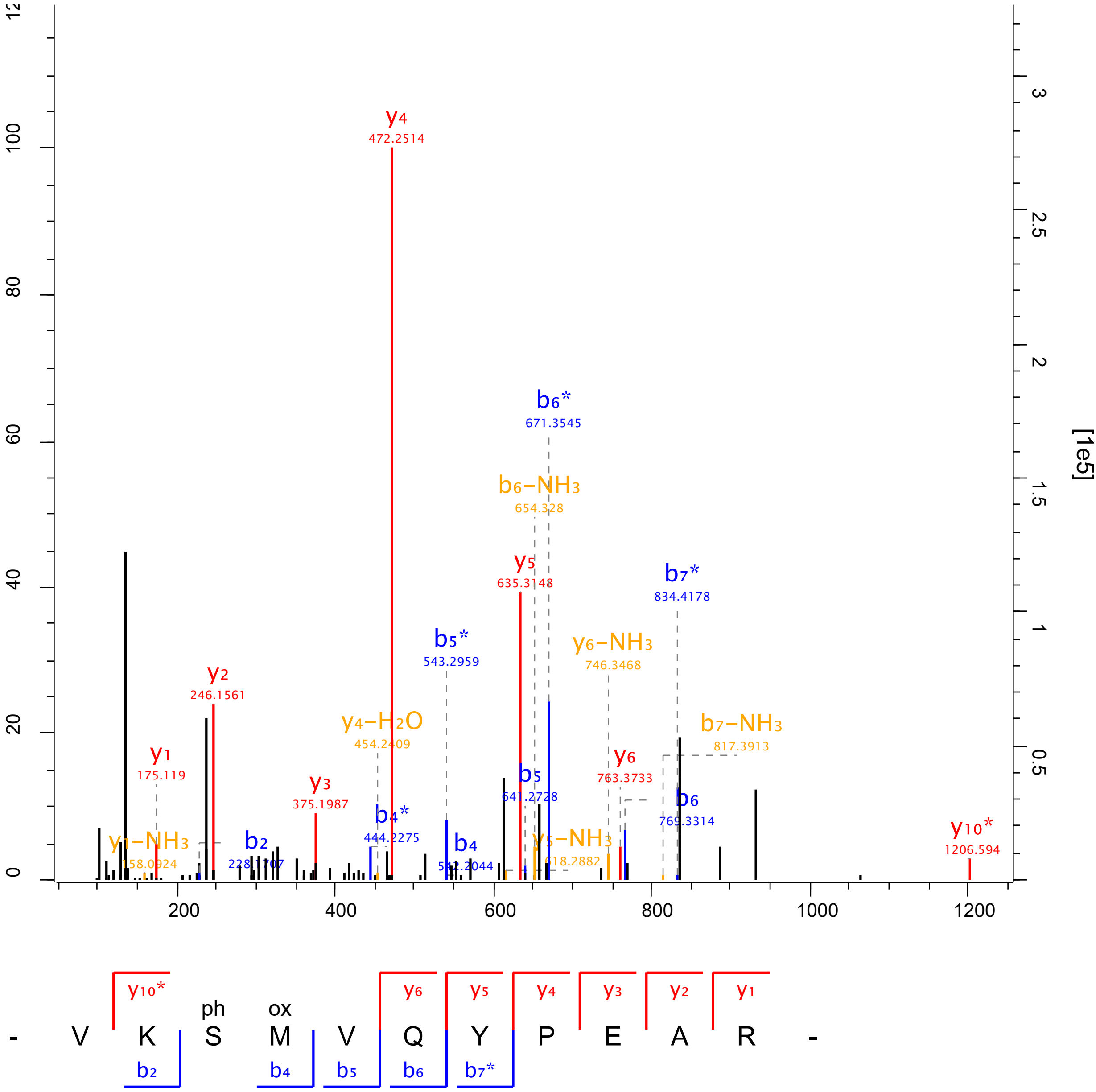

Raw file	Scan	Method	Score	m/z
sys_02_2	5935	FTMS; HCD	68.41	626.24

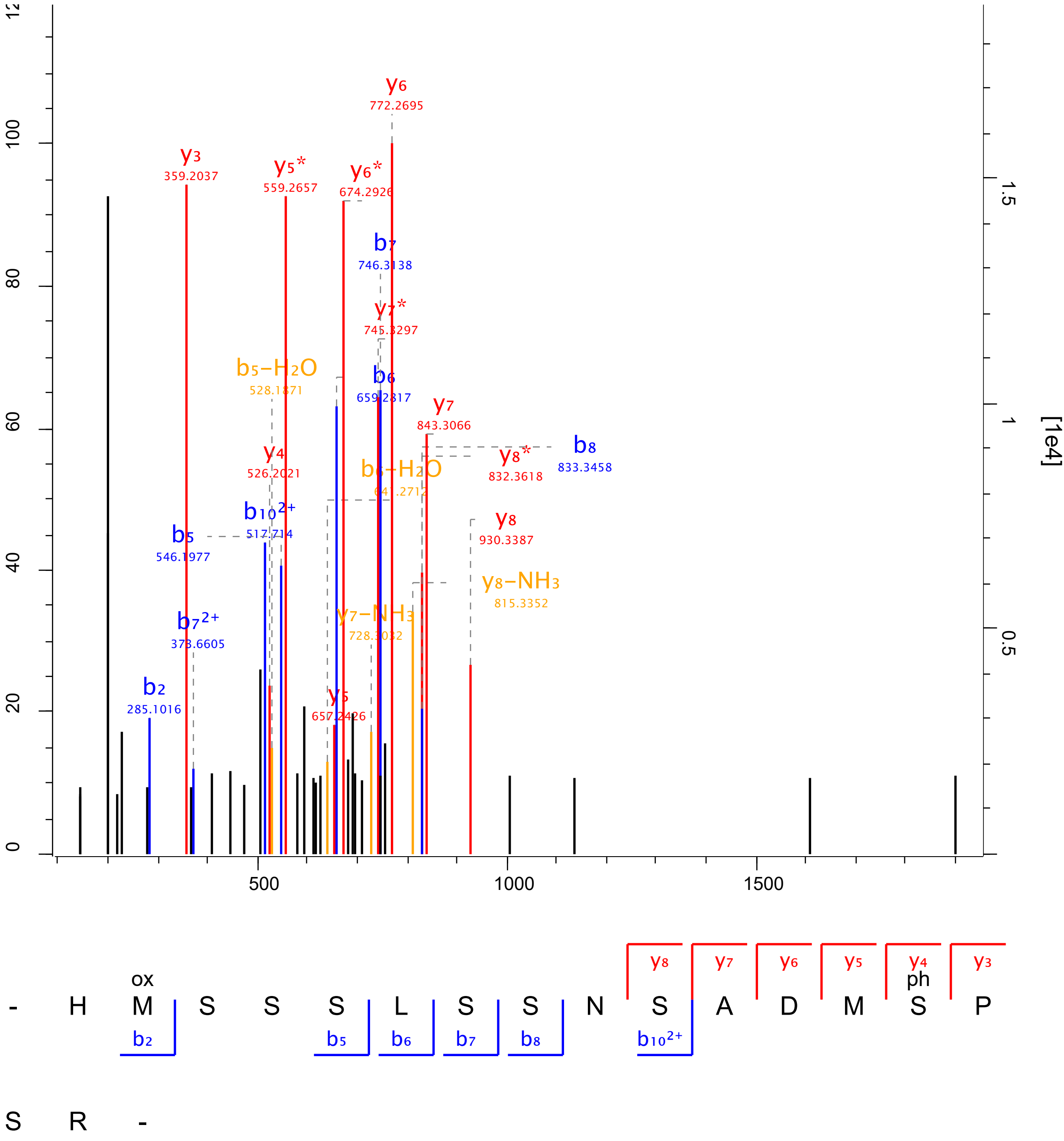

Raw file	Scan	Method	Score	m/z
sys_02_2	5954	FTMS; HCD	209.73	656.26

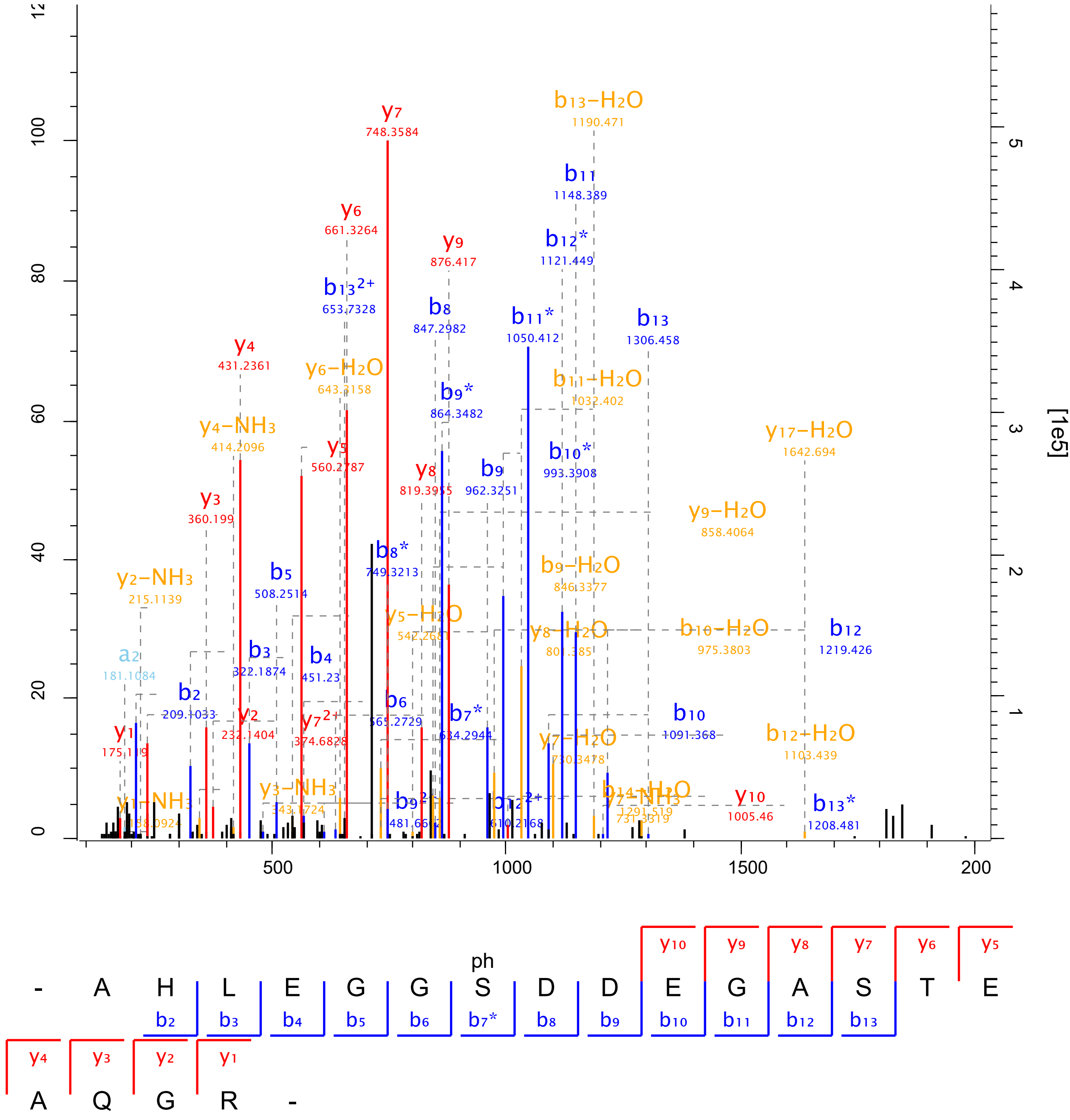

Raw file	Scan	Method	Score	m/z
sys_02_2	5982	FTMS; HCD	76.07	434.87

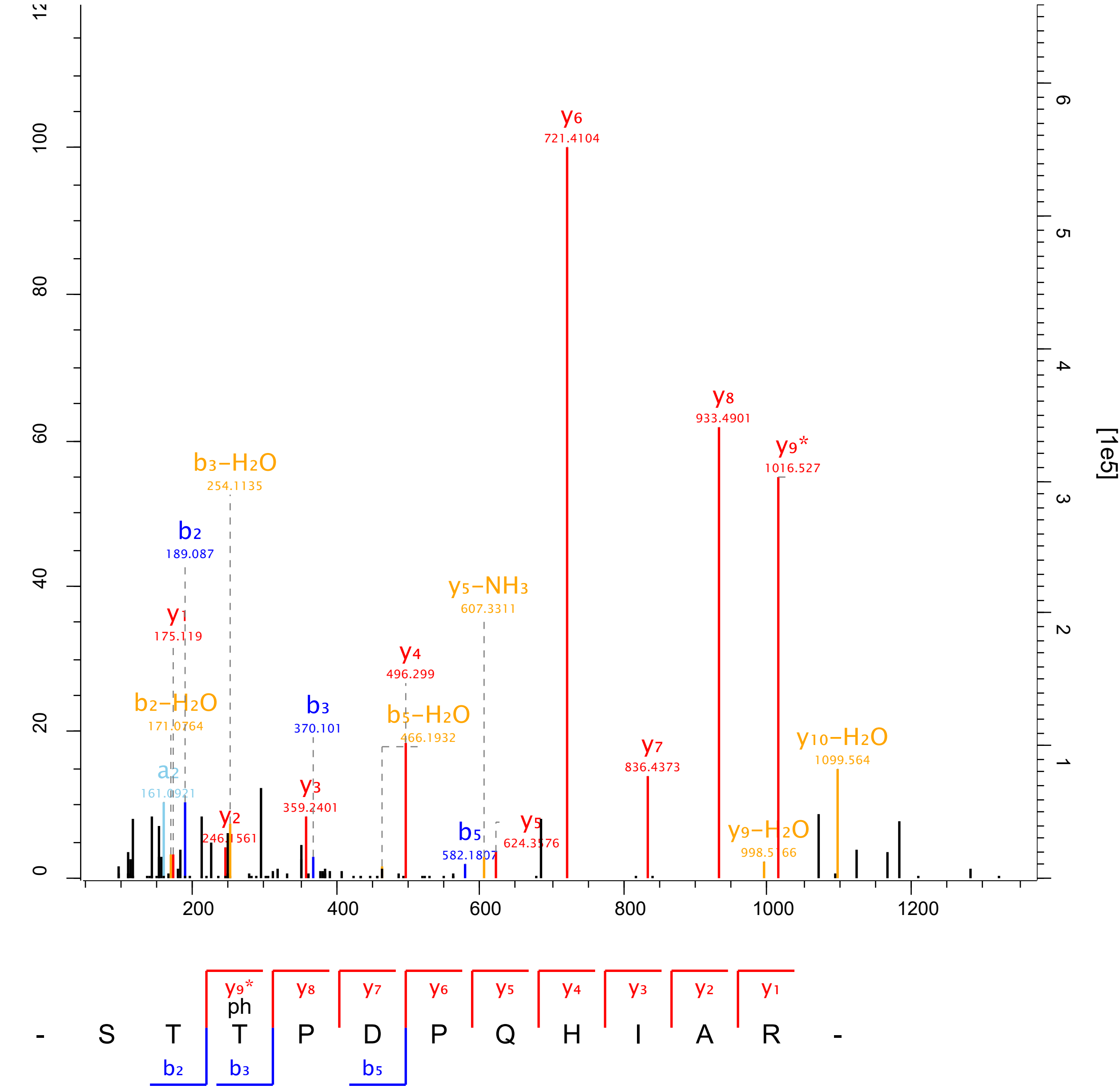

Raw file	Scan	Method	Score	m/z
sys_02_2	6039	FTMS; HCD	44.83	614.23

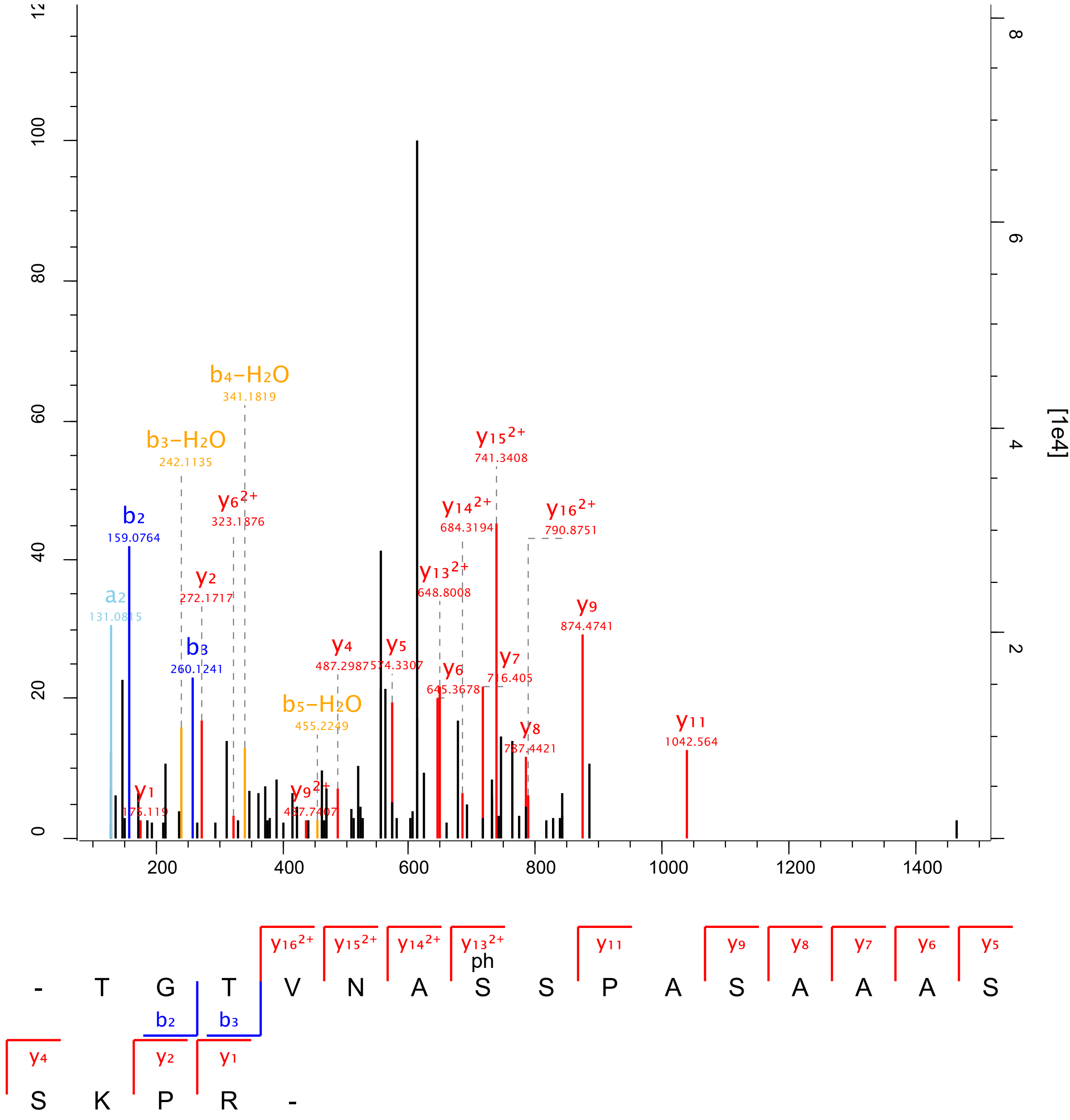

Raw file	Scan	Method	Score	m/z
sys_02_2	6086	FTMS; HCD	211.12	551.56

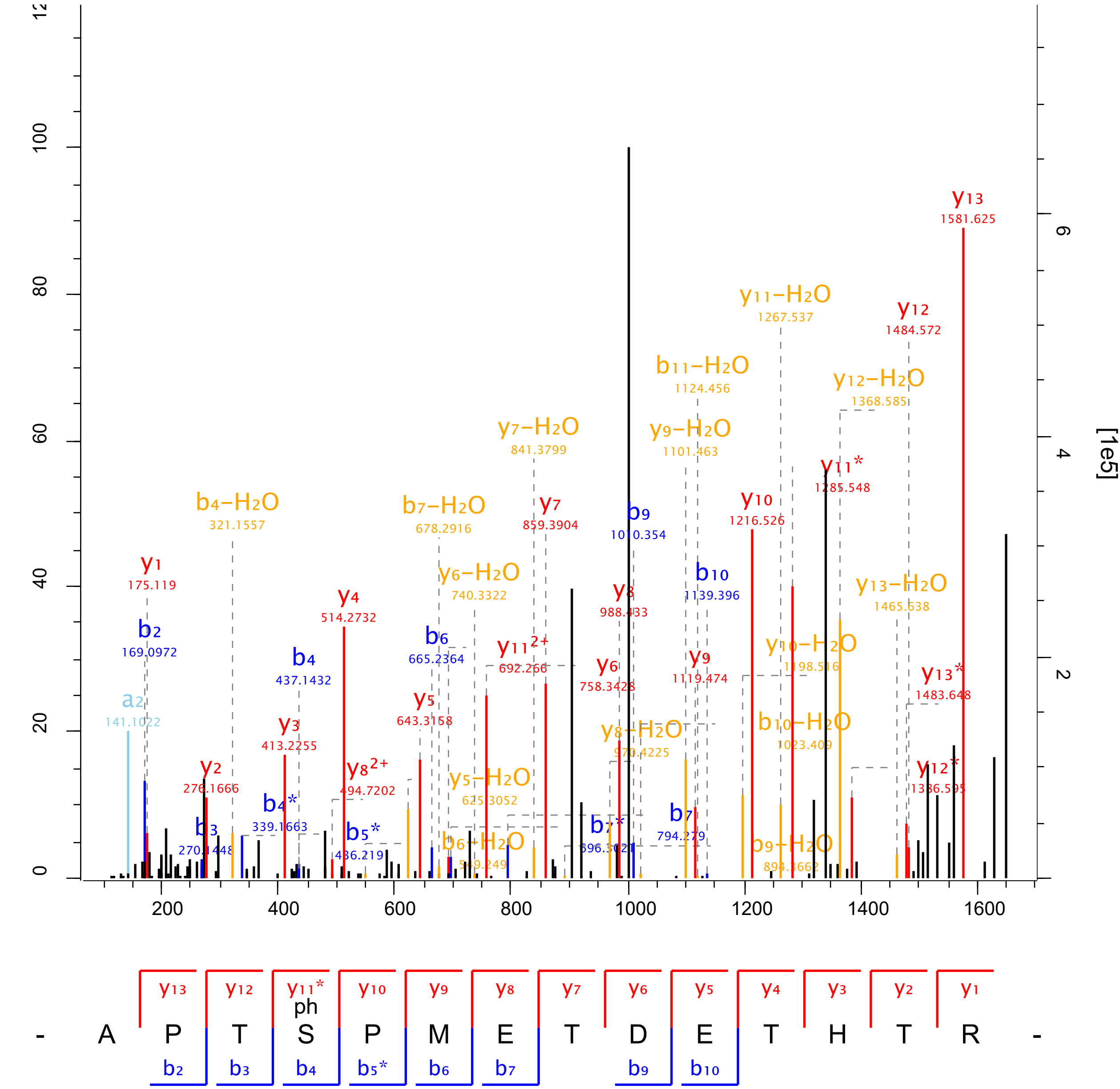

Raw file	Scan	Method	Score	m/z
sys_02_2	6103	FTMS; HCD	84.51	452.2

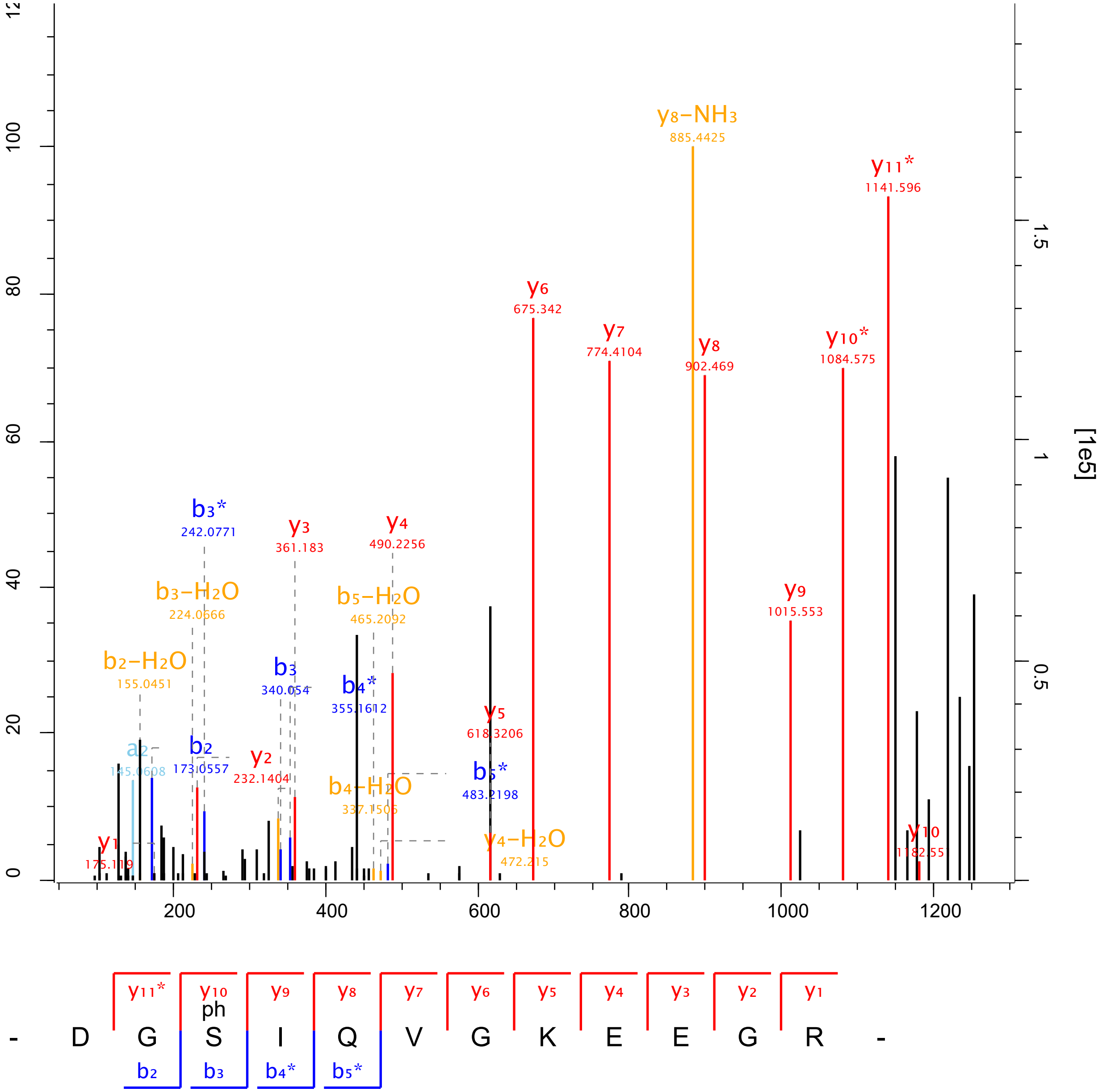

Raw file	Scan	Method	Score	m/z
sys_02_2	6193	FTMS; HCD	159.75	632.63

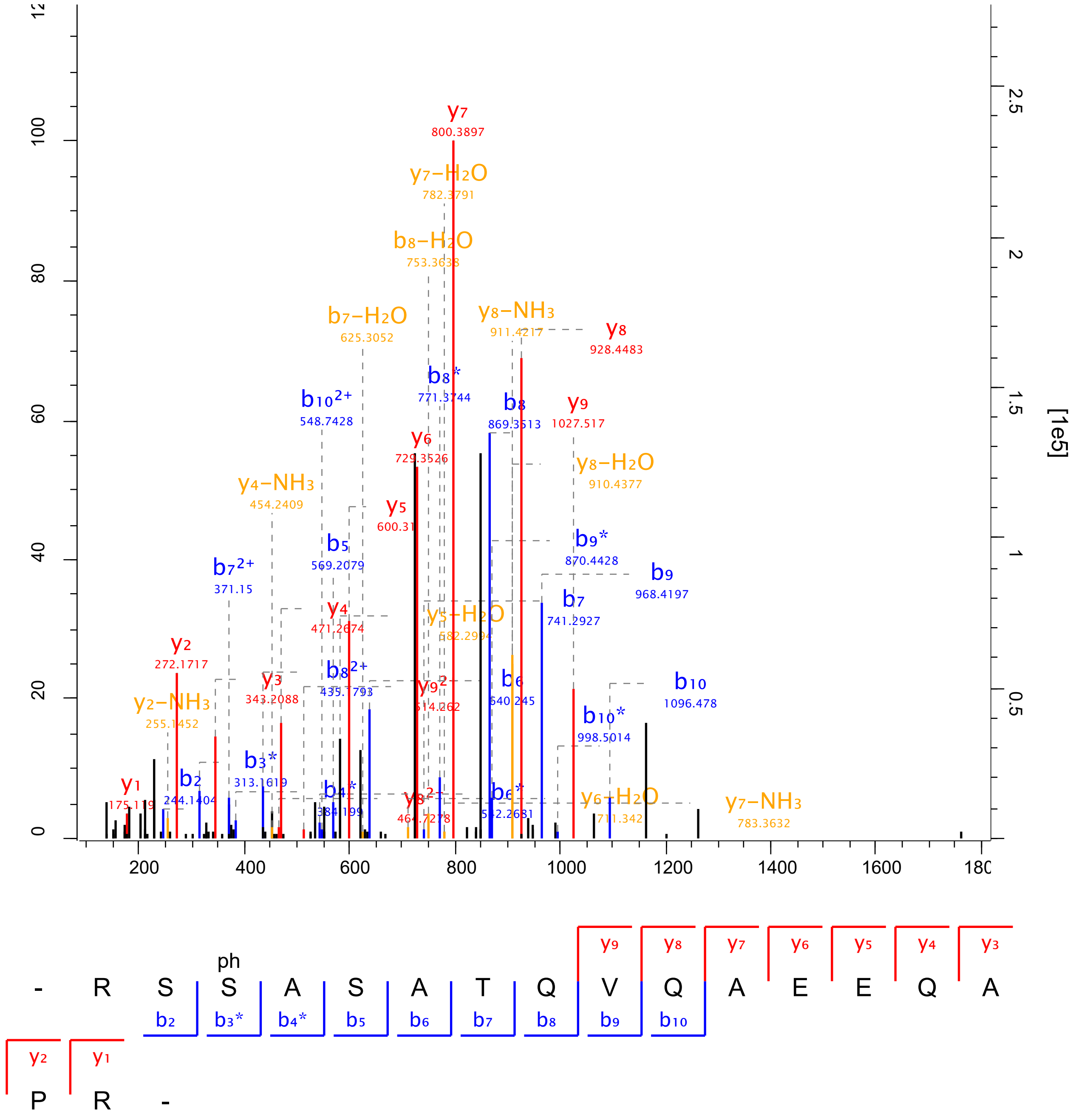

Raw file	Scan	Method	Score	m/z
sys_02_2	6200	FTMS; HCD	115.29	462.73

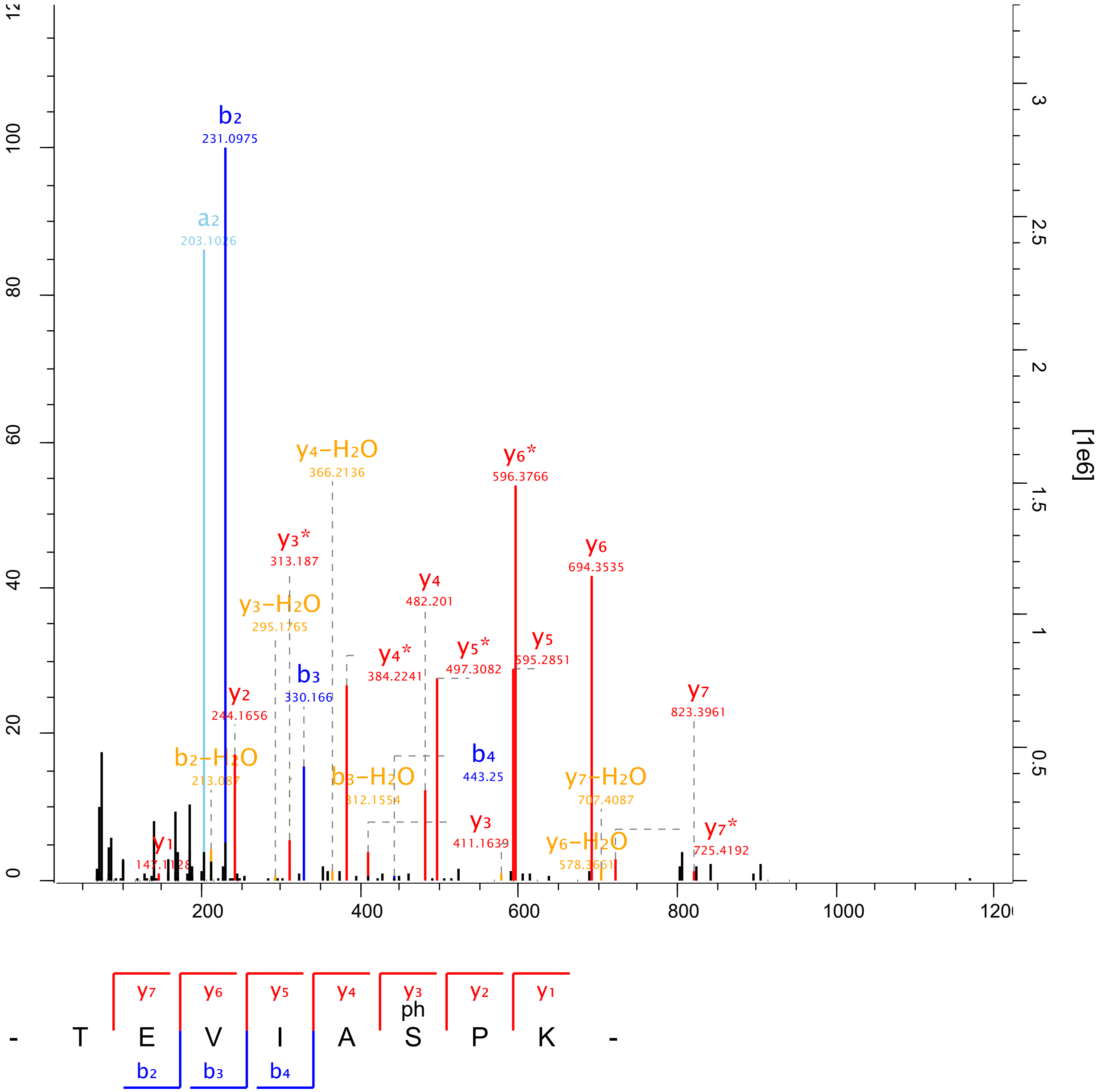

Raw file	Scan	Method	Score	m/z
sys_02_2	6222	FTMS; HCD	132.79	764.82

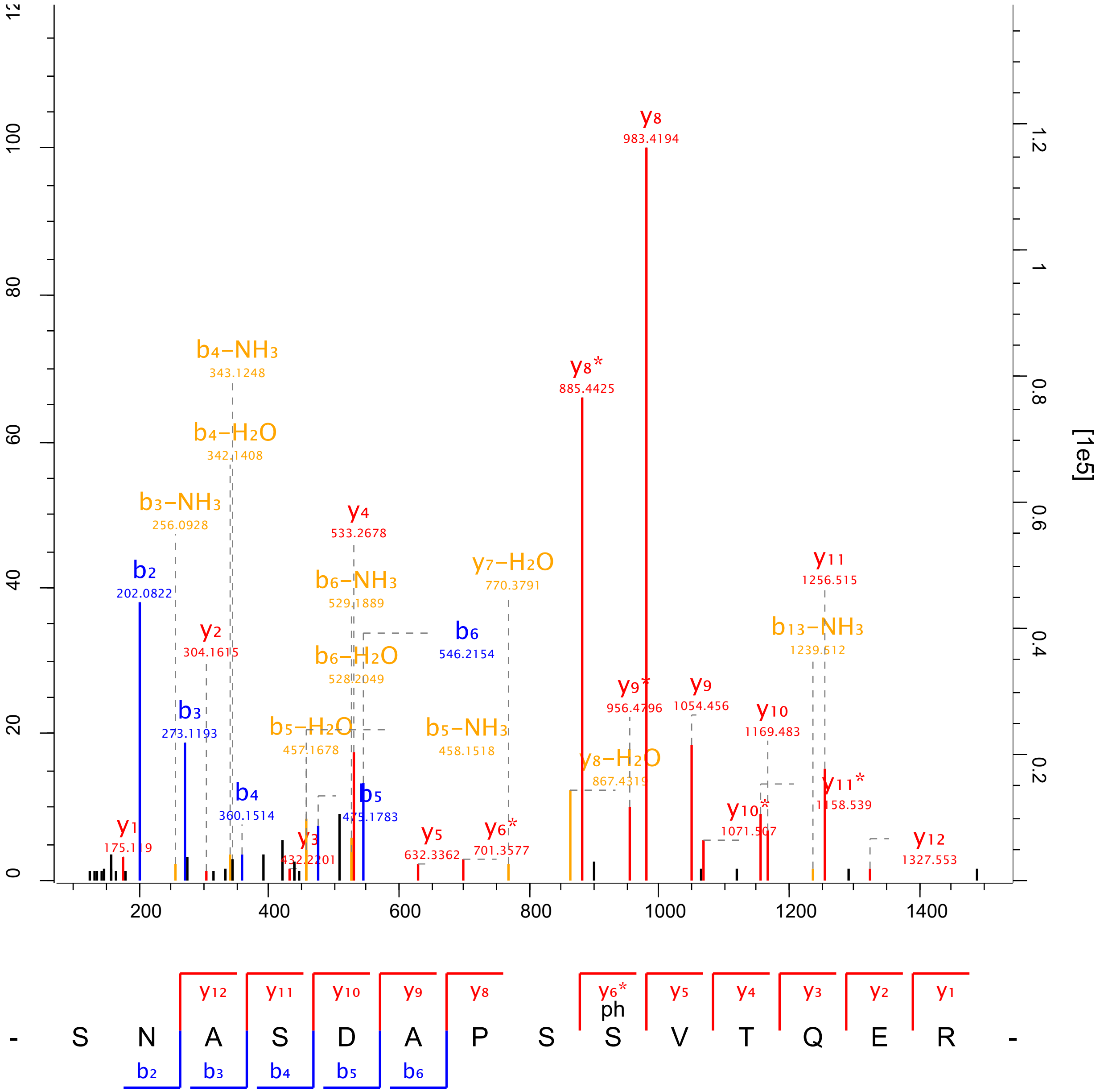

Raw file	Scan	Method	Score	m/z
sys_02_2	6226	FTMS; HCD	107.78	651.95

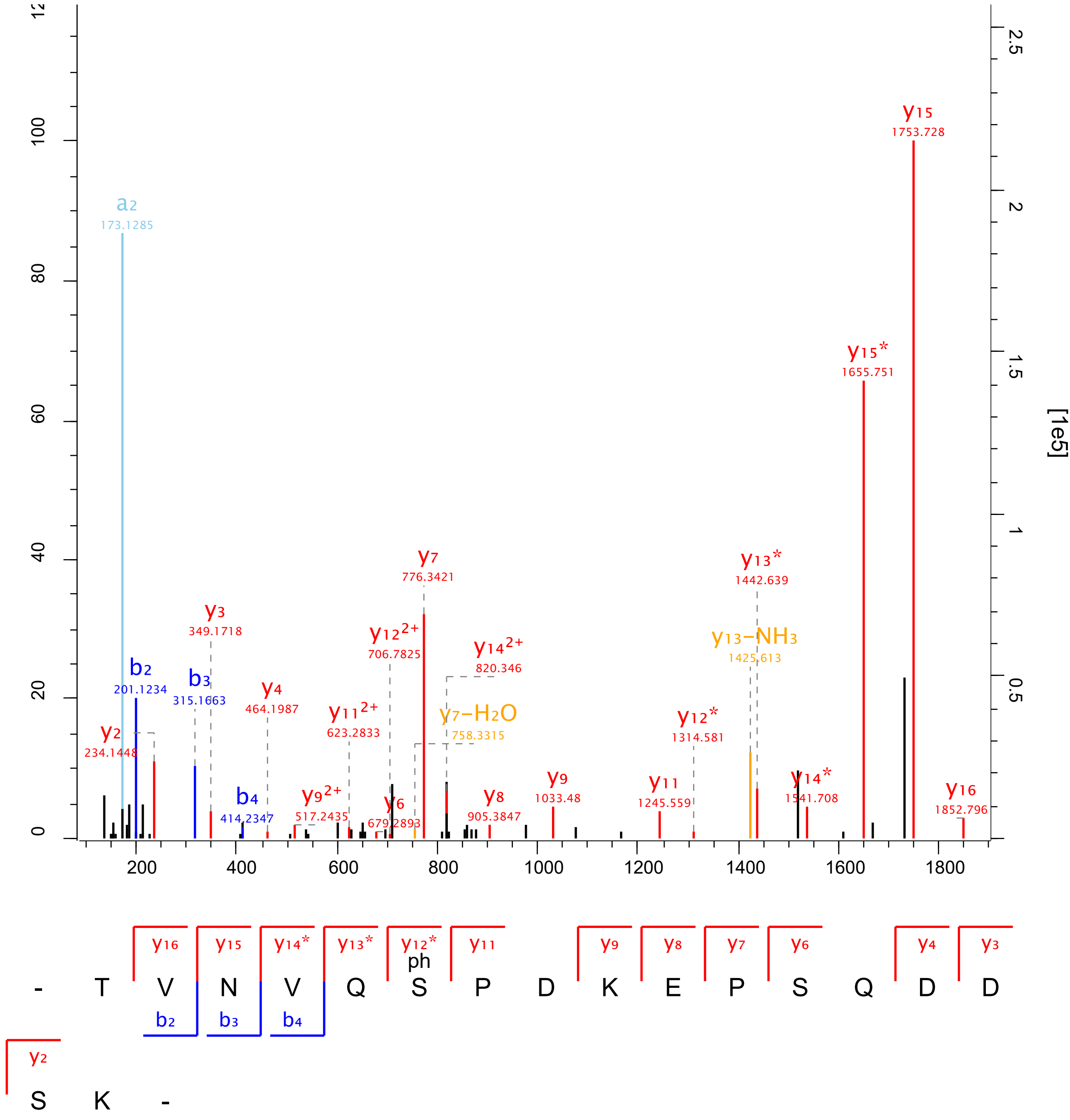

Raw file	Scan	Method	Score	m/z
sys_02_2	6237	FTMS; HCD	90.7	602.77

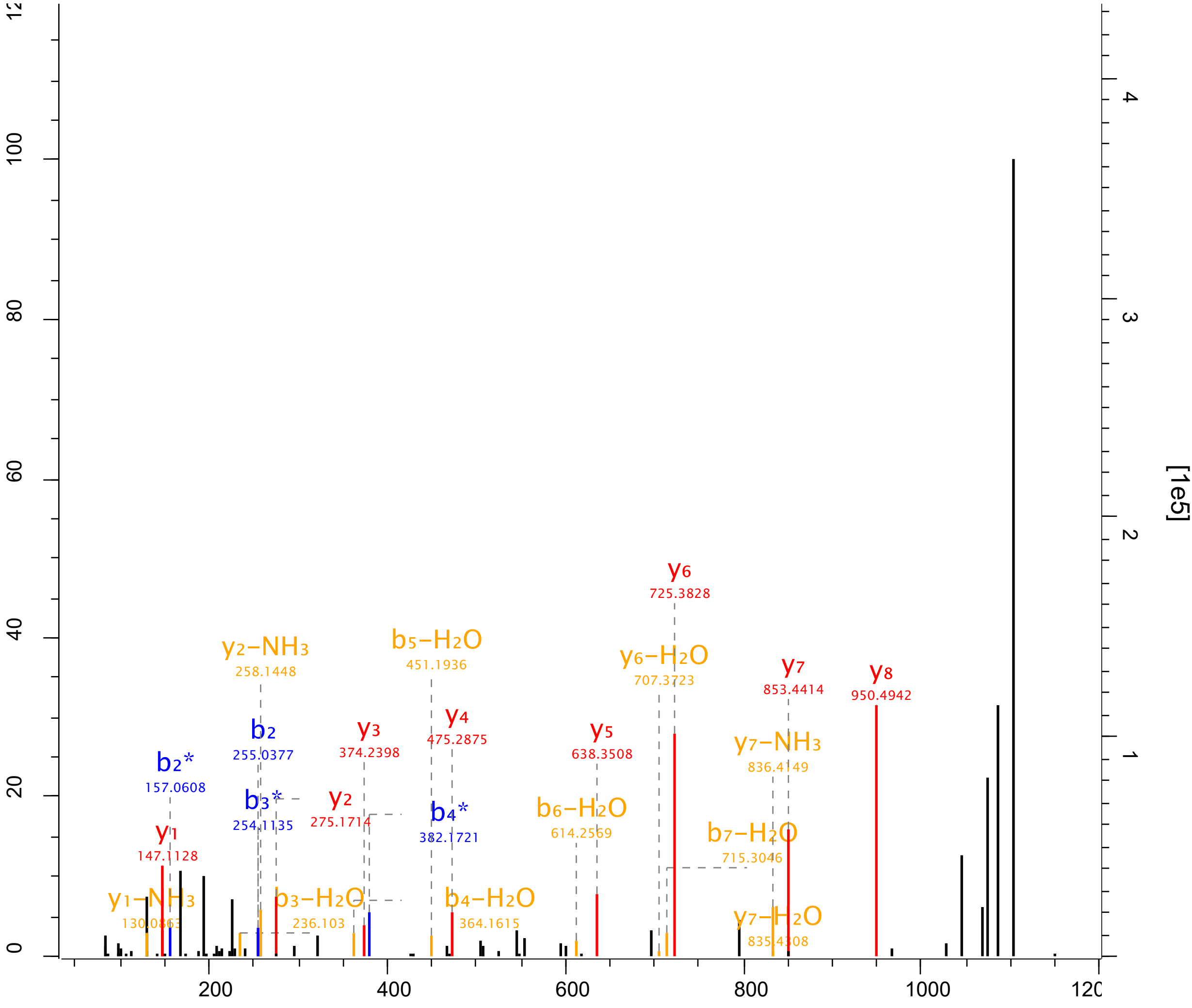

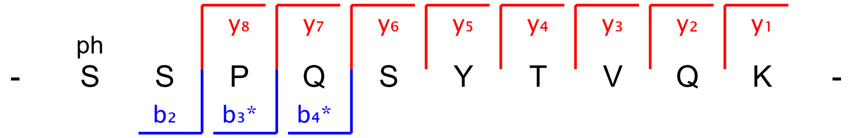

Raw file	Scan	Method	Score	m/z
sys_02_2	6418	FTMS; HCD	42.03	535.23

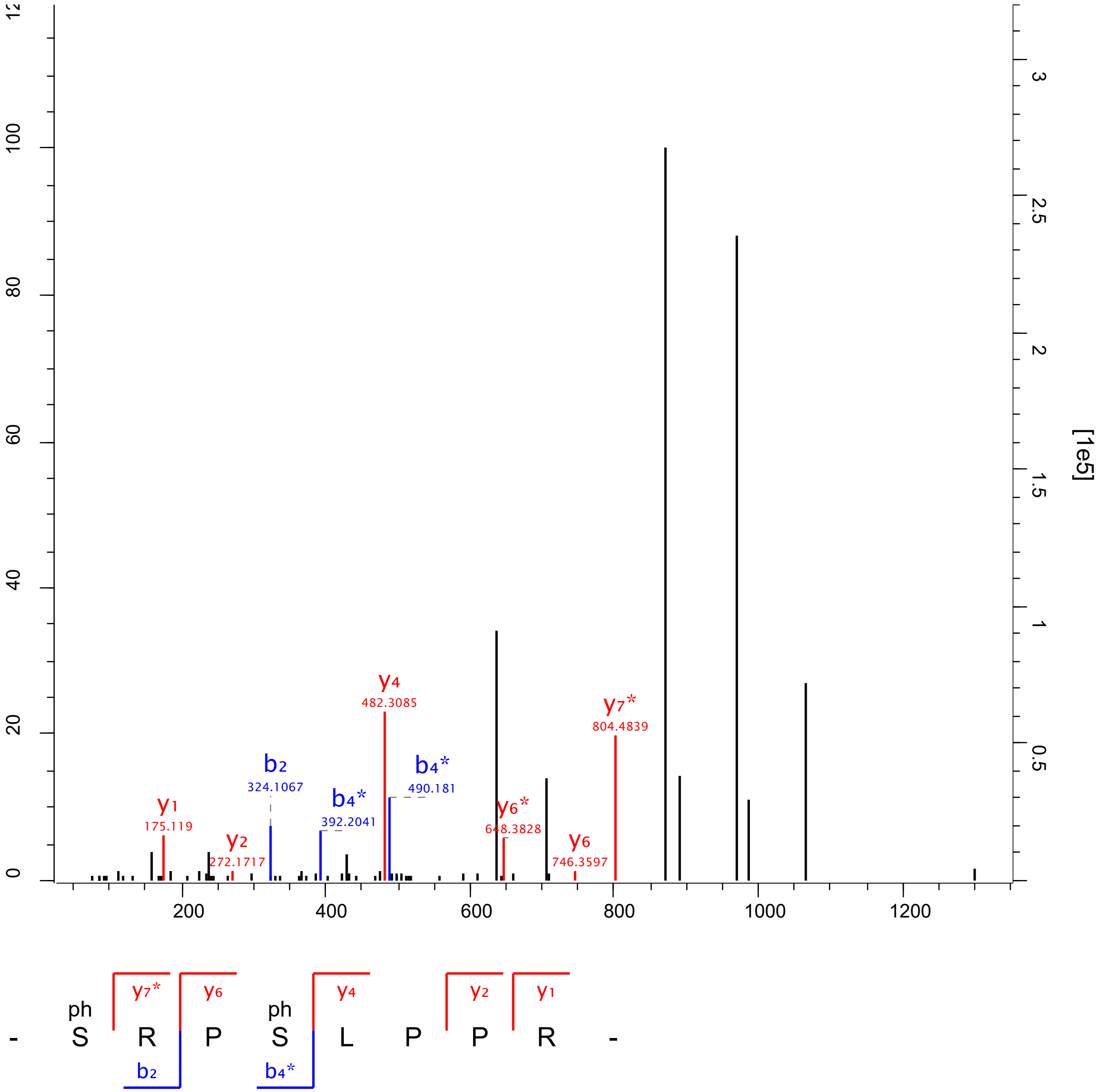

Raw file	Scan	Method	Score	m/z
sys_02_2	6447	FTMS; HCD	78.19	659.77

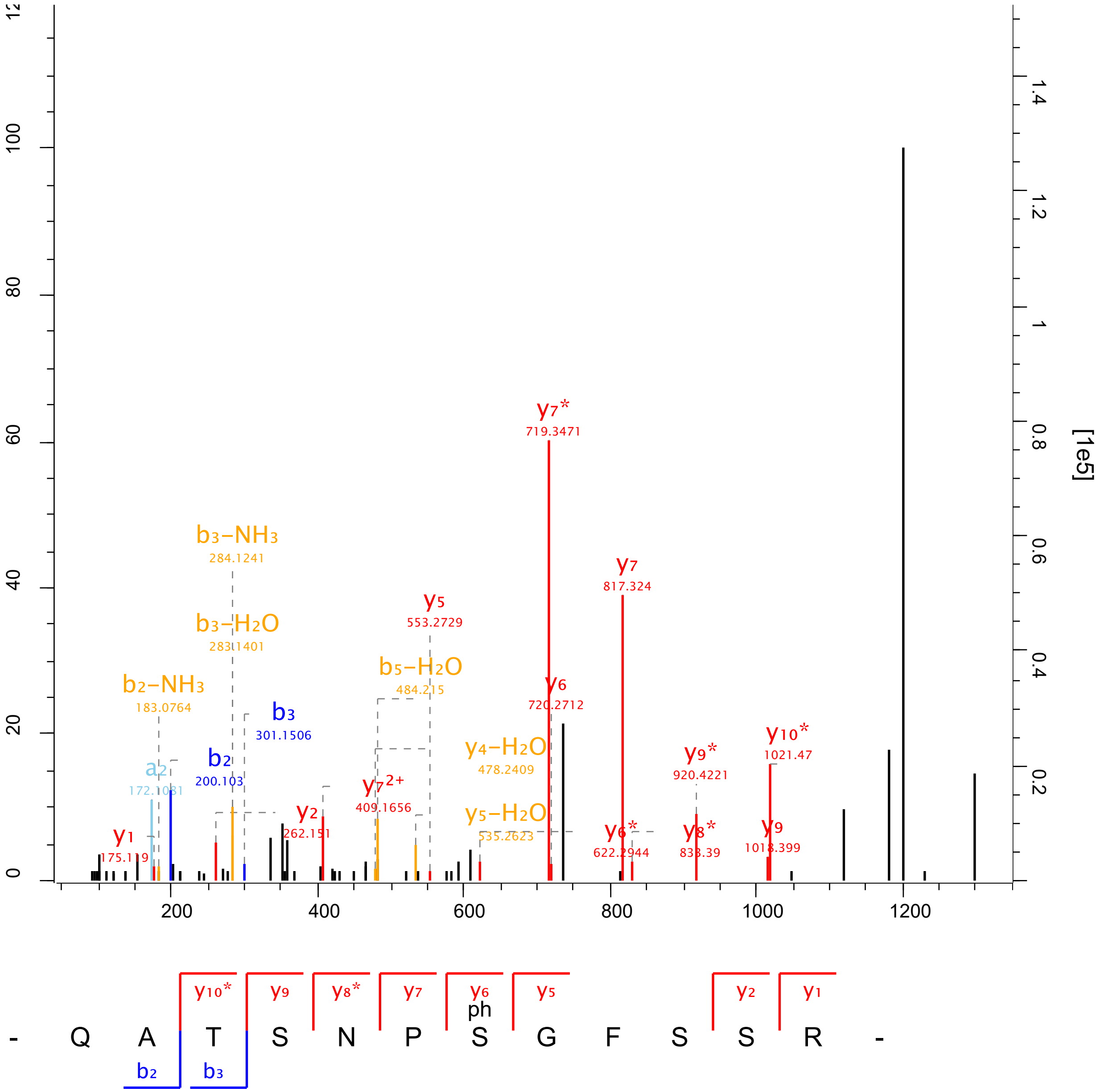

Raw file	Scan	Method	Score	m/z
sys_02_2	6495	FTMS; HCD	98.42	588.26

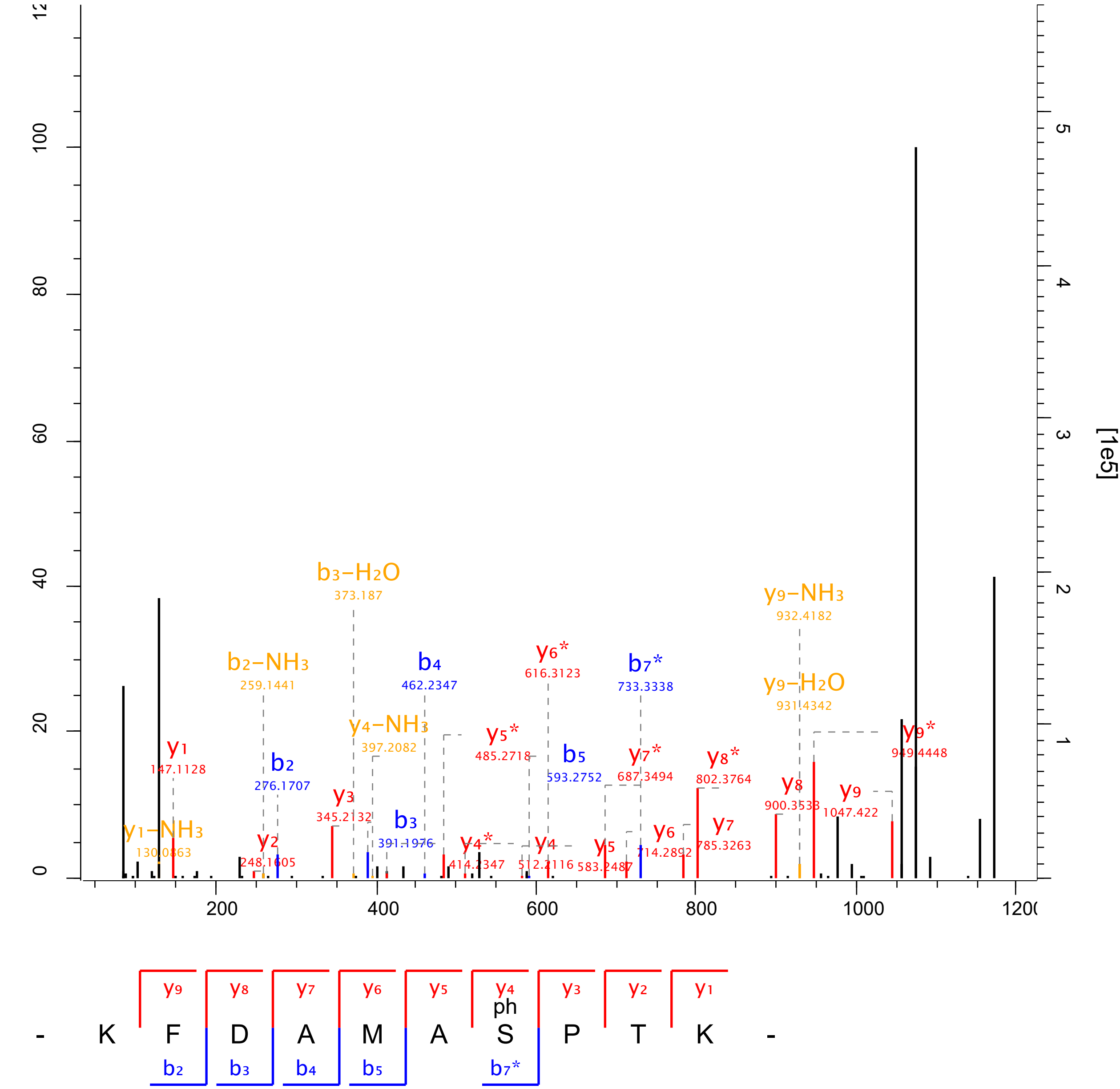

Raw file	Scan	Method	Score	m/z
sys_02_2	6522	FTMS; HCD	62.59	628.29

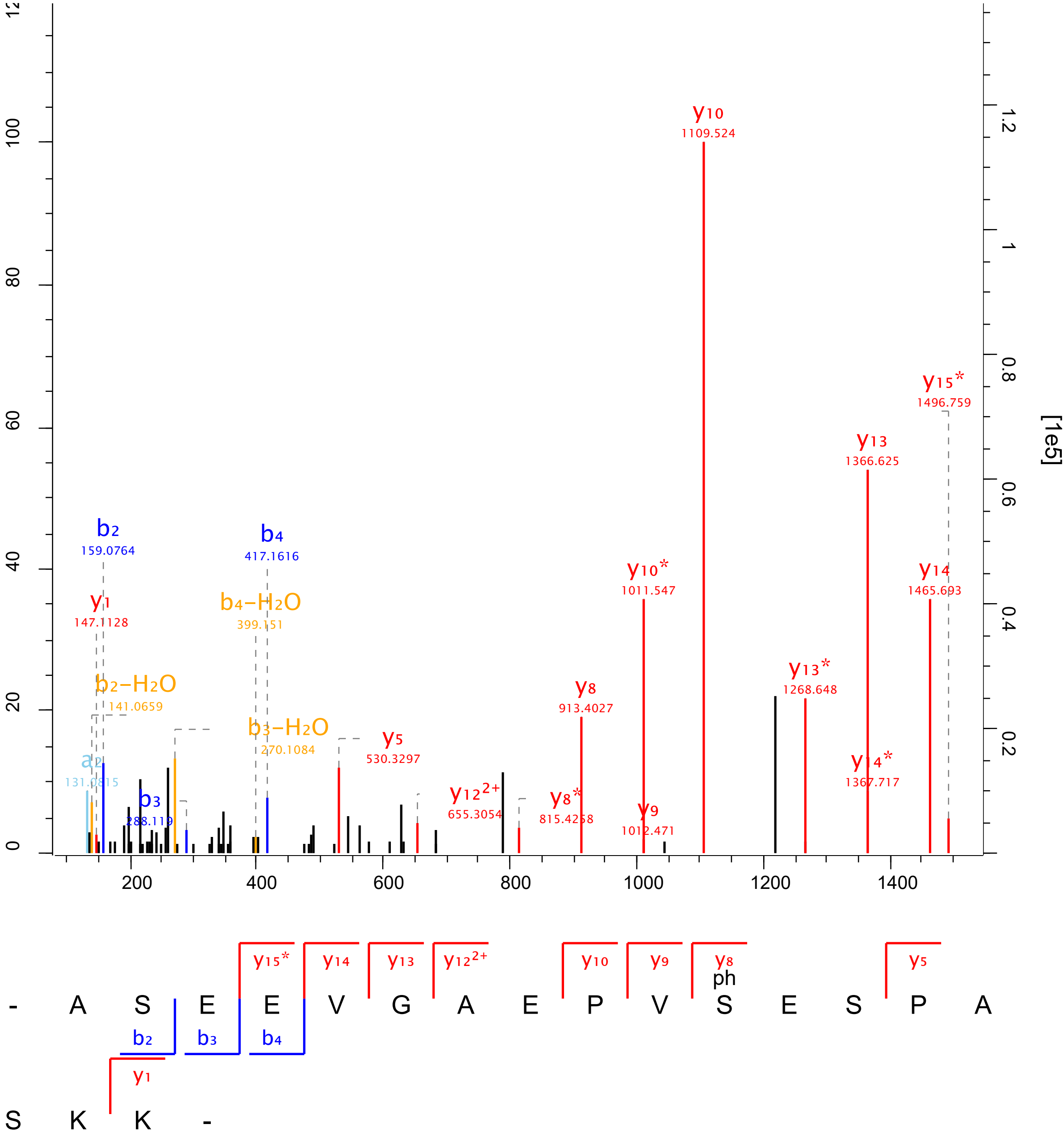

Raw file	Scan	Method	Score	m/z
sys_02_2	6699	FTMS; HCD	102.45	653.95

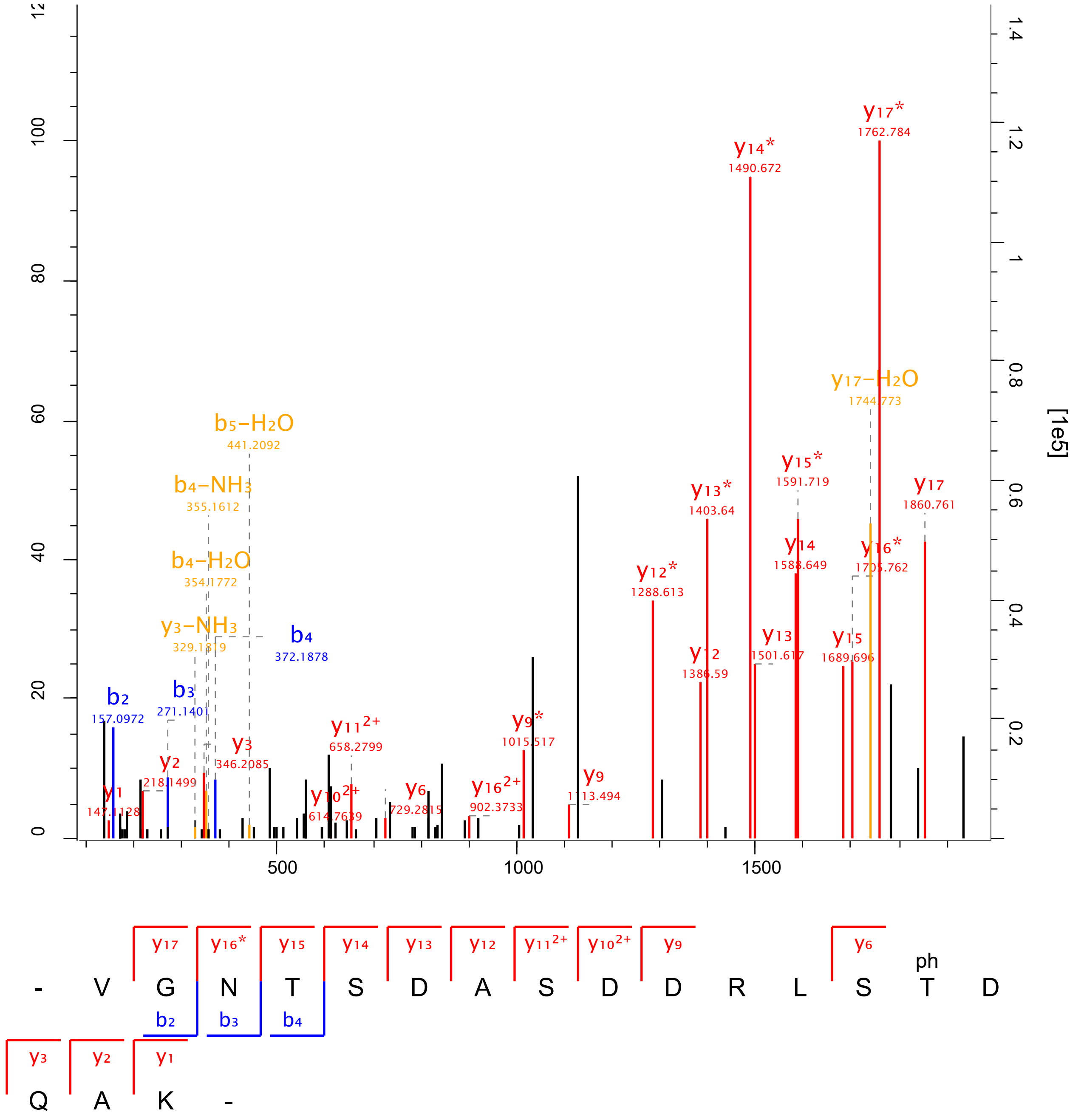

Raw file	Scan	Method	Score	m/z
sys_02_2	6730	FTMS; HCD	47.71	609.78

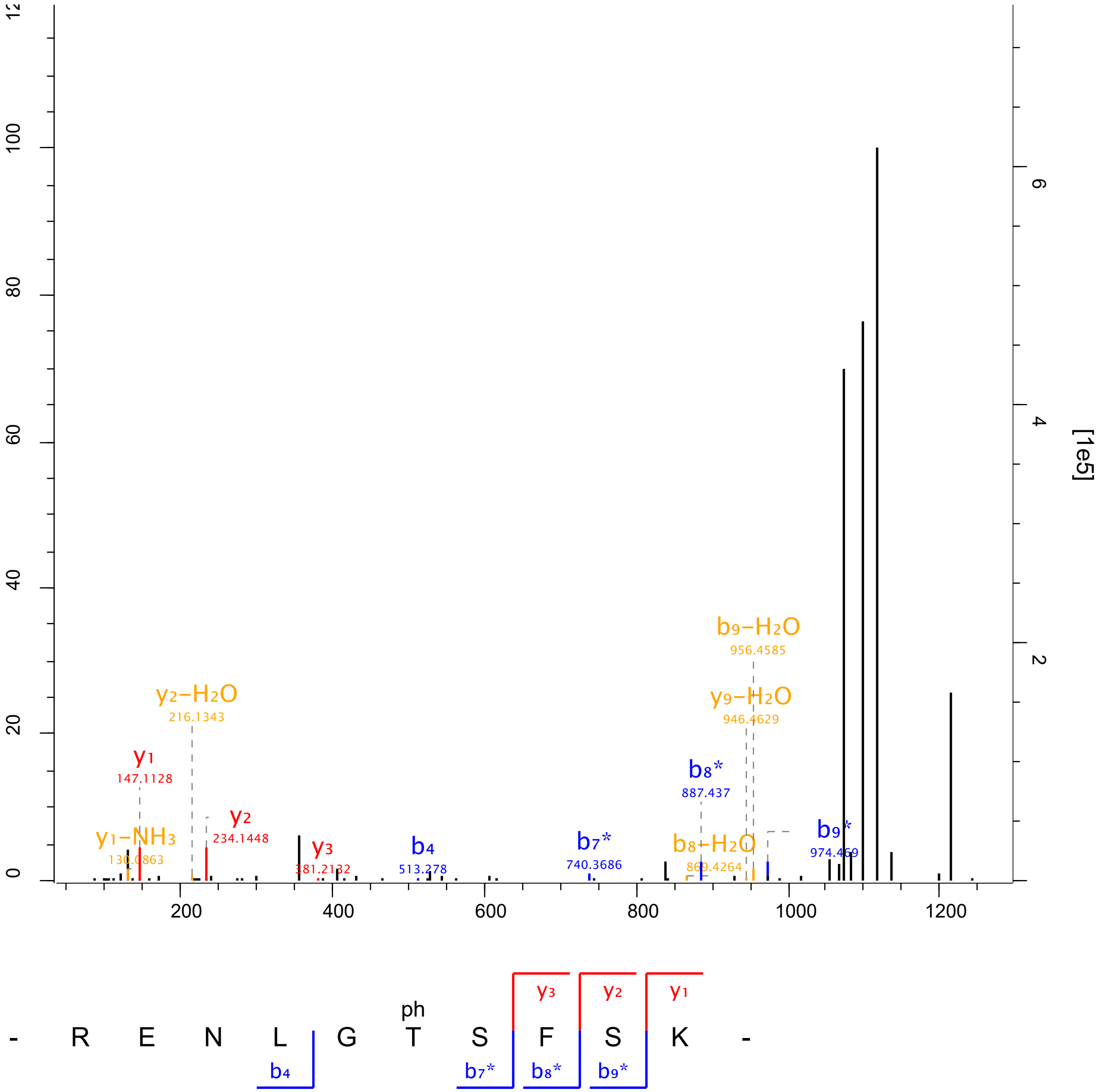

Raw file	Scan	Method	Score	m/z
sys_02_2	6732	FTMS; HCD	130.01	480.71

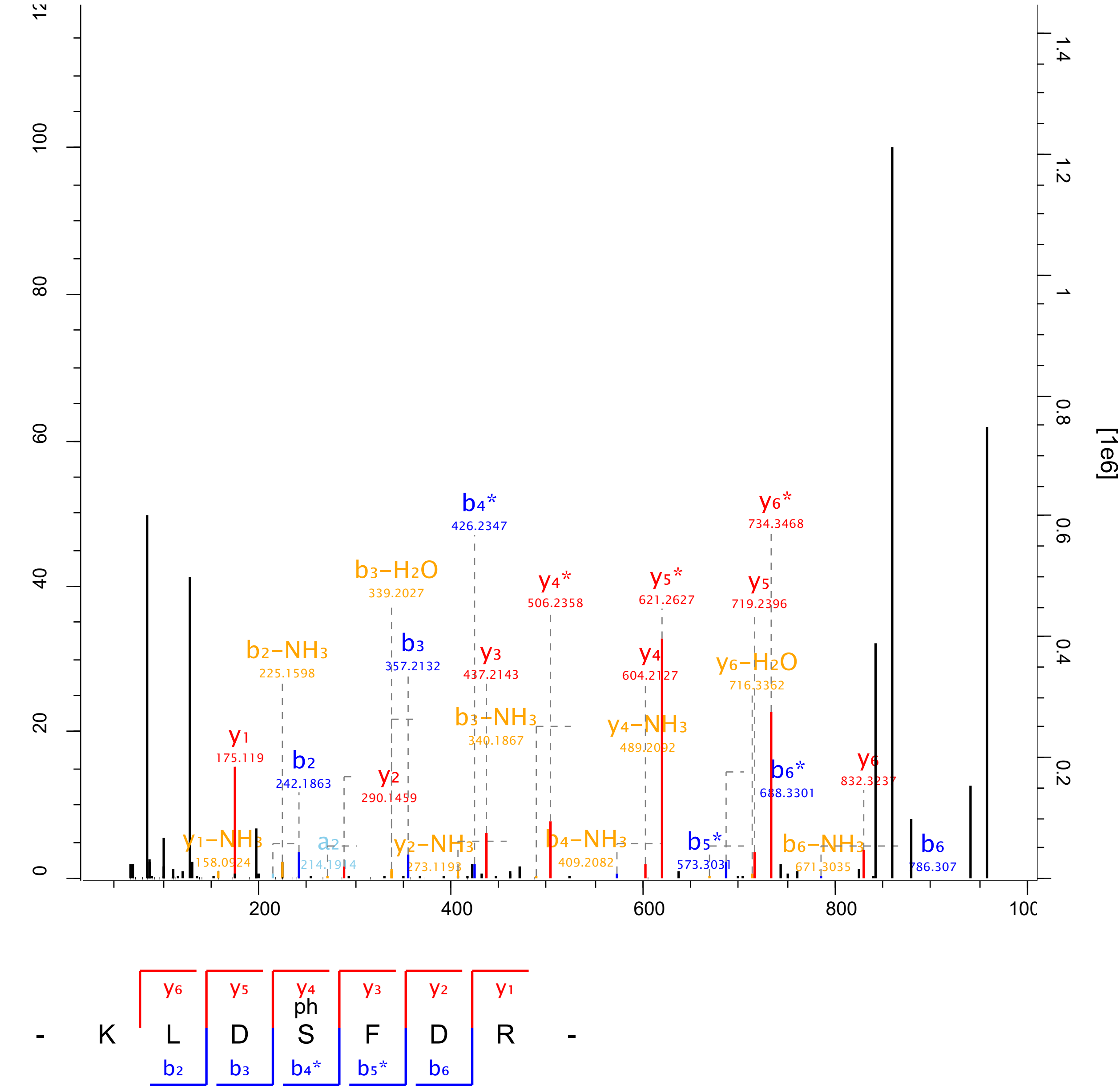

Raw file	Scan	Method	Score	m/z
sys_02_2	6867	FTMS; HCD	107.06	559.74

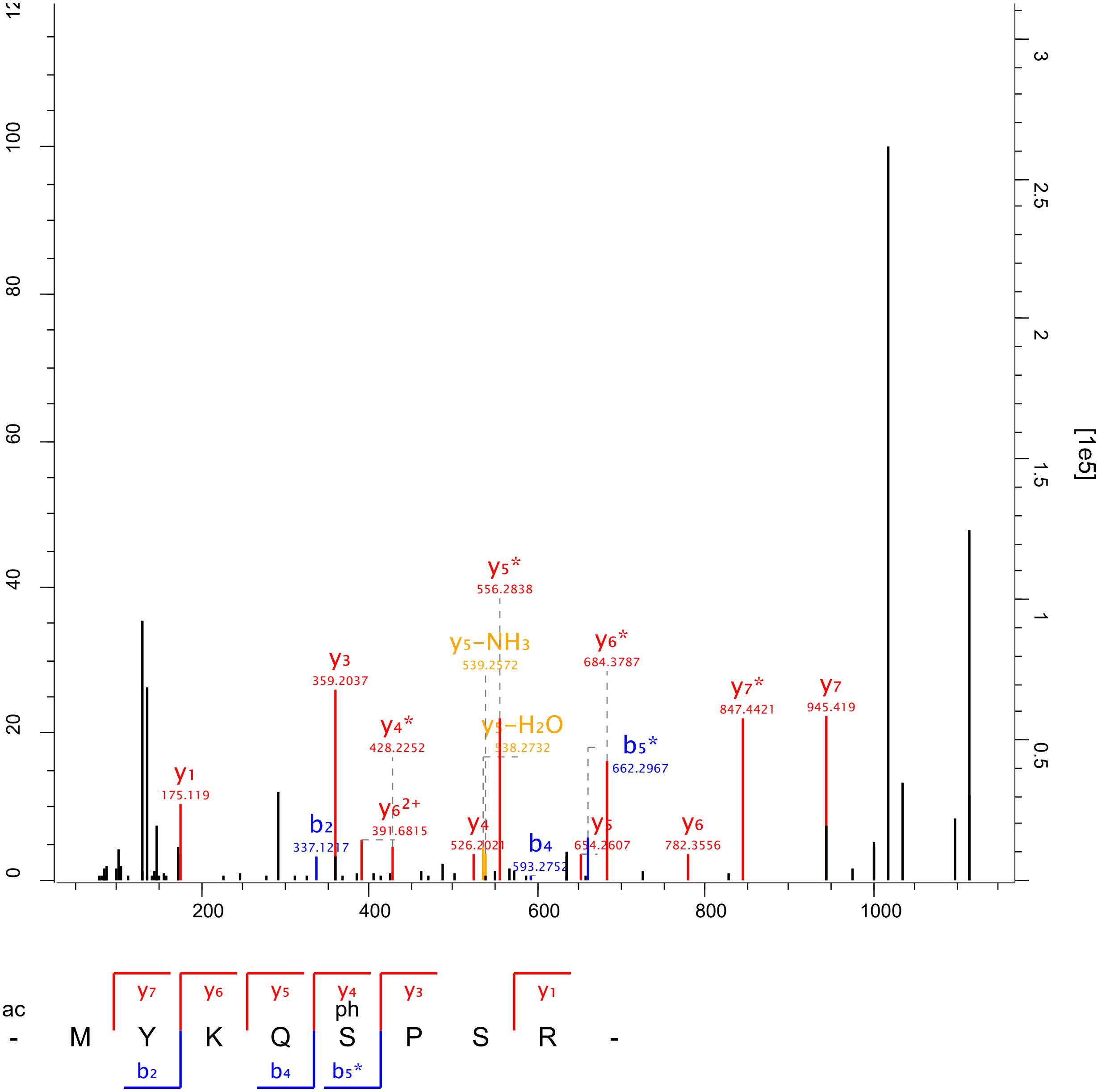

Raw file	Scan	Method	Score	m/z
sys_02_2	7057	FTMS; HCD	45	529.22

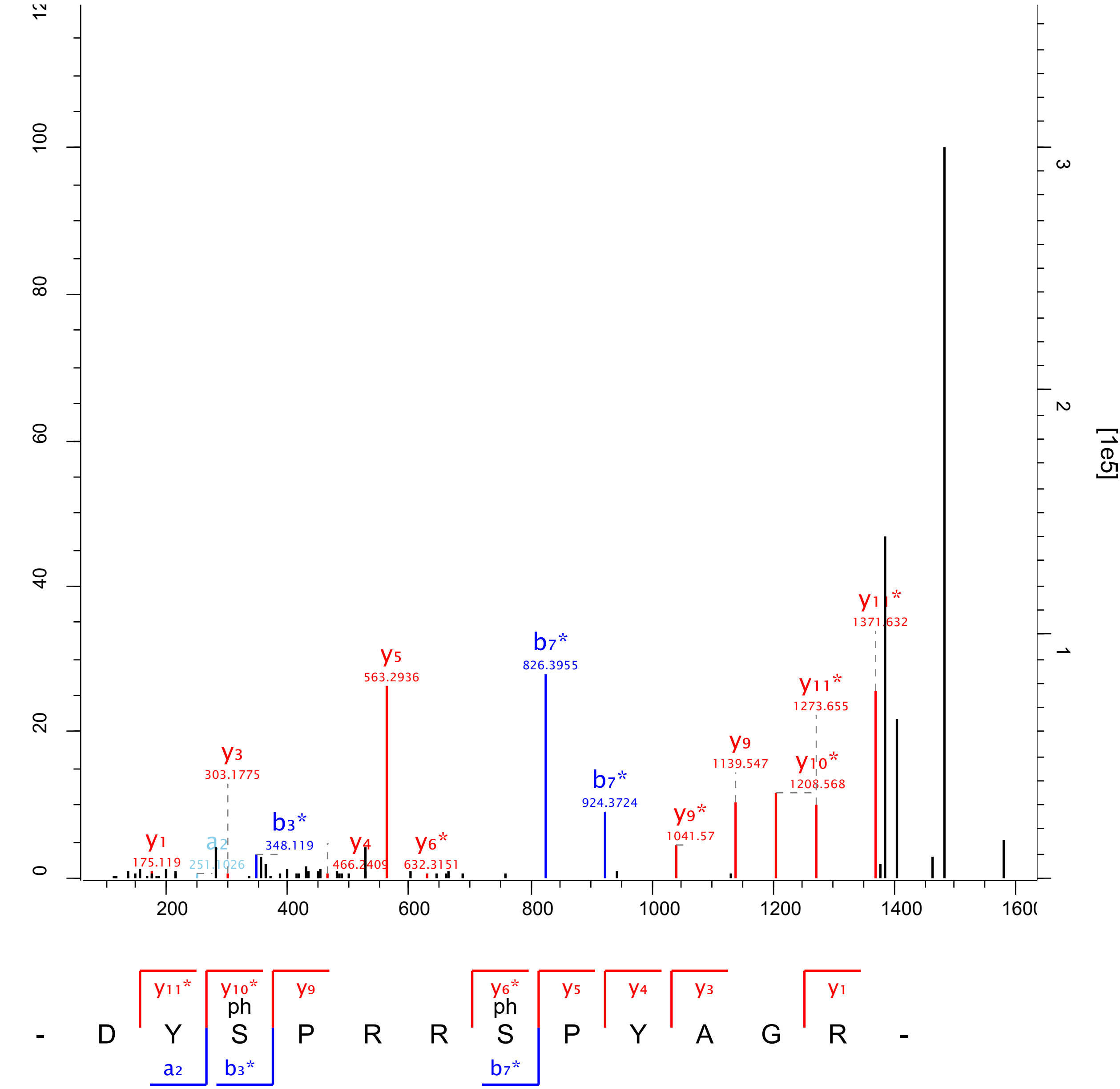

Raw file	Scan	Method	Score	m/z
sys_02_2	7075	FTMS; HCD	89.47	724.29

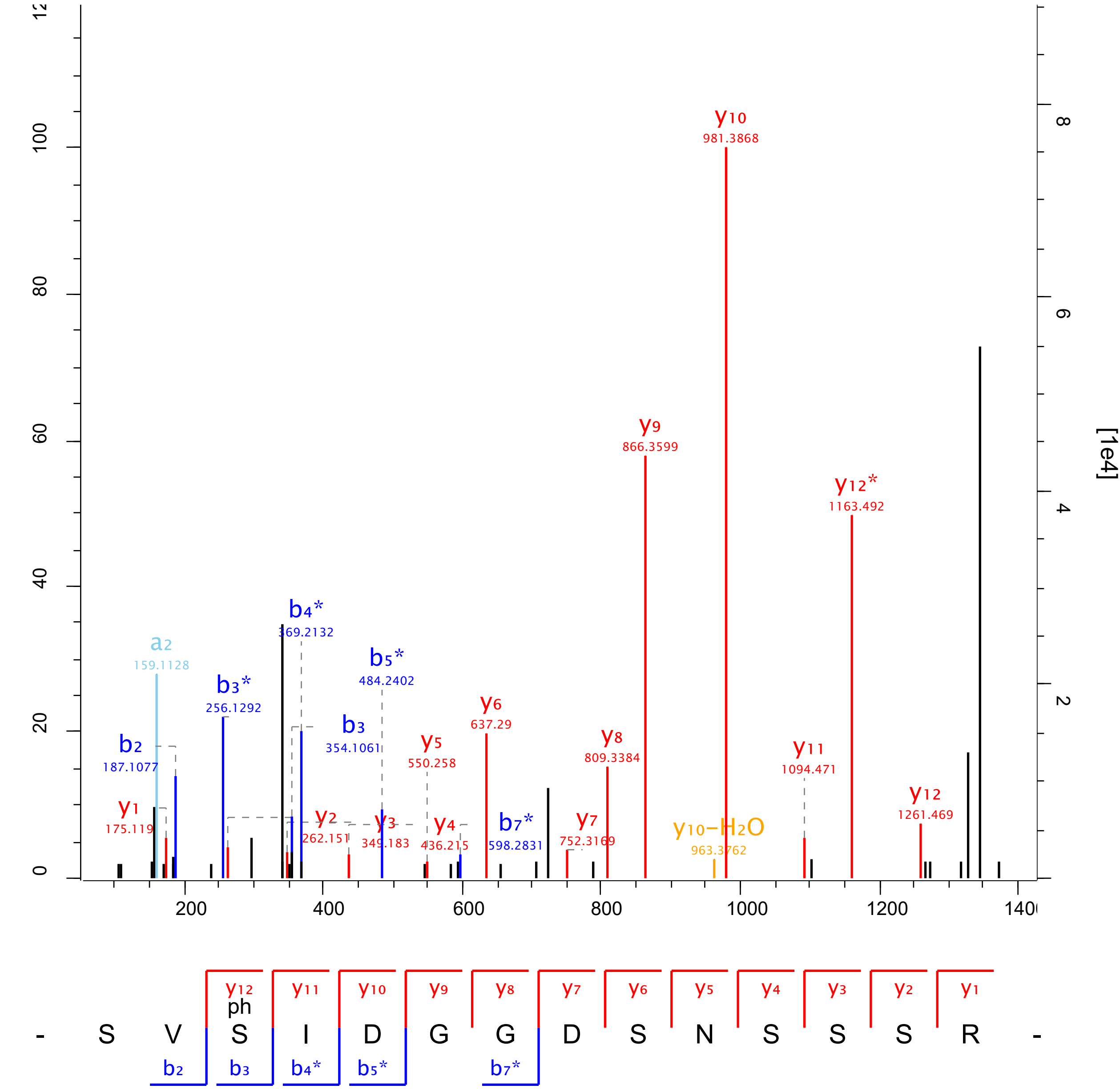

Raw file	Scan	Method	Score	m/z
sys_02_2	7097	FTMS; HCD	67.65	639.93

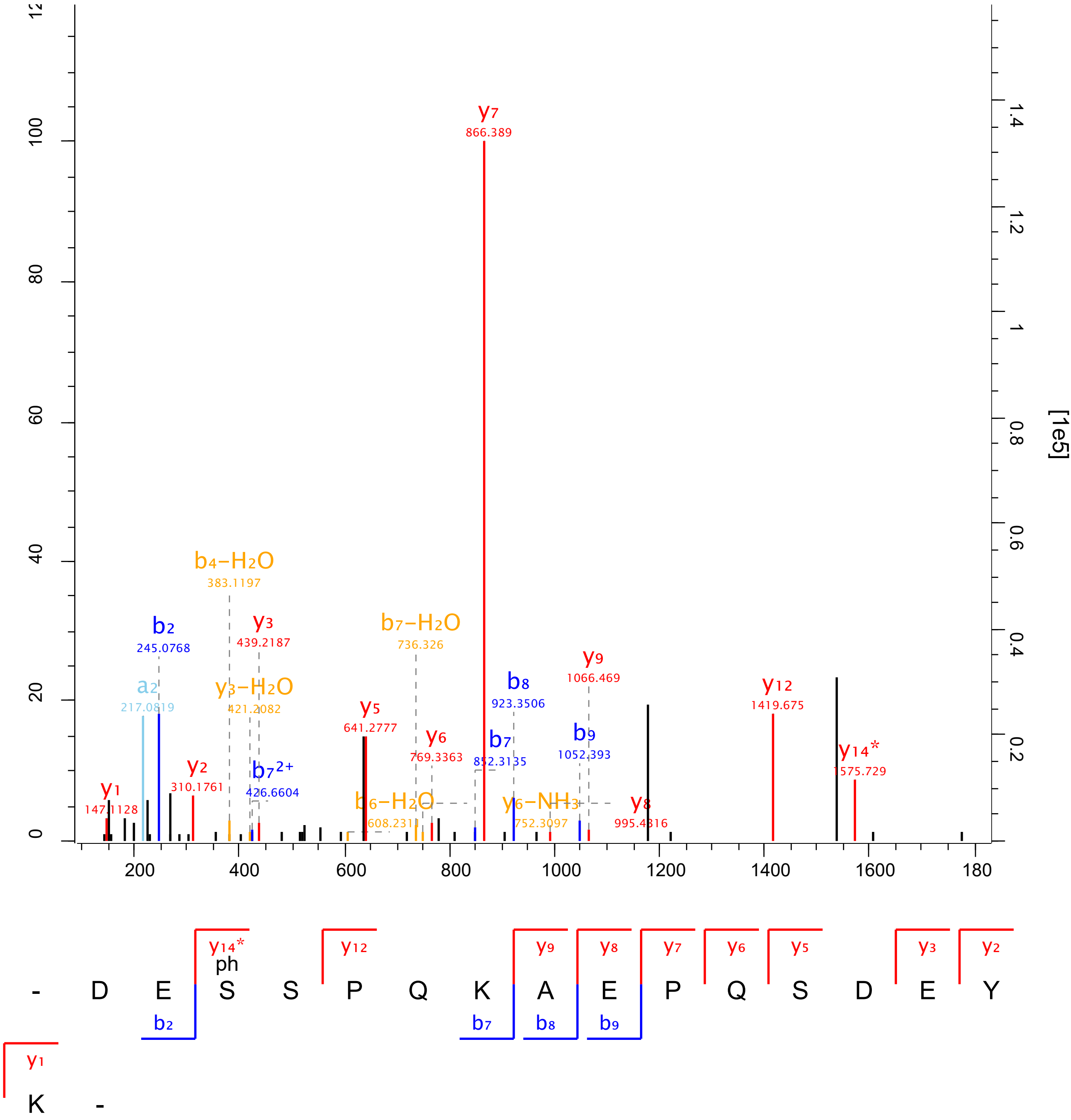

Raw file	Scan	Method	Score	m/z
sys_02_2	7148	FTMS; HCD	106.23	580.9

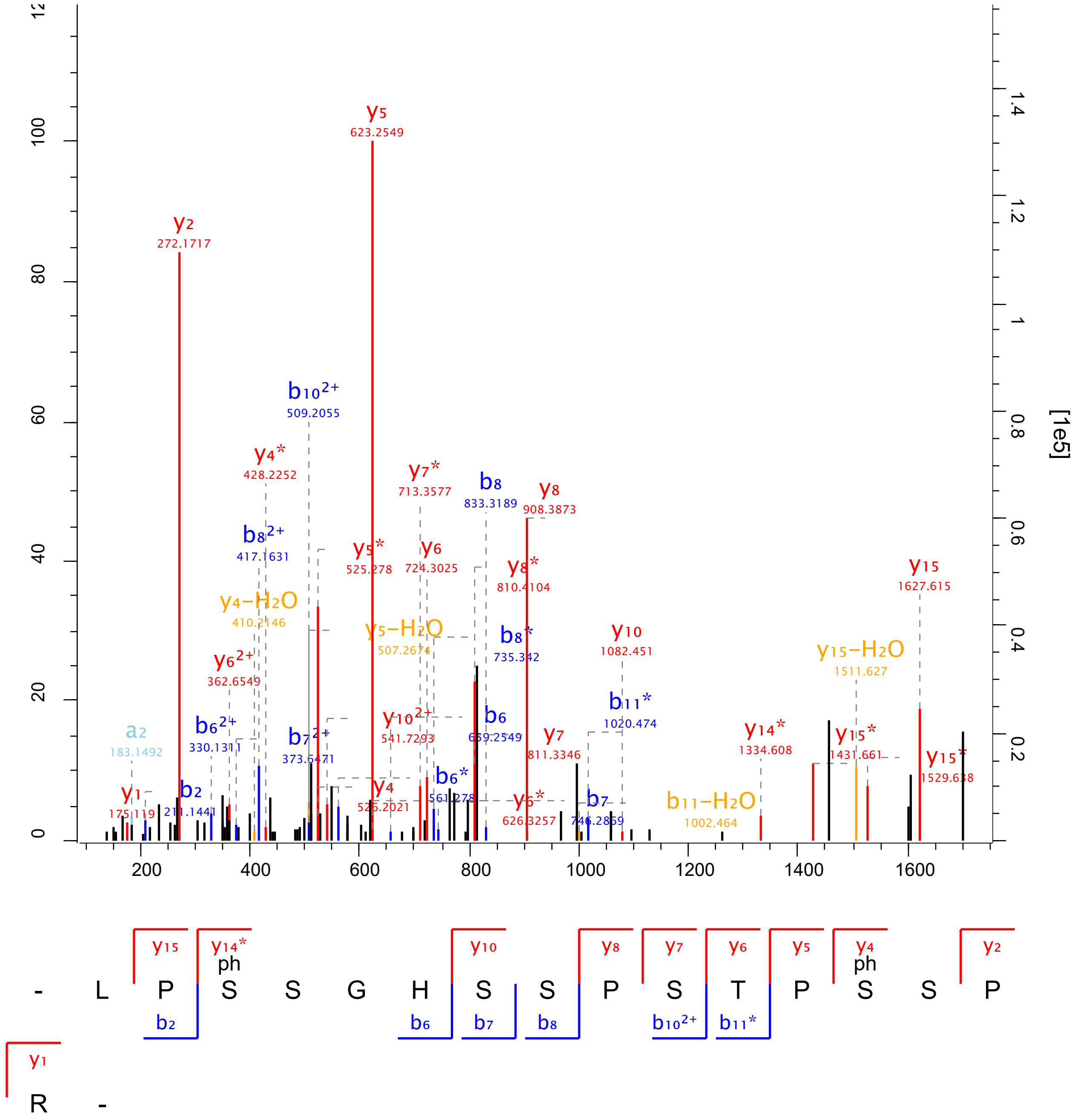

Raw file	Scan	Method	Score	m/z
sys_02_2	7166	FTMS; HCD	255.93	827.86

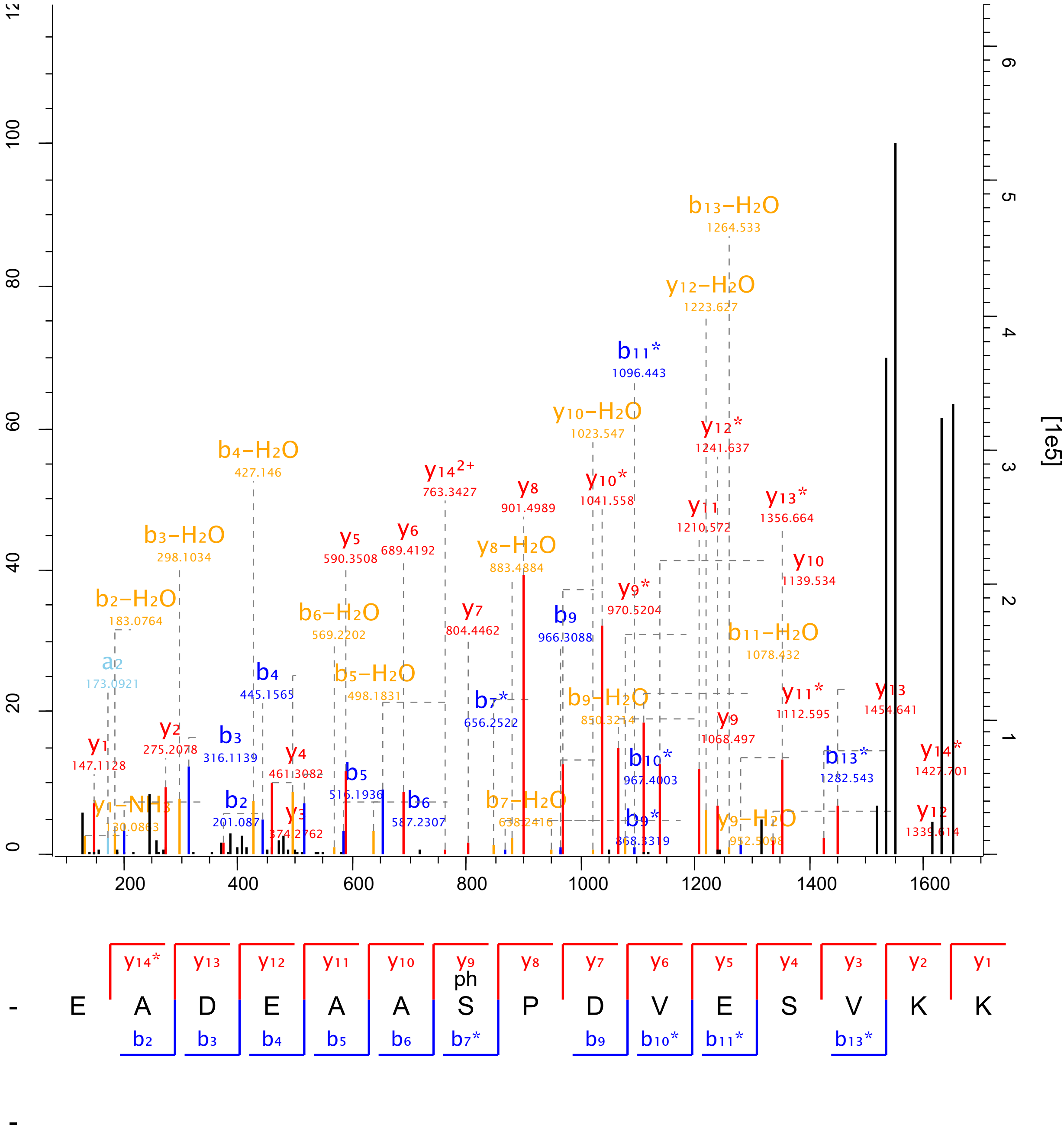

Raw file	Scan	Method	Score	m/z
sys_02_2	7182	FTMS; HCD	82.63	616.62

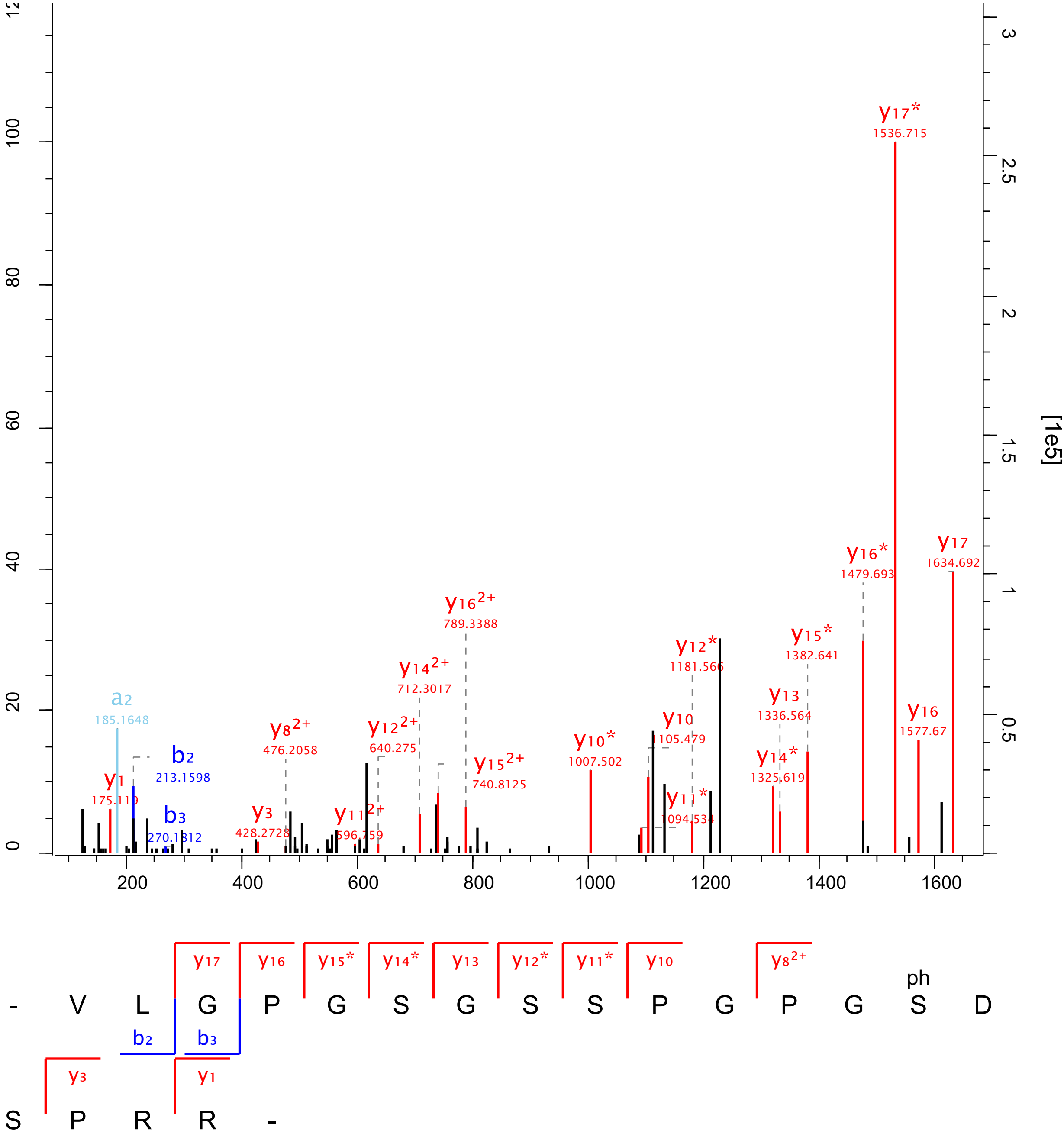

Raw file	Scan	Method	Score	m/z
sys_02_2	7313	FTMS; HCD	50.46	429.85

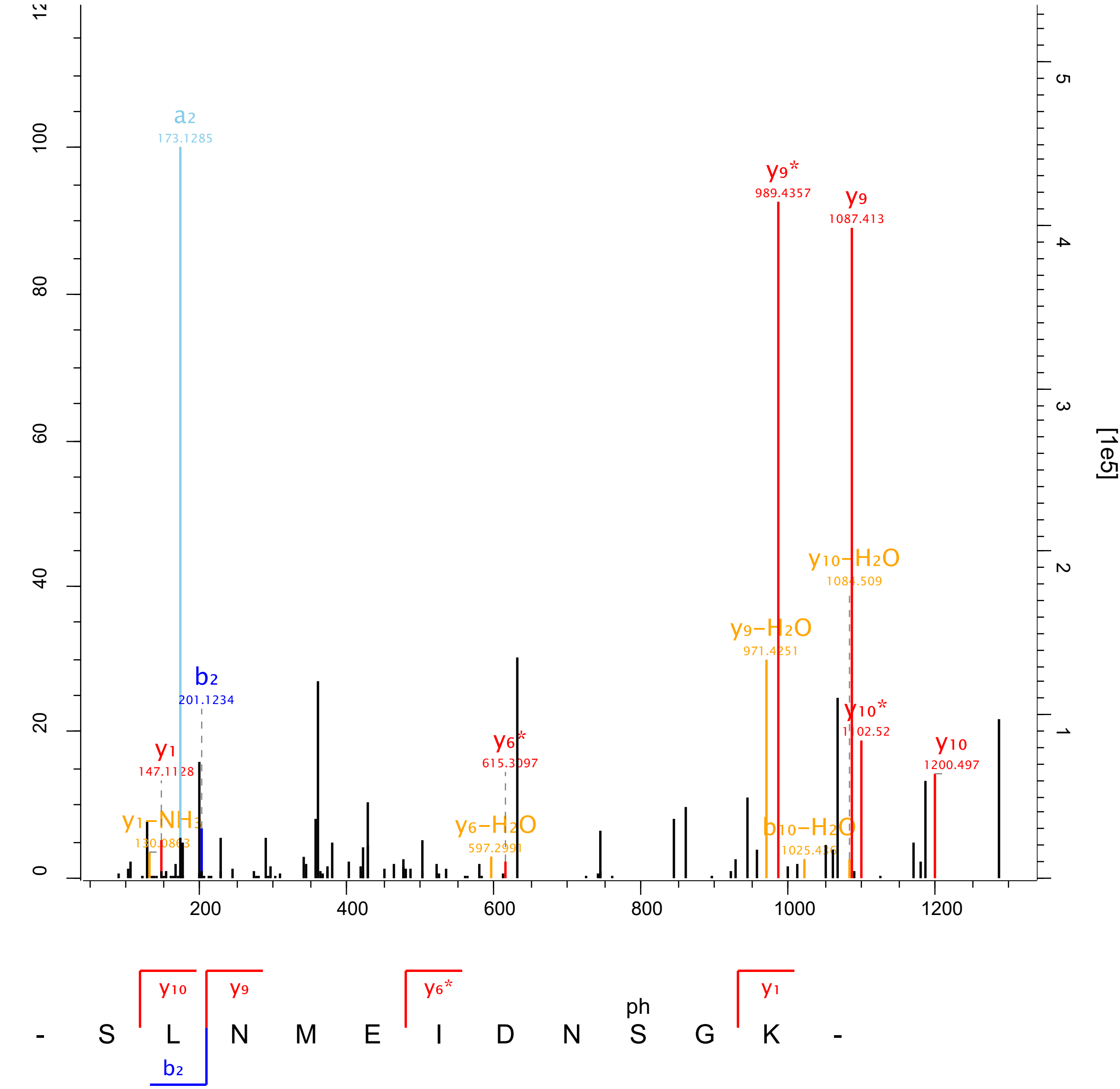

Raw file	Scan	Method	Score	m/z
sys_02_2	7392	FTMS; HCD	182.86	565.27

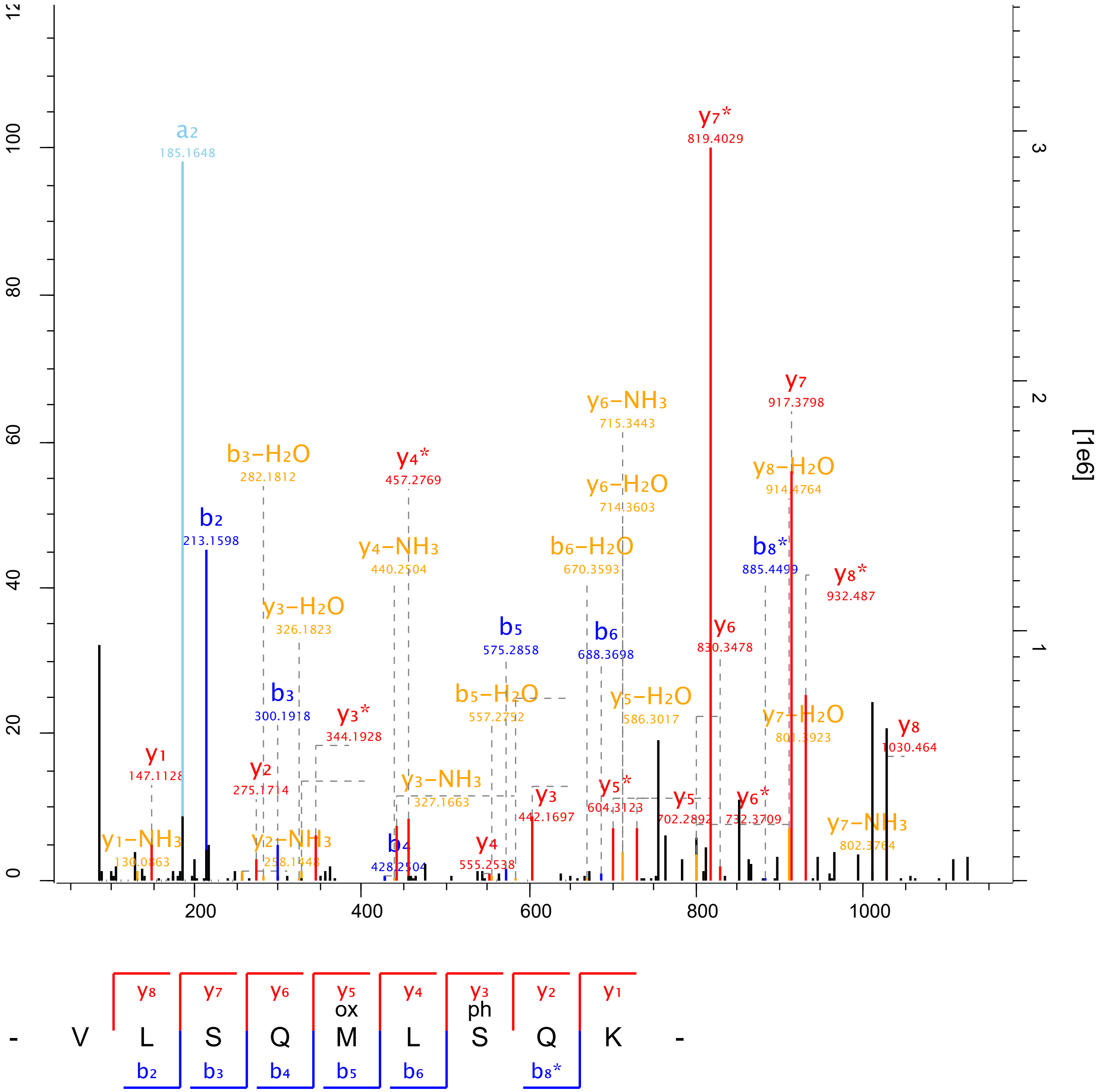

Raw file	Scan	Method	Score	m/z
sys_02_2	7461	FTMS; HCD	189.51	572.76

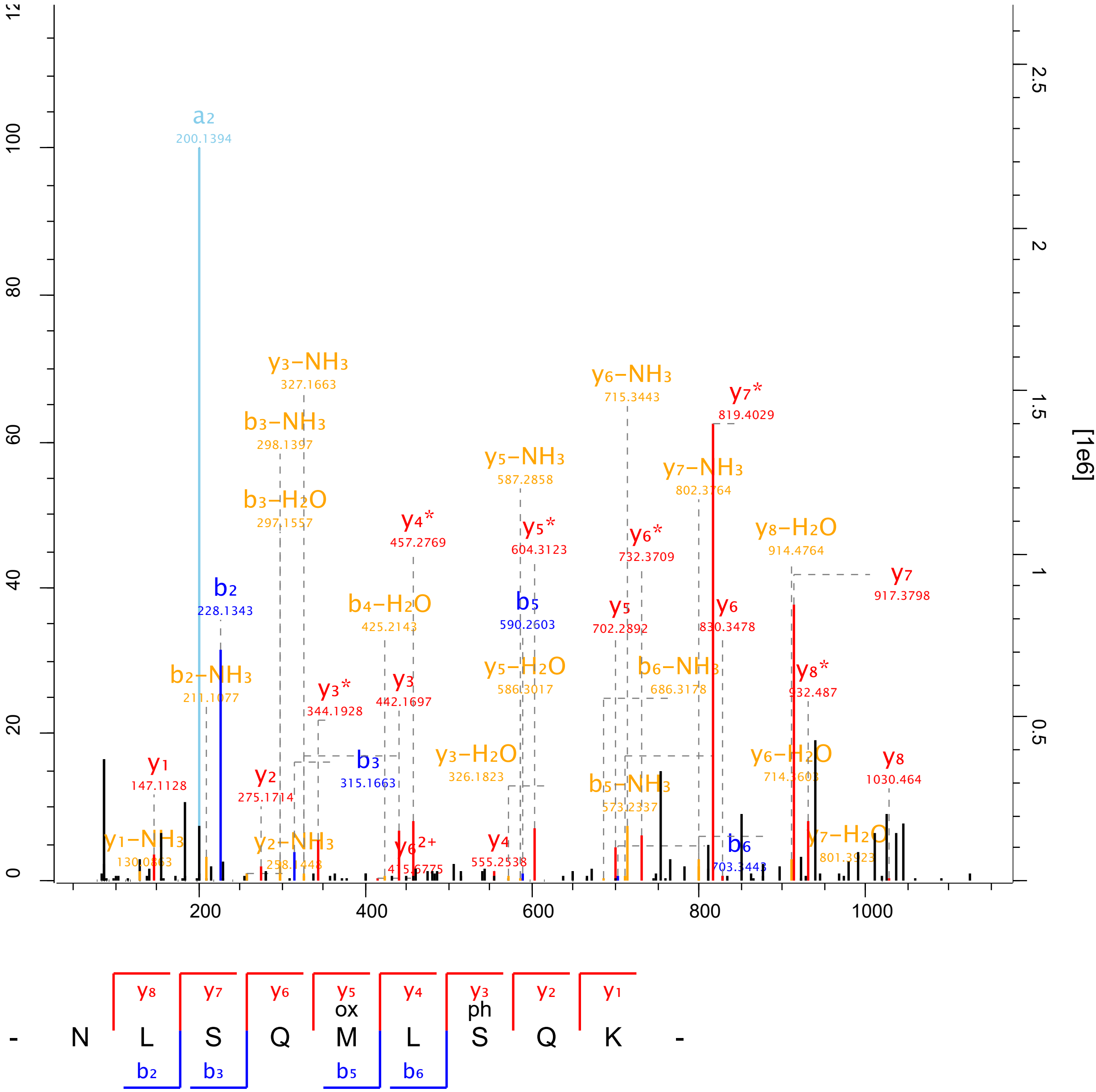

Raw file	Scan	Method	Score	m/z
sys_02_2	7470	FTMS; HCD	83.54	768.79

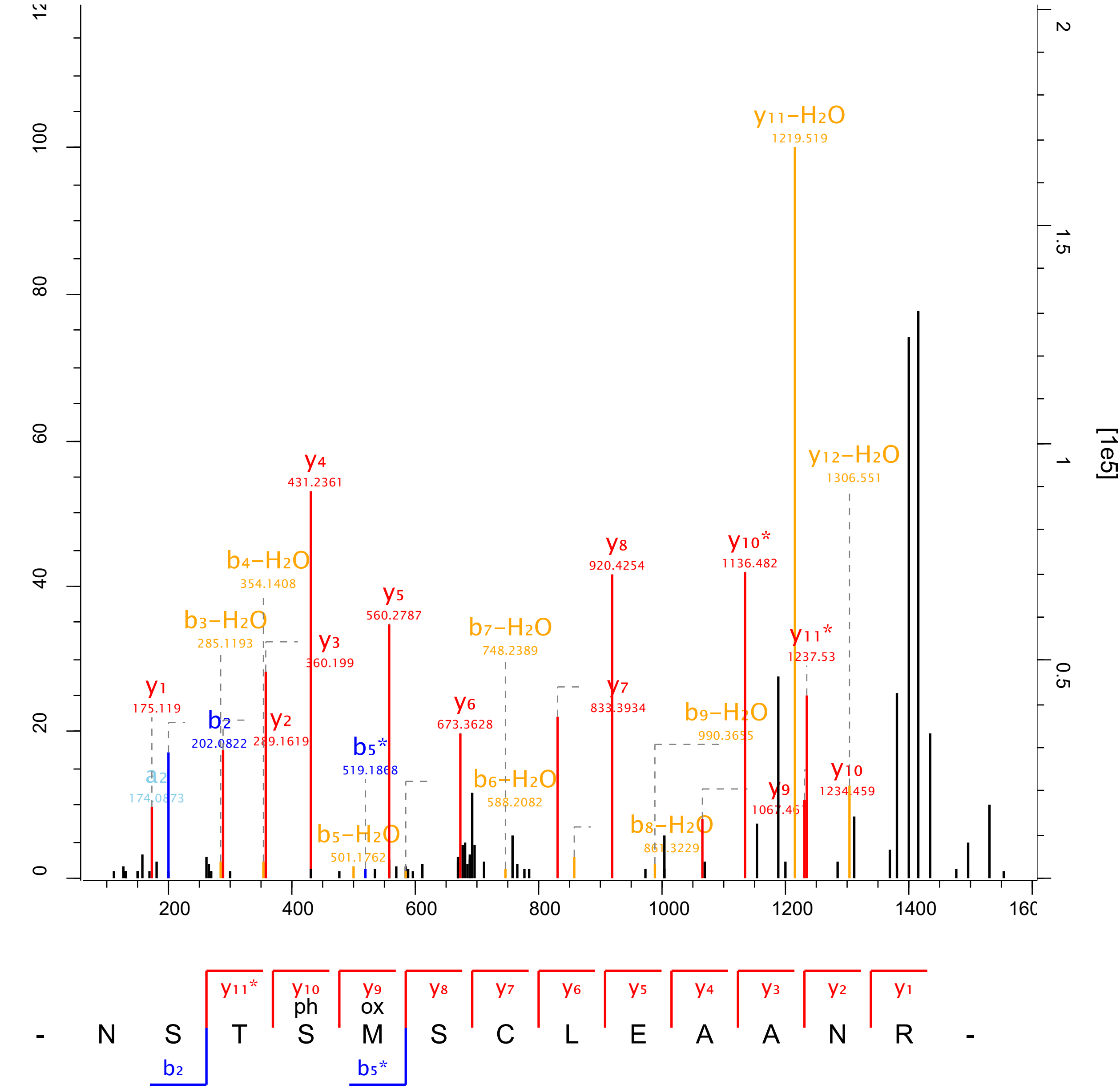

Raw file	Scan	Method	Score	m/z
sys_02_2	7514	FTMS; HCD	195.86	776.85

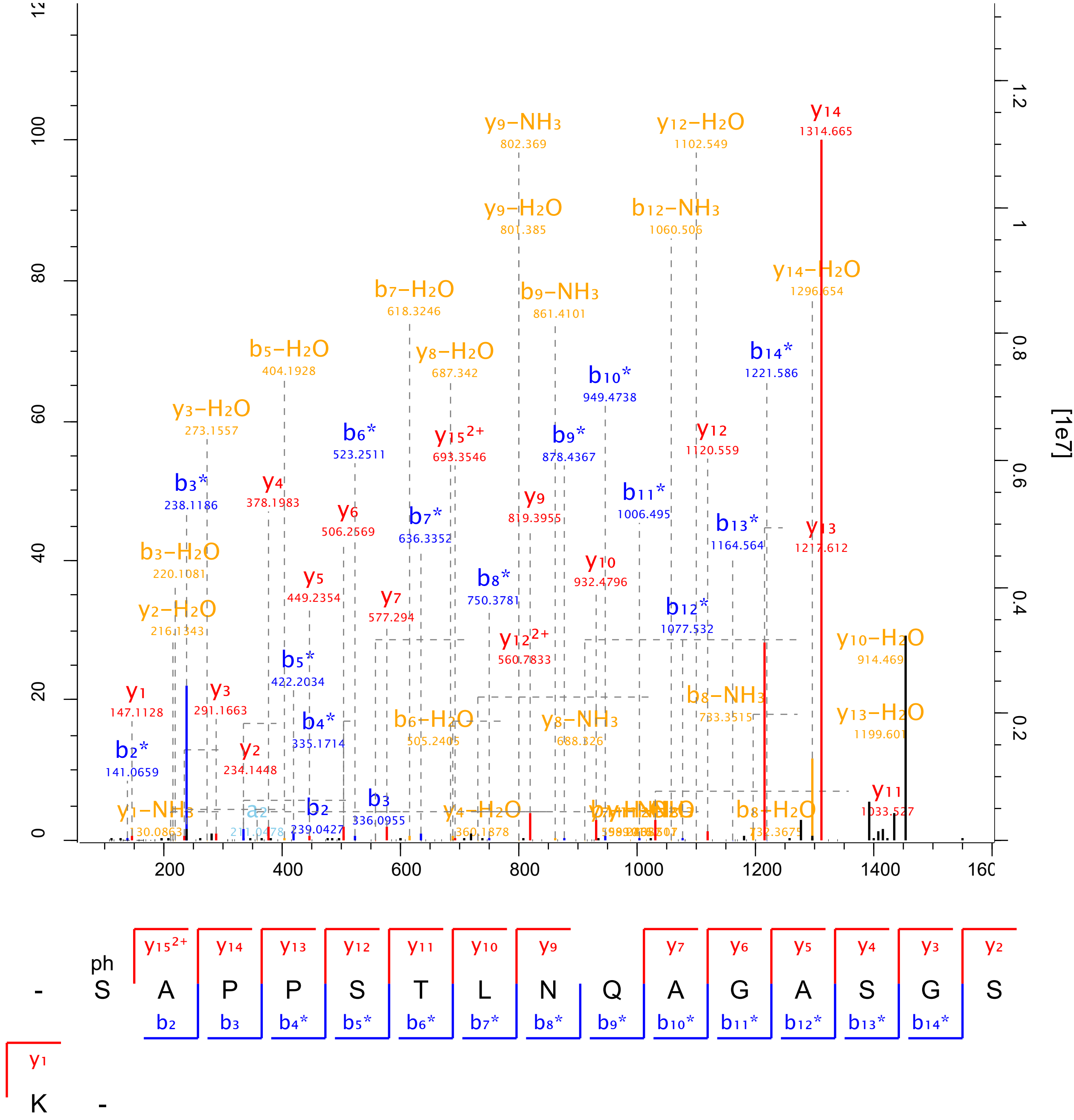

Raw file	Scan	Method	Score	m/z
sys_02_2	7520	FTMS; HCD	86.29	535.72

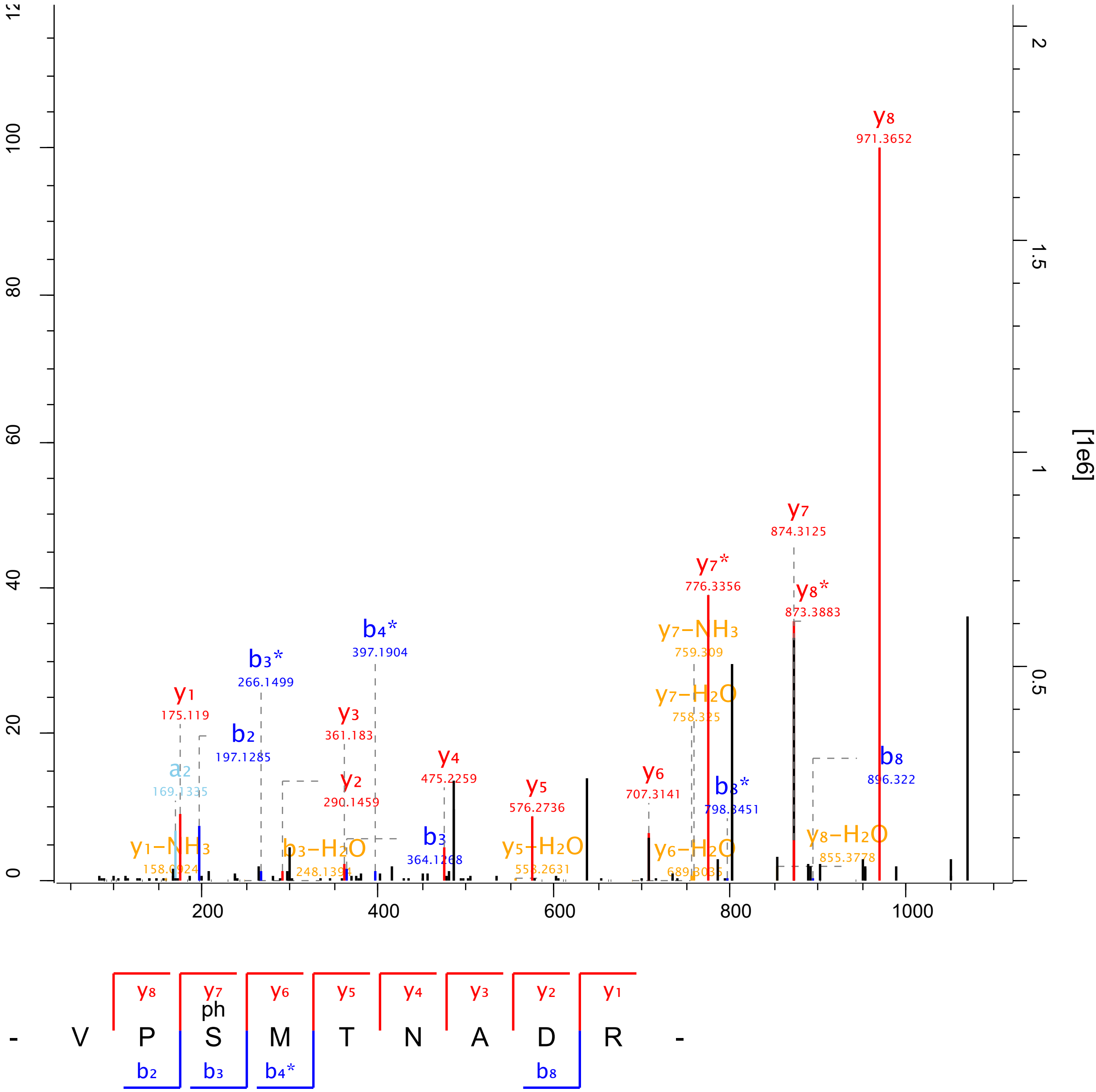

Raw file	Scan	Method	Score	m/z
sys_02_2	7765	FTMS; HCD	102.21	648.94

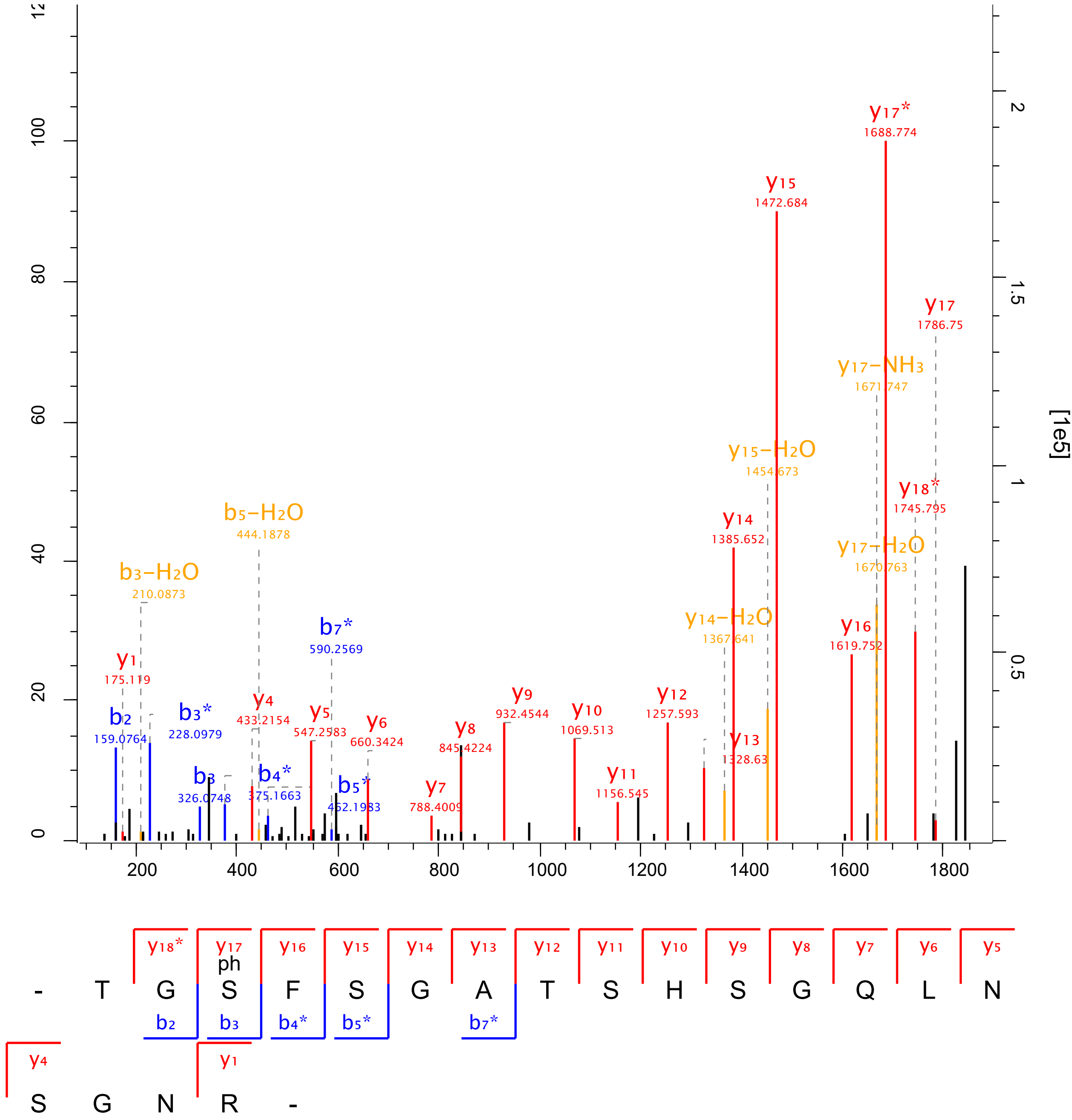

Raw file	Scan	Method	Score	m/z
sys_02_2	7806	FTMS; HCD	83.4	656.26

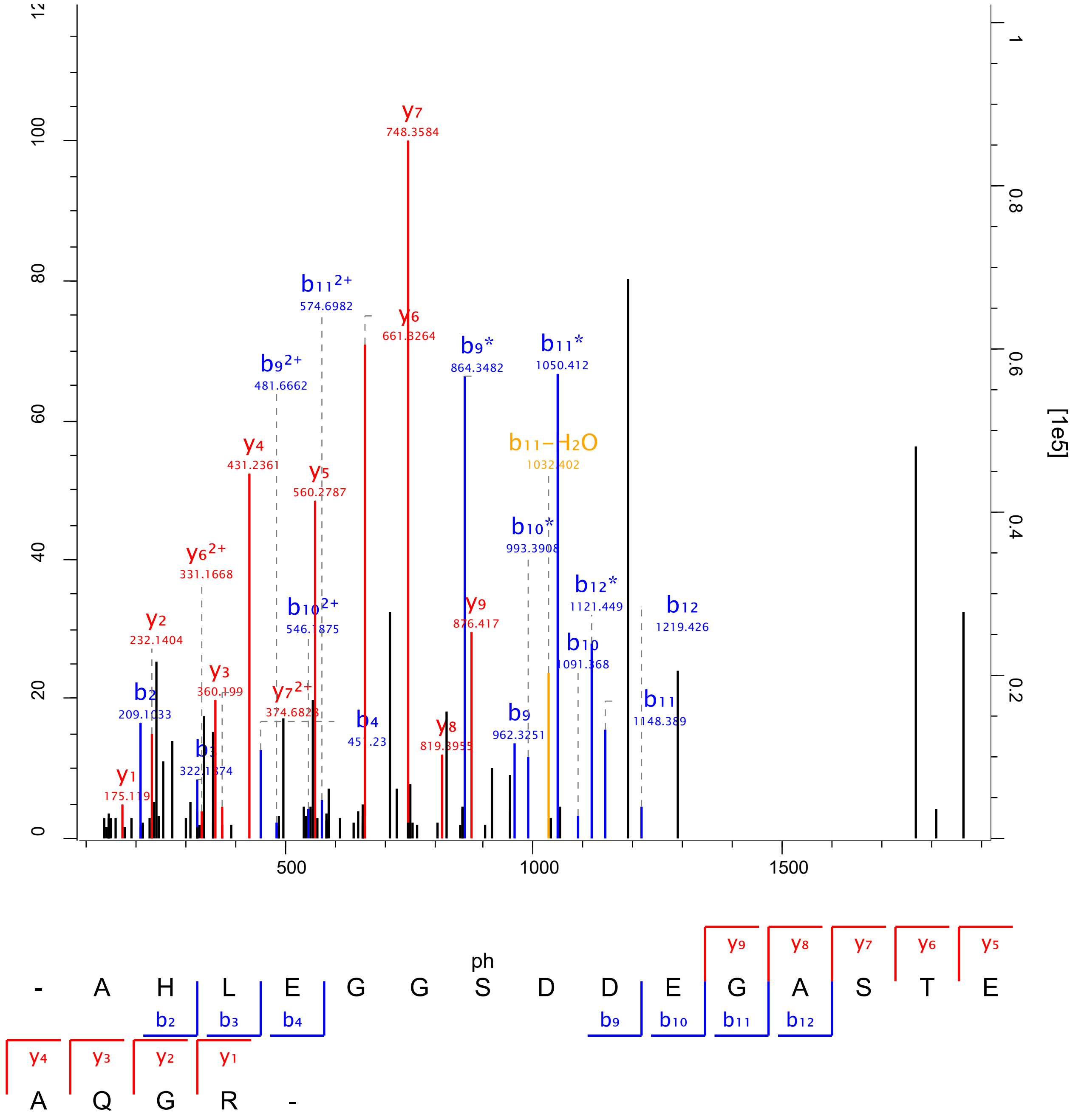

Raw file	Scan	Method	Score	m/z
sys_02_2	7947	FTMS; HCD	41.54	478.56

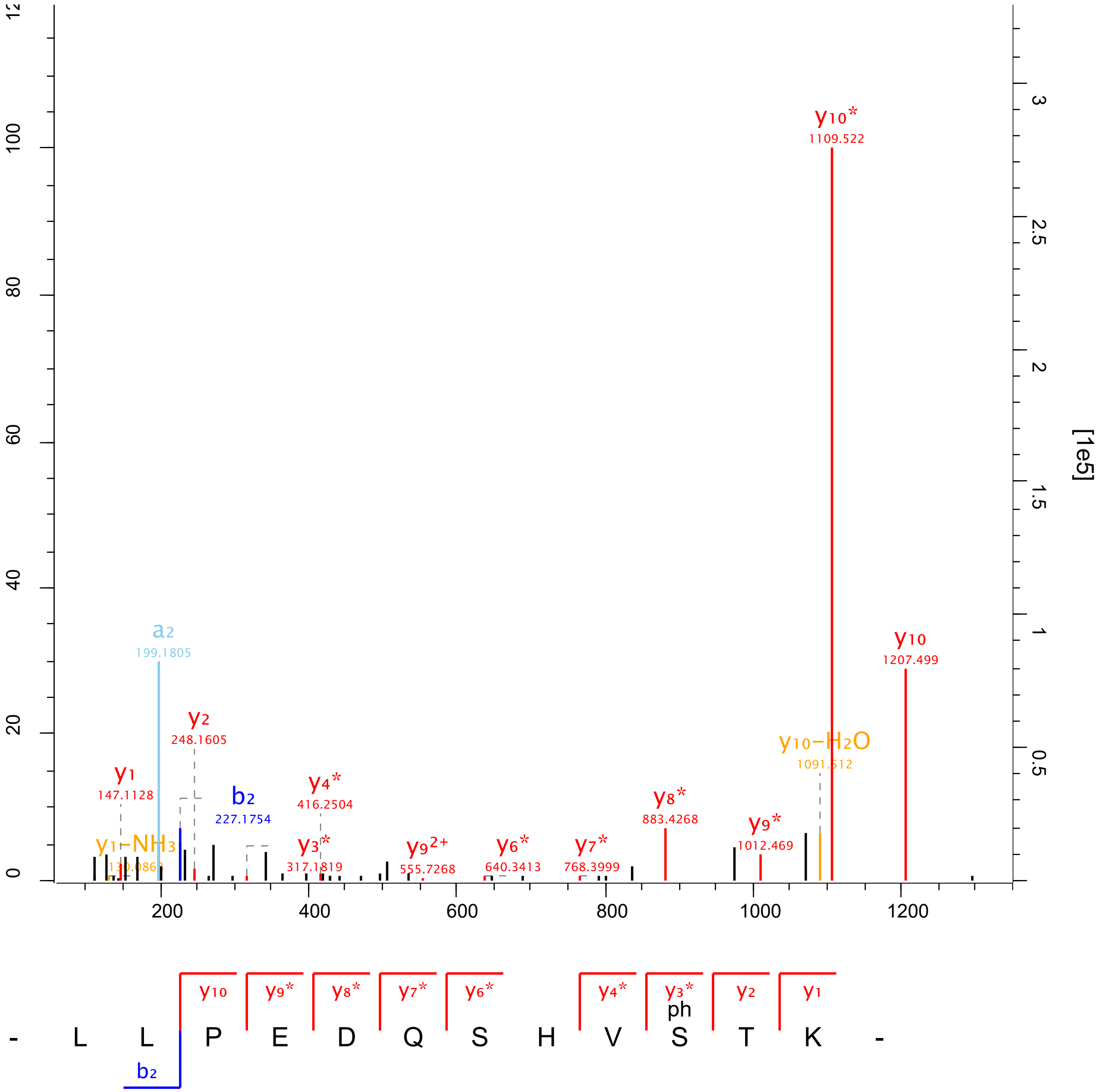

Raw file	Scan	Method	Score	m/z
sys_02_2	8030	FTMS; HCD	46.32	450.87

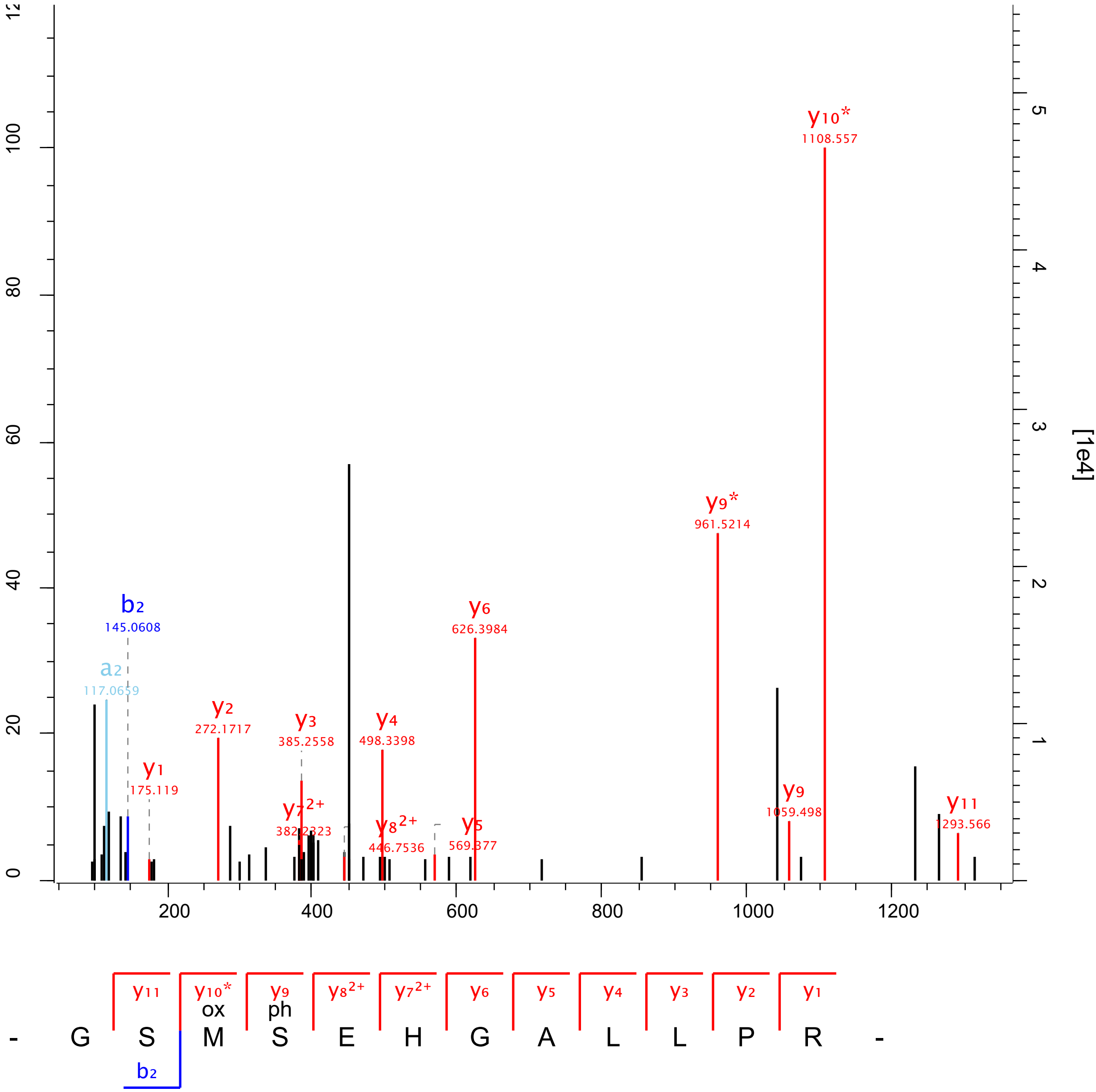

Raw file	Scan	Method	Score	m/z
sys_02_2	8055	FTMS; HCD	66.27	552.75

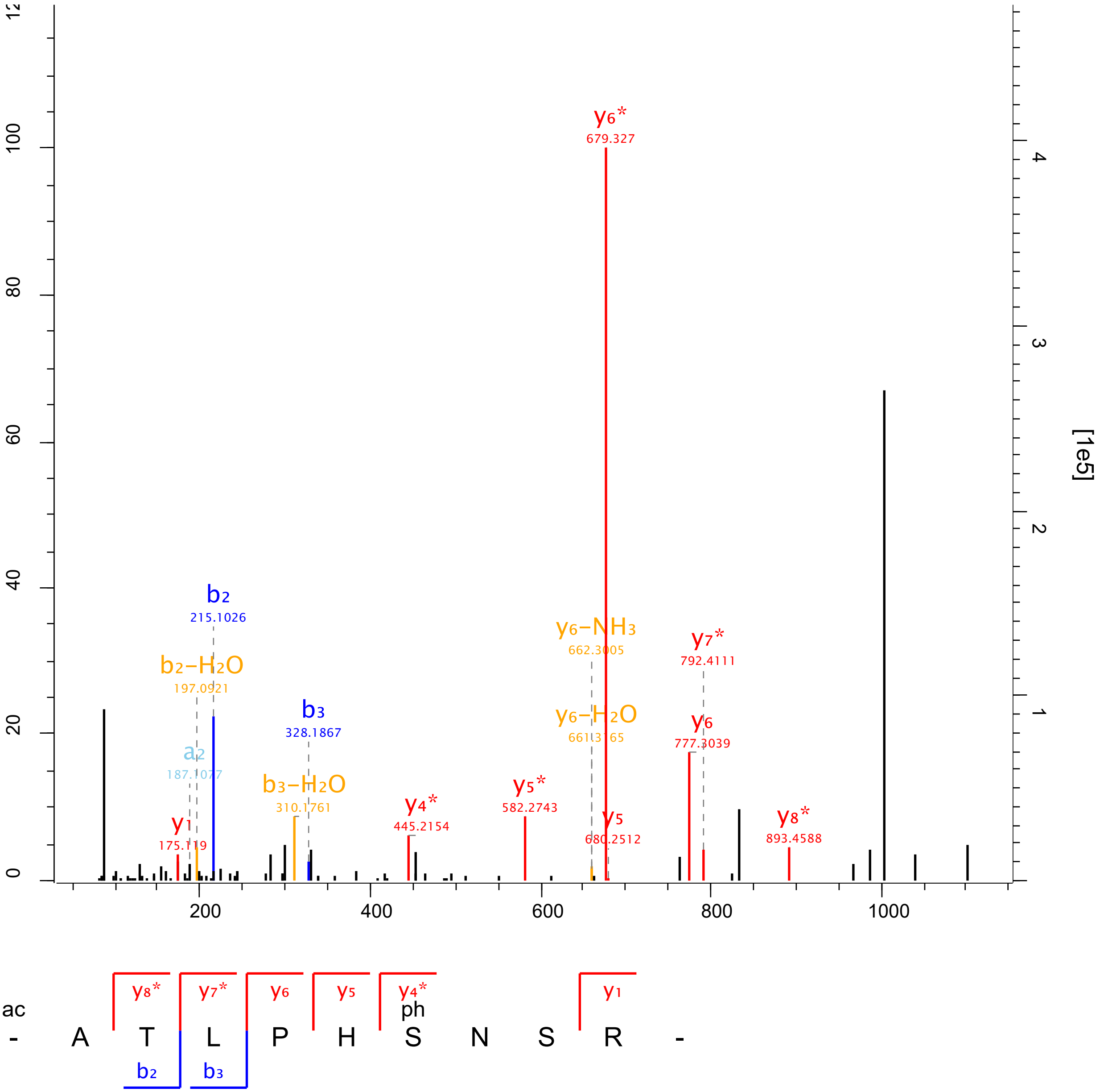

Raw file	Scan	Method	Score	m/z
sys_02_2	8097	FTMS; HCD	104.26	481.23

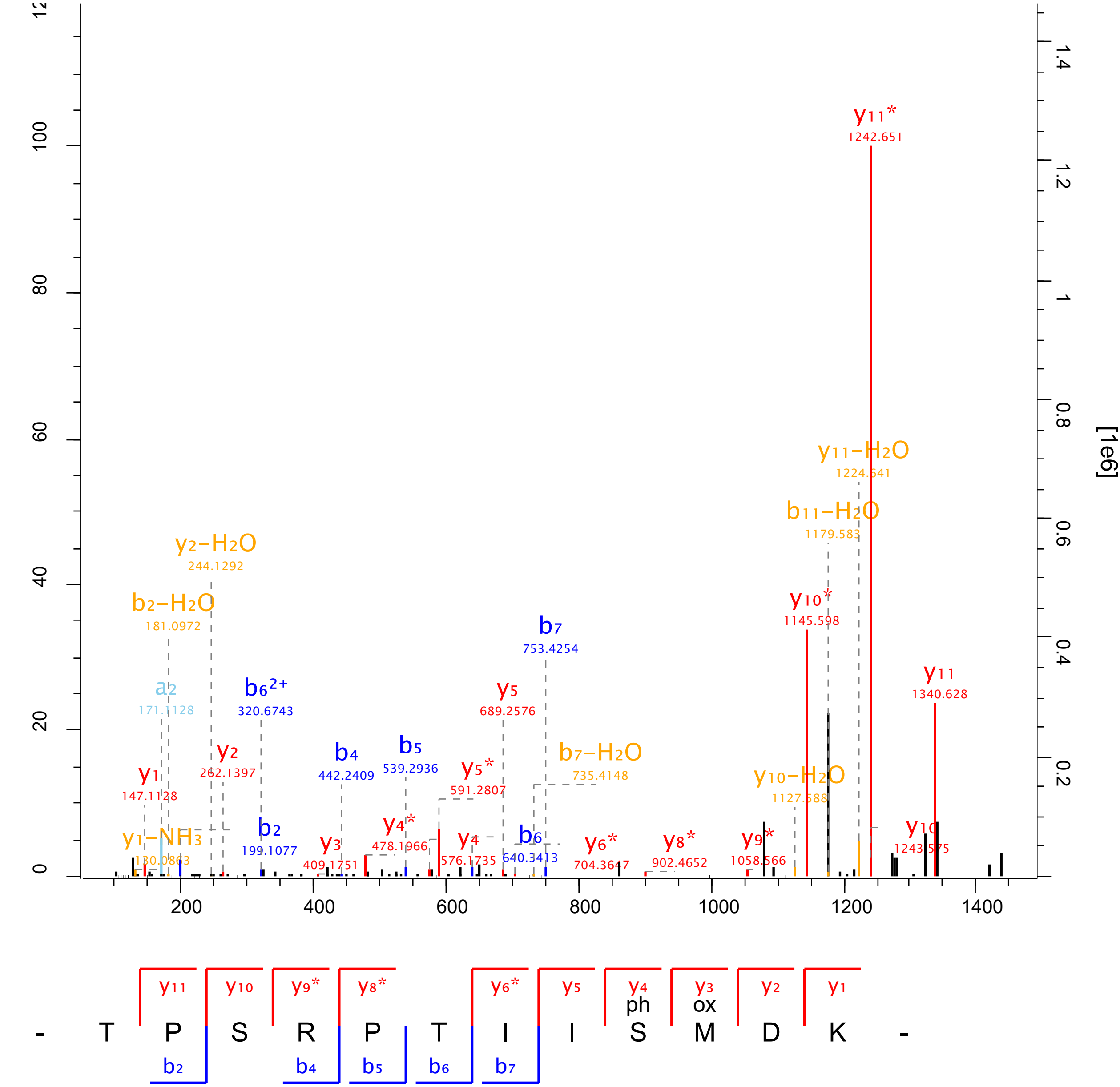

Raw file	Scan	Method	Score	m/z
sys_02_2	8101	FTMS; HCD	42.64	683.6

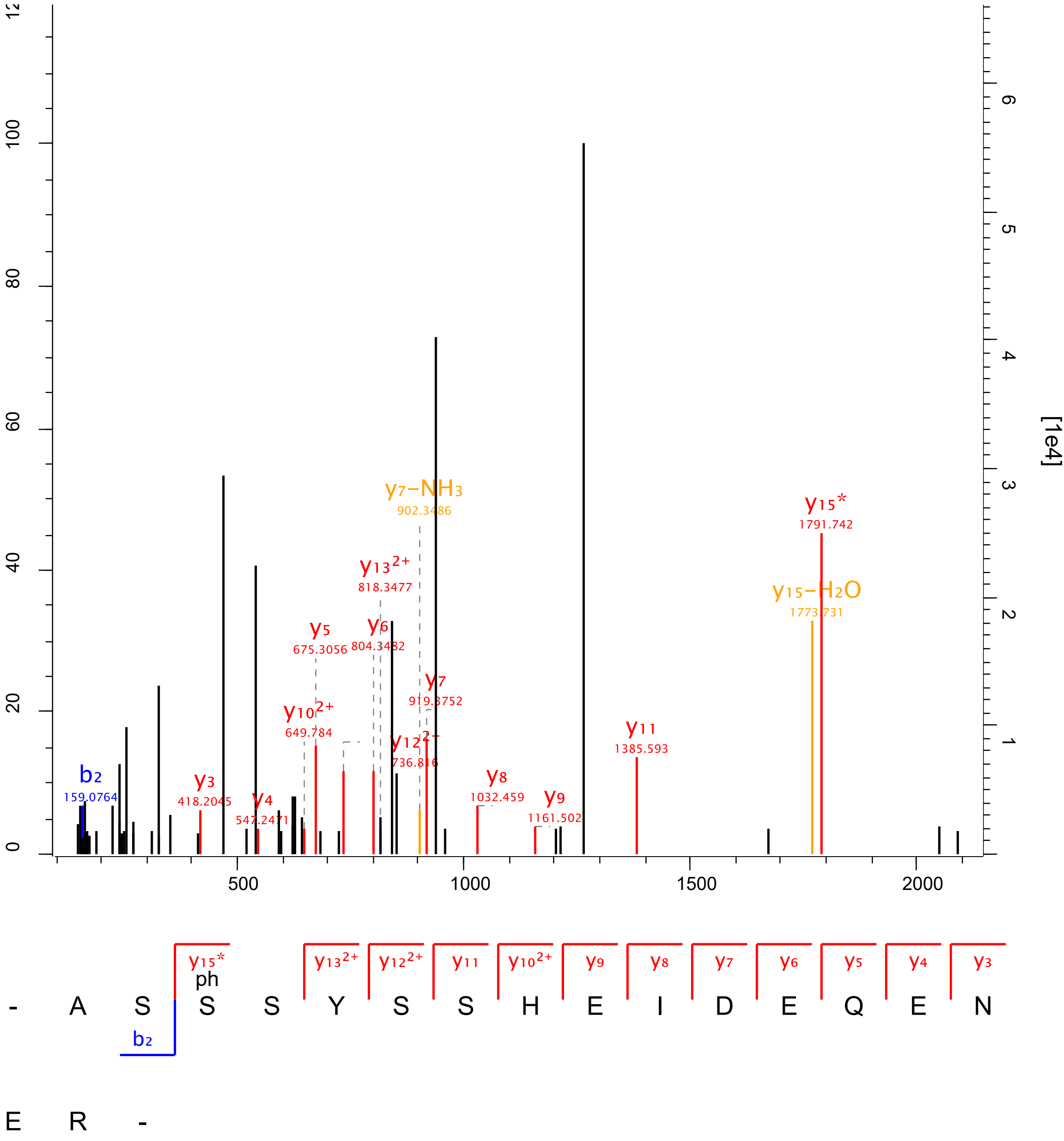

Raw file	Scan	Method	Score	m/z
sys_02_2	8104	FTMS; HCD	181.97	703.82

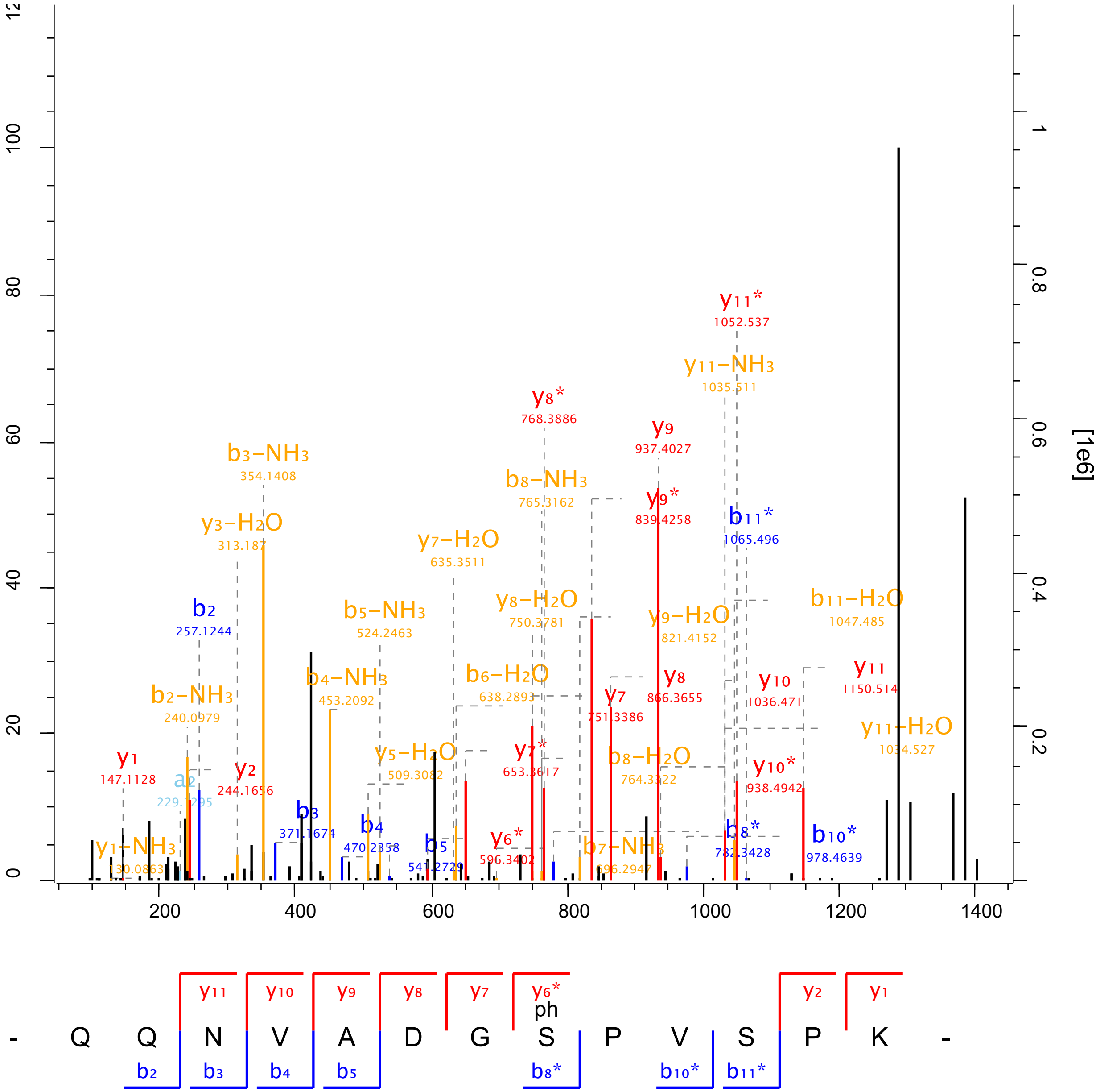

Raw file	Scan	Method	Score	m/z
sys_02_2	8199	FTMS; HCD	115.56	796.86

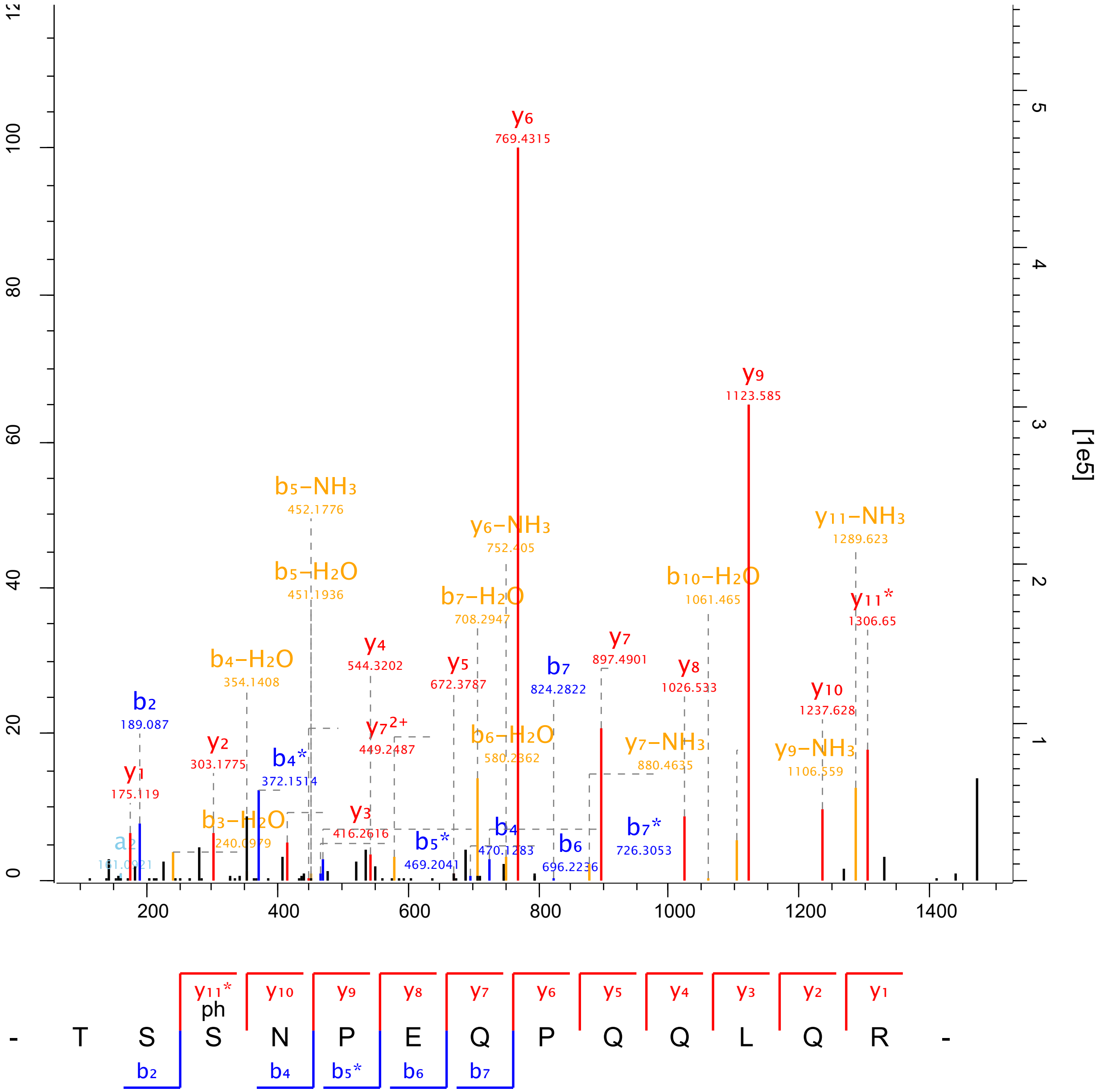

Raw file	Scan	Method	Score	m/z
sys_02_2	8382	FTMS; HCD	46.16	505.57

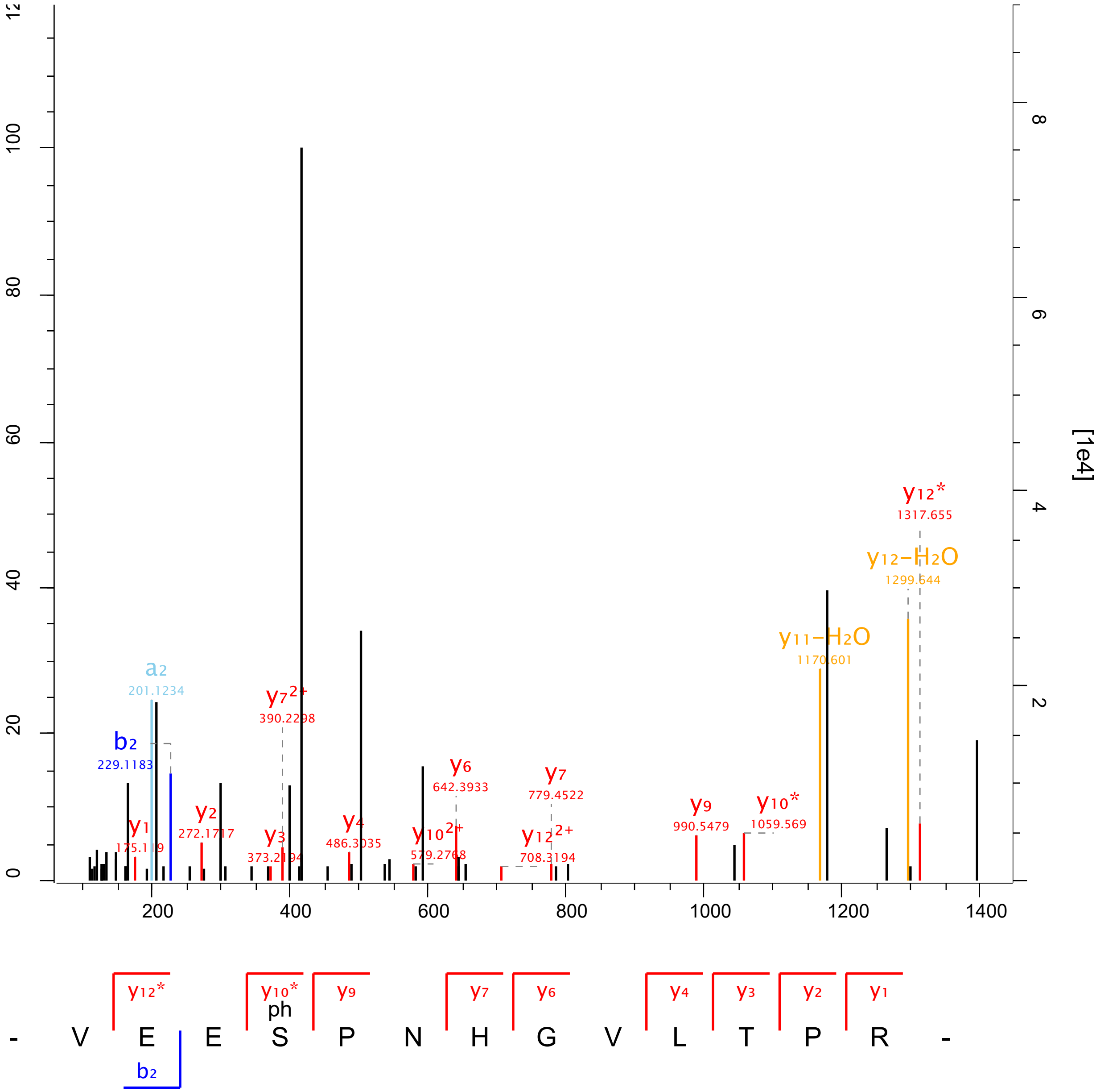

Raw file	Scan	Method	Score	m/z
sys_02_2	8410	FTMS; HCD	233.49	743.82

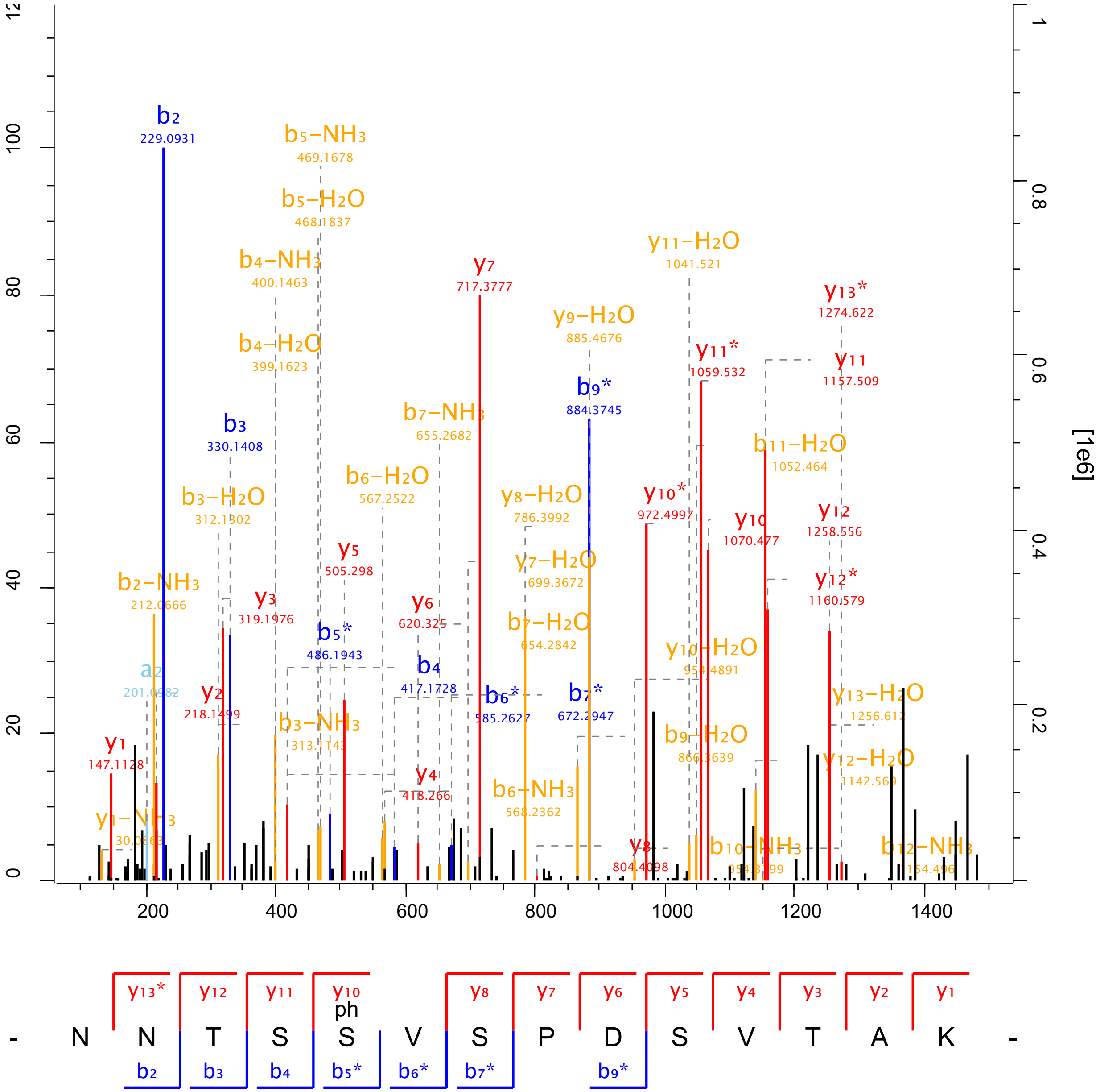

Raw file	Scan	Method	Score	m/z
sys_02_2	8459	FTMS; HCD	132.65	681.64

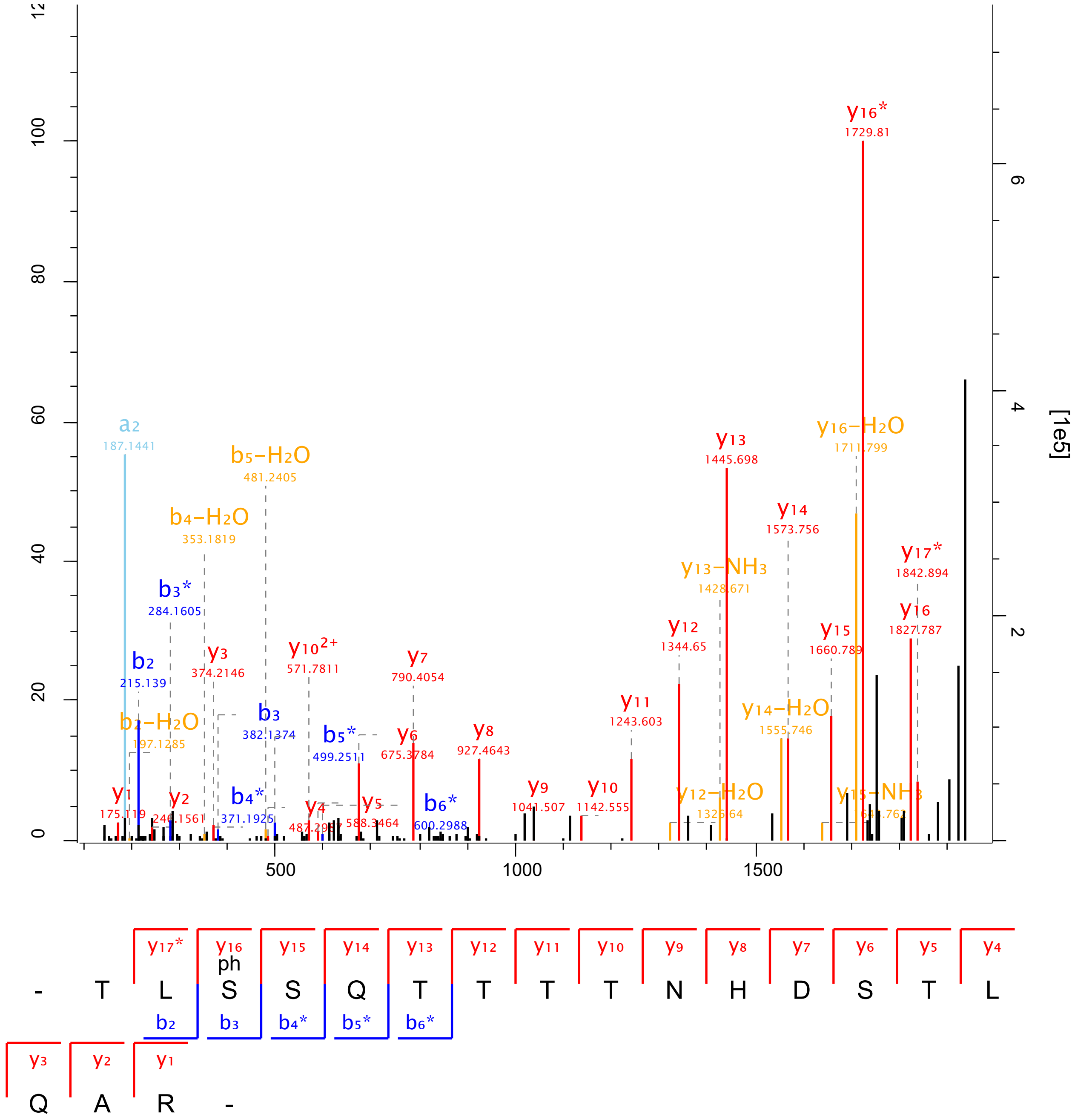

Raw file	Scan	Method	Score	m/z
sys_02_2	8520	FTMS; HCD	206.62	670.34

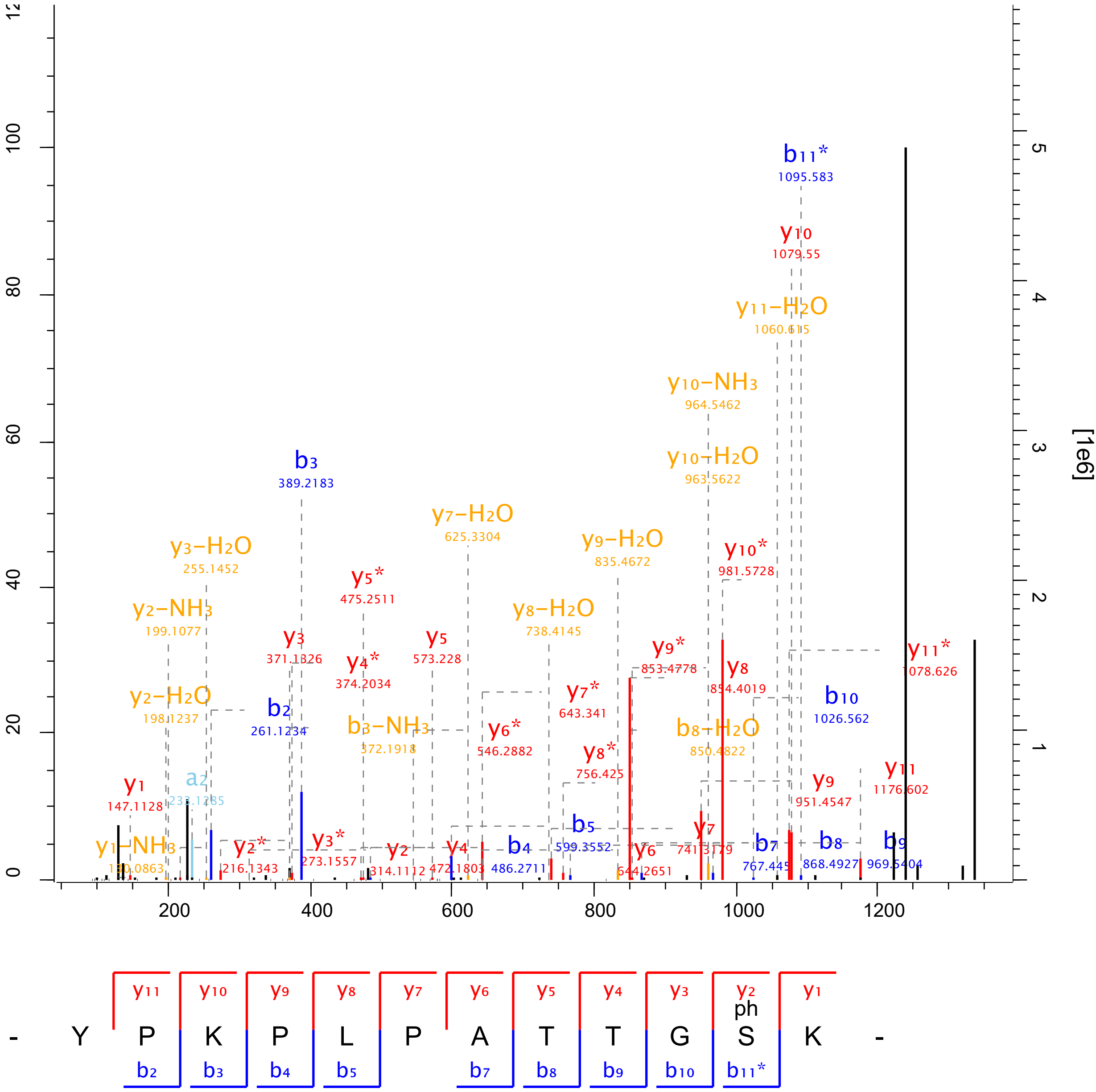

Raw file	Scan	Method	Score	m/z
sys_02_2	8578	FTMS; HCD	41.7	480.24

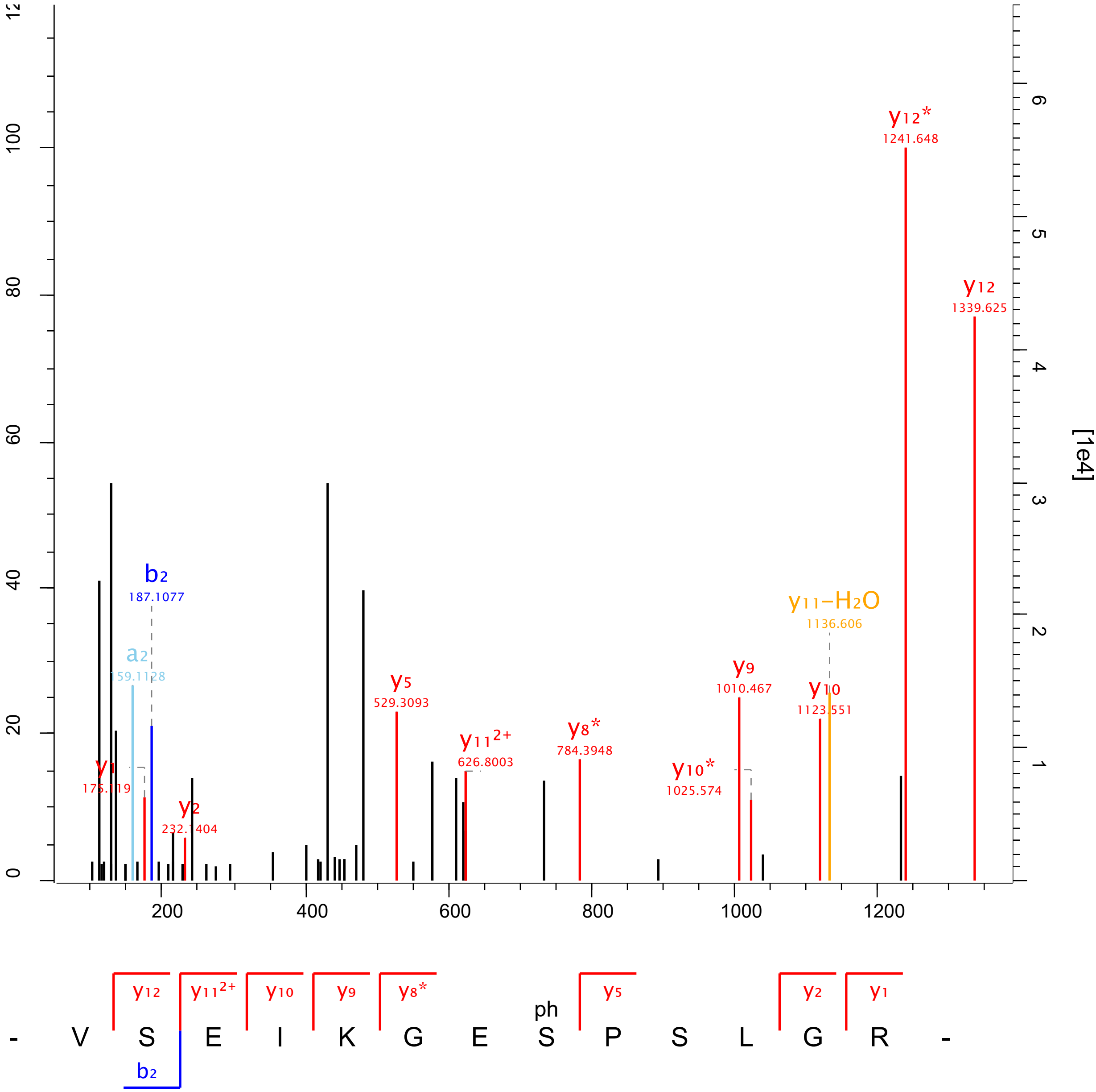

Raw file	Scan	Method	Score	m/z
sys_02_2	8660	FTMS; HCD	57.43	578.26

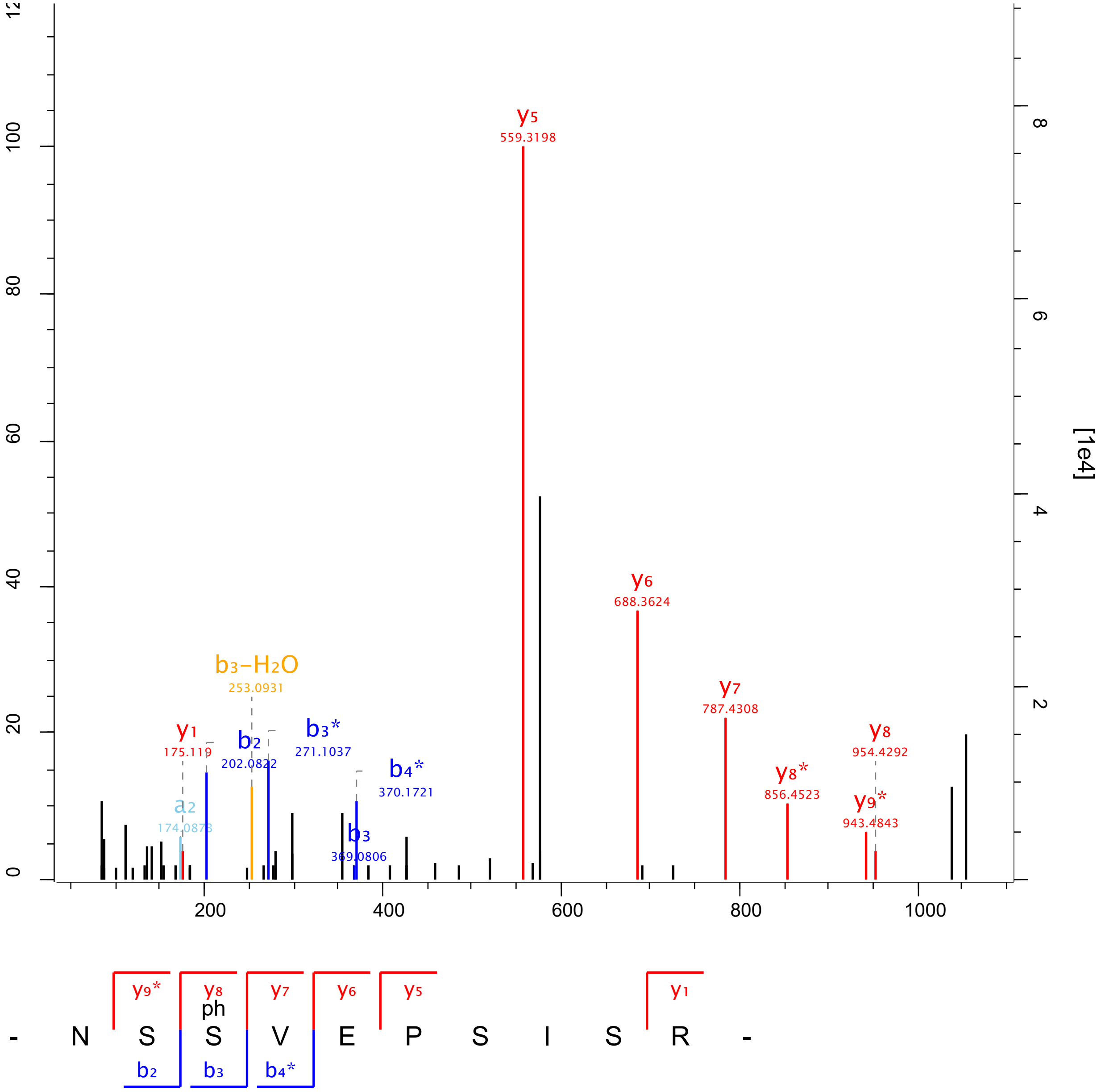

Raw file	Scan	Method	Score	m/z
sys_02_2	8851	FTMS; HCD	104.84	780.35

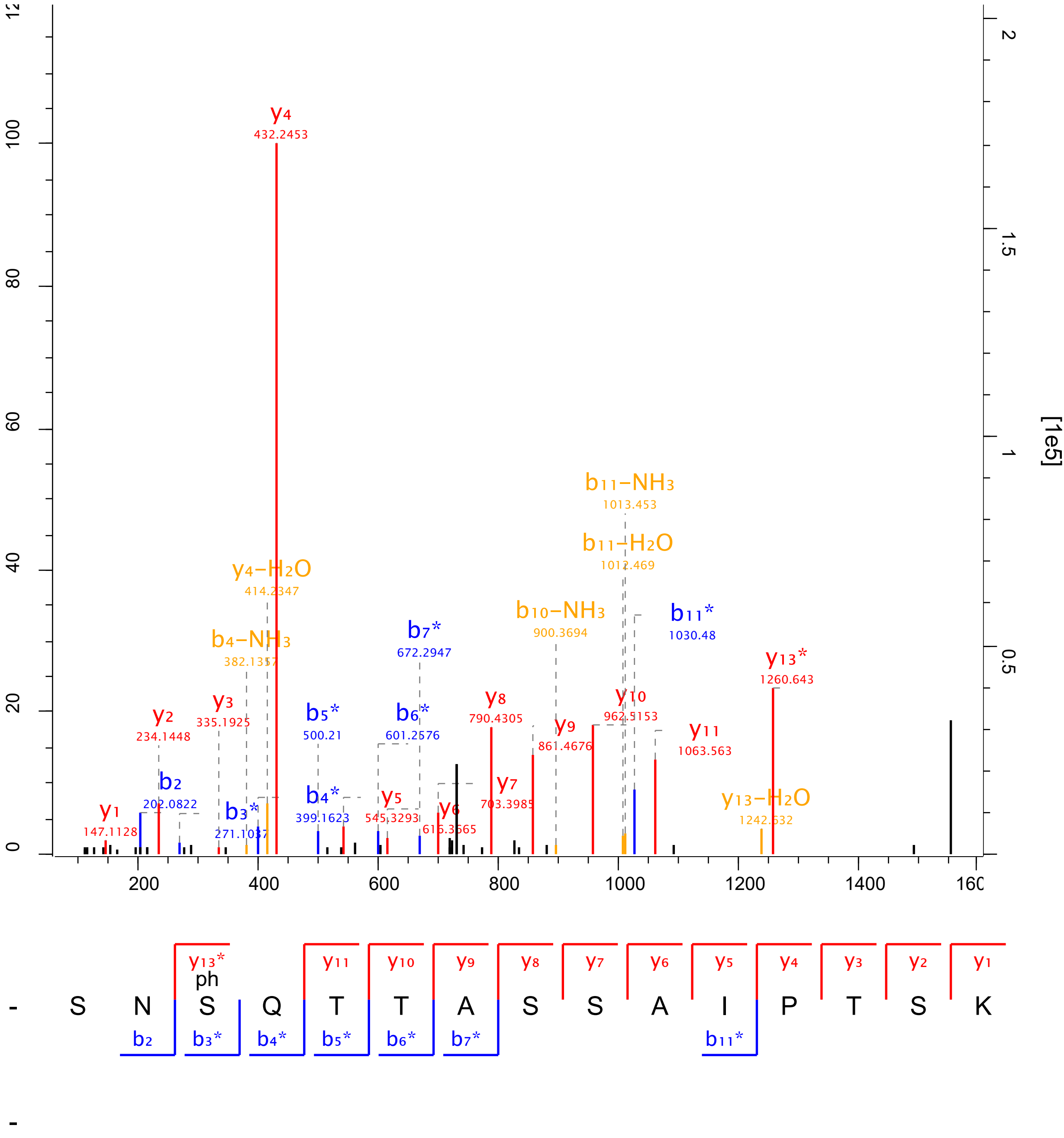

Raw file	Scan	Method	Score	m/z
sys_02_2	8890	FTMS; HCD	66.27	502.22

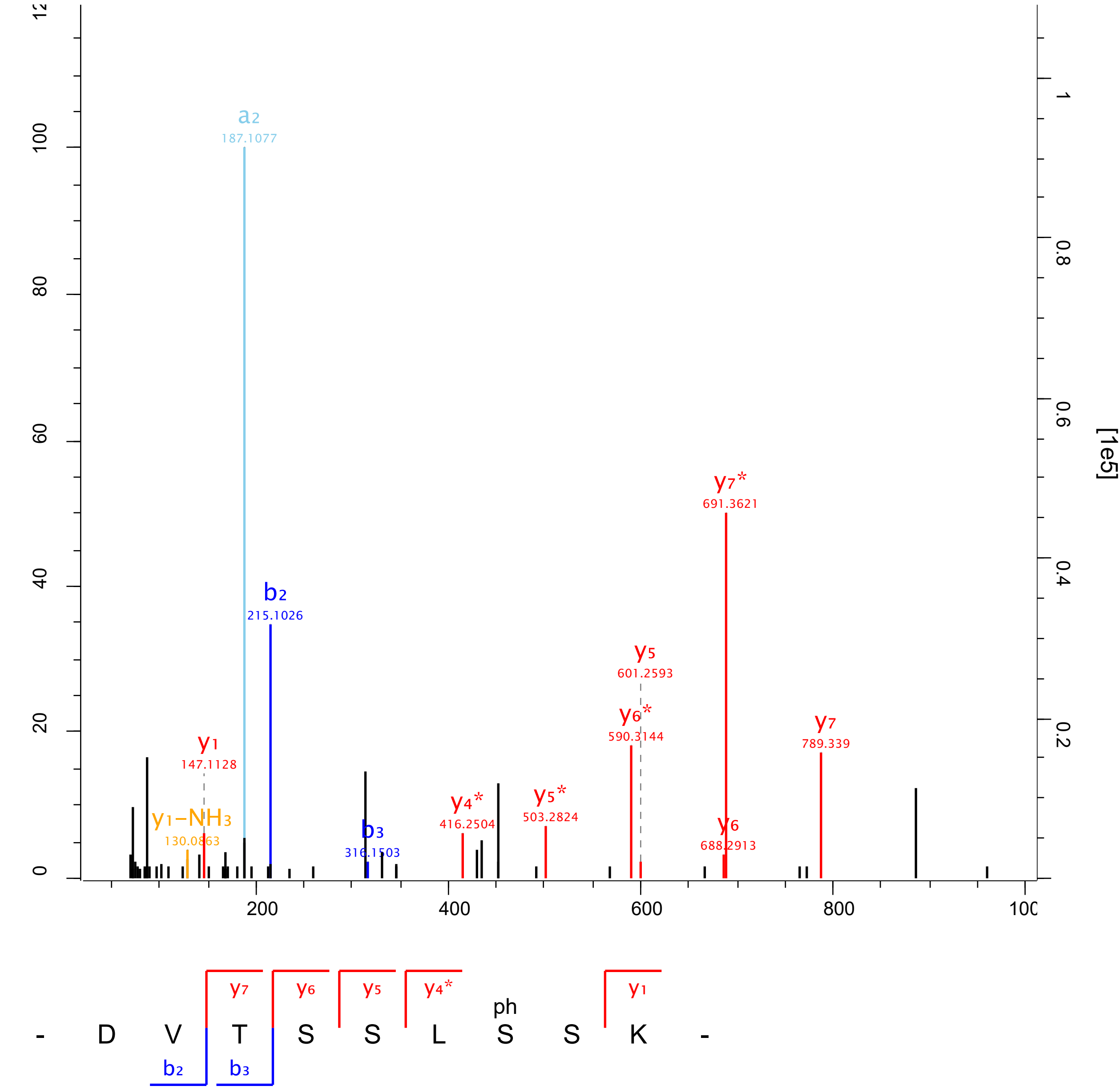

Raw file	Scan	Method	Score	m/z
sys_02_2	8902	FTMS; HCD	76.23	456.73

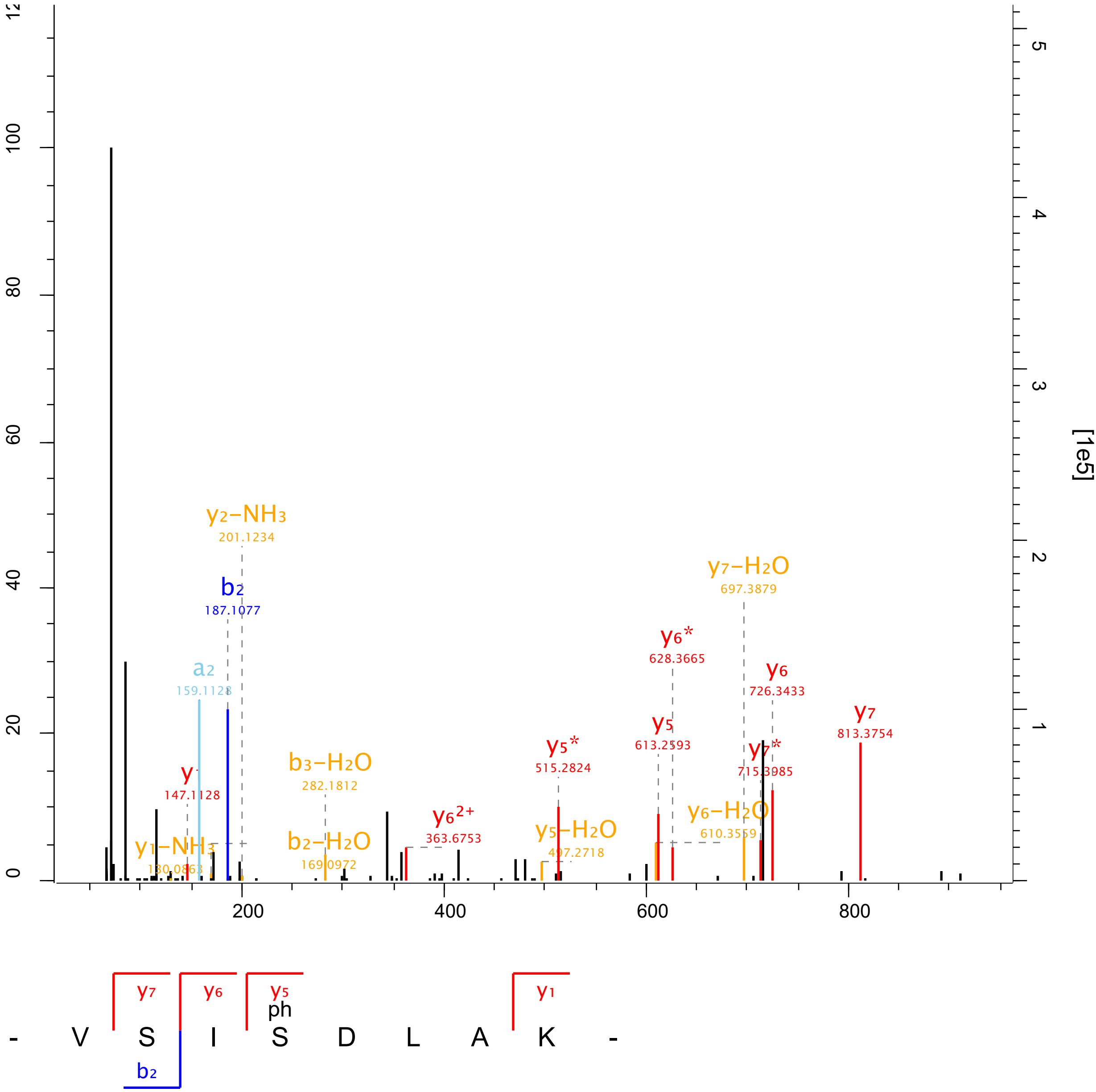

Raw file	Scan	Method	Score	m/z
sys_02_2	8969	FTMS; HCD	67.52	549.74

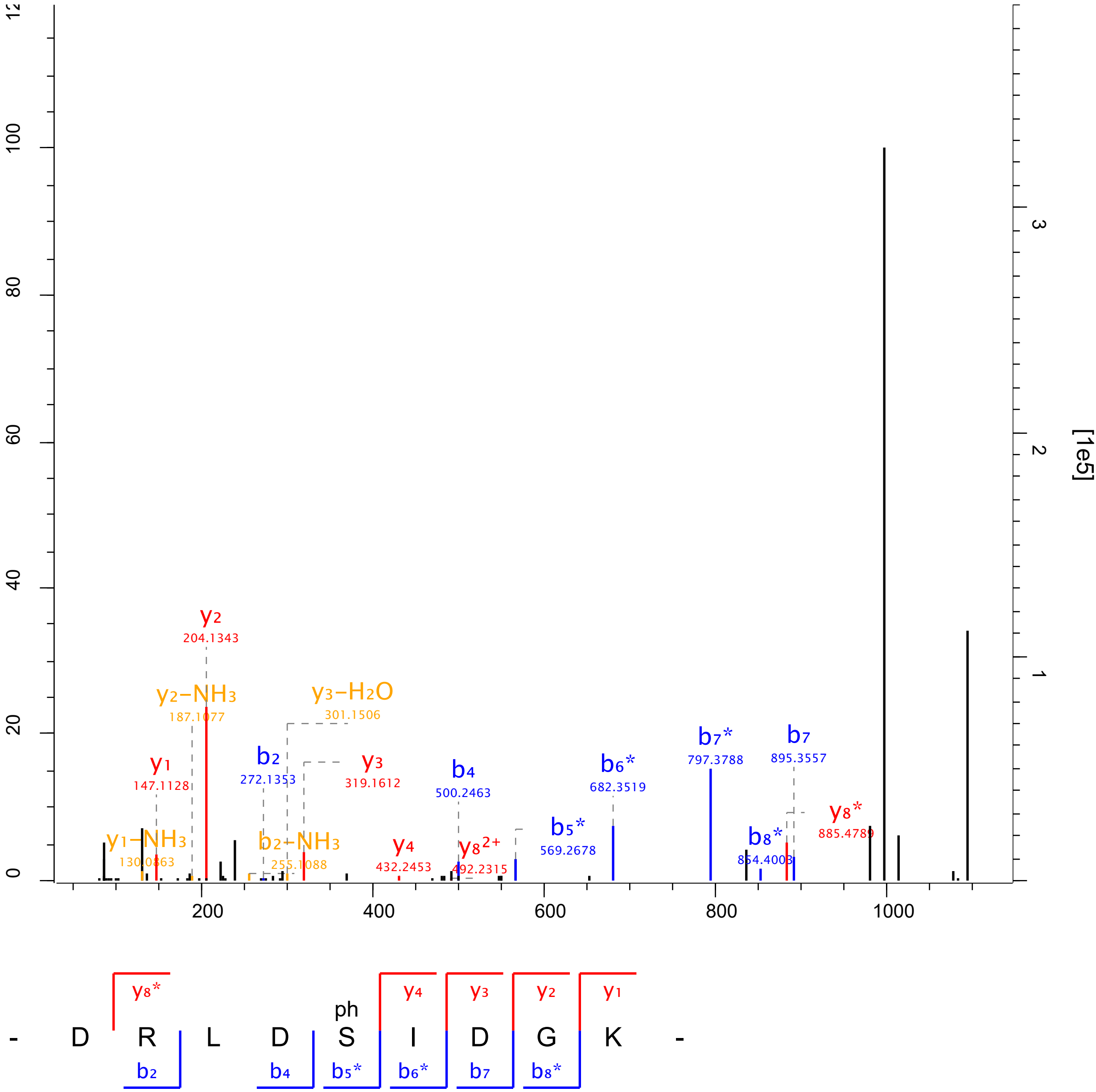

Raw file	Scan	Method	Score	m/z
sys_02_2	8976	FTMS; HCD	233.79	750.81

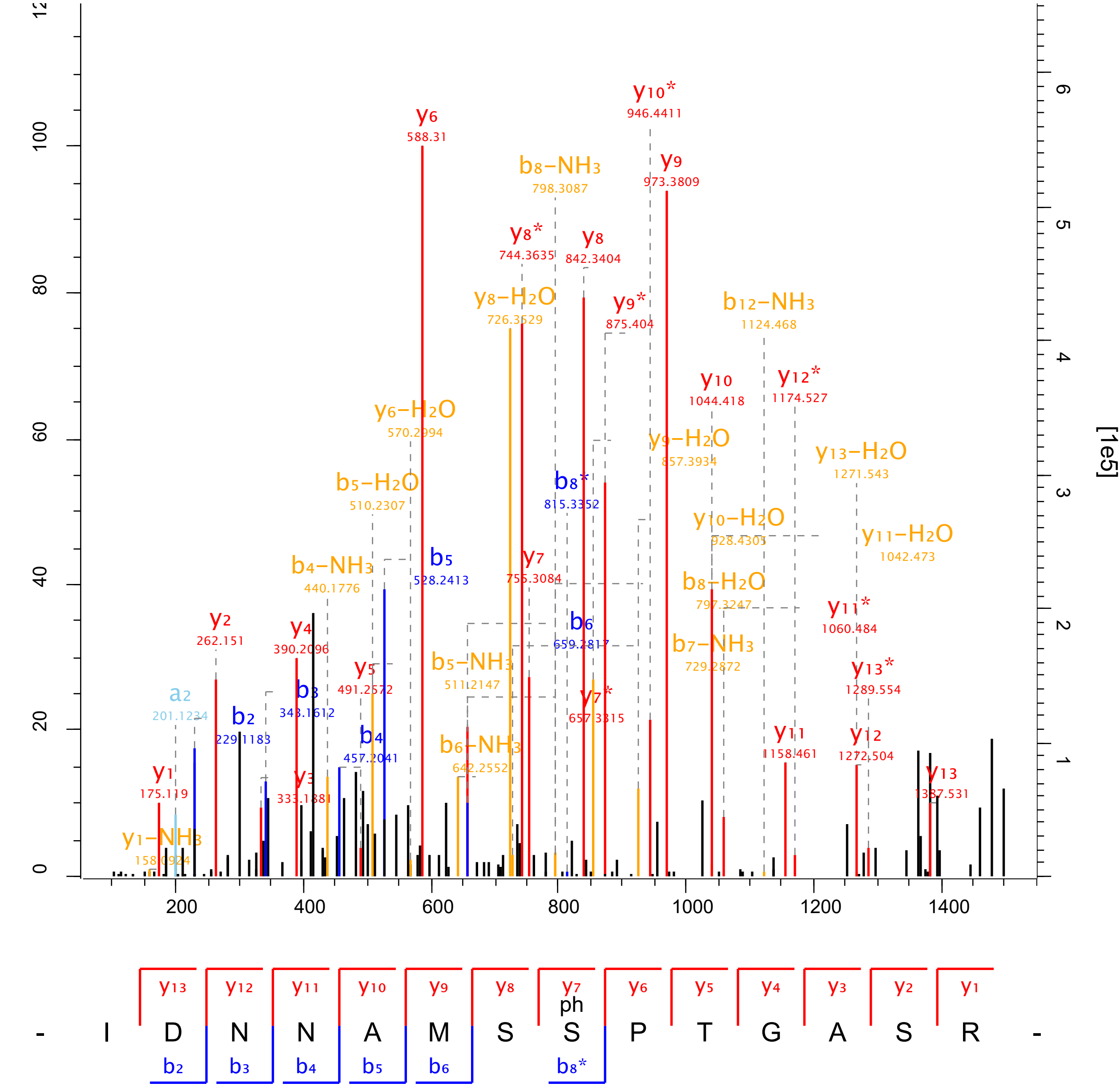

Raw file	Scan	Method	Score	m/z
sys_02_2	8995	FTMS; HCD	123.72	688.28

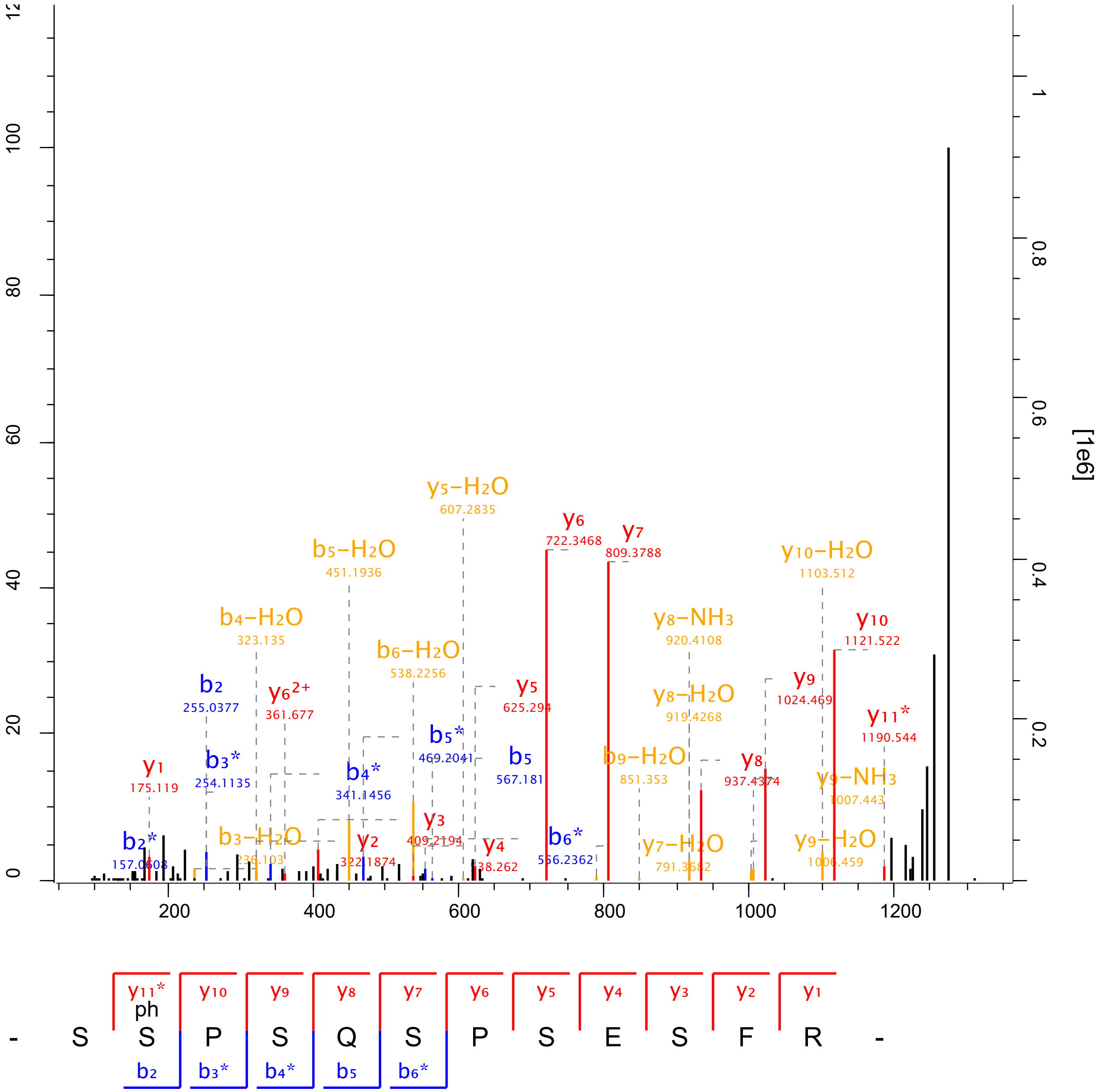

Raw file	Scan	Method	Score	m/z
sys_02_2	9067	FTMS; HCD	47.19	493.72

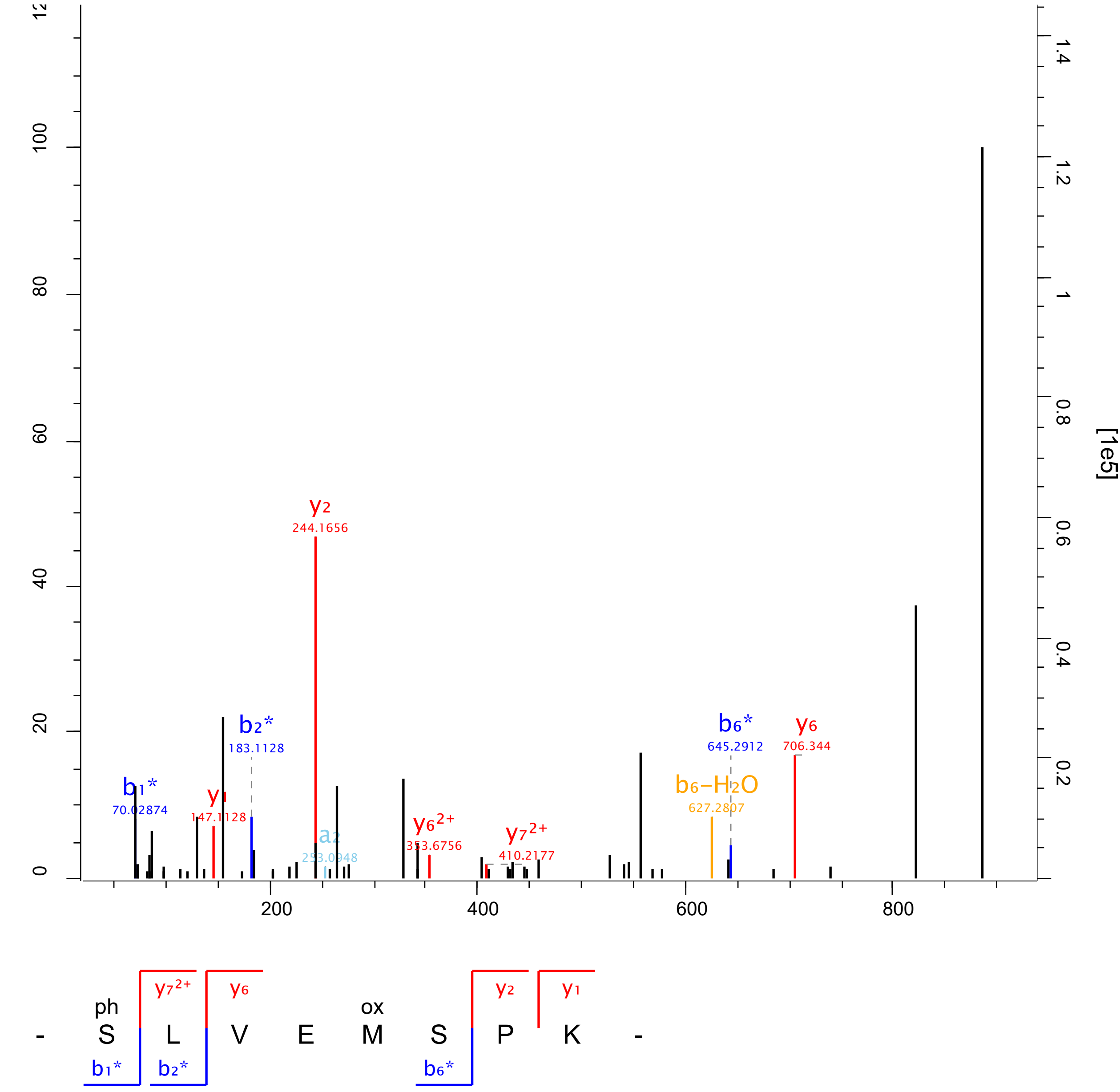

Raw file	Scan	Method	Score	m/z
sys_02_2	9085	FTMS; HCD	49.59	731.77

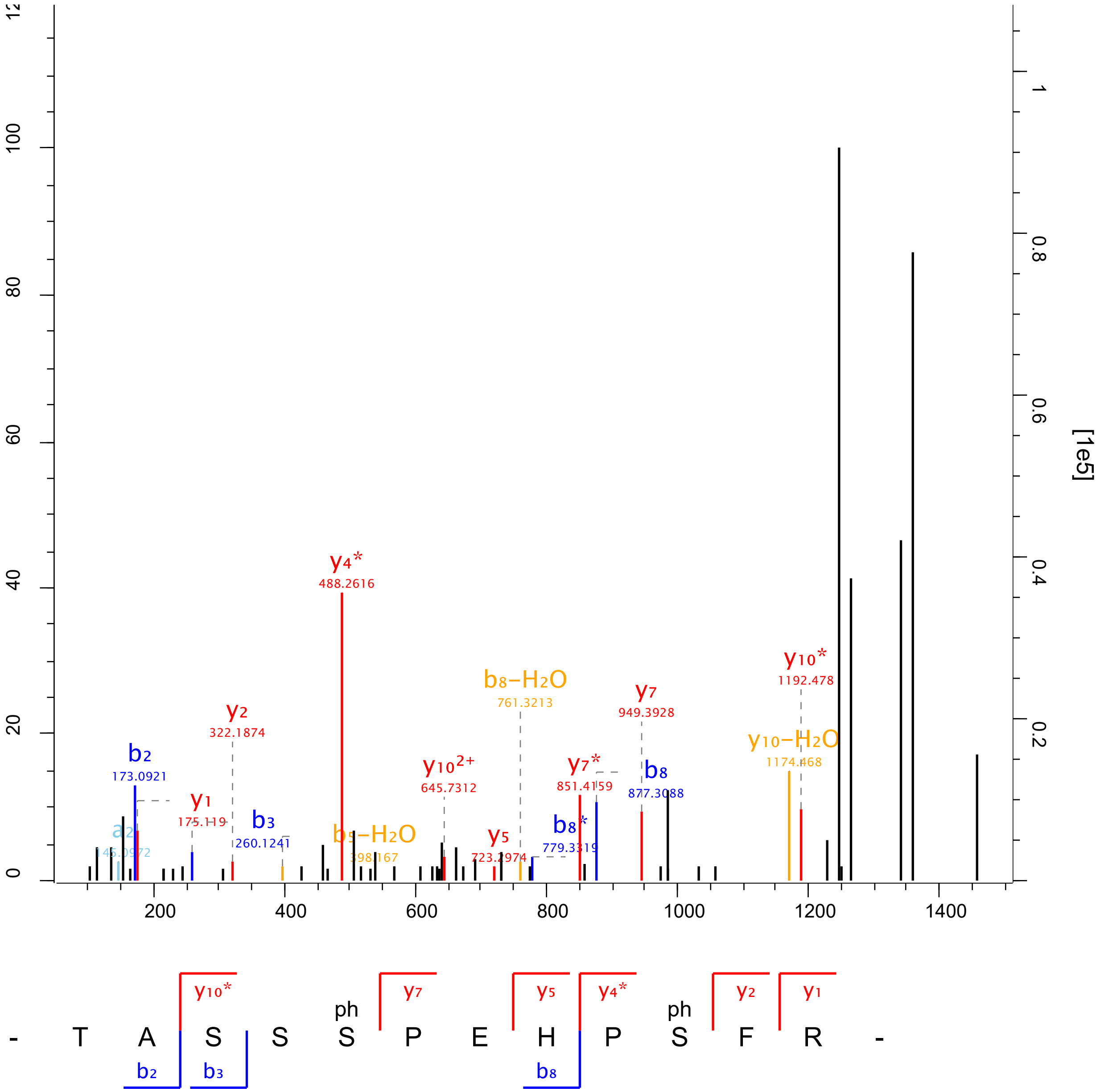

Raw file	Scan	Method	Score	m/z
sys_02_2	9317	FTMS; HCD	51.73	415.54

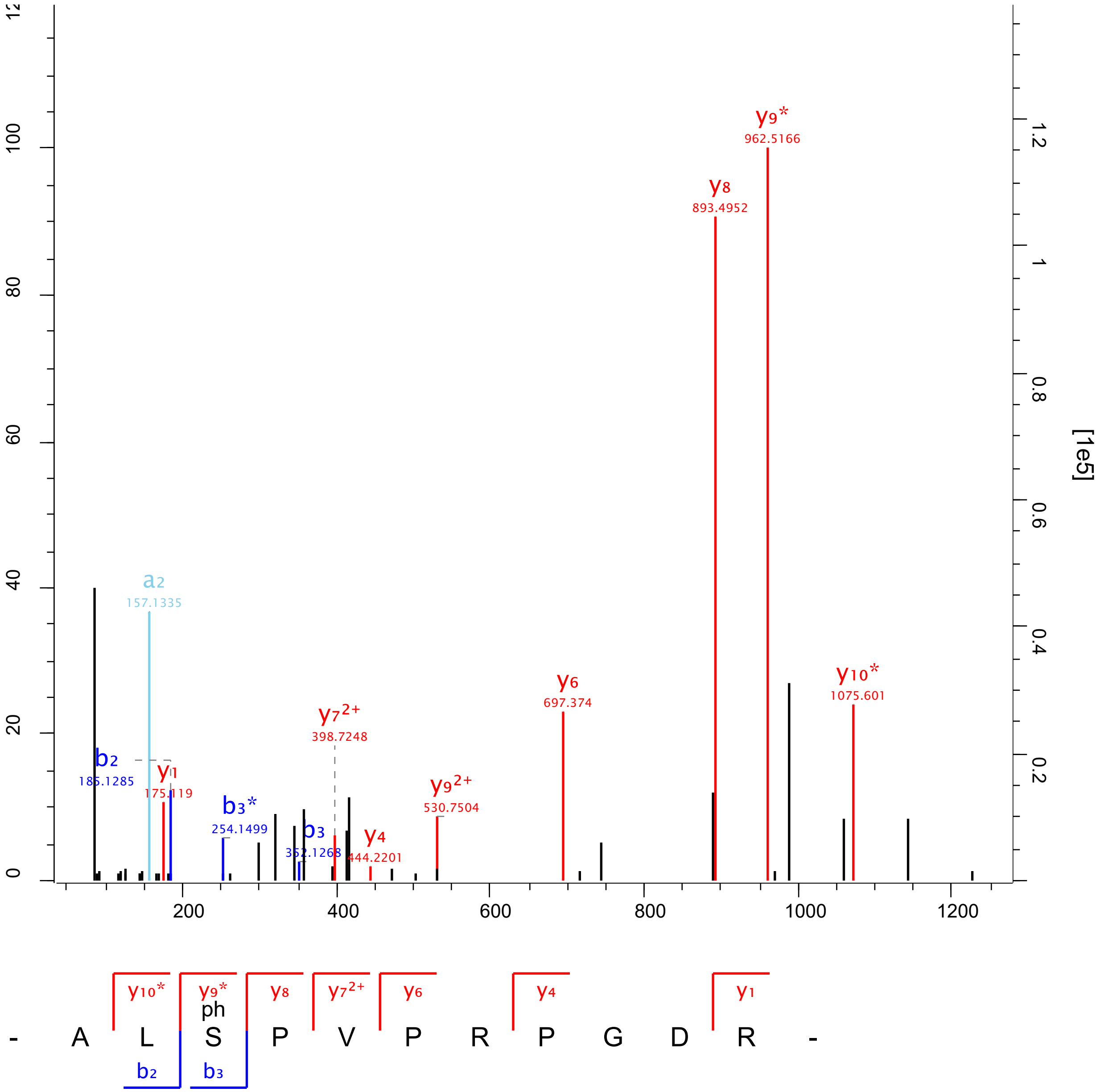

Raw file	Scan	Method	Score	m/z
sys_02_2	9349	FTMS; HCD	62.09	655.29

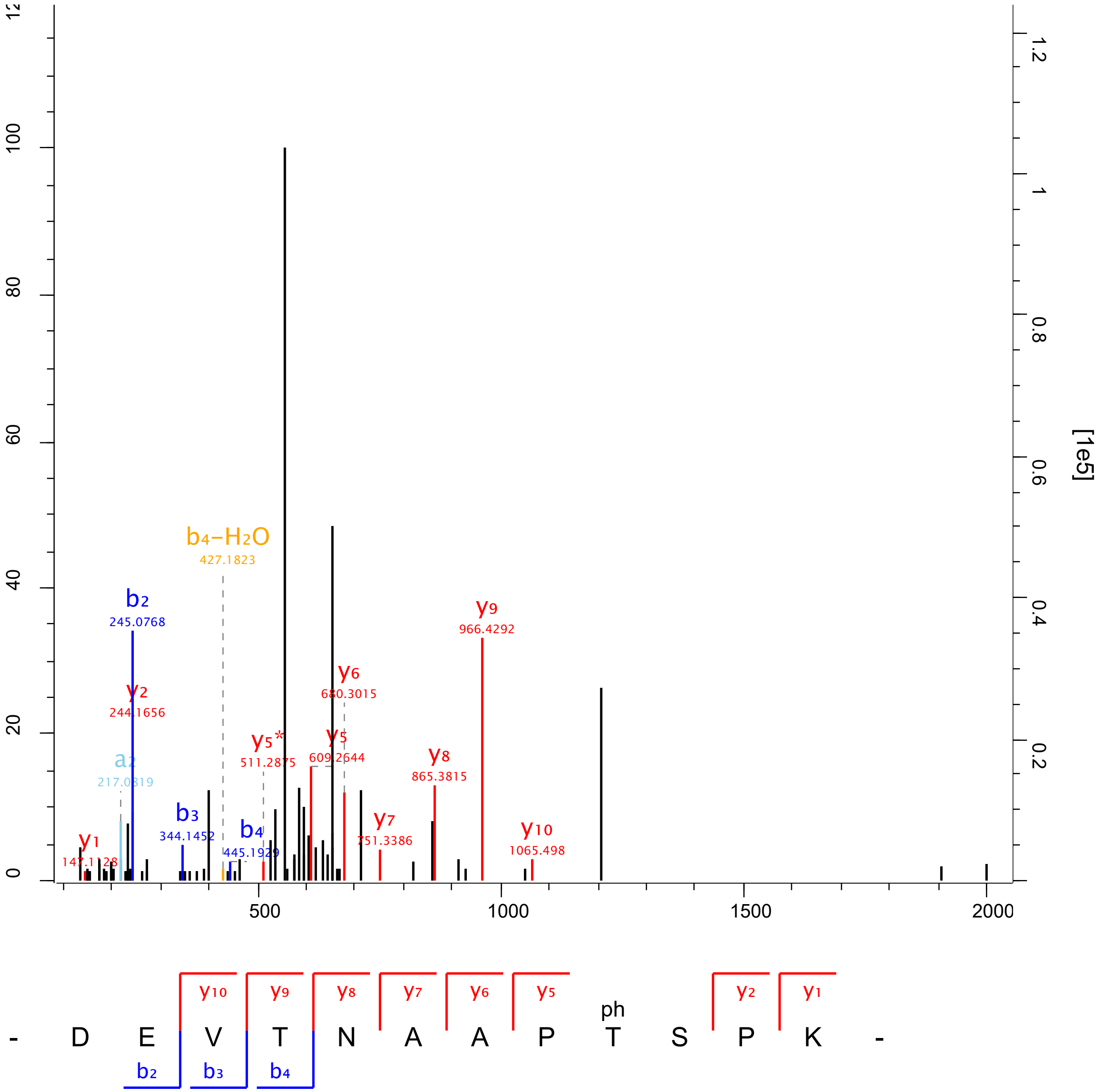

Raw file	Scan	Method	Score	m/z
sys_02_2	9406	FTMS; HCD	197.36	570.75

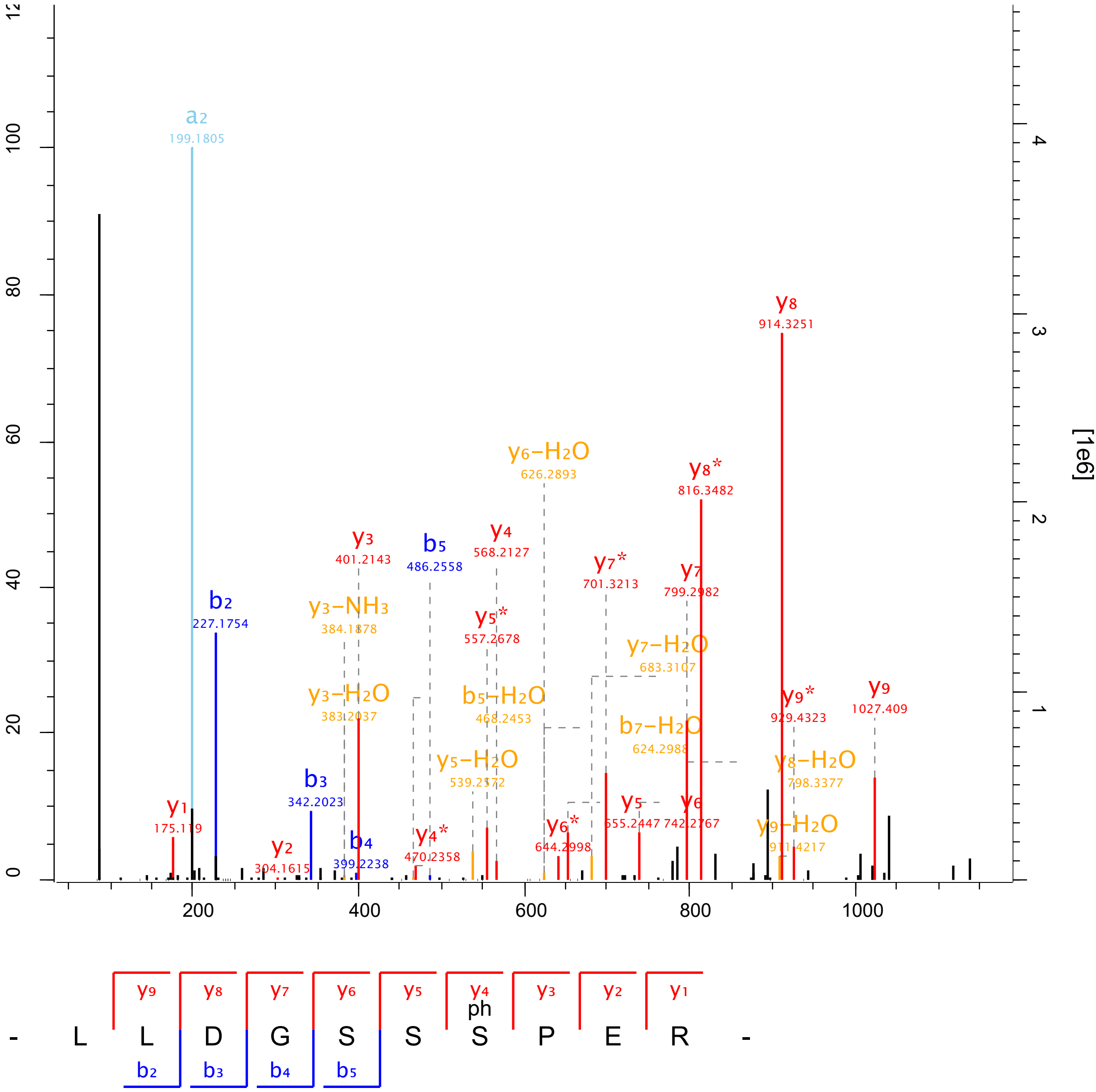

Raw file	Scan	Method	Score	m/z
sys_02_2	9492	FTMS; HCD	41.54	615.26

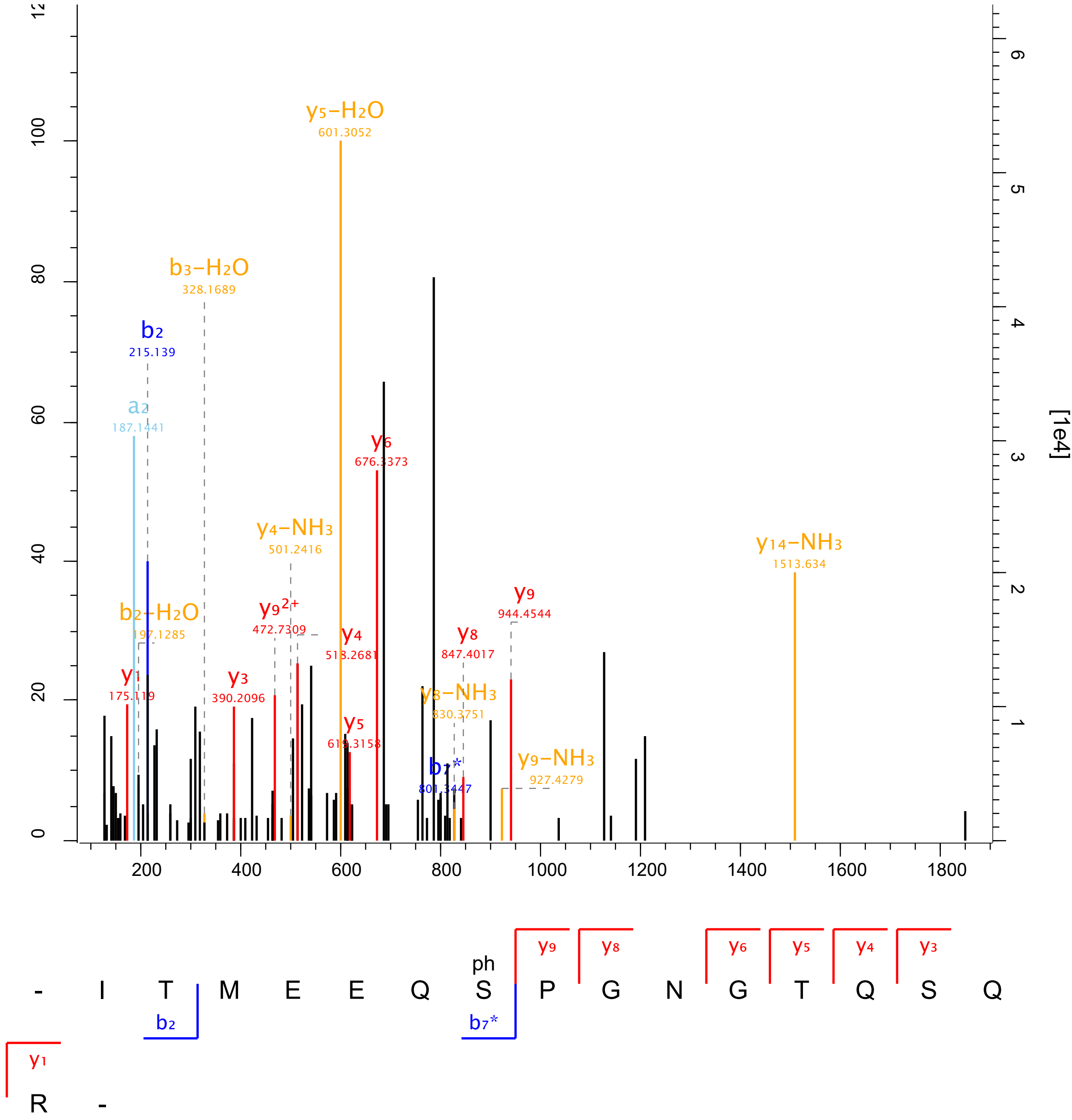

Raw file	Scan	Method	Score	m/z
sys_02_2	9554	FTMS; HCD	106.58	581.28

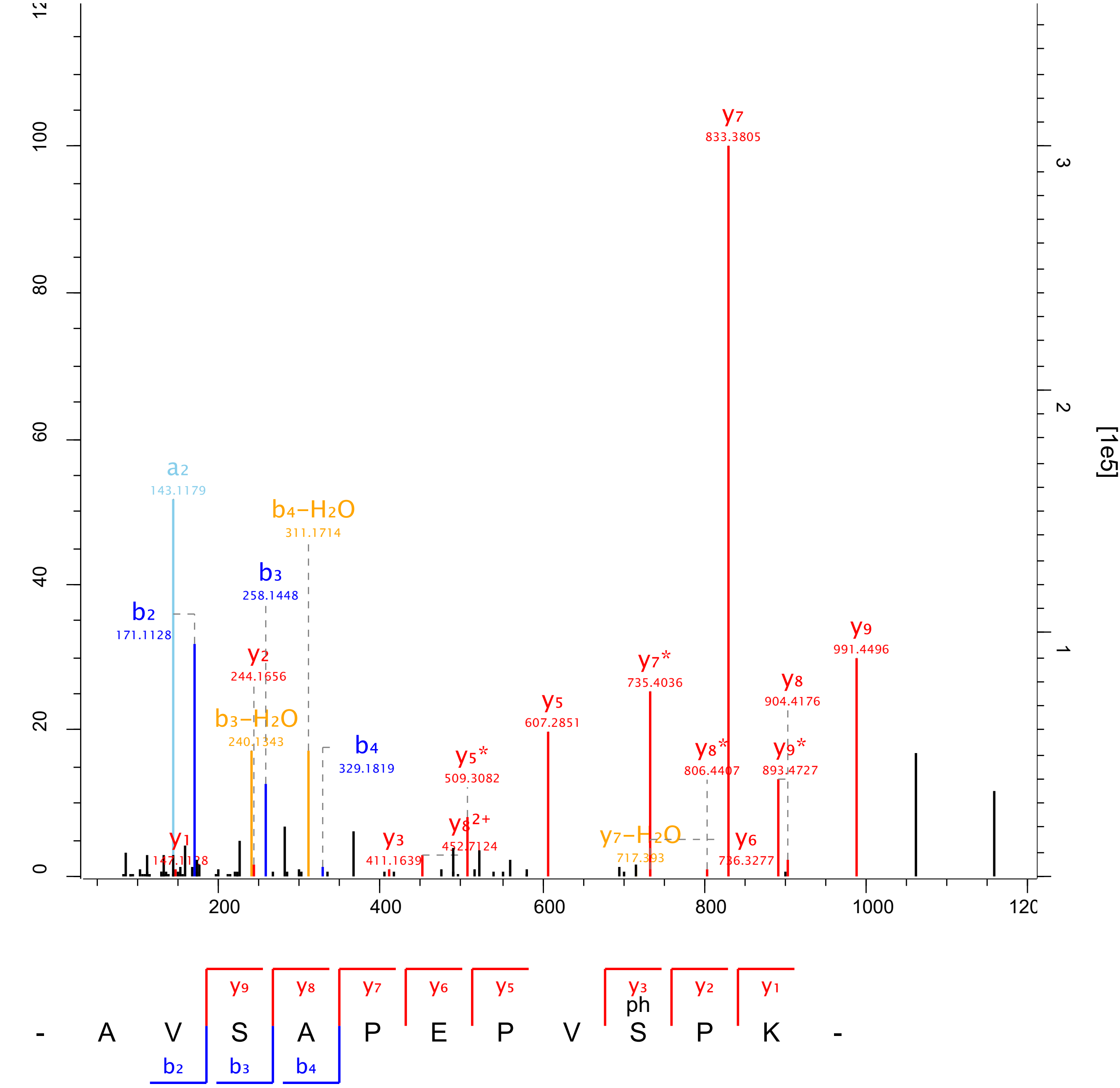

Raw file	Scan	Method	Score	m/z
sys_02_2	9613	FTMS; HCD	170.38	786.34

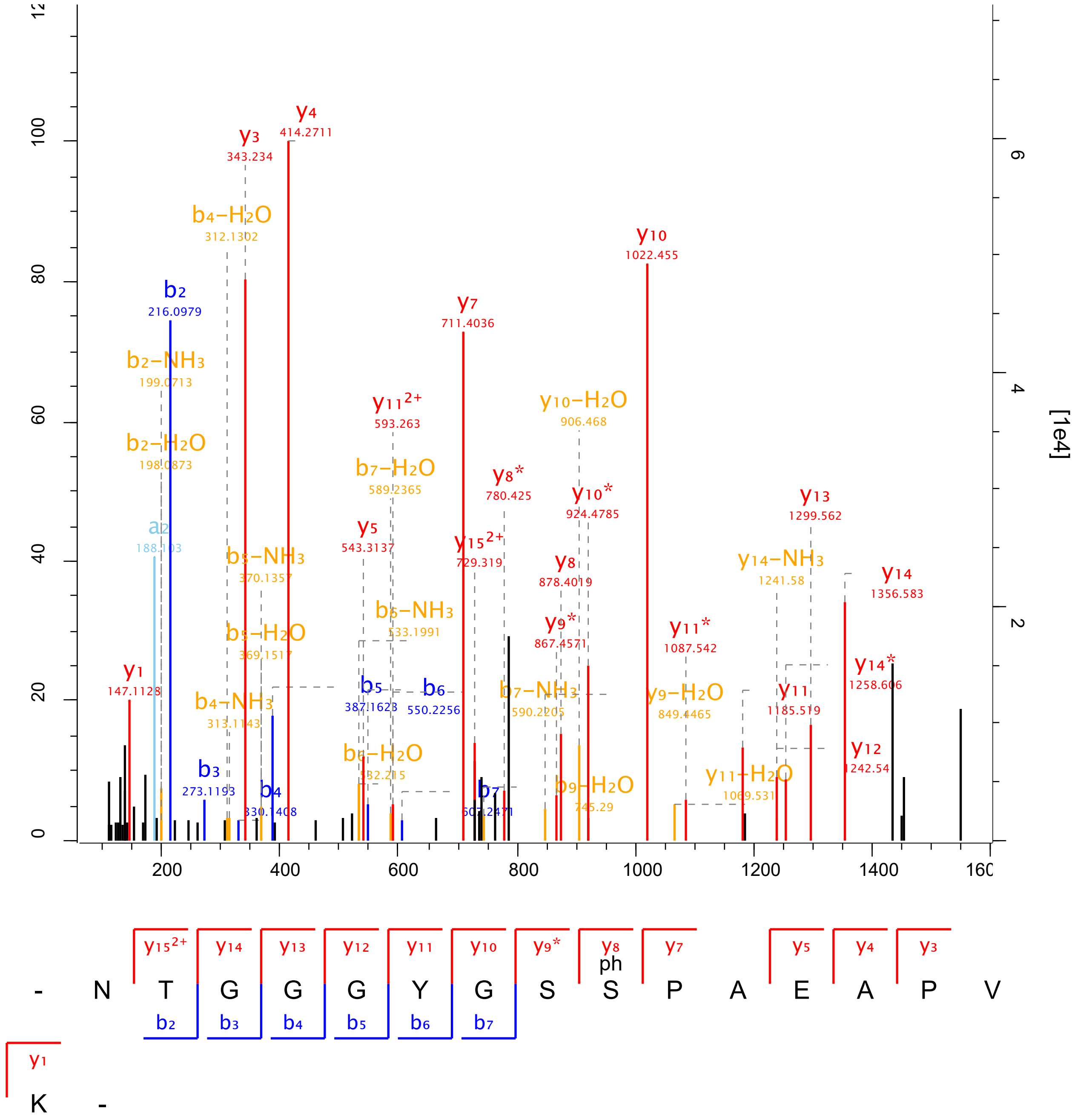

Raw file	Scan	Method	Score	m/z
sys_02_2	9679	FTMS; HCD	198.55	585.76

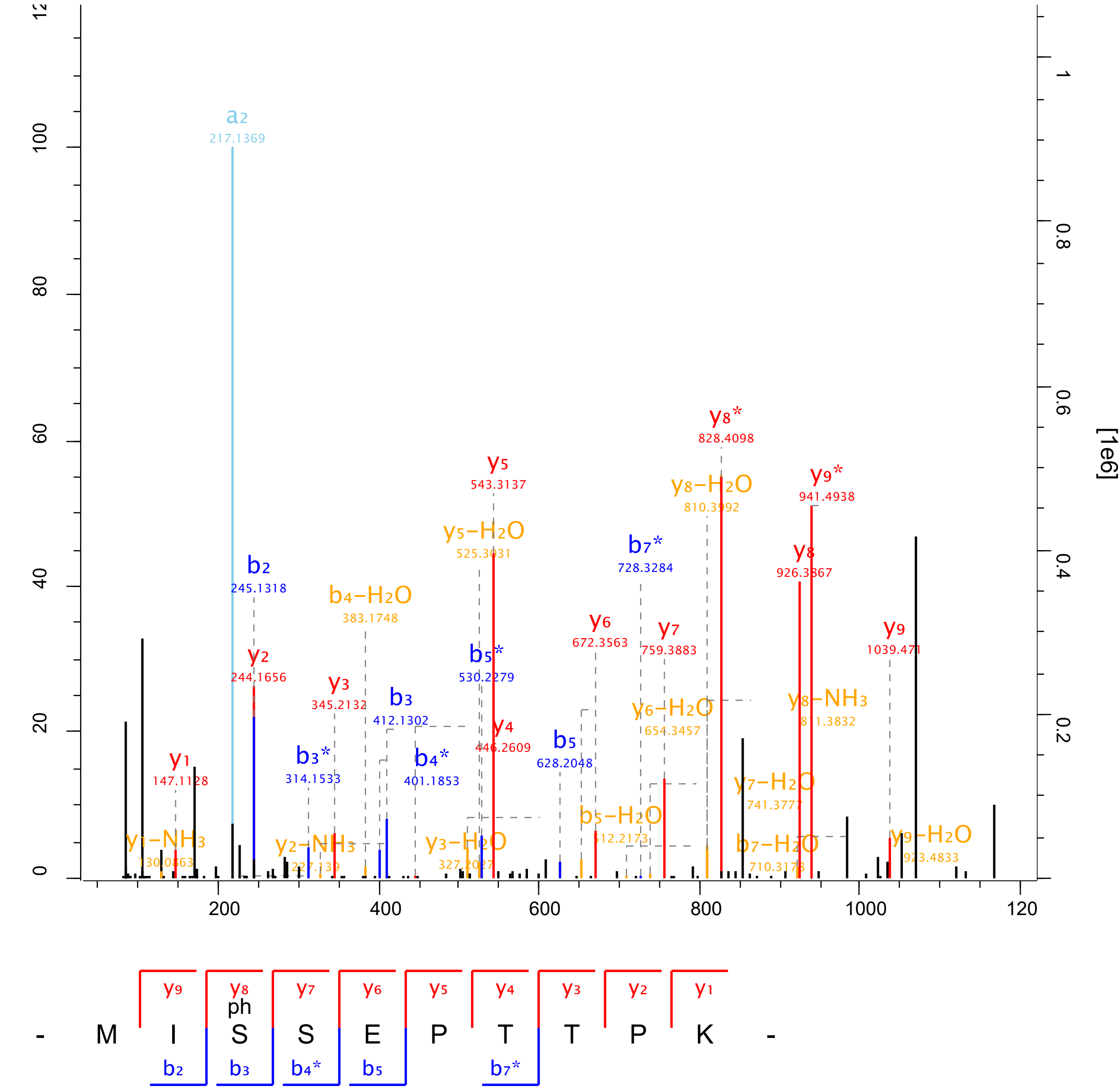

Raw file	Scan	Method	Score	m/z
sys_02_2	9812	FTMS; HCD	94.49	449.57

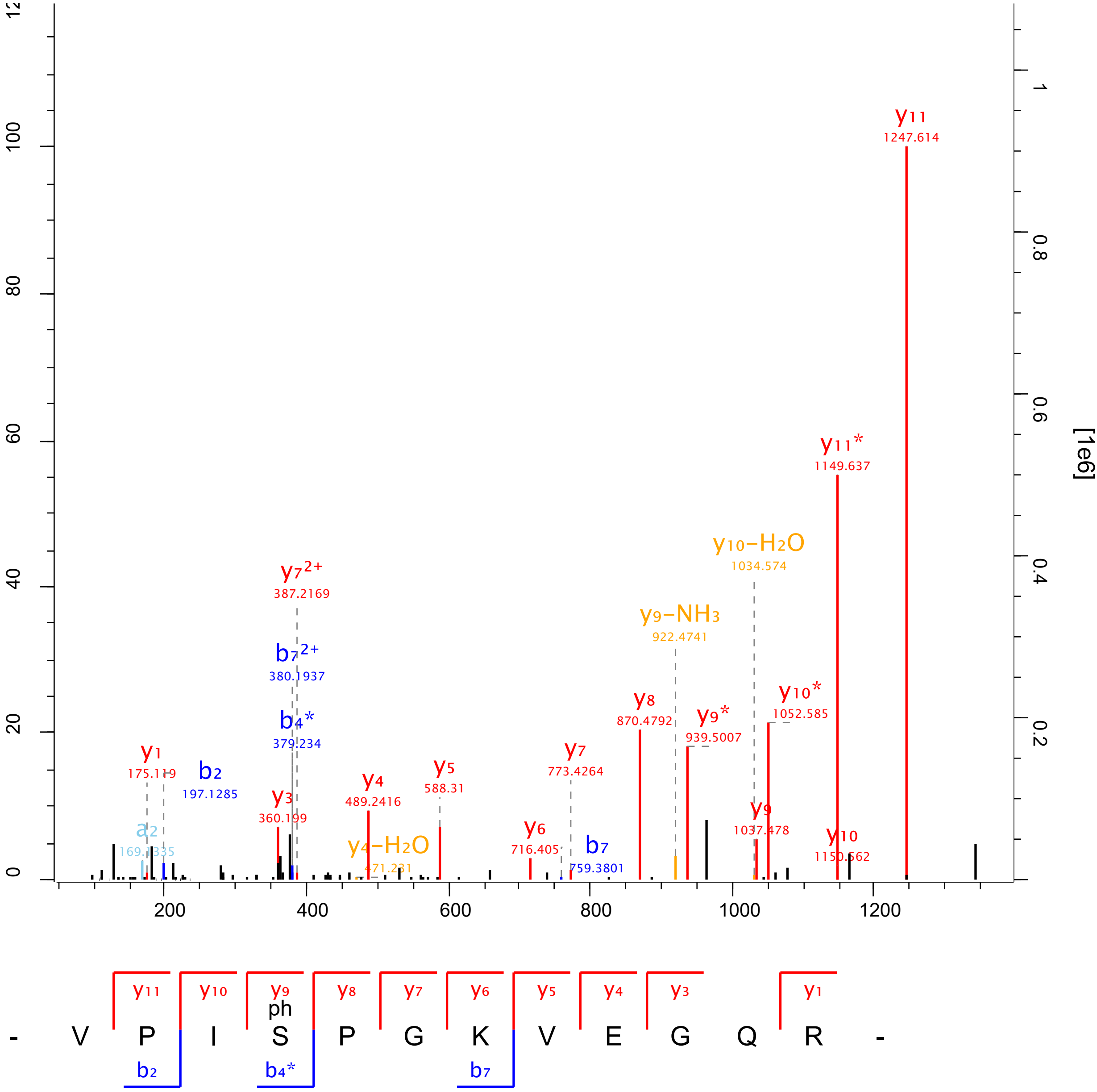

Raw file	Scan	Method	Score	m/z
sys_02_2	9882	FTMS; HCD	85.54	692.3

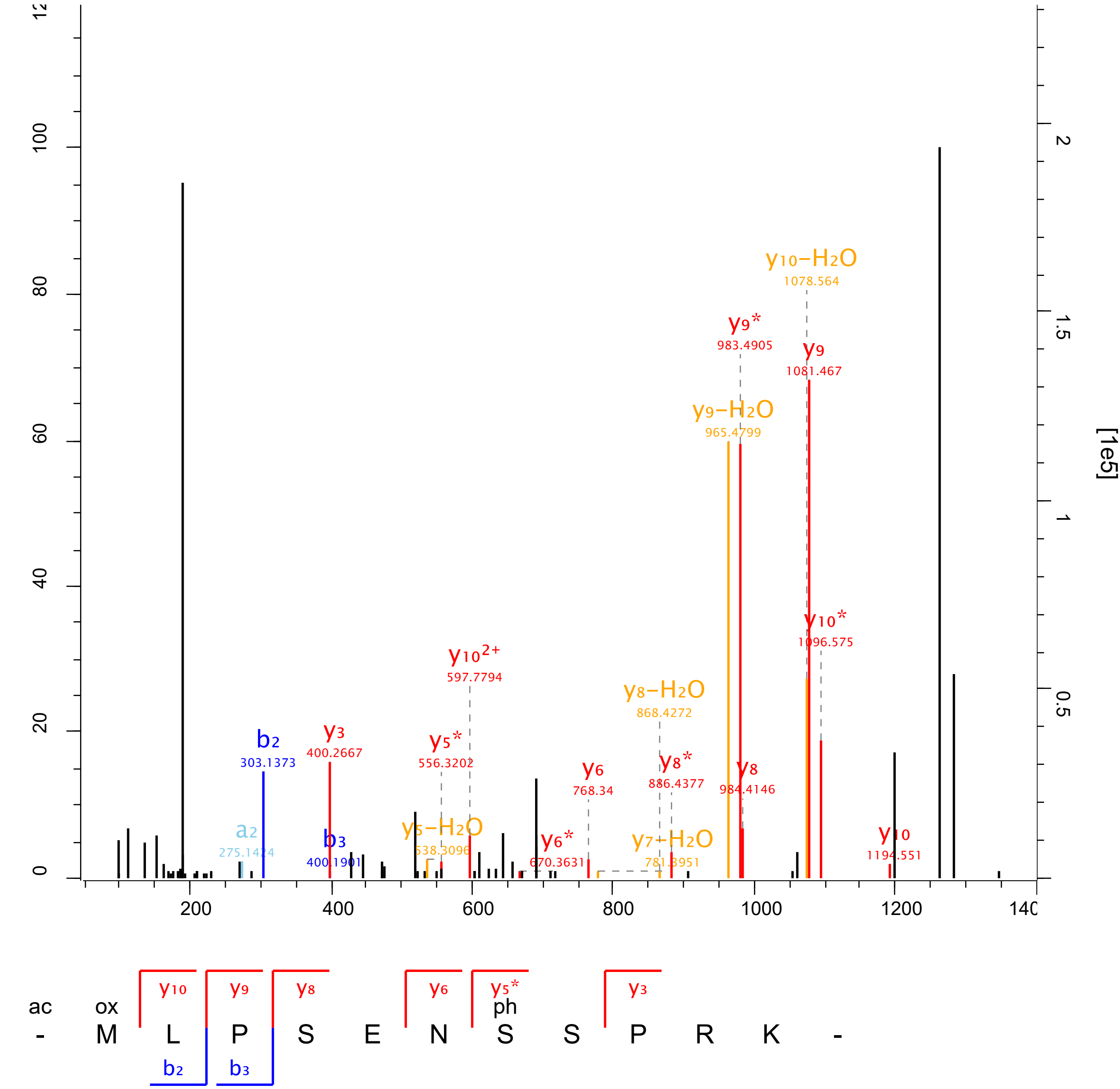

Raw file	Scan	Method	Score	m/z
sys_02_2	9892	FTMS; HCD	100.88	465.72

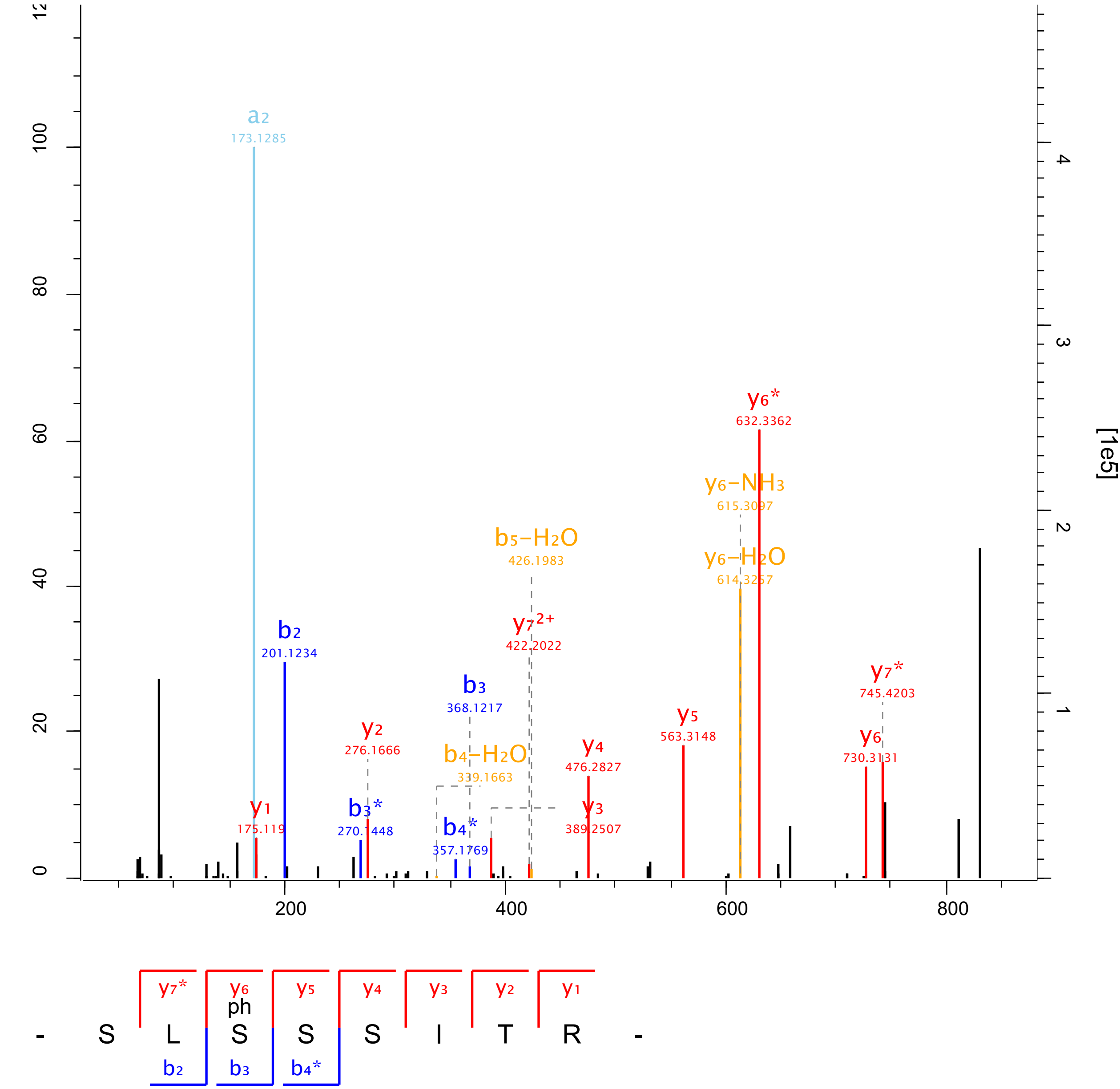

Raw file	Scan	Method	Score	m/z
sys_02_3	10958	FTMS; HCD	113.47	677.31

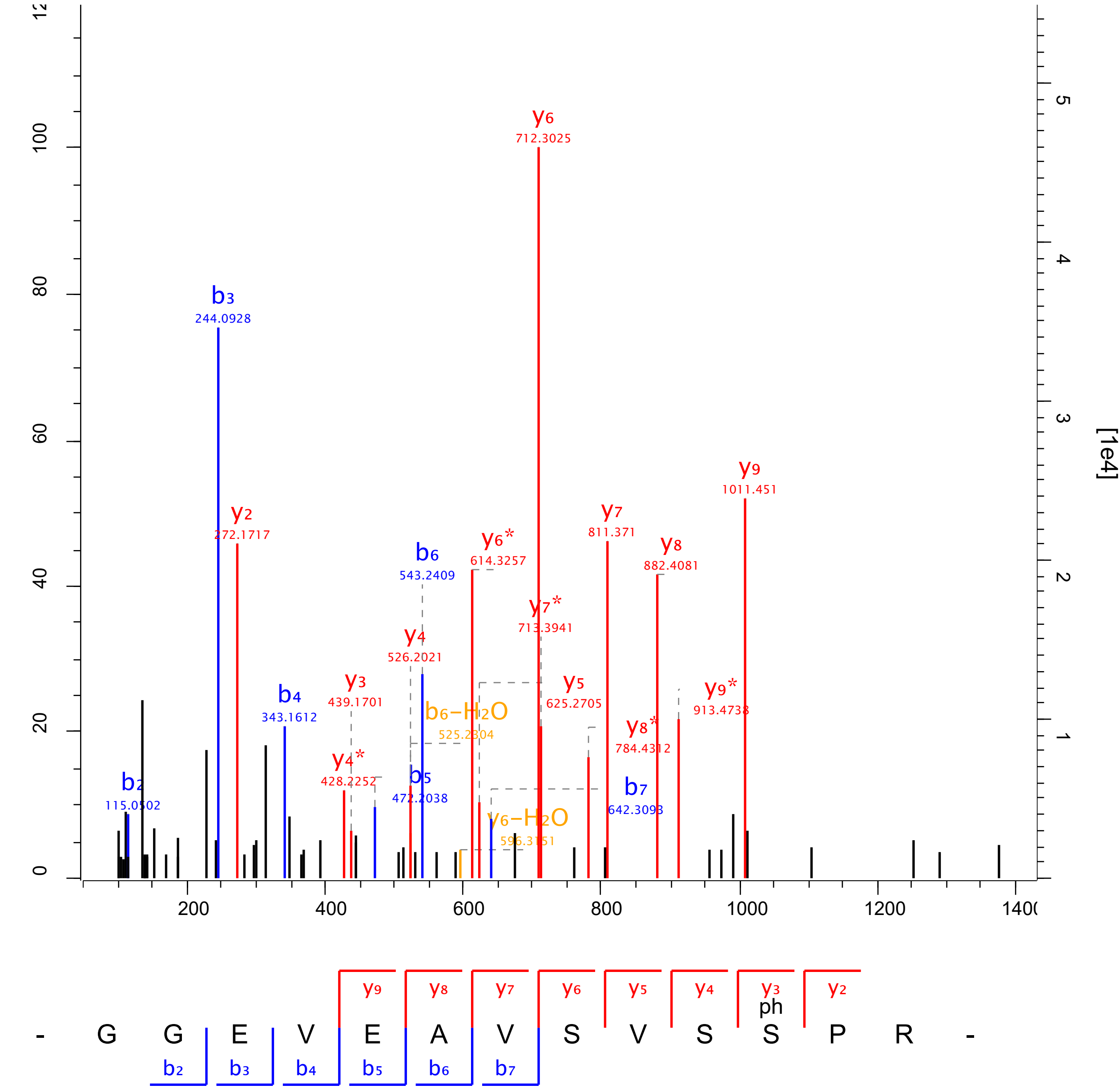

Raw file	Scan	Method	Score	m/z
sys_02_3	14871	FTMS; HCD	47.77	707.33

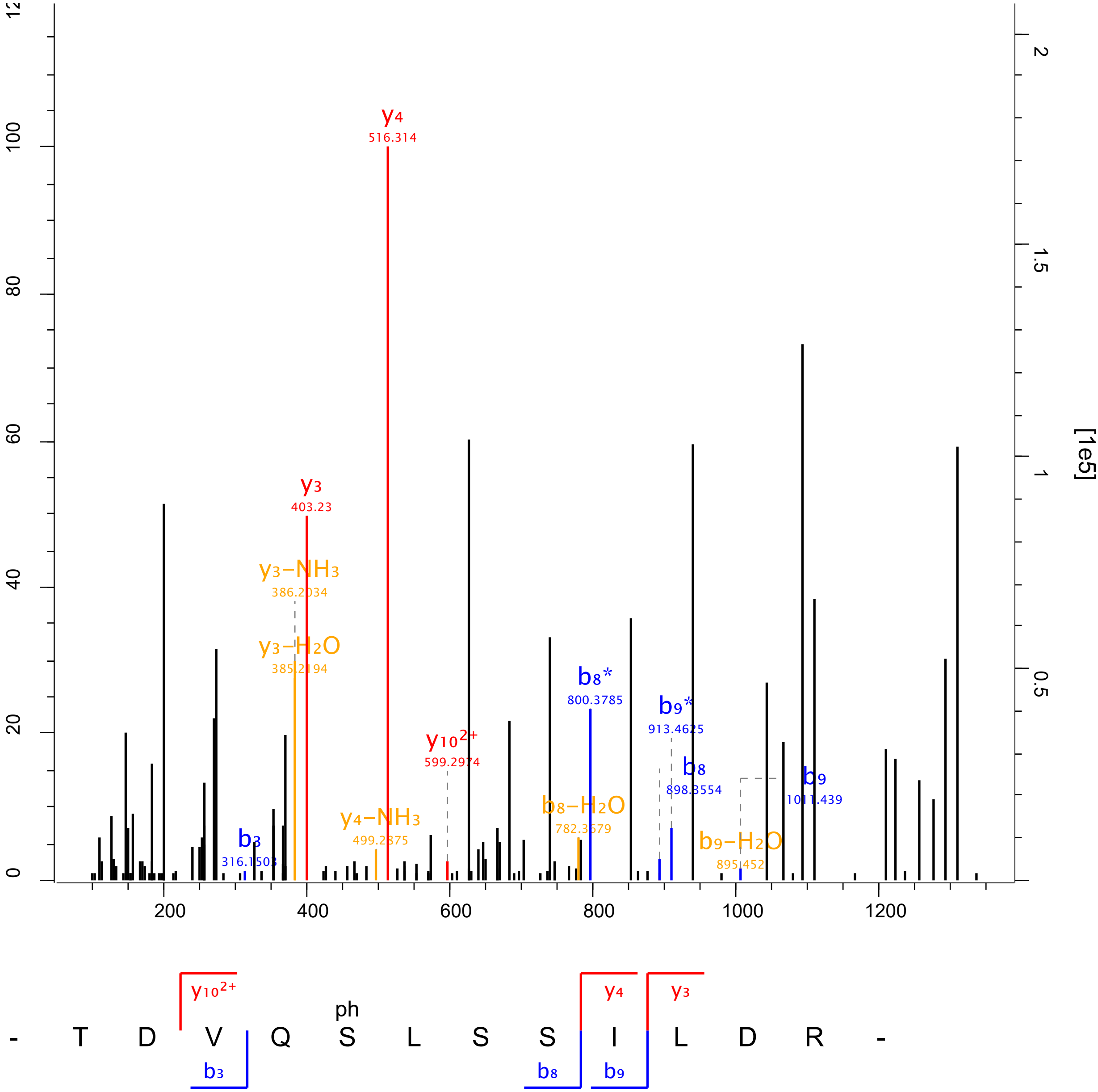

Raw file	Scan	Method	Score	m/z
sys_02_3	18780	FTMS; HCD	43.3	567.25

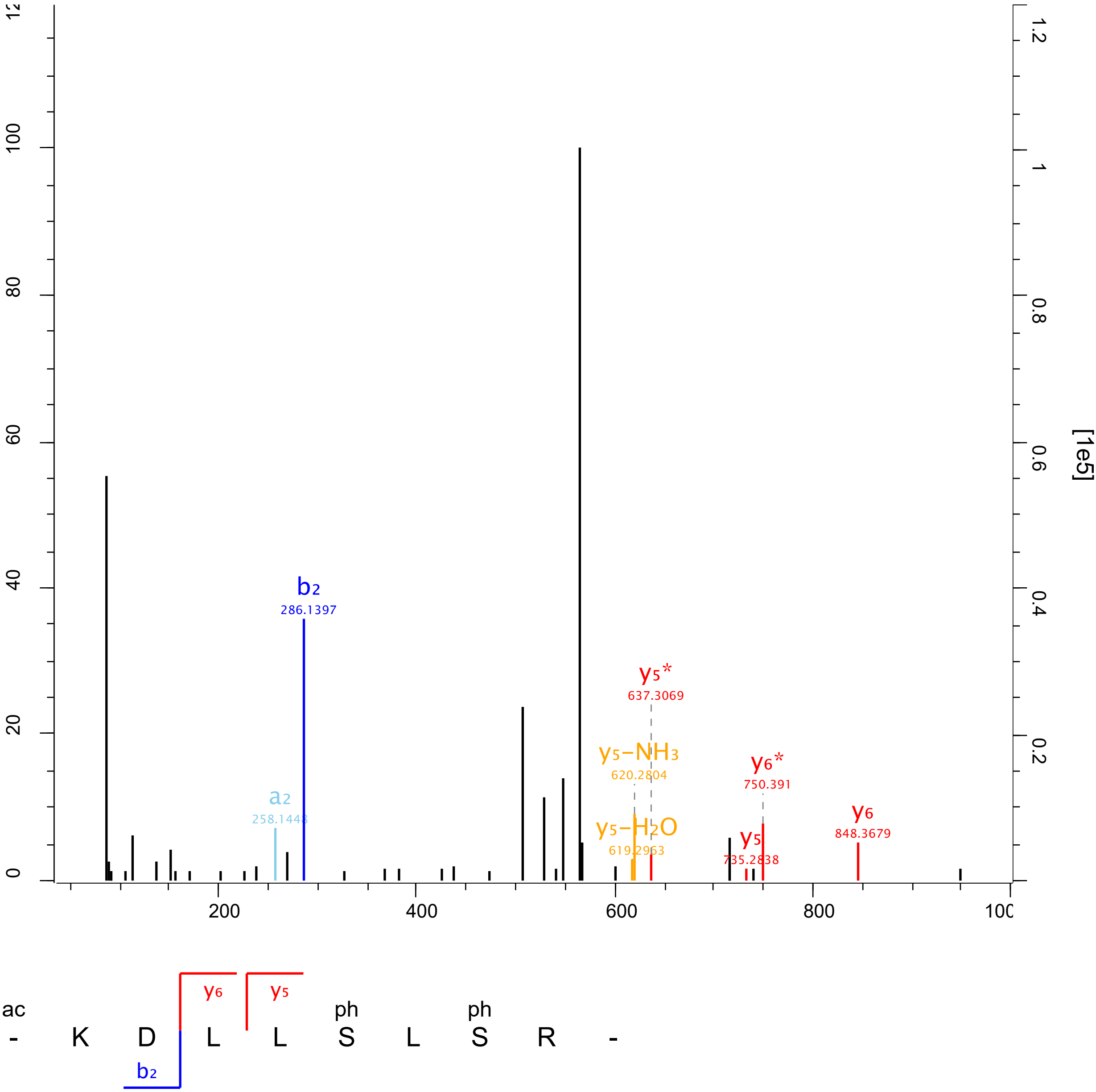

Raw file	Scan	Method	Score	m/z
sys_02_3	19509	FTMS; HCD	45.43	548.24

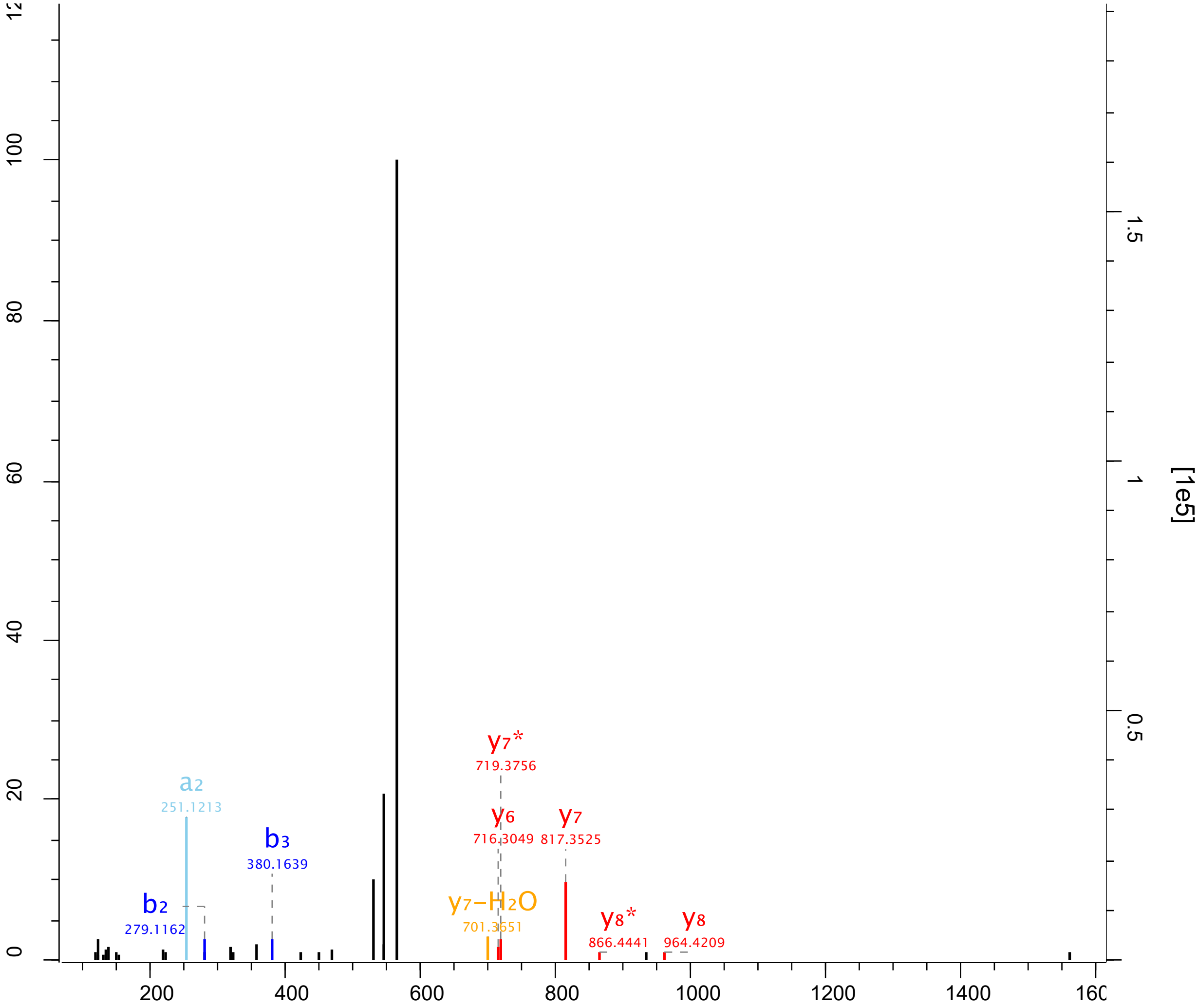

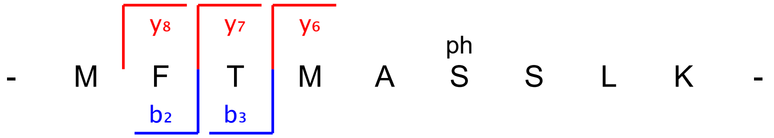

Raw file	Scan	Method	Score	m/z
sys_02_3	2240	FTMS; HCD	42.98	537.19

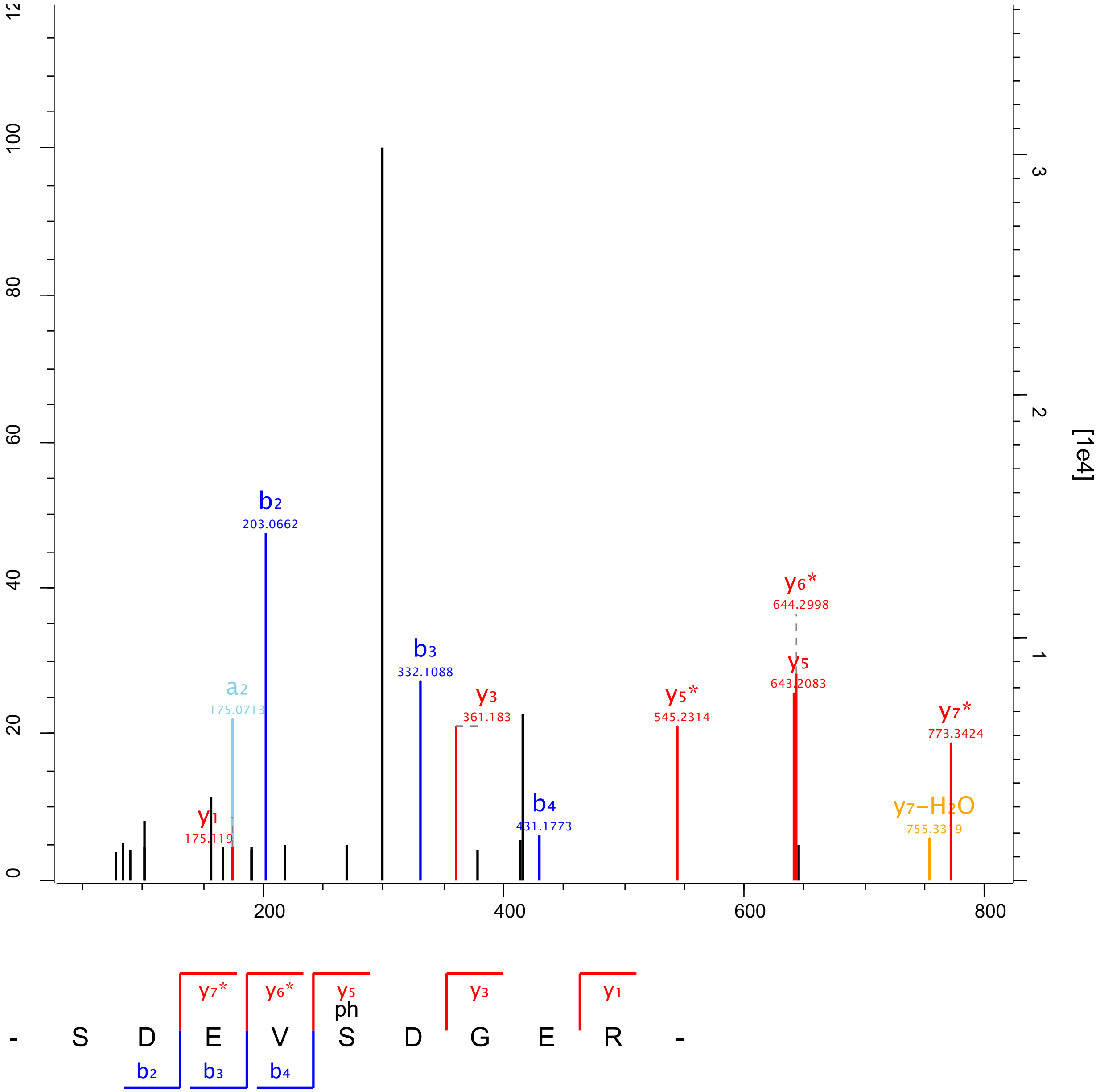

Raw file	Scan	Method	Score	m/z
sys_02_3	23116	FTMS; HCD	154.1	570.29

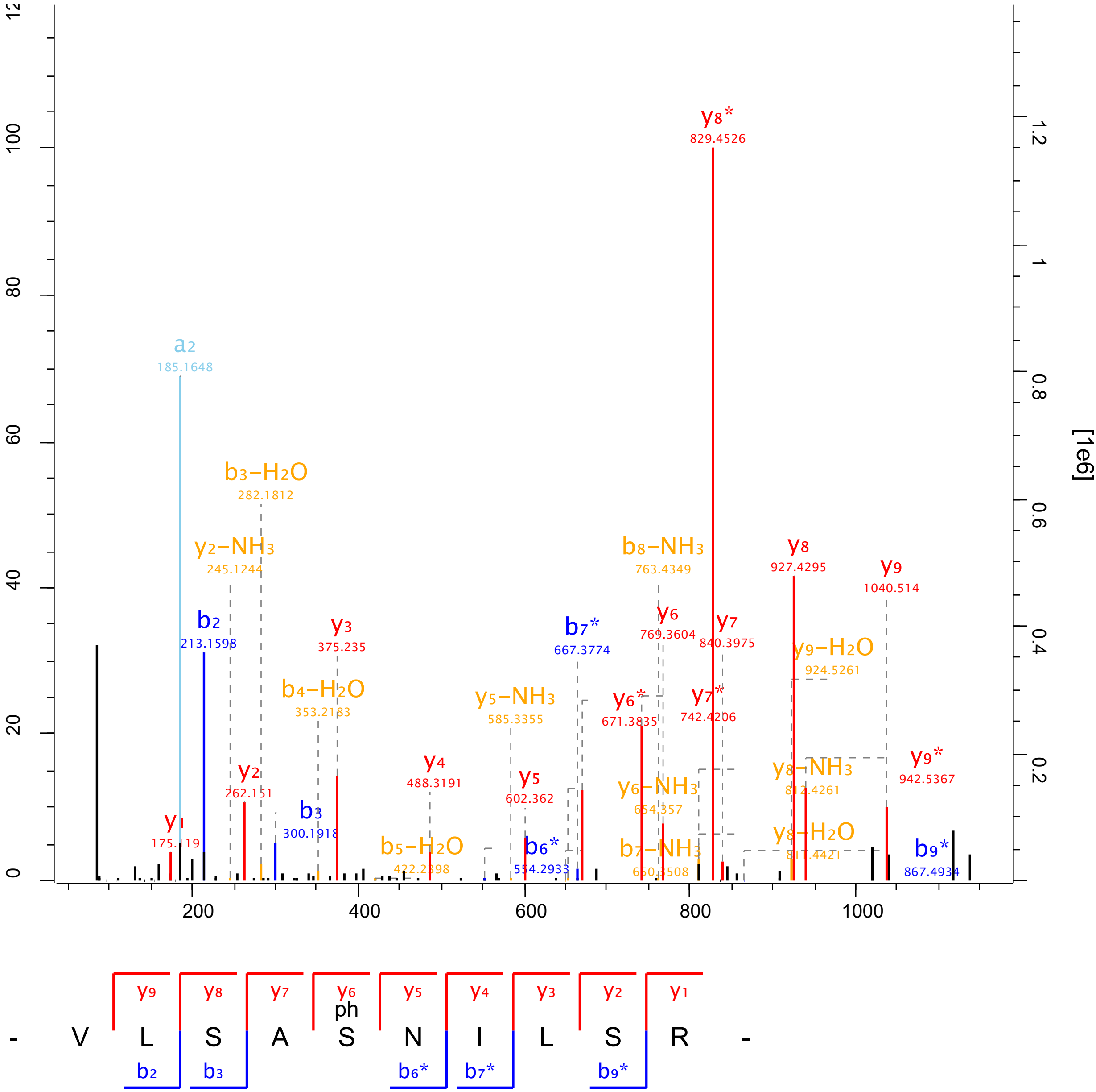

Raw file	Scan	Method	Score	m/z
sys_02_3	23130	FTMS; HCD	41.26	918.42

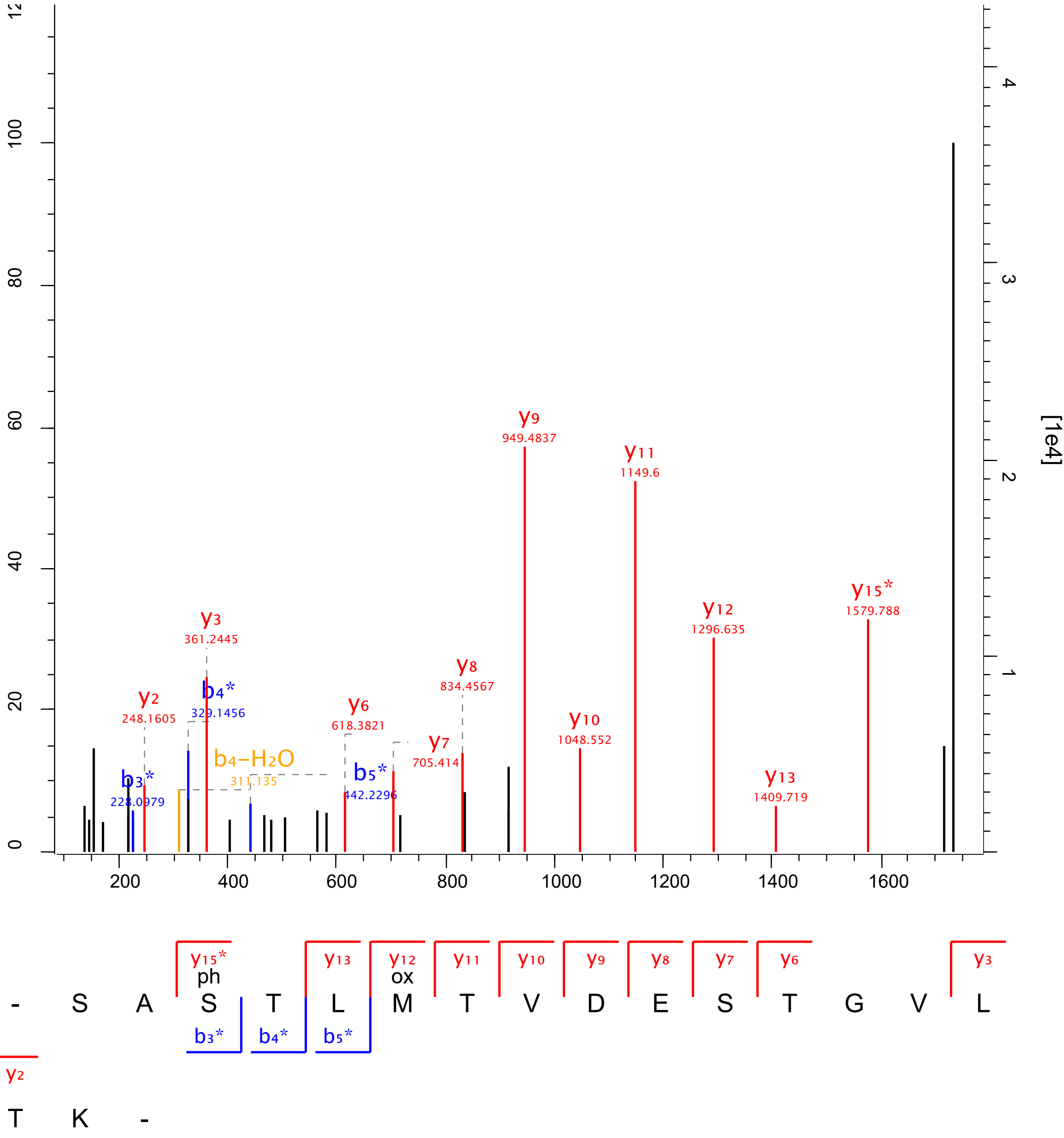

Raw file	Scan	Method	Score	m/z
sys_02_3	2762	FTMS; HCD	56.26	441.74

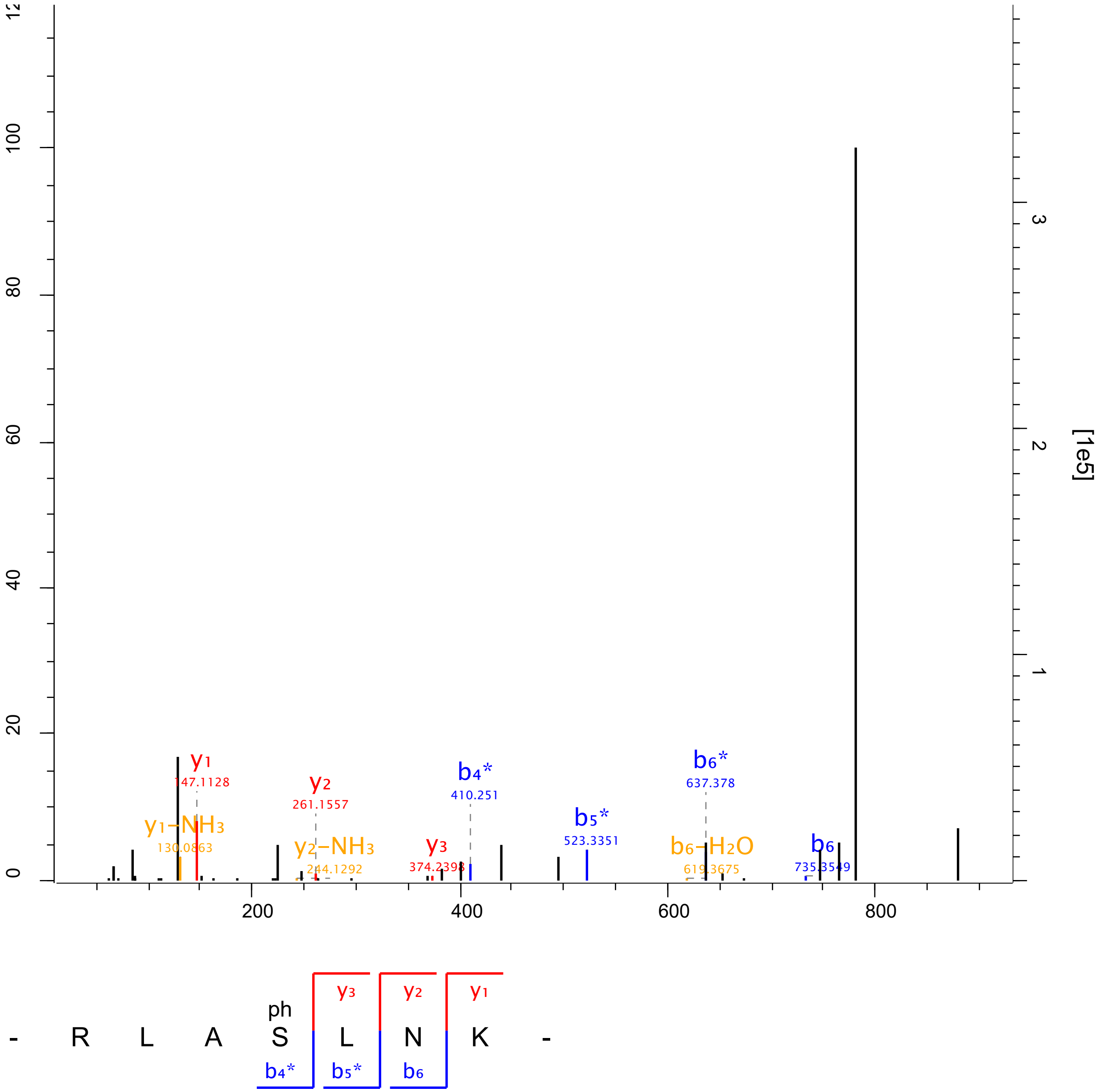

Raw file	Scan	Method	Score	m/z
sys_02_3	3036	FTMS; HCD	48.57	556.89

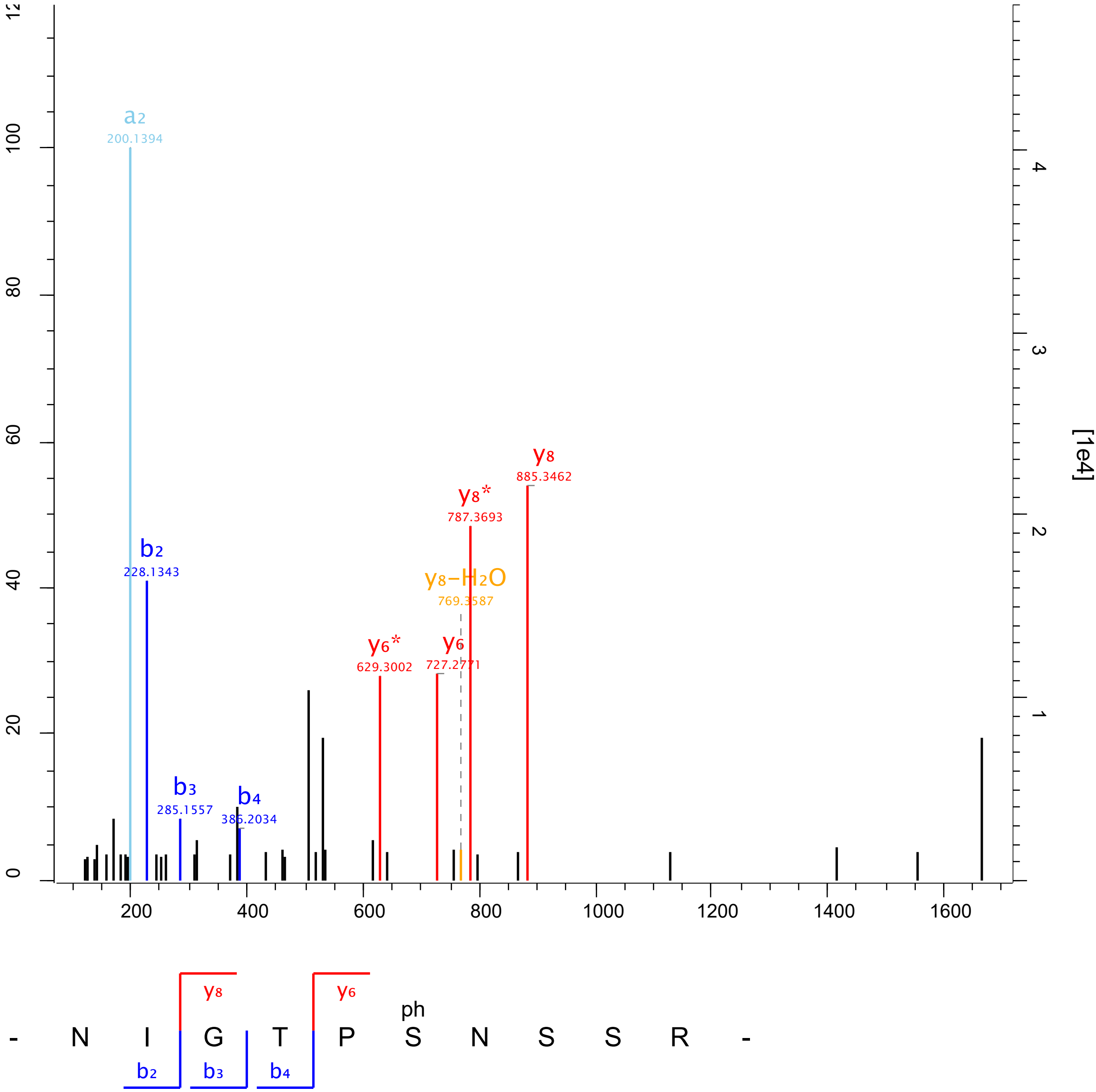

Raw file	Scan	Method	Score	m/z
sys_02_3	36058	FTMS; HCD	75.91	746.31

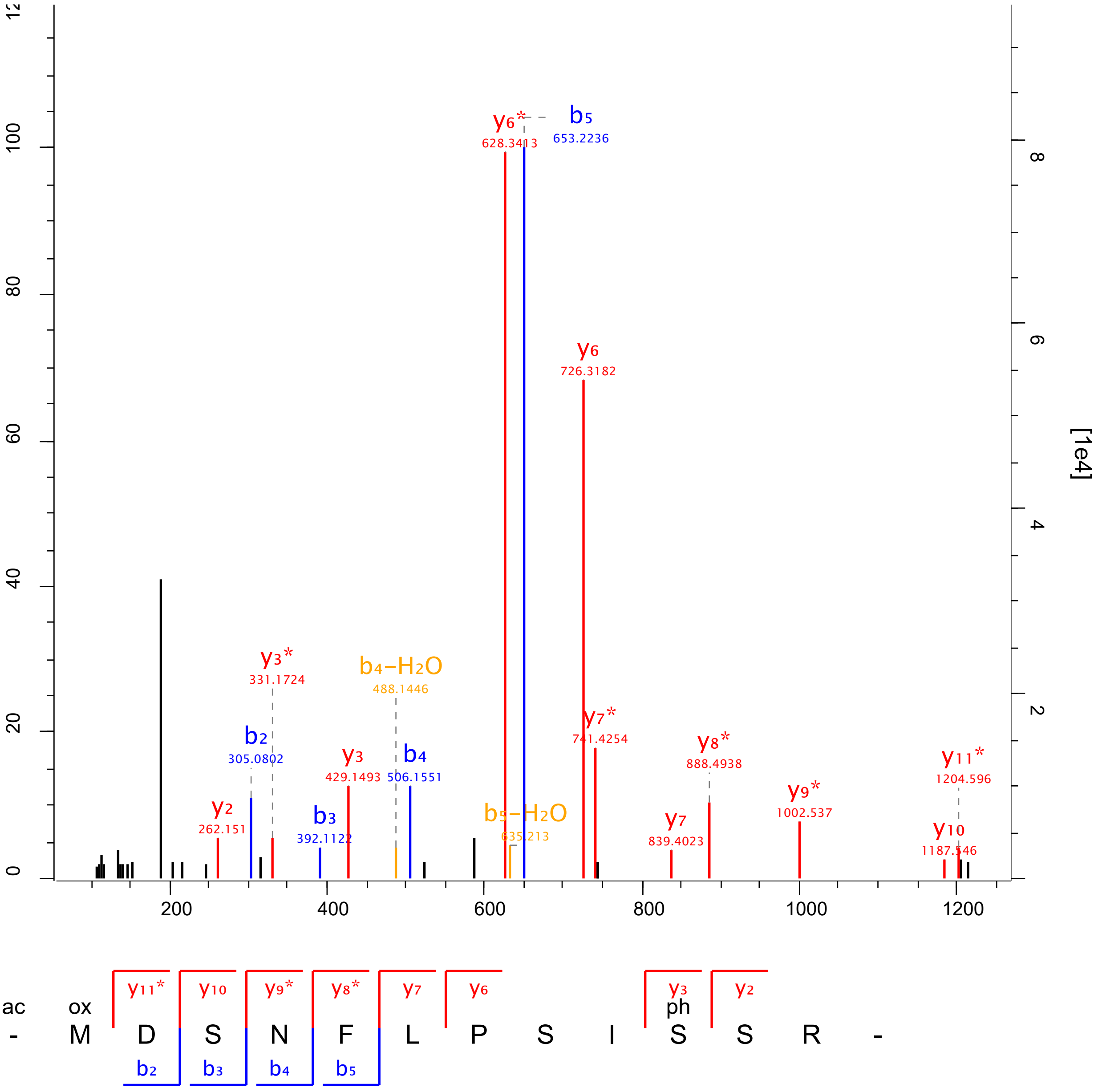

Raw file	Scan	Method	Score	m/z
sys_02_3	3864	FTMS; HCD	144.73	638.73

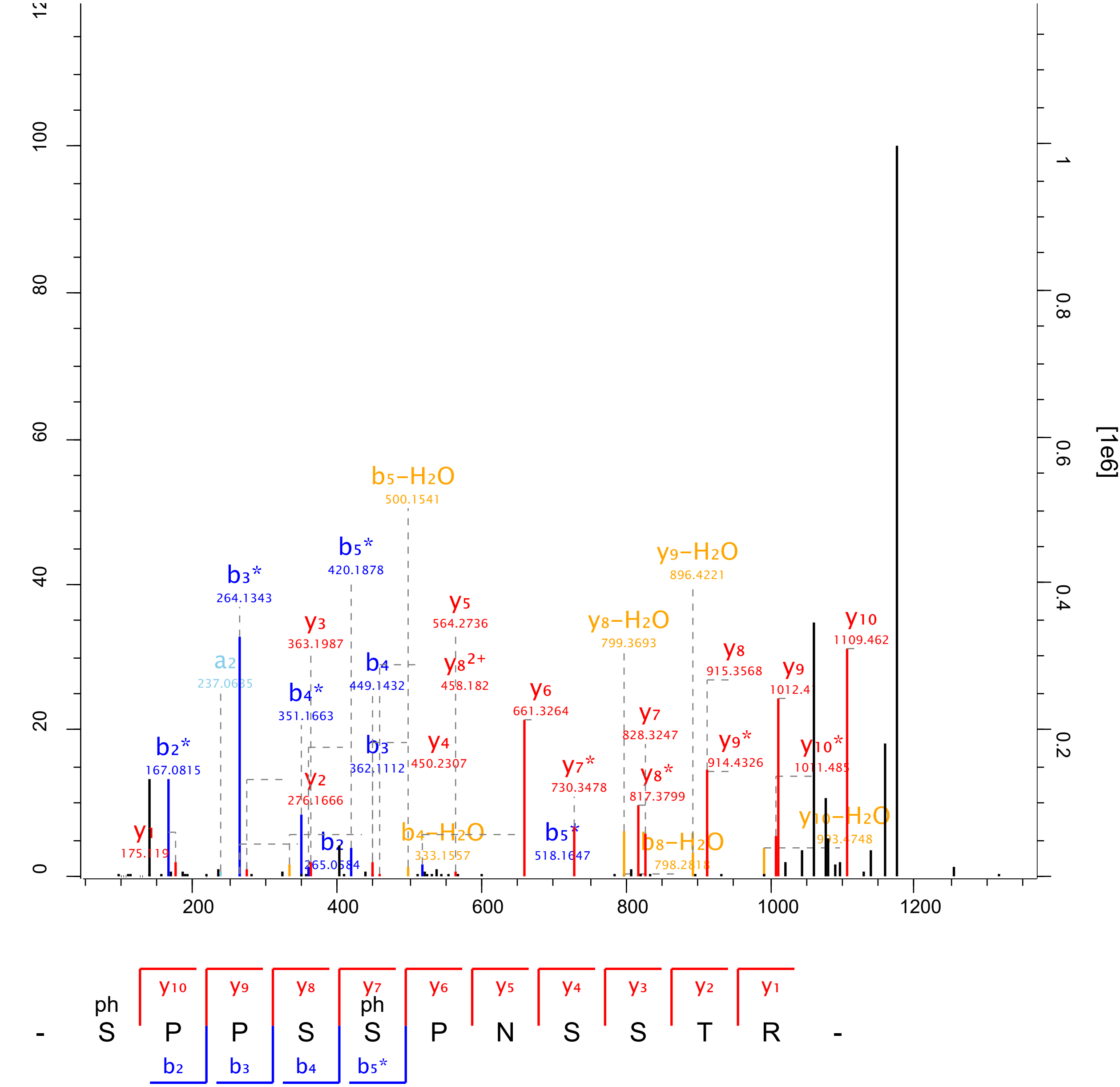

Raw file	Scan	Method	Score	m/z
sys_02_3	4527	FTMS; HCD	138.22	457.22

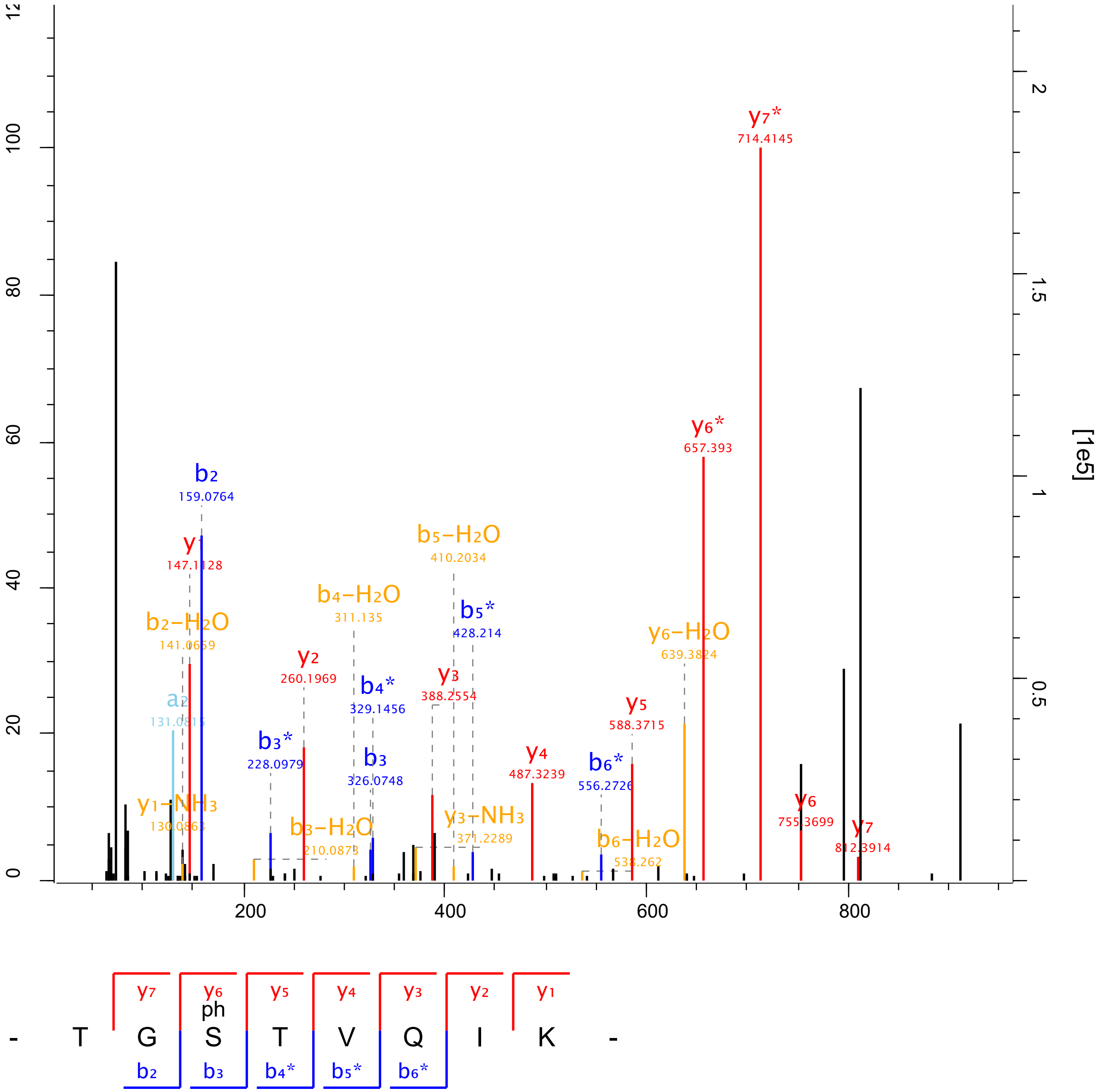

Raw file	Scan	Method	Score	m/z
sys_02_3	5114	FTMS; HCD	92.25	492.74

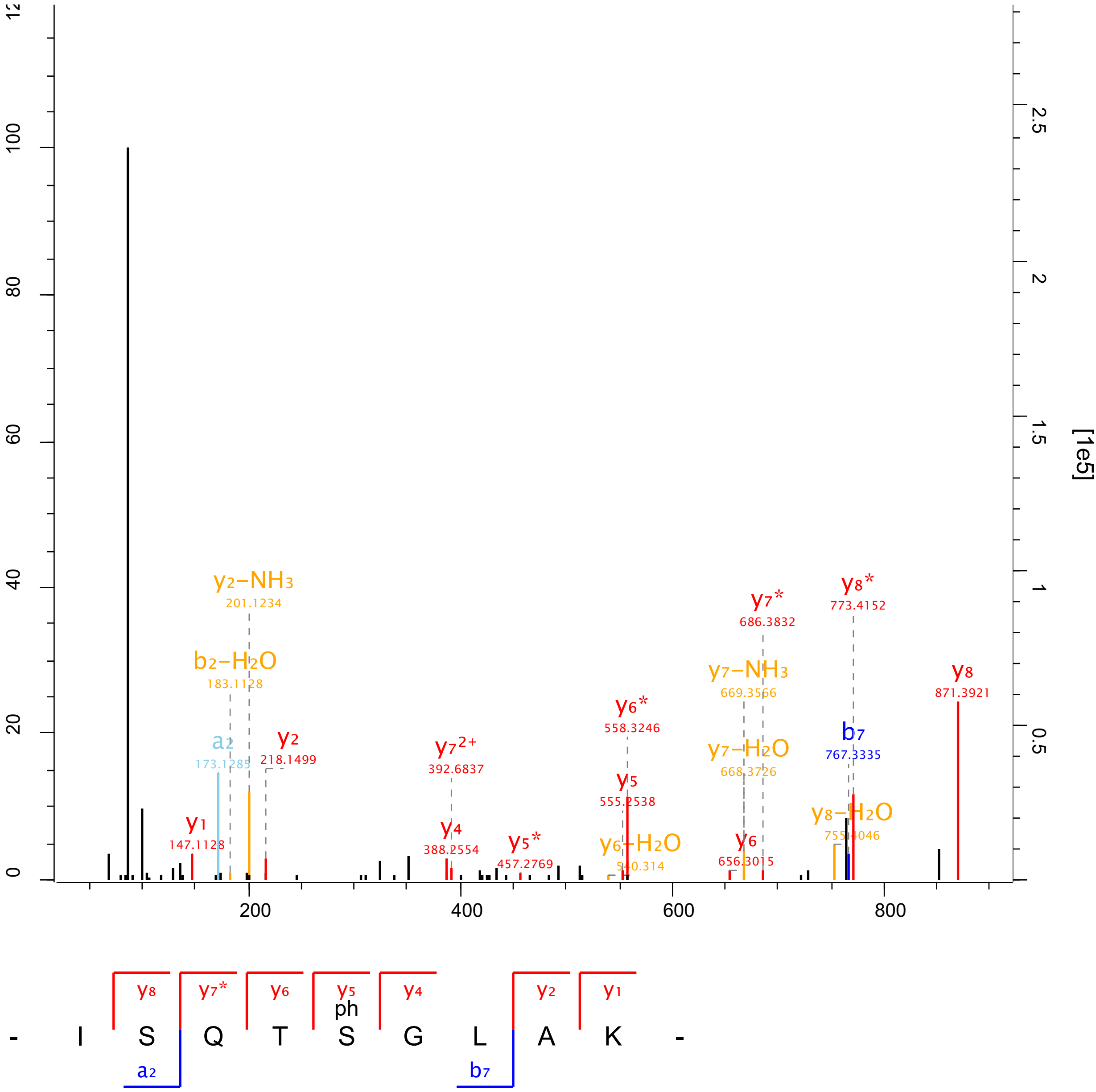

Raw file	Scan	Method	Score	m/z
sys_02_3	7992	FTMS; HCD	78.32	630.78

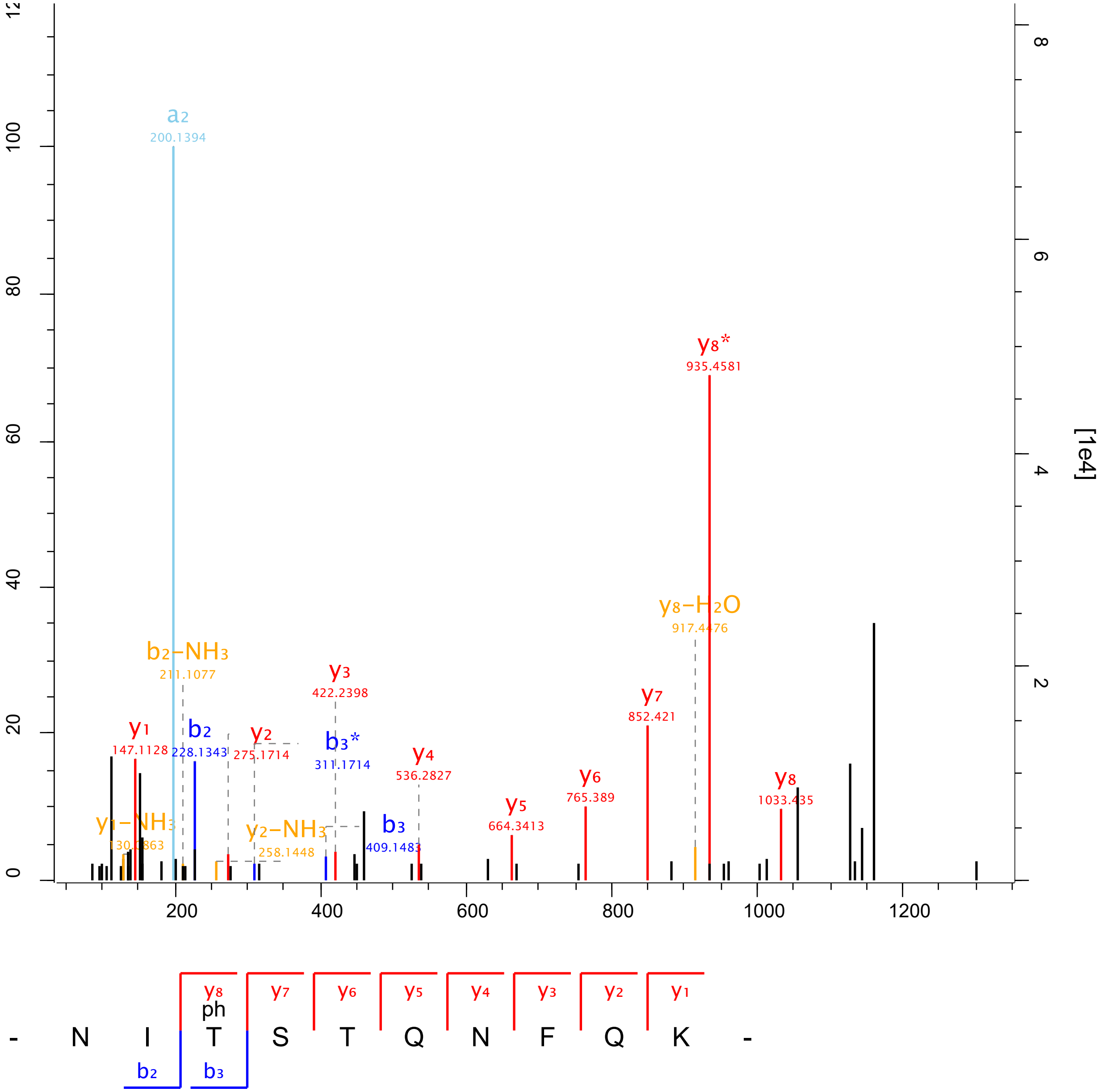

Raw file	Scan	Method	Score	m/z
sys_02_3	8164	FTMS; HCD	60.26	564.23

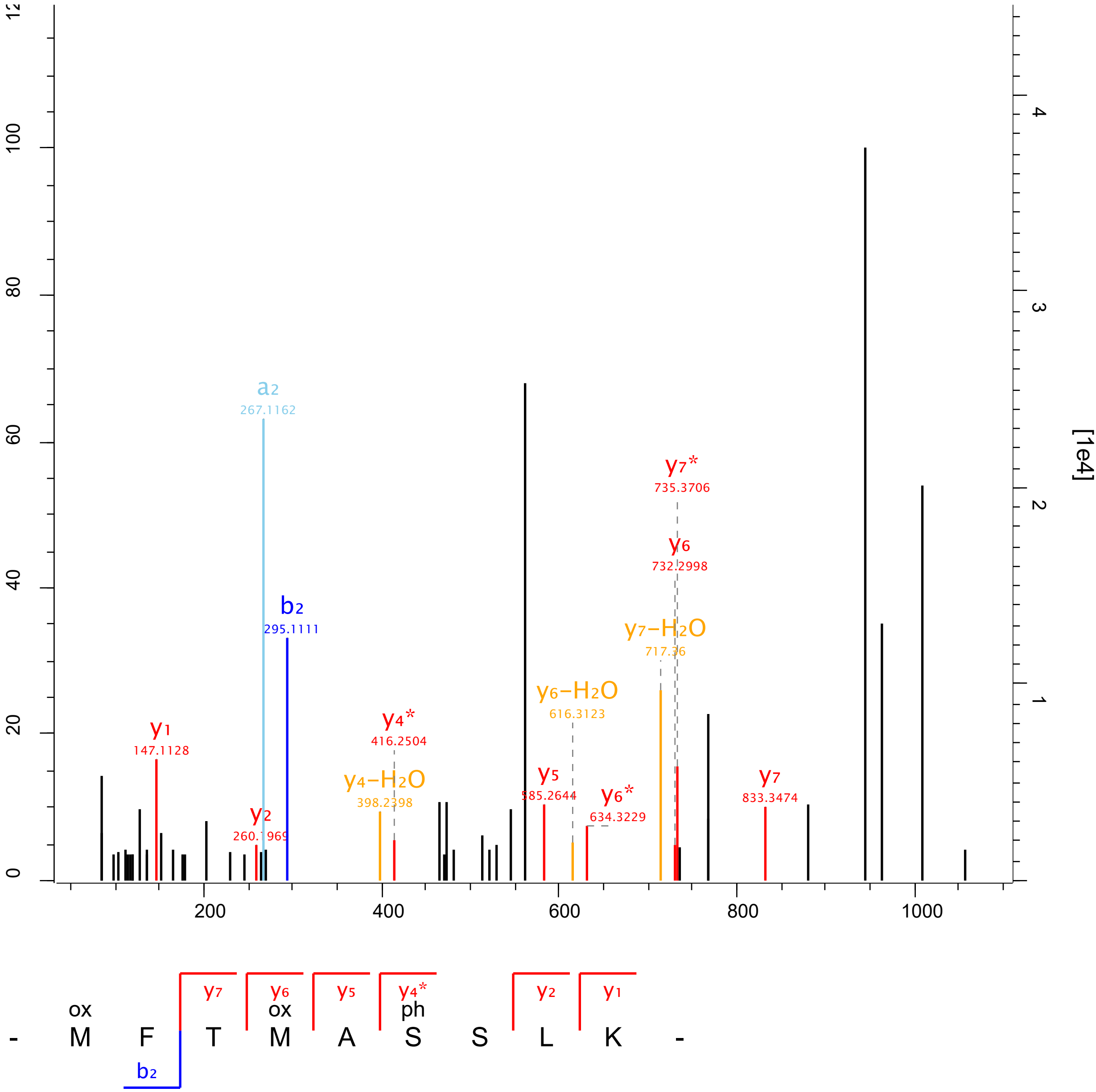

Raw file	Scan	Method	Score	m/z
sys_05_1	13688	FTMS; HCD	136.24	584.22

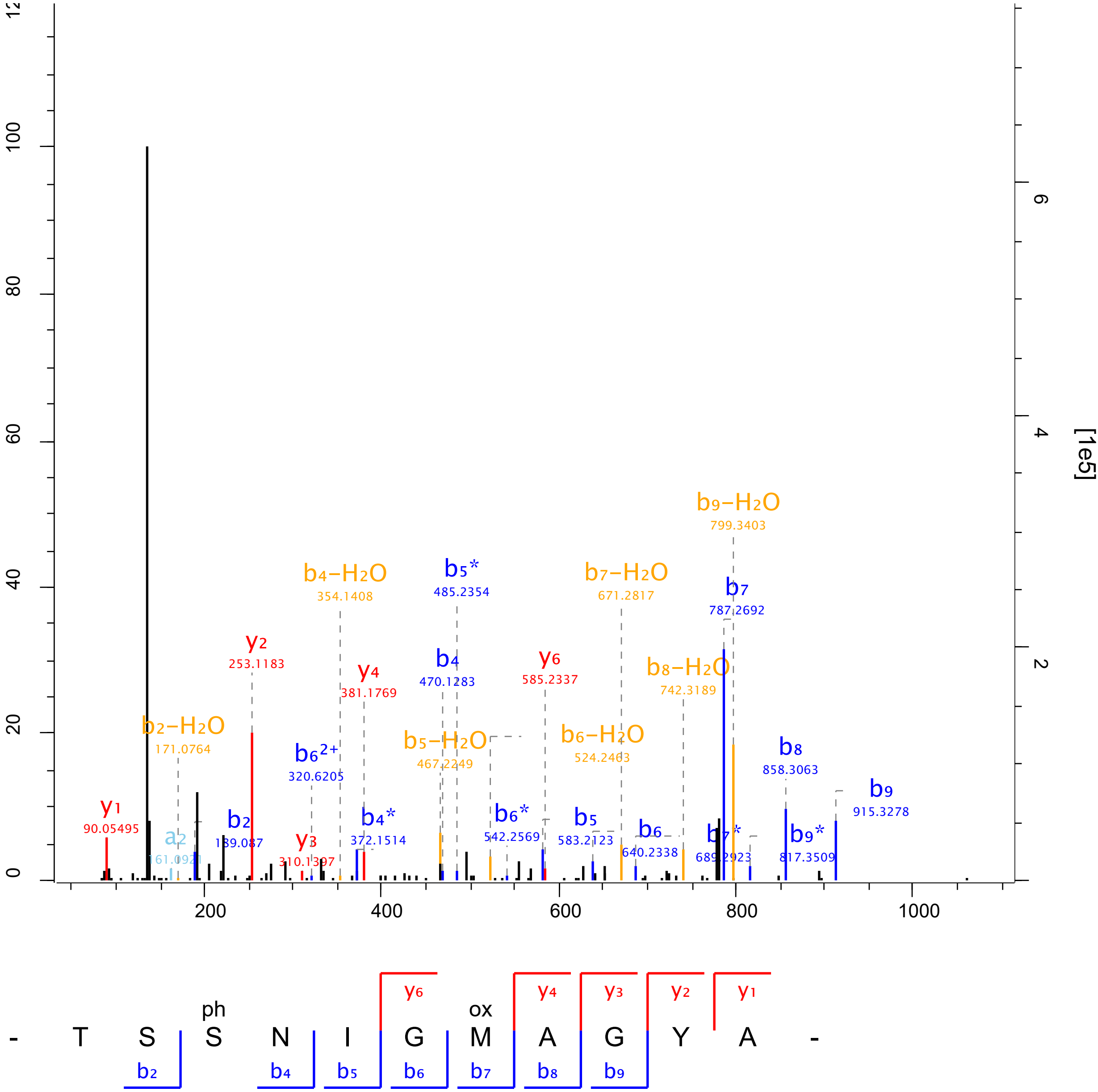

Raw file	Scan	Method	Score	m/z
sys_05_1	21449	FTMS; HCD	50.39	760.82

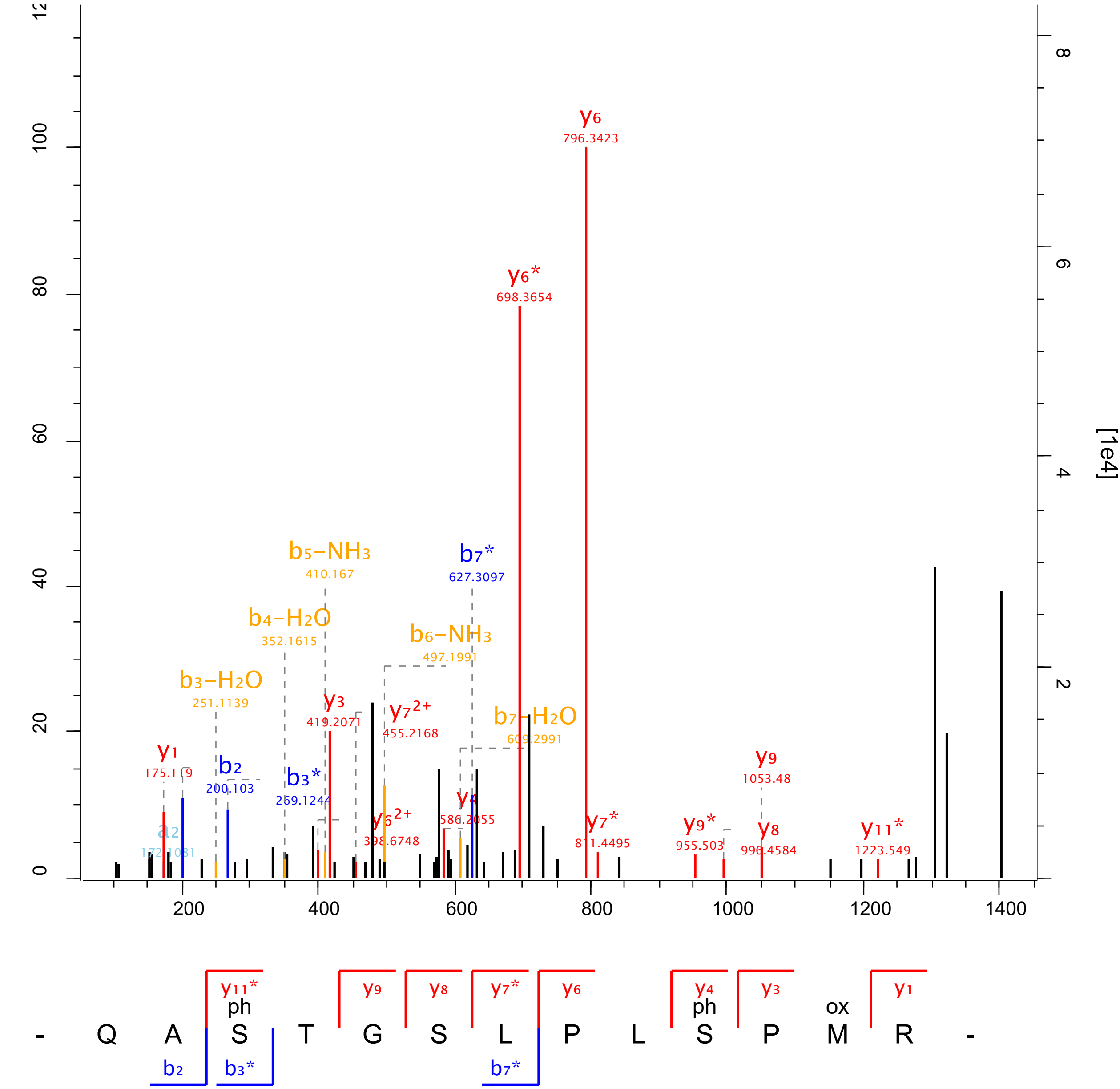

Raw file	Scan	Method	Score	m/z
sys_05_1	23137	FTMS; HCD	89.01	623.95

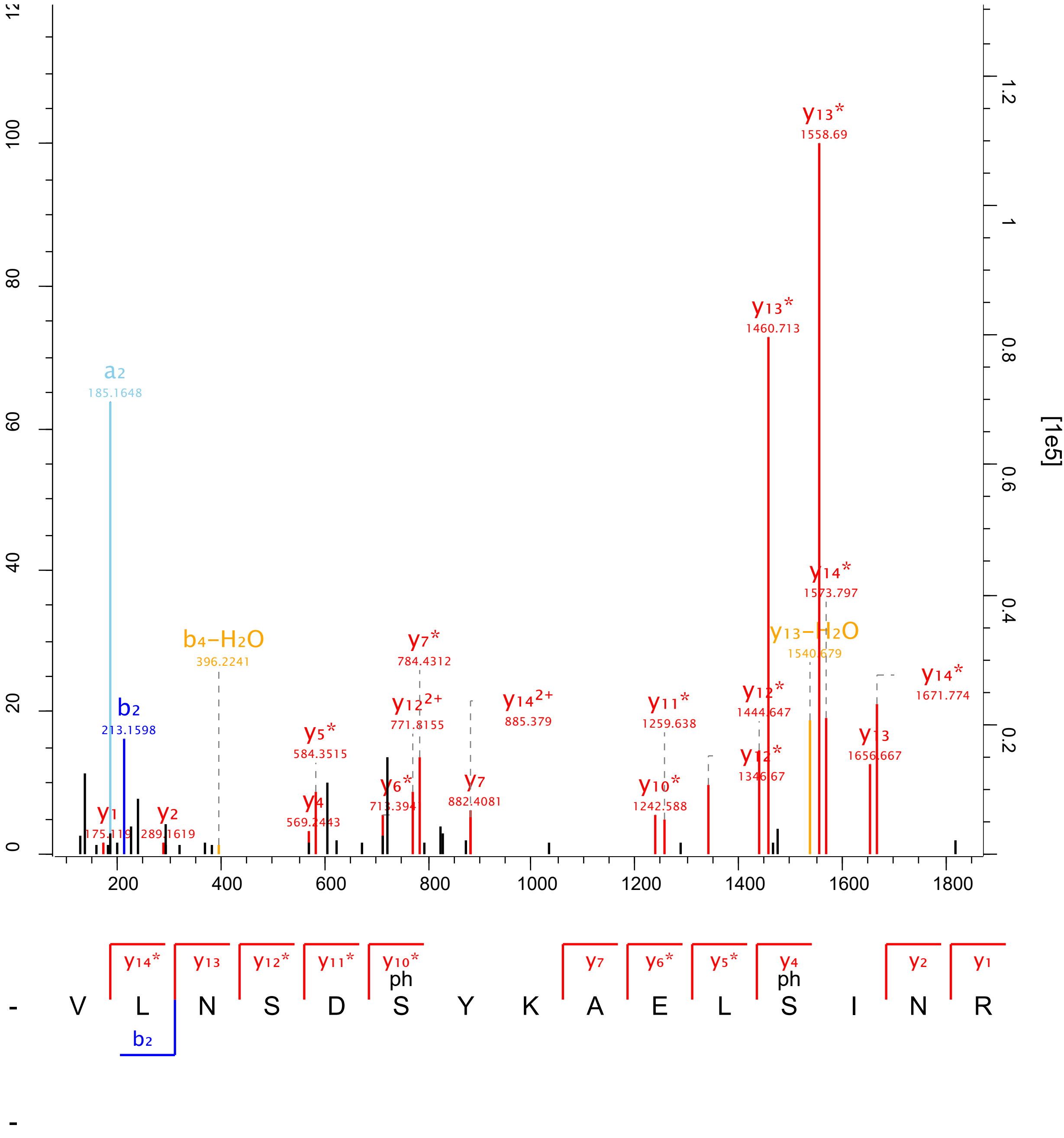

Raw file	Scan	Method	Score	m/z
sys_05_1	24556	FTMS; HCD	77.87	658.33

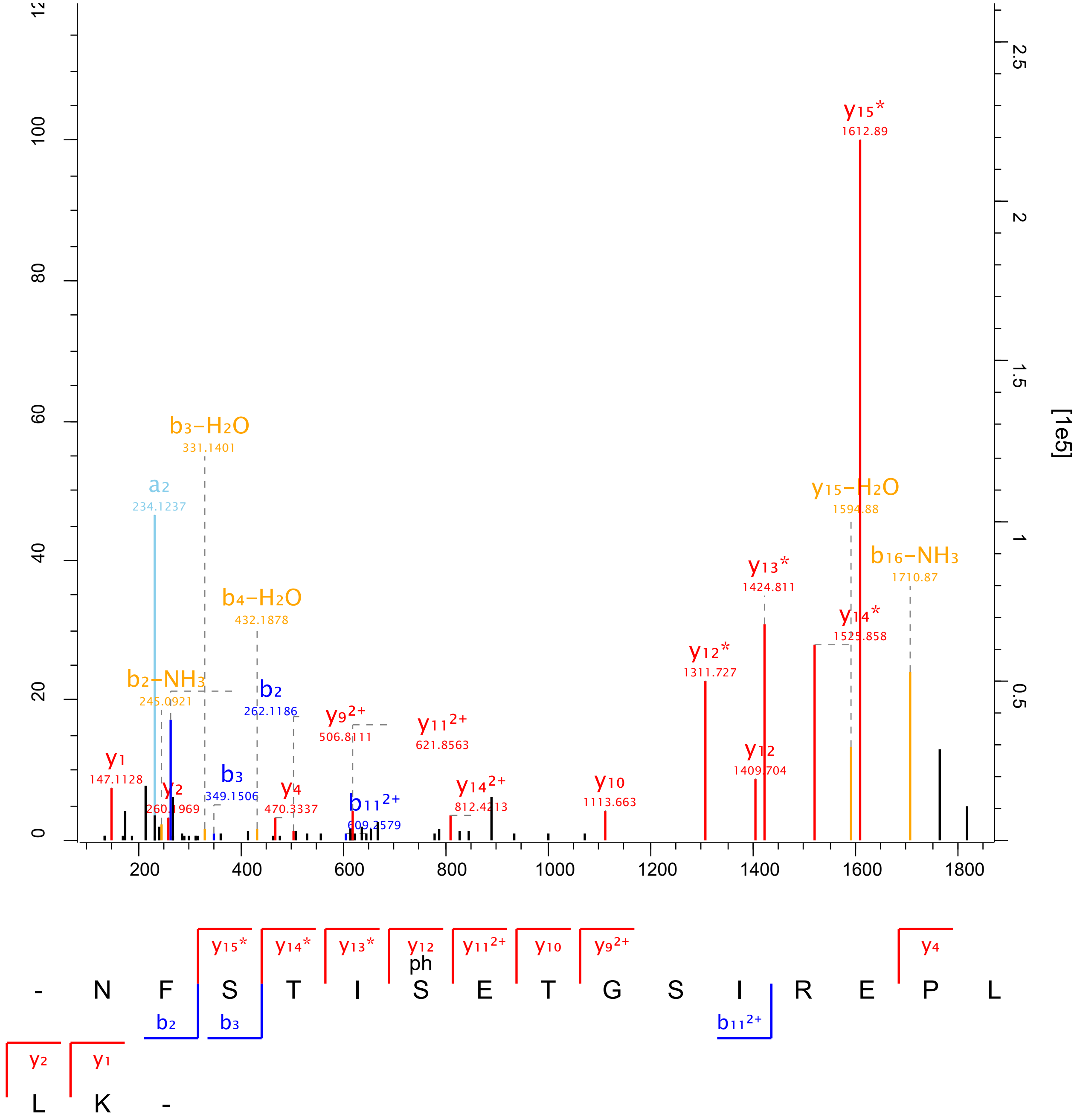

Raw file	Scan	Method	Score	m/z
sys_05_1	2782	FTMS; HCD	75.82	448.21

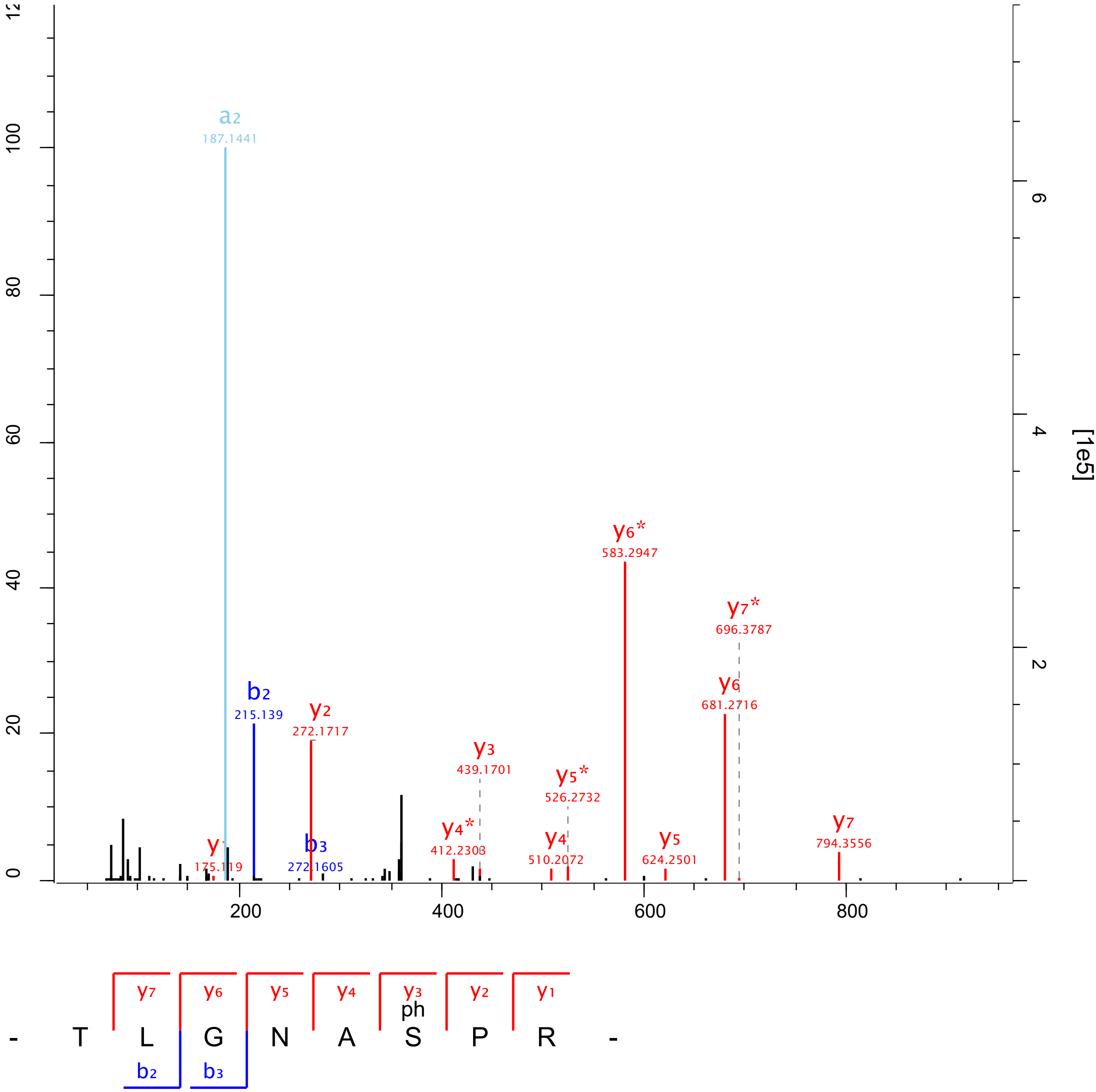

Raw file	Scan	Method	Score	m/z
sys_05_1	30797	FTMS; HCD	78.95	815.87

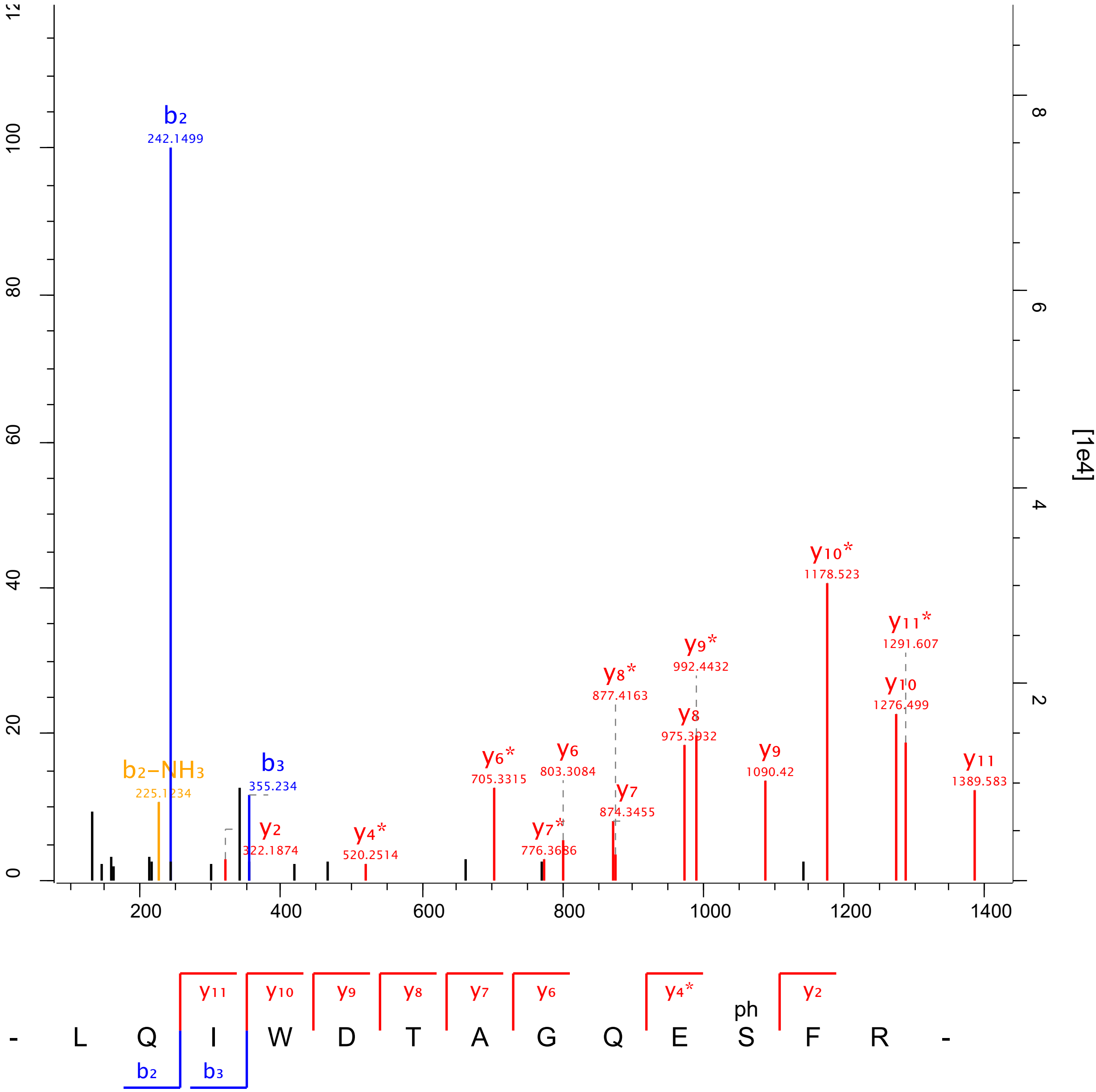

Raw file	Scan	Method	Score	m/z
sys_05_1	31096	FTMS; HCD	130.83	684.65

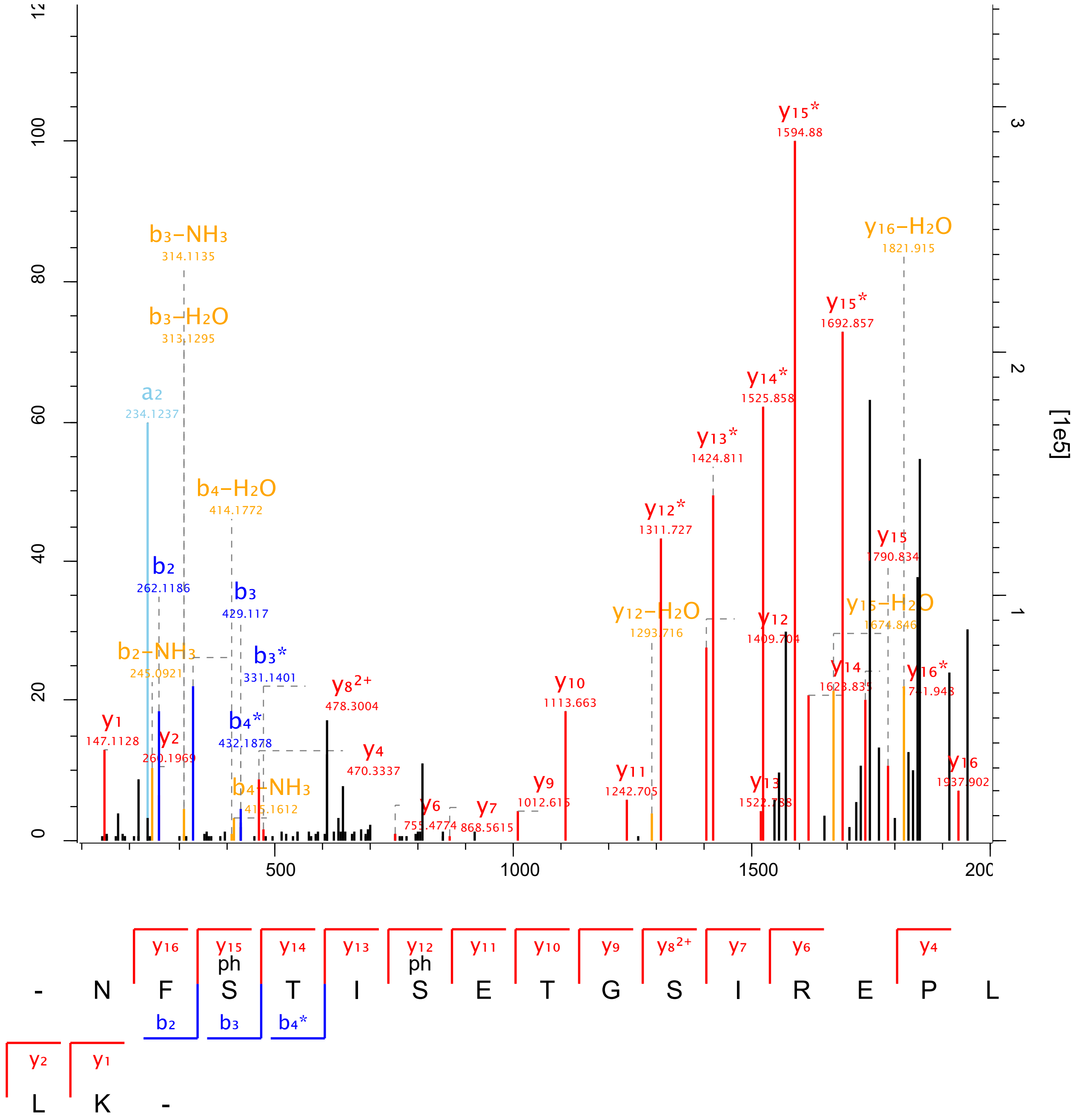

Raw file	Scan	Method	Score	m/z
sys_05_1	32947	FTMS; HCD	48.29	814.36

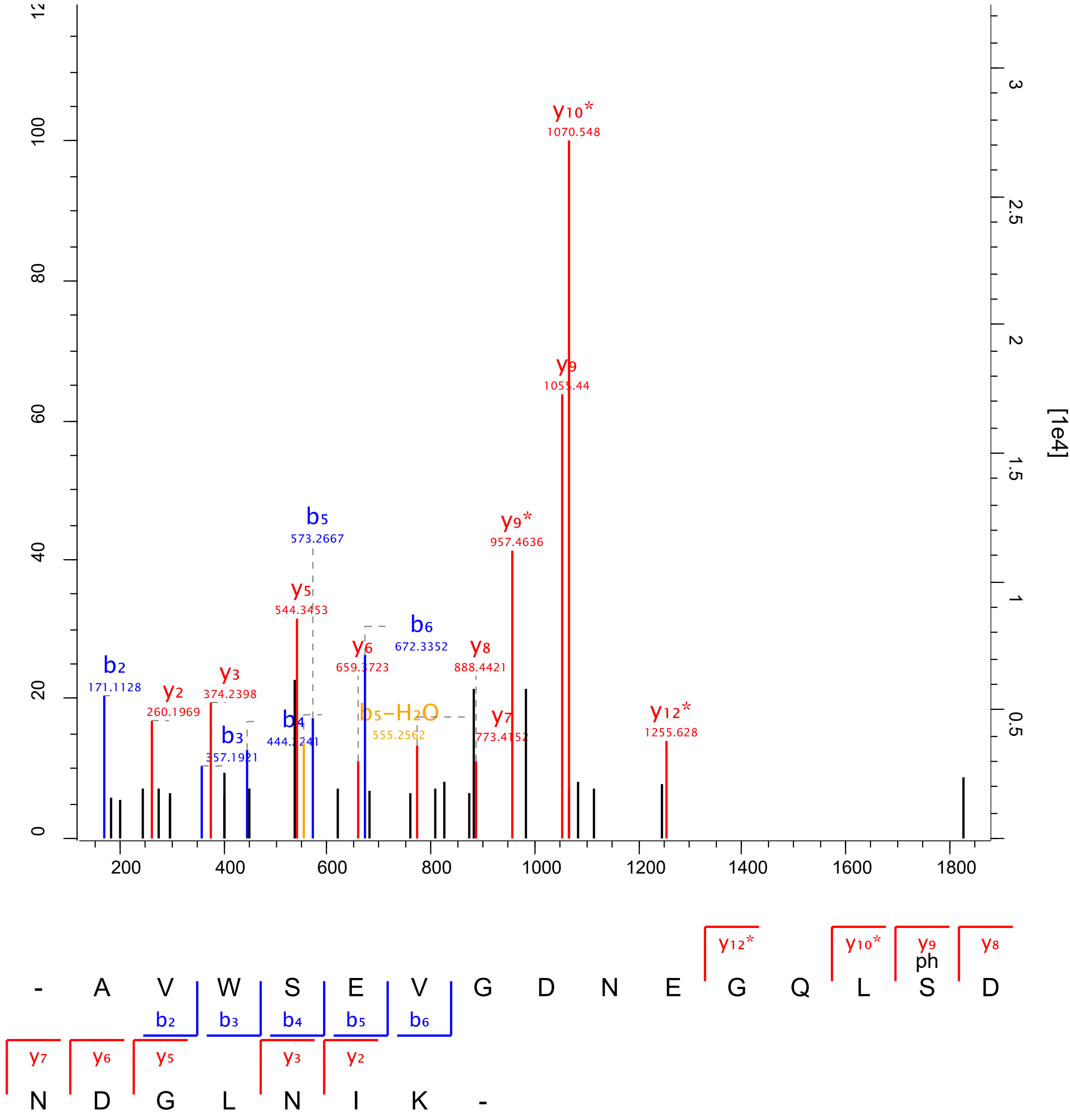

Raw file	Scan	Method	Score	m/z
sys_05_1	3477	FTMS; HCD	113.38	485.7

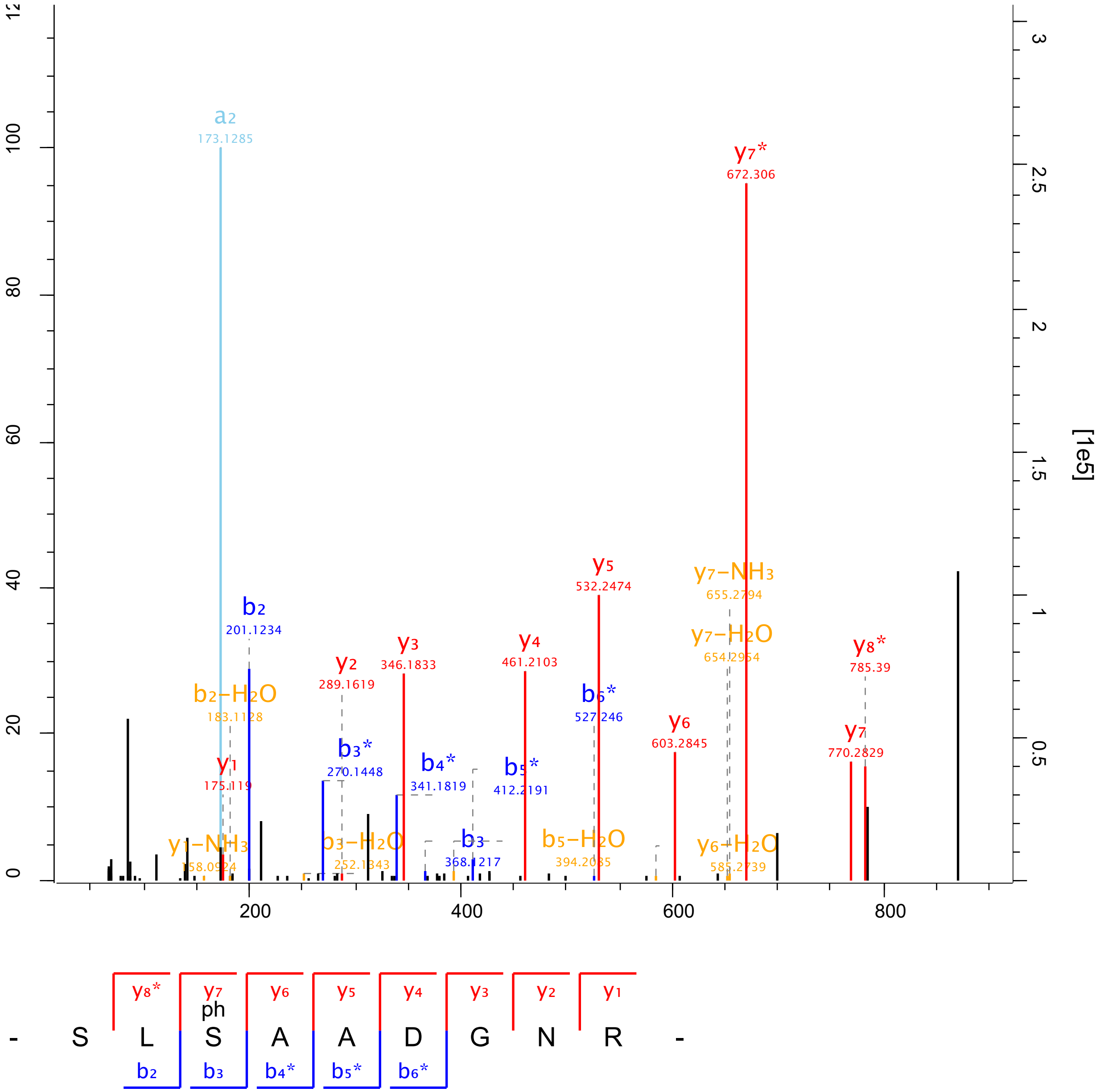

Raw file	Scan	Method	Score	m/z
sys_05_1	39450	FTMS; HCD	146.78	736.8

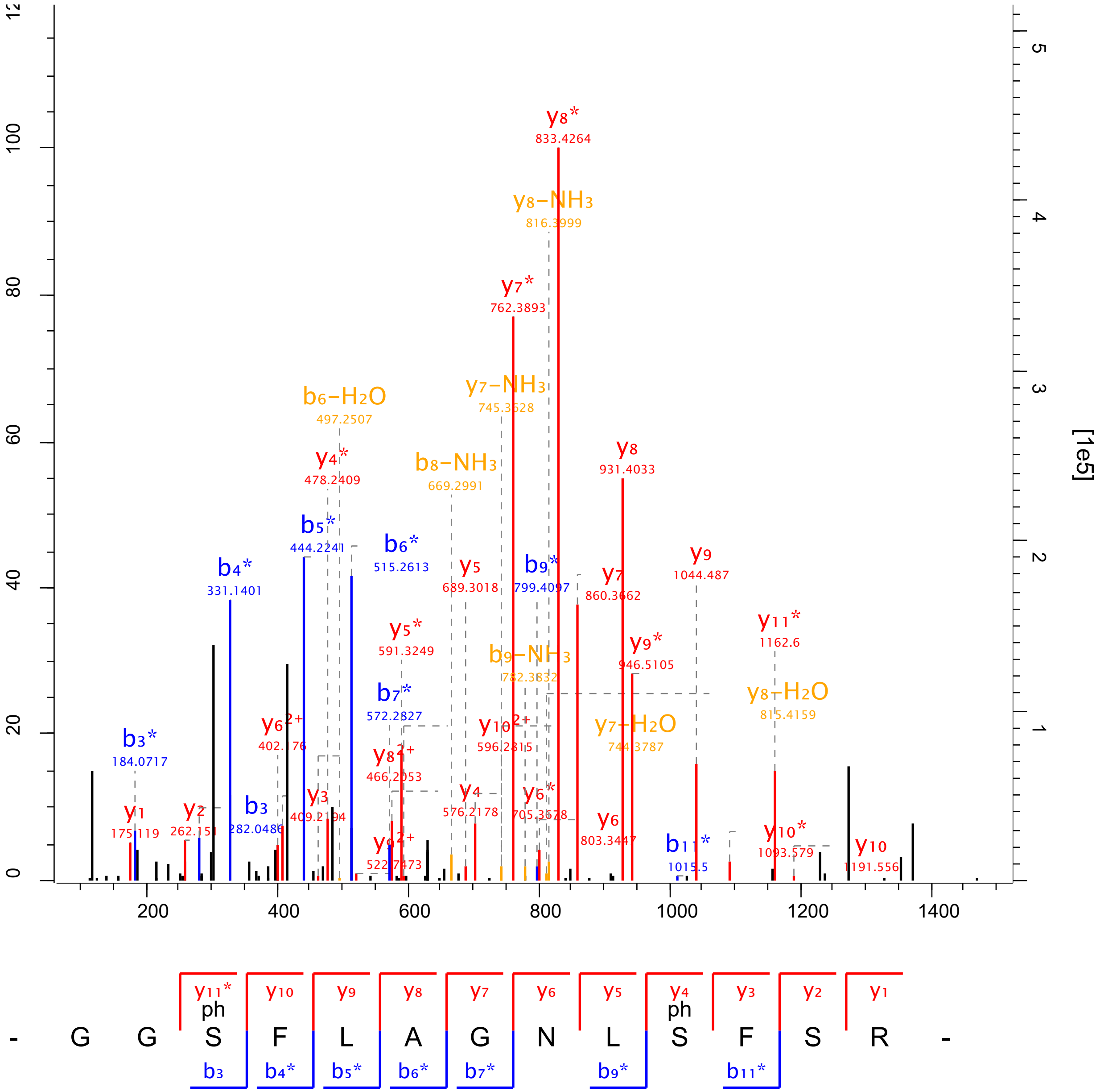

Raw file	Scan	Method	Score	m/z
sys_05_1	3997	FTMS; HCD	107.11	461.7

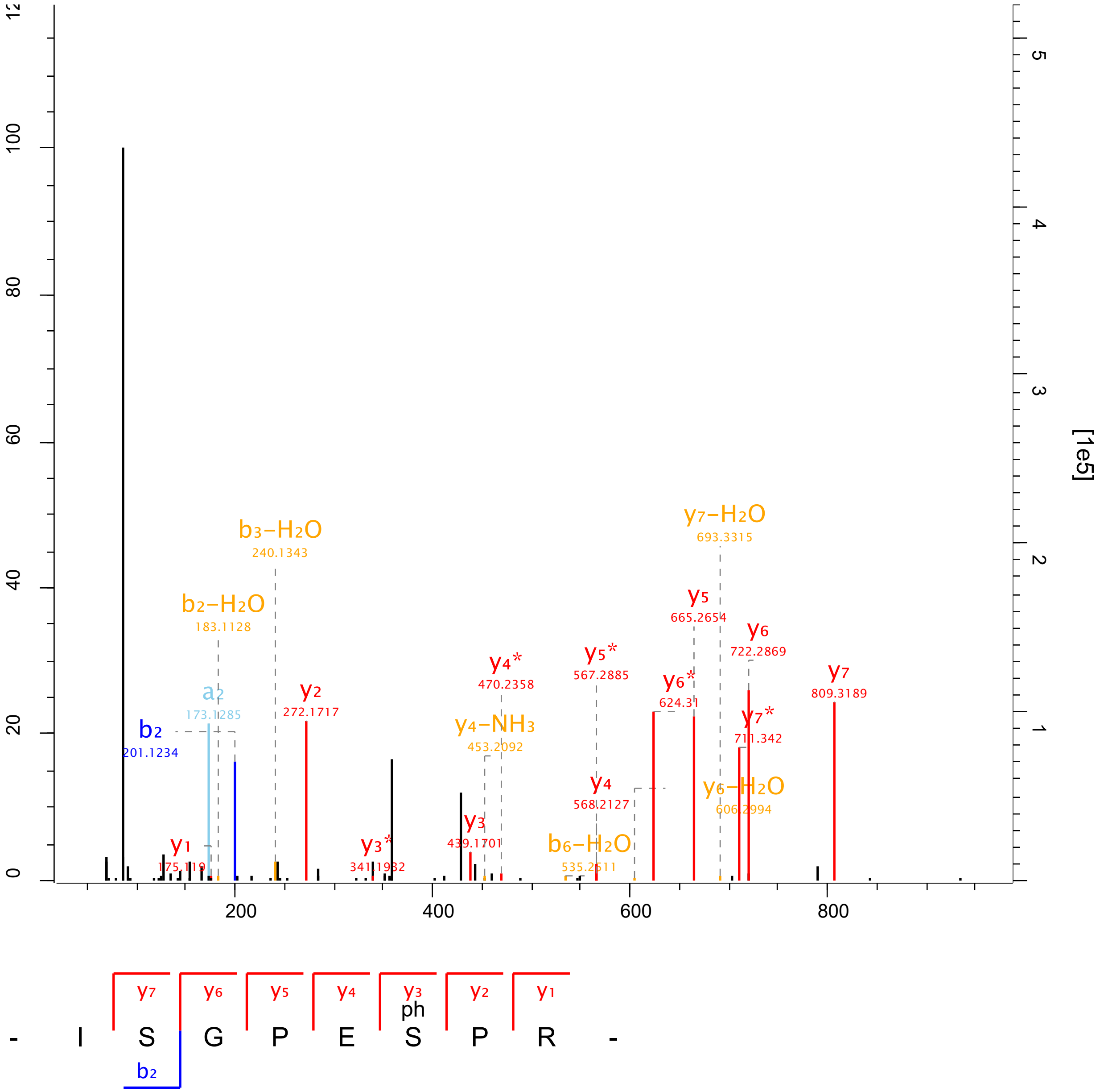

Raw file	Scan	Method	Score	m/z
sys_05_1	40953	FTMS; HCD	77.75	725.34

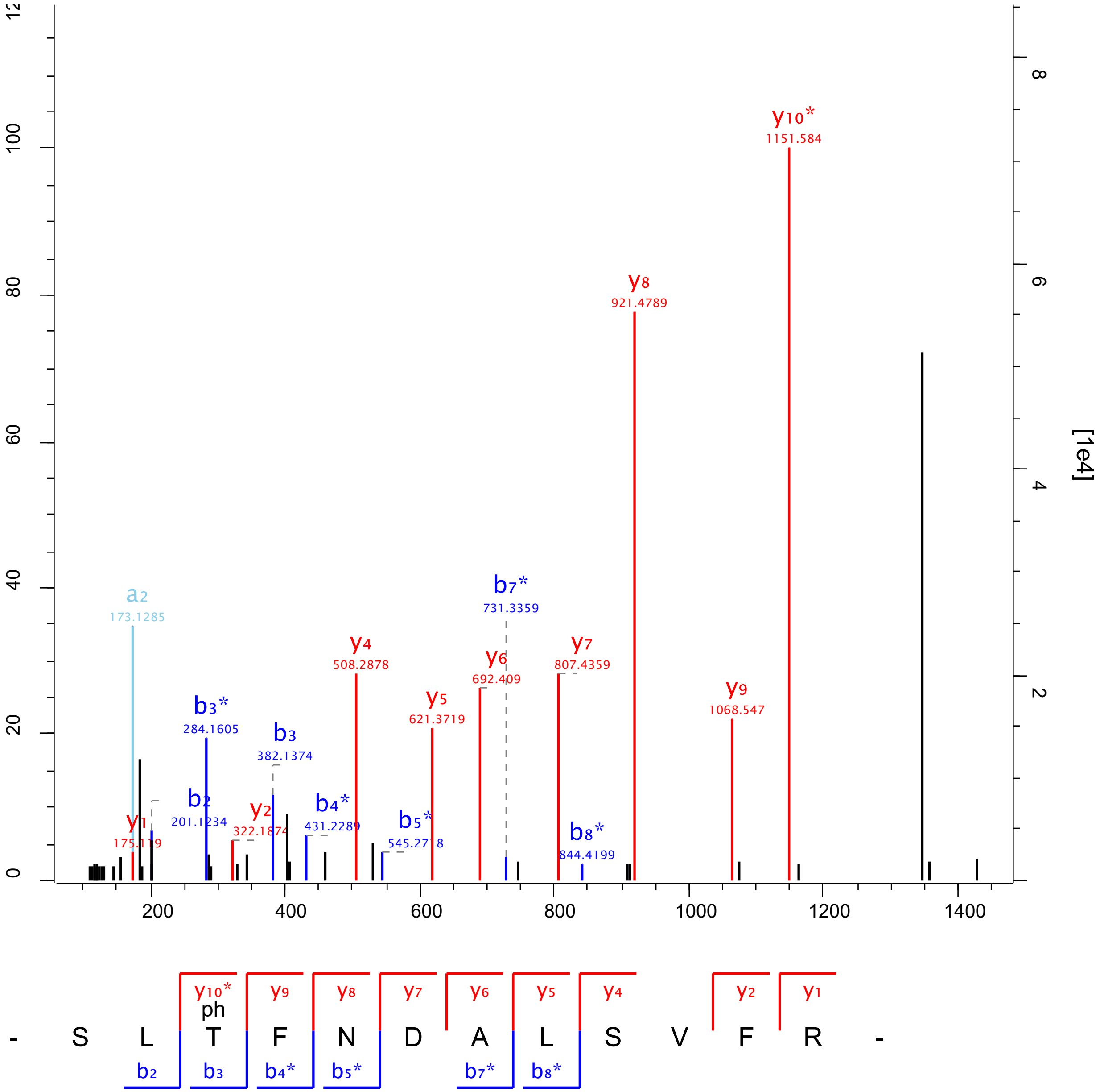

Raw file	Scan	Method	Score	m/z
sys_05_1	42484	FTMS; HCD	138.01	665.3

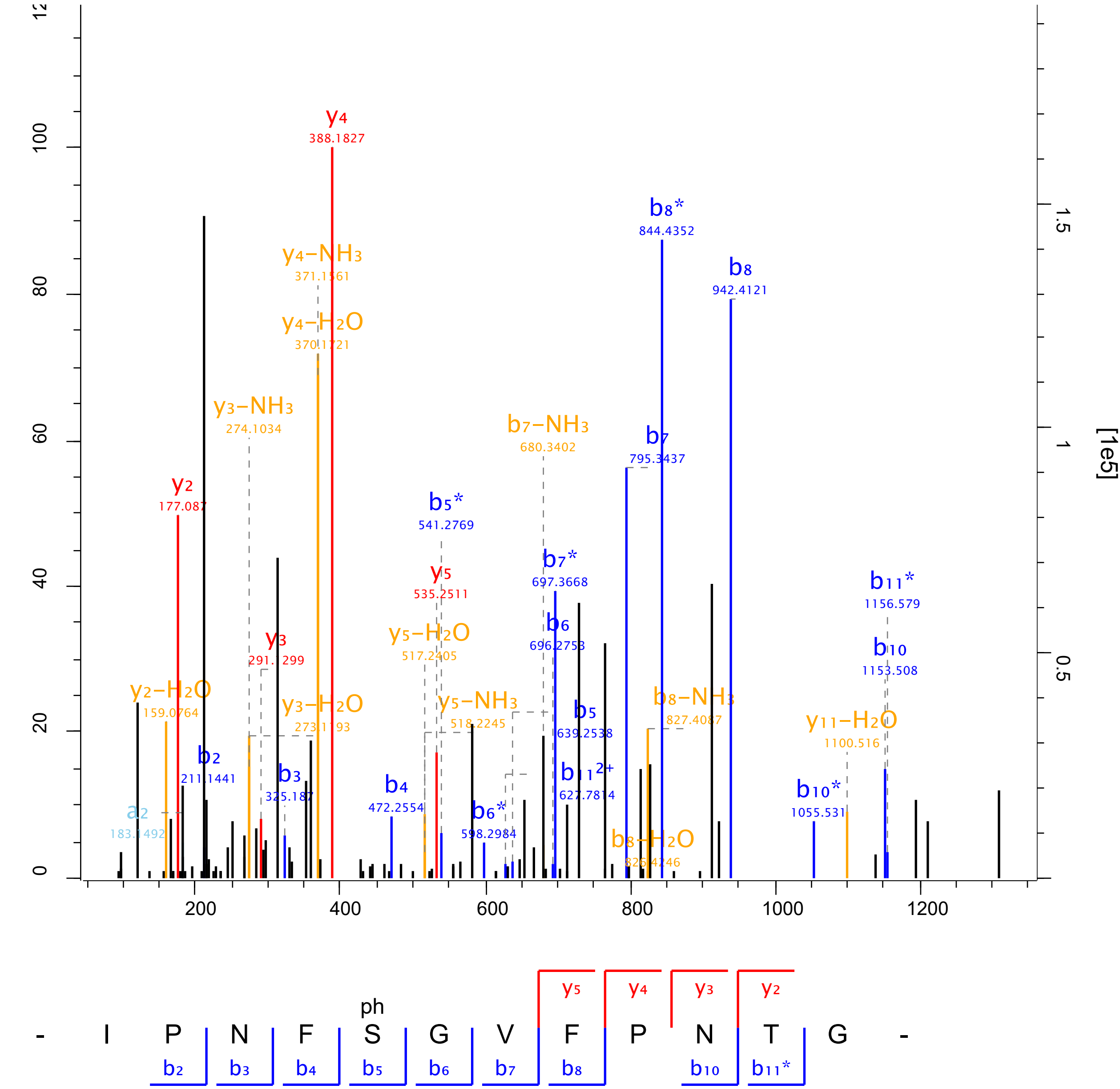

Raw file	Scan	Method	Score	m/z
sys_05_1	43681	FTMS; HCD	75.91	699.81

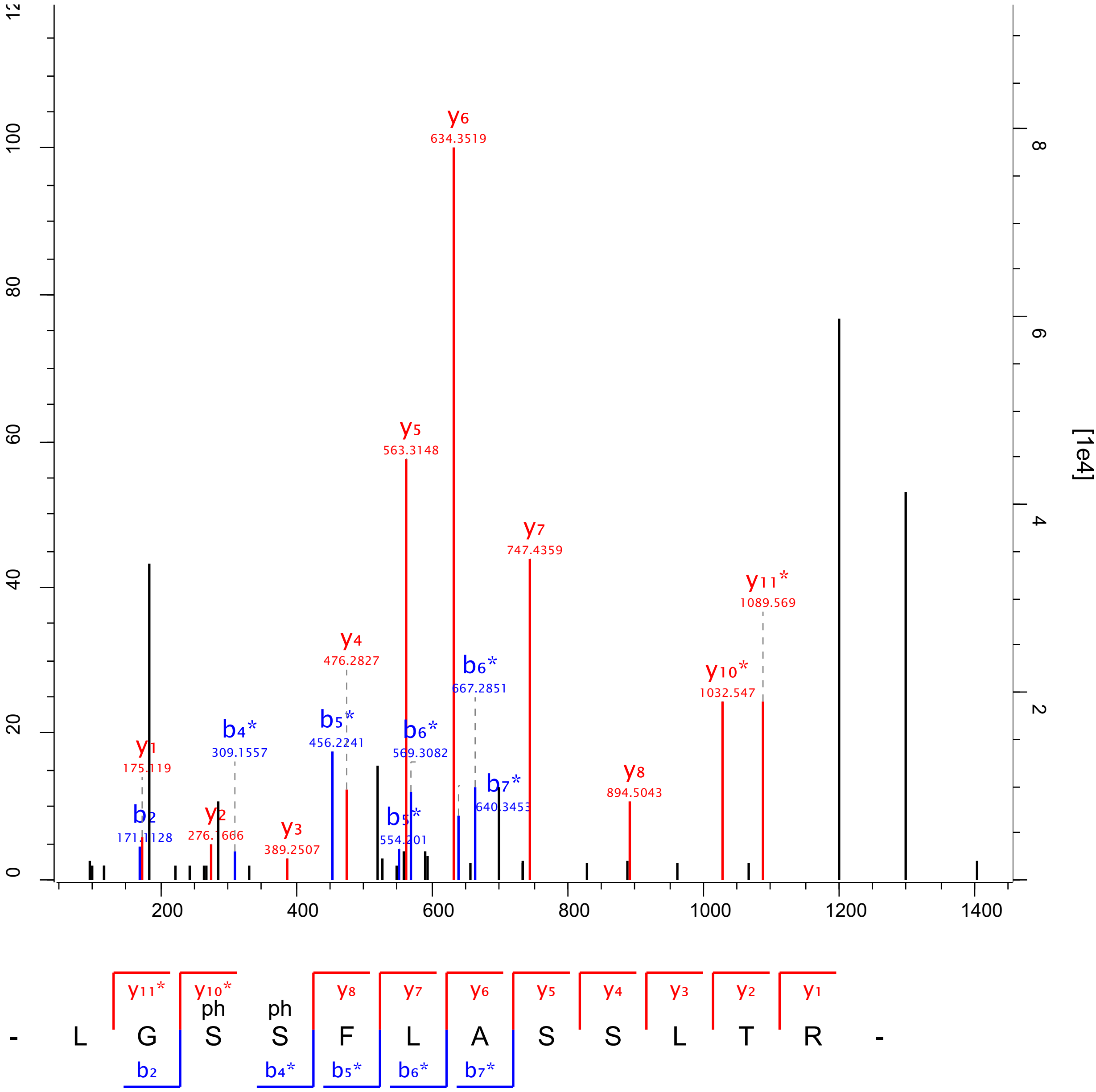

Raw file	Scan	Method	Score	m/z
sys_05_1	44181	FTMS; HCD	45.94	1008.8

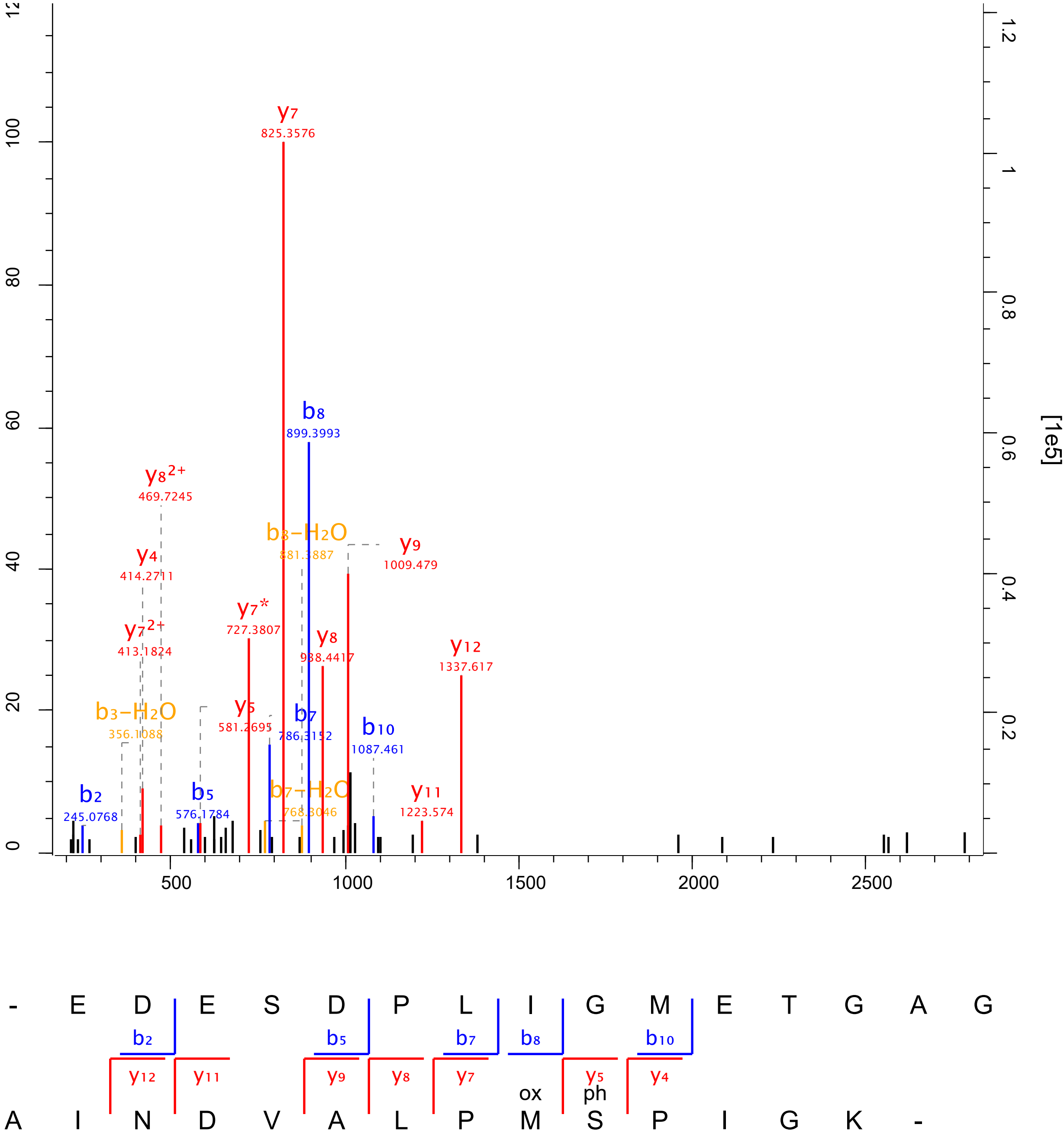

Raw file	Scan	Method	Score	m/z
sys_05_1	45065	FTMS; HCD	58.43	827.38

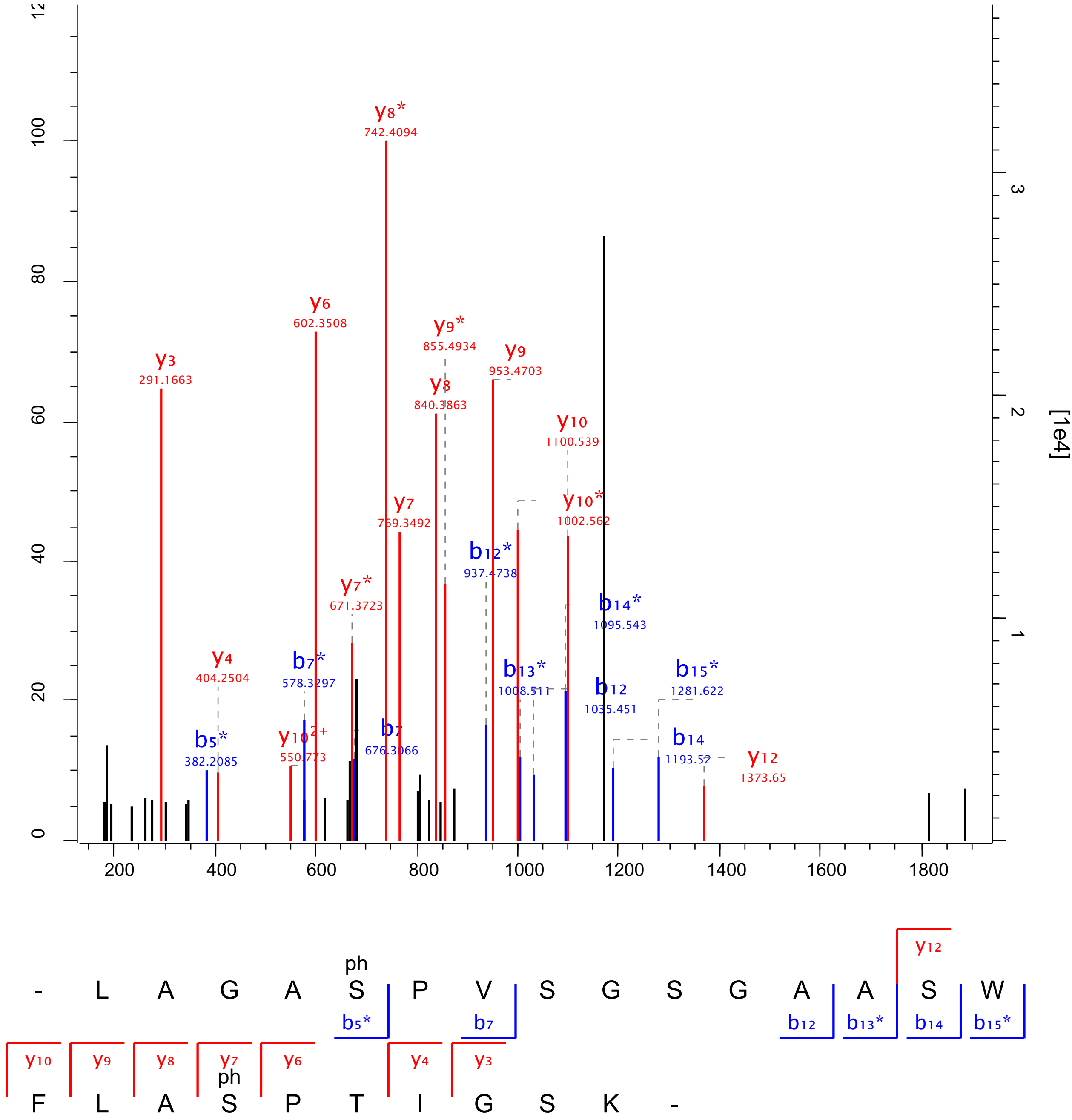

Raw file	Scan	Method	Score	m/z
sys_05_1	45327	FTMS; HCD	181.58	691.78

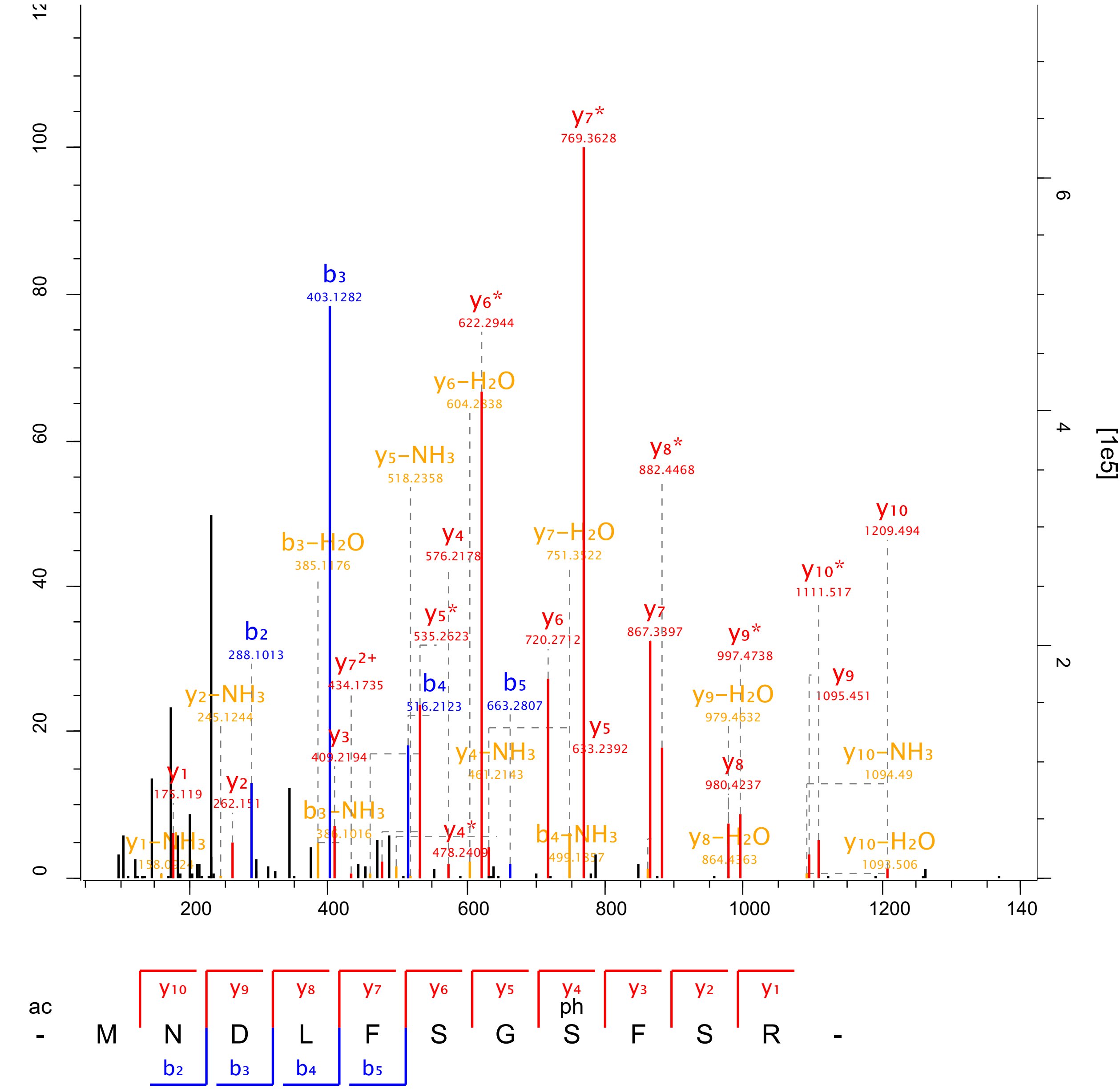

Raw file	Scan	Method	Score	m/z
sys_05_1	45334	FTMS; HCD	127.32	917.86

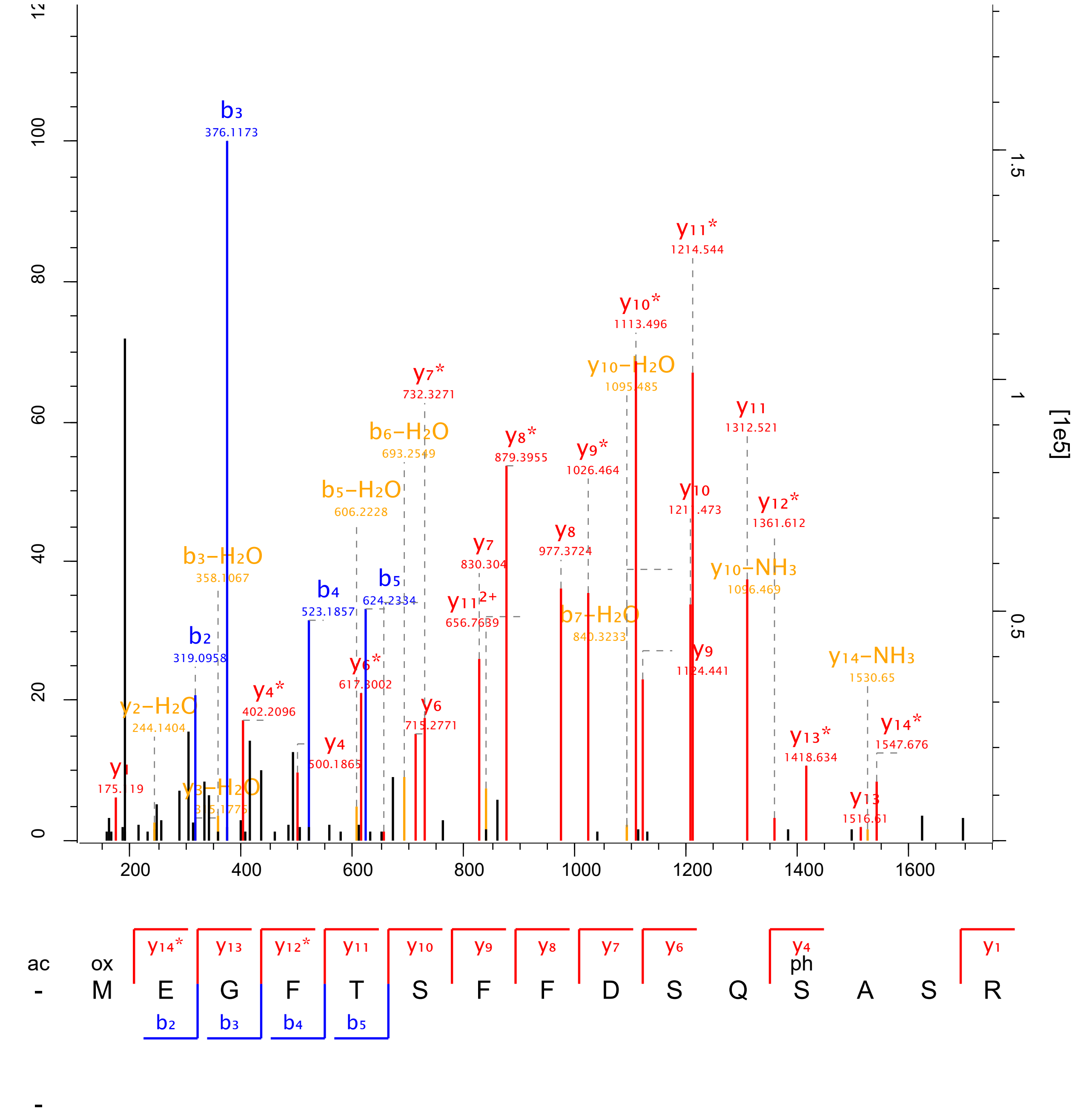

Raw file	Scan	Method	Score	m/z
sys_05_1	7339	FTMS; HCD	92.54	427.71

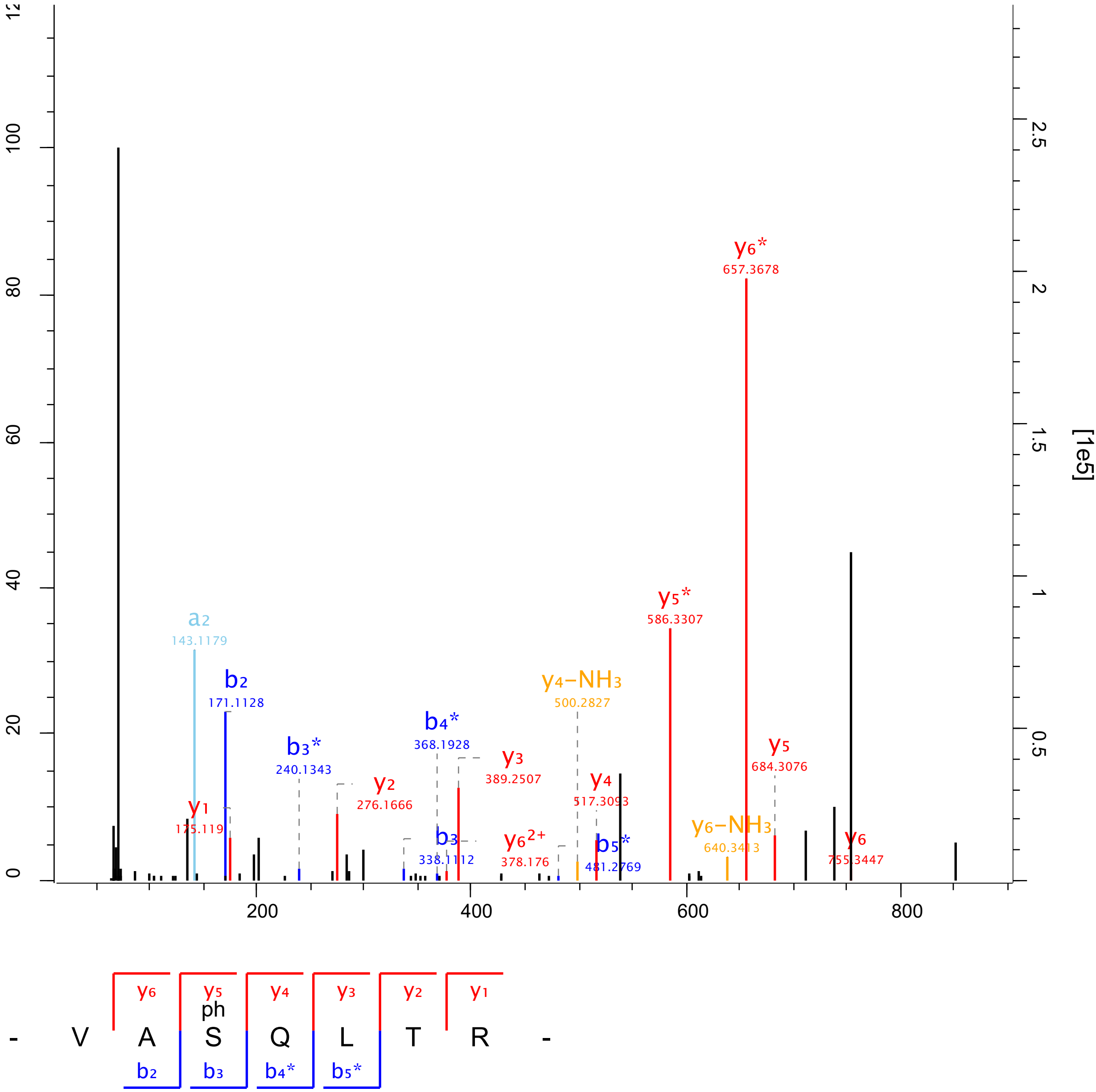

Raw file	Scan	Method	Score	m/z
sys_05_1	7348	FTMS; HCD	64.1	440.88

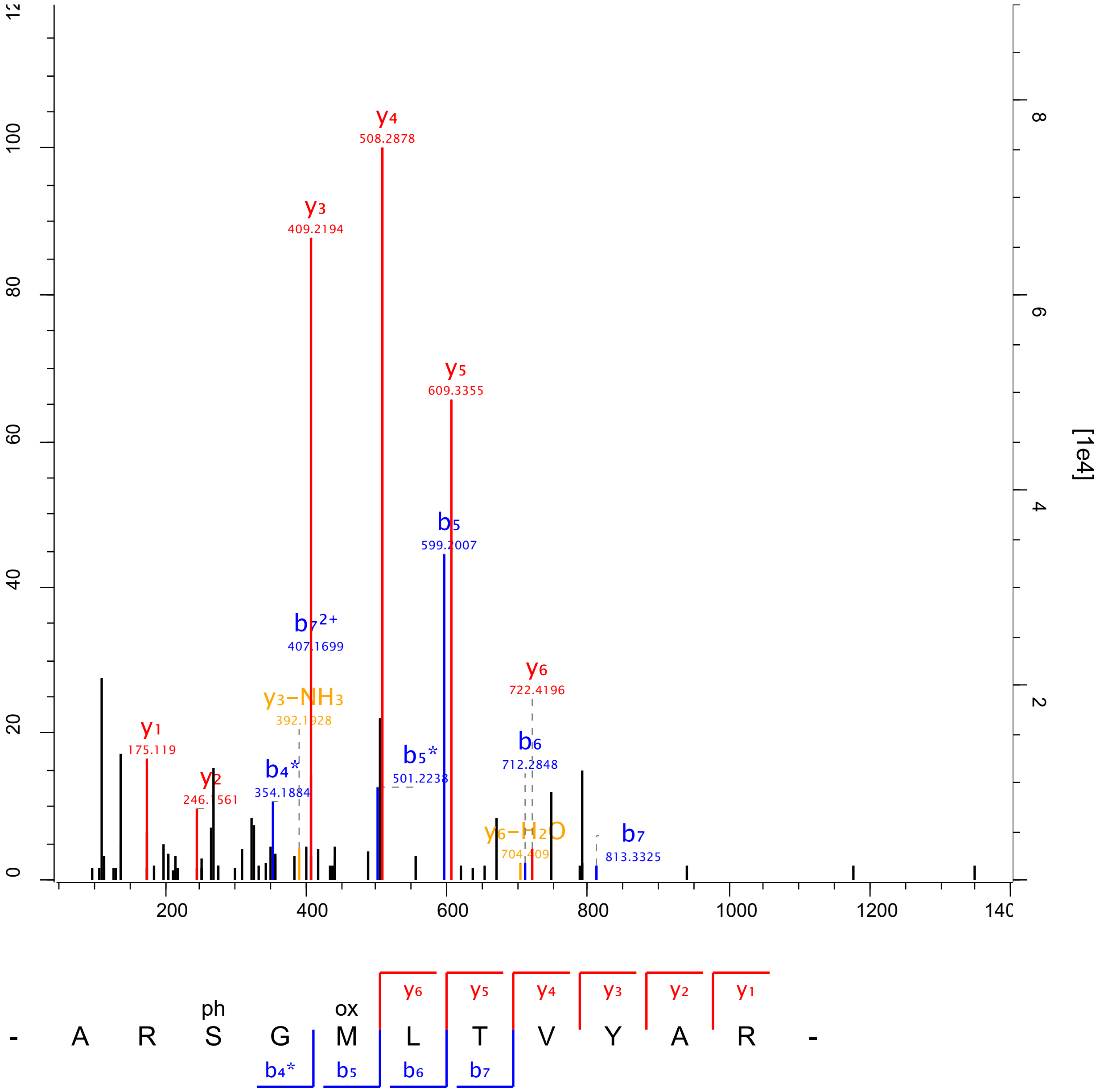

Raw file	Scan	Method	Score	m/z
sys_05_1	8039	FTMS; HCD	174.44	633.74

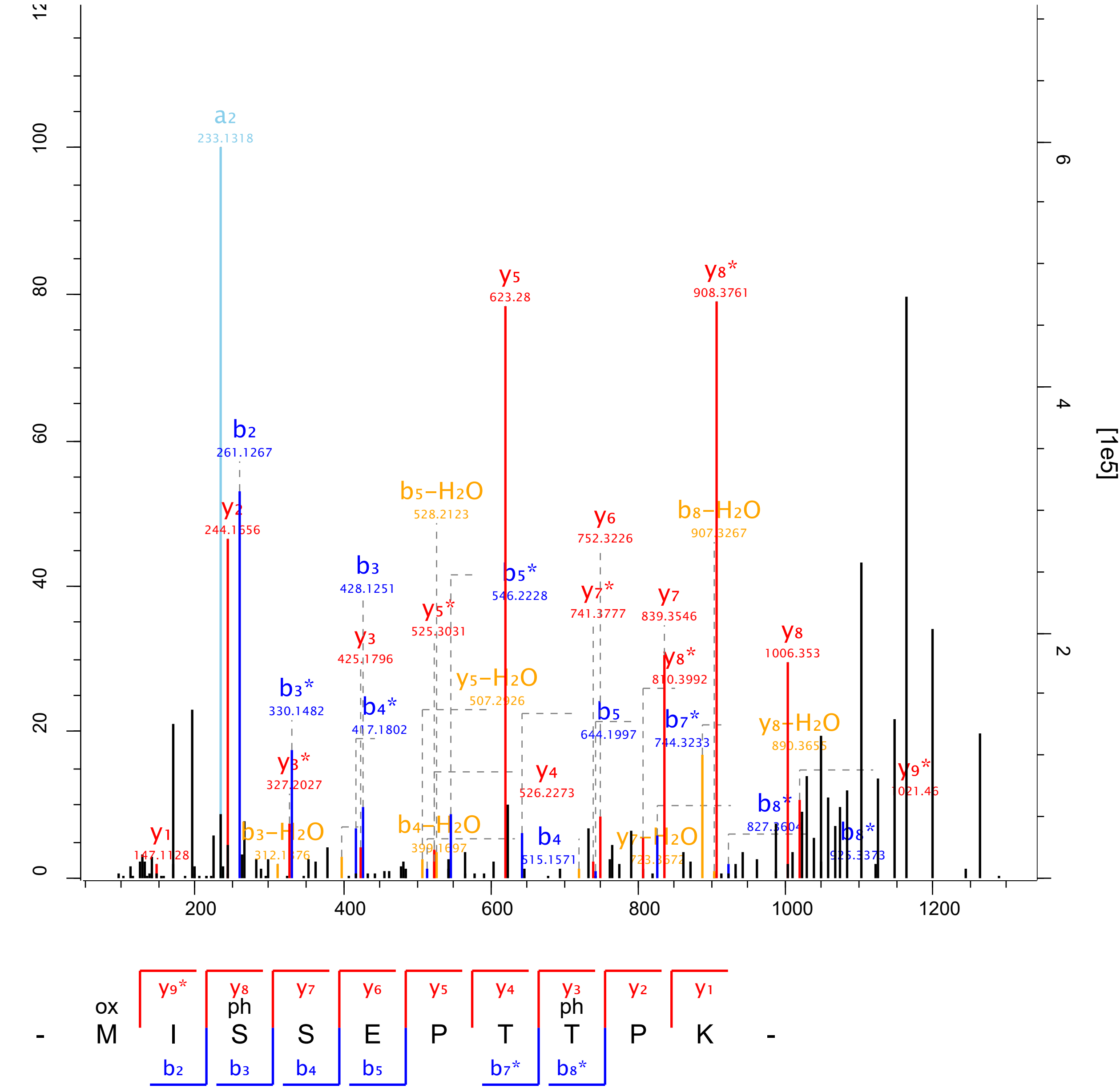

Raw file	Scan	Method	Score	m/z
sys_05_2	10087	FTMS; HCD	74.99	556.75

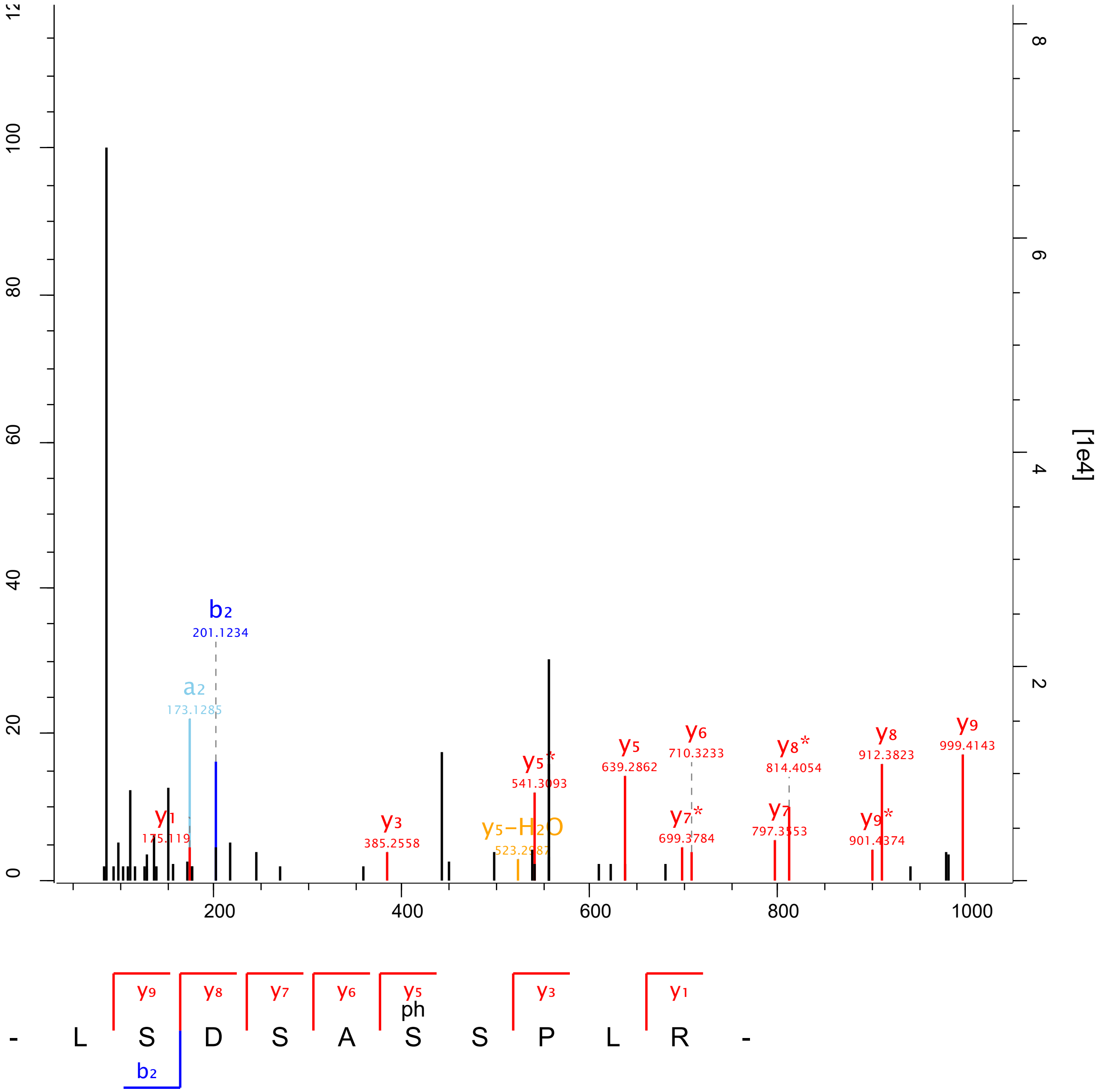

Raw file	Scan	Method	Score	m/z
sys_05_2	10115	FTMS; HCD	114.97	428.7

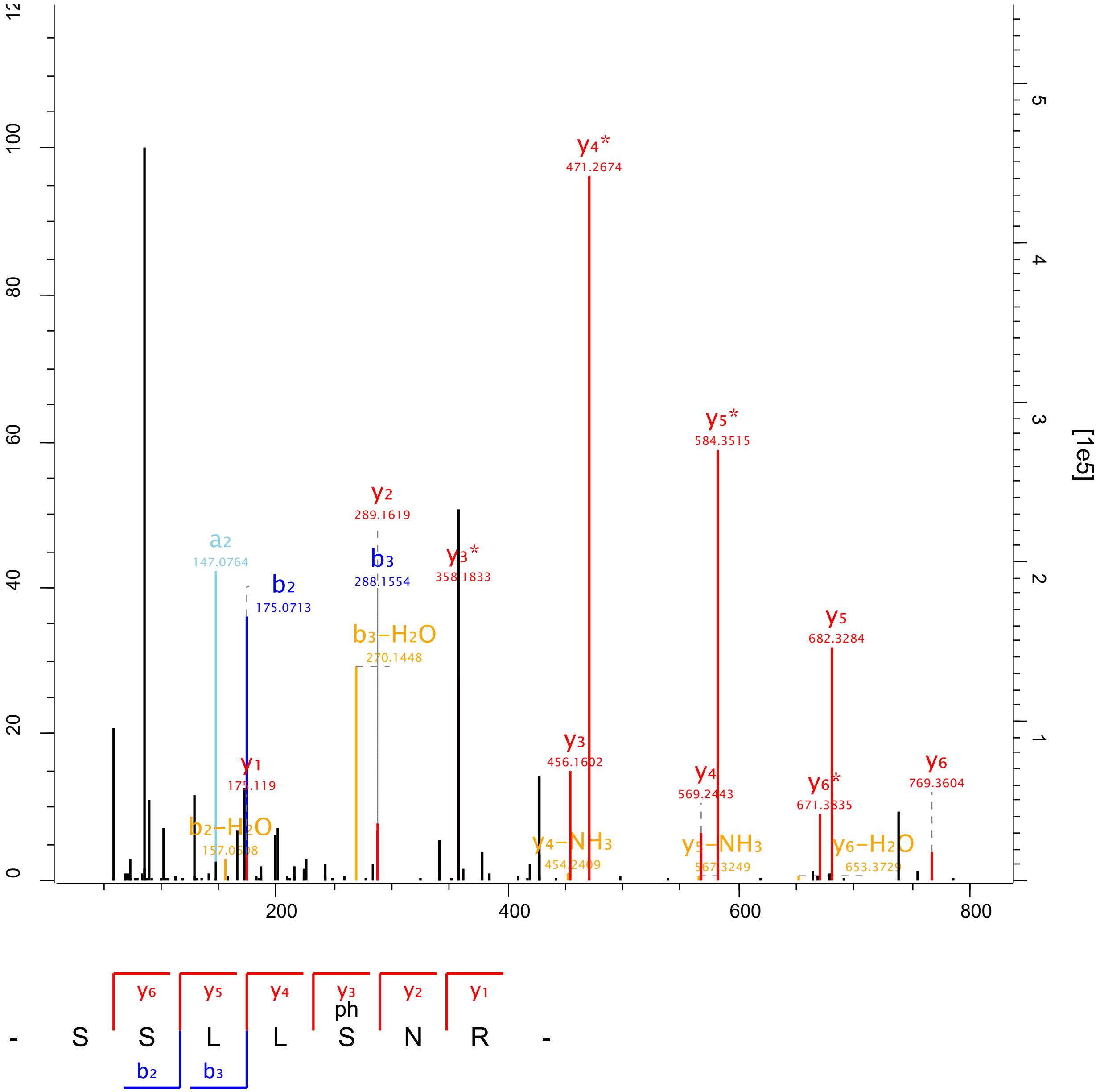

Raw file	Scan	Method	Score	m/z
sys_05_2	10137	FTMS; HCD	81.57	628.27

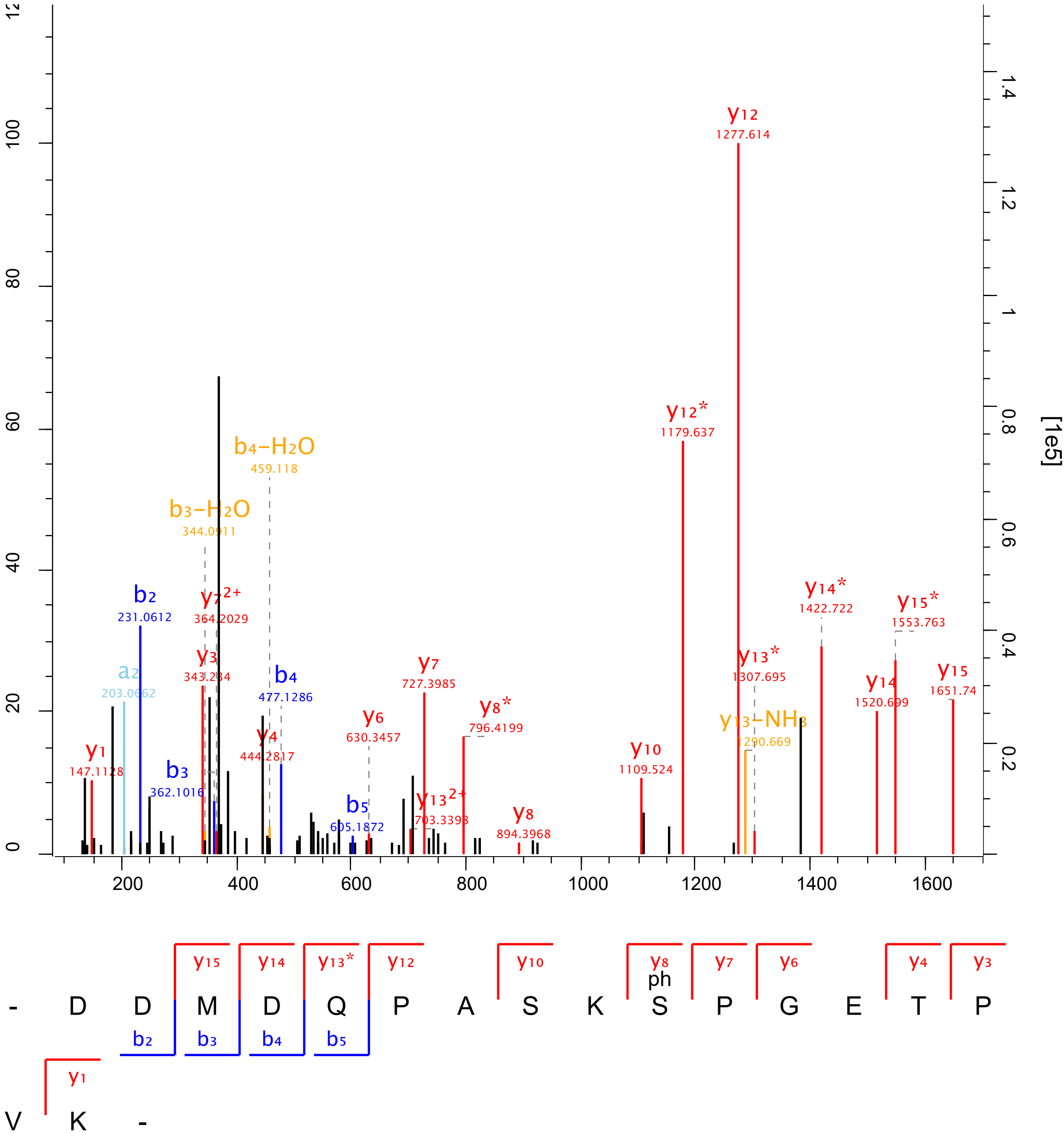

Raw file	Scan	Method	Score	m/z
sys_05_2	10424	FTMS; HCD	96.11	427.72

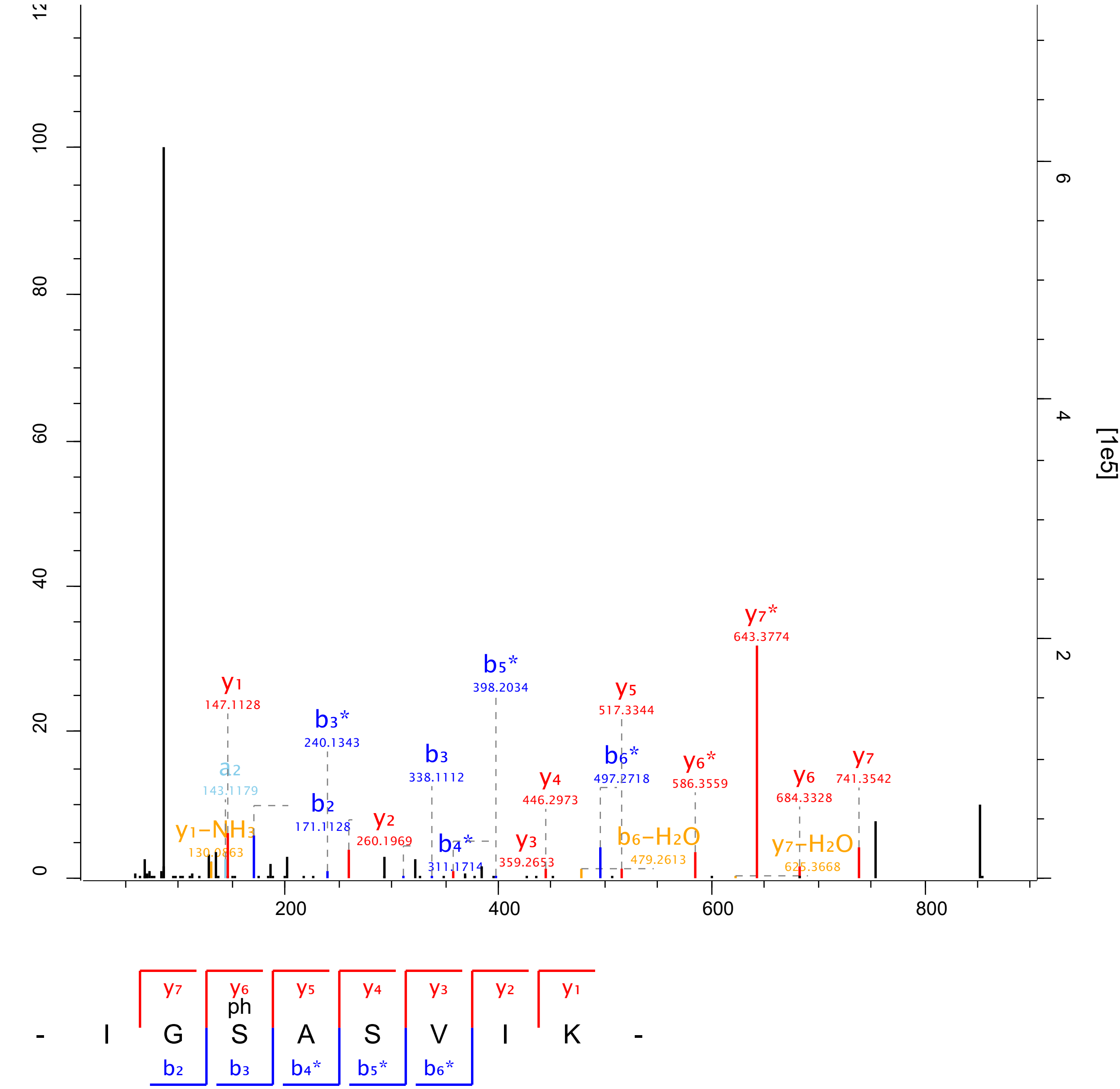

Raw file	Scan	Method	Score	m/z
sys_05_2	10455	FTMS; HCD	189.33	671.79

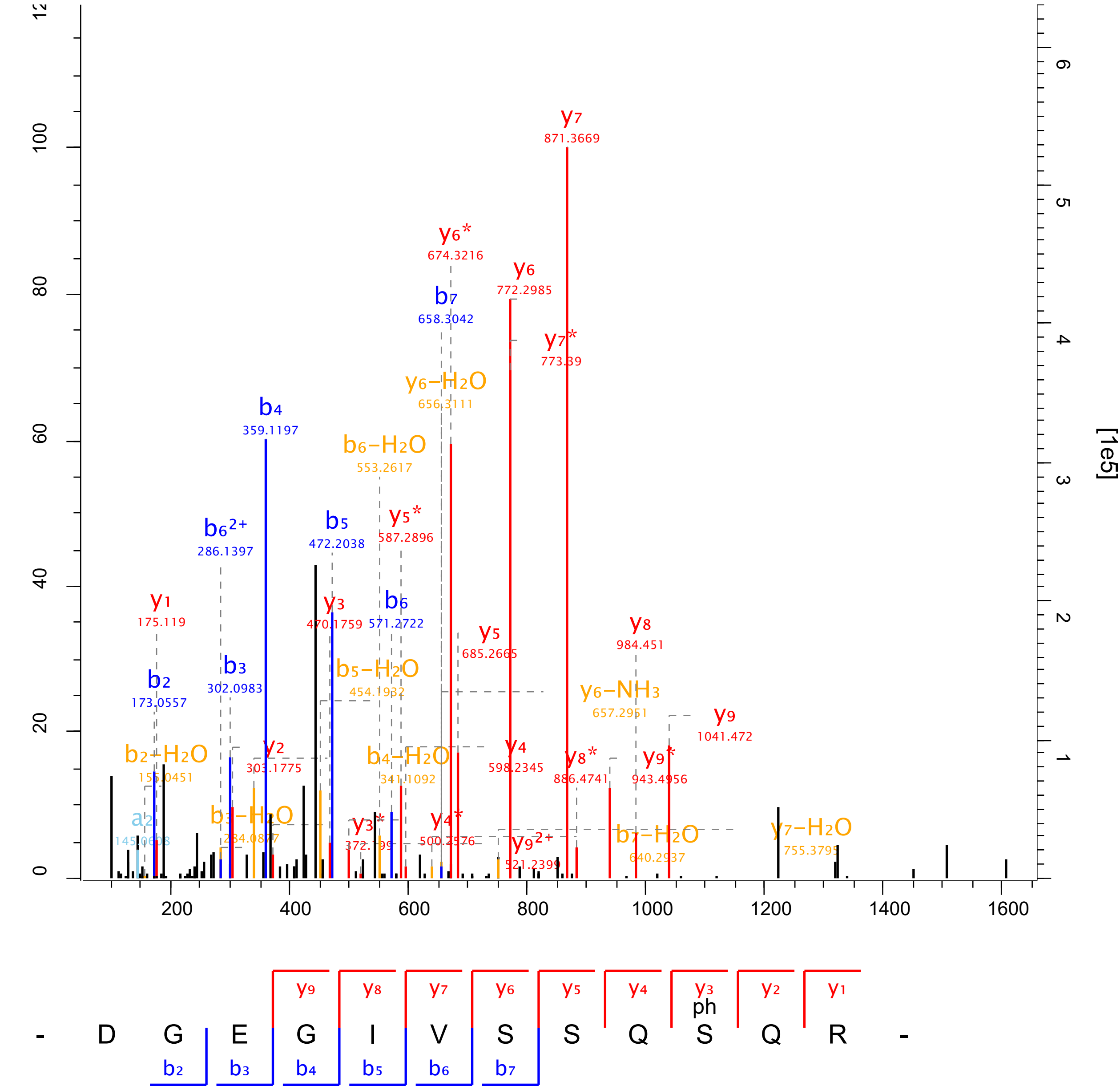

Supplement: Supplementary Figure S2-3 [file 143488_2_supp_334106_ps5hgj.pdf]
