# Supplementary material for: The Systemin Signaling Cascade As Derived from Time Course Analyses of the Systemin-responsive Phosphoproteome
Source: Mol Cell Proteomics. 2019 May 28;18(8):1526–42. doi: 10.1074/mcp.RA119.001367 (PMC6683004; doi:10.1074/mcp.RA119.001367)

Supplementary Figure 2: Representative annotated spectra of identified phosphopeptides under systemin,A17 and water treatment as exported from MaxQuant.

Raw file	Scan	Method	Score	m/z
sys_05_2	10526	FTMS; HCD	275.14	935.84

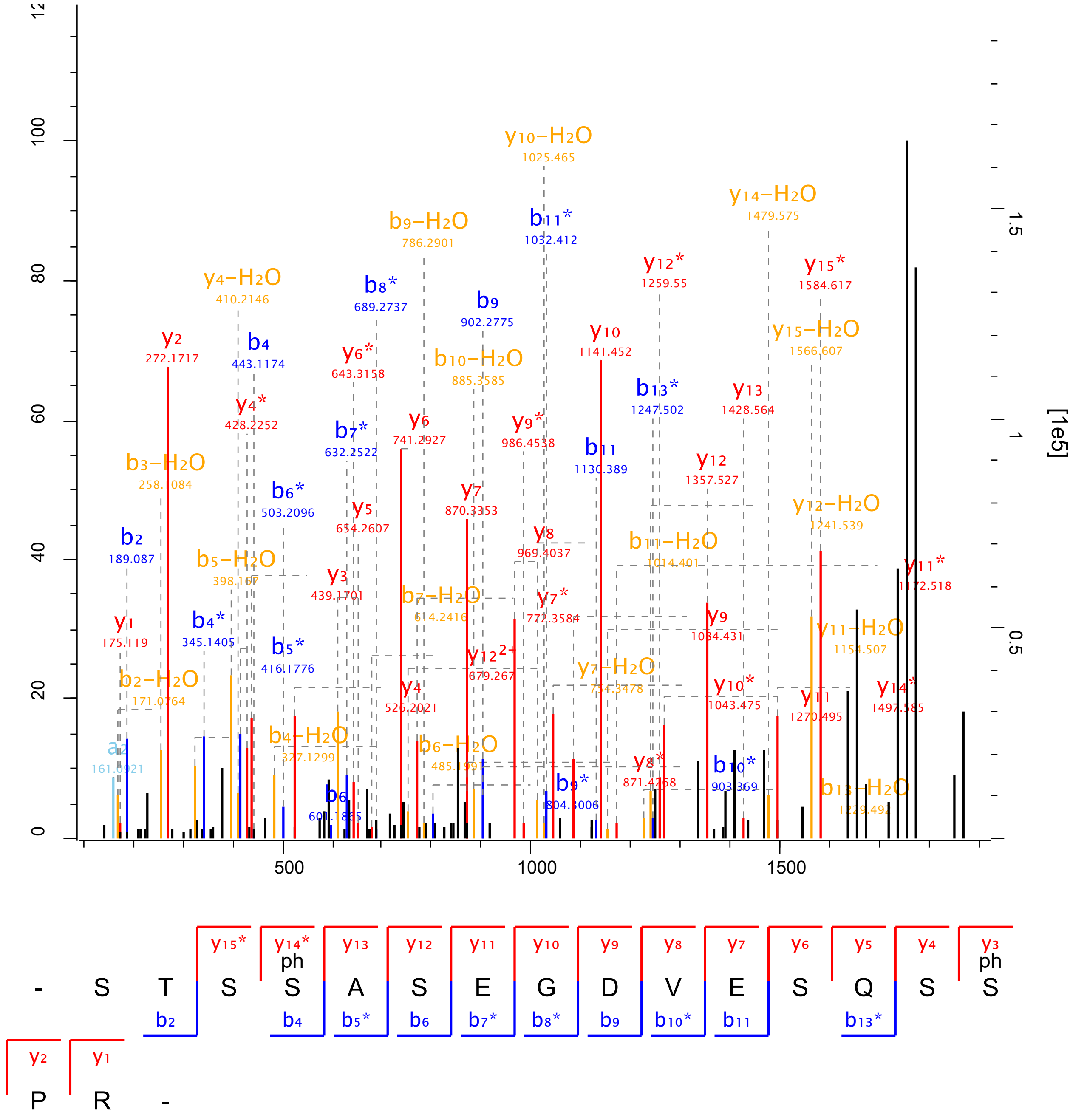

Raw file	Scan	Method	Score	m/z
sys_05_2	10545	FTMS; HCD	184.58	495.72

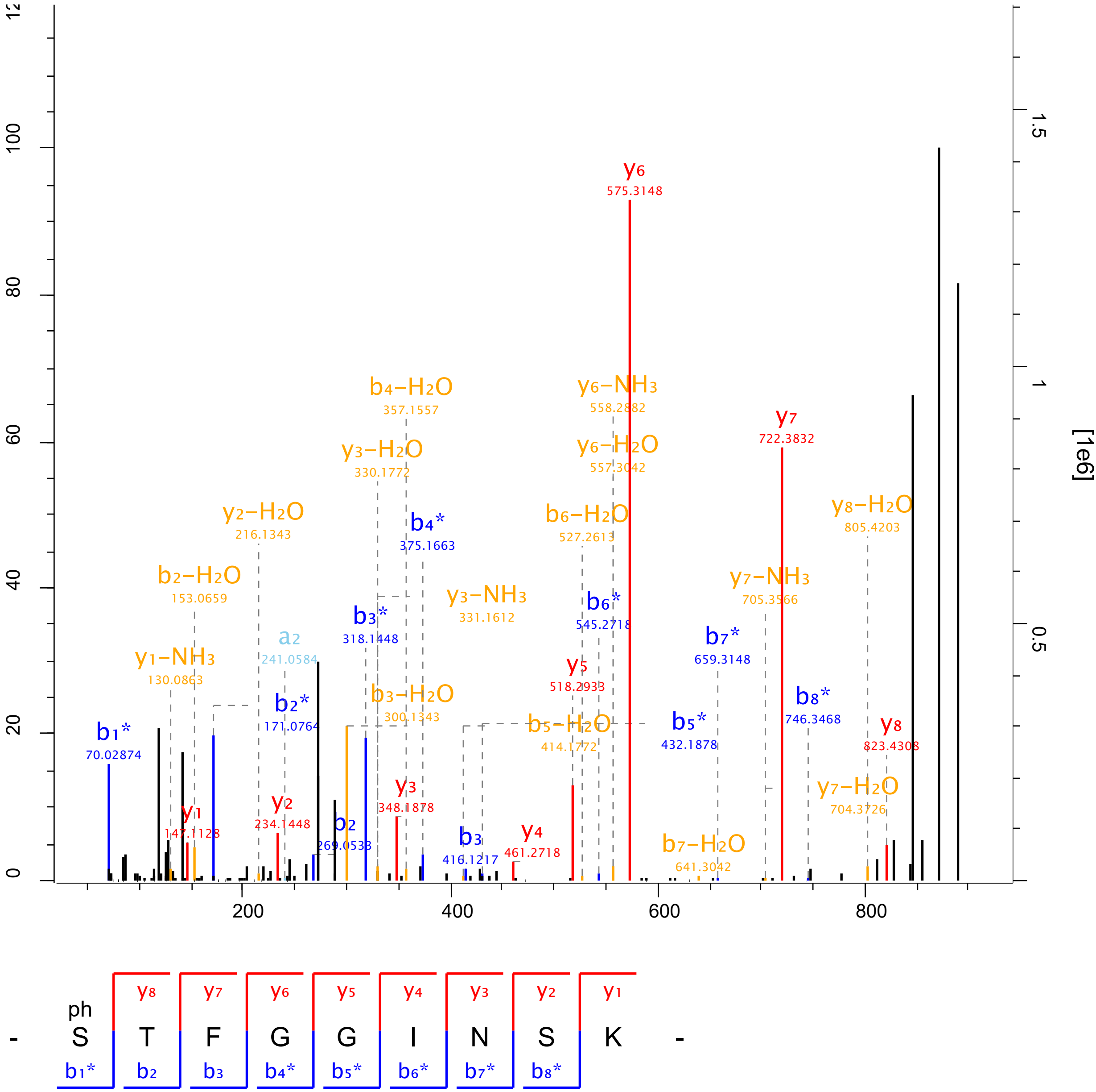

Raw file	Scan	Method	Score	m/z
sys_05_2	10652	FTMS; HCD	54.34	555.2

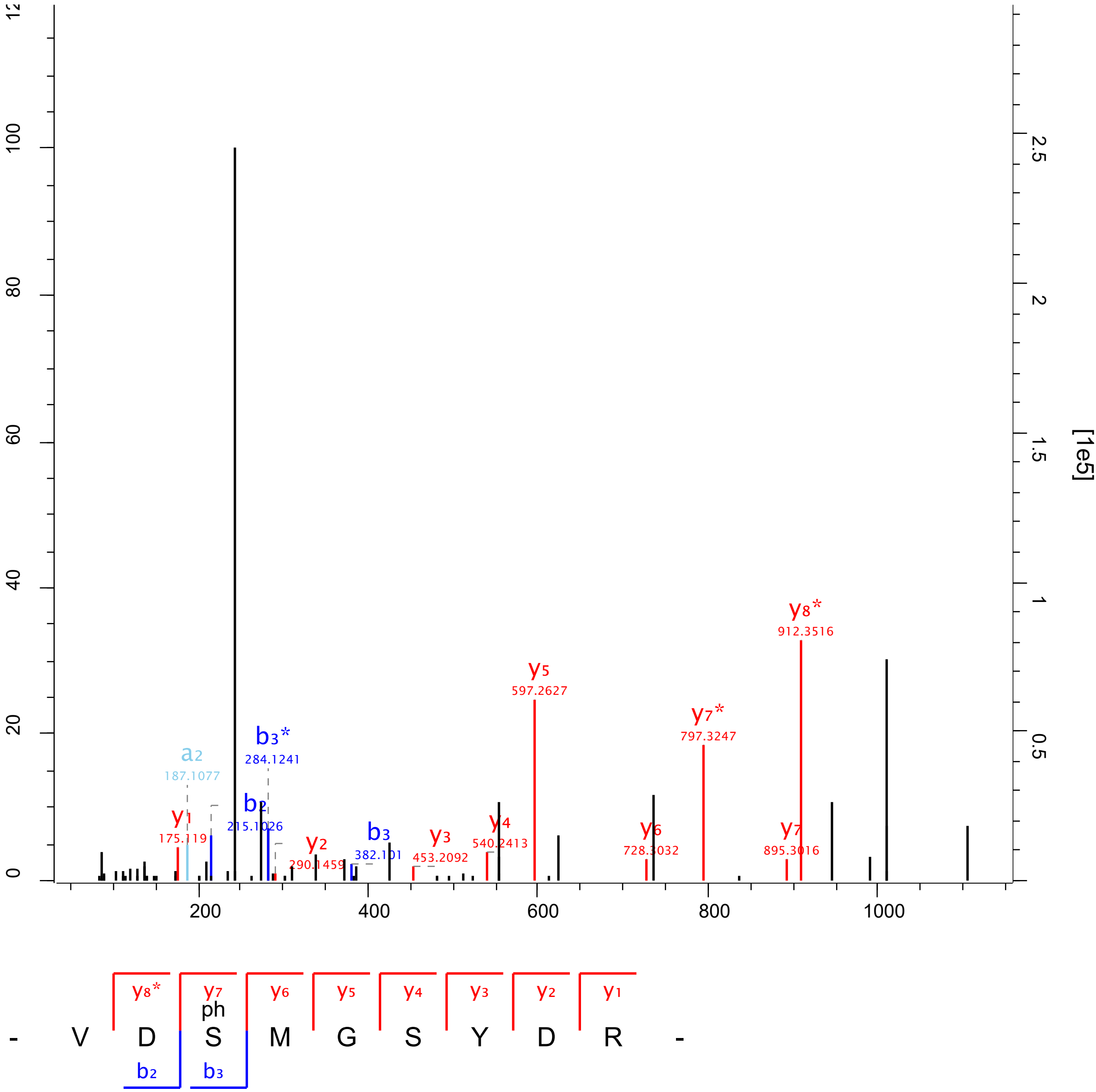

Raw file	Scan	Method	Score	m/z
sys_05_2	10685	FTMS; HCD	83.53	570.77

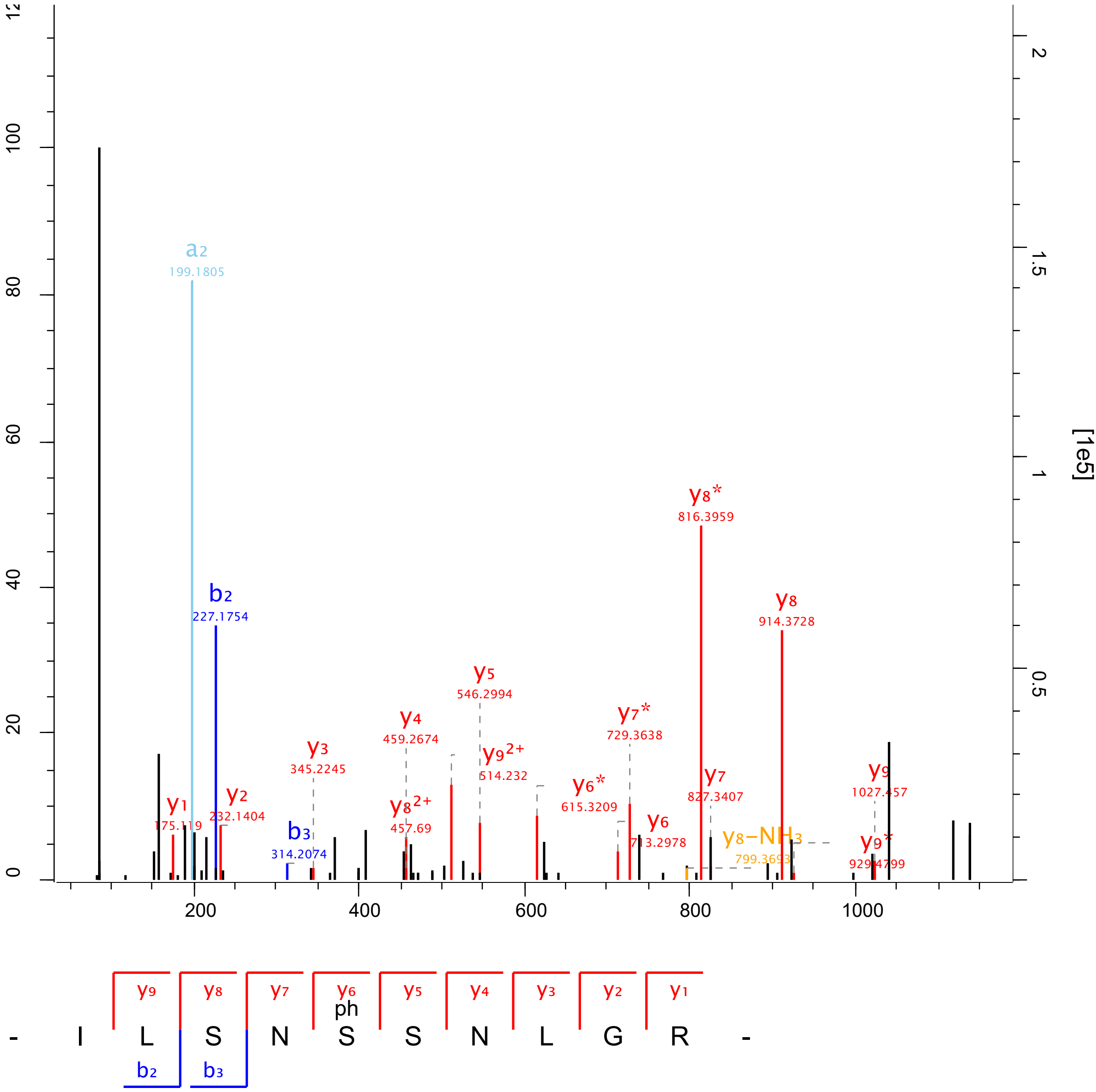

Raw file	Scan	Method	Score	m/z
sys_05_2	10875	FTMS; HCD	184.99	637.27

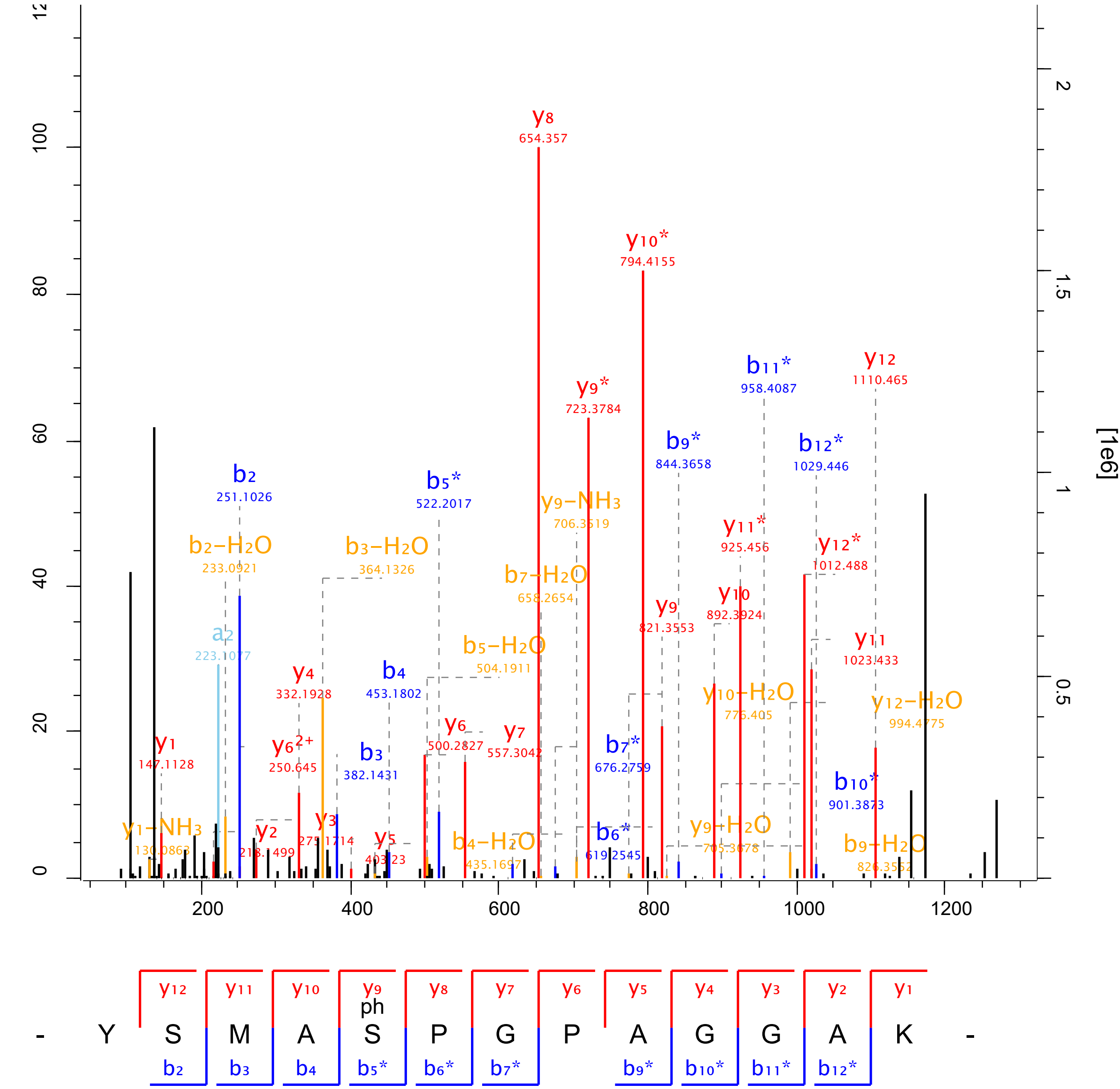

Raw file	Scan	Method	Score	m/z
sys_05_2	11317	FTMS; HCD	62.16	598.73

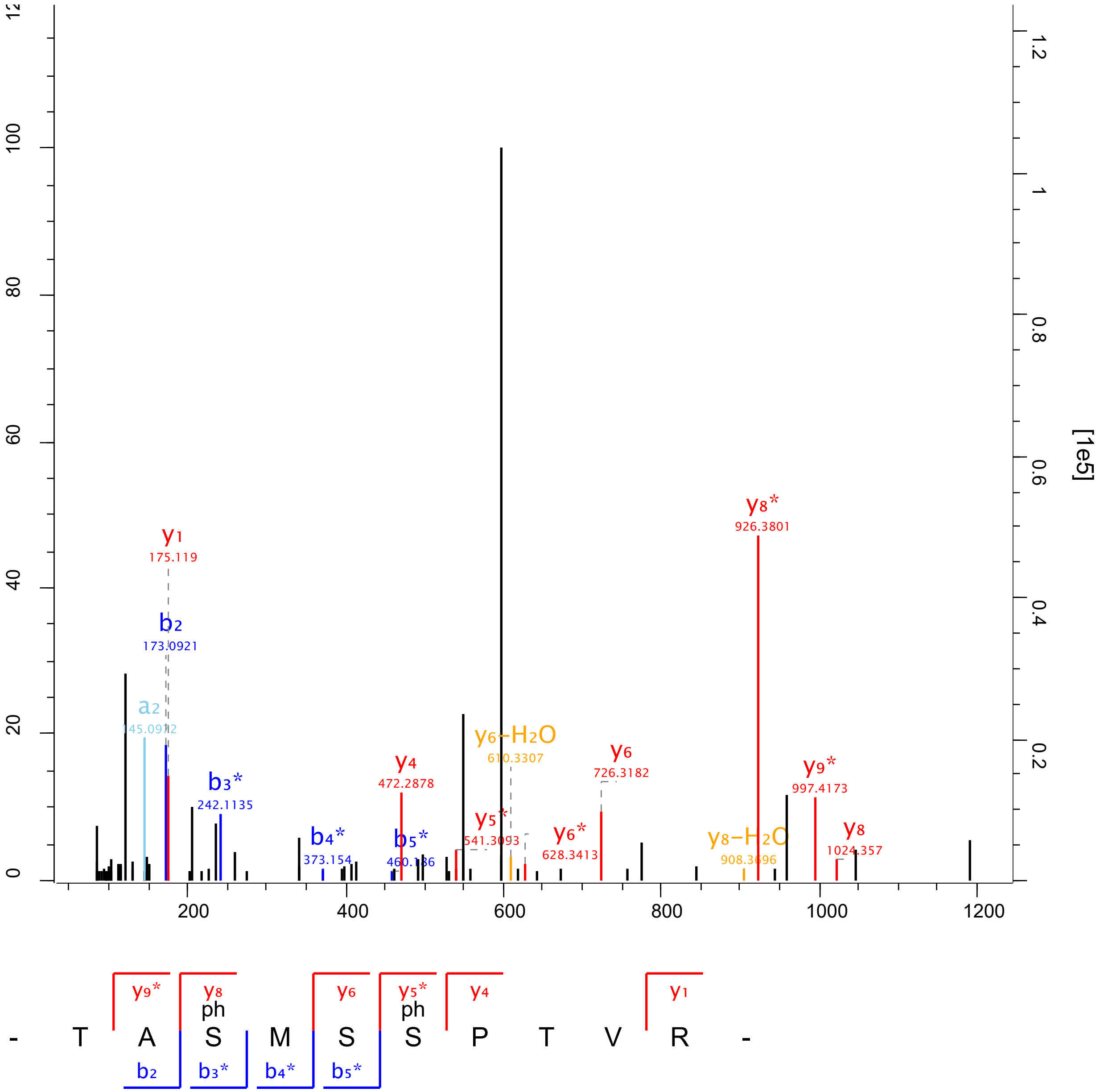

Raw file	Scan	Method	Score	m/z
sys_05_2	11428	FTMS; HCD	229.59	584.22

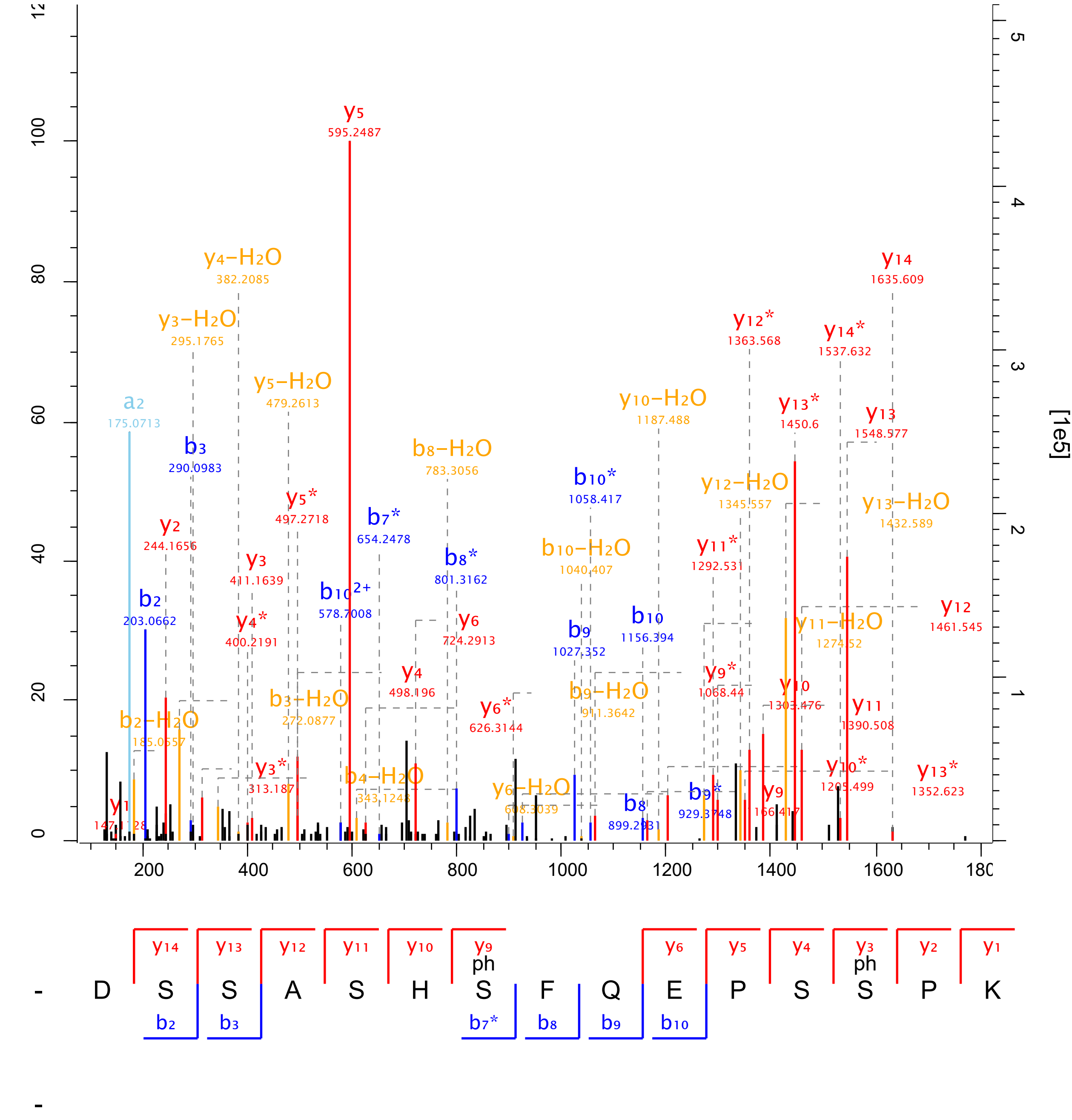

Raw file	Scan	Method	Score	m/z
sys_05_2	11434	FTMS; HCD	196.16	530.25

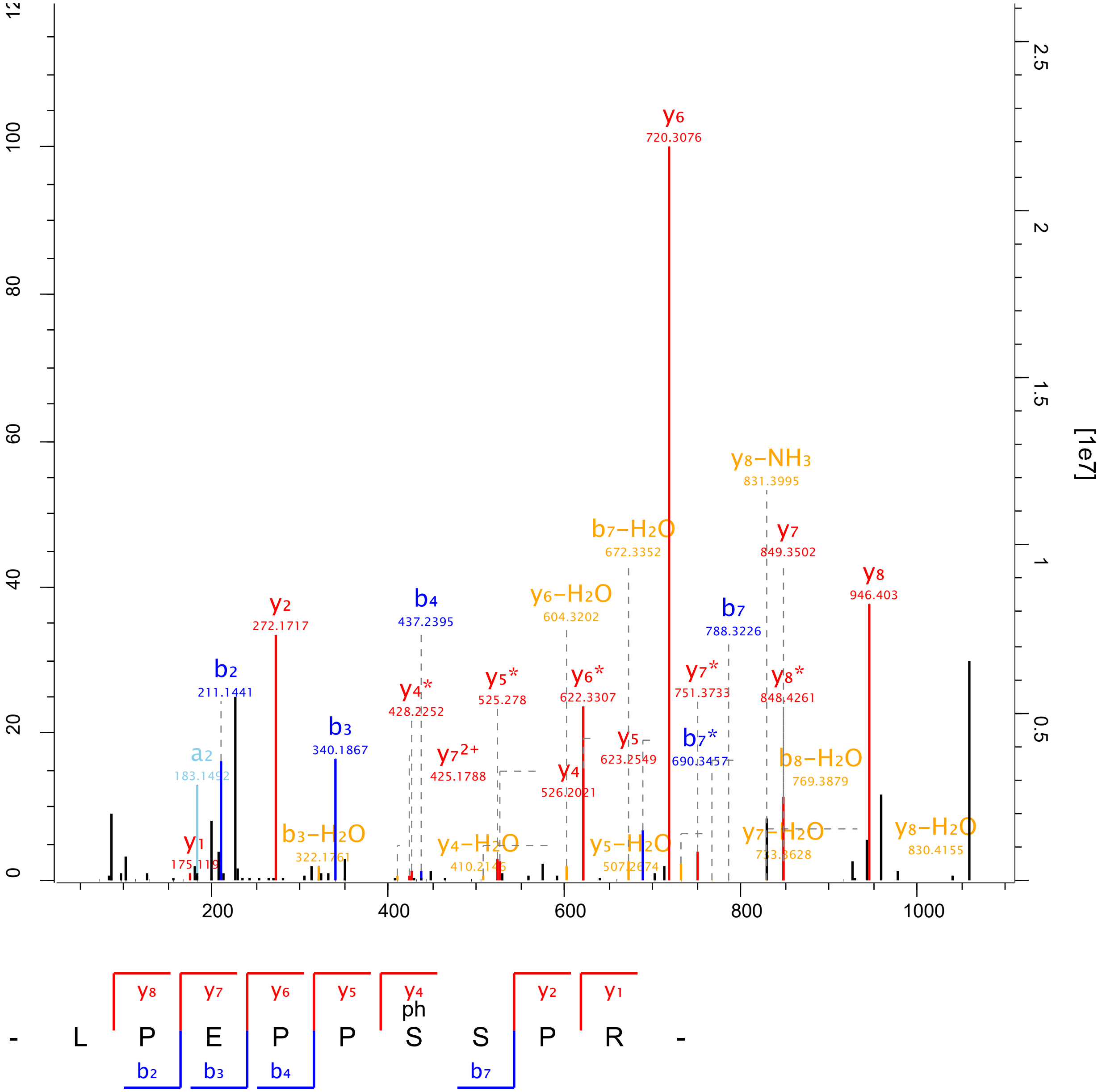

Raw file	Scan	Method	Score	m/z
sys_05_2	11488	FTMS; HCD	220.25	560.24

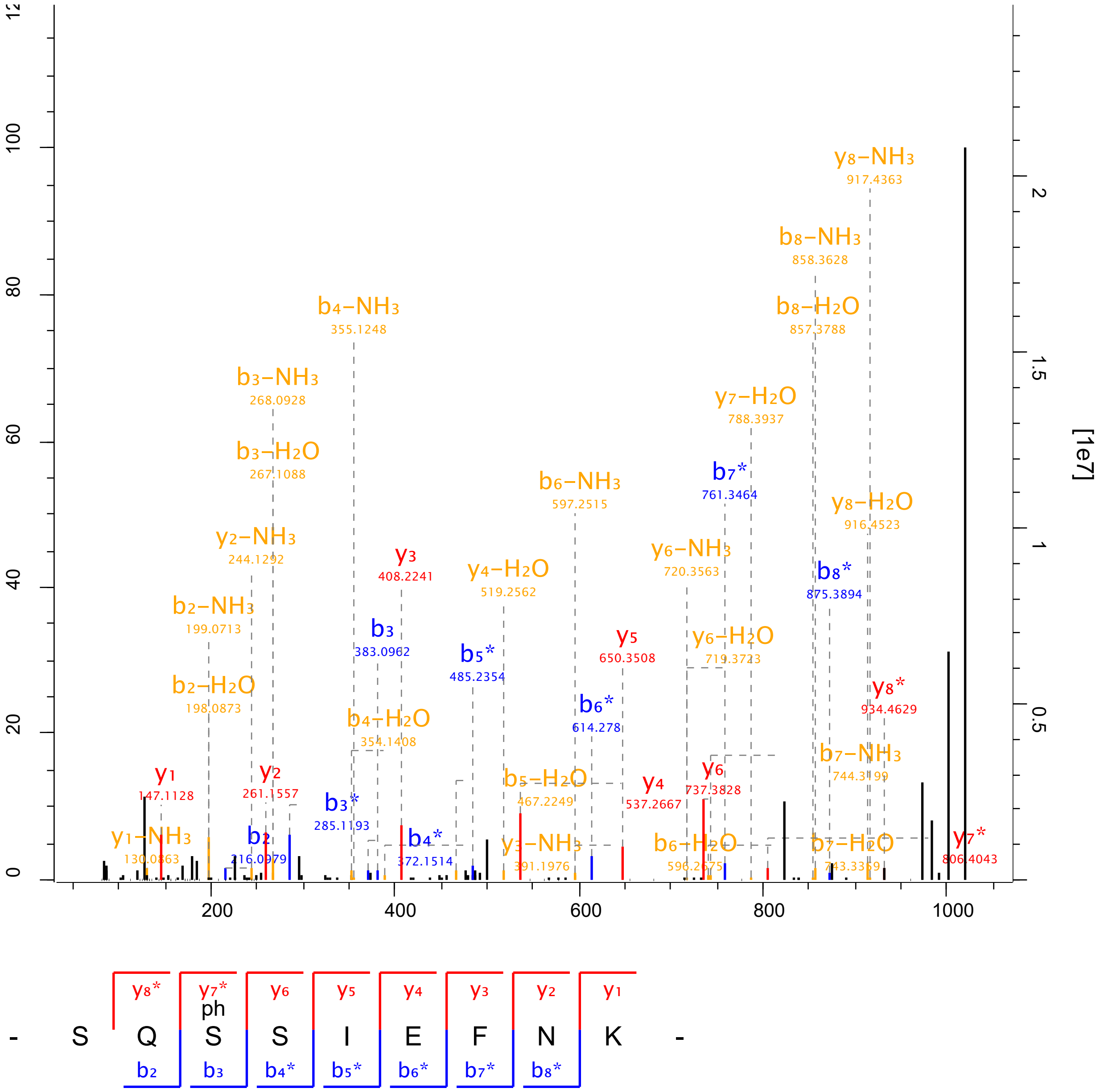

Raw file	Scan	Method	Score	m/z
sys_05_2	11562	FTMS; HCD	155.96	701.79

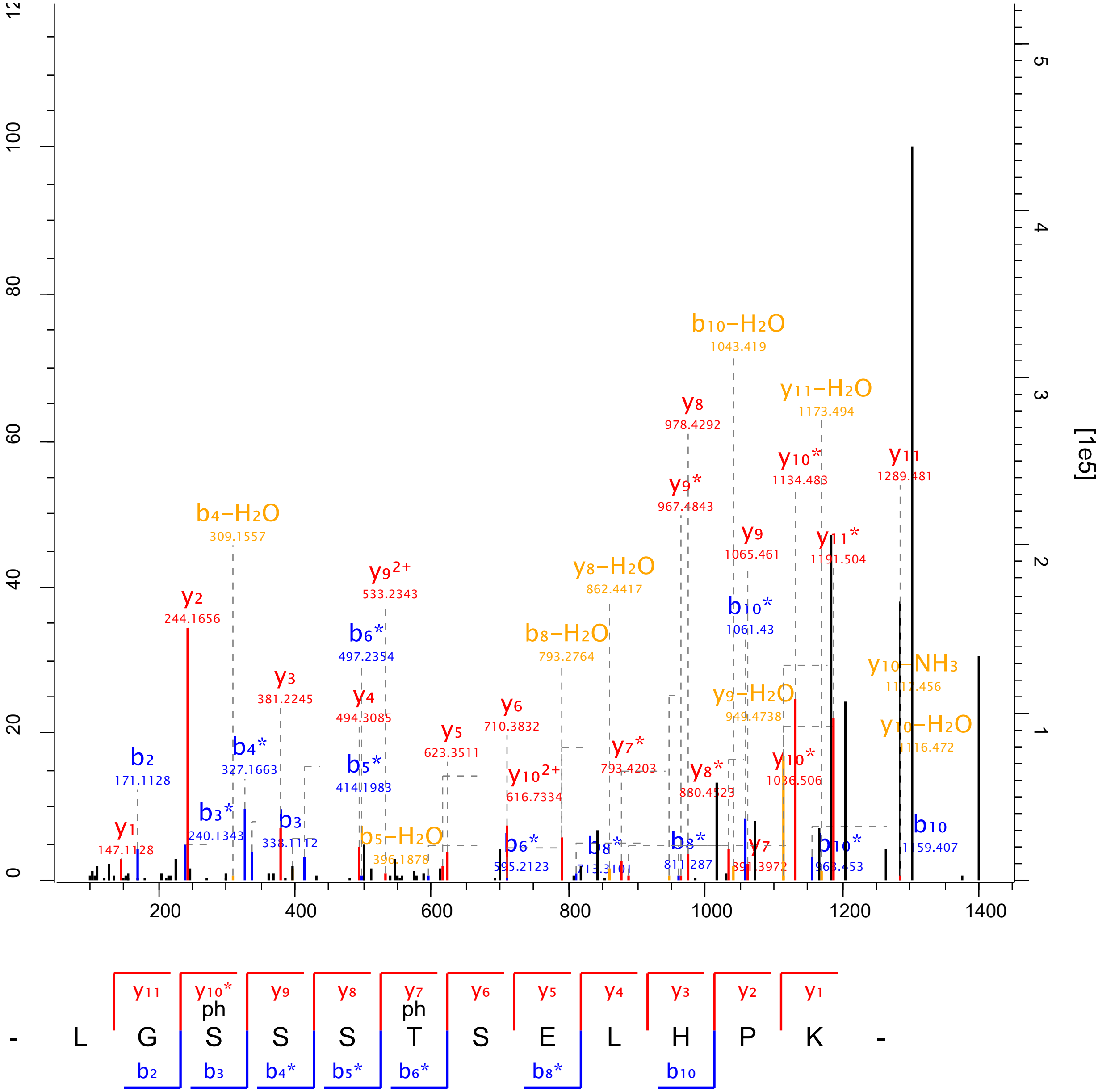

Raw file	Scan	Method	Score	m/z
sys_05_2	11572	FTMS; HCD	59.91	535.24

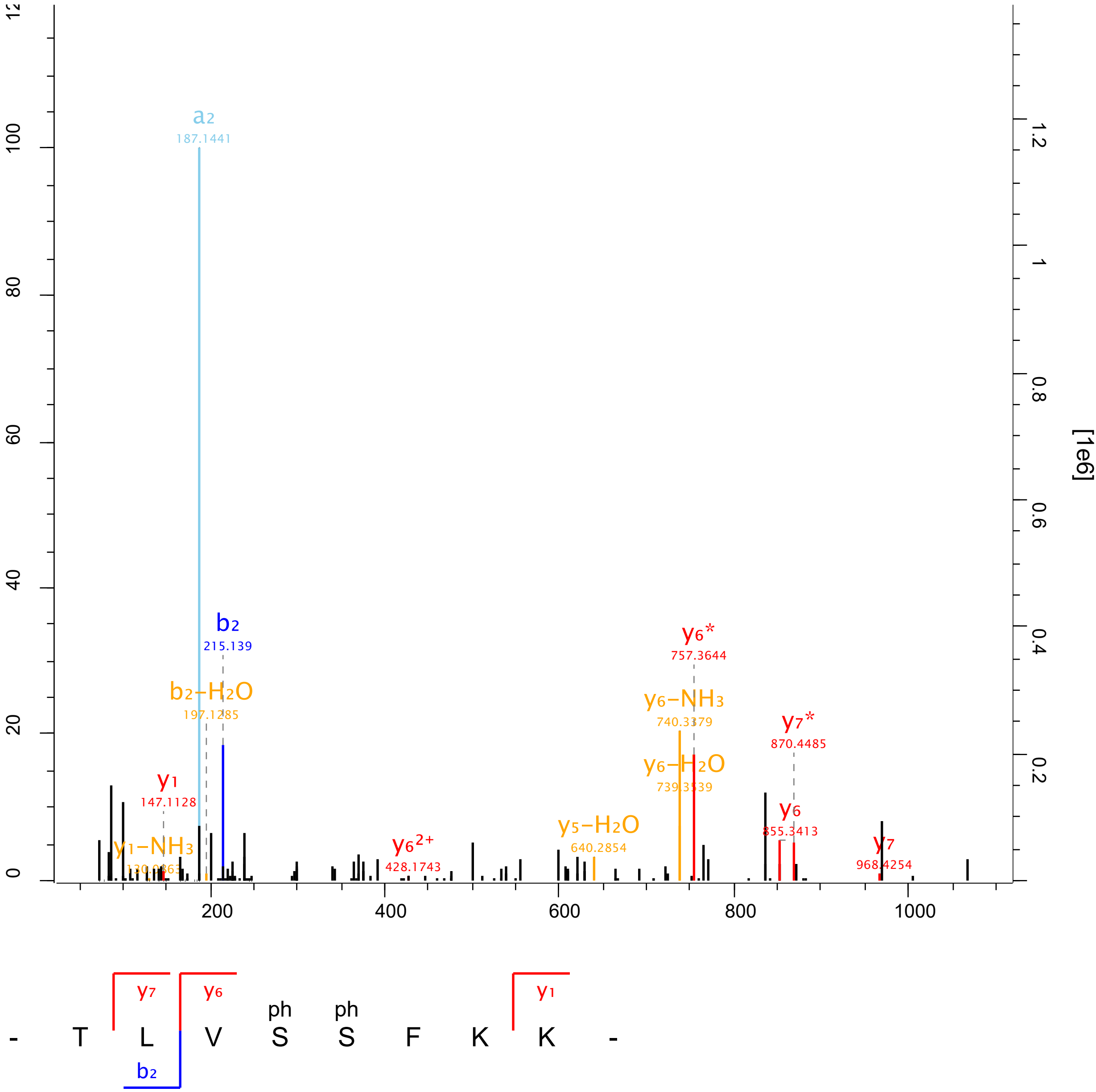

Raw file	Scan	Method	Score	m/z
sys_05_2	11623	FTMS; HCD	119.39	588.79

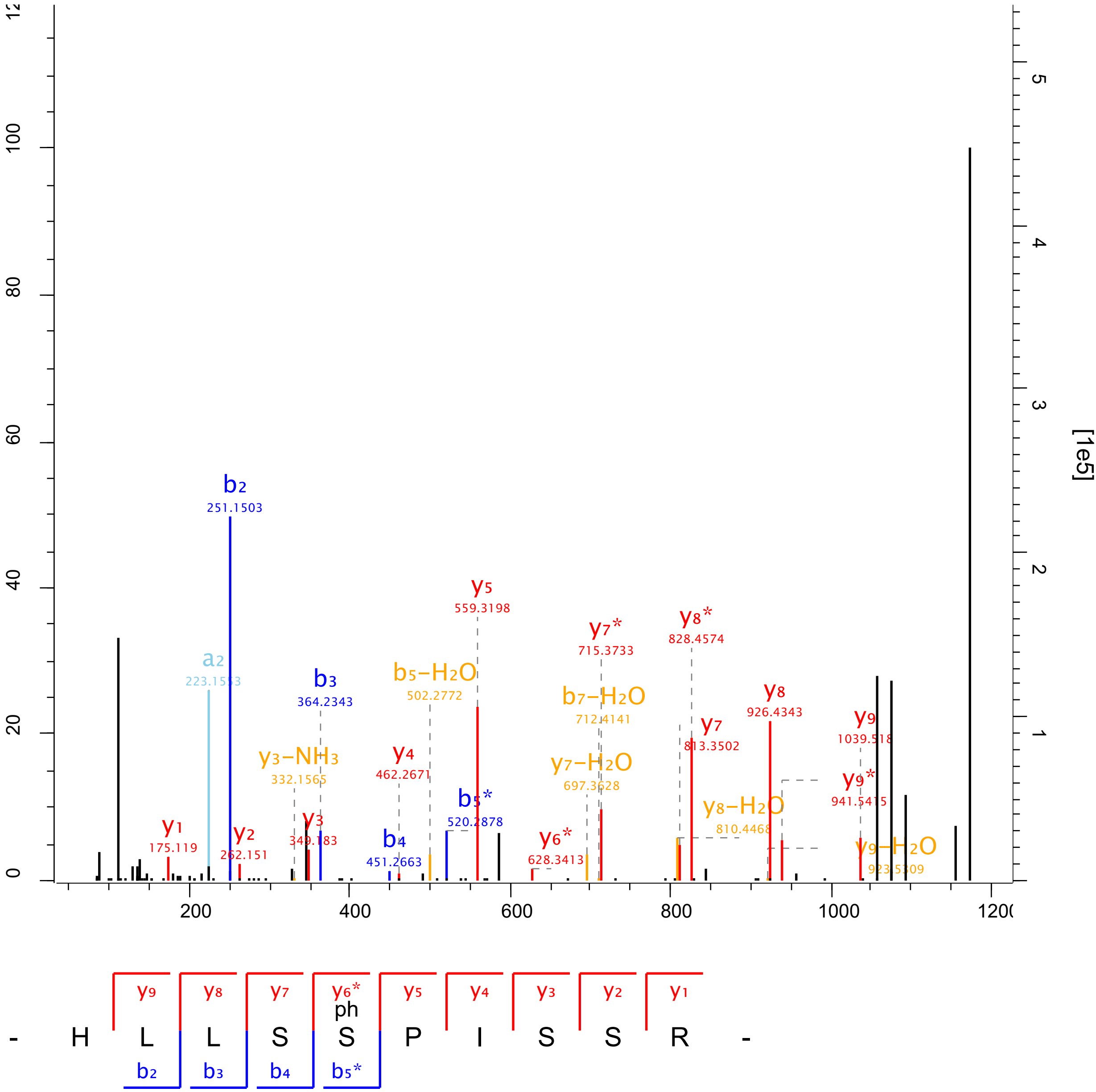

Raw file	Scan	Method	Score	m/z
sys_05_2	11628	FTMS; HCD	81.16	685.82

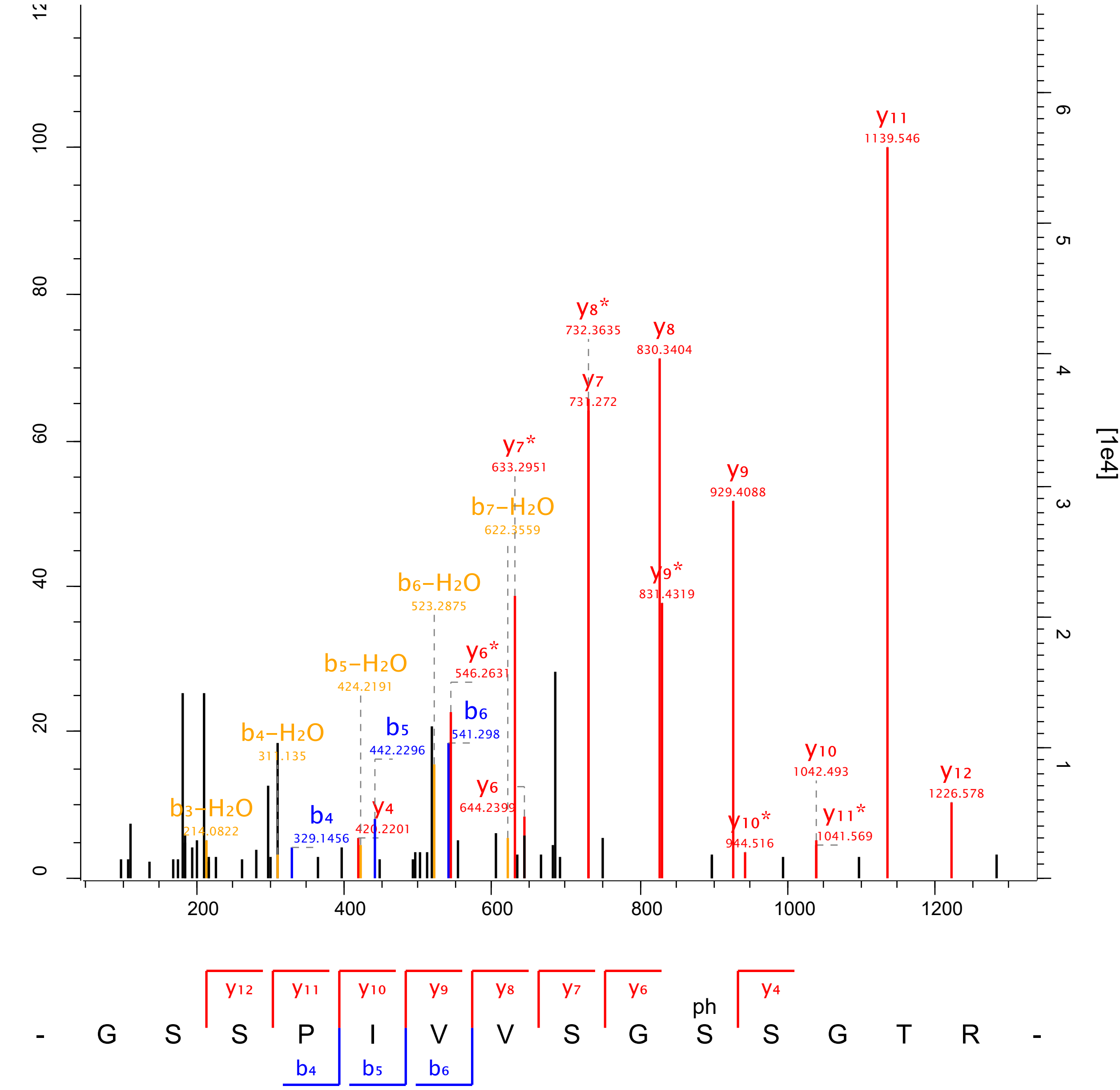

Raw file	Scan	Method	Score	m/z
sys_05_2	11721	FTMS; HCD	163.33	692.26

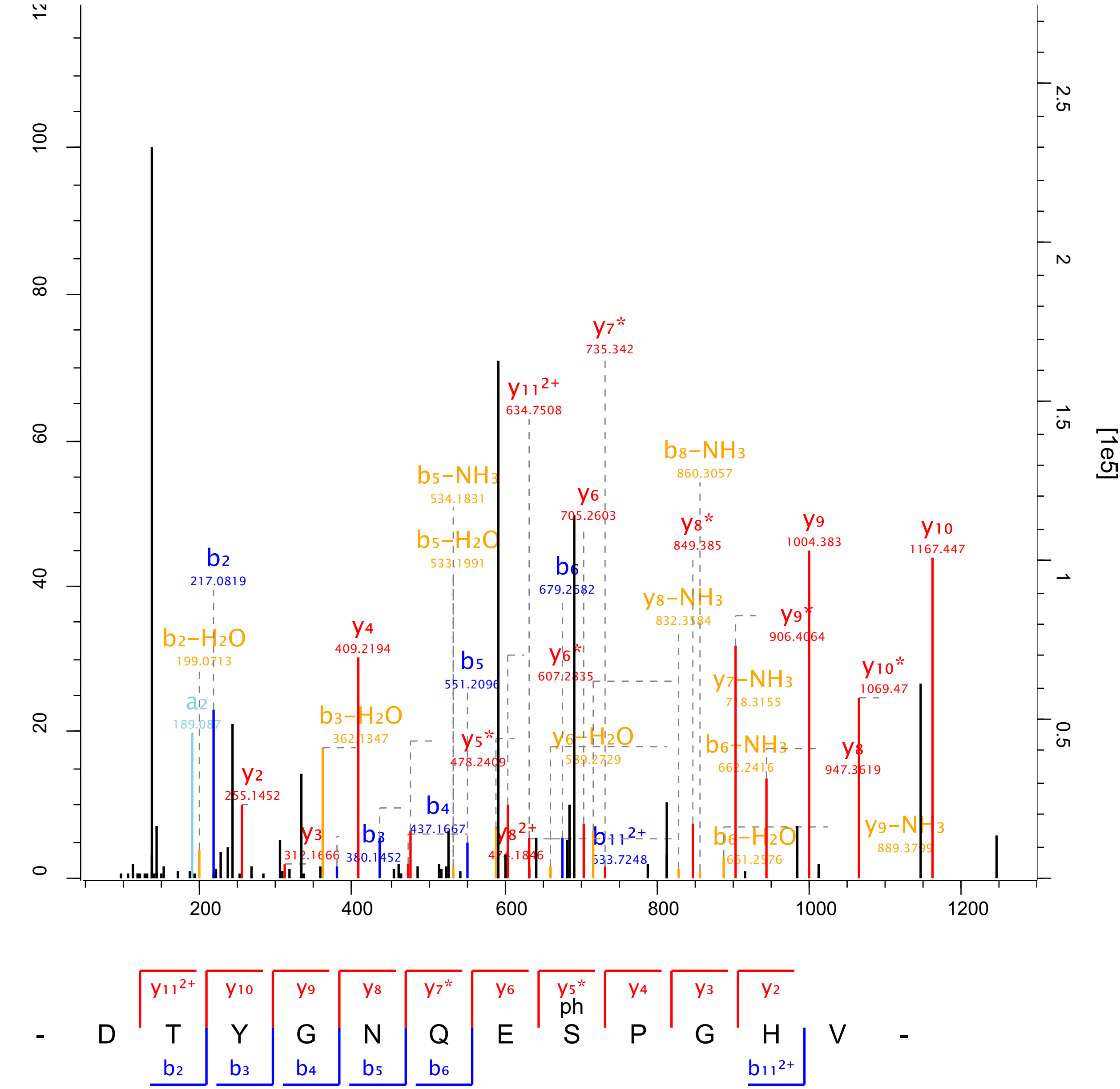

Raw file	Scan	Method	Score	m/z
sys_05_2	11792	FTMS; HCD	132.84	810.78

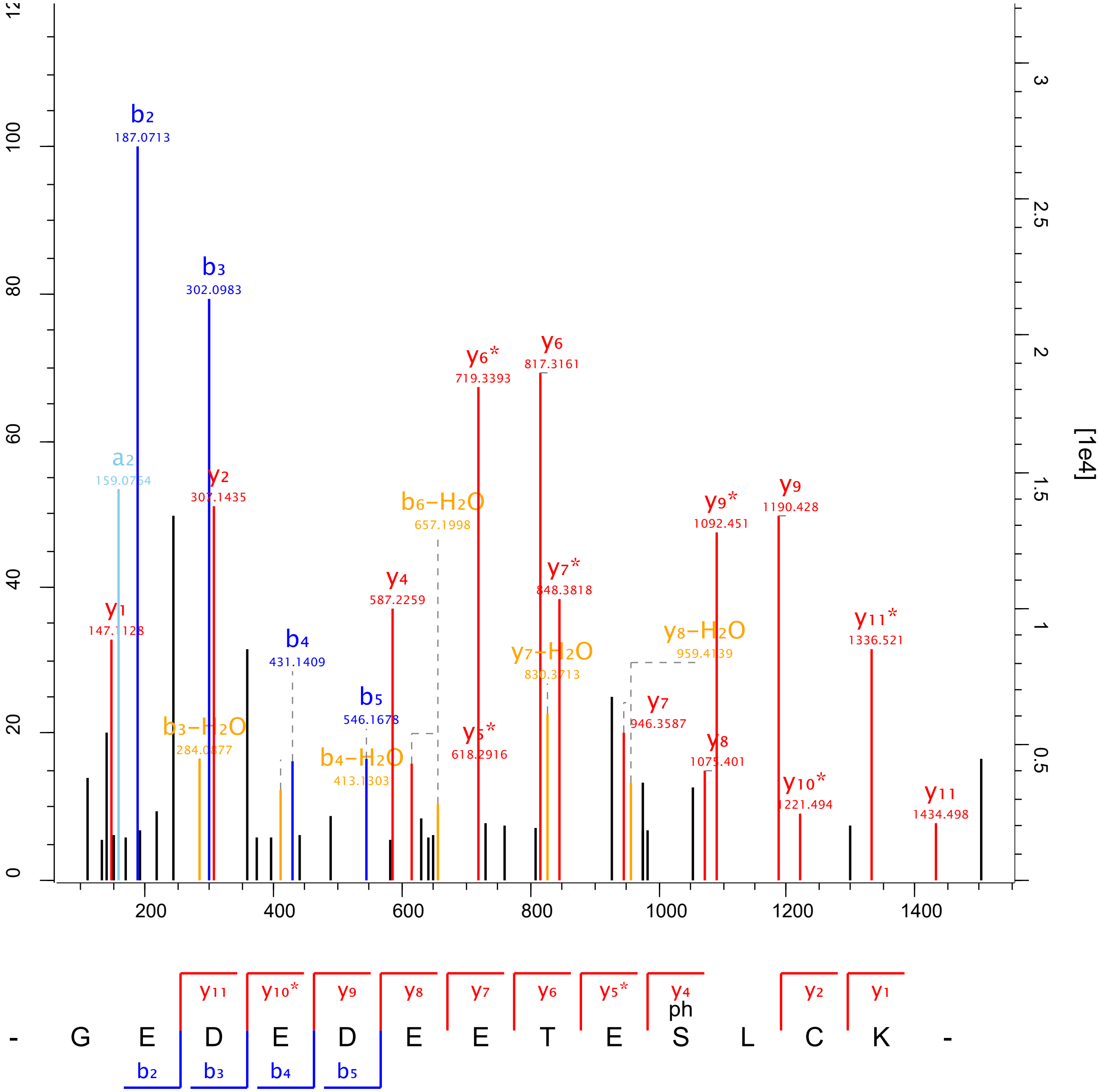

Raw file	Scan	Method	Score	m/z
sys_05_2	11800	FTMS; HCD	74.24	544.24

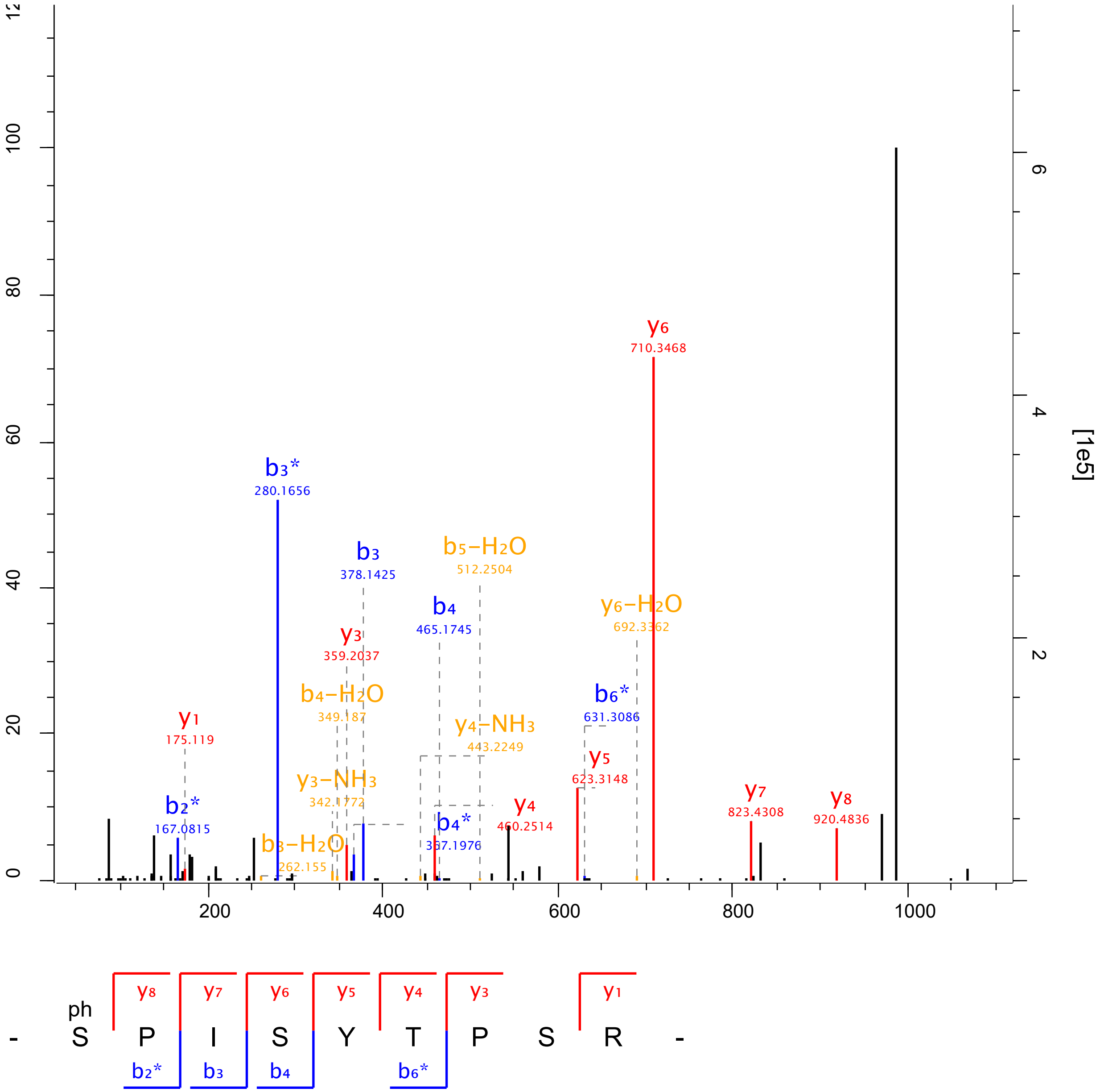

Raw file	Scan	Method	Score	m/z
sys_05_2	12158	FTMS; HCD	76.94	505.72

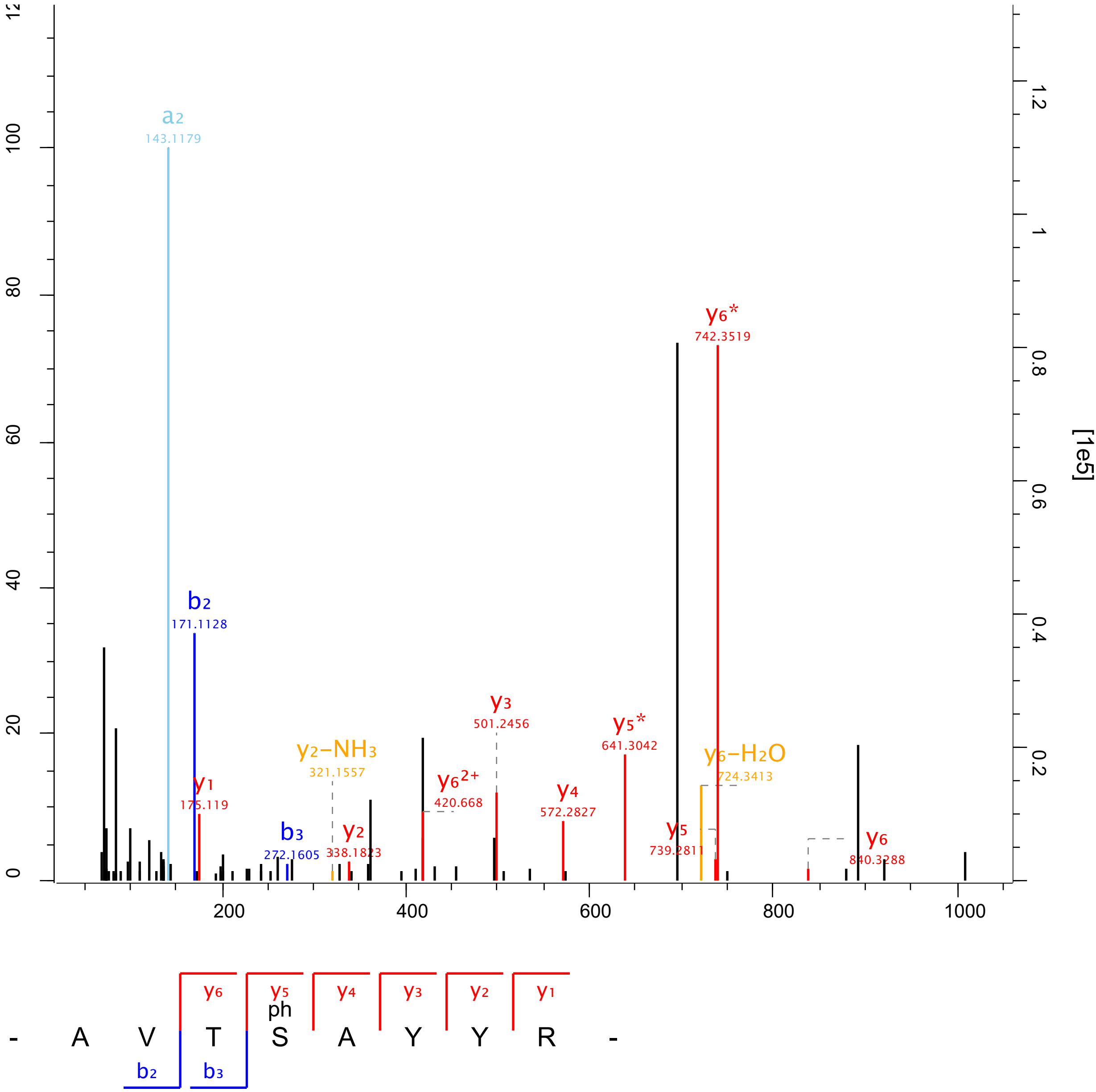

Raw file	Scan	Method	Score	m/z
sys_05_2	12330	FTMS; HCD	112.3	466.18

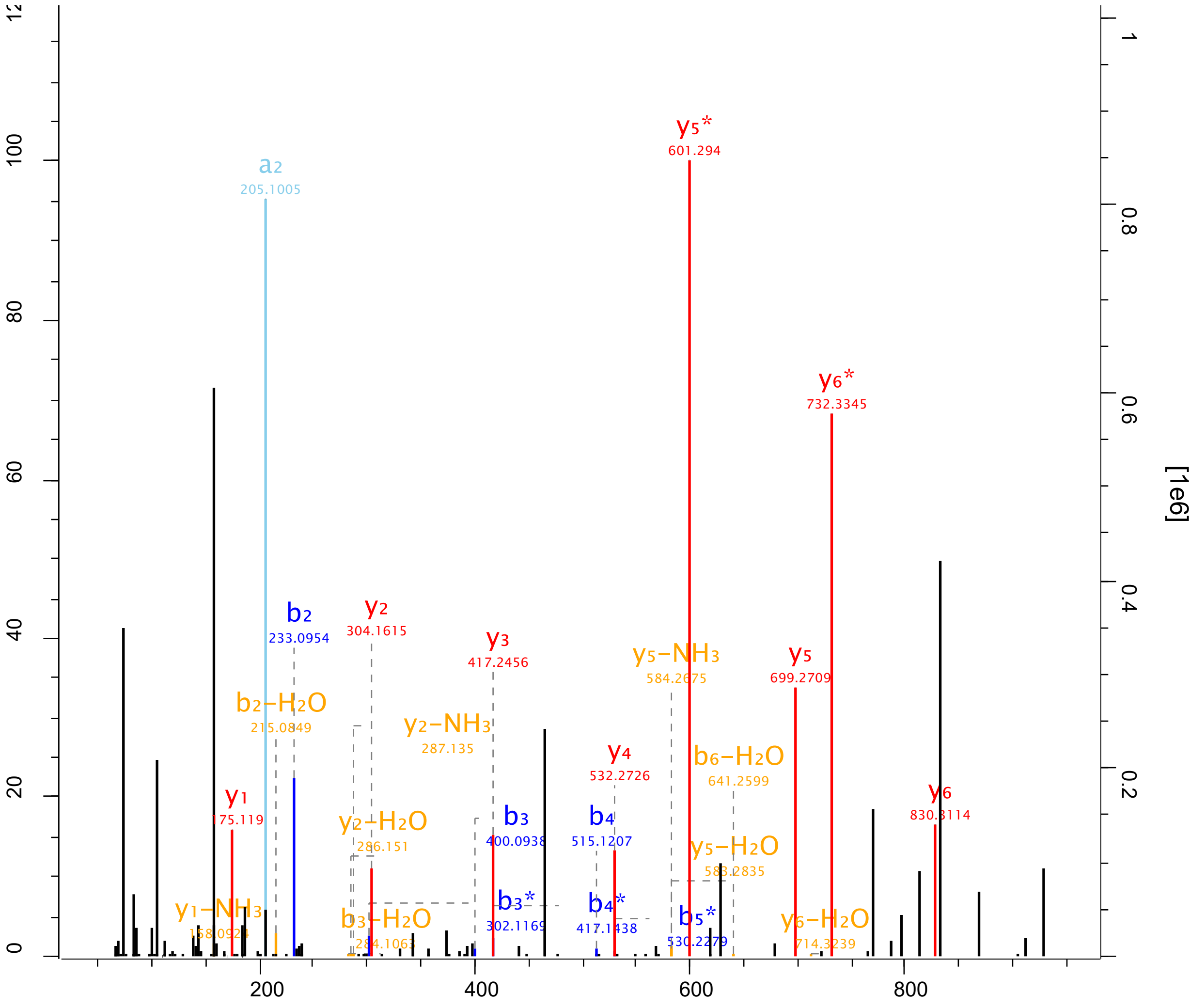

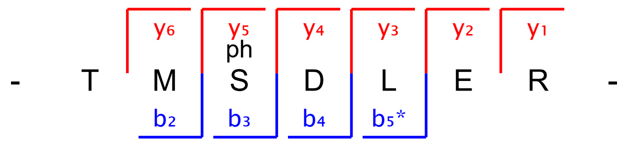

Raw file	Scan	Method	Score	m/z
sys_05_2	12433	FTMS; HCD	73.44	507.23

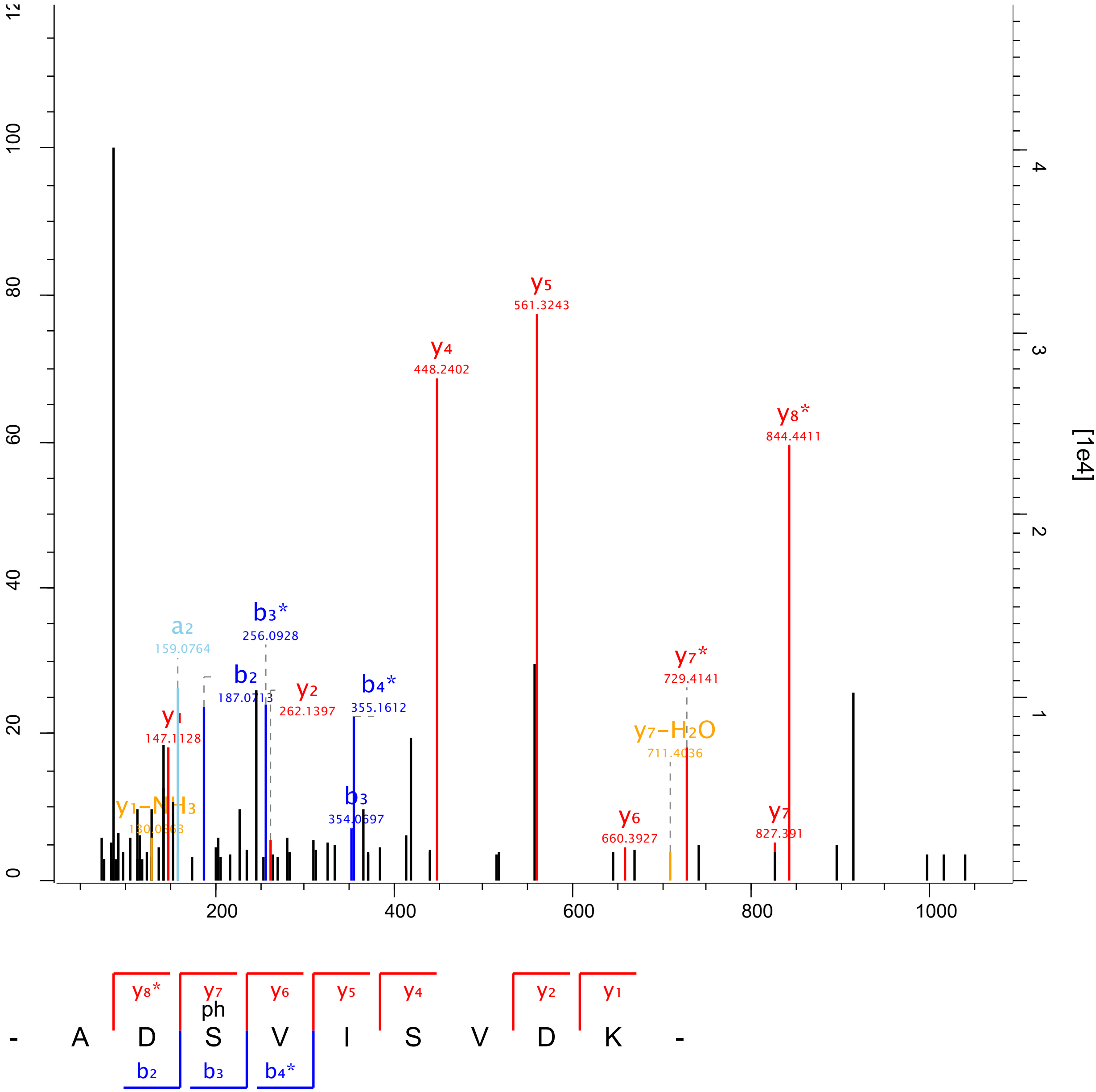

Raw file	Scan	Method	Score	m/z
sys_05_2	12642	FTMS; HCD	145.9	436.7

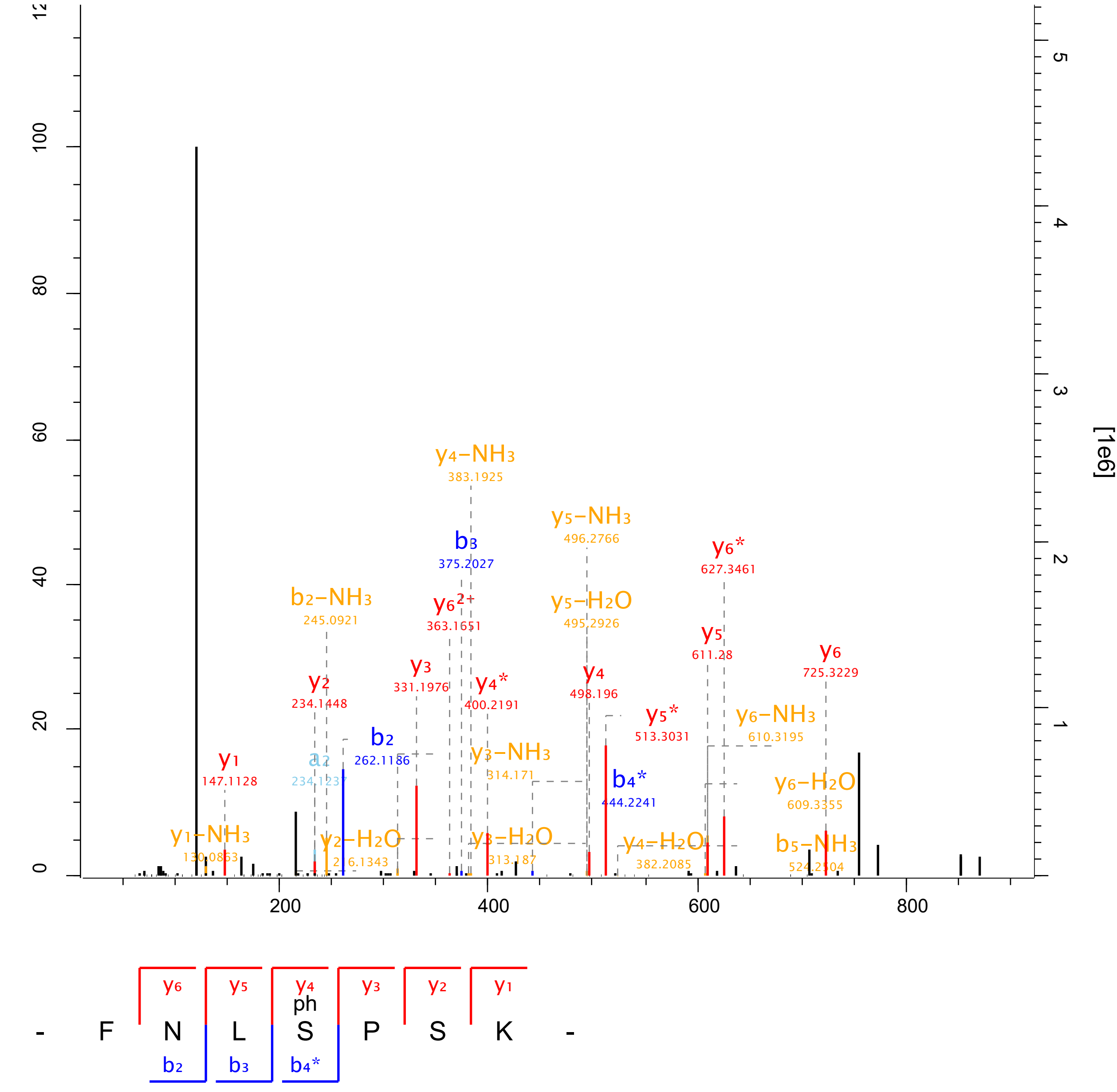

Raw file	Scan	Method	Score	m/z
sys_05_2	12954	FTMS; HCD	140.22	730.31

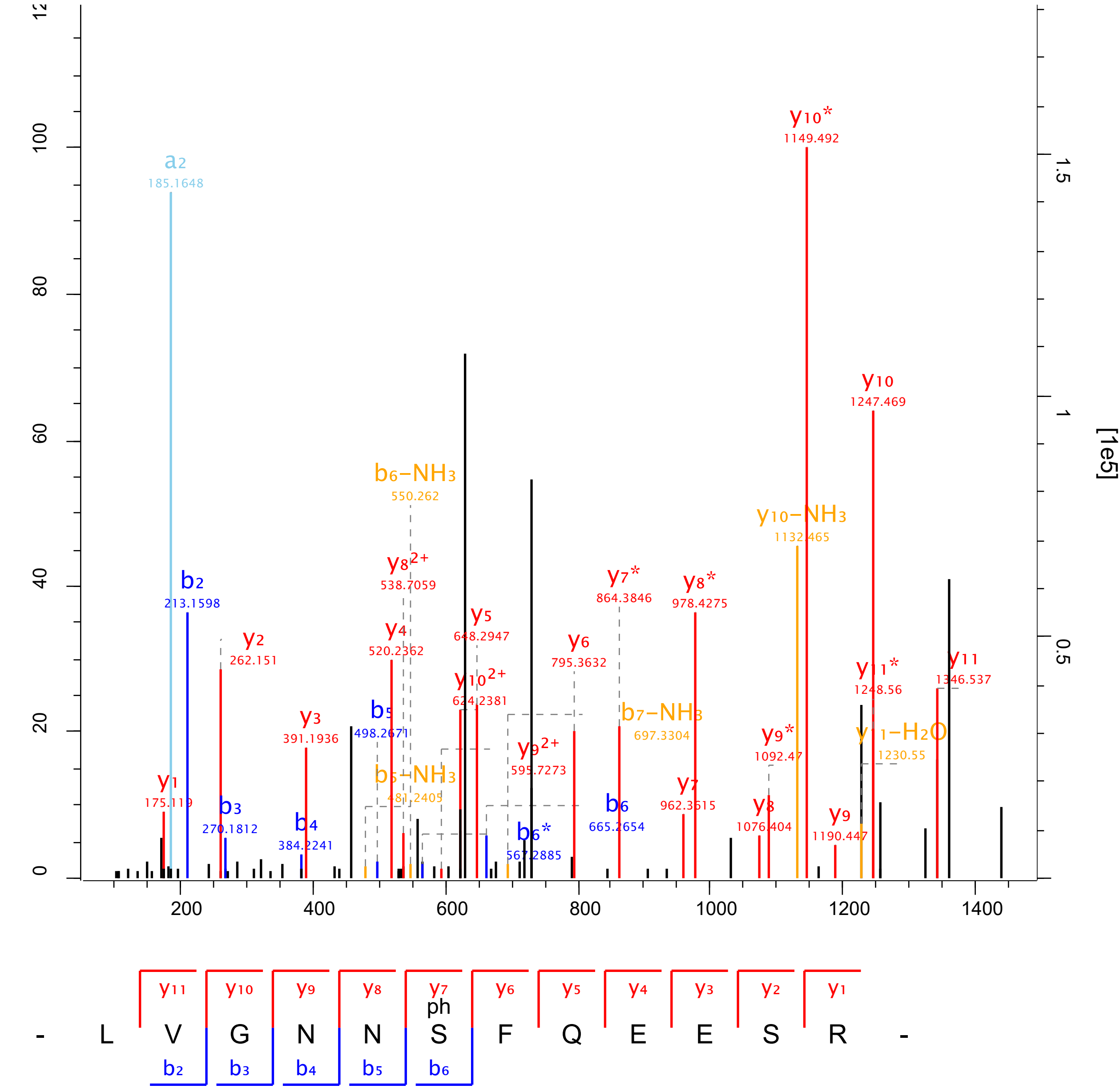

Raw file	Scan	Method	Score	m/z
sys_05_2	12994	FTMS; HCD	109.07	632.81

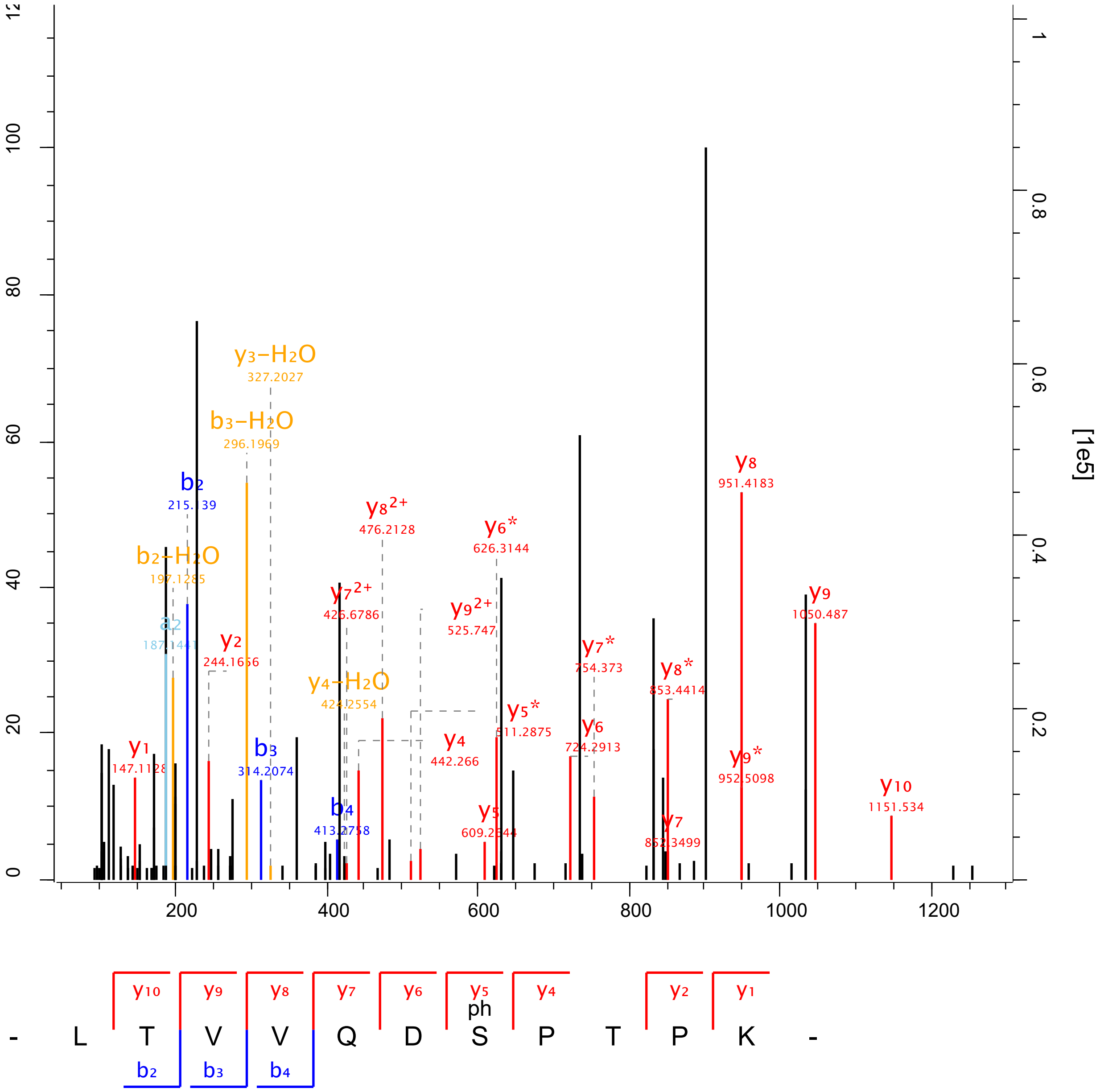

Raw file	Scan	Method	Score	m/z
sys_05_2	13013	FTMS; HCD	118.37	809.35

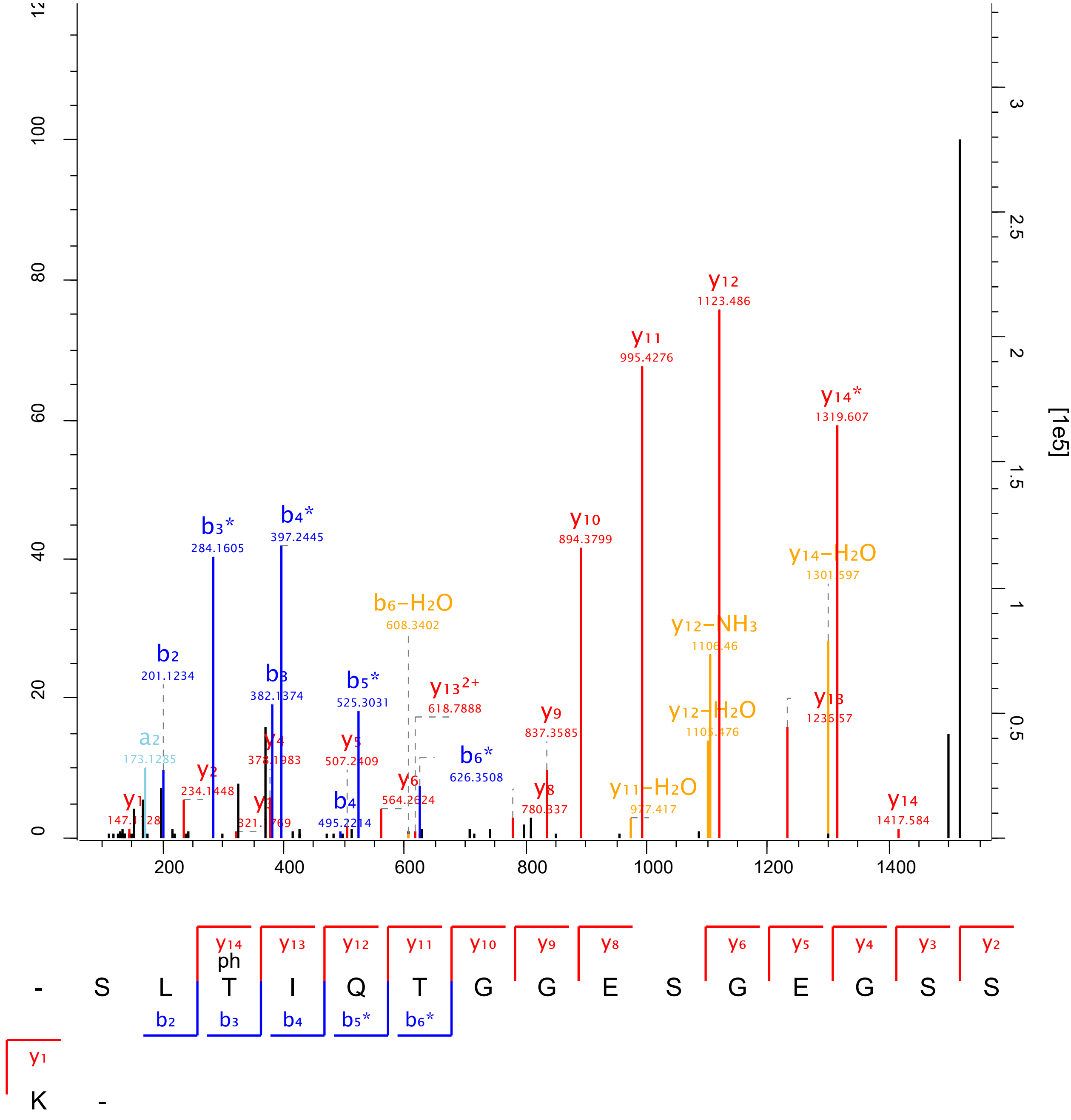

Raw file	Scan	Method	Score	m/z
sys_05_2	13047	FTMS; HCD	117.2	531.73

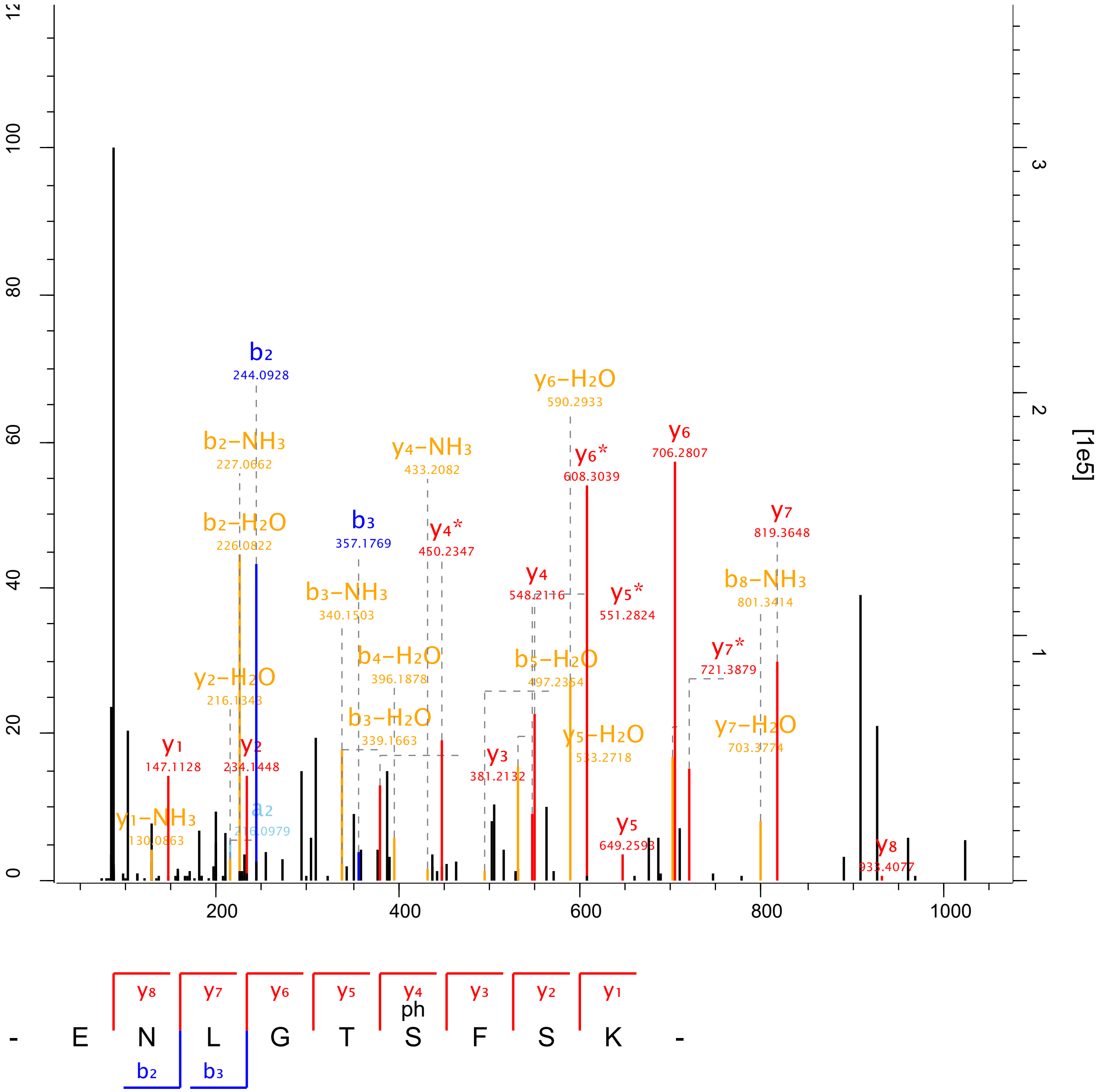

Raw file	Scan	Method	Score	m/z
sys_05_2	13109	FTMS; HCD	188.81	563.24

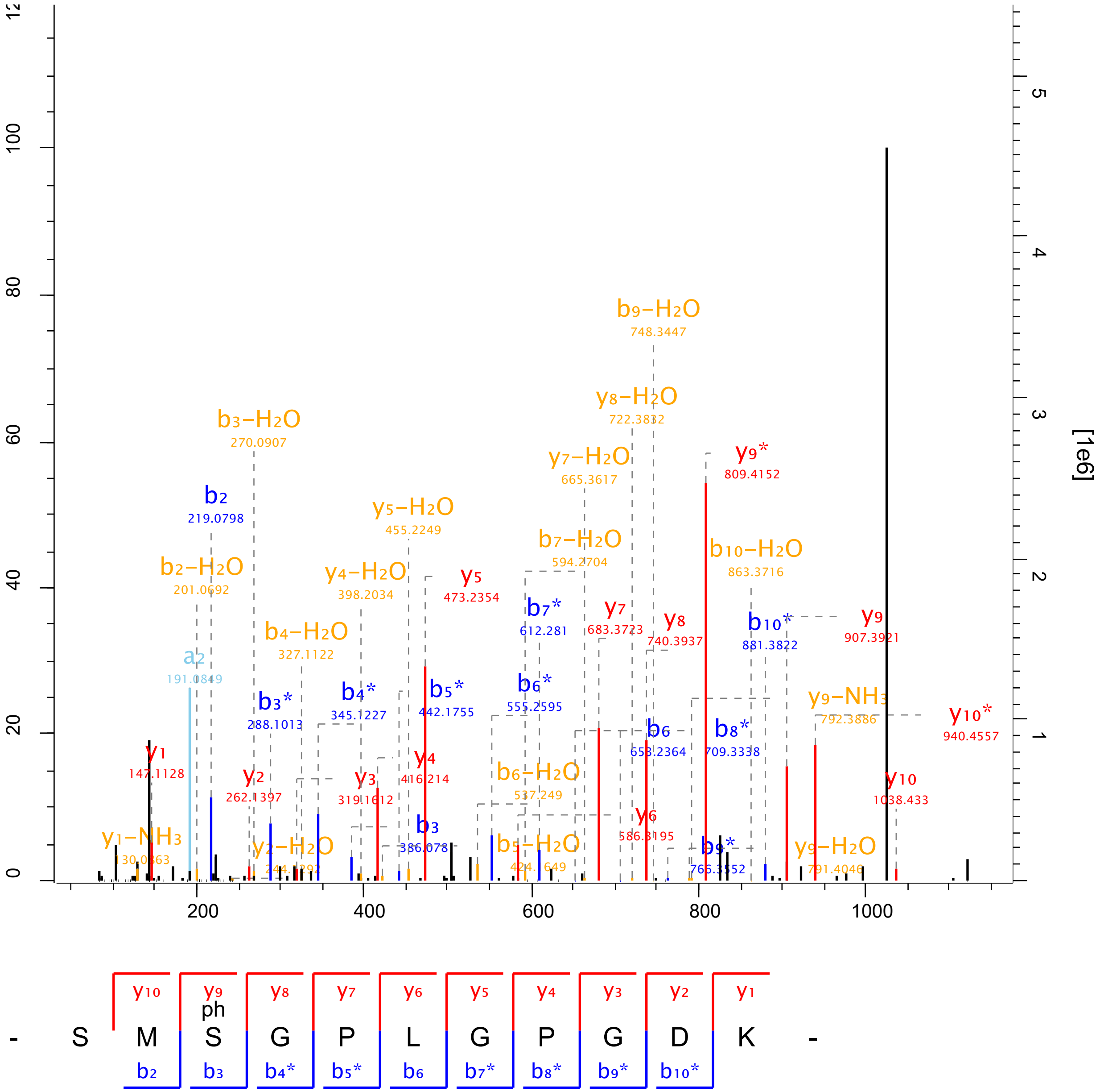

Raw file	Scan	Method	Score	m/z
sys_05_2	13137	FTMS; HCD	206.64	849.39

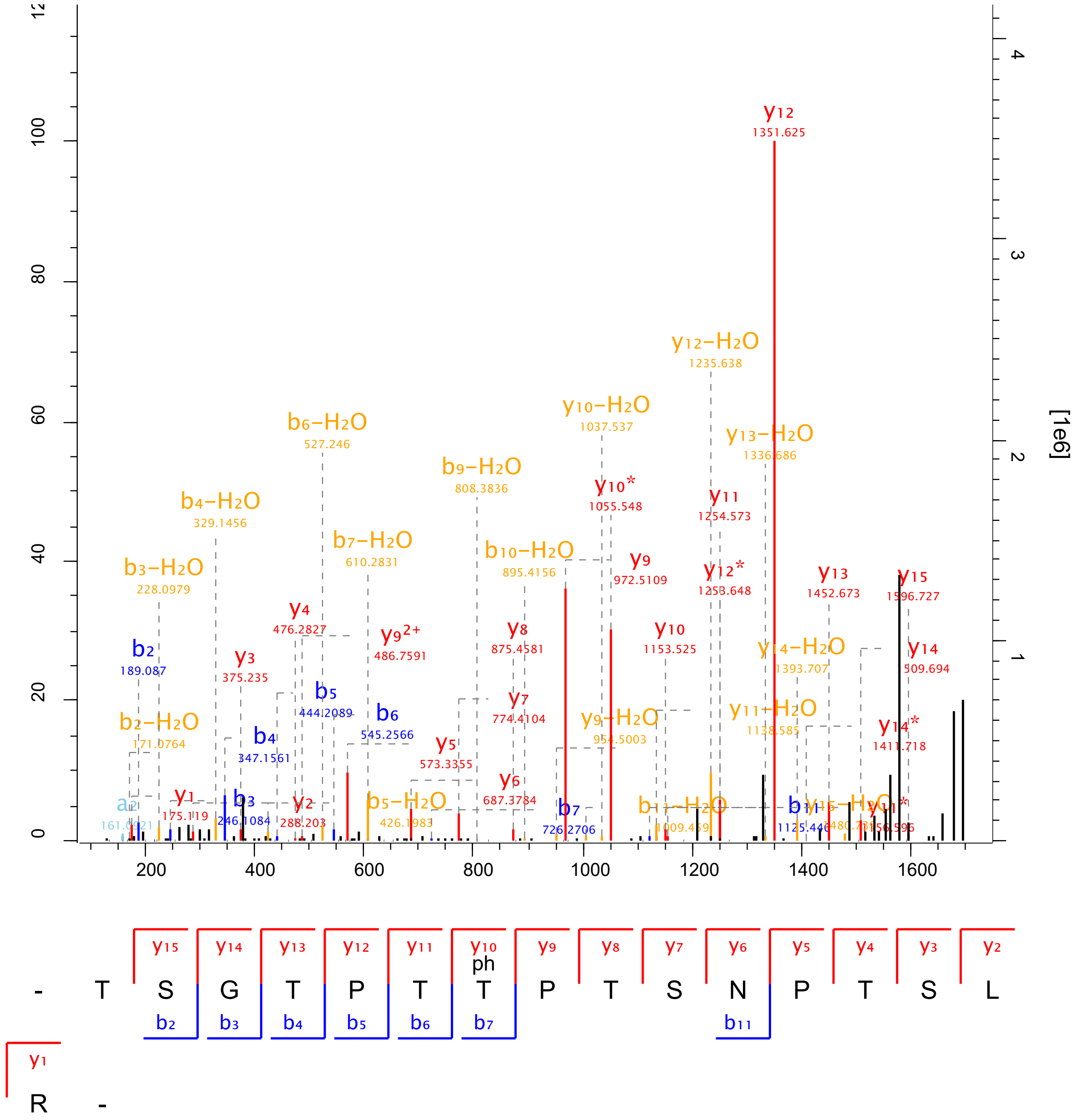

Raw file	Scan	Method	Score	m/z
sys_05_2	13141	FTMS; HCD	55.75	760.77

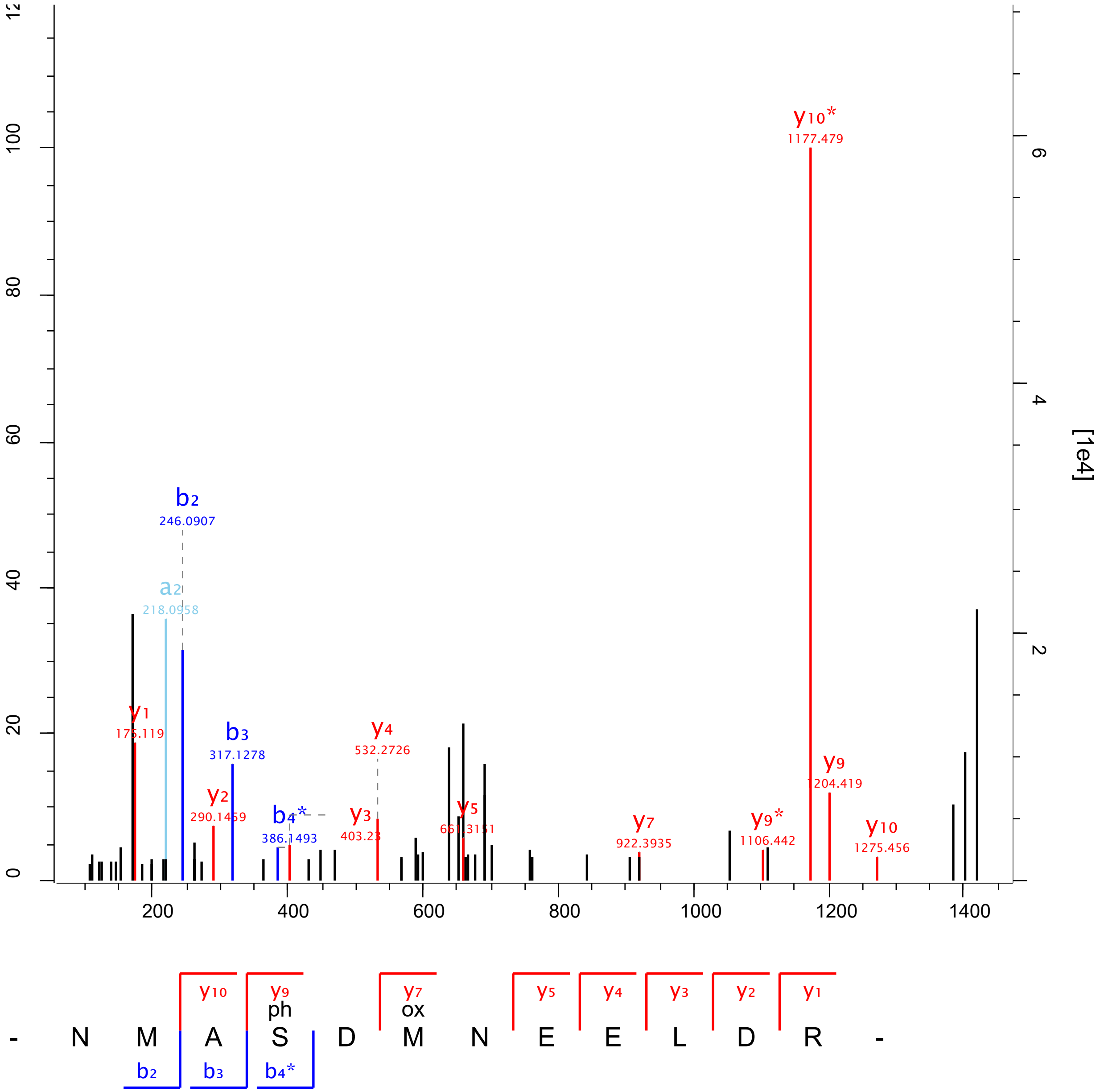

Raw file	Scan	Method	Score	m/z
sys_05_2	13184	FTMS; HCD	142.1	572.24

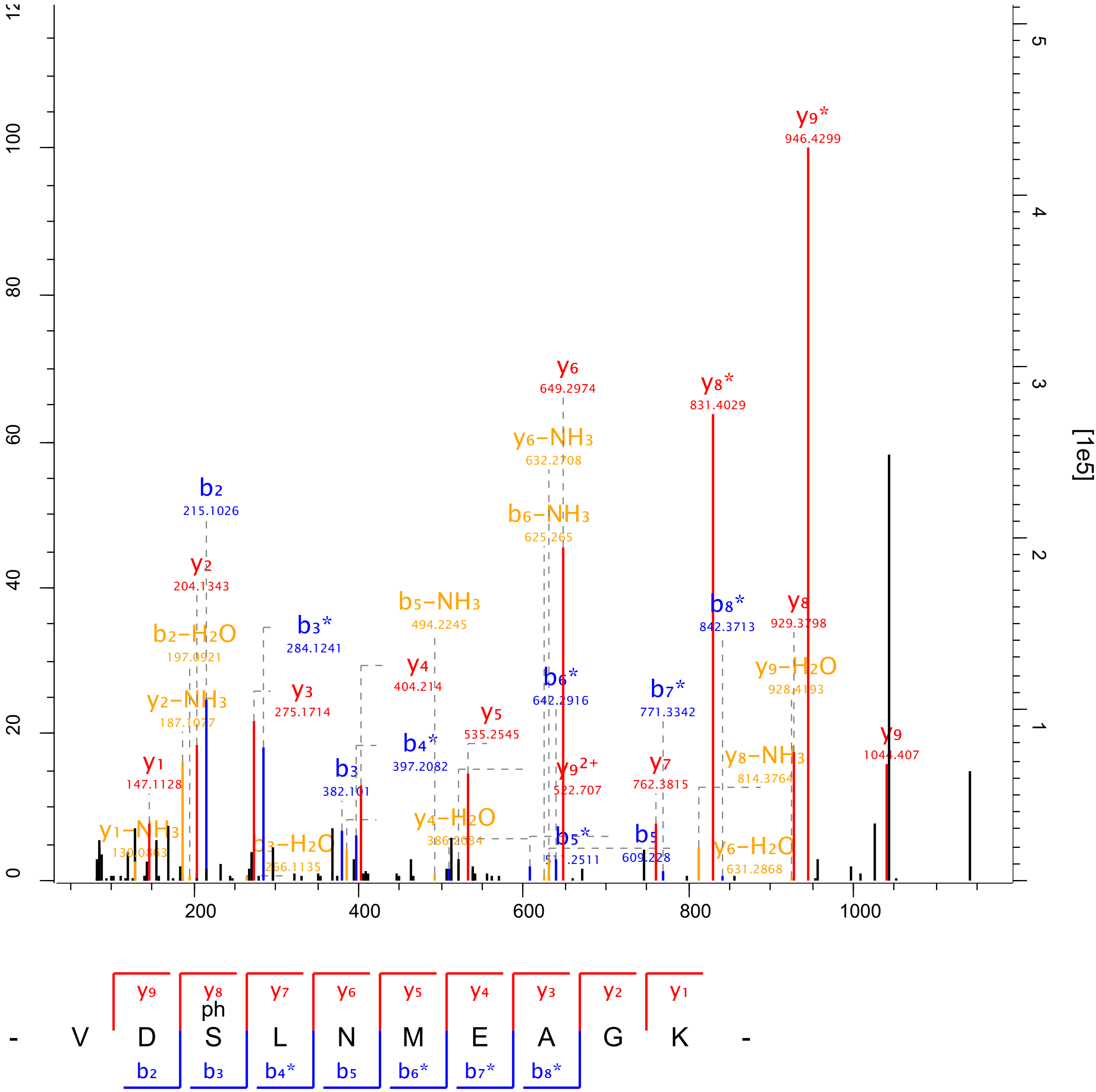

Raw file	Scan	Method	Score	m/z
sys_05_2	13306	FTMS; HCD	113.65	645.78

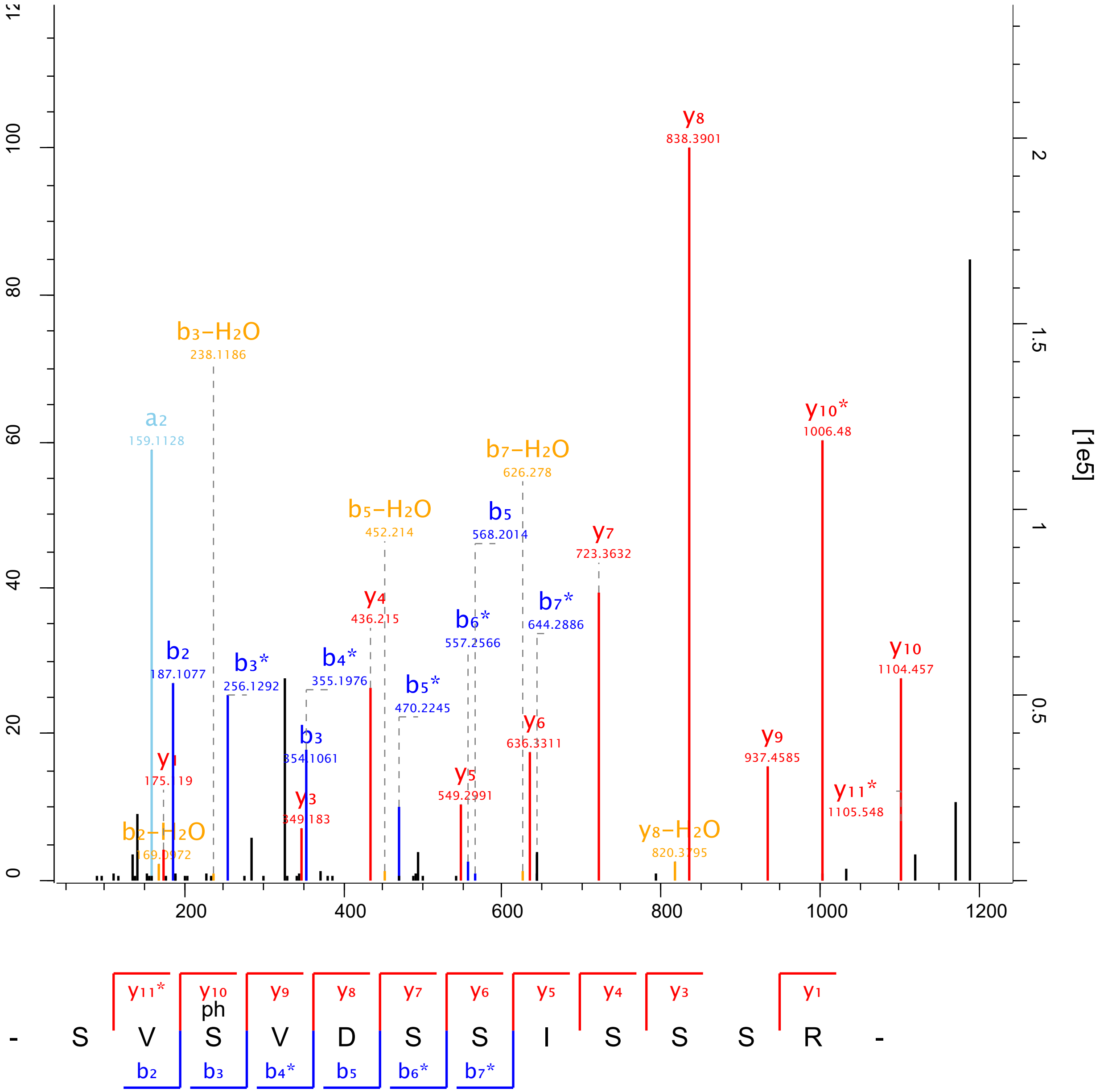

Raw file	Scan	Method	Score	m/z
sys_05_2	13556	FTMS; HCD	81.52	500.22

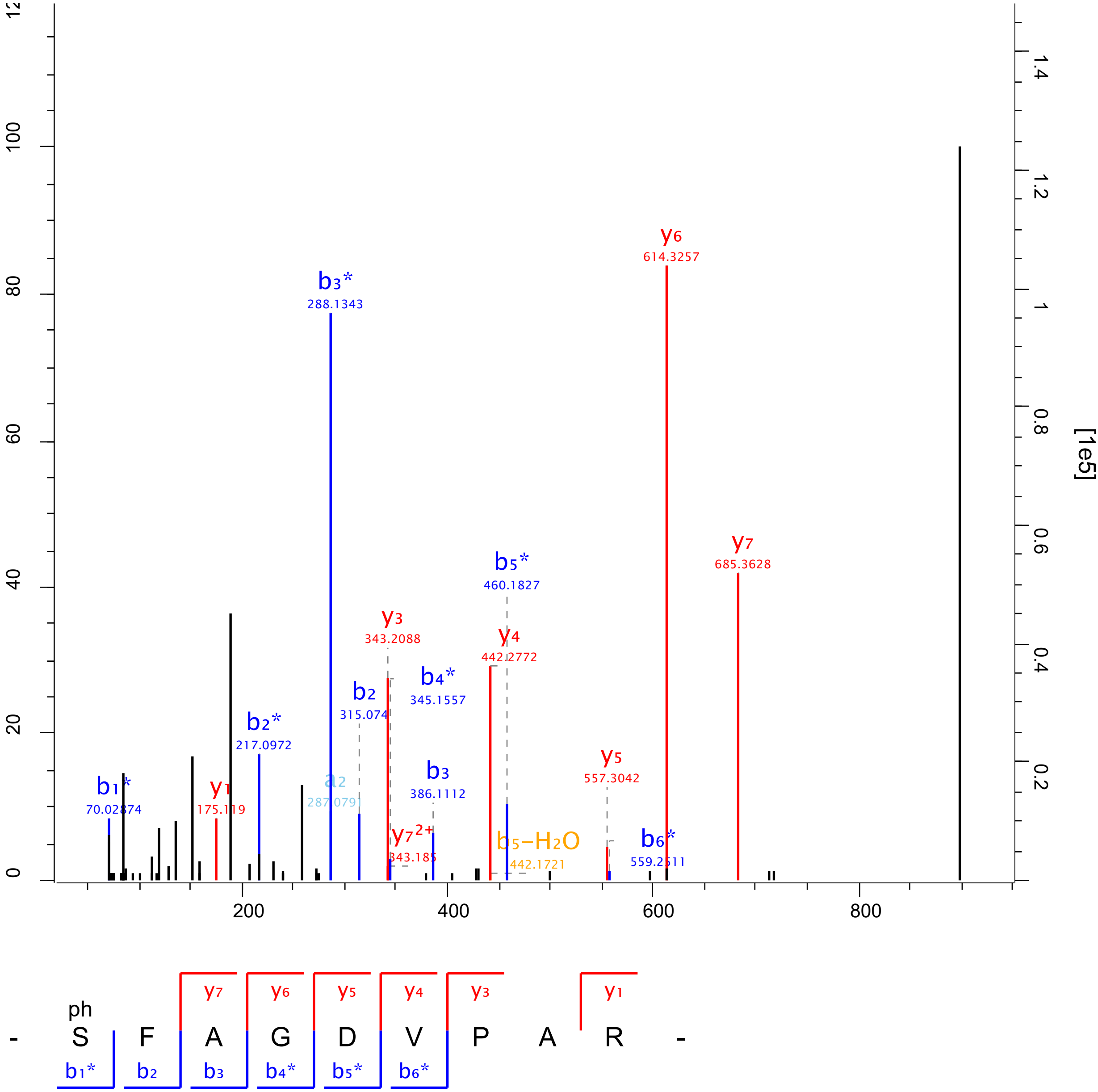

Raw file	Scan	Method	Score	m/z
sys_05_2	13614	FTMS; HCD	94.47	508.74

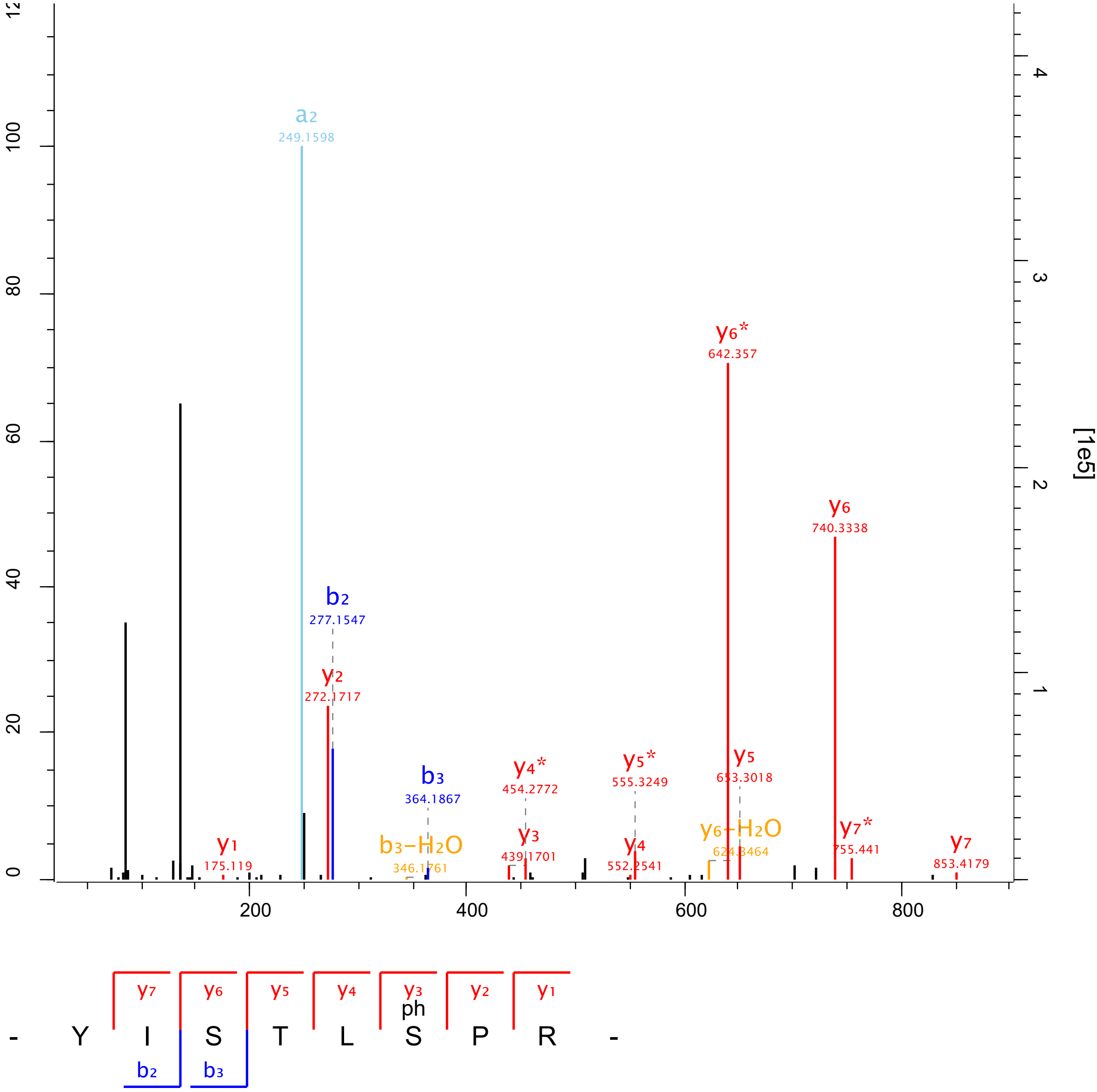

Raw file	Scan	Method	Score	m/z
sys_05_2	13695	FTMS; HCD	116.73	892.87

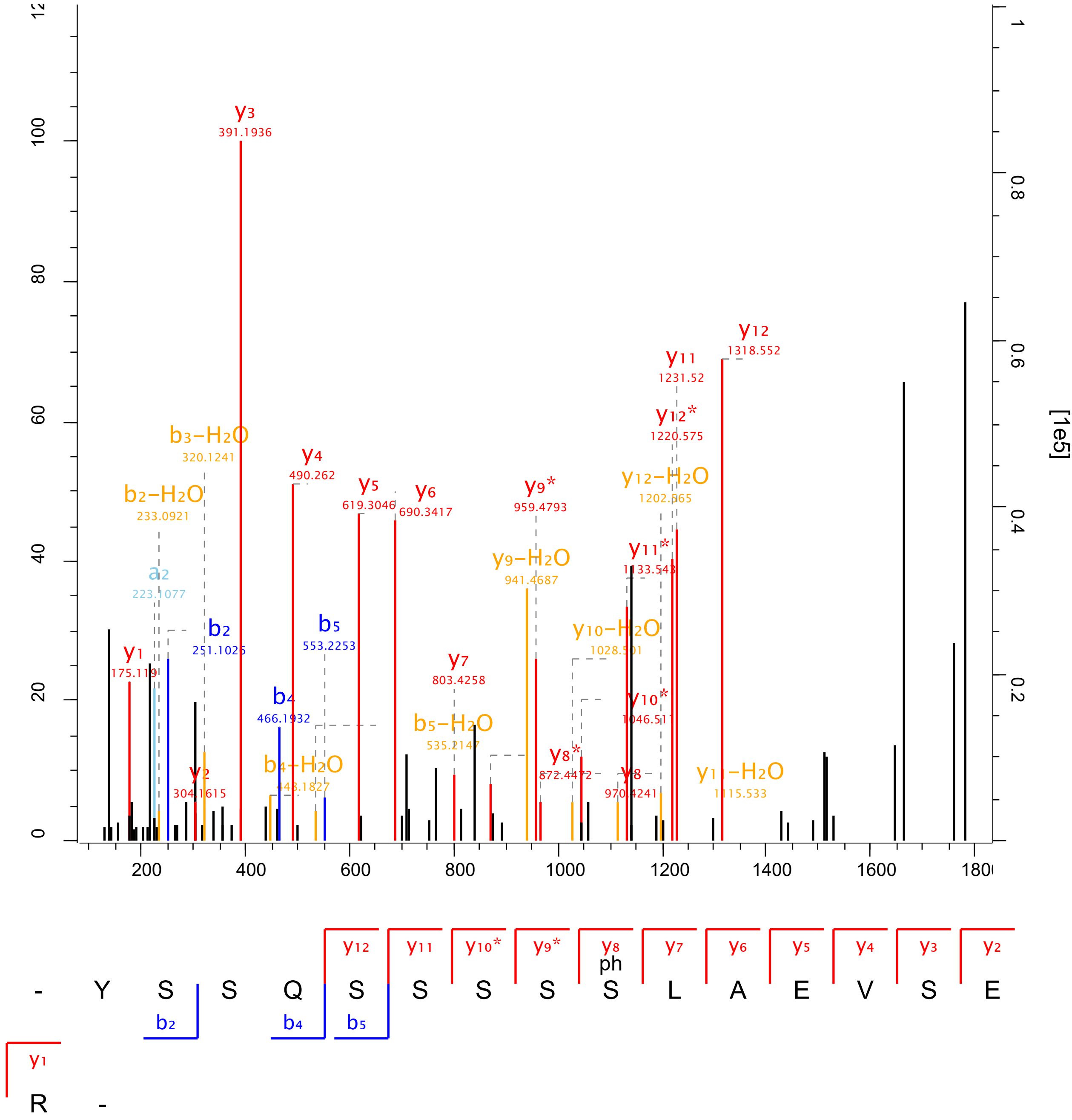

Raw file	Scan	Method	Score	m/z
sys_05_2	13725	FTMS; HCD	108.77	786.33

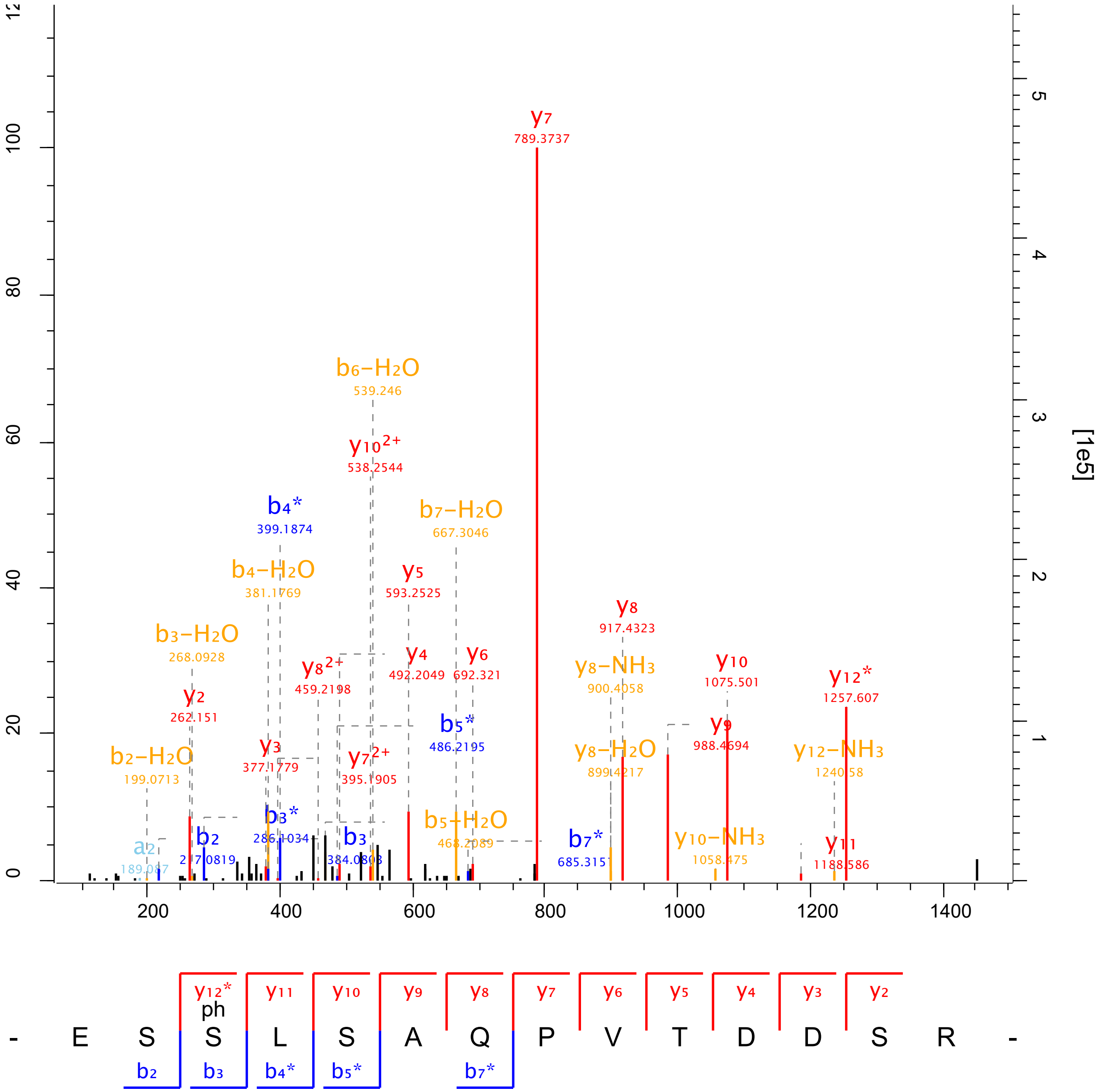

Raw file	Scan	Method	Score	m/z
sys_05_2	13869	FTMS; HCD	107.09	528.24

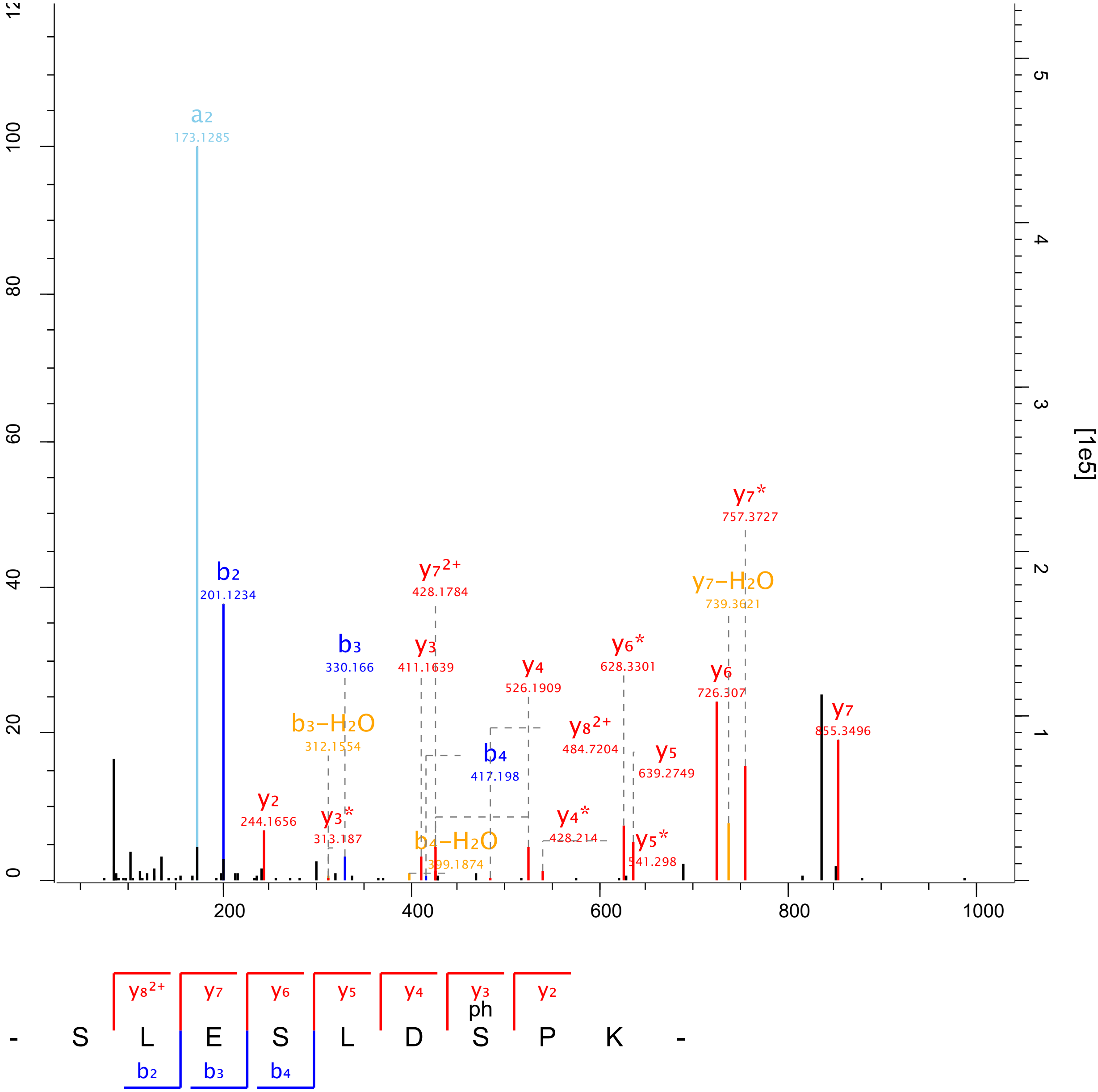

Raw file	Scan	Method	Score	m/z
sys_05_2	13916	FTMS; HCD	47.71	586.24

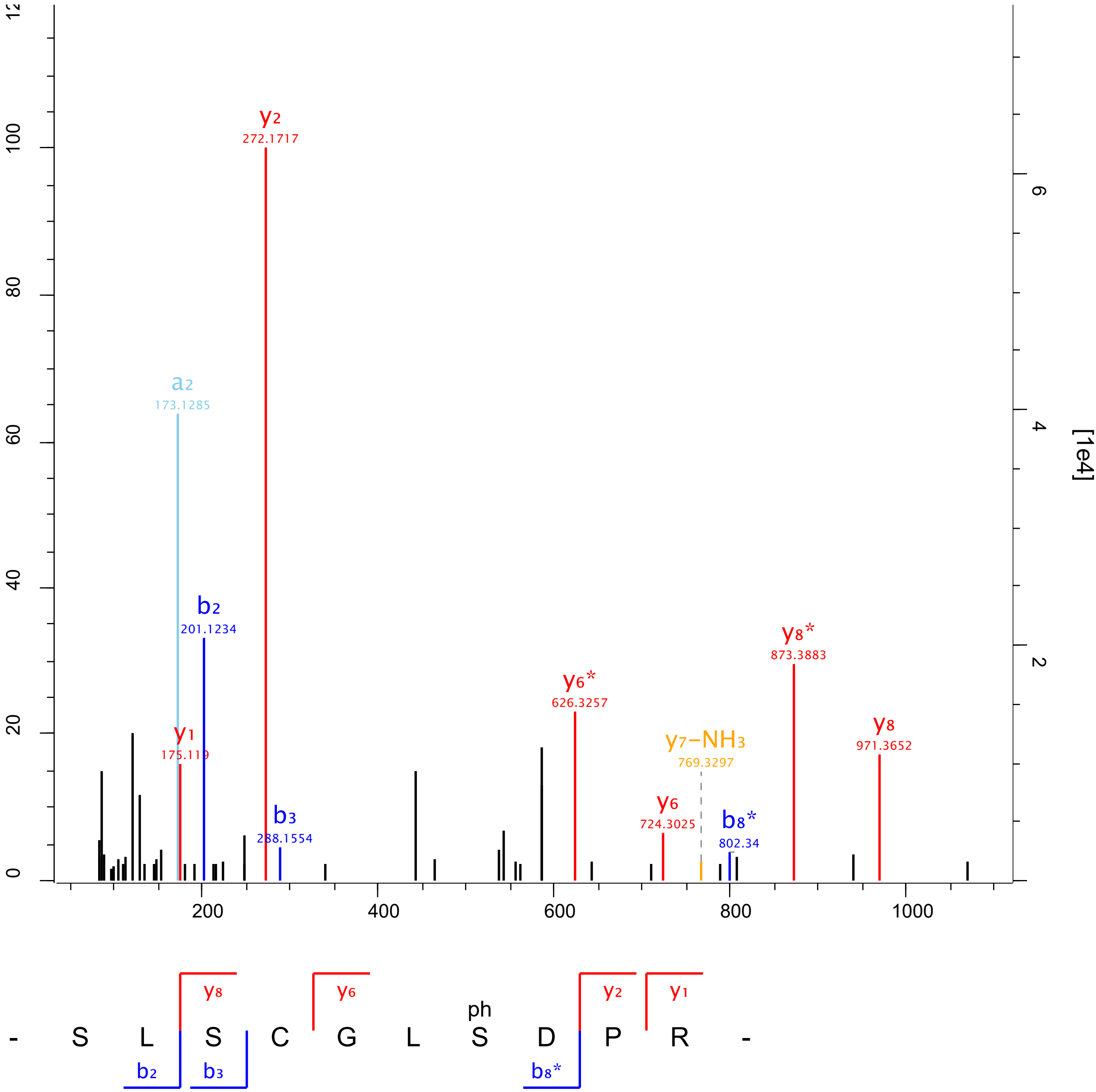

Raw file	Scan	Method	Score	m/z
sys_05_2	13946	FTMS; HCD	231.38	728.34

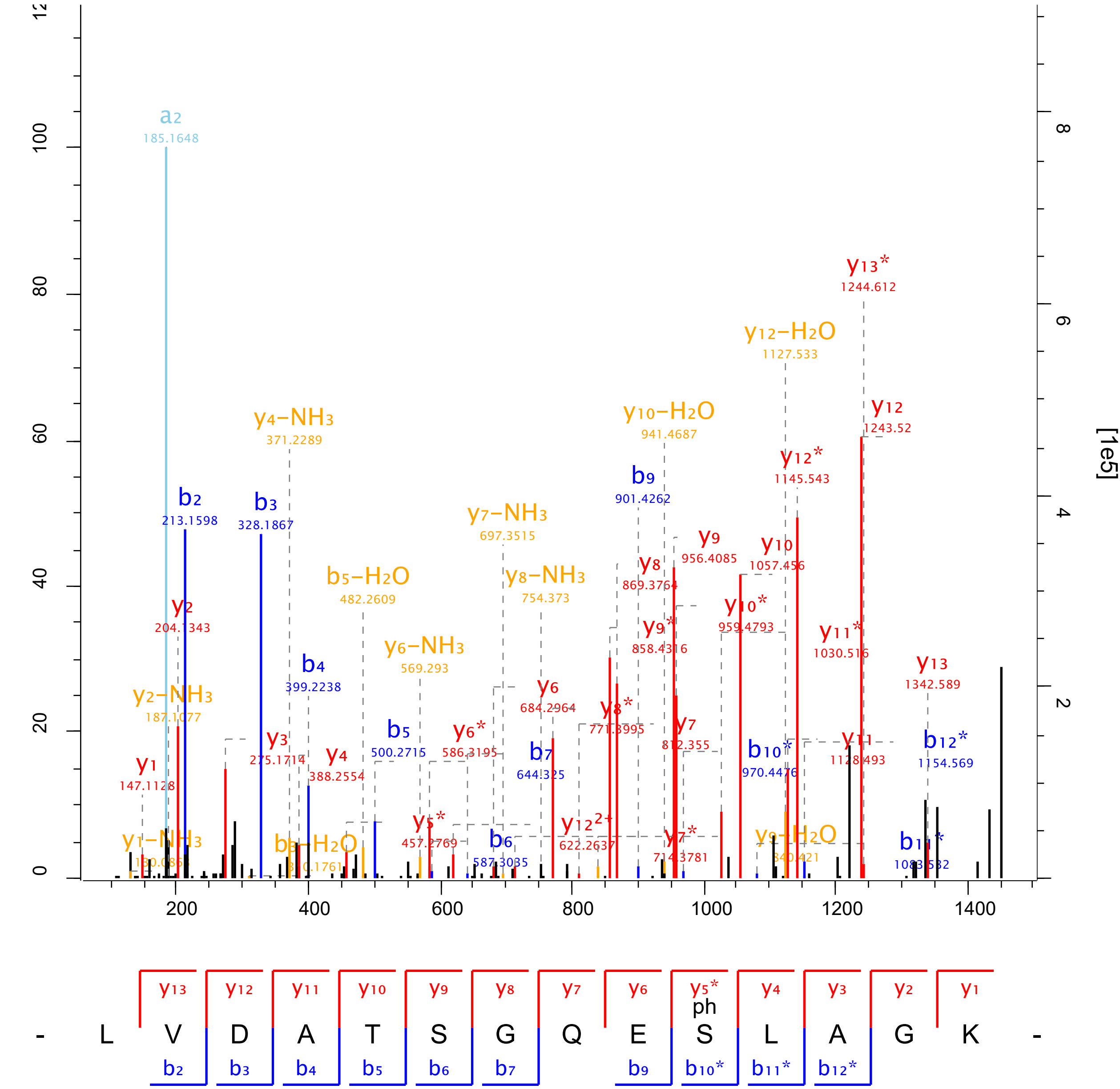

Raw file	Scan	Method	Score	m/z
sys_05_2	13977	FTMS; HCD	225.3	861.35

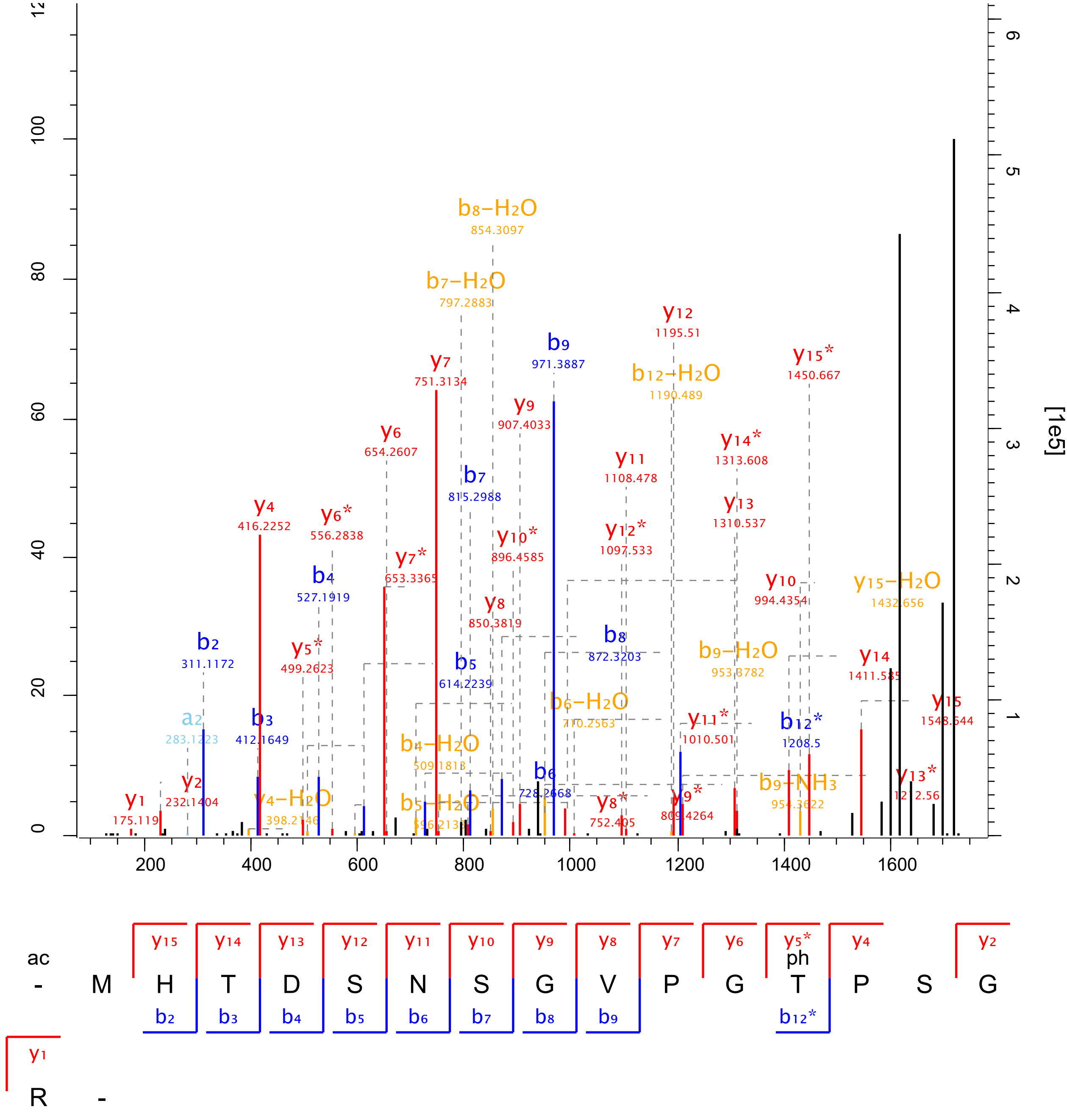

Raw file	Scan	Method	Score	m/z
sys_05_2	13988	FTMS; HCD	186.08	662.27

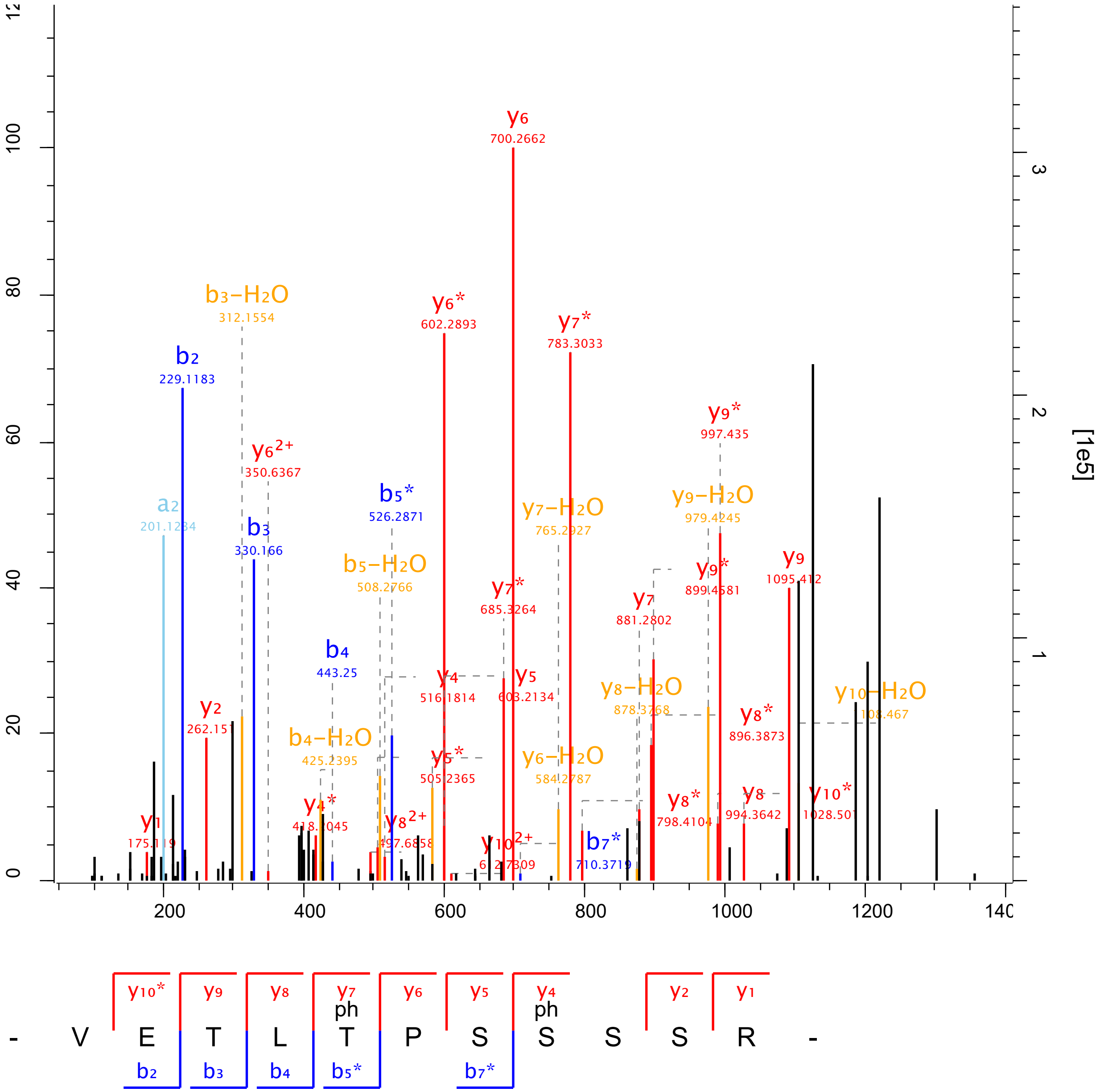

Raw file	Scan	Method	Score	m/z
sys_05_2	14017	FTMS; HCD	49.47	483.71

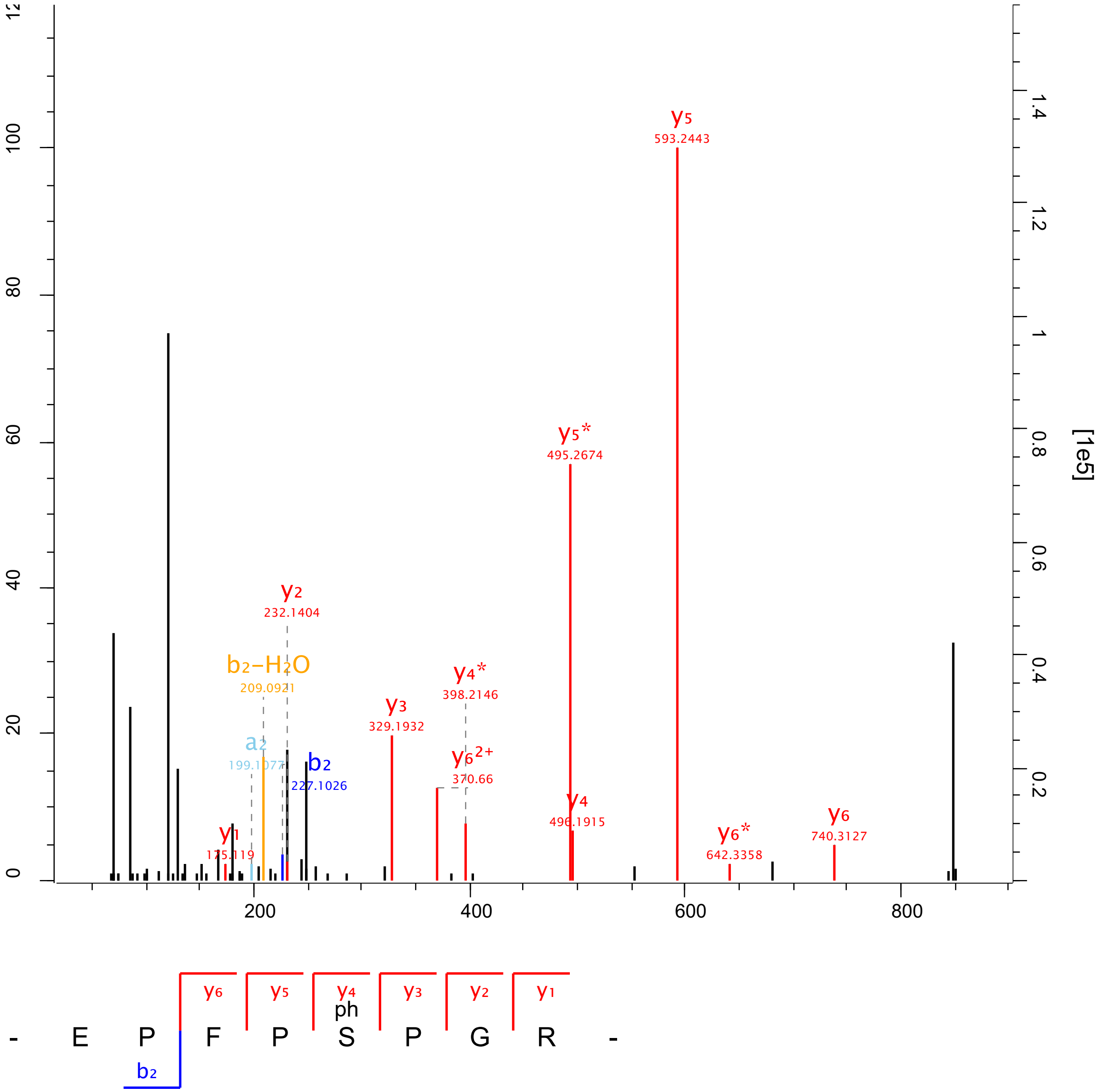

Raw file	Scan	Method	Score	m/z
sys_05_2	14170	FTMS; HCD	192.46	693.31

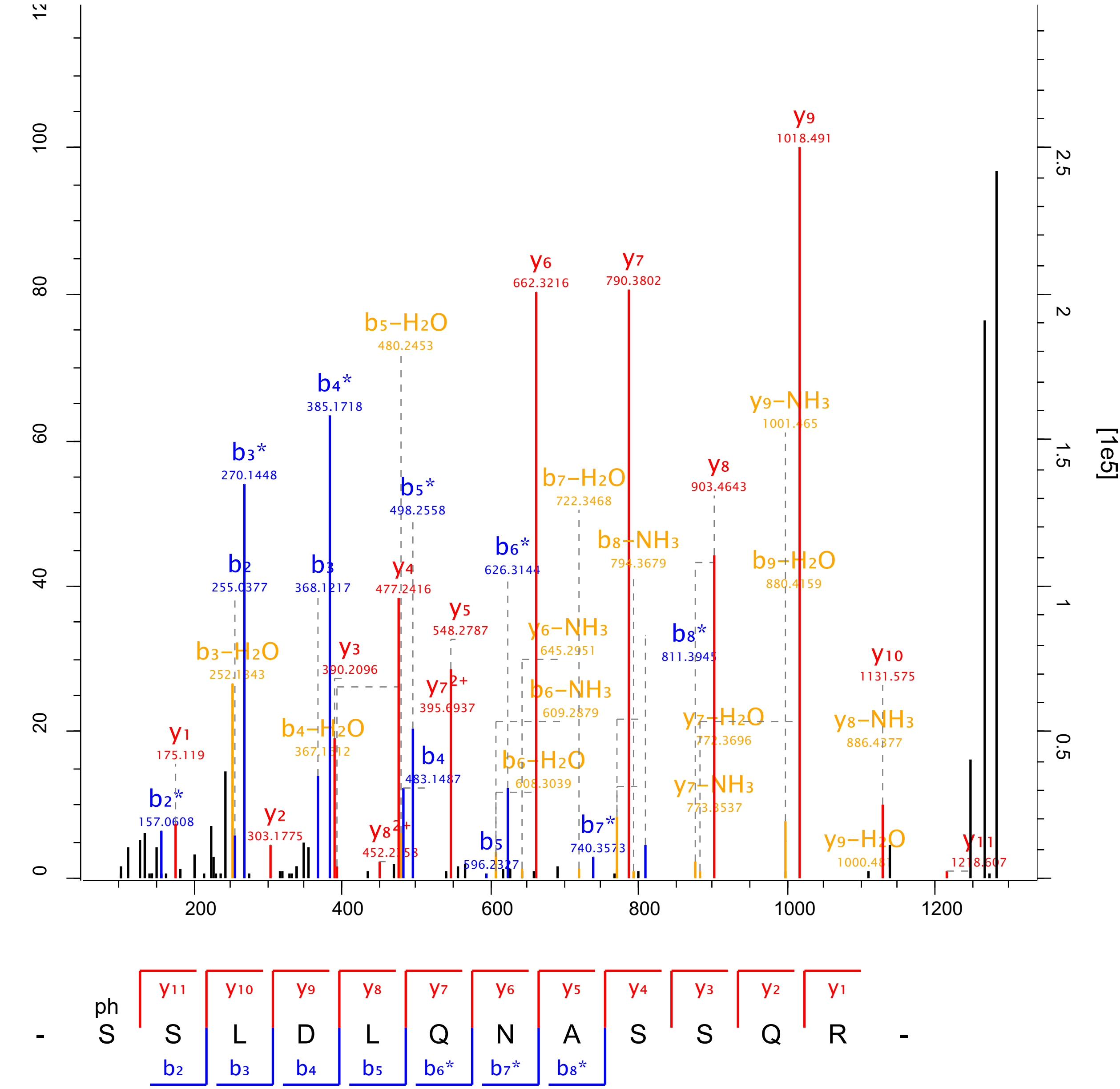

Raw file	Scan	Method	Score	m/z
sys_05_2	14184	FTMS; HCD	71.18	510.69

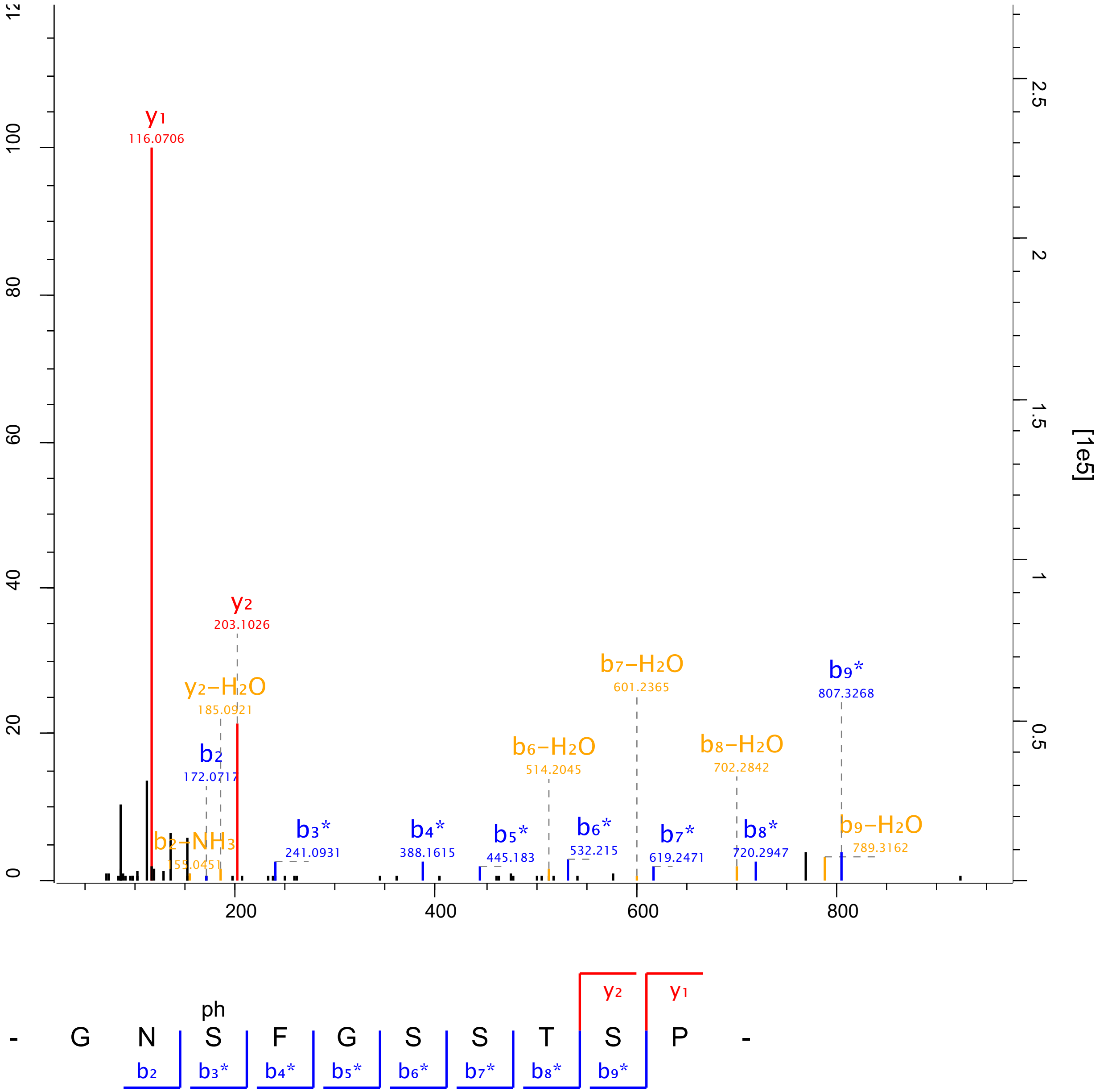

Raw file	Scan	Method	Score	m/z
sys_05_2	14217	FTMS; HCD	103.88	758.83

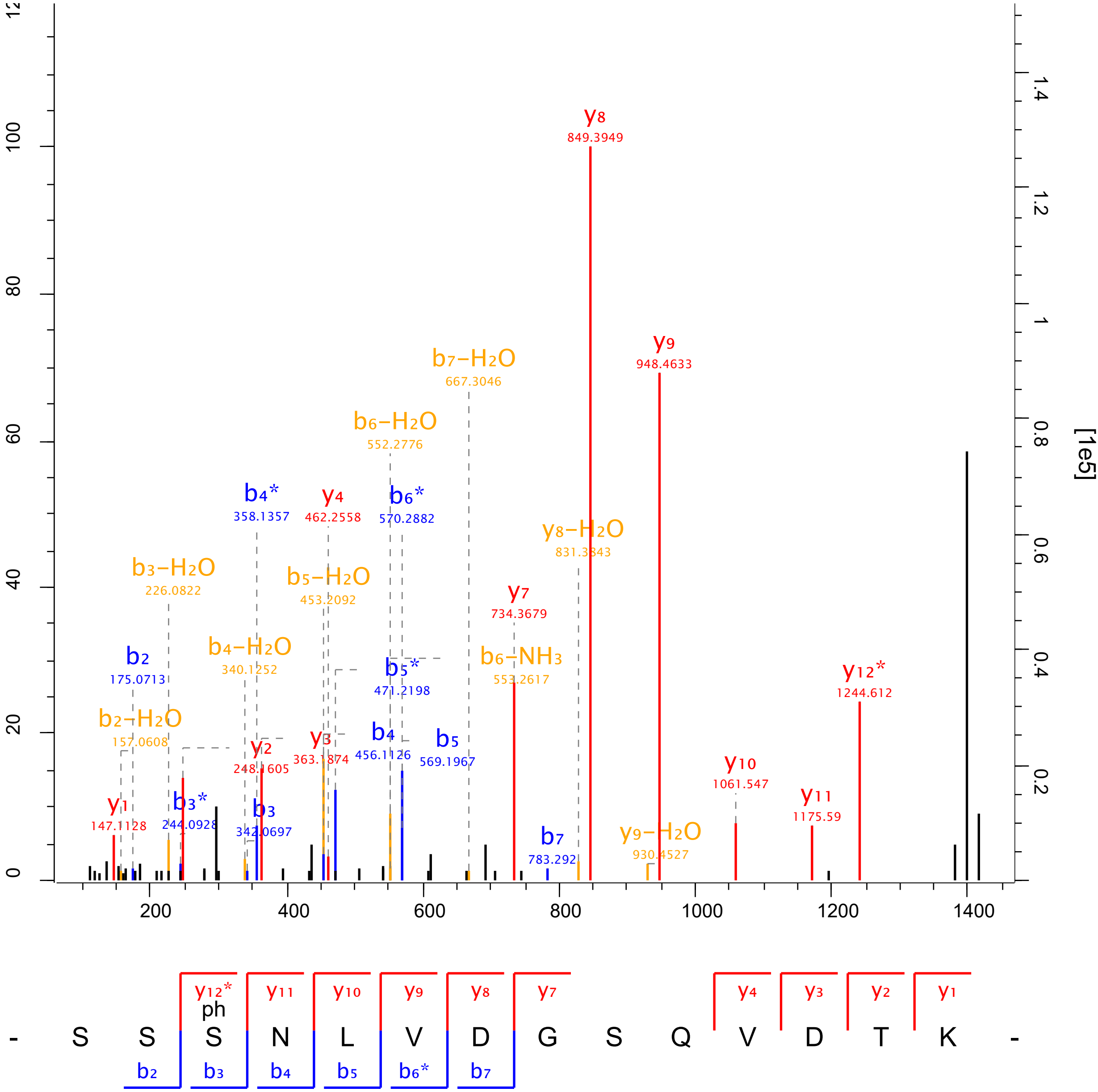

Raw file	Scan	Method	Score	m/z
sys_05_2	14279	FTMS; HCD	84.36	587.76

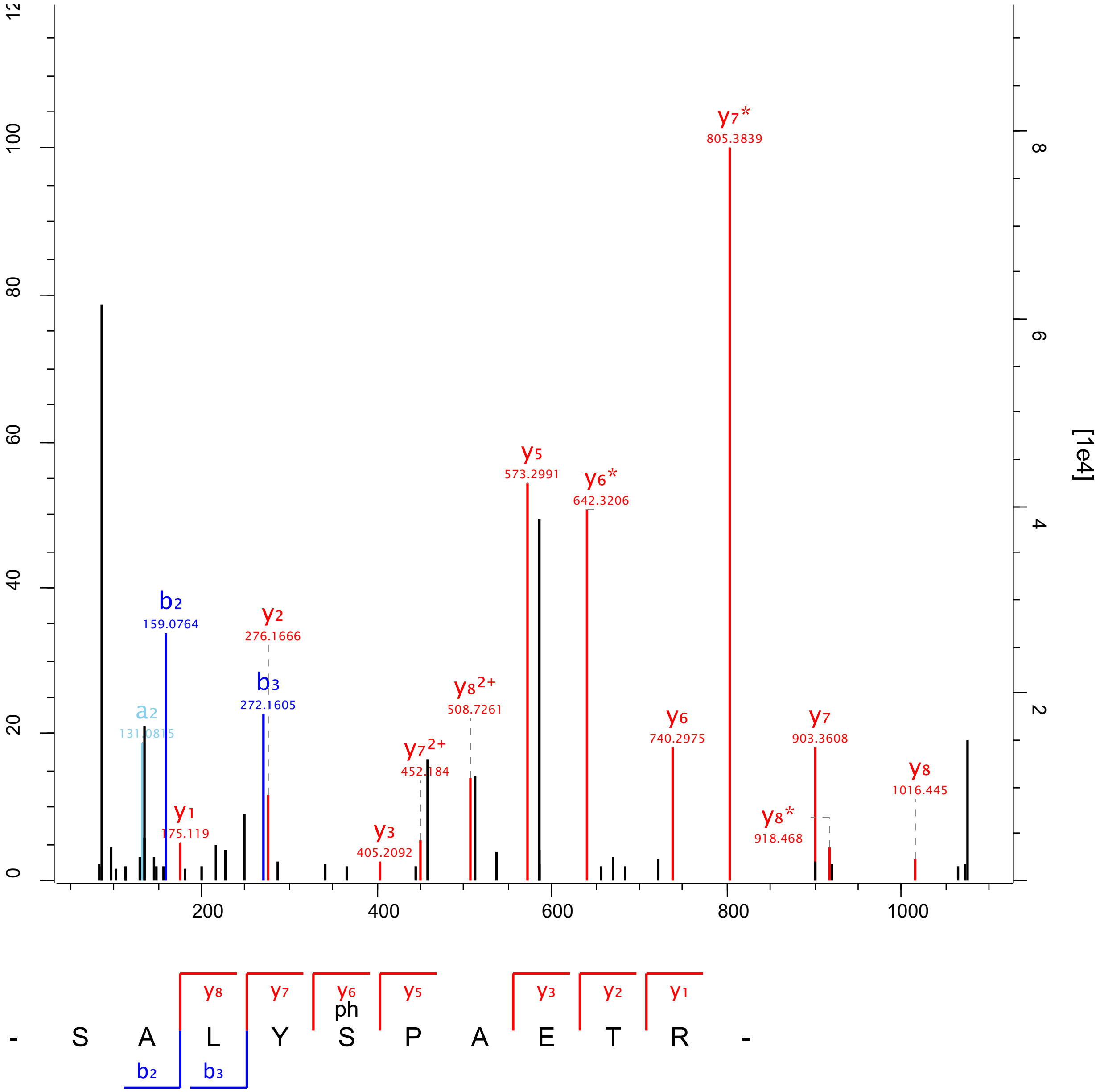

Raw file	Scan	Method	Score	m/z
sys_05_2	14316	FTMS; HCD	108.47	521.73

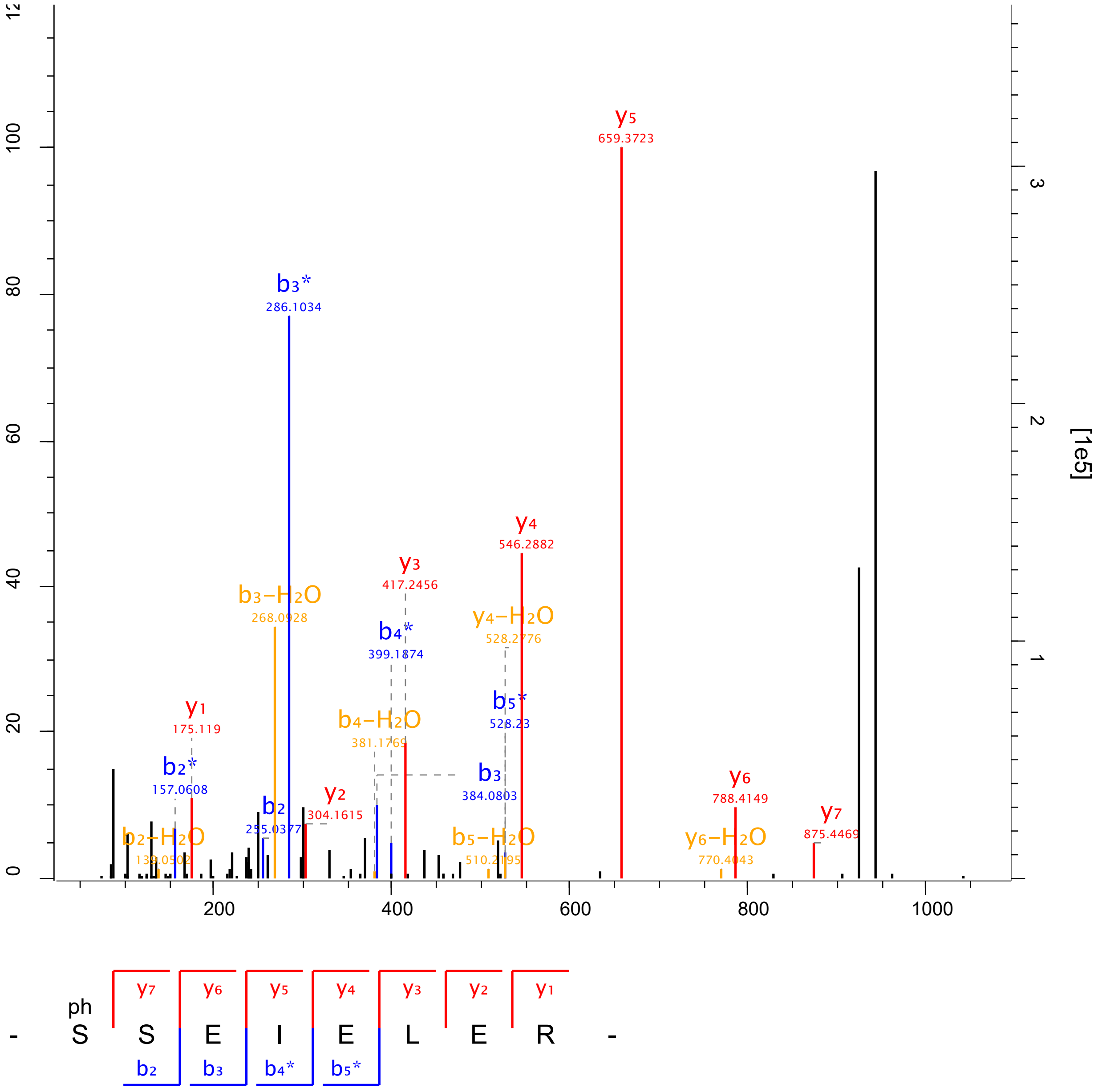

Raw file	Scan	Method	Score	m/z
sys_05_2	14367	FTMS; HCD	152.94	723.82

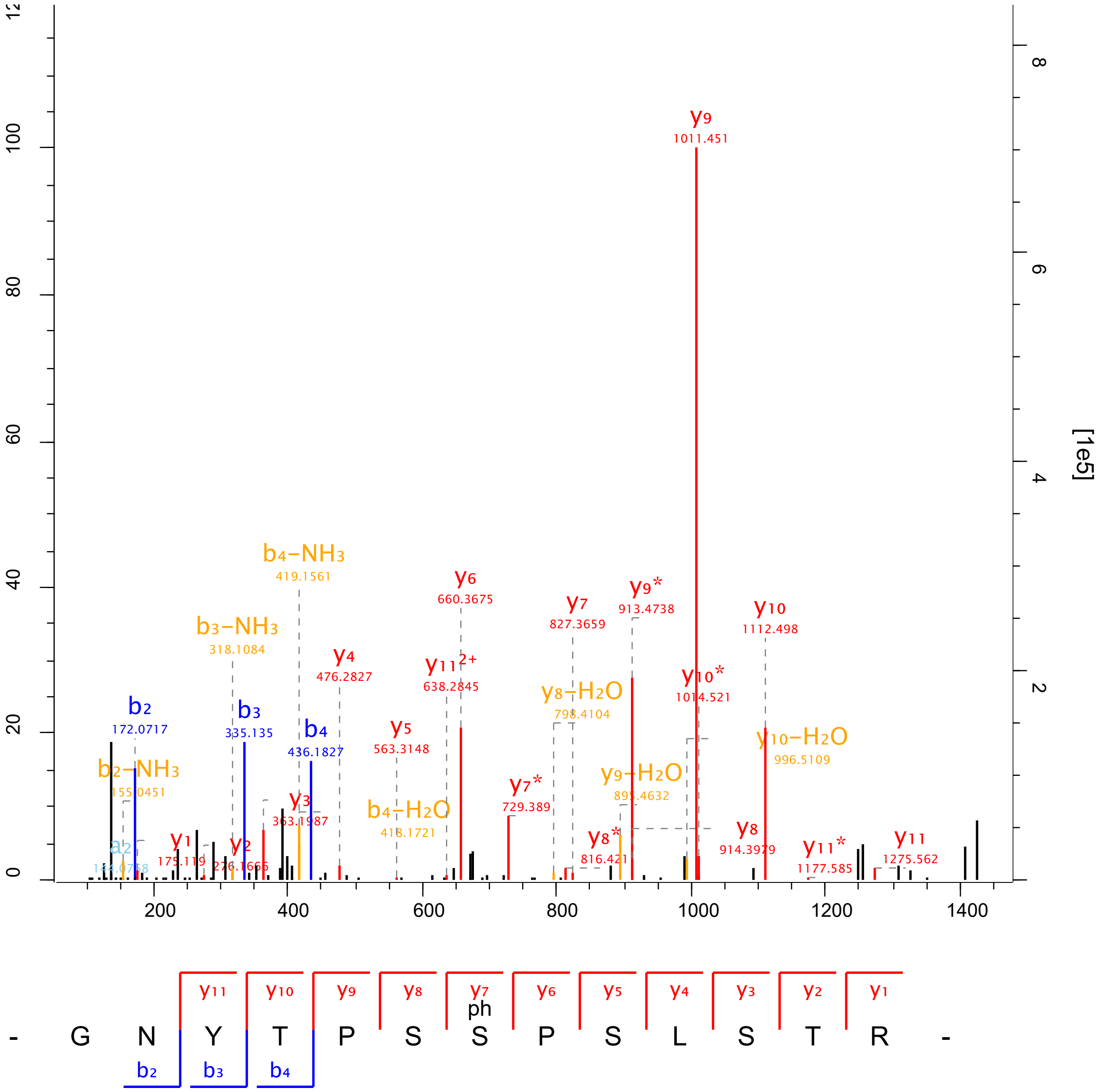

Raw file	Scan	Method	Score	m/z
sys_05_2	14385	FTMS; HCD	75.76	677.76

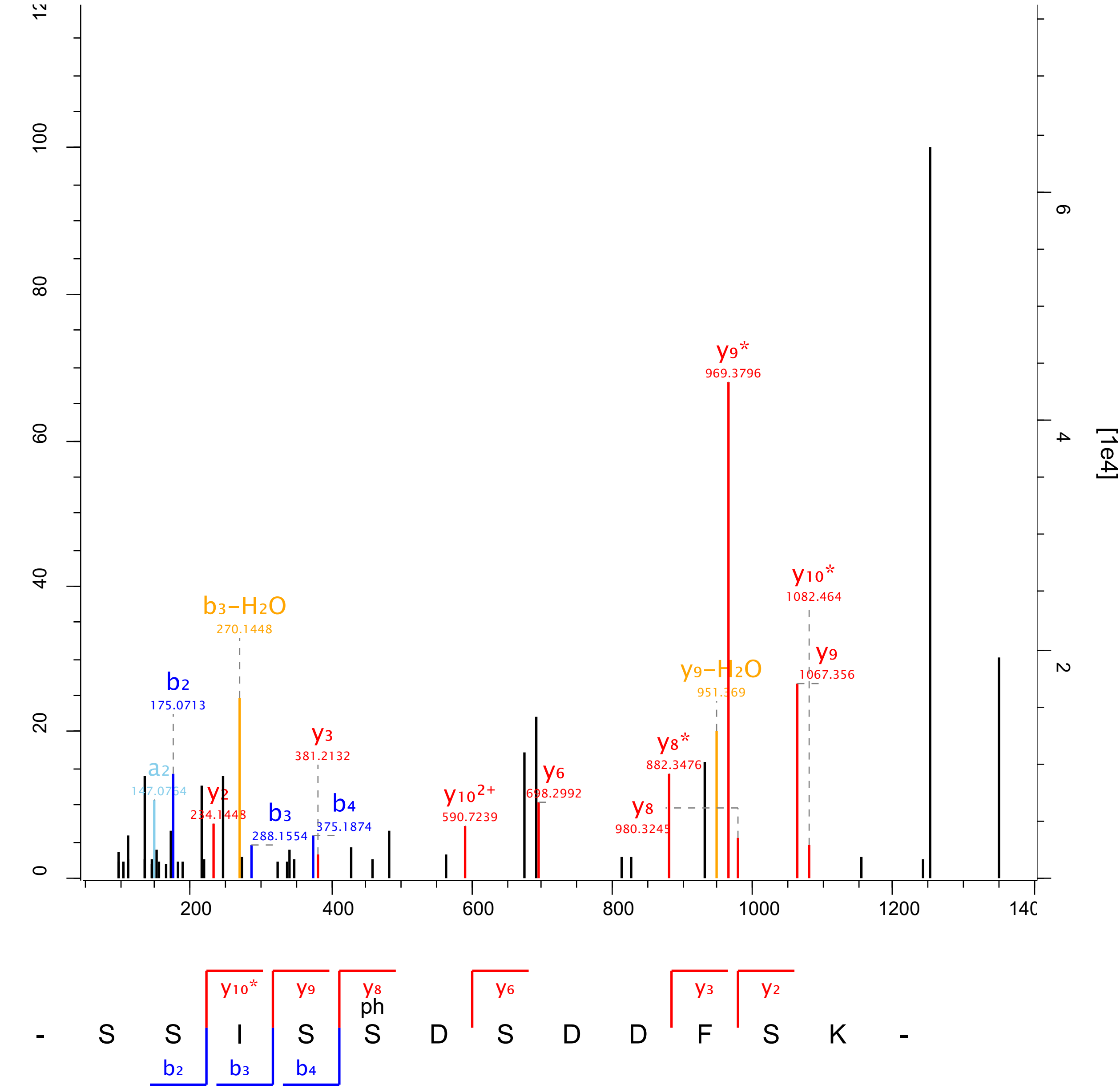

Raw file	Scan	Method	Score	m/z
sys_05_2	14408	FTMS; HCD	51.77	631.76

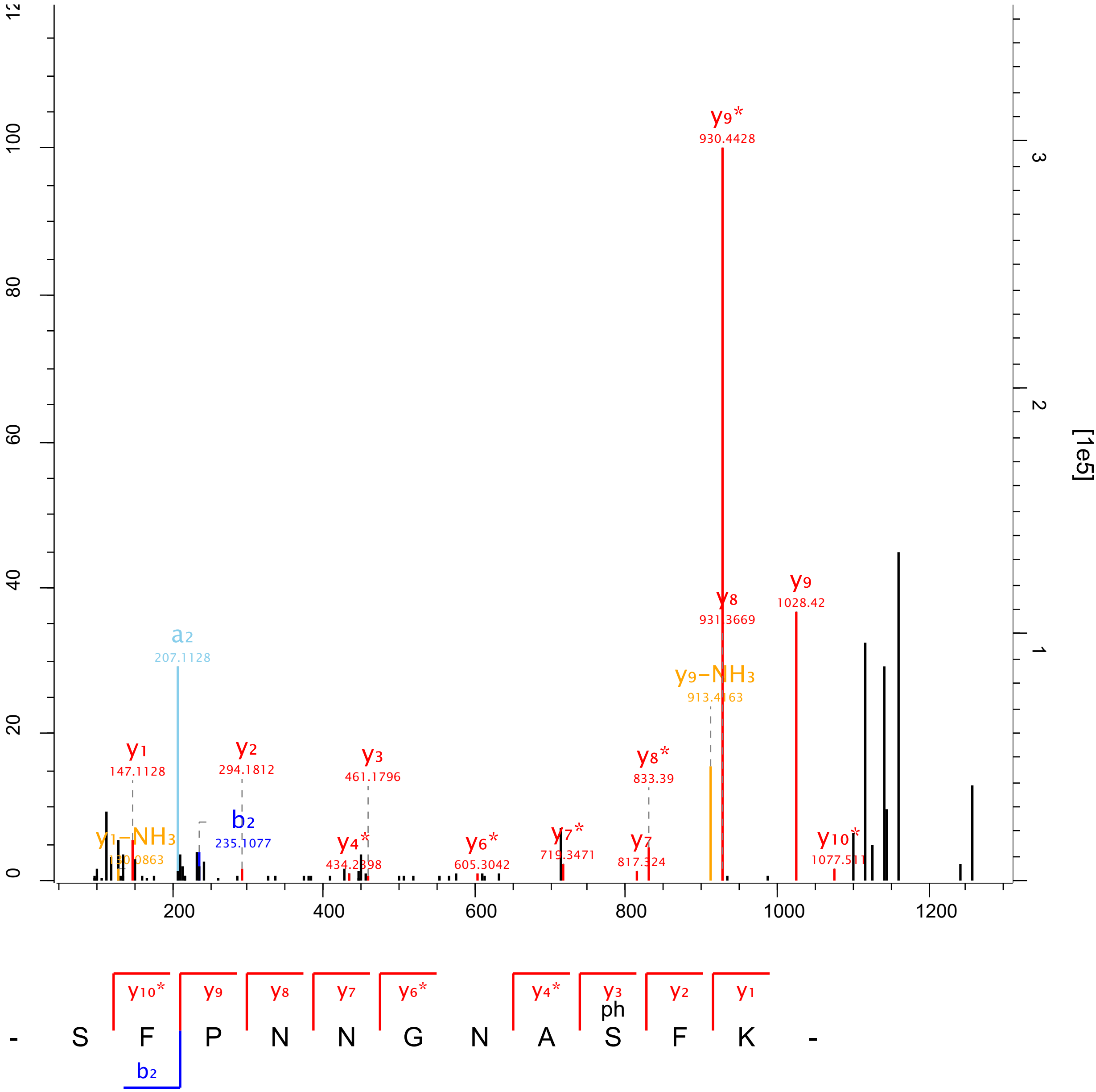

Raw file	Scan	Method	Score	m/z
sys_05_2	14420	FTMS; HCD	197.41	711.81

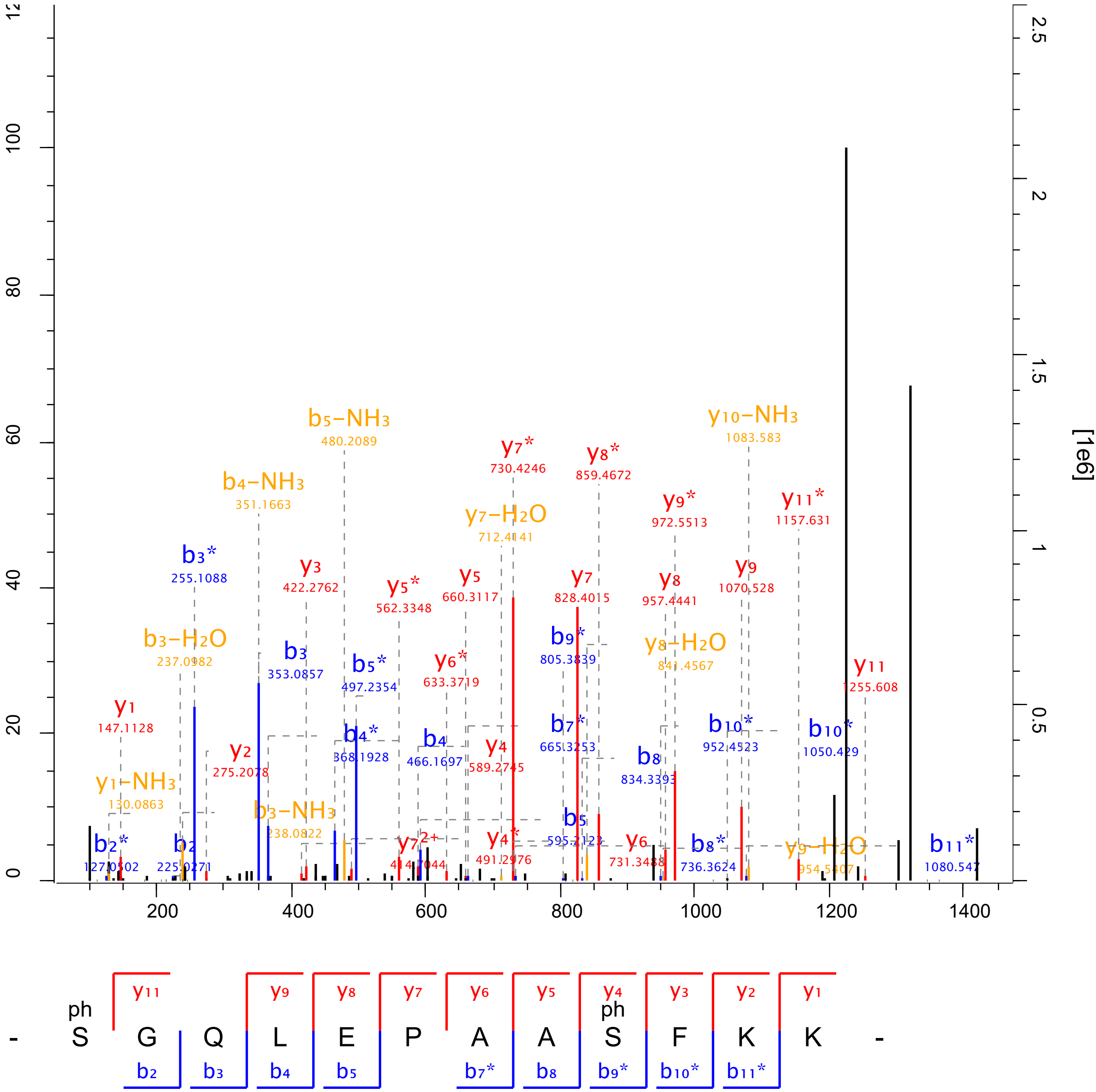

Raw file	Scan	Method	Score	m/z
sys_05_2	14445	FTMS; HCD	84.76	595.78

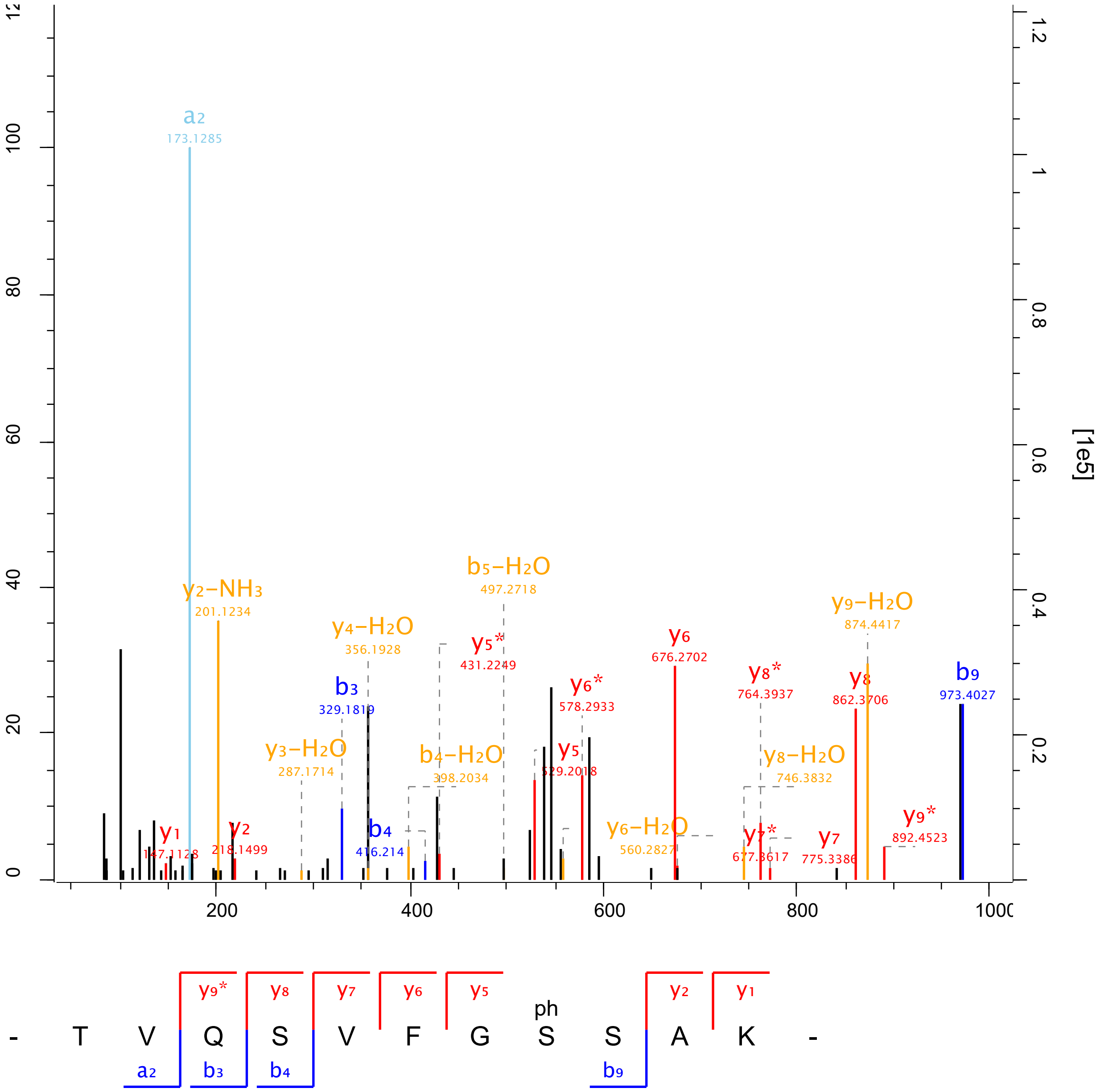

Raw file	Scan	Method	Score	m/z
sys_05_2	14485	FTMS; HCD	97.69	601.26

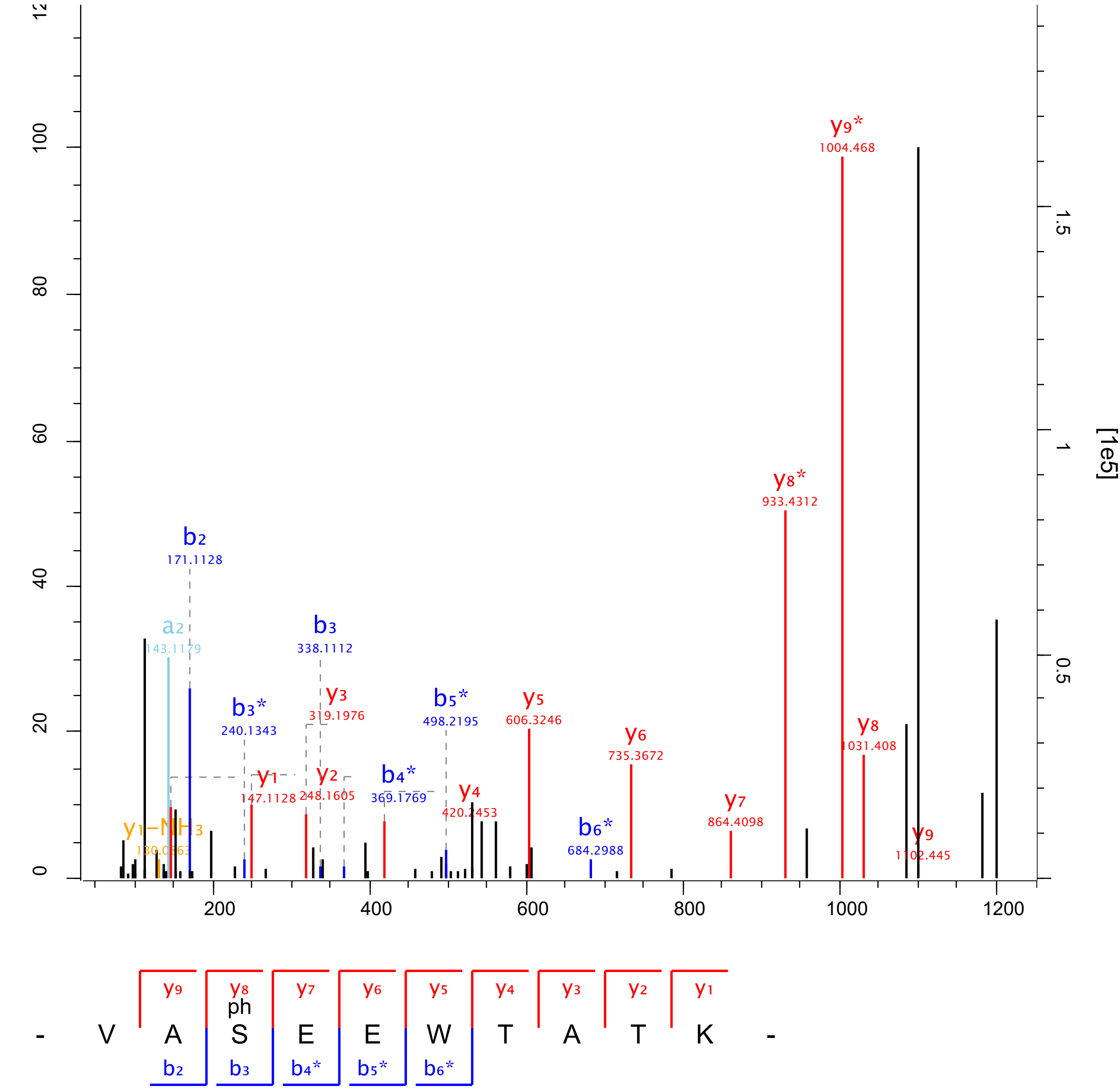

Raw file	Scan	Method	Score	m/z
sys_05_2	14530	FTMS; HCD	77.19	475.9

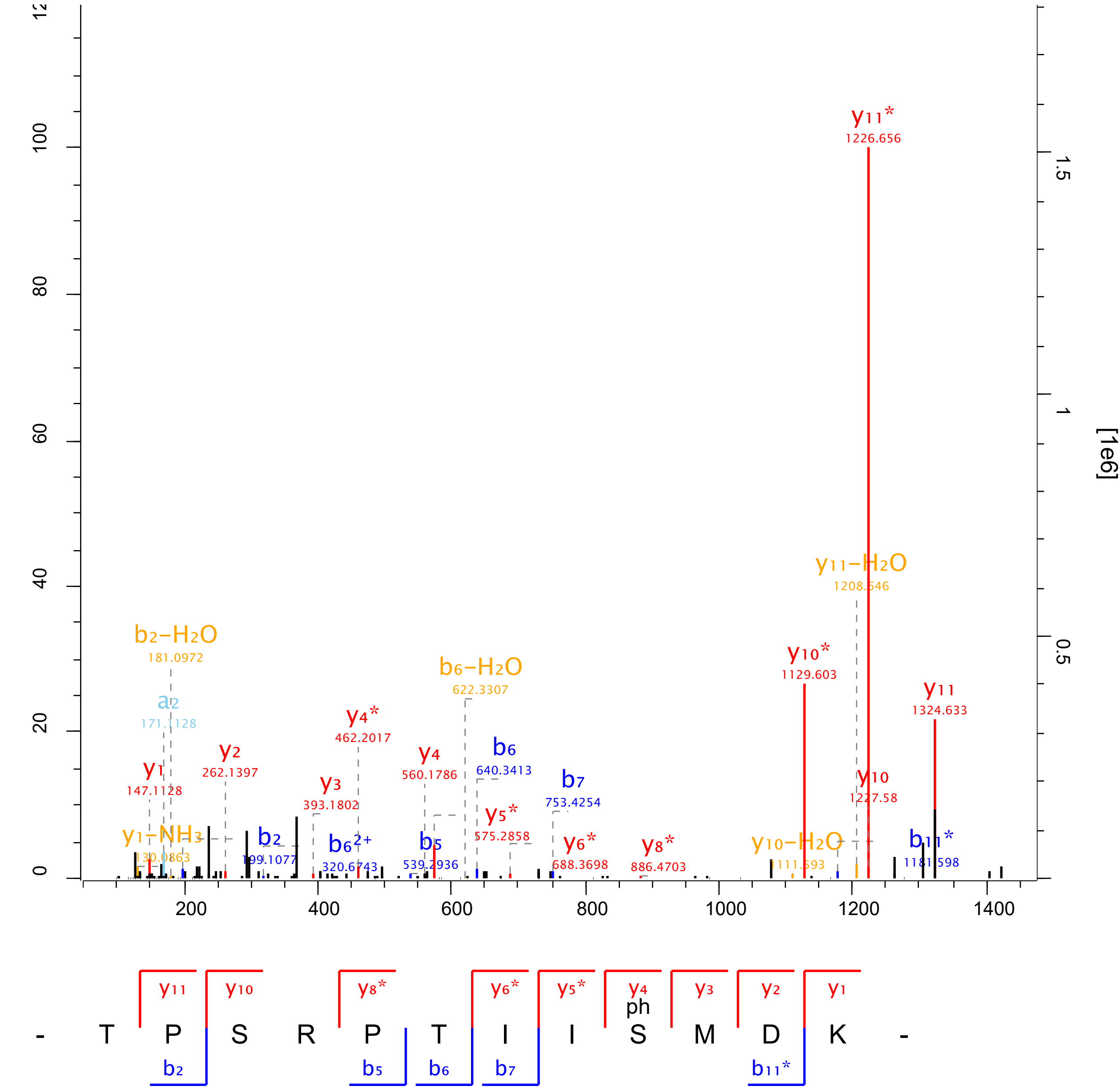

Raw file	Scan	Method	Score	m/z
sys_05_2	14534	FTMS; HCD	192.46	625.74

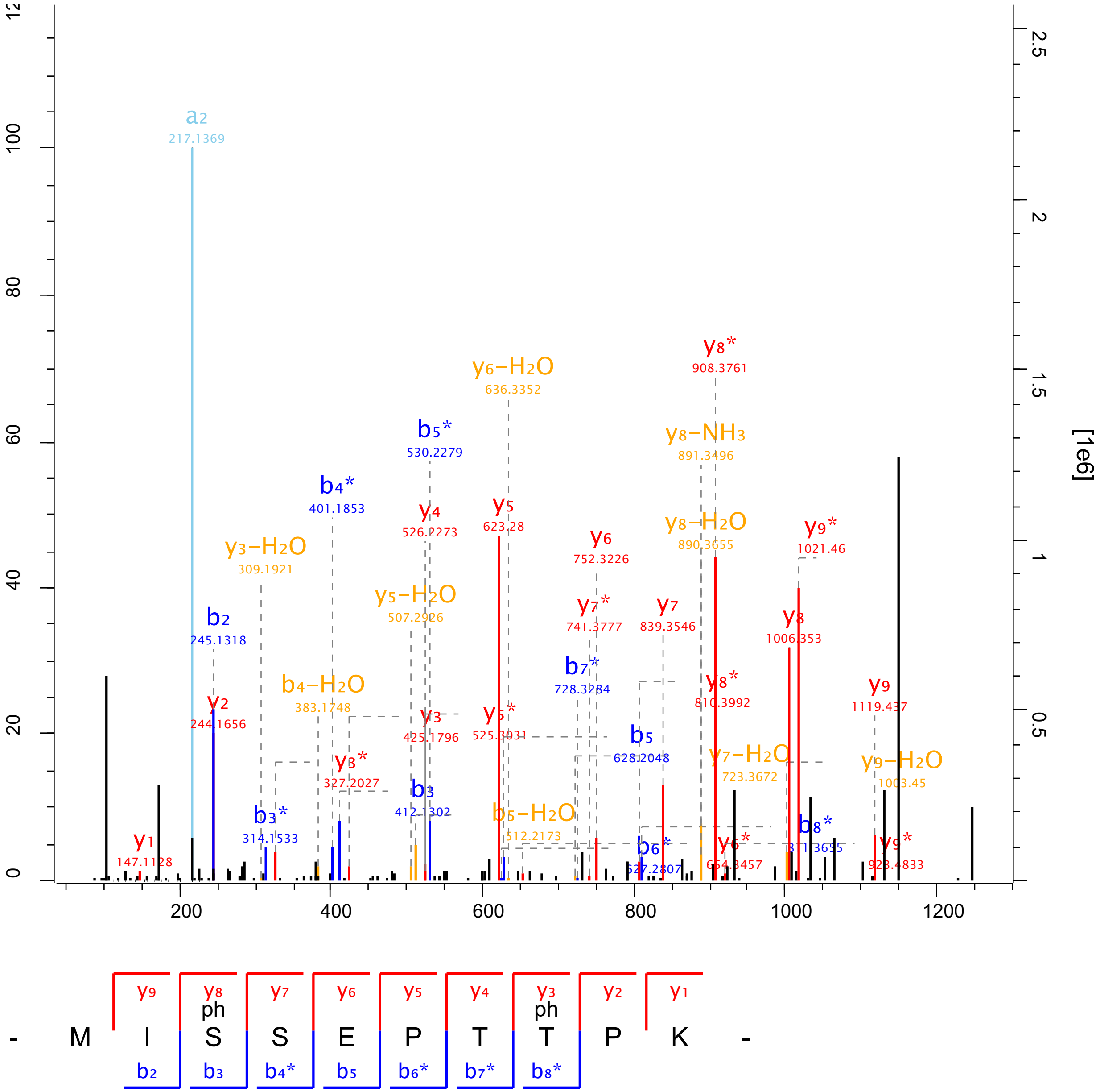

Raw file	Scan	Method	Score	m/z
sys_05_2	14655	FTMS; HCD	49.99	517.9

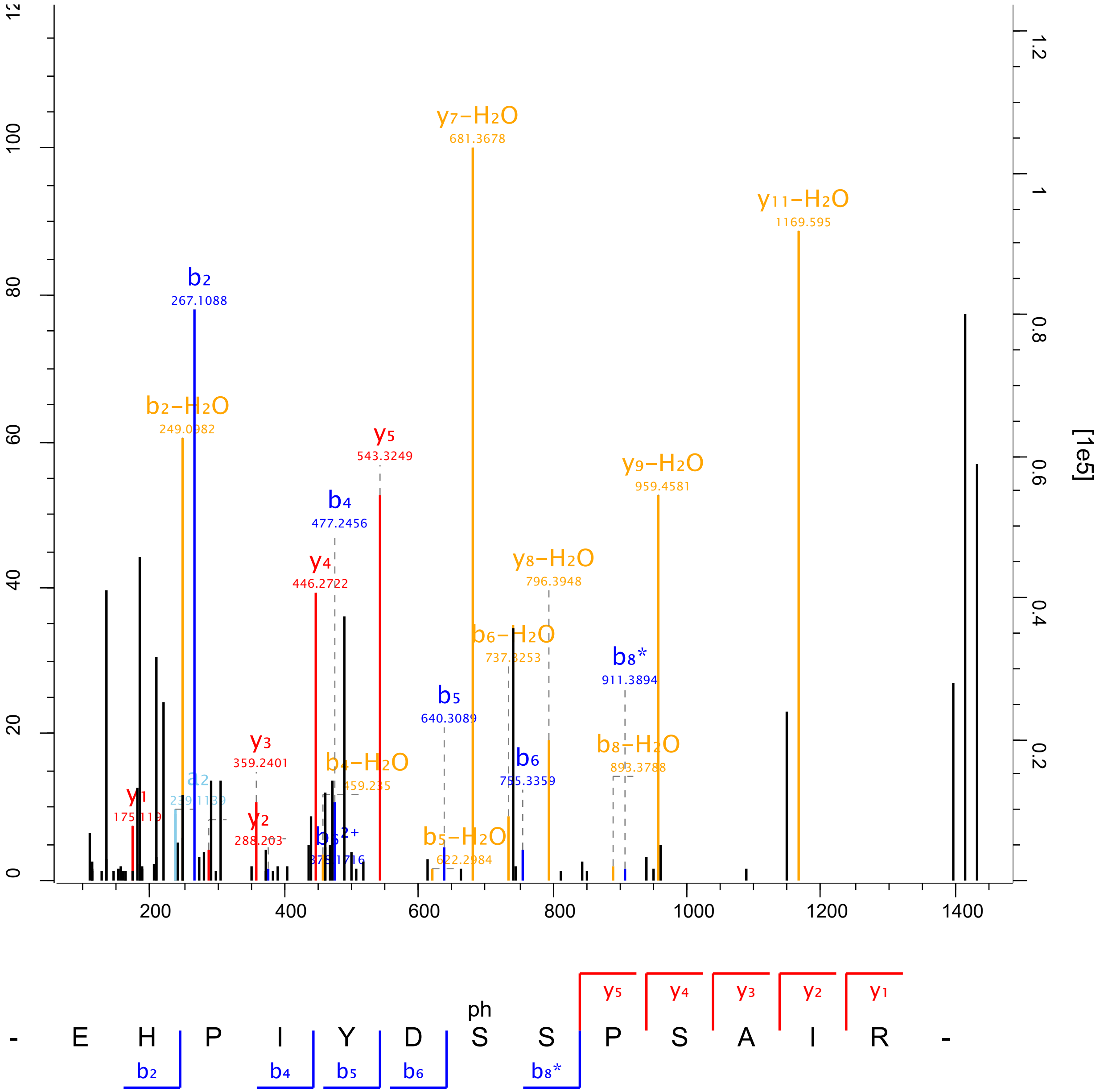

Raw file	Scan	Method	Score	m/z
sys_05_2	14733	FTMS; HCD	46.7	685.29

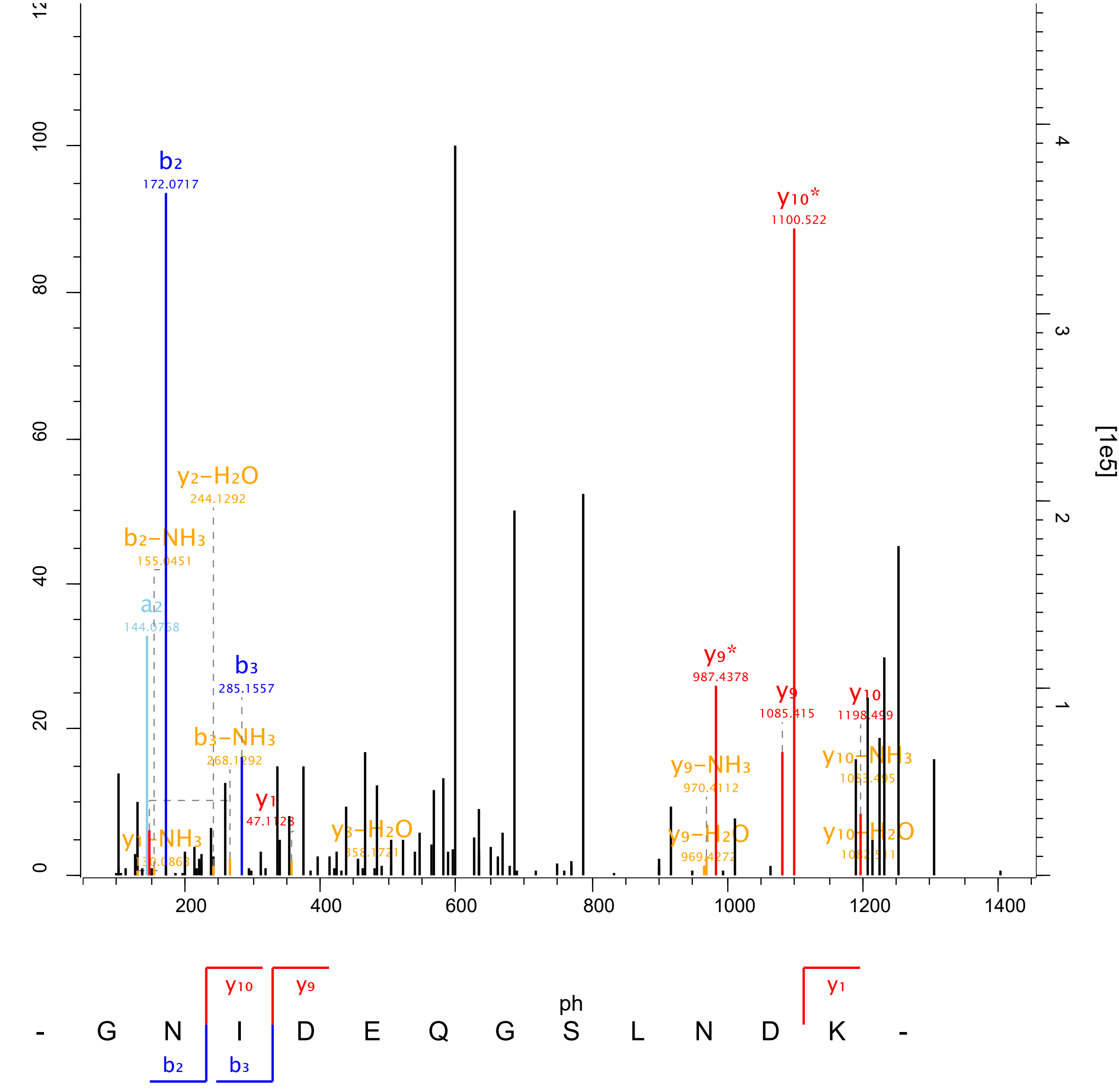

Raw file	Scan	Method	Score	m/z
sys_05_2	14803	FTMS; HCD	119.17	682.29

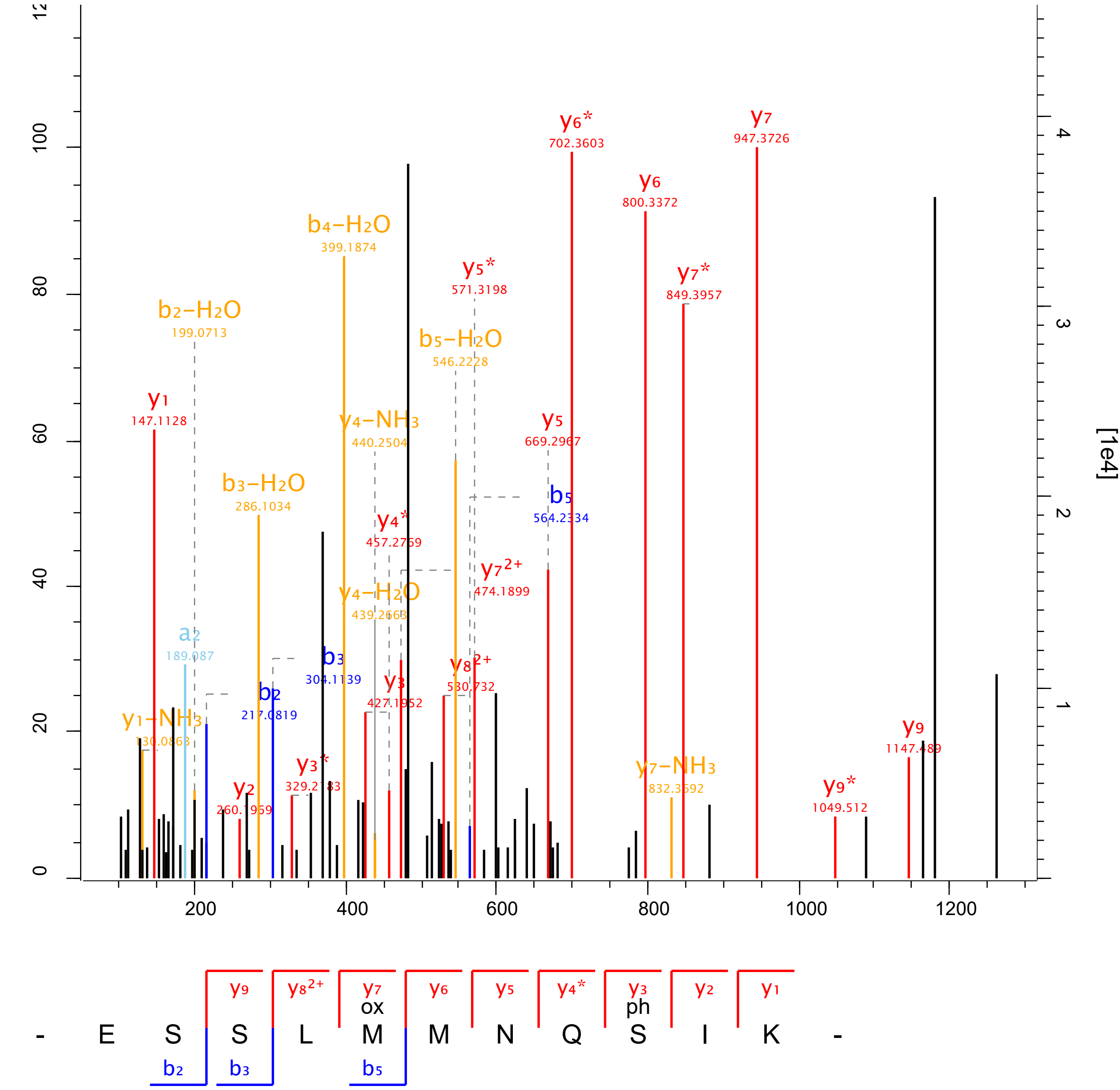

Raw file	Scan	Method	Score	m/z
sys_05_2	14832	FTMS; HCD	61.96	731.83

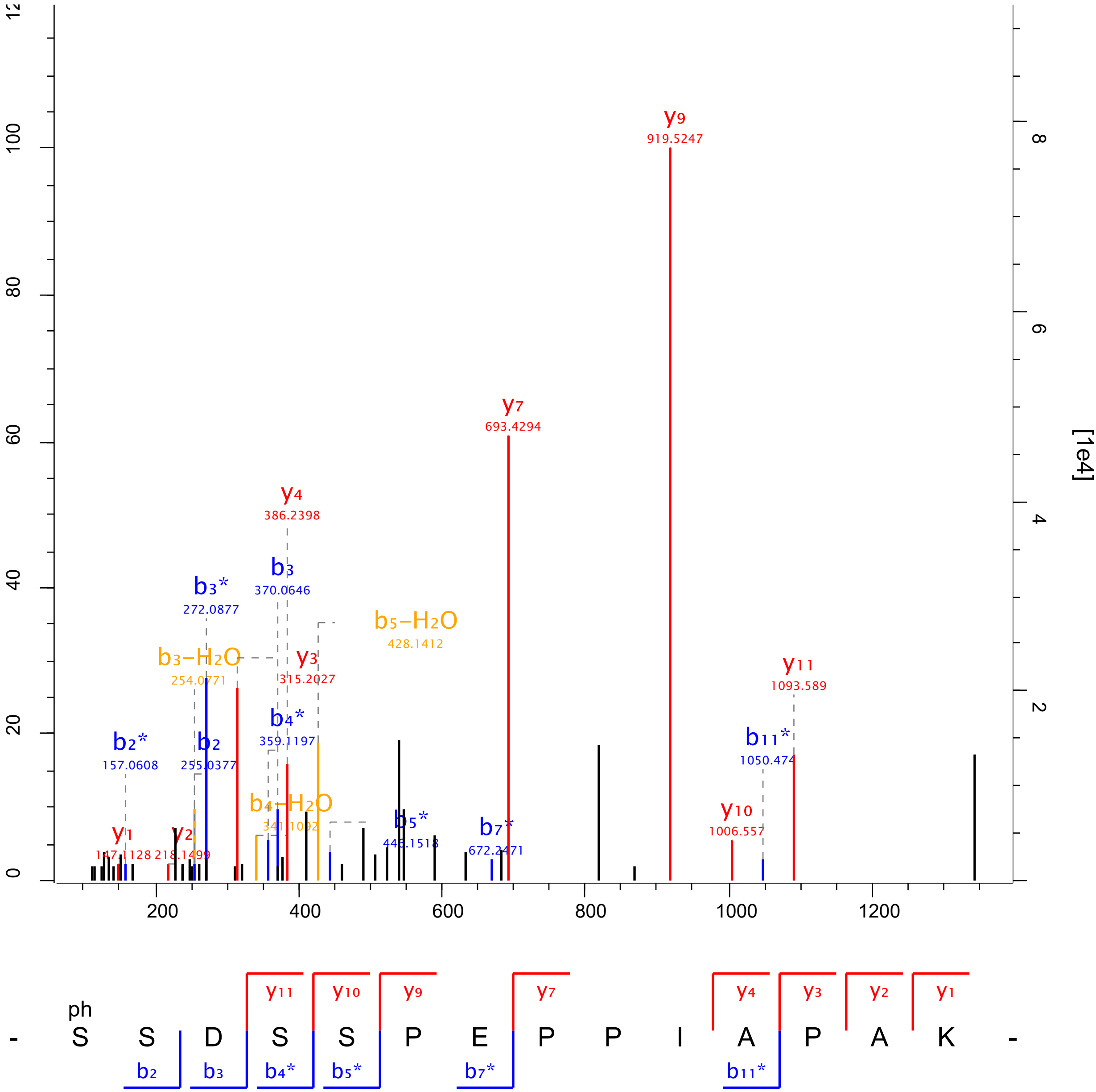

Raw file	Scan	Method	Score	m/z
sys_05_2	14857	FTMS; HCD	57.2	555.78

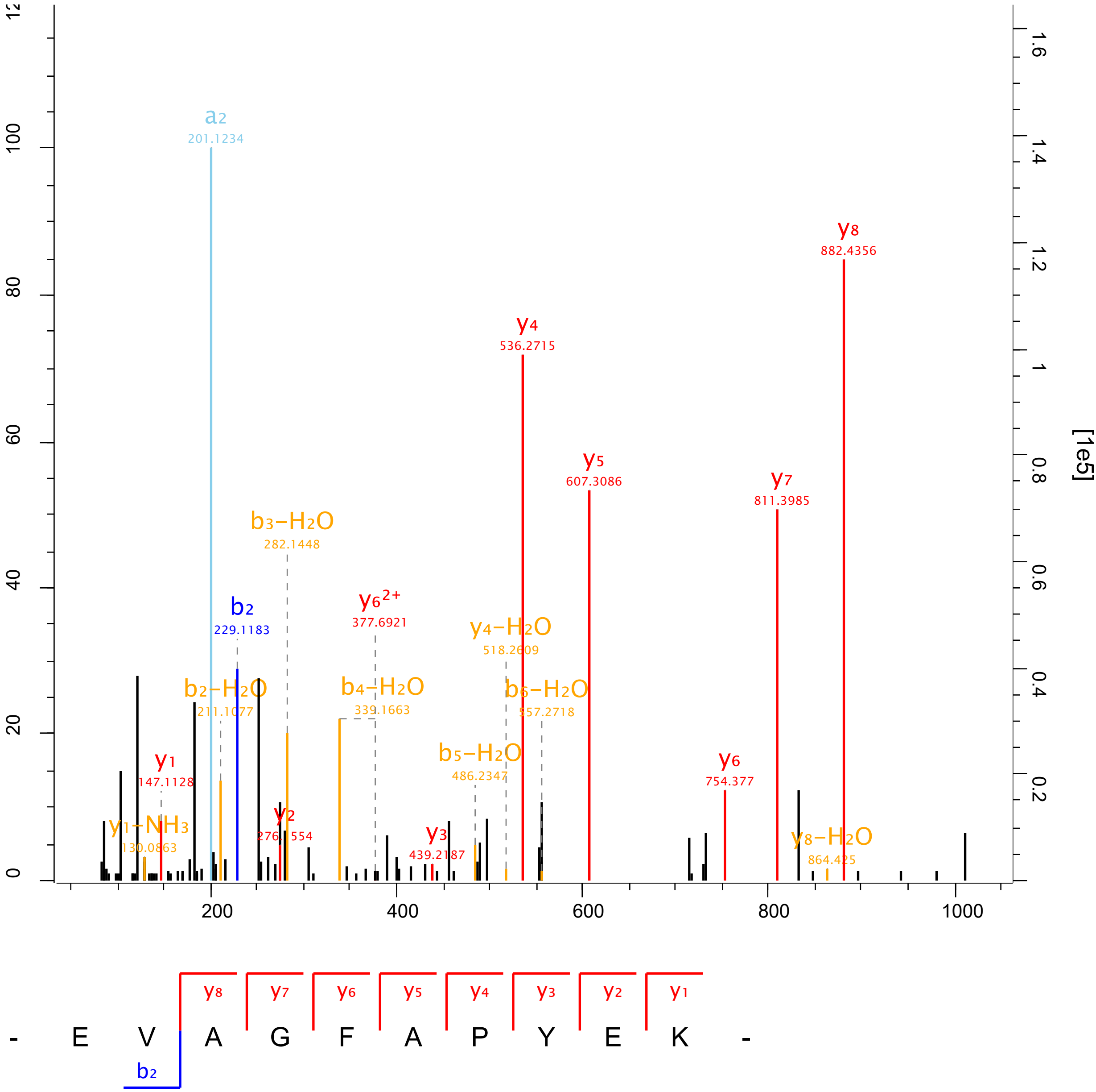

Raw file	Scan	Method	Score	m/z
sys_05_2	14934	FTMS; HCD	108.71	606.93

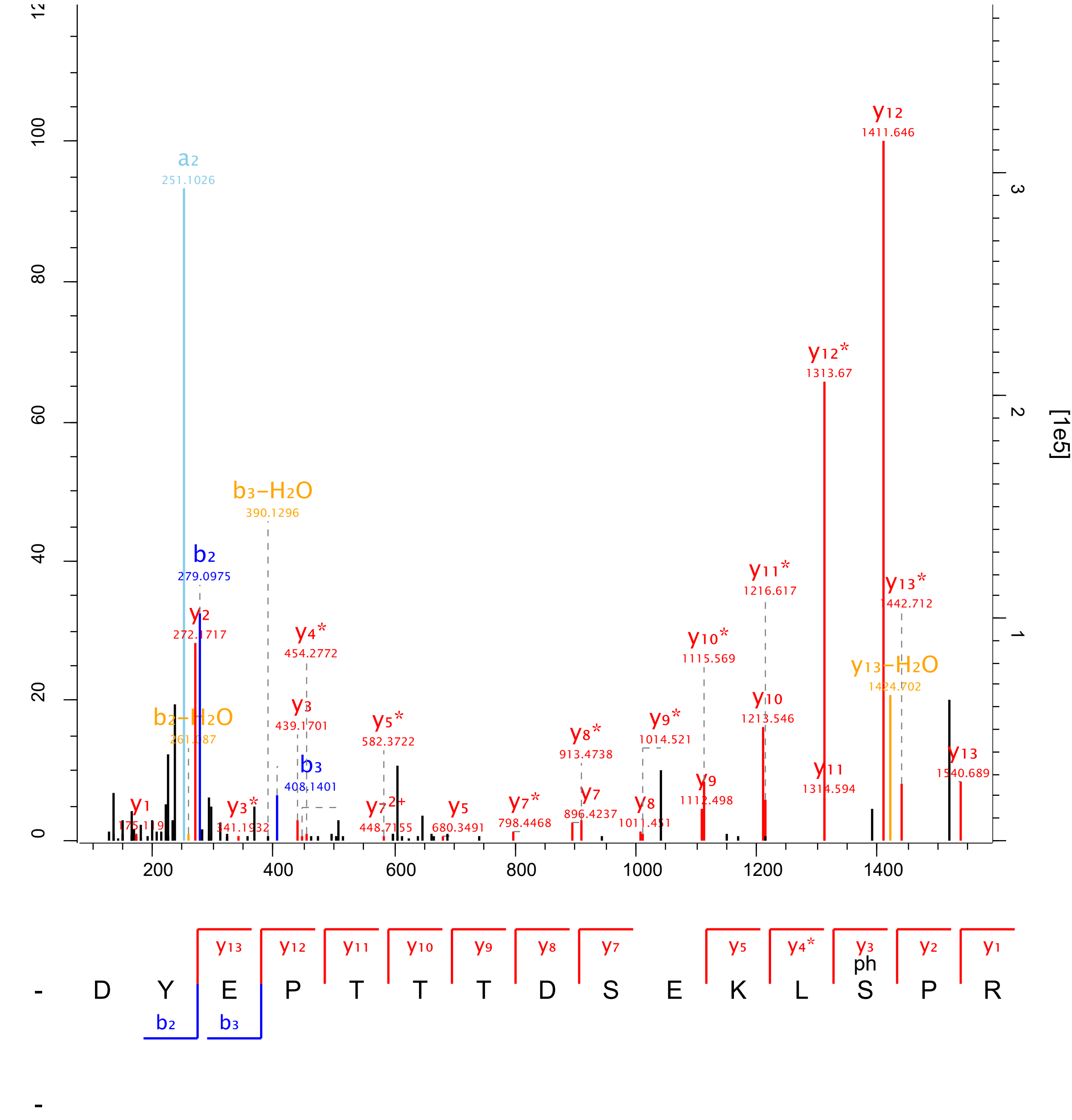

Raw file	Scan	Method	Score	m/z
sys_05_2	1503	FTMS; HCD	66.19	566.74

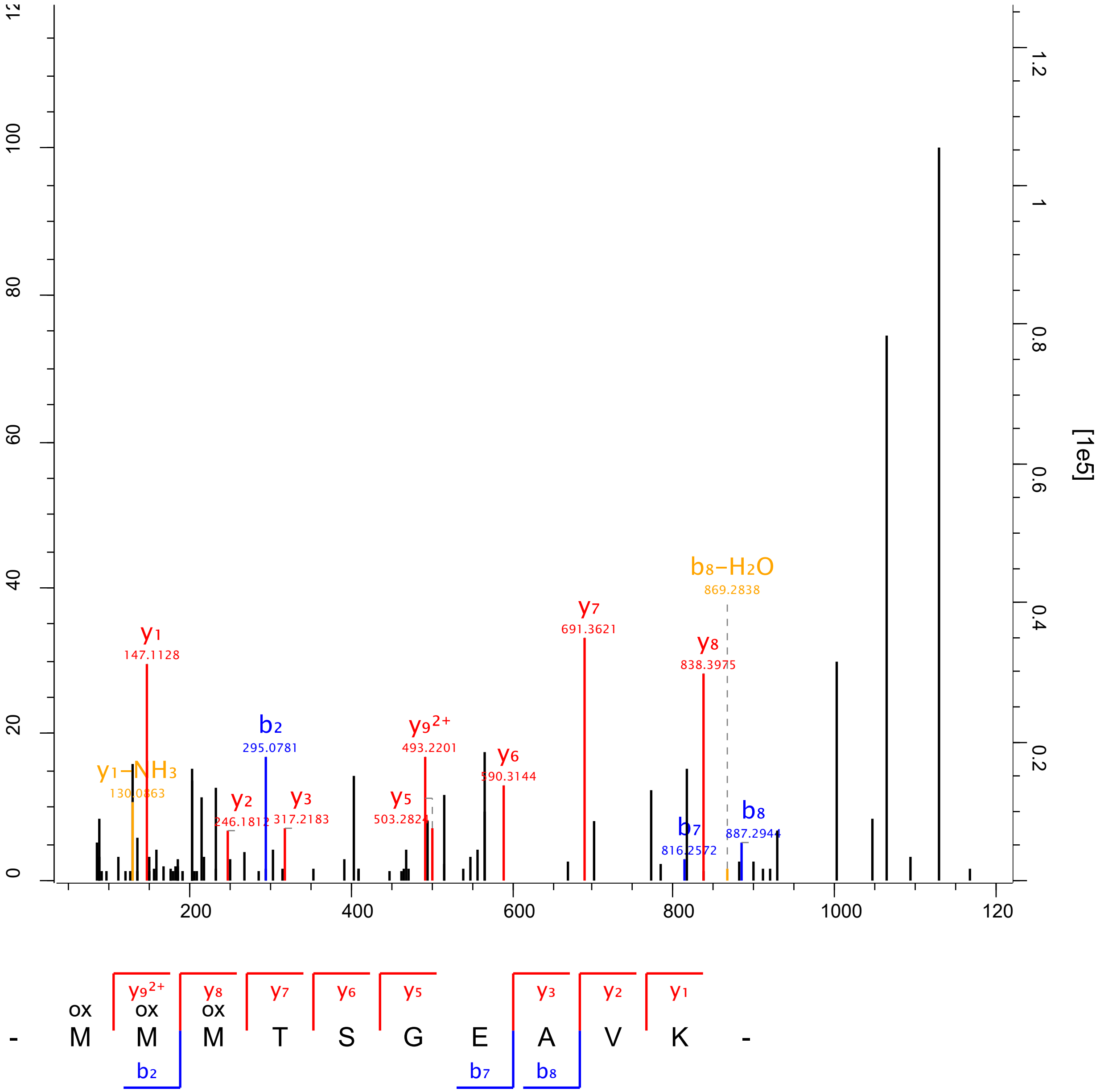

Raw file	Scan	Method	Score	m/z
sys_05_2	15198	FTMS; HCD	120.32	840.88

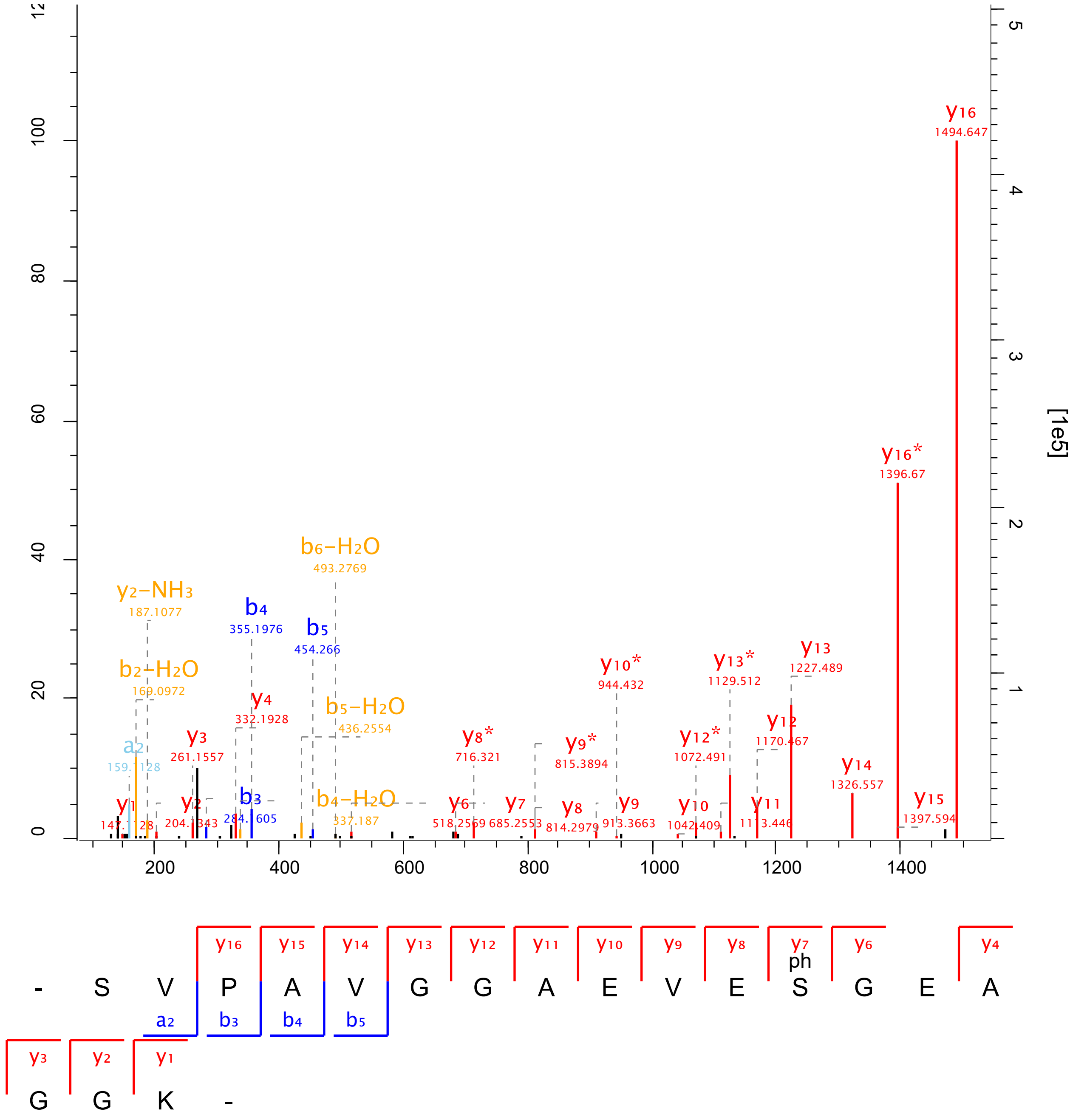

Raw file	Scan	Method	Score	m/z
sys_05_2	15234	FTMS; HCD	54.26	540.73

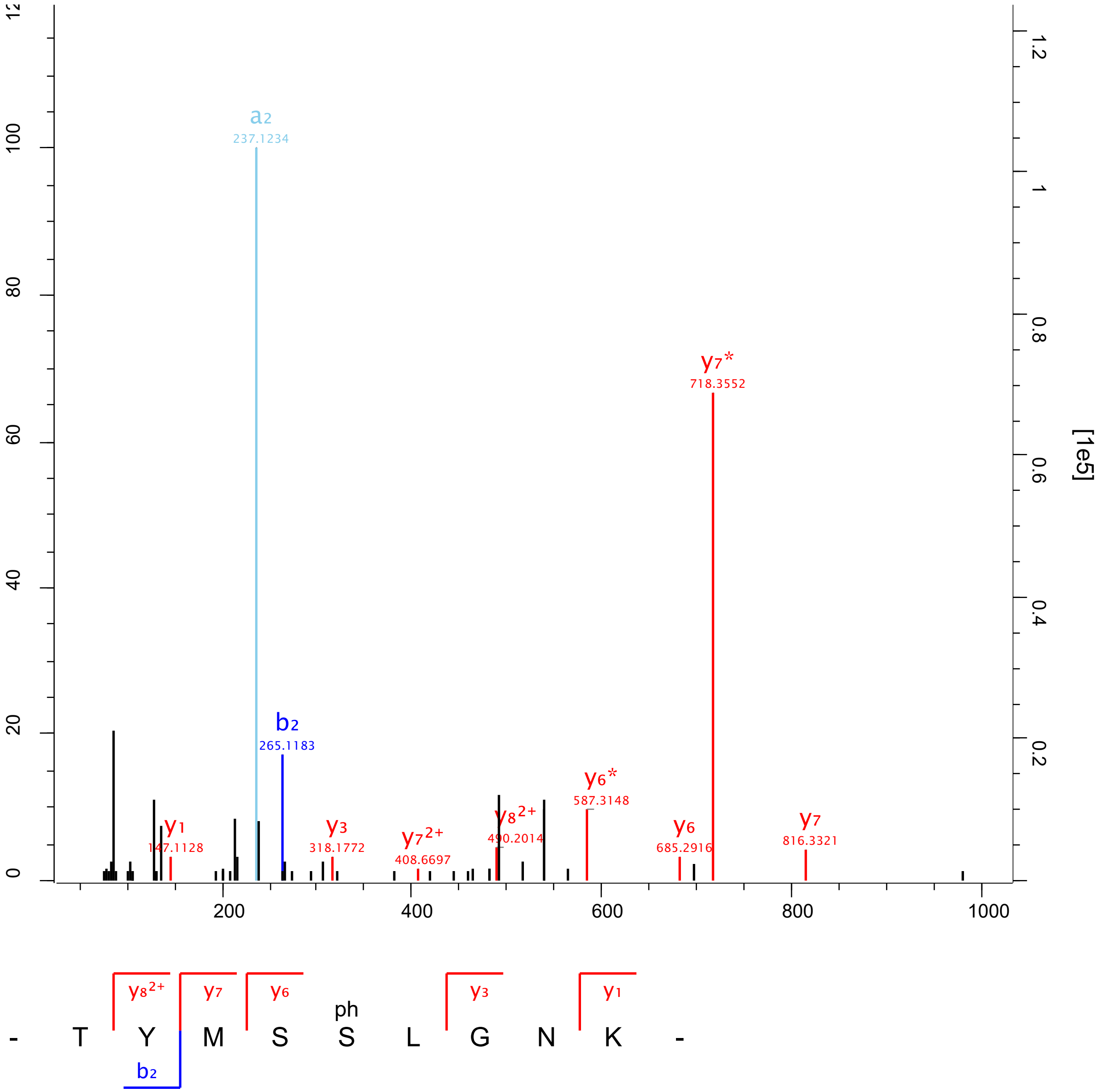

Raw file	Scan	Method	Score	m/z
sys_05_2	15306	FTMS; HCD	139.11	880.84

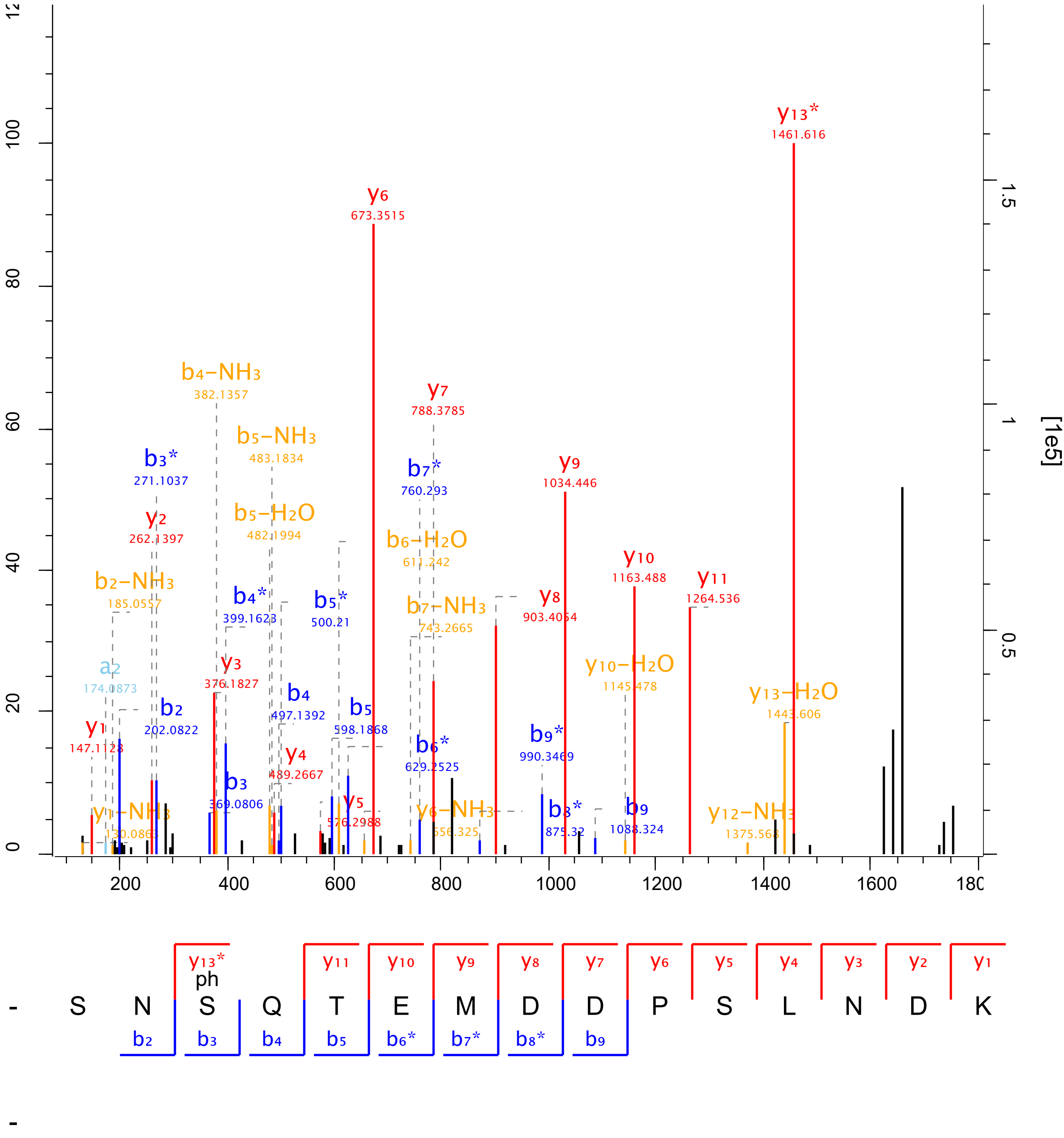

Raw file	Scan	Method	Score	m/z
sys_05_2	15355	FTMS; HCD	84.38	730.83

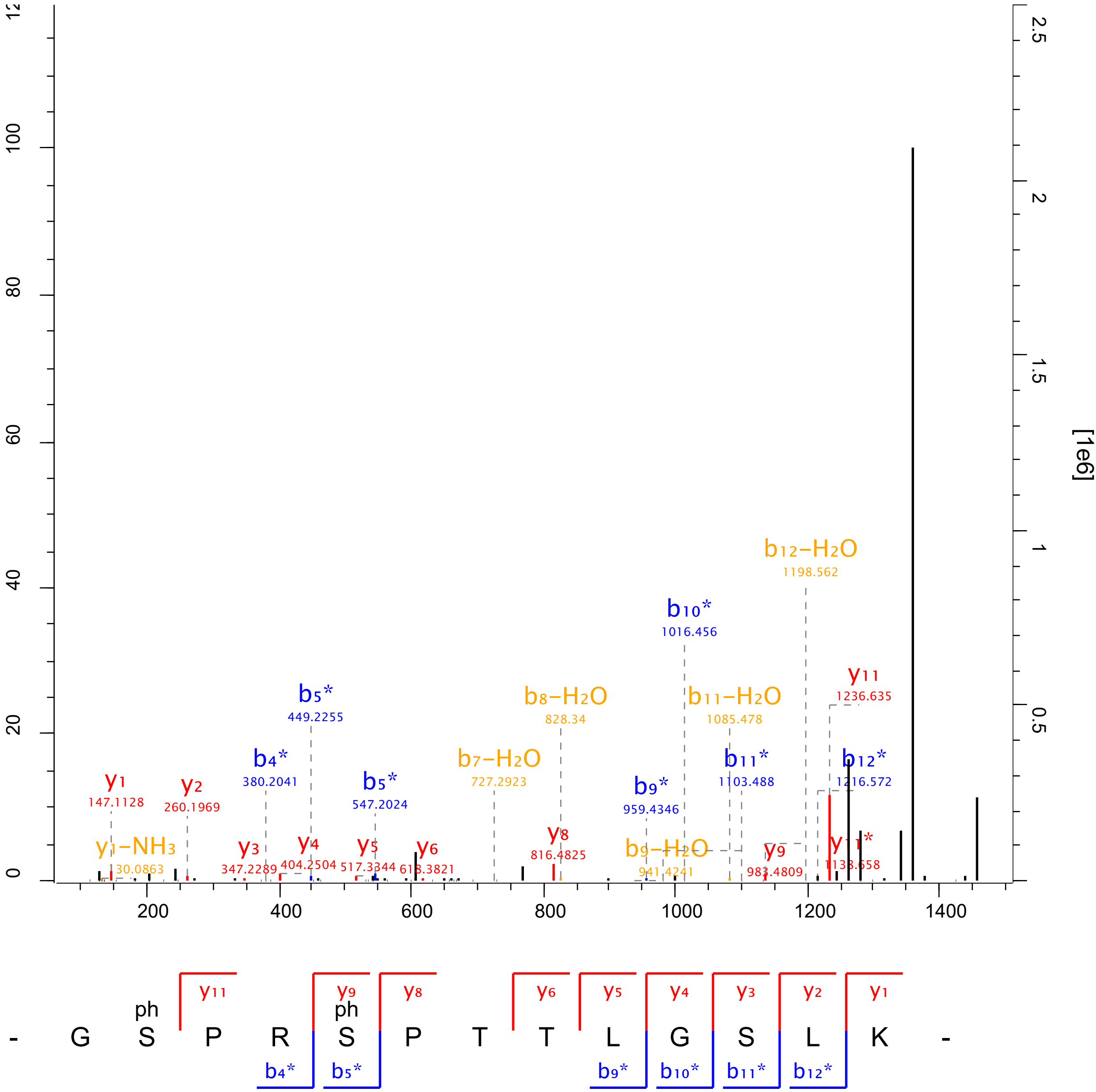

Raw file	Scan	Method	Score	m/z
sys_05_2	15535	FTMS; HCD	50.87	619.29

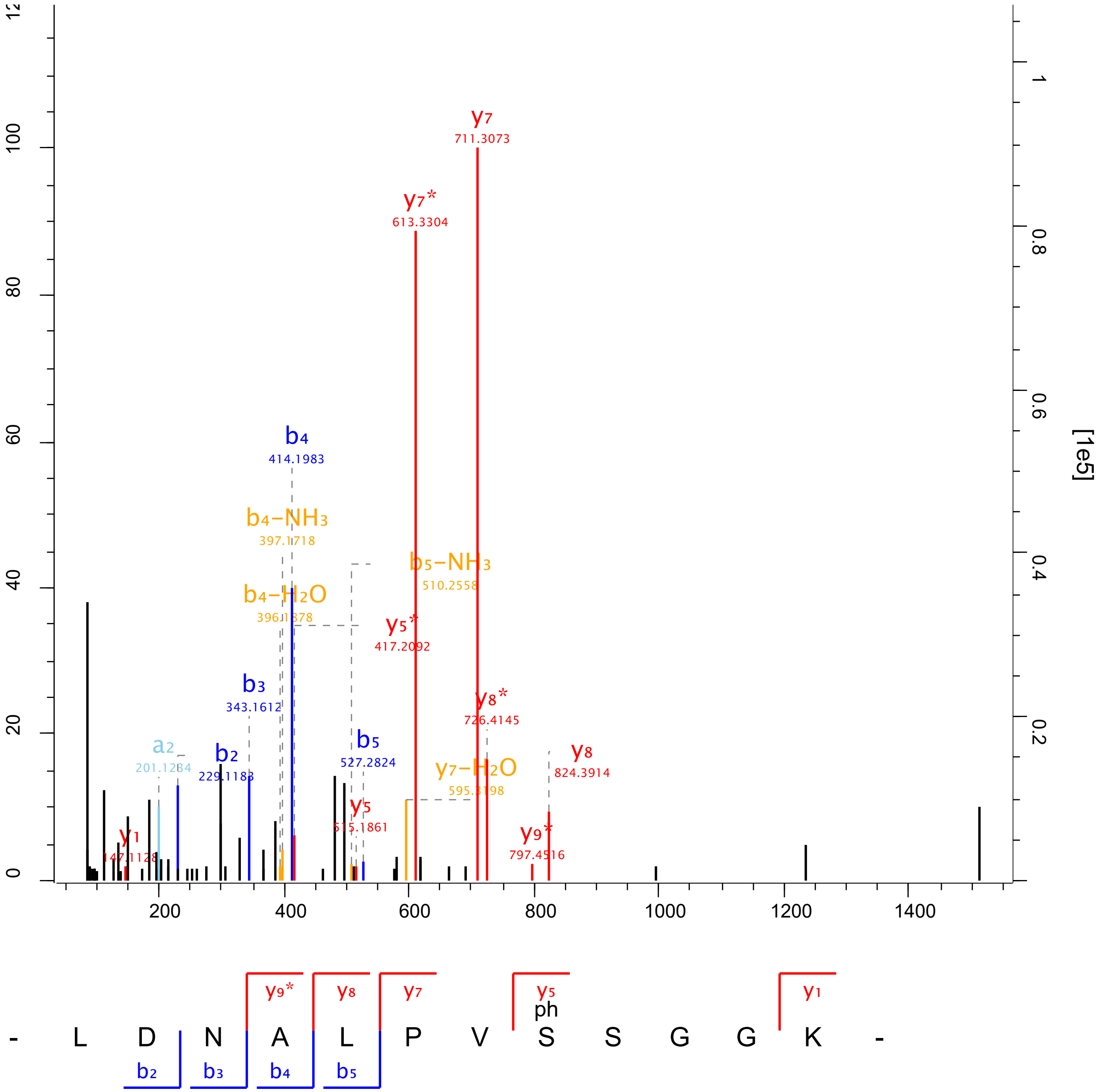

Raw file	Scan	Method	Score	m/z
sys_05_2	15593	FTMS; HCD	40.49	700.31

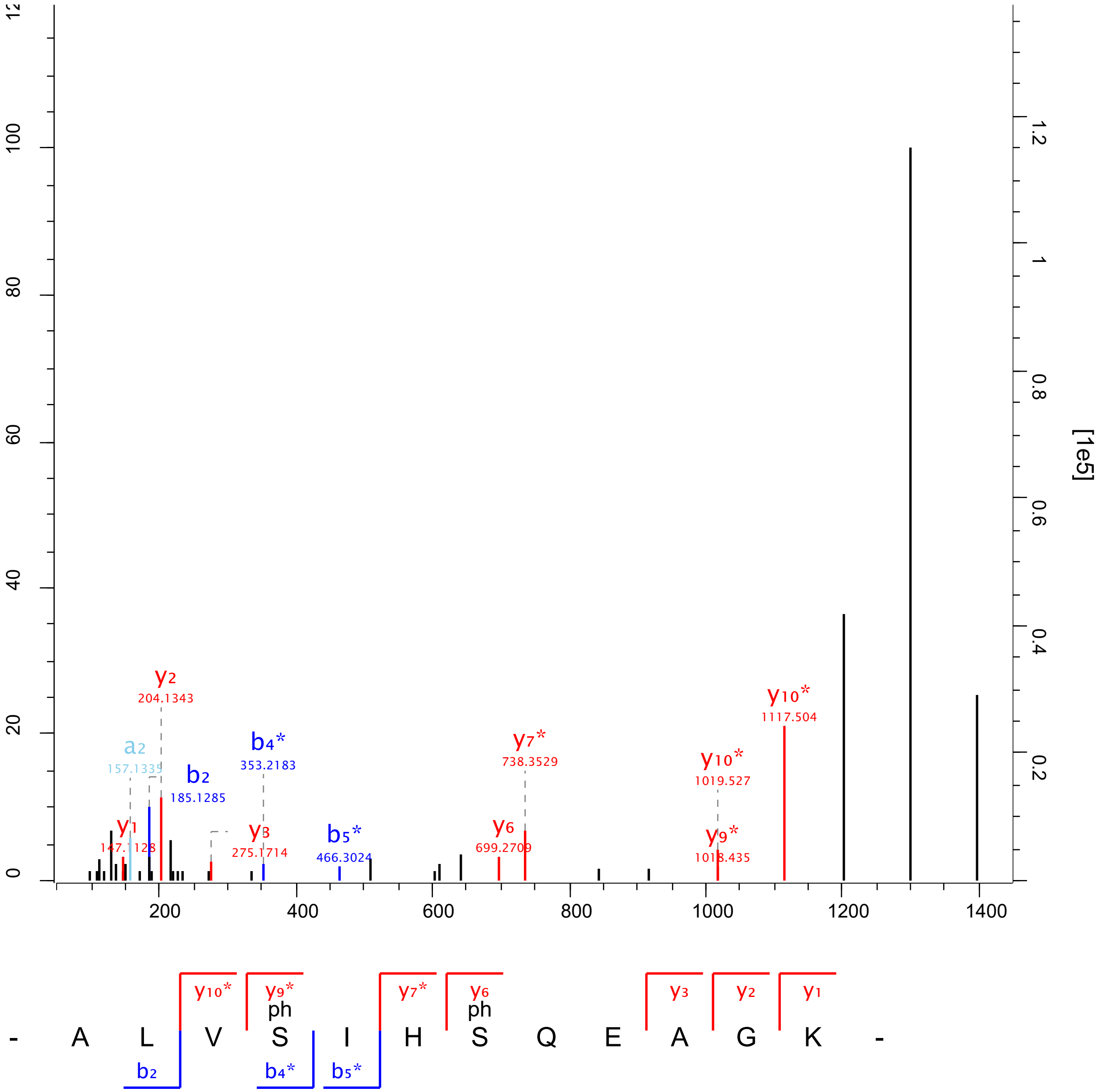

Raw file	Scan	Method	Score	m/z
sys_05_2	15620	FTMS; HCD	74.14	638.34

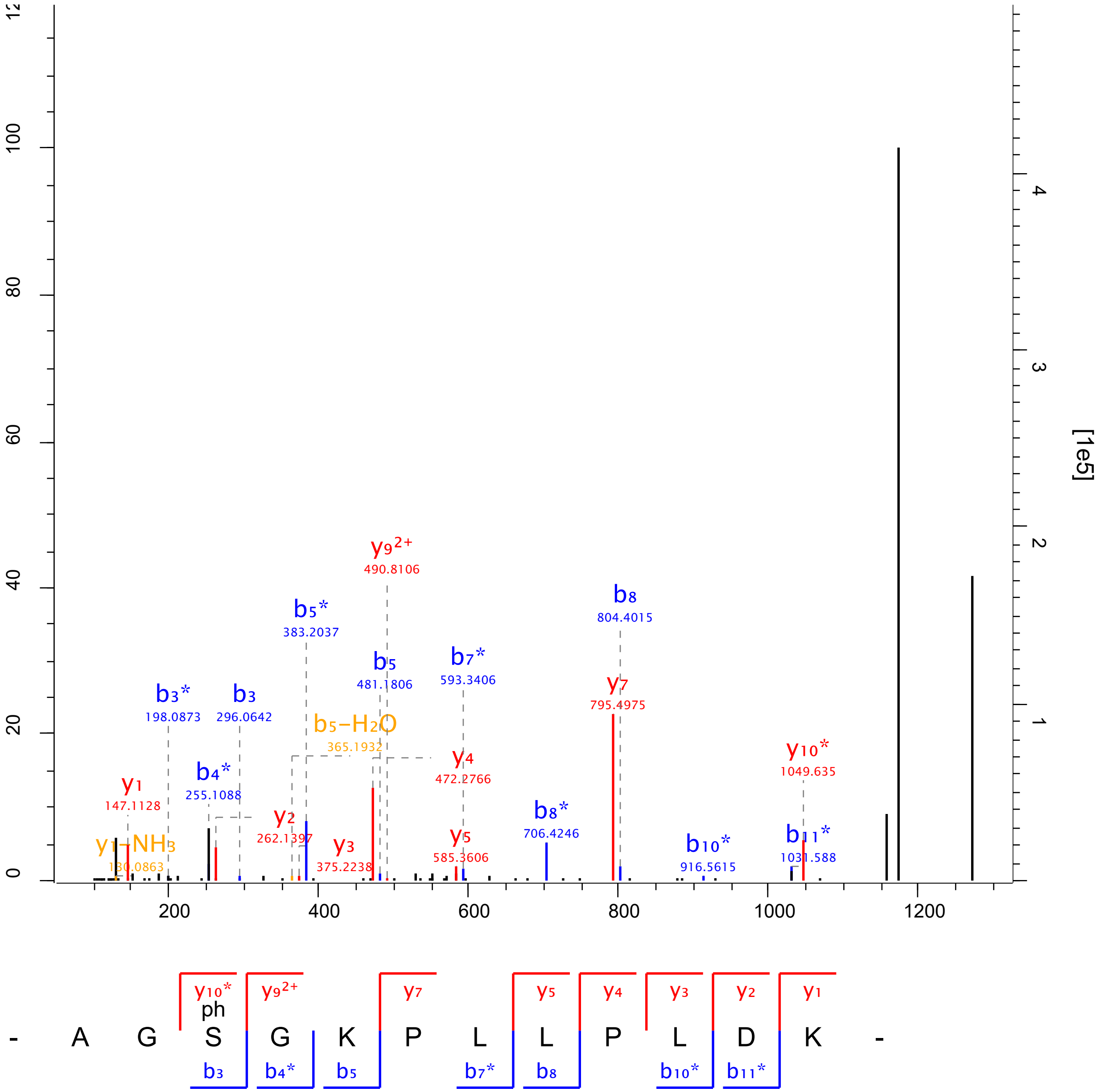

Raw file	Scan	Method	Score	m/z
sys_05_2	15744	FTMS; HCD	59.2	482.24

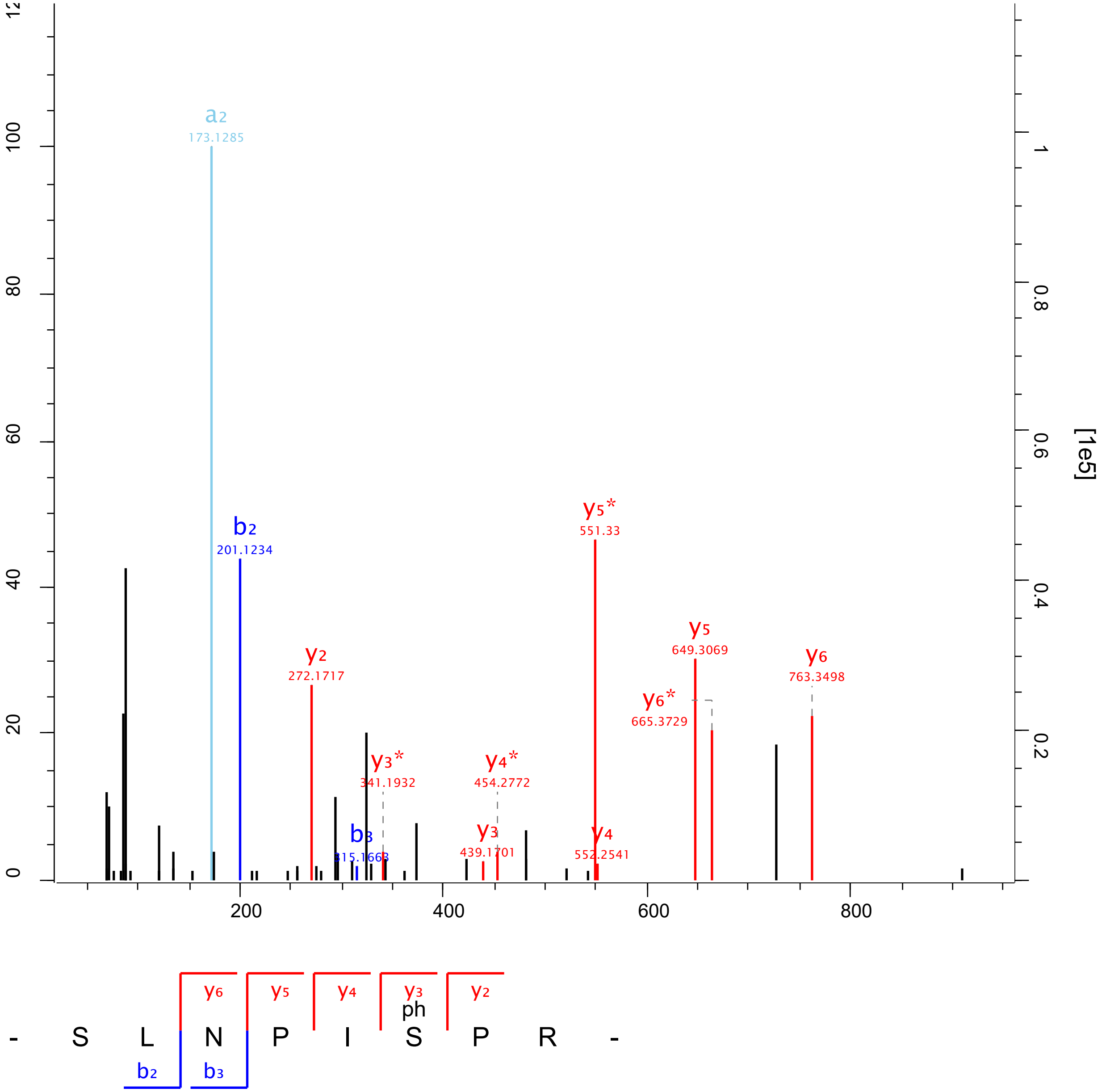

Raw file	Scan	Method	Score	m/z
sys_05_2	15789	FTMS; HCD	64.64	512.26

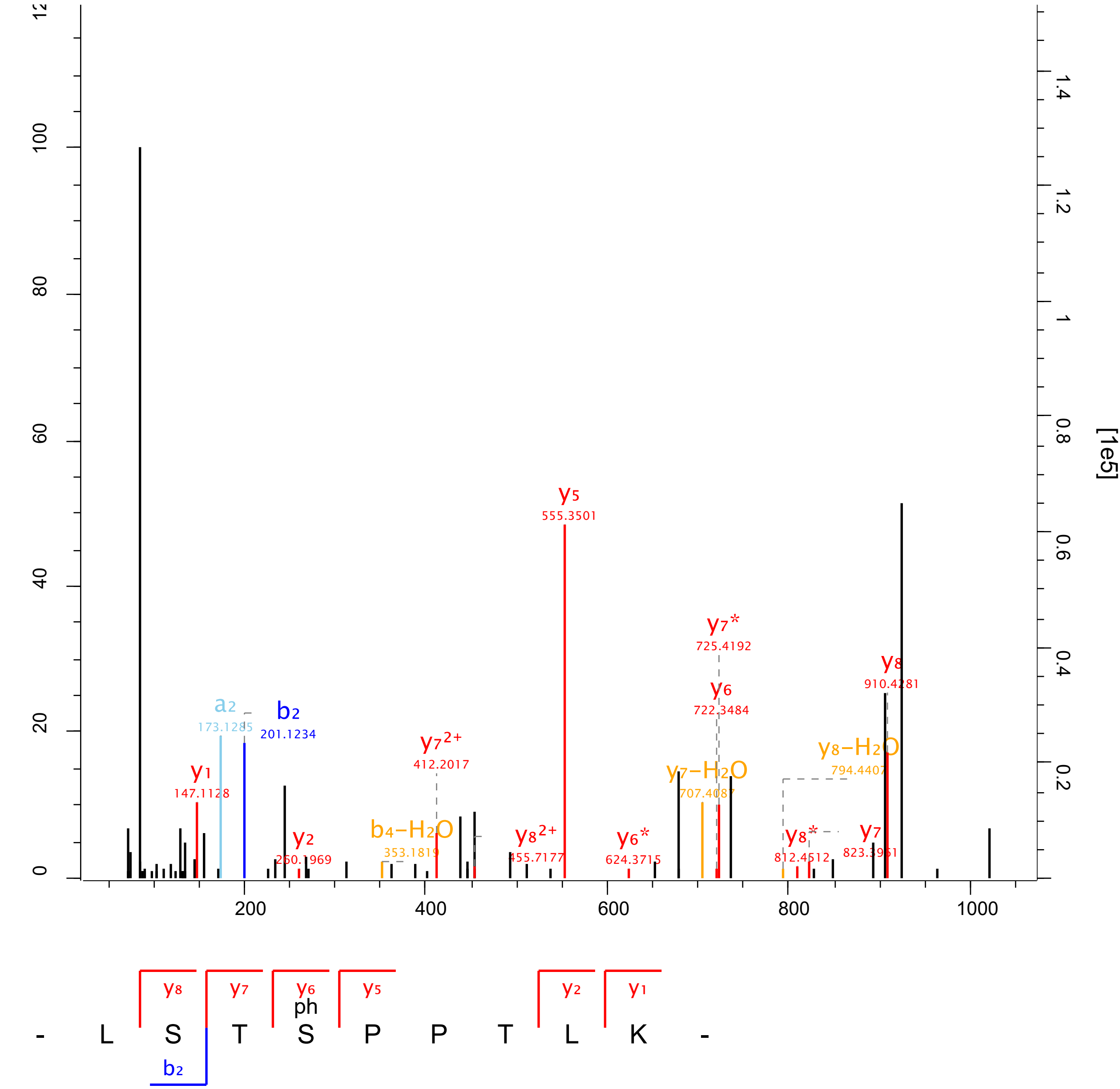

Raw file	Scan	Method	Score	m/z
sys_05_2	15849	FTMS; HCD	225.66	724.83

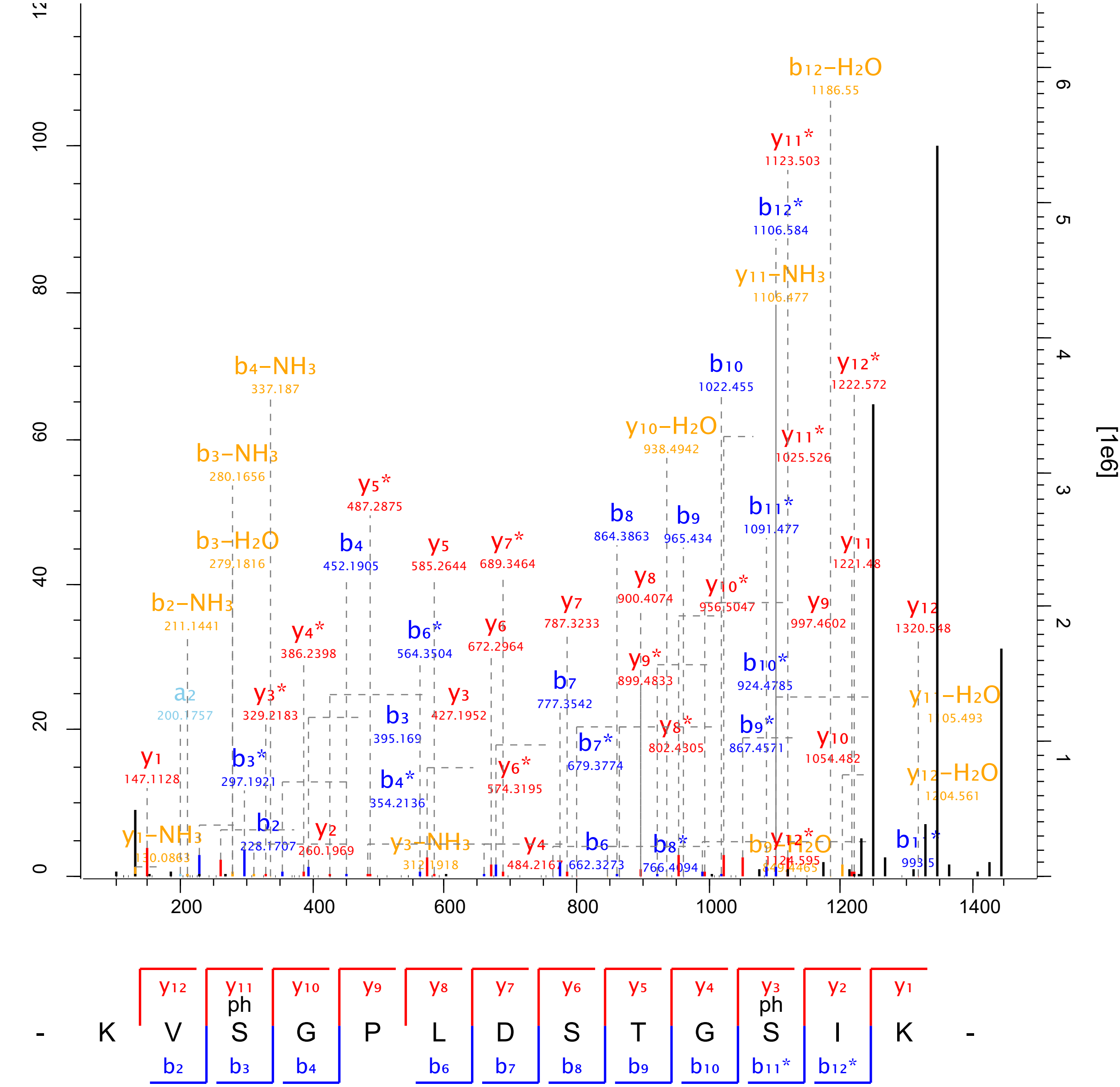

Raw file	Scan	Method	Score	m/z
sys_05_2	15946	FTMS; HCD	67.65	480.23

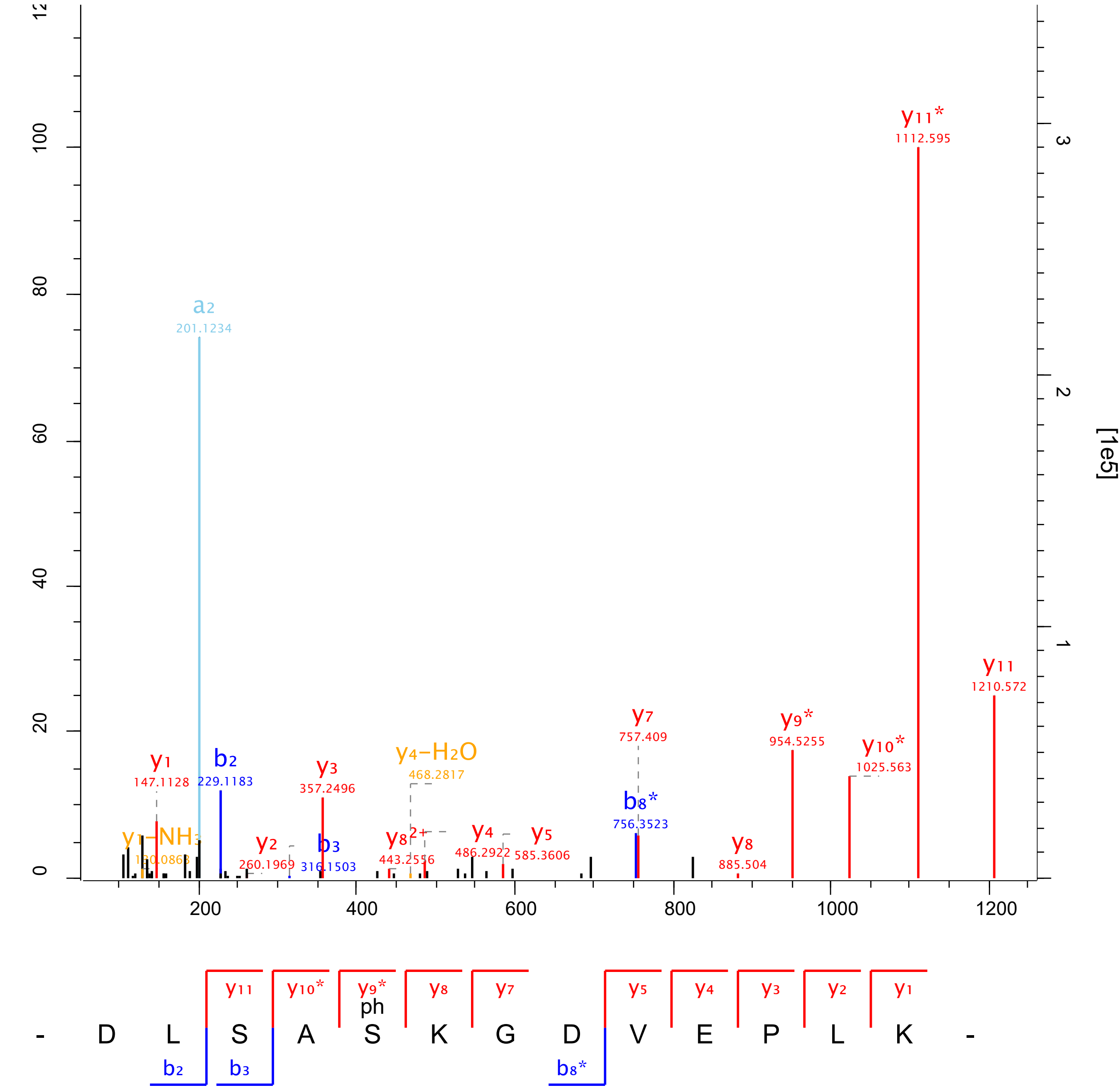

Raw file	Scan	Method	Score	m/z
sys_05_2	16017	FTMS; HCD	111.52	542.26

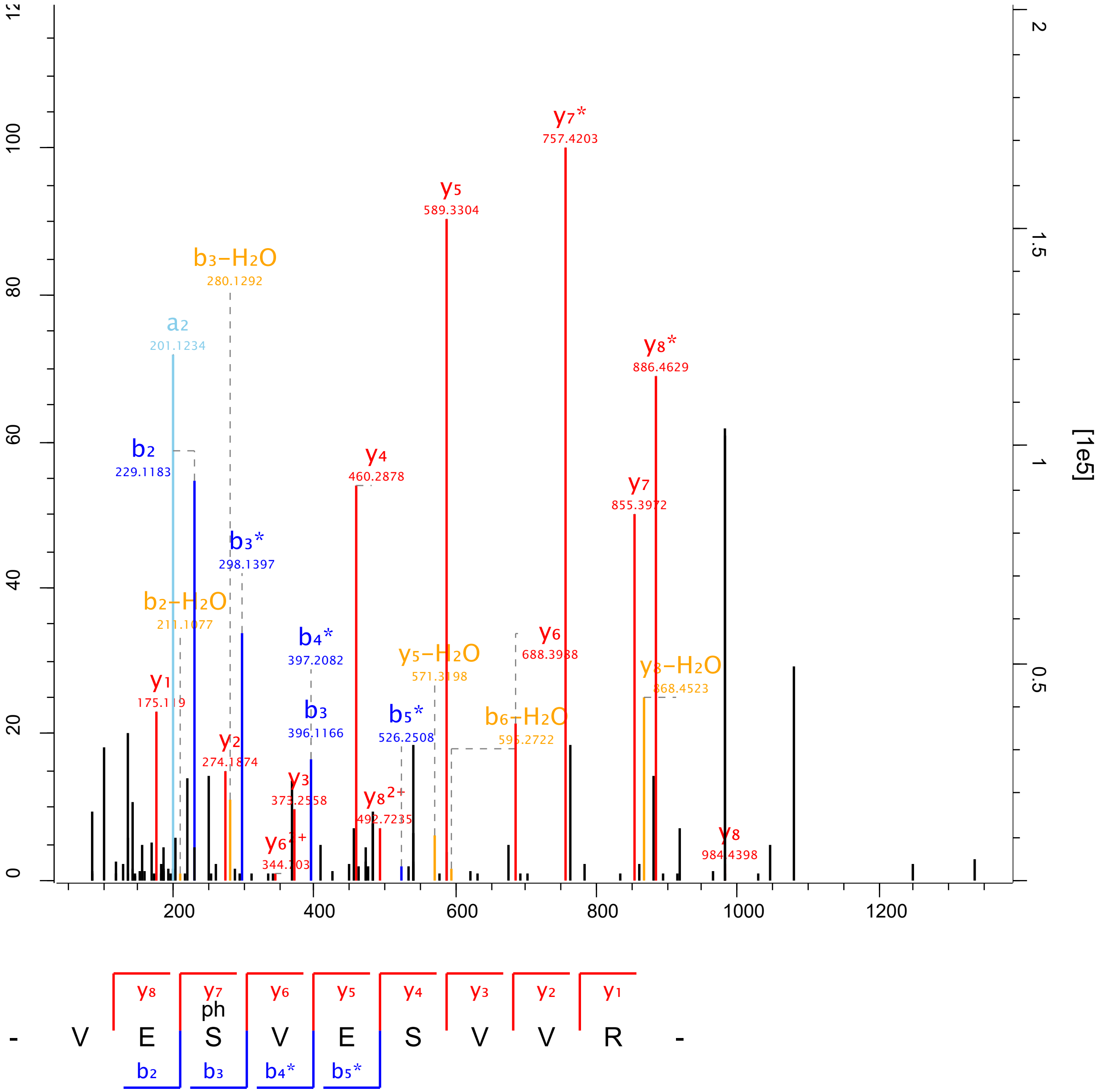

Raw file	Scan	Method	Score	m/z
sys_05_2	16035	FTMS; HCD	254.71	908.41

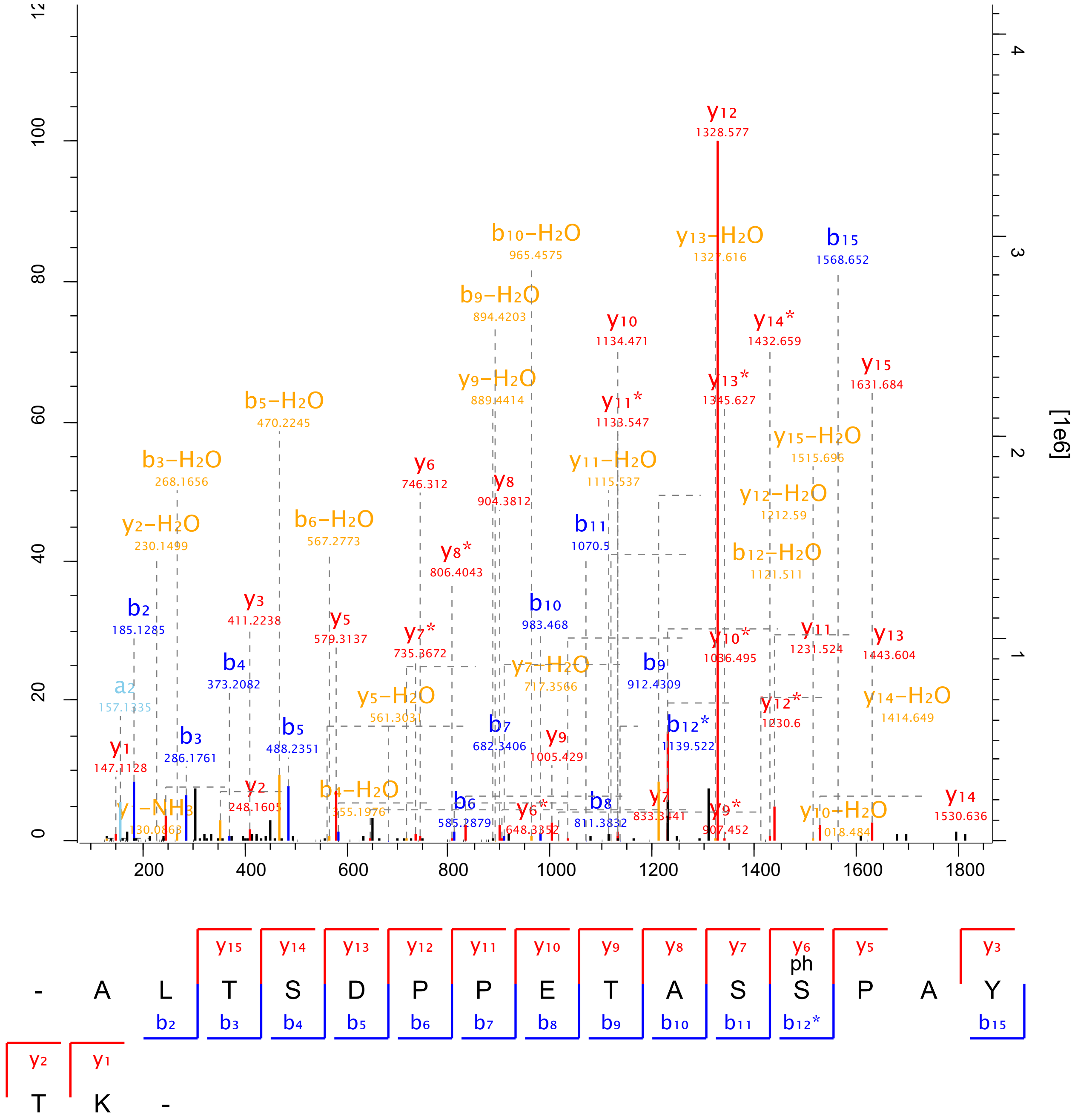

Raw file	Scan	Method	Score	m/z
sys_05_2	16047	FTMS; HCD	103.9	630.25

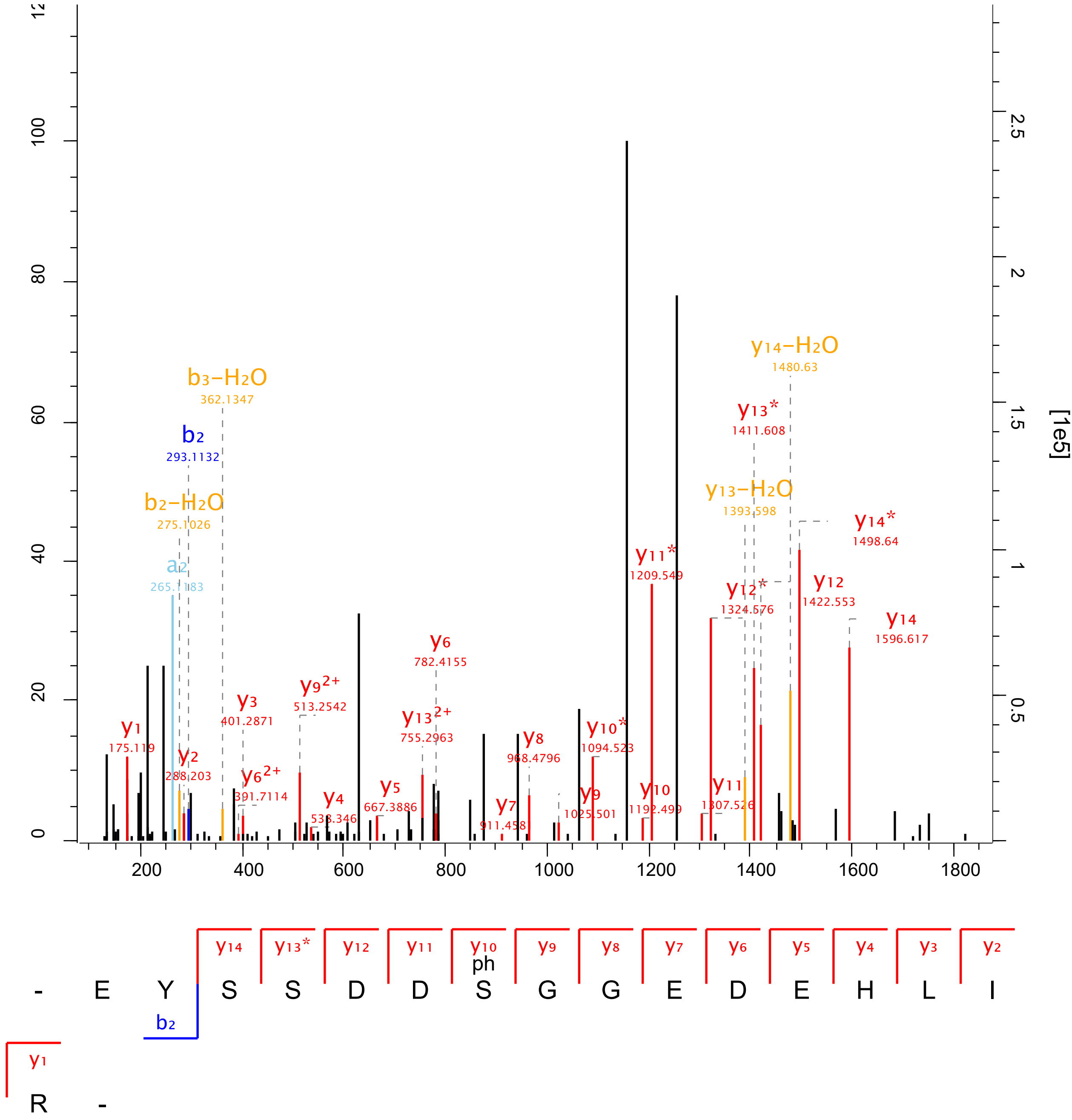

Raw file	Scan	Method	Score	m/z
sys_05_2	16112	FTMS; HCD	42.32	653.62

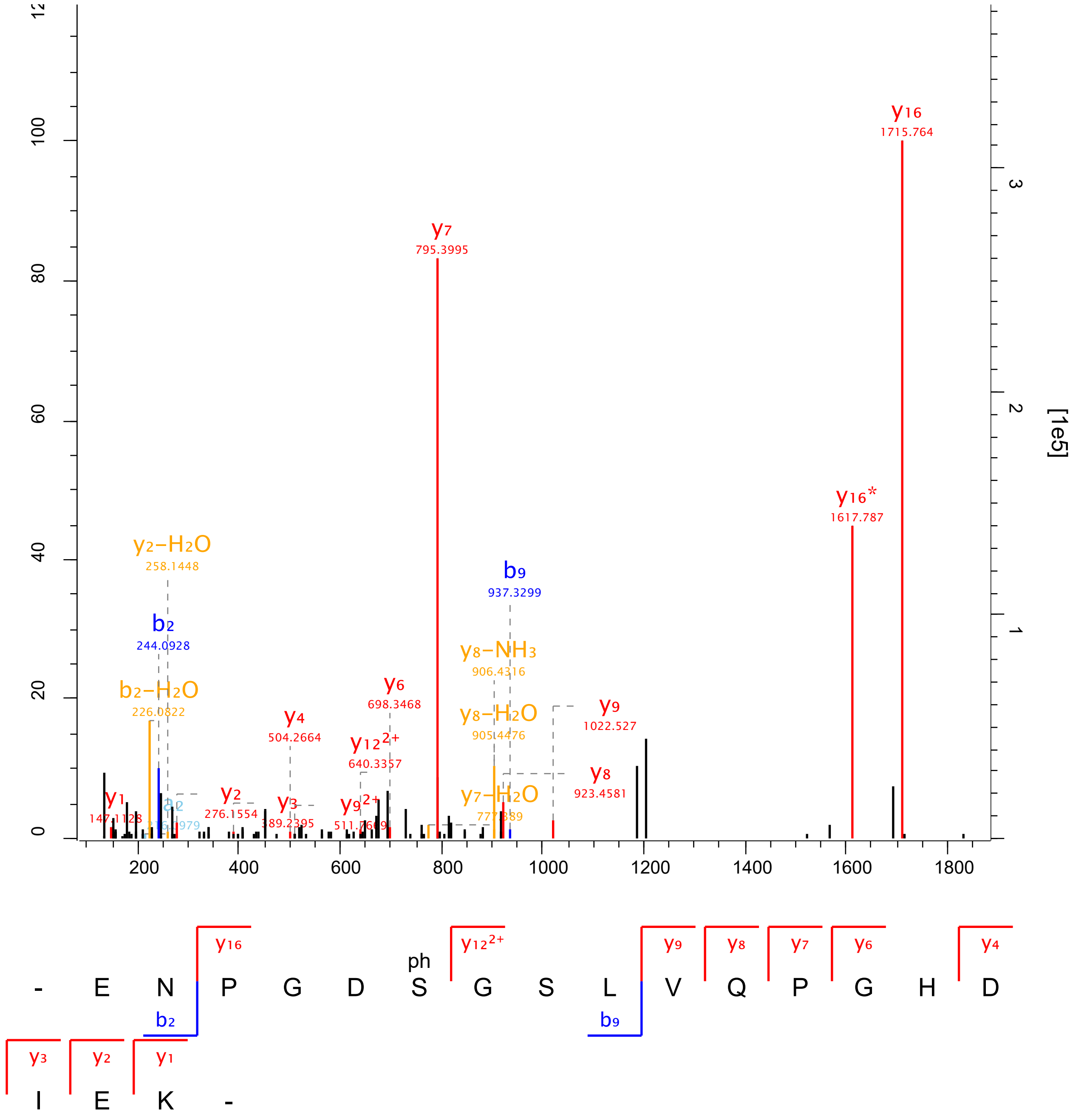

Raw file	Scan	Method	Score	m/z
sys_05_2	16131	FTMS; HCD	95.98	572.74

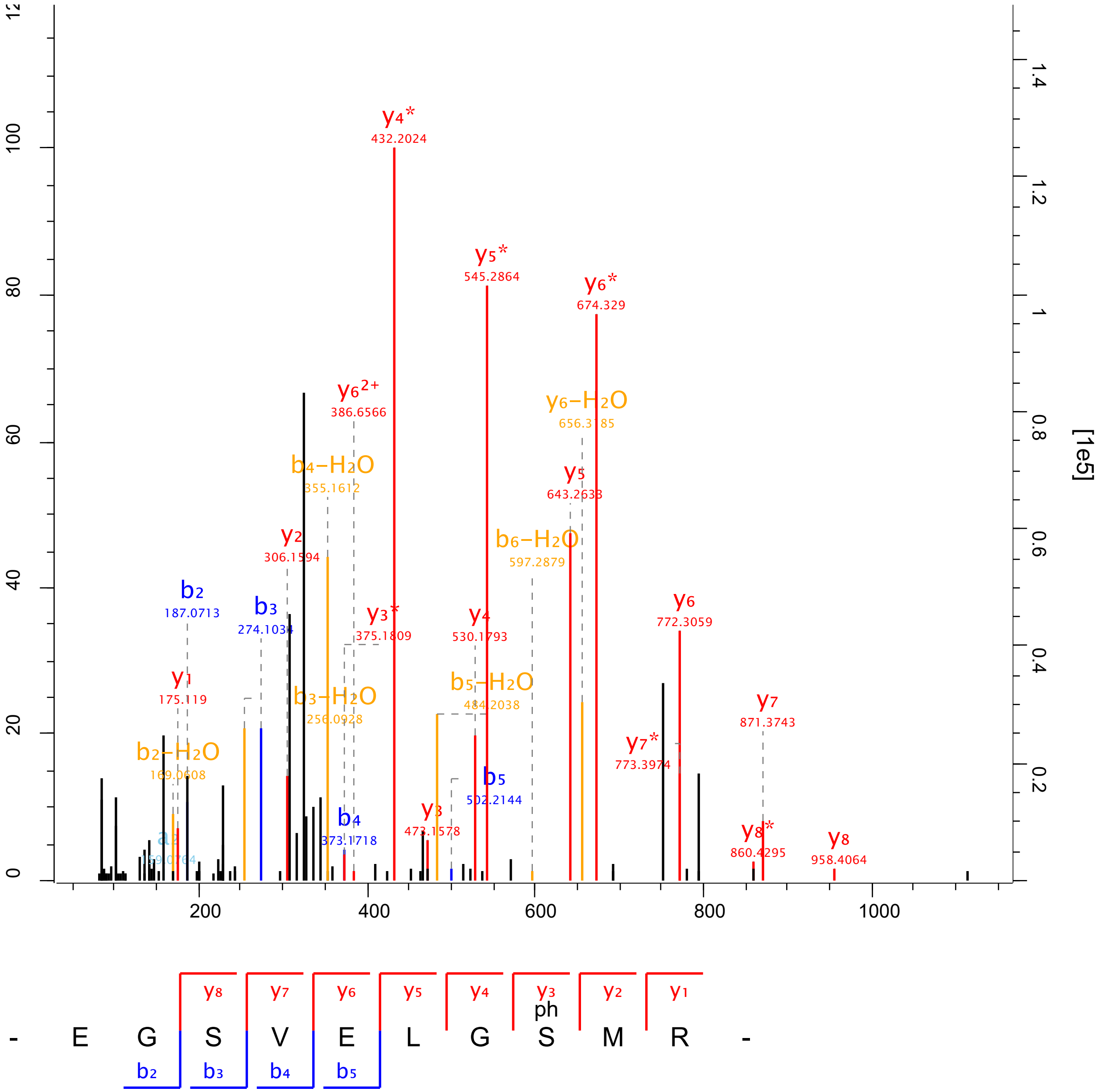

Raw file	Scan	Method	Score	m/z
sys_05_2	16235	FTMS; HCD	114.63	705.78

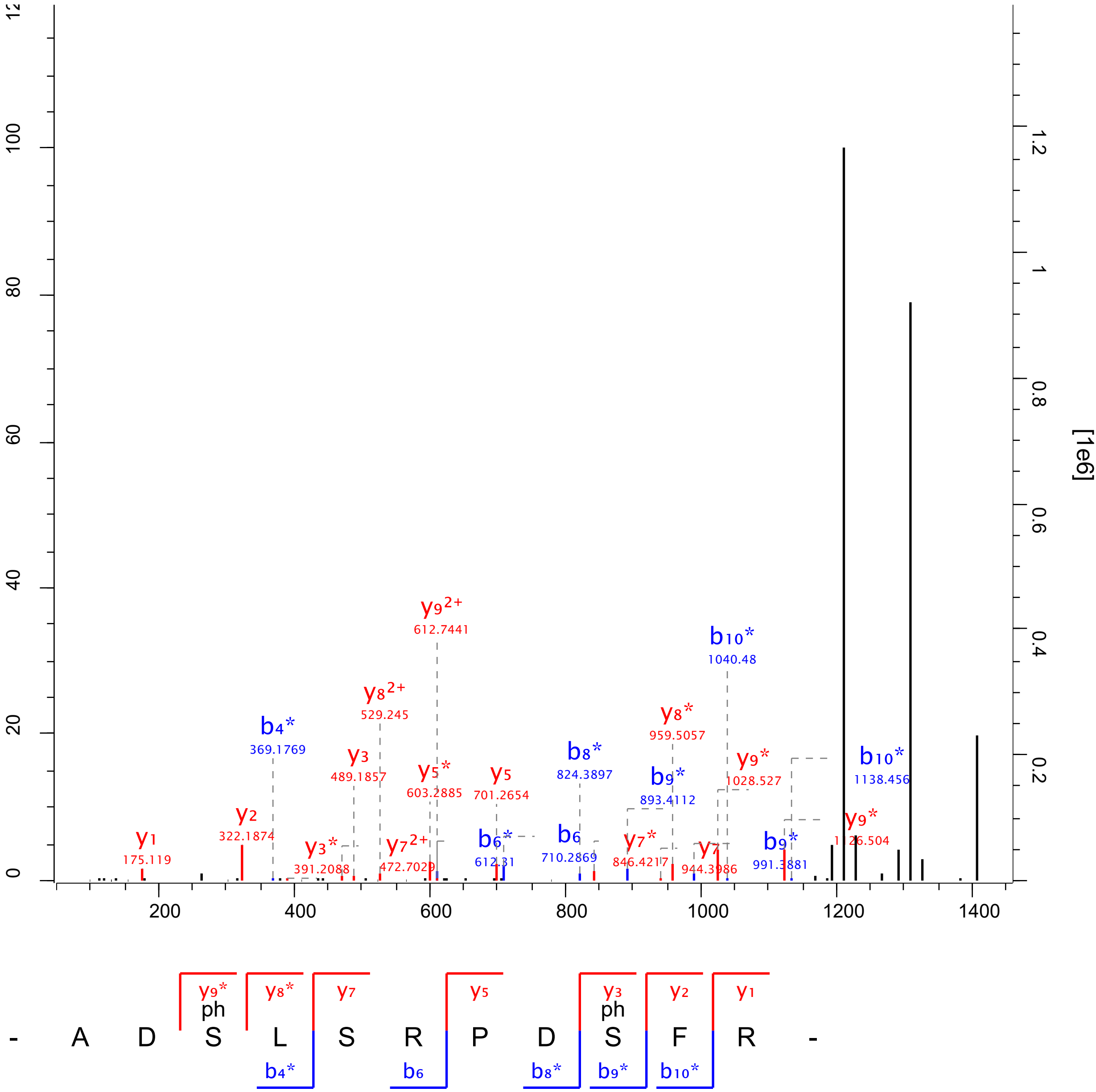

Raw file	Scan	Method	Score	m/z
sys_05_2	16362	FTMS; HCD	100.04	741.31

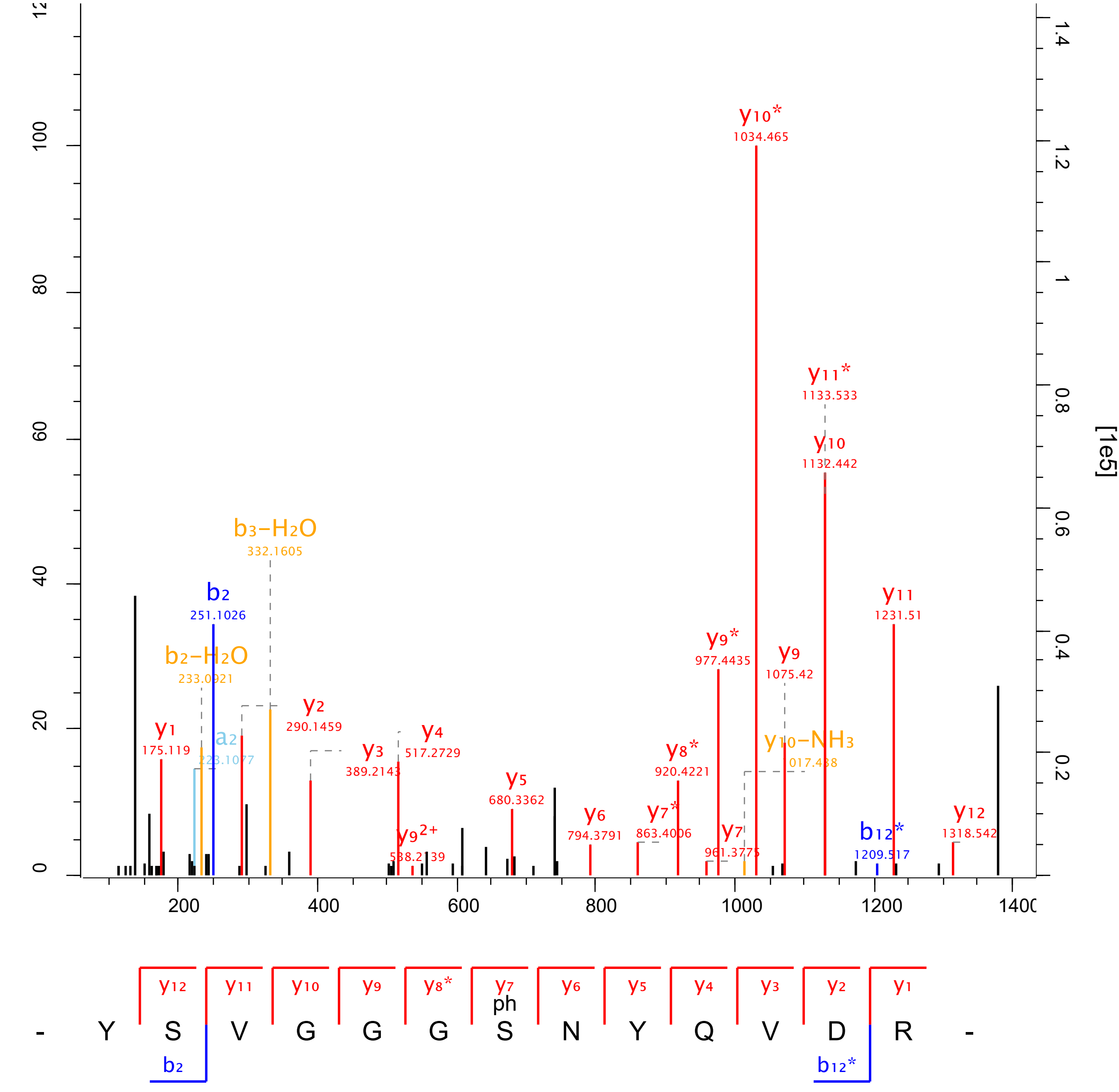

Raw file	Scan	Method	Score	m/z
sys_05_2	16368	FTMS; HCD	259.77	782.85

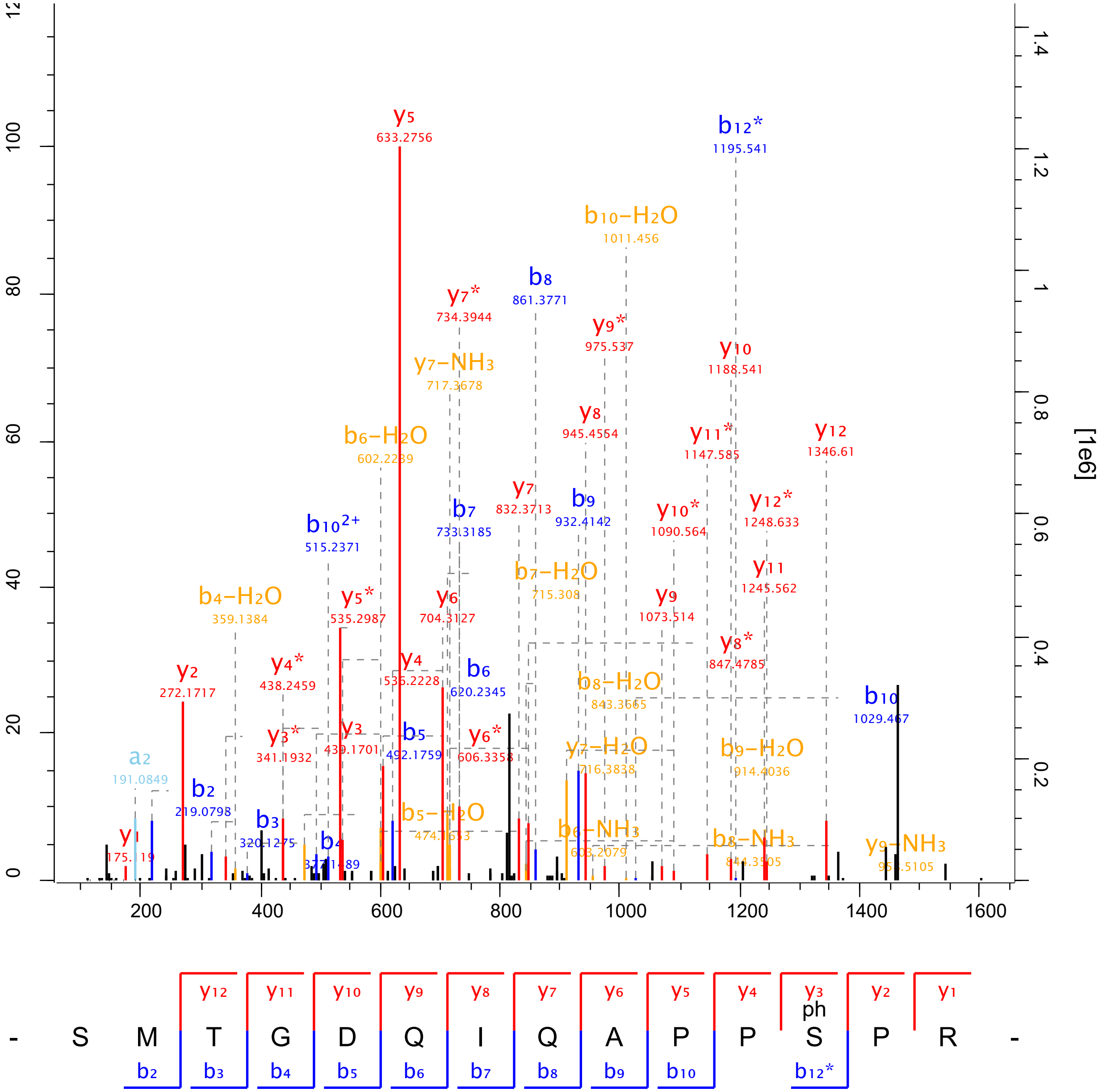

Raw file	Scan	Method	Score	m/z
sys_05_2	16387	FTMS; HCD	77.18	607.73

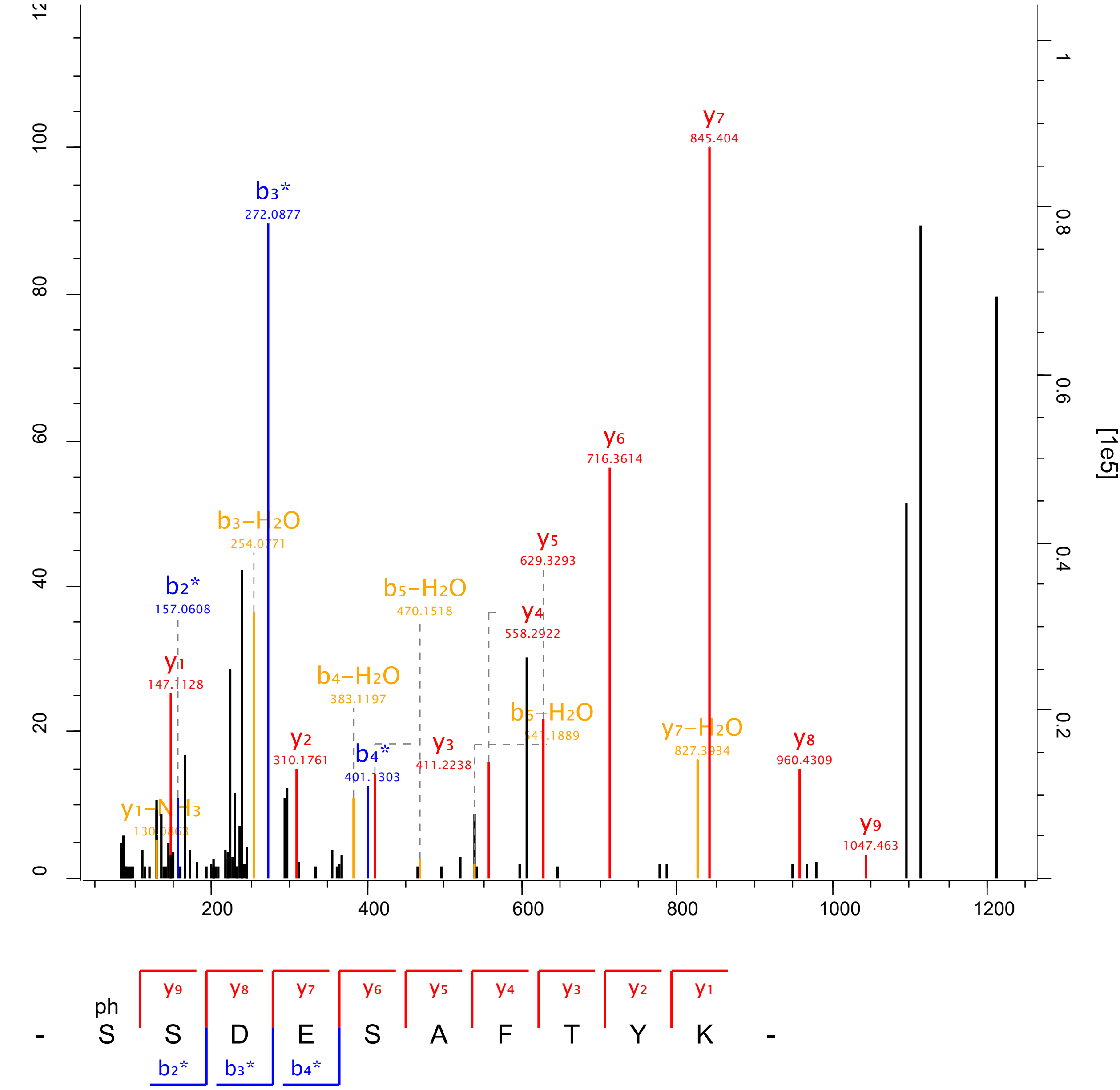

Raw file	Scan	Method	Score	m/z
sys_05_2	16600	FTMS; HCD	124.42	633.81

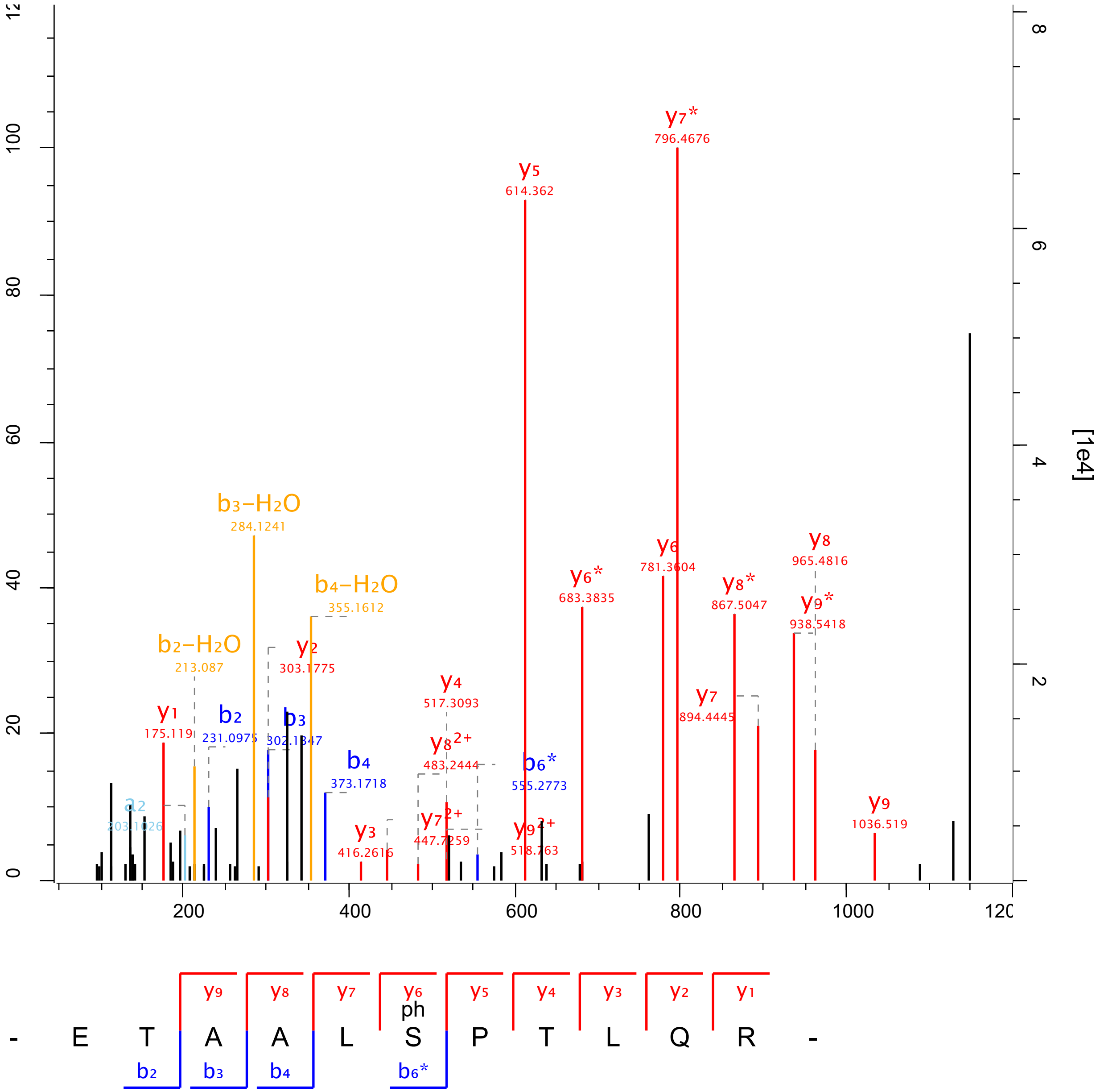

Raw file	Scan	Method	Score	m/z
sys_05_2	16605	FTMS; HCD	214.58	643.79

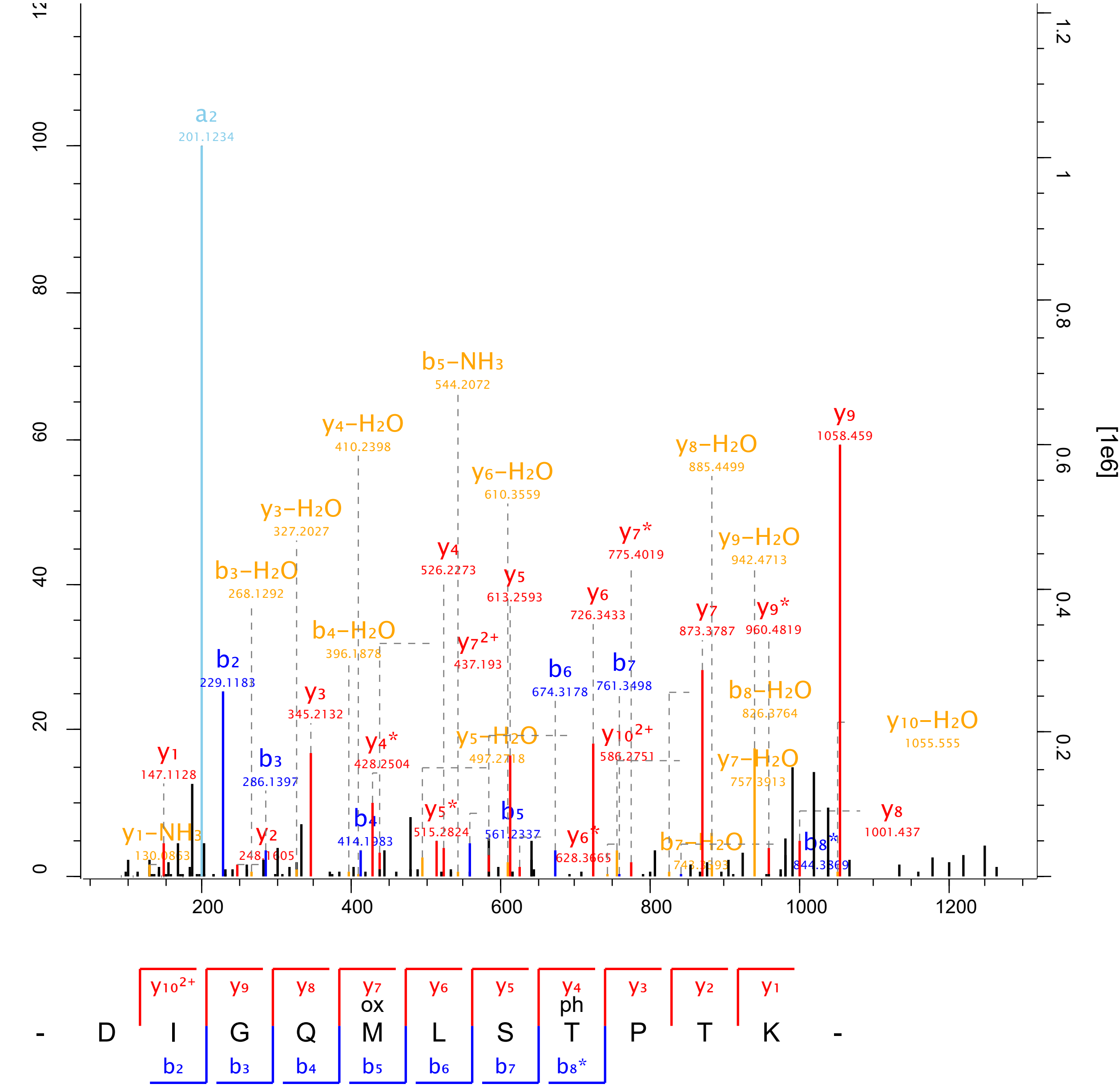

Raw file	Scan	Method	Score	m/z
sys_05_2	16696	FTMS; HCD	162.29	692.81

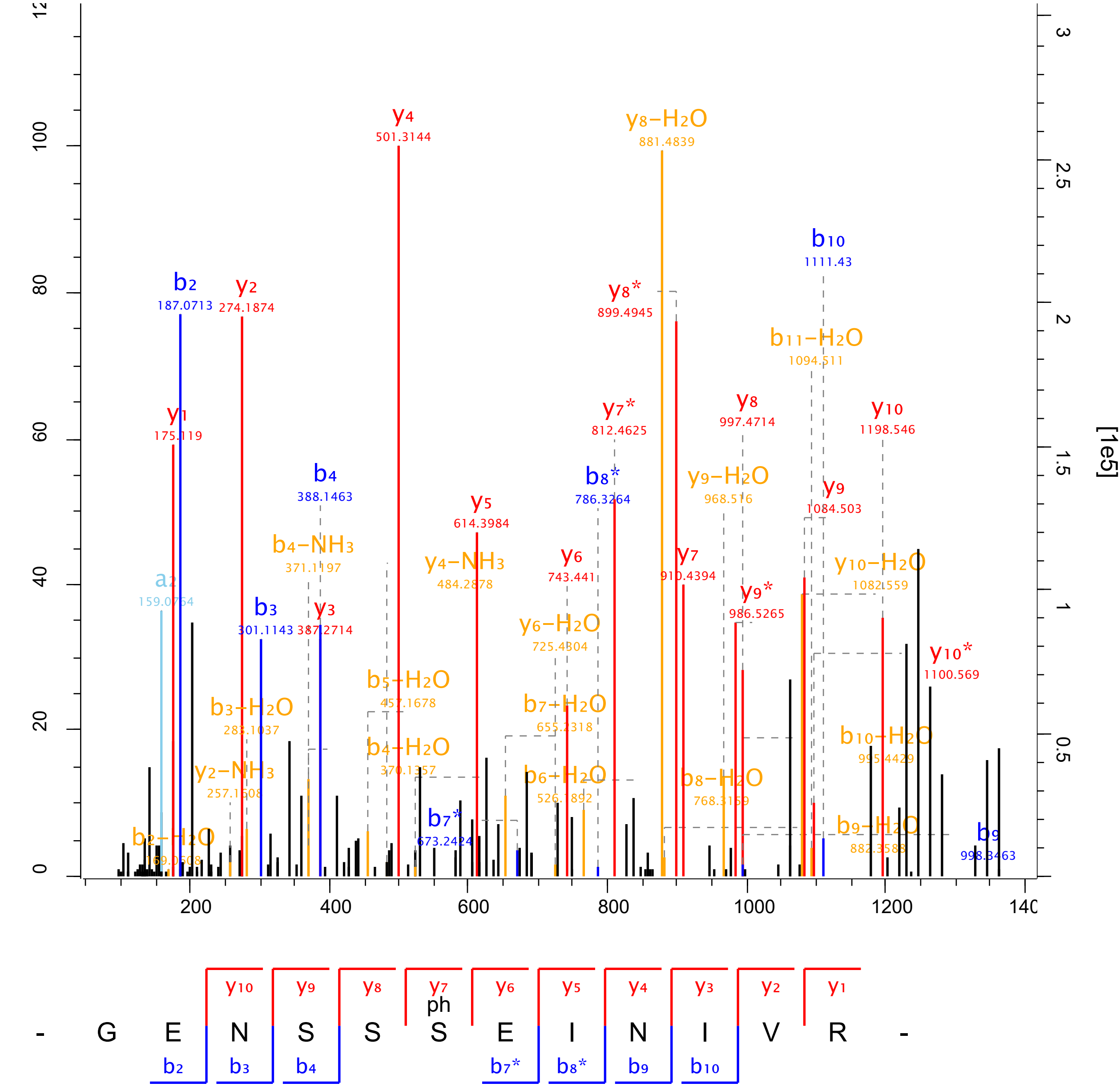

Raw file	Scan	Method	Score	m/z
sys_05_2	16794	FTMS; HCD	68.97	488.91

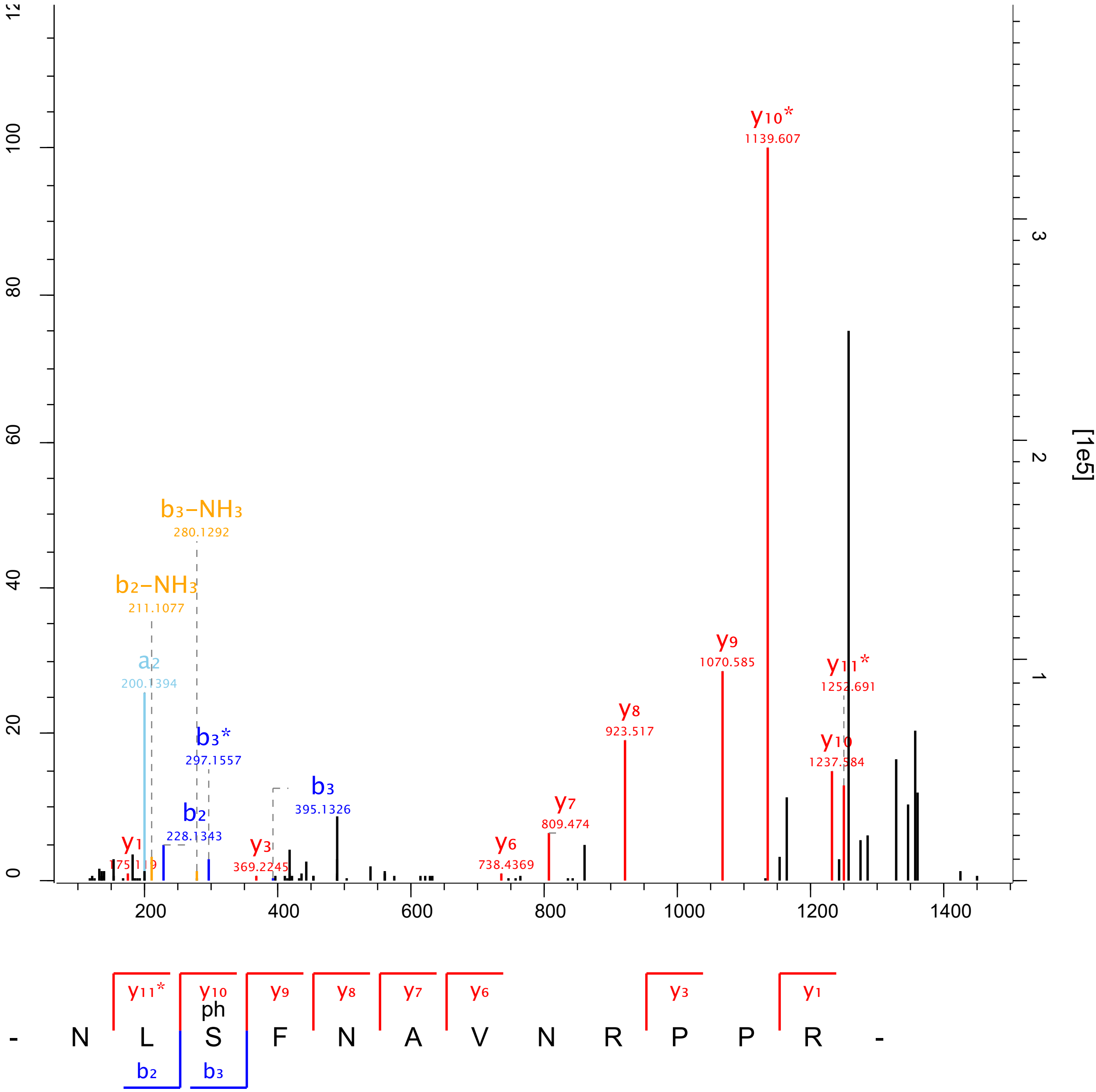

Raw file	Scan	Method	Score	m/z
sys_05_2	16806	FTMS; HCD	82.65	578.22

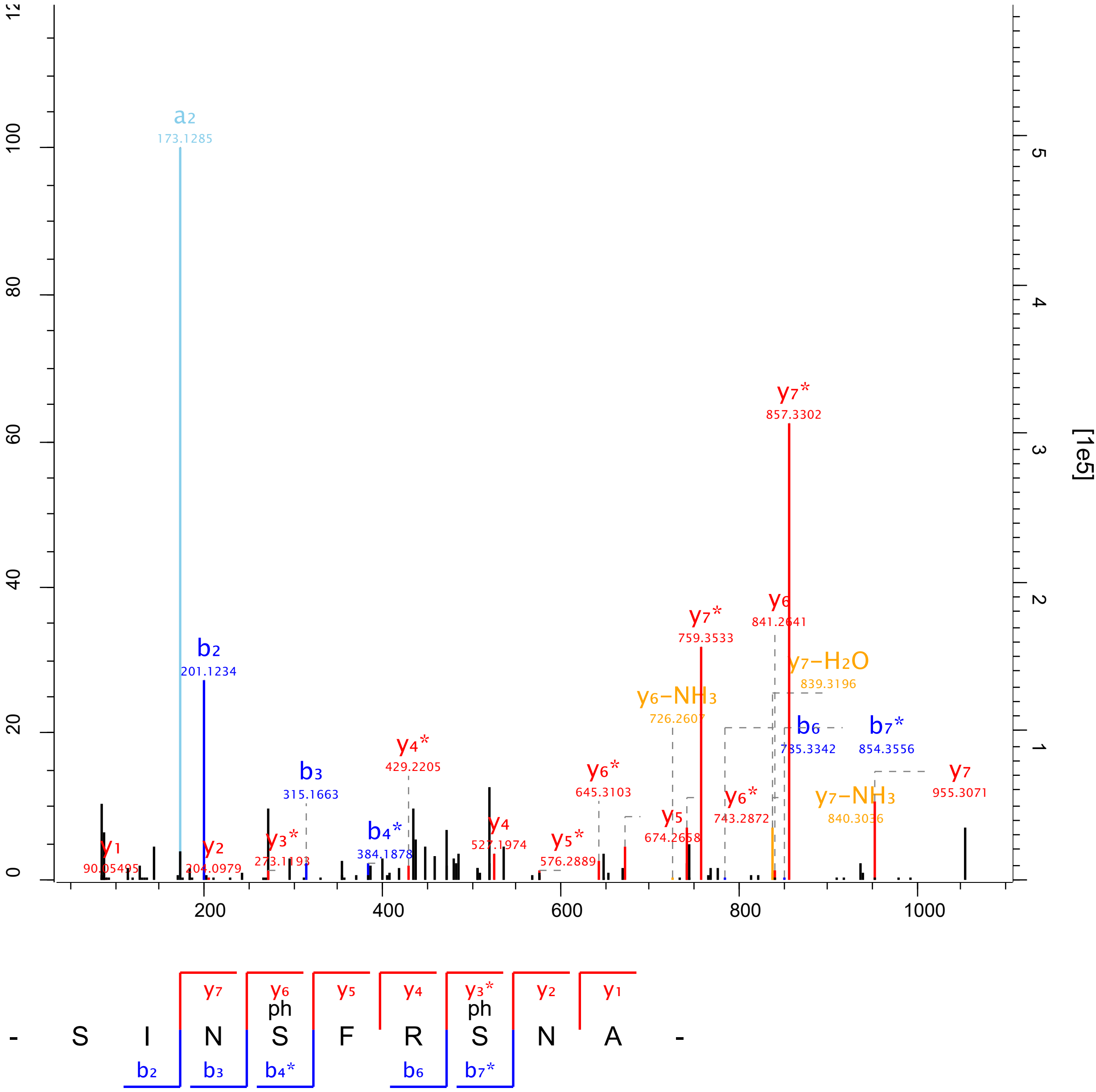

Raw file	Scan	Method	Score	m/z
sys_05_2	16886	FTMS; HCD	194.67	889.32

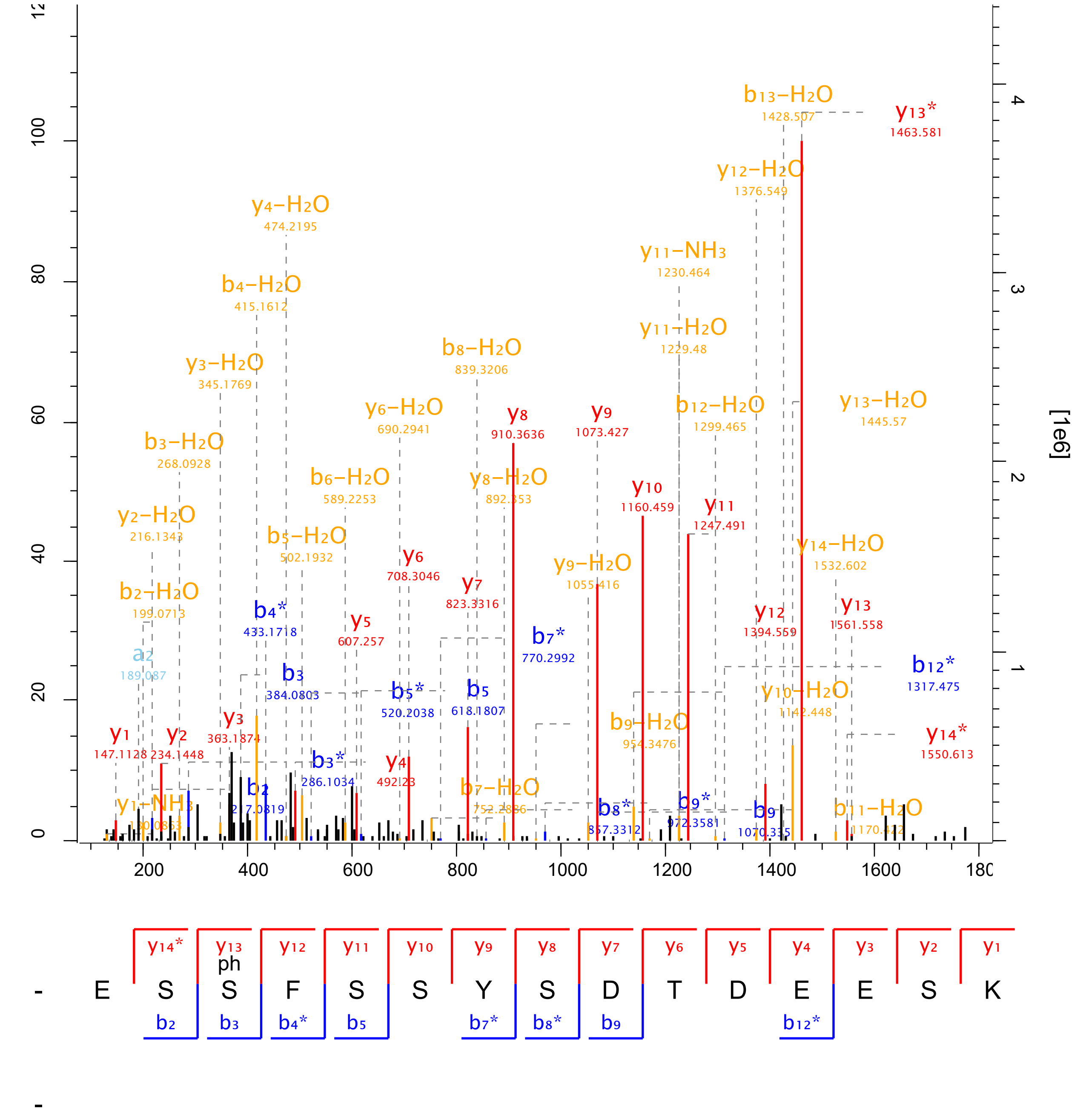

Raw file	Scan	Method	Score	m/z
sys_05_2	16934	FTMS; HCD	50.9	530.58

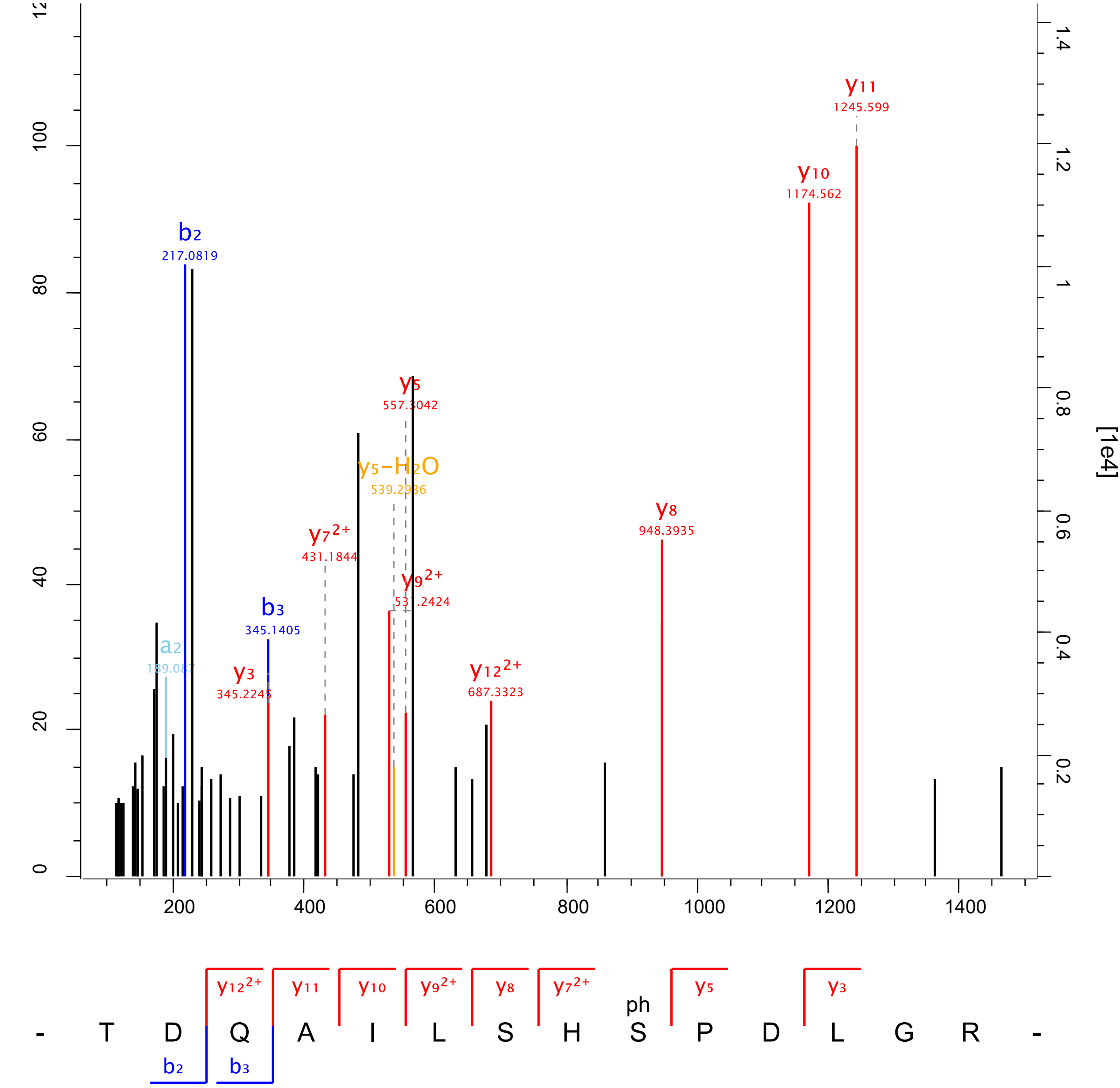

Raw file	Scan	Method	Score	m/z
sys_05_2	16945	FTMS; HCD	171.26	729.82

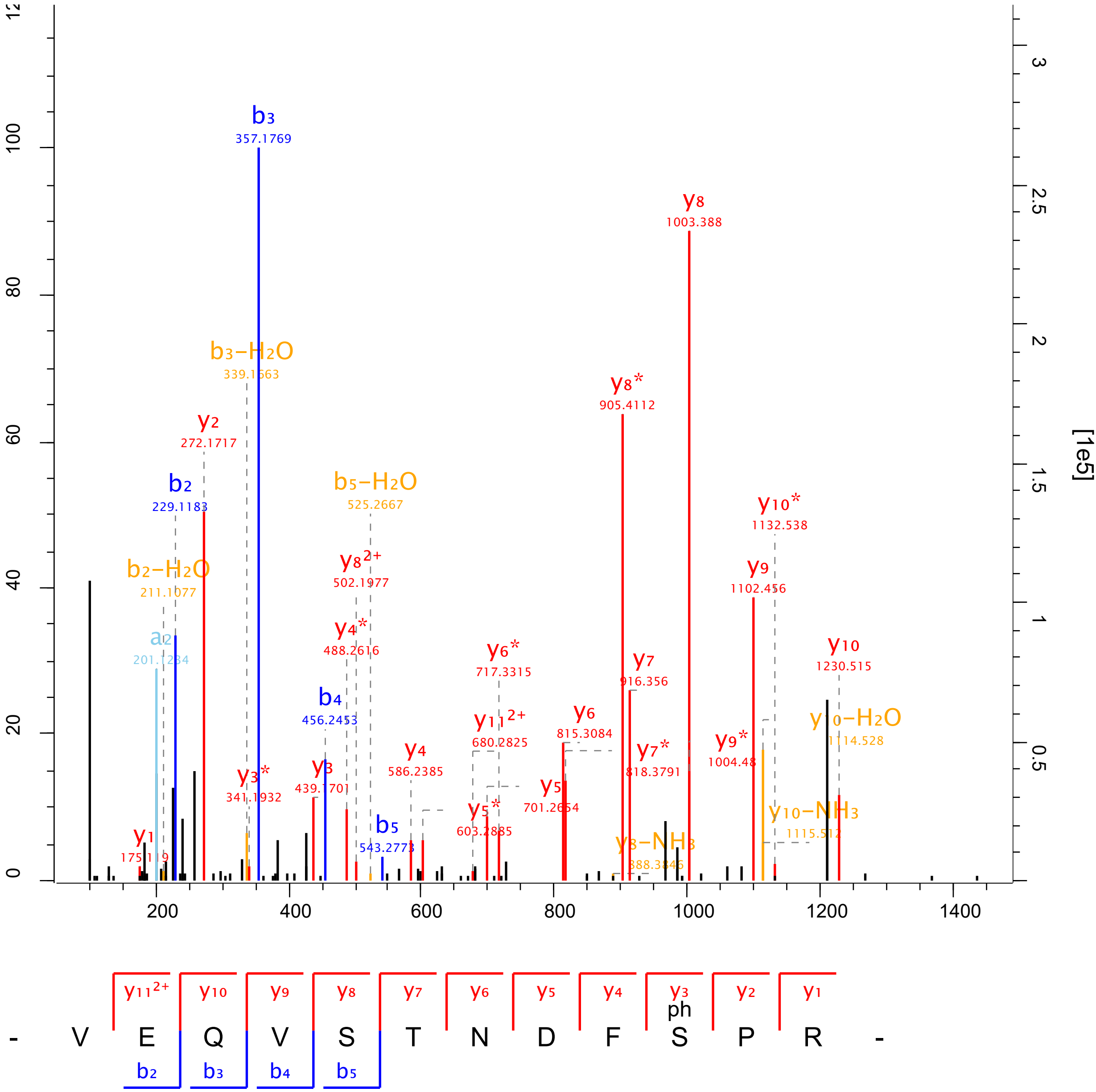

Raw file	Scan	Method	Score	m/z
sys_05_2	16965	FTMS; HCD	49.45	571.27

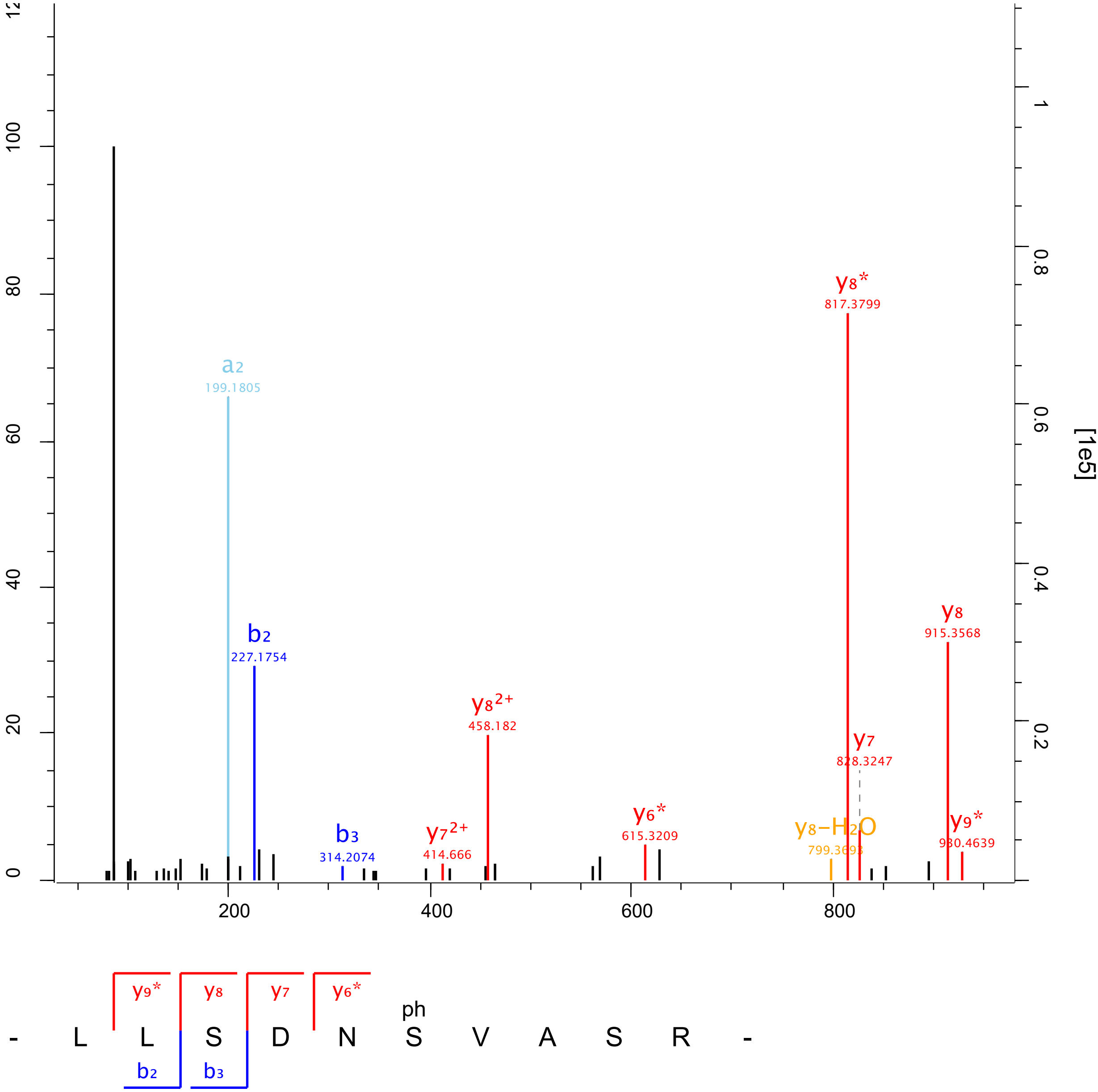

Raw file	Scan	Method	Score	m/z
sys_05_2	16978	FTMS; HCD	197.38	762.33

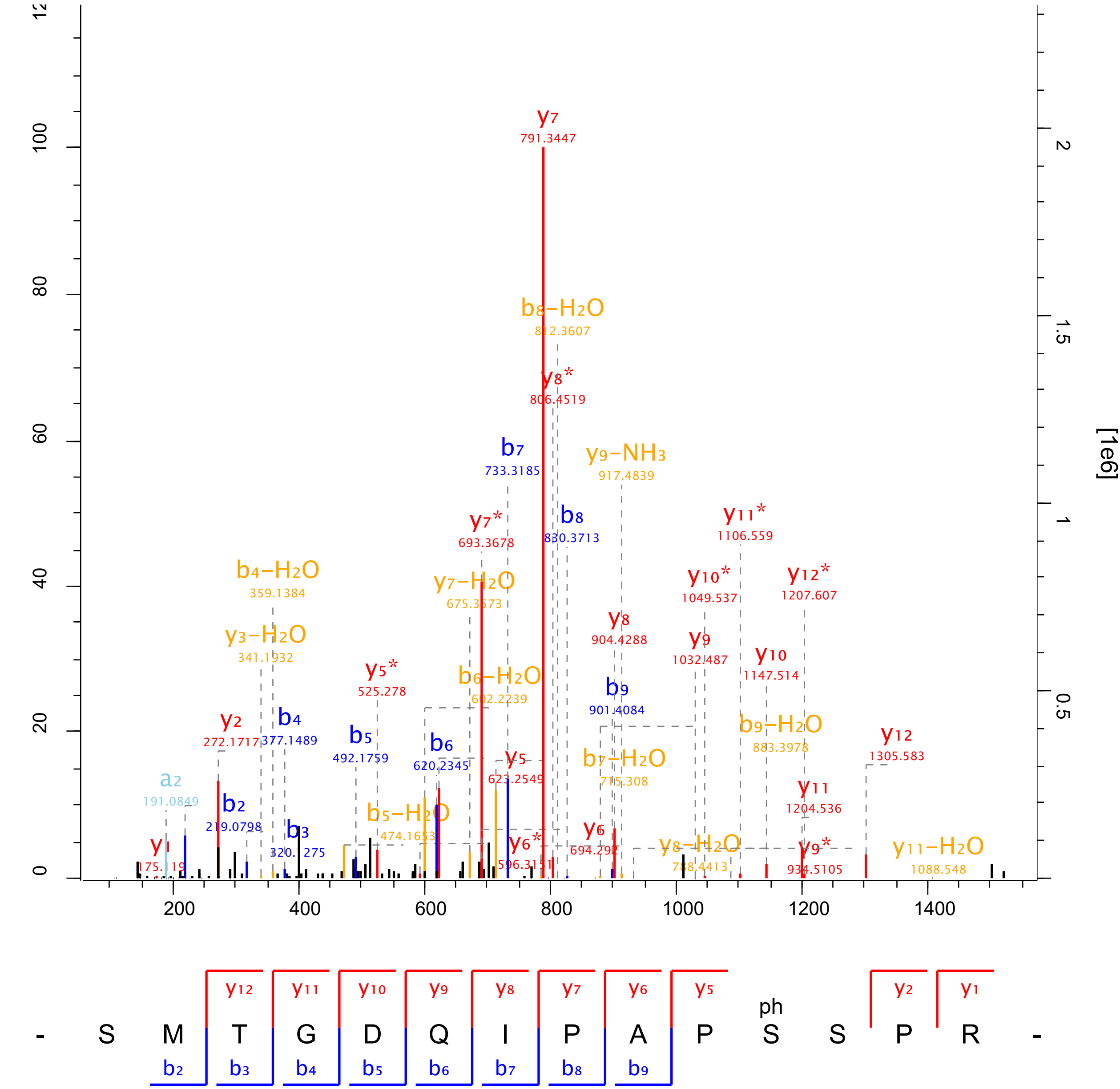

Raw file	Scan	Method	Score	m/z
sys_05_2	17000	FTMS; HCD	144.73	644.77

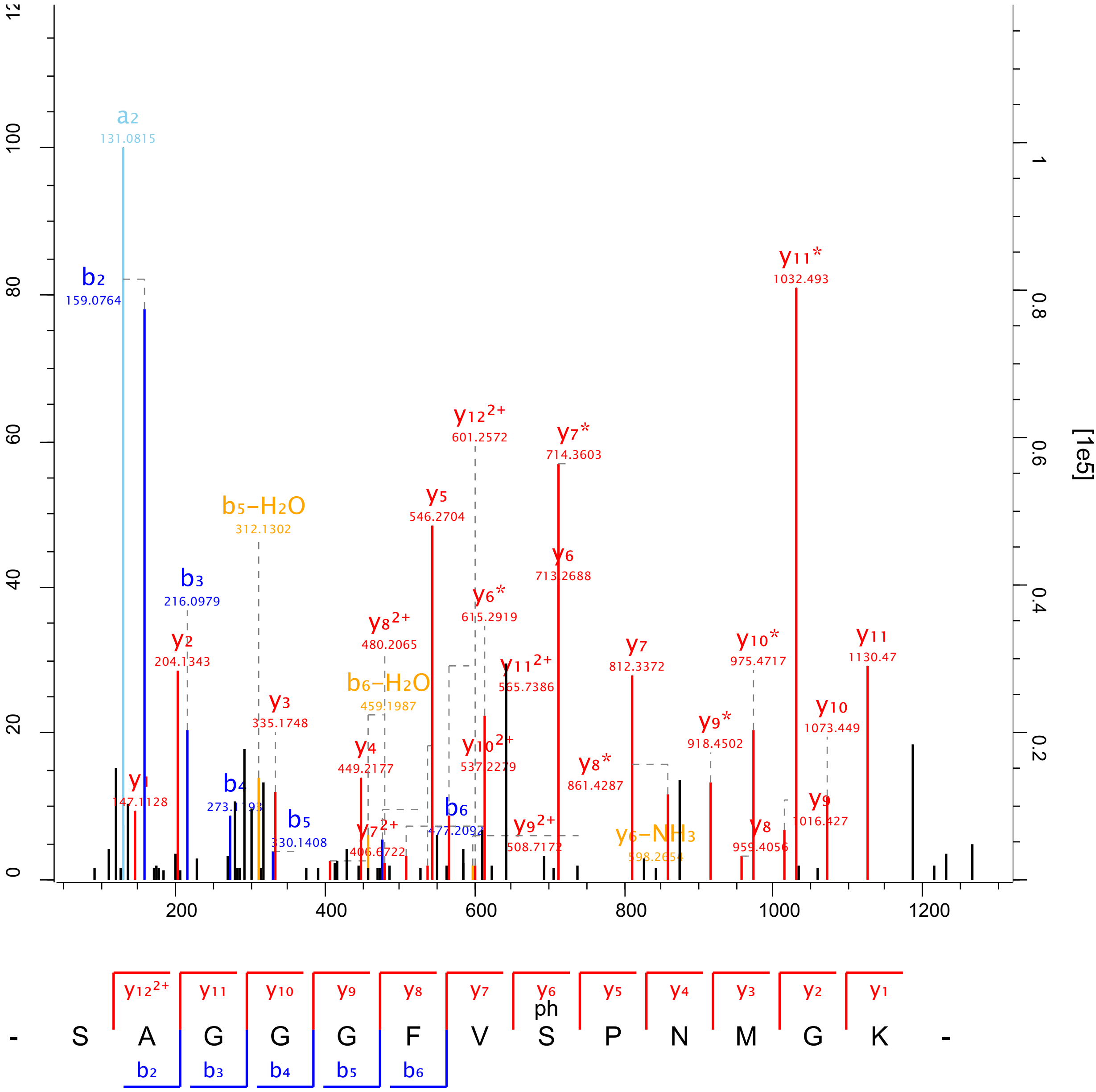

Raw file	Scan	Method	Score	m/z
sys_05_2	17032	FTMS; HCD	186.74	851.35

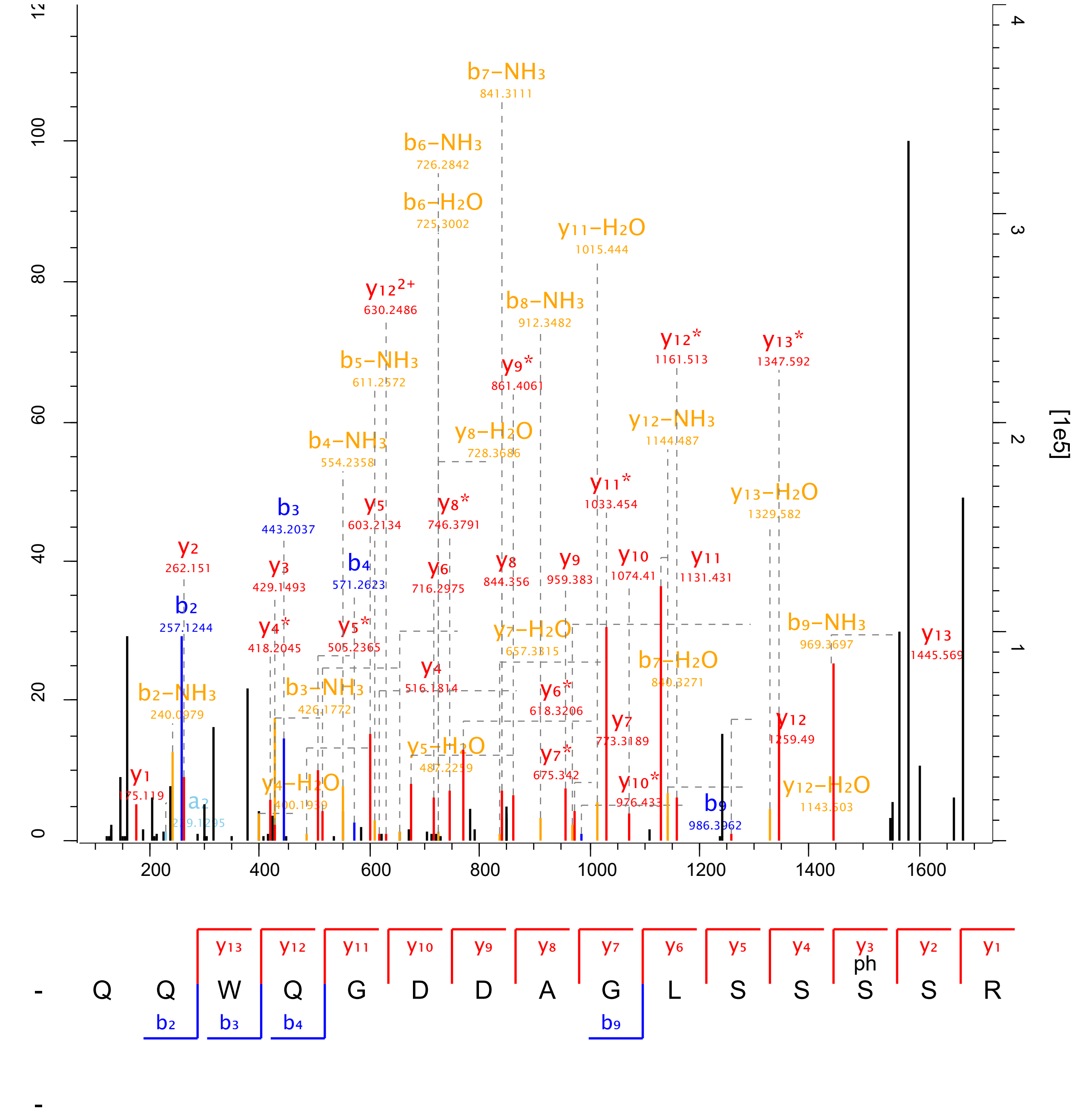

Raw file	Scan	Method	Score	m/z
sys_05_2	17153	FTMS; HCD	64.82	495.71

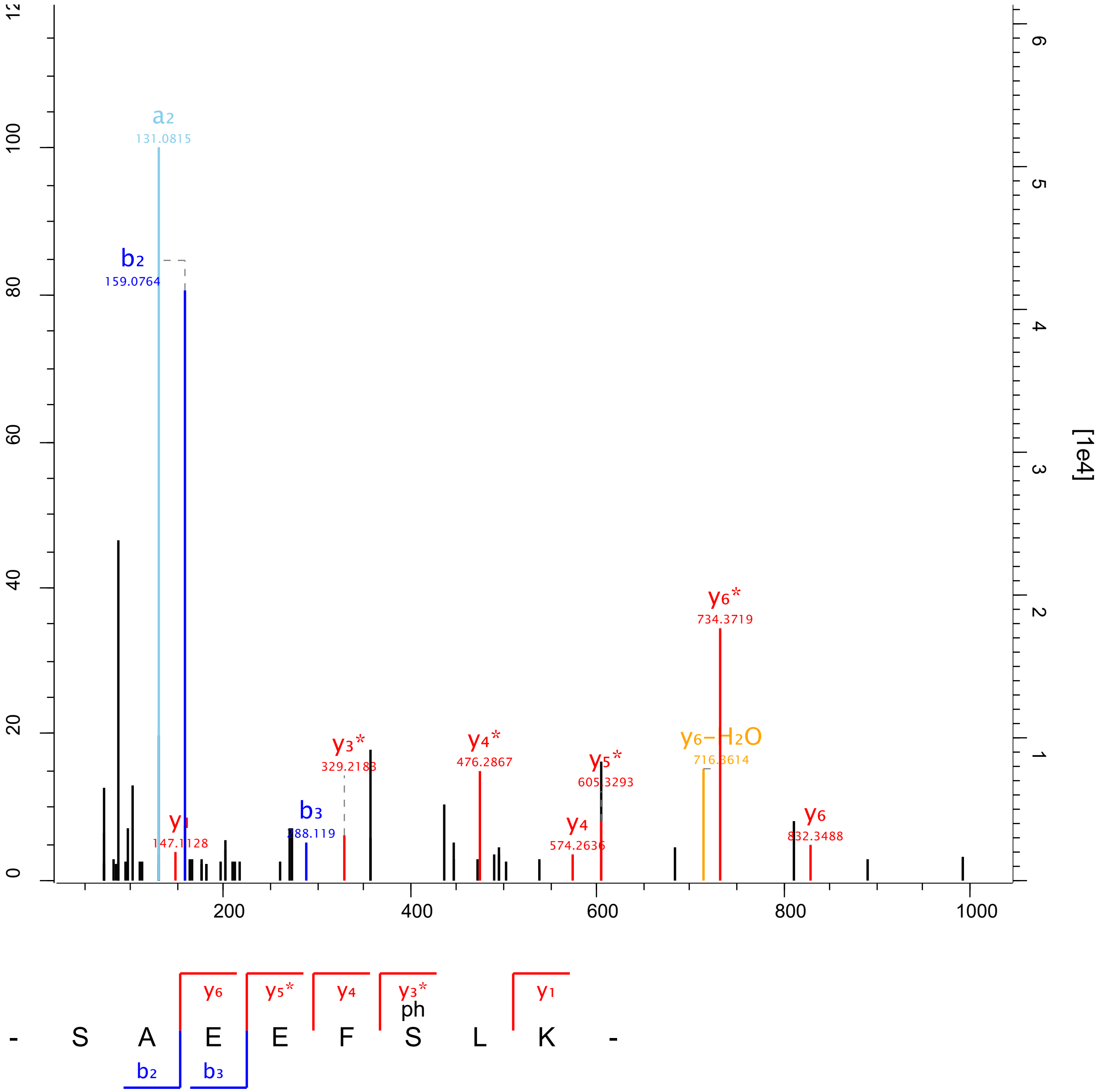

Raw file	Scan	Method	Score	m/z
sys_05_2	17407	FTMS; HCD	115.88	856.4

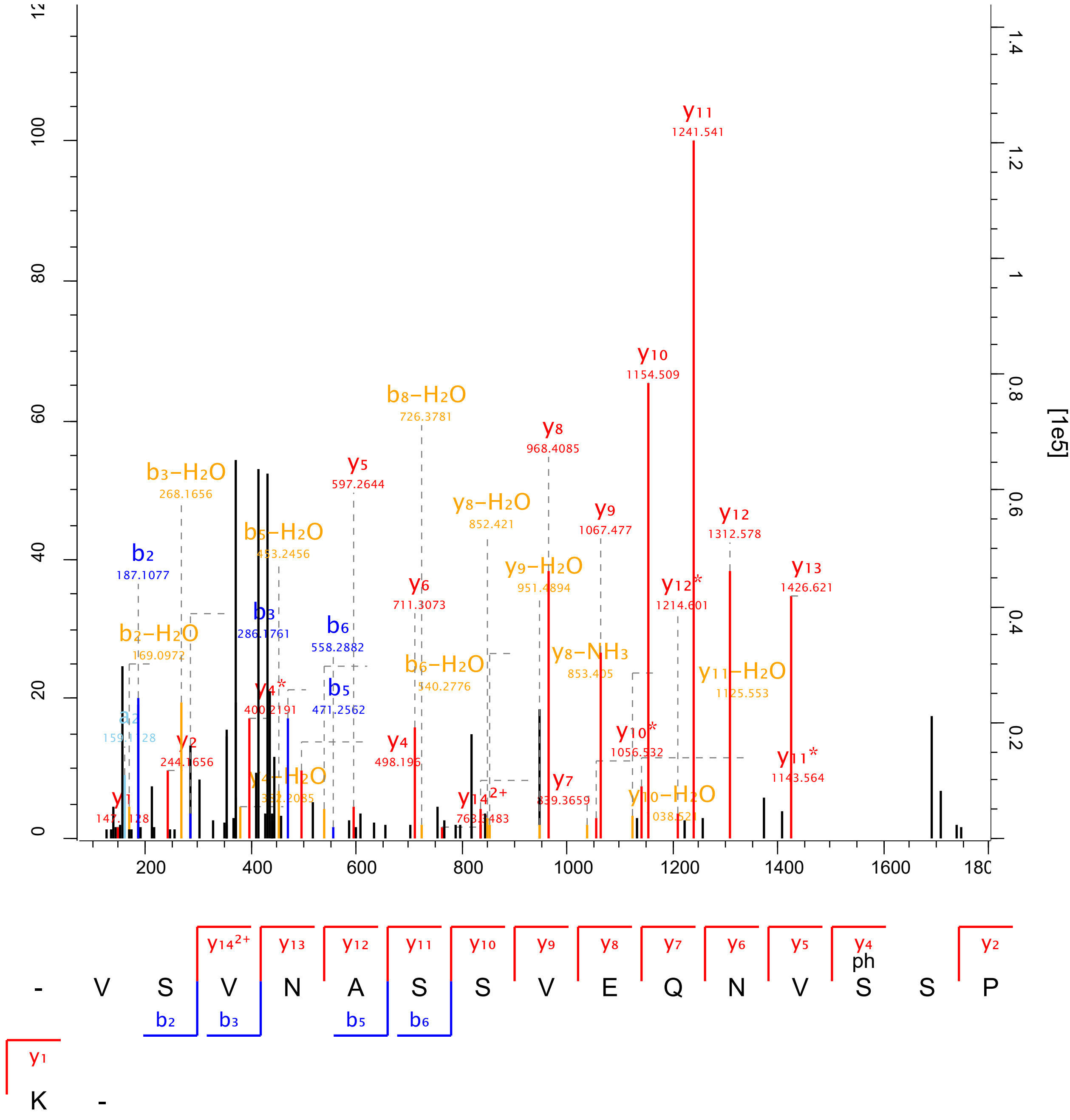

Raw file	Scan	Method	Score	m/z
sys_05_2	17549	FTMS; HCD	163.79	782.84

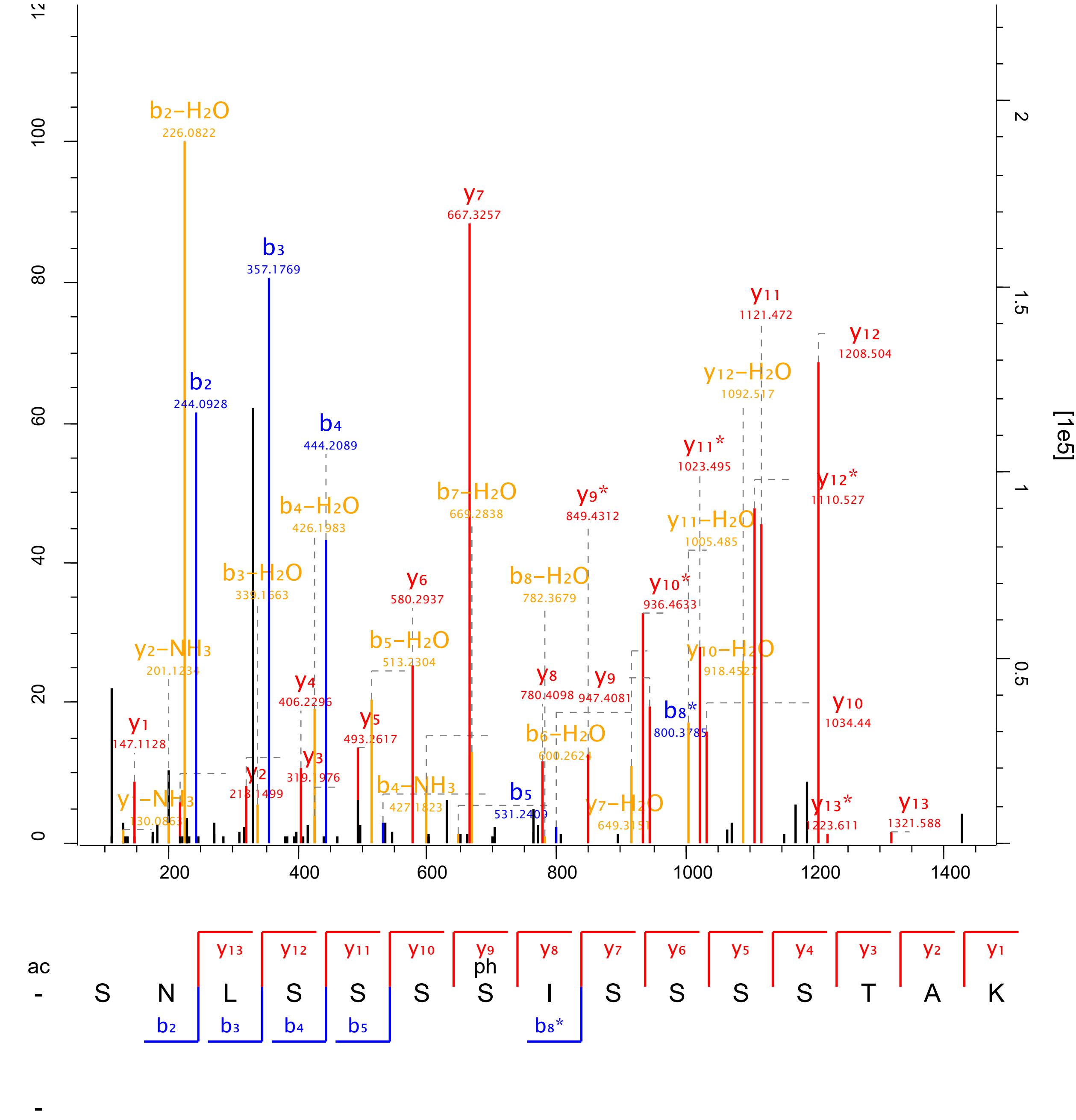

Raw file	Scan	Method	Score	m/z
sys_05_2	17614	FTMS; HCD	157.33	503.86

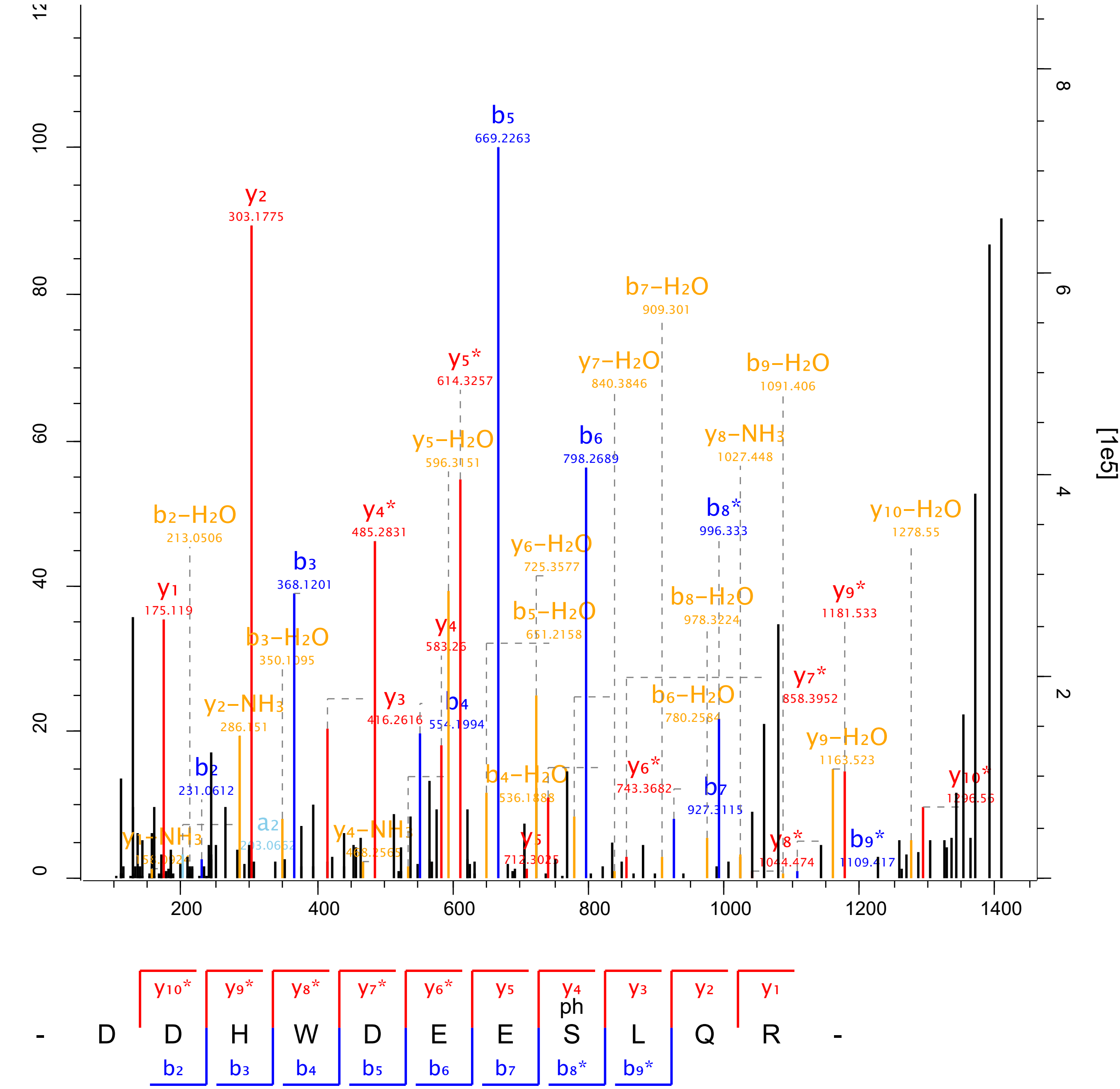

Raw file	Scan	Method	Score	m/z
sys_05_2	17651	FTMS; HCD	171.79	794.35

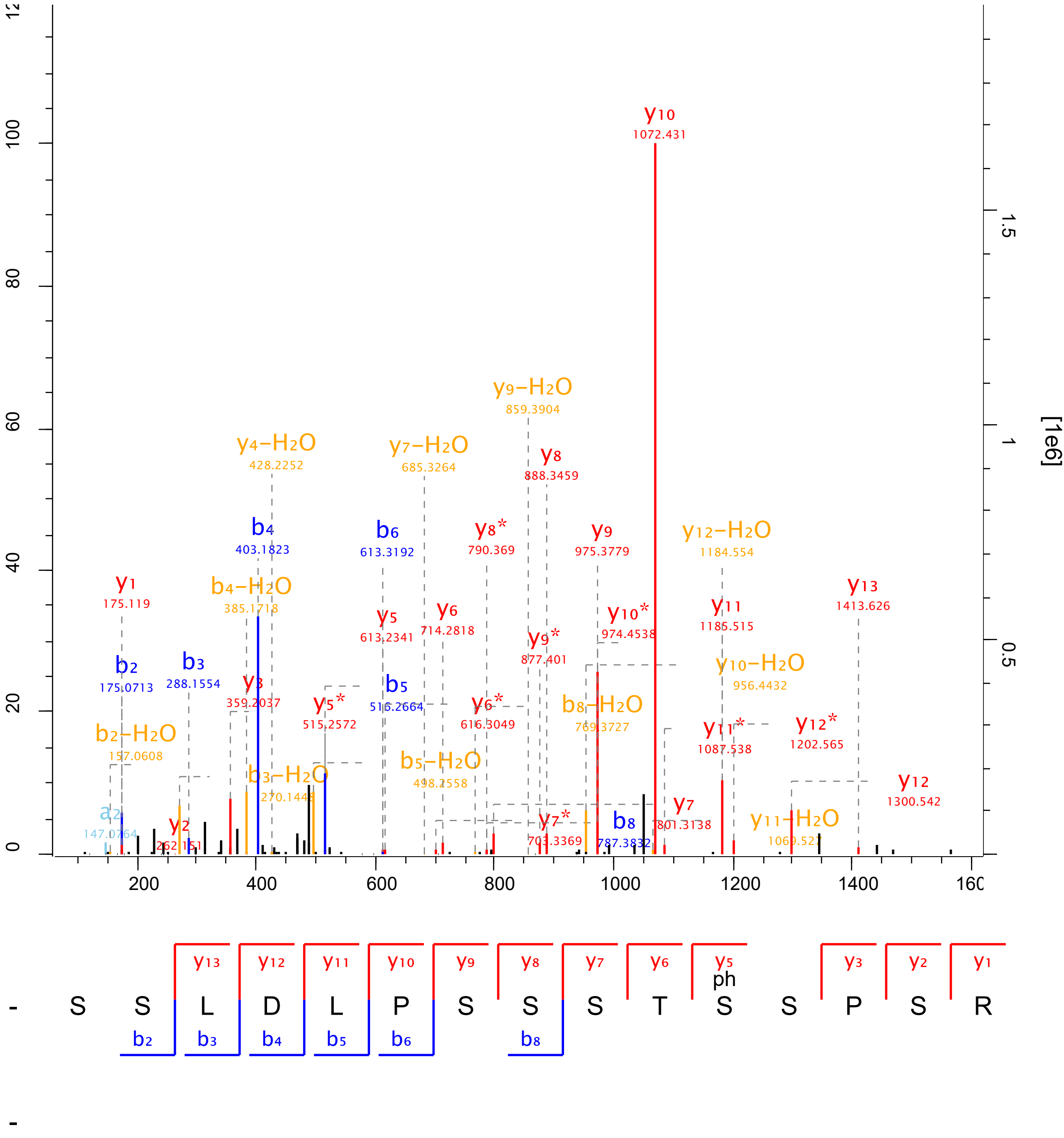

Raw file	Scan	Method	Score	m/z
sys_05_2	17727	FTMS; HCD	58.08	744.8

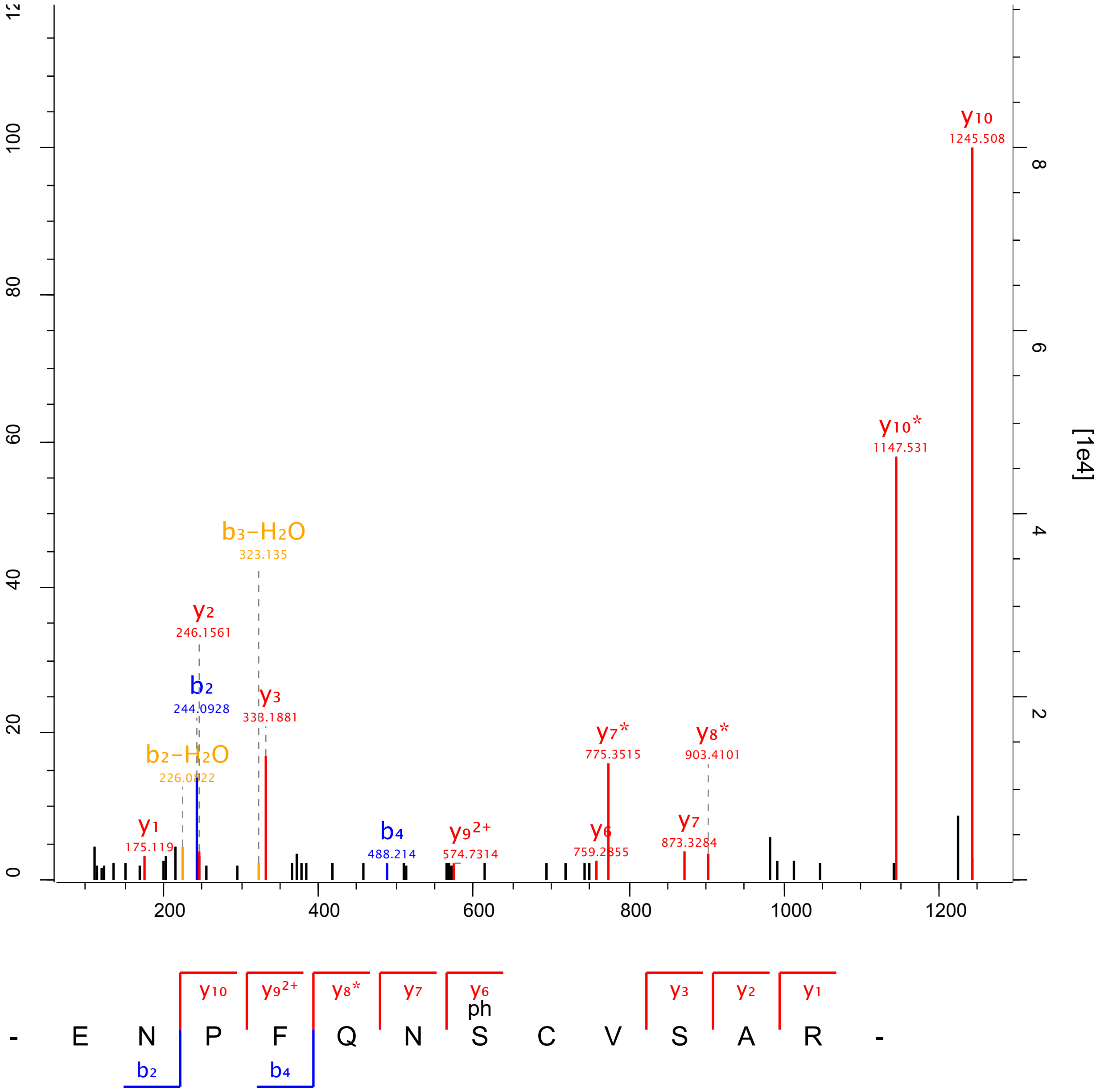

Raw file	Scan	Method	Score	m/z
sys_05_2	18324	FTMS; HCD	129.38	695.34

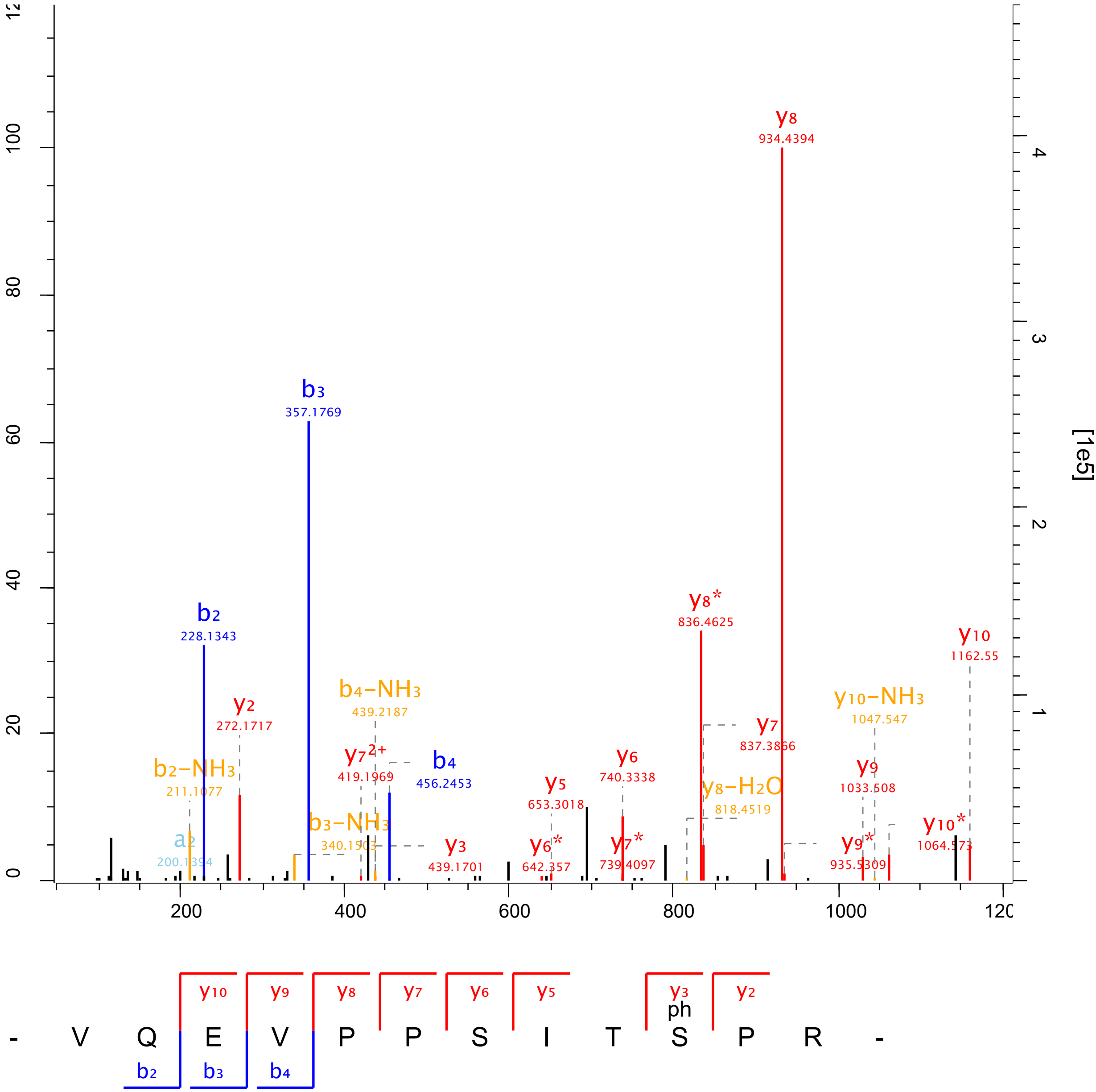

Raw file	Scan	Method	Score	m/z
sys_05_2	18795	FTMS; HCD	213.72	564.76

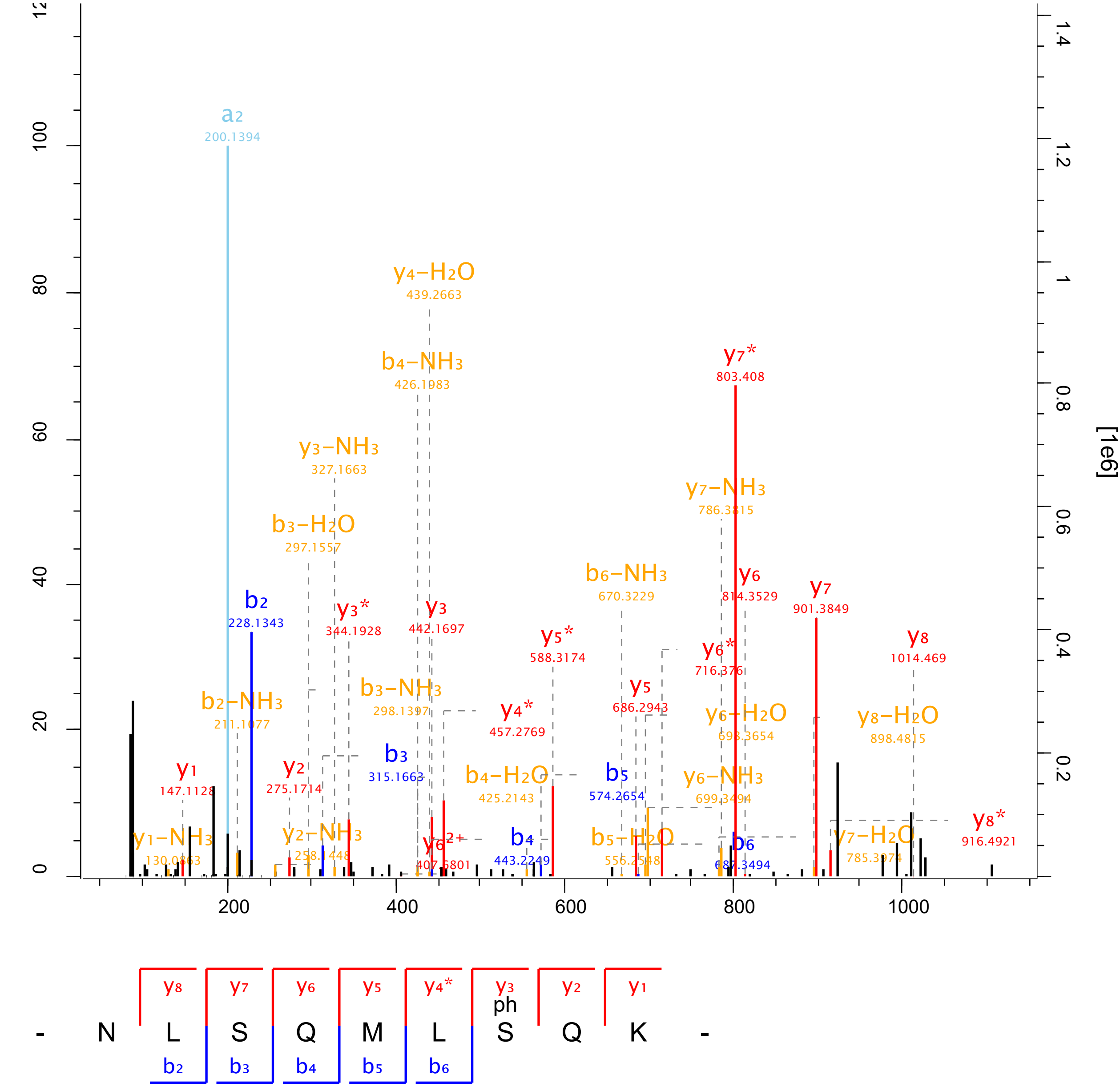

Raw file	Scan	Method	Score	m/z
sys_05_2	18826	FTMS; HCD	128.76	454.52

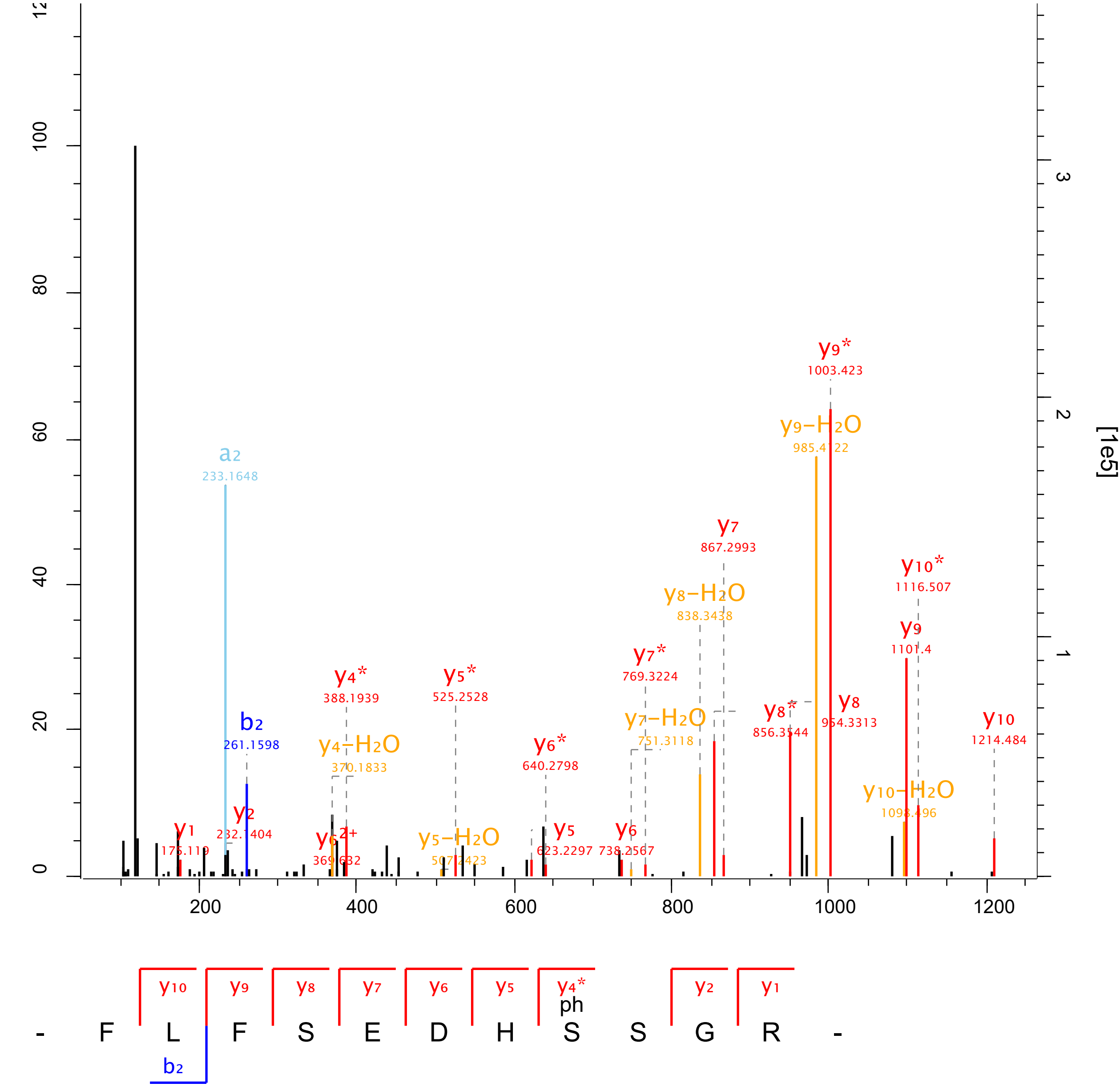

Raw file	Scan	Method	Score	m/z
sys_05_2	19087	FTMS; HCD	55.45	840.87

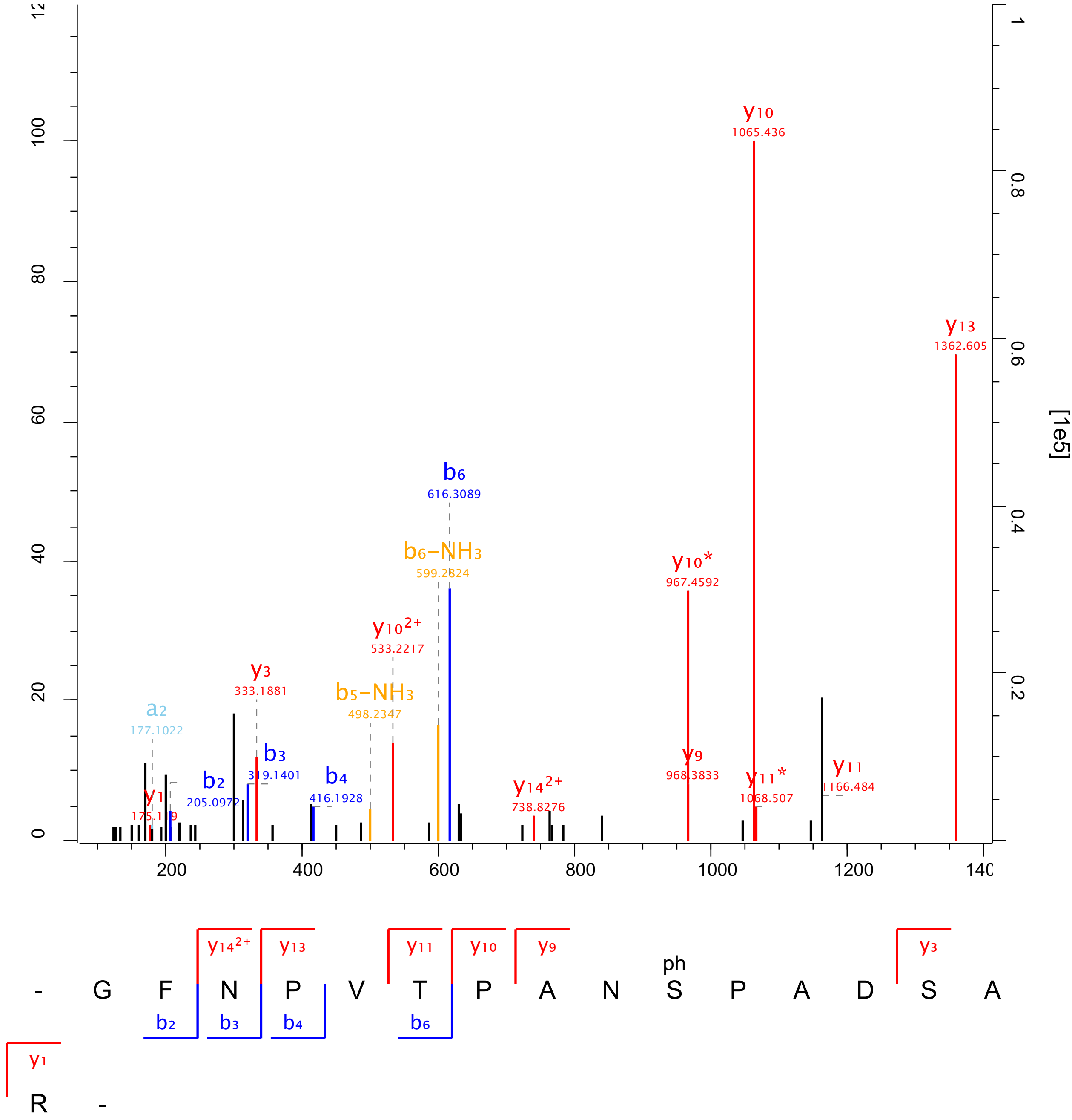

Raw file	Scan	Method	Score	m/z
sys_05_2	19112	FTMS; HCD	97.27	643.3

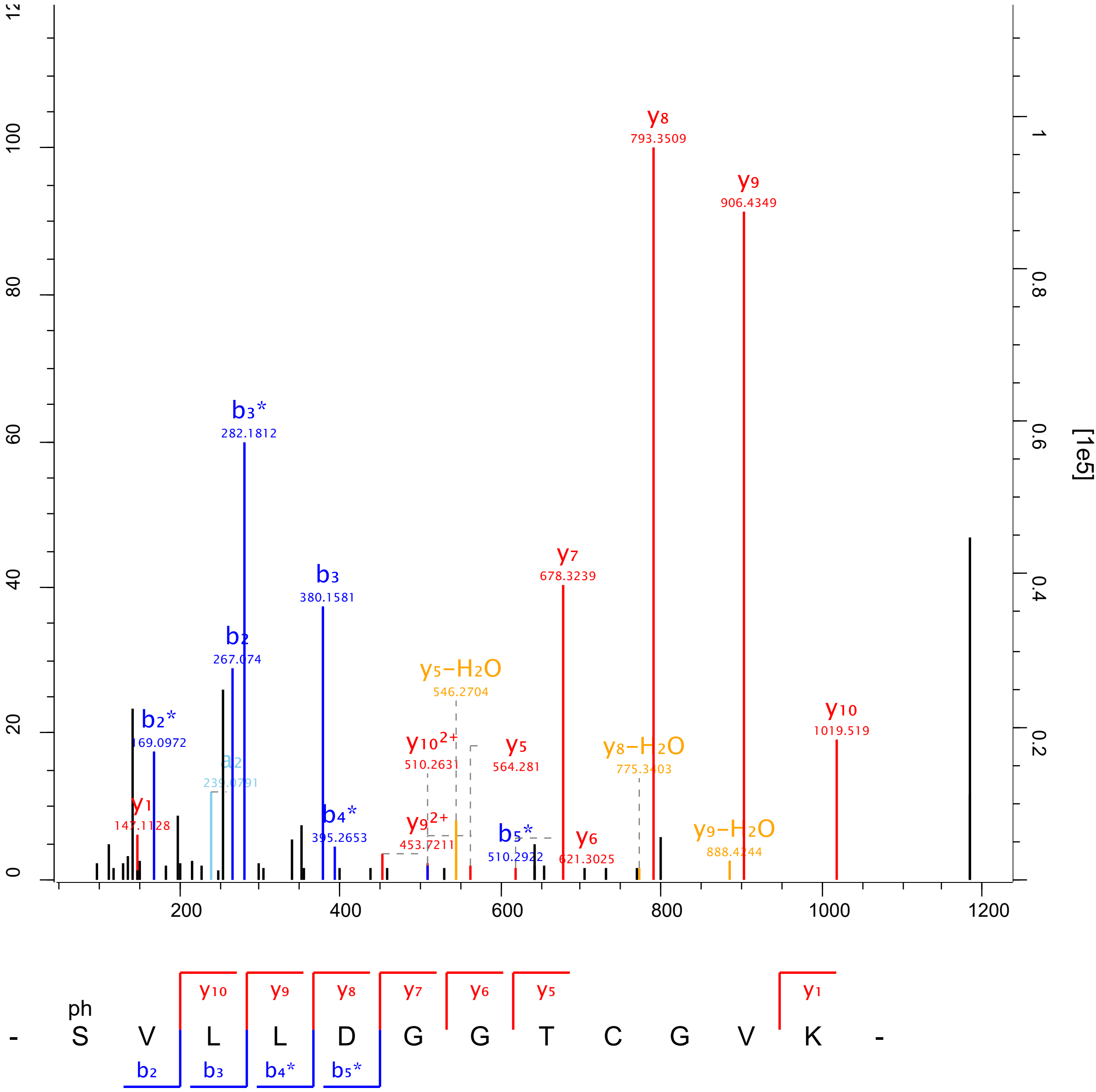

Raw file	Scan	Method	Score	m/z
sys_05_2	19232	FTMS; HCD	135.73	519.2

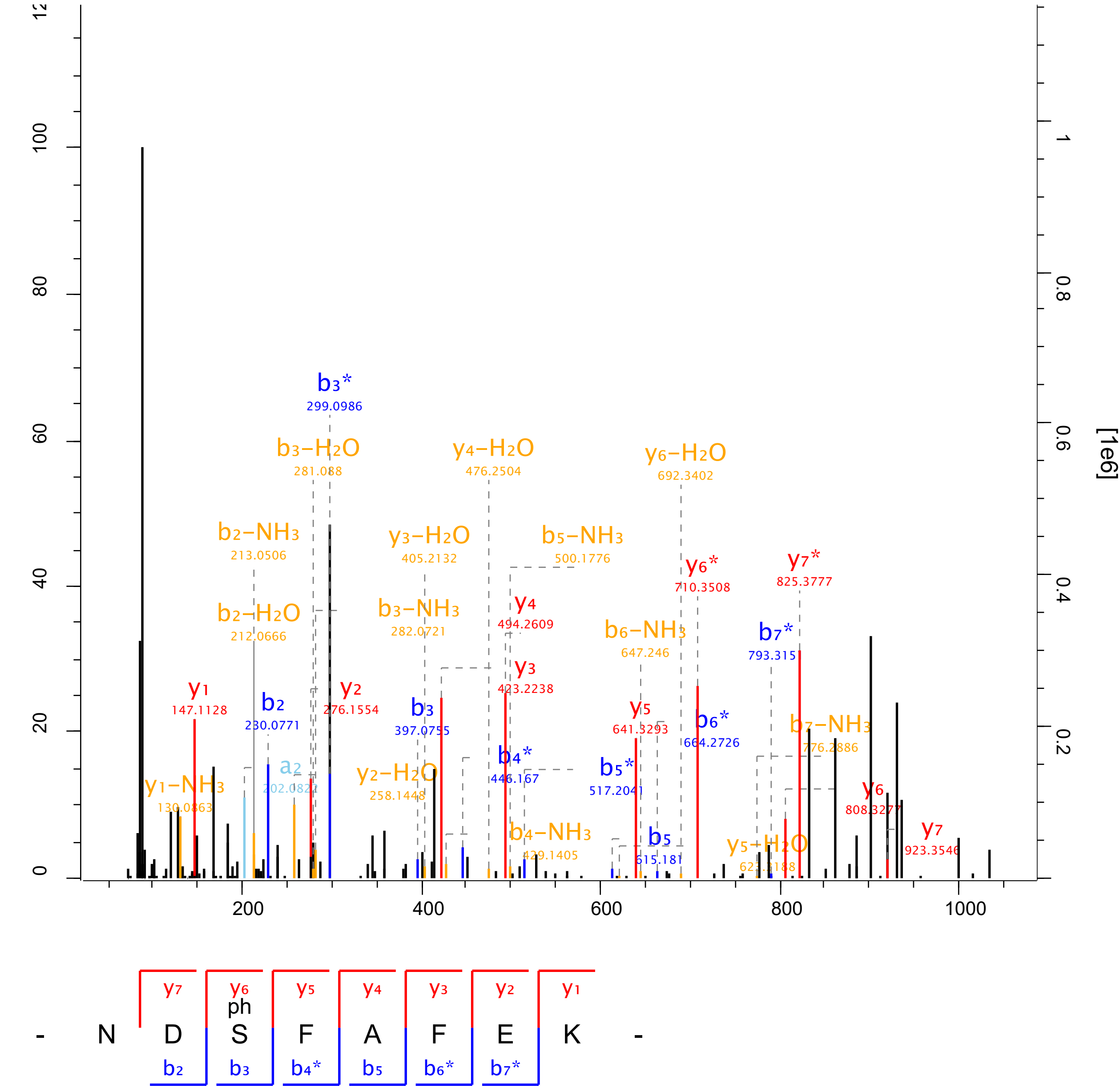

Raw file	Scan	Method	Score	m/z
sys_05_2	19594	FTMS; HCD	177.32	914.88

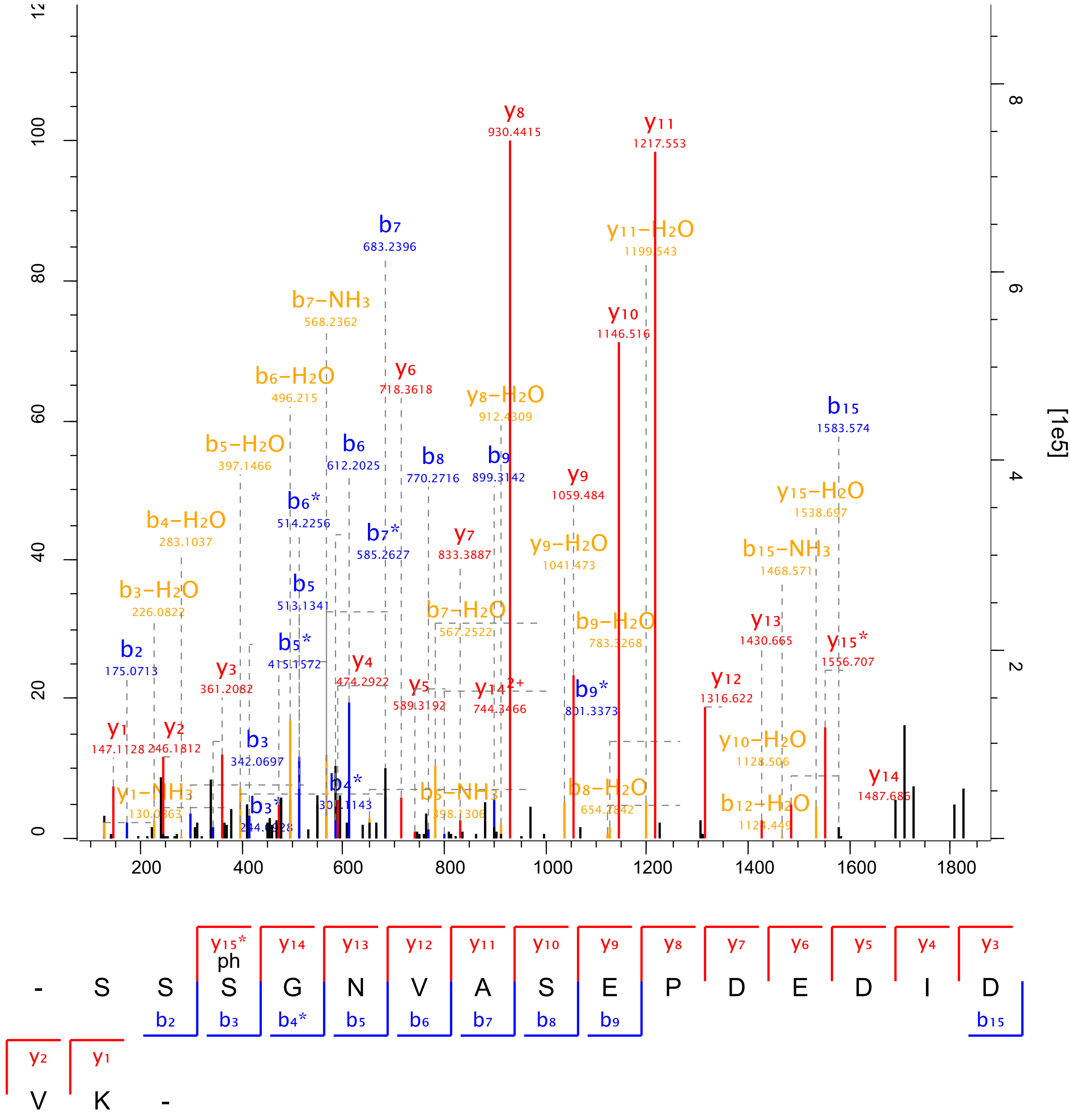

Raw file	Scan	Method	Score	m/z
sys_05_2	19781	FTMS; HCD	110.44	795.85

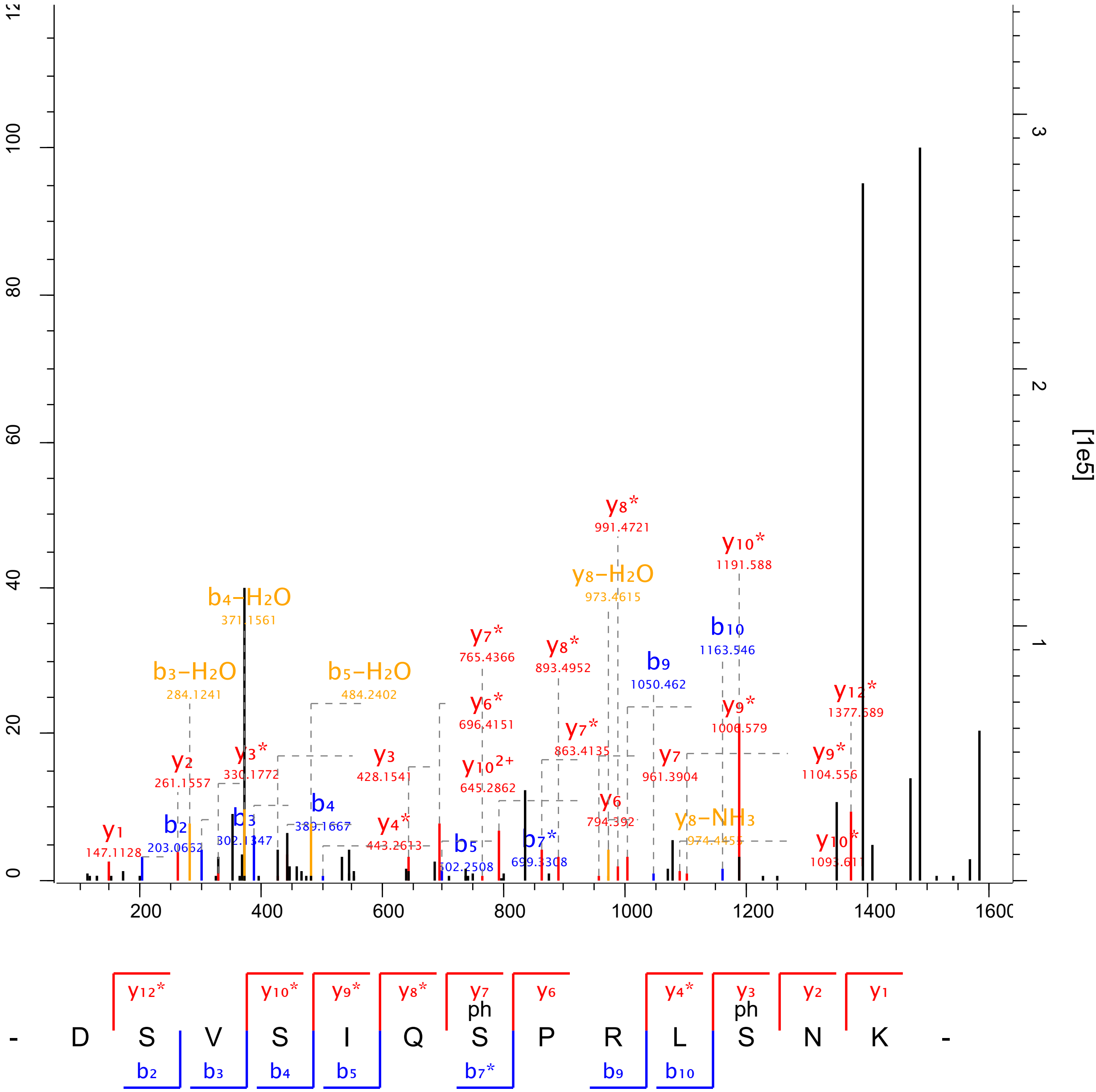

Raw file	Scan	Method	Score	m/z
sys_05_2	20126	FTMS; HCD	202.81	707.84

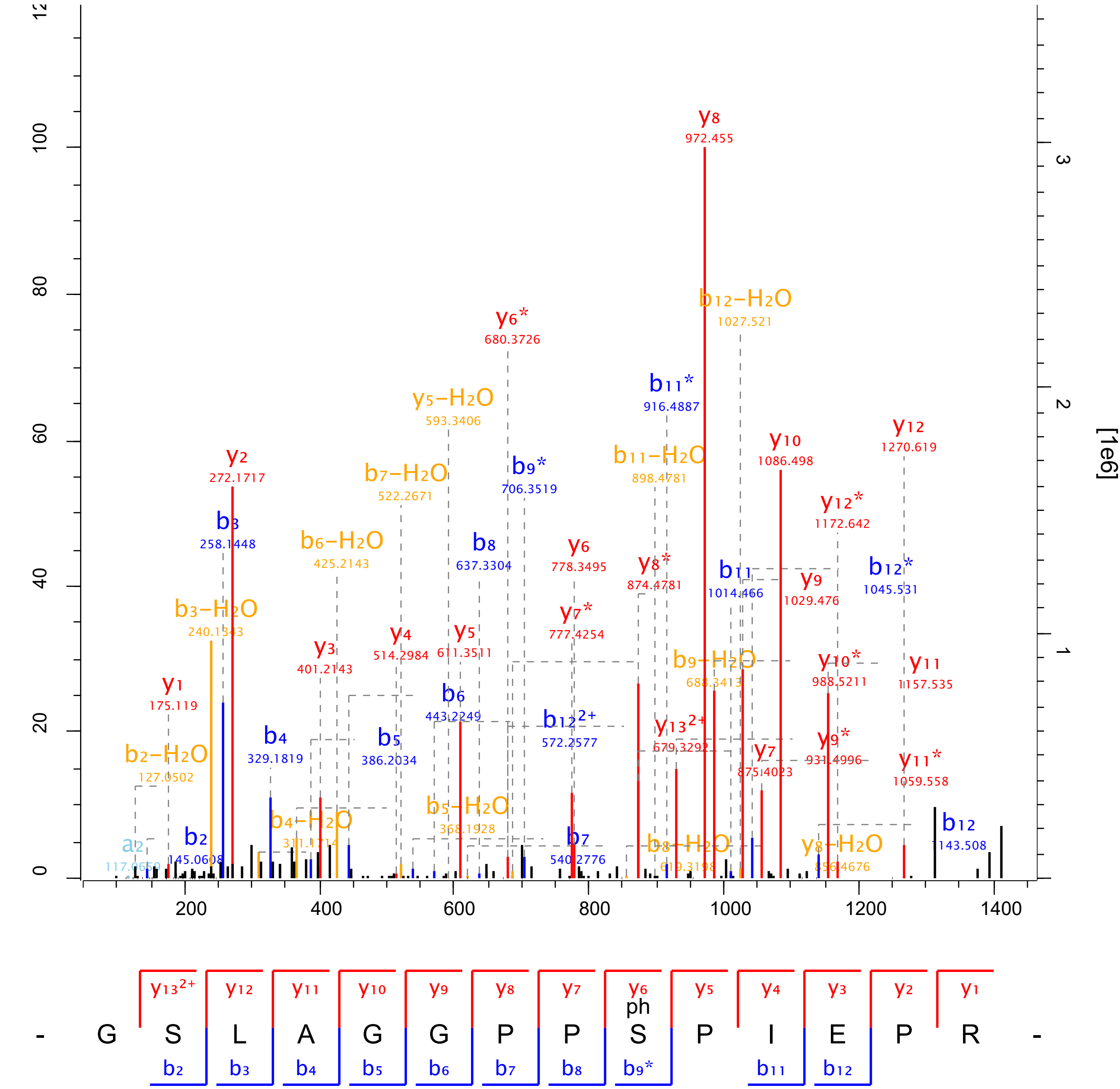

Raw file	Scan	Method	Score	m/z
sys_05_2	20341	FTMS; HCD	86.21	674.29

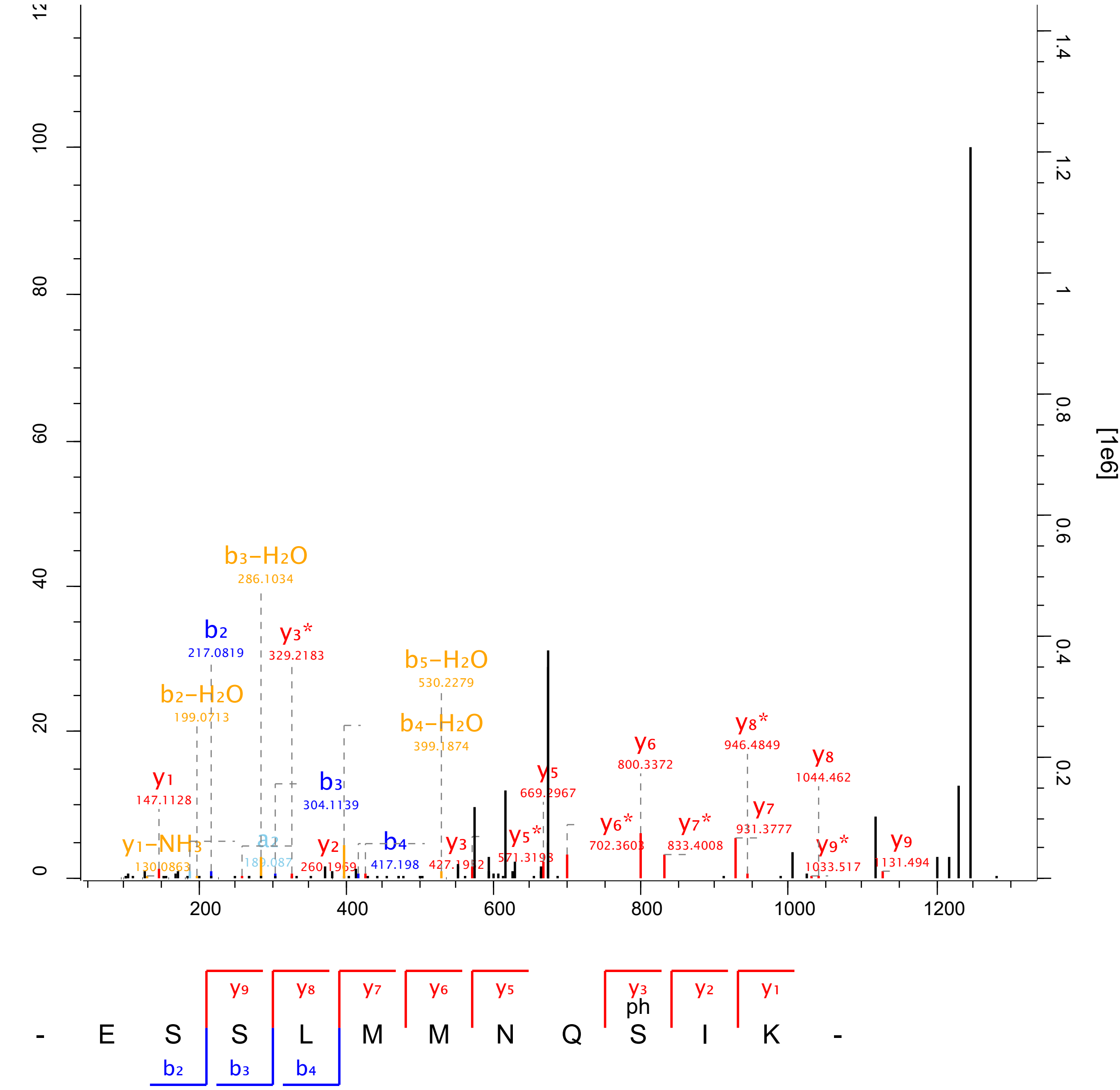

Raw file	Scan	Method	Score	m/z
sys_05_2	20520	FTMS; HCD	93.65	433.23

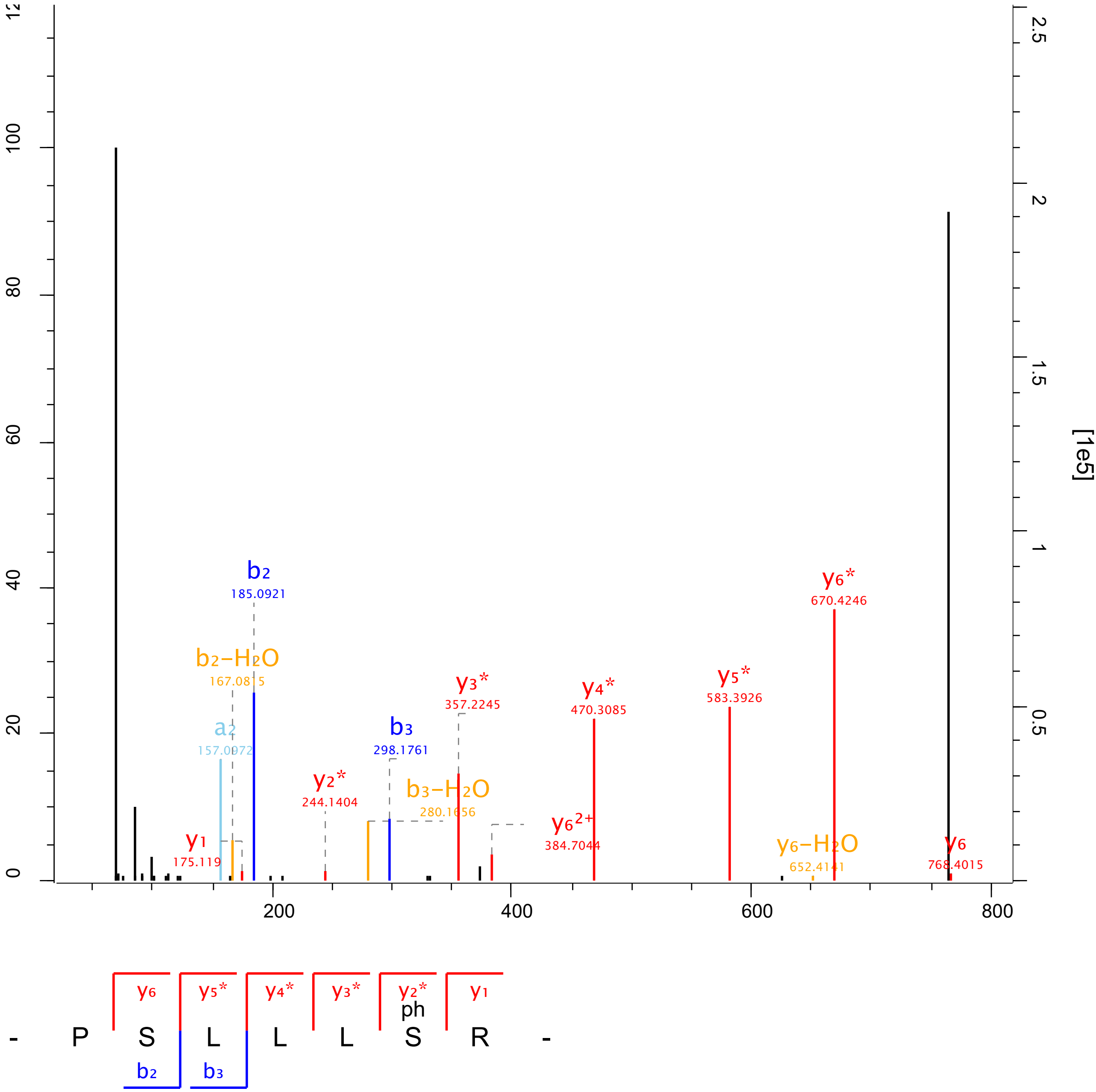

Raw file	Scan	Method	Score	m/z
sys_05_2	20773	FTMS; HCD	199.91	787.34

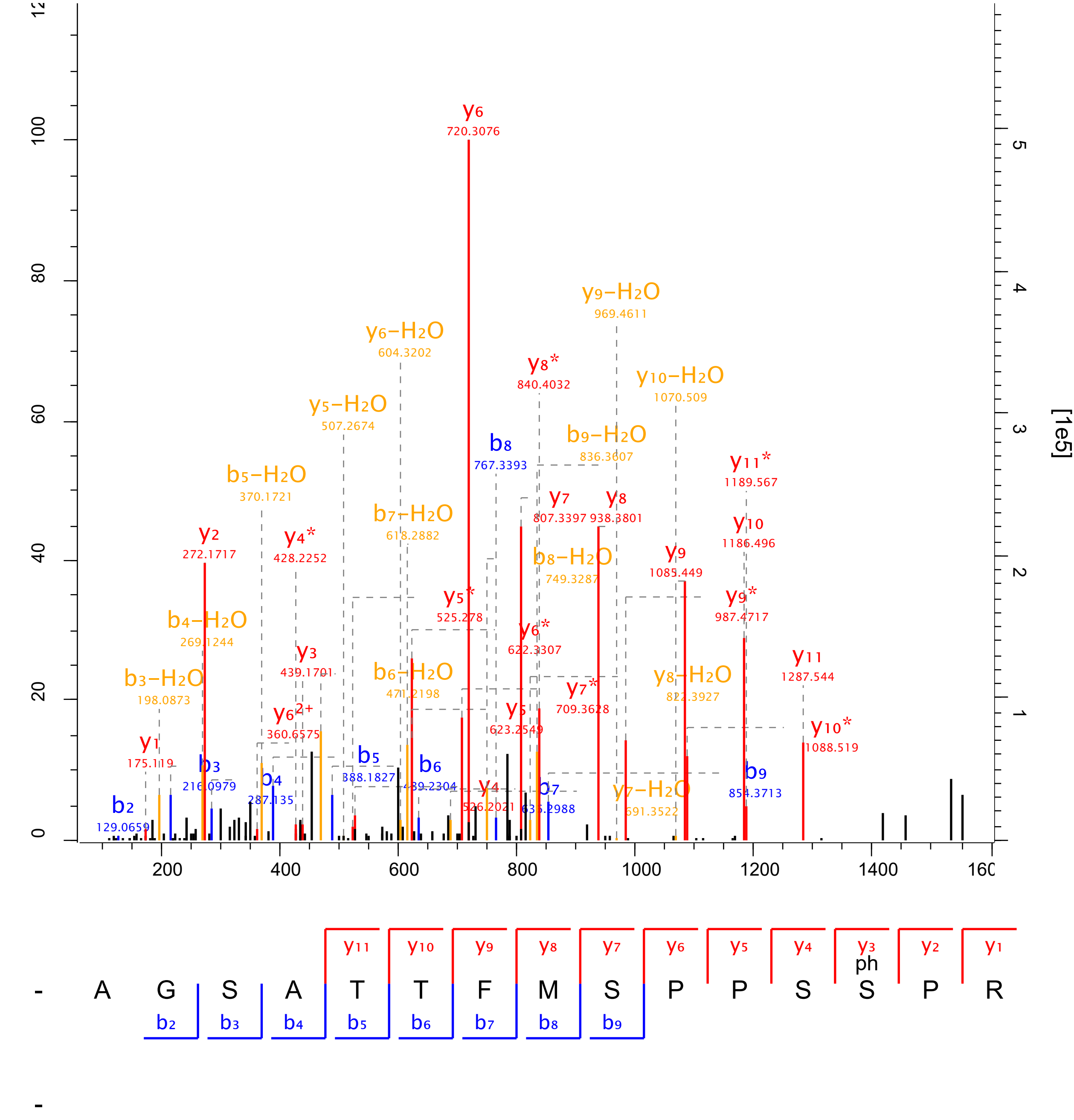

Raw file	Scan	Method	Score	m/z
sys_05_2	20939	FTMS; HCD	181.11	889.72

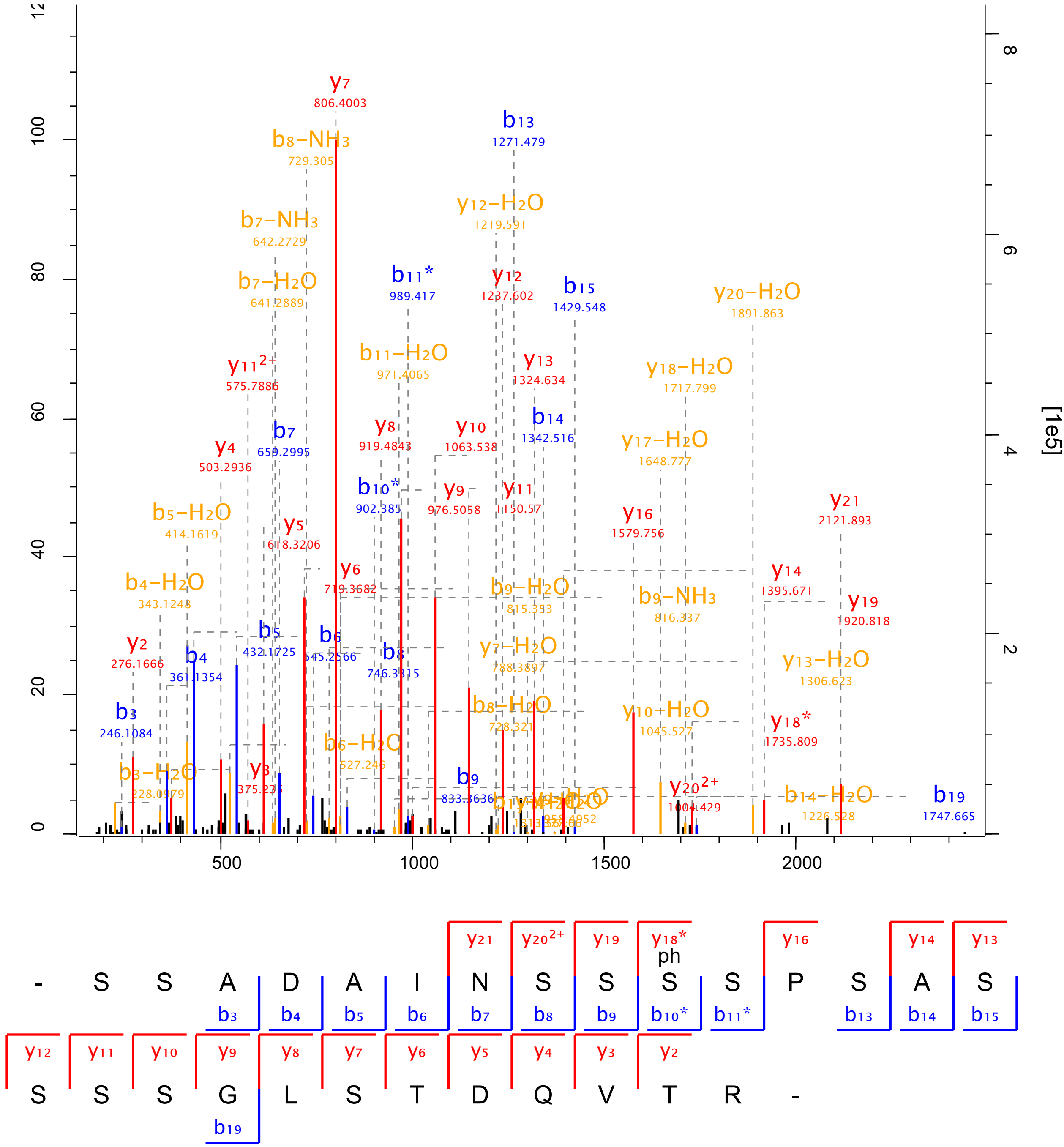

Raw file	Scan	Method	Score	m/z
sys_05_2	21084	FTMS; HCD	138.91	777.82

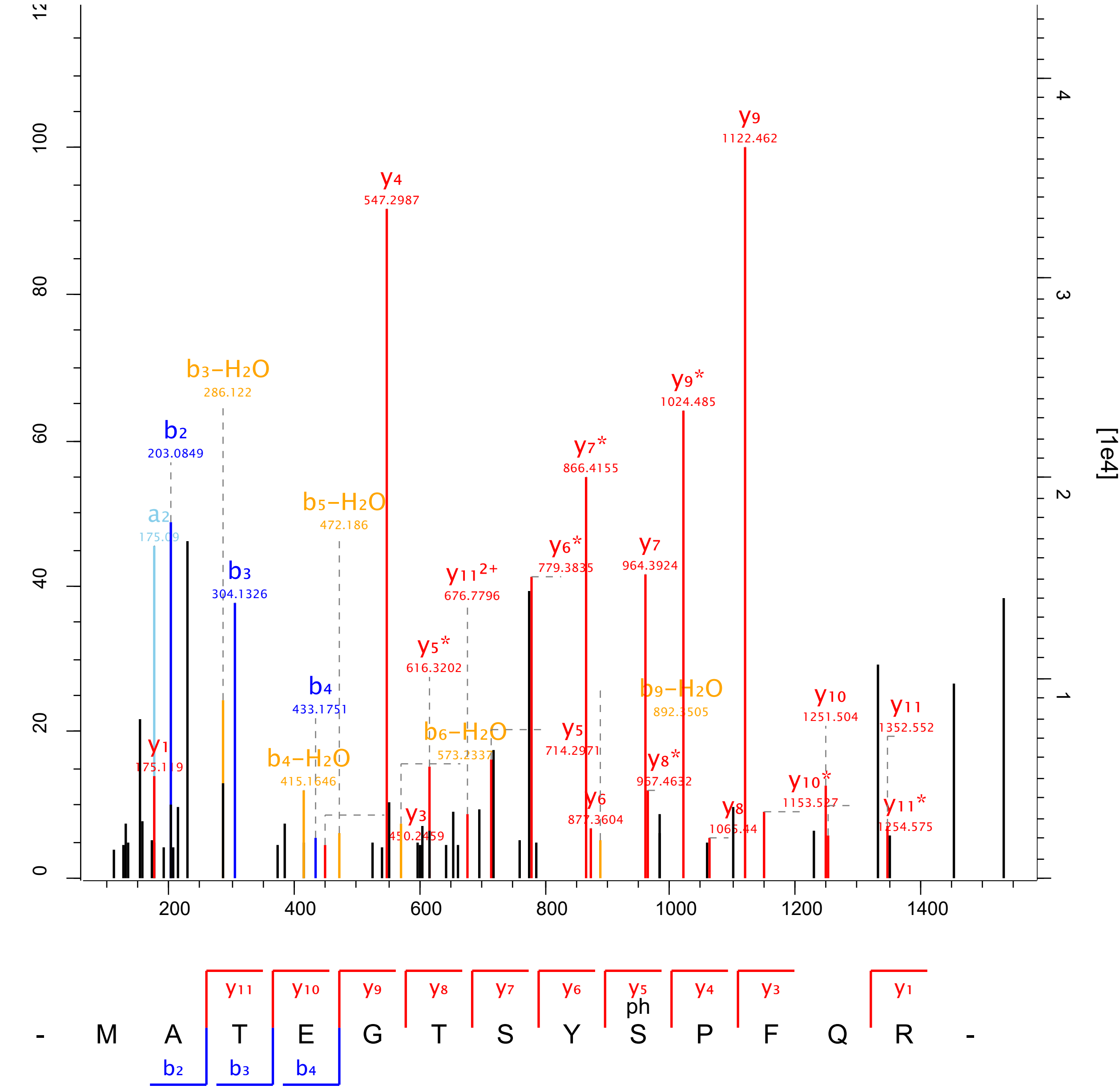

Raw file	Scan	Method	Score	m/z
sys_05_2	21250	FTMS; HCD	298	857.86

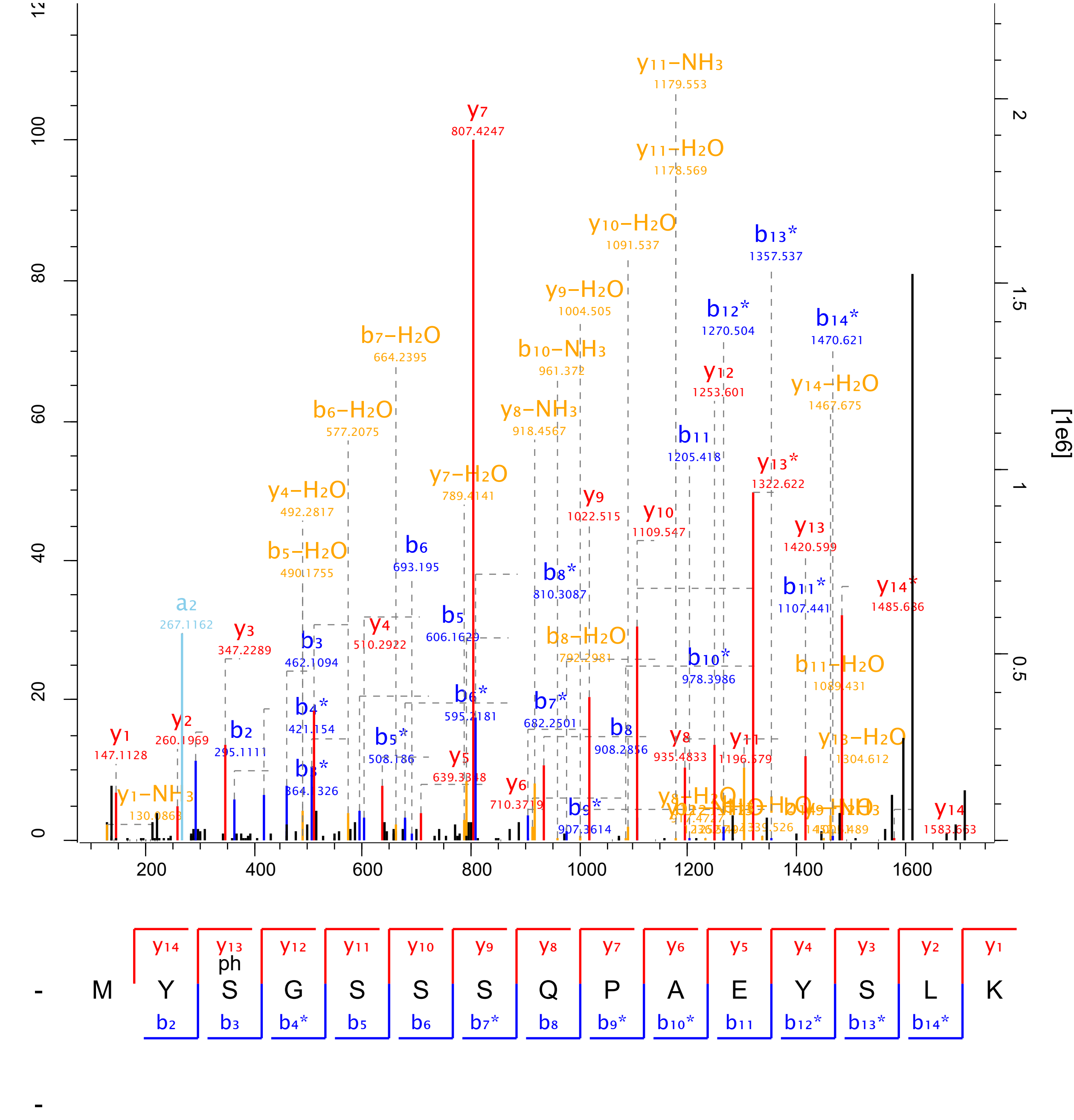

Raw file	Scan	Method	Score	m/z
sys_05_2	21297	FTMS; HCD	76.82	932.35

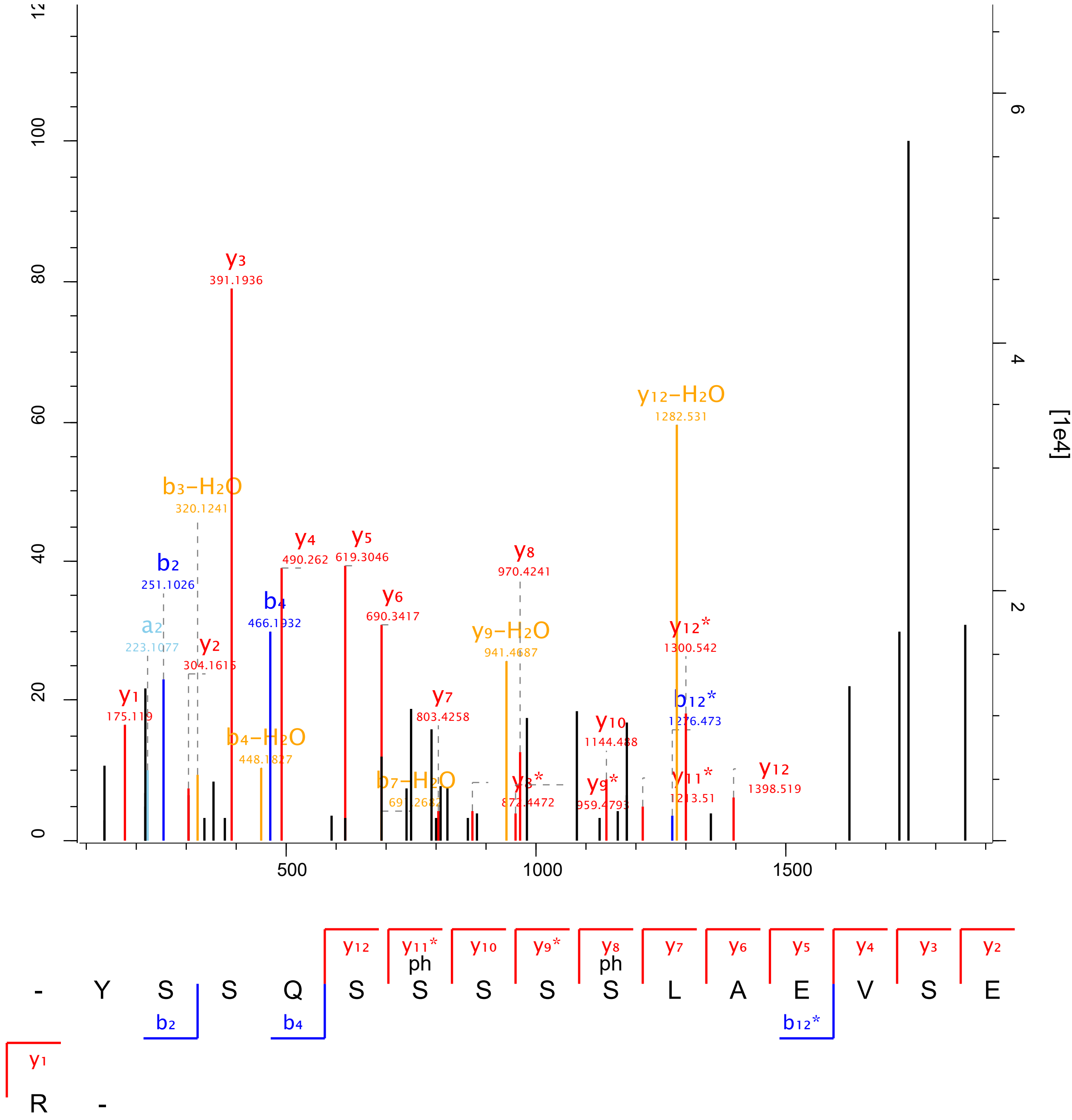

Raw file	Scan	Method	Score	m/z
sys_05_2	21658	FTMS; HCD	112.34	768.31

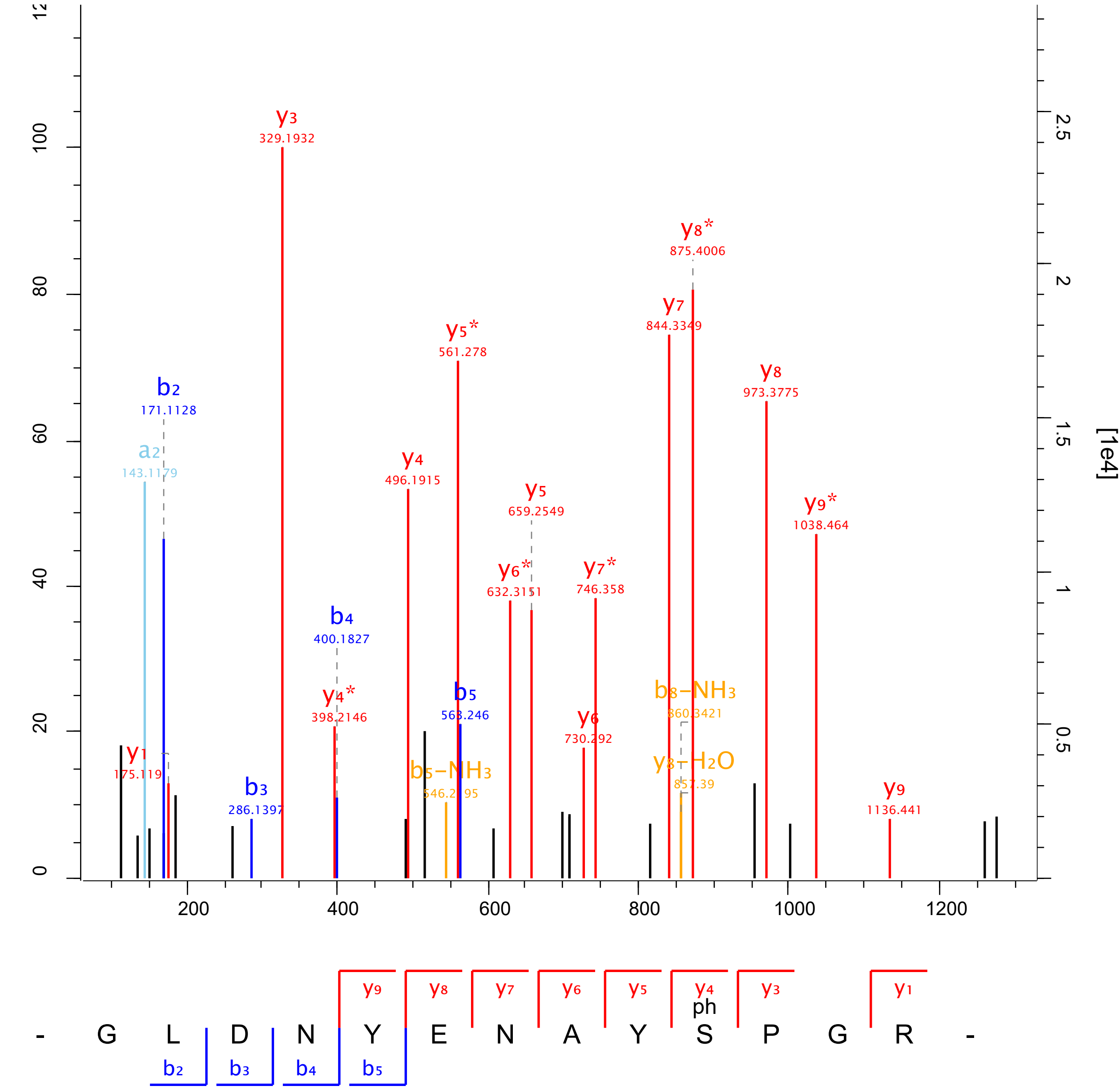

Raw file	Scan	Method	Score	m/z
sys_05_2	21695	FTMS; HCD	49.19	617.27

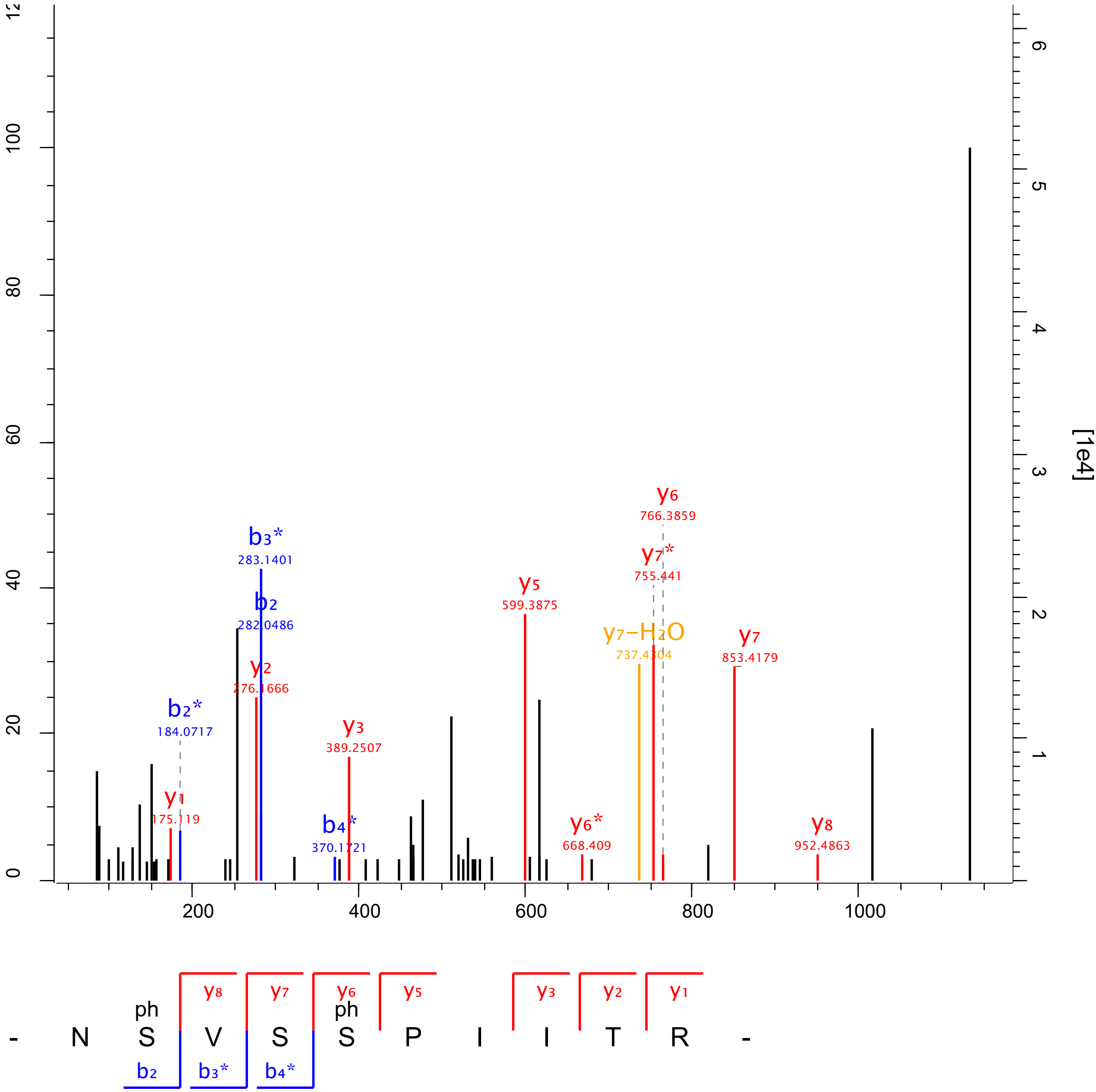

Raw file	Scan	Method	Score	m/z
sys_05_2	21873	FTMS; HCD	136.66	804.86

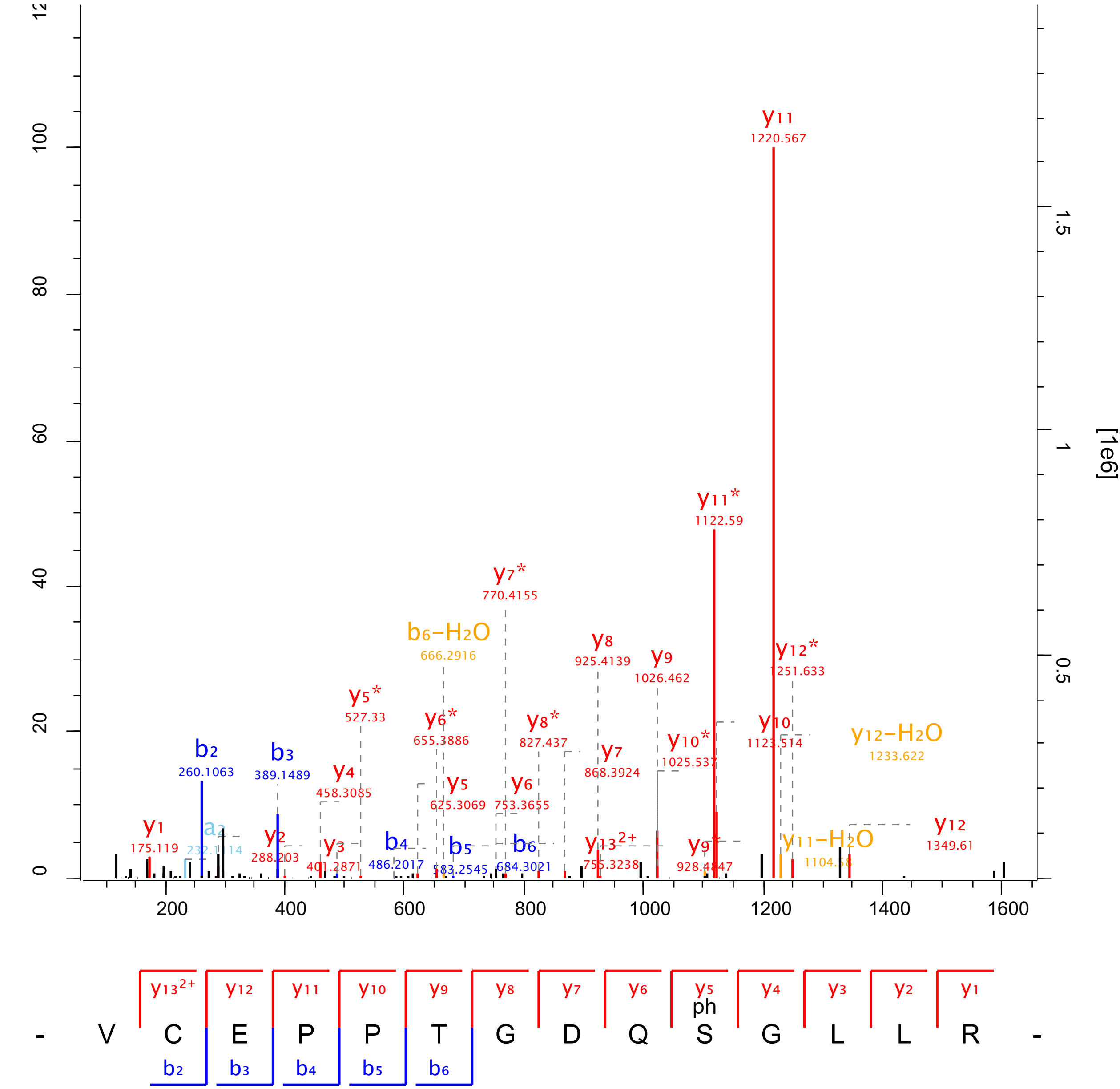

Raw file	Scan	Method	Score	m/z
sys_05_2	21880	FTMS; HCD	104.21	766.83

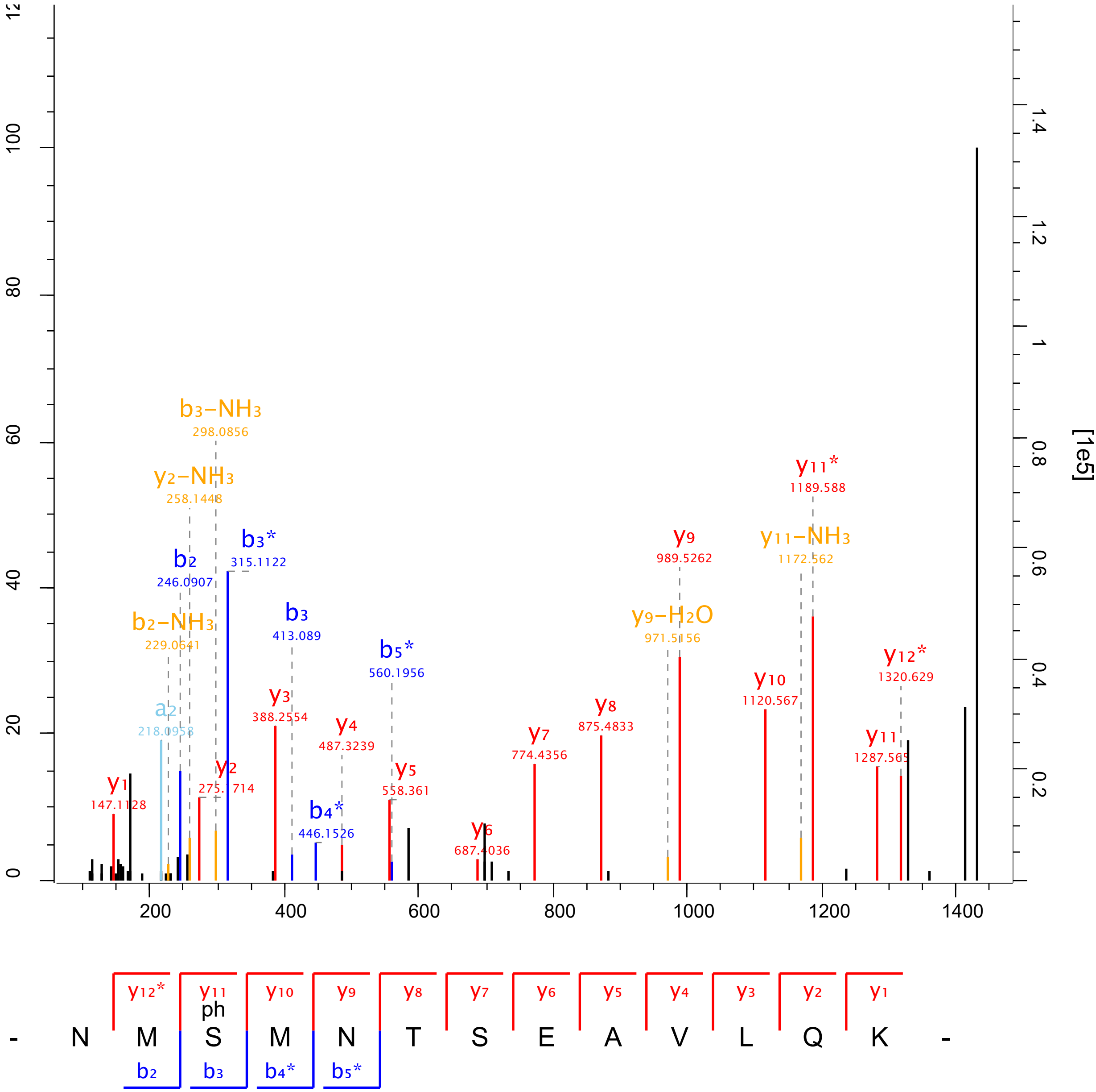

Raw file	Scan	Method	Score	m/z
sys_05_2	21887	FTMS; HCD	179.67	582.22

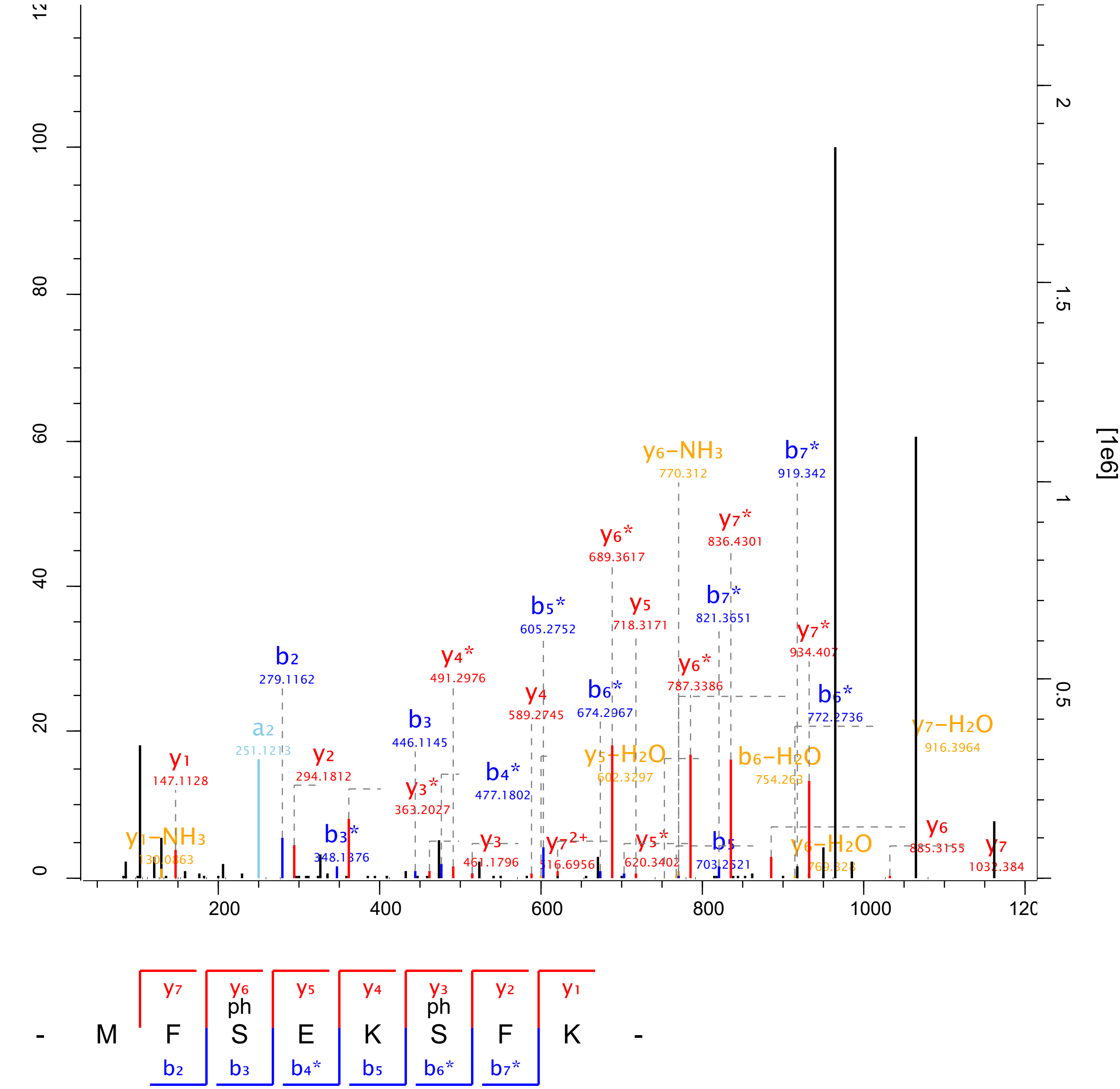

Raw file	Scan	Method	Score	m/z
sys_05_2	21922	FTMS; HCD	75.06	637.29

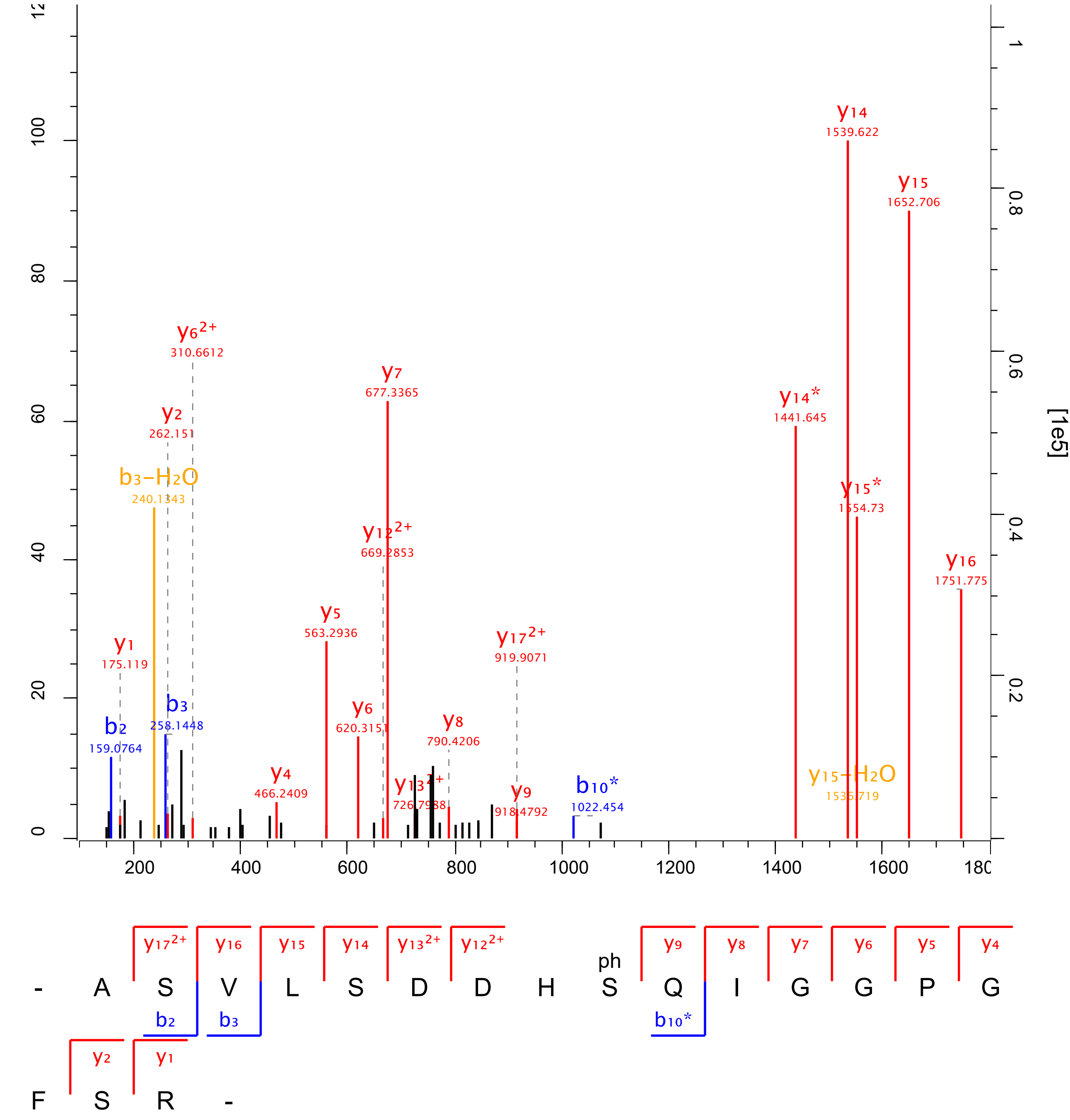

Raw file	Scan	Method	Score	m/z
sys_05_2	22060	FTMS; HCD	255.83	669.62

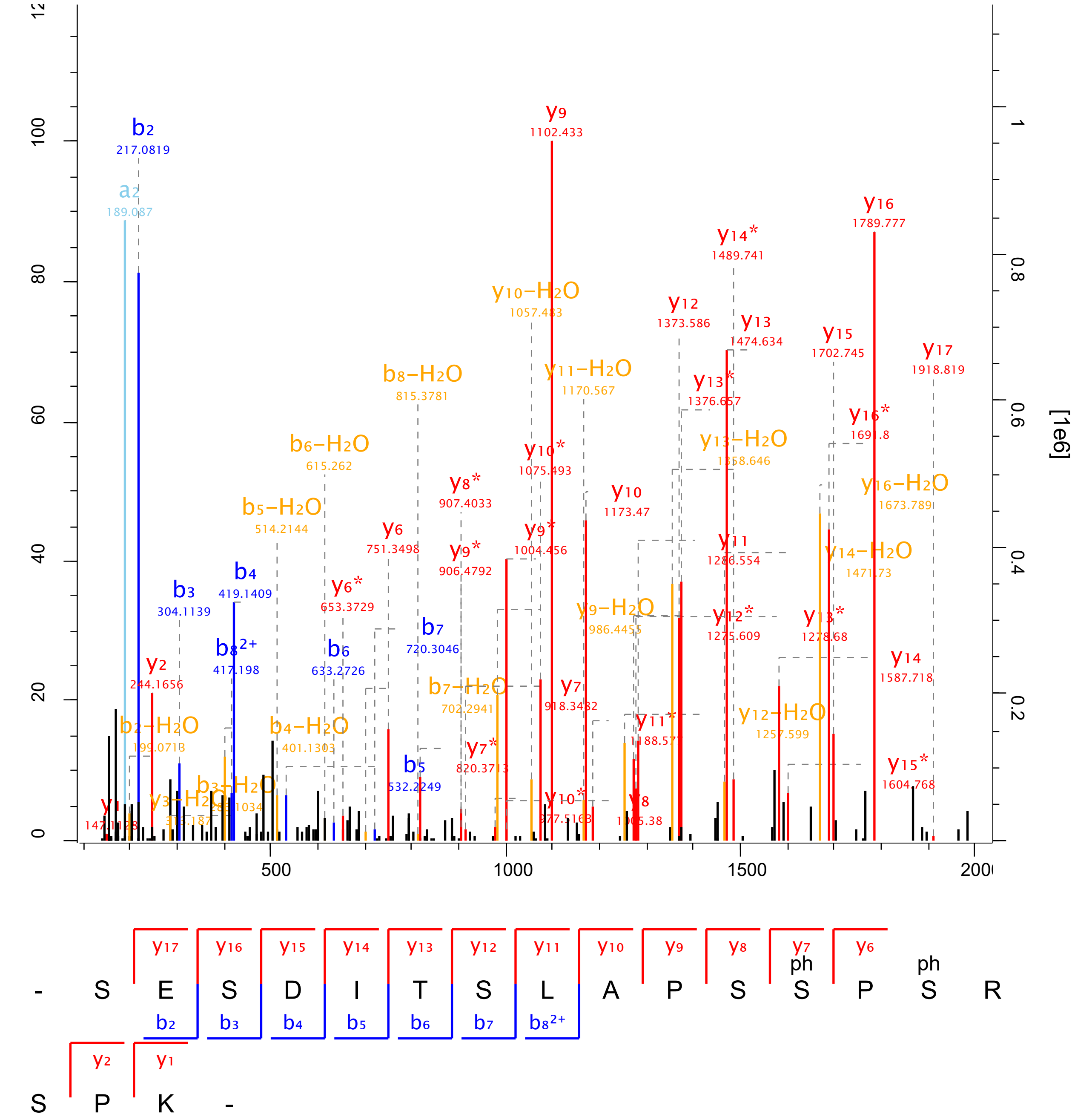

Raw file	Scan	Method	Score	m/z
sys_05_2	22298	FTMS; HCD	67.08	524.72

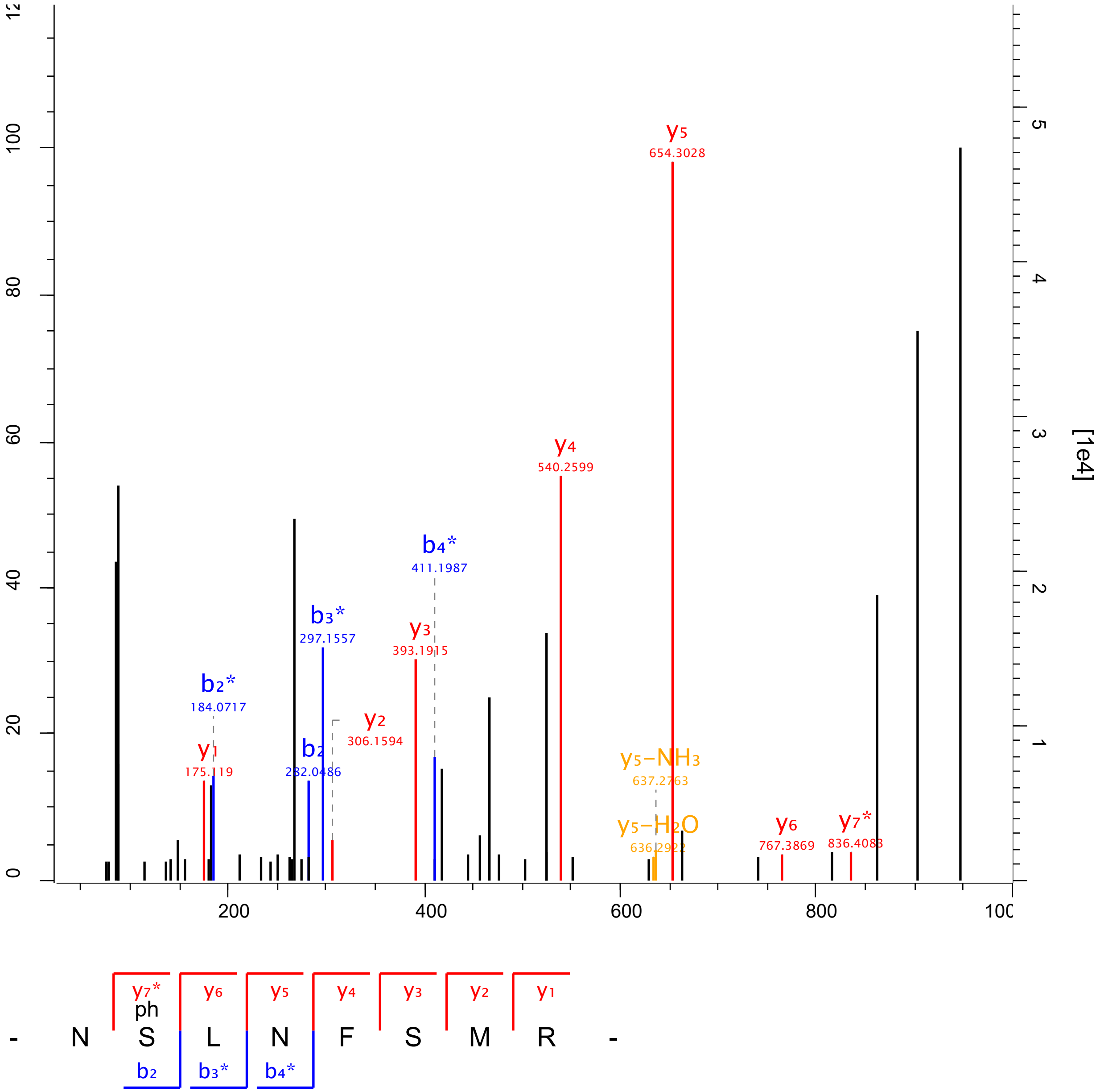

Raw file	Scan	Method	Score	m/z
sys_05_2	22371	FTMS; HCD	147.64	782.34

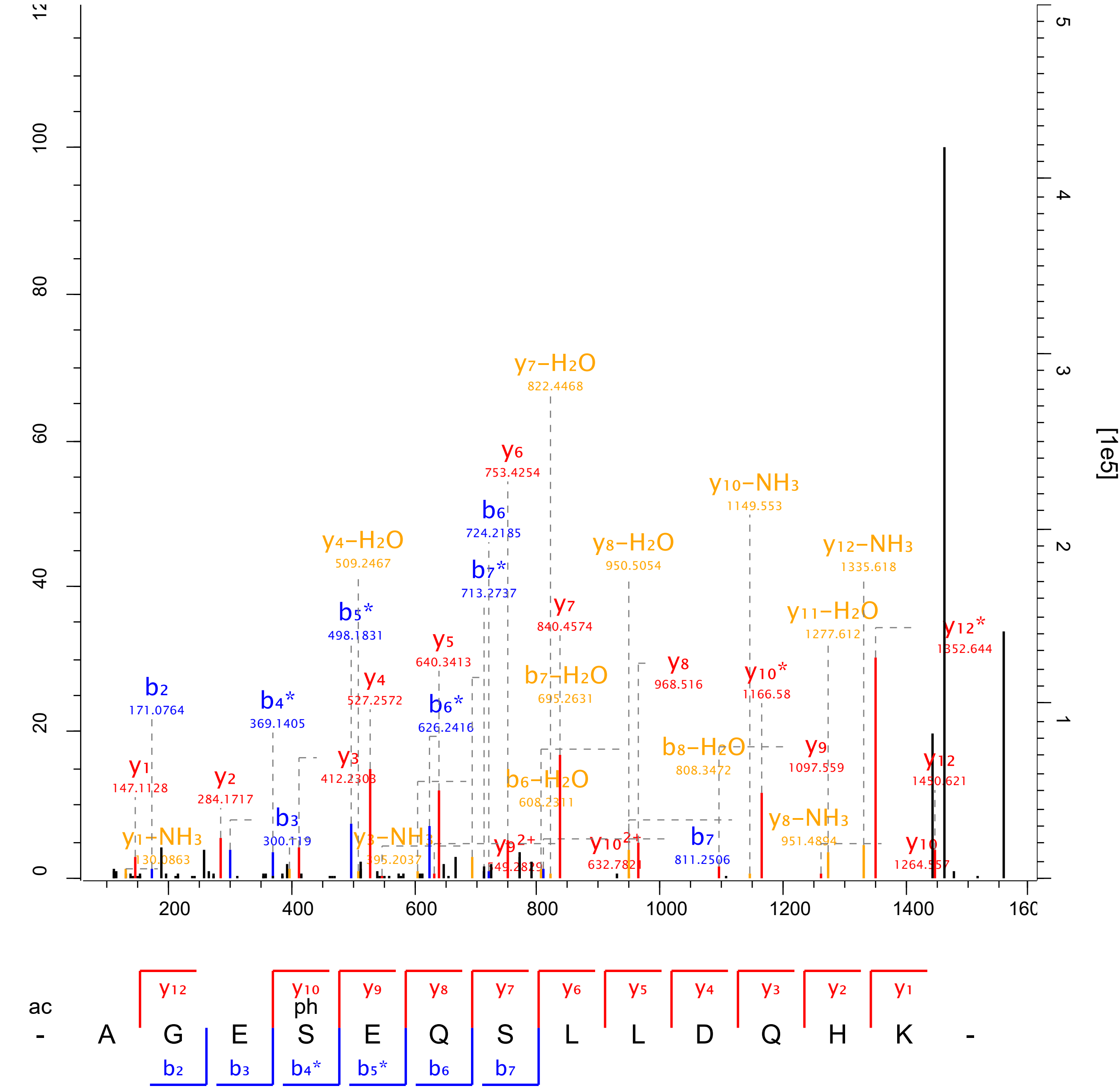

Raw file	Scan	Method	Score	m/z
sys_05_2	22737	FTMS; HCD	113.65	685.82

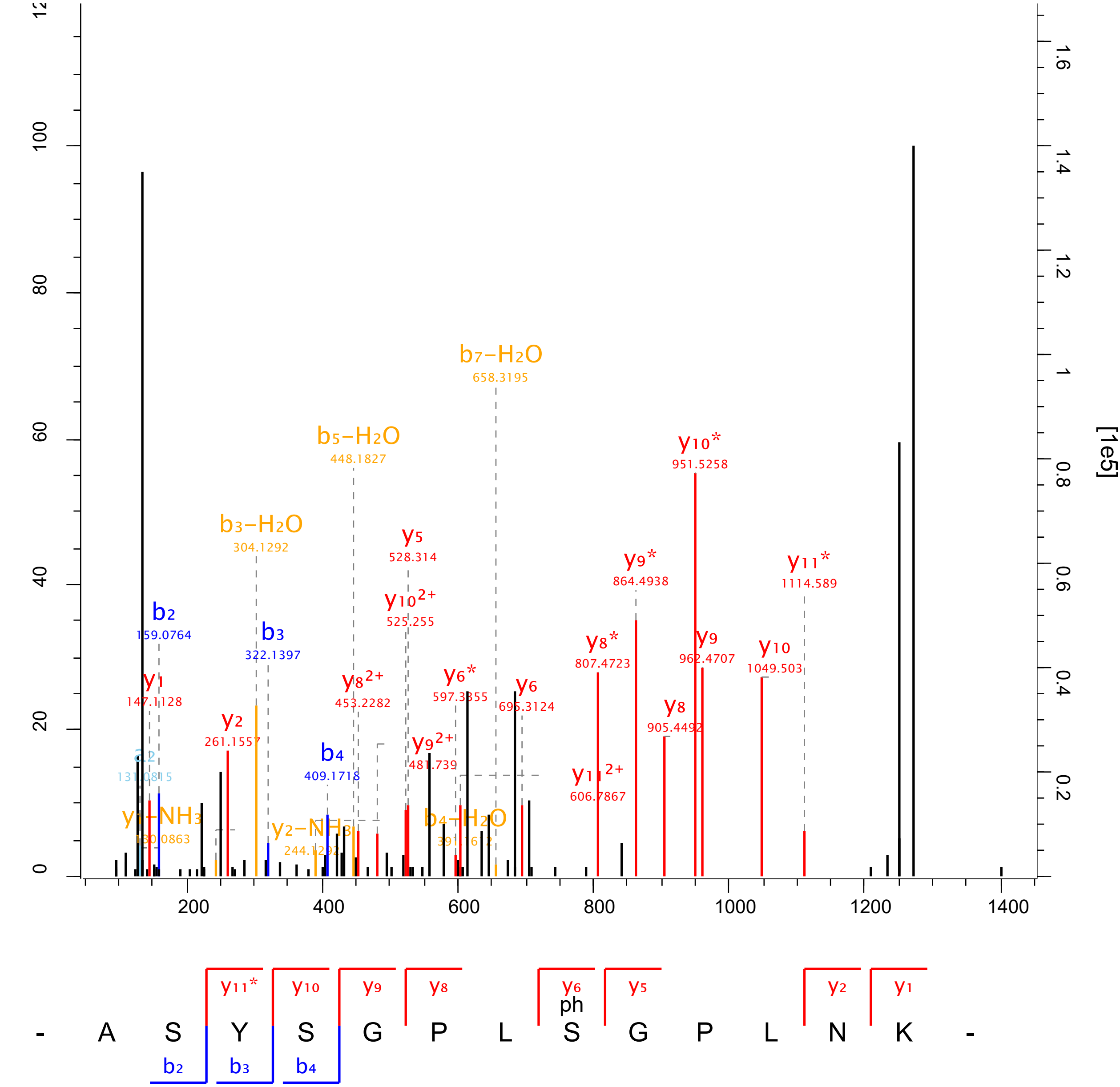

Raw file	Scan	Method	Score	m/z
sys_05_2	23189	FTMS; HCD	104.84	697.29

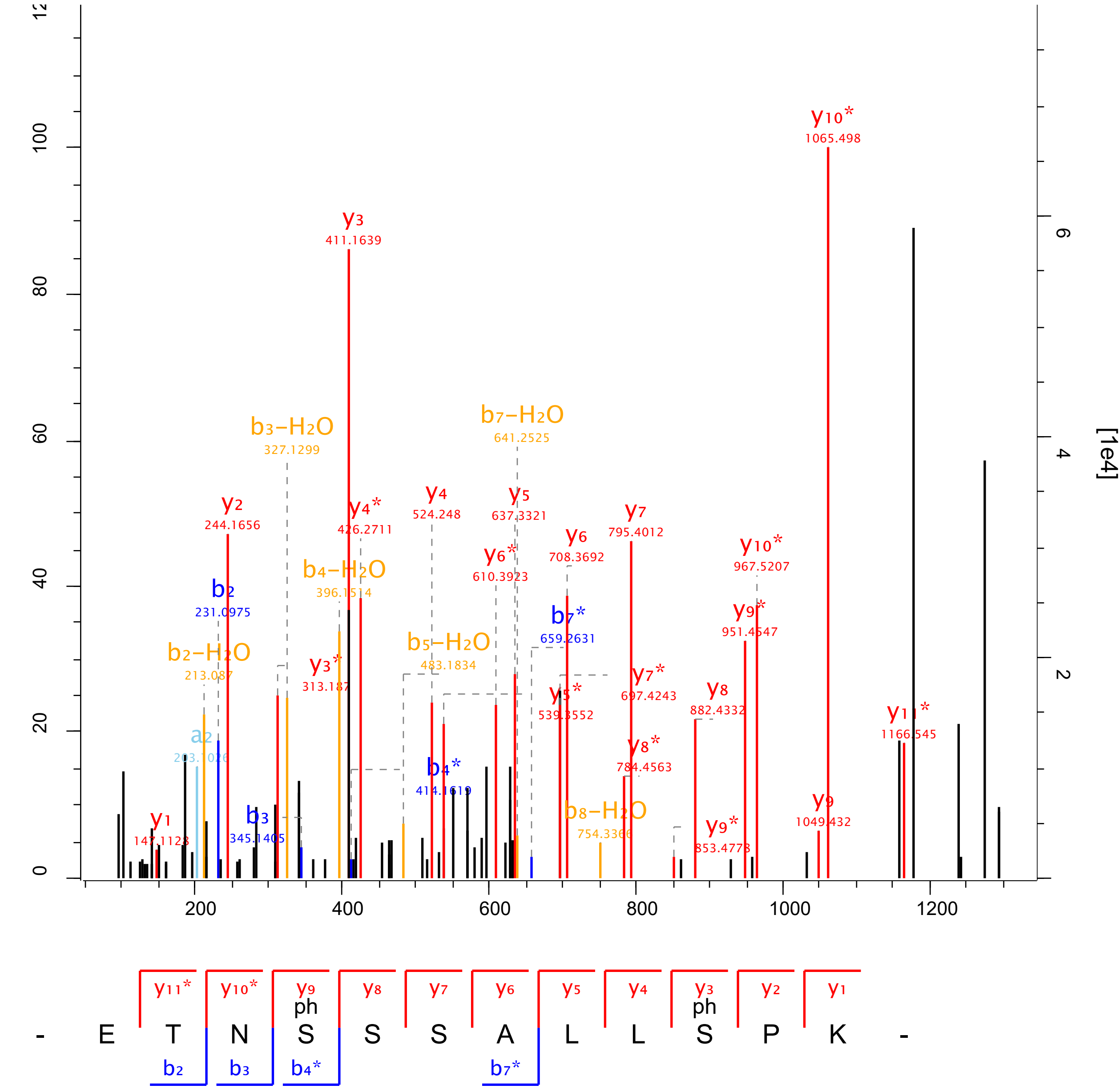

Raw file	Scan	Method	Score	m/z
sys_05_2	23202	FTMS; HCD	120.92	595.27

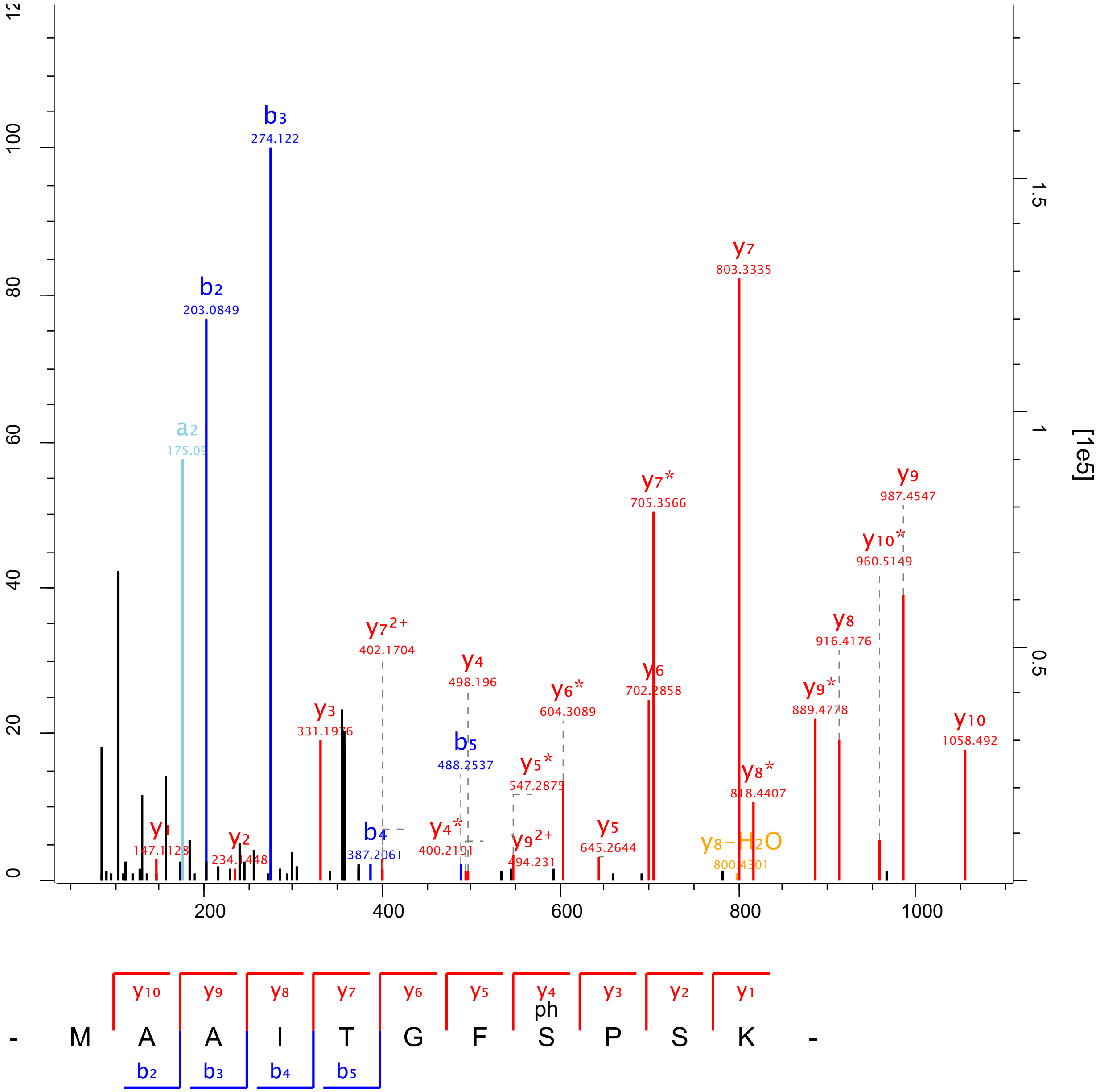

Raw file	Scan	Method	Score	m/z
sys_05_2	23420	FTMS; HCD	141.82	639.27

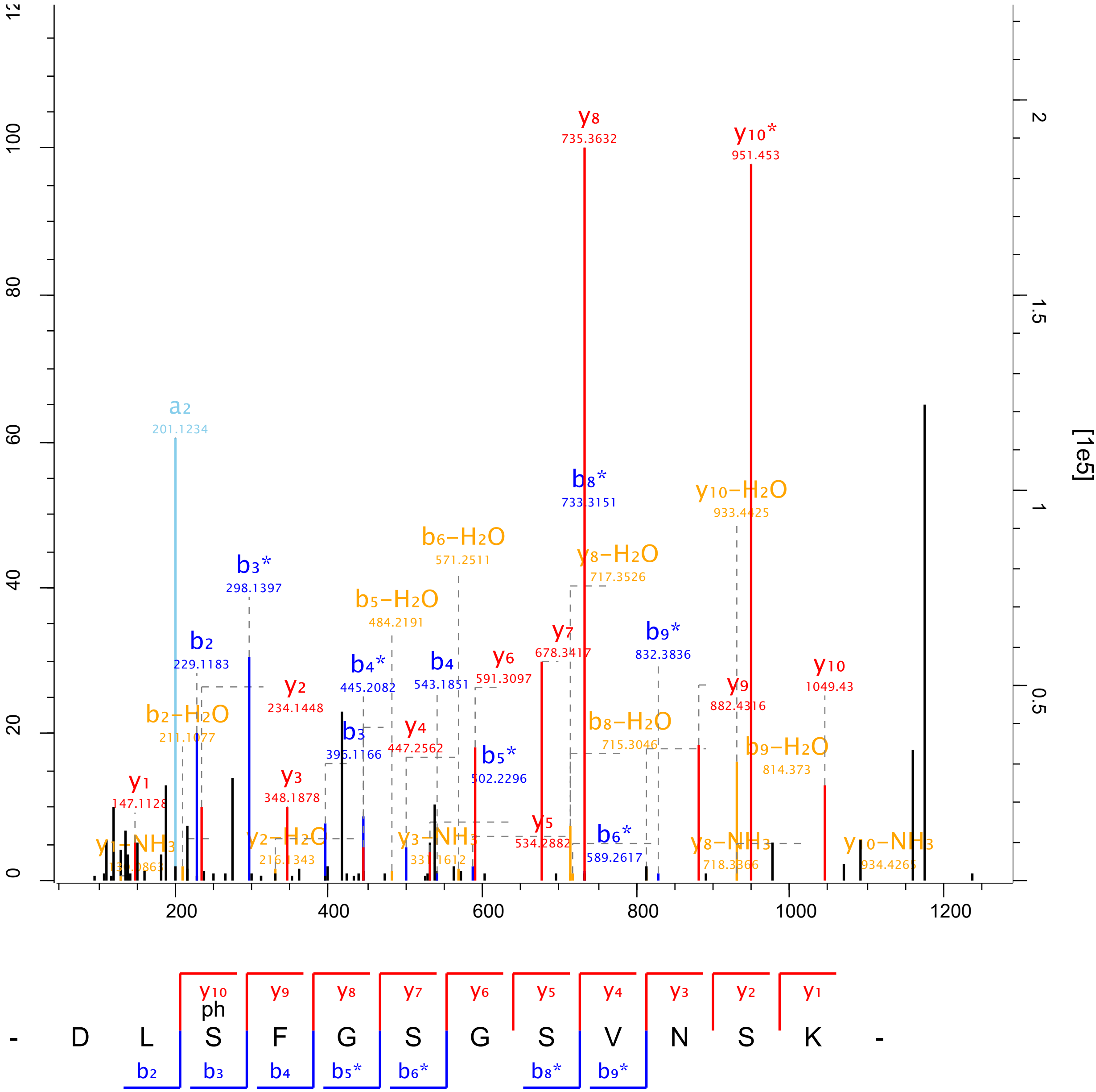

Raw file	Scan	Method	Score	m/z
sys_05_2	23525	FTMS; HCD	176.46	738.32

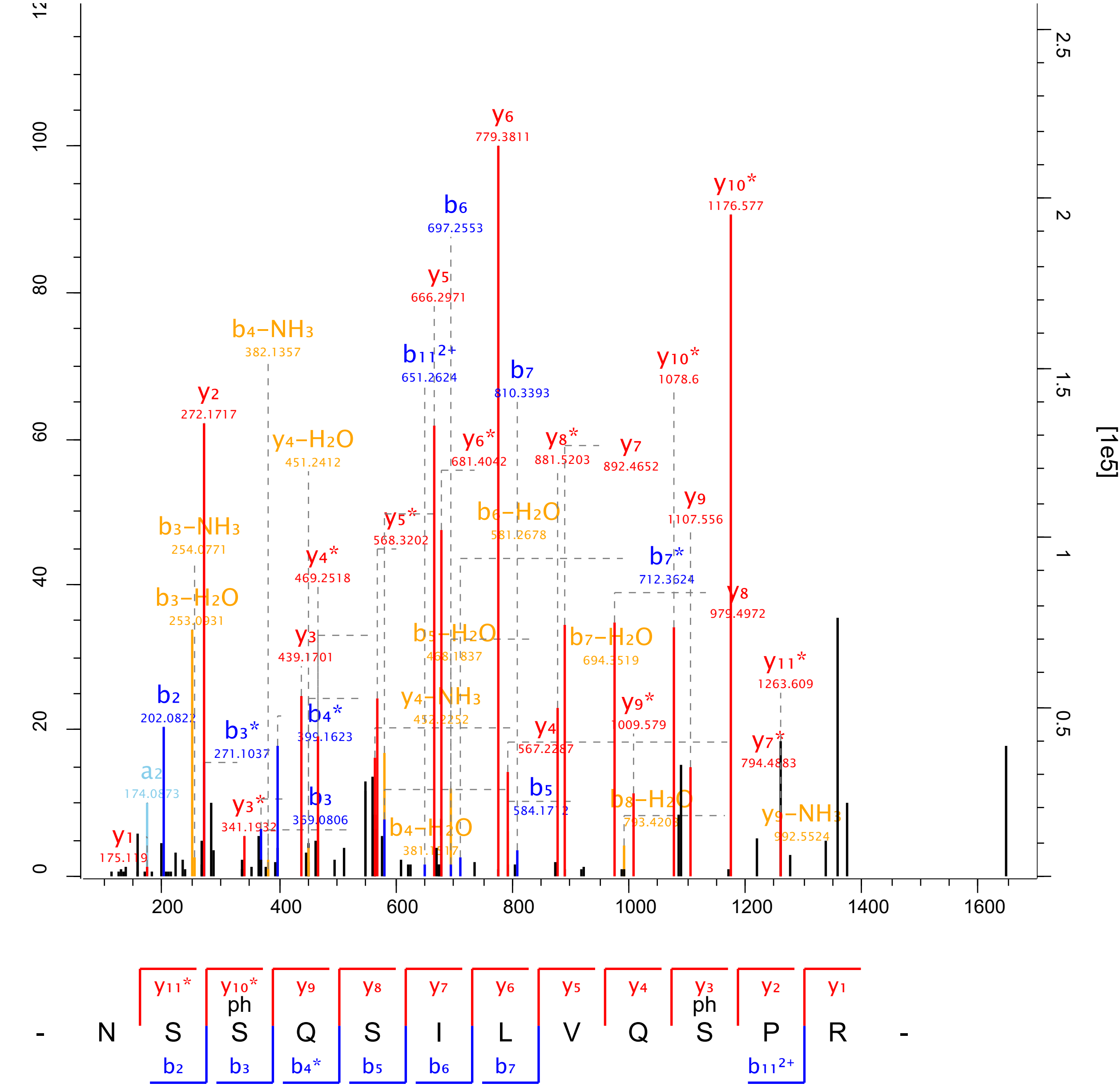

Raw file	Scan	Method	Score	m/z
sys_05_2	23608	FTMS; HCD	359.08	913.35

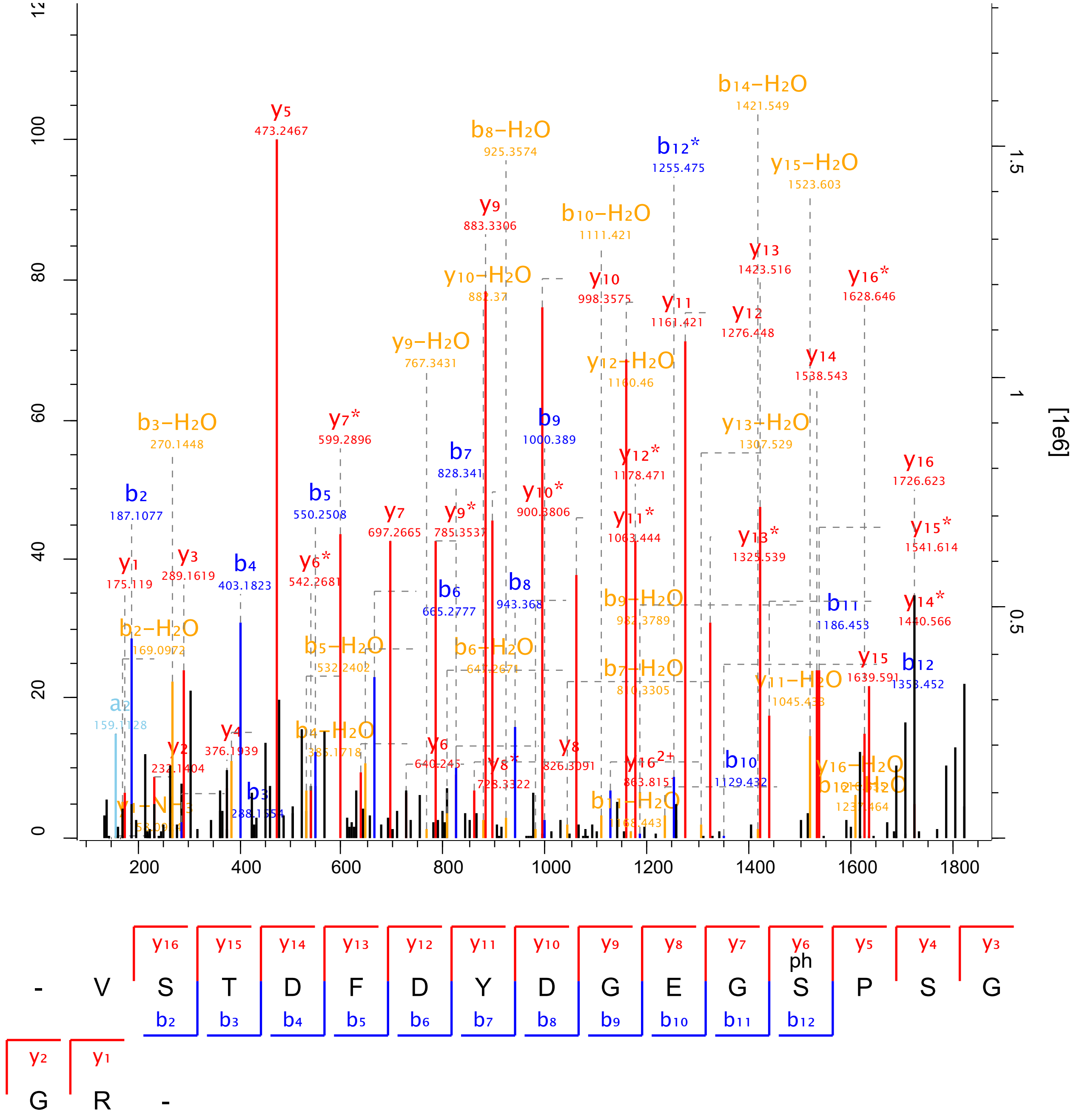

Raw file	Scan	Method	Score	m/z
sys_05_2	23651	FTMS; HCD	71.88	681.81

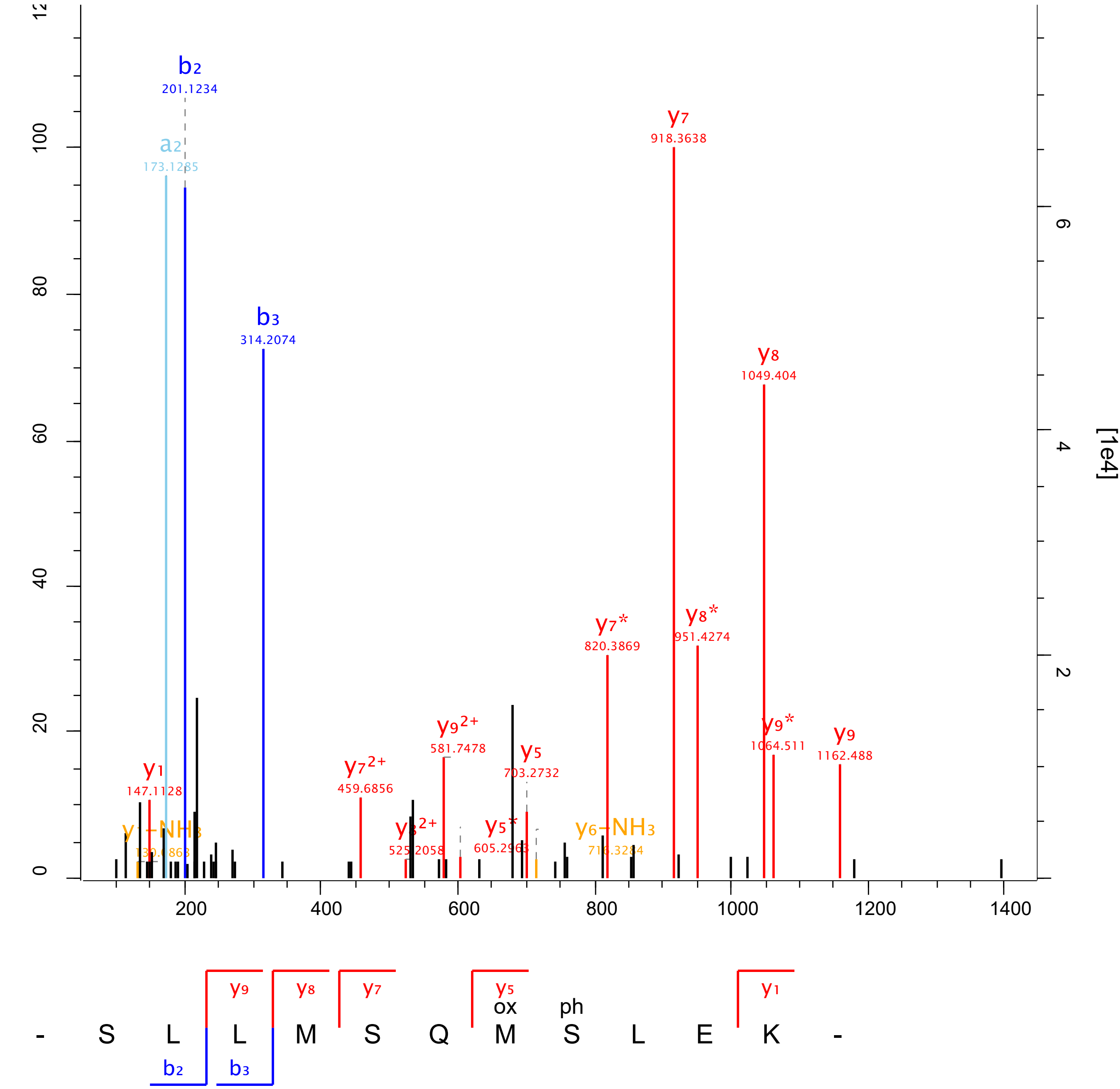

Raw file	Scan	Method	Score	m/z
sys_05_2	23668	FTMS; HCD	177.57	539.24

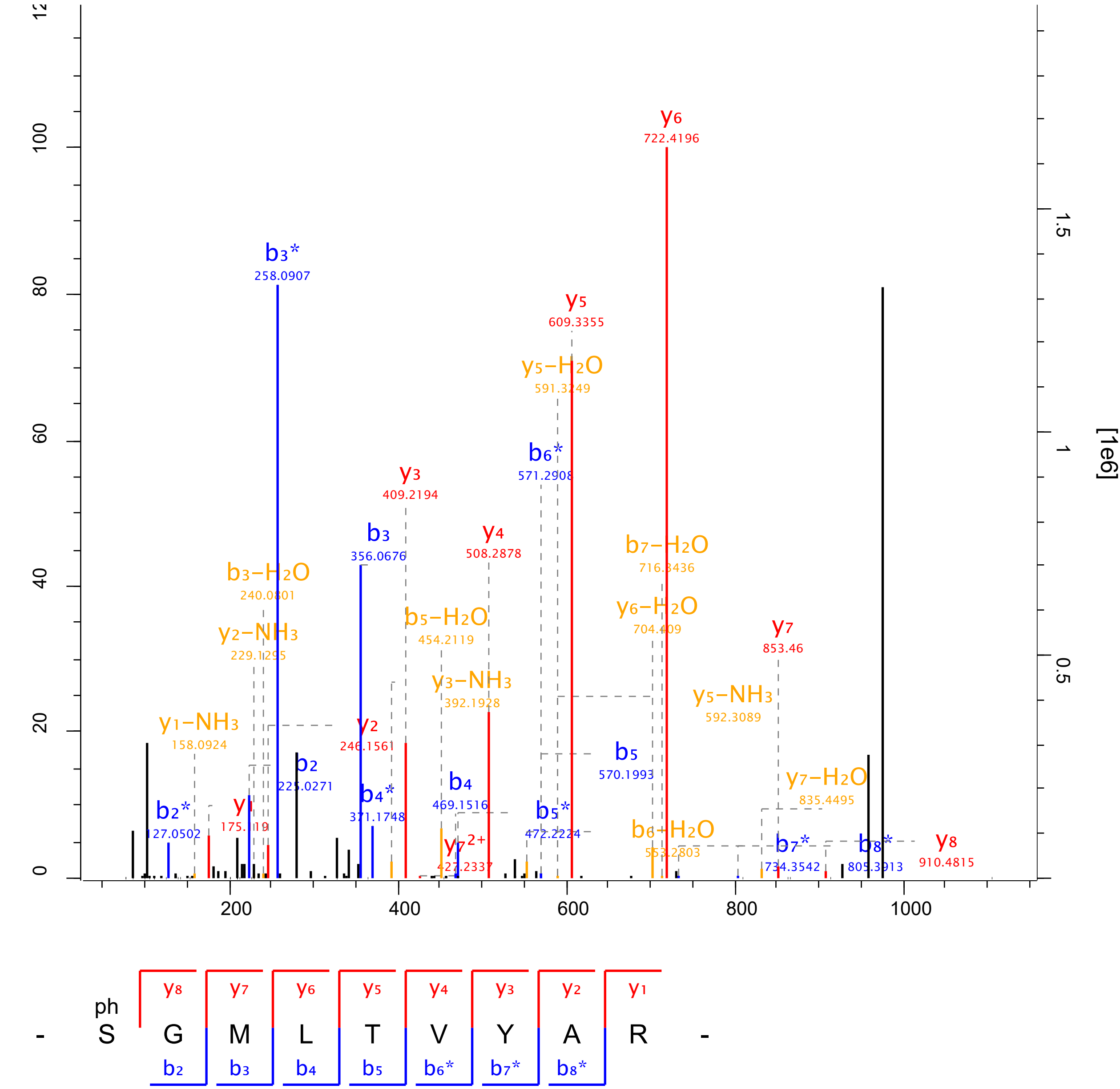

Raw file	Scan	Method	Score	m/z
sys_05_2	23760	FTMS; HCD	48.53	620.26

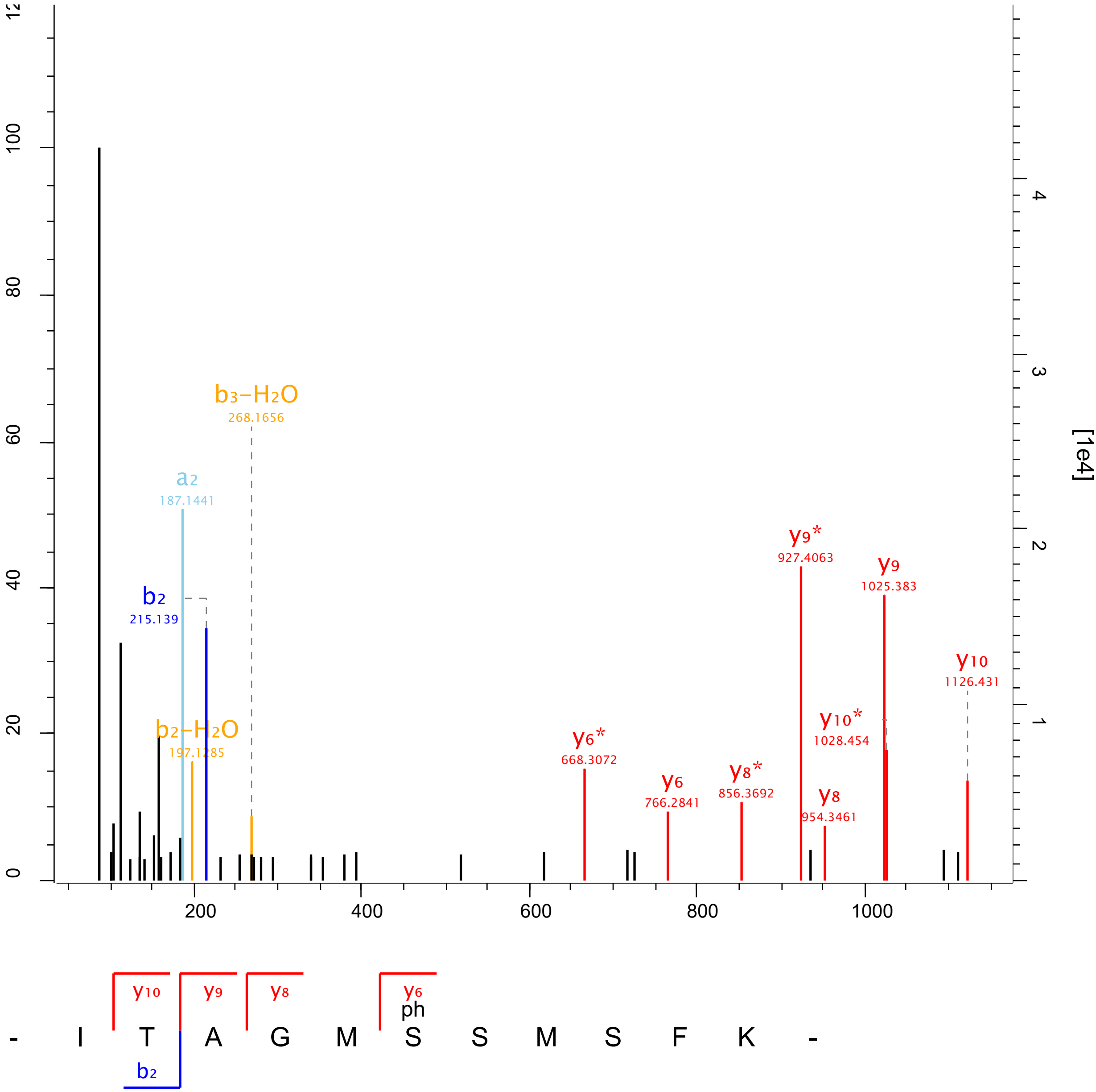

Raw file	Scan	Method	Score	m/z
sys_05_2	23844	FTMS; HCD	159.26	775.38

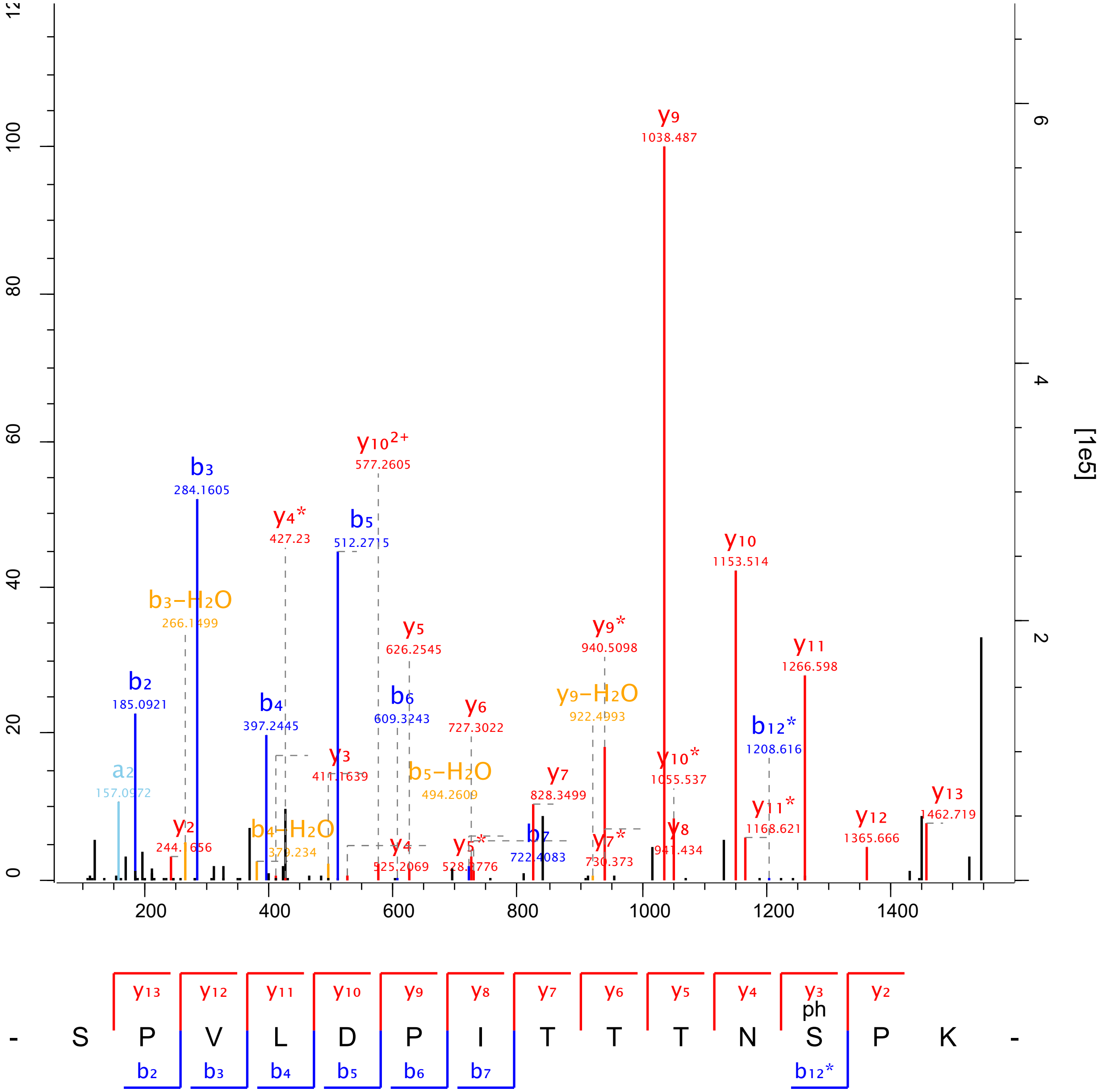

Raw file	Scan	Method	Score	m/z
sys_05_2	23852	FTMS; HCD	138.05	843.89

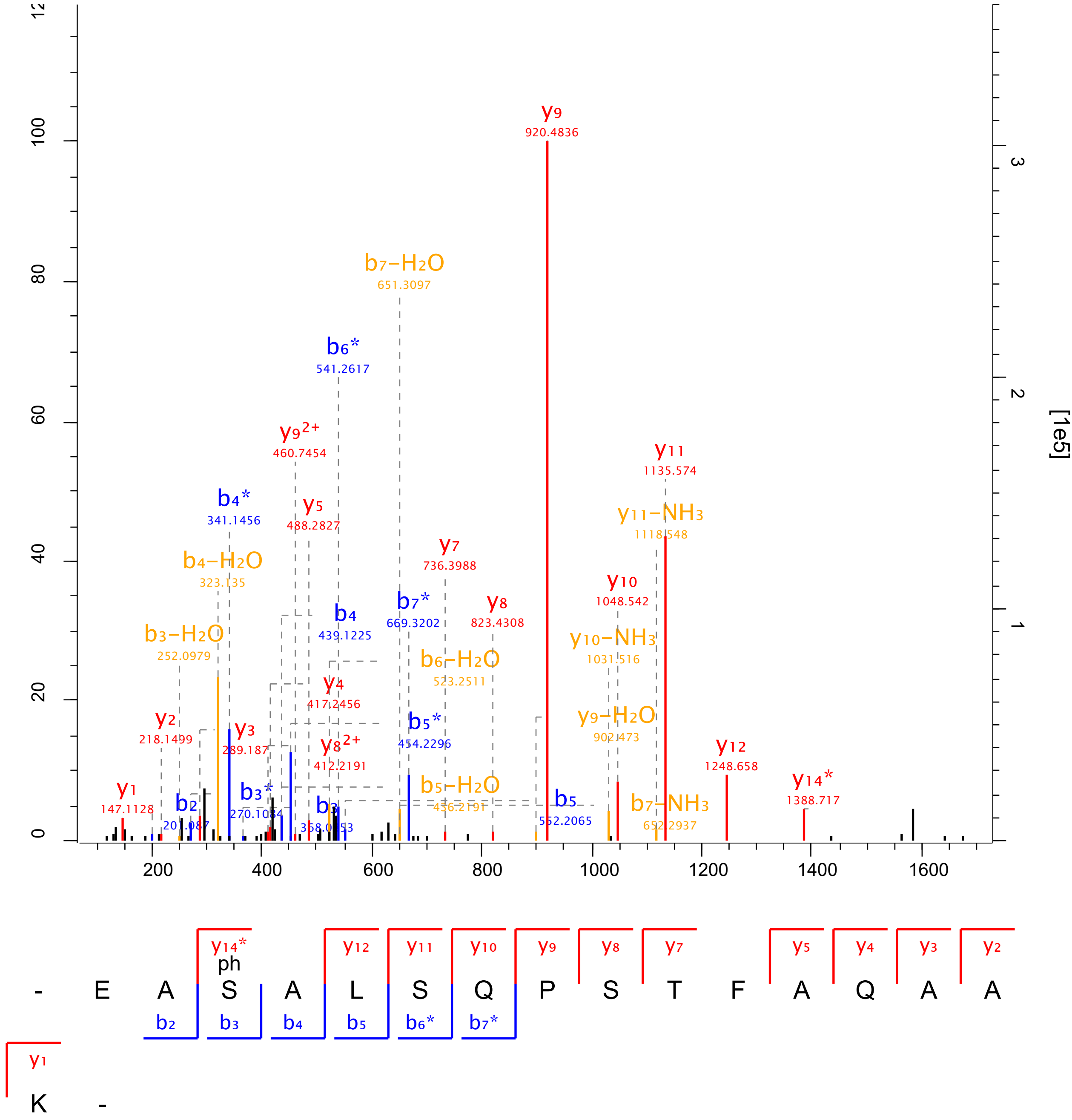

Raw file	Scan	Method	Score	m/z
sys_05_2	24230	FTMS; HCD	123.19	701.31

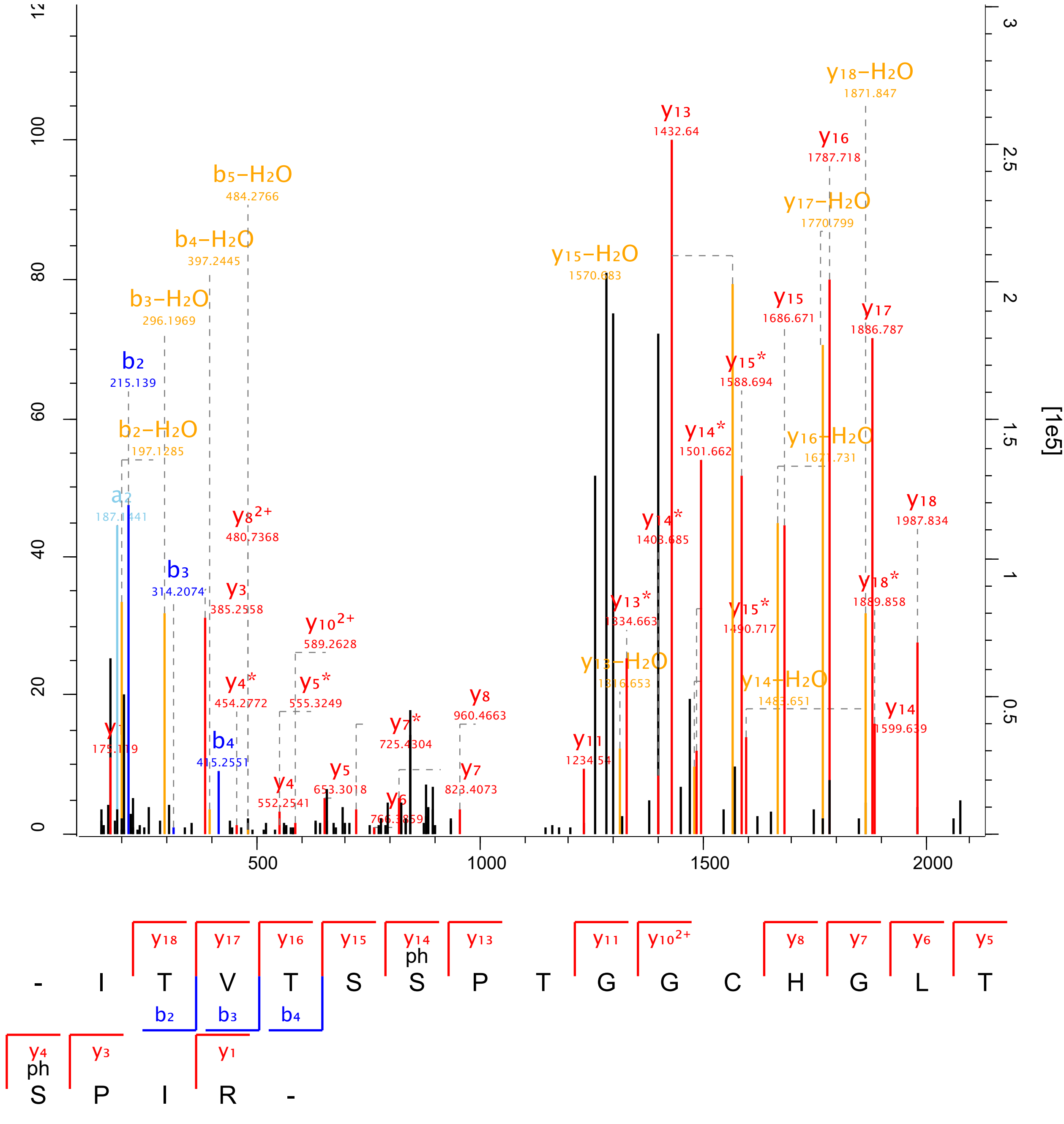

Raw file	Scan	Method	Score	m/z
sys_05_2	24402	FTMS; HCD	127.01	885.91

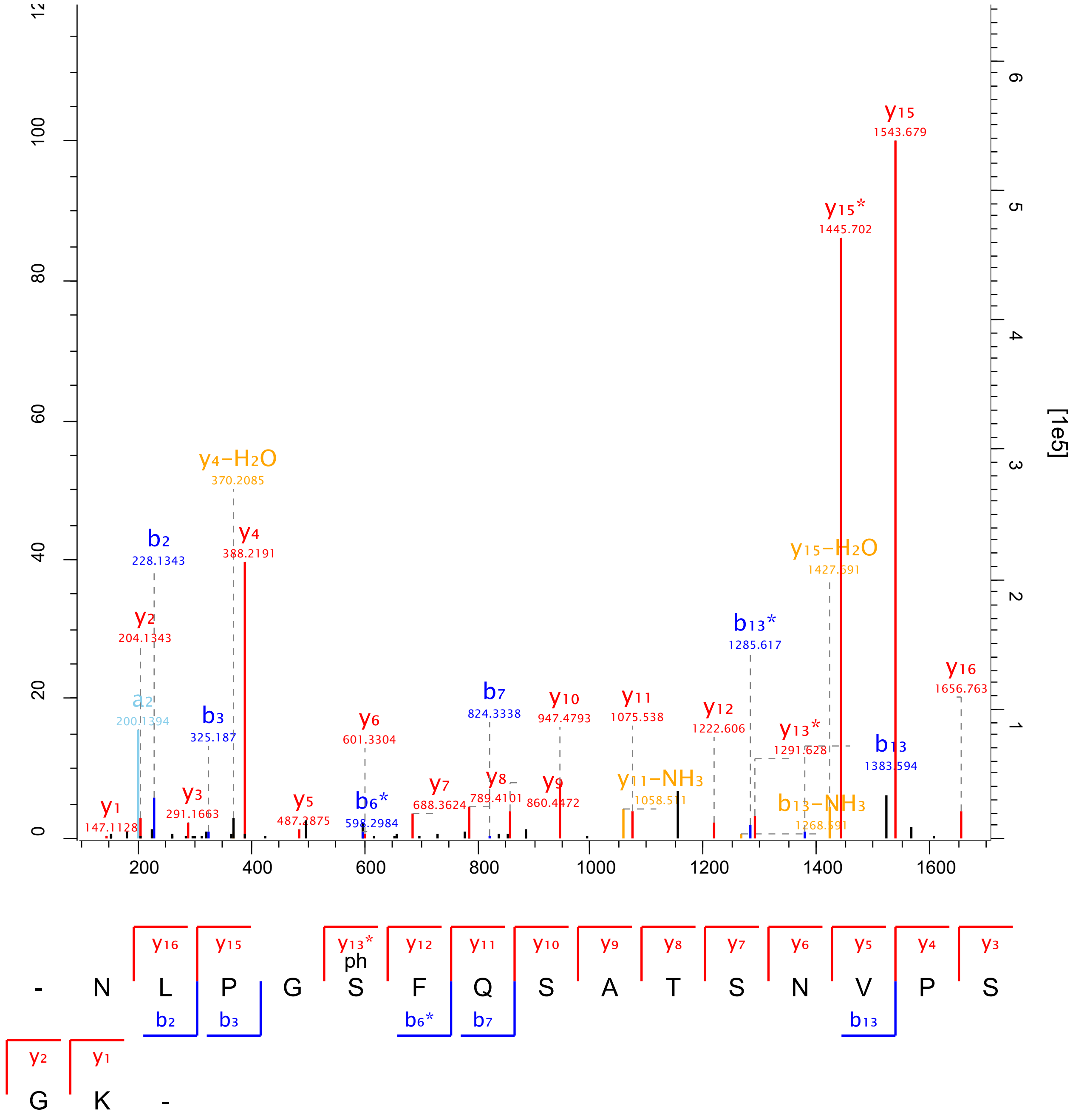

Raw file	Scan	Method	Score	m/z
sys_05_2	24423	FTMS; HCD	46.07	599.78

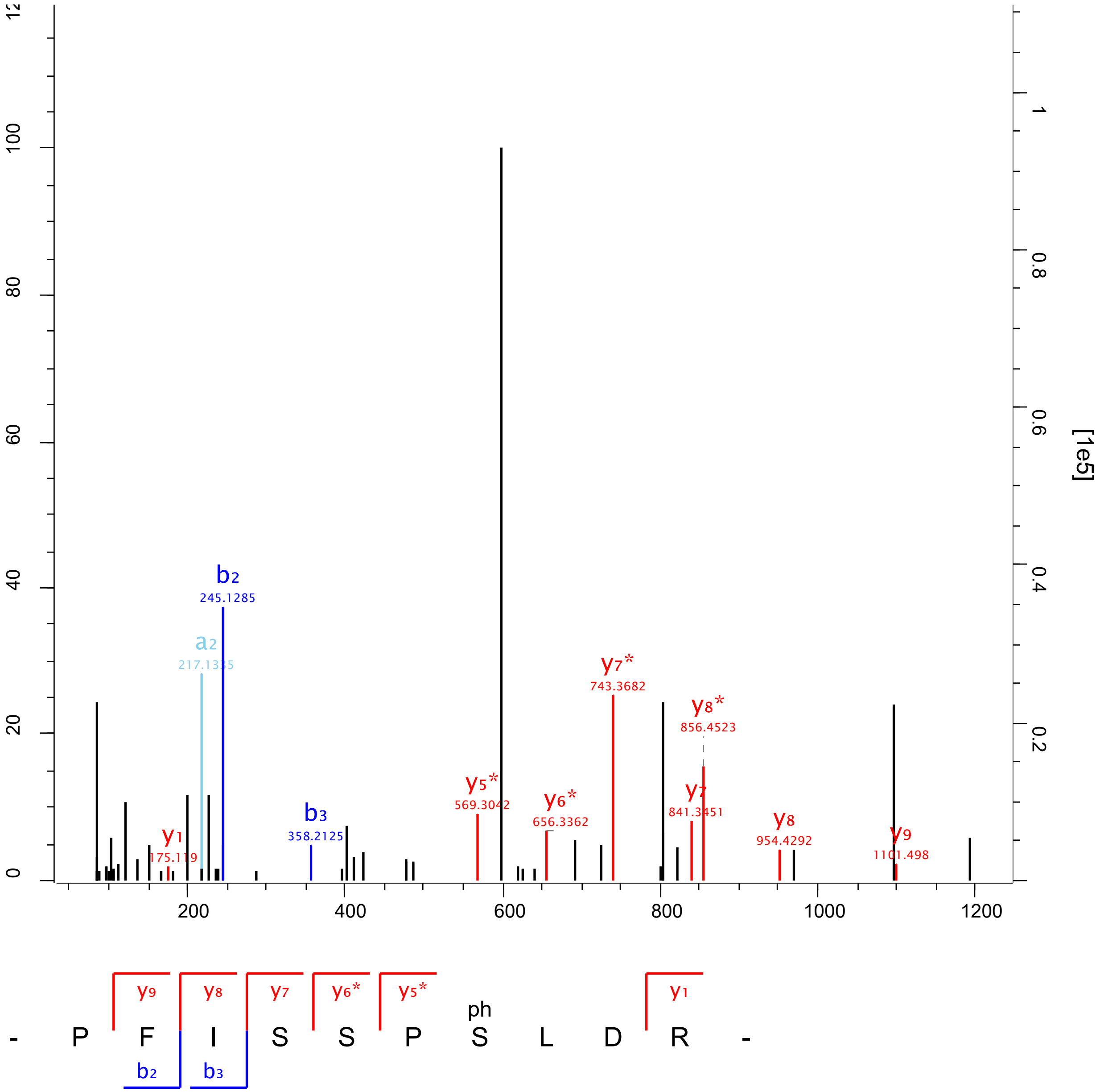

Raw file	Scan	Method	Score	m/z
sys_05_2	24616	FTMS; HCD	224.42	627.3

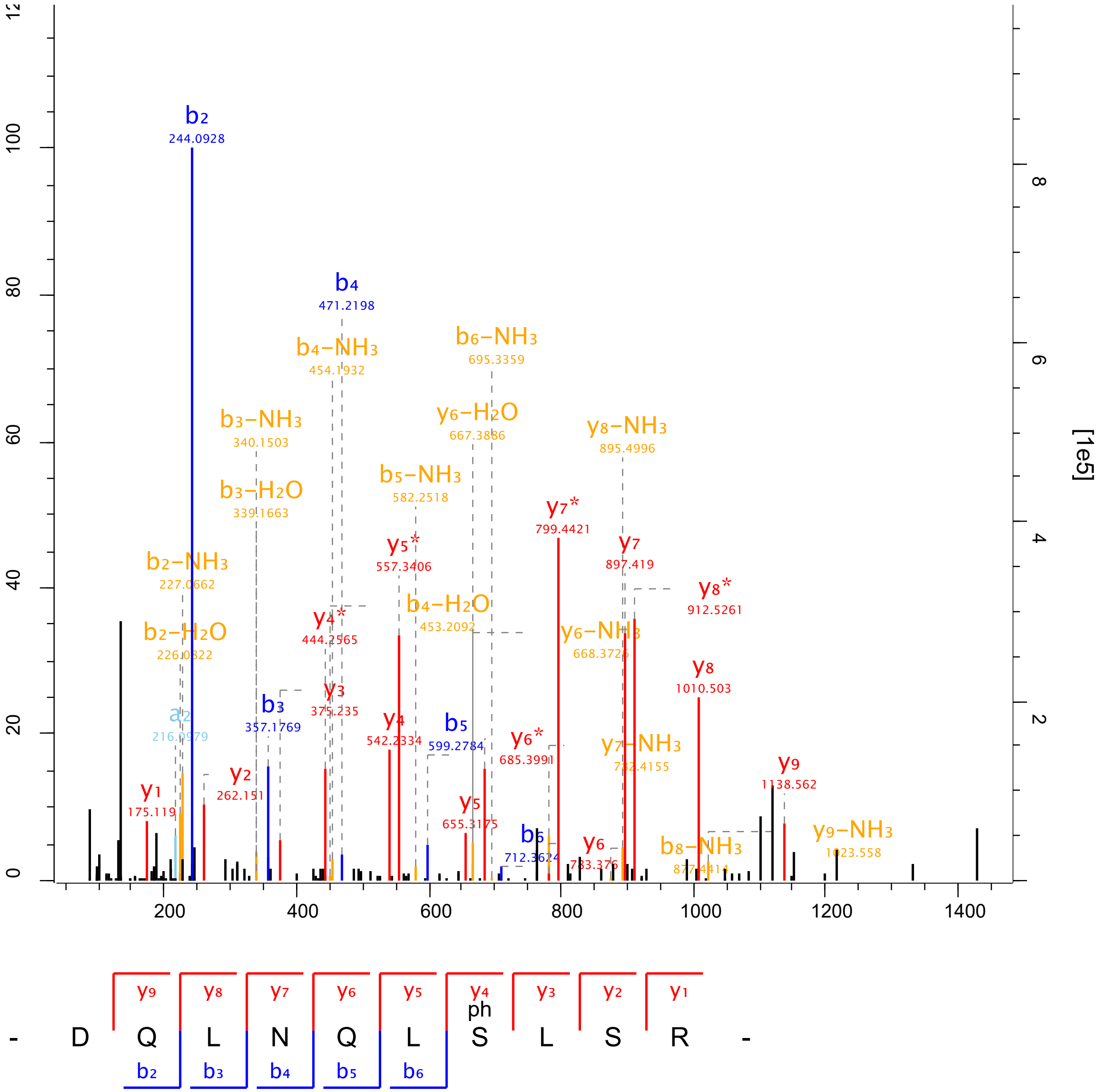

Raw file	Scan	Method	Score	m/z
sys_05_2	24877	FTMS; HCD	105.79	890.41

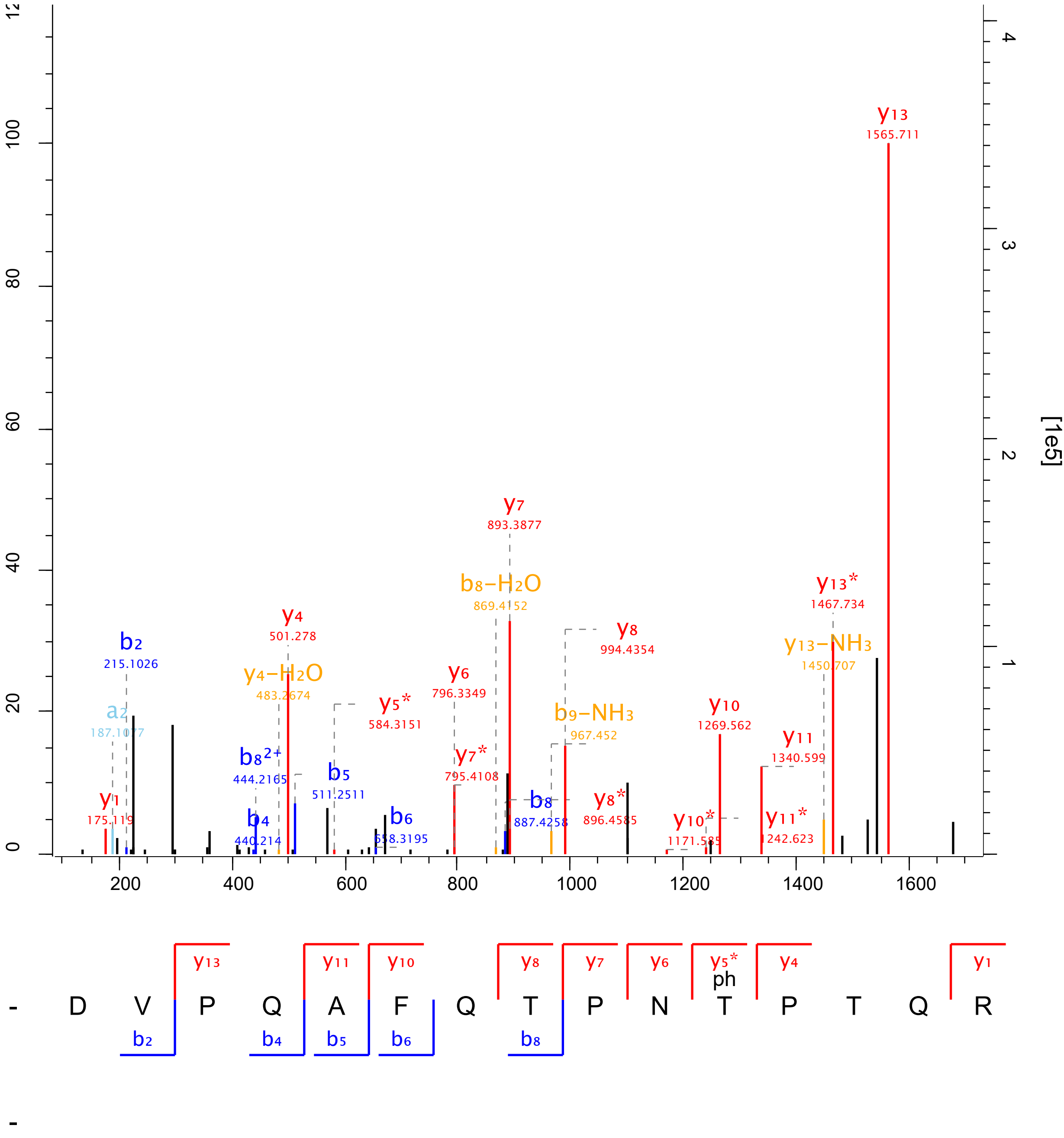

Raw file	Scan	Method	Score	m/z
sys_05_2	24919	FTMS; HCD	121.82	766.32

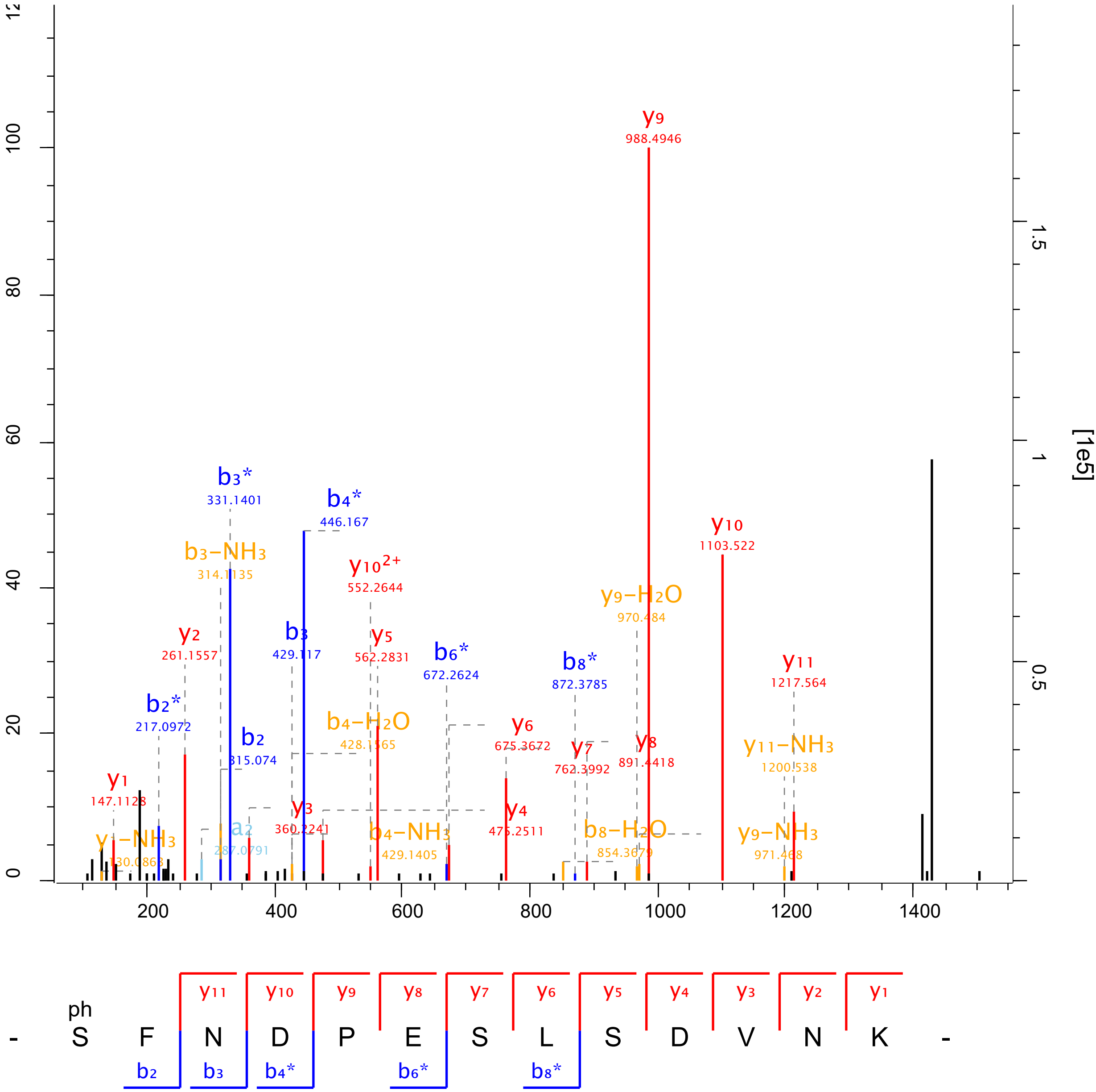

Raw file	Scan	Method	Score	m/z
sys_05_2	25152	FTMS; HCD	66.81	697.86

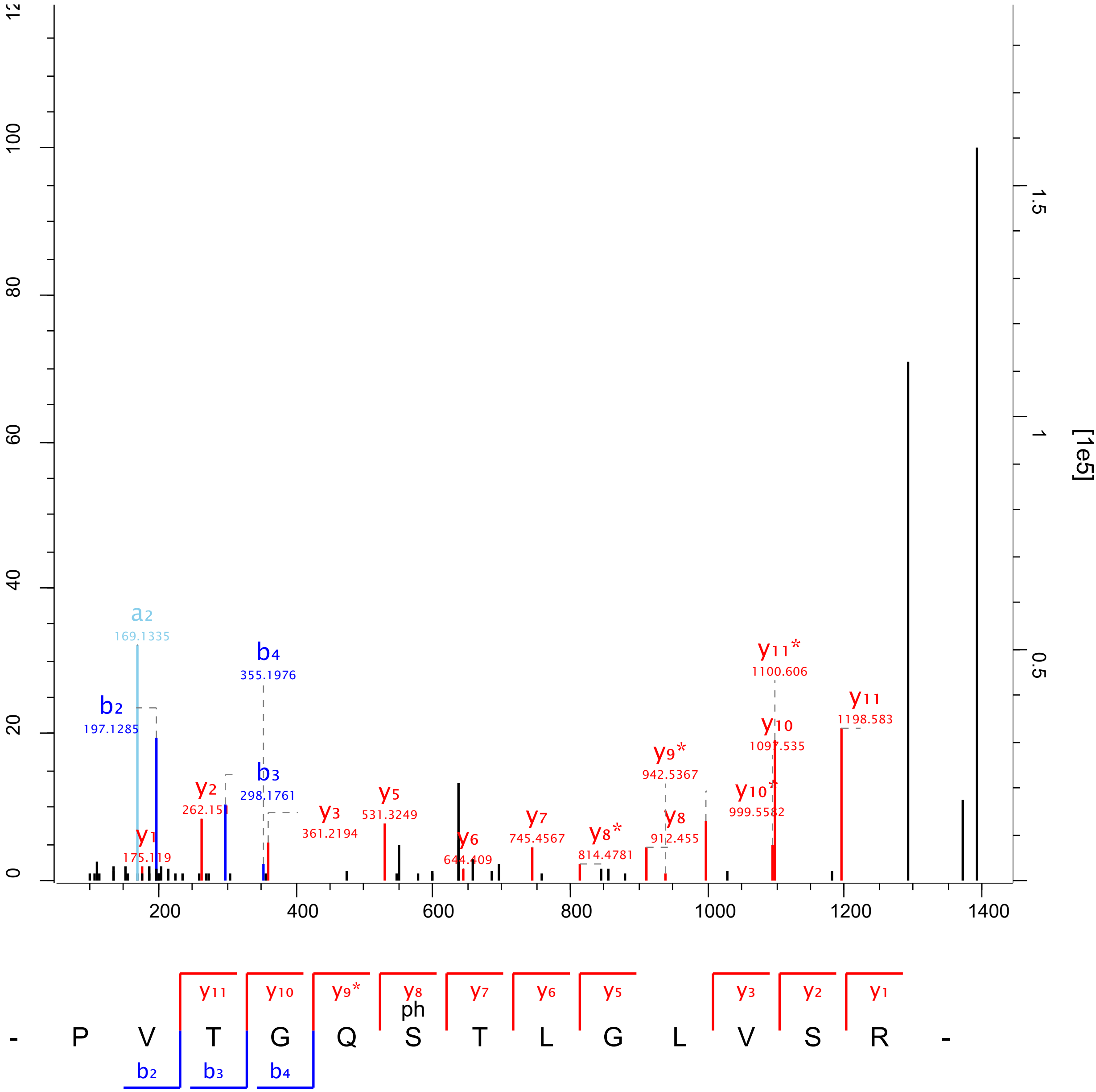

Raw file	Scan	Method	Score	m/z
sys_05_2	25263	FTMS; HCD	287.48	919.35

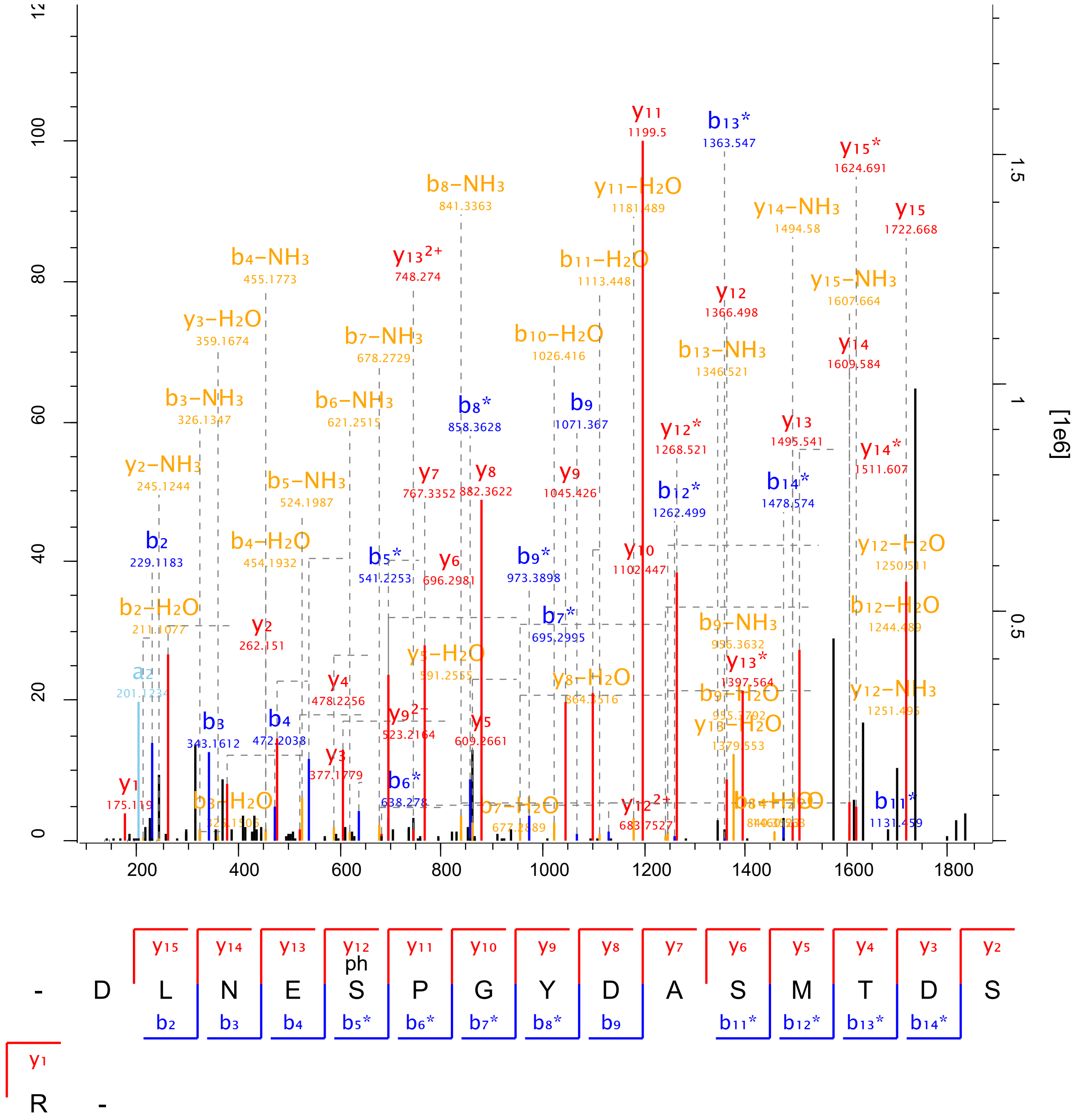

Raw file	Scan	Method	Score	m/z
sys_05_2	25469	FTMS; HCD	41.62	708.66

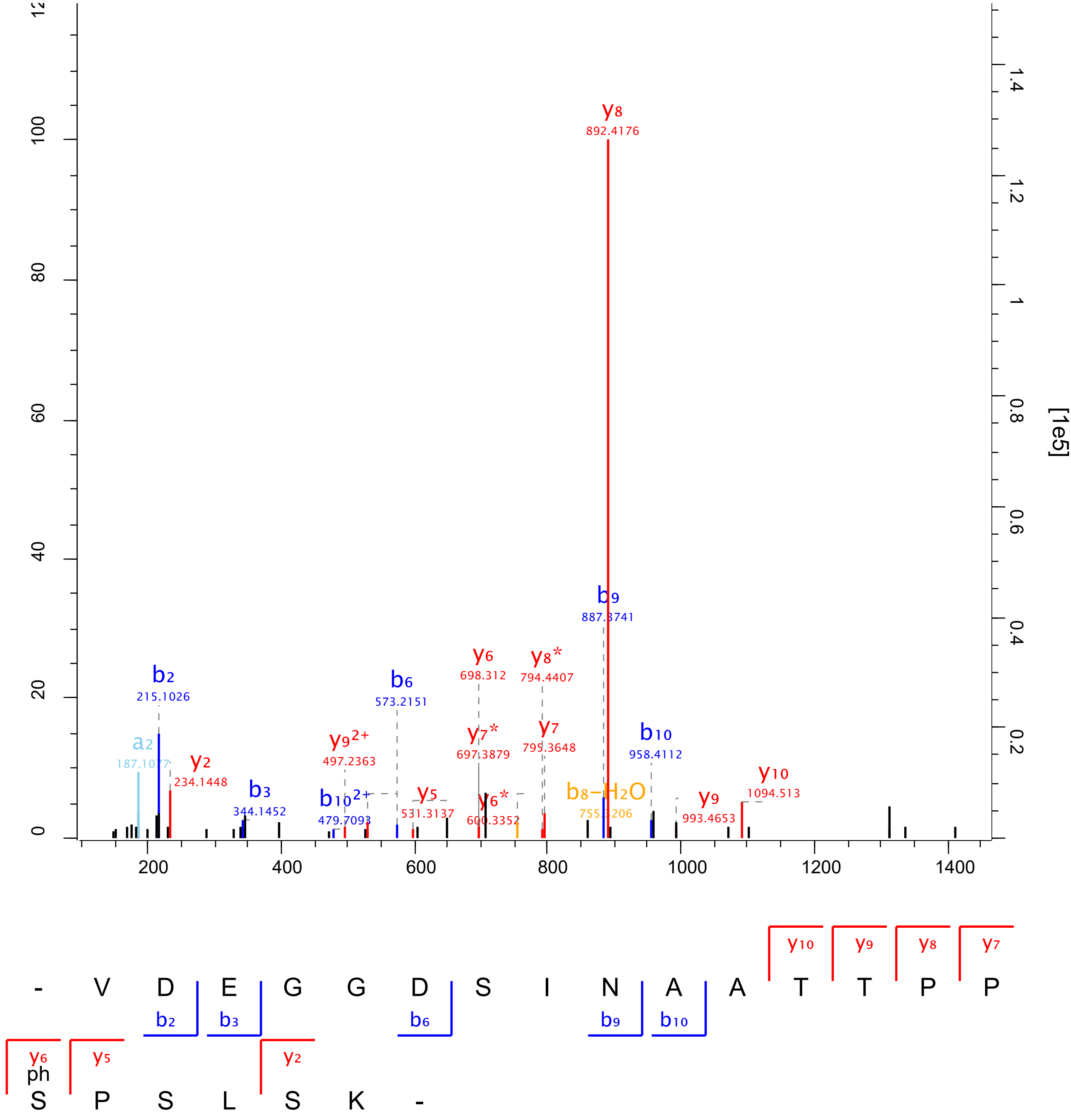

Raw file	Scan	Method	Score	m/z
sys_05_2	25476	FTMS; HCD	198.67	749.36

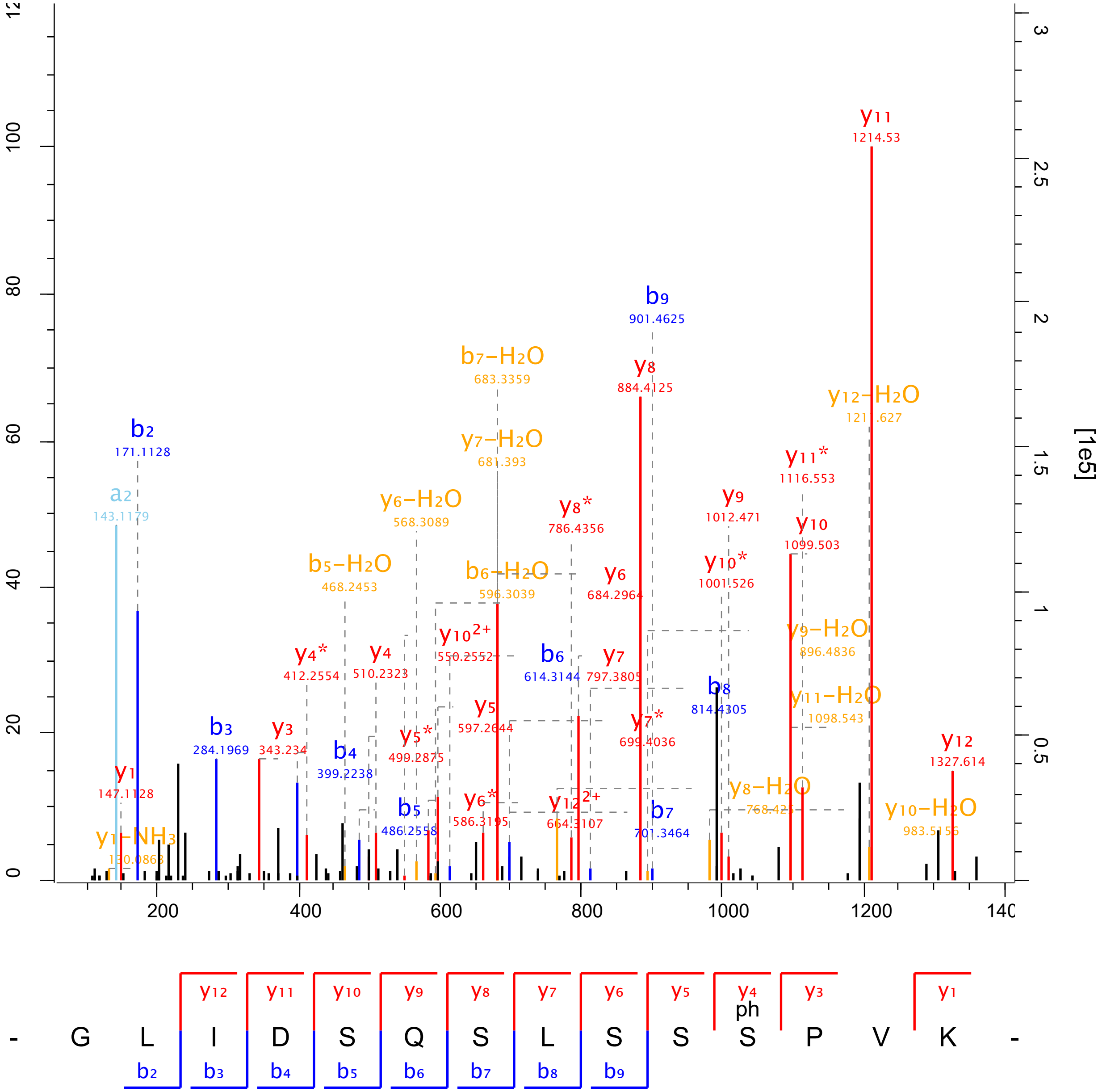

Raw file	Scan	Method	Score	m/z
sys_05_2	25726	FTMS; HCD	103.88	682.3

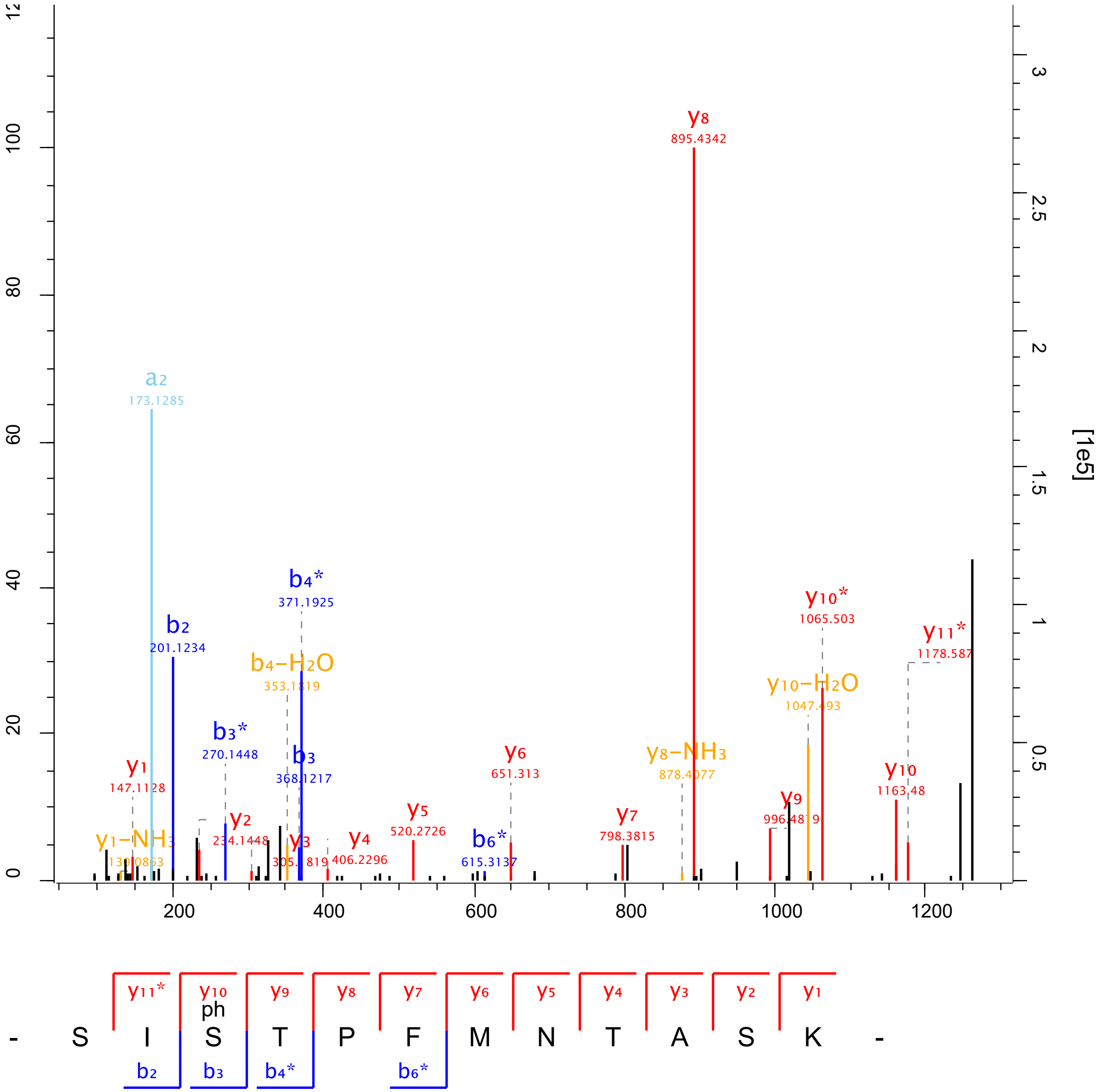

Raw file	Scan	Method	Score	m/z
sys_05_2	25902	FTMS; HCD	154.72	564.76

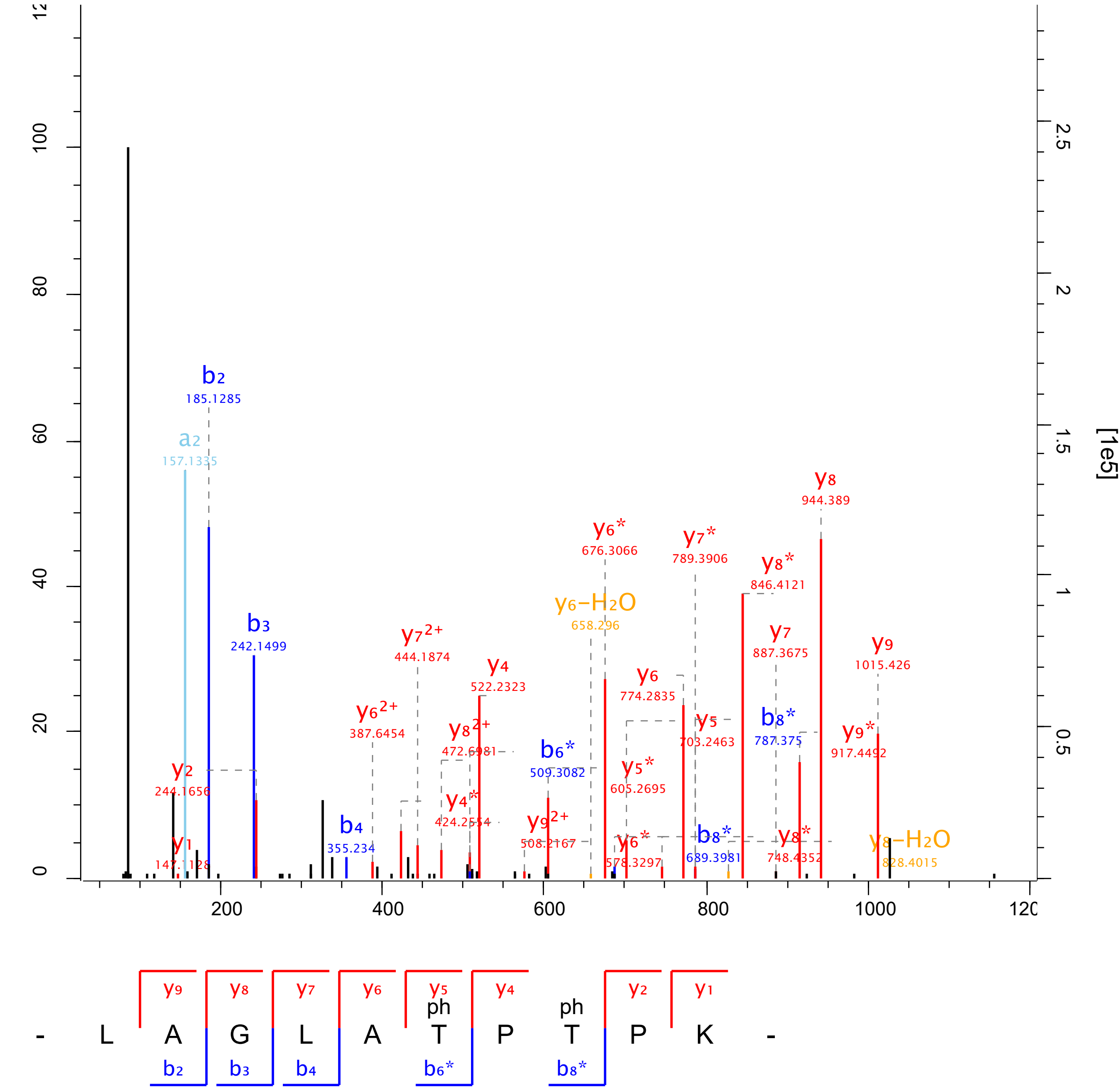

Raw file	Scan	Method	Score	m/z
sys_05_2	25944	FTMS; HCD	96.77	647.76

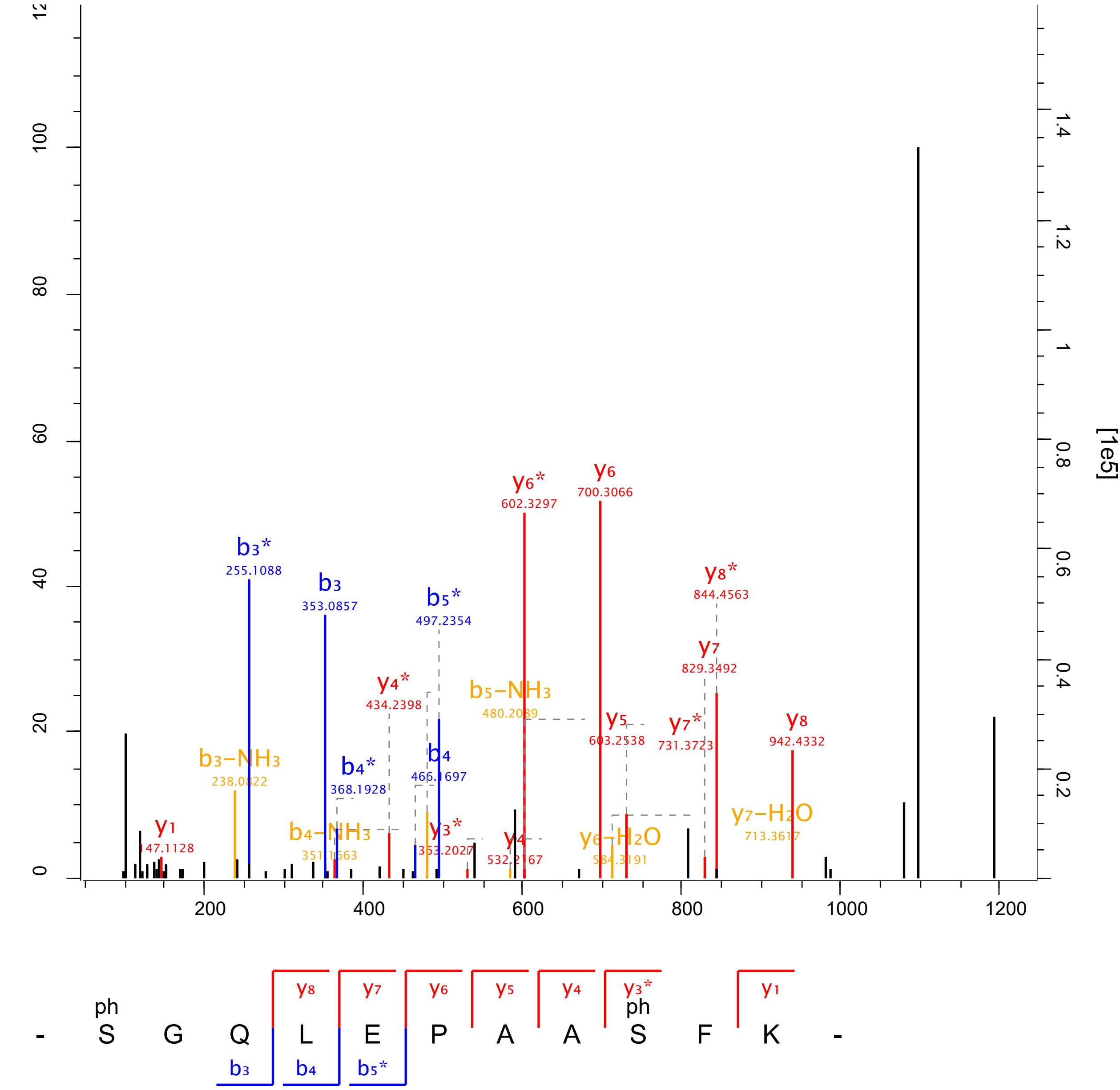

Raw file	Scan	Method	Score	m/z
sys_05_2	26024	FTMS; HCD	67.58	967.93

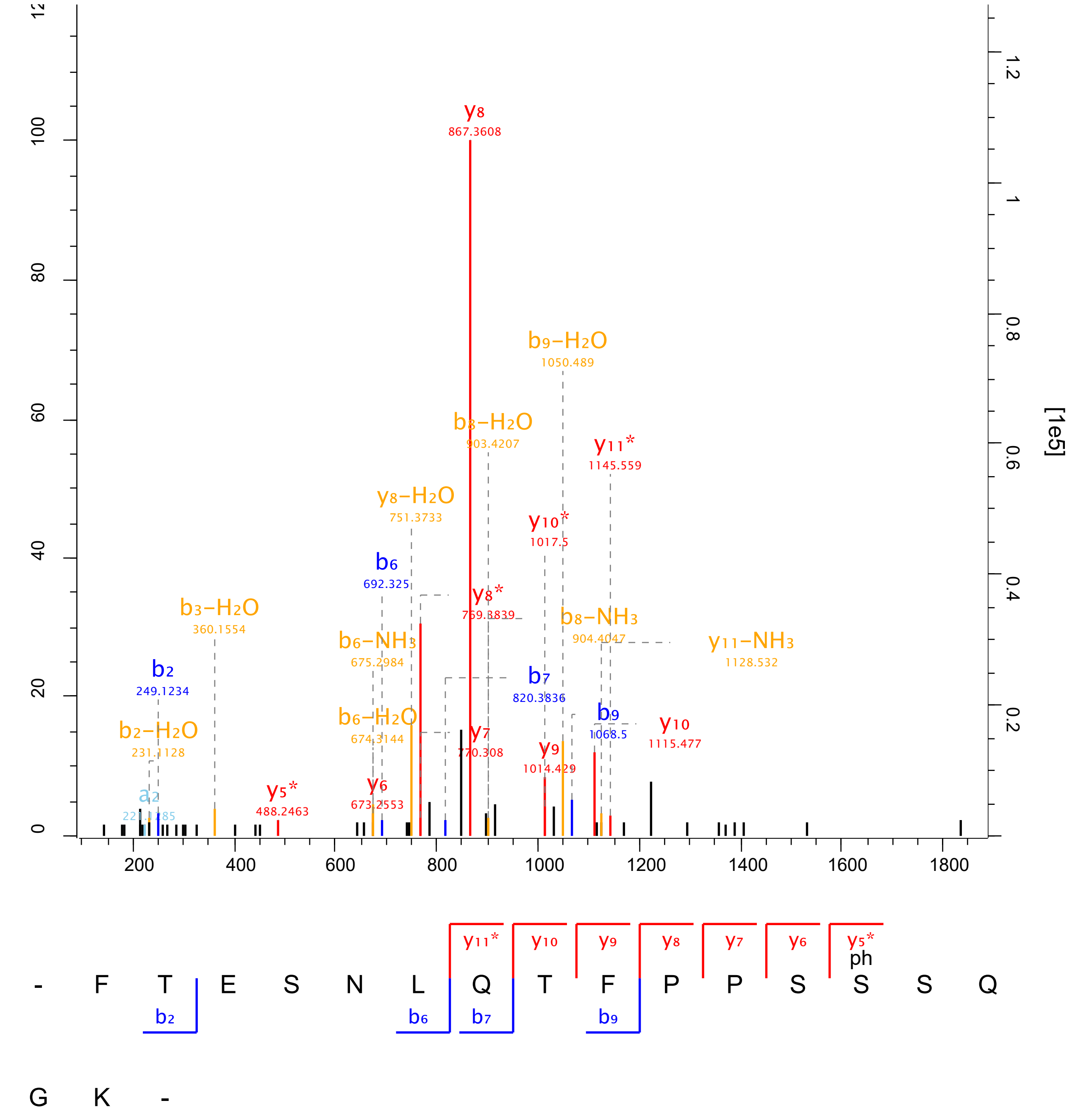

Raw file	Scan	Method	Score	m/z
sys_05_2	26246	FTMS; HCD	251.02	555.78

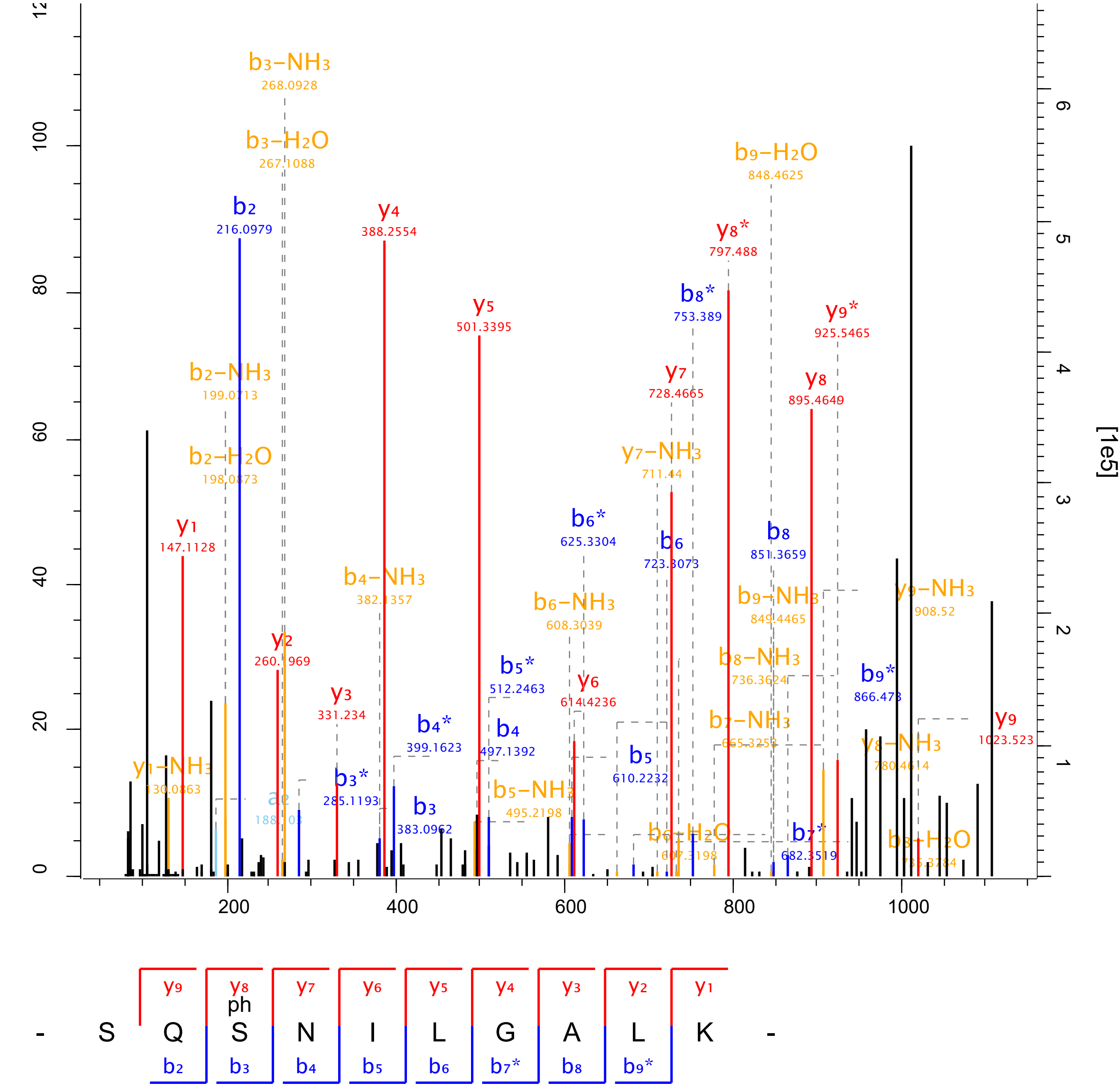

Raw file	Scan	Method	Score	m/z
sys_05_2	26258	FTMS; HCD	247.5	832.36

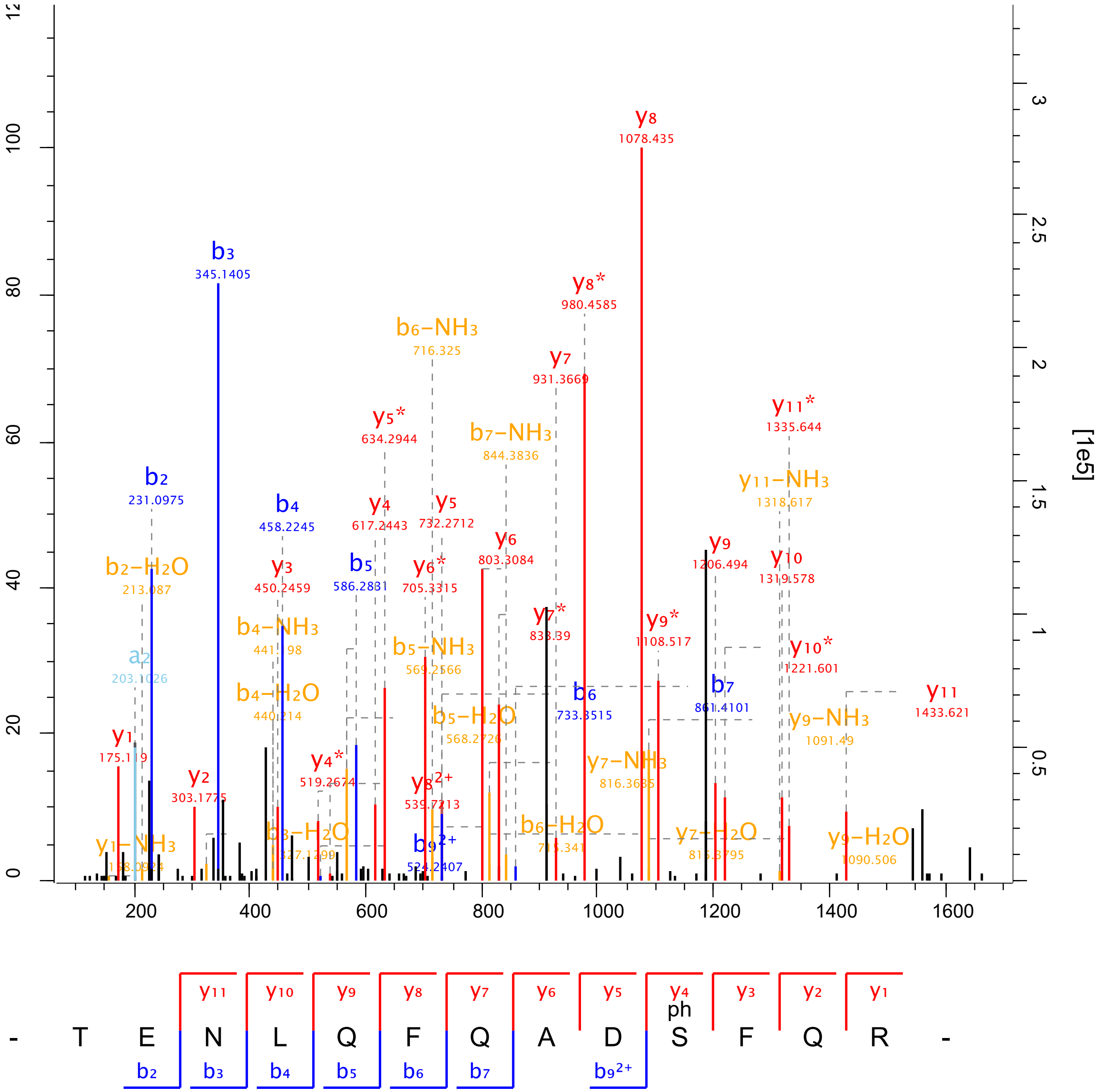

Raw file	Scan	Method	Score	m/z
sys_05_2	26297	FTMS; HCD	127.37	526.2

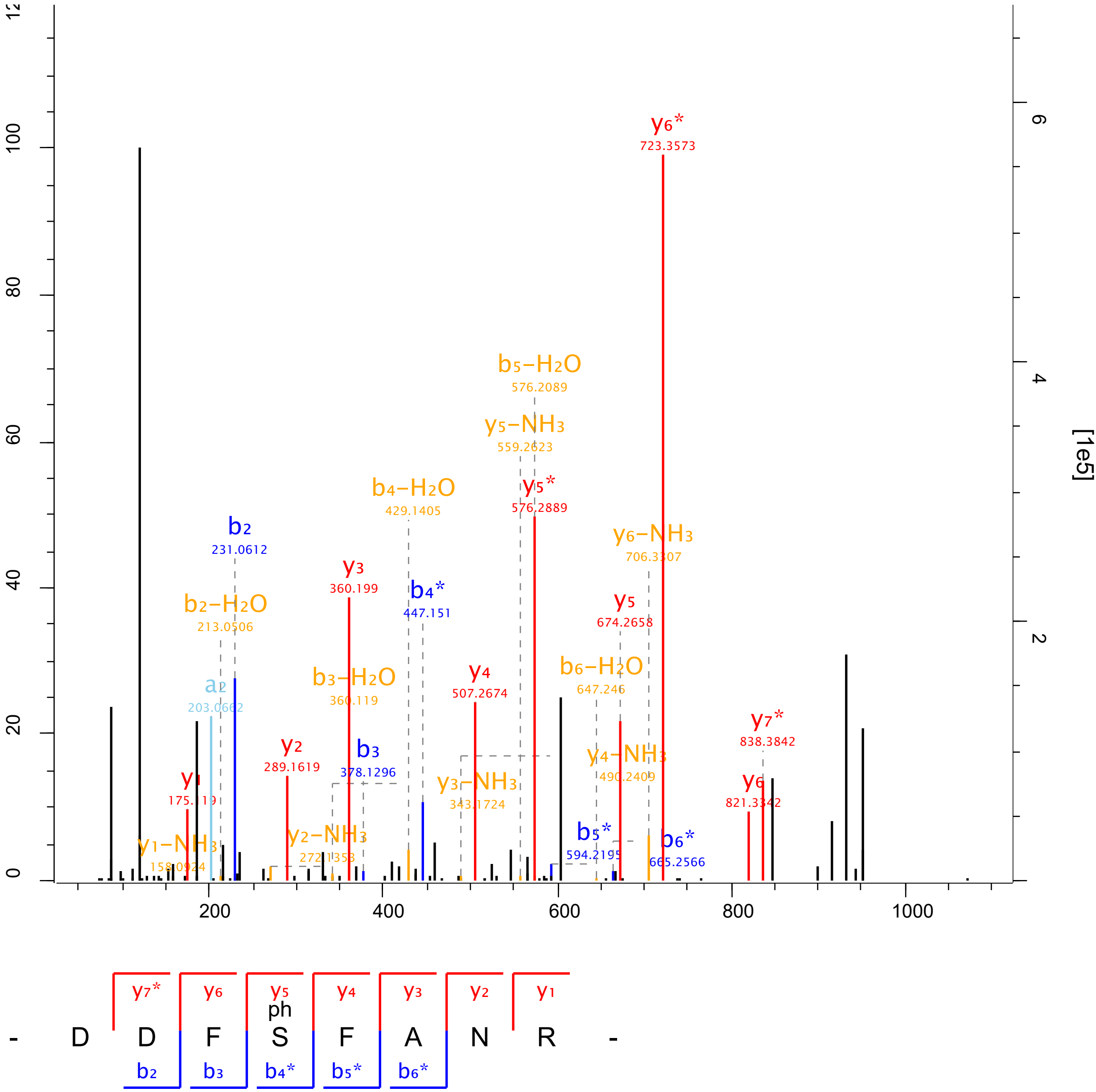

Raw file	Scan	Method	Score	m/z
sys_05_2	26304	FTMS; HCD	180.86	889.84

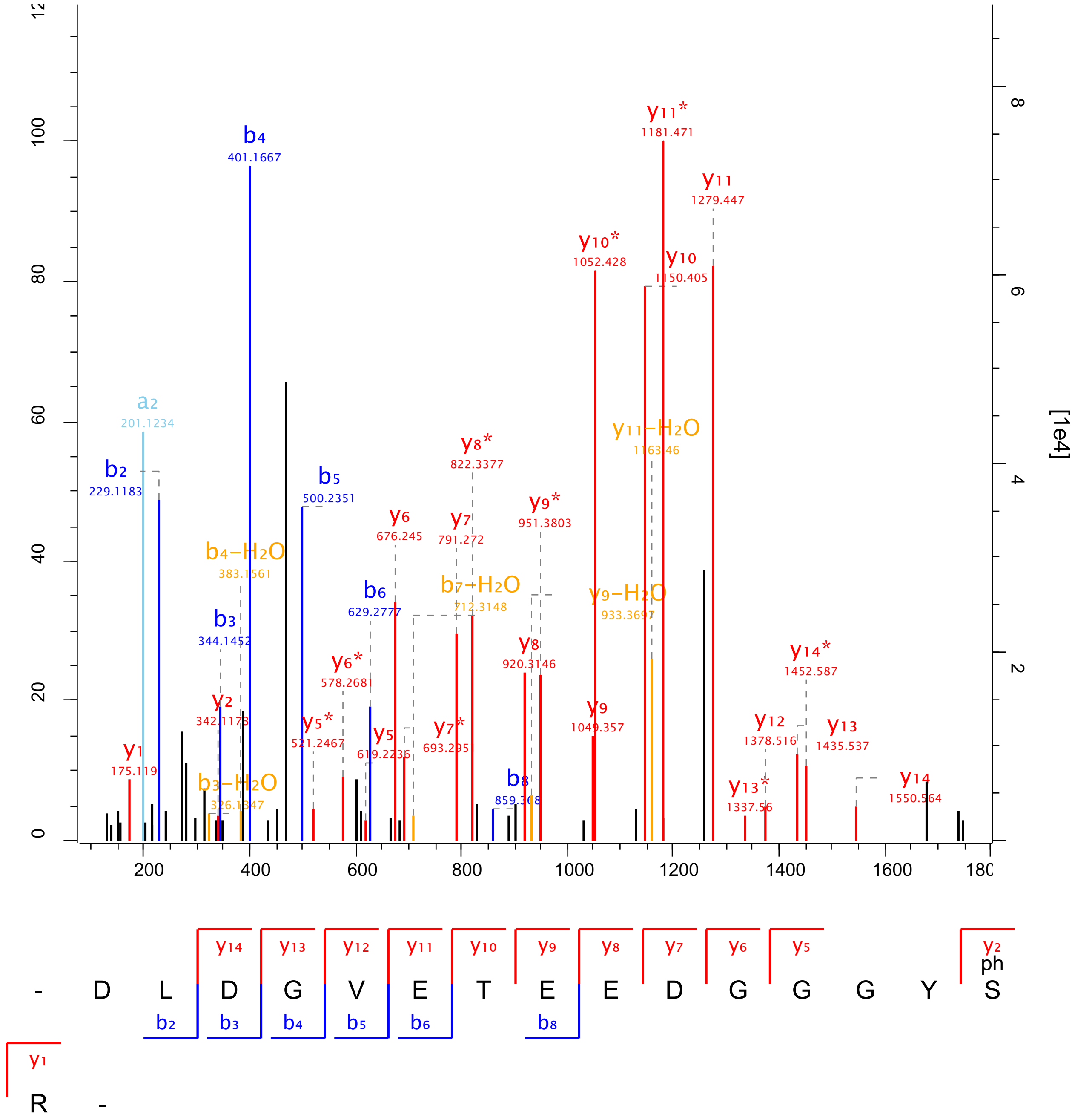

Raw file	Scan	Method	Score	m/z
sys_05_2	26667	FTMS; HCD	42	583.75

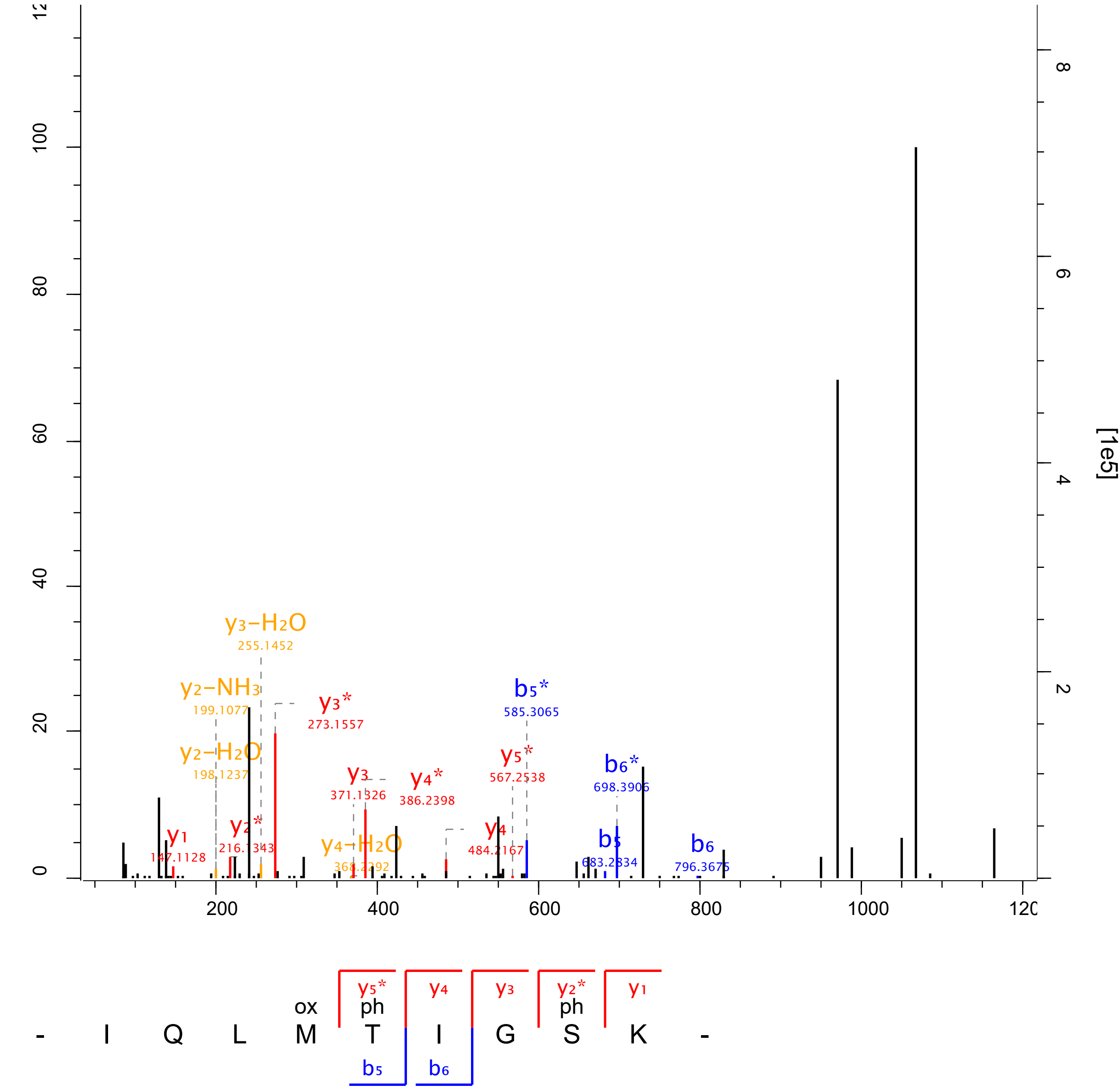

Raw file	Scan	Method	Score	m/z
sys_05_2	26767	FTMS; HCD	97.64	478.71

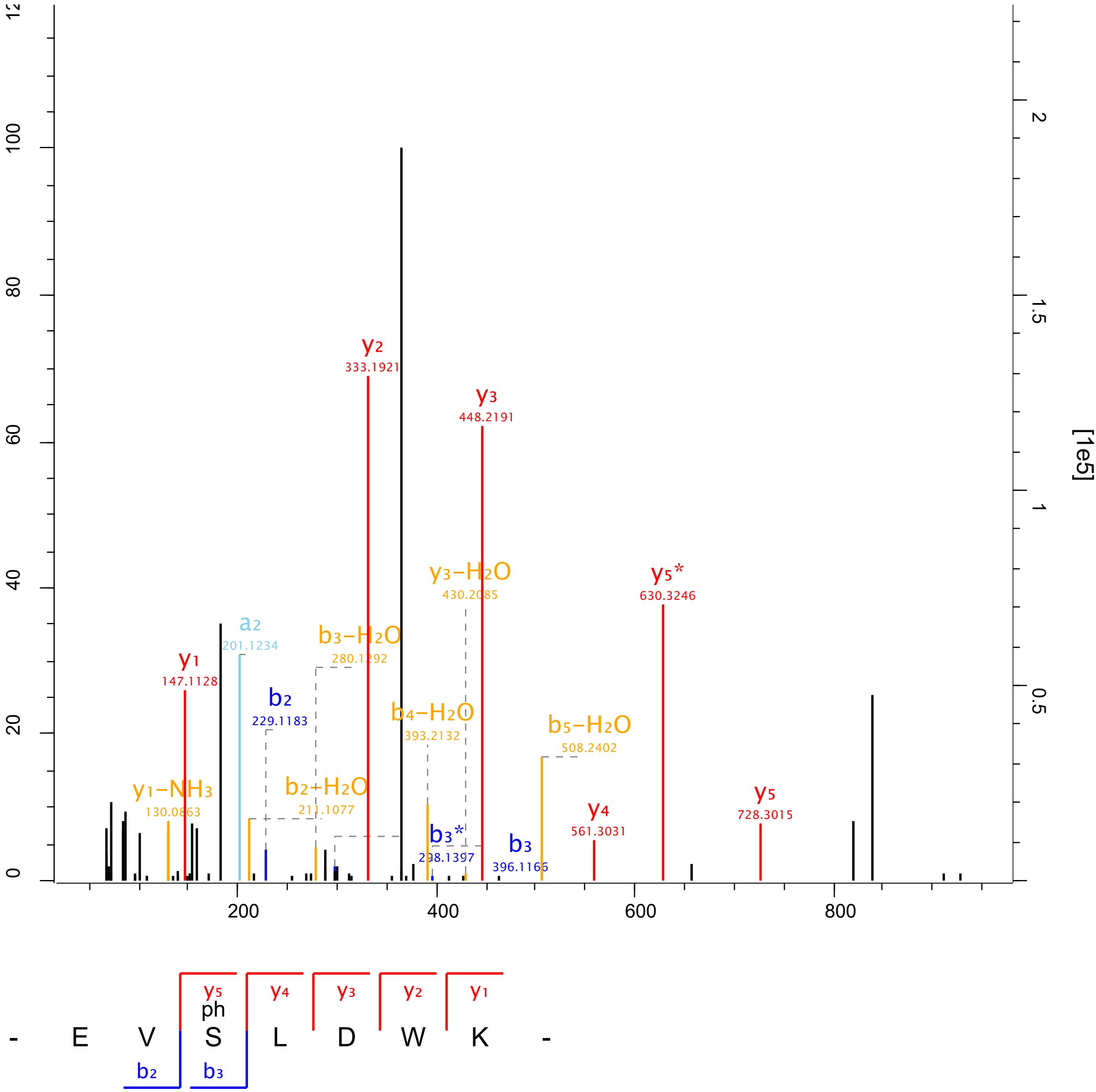

Raw file	Scan	Method	Score	m/z
sys_05_2	26806	FTMS; HCD	40.28	671.83

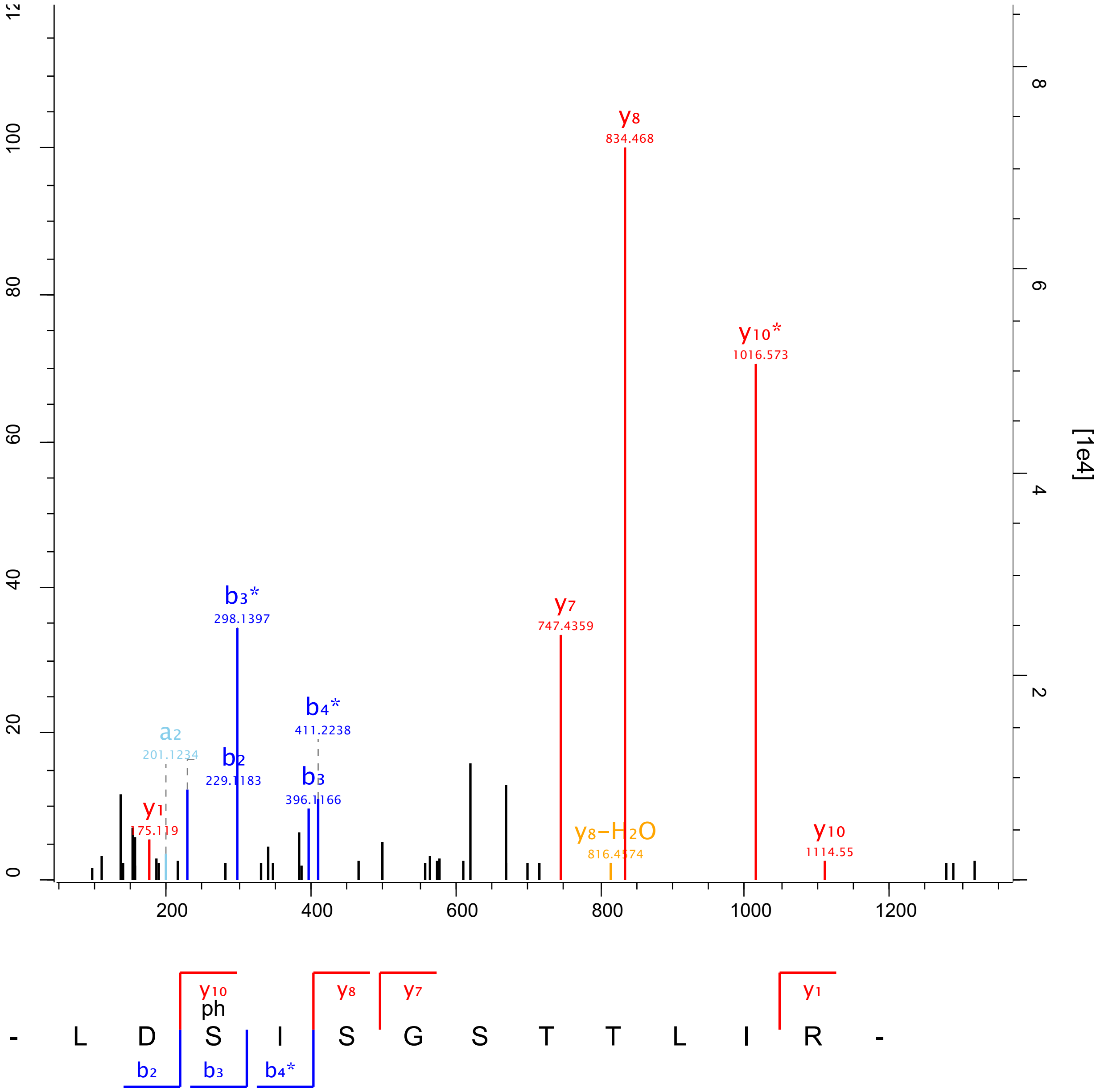

Raw file	Scan	Method	Score	m/z
sys_05_2	26856	FTMS; HCD	106.38	752.78

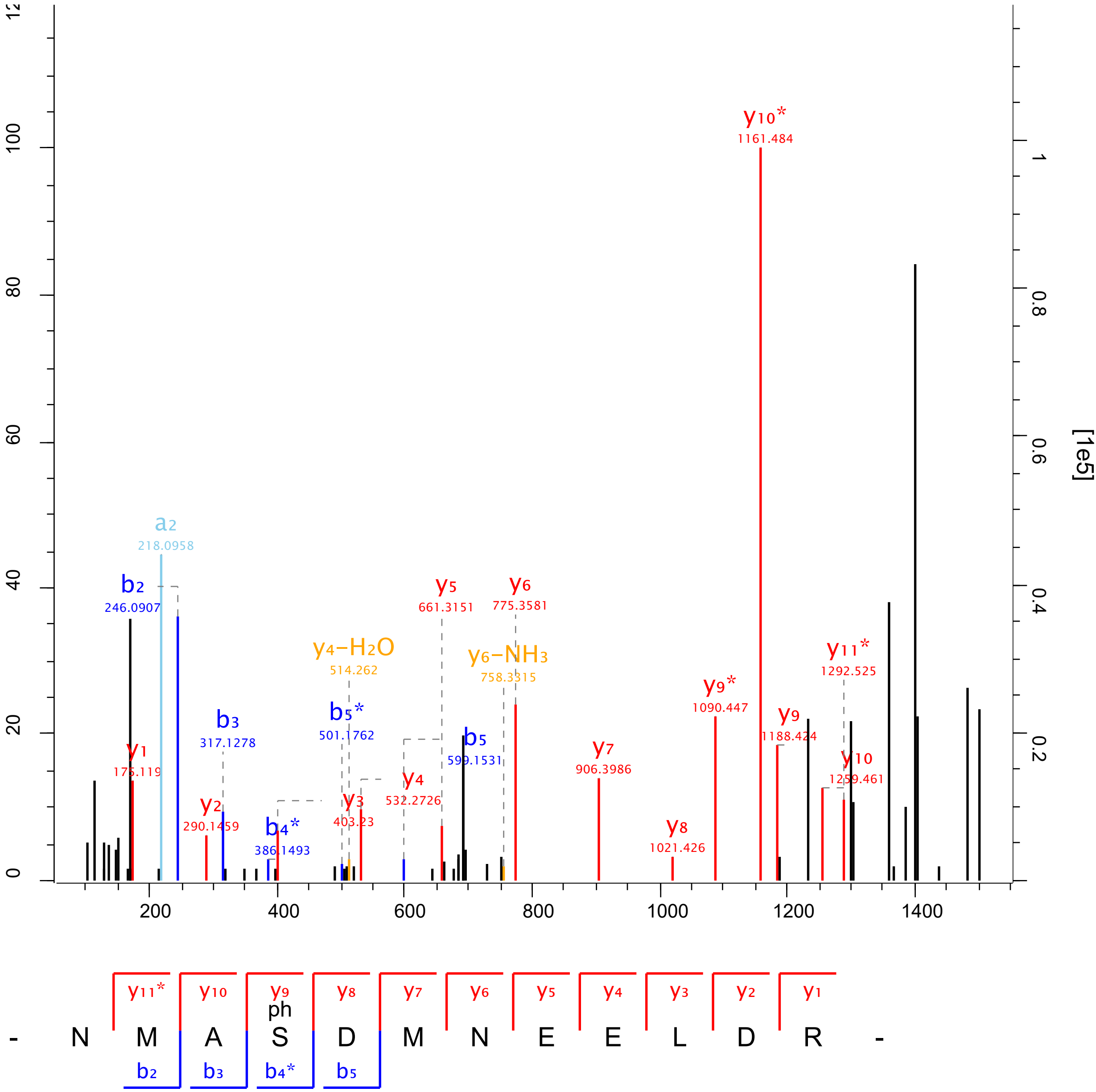

Raw file	Scan	Method	Score	m/z
sys_05_2	26898	FTMS; HCD	118.54	871.4

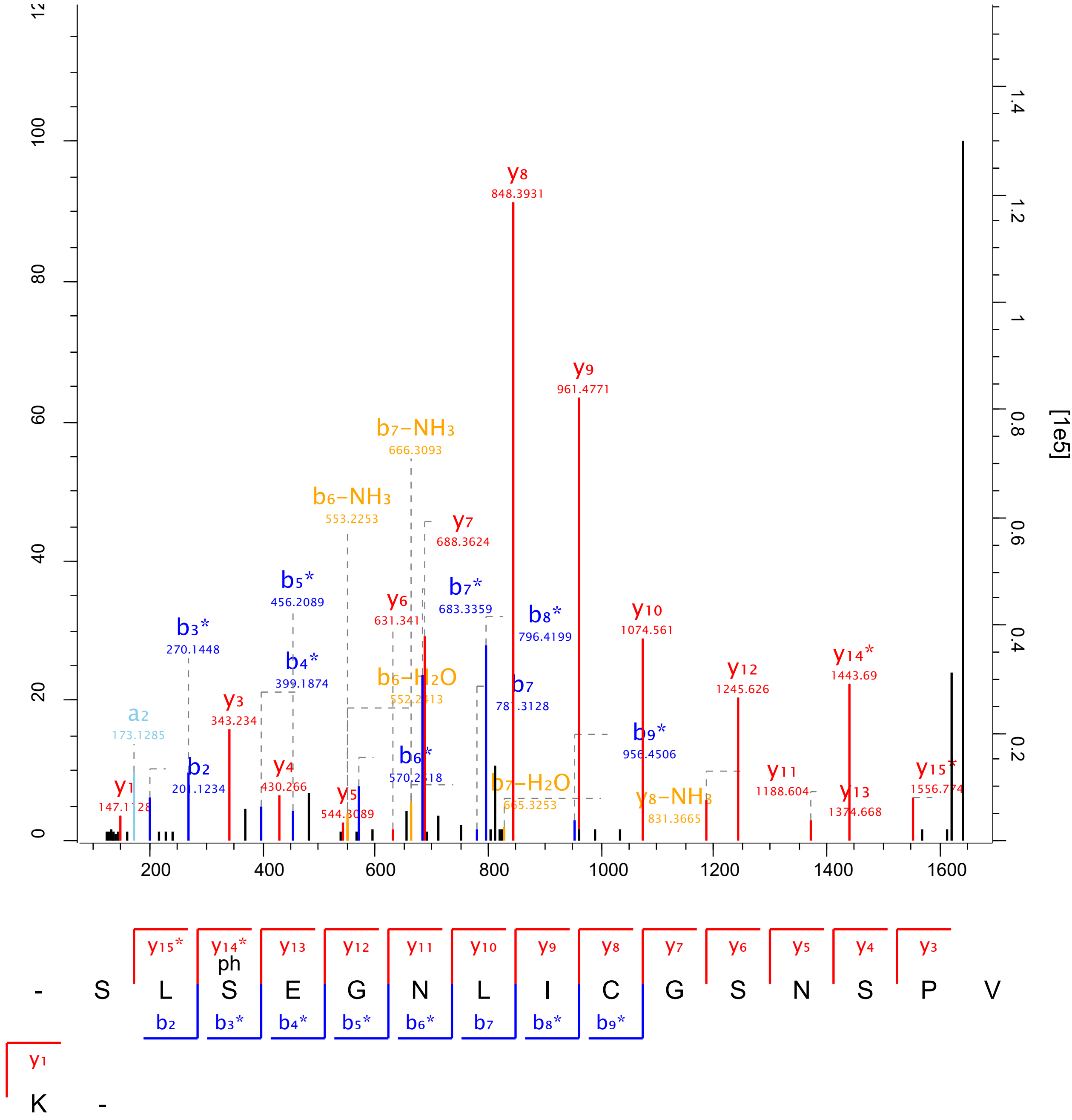

Raw file	Scan	Method	Score	m/z
sys_05_2	27043	FTMS; HCD	204.09	1151.45

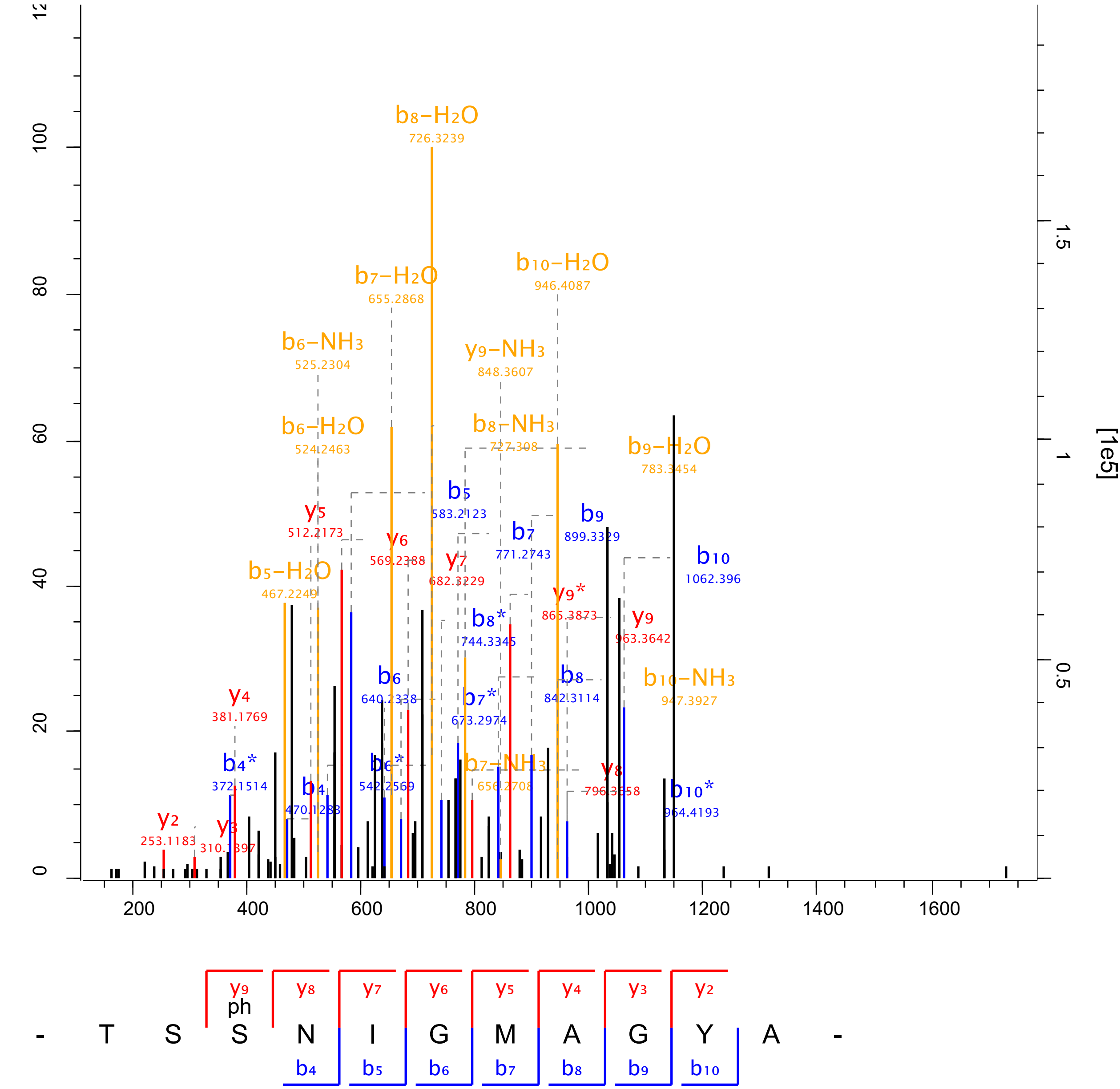

Raw file	Scan	Method	Score	m/z
sys_05_2	27224	FTMS; HCD	199.41	934.92

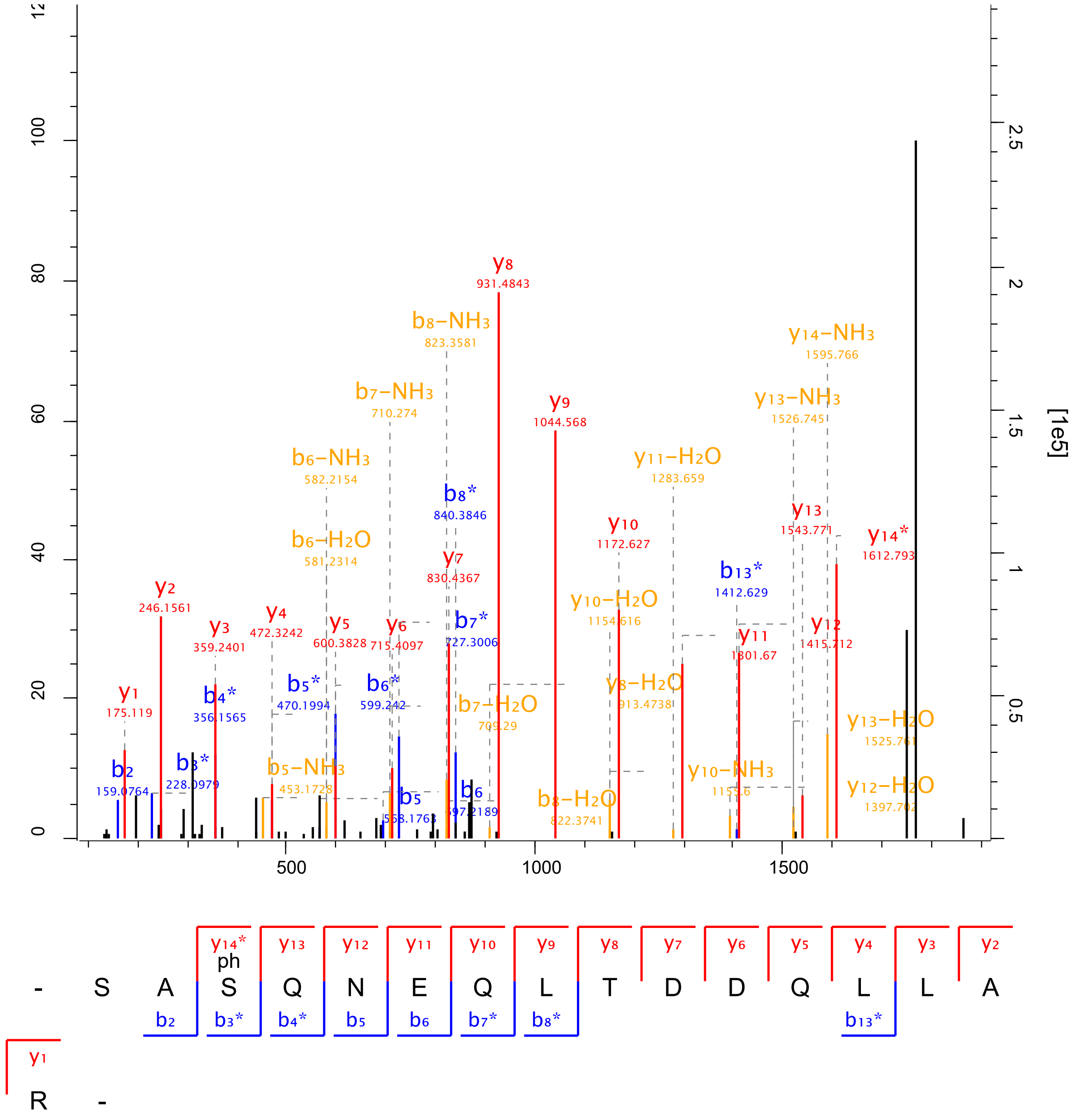

Raw file	Scan	Method	Score	m/z
sys_05_2	27318	FTMS; HCD	220.06	635.79

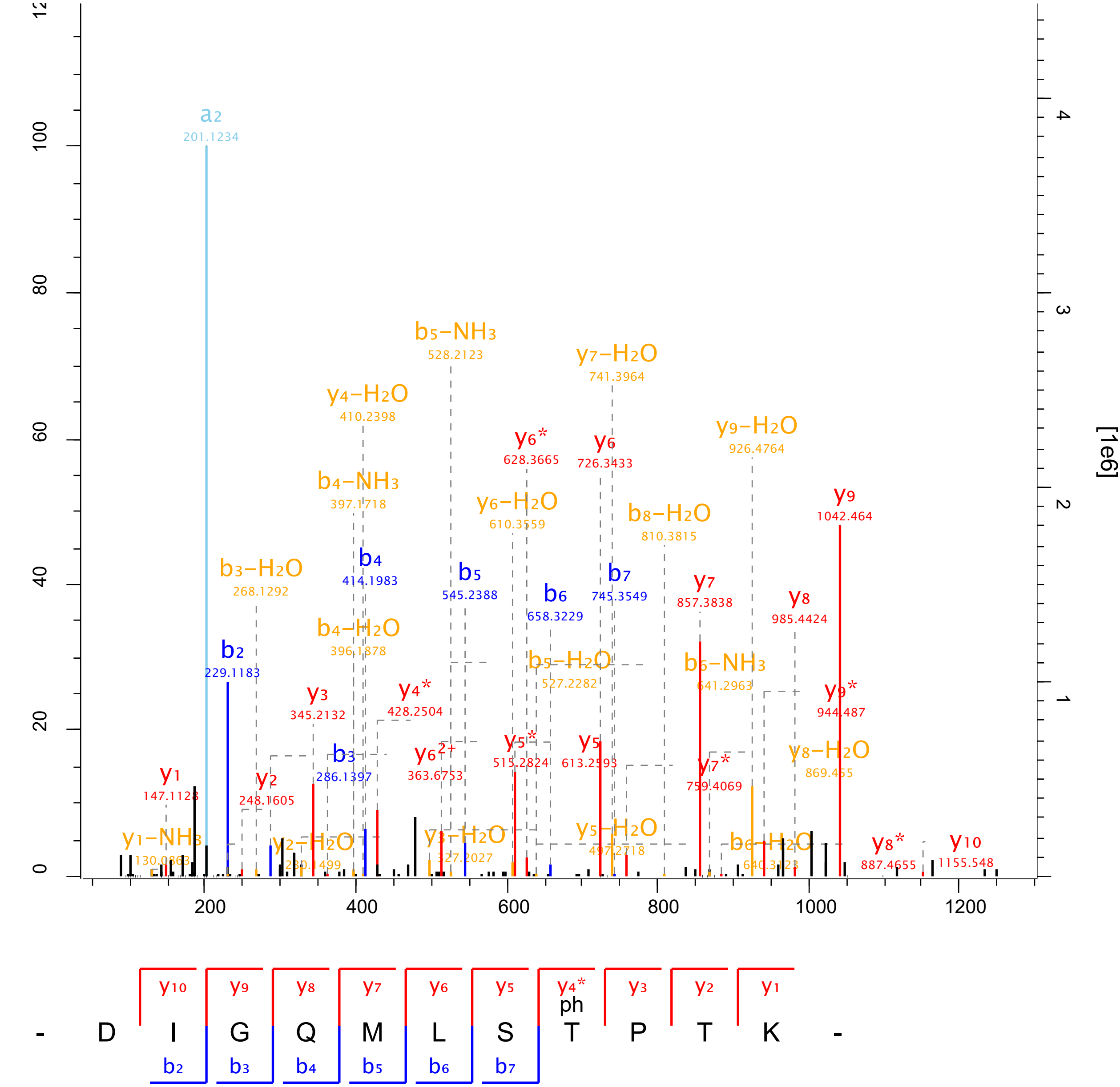

Raw file	Scan	Method	Score	m/z
sys_05_2	27838	FTMS; HCD	99.27	912.43

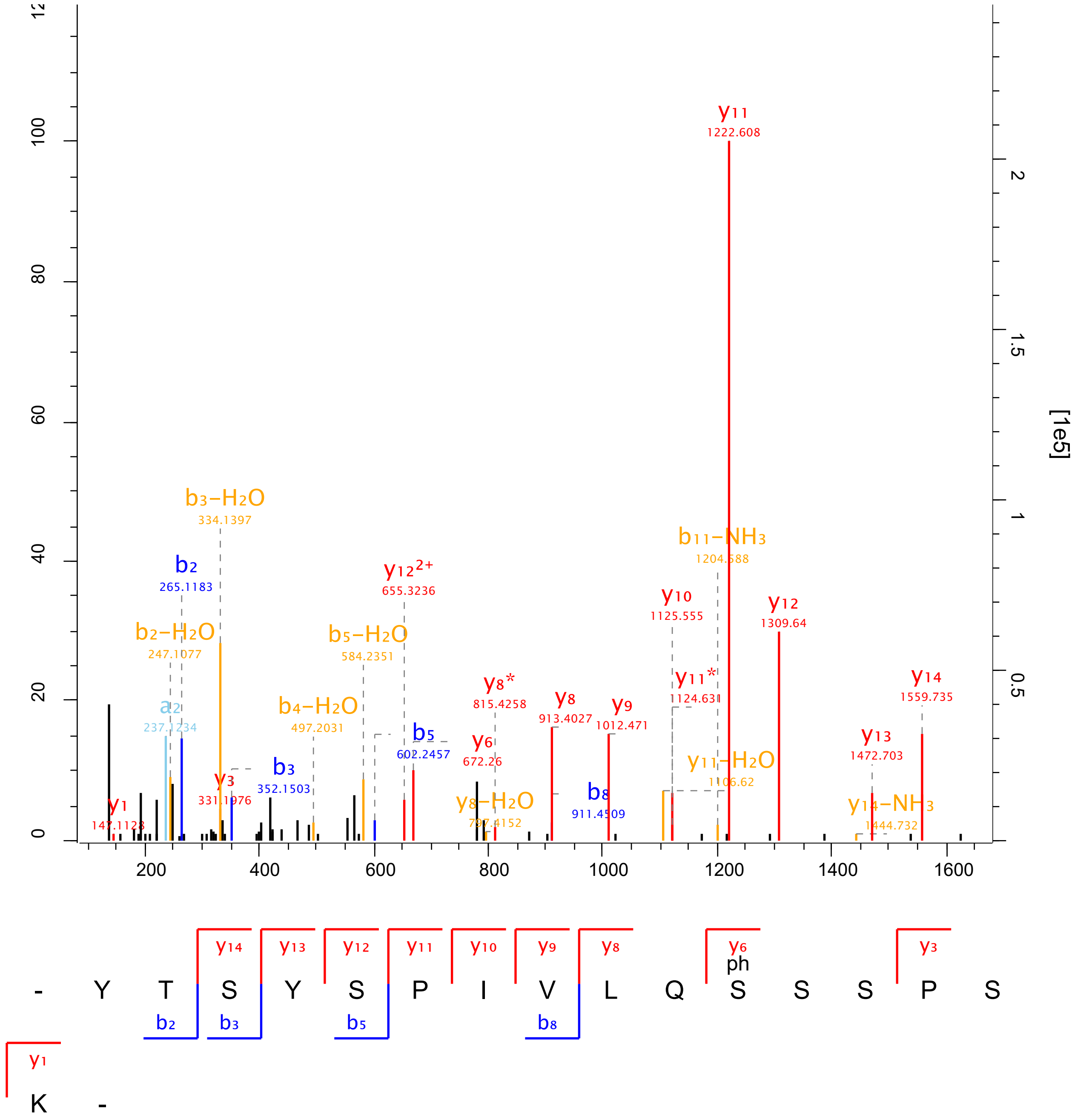

Raw file	Scan	Method	Score	m/z
sys_05_2	27987	FTMS; HCD	122.33	606.77

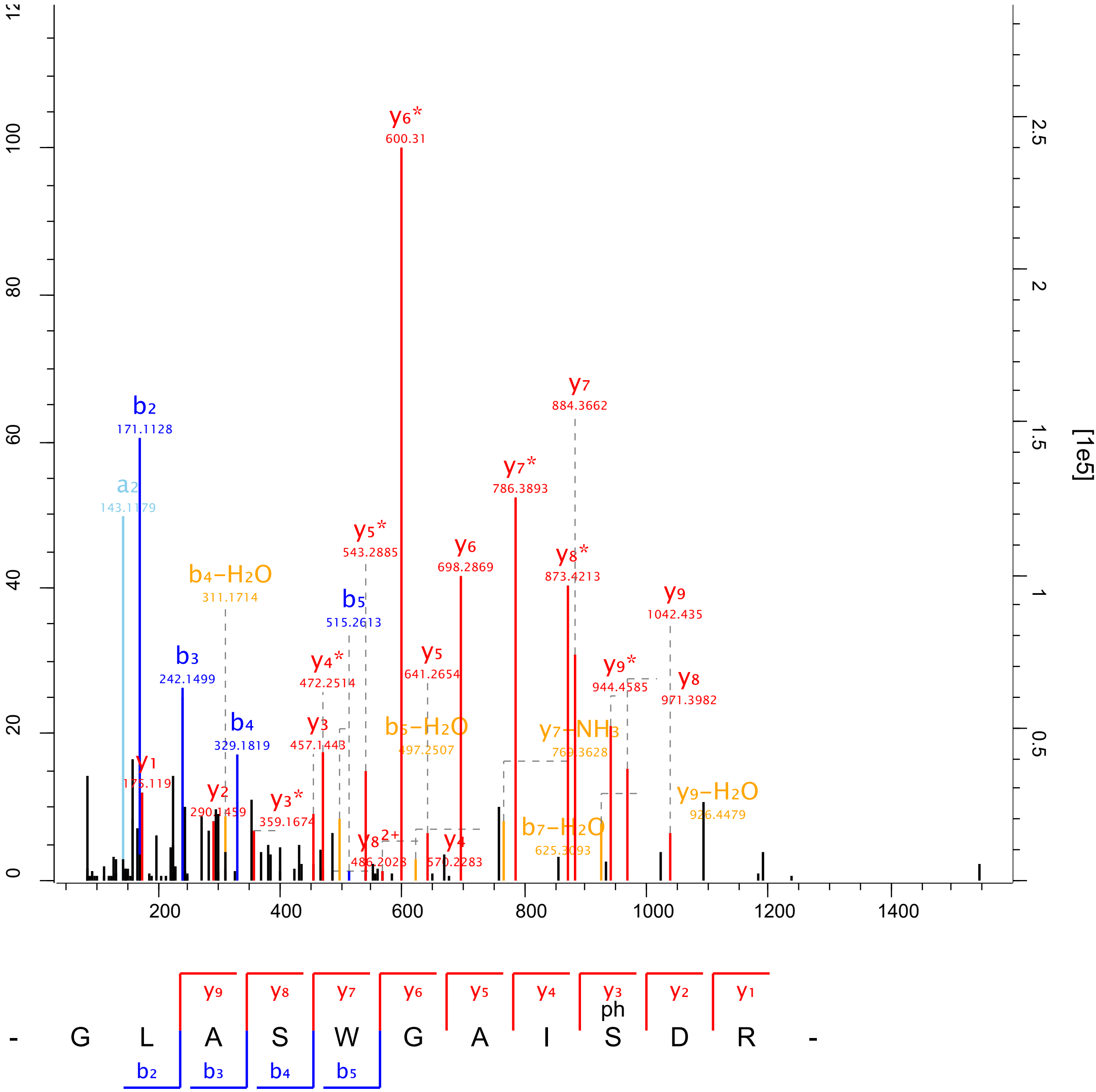

Raw file	Scan	Method	Score	m/z
sys_05_2	28580	FTMS; HCD	207.66	686.79

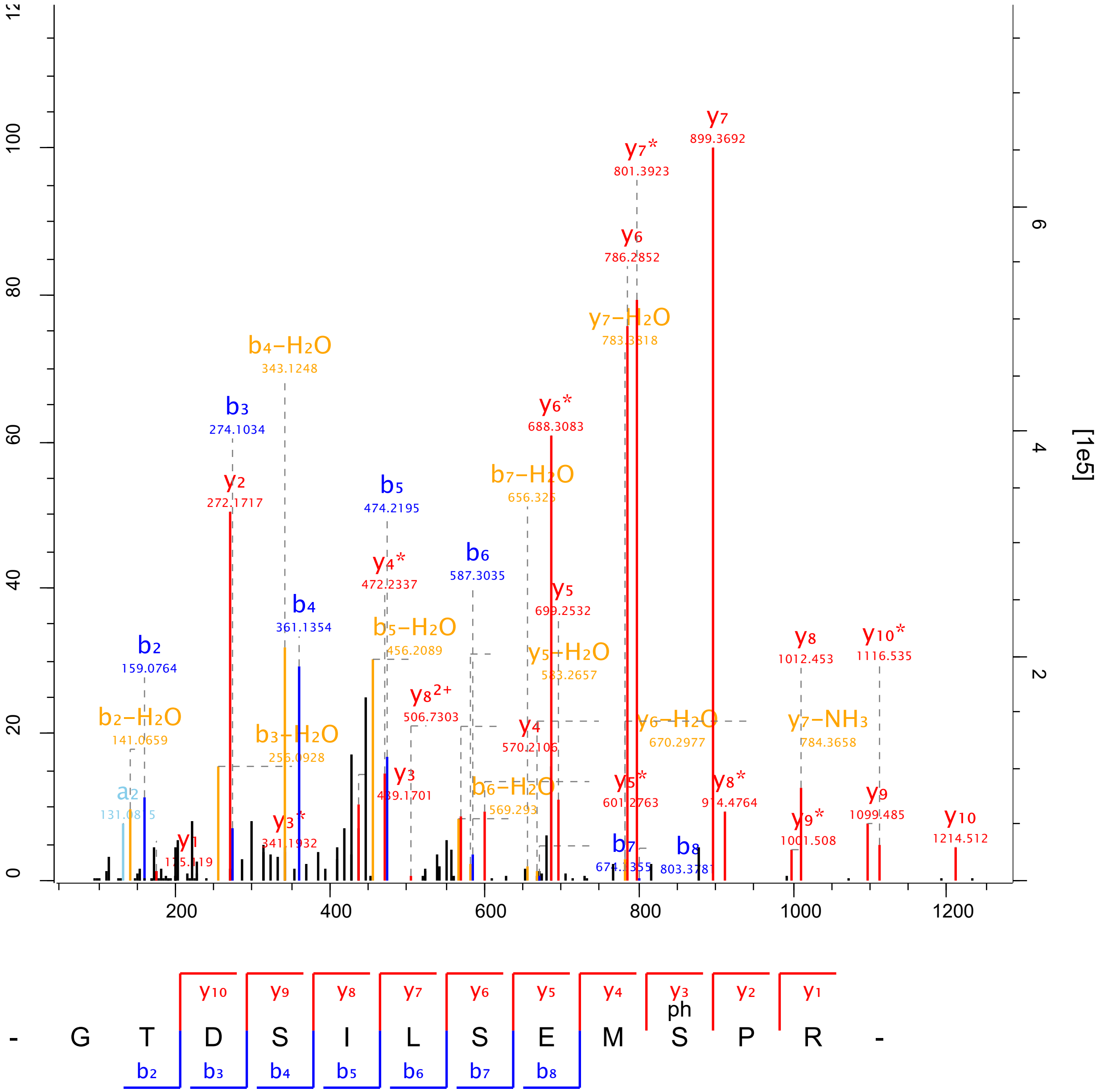

Raw file	Scan	Method	Score	m/z
sys_05_2	28619	FTMS; HCD	52.5	607.75

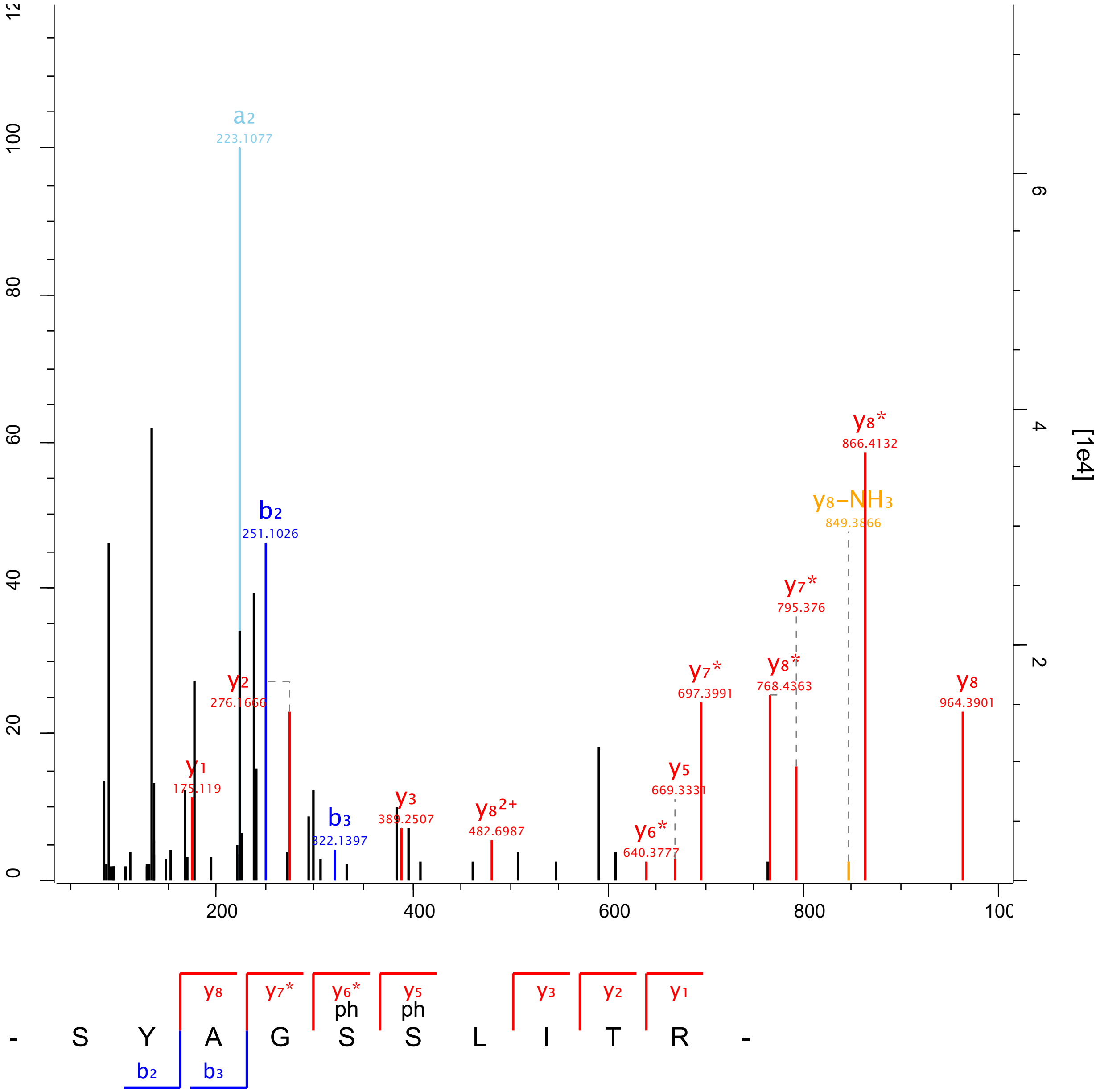

Raw file	Scan	Method	Score	m/z
sys_05_2	28630	FTMS; HCD	99.32	894.38

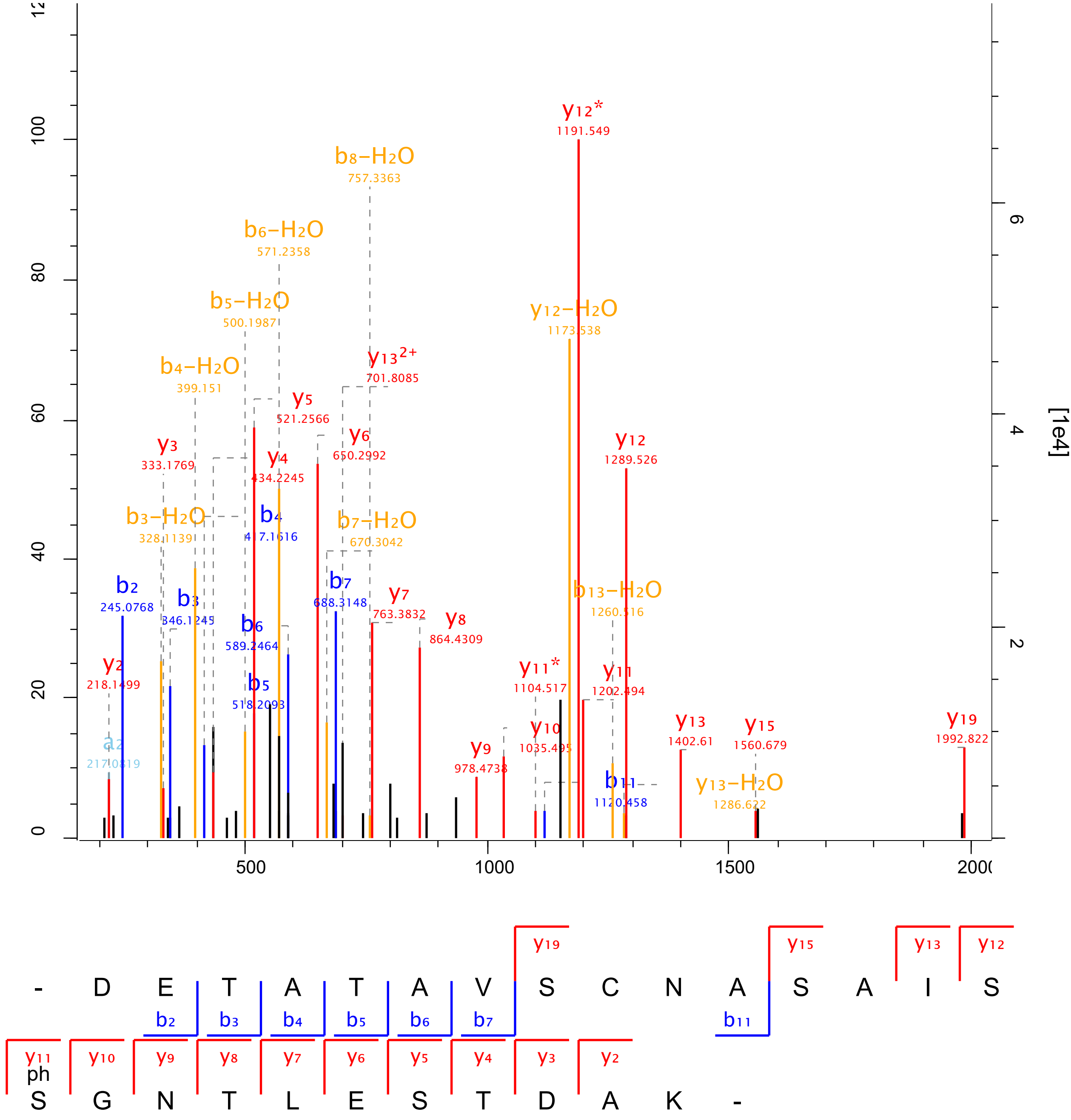

Raw file	Scan	Method	Score	m/z
sys_05_2	28908	FTMS; HCD	274.91	827.32

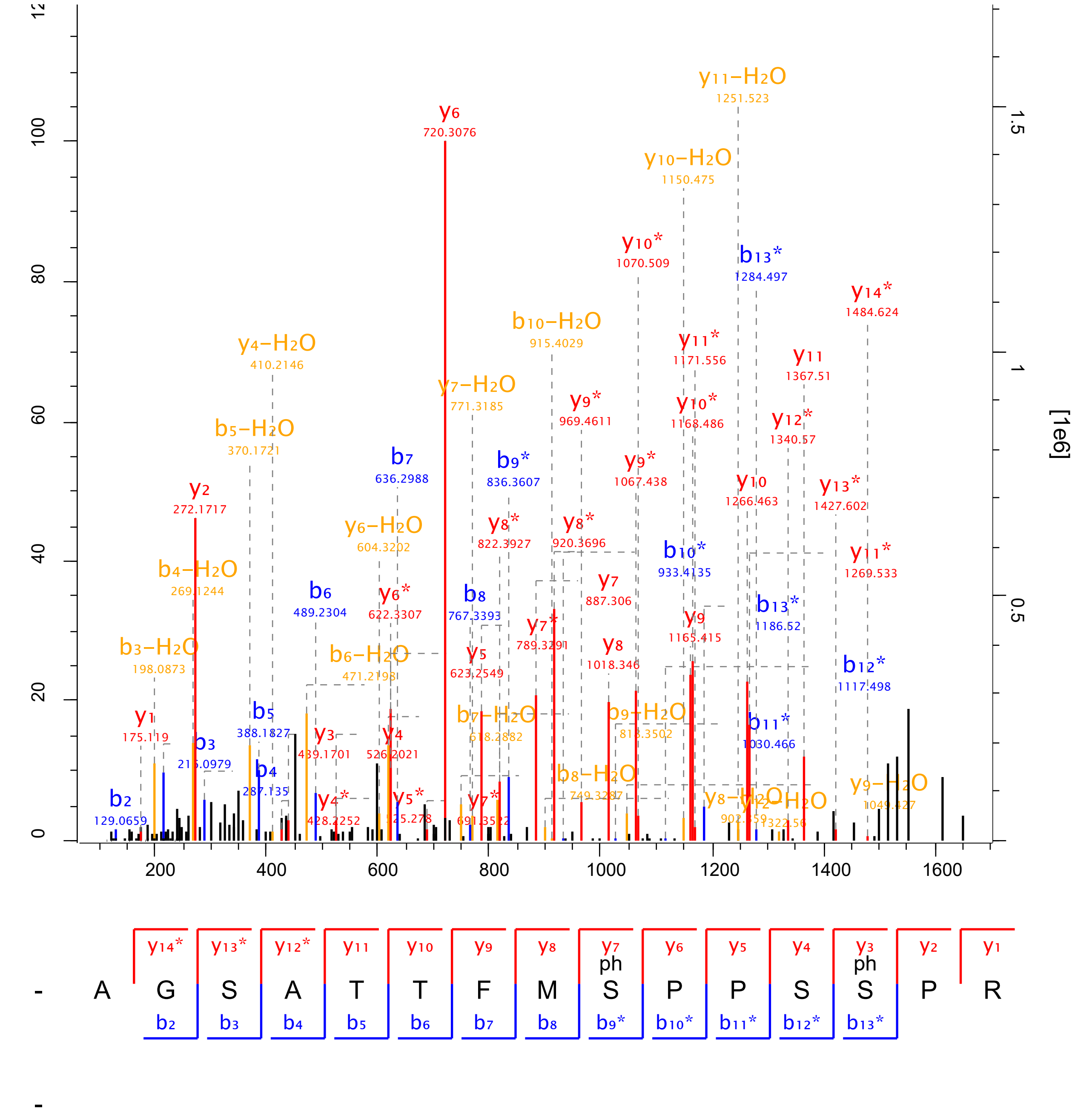

Raw file	Scan	Method	Score	m/z
sys_05_2	28958	FTMS; HCD	50.9	643.79

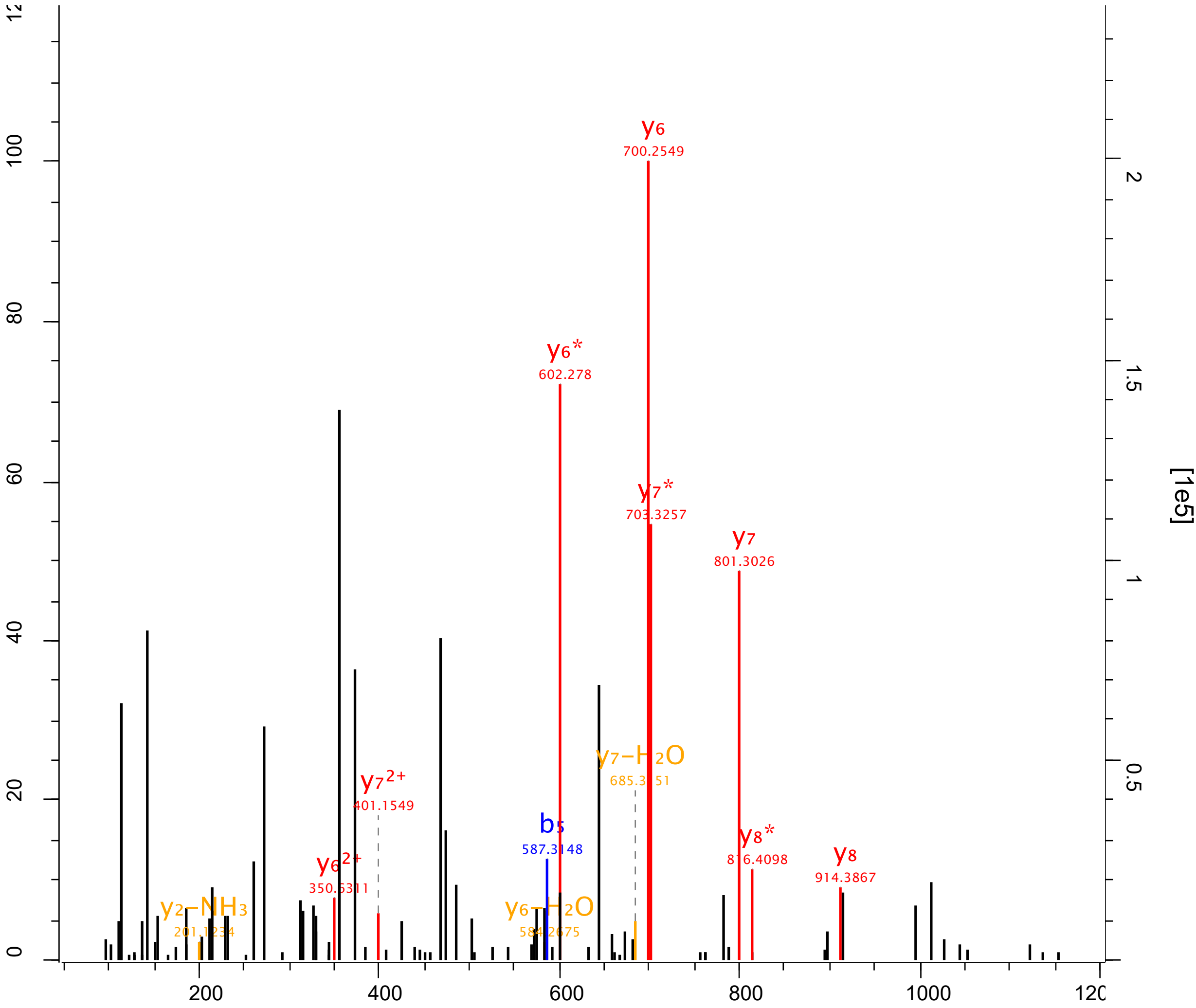

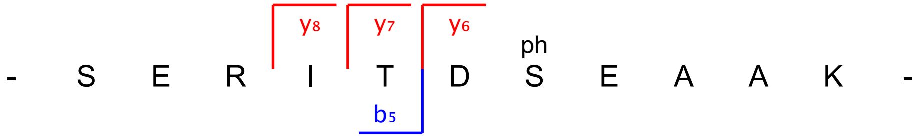

Raw file	Scan	Method	Score	m/z
sys_05_2	29072	FTMS; HCD	92.44	804.35

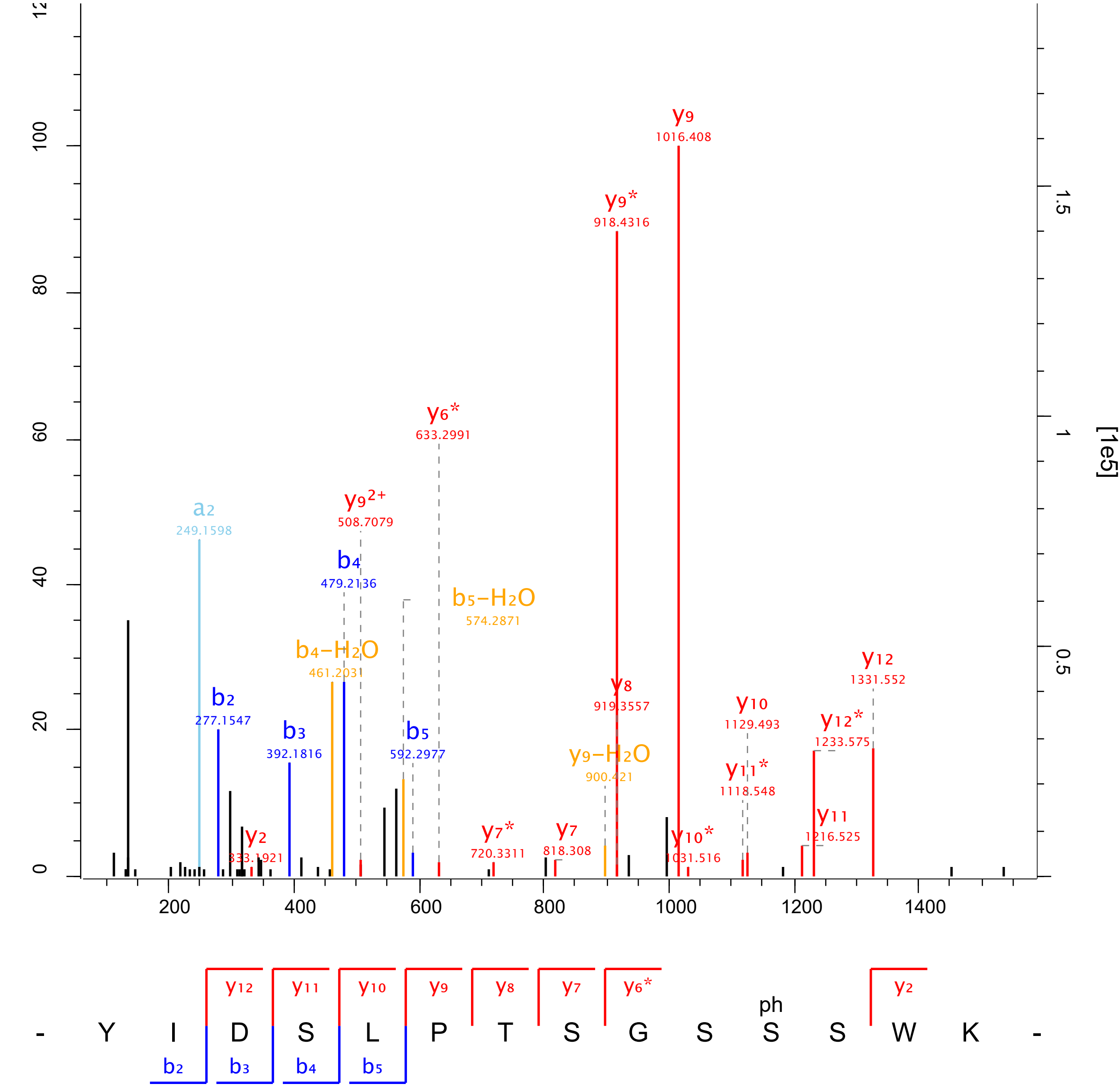

Raw file	Scan	Method	Score	m/z
sys_05_2	29132	FTMS; HCD	104.88	867.39

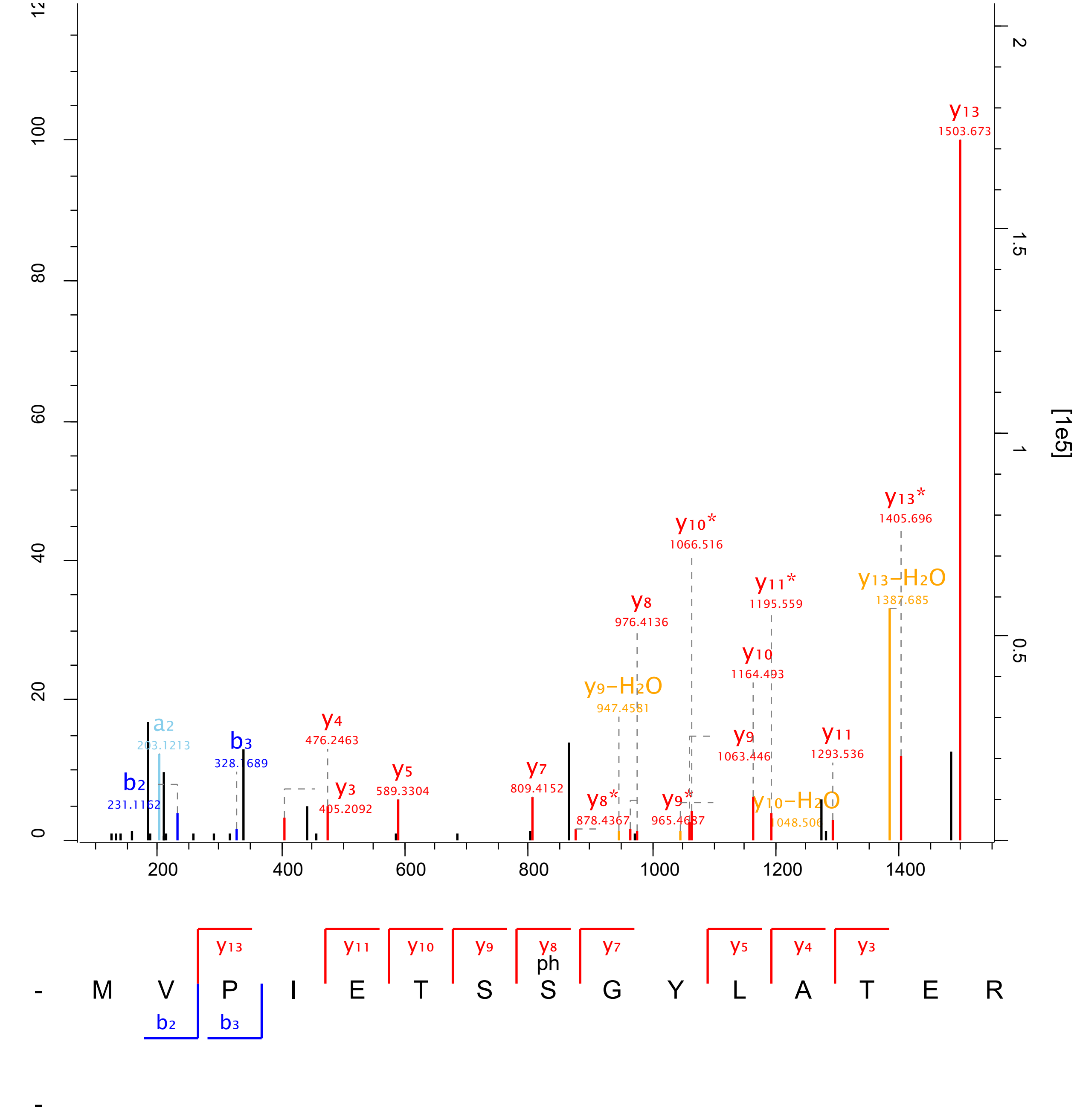

Raw file	Scan	Method	Score	m/z
sys_05_2	29141	FTMS; HCD	131.18	854.9

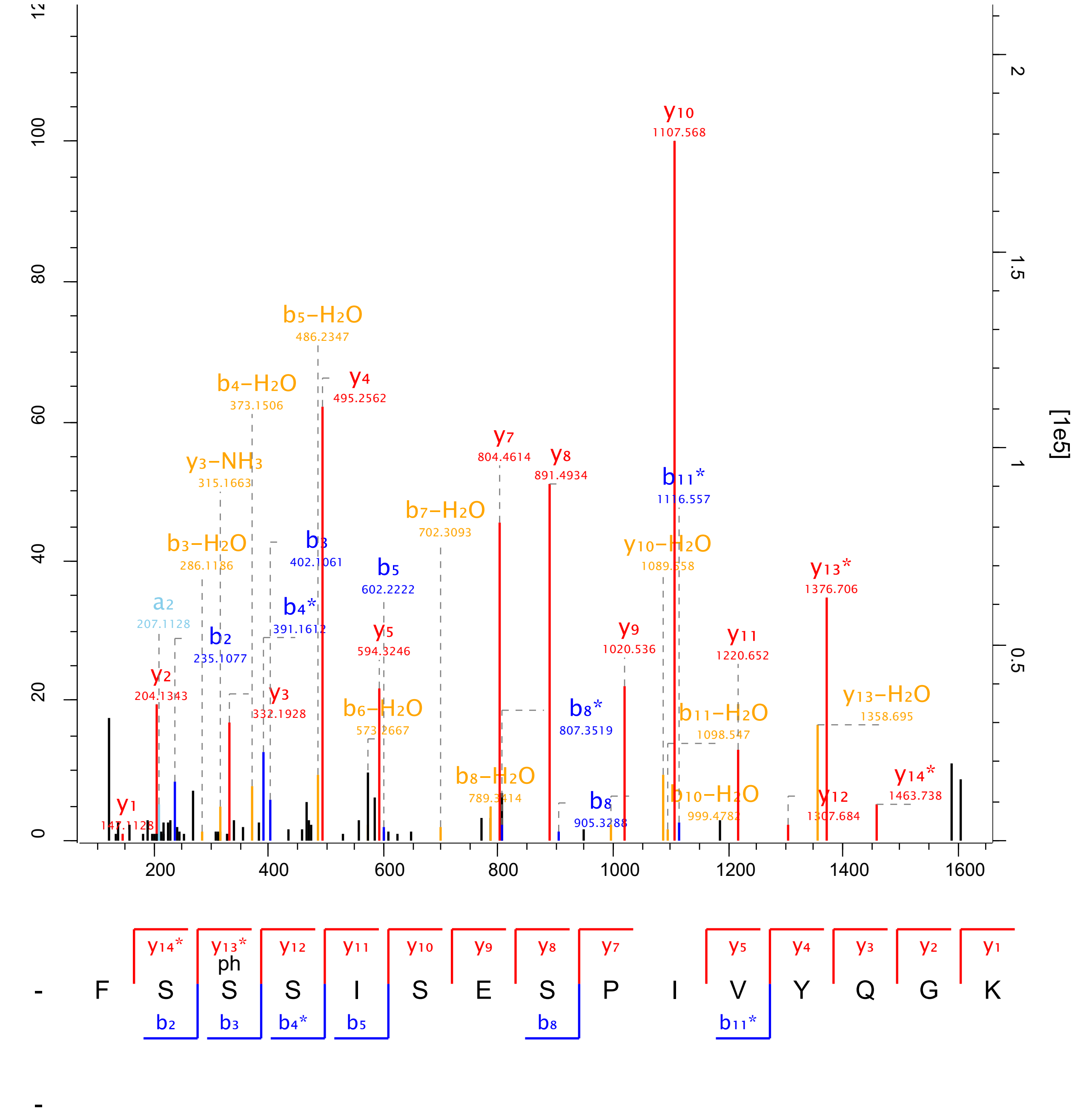

Raw file	Scan	Method	Score	m/z
sys_05_2	29684	FTMS; HCD	79.36	1089.5

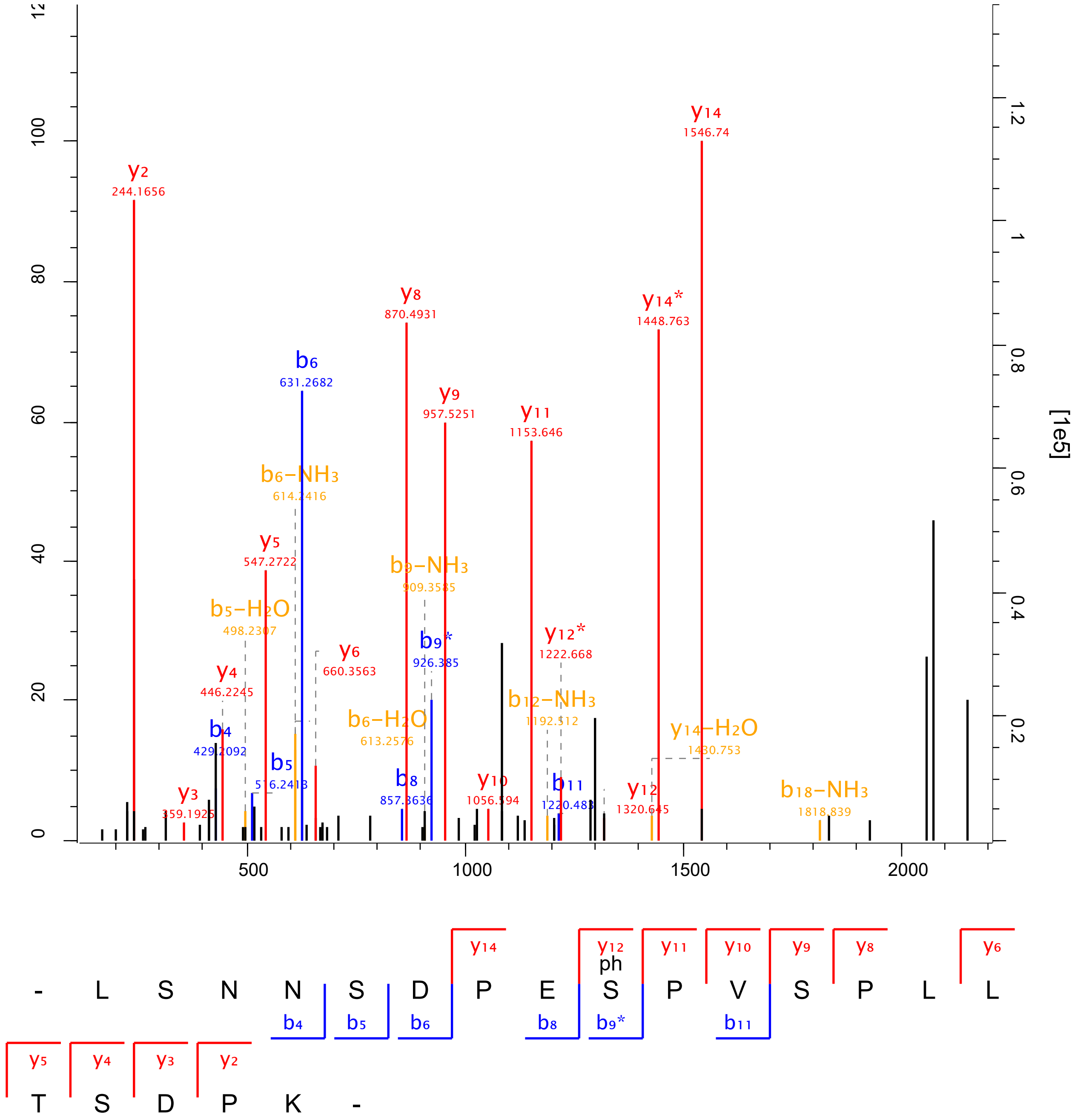

Raw file	Scan	Method	Score	m/z
sys_05_2	30131	FTMS; HCD	217.89	871.36

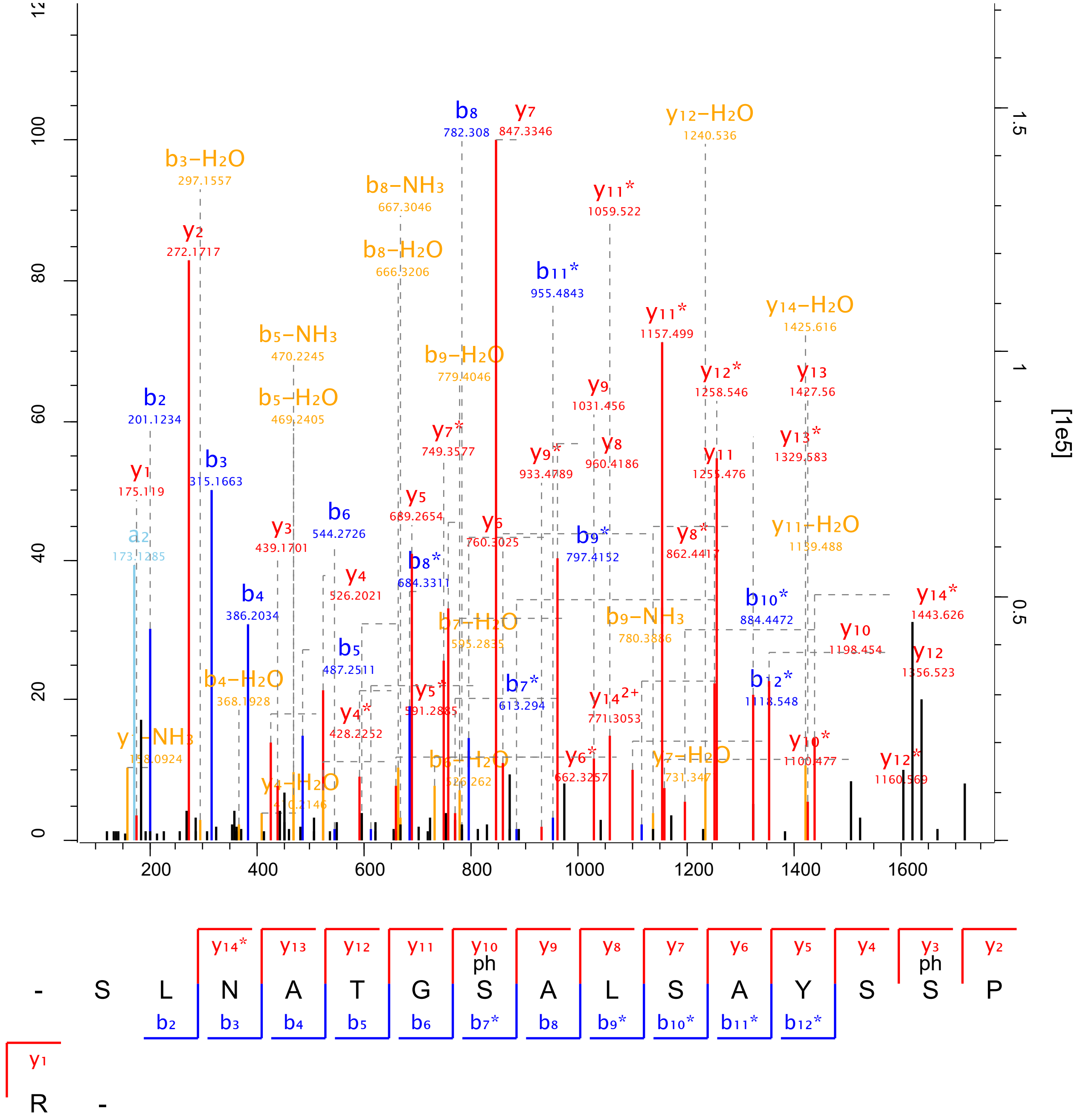

Raw file	Scan	Method	Score	m/z
sys_05_2	30162	FTMS; HCD	181.87	912.39

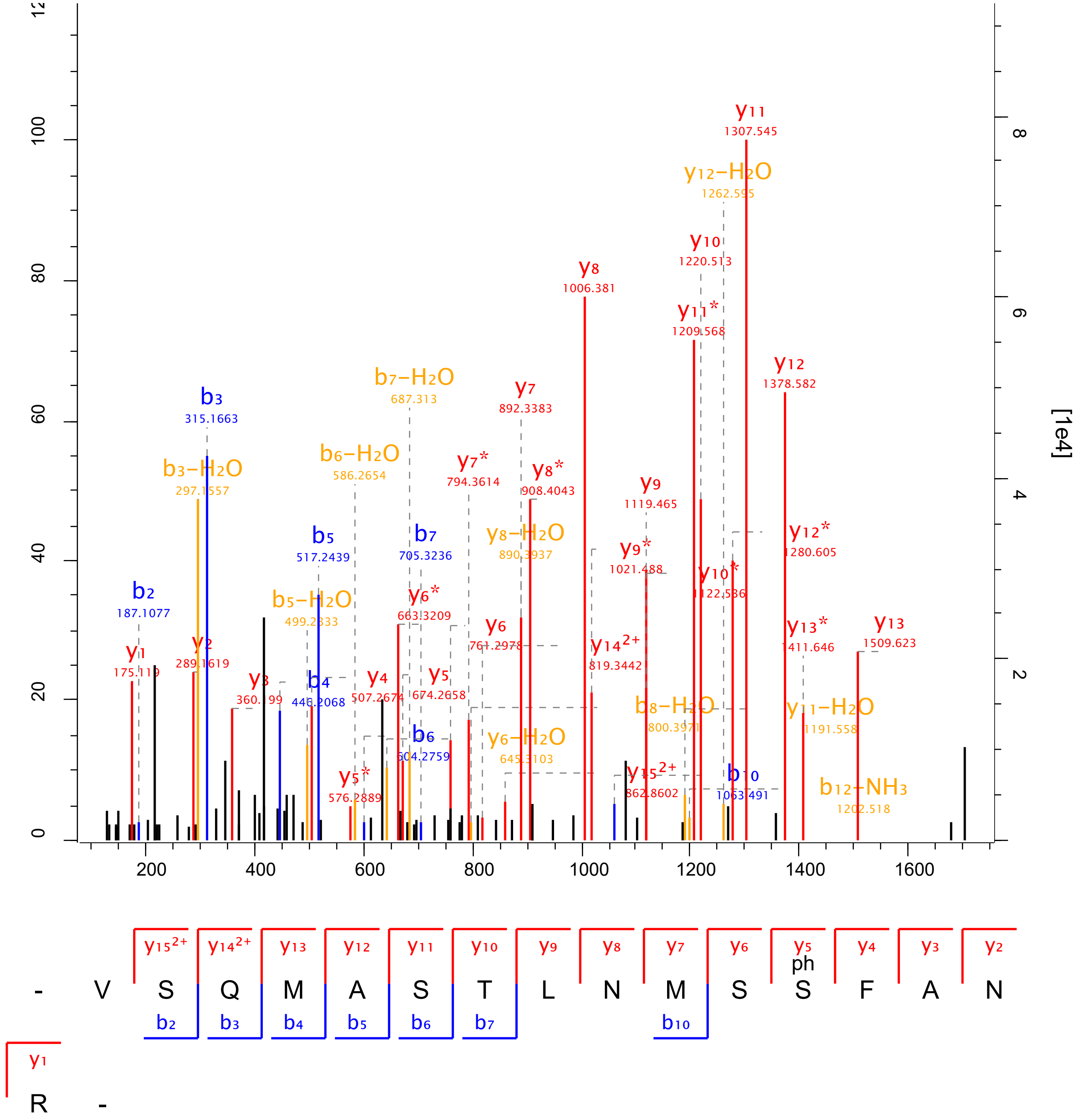

Raw file	Scan	Method	Score	m/z
sys_05_2	30283	FTMS; HCD	42.31	745.87

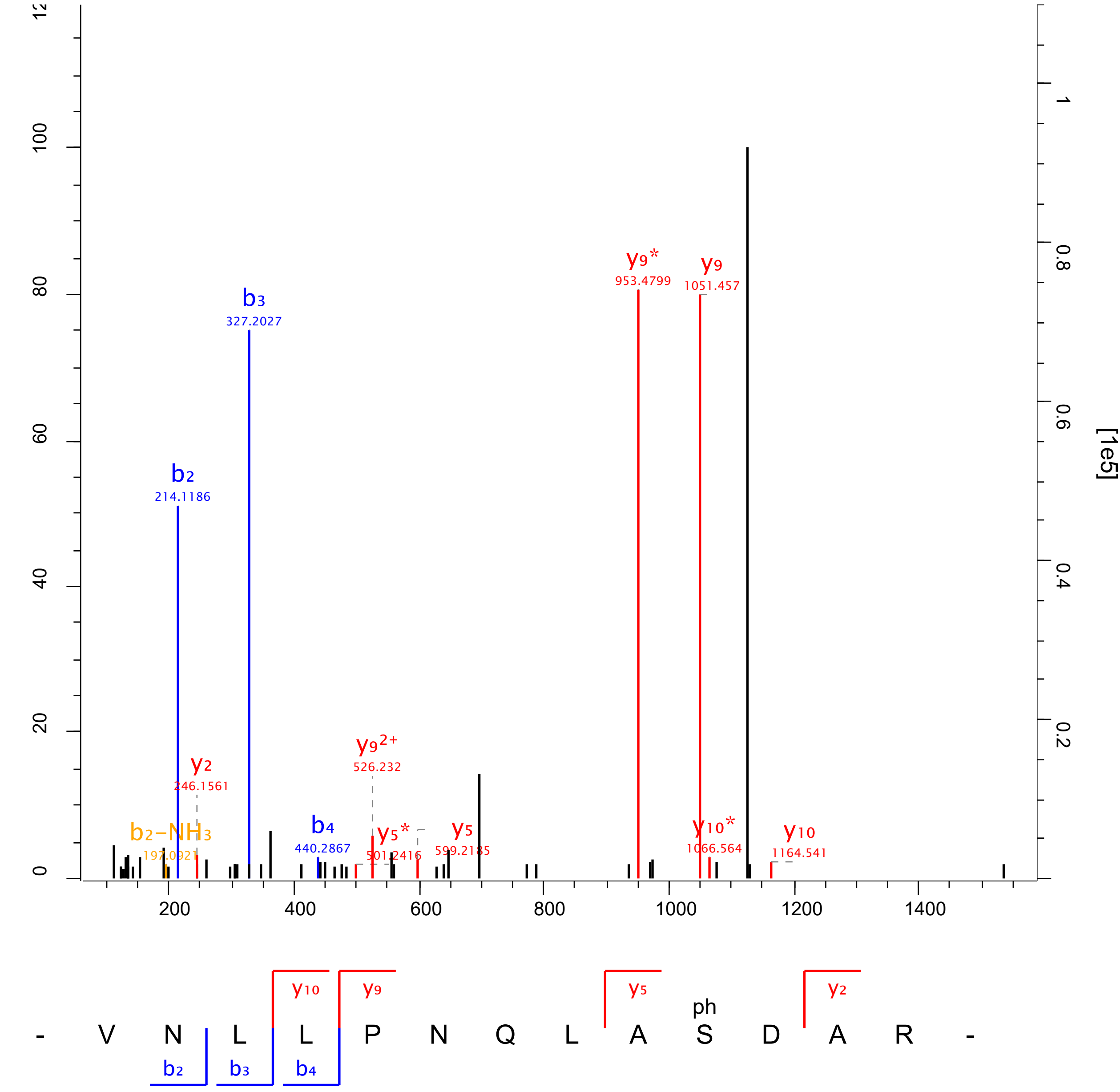

Raw file	Scan	Method	Score	m/z
sys_05_2	30398	FTMS; HCD	72.66	663.27

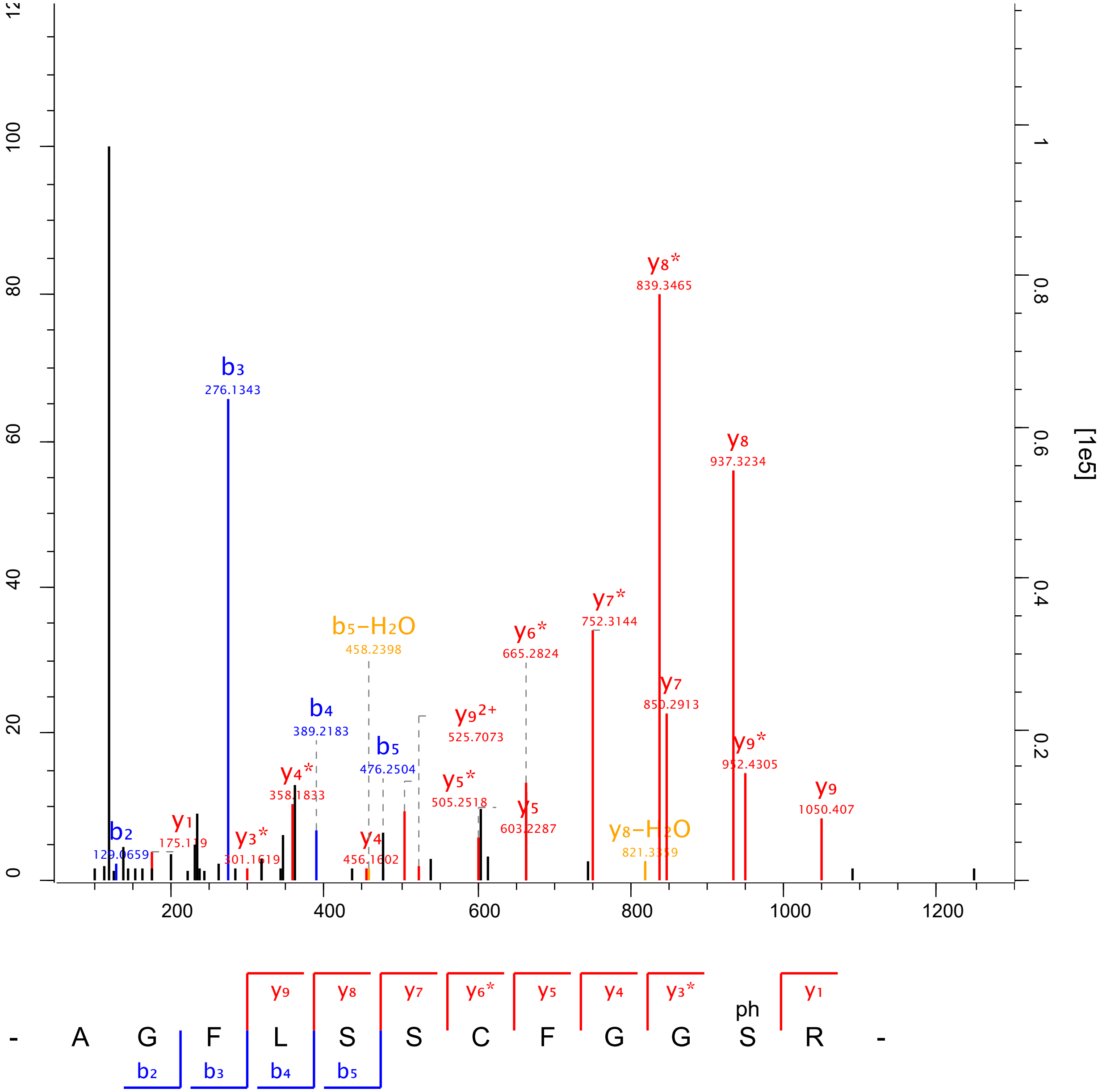

Raw file	Scan	Method	Score	m/z
sys_05_2	30514	FTMS; HCD	162.59	730.33

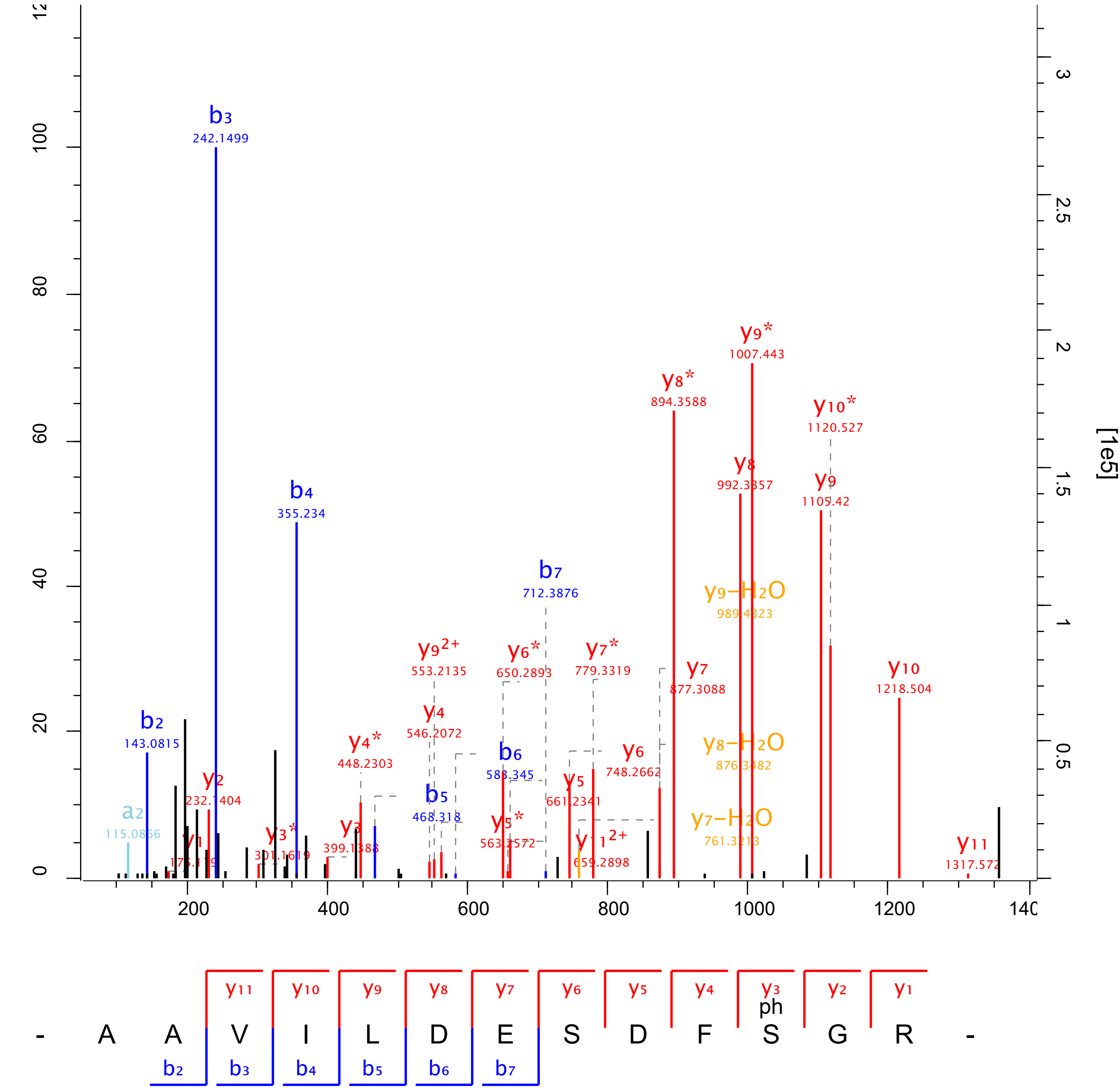

Raw file	Scan	Method	Score	m/z
sys_05_2	30700	FTMS; HCD	52.58	635.79

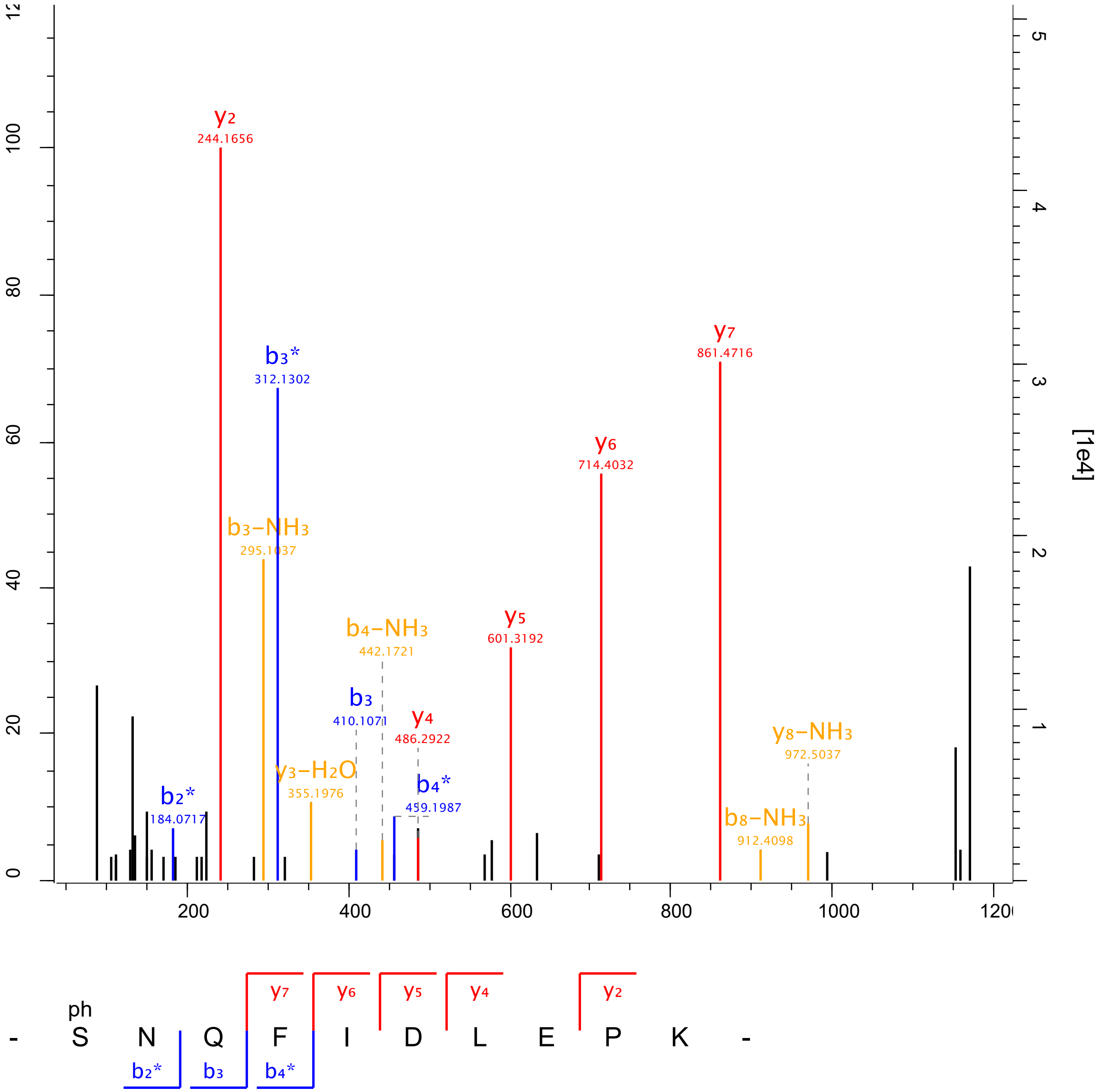

Raw file	Scan	Method	Score	m/z
sys_05_2	30743	FTMS; HCD	89.3	574.78

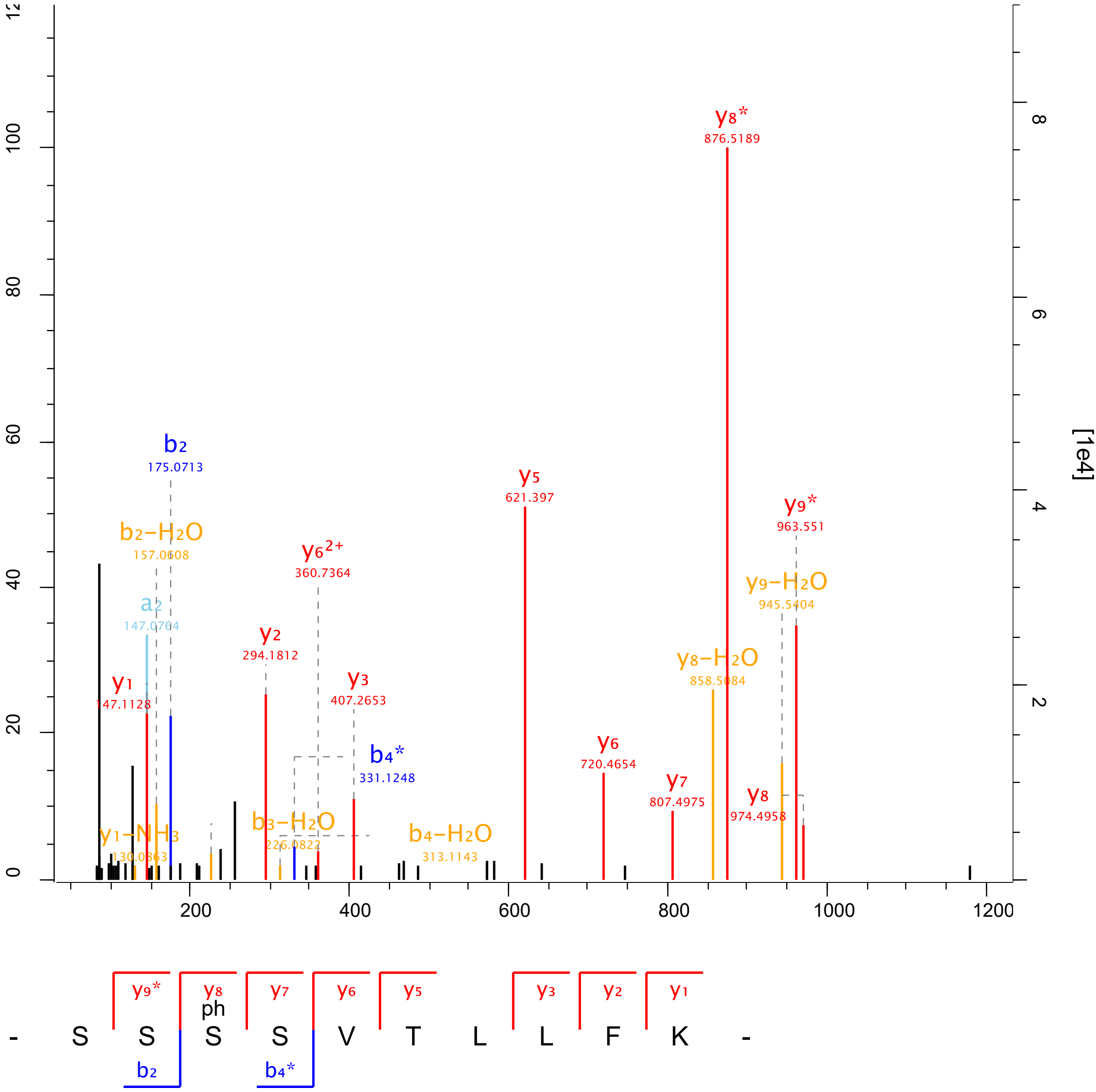

Raw file	Scan	Method	Score	m/z
sys_05_2	30774	FTMS; HCD	84.95	931.41

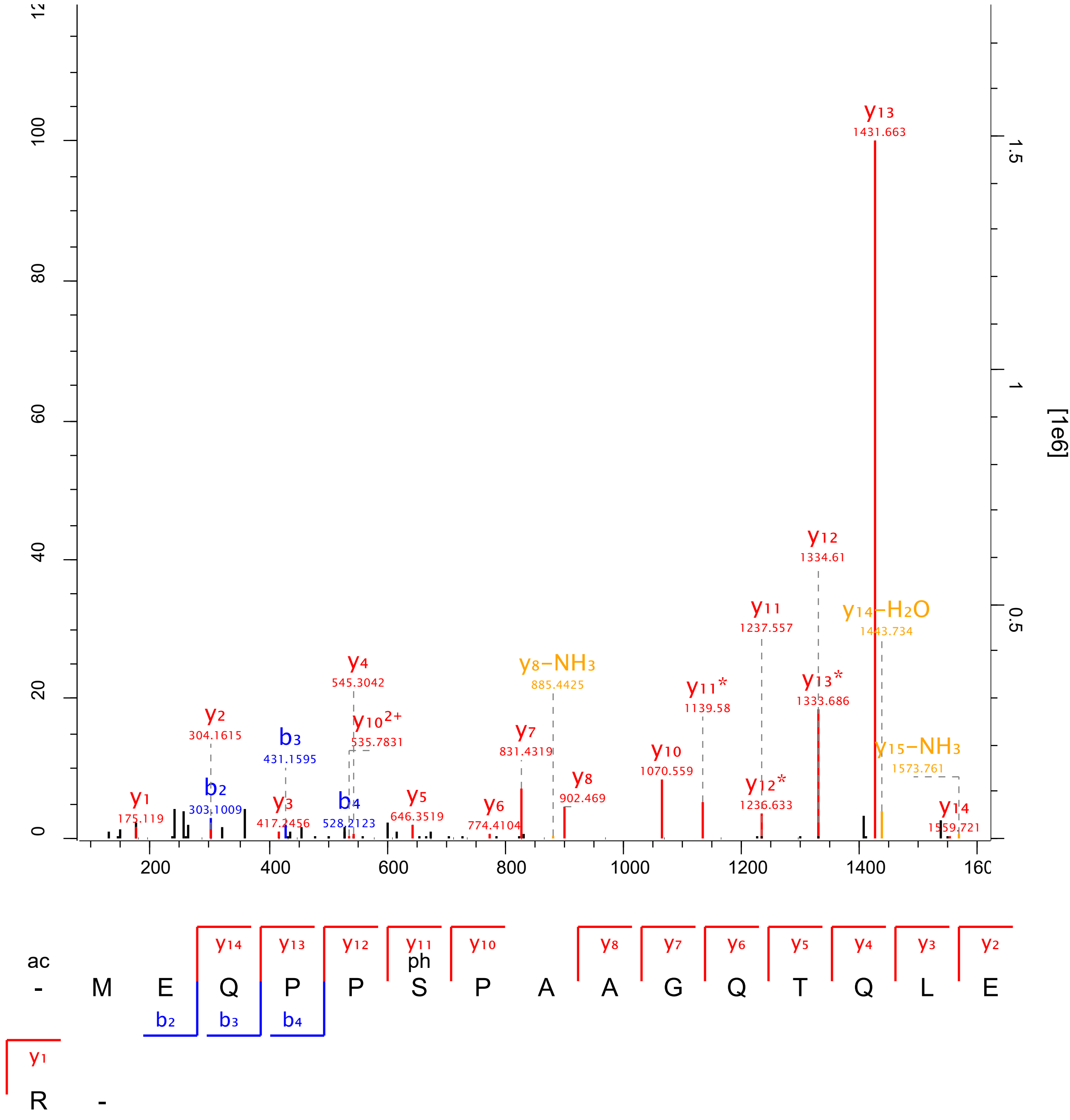

Raw file	Scan	Method	Score	m/z
sys_05_2	3081	FTMS; HCD	115.91	628.27

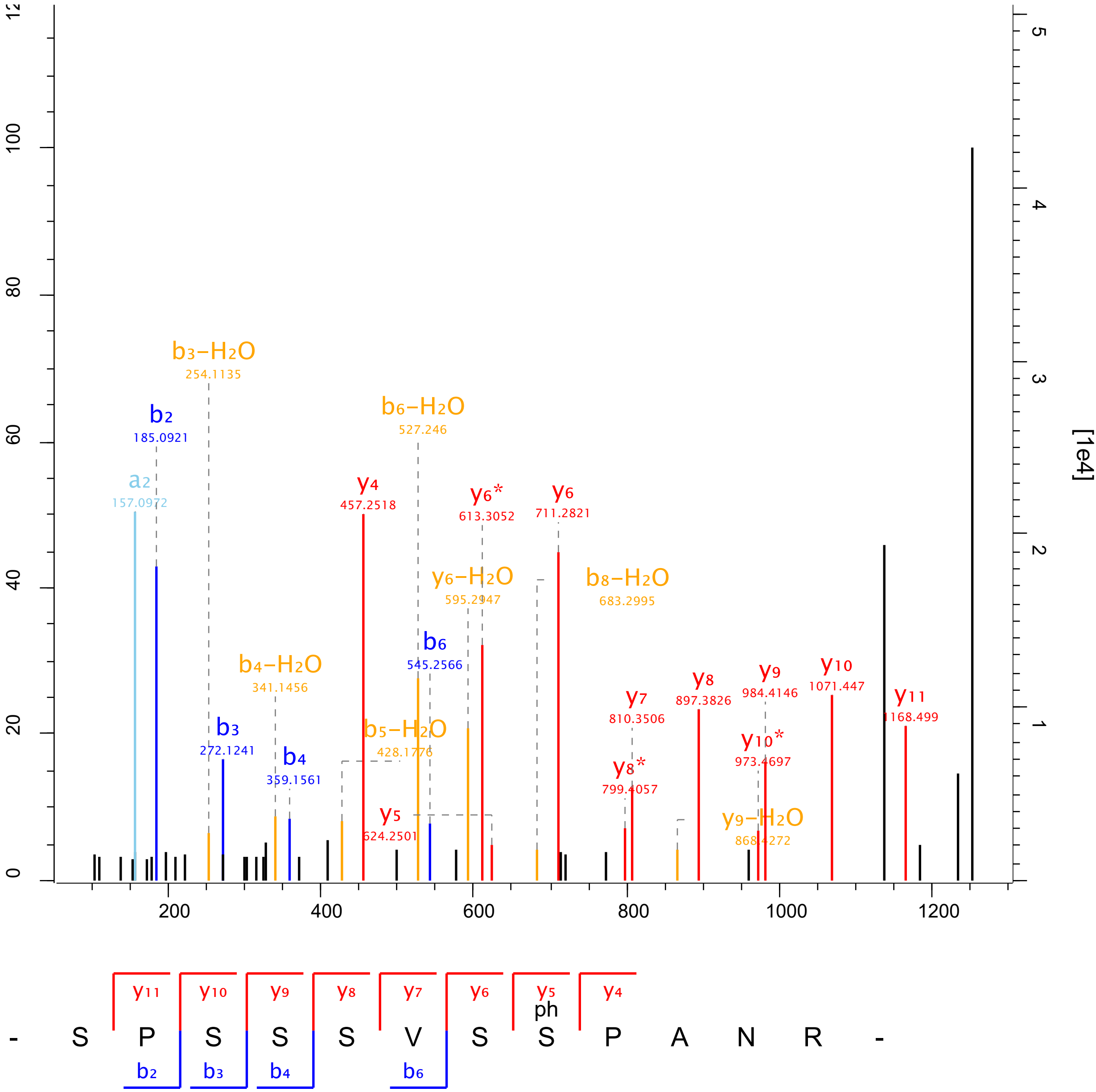

Raw file	Scan	Method	Score	m/z
sys_05_2	30905	FTMS; HCD	54.09	779.89

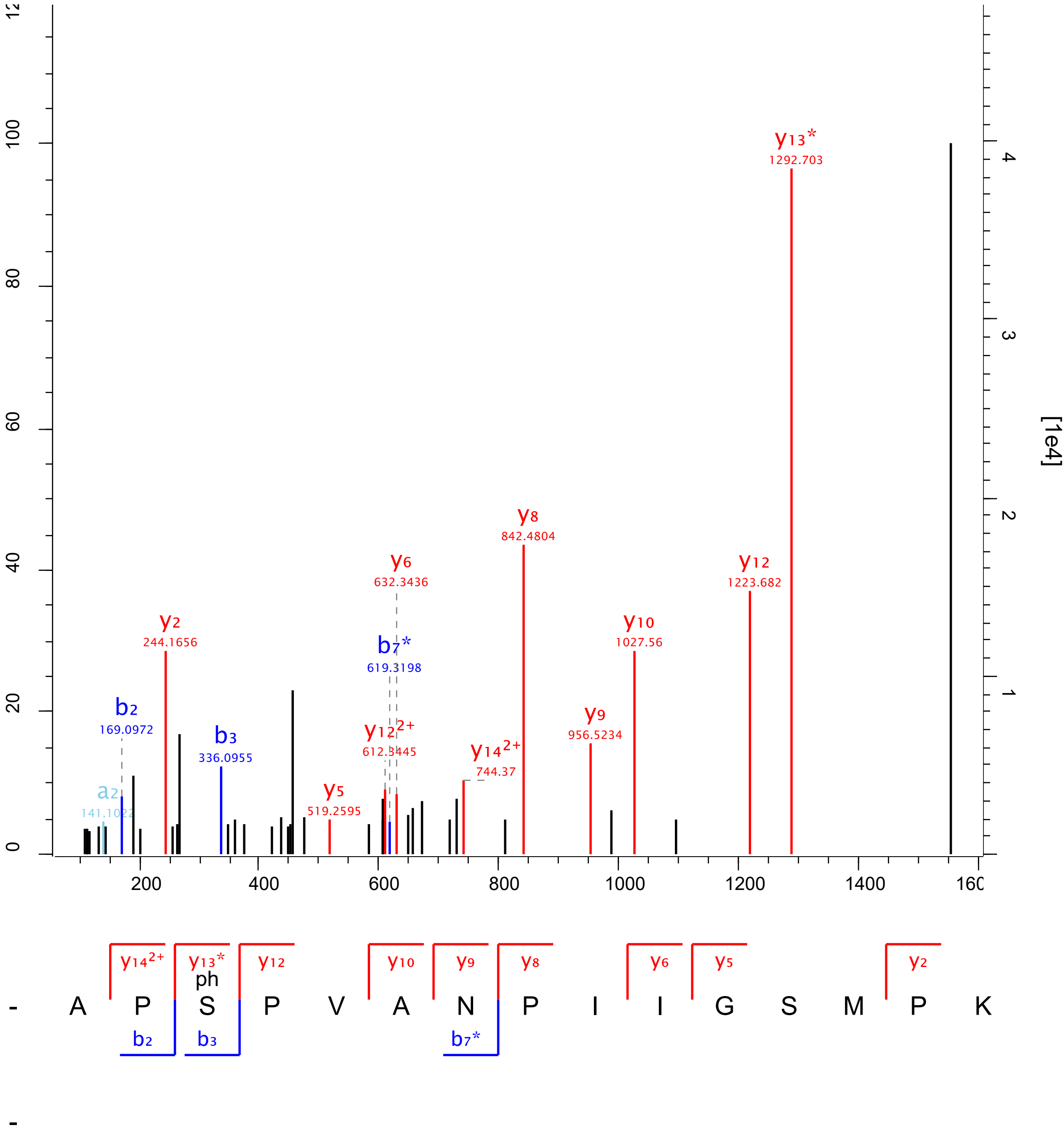

Raw file	Scan	Method	Score	m/z
sys_05_2	31025	FTMS; HCD	248.67	698.82

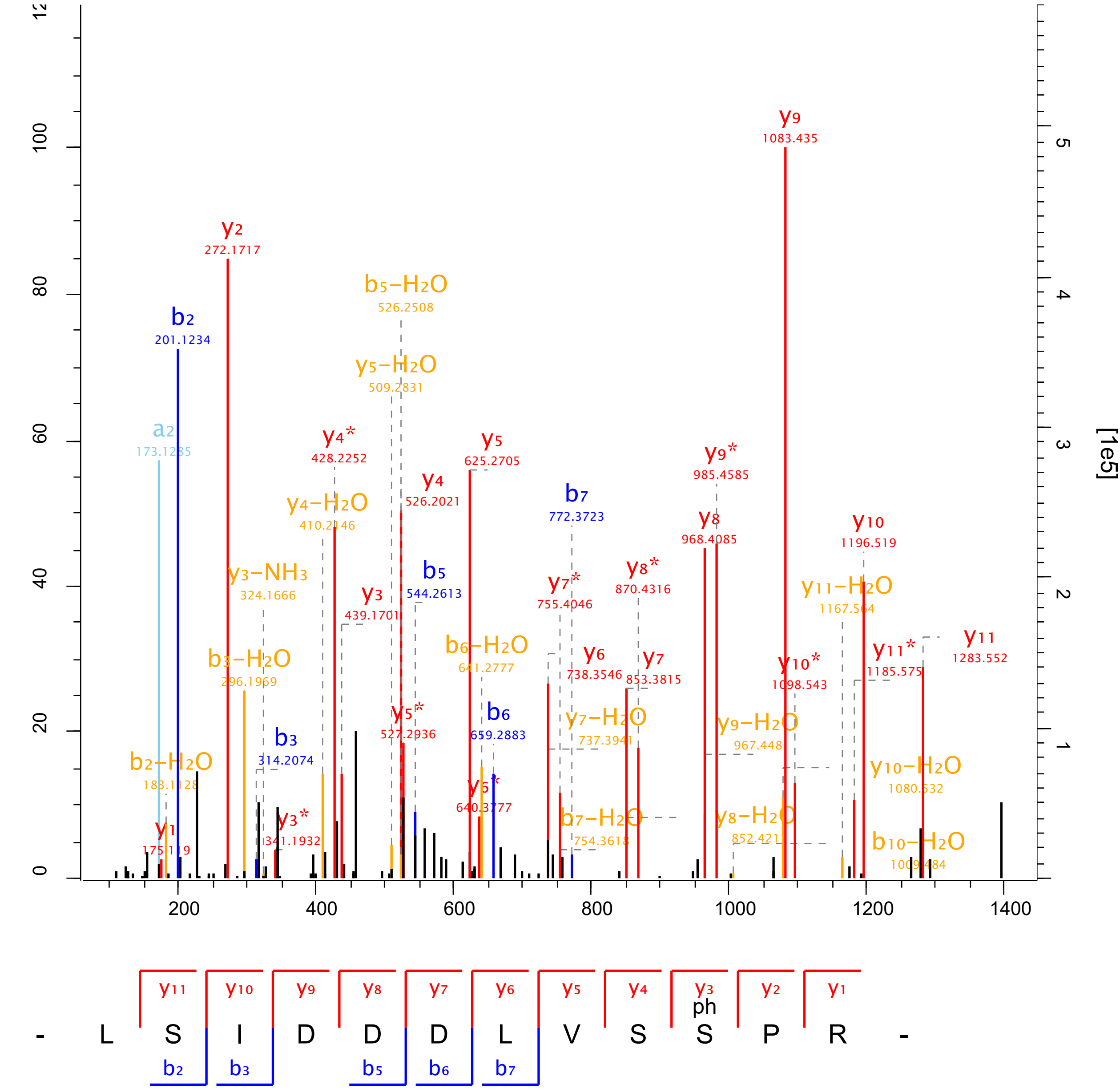

Raw file	Scan	Method	Score	m/z
sys_05_2	31109	FTMS; HCD	84.75	673.81

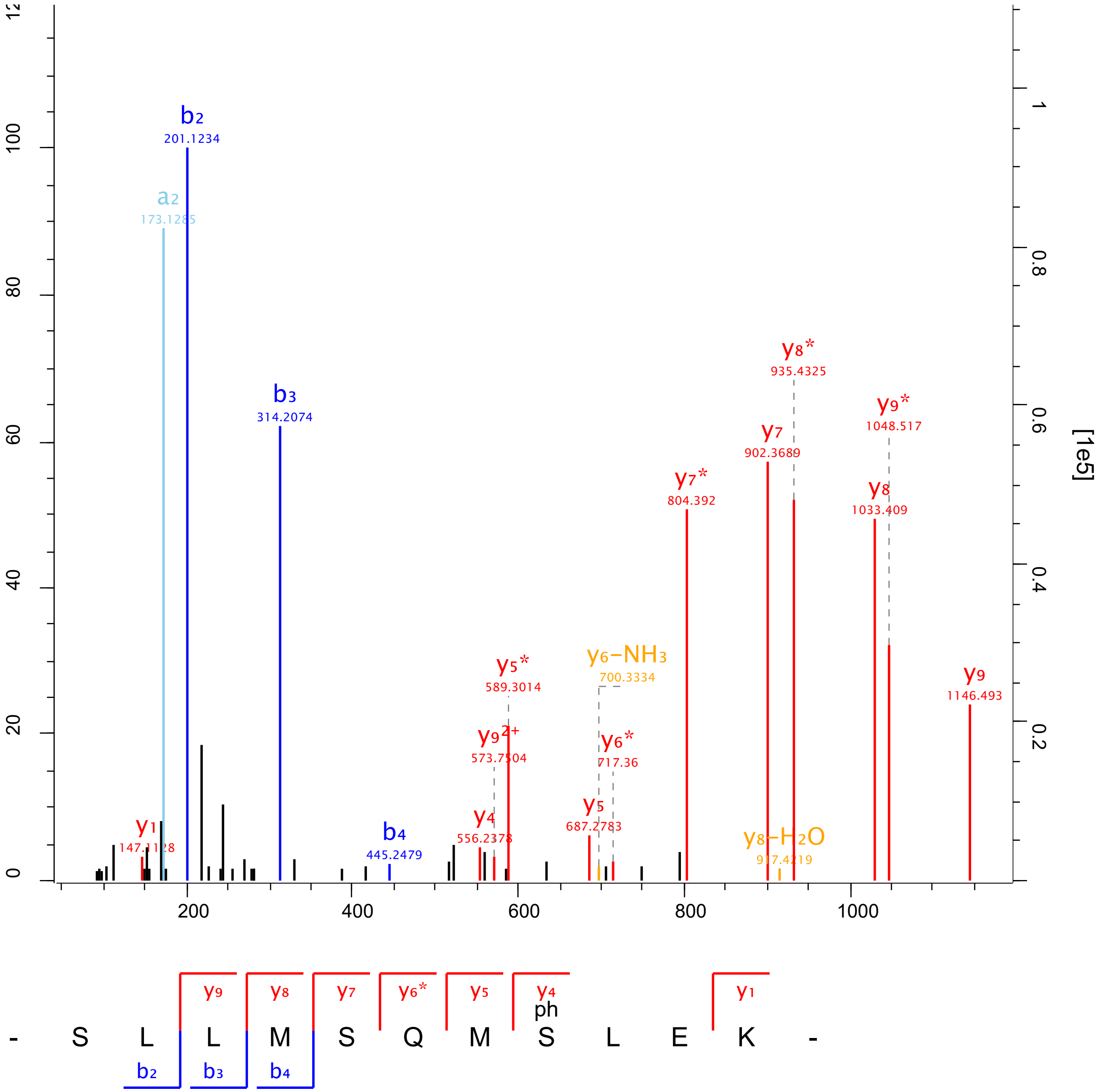

Raw file	Scan	Method	Score	m/z
sys_05_2	31487	FTMS; HCD	109.79	667.26

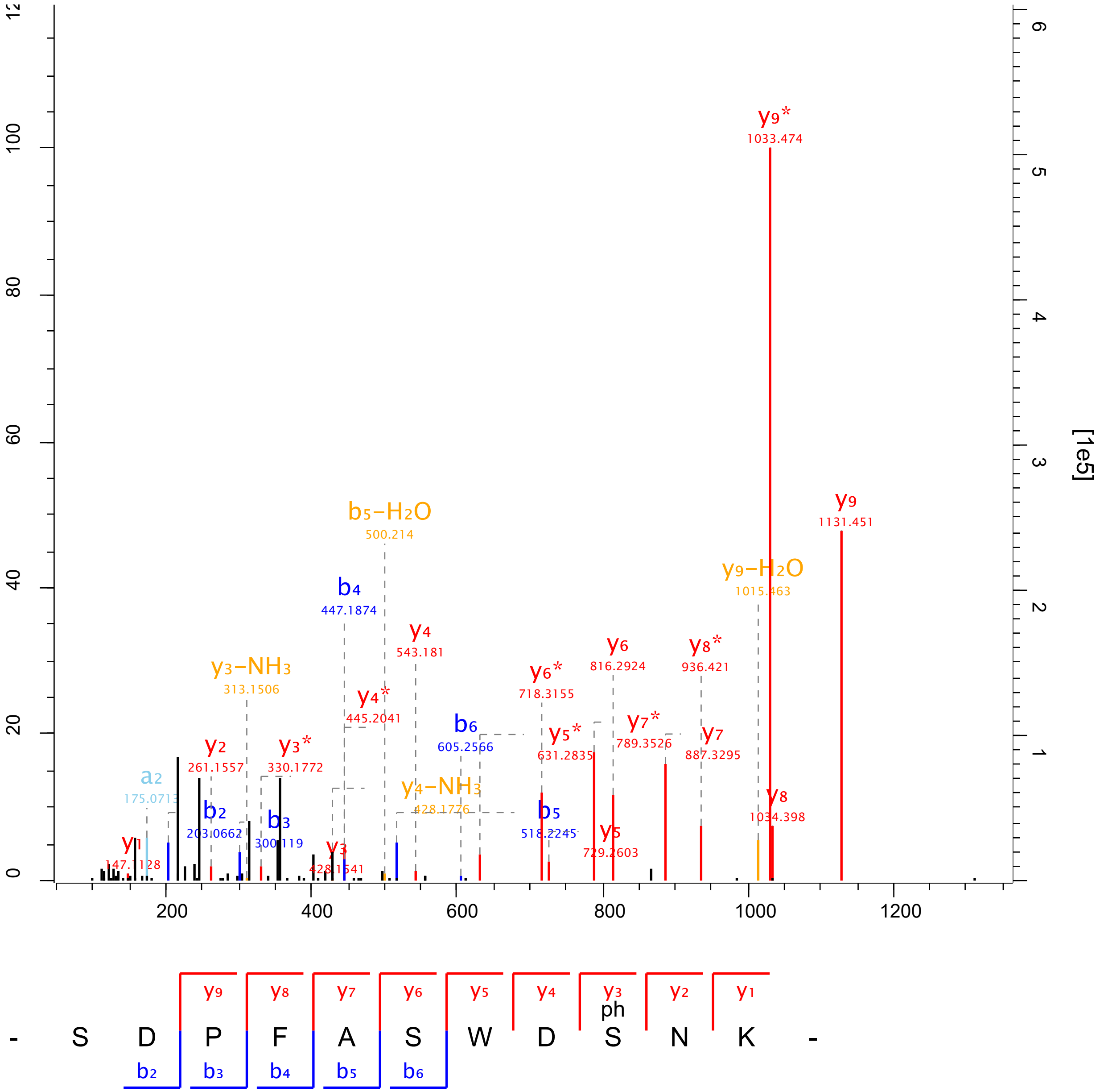

Raw file	Scan	Method	Score	m/z
sys_05_2	31591	FTMS; HCD	47.55	732.83

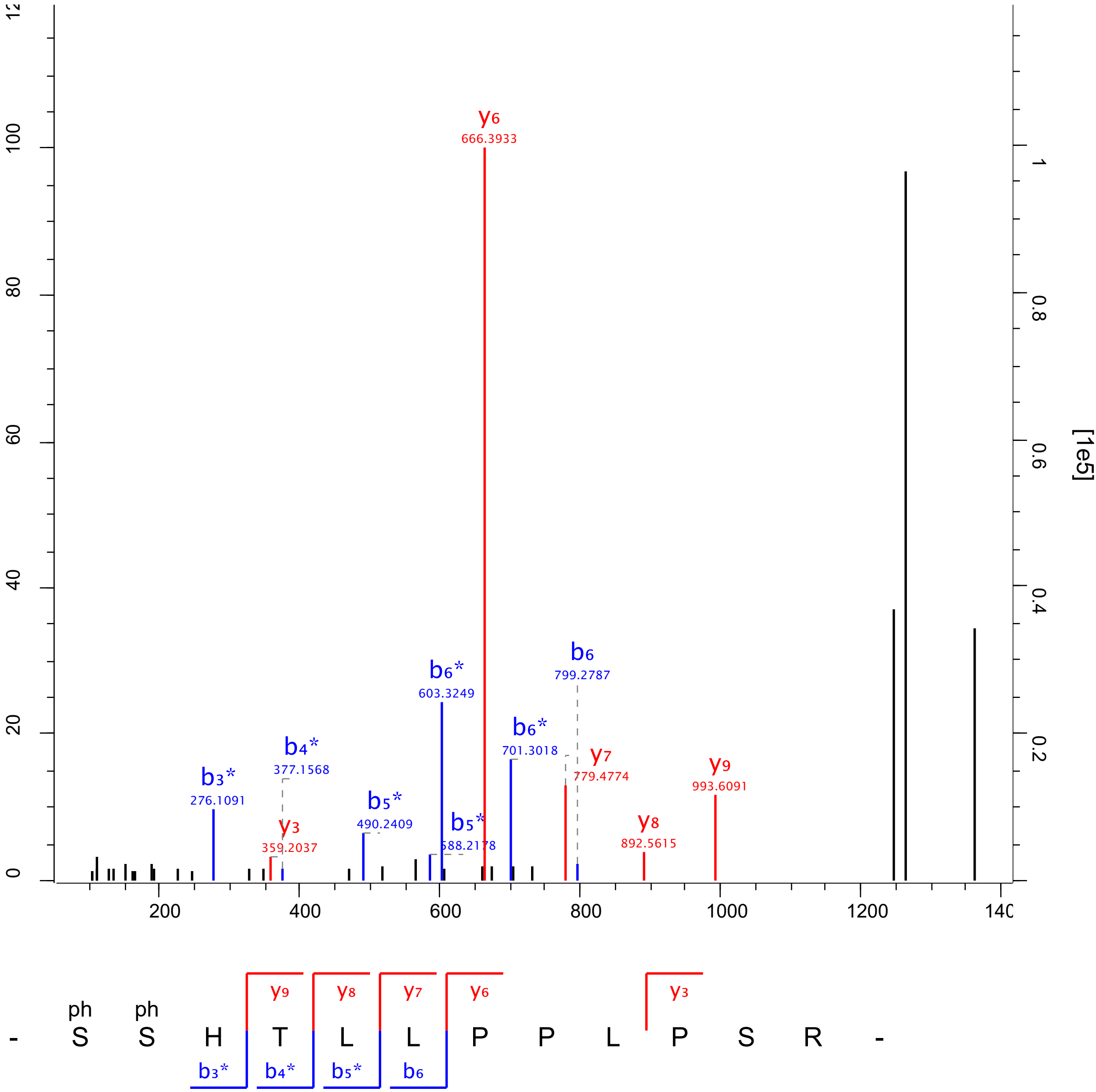

Raw file	Scan	Method	Score	m/z
sys_05_2	31802	FTMS; HCD	66.02	664.3

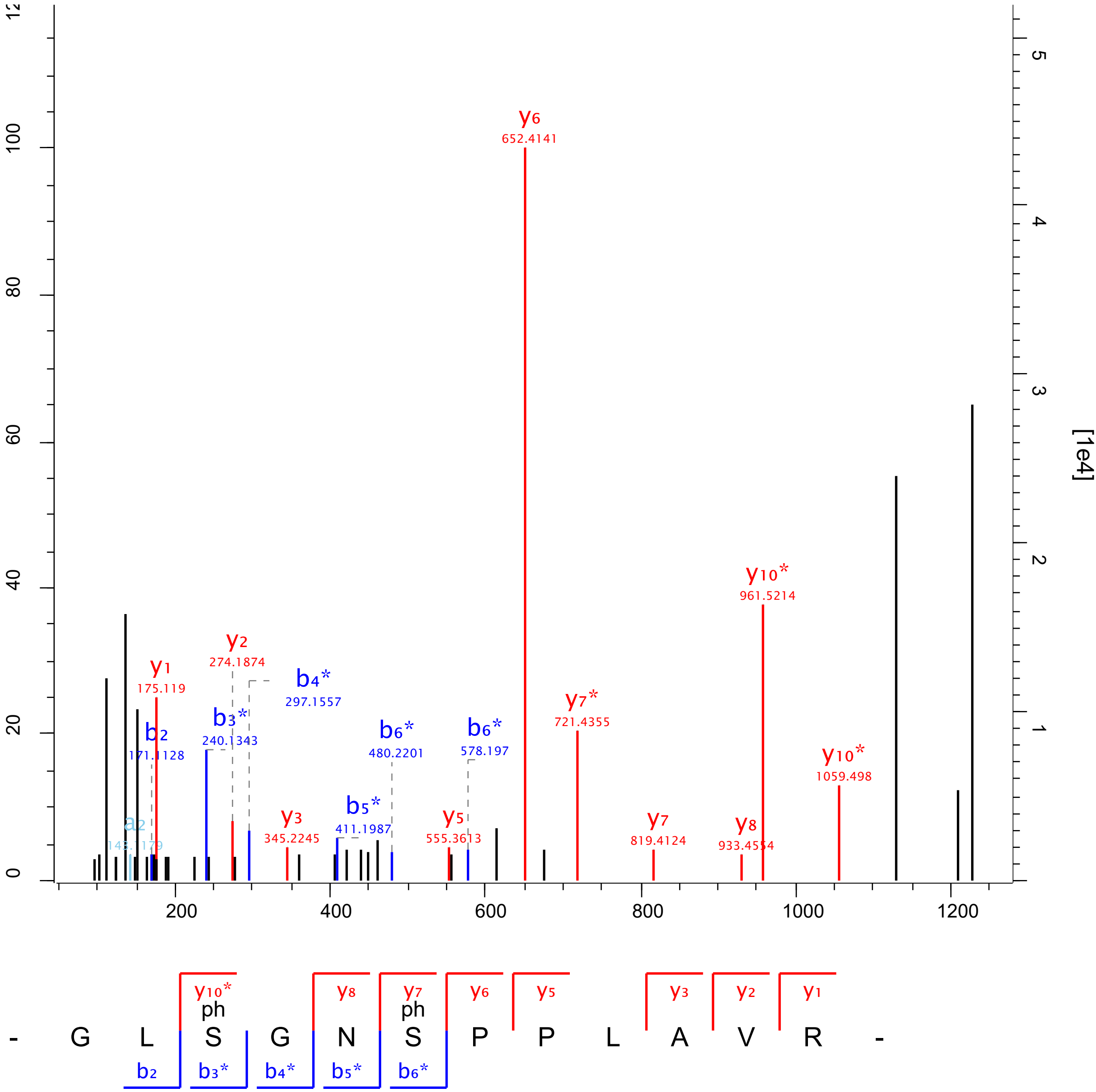

Supplement: Supplementary Figure S2-4 [file 143488_2_supp_337932_ps5hgk.pdf]
