# Supplementary material for: The Systemin Signaling Cascade As Derived from Time Course Analyses of the Systemin-responsive Phosphoproteome
Source: Mol Cell Proteomics. 2019 May 28;18(8):1526–42. doi: 10.1074/mcp.RA119.001367 (PMC6683004; doi:10.1074/mcp.RA119.001367)

Supplementary Figure 2: Representative annotated spectra of identified phosphopeptides under systemin,A17 and water treatment as exported from MaxQuant.

Raw file	Scan	Method	Score	m/z
sys_05_2	31849	FTMS; HCD	160.81	696.34

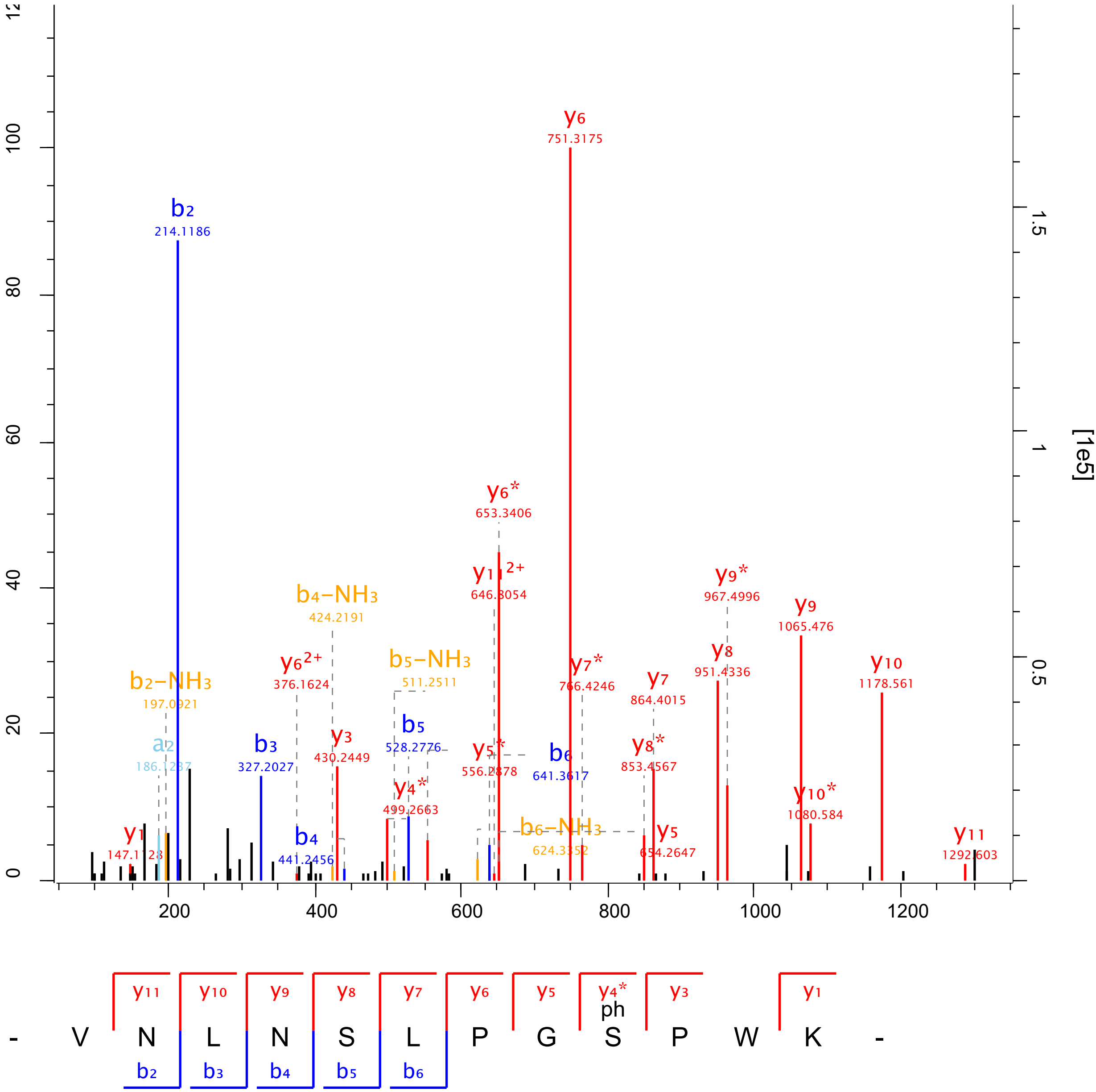

Raw file	Scan	Method	Score	m/z
sys_05_2	31871	FTMS; HCD	62.2	813.86

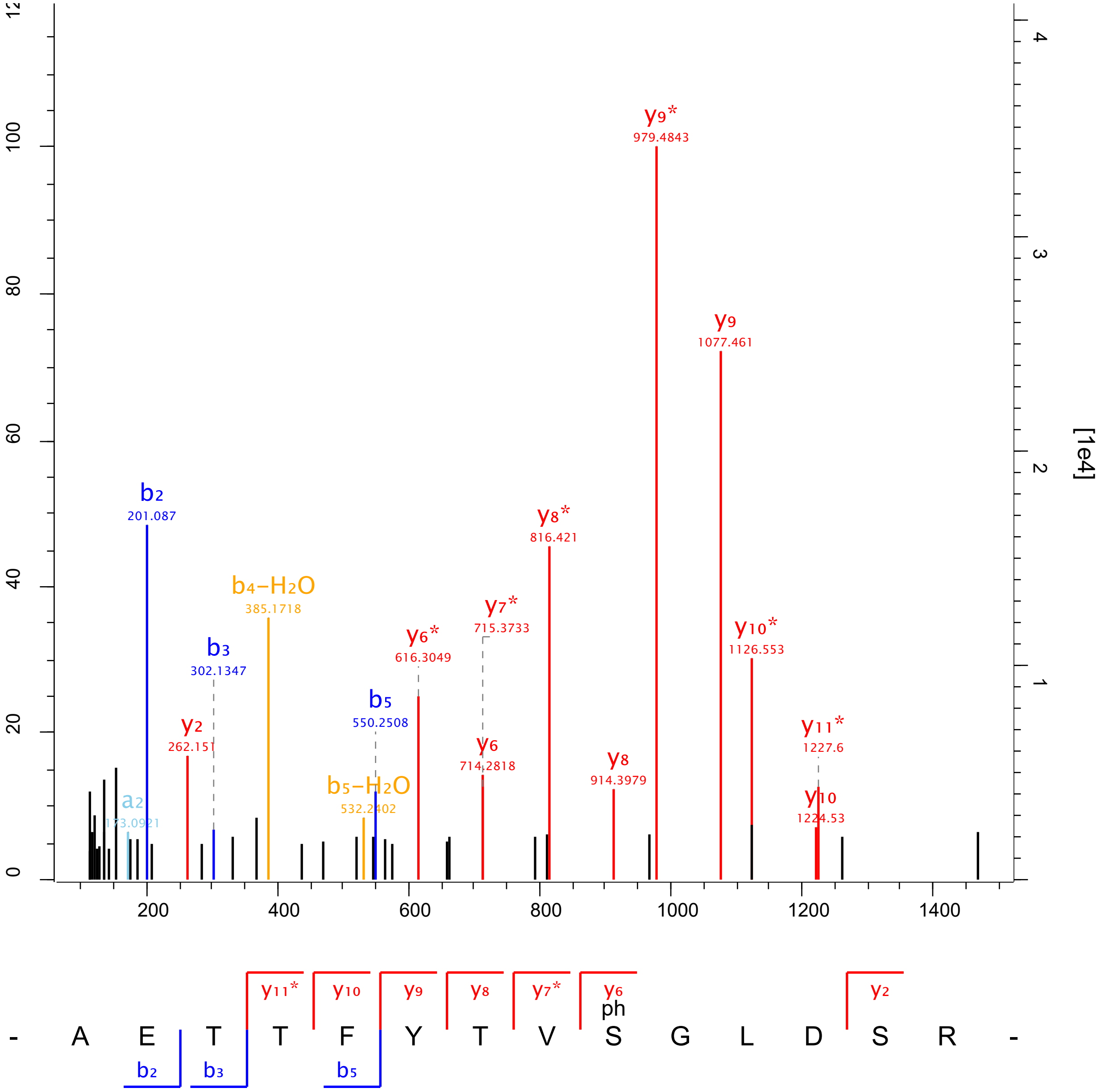

Raw file	Scan	Method	Score	m/z
sys_05_2	32079	FTMS; HCD	72.68	677.98

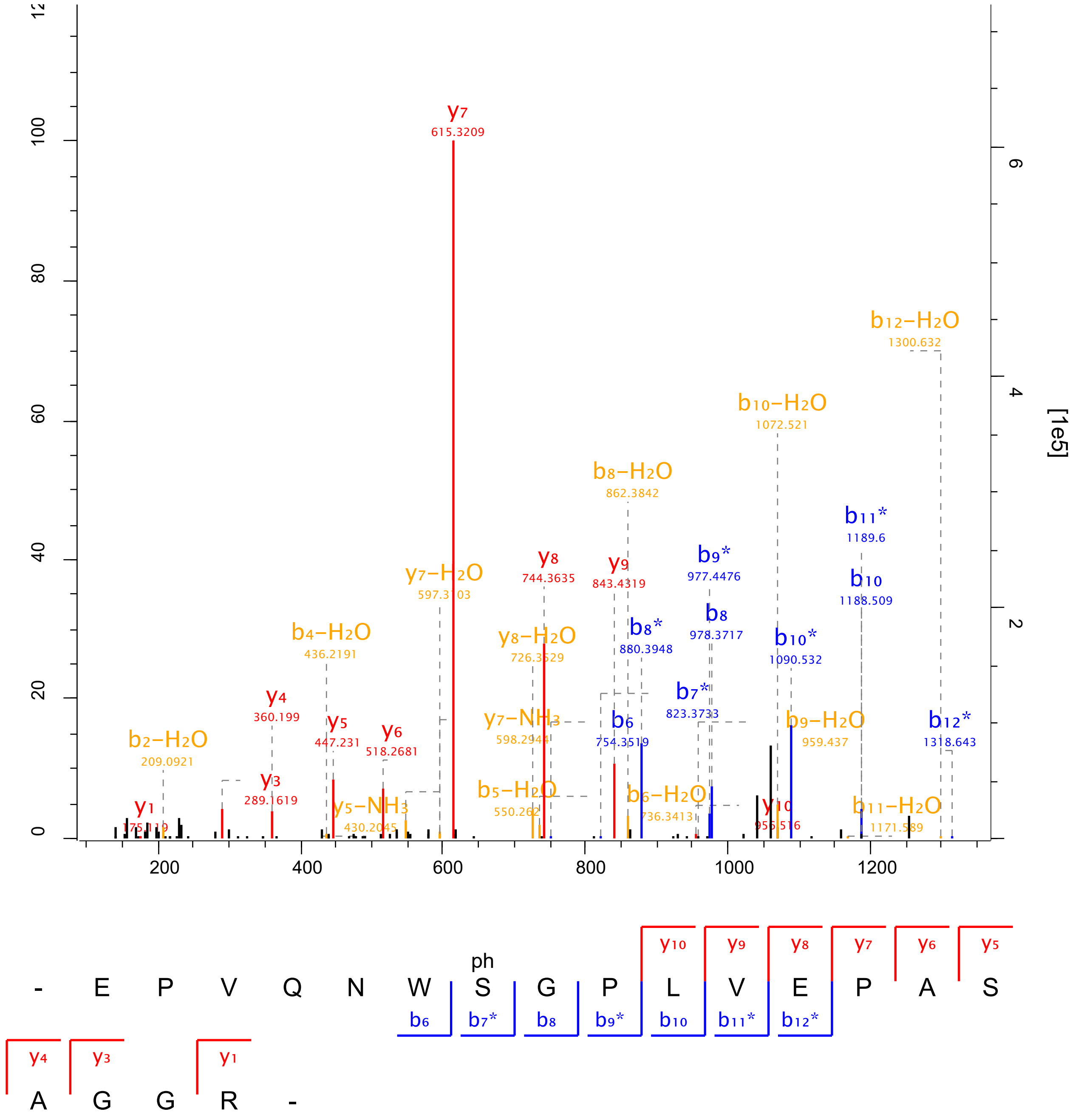

Raw file	Scan	Method	Score	m/z
sys_05_2	32203	FTMS; HCD	223.93	802.34

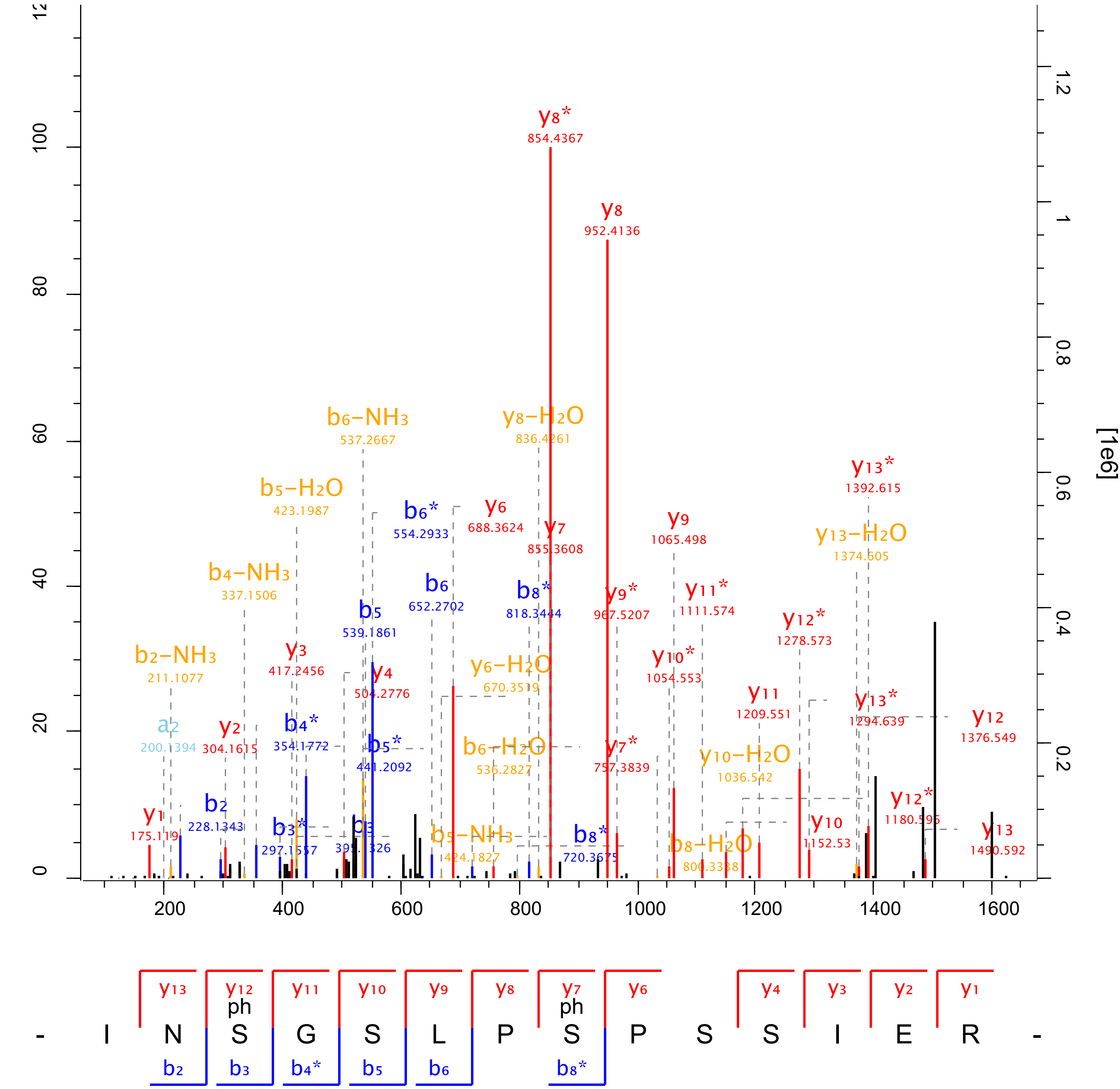

Raw file	Scan	Method	Score	m/z
sys_05_2	32263	FTMS; HCD	215.51	722.33

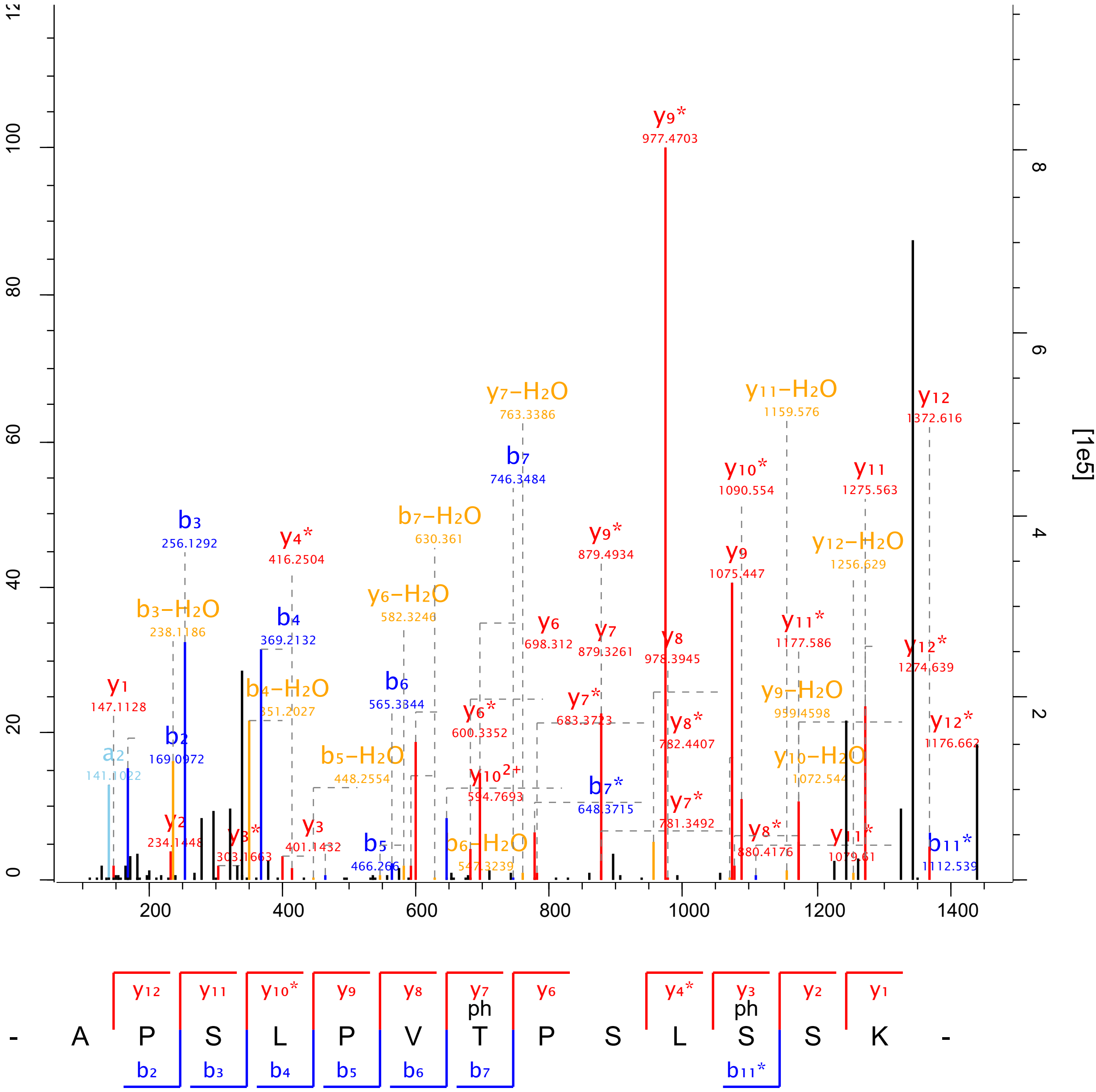

Raw file	Scan	Method	Score	m/z
sys_05_2	32348	FTMS; HCD	125.41	719.82

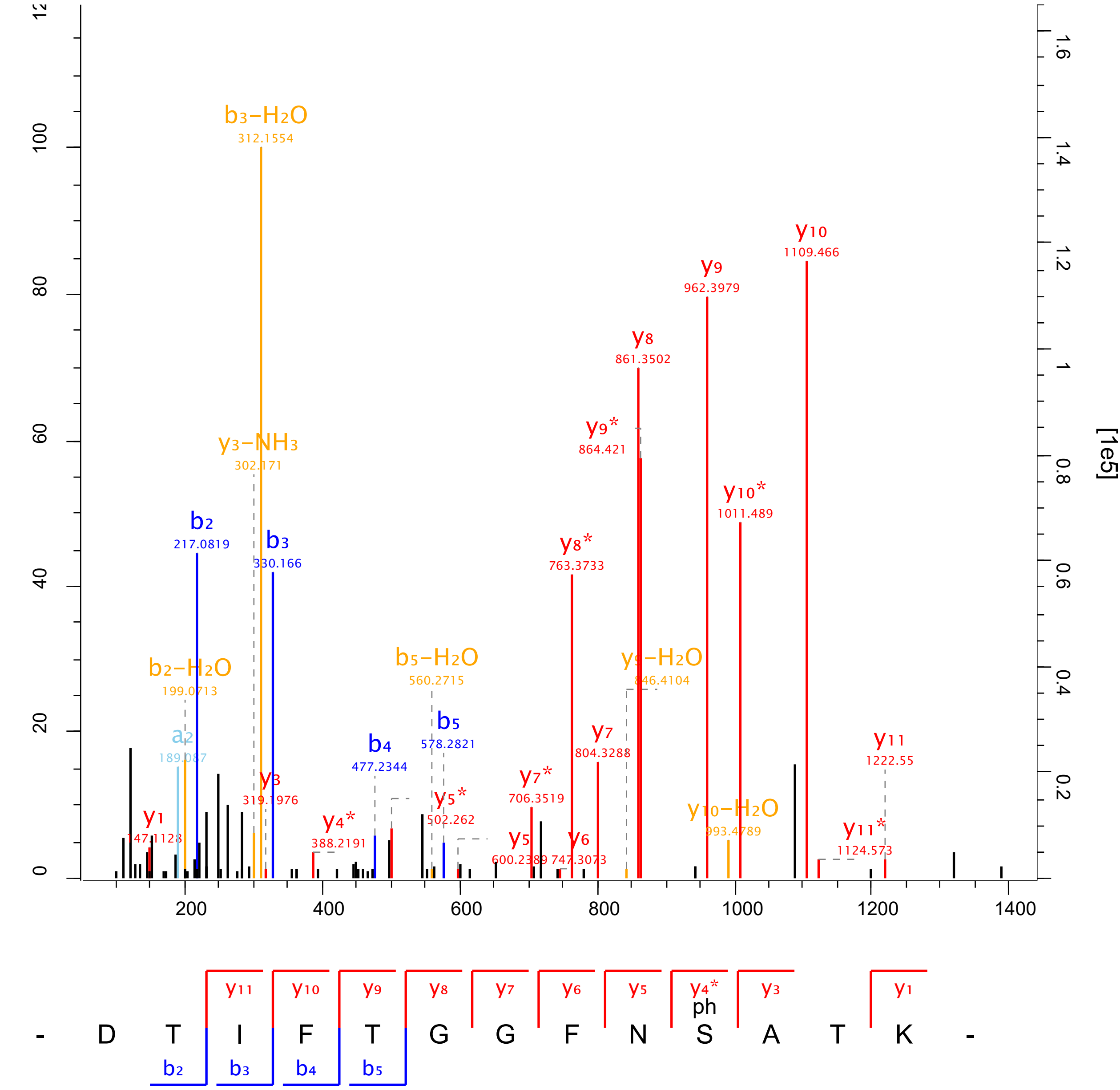

Raw file	Scan	Method	Score	m/z
sys_05_2	32375	FTMS; HCD	44.44	696.81

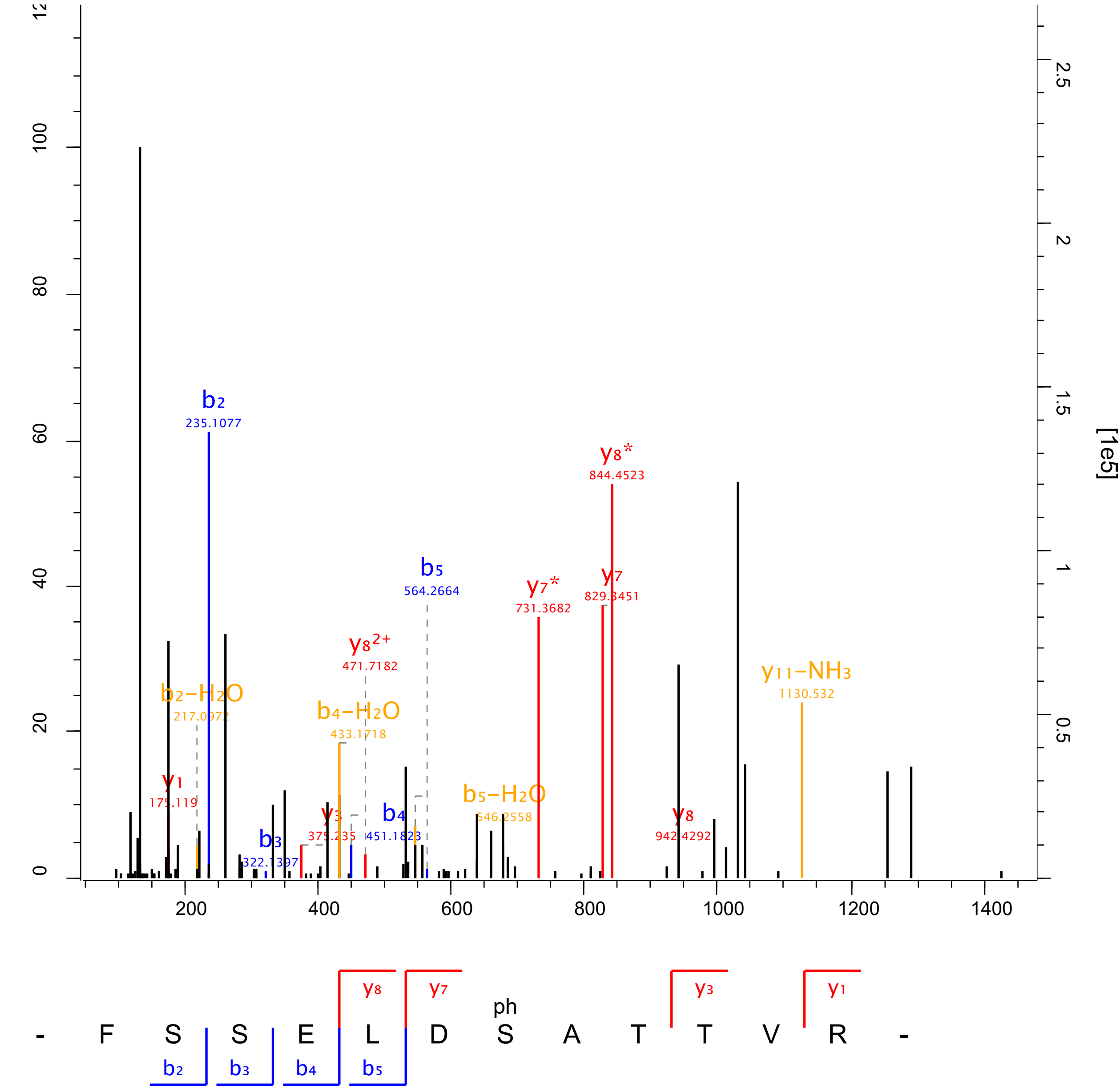

Raw file	Scan	Method	Score	m/z
sys_05_2	32652	FTMS; HCD	65.02	958.92

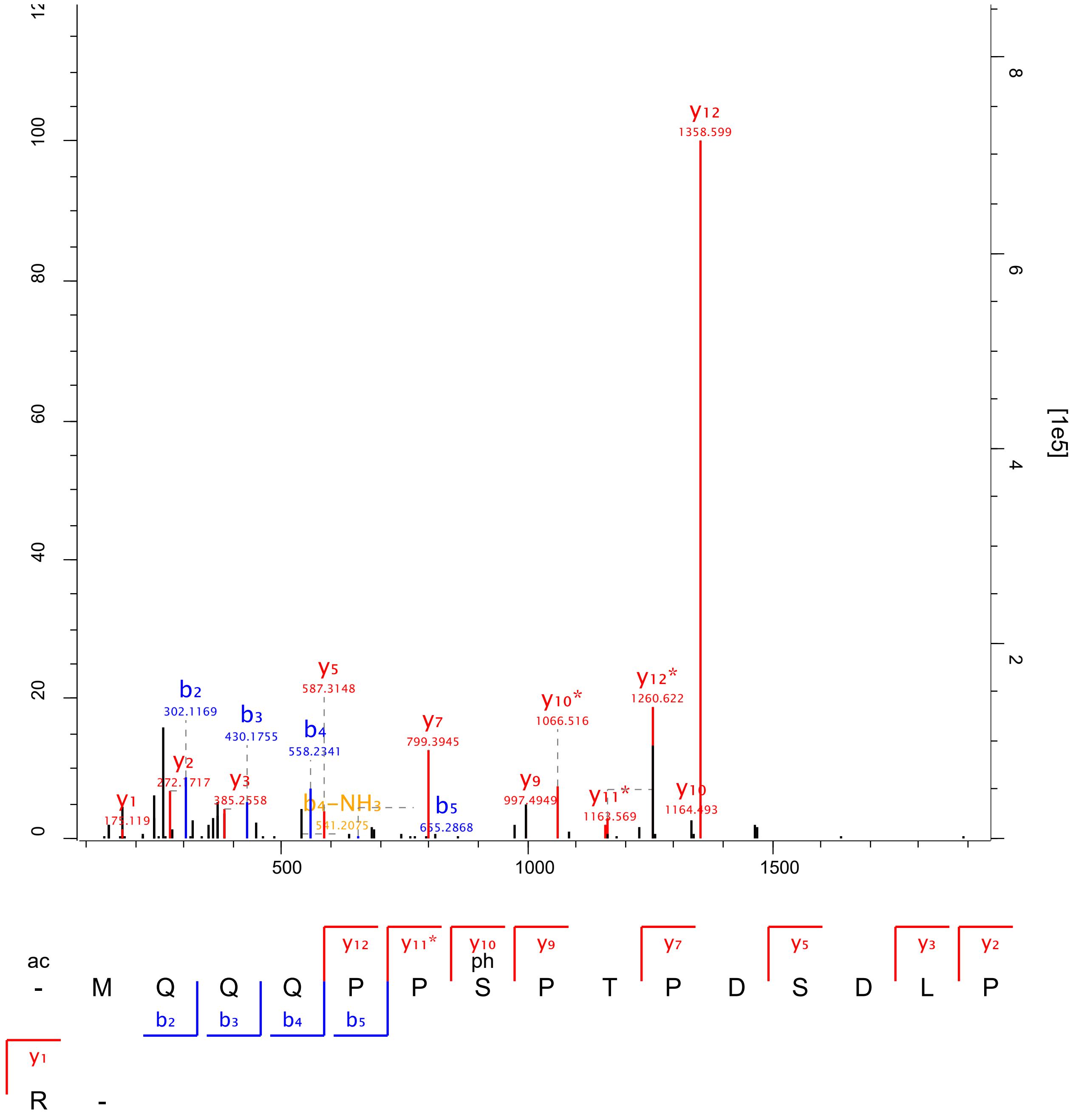

Raw file	Scan	Method	Score	m/z
sys_05_2	32749	FTMS; HCD	54.76	789.35

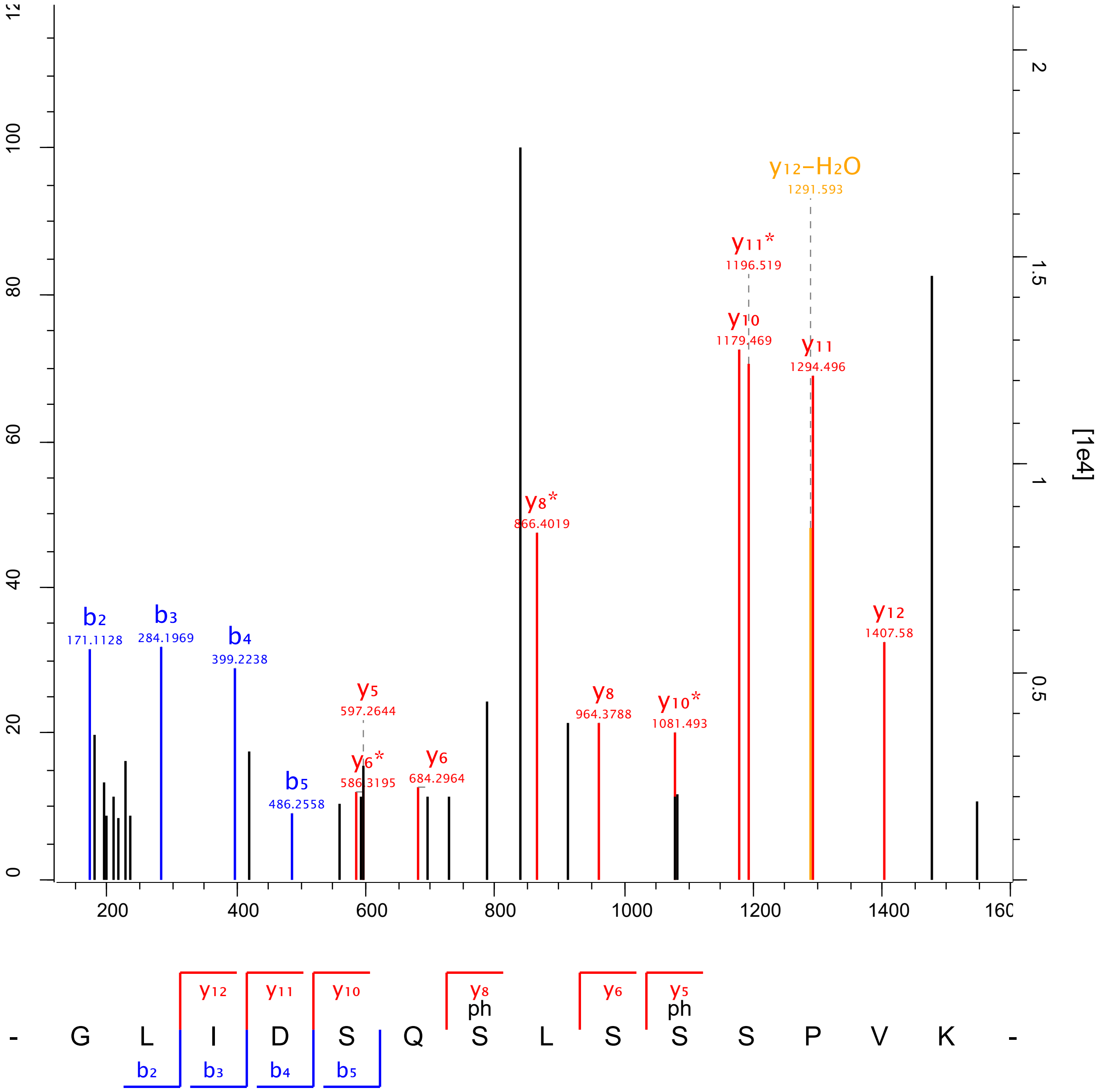

Raw file	Scan	Method	Score	m/z
sys_05_2	32799	FTMS; HCD	110.44	729.84

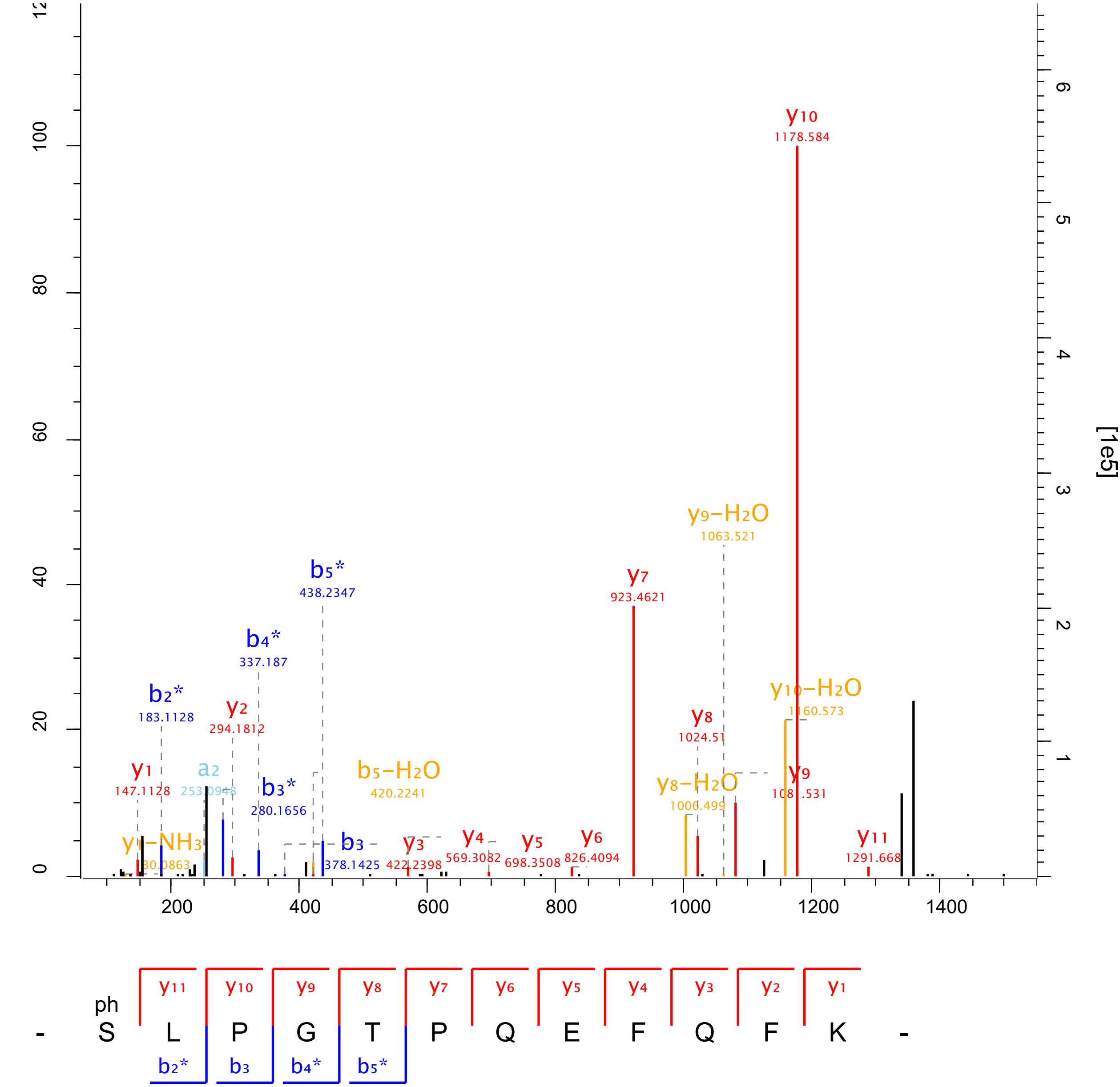

Raw file	Scan	Method	Score	m/z
sys_05_2	3295	FTMS; HCD	52.58	633.79

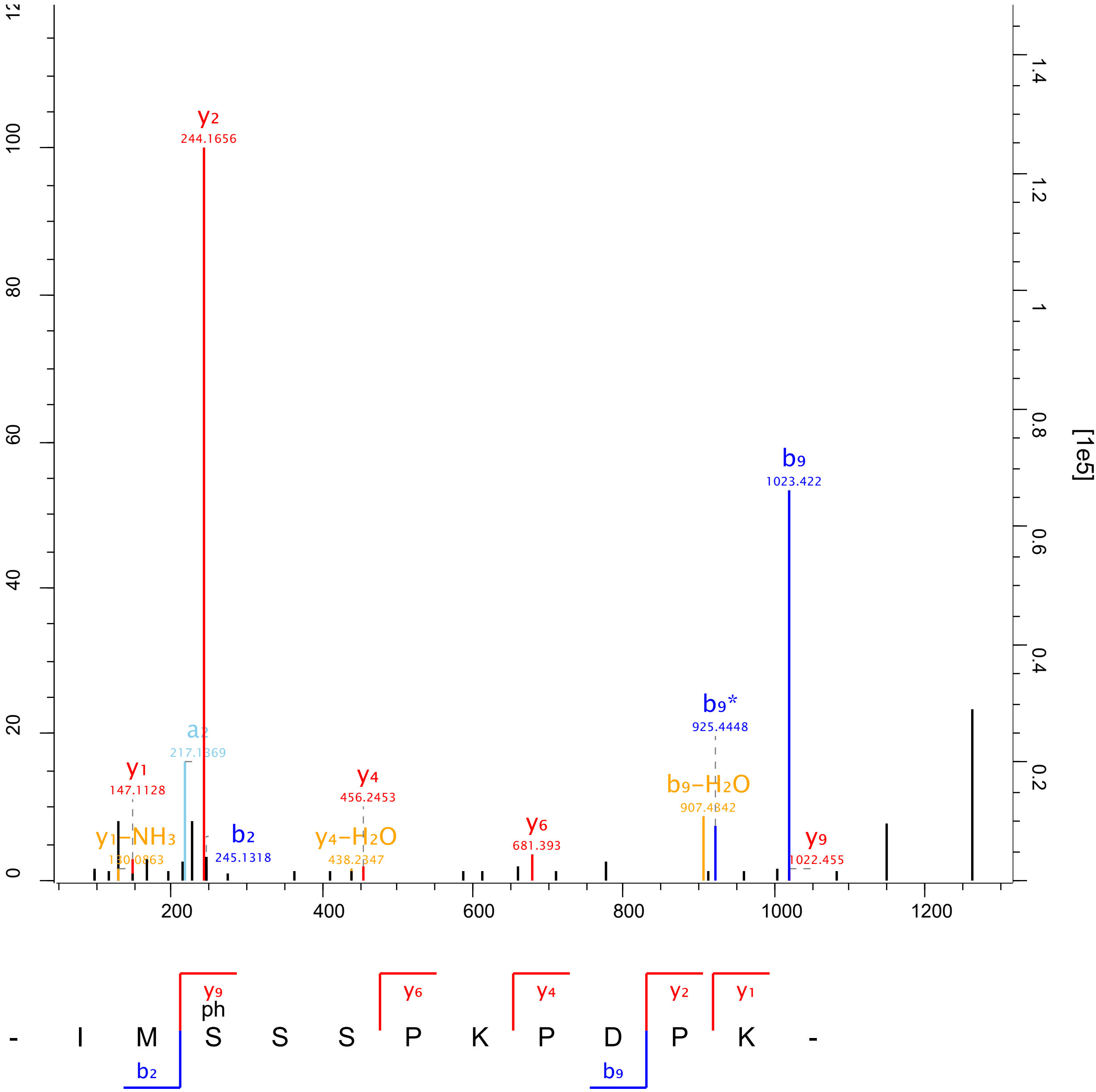

Raw file	Scan	Method	Score	m/z
sys_05_2	33063	FTMS; HCD	105.79	752.82

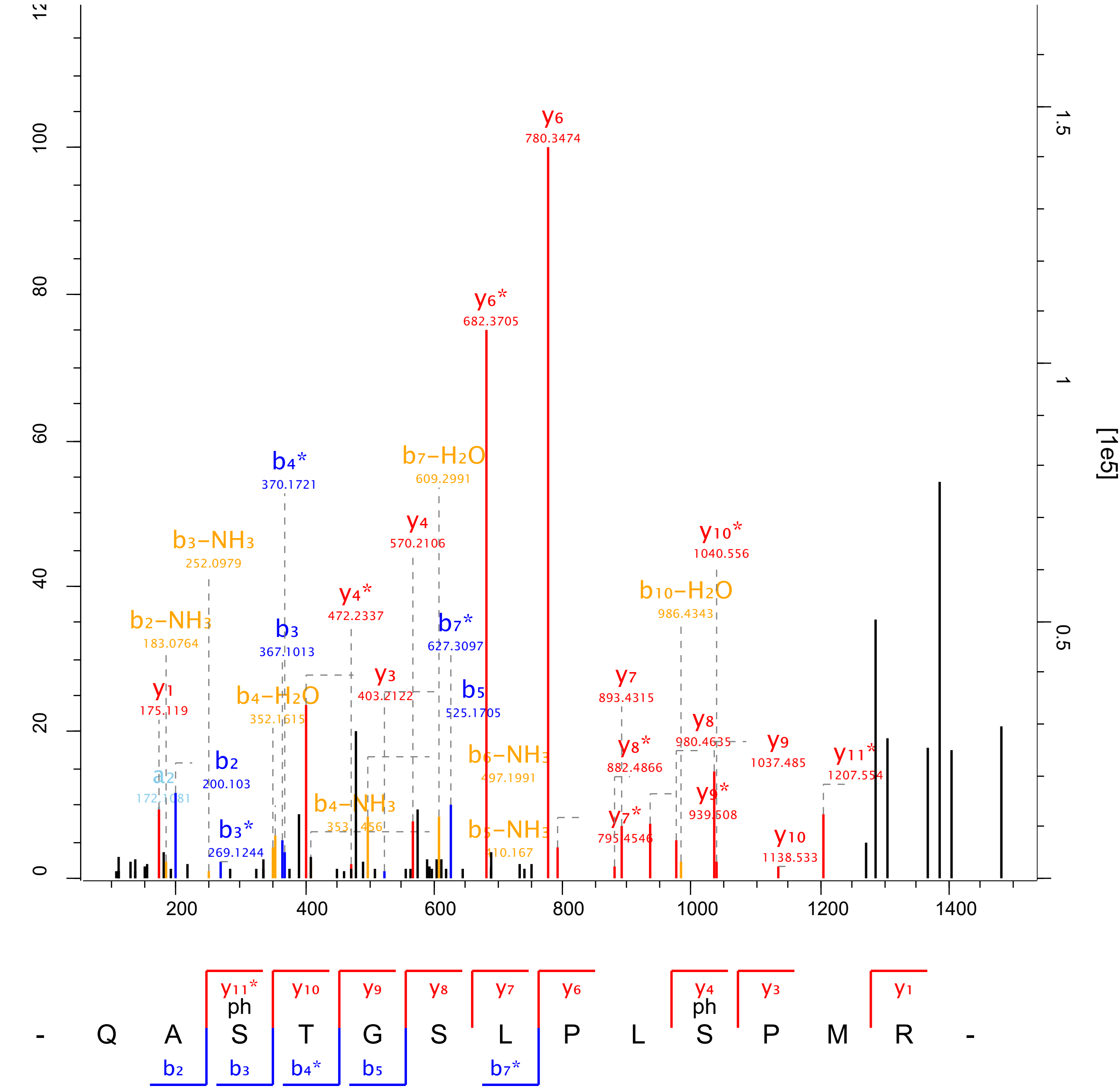

Raw file	Scan	Method	Score	m/z
sys_05_2	33199	FTMS; HCD	163.18	1023.42

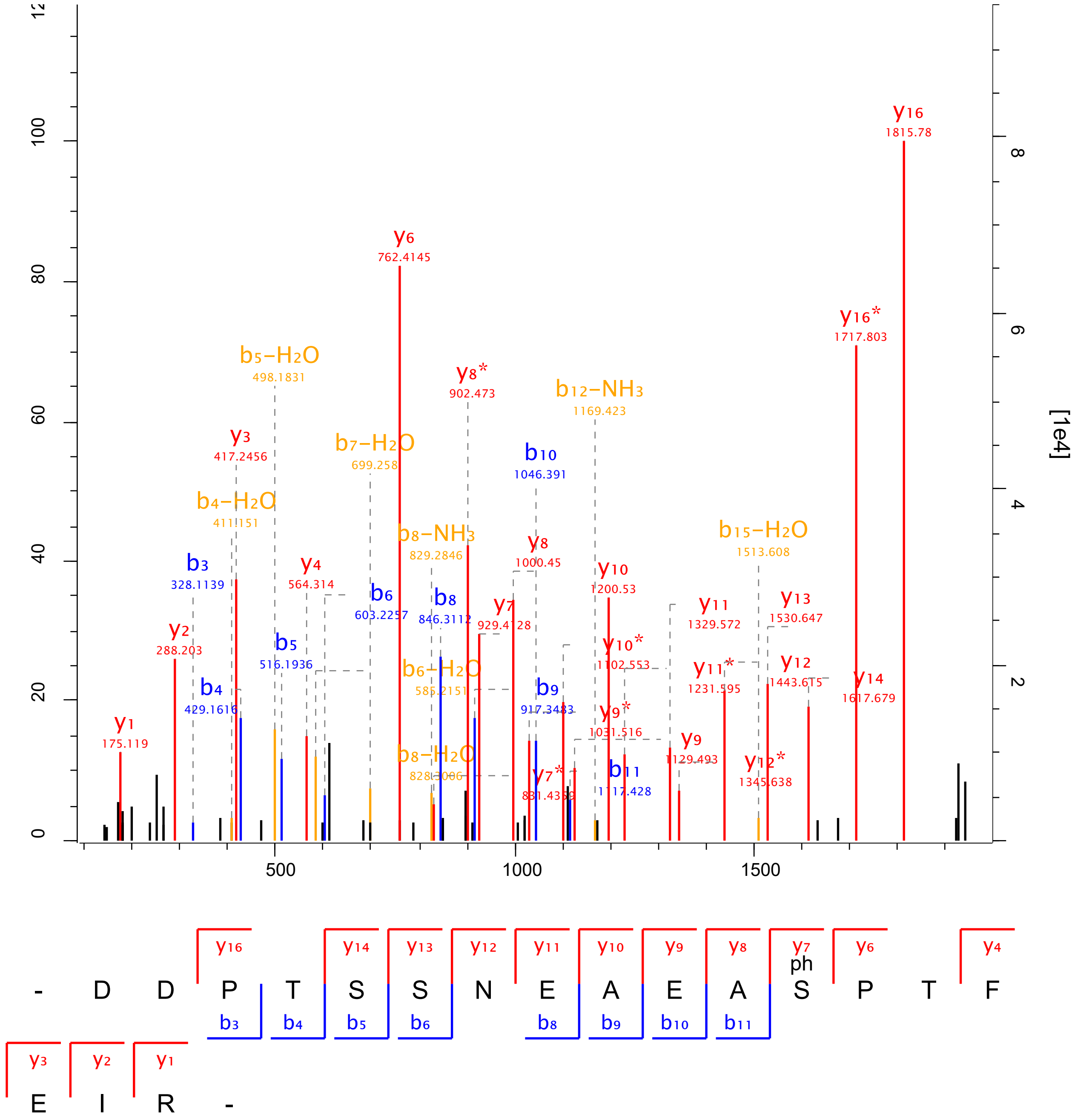

Raw file	Scan	Method	Score	m/z
sys_05_2	33301	FTMS; HCD	218.2	742.34

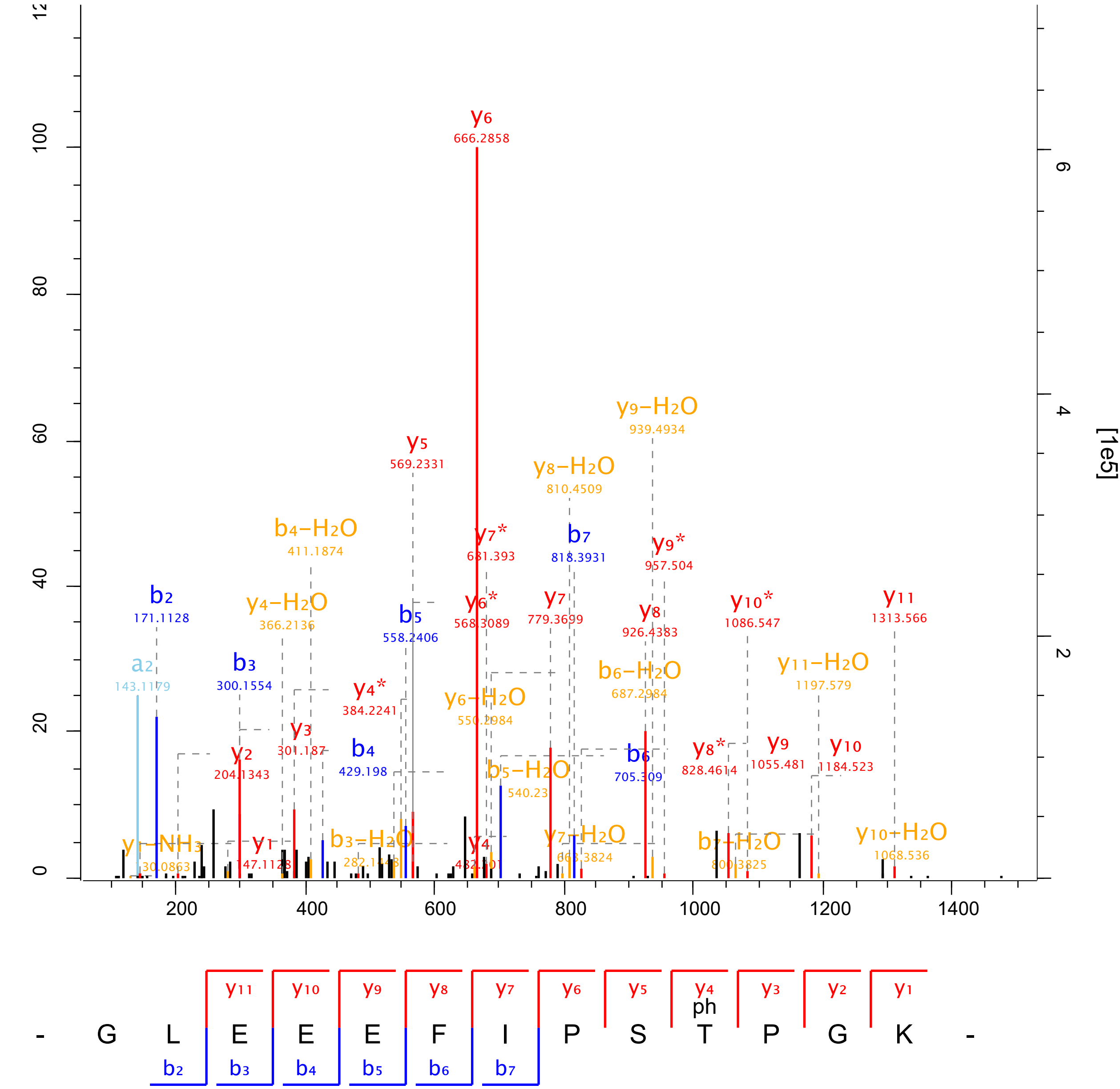

Raw file	Scan	Method	Score	m/z
sys_05_2	33521	FTMS; HCD	47.04	727.29

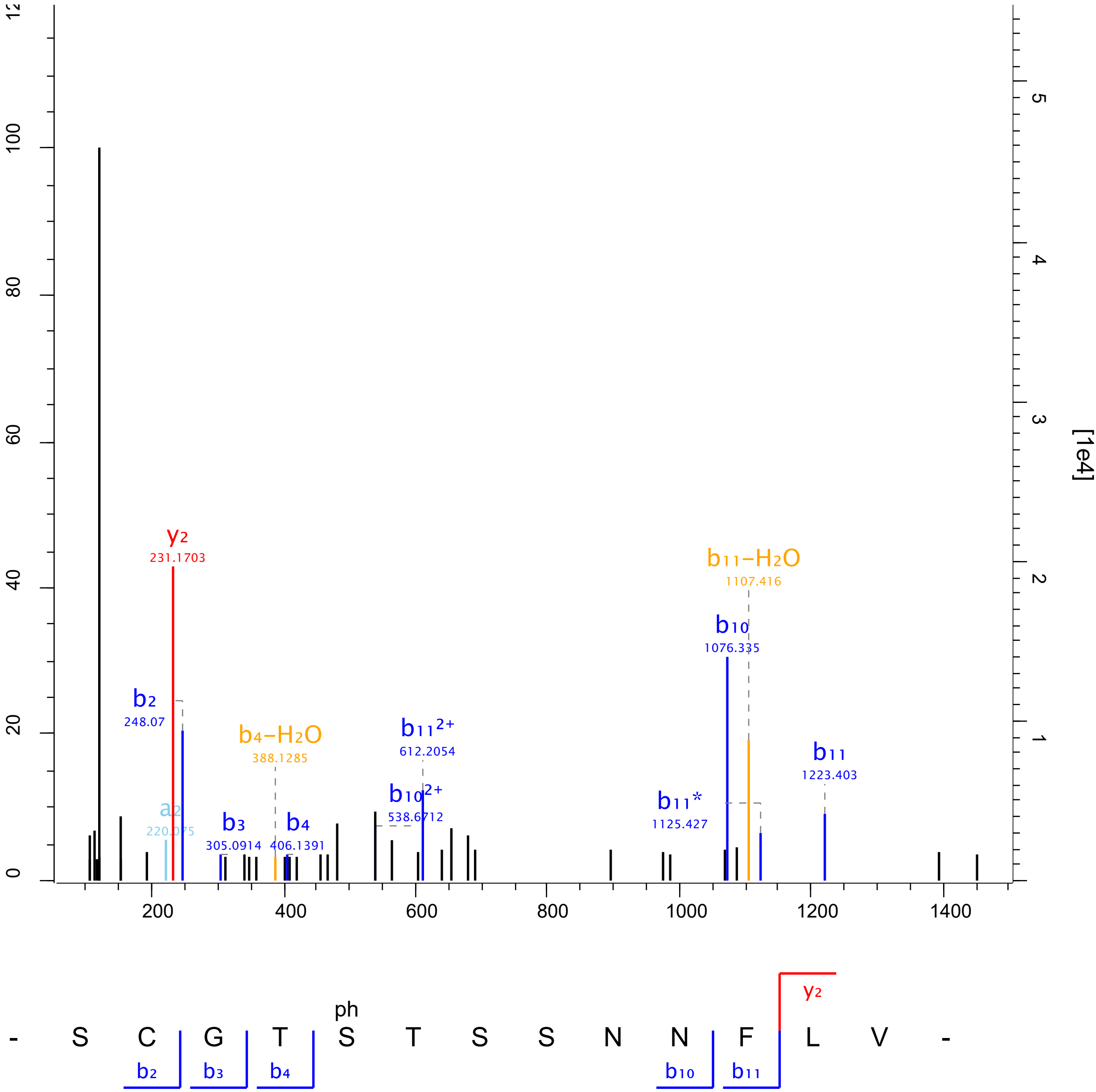

Raw file	Scan	Method	Score	m/z
sys_05_2	33540	FTMS; HCD	104.26	741.35

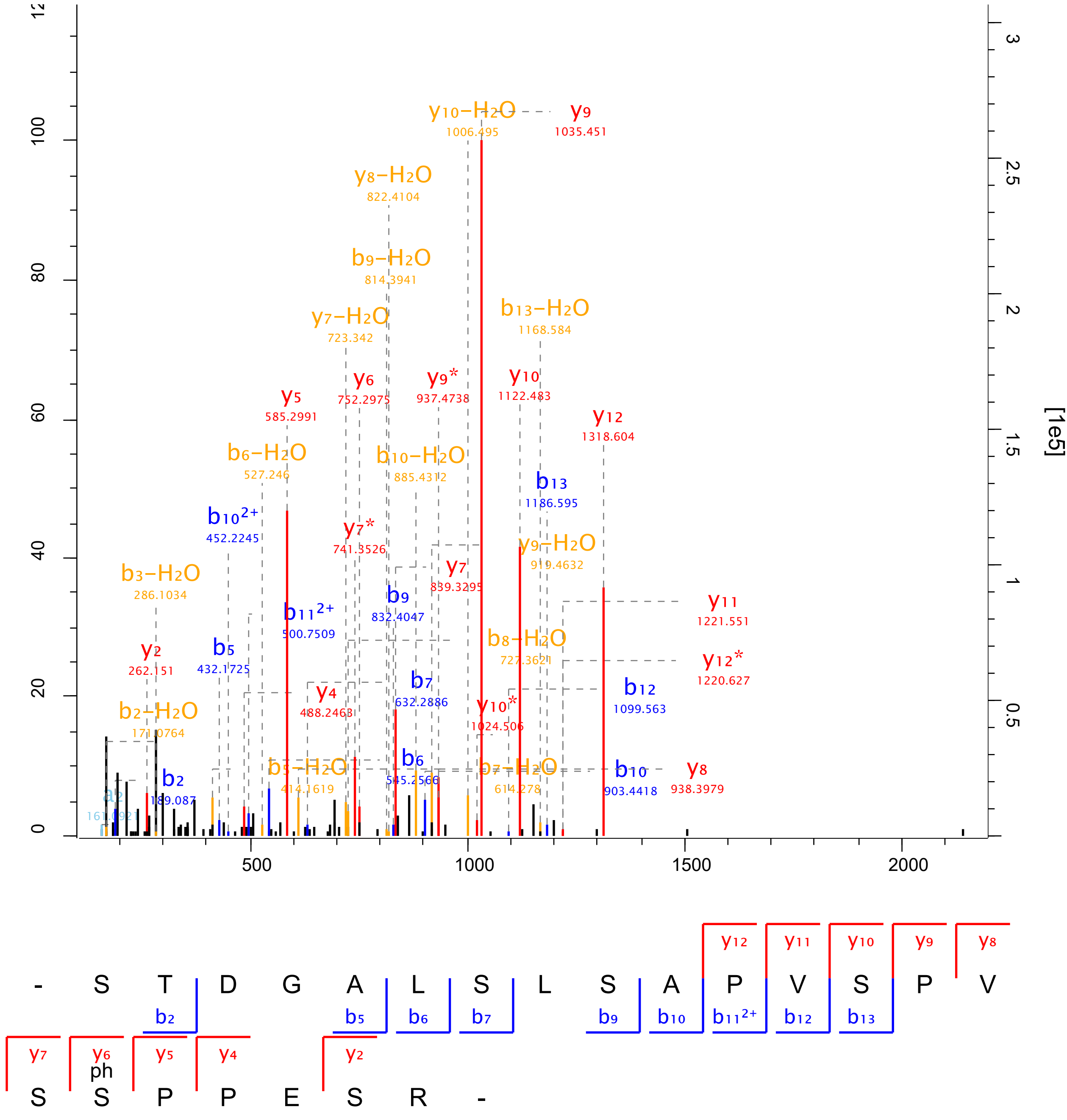

Raw file	Scan	Method	Score	m/z
sys_05_2	33583	FTMS; HCD	41.7	757.82

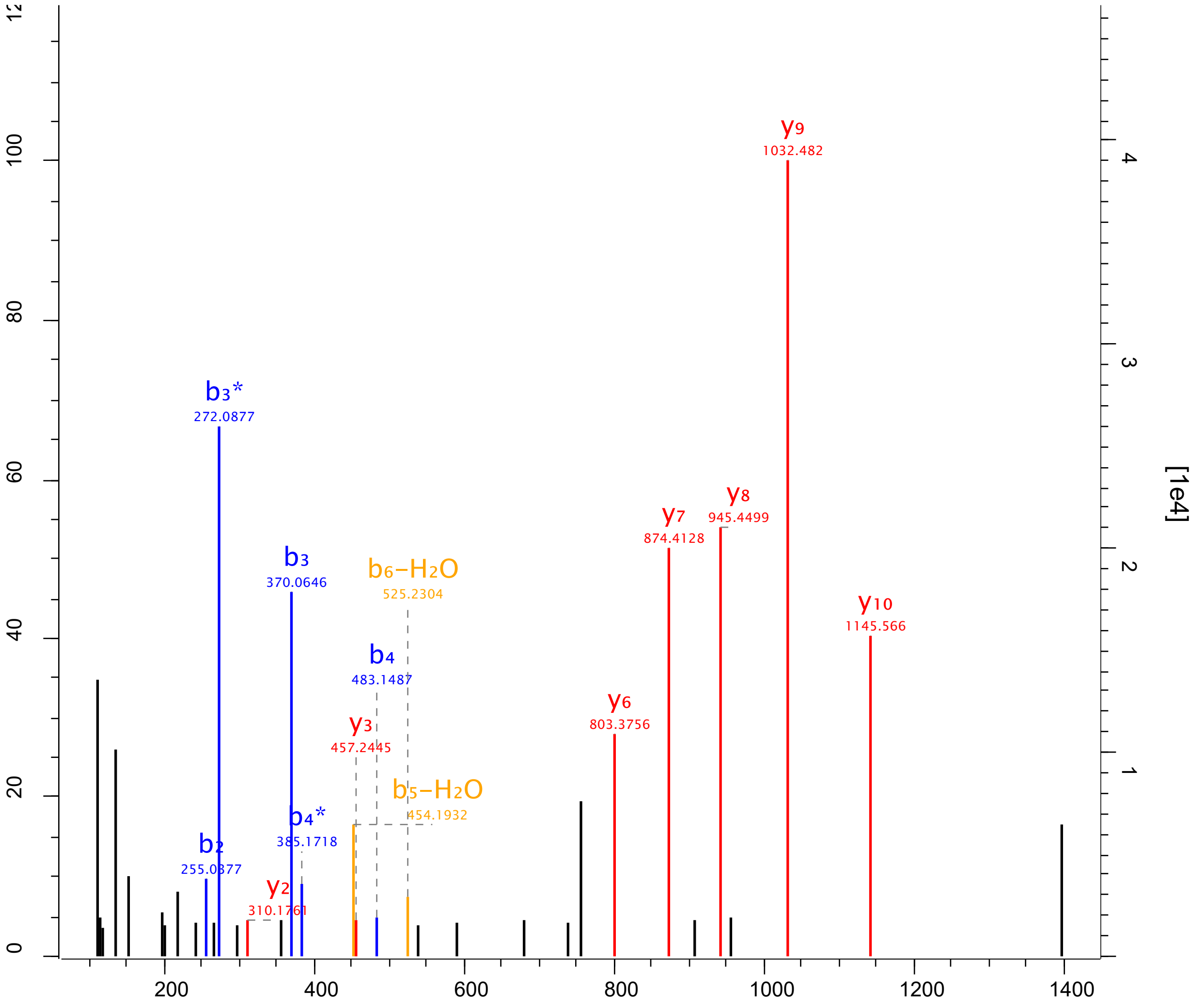

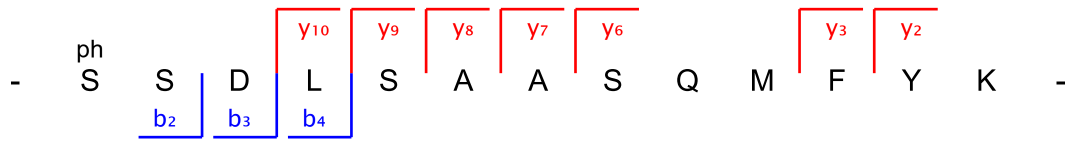

Raw file	Scan	Method	Score	m/z
sys_05_2	34587	FTMS; HCD	106.67	580.73

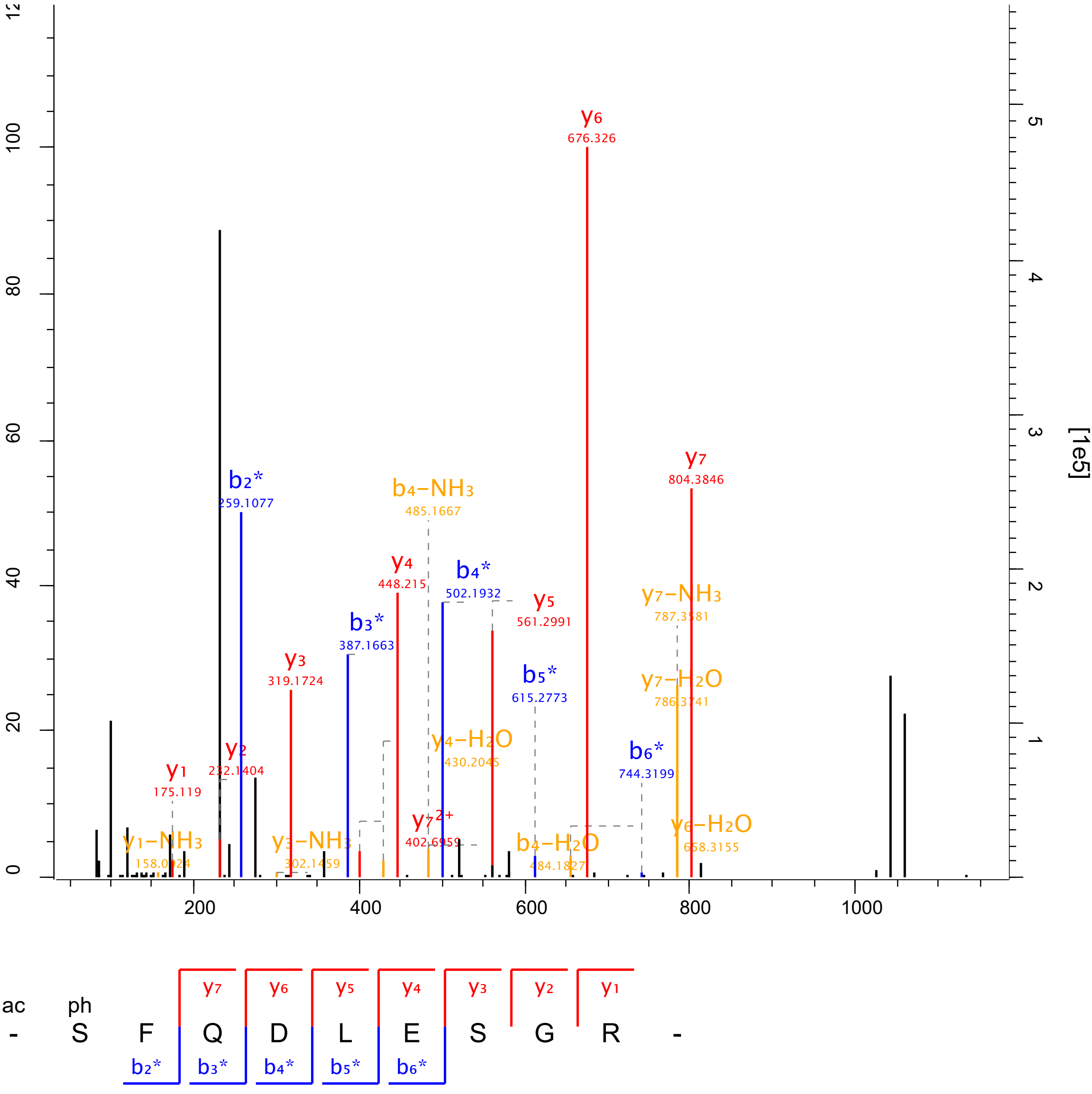

Raw file	Scan	Method	Score	m/z
sys_05_2	34708	FTMS; HCD	247.91	731.78

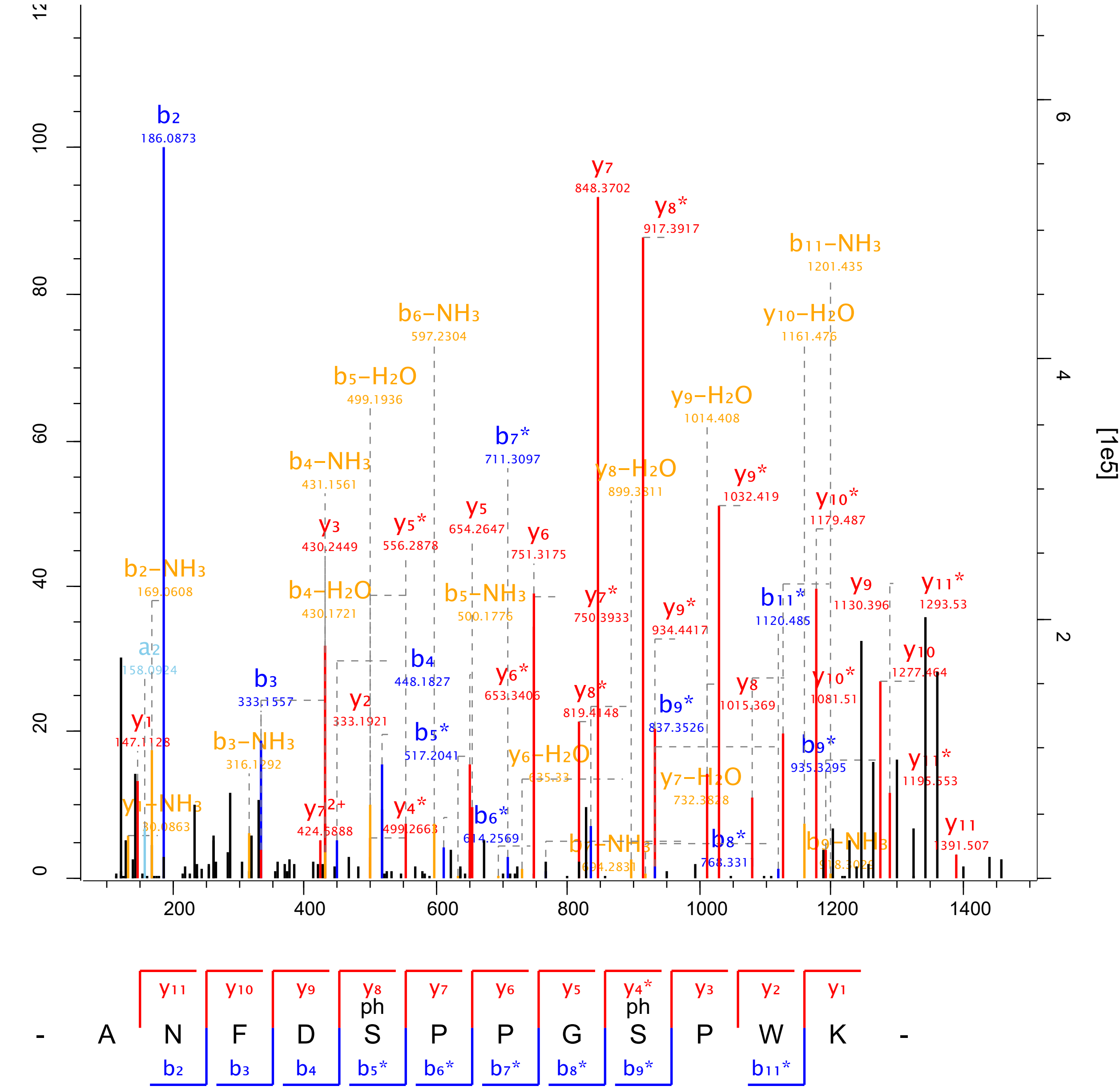

Raw file	Scan	Method	Score	m/z
sys_05_2	35017	FTMS; HCD	68.68	662.33

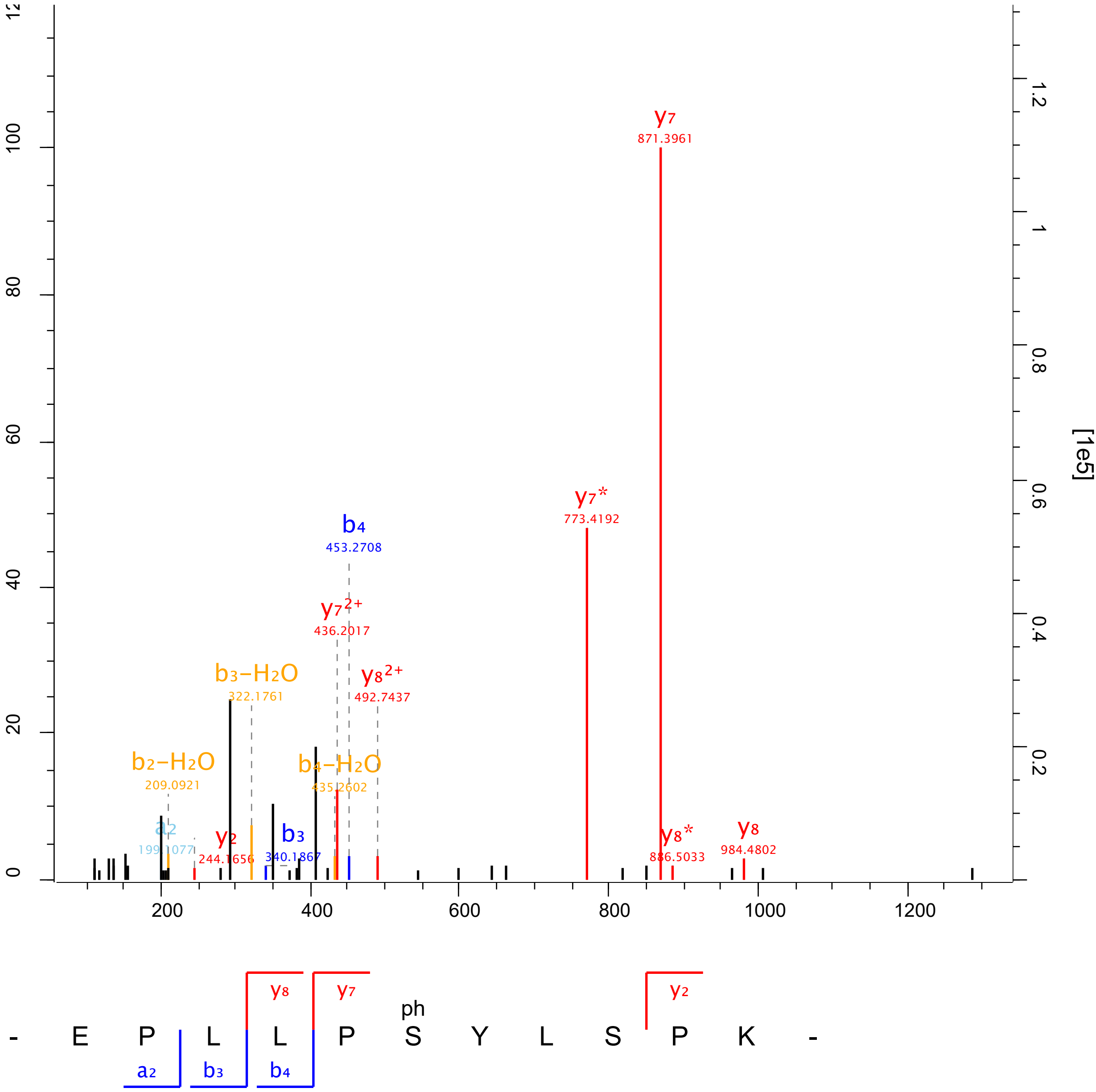

Raw file	Scan	Method	Score	m/z
sys_05_2	35388	FTMS; HCD	185.66	597.26

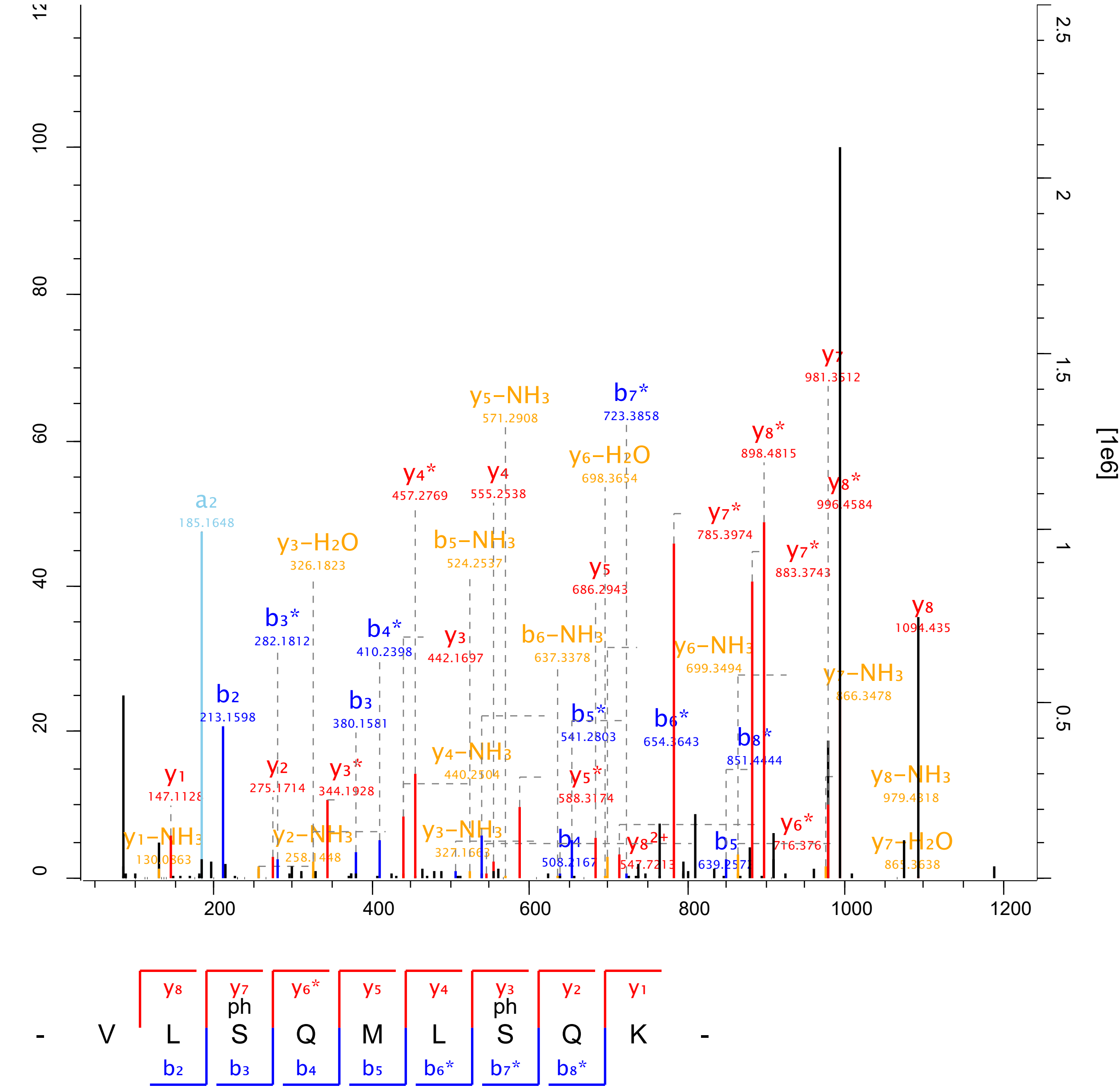

Raw file	Scan	Method	Score	m/z
sys_05_2	3551	FTMS; HCD	80.69	606.25

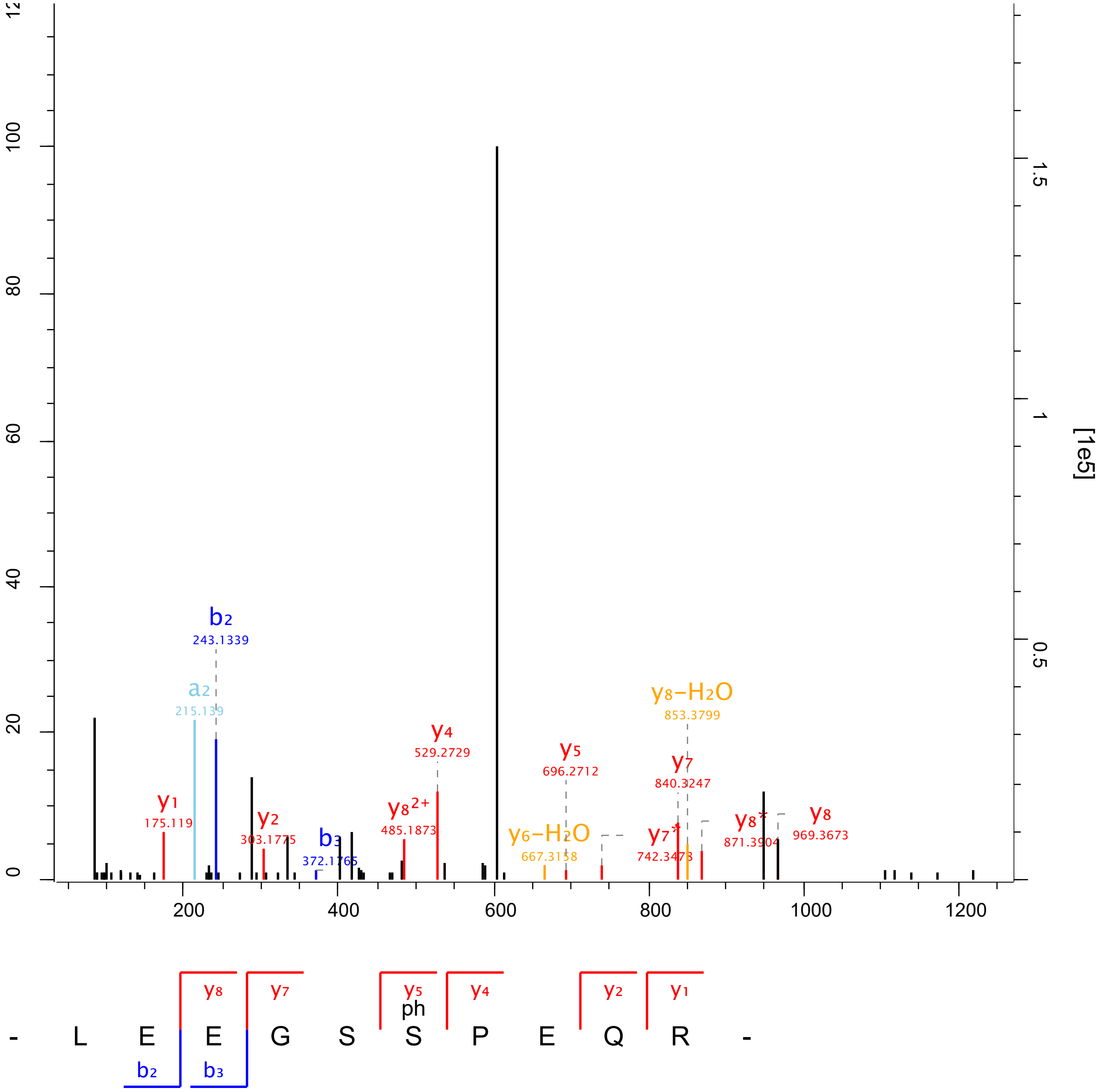

Raw file	Scan	Method	Score	m/z
sys_05_2	3575	FTMS; HCD	61.24	545.73

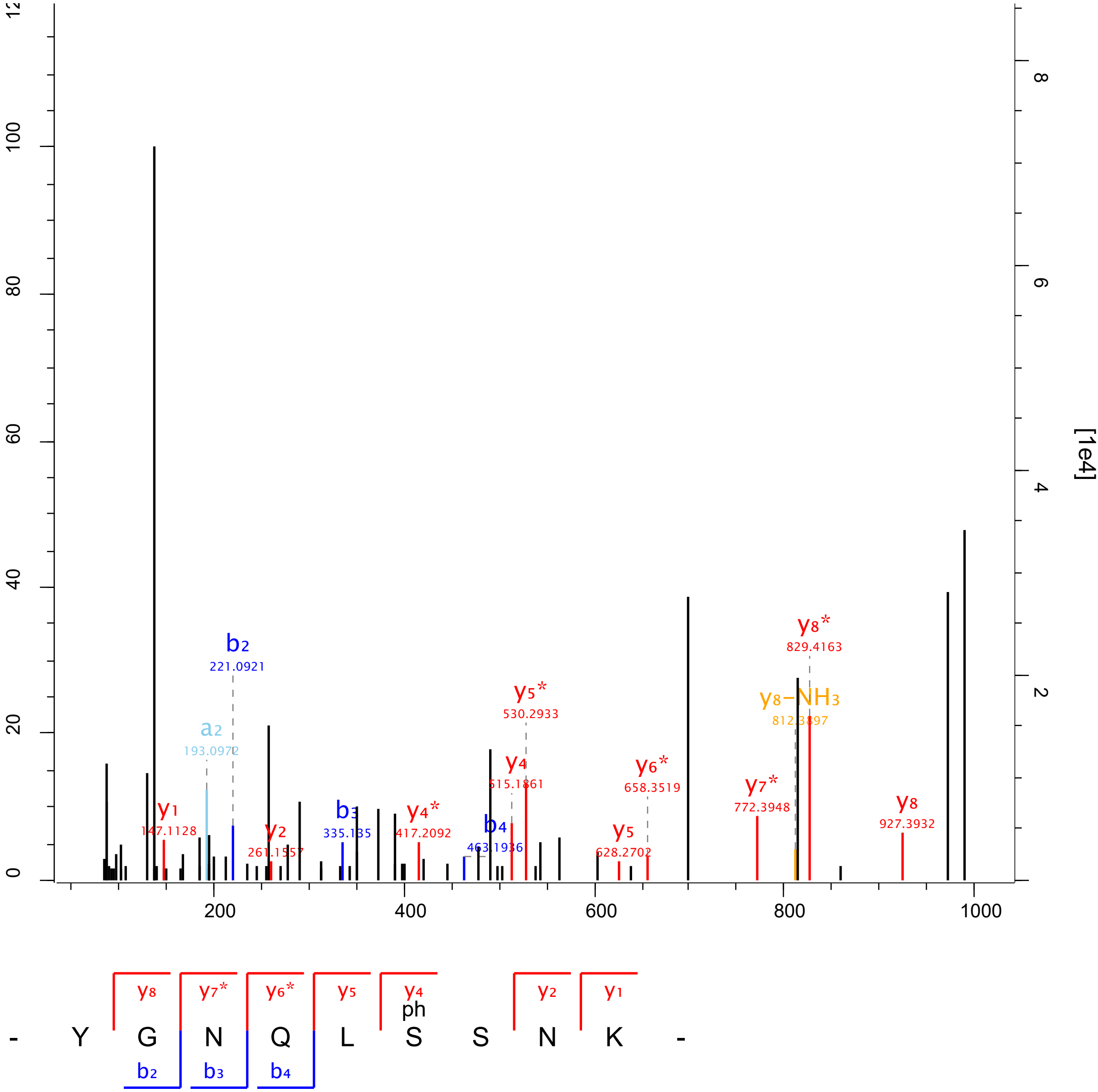

Raw file	Scan	Method	Score	m/z
sys_05_2	35983	FTMS; HCD	68.58	946.92

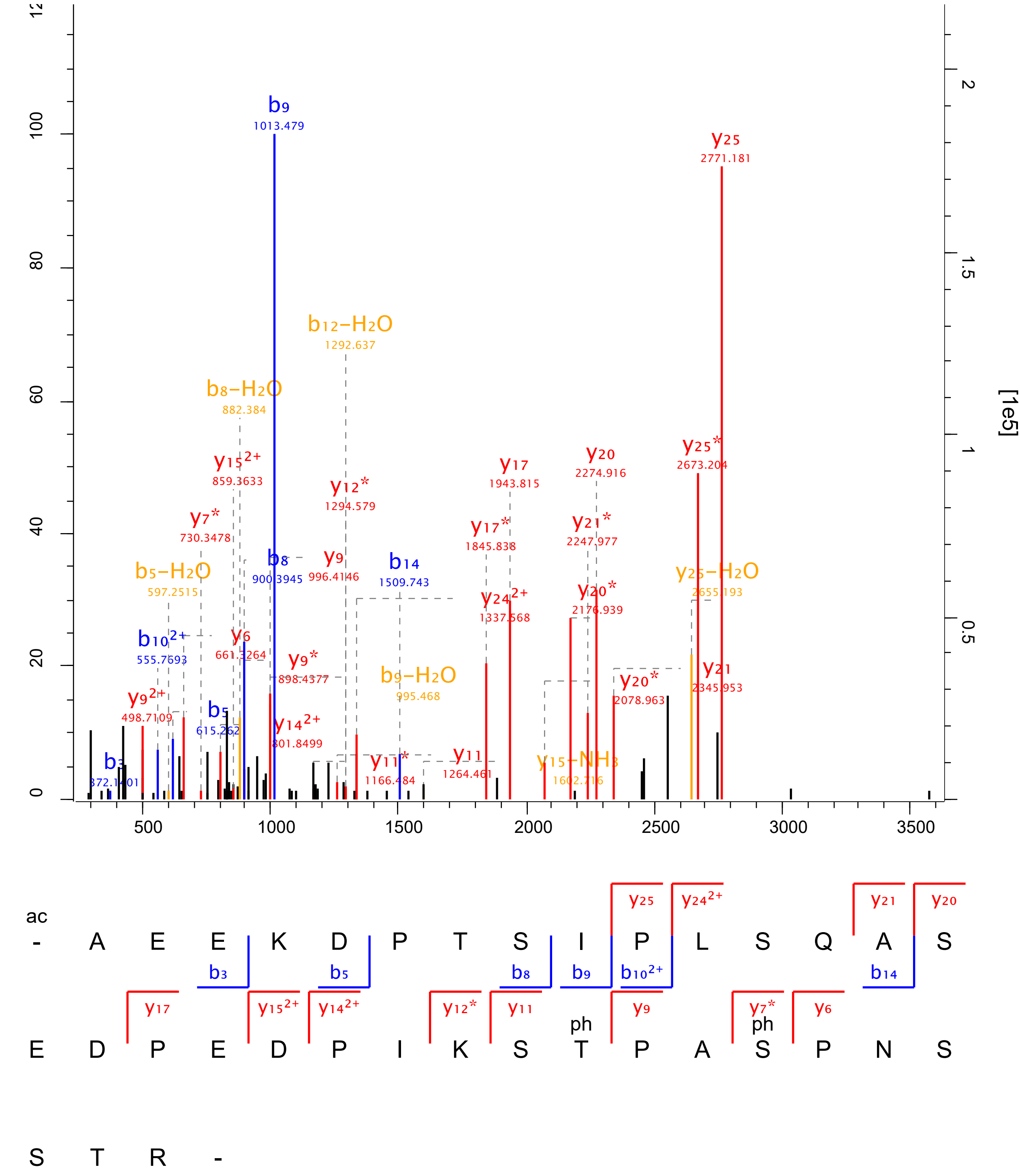

Raw file	Scan	Method	Score	m/z
sys_05_2	36133	FTMS; HCD	170.09	799.37

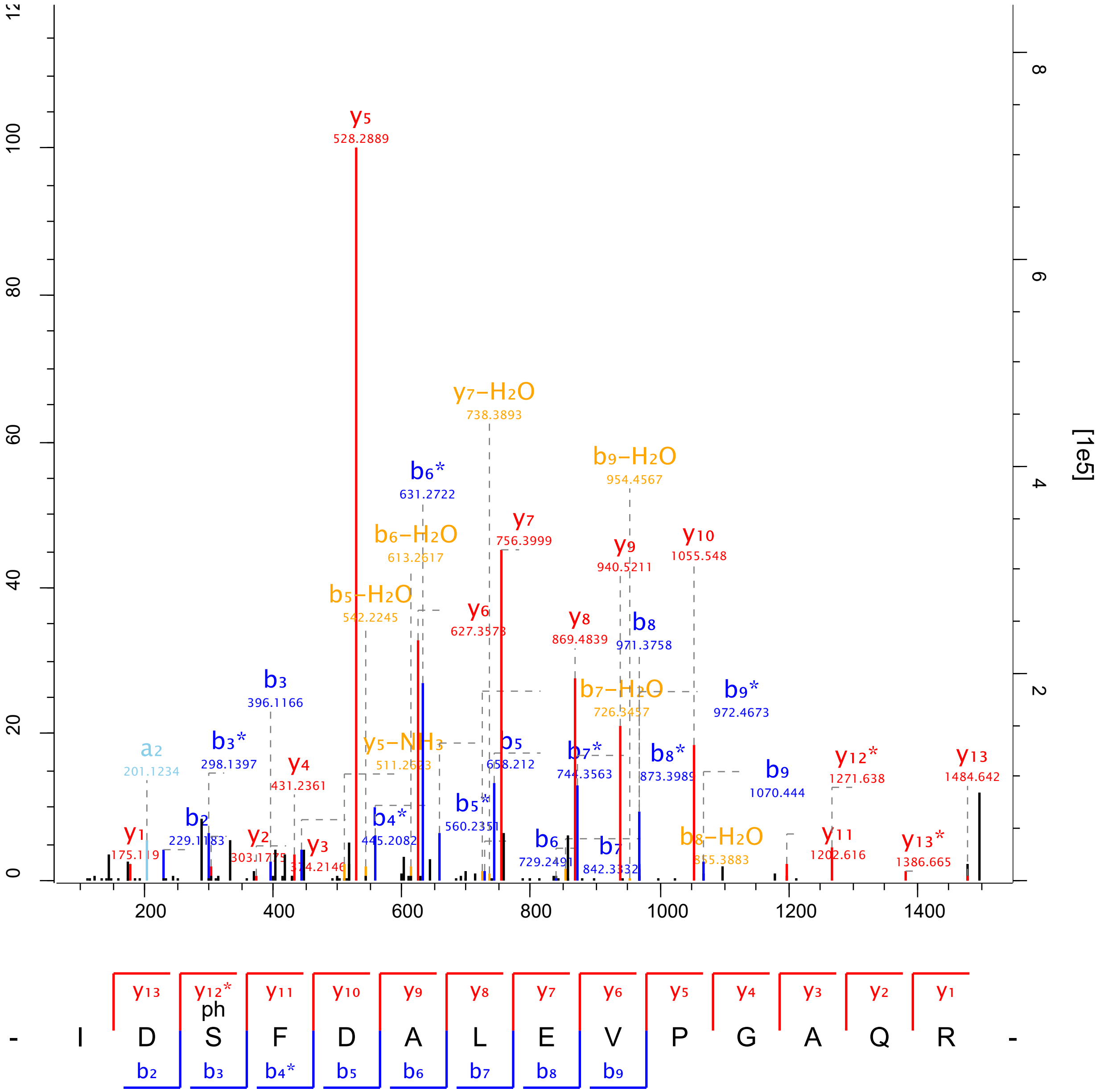

Raw file	Scan	Method	Score	m/z
sys_05_2	3714	FTMS; HCD	83.88	507.22

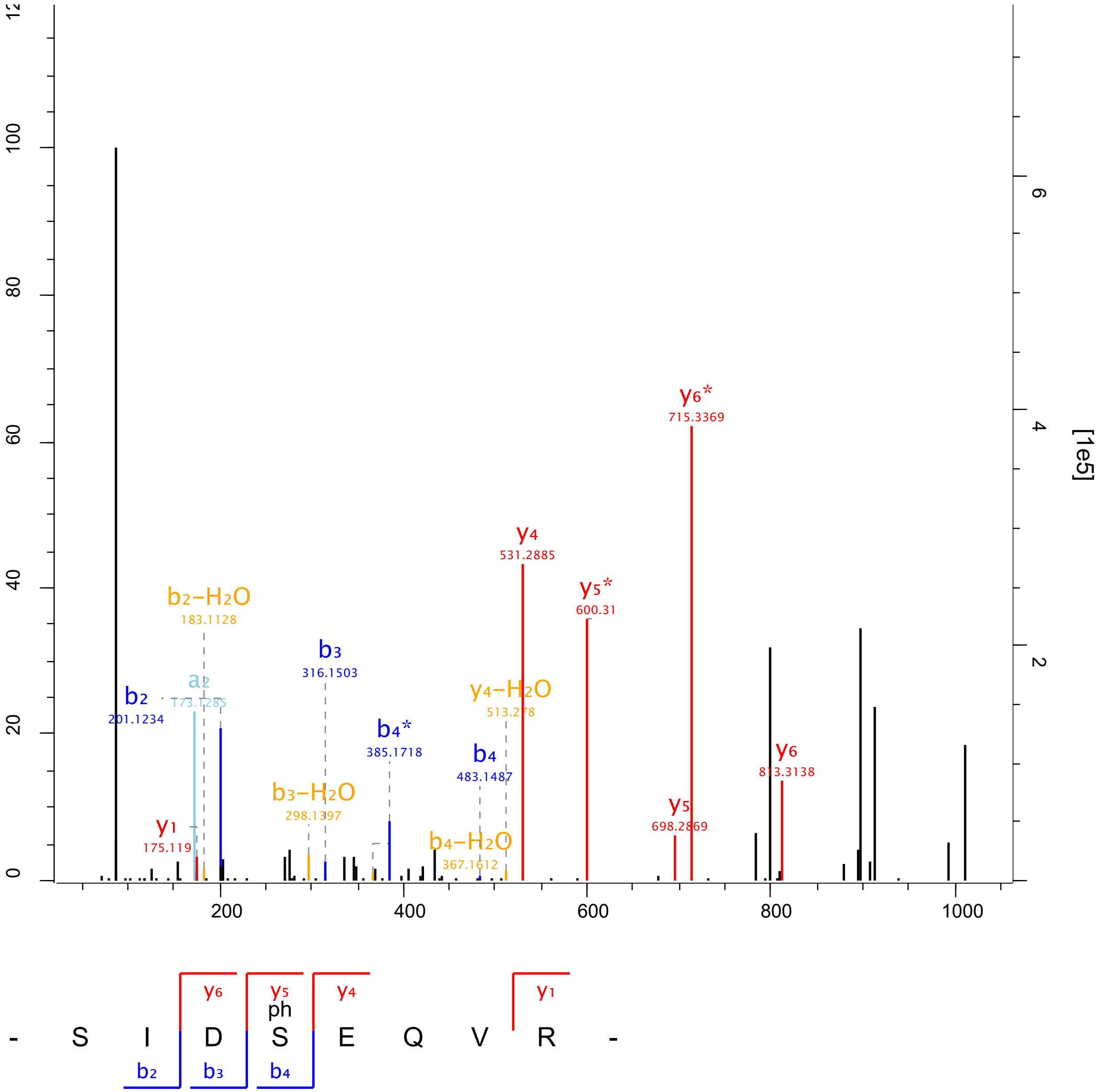

Raw file	Scan	Method	Score	m/z
sys_05_2	38471	FTMS; HCD	47.97	550.7

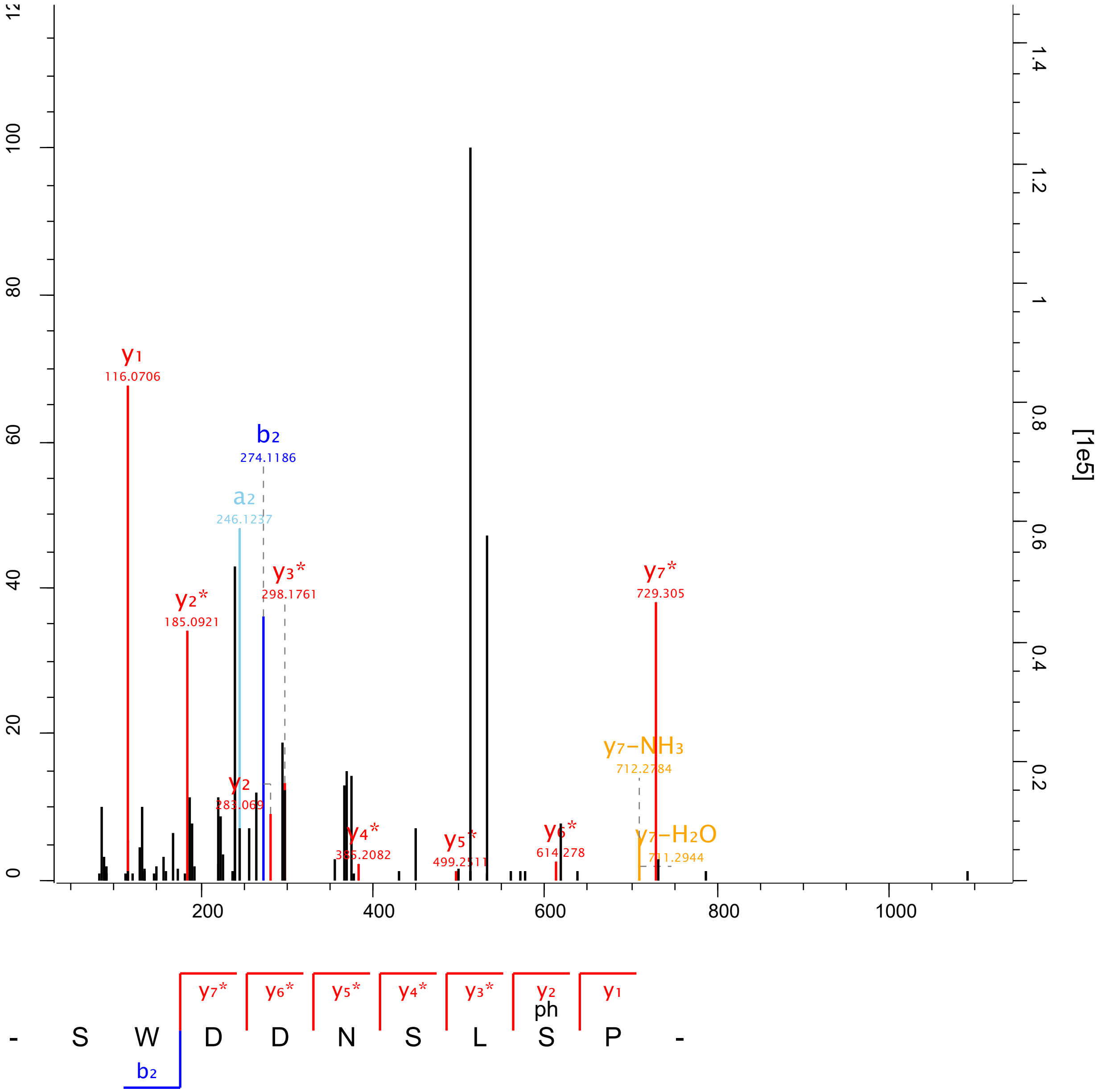

Raw file	Scan	Method	Score	m/z
sys_05_2	38501	FTMS; HCD	313.45	819.86

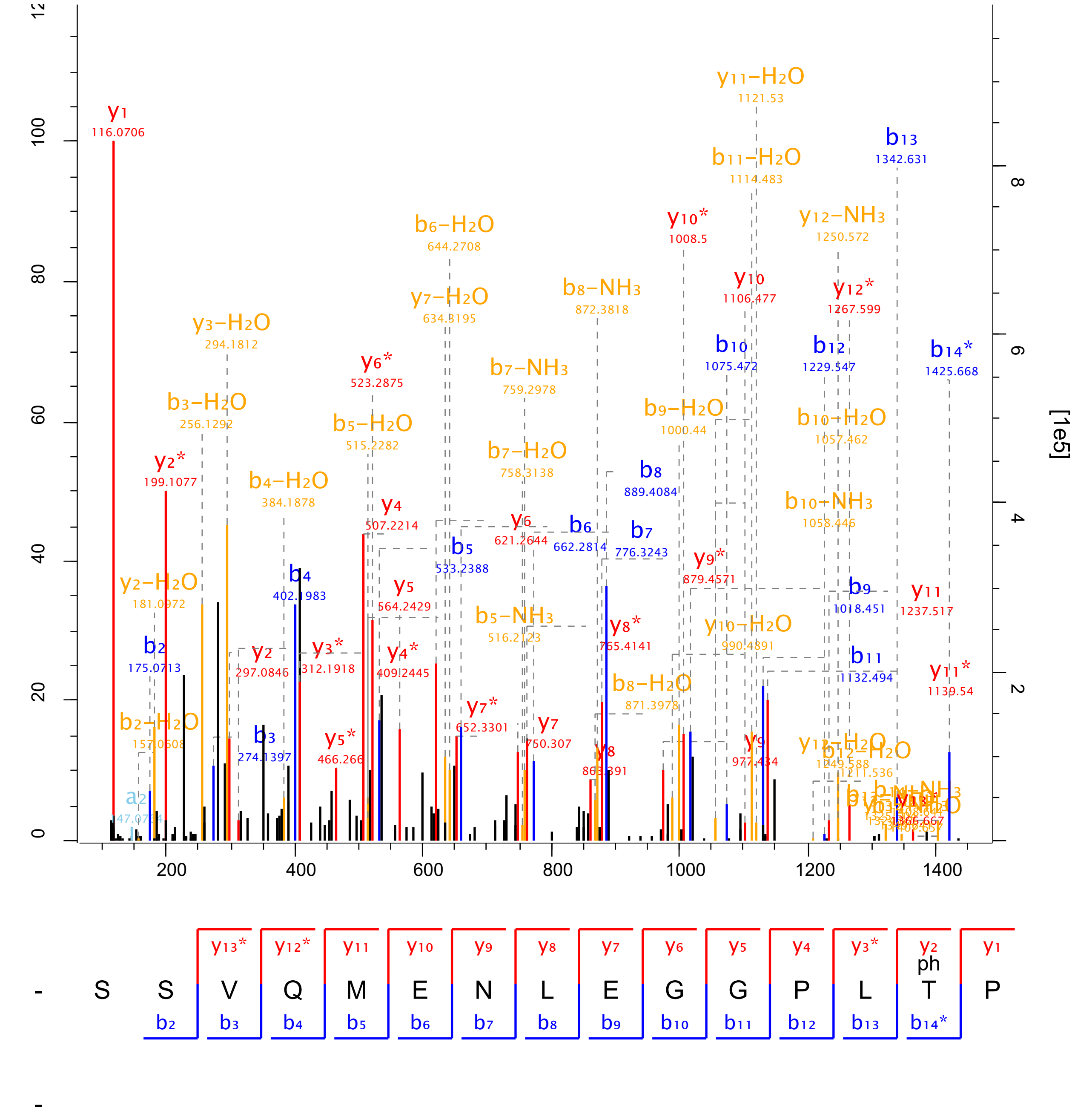

Raw file	Scan	Method	Score	m/z
sys_05_2	3904	FTMS; HCD	86.77	548.74

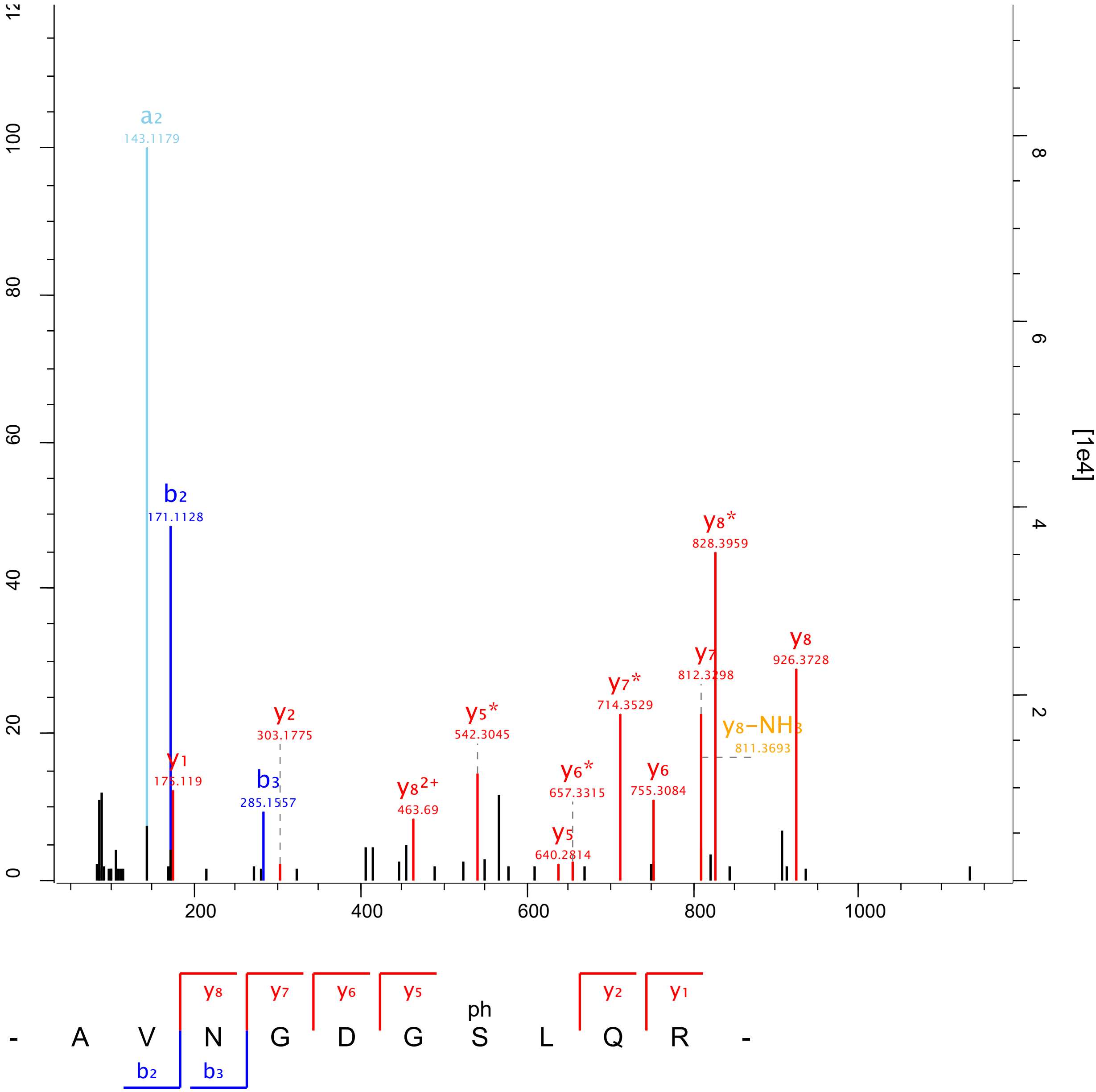

Raw file	Scan	Method	Score	m/z
sys_05_2	39396	FTMS; HCD	60.47	704.64

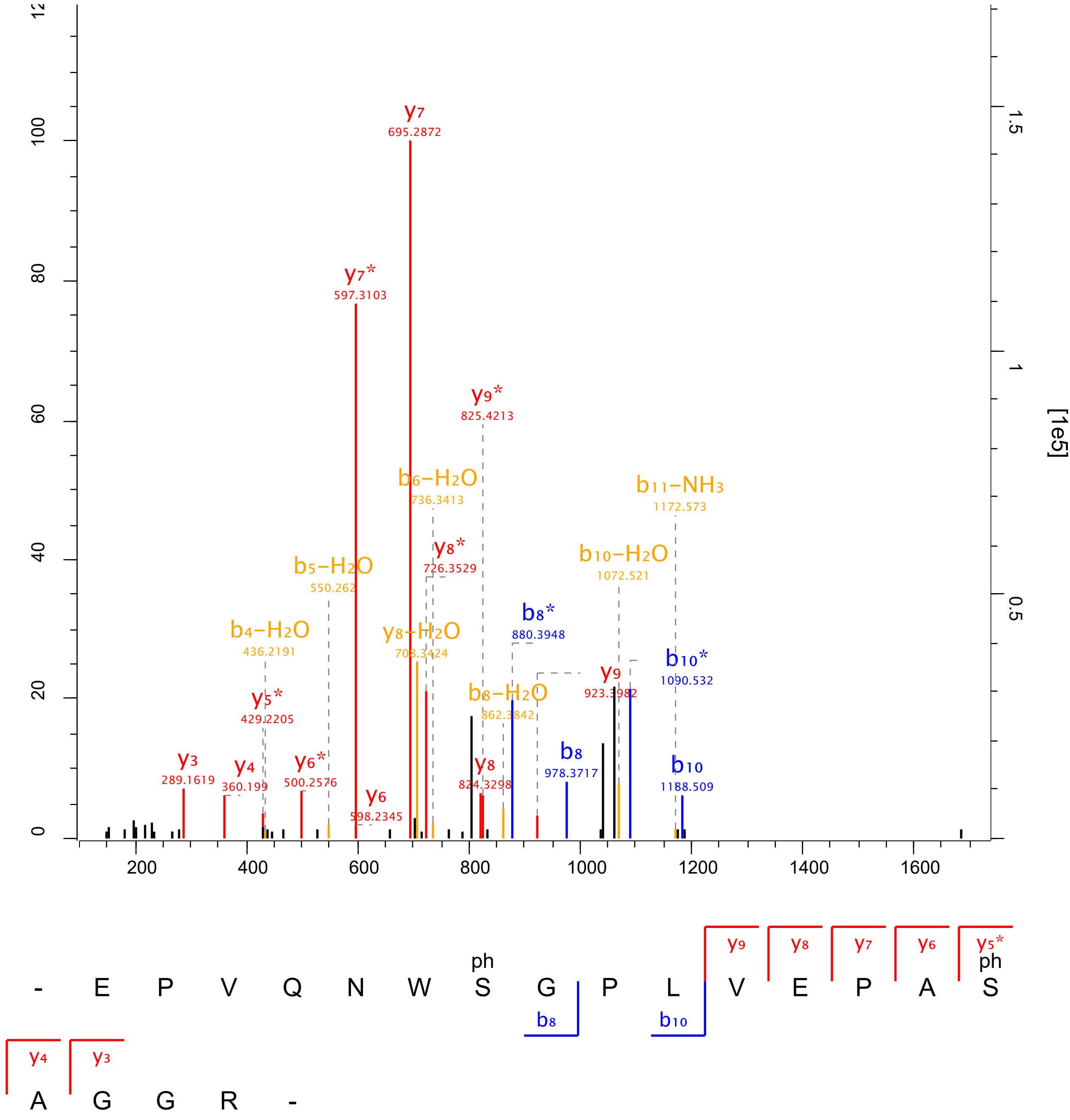

Raw file	Scan	Method	Score	m/z
sys_05_2	39835	FTMS; HCD	104.67	856.35

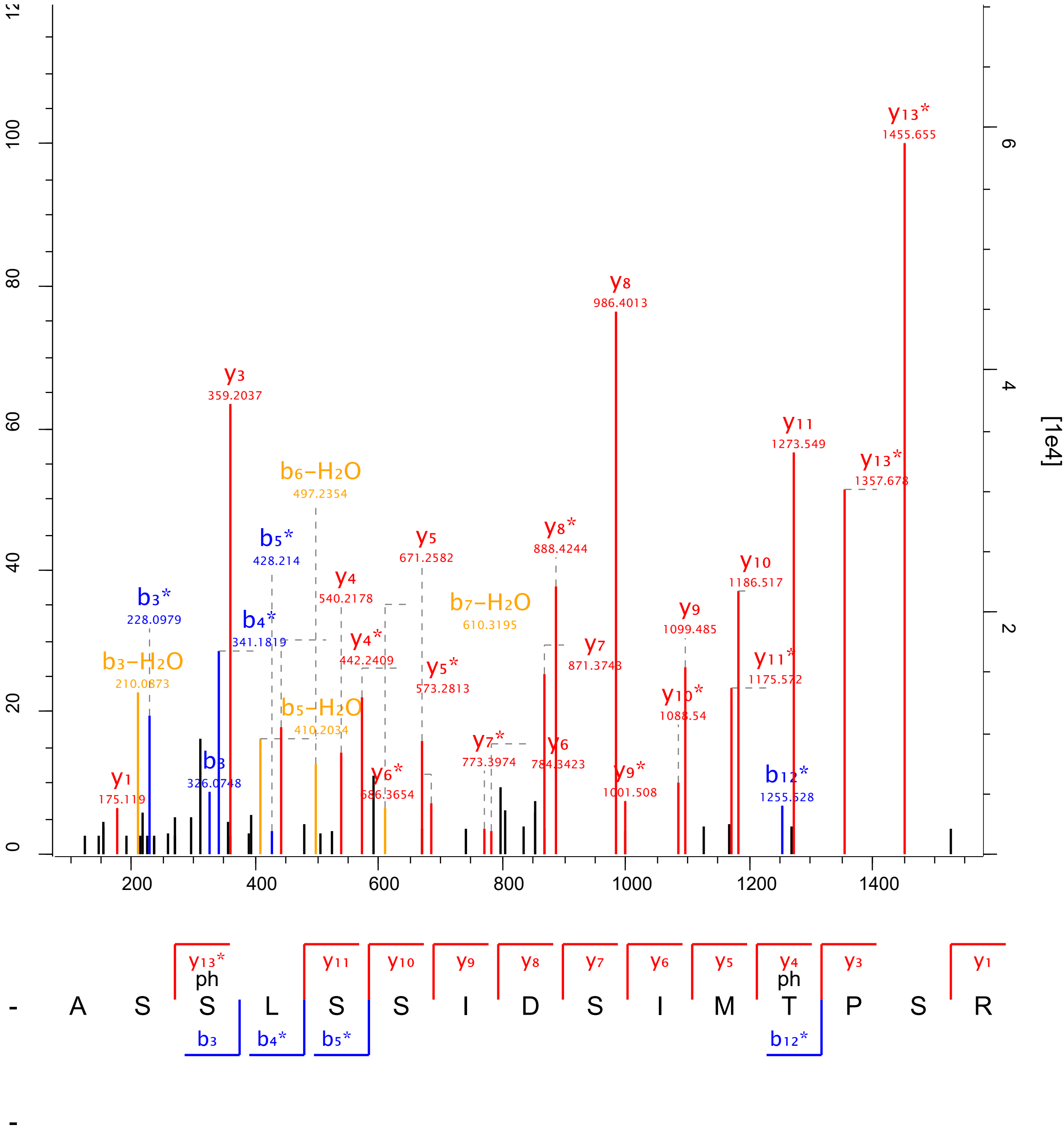

Raw file	Scan	Method	Score	m/z
sys_05_2	40238	FTMS; HCD	110.97	732.32

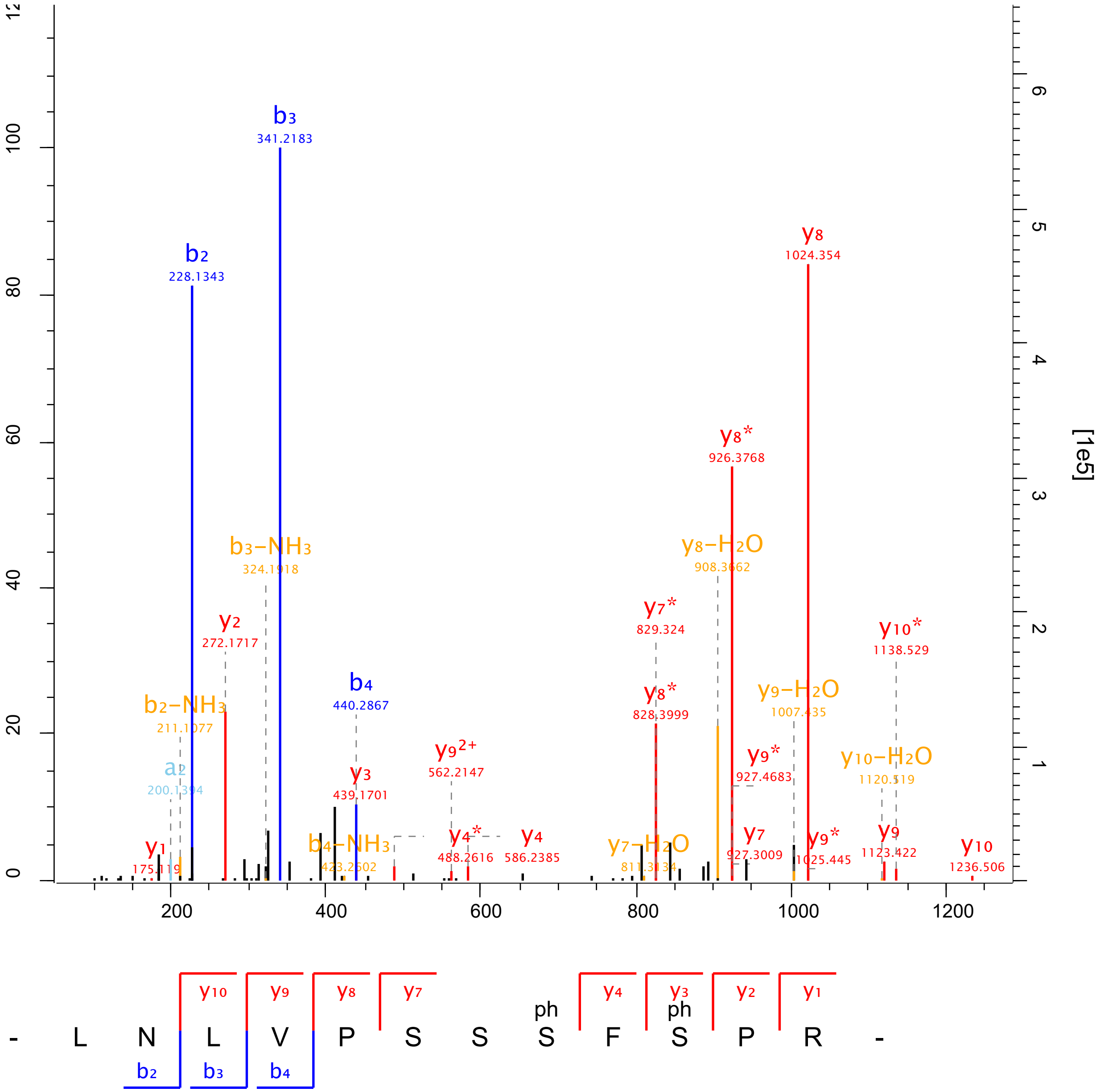

Raw file	Scan	Method	Score	m/z
sys_05_2	4074	FTMS; HCD	79.66	513.26

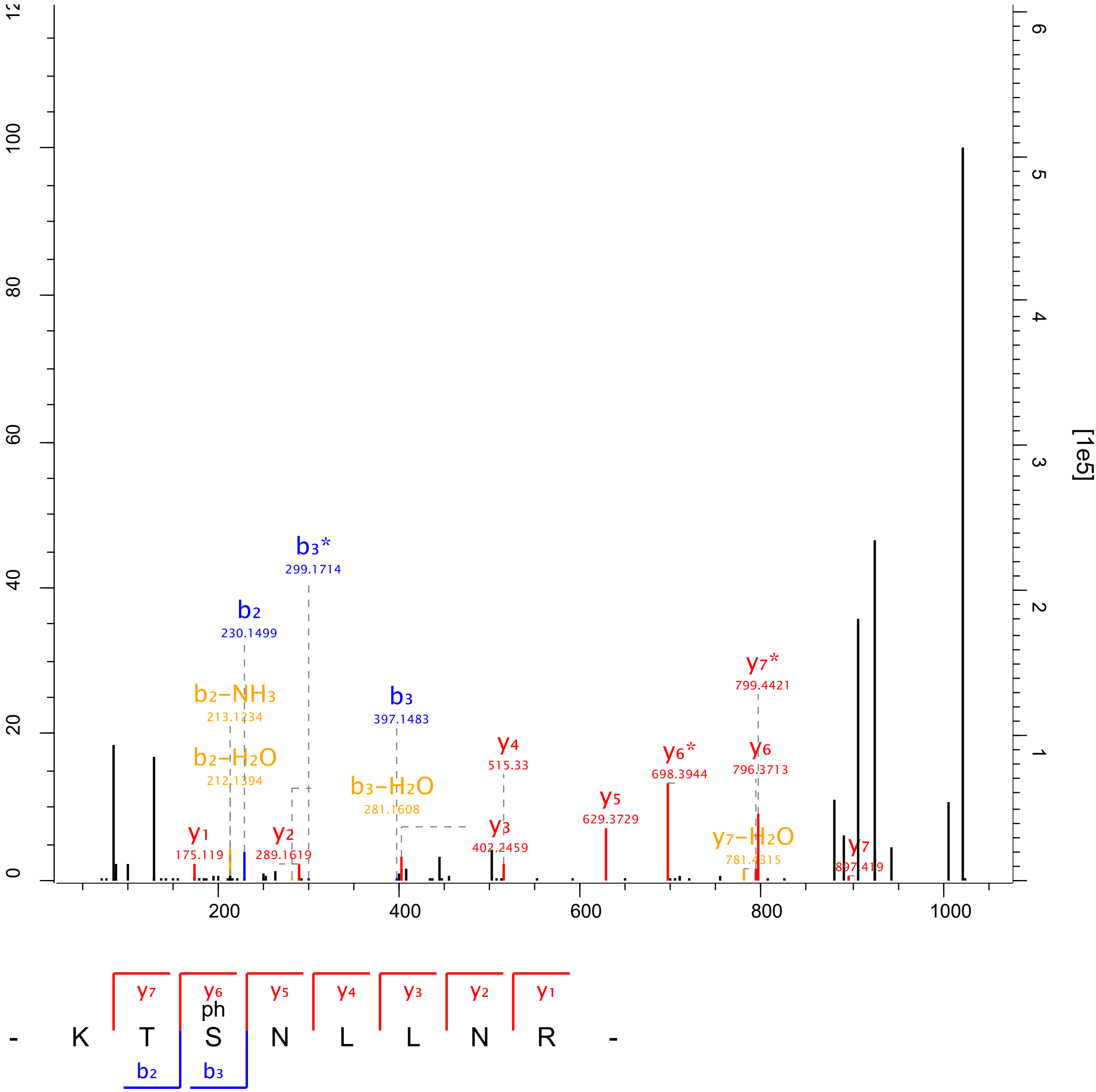

Raw file	Scan	Method	Score	m/z
sys_05_2	41352	FTMS; HCD	117.18	691.31

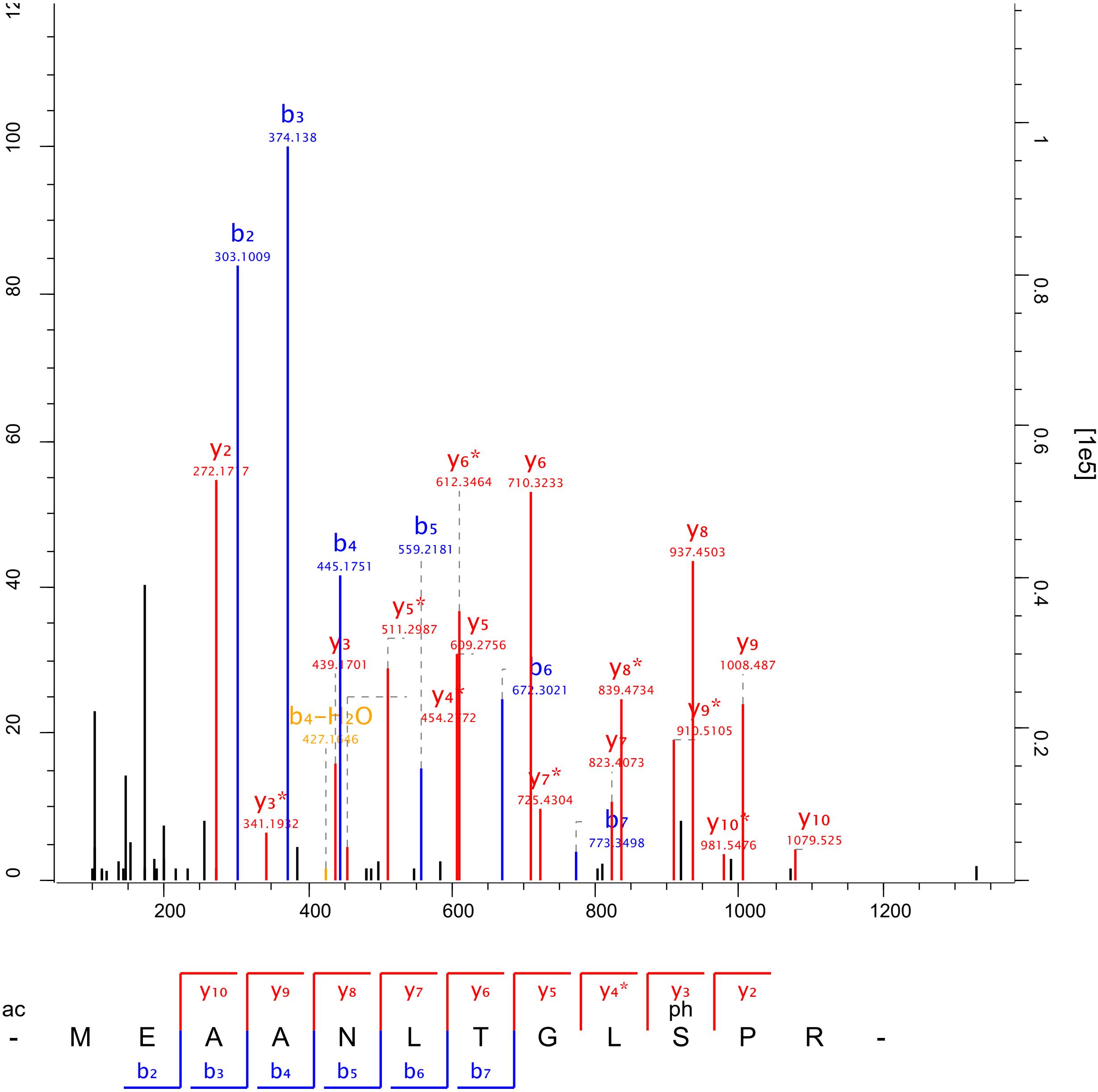

Raw file	Scan	Method	Score	m/z
sys_05_2	41402	FTMS; HCD	155.1	809.88

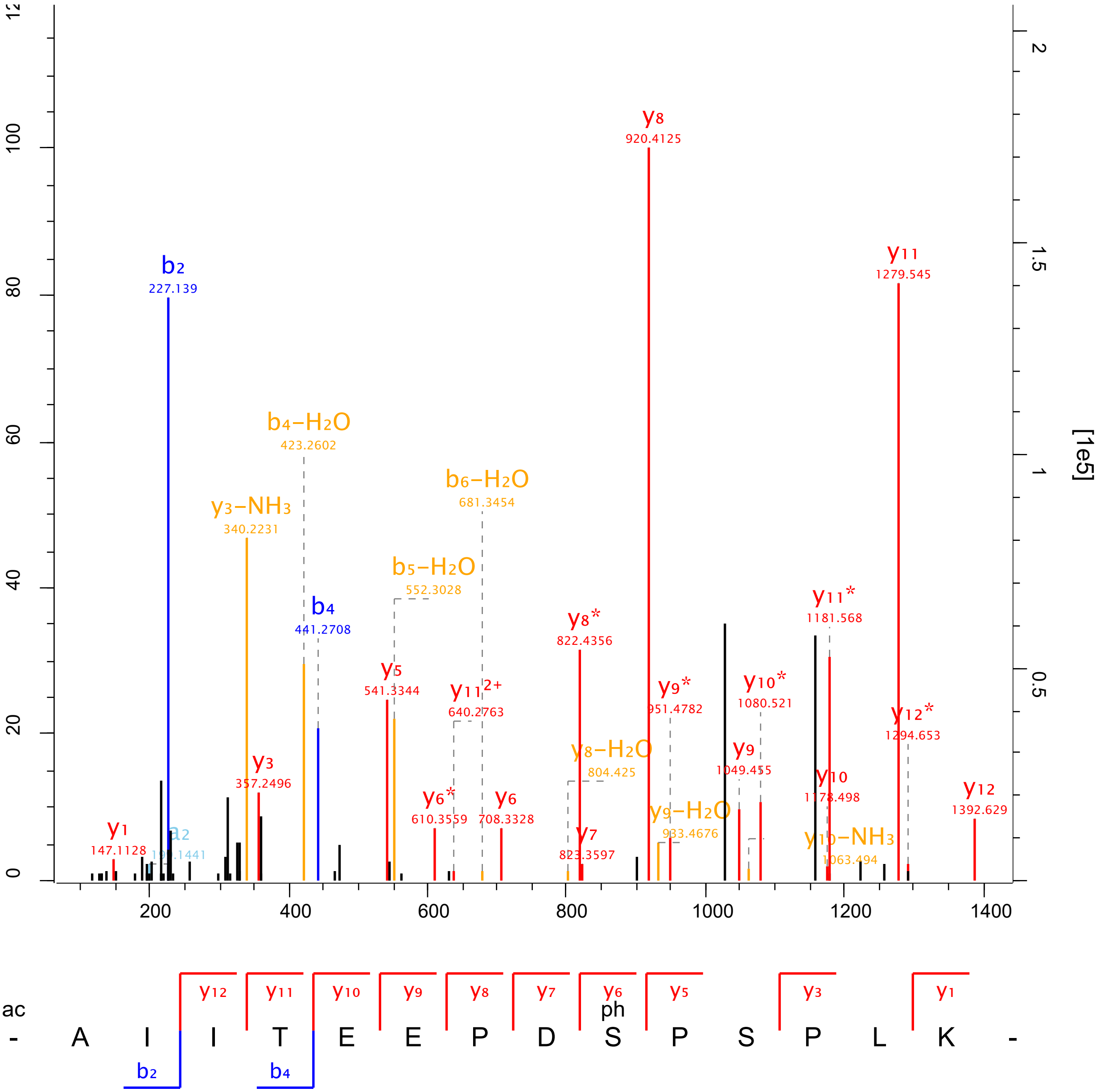

Raw file	Scan	Method	Score	m/z
sys_05_2	42234	FTMS; HCD	108.75	790.86

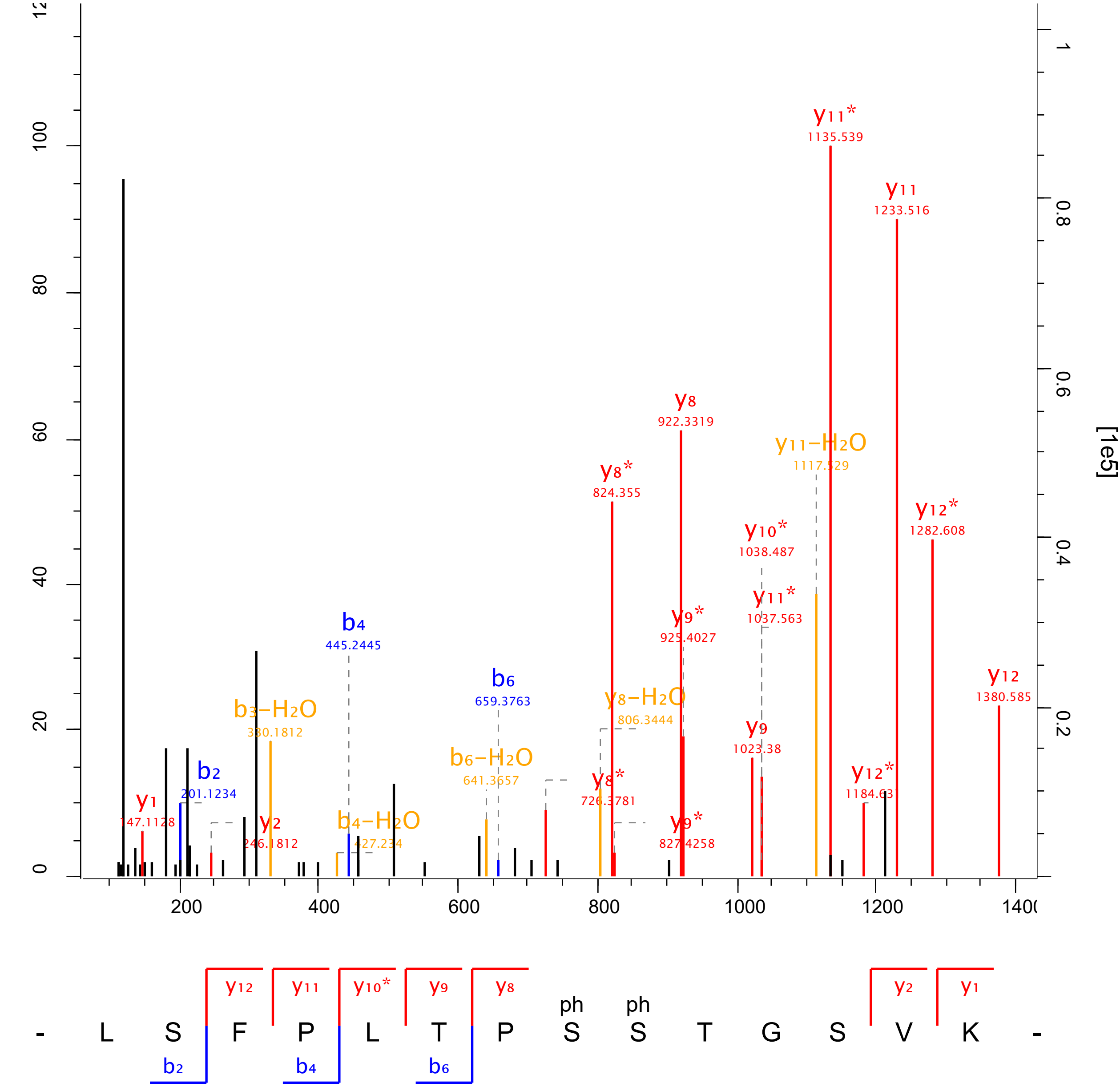

Raw file	Scan	Method	Score	m/z
sys_05_2	42536	FTMS; HCD	273.25	993.42

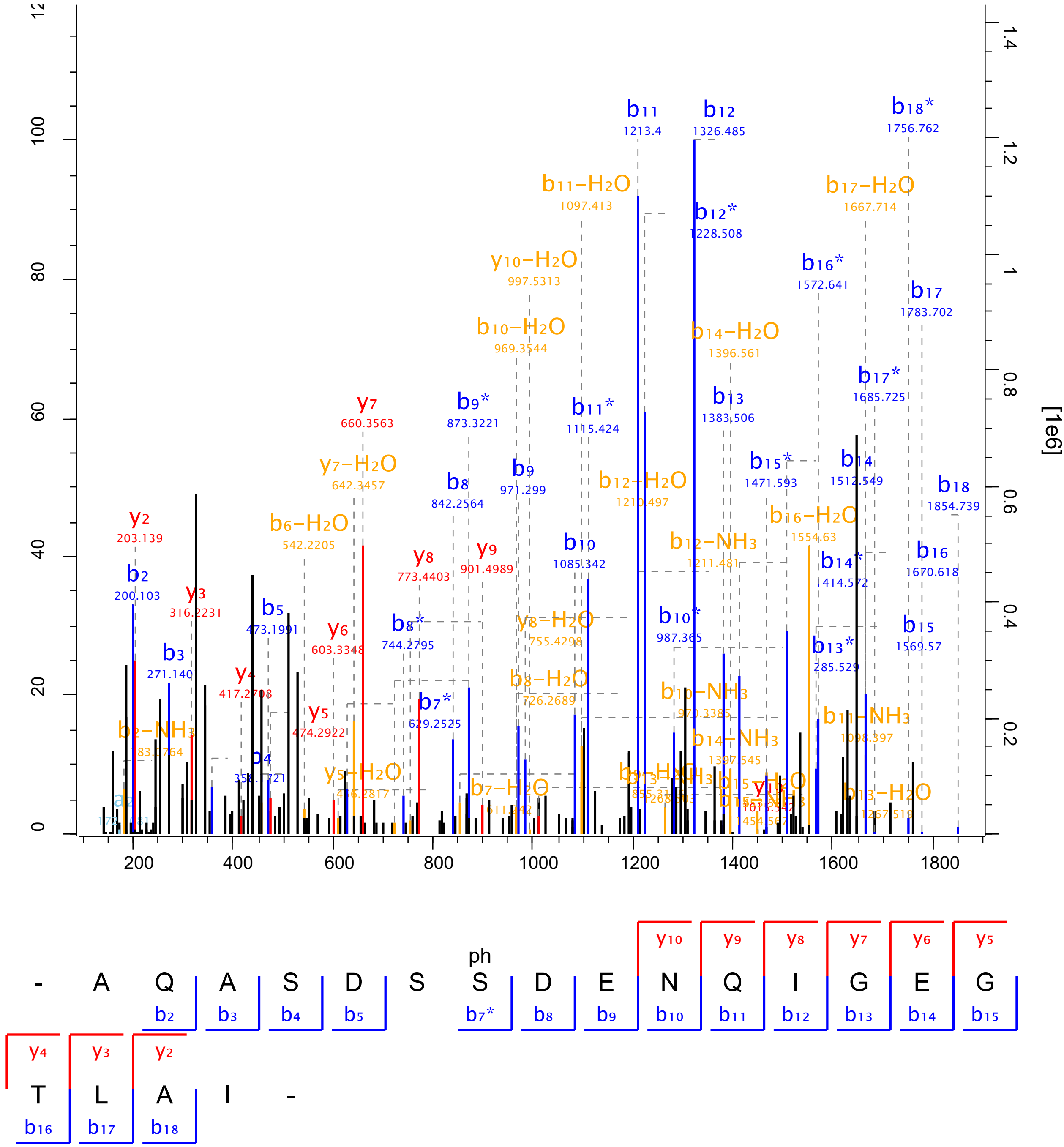

Raw file	Scan	Method	Score	m/z
sys_05_2	42764	FTMS; HCD	264.01	835.87

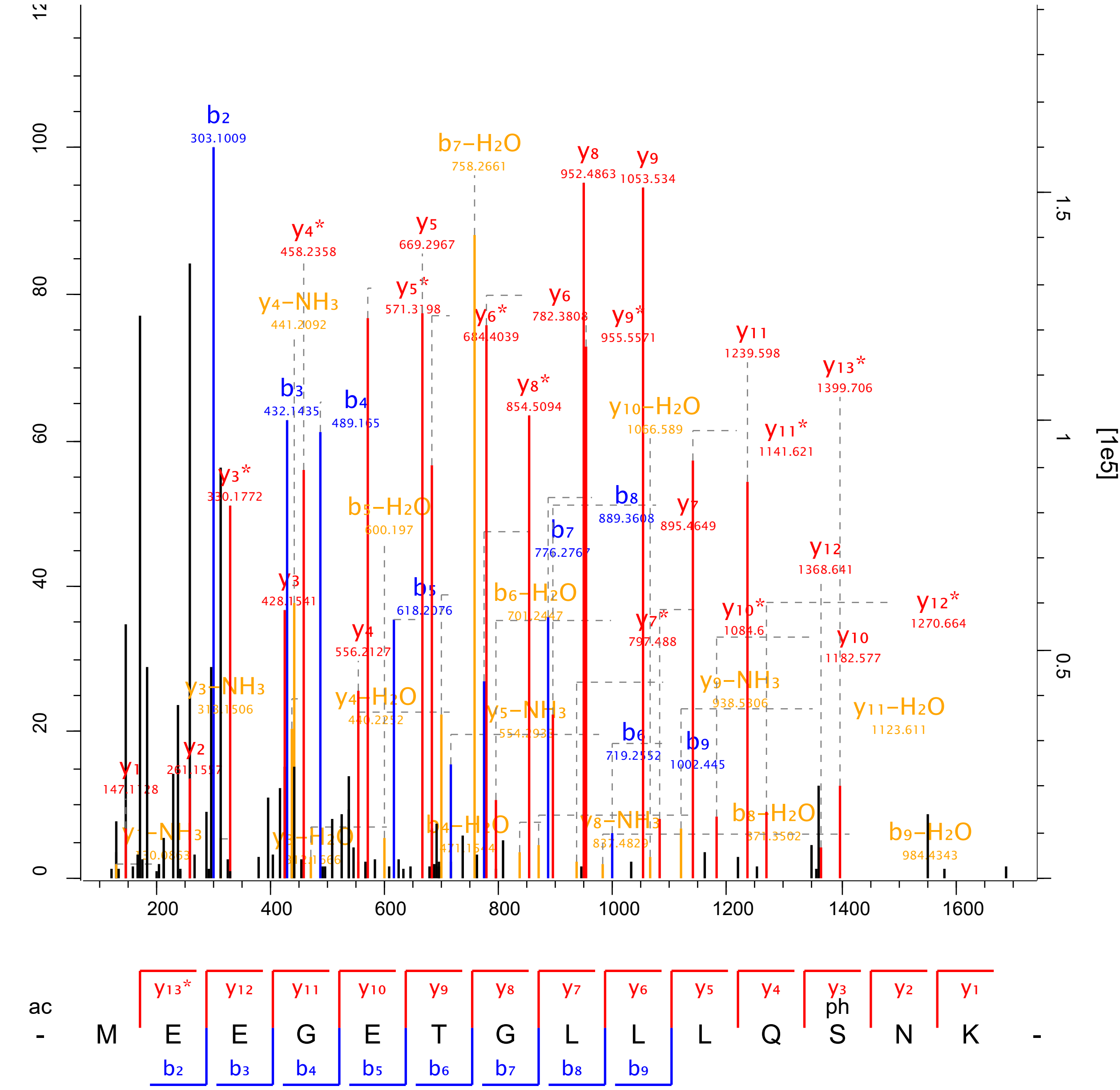

Raw file	Scan	Method	Score	m/z
sys_05_2	4327	FTMS; HCD	167.89	585.26

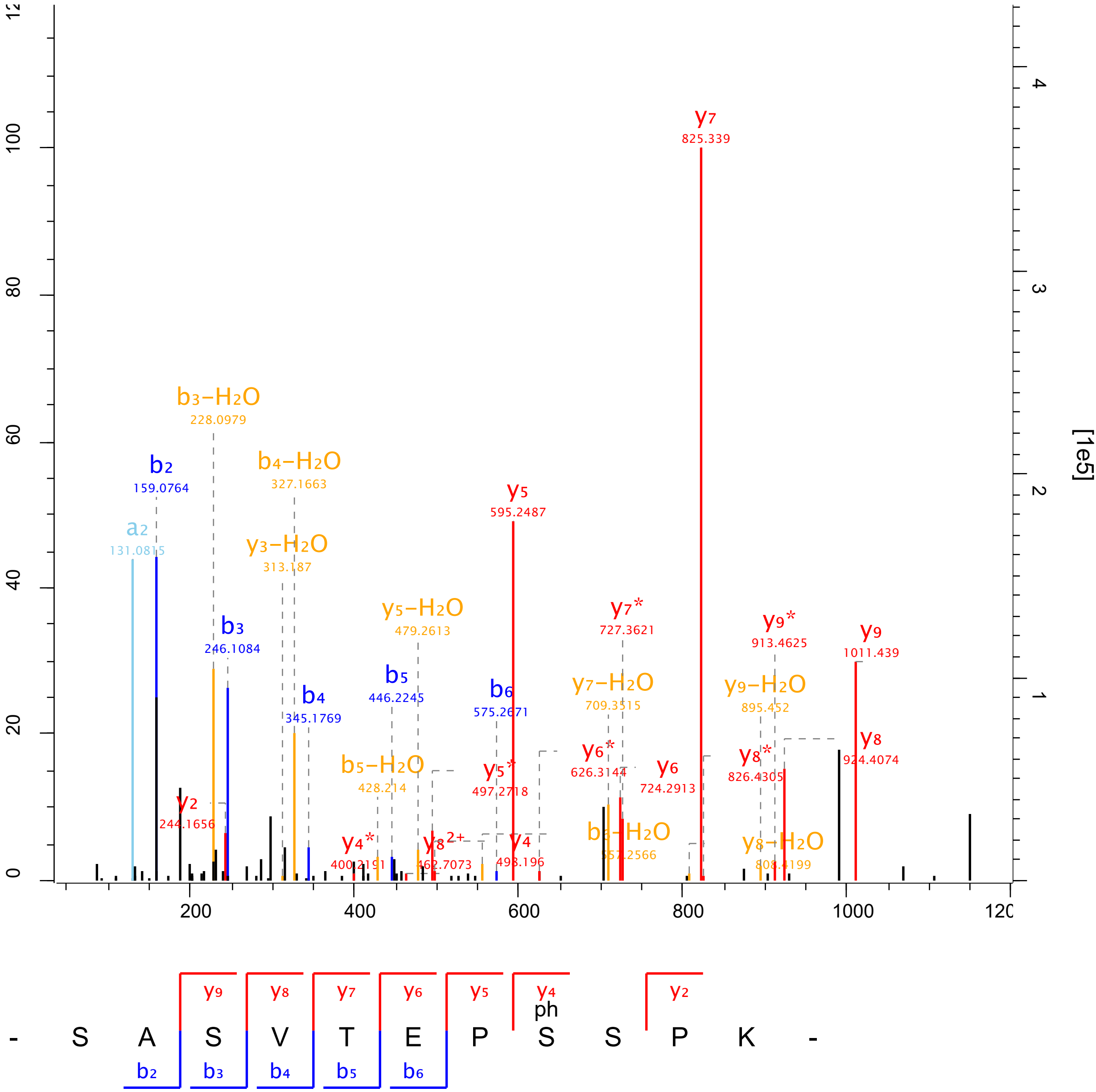

Raw file	Scan	Method	Score	m/z
sys_05_2	43787	FTMS; HCD	48.9	722.31

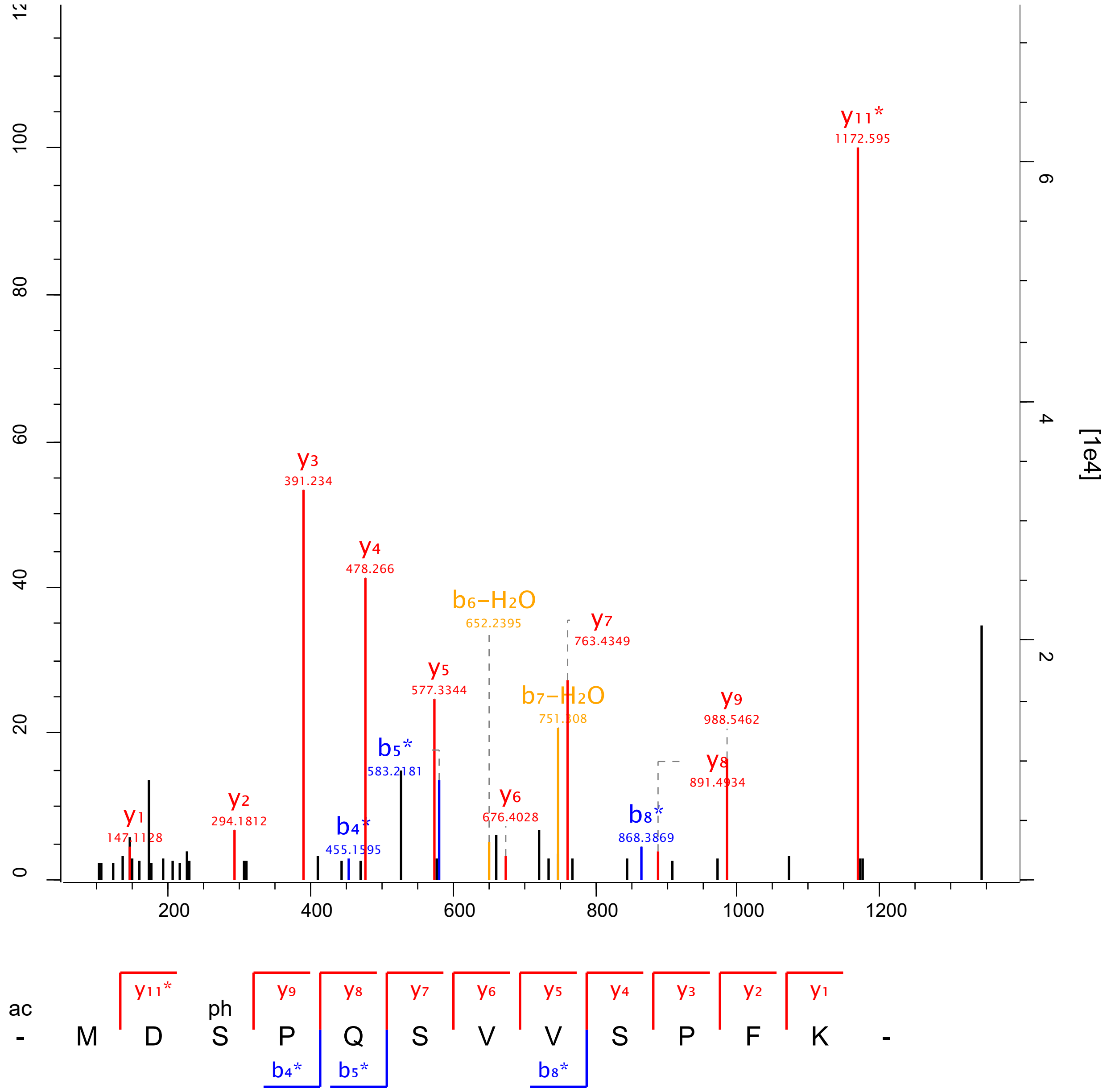

Raw file	Scan	Method	Score	m/z
sys_05_2	45695	FTMS; HCD	130.01	534.76

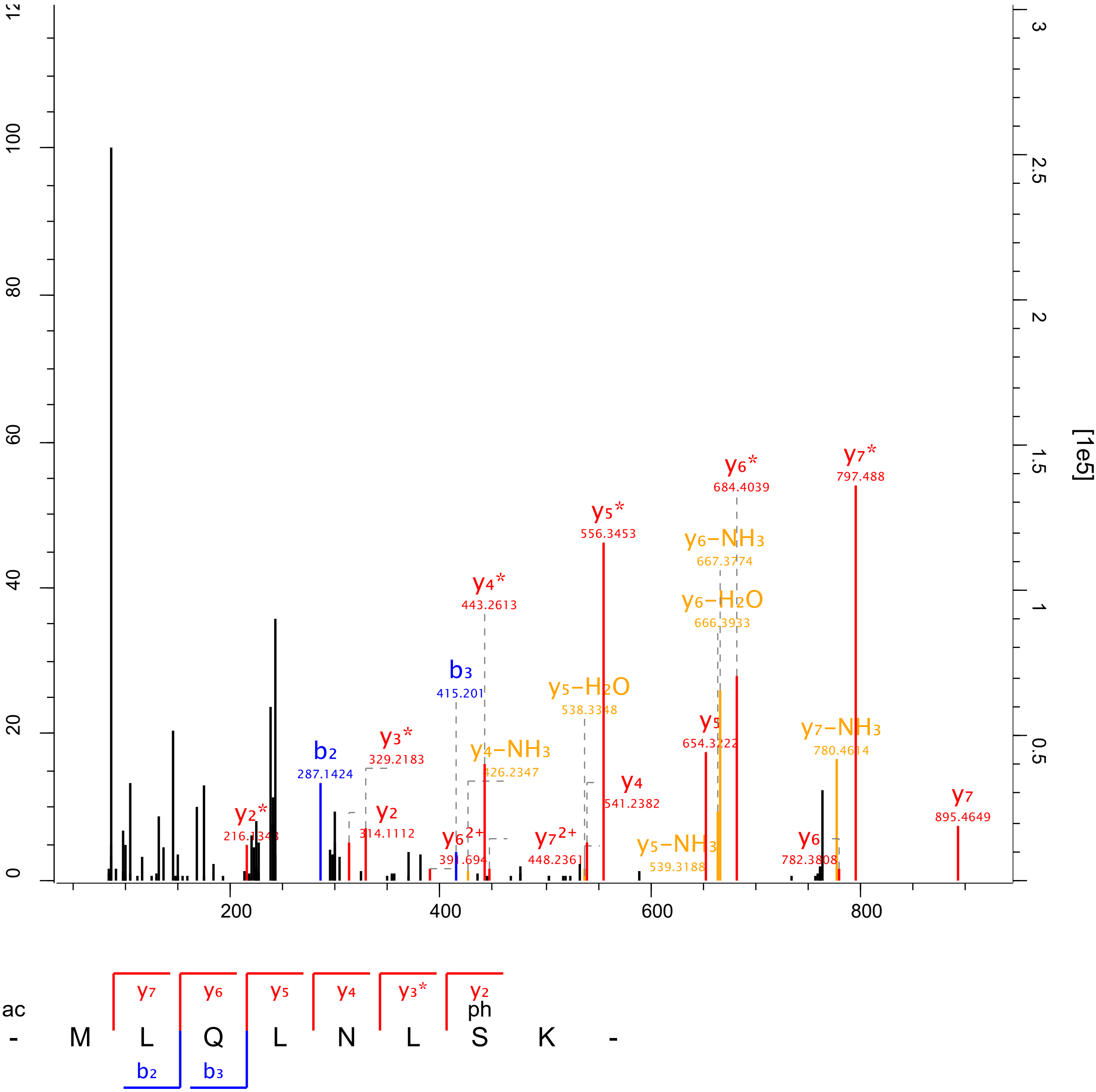

Raw file	Scan	Method	Score	m/z
sys_05_2	4607	FTMS; HCD	91.62	557.76

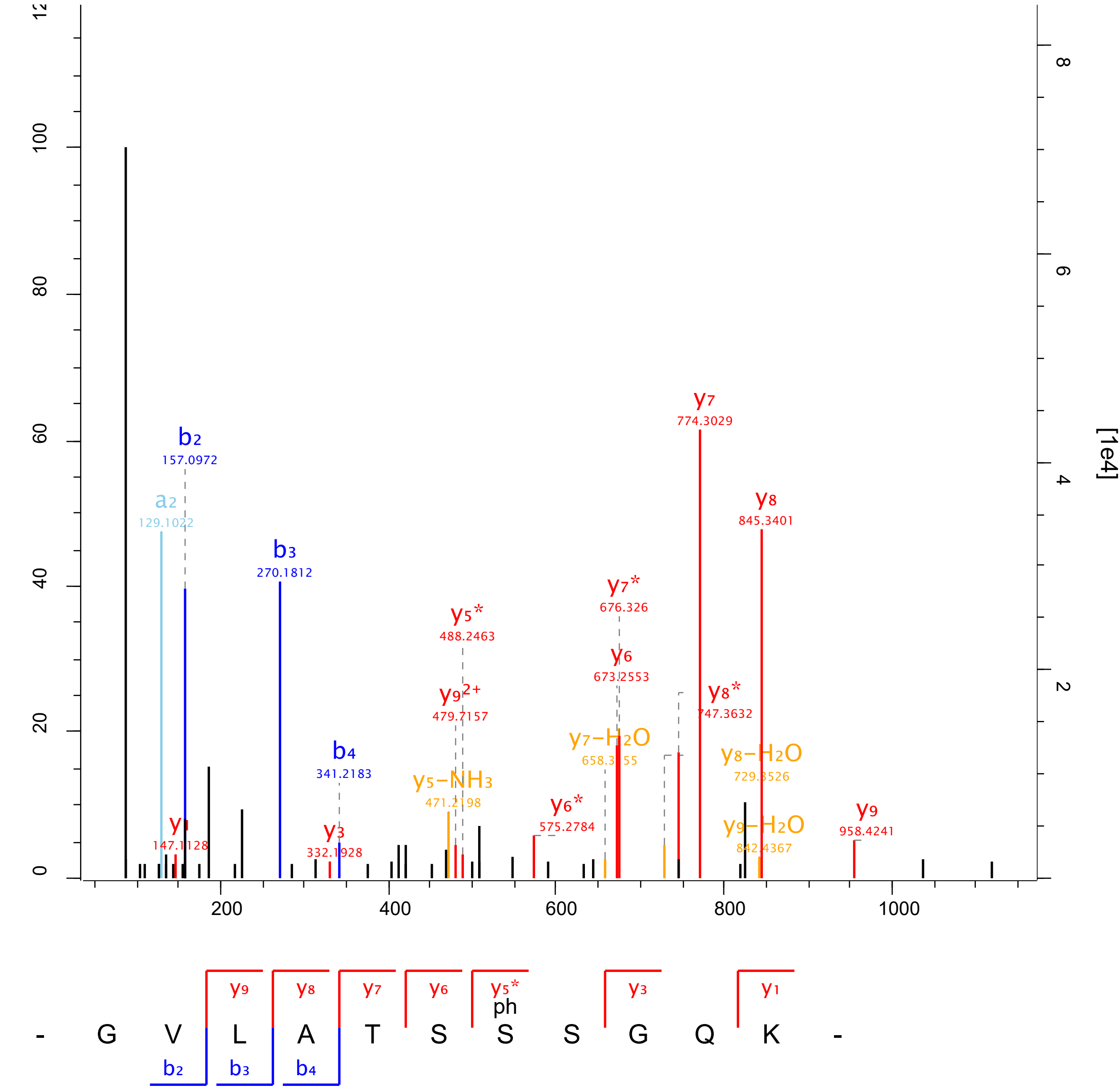

Raw file	Scan	Method	Score	m/z
sys_05_2	4614	FTMS; HCD	44.93	632.76

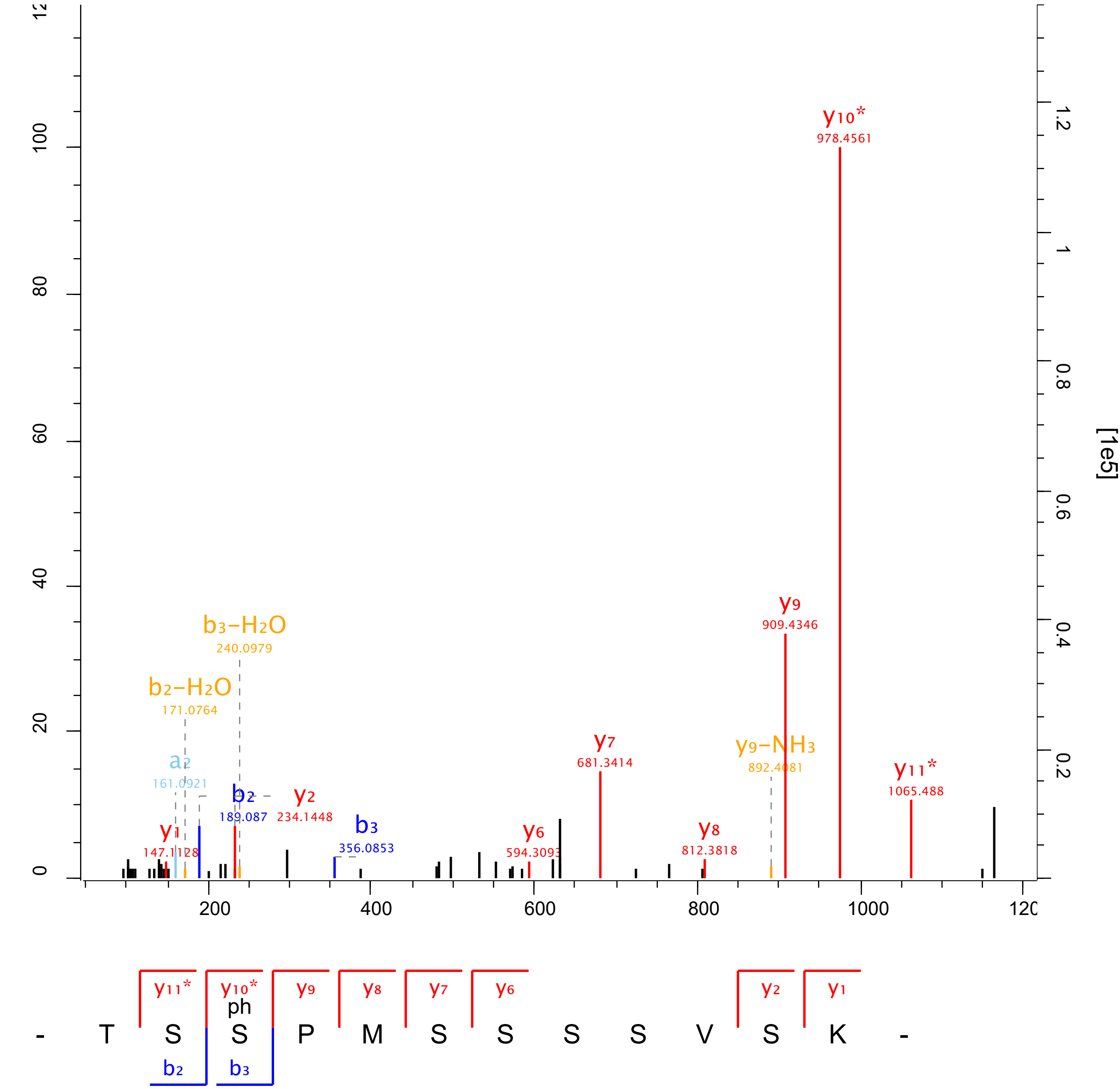

Raw file	Scan	Method	Score	m/z
sys_05_2	4635	FTMS; HCD	140.78	506.73

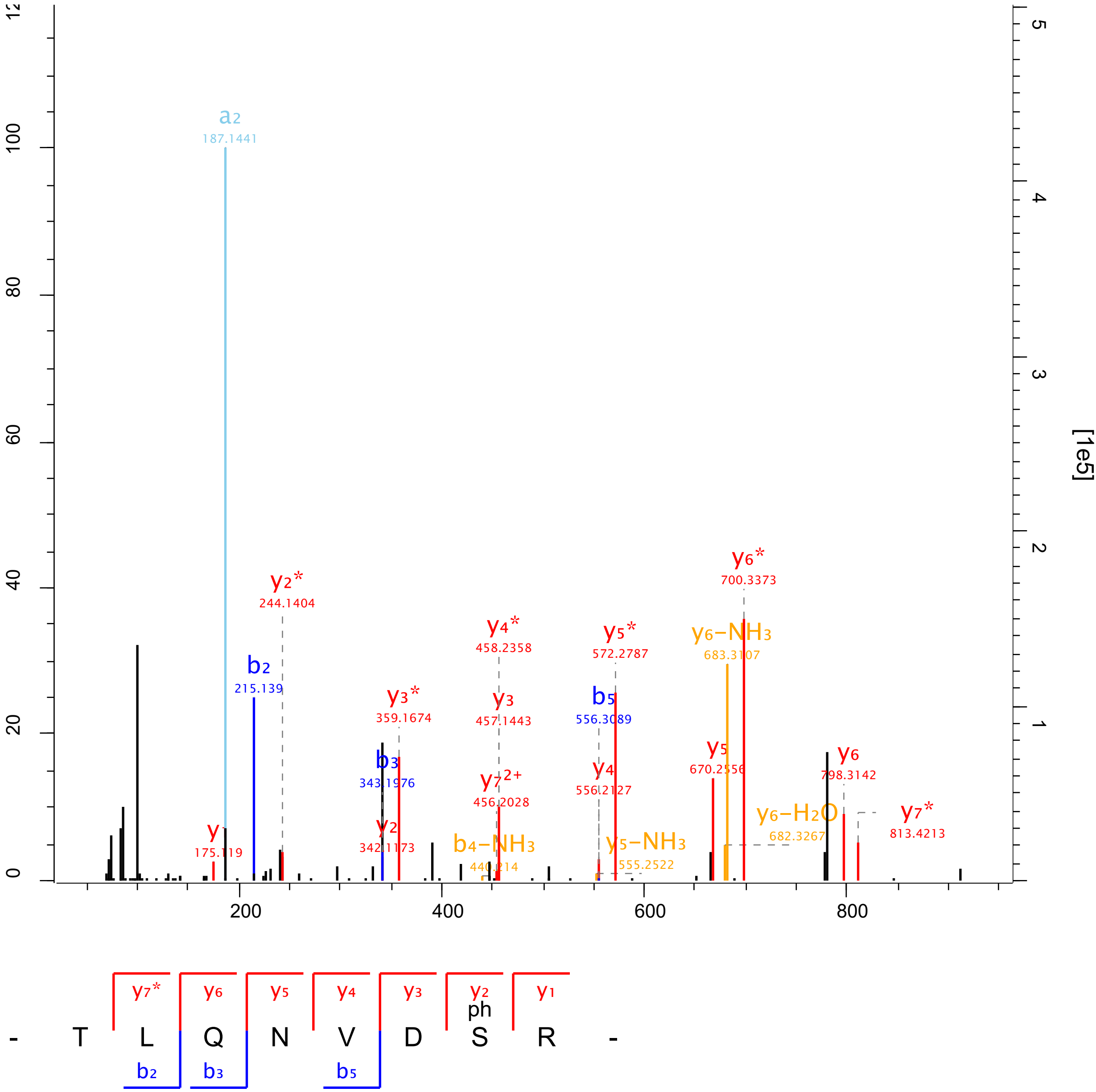

Raw file	Scan	Method	Score	m/z
sys_05_2	4649	FTMS; HCD	108.47	485.72

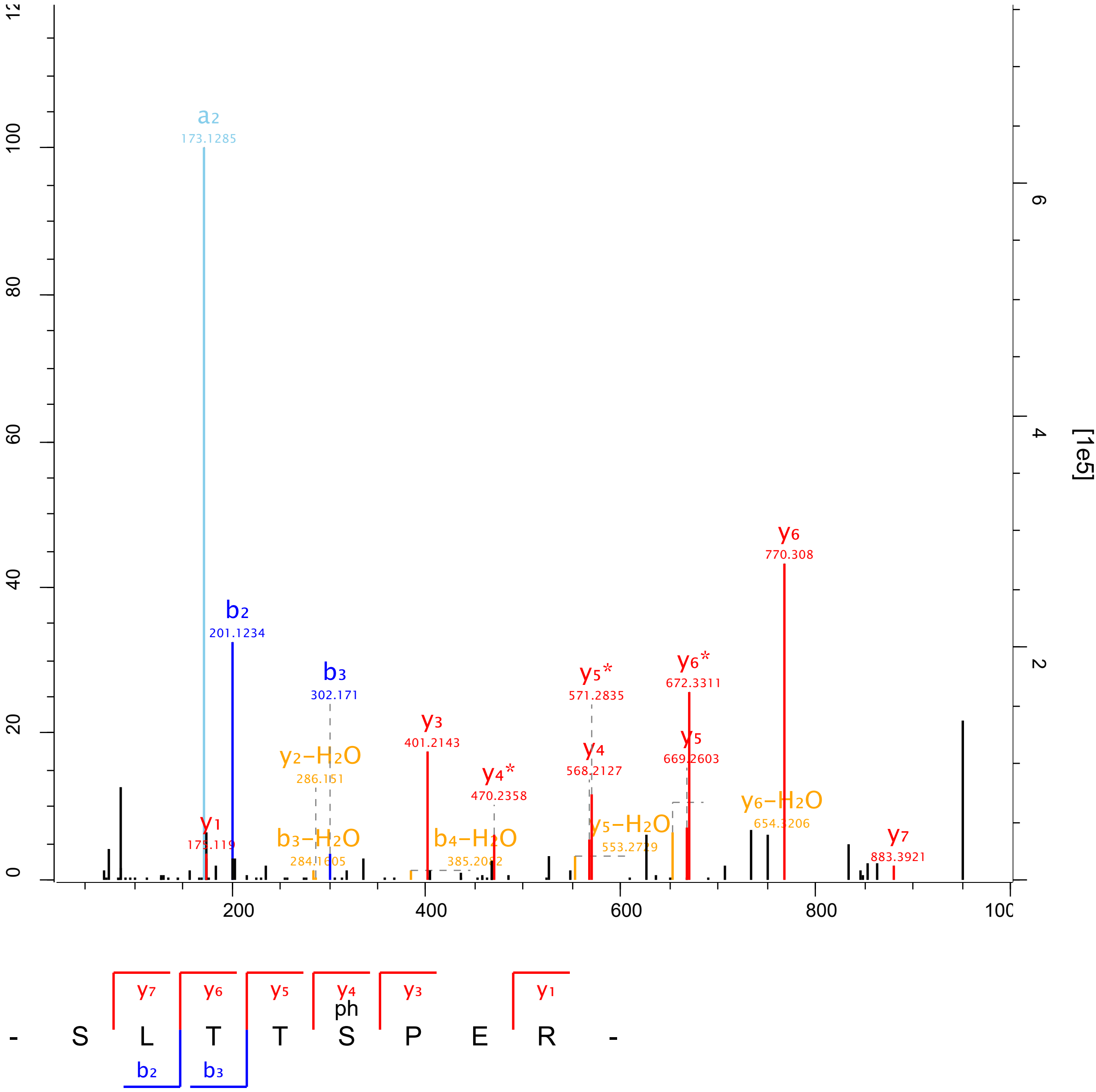

Raw file	Scan	Method	Score	m/z
sys_05_2	4729	FTMS; HCD	203.76	658.28

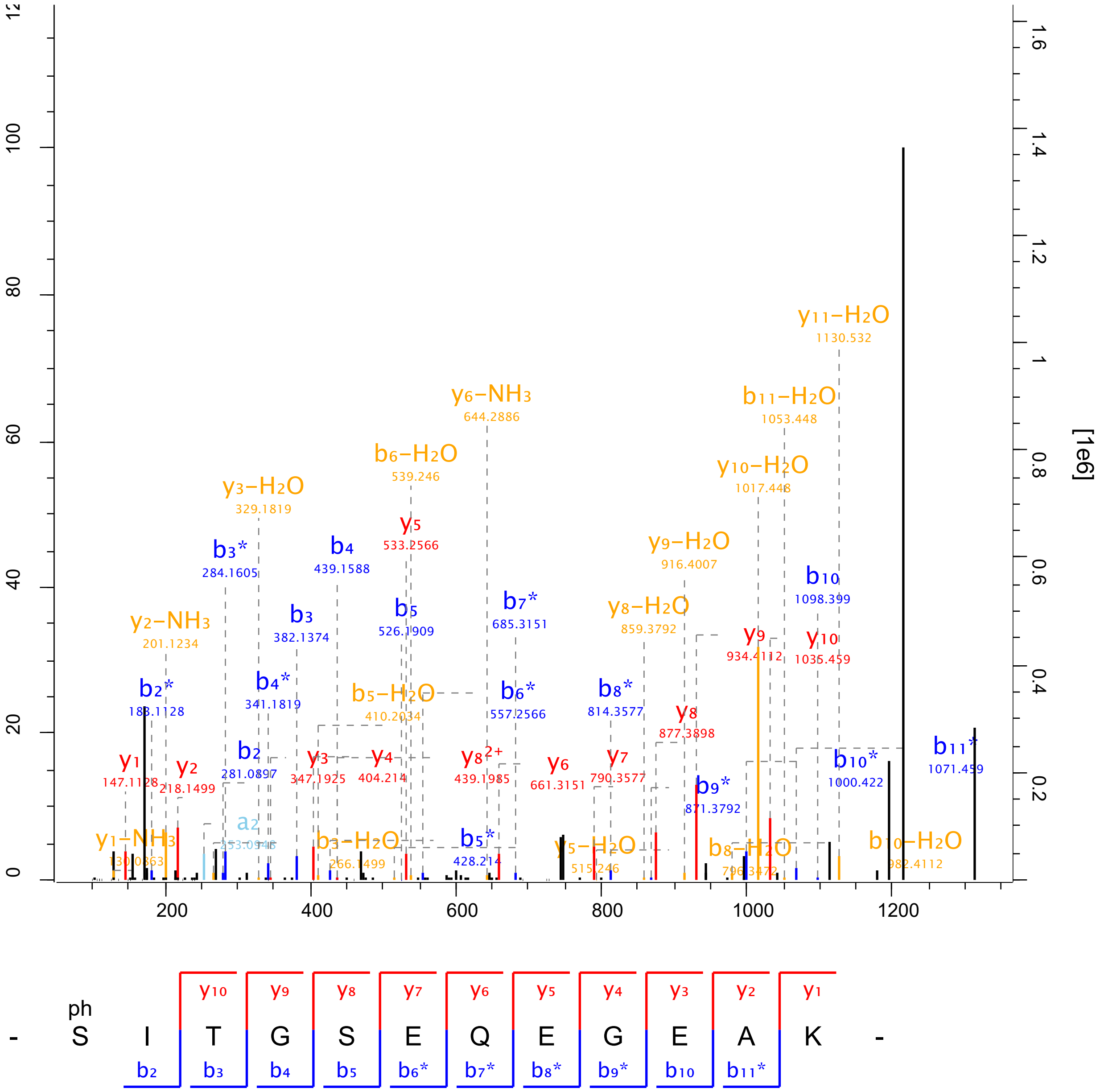

Raw file	Scan	Method	Score	m/z
sys_05_2	4834	FTMS; HCD	68.68	599.74

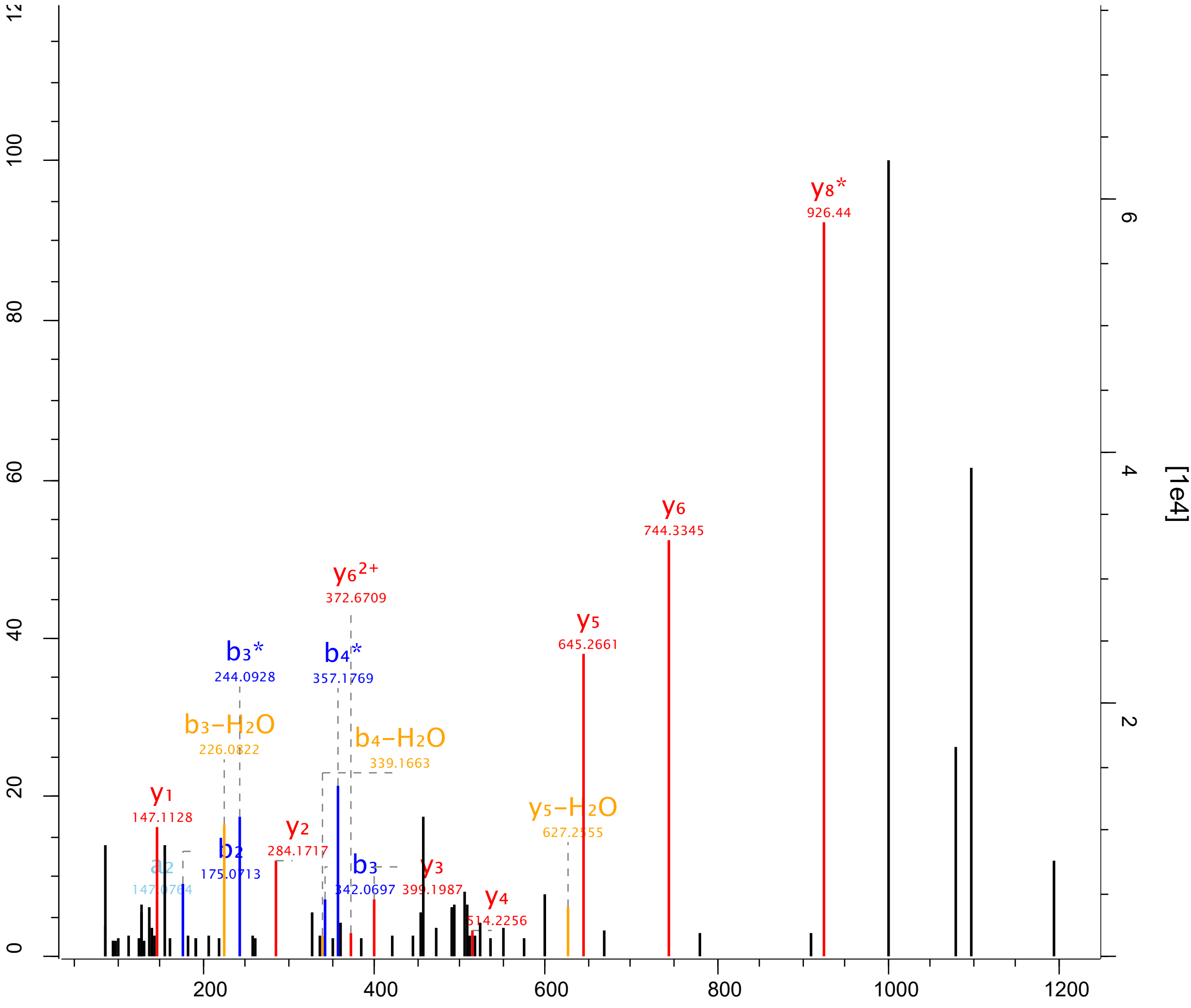

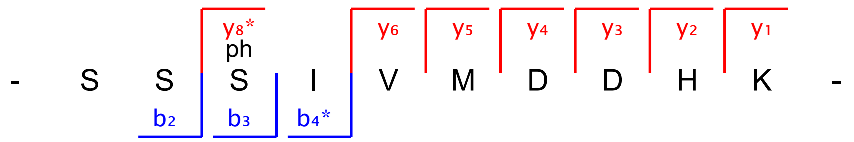

Raw file	Scan	Method	Score	m/z
sys_05_2	4870	FTMS; HCD	179.6	507.72

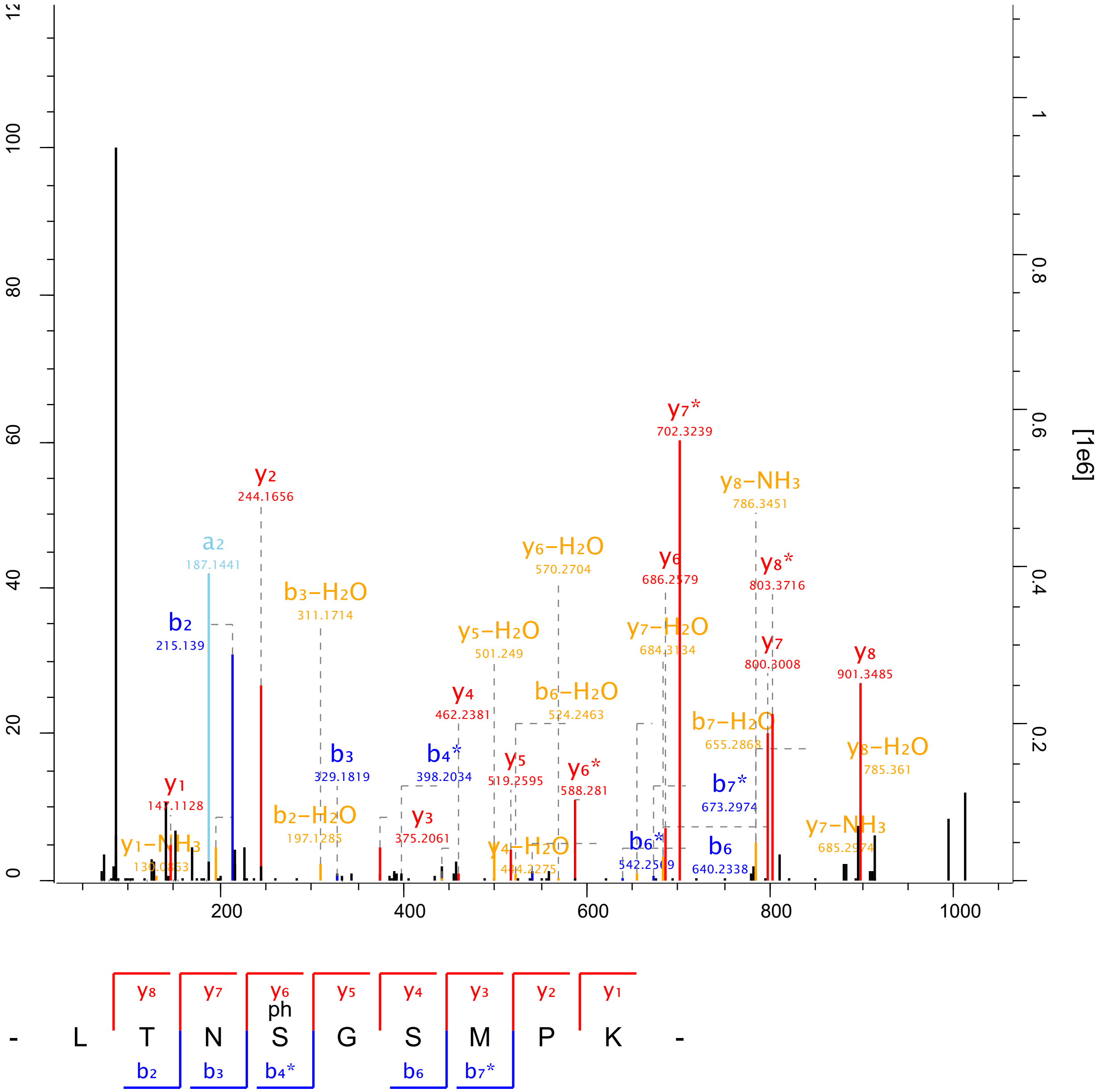

Raw file	Scan	Method	Score	m/z
sys_05_2	49176	FTMS; HCD	111.57	994.41

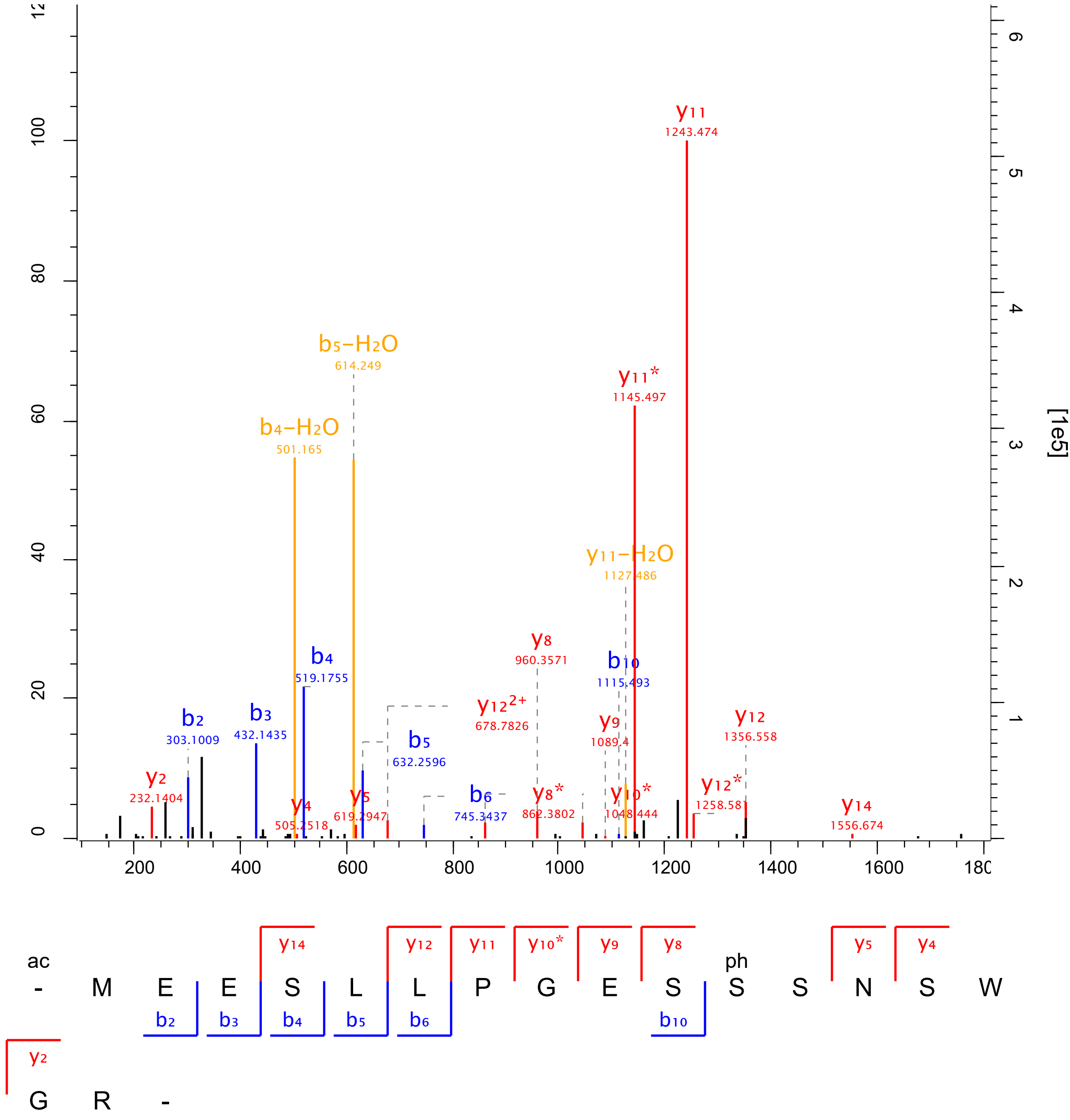

Raw file	Scan	Method	Score	m/z
sys_05_2	49177	FTMS; HCD	50.3	738.31

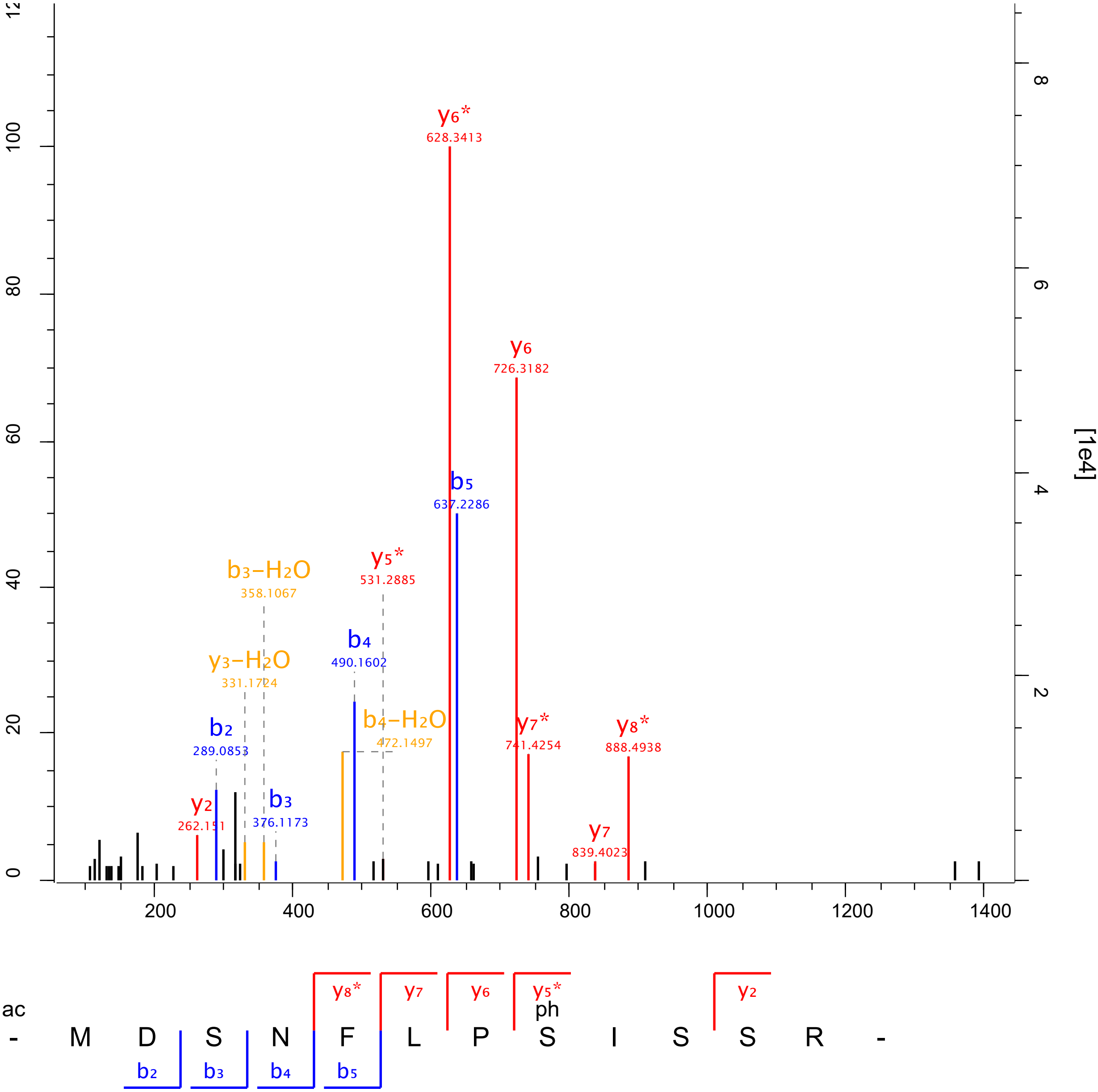

Raw file	Scan	Method	Score	m/z
sys_05_2	5204	FTMS; HCD	67.33	554.73

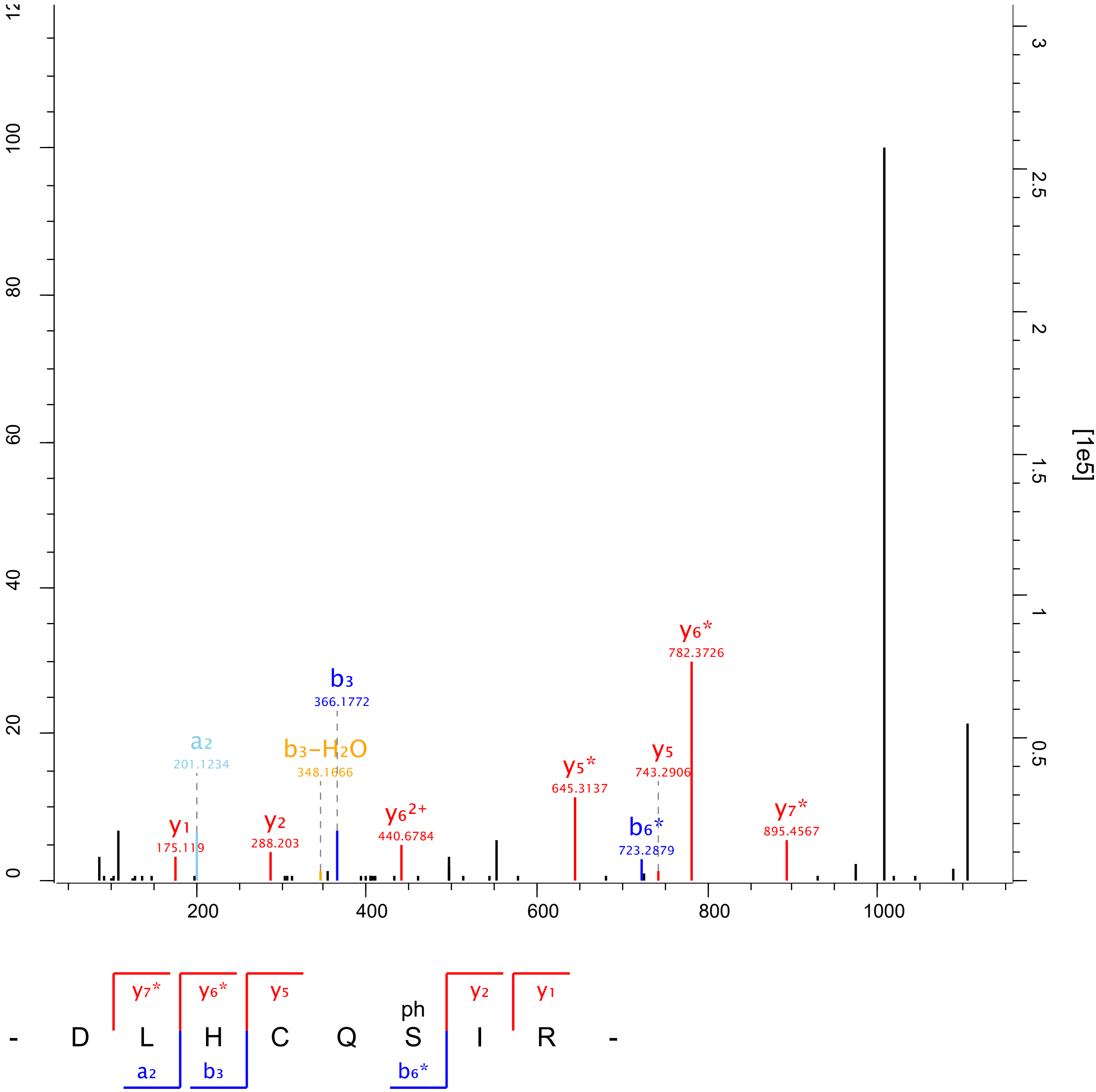

Raw file	Scan	Method	Score	m/z
sys_05_2	5273	FTMS; HCD	68.66	508.75

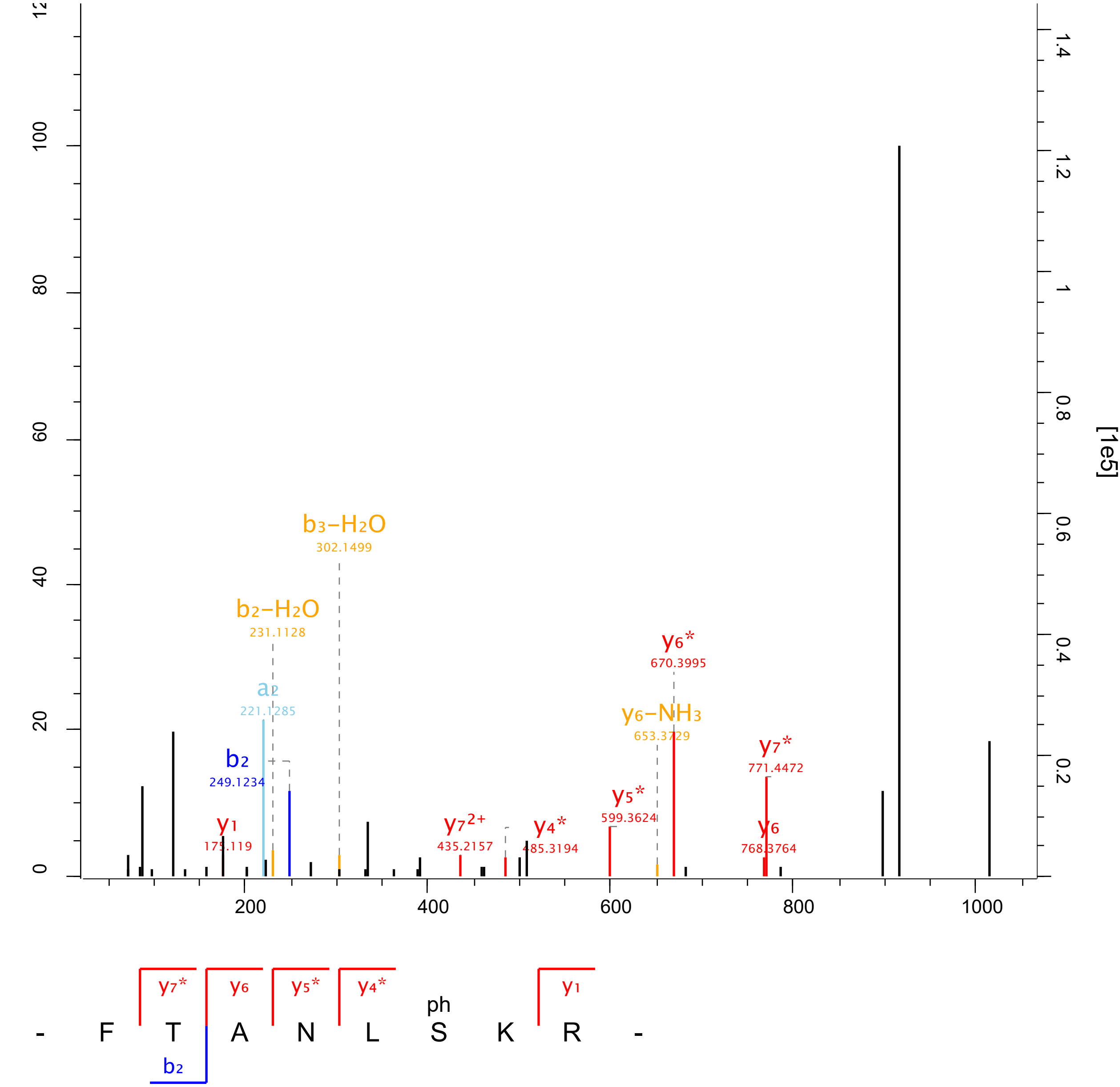

Raw file	Scan	Method	Score	m/z
sys_05_2	5406	FTMS; HCD	107.35	684.28

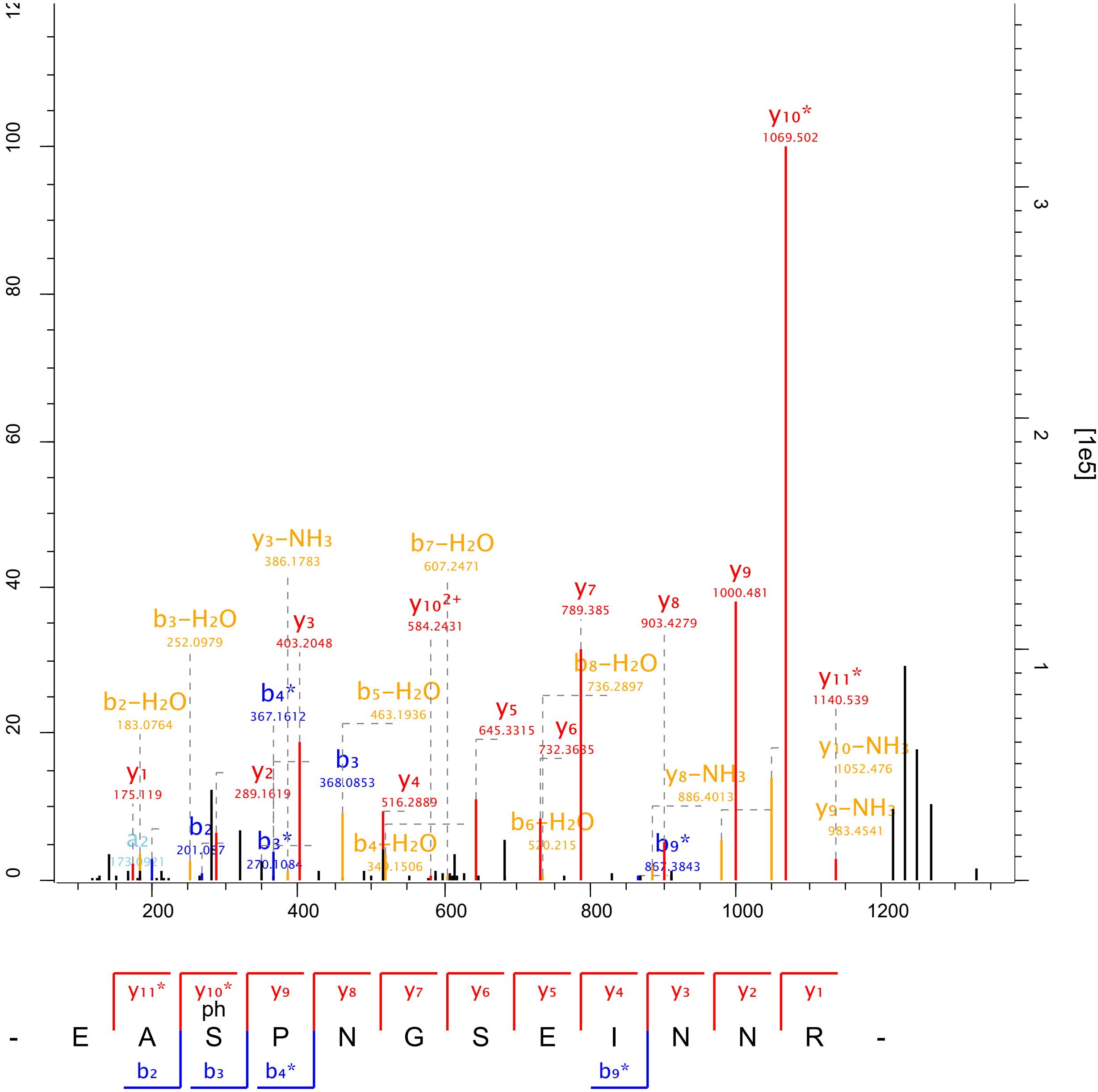

Raw file	Scan	Method	Score	m/z
sys_05_2	5611	FTMS; HCD	189.09	829.32

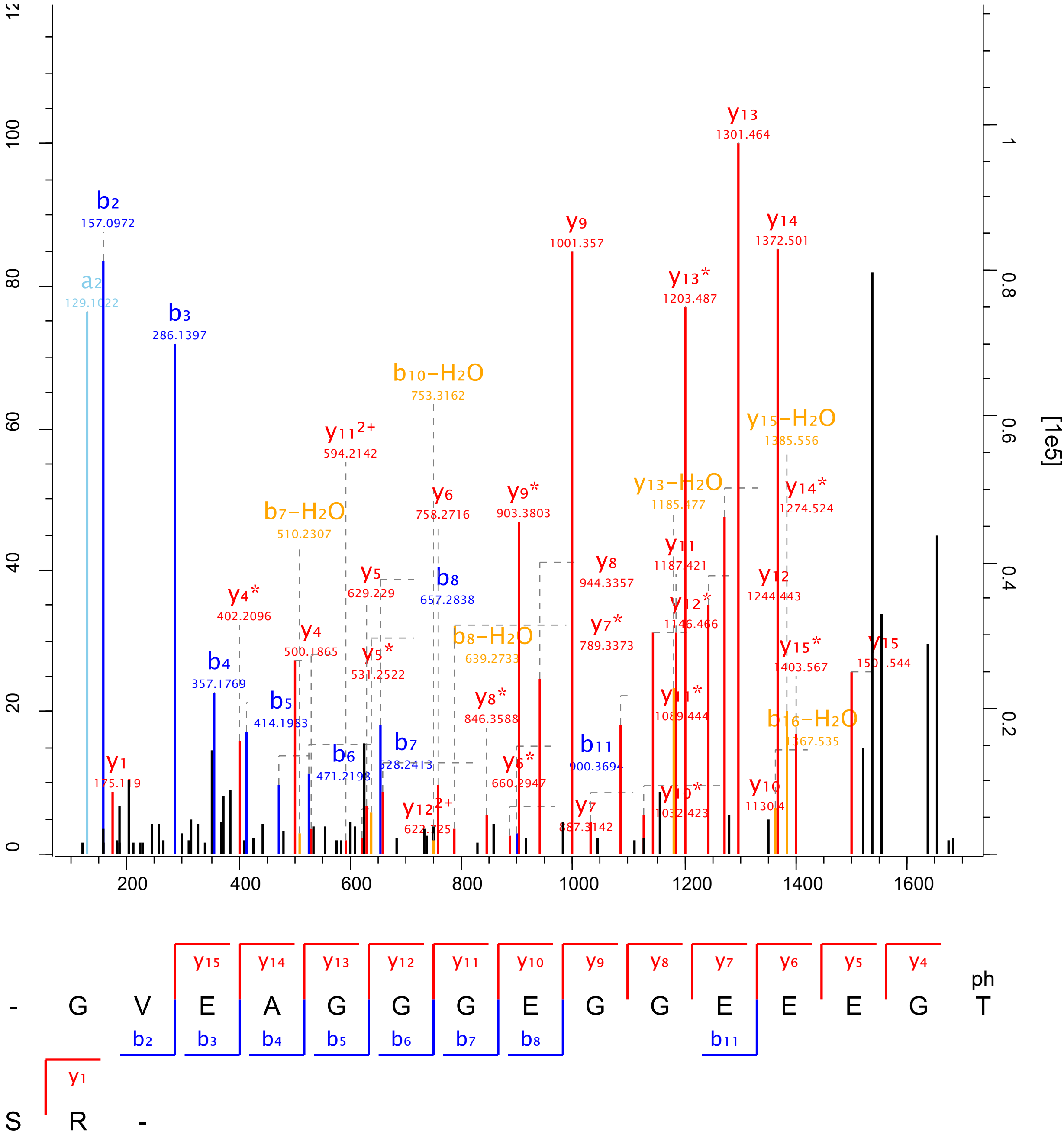

Raw file	Scan	Method	Score	m/z
sys_05_2	5630	FTMS; HCD	68.54	452.69

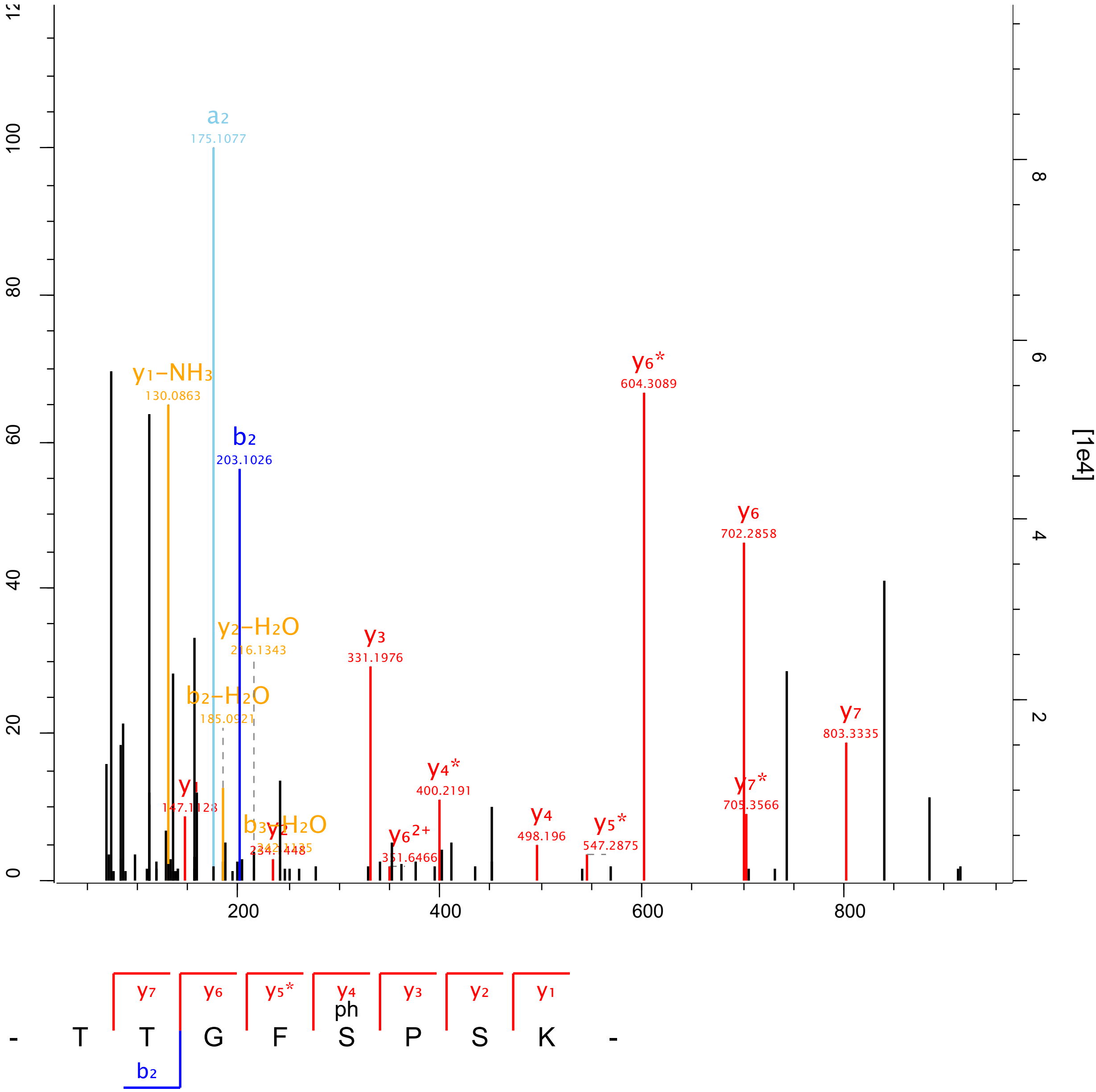

Raw file	Scan	Method	Score	m/z
sys_05_2	5681	FTMS; HCD	311.16	895.86

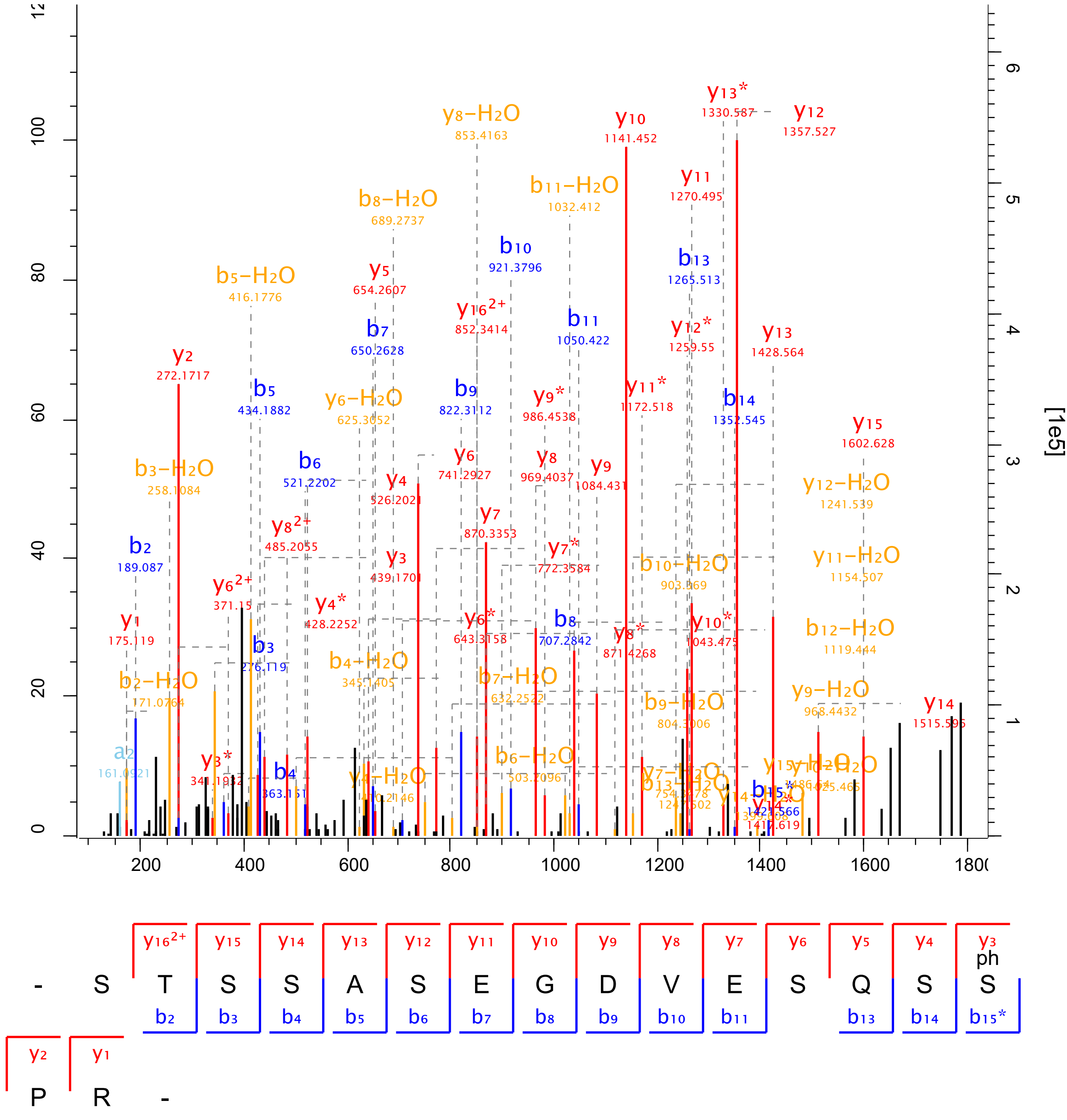

Raw file	Scan	Method	Score	m/z
sys_05_2	5694	FTMS; HCD	194.58	769.81

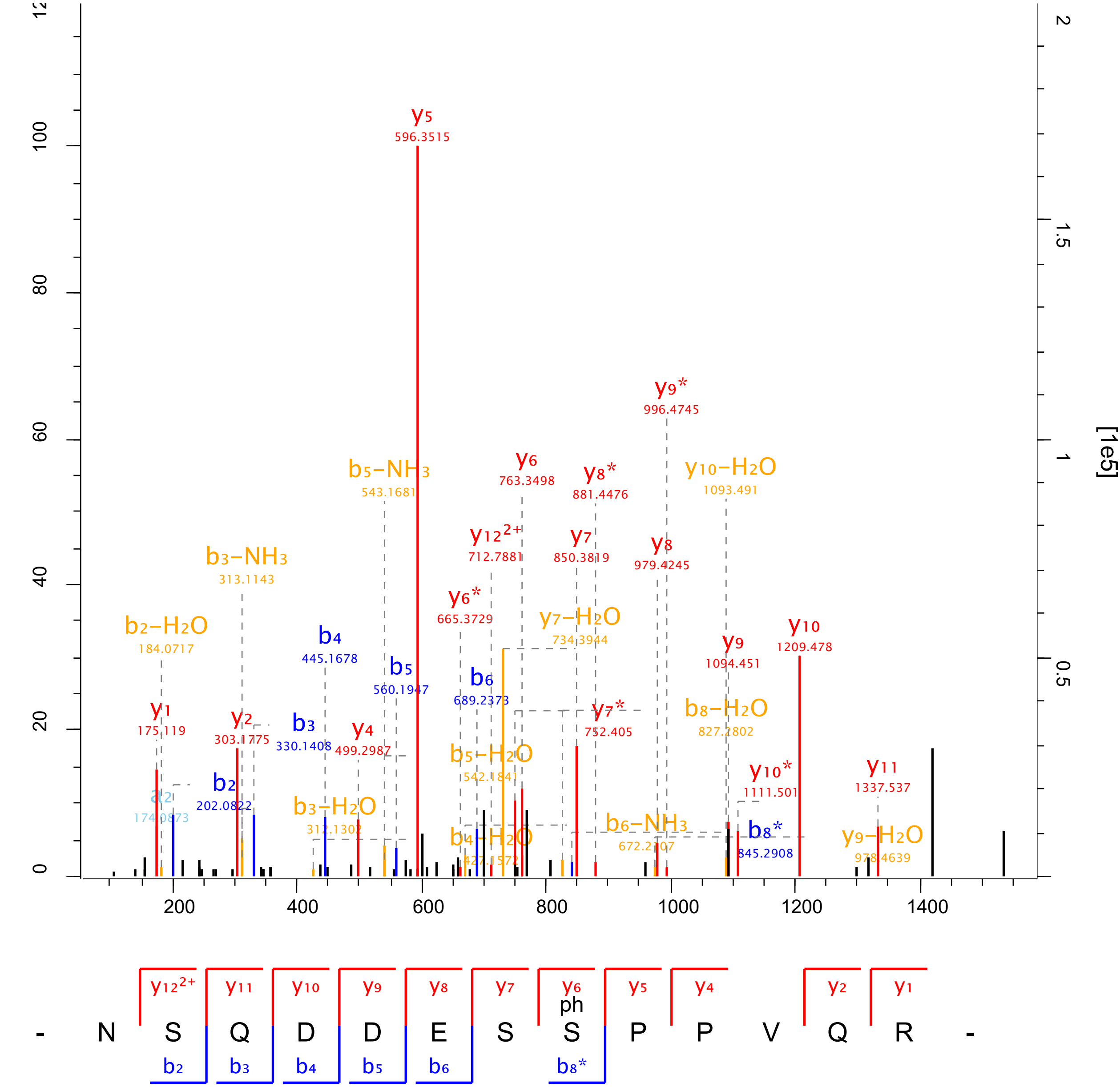

Raw file	Scan	Method	Score	m/z
sys_05_2	5859	FTMS; HCD	206.63	684.3

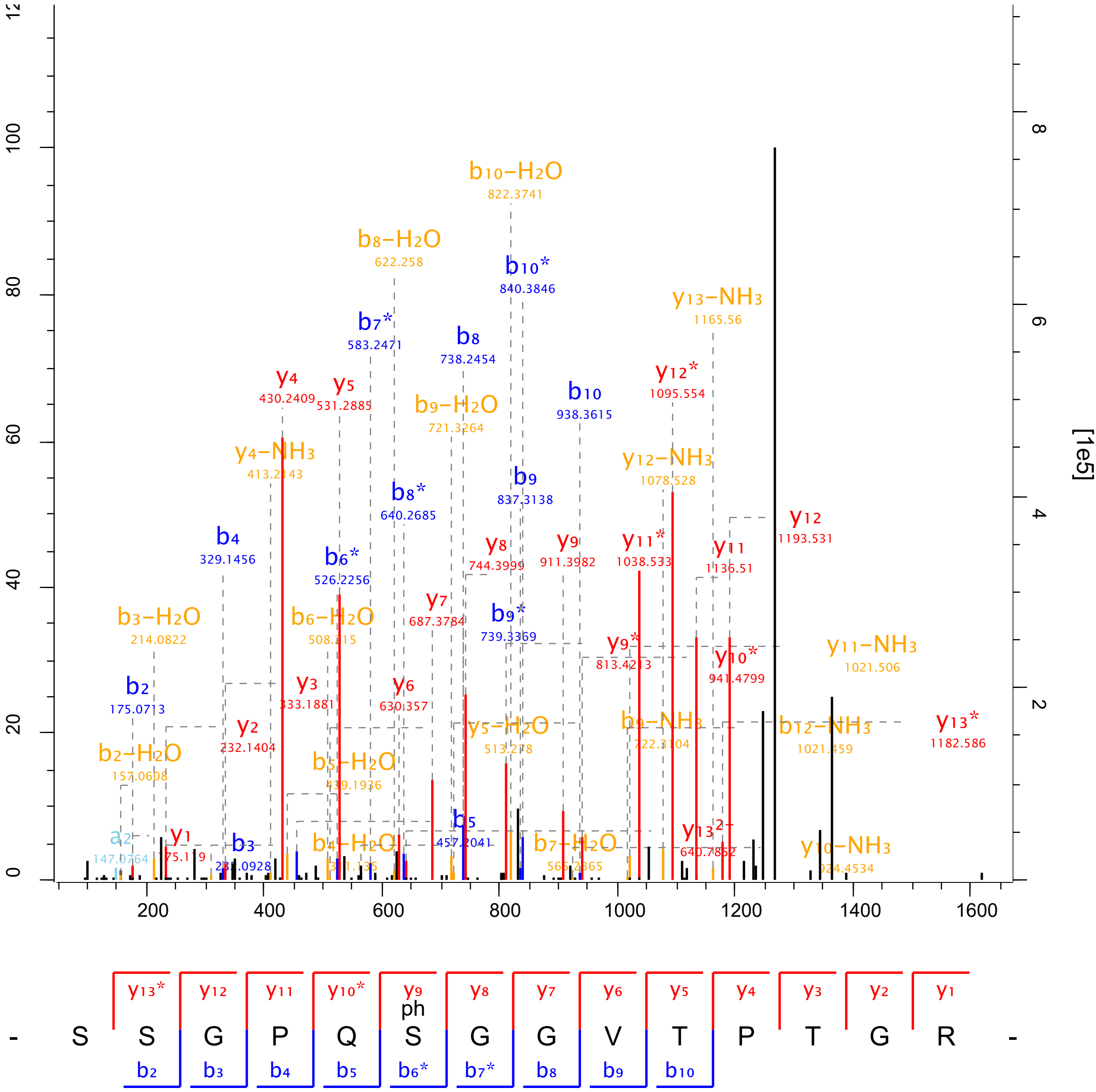

Raw file	Scan	Method	Score	m/z
sys_05_2	6060	FTMS; HCD	123.76	618.77

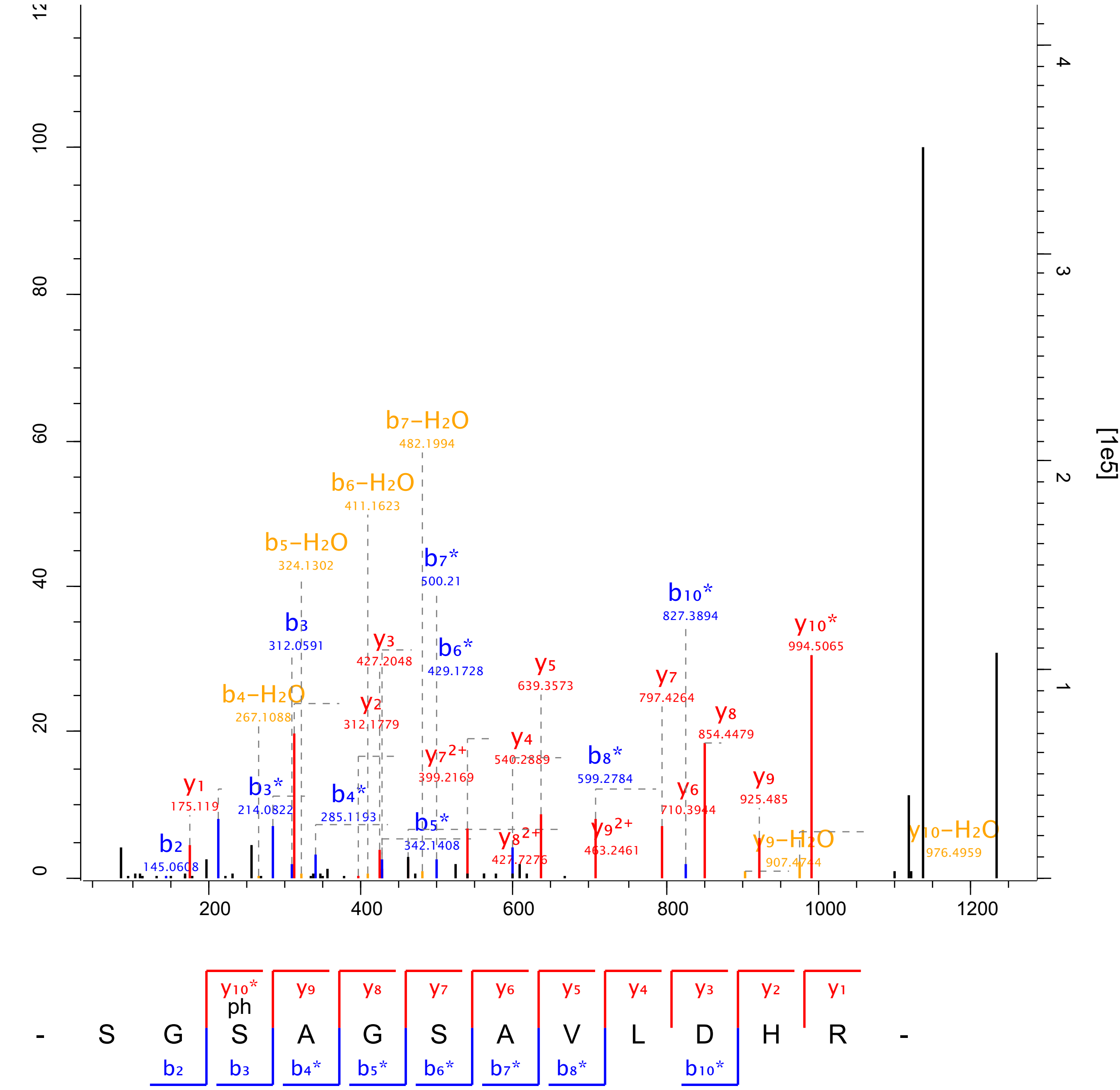

Raw file	Scan	Method	Score	m/z
sys_05_2	6073	FTMS; HCD	151.18	676.6

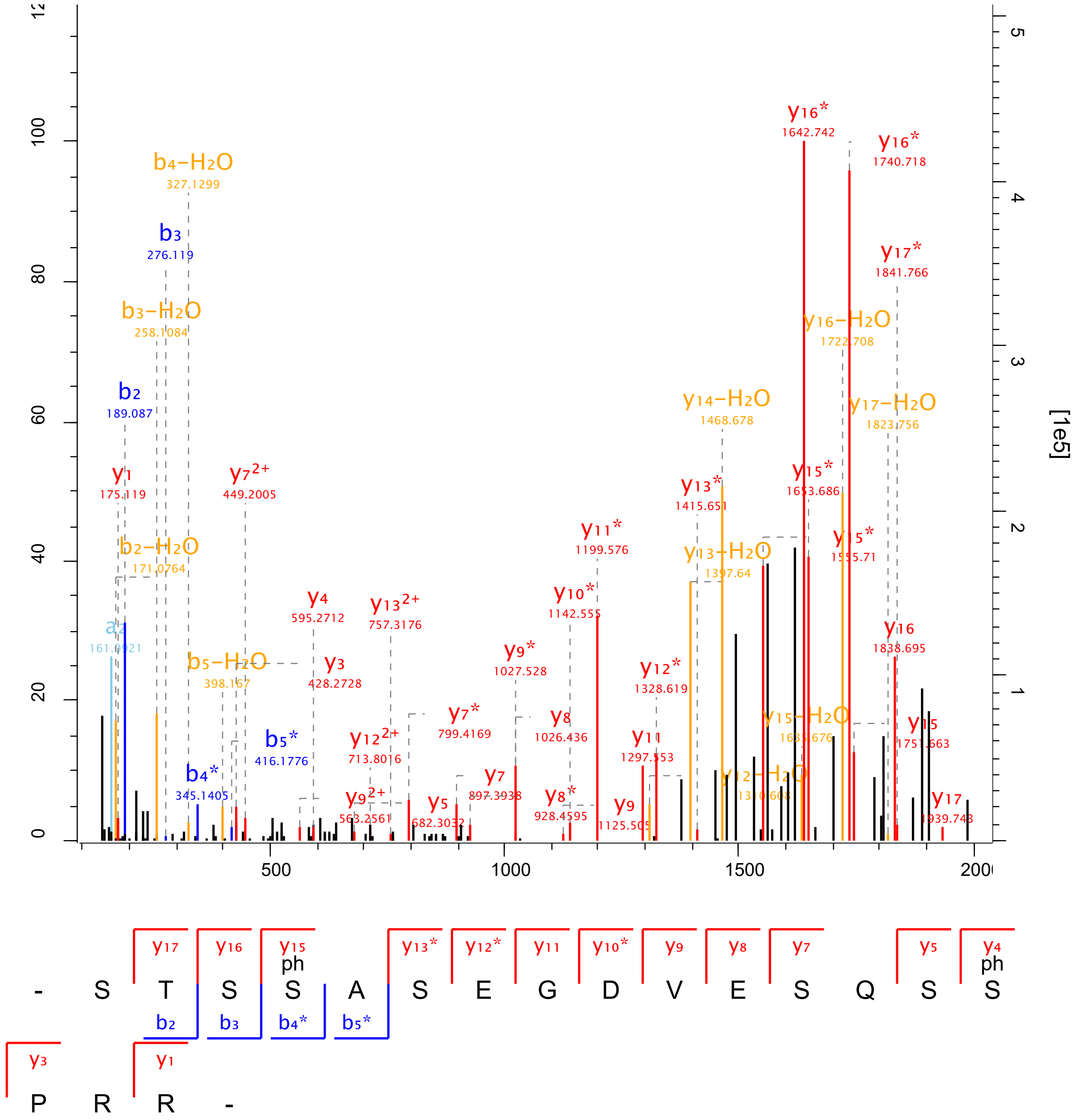

Raw file	Scan	Method	Score	m/z
sys_05_2	6121	FTMS; HCD	85.68	431.71

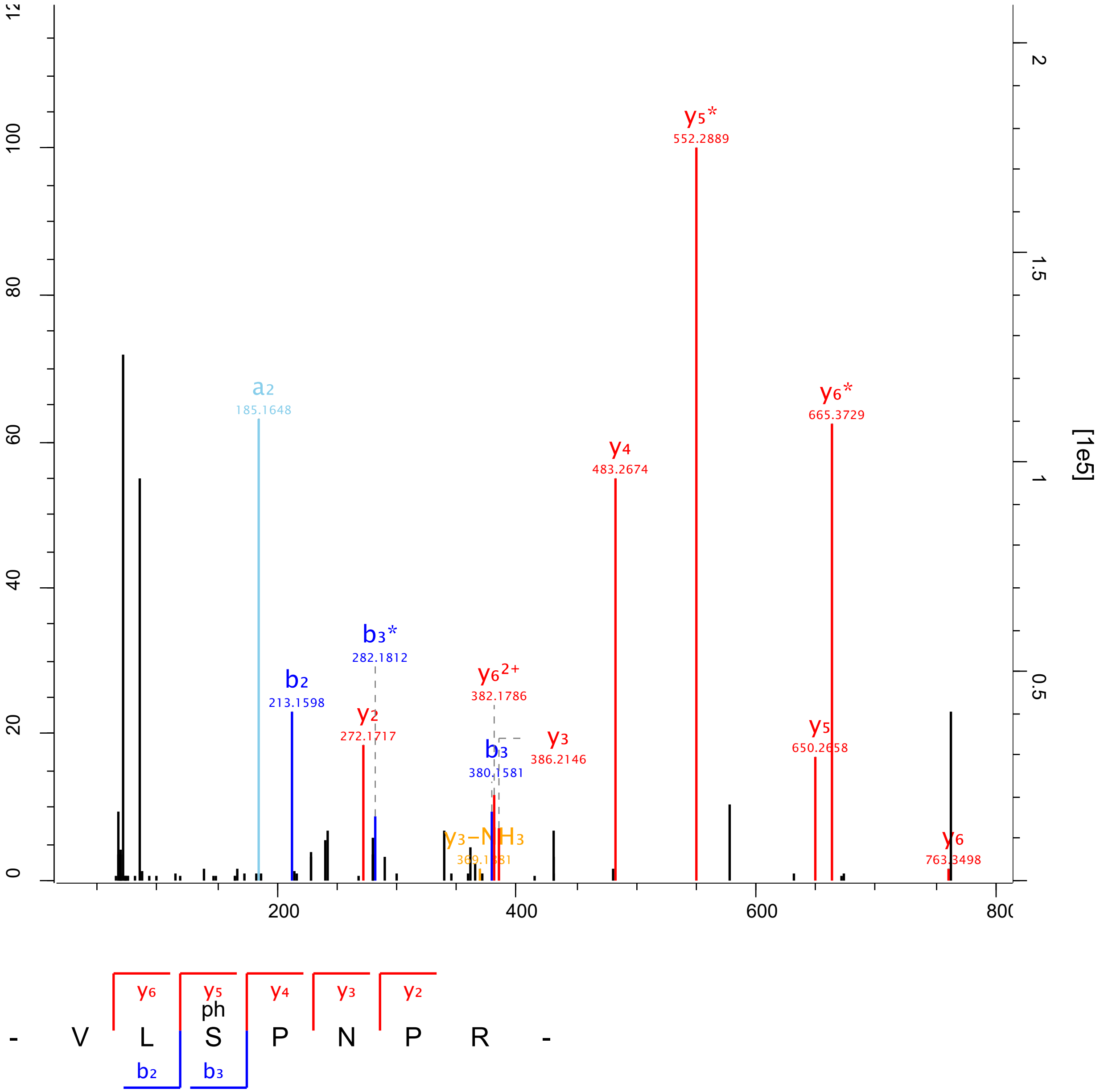

Raw file	Scan	Method	Score	m/z
sys_05_2	6151	FTMS; HCD	144.97	739.83

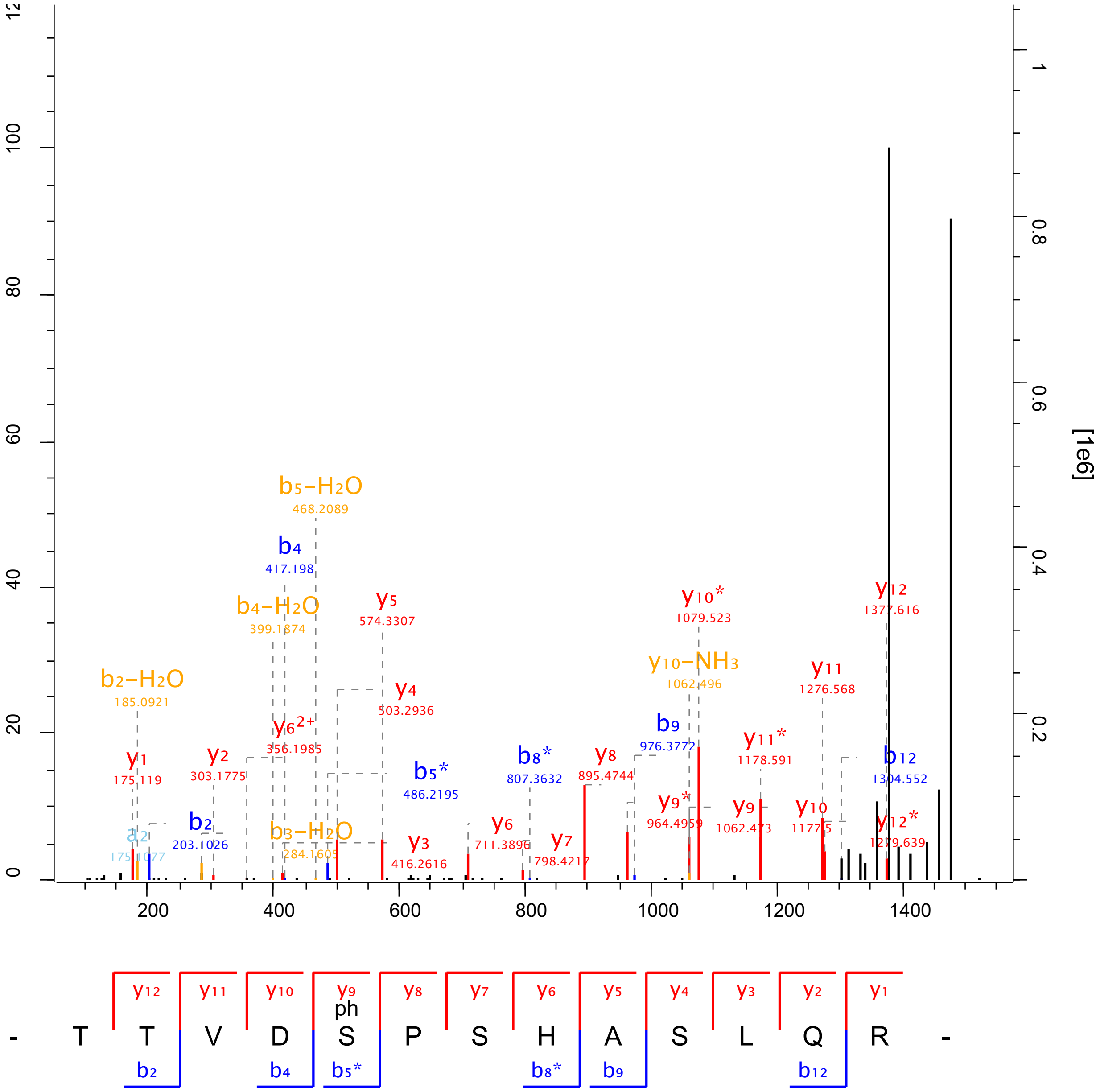

Raw file	Scan	Method	Score	m/z
sys_05_2	6228	FTMS; HCD	95.48	585.77

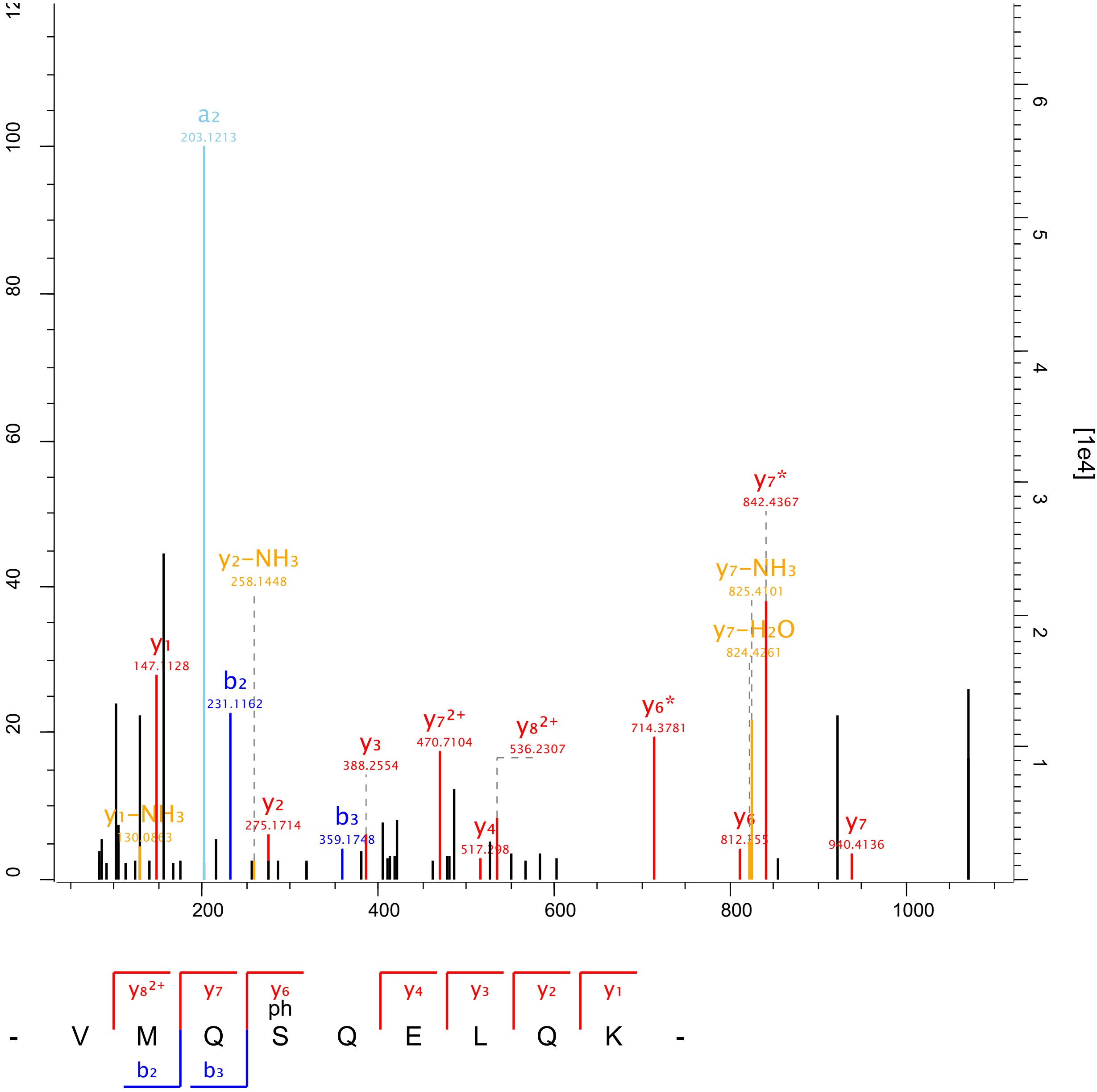

Raw file	Scan	Method	Score	m/z
sys_05_2	6346	FTMS; HCD	204.63	736.8

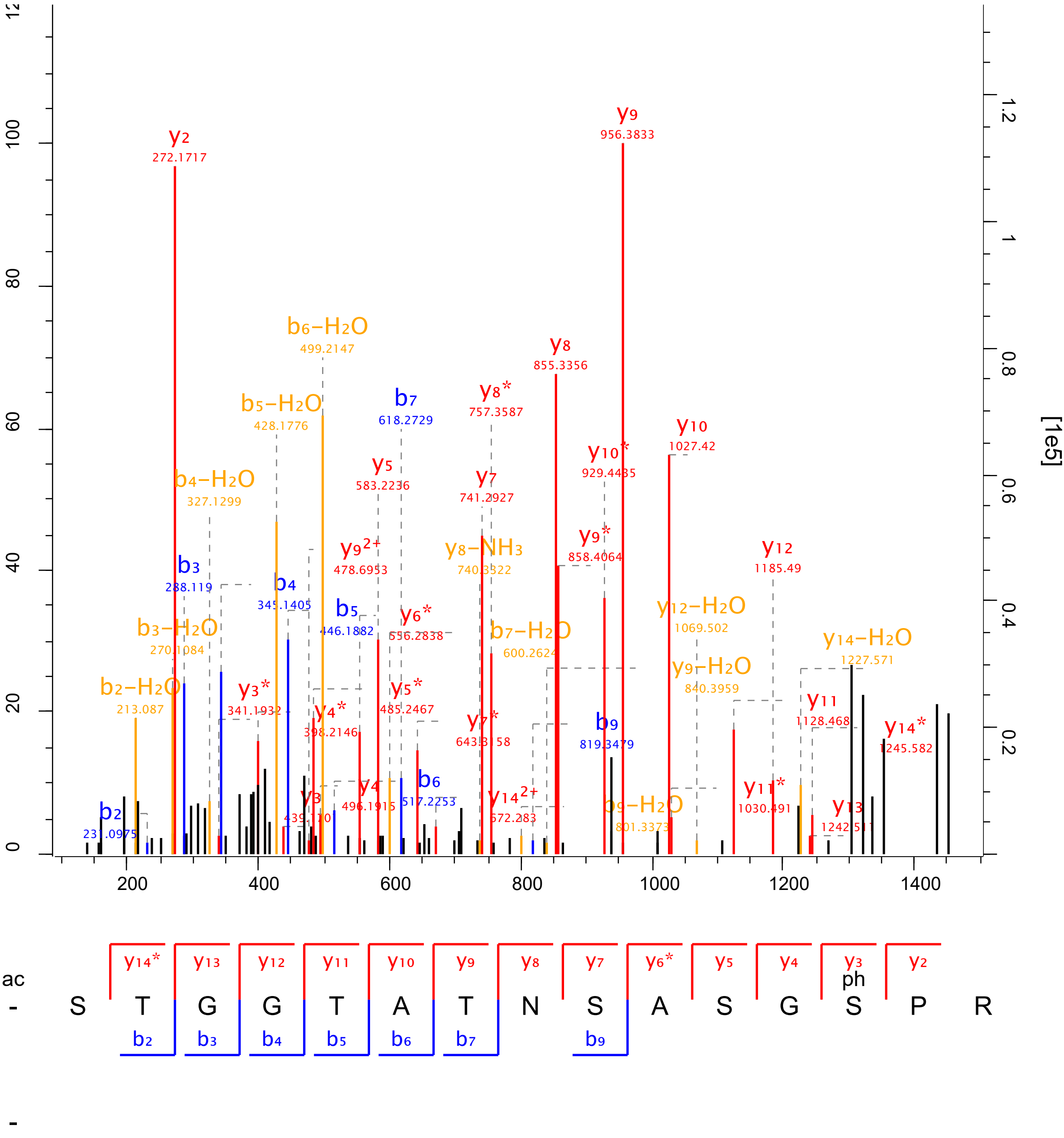

Raw file	Scan	Method	Score	m/z
sys_05_2	6385	FTMS; HCD	127.32	622.29

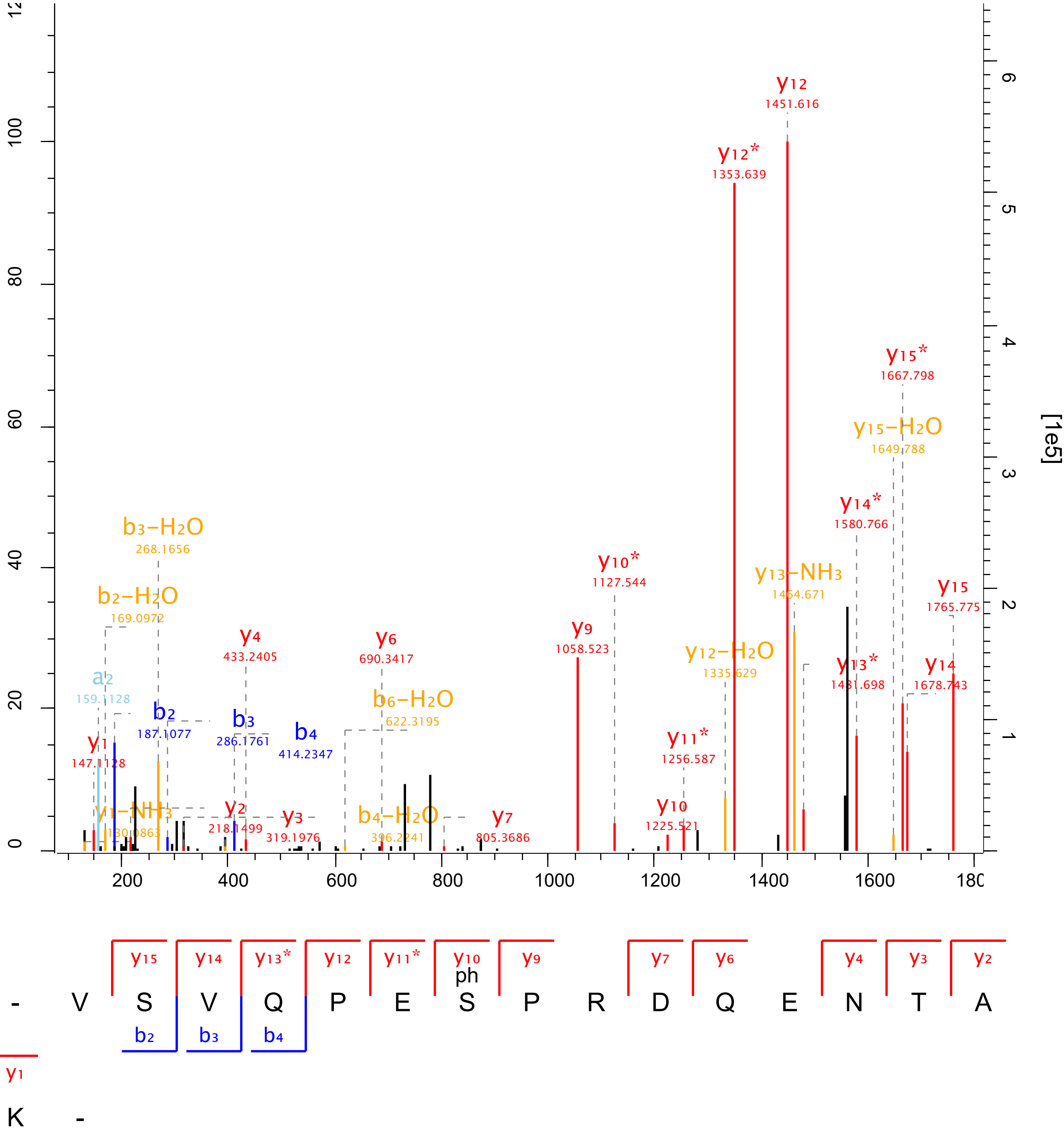

Raw file	Scan	Method	Score	m/z
sys_05_2	6423	FTMS; HCD	140.93	515.88

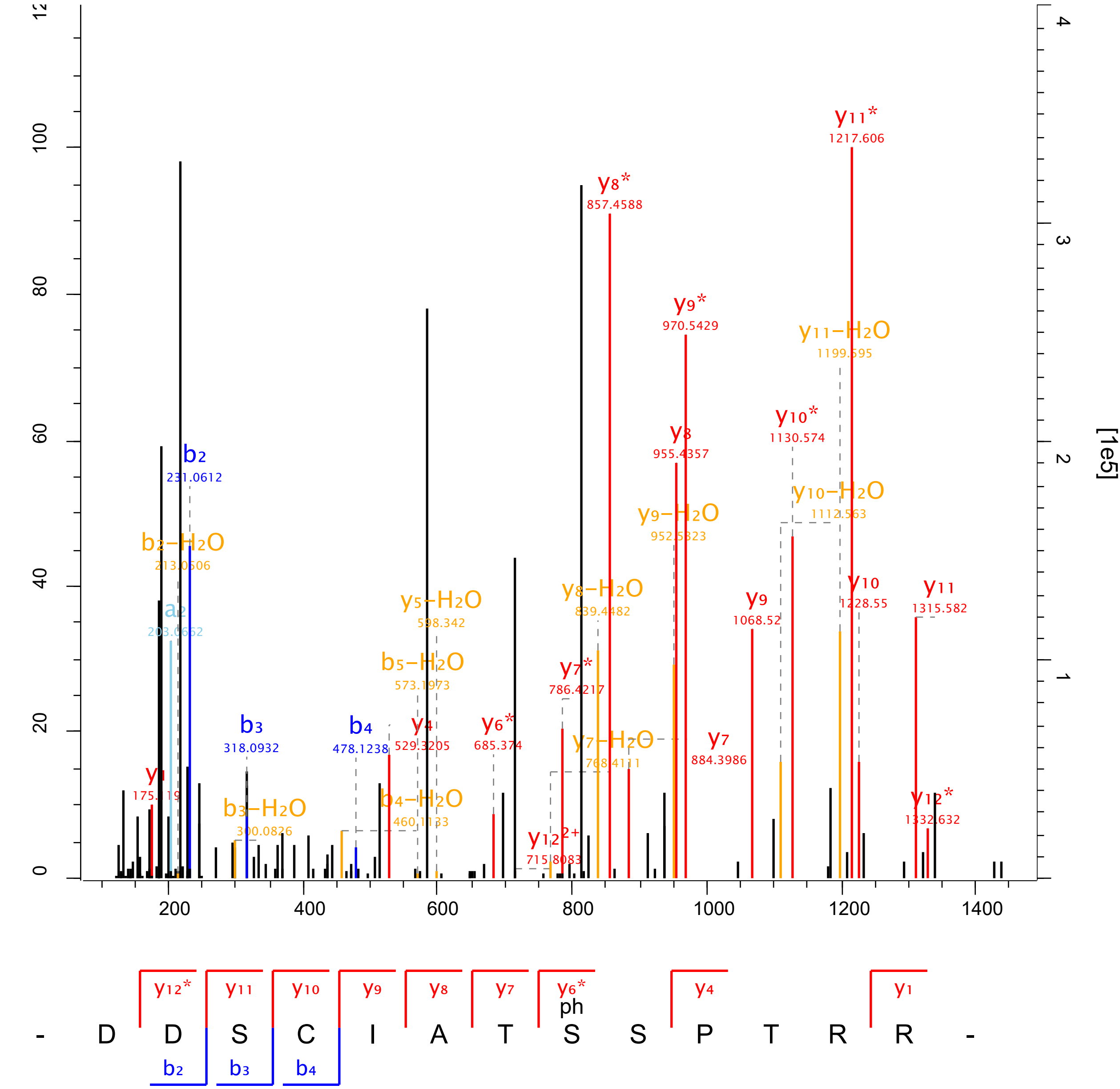

Raw file	Scan	Method	Score	m/z
sys_05_2	6453	FTMS; HCD	137.45	789.3

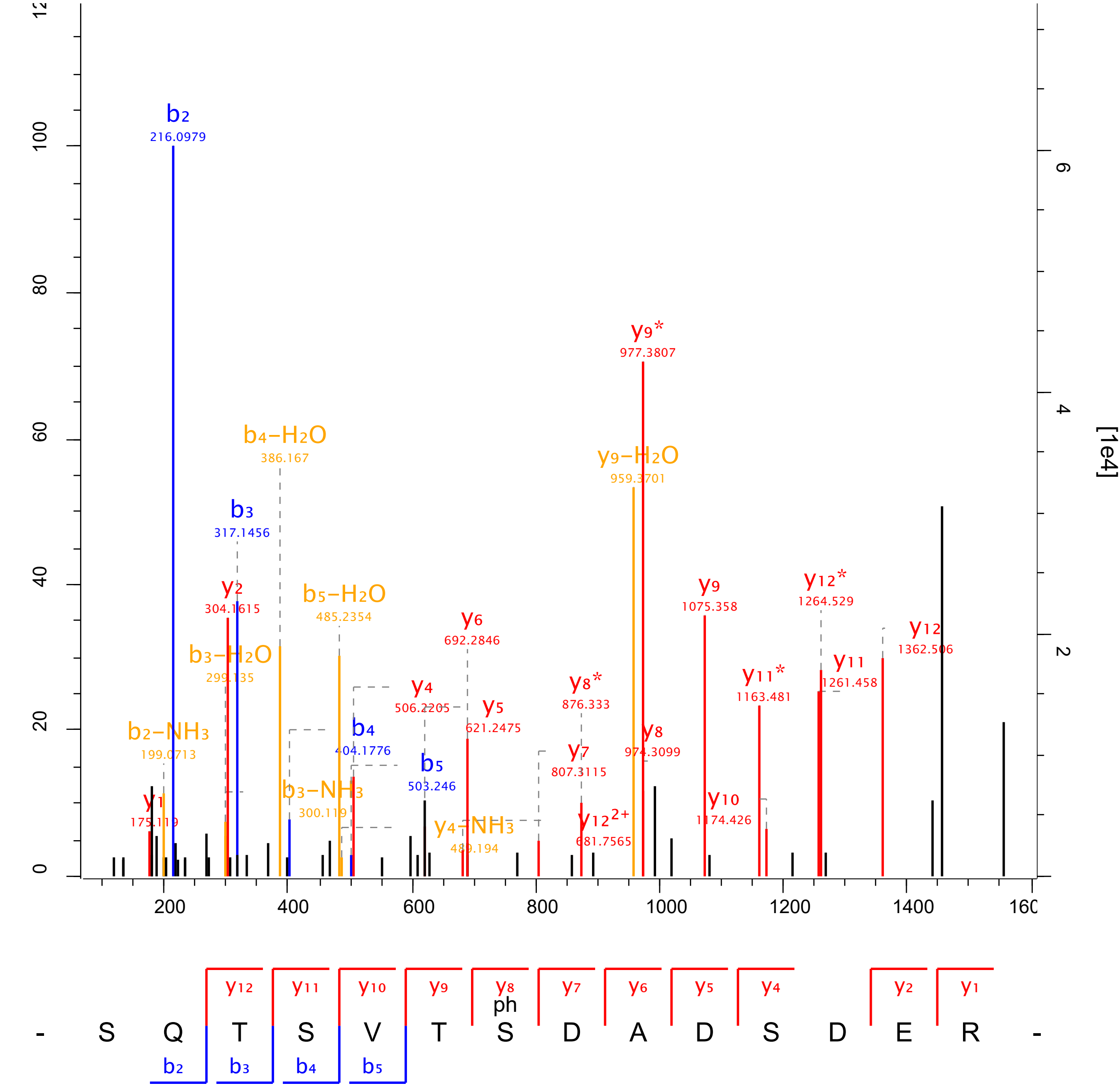

Raw file	Scan	Method	Score	m/z
sys_05_2	6524	FTMS; HCD	95.85	585.76

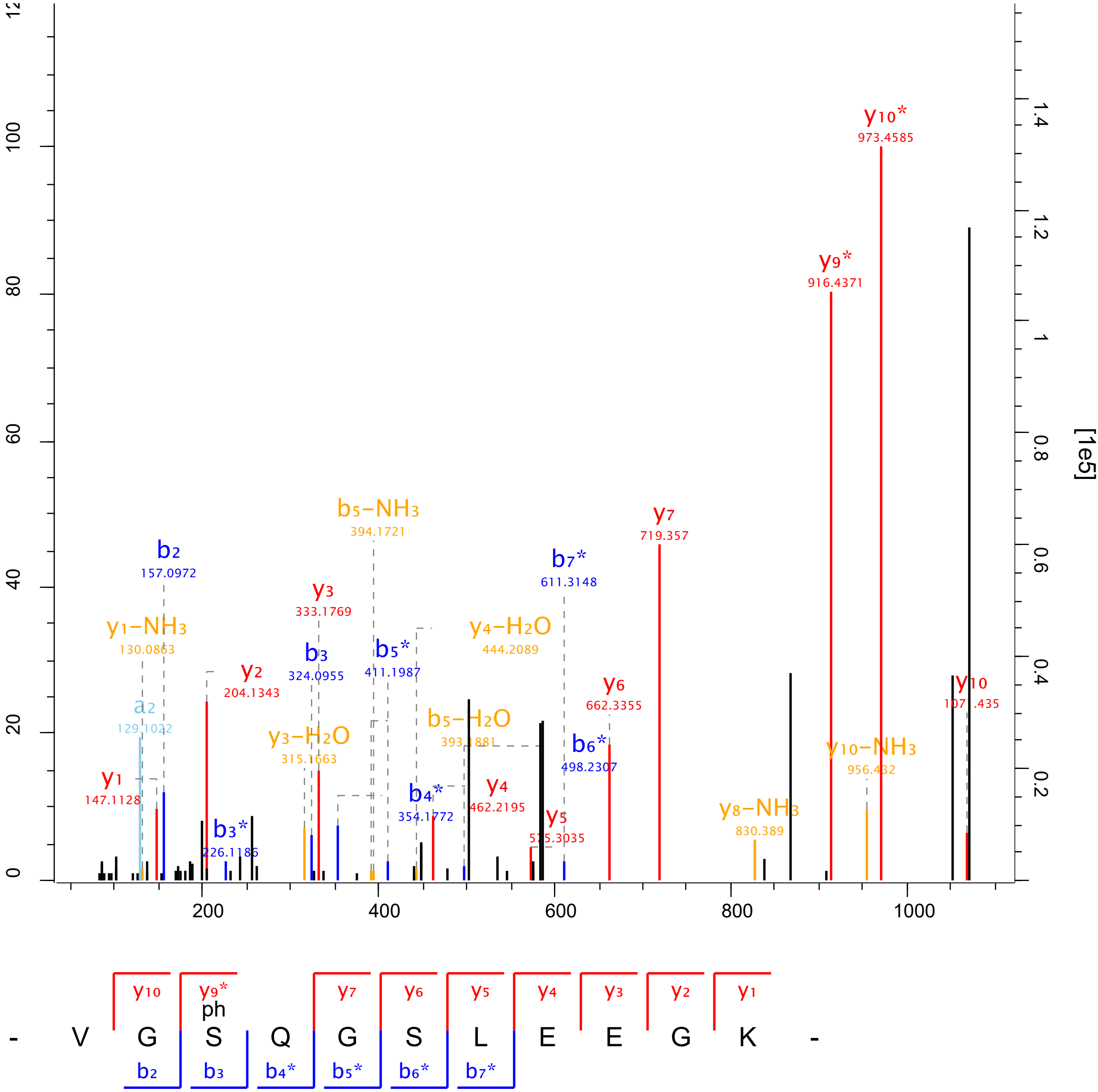

Raw file	Scan	Method	Score	m/z
sys_05_2	6539	FTMS; HCD	200.93	456.17

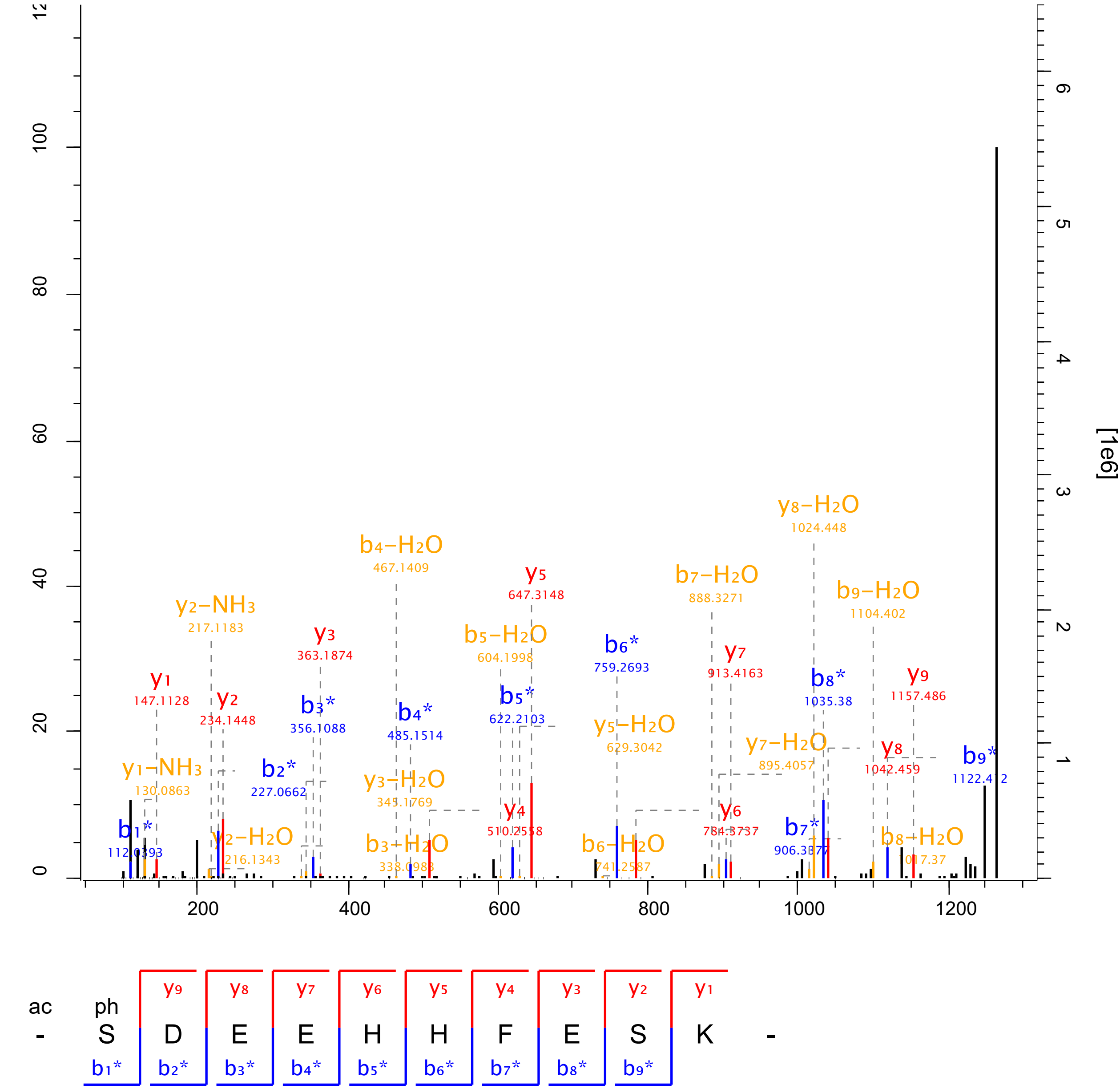

Raw file	Scan	Method	Score	m/z
sys_05_2	6588	FTMS; HCD	133.89	503.22

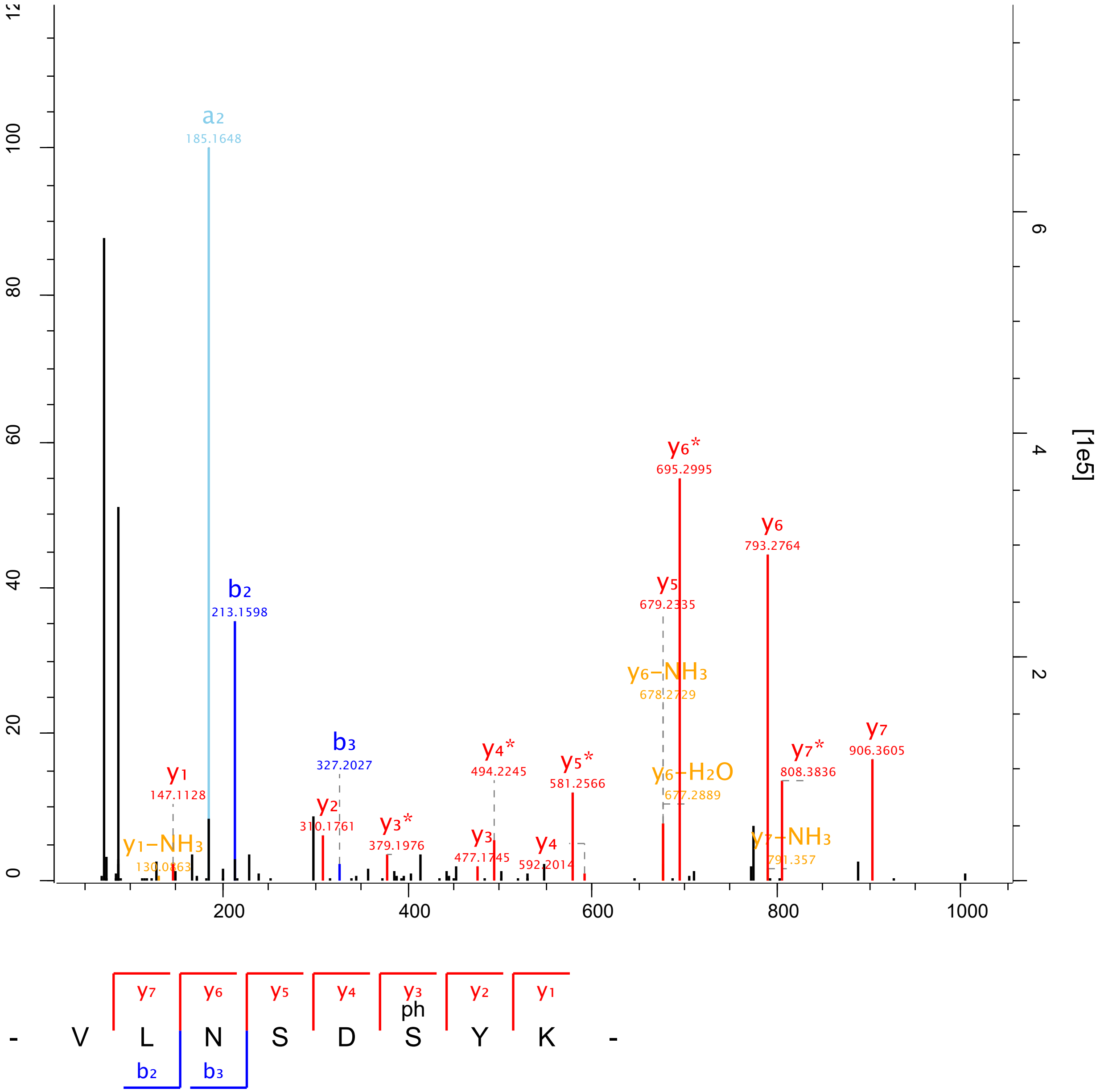

Raw file	Scan	Method	Score	m/z
sys_05_2	6614	FTMS; HCD	140.11	434.7

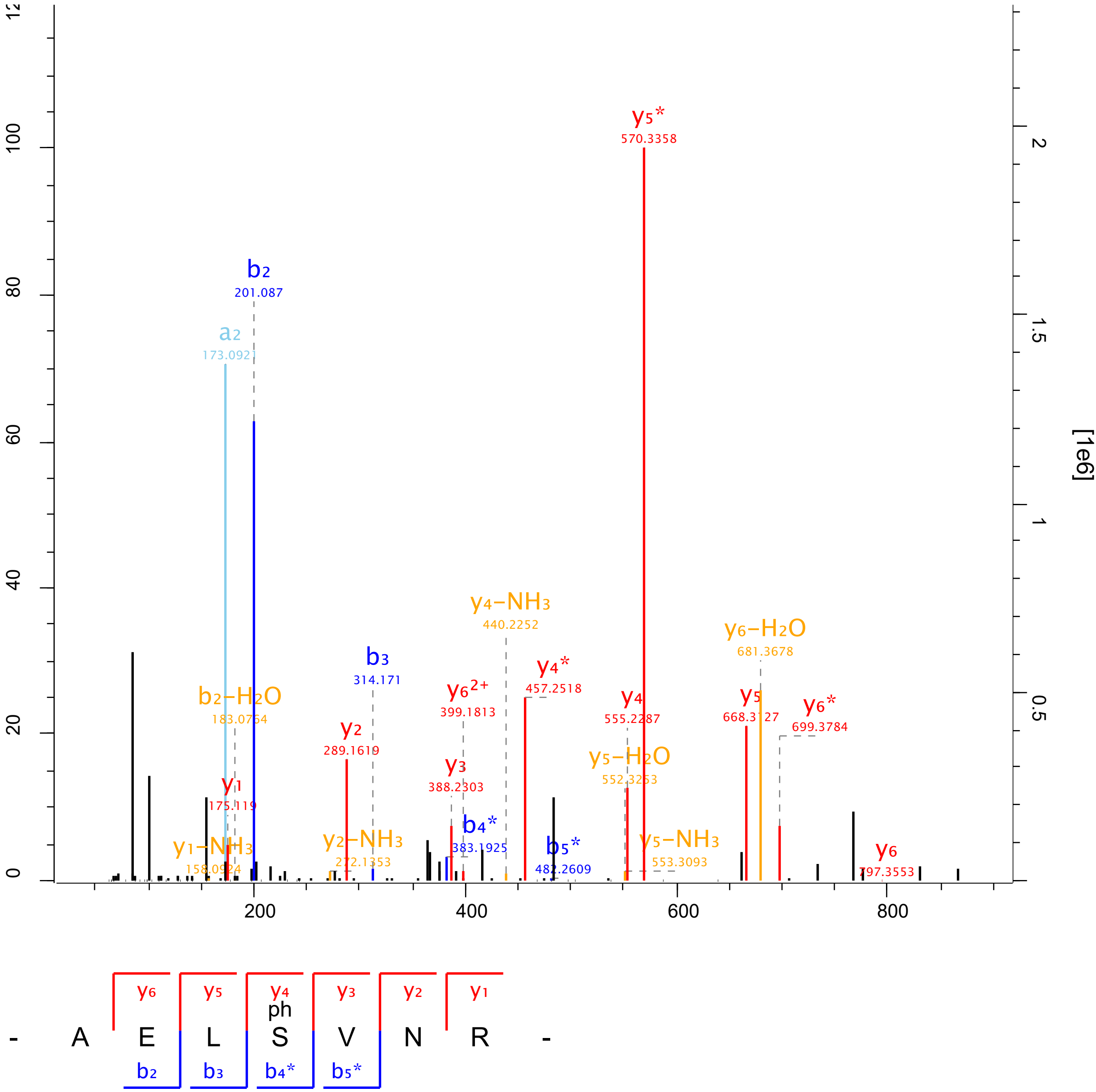

Raw file	Scan	Method	Score	m/z
sys_05_2	6624	FTMS; HCD	177.63	548.28

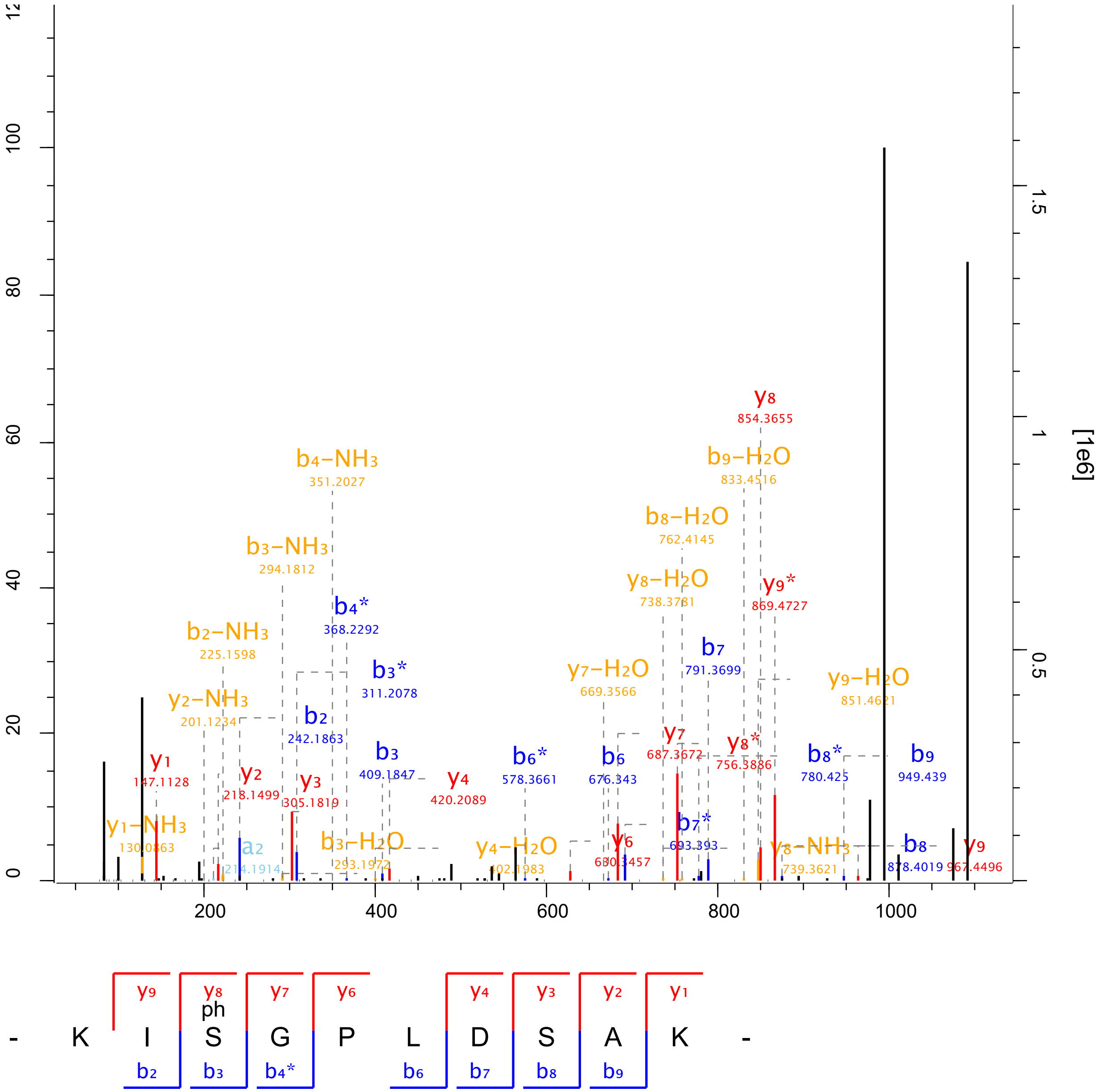

Raw file	Scan	Method	Score	m/z
sys_05_2	6638	FTMS; HCD	85.54	579.26

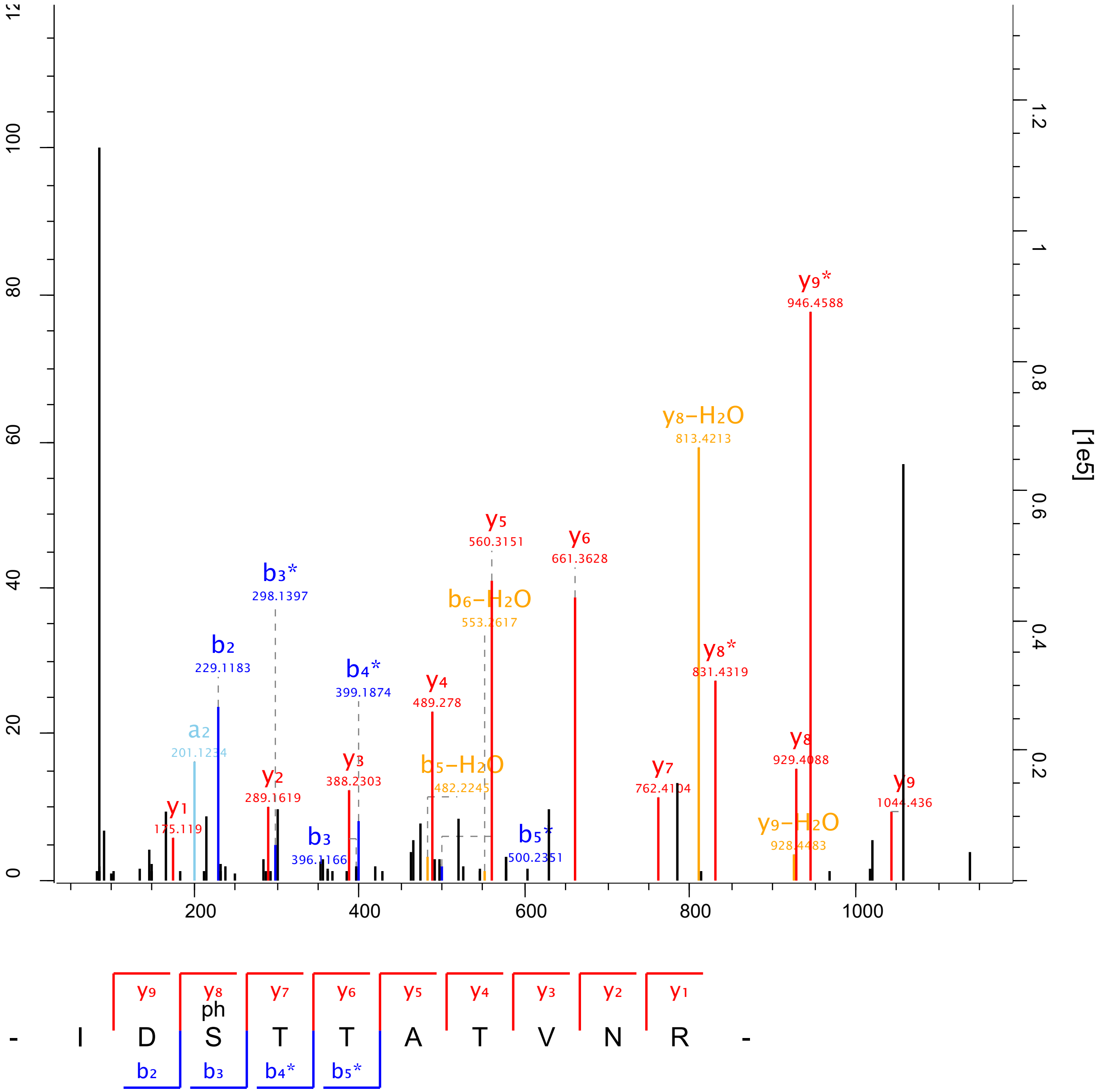

Raw file	Scan	Method	Score	m/z
sys_05_2	6780	FTMS; HCD	43.37	706.3

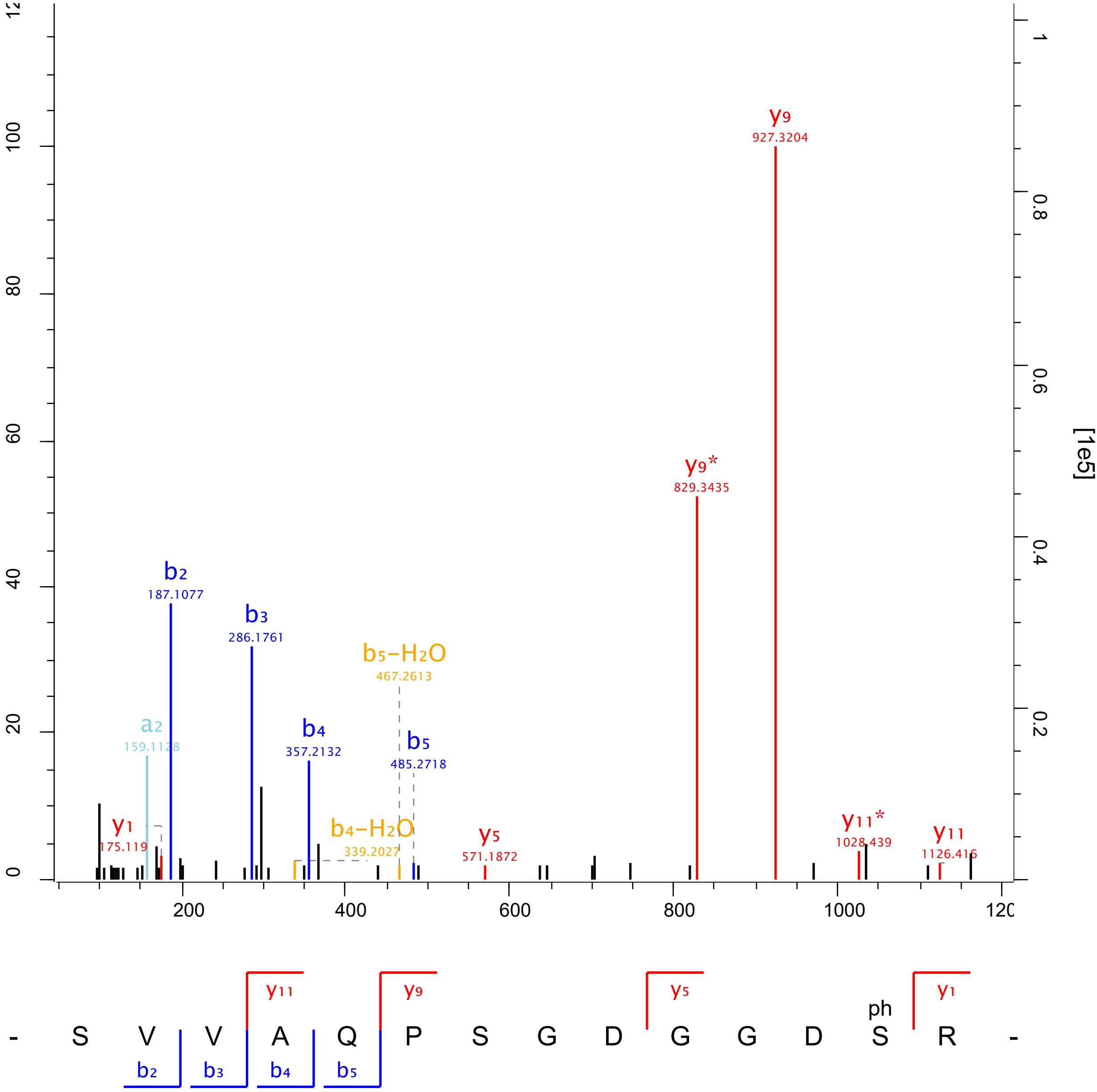

Raw file	Scan	Method	Score	m/z
sys_05_2	6827	FTMS; HCD	119.77	595.24

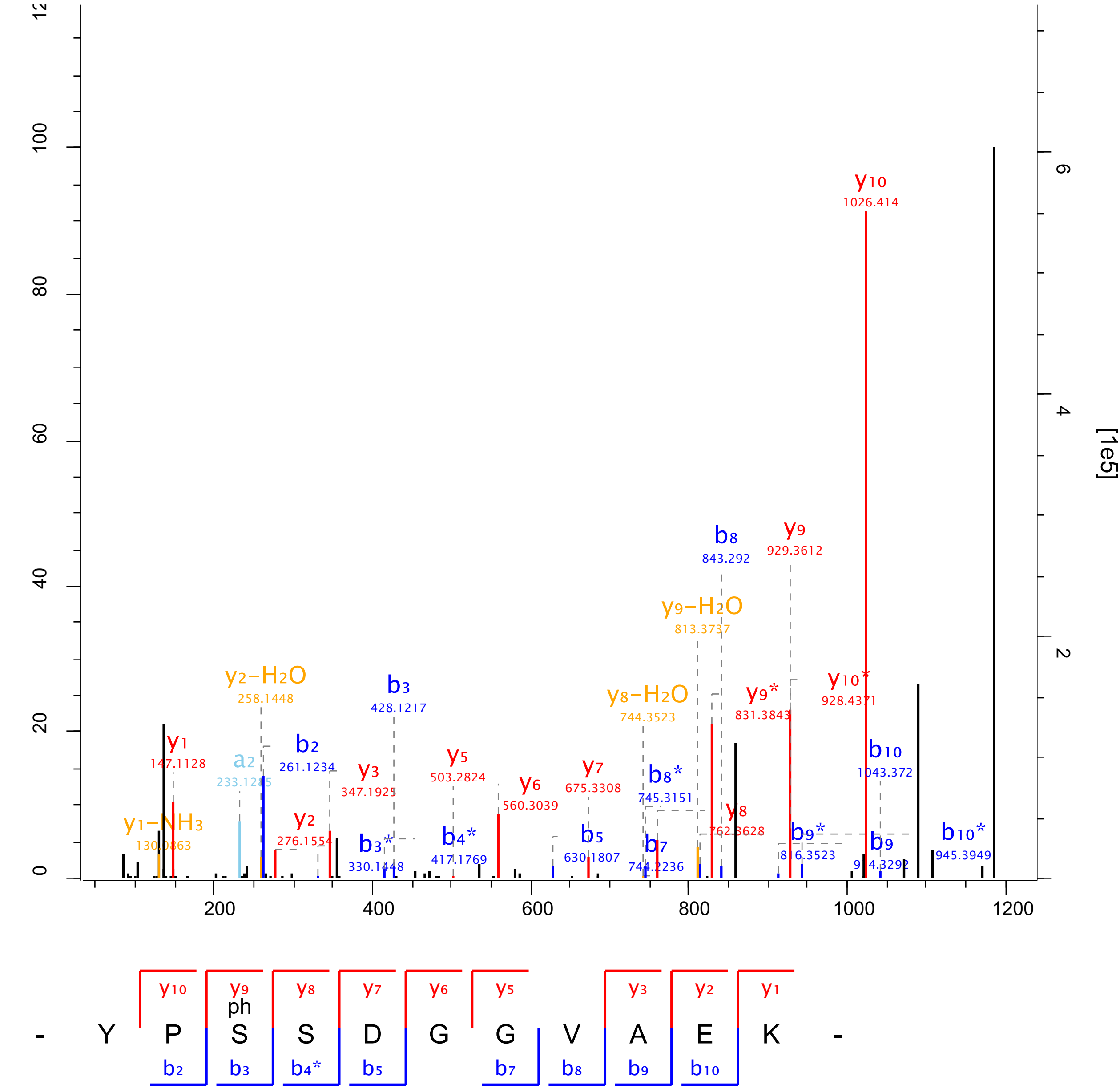

Raw file	Scan	Method	Score	m/z
sys_05_2	6918	FTMS; HCD	120.53	627.25

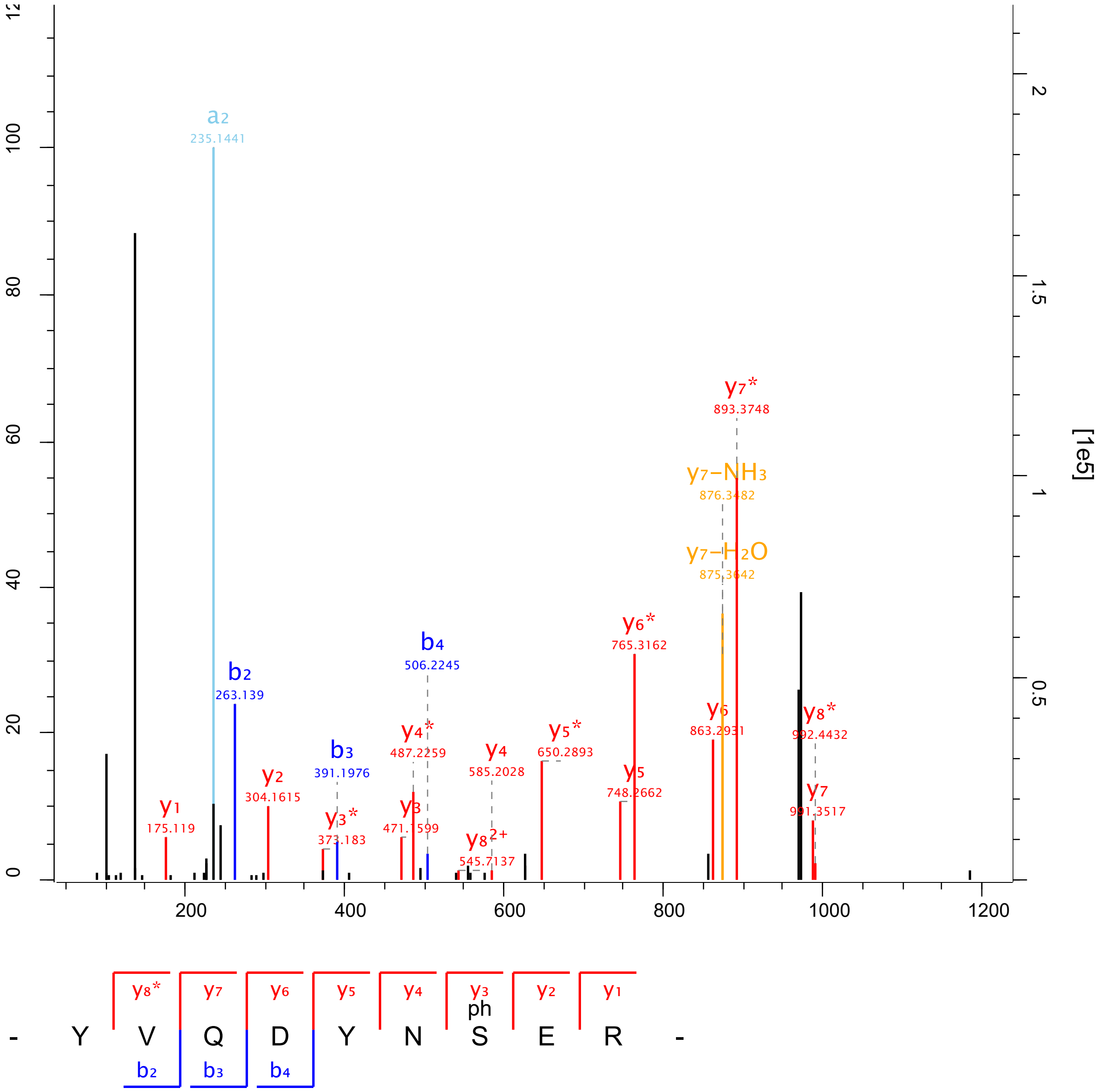

Raw file	Scan	Method	Score	m/z
sys_05_2	6972	FTMS; HCD	192.39	661.8

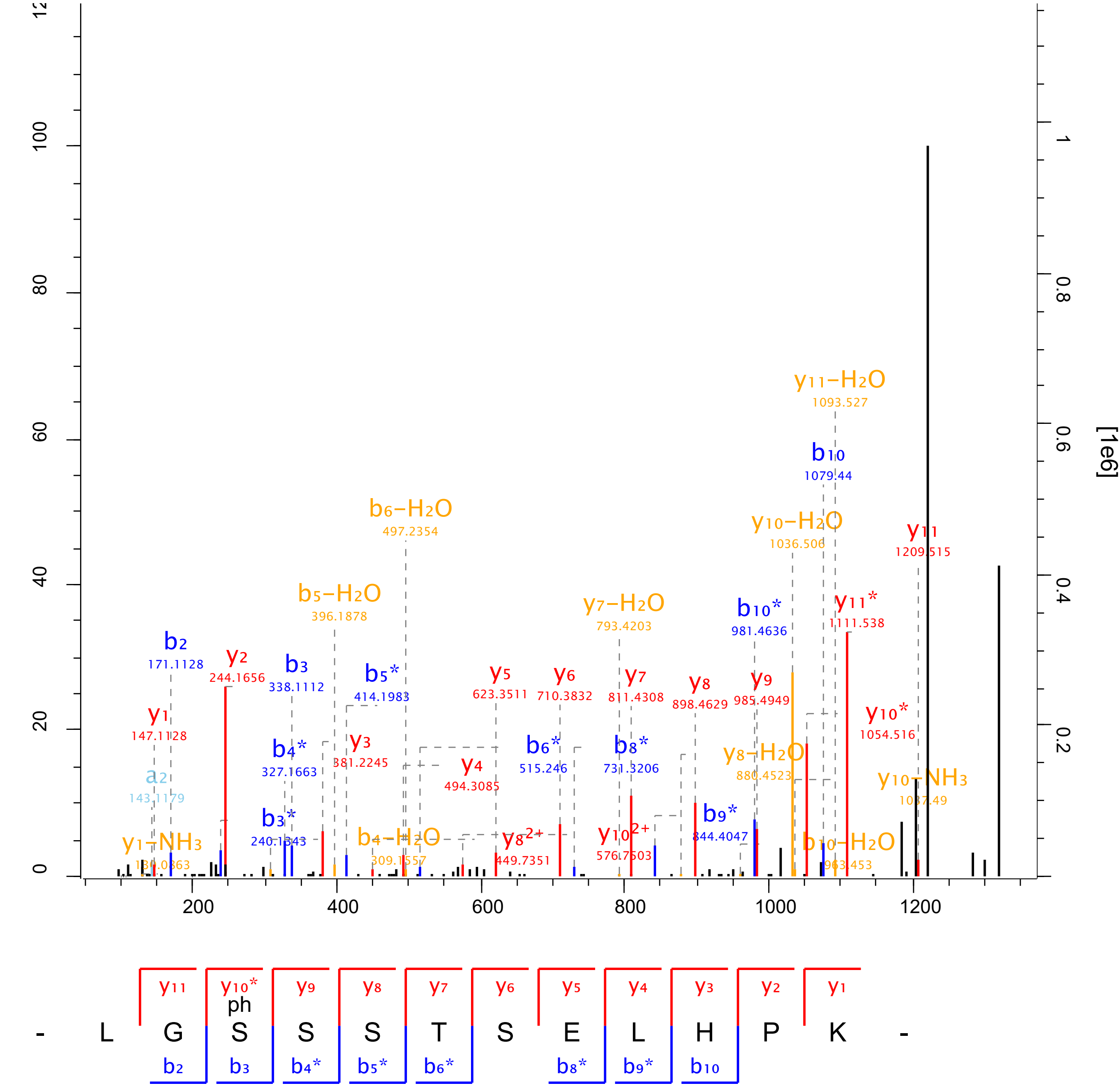

Raw file	Scan	Method	Score	m/z
sys_05_2	7017	FTMS; HCD	84.51	476.71

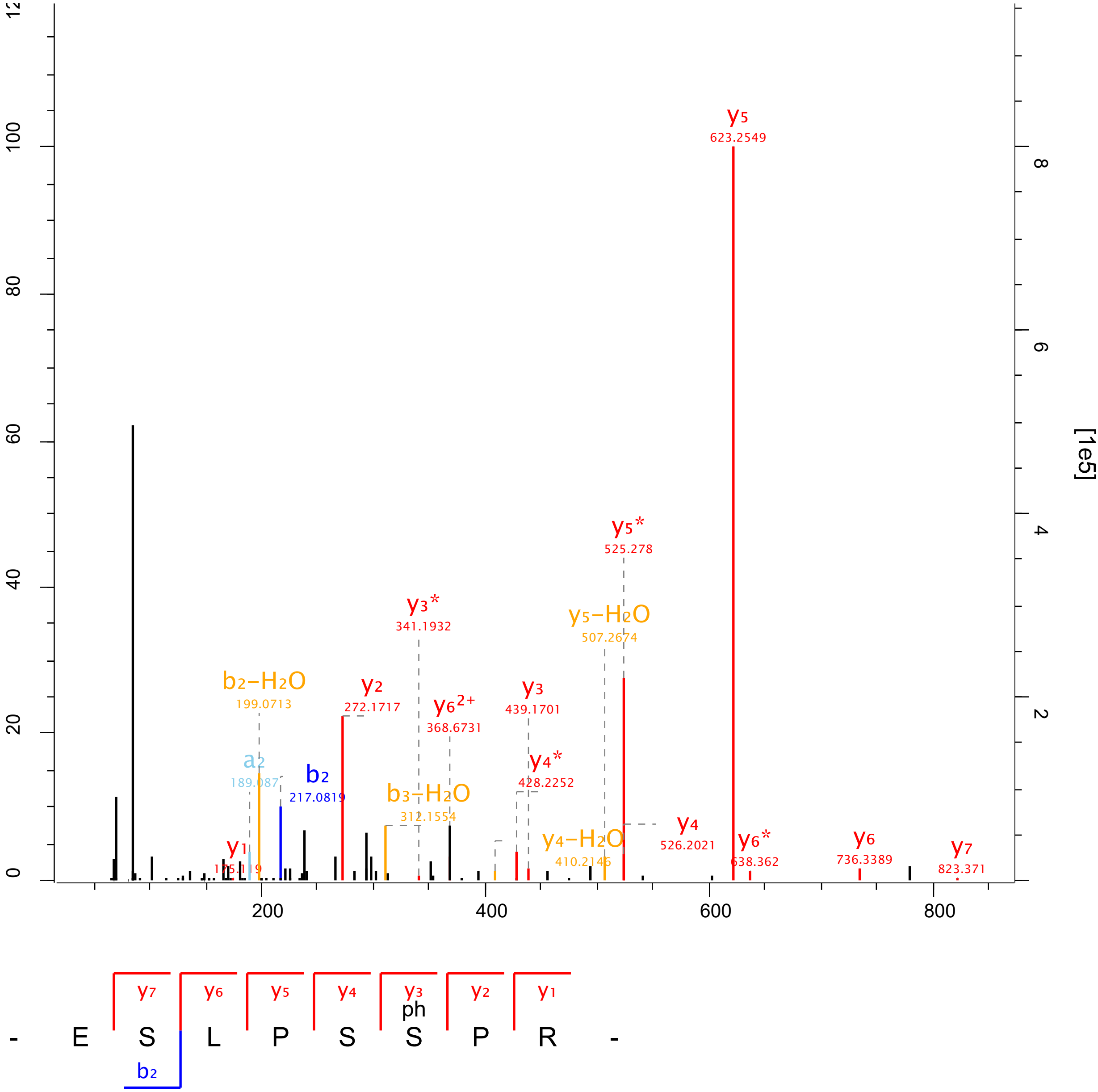

Raw file	Scan	Method	Score	m/z
sys_05_2	7133	FTMS; HCD	129.53	566.73

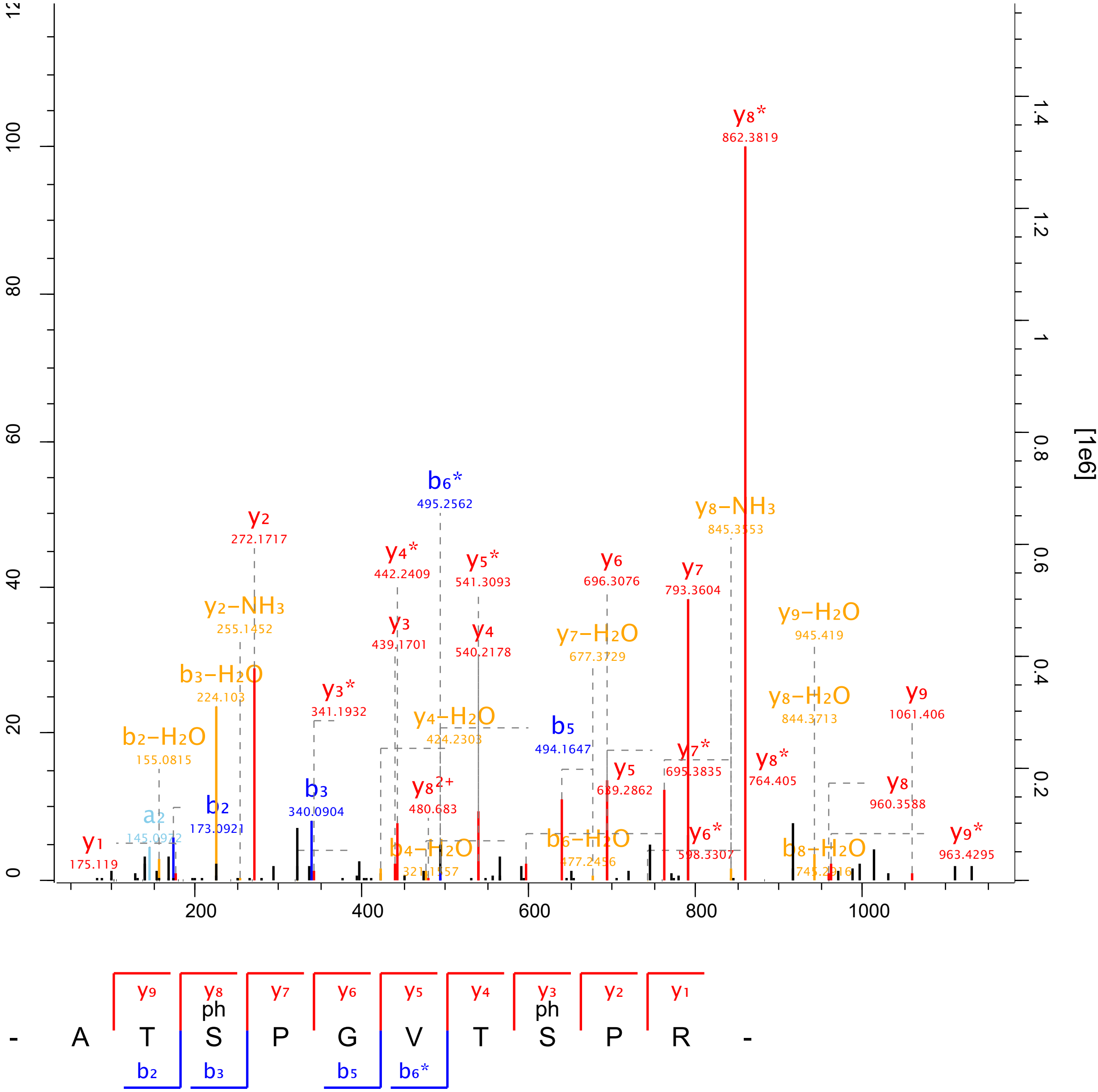

Raw file	Scan	Method	Score	m/z
sys_05_2	7161	FTMS; HCD	92.01	762.65

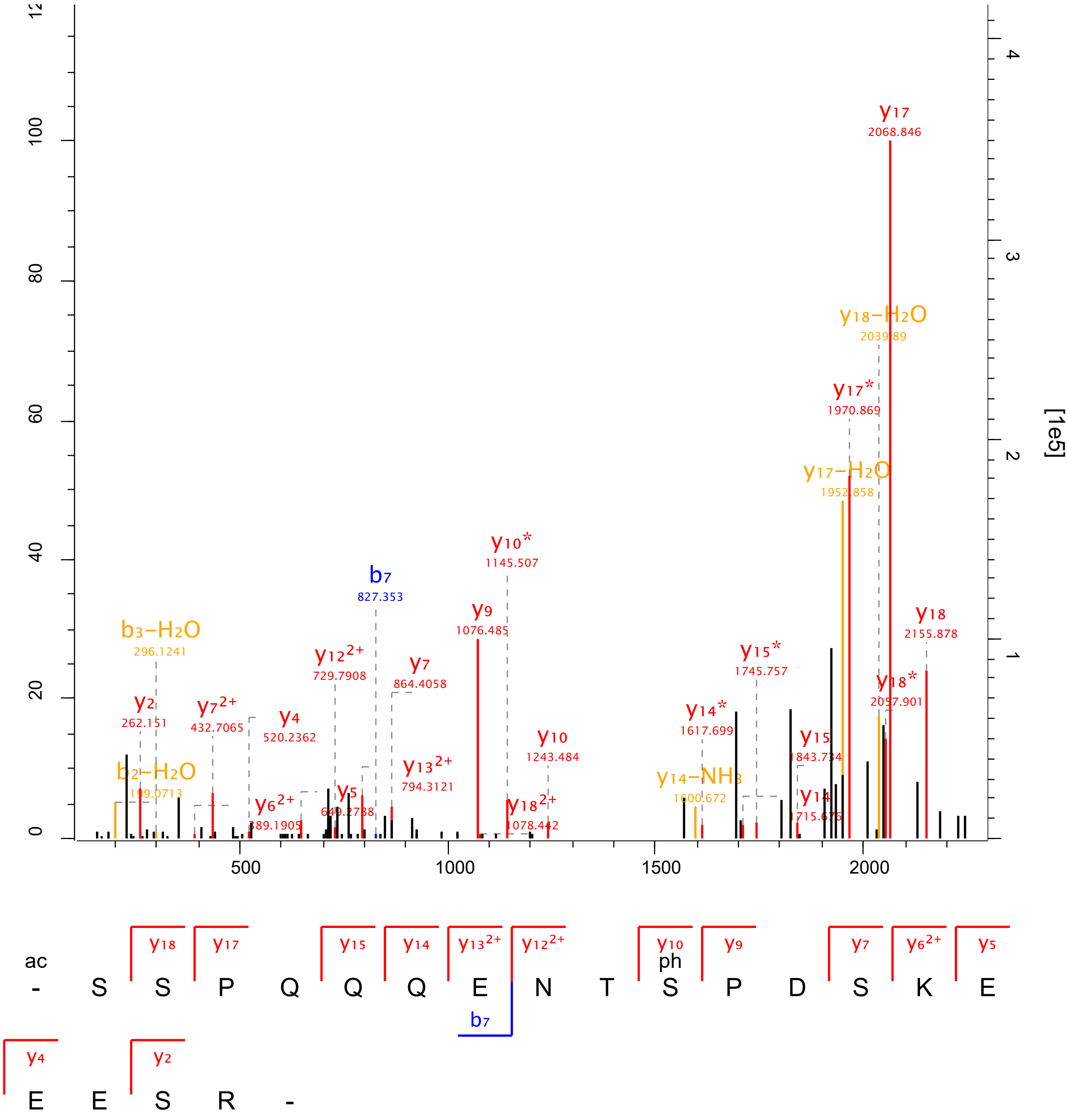

Raw file	Scan	Method	Score	m/z
sys_05_2	7209	FTMS; HCD	56.57	646.8

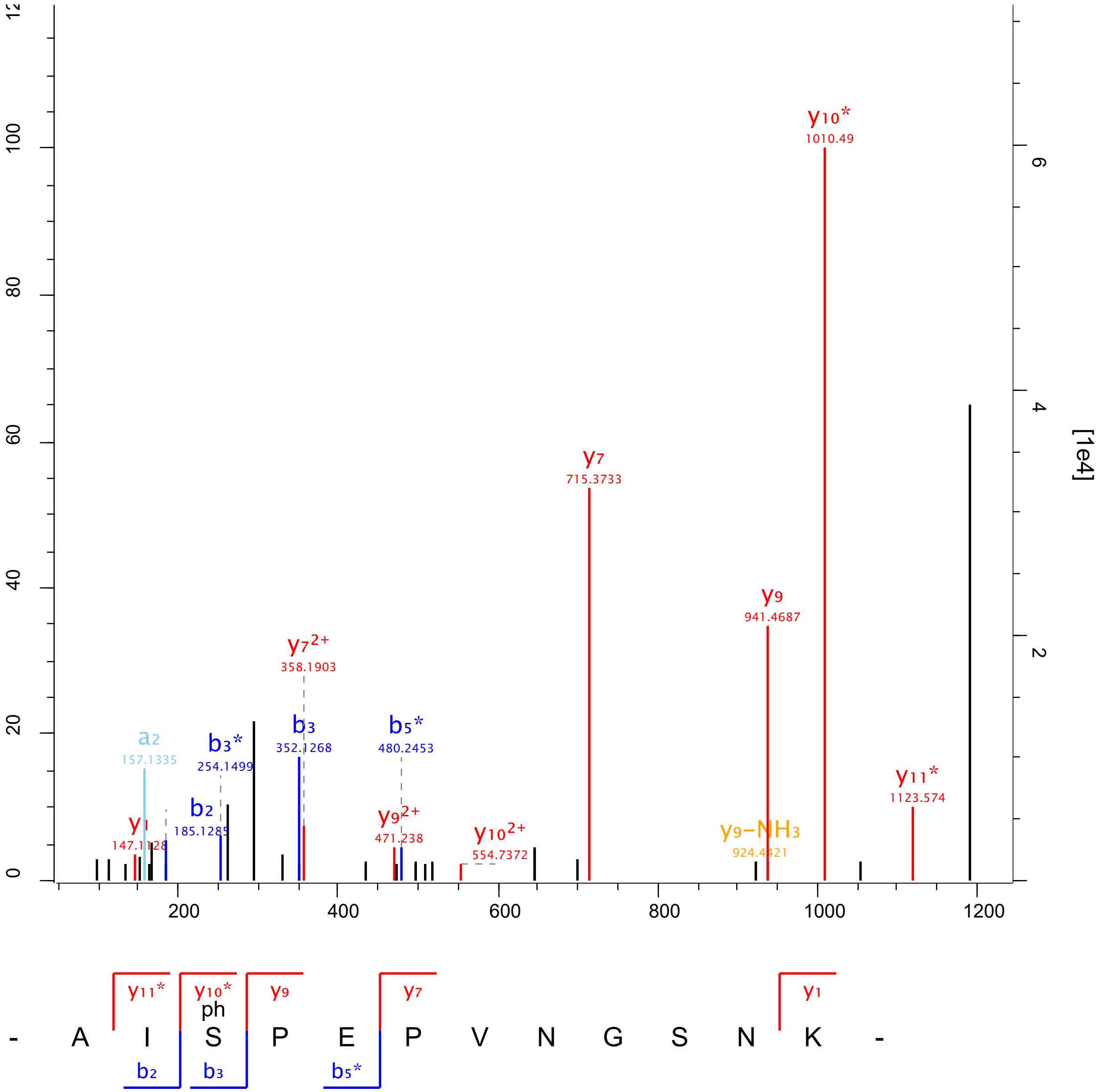

Raw file	Scan	Method	Score	m/z
sys_05_2	7267	FTMS; HCD	90.83	576.79

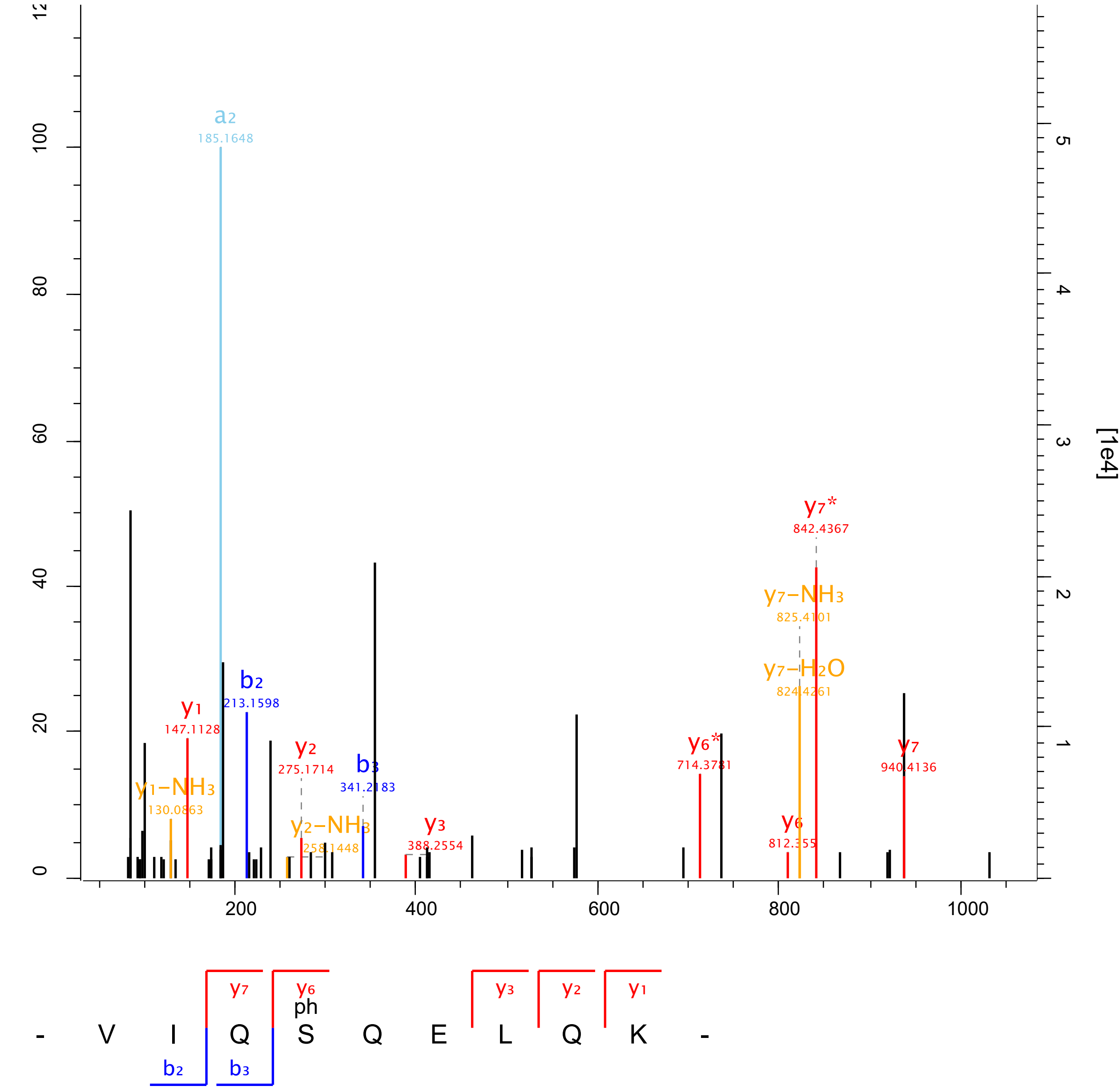

Raw file	Scan	Method	Score	m/z
sys_05_2	7308	FTMS; HCD	93.37	839.83

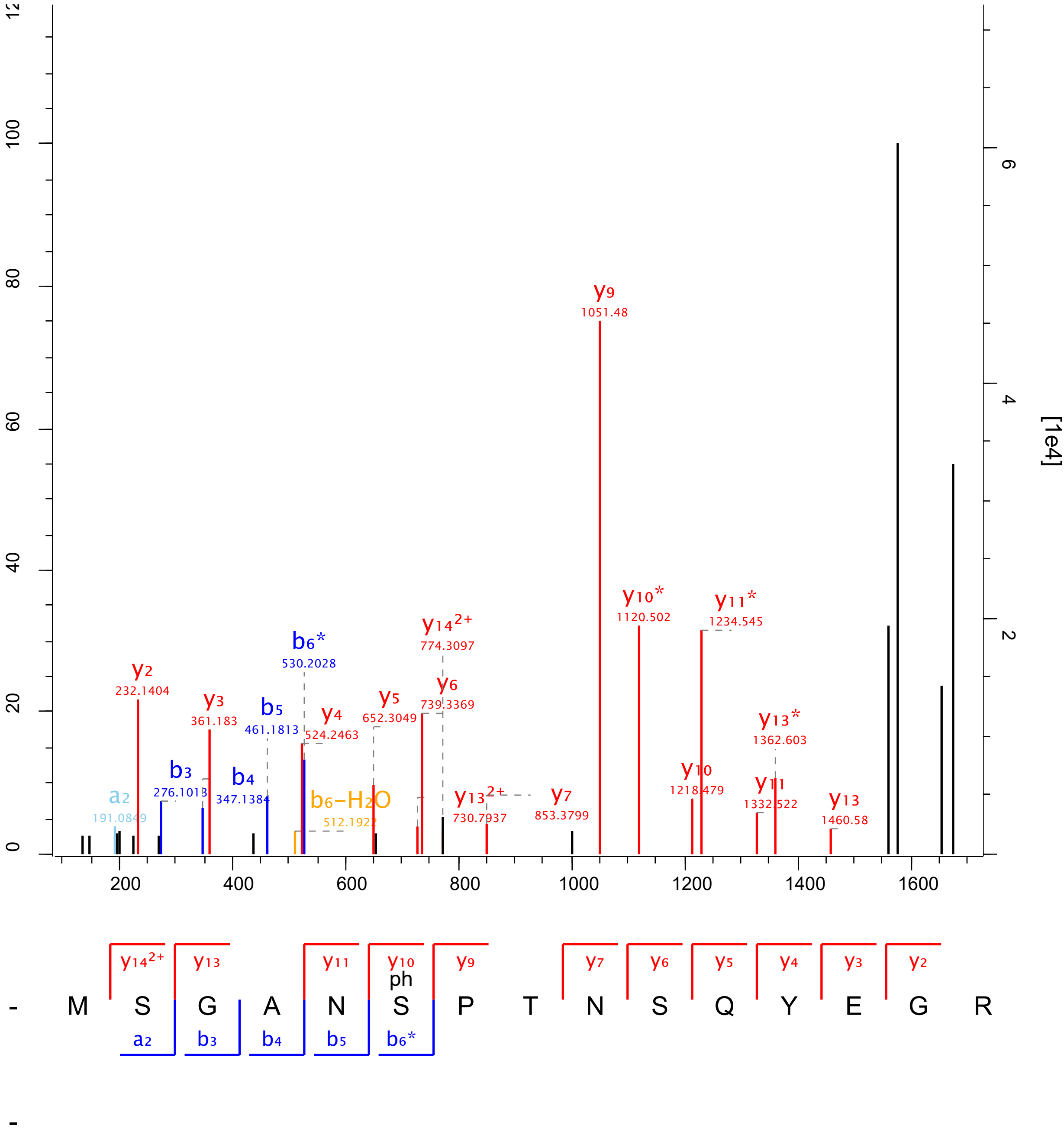

Raw file	Scan	Method	Score	m/z
sys_05_2	7373	FTMS; HCD	116.37	412.22

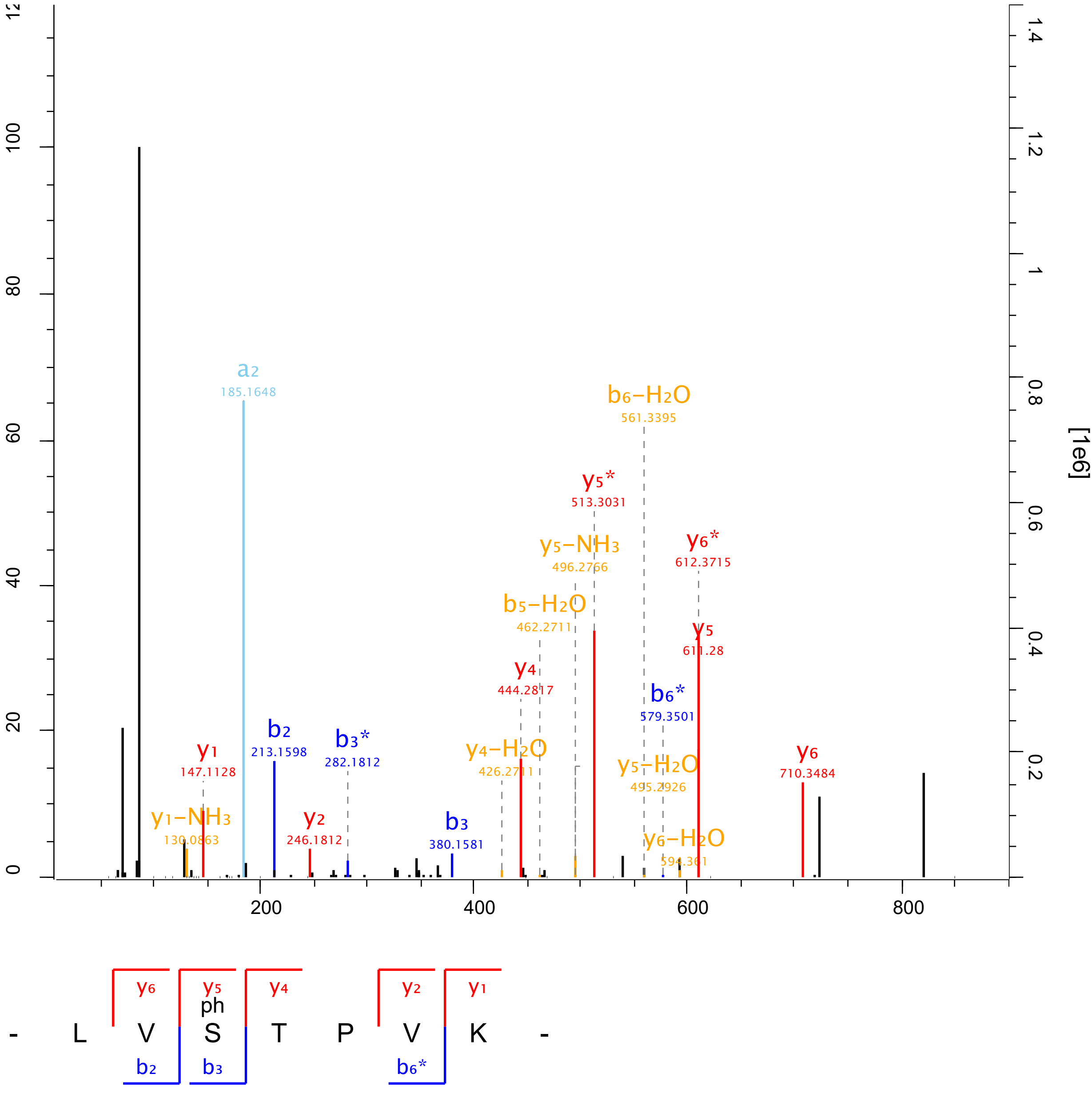

Raw file	Scan	Method	Score	m/z
sys_05_2	7474	FTMS; HCD	280.5	663.27

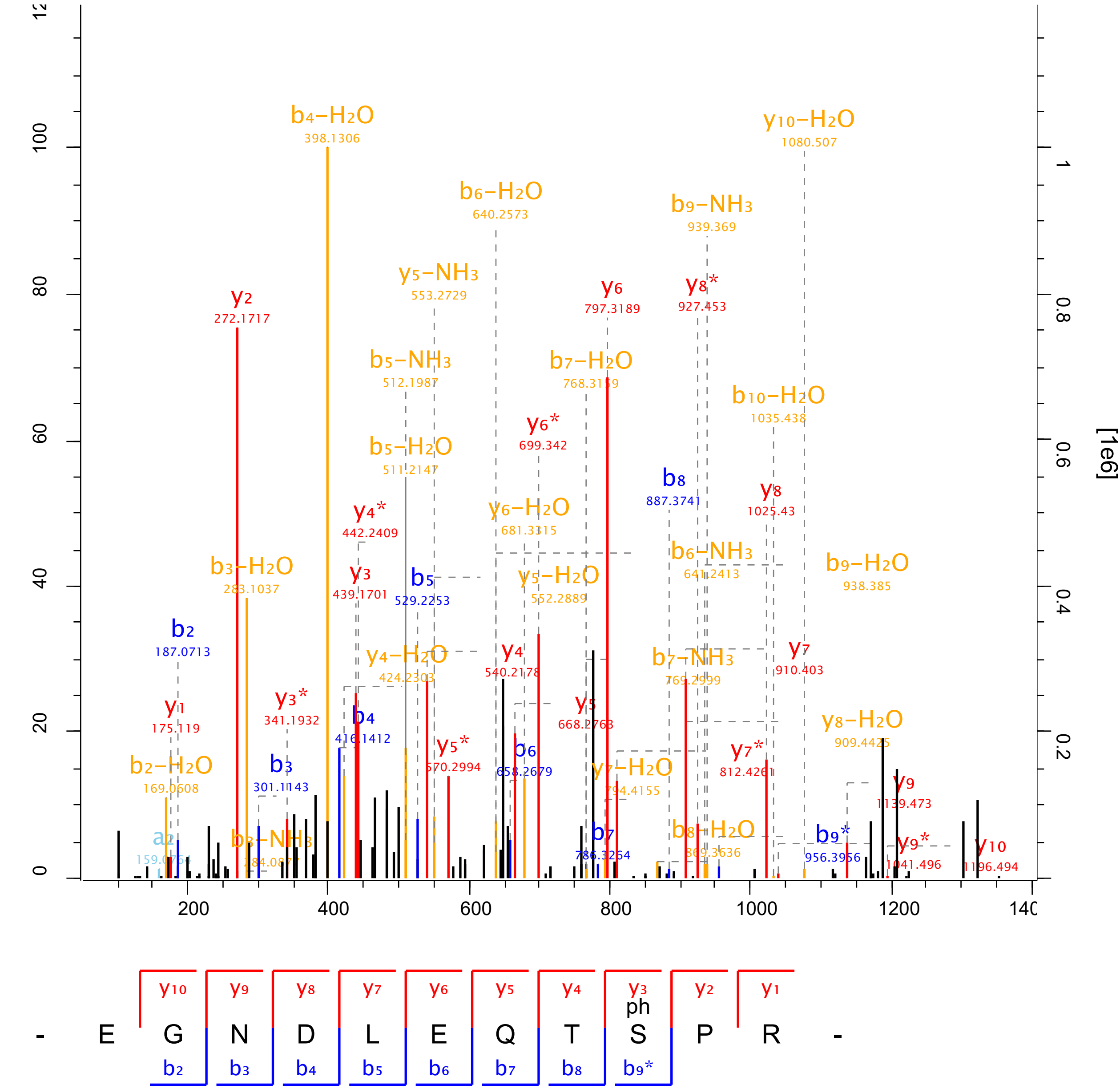

Raw file	Scan	Method	Score	m/z
sys_05_2	7489	FTMS; HCD	69.23	591.26

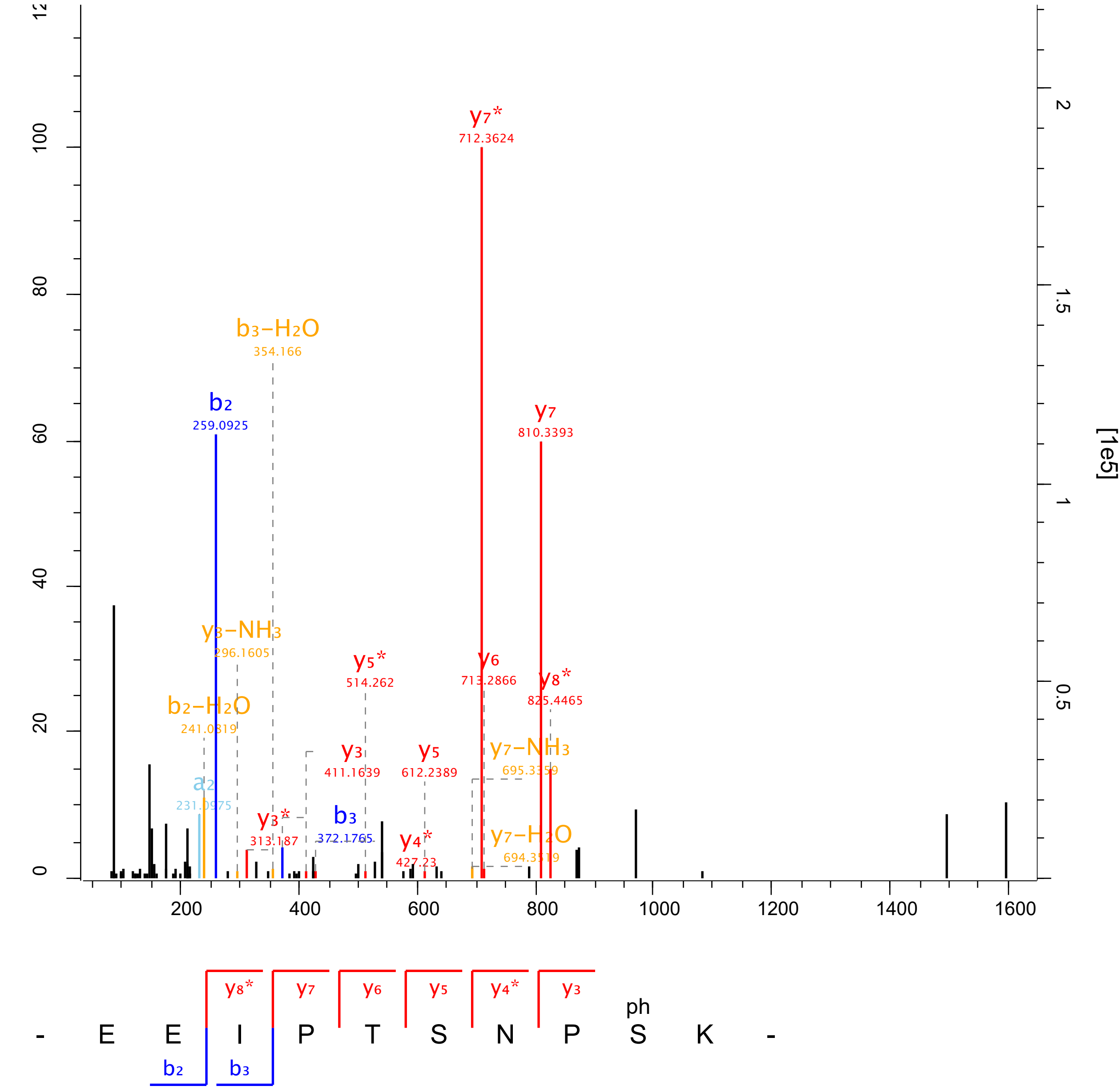

Raw file	Scan	Method	Score	m/z
sys_05_2	7502	FTMS; HCD	82.07	558.74

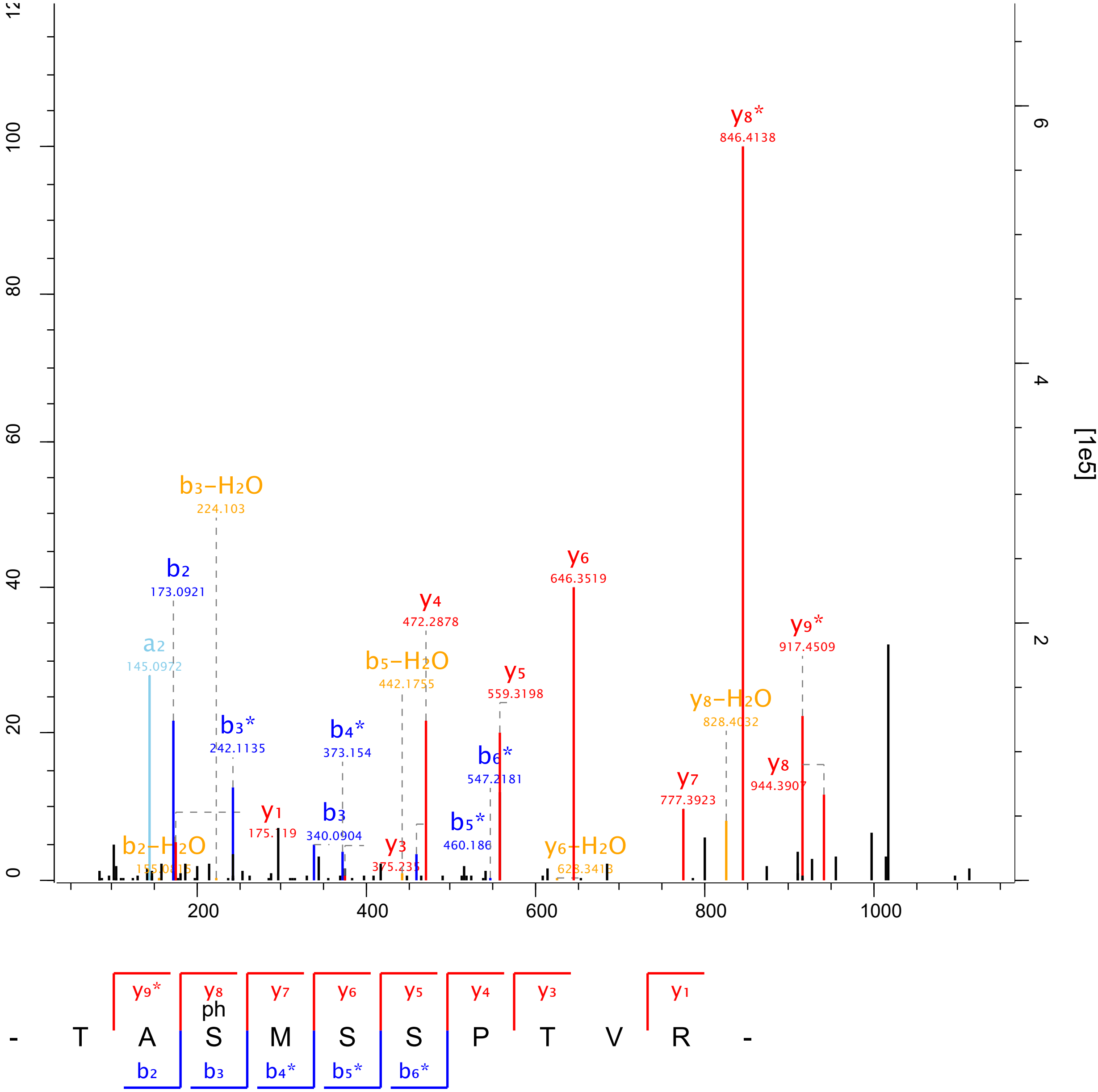

Raw file	Scan	Method	Score	m/z
sys_05_2	7587	FTMS; HCD	109.1	573.75

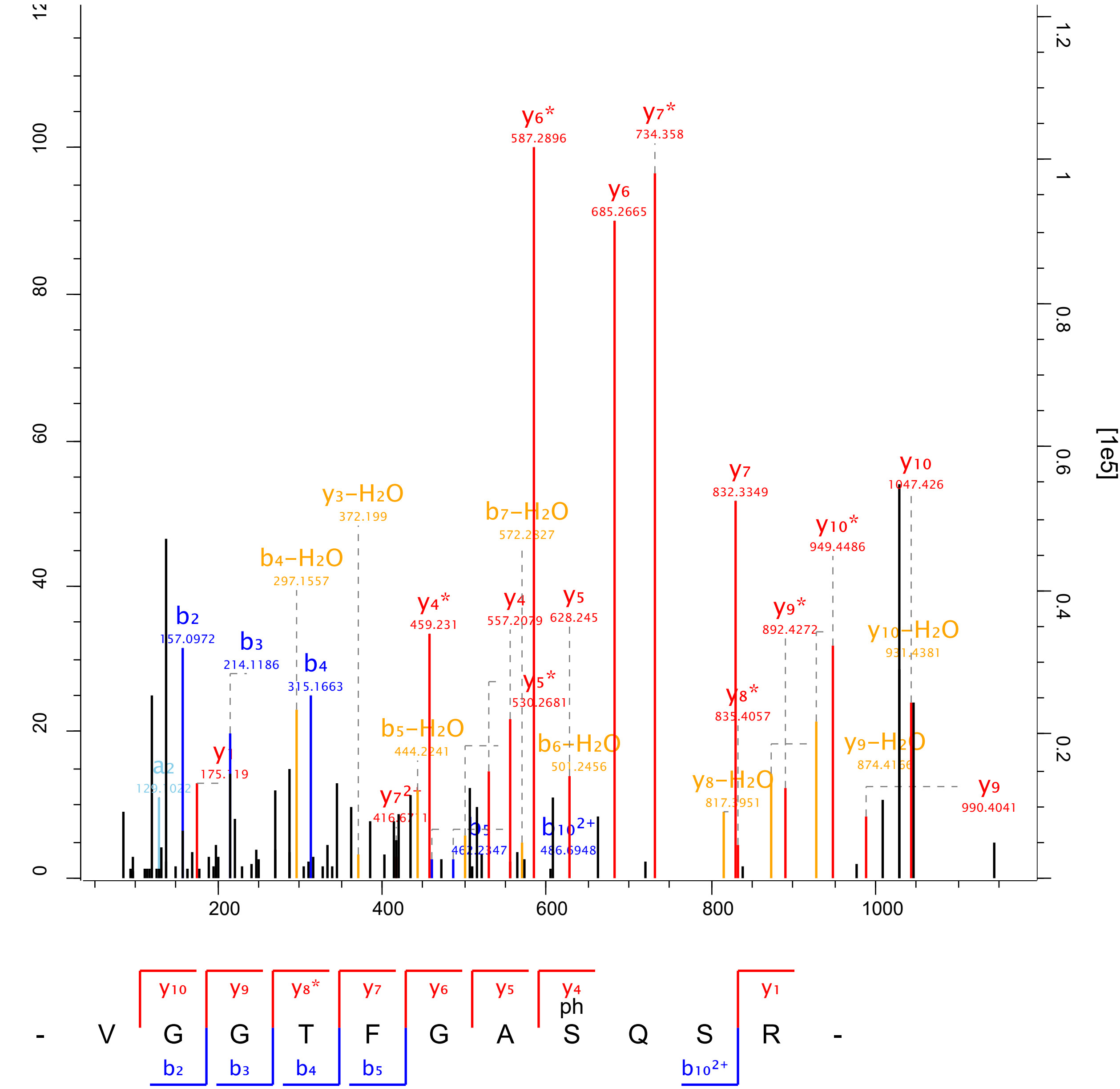

Raw file	Scan	Method	Score	m/z
sys_05_2	7605	FTMS; HCD	123.19	627.24

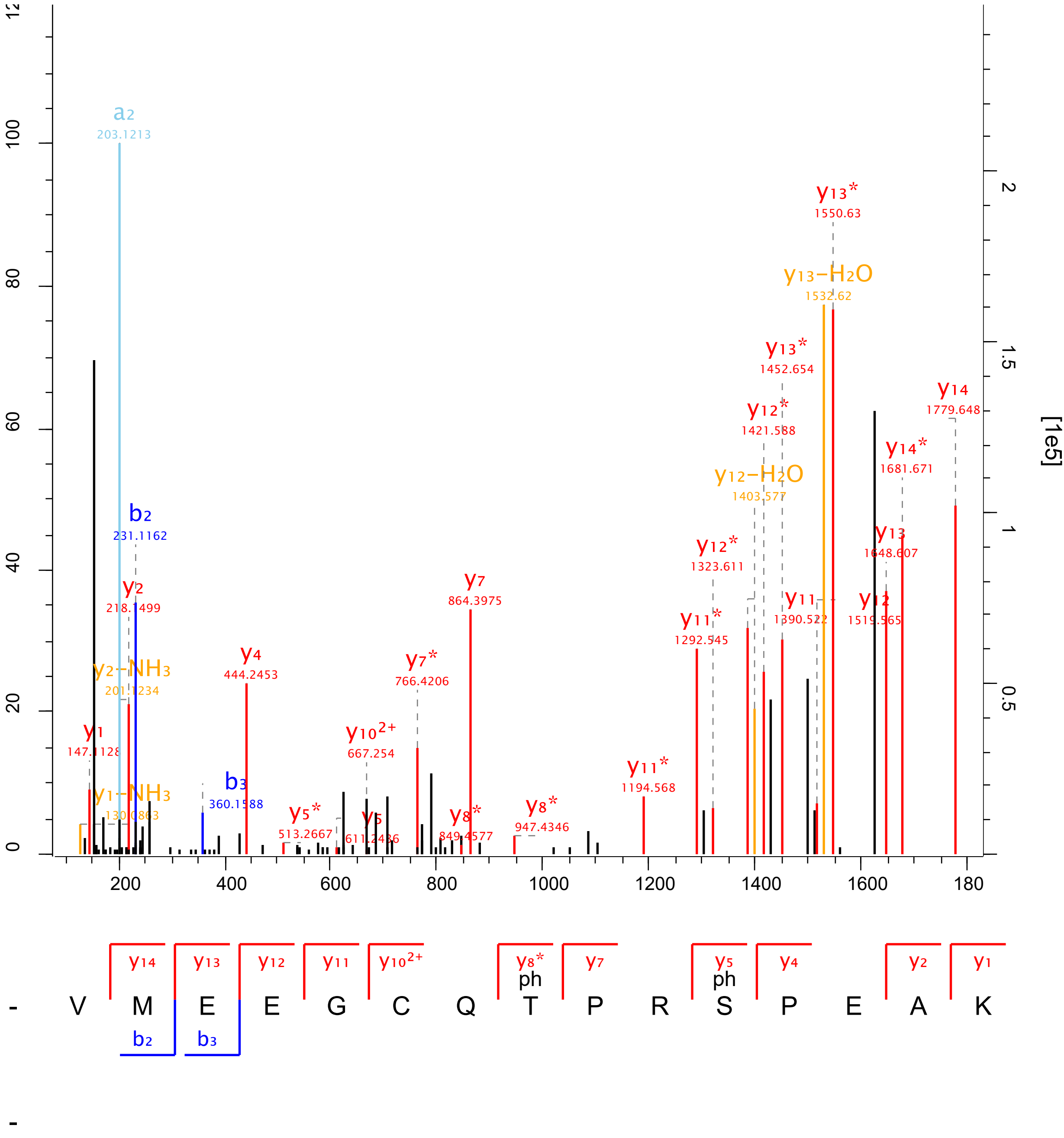

Raw file	Scan	Method	Score	m/z
sys_05_2	7753	FTMS; HCD	228.38	557.56

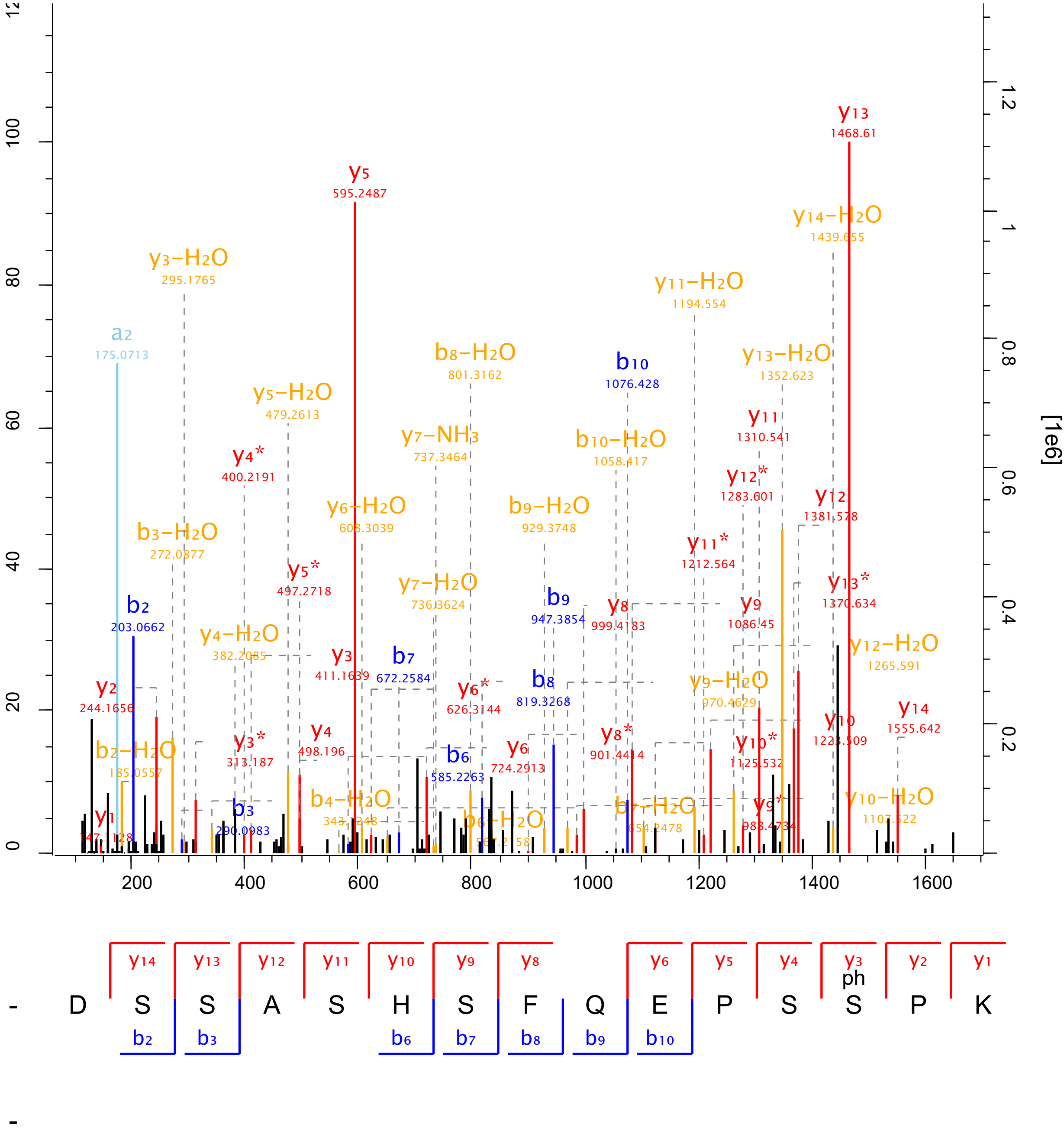

Raw file	Scan	Method	Score	m/z
sys_05_2	7769	FTMS; HCD	66.02	526.74

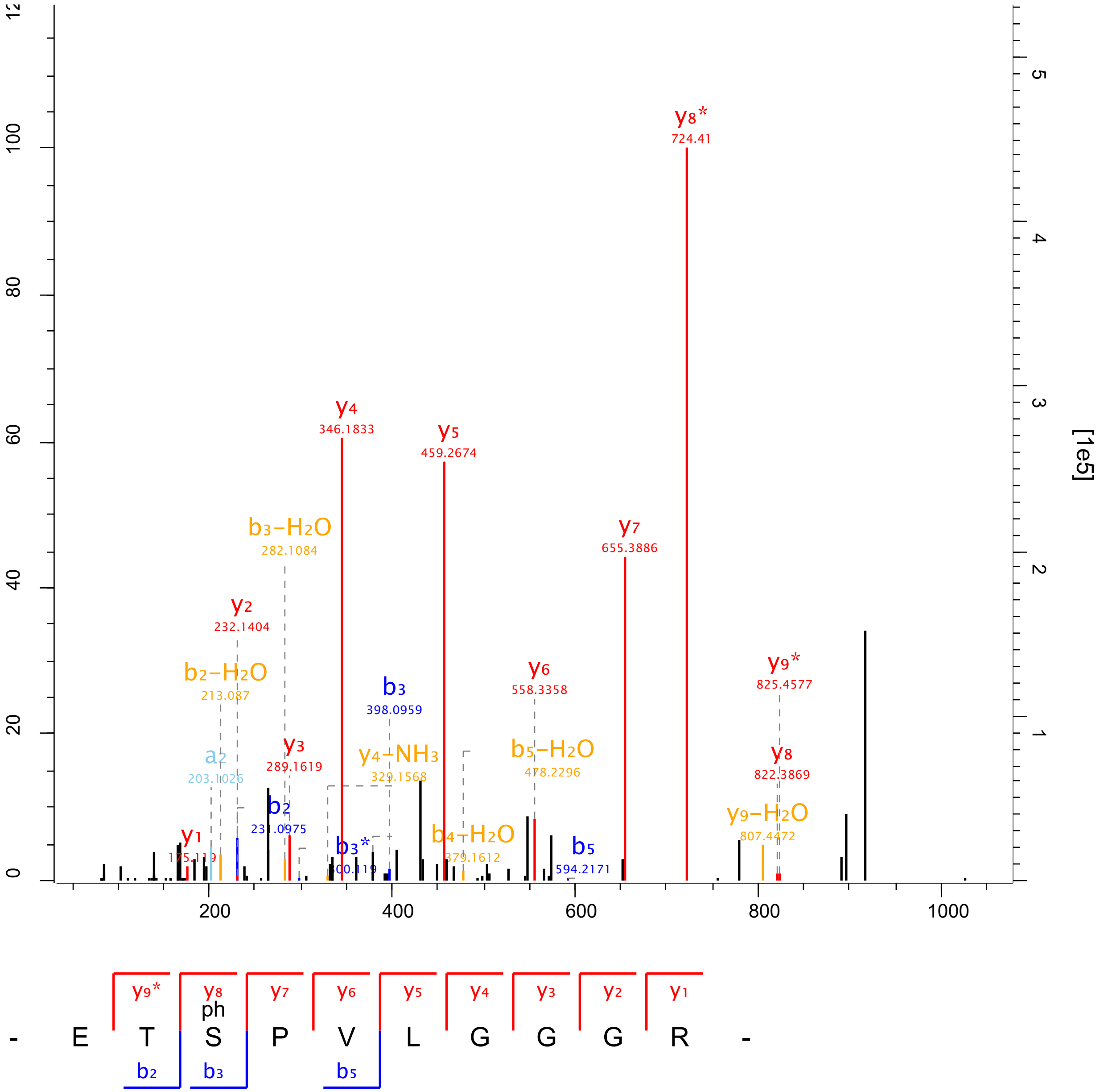

Raw file	Scan	Method	Score	m/z
sys_05_2	7897	FTMS; HCD	97.16	390.52

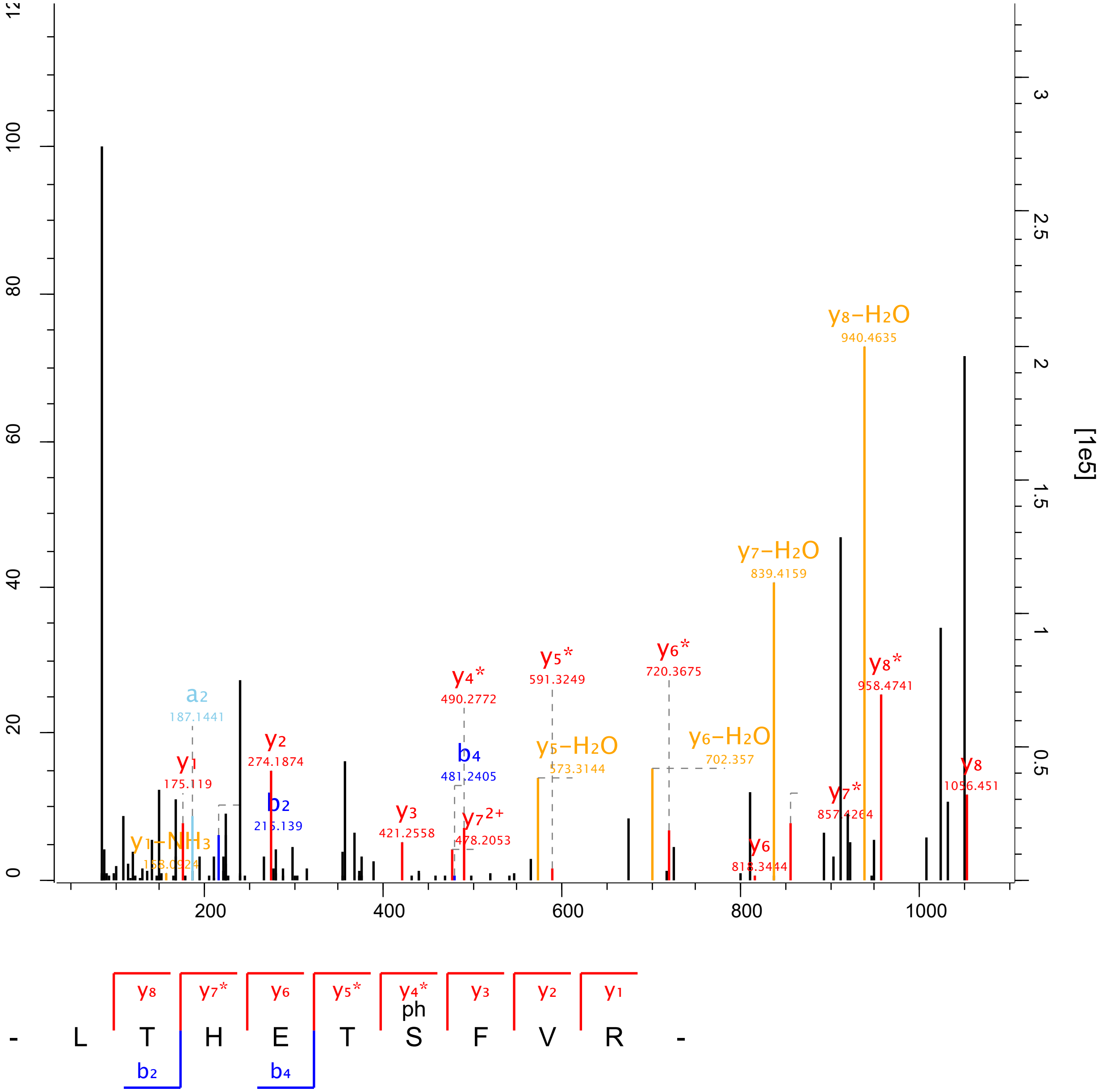

Raw file	Scan	Method	Score	m/z
sys_05_2	8021	FTMS; HCD	46.32	440.55

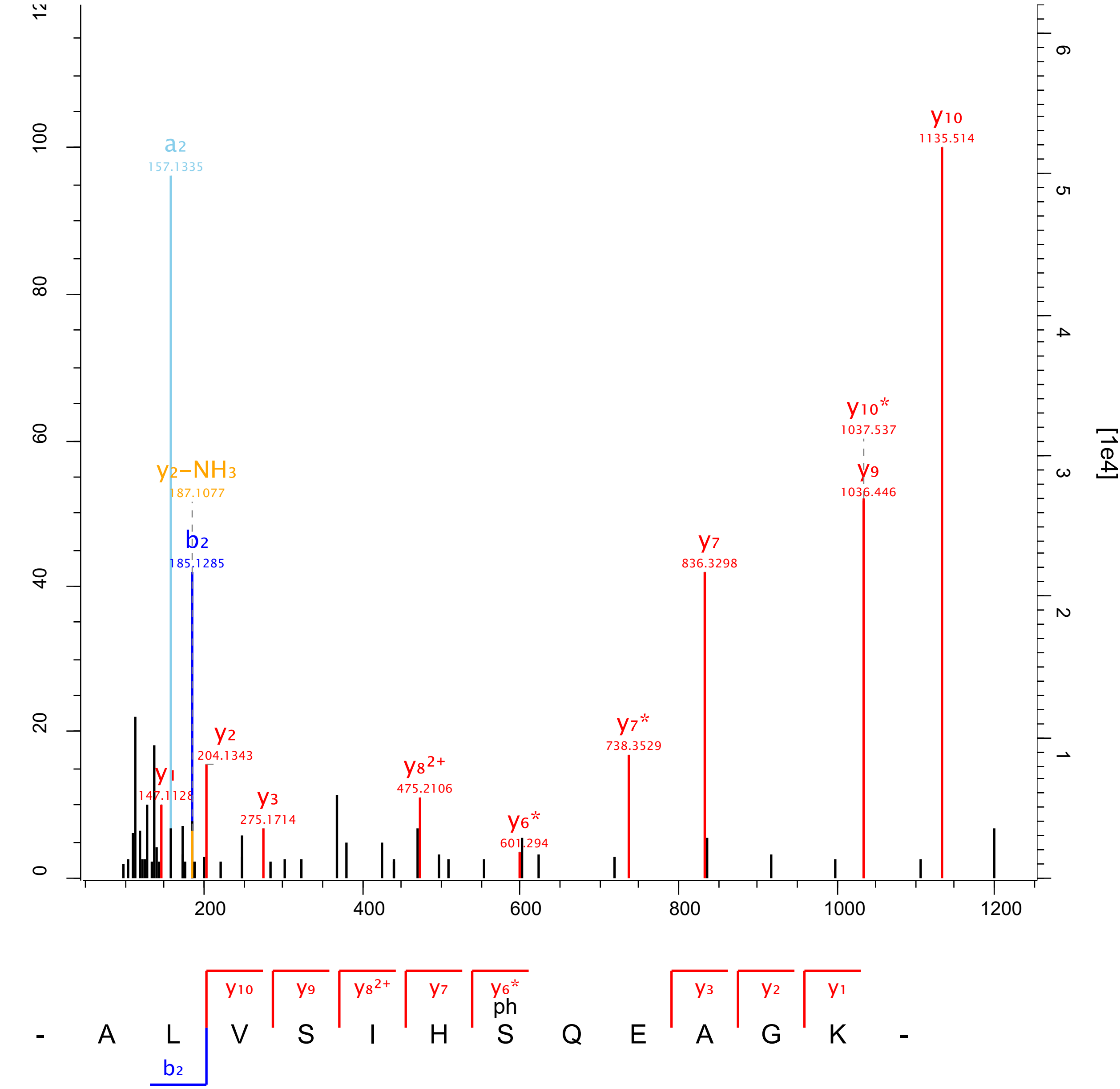

Raw file	Scan	Method	Score	m/z
sys_05_2	8192	FTMS; HCD	194.19	726.79

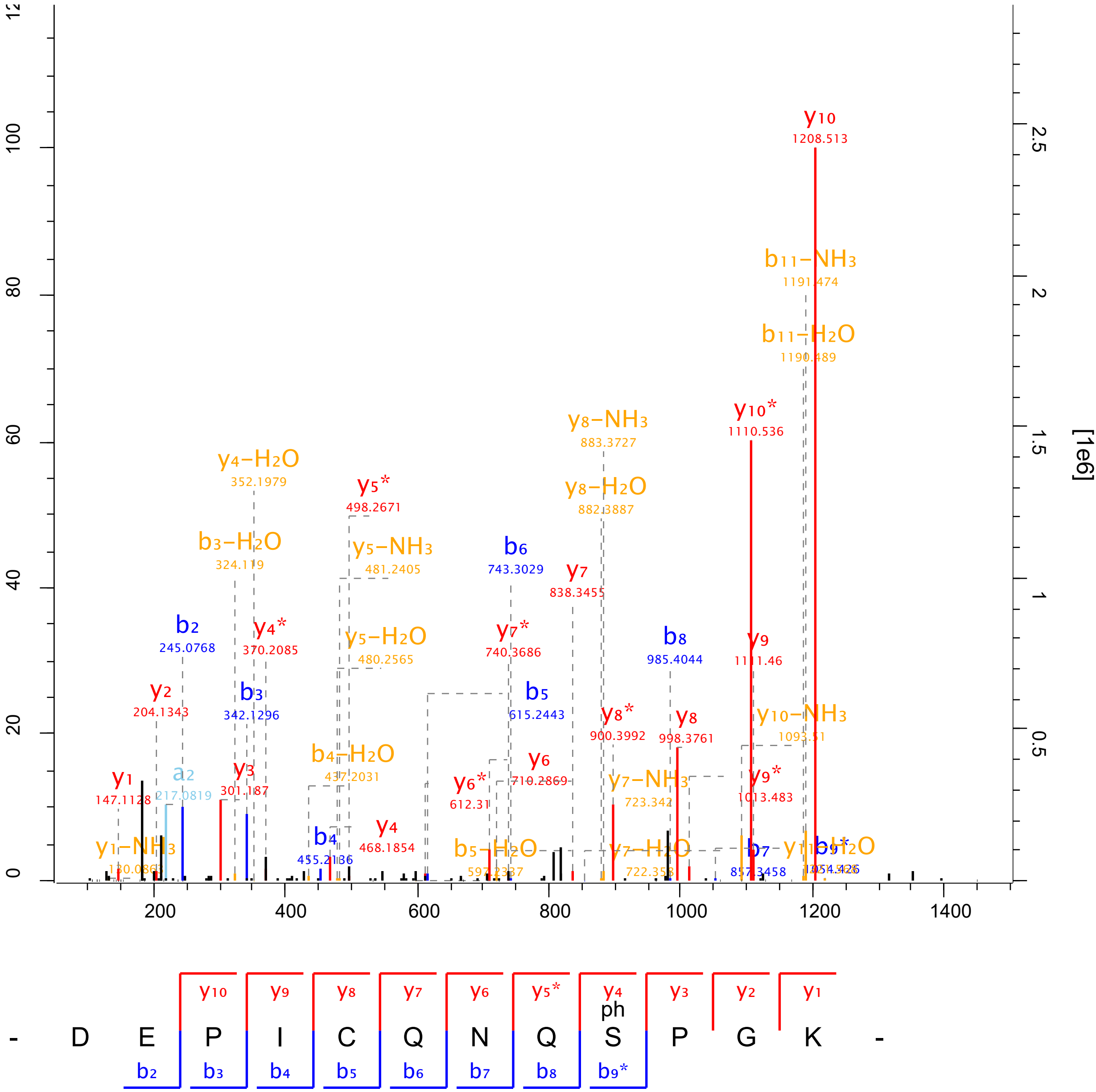

Raw file	Scan	Method	Score	m/z
sys_05_2	8483	FTMS; HCD	90.56	678.77

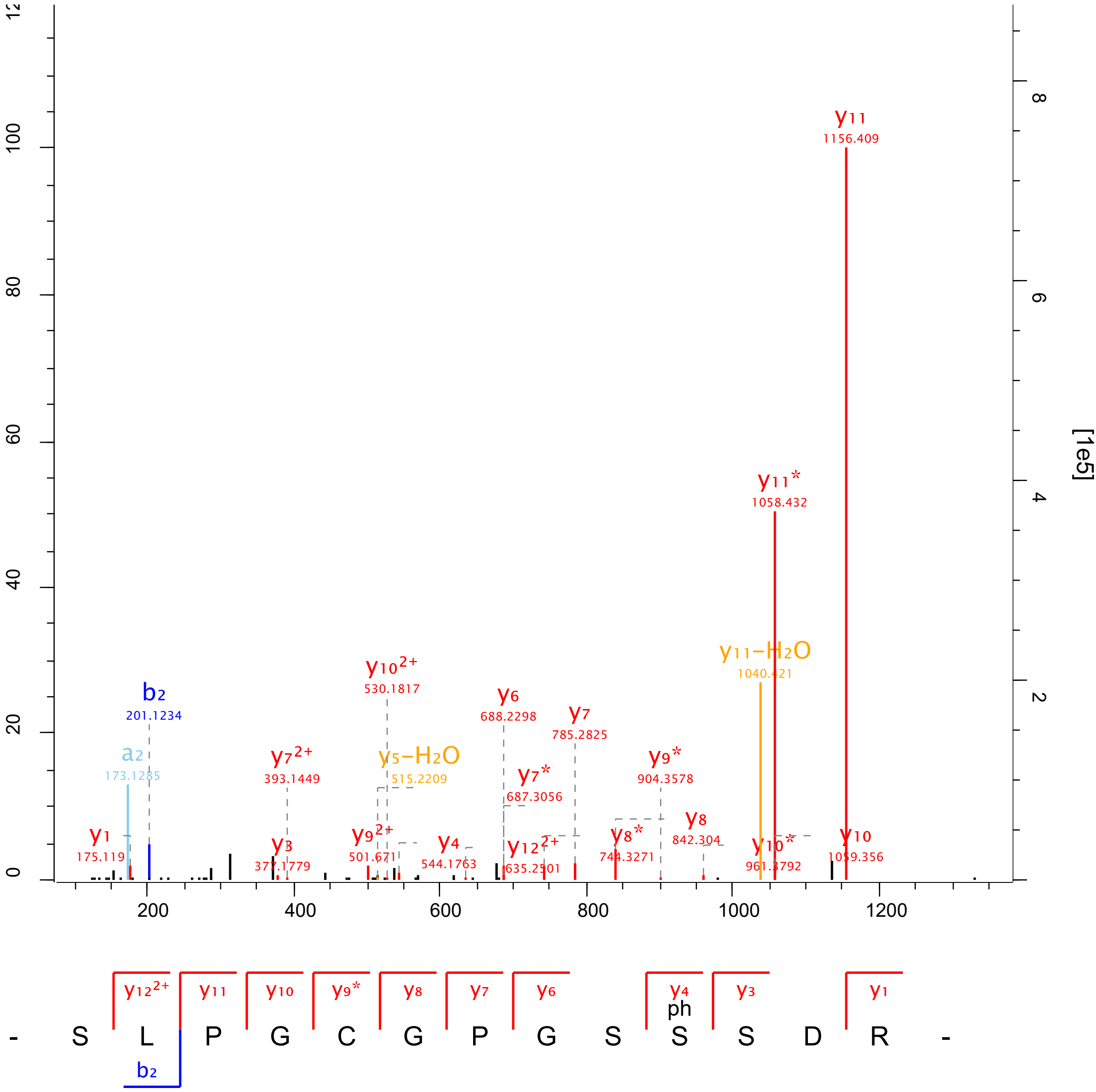

Raw file	Scan	Method	Score	m/z
sys_05_2	8666	FTMS; HCD	103.88	640.29

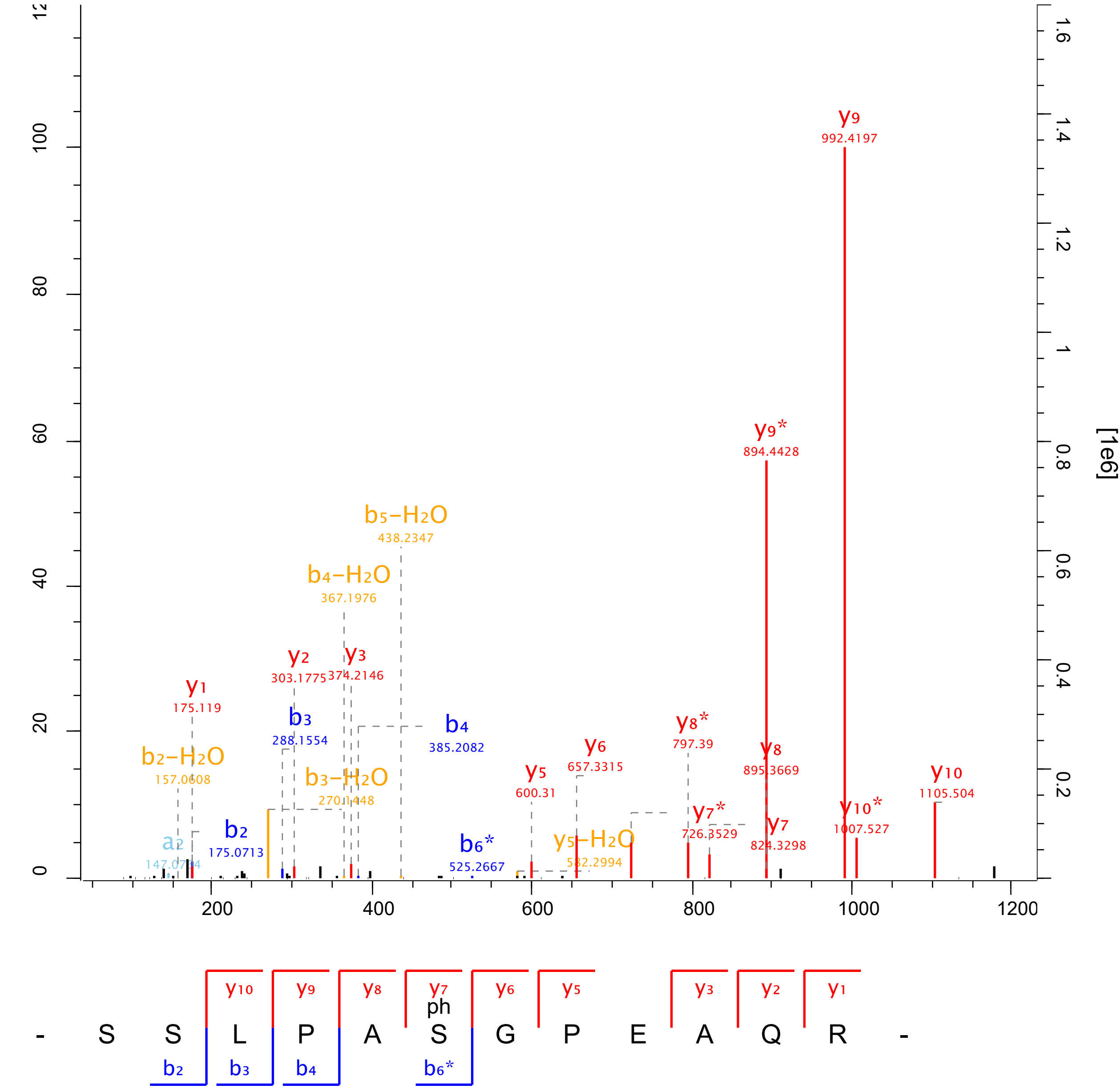

Raw file	Scan	Method	Score	m/z
sys_05_2	8807	FTMS; HCD	68.16	541.57

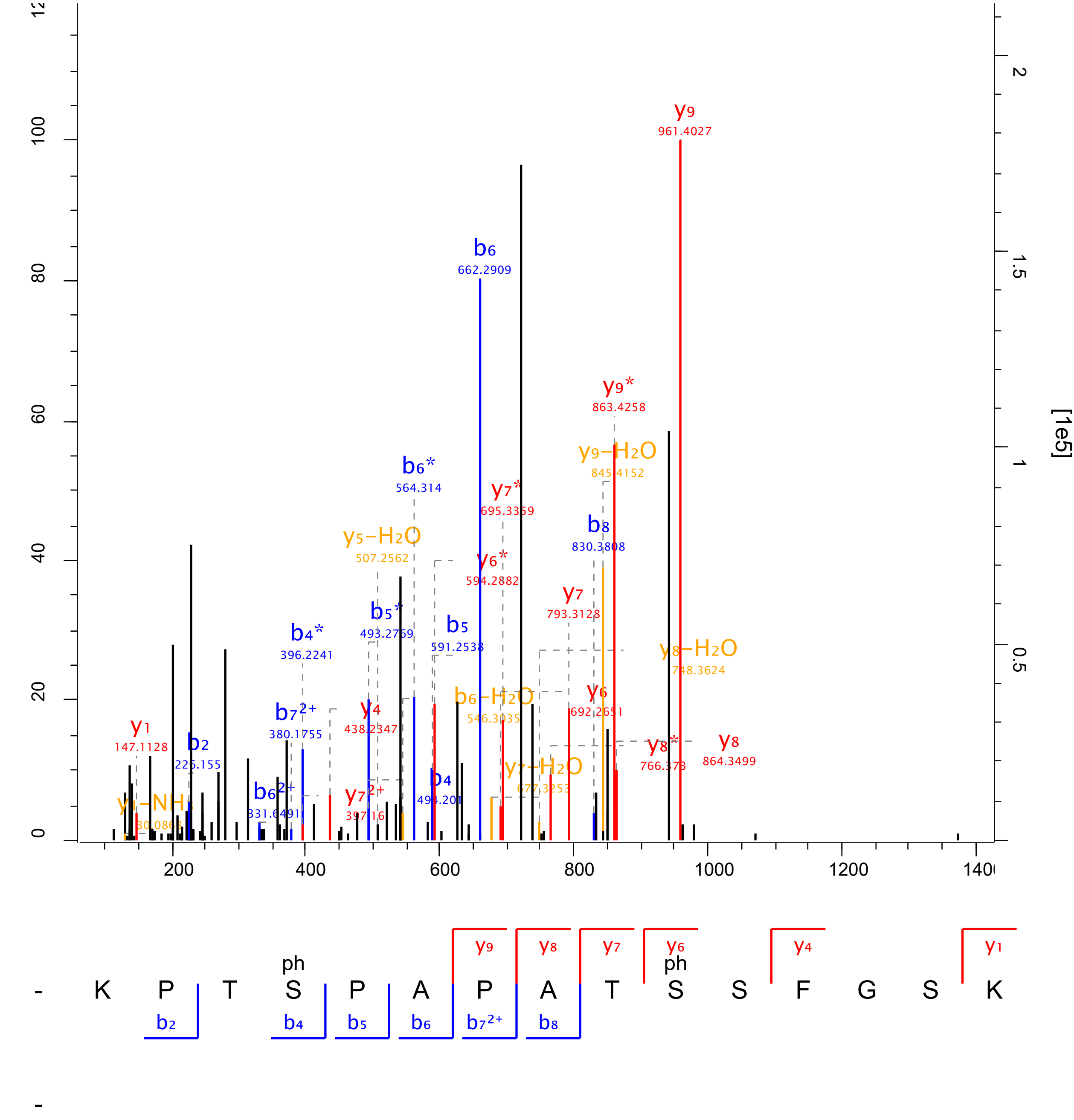

Raw file	Scan	Method	Score	m/z
sys_05_2	8887	FTMS; HCD	91.62	580.74

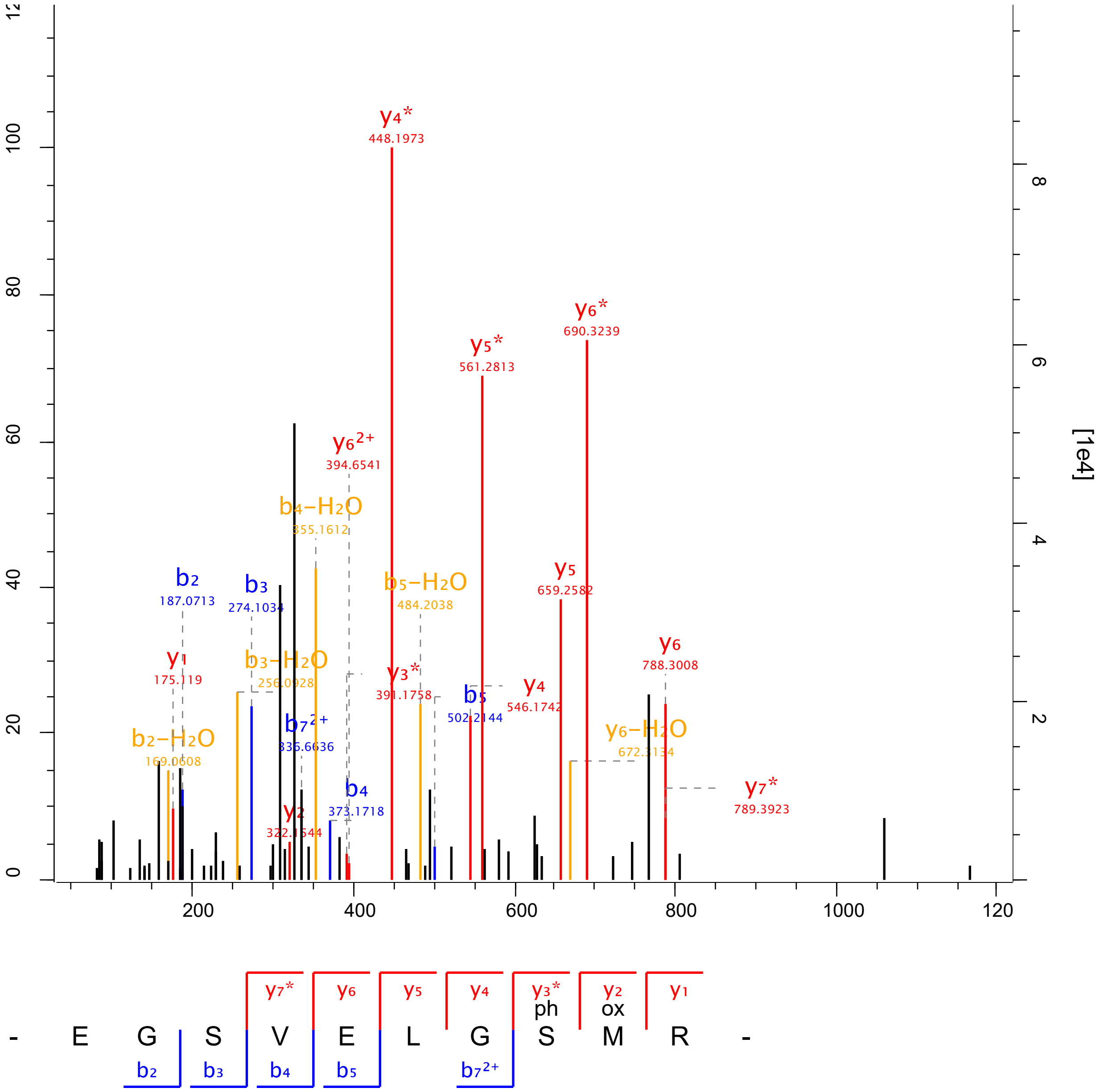

Raw file	Scan	Method	Score	m/z
sys_05_2	8974	FTMS; HCD	287.16	826.89

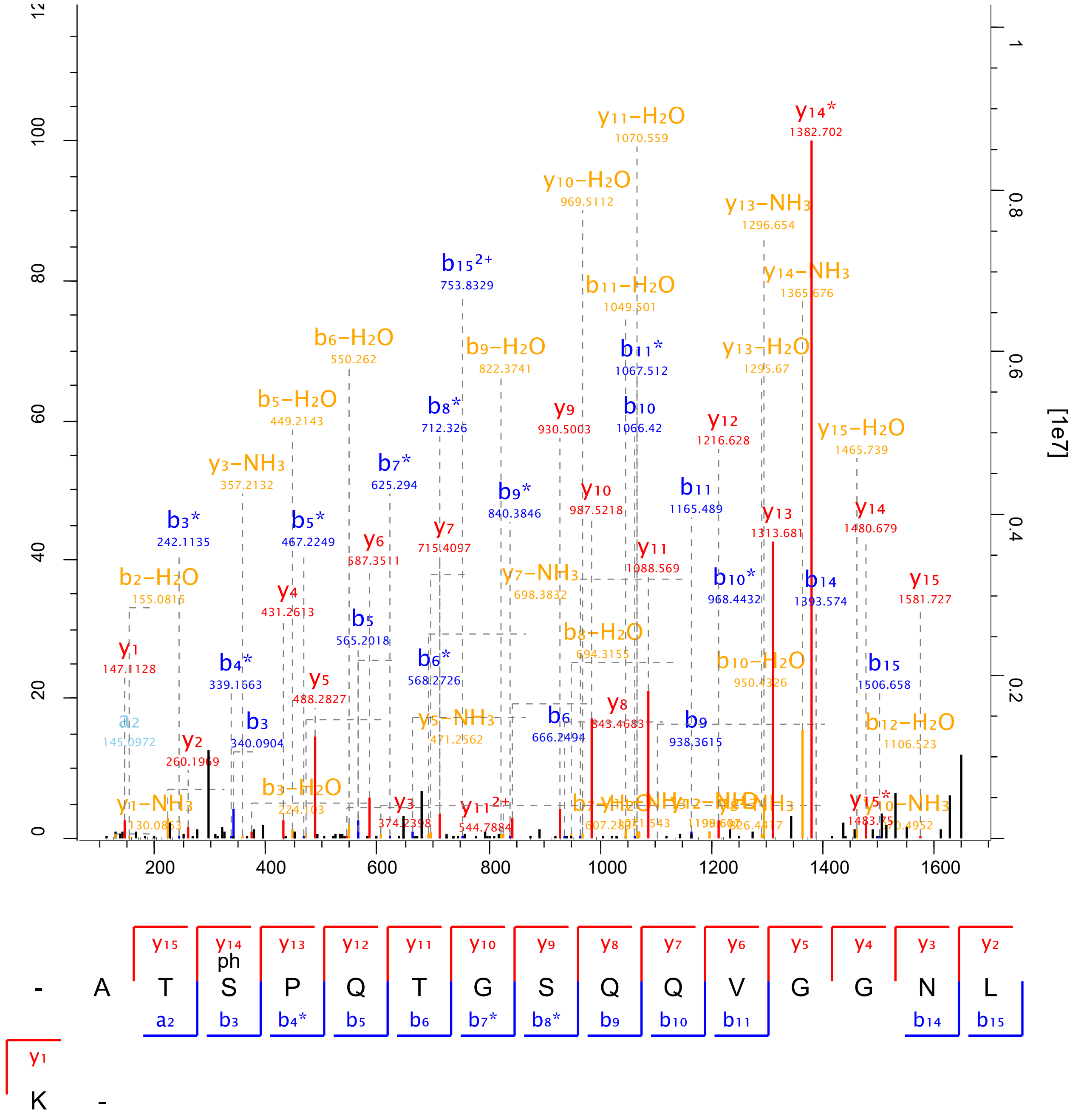

Raw file	Scan	Method	Score	m/z
sys_05_2	9095	FTMS; HCD	74.78	572.6

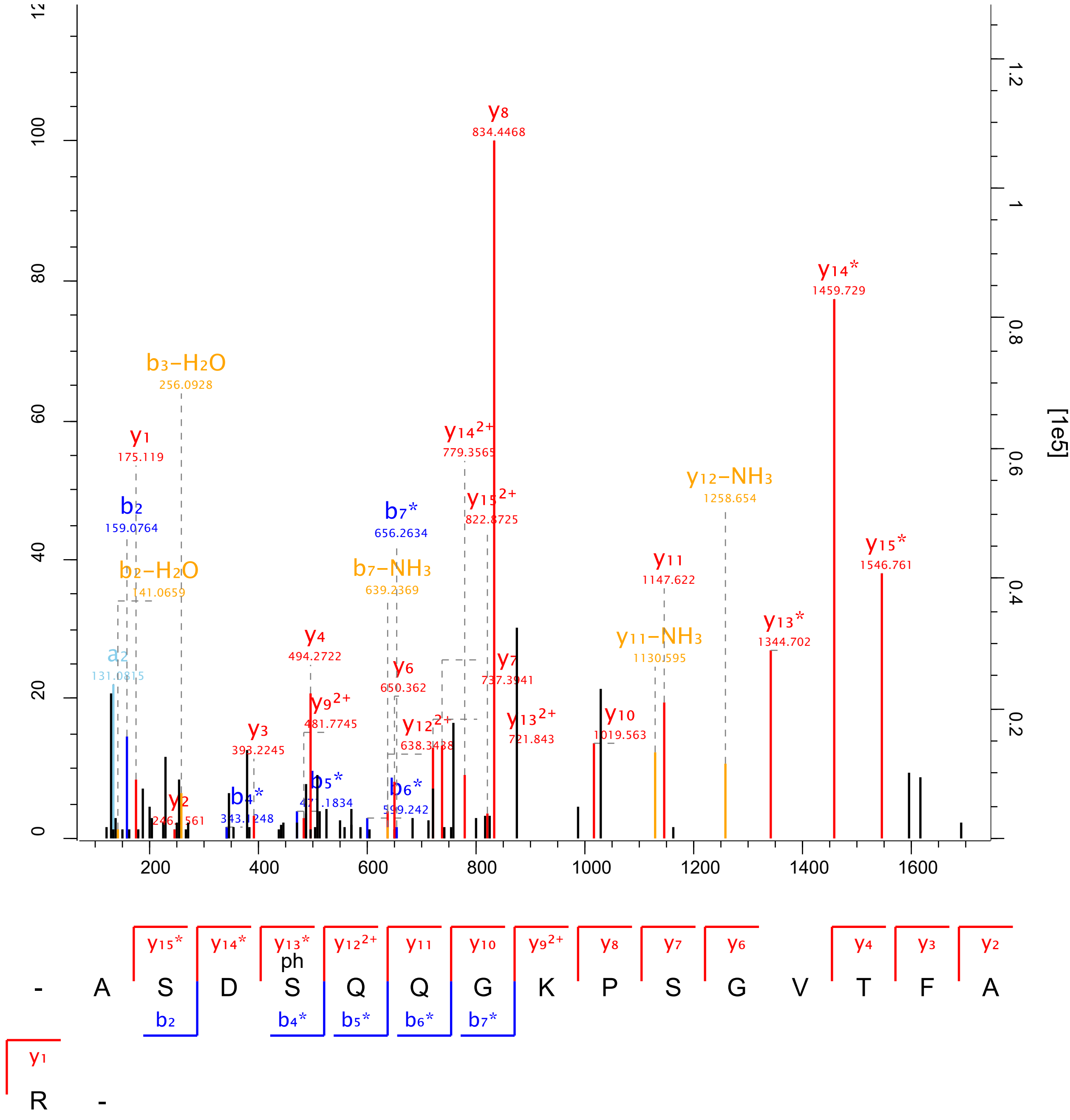

Raw file	Scan	Method	Score	m/z
sys_05_2	9241	FTMS; HCD	181.35	701.79

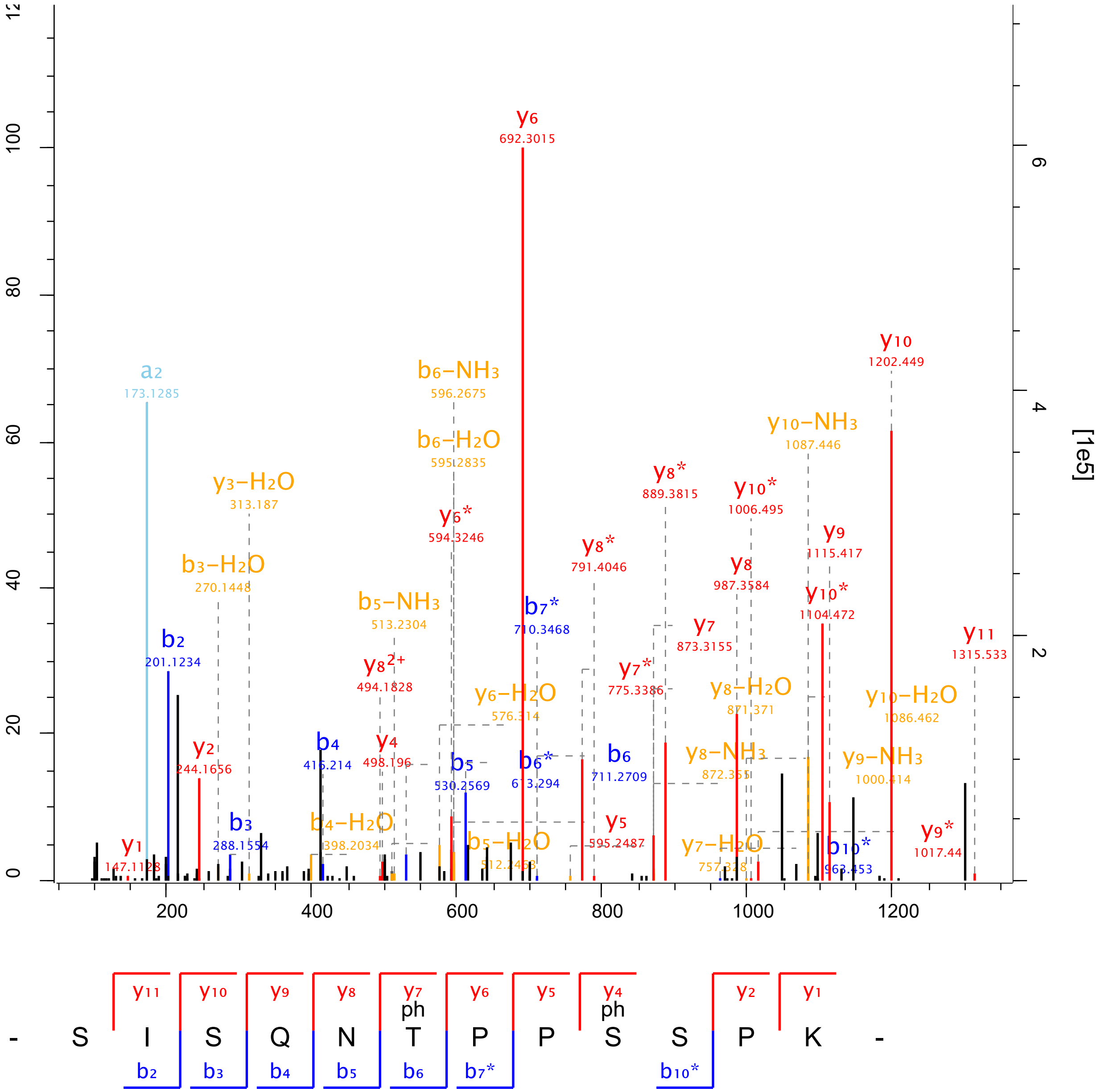

Raw file	Scan	Method	Score	m/z
sys_05_2	9347	FTMS; HCD	79.49	676.74

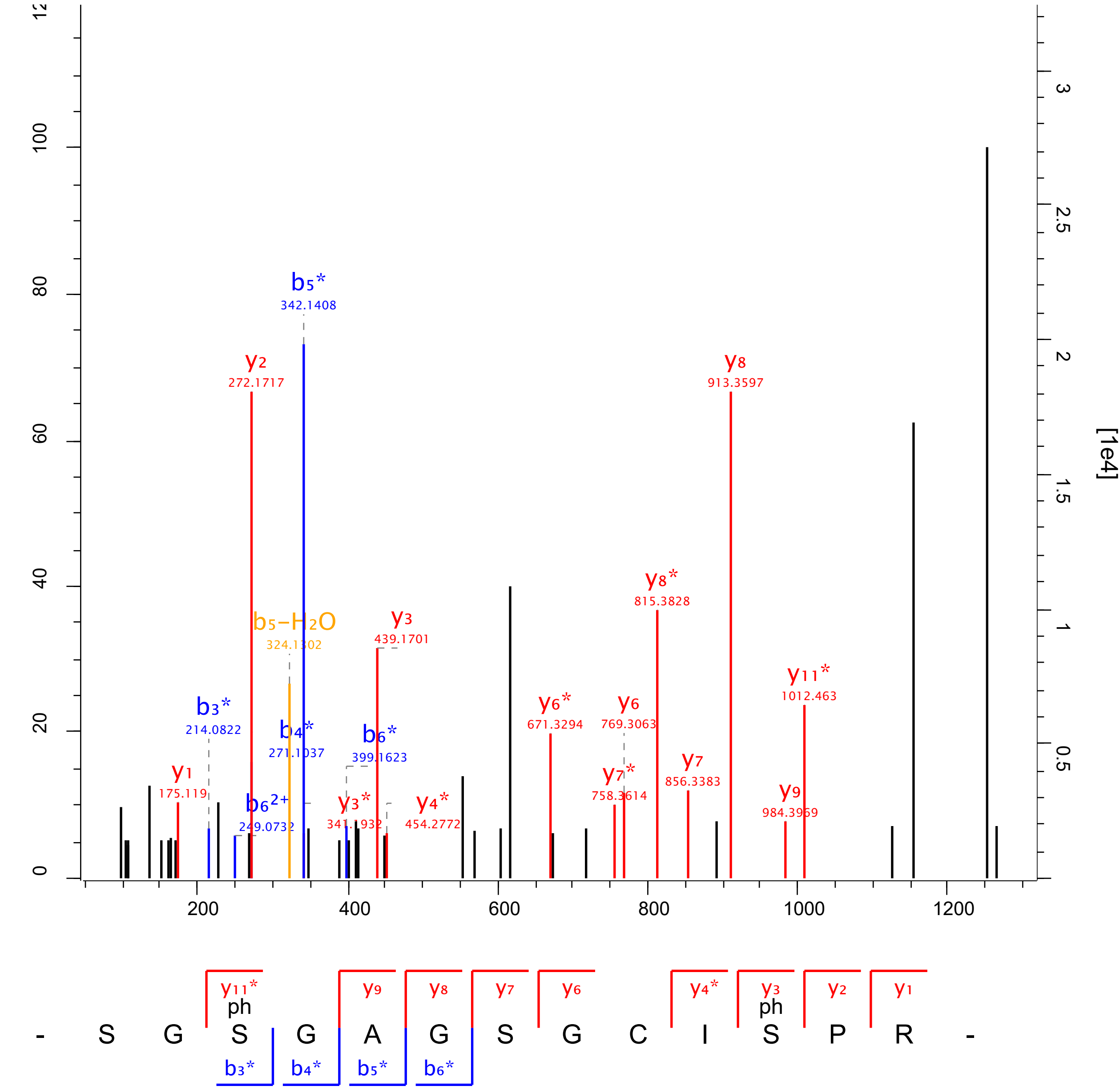

Raw file	Scan	Method	Score	m/z
sys_05_2	9355	FTMS; HCD	63.09	511.69

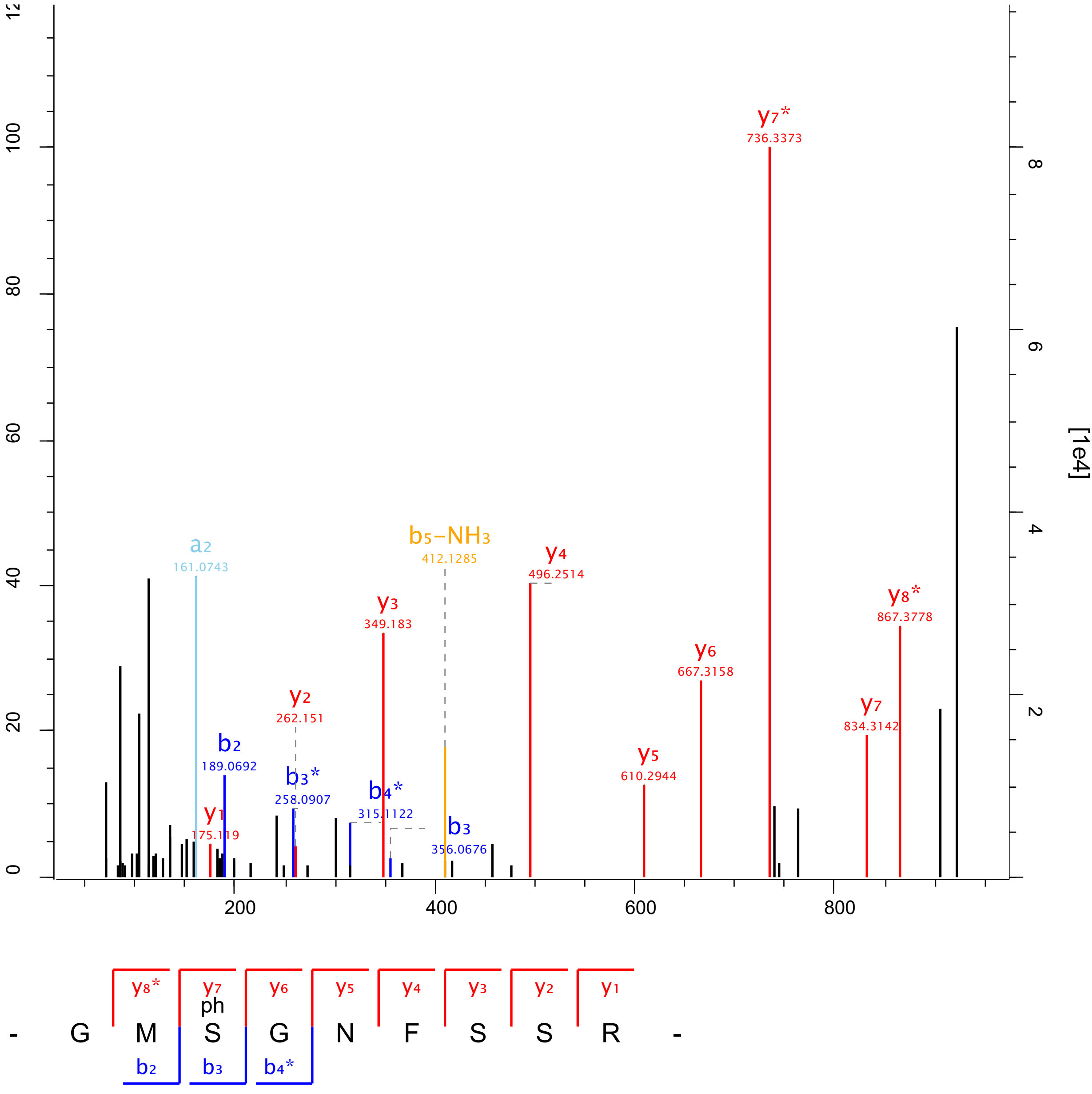

Raw file	Scan	Method	Score	m/z
sys_05_2	9458	FTMS; HCD	40.97	561.22

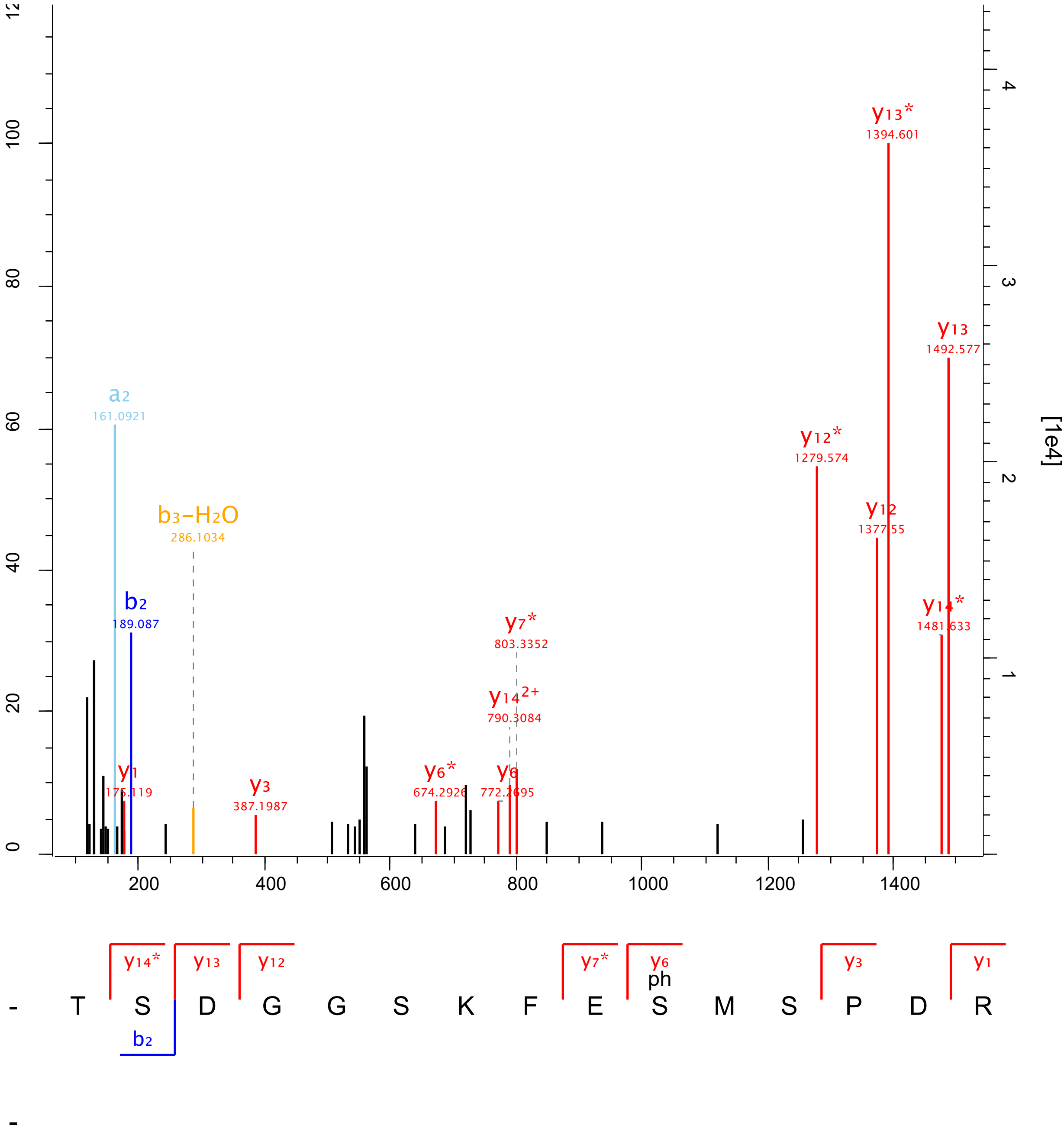

Raw file	Scan	Method	Score	m/z
sys_05_2	9492	FTMS; HCD	102.73	547.7

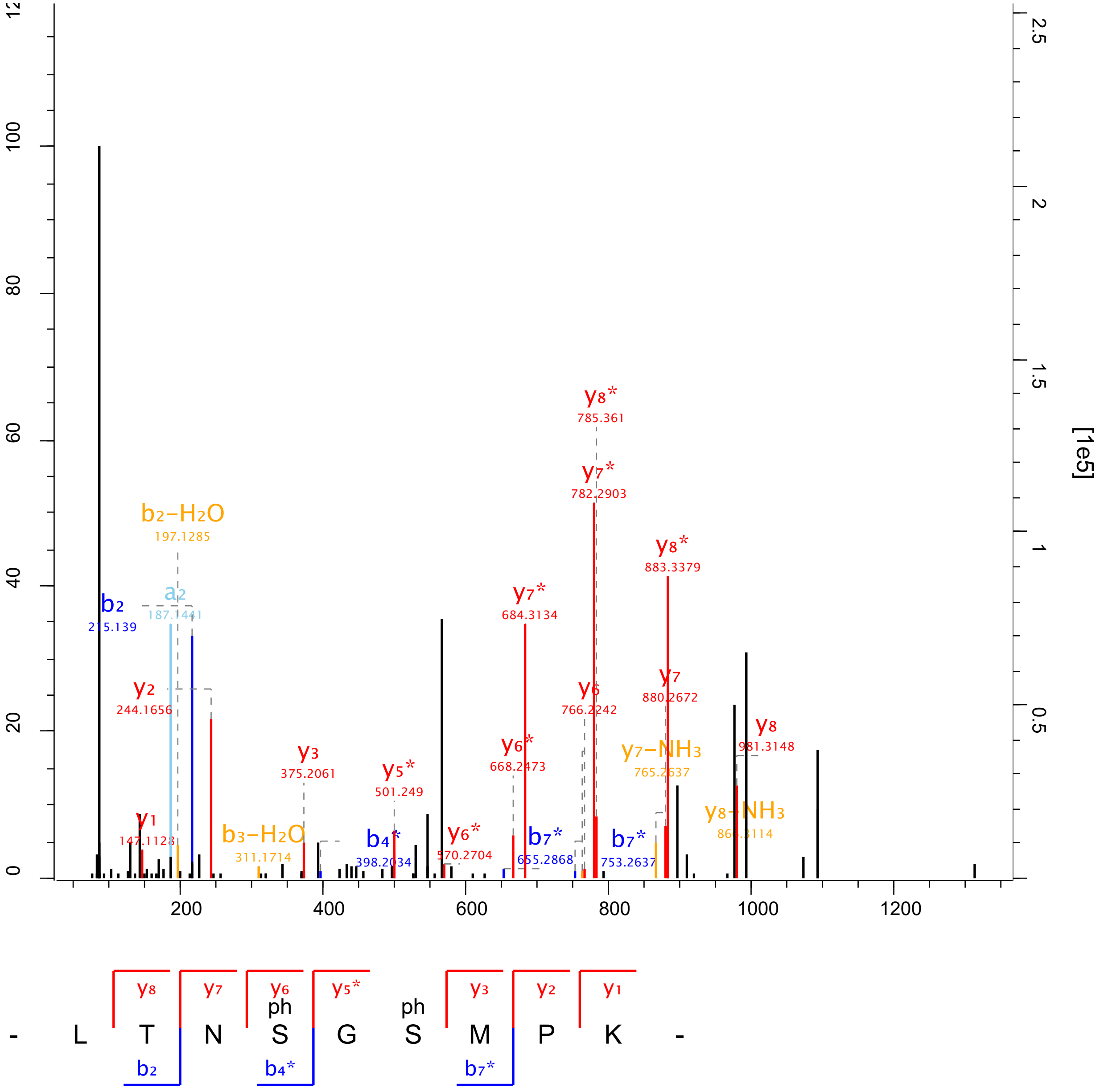

Raw file	Scan	Method	Score	m/z
sys_05_2	9617	FTMS; HCD	127.83	528.73

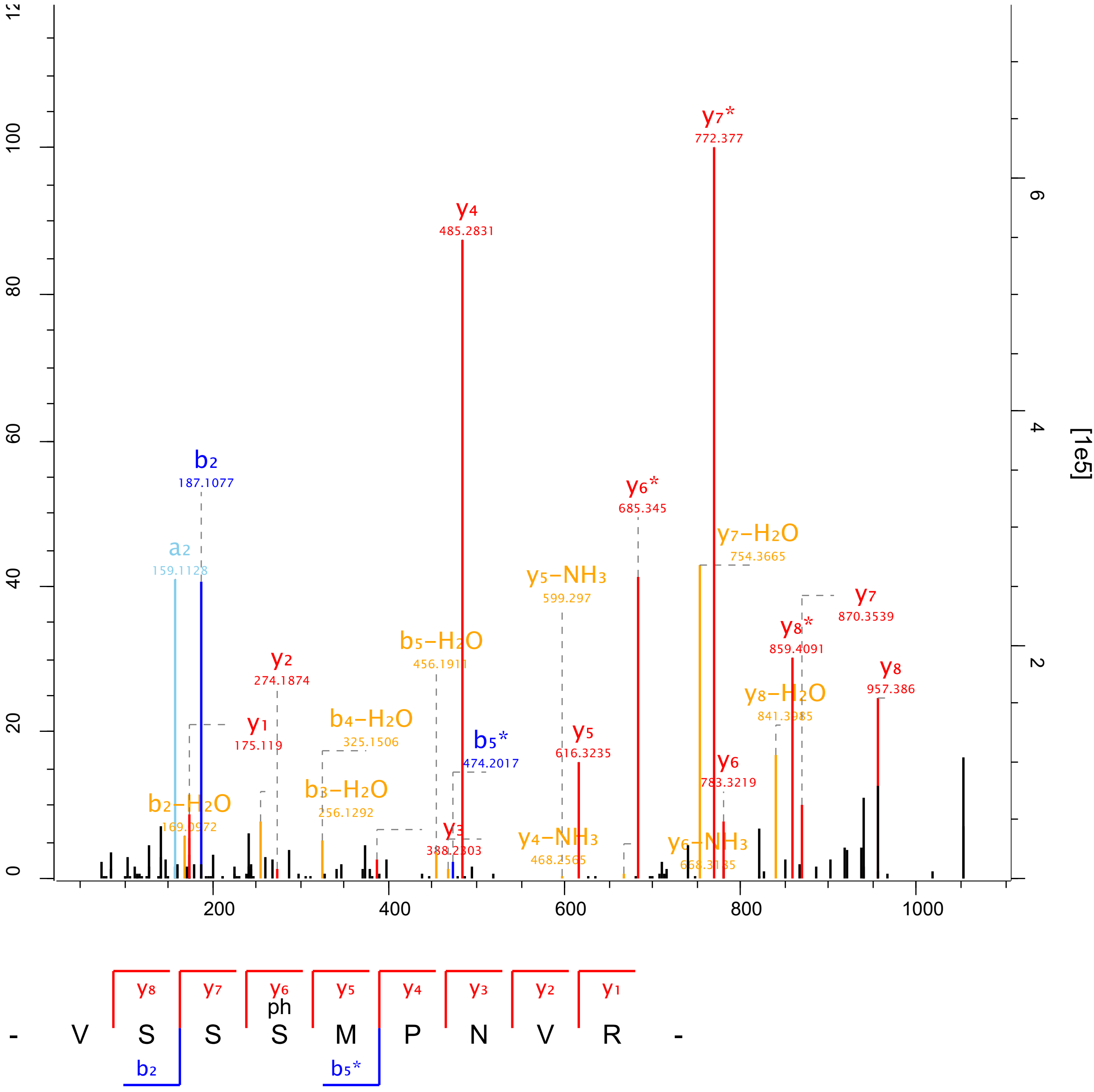

Raw file	Scan	Method	Score	m/z
sys_05_2	9646	FTMS; HCD	43.5	492.21

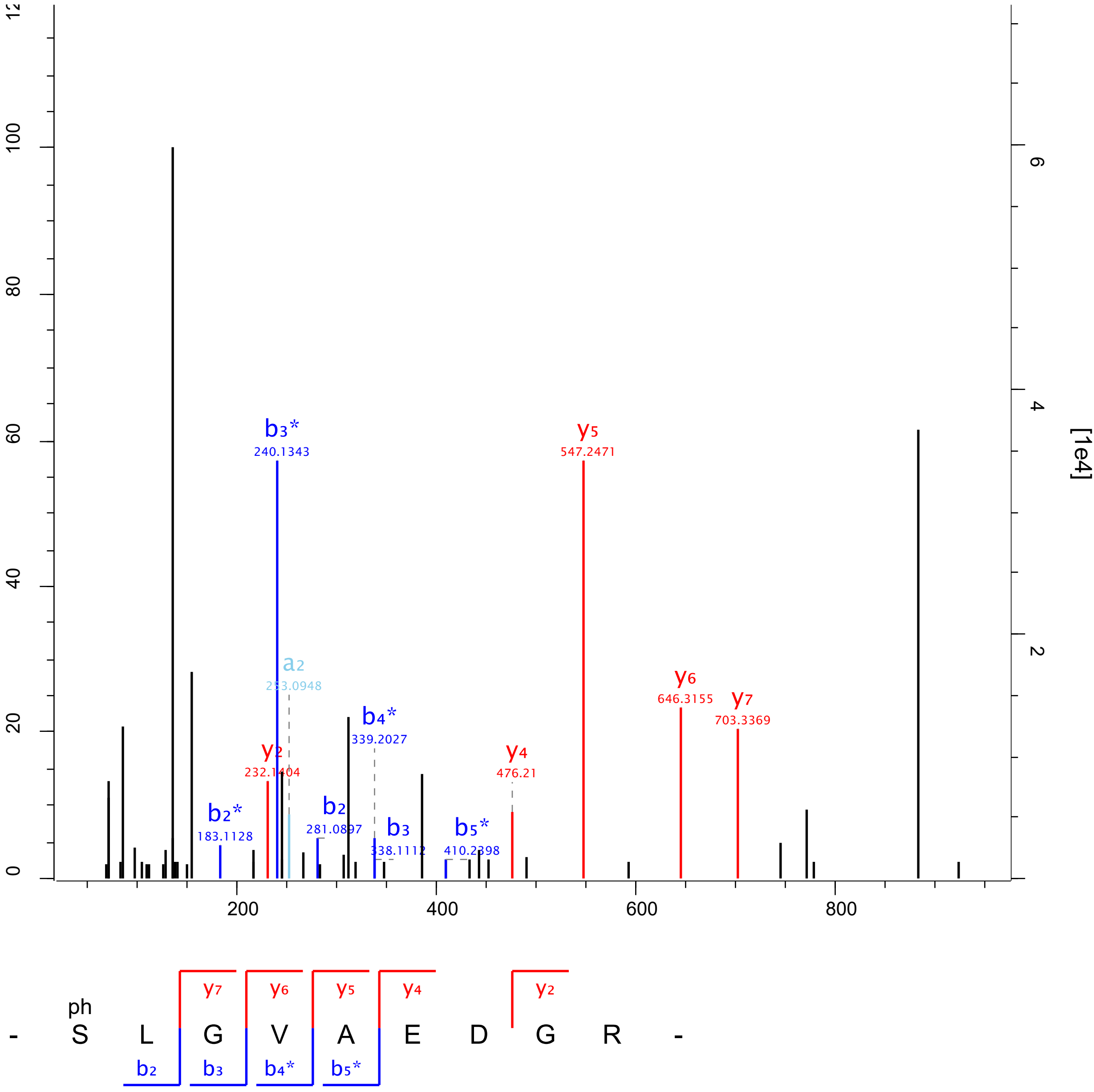

Raw file	Scan	Method	Score	m/z
sys_05_2	9769	FTMS; HCD	59.42	506.73

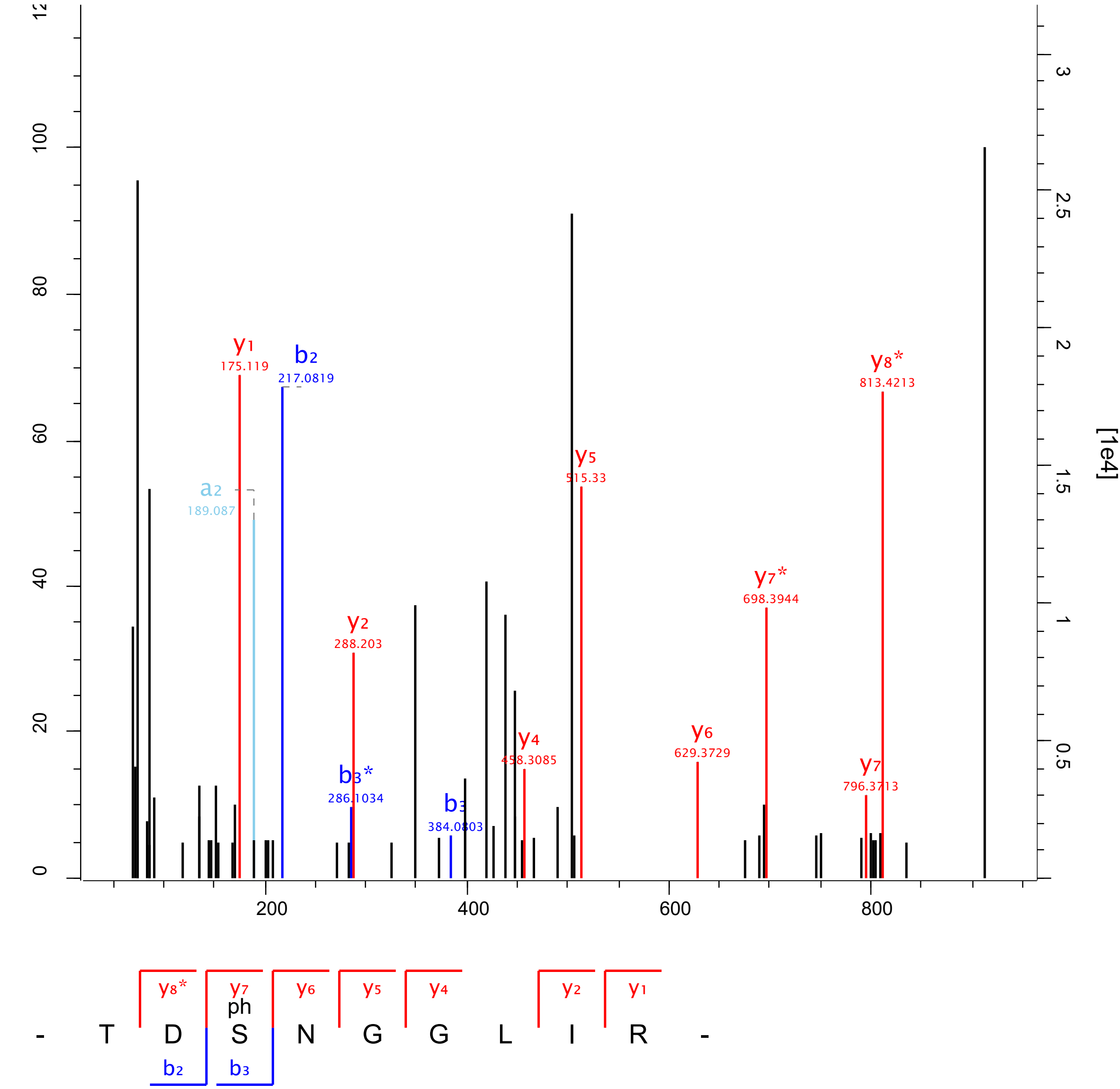

Raw file	Scan	Method	Score	m/z
sys_05_2	9902	FTMS; HCD	246.8	676.3

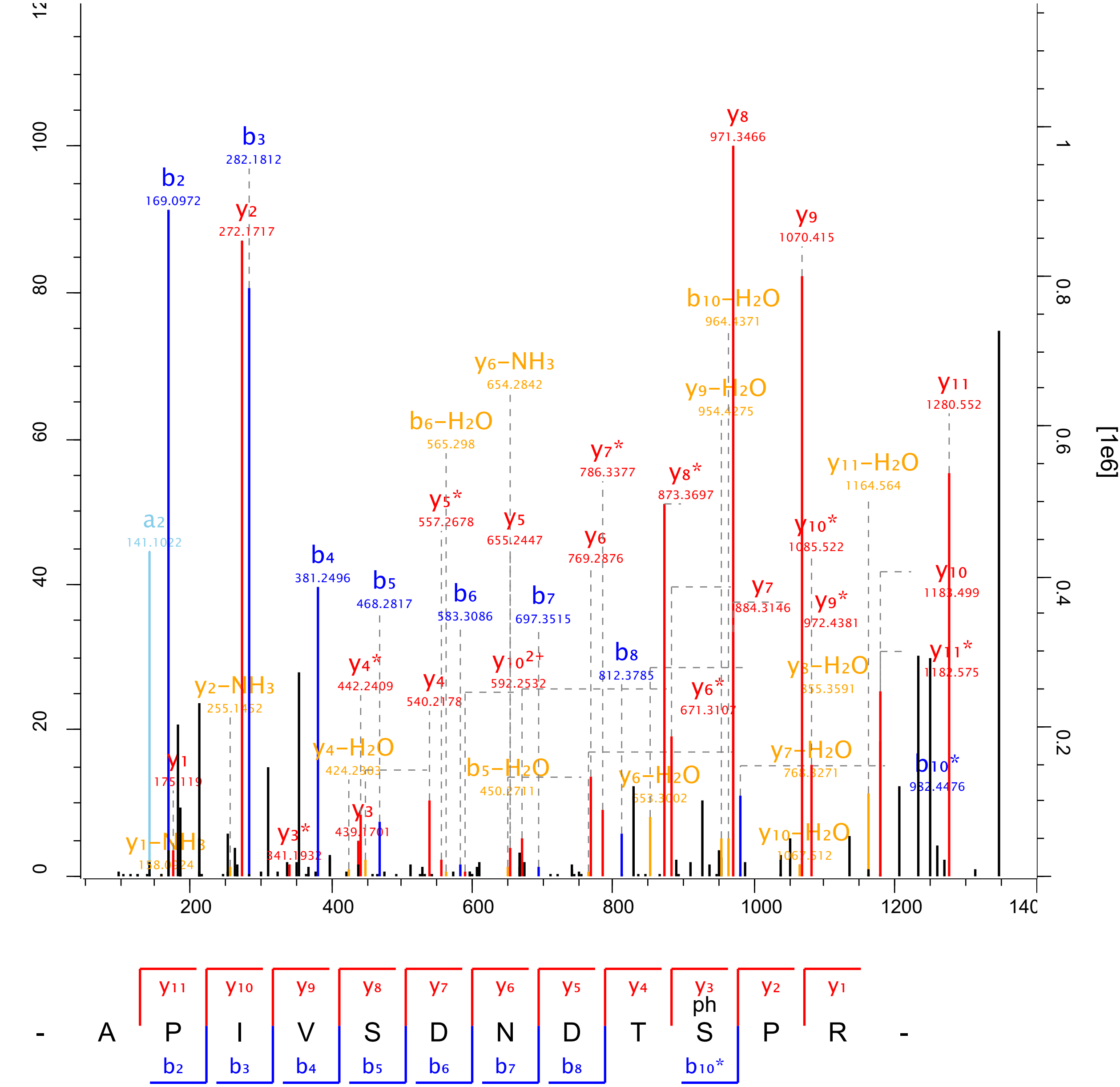

Raw file	Scan	Method	Score	m/z
sys_05_2	9997	FTMS; HCD	41.7	785.32

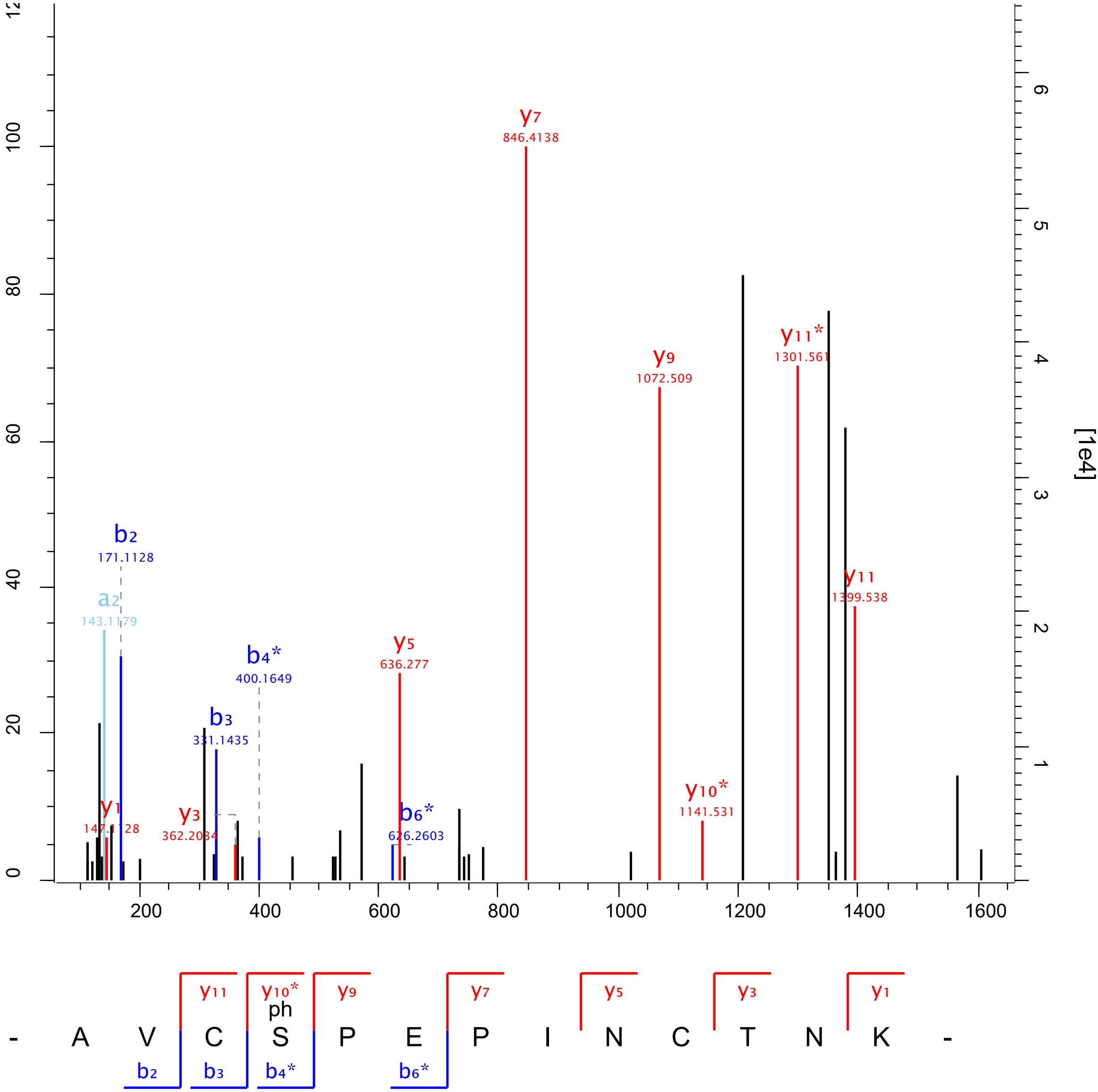

Raw file	Scan	Method	Score	m/z
sys_05_3	13608	FTMS; HCD	93.35	503.77

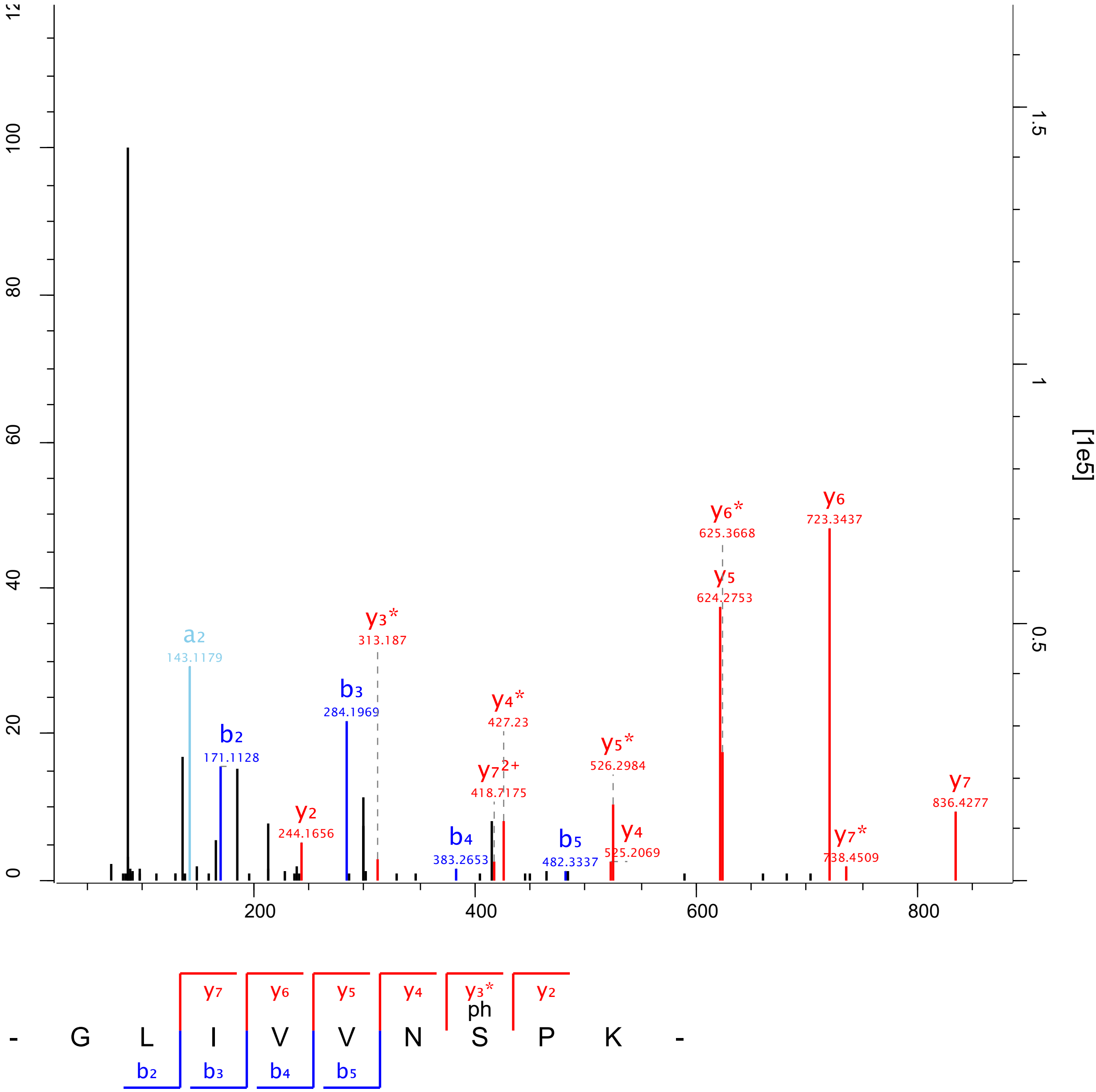

Raw file	Scan	Method	Score	m/z
sys_05_3	4381	FTMS; HCD	172.06	634.26

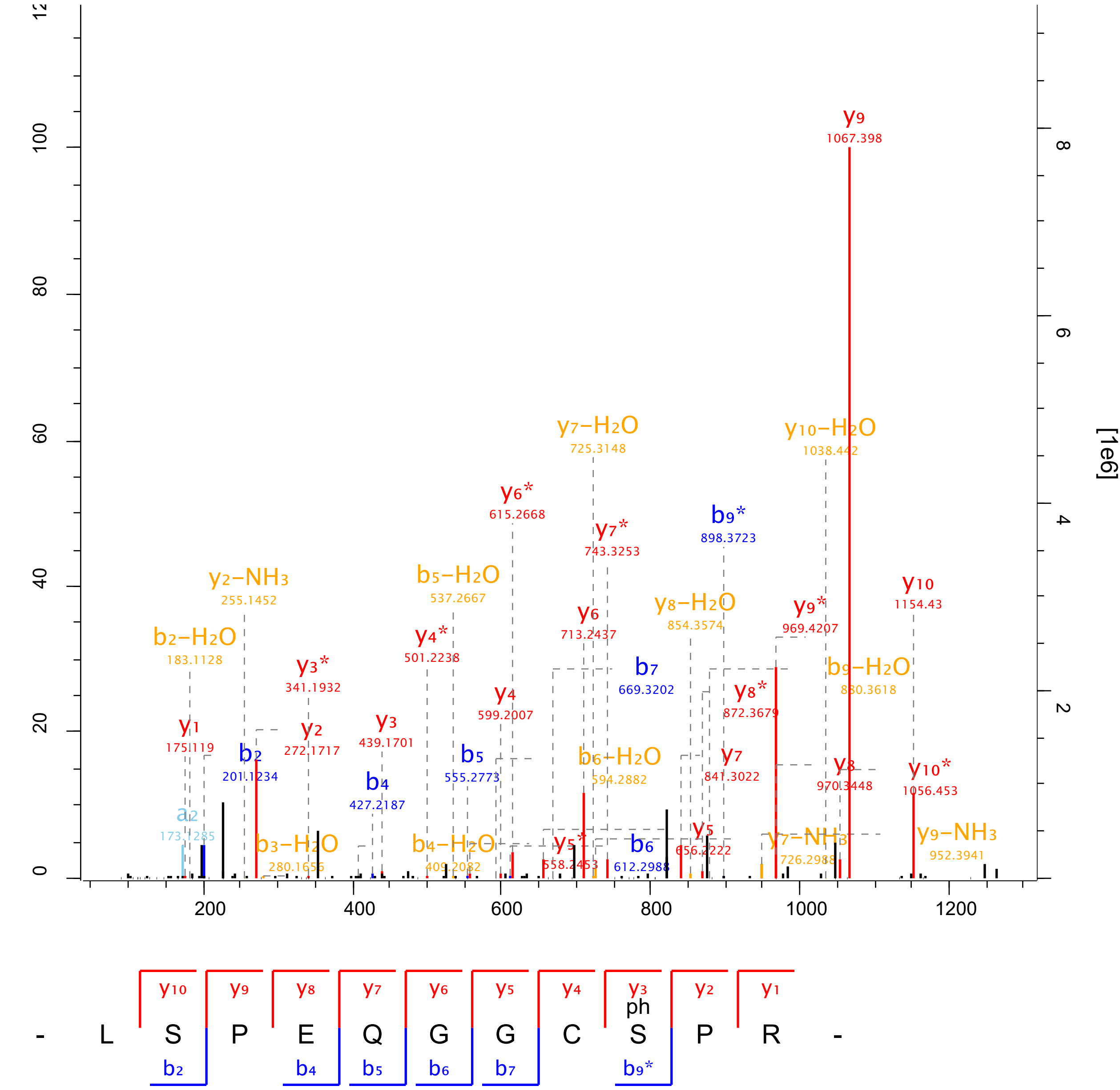

Raw file	Scan	Method	Score	m/z
sys_05_3	8633	FTMS; HCD	57.79	443.69

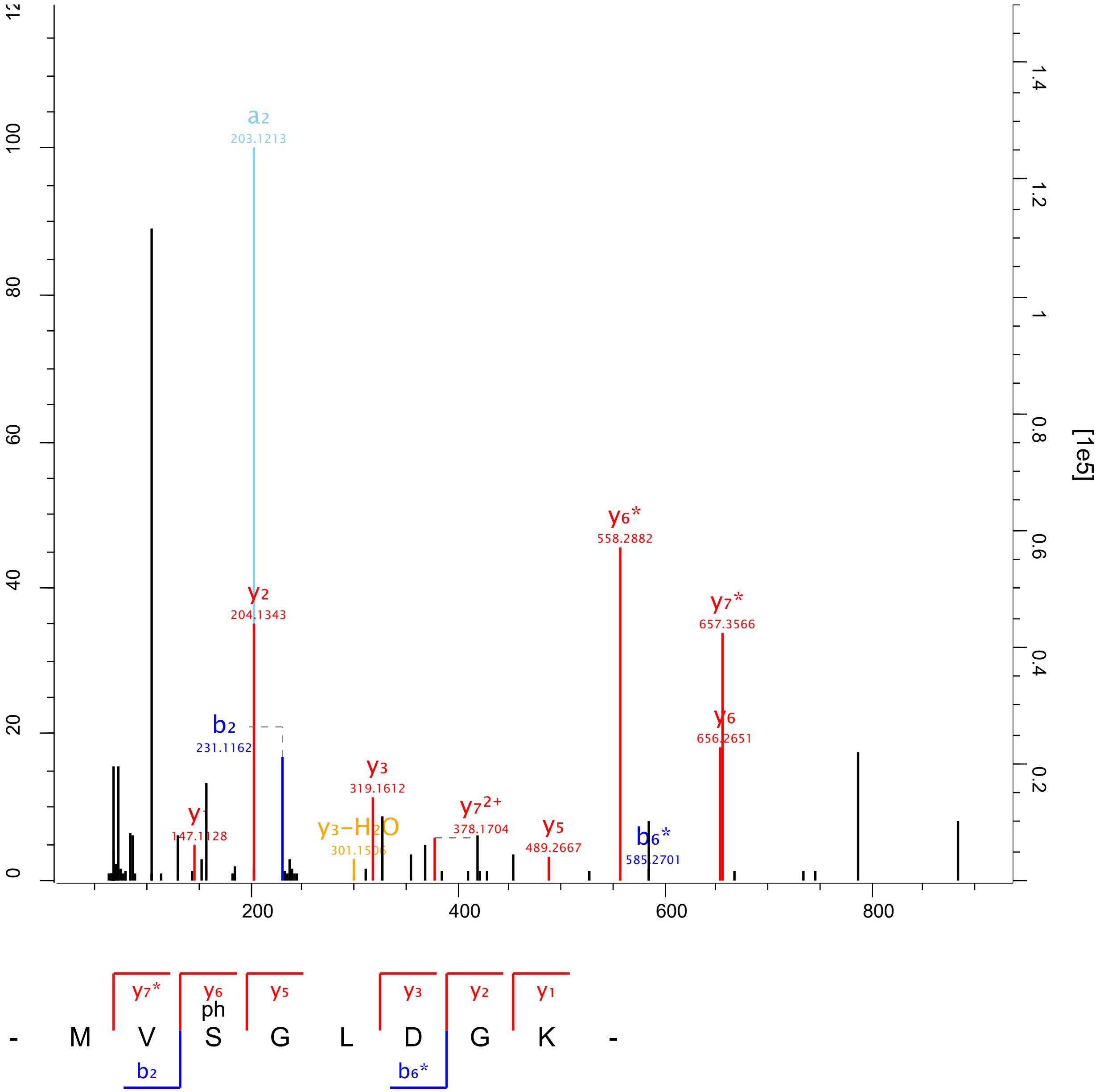

Raw file	Scan	Method	Score	m/z
sys_15_1	10064	FTMS; HCD	109.44	684.28

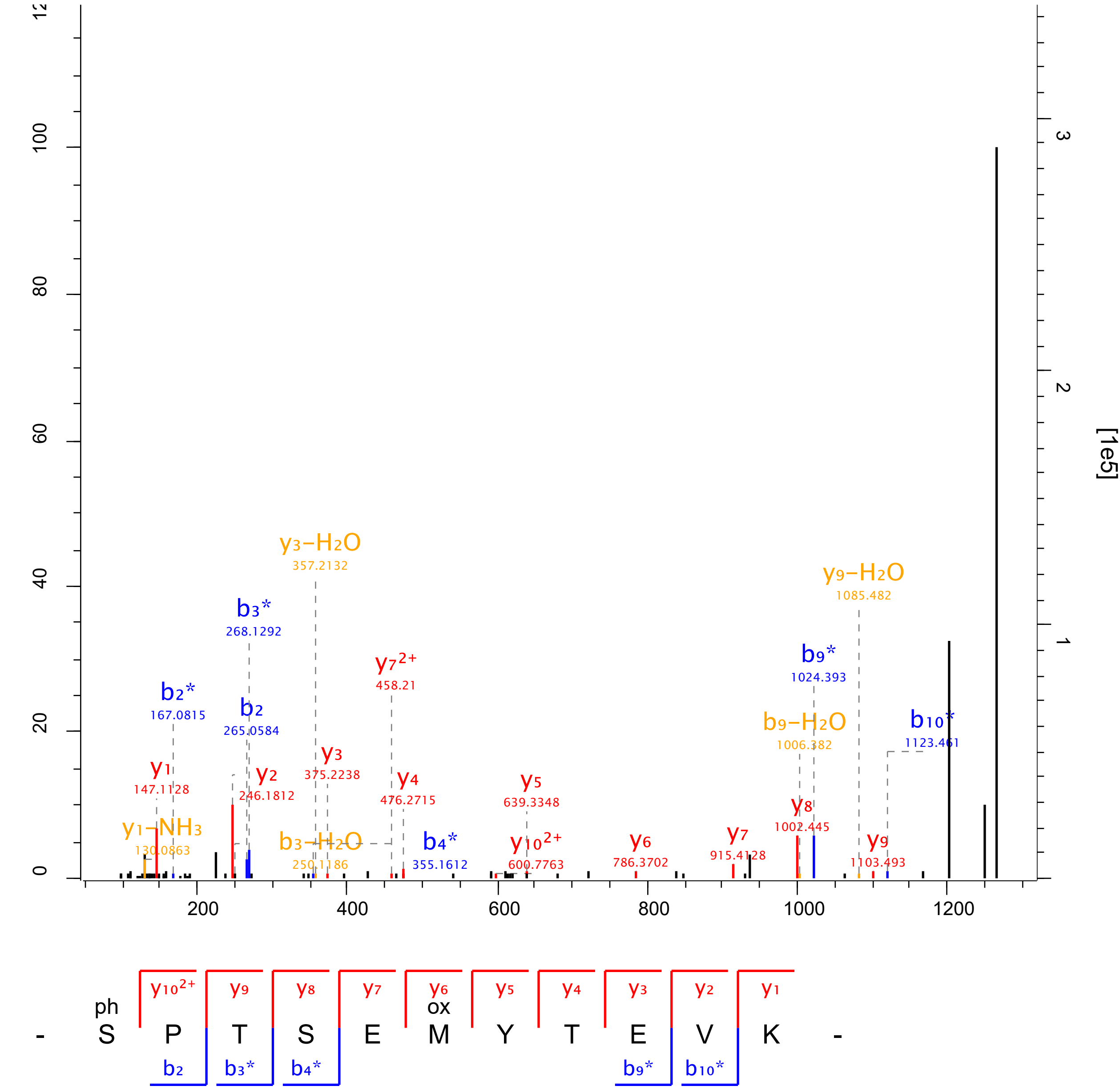

Raw file	Scan	Method	Score	m/z
sys_15_1	10104	FTMS; HCD	111.95	421.7

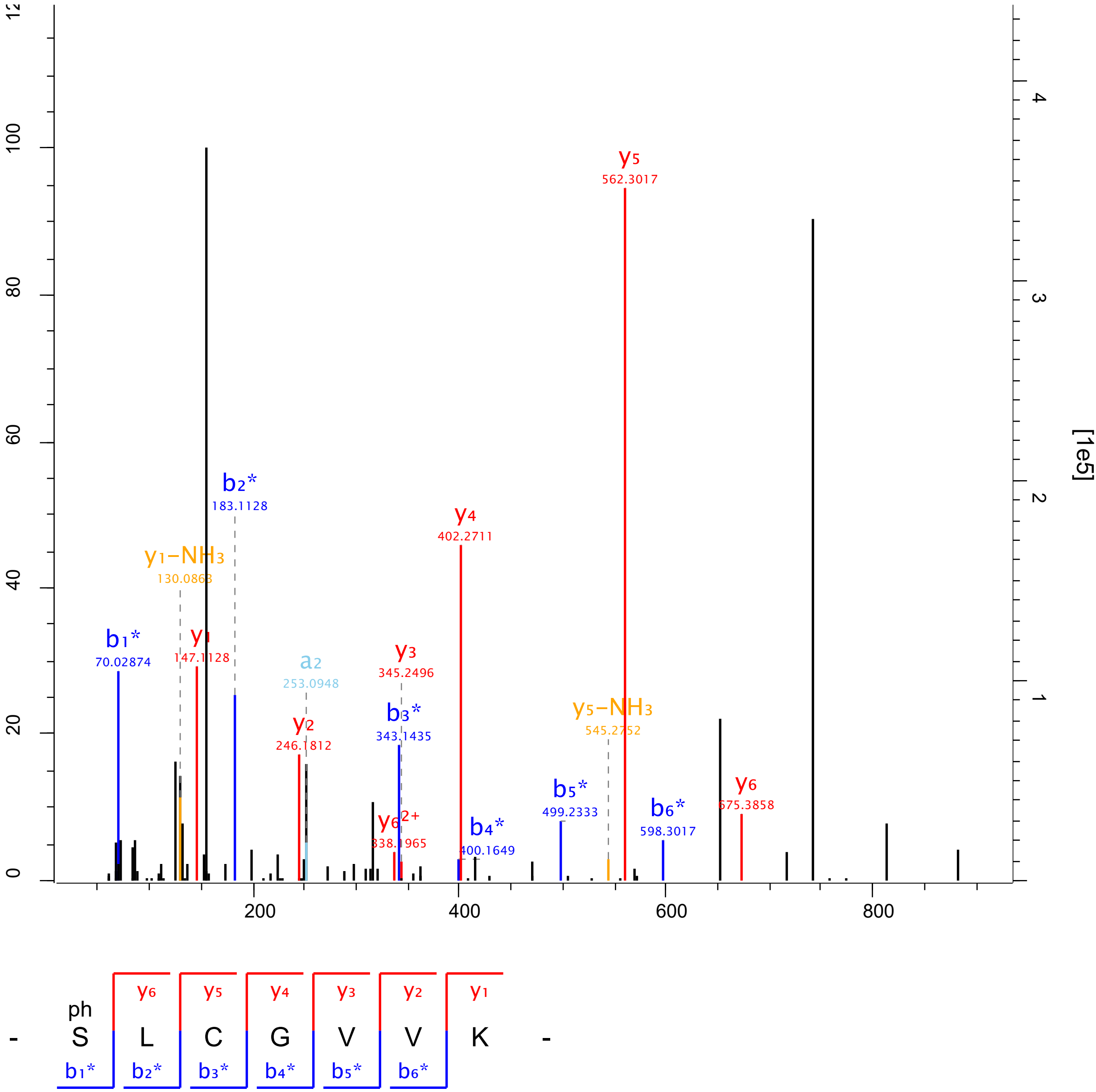

Raw file	Scan	Method	Score	m/z
sys_15_1	10119	FTMS; HCD	117.02	517.71

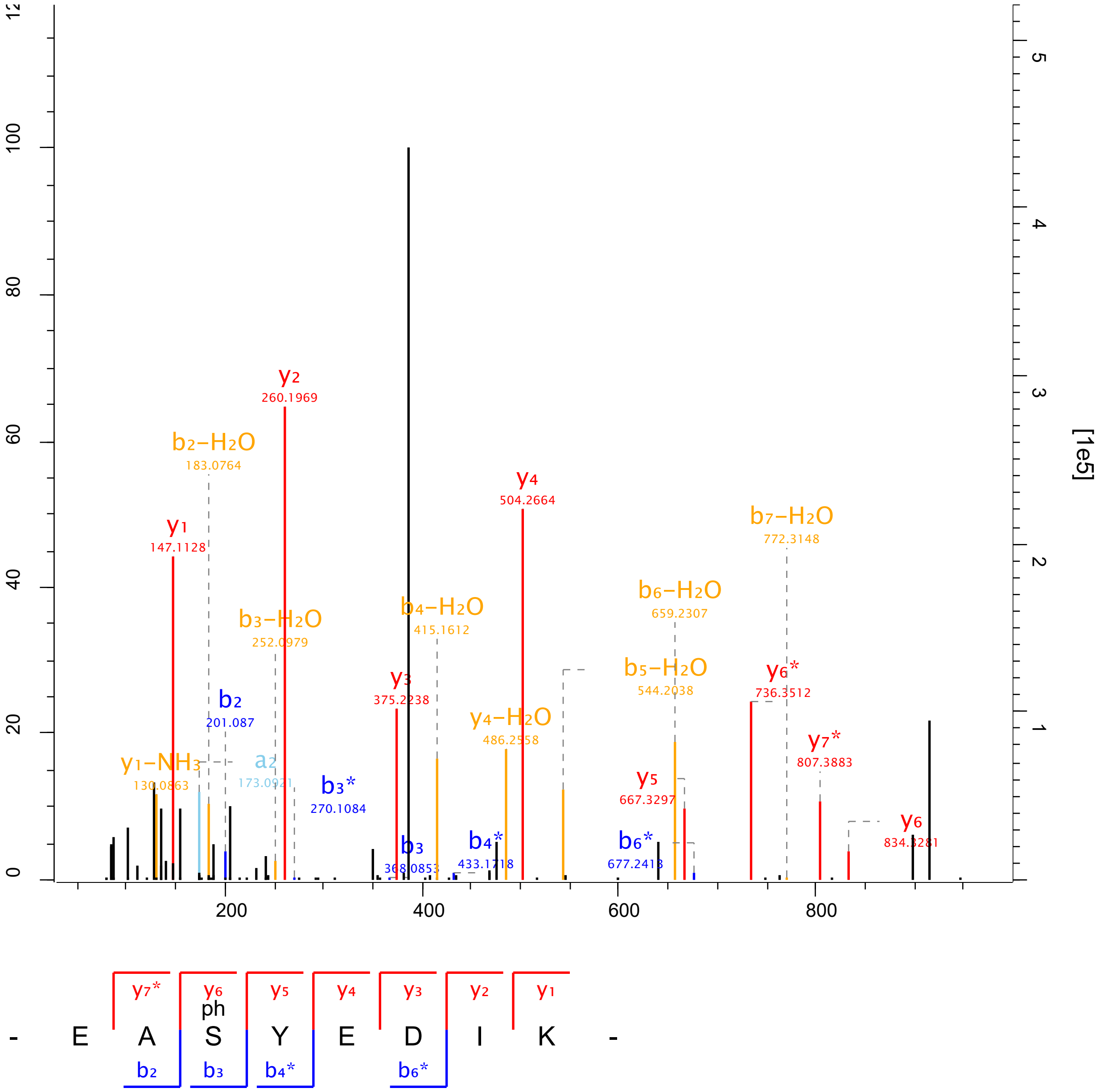

Raw file	Scan	Method	Score	m/z
sys_15_1	10136	FTMS; HCD	139.81	853.3

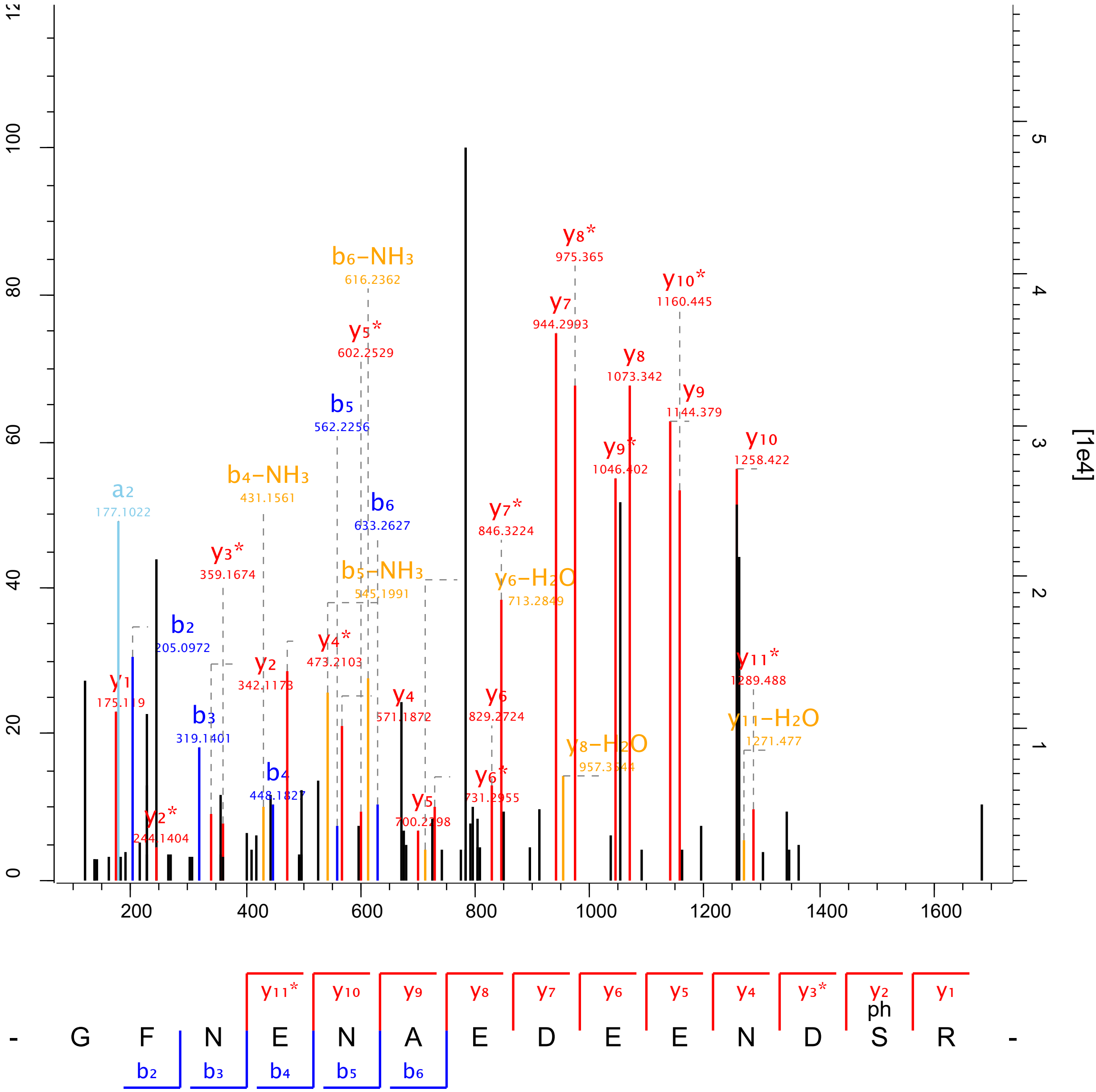

Raw file	Scan	Method	Score	m/z
sys_15_1	10175	FTMS; HCD	281.96	834.34

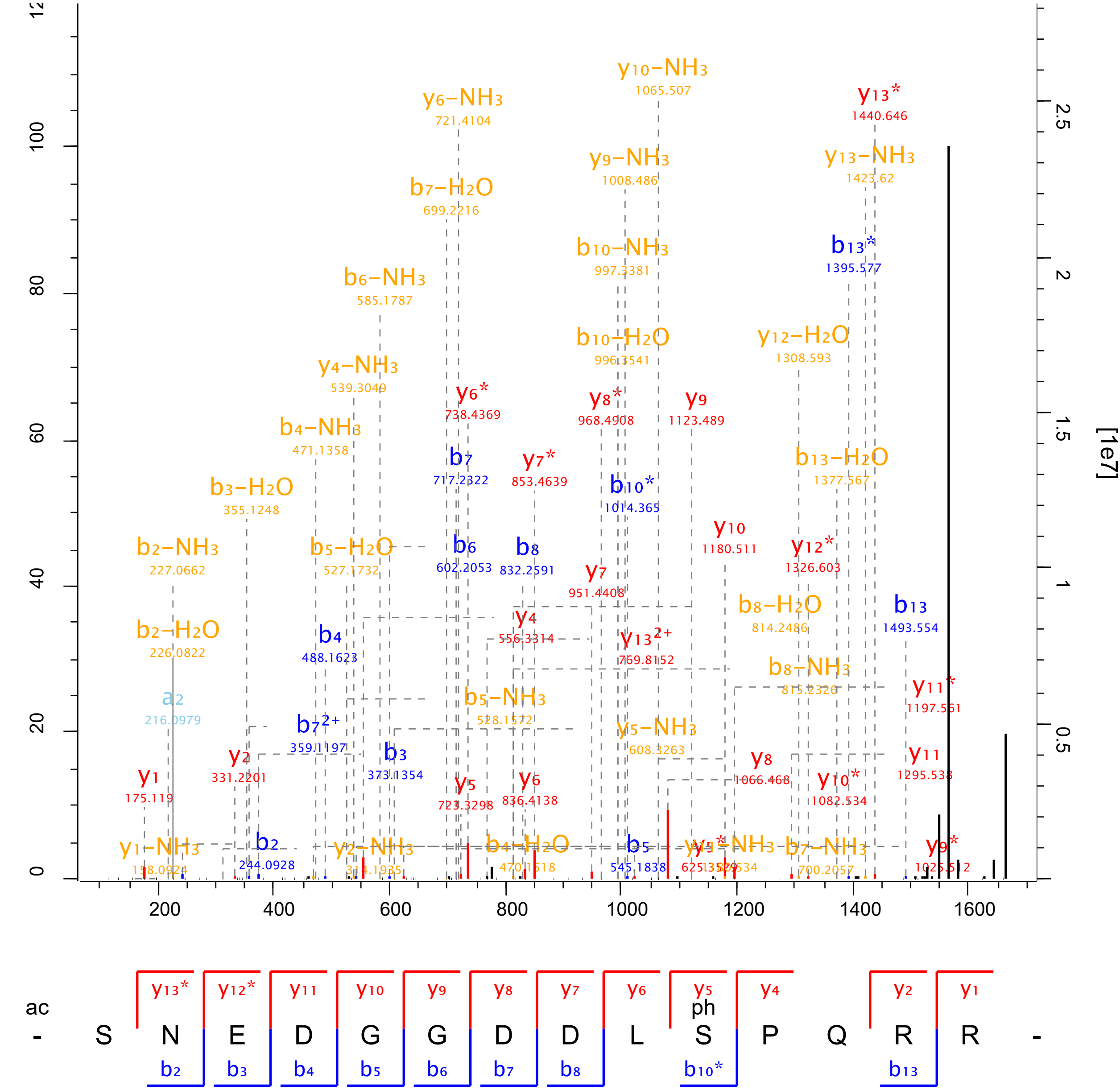

Raw file	Scan	Method	Score	m/z
sys_15_1	10178	FTMS; HCD	70.44	540.75

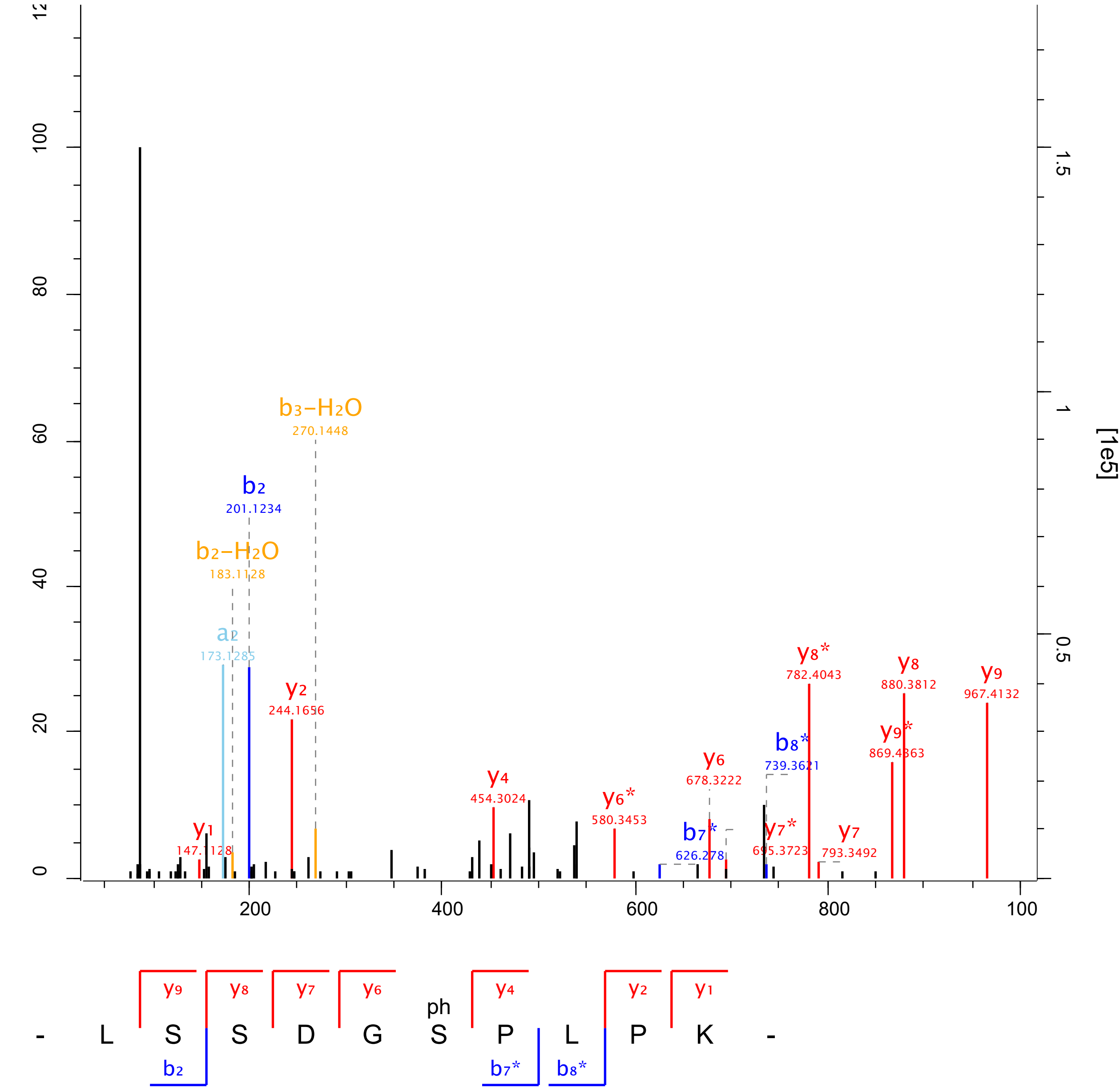

Raw file	Scan	Method	Score	m/z
sys_15_1	10190	FTMS; HCD	81.85	768.26

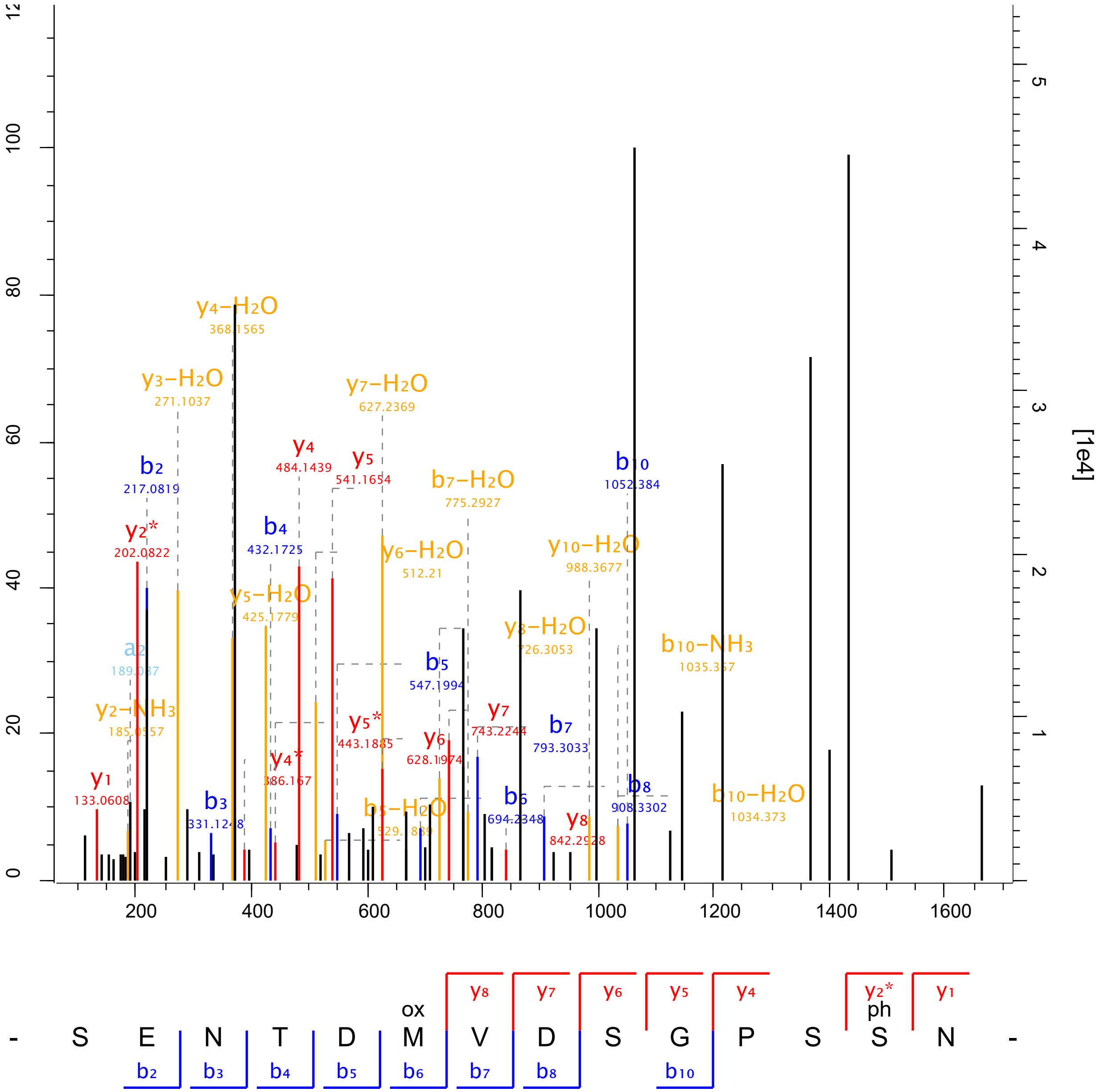

Raw file	Scan	Method	Score	m/z
sys_15_1	10226	FTMS; HCD	214.89	713.83

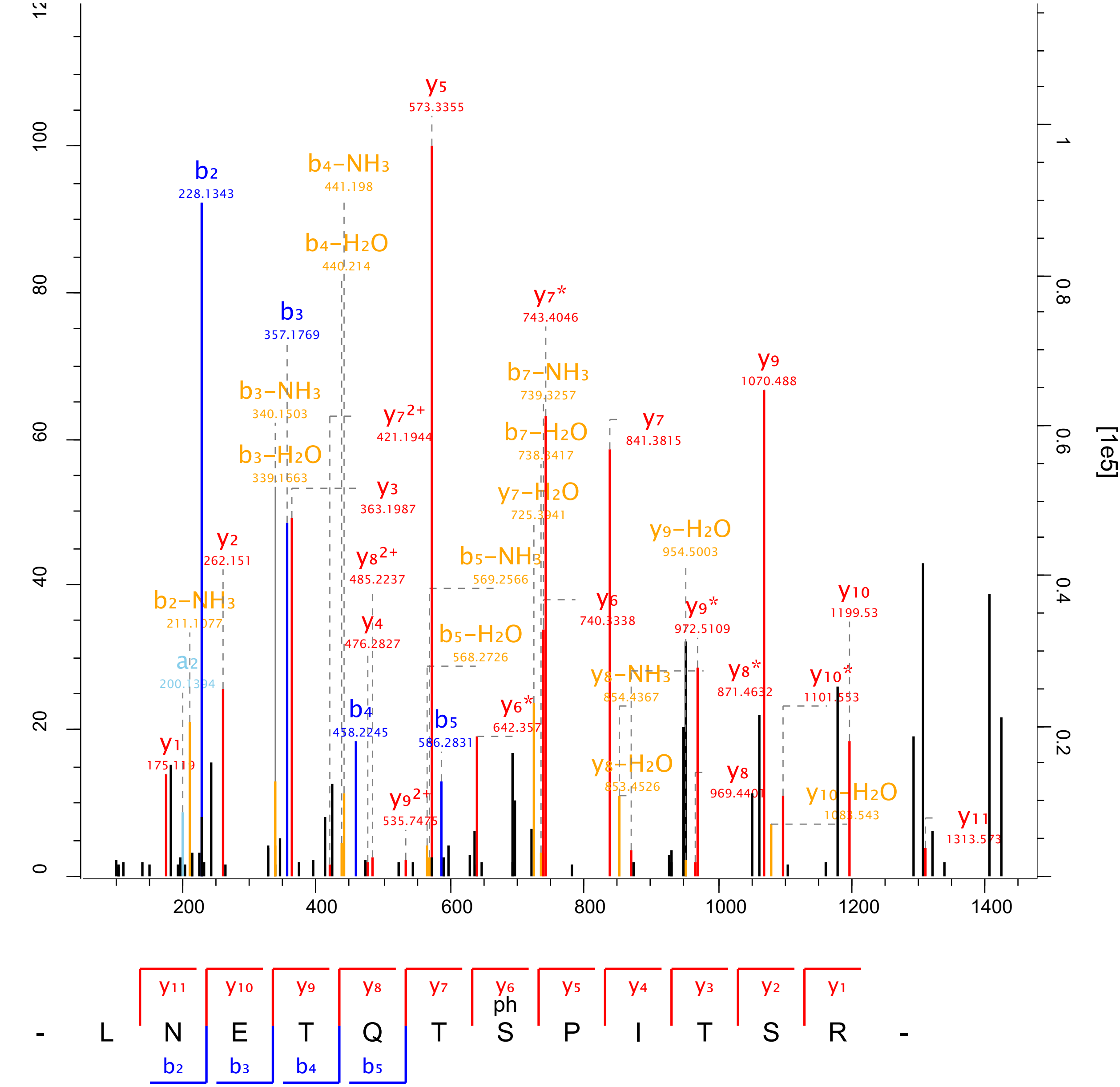

Raw file	Scan	Method	Score	m/z
sys_15_1	10242	FTMS; HCD	101.2	645.8

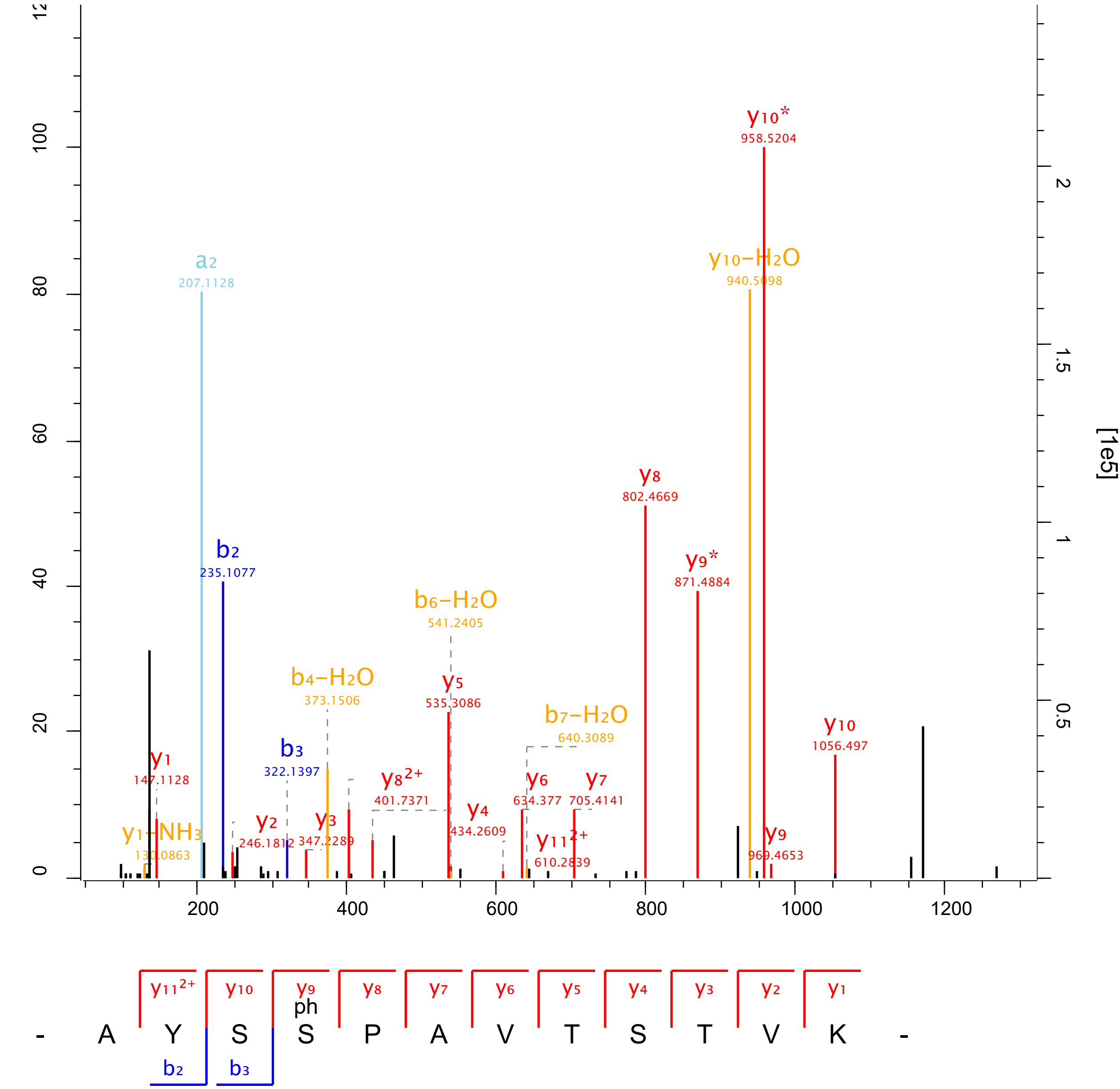

Raw file	Scan	Method	Score	m/z
sys_15_1	10313	FTMS; HCD	110.12	672.3

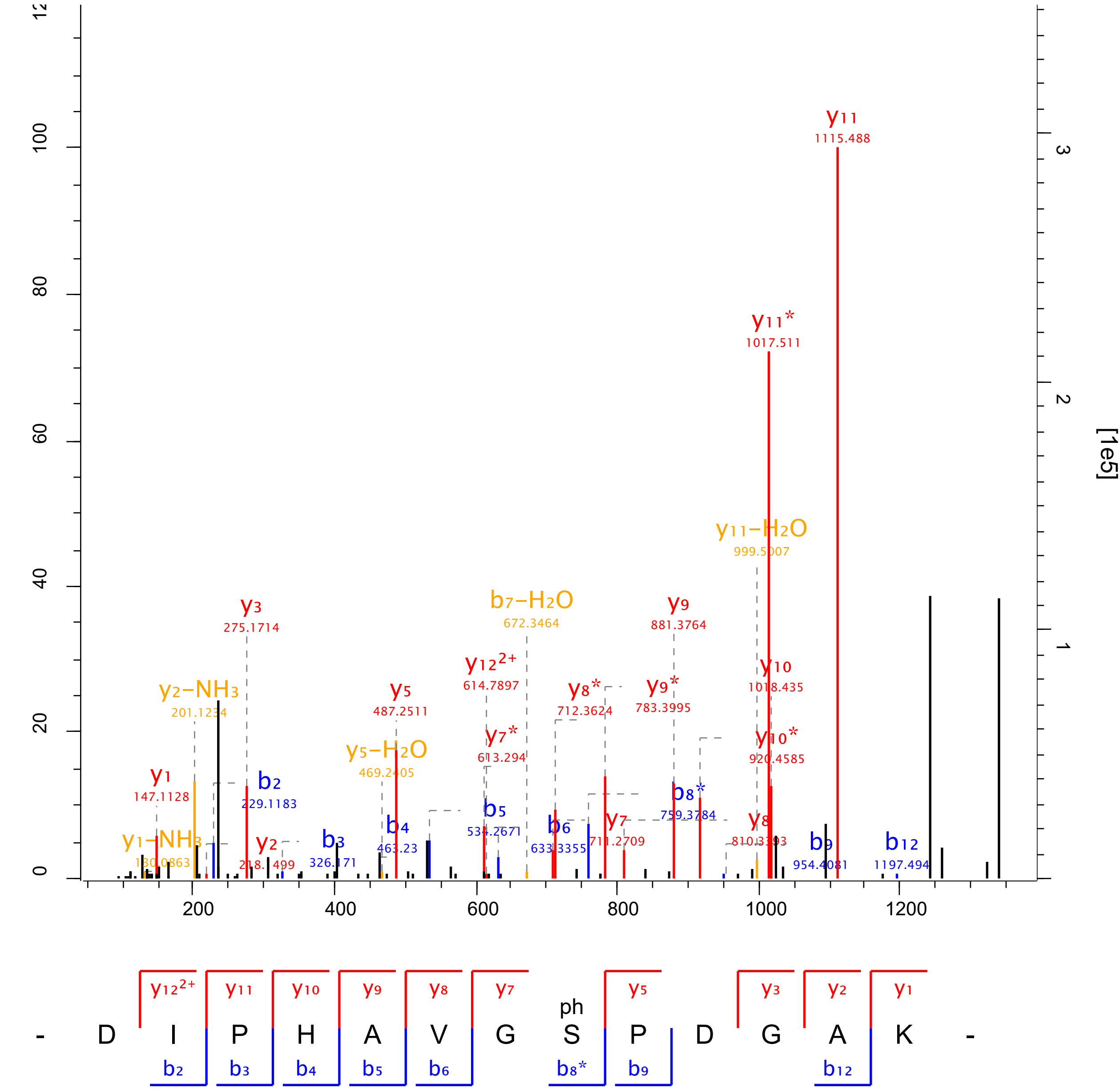

Raw file	Scan	Method	Score	m/z
sys_15_1	10314	FTMS; HCD	73.5	516.71

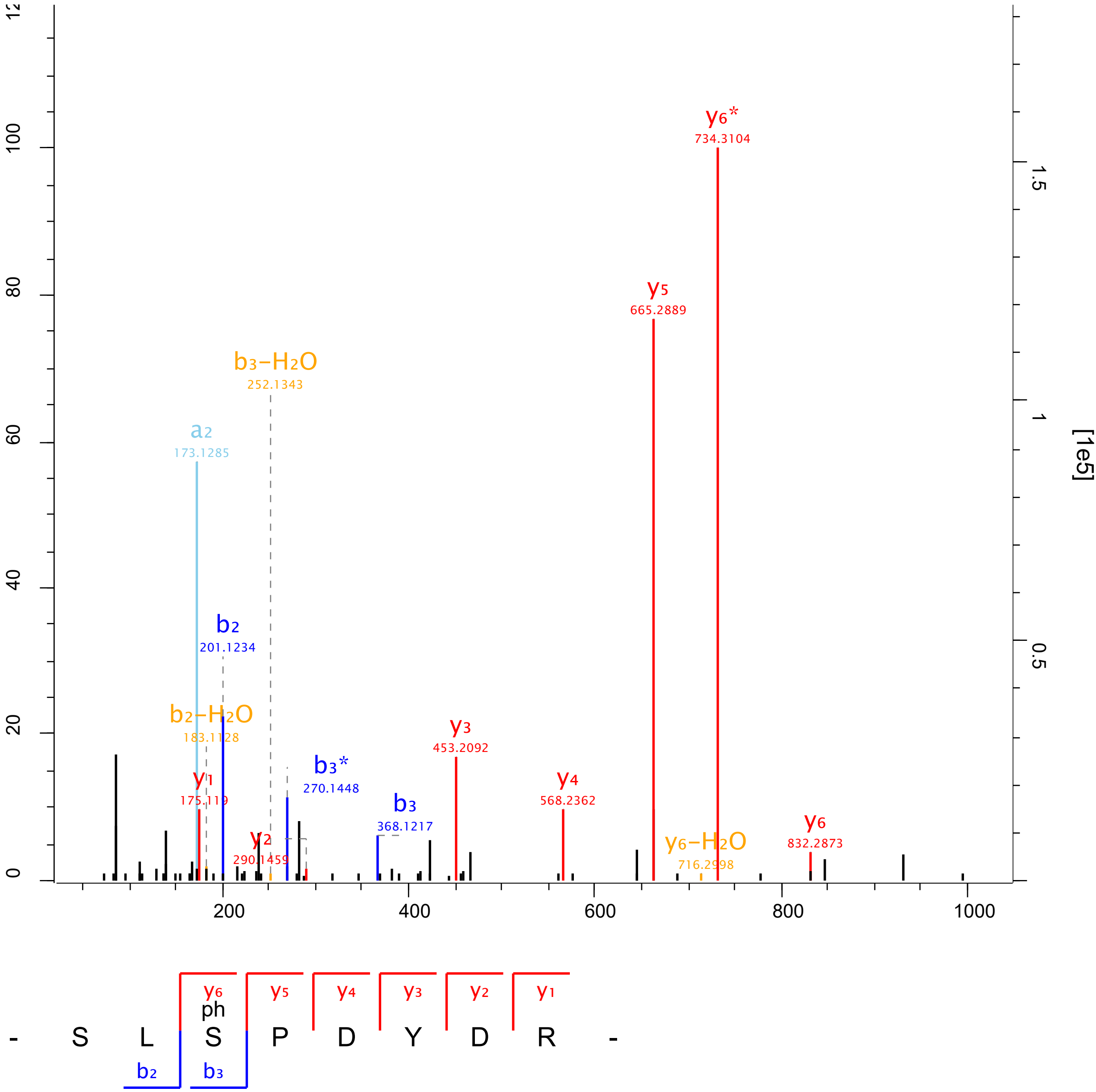

Raw file	Scan	Method	Score	m/z
sys_15_1	10355	FTMS; HCD	46.07	586.72

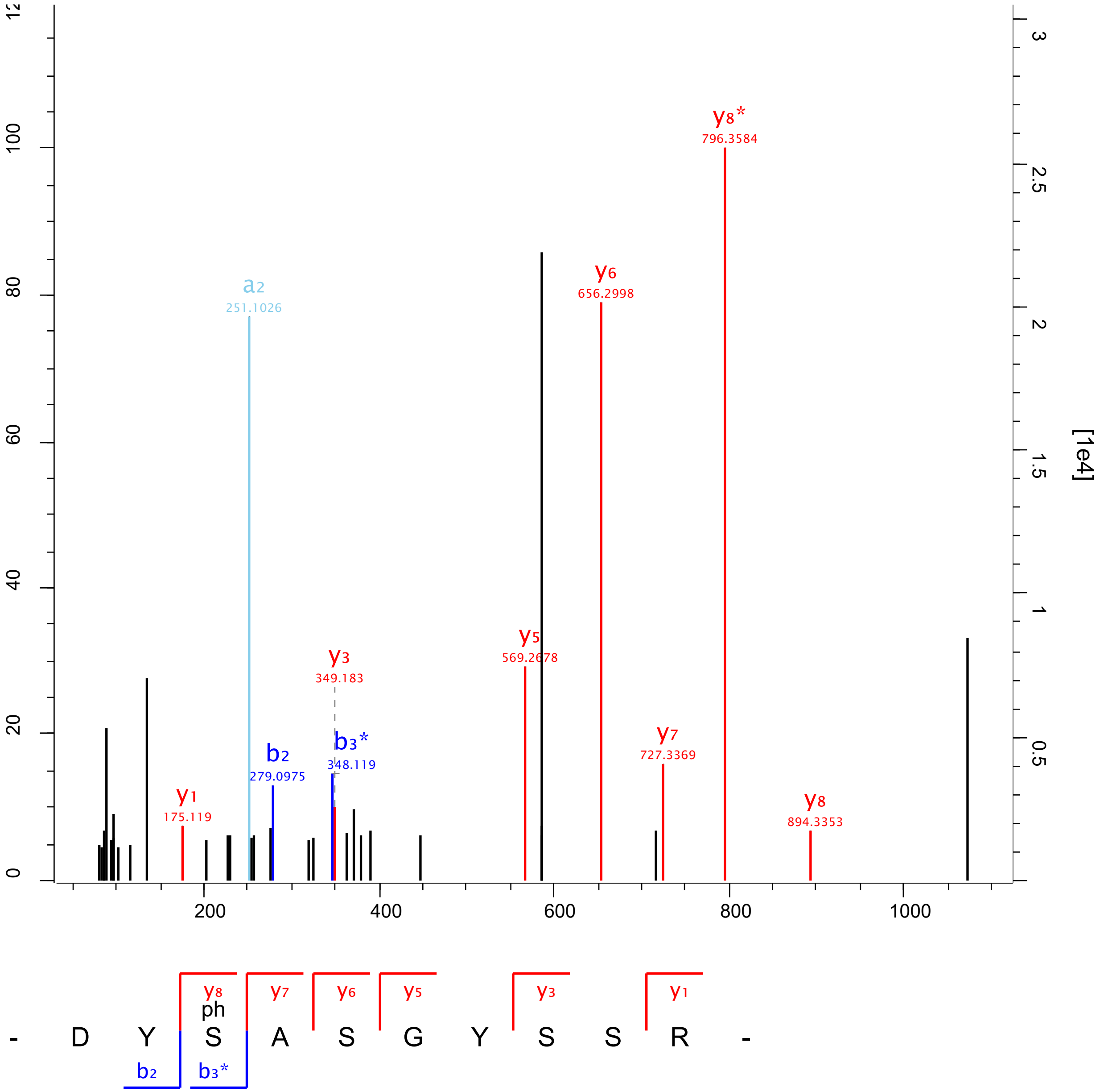

Raw file	Scan	Method	Score	m/z
sys_15_1	10454	FTMS; HCD	41.45	600.24

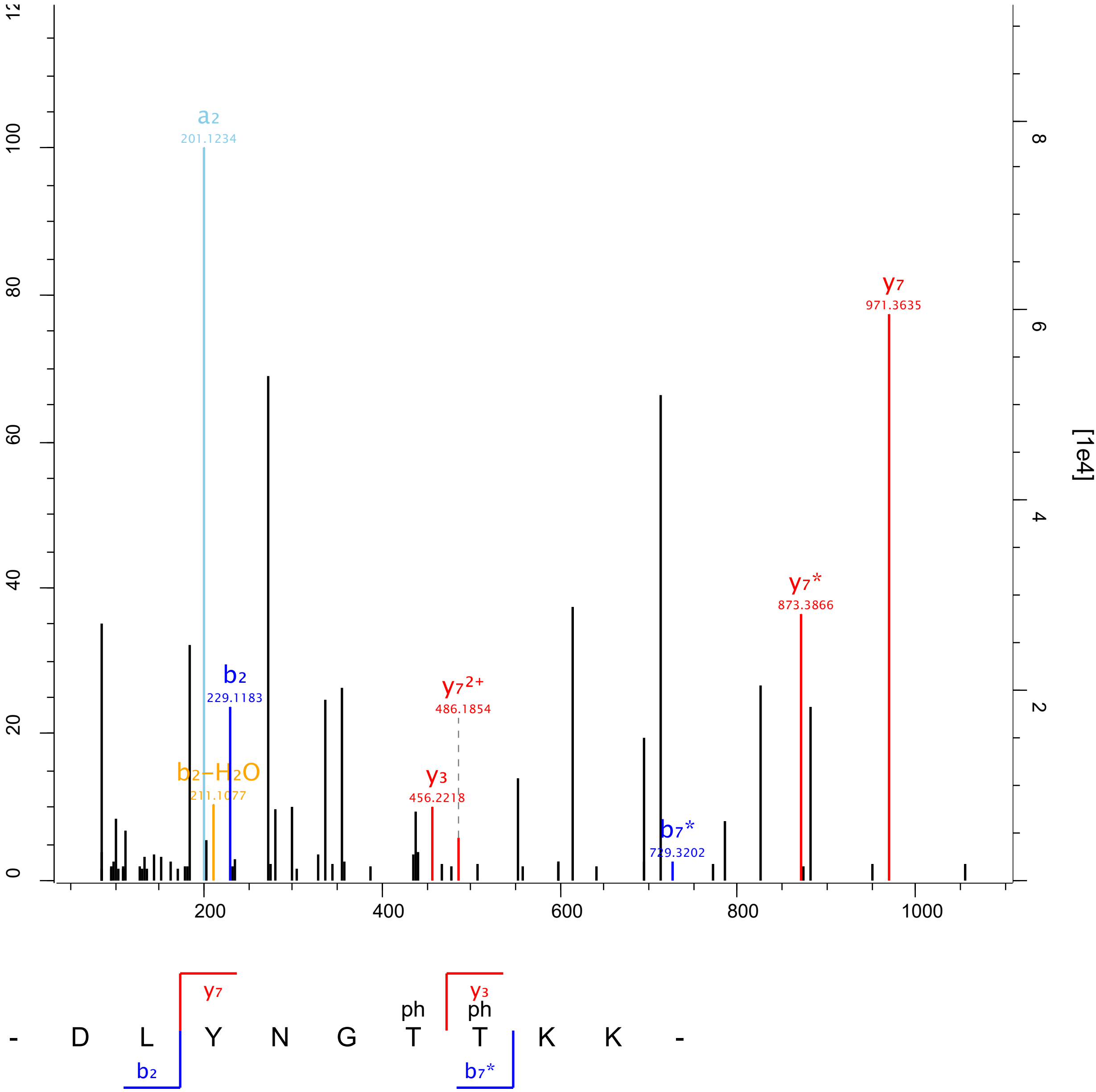

Raw file	Scan	Method	Score	m/z
sys_15_1	10480	FTMS; HCD	205.31	557.76

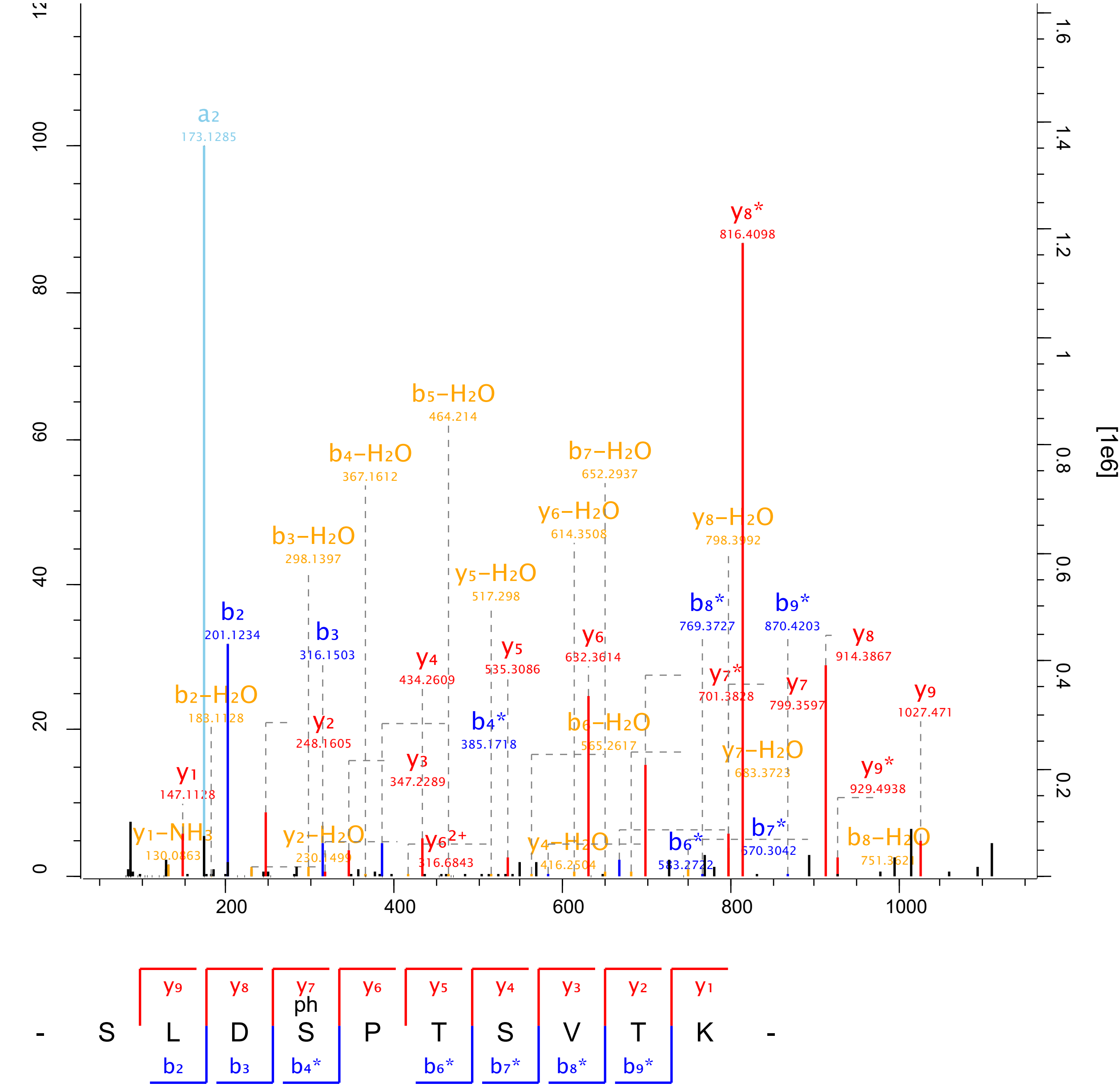

Raw file	Scan	Method	Score	m/z
sys_15_1	10614	FTMS; HCD	40.14	887.38

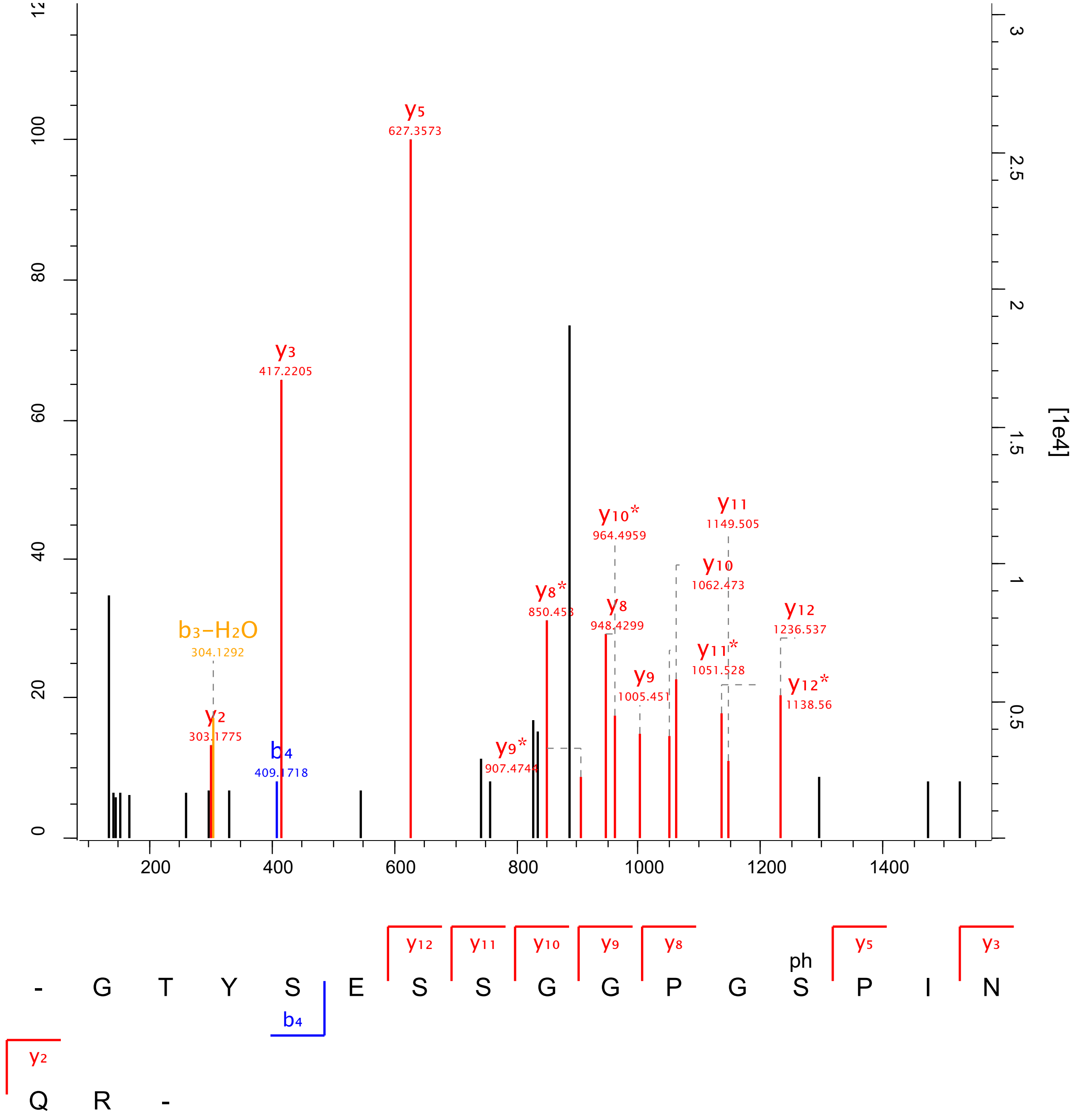

Raw file	Scan	Method	Score	m/z
sys_15_1	10712	FTMS; HCD	93.24	620.26

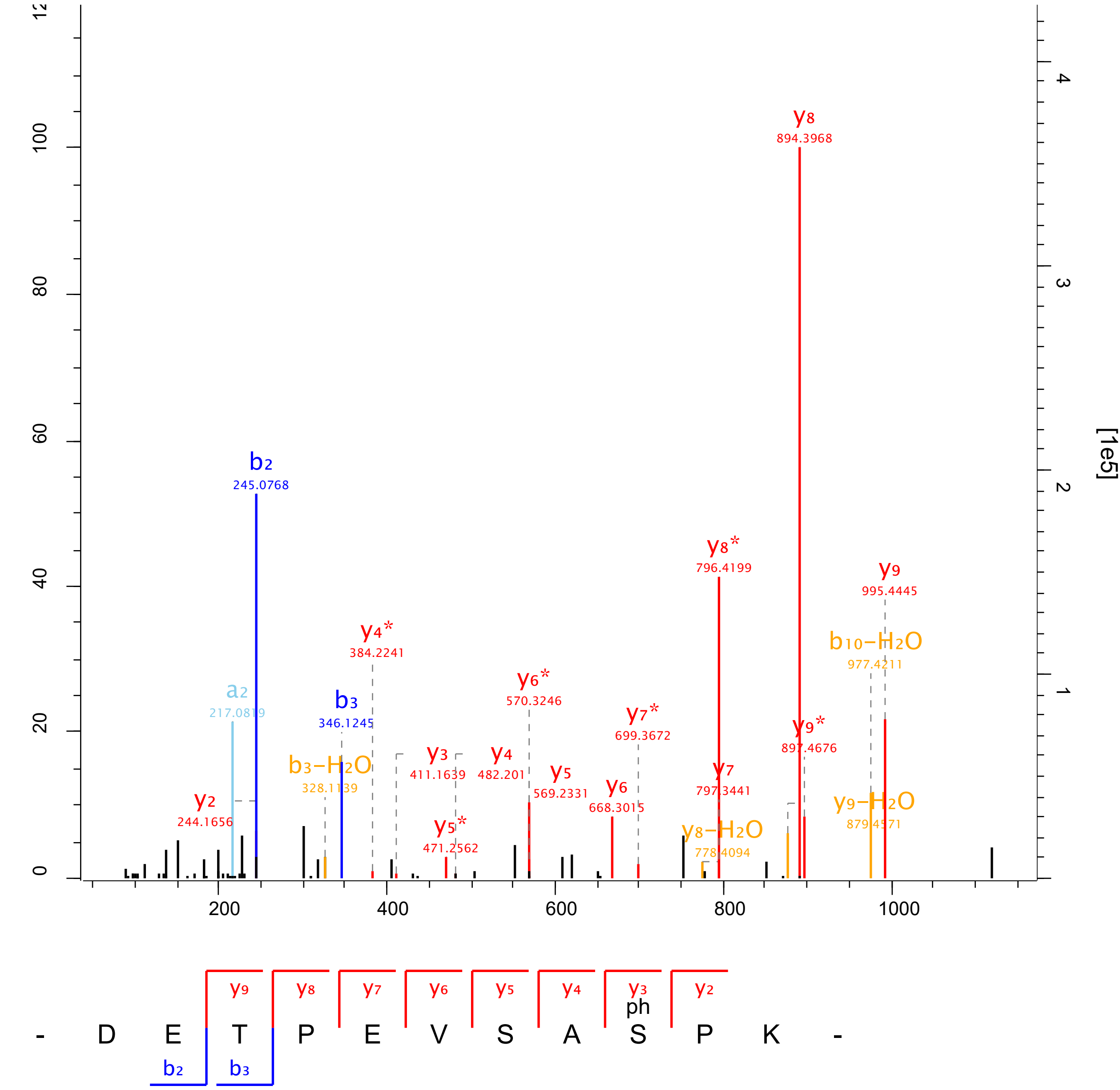

Raw file	Scan	Method	Score	m/z
sys_15_1	10777	FTMS; HCD	59.12	616.78

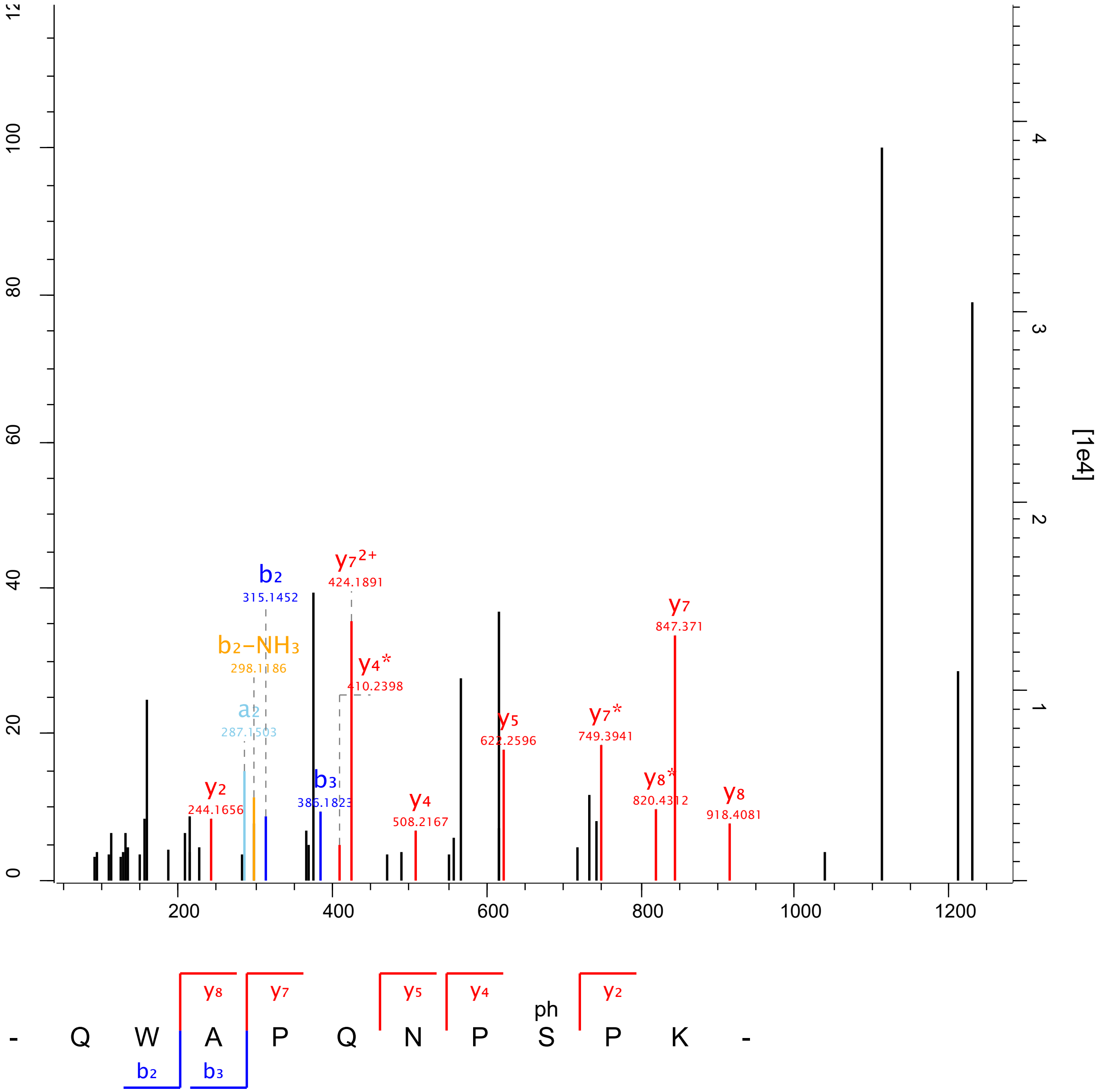

Raw file	Scan	Method	Score	m/z
sys_15_1	10780	FTMS; HCD	106.12	750.64

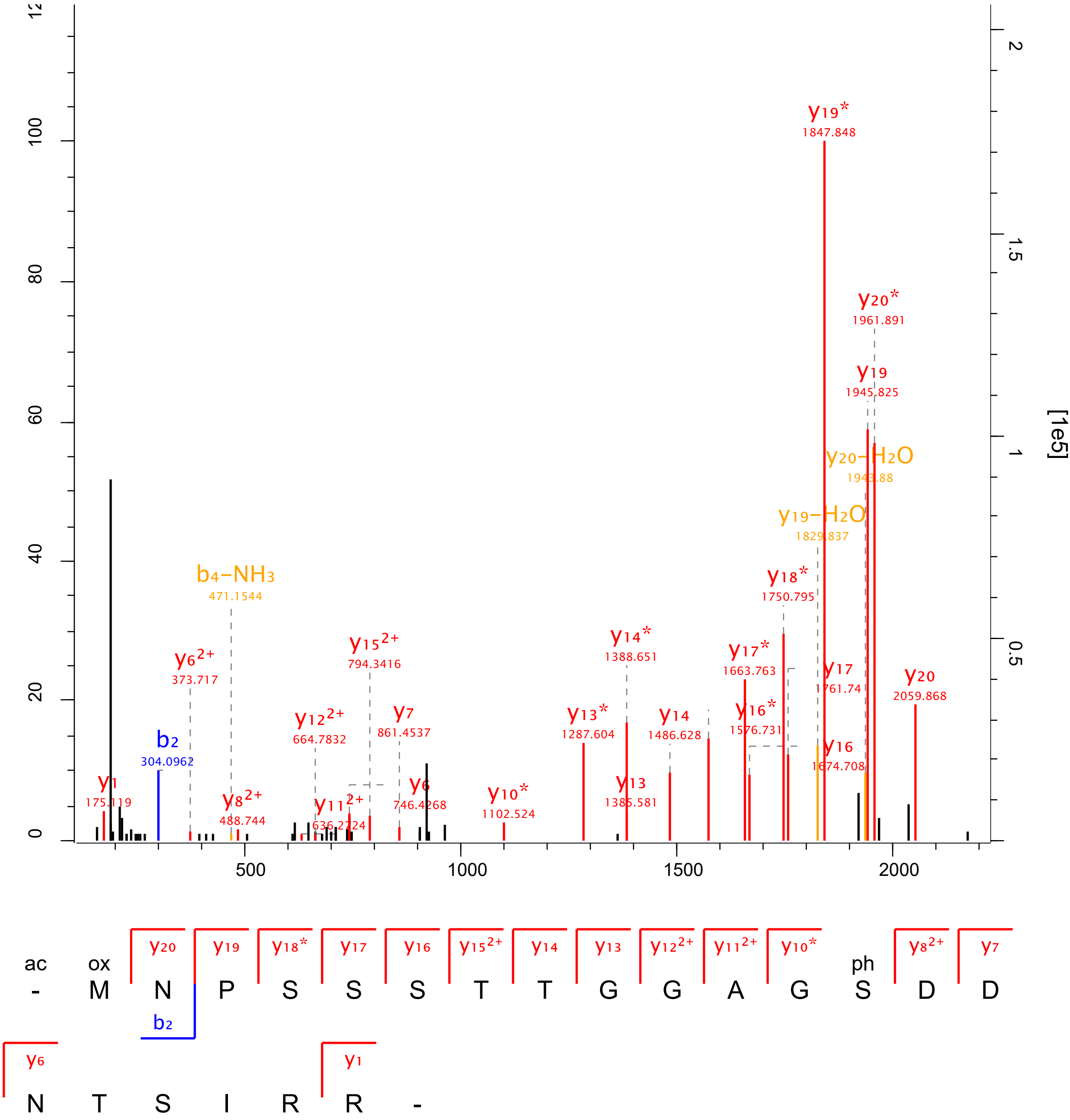

Raw file	Scan	Method	Score	m/z
sys_15_1	10830	FTMS; HCD	48.4	525.21

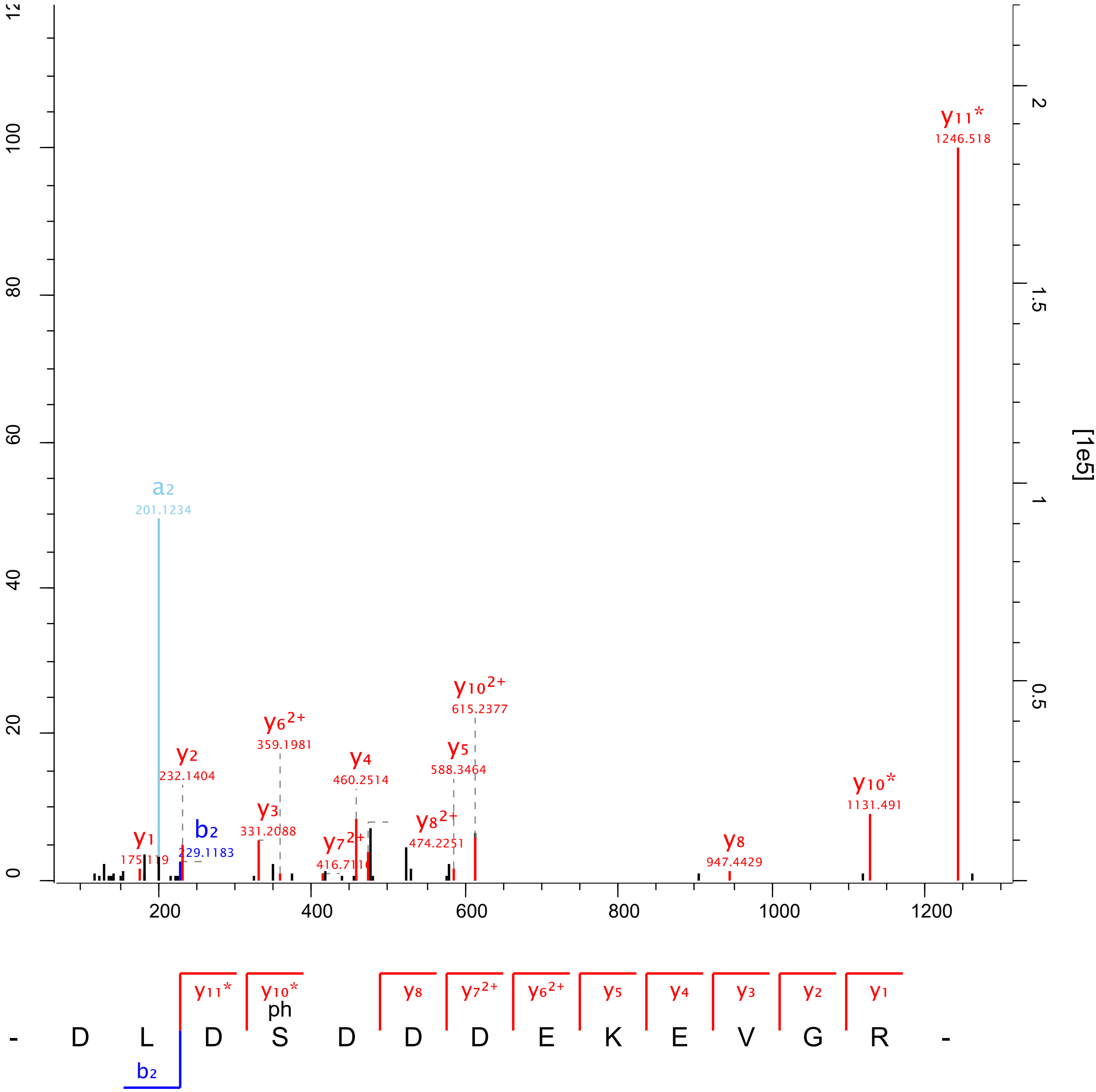

Raw file	Scan	Method	Score	m/z
sys_15_1	10865	FTMS; HCD	89.48	753.81

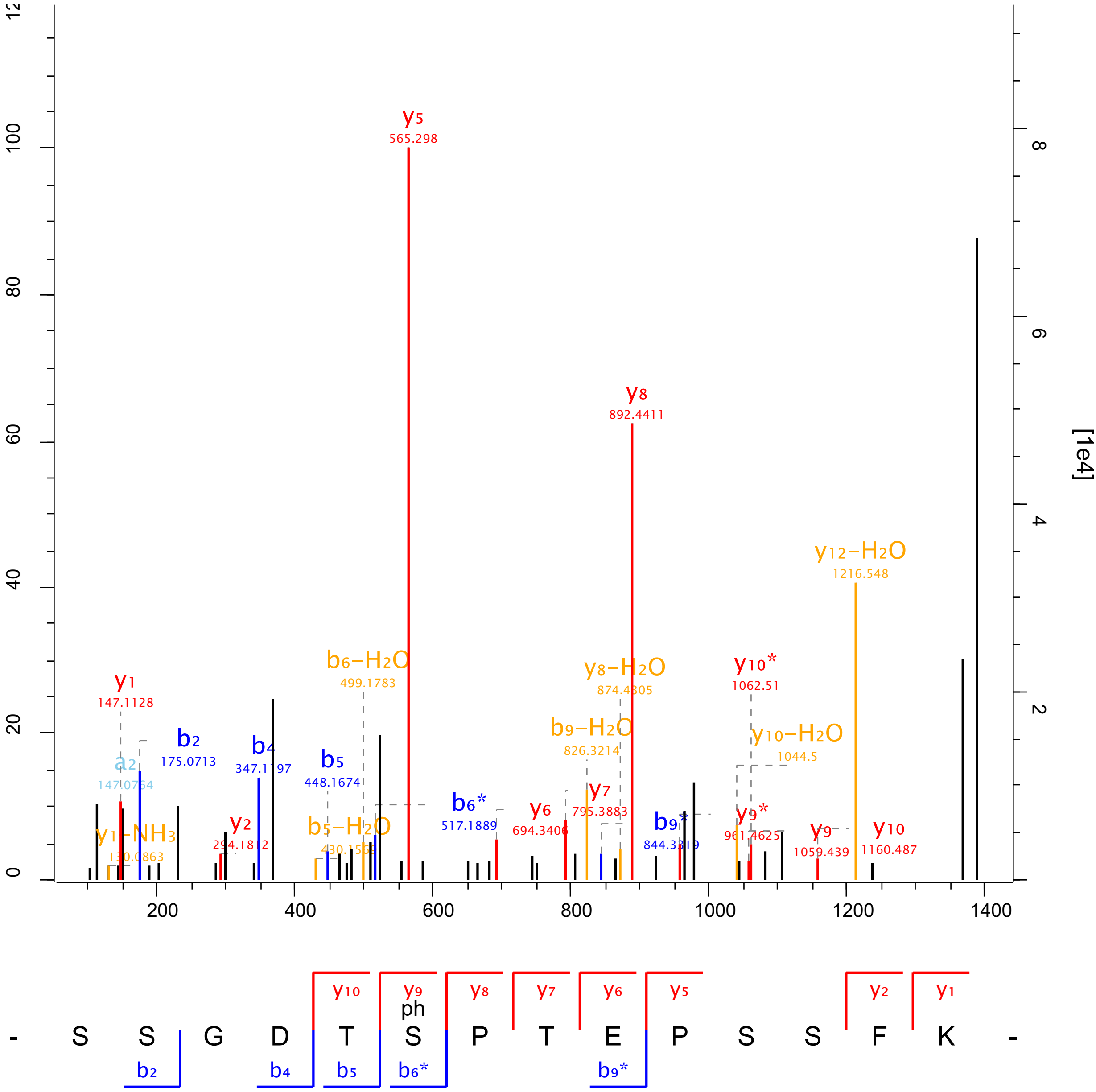

Raw file	Scan	Method	Score	m/z
sys_15_1	10870	FTMS; HCD	133.91	519.22

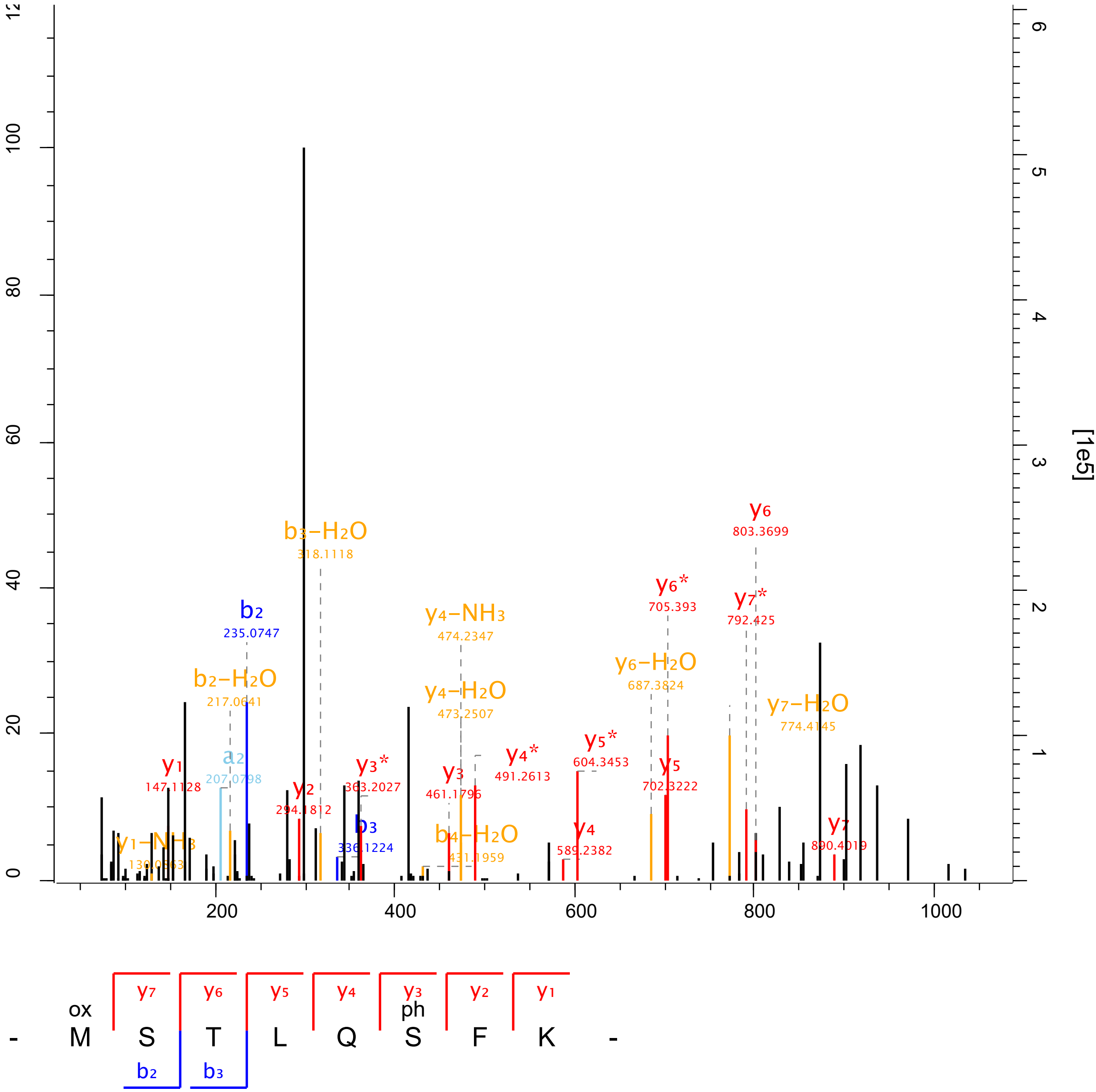

Raw file	Scan	Method	Score	m/z
sys_15_1	10878	FTMS; HCD	139.74	573.76

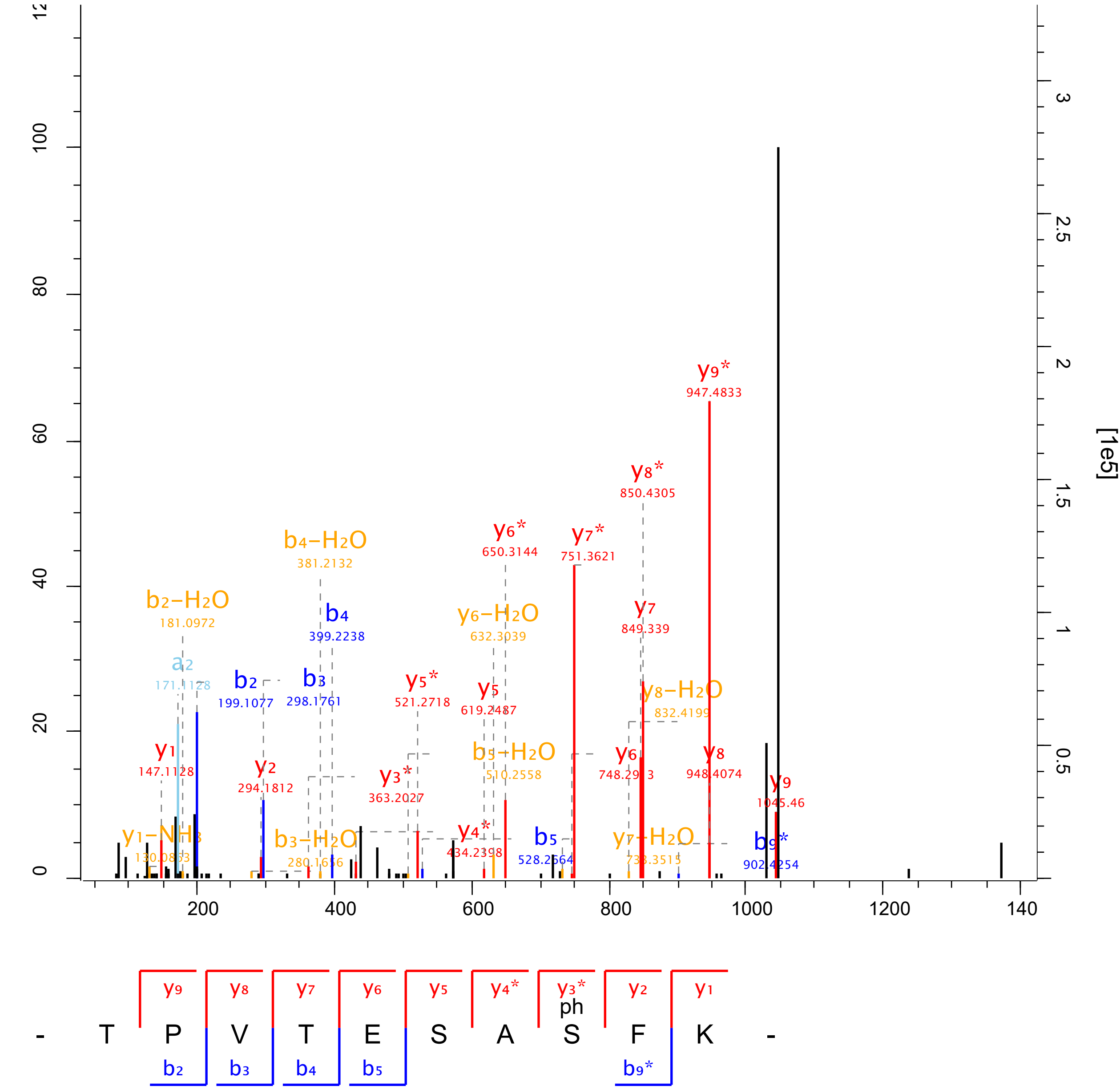

Raw file	Scan	Method	Score	m/z
sys_15_1	10949	FTMS; HCD	86.49	678.81

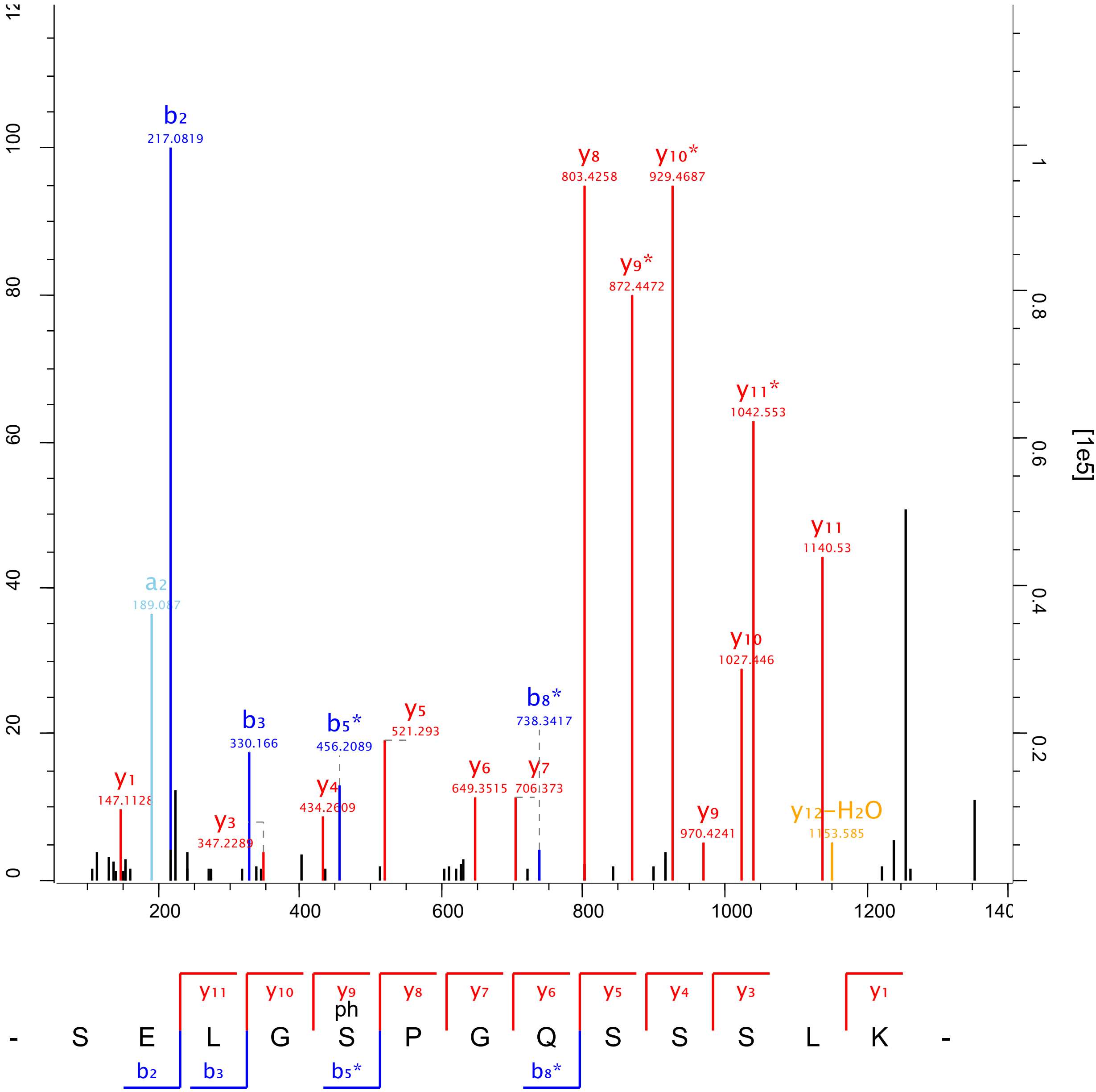

Raw file	Scan	Method	Score	m/z
sys_15_1	10962	FTMS; HCD	97.21	658.77

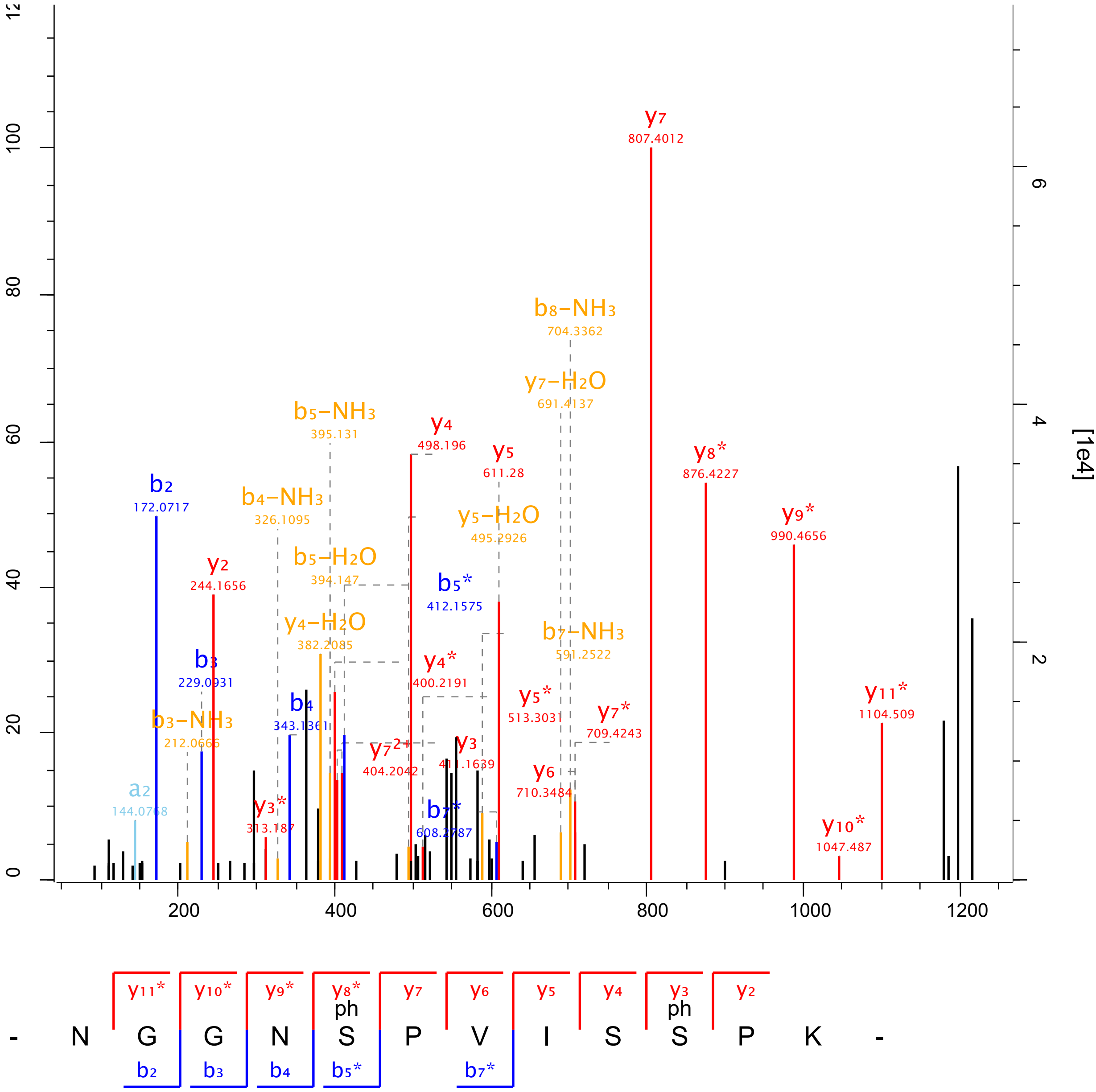

Raw file	Scan	Method	Score	m/z
sys_15_1	11046	FTMS; HCD	91.59	753.82

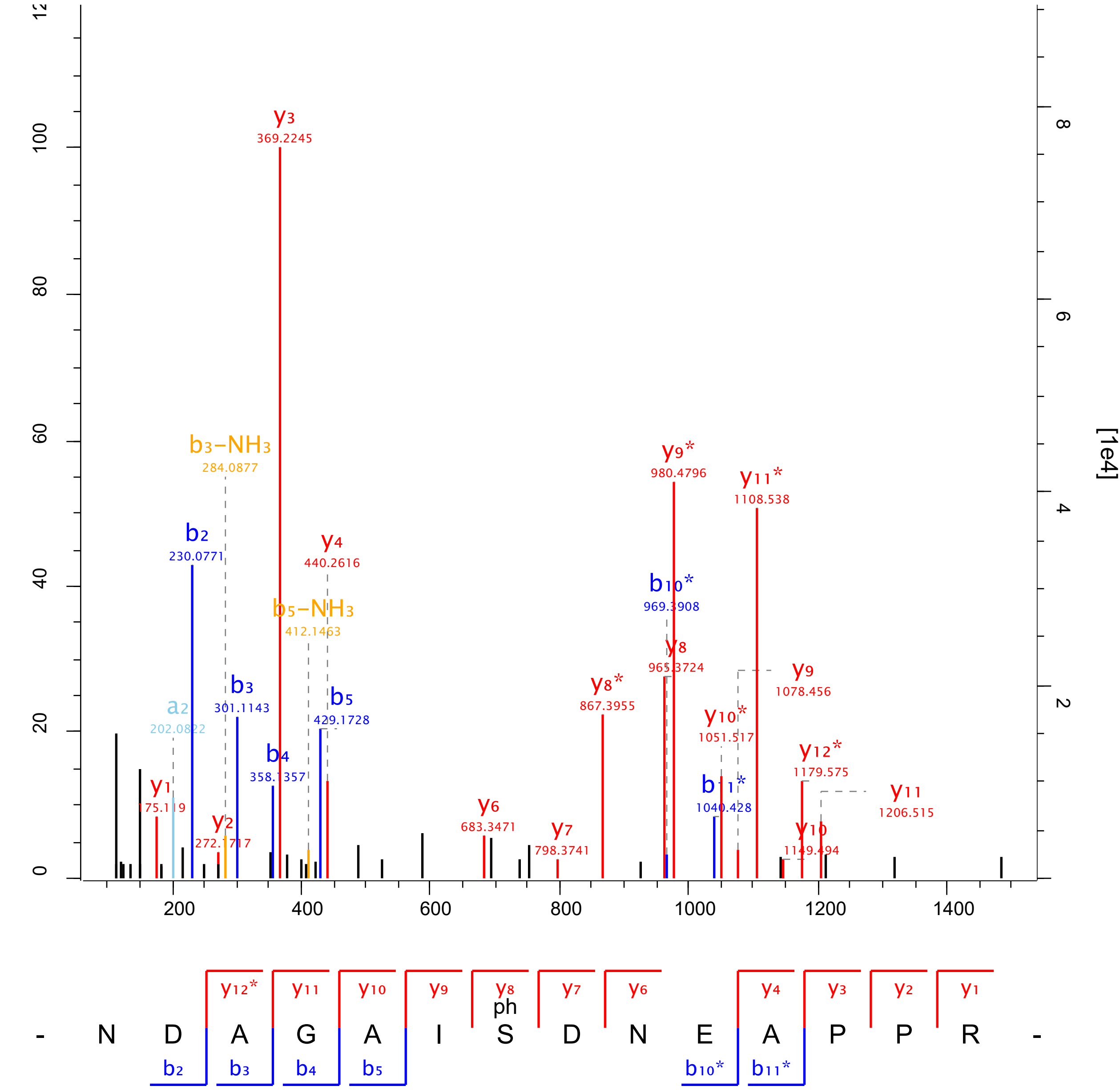

Raw file	Scan	Method	Score	m/z
sys_15_1	11068	FTMS; HCD	136.33	608.24

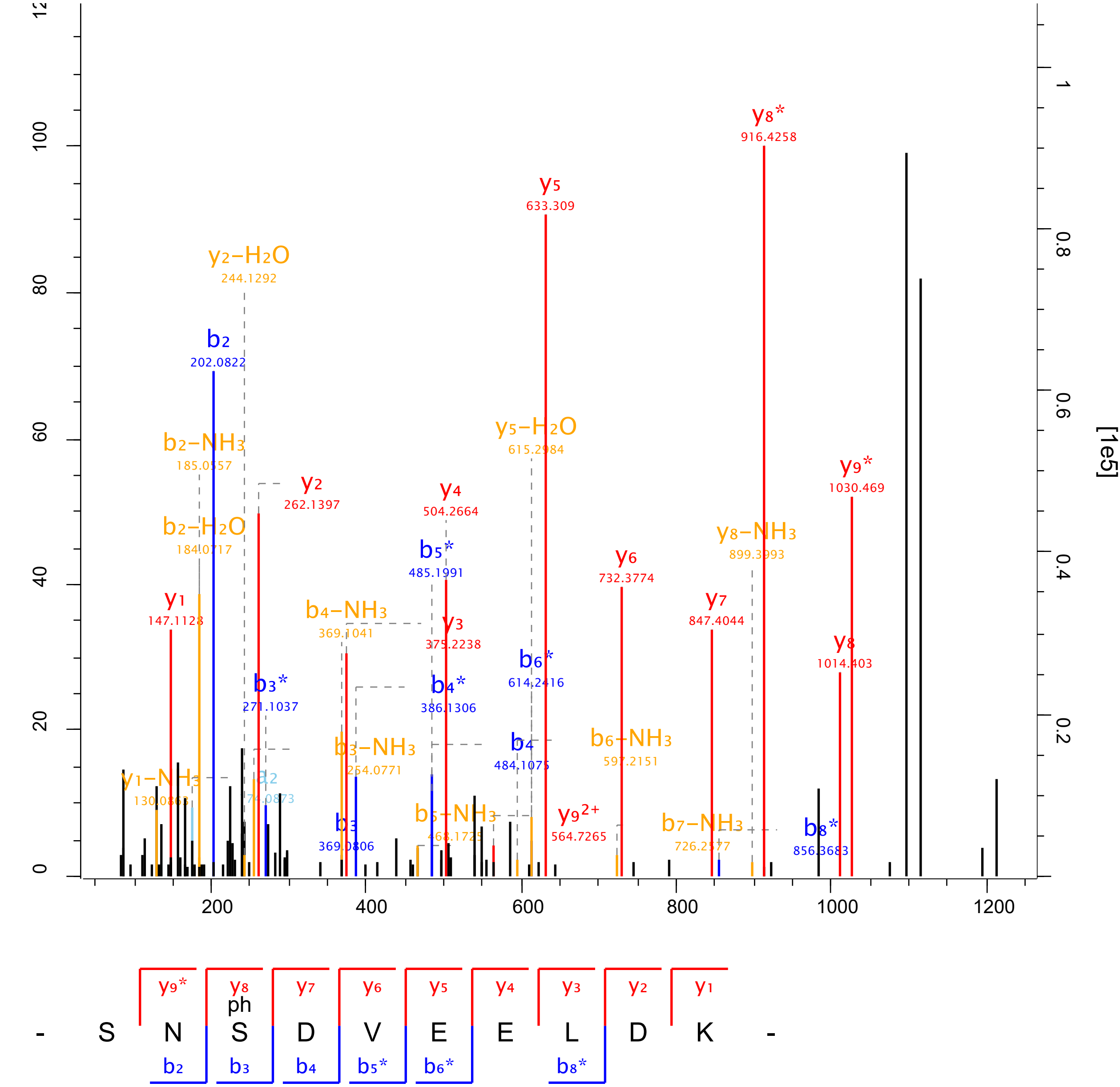

Raw file	Scan	Method	Score	m/z
sys_15_1	11078	FTMS; HCD	98.06	556.24

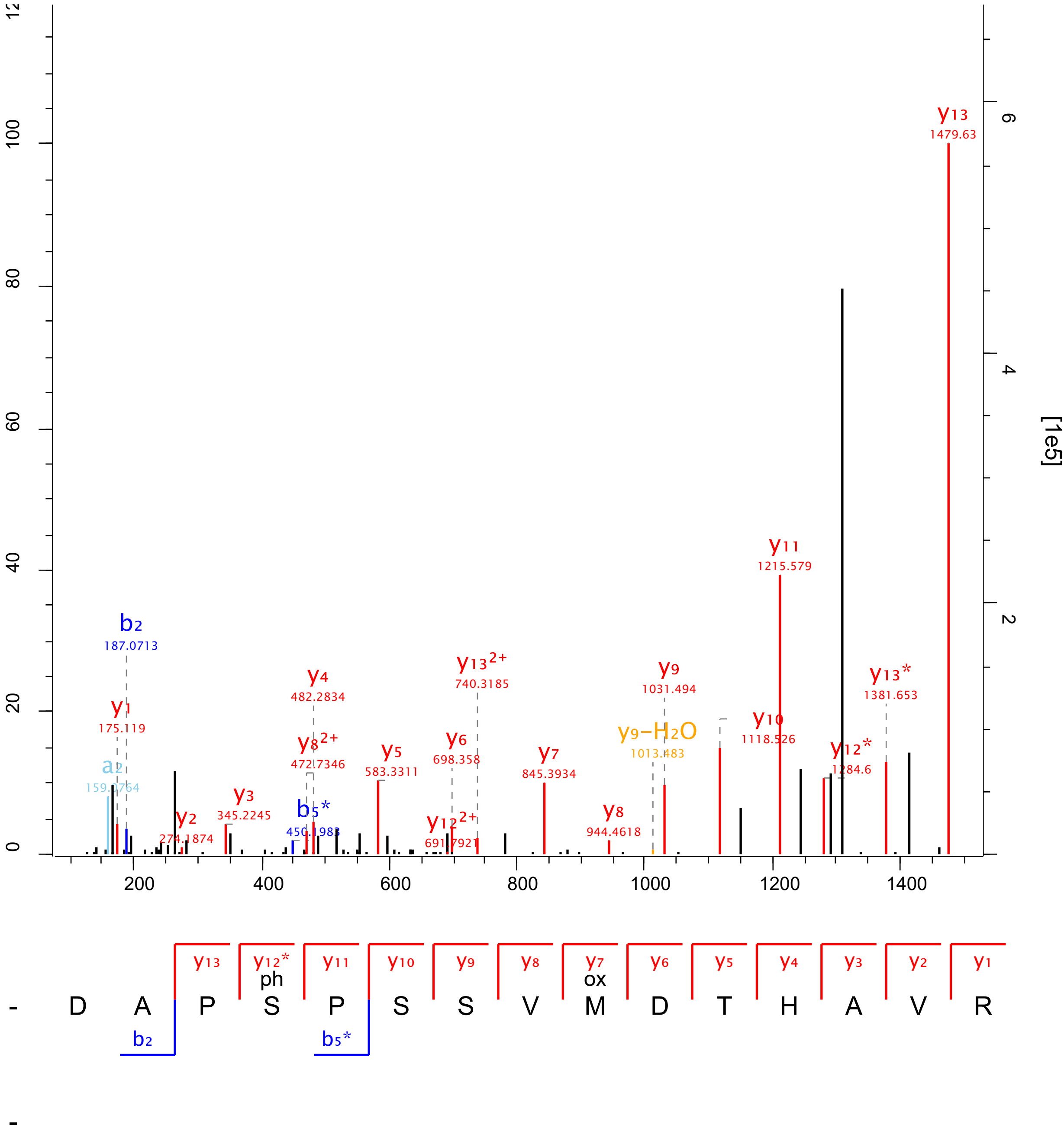

Raw file	Scan	Method	Score	m/z
sys_15_1	11081	FTMS; HCD	186.56	681.81

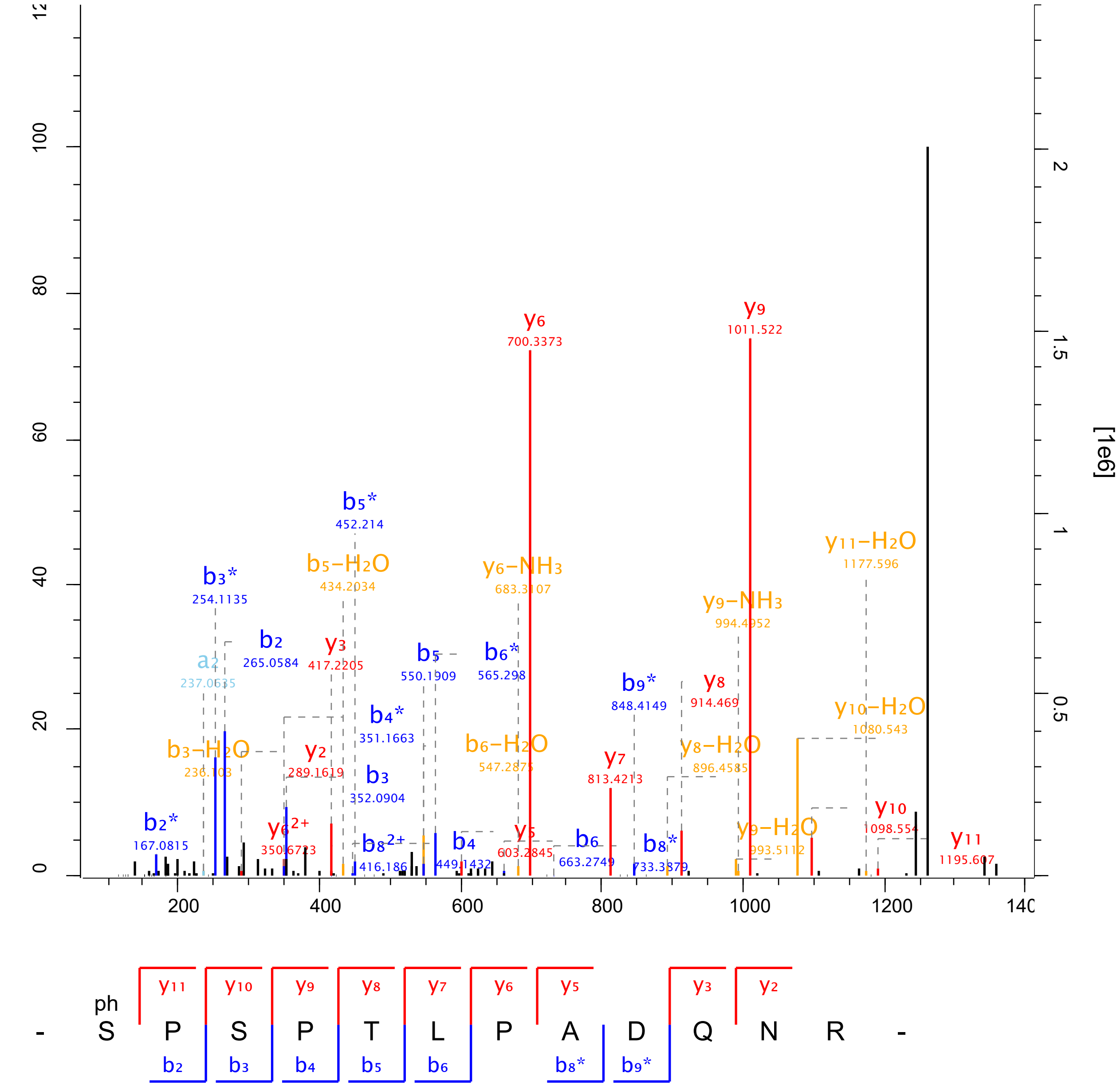

Raw file	Scan	Method	Score	m/z
sys_15_1	11082	FTMS; HCD	78.9	720.33

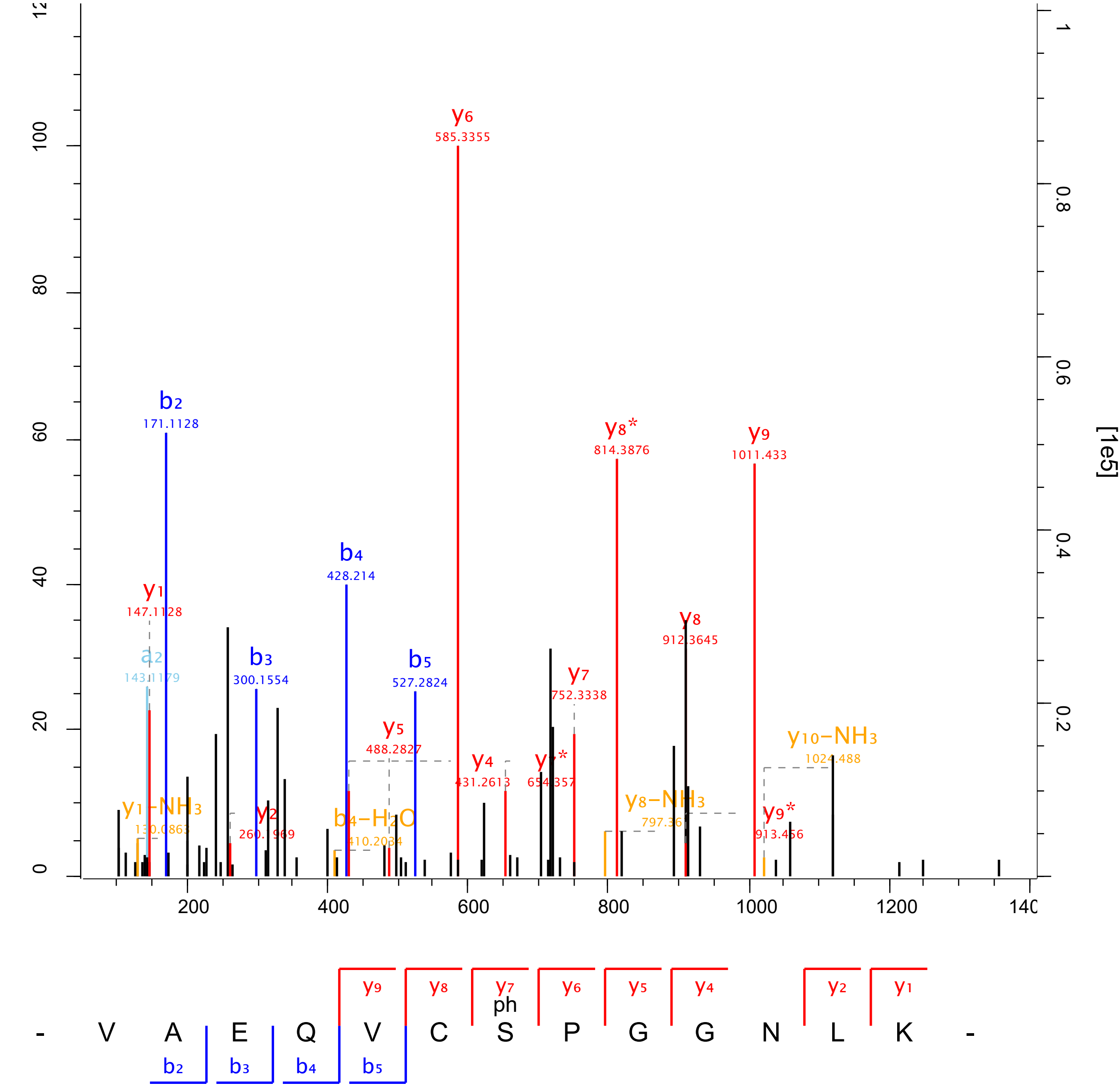

Raw file	Scan	Method	Score	m/z
sys_15_1	11115	FTMS; HCD	58.78	495.22

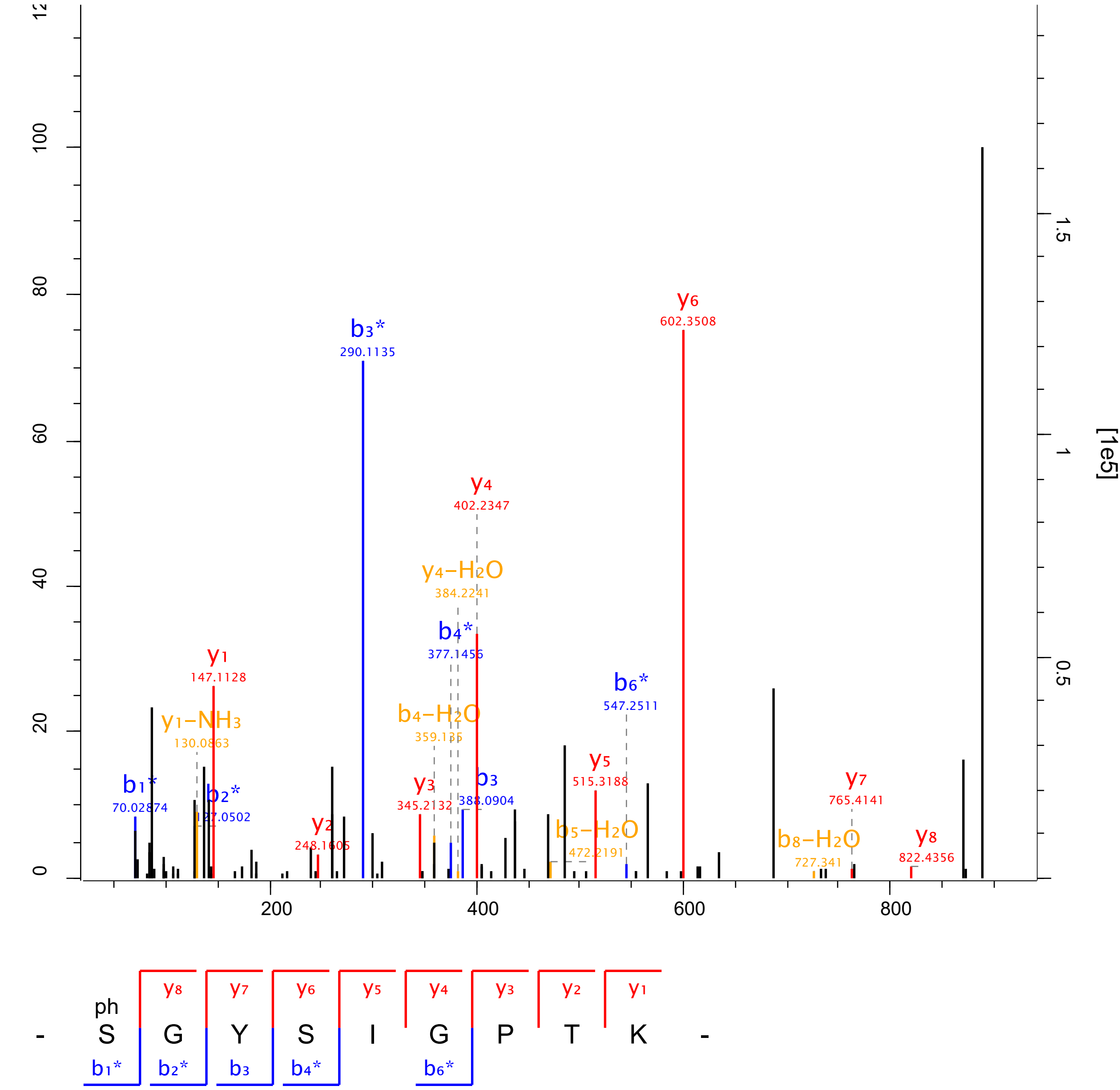

Raw file	Scan	Method	Score	m/z
sys_15_1	11218	FTMS; HCD	89.23	650.27

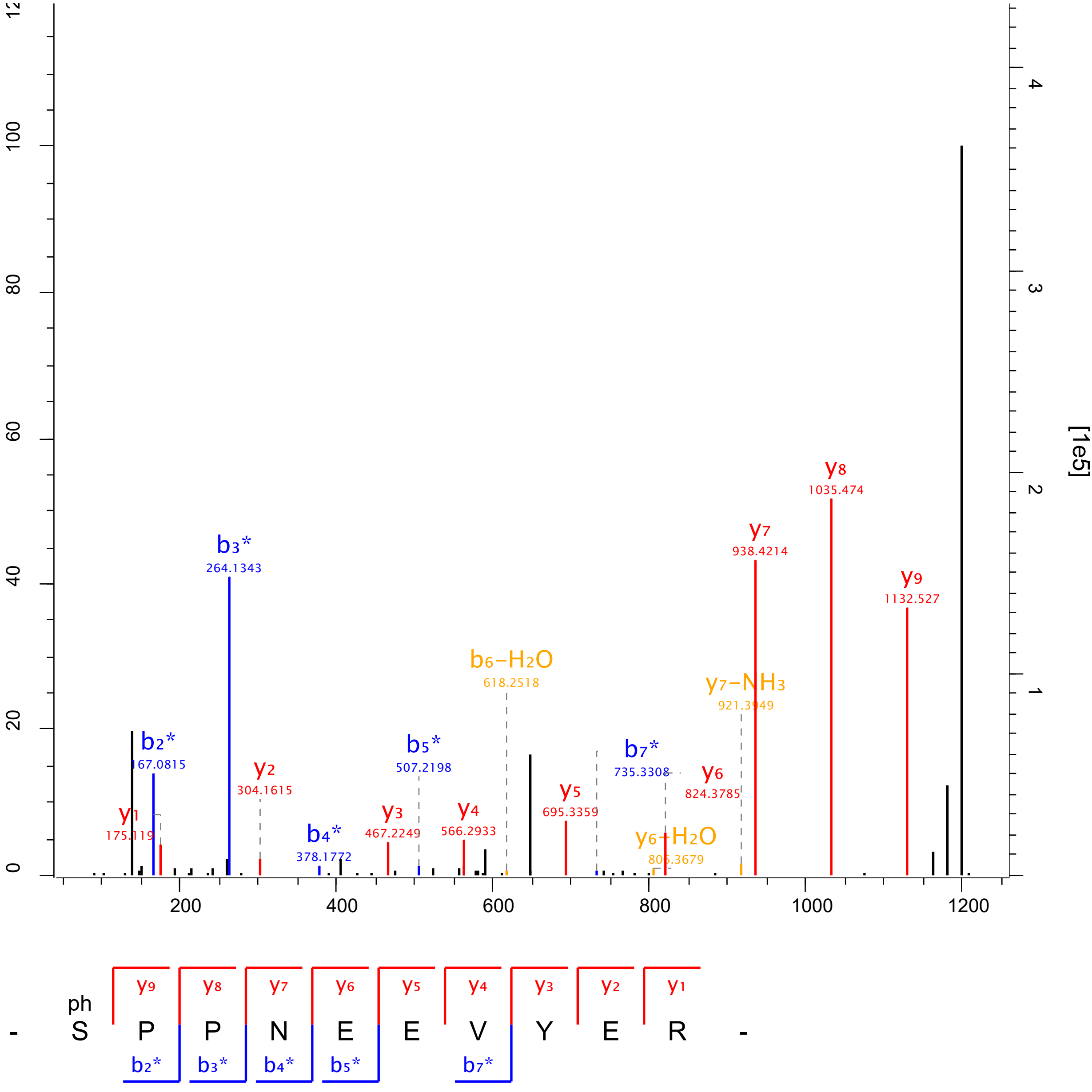

Raw file	Scan	Method	Score	m/z
sys_15_1	11314	FTMS; HCD	93.35	575.74

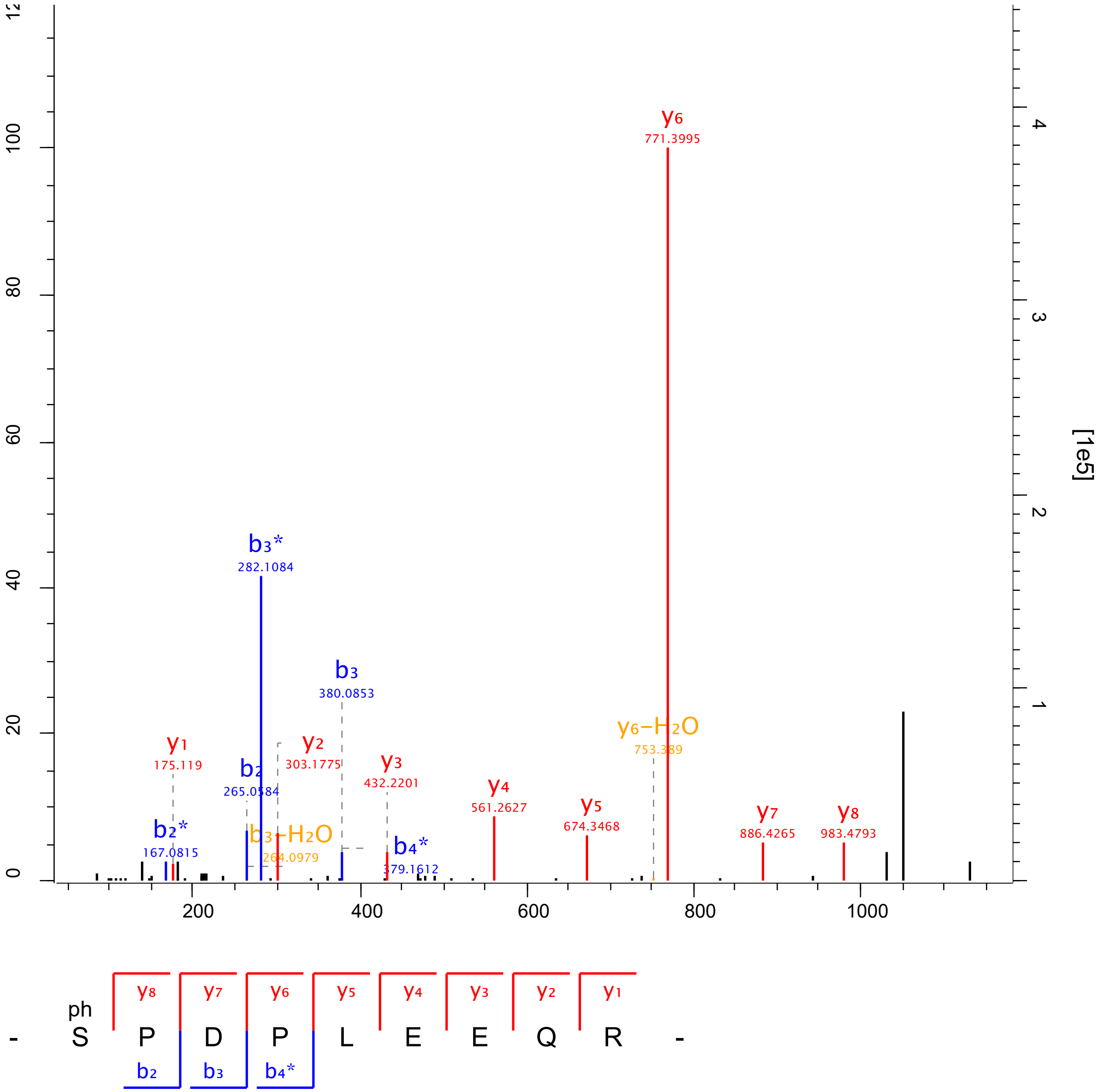

Raw file	Scan	Method	Score	m/z
sys_15_1	11322	FTMS; HCD	76.36	627.78

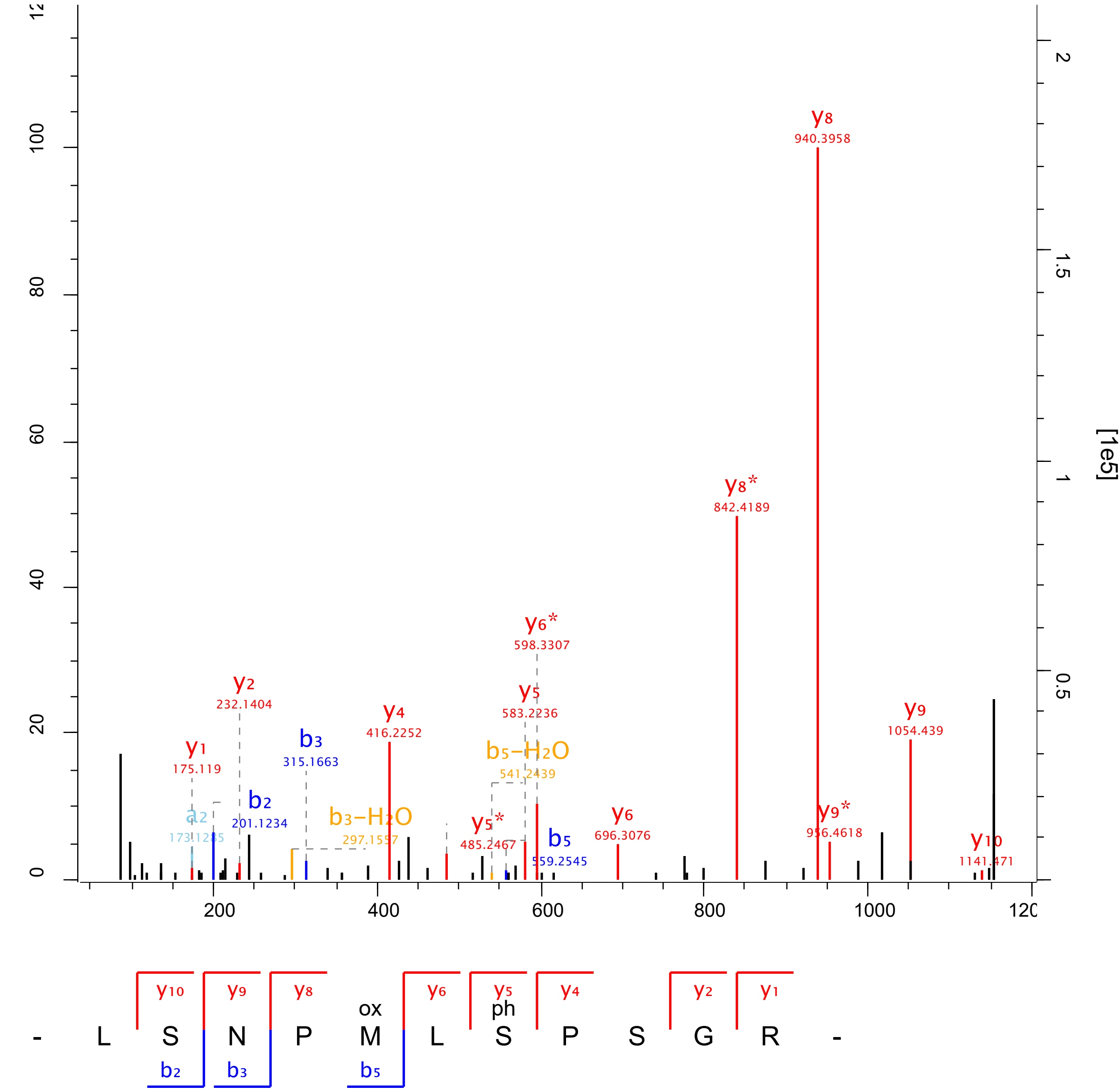

Raw file	Scan	Method	Score	m/z
sys_15_1	11357	FTMS; HCD	53.45	748.33

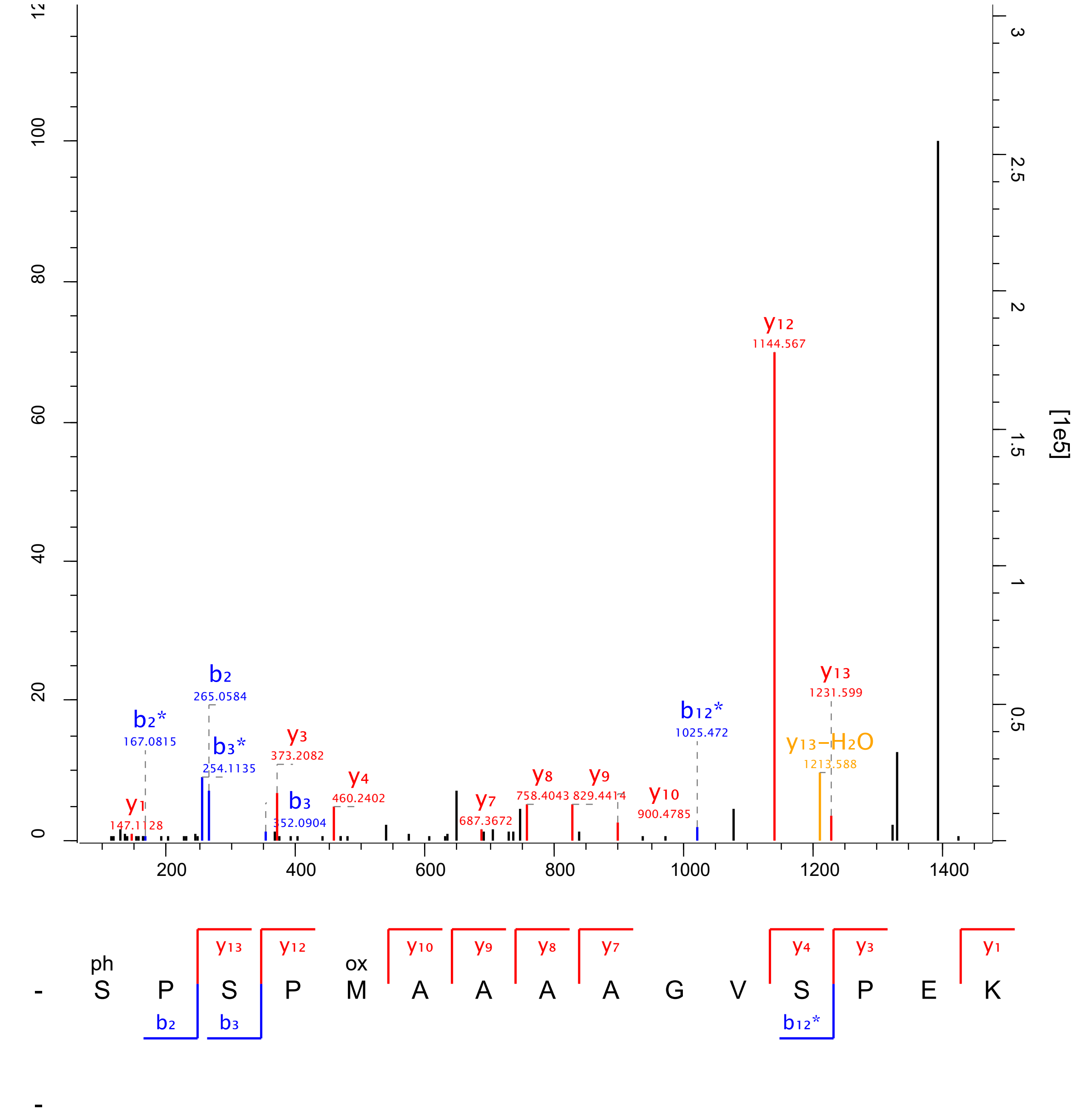

Raw file	Scan	Method	Score	m/z
sys_15_1	11360	FTMS; HCD	51.77	727.3

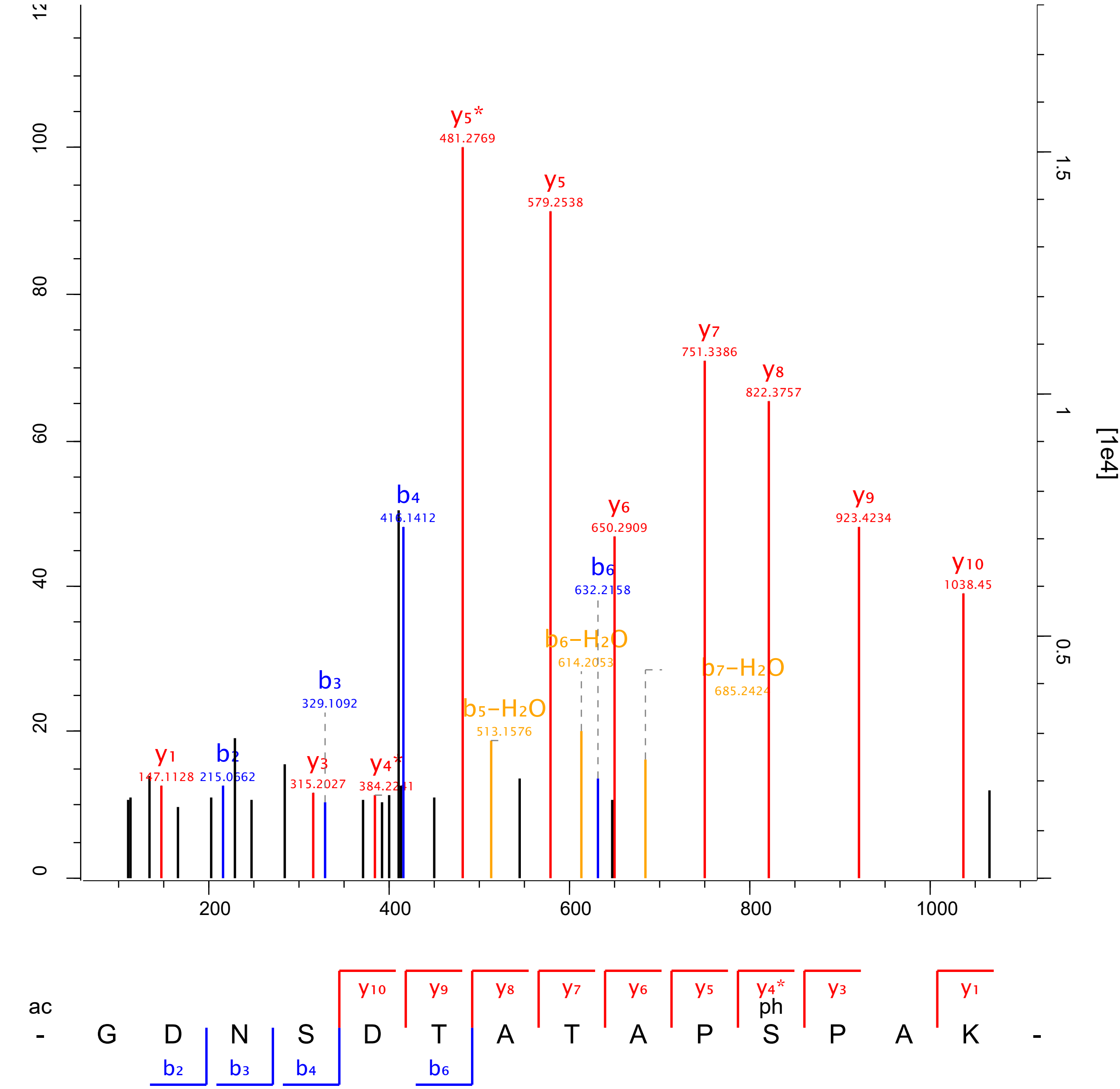

Raw file	Scan	Method	Score	m/z
sys_15_1	11429	FTMS; HCD	198.77	788.85

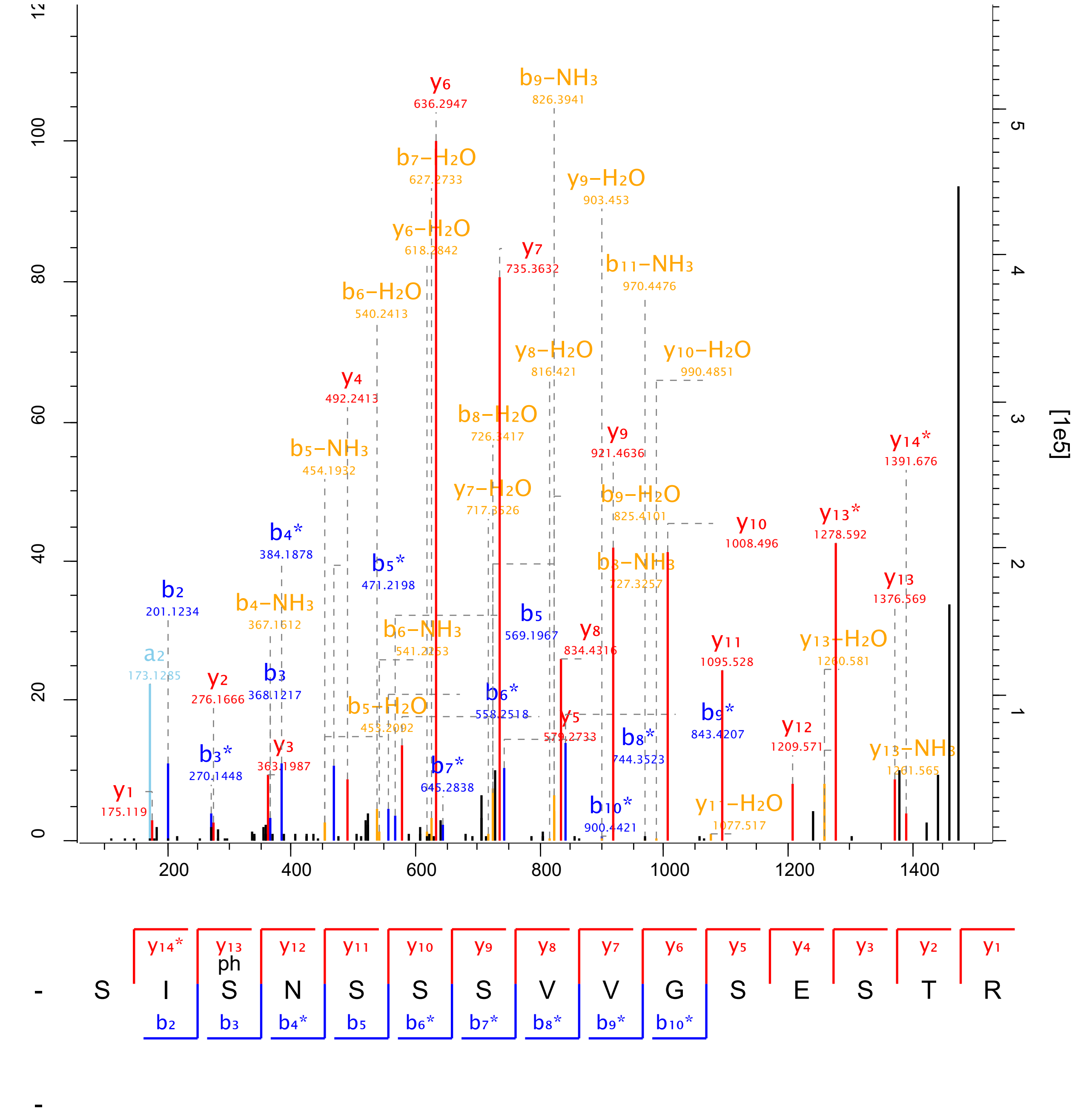

Raw file	Scan	Method	Score	m/z
sys_15_1	11688	FTMS; HCD	126.26	898.33

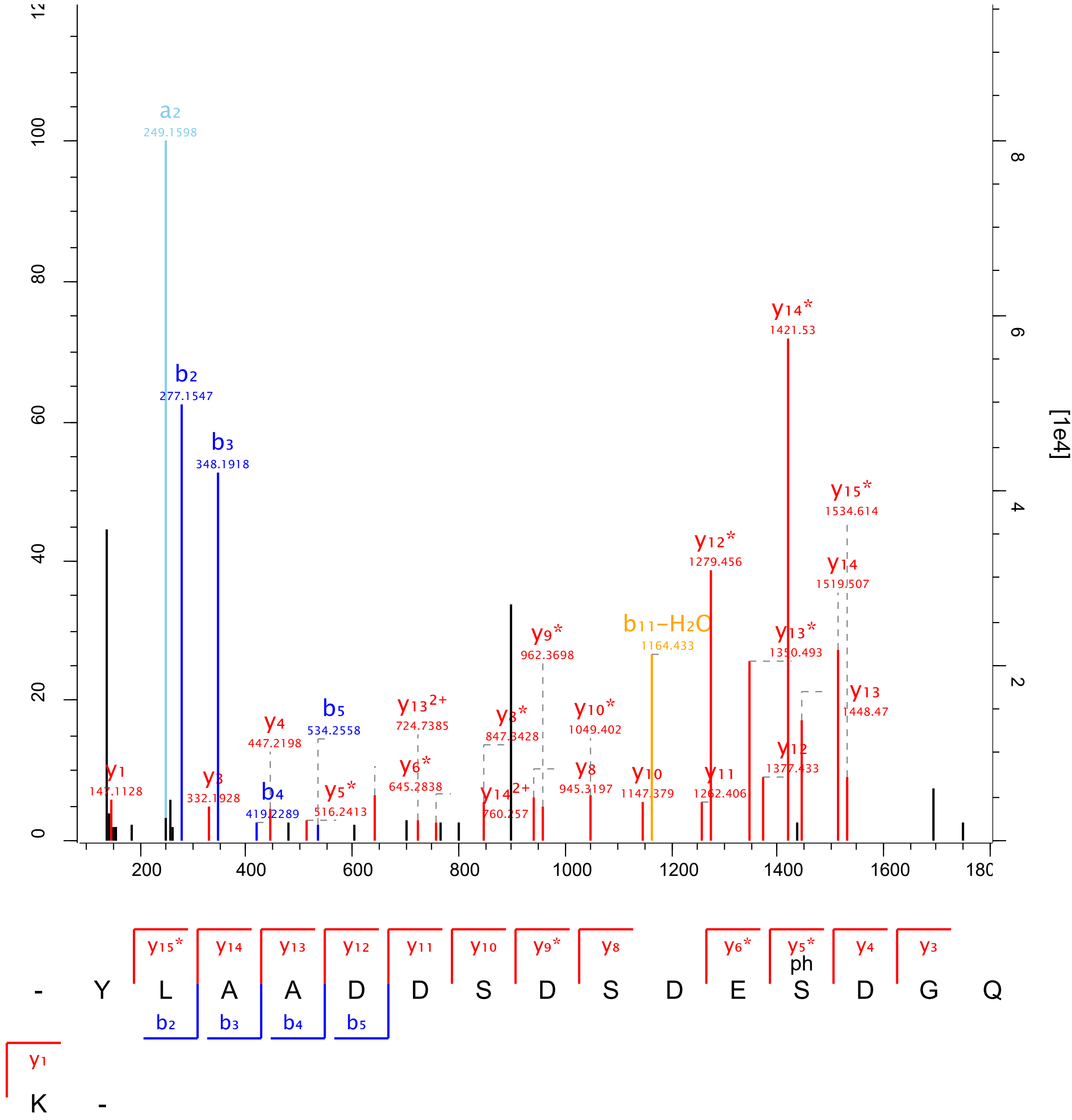

Raw file	Scan	Method	Score	m/z
sys_15_1	11860	FTMS; HCD	107.09	617.77

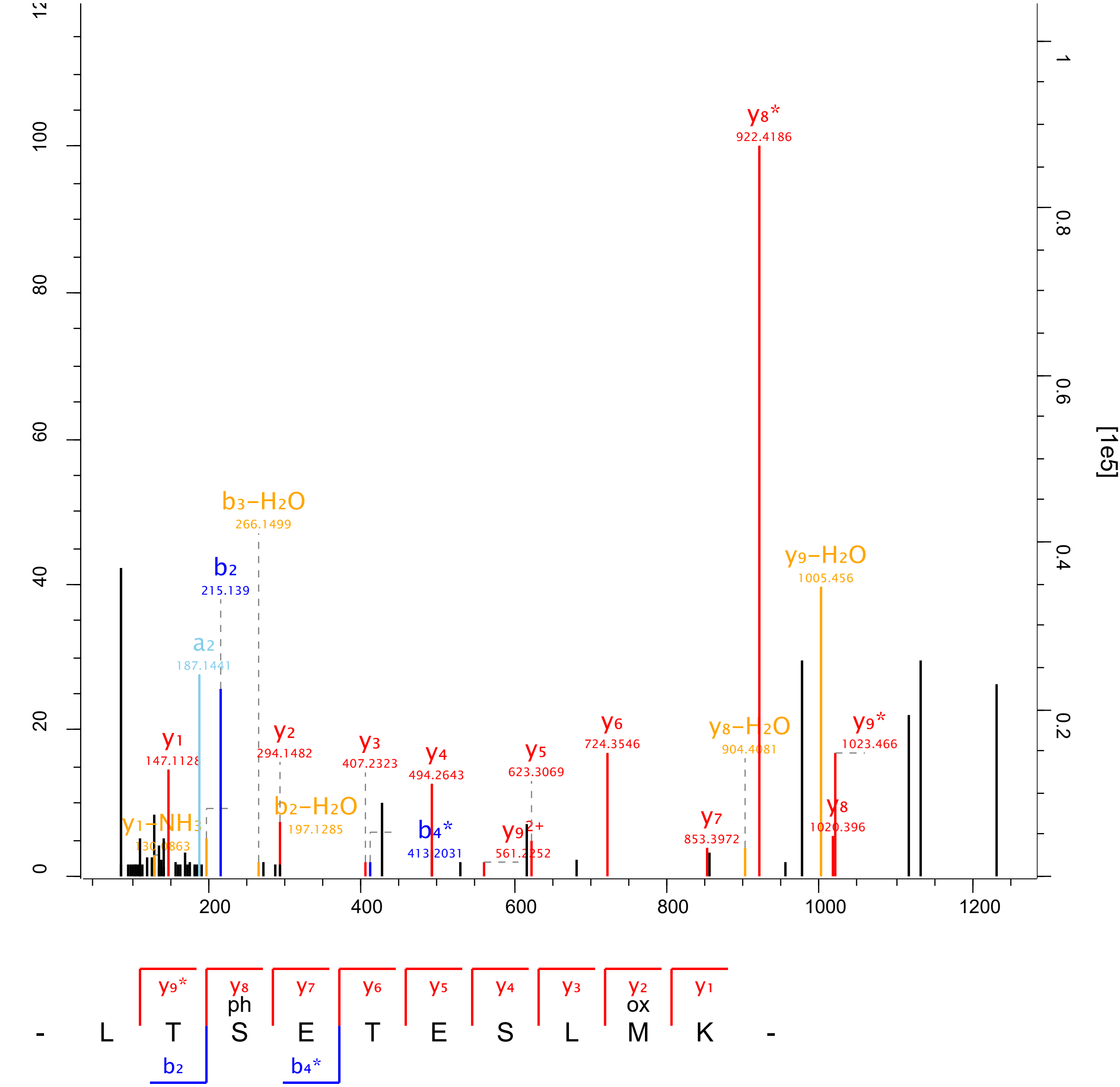

Raw file	Scan	Method	Score	m/z
sys_15_1	11872	FTMS; HCD	269.61	790.84

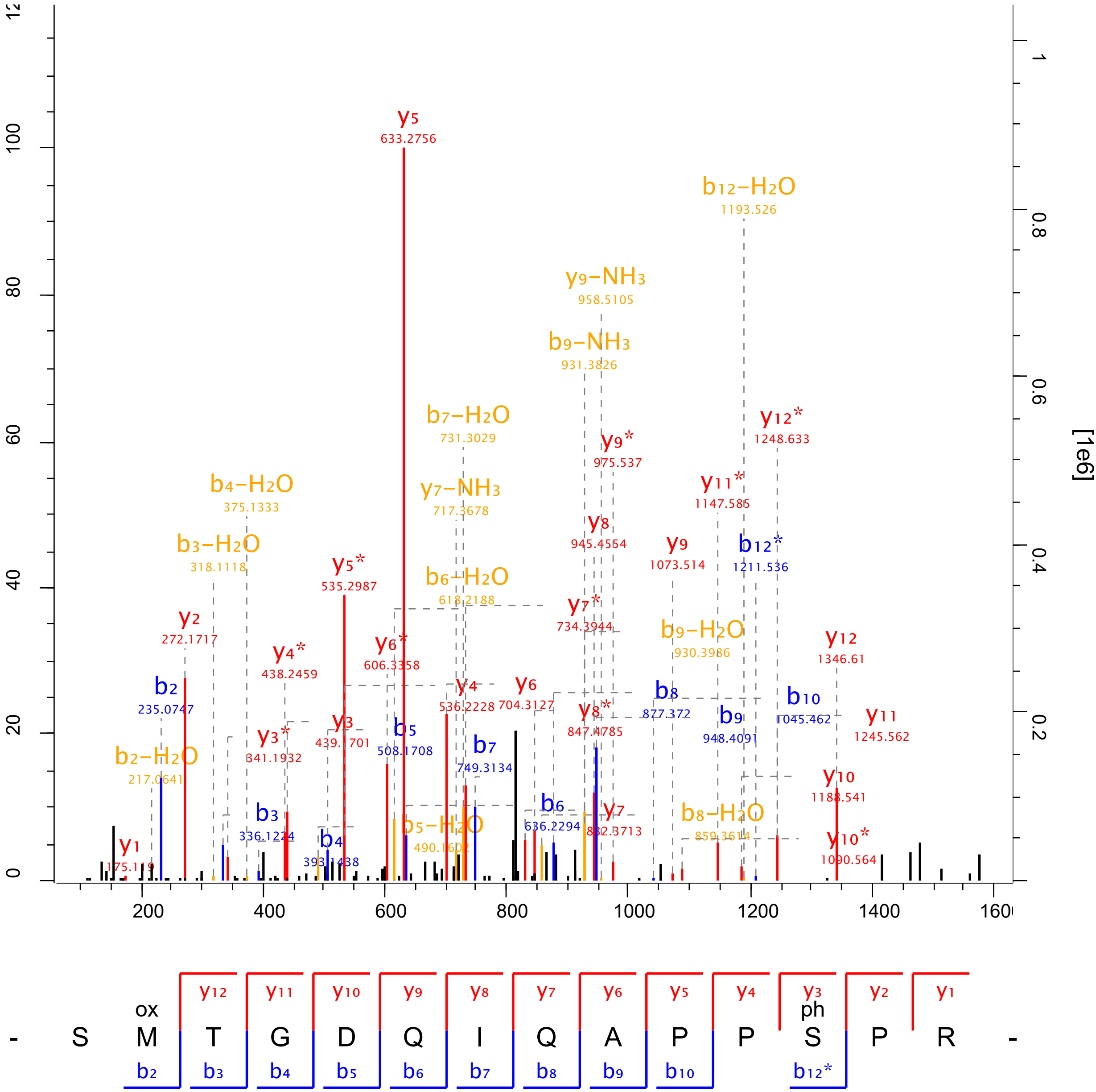

Raw file	Scan	Method	Score	m/z
sys_15_1	11873	FTMS; HCD	112.44	595.75

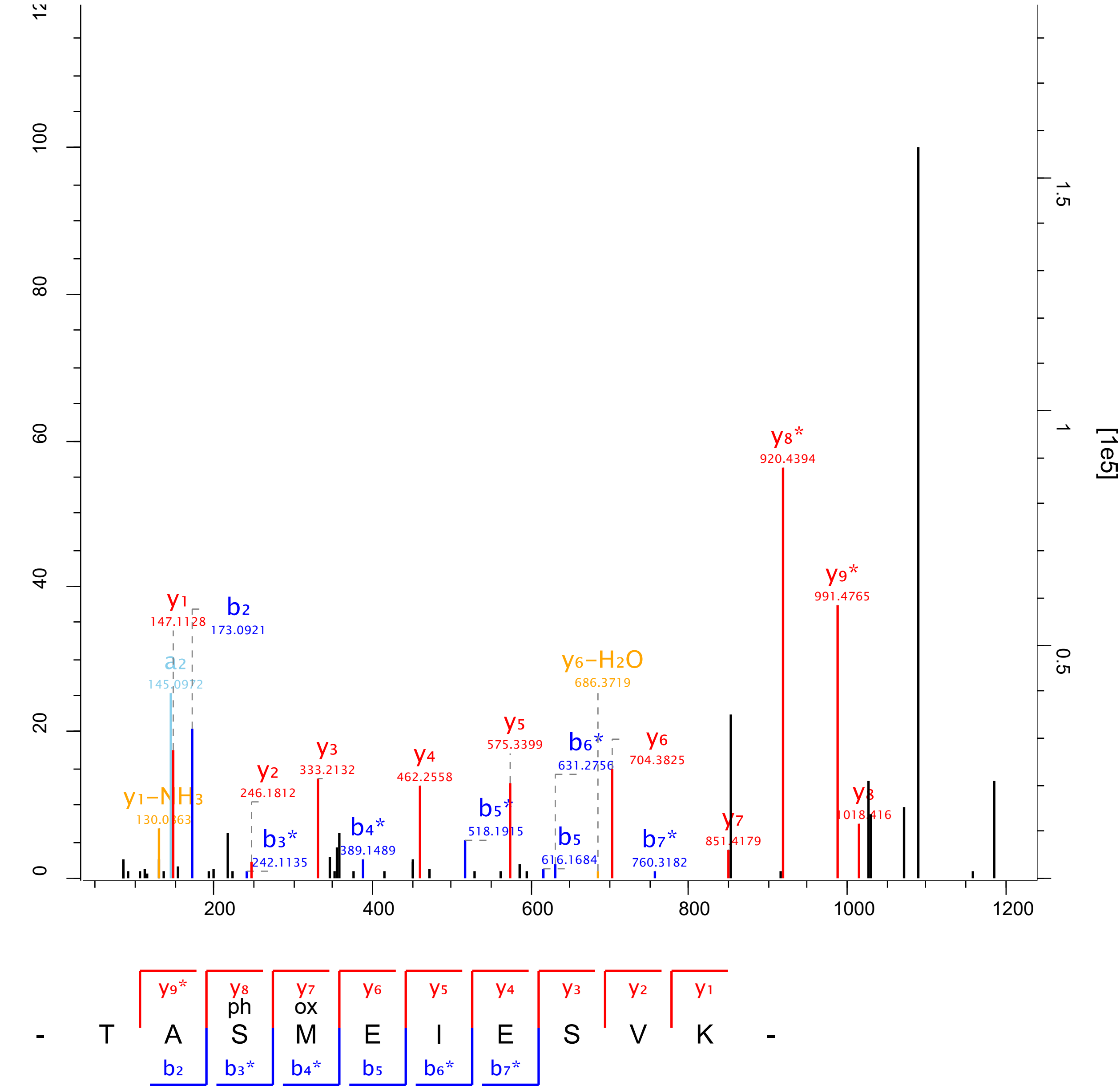

Raw file	Scan	Method	Score	m/z
sys_15_1	11896	FTMS; HCD	251	829.35

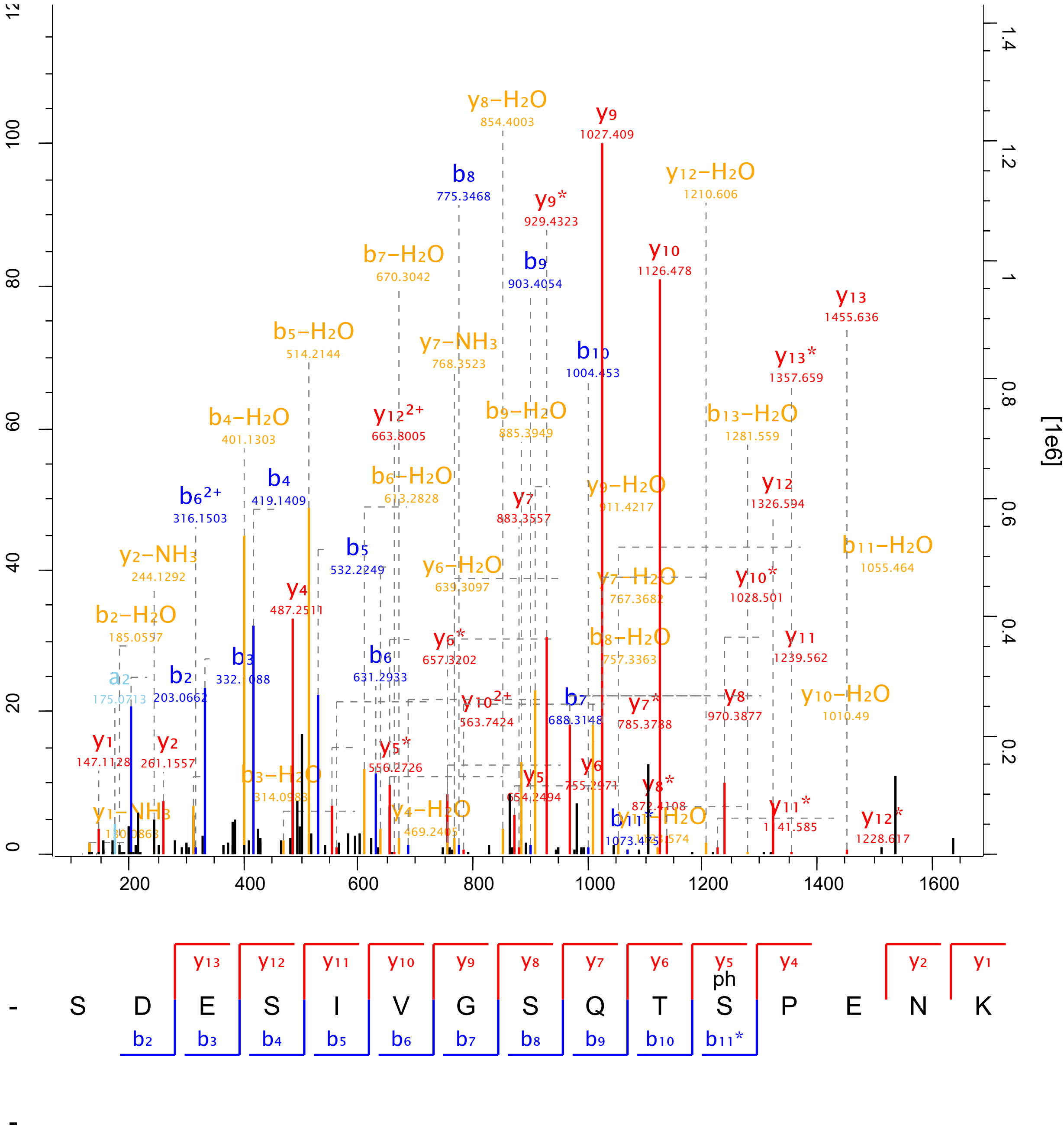

Raw file	Scan	Method	Score	m/z
sys_15_1	12078	FTMS; HCD	107.65	675.81

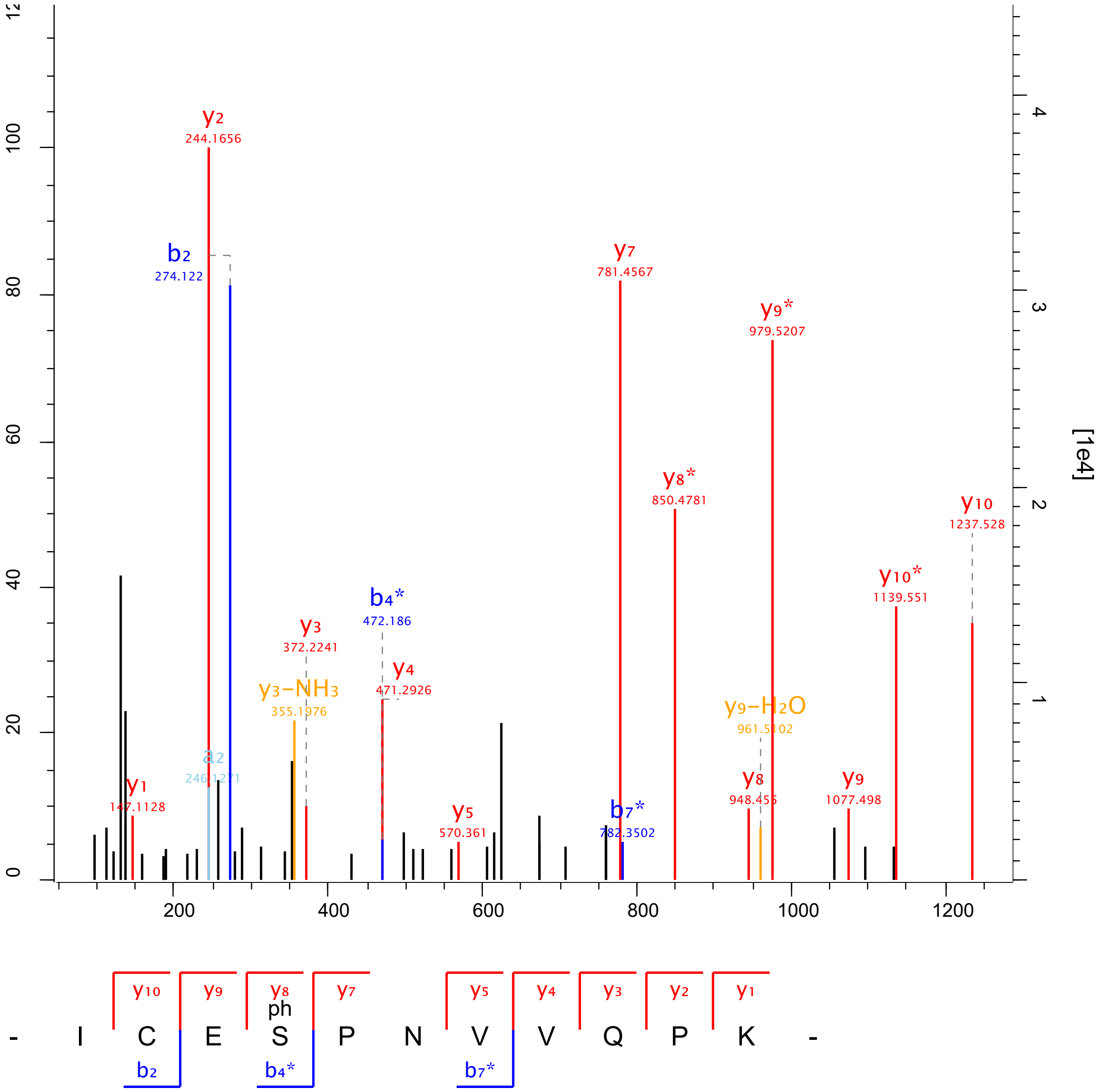

Raw file	Scan	Method	Score	m/z
sys_15_1	12117	FTMS; HCD	190.1	693.81

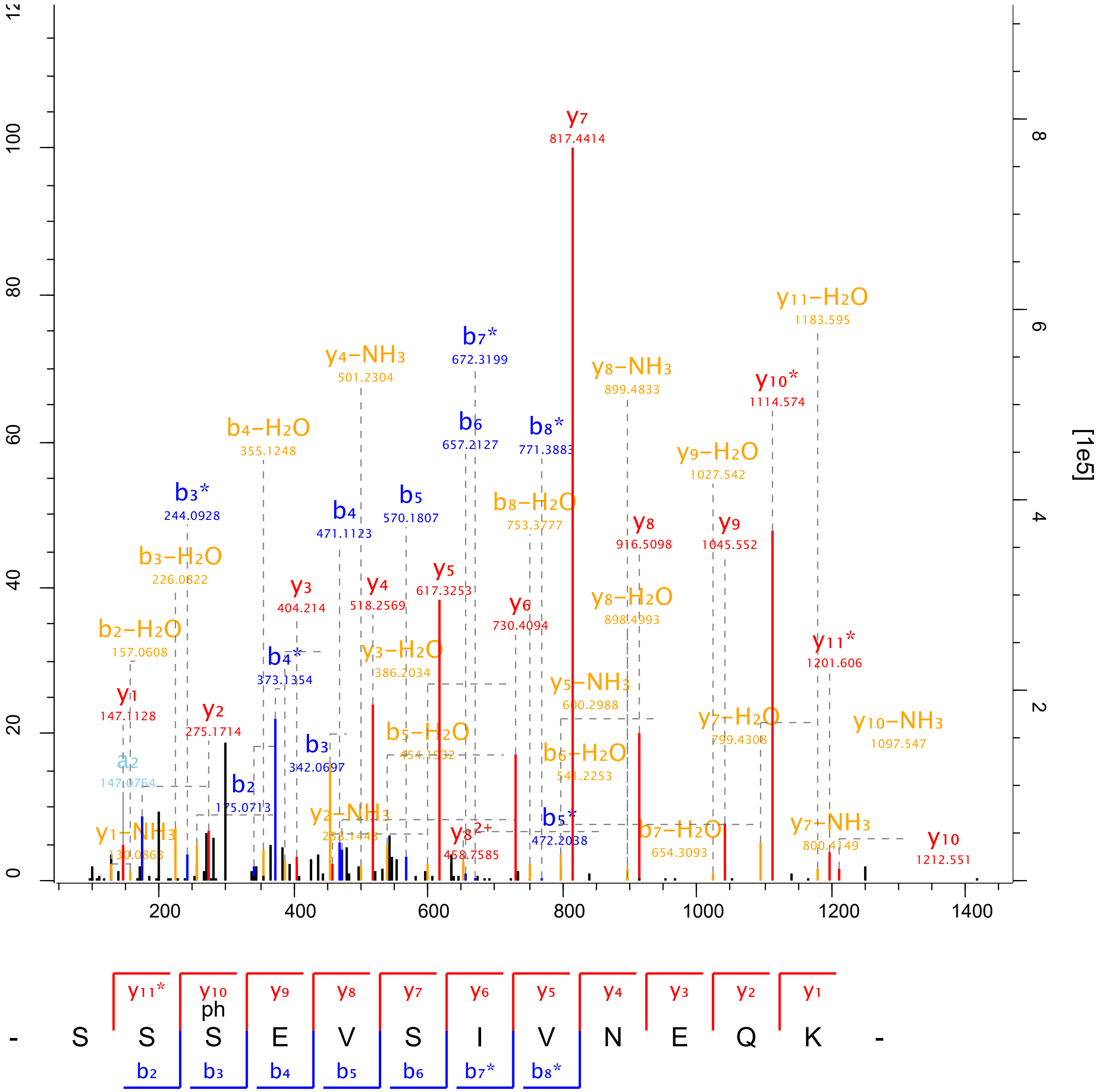

Raw file	Scan	Method	Score	m/z
sys_15_1	12148	FTMS; HCD	116.19	556.26

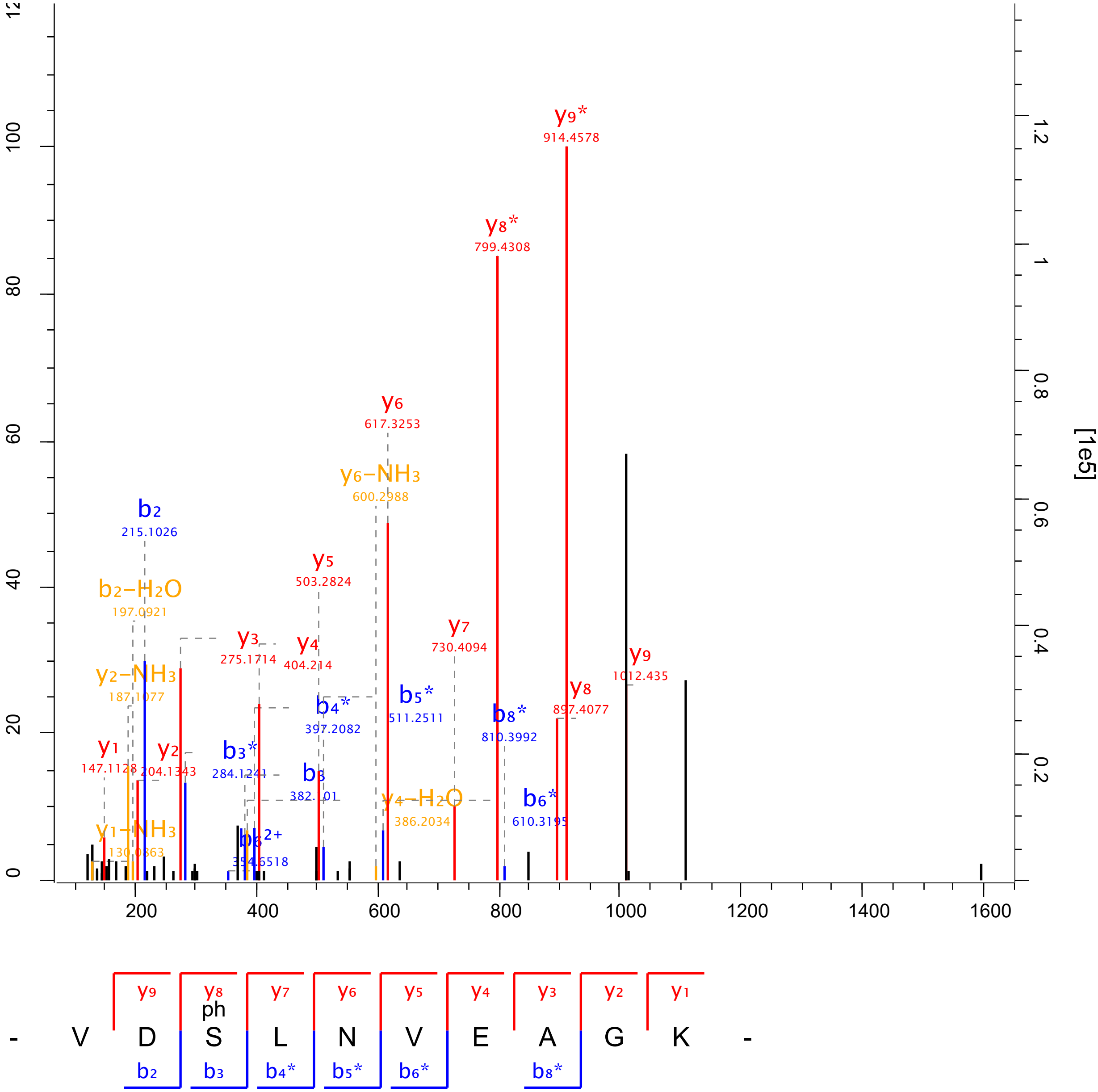

Raw file	Scan	Method	Score	m/z
sys_15_1	12158	FTMS; HCD	99.28	455.72

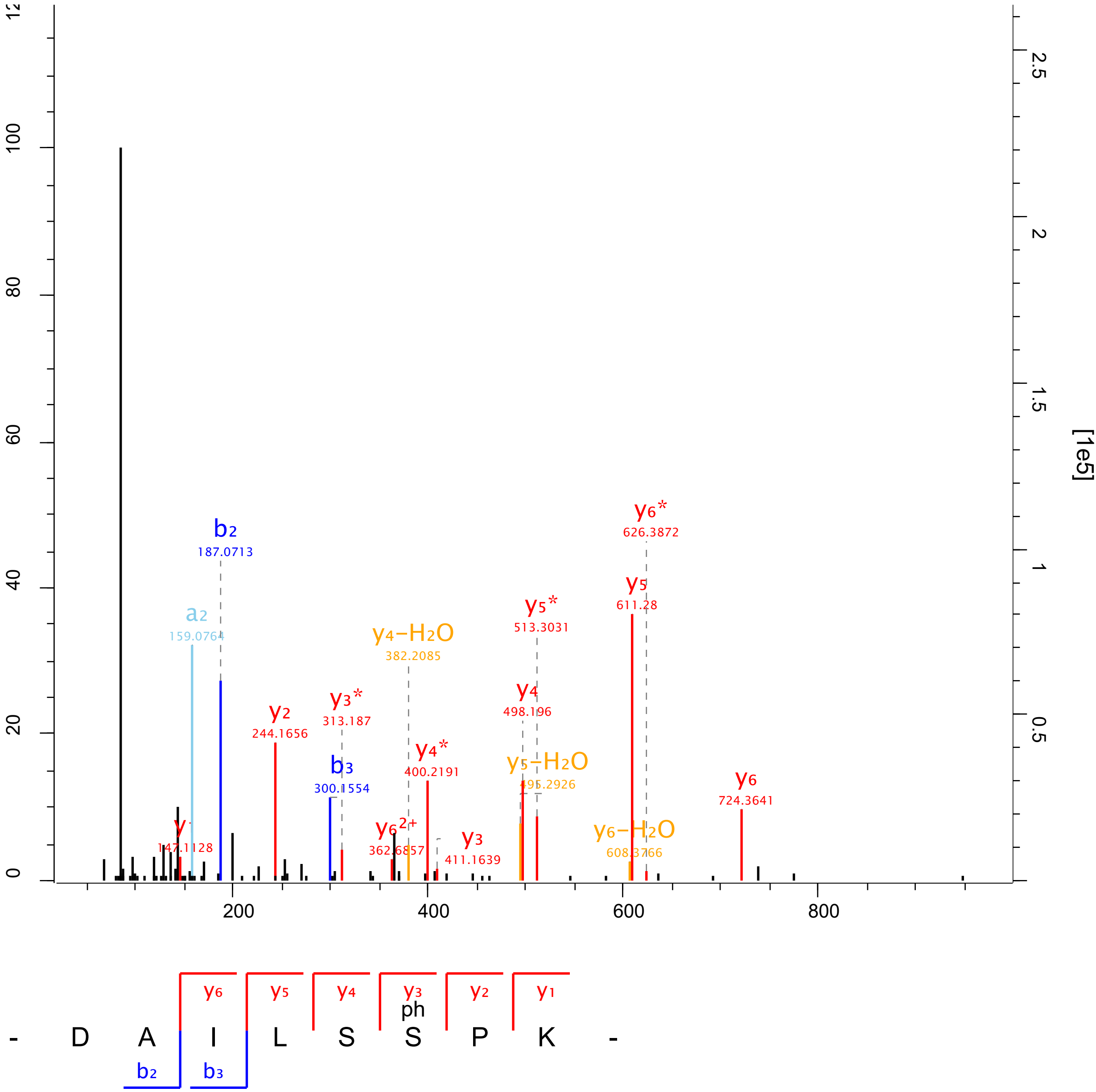

Raw file	Scan	Method	Score	m/z
sys_15_1	12159	FTMS; HCD	109.04	849.3

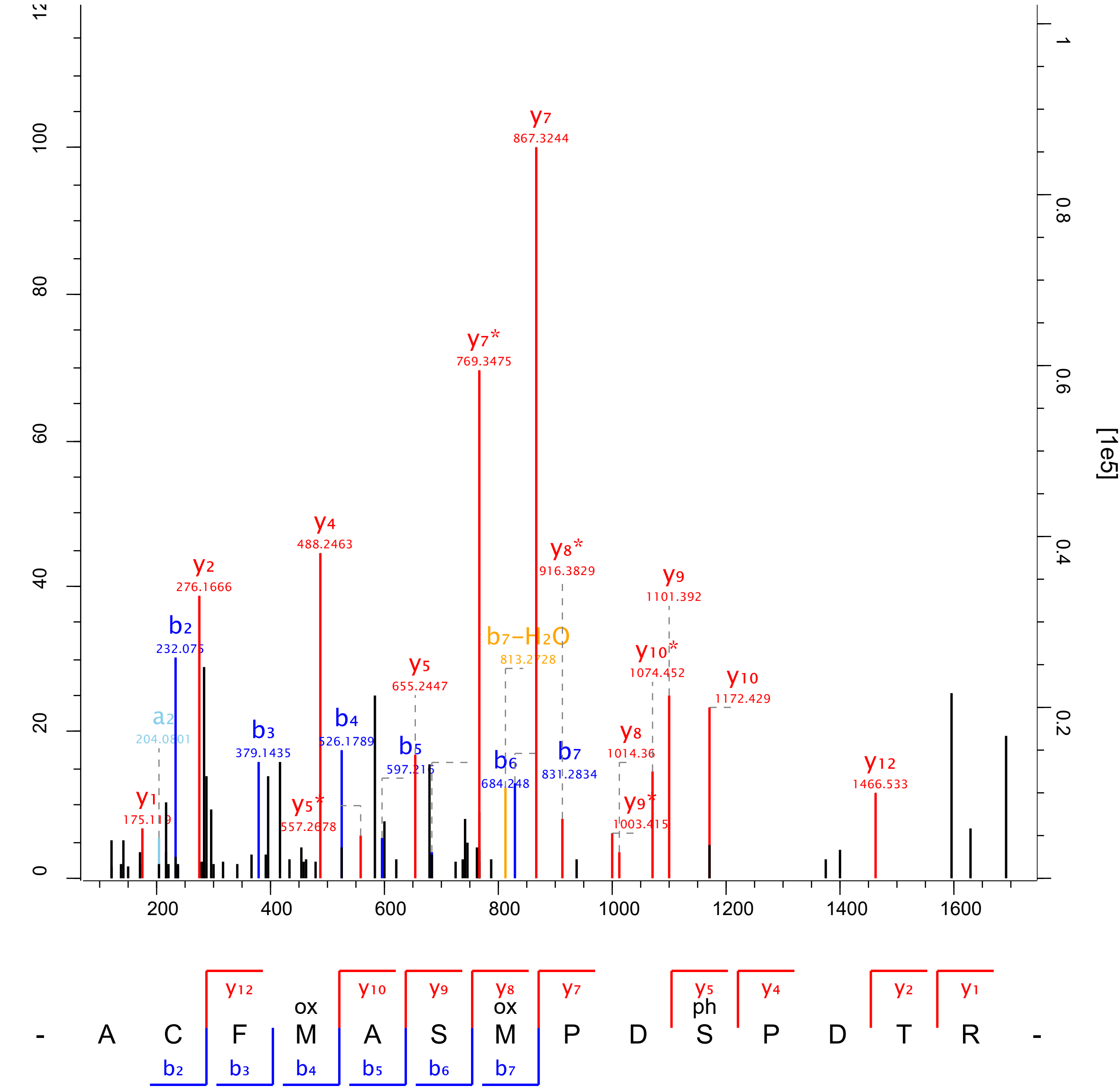

Raw file	Scan	Method	Score	m/z
sys_15_1	12182	FTMS; HCD	50.04	432.7

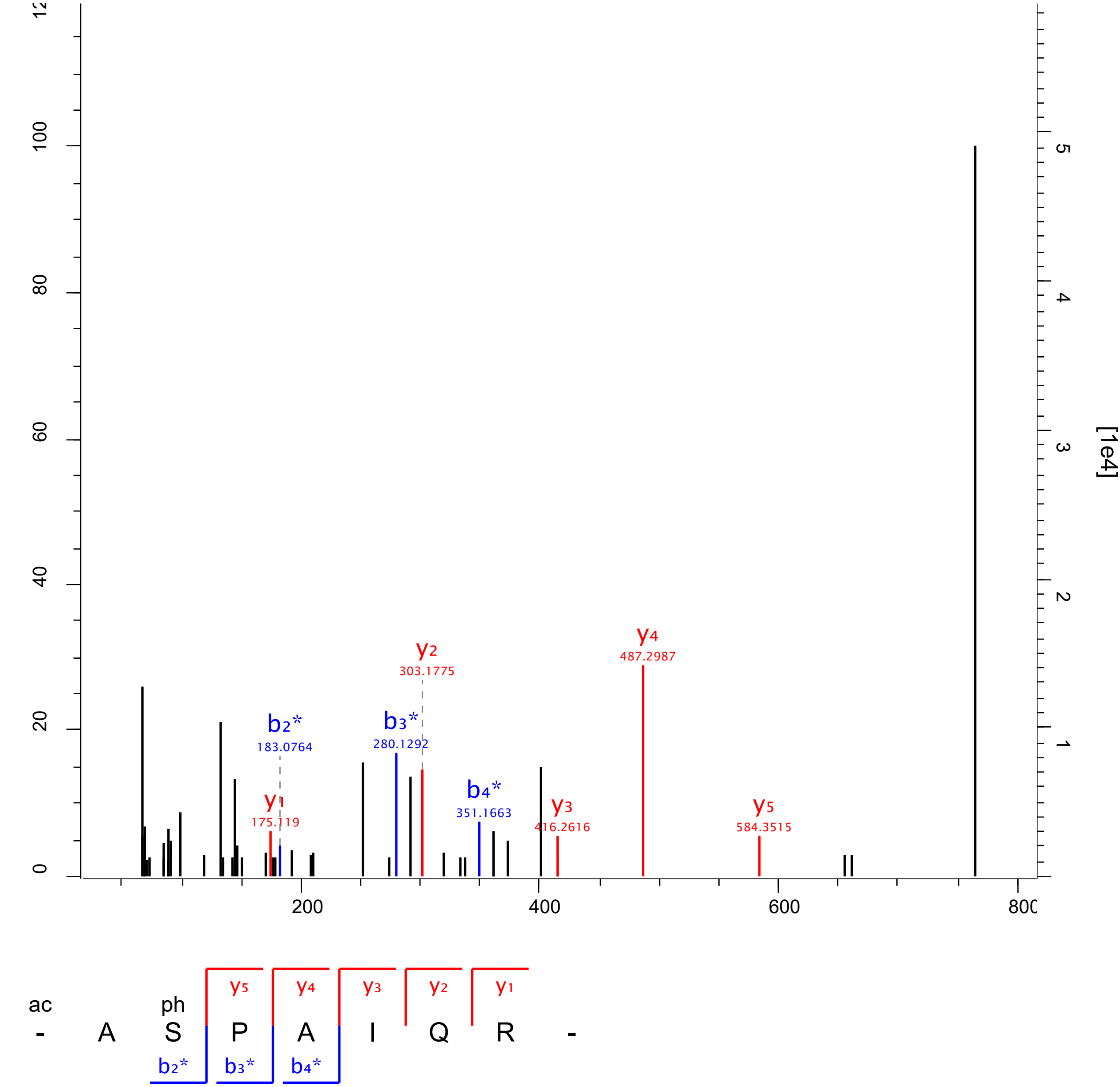

Raw file	Scan	Method	Score	m/z
sys_15_1	12236	FTMS; HCD	104.43	477.23

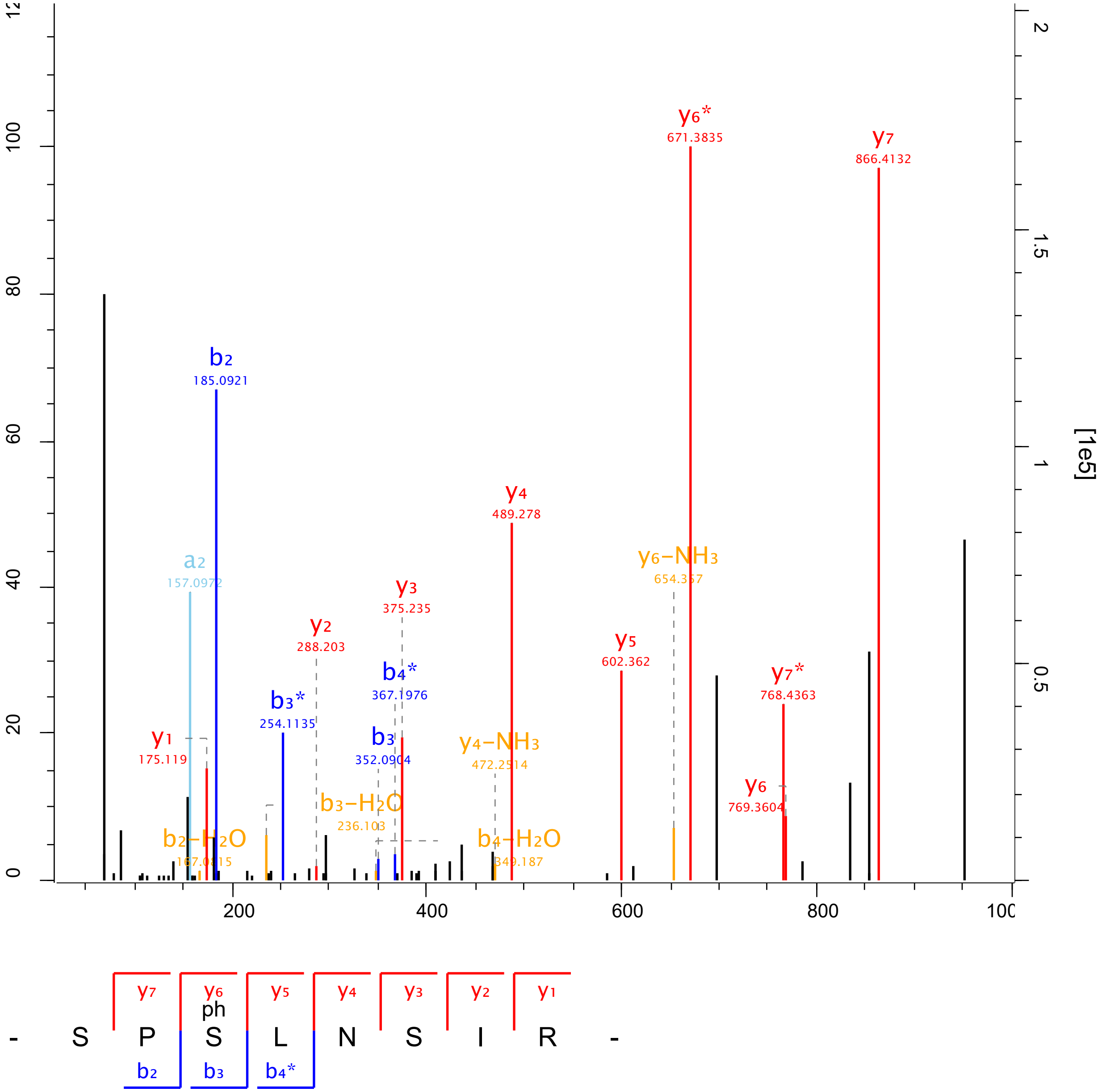

Raw file	Scan	Method	Score	m/z
sys_15_1	12327	FTMS; HCD	137.64	841.87

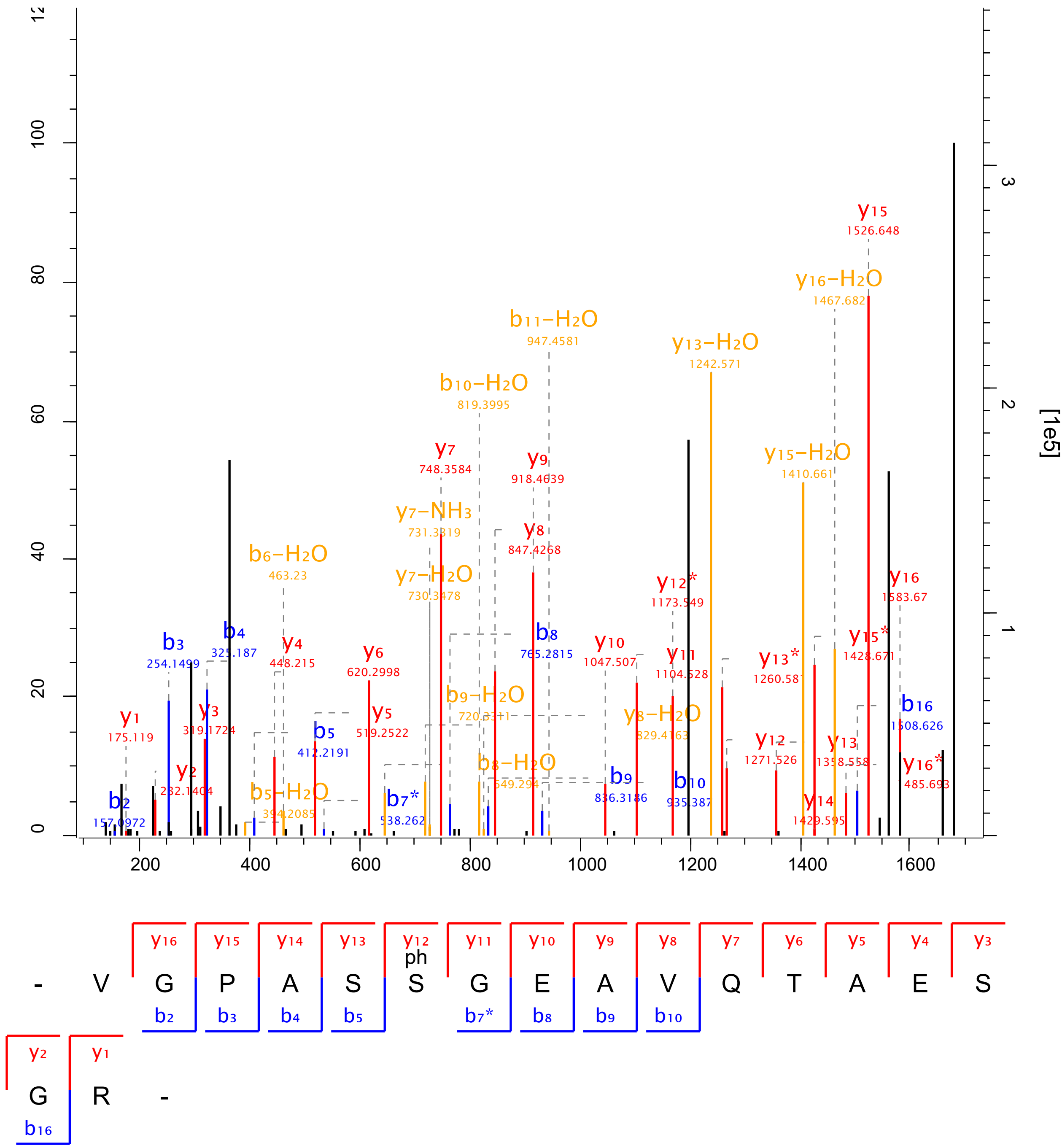

Raw file	Scan	Method	Score	m/z
sys_15_1	12357	FTMS; HCD	69.7	949.06

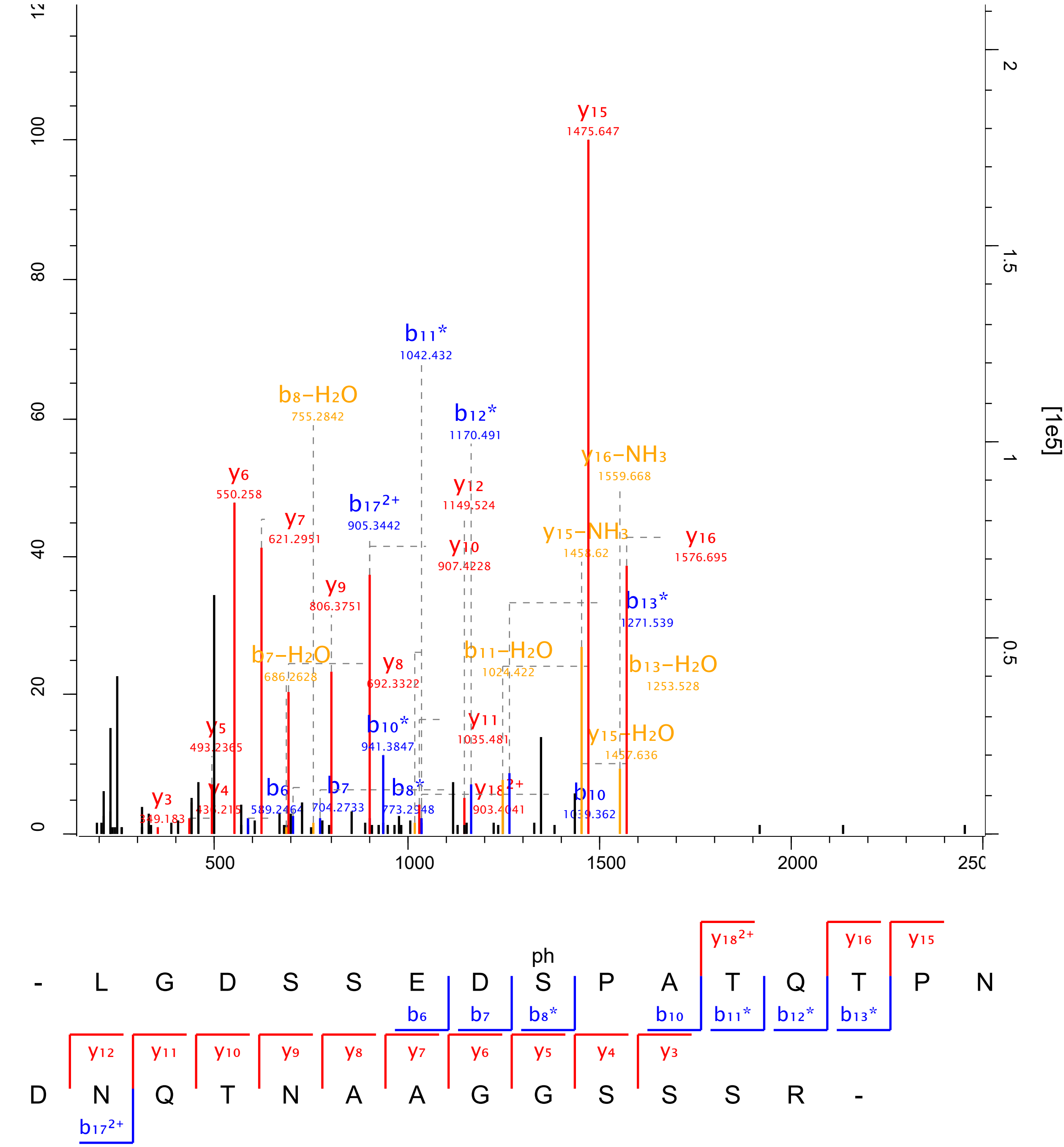

Raw file	Scan	Method	Score	m/z
sys_15_1	12376	FTMS; HCD	186.45	833.82

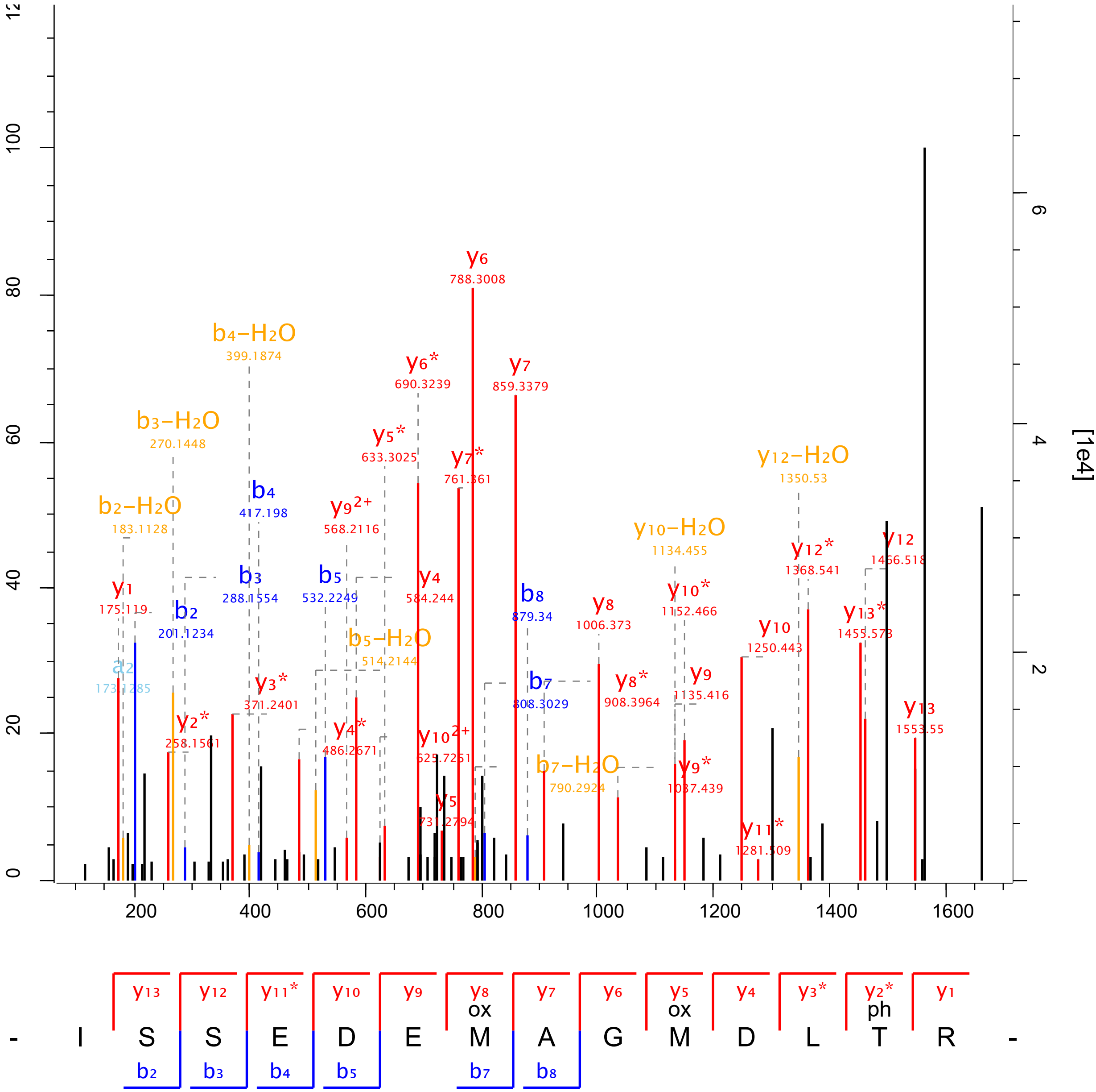

Raw file	Scan	Method	Score	m/z
sys_15_1	12564	FTMS; HCD	108.63	702.28

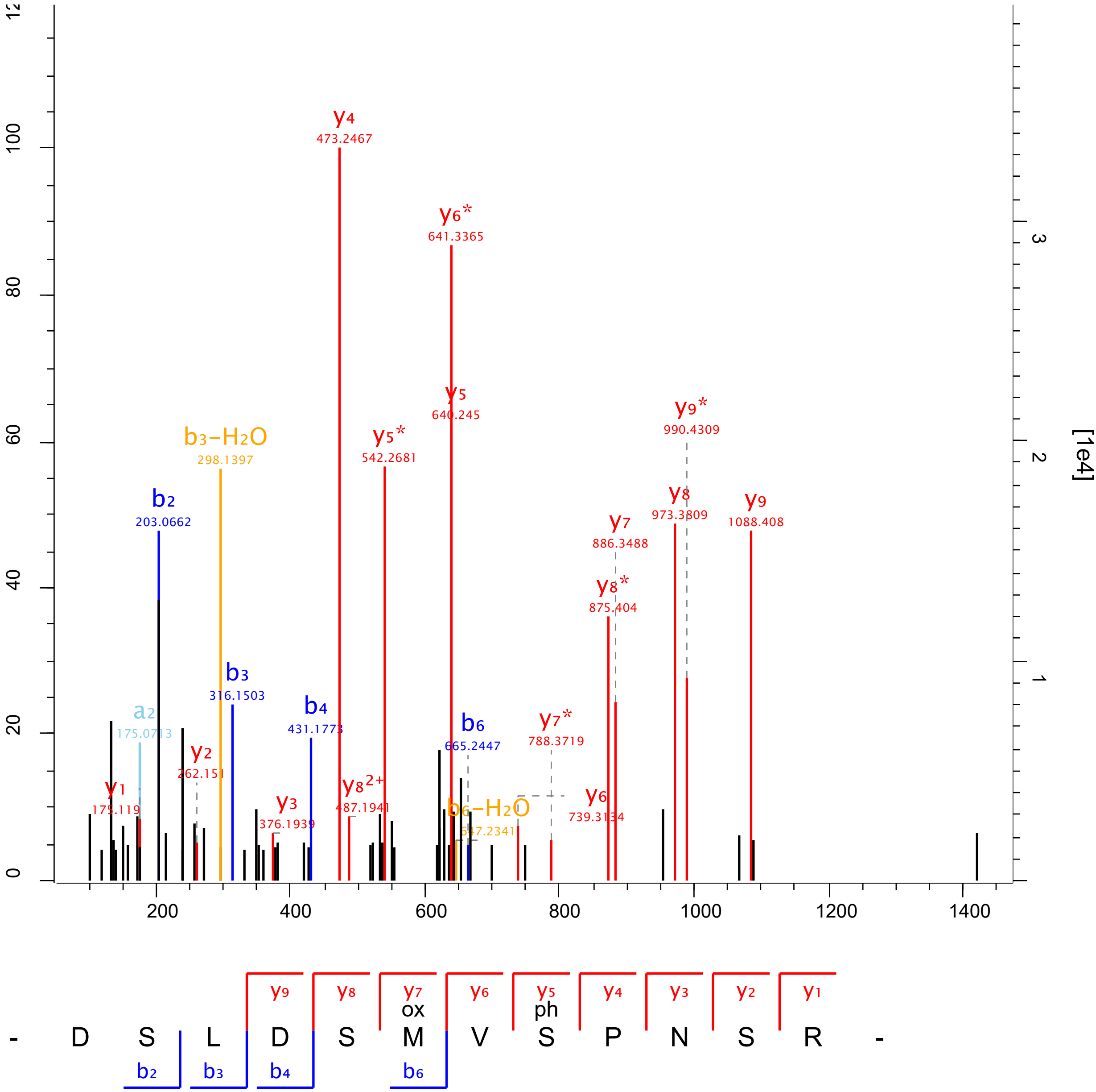

Raw file	Scan	Method	Score	m/z
sys_15_1	12592	FTMS; HCD	203.93	776.32

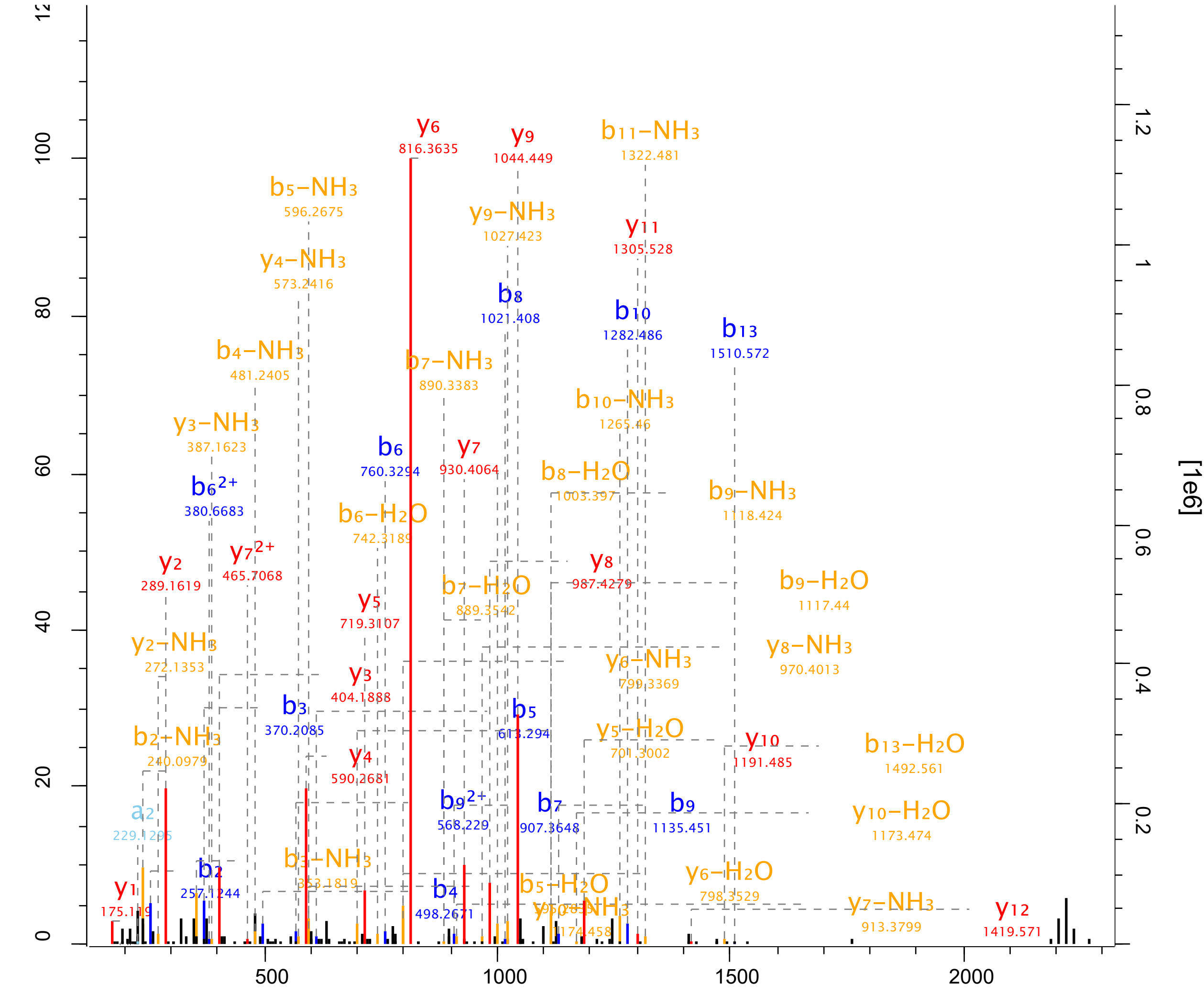

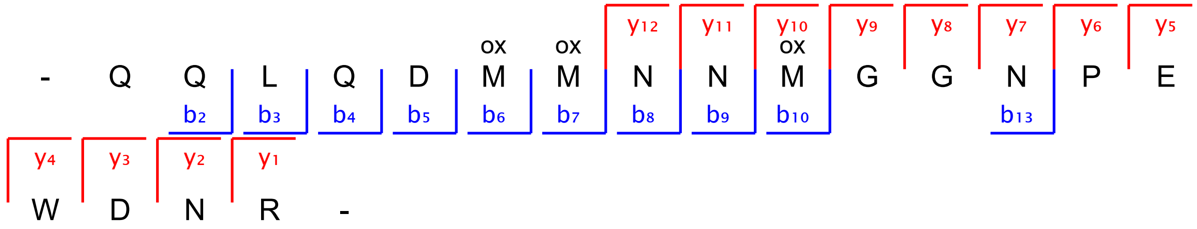

Raw file	Scan	Method	Score	m/z
sys_15_1	12713	FTMS; HCD	54.27	532.72

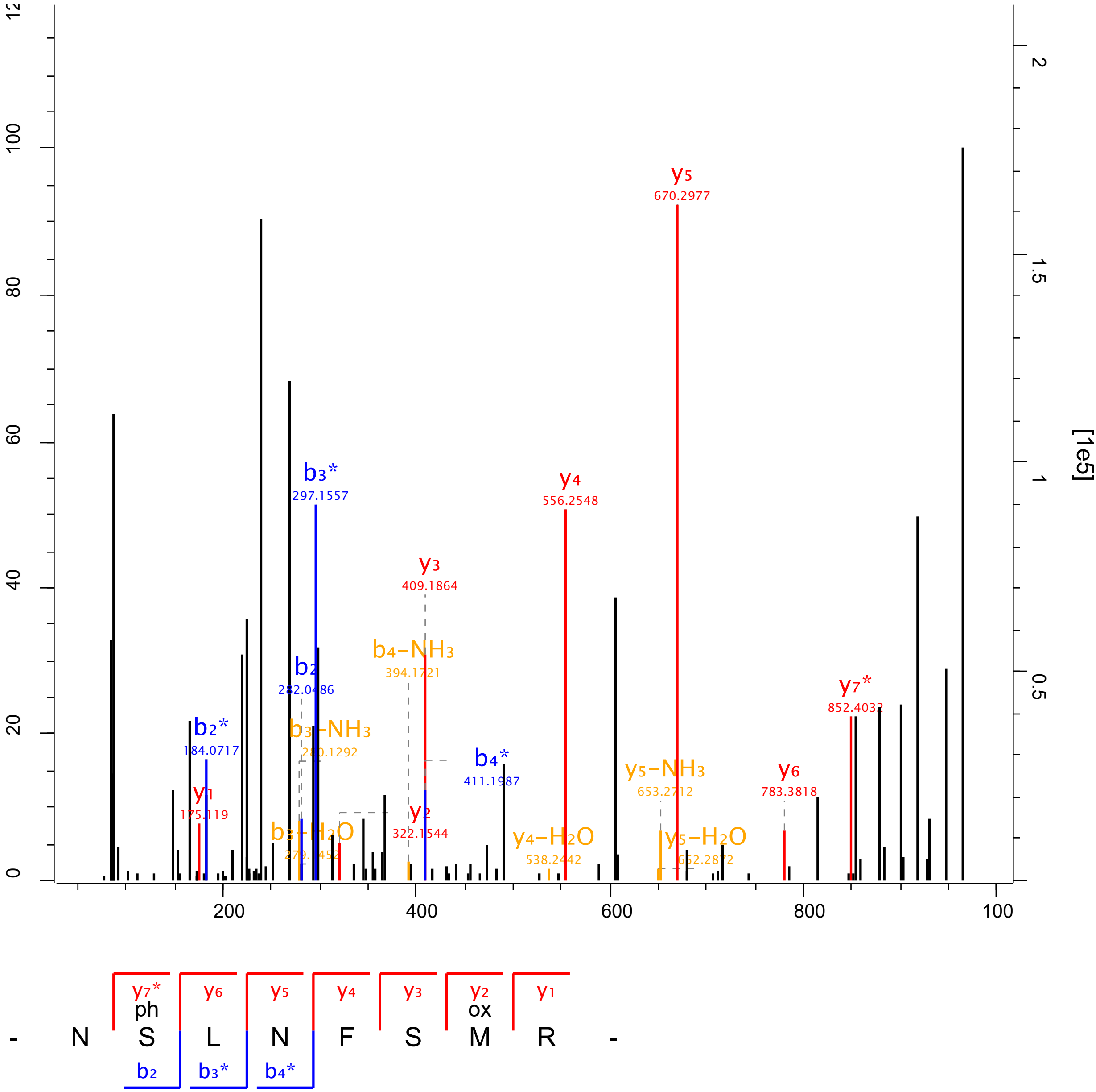

Raw file	Scan	Method	Score	m/z
sys_15_1	12735	FTMS; HCD	92.65	635.27

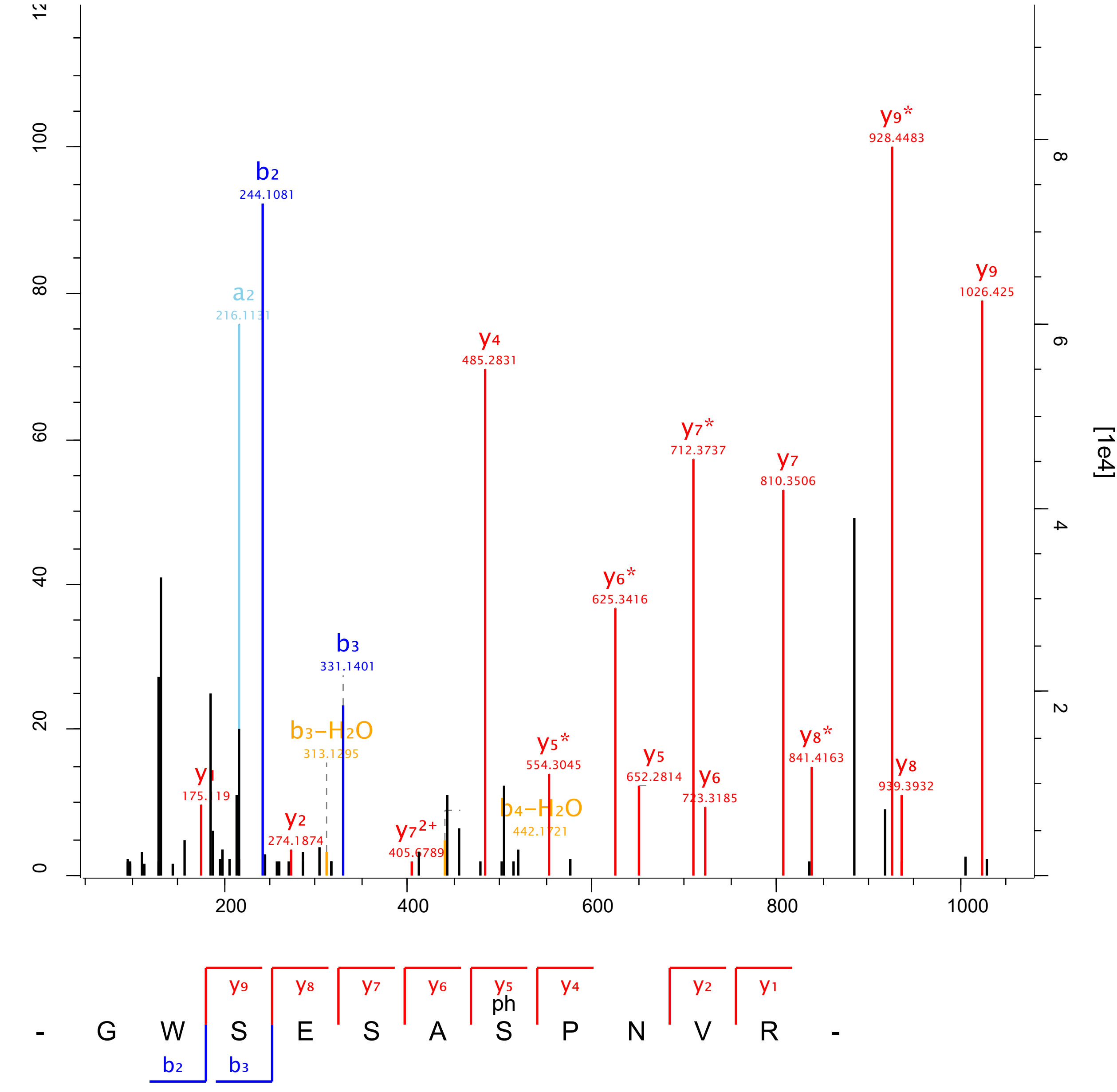

Raw file	Scan	Method	Score	m/z
sys_15_1	12905	FTMS; HCD	157.3	671.63

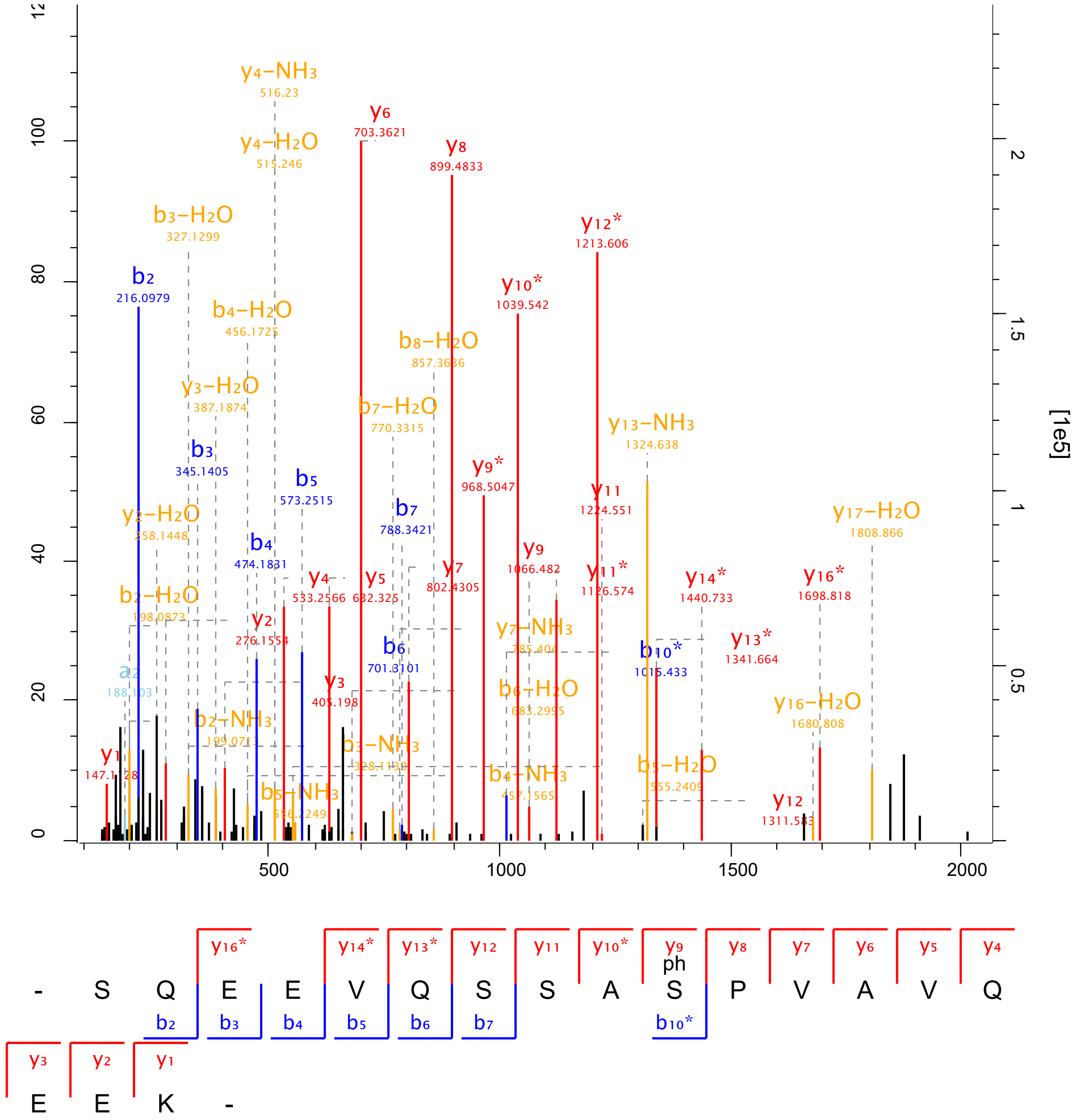

Raw file	Scan	Method	Score	m/z
sys_15_1	12913	FTMS; HCD	48.22	737.29

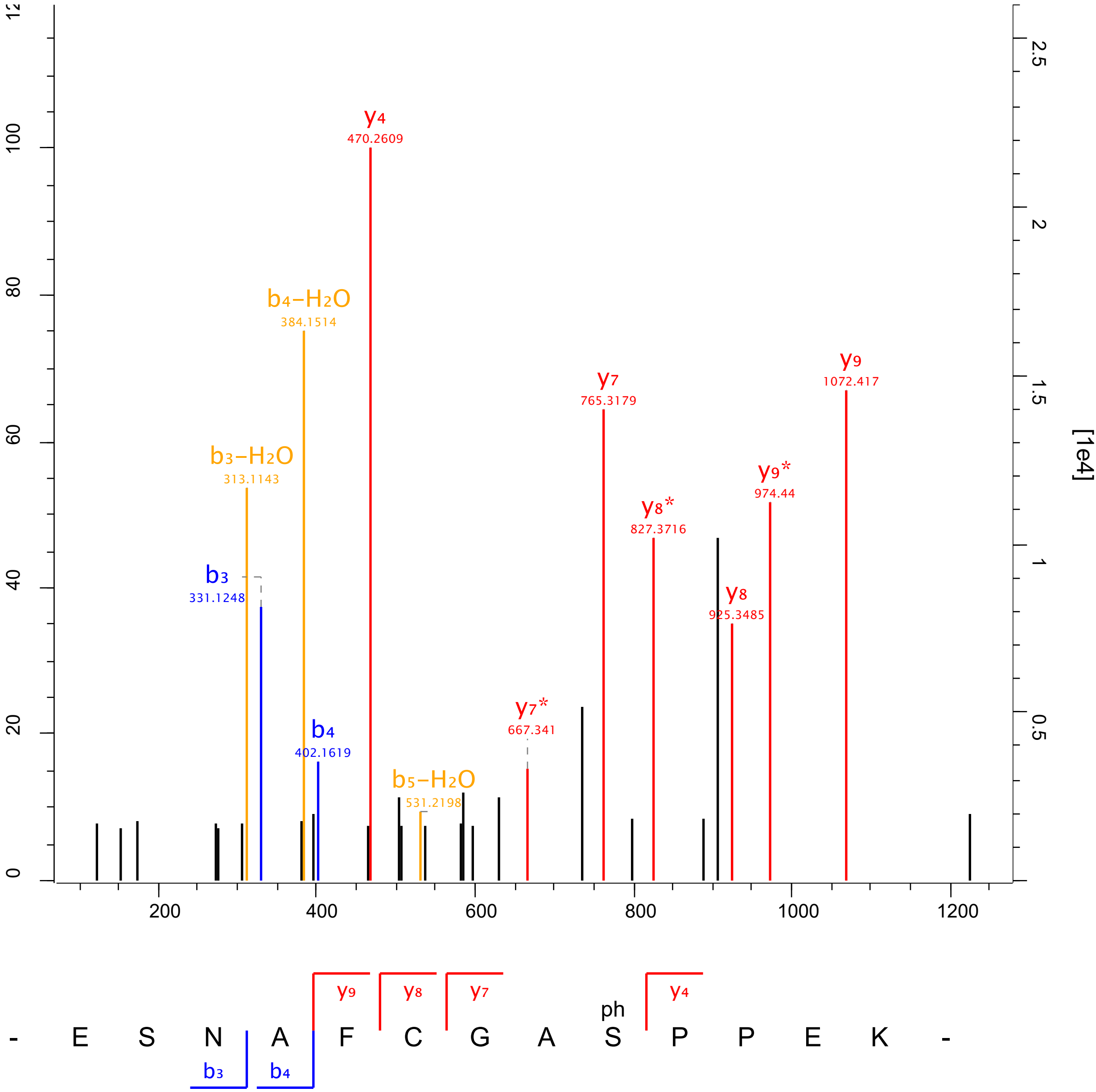

Raw file	Scan	Method	Score	m/z
sys_15_1	12918	FTMS; HCD	40.94	608.8

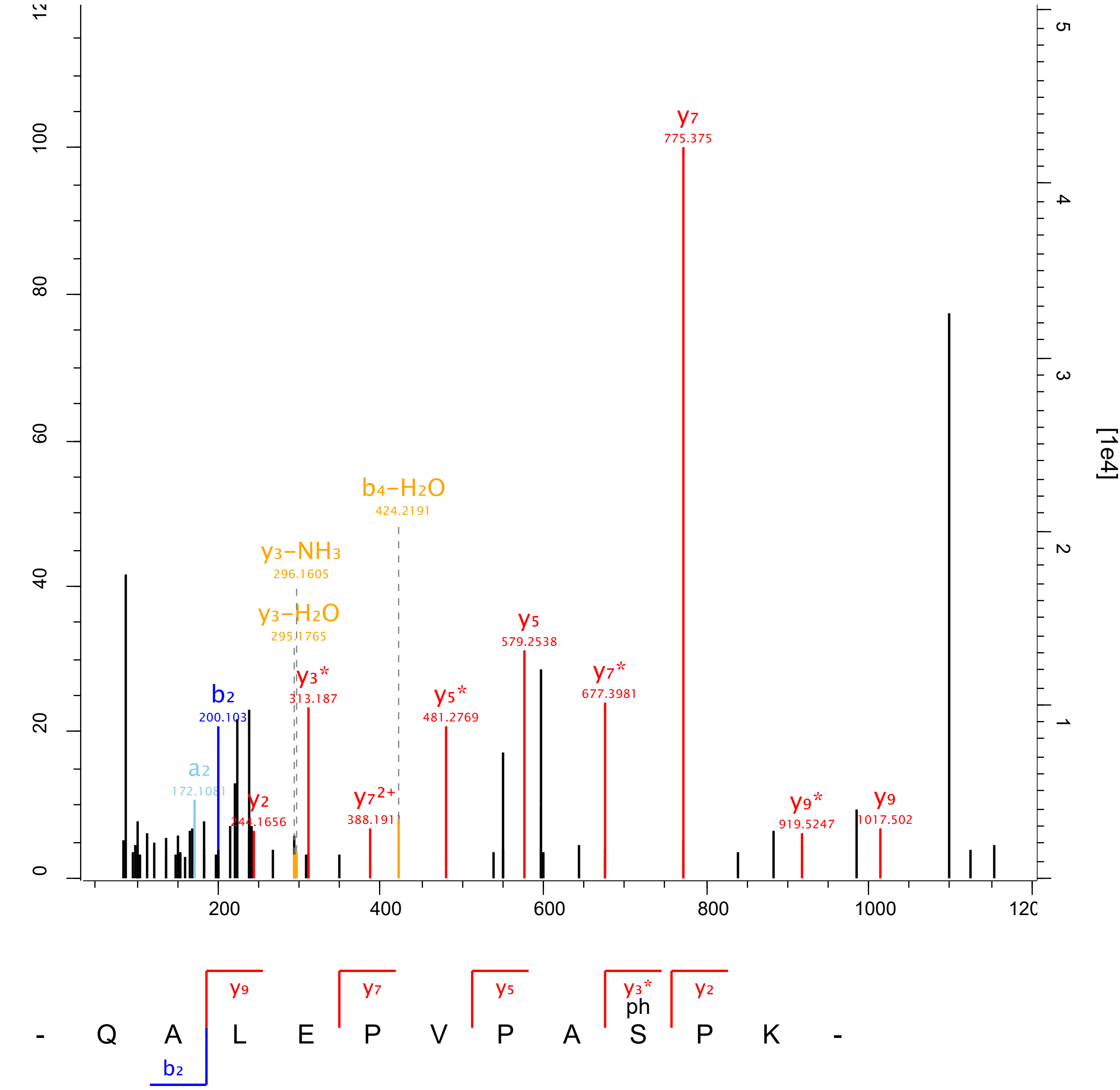

Raw file	Scan	Method	Score	m/z
sys_15_1	12998	FTMS; HCD	146.86	728.78

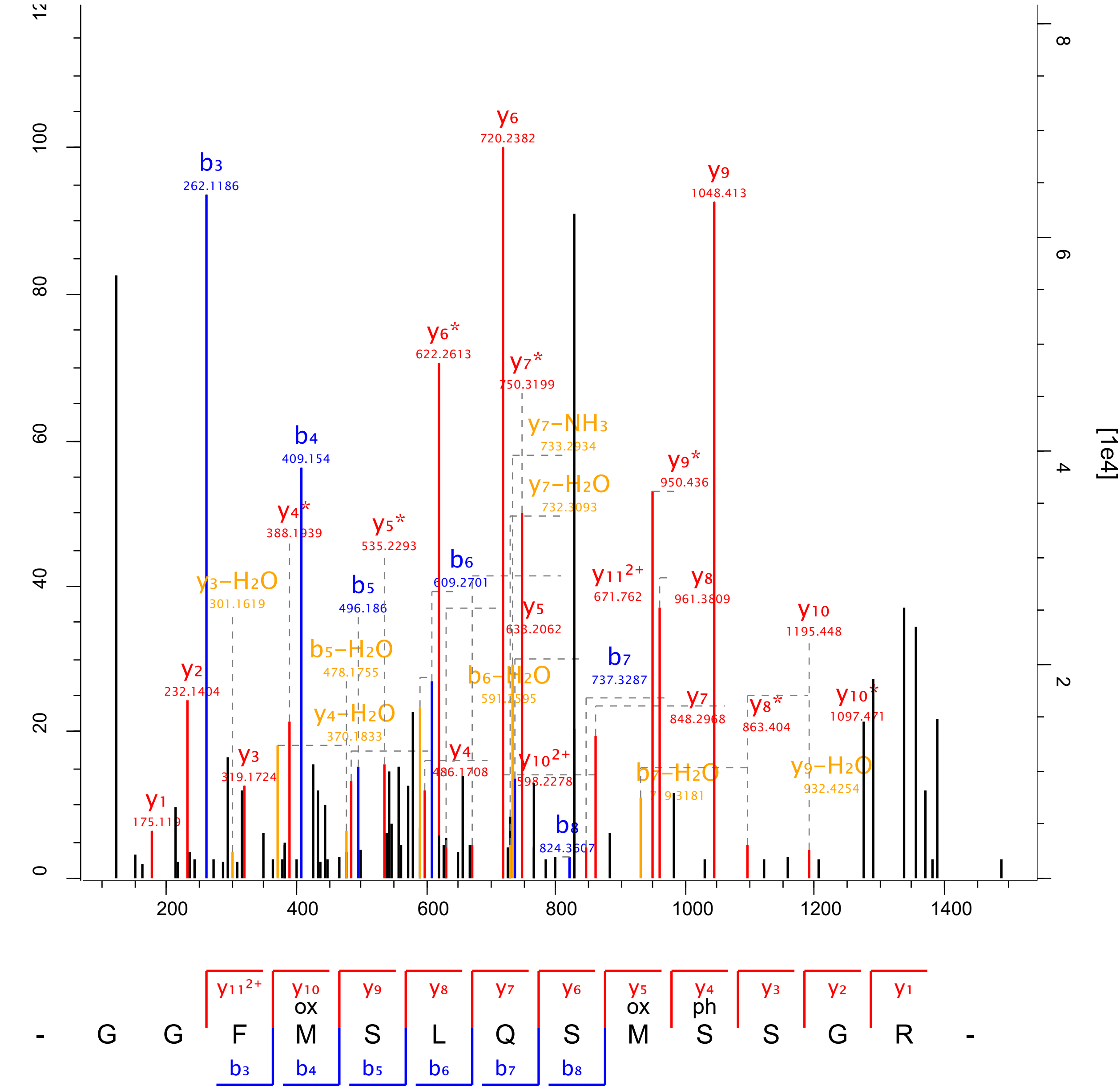

Raw file	Scan	Method	Score	m/z
sys_15_1	13068	FTMS; HCD	79.66	418.7

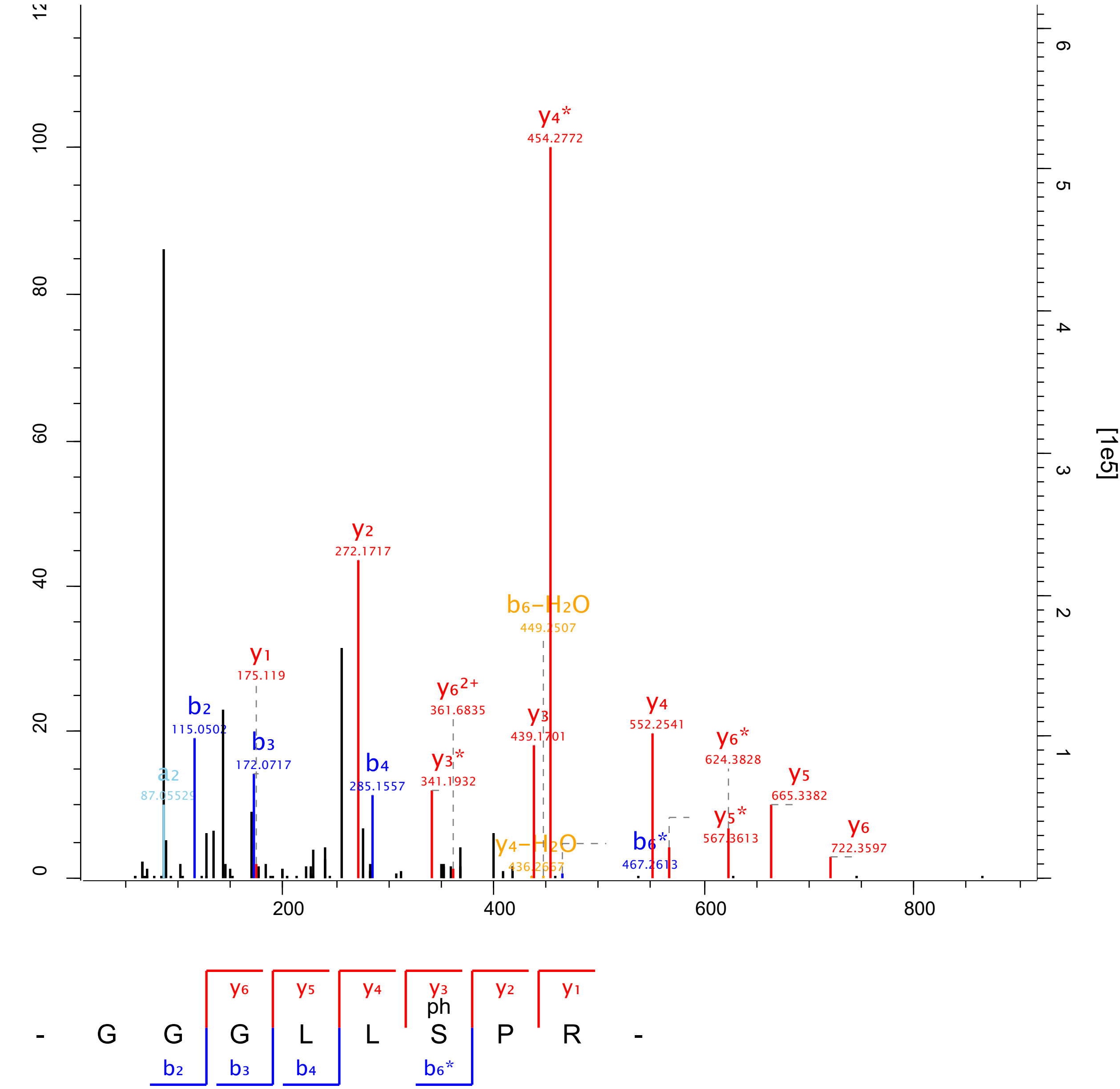

Raw file	Scan	Method	Score	m/z
sys_15_1	13098	FTMS; HCD	45.92	830.36

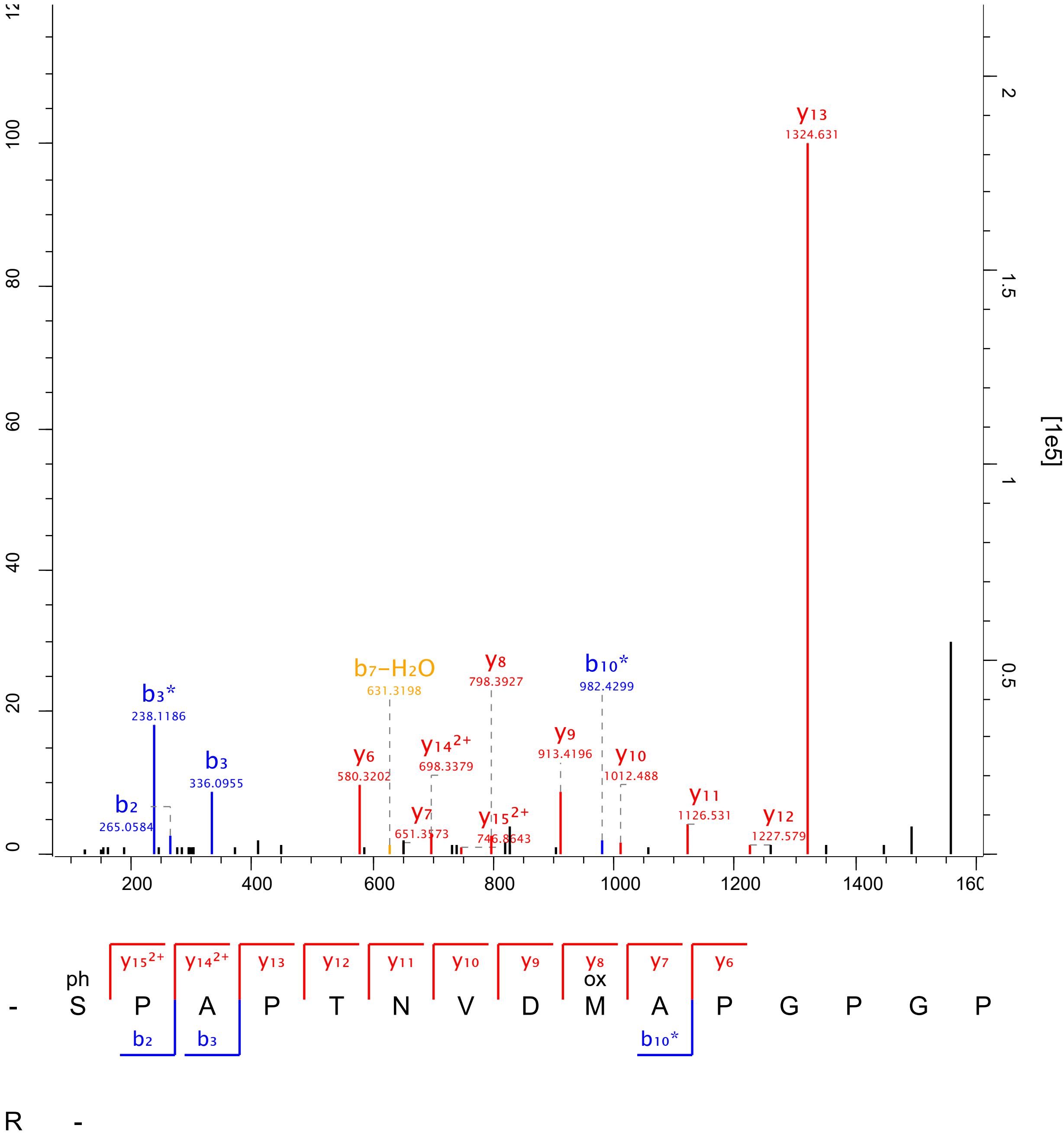

Raw file	Scan	Method	Score	m/z
sys_15_1	13100	FTMS; HCD	149.3	724.8

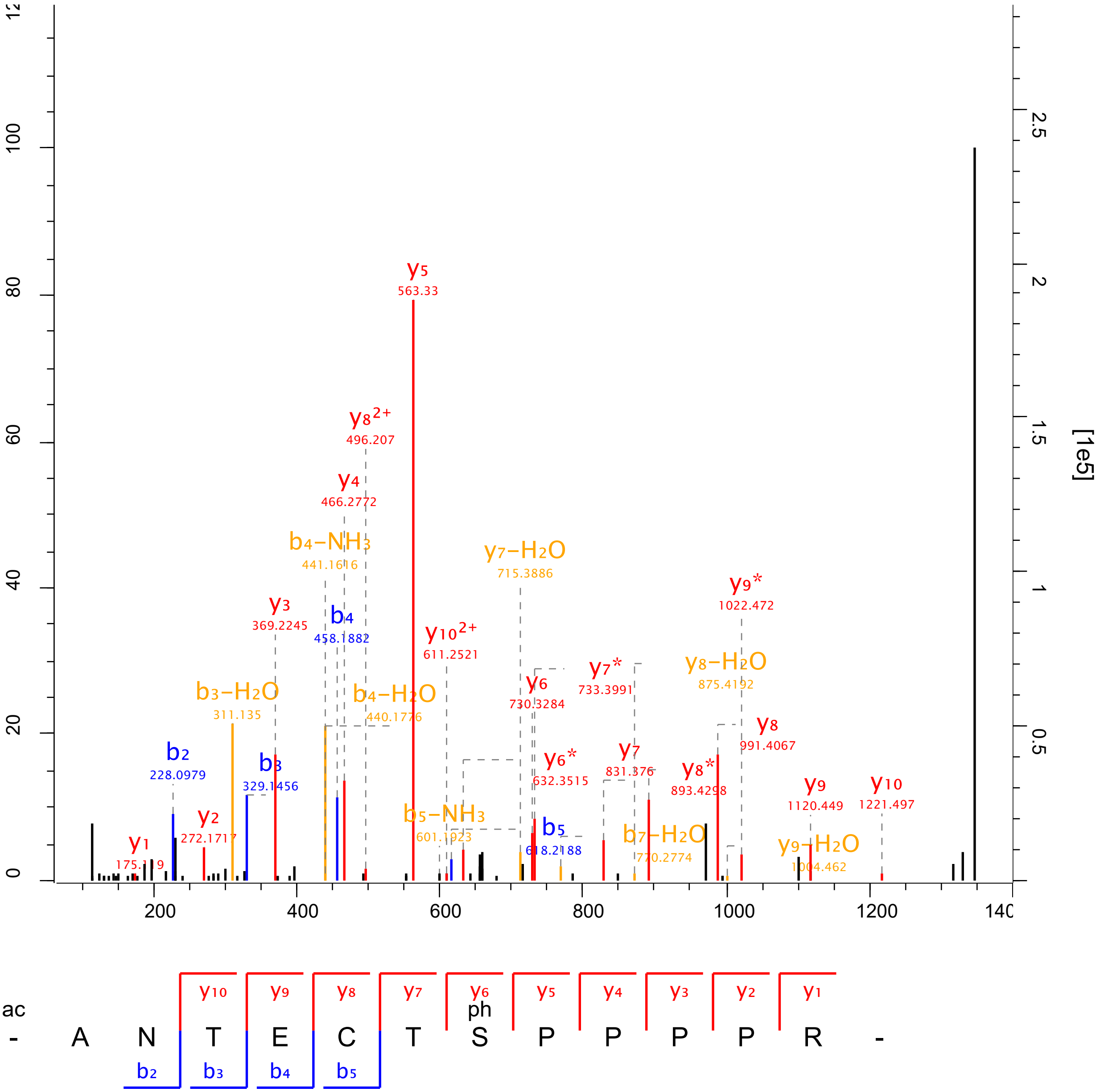

Raw file	Scan	Method	Score	m/z
sys_15_1	13106	FTMS; HCD	104.2	730.82

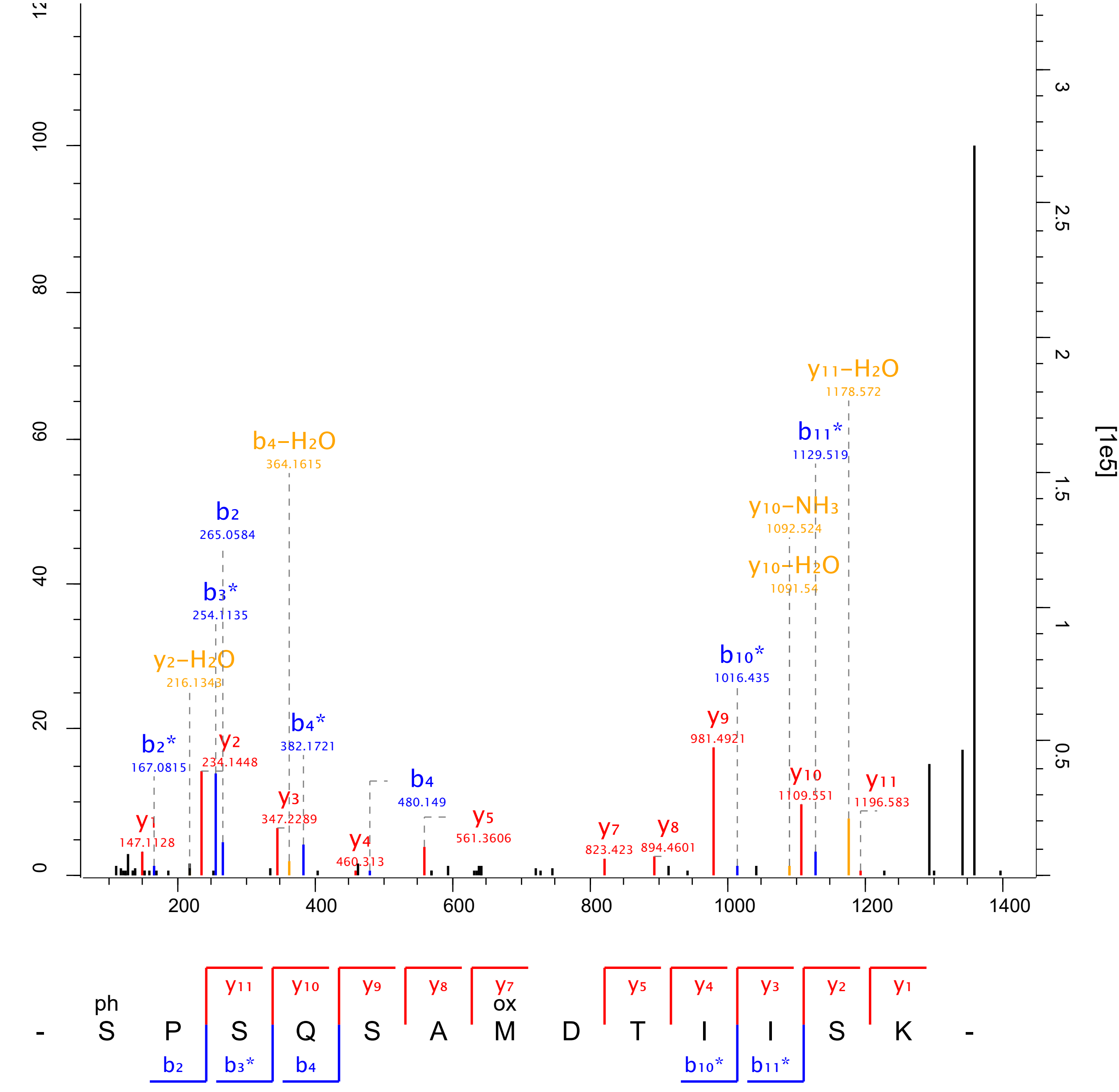

Raw file	Scan	Method	Score	m/z
sys_15_1	13210	FTMS; HCD	55.35	526.21

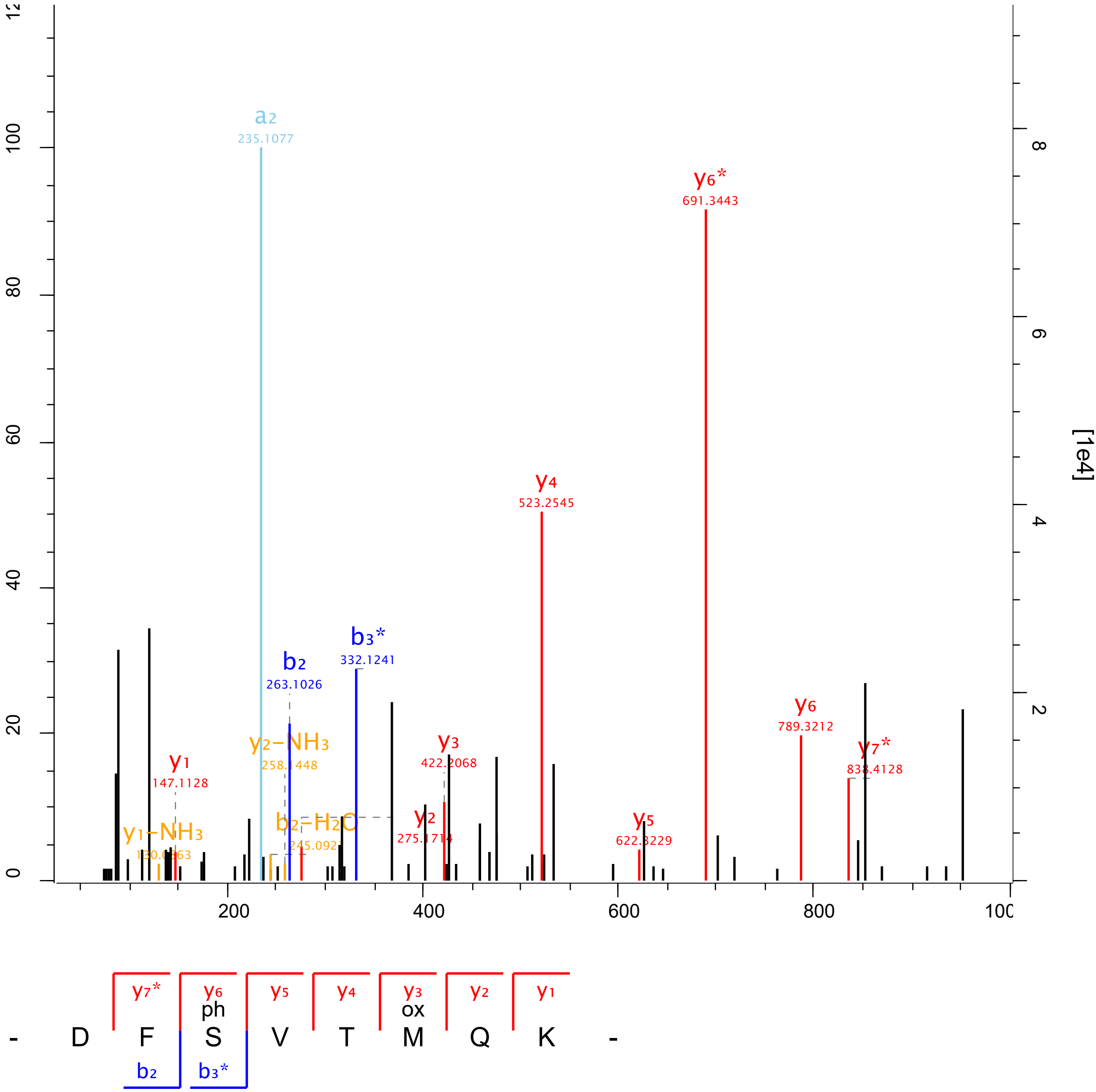

Raw file	Scan	Method	Score	m/z
sys_15_1	13283	FTMS; HCD	179.5	577.28

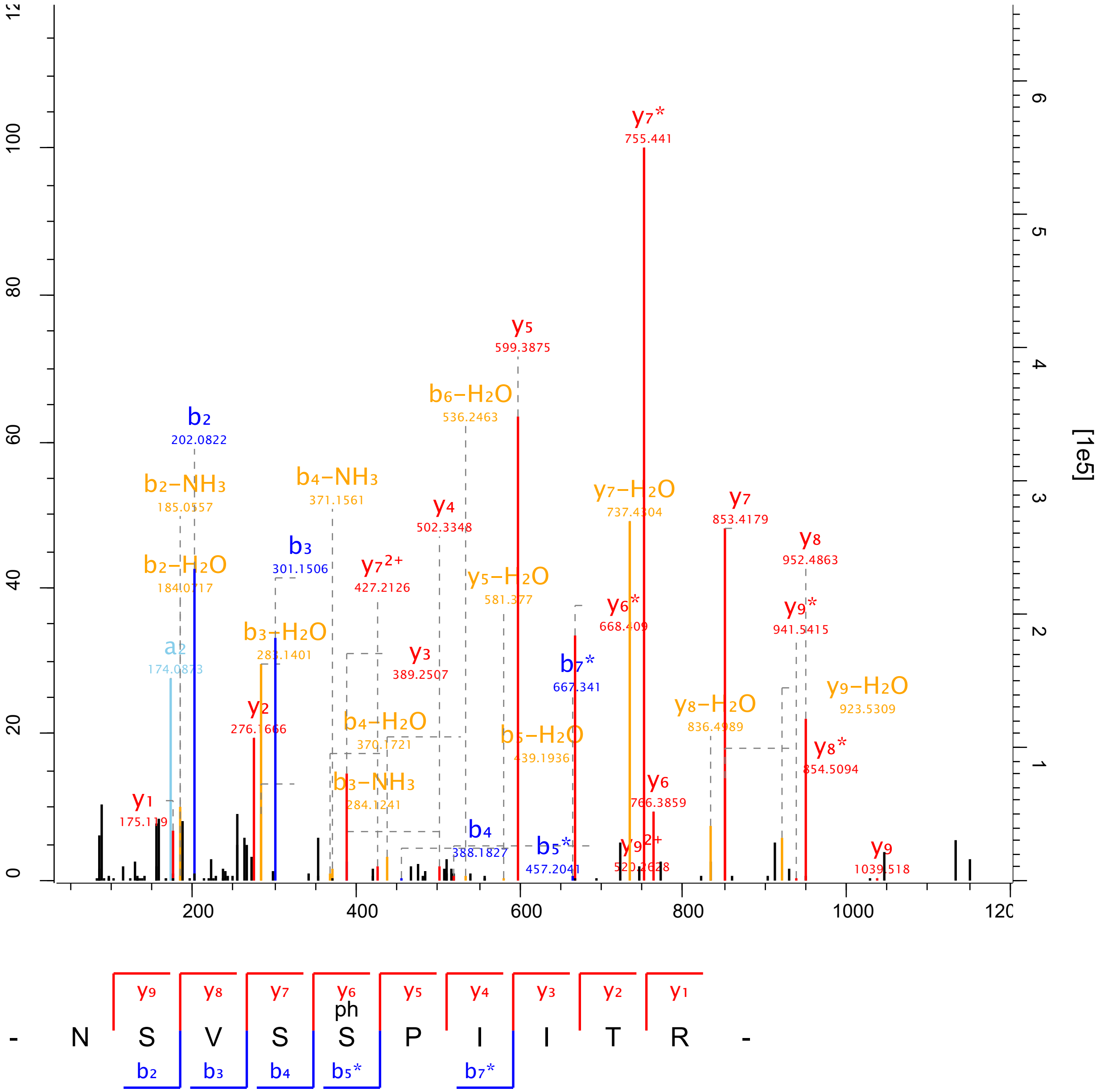

Raw file	Scan	Method	Score	m/z
sys_15_1	13304	FTMS; HCD	100.93	535.25

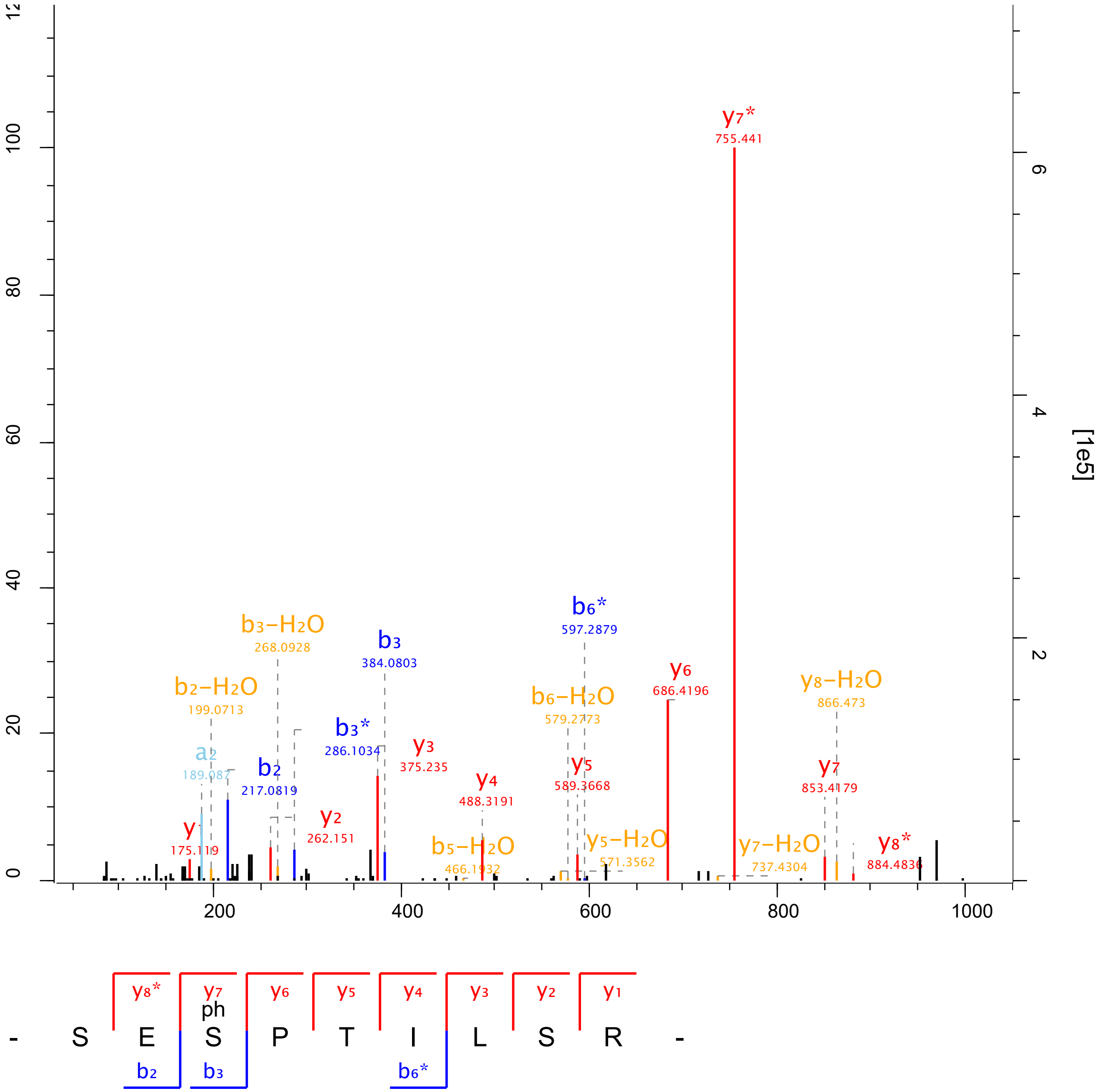

Raw file	Scan	Method	Score	m/z
sys_15_1	13322	FTMS; HCD	146.15	721.27

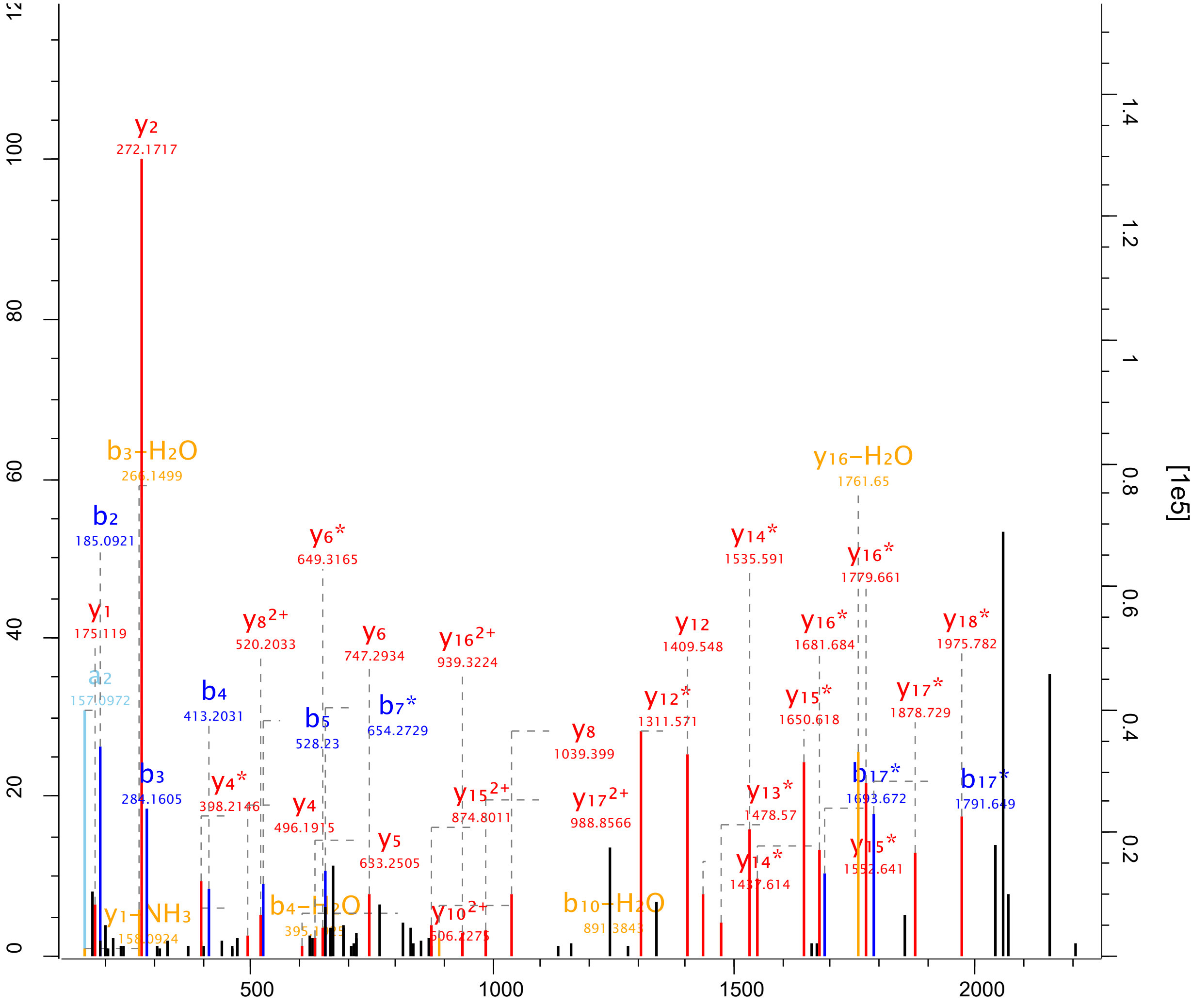

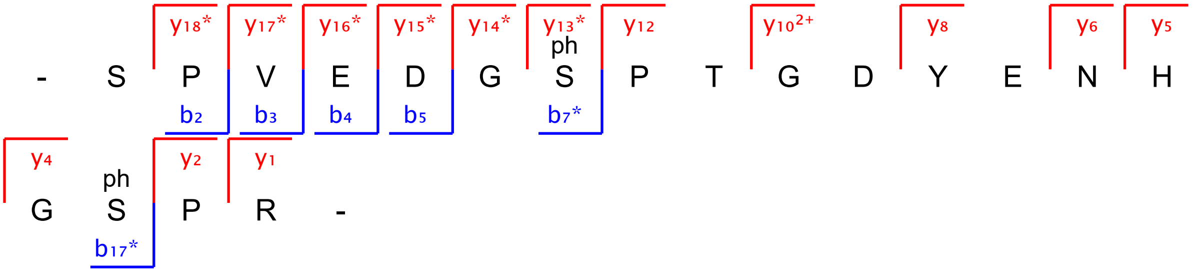

Raw file	Scan	Method	Score	m/z
sys_15_1	13388	FTMS; HCD	92.38	683.29

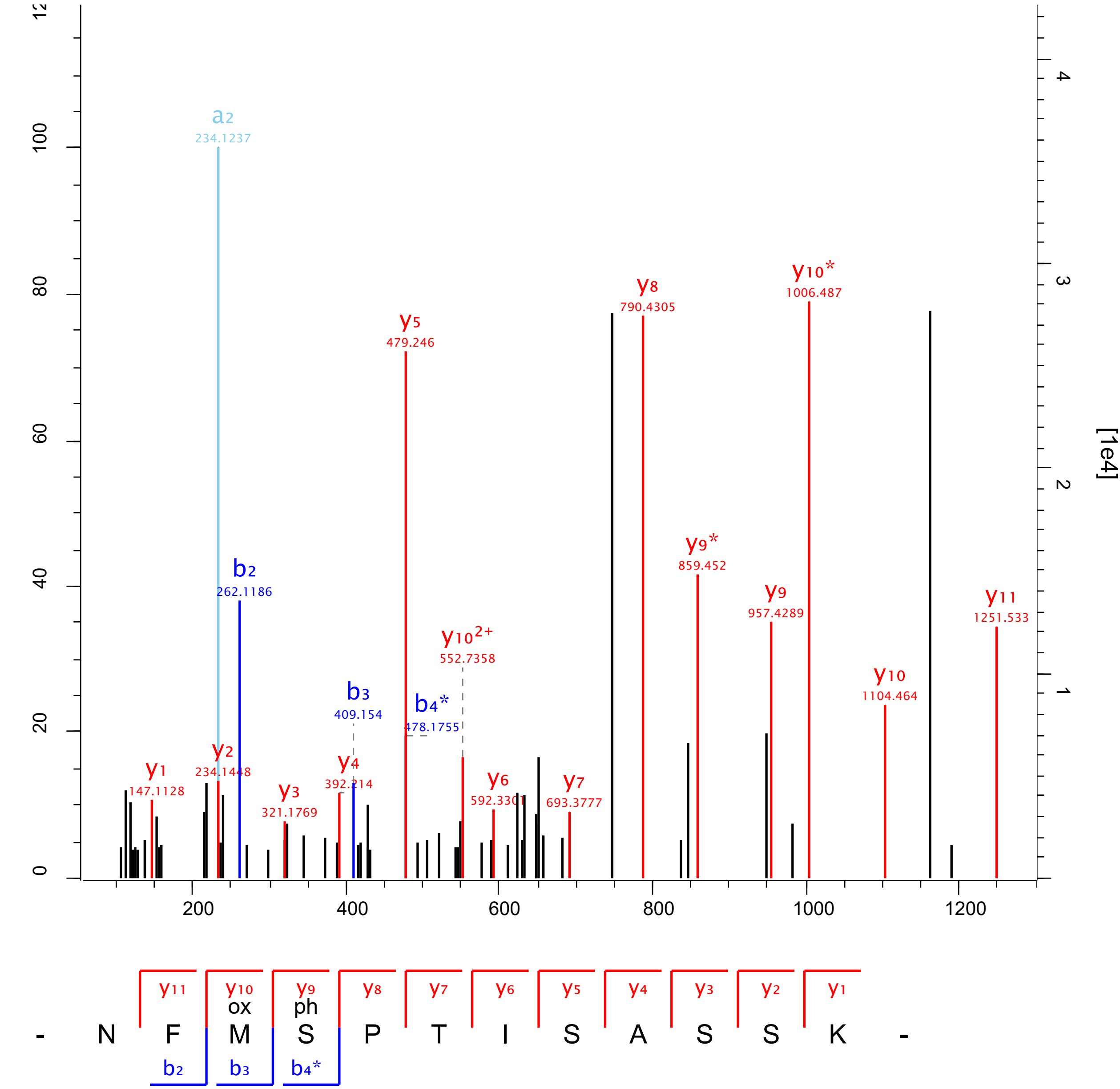

Raw file	Scan	Method	Score	m/z
sys_15_1	13479	FTMS; HCD	126.8	898.4

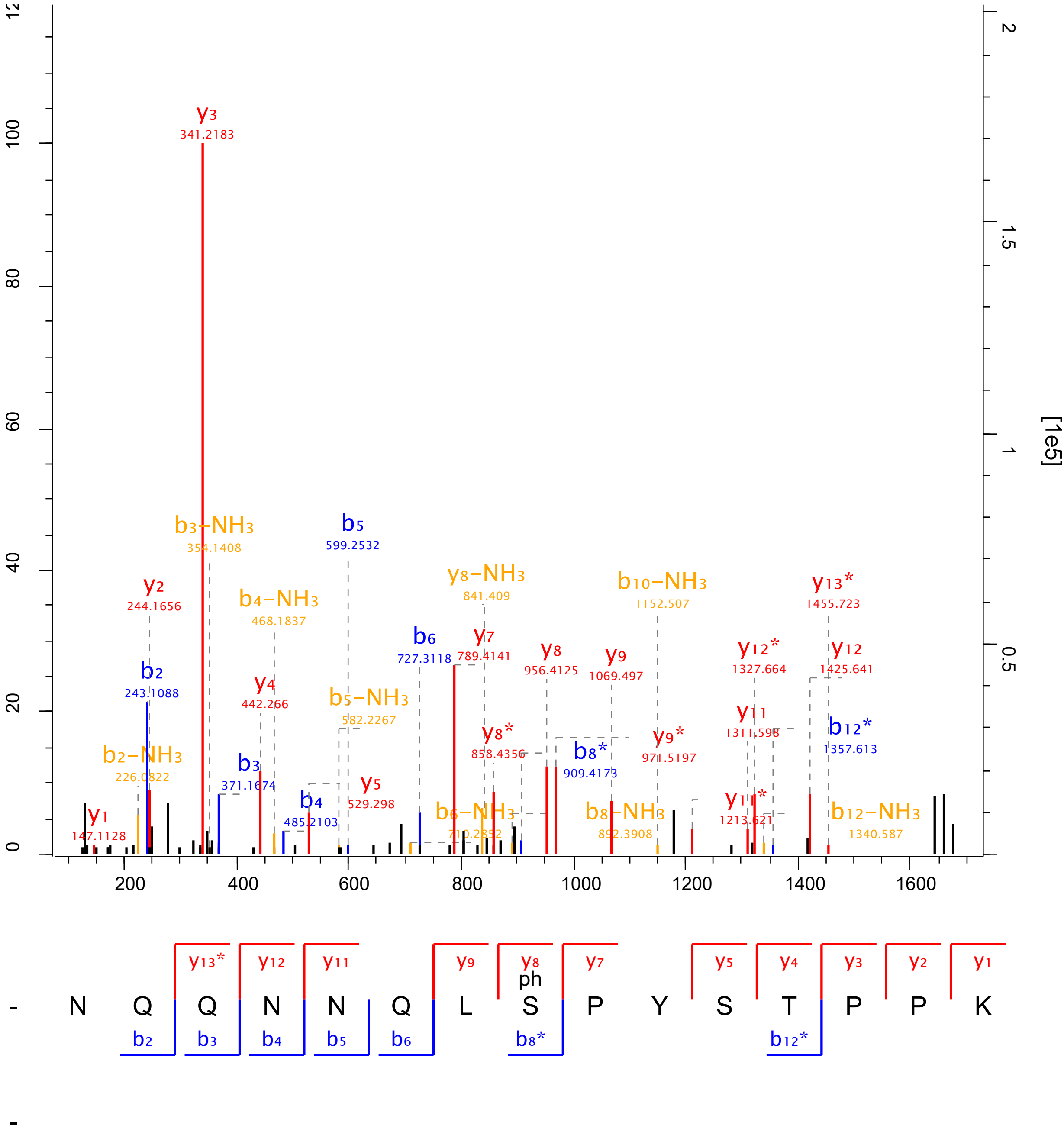

Raw file	Scan	Method	Score	m/z
sys_15_1	13716	FTMS; HCD	50.15	488.23

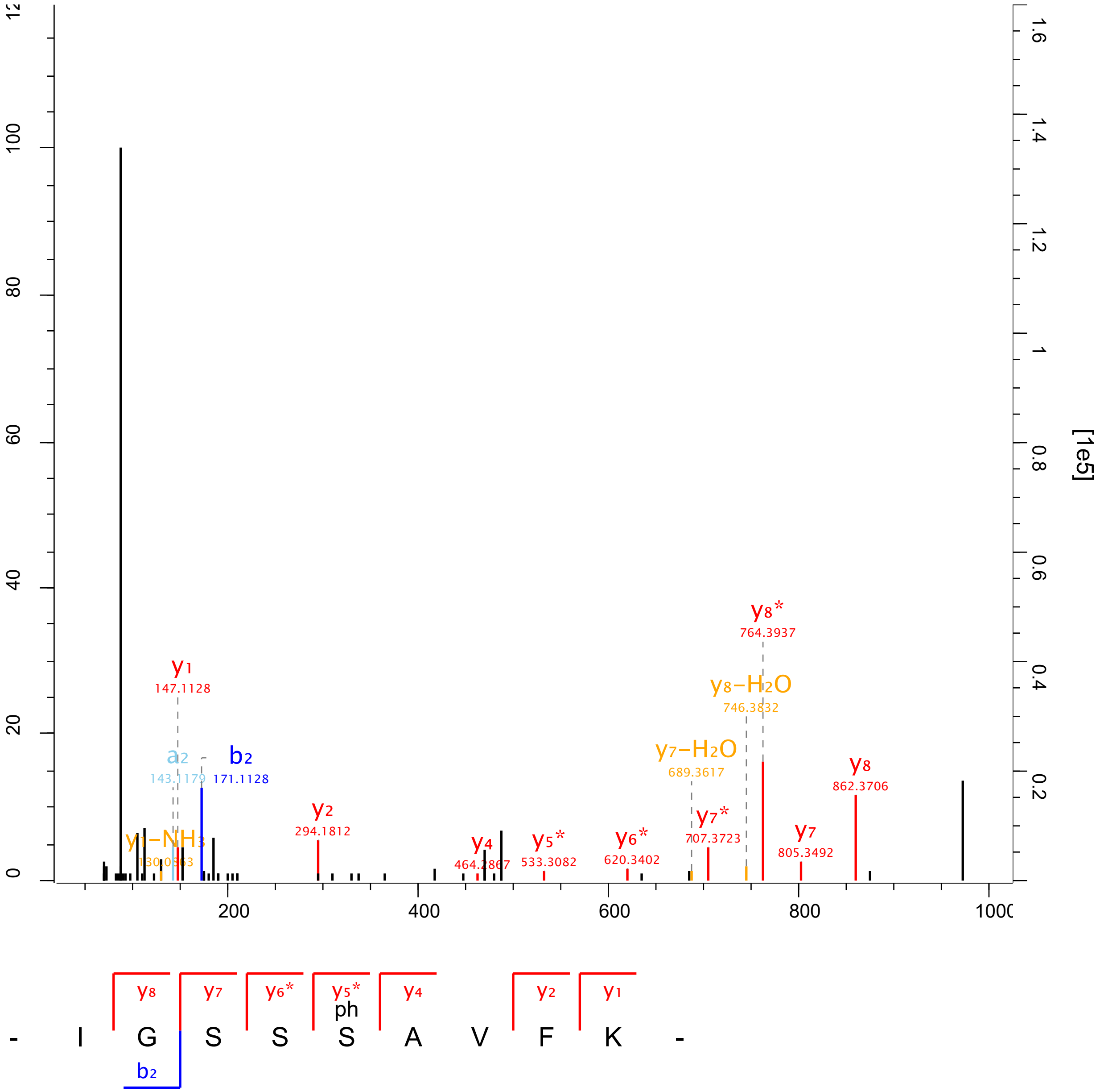

Raw file	Scan	Method	Score	m/z
sys_15_1	13820	FTMS; HCD	144.8	662.24

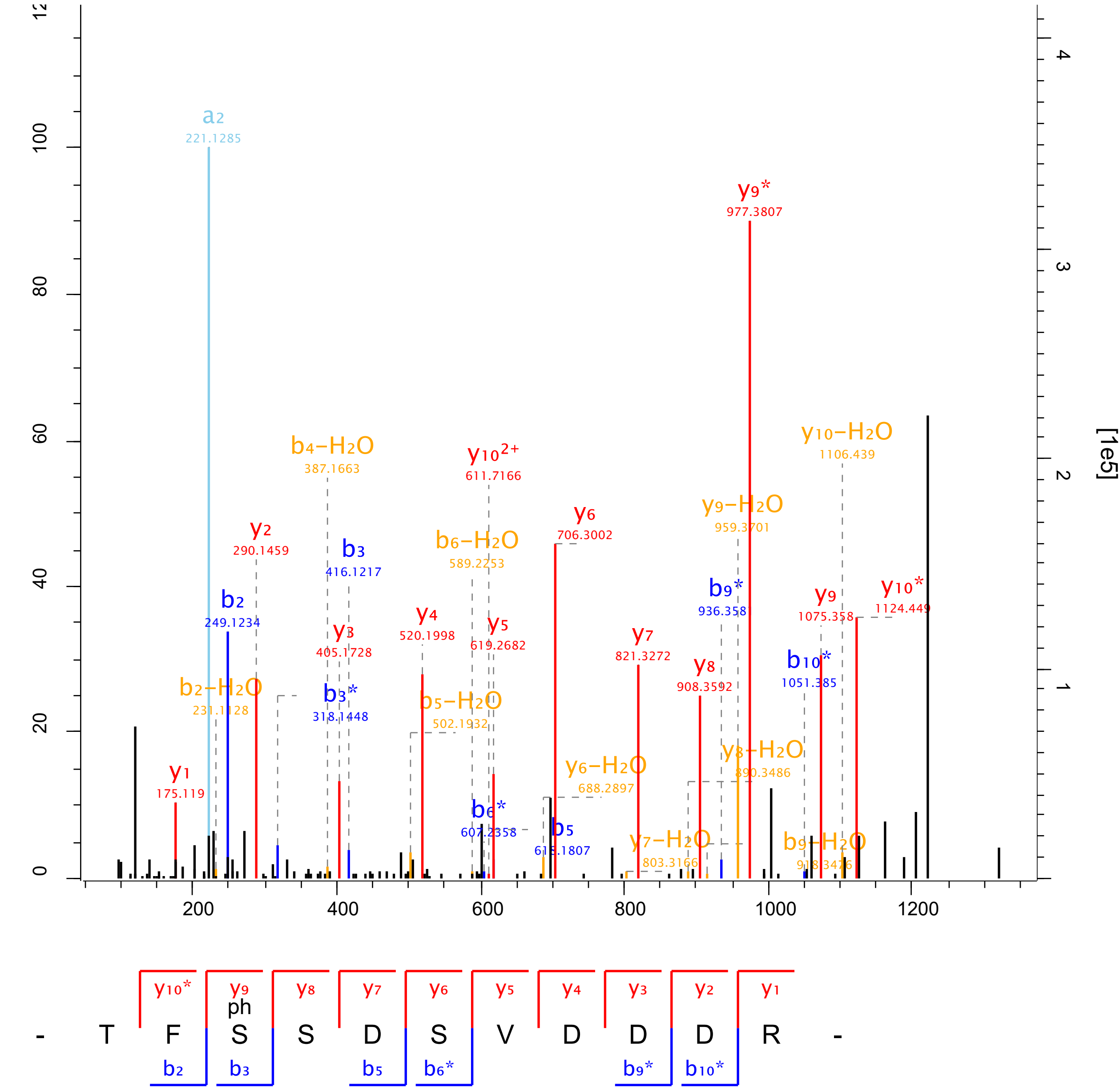

Raw file	Scan	Method	Score	m/z
sys_15_1	13846	FTMS; HCD	97.77	577.27

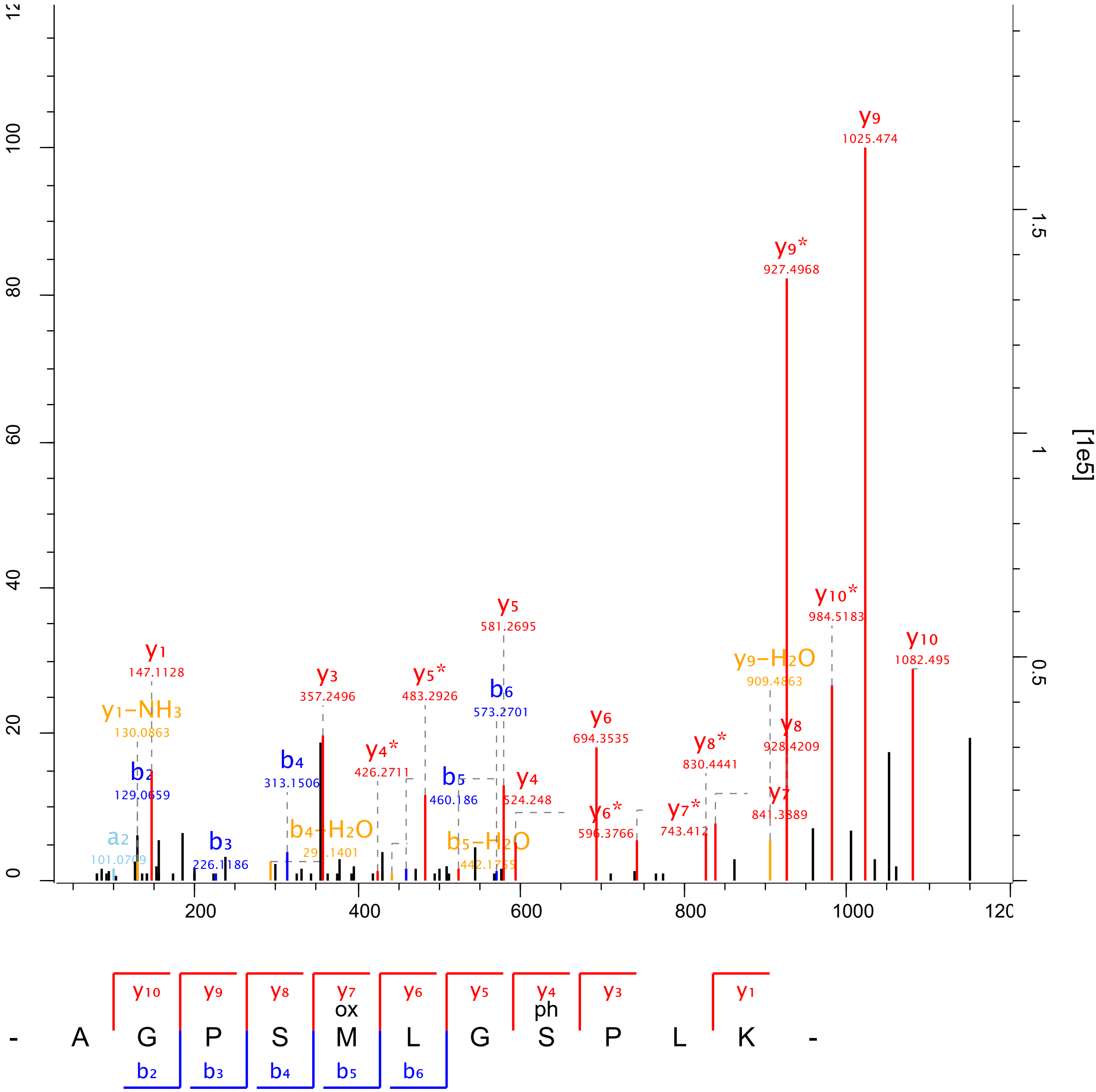

Supplement: Supplementary Figure S2-5 [file 143488_2_supp_337933_ps5hgl.pdf]
