# Supplementary material for: The Systemin Signaling Cascade As Derived from Time Course Analyses of the Systemin-responsive Phosphoproteome
Source: Mol Cell Proteomics. 2019 May 28;18(8):1526–42. doi: 10.1074/mcp.RA119.001367 (PMC6683004; doi:10.1074/mcp.RA119.001367)

Supplementary Figure 2: Representative annotated spectra of identified phosphopeptides under systemin,A17 and water treatment as exported from MaxQuant.

Raw file	Scan	Method	Score	m/z
sys_15_1	13874	FTMS; HCD	139.7	579.76

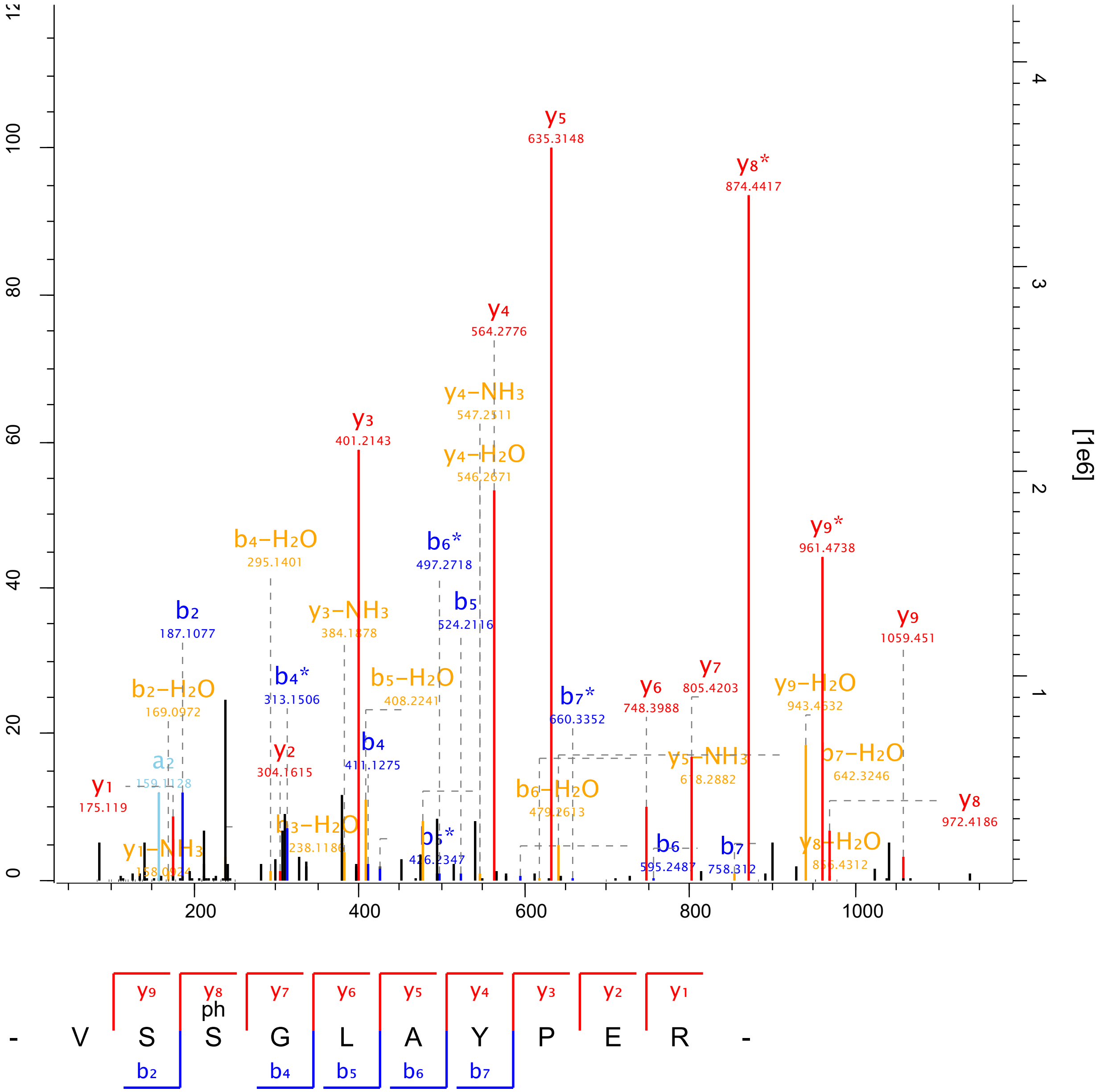

Raw file	Scan	Method	Score	m/z
sys_15_1	13875	FTMS; HCD	325.18	946.36

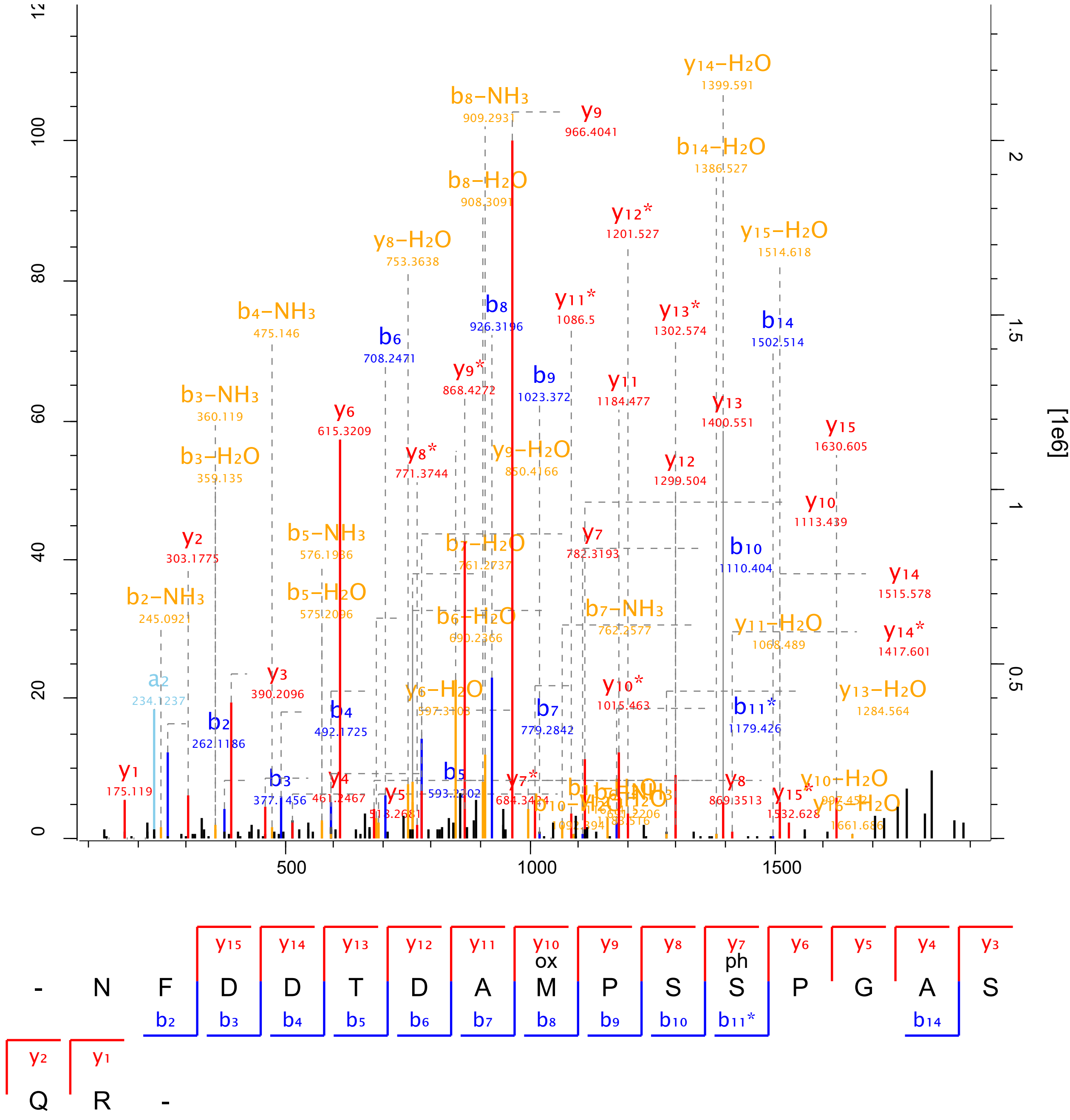

Raw file	Scan	Method	Score	m/z
sys_15_1	13948	FTMS; HCD	50.18	803.33

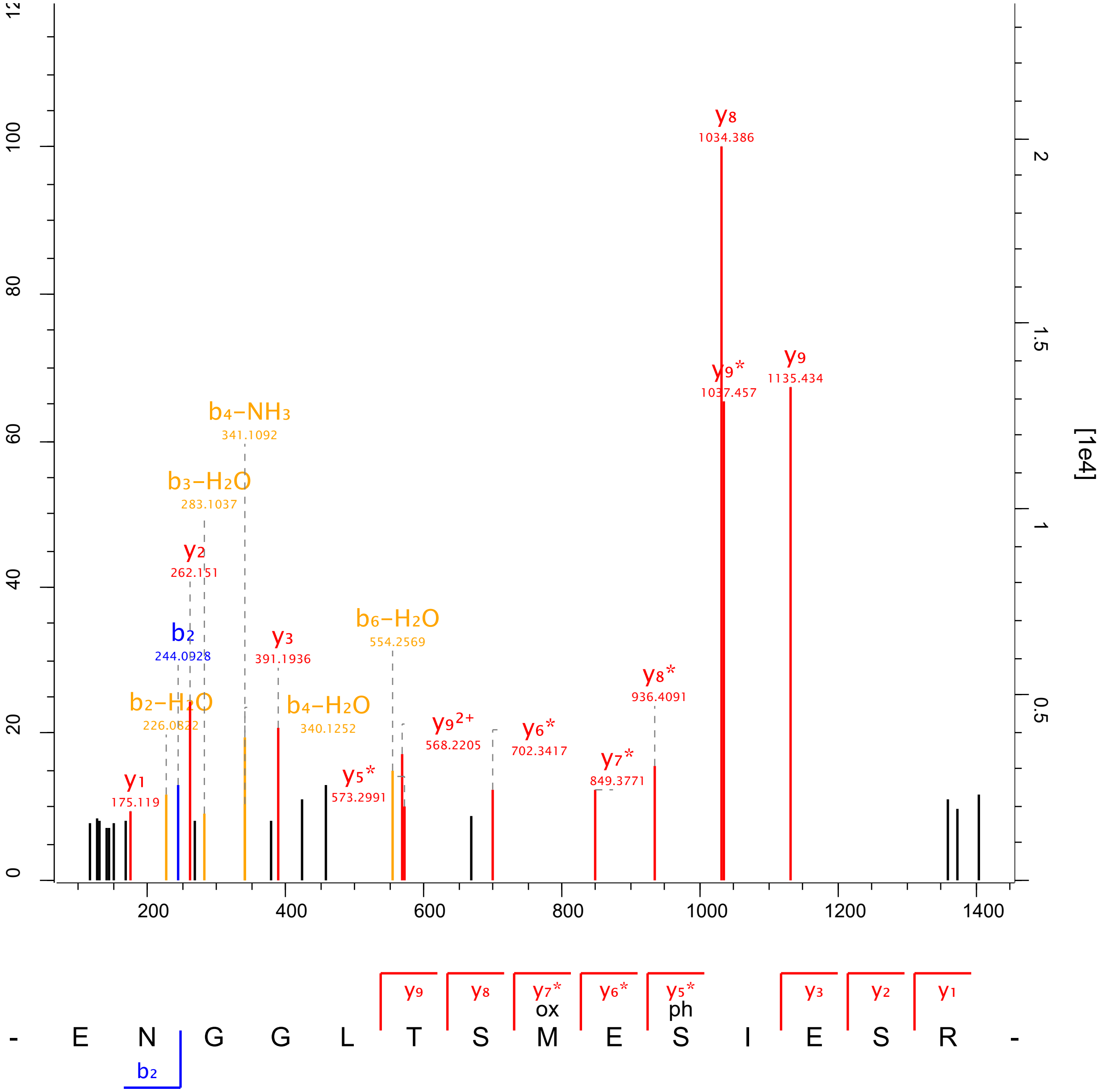

Raw file	Scan	Method	Score	m/z
sys_15_1	14016	FTMS; HCD	45.37	559.69

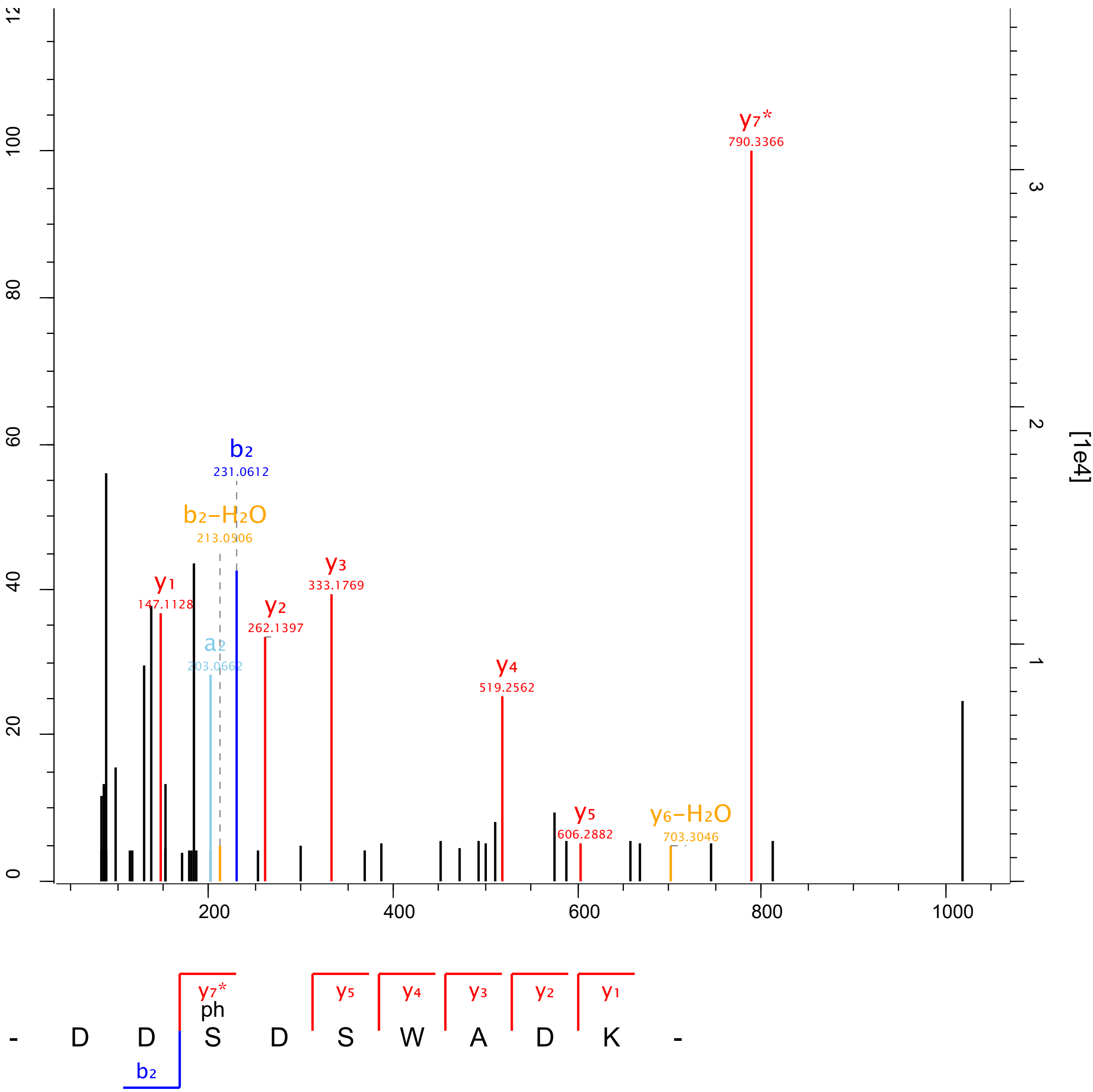

Raw file	Scan	Method	Score	m/z
sys_15_1	14039	FTMS; HCD	95.57	610.79

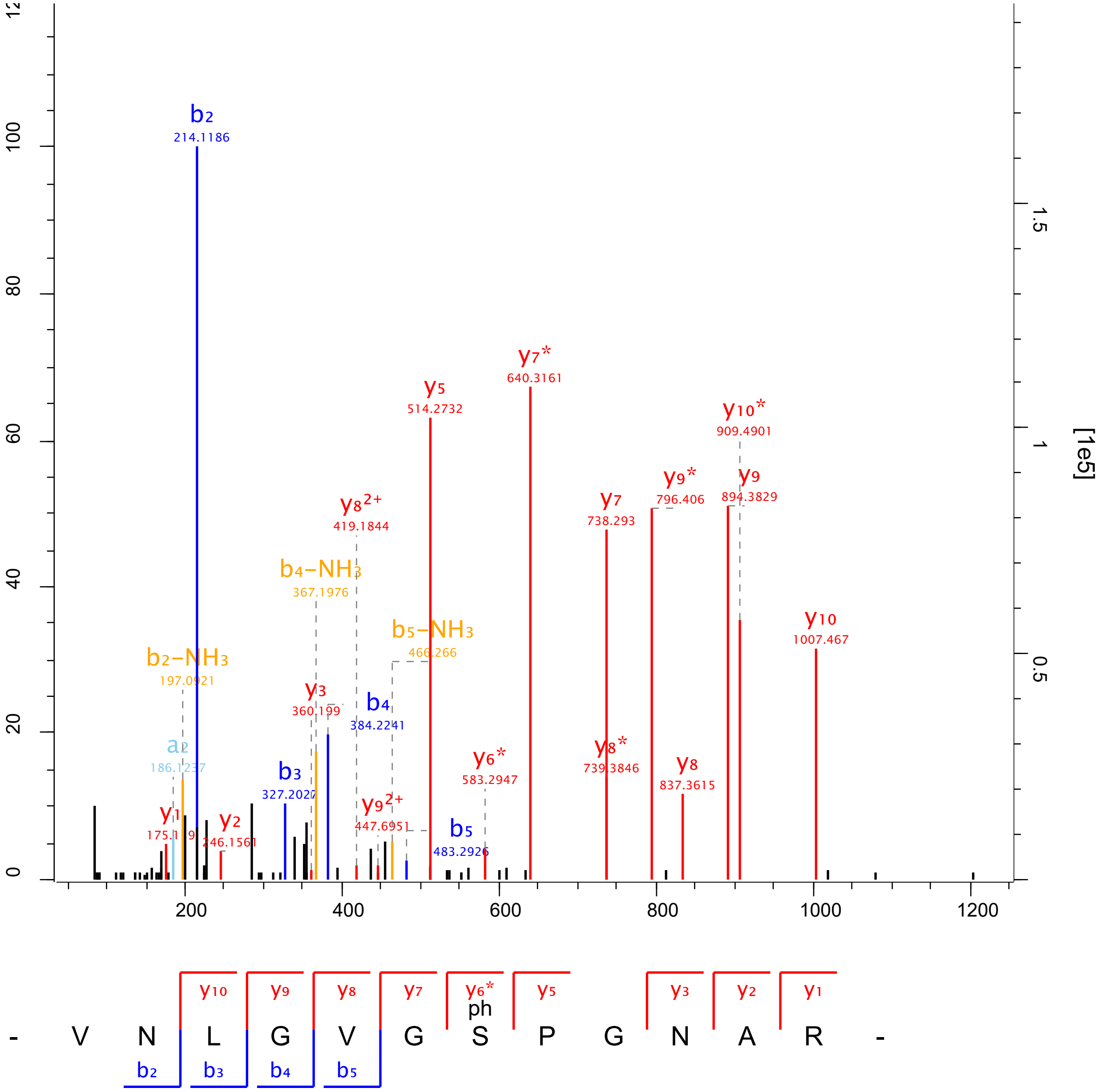

Raw file	Scan	Method	Score	m/z
sys_15_1	14217	FTMS; HCD	136.14	547.27

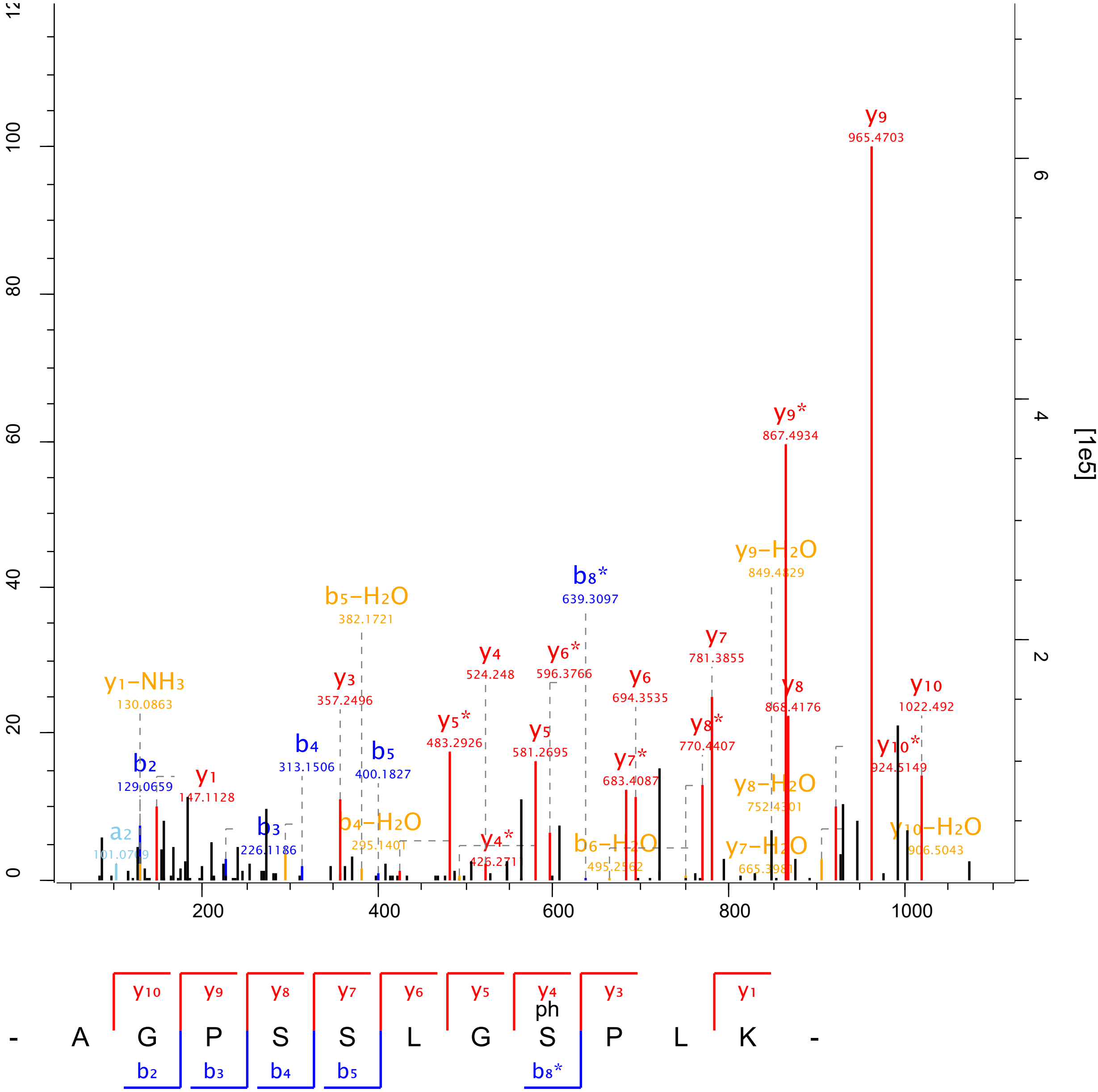

Raw file	Scan	Method	Score	m/z
sys_15_1	14263	FTMS; HCD	248.23	891.87

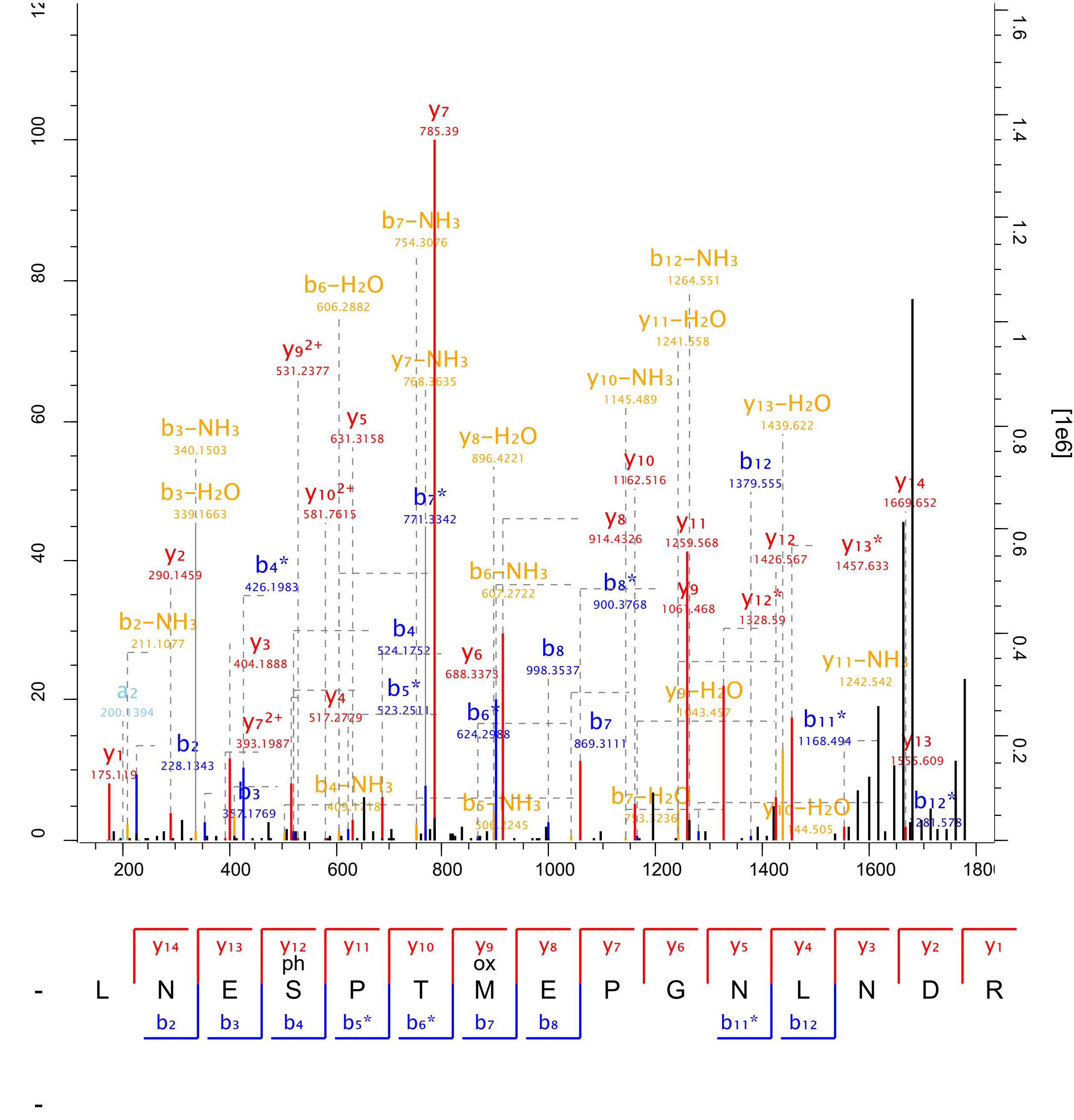

Raw file	Scan	Method	Score	m/z
sys_15_1	14334	FTMS; HCD	136.43	665.28

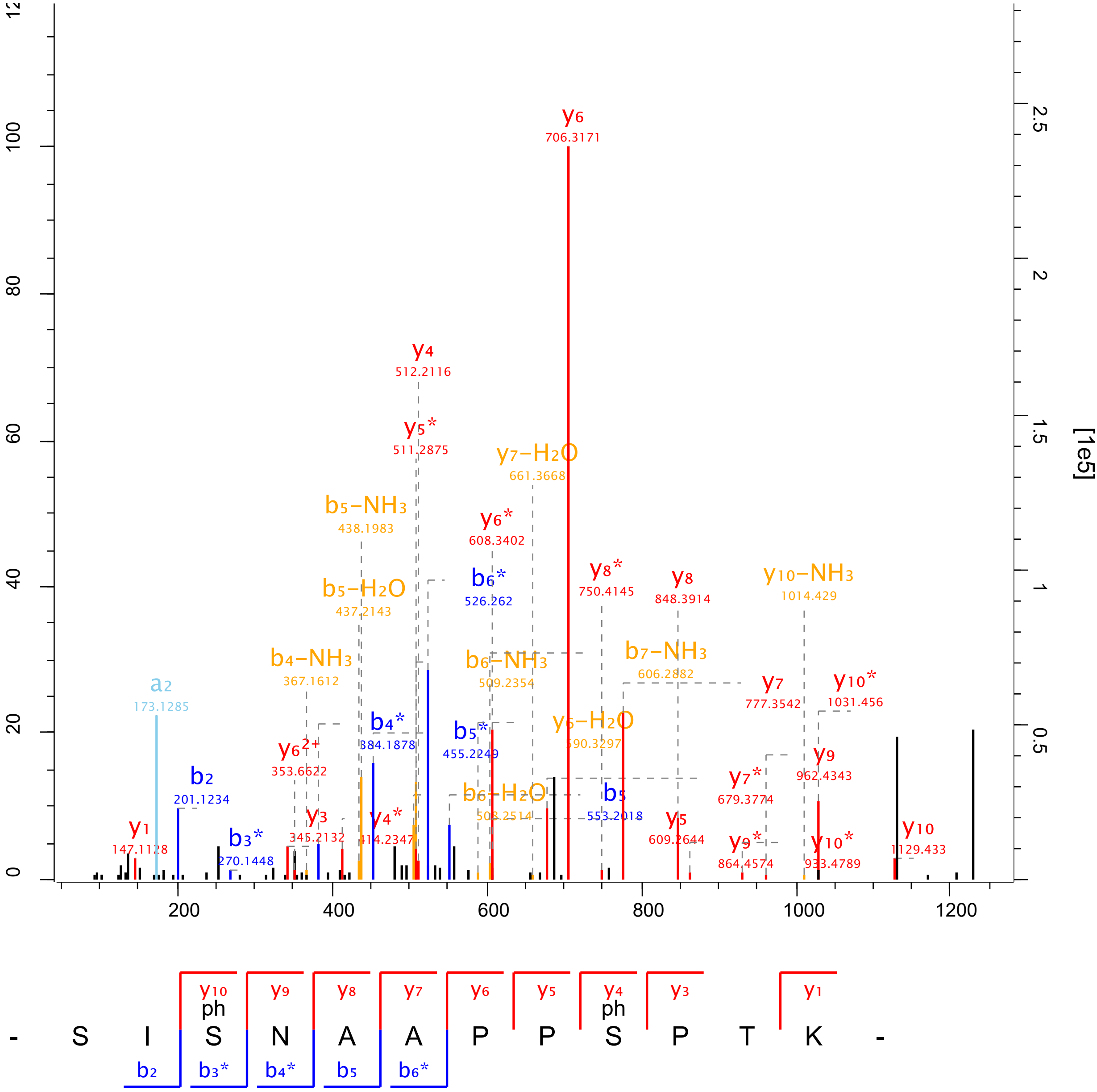

Raw file	Scan	Method	Score	m/z
sys_15_1	14339	FTMS; HCD	167.18	742.28

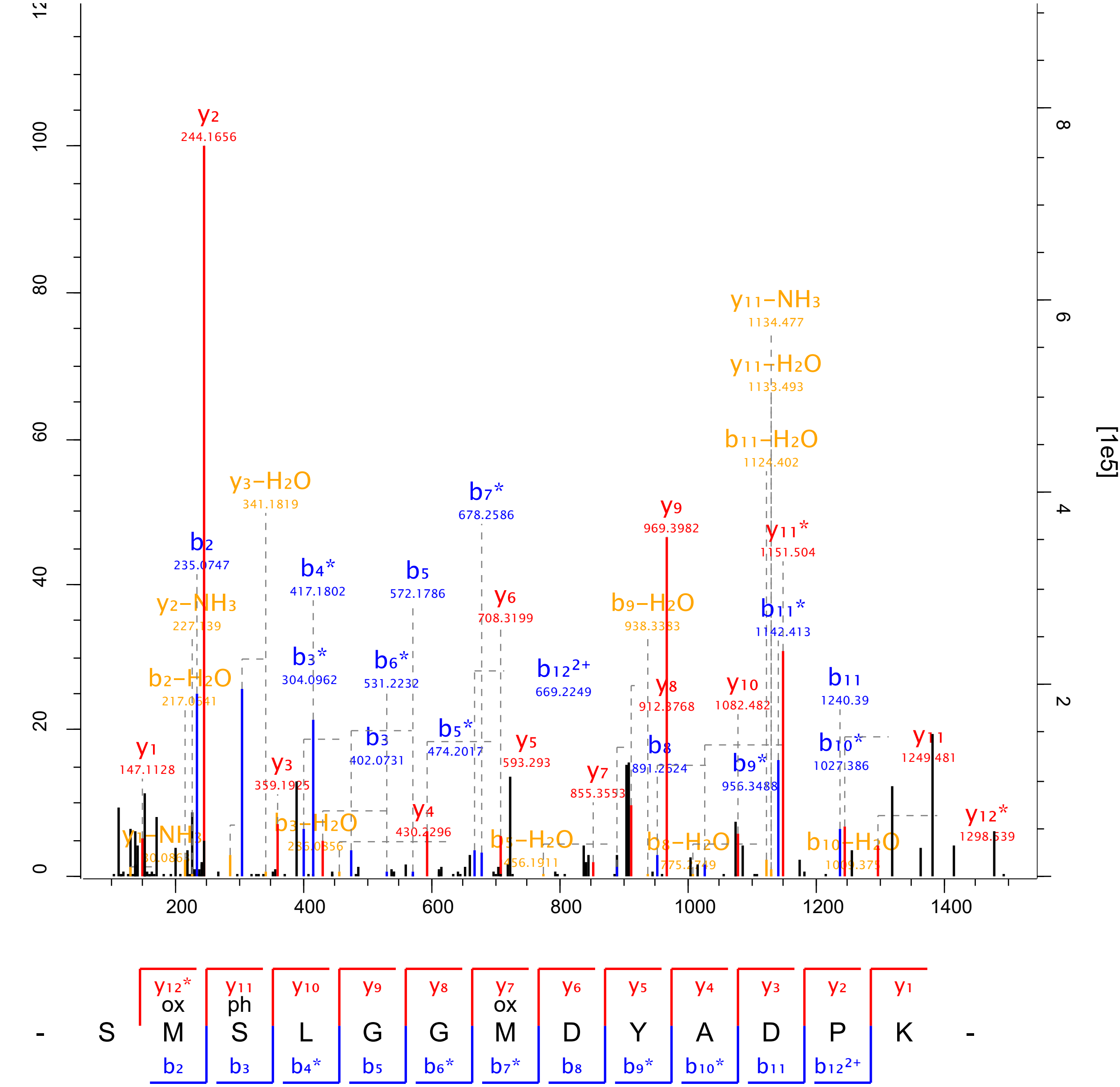

Raw file	Scan	Method	Score	m/z
sys_15_1	14353	FTMS; HCD	267.38	728.82

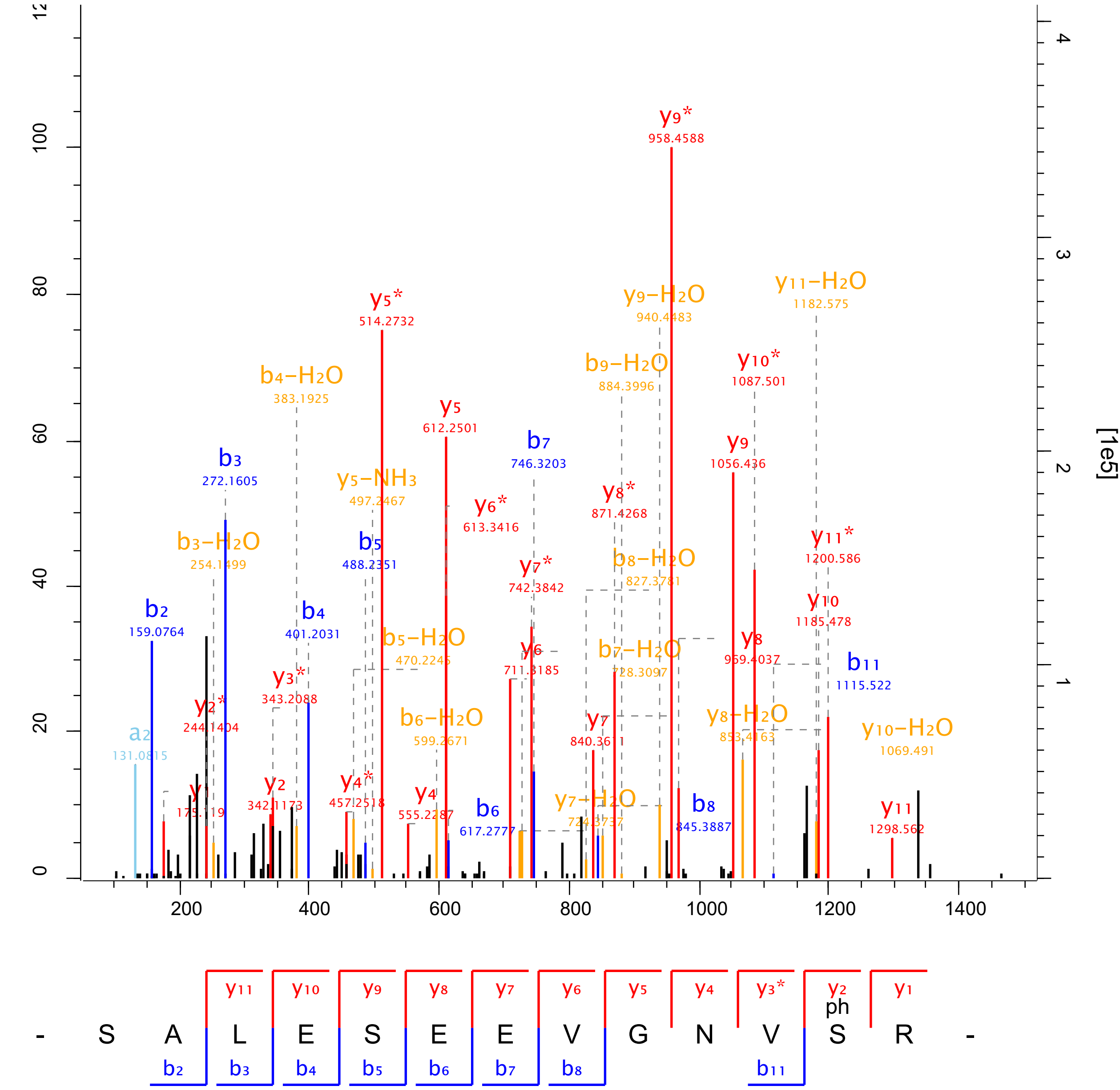

Raw file	Scan	Method	Score	m/z
sys_15_1	14357	FTMS; HCD	77.22	816.8

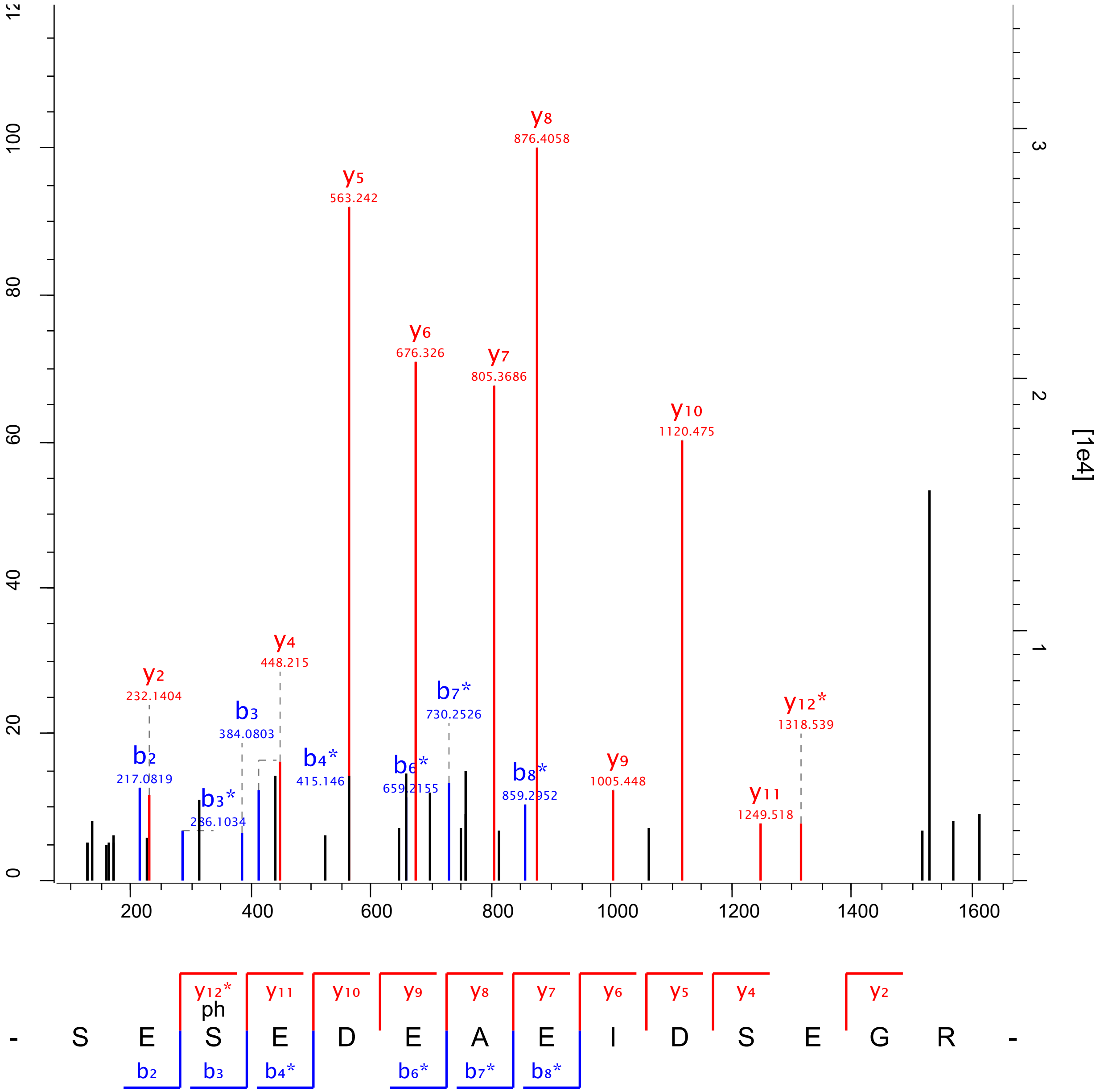

Raw file	Scan	Method	Score	m/z
sys_15_1	14392	FTMS; HCD	72.9	755.29

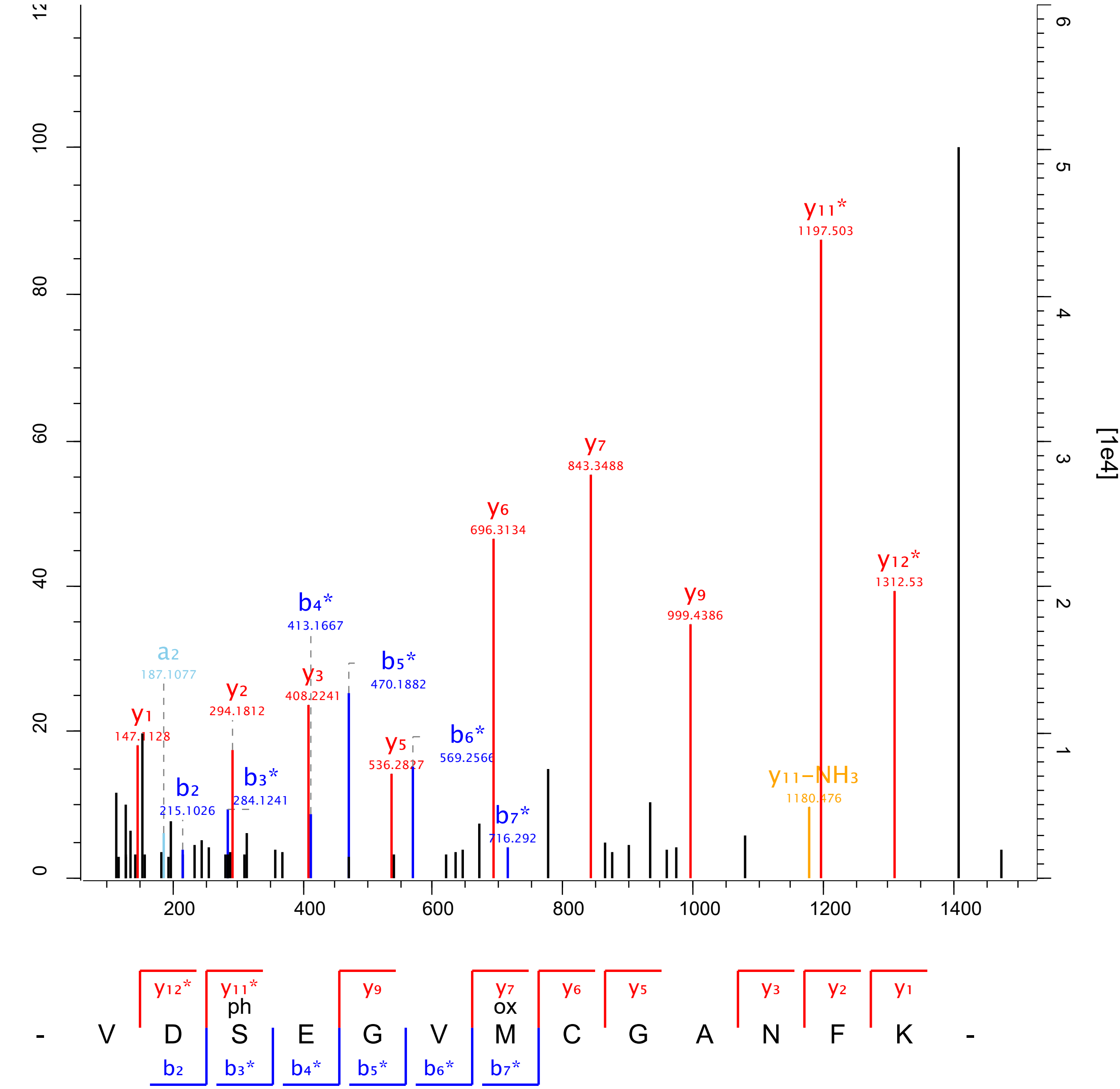

Raw file	Scan	Method	Score	m/z
sys_15_1	14453	FTMS; HCD	237.5	928.39

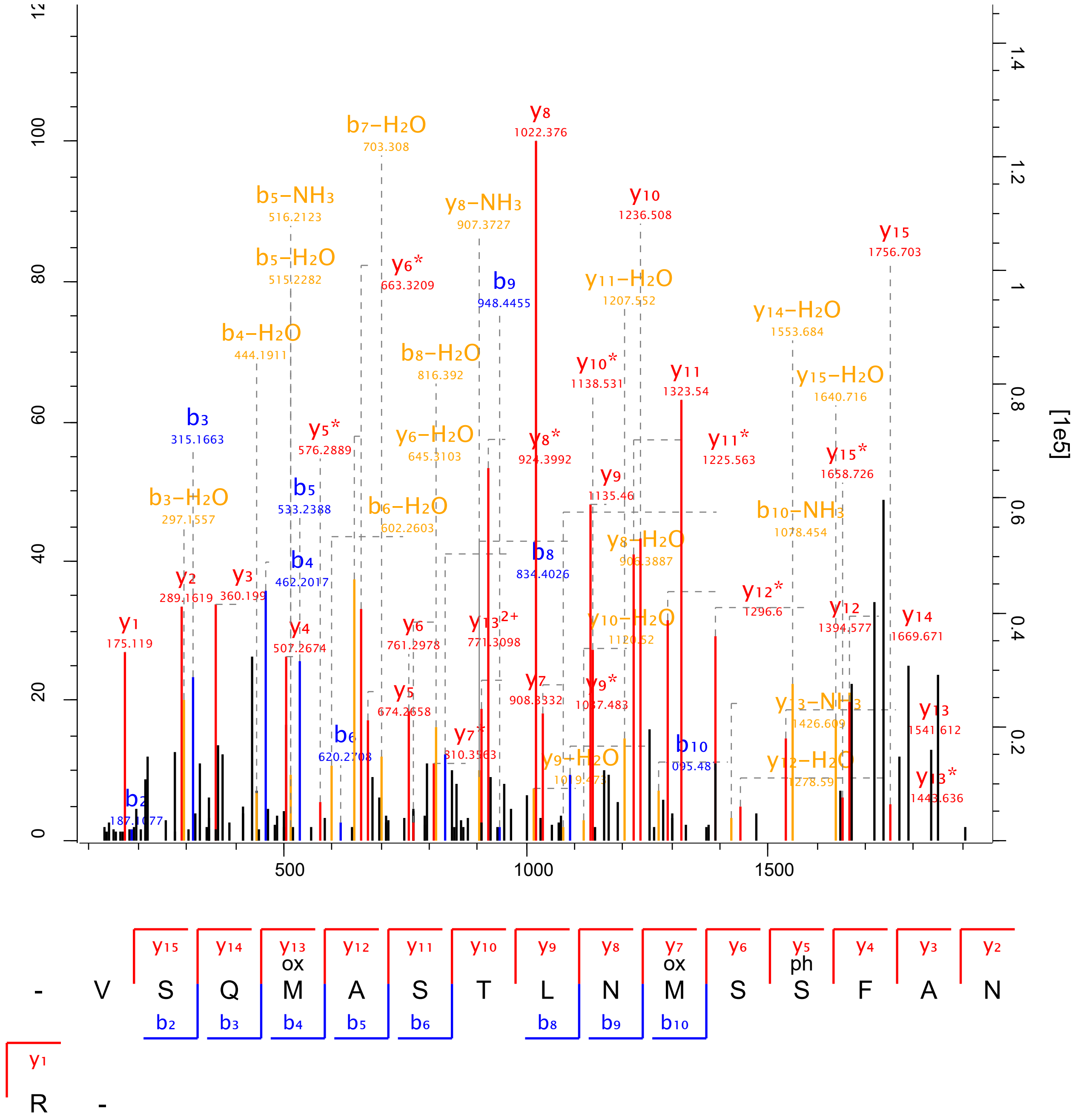

Raw file	Scan	Method	Score	m/z
sys_15_1	14462	FTMS; HCD	119.39	556.75

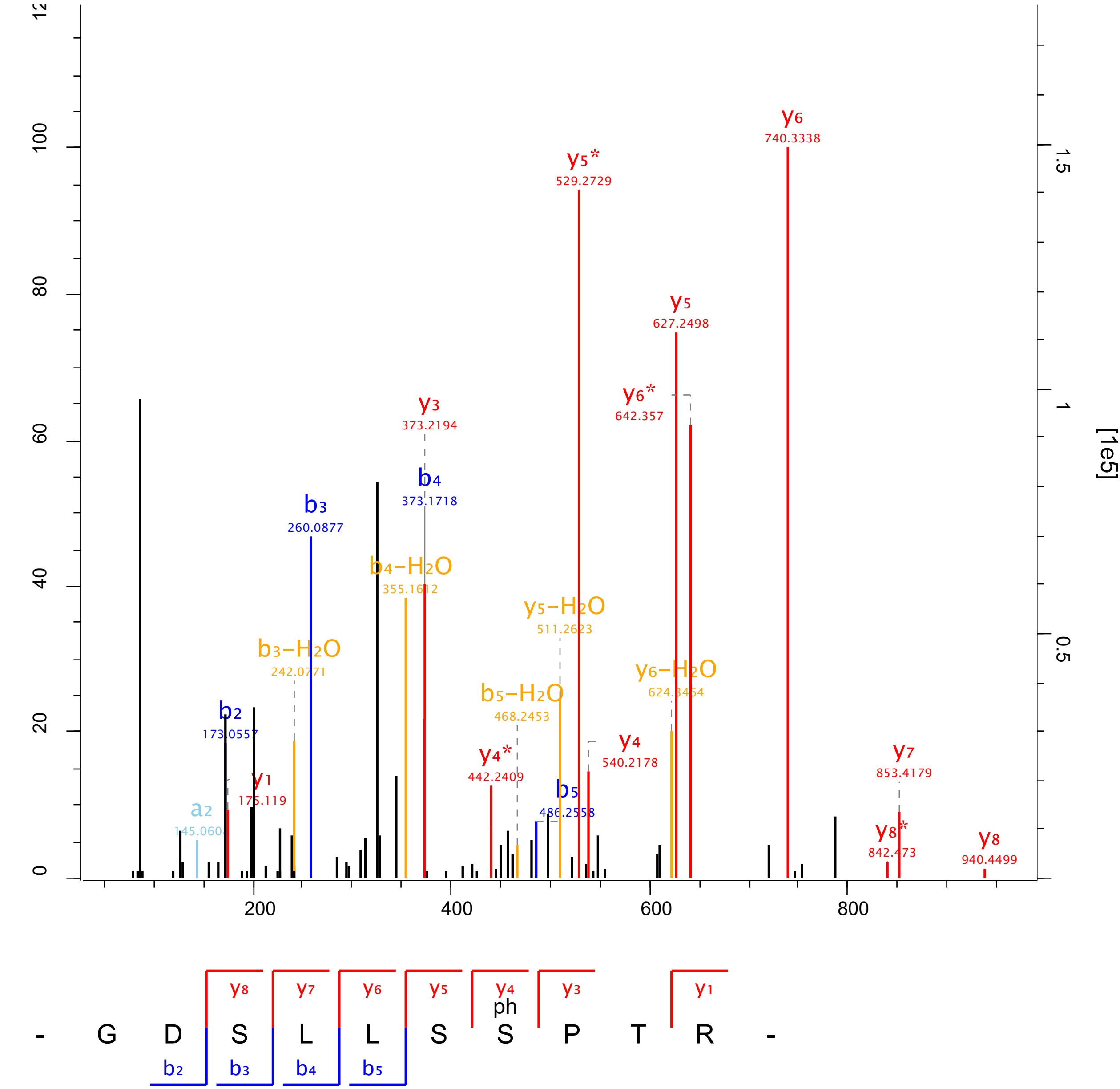

Raw file	Scan	Method	Score	m/z
sys_15_1	14488	FTMS; HCD	242.71	1012.43

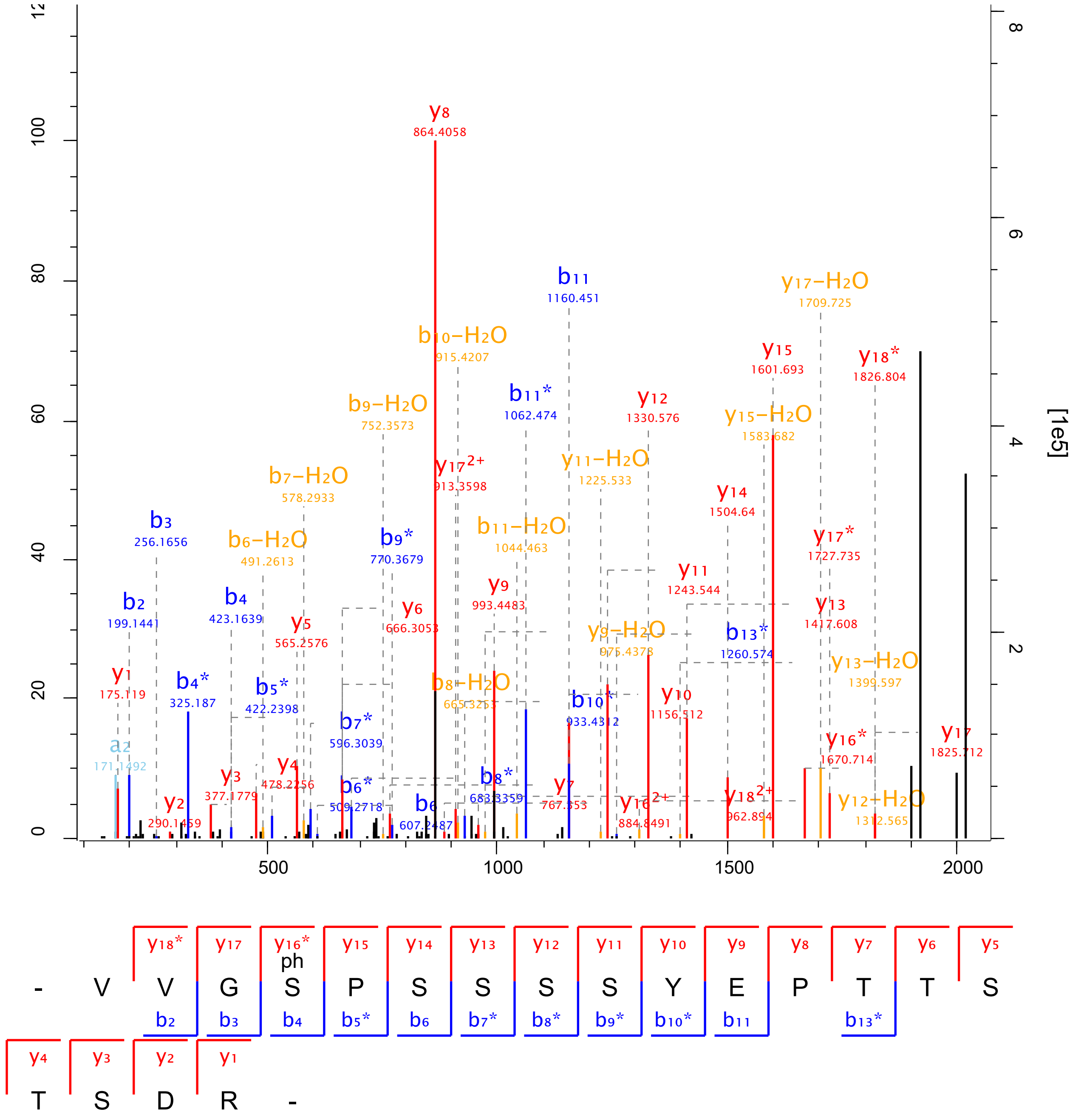

Raw file	Scan	Method	Score	m/z
sys_15_1	14562	FTMS; HCD	67.65	806.36

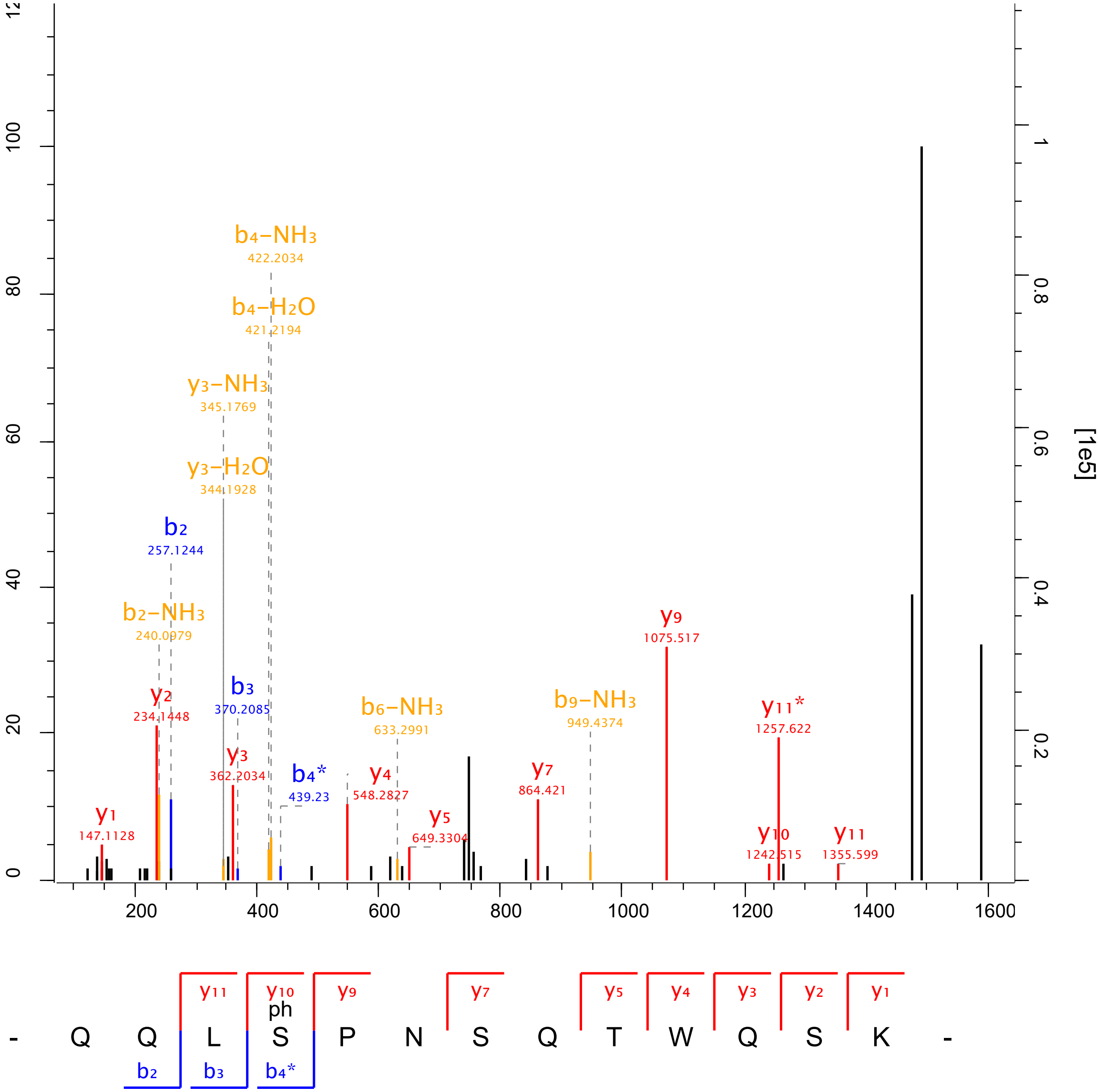

Raw file	Scan	Method	Score	m/z
sys_15_1	14567	FTMS; HCD	80.01	565.73

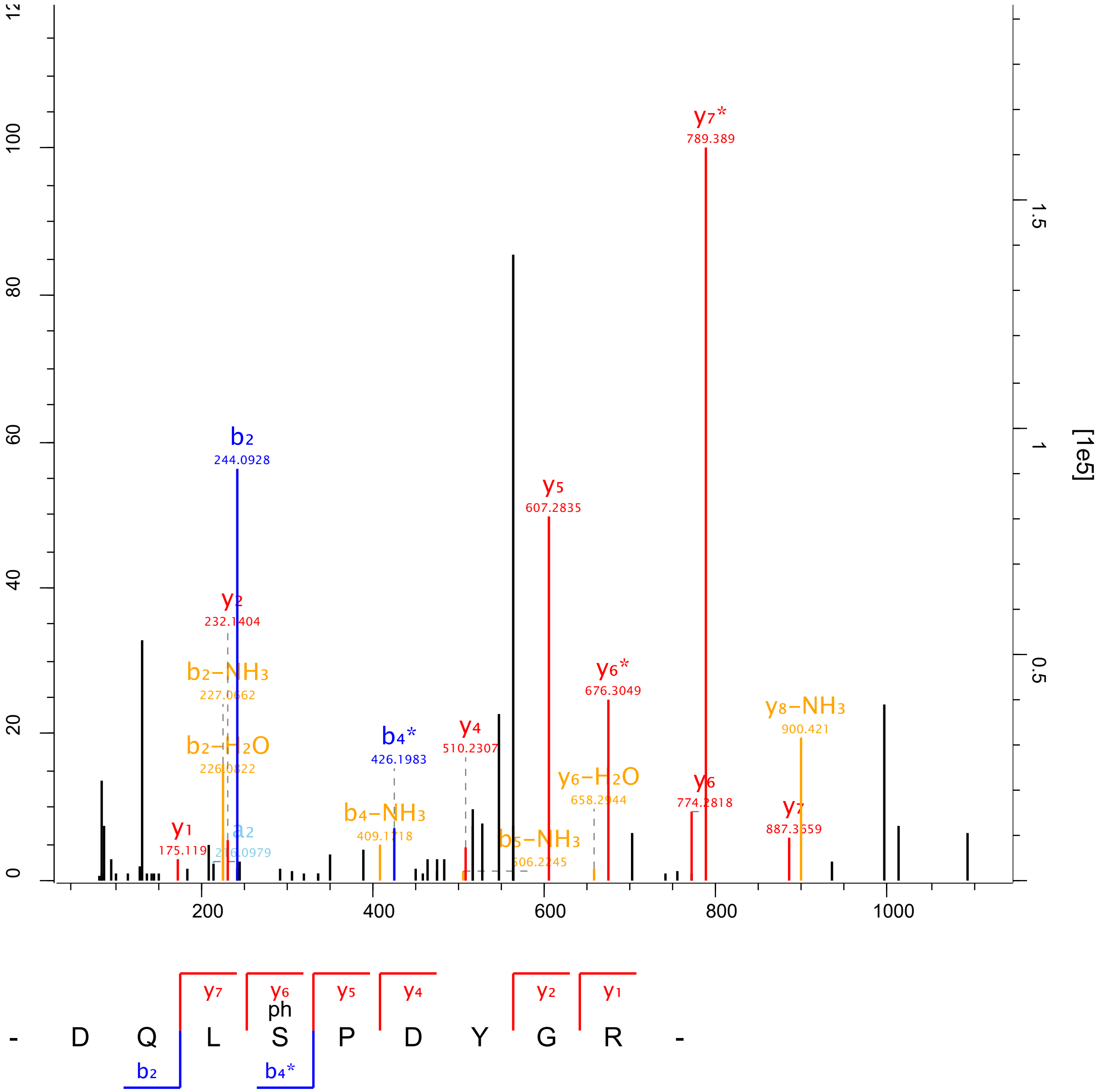

Raw file	Scan	Method	Score	m/z
sys_15_1	14675	FTMS; HCD	254.72	725.29

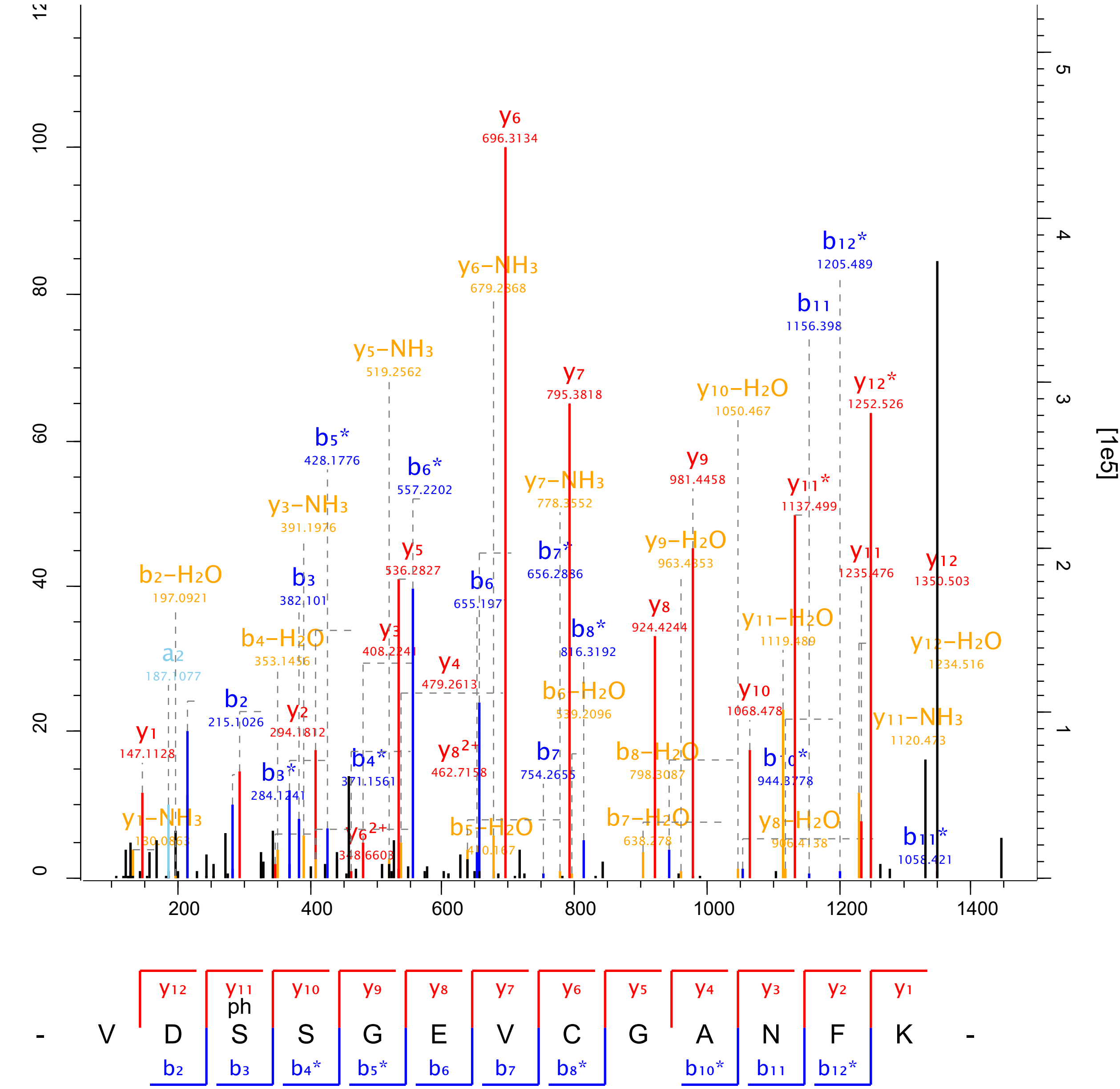

Raw file	Scan	Method	Score	m/z
sys_15_1	14695	FTMS; HCD	41.51	960.94

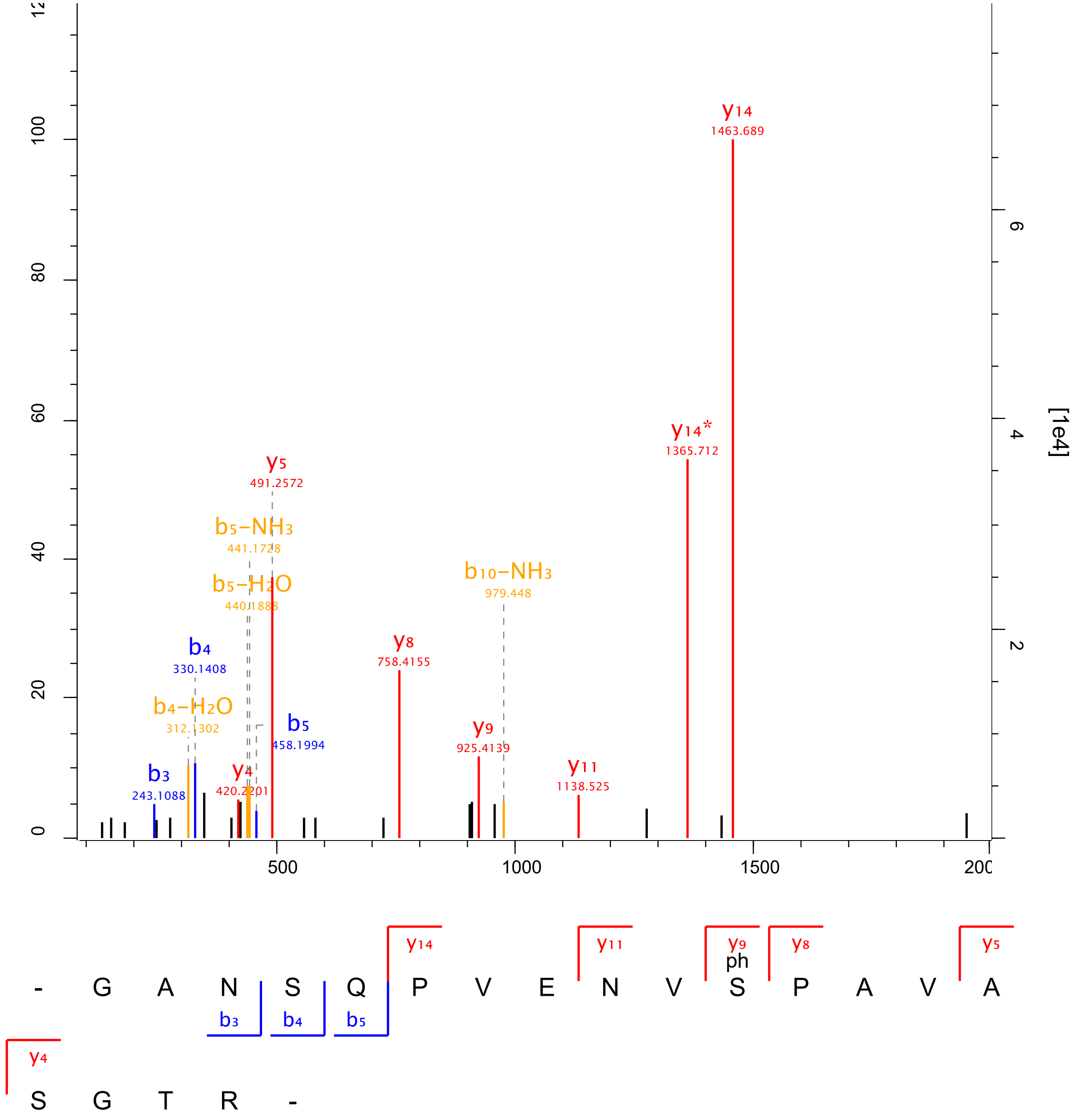

Raw file	Scan	Method	Score	m/z
sys_15_1	14850	FTMS; HCD	94.09	679.27

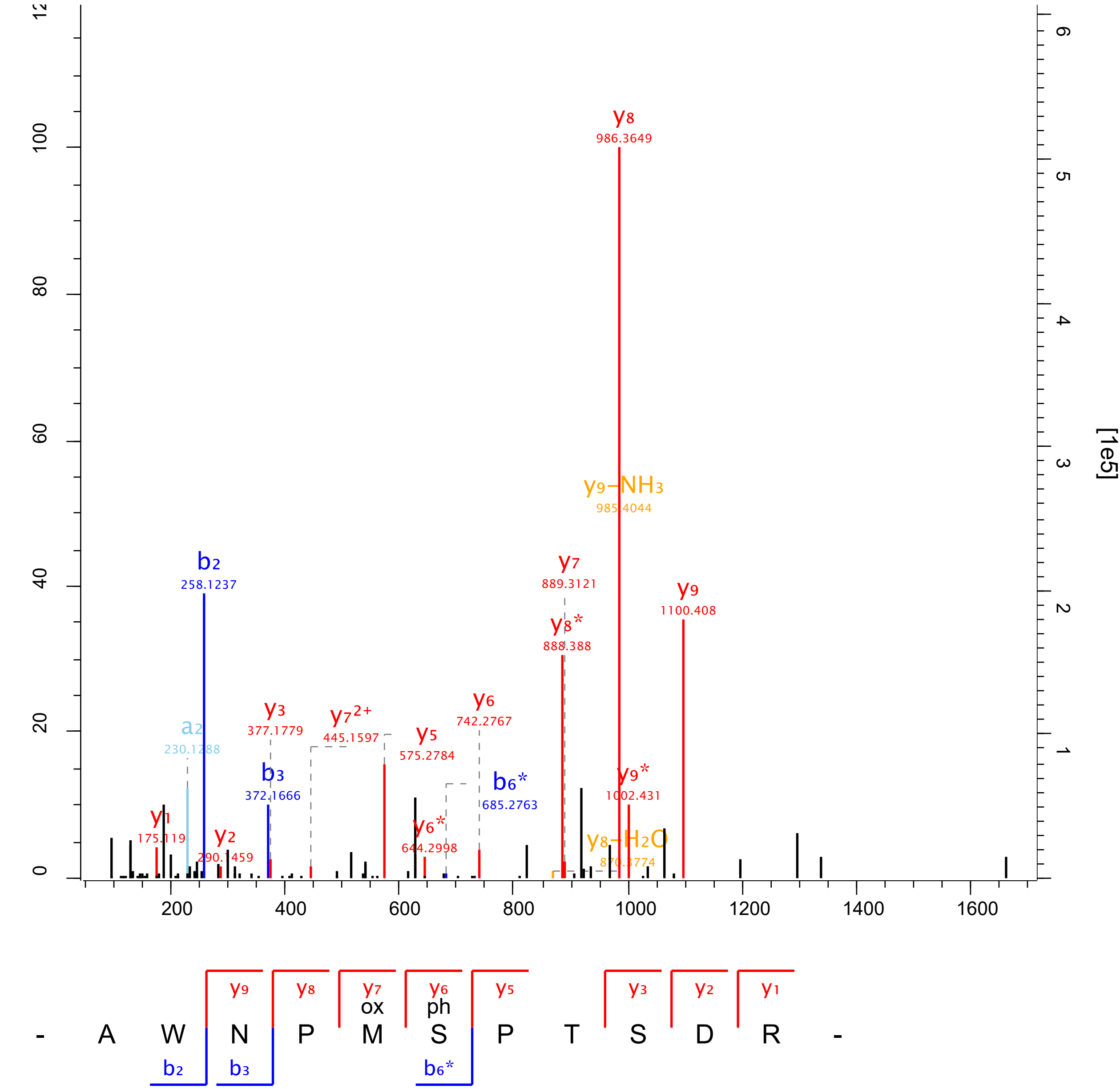

Raw file	Scan	Method	Score	m/z
sys_15_1	14894	FTMS; HCD	118.2	670.3

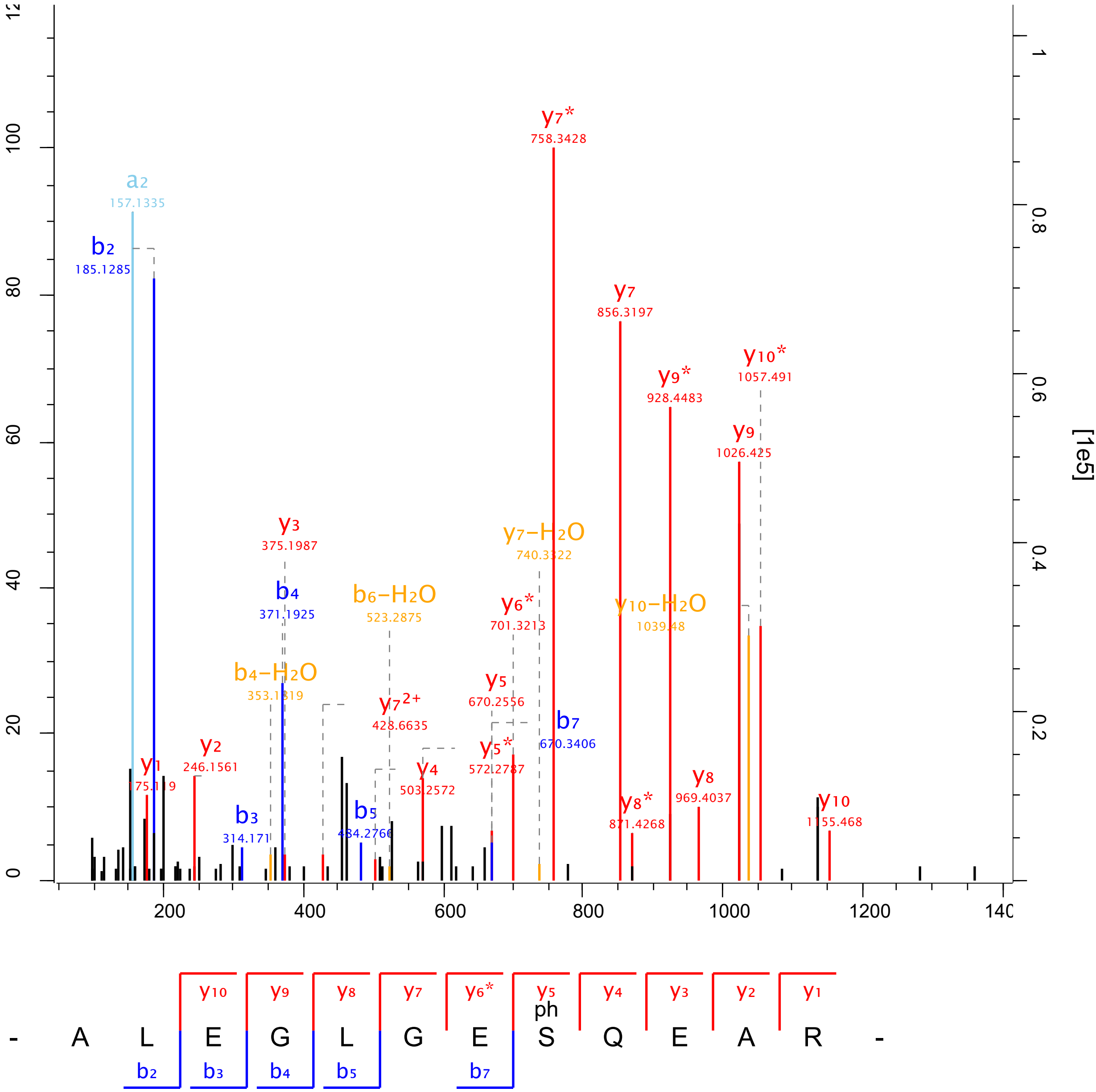

Raw file	Scan	Method	Score	m/z
sys_15_1	14948	FTMS; HCD	111.4	926.4

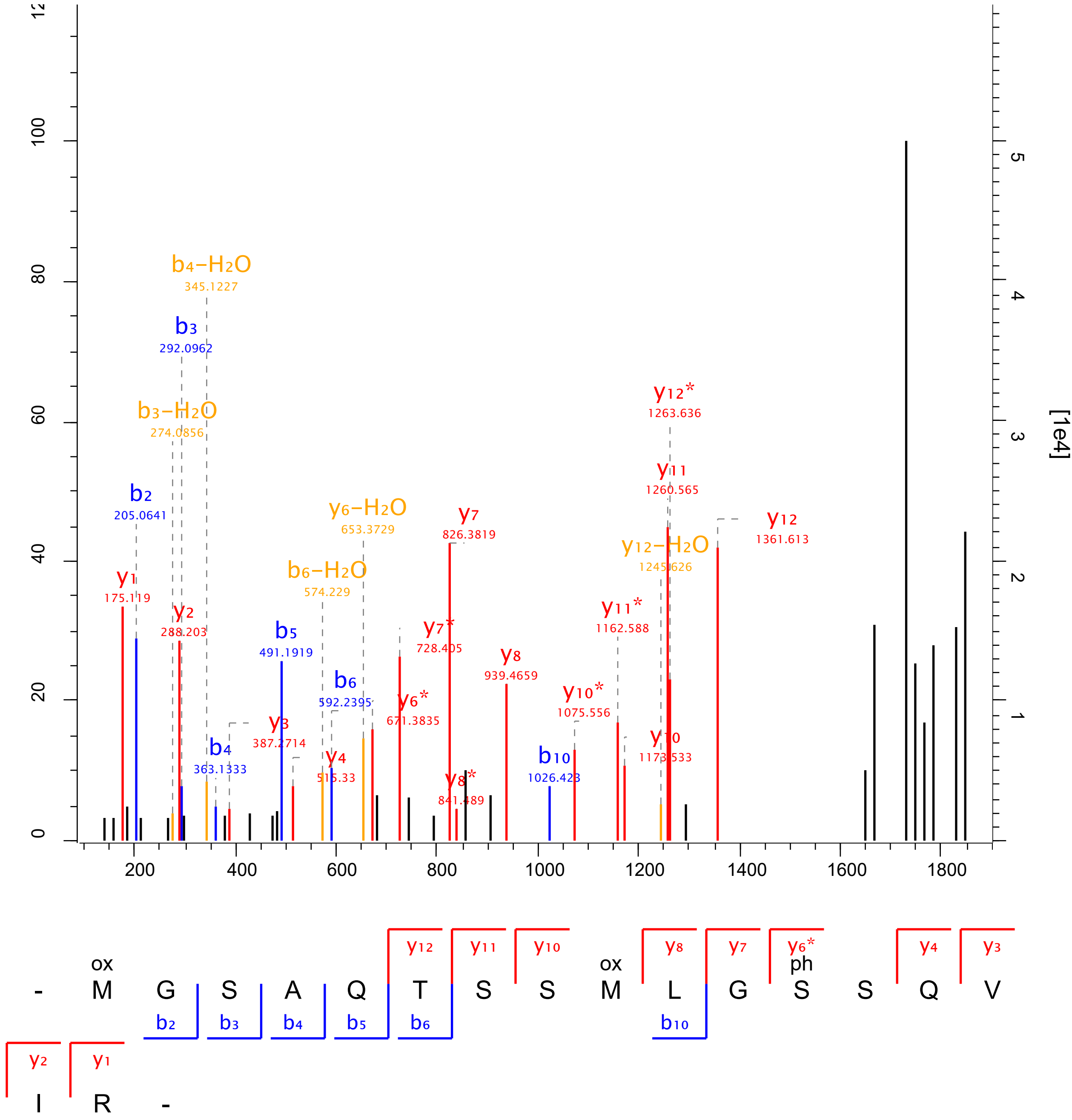

Raw file	Scan	Method	Score	m/z
sys_15_1	14991	FTMS; HCD	162.59	751.33

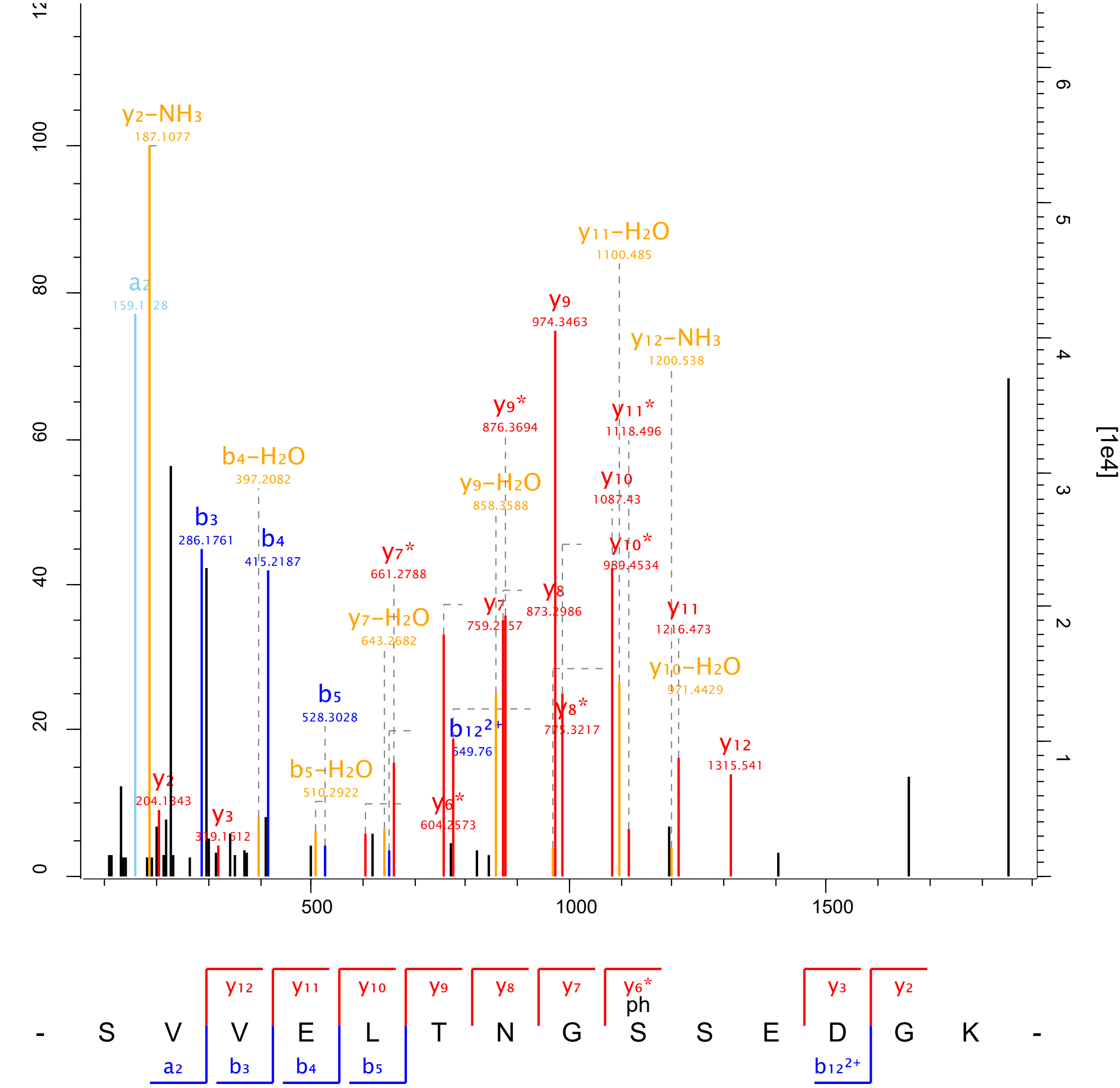

Raw file	Scan	Method	Score	m/z
sys_15_1	15005	FTMS; HCD	49.3	634.79

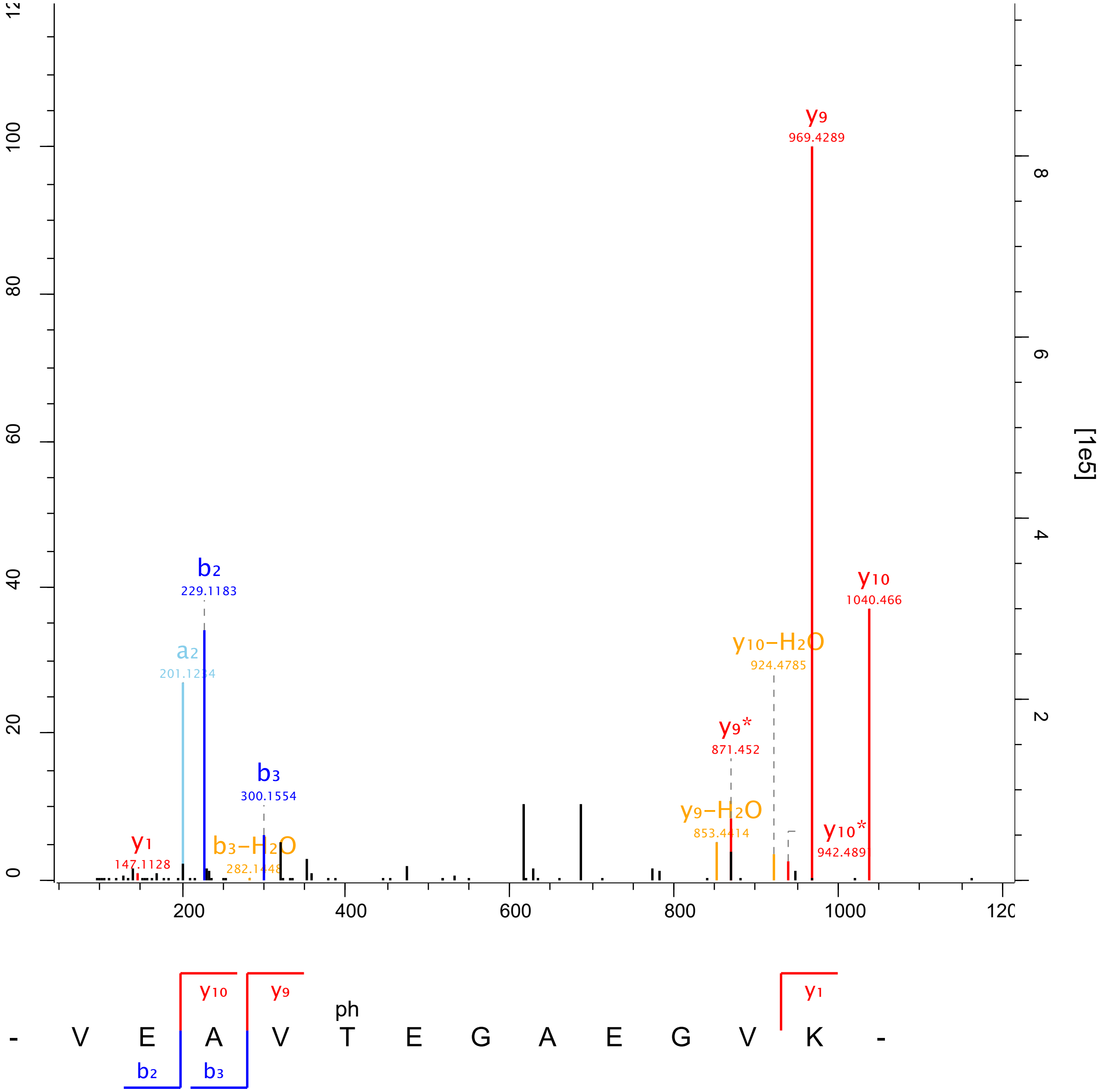

Raw file	Scan	Method	Score	m/z
sys_15_1	15127	FTMS; HCD	63.69	877.35

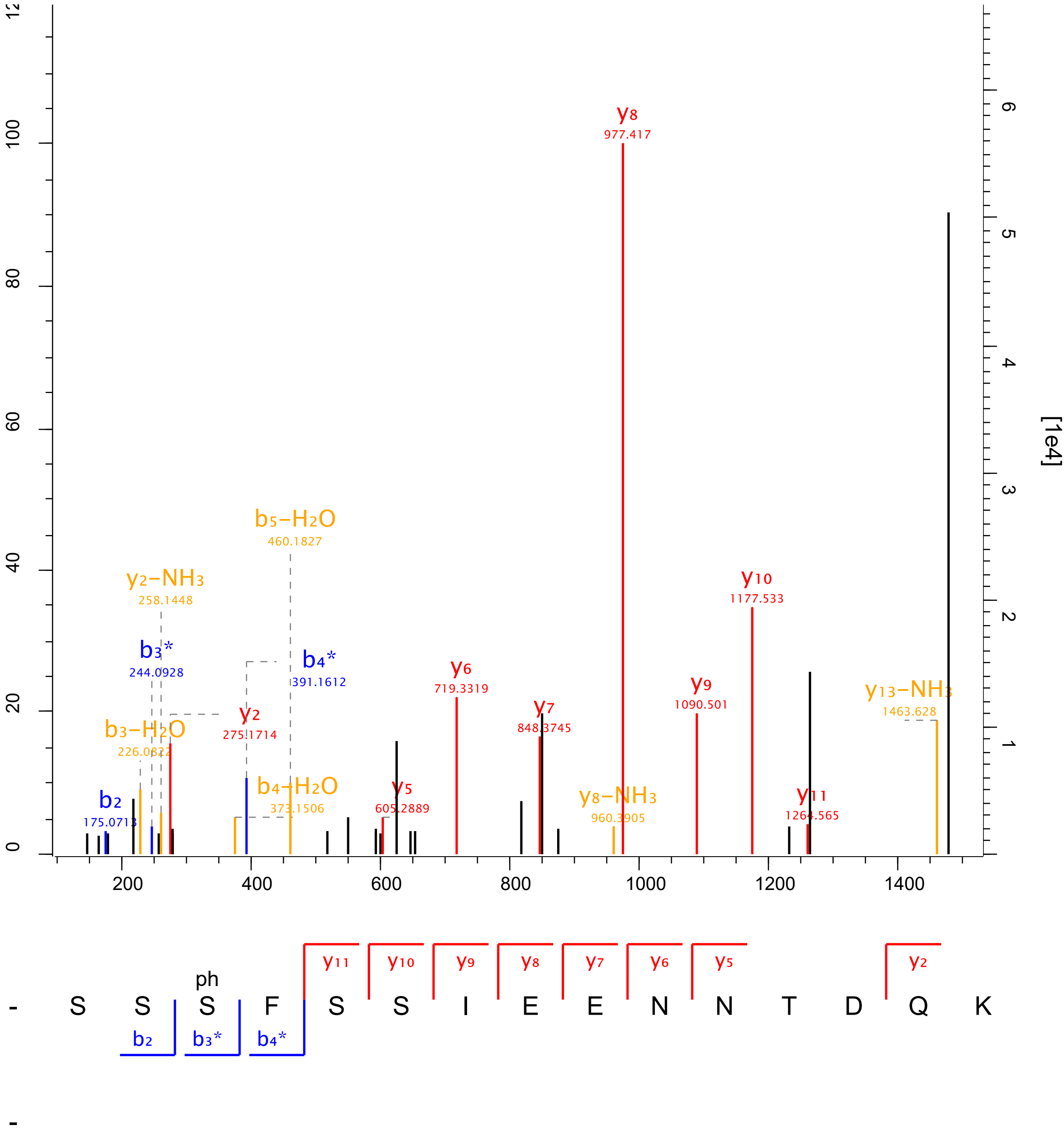

Raw file	Scan	Method	Score	m/z
sys_15_1	15152	FTMS; HCD	96.25	701.28

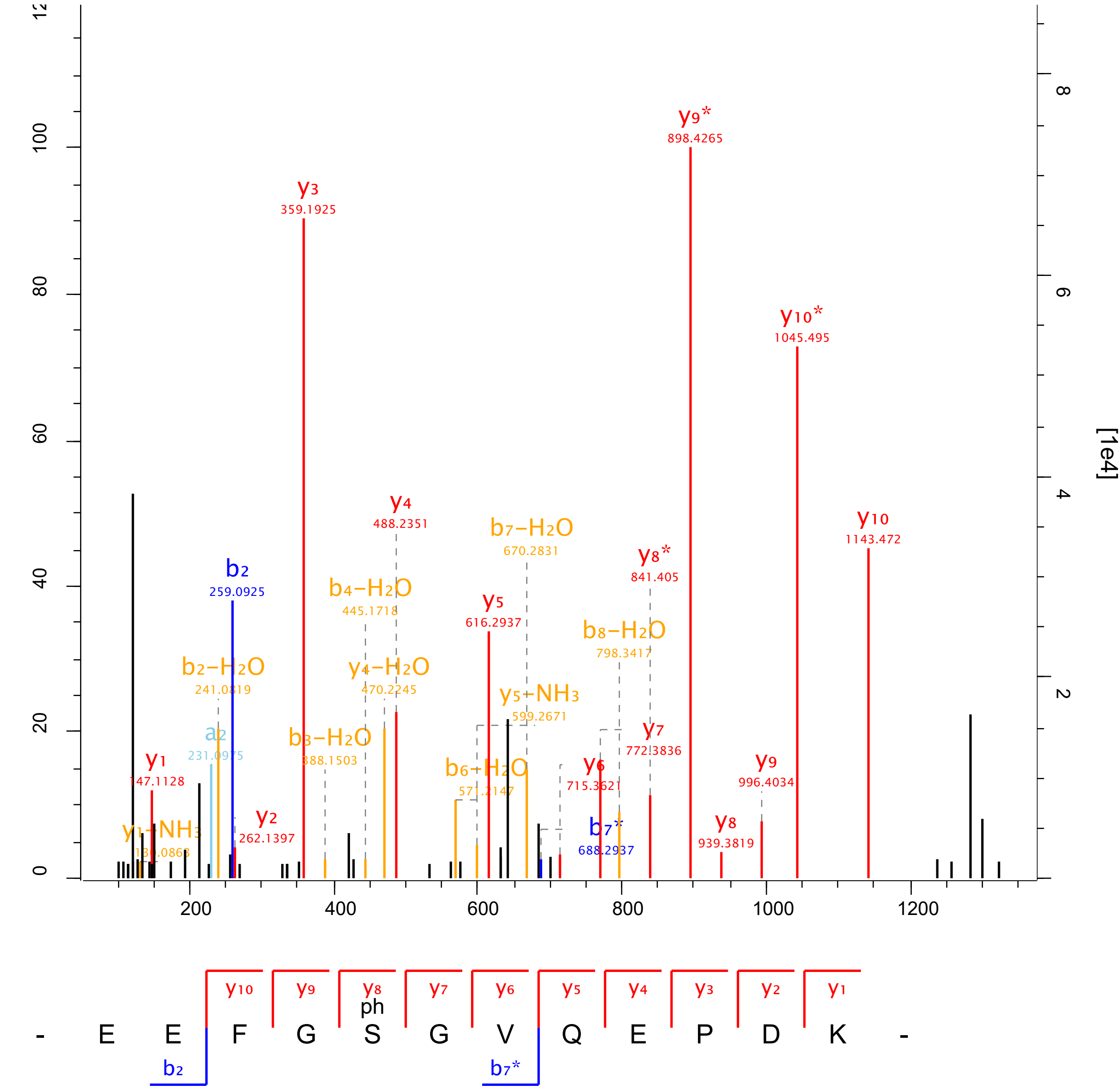

Raw file	Scan	Method	Score	m/z
sys_15_1	15390	FTMS; HCD	288.25	840.79

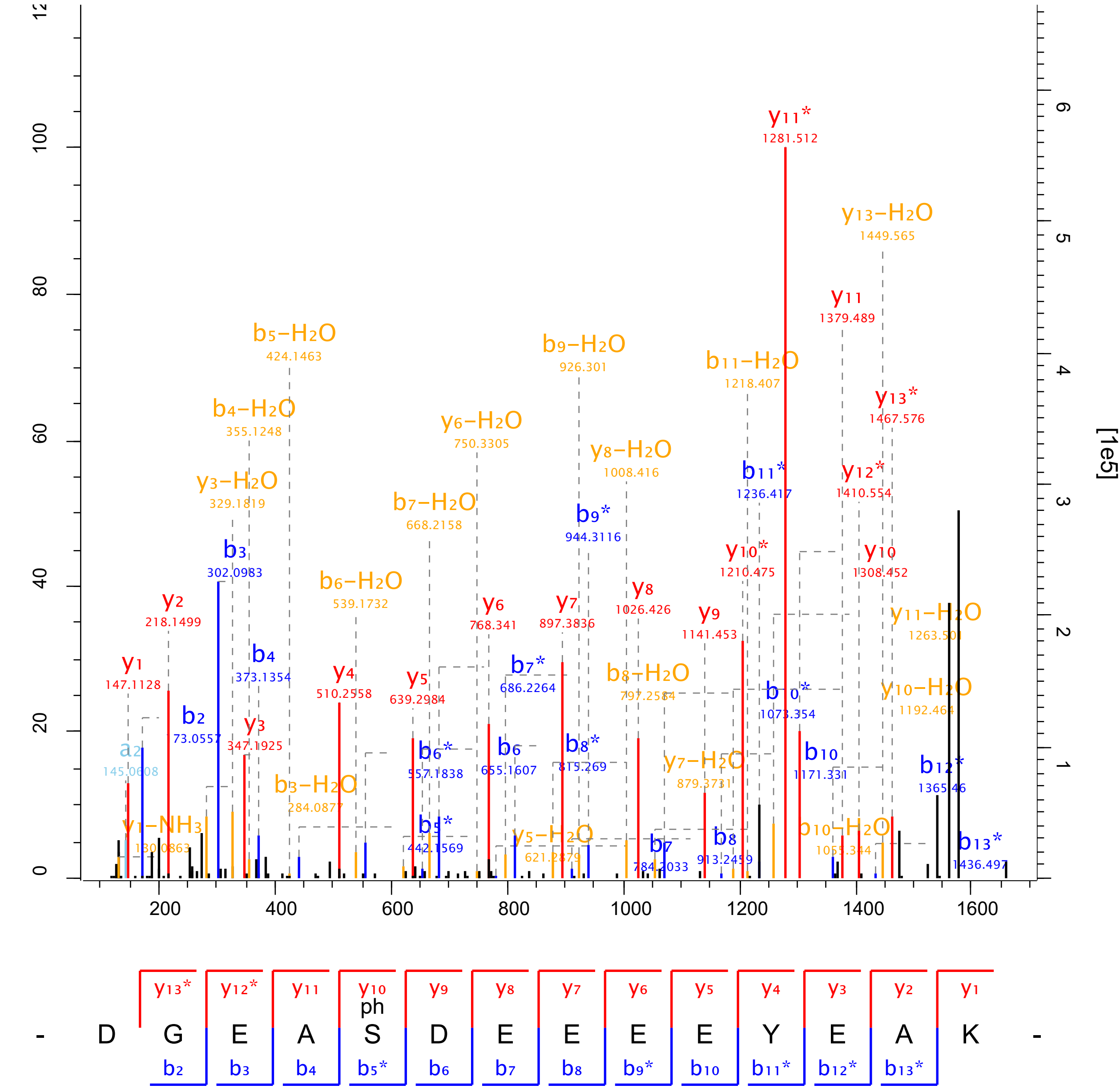

Raw file	Scan	Method	Score	m/z
sys_15_1	15393	FTMS; HCD	134.14	669.73

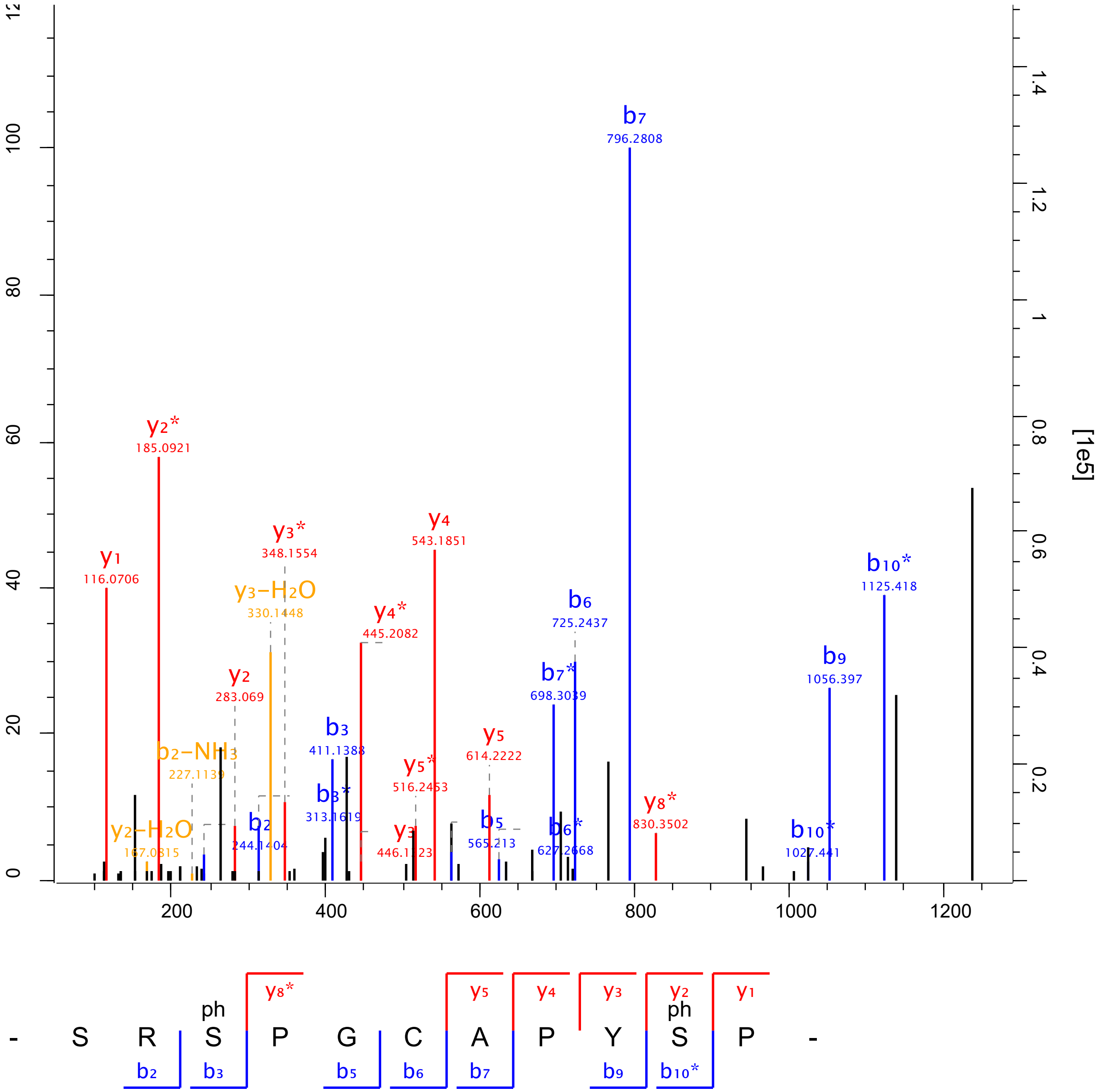

Raw file	Scan	Method	Score	m/z
sys_15_1	15435	FTMS; HCD	102.52	676.3

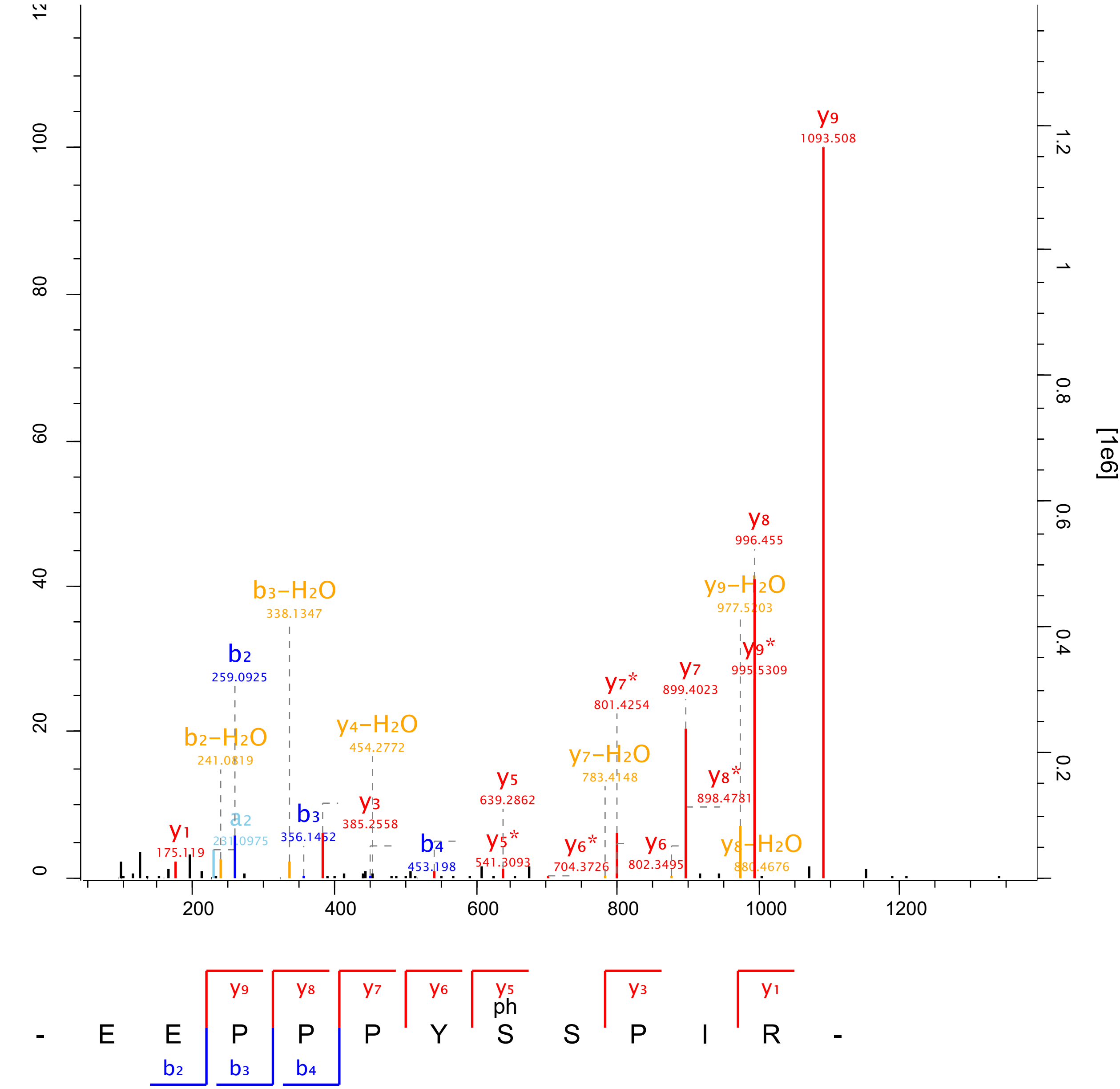

Raw file	Scan	Method	Score	m/z
sys_15_1	15475	FTMS; HCD	68.48	664.27

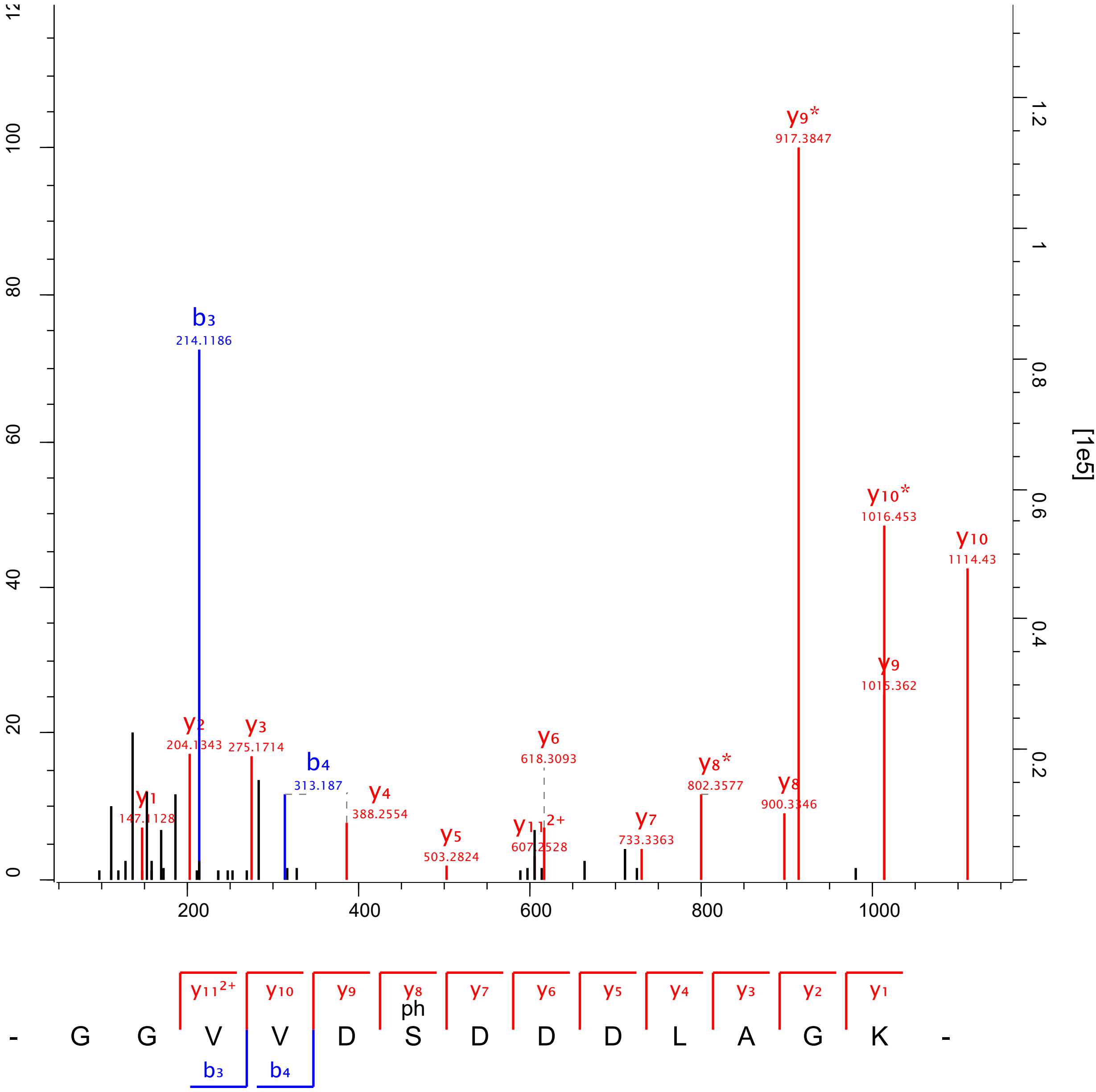

Raw file	Scan	Method	Score	m/z
sys_15_1	15551	FTMS; HCD	66.15	646.25

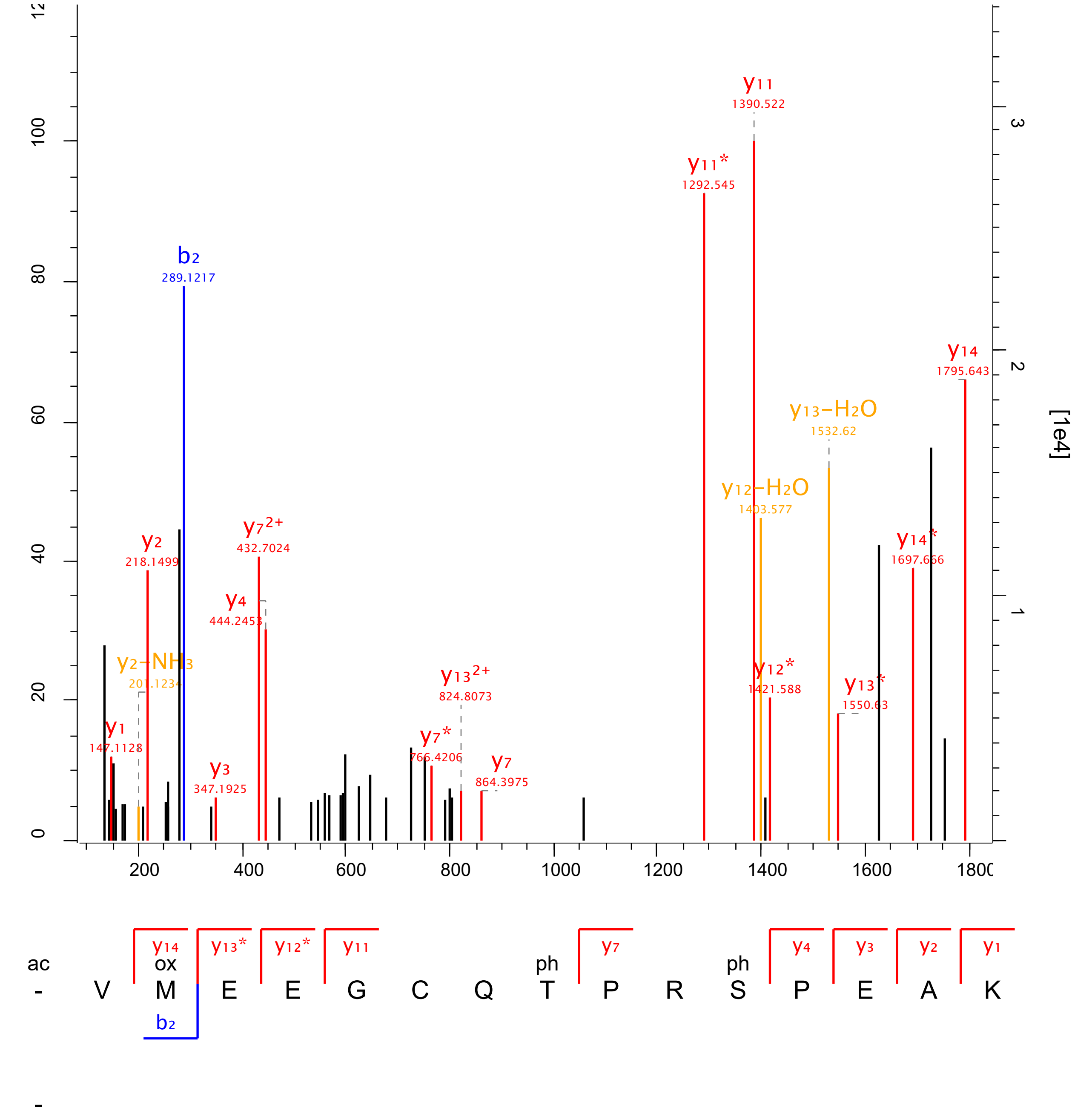

Raw file	Scan	Method	Score	m/z
sys_15_1	1558	FTMS; HCD	81.95	421.17

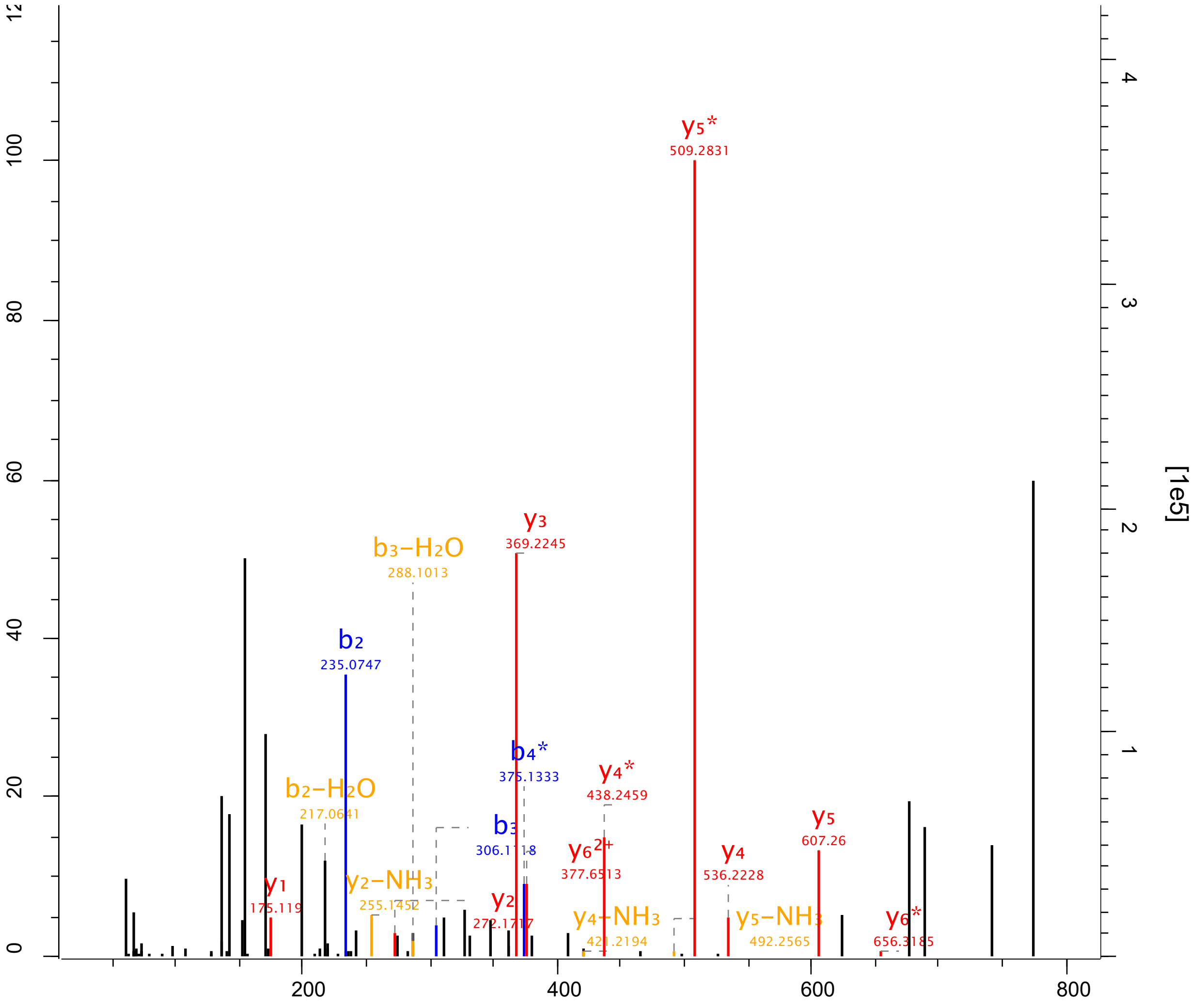

- S M A S P P R -

ox M, ph S; y6* y5 y4 y3 y2 y1; b2 b3 b4*

Raw file	Scan	Method	Score	m/z
sys_15_1	15622	FTMS; HCD	54.34	593.75

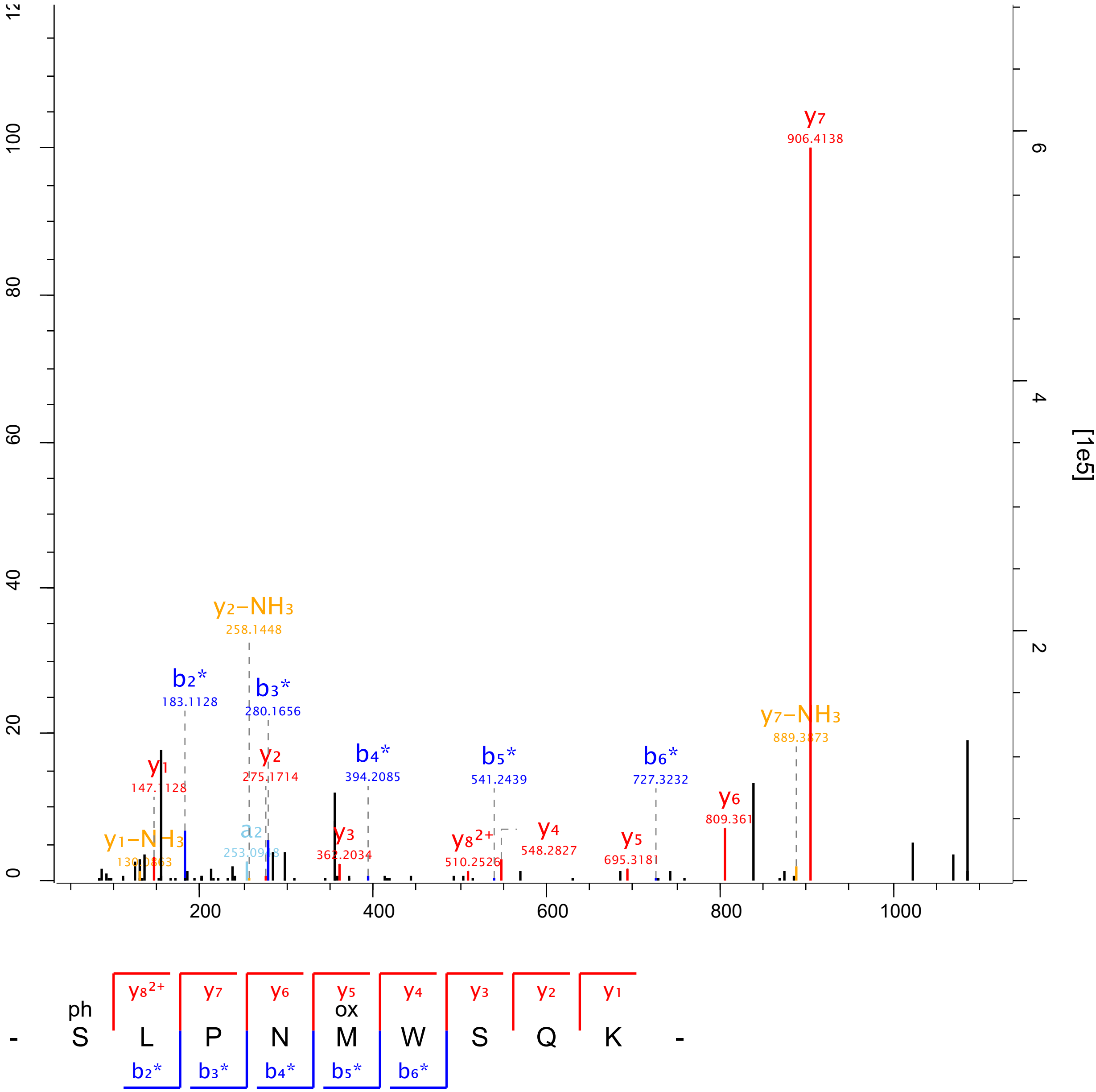

Raw file	Scan	Method	Score	m/z
sys_15_1	15645	FTMS; HCD	48.22	728.81

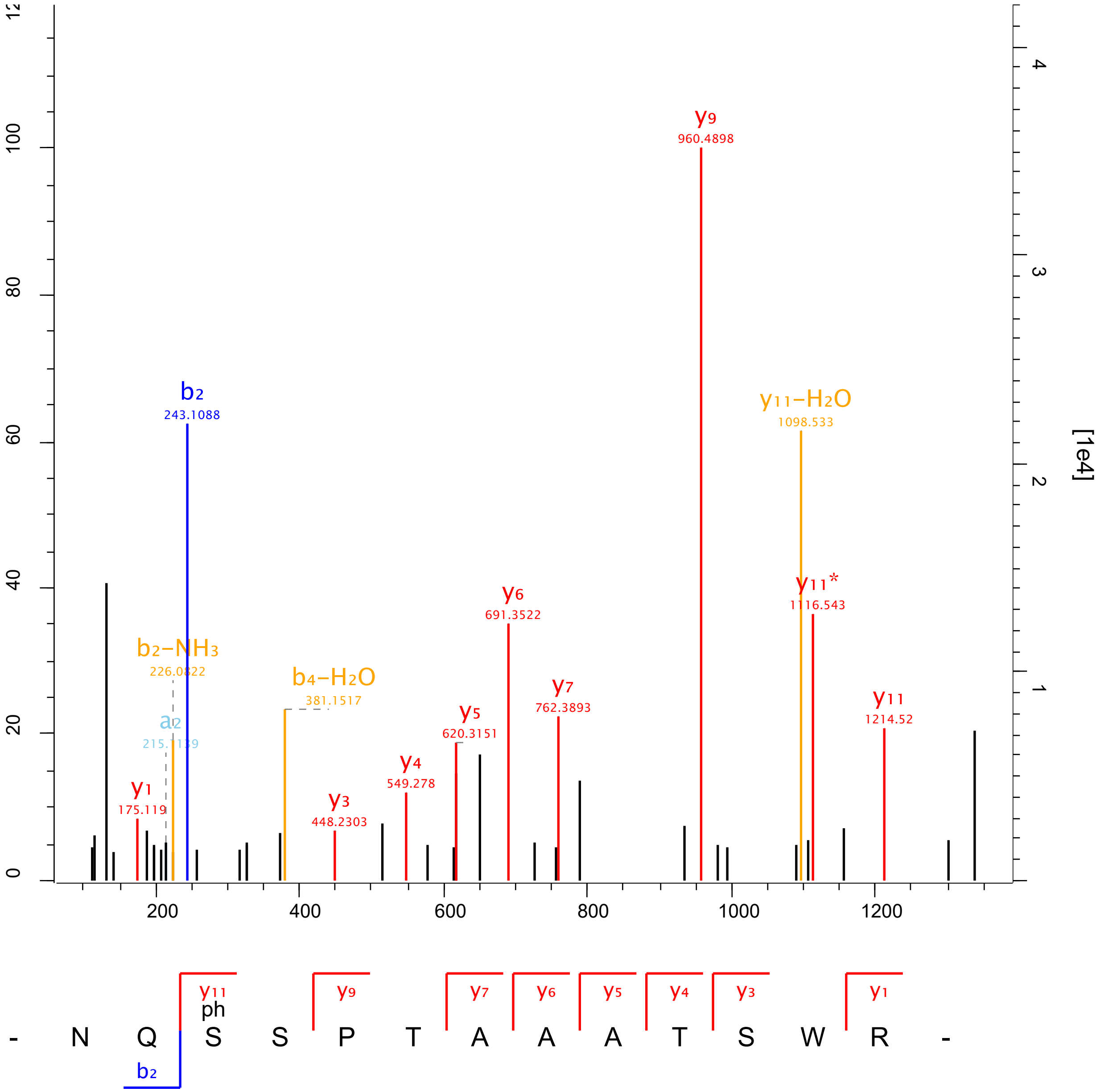

Raw file	Scan	Method	Score	m/z
sys_15_1	15684	FTMS; HCD	118.05	1013.46

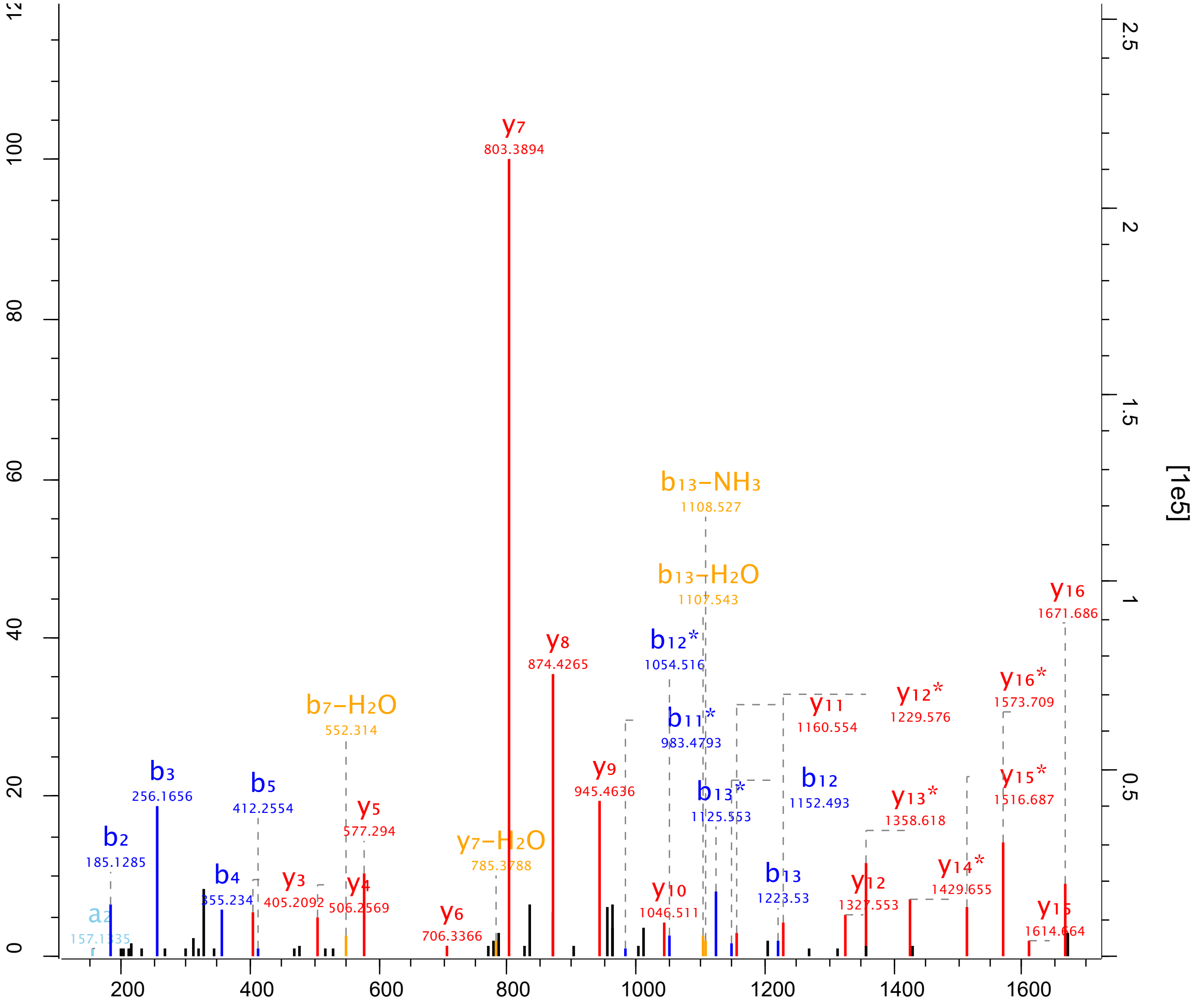

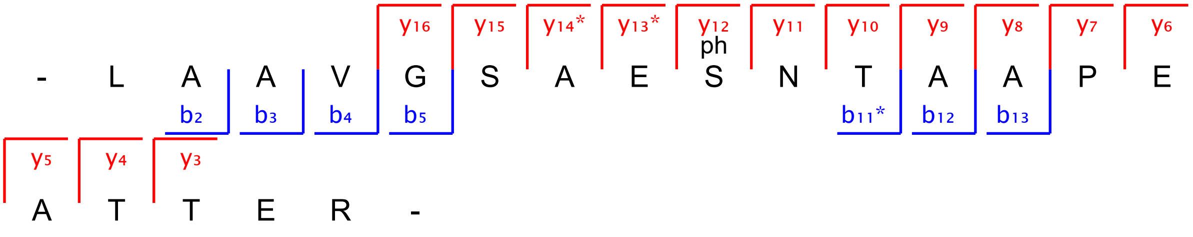

Raw file	Scan	Method	Score	m/z
sys_15_1	15880	FTMS; HCD	133.47	620.24

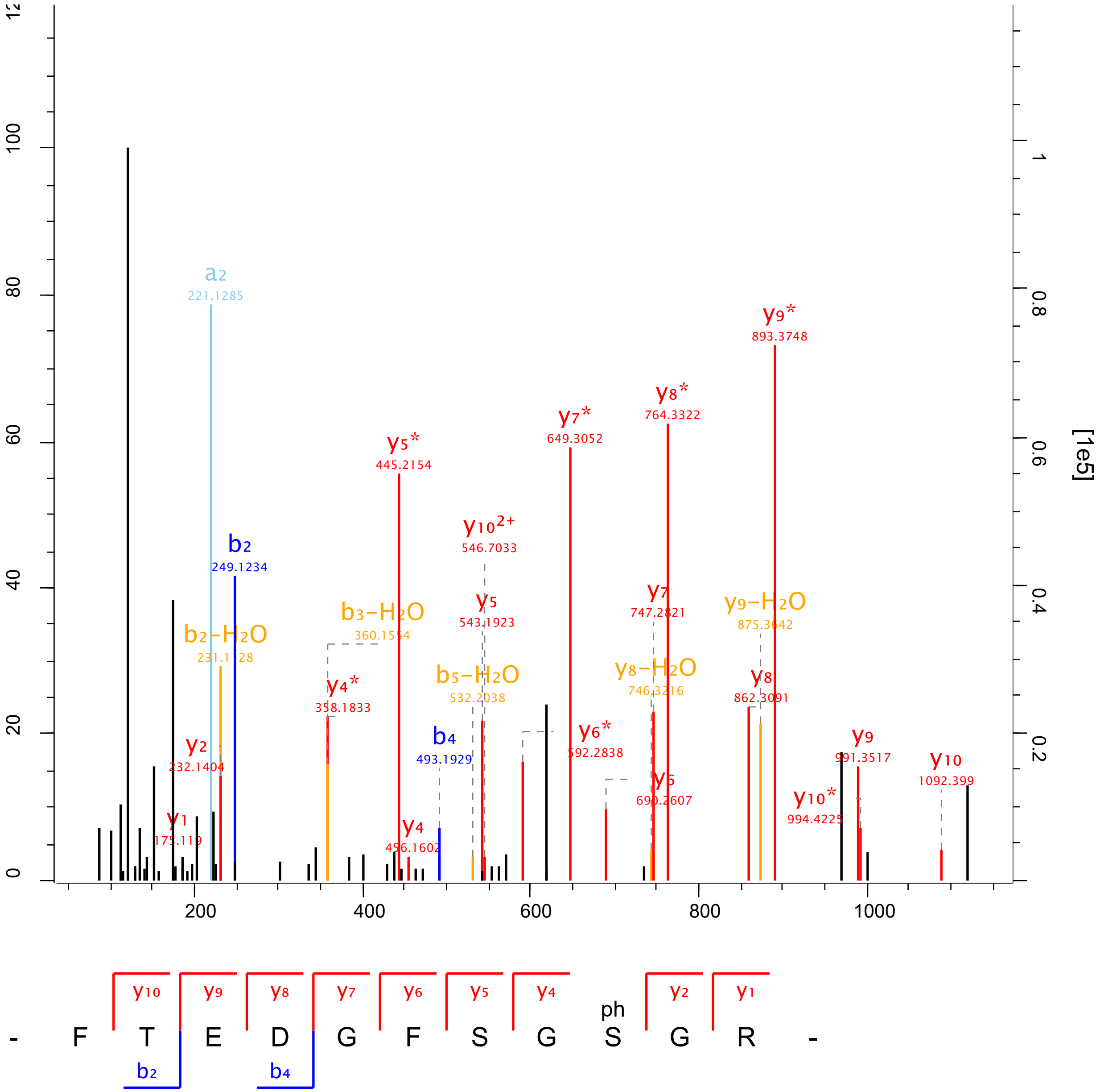

Raw file	Scan	Method	Score	m/z
sys_15_1	15968	FTMS; HCD	61.28	624.93

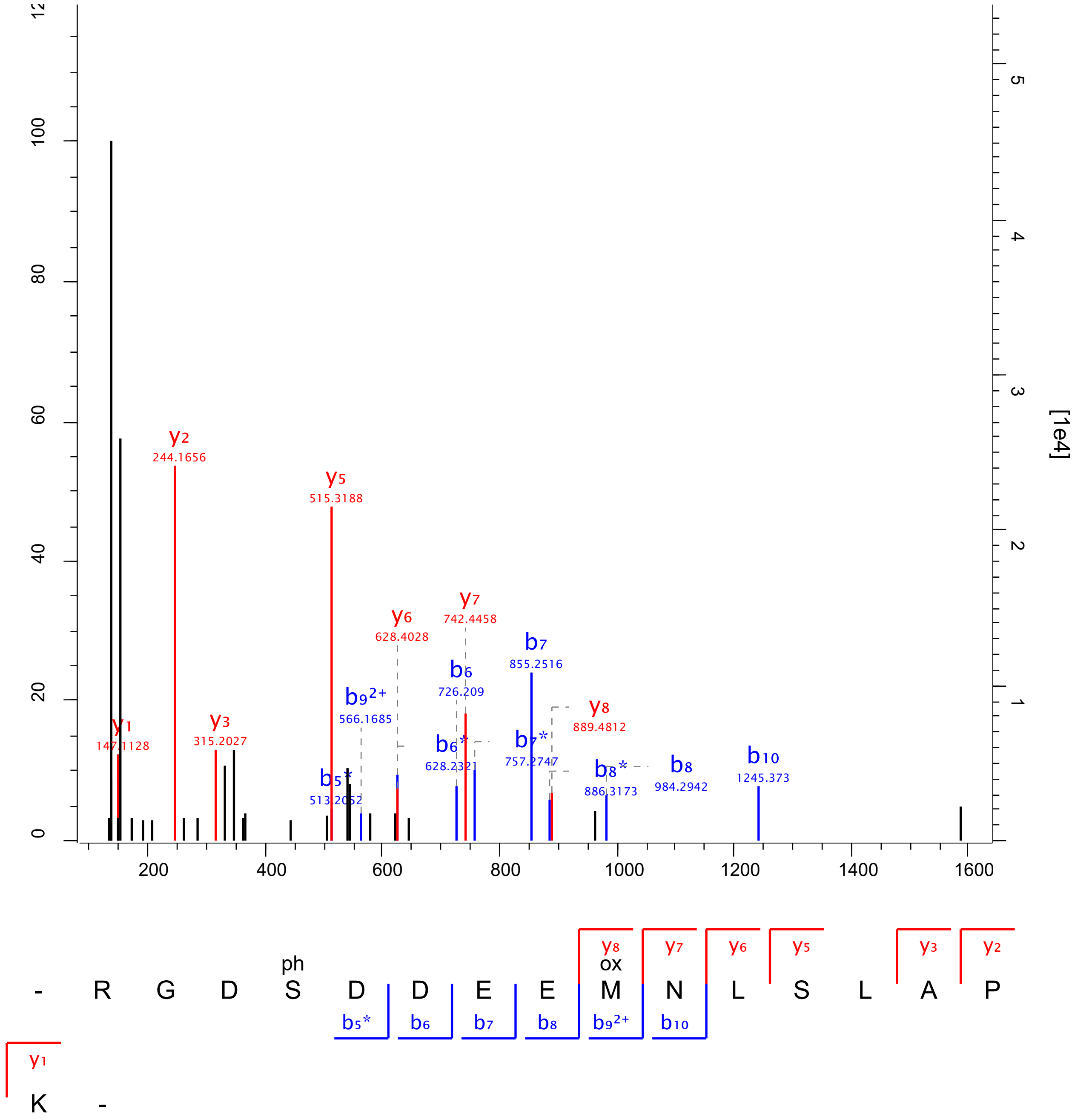

Raw file	Scan	Method	Score	m/z
sys_15_1	16033	FTMS; HCD	75.09	681.28

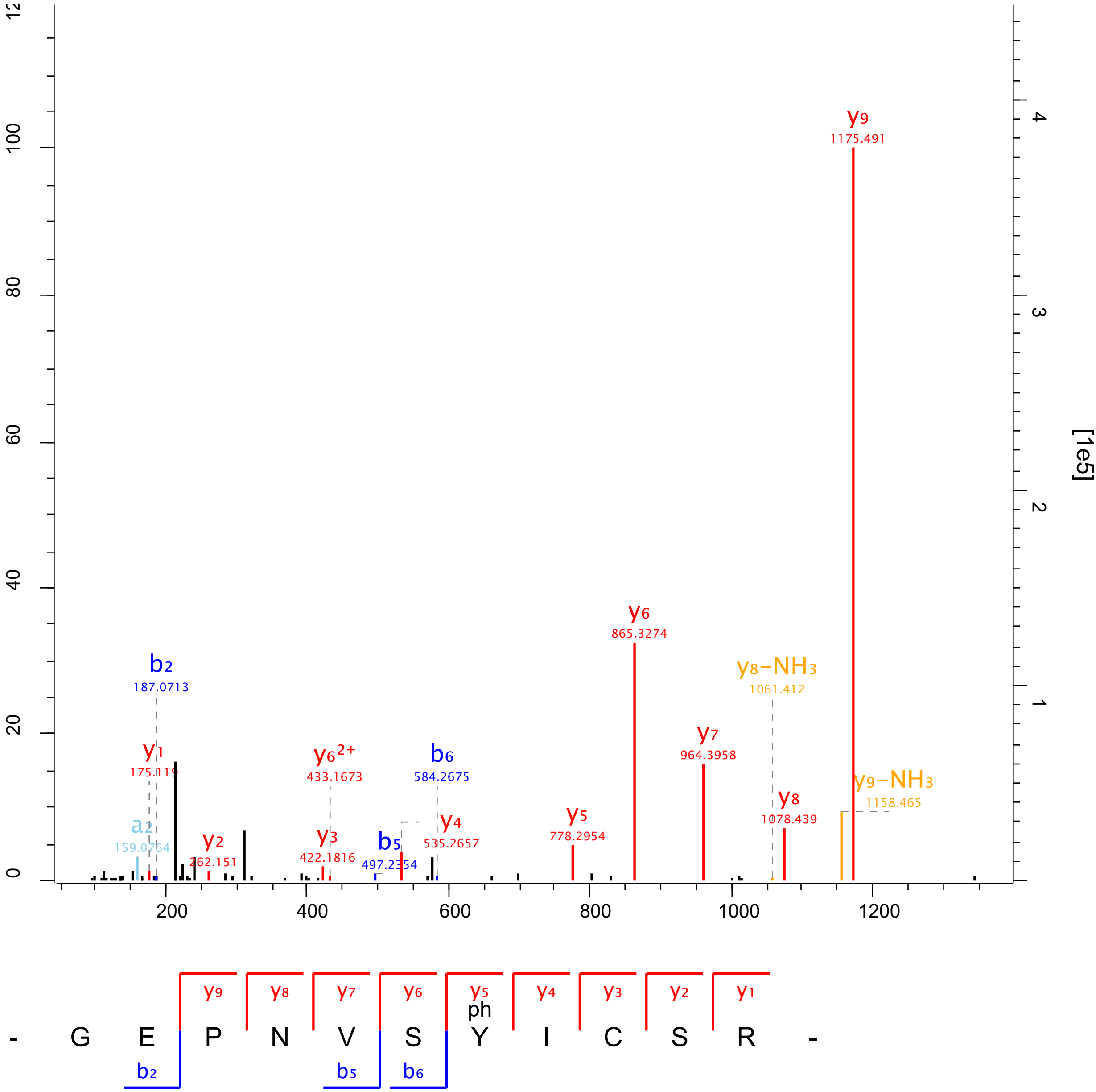

Raw file	Scan	Method	Score	m/z
sys_15_1	16217	FTMS; HCD	185.86	723.29

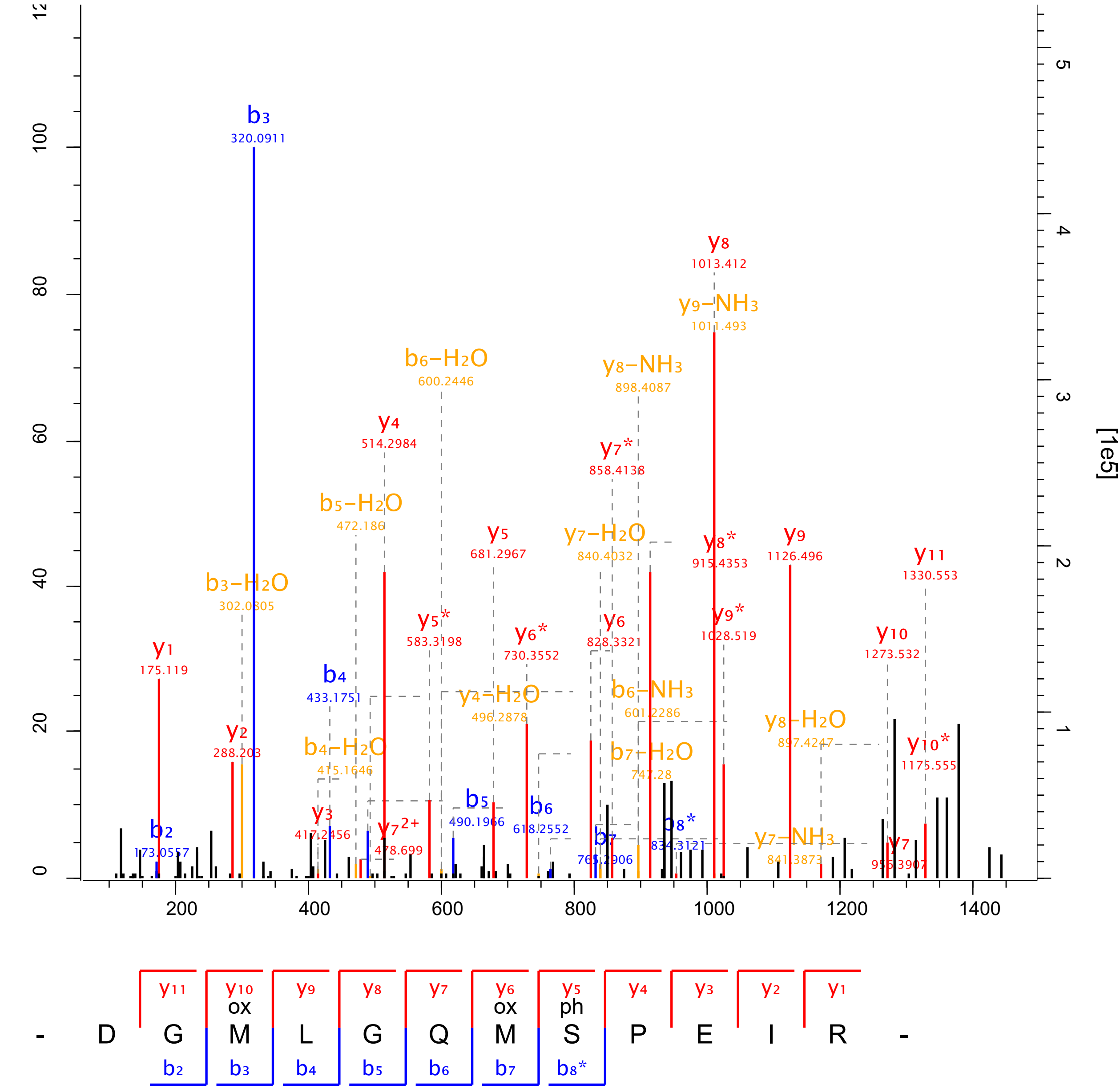

Raw file	Scan	Method	Score	m/z
sys_15_1	16236	FTMS; HCD	92.46	780.67

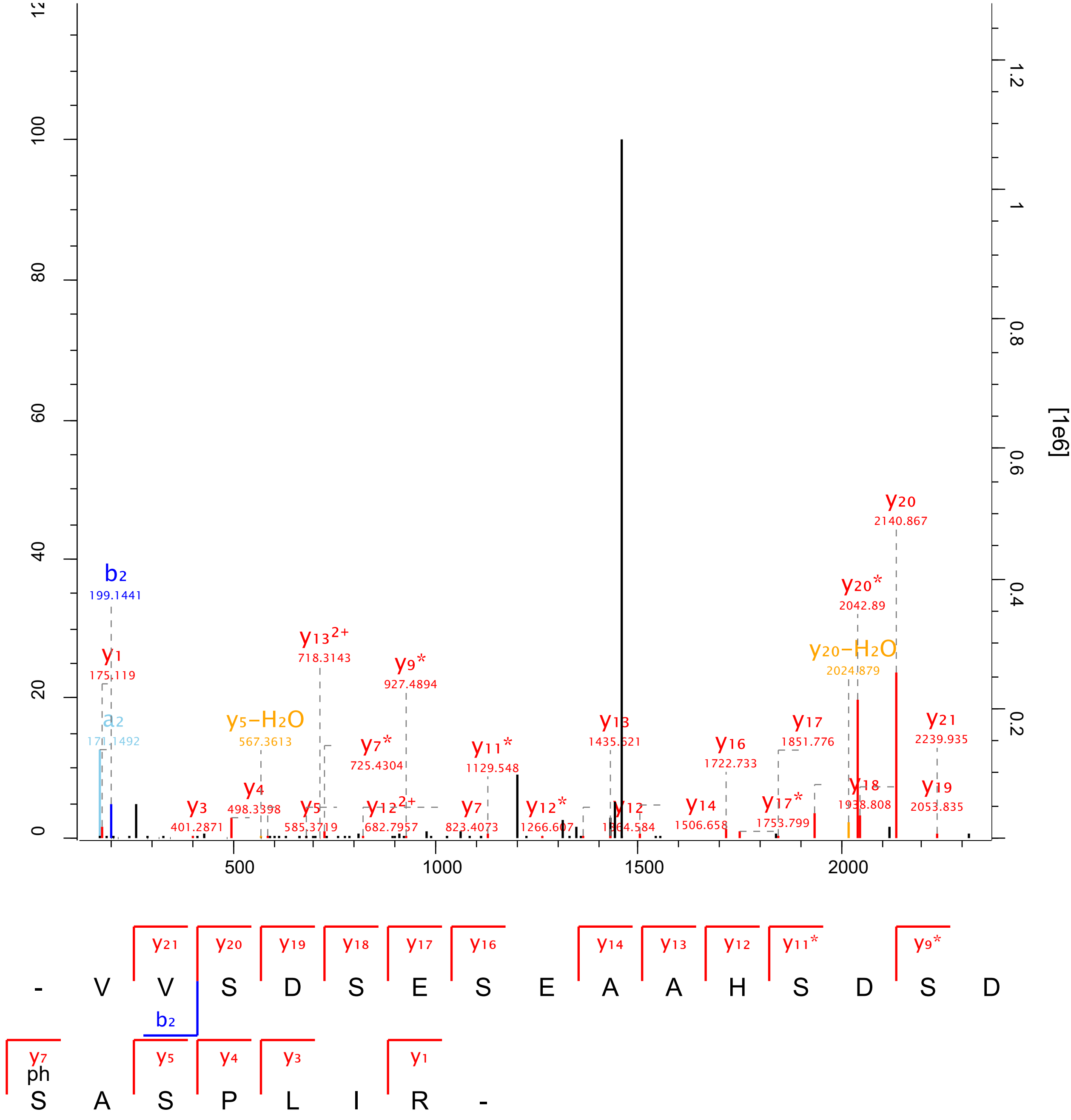

Raw file	Scan	Method	Score	m/z
sys_15_1	16367	FTMS; HCD	152.34	686.29

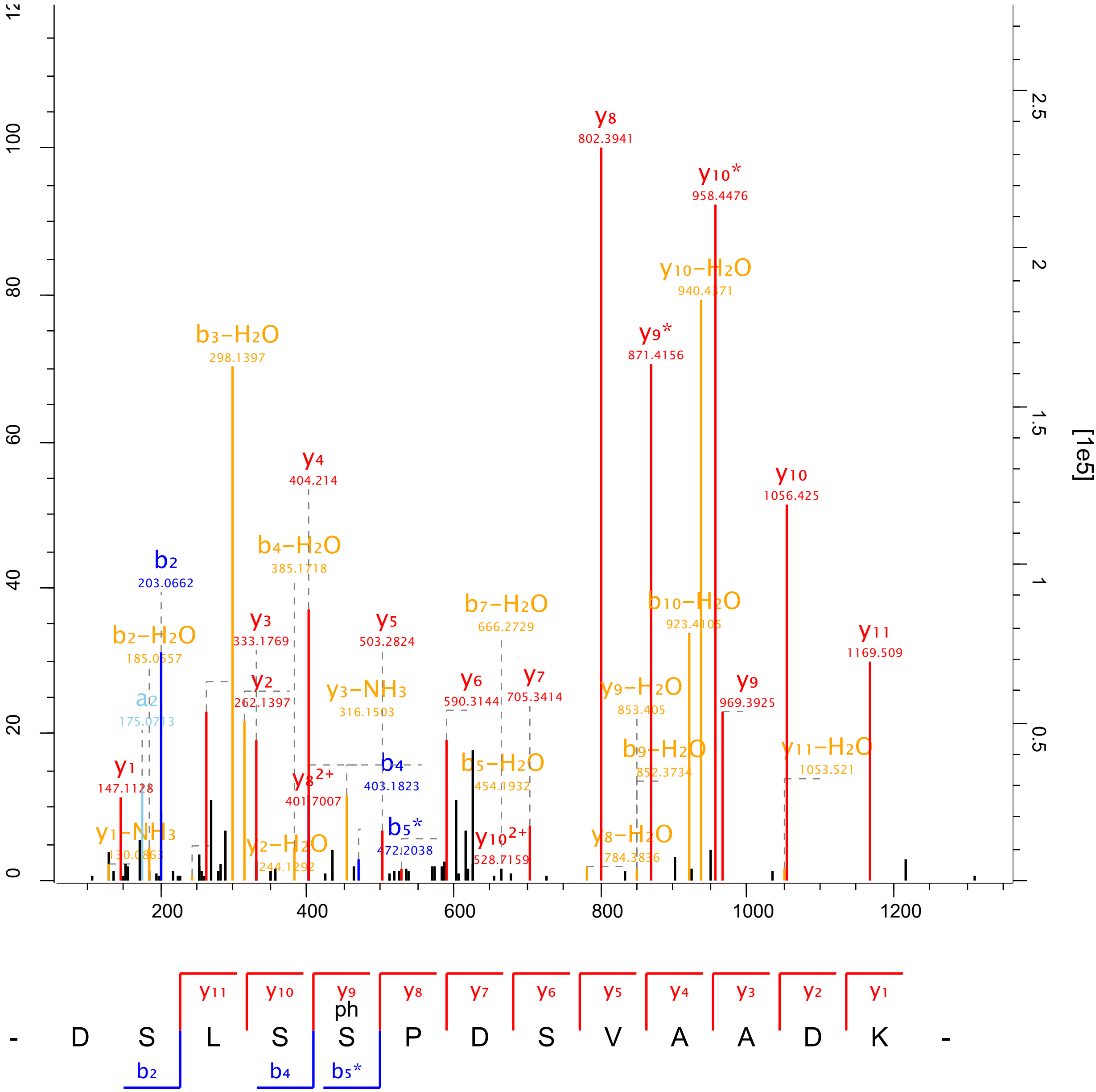

Raw file	Scan	Method	Score	m/z
sys_15_1	16386	FTMS; HCD	60.16	615.27

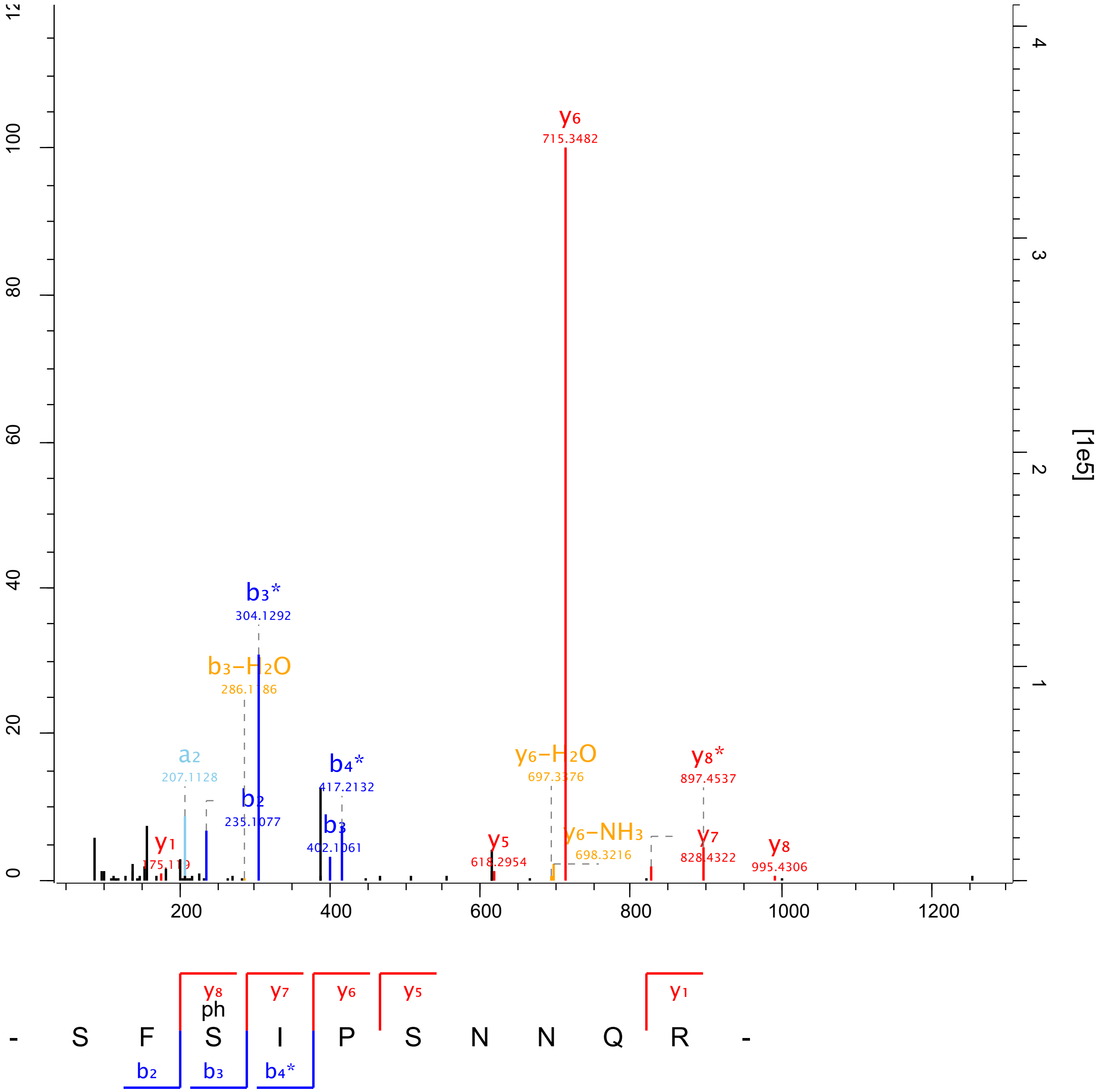

Raw file	Scan	Method	Score	m/z
sys_15_1	16413	FTMS; HCD	47.55	698.24

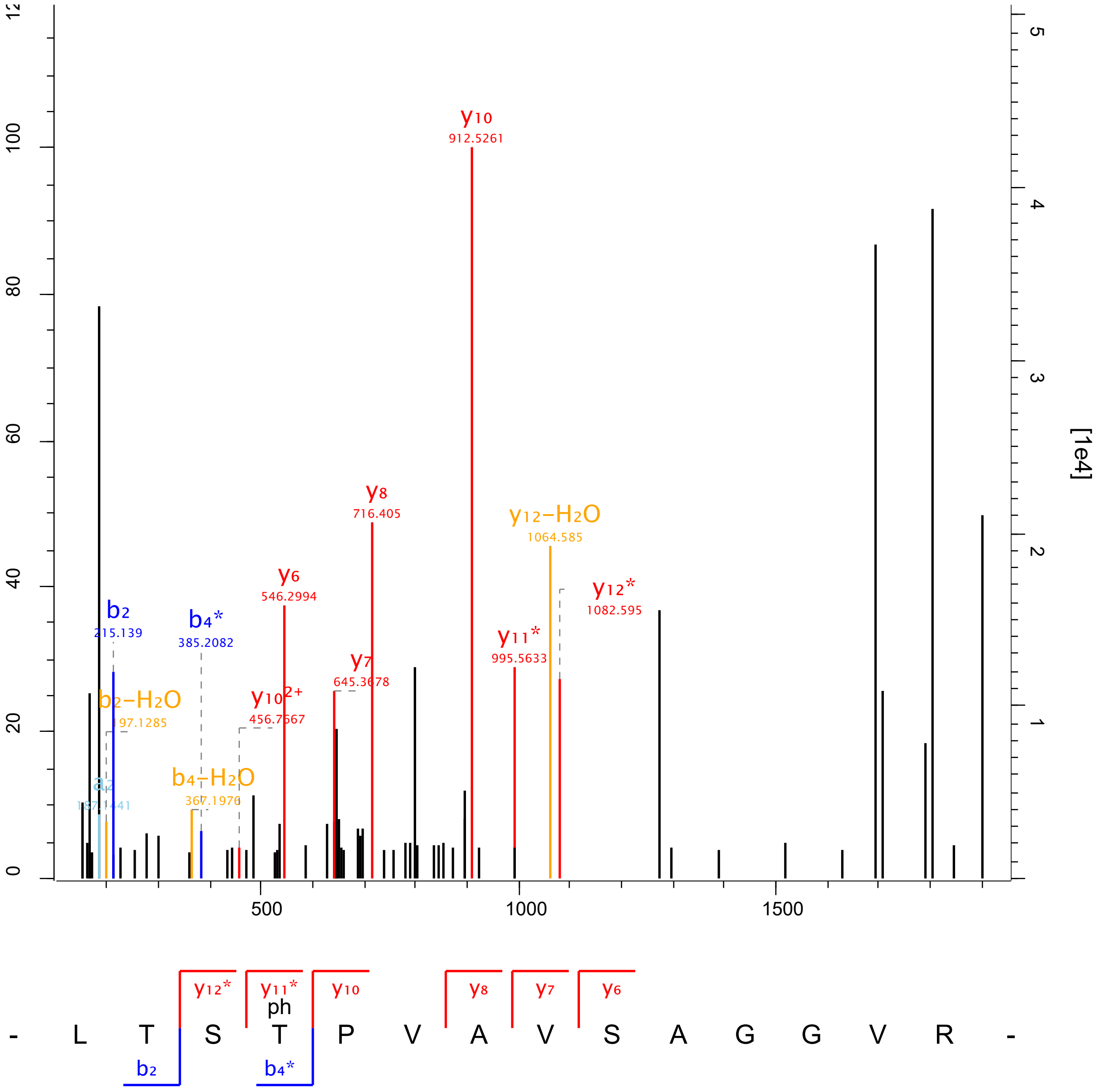

Raw file	Scan	Method	Score	m/z
sys_15_1	16442	FTMS; HCD	182.22	873.36

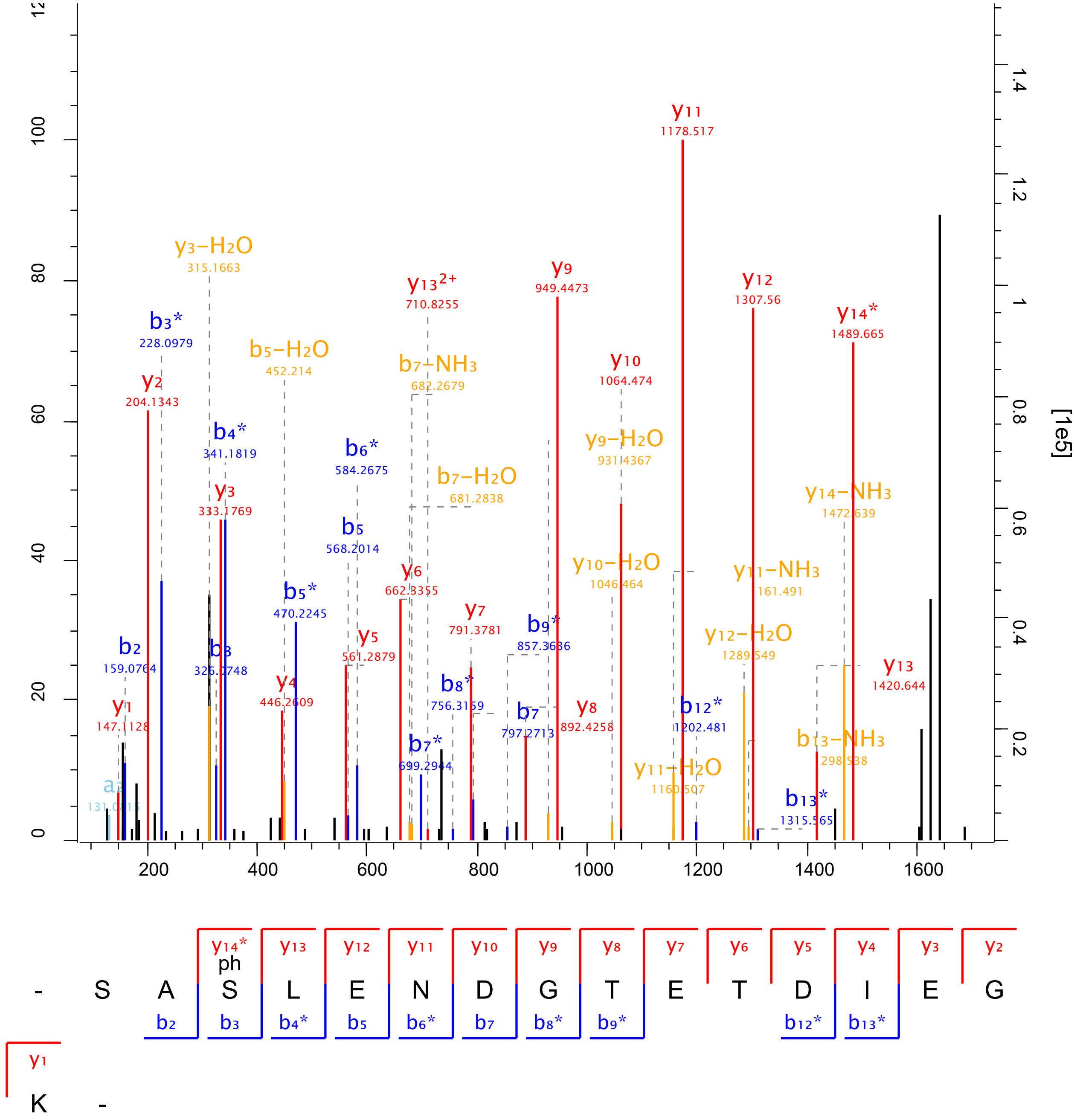

Raw file	Scan	Method	Score	m/z
sys_15_1	16502	FTMS; HCD	116.87	720.83

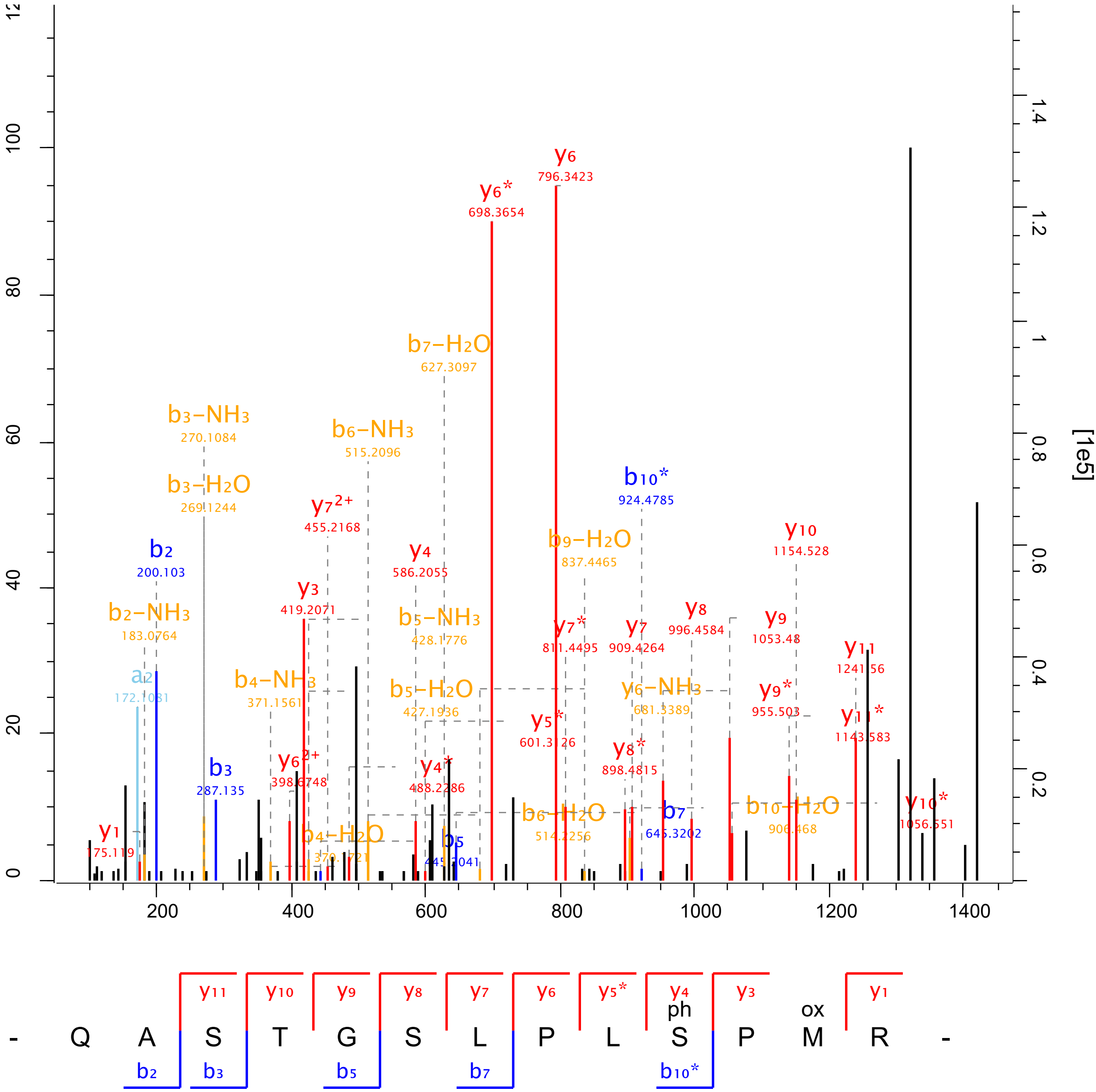

Raw file	Scan	Method	Score	m/z
sys_15_1	16533	FTMS; HCD	80.31	540.76

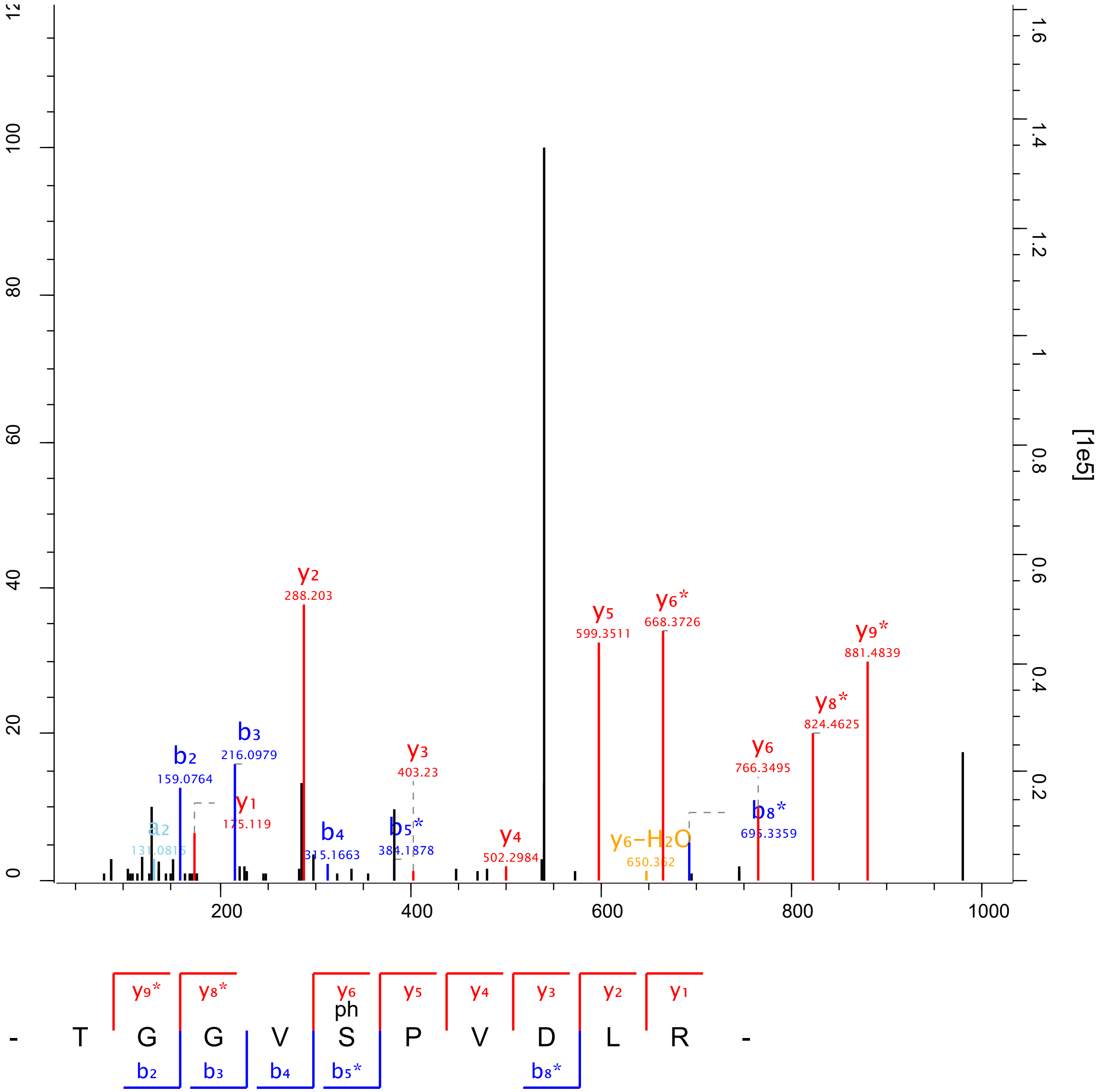

Raw file	Scan	Method	Score	m/z
sys_15_1	16536	FTMS; HCD	142.85	656.64

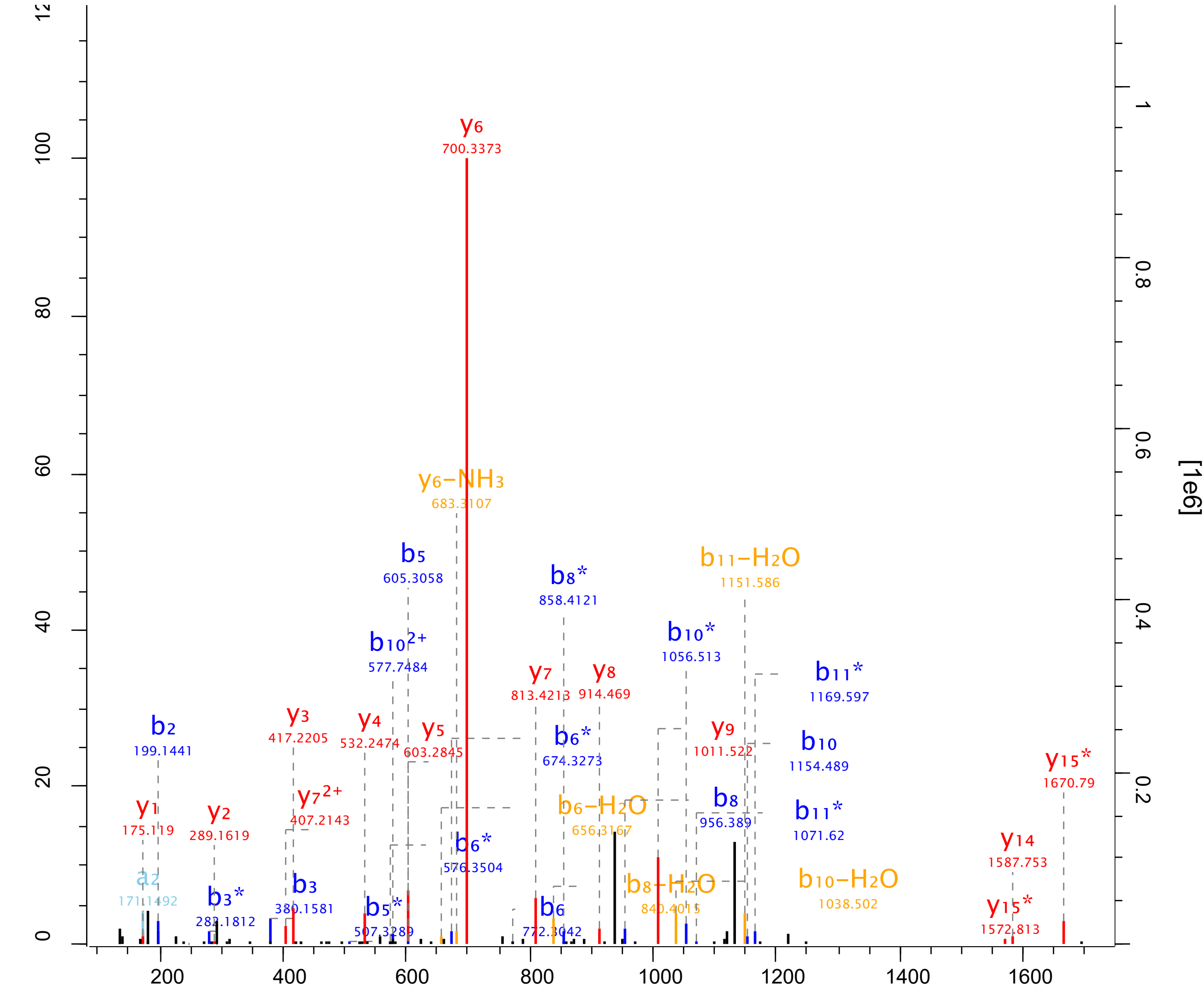

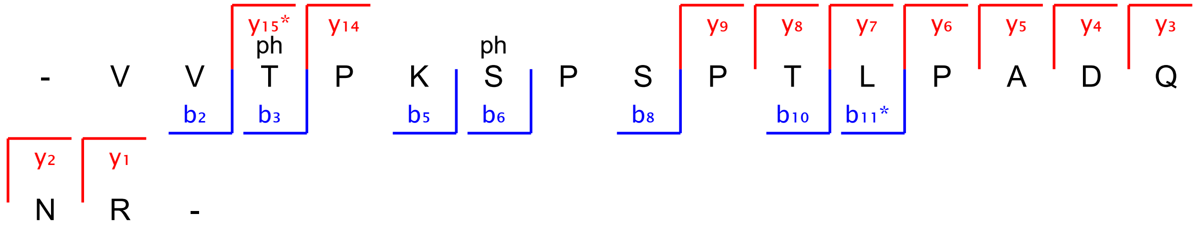

Raw file	Scan	Method	Score	m/z
sys_15_1	16584	FTMS; HCD	92.94	635.28

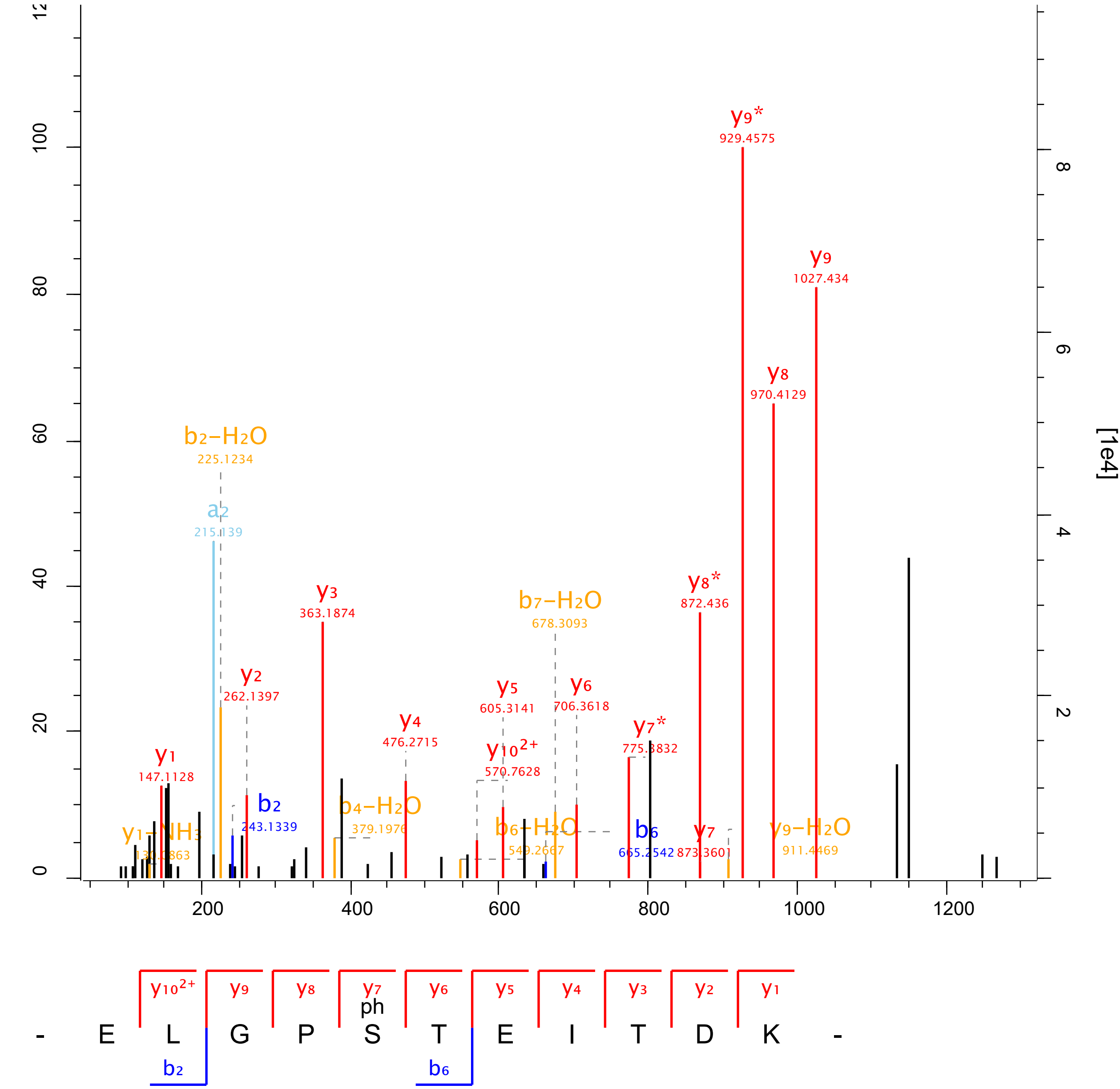

Raw file	Scan	Method	Score	m/z
sys_15_1	16585	FTMS; HCD	42.86	732.67

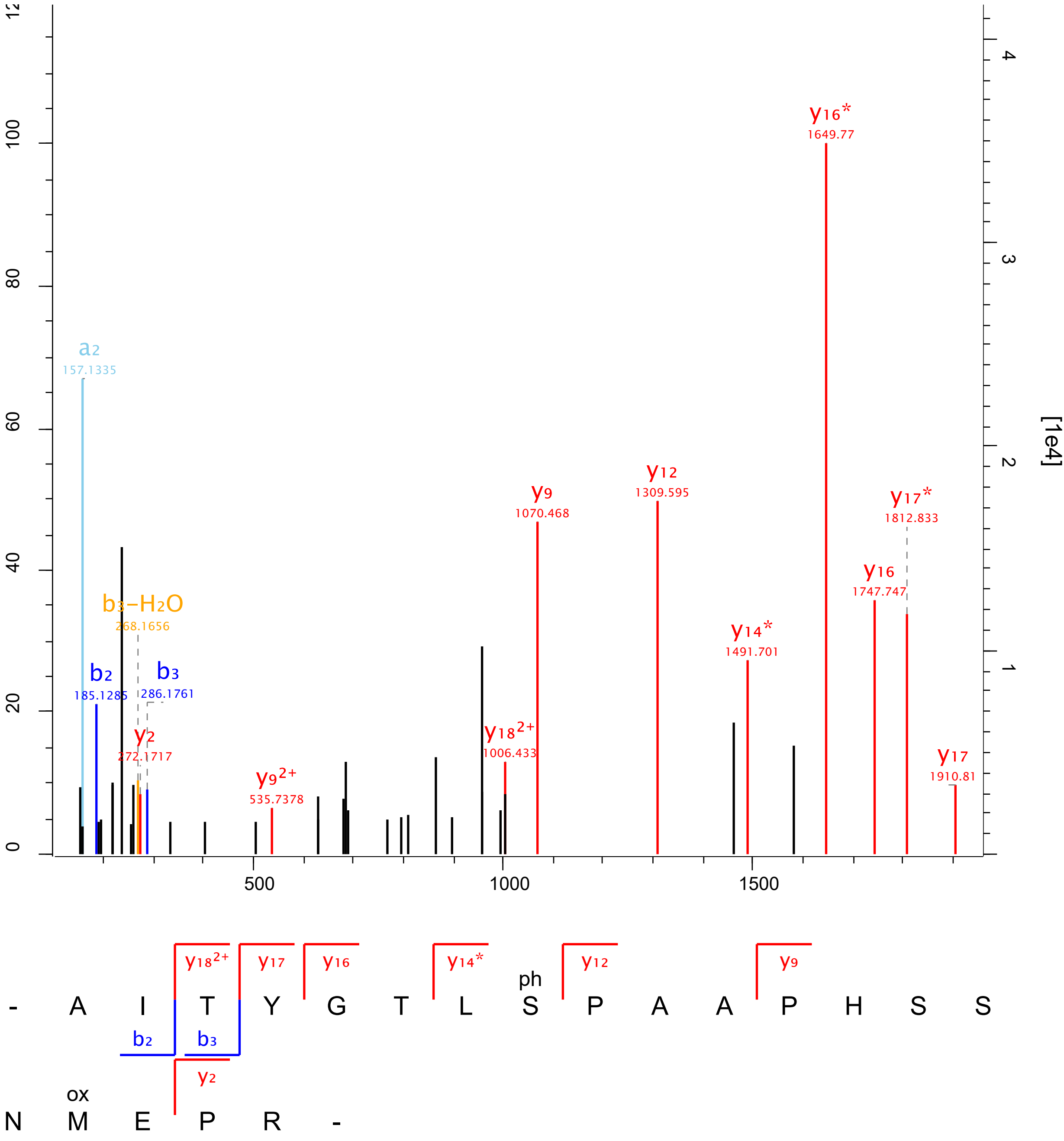

Raw file	Scan	Method	Score	m/z
sys_15_1	16667	FTMS; HCD	107.78	883.87

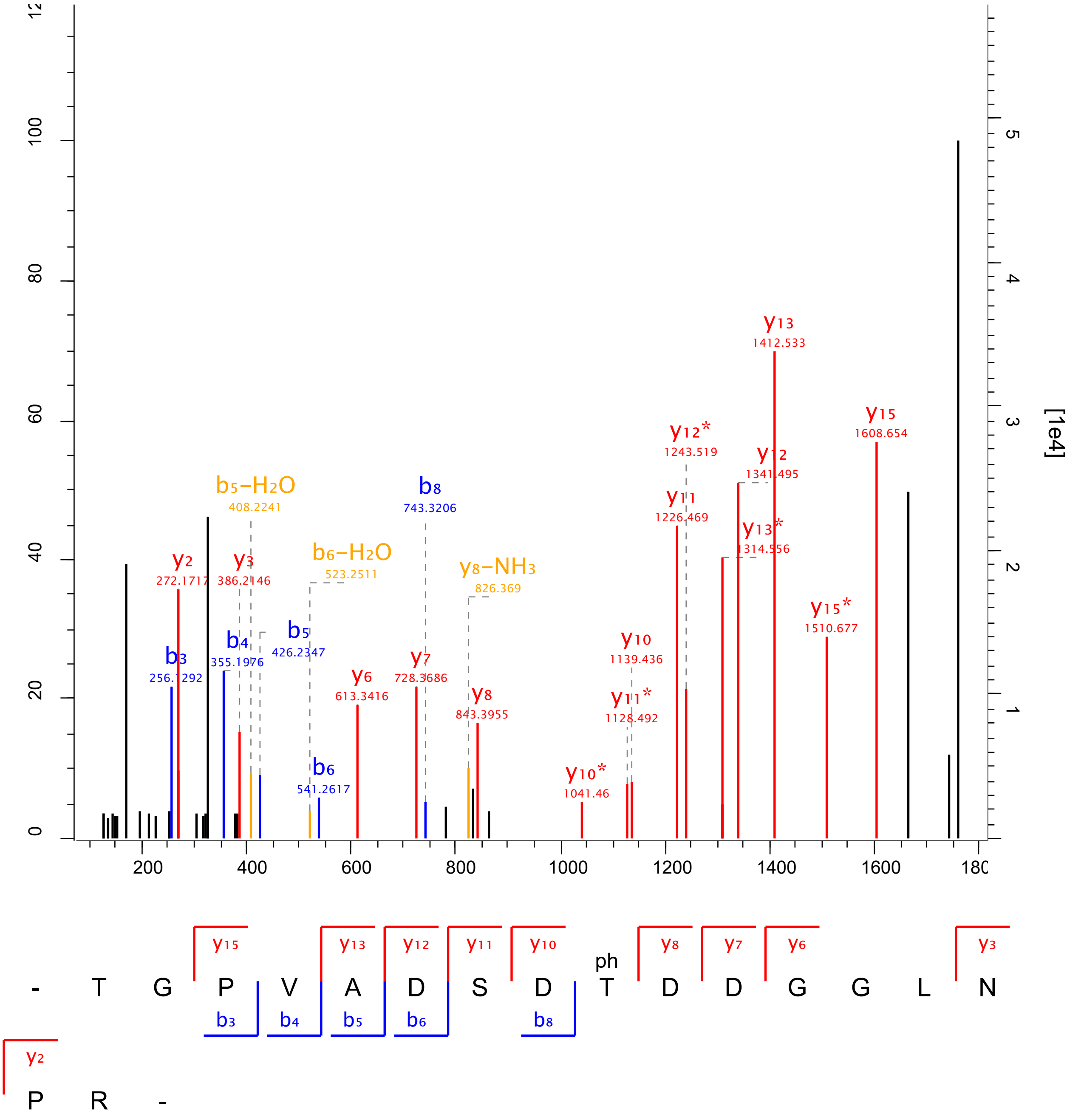

Raw file	Scan	Method	Score	m/z
sys_15_1	16679	FTMS; HCD	151.96	841.34

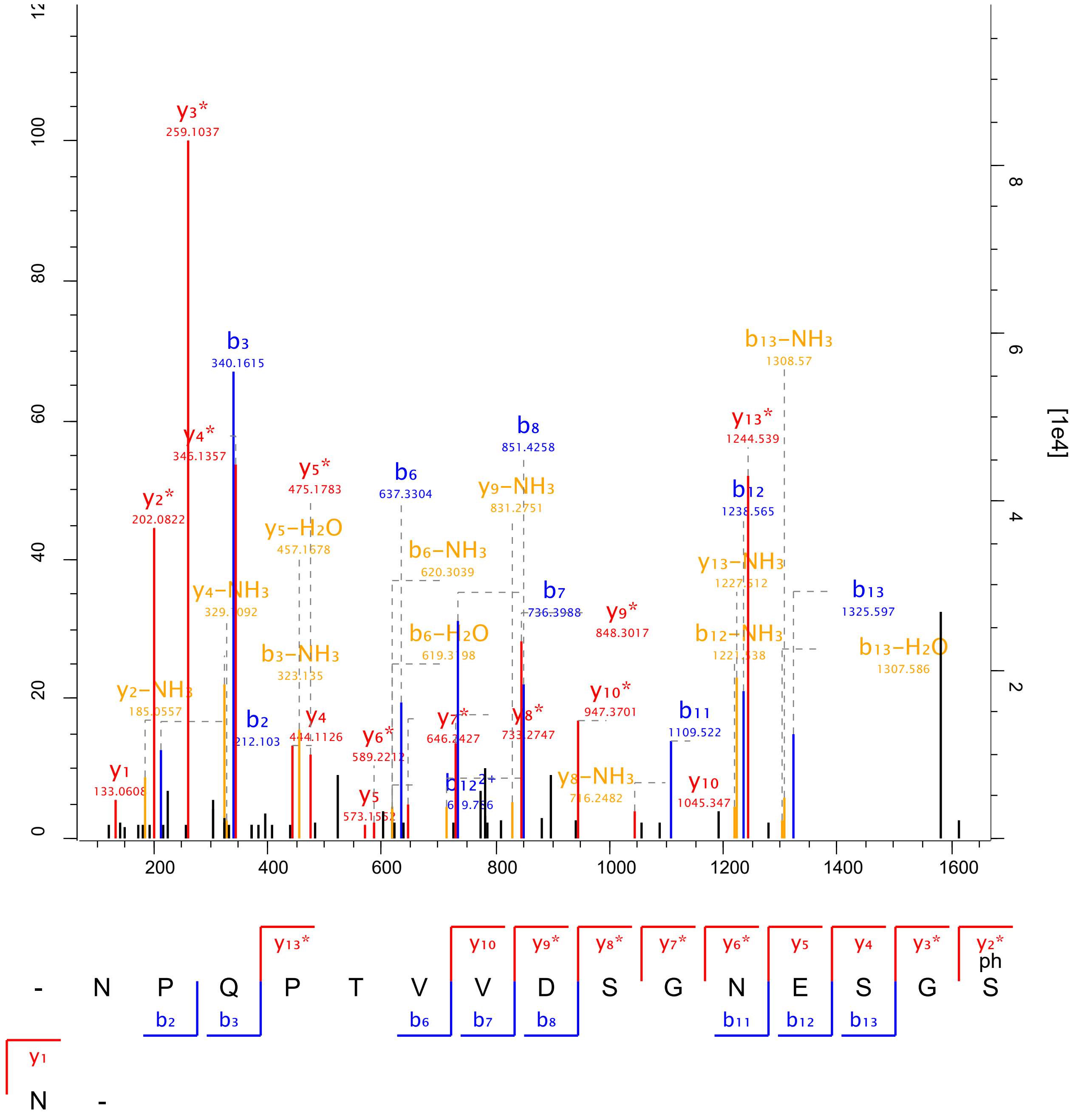

Raw file	Scan	Method	Score	m/z
sys_15_1	16695	FTMS; HCD	45	746.86

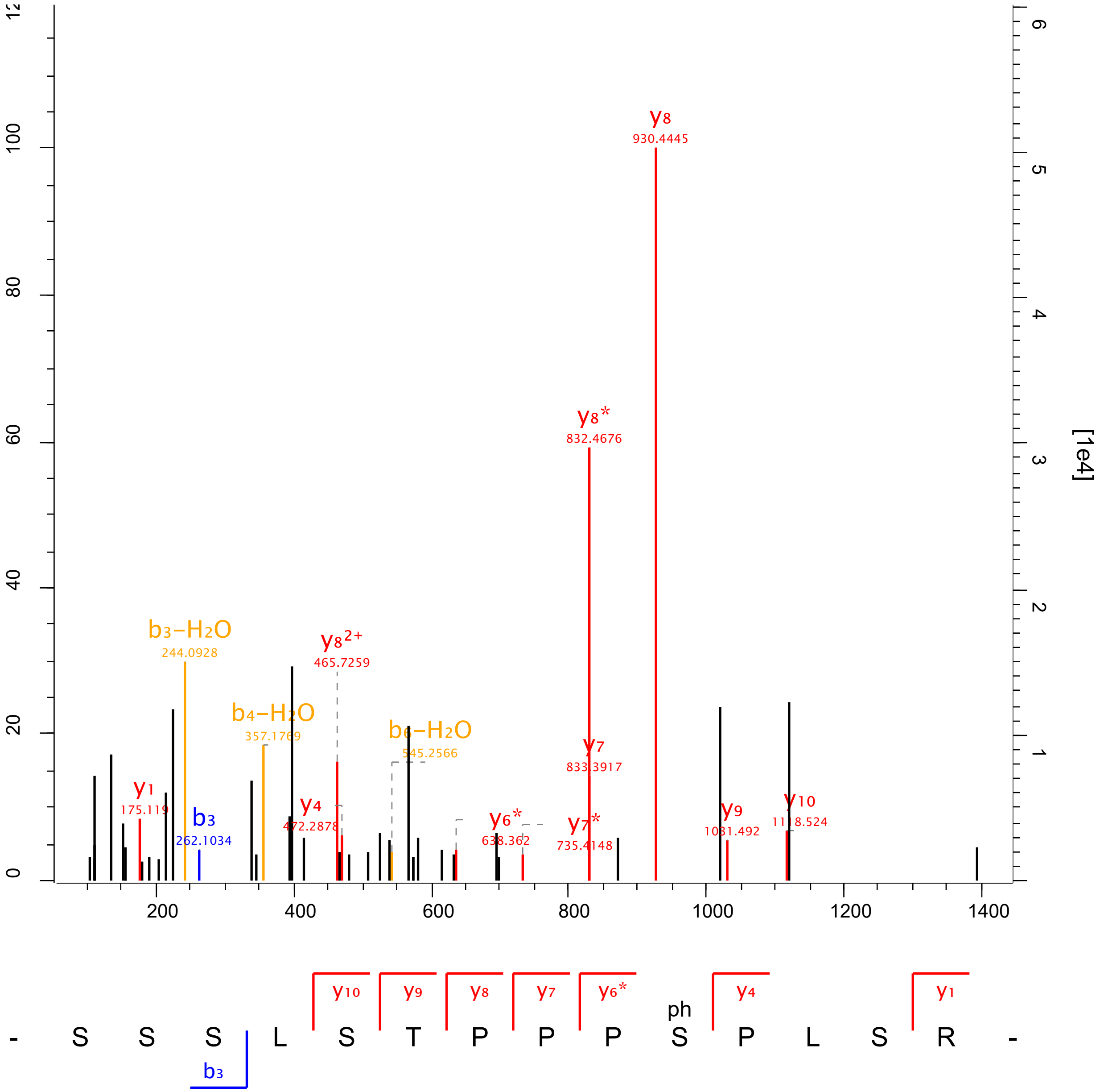

Raw file	Scan	Method	Score	m/z
sys_15_1	16709	FTMS; HCD	117.08	603.27

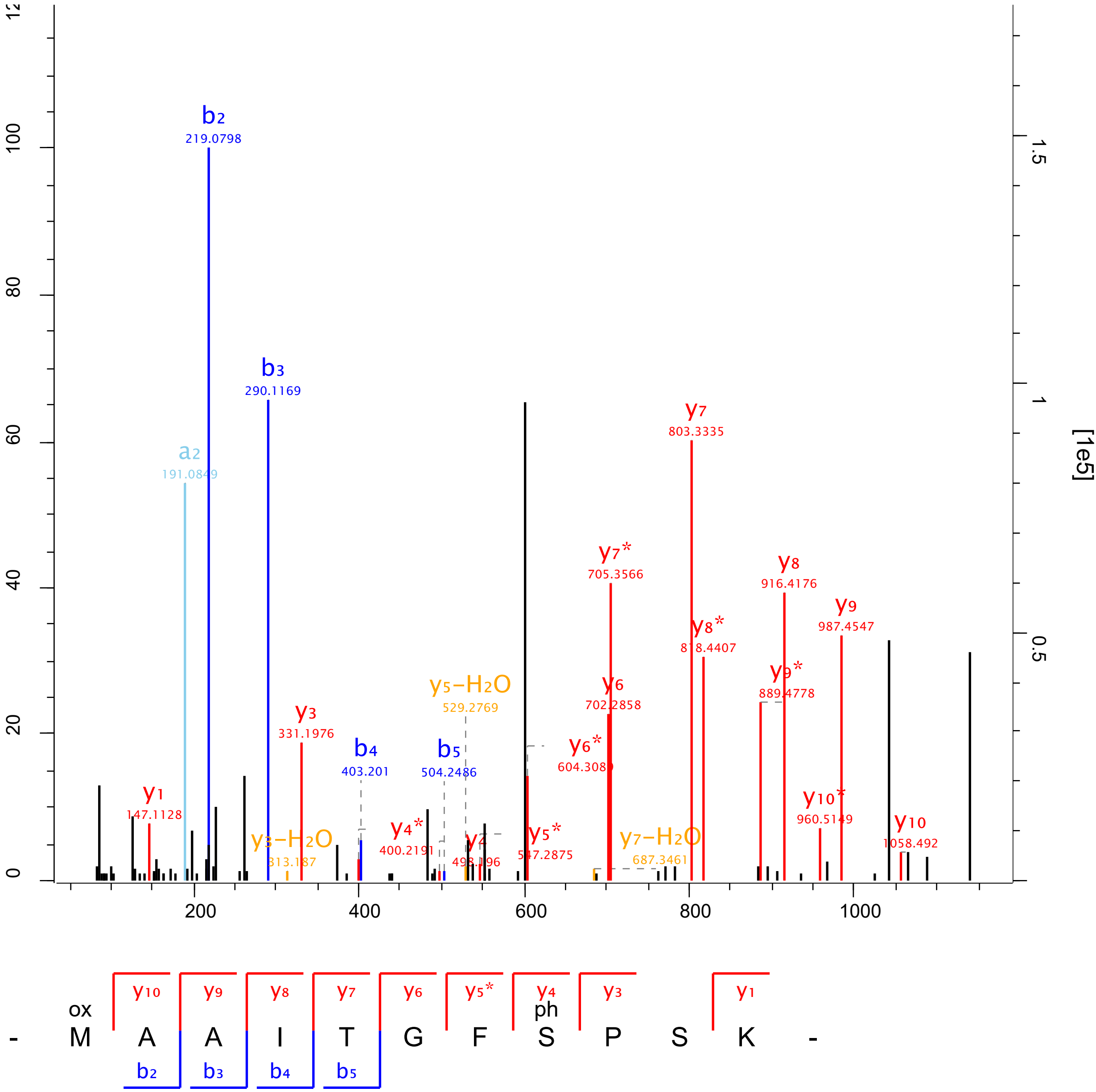

Raw file	Scan	Method	Score	m/z
sys_15_1	16718	FTMS; HCD	46.07	785.31

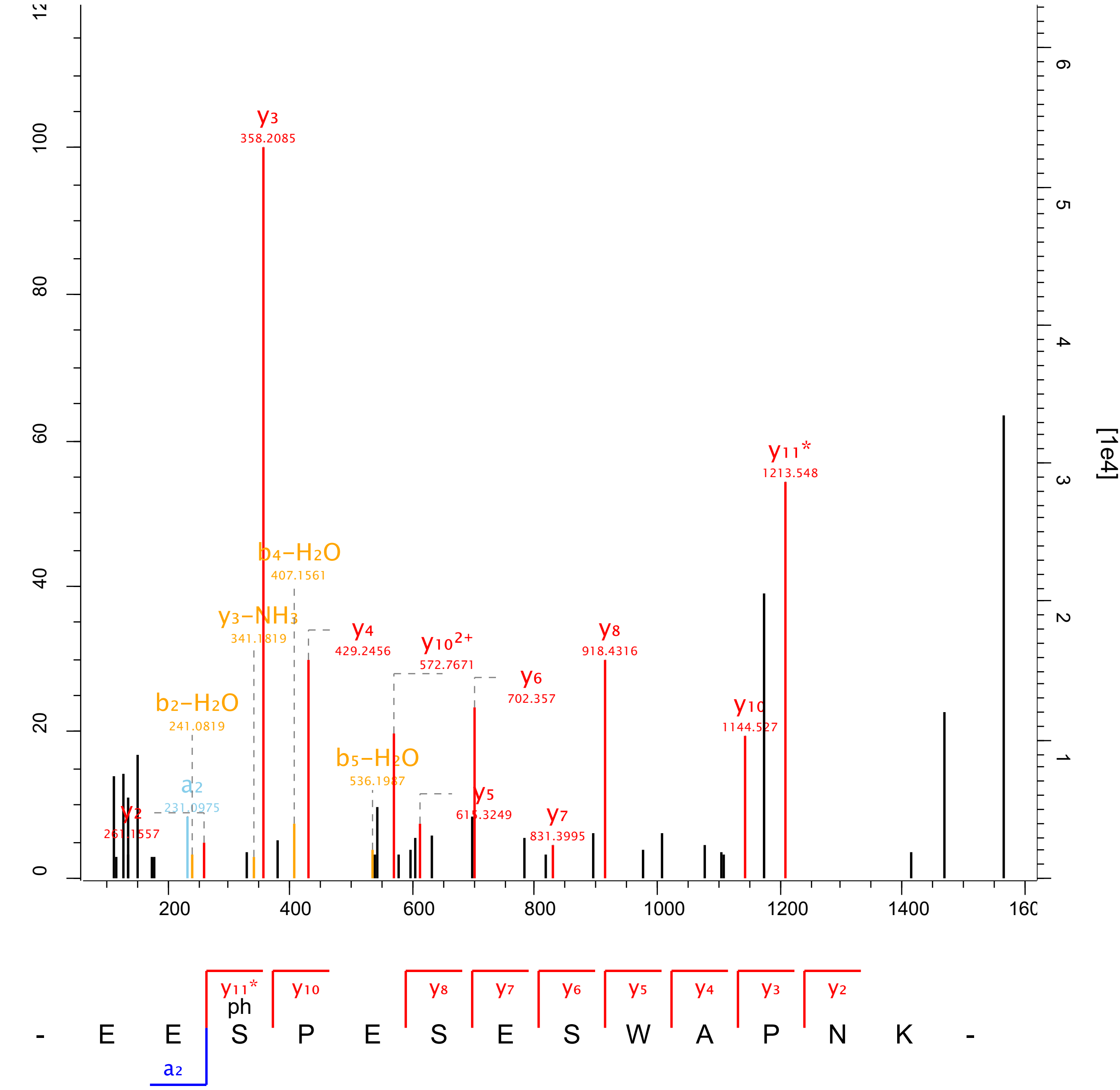

Raw file	Scan	Method	Score	m/z
sys_15_1	16829	FTMS; HCD	107.09	542.23

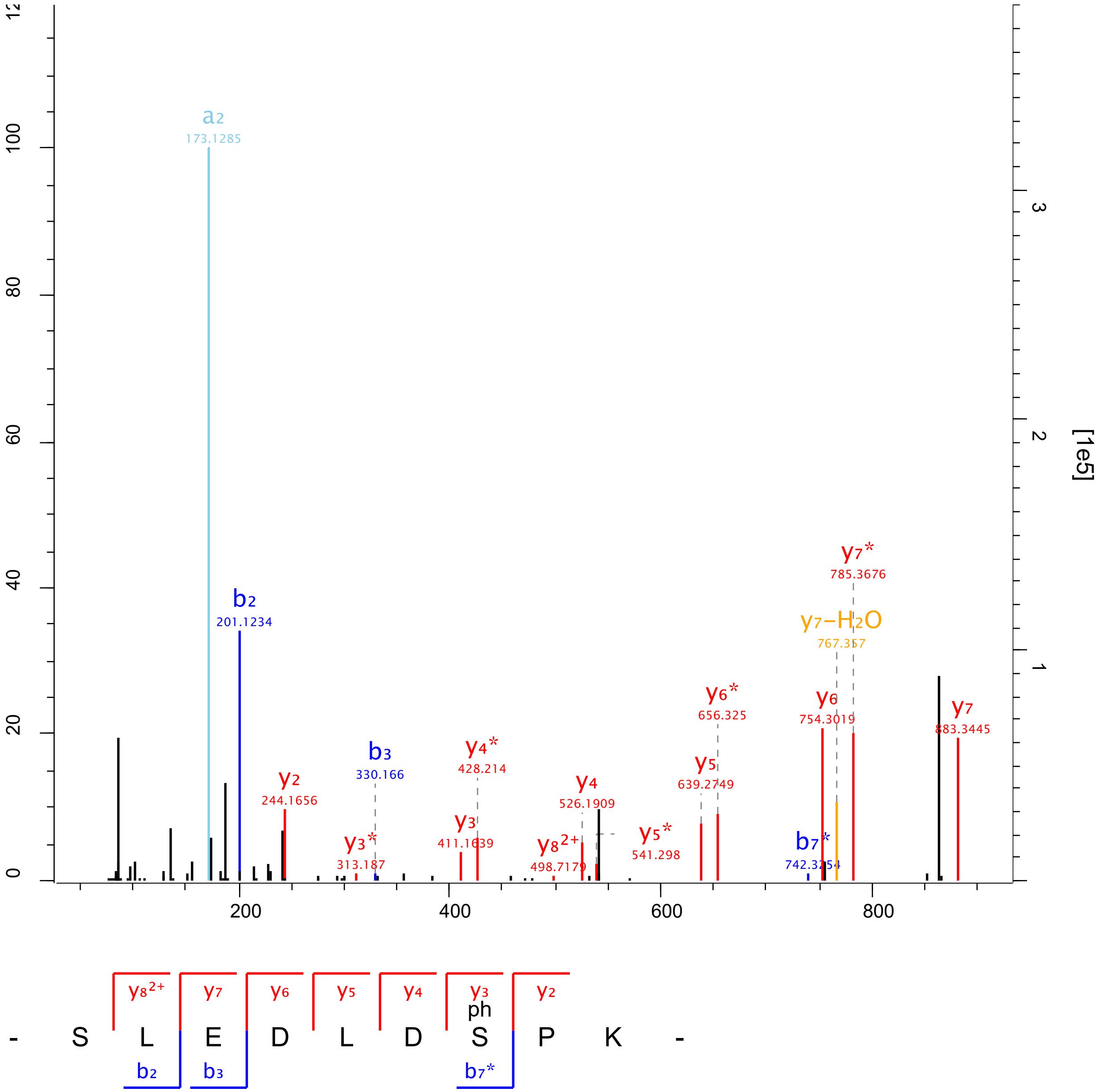

Raw file	Scan	Method	Score	m/z
sys_15_1	16844	FTMS; HCD	44.93	512.25

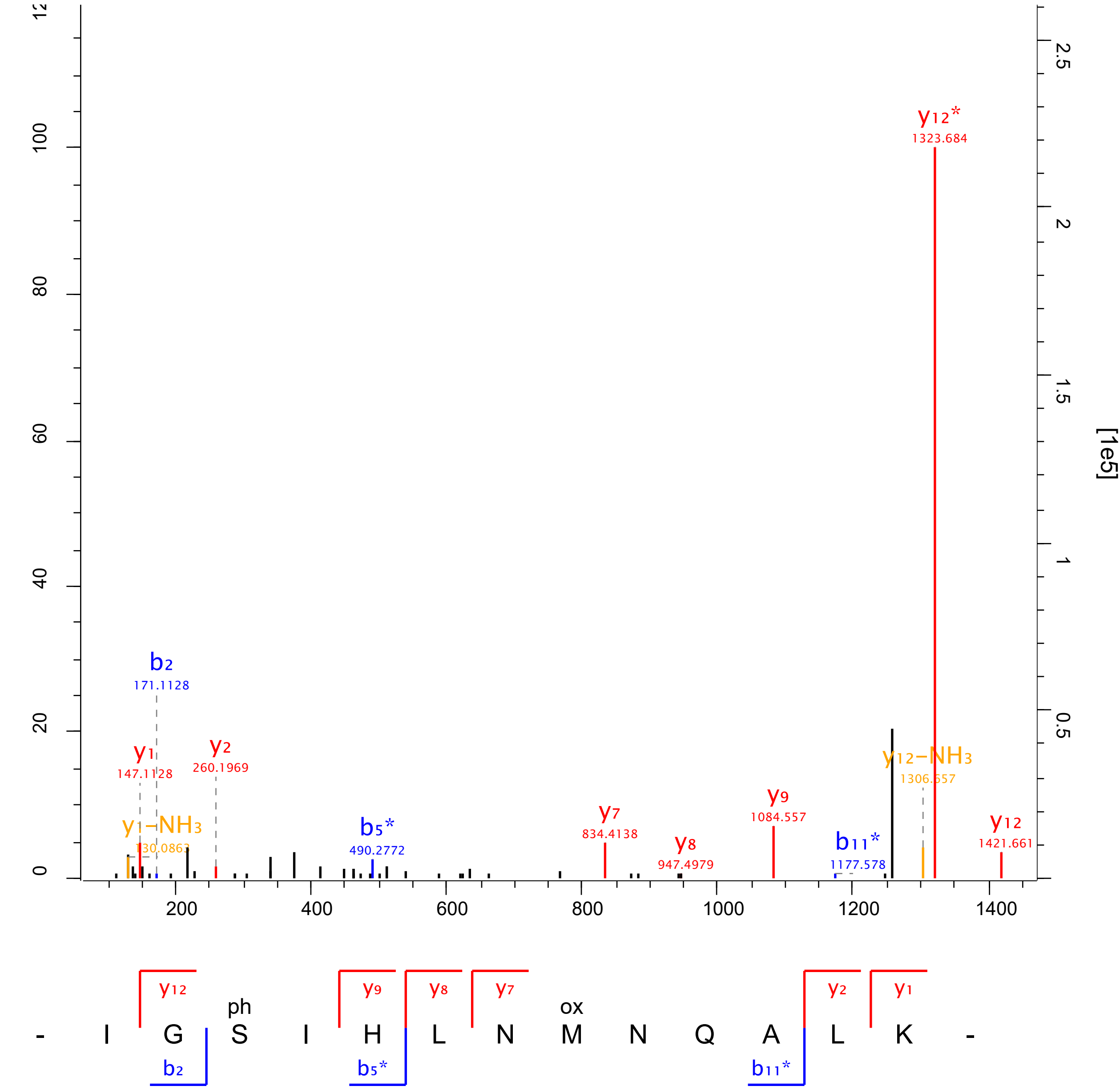

Raw file	Scan	Method	Score	m/z
sys_15_1	1685	FTMS; HCD	91.55	365.18

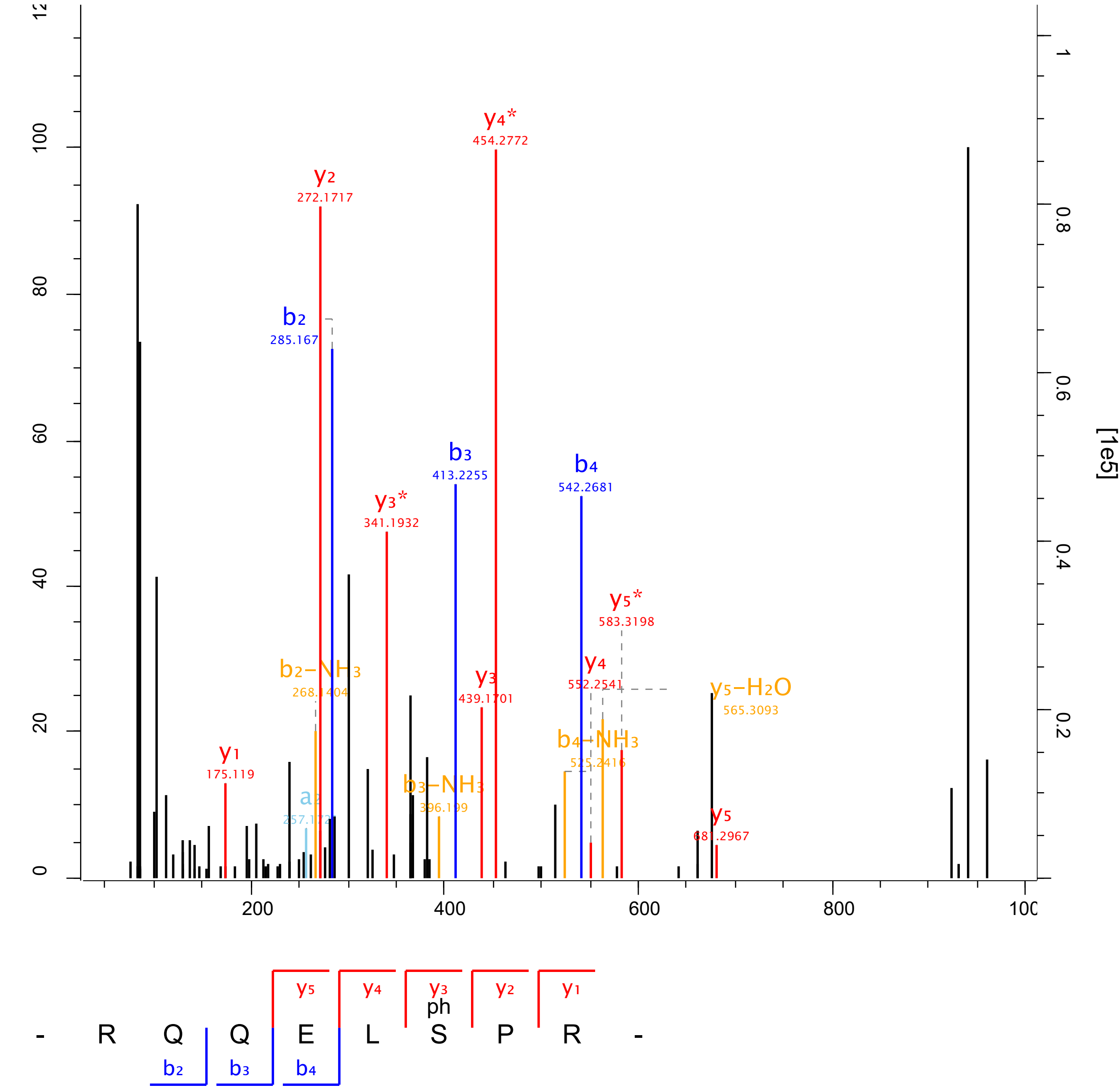

Raw file	Scan	Method	Score	m/z
sys_15_1	16850	FTMS; HCD	44.56	651.81

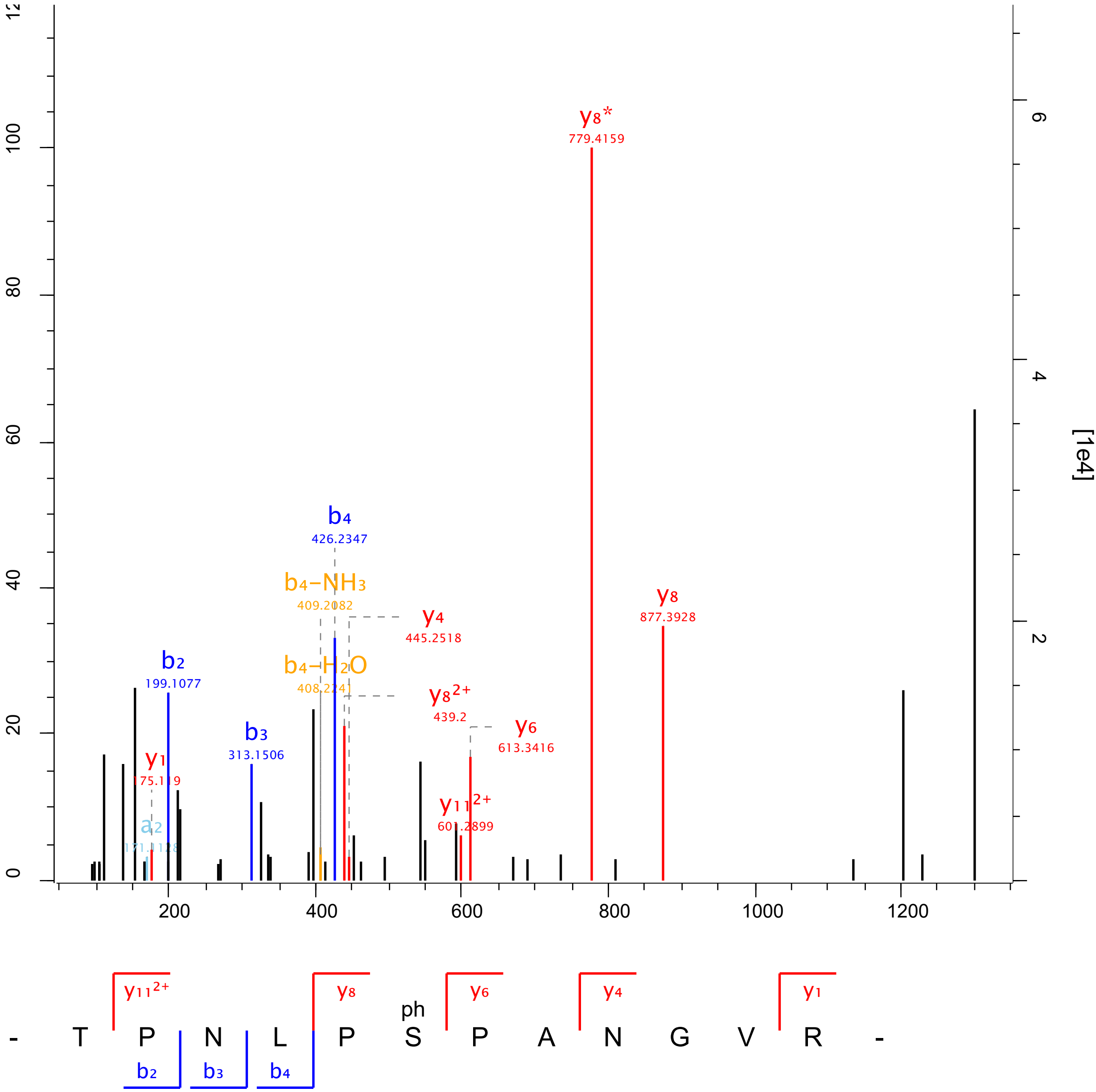

Raw file	Scan	Method	Score	m/z
sys_15_1	16904	FTMS; HCD	162.81	693.32

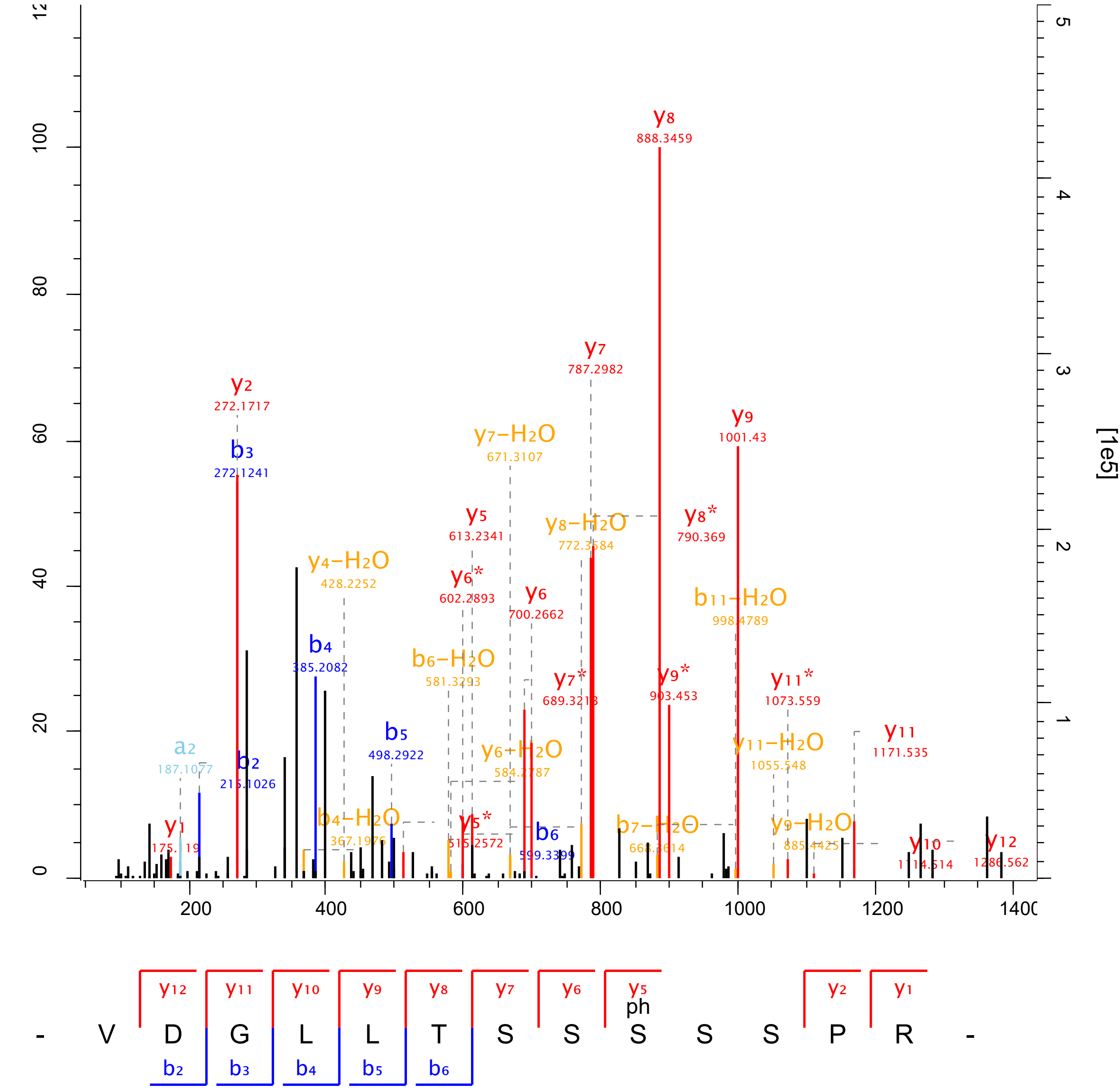

Raw file	Scan	Method	Score	m/z
sys_15_1	17036	FTMS; HCD	234.74	926.71

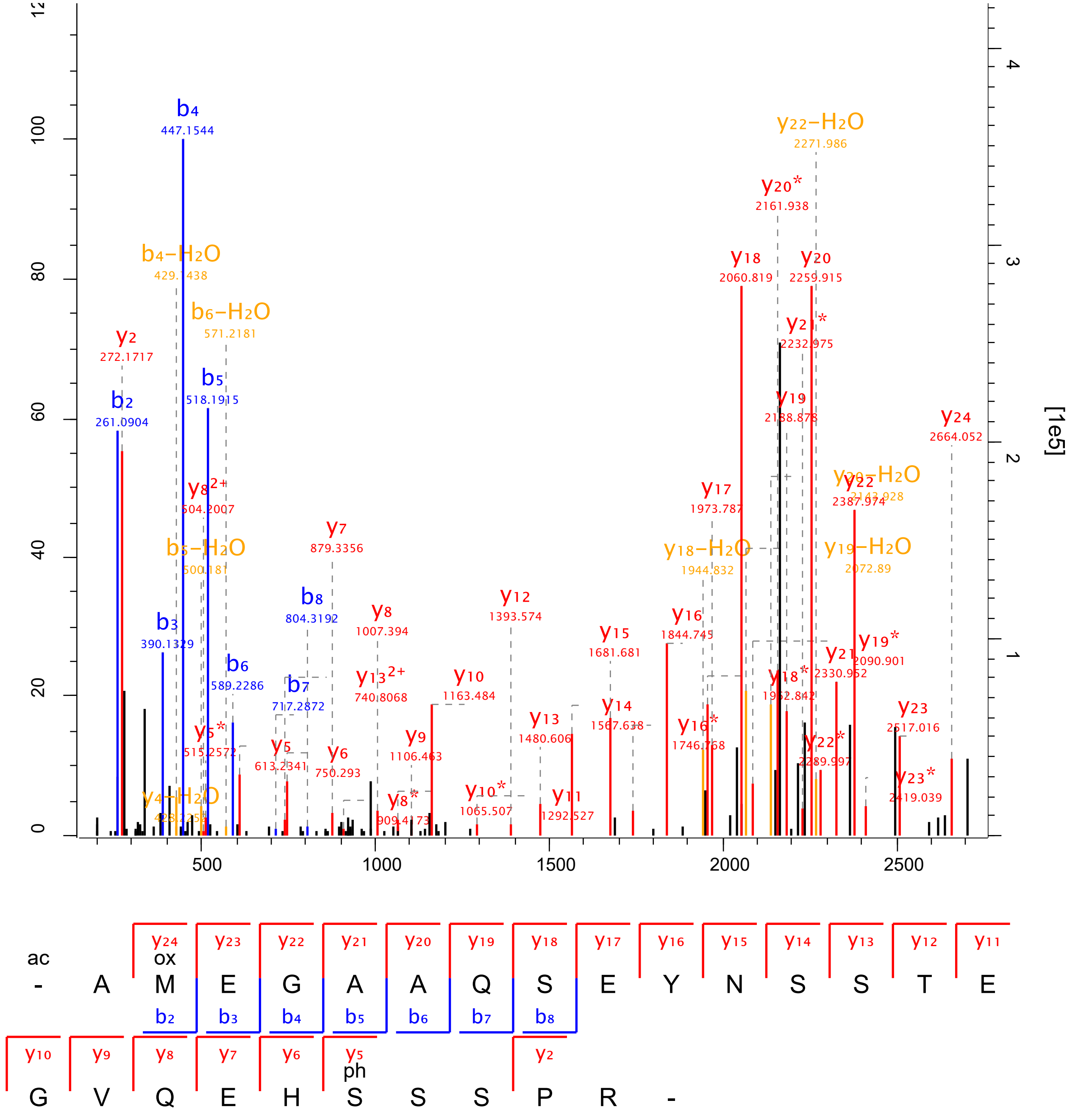

Raw file	Scan	Method	Score	m/z
sys_15_1	17048	FTMS; HCD	135.14	689.8

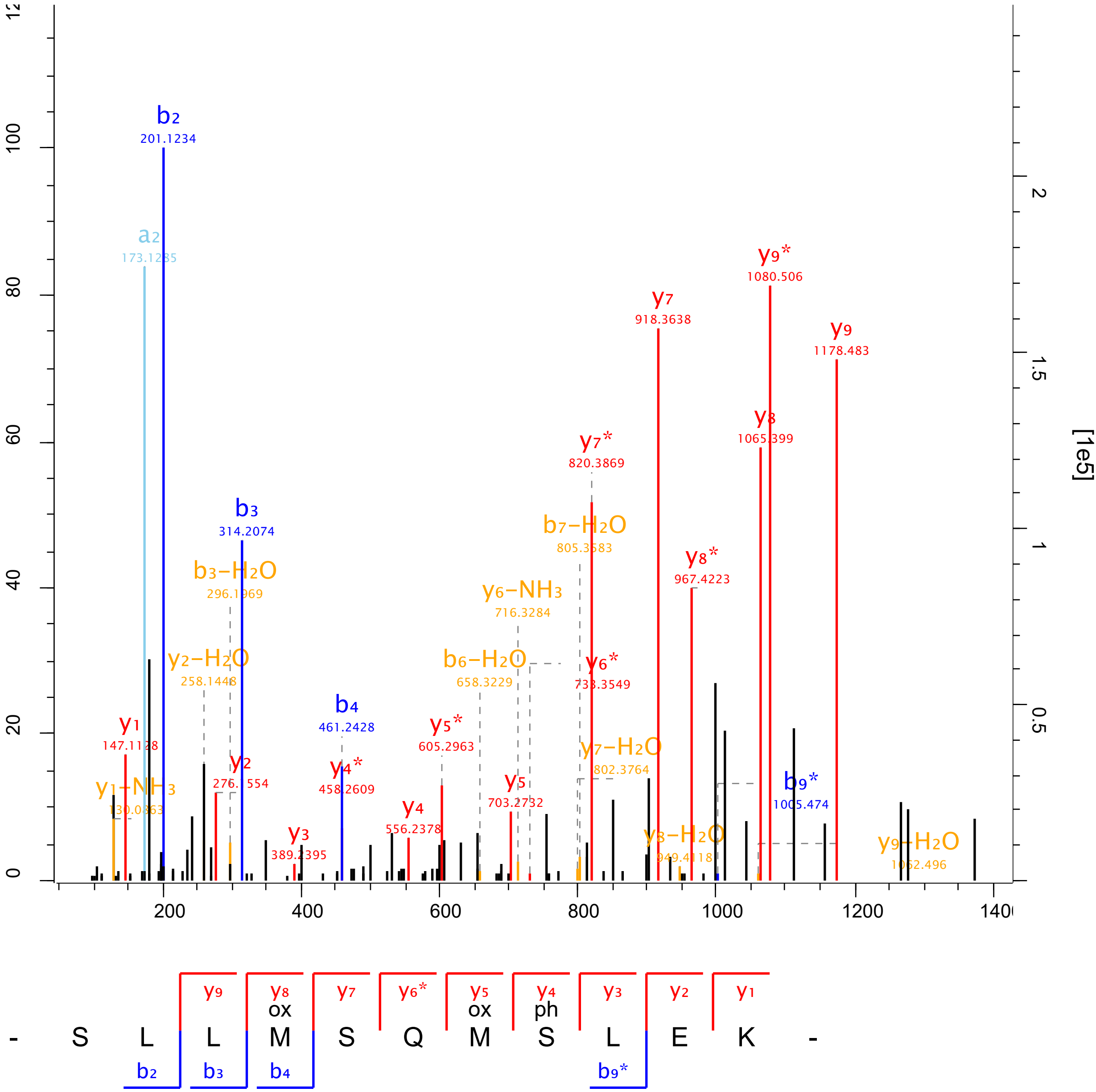

Raw file	Scan	Method	Score	m/z
sys_15_1	17081	FTMS; HCD	89.23	642.8

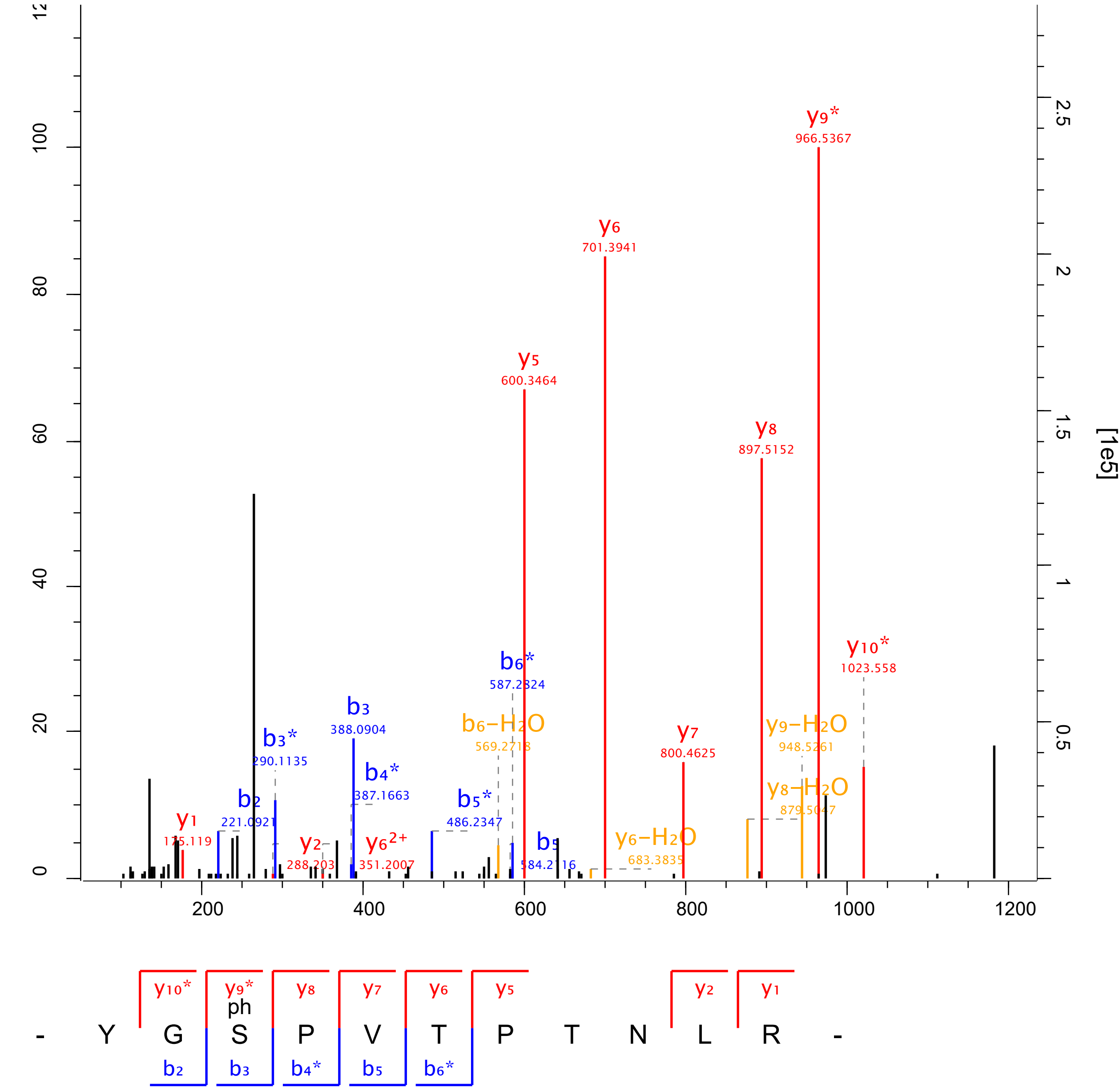

Raw file	Scan	Method	Score	m/z
sys_15_1	17228	FTMS; HCD	43.84	979.92

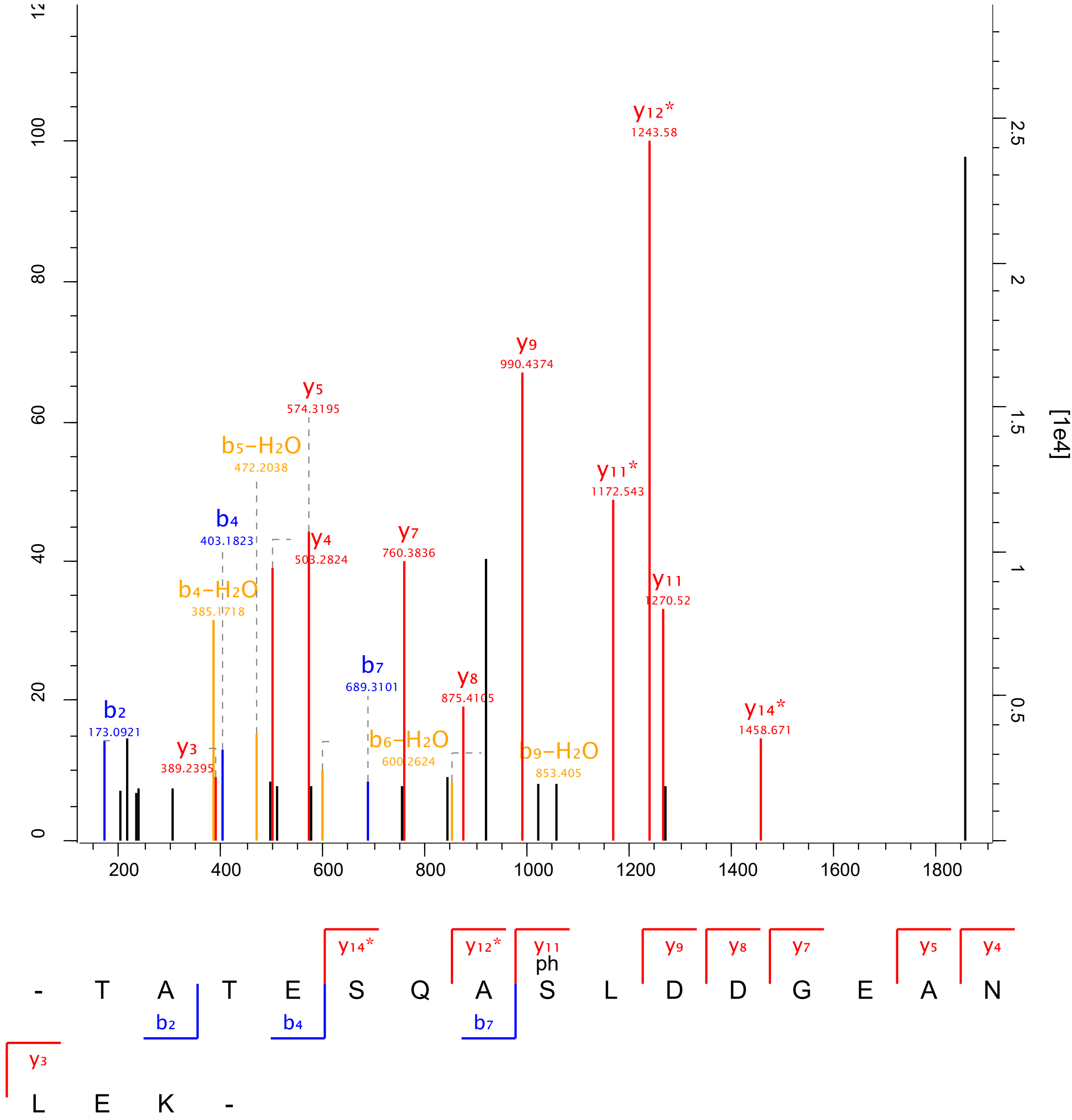

Raw file	Scan	Method	Score	m/z
sys_15_1	17289	FTMS; HCD	100.97	839.84

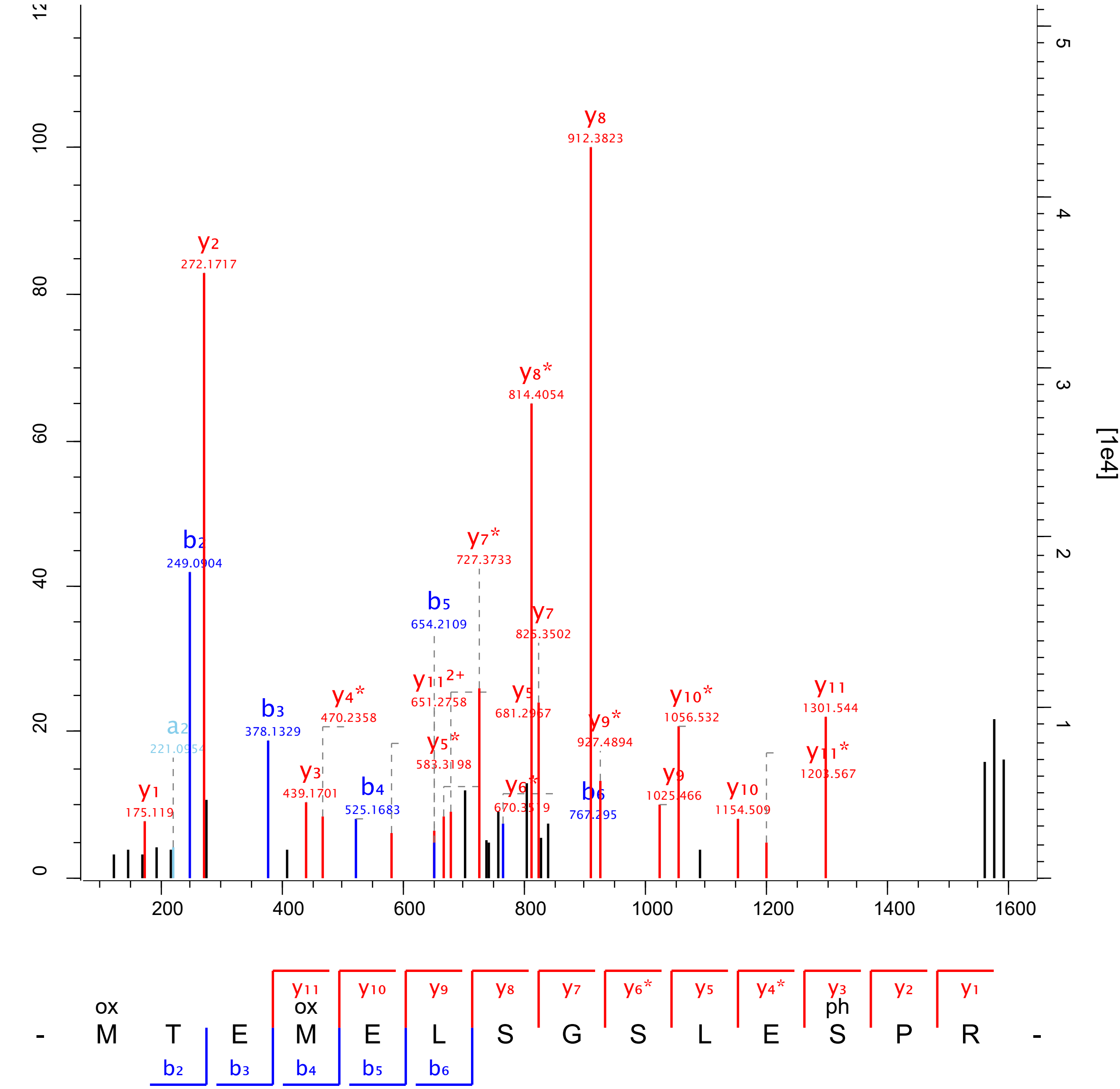

Raw file	Scan	Method	Score	m/z
sys_15_1	17319	FTMS; HCD	178.15	1046.91

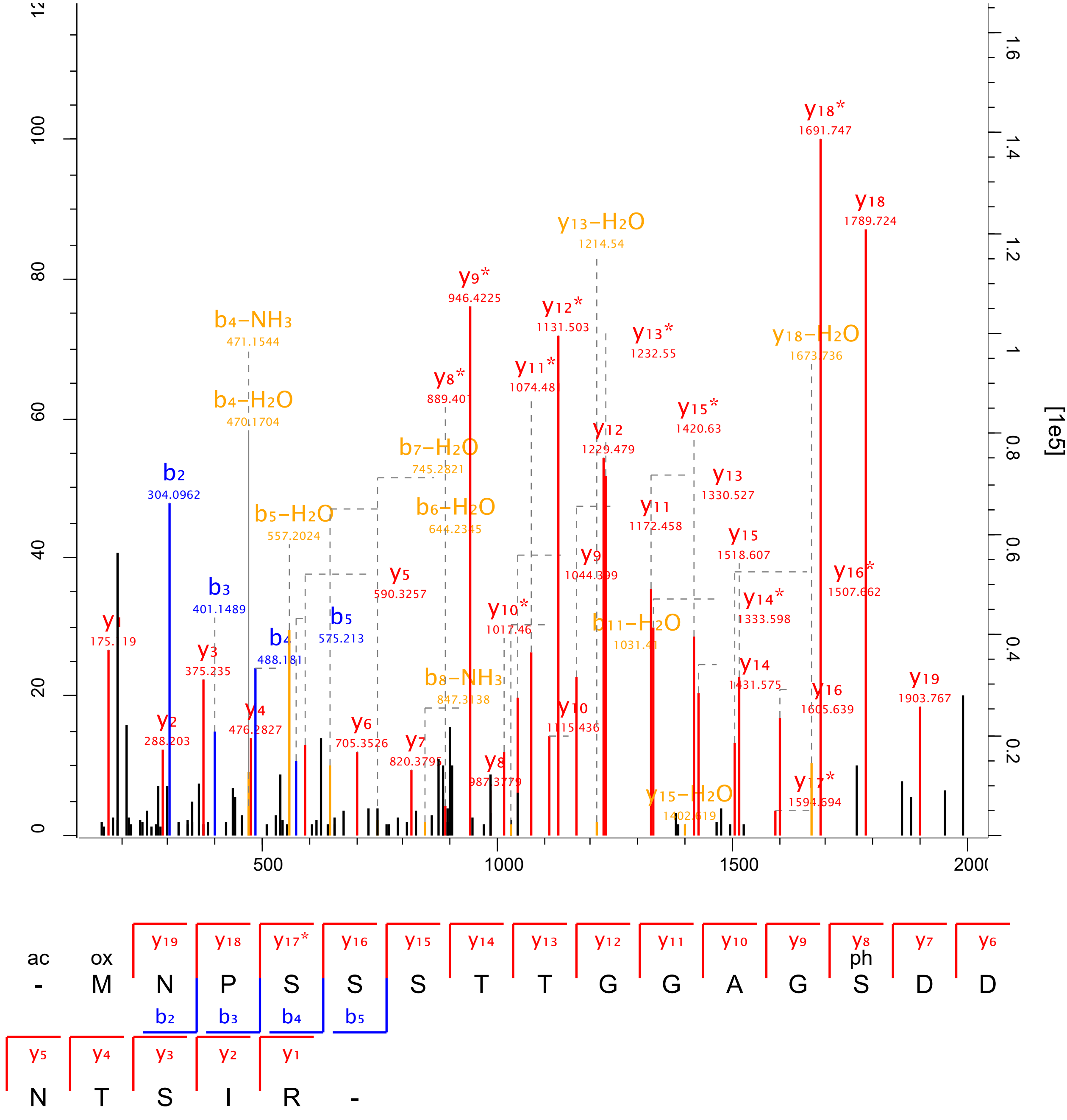

Raw file	Scan	Method	Score	m/z
sys_15_1	17338	FTMS; HCD	195.92	872.35

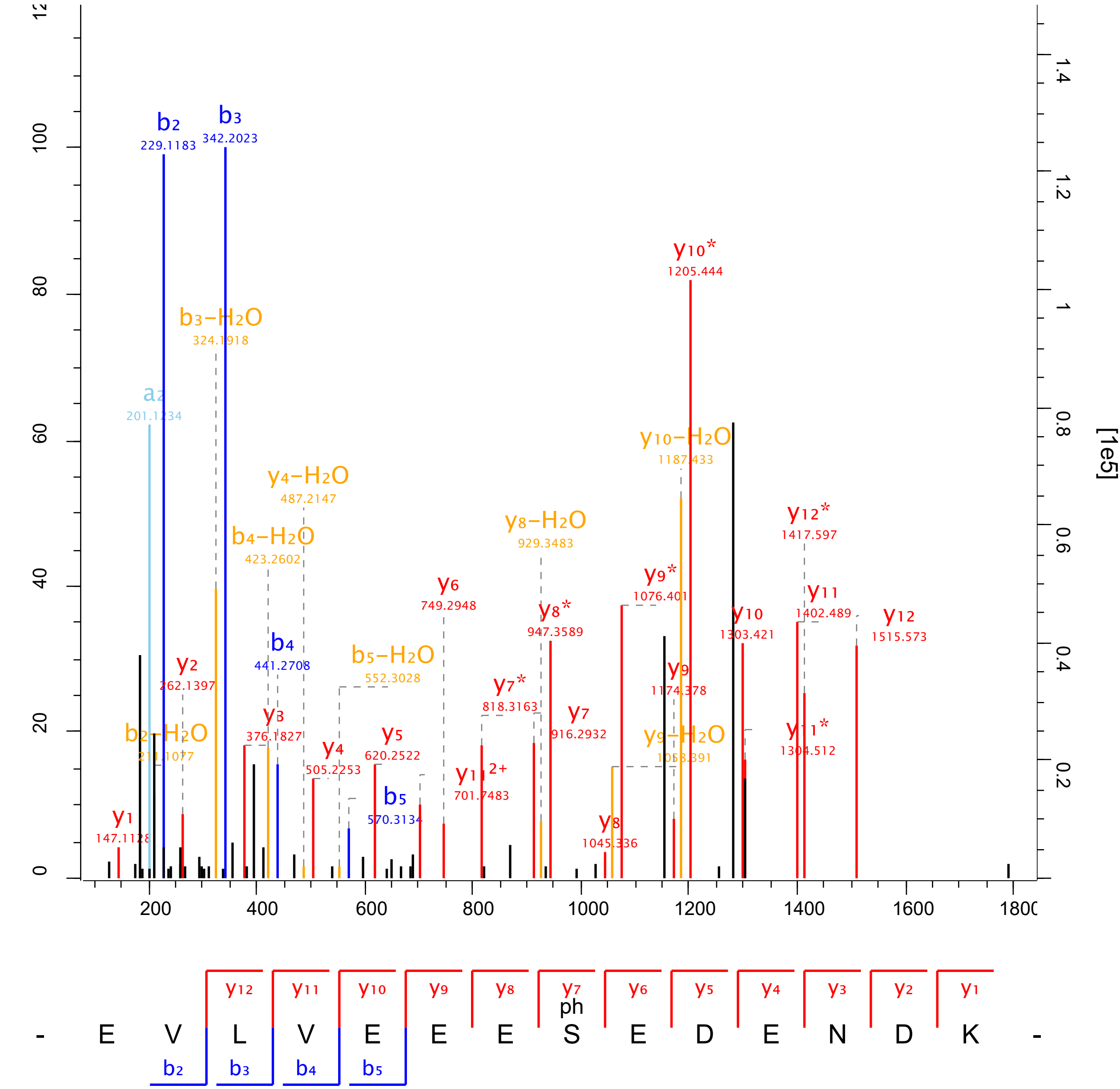

Raw file	Scan	Method	Score	m/z
sys_15_1	17496	FTMS; HCD	172.13	576.26

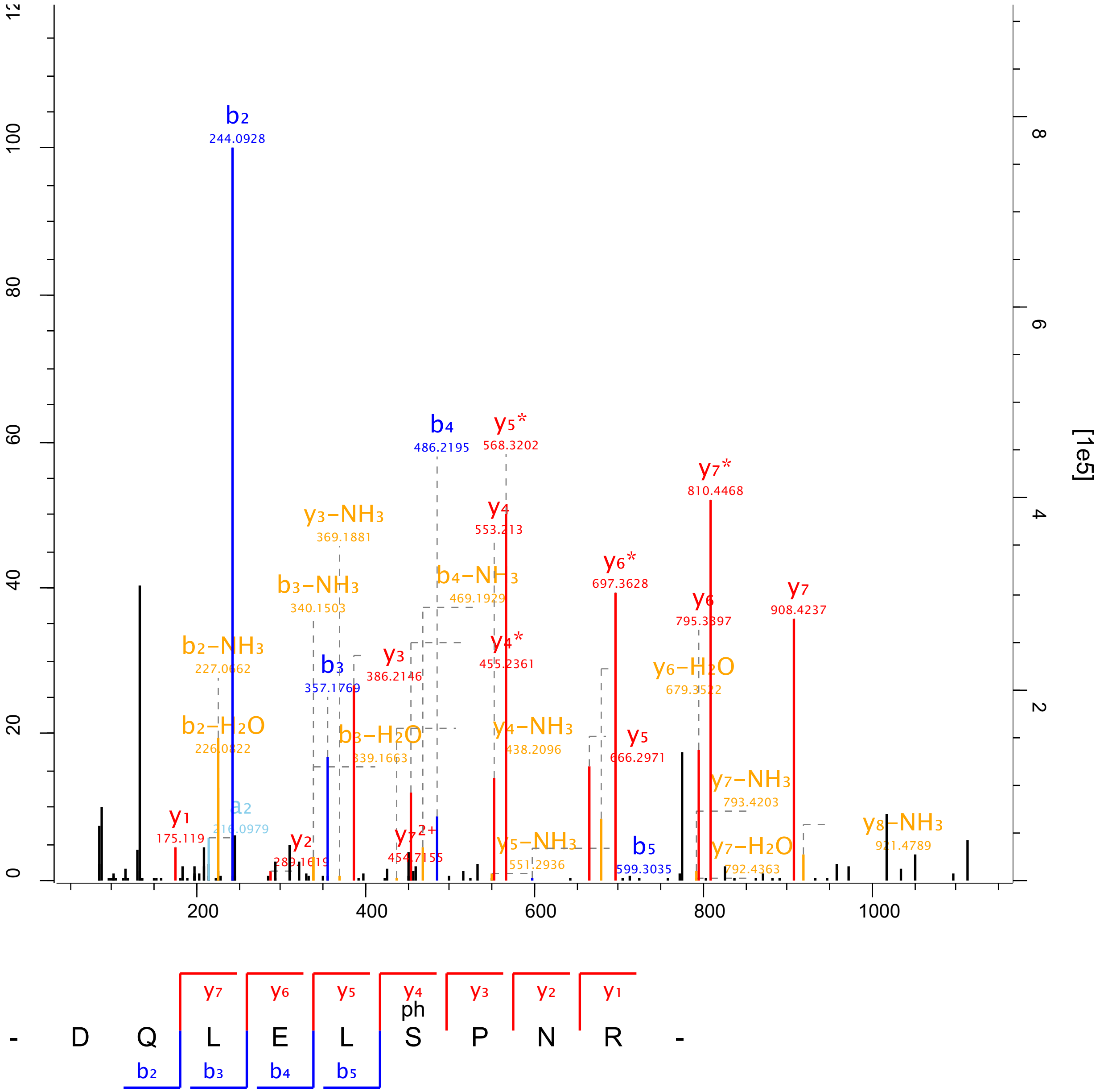

Raw file	Scan	Method	Score	m/z
sys_15_1	17509	FTMS; HCD	69.98	848.86

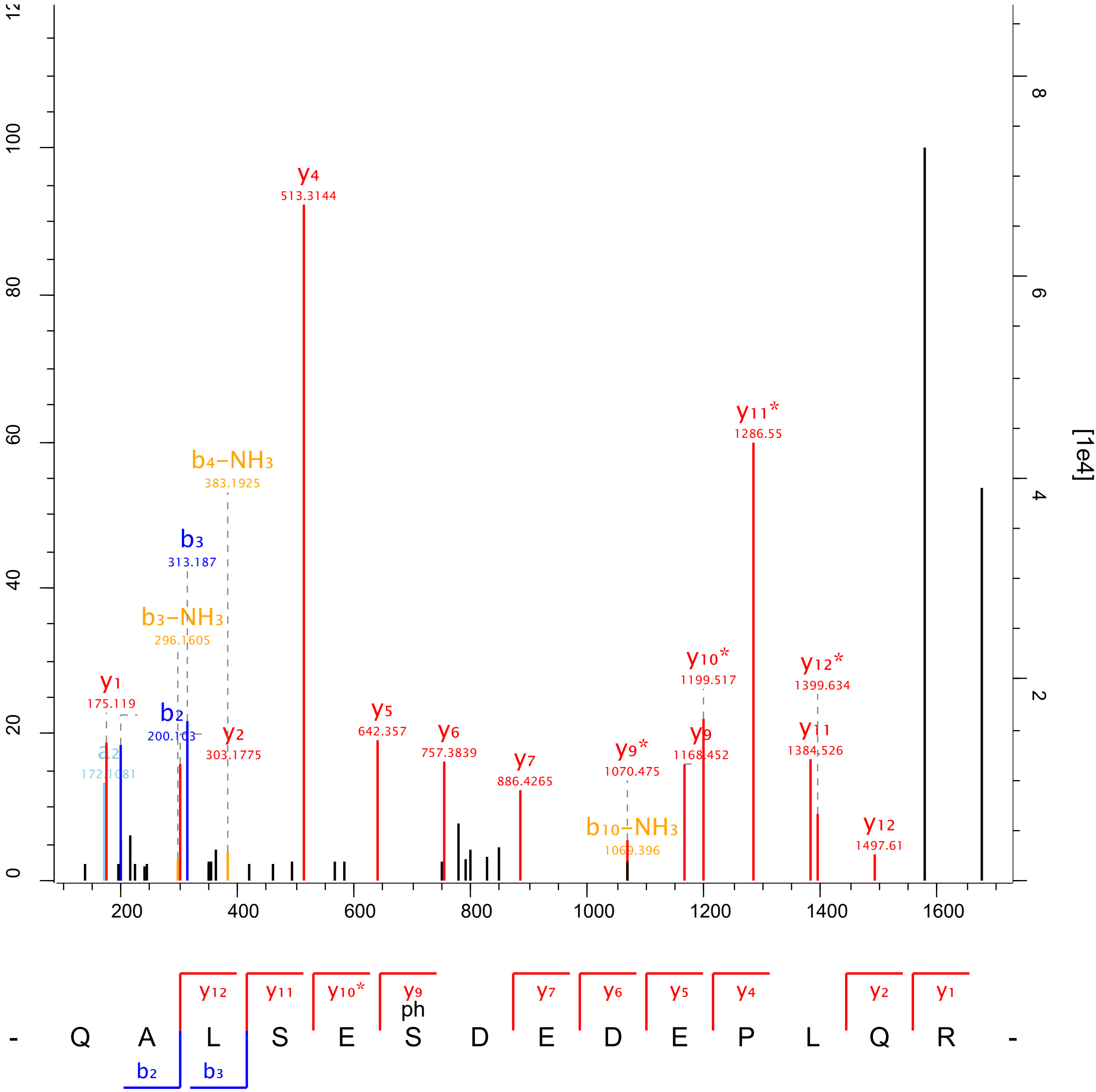

Raw file	Scan	Method	Score	m/z
sys_15_1	17529	FTMS; HCD	148.96	580.75

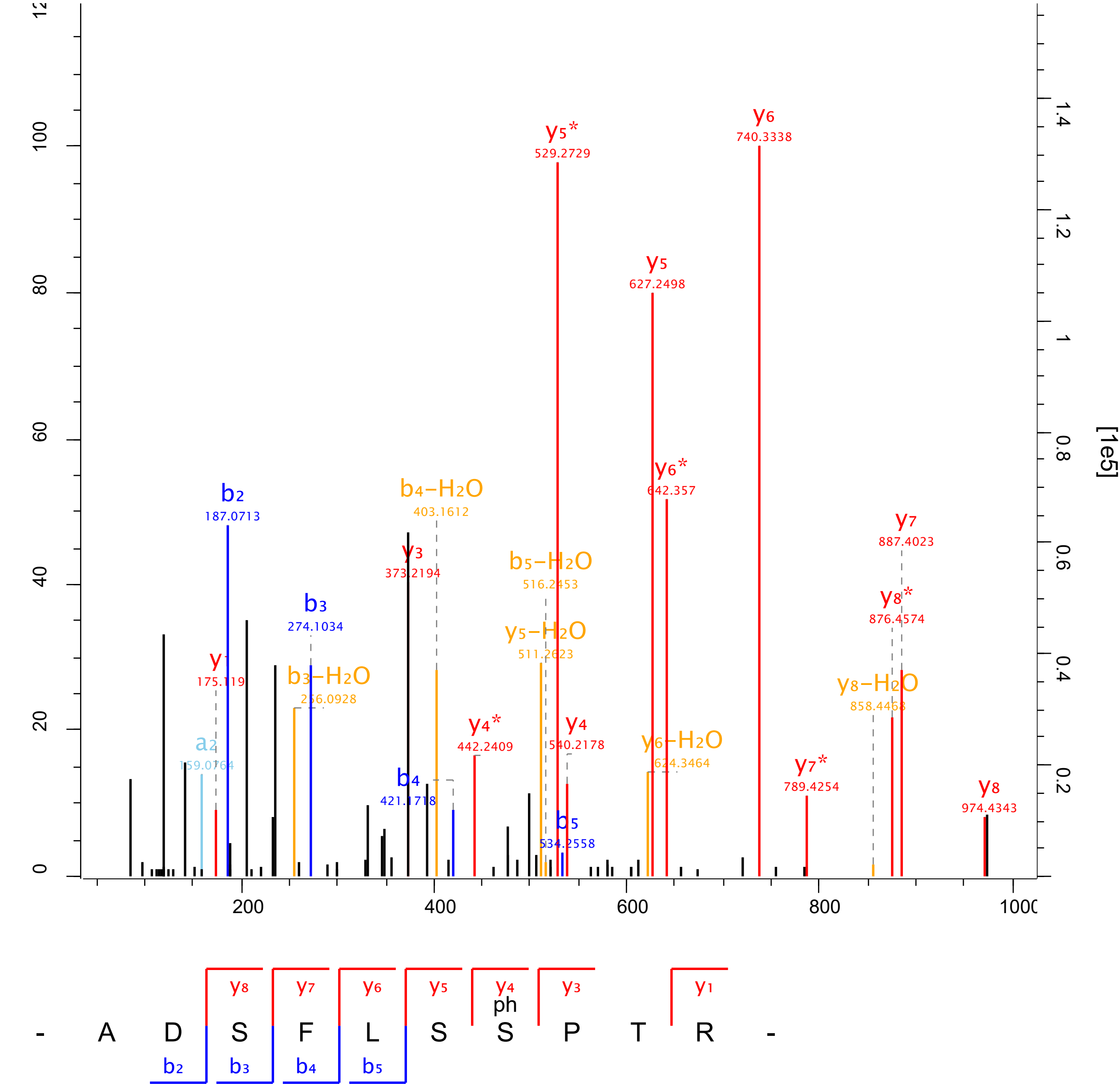

Raw file	Scan	Method	Score	m/z
sys_15_1	17623	FTMS; HCD	111.34	697.83

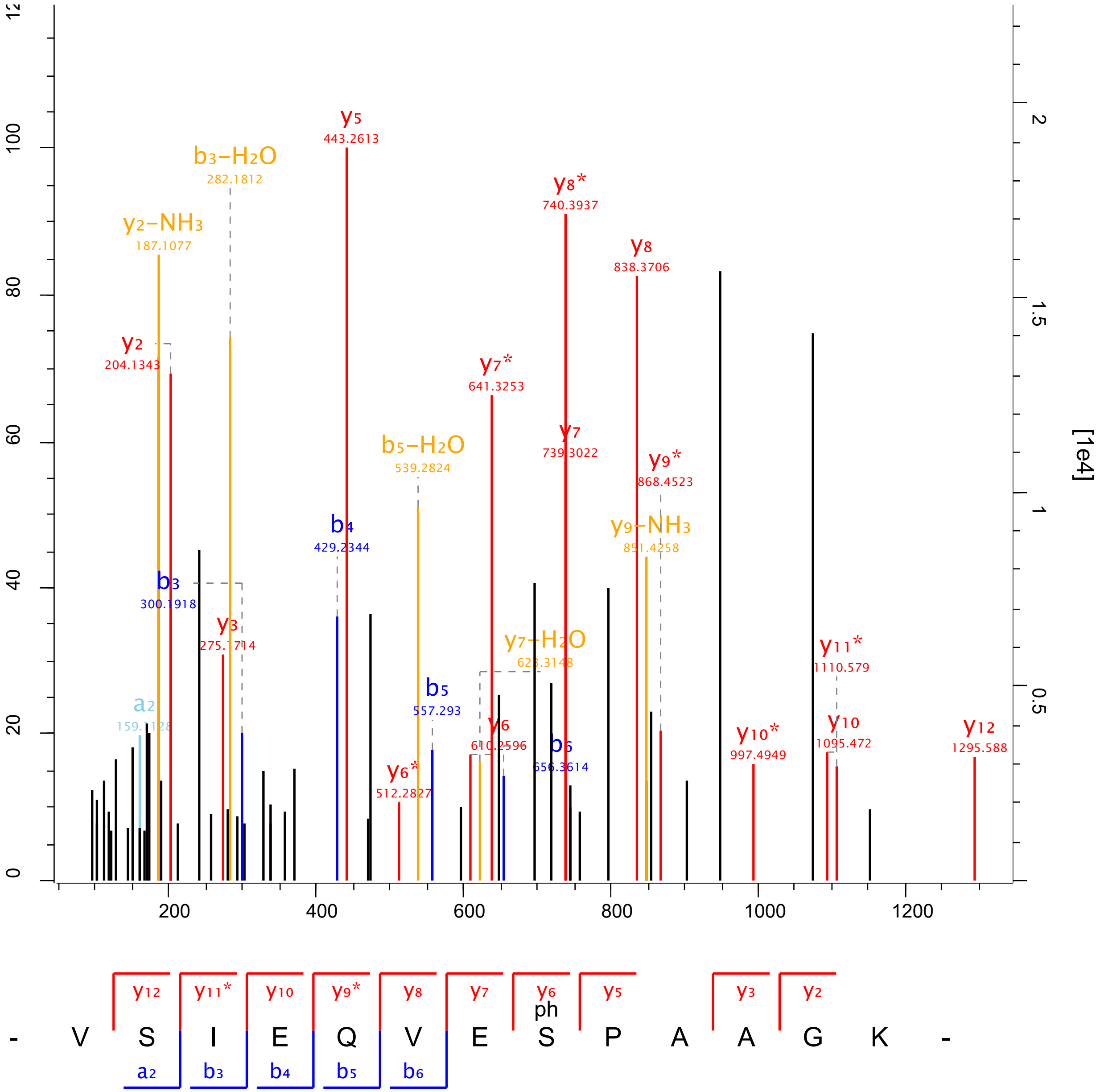

Raw file	Scan	Method	Score	m/z
sys_15_1	17648	FTMS; HCD	79.68	591.93

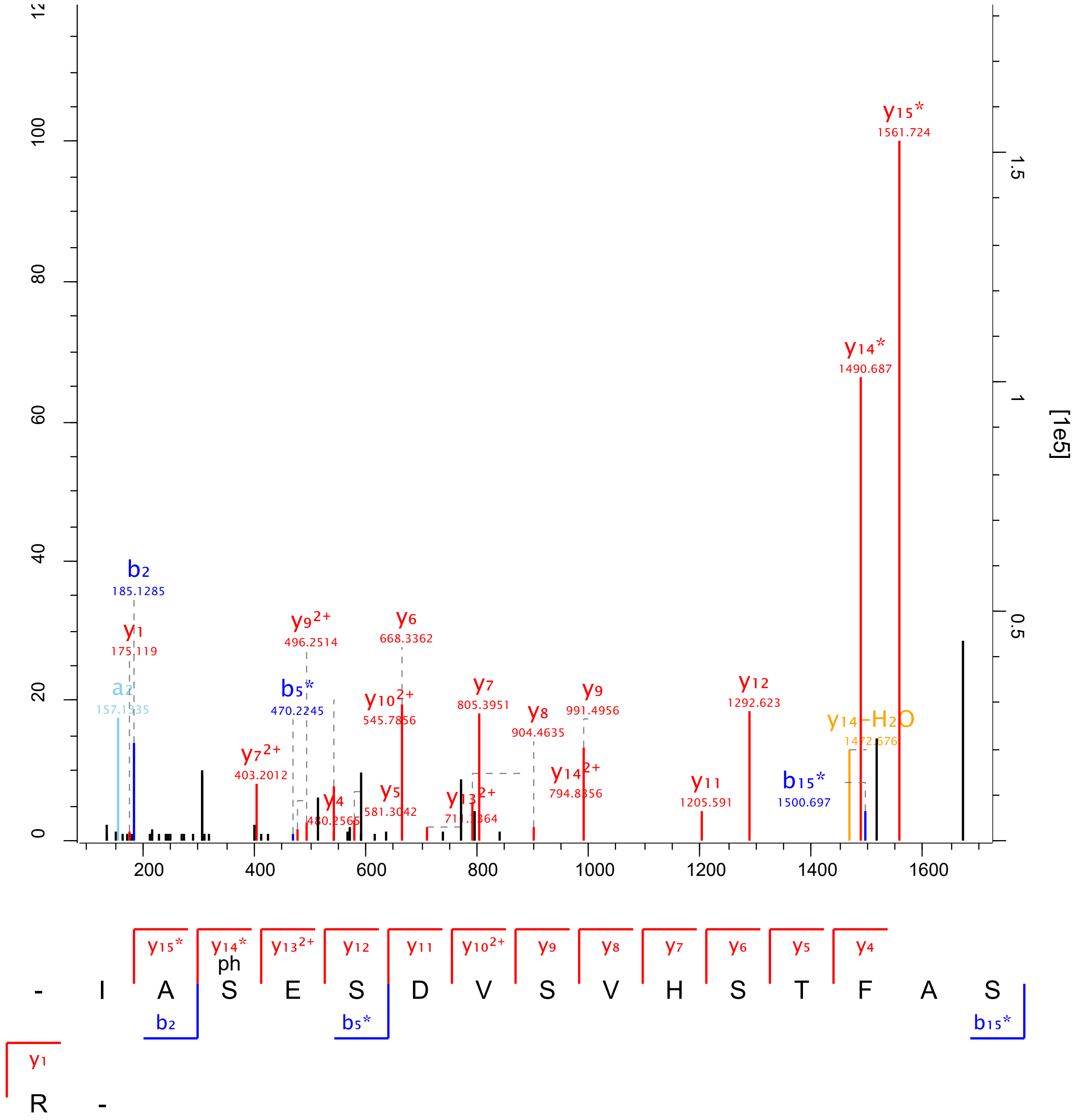

Raw file	Scan	Method	Score	m/z
sys_15_1	17651	FTMS; HCD	97.69	598.27

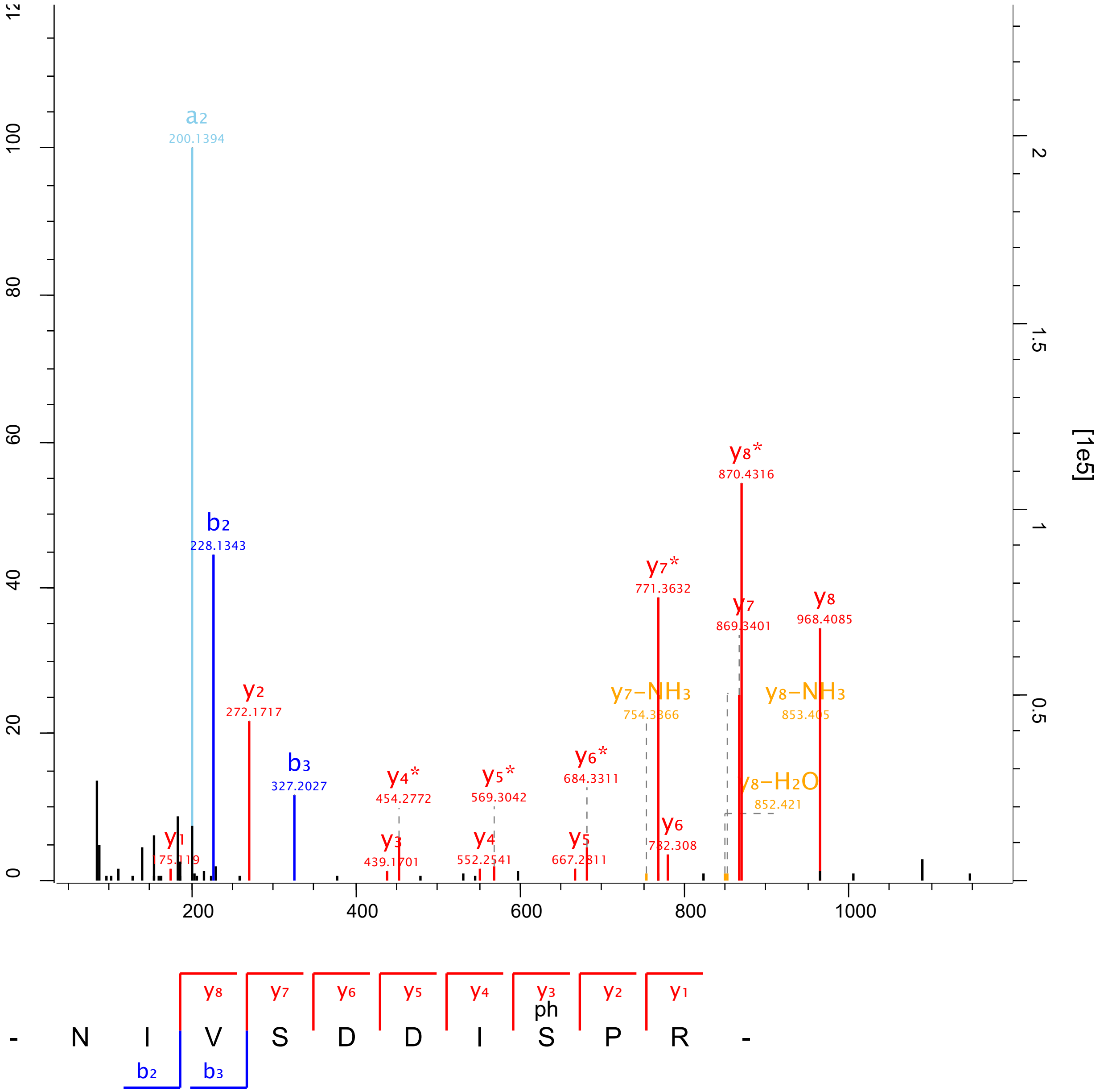

Raw file	Scan	Method	Score	m/z
sys_15_1	17772	FTMS; HCD	91.81	657.74

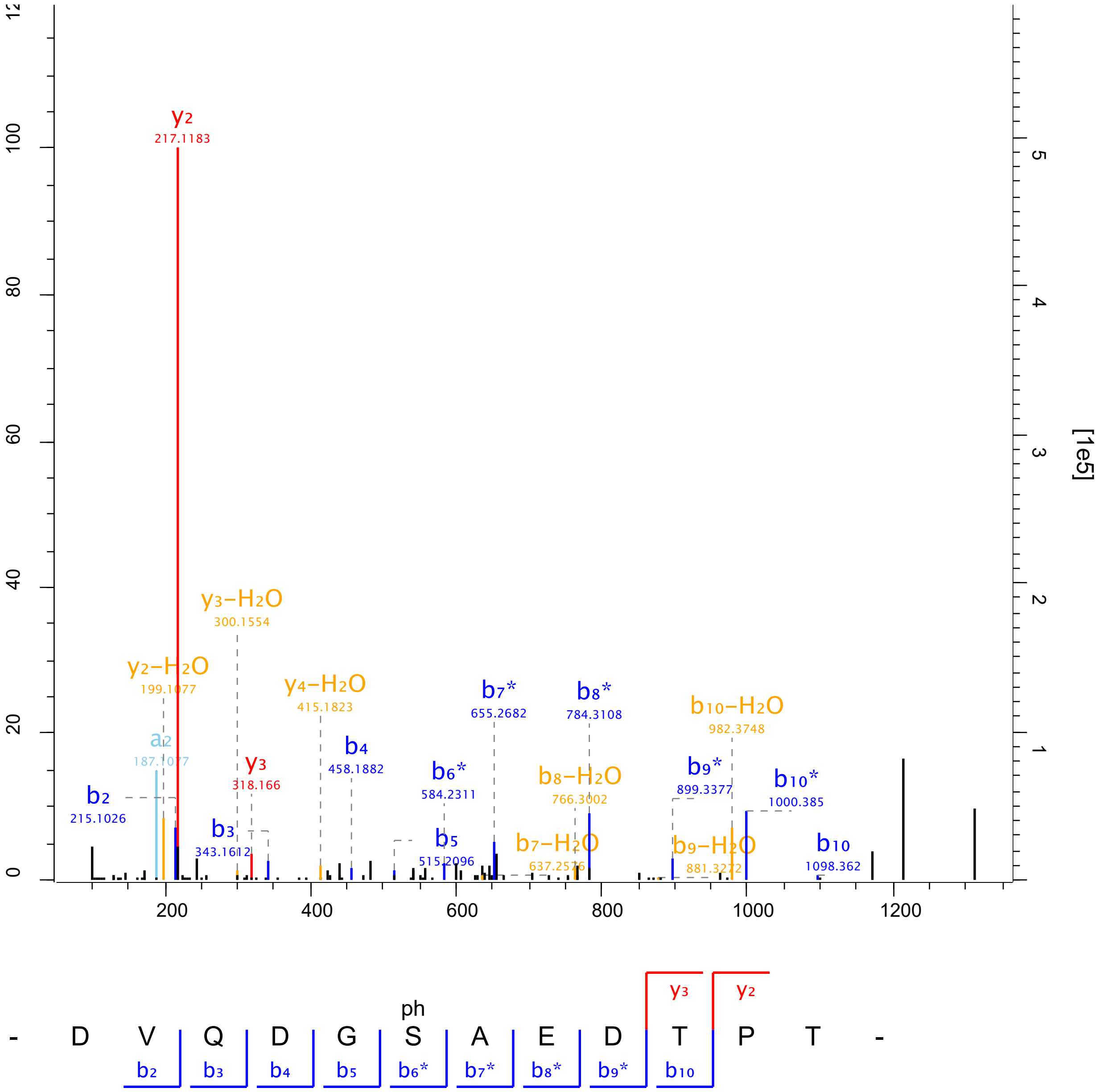

Raw file	Scan	Method	Score	m/z
sys_15_1	17862	FTMS; HCD	69.7	885.86

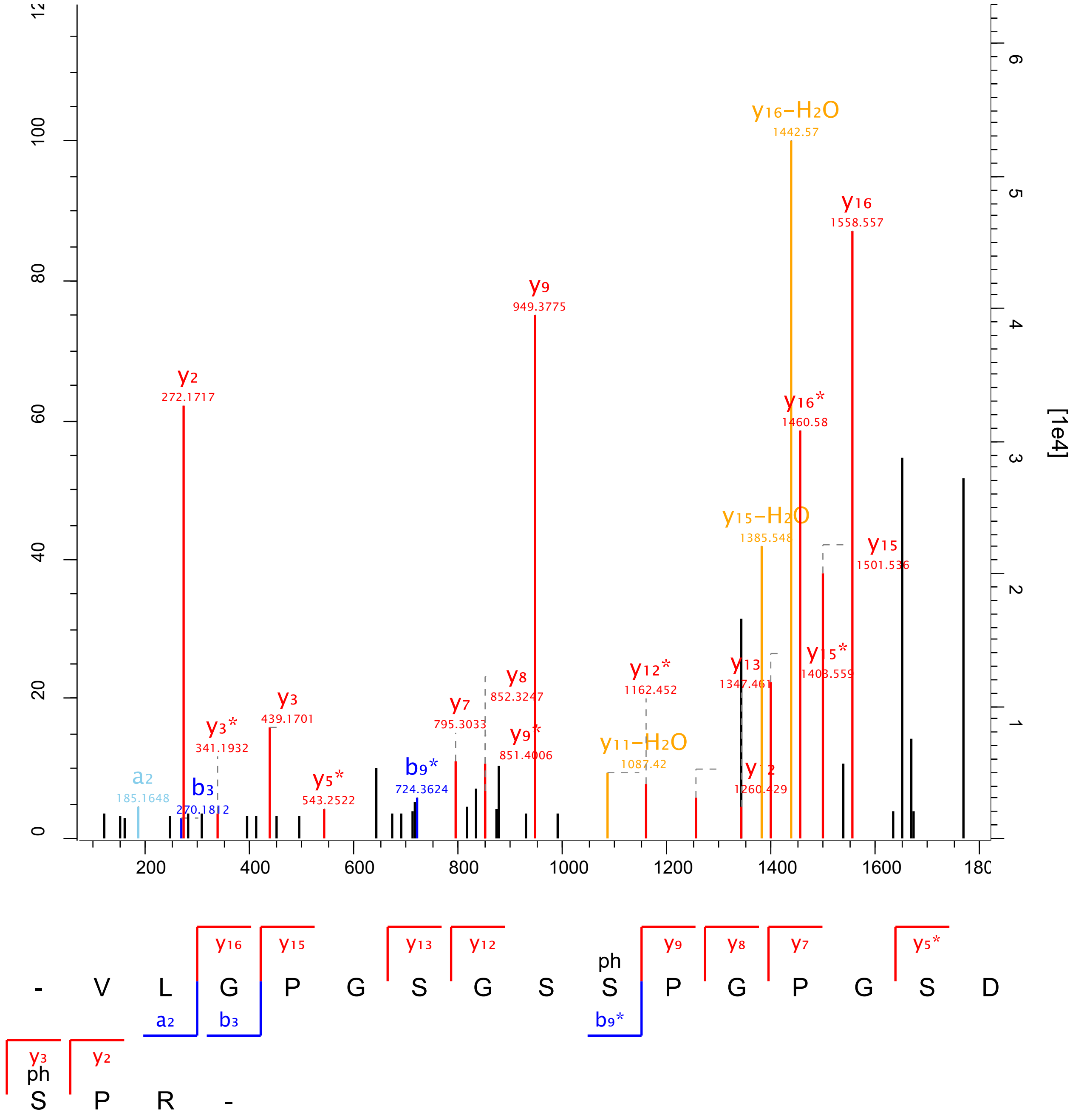

Raw file	Scan	Method	Score	m/z
sys_15_1	17892	FTMS; HCD	62.62	795.01

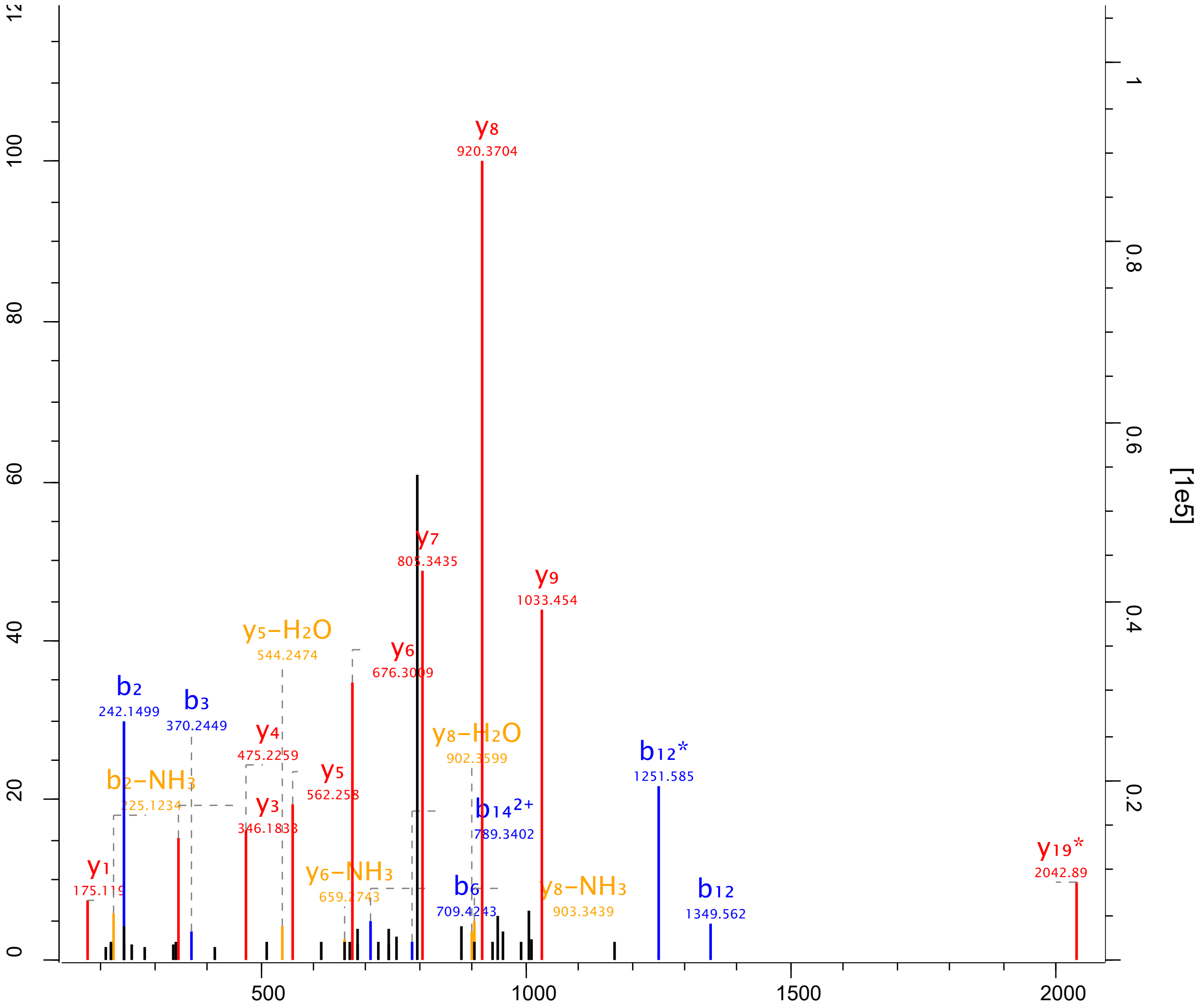

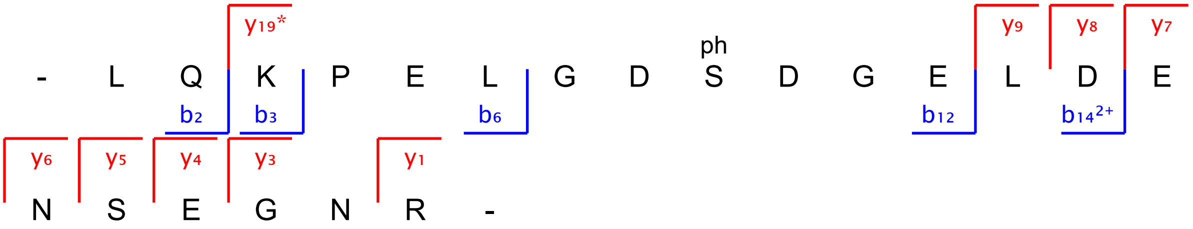

Raw file	Scan	Method	Score	m/z
sys_15_1	17953	FTMS; HCD	125.79	888.9

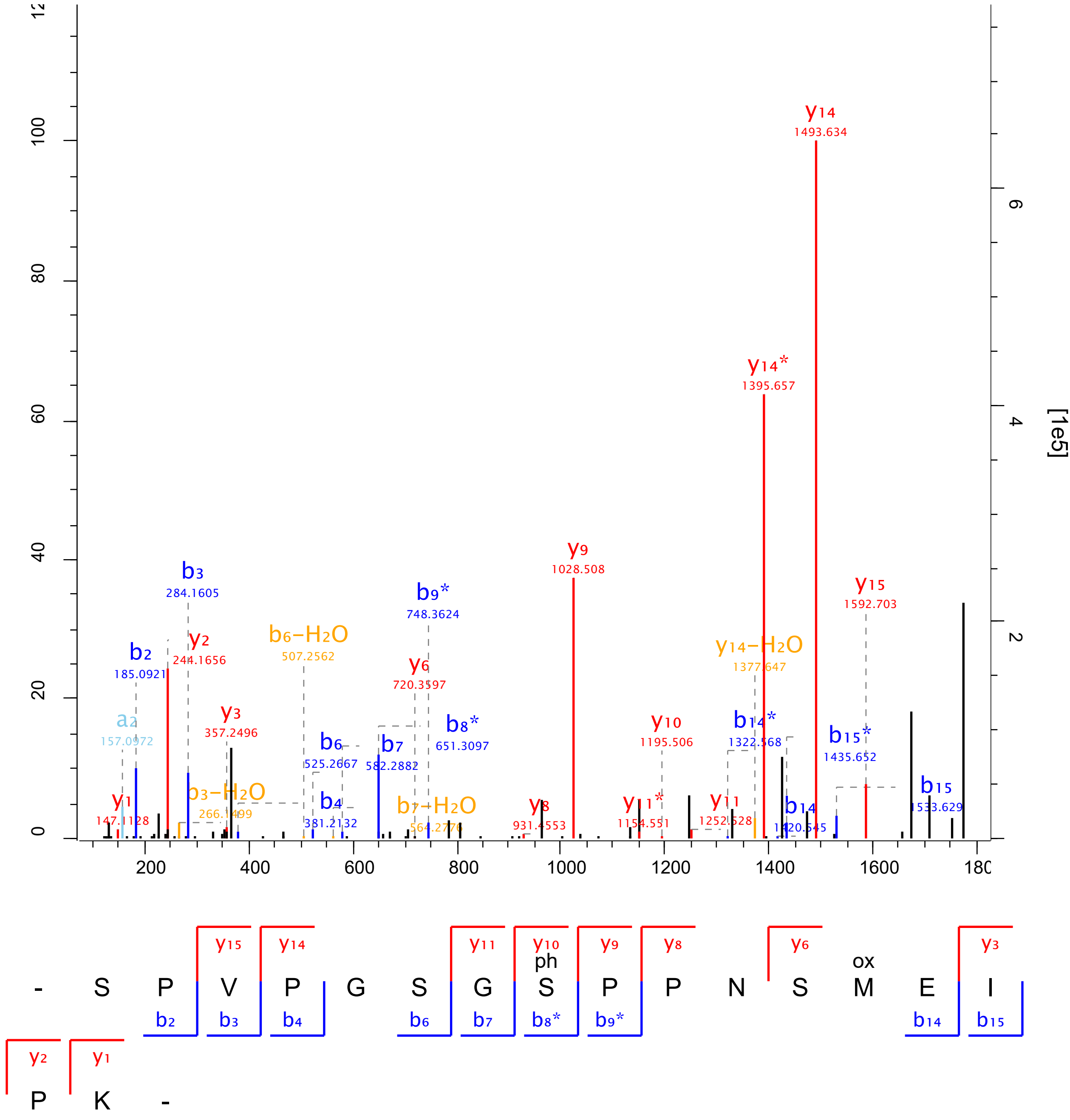

Raw file	Scan	Method	Score	m/z
sys_15_1	18008	FTMS; HCD	51.94	563.78

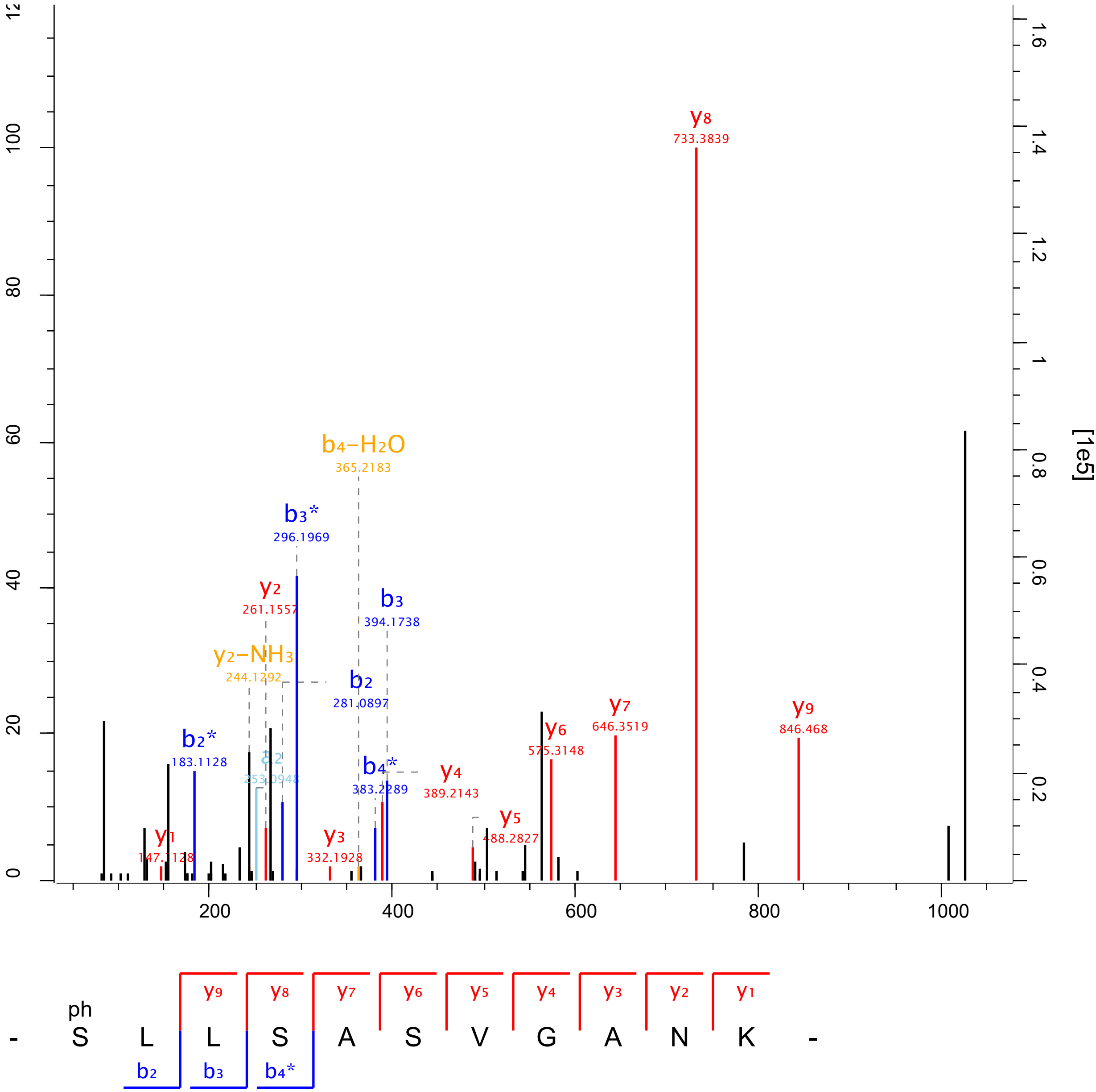

Raw file	Scan	Method	Score	m/z
sys_15_1	18070	FTMS; HCD	211.45	705.82

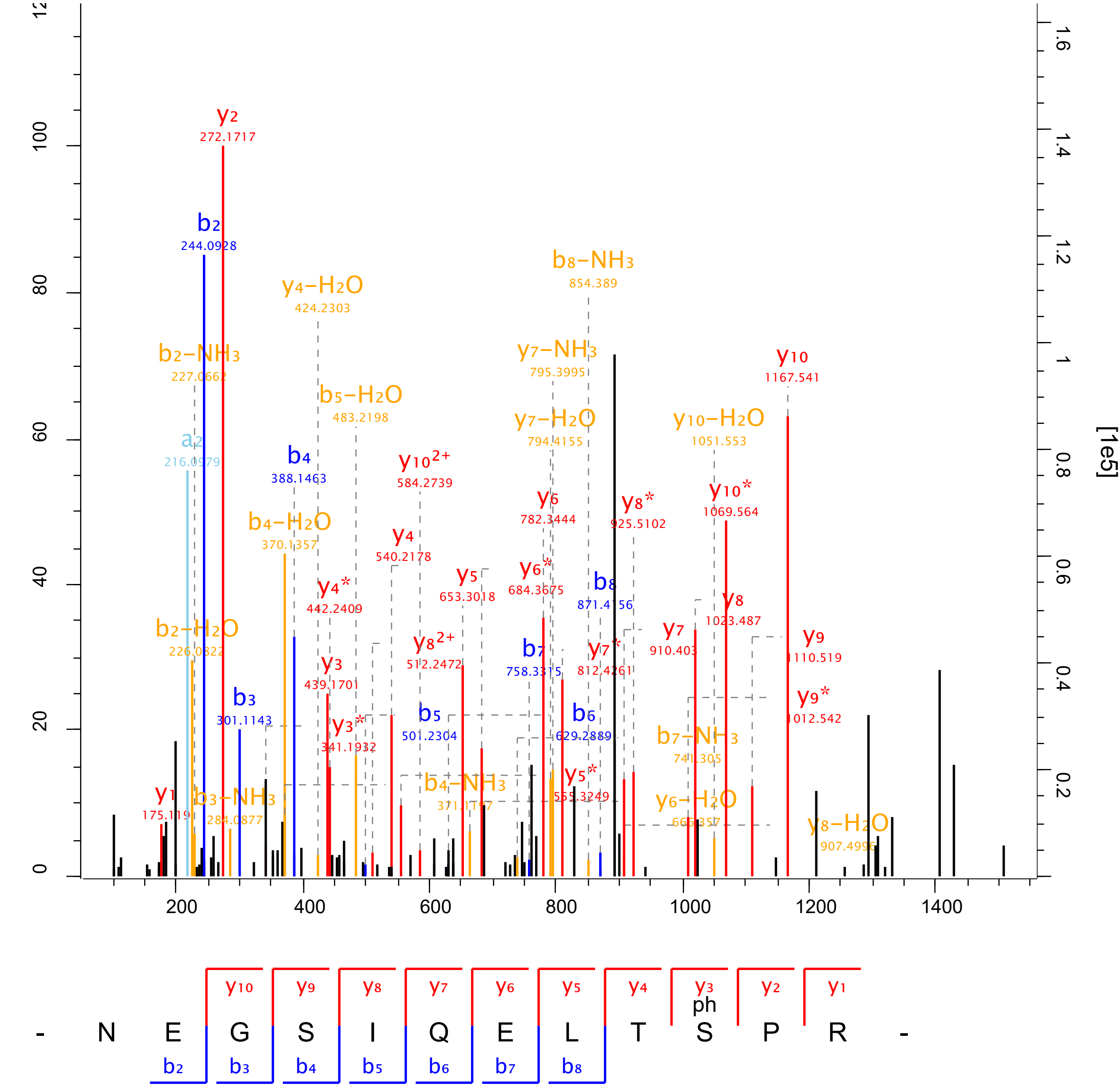

Raw file	Scan	Method	Score	m/z
sys_15_1	1810	FTMS; HCD	56.57	641.79

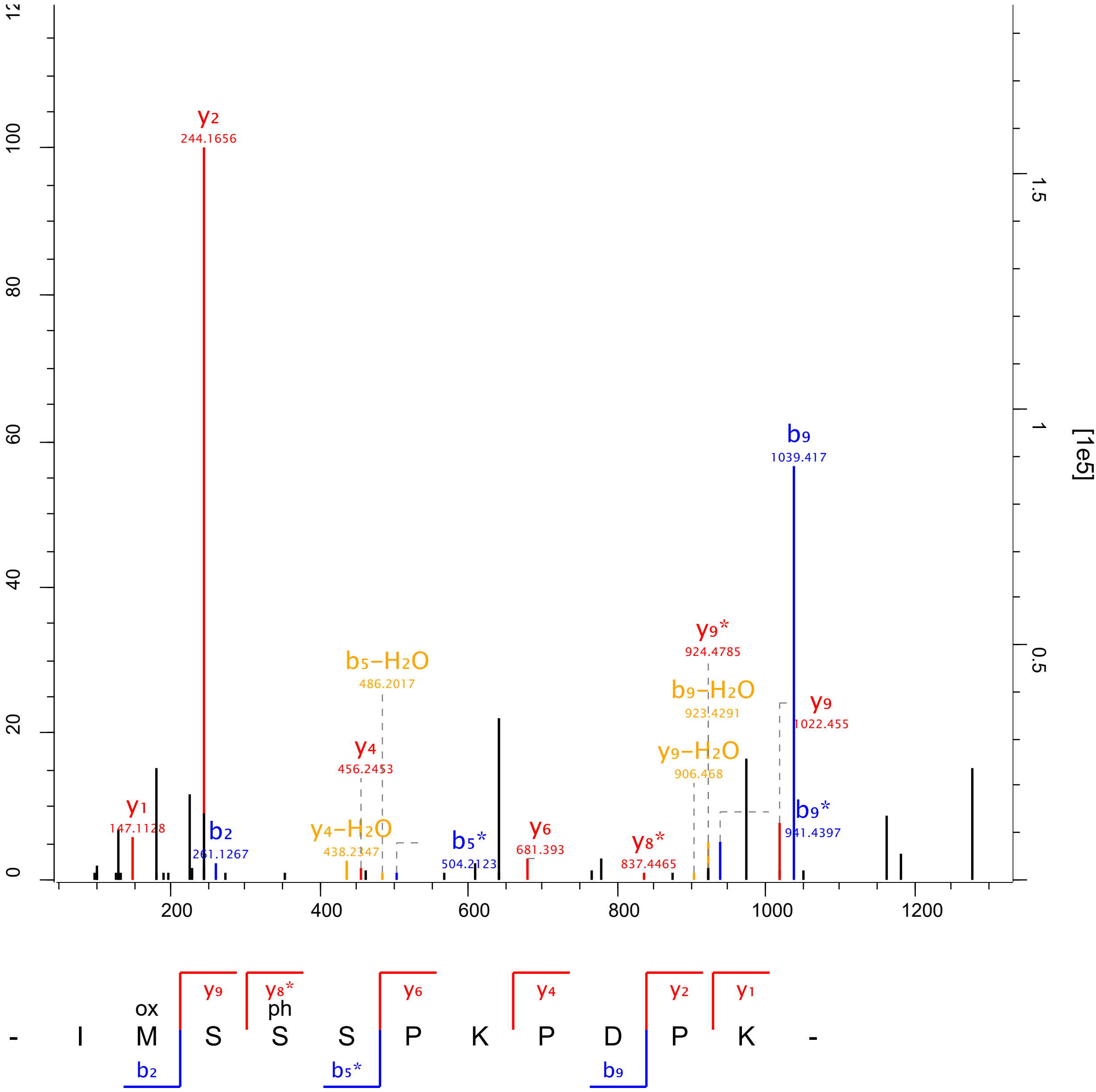

Raw file	Scan	Method	Score	m/z
sys_15_1	18134	FTMS; HCD	135.8	527.73

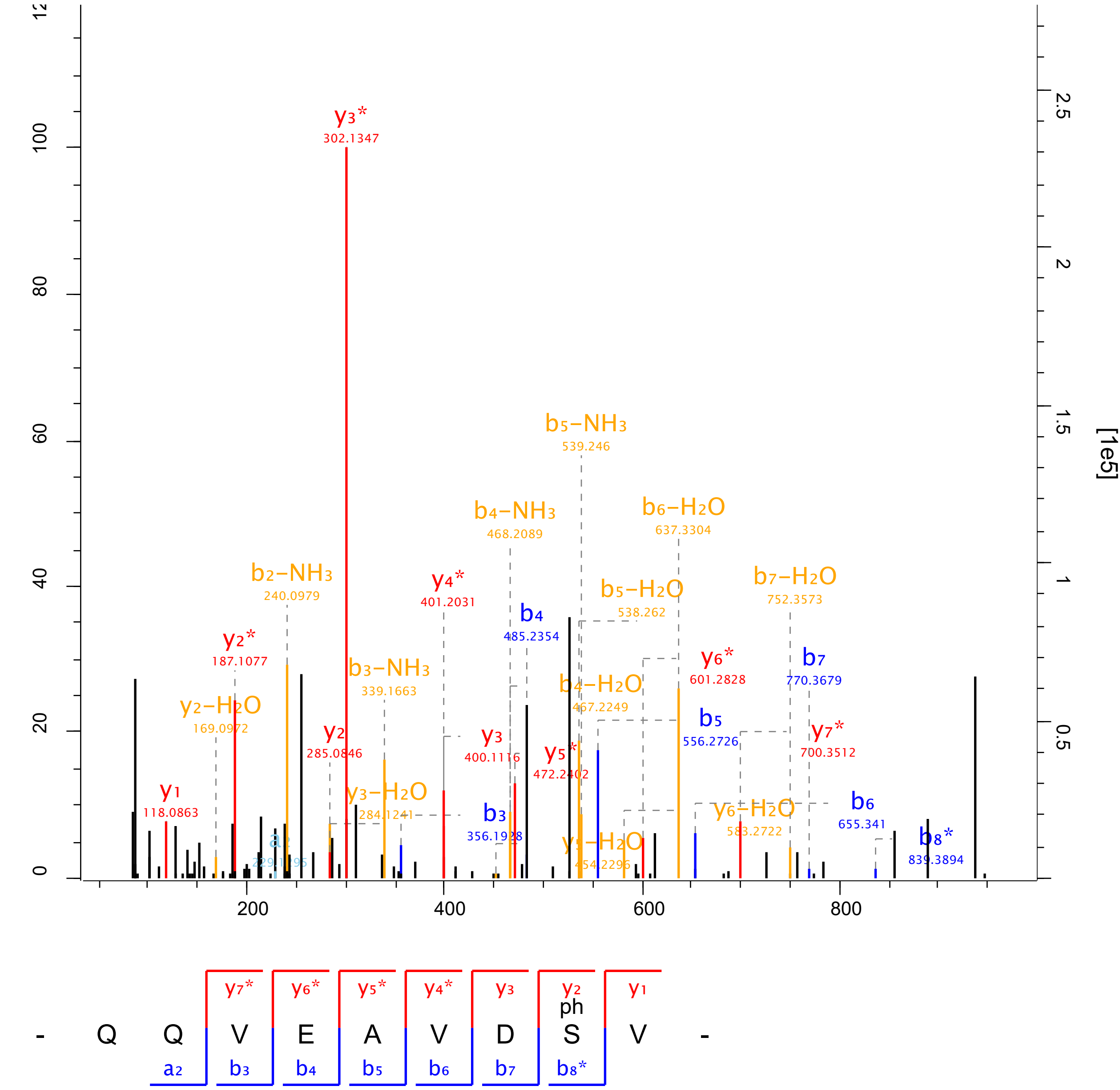

Raw file	Scan	Method	Score	m/z
sys_15_1	18194	FTMS; HCD	50.86	904.38

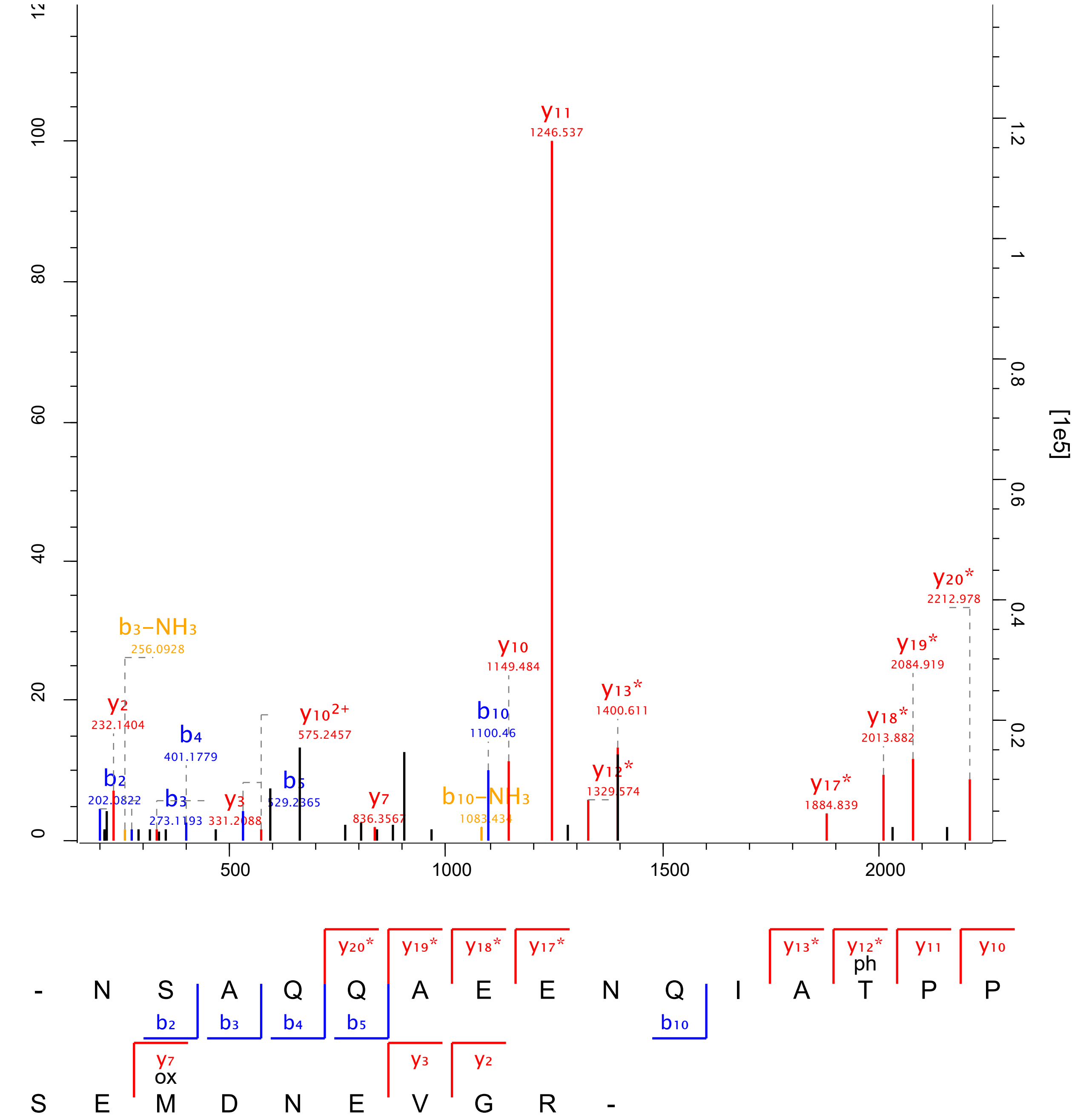

Raw file	Scan	Method	Score	m/z
sys_15_1	18273	FTMS; HCD	159.66	557.27

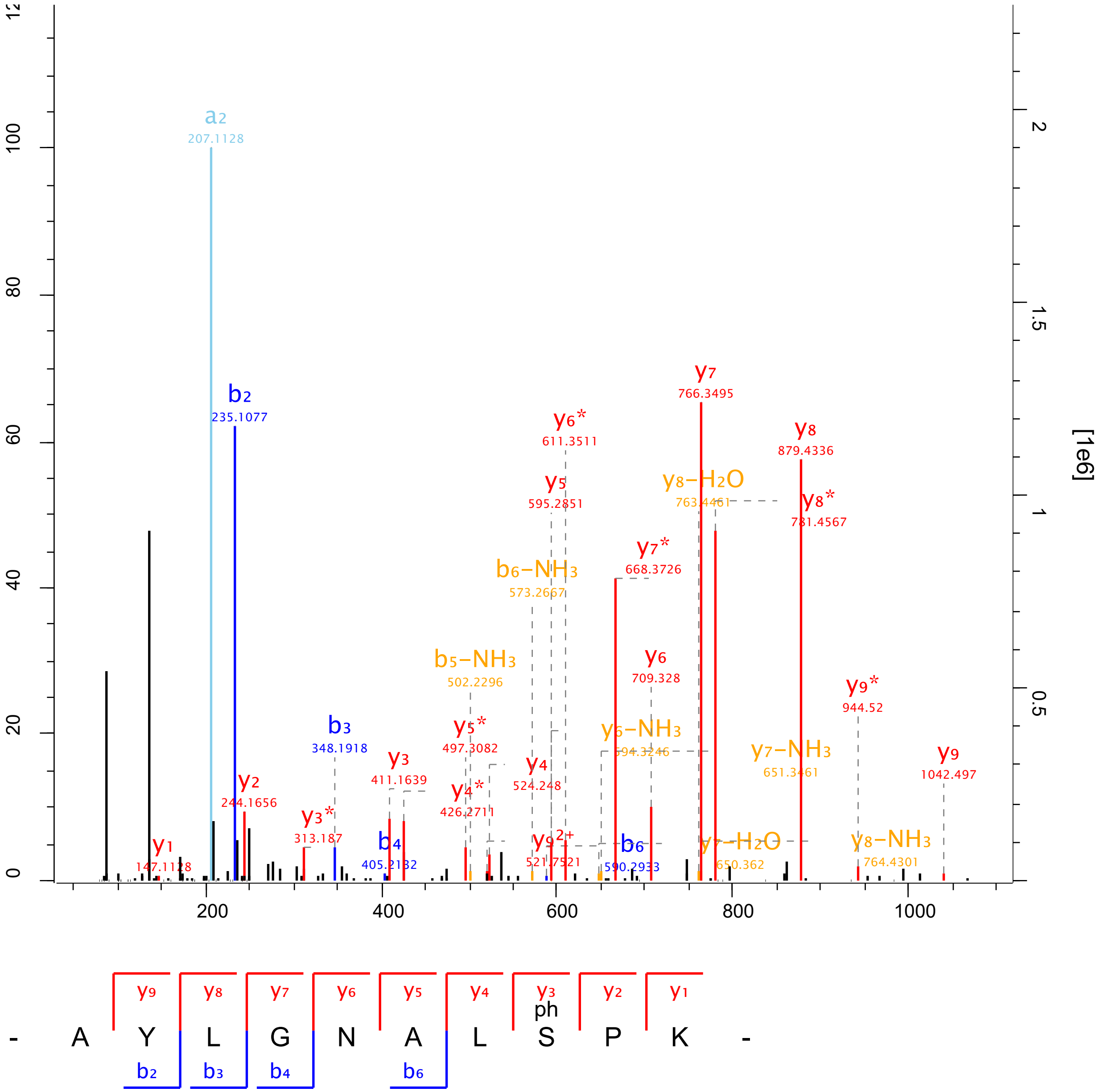

Raw file	Scan	Method	Score	m/z
sys_15_1	18285	FTMS; HCD	64.12	553.73

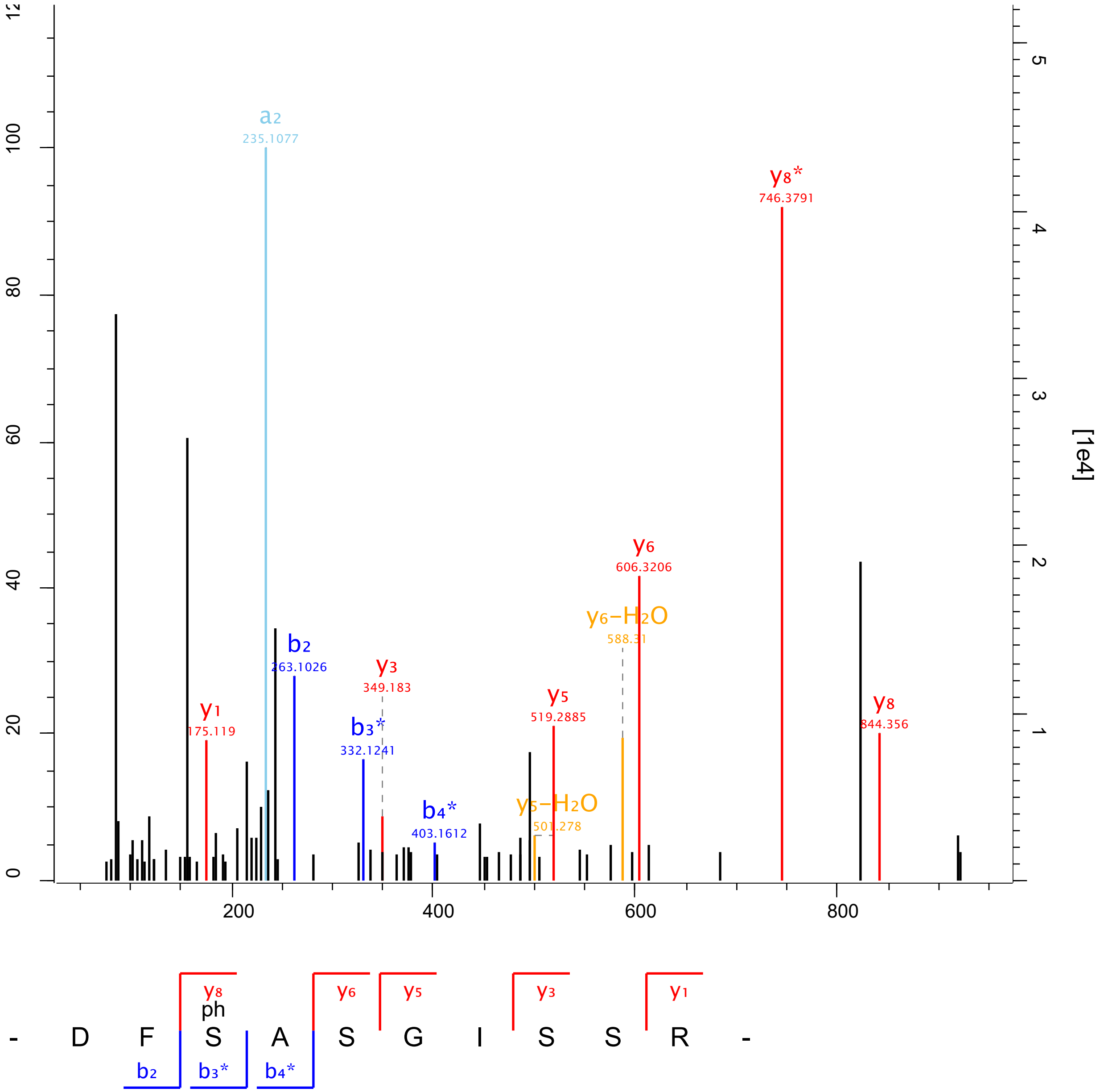

Raw file	Scan	Method	Score	m/z
sys_15_1	18288	FTMS; HCD	261.76	761.88

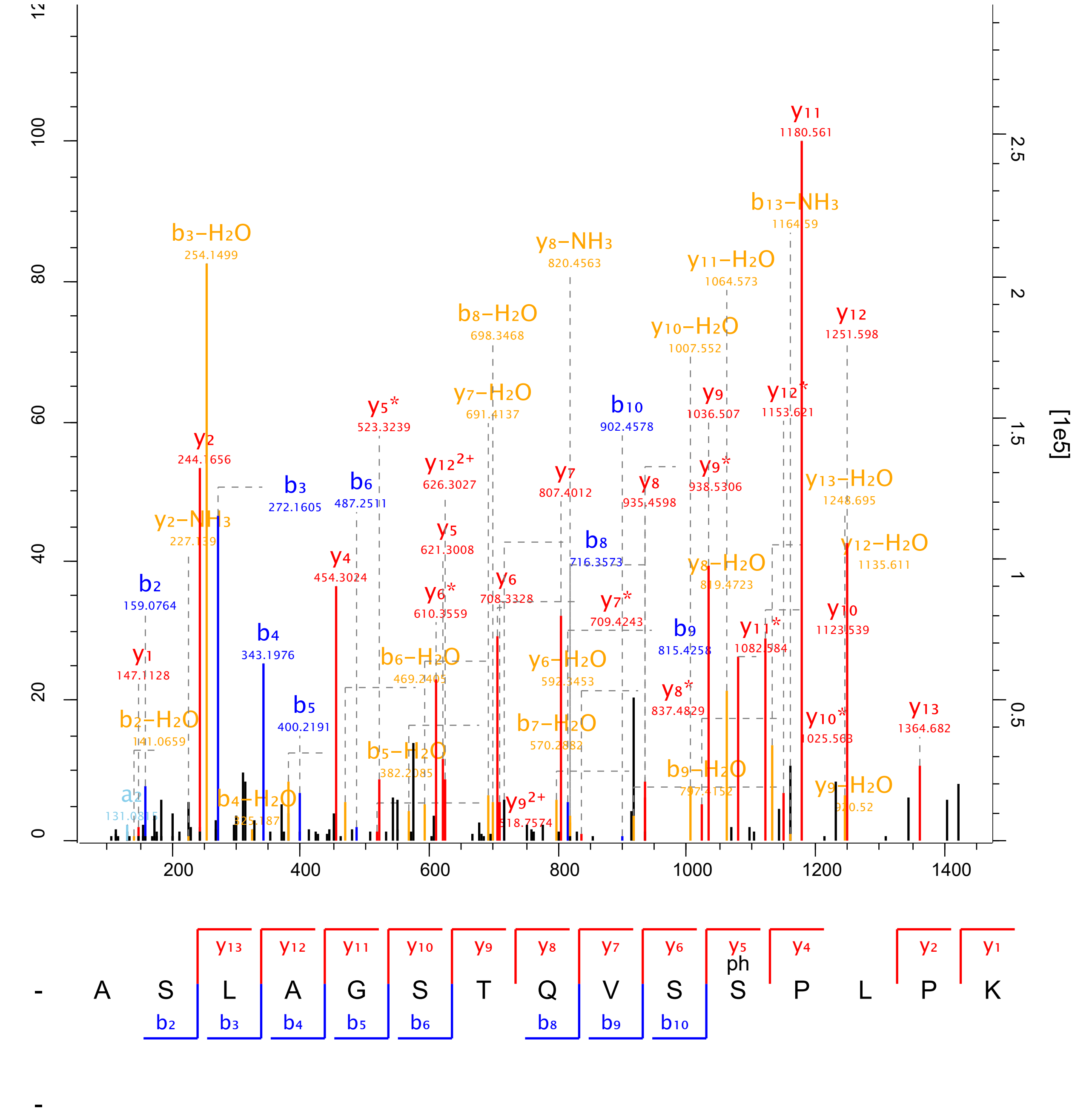

Raw file	Scan	Method	Score	m/z
sys_15_1	18394	FTMS; HCD	41.8	950.41

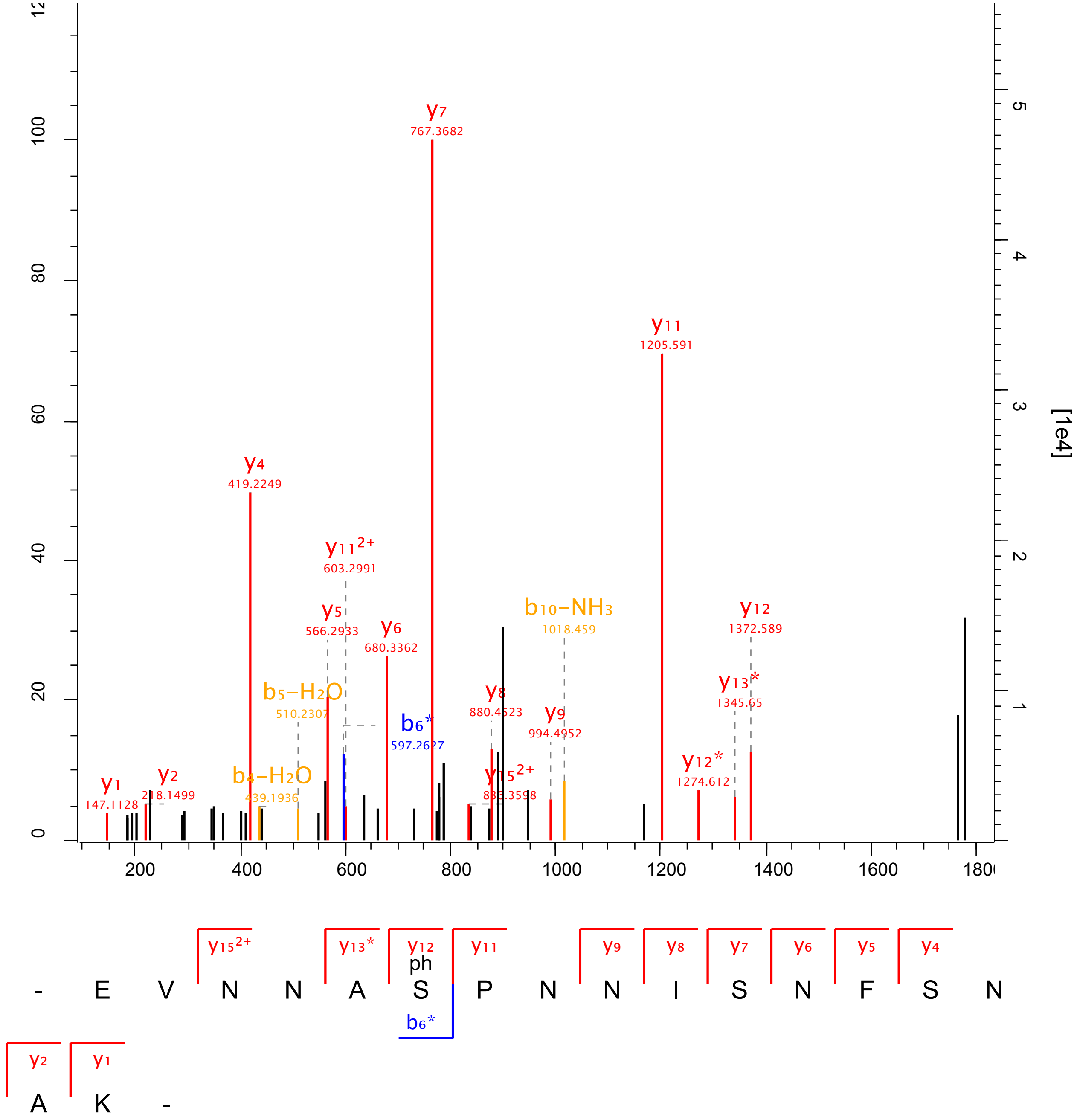

Raw file	Scan	Method	Score	m/z
sys_15_1	18410	FTMS; HCD	42.95	511.57

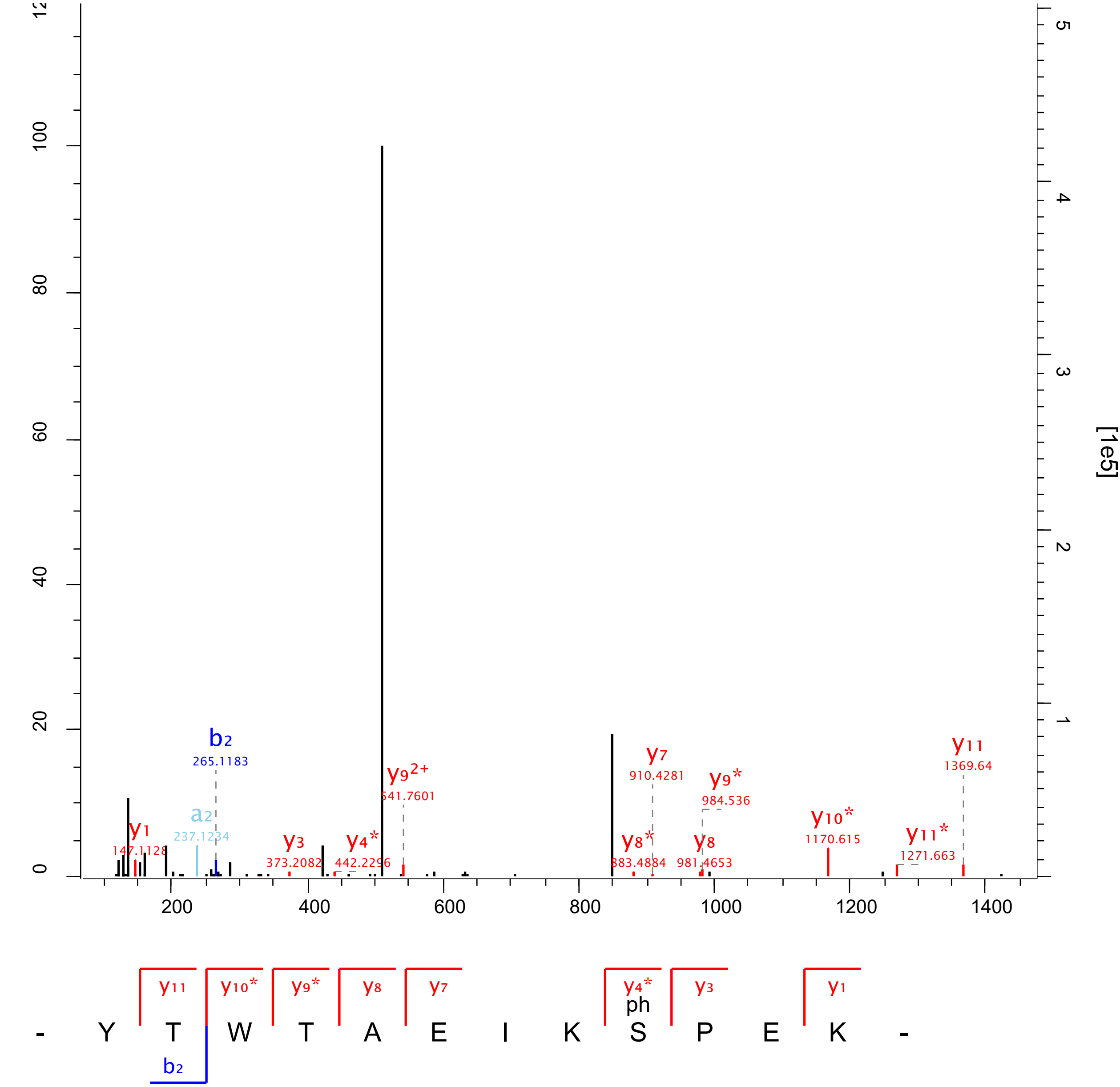

Raw file	Scan	Method	Score	m/z
sys_15_1	18432	FTMS; HCD	105.65	552.78

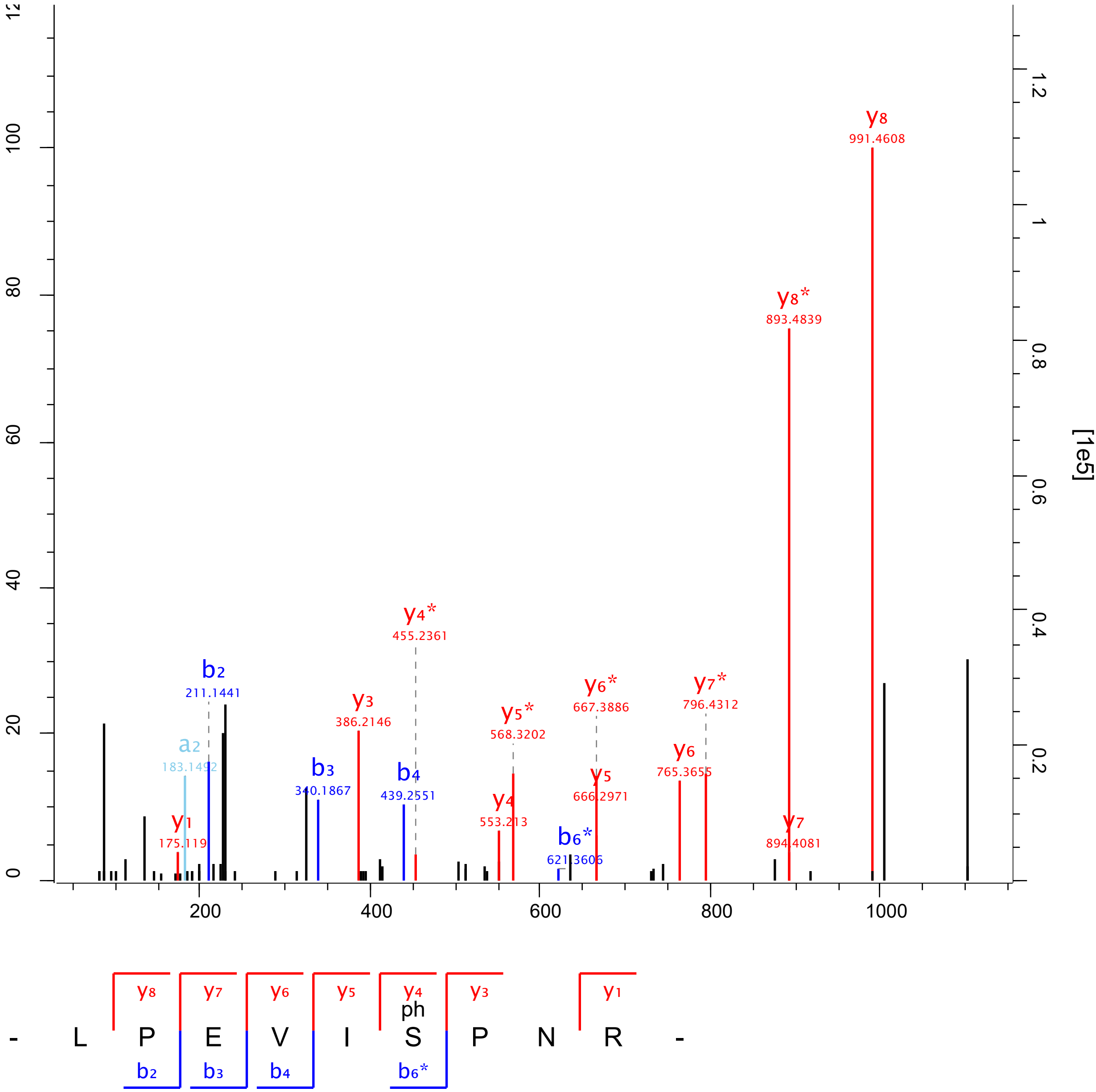

Raw file	Scan	Method	Score	m/z
sys_15_1	18537	FTMS; HCD	120.4	889.37

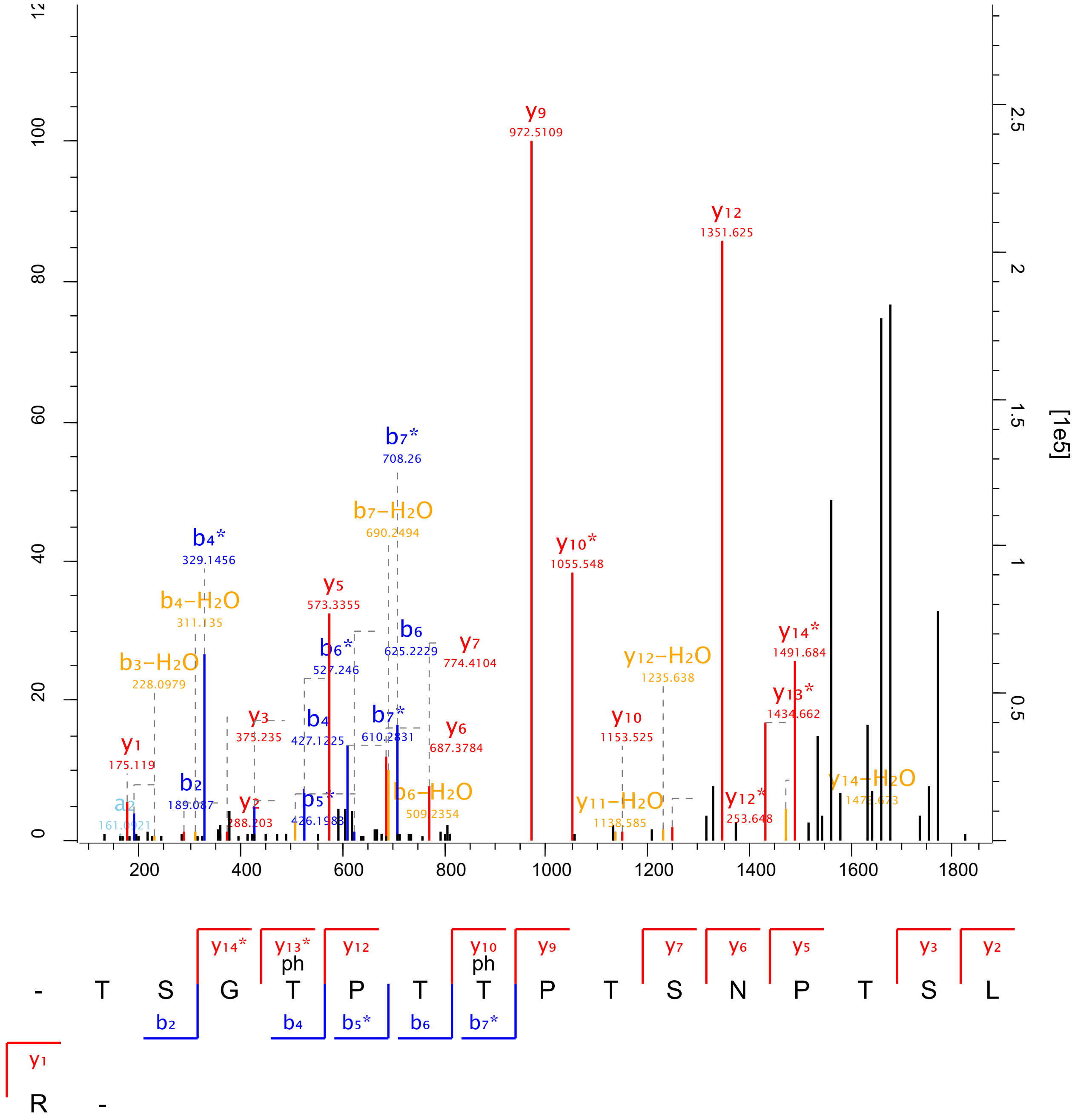

Raw file	Scan	Method	Score	m/z
sys_15_1	18559	FTMS; HCD	86.54	828.83

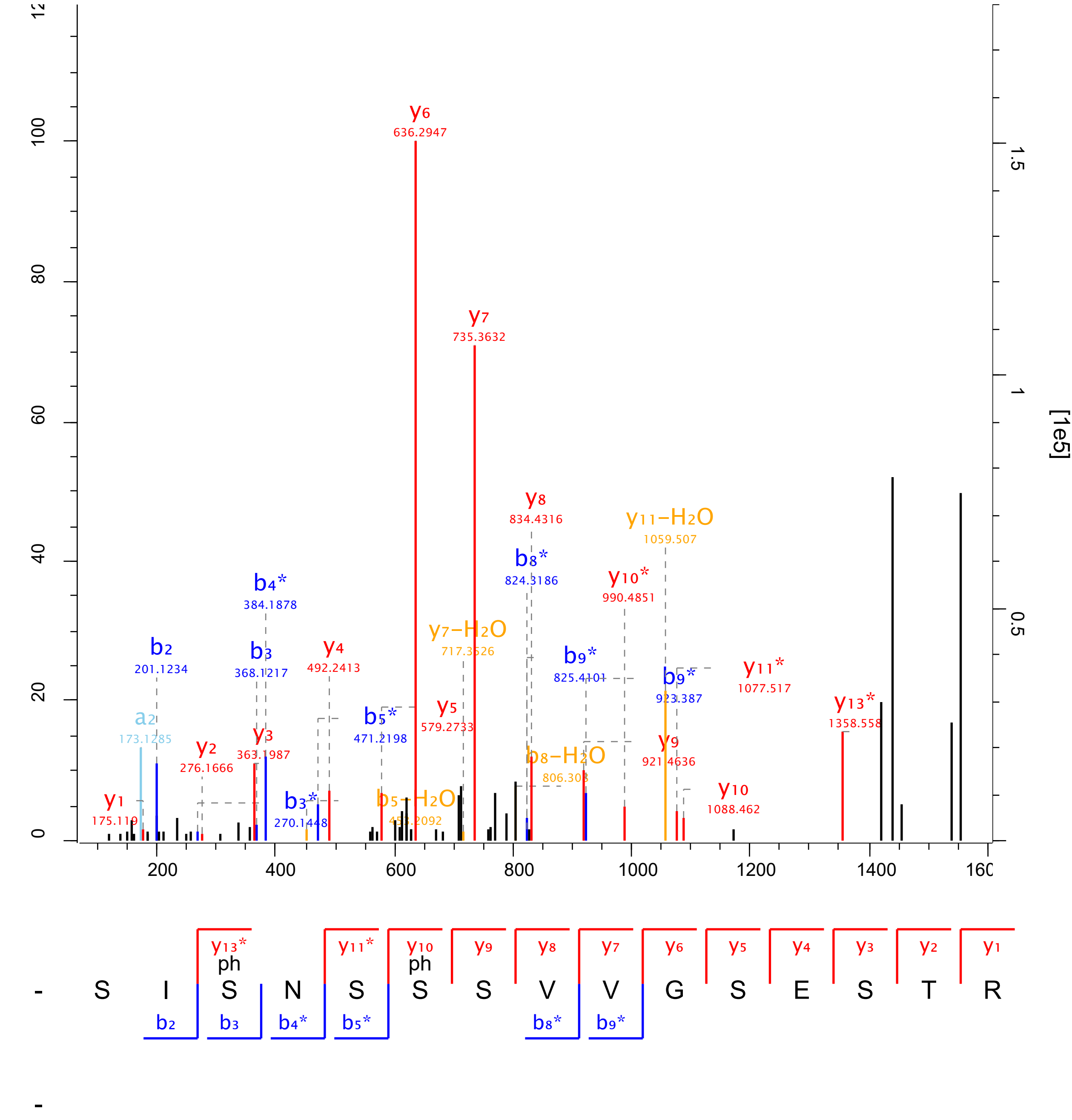

Raw file	Scan	Method	Score	m/z
sys_15_1	18582	FTMS; HCD	53.24	481.75

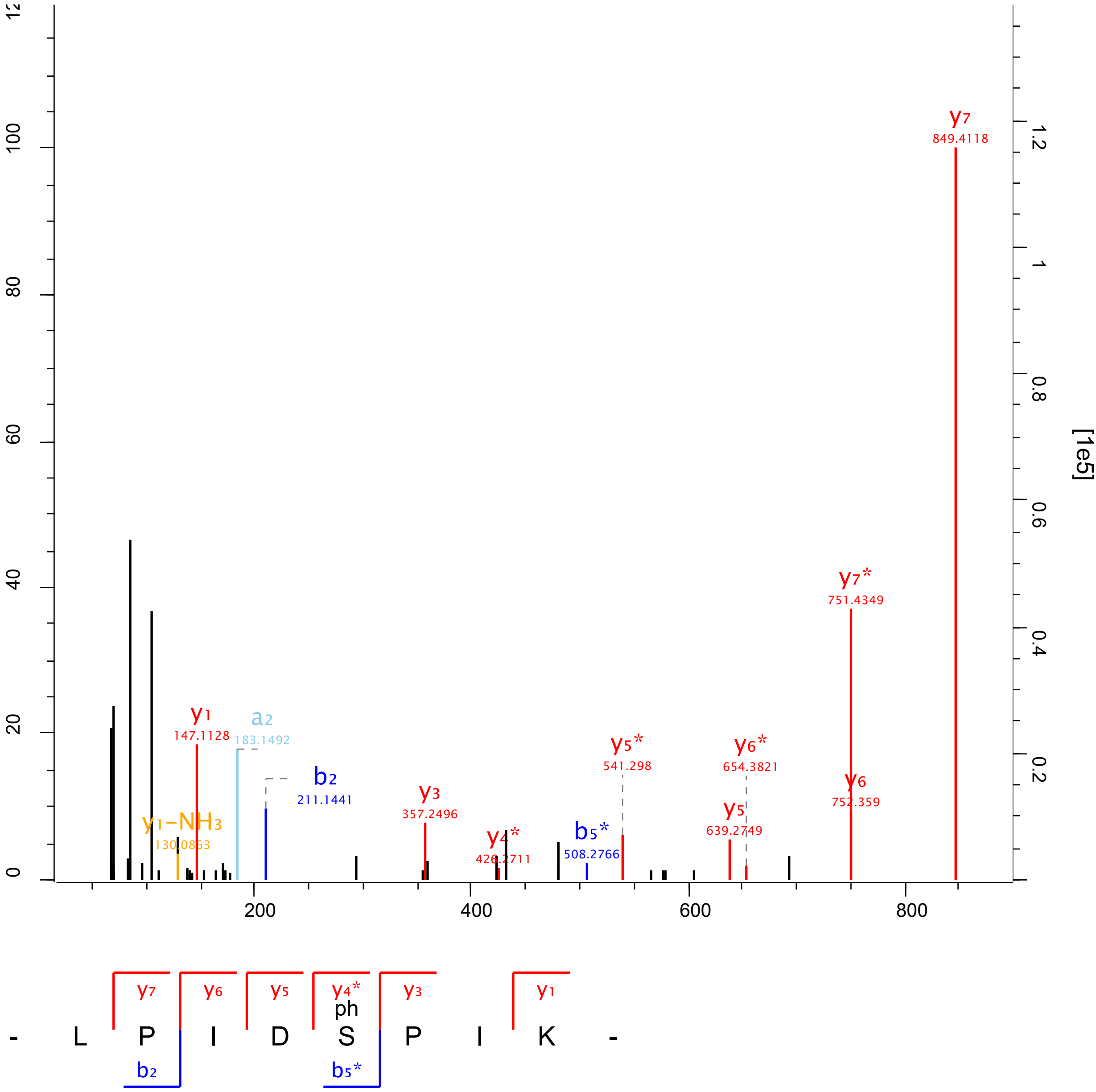

Raw file	Scan	Method	Score	m/z
sys_15_1	18646	FTMS; HCD	286.81	835.32

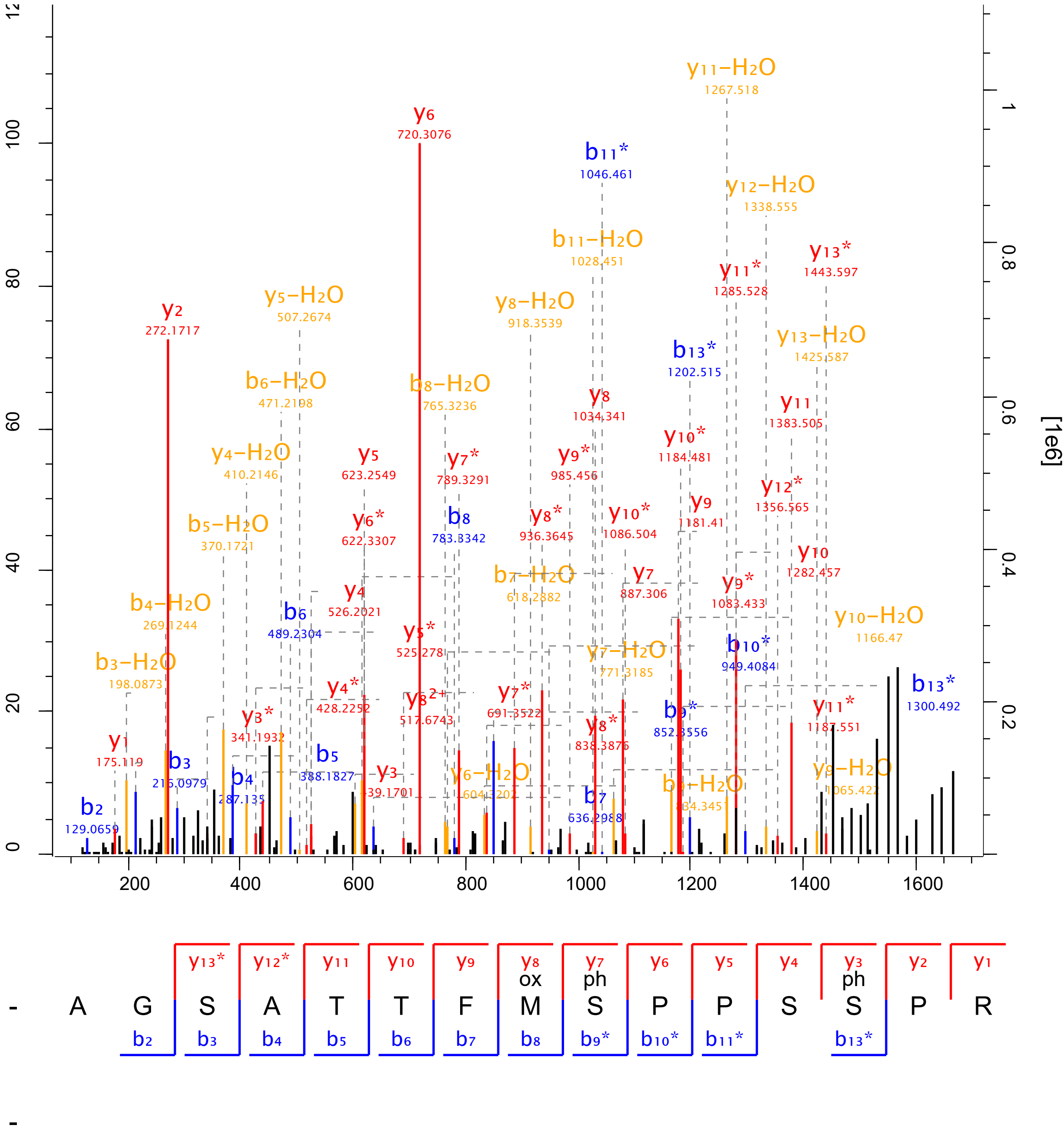

Raw file	Scan	Method	Score	m/z
sys_15_1	18746	FTMS; HCD	46.42	1003.38

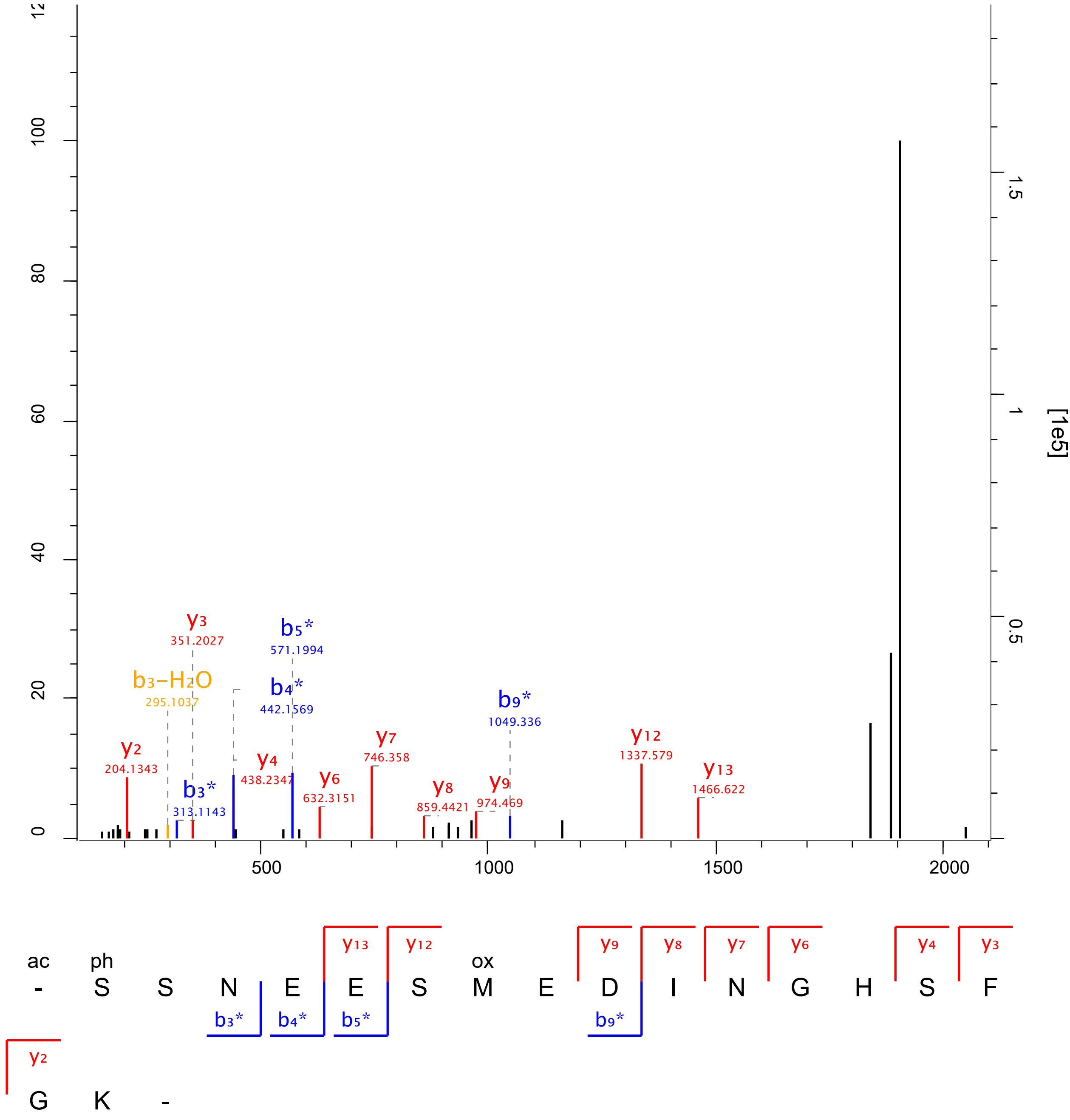

Raw file	Scan	Method	Score	m/z
sys_15_1	18776	FTMS; HCD	107.35	708.28

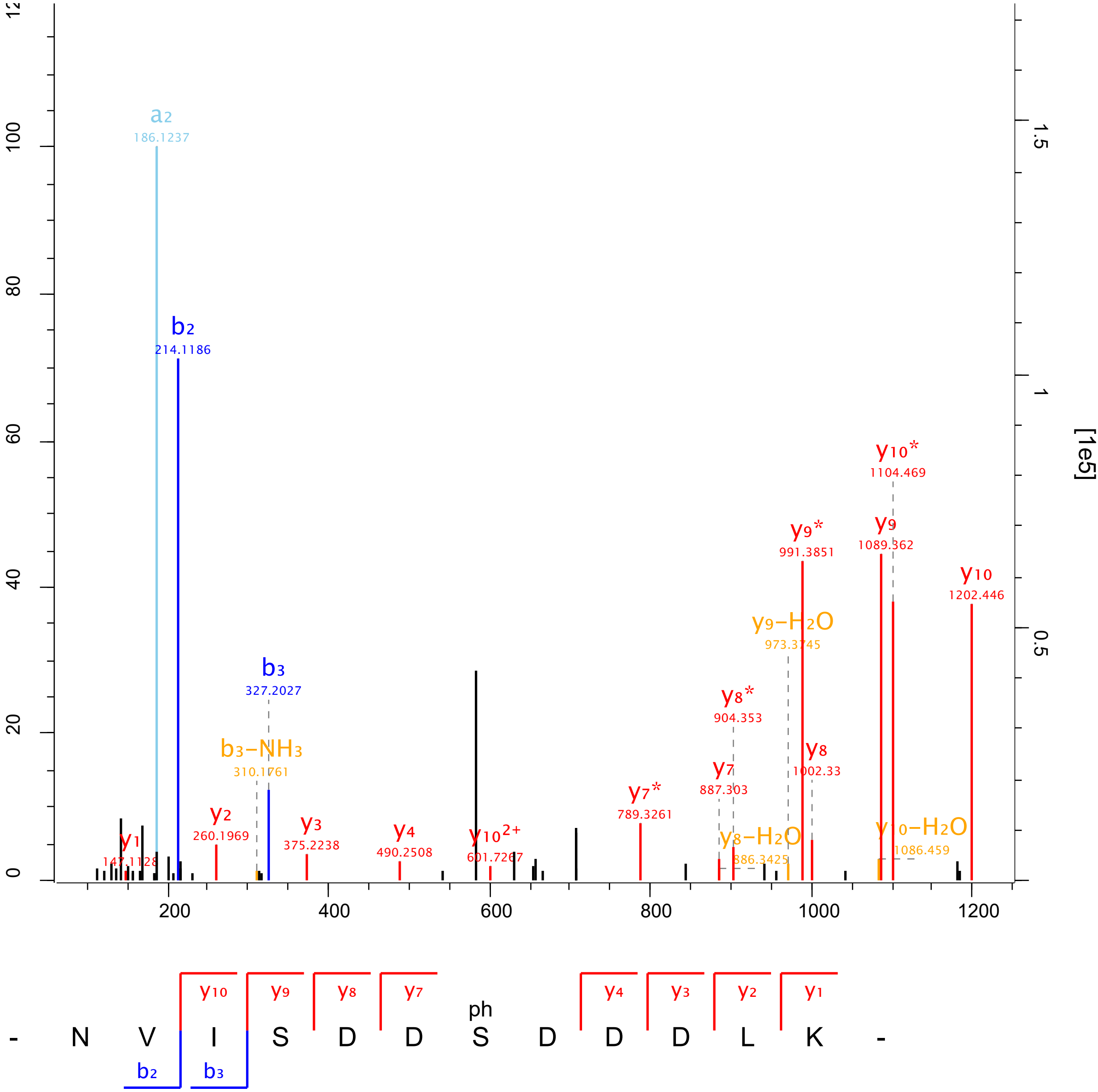

Raw file	Scan	Method	Score	m/z
sys_15_1	18830	FTMS; HCD	193.45	652.27

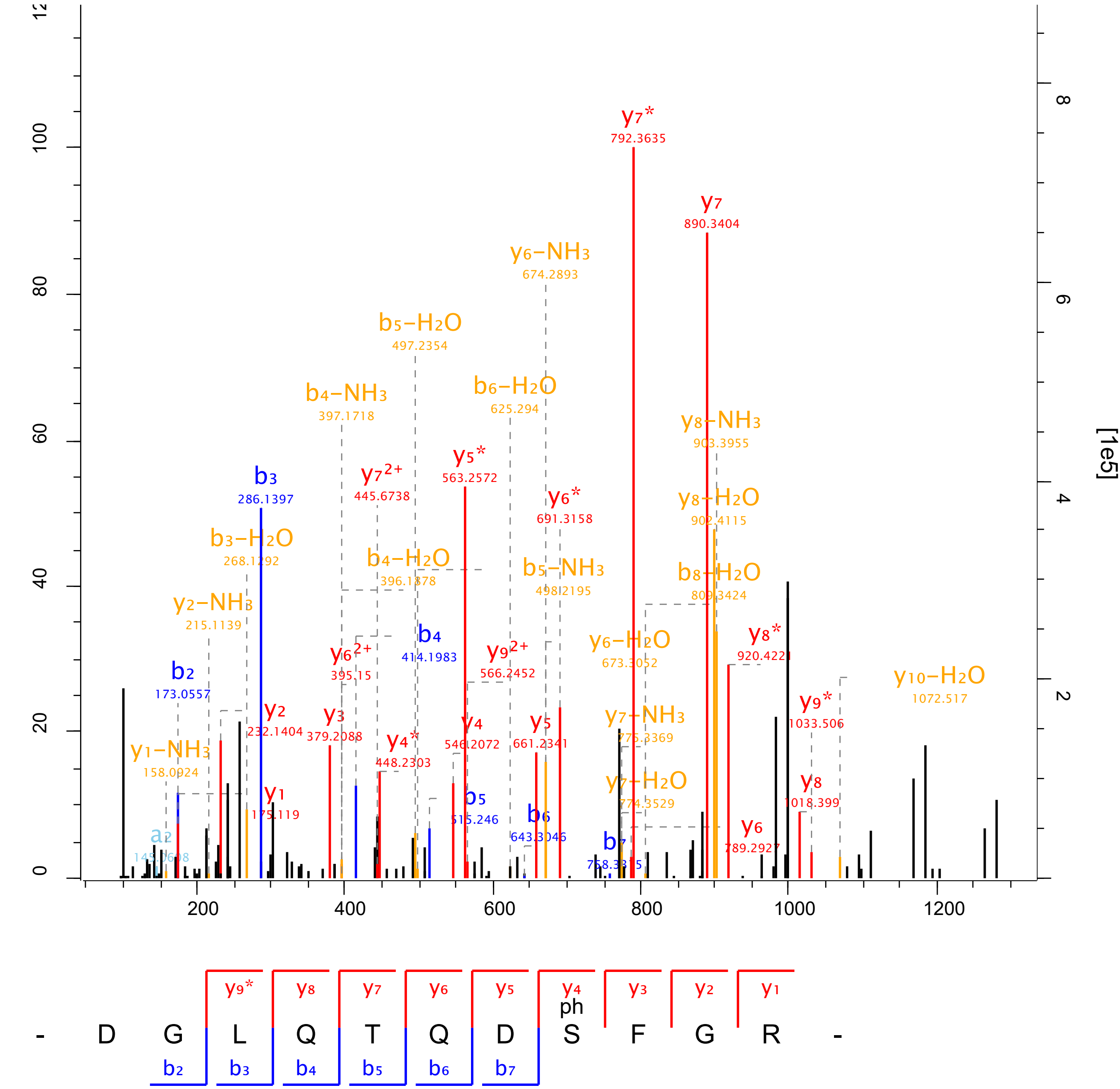

Raw file	Scan	Method	Score	m/z
sys_15_1	18840	FTMS; HCD	103.83	667.29

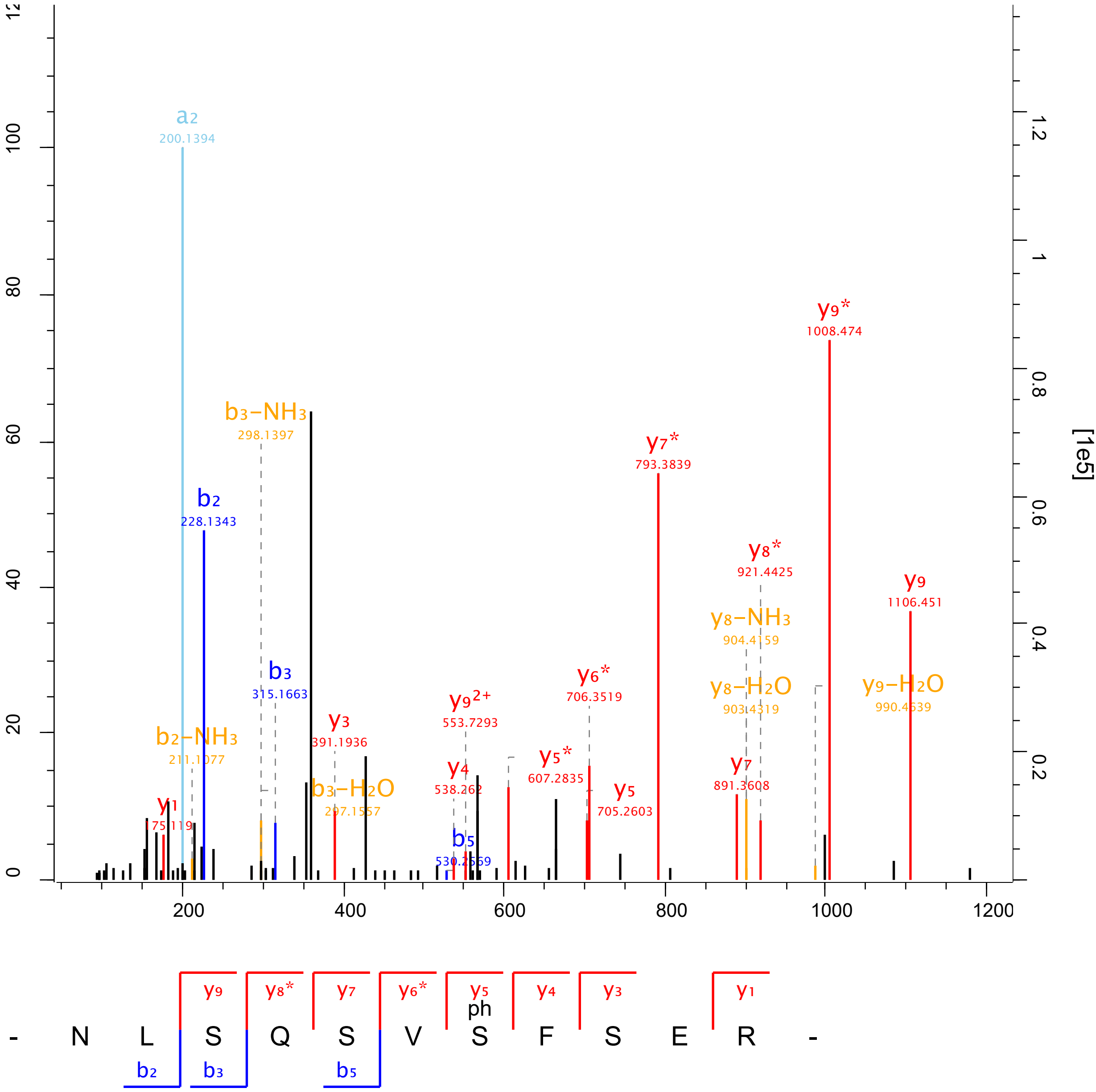

Raw file	Scan	Method	Score	m/z
sys_15_1	18844	FTMS; HCD	140.77	624.31

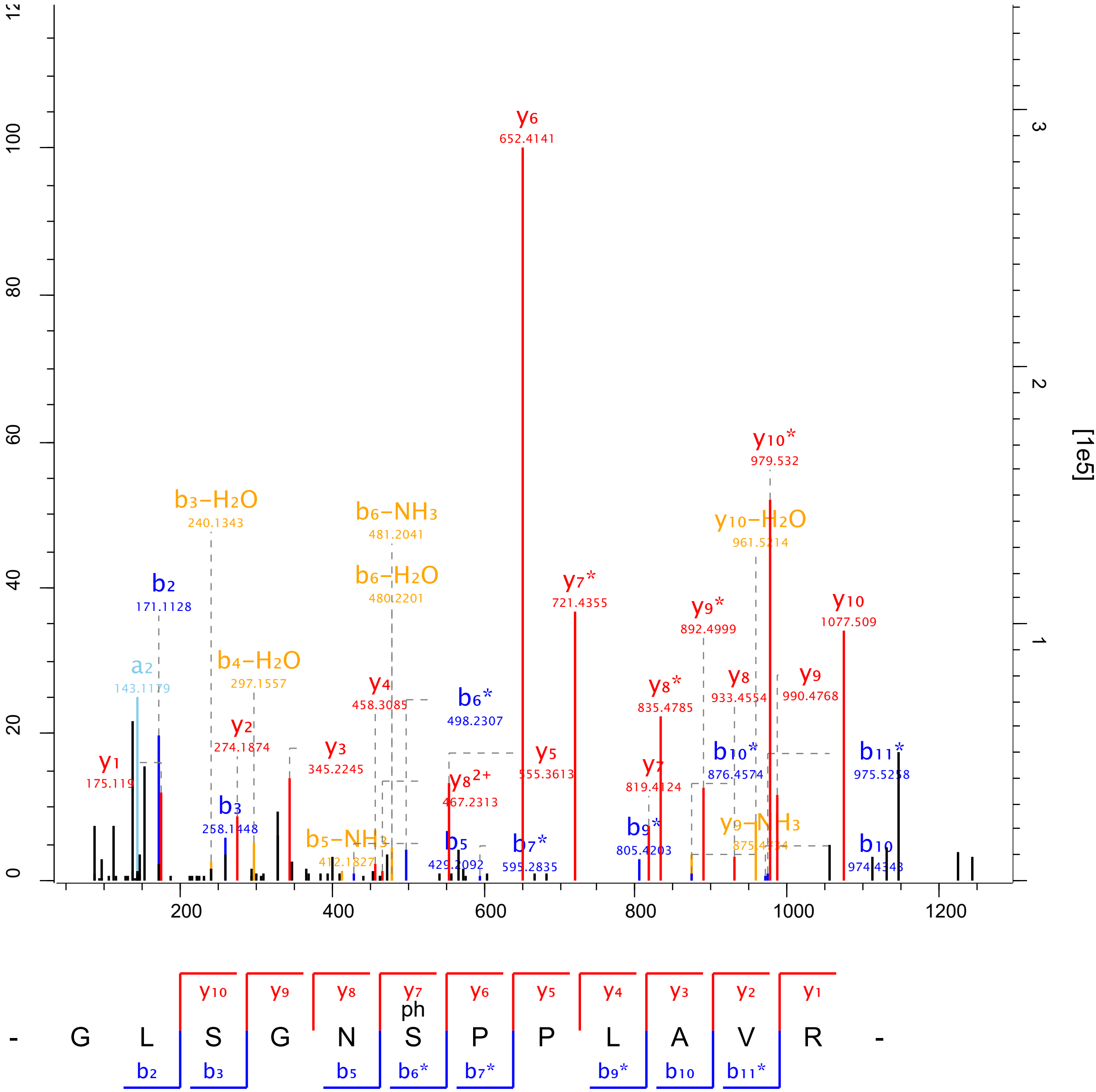

Raw file	Scan	Method	Score	m/z
sys_15_1	18956	FTMS; HCD	136.92	739.65

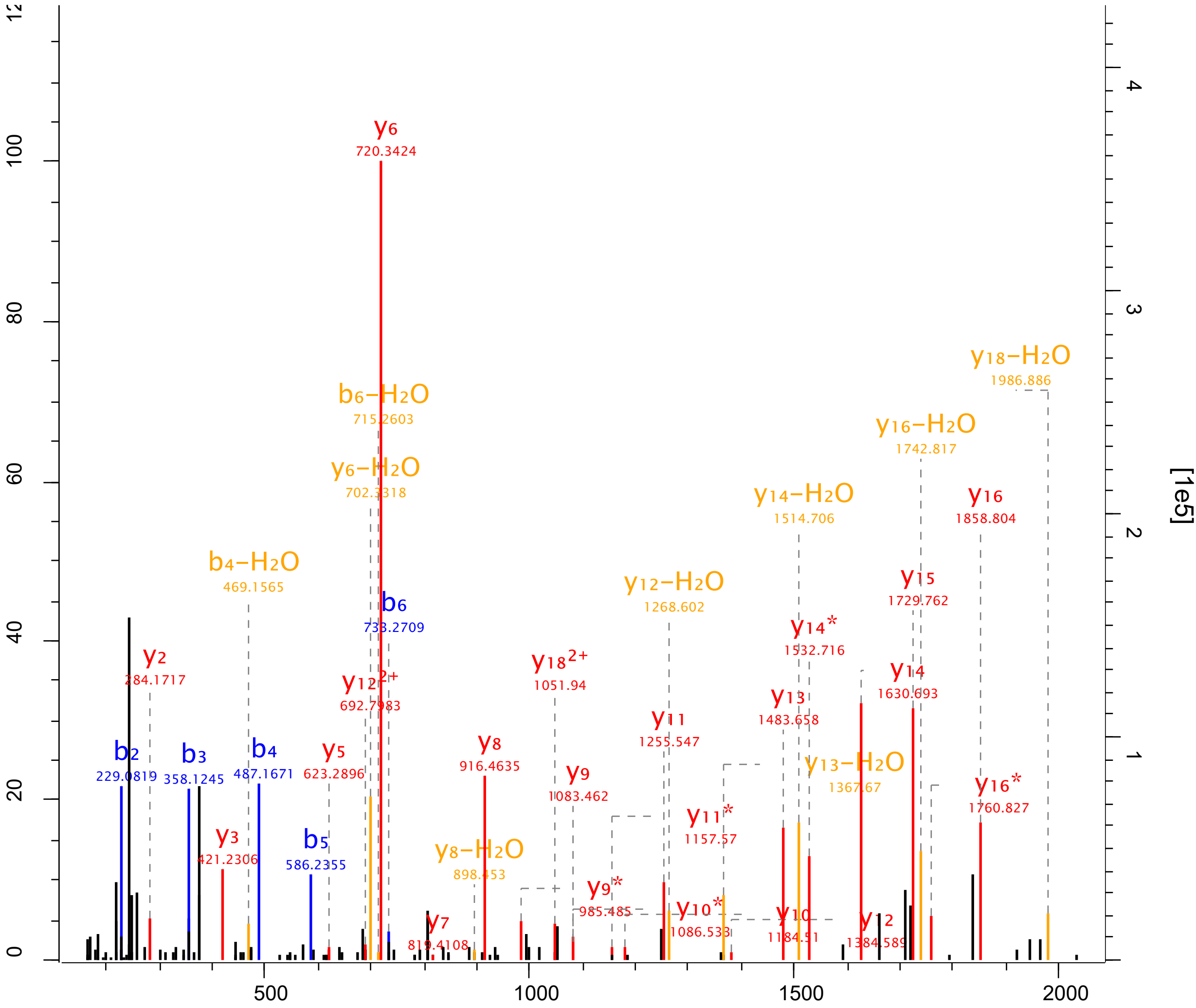

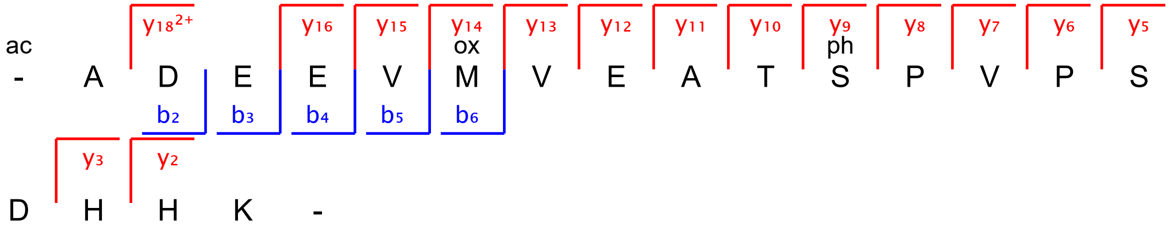

Raw file	Scan	Method	Score	m/z
sys_15_1	19027	FTMS; HCD	51.29	542.76

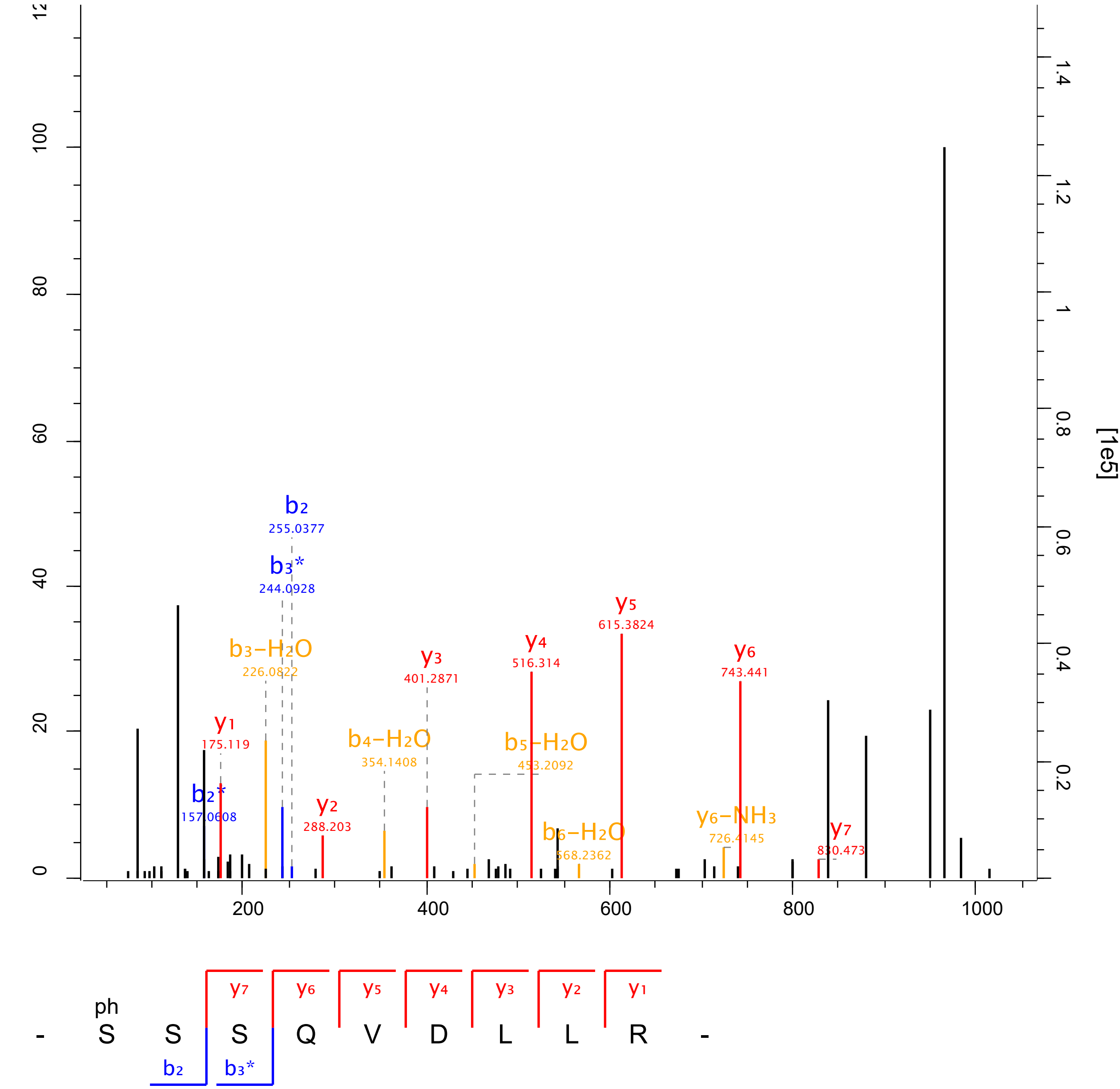

Raw file	Scan	Method	Score	m/z
sys_15_1	19043	FTMS; HCD	219.41	645.32

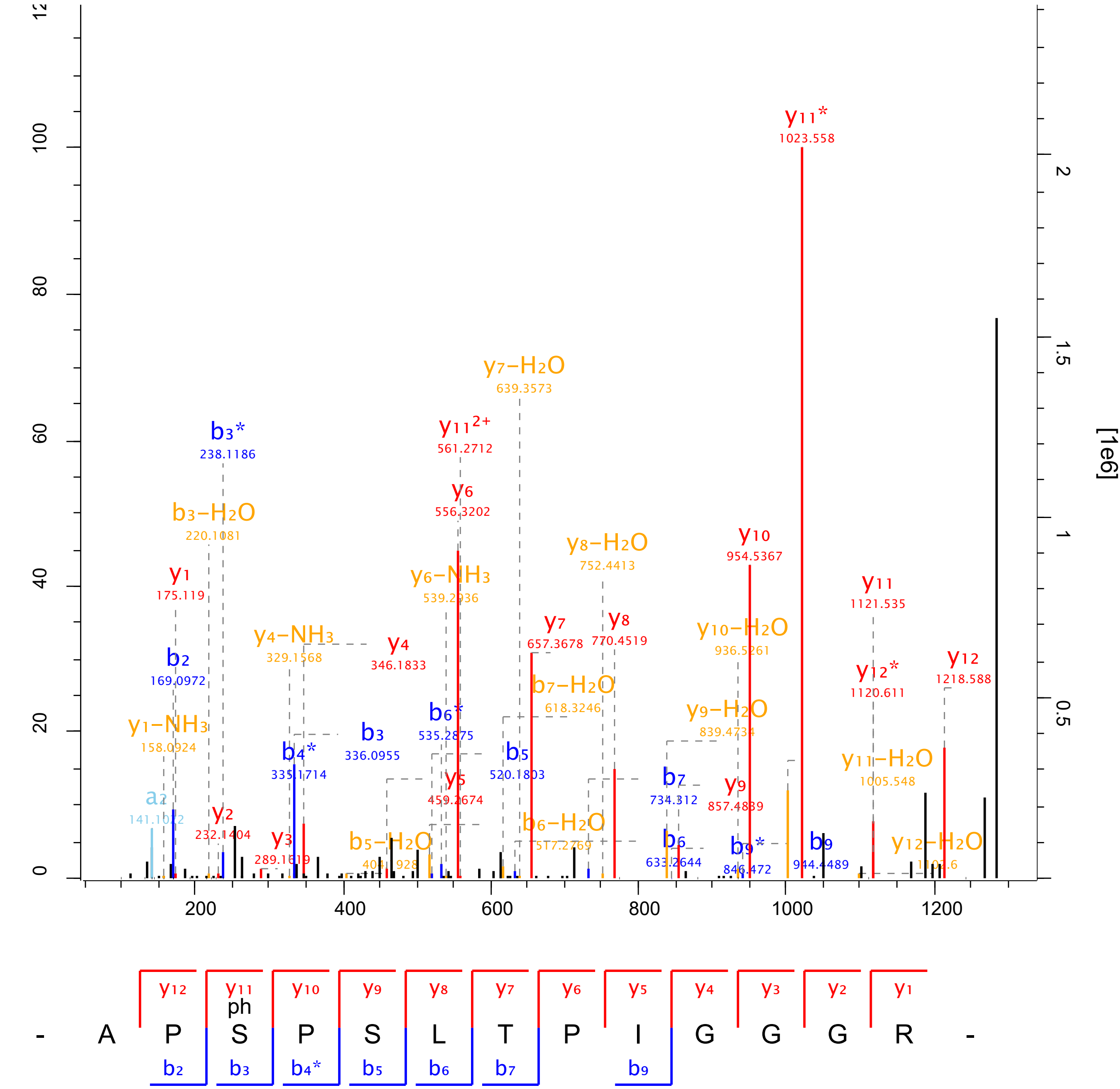

Raw file	Scan	Method	Score	m/z
sys_15_1	19057	FTMS; HCD	70.2	503.91

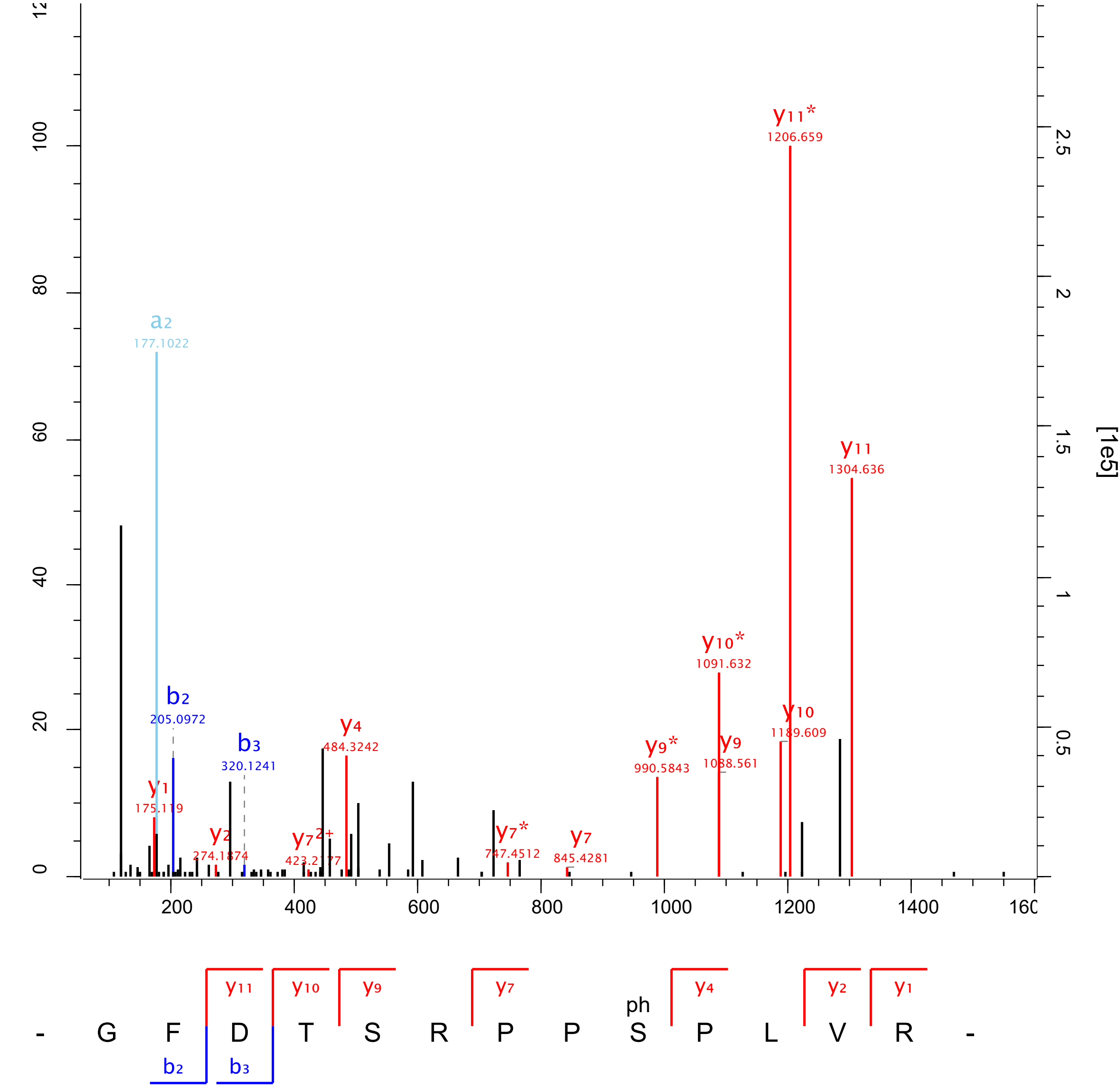

Raw file	Scan	Method	Score	m/z
sys_15_1	19091	FTMS; HCD	109.07	626.81

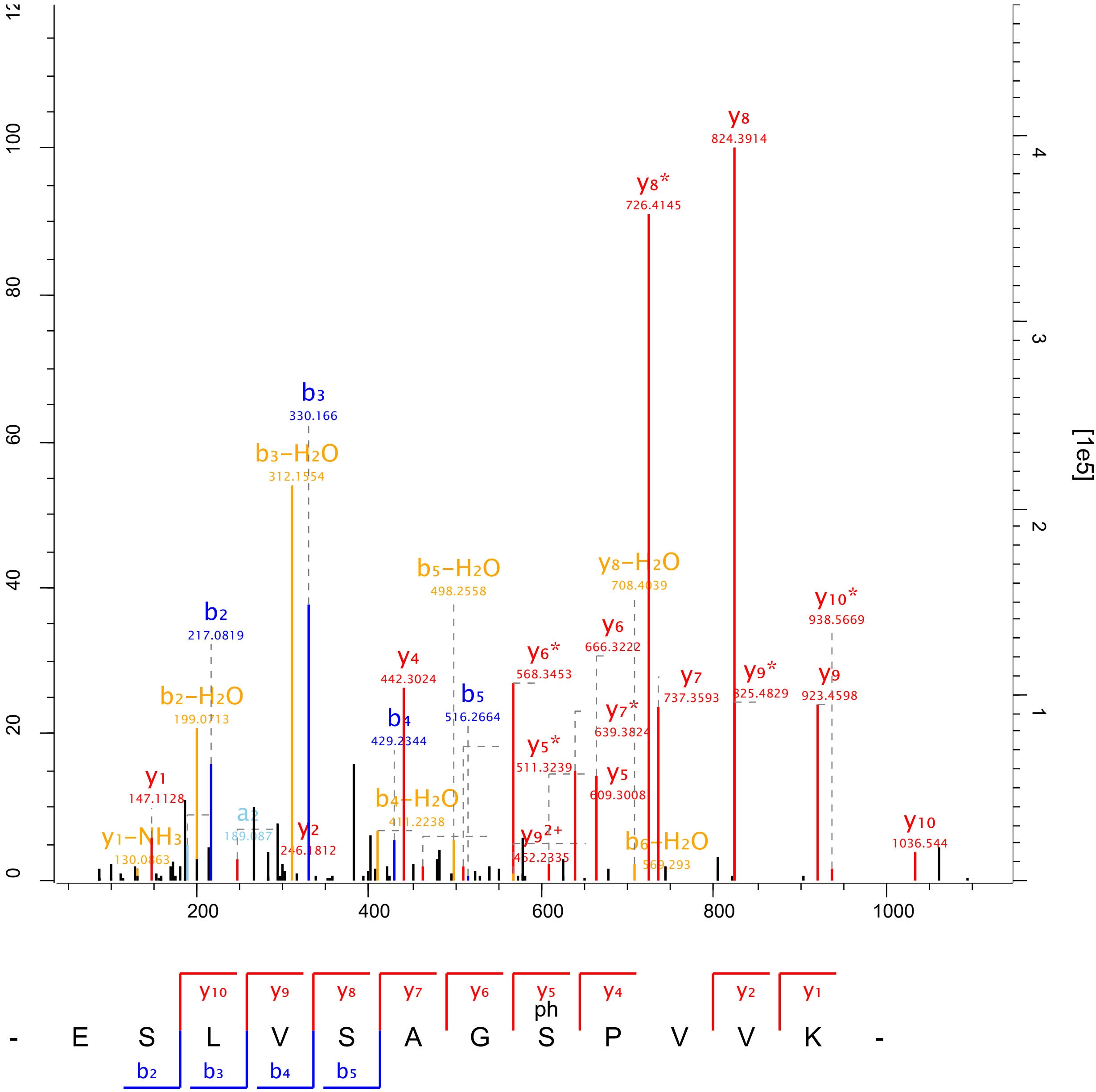

Raw file	Scan	Method	Score	m/z
sys_15_1	19198	FTMS; HCD	63.22	670.01

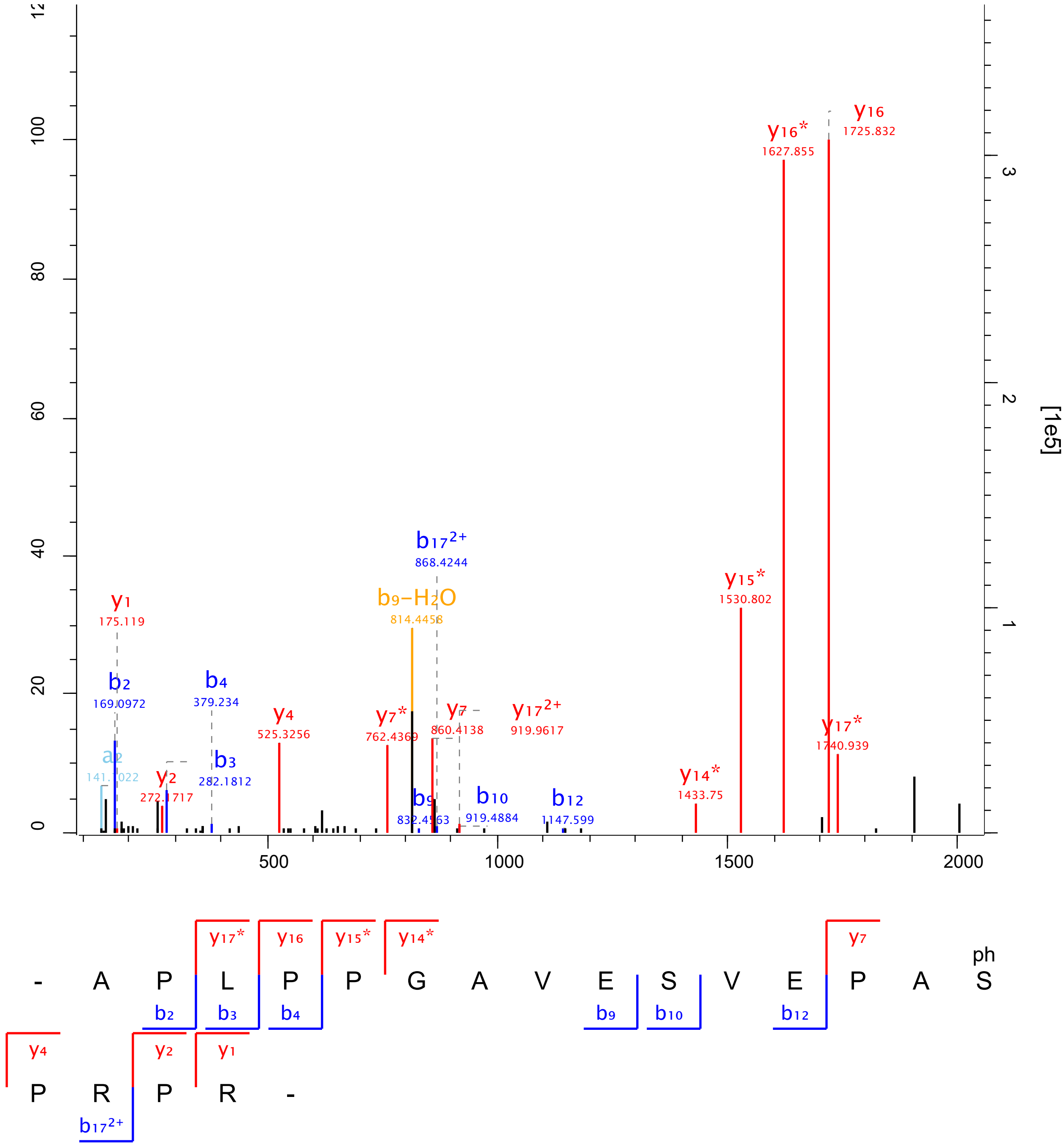

Raw file	Scan	Method	Score	m/z
sys_15_1	19204	FTMS; HCD	110.26	544.57

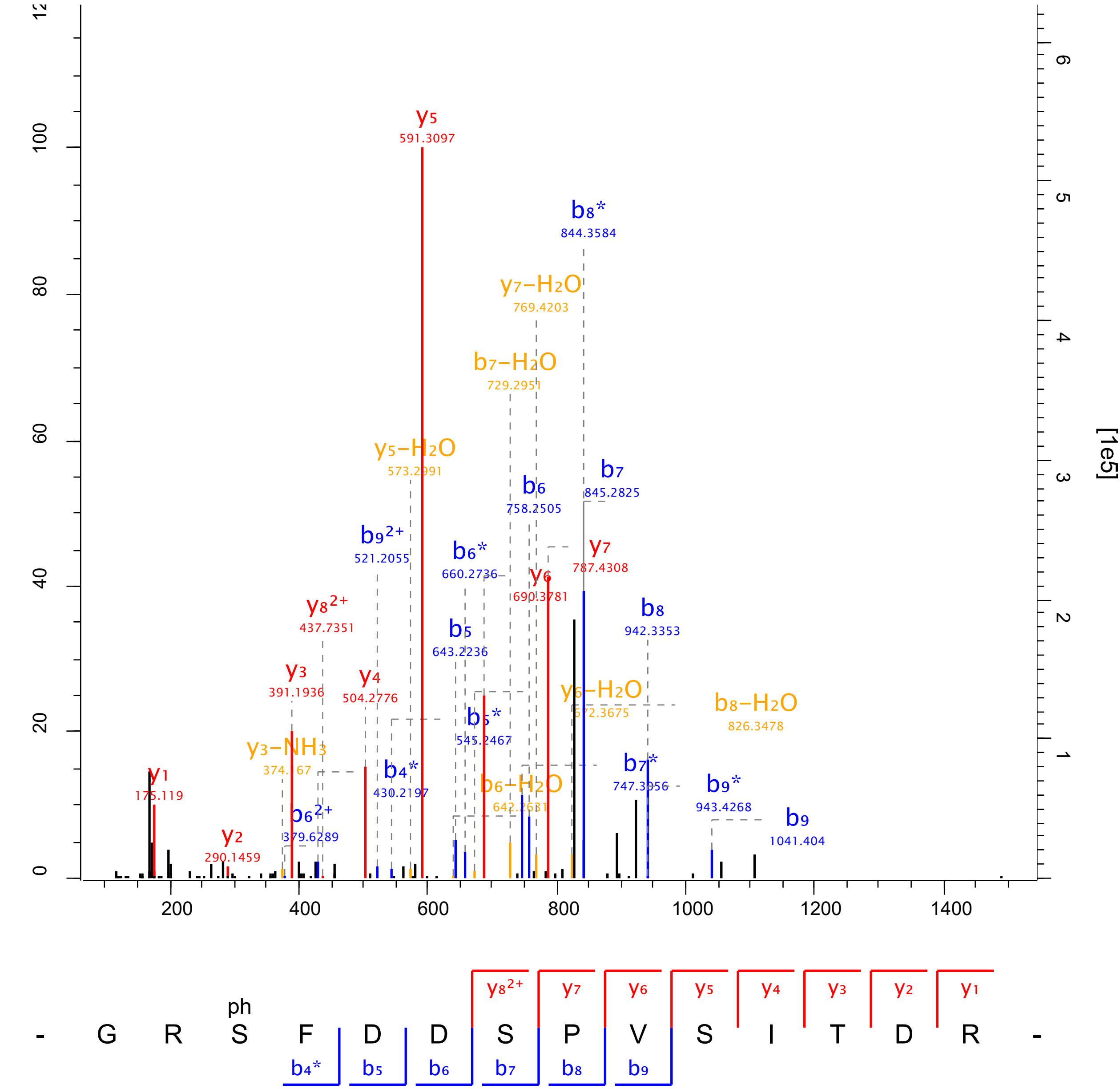

Raw file	Scan	Method	Score	m/z
sys_15_1	19254	FTMS; HCD	63.98	822.28

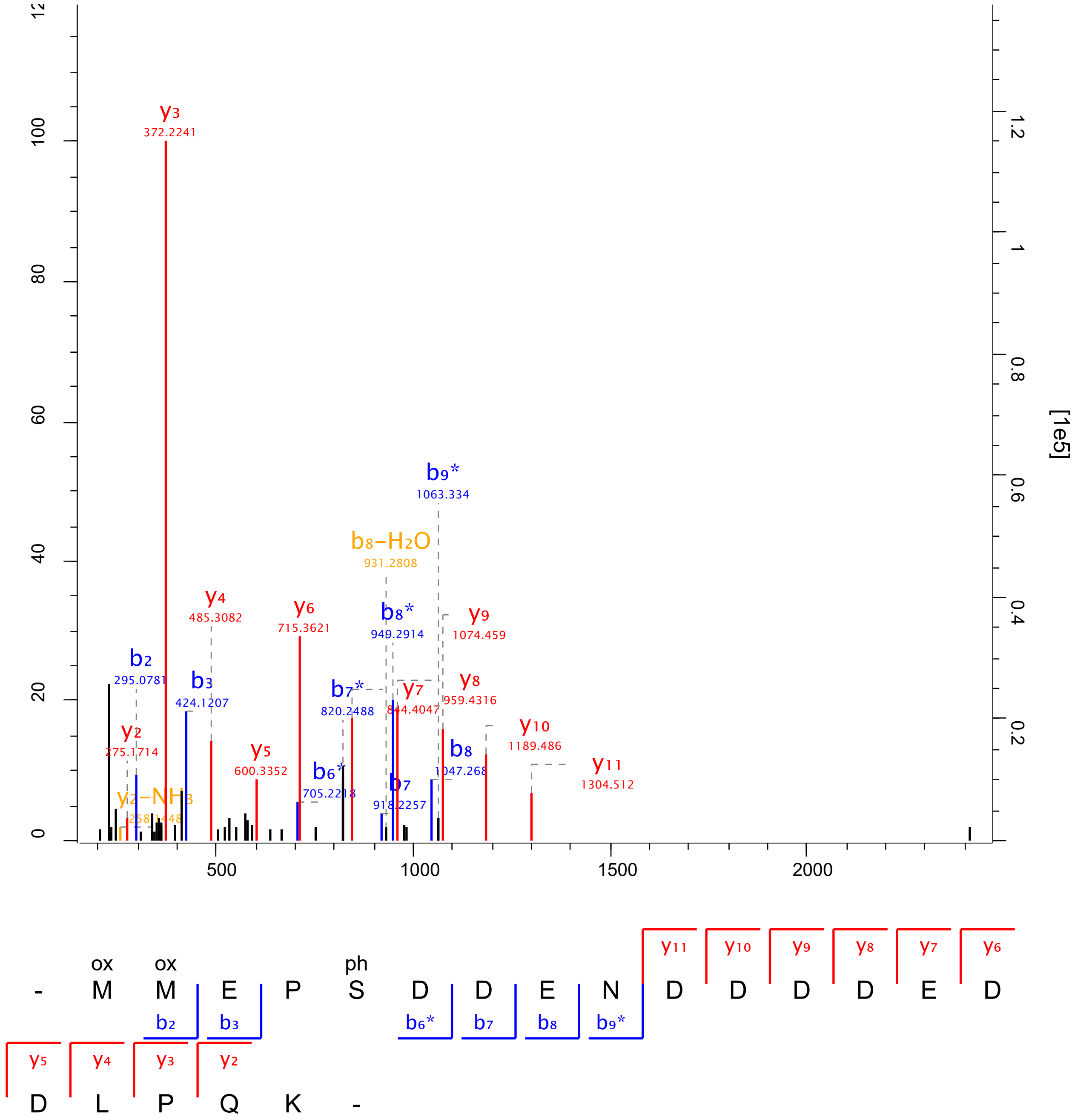

Raw file	Scan	Method	Score	m/z
sys_15_1	19271	FTMS; HCD	194.24	1031.42

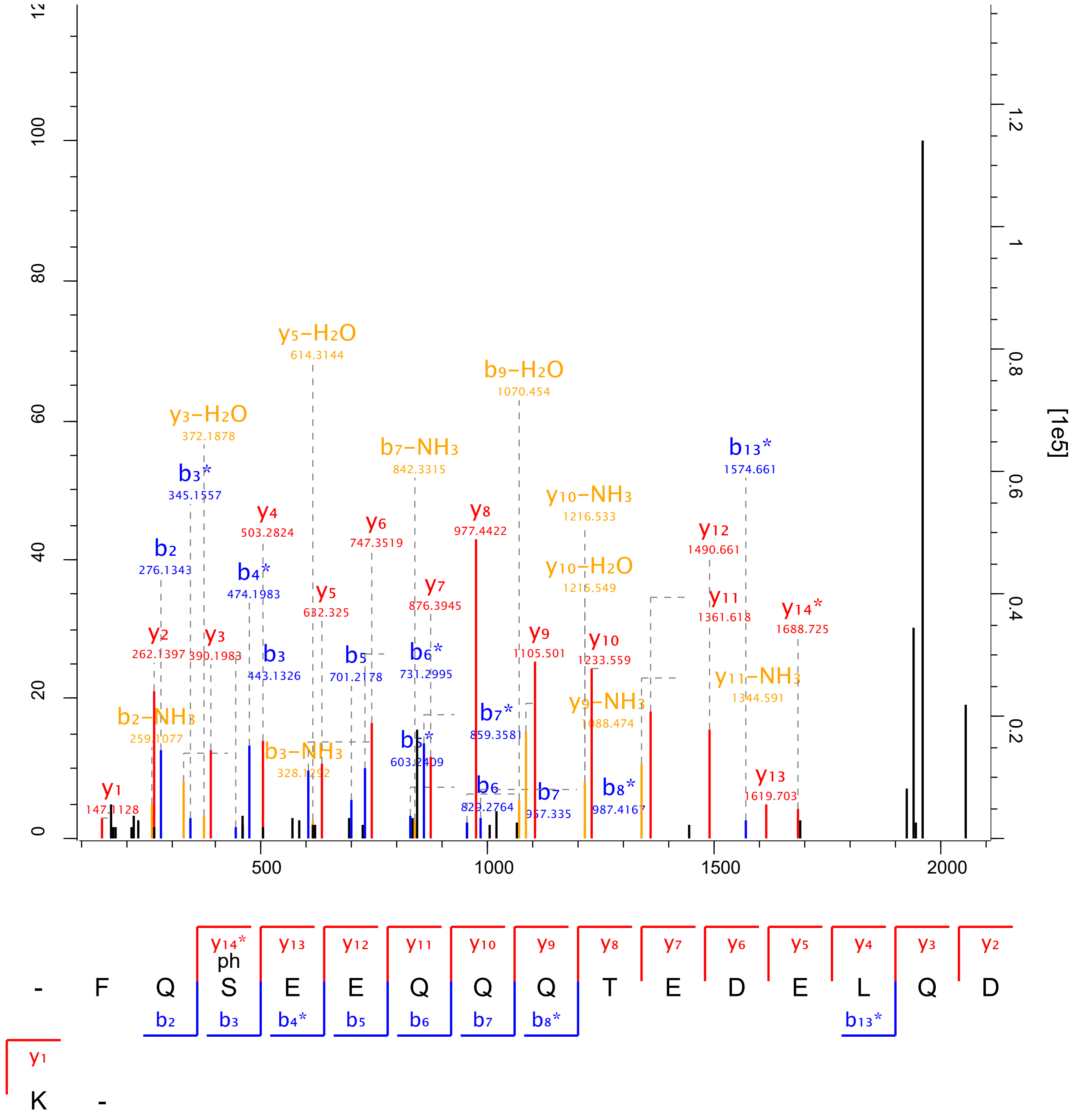

Raw file	Scan	Method	Score	m/z
sys_15_1	19278	FTMS; HCD	166.3	769.37

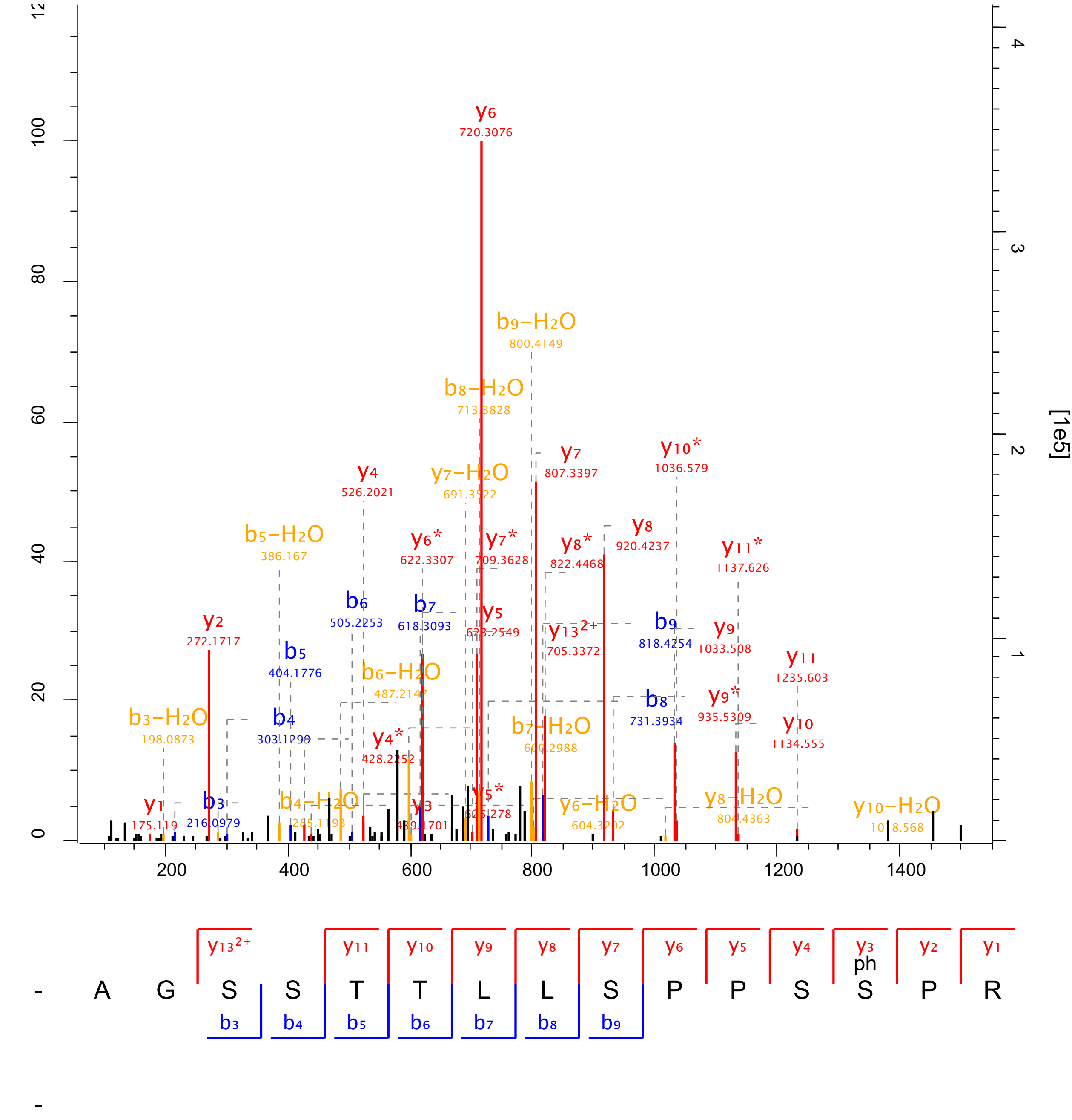

Raw file	Scan	Method	Score	m/z
sys_15_1	19283	FTMS; HCD	47.55	703.28

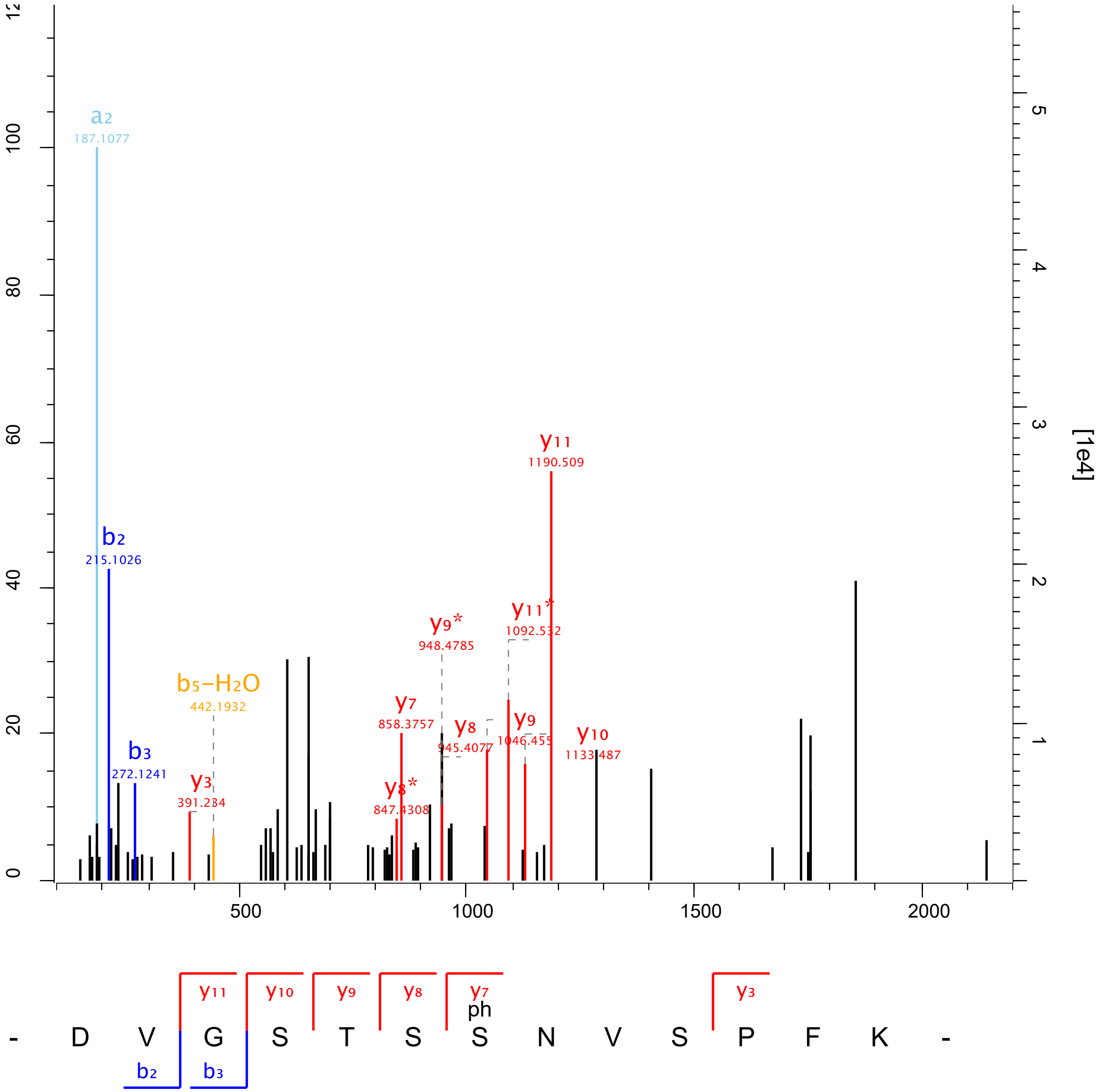

Raw file	Scan	Method	Score	m/z
sys_15_1	19329	FTMS; HCD	133.16	650.26

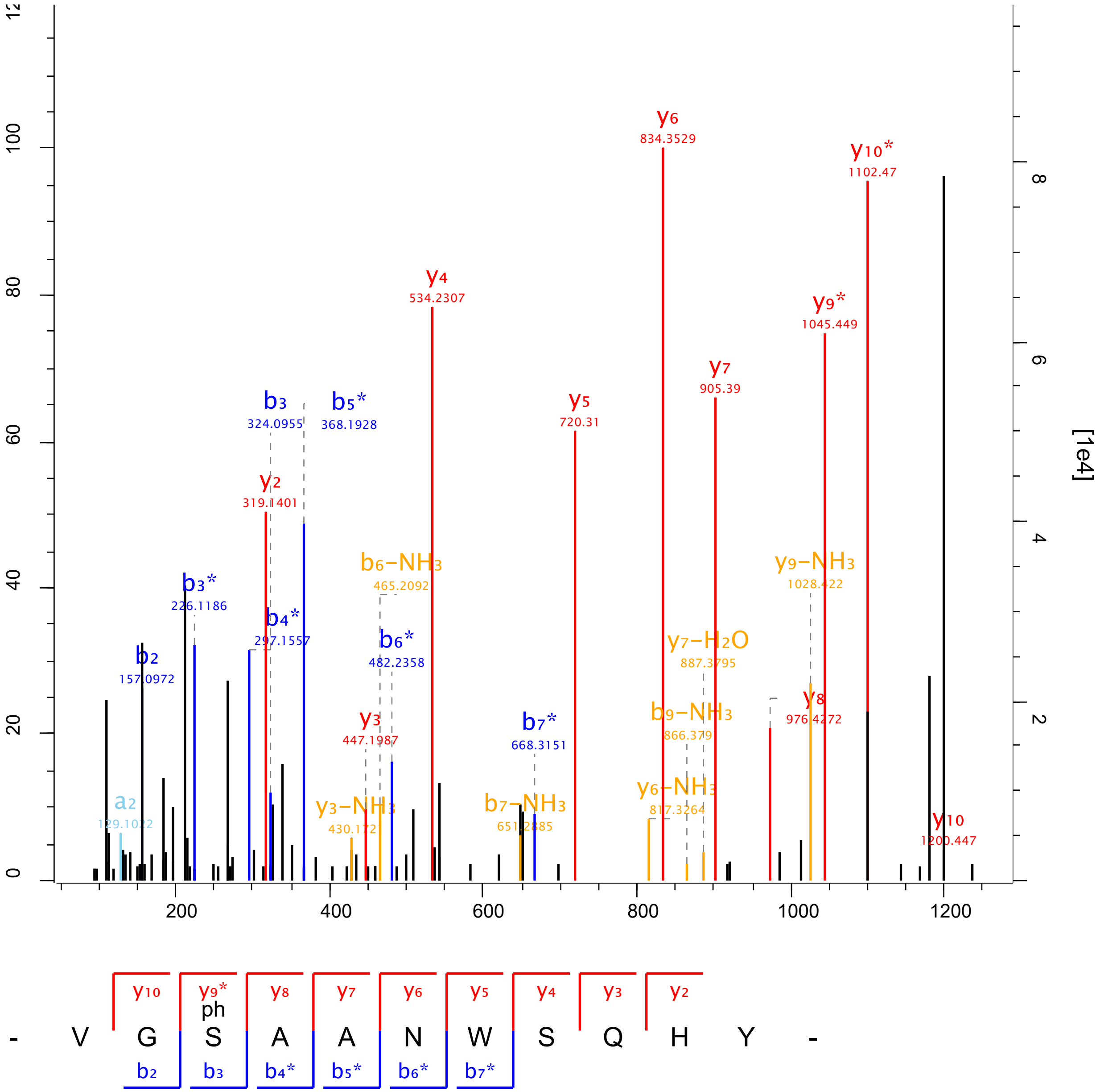

Raw file	Scan	Method	Score	m/z
sys_15_1	1935	FTMS; HCD	57.59	437.18

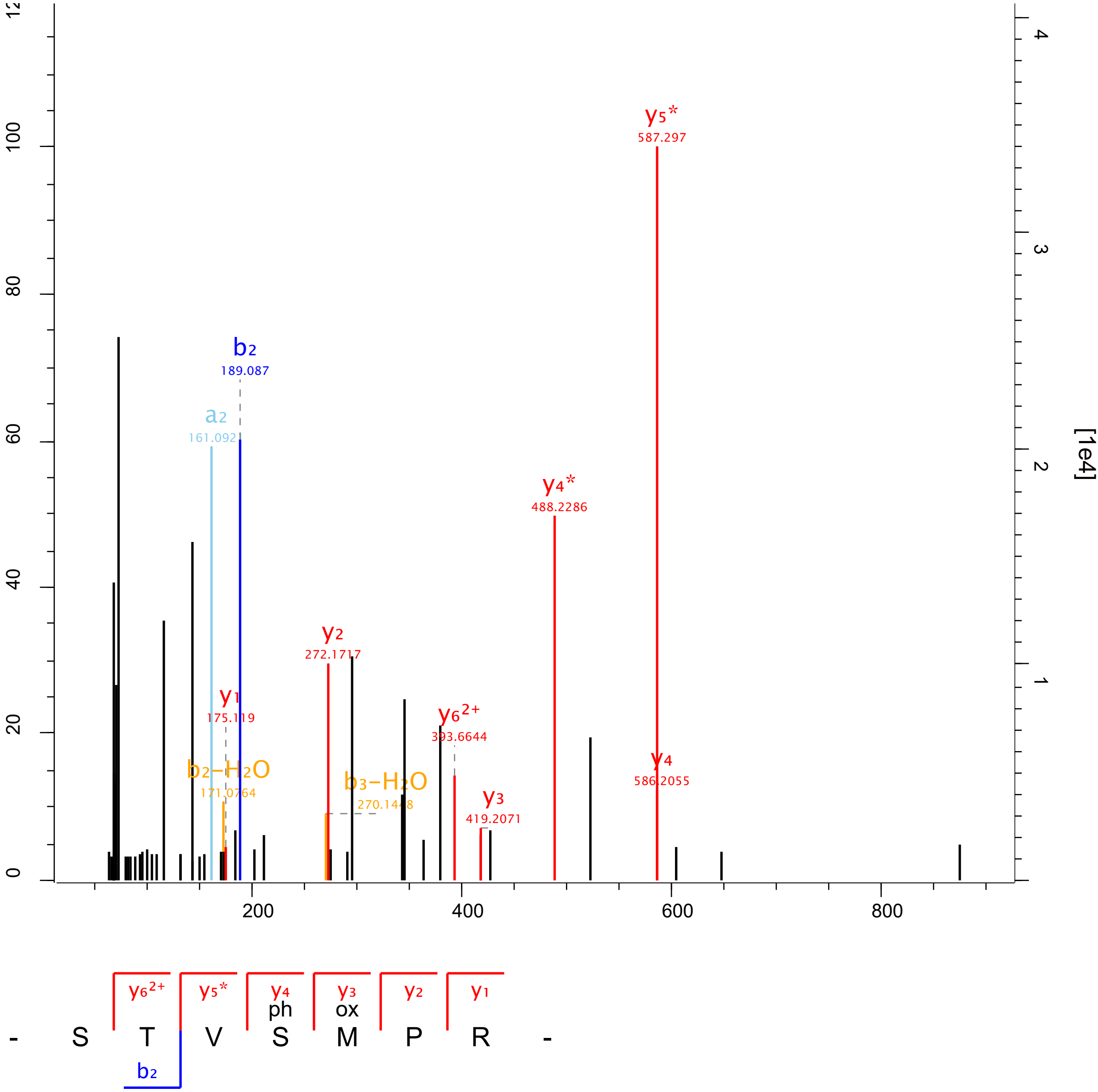

Raw file	Scan	Method	Score	m/z
sys_15_1	19441	FTMS; HCD	41.24	628.25

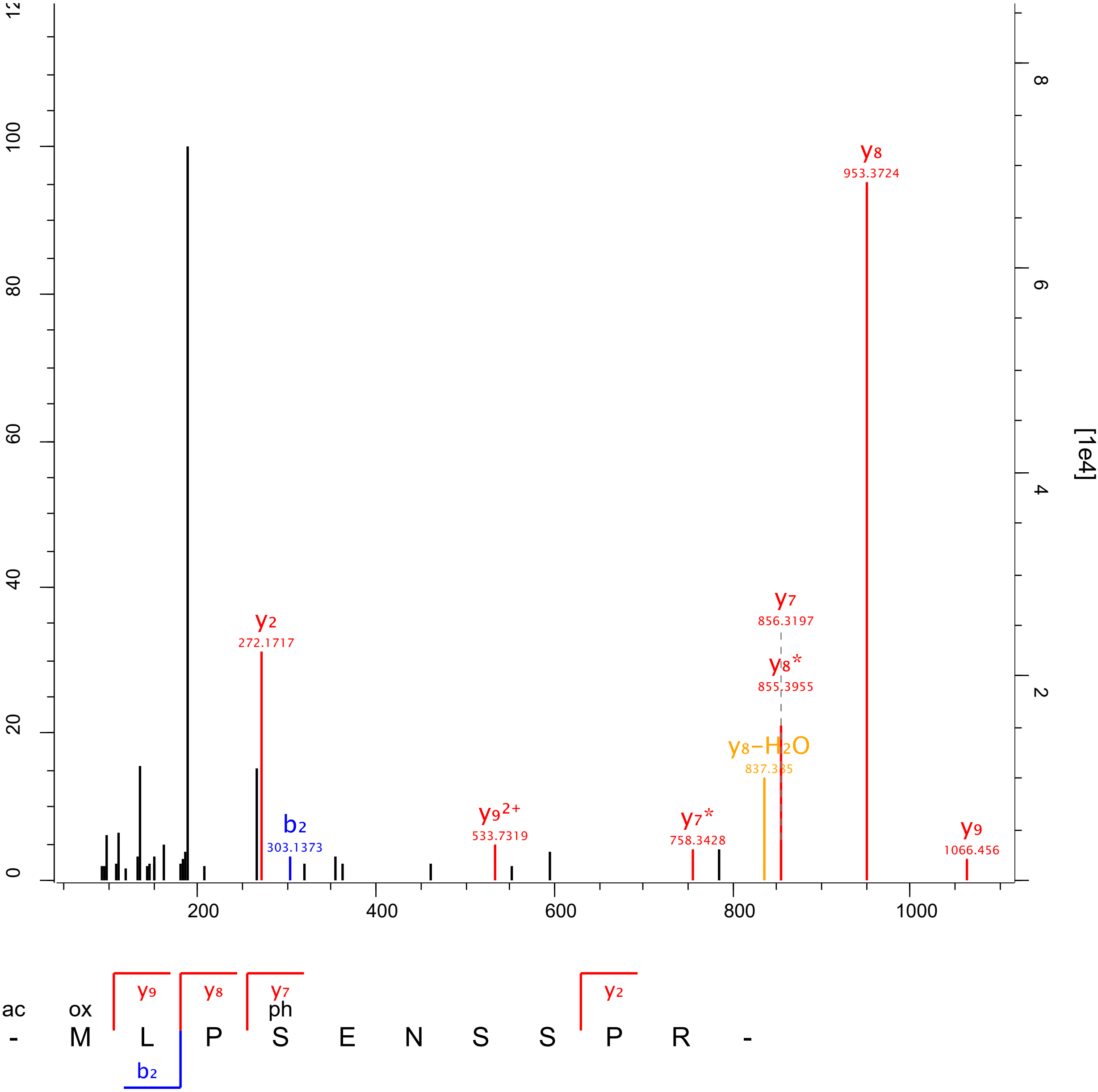

Raw file	Scan	Method	Score	m/z
sys_15_1	19541	FTMS; HCD	63.91	740.29

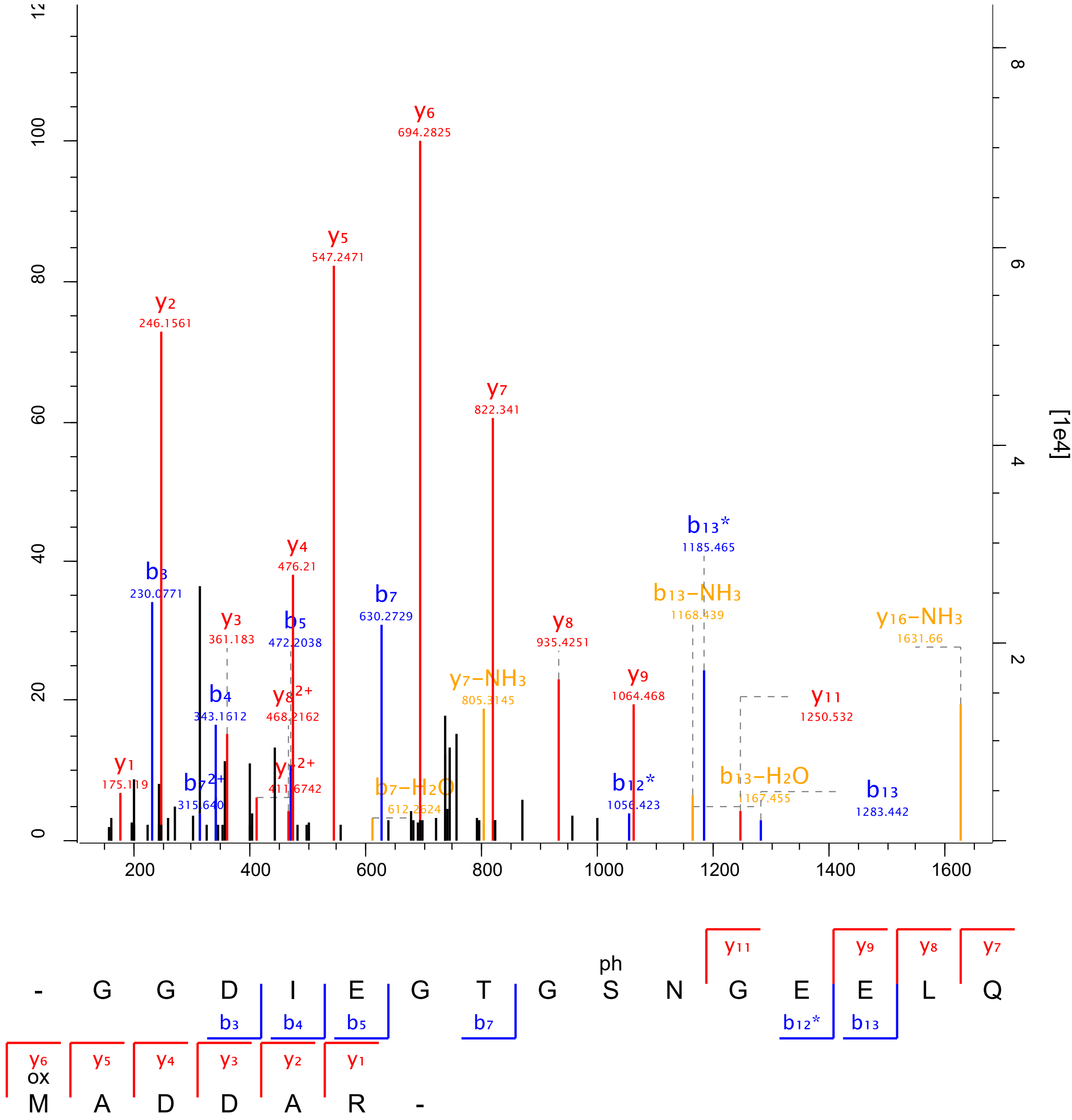

Raw file	Scan	Method	Score	m/z
sys_15_1	19597	FTMS; HCD	166.05	780.97

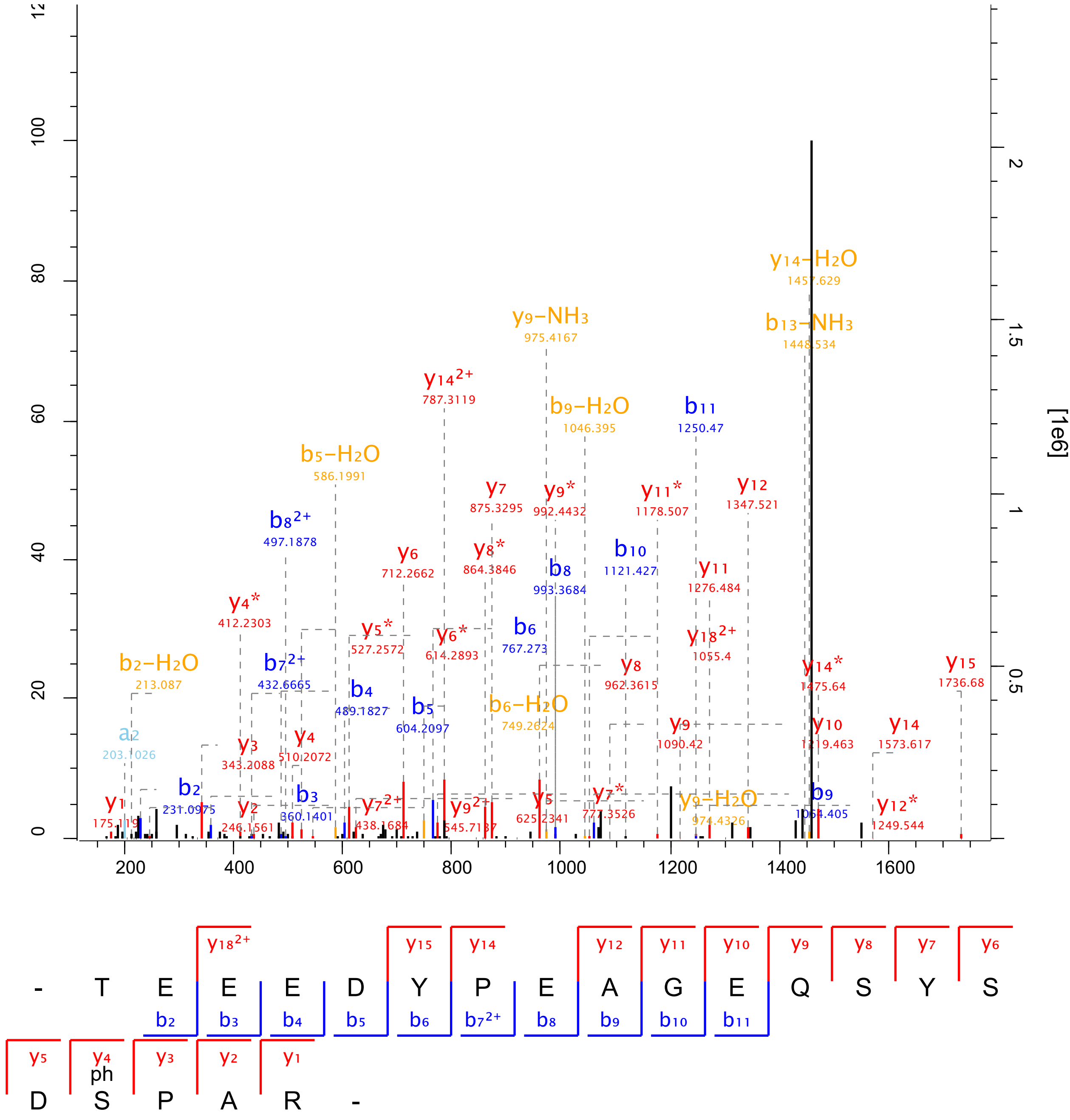

Raw file	Scan	Method	Score	m/z
sys_15_1	19668	FTMS; HCD	93.63	959.38

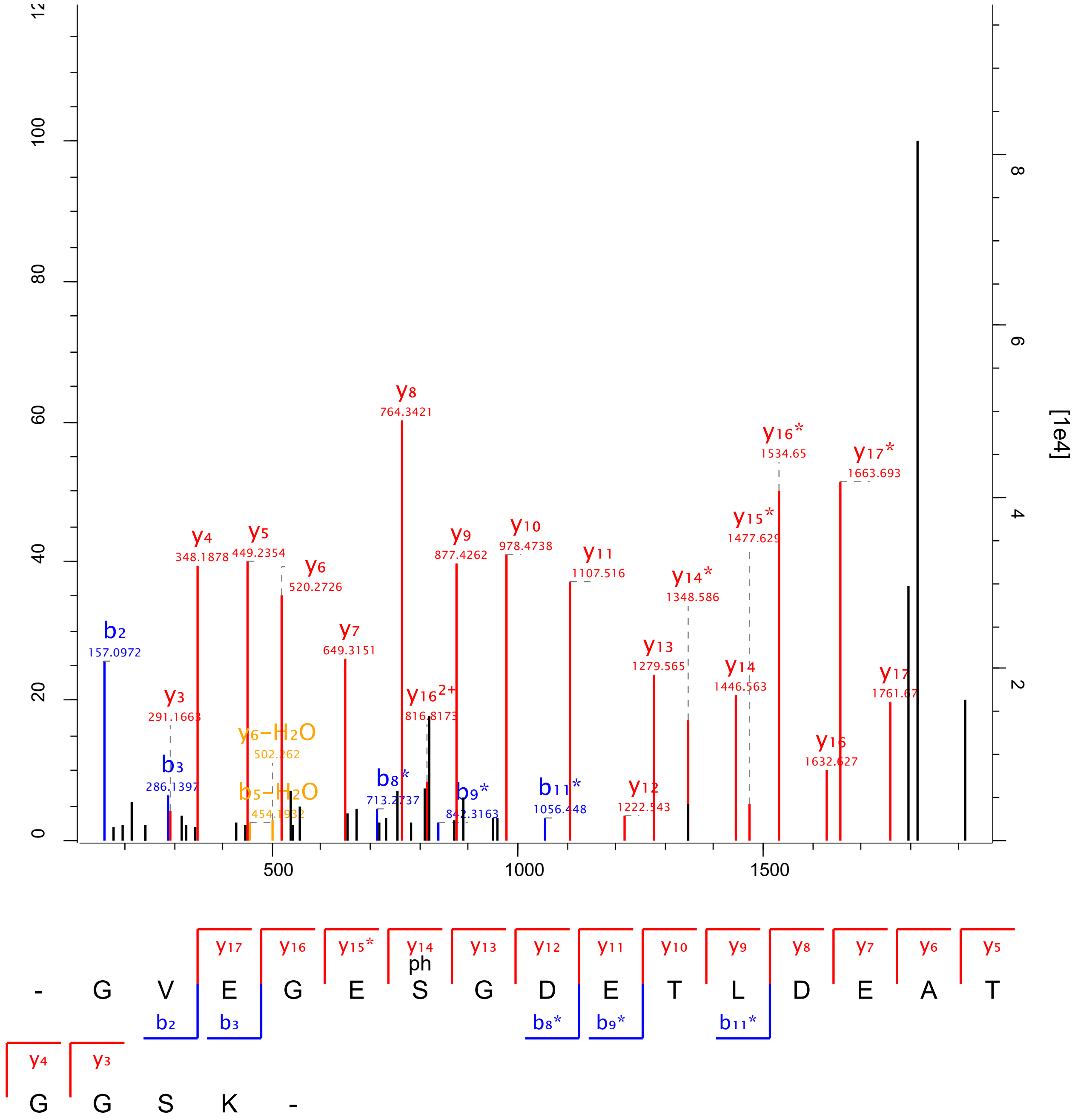

Raw file	Scan	Method	Score	m/z
sys_15_1	1967	FTMS; HCD	51.28	487.2

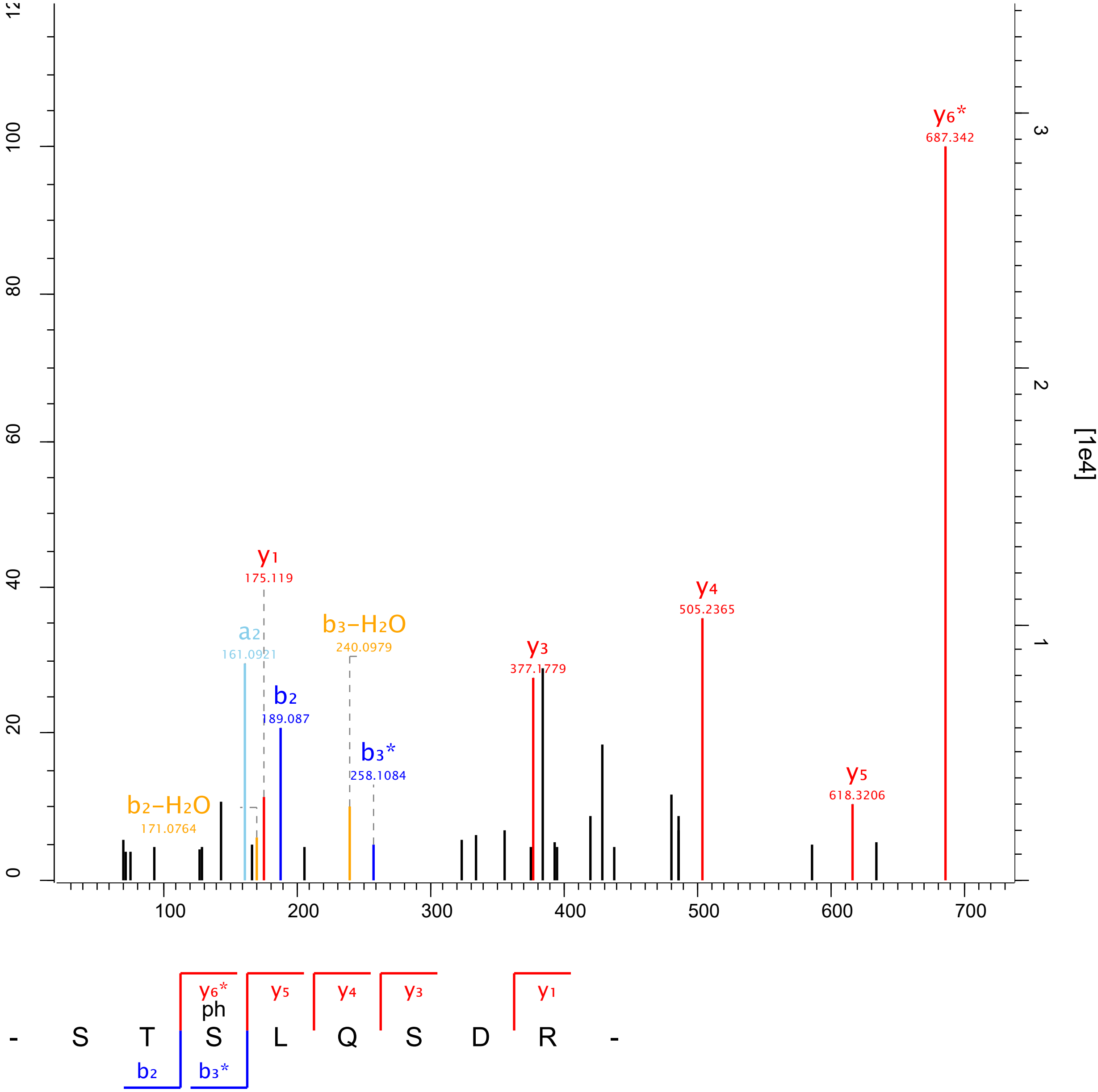

Raw file	Scan	Method	Score	m/z
sys_15_1	19786	FTMS; HCD	79.84	549.91

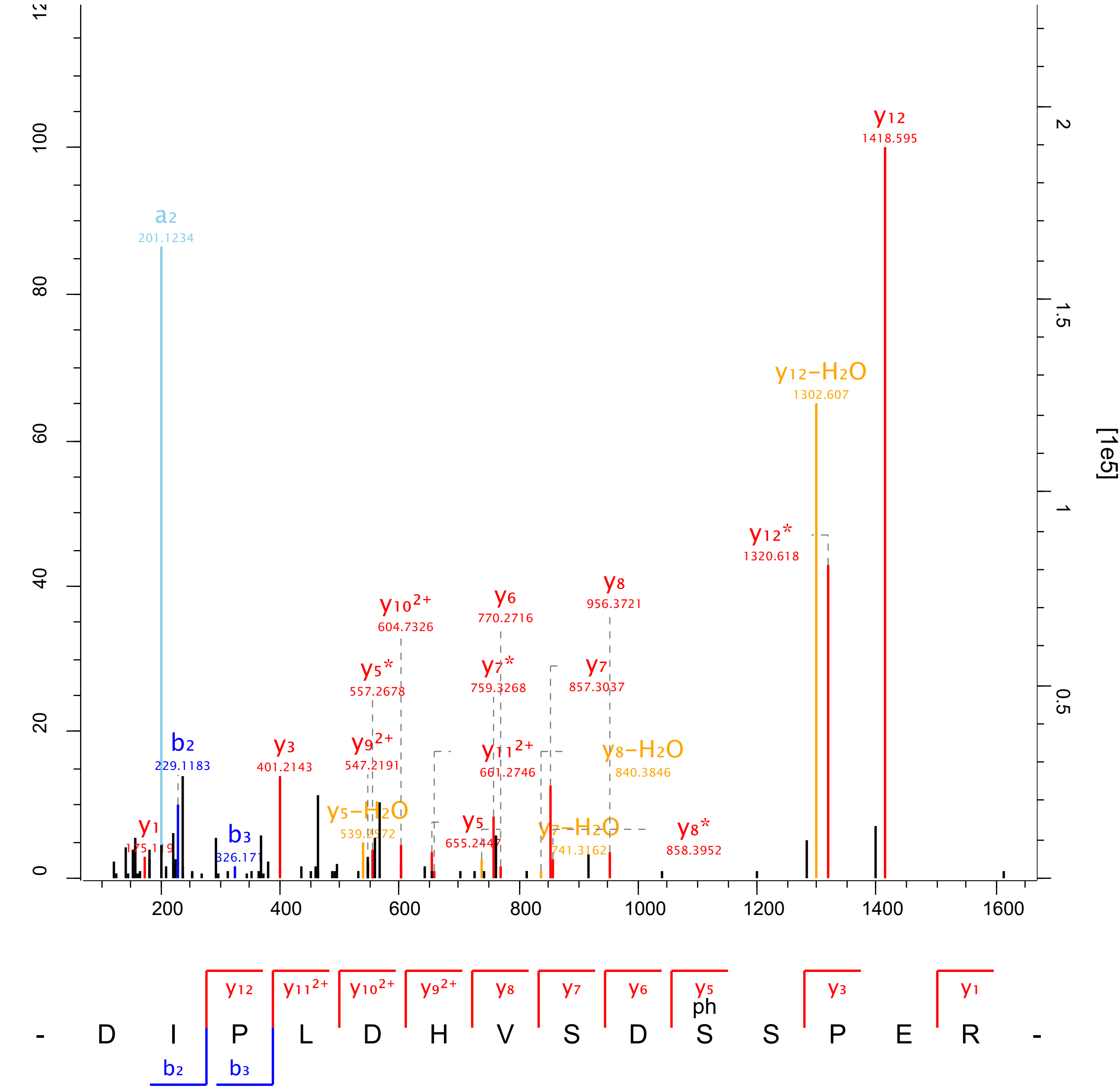

Raw file	Scan	Method	Score	m/z
sys_15_1	1984	FTMS; HCD	69.72	546.23

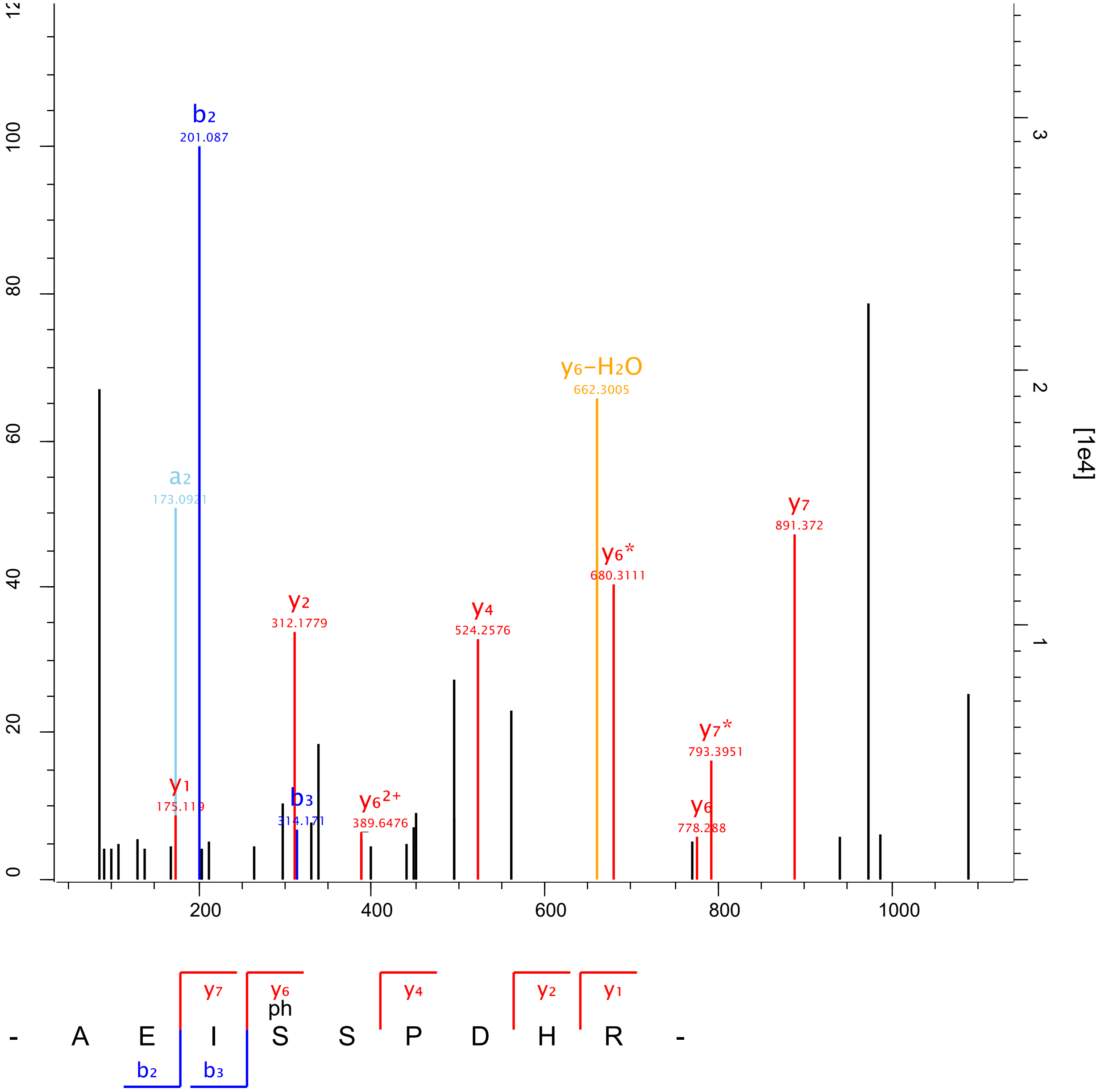

Raw file	Scan	Method	Score	m/z
sys_15_1	19935	FTMS; HCD	52.49	515.74

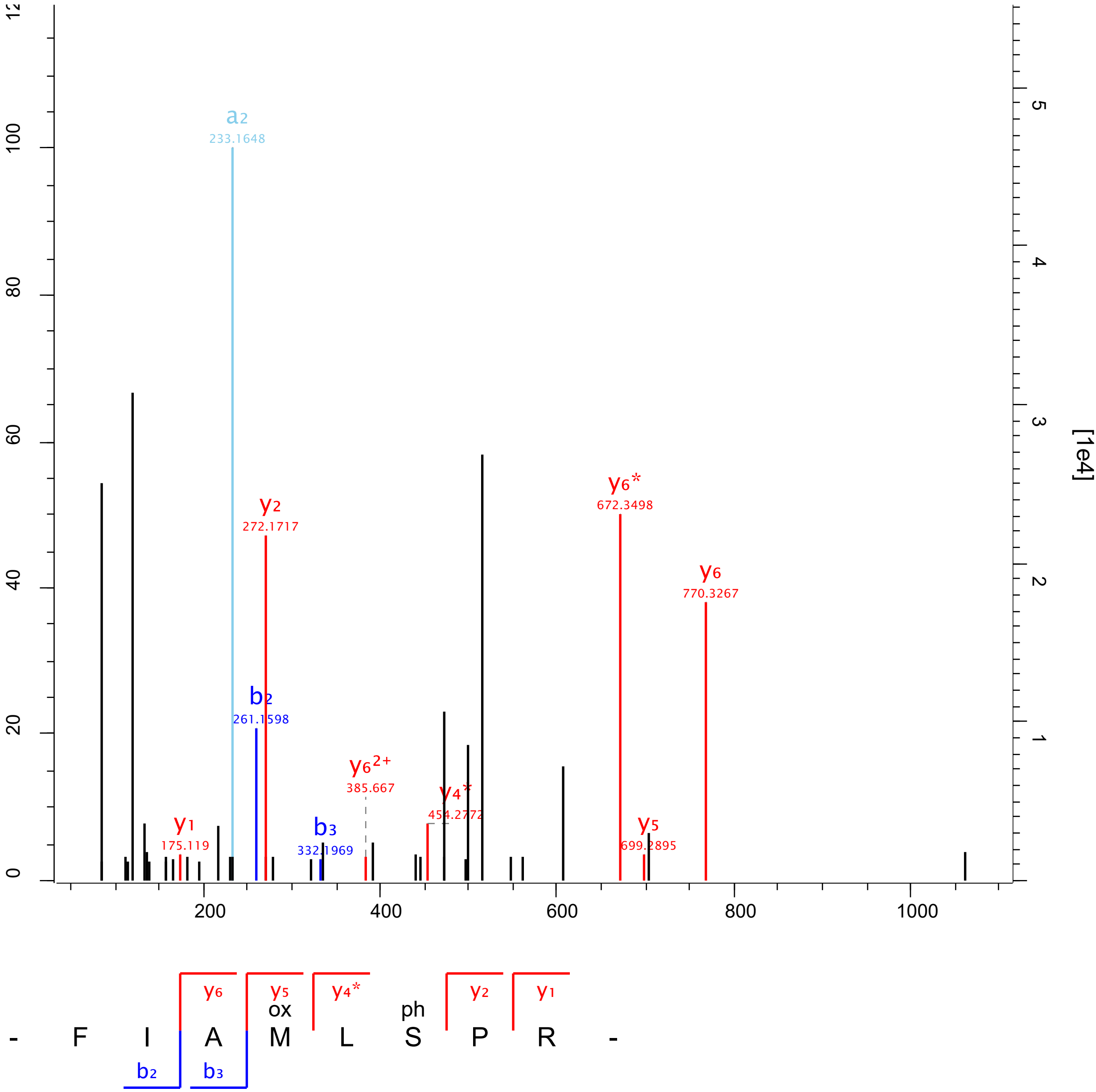

Raw file	Scan	Method	Score	m/z
sys_15_1	19985	FTMS; HCD	80.07	645.28

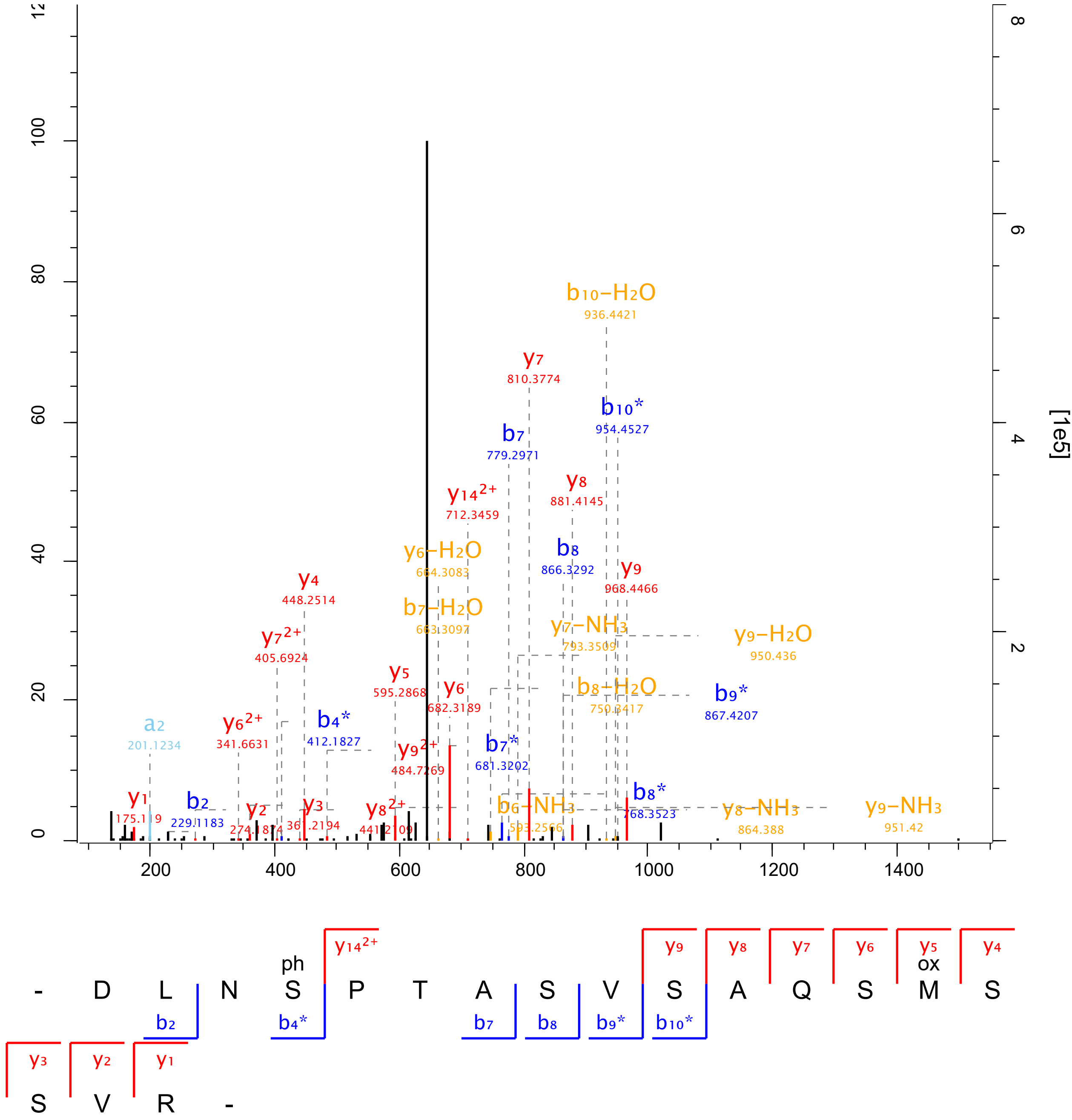

Raw file	Scan	Method	Score	m/z
sys_15_1	20120	FTMS; HCD	69.33	727.33

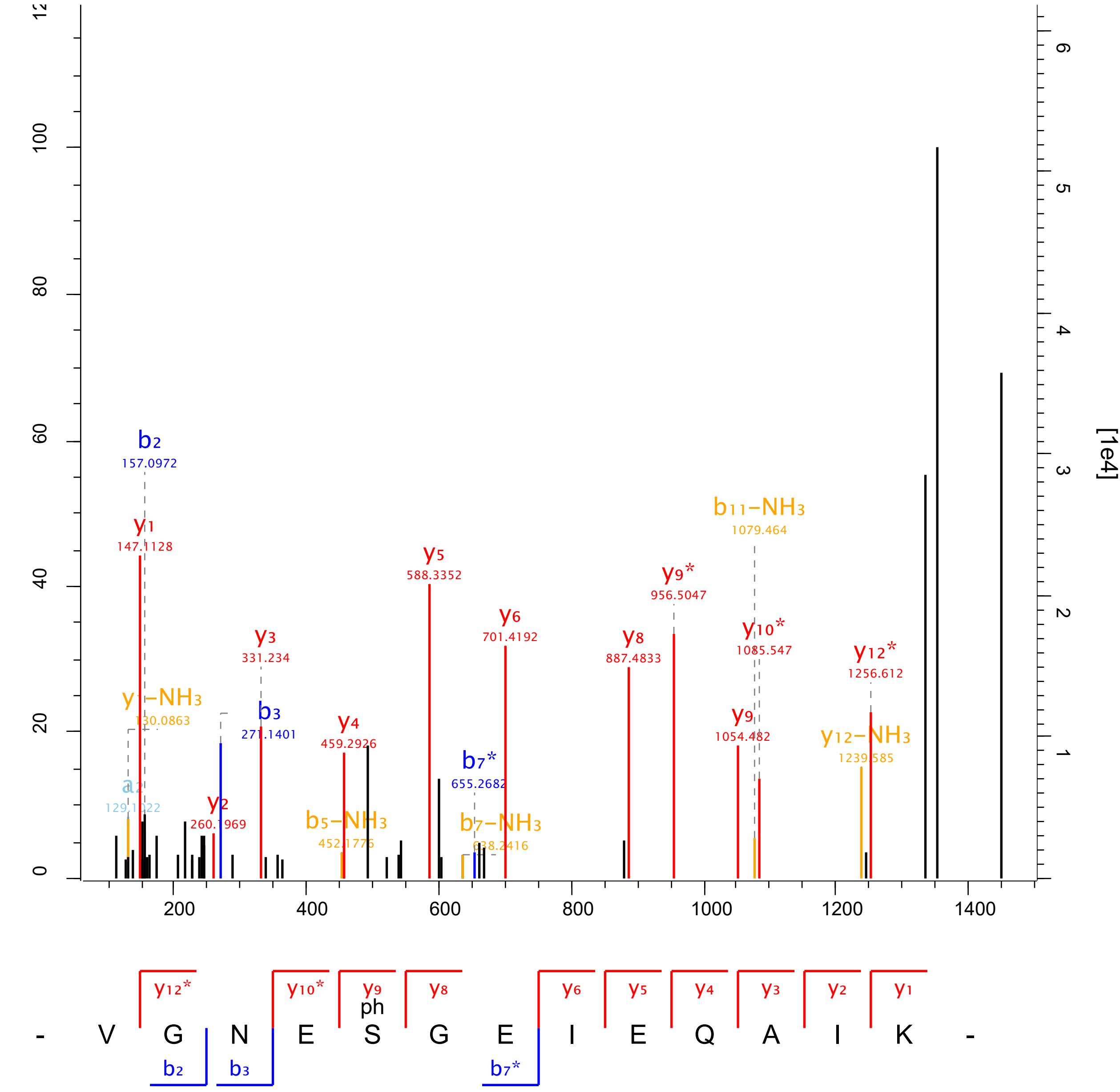

Raw file	Scan	Method	Score	m/z
sys_15_1	20130	FTMS; HCD	52.17	768.31

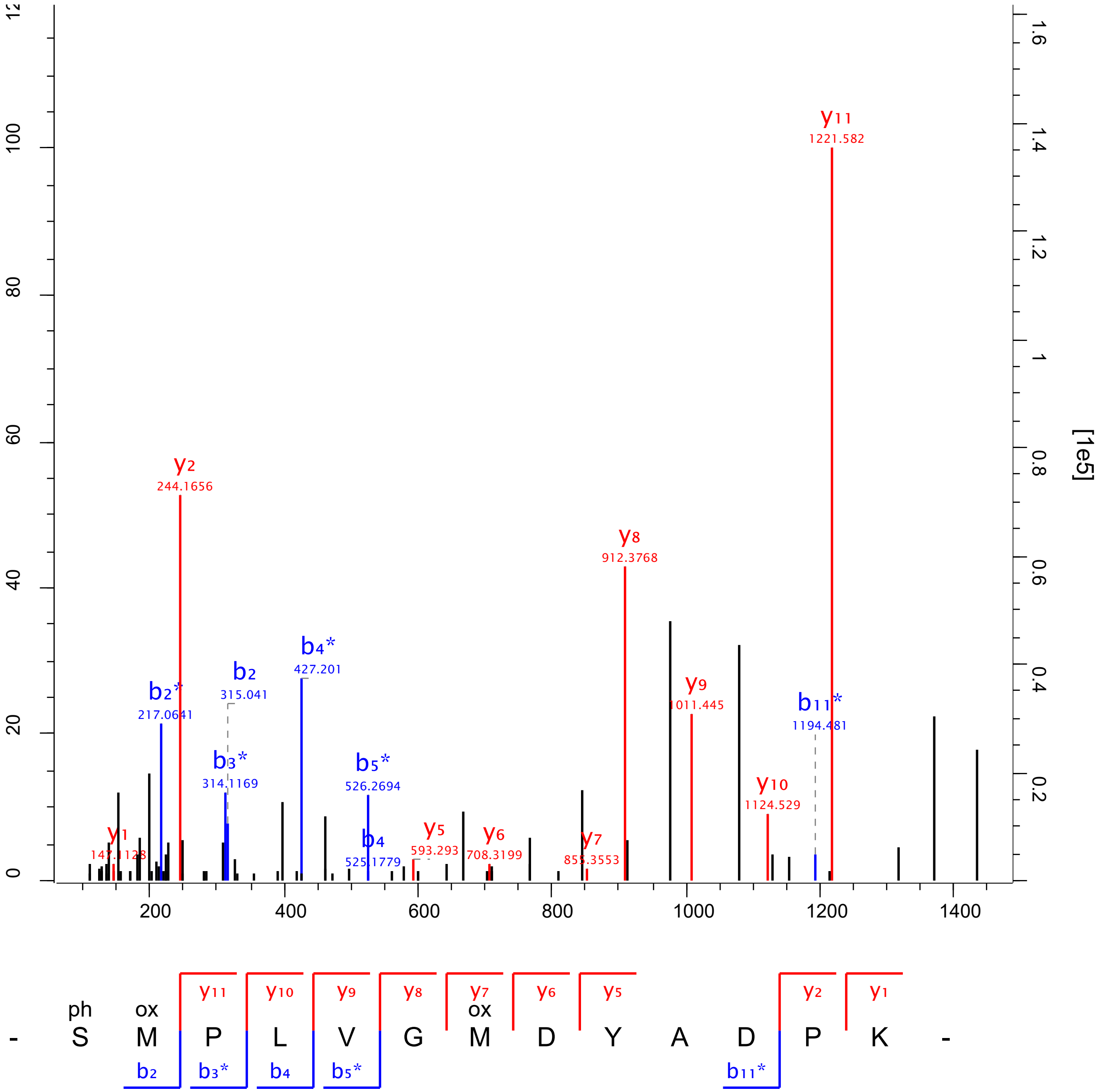

Raw file	Scan	Method	Score	m/z
sys_15_1	20134	FTMS; HCD	58.32	439.86

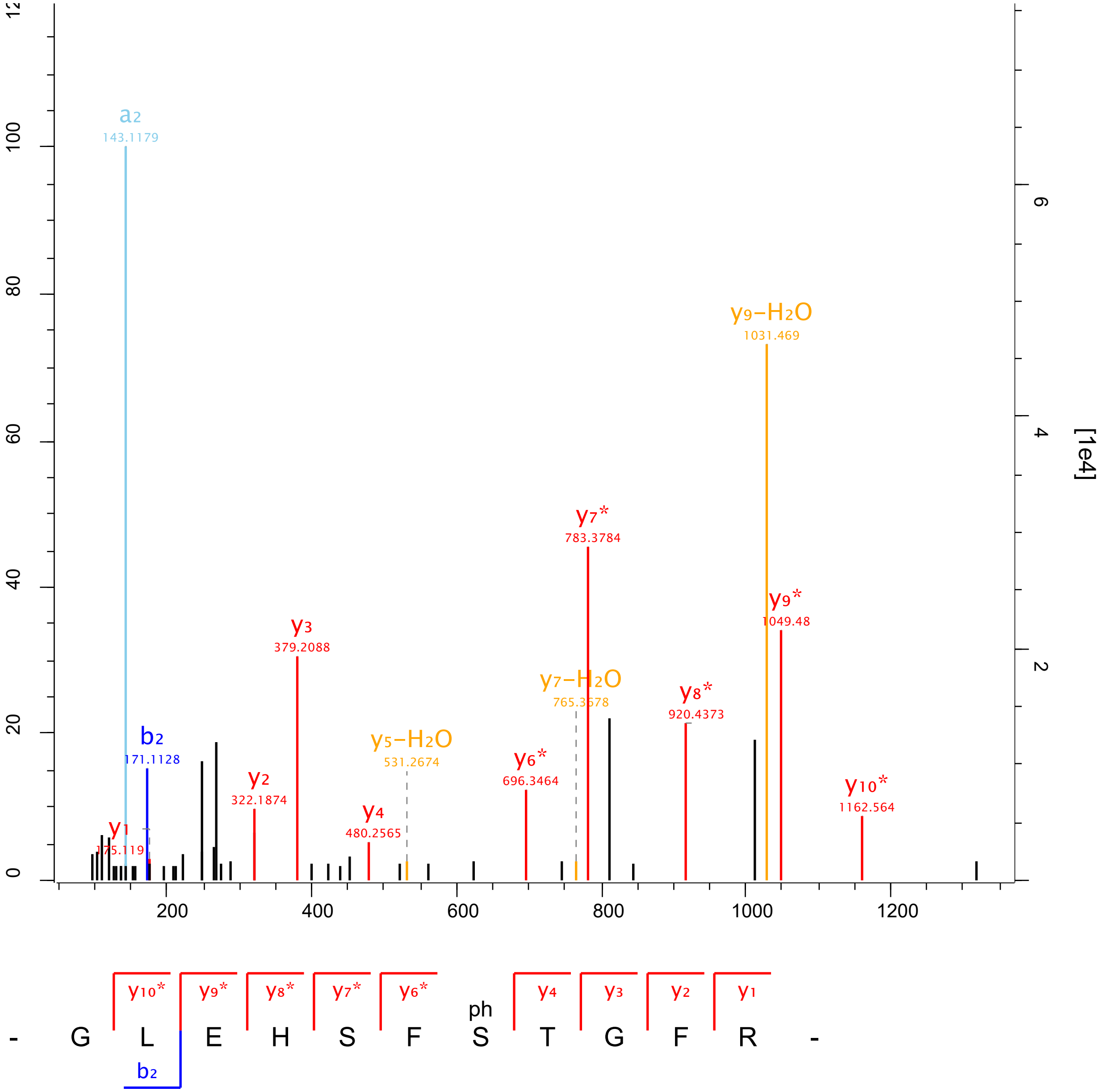

Raw file	Scan	Method	Score	m/z
sys_15_1	20154	FTMS; HCD	105.79	822.87

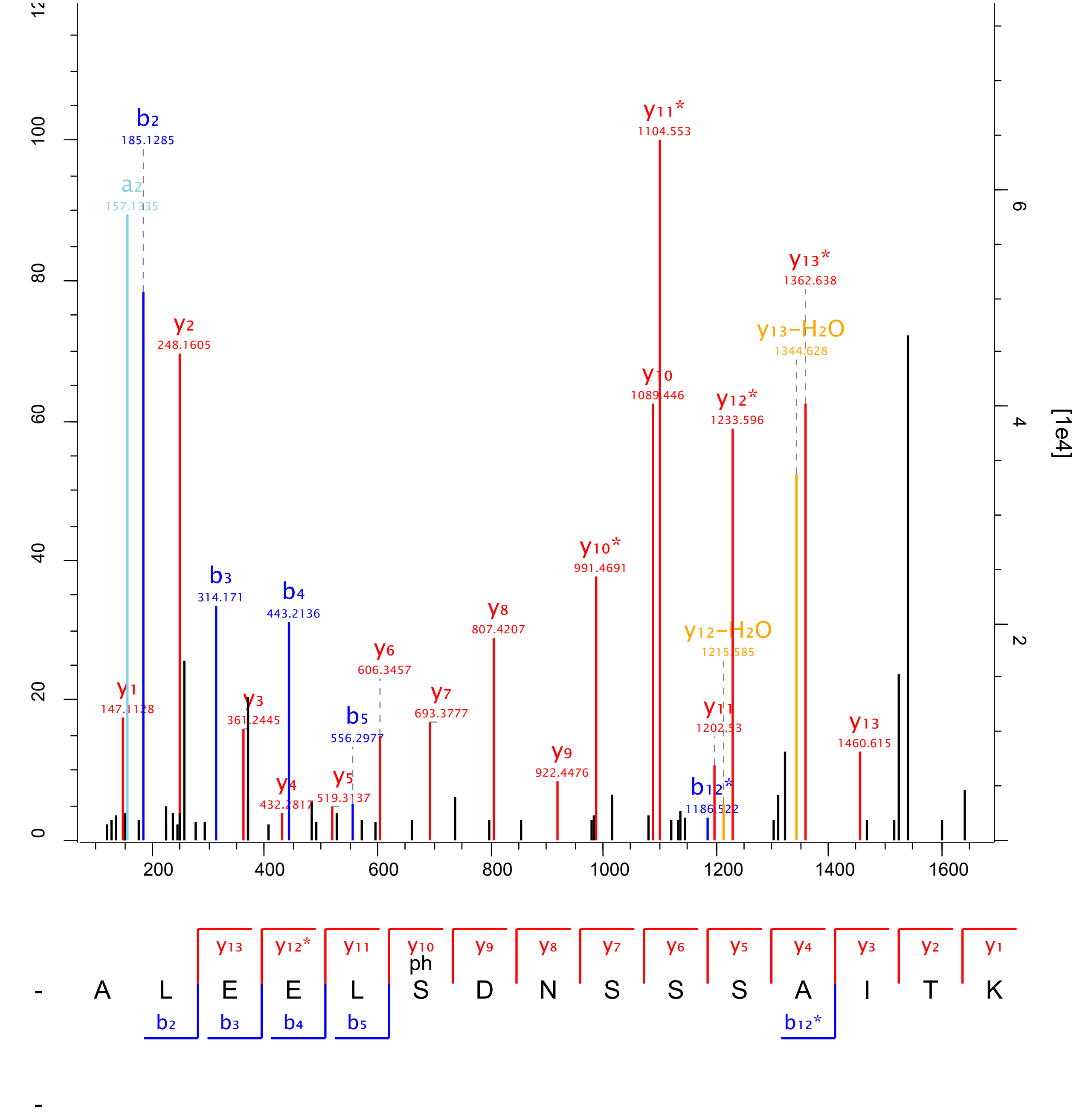

Raw file	Scan	Method	Score	m/z
sys_15_1	20564	FTMS; HCD	162.81	632.25

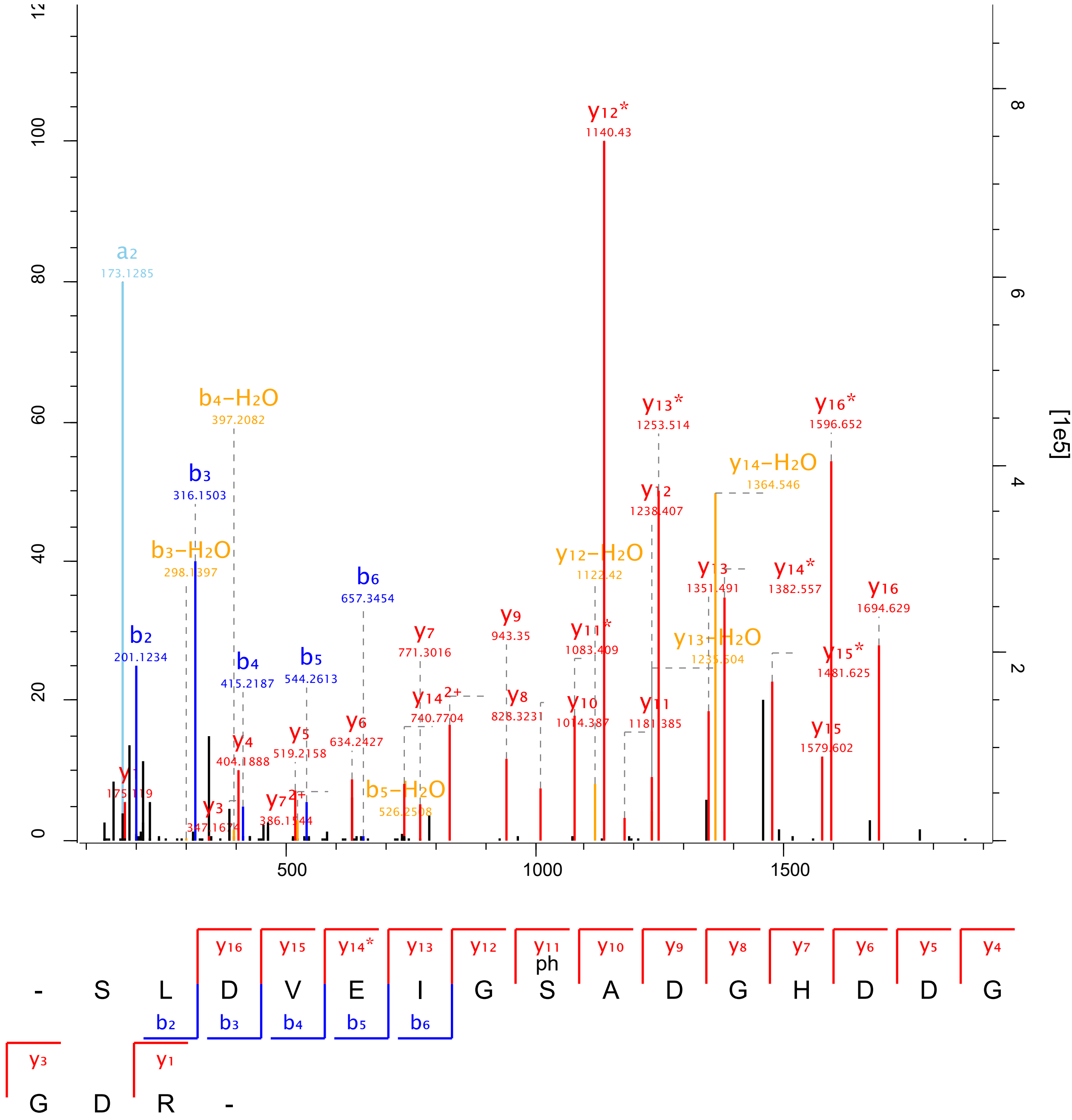

Raw file	Scan	Method	Score	m/z
sys_15_1	20596	FTMS; HCD	119.74	688.29

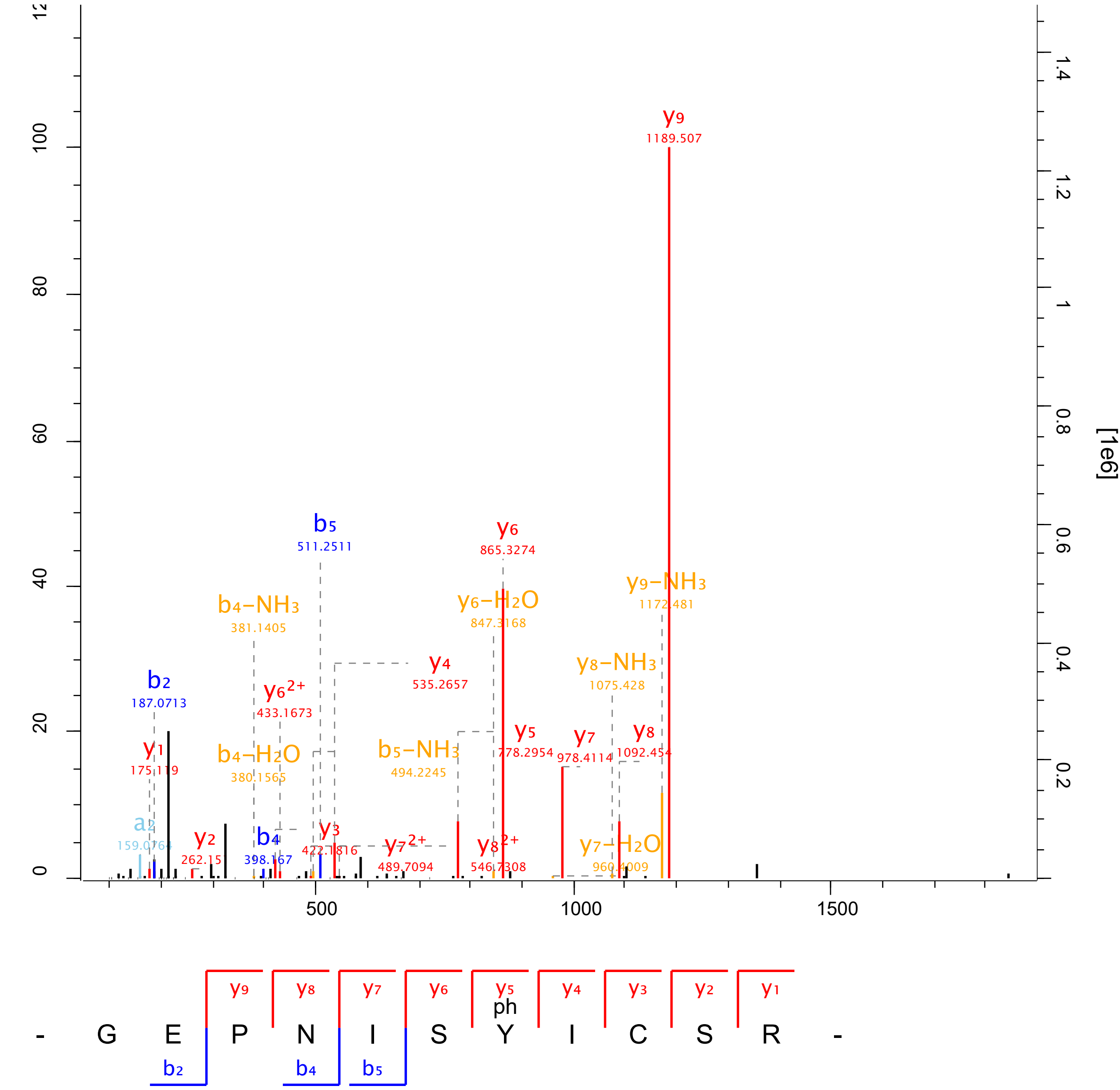

Raw file	Scan	Method	Score	m/z
sys_15_1	20659	FTMS; HCD	88.34	517.27

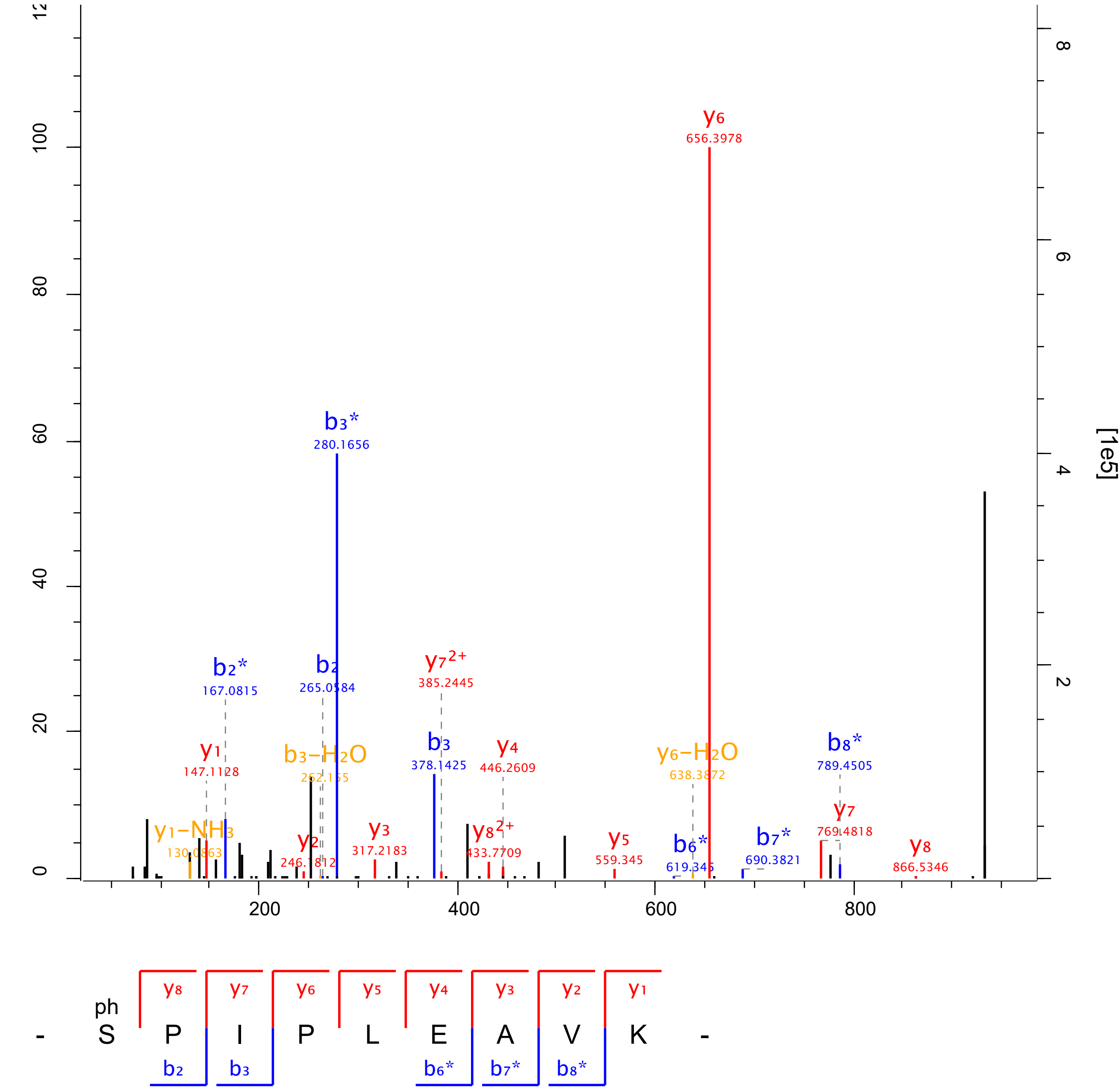

Raw file	Scan	Method	Score	m/z
sys_15_1	20683	FTMS; HCD	189.77	806.88

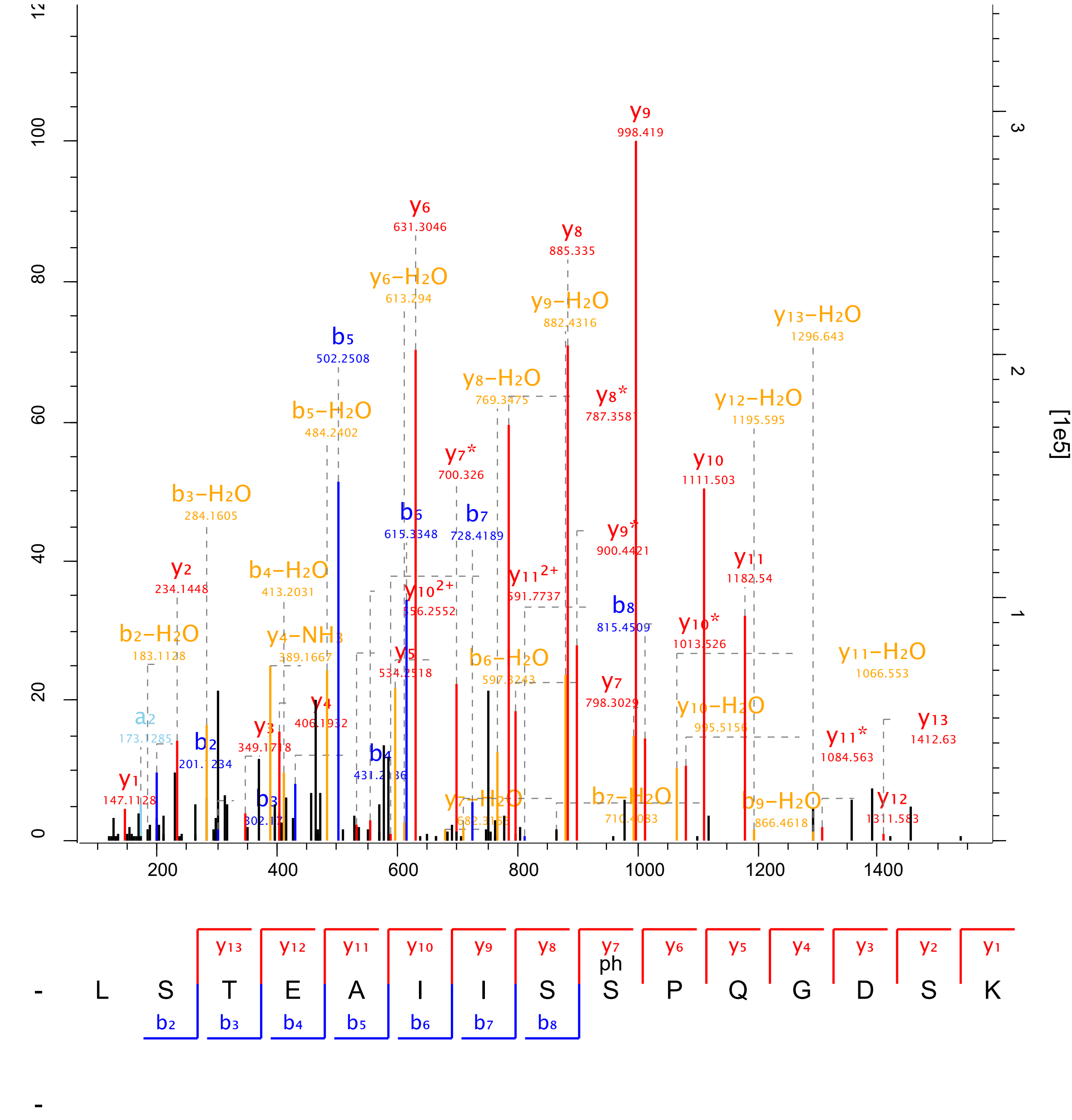

Raw file	Scan	Method	Score	m/z
sys_15_1	20702	FTMS; HCD	263.55	894.36

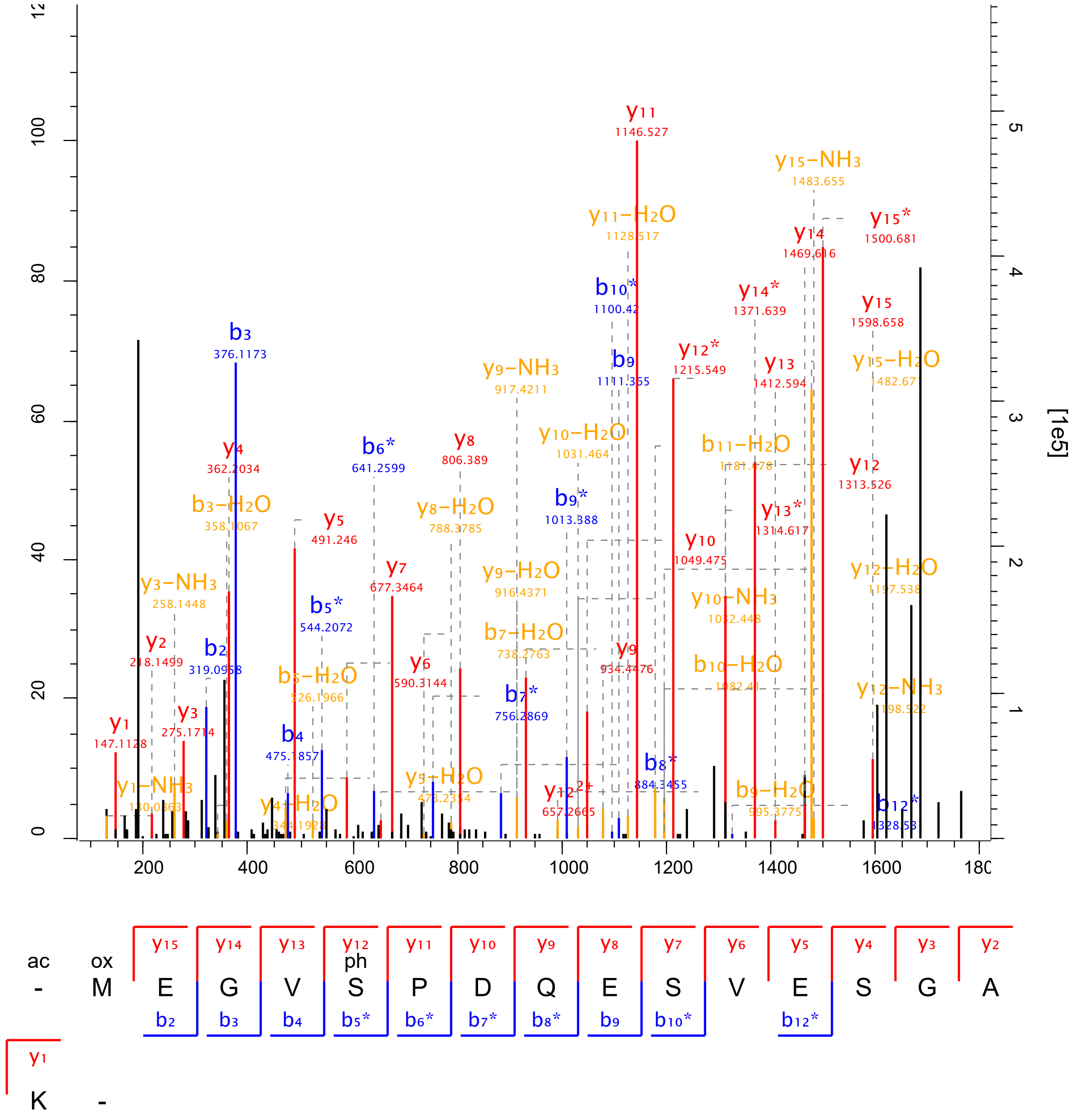

Raw file	Scan	Method	Score	m/z
sys_15_1	20782	FTMS; HCD	101.6	694.63

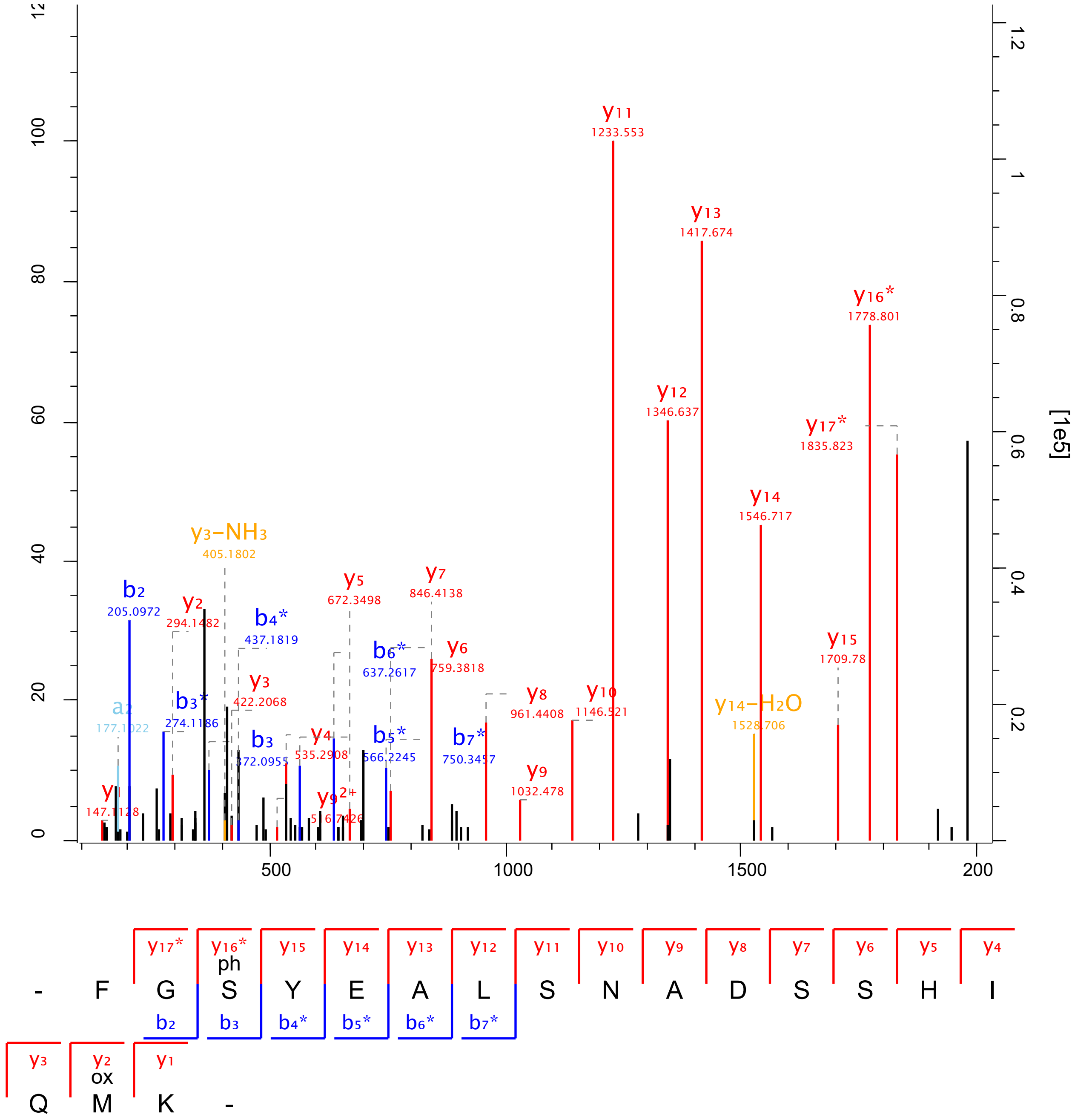

Raw file	Scan	Method	Score	m/z
sys_15_1	20799	FTMS; HCD	75.54	883.73

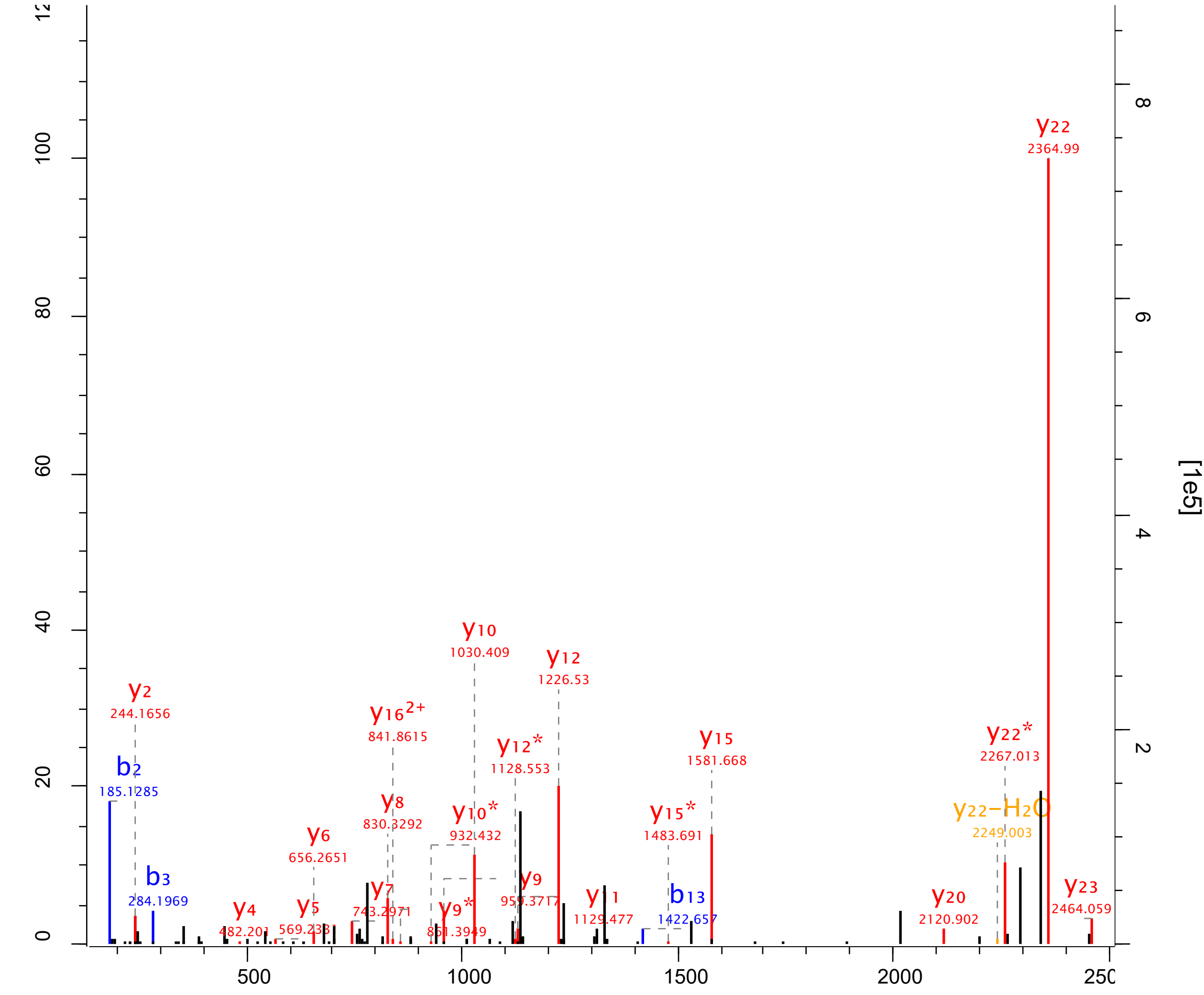

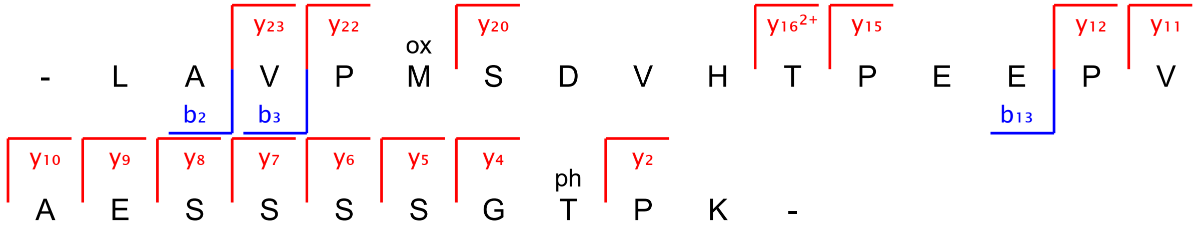

Raw file	Scan	Method	Score	m/z
sys_15_1	20955	FTMS; HCD	62.09	615.76

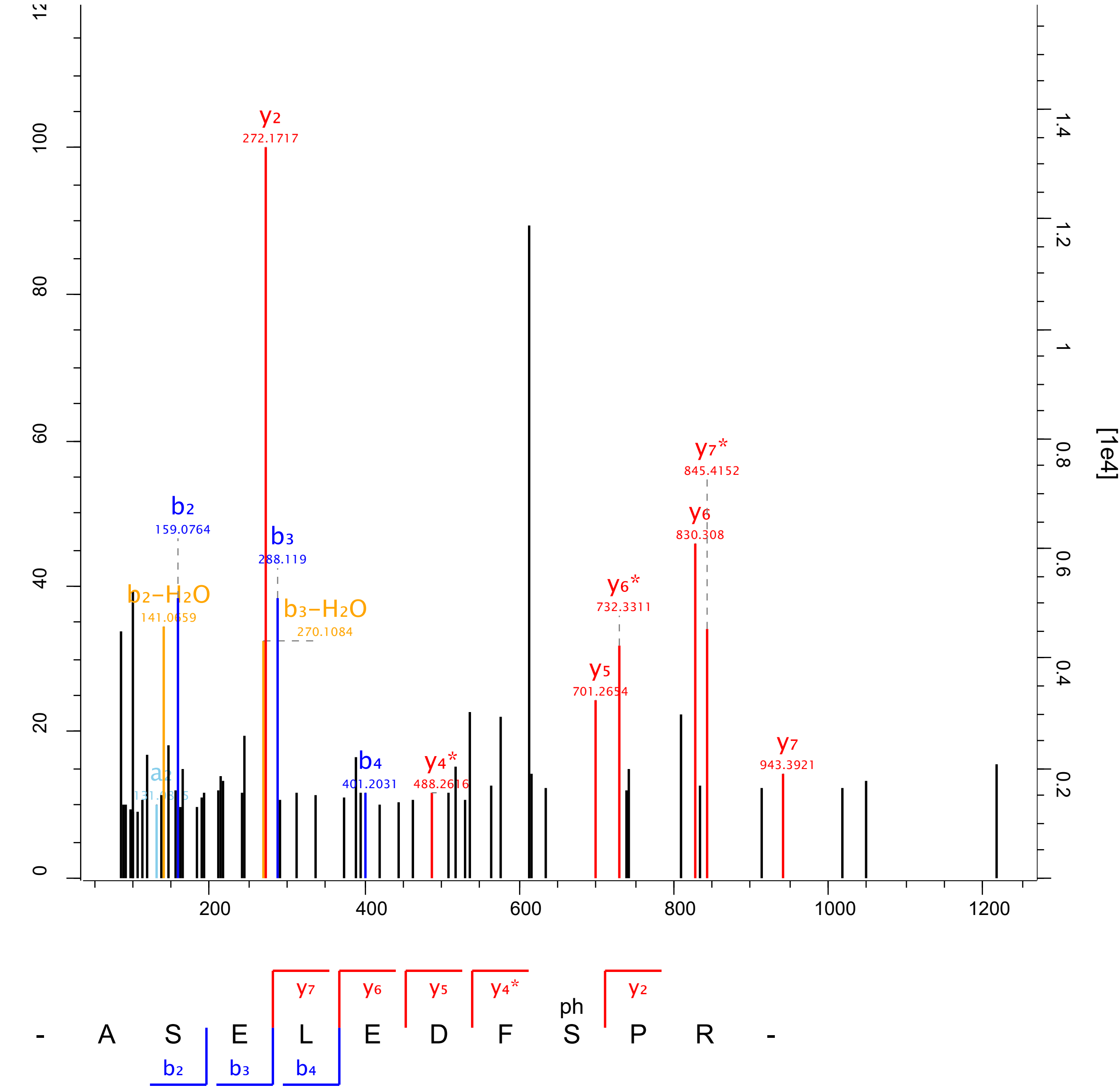

Raw file	Scan	Method	Score	m/z
sys_15_1	21058	FTMS; HCD	71.12	847.37

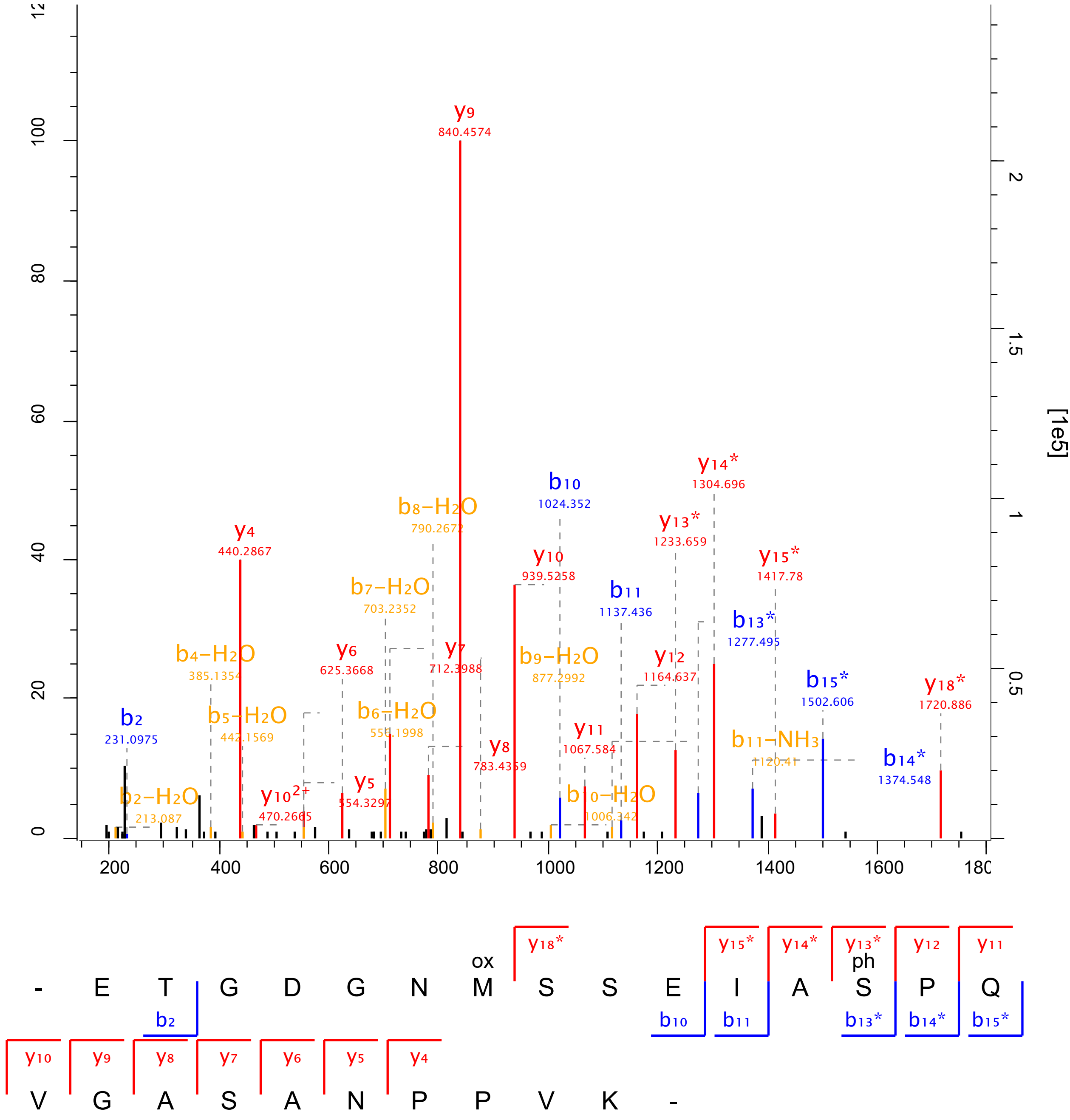

Raw file	Scan	Method	Score	m/z
sys_15_1	21078	FTMS; HCD	148.15	871.91

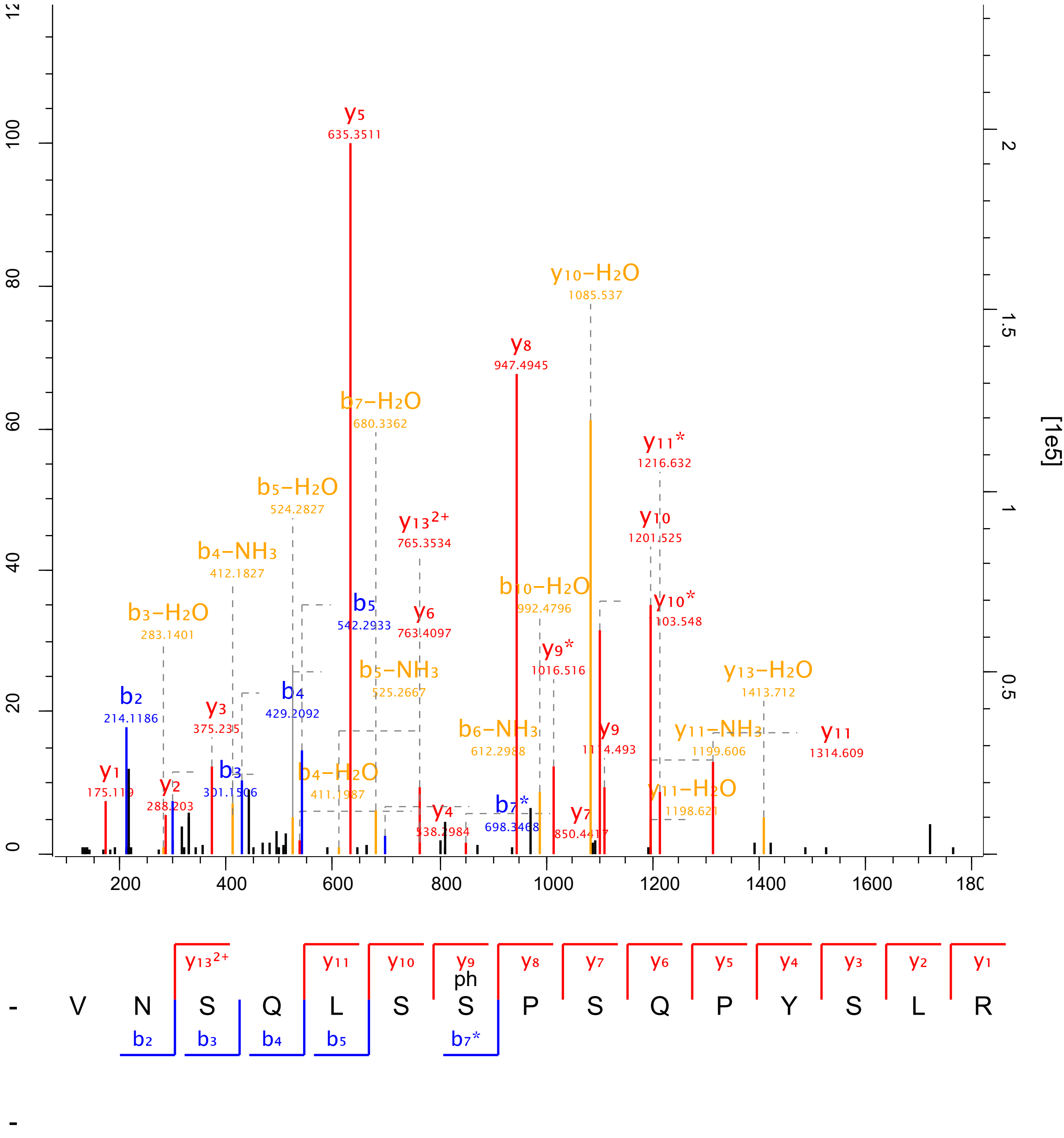

Raw file	Scan	Method	Score	m/z
sys_15_1	21530	FTMS; HCD	279.97	831.38

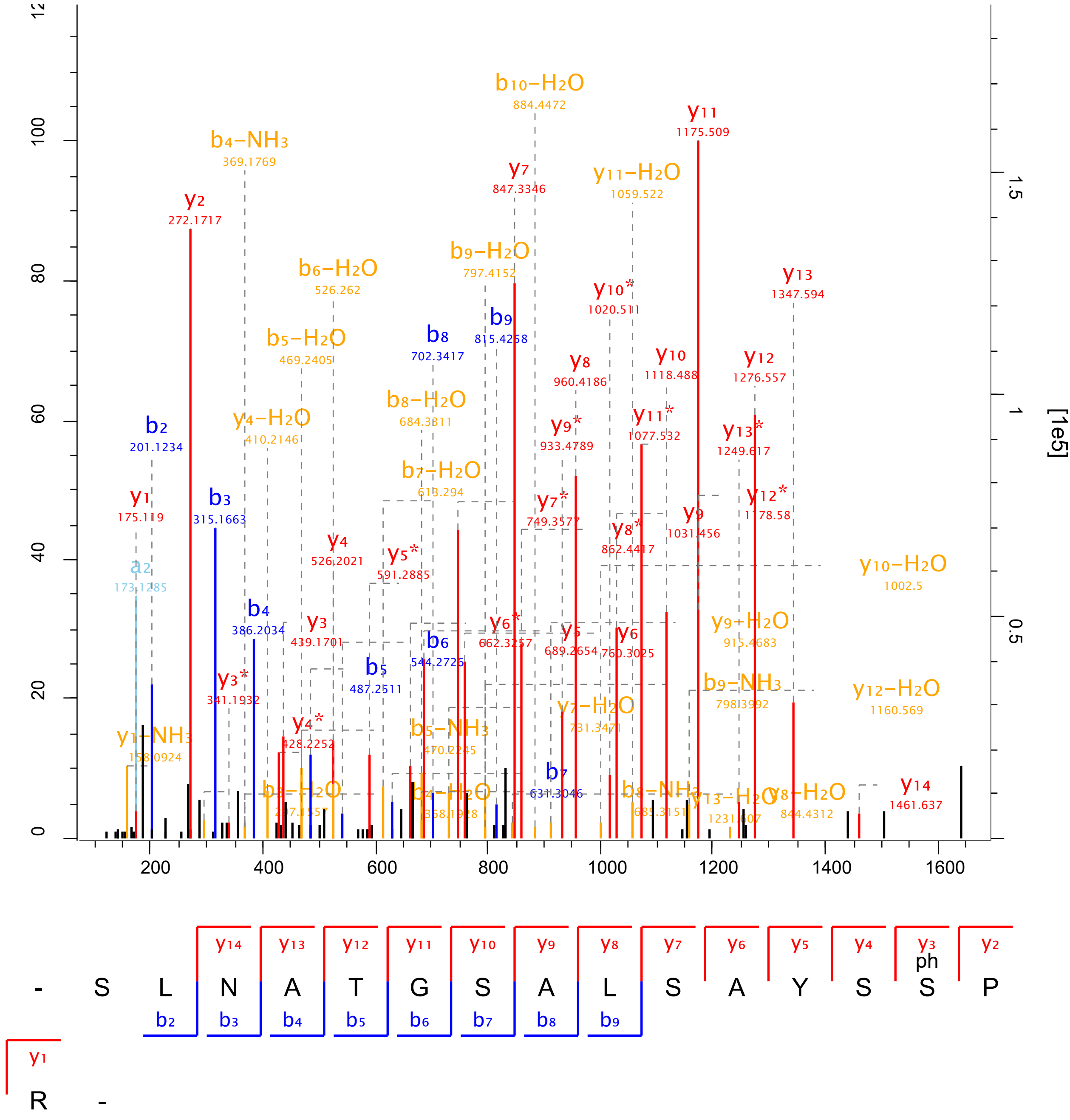

Raw file	Scan	Method	Score	m/z
sys_15_1	21543	FTMS; HCD	164.2	851.38

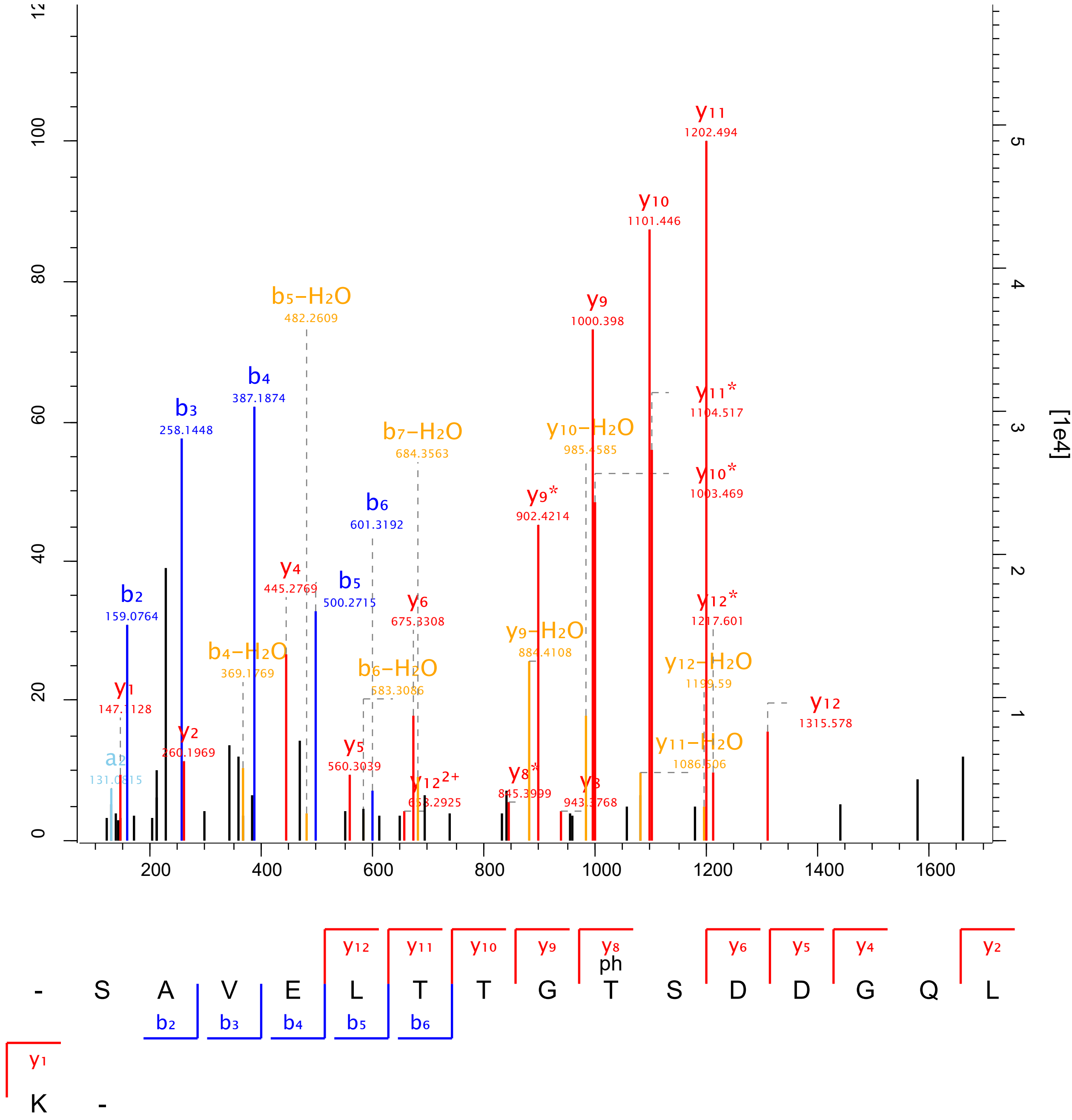

Raw file	Scan	Method	Score	m/z
sys_15_1	21660	FTMS; HCD	104.84	865.87

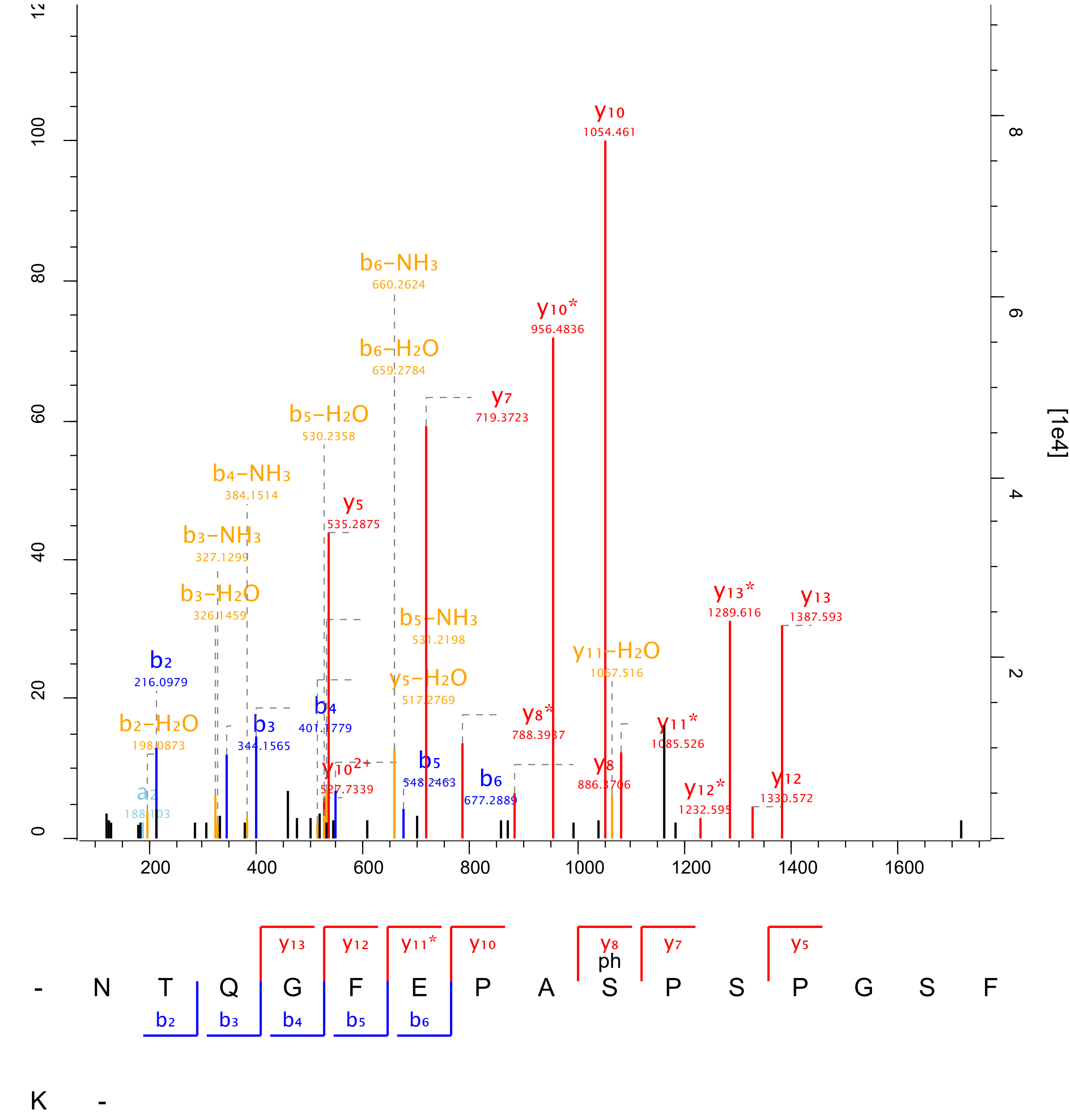

Raw file	Scan	Method	Score	m/z
sys_15_1	21760	FTMS; HCD	94.36	501.23

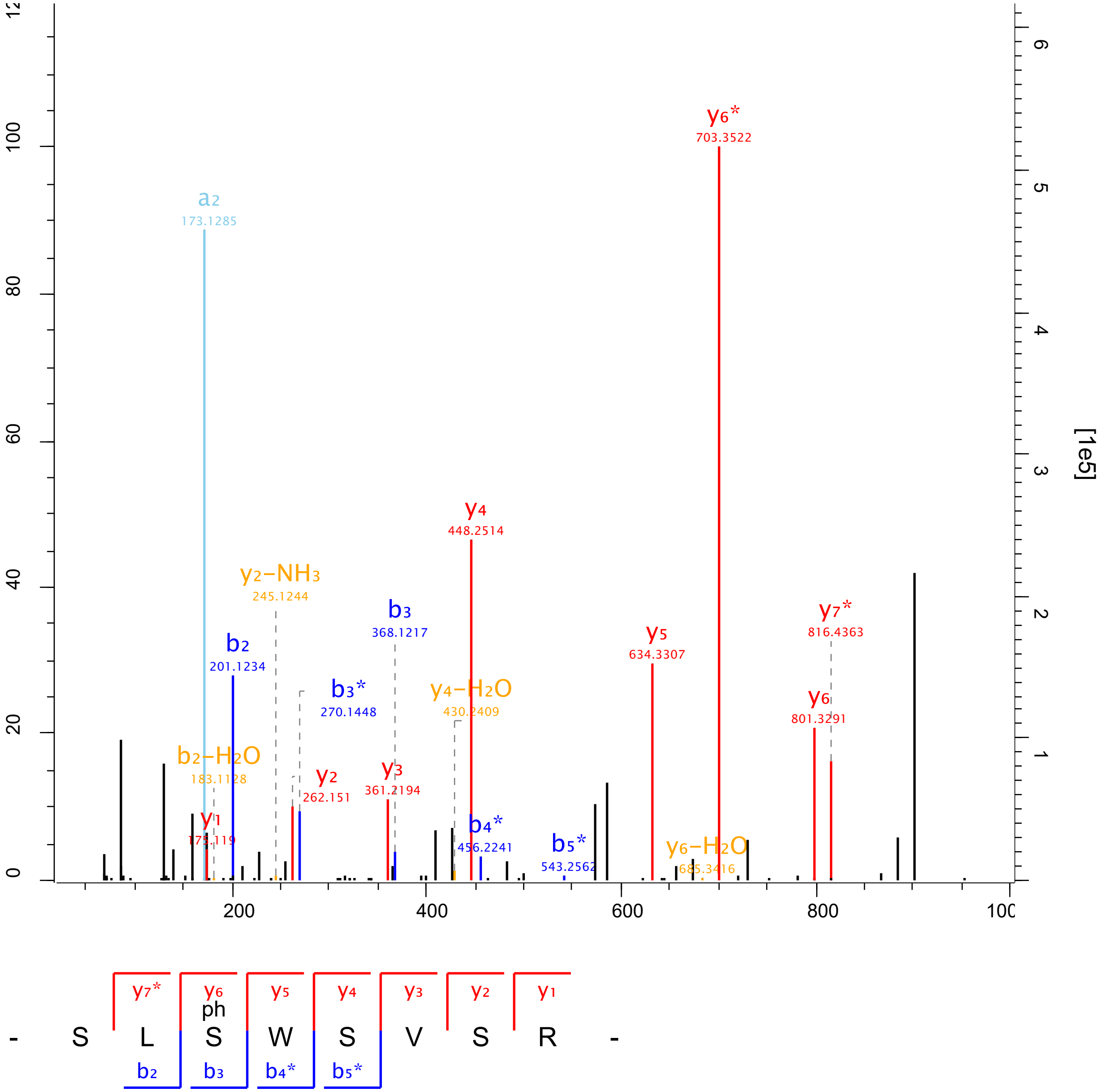

Raw file	Scan	Method	Score	m/z
sys_15_1	21772	FTMS; HCD	64.71	707.3

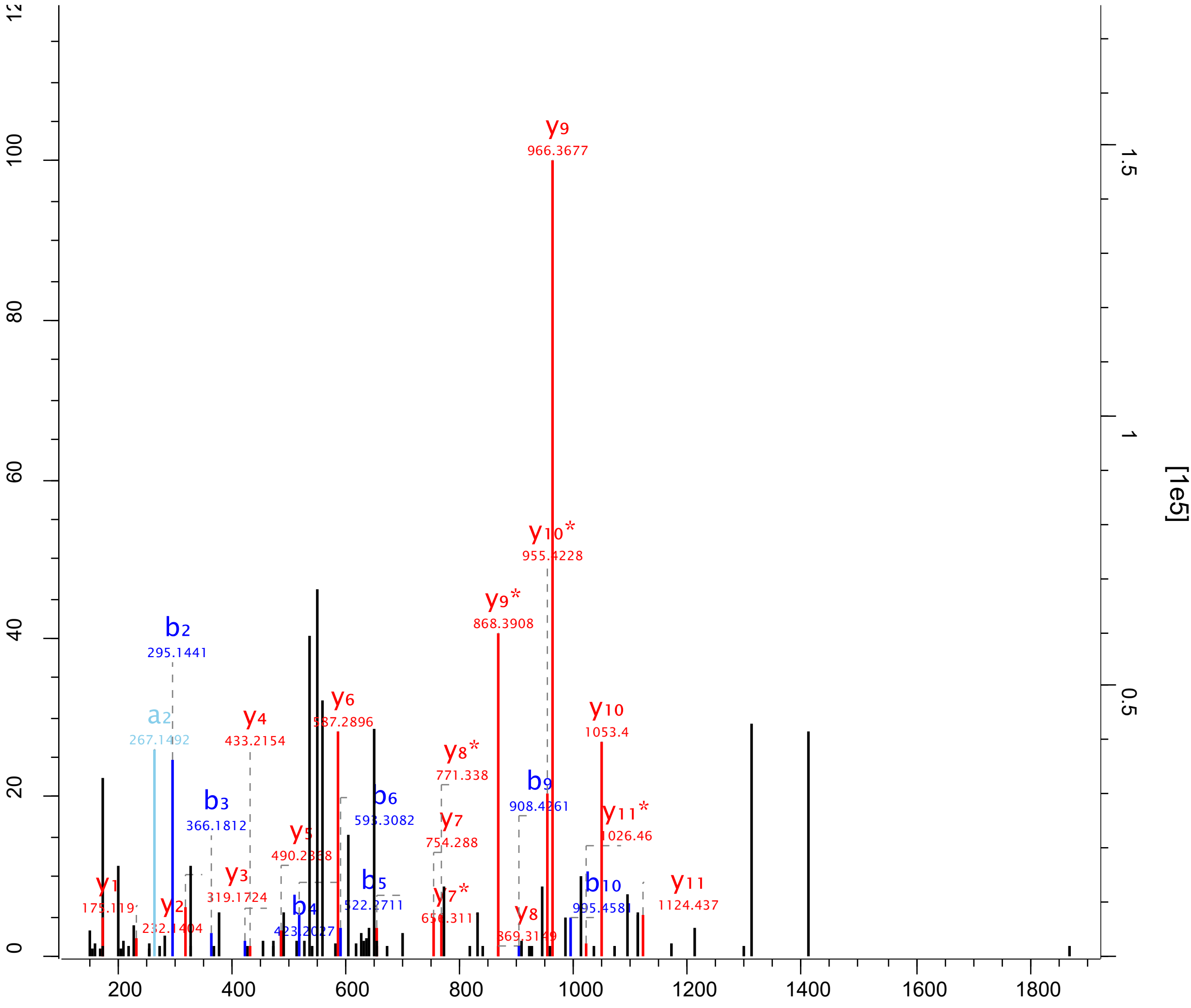

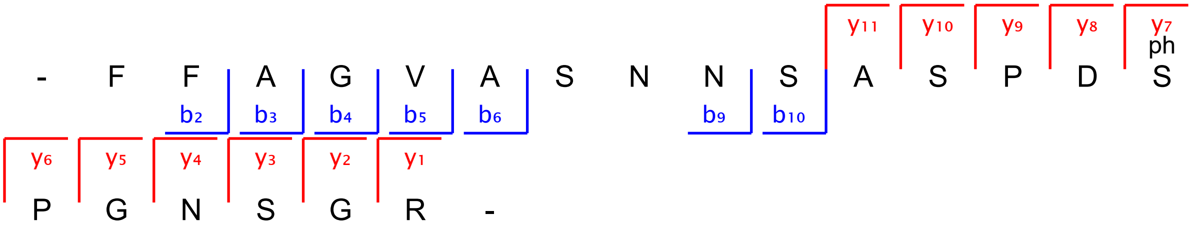

Raw file	Scan	Method	Score	m/z
sys_15_1	21780	FTMS; HCD	165.4	679.98

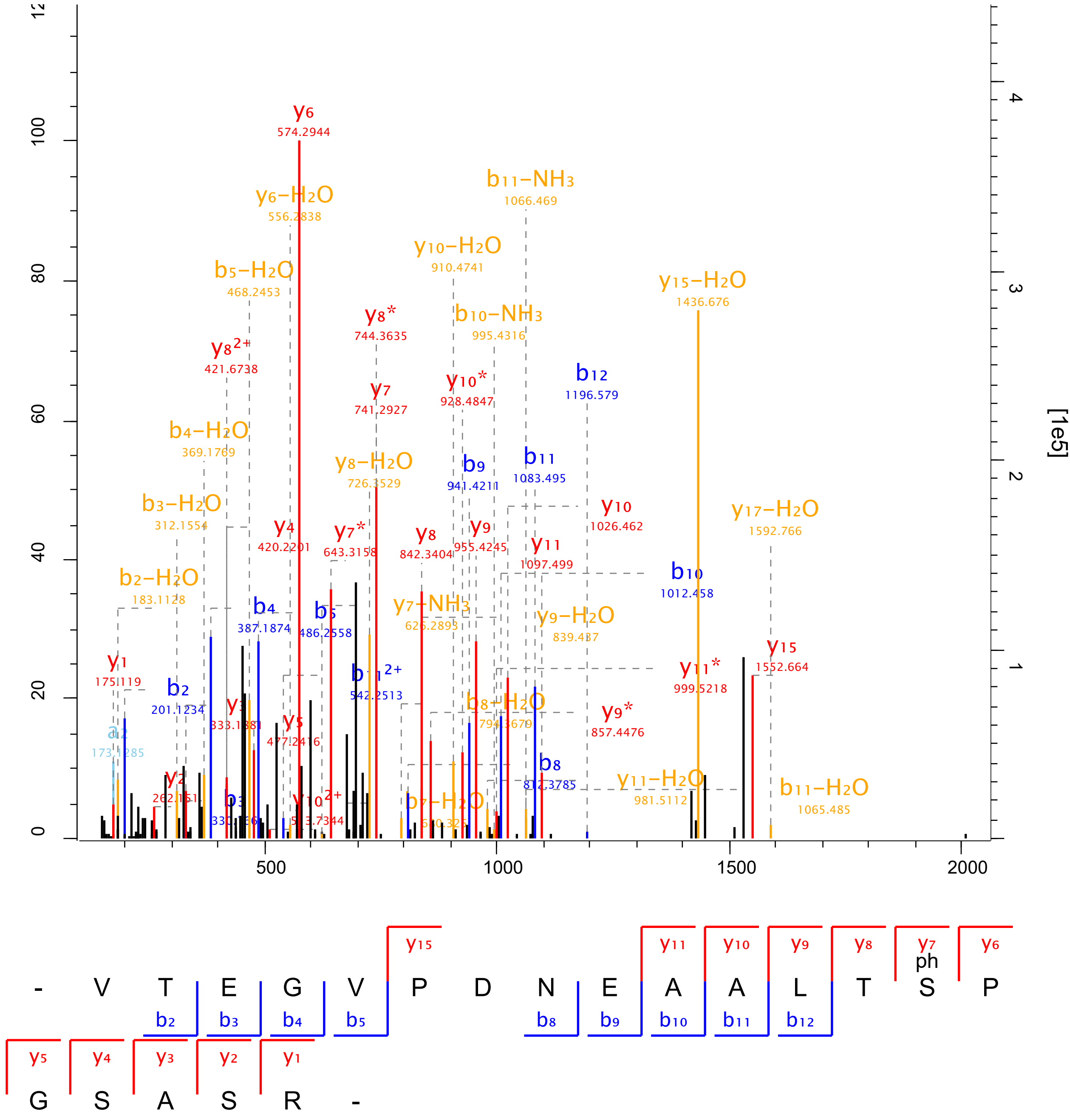

Raw file	Scan	Method	Score	m/z
sys_15_1	21863	FTMS; HCD	108.63	750.82

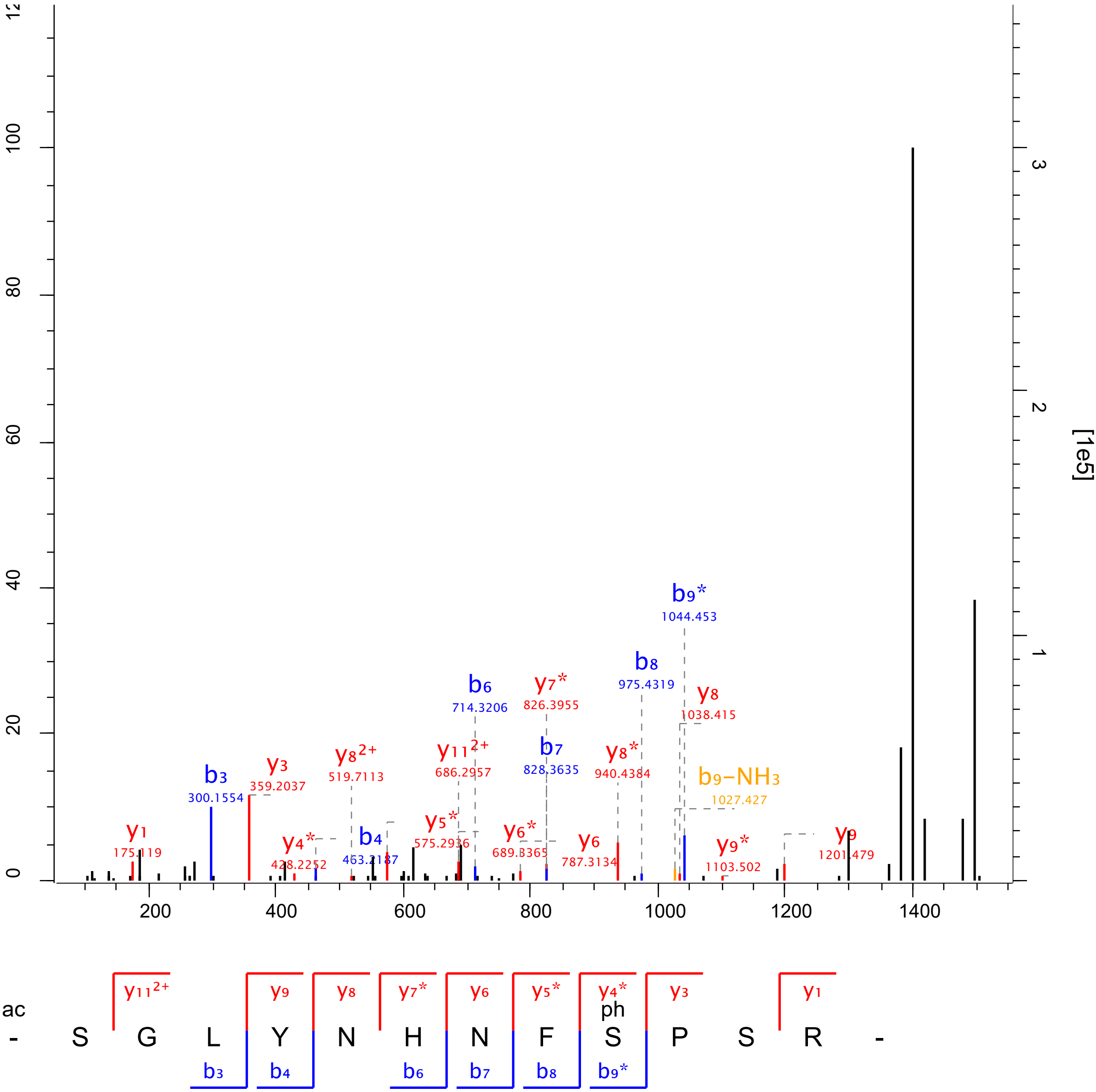

Raw file	Scan	Method	Score	m/z
sys_15_1	21888	FTMS; HCD	70.09	587.29

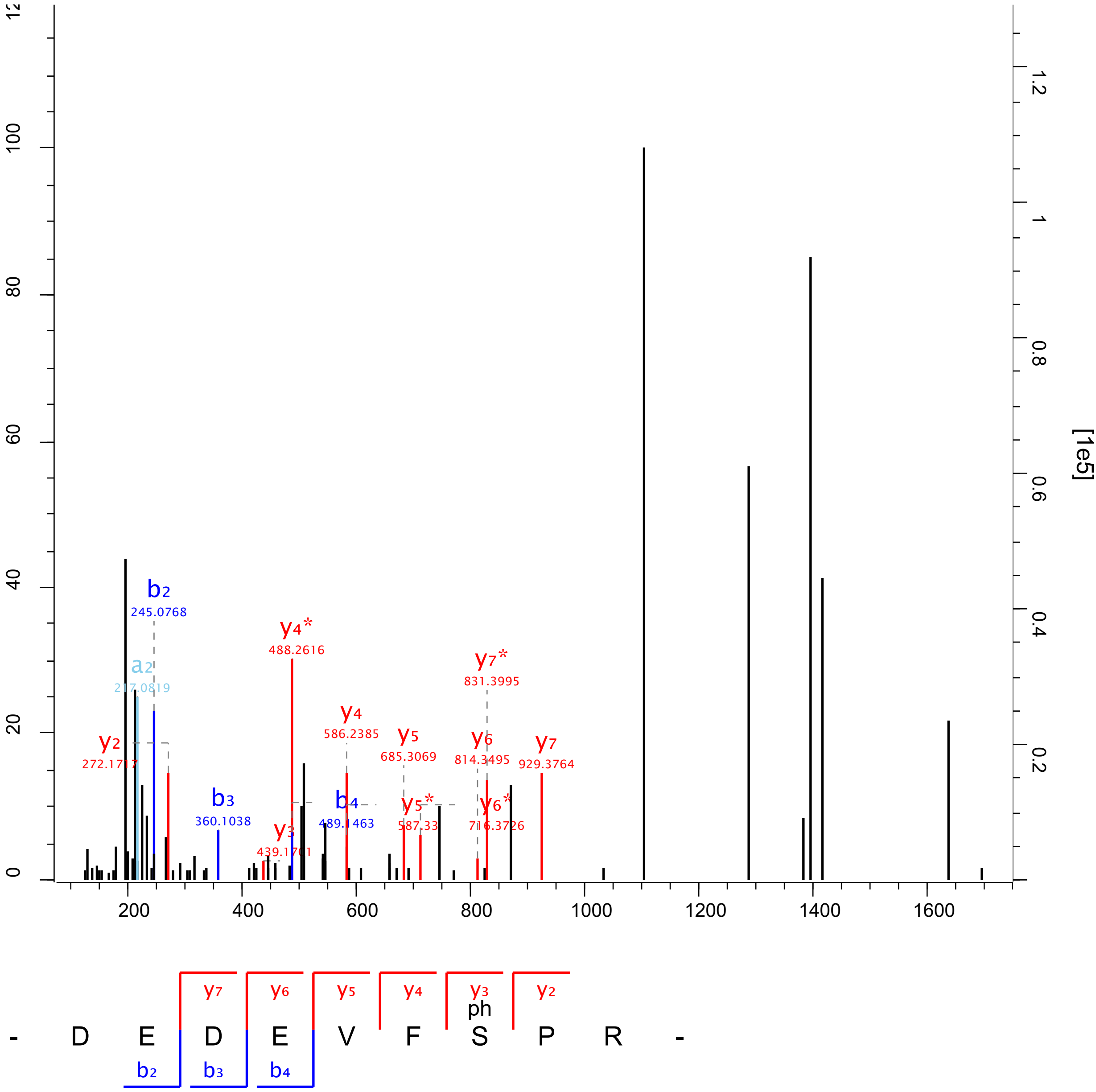

Raw file	Scan	Method	Score	m/z
sys_15_1	21920	FTMS; HCD	58.32	761.29

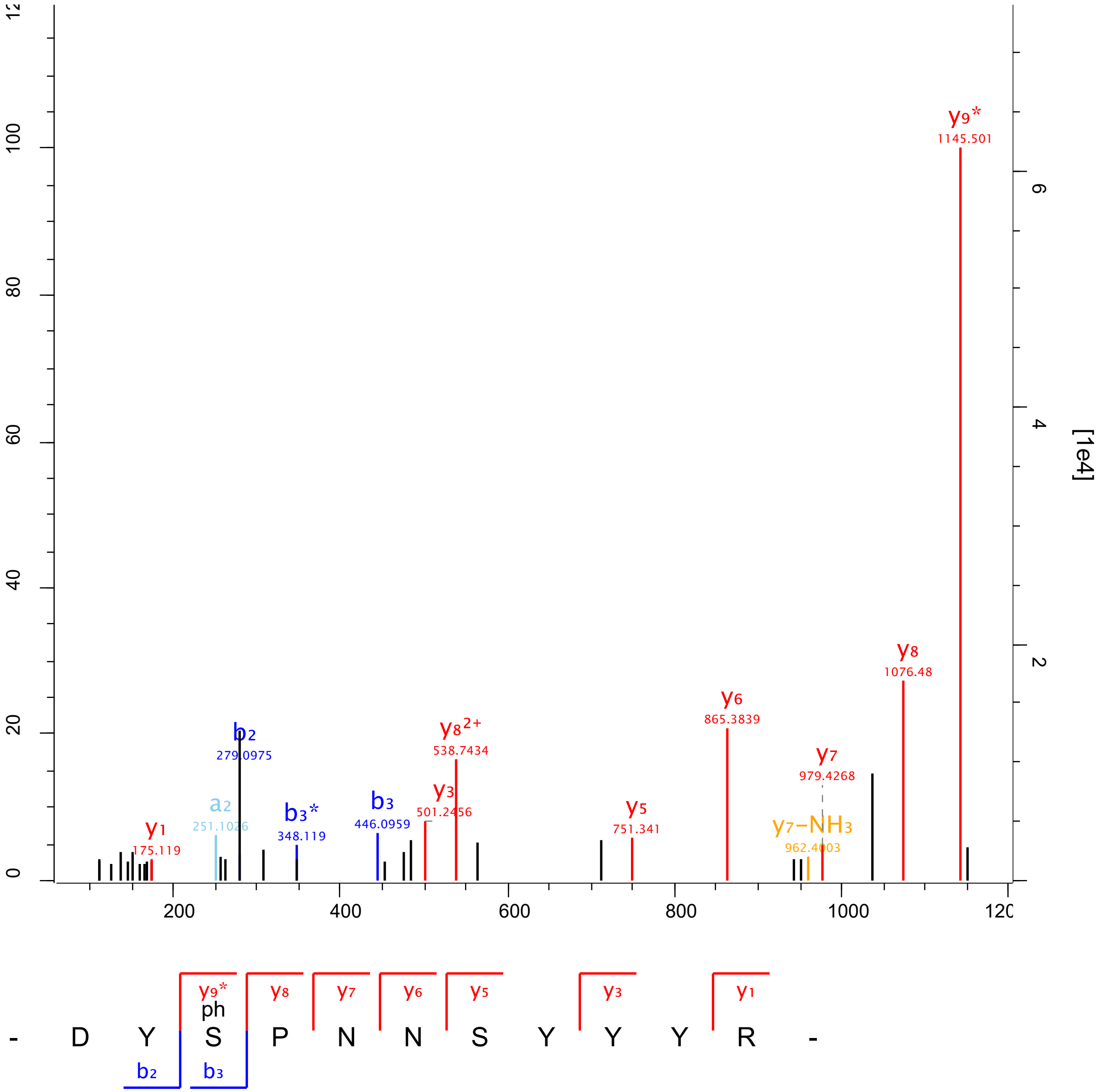

Raw file	Scan	Method	Score	m/z
sys_15_1	21946	FTMS; HCD	96.21	796.35

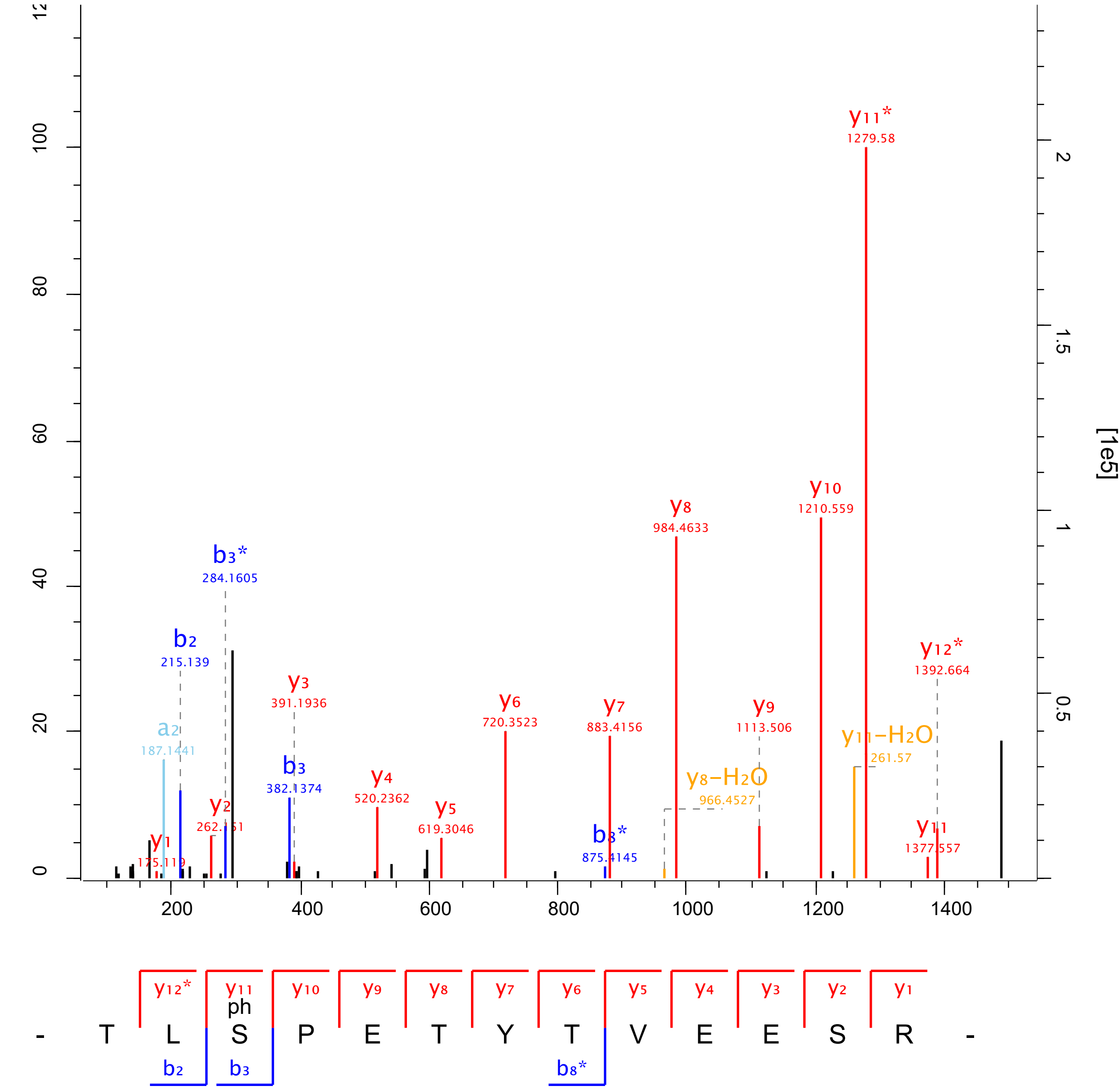

Raw file	Scan	Method	Score	m/z
sys_15_1	22049	FTMS; HCD	69.72	480.73

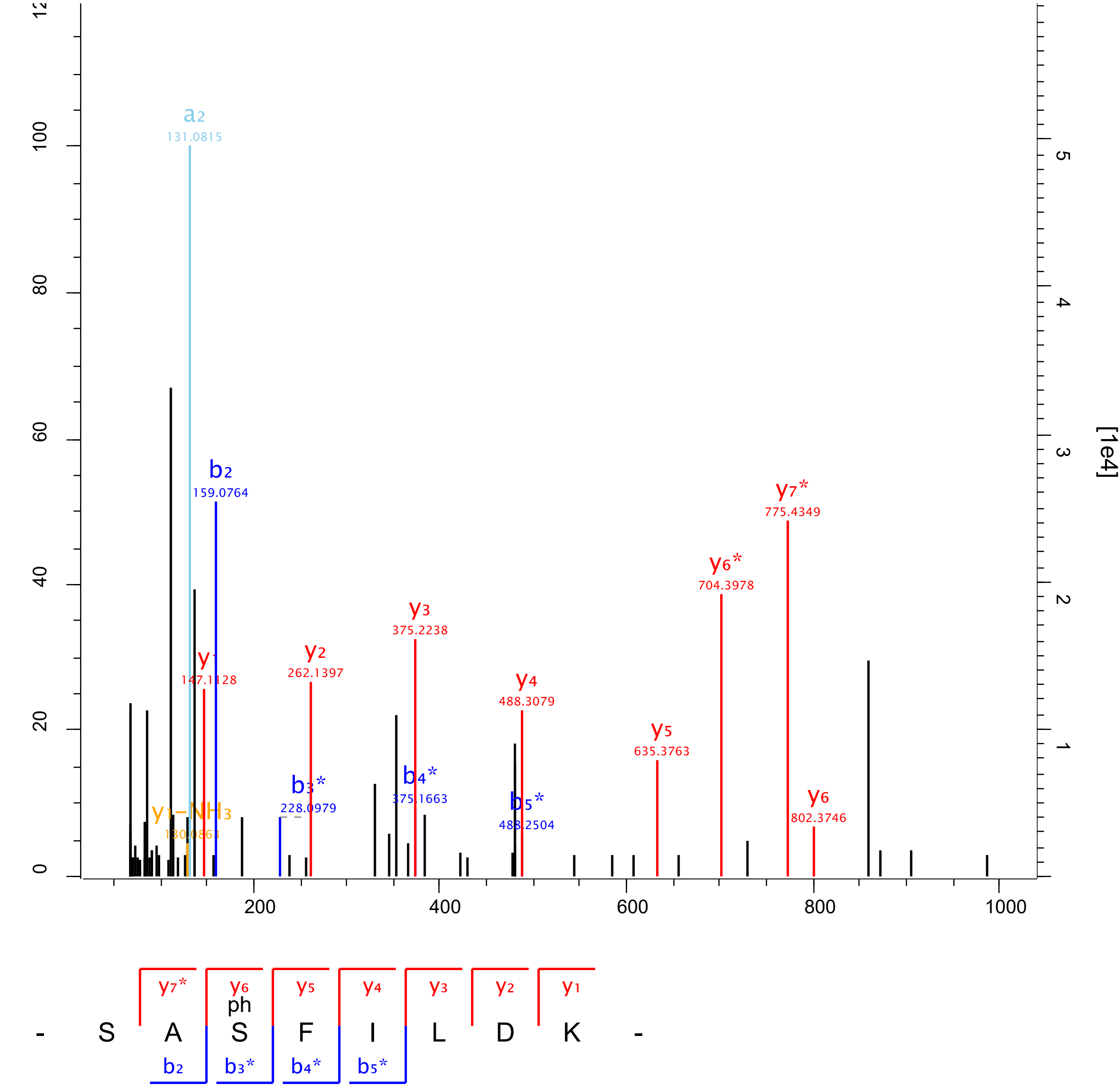

Raw file	Scan	Method	Score	m/z
sys_15_1	22102	FTMS; HCD	107.99	649.82

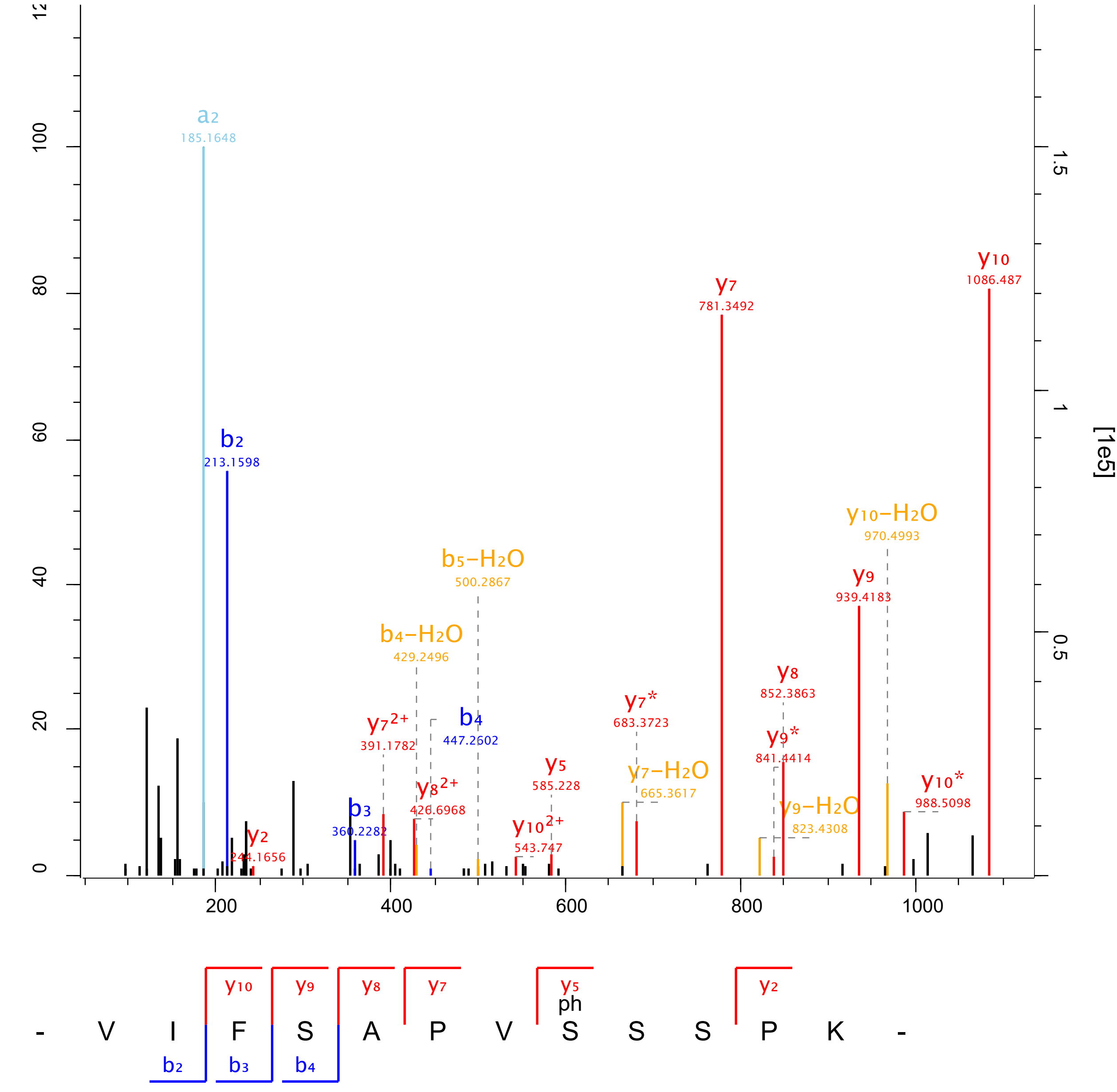

Raw file	Scan	Method	Score	m/z
sys_15_1	22126	FTMS; HCD	86.8	1005.38

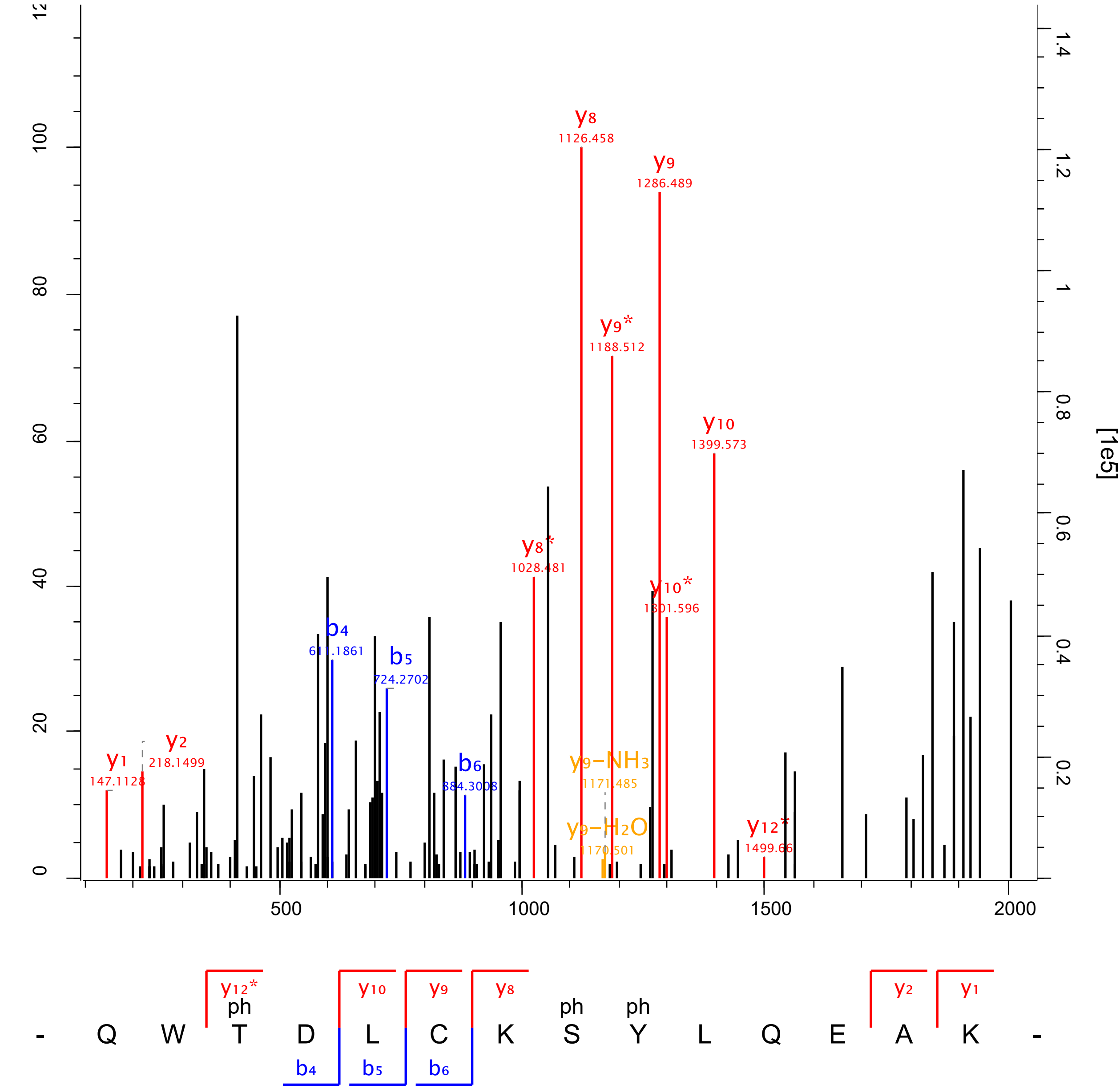

Raw file	Scan	Method	Score	m/z
sys_15_1	22130	FTMS; HCD	174.78	810.89

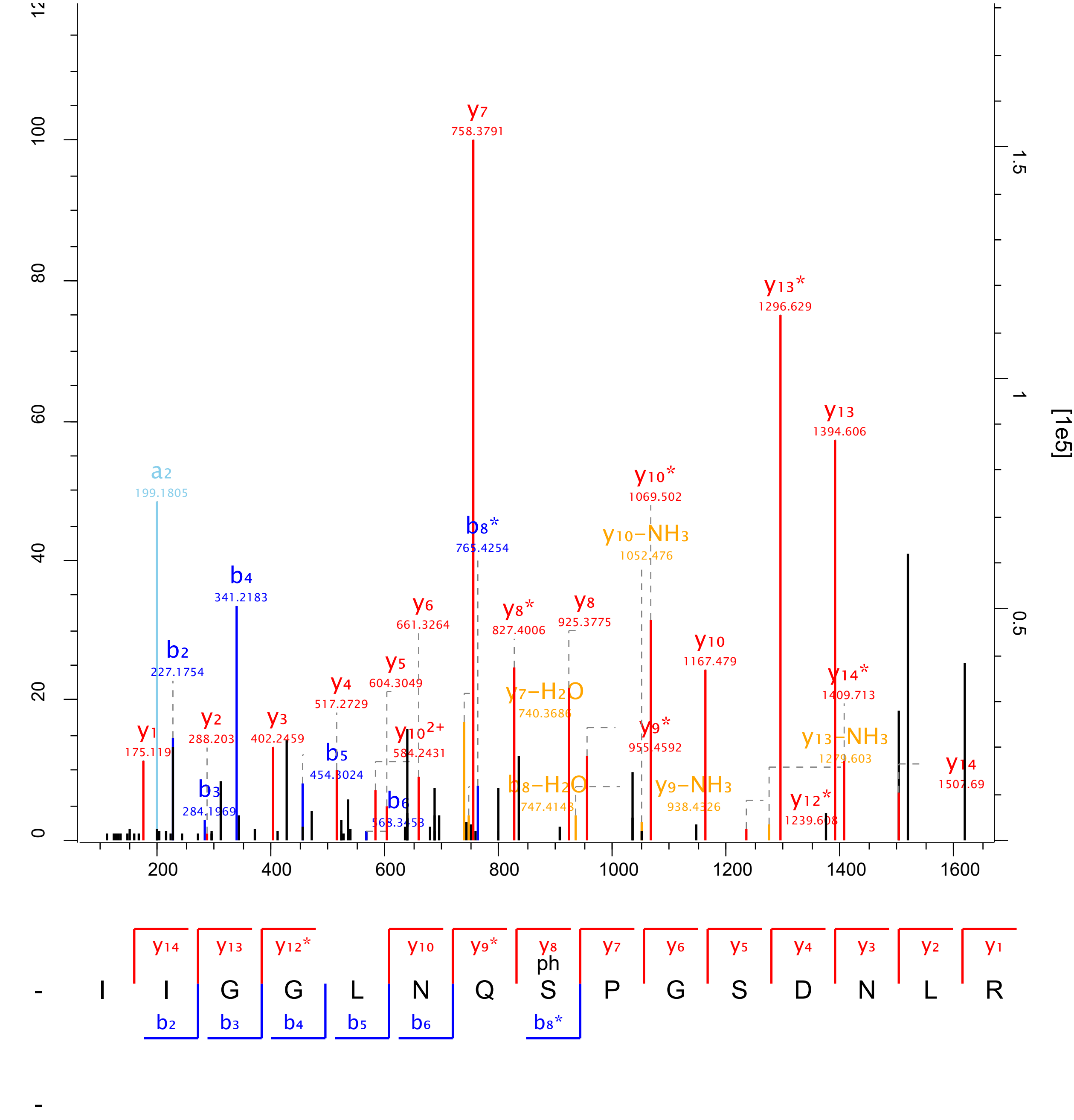

Raw file	Scan	Method	Score	m/z
sys_15_1	22140	FTMS; HCD	55.01	599.6

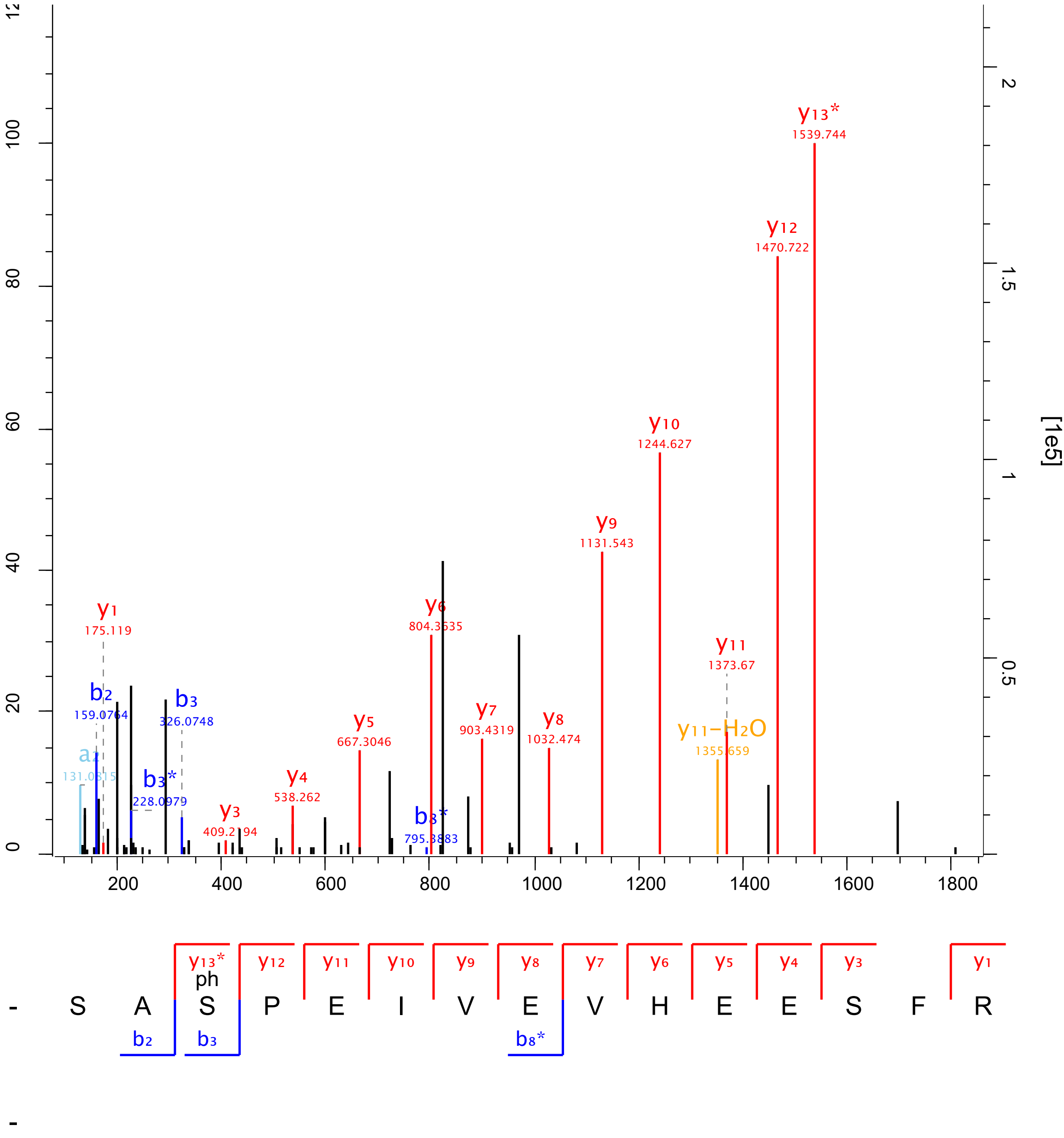

Raw file	Scan	Method	Score	m/z
sys_15_1	22159	FTMS; HCD	70.45	909.39

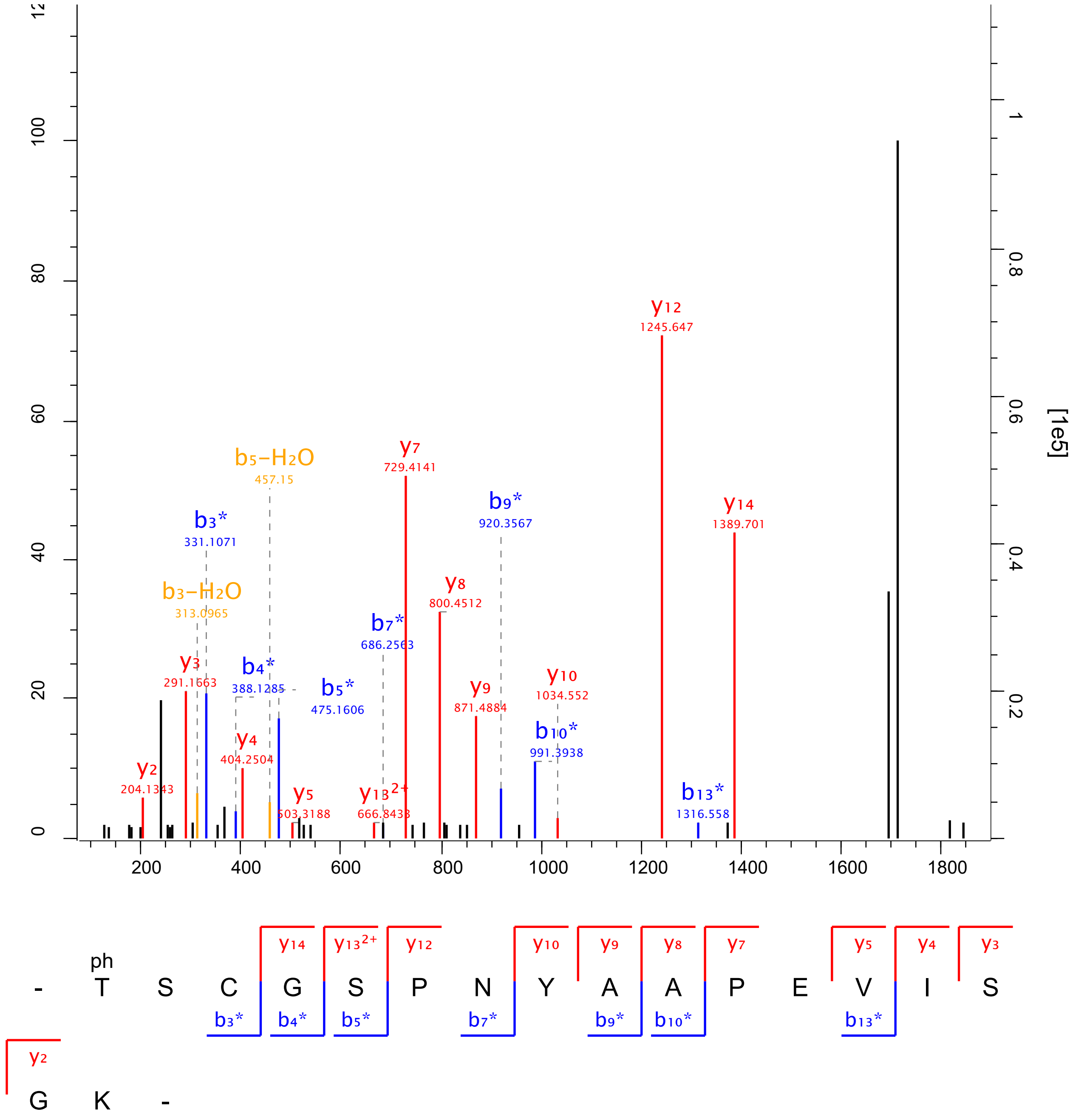

Raw file	Scan	Method	Score	m/z
sys_15_1	22245	FTMS; HCD	150.34	723.33

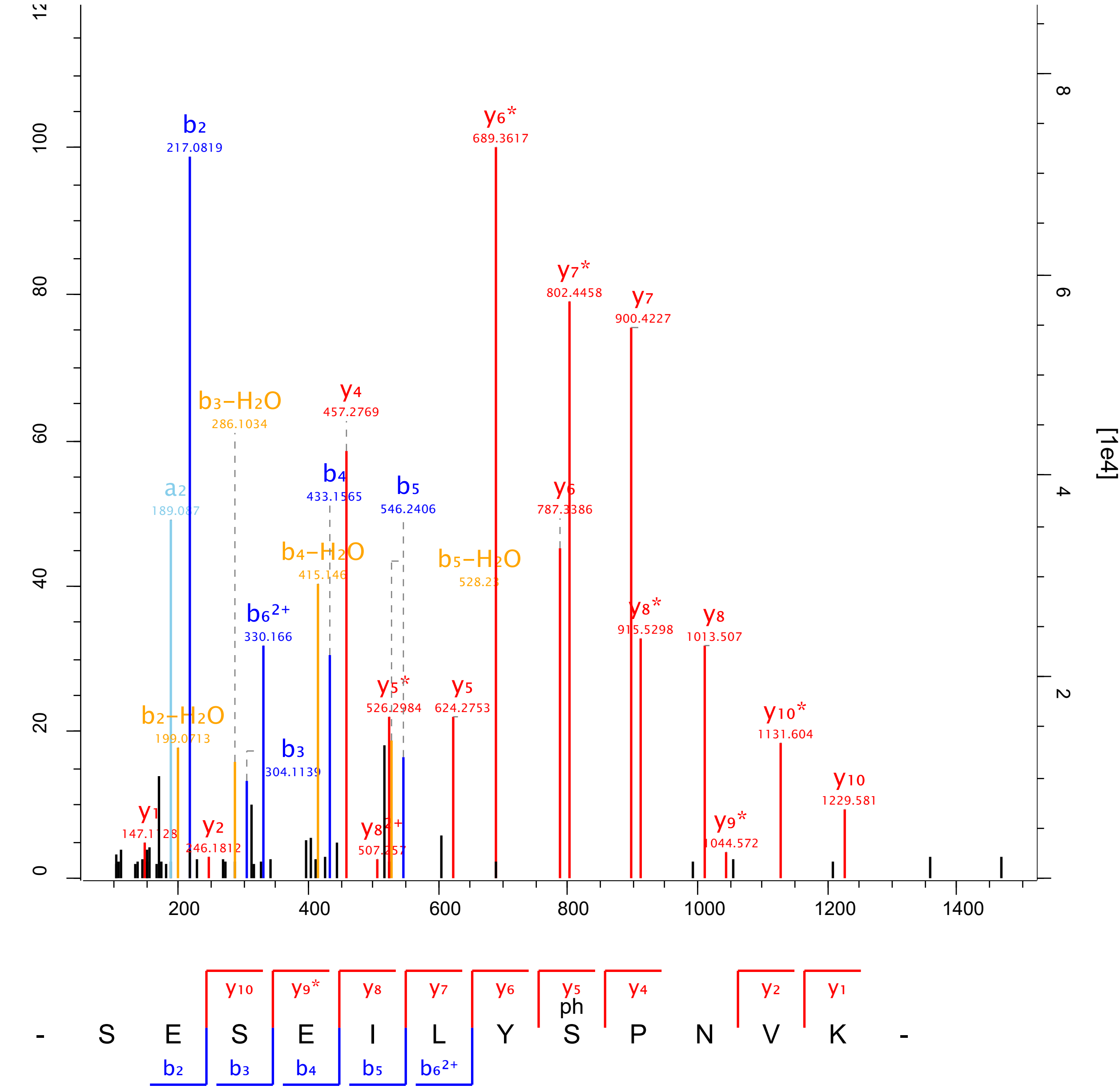

Raw file	Scan	Method	Score	m/z
sys_15_1	22365	FTMS; HCD	134.52	827.82

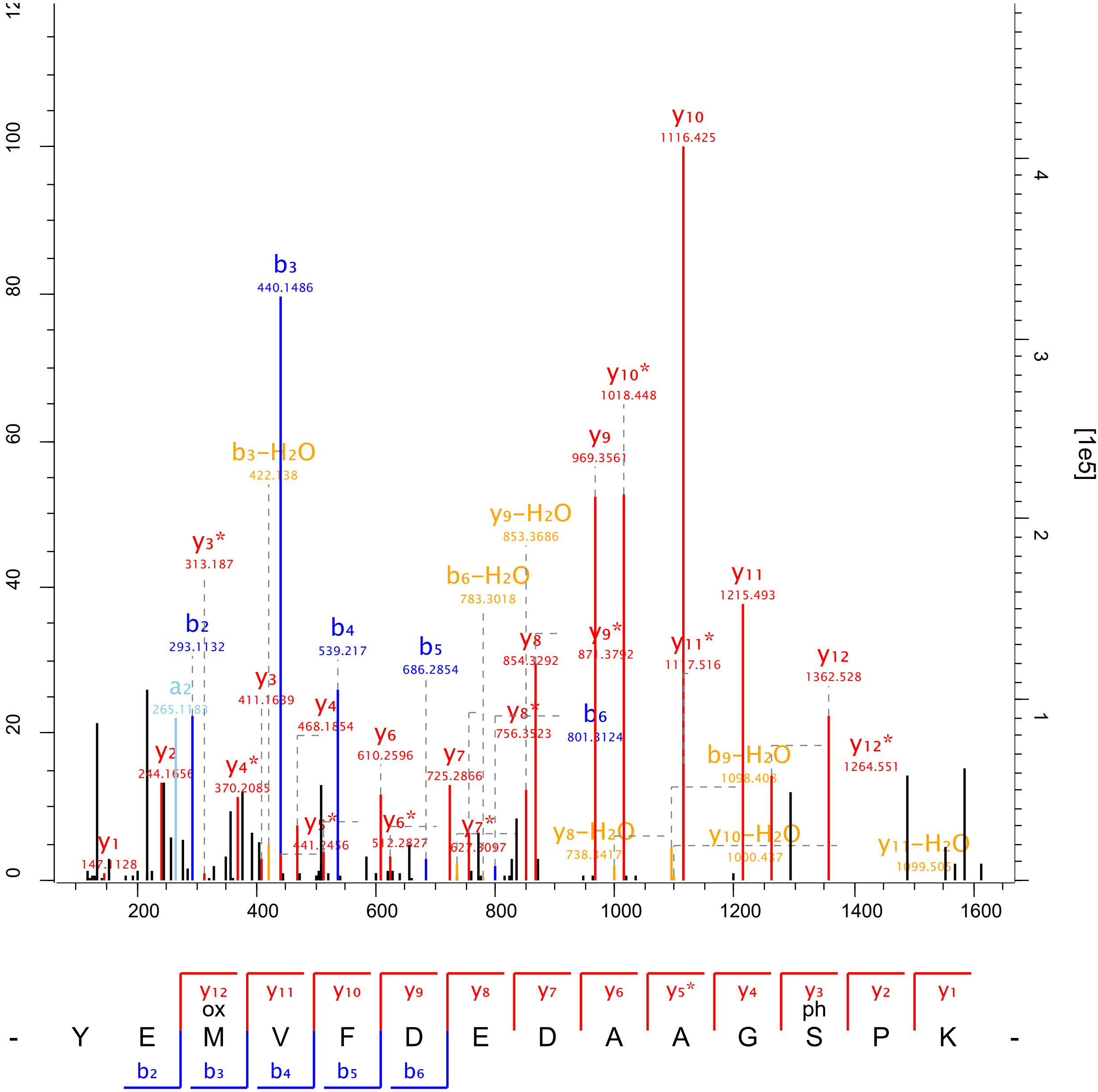

Raw file	Scan	Method	Score	m/z
sys_15_1	2250	FTMS; HCD	48.57	405.5

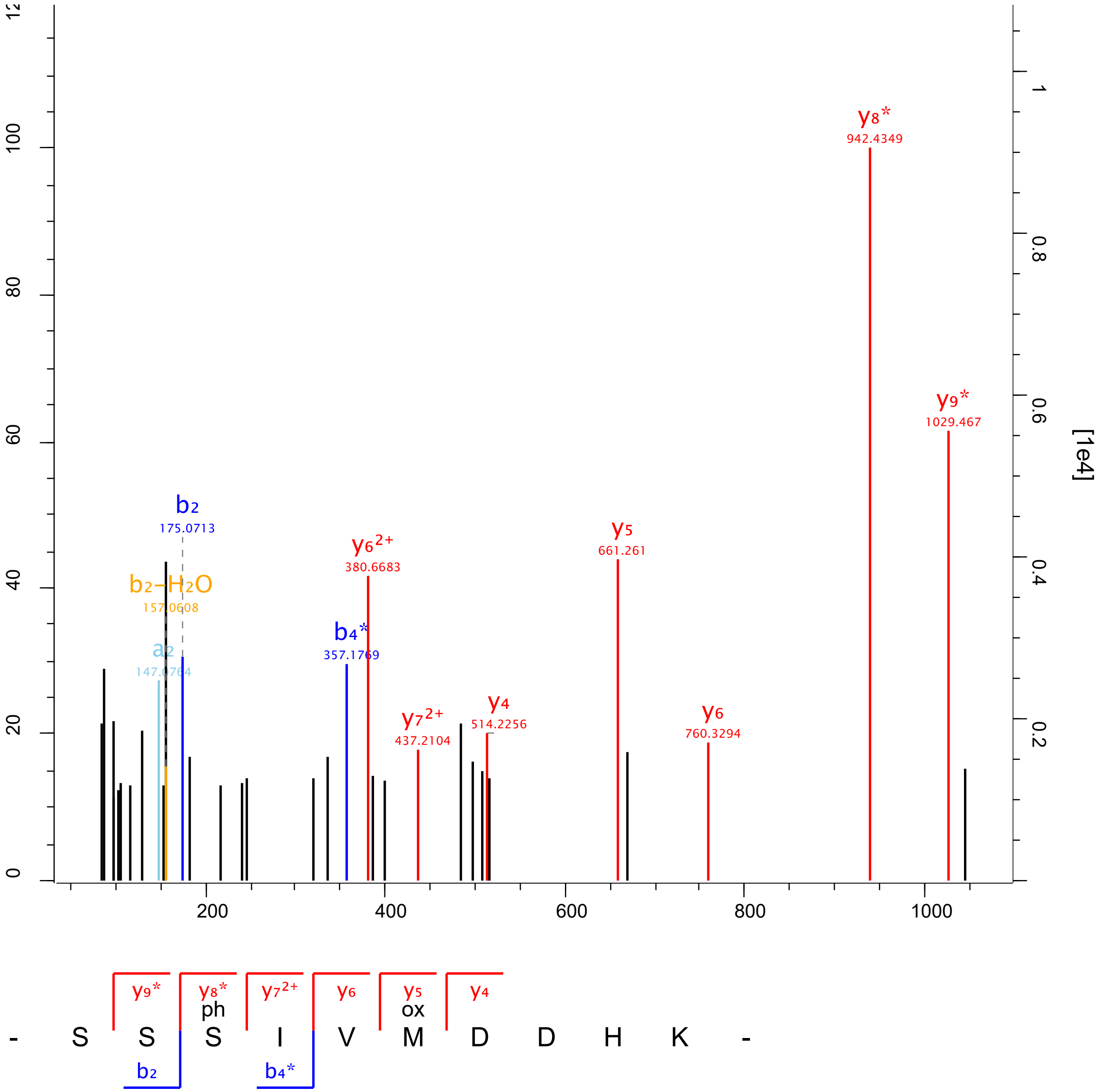

Raw file	Scan	Method	Score	m/z
sys_15_1	22527	FTMS; HCD	59.71	636.8

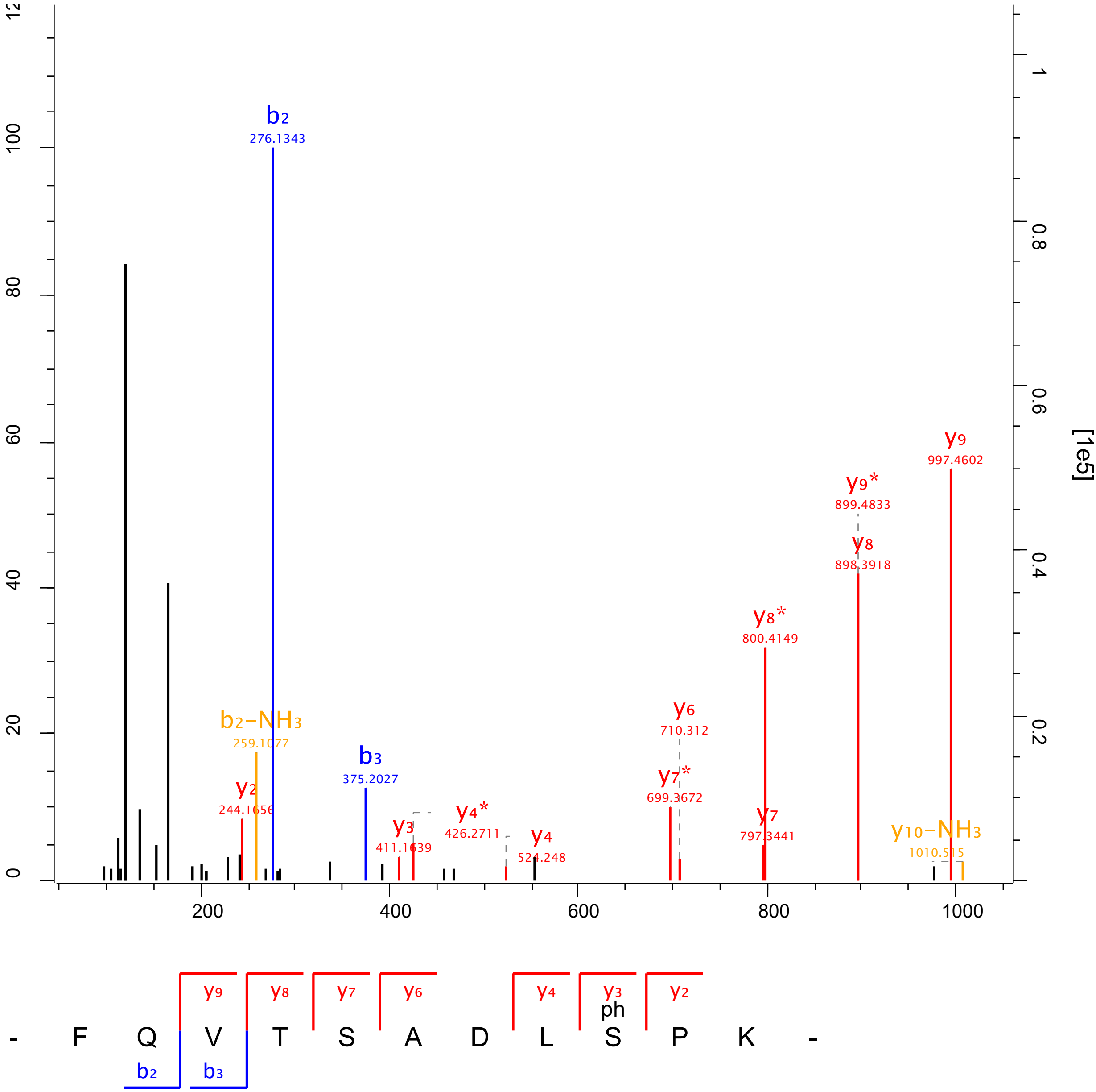

Raw file	Scan	Method	Score	m/z
sys_15_1	22543	FTMS; HCD	63.19	436.91

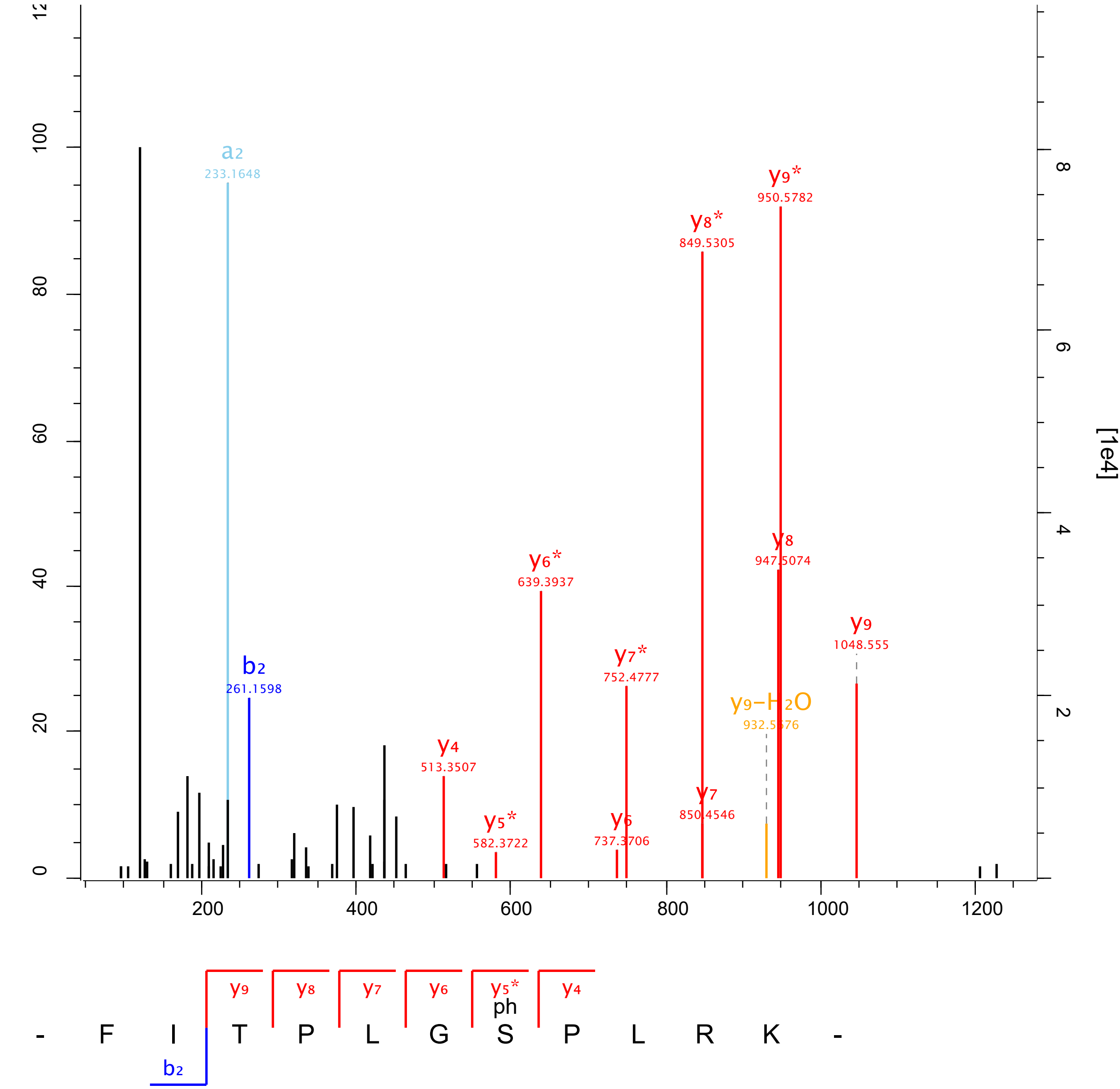

Raw file	Scan	Method	Score	m/z
sys_15_1	22596	FTMS; HCD	42.47	581.74

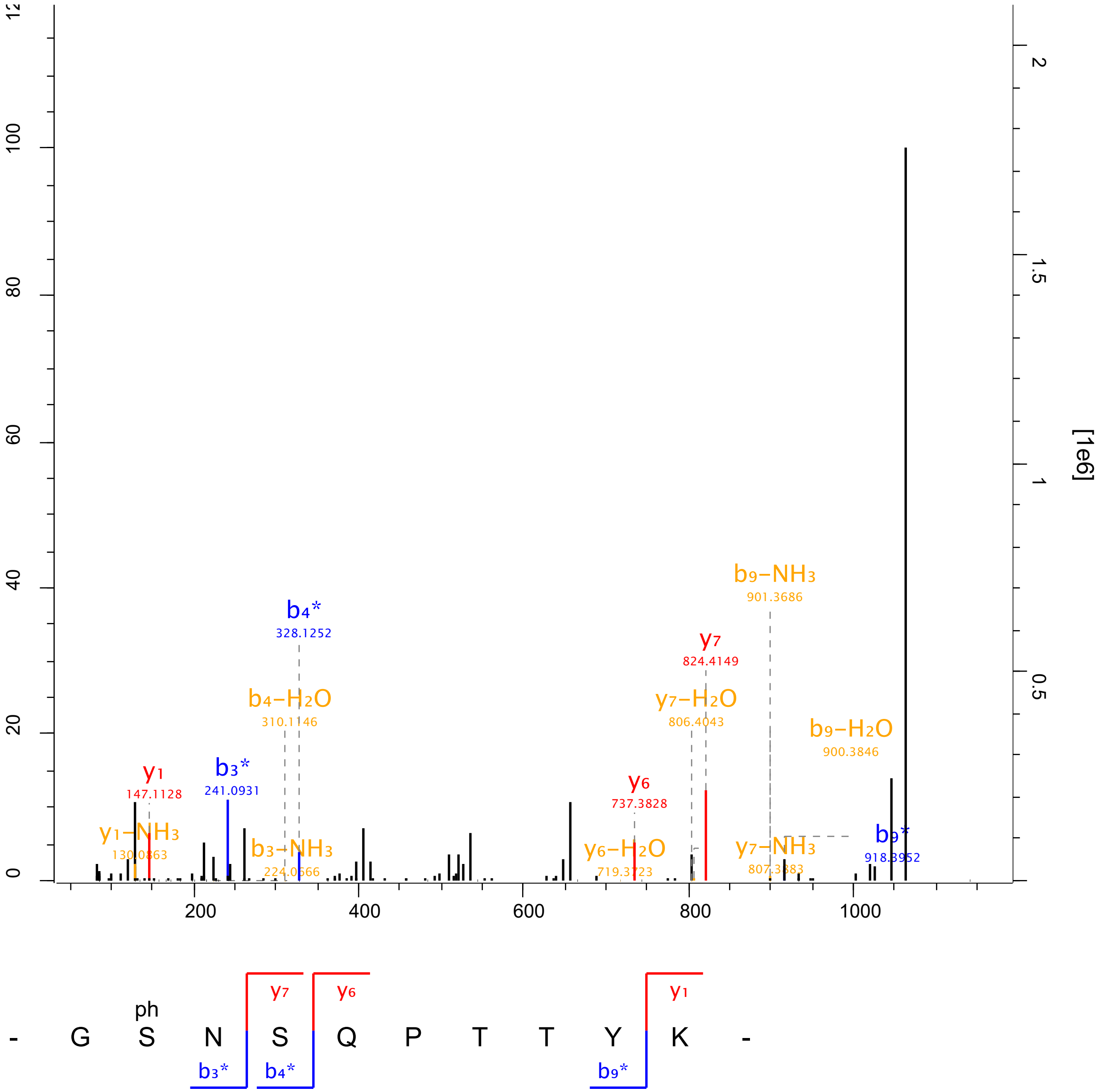

Raw file	Scan	Method	Score	m/z
sys_15_1	22703	FTMS; HCD	151.42	626.96

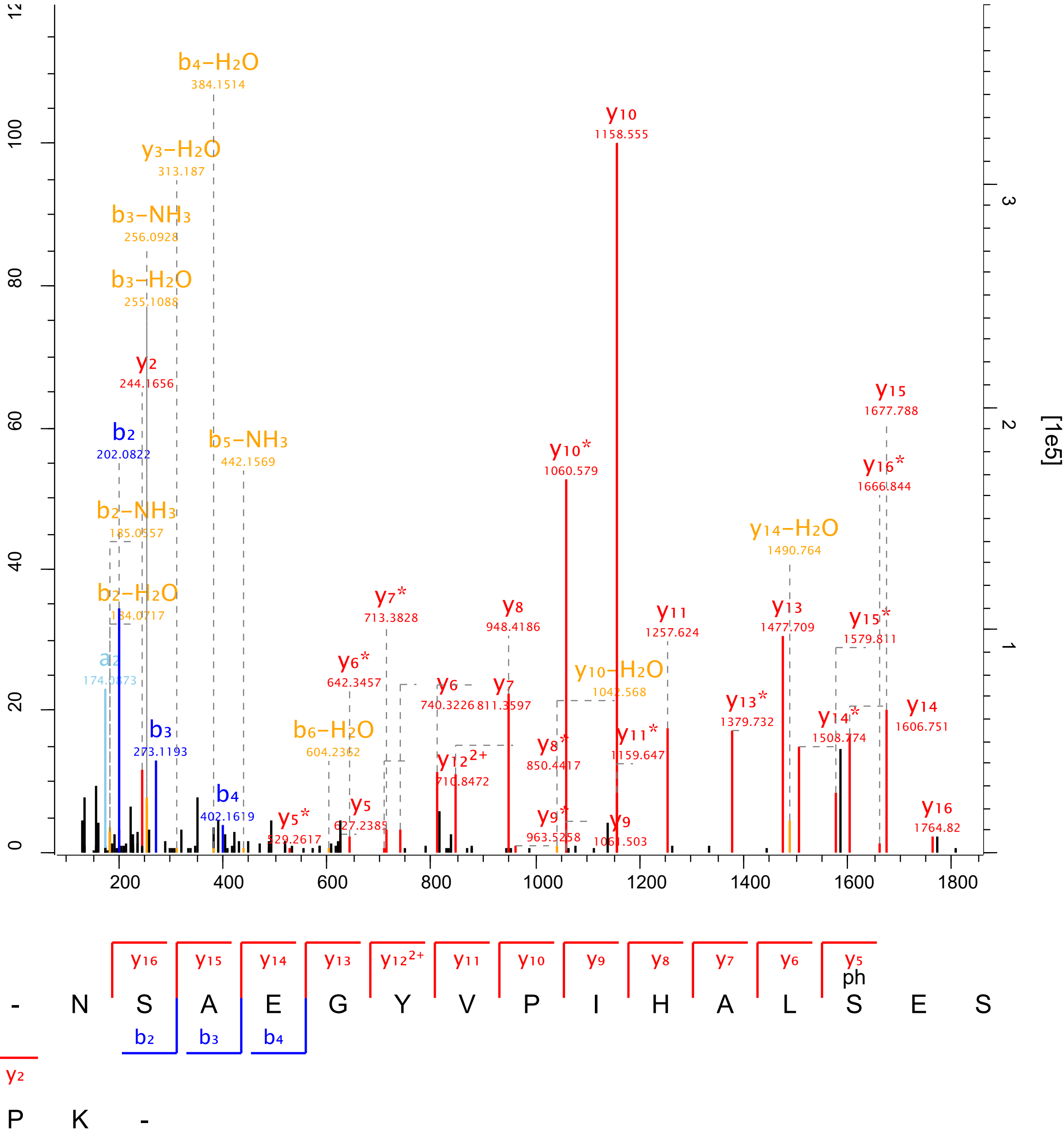

Raw file	Scan	Method	Score	m/z
sys_15_1	22916	FTMS; HCD	71.65	735.98

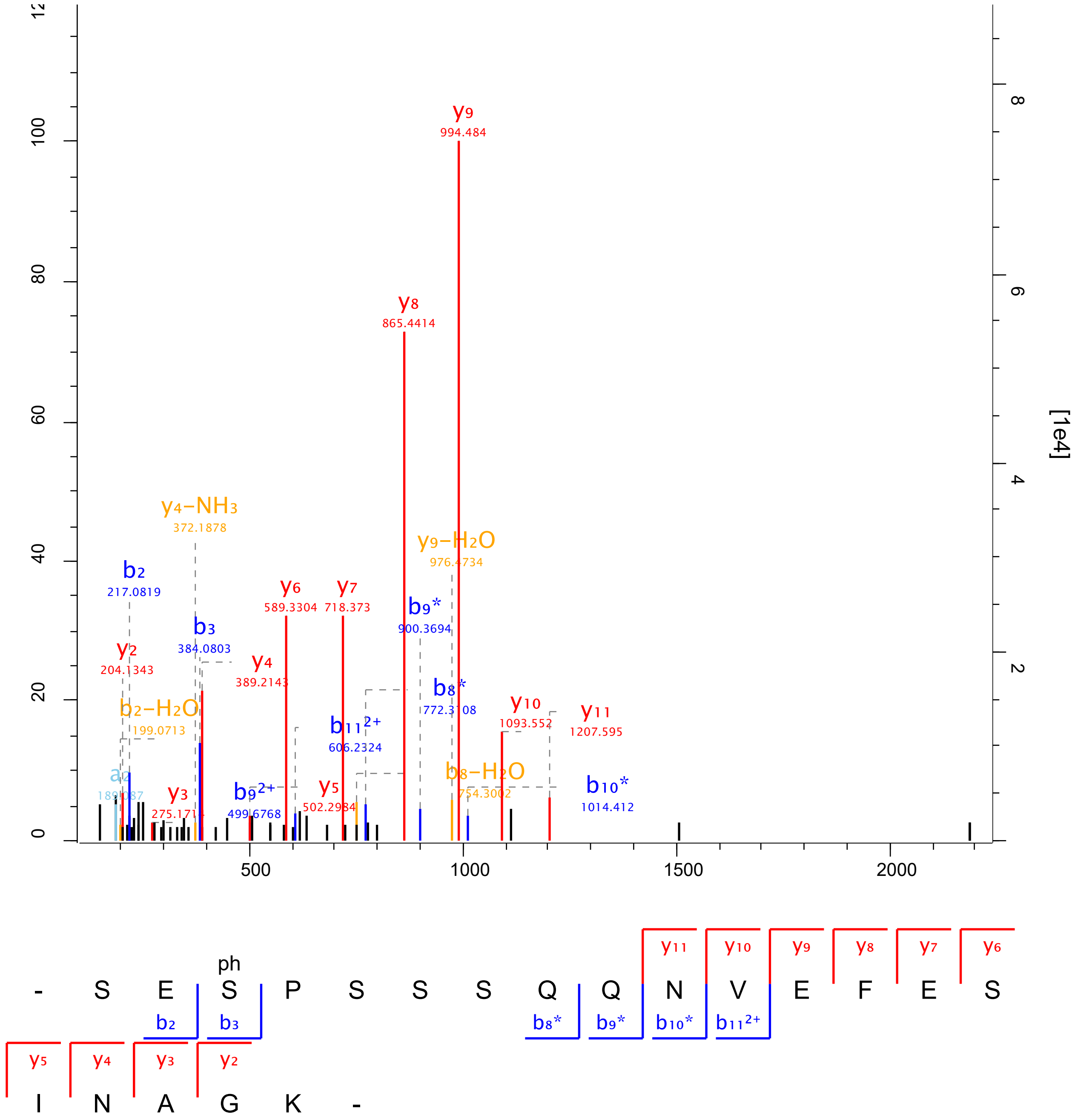

Raw file	Scan	Method	Score	m/z
sys_15_1	22976	FTMS; HCD	68.41	618.63

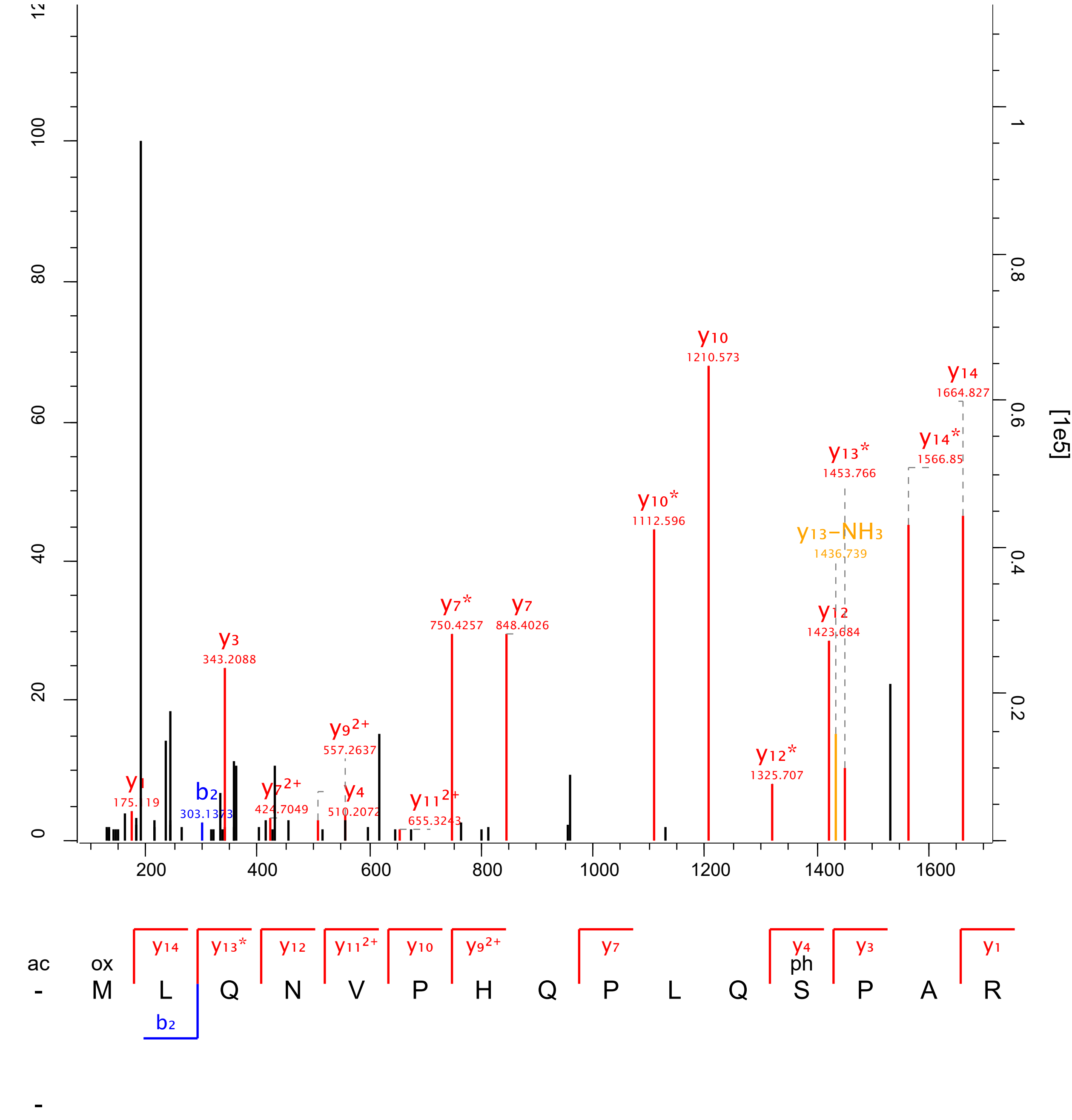

Raw file	Scan	Method	Score	m/z
sys_15_1	23027	FTMS; HCD	145.46	509.73

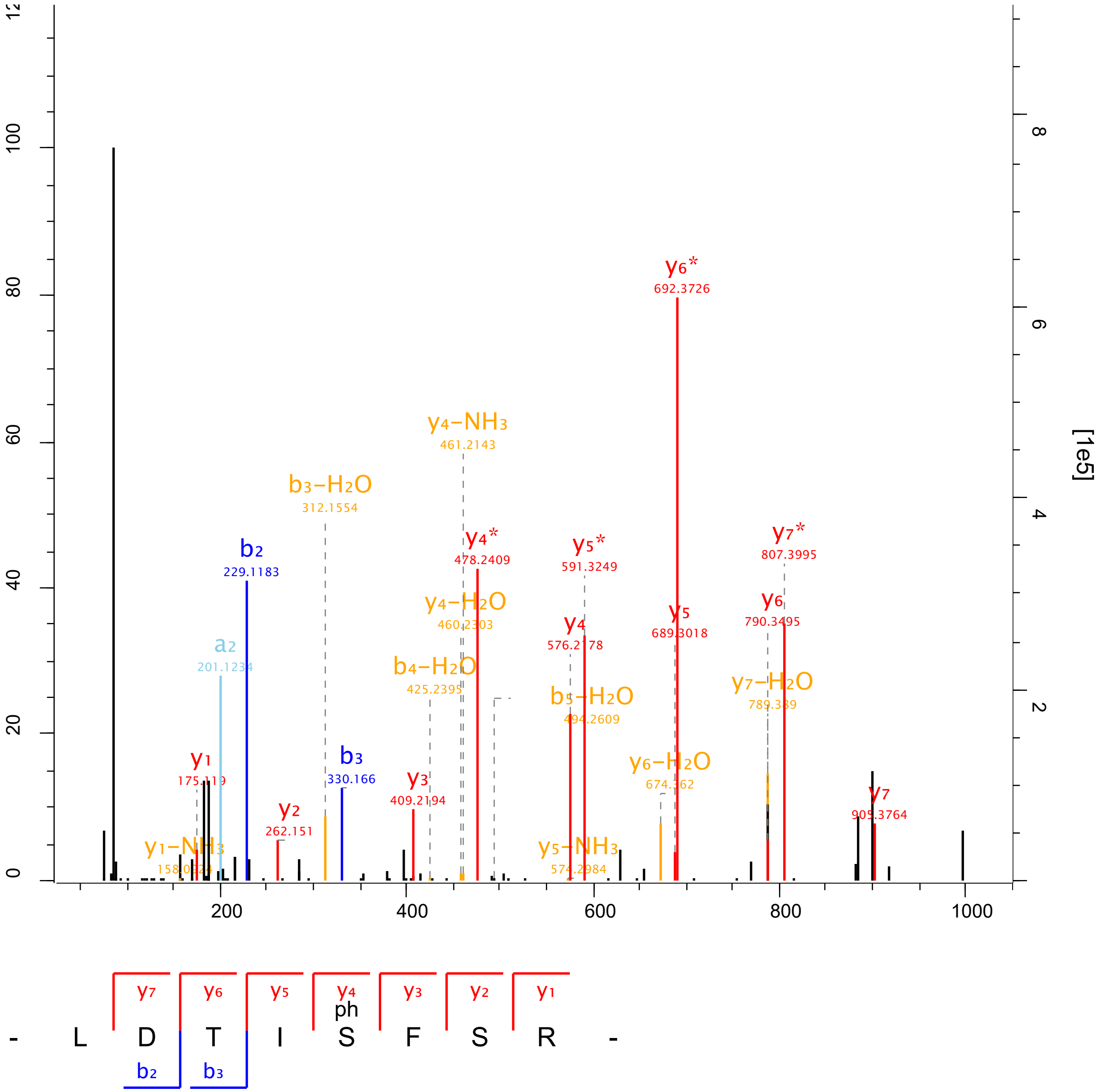

Raw file	Scan	Method	Score	m/z
sys_15_1	23150	FTMS; HCD	224.86	827.89

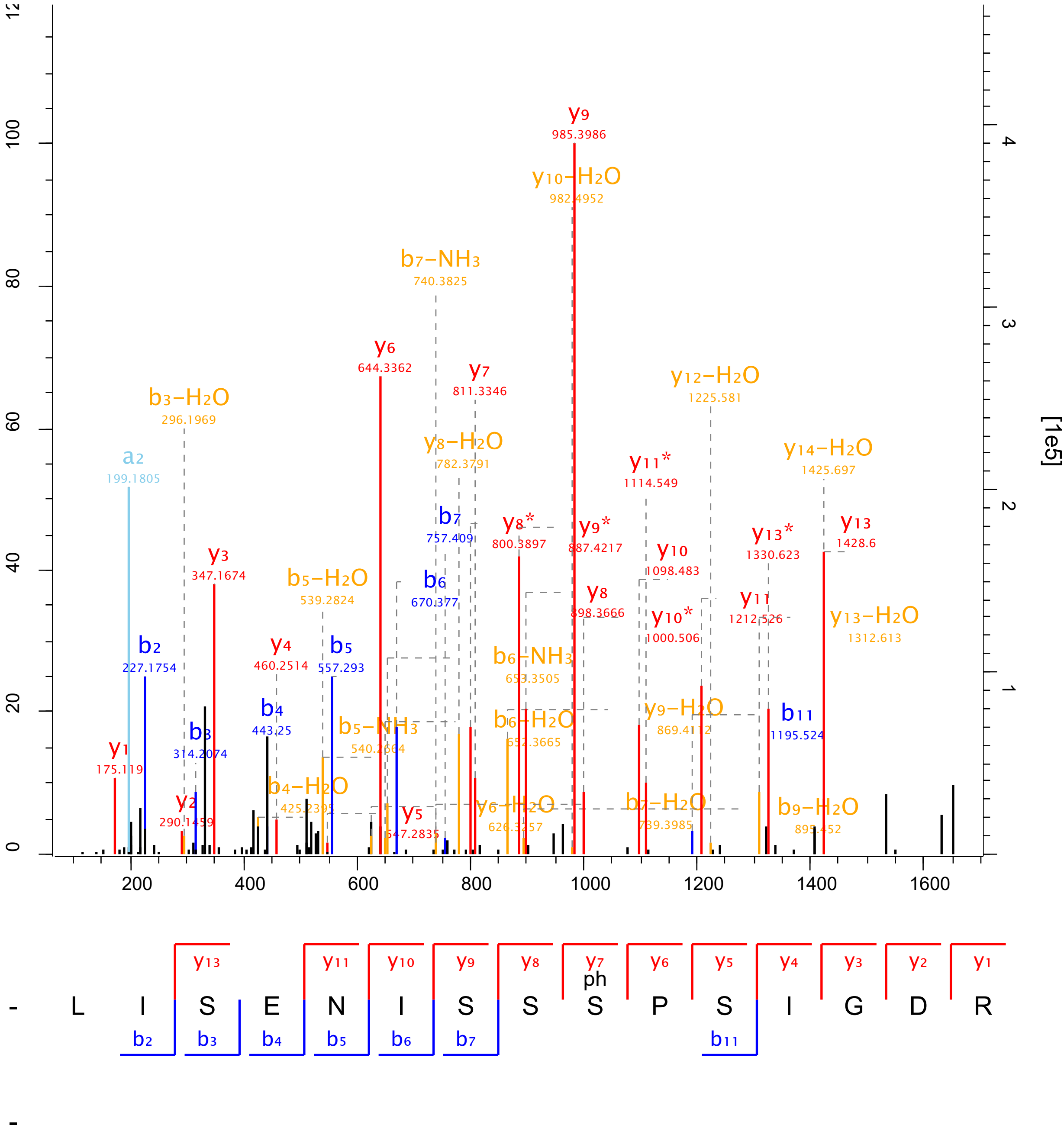

Raw file	Scan	Method	Score	m/z
sys_15_1	2320	FTMS; HCD	85.55	555.7

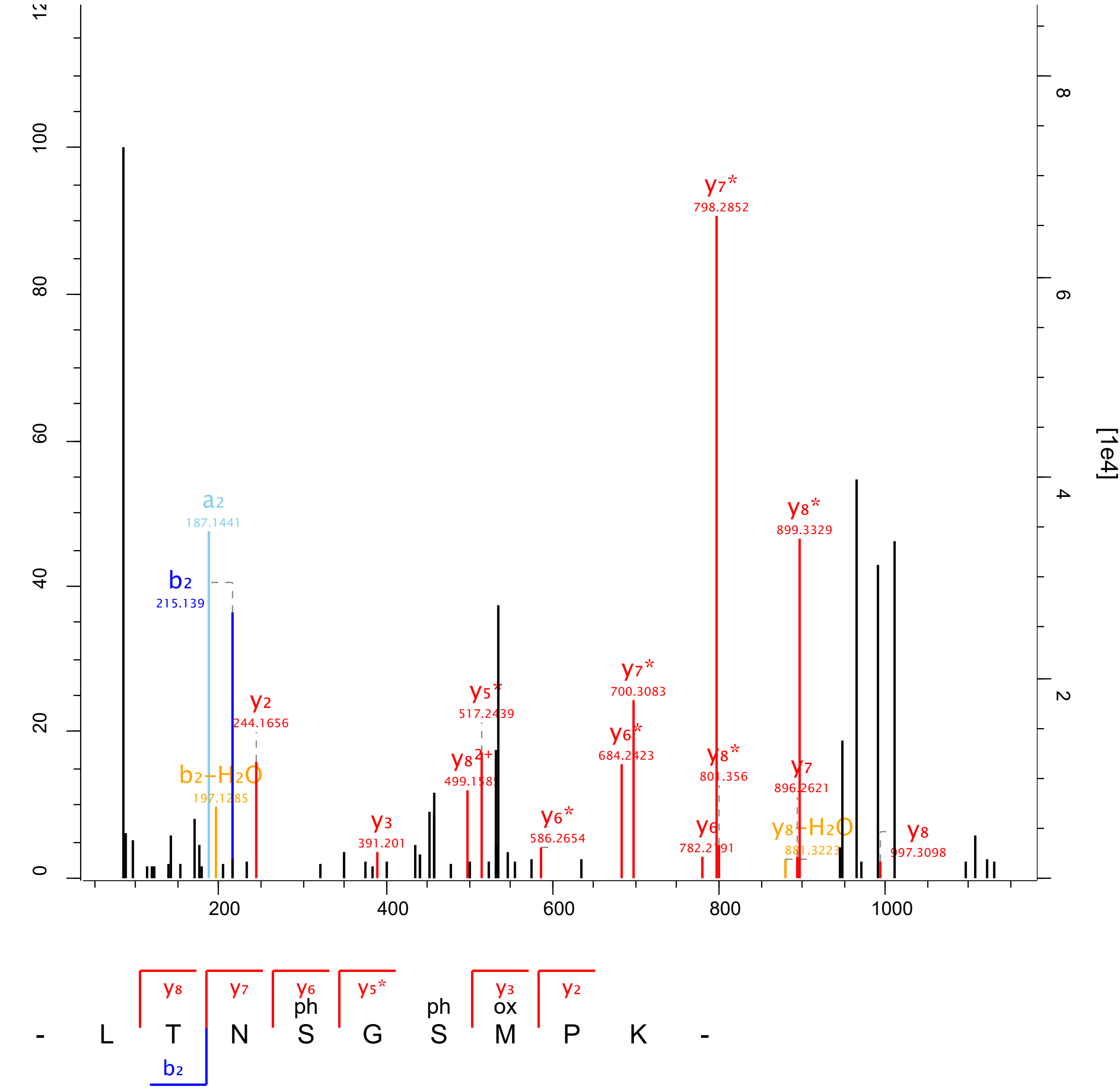

Raw file	Scan	Method	Score	m/z
sys_15_1	23221	FTMS; HCD	127.3	768.32

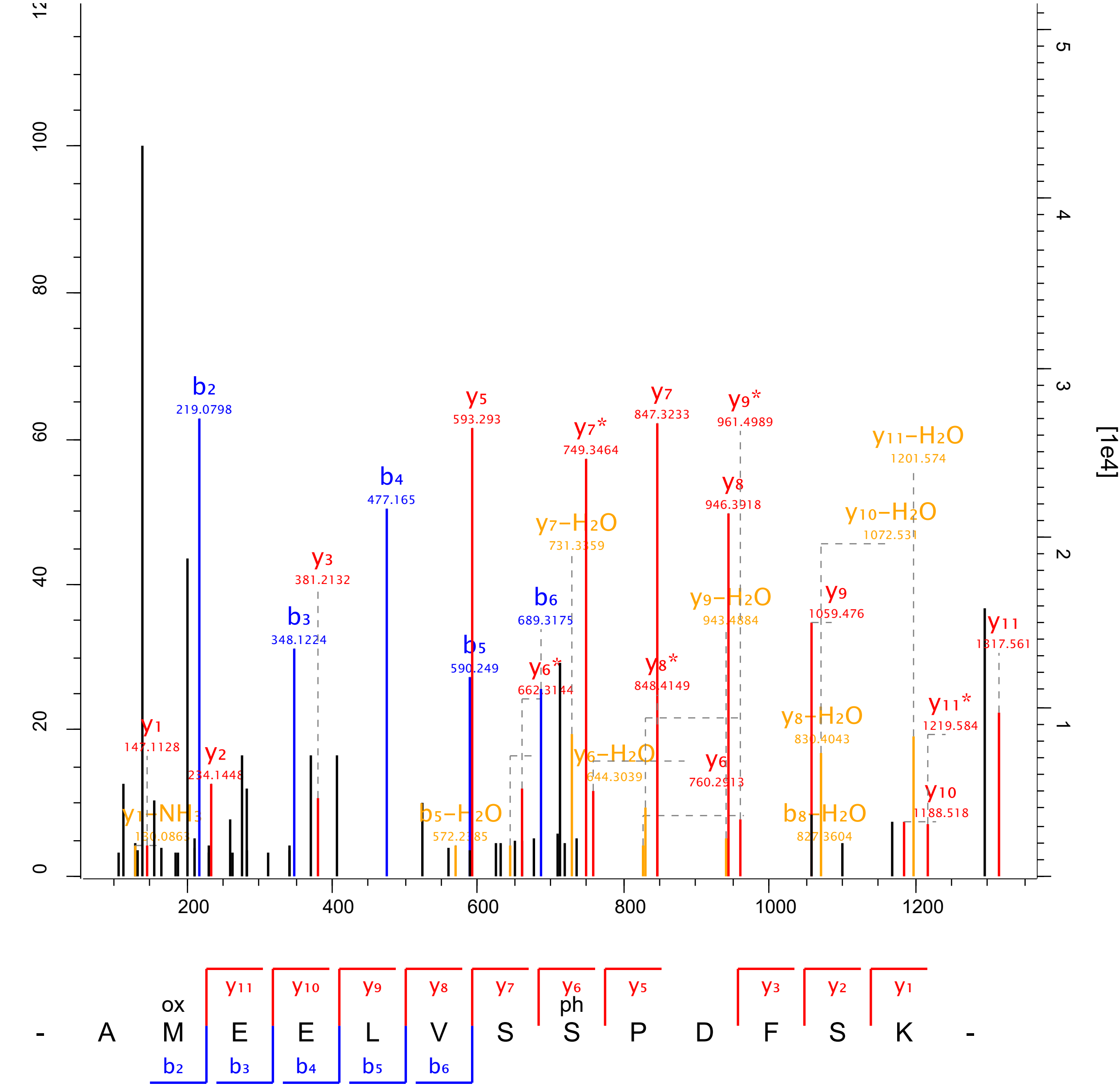

Raw file	Scan	Method	Score	m/z
sys_15_1	23270	FTMS; HCD	52.86	999.93

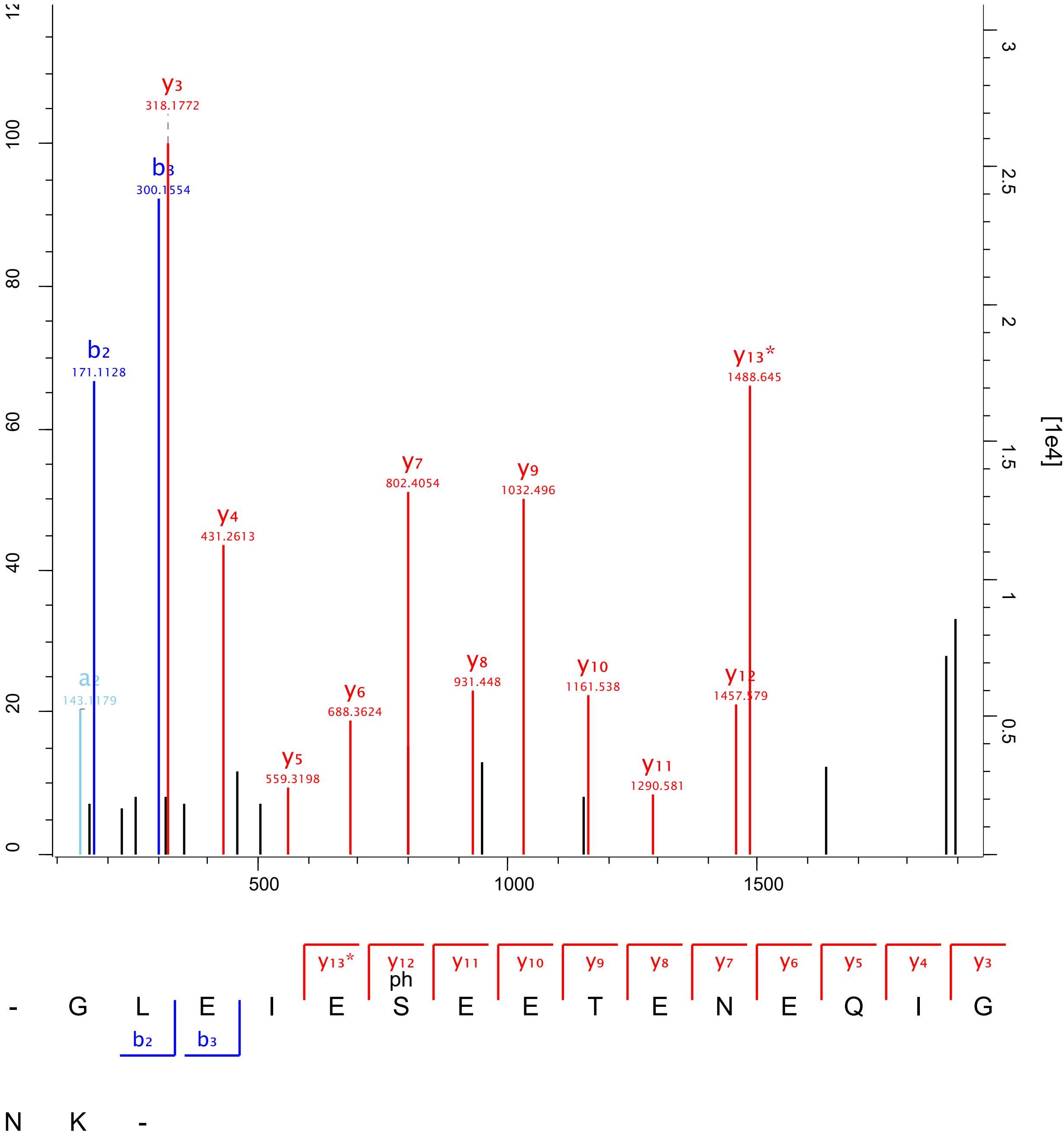

Raw file	Scan	Method	Score	m/z
sys_15_1	23342	FTMS; HCD	263.1	955.41

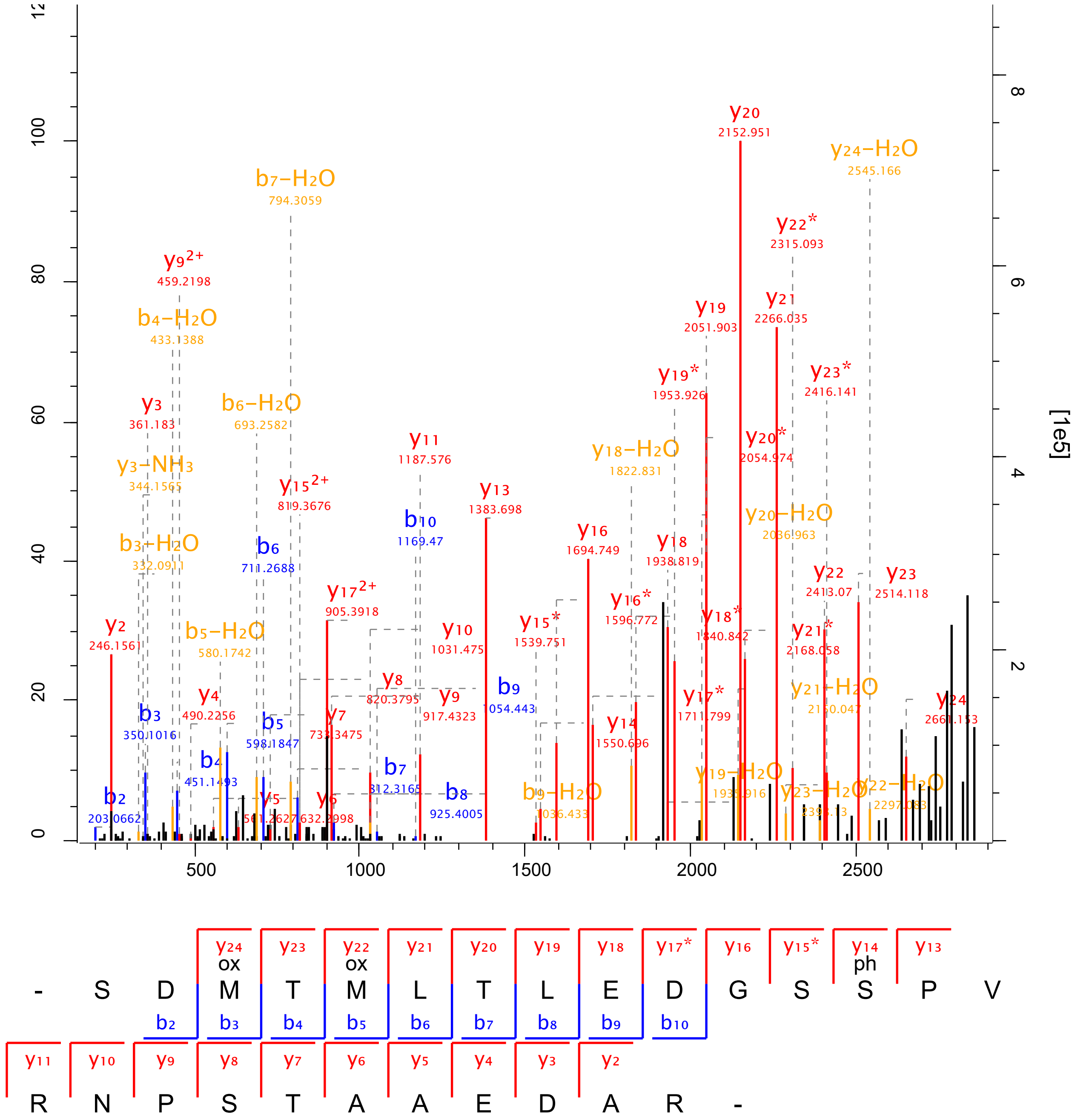

Raw file	Scan	Method	Score	m/z
sys_15_1	23369	FTMS; HCD	100.22	576.31

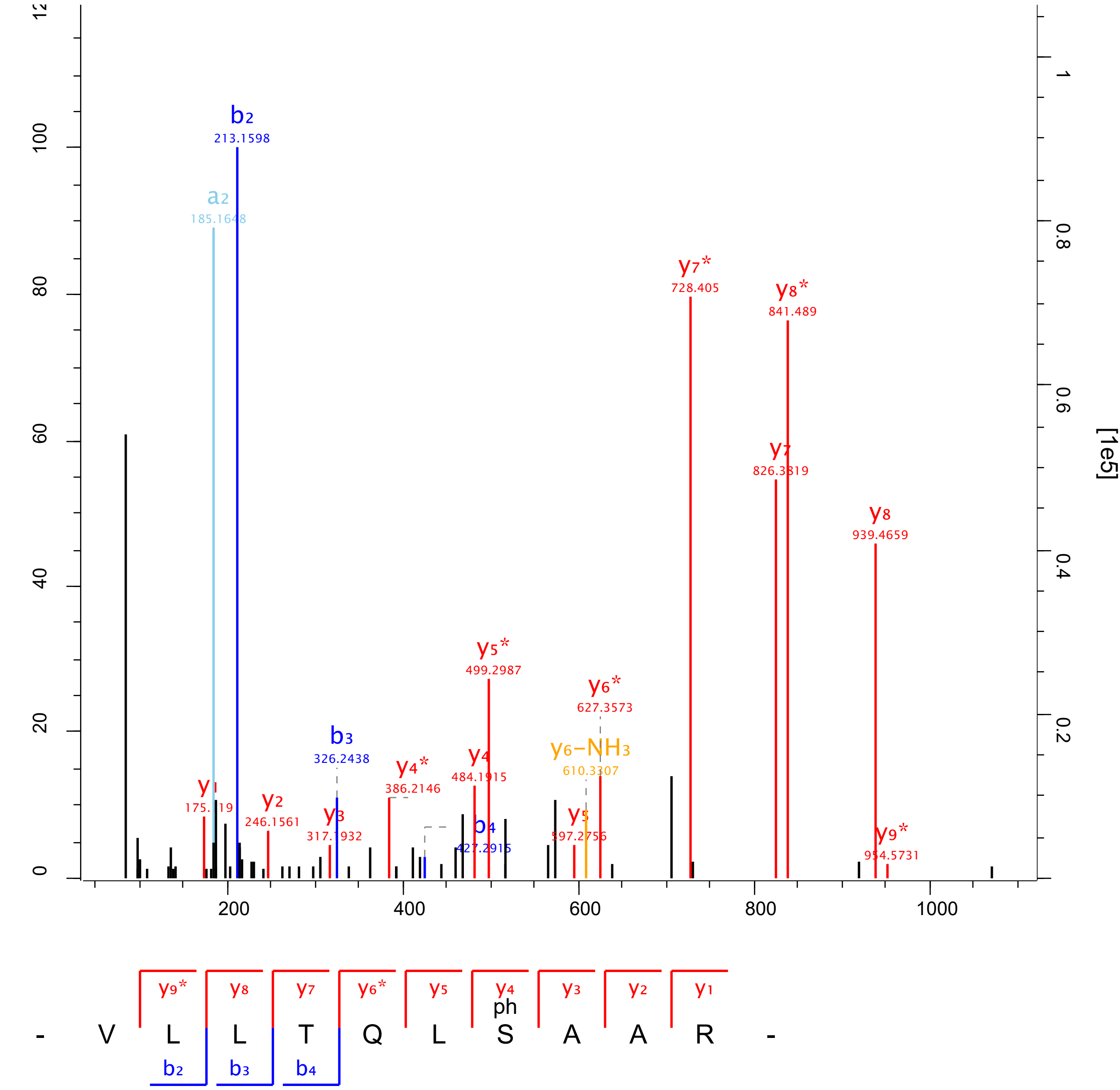

Raw file	Scan	Method	Score	m/z
sys_15_1	23406	FTMS; HCD	51.57	632.63

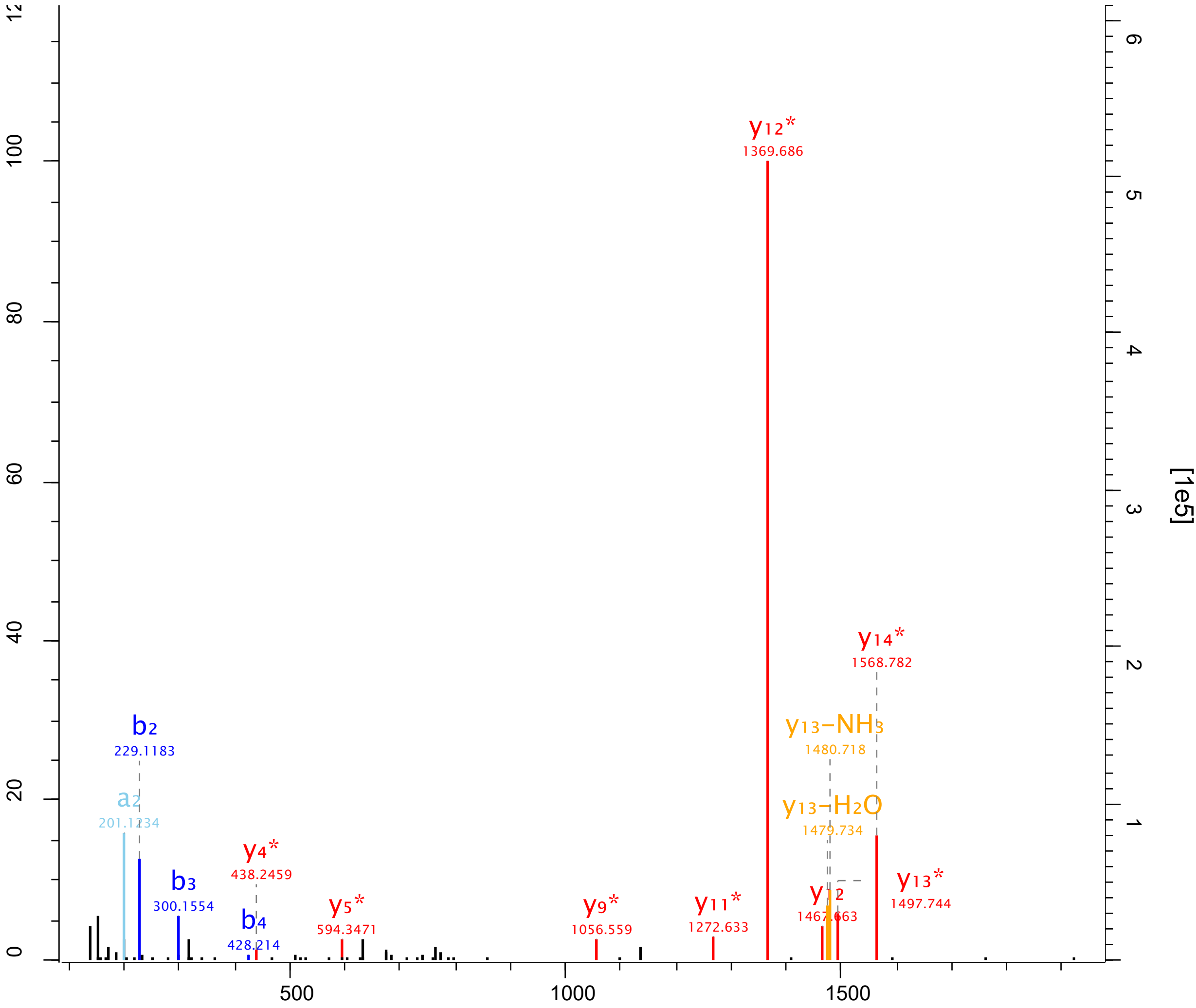

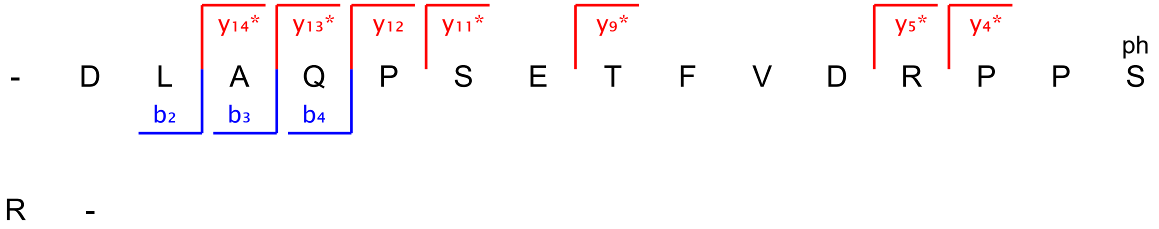

Raw file	Scan	Method	Score	m/z
sys_15_1	23436	FTMS; HCD	43.9	524.25

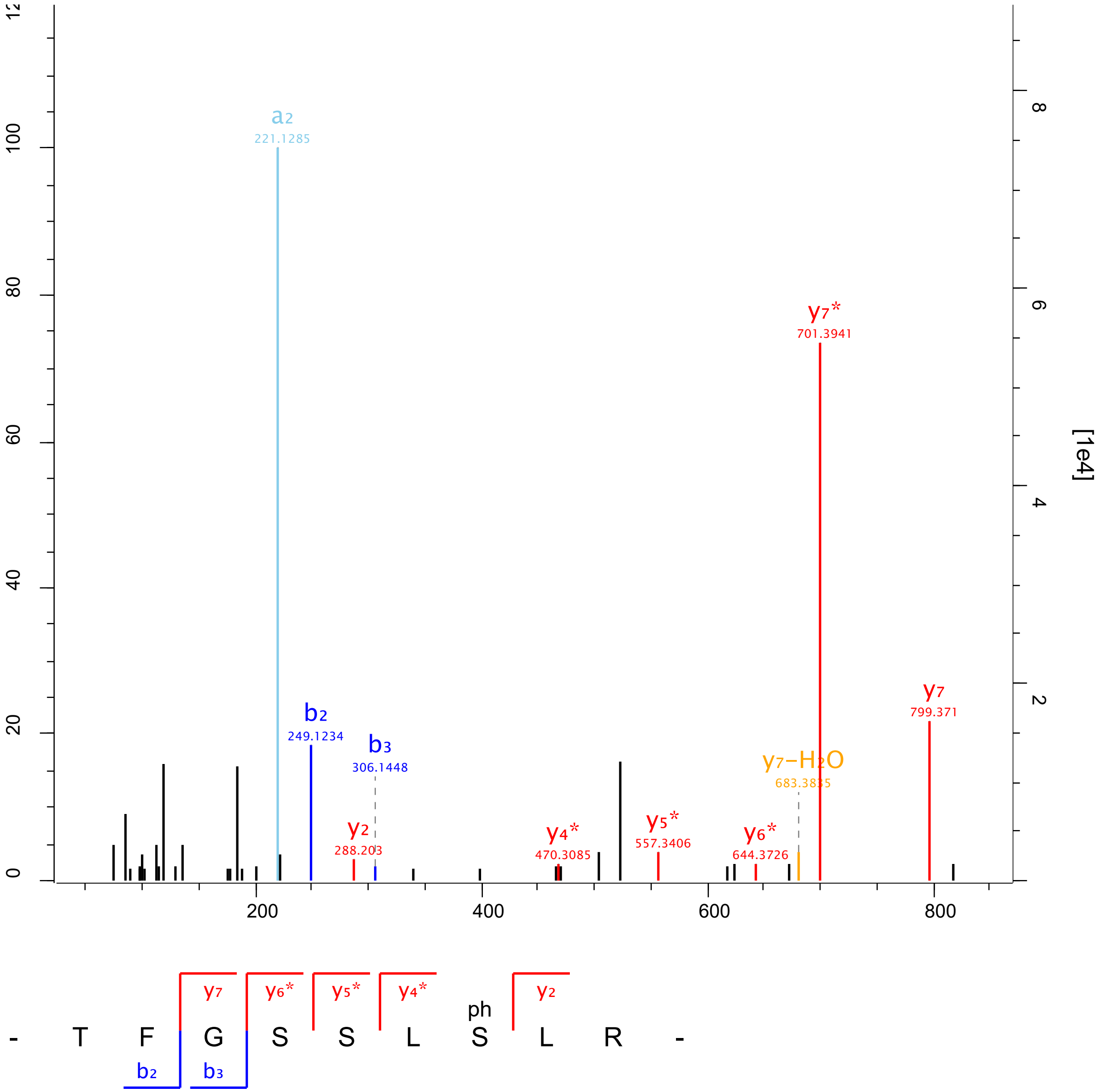

Raw file	Scan	Method	Score	m/z
sys_15_1	23453	FTMS; HCD	139.3	559.27

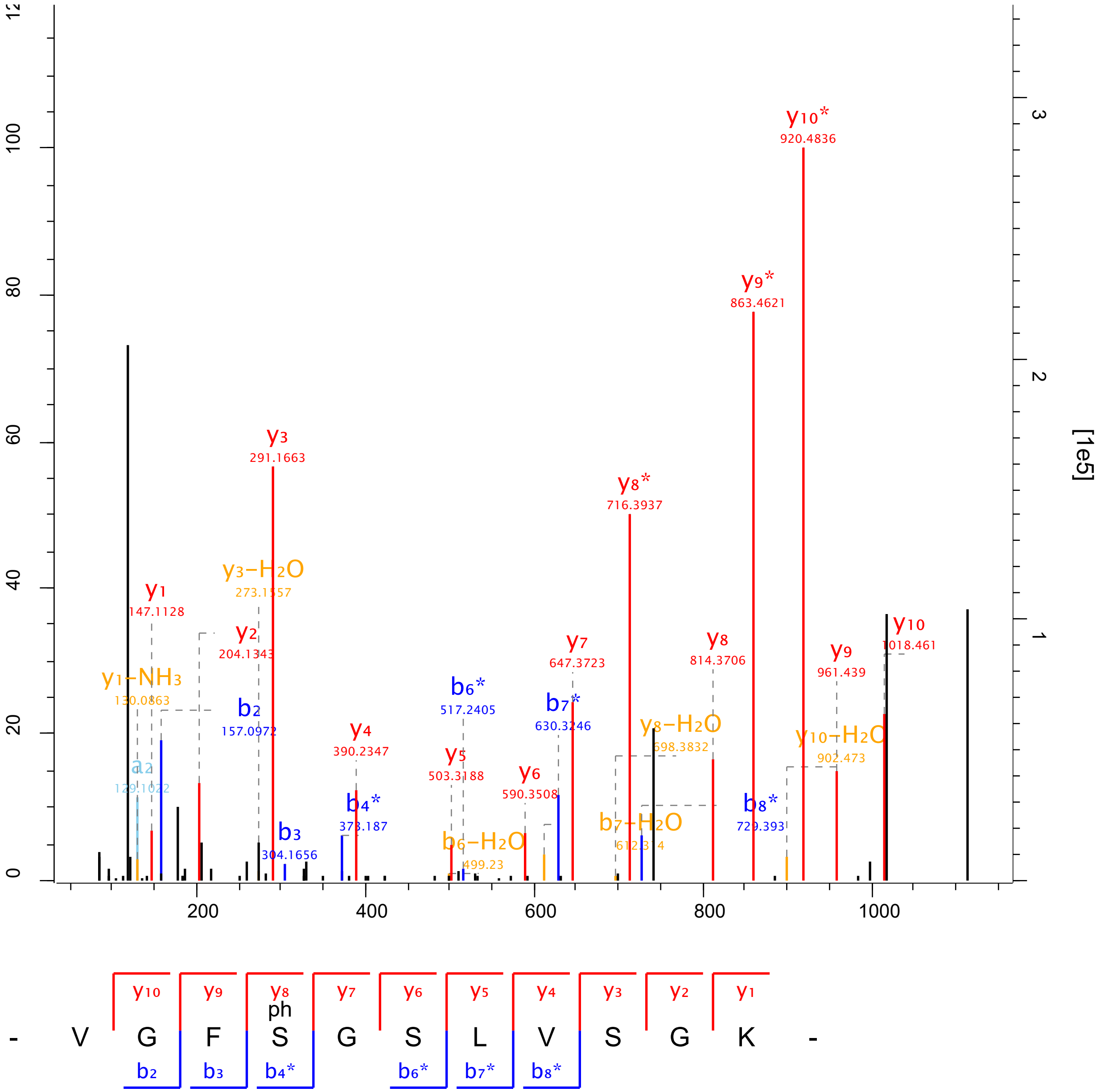

Raw file	Scan	Method	Score	m/z
sys_15_1	23465	FTMS; HCD	77.44	748.04

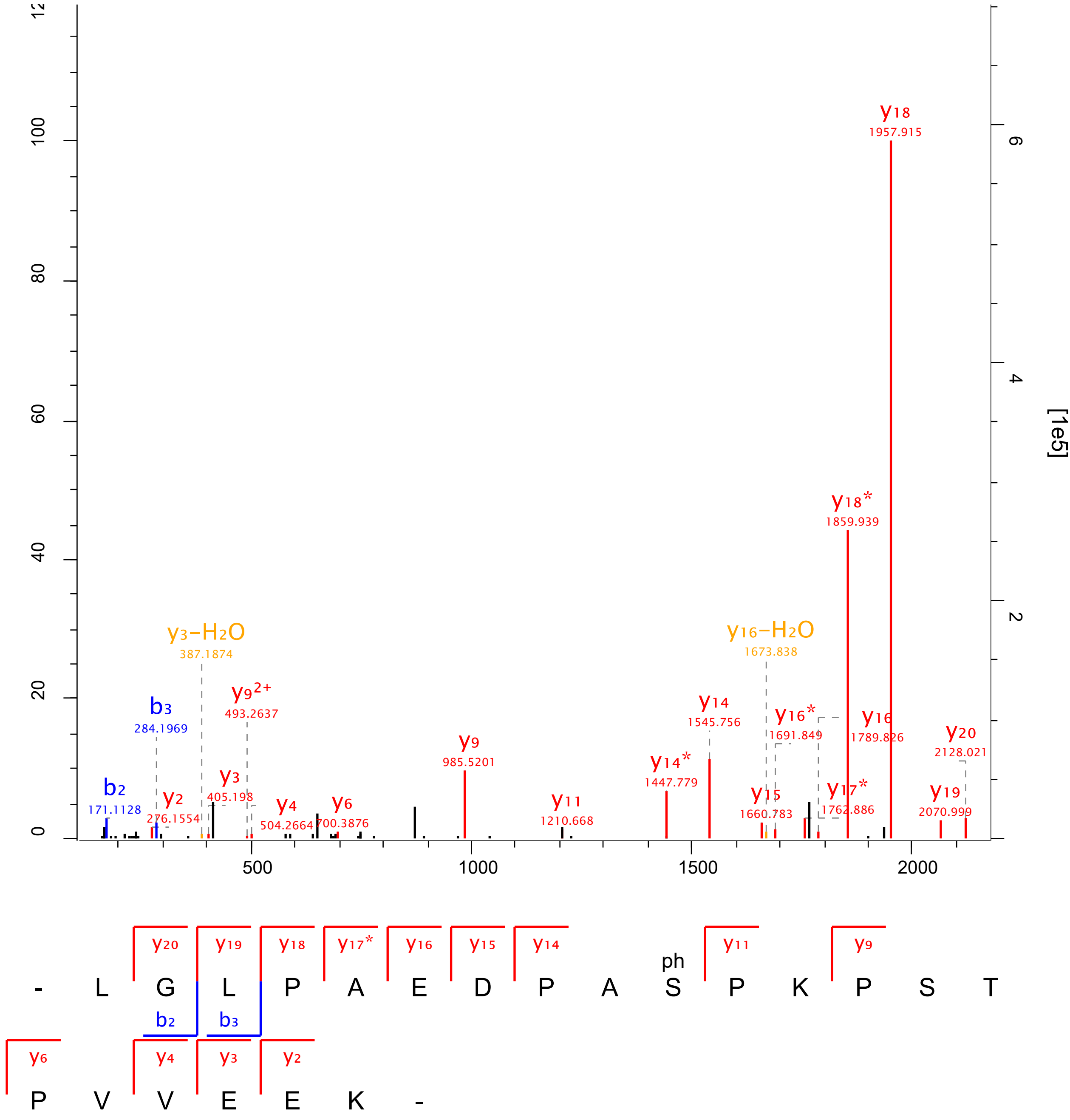

Raw file	Scan	Method	Score	m/z
sys_15_1	23501	FTMS; HCD	100.22	581.76

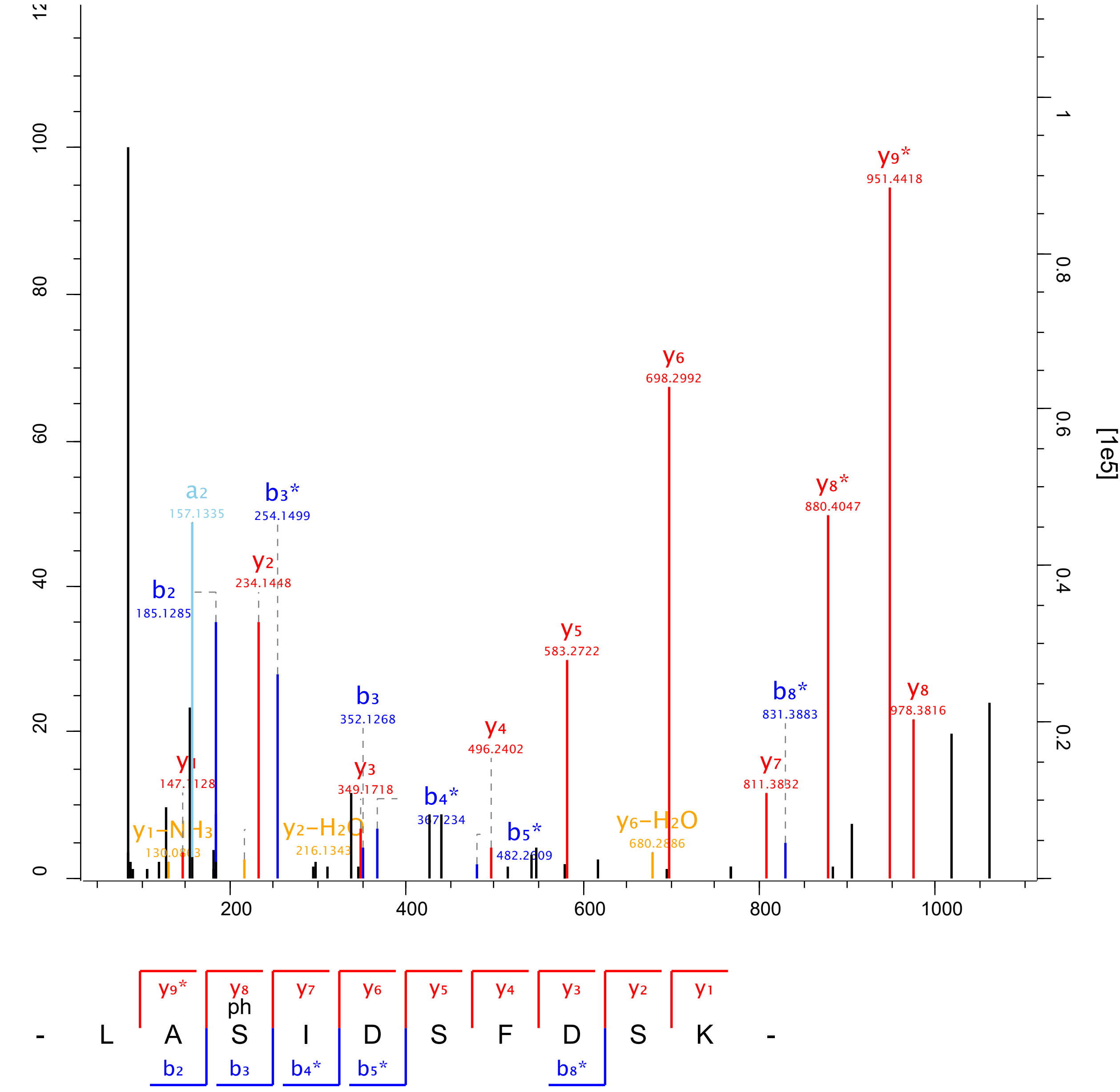

Raw file	Scan	Method	Score	m/z
sys_15_1	23512	FTMS; HCD	134.57	532.73

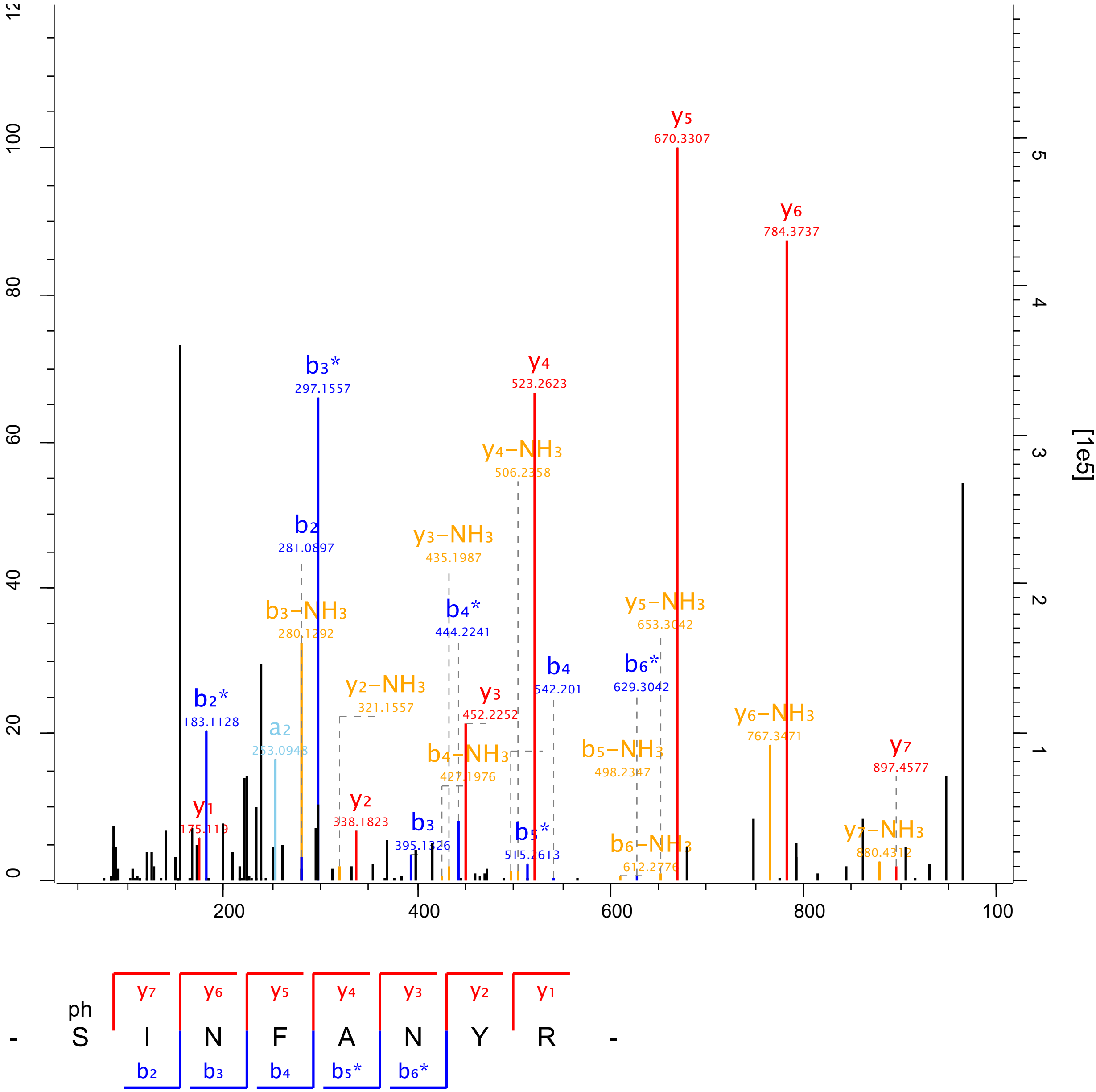

Raw file	Scan	Method	Score	m/z
sys_15_1	23579	FTMS; HCD	100.23	707.79

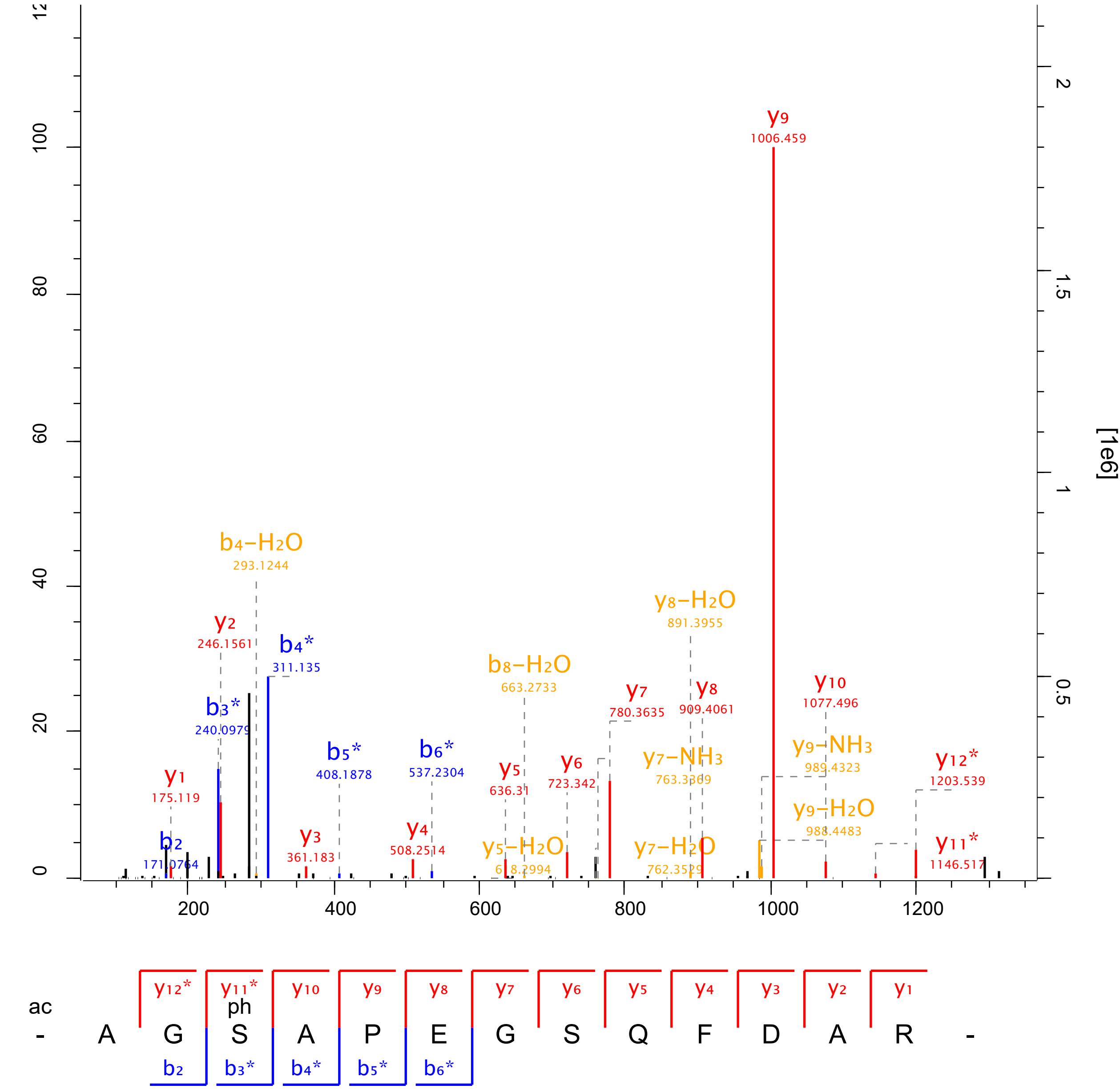

Raw file	Scan	Method	Score	m/z
sys_15_1	23587	FTMS; HCD	106.93	496.21

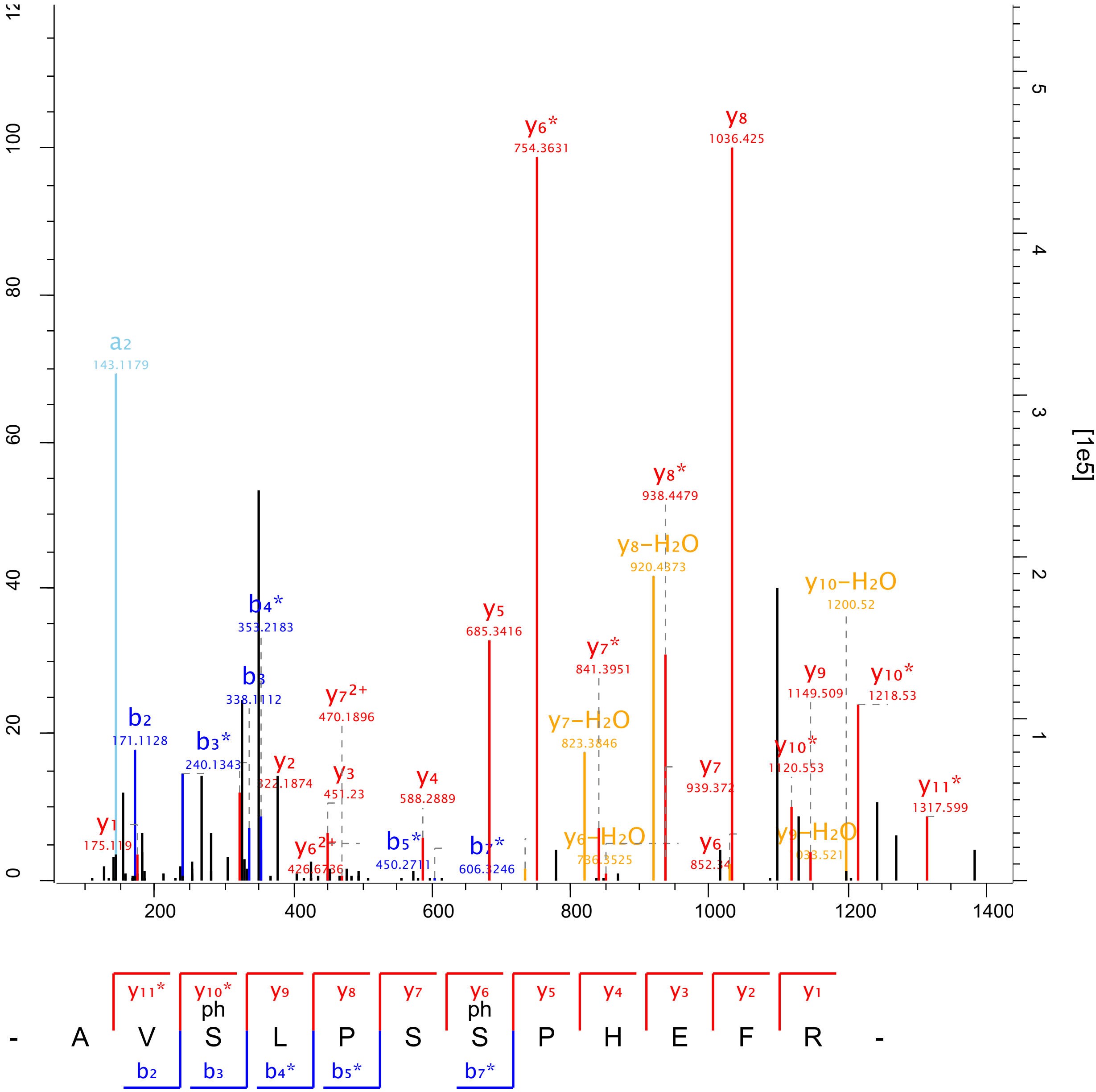

Raw file	Scan	Method	Score	m/z
sys_15_1	23727	FTMS; HCD	209.43	784.84

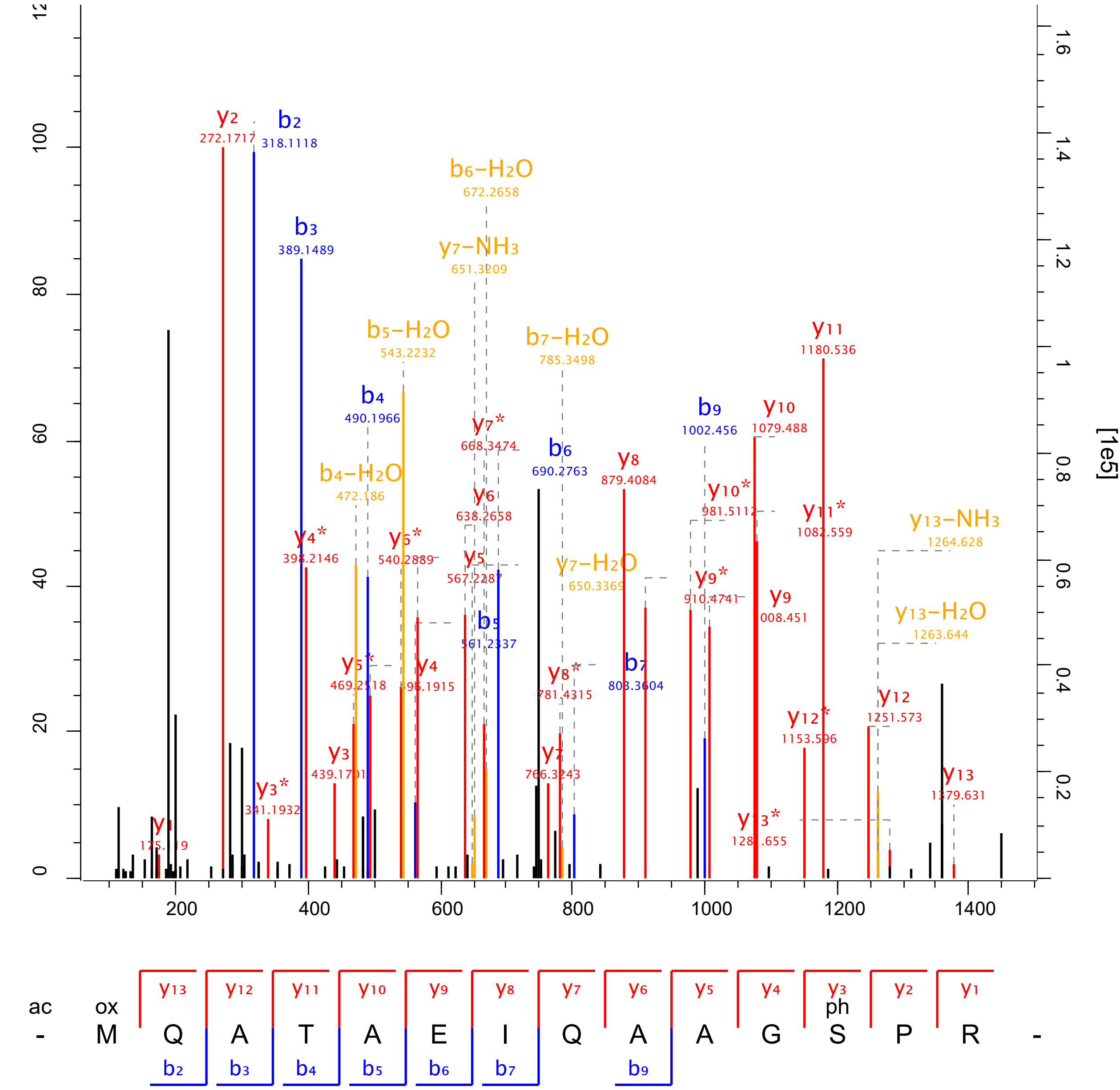

Raw file	Scan	Method	Score	m/z
sys_15_1	23736	FTMS; HCD	166.29	925.88

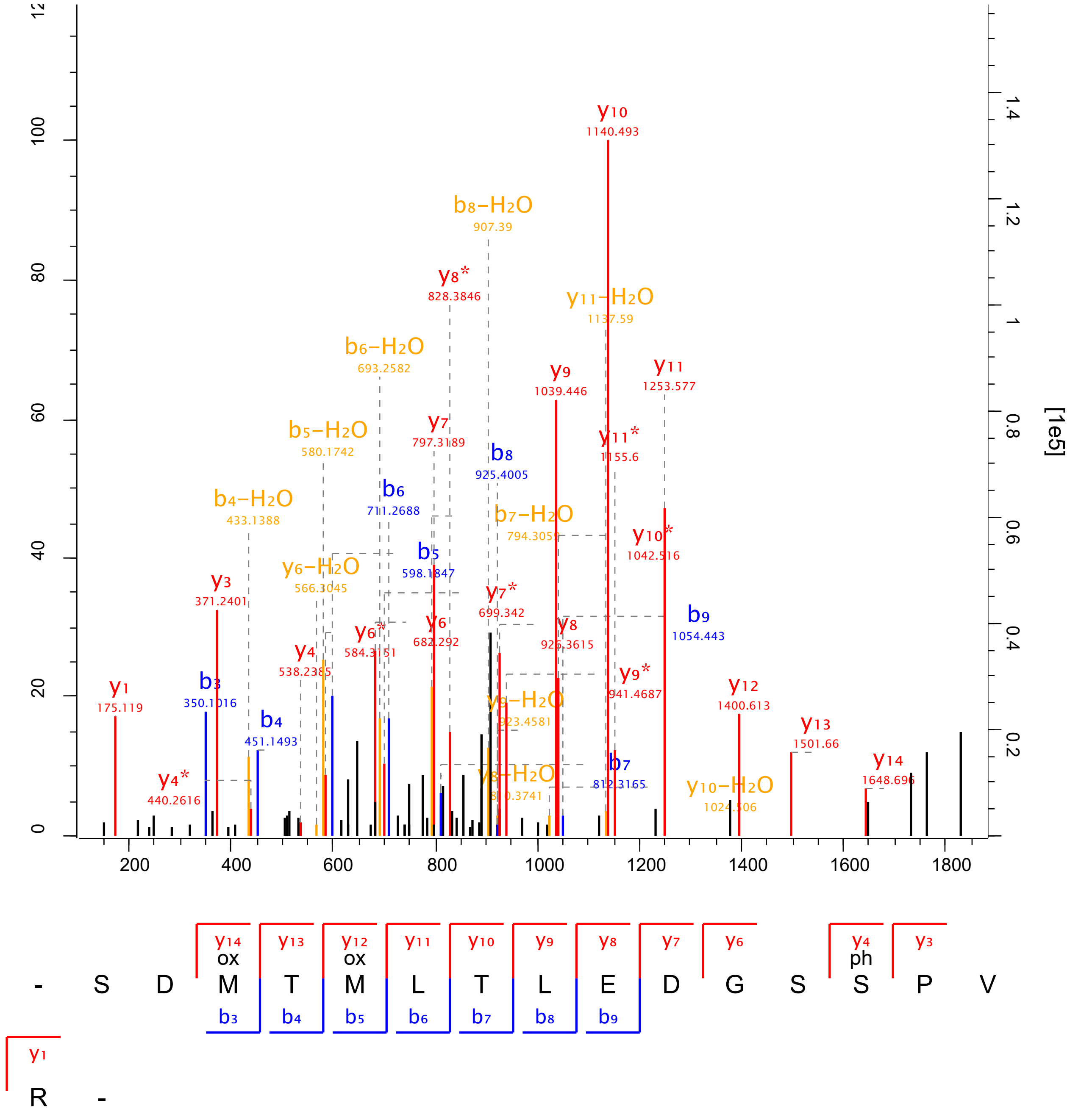

Raw file	Scan	Method	Score	m/z
sys_15_1	23777	FTMS; HCD	41.09	756.33

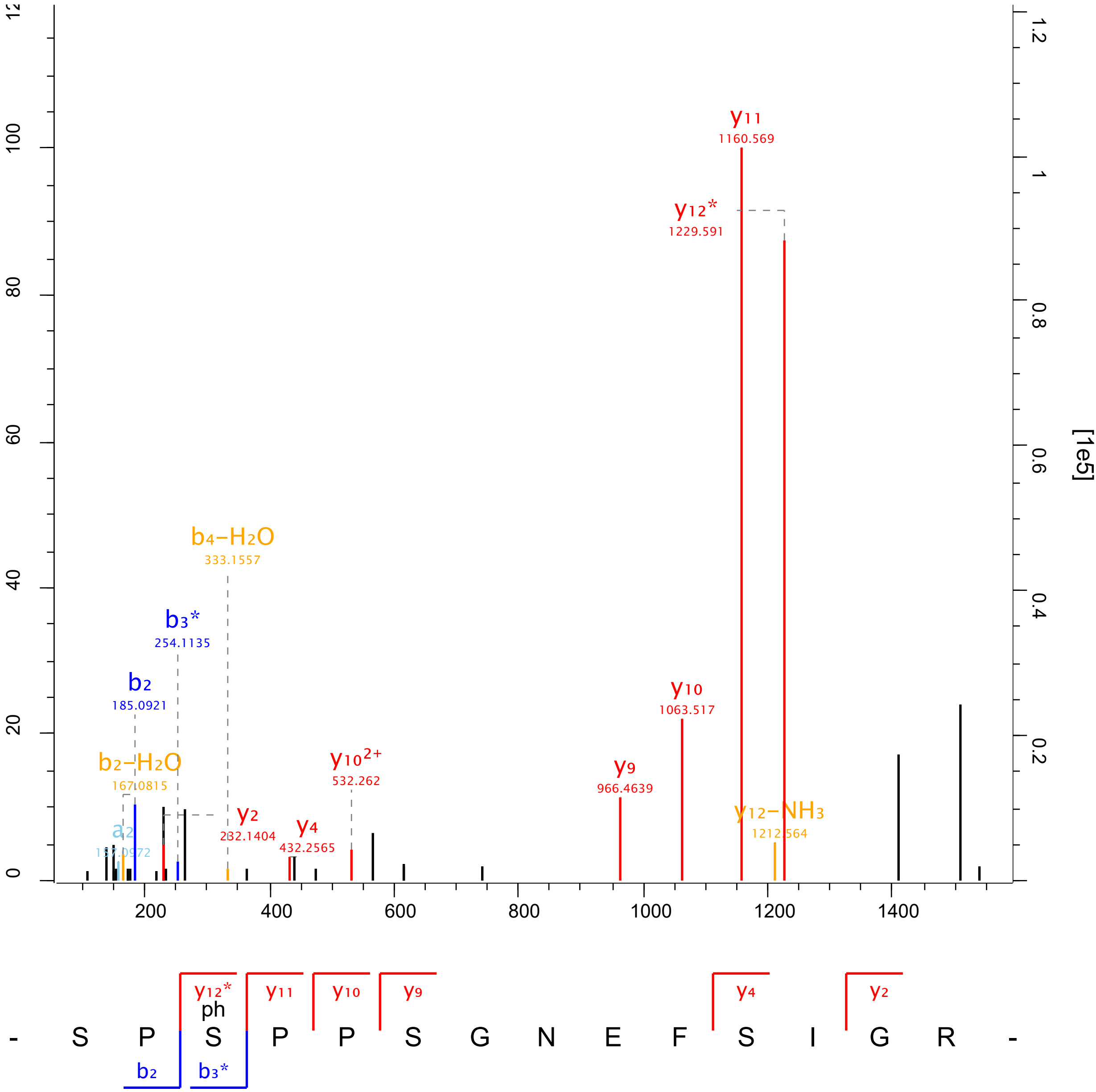

Raw file	Scan	Method	Score	m/z
sys_15_1	23780	FTMS; HCD	58.04	585.79

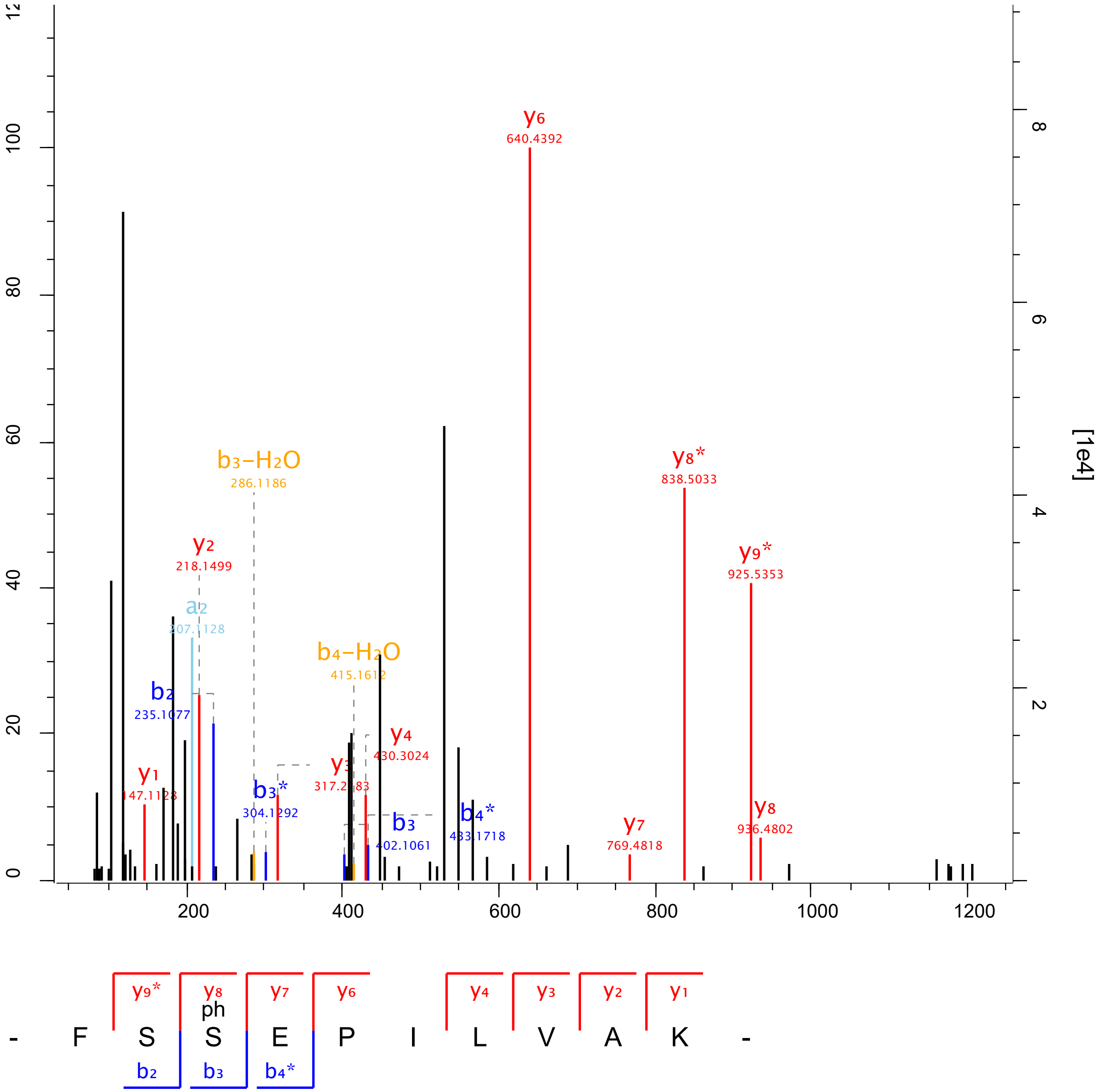

Raw file	Scan	Method	Score	m/z
sys_15_1	23894	FTMS; HCD	53.45	607.79

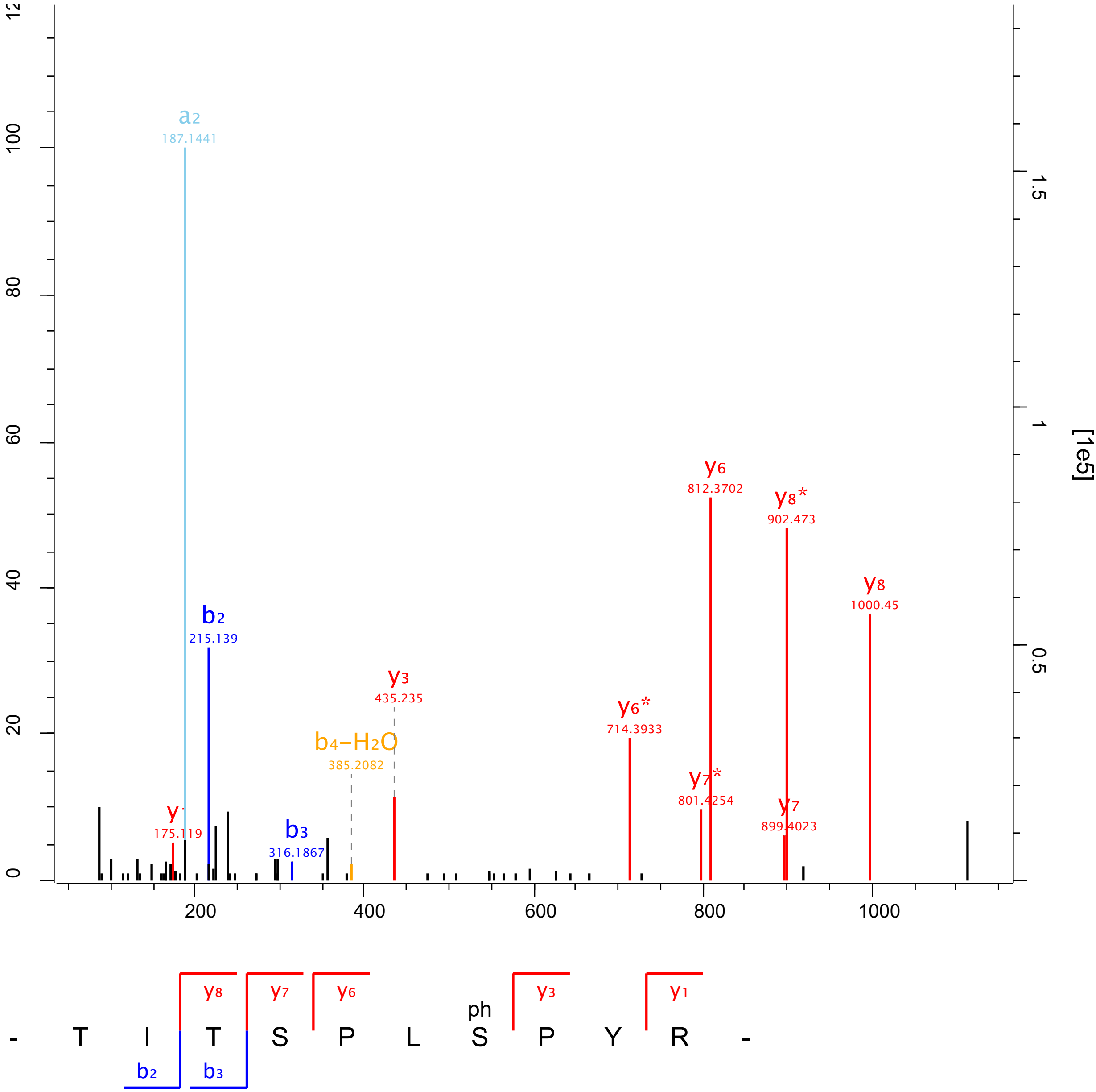

Raw file	Scan	Method	Score	m/z
sys_15_1	24073	FTMS; HCD	57.53	558.62

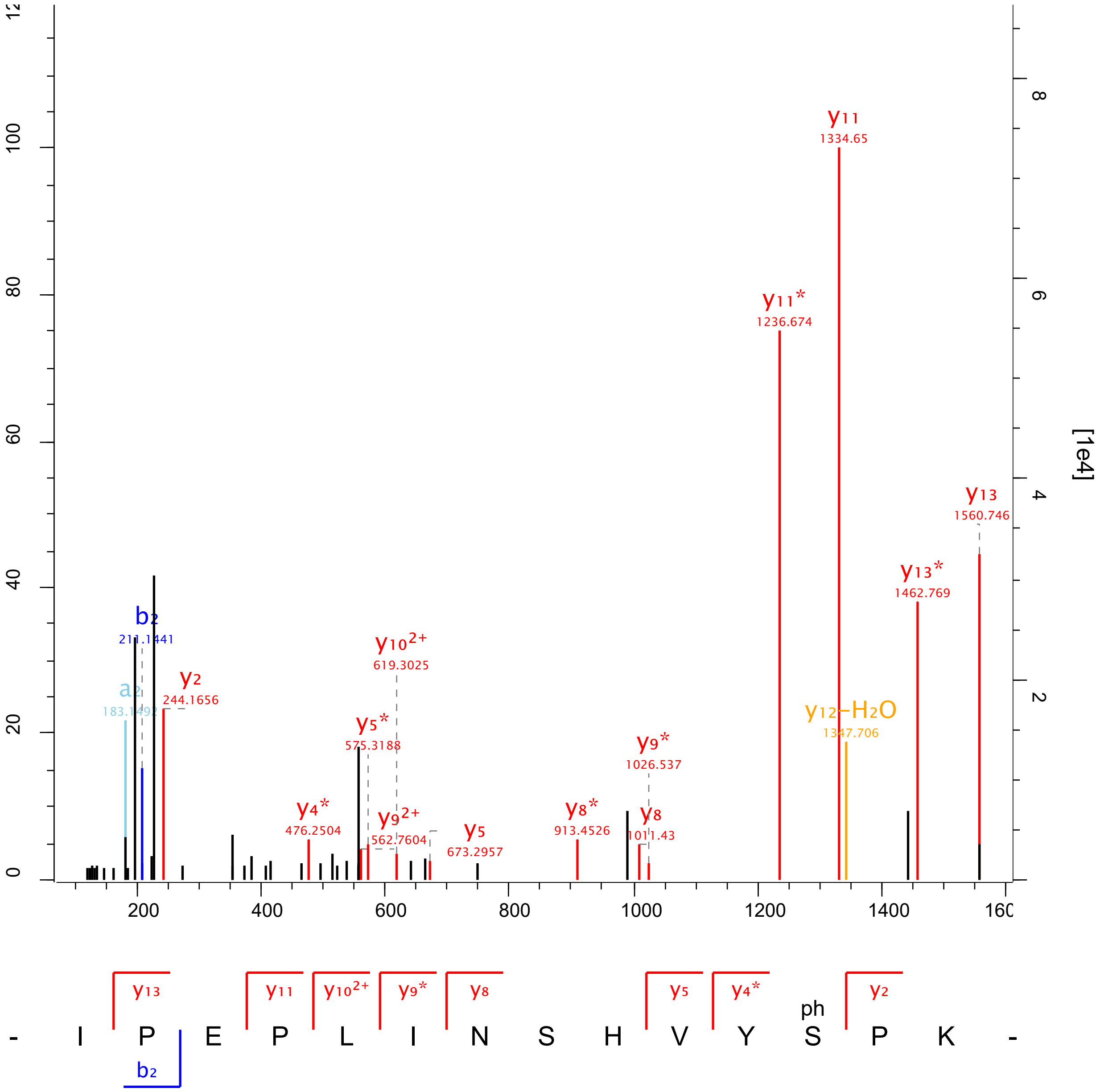

Raw file	Scan	Method	Score	m/z
sys_15_1	24104	FTMS; HCD	95.67	775.03

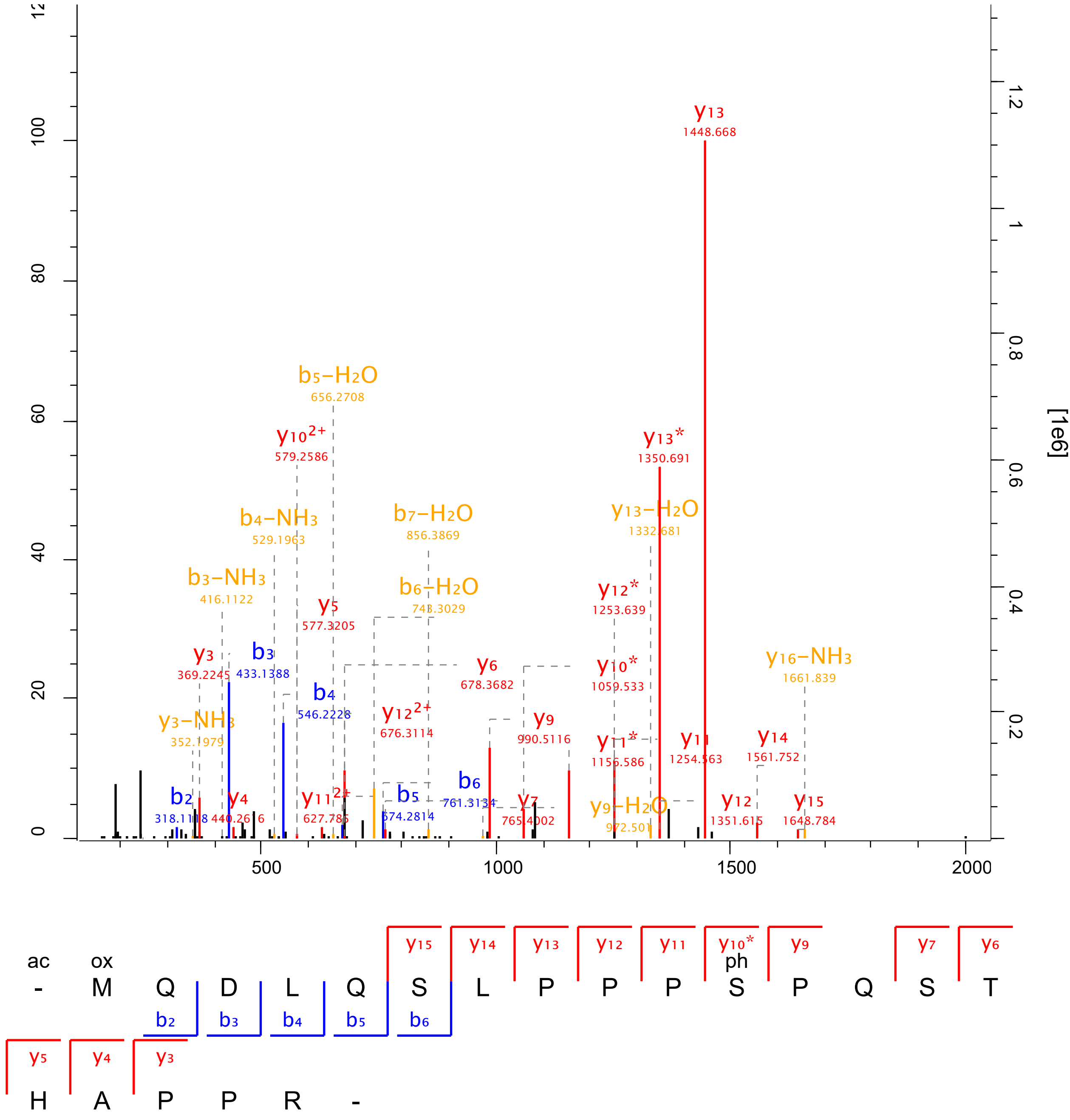

Raw file	Scan	Method	Score	m/z
sys_15_1	24237	FTMS; HCD	57.29	749.33

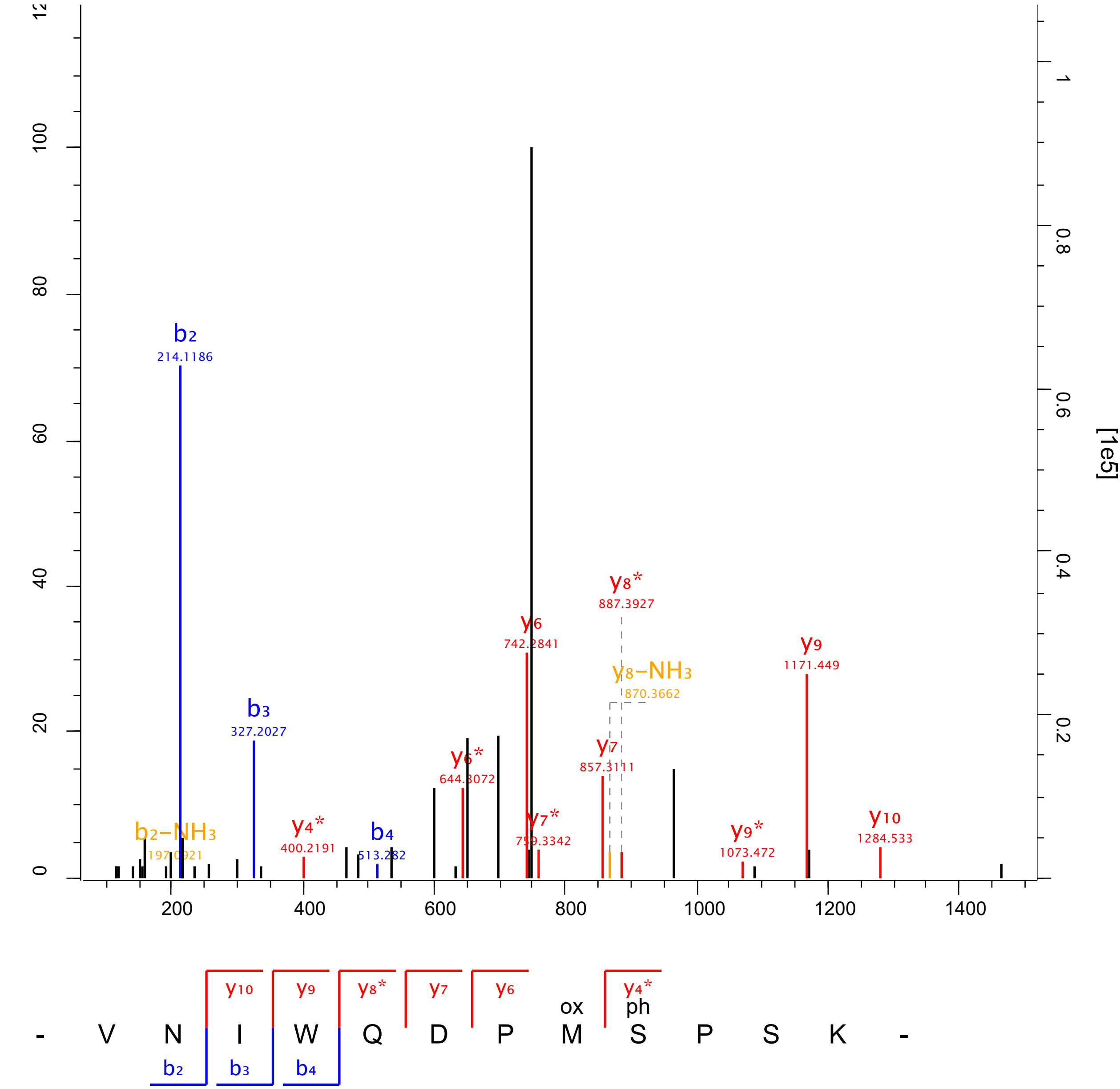

Raw file	Scan	Method	Score	m/z
sys_15_1	24322	FTMS; HCD	78.81	674.8

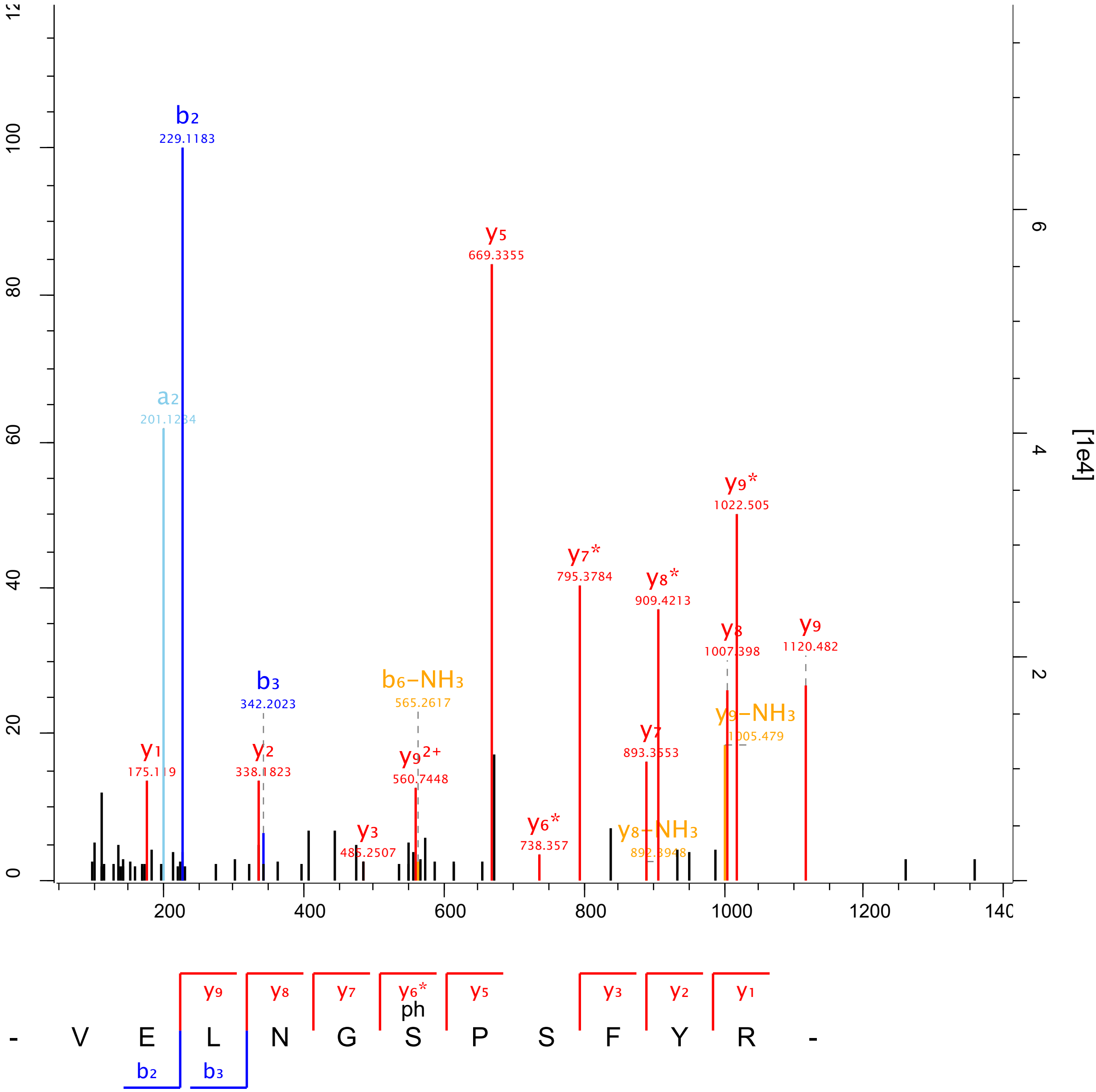

Raw file	Scan	Method	Score	m/z
sys_15_1	24333	FTMS; HCD	65.23	794.84

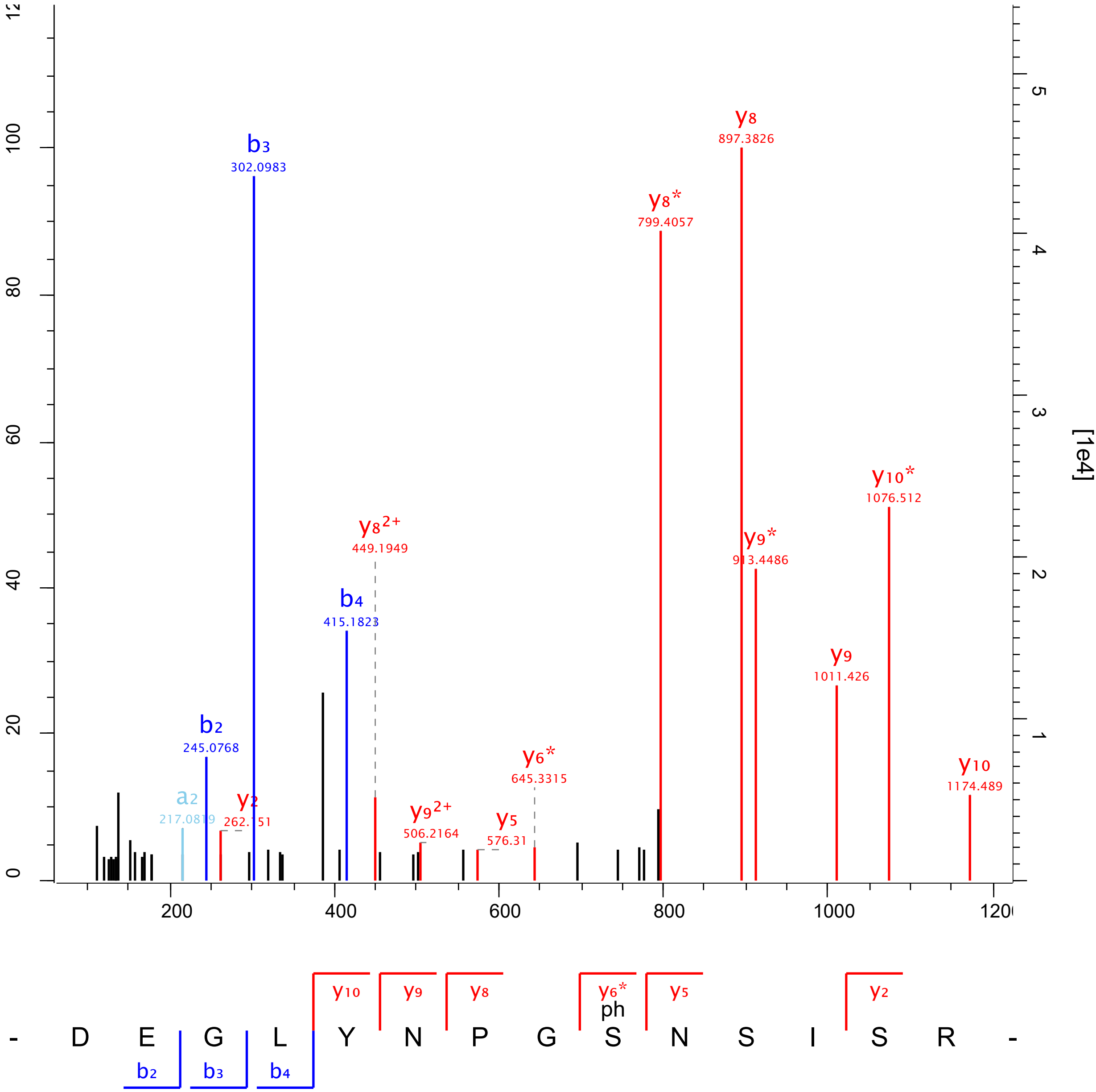

Raw file	Scan	Method	Score	m/z
sys_15_1	24374	FTMS; HCD	71.88	803.69

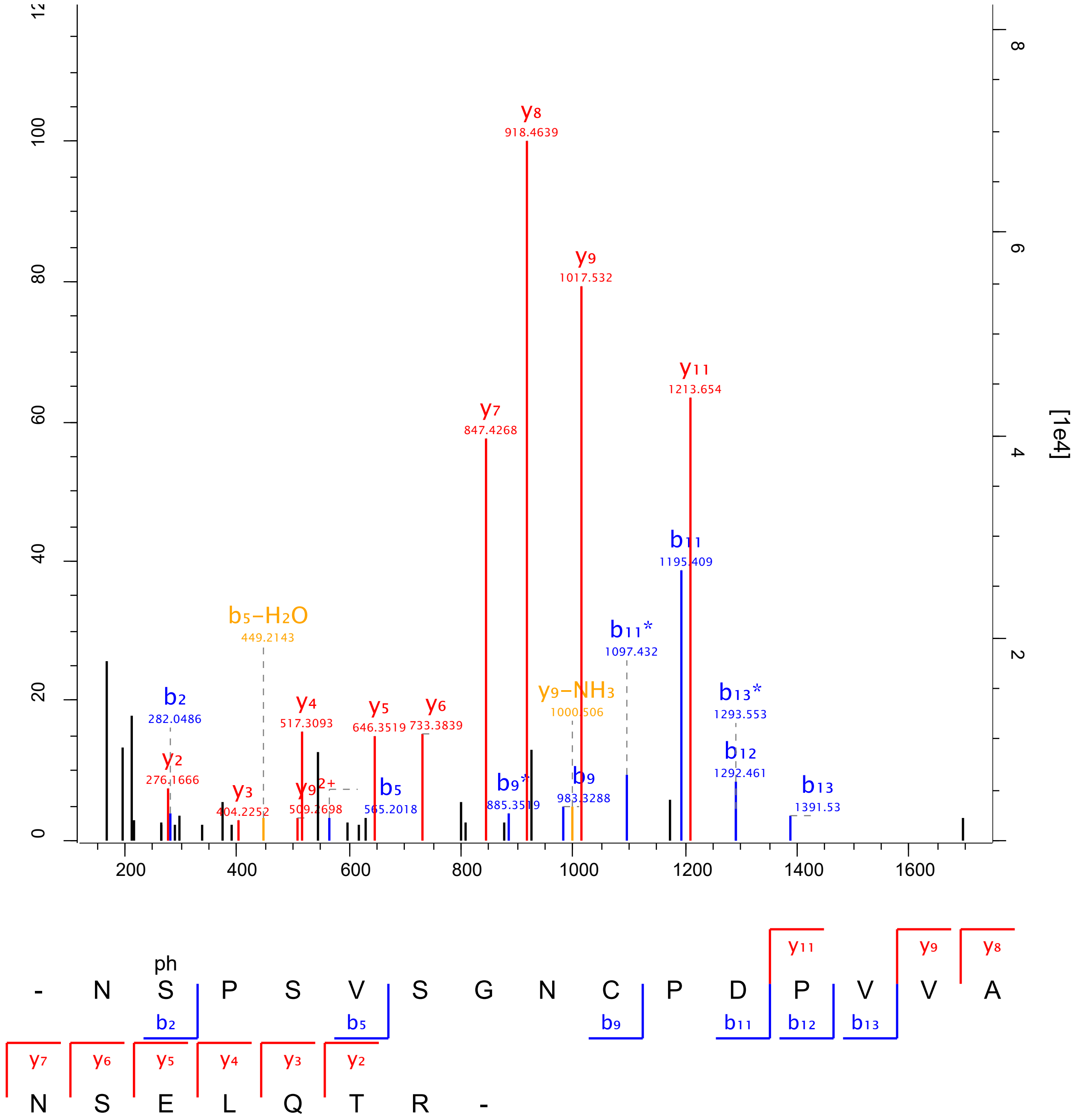

Raw file	Scan	Method	Score	m/z
sys_15_1	24464	FTMS; HCD	238.88	713.84

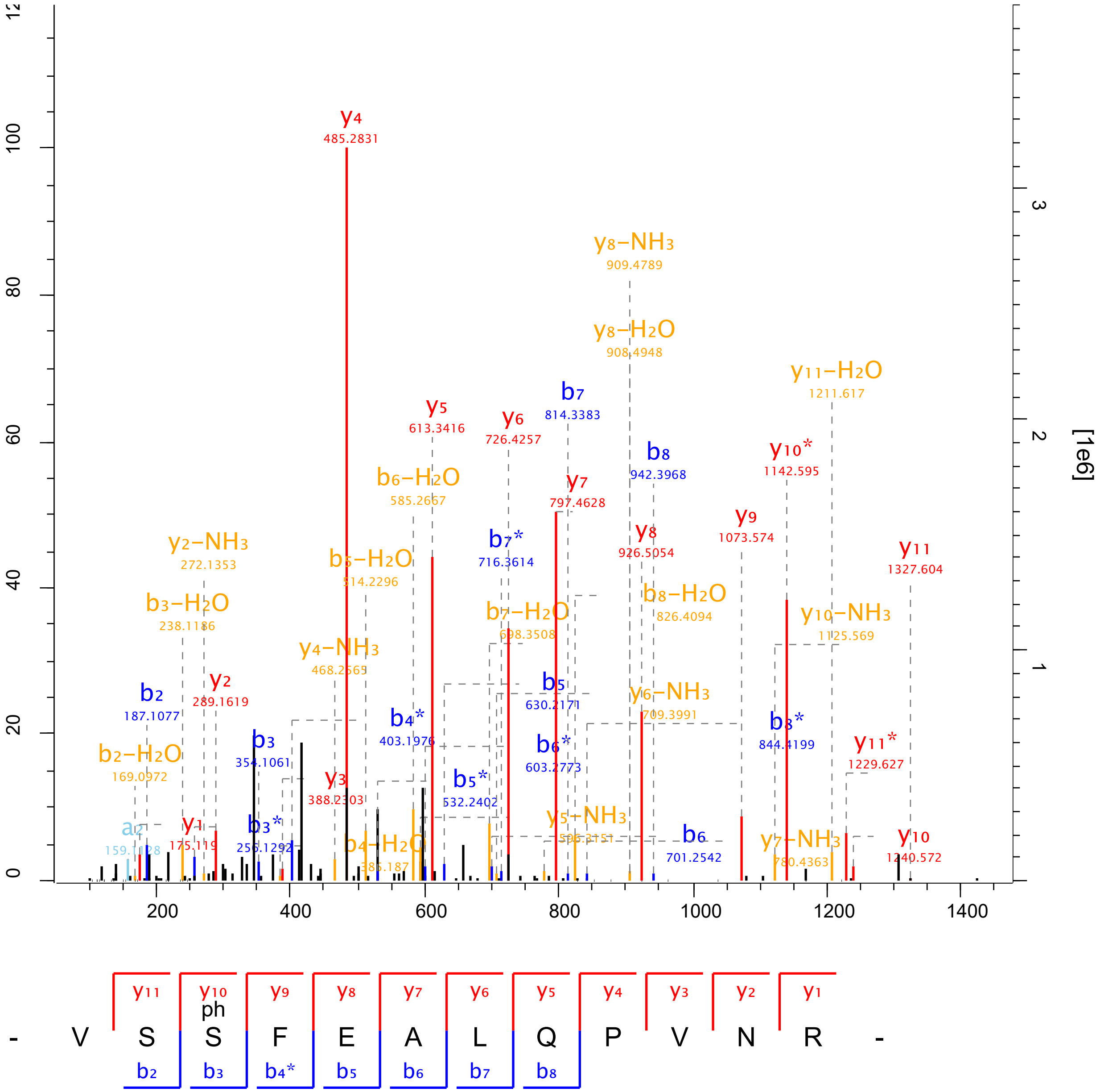

Raw file	Scan	Method	Score	m/z
sys_15_1	24523	FTMS; HCD	127.83	740.27

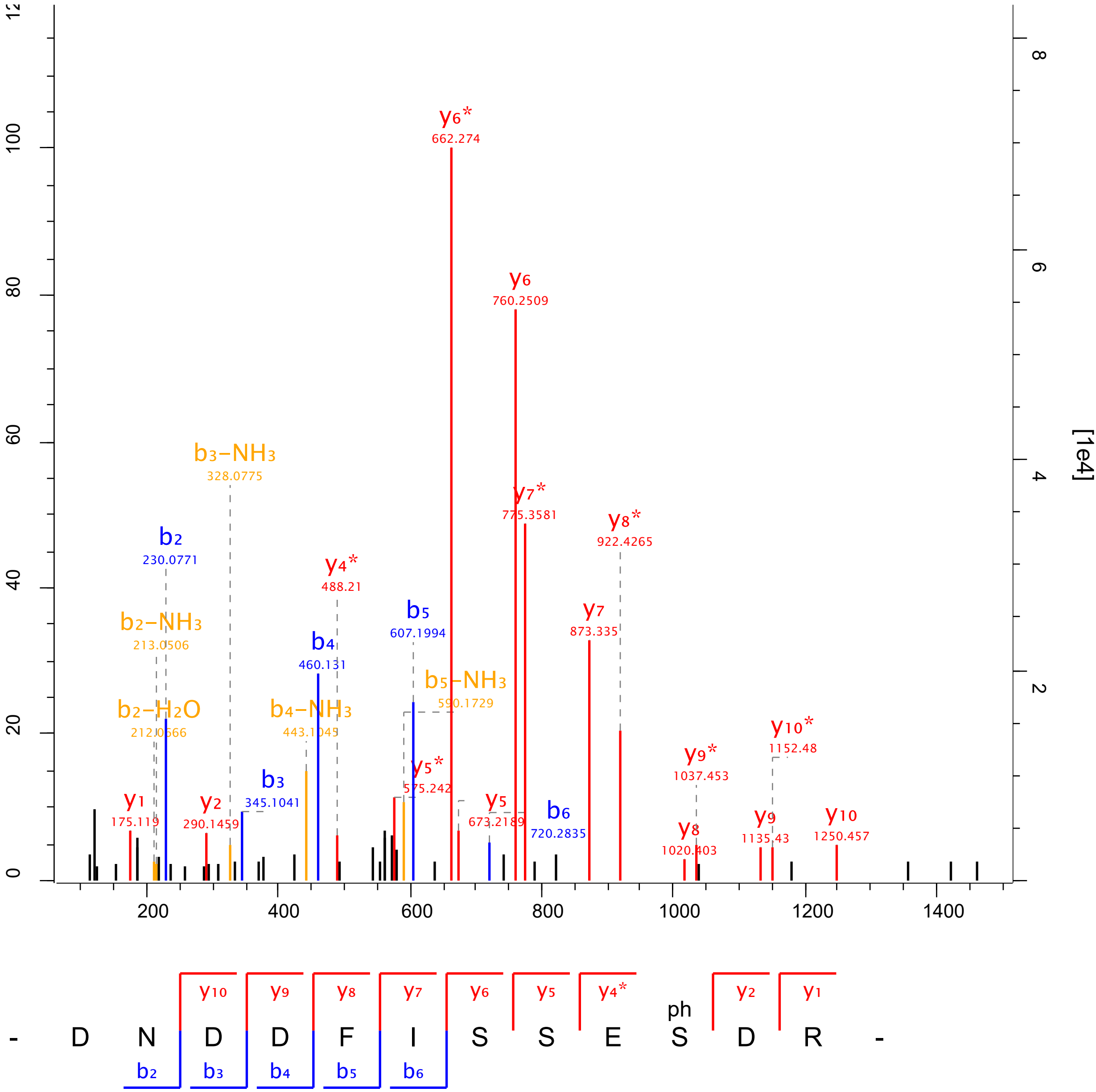

Supplement: Supplementary Figure S2-6 [file 143488_2_supp_337928_ps5hgj.pdf]
