# Supplementary material for: The Systemin Signaling Cascade As Derived from Time Course Analyses of the Systemin-responsive Phosphoproteome
Source: Mol Cell Proteomics. 2019 May 28;18(8):1526–42. doi: 10.1074/mcp.RA119.001367 (PMC6683004; doi:10.1074/mcp.RA119.001367)

Supplementary Figure 2: Representative annotated spectra of identified phosphopeptides under systemin,A17 and water treatment as exported from MaxQuant.

Raw file	Scan	Method	Score	m/z
sys_15_1	24677	FTMS; HCD	46.42	743.68

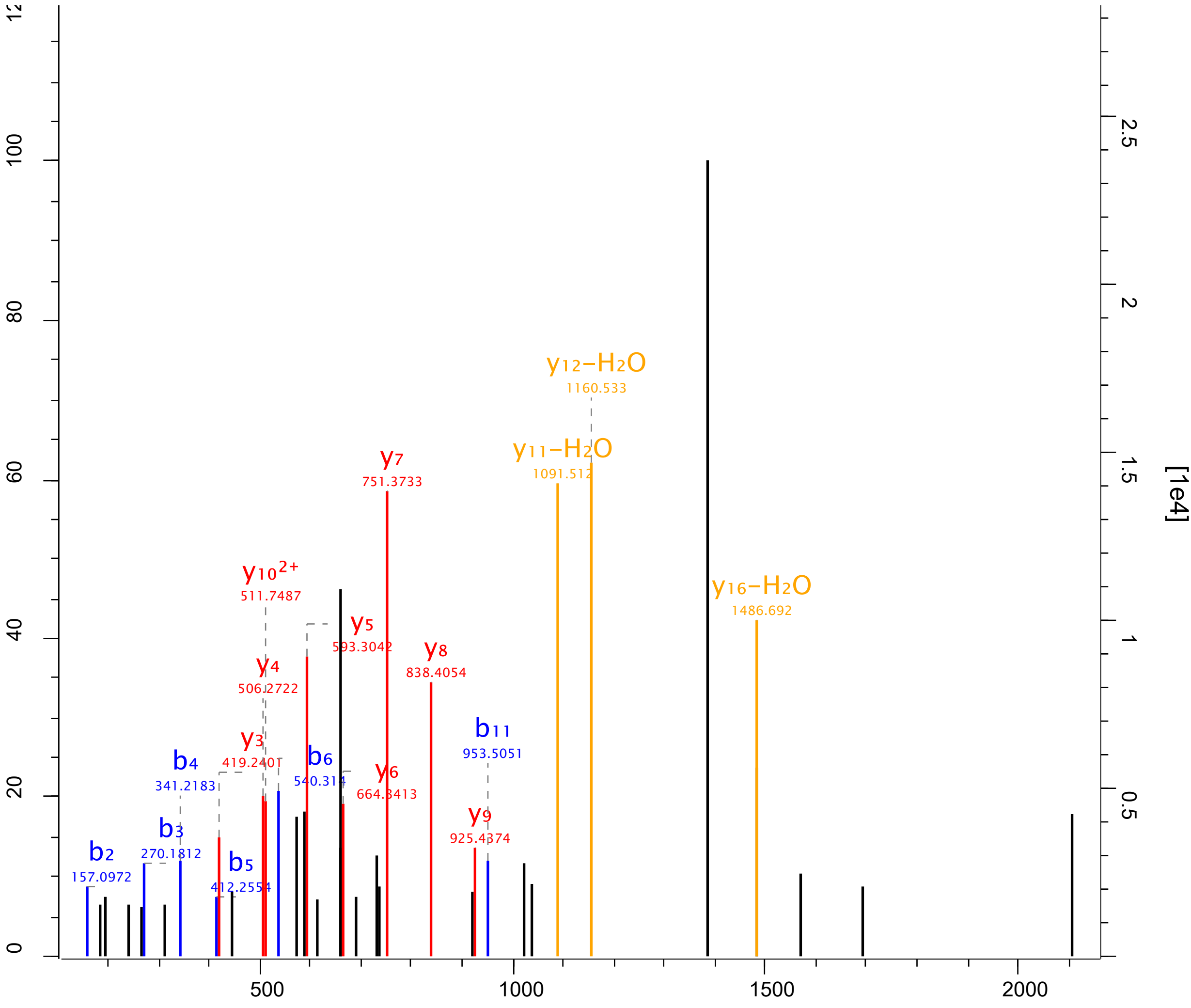

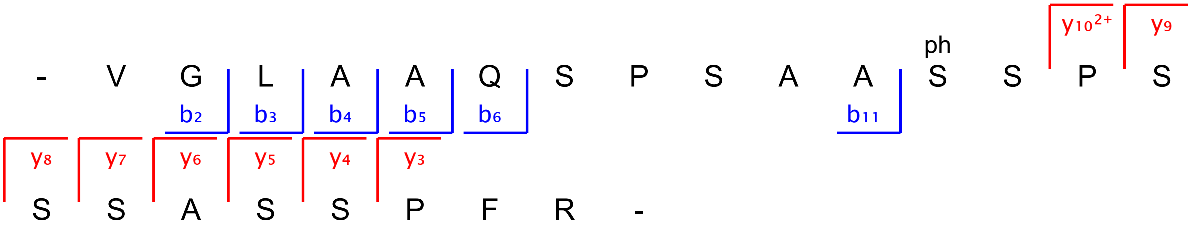

Raw file	Scan	Method	Score	m/z
sys_15_1	24740	FTMS; HCD	116.08	832.02

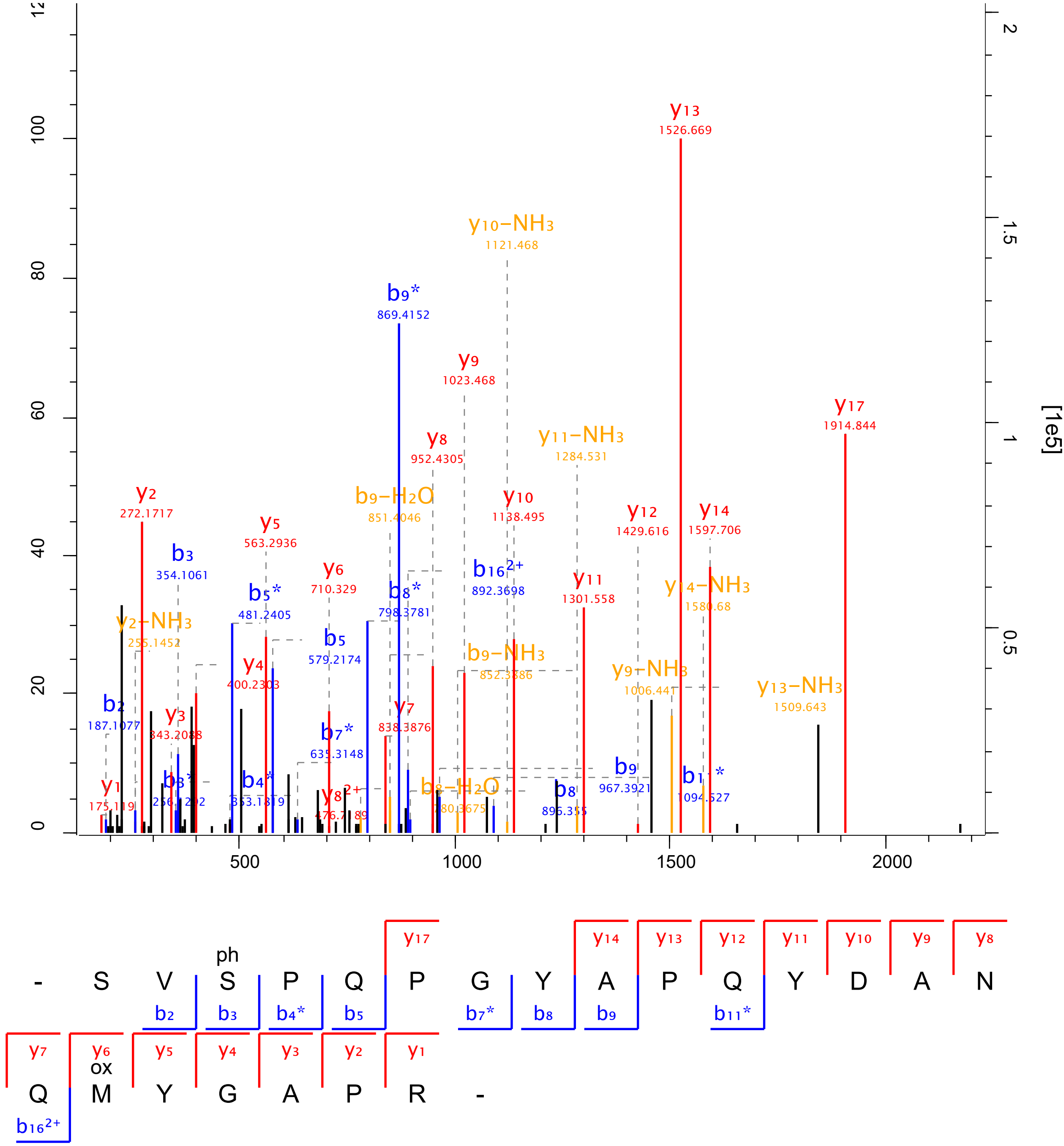

Raw file	Scan	Method	Score	m/z
sys_15_1	24741	FTMS; HCD	99.09	895.88

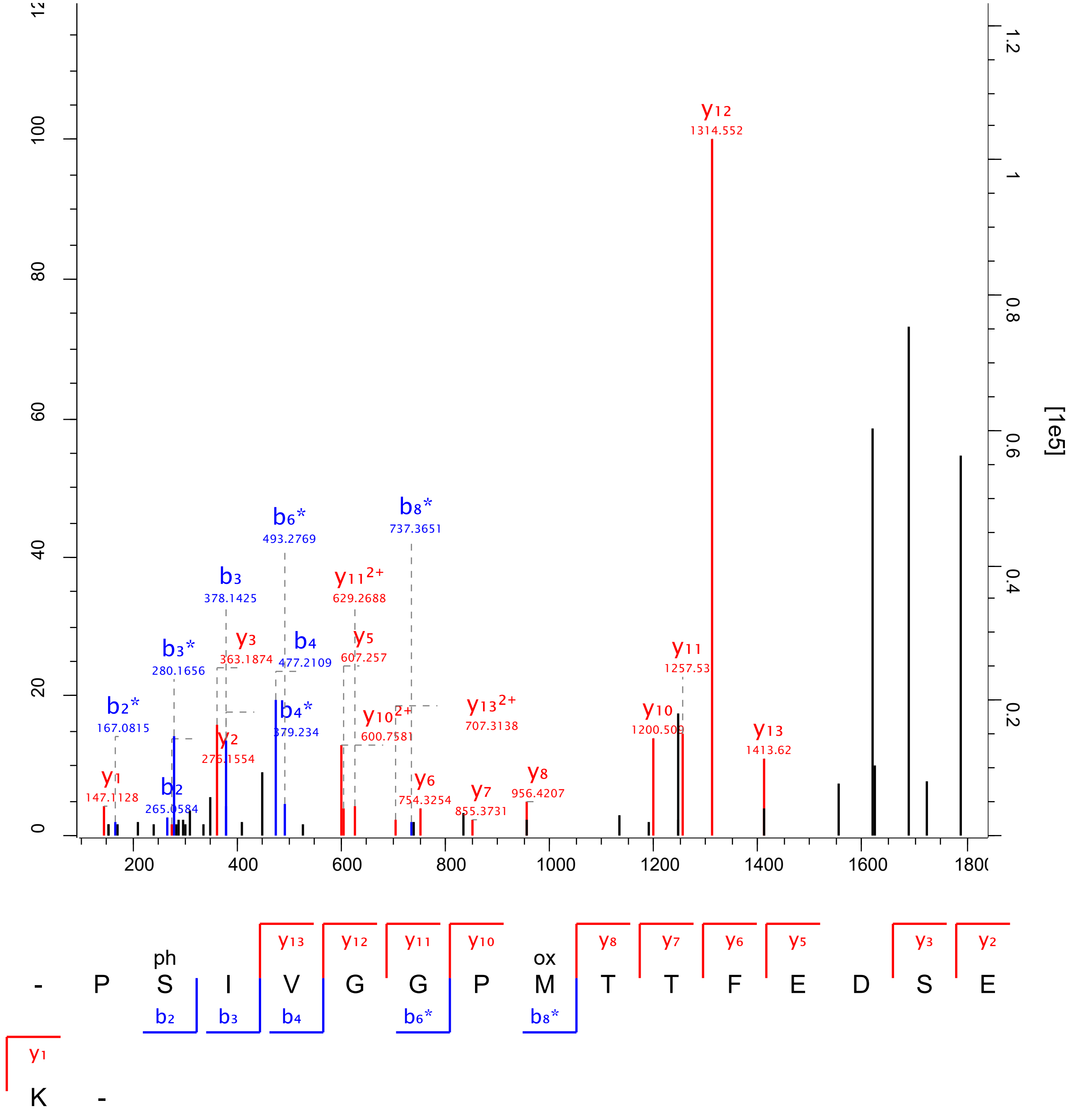

Raw file	Scan	Method	Score	m/z
sys_15_1	24756	FTMS; HCD	73.29	550.61

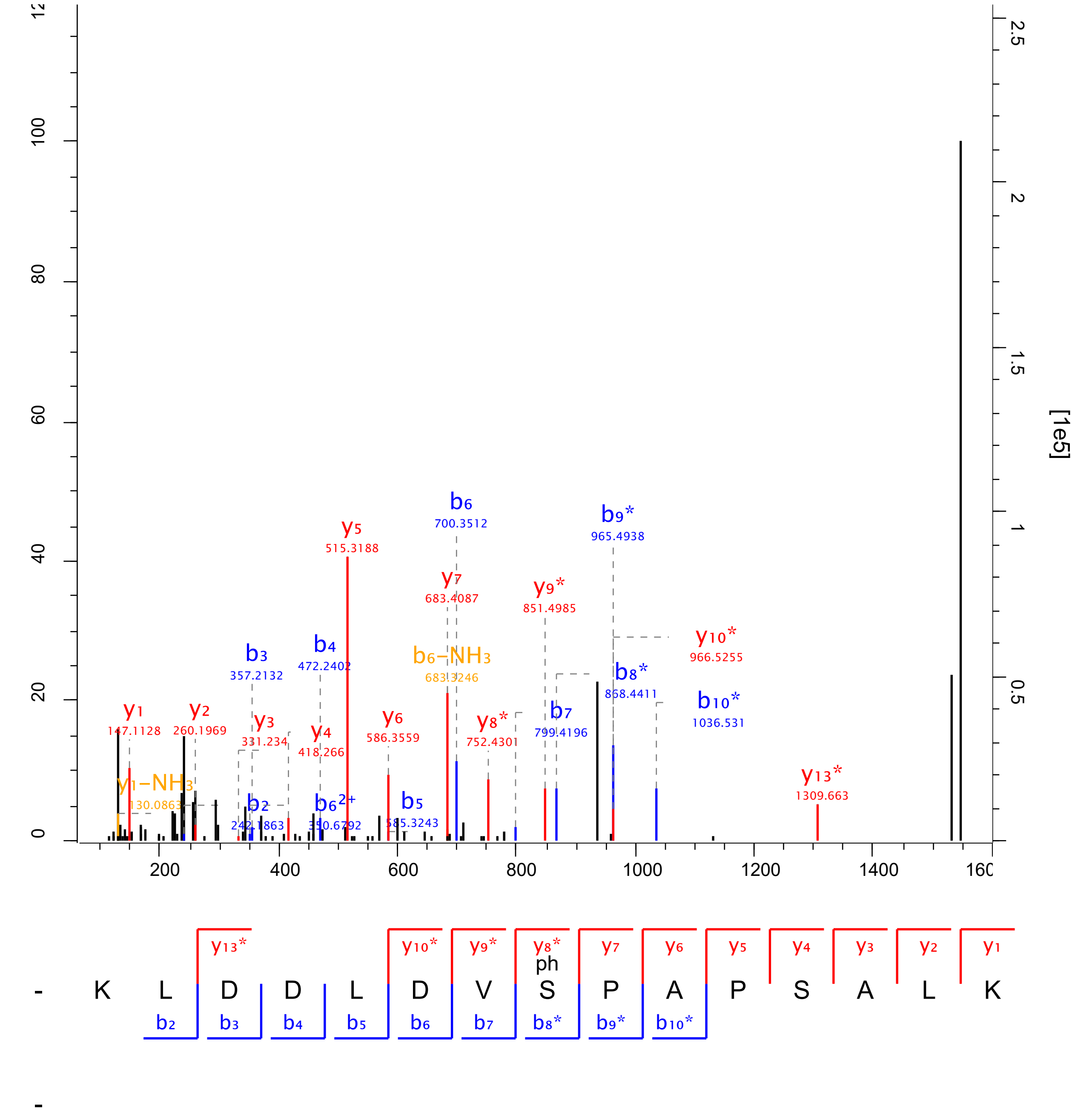

Raw file	Scan	Method	Score	m/z
sys_15_1	24885	FTMS; HCD	66.89	875.39

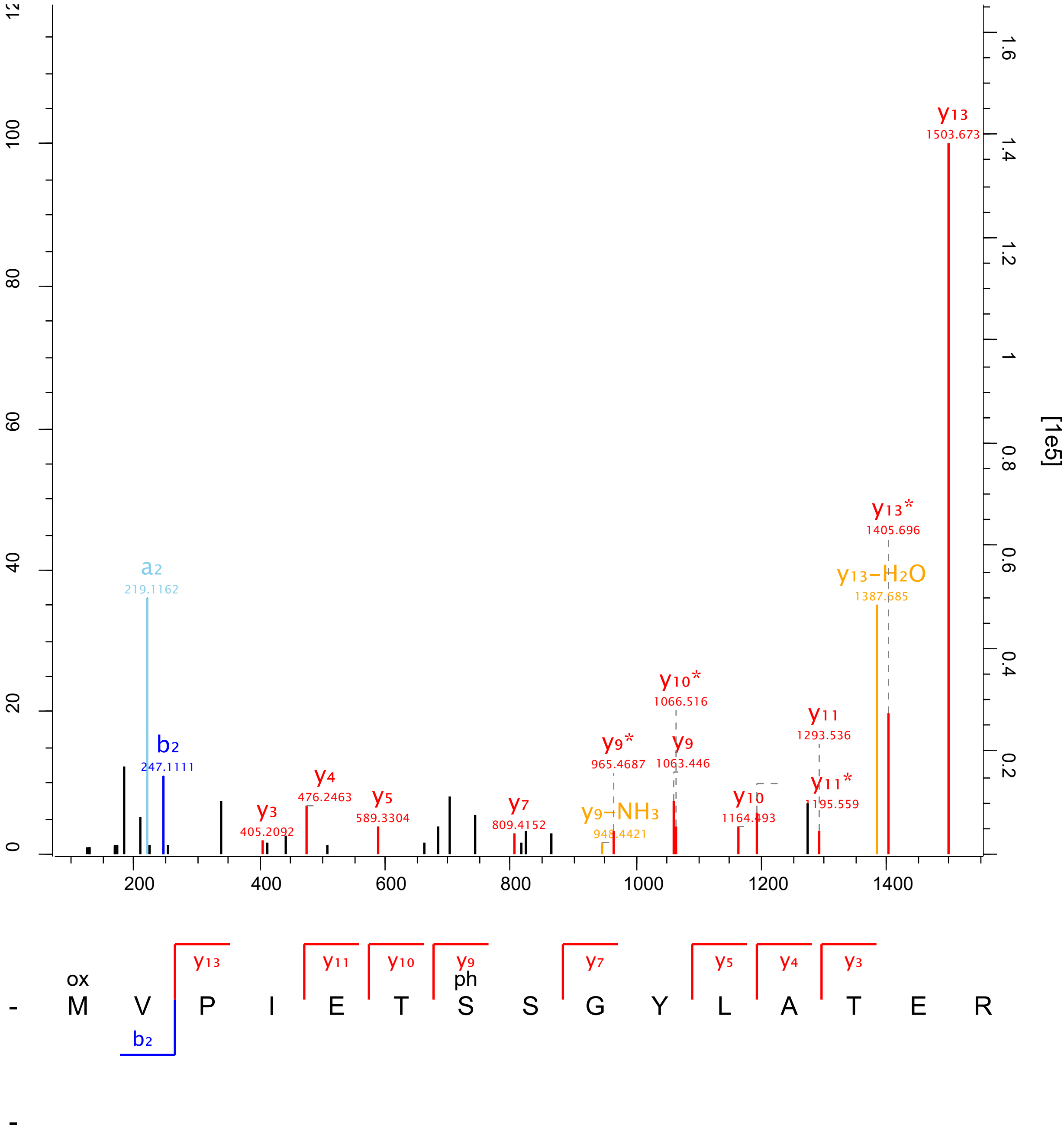

Raw file	Scan	Method	Score	m/z
sys_15_1	24947	FTMS; HCD	100.11	696.28

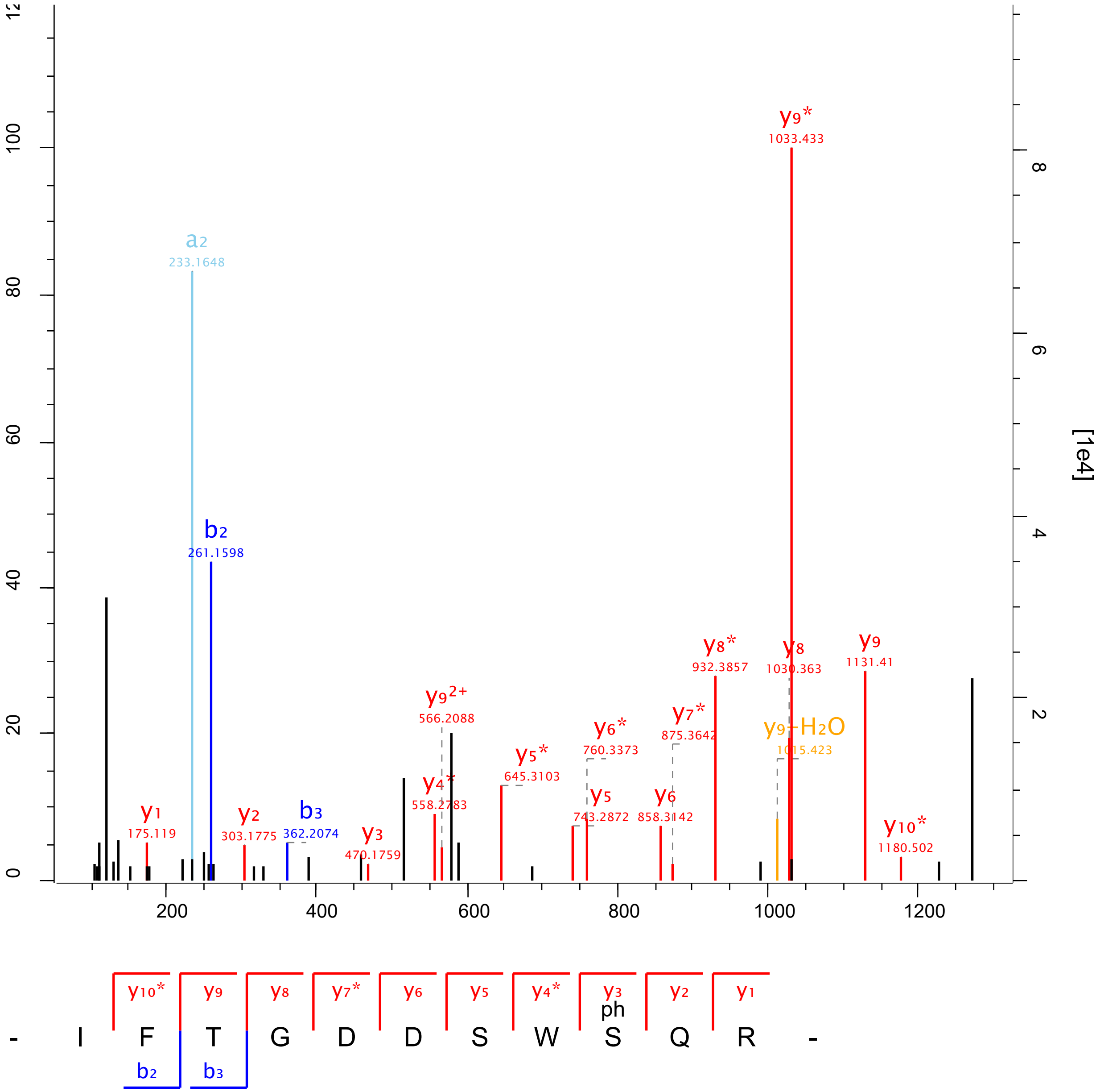

Raw file	Scan	Method	Score	m/z
sys_15_1	25000	FTMS; HCD	42.03	642.31

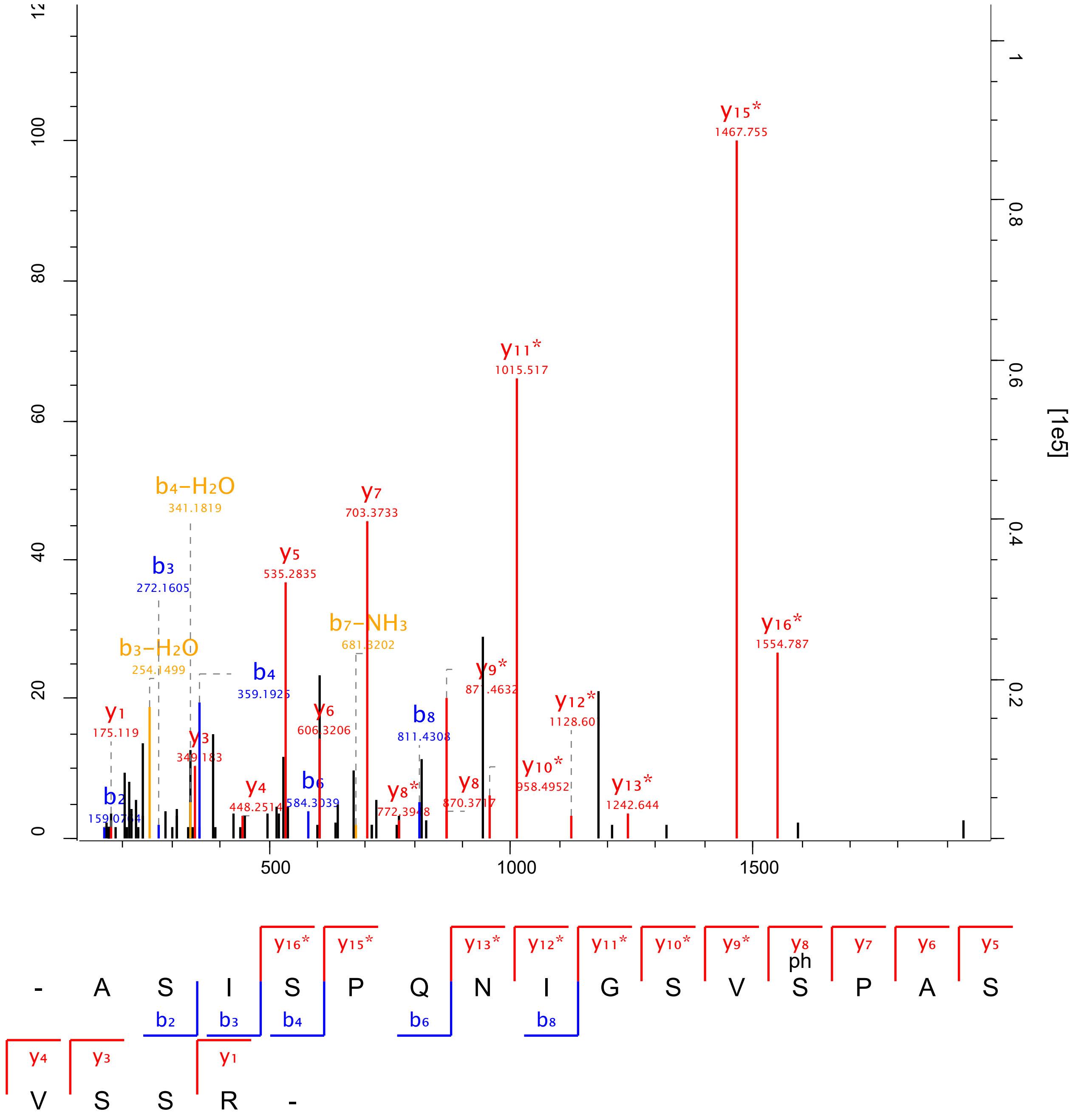

Raw file	Scan	Method	Score	m/z
sys_15_1	25112	FTMS; HCD	58.95	691.63

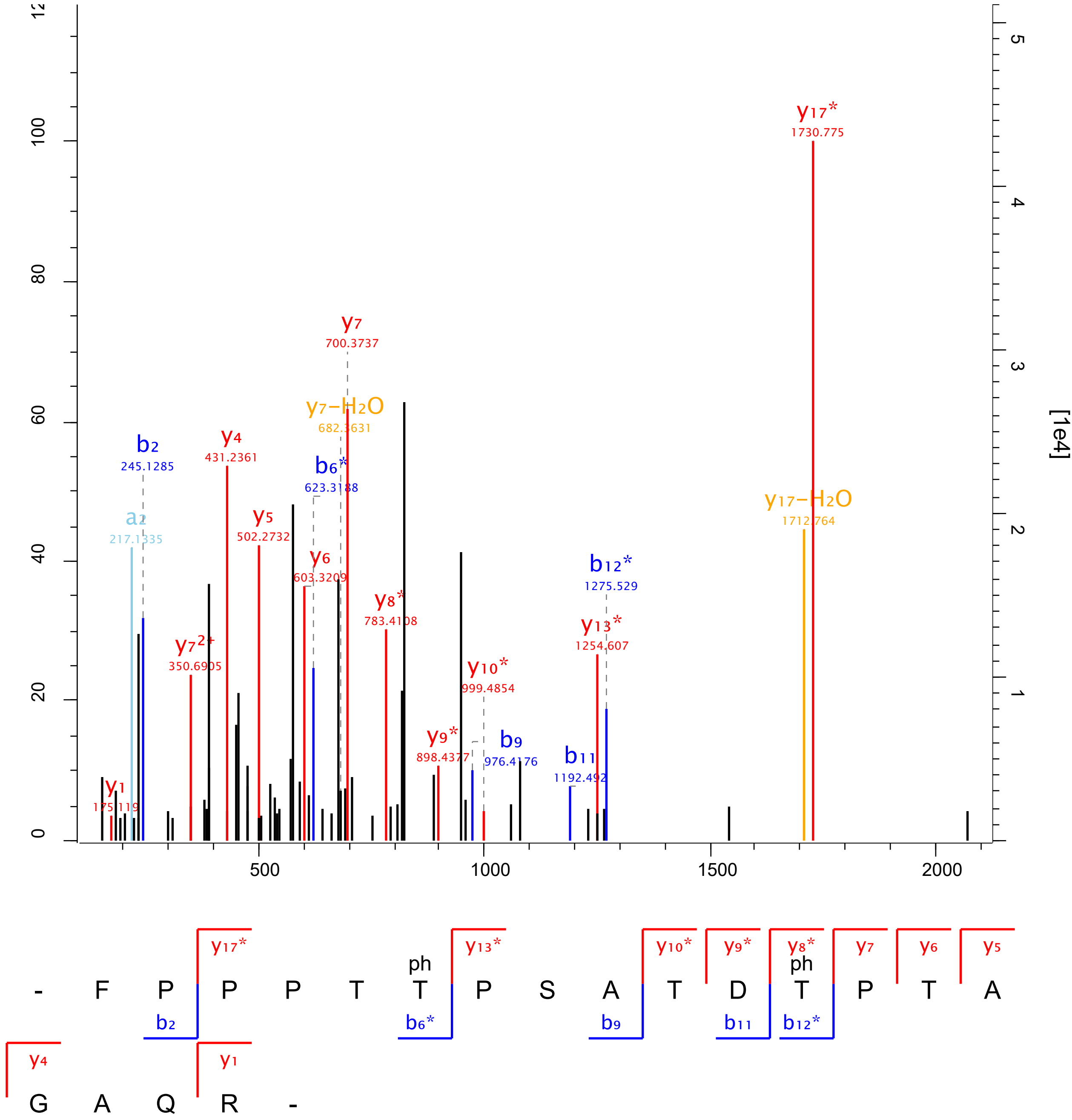

Raw file	Scan	Method	Score	m/z
sys_15_1	25171	FTMS; HCD	288.96	949.39

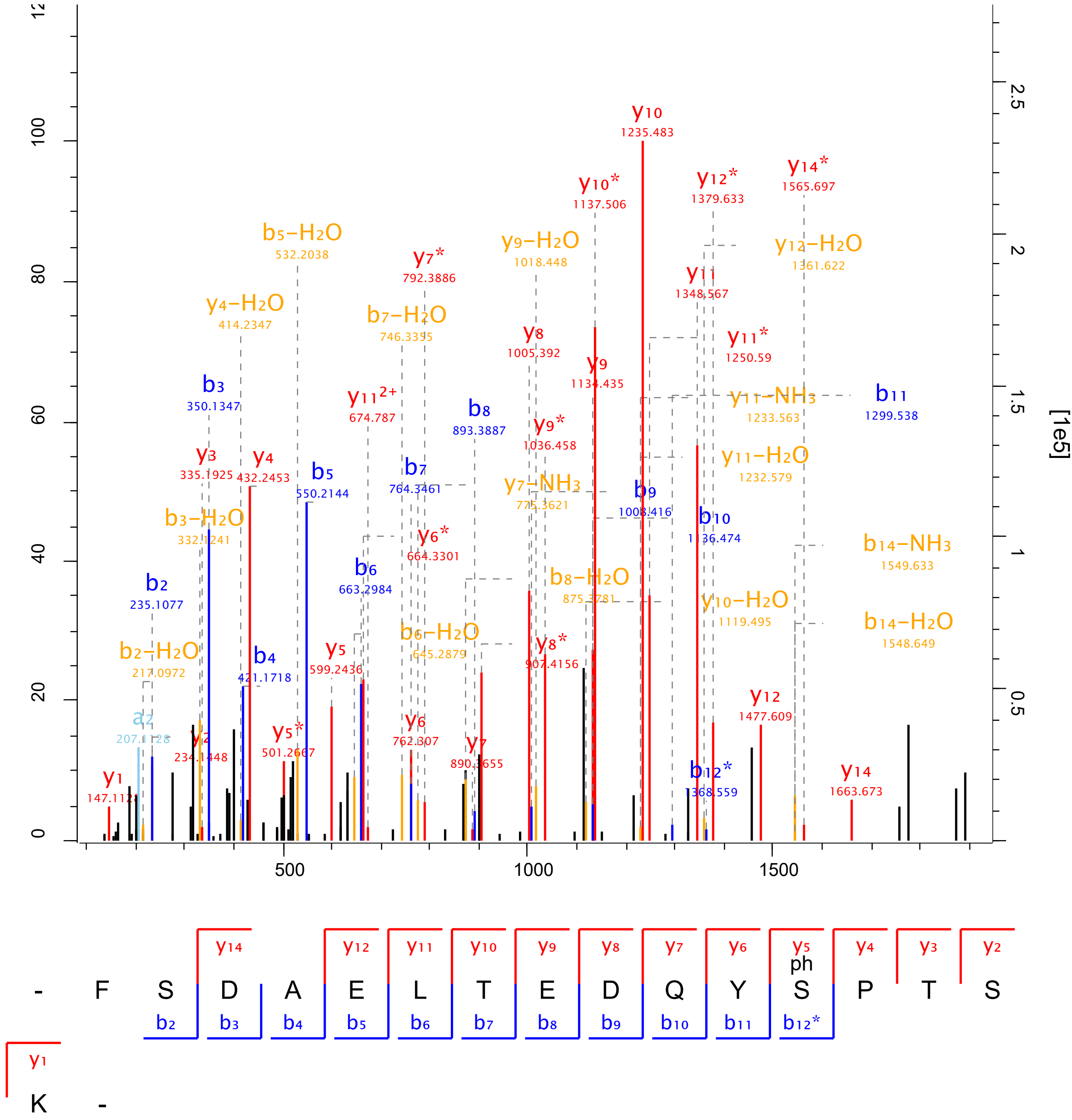

Raw file	Scan	Method	Score	m/z
sys_15_1	25239	FTMS; HCD	159.18	643.81

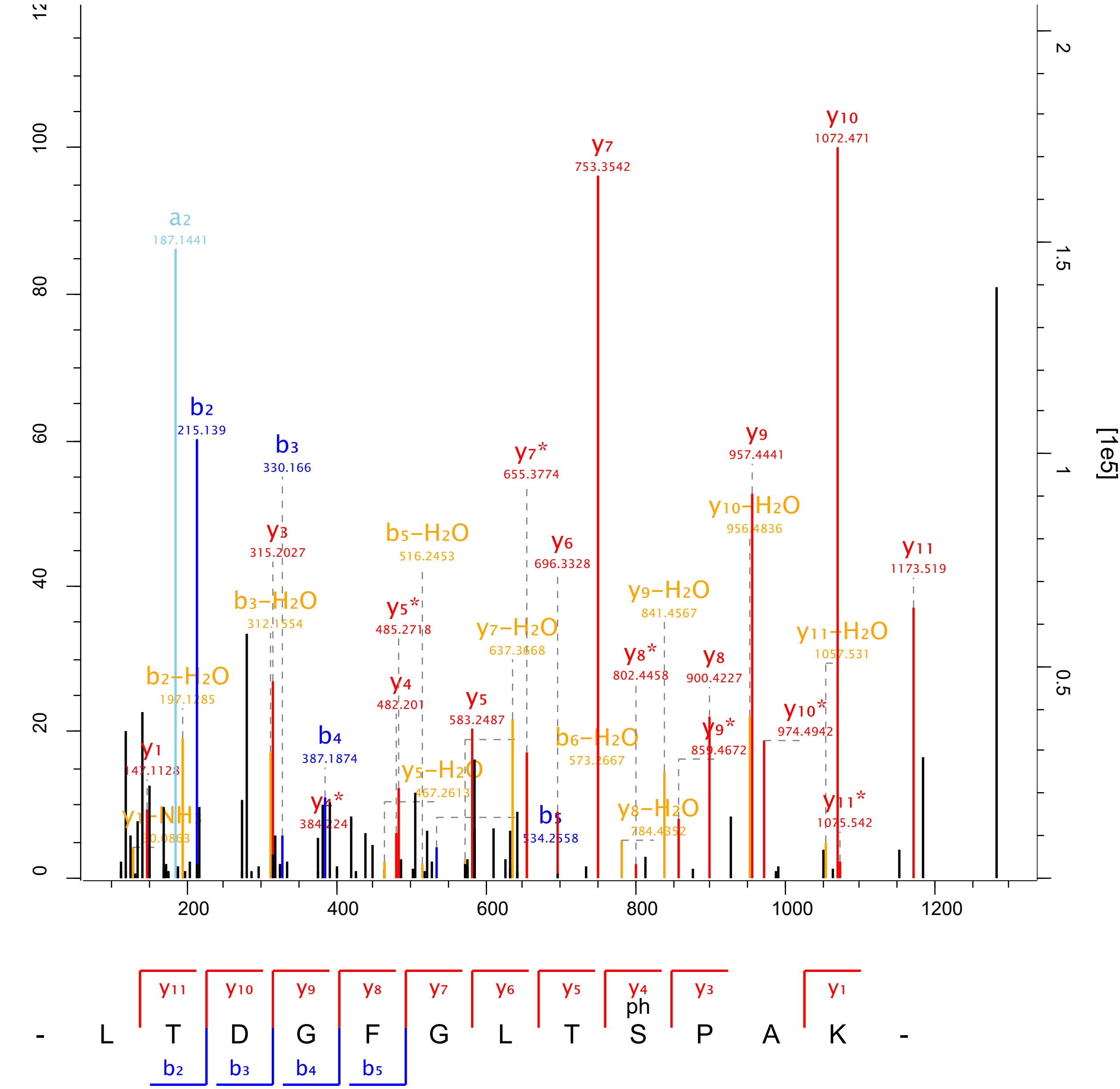

Raw file	Scan	Method	Score	m/z
sys_15_1	25246	FTMS; HCD	106.93	748.84

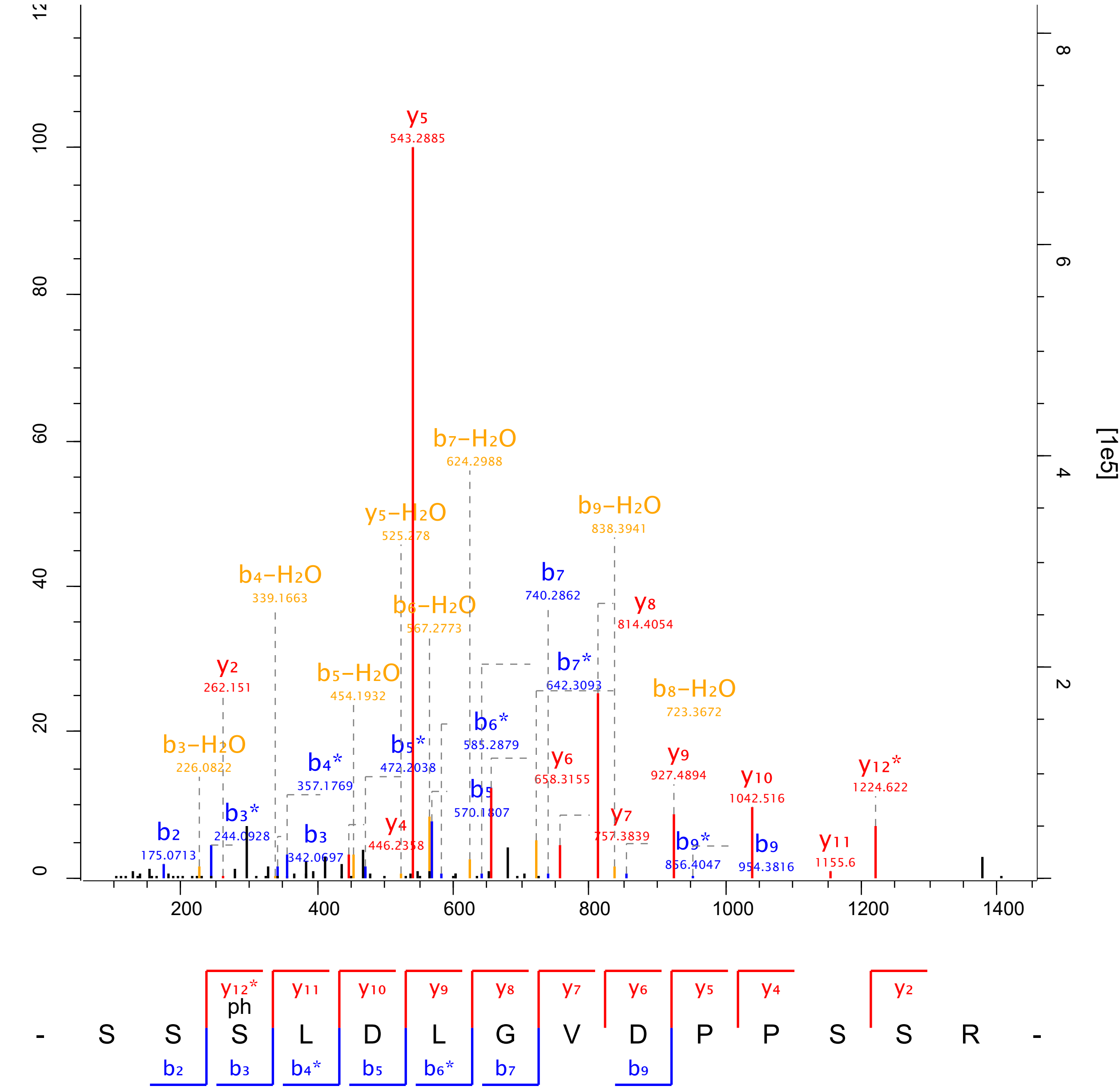

Raw file	Scan	Method	Score	m/z
sys_15_1	25285	FTMS; HCD	101.53	814.85

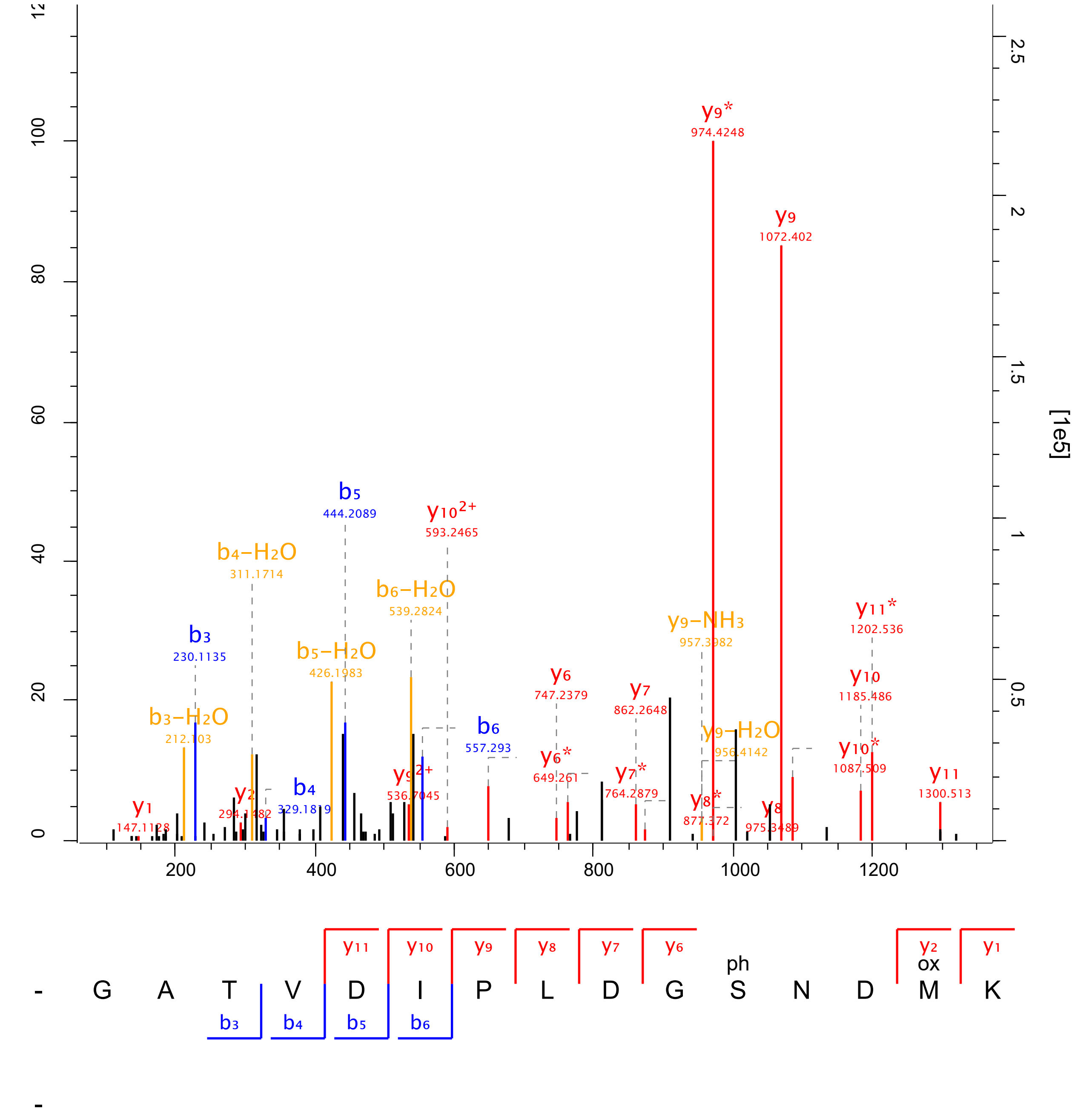

Raw file	Scan	Method	Score	m/z
sys_15_1	25499	FTMS; HCD	82.89	1159.97

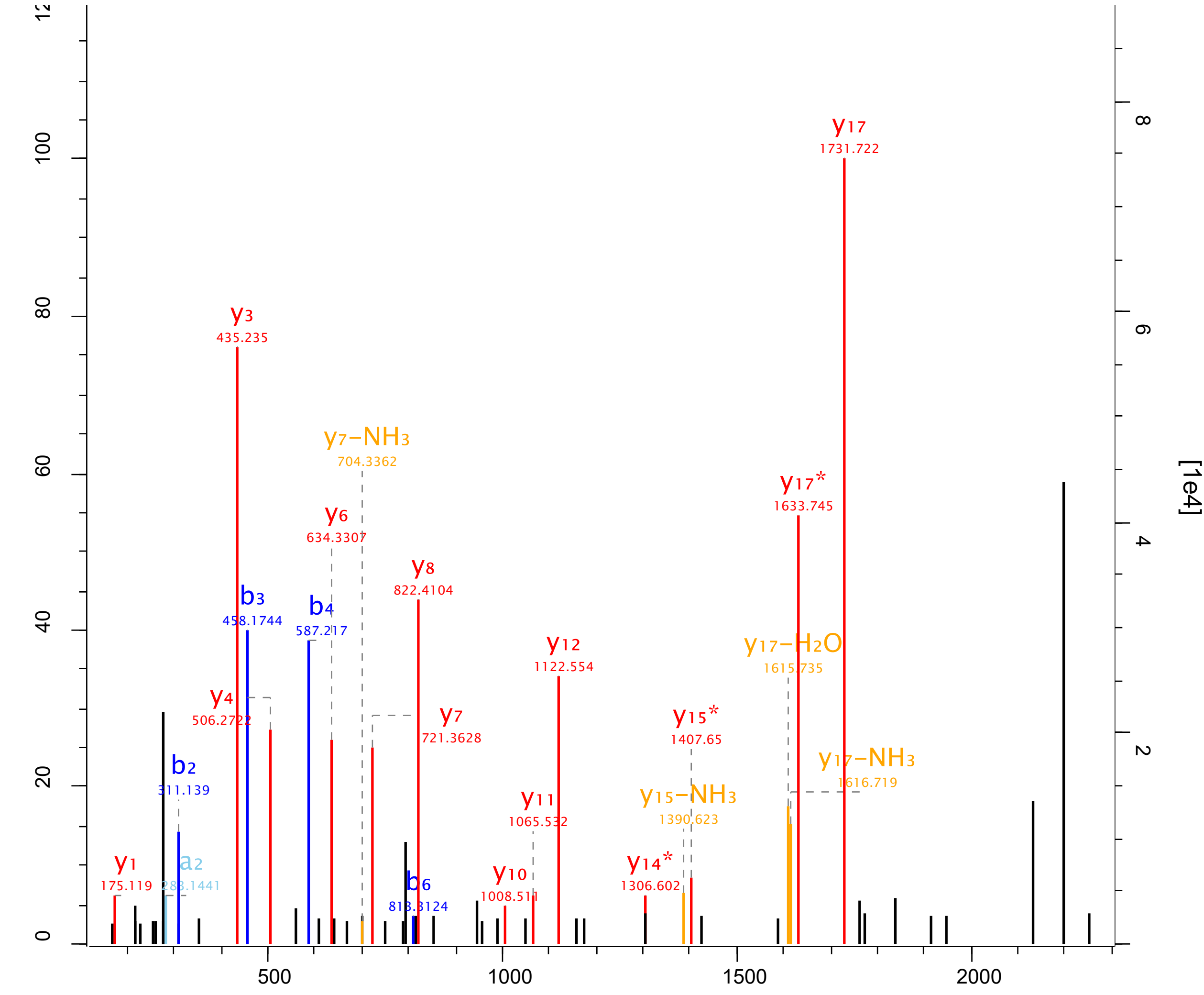

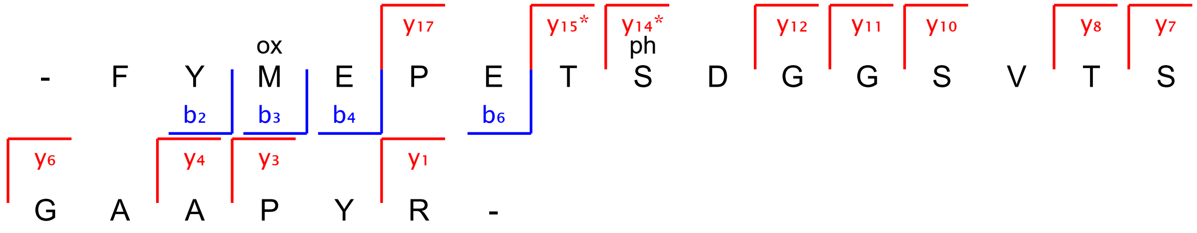

Raw file	Scan	Method	Score	m/z
sys_15_1	25504	FTMS; HCD	110.97	765.81

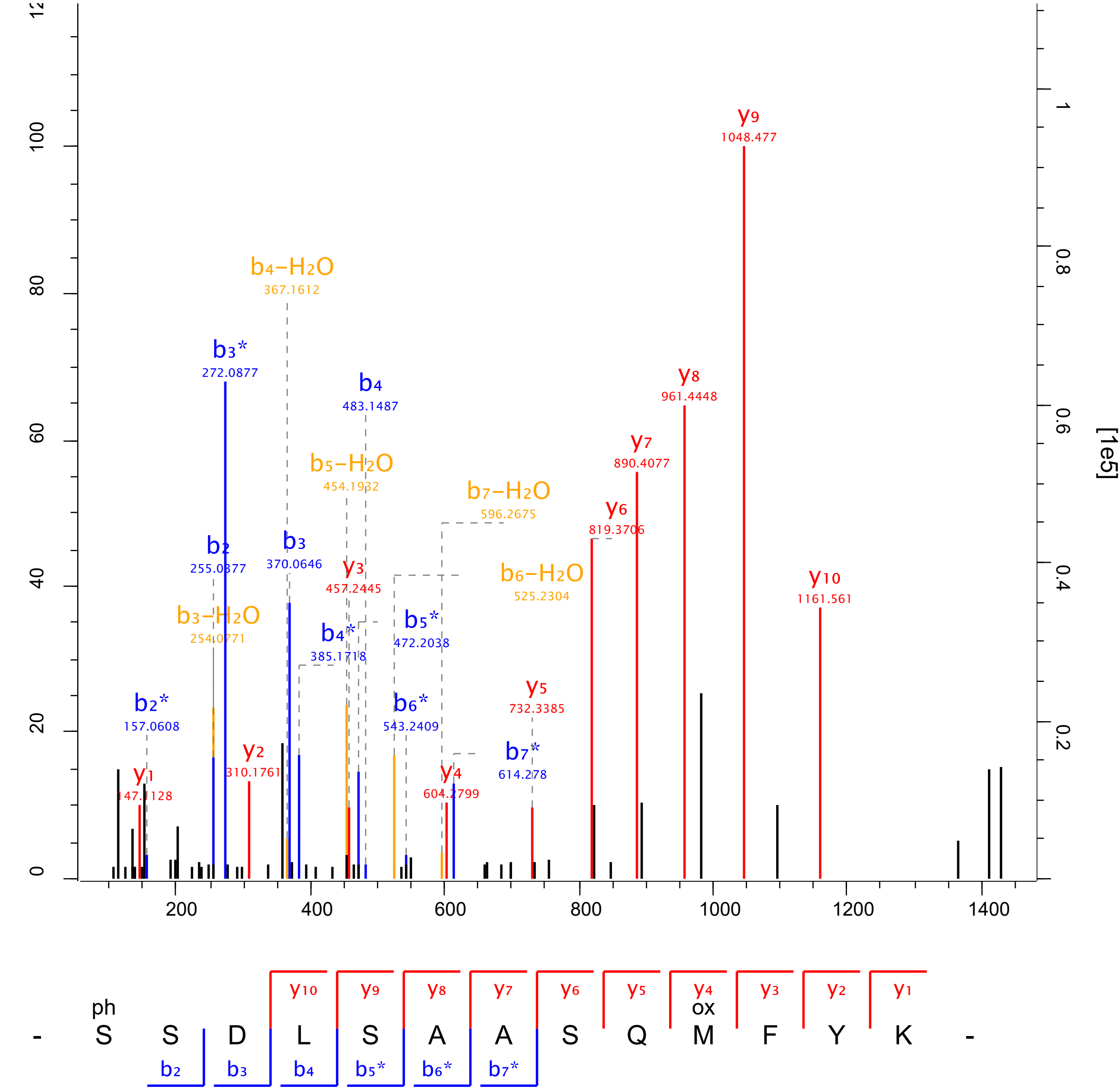

Raw file	Scan	Method	Score	m/z
sys_15_1	25545	FTMS; HCD	190.86	937.4

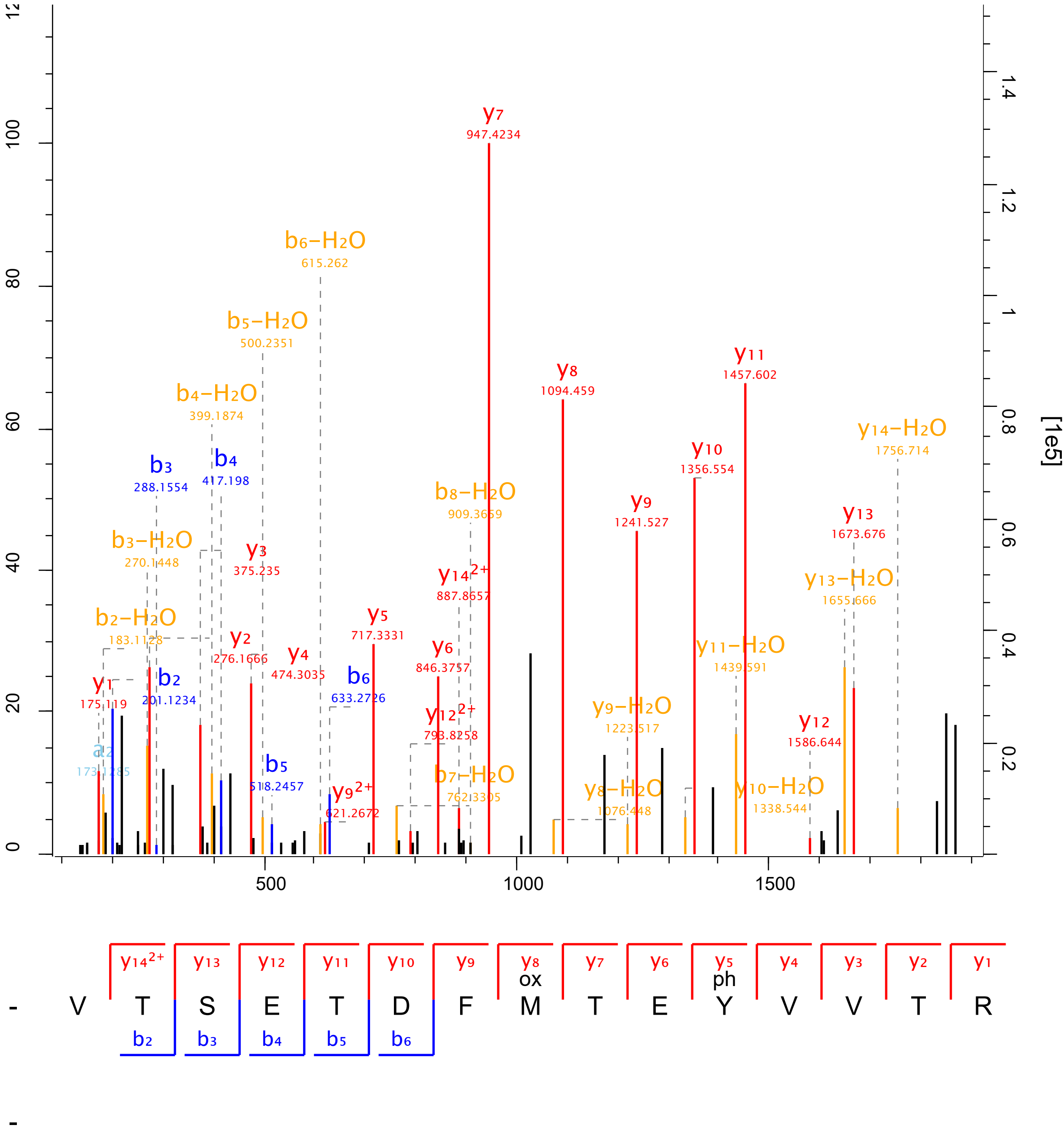

Raw file	Scan	Method	Score	m/z
sys_15_1	25788	FTMS; HCD	43.68	599.77

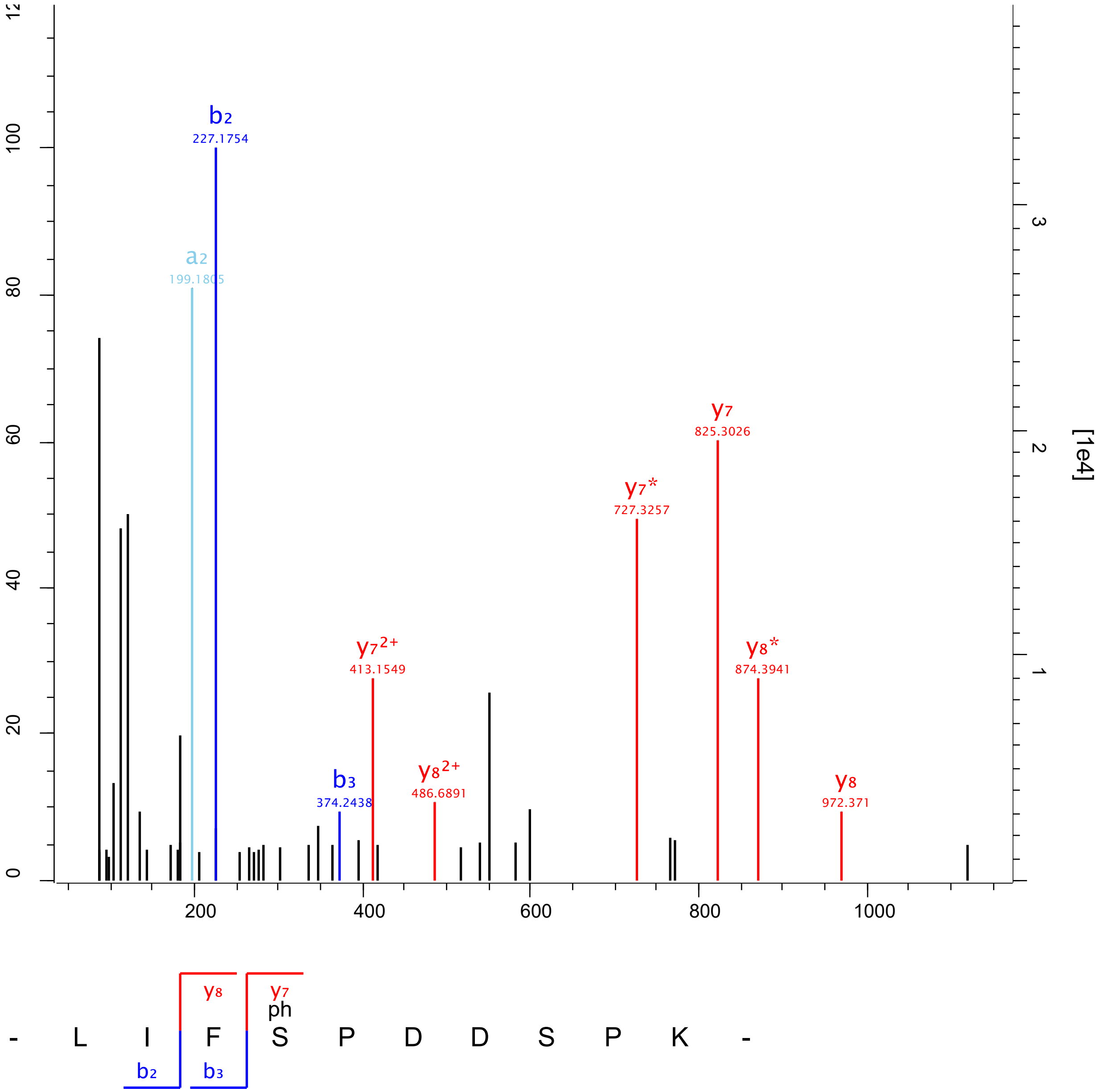

Raw file	Scan	Method	Score	m/z
sys_15_1	25982	FTMS; HCD	118.48	729.85

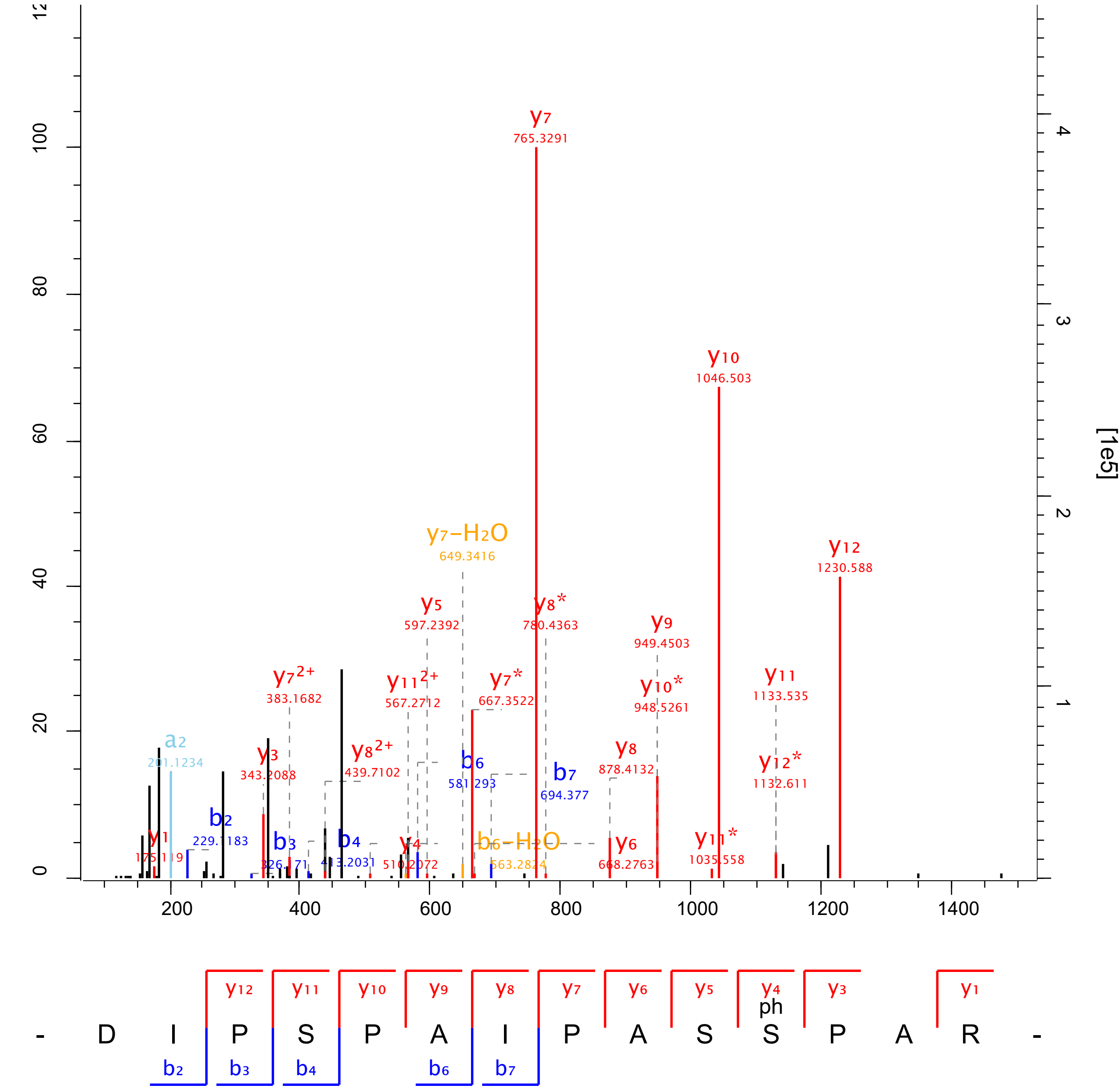

Raw file	Scan	Method	Score	m/z
sys_15_1	26076	FTMS; HCD	80.96	692.81

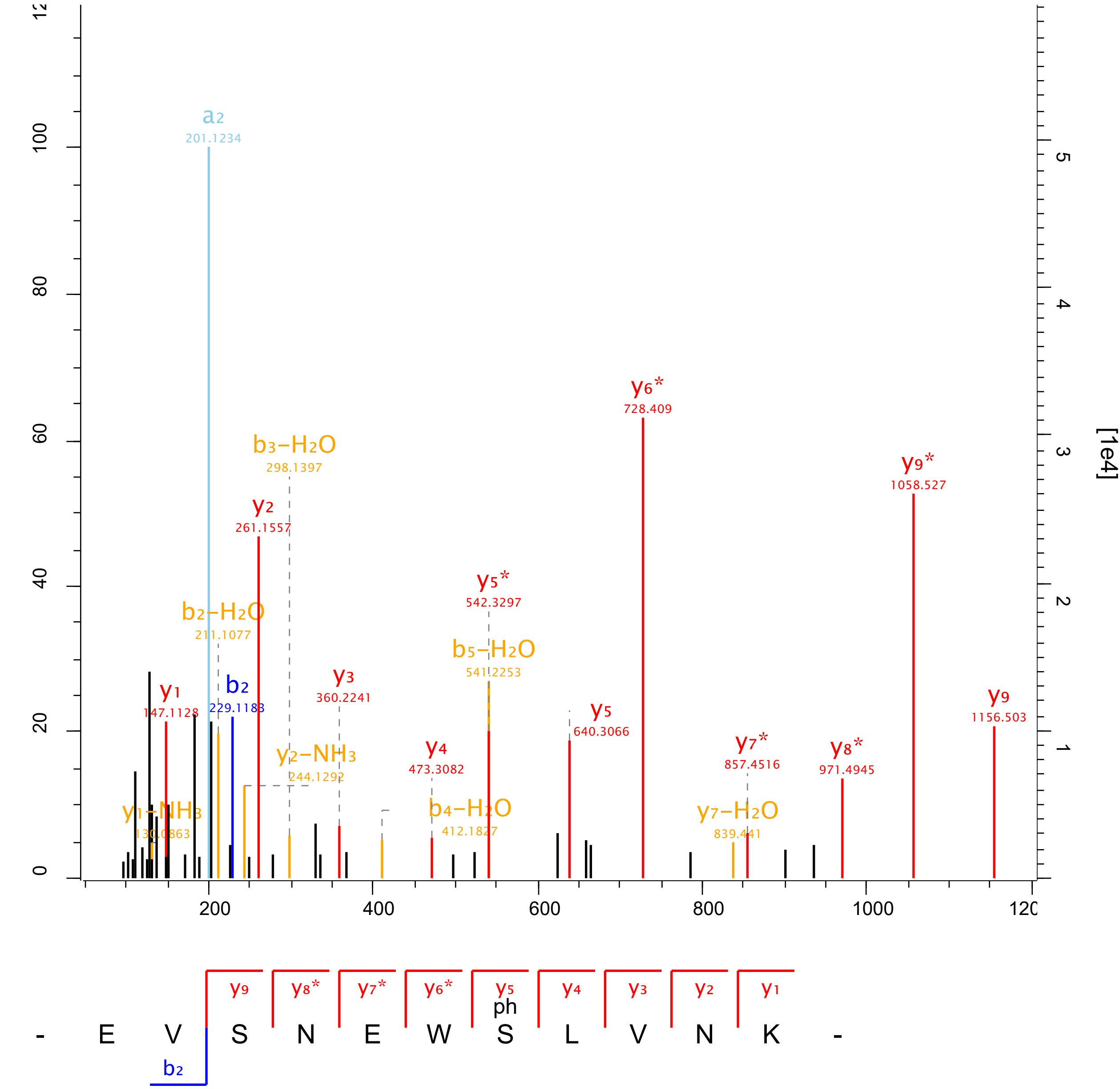

Raw file	Scan	Method	Score	m/z
sys_15_1	26147	FTMS; HCD	61.11	544.23

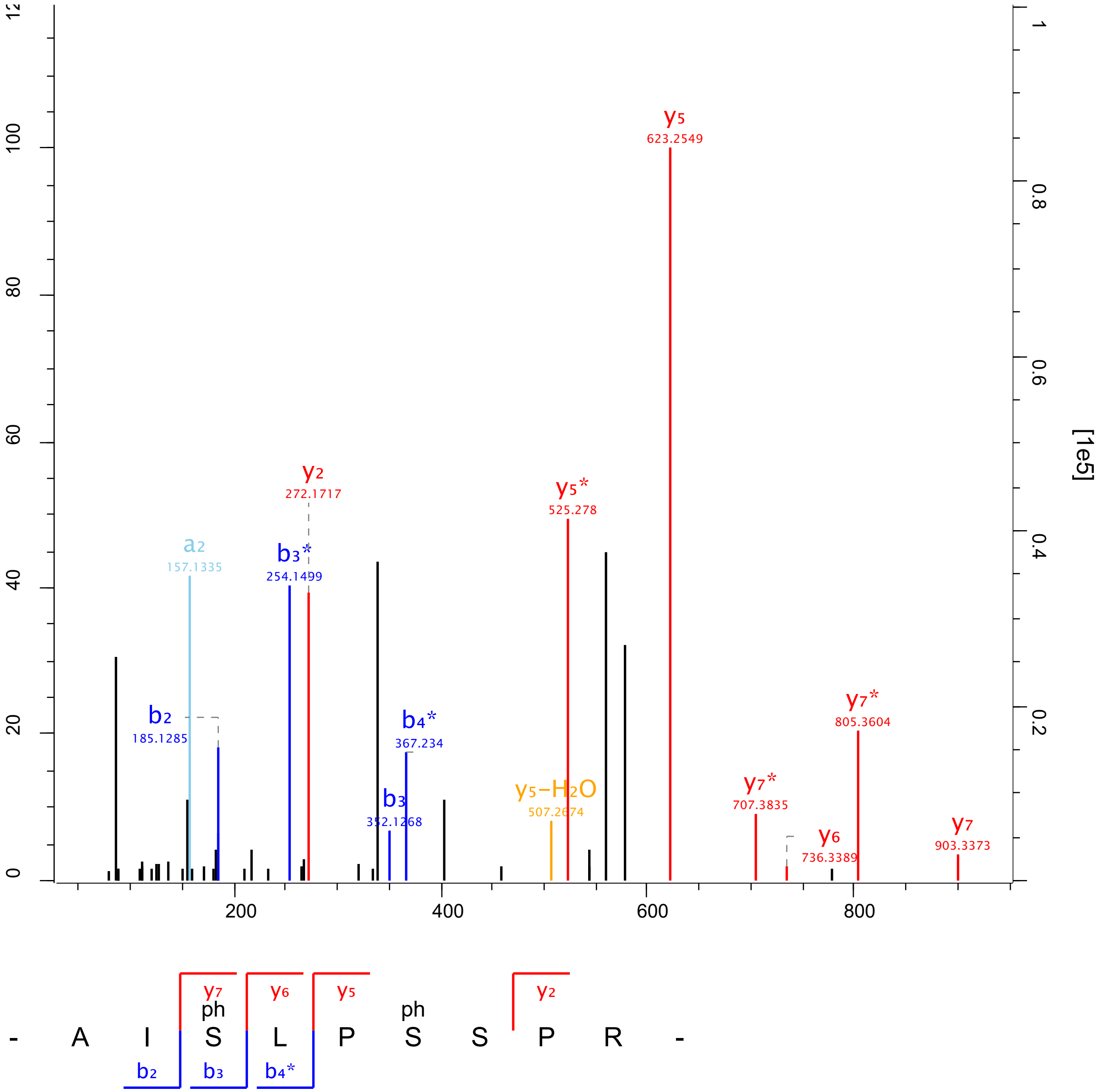

Raw file	Scan	Method	Score	m/z
sys_15_1	26149	FTMS; HCD	144.72	951.87

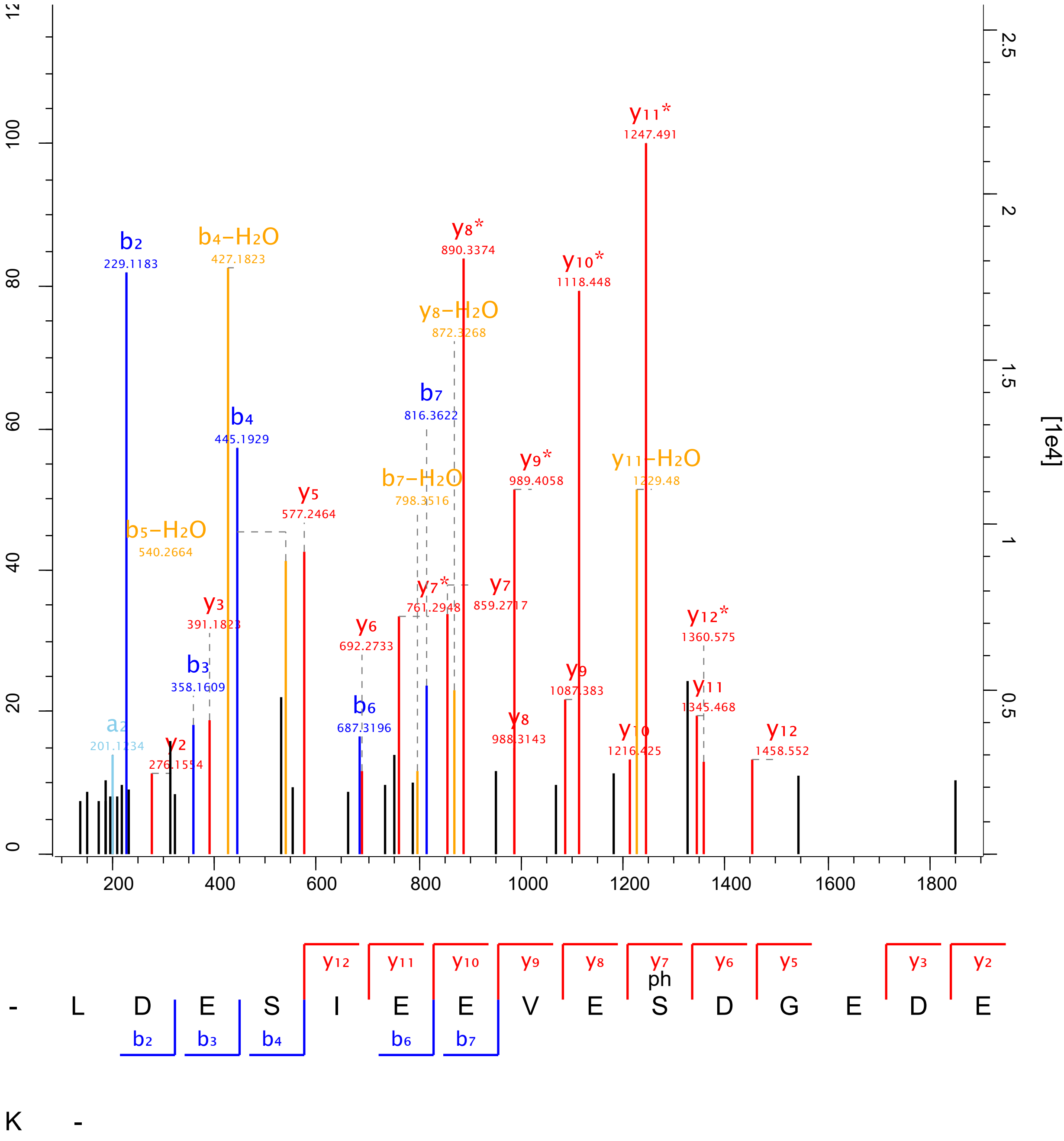

Raw file	Scan	Method	Score	m/z
sys_15_1	26250	FTMS; HCD	150.36	623.97

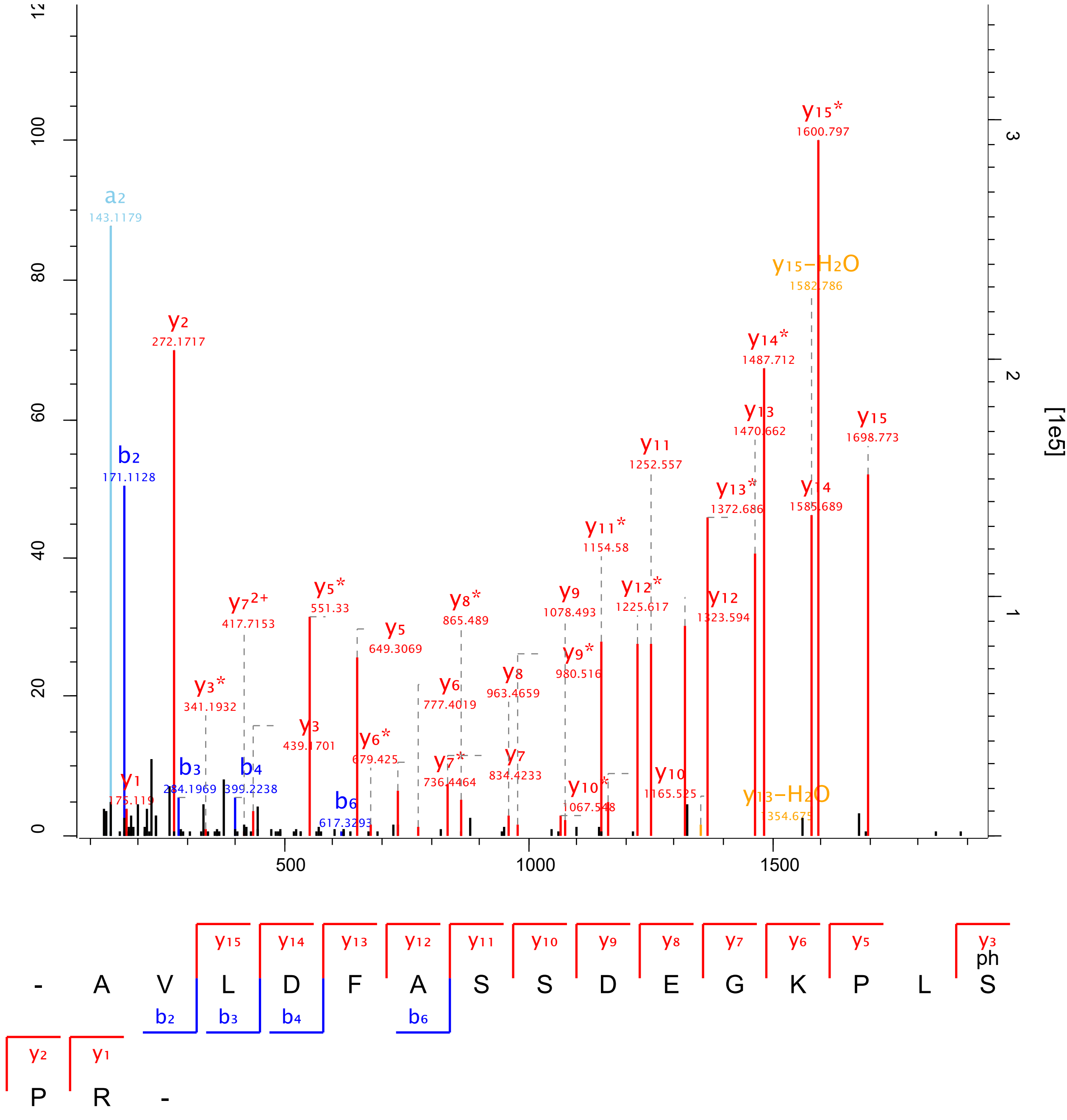

Raw file	Scan	Method	Score	m/z
sys_15_1	26314	FTMS; HCD	161.68	1039.82

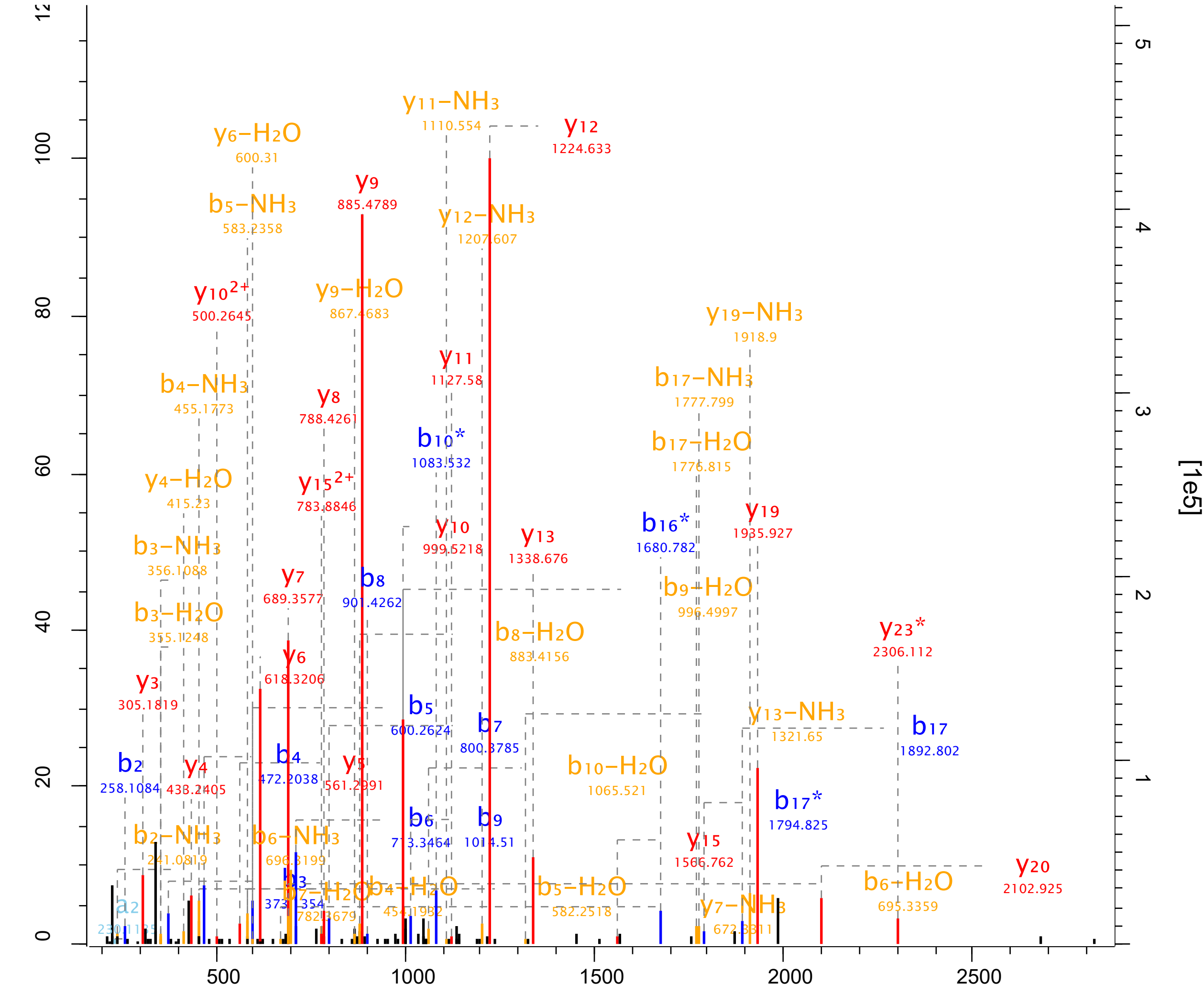

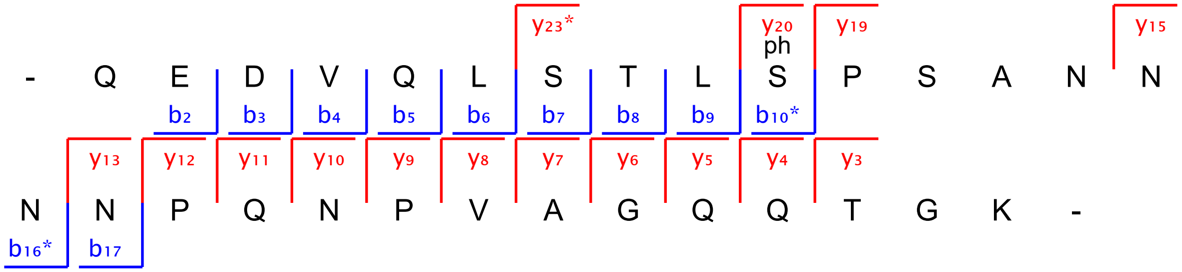

Raw file	Scan	Method	Score	m/z
sys_15_1	26419	FTMS; HCD	165.3	801.04

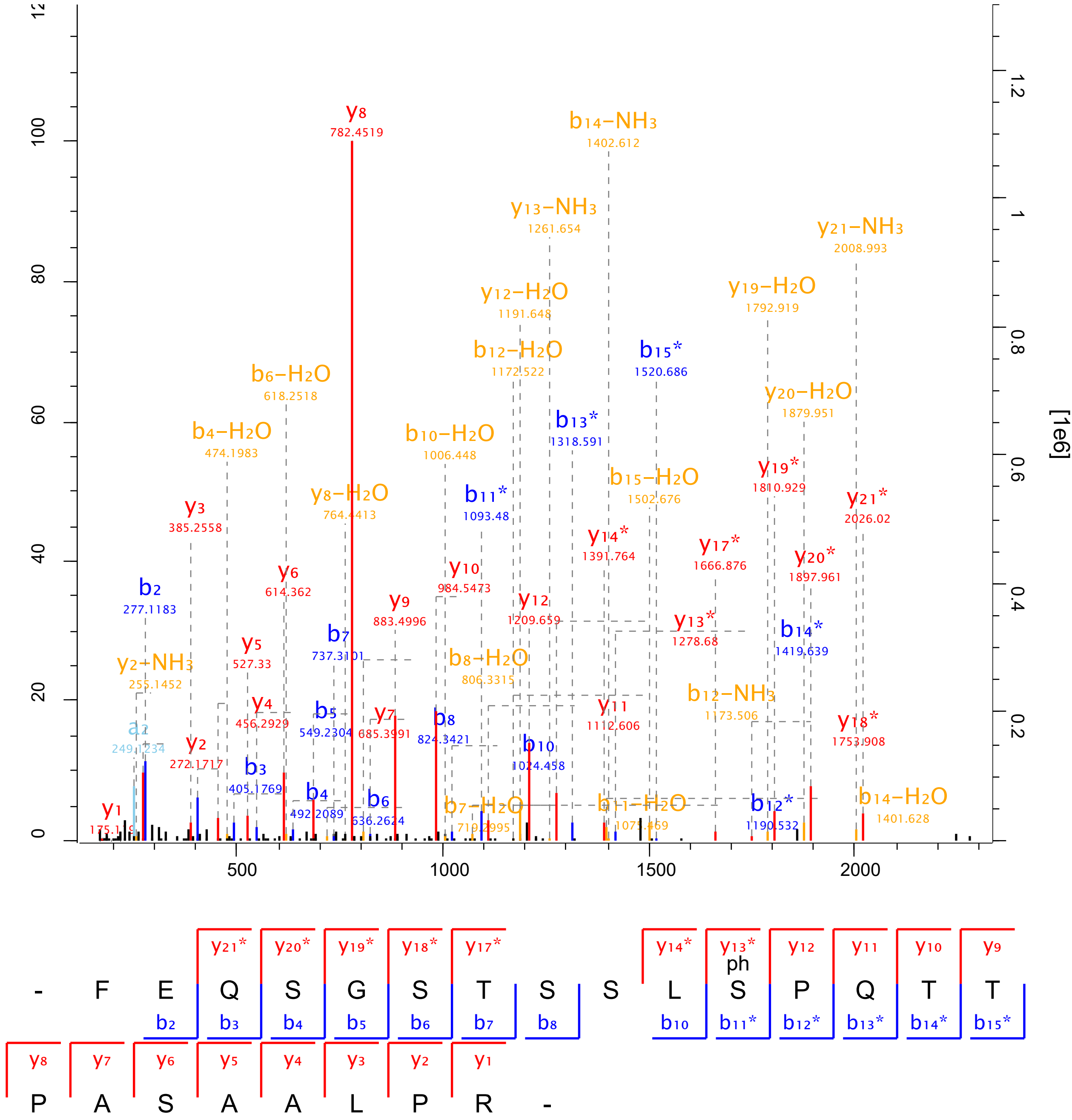

Raw file	Scan	Method	Score	m/z
sys_15_1	26467	FTMS; HCD	63.68	714.81

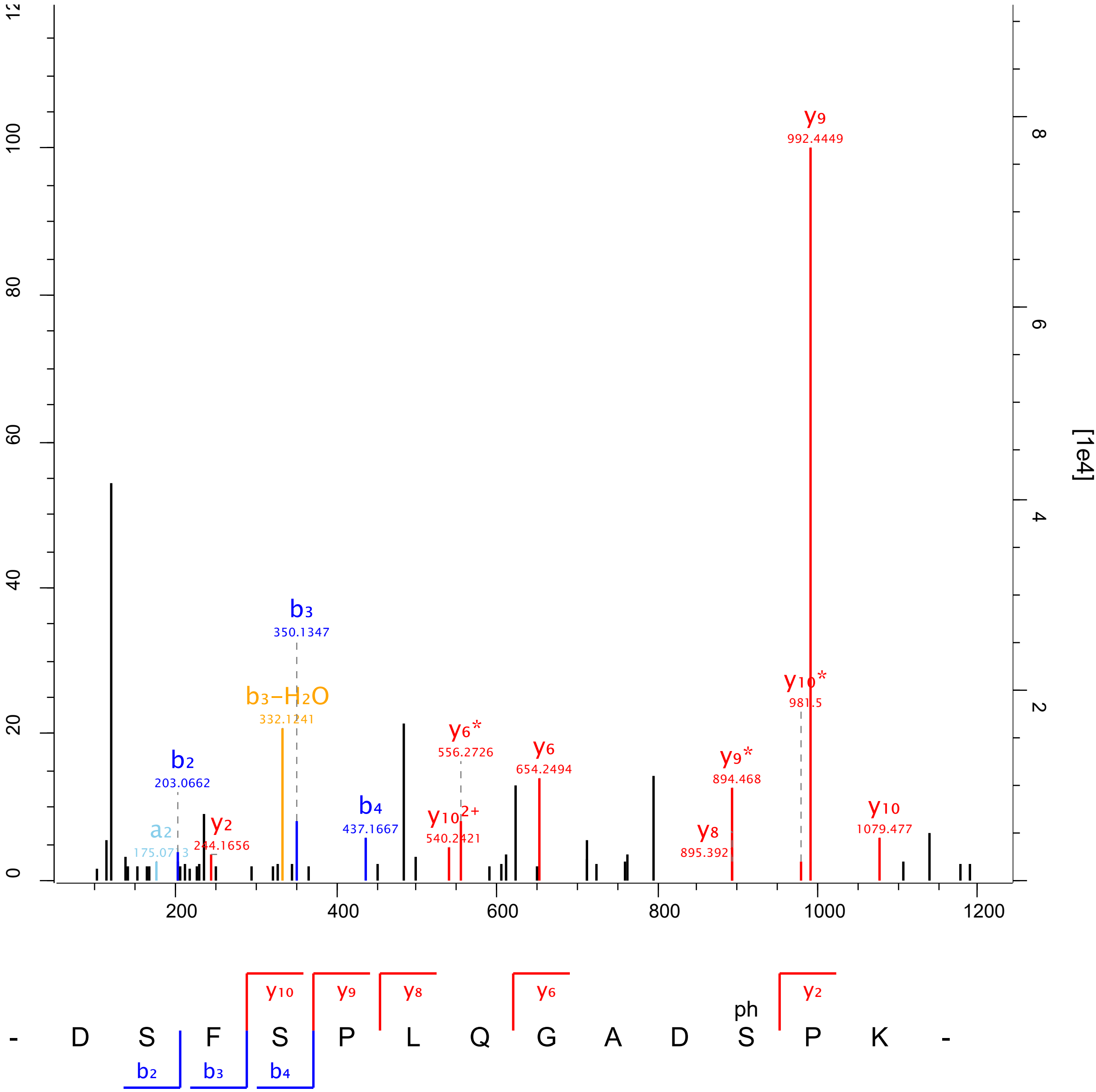

Raw file	Scan	Method	Score	m/z
sys_15_1	2650	FTMS; HCD	198.98	697.78

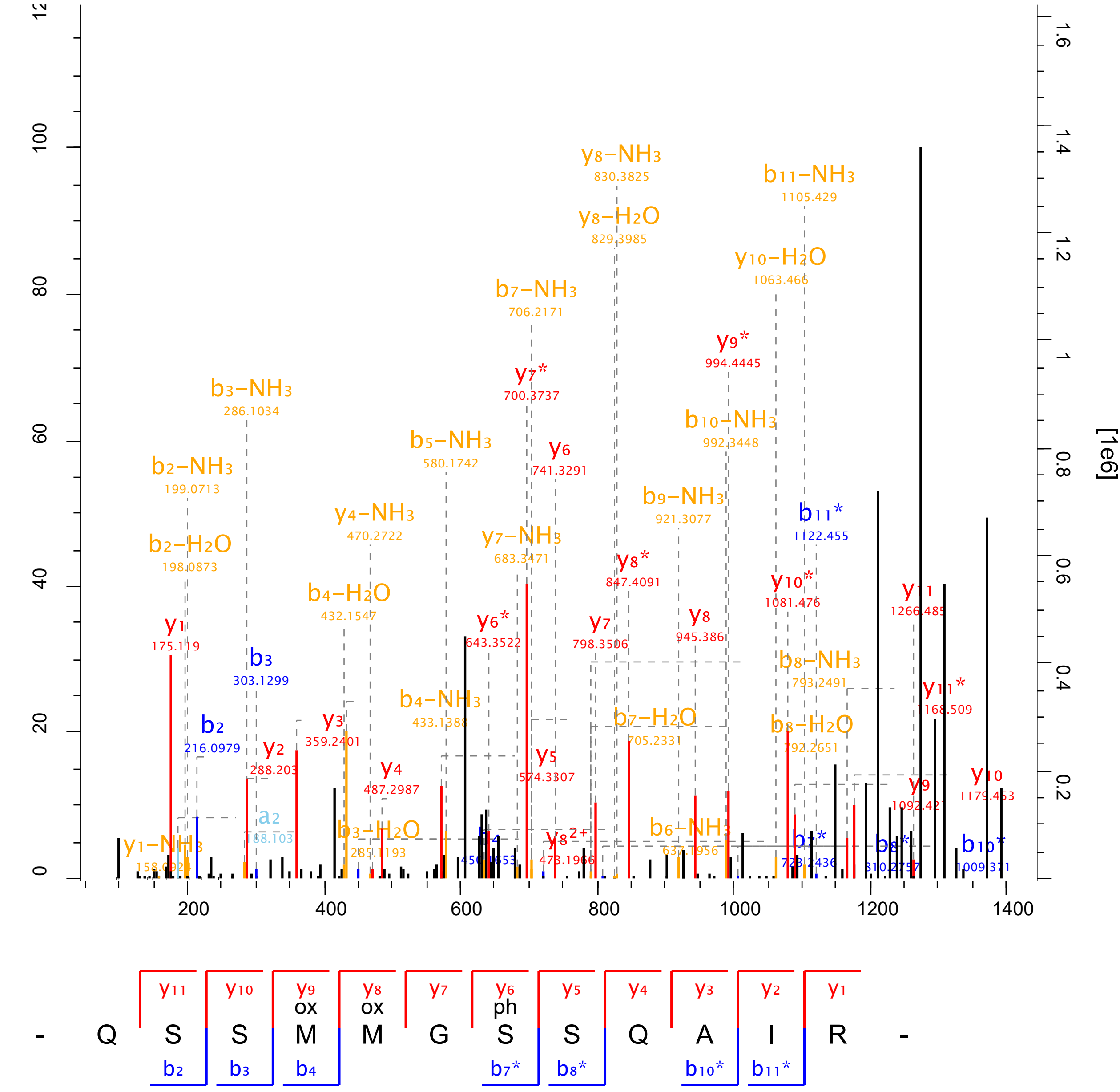

Raw file	Scan	Method	Score	m/z
sys_15_1	26518	FTMS; HCD	88.54	749

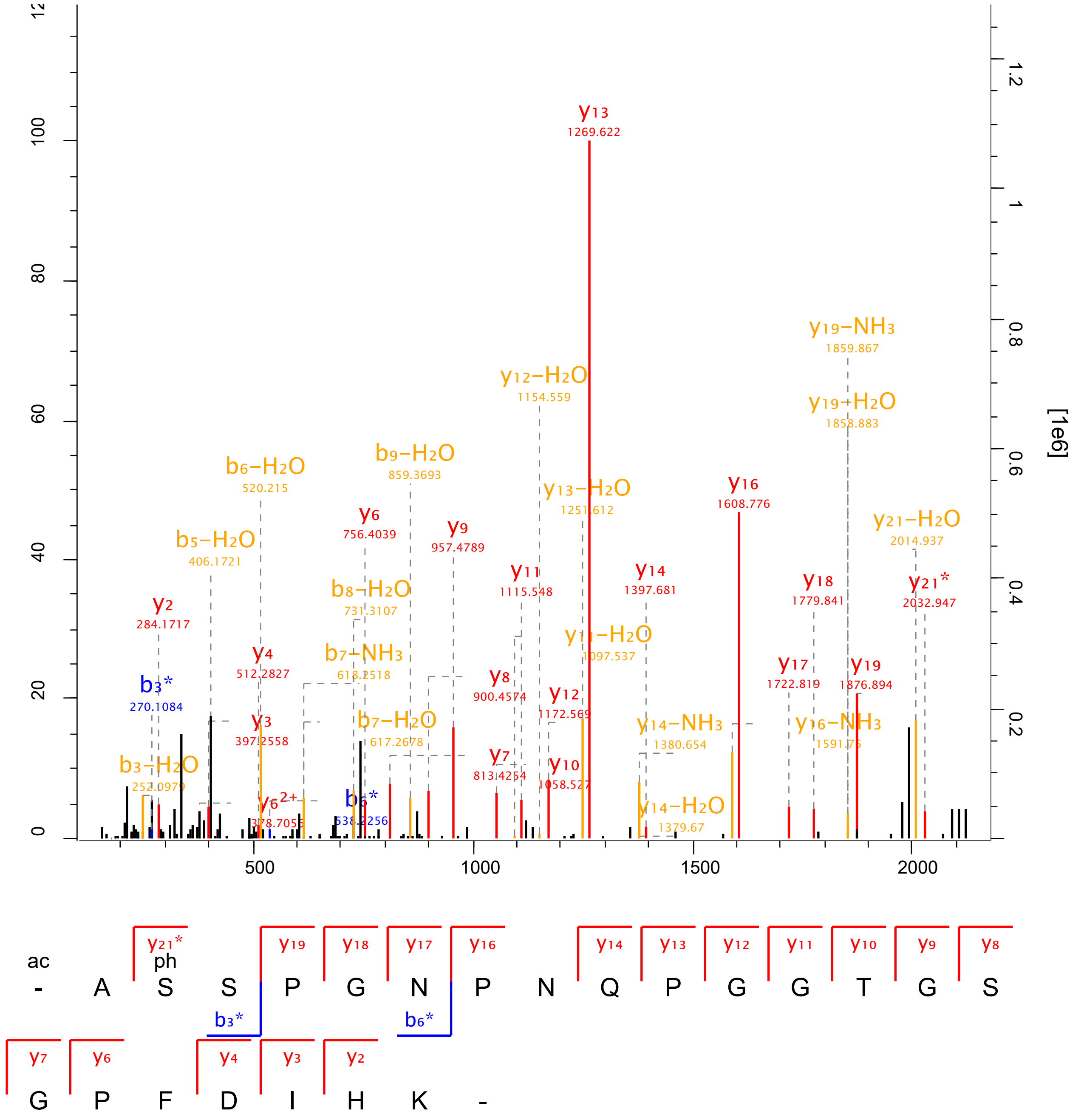

Raw file	Scan	Method	Score	m/z
sys_15_1	26601	FTMS; HCD	139.86	607.76

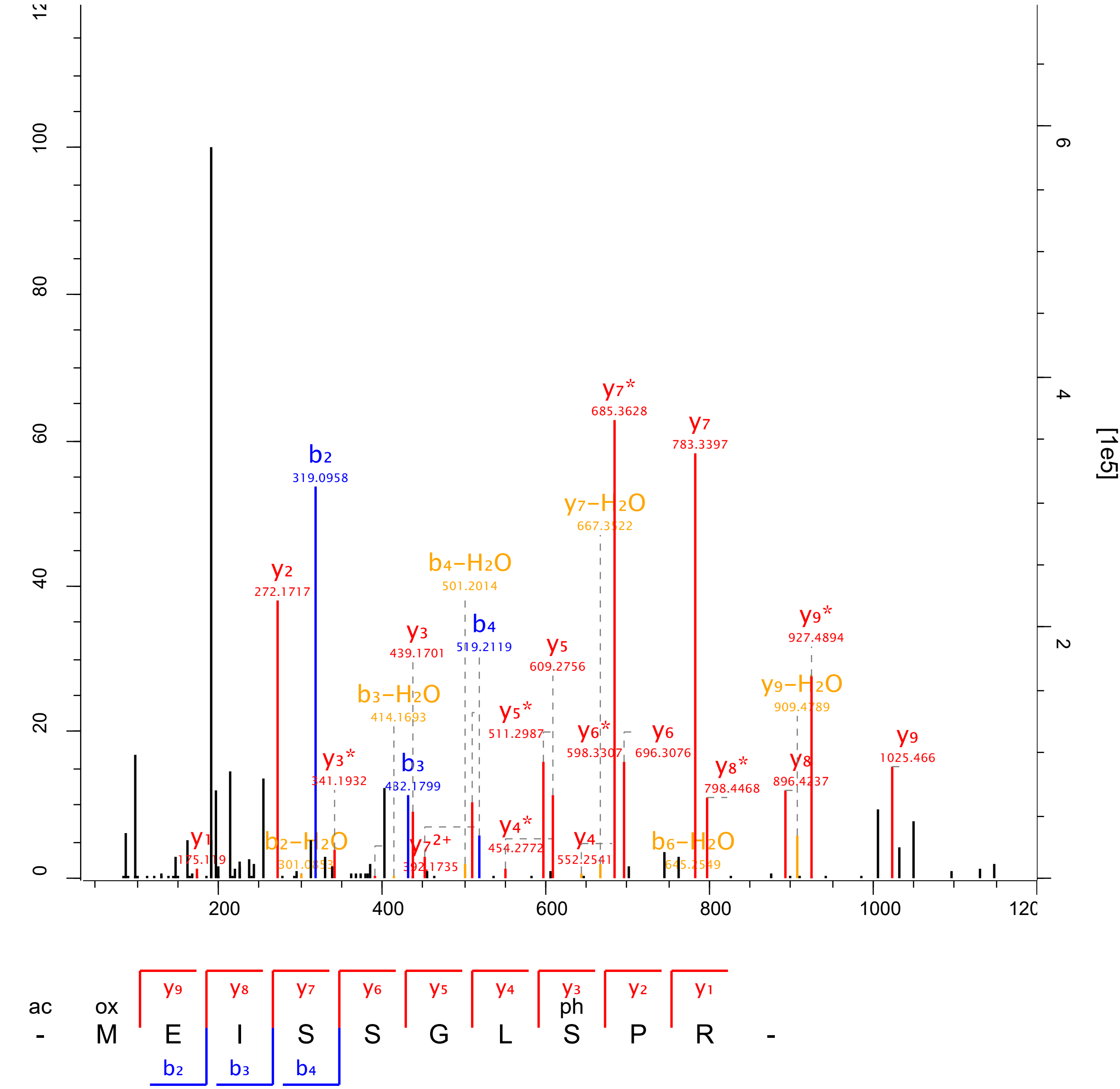

Raw file	Scan	Method	Score	m/z
sys_15_1	26741	FTMS; HCD	100.97	871.87

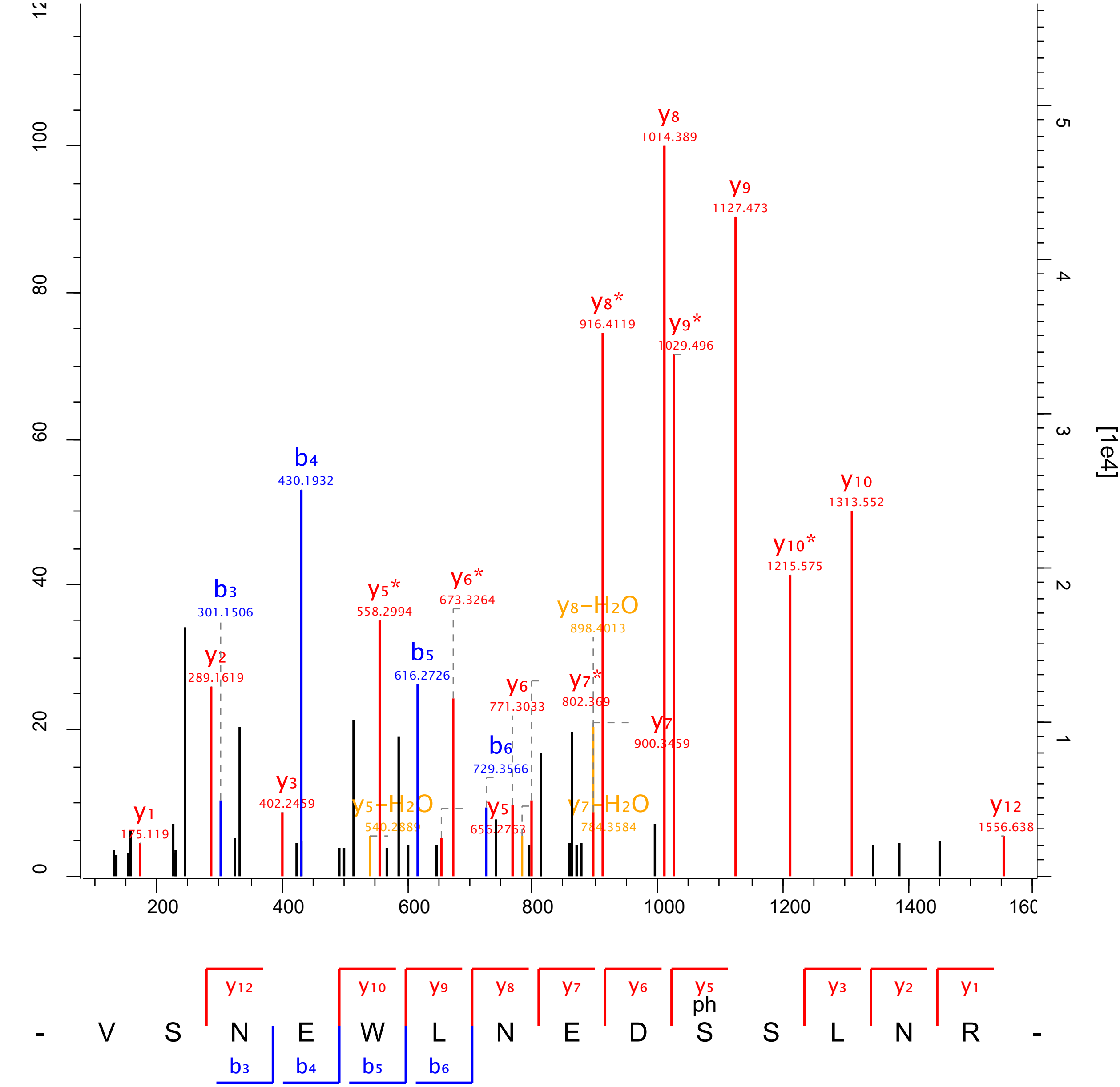

Raw file	Scan	Method	Score	m/z
sys_15_1	26783	FTMS; HCD	112.96	755.79

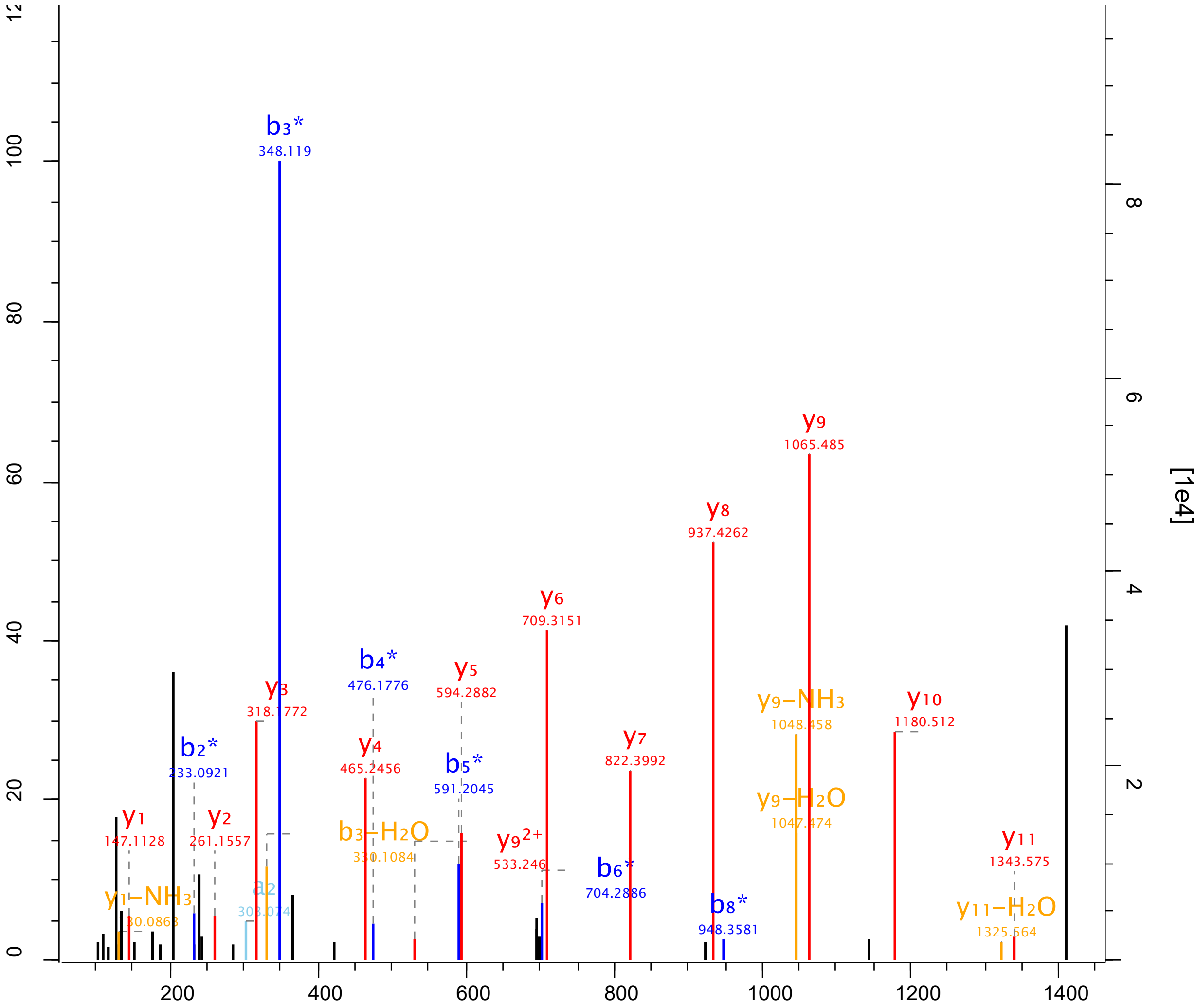

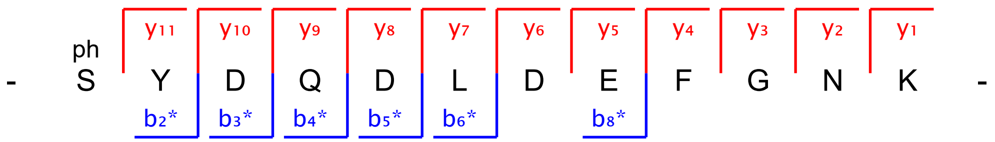

Raw file	Scan	Method	Score	m/z
sys_15_1	26808	FTMS; HCD	118.15	966.41

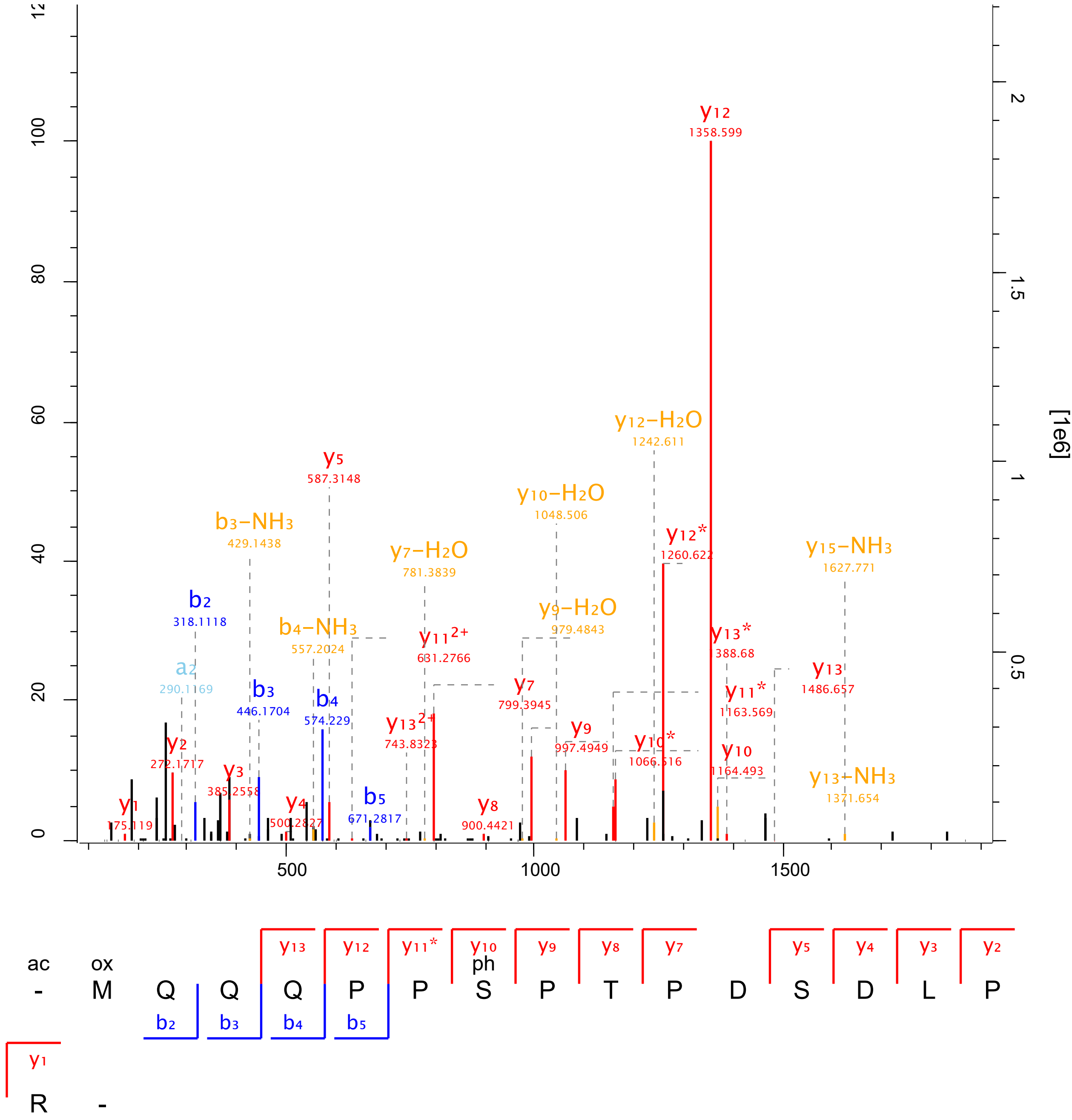

Raw file	Scan	Method	Score	m/z
sys_15_1	26823	FTMS; HCD	52.36	761.03

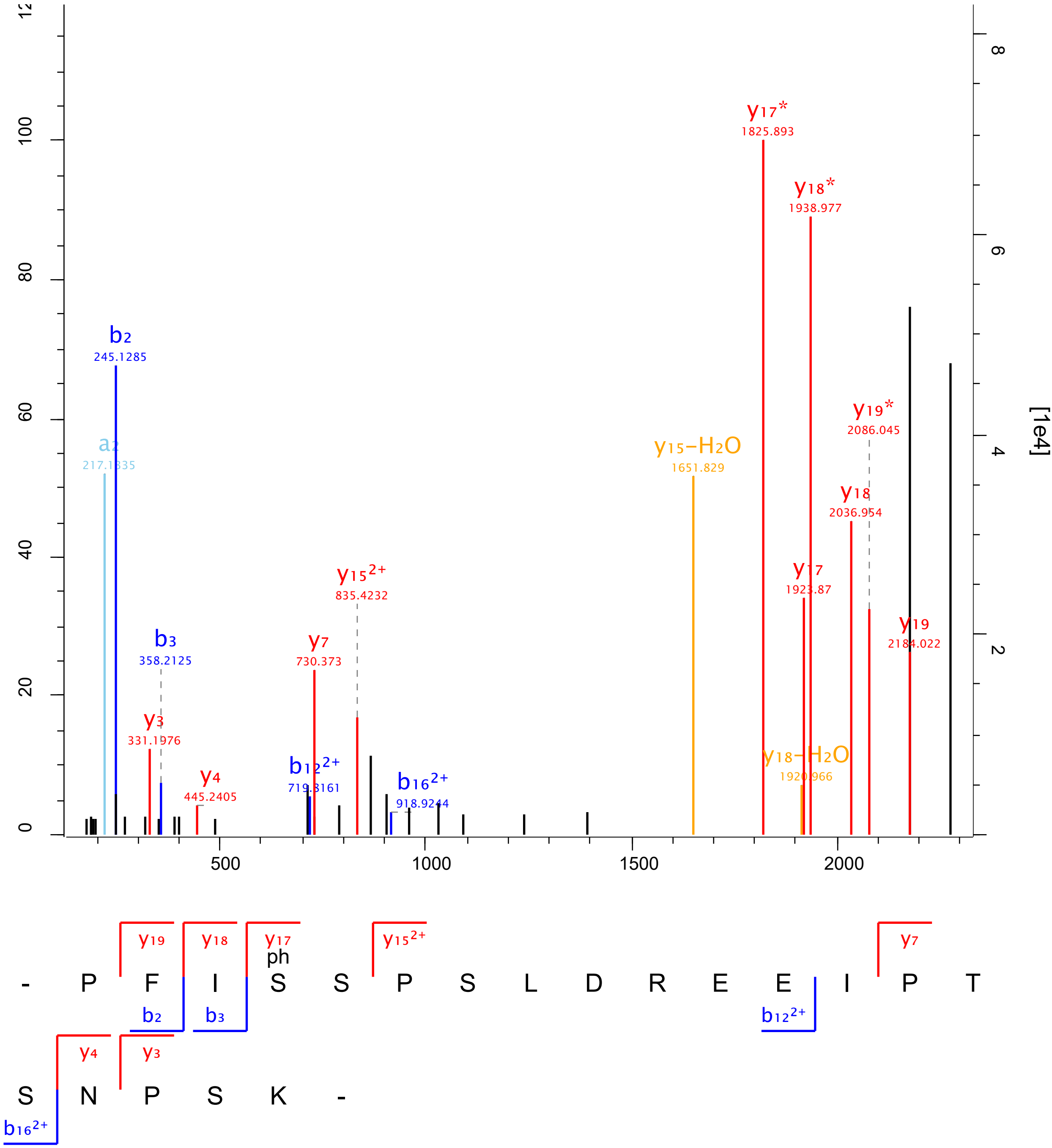

Raw file	Scan	Method	Score	m/z
sys_15_1	26825	FTMS; HCD	86.8	847.39

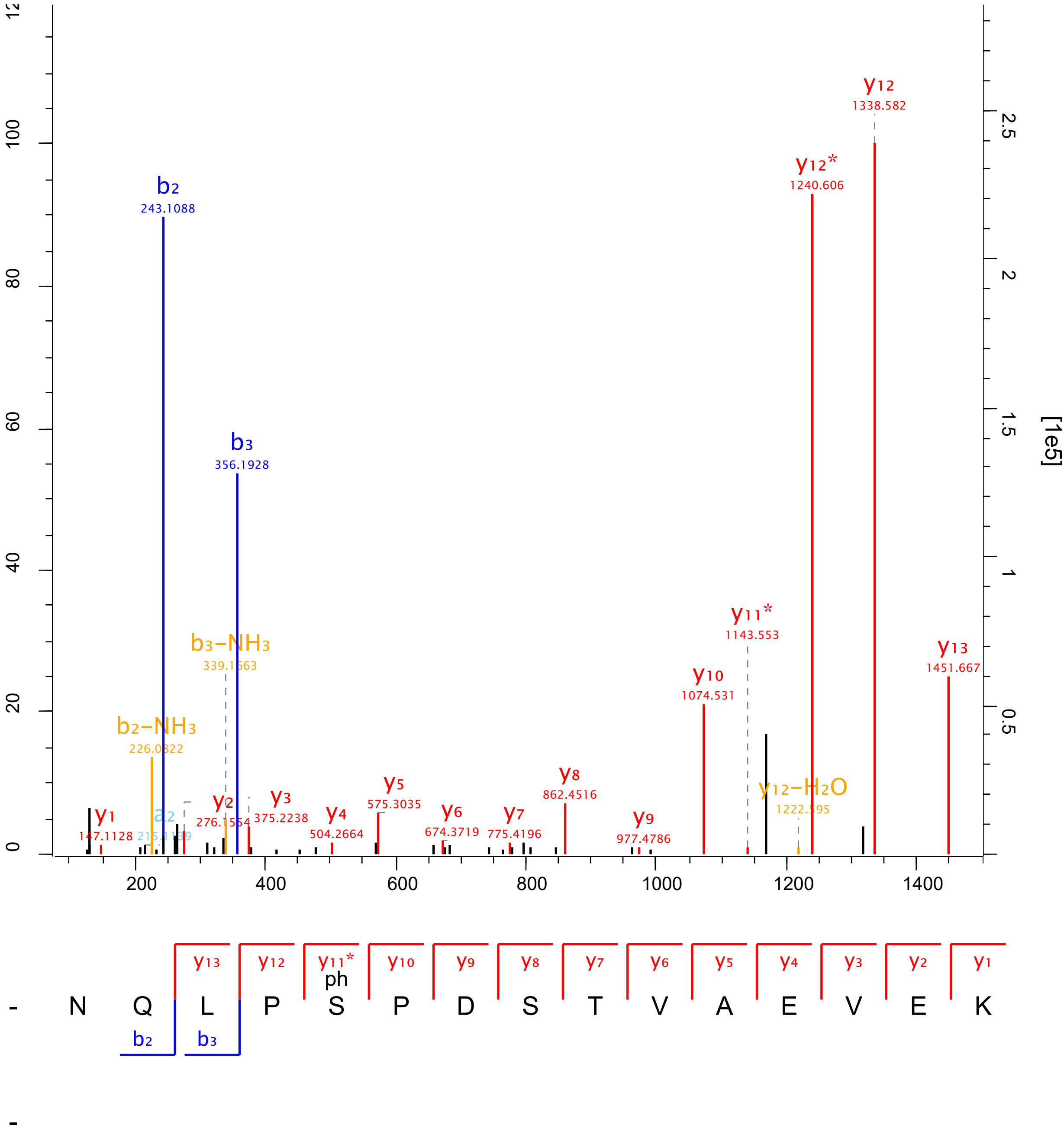

Raw file	Scan	Method	Score	m/z
sys_15_1	26888	FTMS; HCD	42.35	913.74

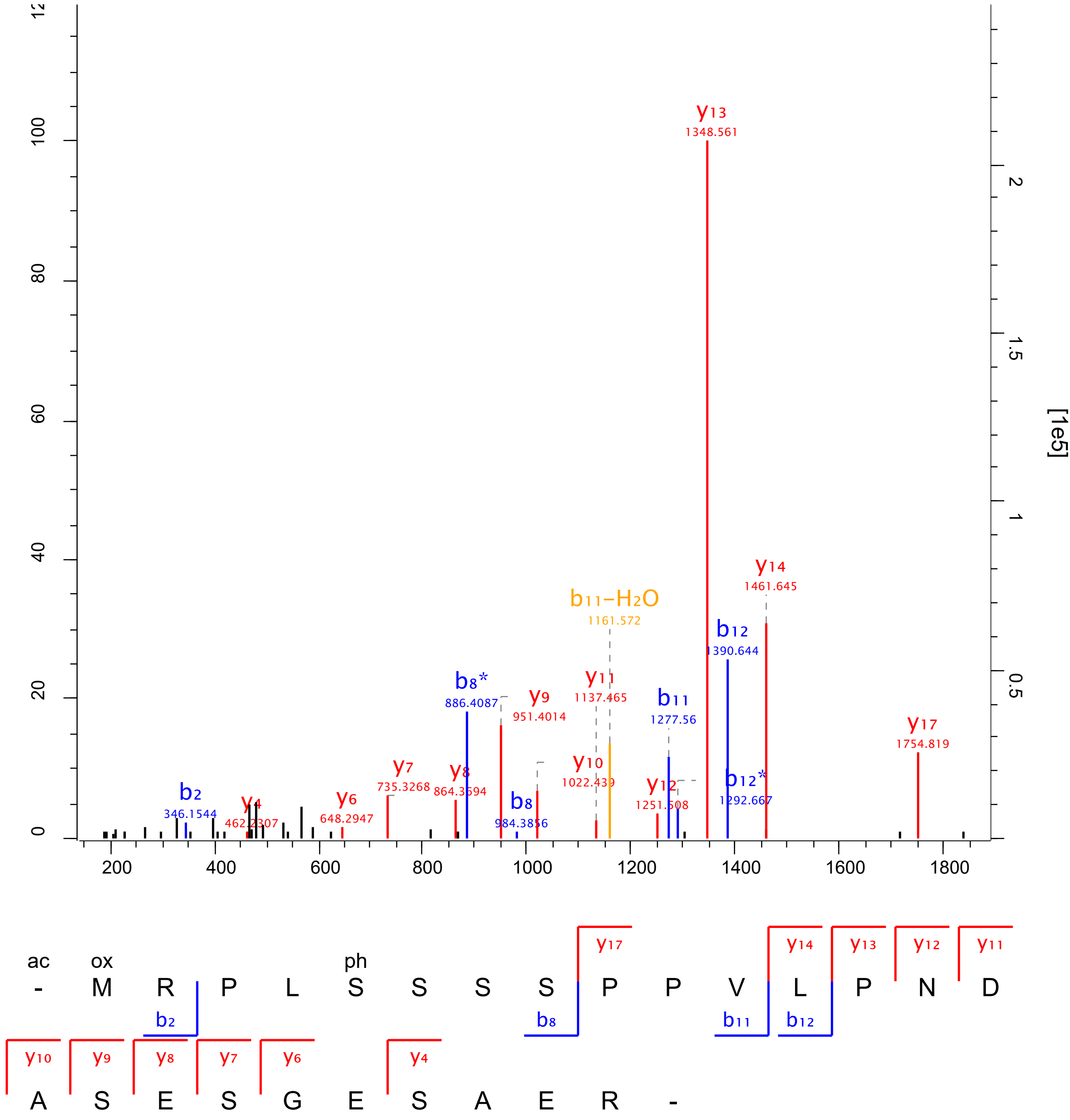

Raw file	Scan	Method	Score	m/z
sys_15_1	26921	FTMS; HCD	56.51	728.36

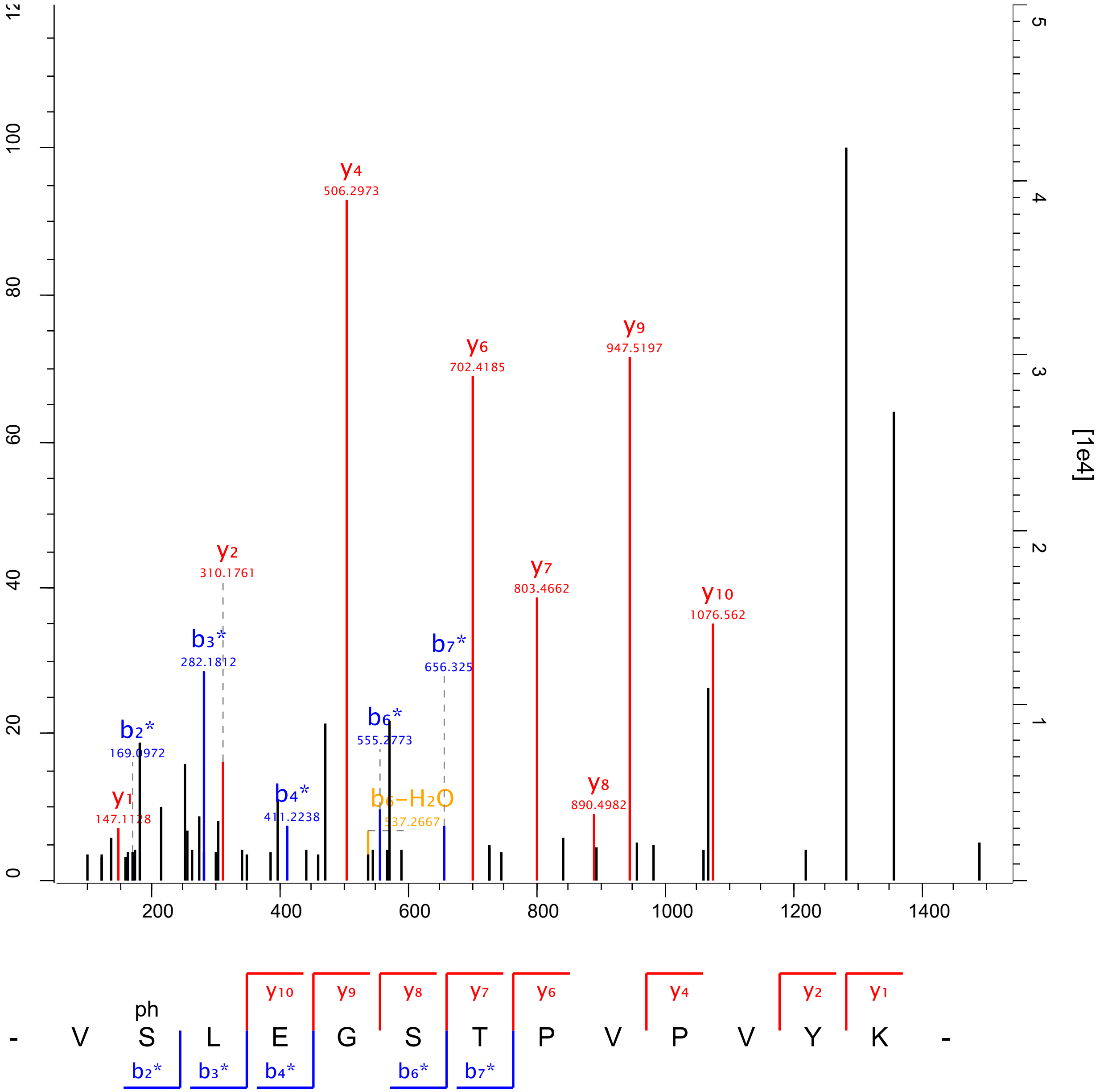

Raw file	Scan	Method	Score	m/z
sys_15_1	2696	FTMS; HCD	170.74	599.76

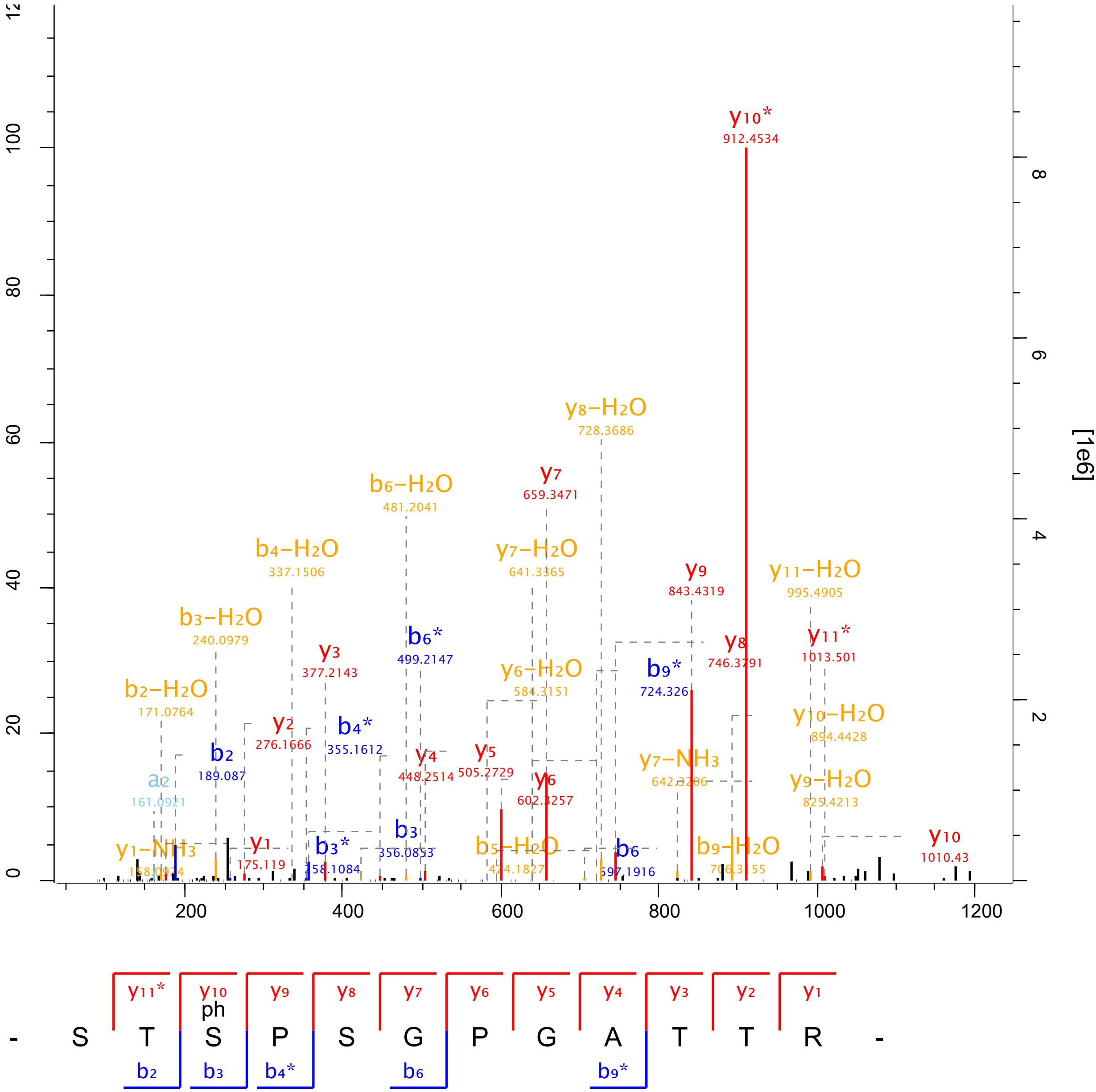

Raw file	Scan	Method	Score	m/z
sys_15_1	26962	FTMS; HCD	242.64	774.65

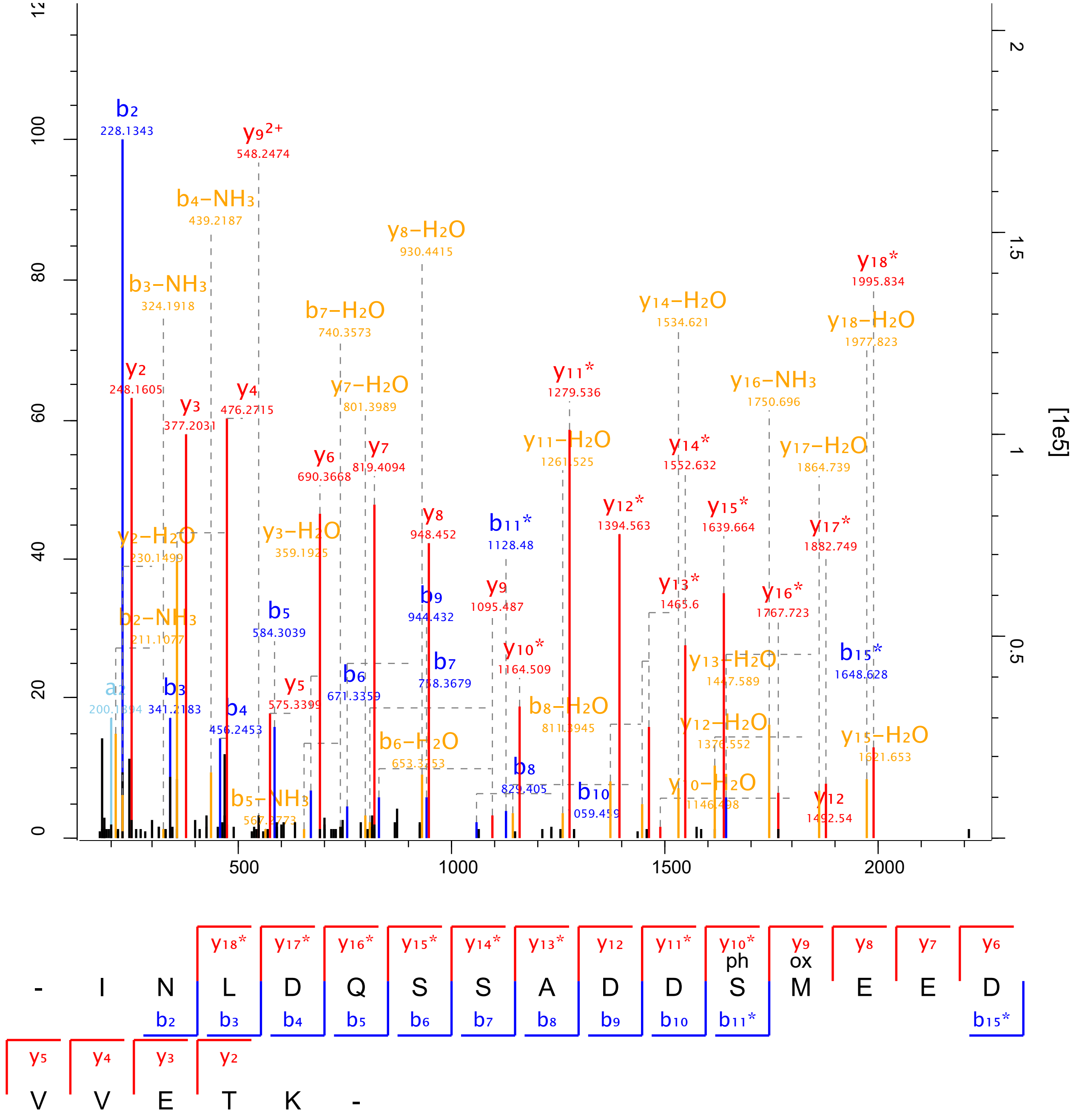

Raw file	Scan	Method	Score	m/z
sys_15_1	26965	FTMS; HCD	84.36	625.97

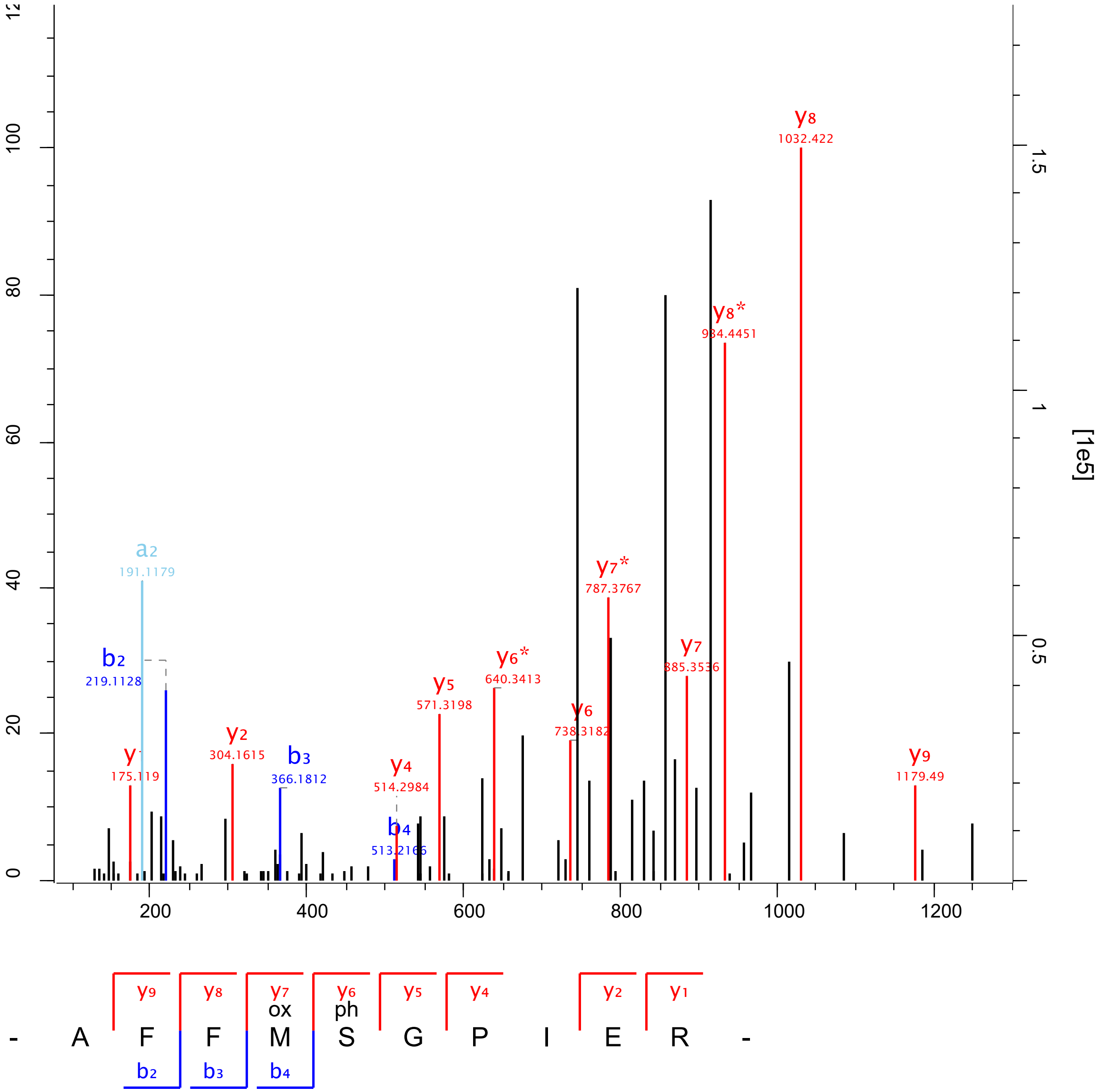

Raw file	Scan	Method	Score	m/z
sys_15_1	2704	FTMS; HCD	70.41	447.7

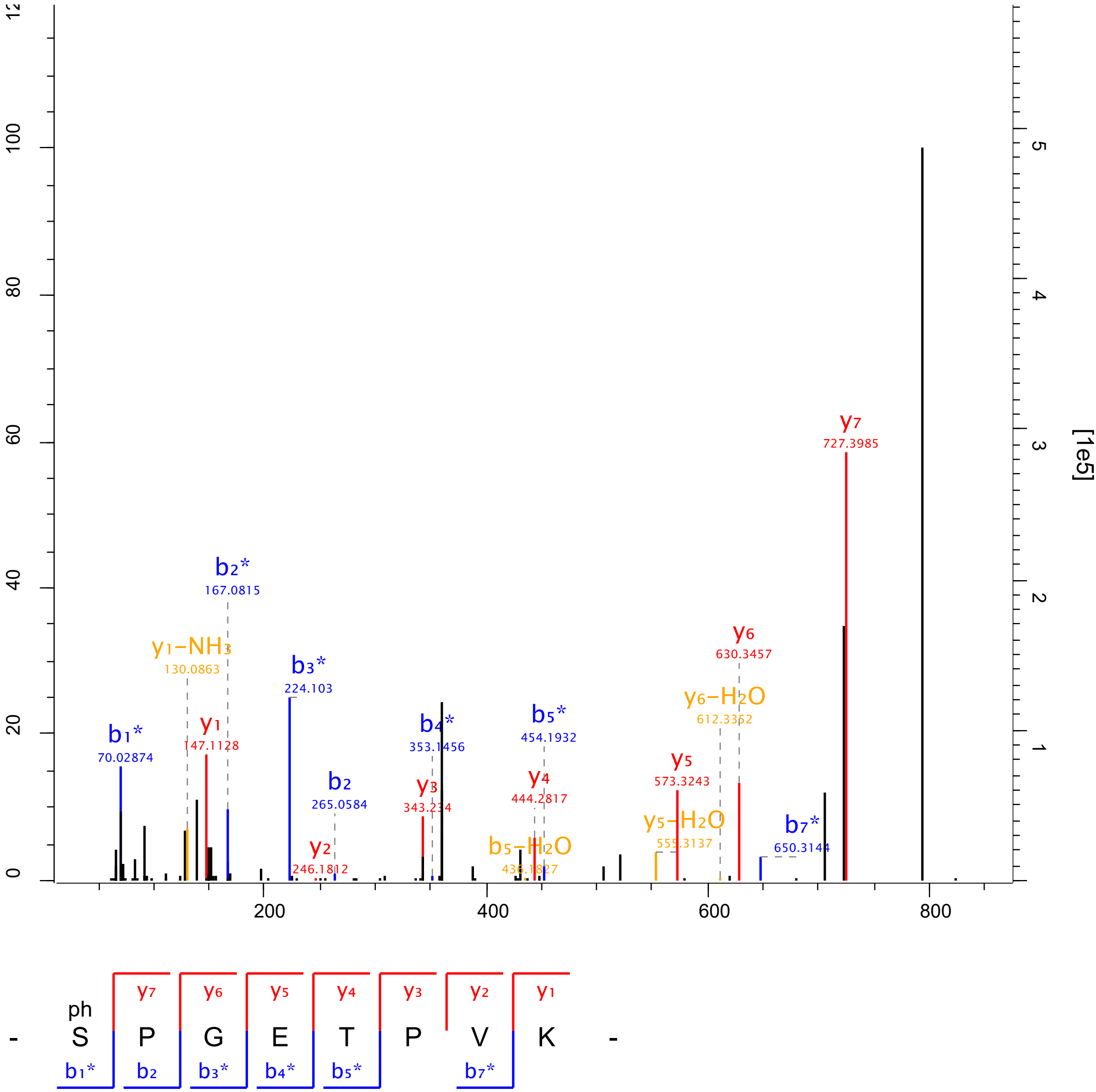

Raw file	Scan	Method	Score	m/z
sys_15_1	27054	FTMS; HCD	86.54	941.93

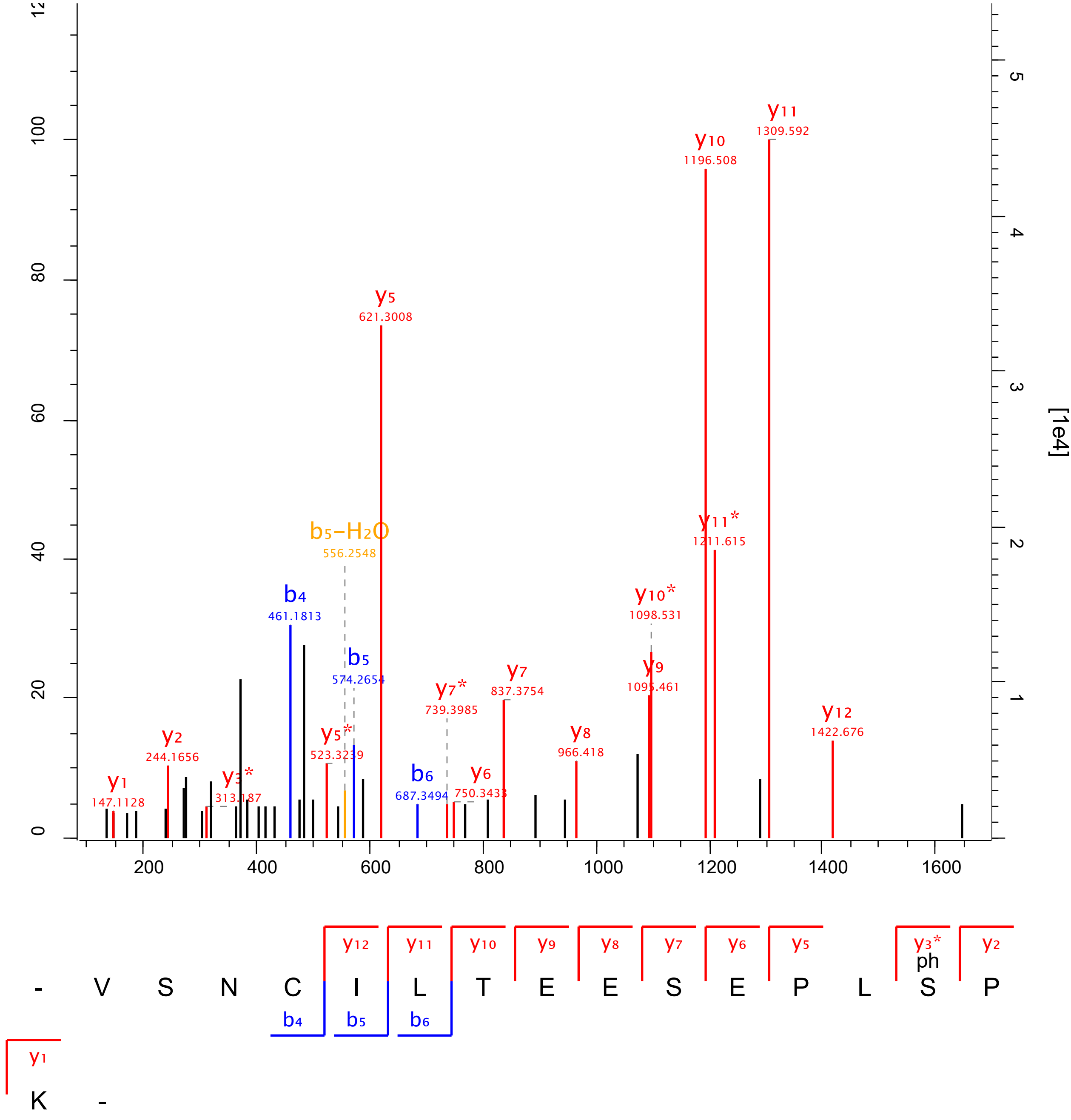

Raw file	Scan	Method	Score	m/z
sys_15_1	27078	FTMS; HCD	51.03	724.88

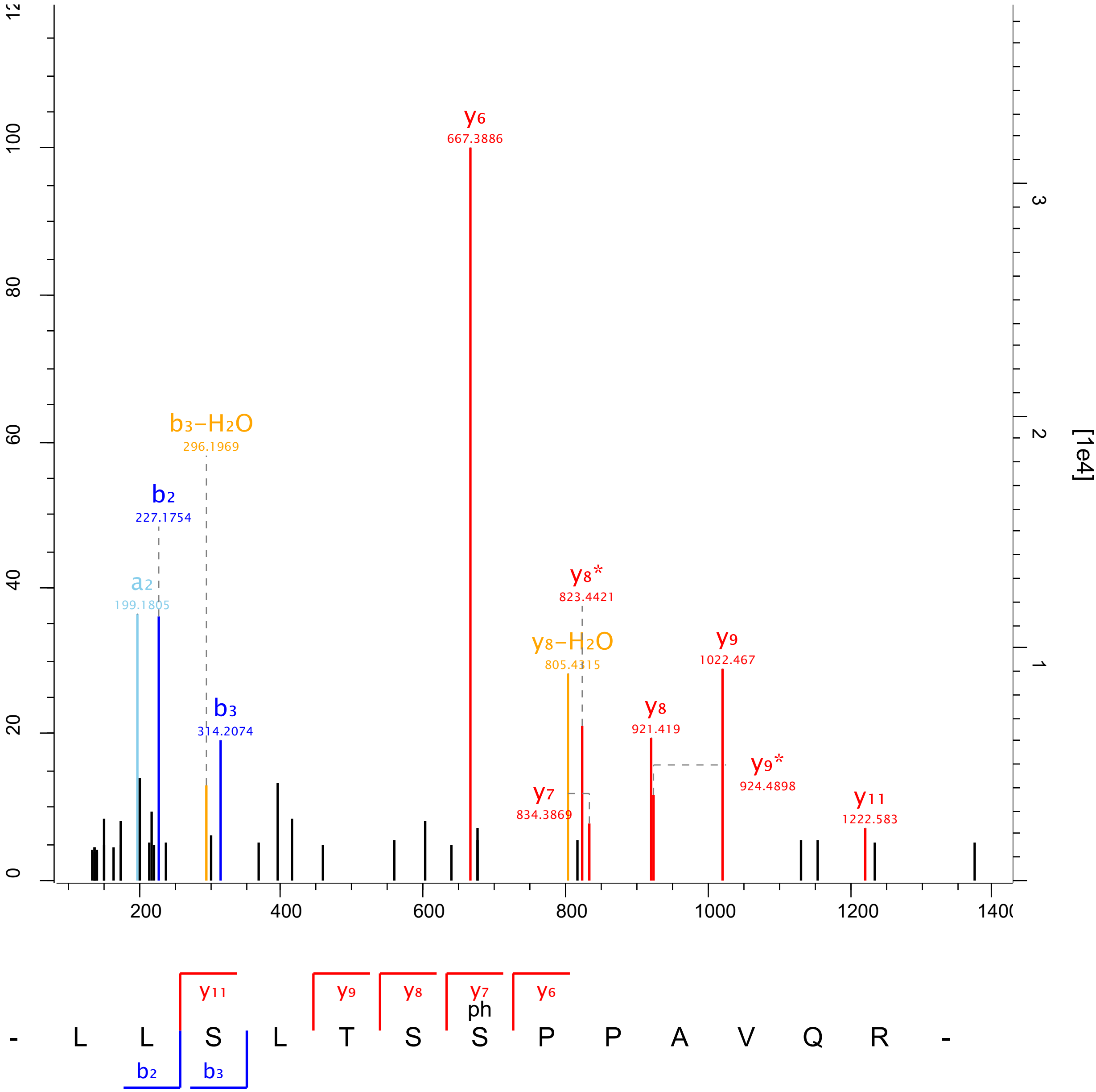

Raw file	Scan	Method	Score	m/z
sys_15_1	27132	FTMS; HCD	178.35	784.68

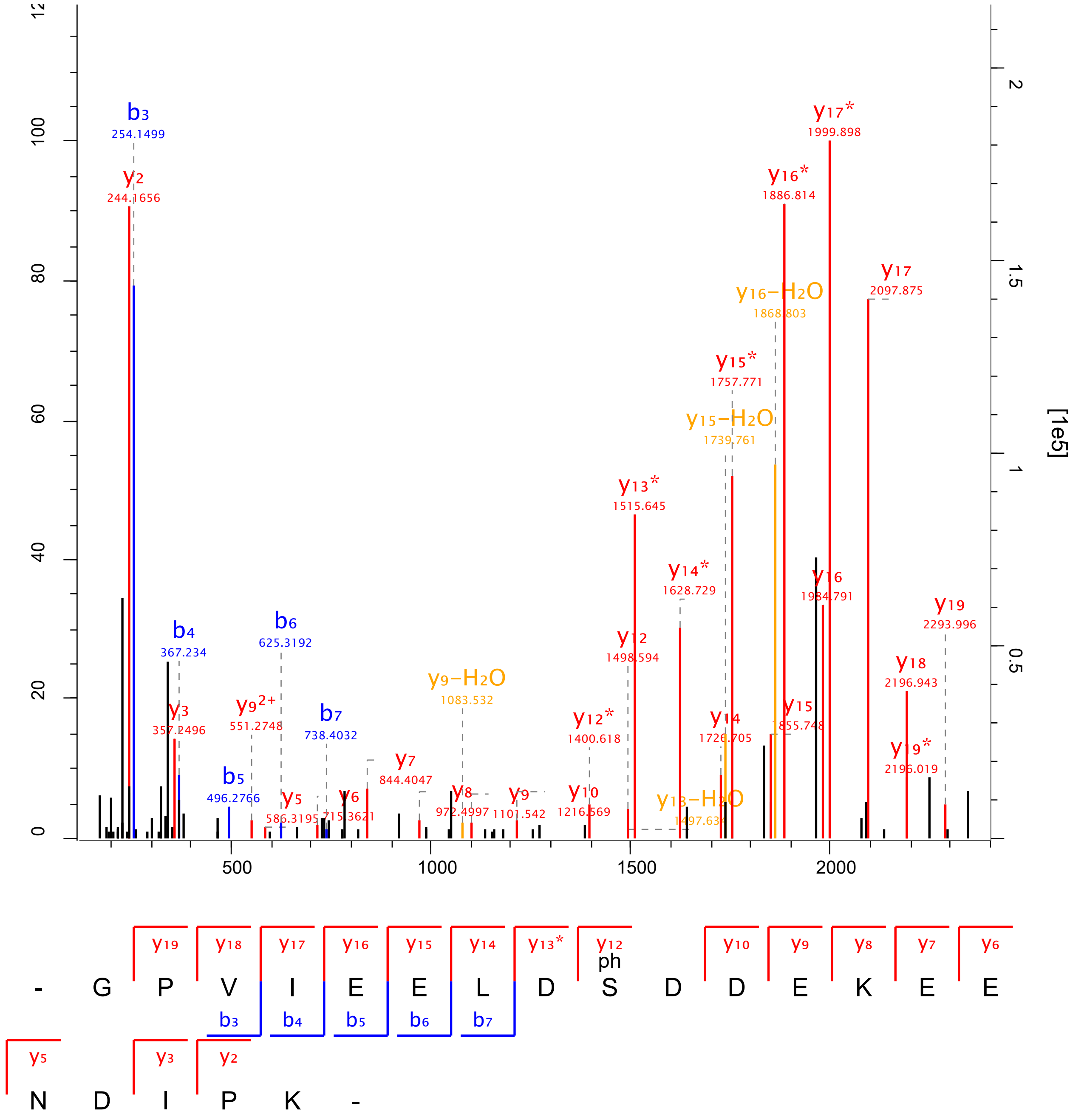

Raw file	Scan	Method	Score	m/z
sys_15_1	27308	FTMS; HCD	86.56	626.62

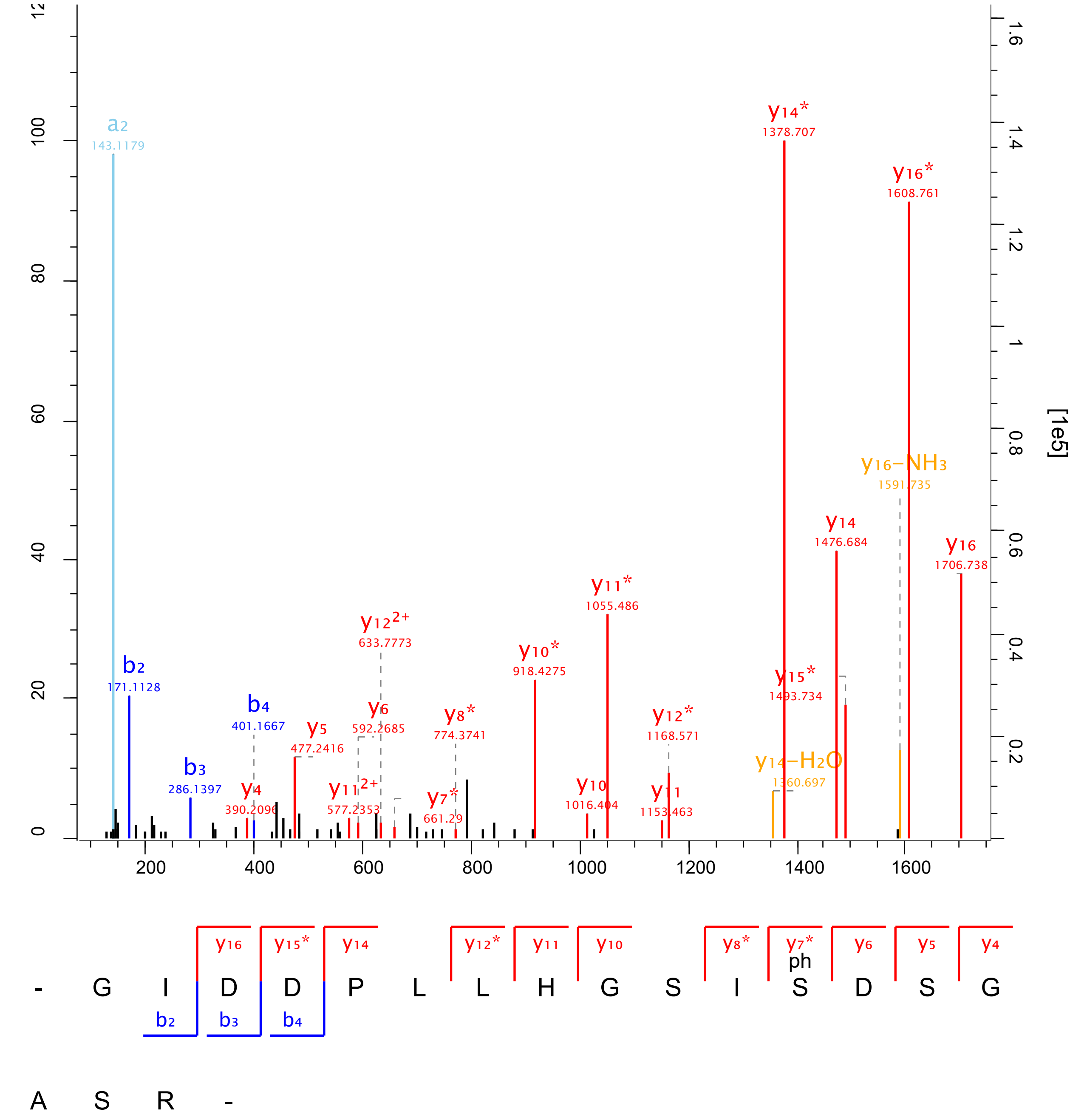

Raw file	Scan	Method	Score	m/z
sys_15_1	27335	FTMS; HCD	40.86	1031.08

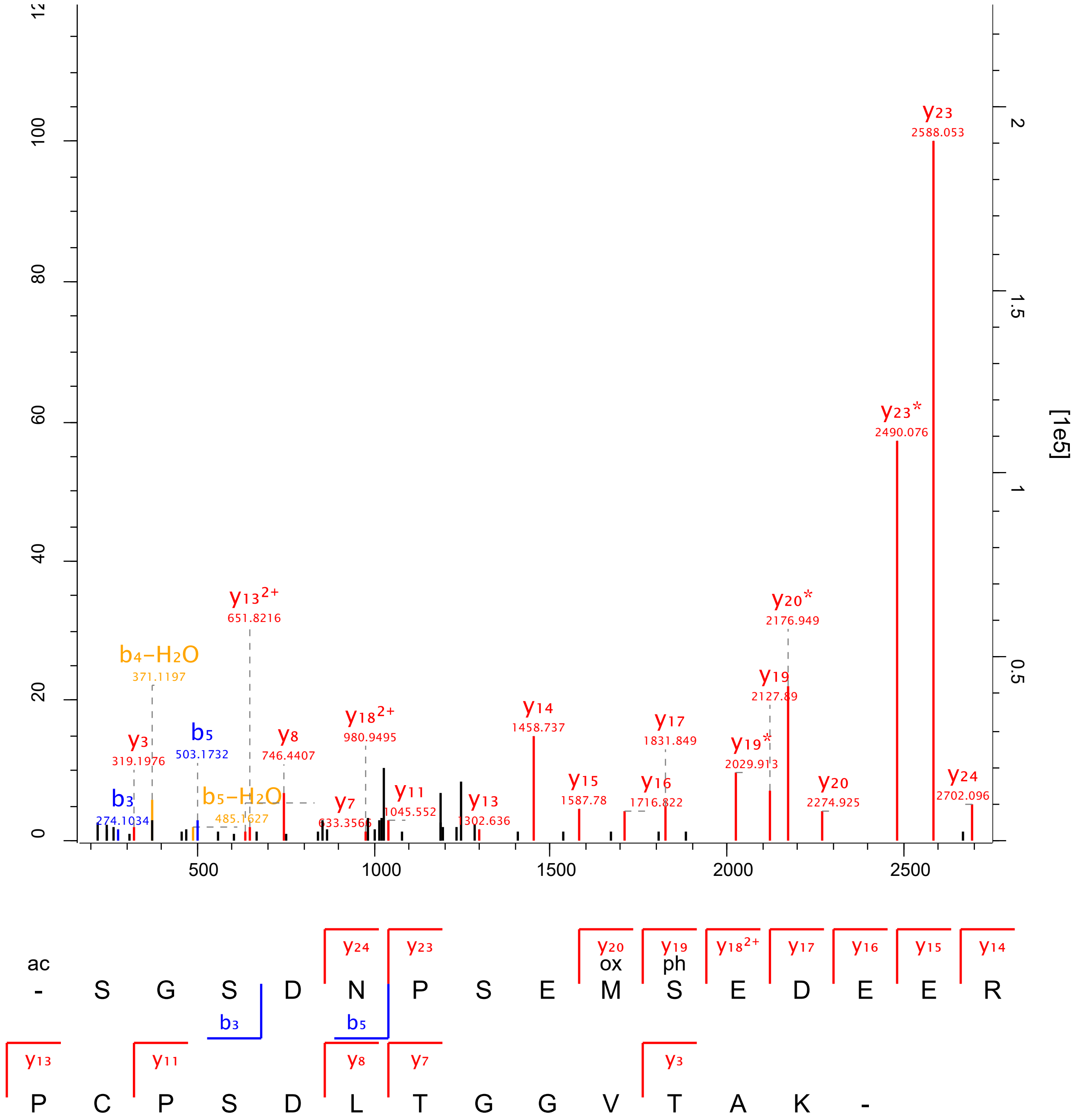

Raw file	Scan	Method	Score	m/z
sys_15_1	2737	FTMS; HCD	59.07	497.69

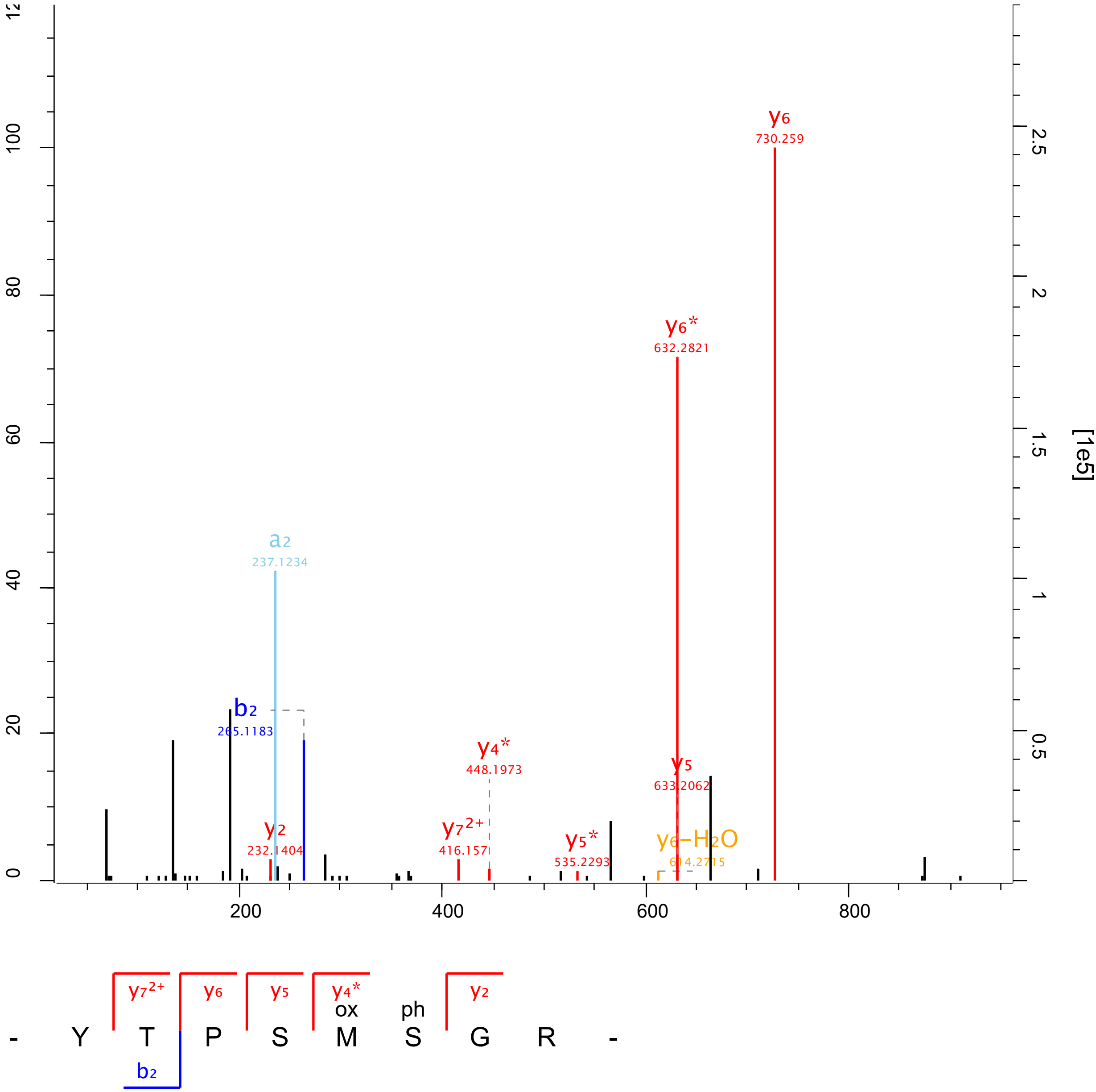

Raw file	Scan	Method	Score	m/z
sys_15_1	27442	FTMS; HCD	94.38	709.68

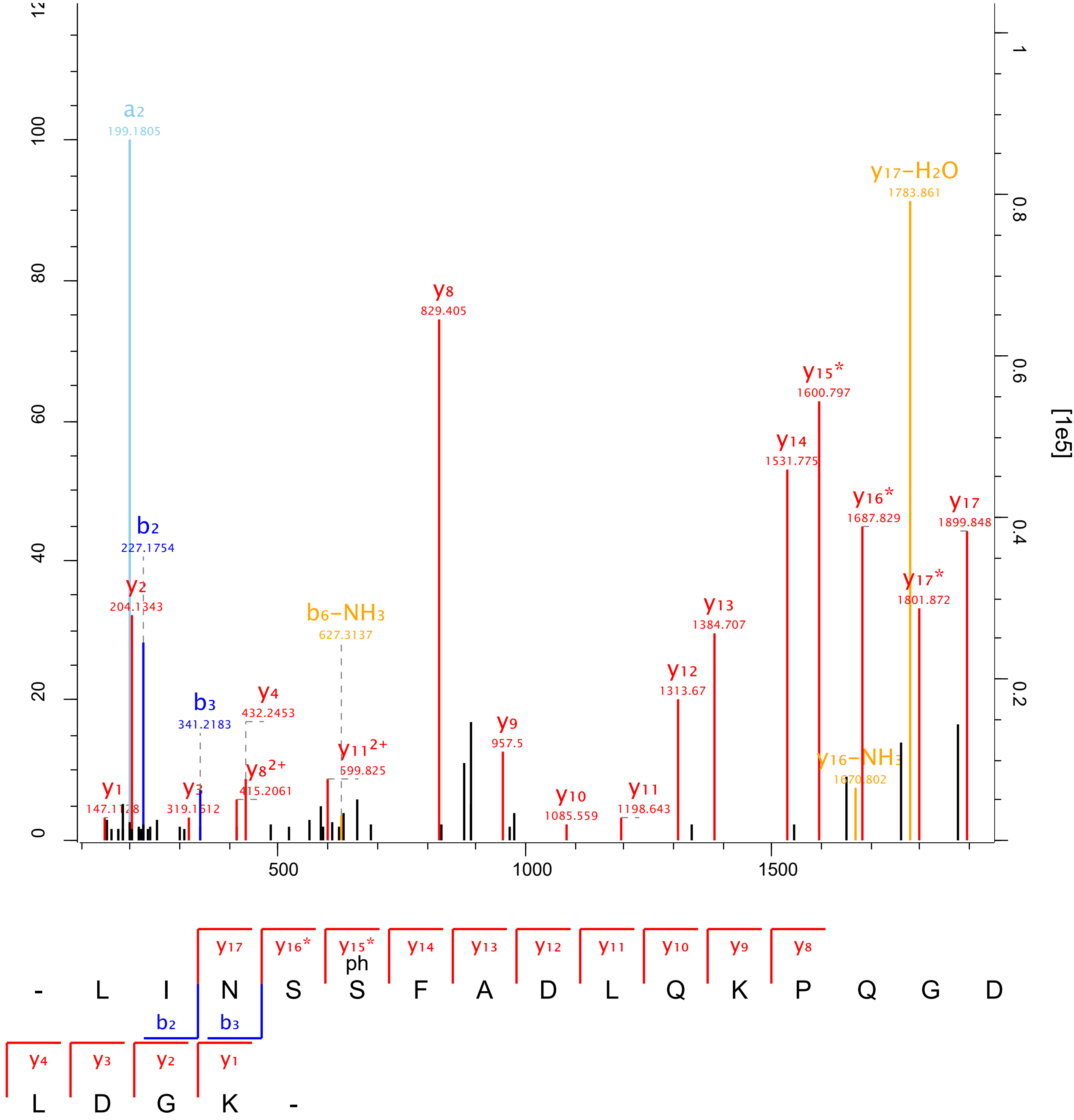

Raw file	Scan	Method	Score	m/z
sys_15_1	27502	FTMS; HCD	149.71	896.89

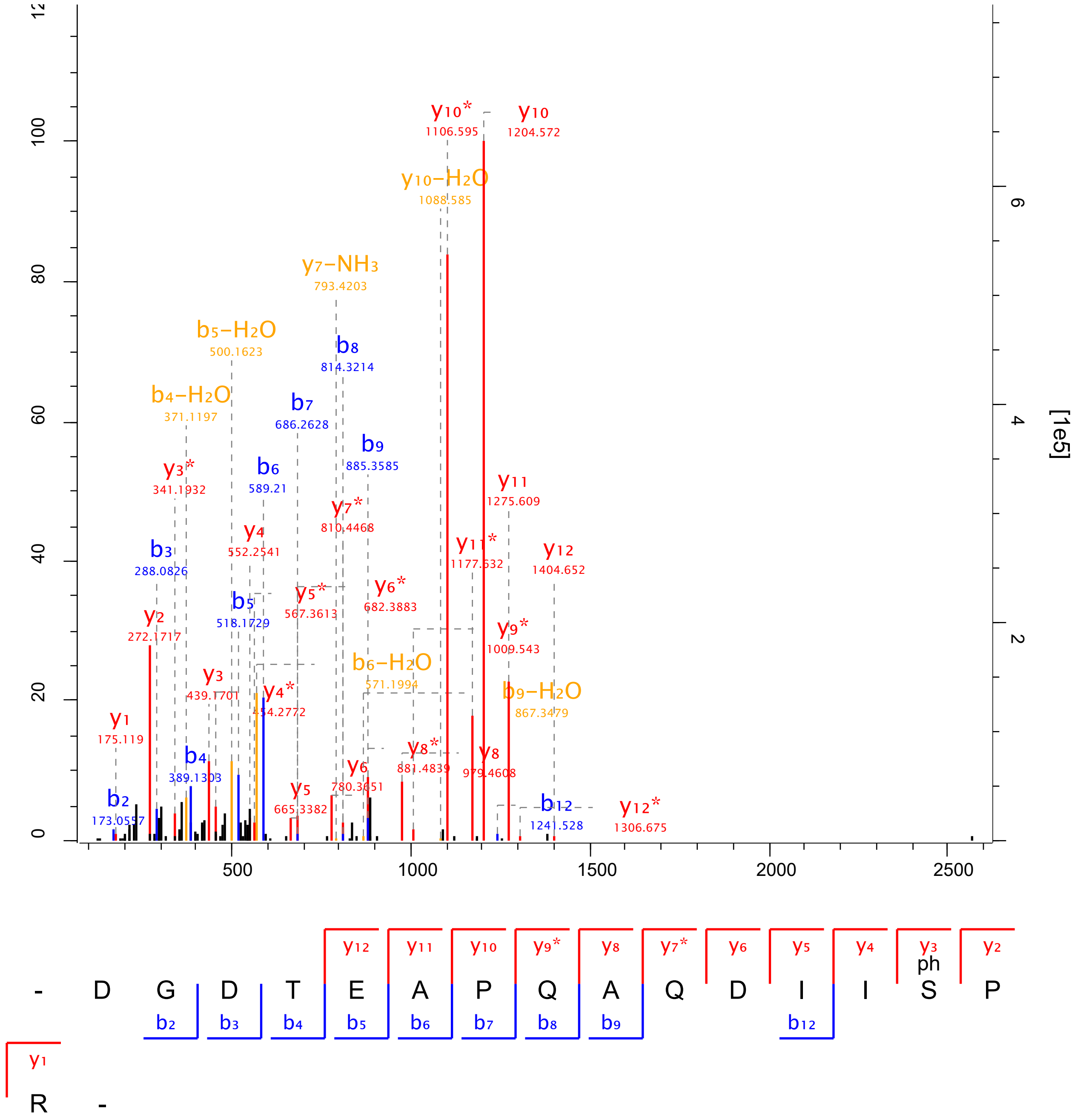

Raw file	Scan	Method	Score	m/z
sys_15_1	27569	FTMS; HCD	172.98	954.93

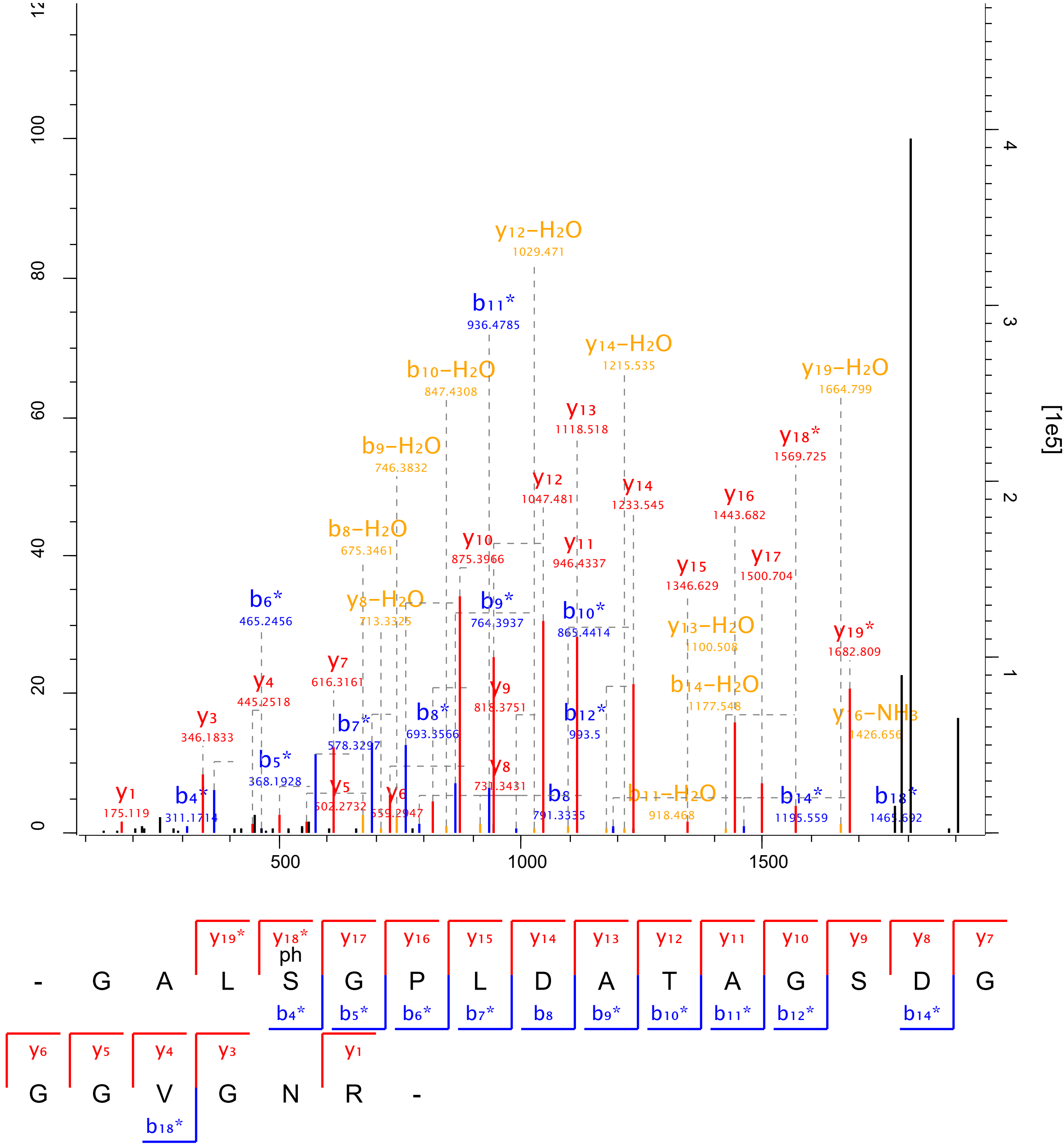

Raw file	Scan	Method	Score	m/z
sys_15_1	27604	FTMS; HCD	82.07	596.77

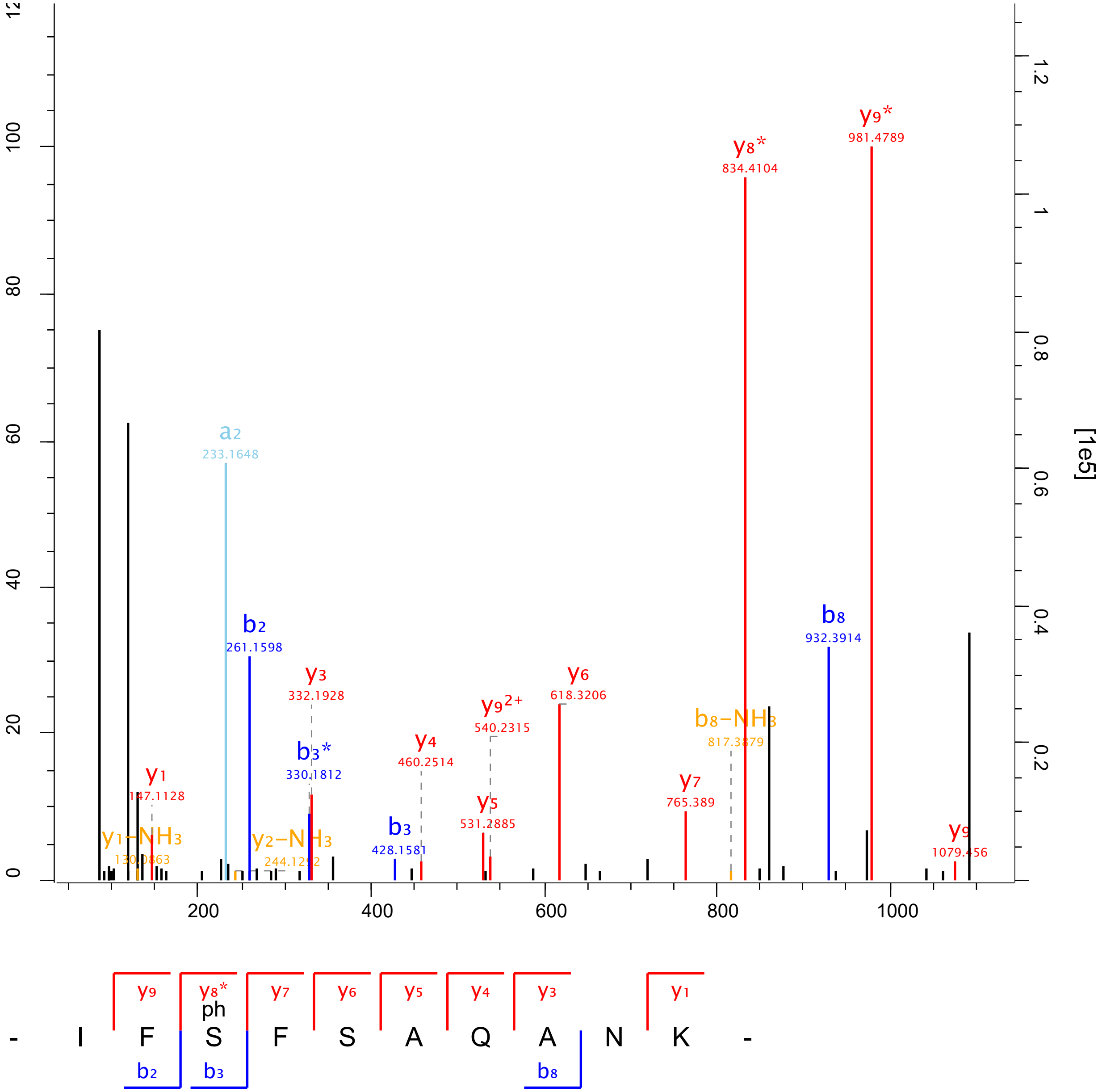

Raw file	Scan	Method	Score	m/z
sys_15_1	27623	FTMS; HCD	139.78	444.71

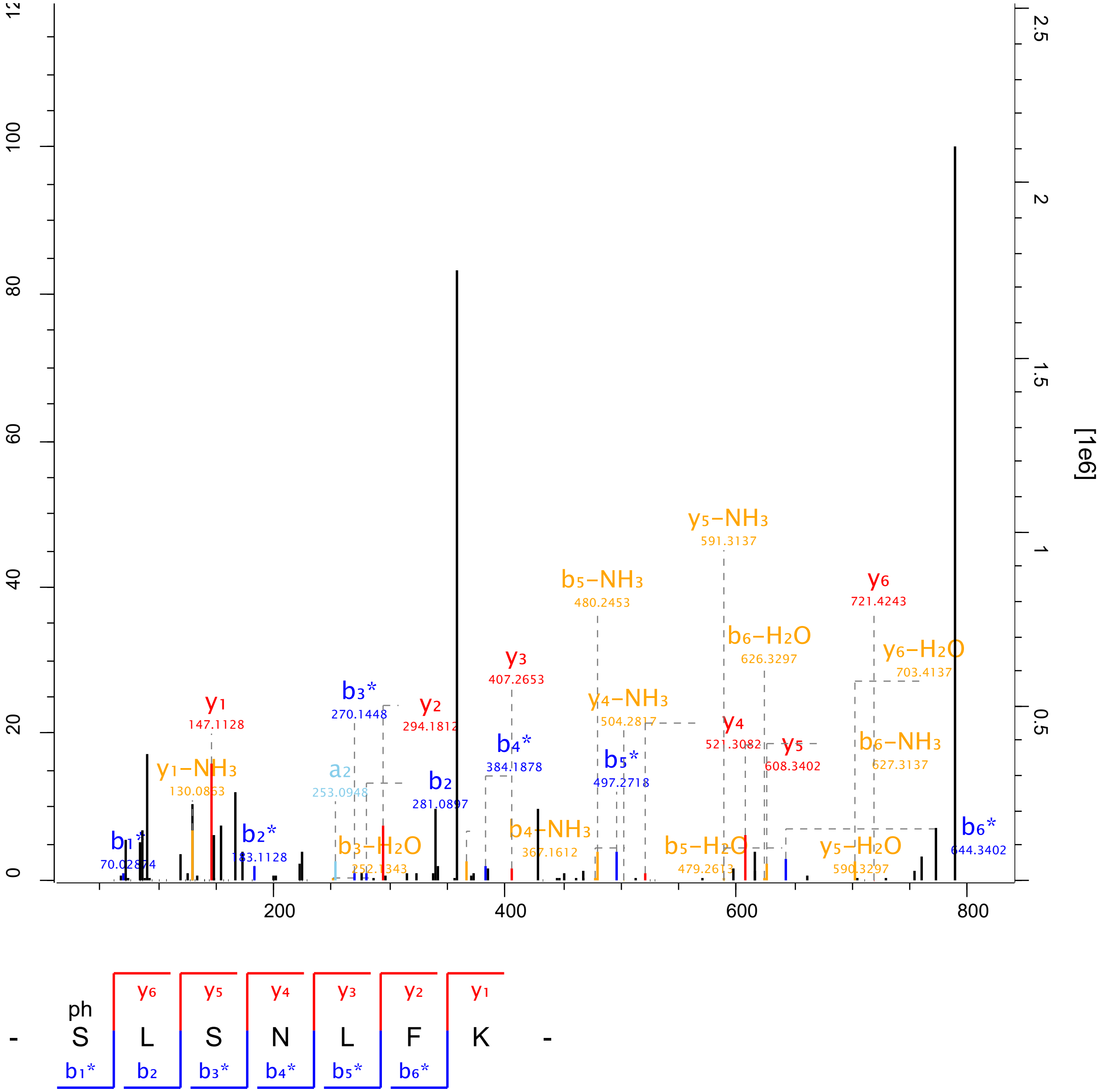

Raw file	Scan	Method	Score	m/z
sys_15_1	27796	FTMS; HCD	60.49	693.82

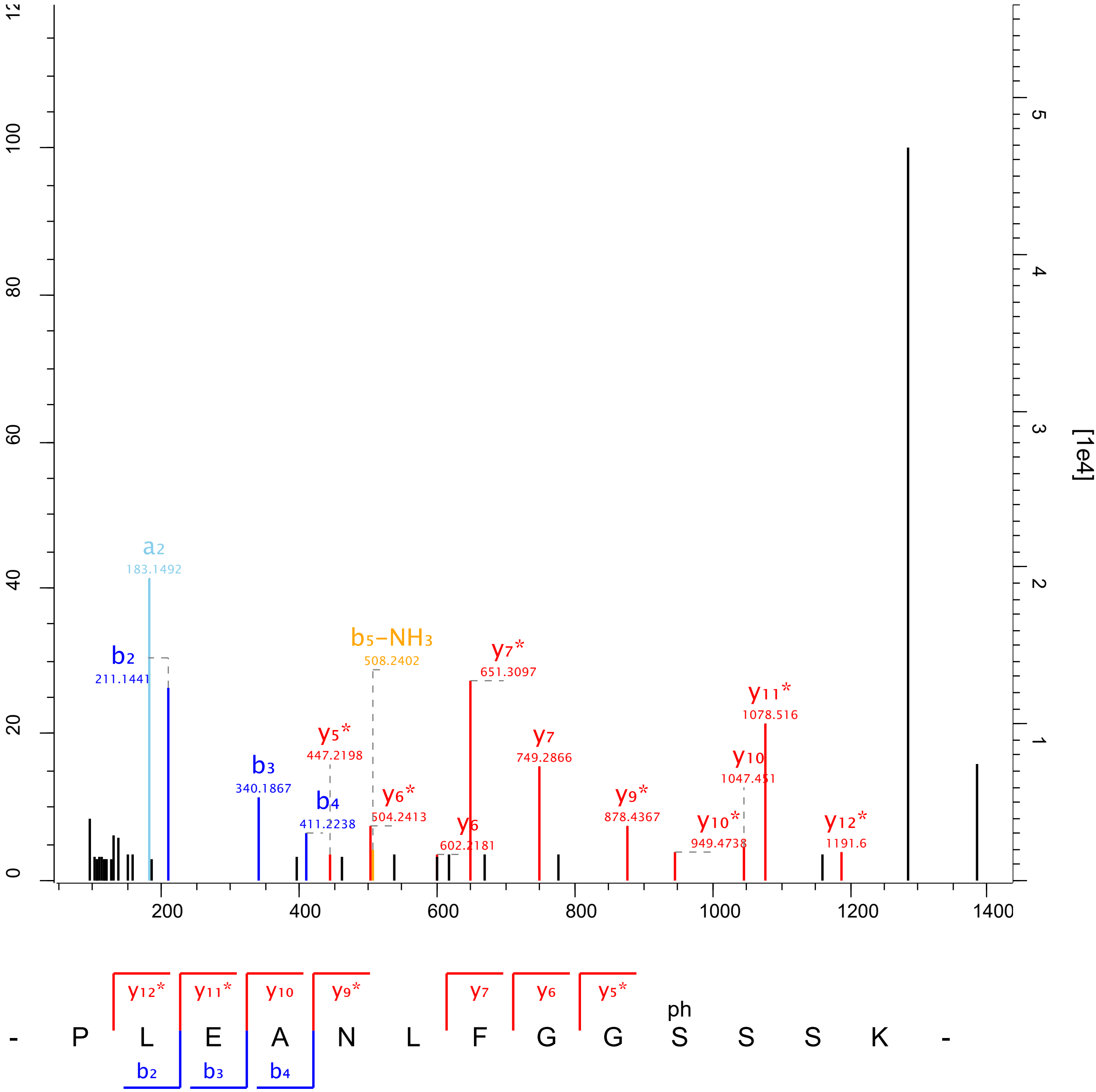

Raw file	Scan	Method	Score	m/z
sys_15_1	27899	FTMS; HCD	110.26	961.91

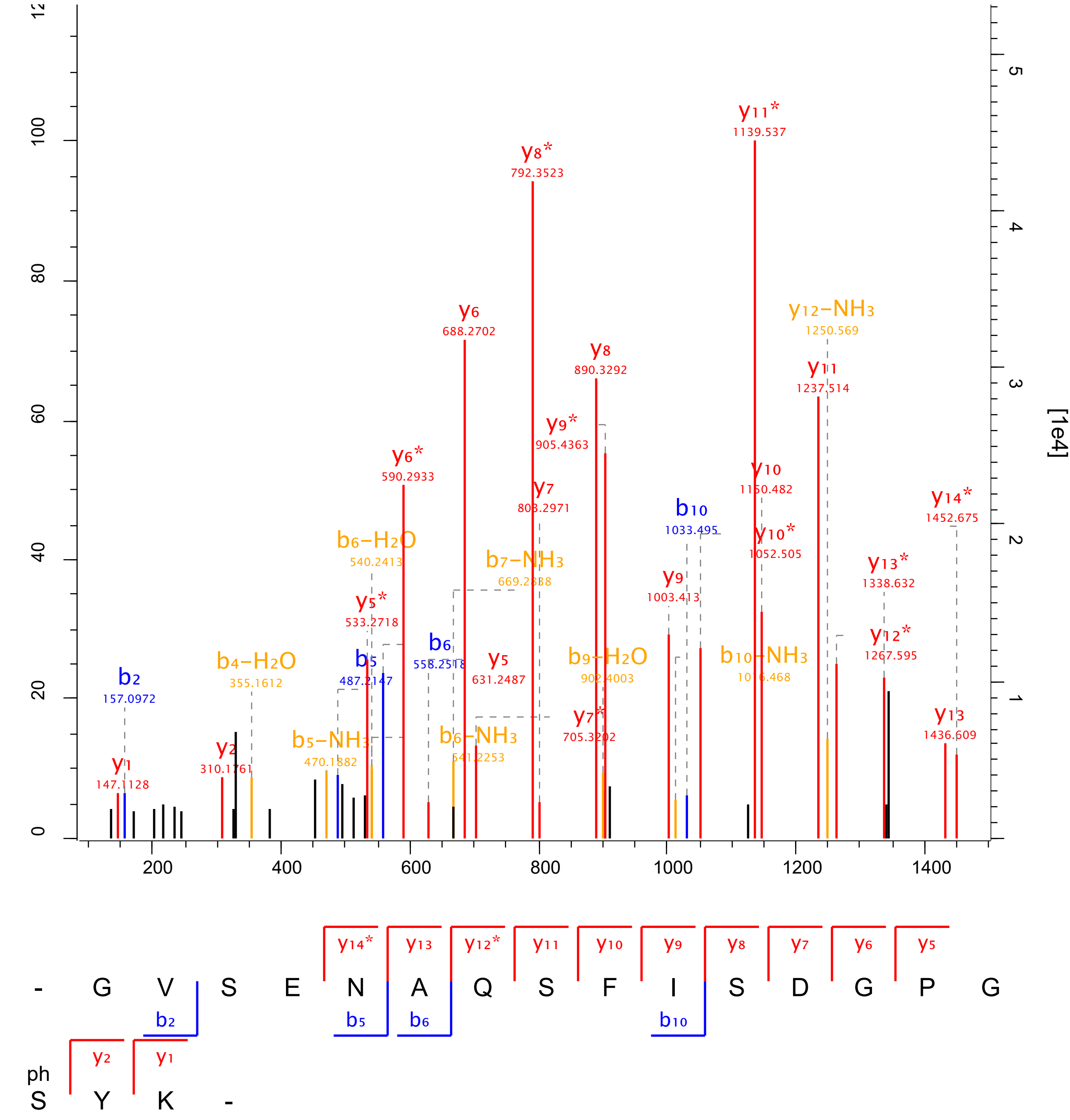

Raw file	Scan	Method	Score	m/z
sys_15_1	27936	FTMS; HCD	77.22	740.02

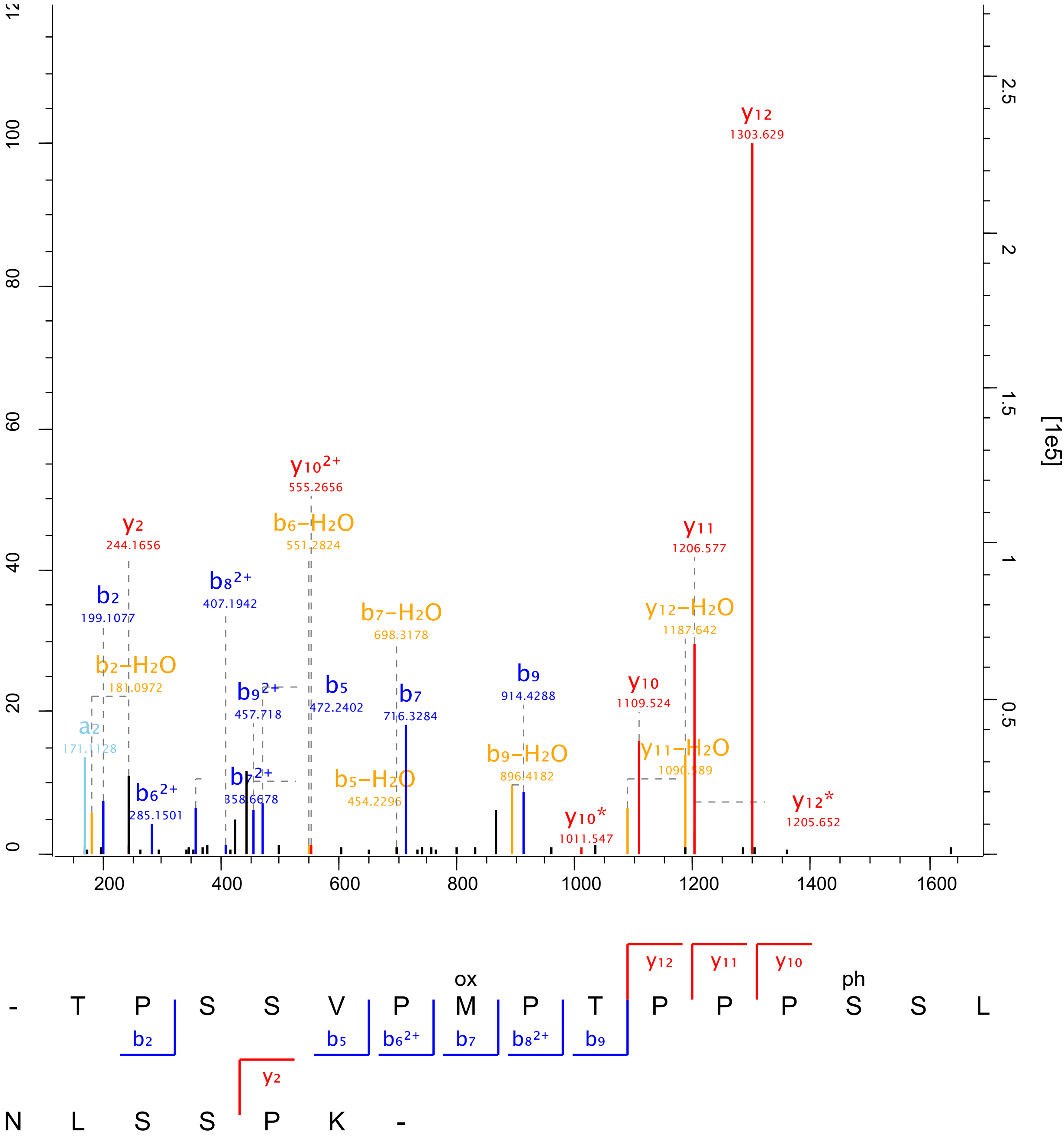

Raw file	Scan	Method	Score	m/z
sys_15_1	27985	FTMS; HCD	49.66	667.34

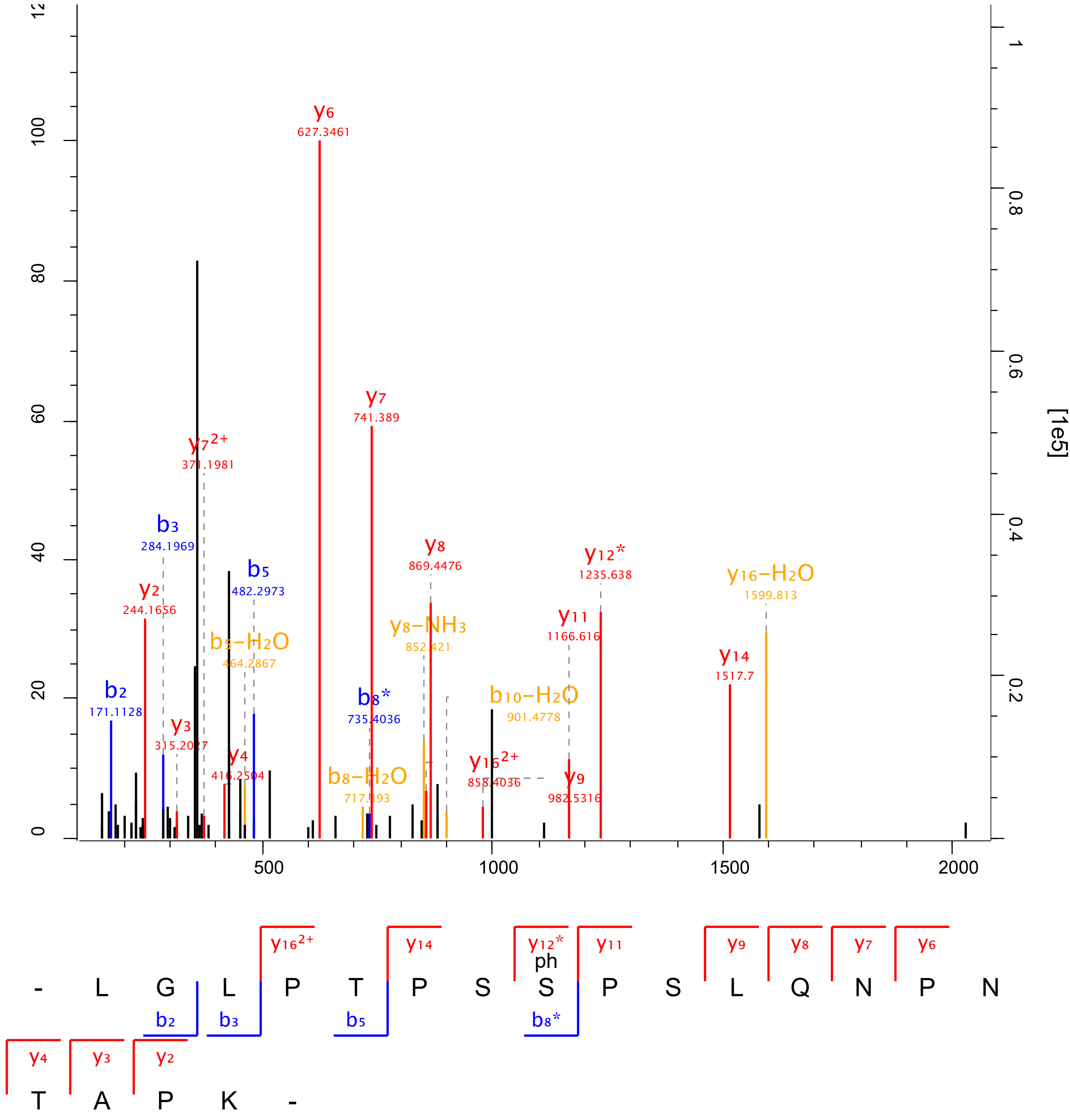

Raw file	Scan	Method	Score	m/z
sys_15_1	28118	FTMS; HCD	105.46	587.26

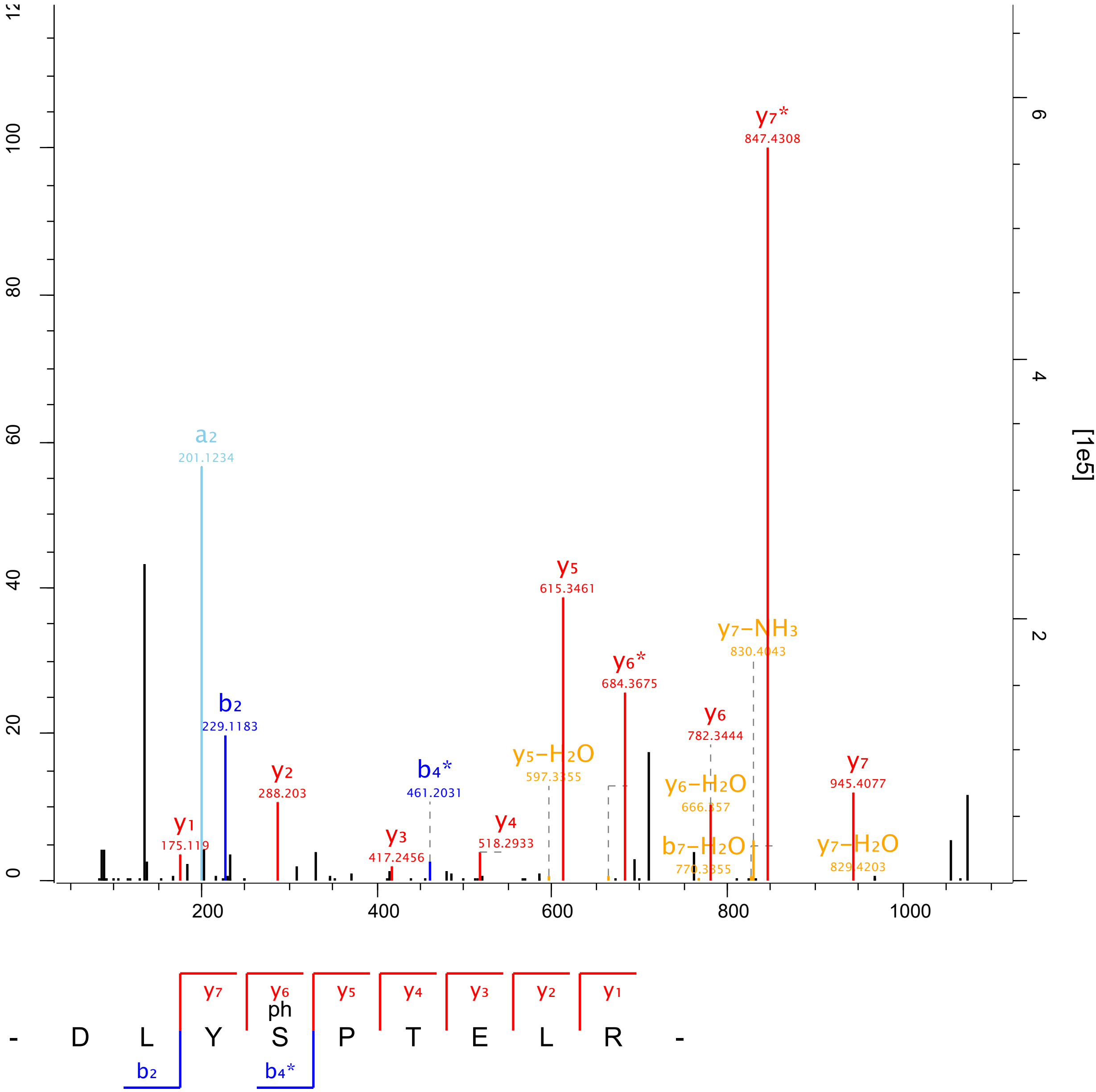

Raw file	Scan	Method	Score	m/z
sys_15_1	28139	FTMS; HCD	50.5	792.35

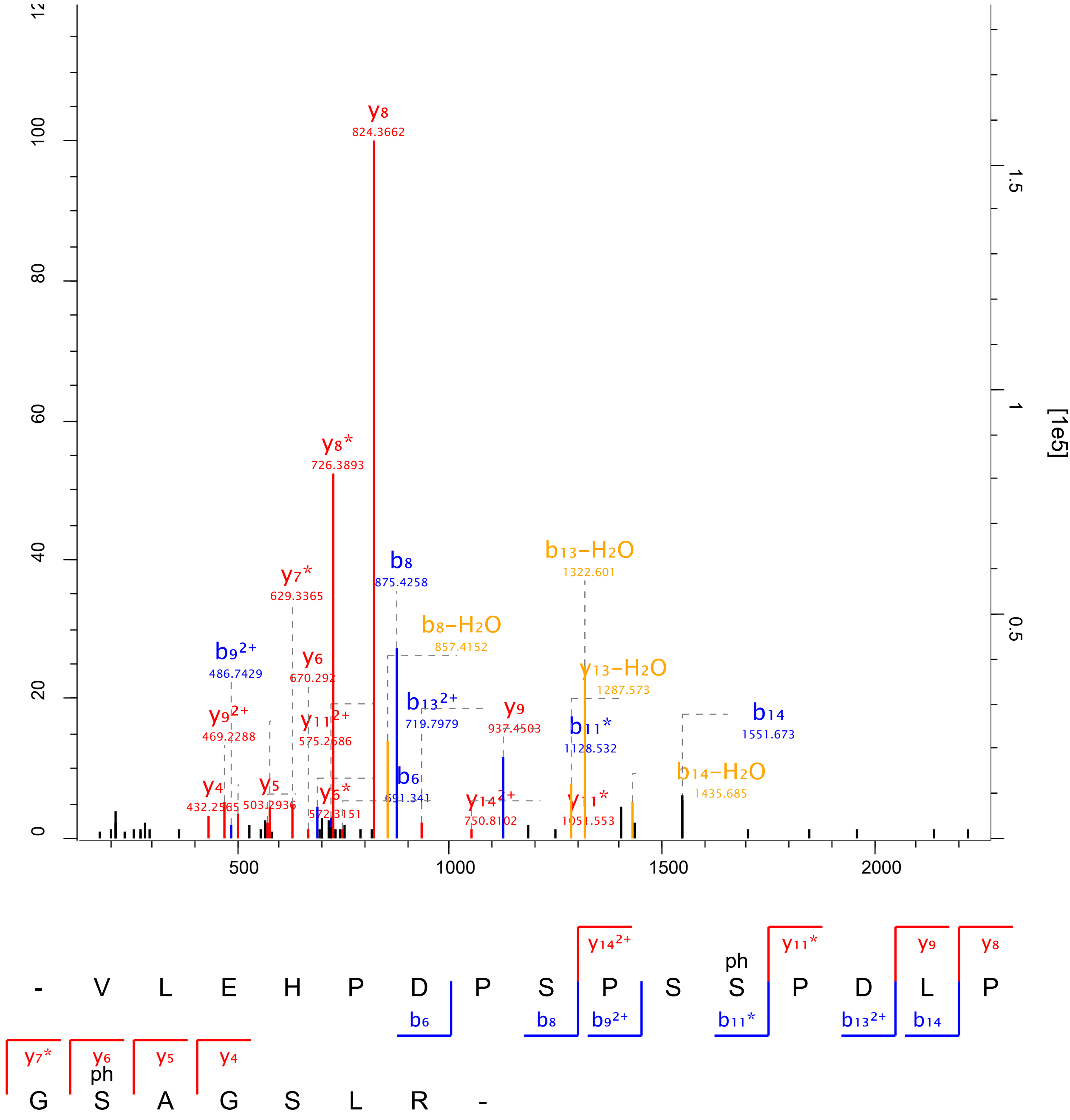

Raw file	Scan	Method	Score	m/z
sys_15_1	28290	FTMS; HCD	204.17	709.8

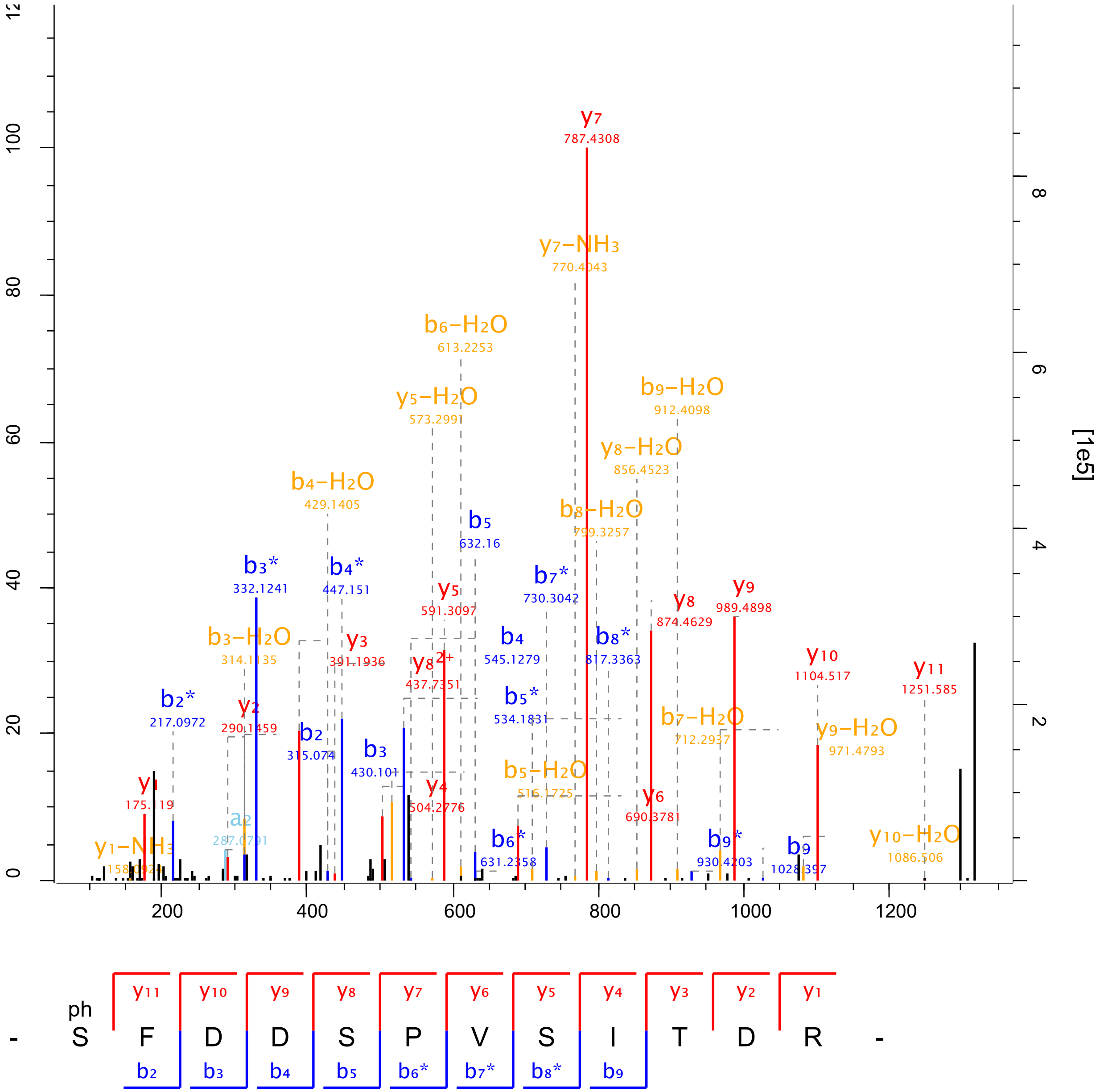

Raw file	Scan	Method	Score	m/z
sys_15_1	28328	FTMS; HCD	131.35	694.81

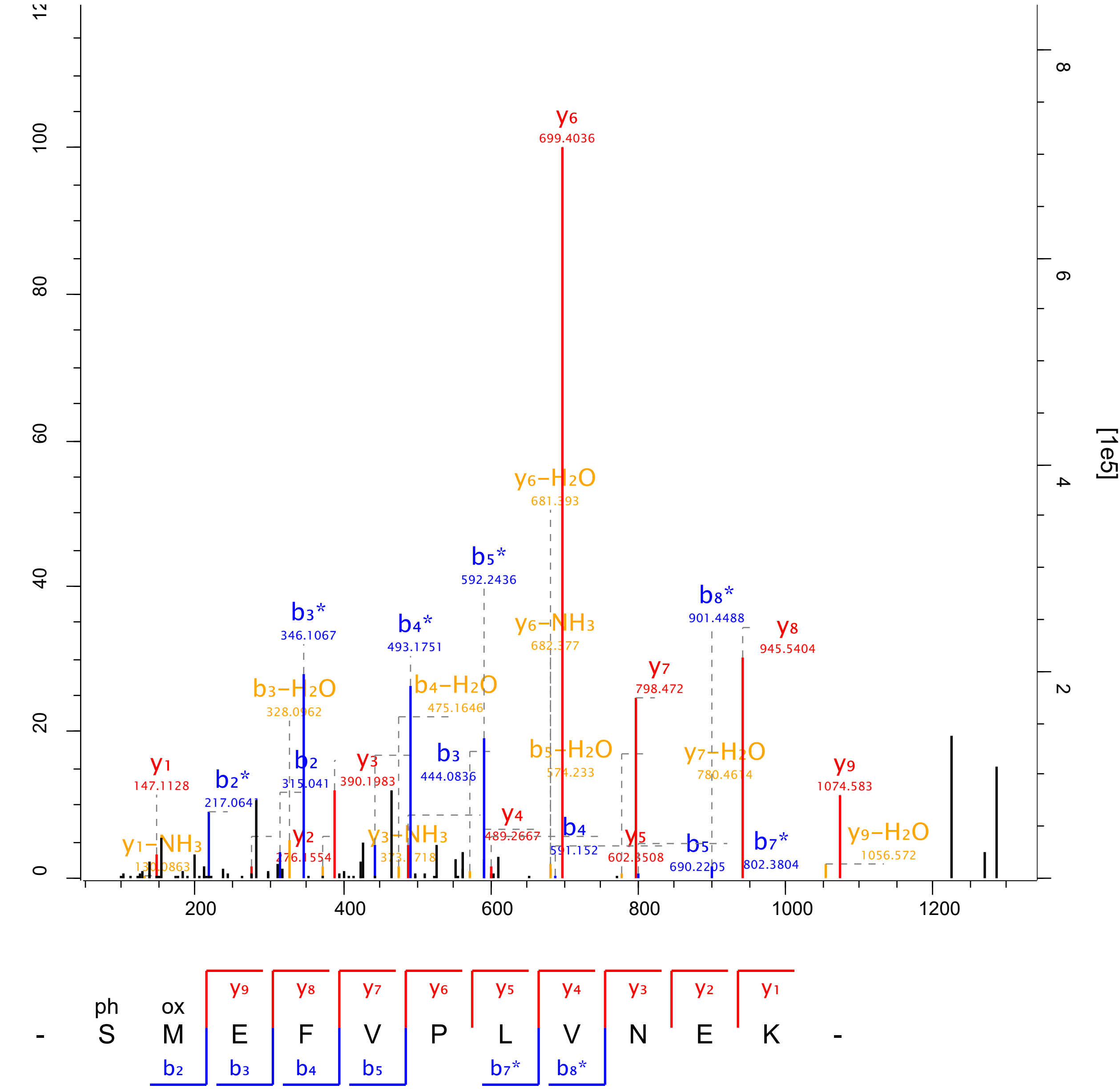

Raw file	Scan	Method	Score	m/z
sys_15_1	28403	FTMS; HCD	173.09	801.38

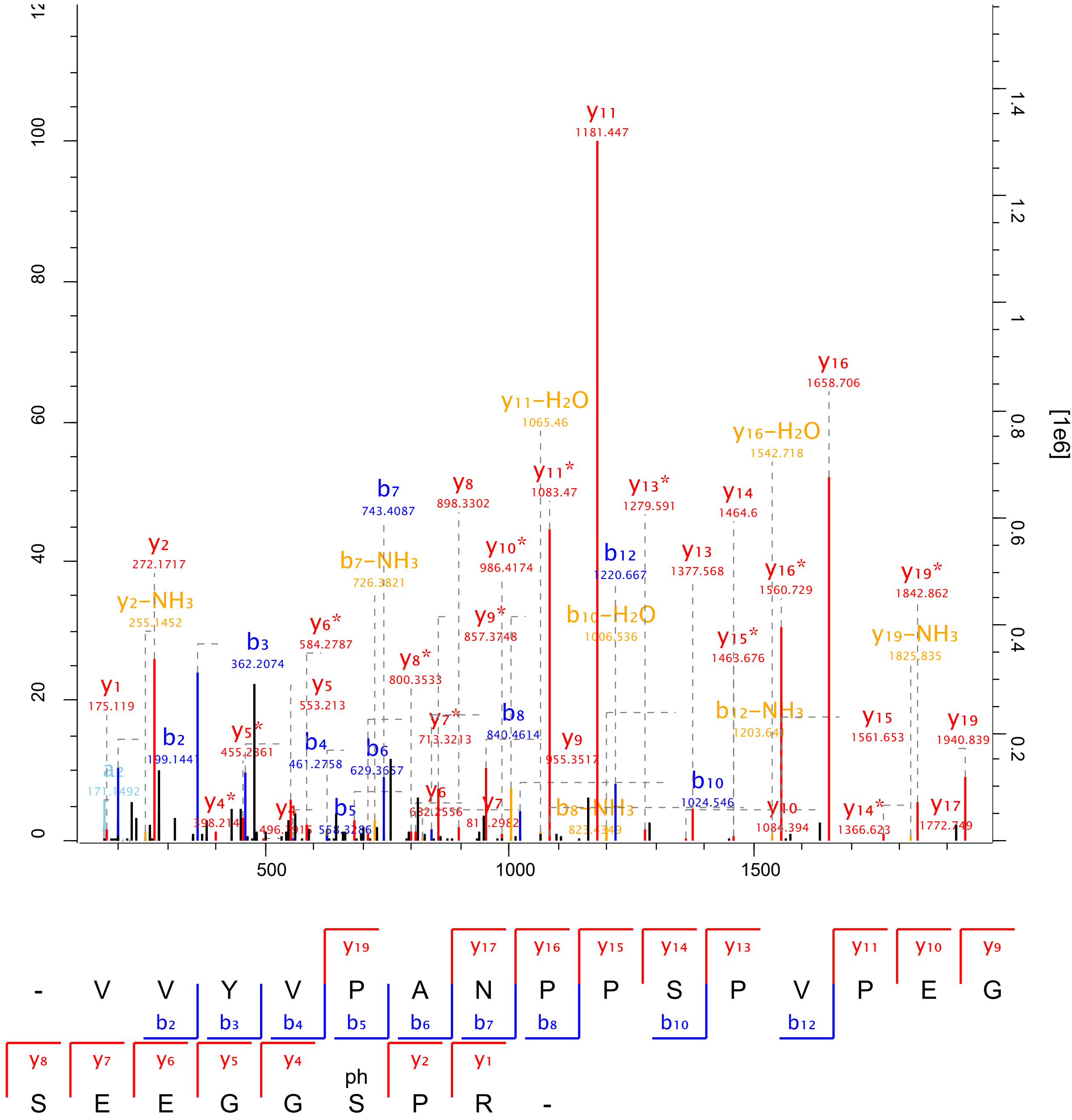

Raw file	Scan	Method	Score	m/z
sys_15_1	28460	FTMS; HCD	109.79	735.33

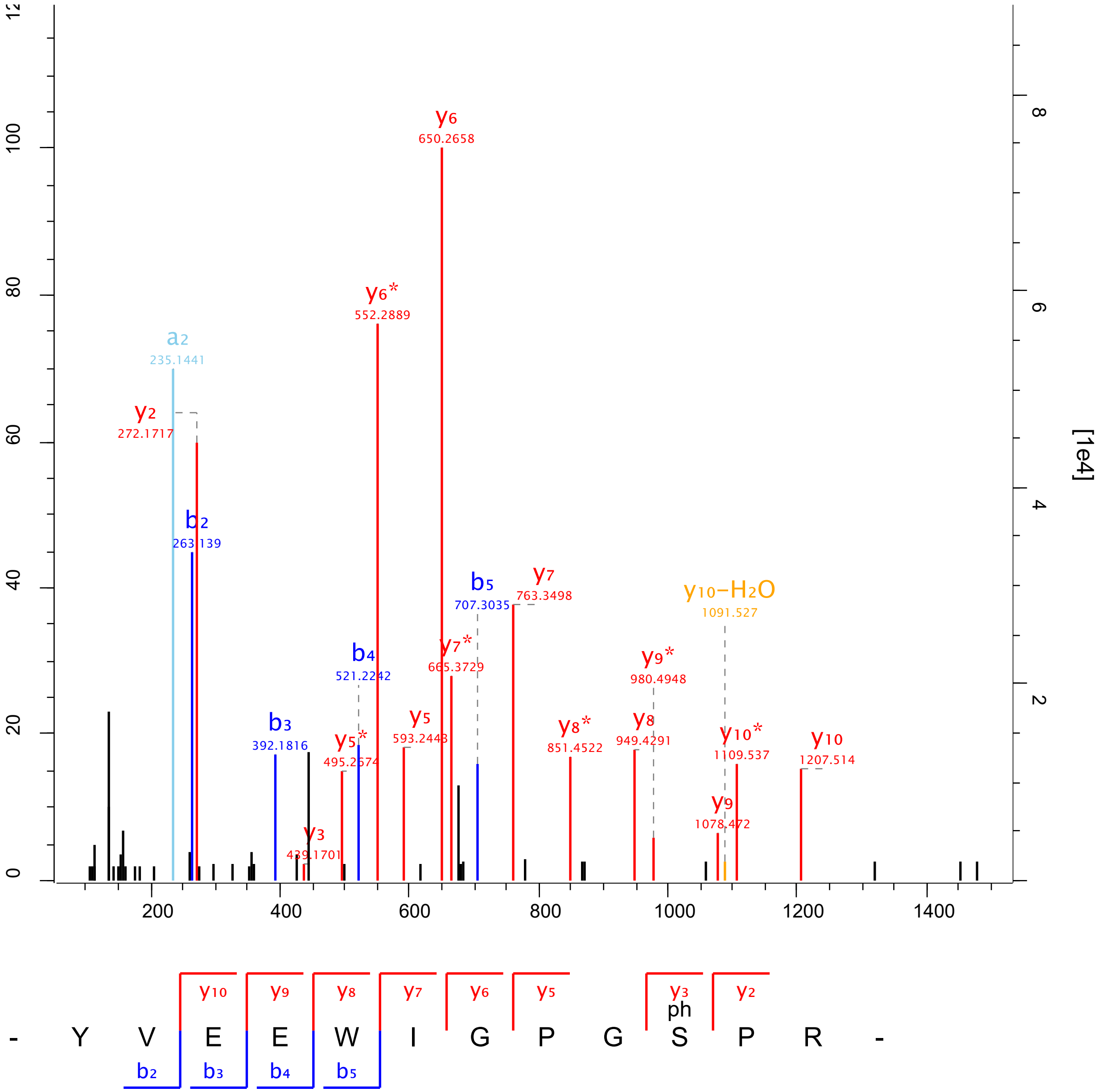

Raw file	Scan	Method	Score	m/z
sys_15_1	28491	FTMS; HCD	123.29	781.31

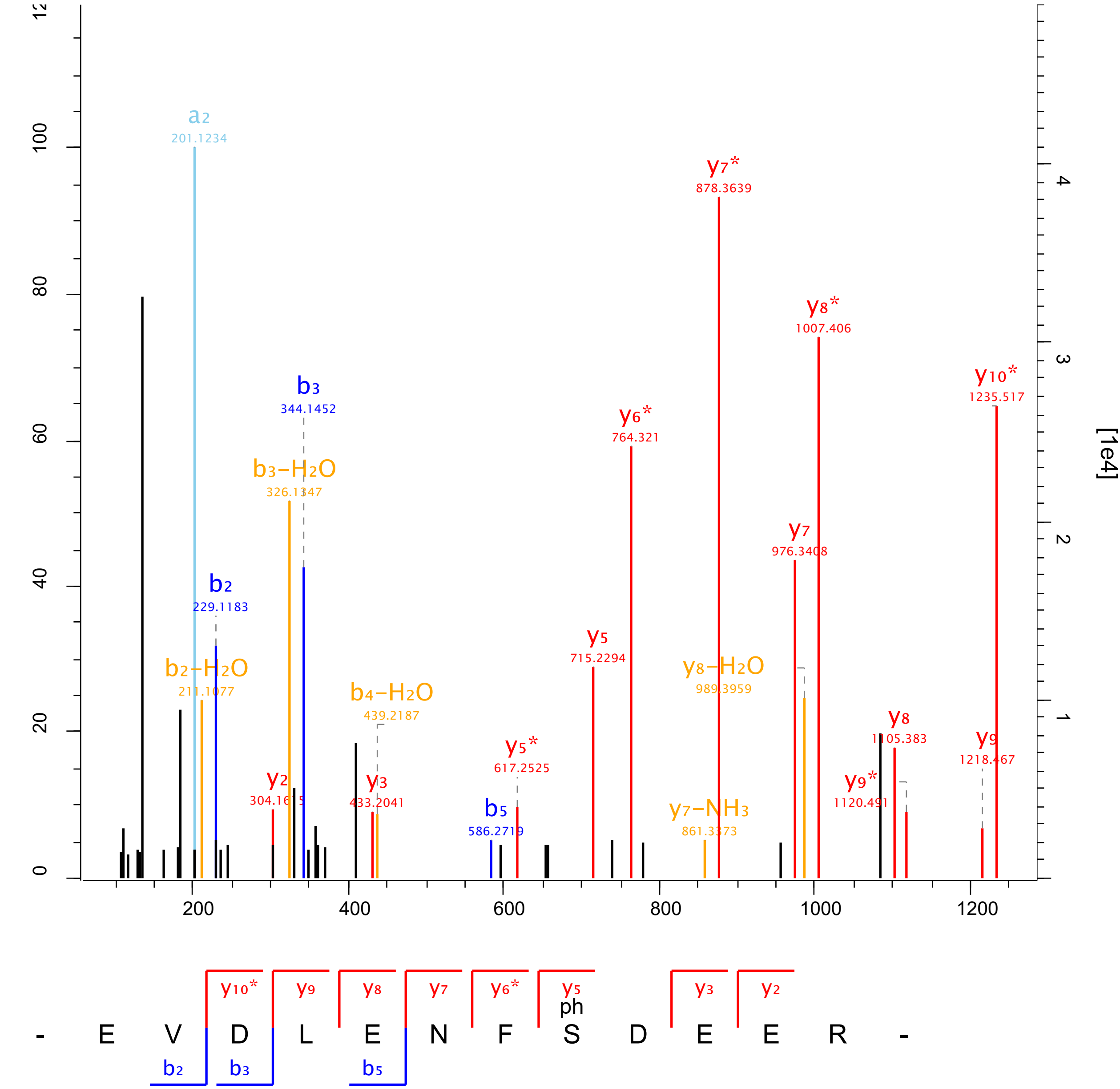

Raw file	Scan	Method	Score	m/z
sys_15_1	28549	FTMS; HCD	97.71	757.87

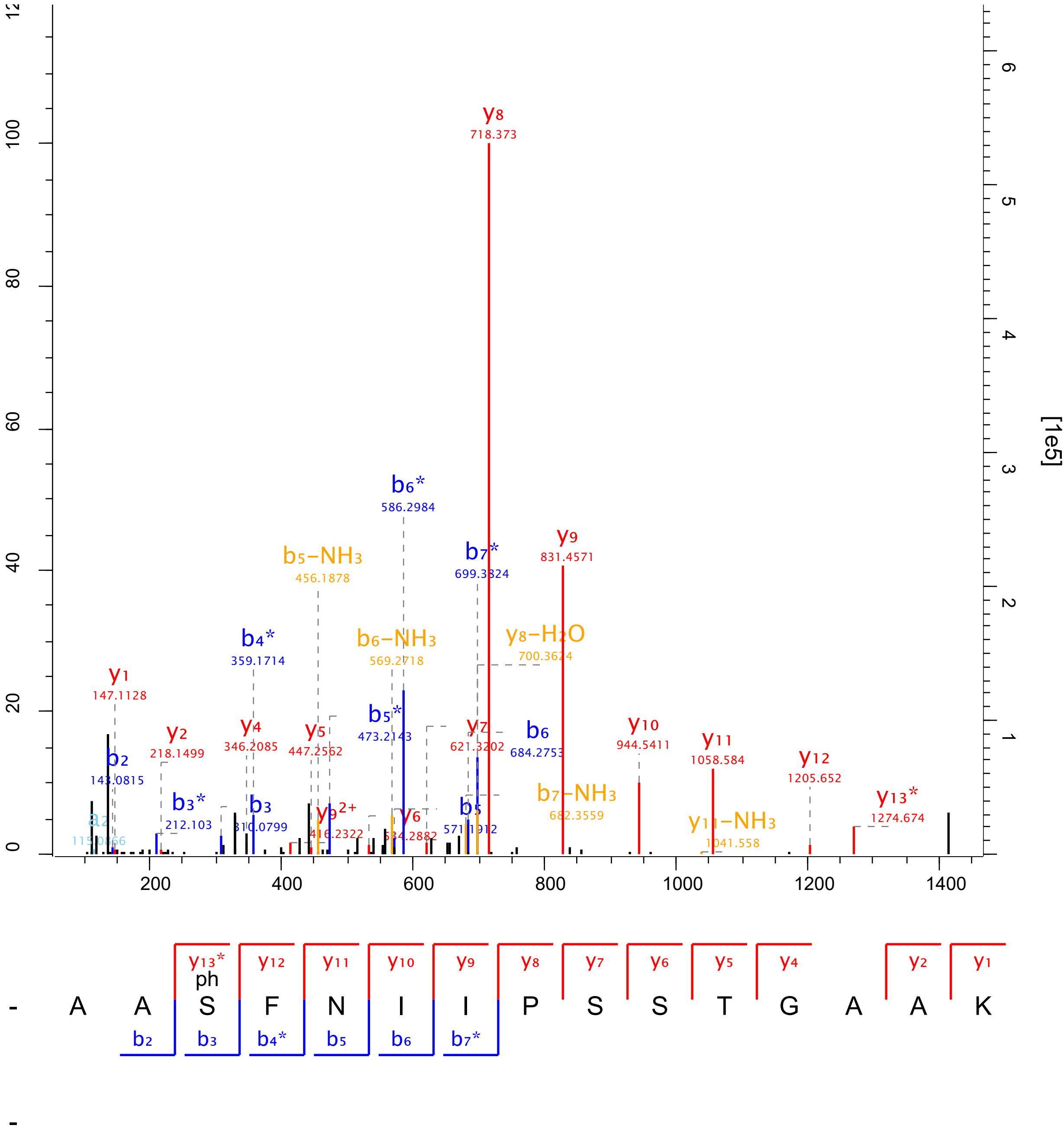

Raw file	Scan	Method	Score	m/z
sys_15_1	28886	FTMS; HCD	77.07	797.83

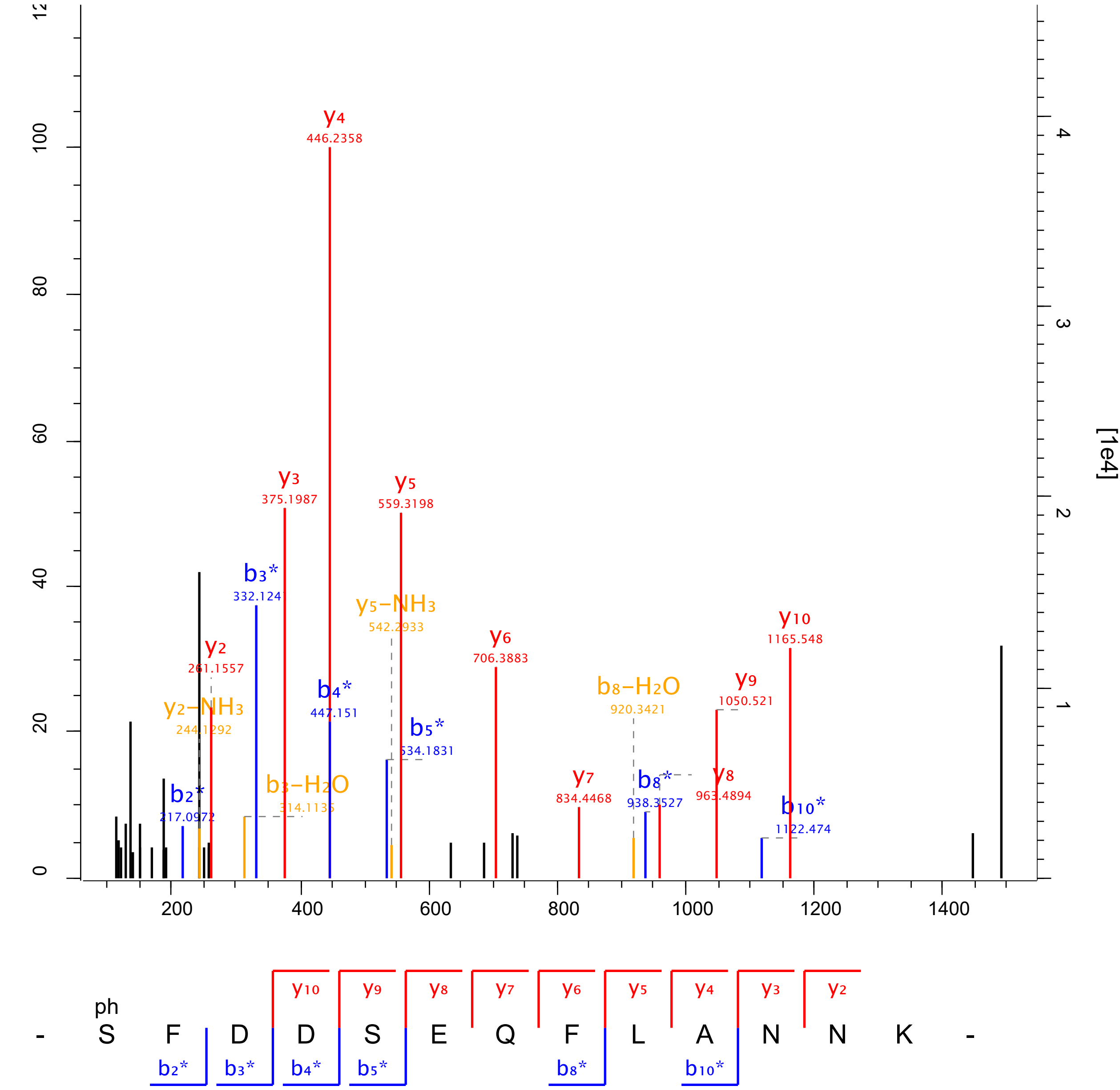

Raw file	Scan	Method	Score	m/z
sys_15_1	28929	FTMS; HCD	108.57	764.05

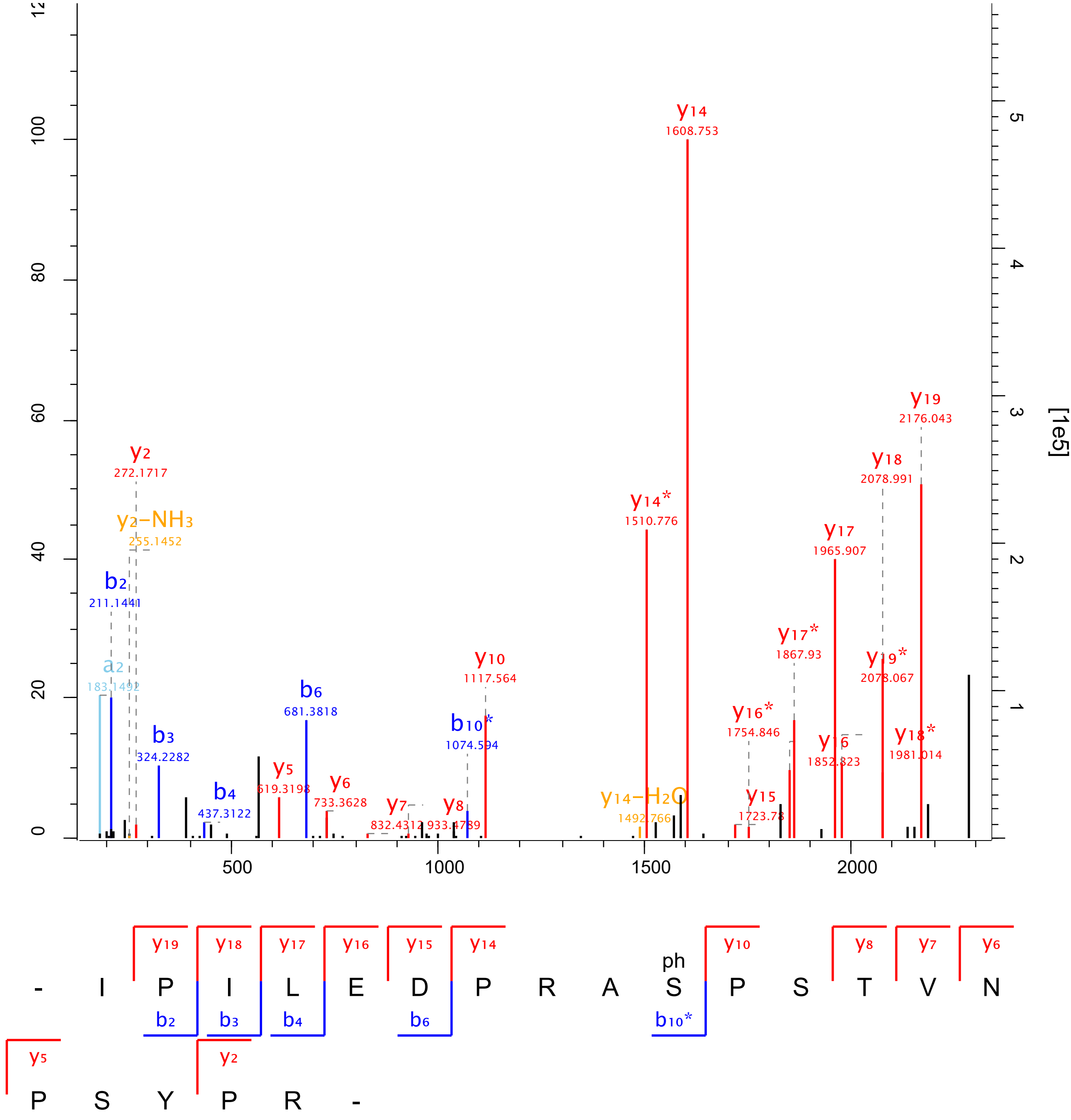

Raw file	Scan	Method	Score	m/z
sys_15_1	28942	FTMS; HCD	76.76	678.77

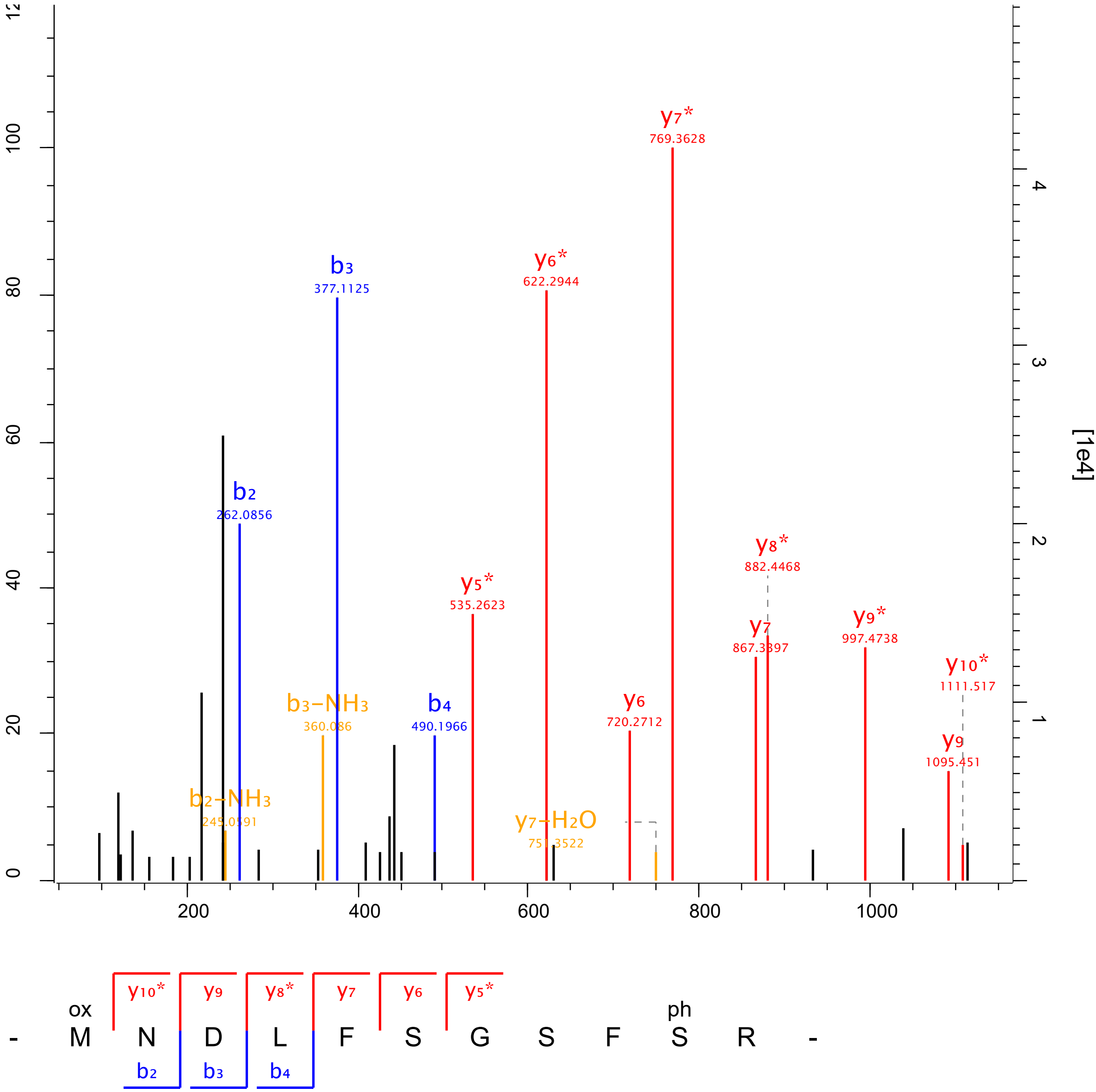

Raw file	Scan	Method	Score	m/z
sys_15_1	29114	FTMS; HCD	48.57	548.28

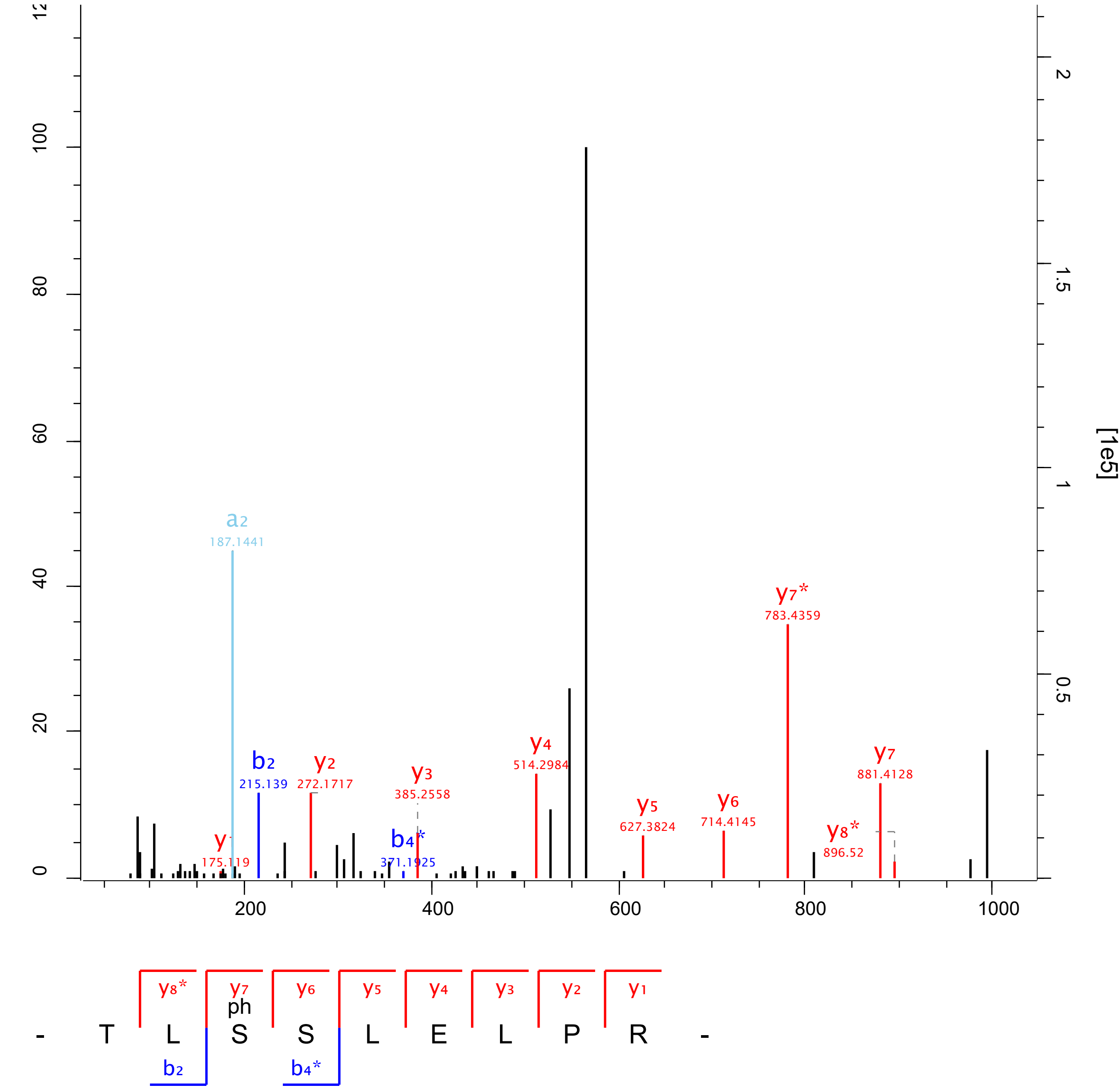

Raw file	Scan	Method	Score	m/z
sys_15_1	29185	FTMS; HCD	50.3	710.3

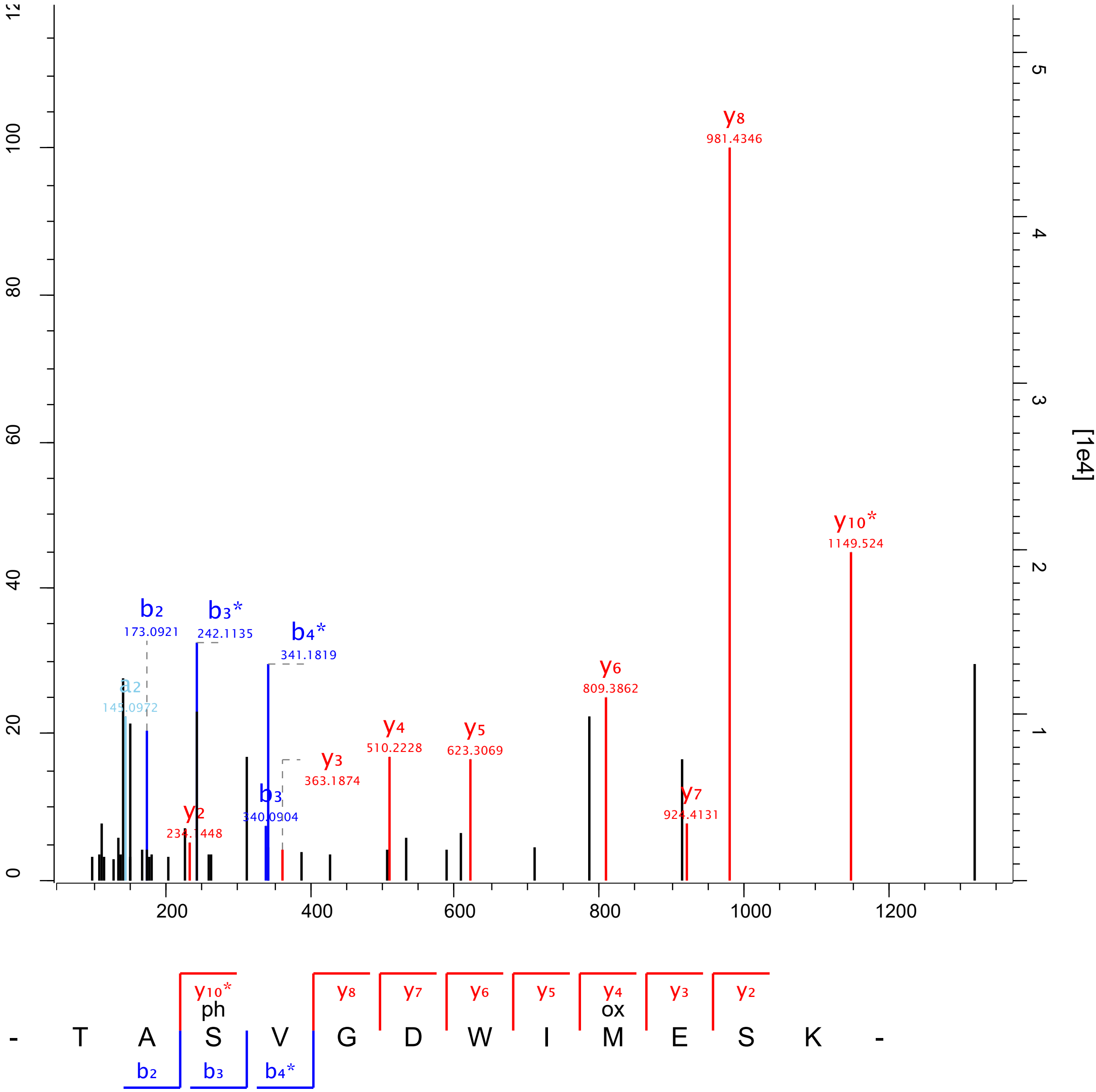

Raw file	Scan	Method	Score	m/z
sys_15_1	29224	FTMS; HCD	191.93	887.88

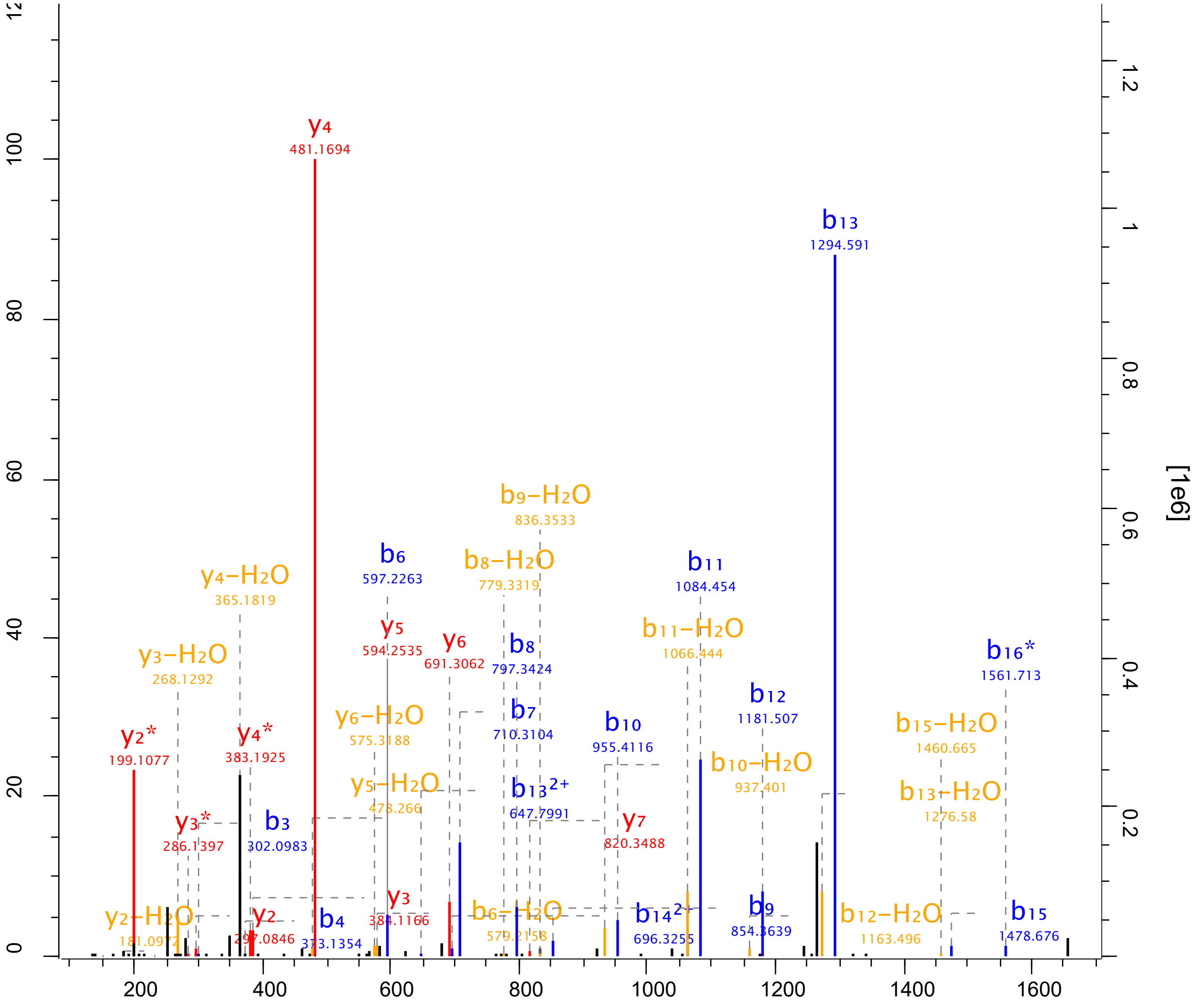

- D D A A S H I S G T E P I P S
b3 b4 b6 b7 b8 b9 b10 b11 b12 b13 b14 2+ b15
y7 y6 y5 y4 y3

y2
ph
T P -
b16*

Raw file	Scan	Method	Score	m/z
sys_15_1	2931	FTMS; HCD	42.03	711.32

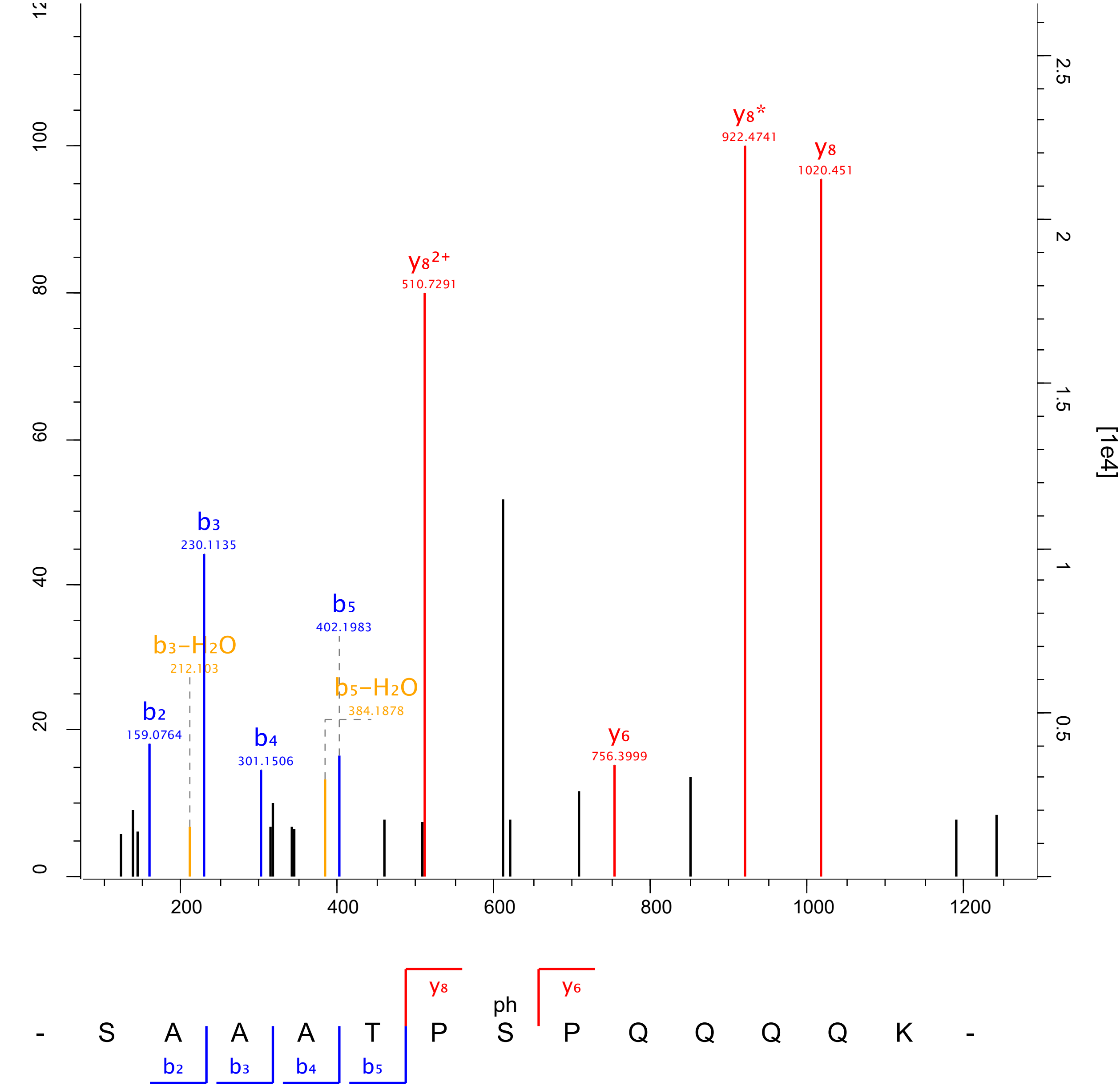

Raw file	Scan	Method	Score	m/z
sys_15_1	29353	FTMS; HCD	44.34	723.96

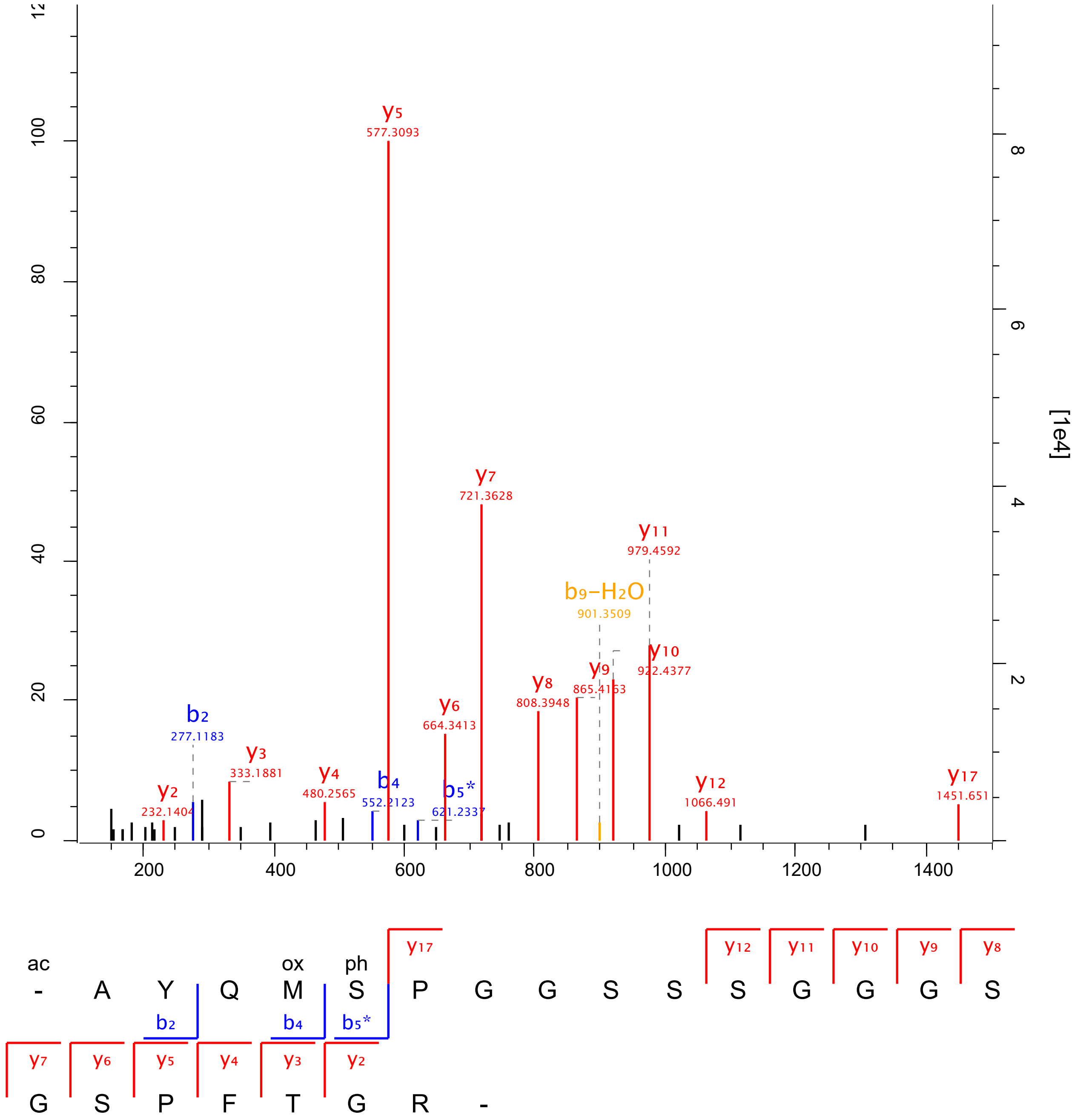

Raw file	Scan	Method	Score	m/z
sys_15_1	2937	FTMS; HCD	43.76	659.27

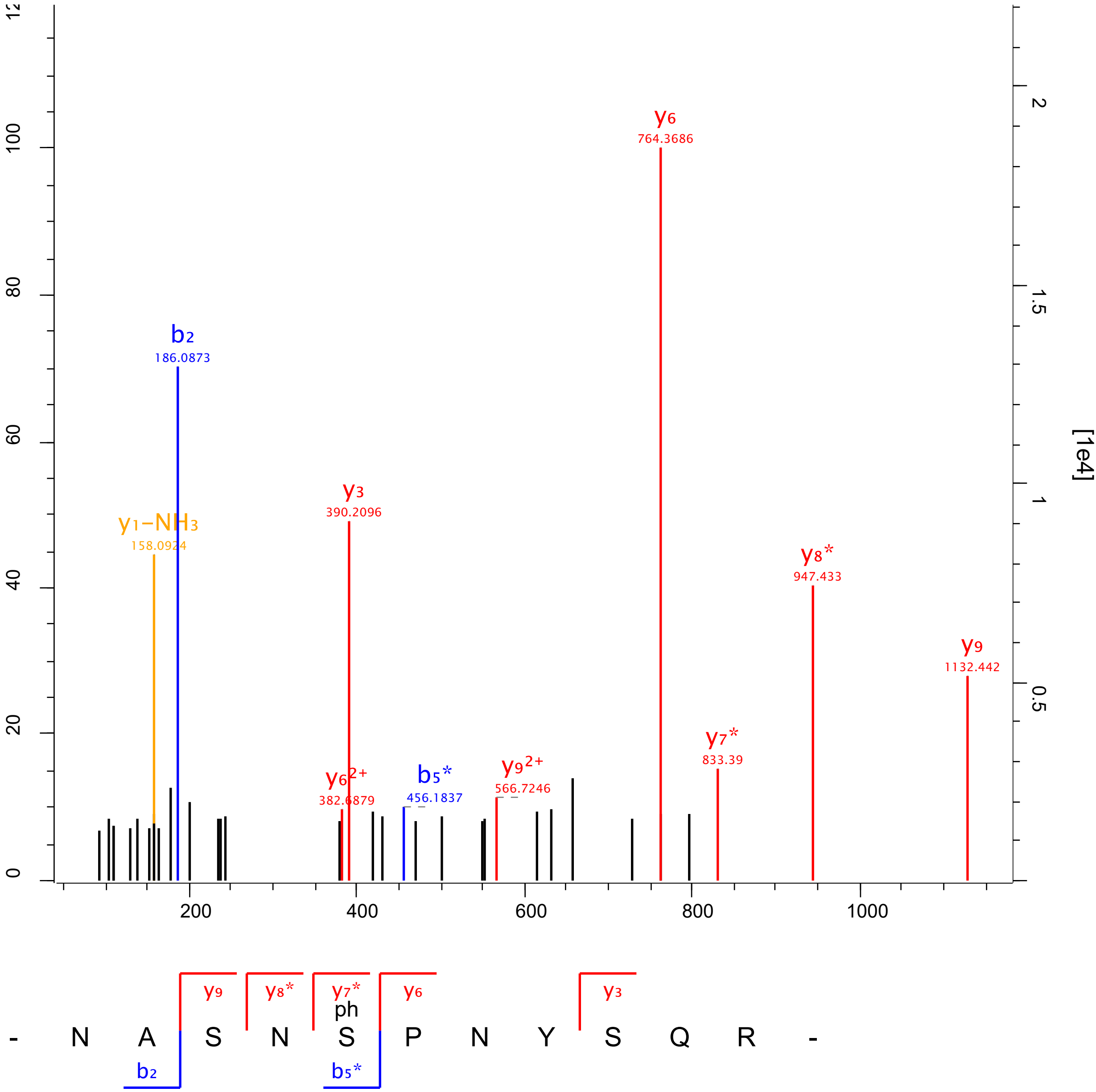

Raw file	Scan	Method	Score	m/z
sys_15_1	2946	FTMS; HCD	86.34	498.73

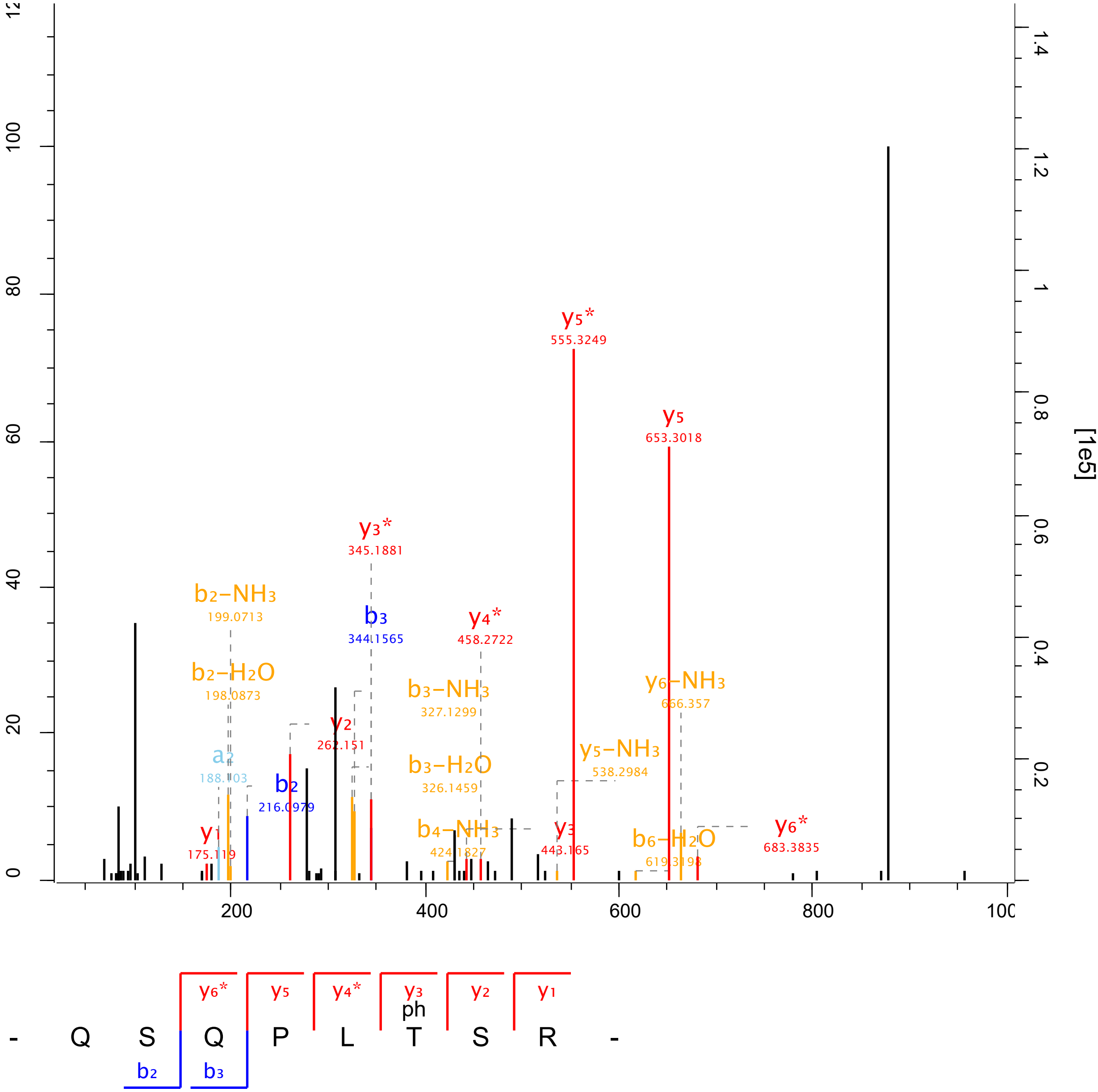

Raw file	Scan	Method	Score	m/z
sys_15_1	29469	FTMS; HCD	159.76	1079.98

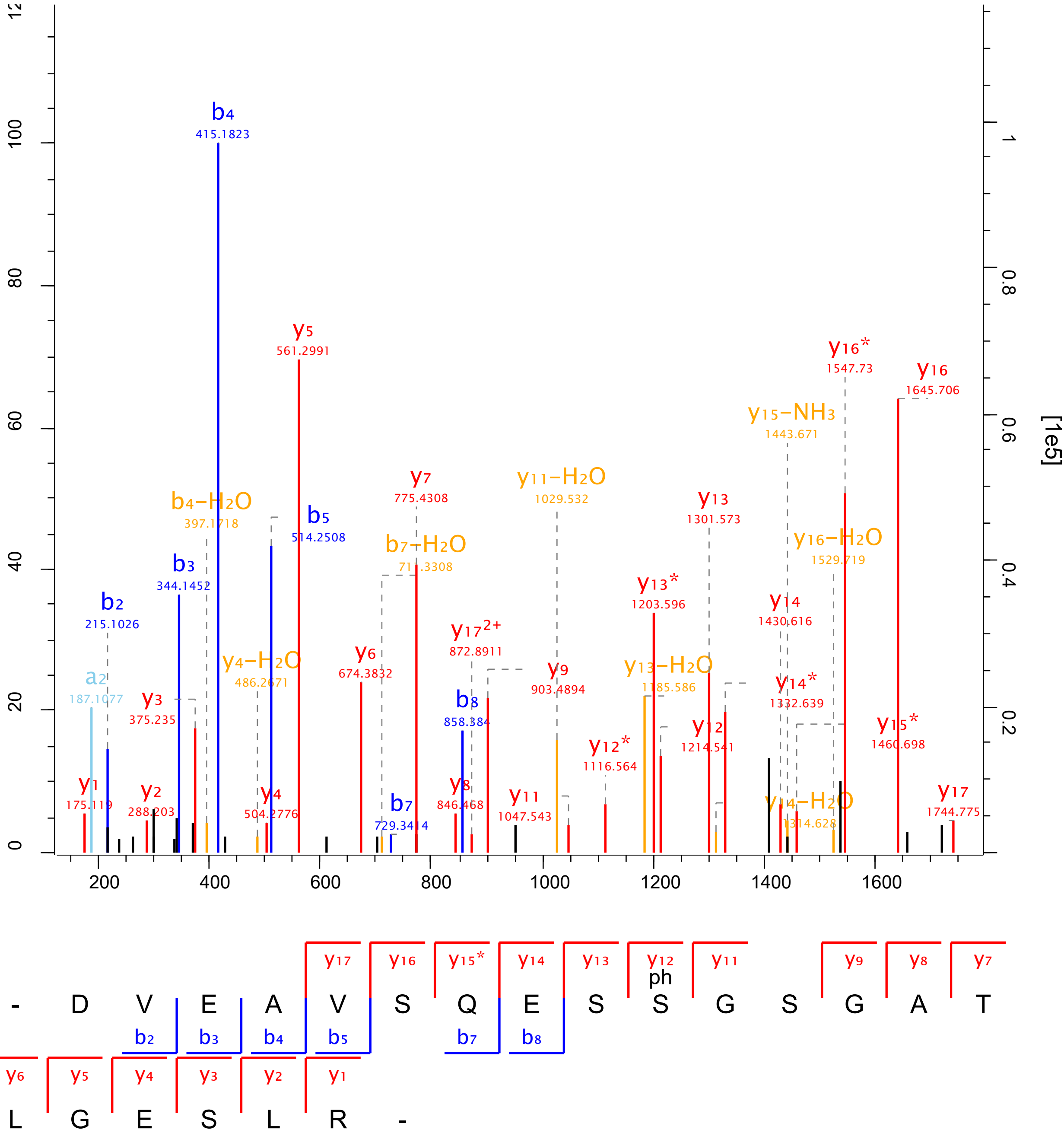

Raw file	Scan	Method	Score	m/z
sys_15_1	29561	FTMS; HCD	72.99	728.69

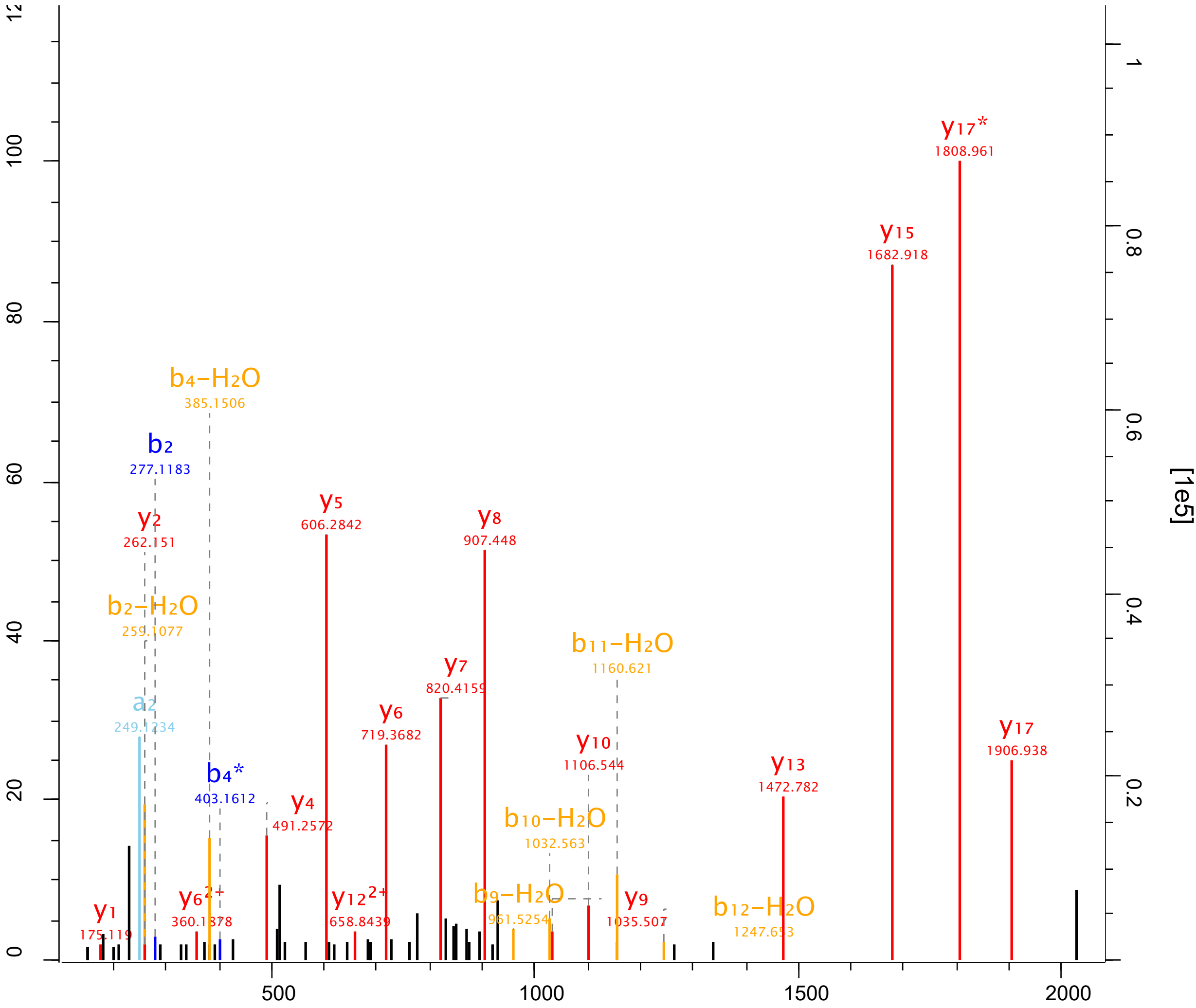

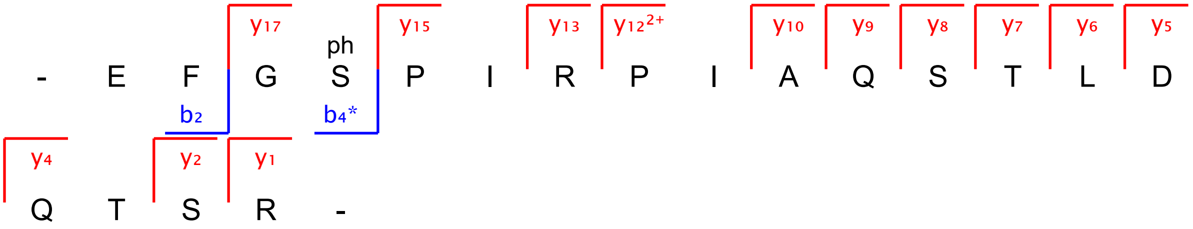

Raw file	Scan	Method	Score	m/z
sys_15_1	2965	FTMS; HCD	50.35	506.71

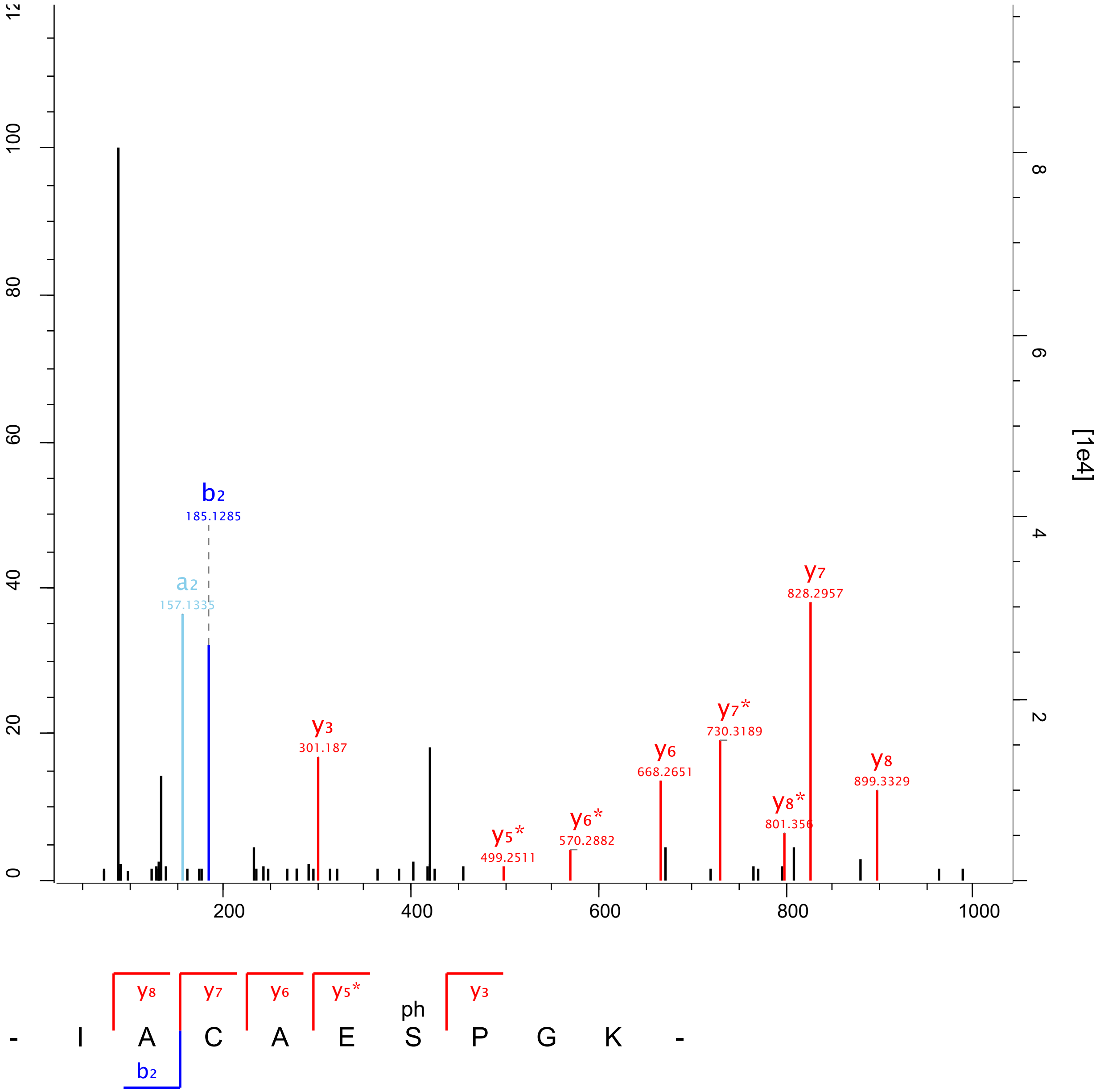

Raw file	Scan	Method	Score	m/z
sys_15_1	29675	FTMS; HCD	51.73	583.25

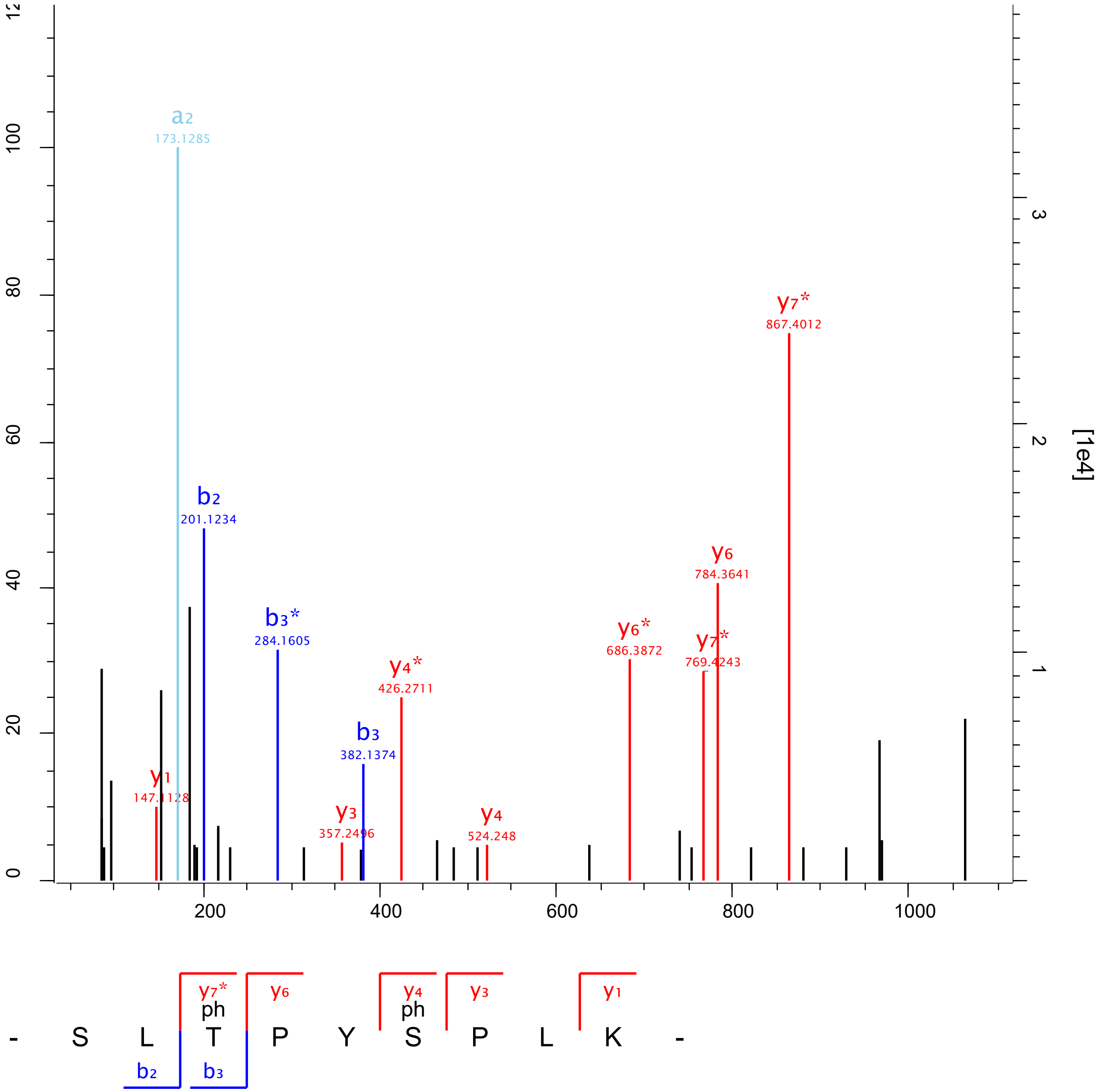

Raw file	Scan	Method	Score	m/z
sys_15_1	29855	FTMS; HCD	42.06	657.64

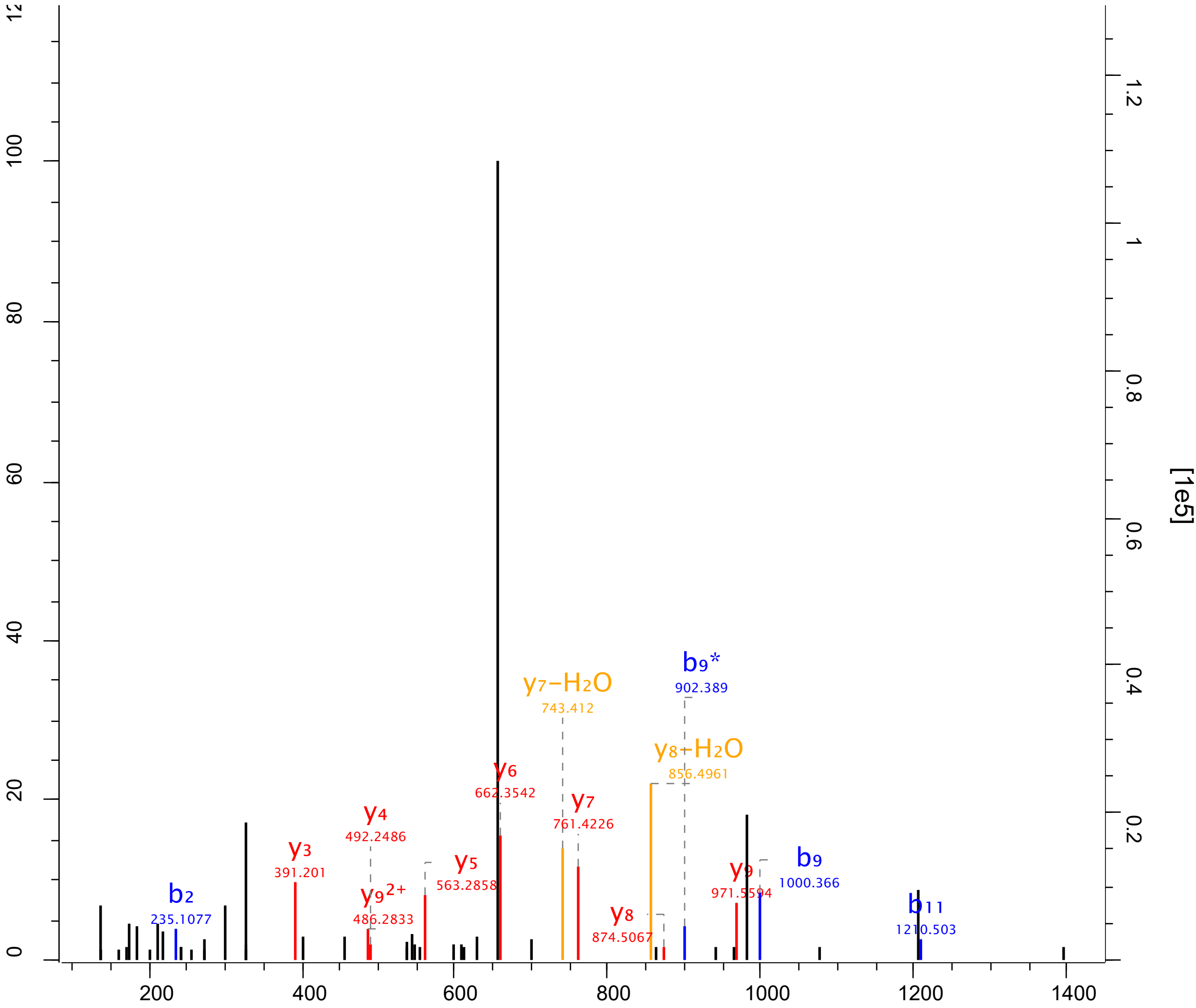

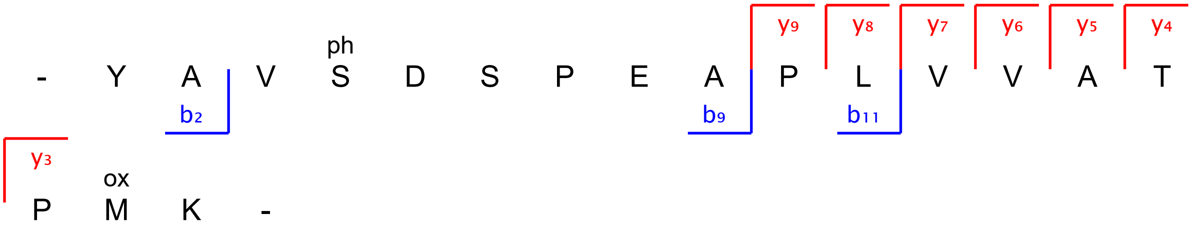

Raw file	Scan	Method	Score	m/z
sys_15_1	29863	FTMS; HCD	113.4	758.33

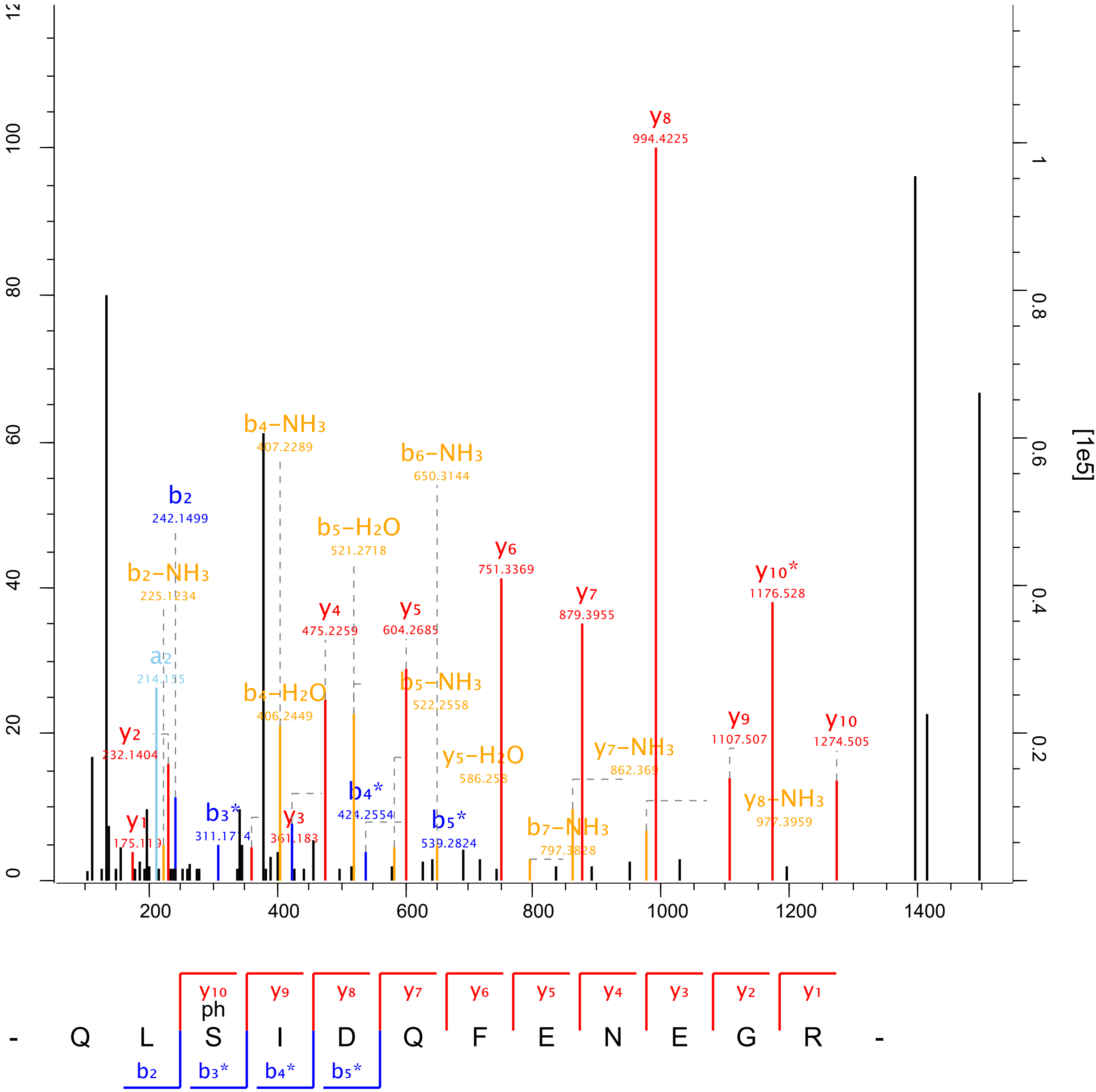

Raw file	Scan	Method	Score	m/z
sys_15_1	29954	FTMS; HCD	166.28	854.89

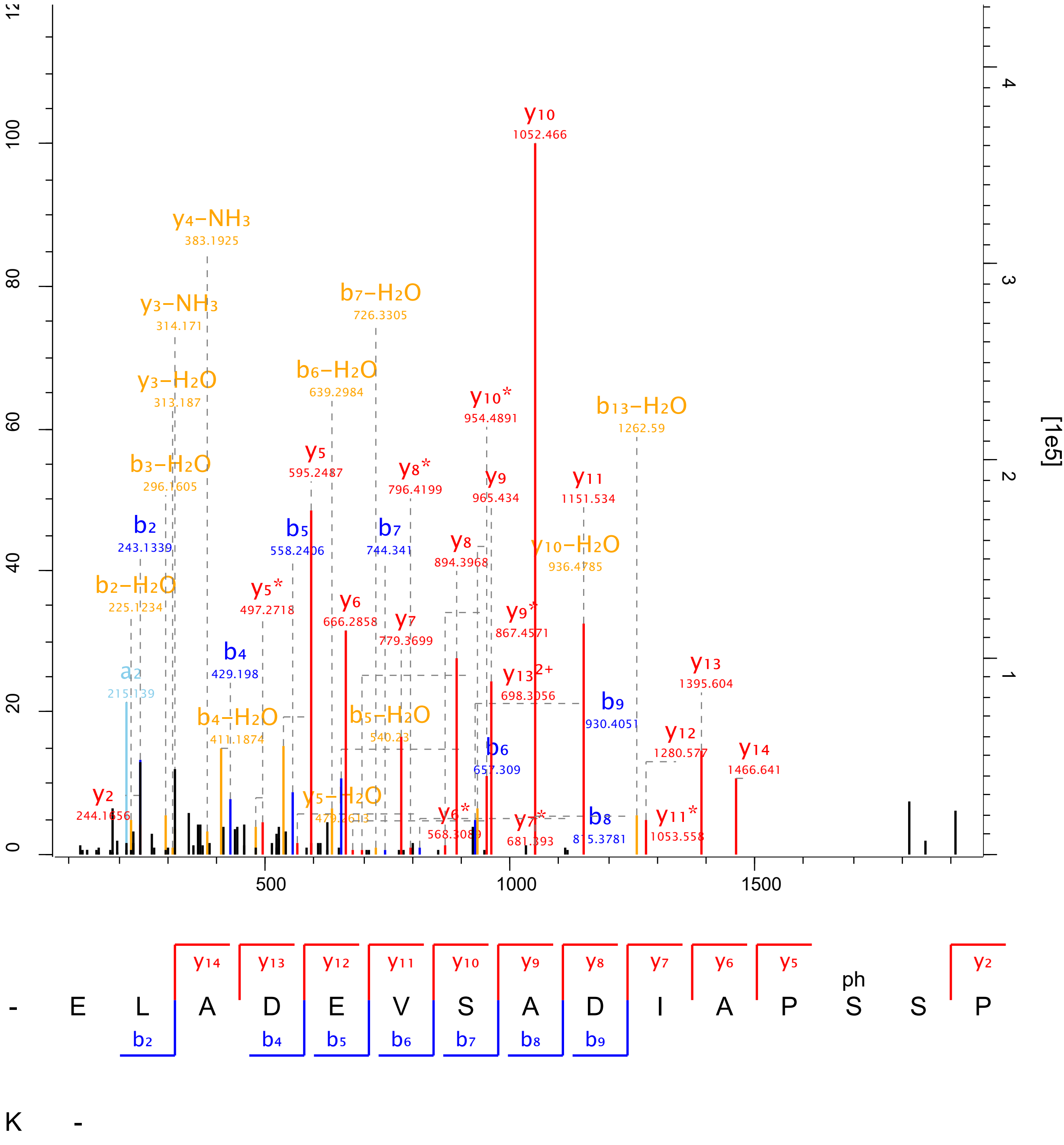

Raw file	Scan	Method	Score	m/z
sys_15_1	29977	FTMS; HCD	98.16	549.23

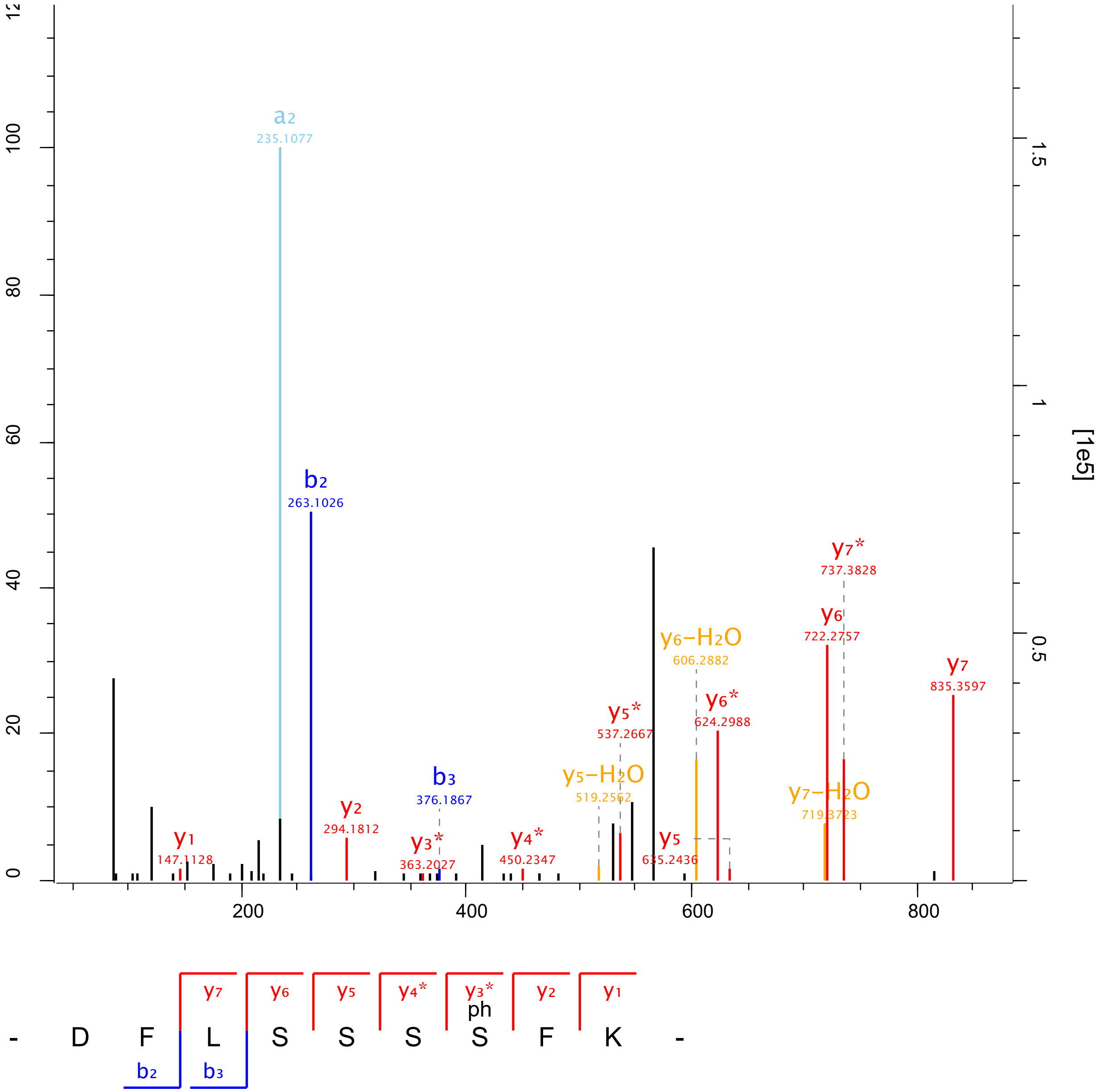

Raw file	Scan	Method	Score	m/z
sys_15_1	3002	FTMS; HCD	93.78	871.78

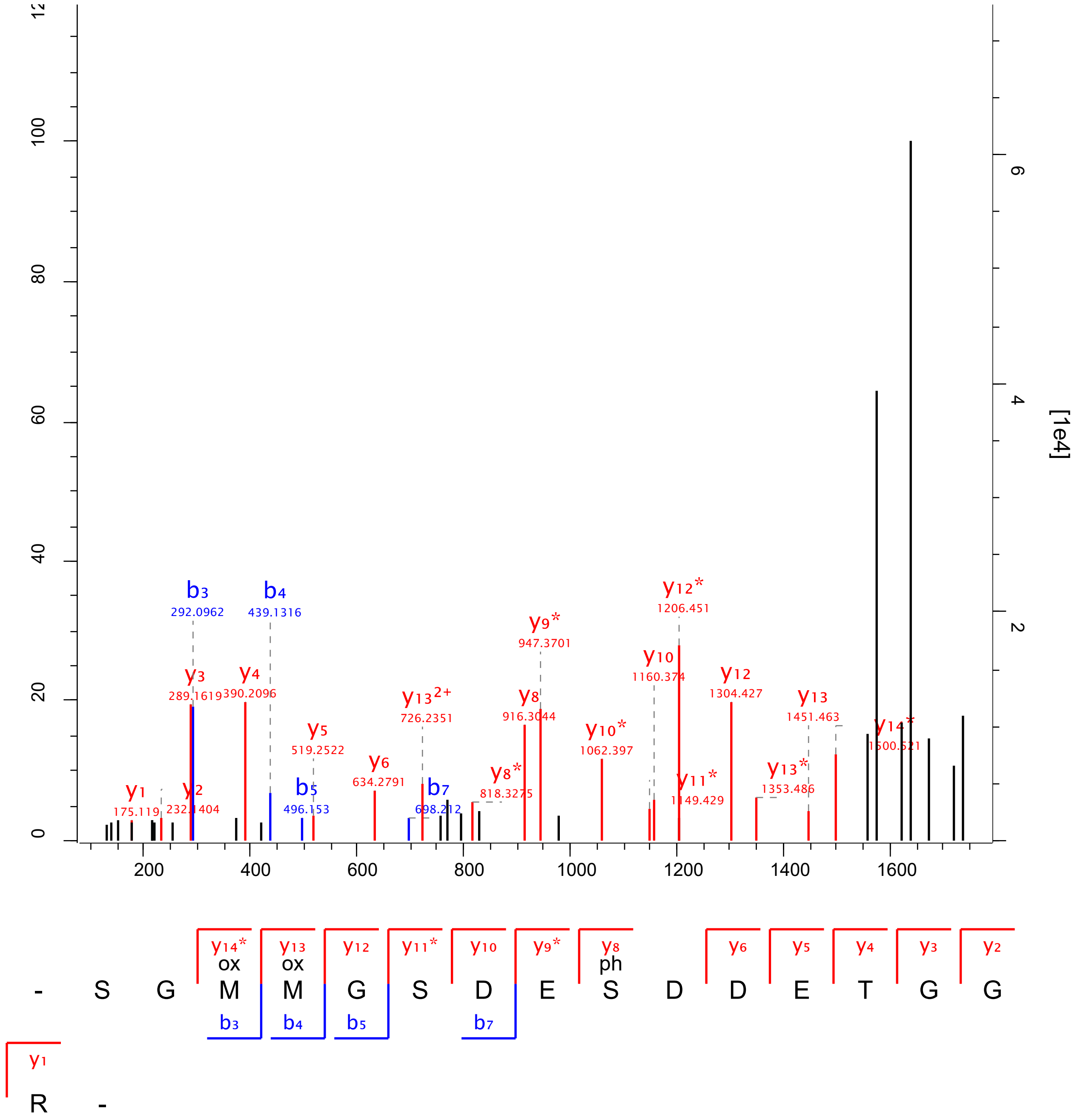

Raw file	Scan	Method	Score	m/z
sys_15_1	30040	FTMS; HCD	50.75	724.65

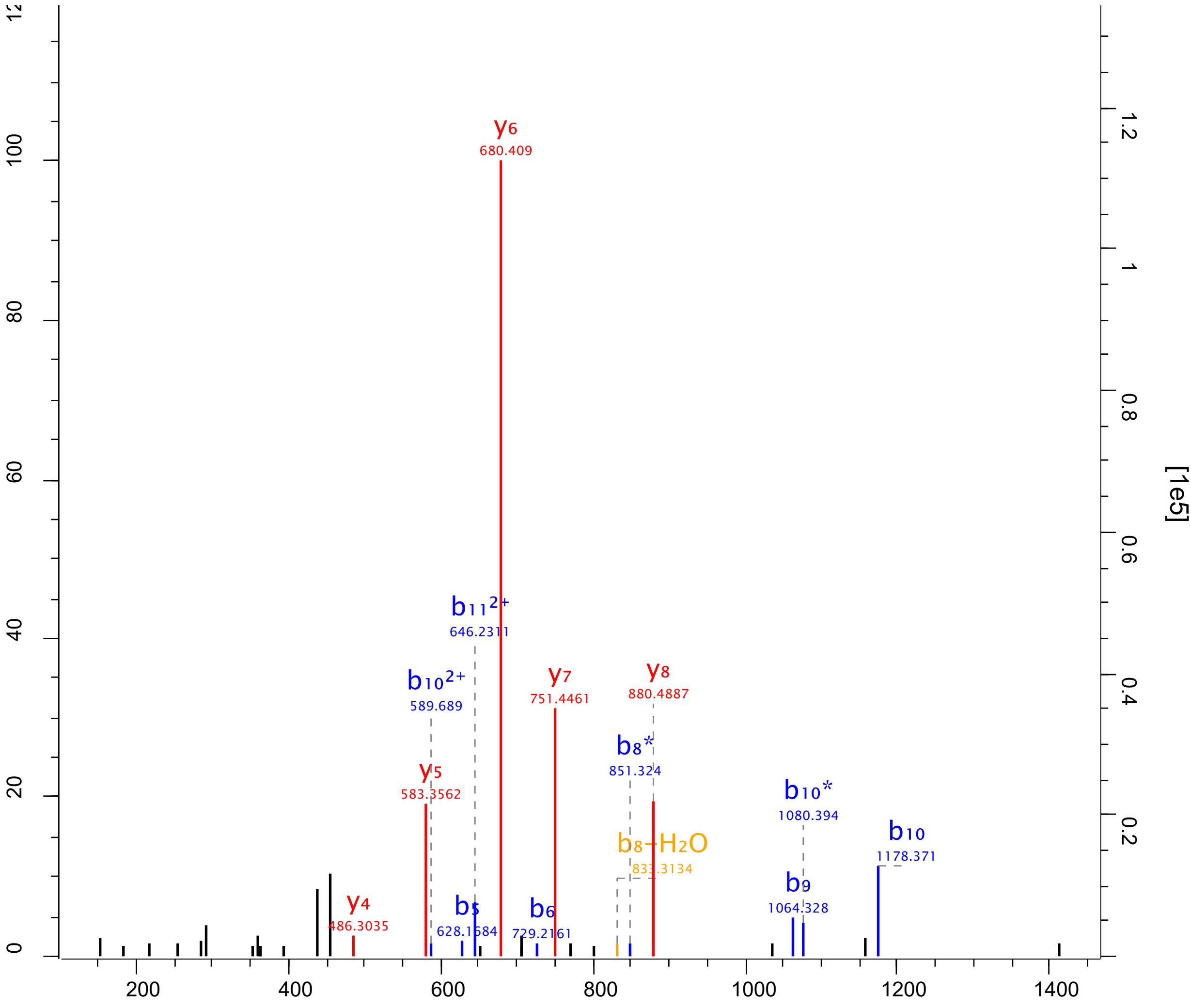

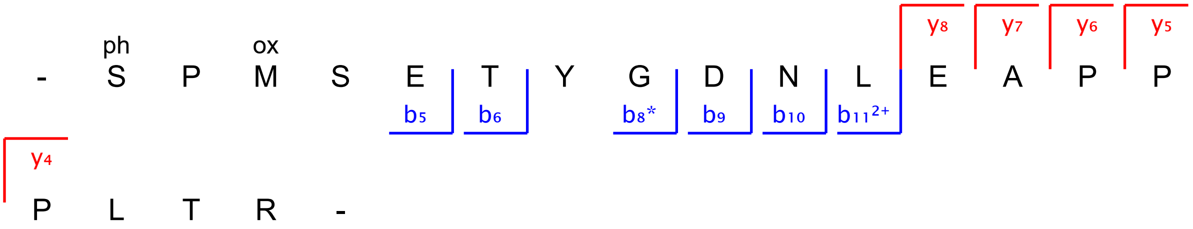

Raw file	Scan	Method	Score	m/z
sys_15_1	3006	FTMS; HCD	121.82	769.76

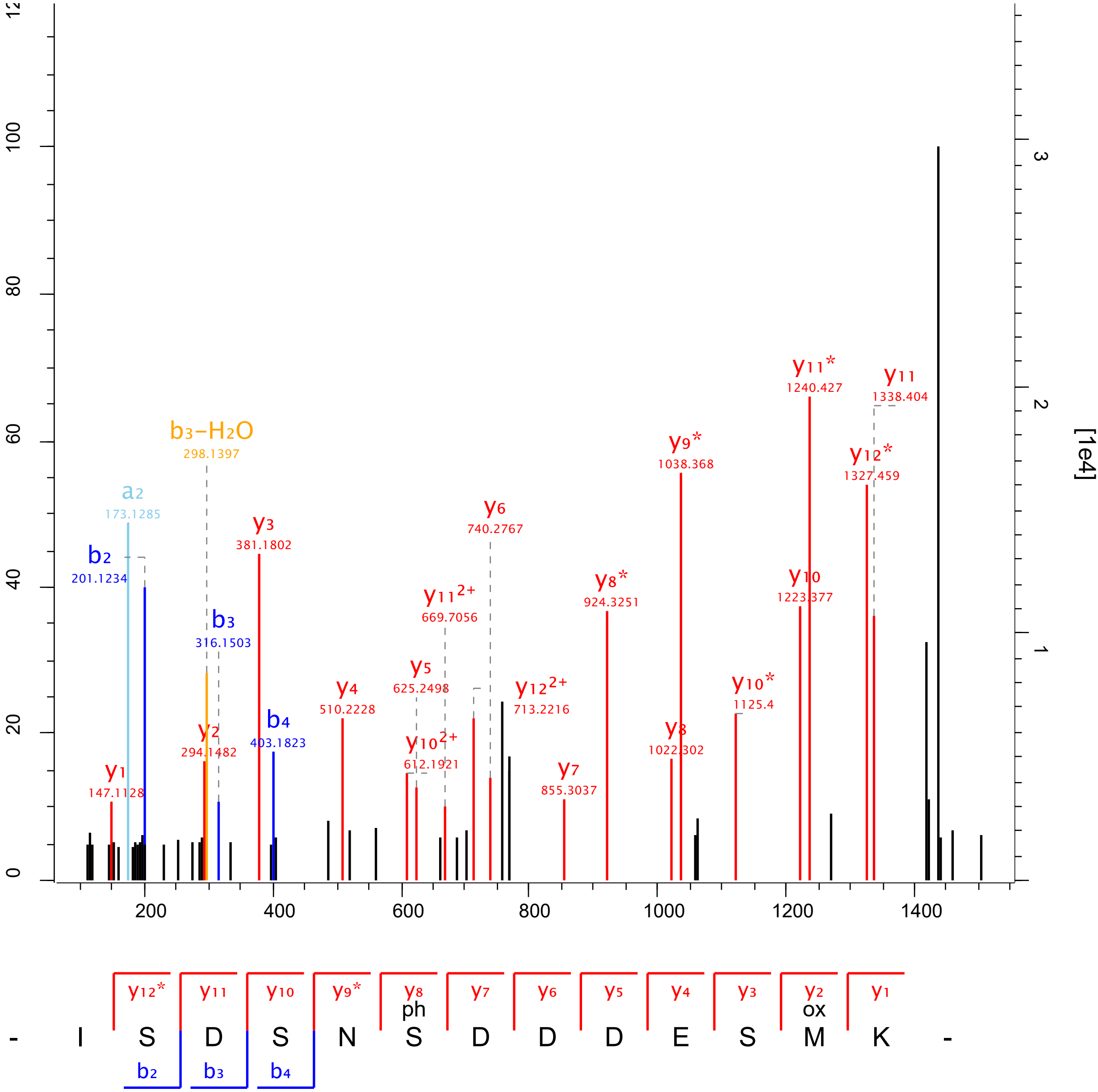

Raw file	Scan	Method	Score	m/z
sys_15_1	30146	FTMS; HCD	285.5	827.85

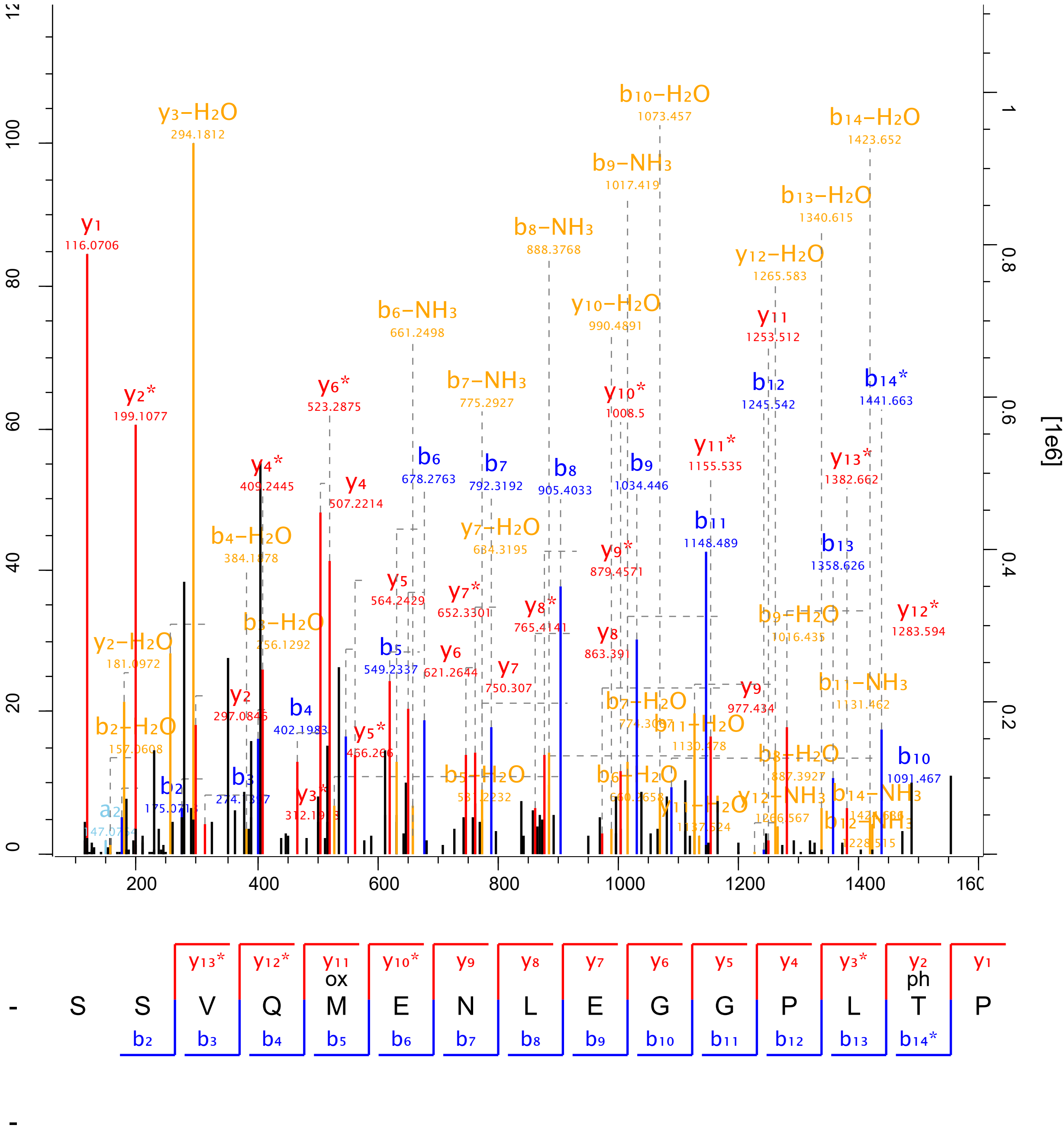

Raw file	Scan	Method	Score	m/z
sys_15_1	3021	FTMS; HCD	46.84	593.75

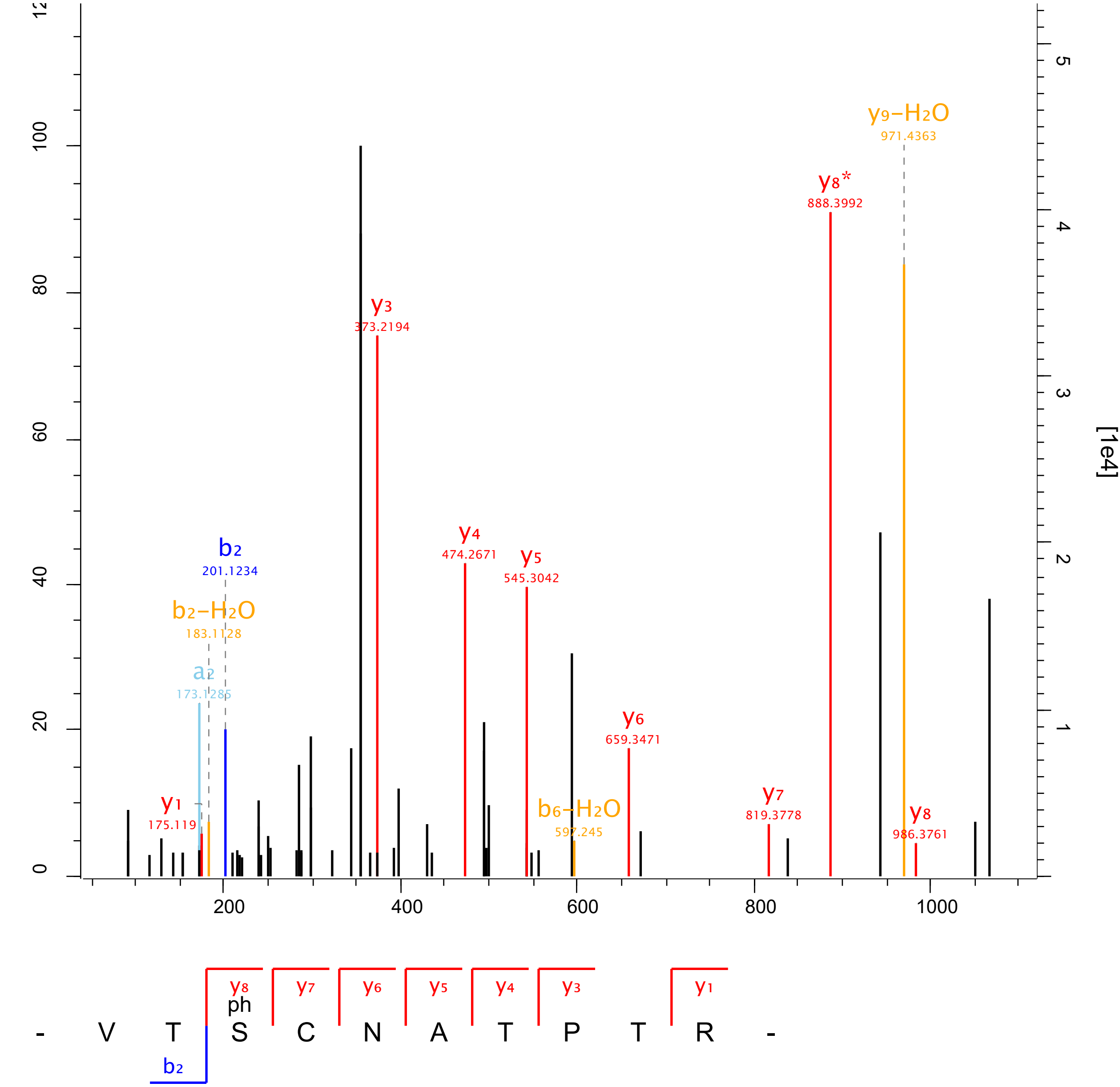

Raw file	Scan	Method	Score	m/z
sys_15_1	30232	FTMS; HCD	141.43	805.67

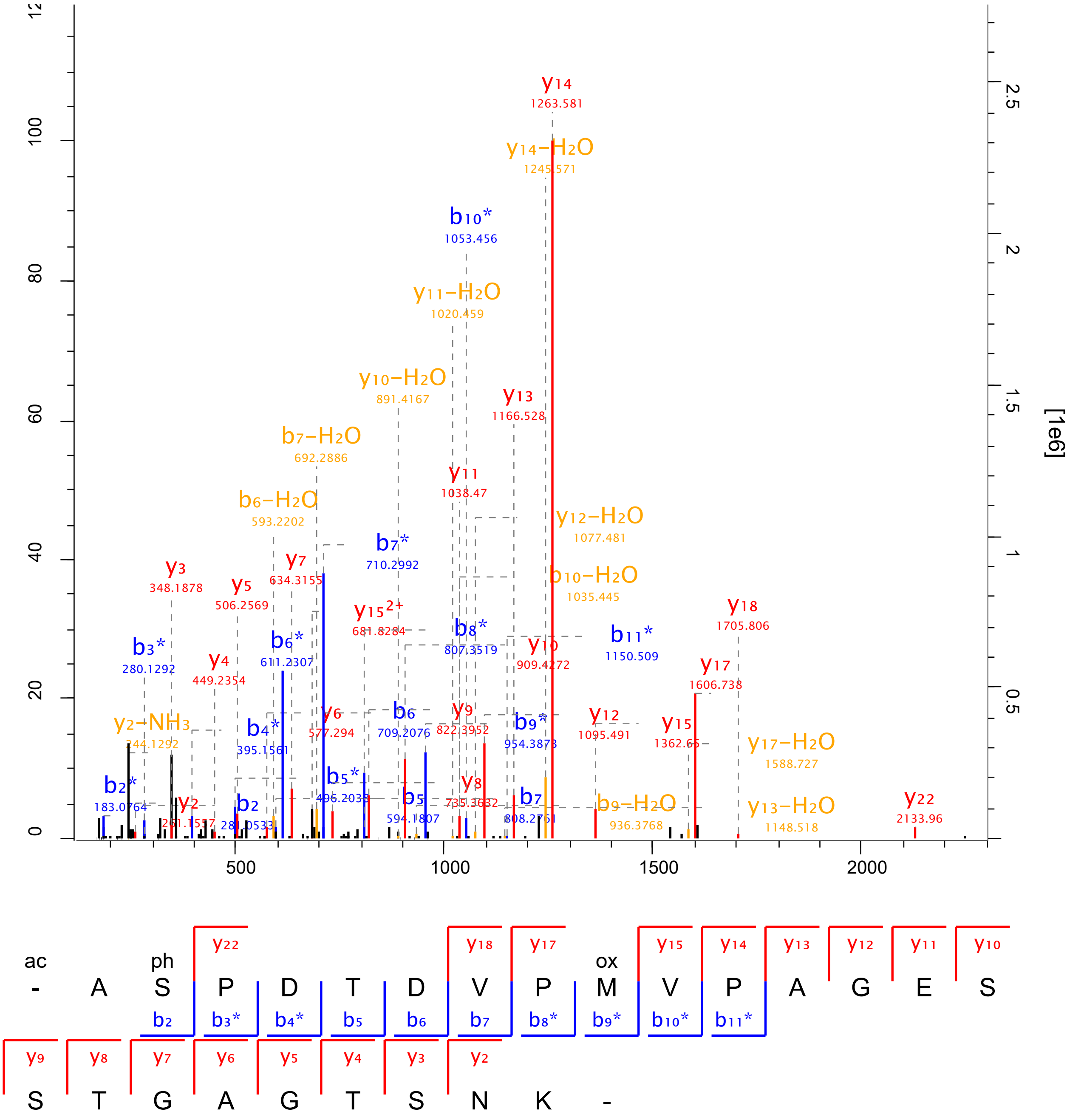

Raw file	Scan	Method	Score	m/z
sys_15_1	30420	FTMS; HCD	84.16	653.31

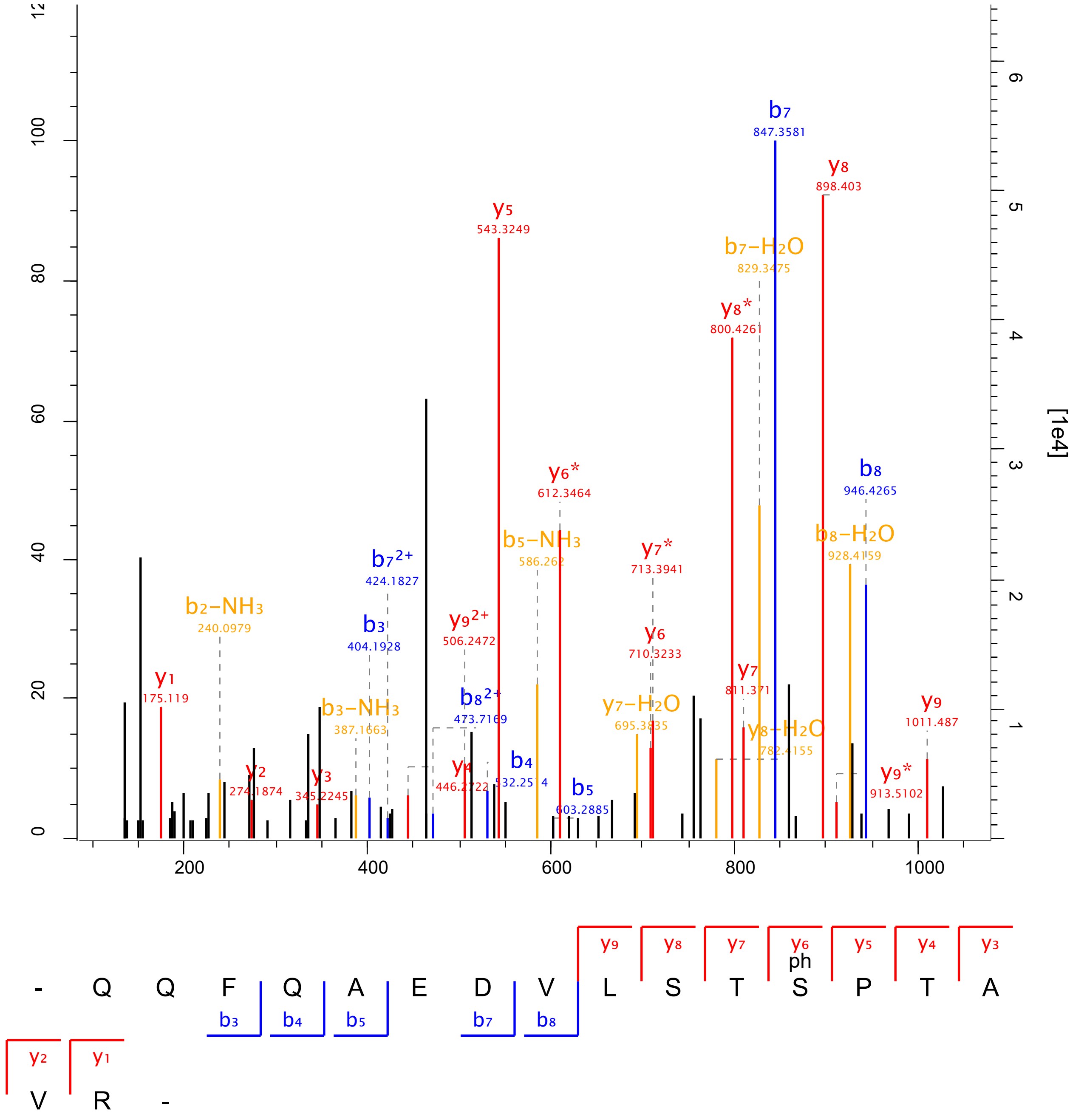

Raw file	Scan	Method	Score	m/z
sys_15_1	30426	FTMS; HCD	87.35	948.9

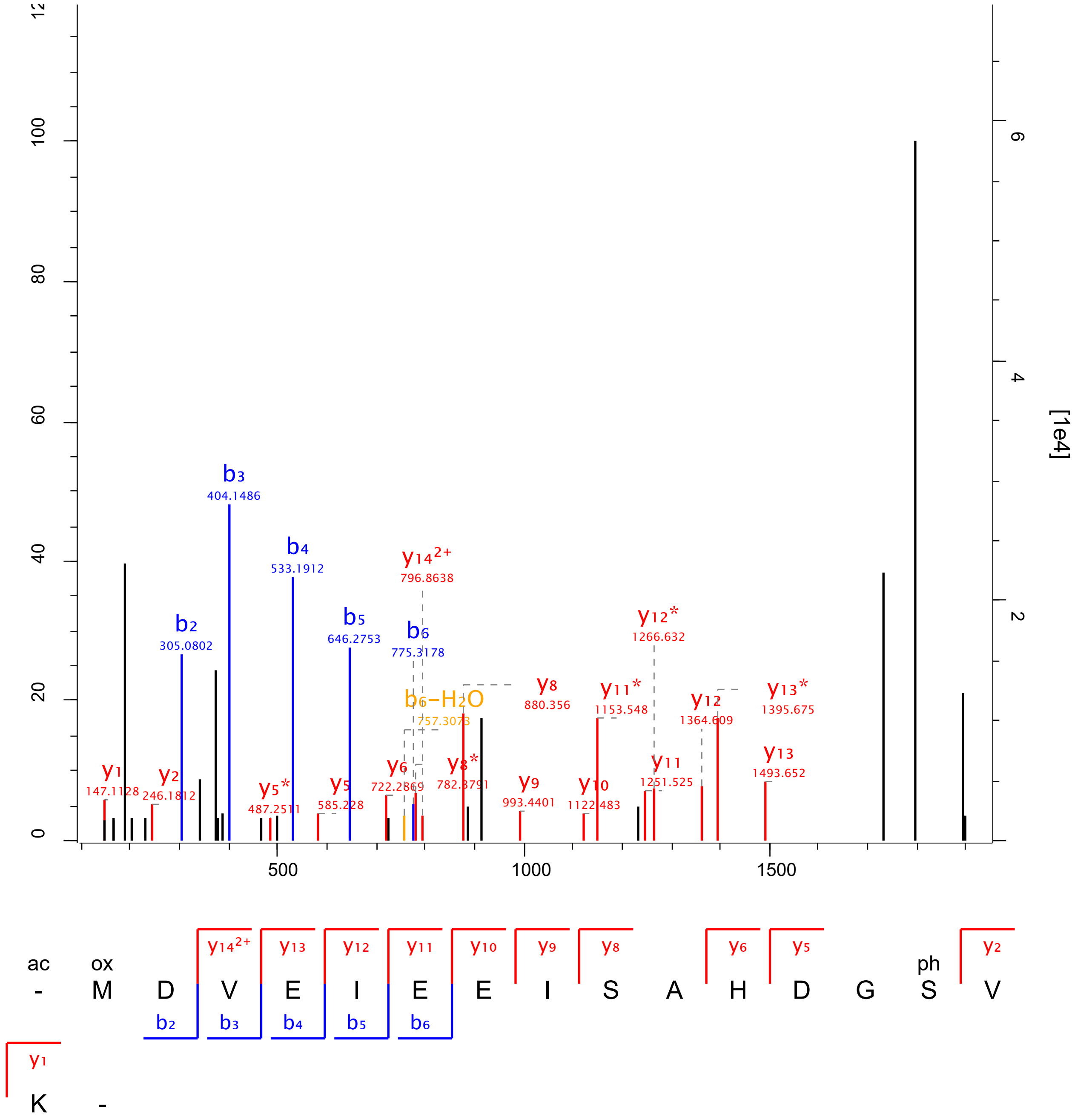

Raw file	Scan	Method	Score	m/z
sys_15_1	30464	FTMS; HCD	55.71	926.41

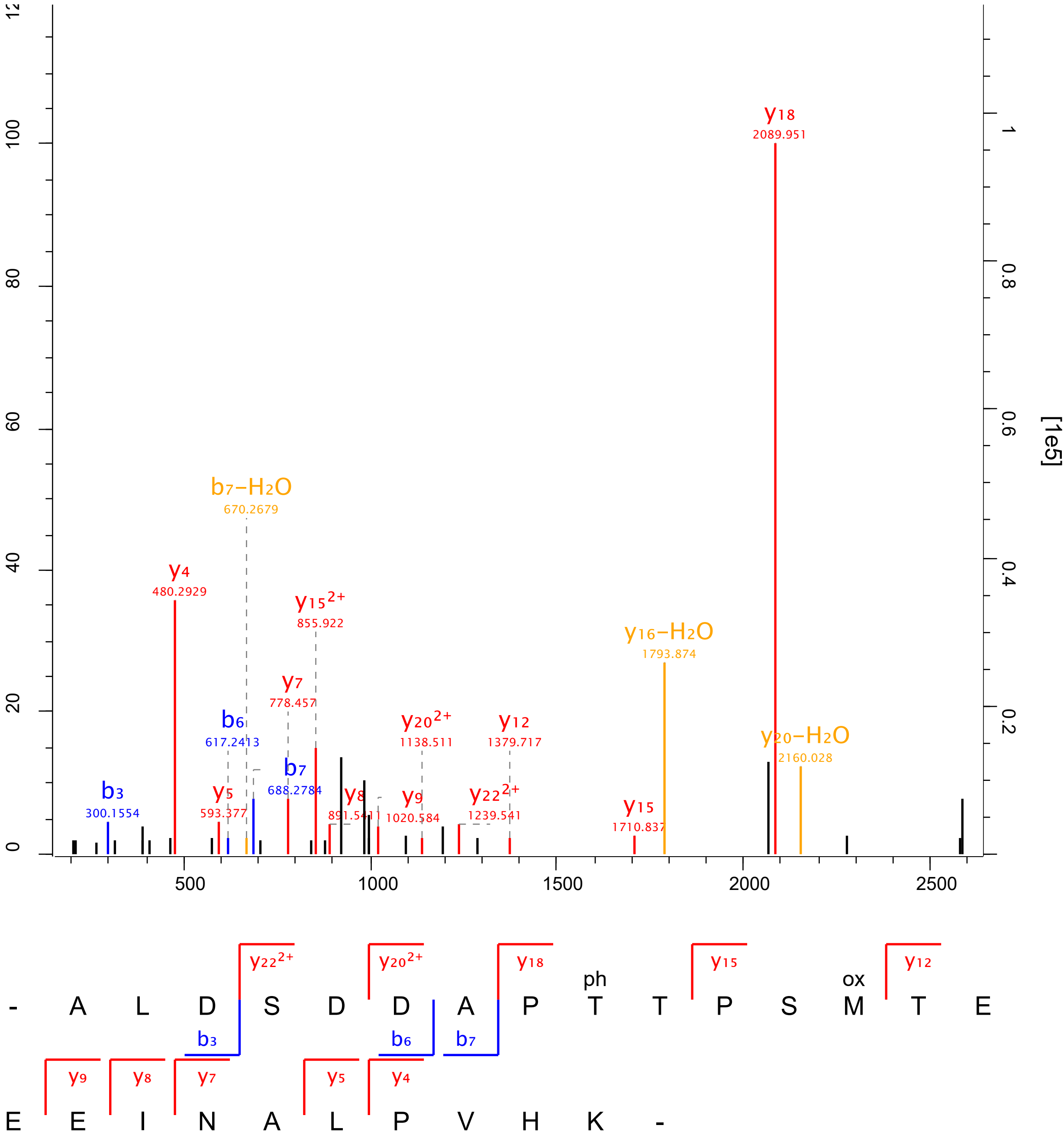

Raw file	Scan	Method	Score	m/z
sys_15_1	30524	FTMS; HCD	91.62	755.87

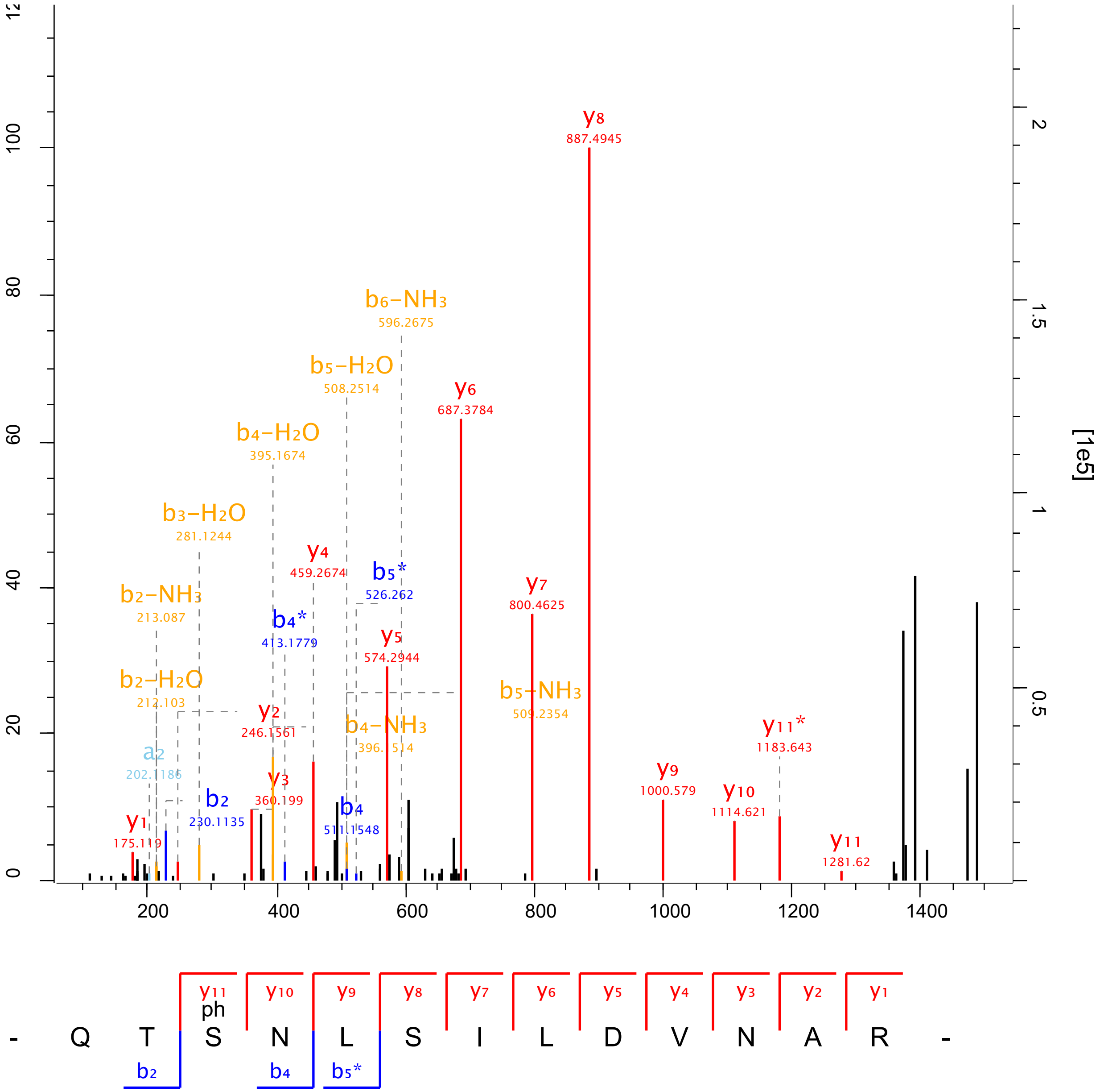

Raw file	Scan	Method	Score	m/z
sys_15_1	30549	FTMS; HCD	114.46	901.91

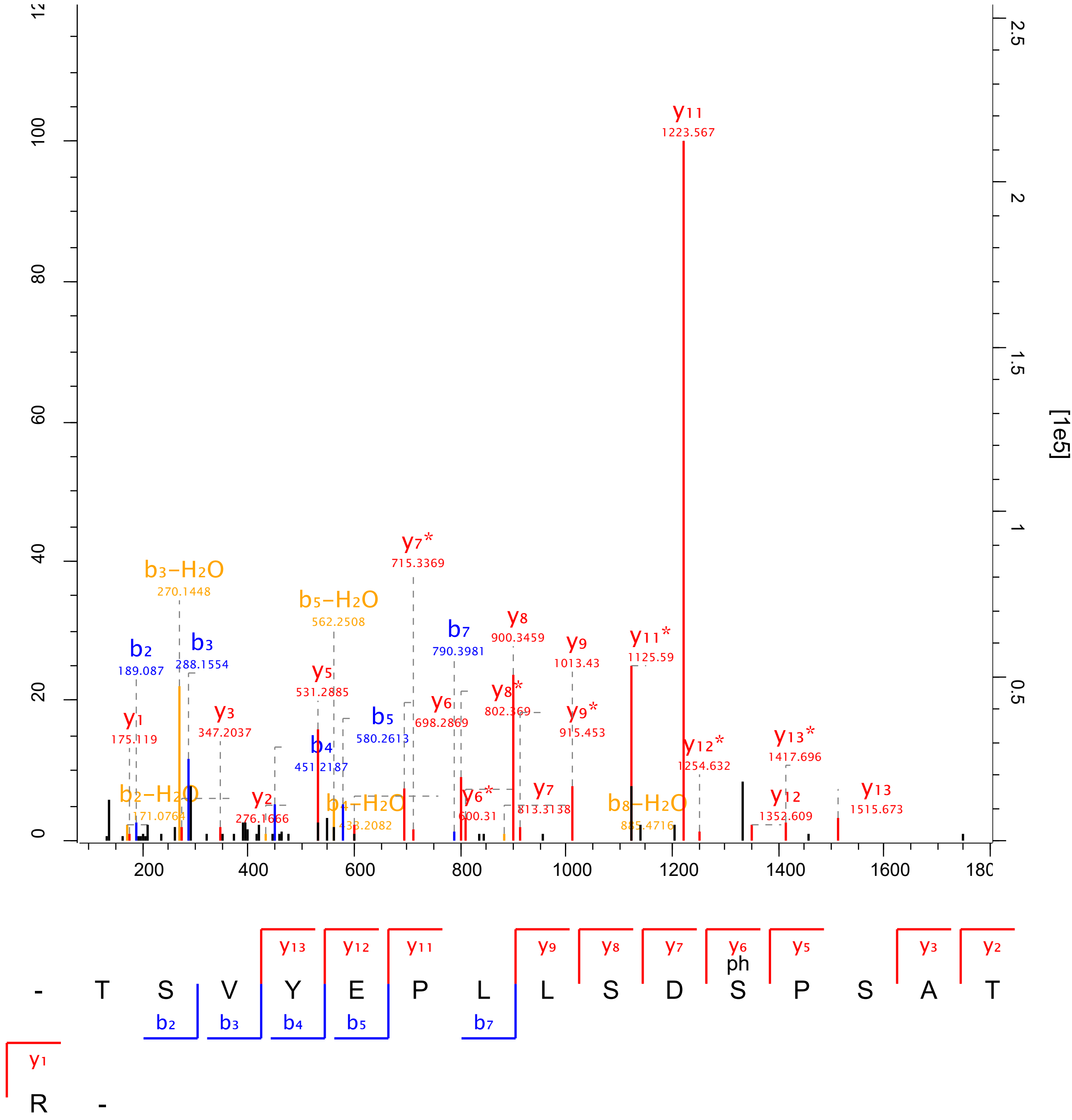

Raw file	Scan	Method	Score	m/z
sys_15_1	30603	FTMS; HCD	46.88	607.78

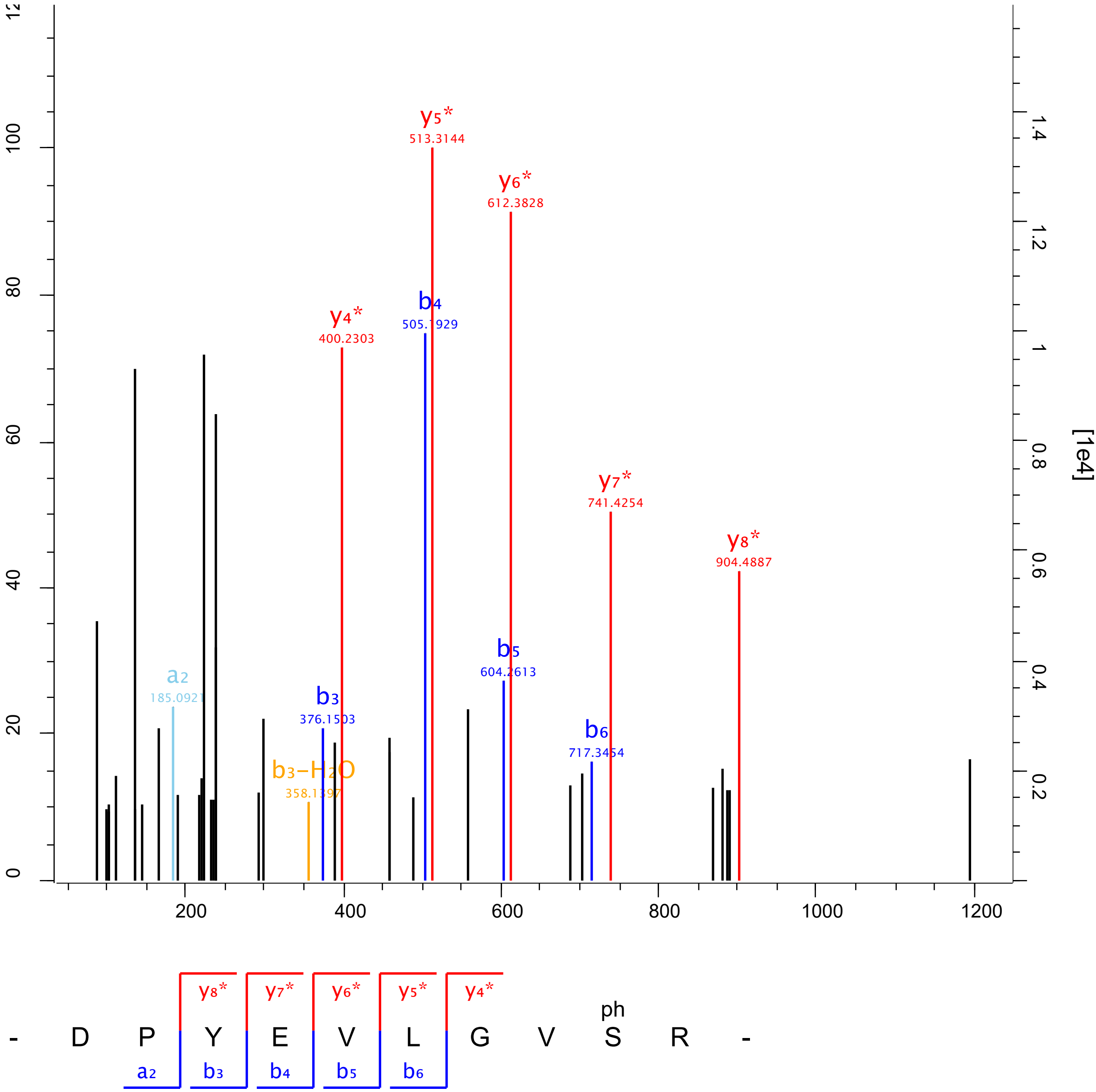

Raw file	Scan	Method	Score	m/z
sys_15_1	30651	FTMS; HCD	208.28	642.29

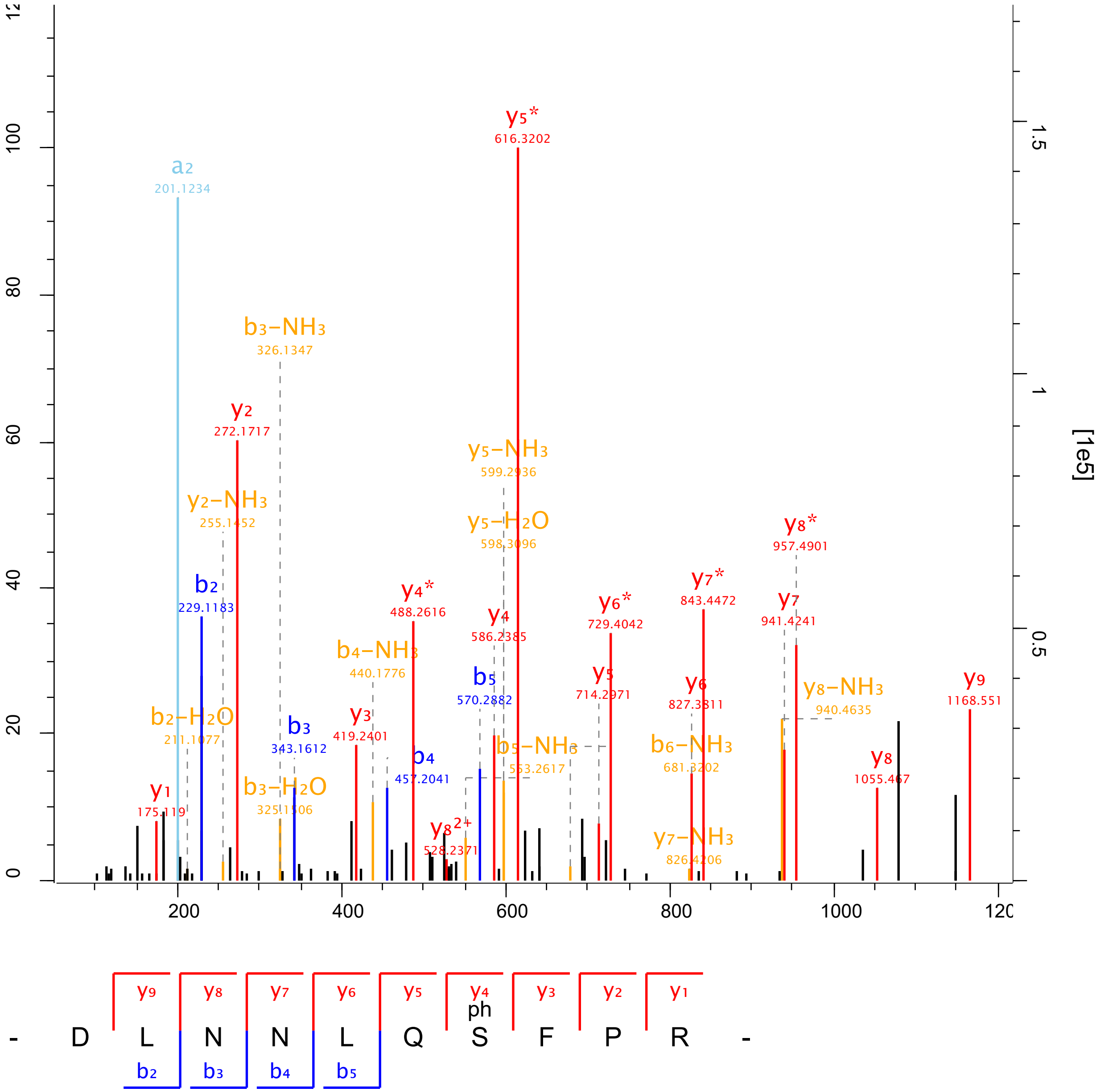

Raw file	Scan	Method	Score	m/z
sys_15_1	30662	FTMS; HCD	181.4	924.35

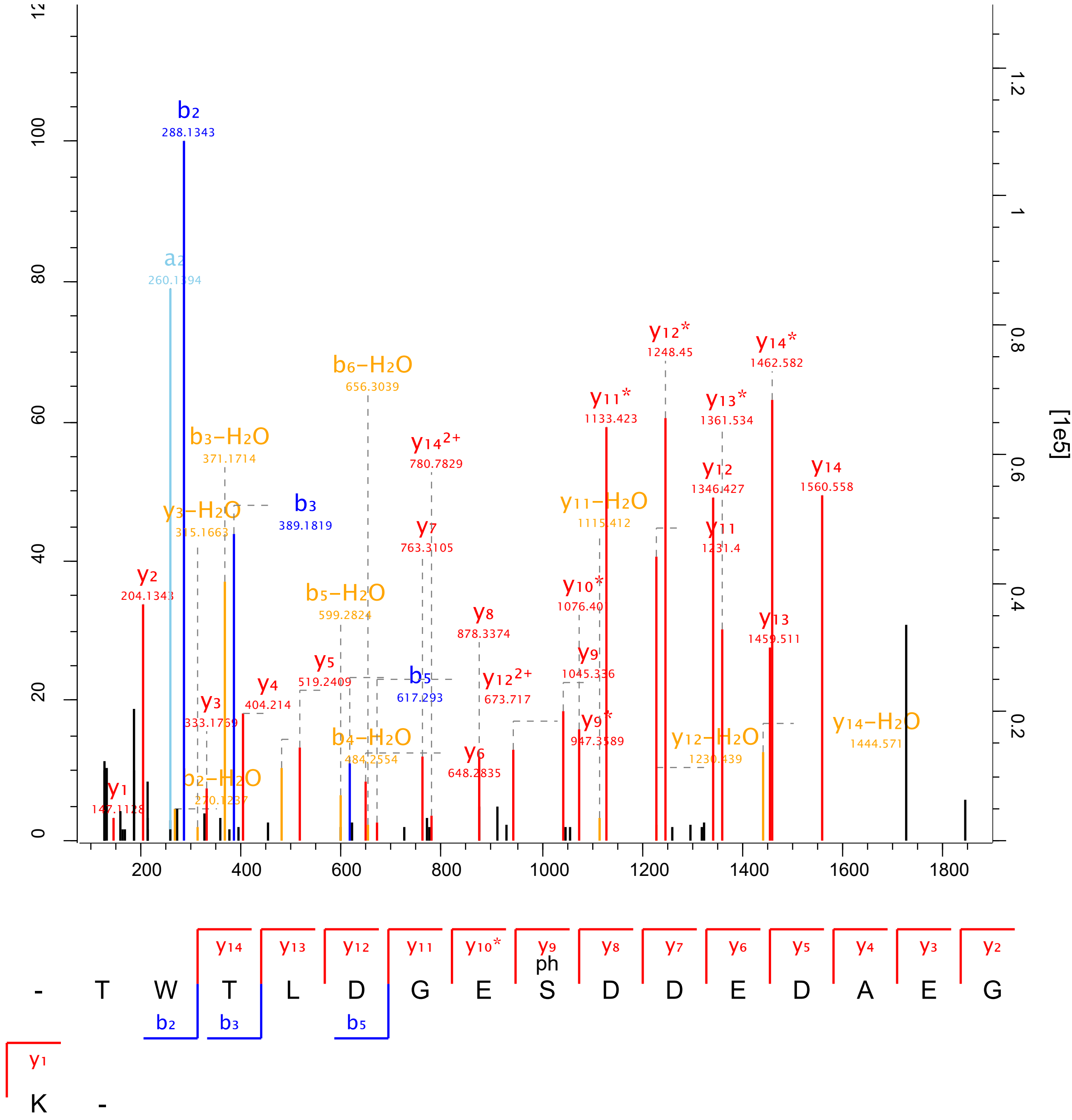

Raw file	Scan	Method	Score	m/z
sys_15_1	30668	FTMS; HCD	91.96	545.76

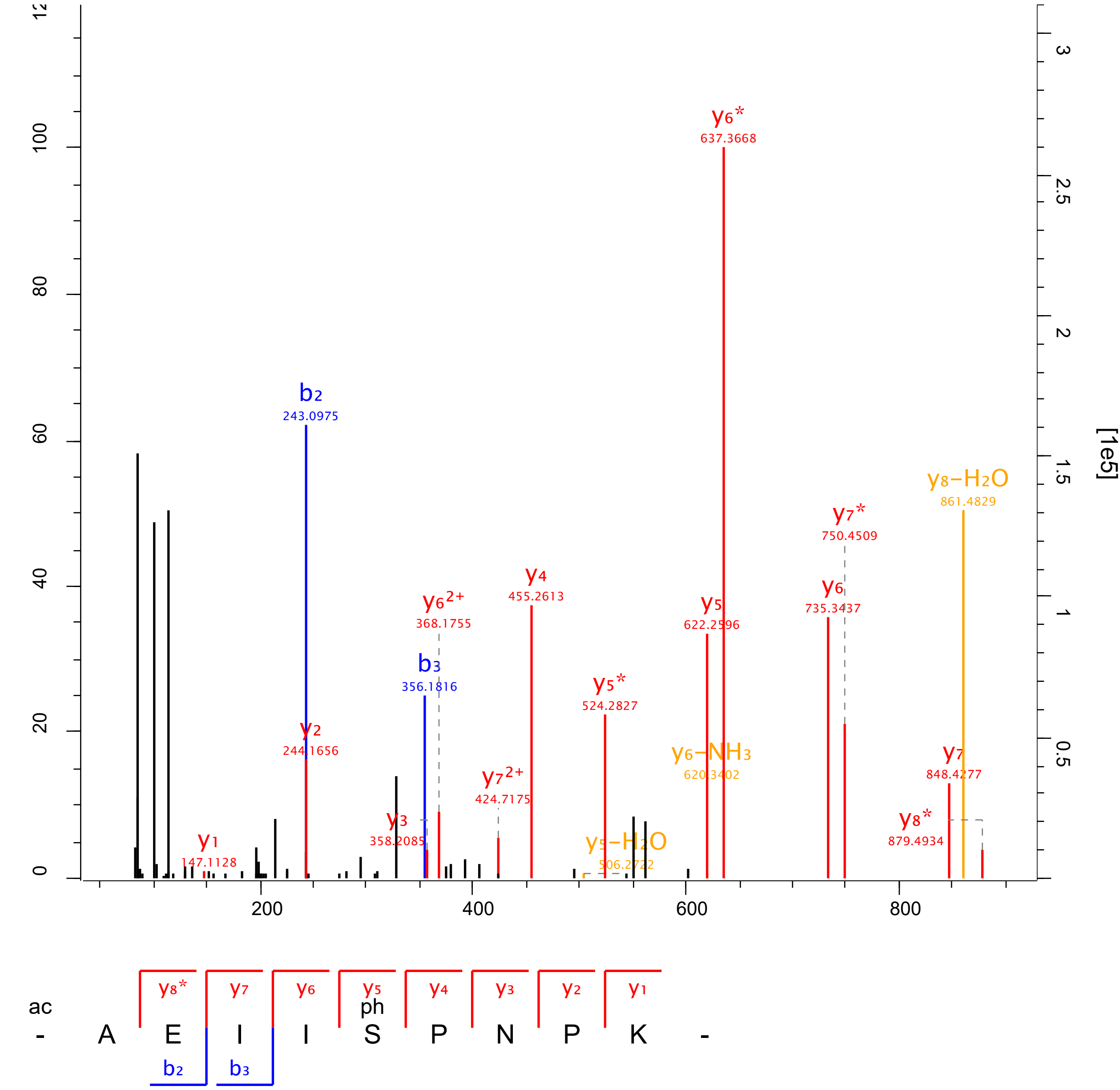

Raw file	Scan	Method	Score	m/z
sys_15_1	30685	FTMS; HCD	64.22	583.81

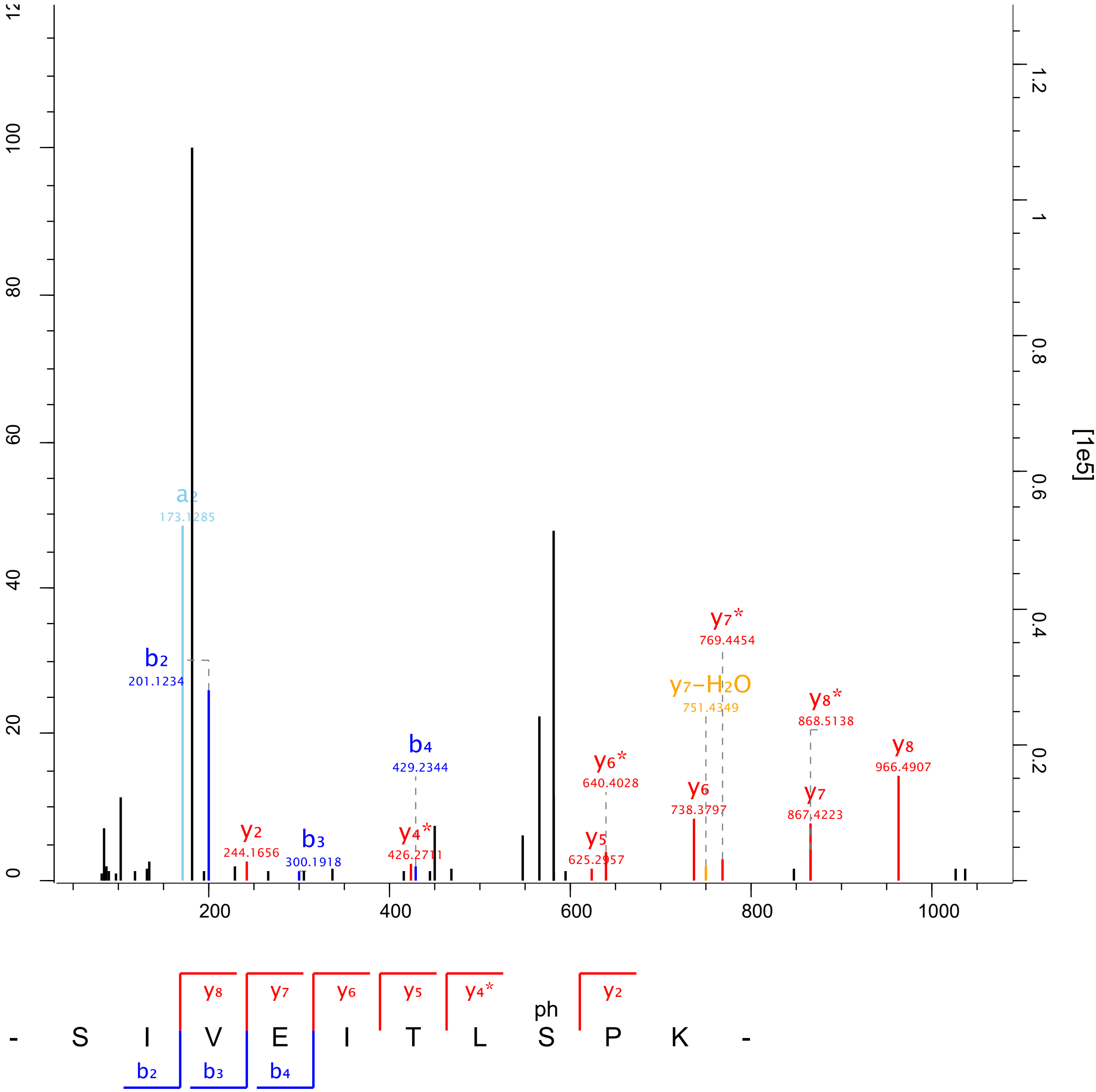

Raw file	Scan	Method	Score	m/z
sys_15_1	30778	FTMS; HCD	136.64	851.37

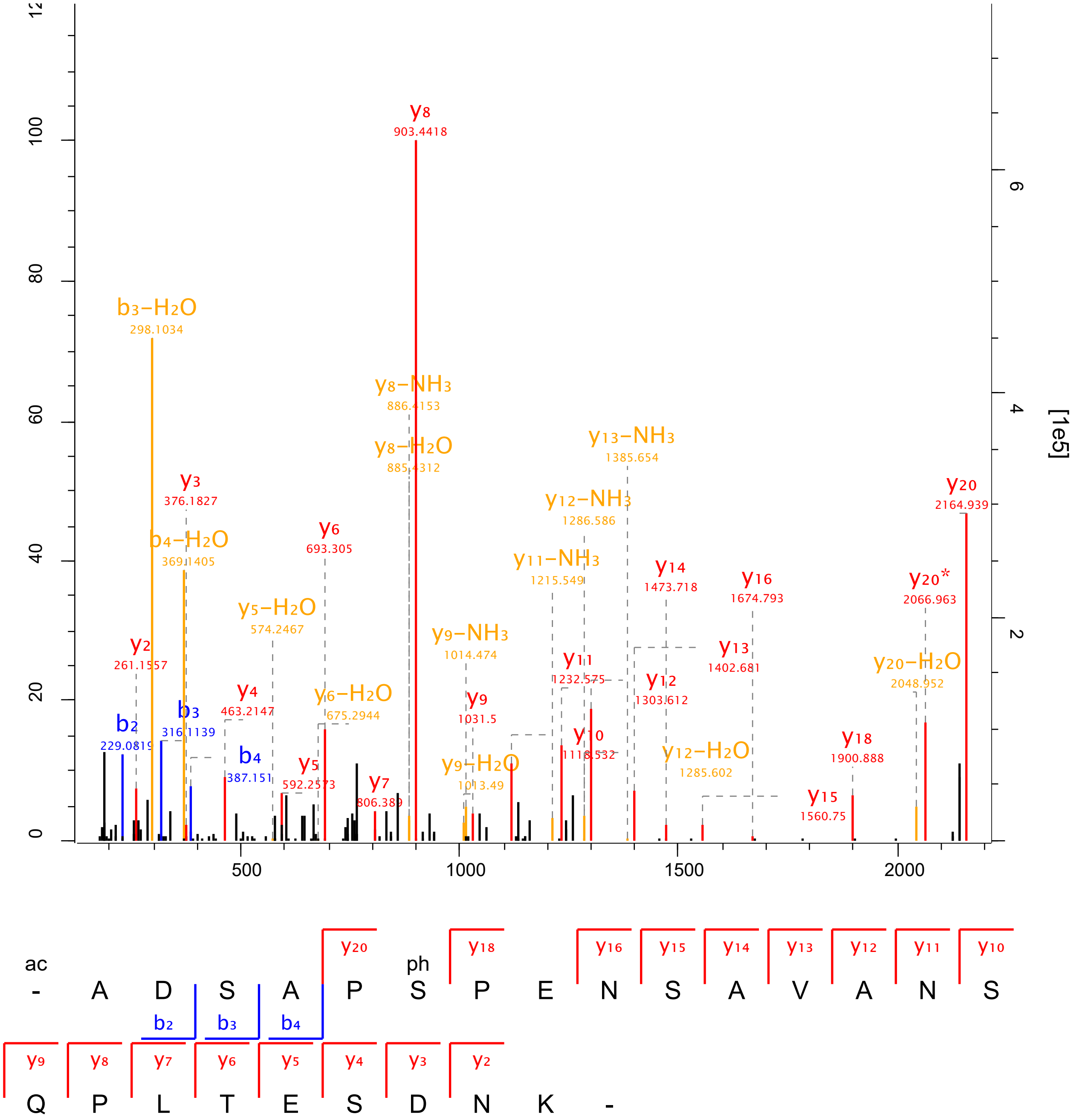

Raw file	Scan	Method	Score	m/z
sys_15_1	30825	FTMS; HCD	154.69	773.33

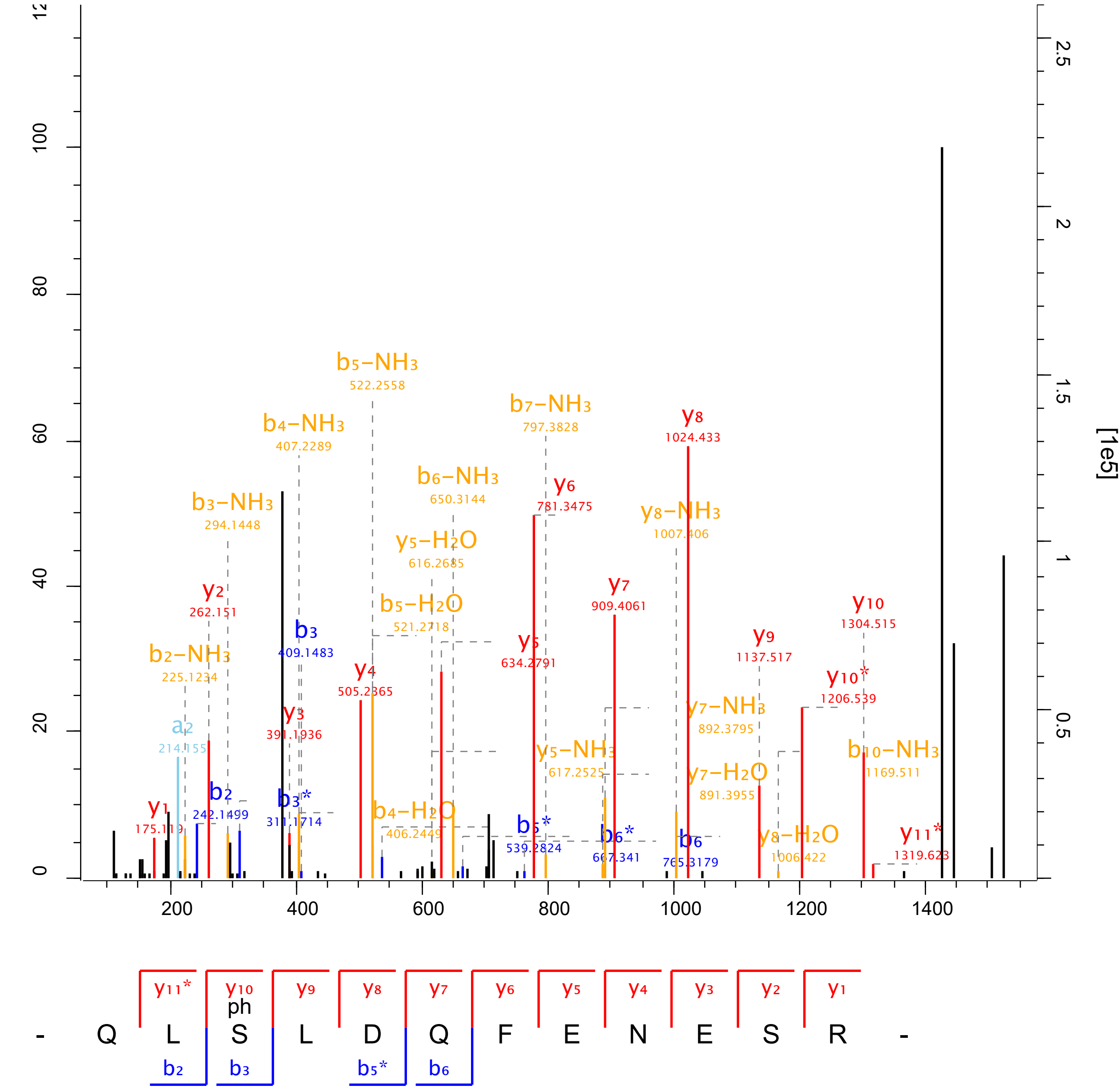

Raw file	Scan	Method	Score	m/z
sys_15_1	30853	FTMS; HCD	140.73	864.35

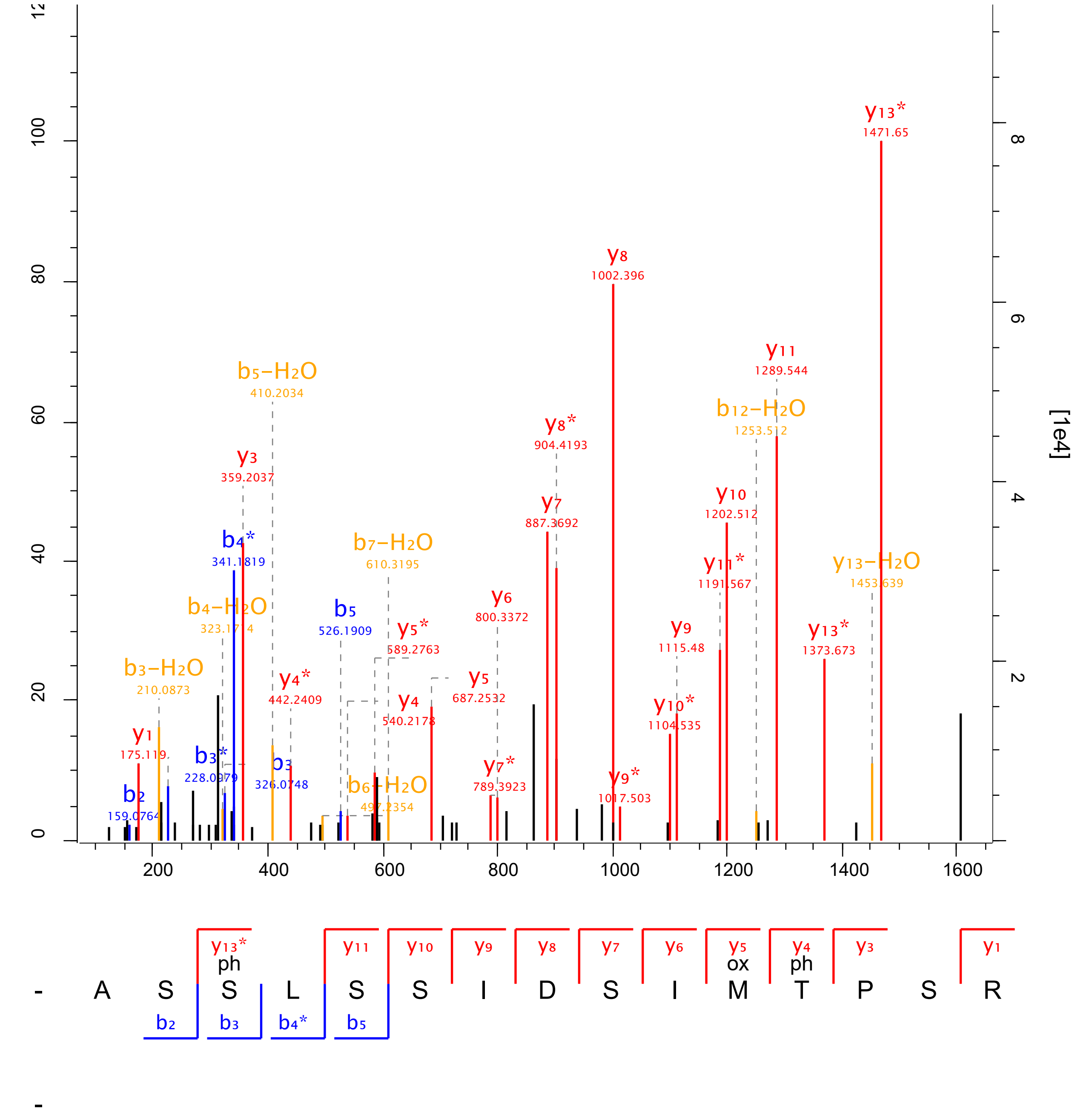

Raw file	Scan	Method	Score	m/z
sys_15_1	30880	FTMS; HCD	275.69	977.38

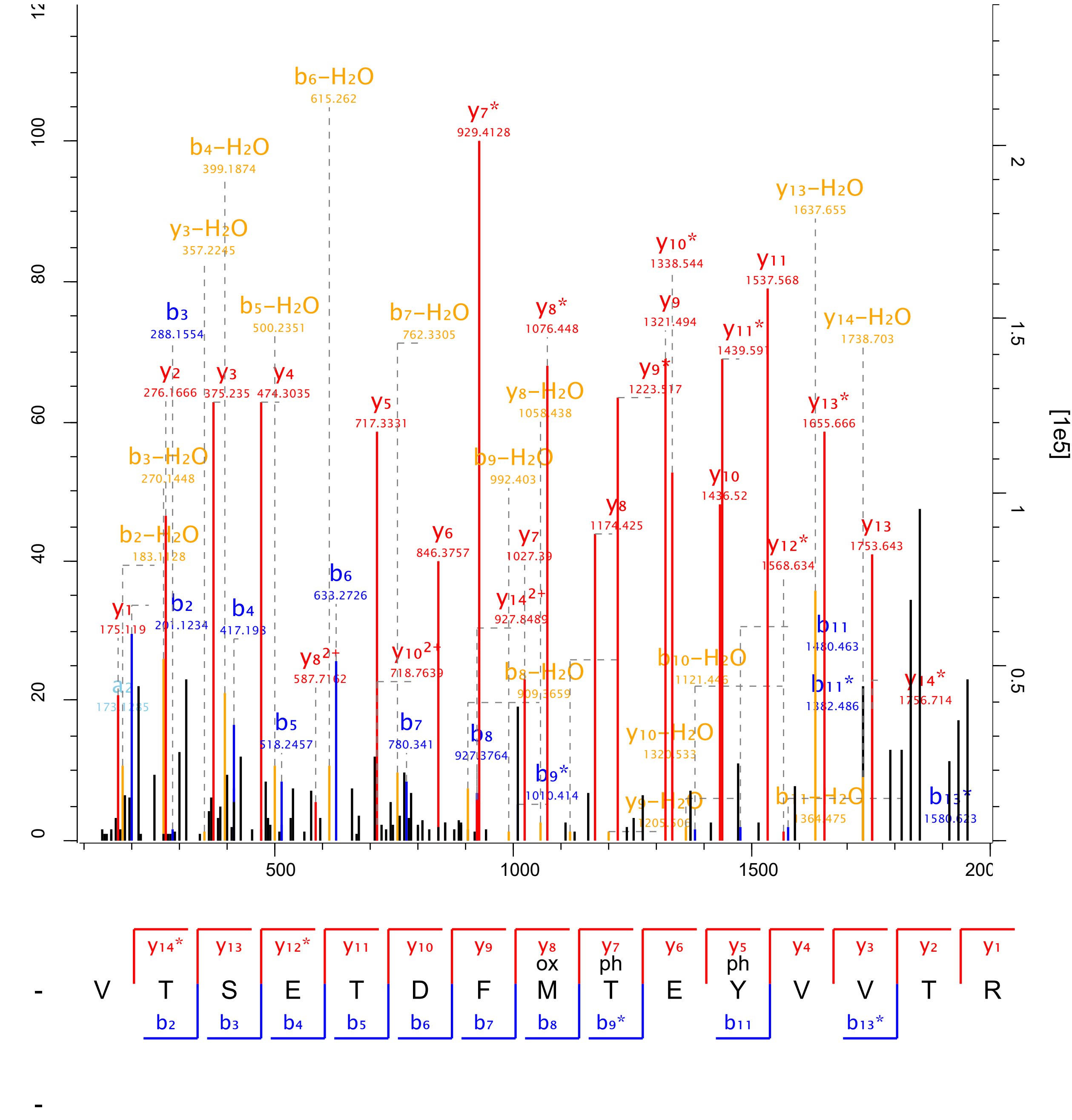

Raw file	Scan	Method	Score	m/z
sys_15_1	3111	FTMS; HCD	66.06	451.69

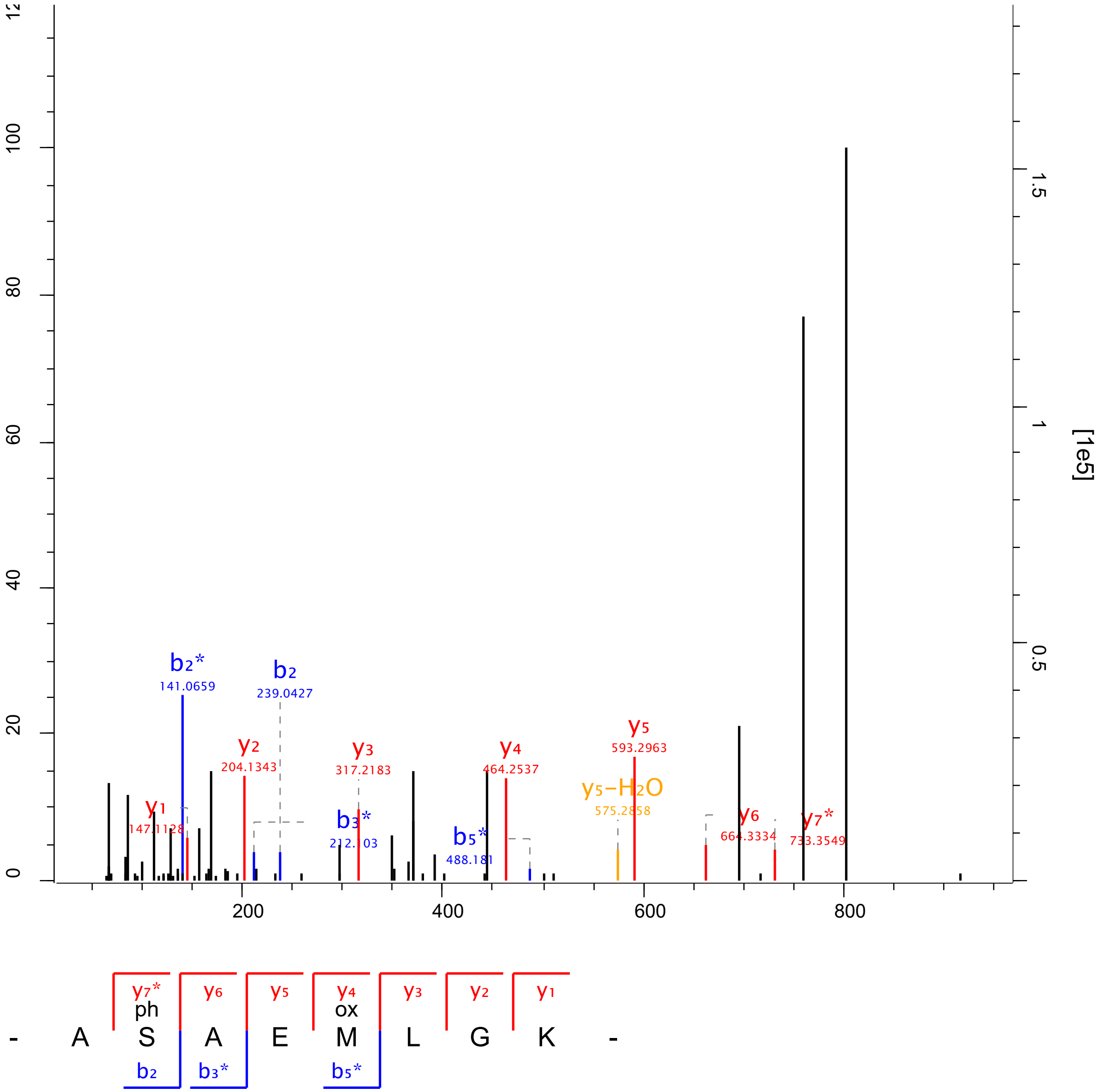

Raw file	Scan	Method	Score	m/z
sys_15_1	31129	FTMS; HCD	152.4	674.27

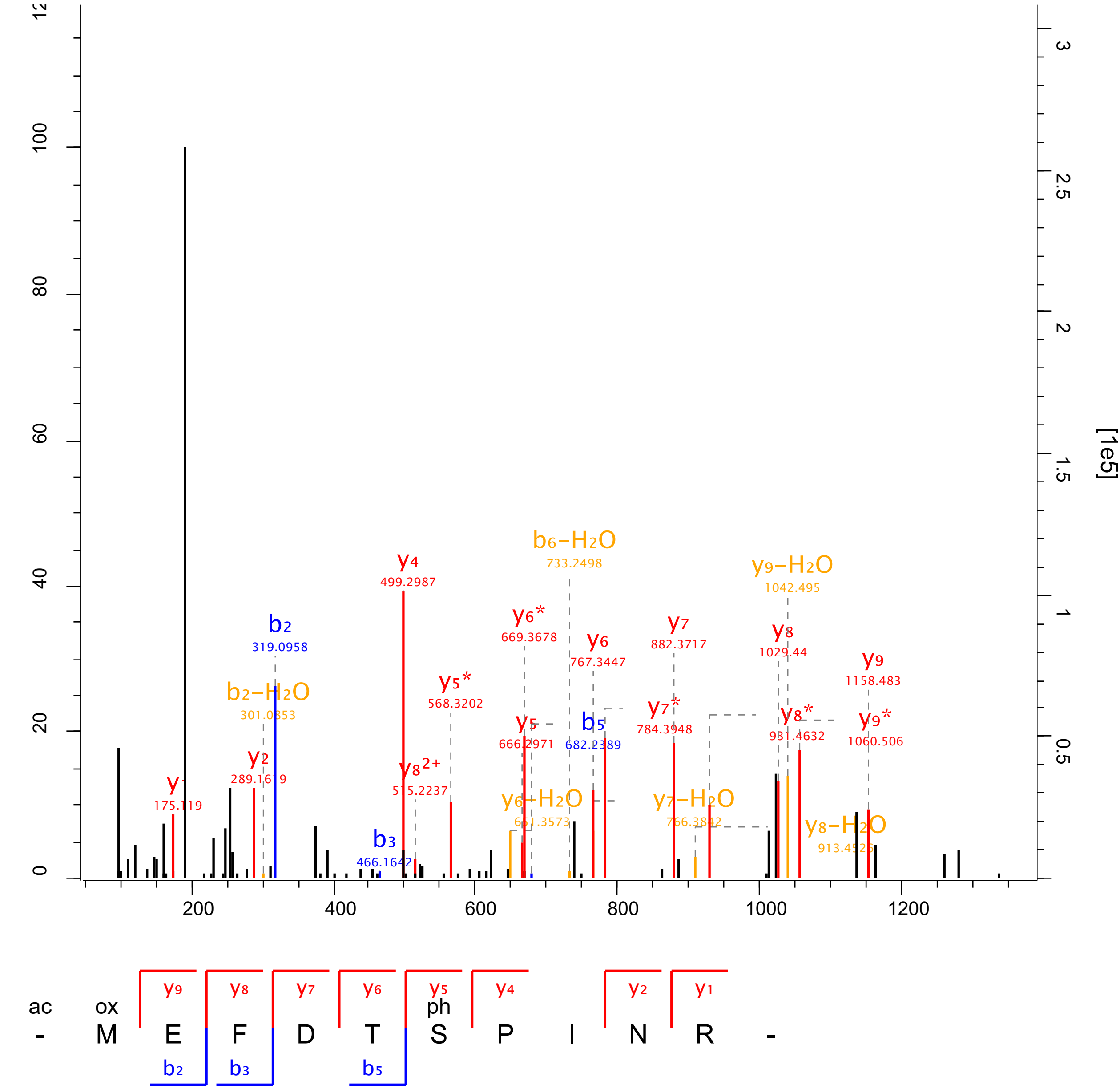

Raw file	Scan	Method	Score	m/z
sys_15_1	31200	FTMS; HCD	139.9	820.9

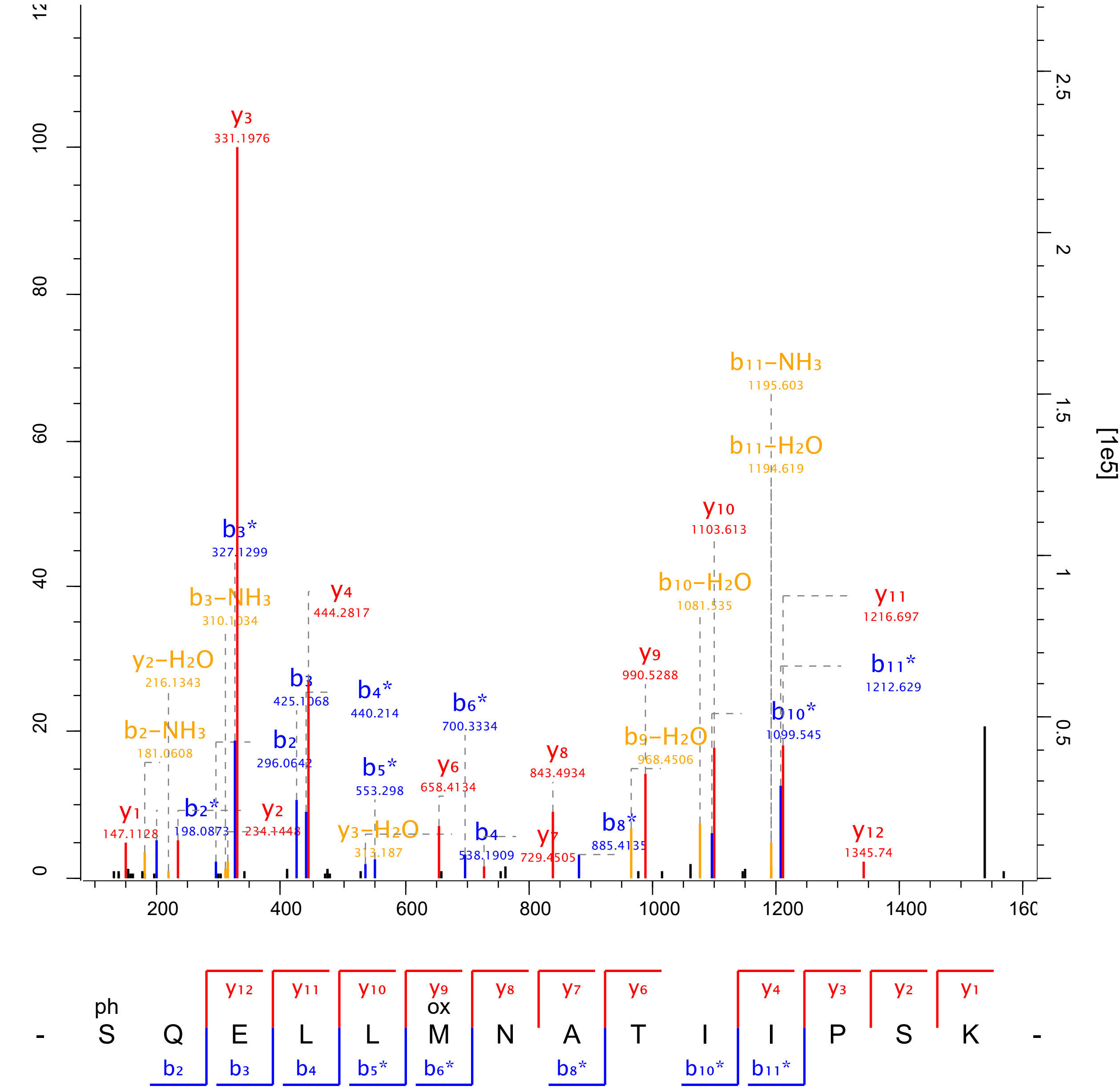

Raw file	Scan	Method	Score	m/z
sys_15_1	31232	FTMS; HCD	91.32	803.42

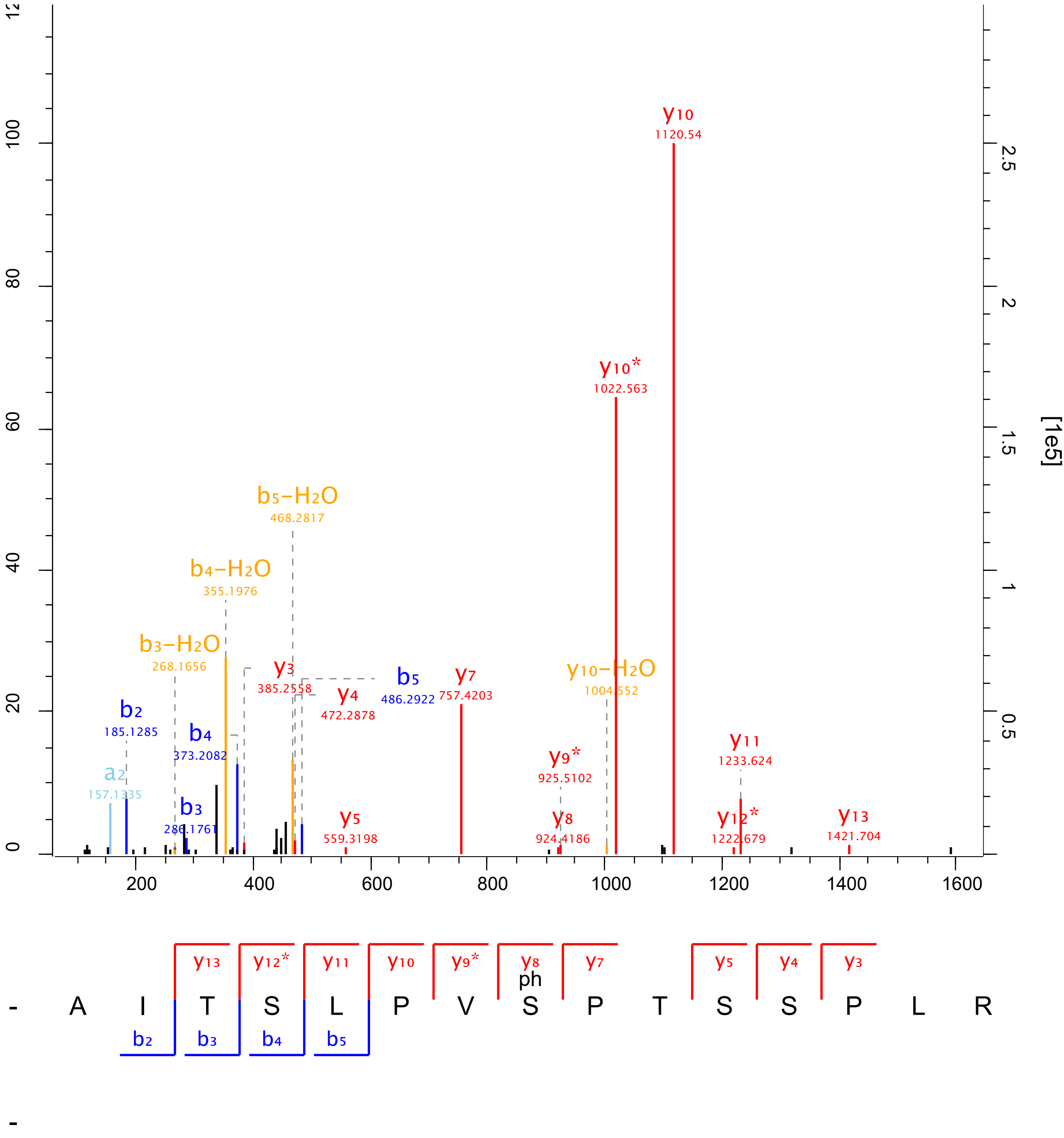

Raw file	Scan	Method	Score	m/z
sys_15_1	31373	FTMS; HCD	41.45	639.31

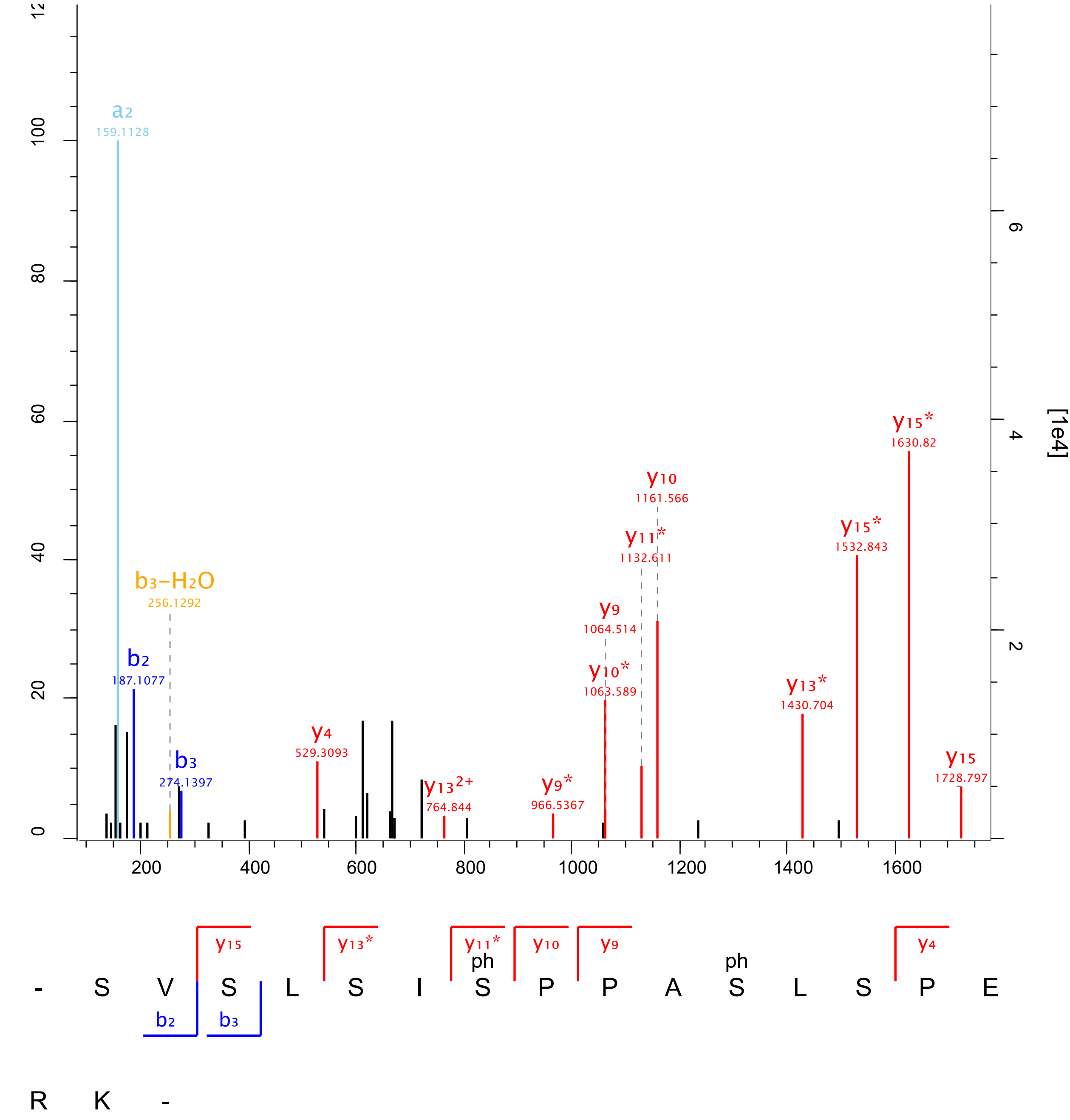

Raw file	Scan	Method	Score	m/z
sys_15_1	31482	FTMS; HCD	227.95	920.88

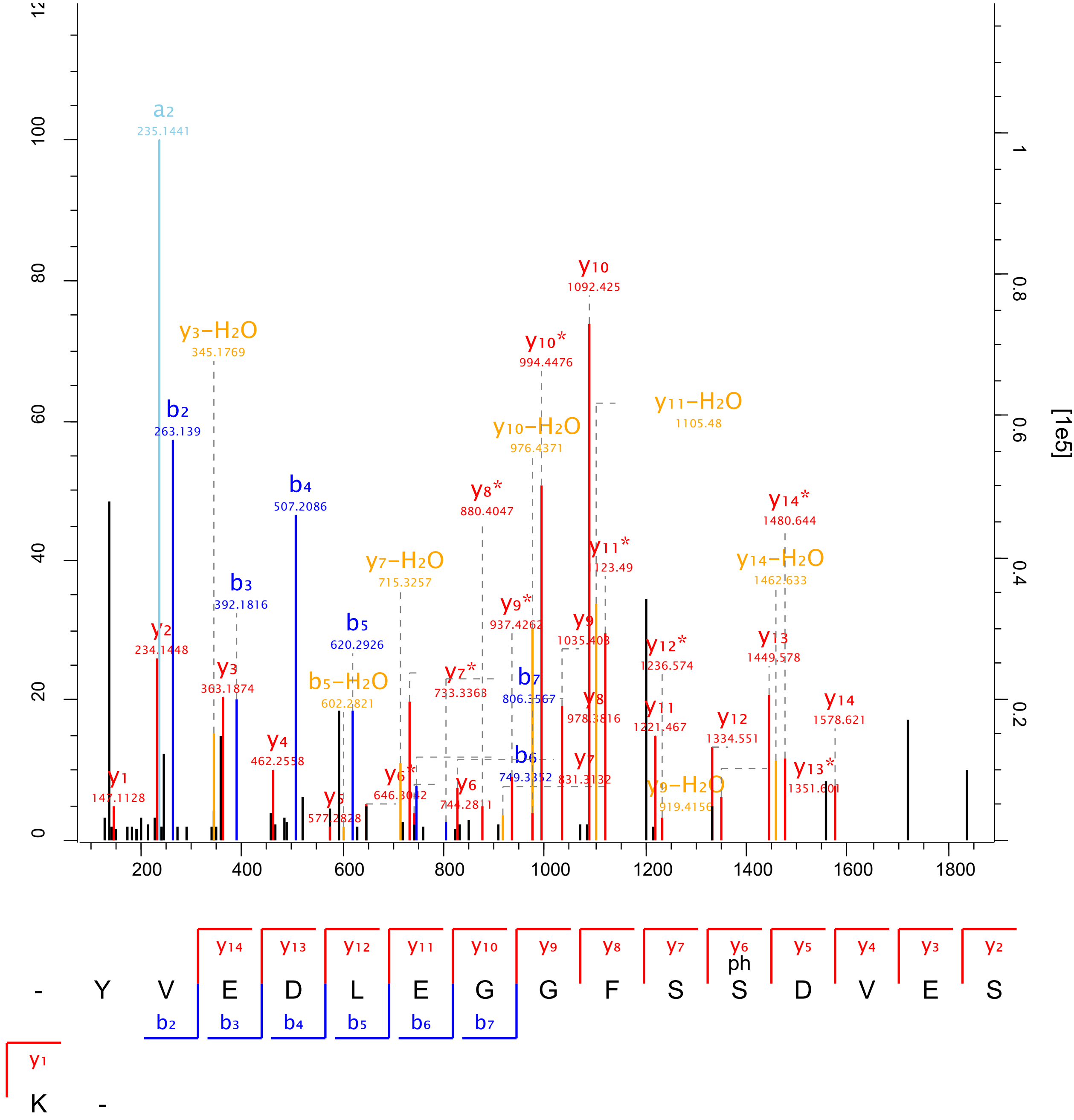

Raw file	Scan	Method	Score	m/z
sys_15_1	31524	FTMS; HCD	58.95	751.01

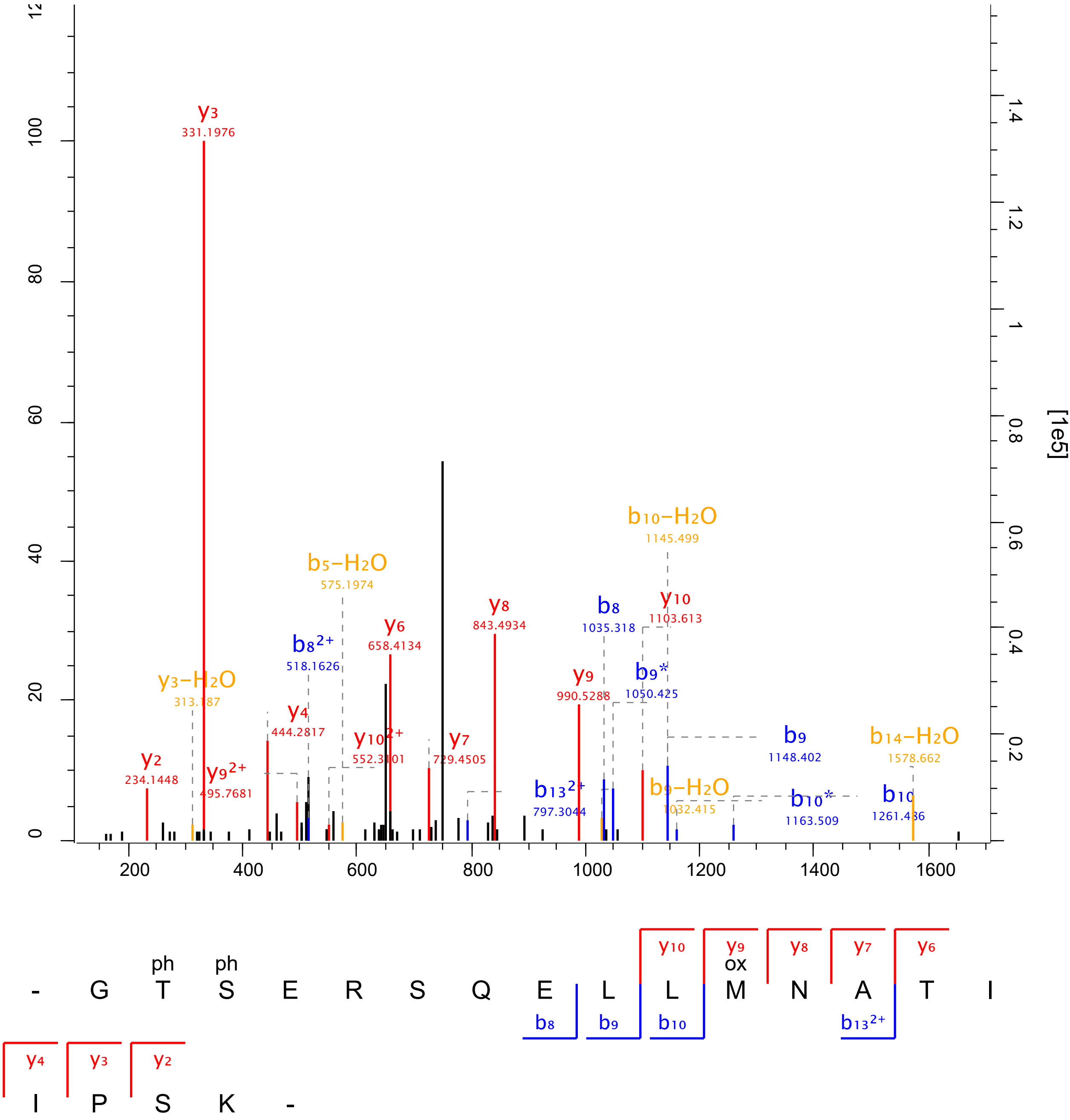

Raw file	Scan	Method	Score	m/z
sys_15_1	31552	FTMS; HCD	41.65	668.97

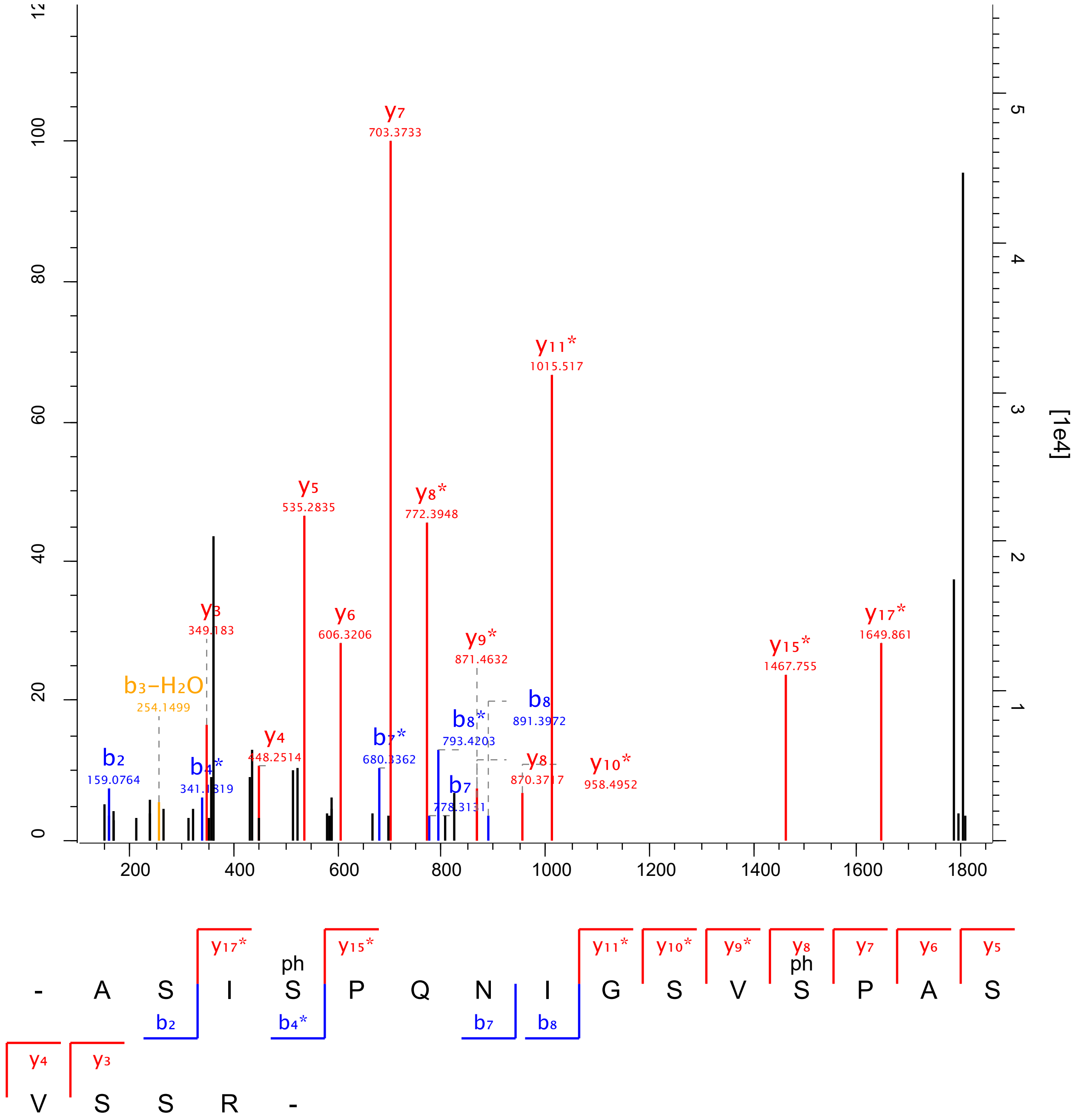

Raw file	Scan	Method	Score	m/z
sys_15_1	31651	FTMS; HCD	100.97	904.39

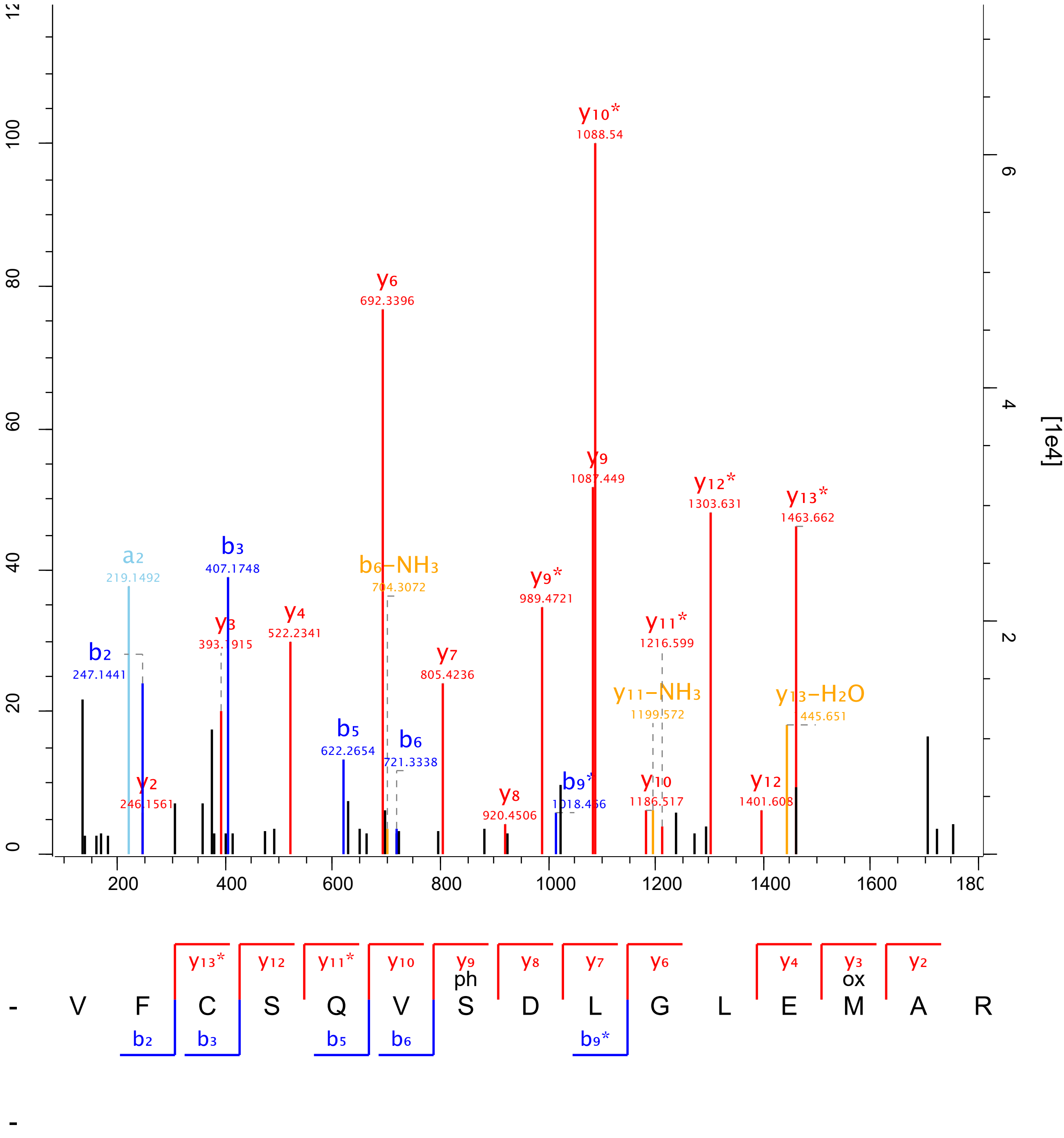

Raw file	Scan	Method	Score	m/z
sys_15_1	3181	FTMS; HCD	68.97	453.54

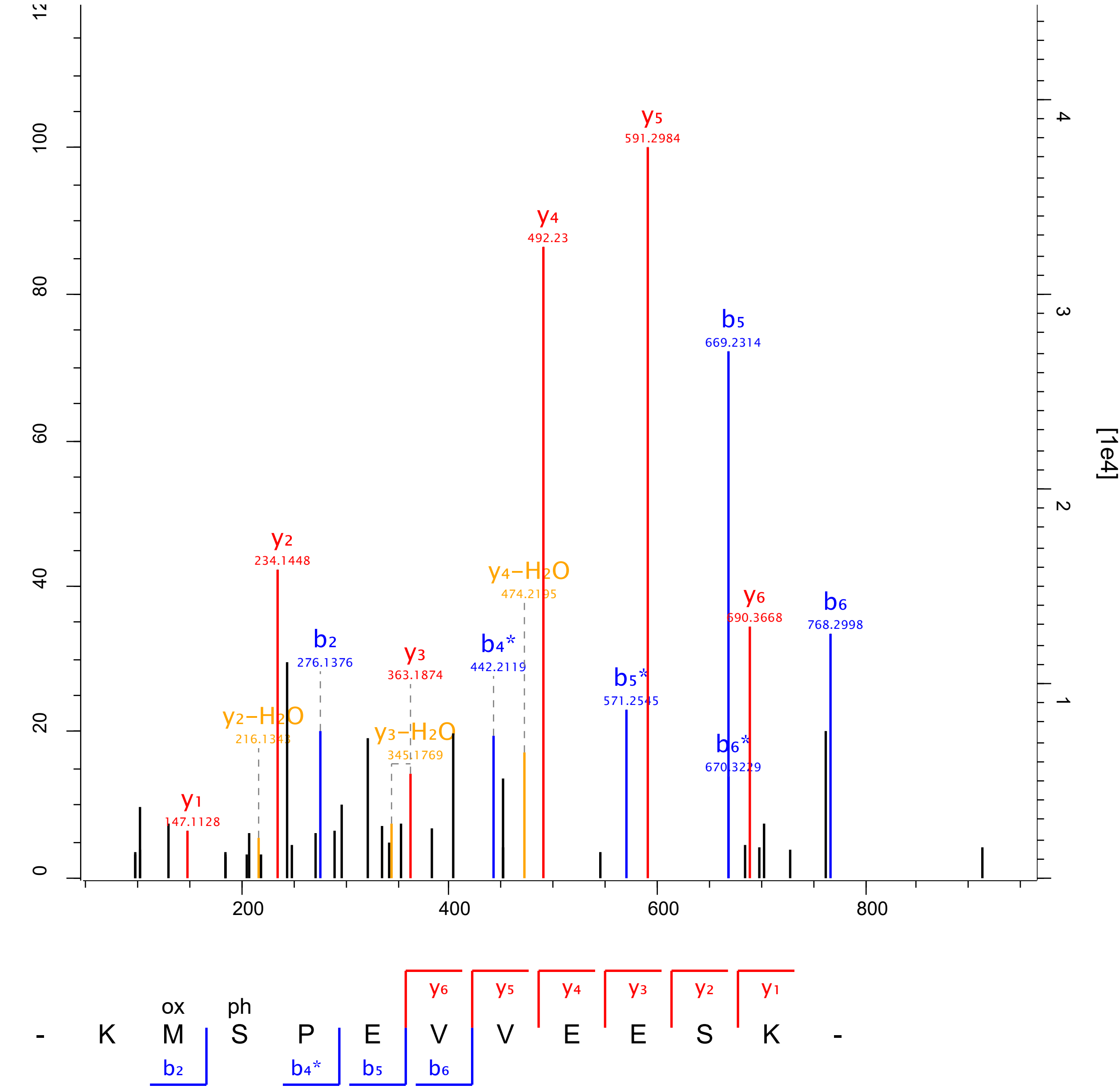

Raw file	Scan	Method	Score	m/z
sys_15_1	31811	FTMS; HCD	171.47	1005.38

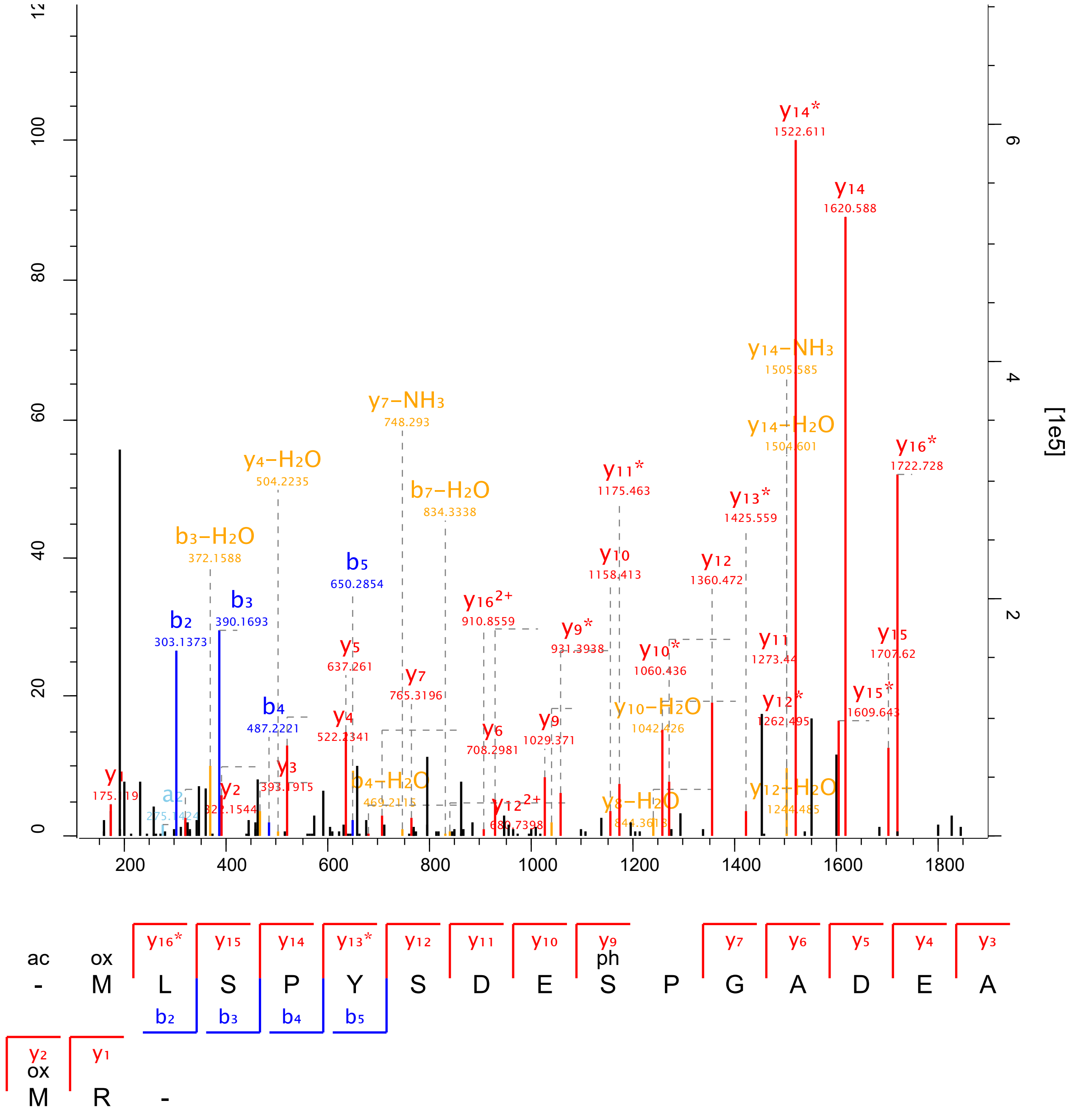

Raw file	Scan	Method	Score	m/z
sys_15_1	32081	FTMS; HCD	112.26	941.77

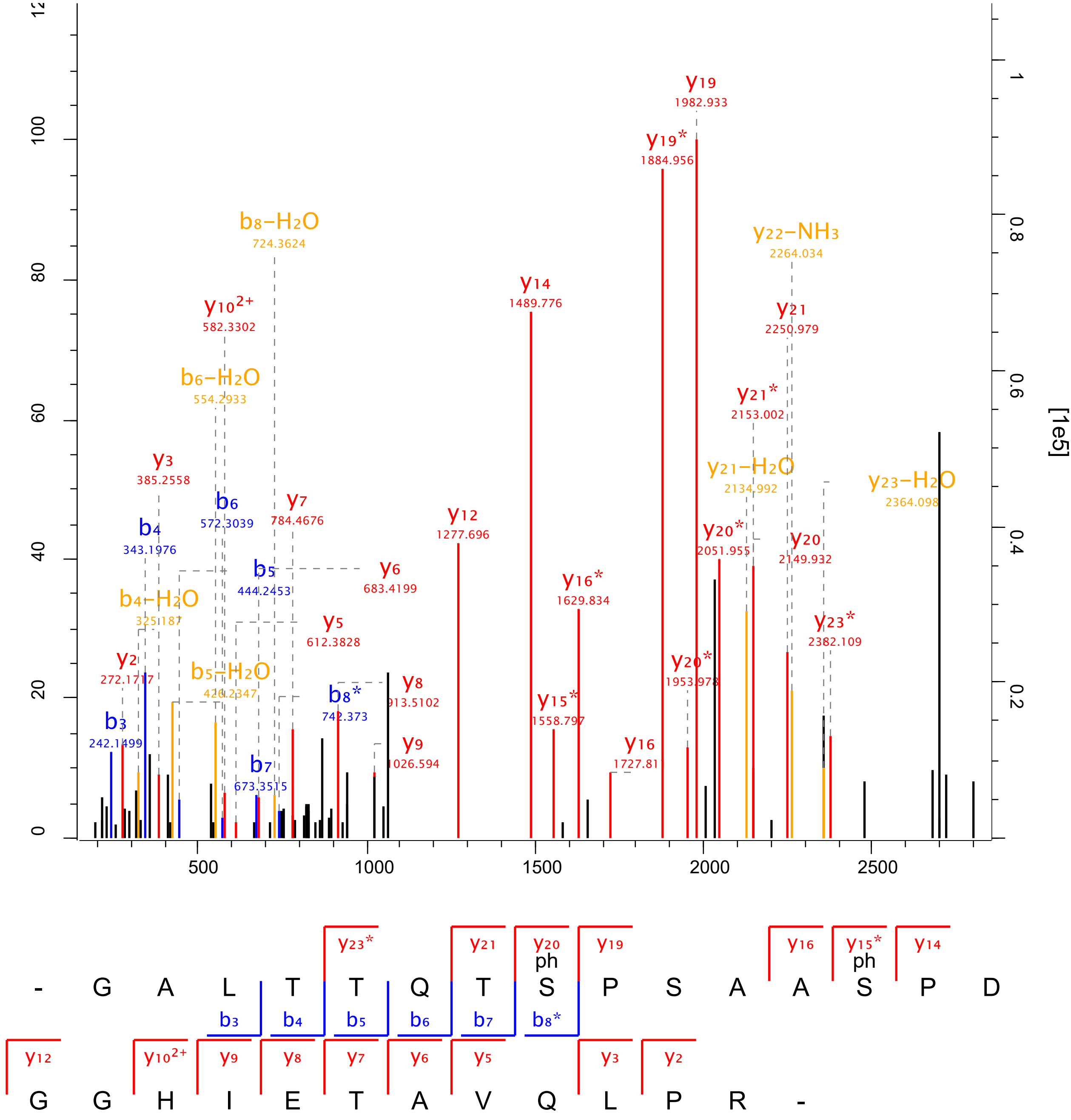

Raw file	Scan	Method	Score	m/z
sys_15_1	32091	FTMS; HCD	49.99	916.41

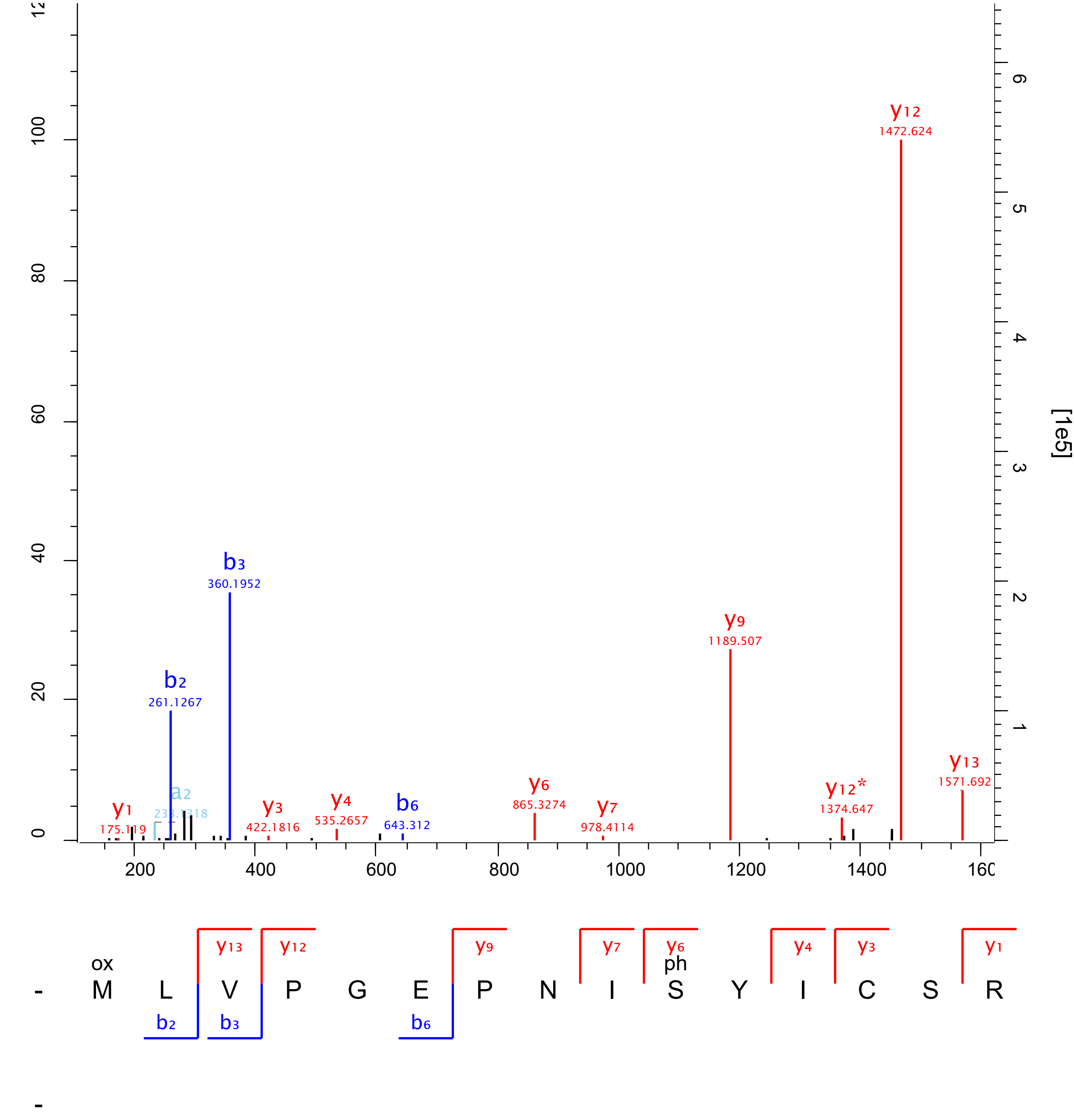

Raw file	Scan	Method	Score	m/z
sys_15_1	32151	FTMS; HCD	109	632.78

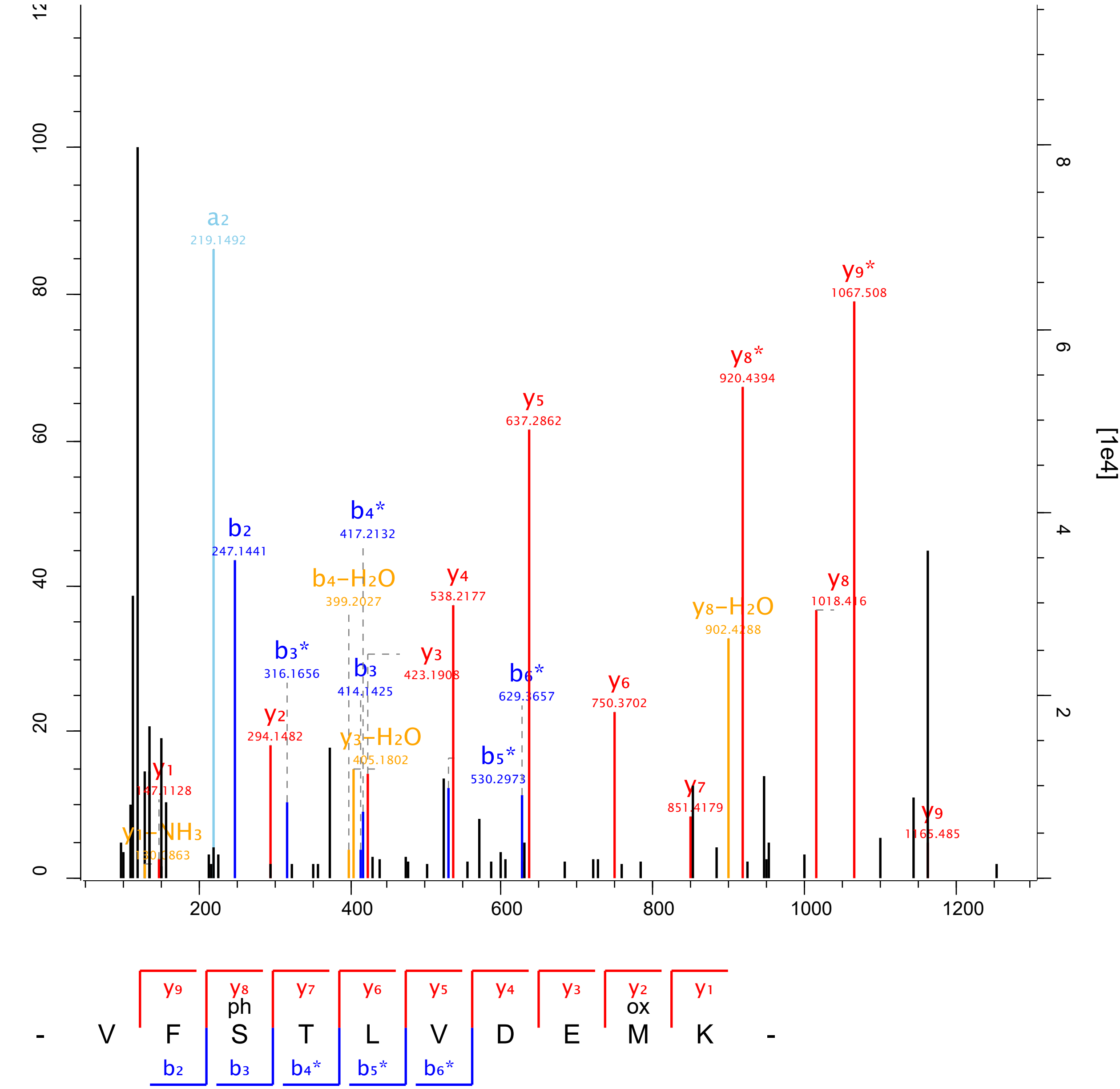

Raw file	Scan	Method	Score	m/z
sys_15_1	32255	FTMS; HCD	41.91	688.65

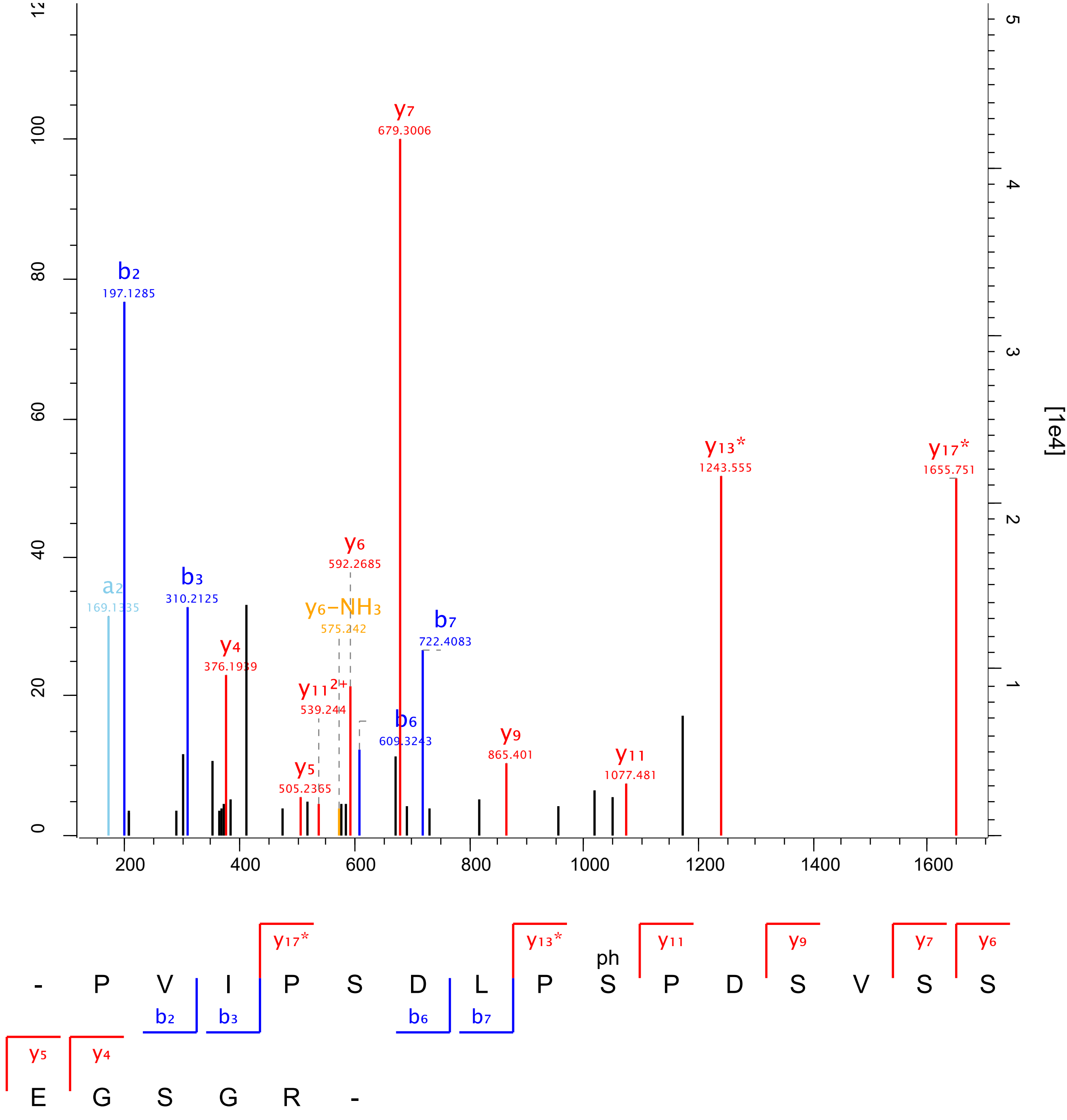

Raw file	Scan	Method	Score	m/z
sys_15_1	32257	FTMS; HCD	166.6	853.43

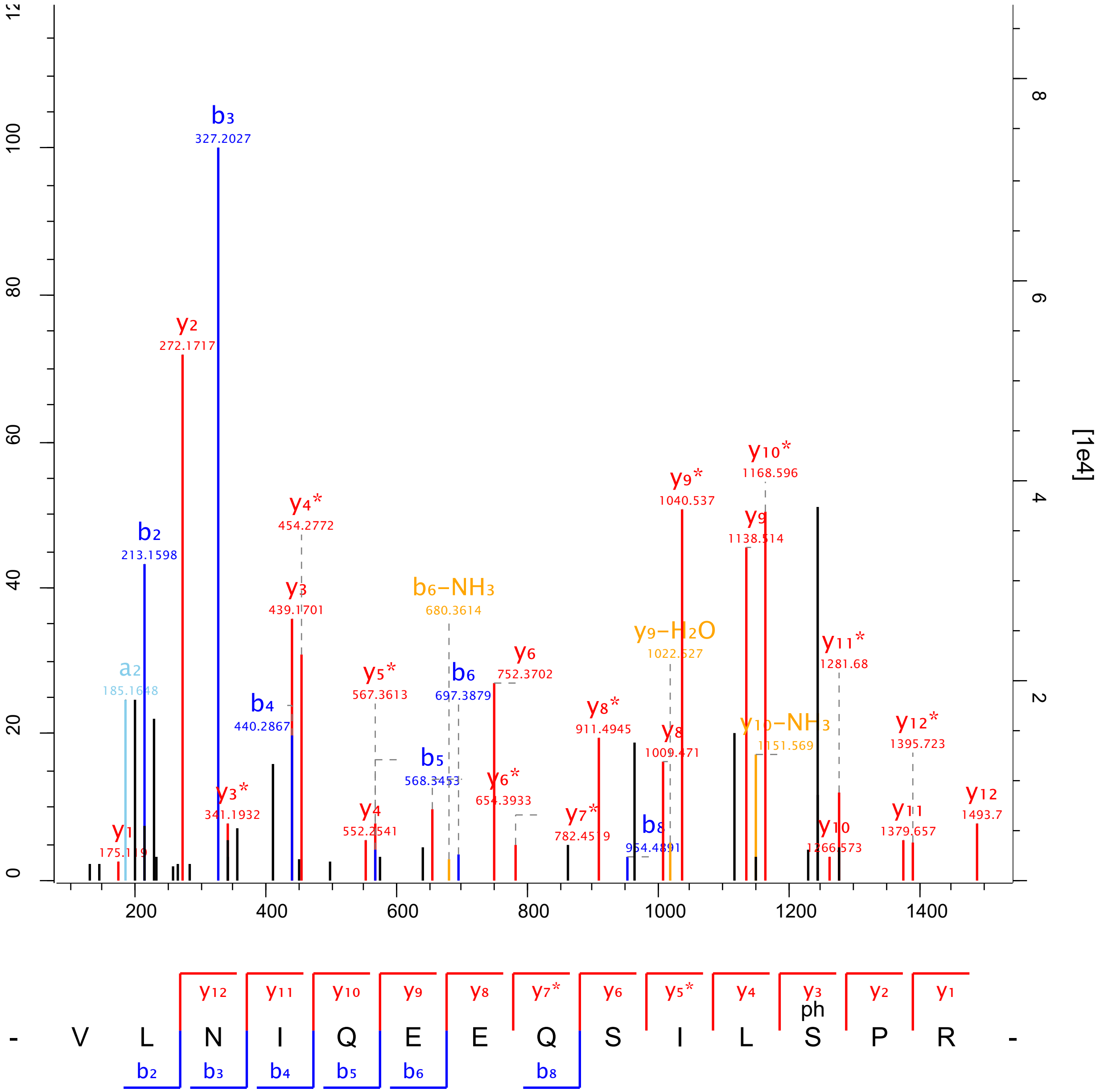

Raw file	Scan	Method	Score	m/z
sys_15_1	32262	FTMS; HCD	45.58	741.65

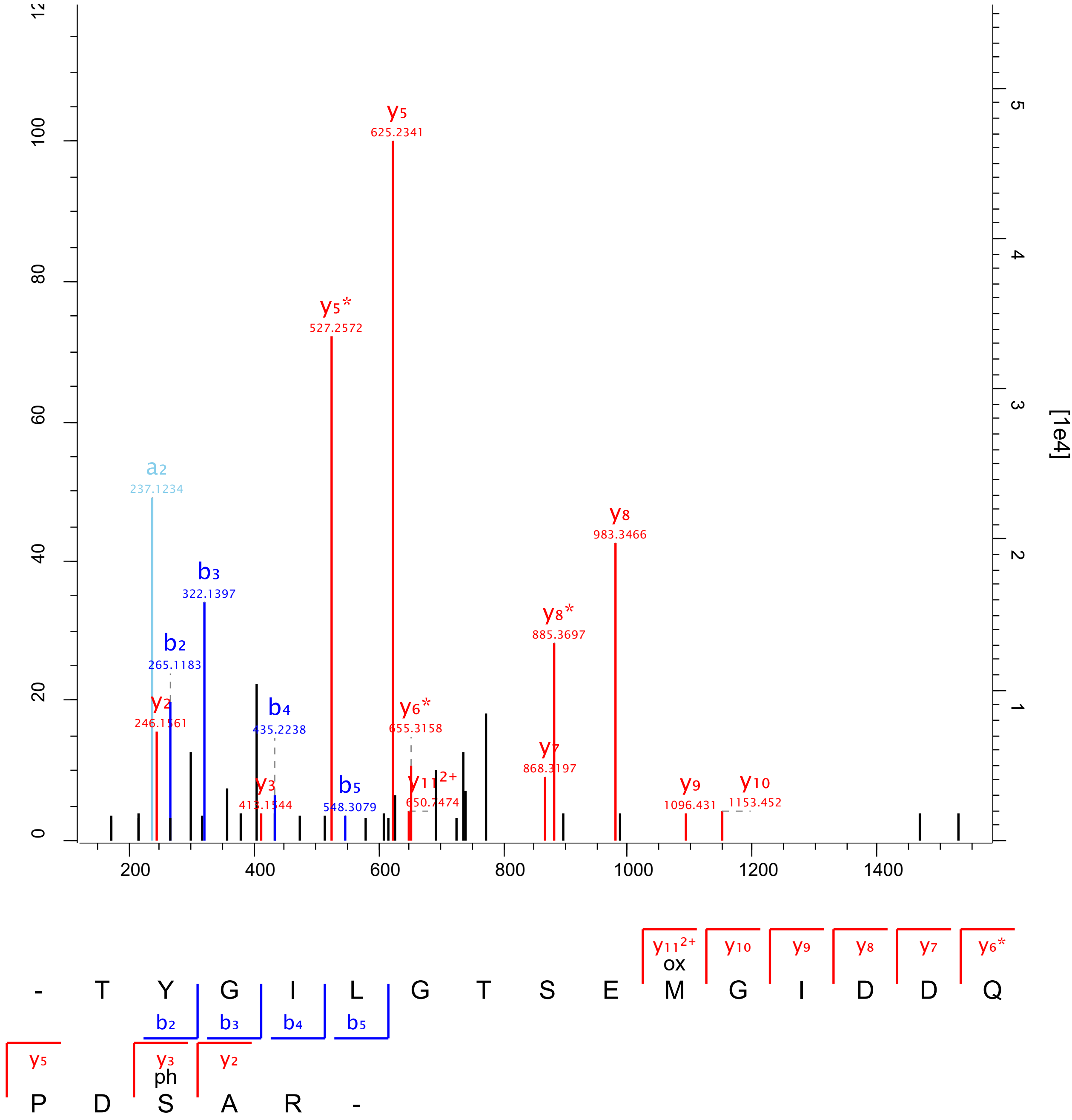

Raw file	Scan	Method	Score	m/z
sys_15_1	32282	FTMS; HCD	110.33	1108.45

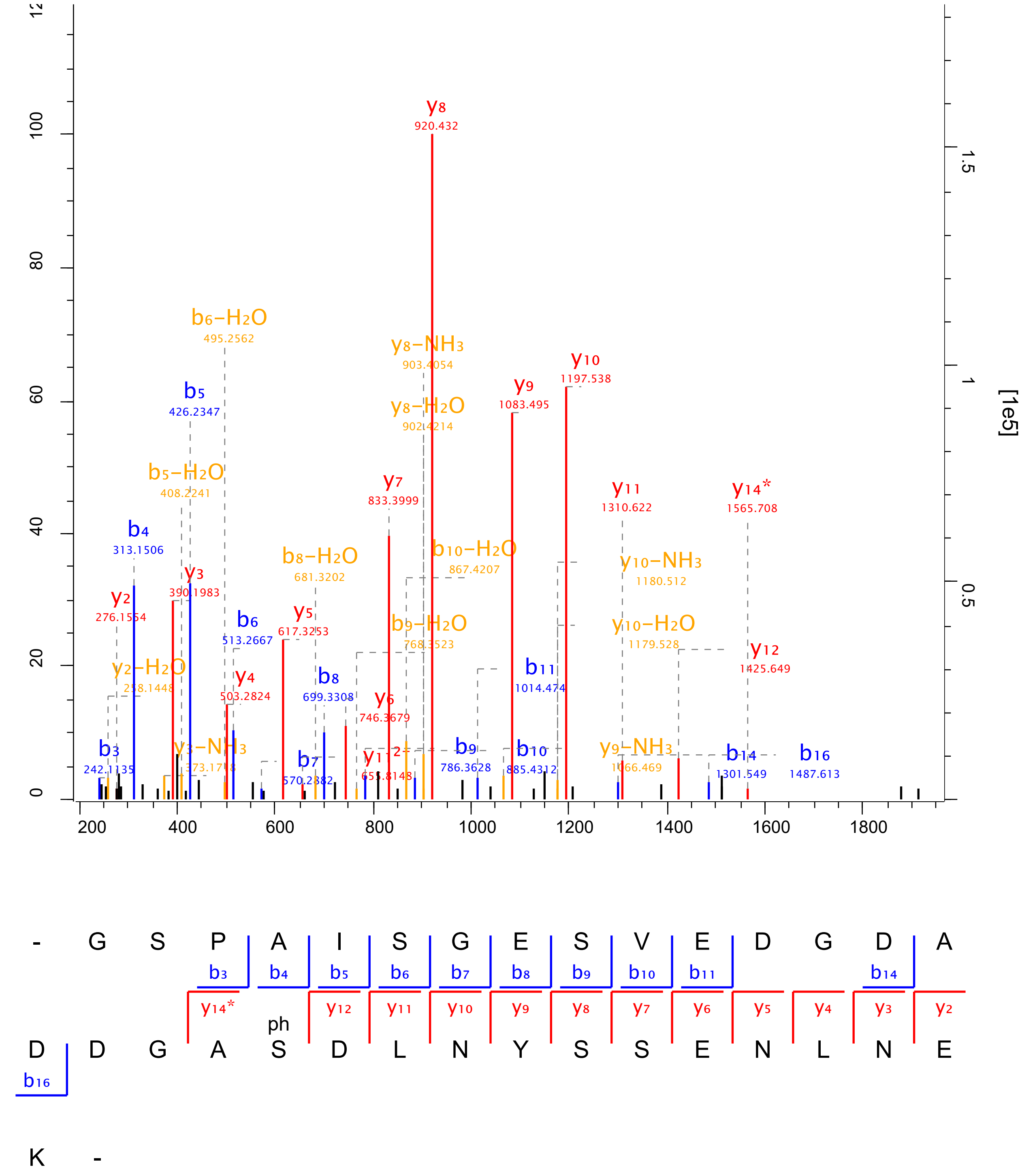

Raw file	Scan	Method	Score	m/z
sys_15_1	32313	FTMS; HCD	56.69	957.76

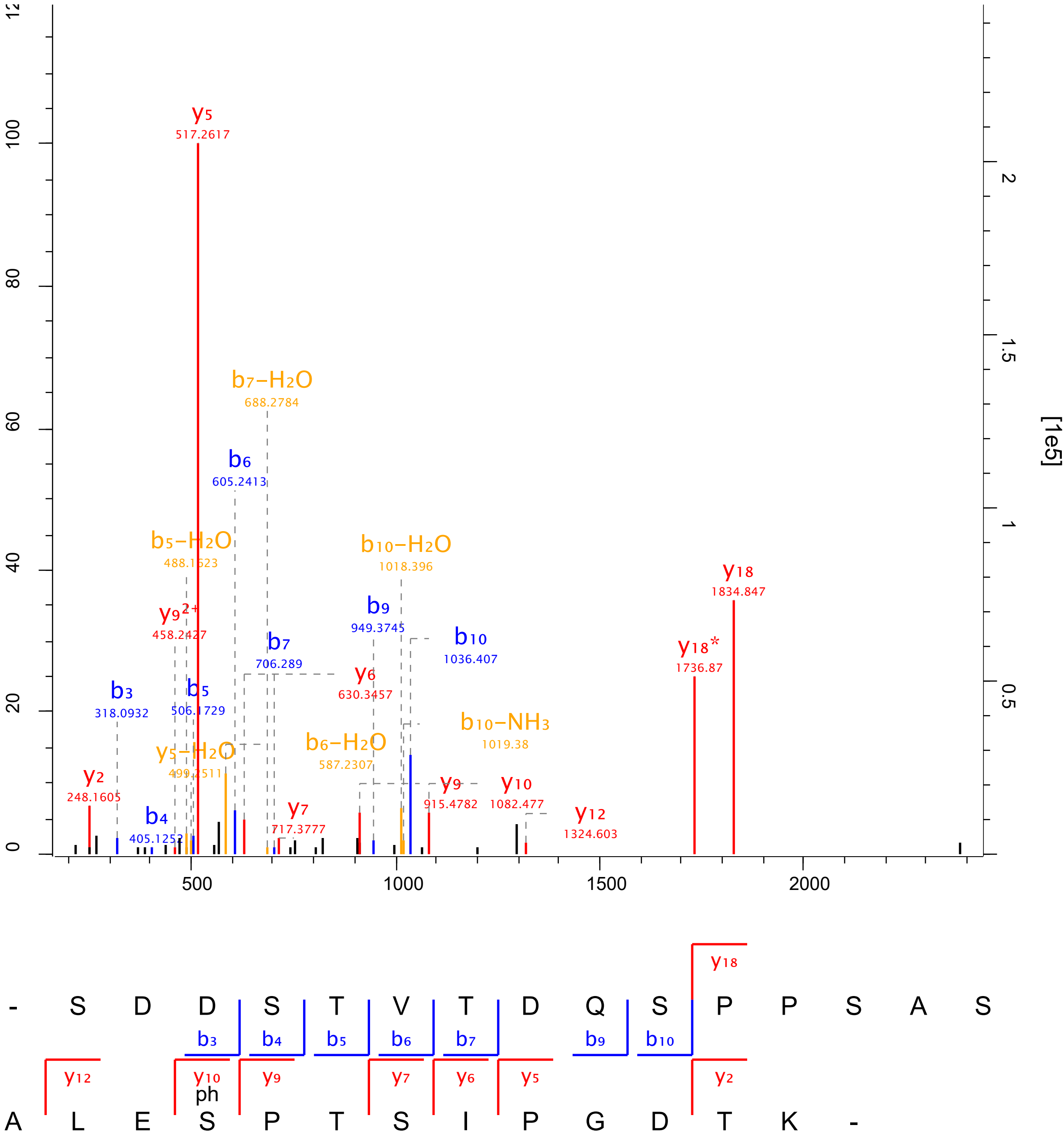

Raw file	Scan	Method	Score	m/z
sys_15_1	32356	FTMS; HCD	101.41	755.33

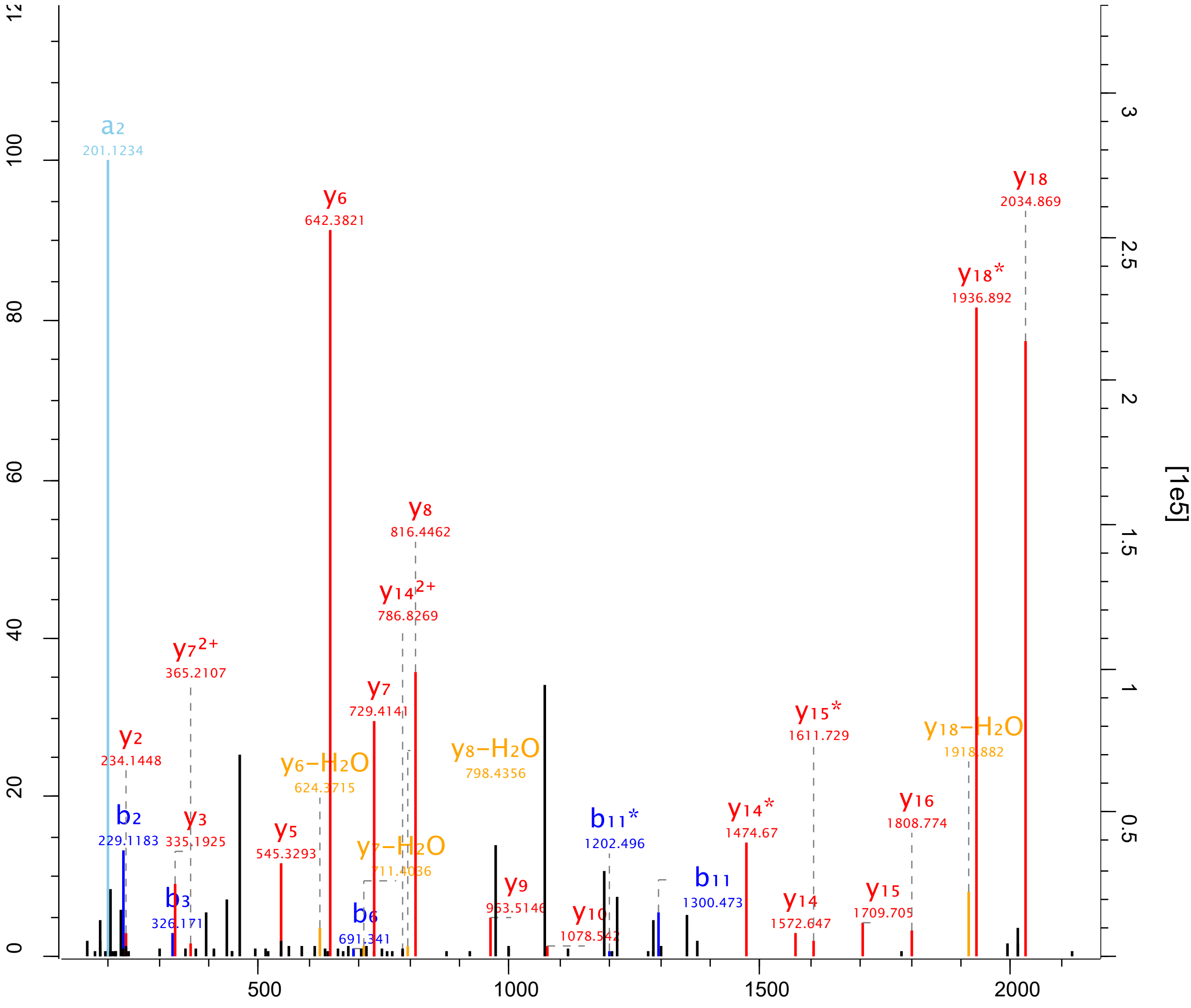

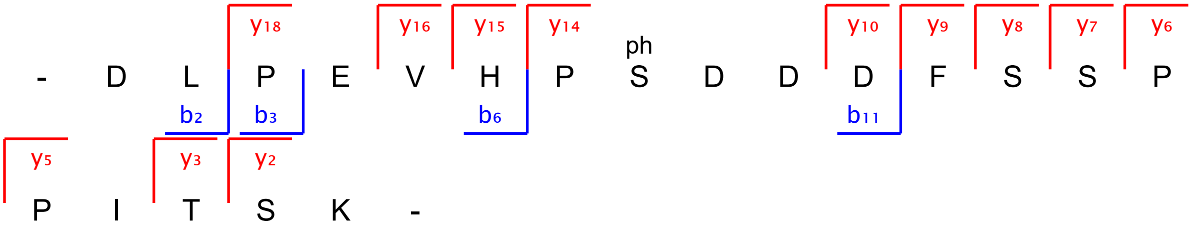

Raw file	Scan	Method	Score	m/z
sys_15_1	32391	FTMS; HCD	71.38	769.84

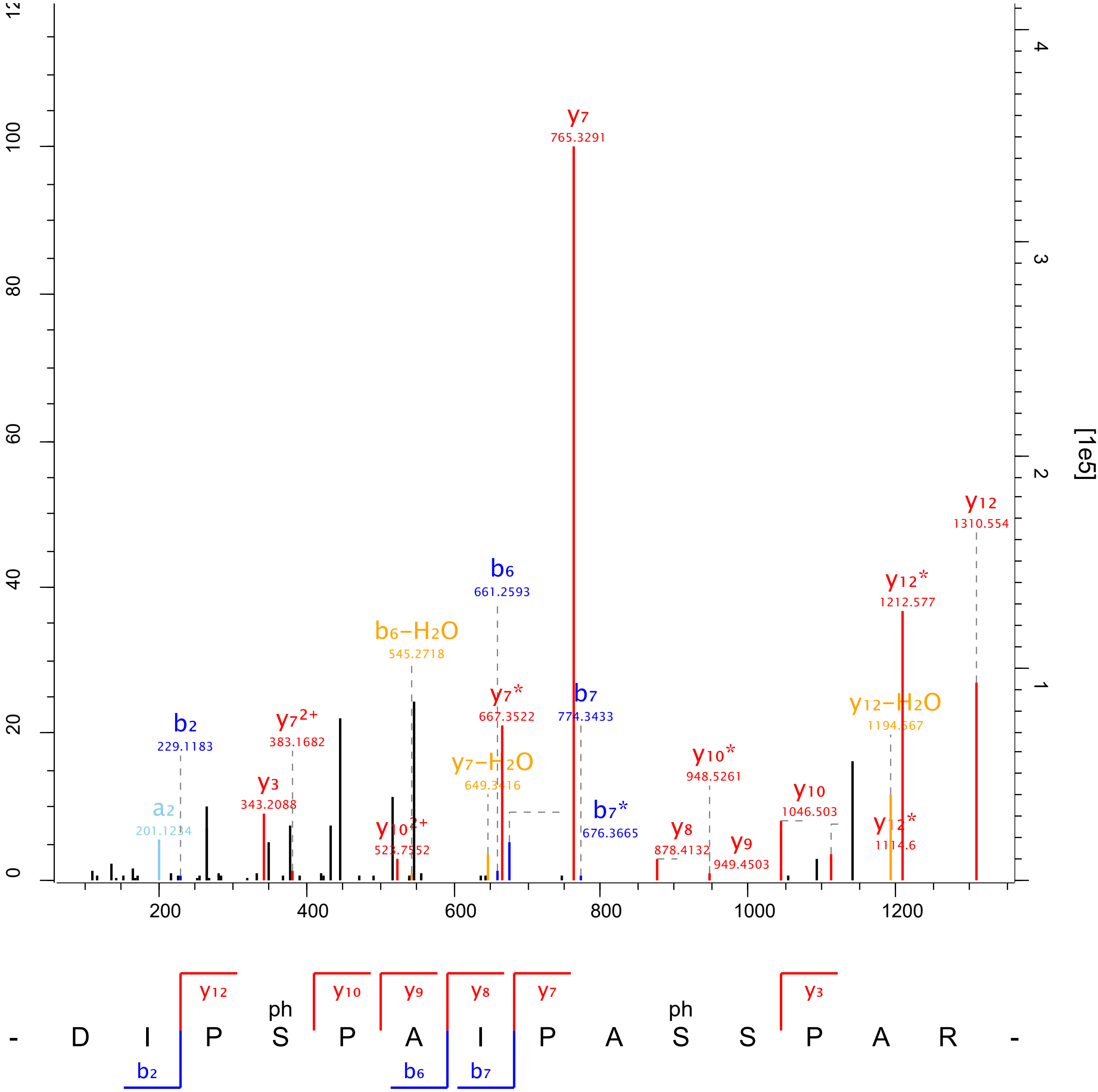

Raw file	Scan	Method	Score	m/z
sys_15_1	32427	FTMS; HCD	159.81	699.31

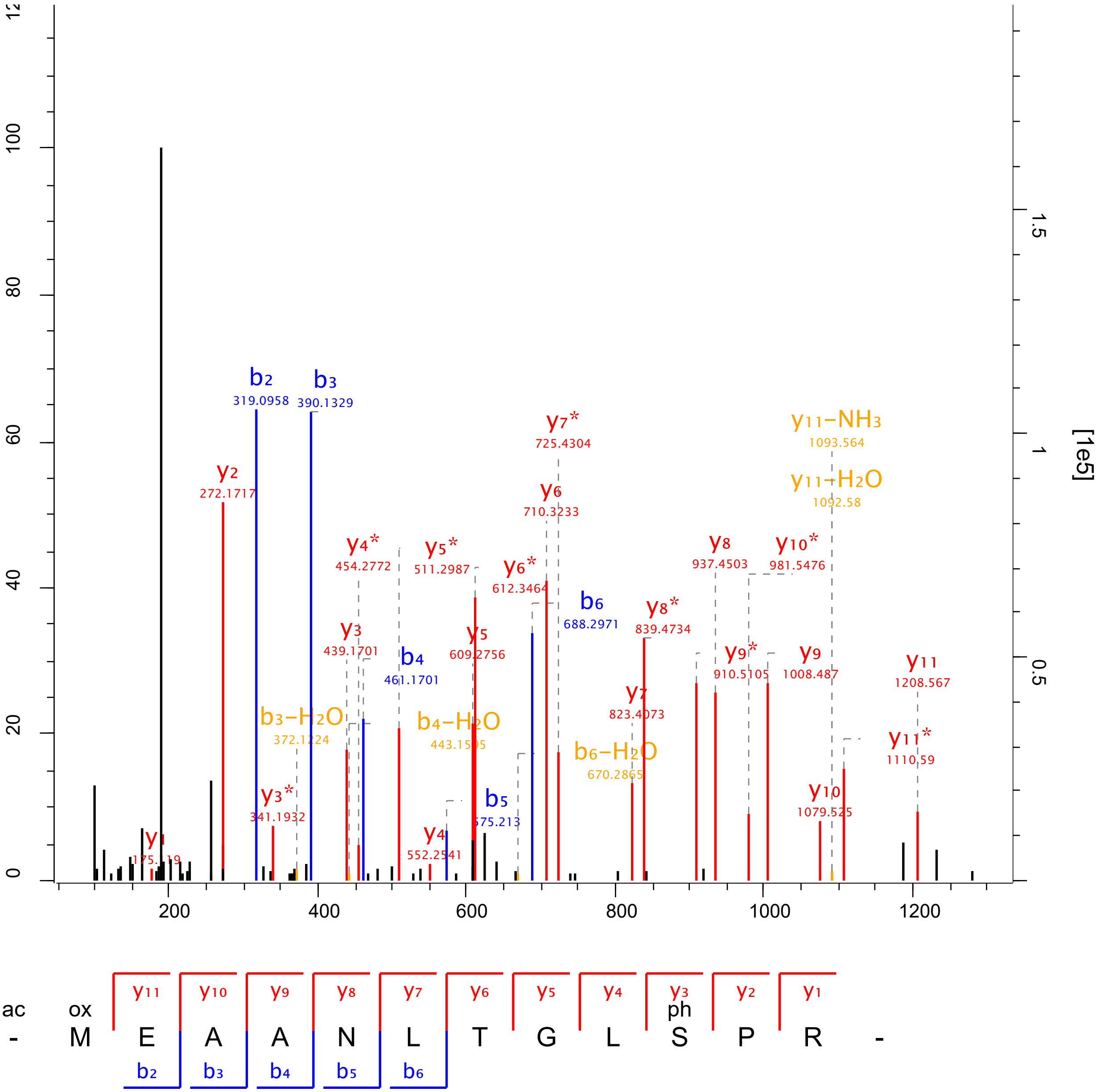

Raw file	Scan	Method	Score	m/z
sys_15_1	32593	FTMS; HCD	142.94	869.4

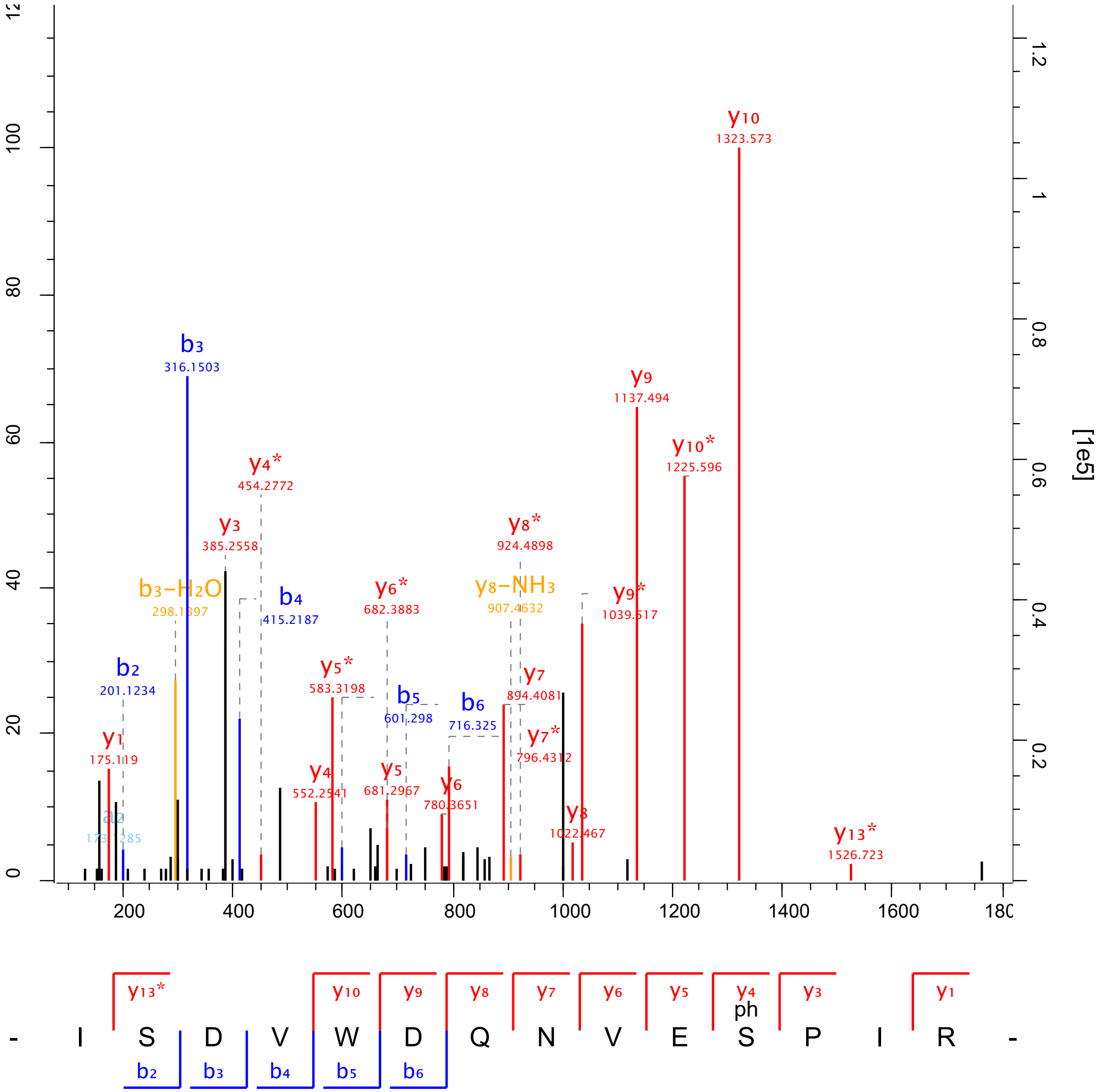

Raw file	Scan	Method	Score	m/z
sys_15_1	32823	FTMS; HCD	108.72	876.41

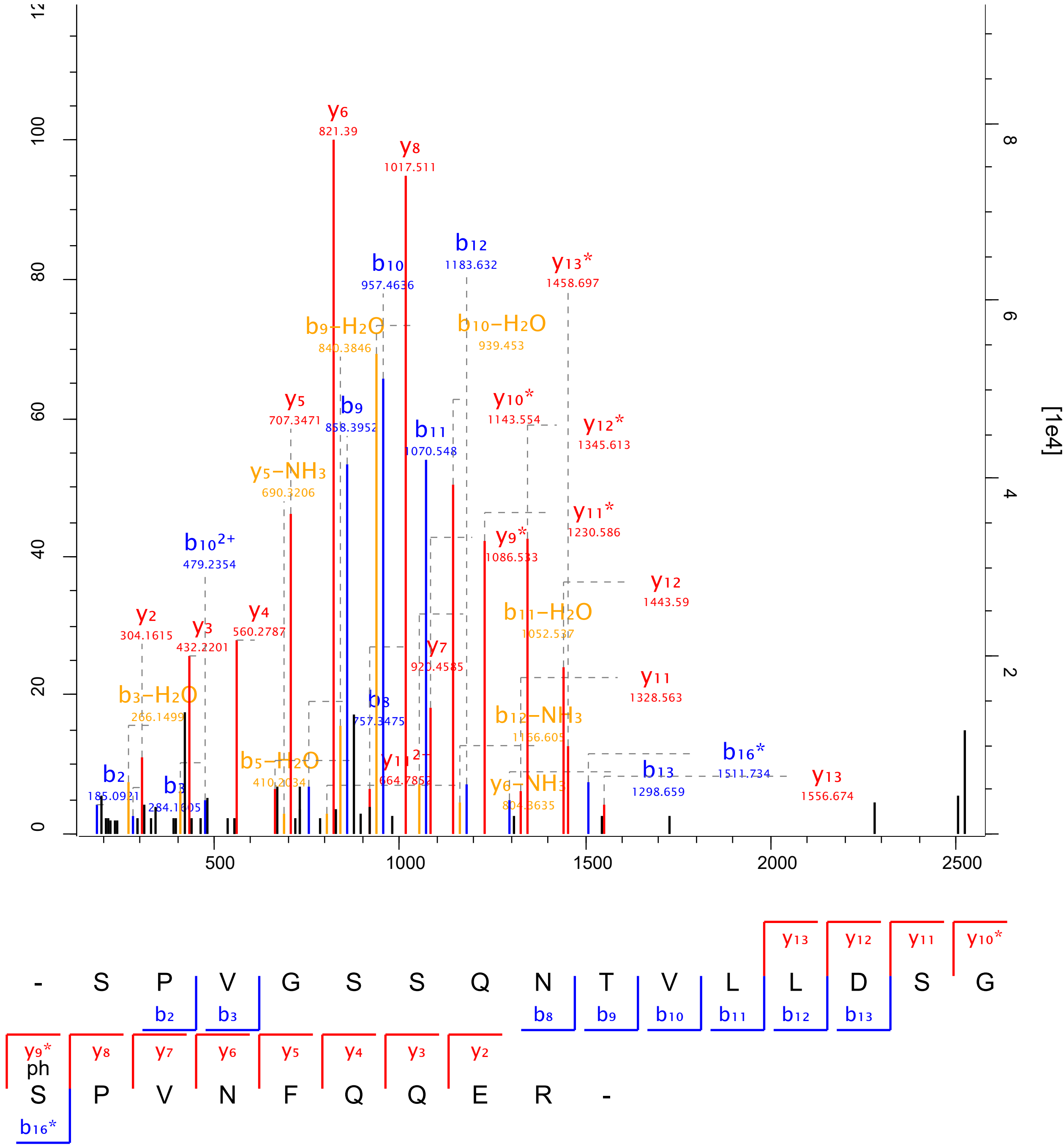

Raw file	Scan	Method	Score	m/z
sys_15_1	32848	FTMS; HCD	67.14	881.37

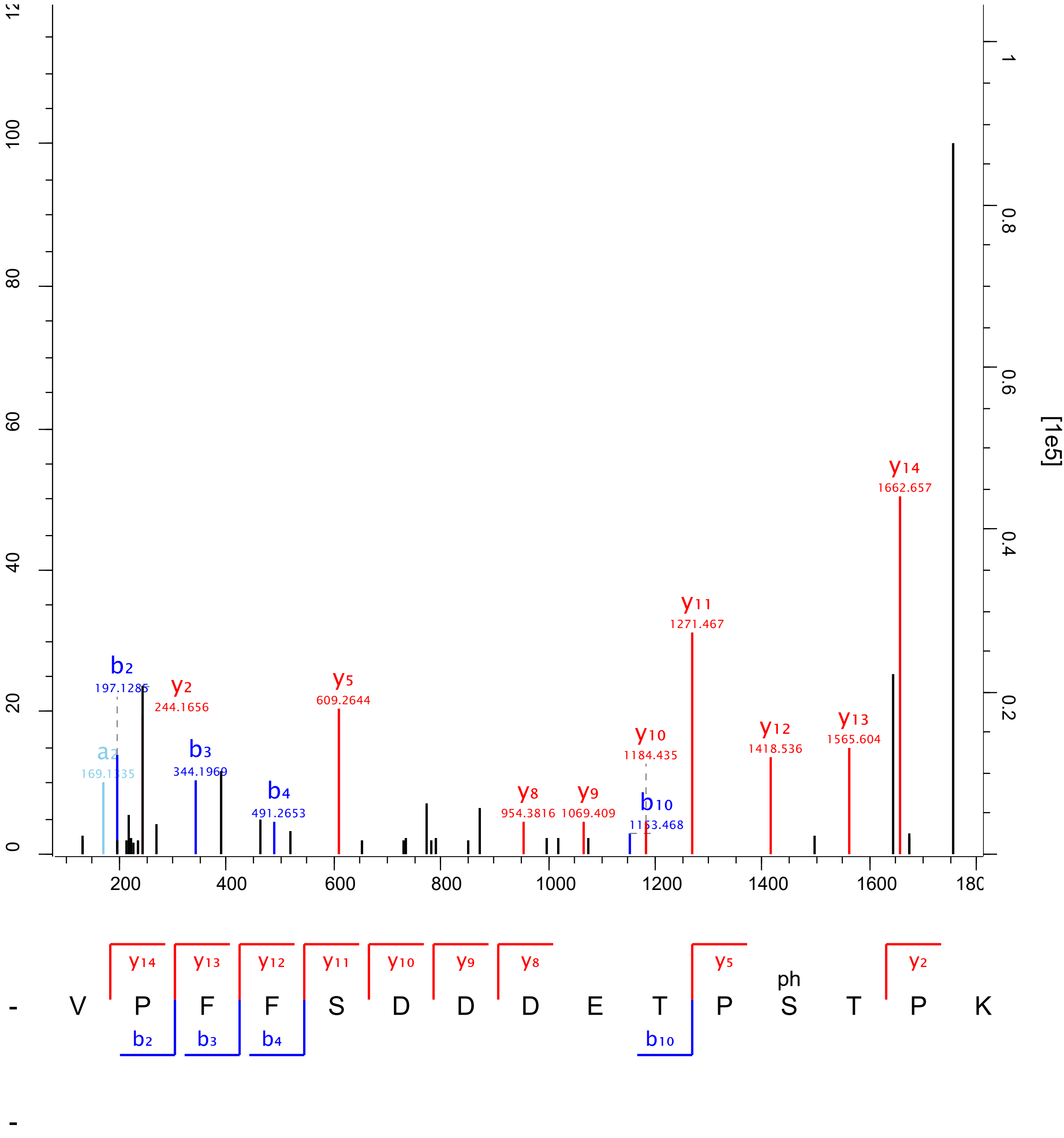

Raw file	Scan	Method	Score	m/z
sys_15_1	32999	FTMS; HCD	88.28	815.83

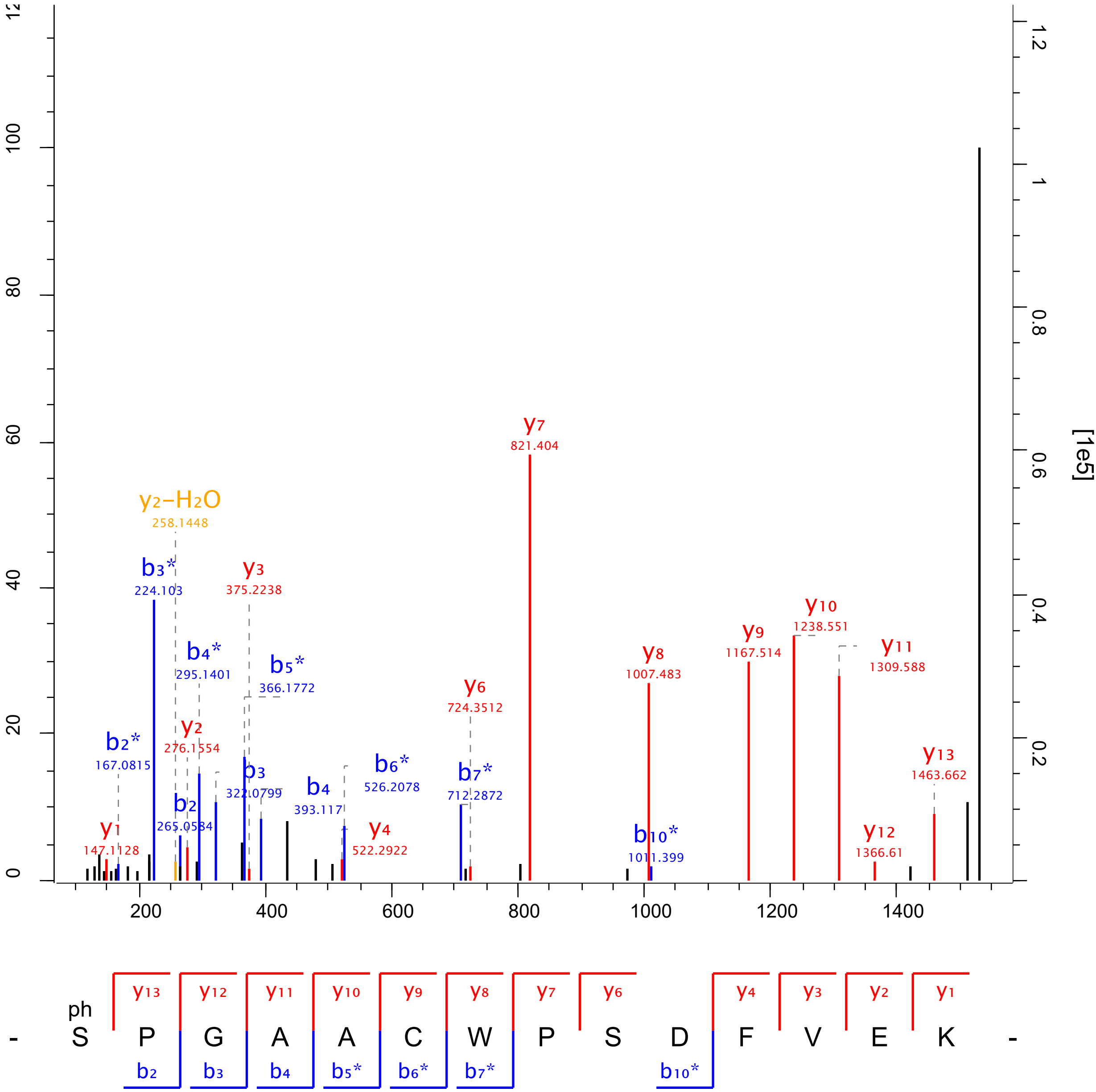

Raw file	Scan	Method	Score	m/z
sys_15_1	3321	FTMS; HCD	103.01	670.3

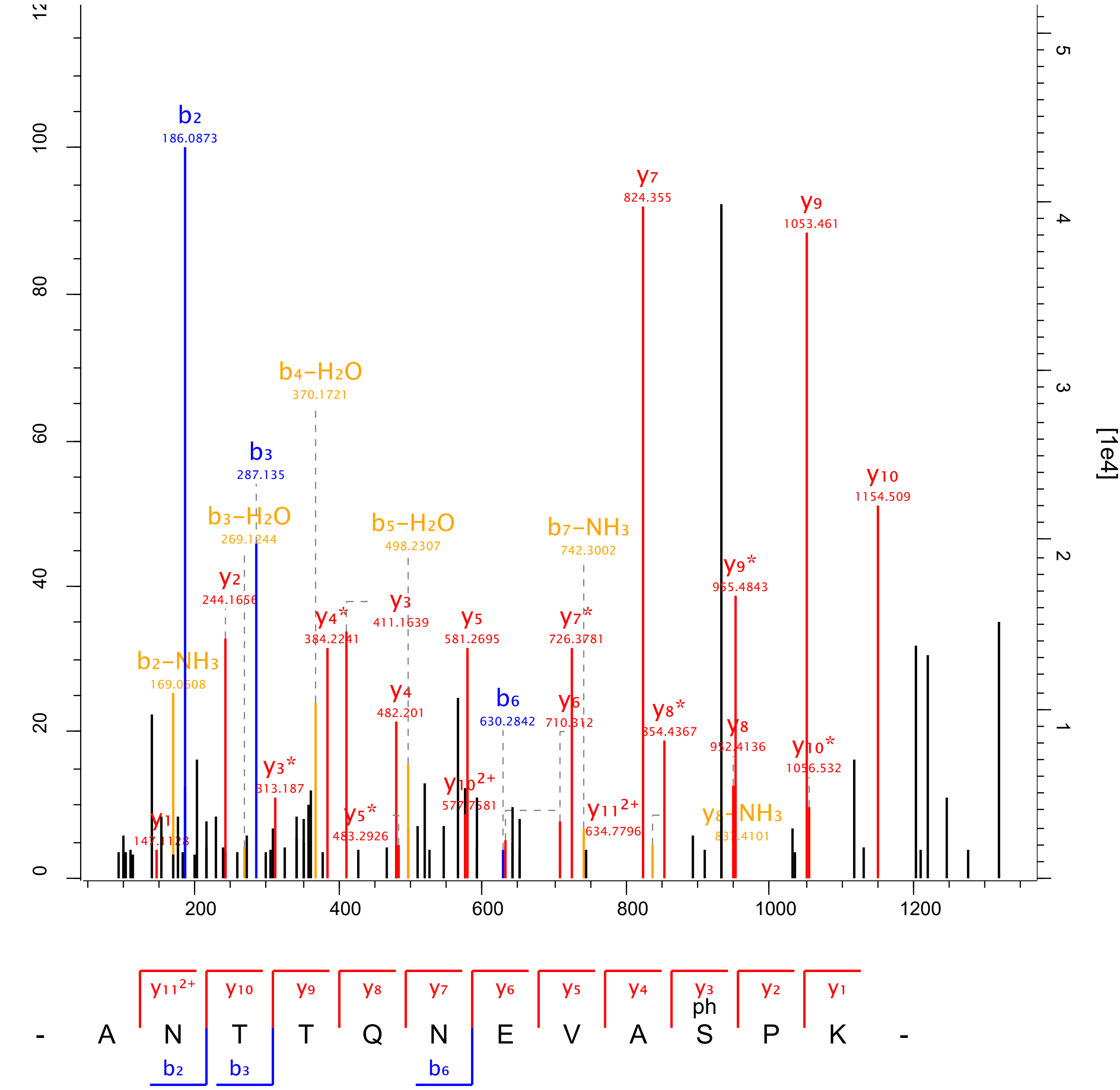

Raw file	Scan	Method	Score	m/z
sys_15_1	33364	FTMS; HCD	250.94	818.37

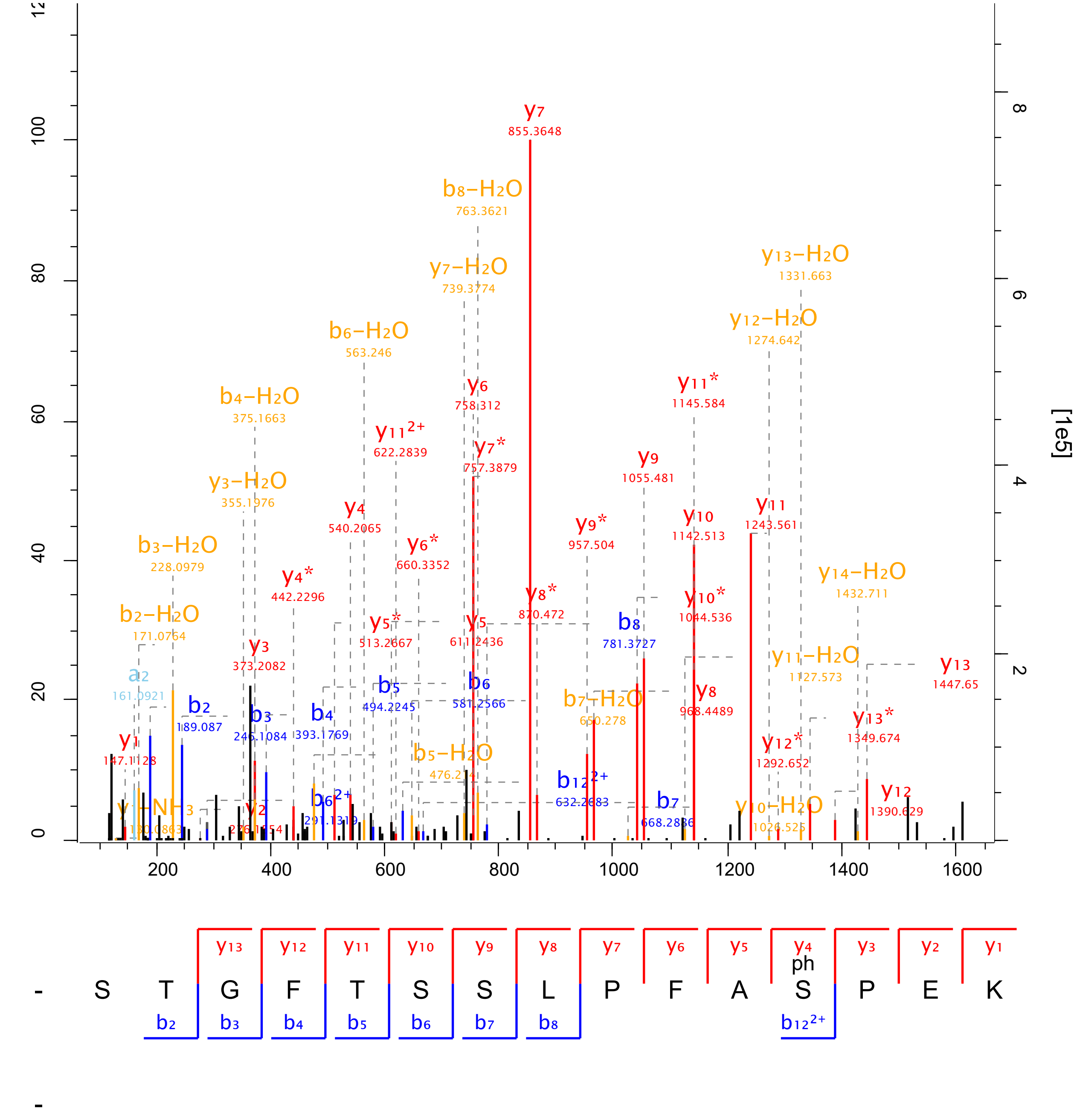

Raw file	Scan	Method	Score	m/z
sys_15_1	3348	FTMS; HCD	87.12	474.18

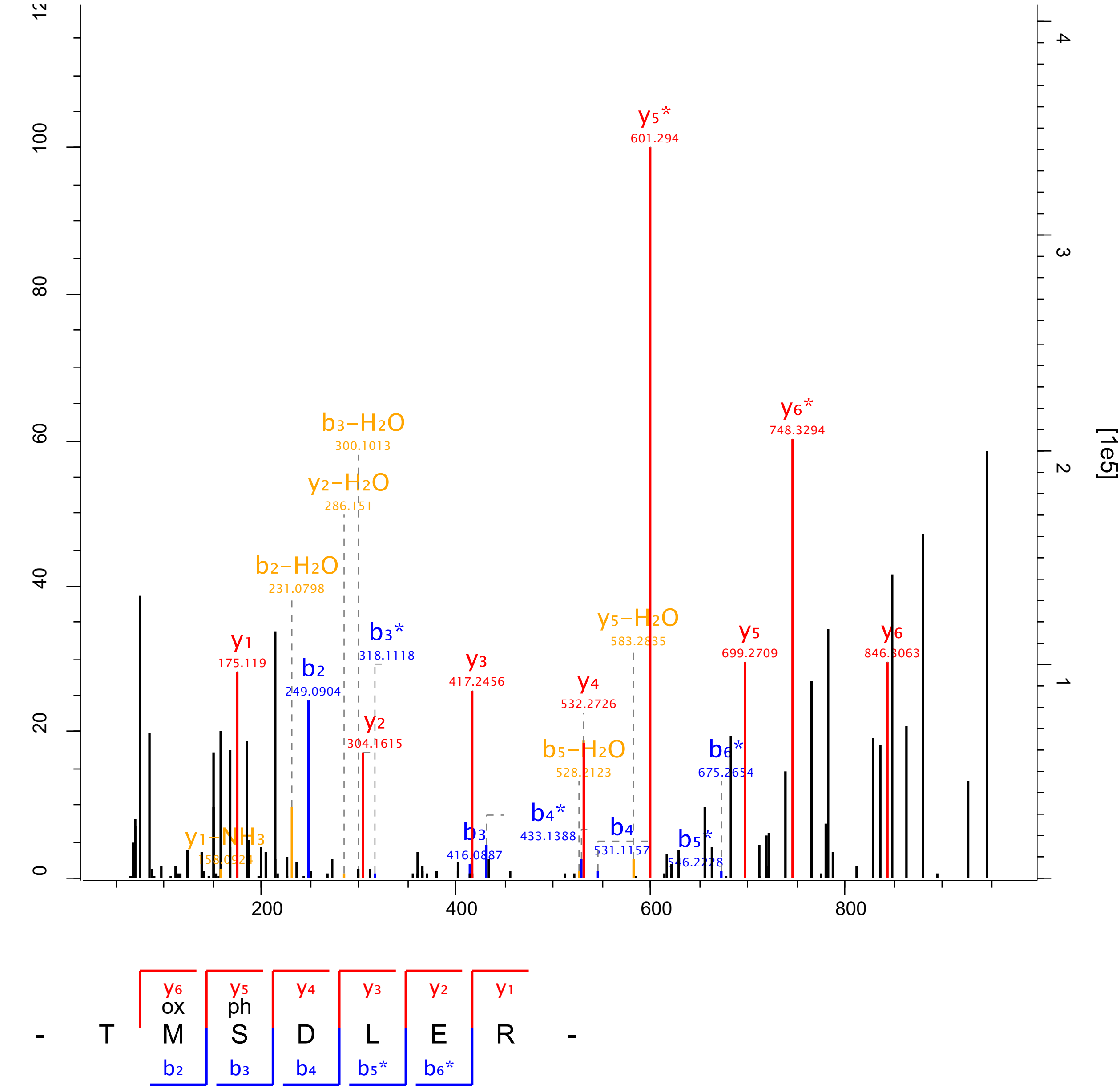

Raw file	Scan	Method	Score	m/z
sys_15_1	3351	FTMS; HCD	122.33	593.76

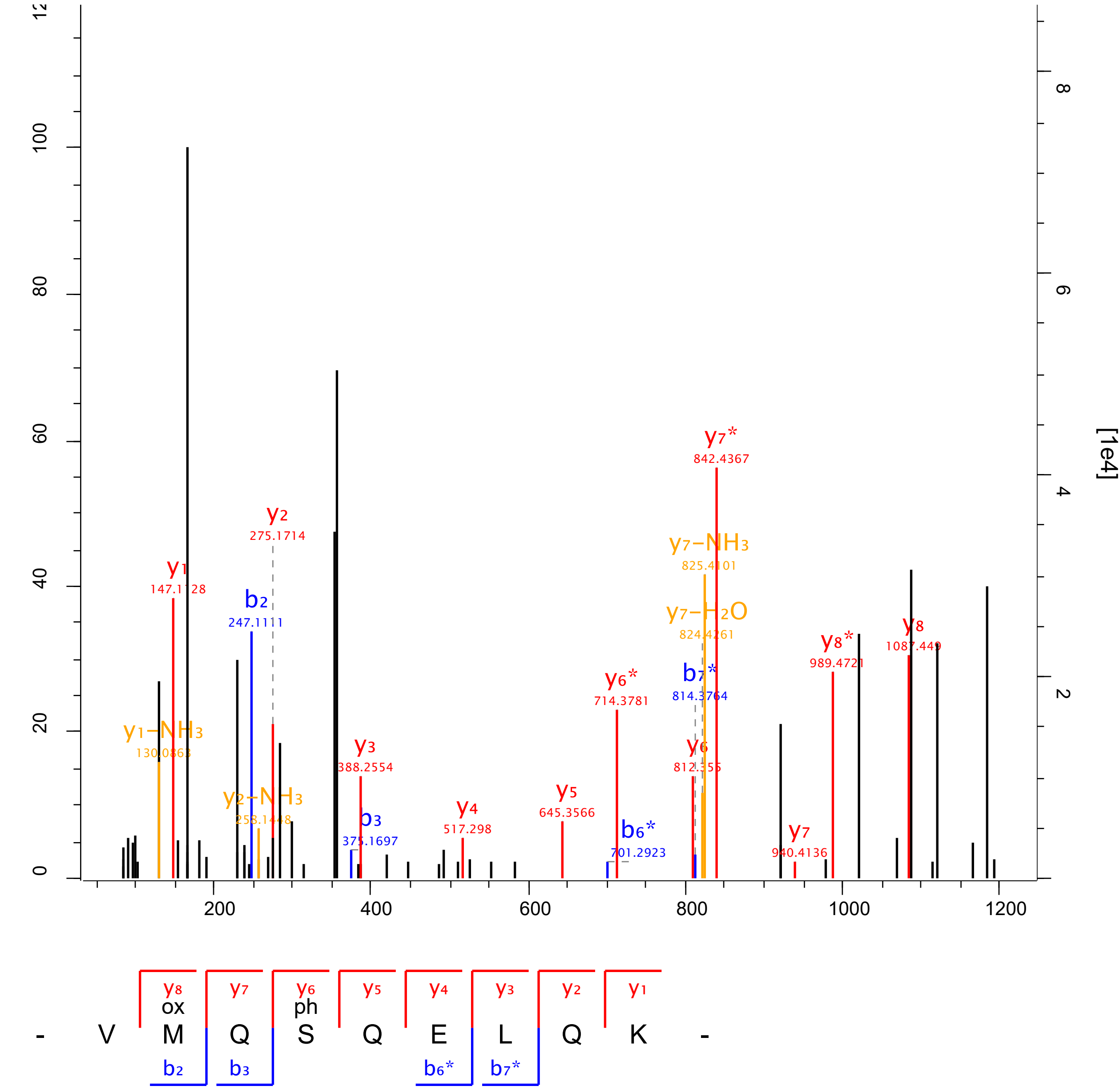

Raw file	Scan	Method	Score	m/z
sys_15_1	33585	FTMS; HCD	46.07	642.78

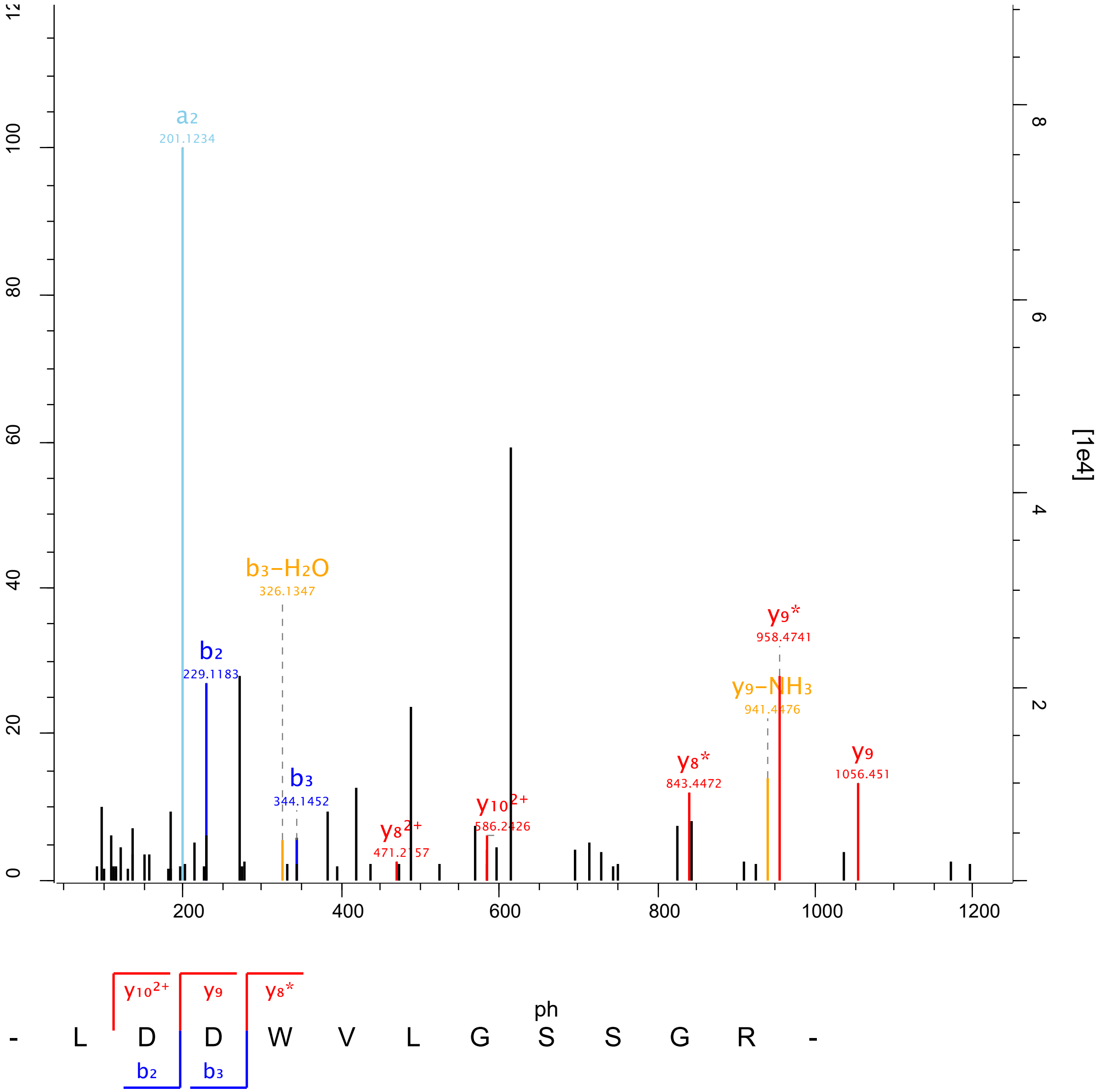

Raw file	Scan	Method	Score	m/z
sys_15_1	3379	FTMS; HCD	62.17	533.73

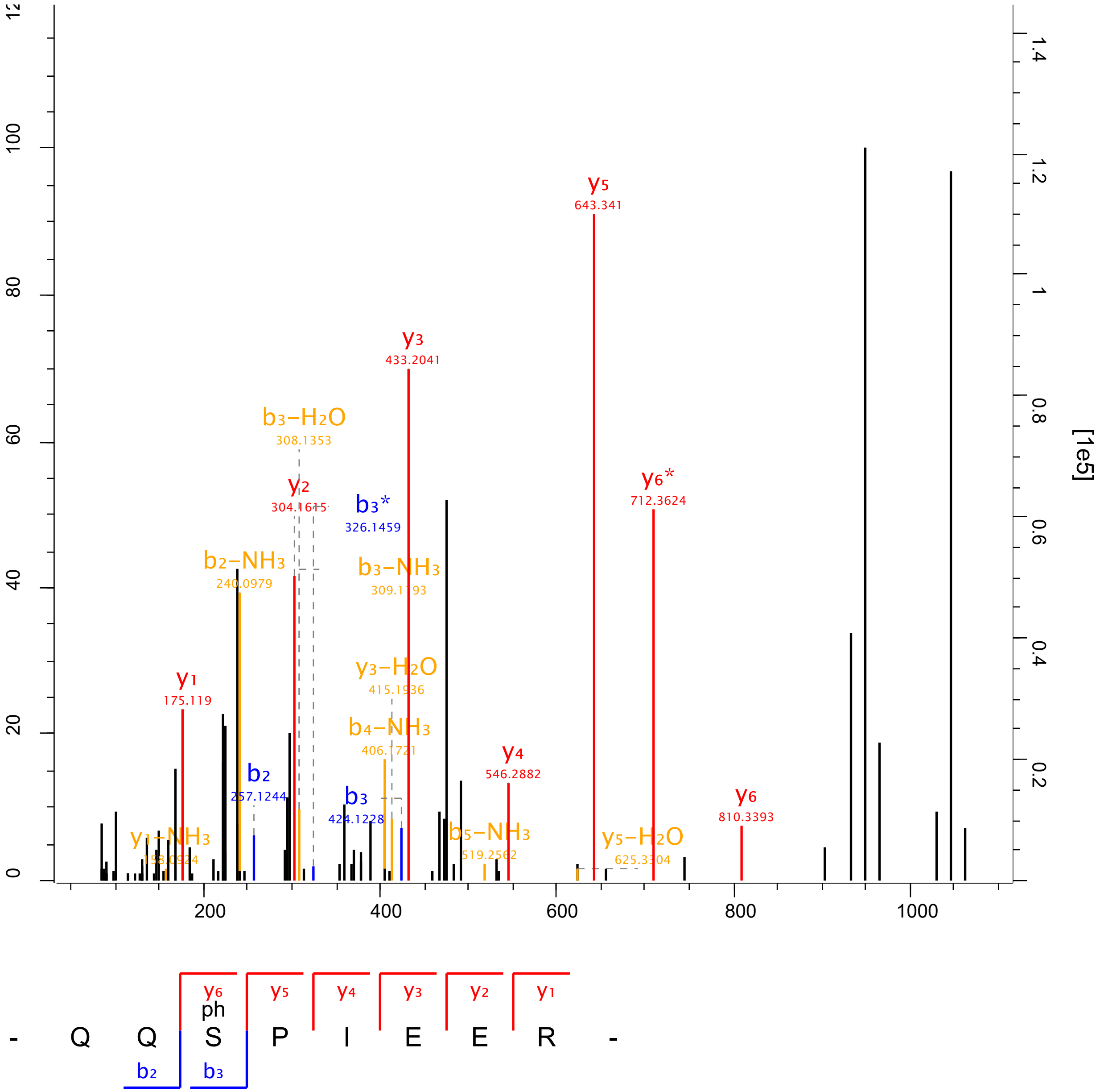

Raw file	Scan	Method	Score	m/z
sys_15_1	34031	FTMS; HCD	117	804.36

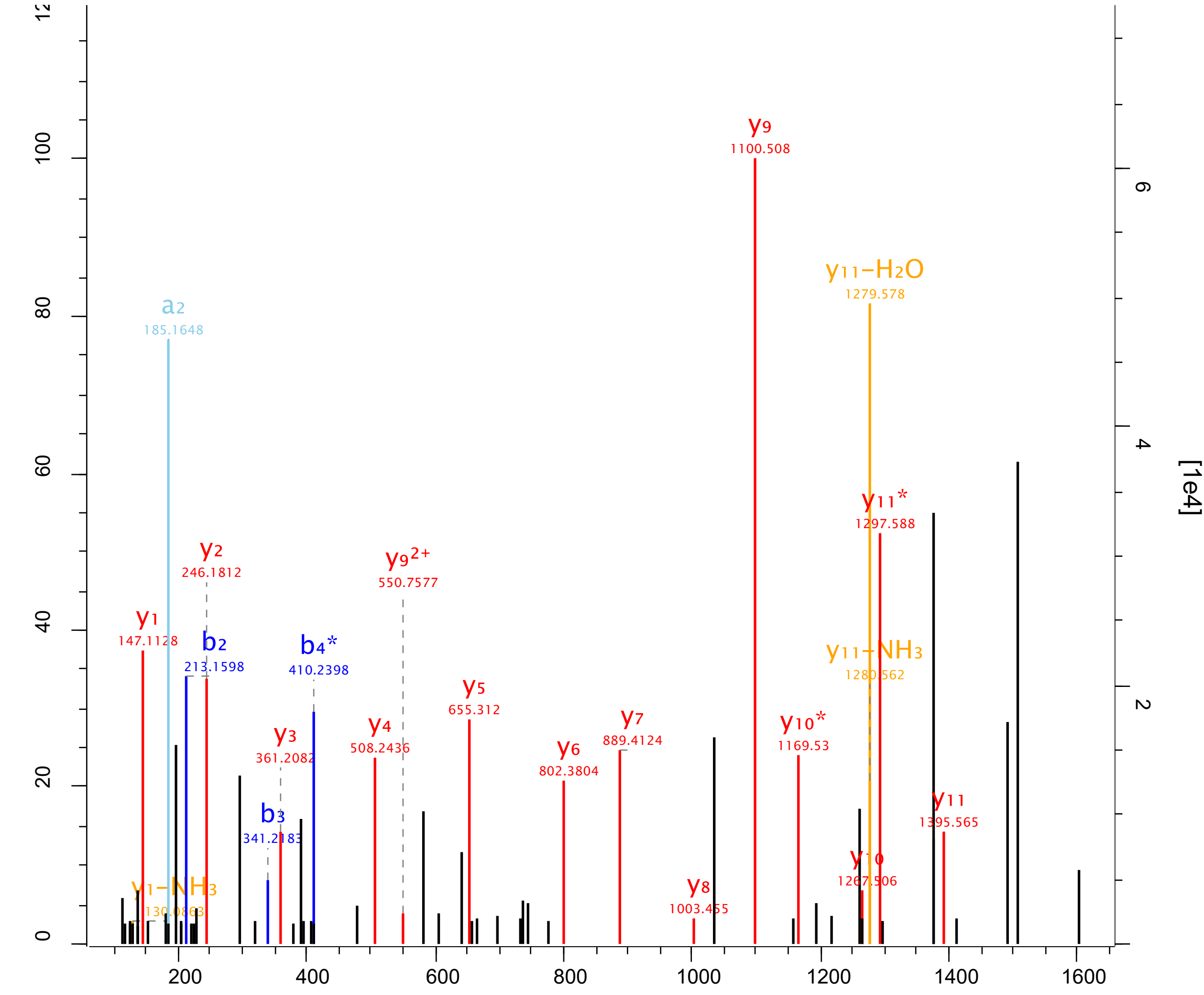

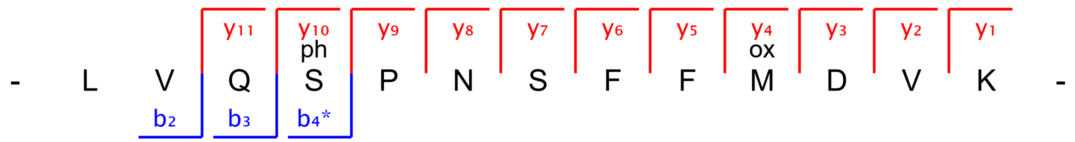

Raw file	Scan	Method	Score	m/z
sys_15_1	3430	FTMS; HCD	64.71	435.19

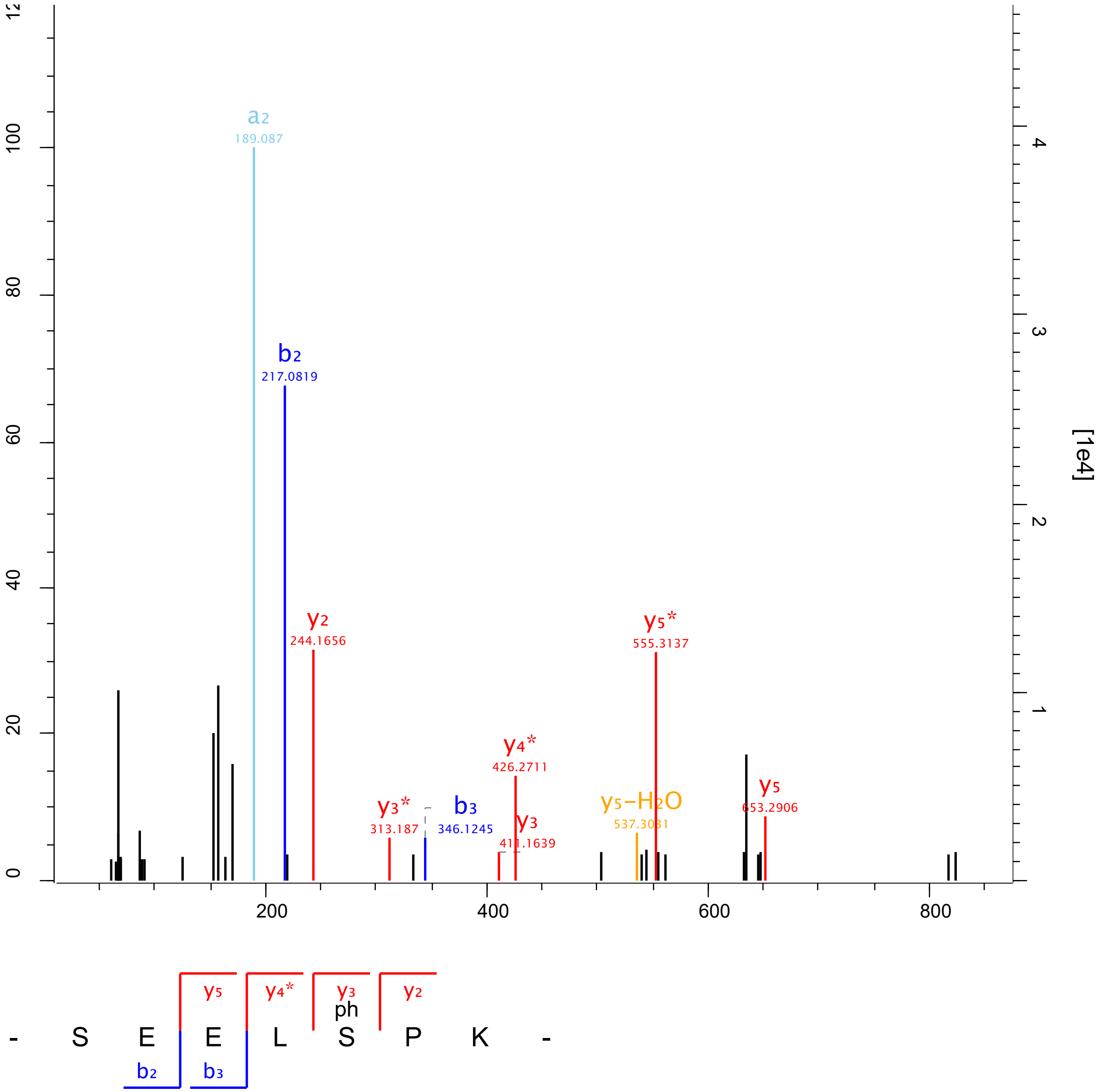

Raw file	Scan	Method	Score	m/z
sys_15_1	34334	FTMS; HCD	62.05	881.69

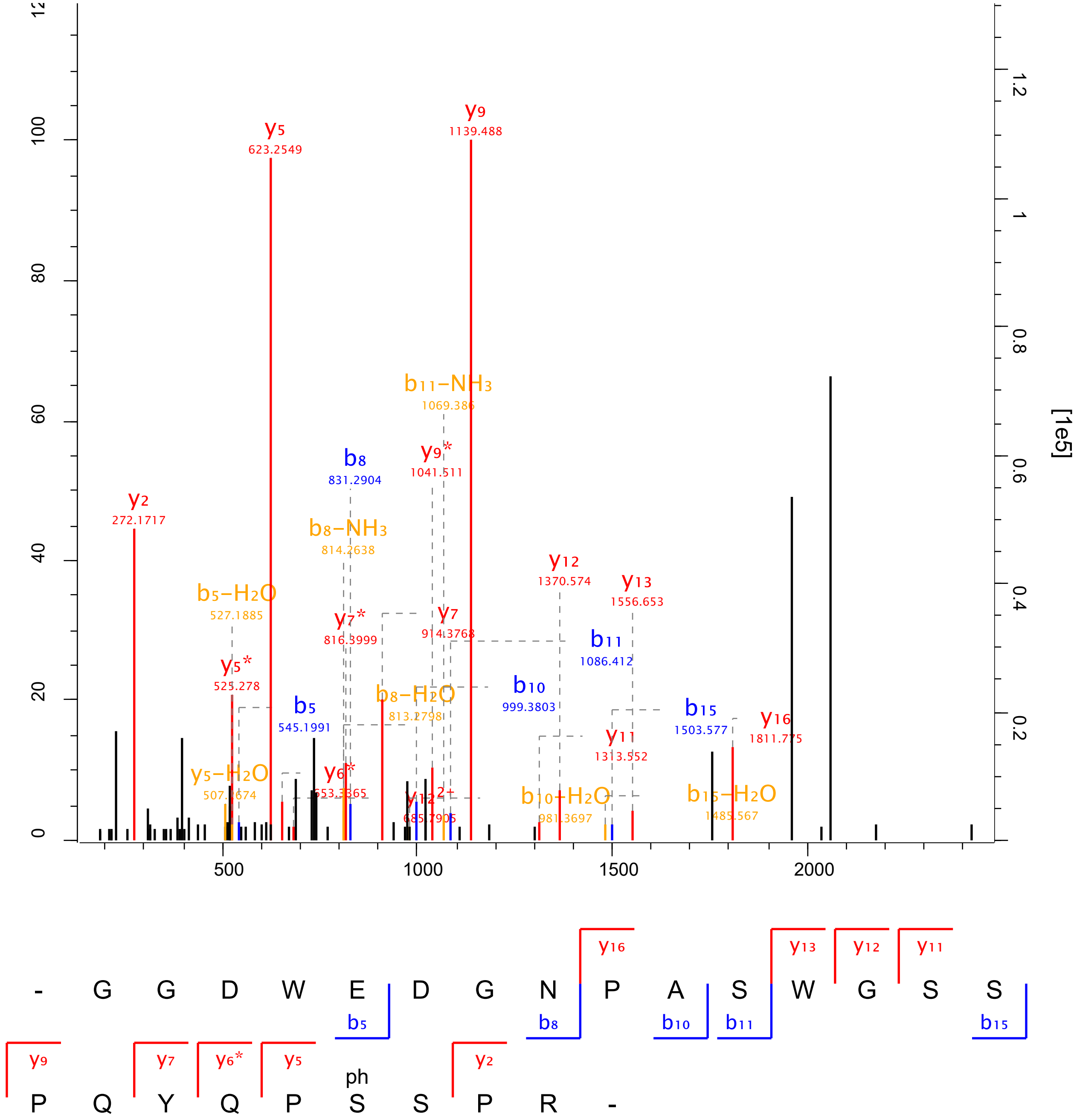

Raw file	Scan	Method	Score	m/z
sys_15_1	34340	FTMS; HCD	79.69	1104.43

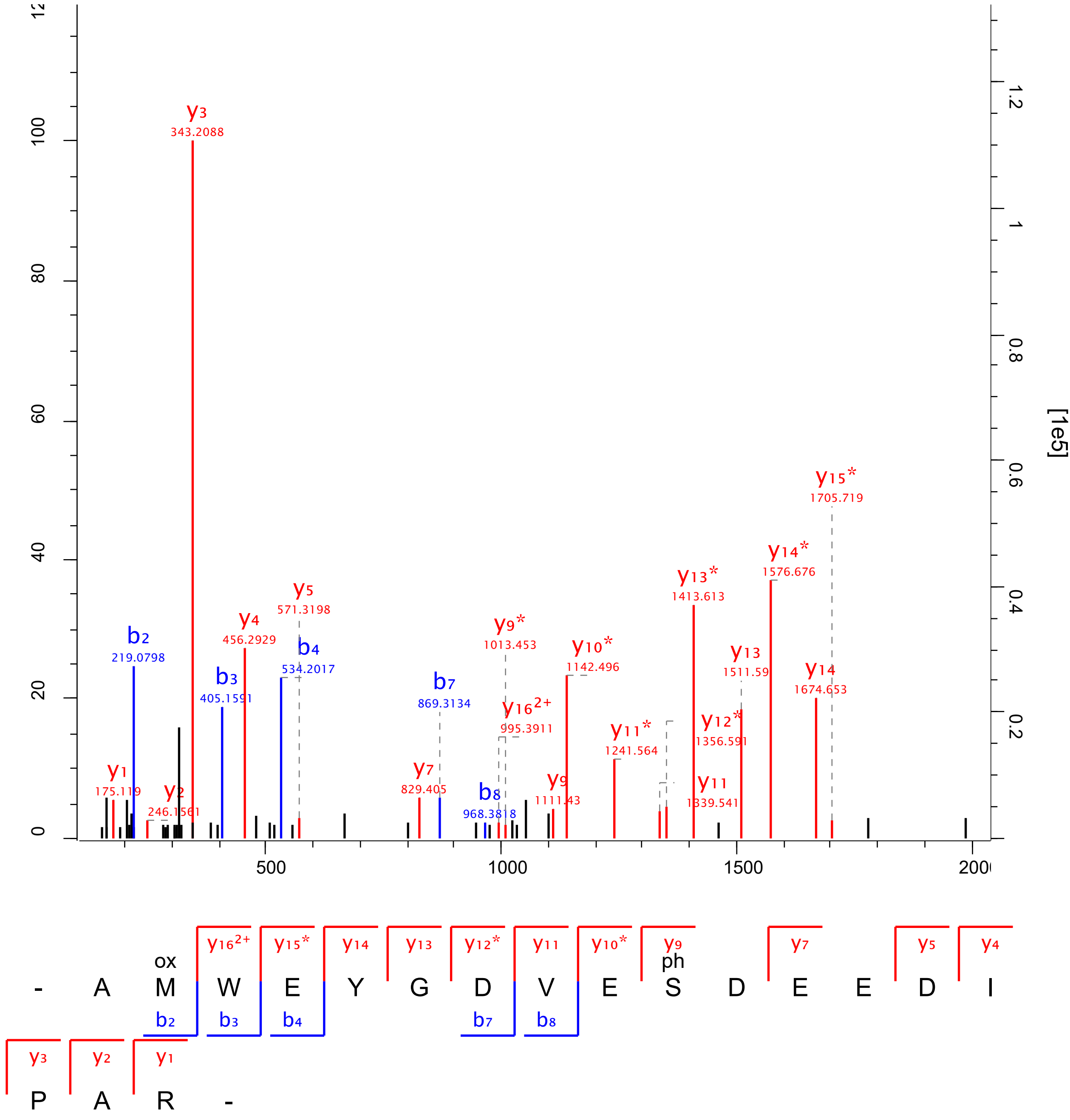

Raw file	Scan	Method	Score	m/z
sys_15_1	34631	FTMS; HCD	42.71	884.07

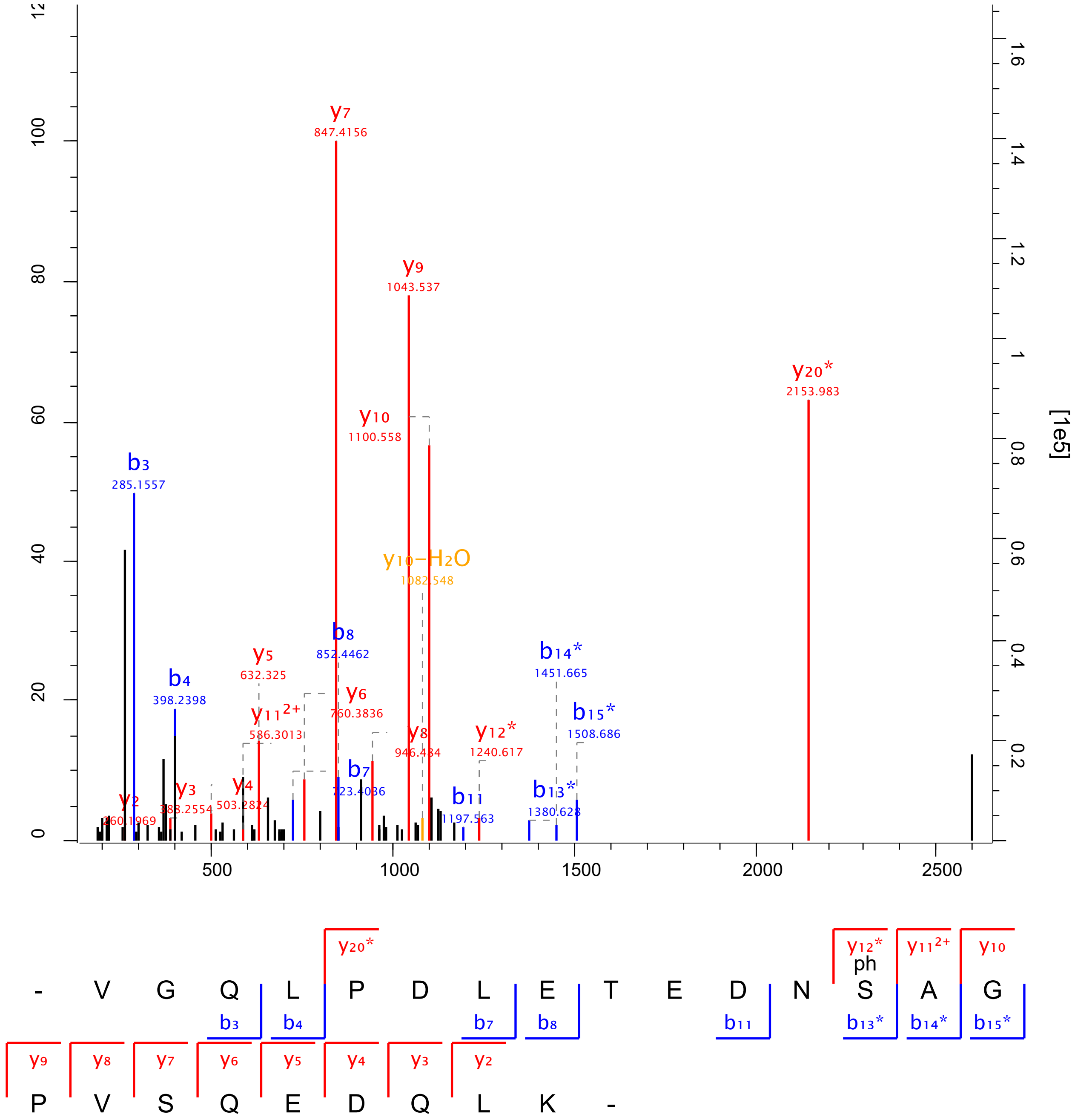

Raw file	Scan	Method	Score	m/z
sys_15_1	34723	FTMS; HCD	40.68	1030.13

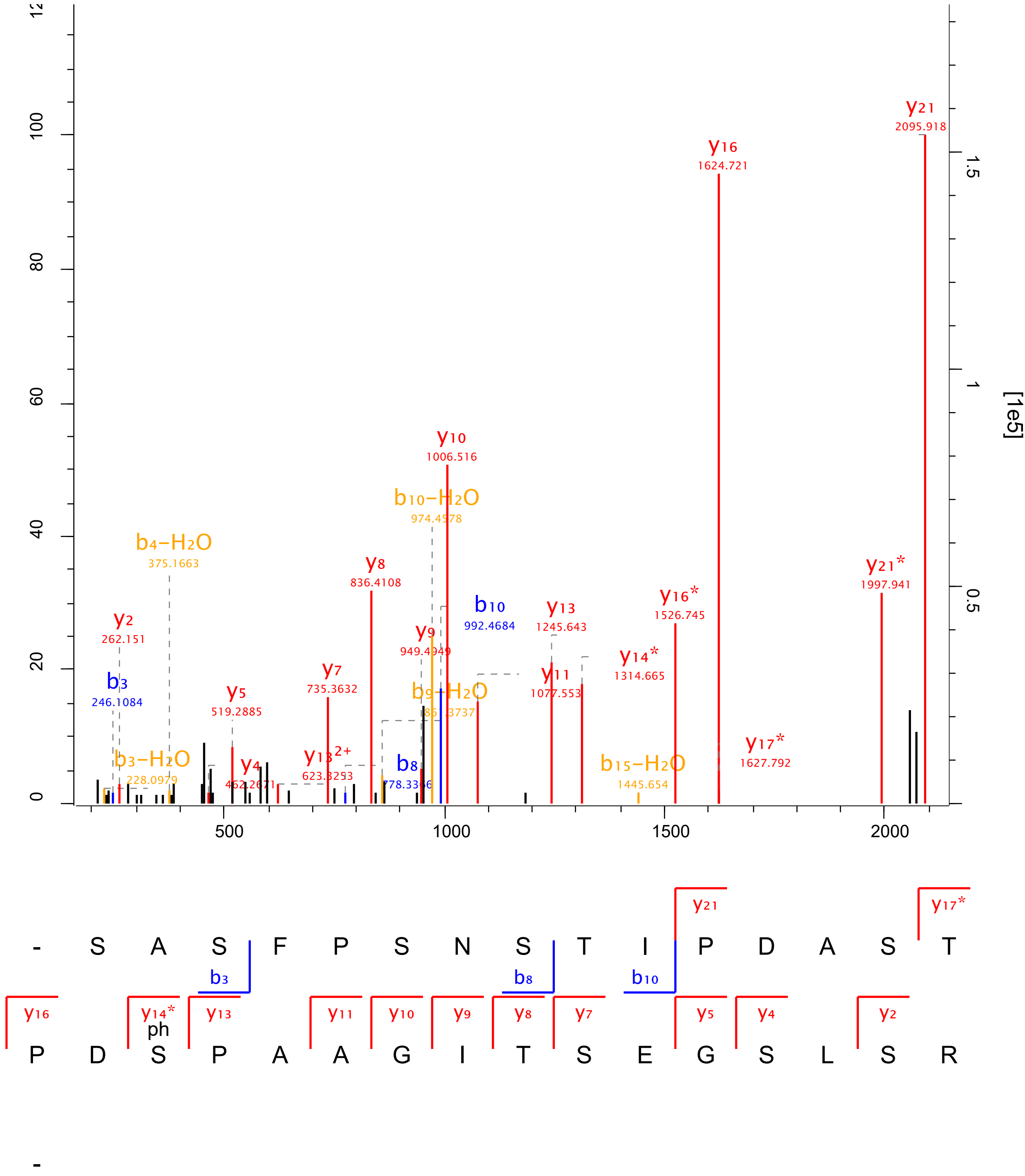

Raw file	Scan	Method	Score	m/z
sys_15_1	34929	FTMS; HCD	182.76	819.88

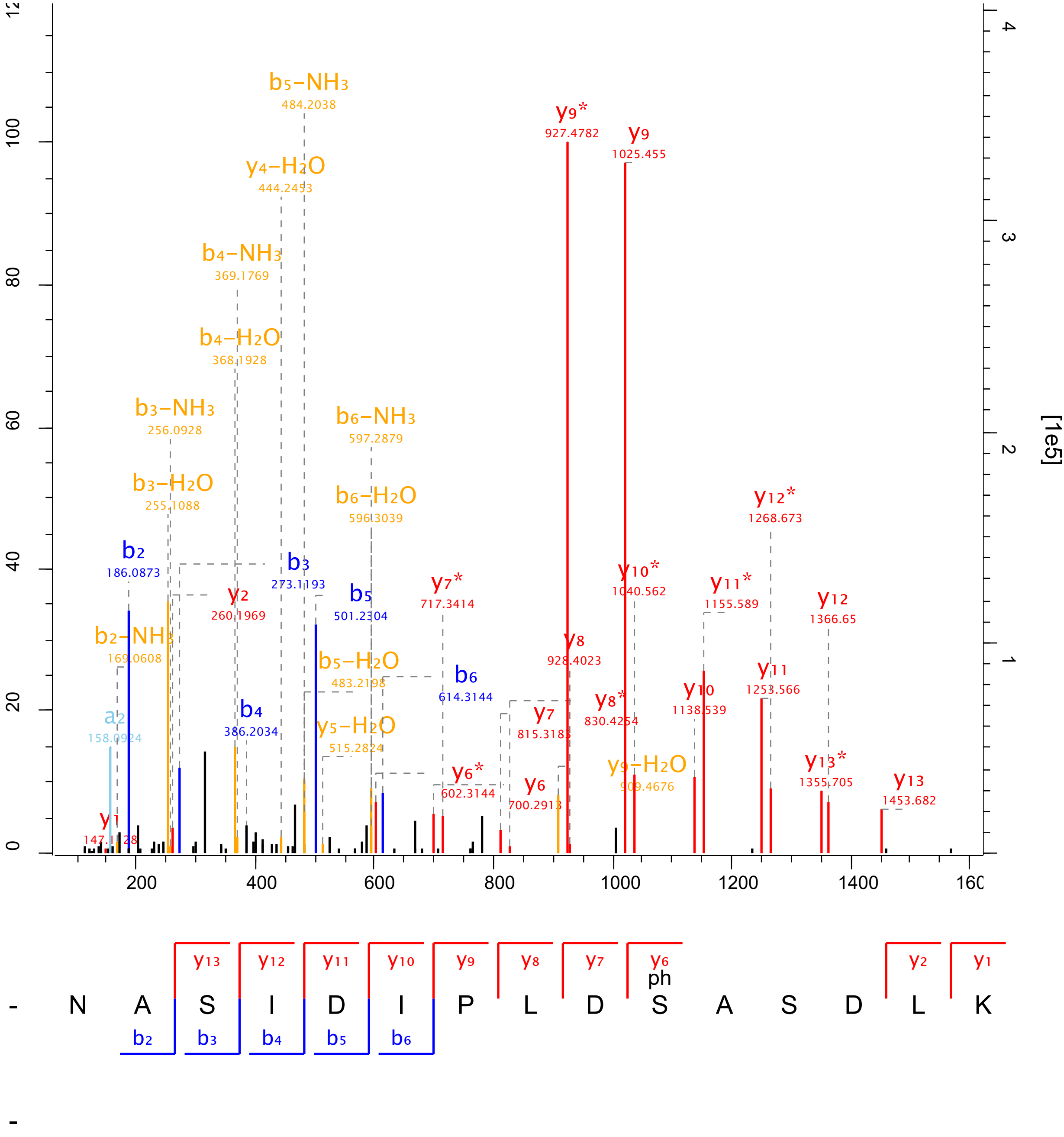

Raw file	Scan	Method	Score	m/z
sys_15_1	34951	FTMS; HCD	134.64	906.4

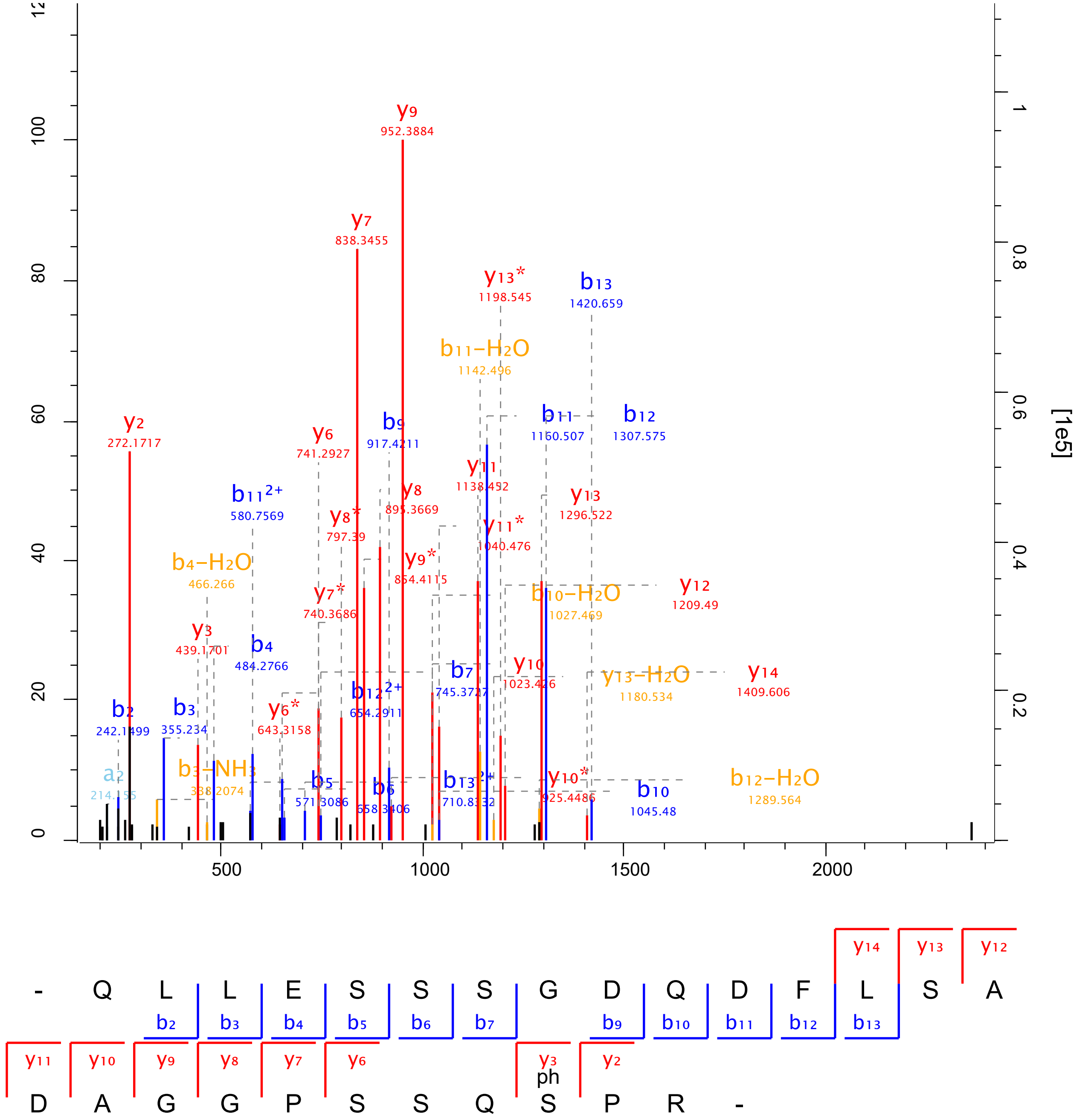

Raw file	Scan	Method	Score	m/z
sys_15_1	35311	FTMS; HCD	50.83	842.91

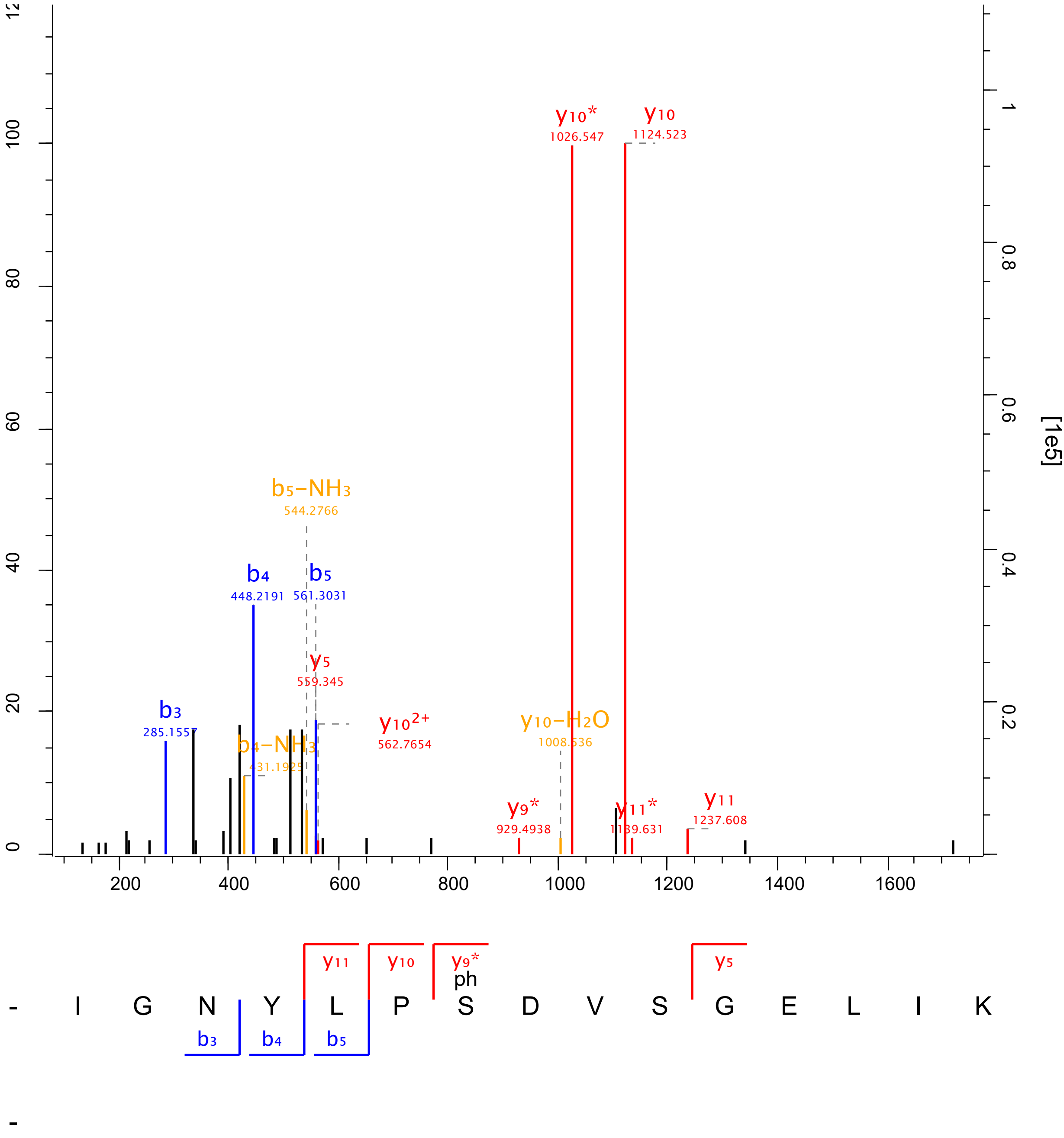

Raw file	Scan	Method	Score	m/z
sys_15_1	35426	FTMS; HCD	109.65	926.92

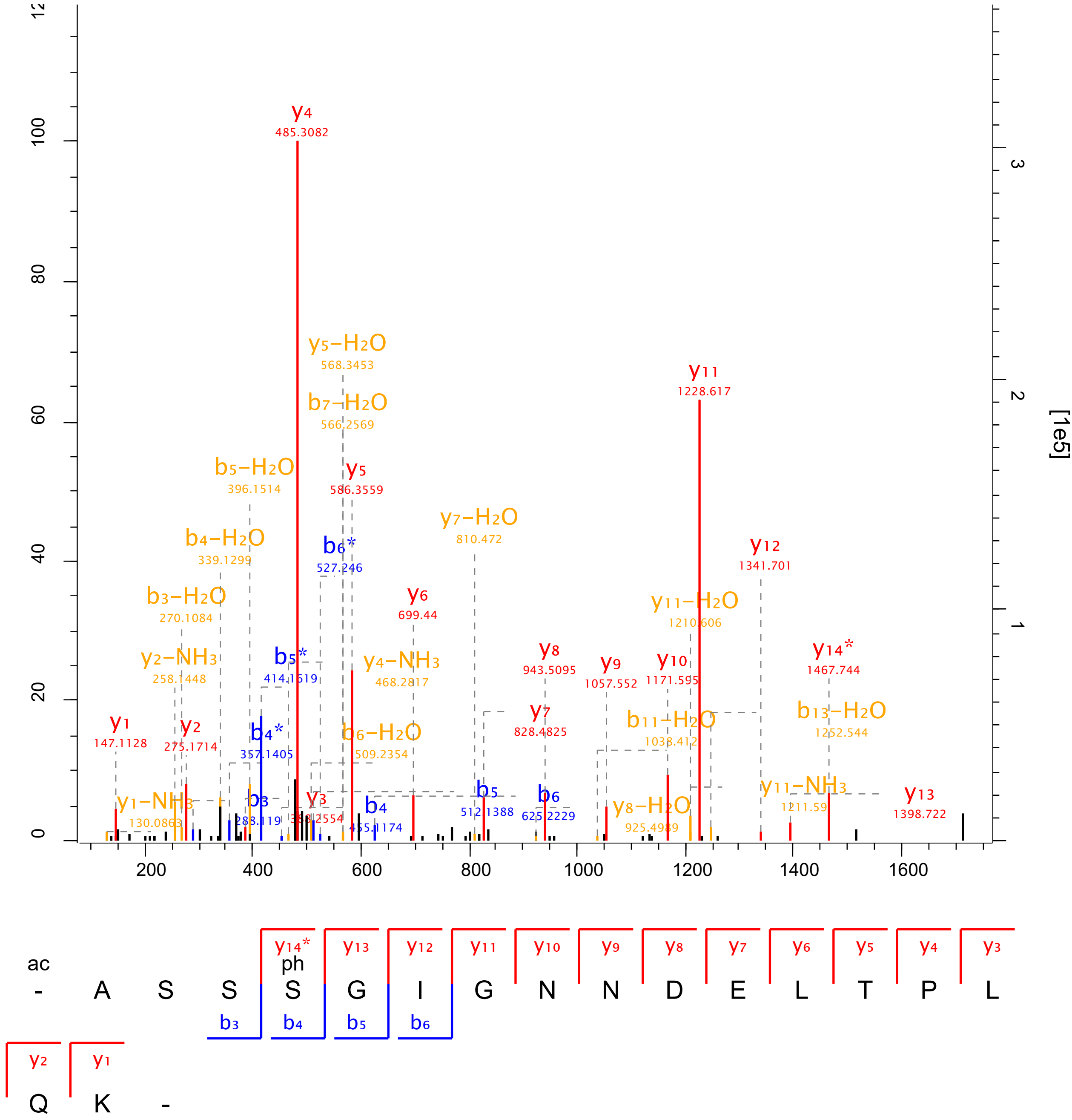

Raw file	Scan	Method	Score	m/z
sys_15_1	35427	FTMS; HCD	57.43	647.78

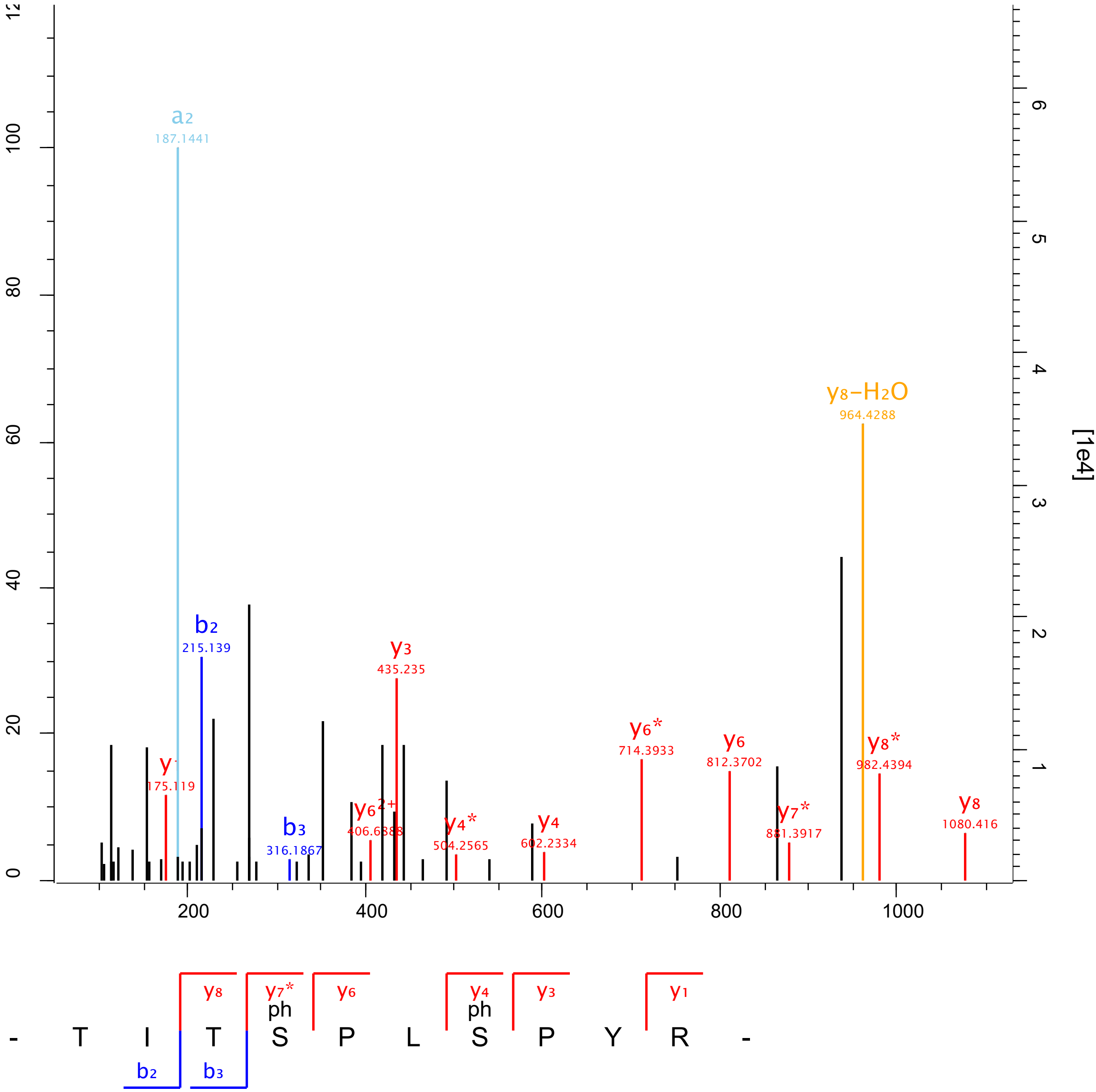

Raw file	Scan	Method	Score	m/z
sys_15_1	35597	FTMS; HCD	159.46	1010.92

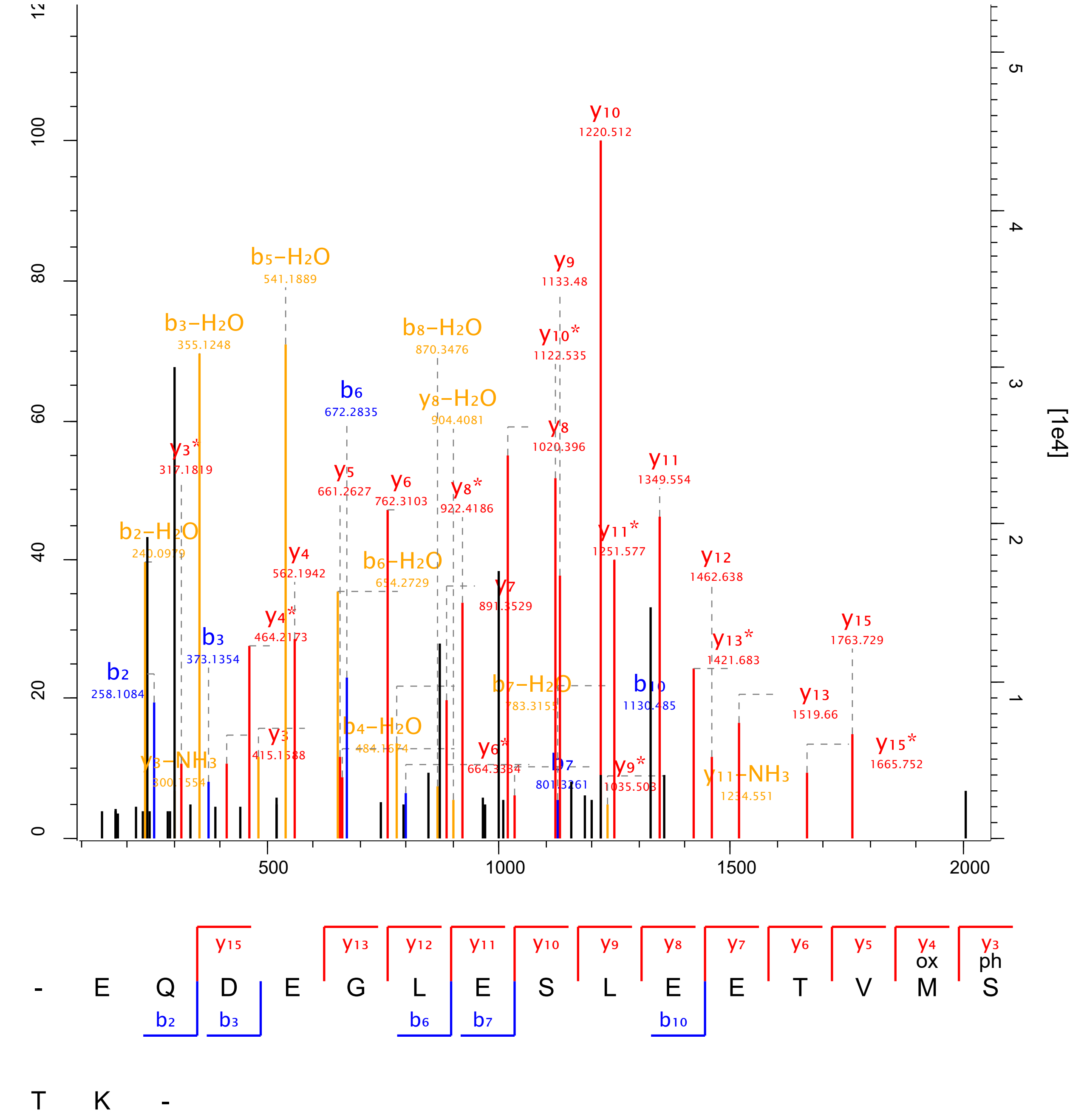

Raw file	Scan	Method	Score	m/z
sys_15_1	3560	FTMS; HCD	271.97	556.89

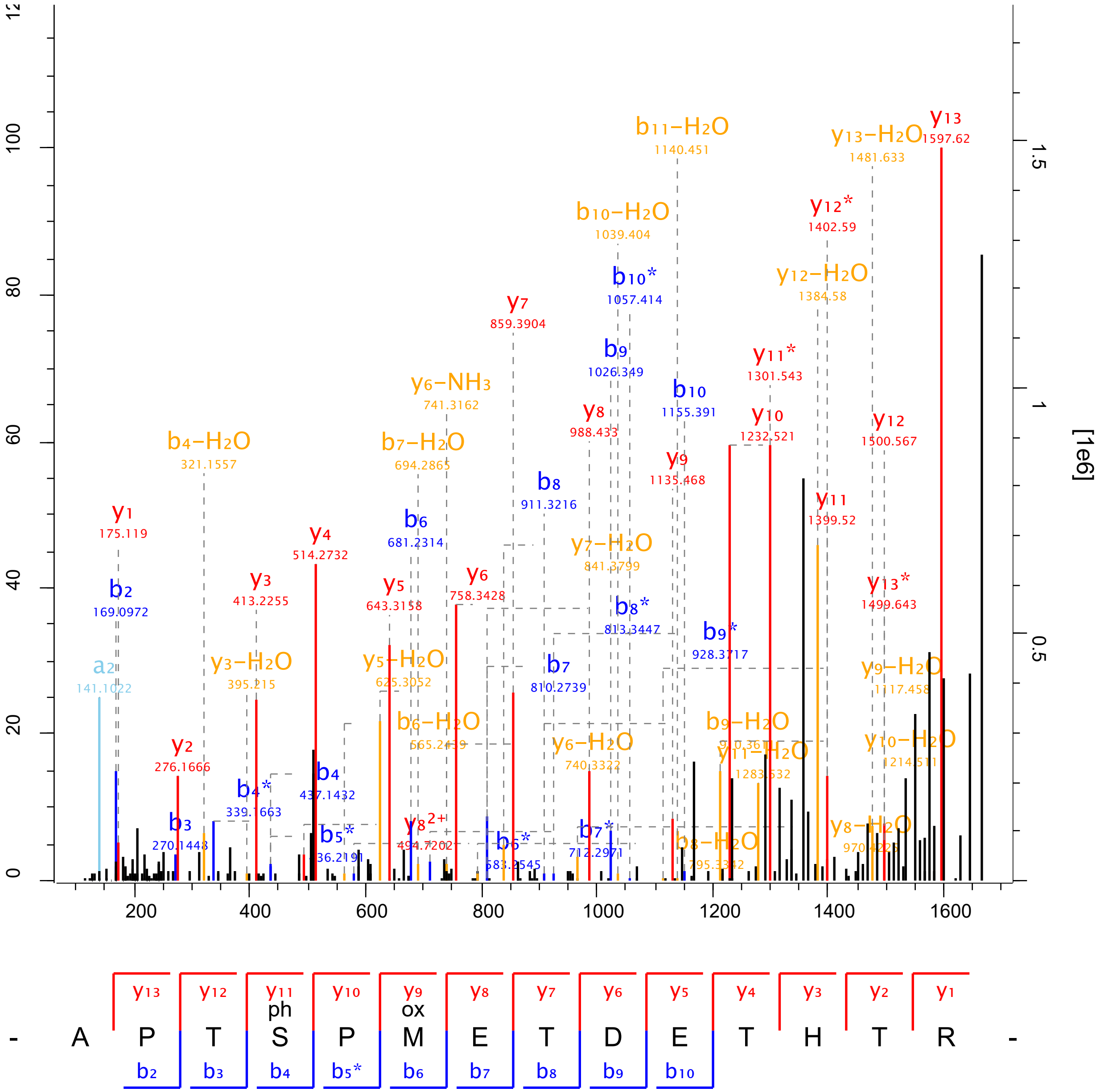

Raw file	Scan	Method	Score	m/z
sys_15_1	35600	FTMS; HCD	210.98	882.42

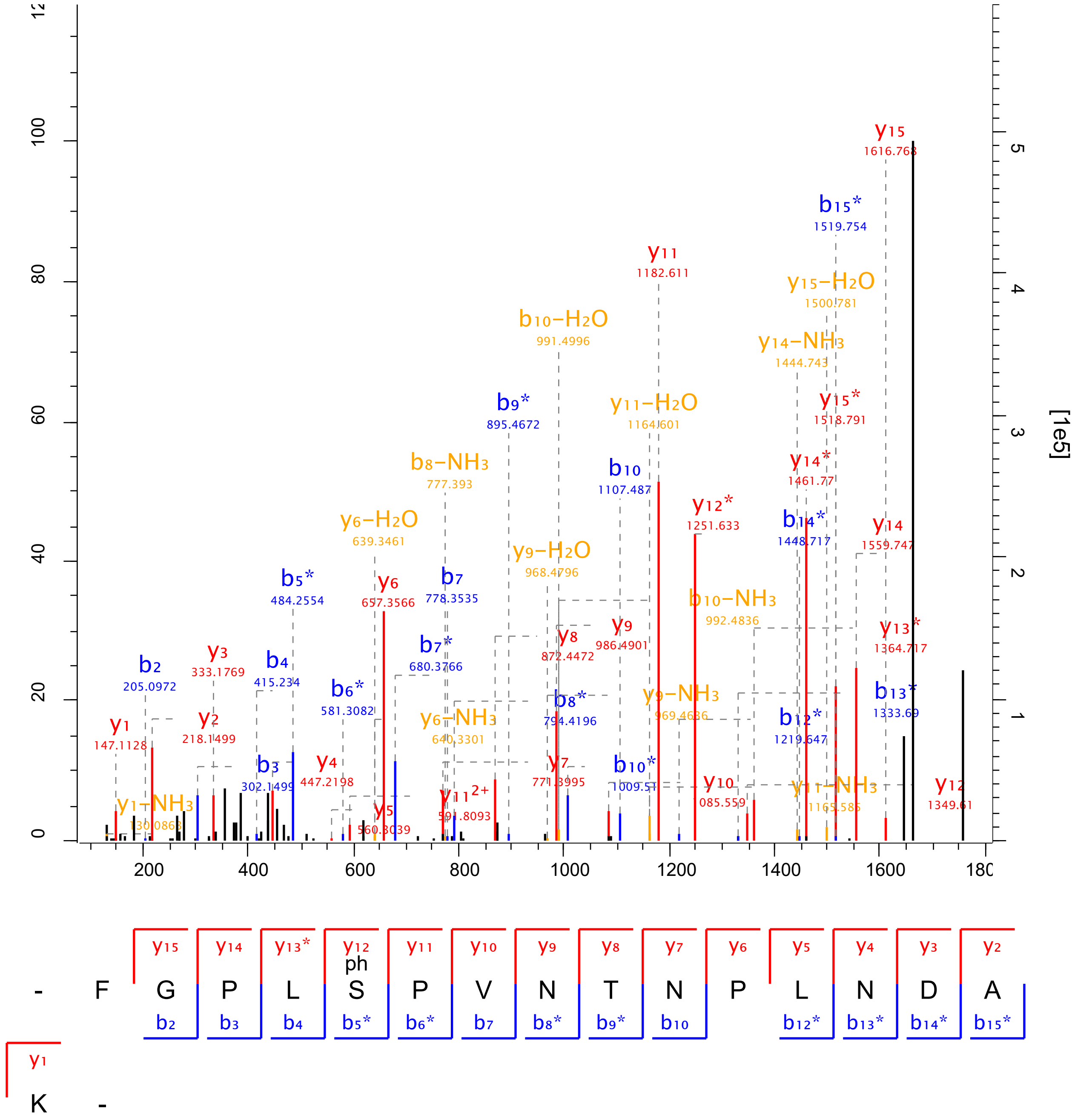

Raw file	Scan	Method	Score	m/z
sys_15_1	35649	FTMS; HCD	43.79	975.44

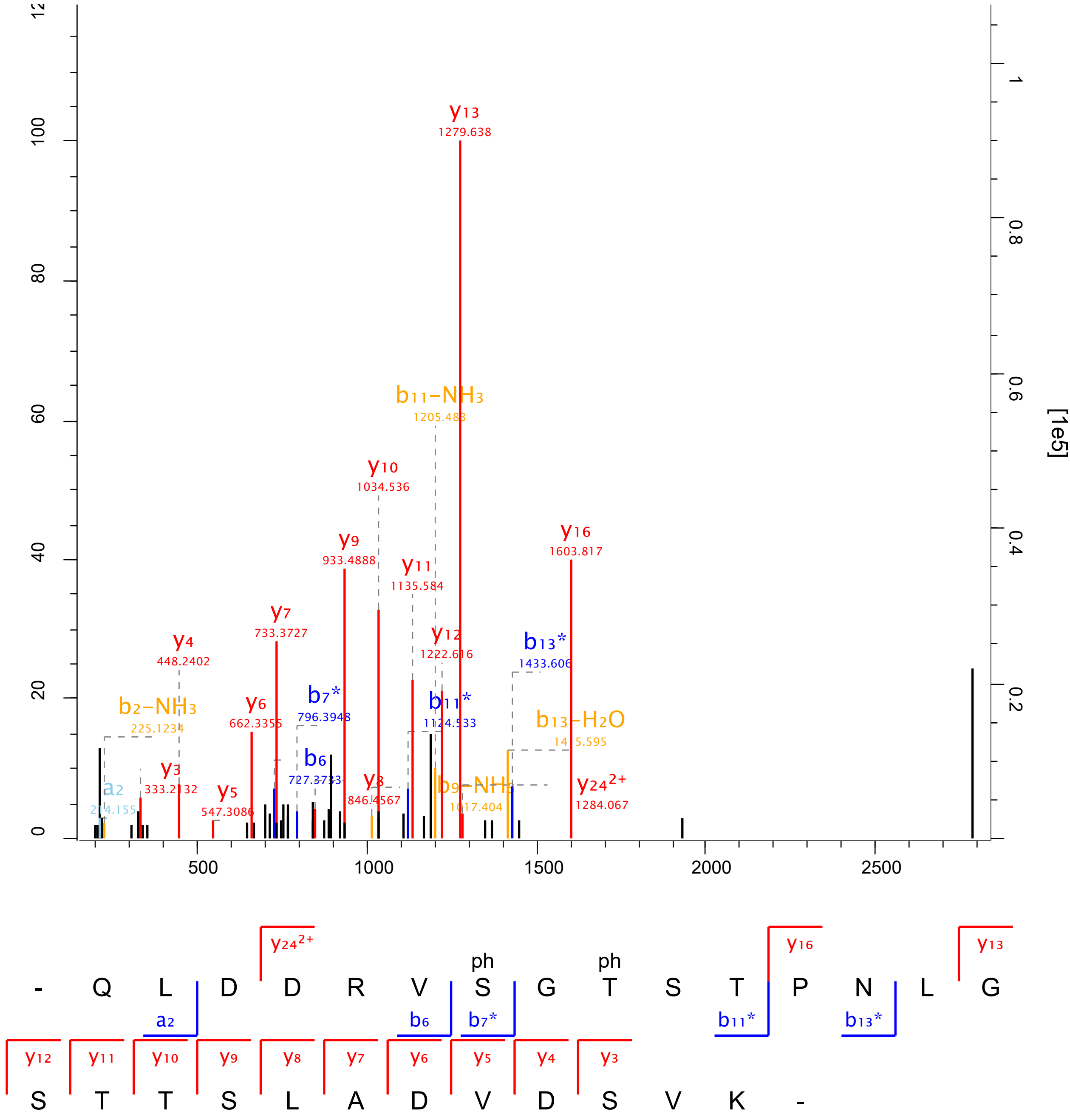

Raw file	Scan	Method	Score	m/z
sys_15_1	35985	FTMS; HCD	56.57	656.87

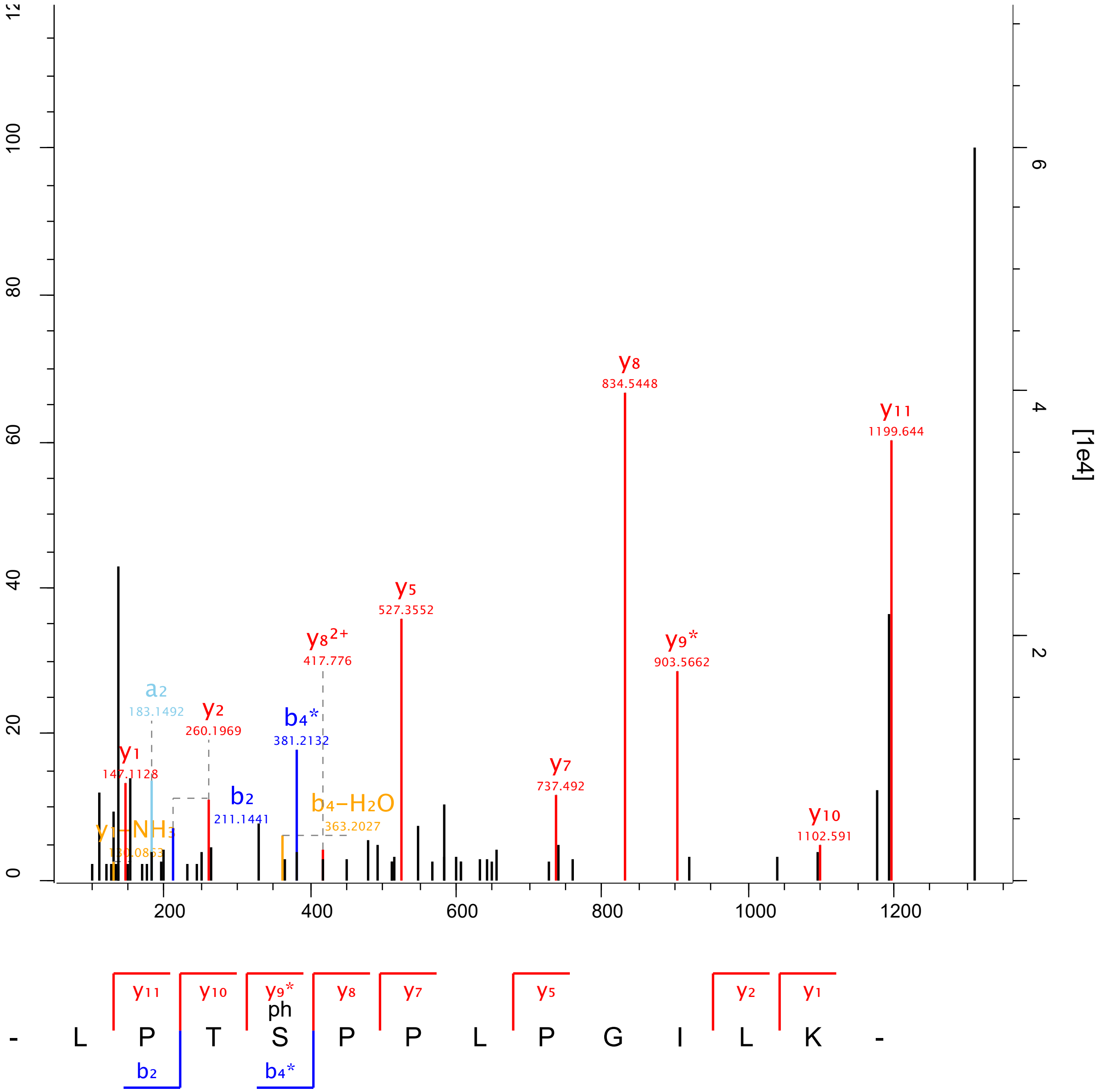

Raw file	Scan	Method	Score	m/z
sys_15_1	36049	FTMS; HCD	74.78	923.86

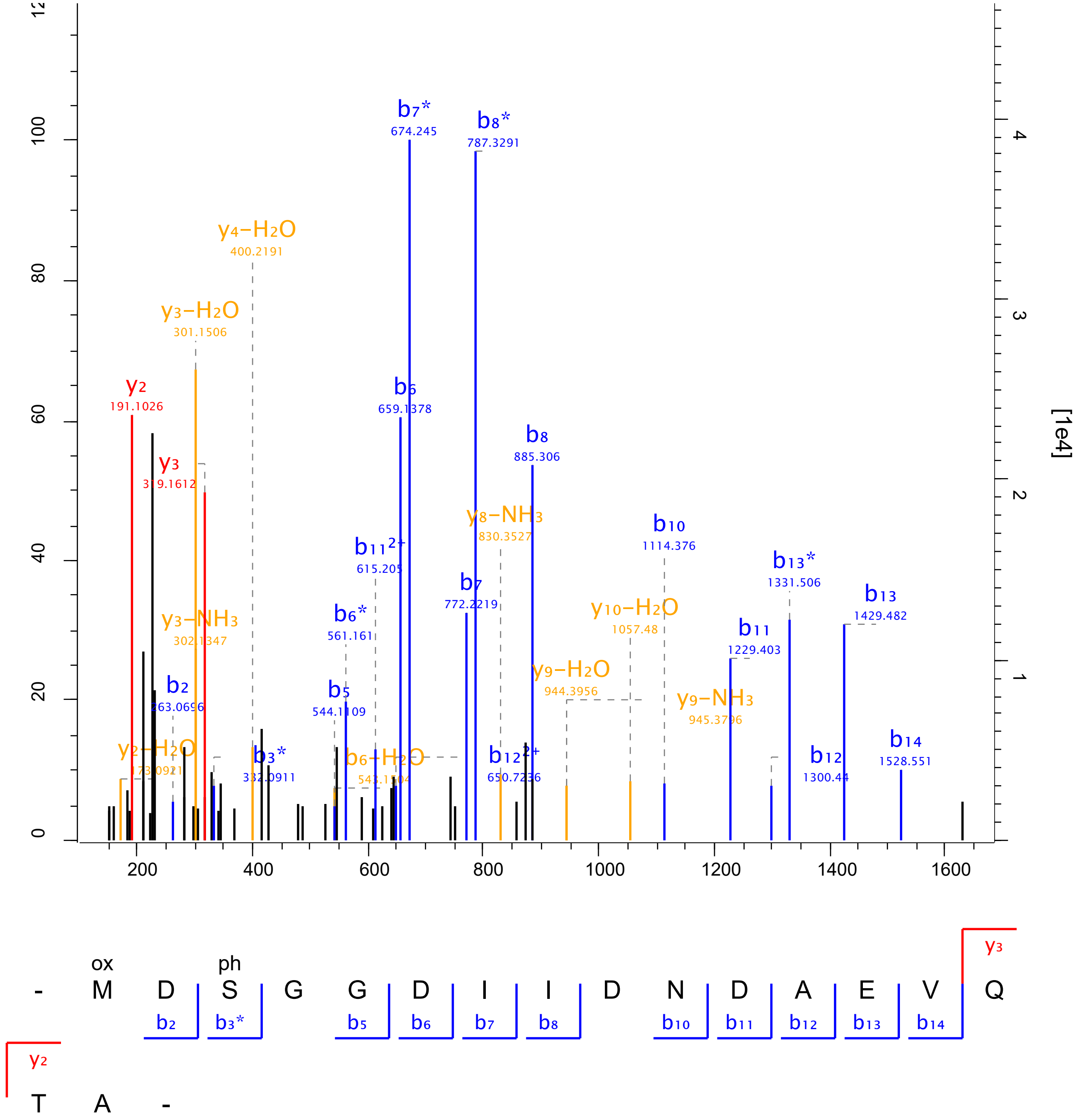

Raw file	Scan	Method	Score	m/z
sys_15_1	36225	FTMS; HCD	50.36	750.65

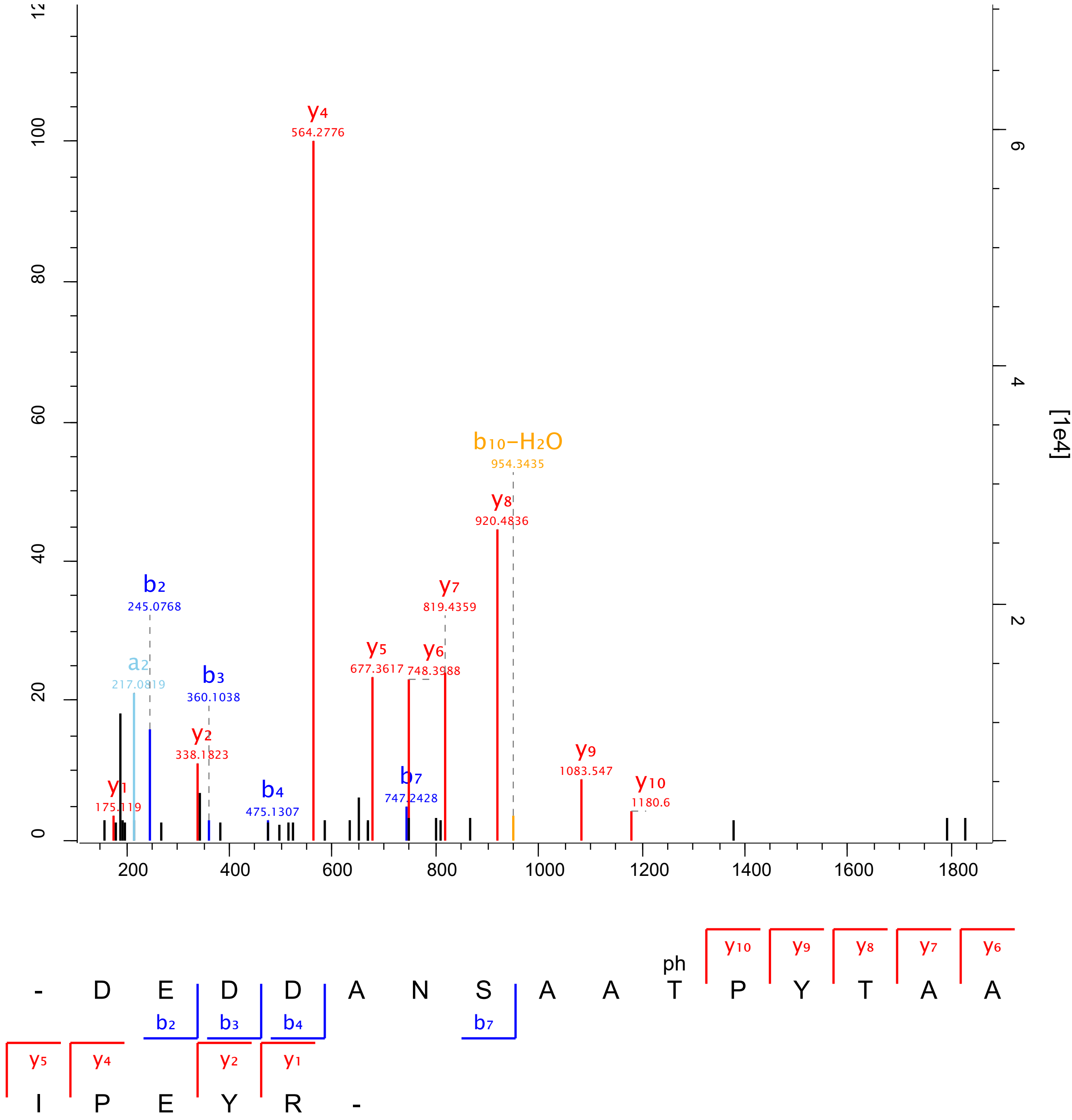

Raw file	Scan	Method	Score	m/z
sys_15_1	36288	FTMS; HCD	79.51	823.06

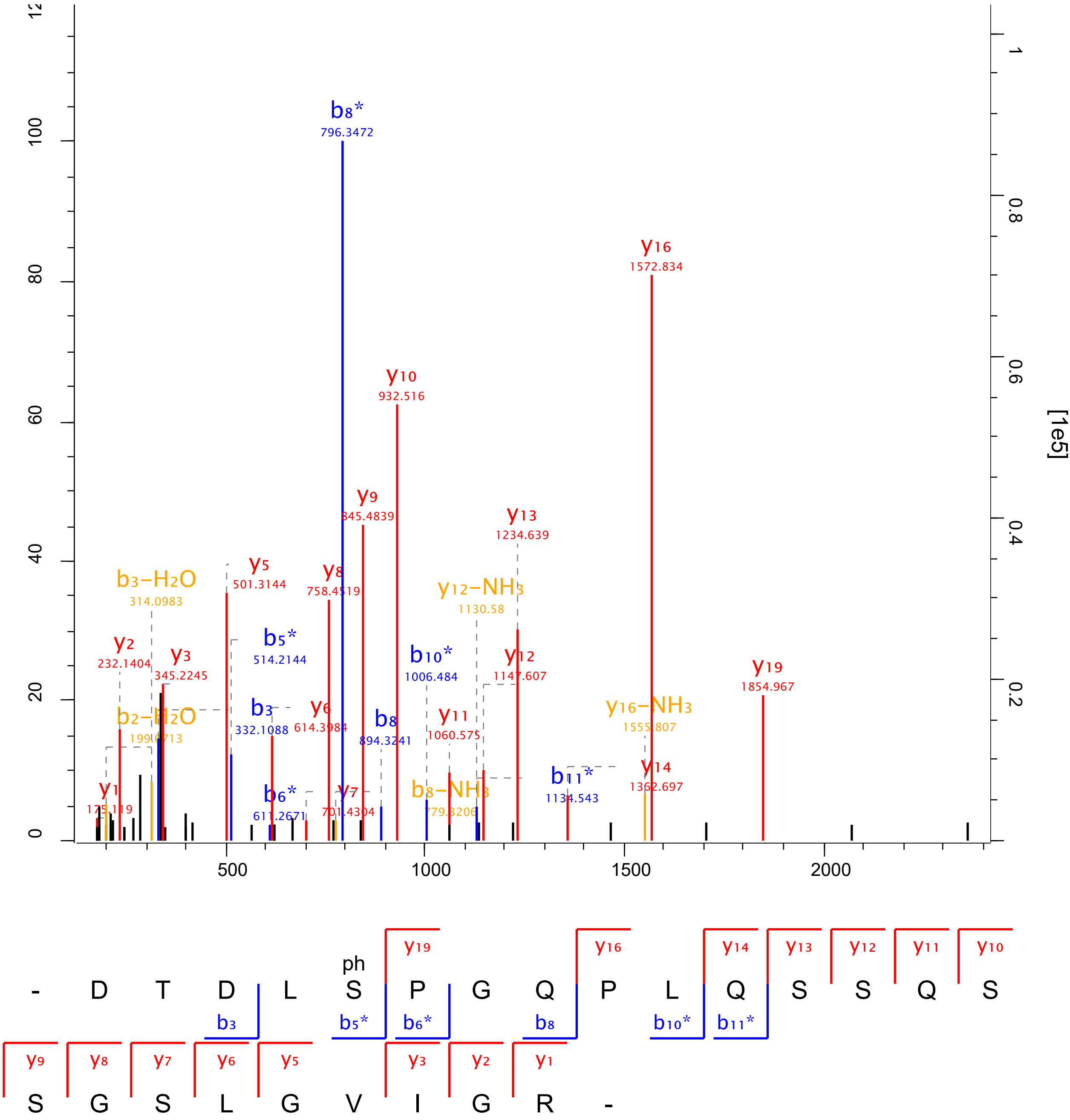

Raw file	Scan	Method	Score	m/z
sys_15_1	36340	FTMS; HCD	144.7	542.76

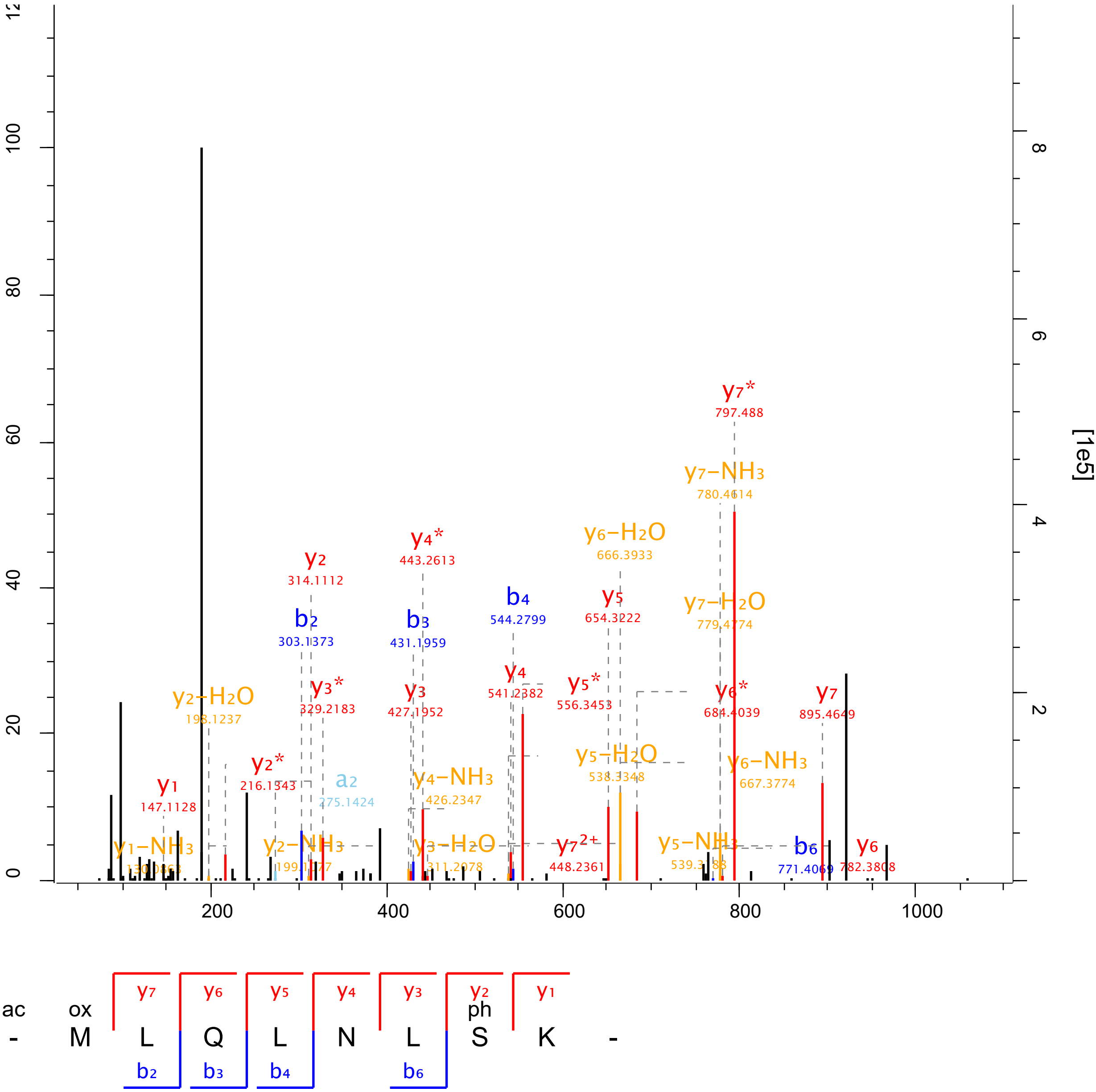

Raw file	Scan	Method	Score	m/z
sys_15_1	36373	FTMS; HCD	55.13	848.4

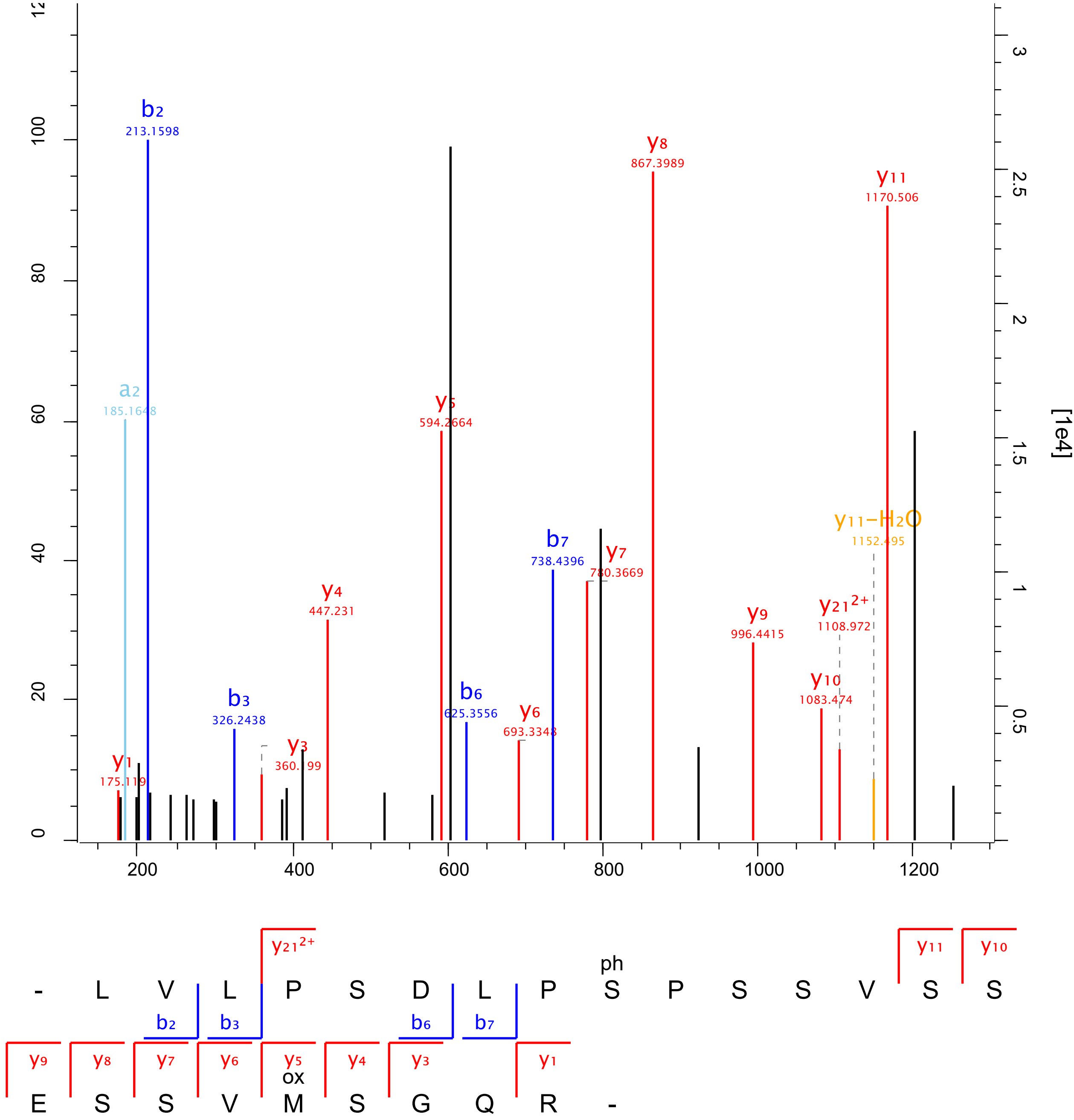

Raw file	Scan	Method	Score	m/z
sys_15_1	3644	FTMS; HCD	115.29	536.73

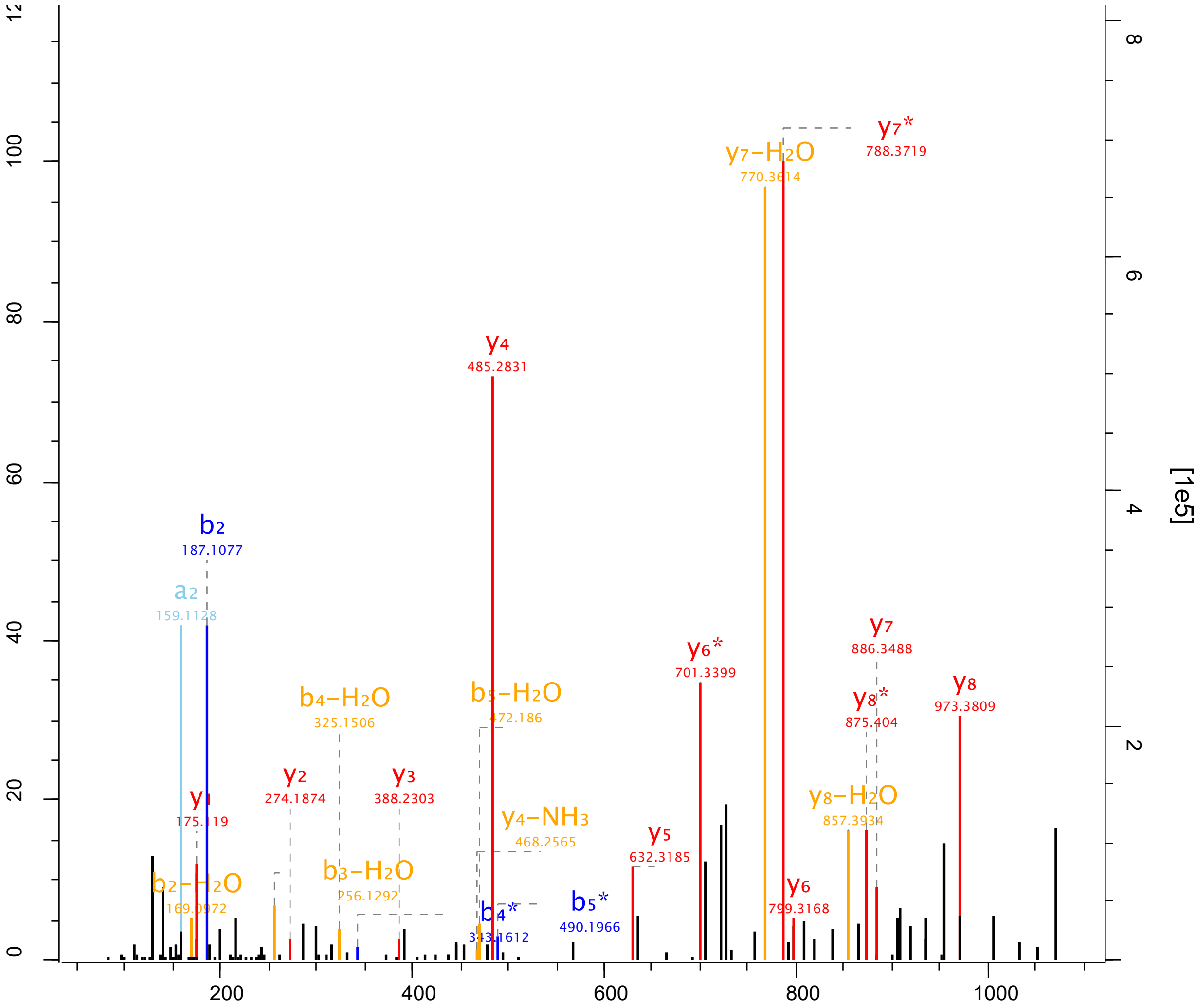

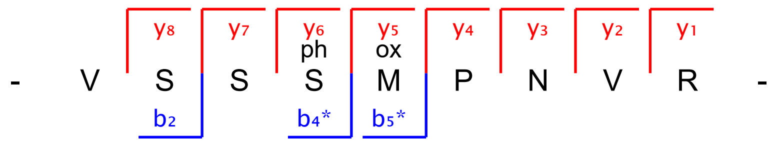

Raw file	Scan	Method	Score	m/z
sys_15_1	36442	FTMS; HCD	55.59	956.45

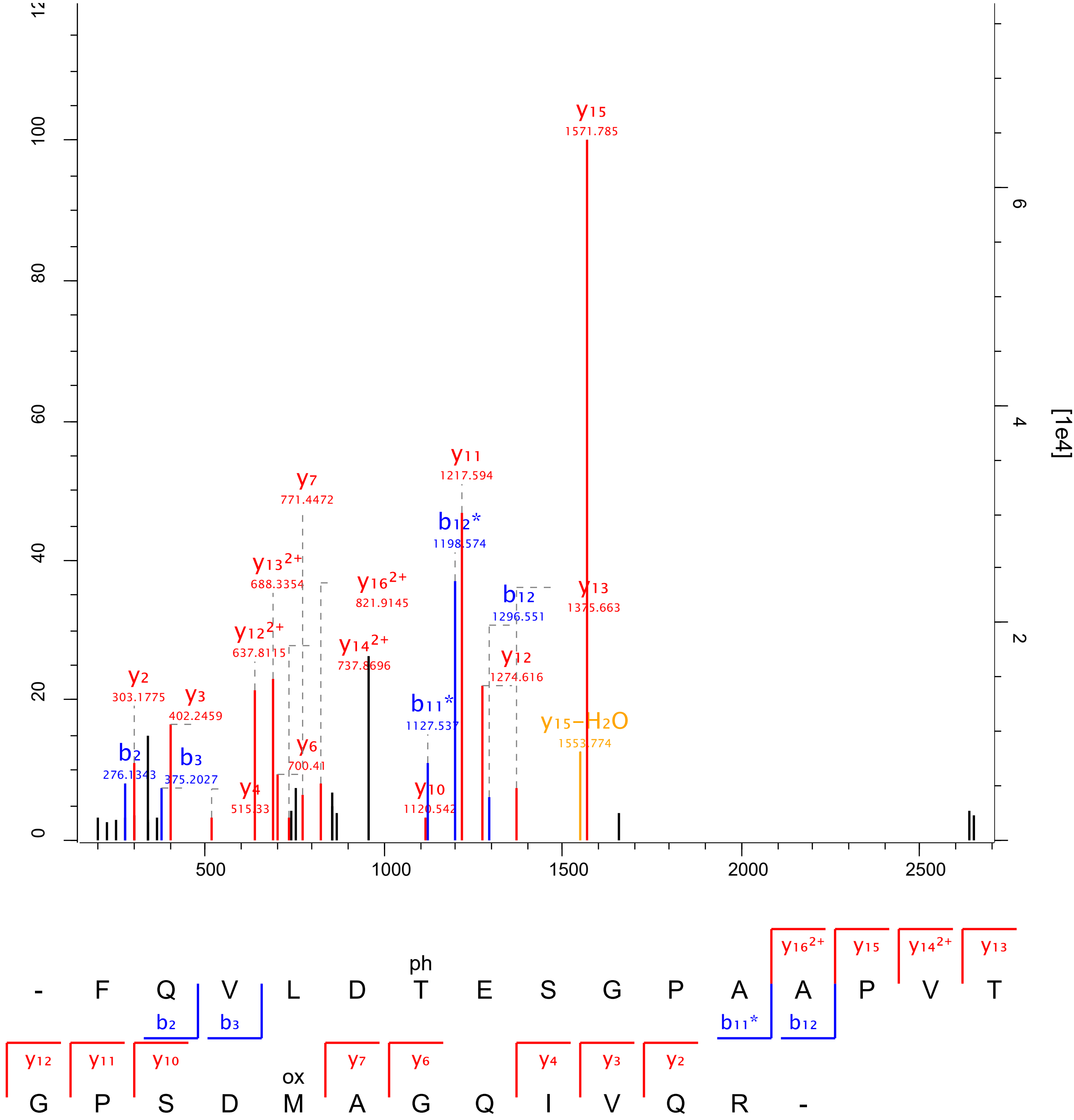

Raw file	Scan	Method	Score	m/z
sys_15_1	36610	FTMS; HCD	102.65	722.82

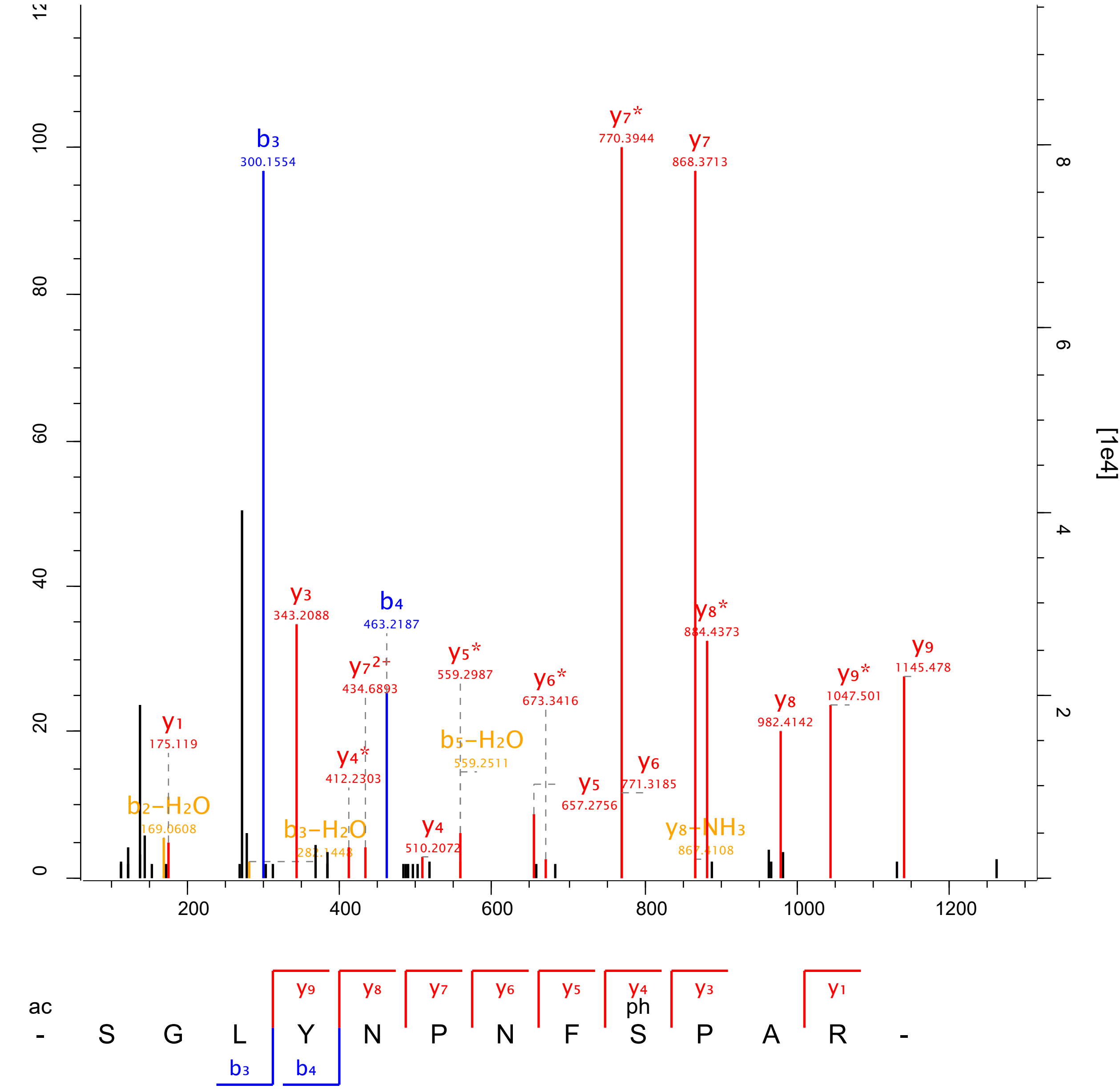

Raw file	Scan	Method	Score	m/z
sys_15_1	3663	FTMS; HCD	76.36	583.75

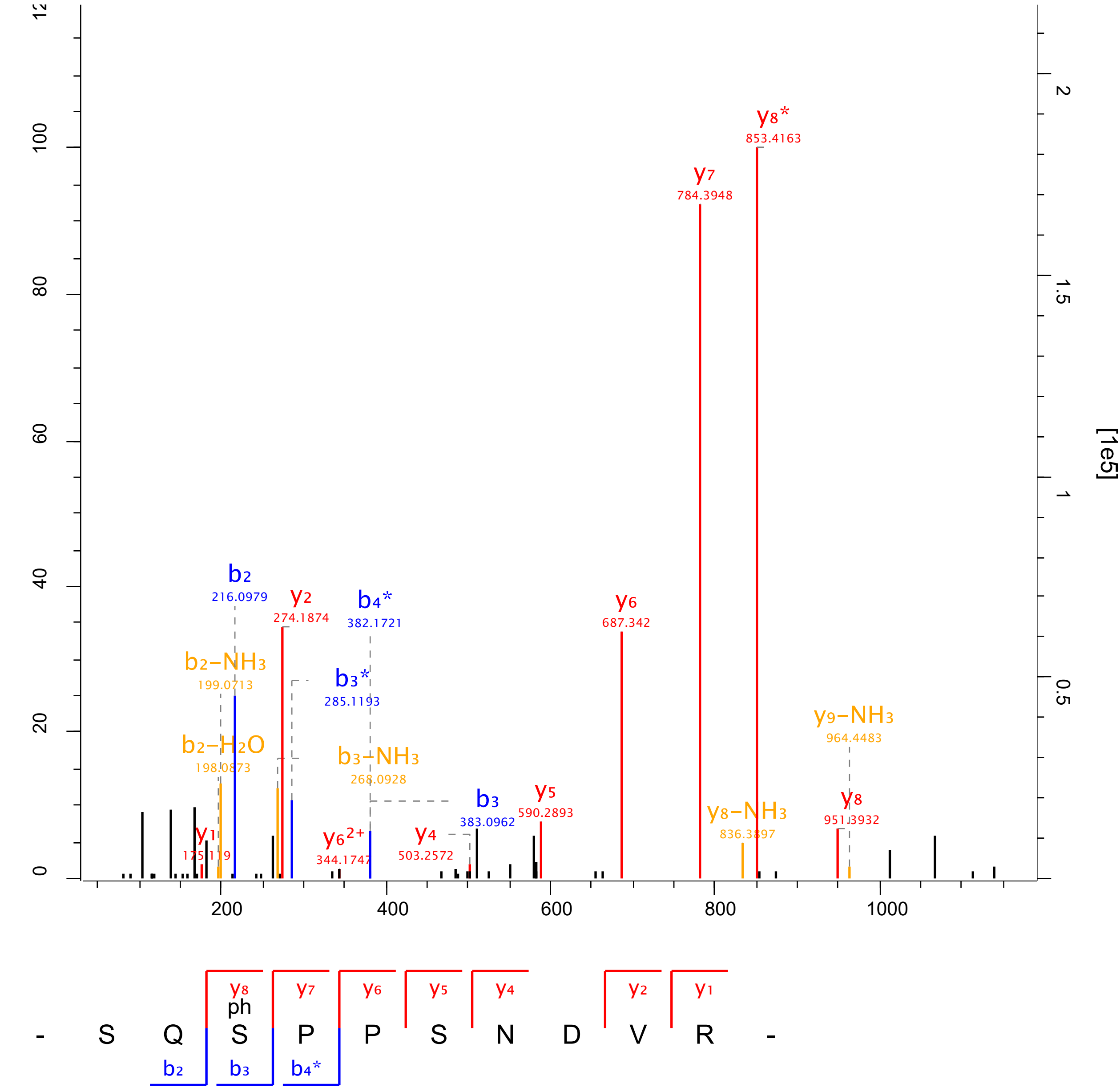

Raw file	Scan	Method	Score	m/z
sys_15_1	36782	FTMS; HCD	85.36	778.89

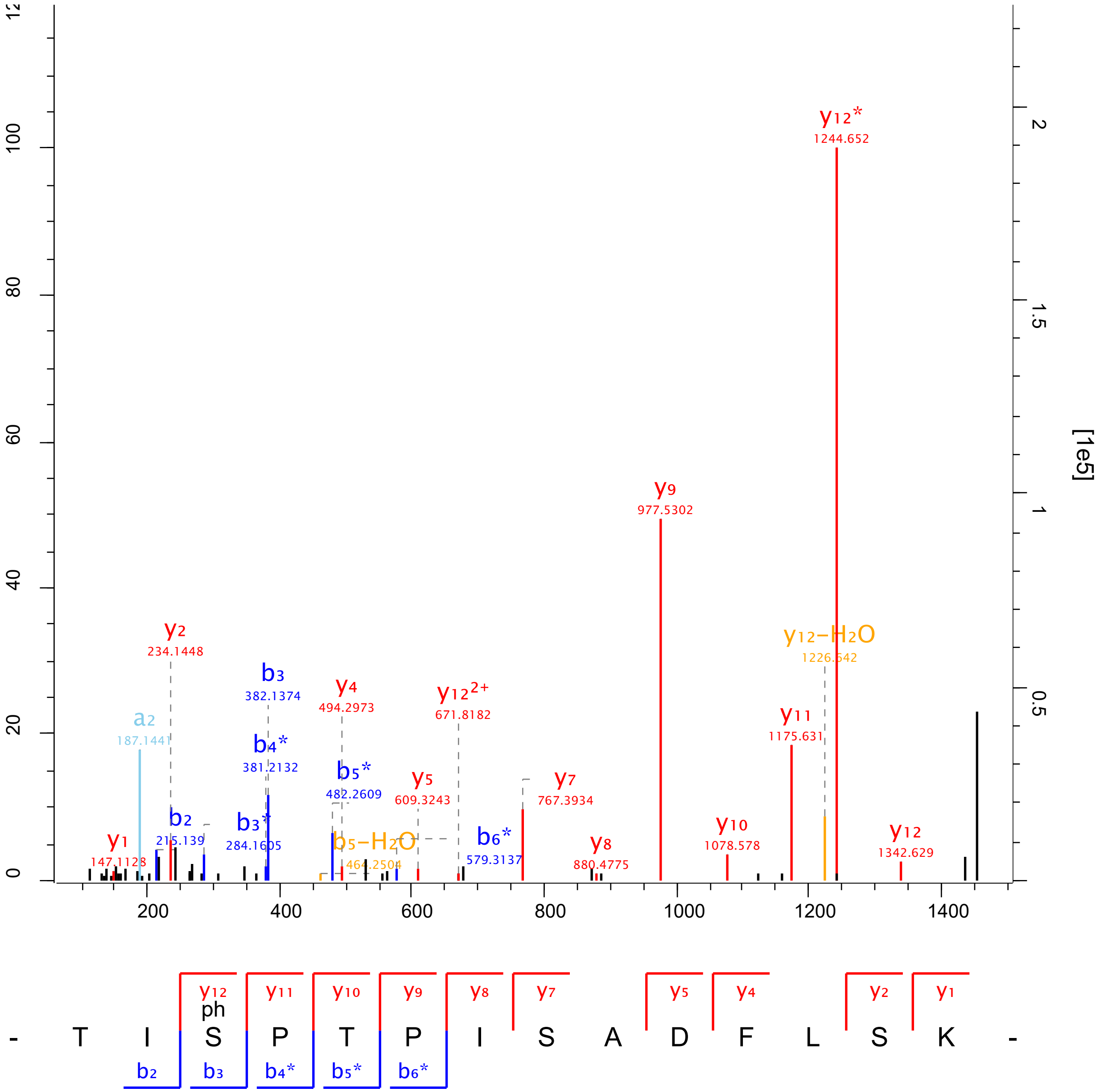

Raw file	Scan	Method	Score	m/z
sys_15_1	36785	FTMS; HCD	227.49	889.69

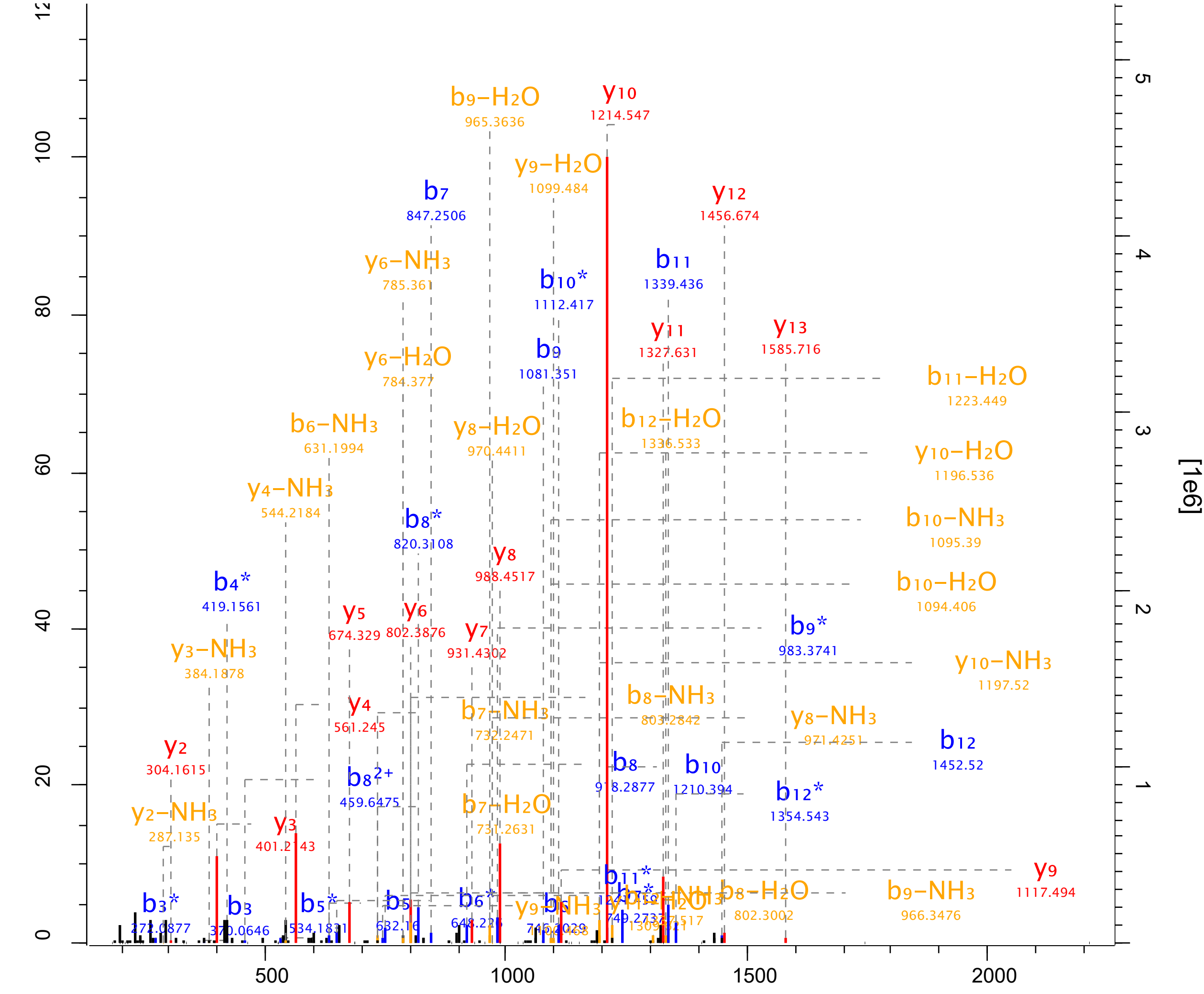

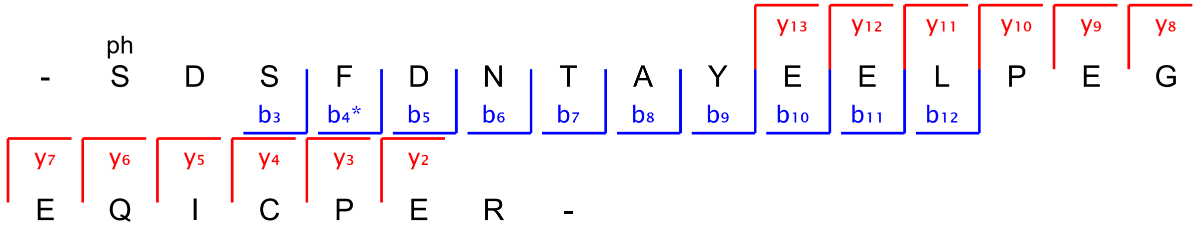

Raw file	Scan	Method	Score	m/z
sys_15_1	36852	FTMS; HCD	104.18	858.39

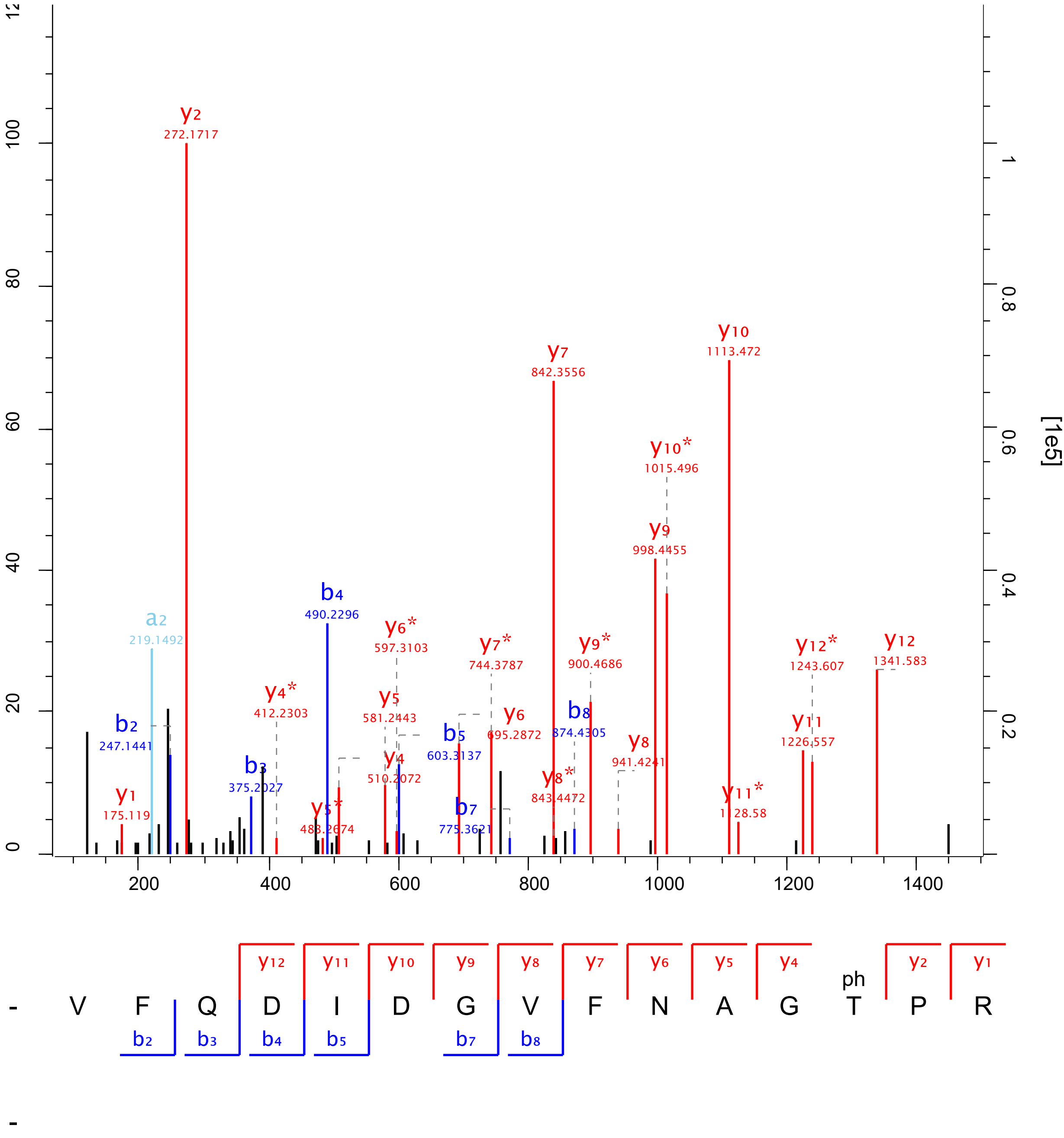

Raw file	Scan	Method	Score	m/z
sys_15_1	36975	FTMS; HCD	137.73	843.4

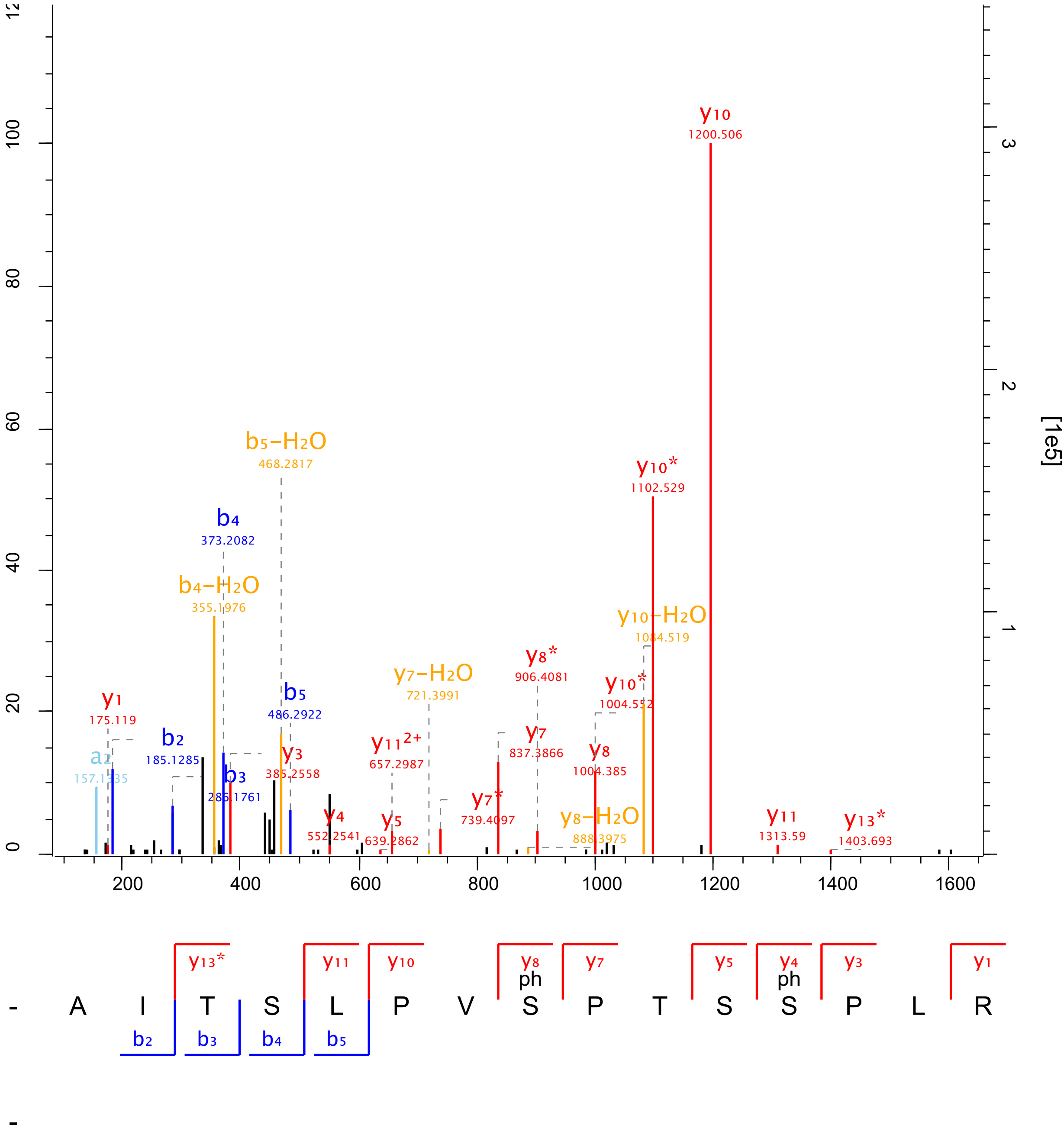

Raw file	Scan	Method	Score	m/z
sys_15_1	3705	FTMS; HCD	50.35	476.72

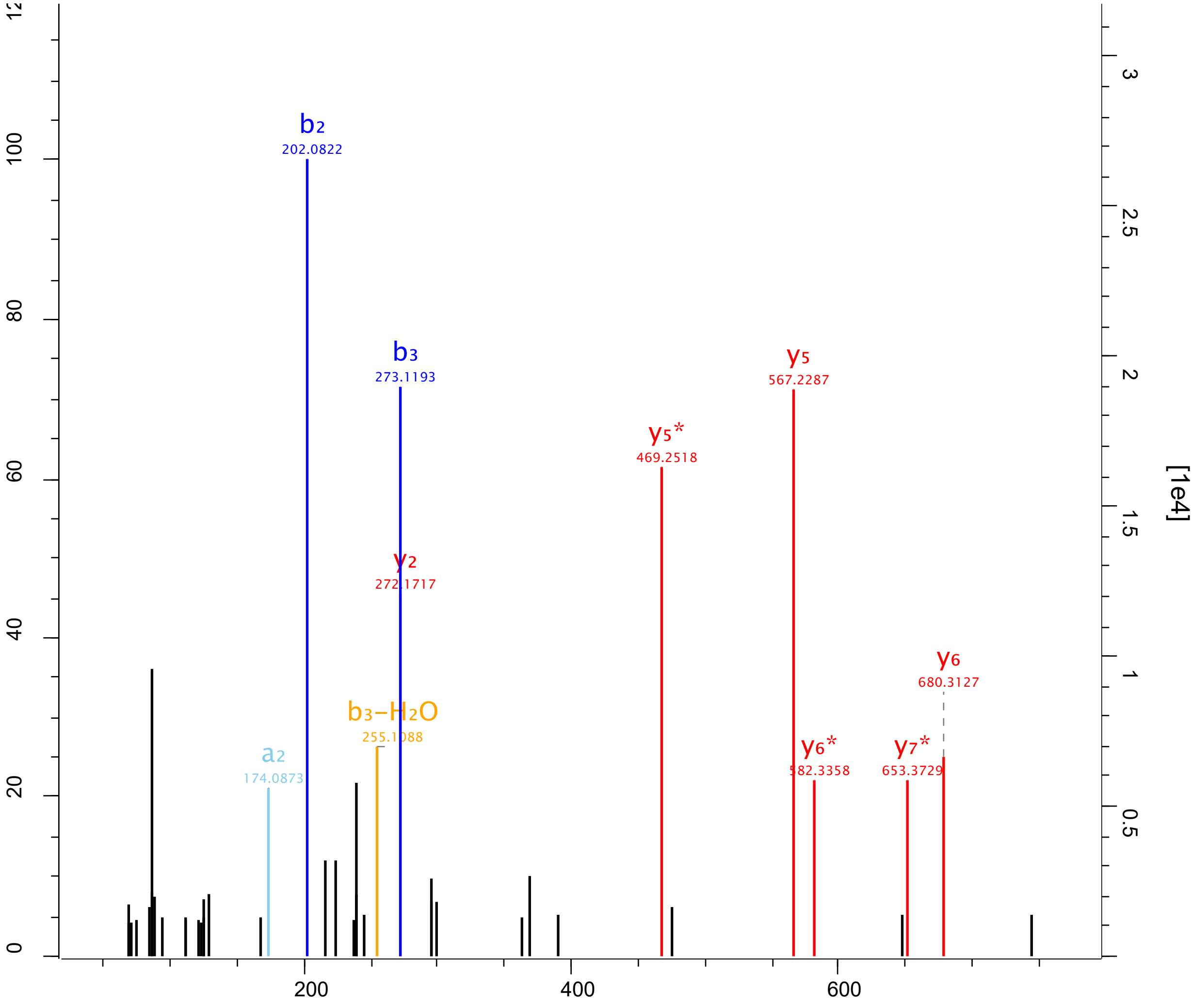

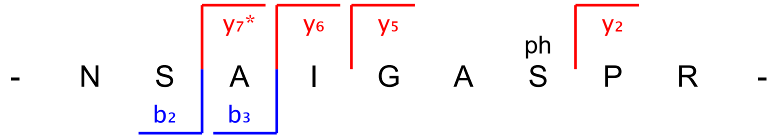

Raw file	Scan	Method	Score	m/z
sys_15_1	37280	FTMS; HCD	141.45	986

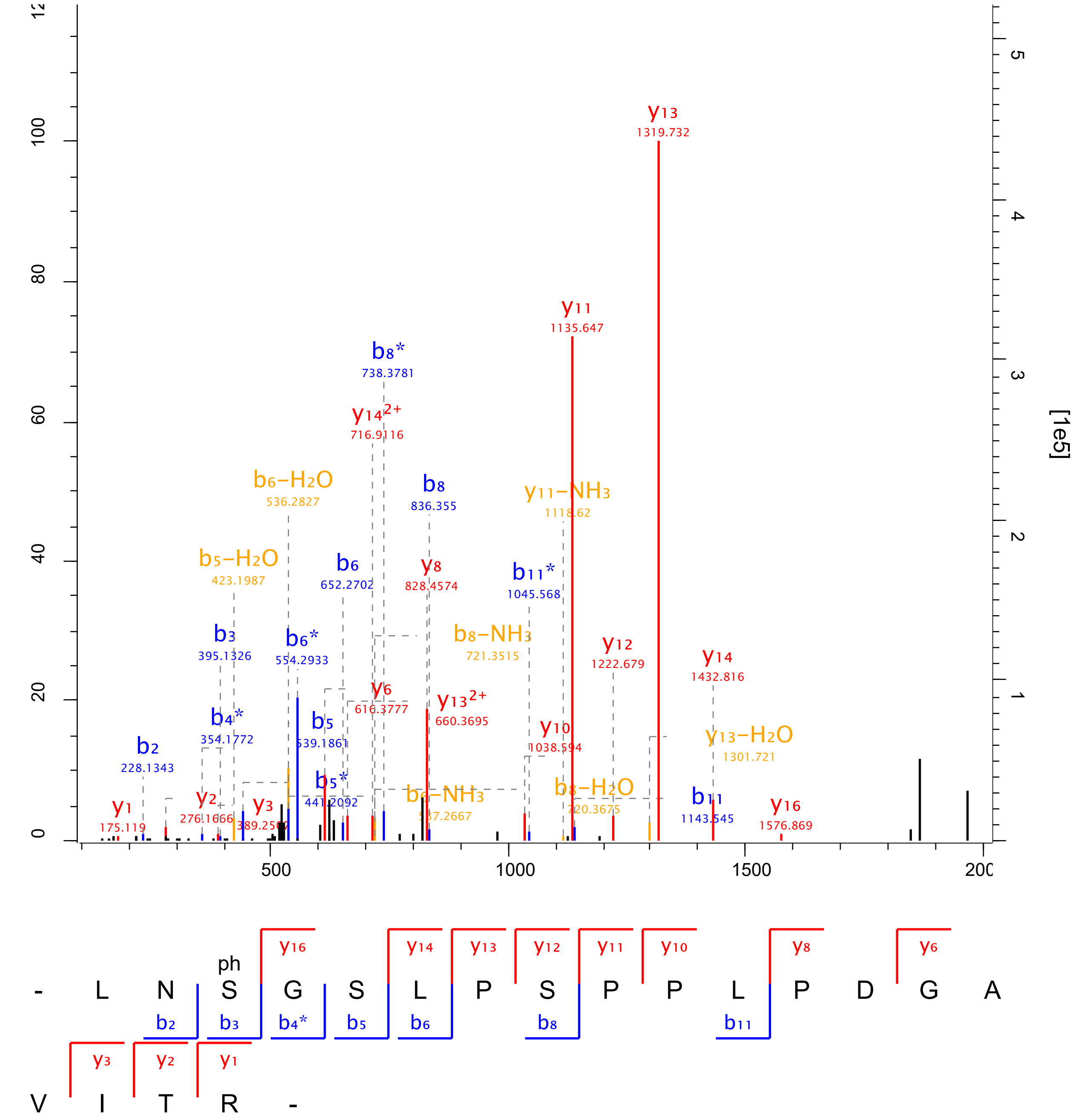

Raw file	Scan	Method	Score	m/z
sys_15_1	37388	FTMS; HCD	171.46	809.36

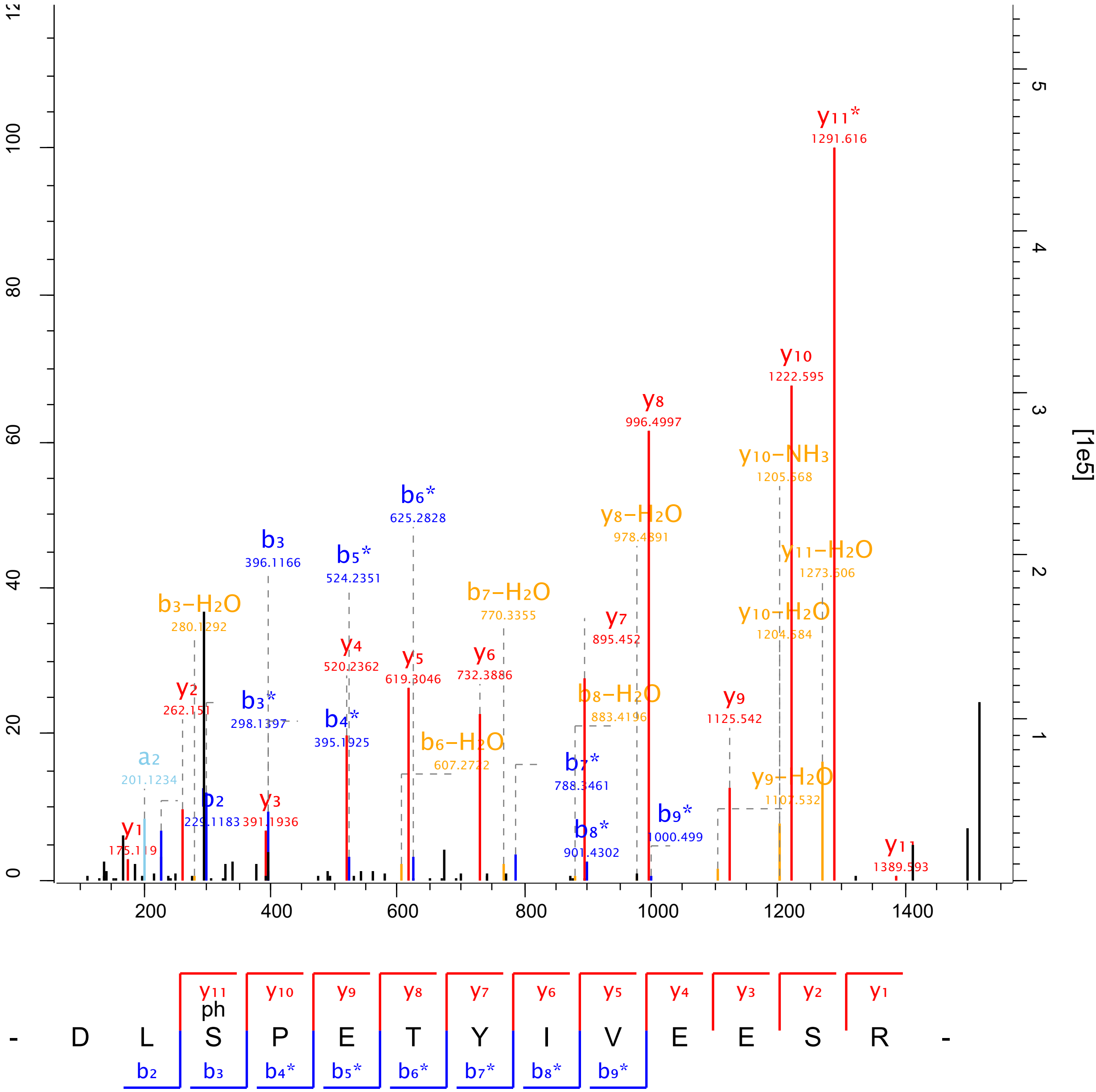

Raw file	Scan	Method	Score	m/z
sys_15_1	37469	FTMS; HCD	129.05	797.89

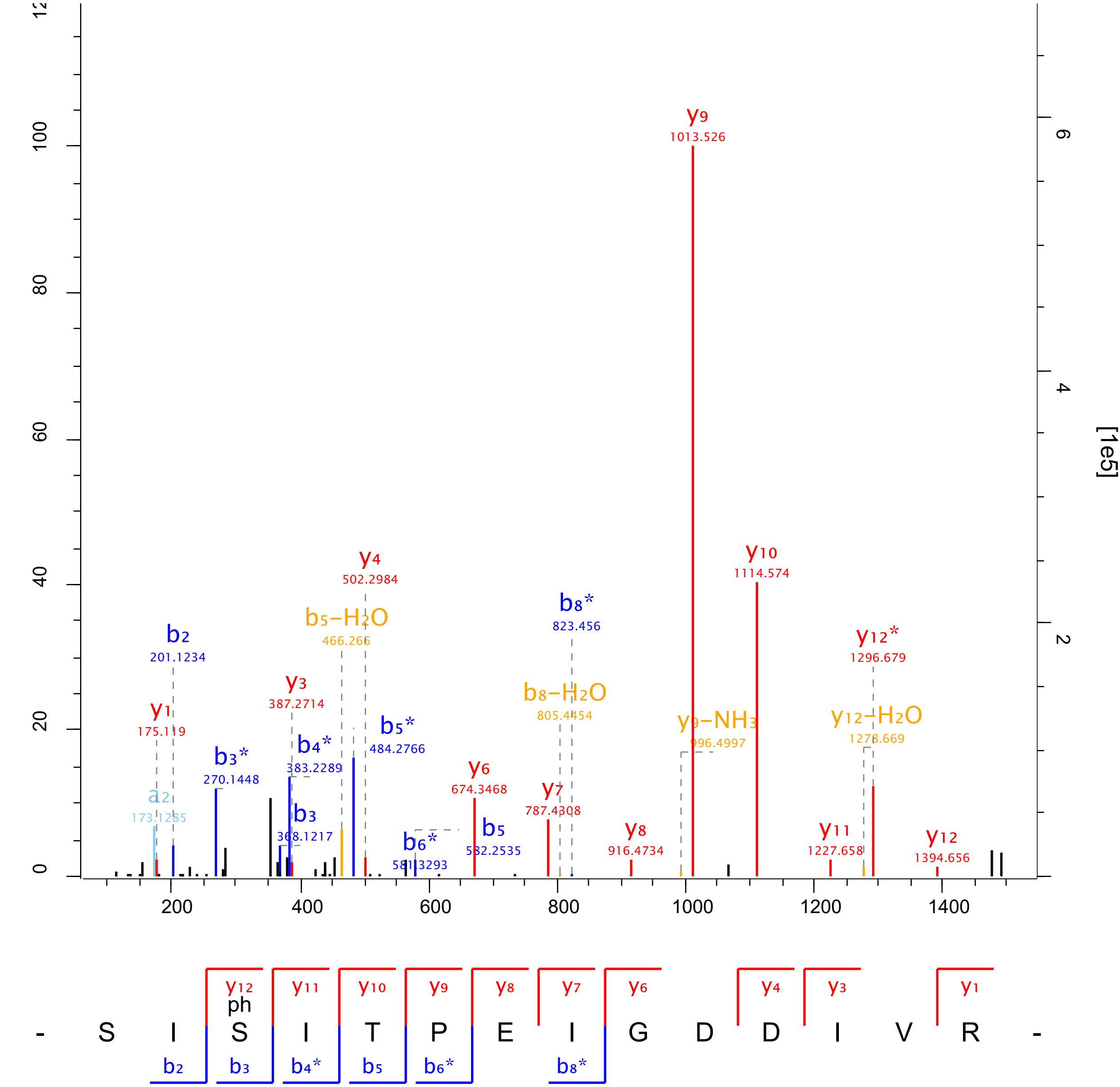

Raw file	Scan	Method	Score	m/z
sys_15_1	37610	FTMS; HCD	106.35	730.31

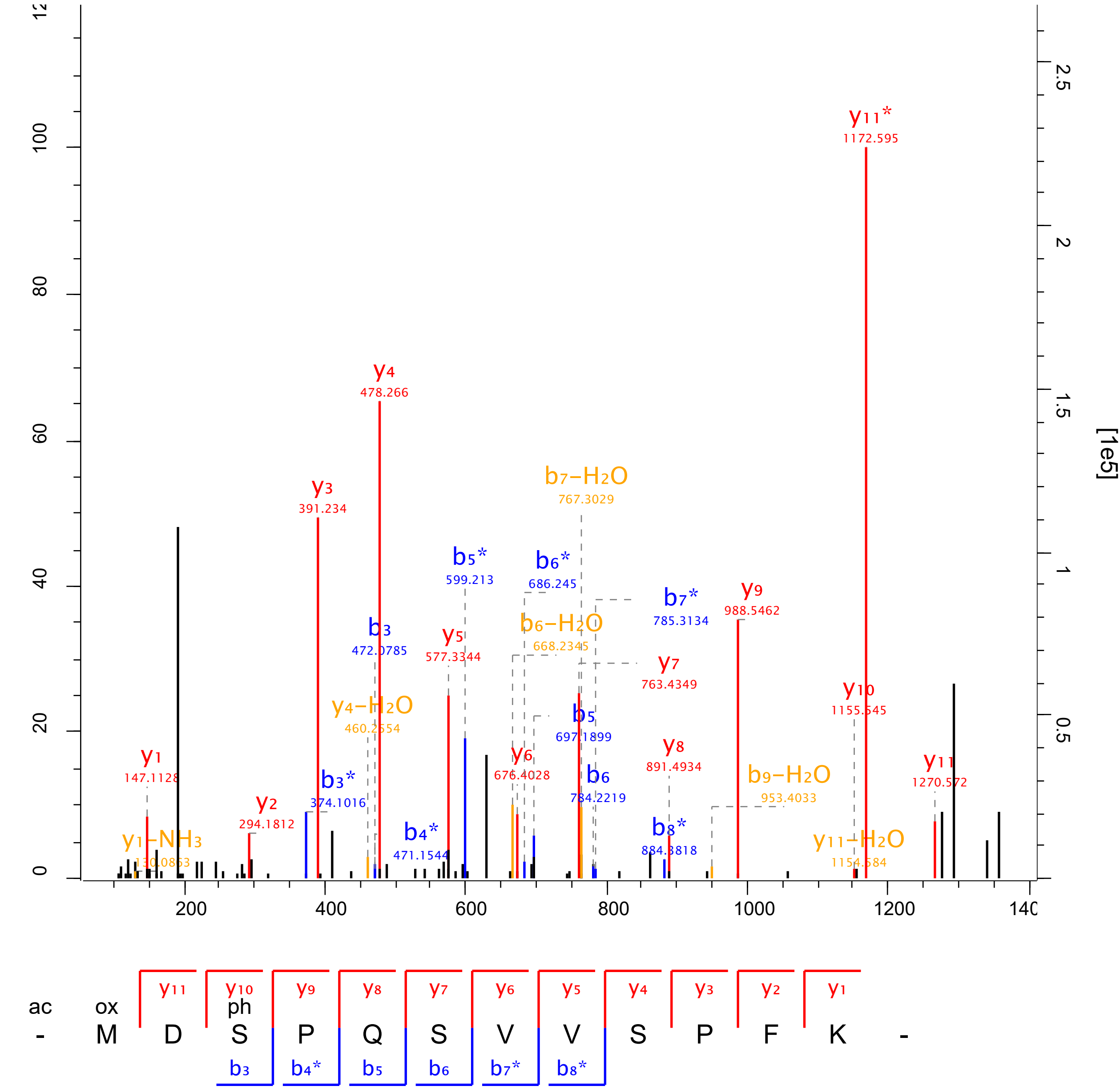

Raw file	Scan	Method	Score	m/z
sys_15_1	3780	FTMS; HCD	107.5	641.79

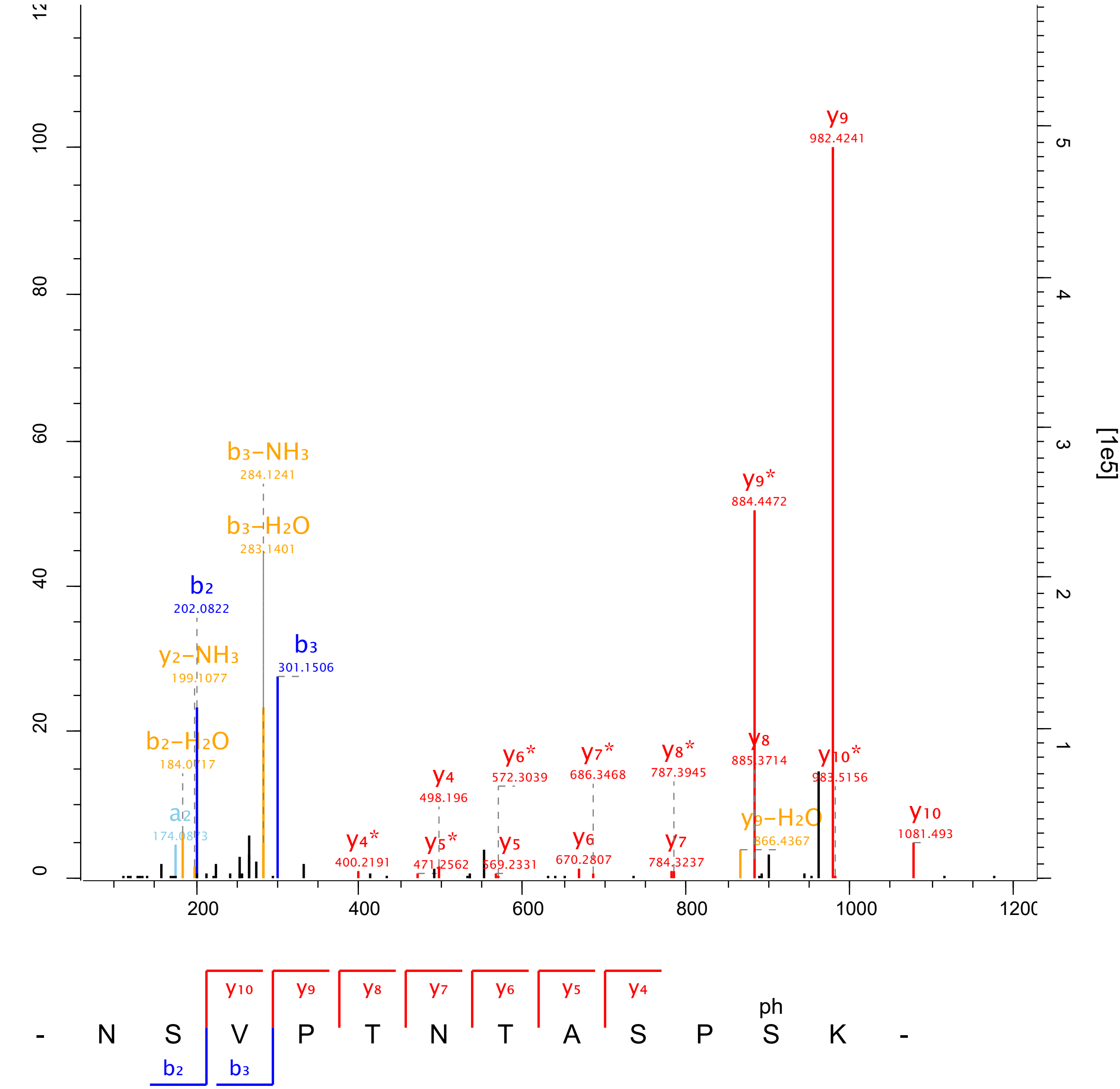

Raw file	Scan	Method	Score	m/z
sys_15_1	37883	FTMS; HCD	95.2	958.93

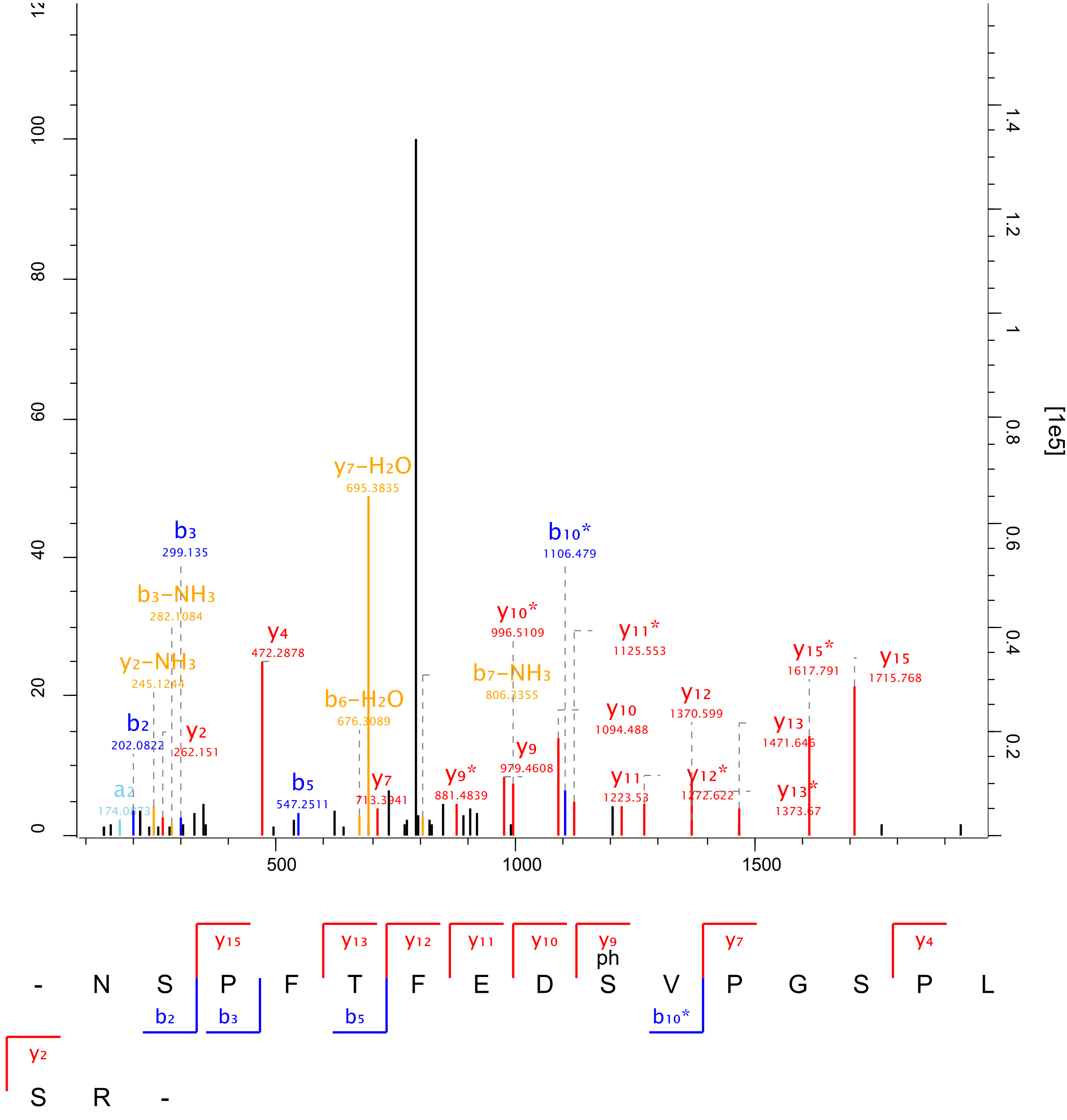

Raw file	Scan	Method	Score	m/z
sys_15_1	38118	FTMS; HCD	142.26	876.84

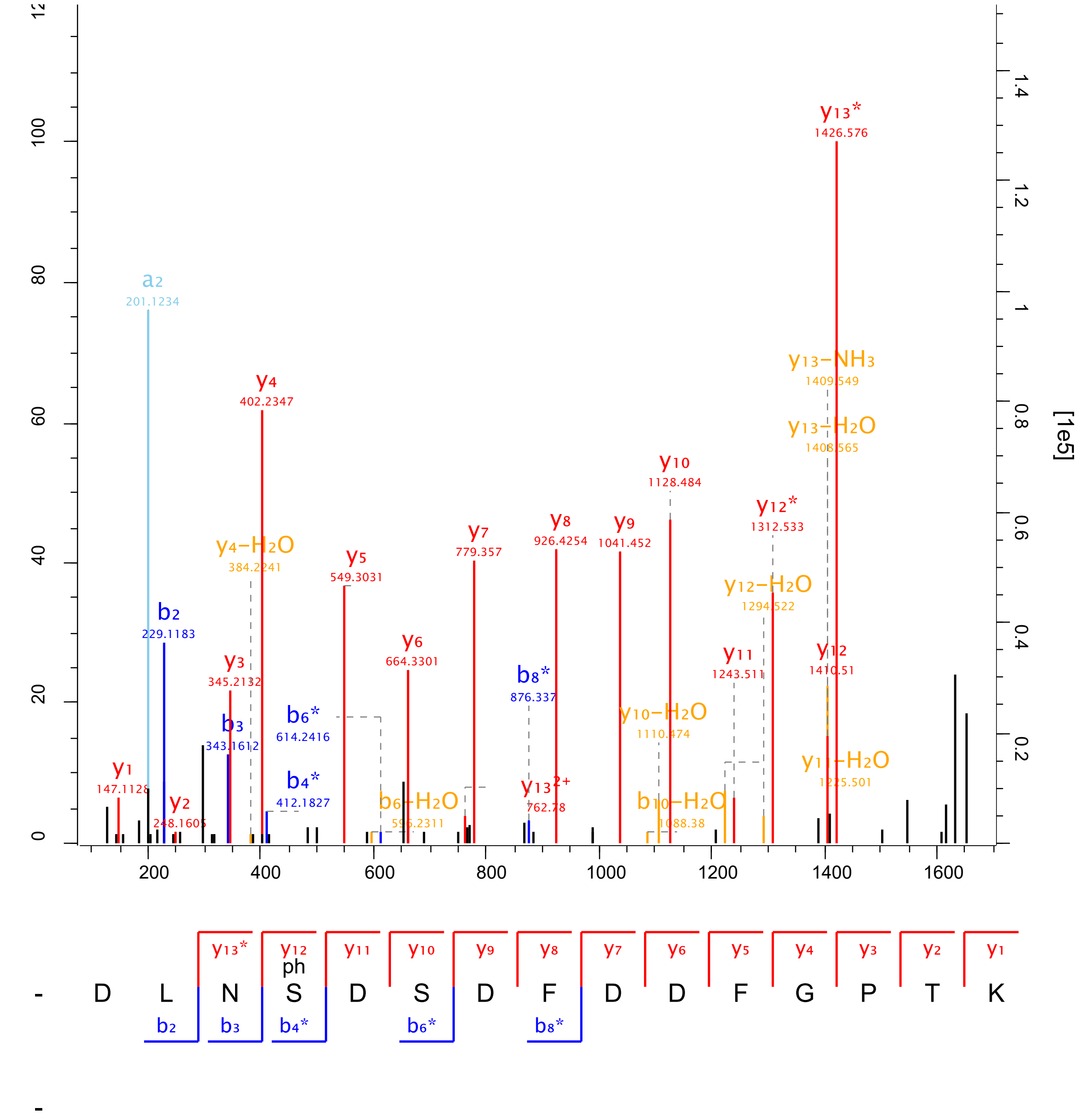

Raw file	Scan	Method	Score	m/z
sys_15_1	3815	FTMS; HCD	226.99	870.37

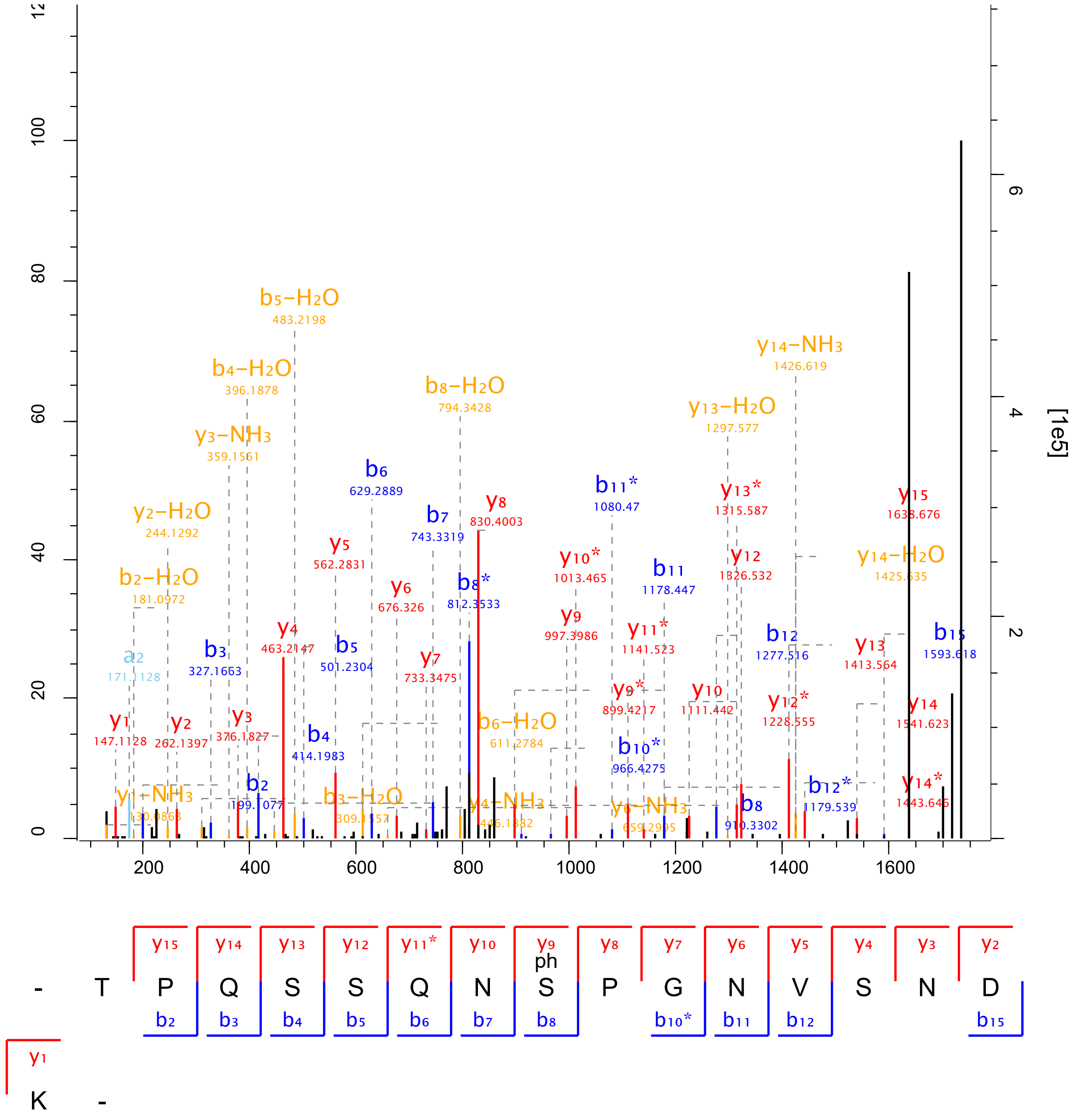

Raw file	Scan	Method	Score	m/z
sys_15_1	38231	FTMS; HCD	131.33	718.35

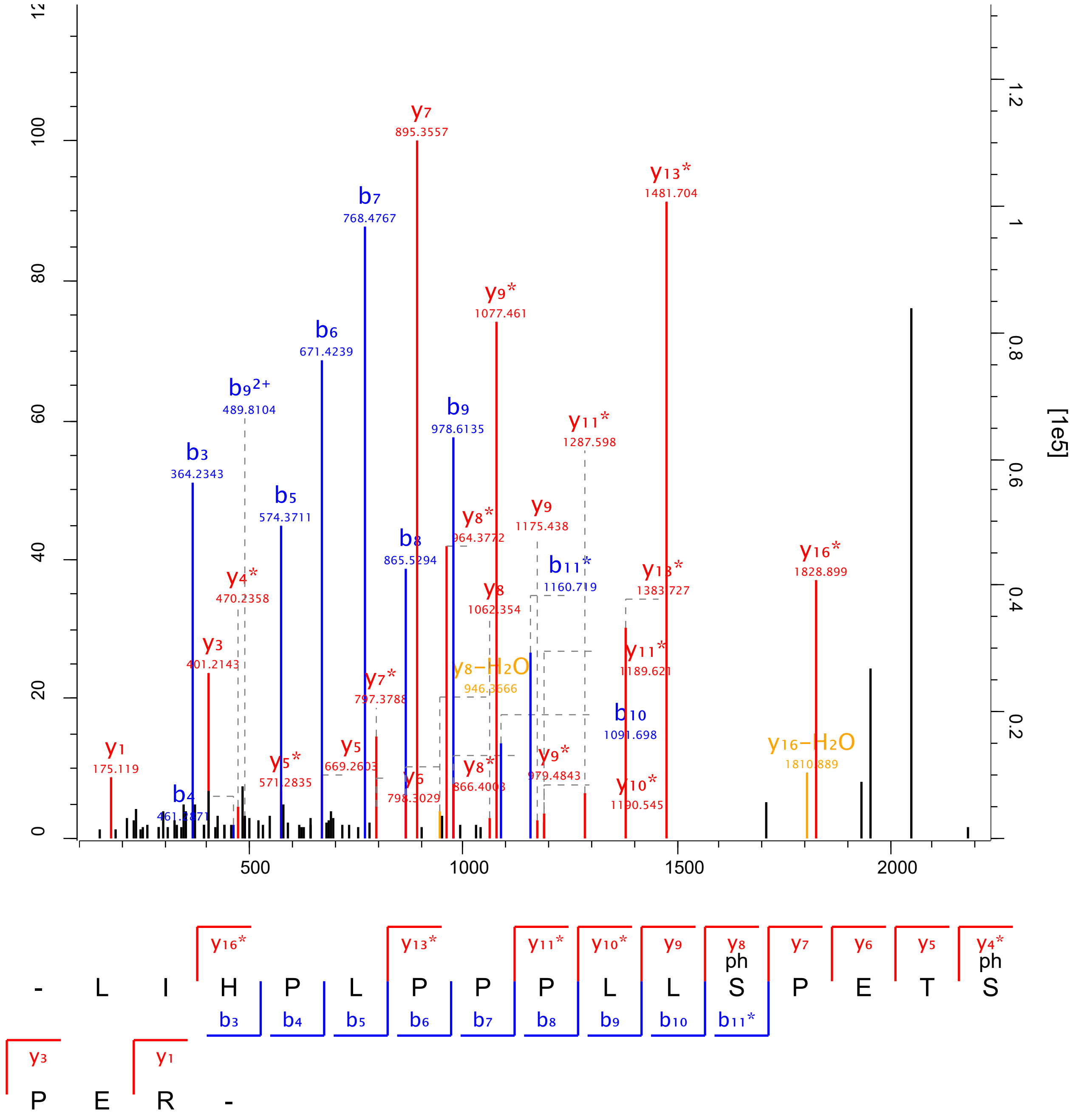

Raw file	Scan	Method	Score	m/z
sys_15_1	3835	FTMS; HCD	58.69	802.78

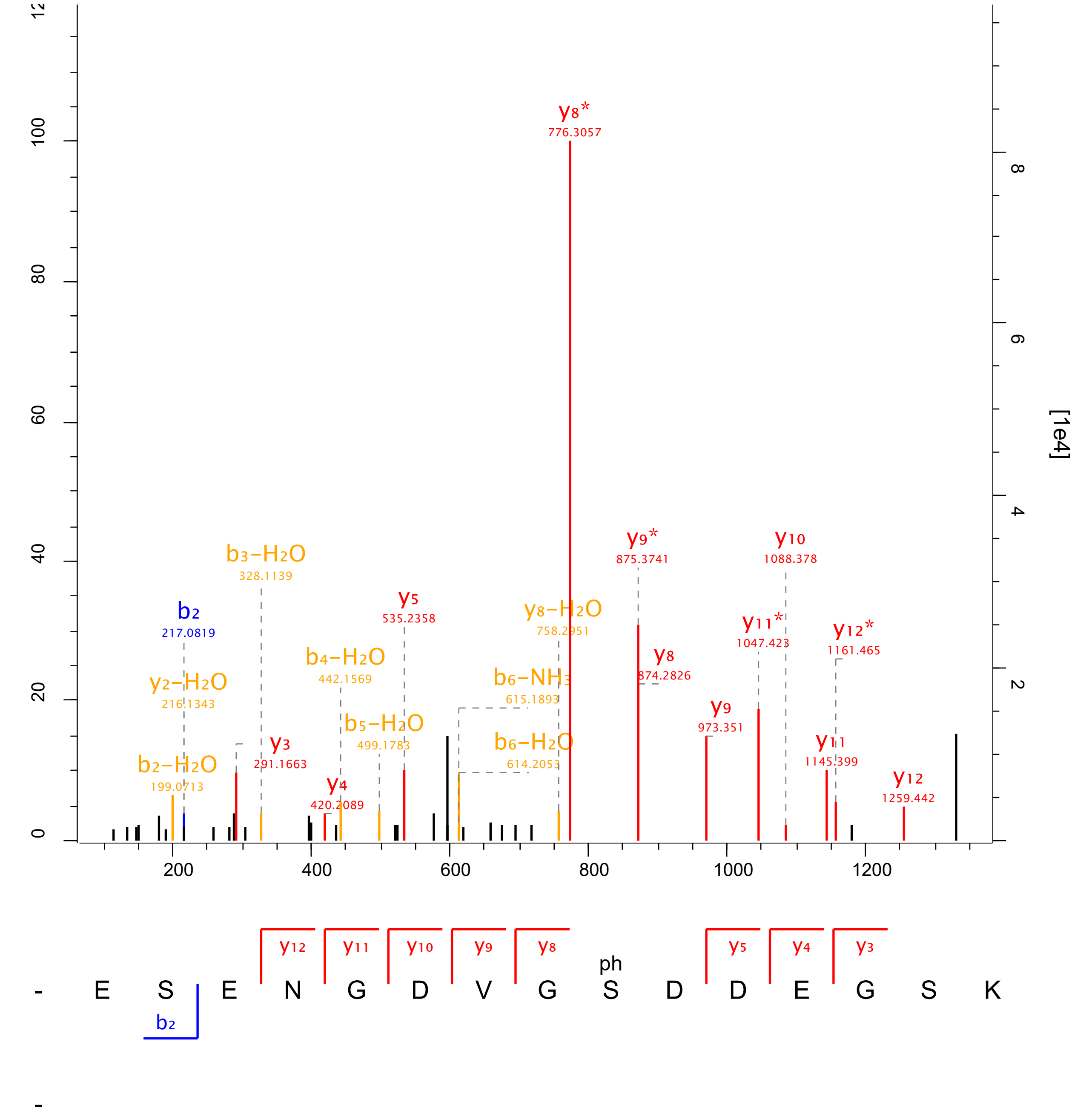

Raw file	Scan	Method	Score	m/z
sys_15_1	38425	FTMS; HCD	159.14	1085.99

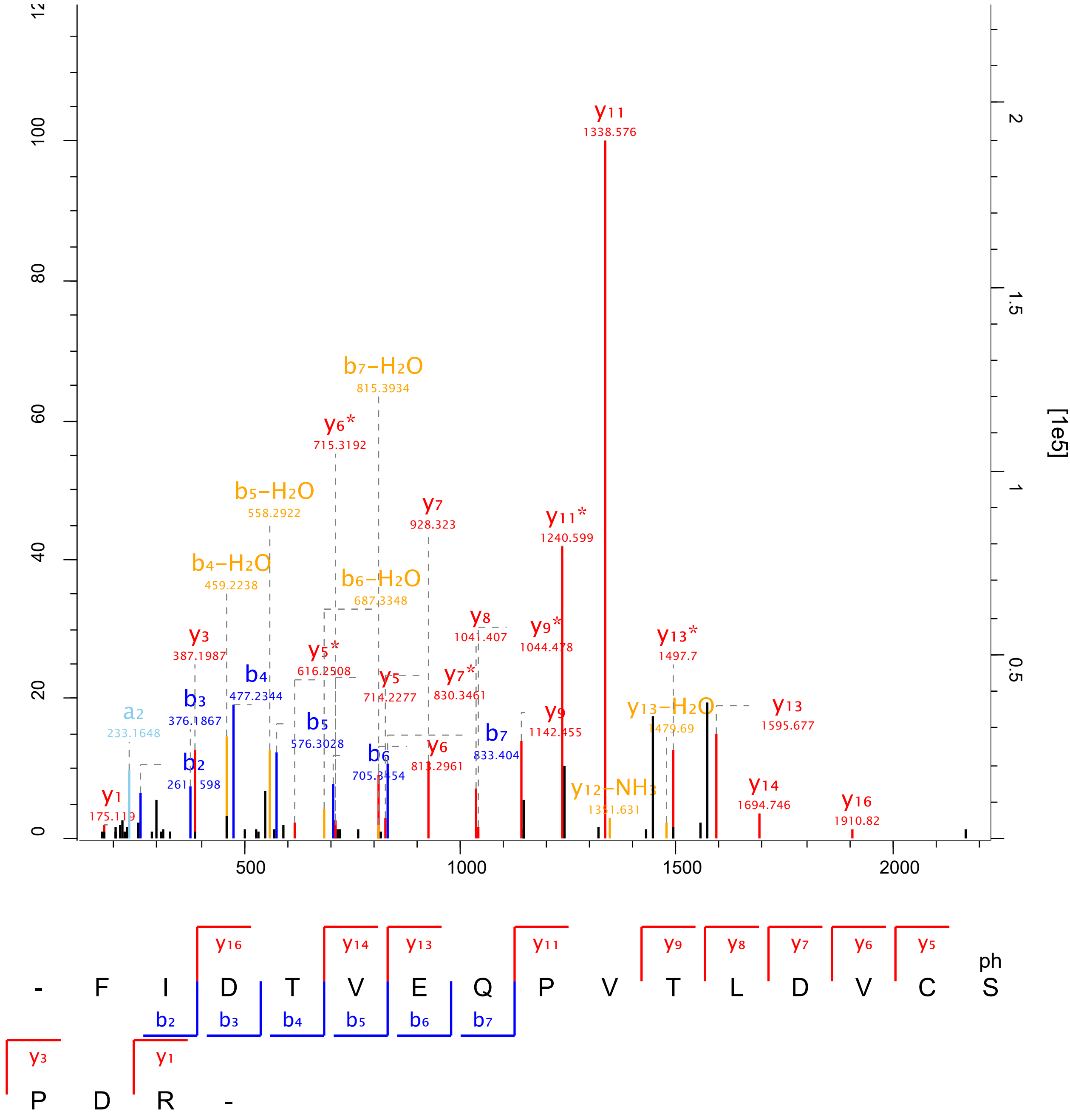

Raw file	Scan	Method	Score	m/z
sys_15_1	38484	FTMS; HCD	124.51	1138.46

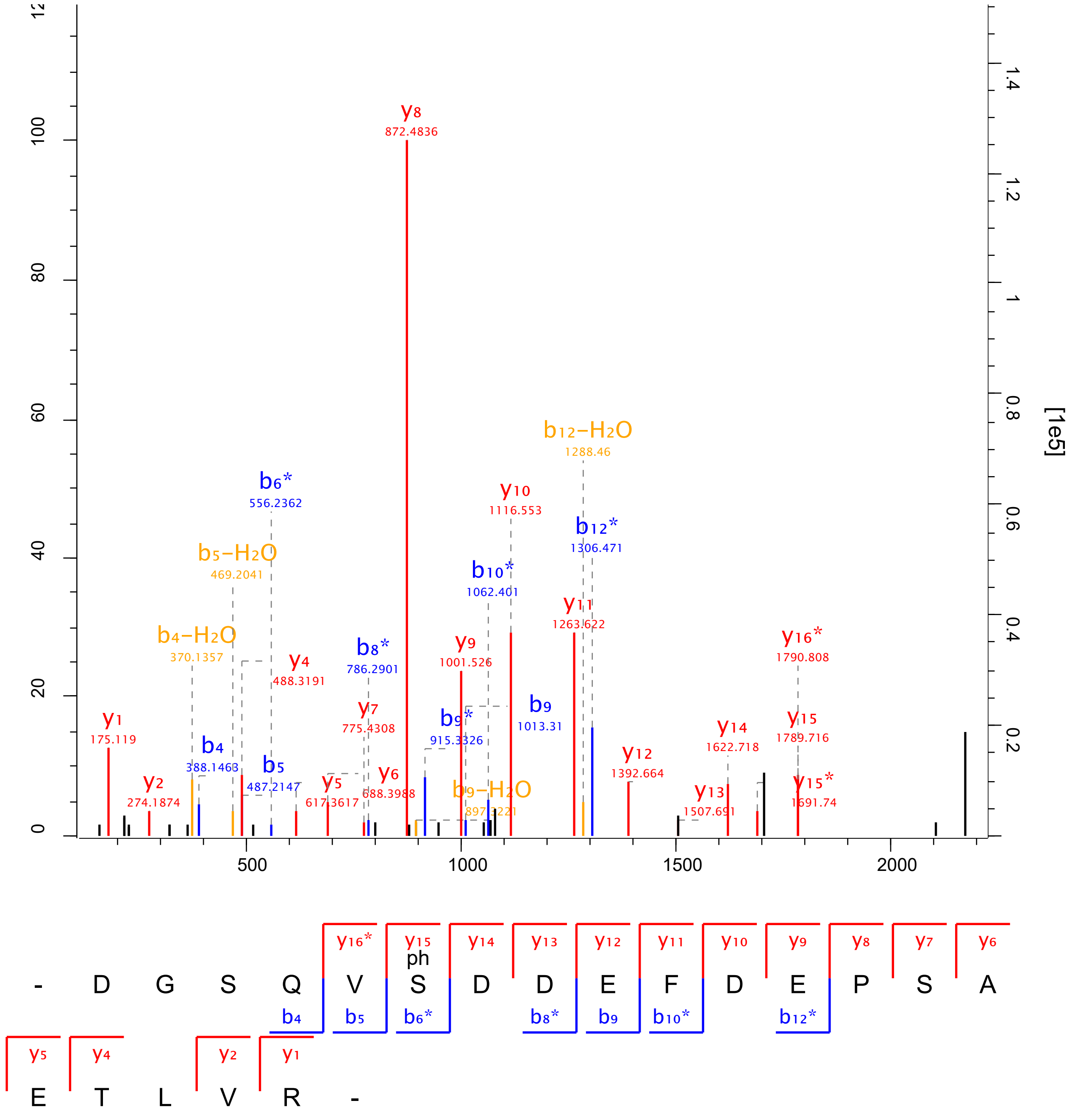

Raw file	Scan	Method	Score	m/z
sys_15_1	3851	FTMS; HCD	88.71	594.76

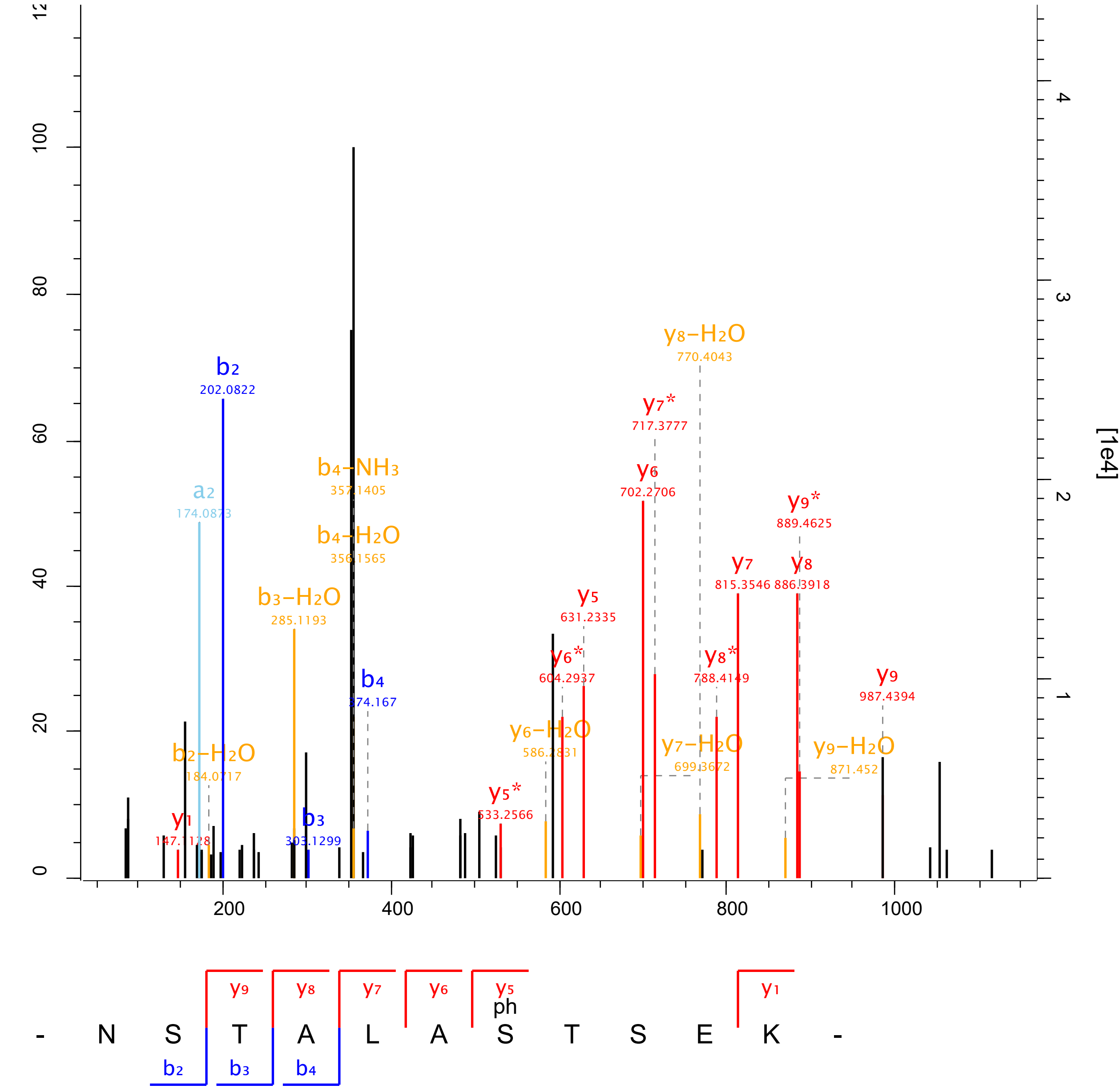

Raw file	Scan	Method	Score	m/z
sys_15_1	38596	FTMS; HCD	111.01	887.03

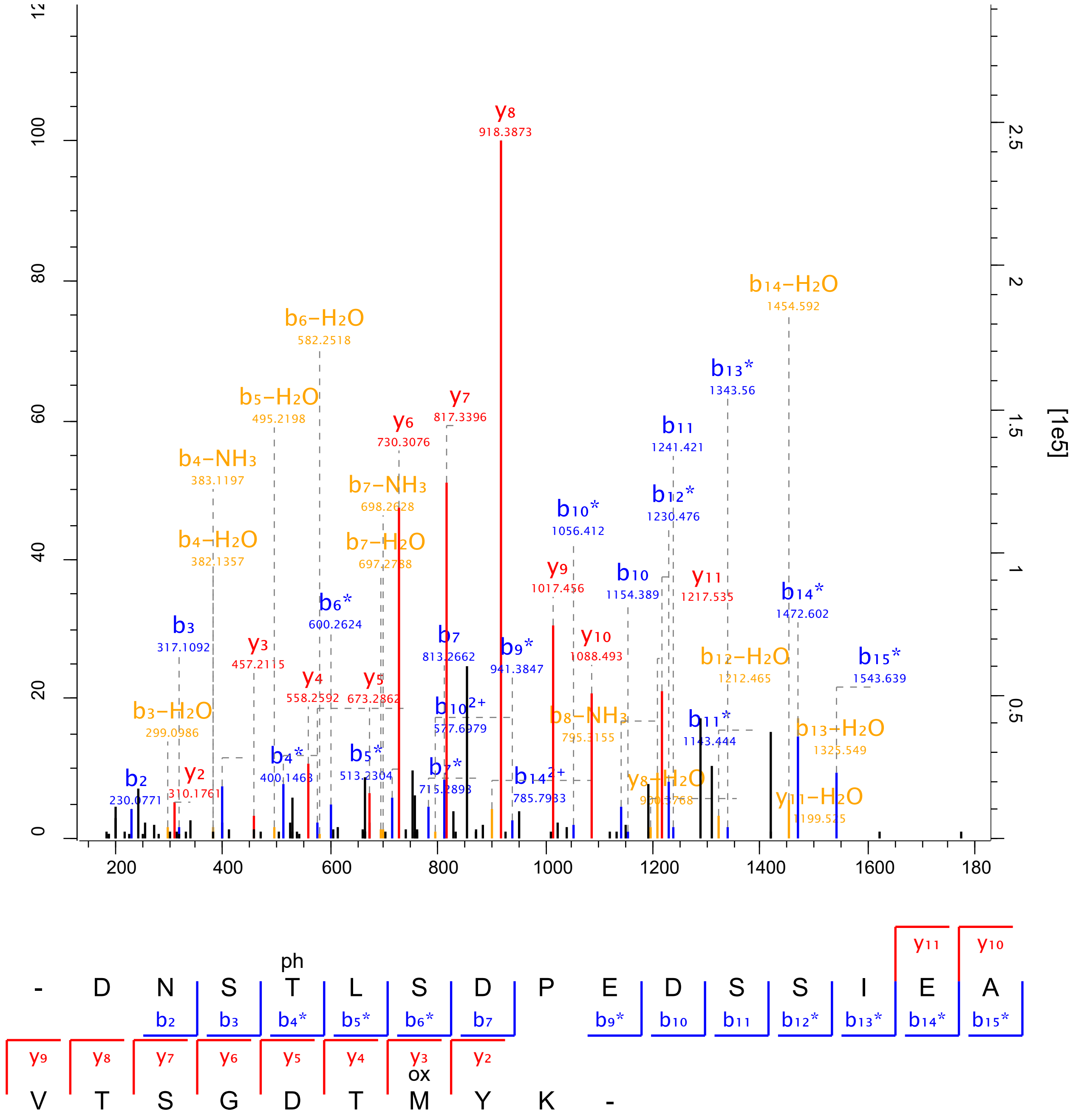

Raw file	Scan	Method	Score	m/z
sys_15_1	38621	FTMS; HCD	203.19	902.03

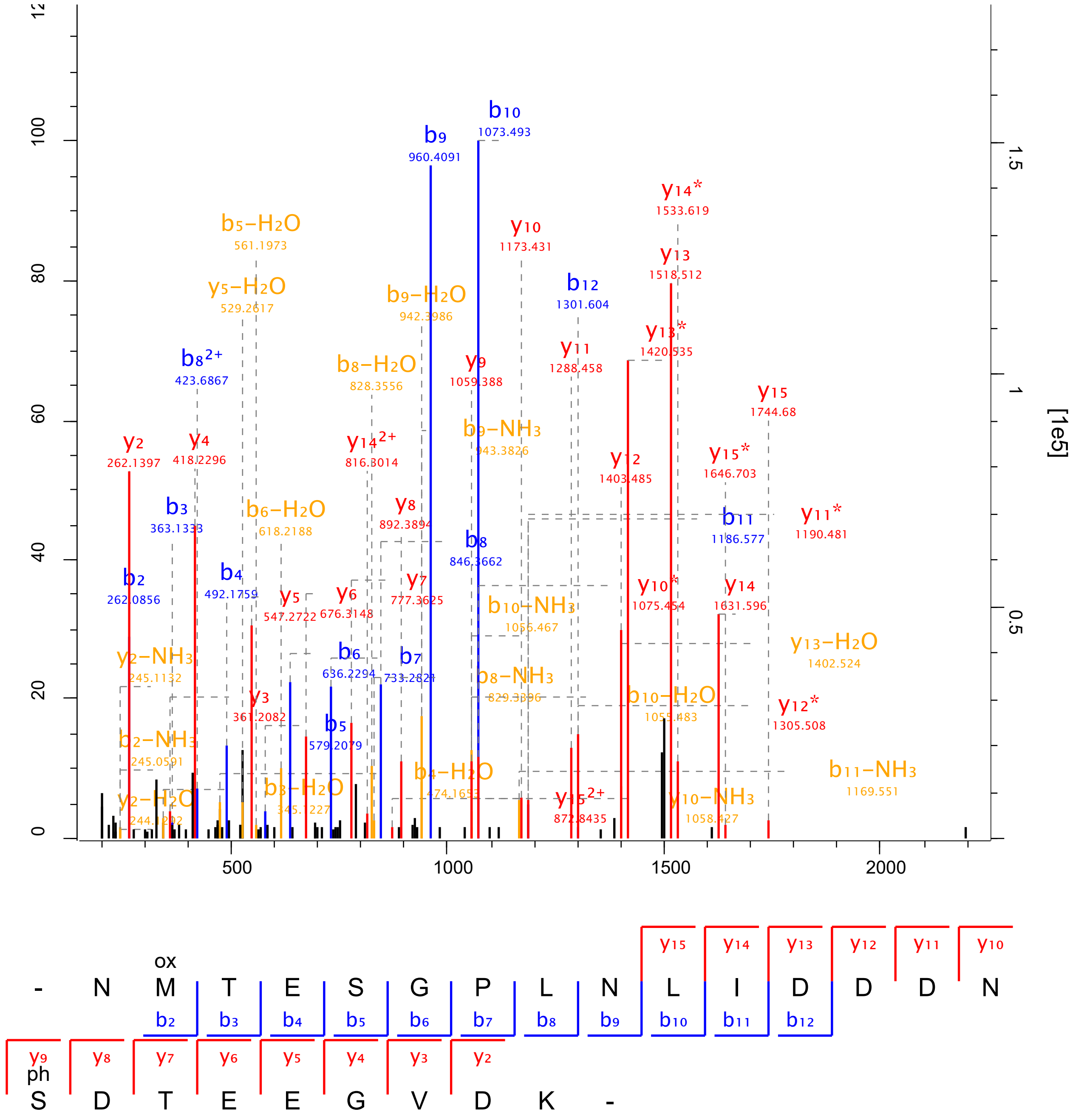

Raw file	Scan	Method	Score	m/z
sys_15_1	38623	FTMS; HCD	205.95	805.87

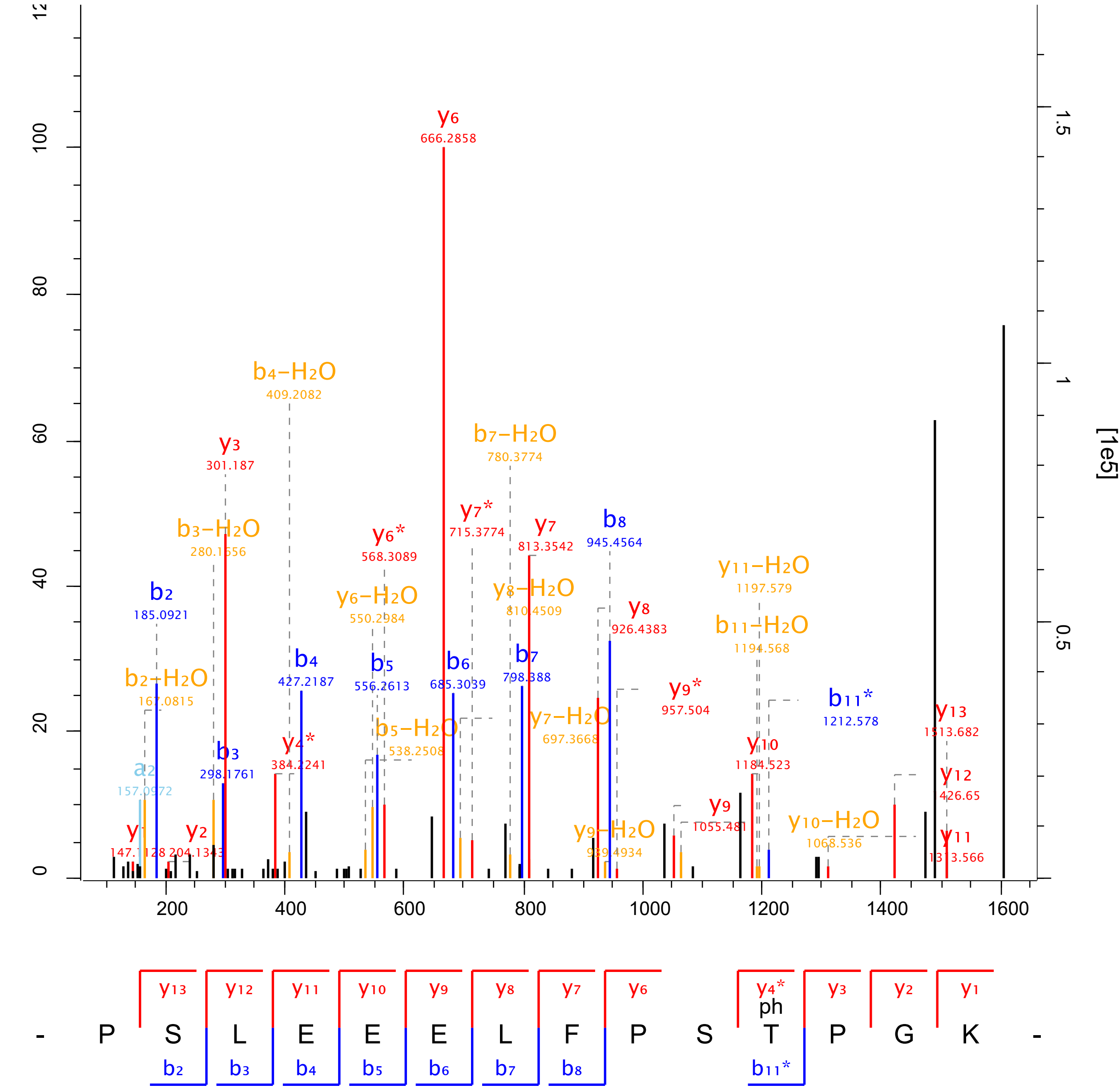

Raw file	Scan	Method	Score	m/z
sys_15_1	38872	FTMS; HCD	98.3	761.03

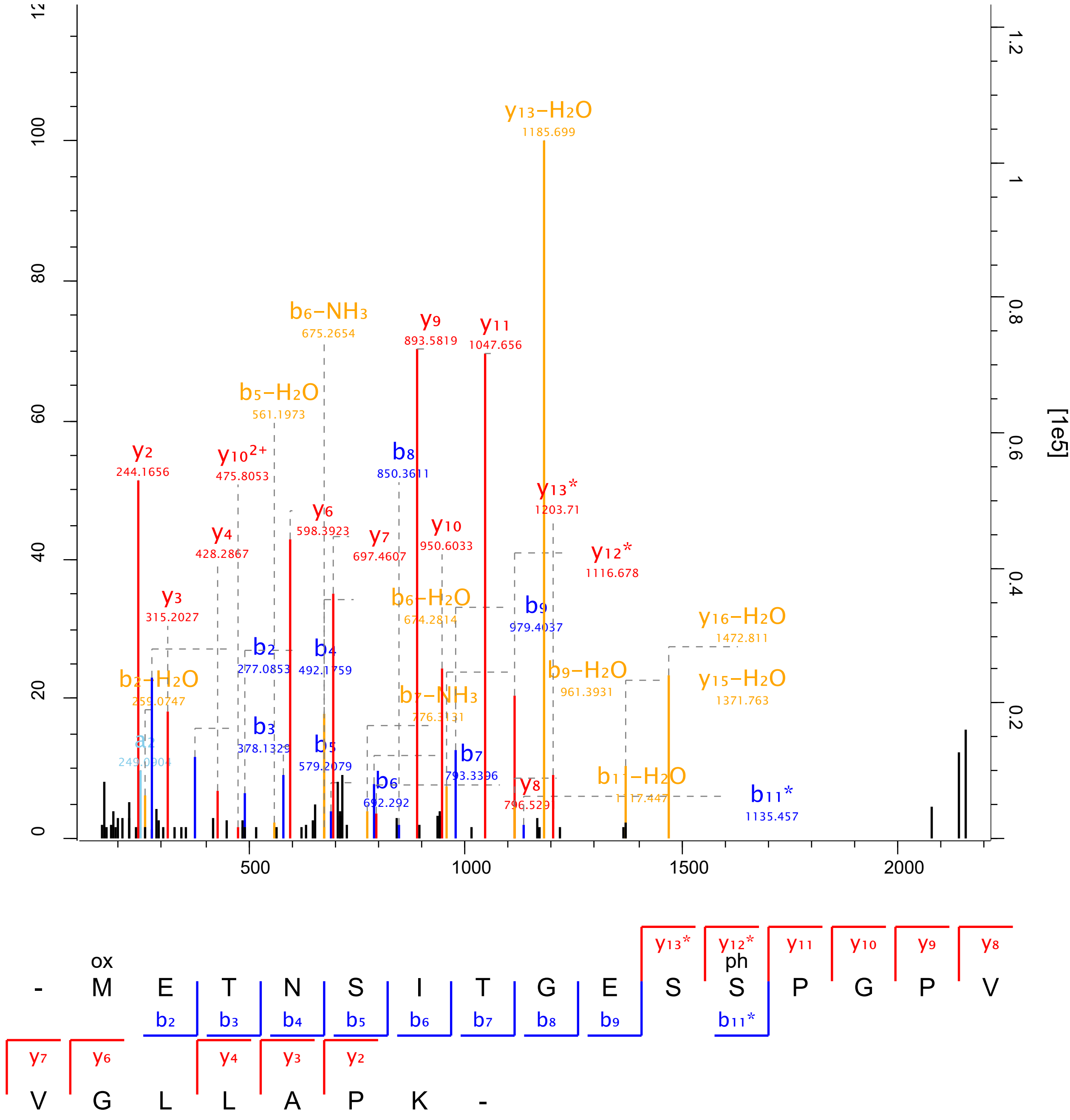

Raw file	Scan	Method	Score	m/z
sys_15_1	3888	FTMS; HCD	61.78	462.69

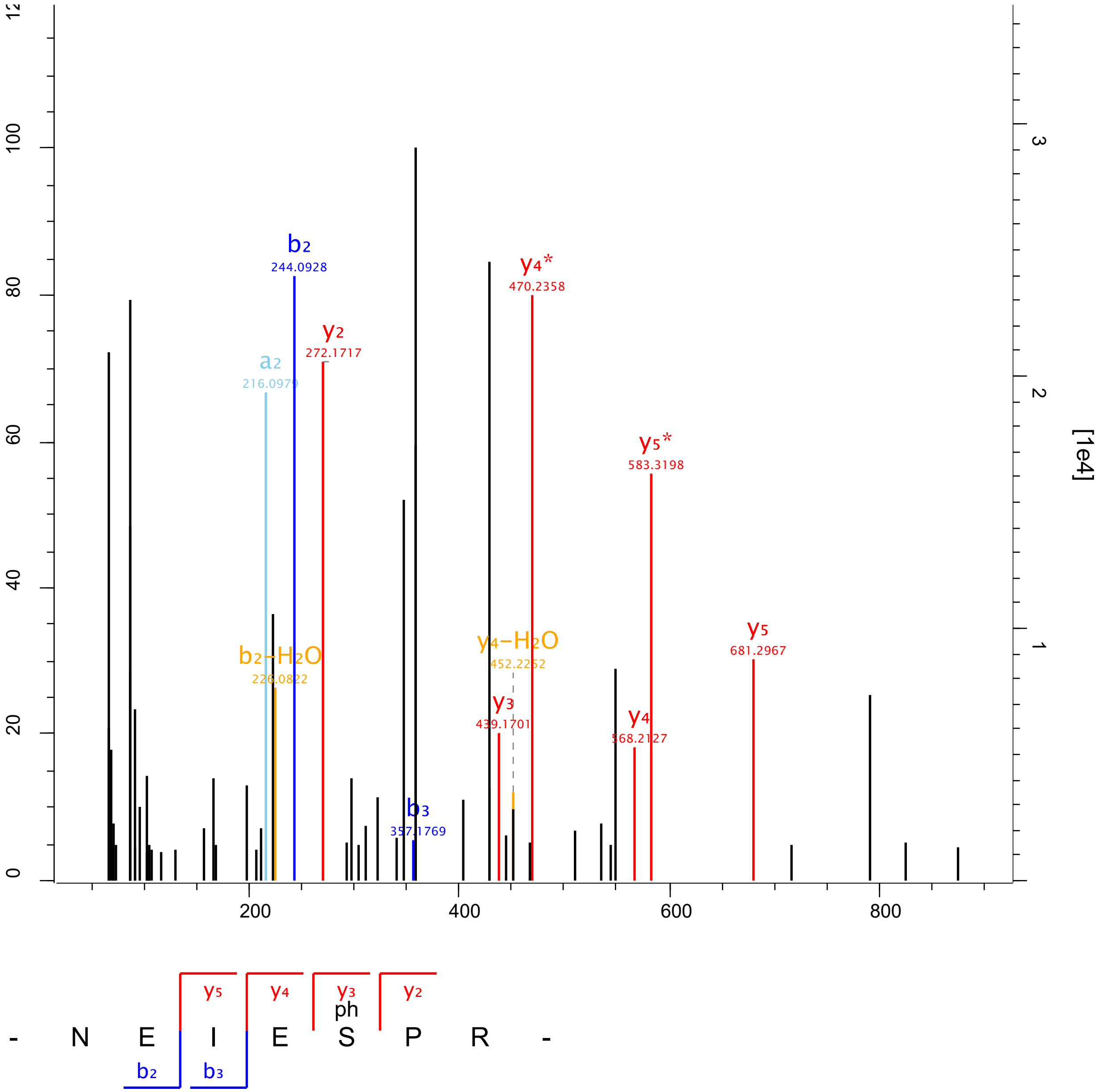

Raw file	Scan	Method	Score	m/z
sys_15_1	39109	FTMS; HCD	45.92	837.88

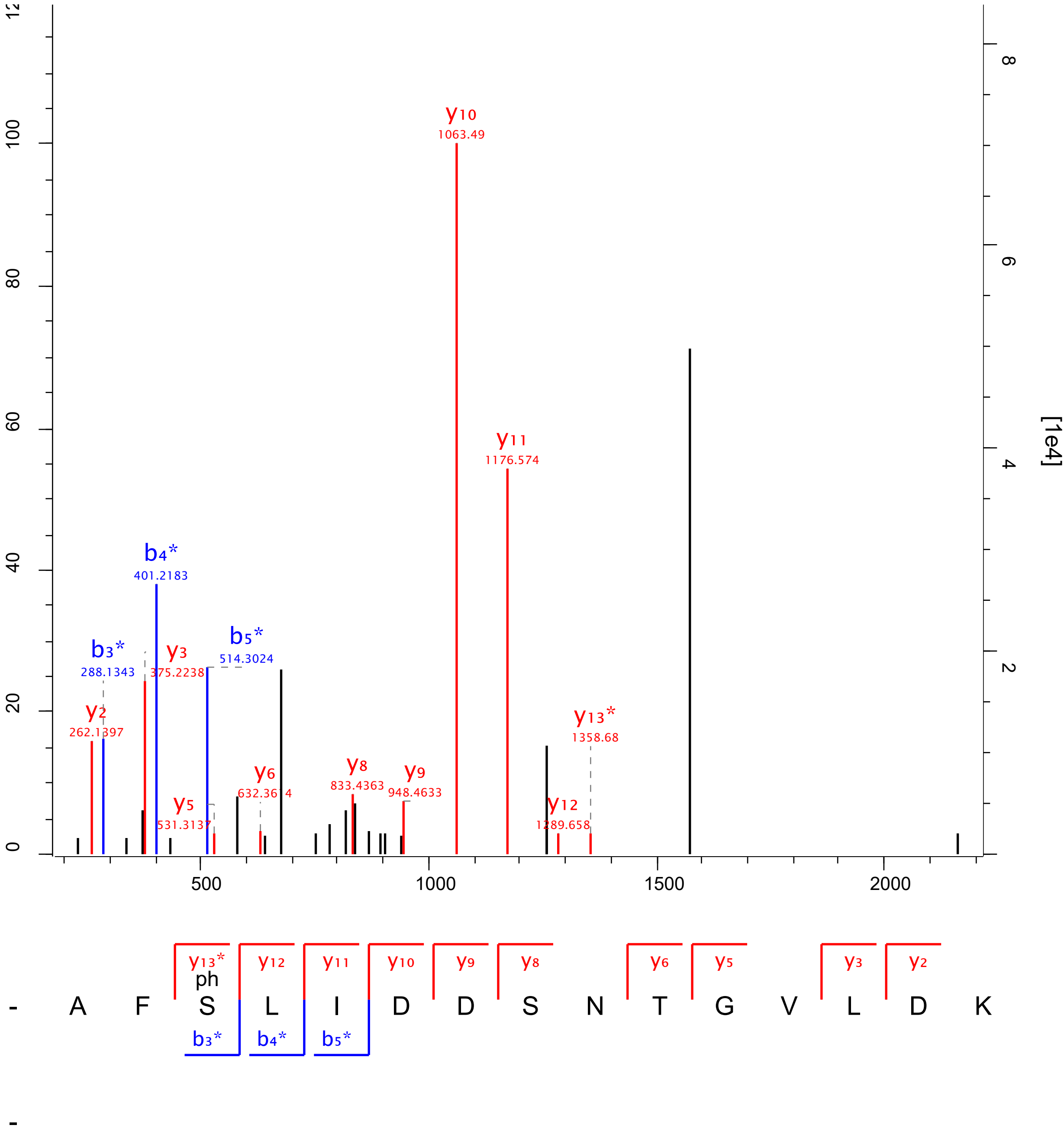

Raw file	Scan	Method	Score	m/z
sys_15_1	39289	FTMS; HCD	64.7	838.42

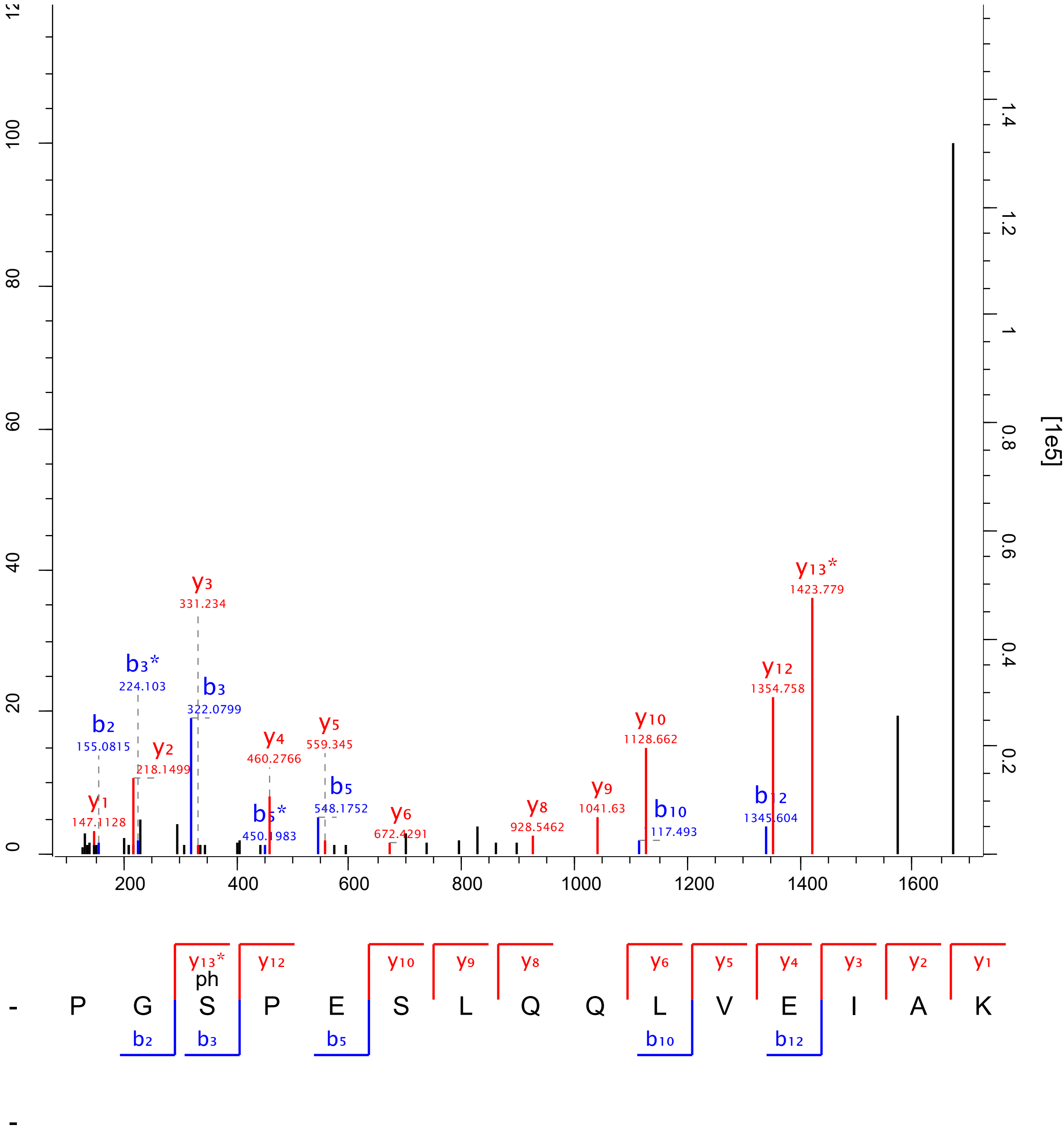

Raw file	Scan	Method	Score	m/z
sys_15_1	39425	FTMS; HCD	218.12	747.88

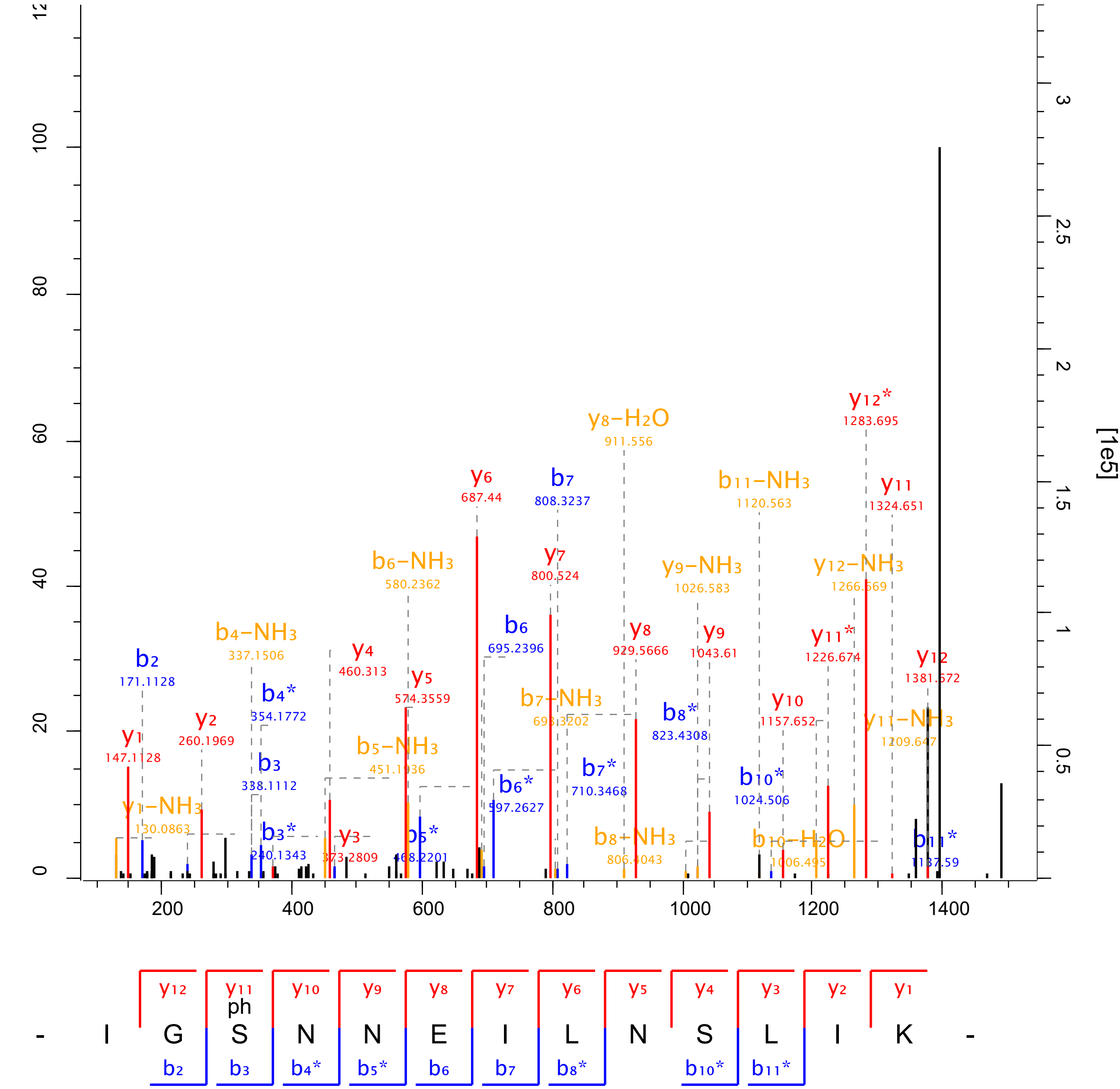

Raw file	Scan	Method	Score	m/z
sys_15_1	3966	FTMS; HCD	120.77	532.69

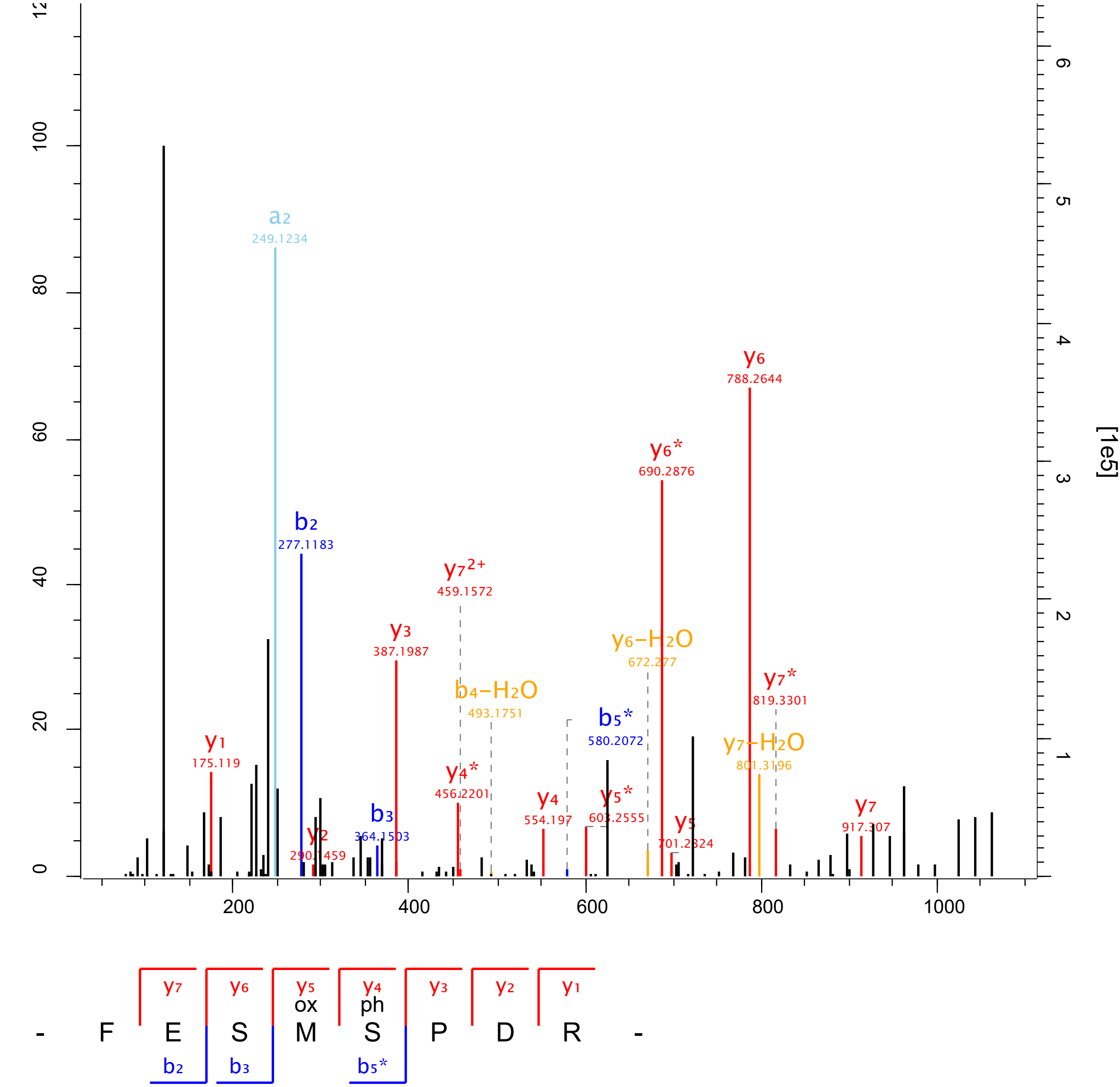

Raw file	Scan	Method	Score	m/z
sys_15_1	39821	FTMS; HCD	43.7	747.31

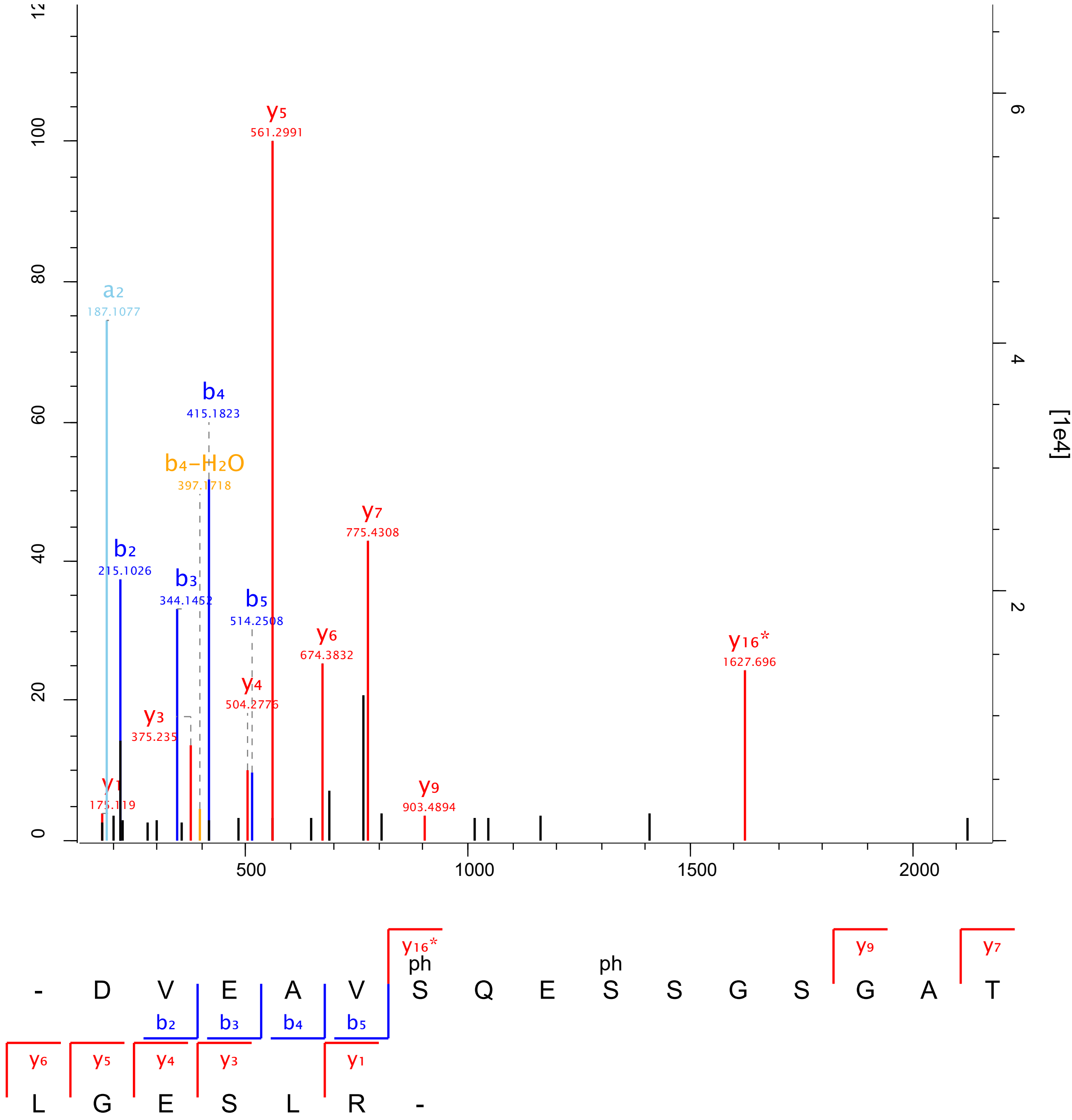

Raw file	Scan	Method	Score	m/z
sys_15_1	39897	FTMS; HCD	166.47	869.87

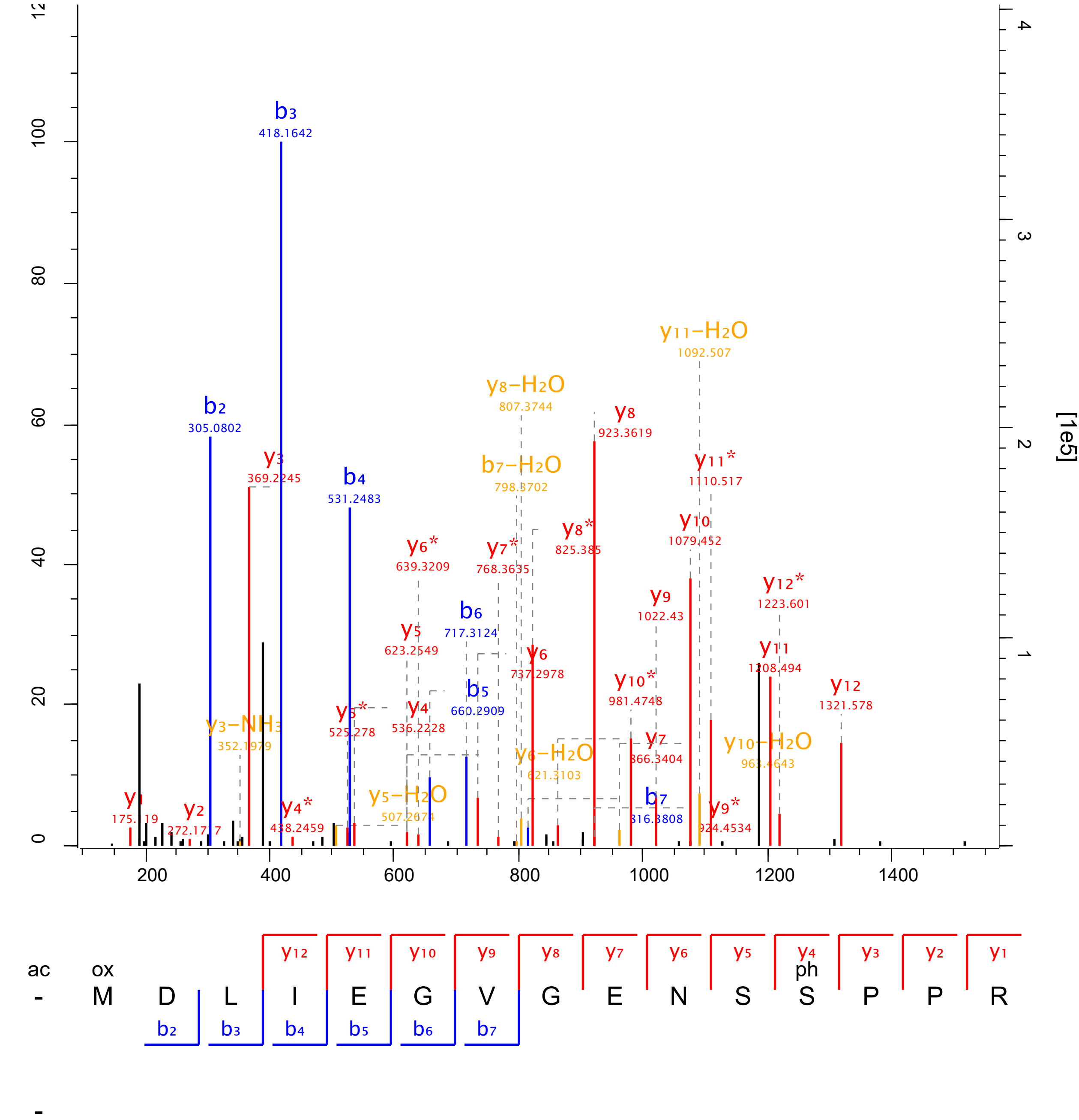

Supplement: Supplementary Figure S2-7 [file 143488_2_supp_337929_ps5hgj.pdf]
