# Supplementary material for: The Systemin Signaling Cascade As Derived from Time Course Analyses of the Systemin-responsive Phosphoproteome
Source: Mol Cell Proteomics. 2019 May 28;18(8):1526–42. doi: 10.1074/mcp.RA119.001367 (PMC6683004; doi:10.1074/mcp.RA119.001367)

Supplementary Figure 2: Representative annotated spectra of identified phosphopeptides under systemin,A17 and water treatment as exported from MaxQuant.

Raw file	Scan	Method	Score	m/z
sys_15_1	4012	FTMS; HCD	51.73	556.18

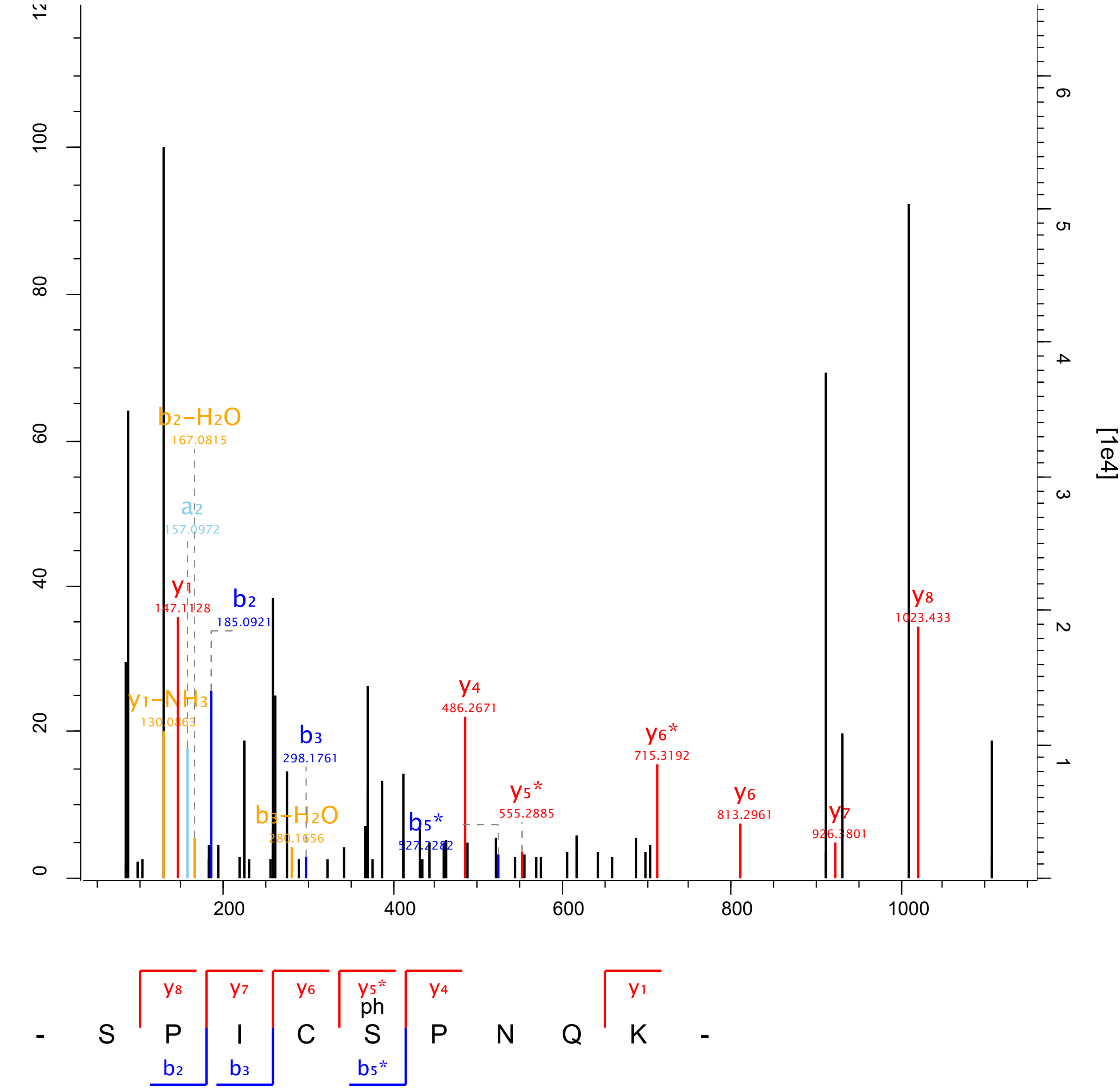

Raw file	Scan	Method	Score	m/z
sys_15_1	40251	FTMS; HCD	162.87	930.74

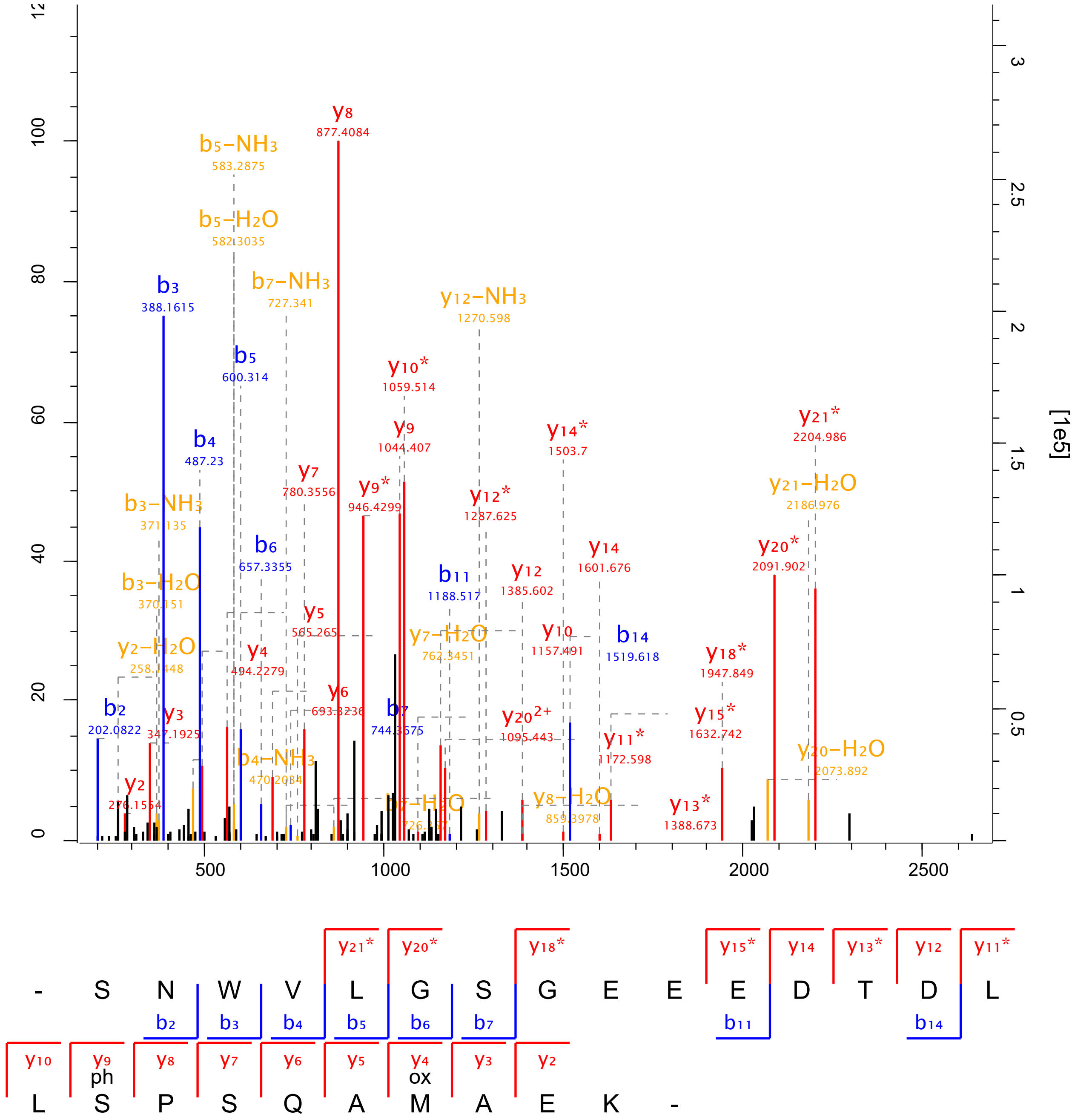

Raw file	Scan	Method	Score	m/z
sys_15_1	4031	FTMS; HCD	160.44	578.74

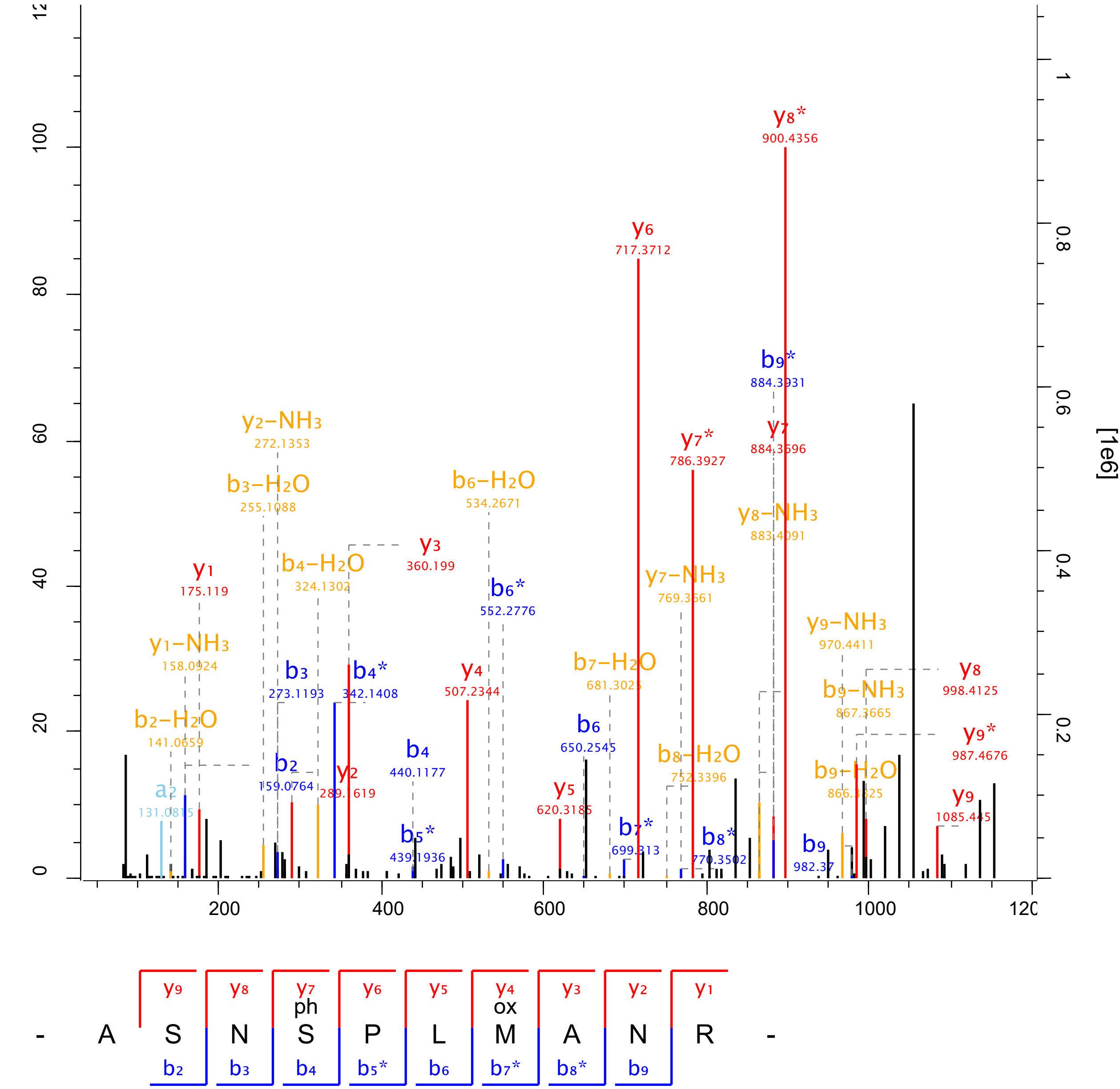

Raw file	Scan	Method	Score	m/z
sys_15_1	40941	FTMS; HCD	42	740.36

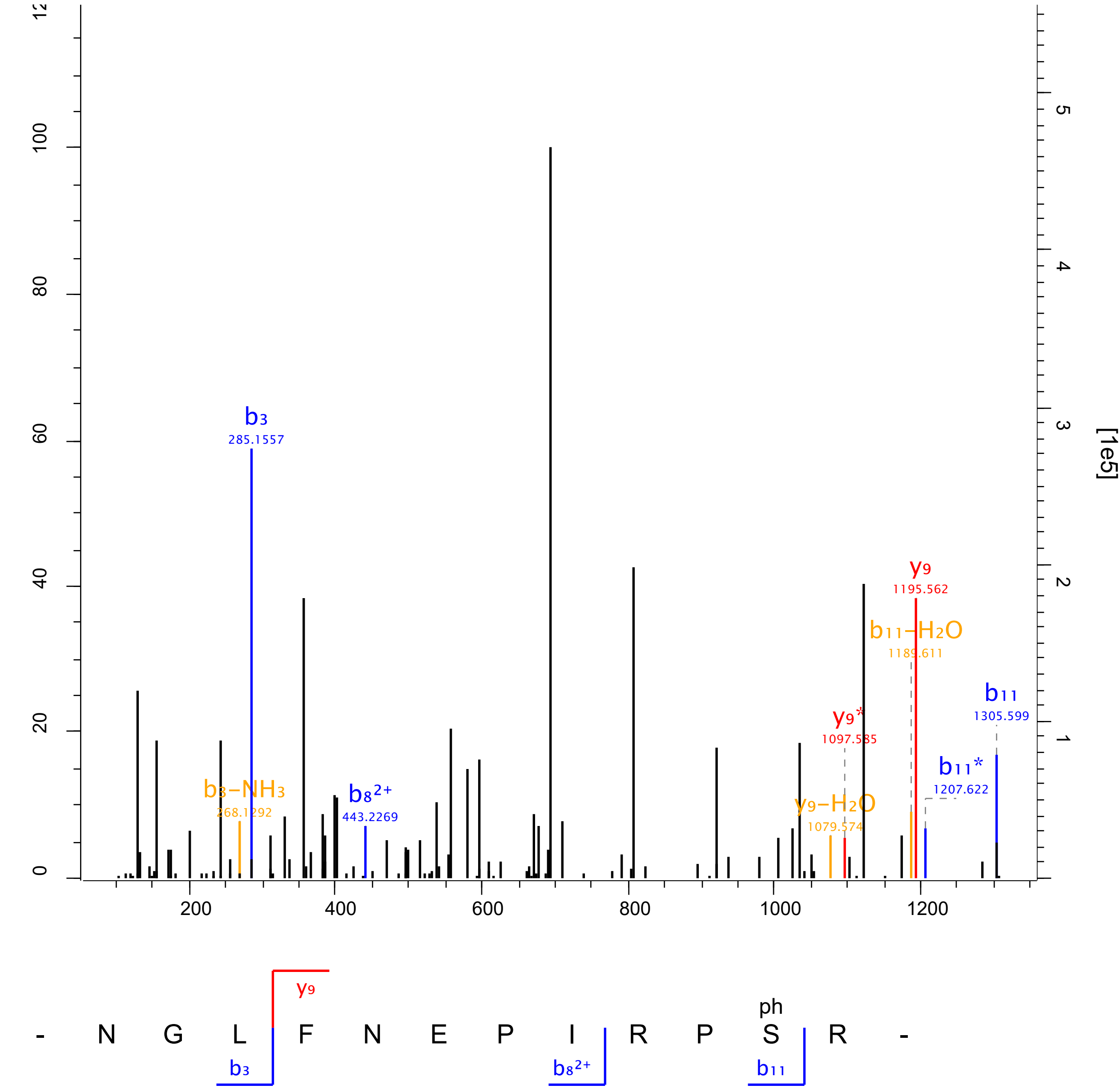

Raw file	Scan	Method	Score	m/z
sys_15_1	41018	FTMS; HCD	154.48	847.4

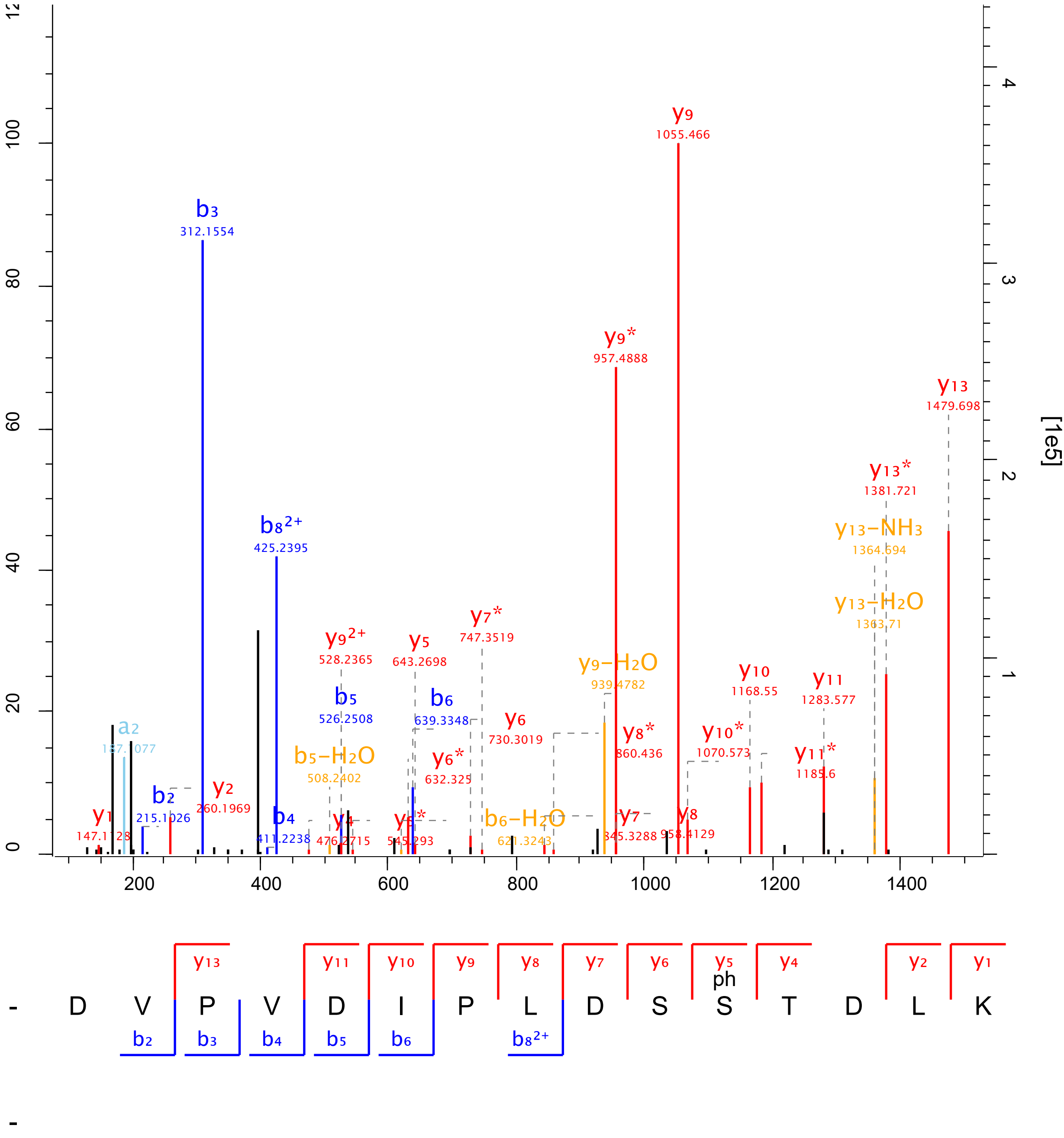

Raw file	Scan	Method	Score	m/z
sys_15_1	41058	FTMS; HCD	72.43	816.35

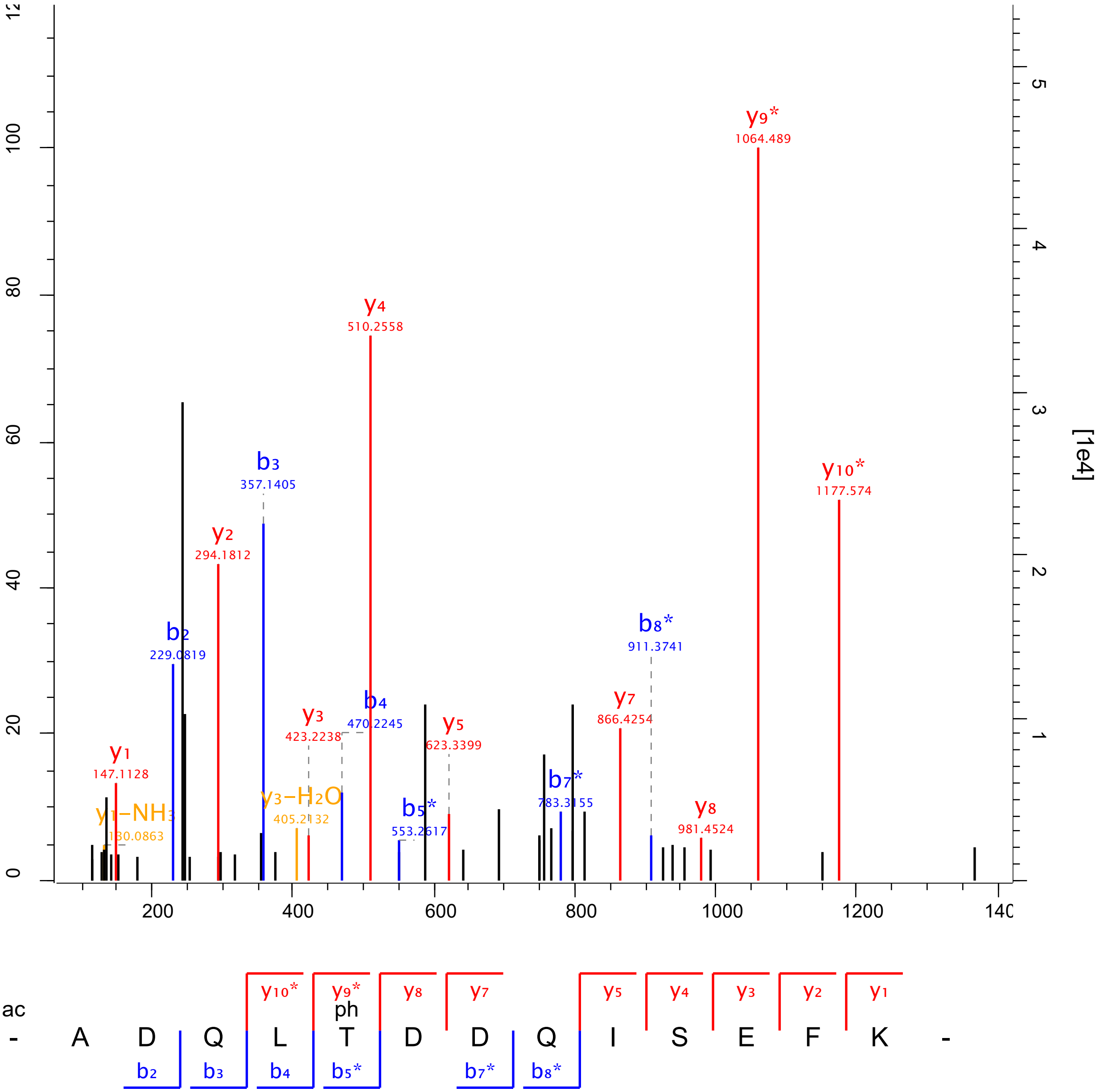

Raw file	Scan	Method	Score	m/z
sys_15_1	41078	FTMS; HCD	96.79	800.41

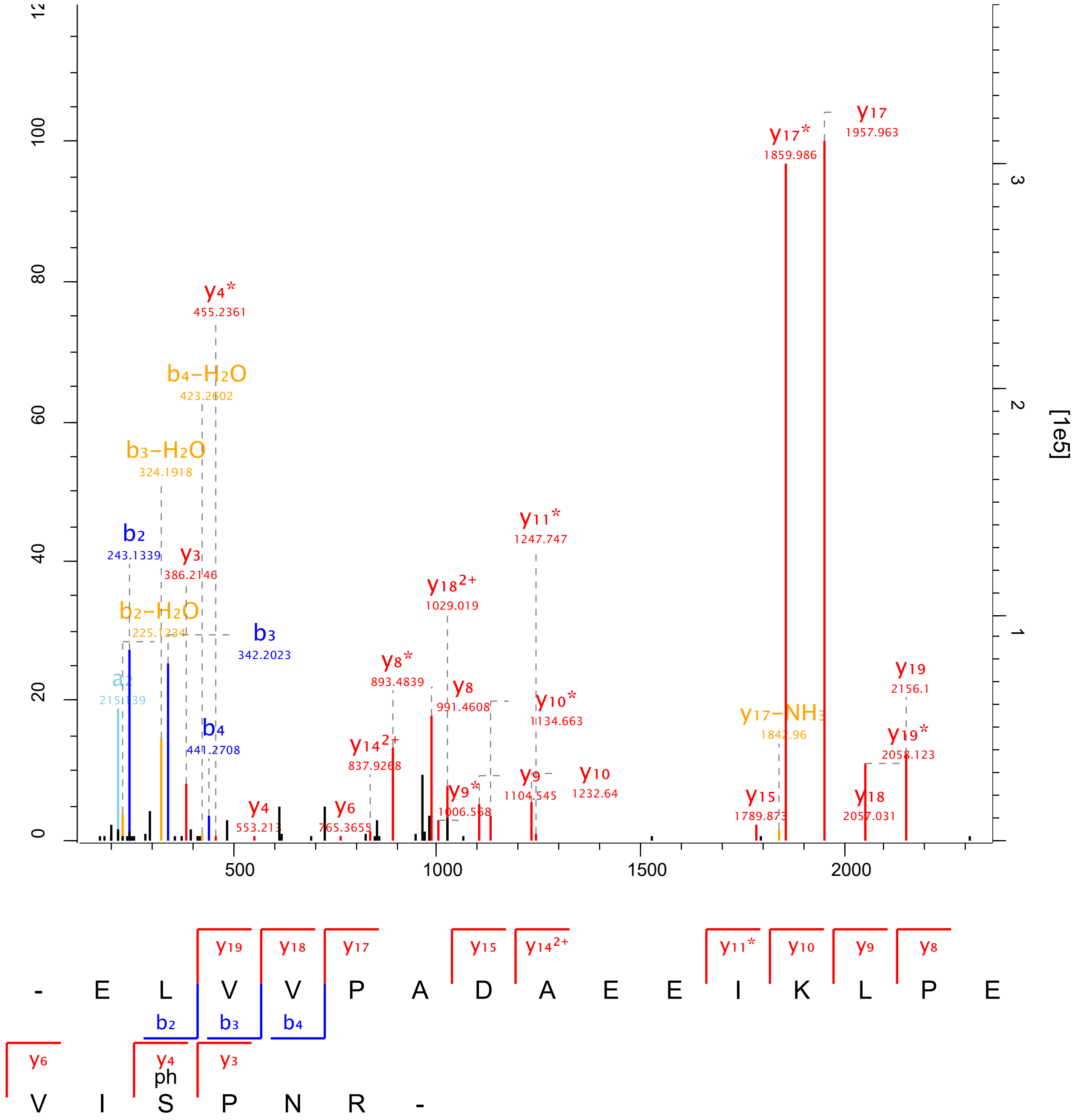

Raw file	Scan	Method	Score	m/z
sys_15_1	41706	FTMS; HCD	68.58	1056.79

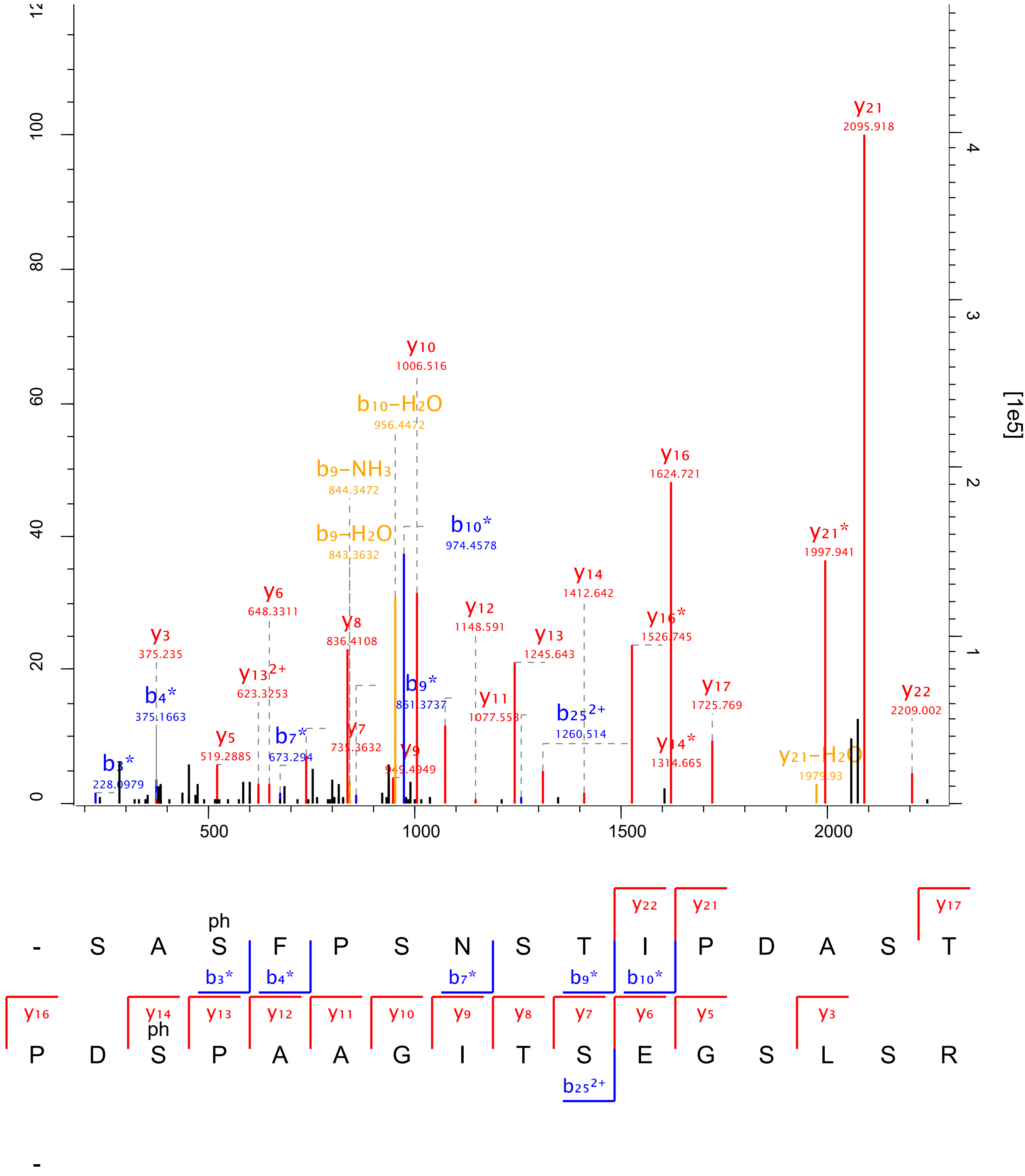

Raw file	Scan	Method	Score	m/z
sys_15_1	41892	FTMS; HCD	107.21	978

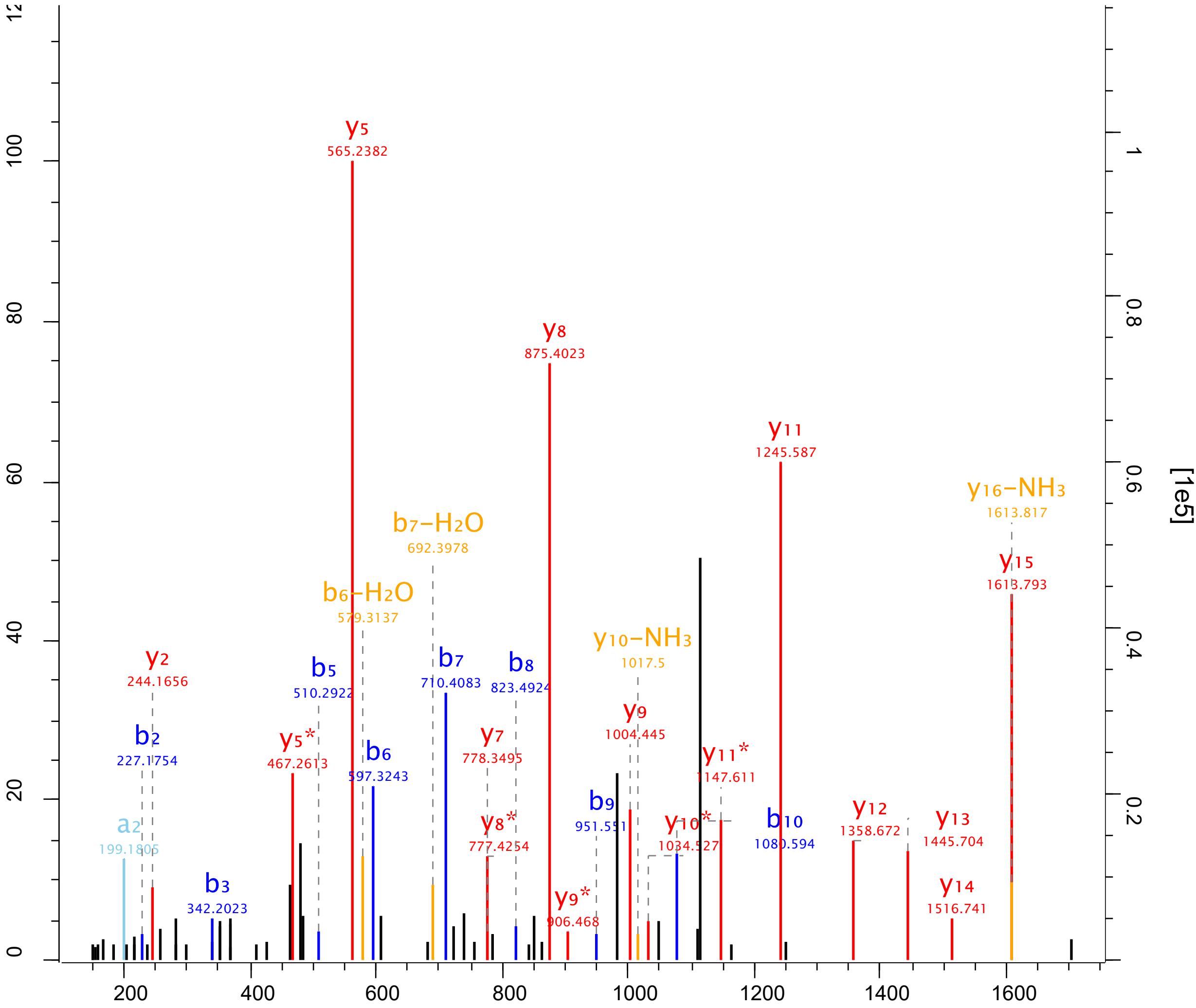

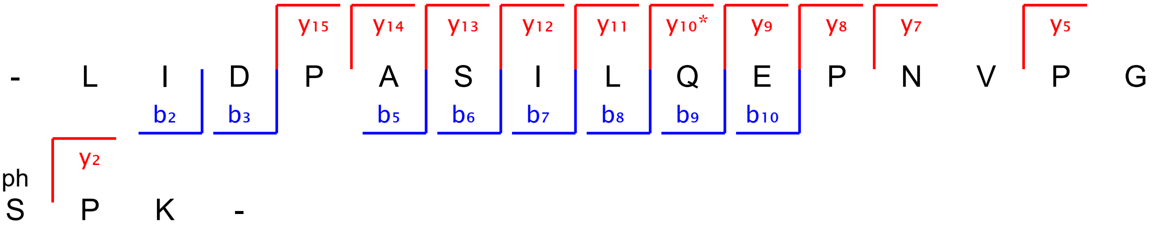

Raw file	Scan	Method	Score	m/z
sys_15_1	4205	FTMS; HCD	102.73	570.76

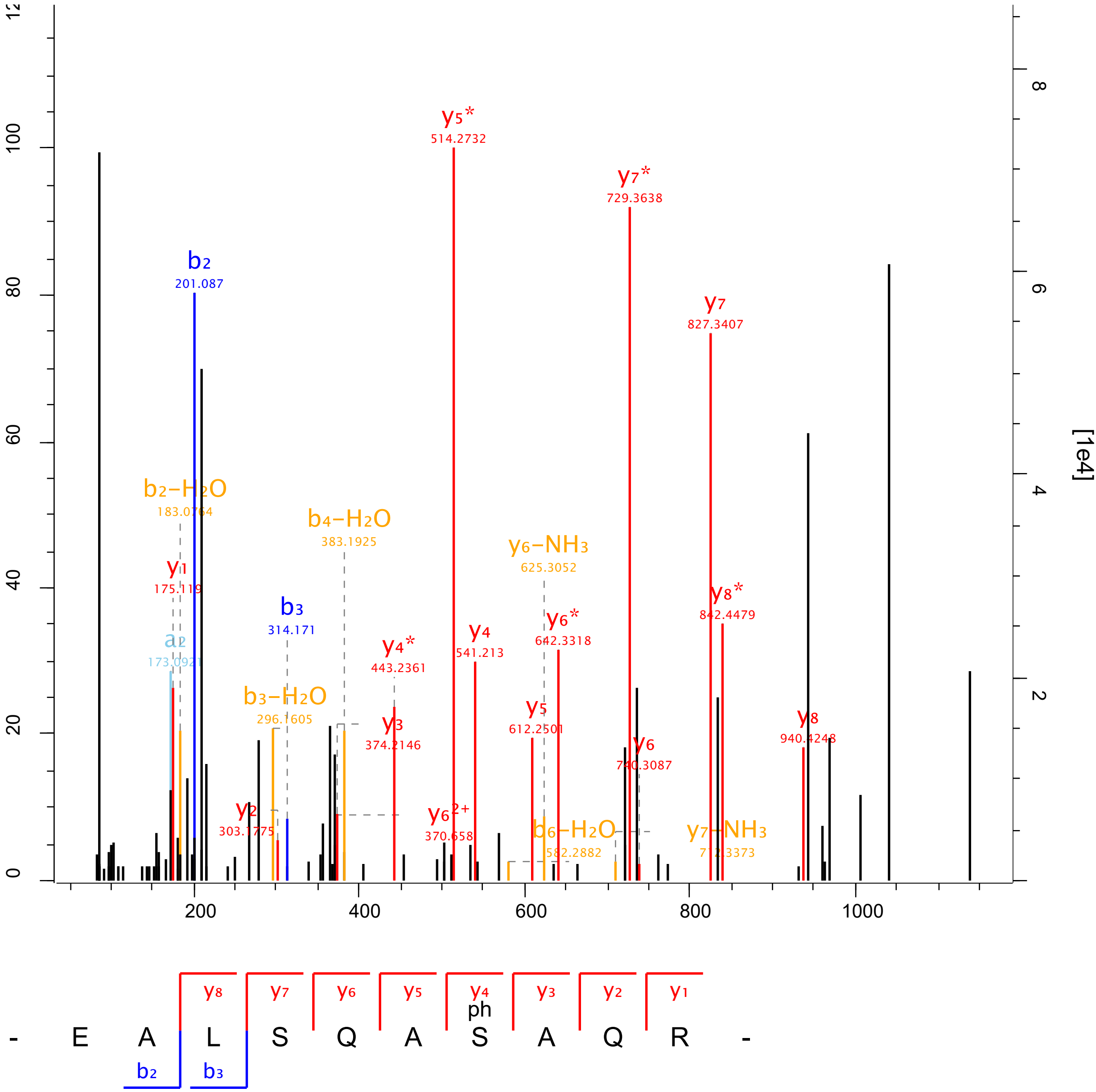

Raw file	Scan	Method	Score	m/z
sys_15_1	4227	FTMS; HCD	68.04	580.72

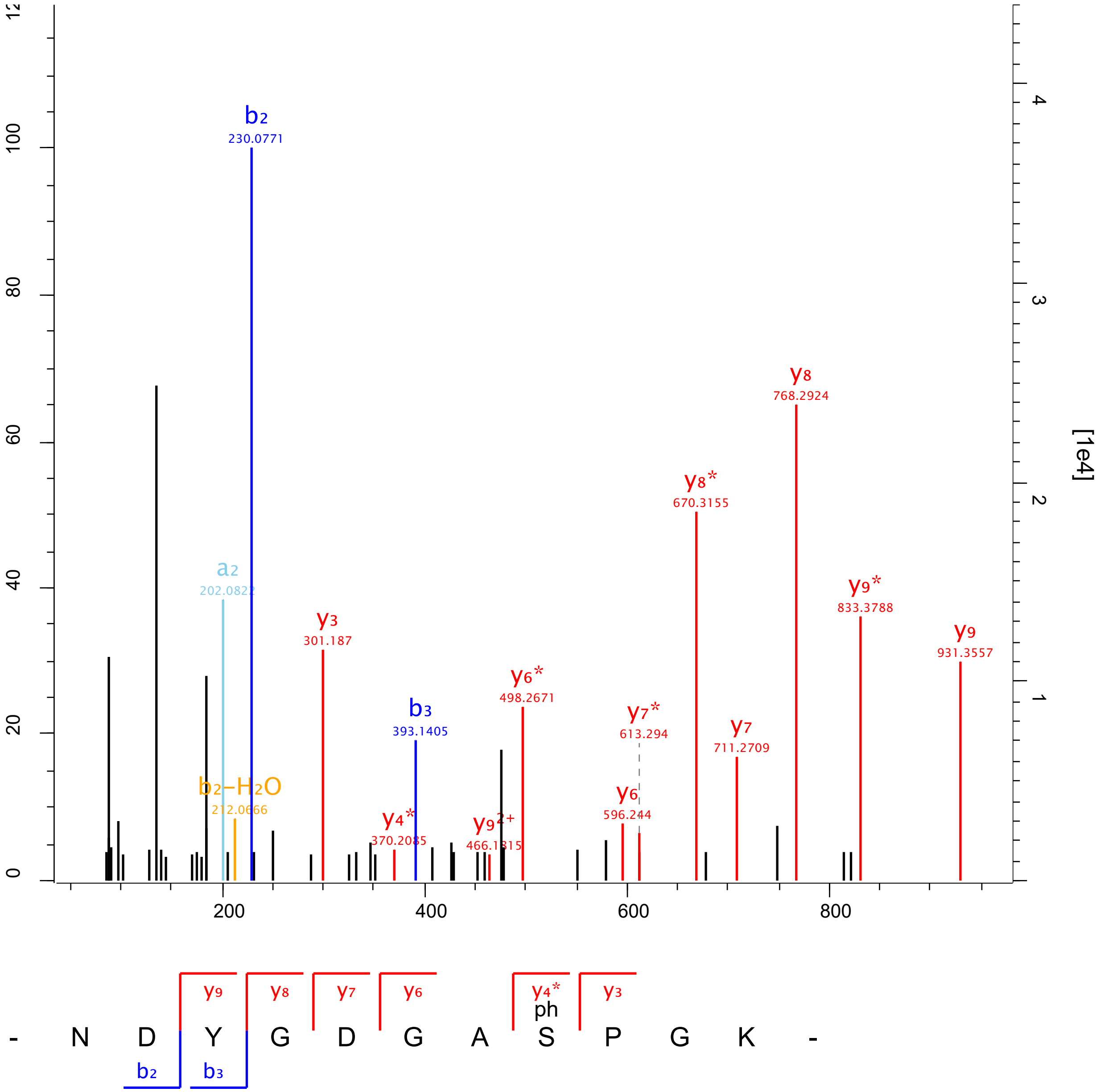

Raw file	Scan	Method	Score	m/z
sys_15_1	42328	FTMS; HCD	134.26	771.75

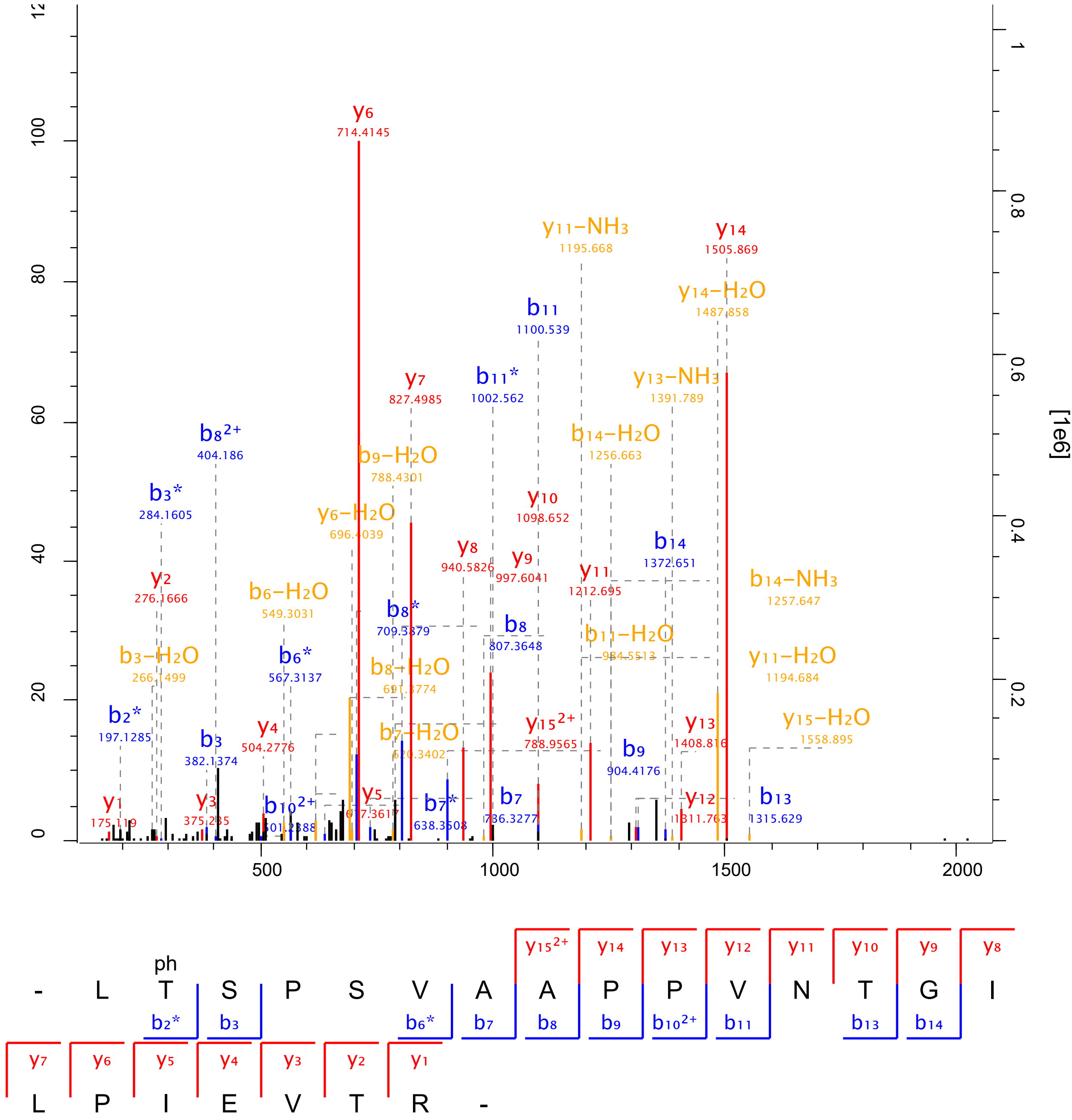

Raw file	Scan	Method	Score	m/z
sys_15_1	4244	FTMS; HCD	49.3	549.23

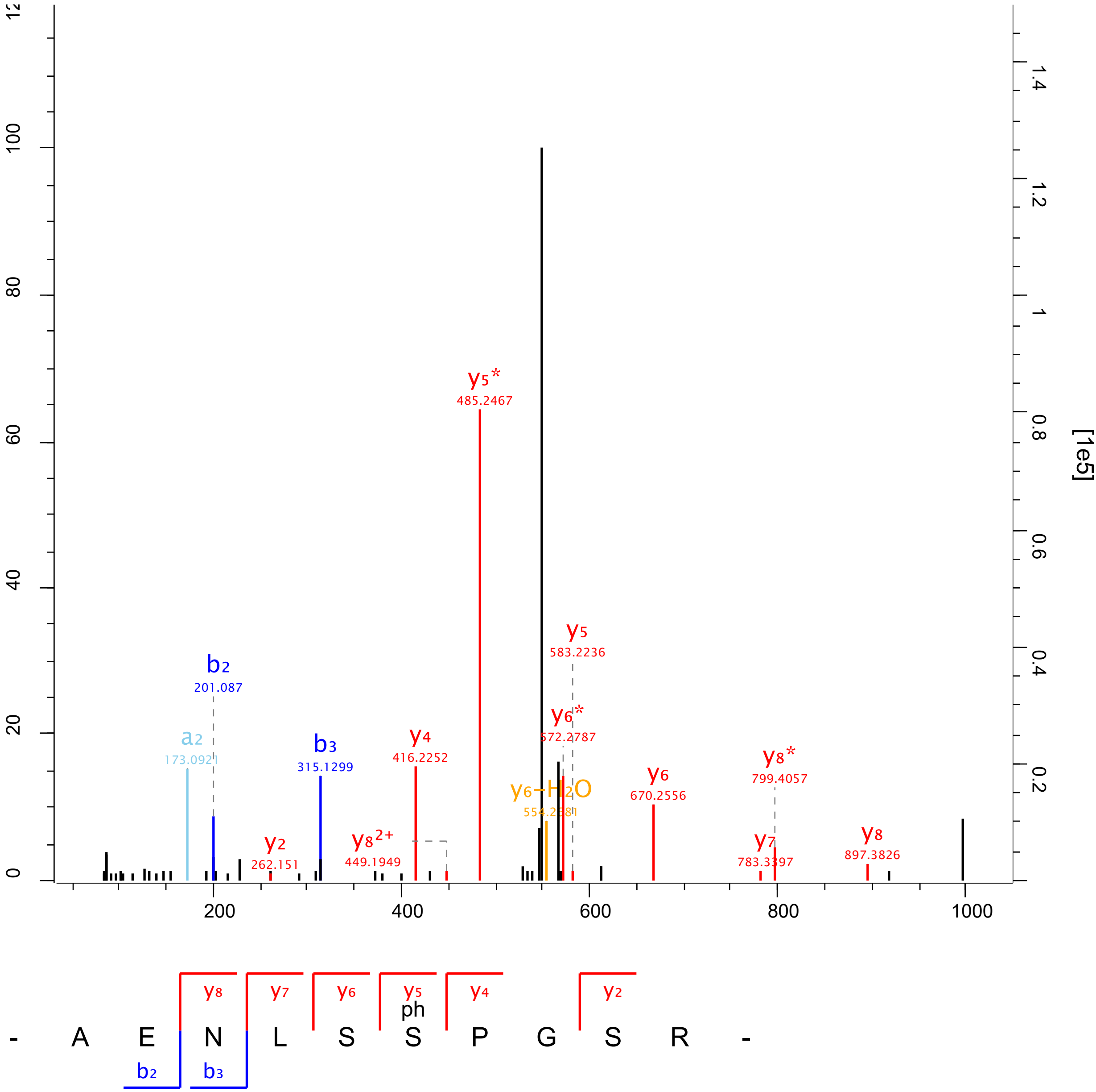

Raw file	Scan	Method	Score	m/z
sys_15_1	4256	FTMS; HCD	43.59	688.26

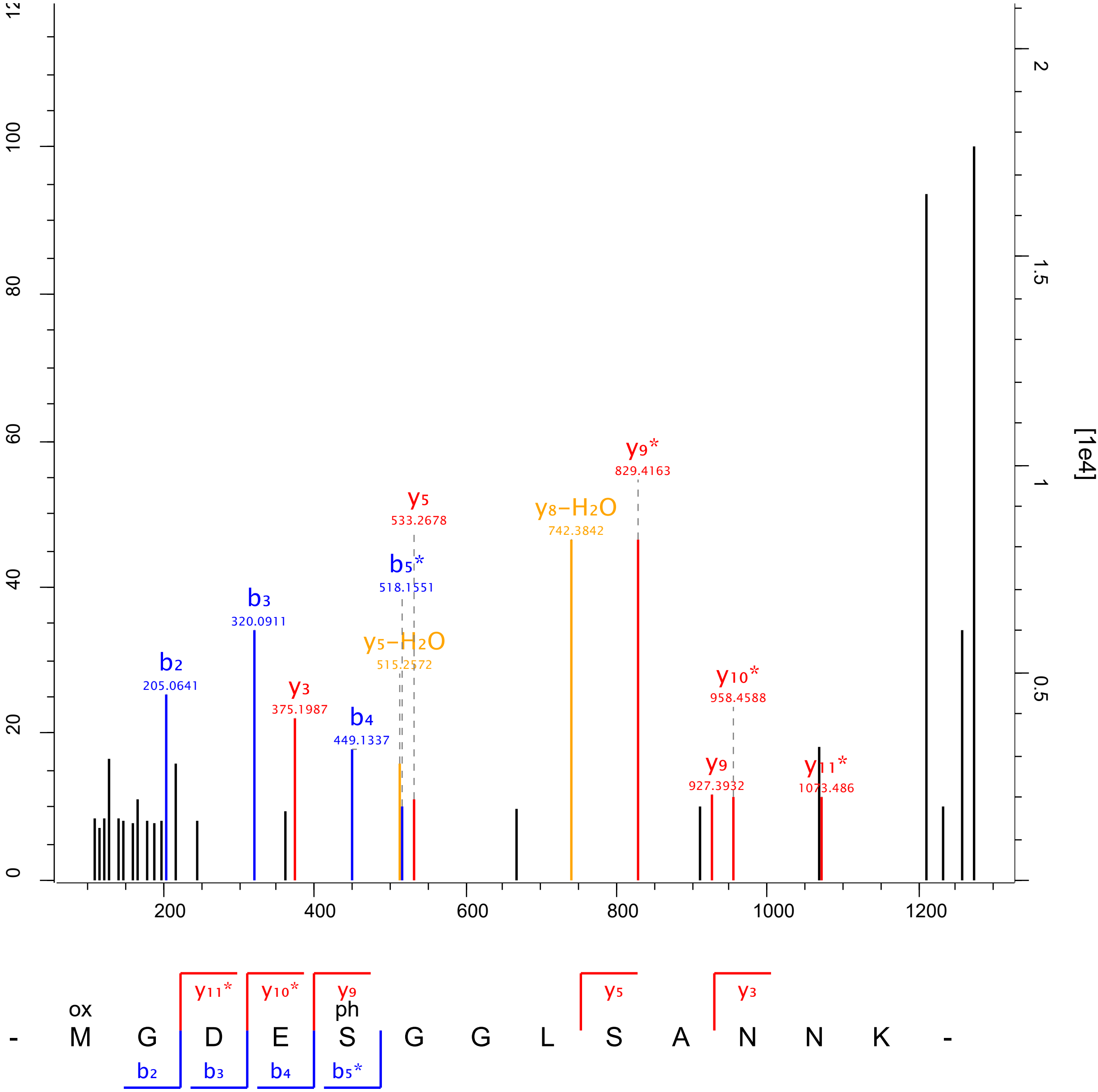

Raw file	Scan	Method	Score	m/z
sys_15_1	42613	FTMS; HCD	63.14	710.01

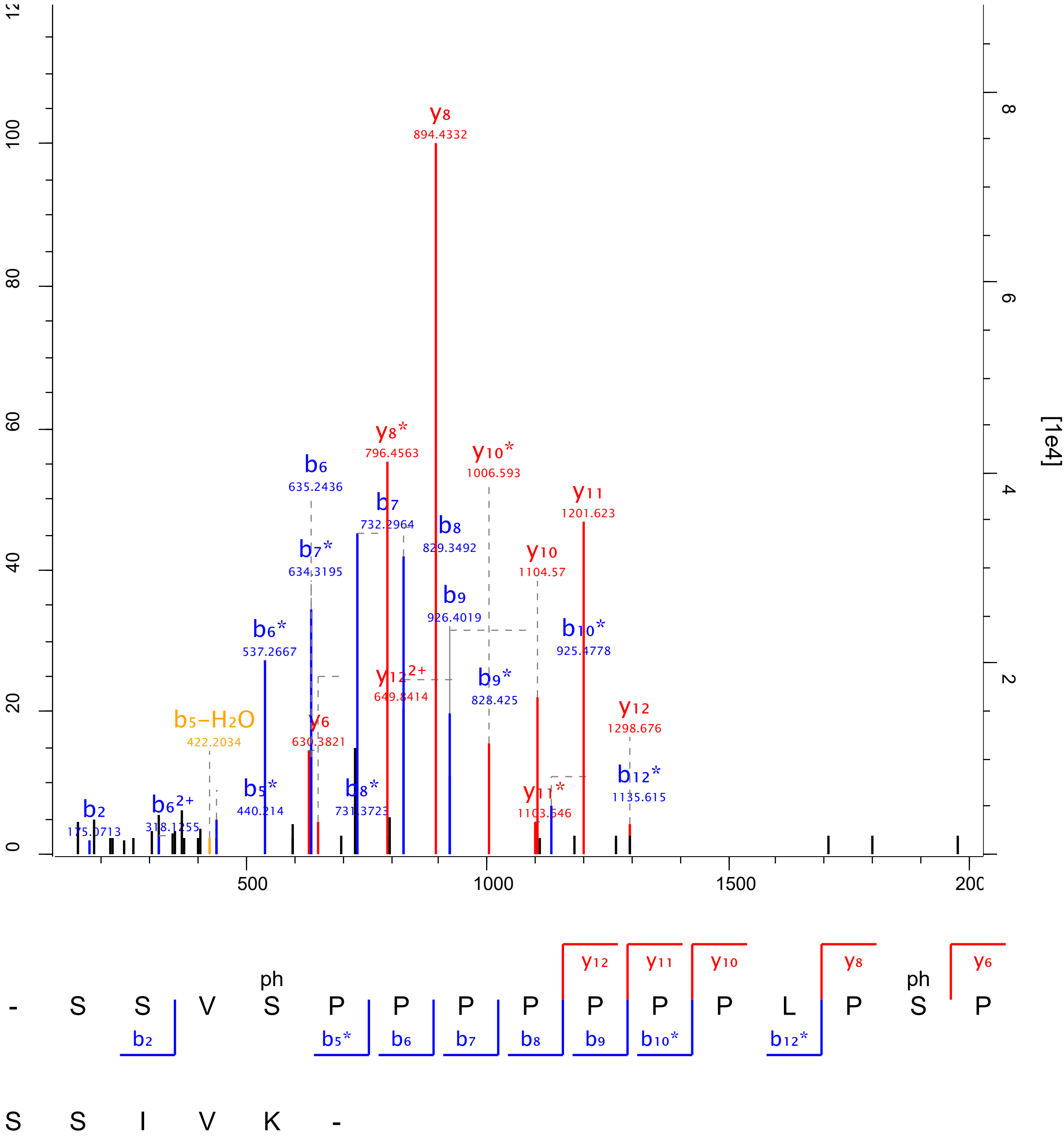

Raw file	Scan	Method	Score	m/z
sys_15_1	4294	FTMS; HCD	81.79	645.76

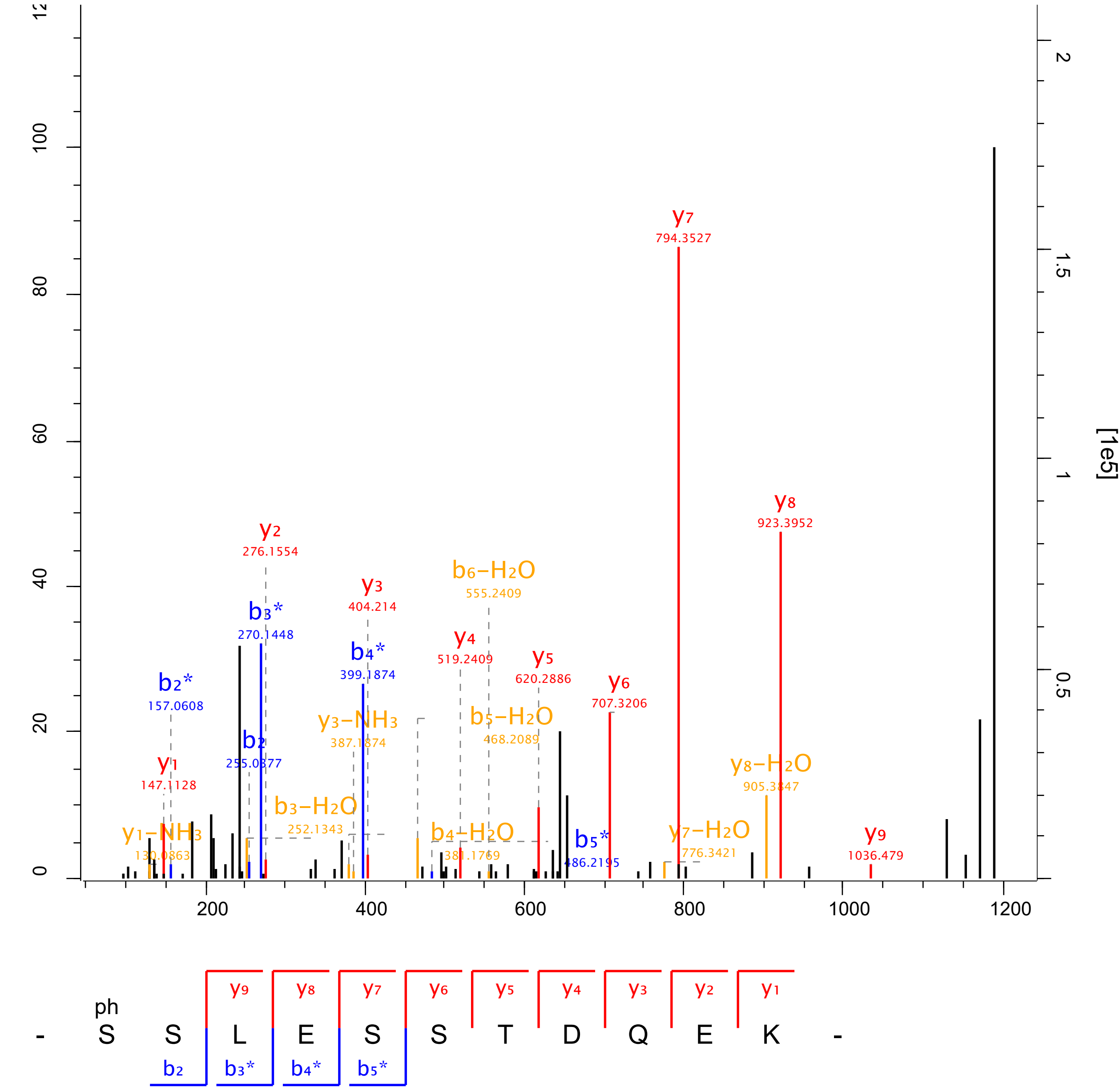

Raw file	Scan	Method	Score	m/z
sys_15_1	42954	FTMS; HCD	74.3	770.83

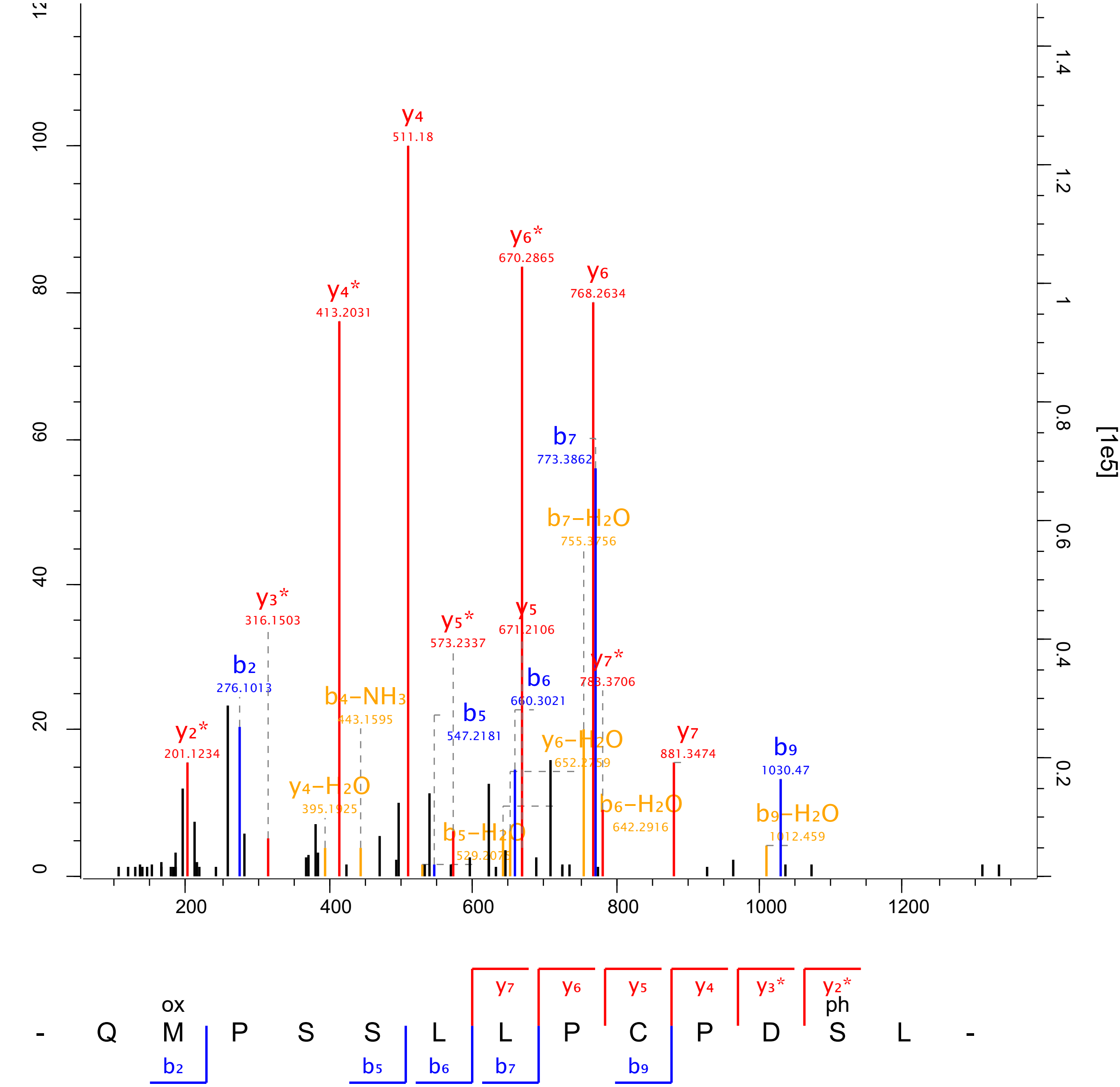

Raw file	Scan	Method	Score	m/z
sys_15_1	43087	FTMS; HCD	178.32	1002.41

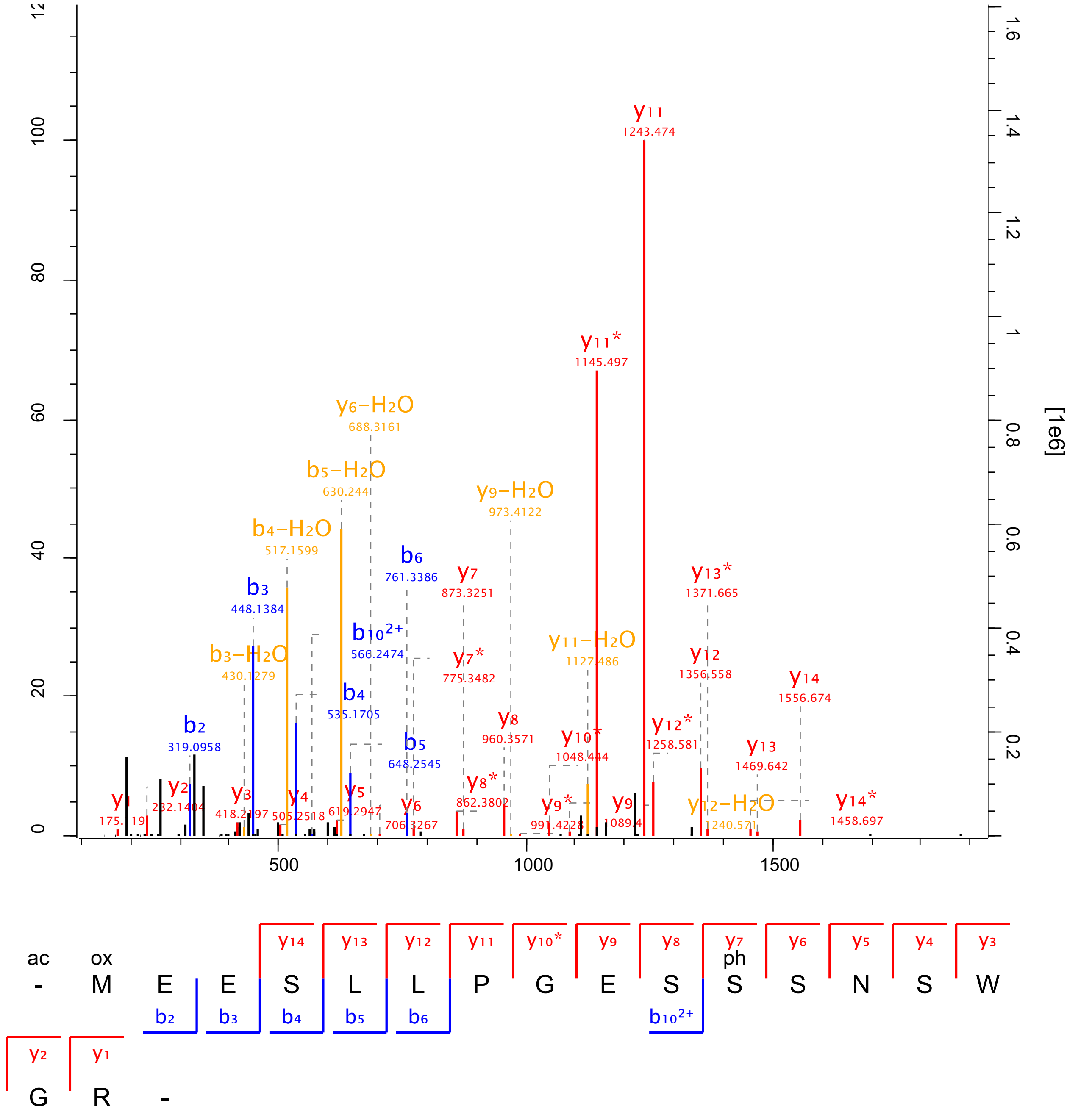

Raw file	Scan	Method	Score	m/z
sys_15_1	4332	FTMS; HCD	80.69	563.2

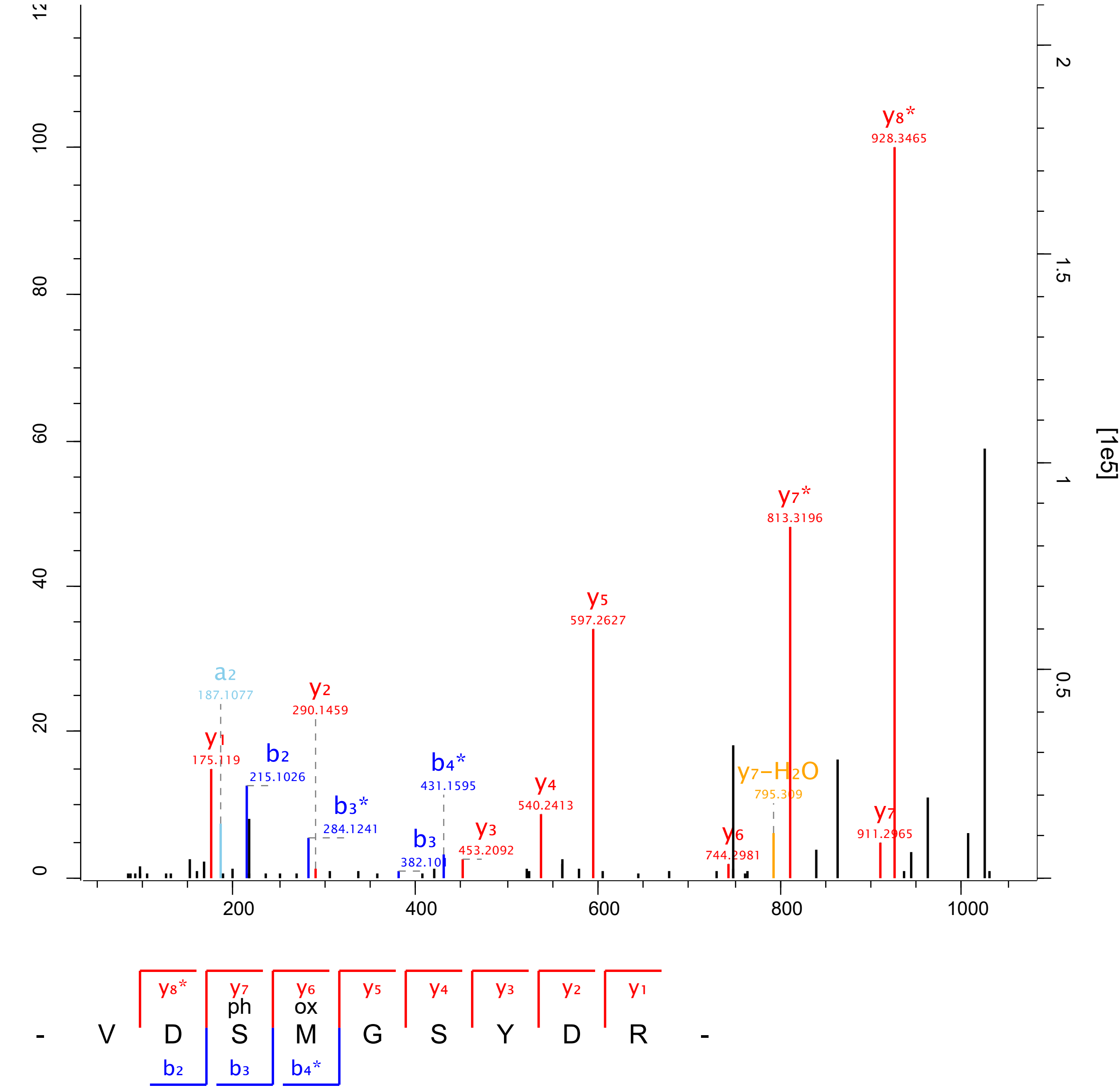

Raw file	Scan	Method	Score	m/z
sys_15_1	4338	FTMS; HCD	90.61	548.74

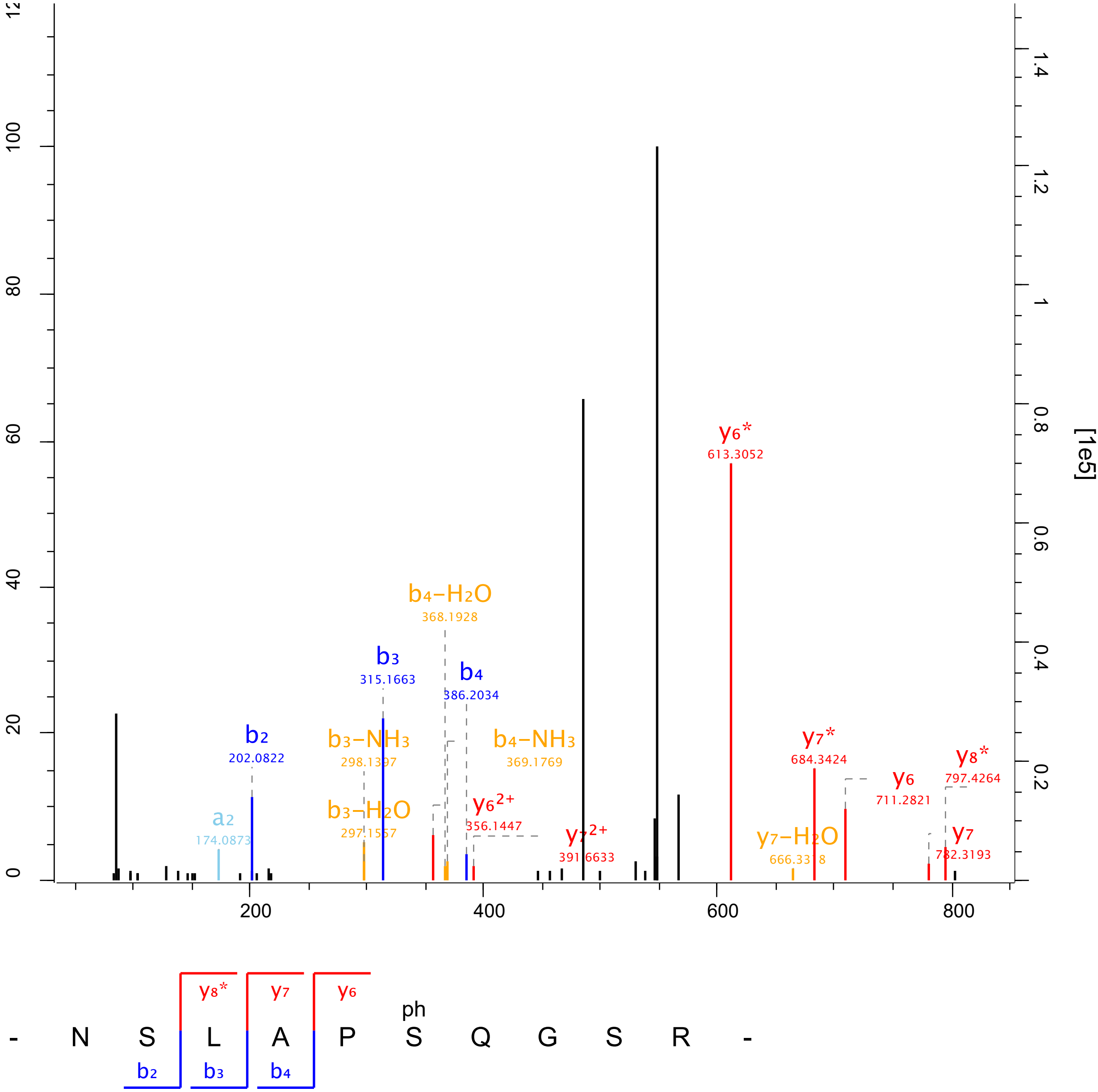

Raw file	Scan	Method	Score	m/z
sys_15_1	43423	FTMS; HCD	142.45	847.06

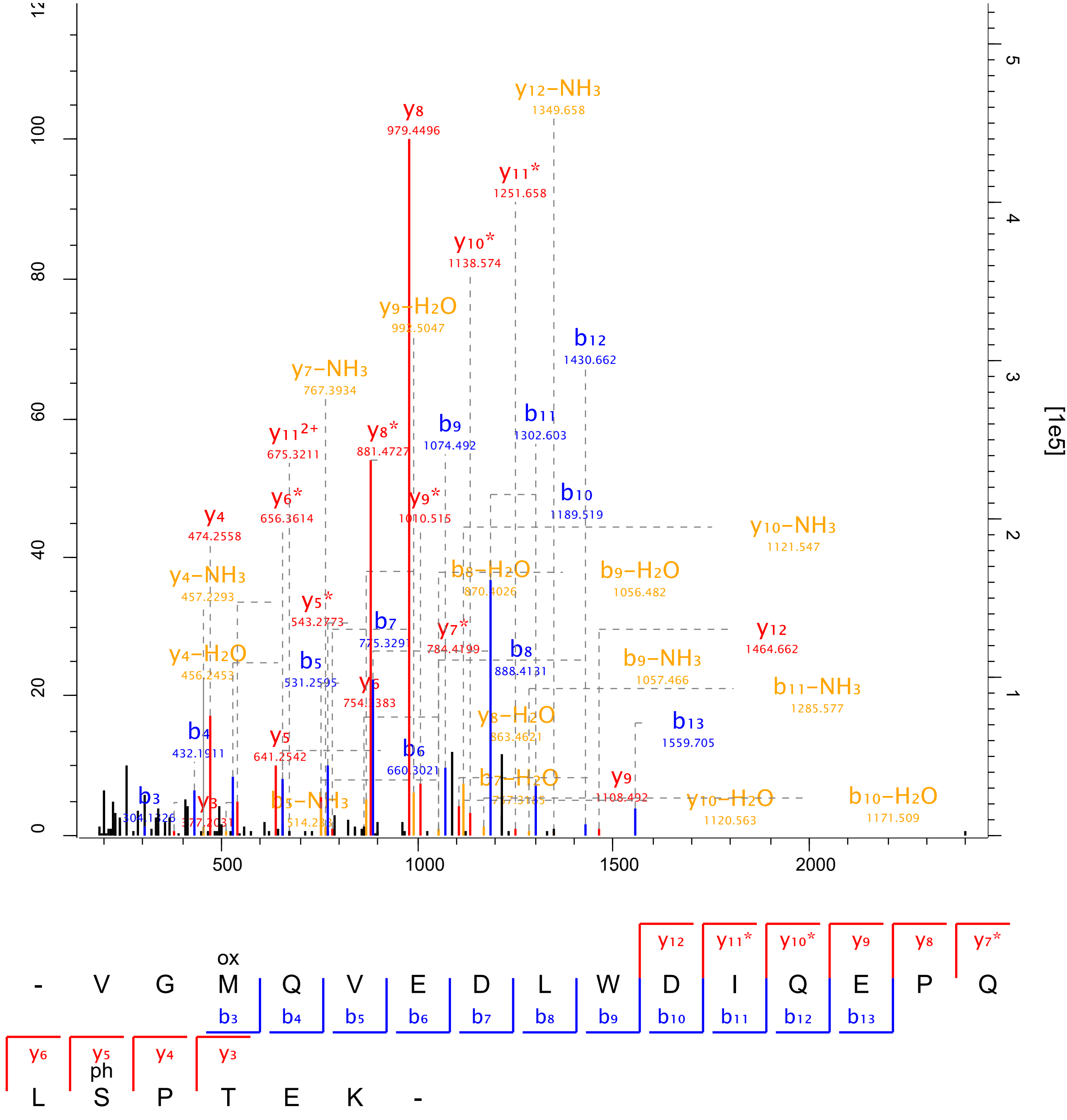

Raw file	Scan	Method	Score	m/z
sys_15_1	44236	FTMS; HCD	120.84	801.76

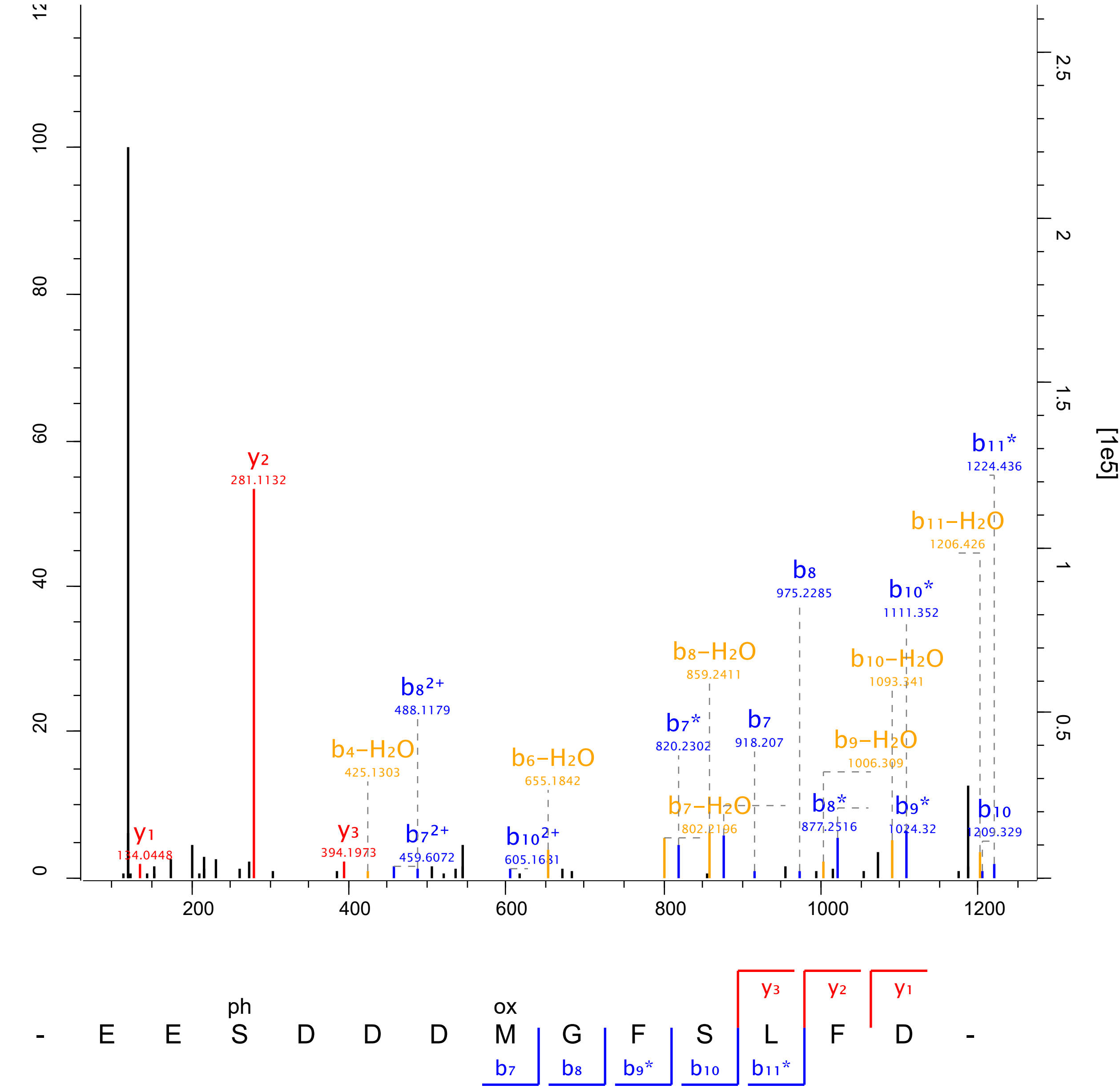

Raw file	Scan	Method	Score	m/z
sys_15_1	44495	FTMS; HCD	157.5	1014.13

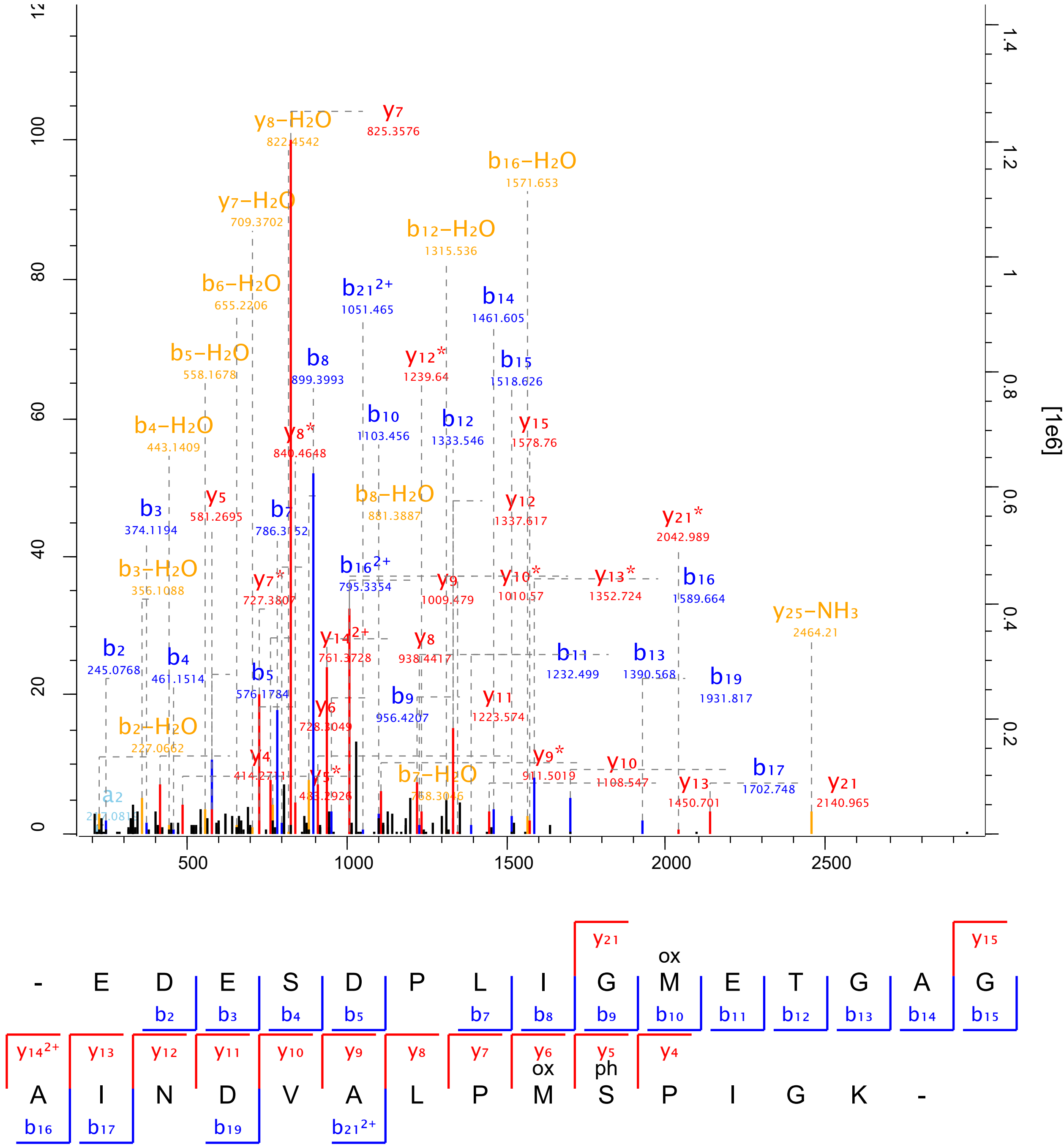

Raw file	Scan	Method	Score	m/z
sys_15_1	4501	FTMS; HCD	101.71	543.21

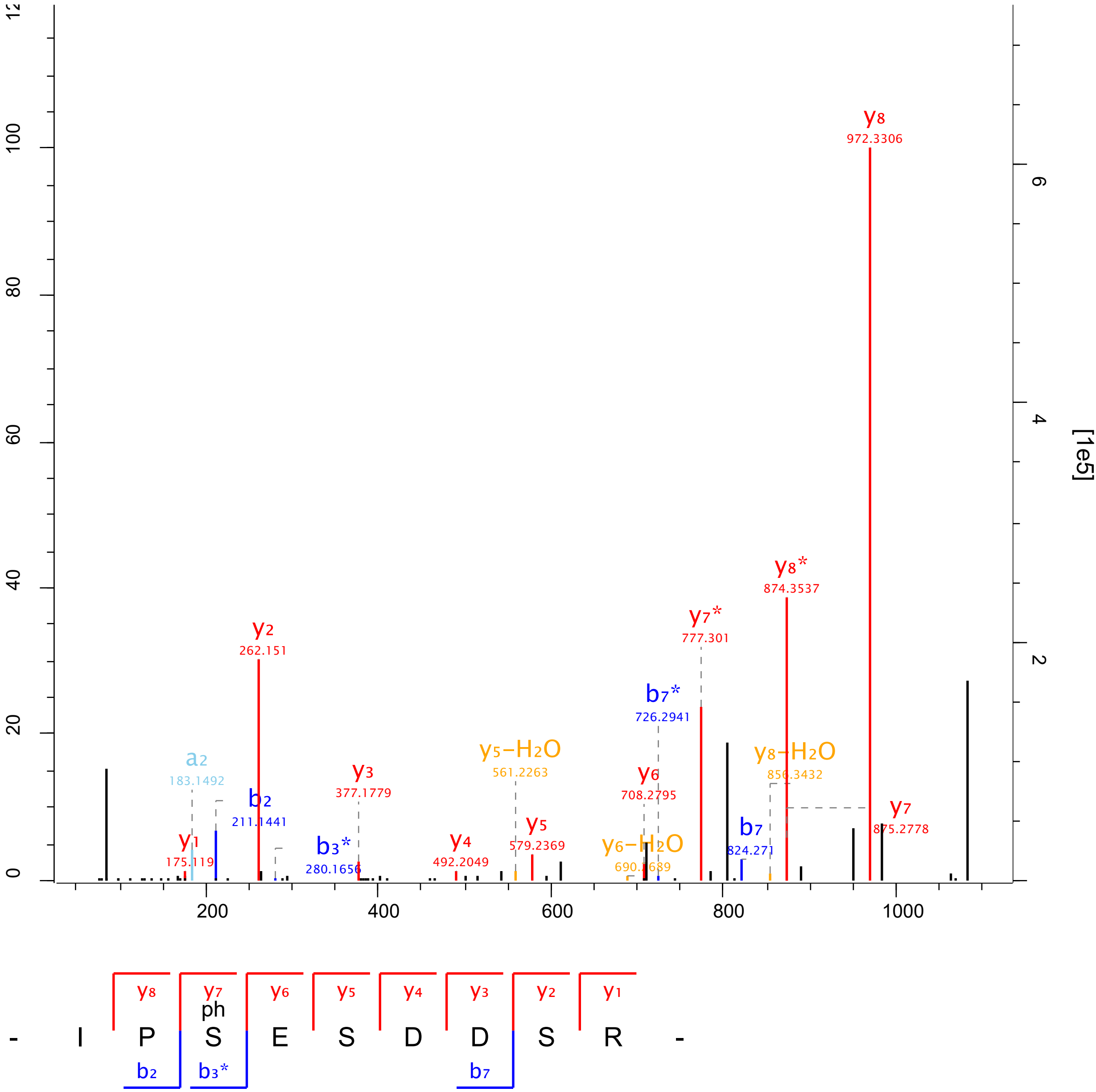

Raw file	Scan	Method	Score	m/z
sys_15_1	4553	FTMS; HCD	77.18	414.84

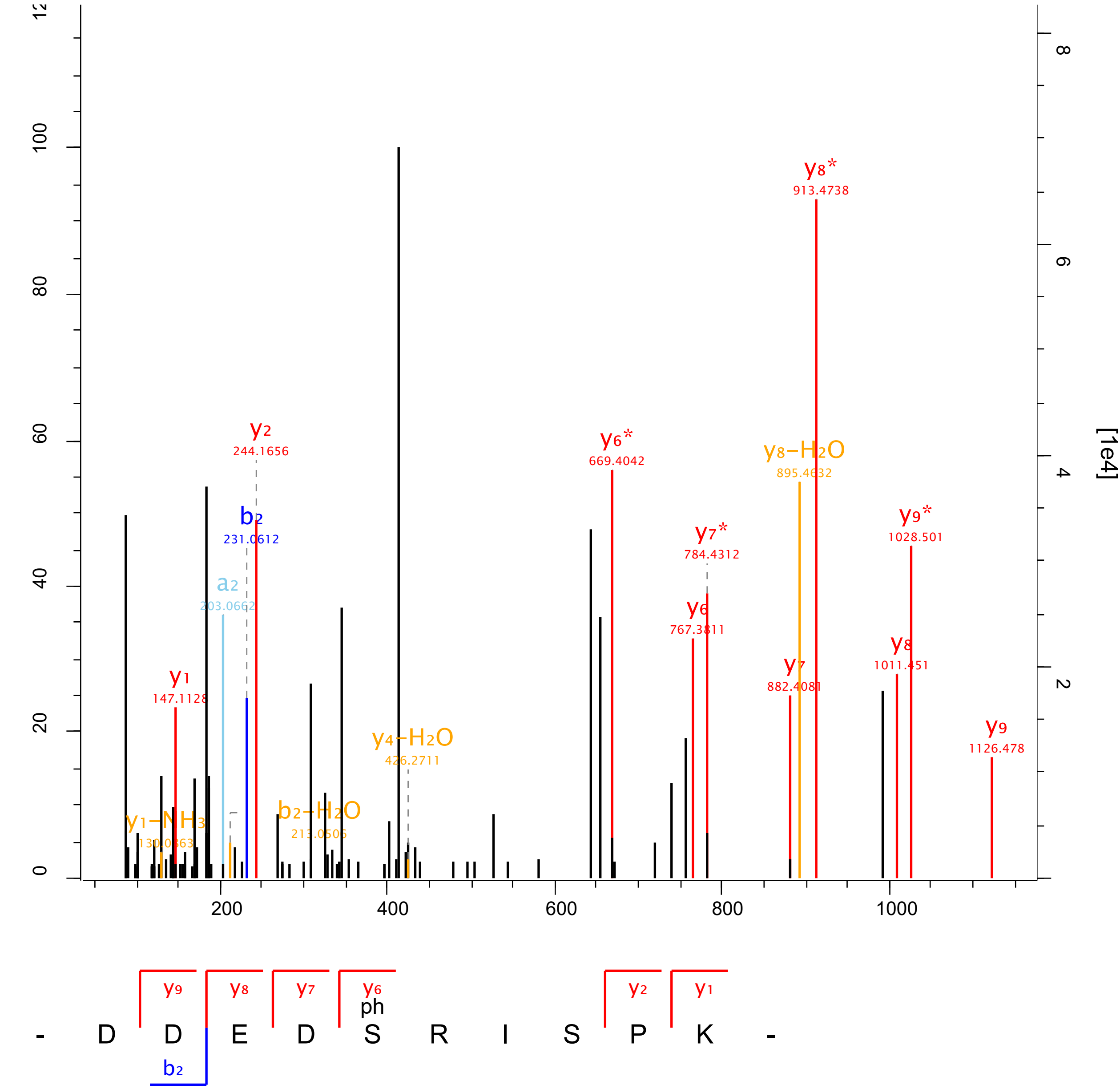

Raw file	Scan	Method	Score	m/z
sys_15_1	45702	FTMS; HCD	106.86	1044.39

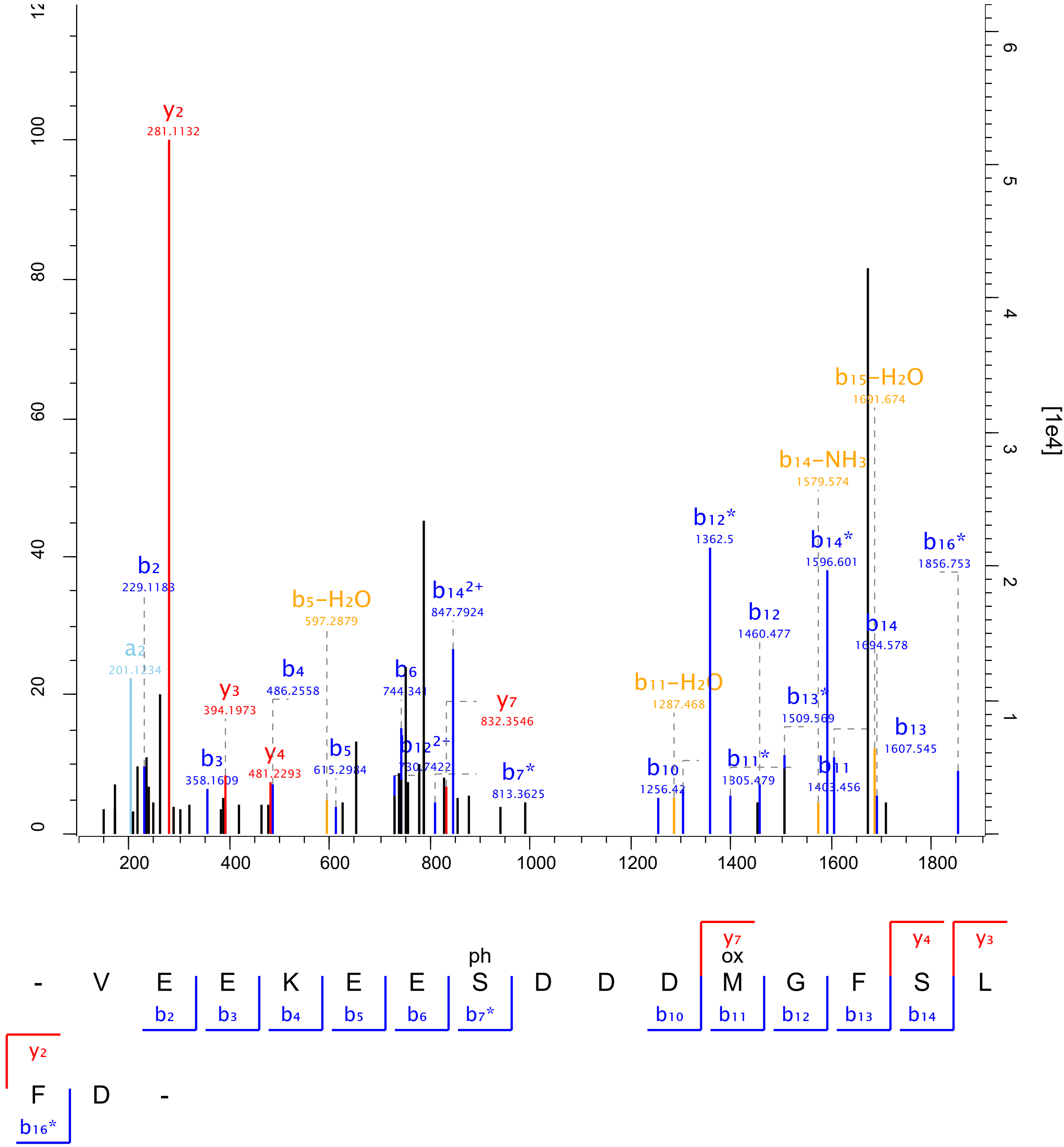

Raw file	Scan	Method	Score	m/z
sys_15_1	46699	FTMS; HCD	56.72	903.89

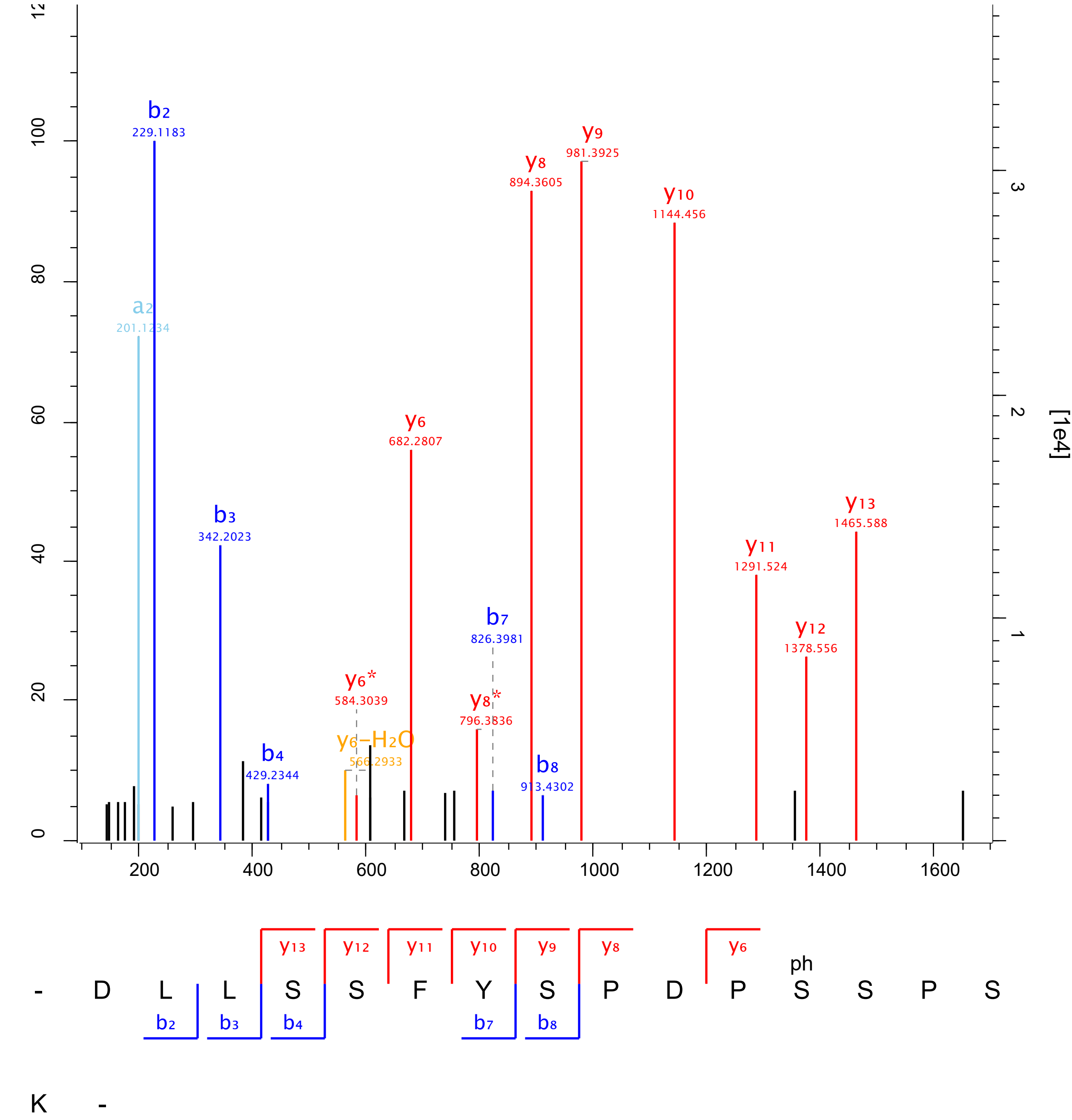

Raw file	Scan	Method	Score	m/z
sys_15_1	46904	FTMS; HCD	52.17	824.9

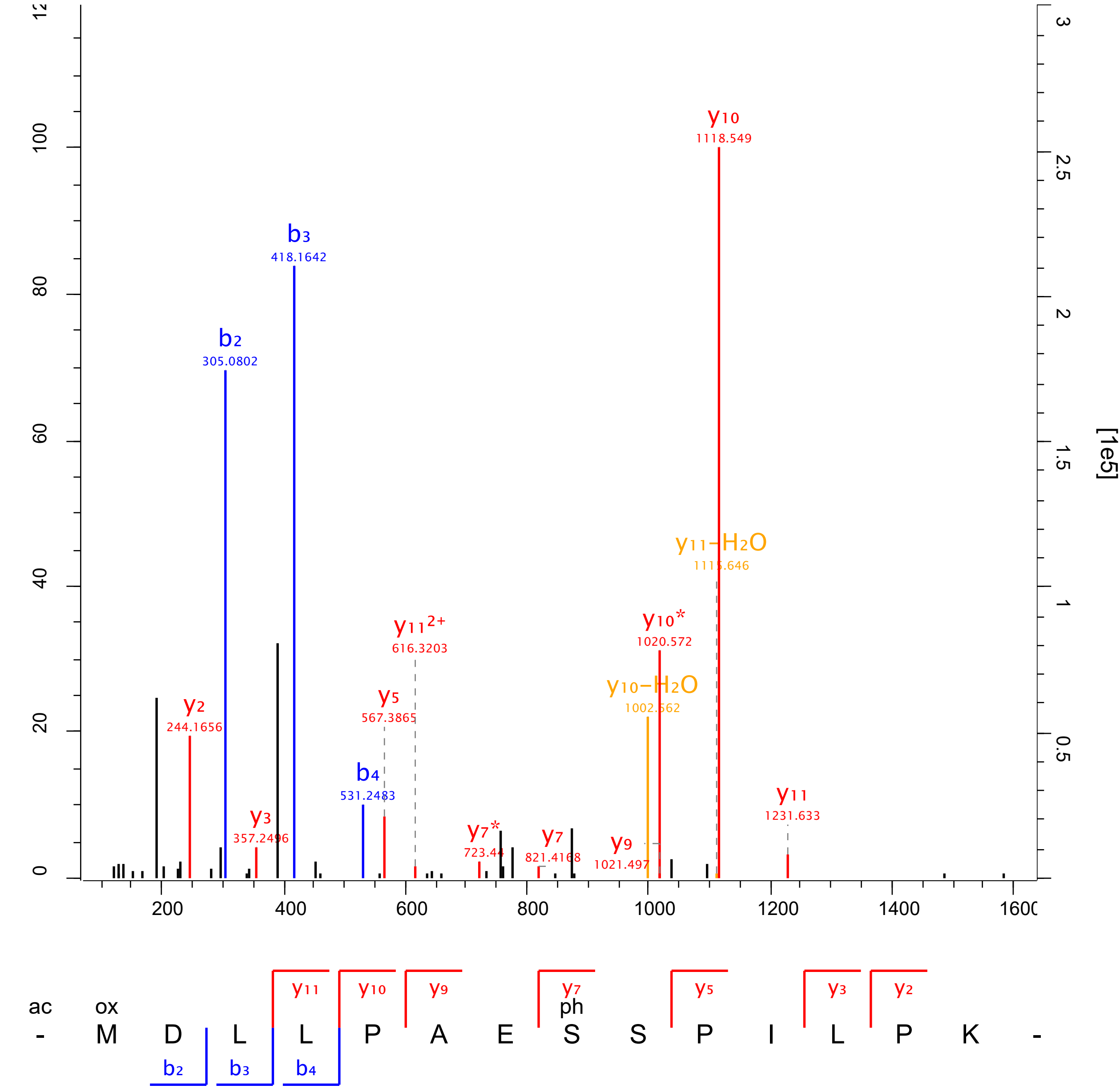

Raw file	Scan	Method	Score	m/z
sys_15_1	46923	FTMS; HCD	190.55	885.89

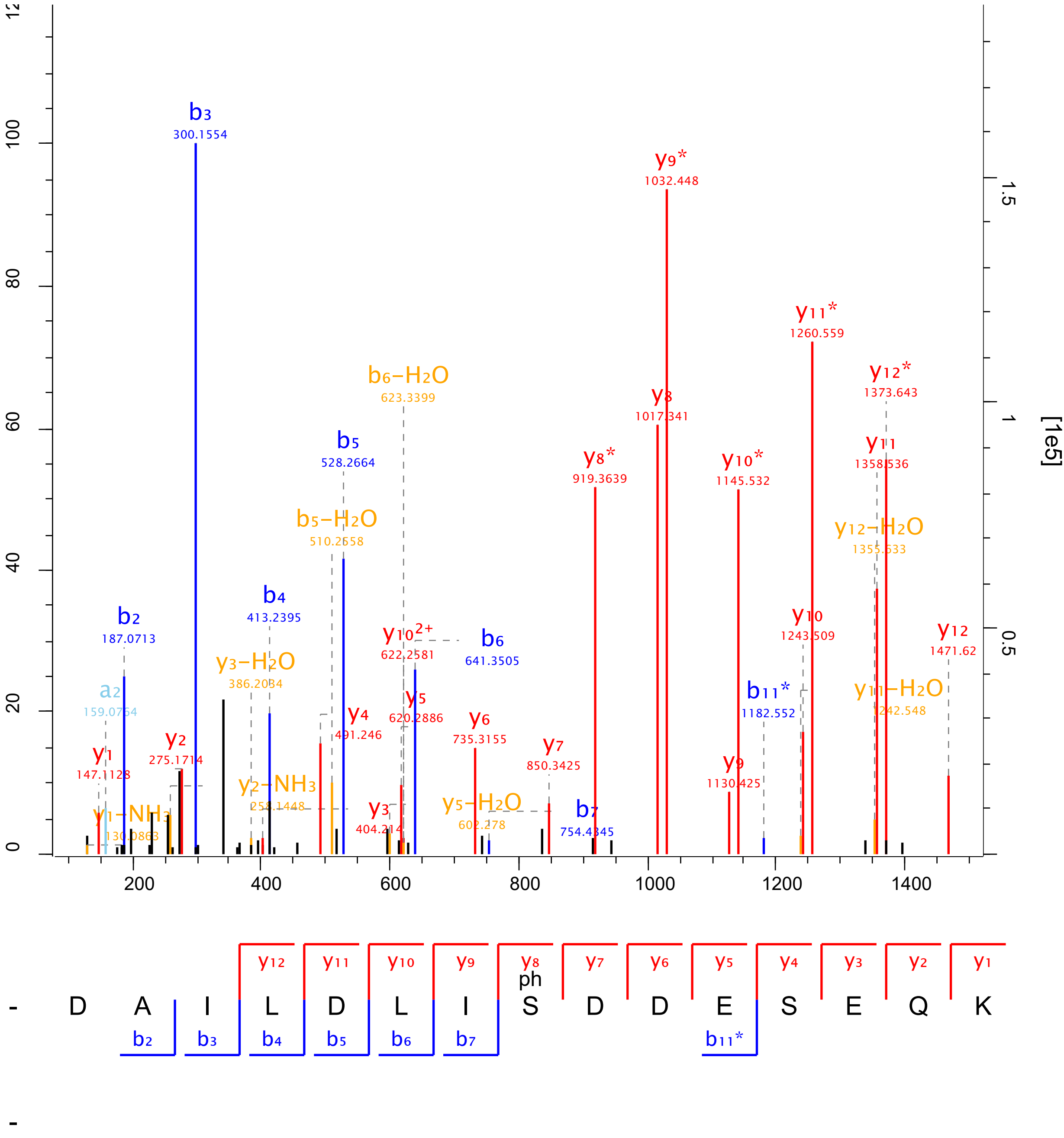

Raw file	Scan	Method	Score	m/z
sys_15_1	4711	FTMS; HCD	188.48	886.85

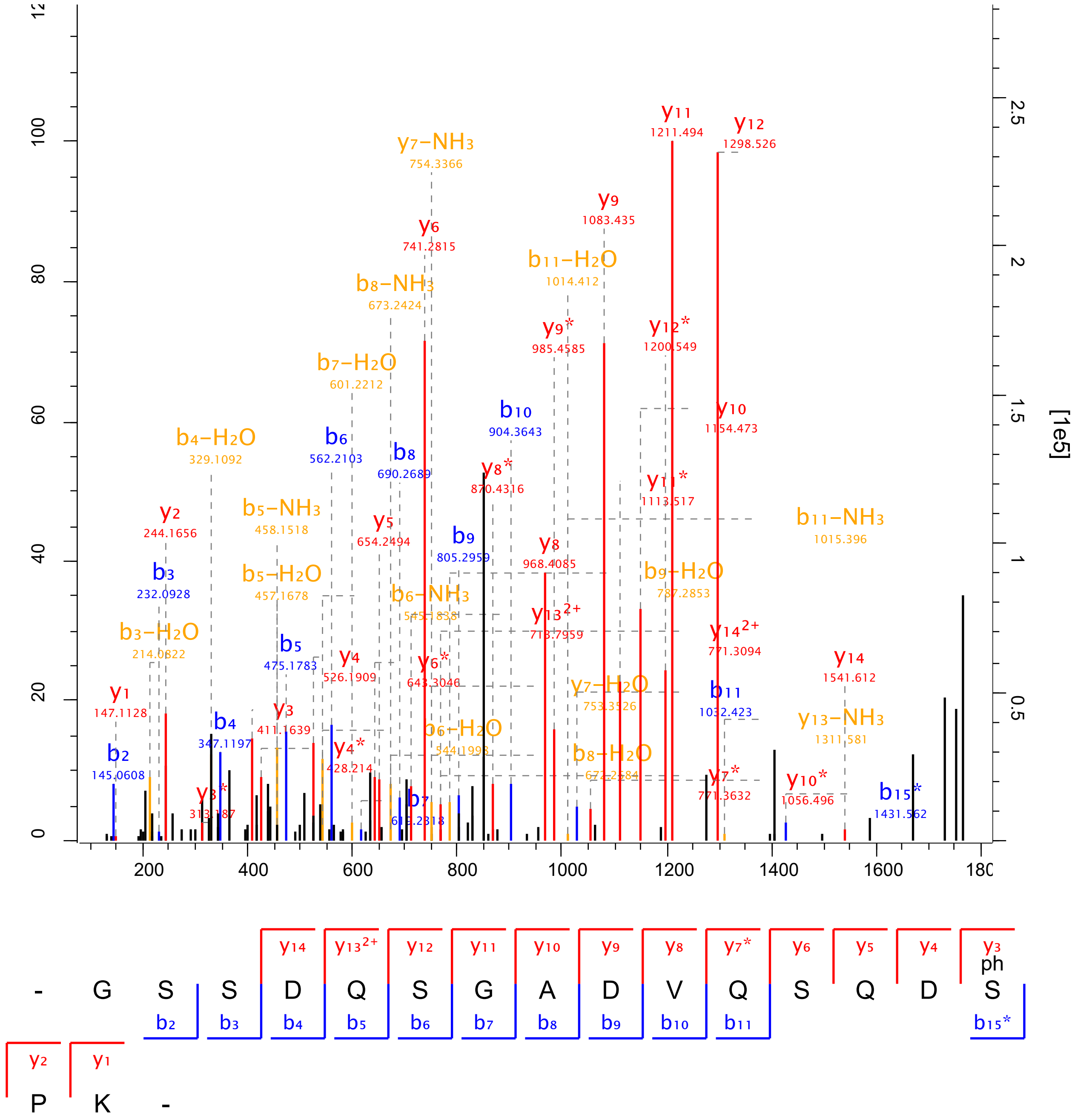

Raw file	Scan	Method	Score	m/z
sys_15_1	4723	FTMS; HCD	63.3	620.26

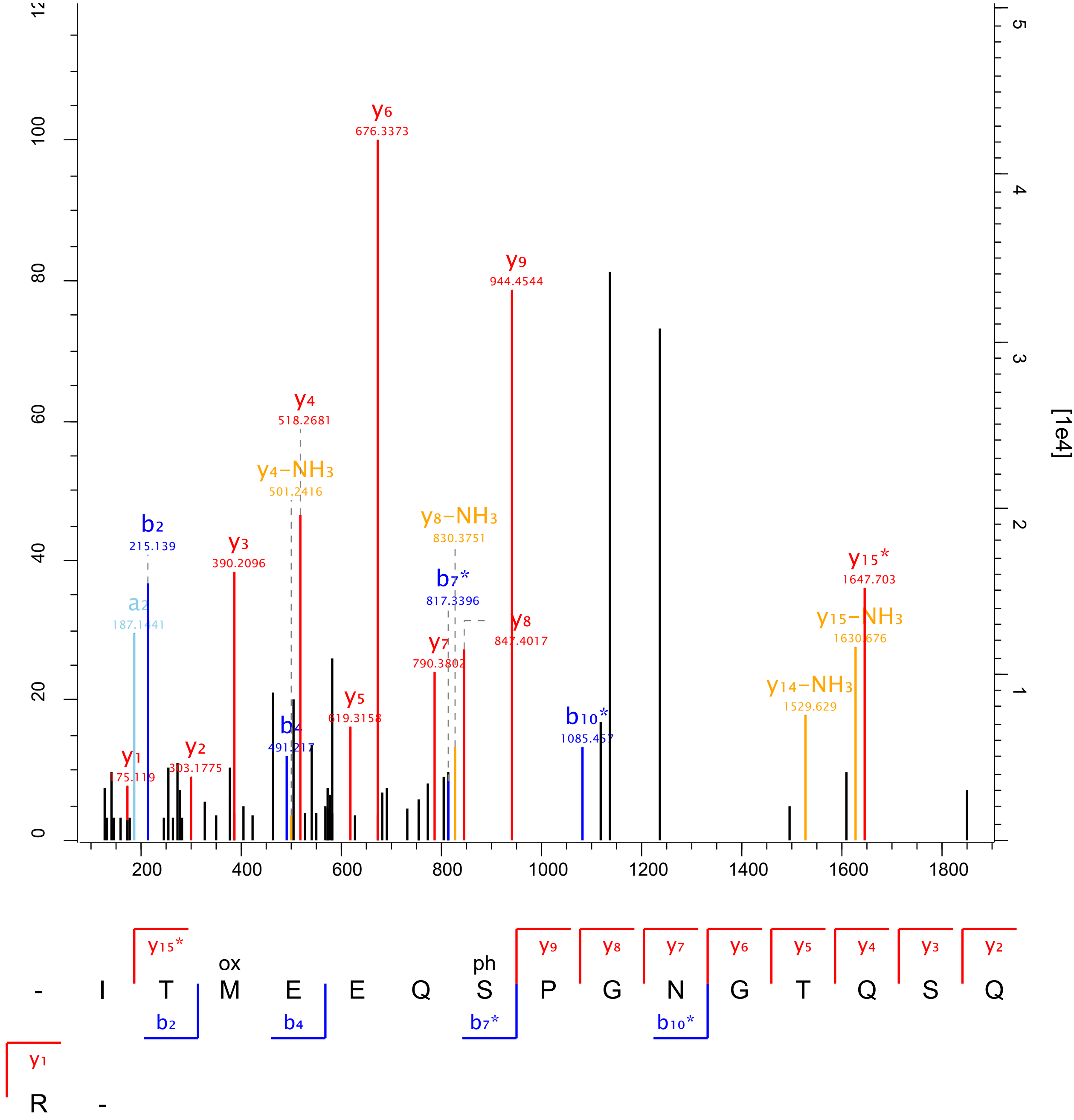

Raw file	Scan	Method	Score	m/z
sys_15_1	47430	FTMS; HCD	67.11	701.68

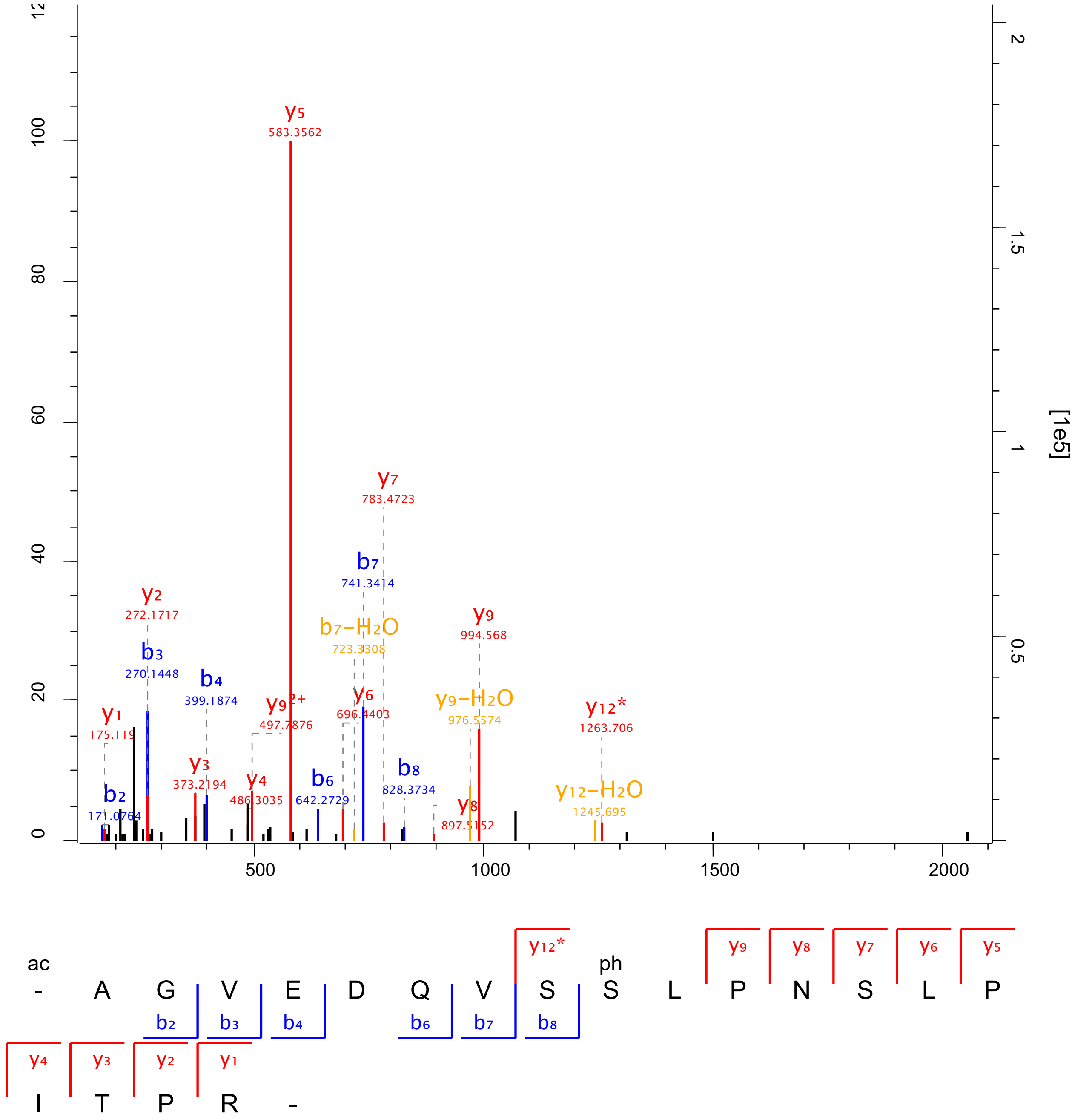

Raw file	Scan	Method	Score	m/z
sys_15_1	47474	FTMS; HCD	134.04	1043.38

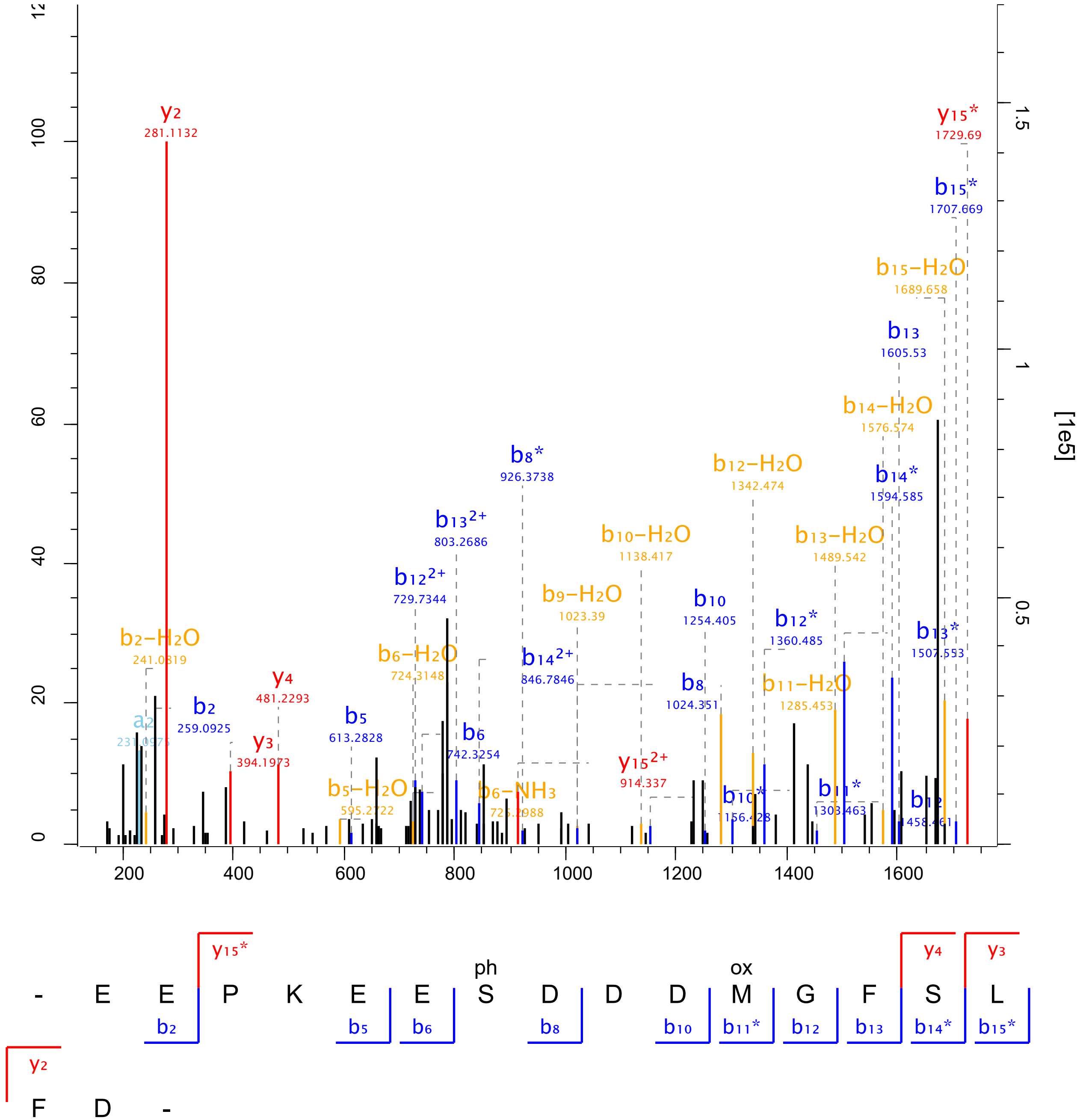

- E E P K E E S D D D M G F S L
F D -

Raw file	Scan	Method	Score	m/z
sys_15_1	4748	FTMS; HCD	78.33	668.78

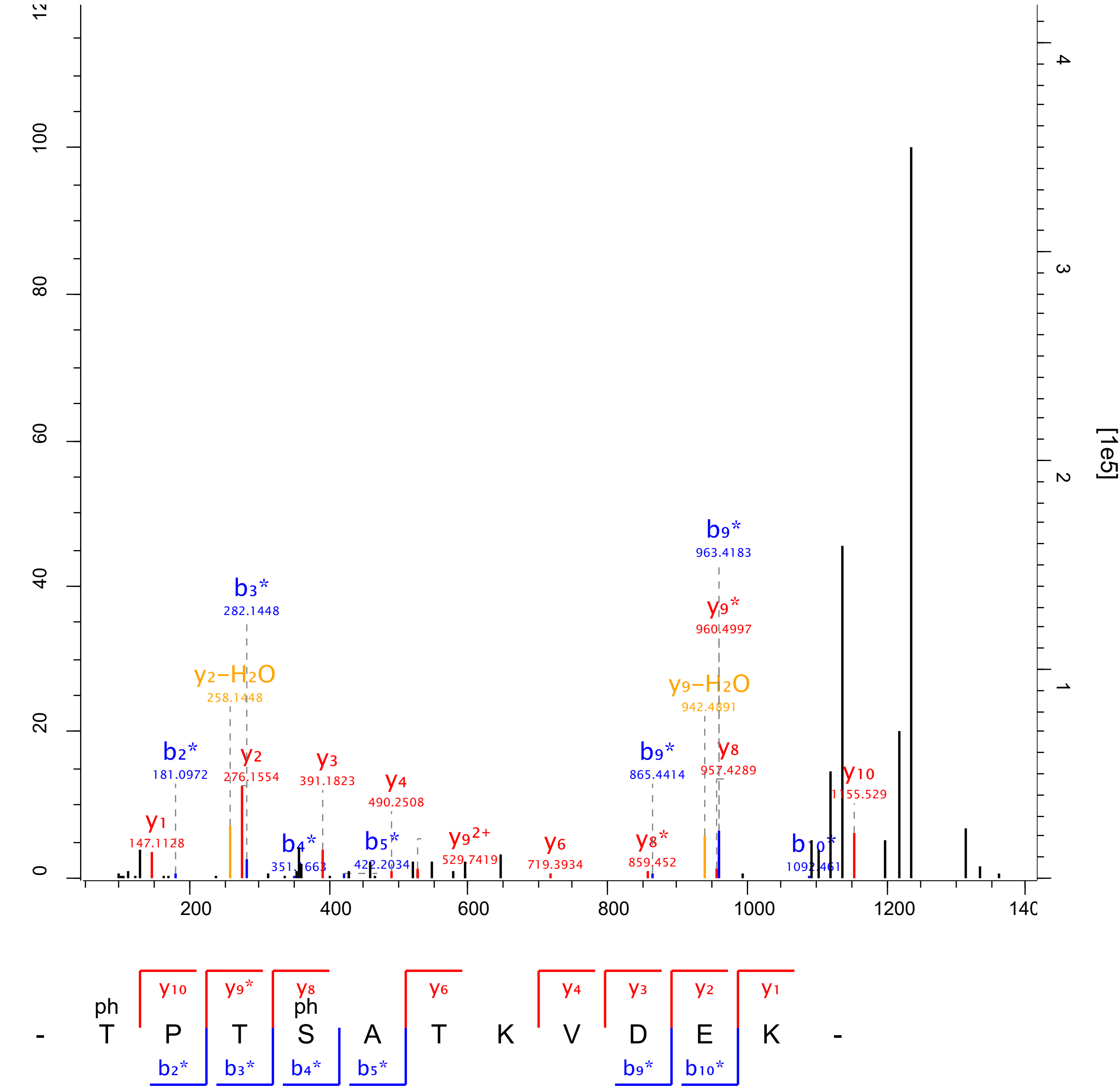

Raw file	Scan	Method	Score	m/z
sys_15_1	48276	FTMS; HCD	149.44	944.42

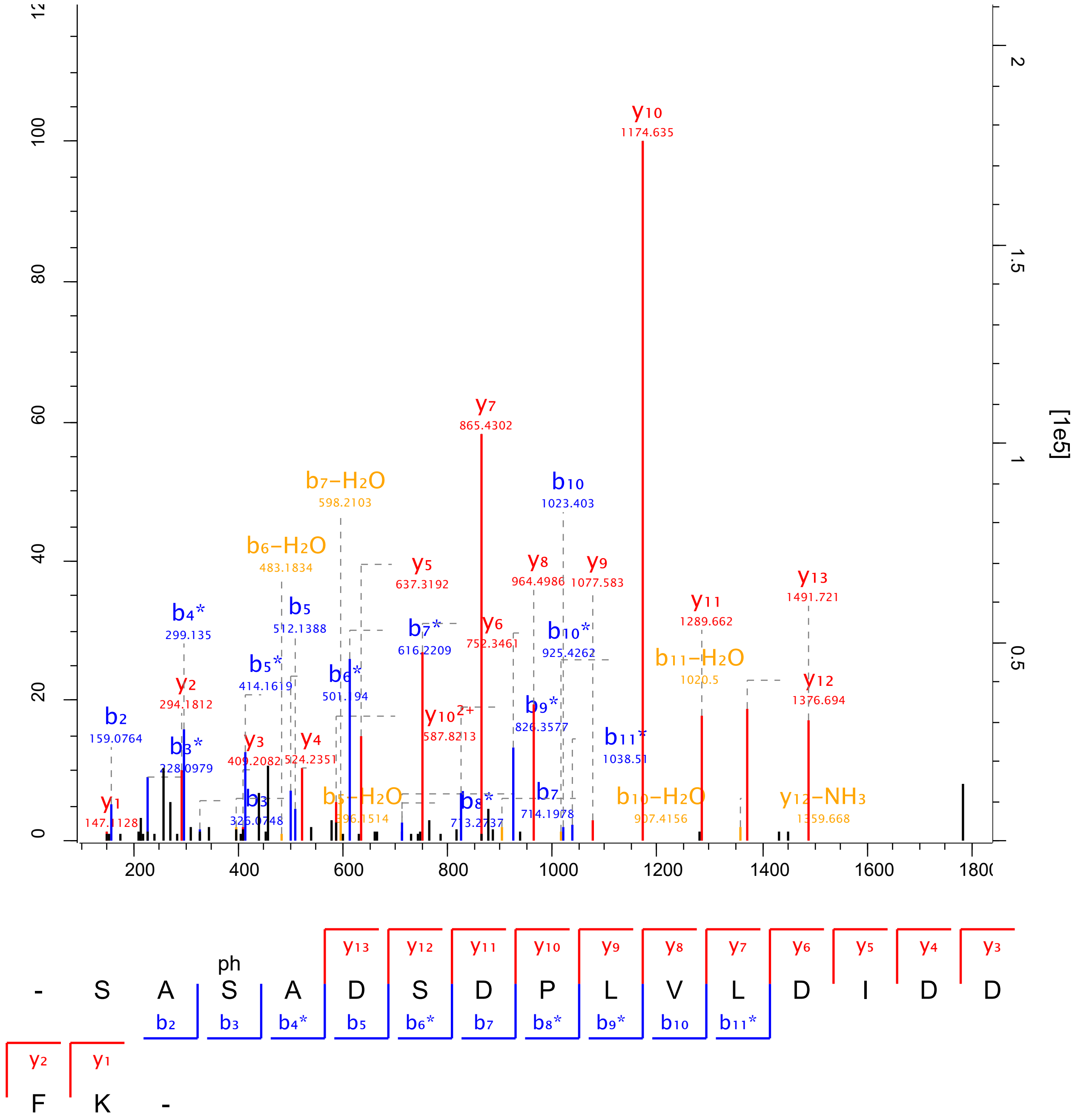

Raw file	Scan	Method	Score	m/z
sys_15_1	4858	FTMS; HCD	66.27	604.77

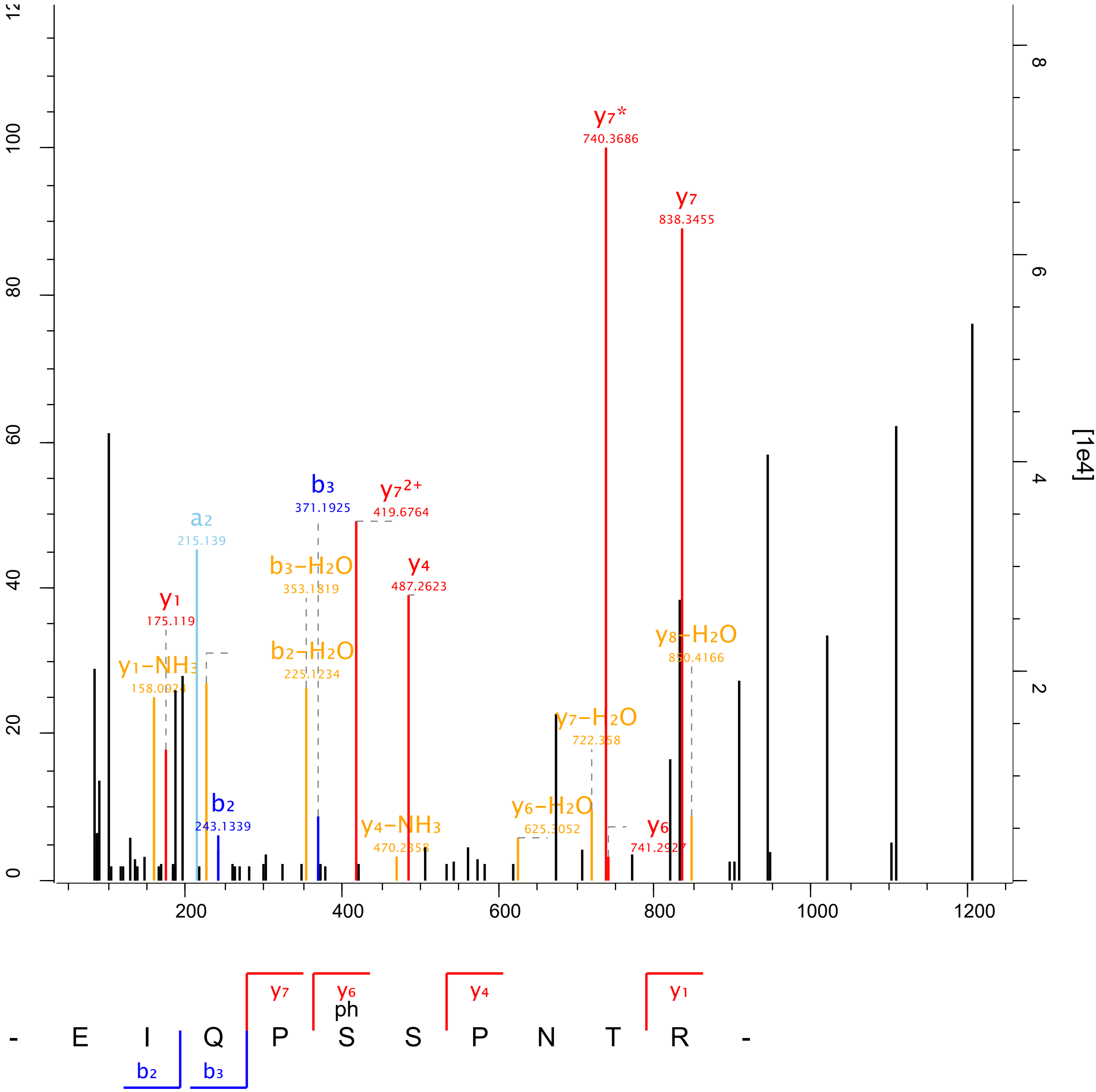

Raw file	Scan	Method	Score	m/z
sys_15_1	48906	FTMS; HCD	112.26	776.38

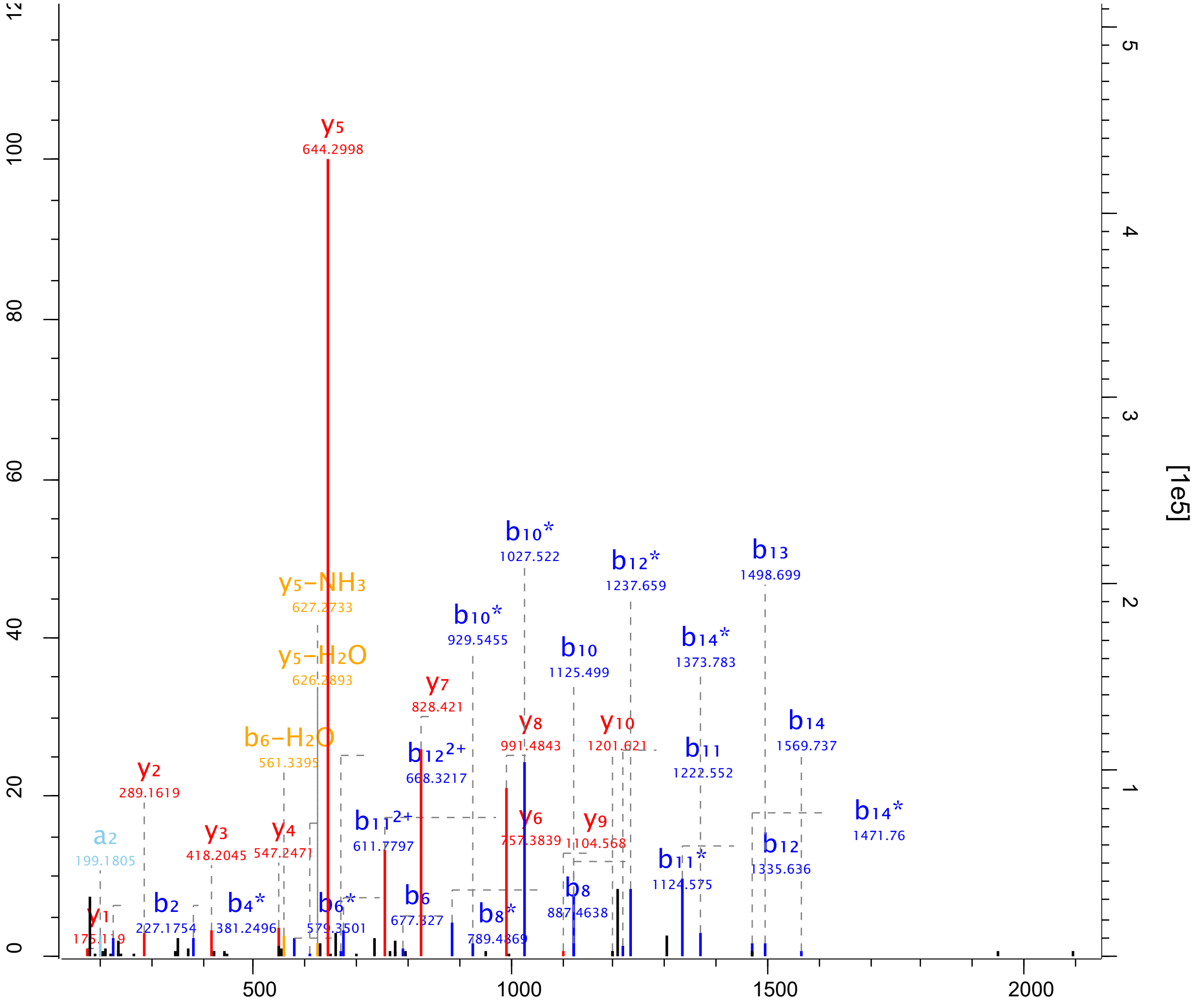

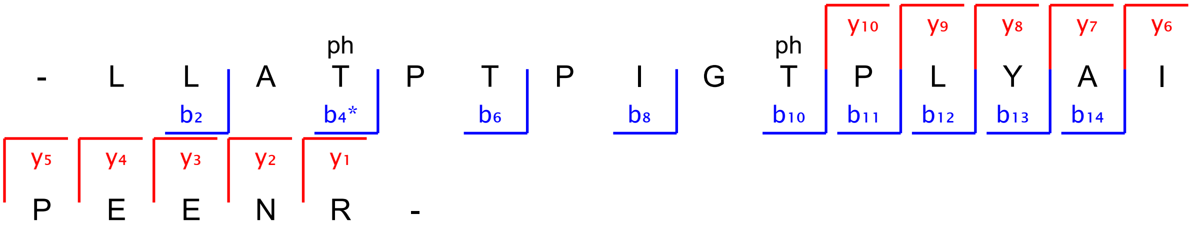

Raw file	Scan	Method	Score	m/z
sys_15_1	48919	FTMS; HCD	77.08	889.44

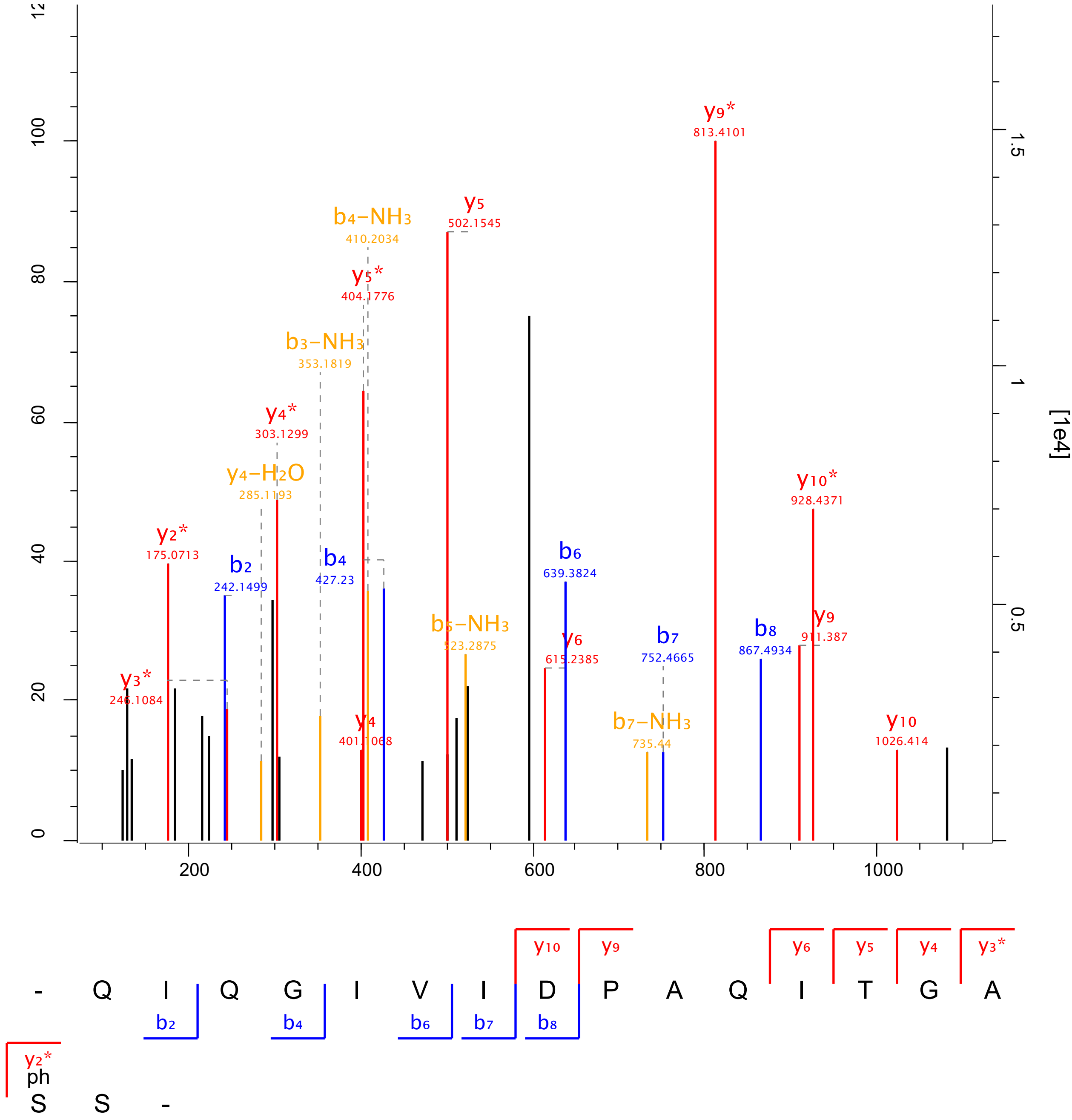

Raw file	Scan	Method	Score	m/z
sys_15_1	49508	FTMS; HCD	46.89	853.39

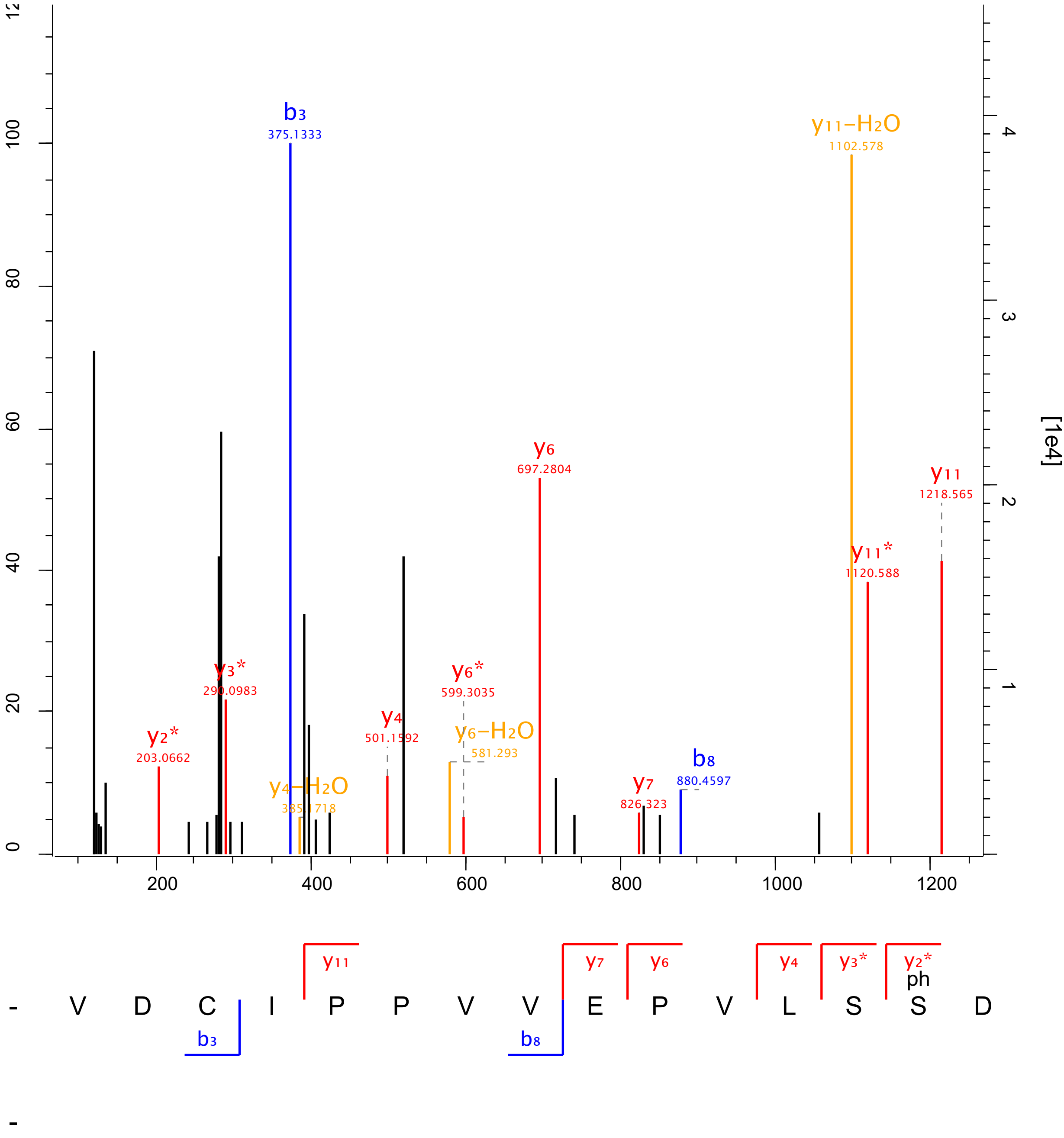

Raw file	Scan	Method	Score	m/z
sys_15_1	4959	FTMS; HCD	166.65	629.28

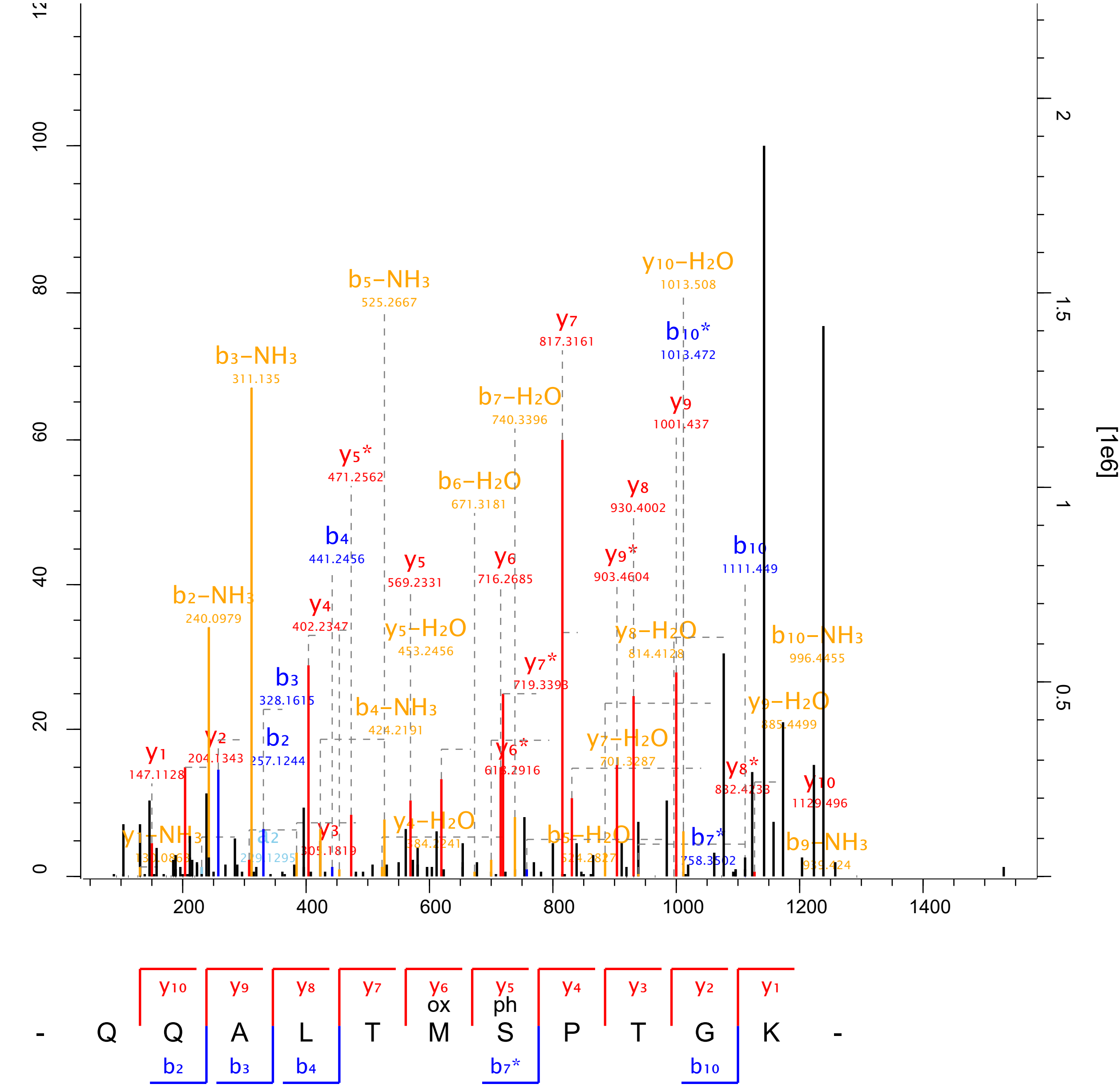

Raw file	Scan	Method	Score	m/z
sys_15_1	50021	FTMS; HCD	44.79	969.41

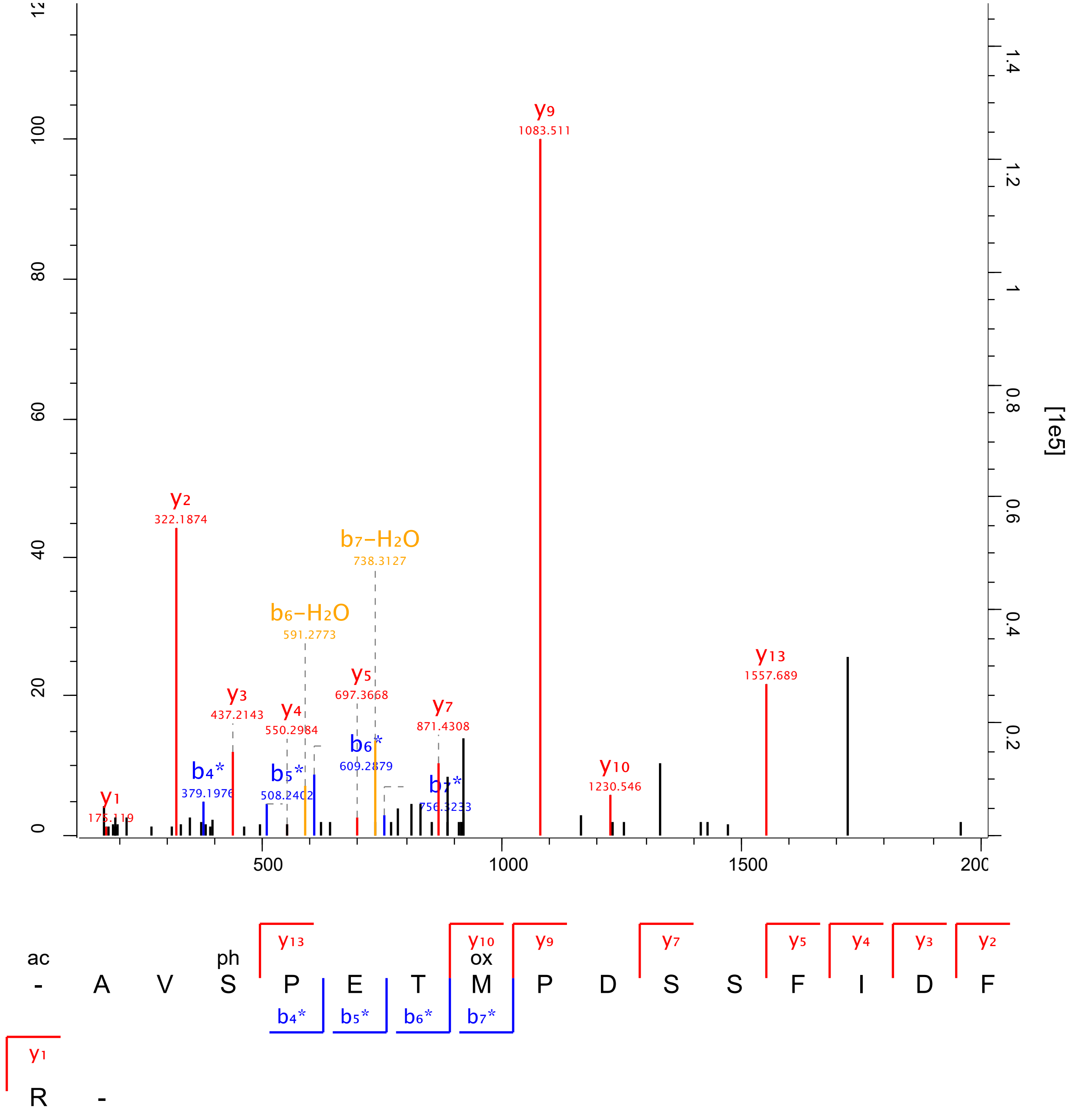

Raw file	Scan	Method	Score	m/z
sys_15_1	5021	FTMS; HCD	131.41	632.24

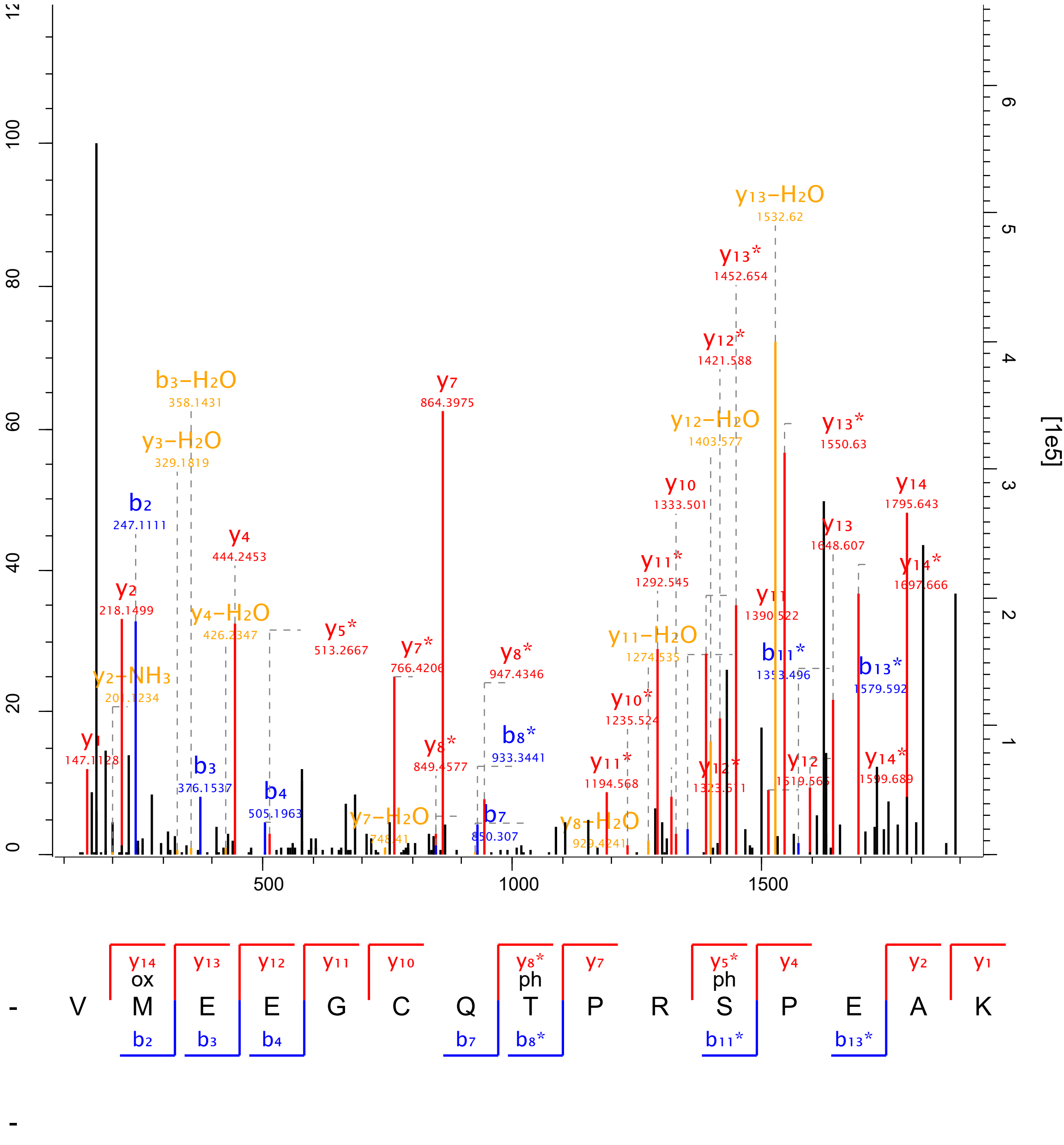

Raw file	Scan	Method	Score	m/z
sys_15_1	5055	FTMS; HCD	66.27	582.72

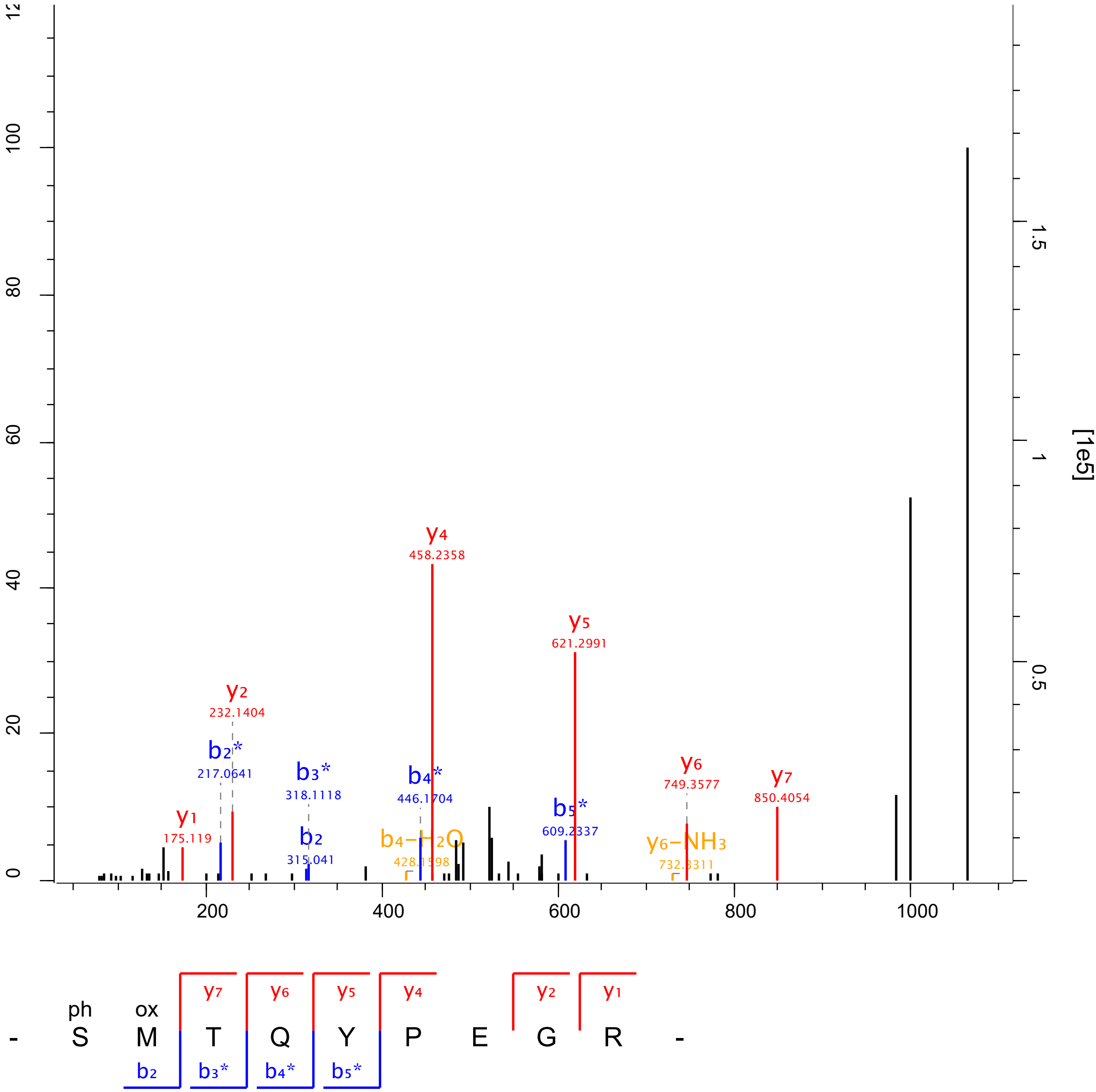

Raw file	Scan	Method	Score	m/z
sys_15_1	50600	FTMS; HCD	108.08	1018.01

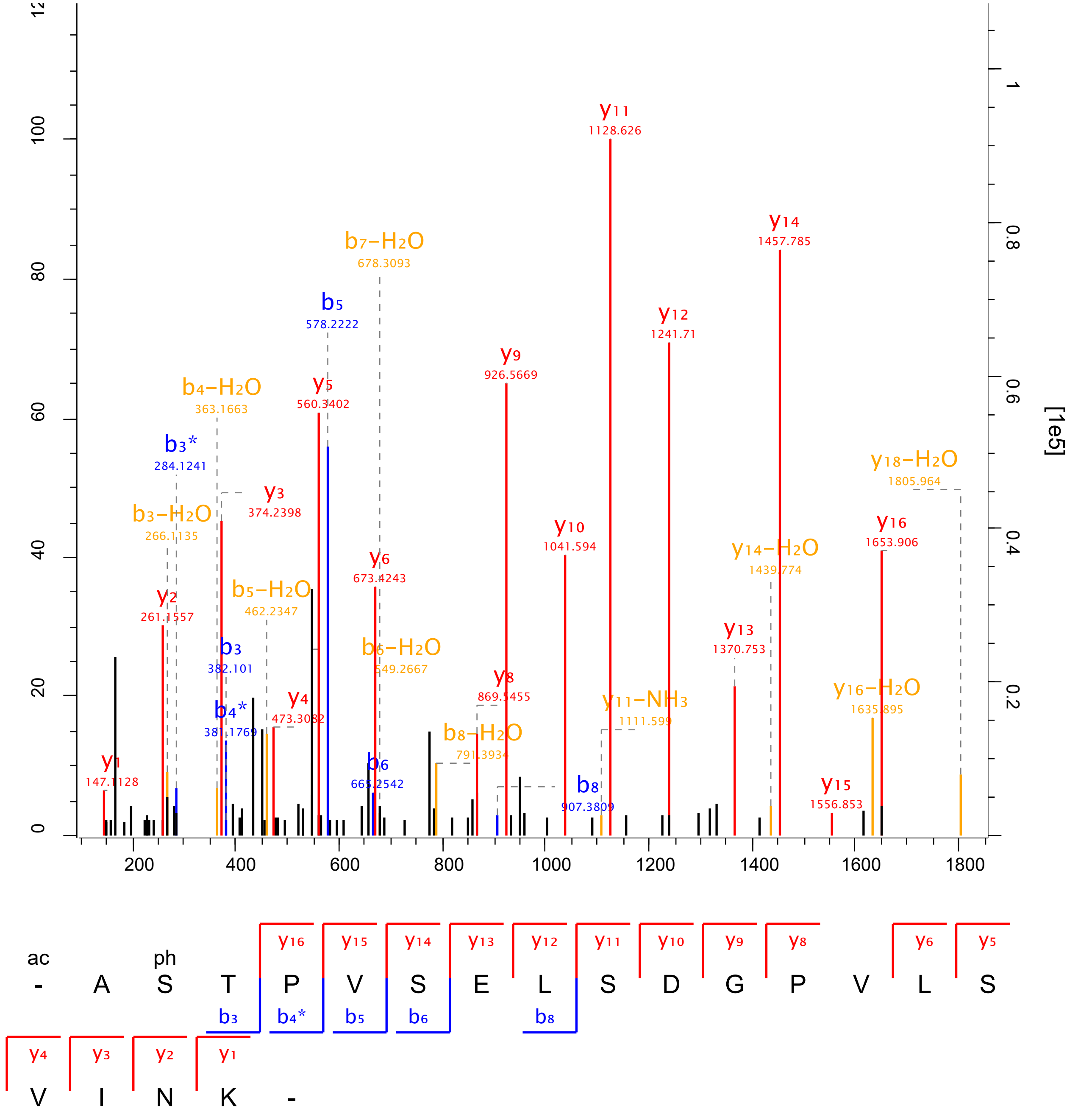

Raw file	Scan	Method	Score	m/z
sys_15_1	5077	FTMS; HCD	80.71	461.72

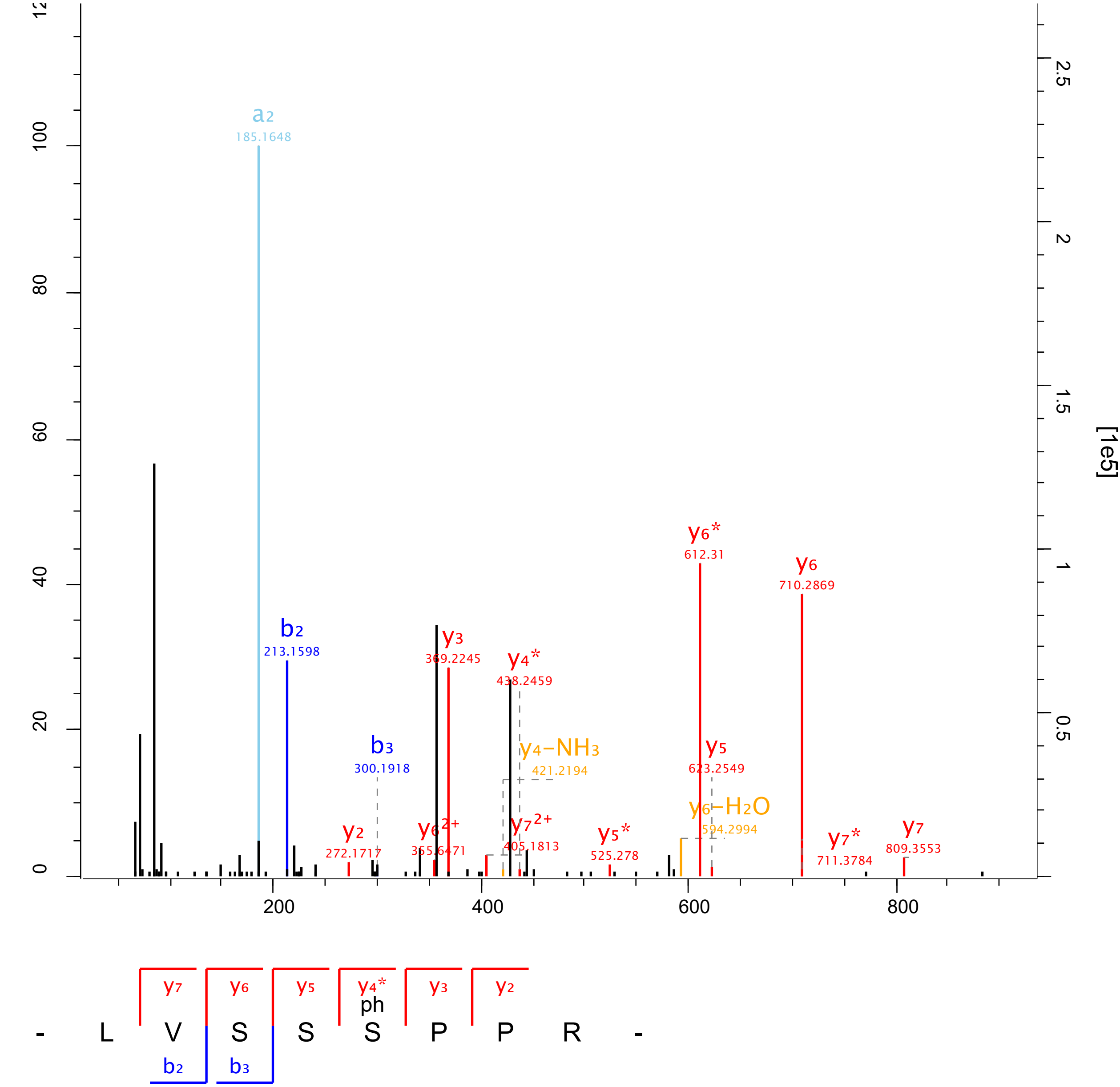

Raw file	Scan	Method	Score	m/z
sys_15_1	5107	FTMS; HCD	108.74	472.7

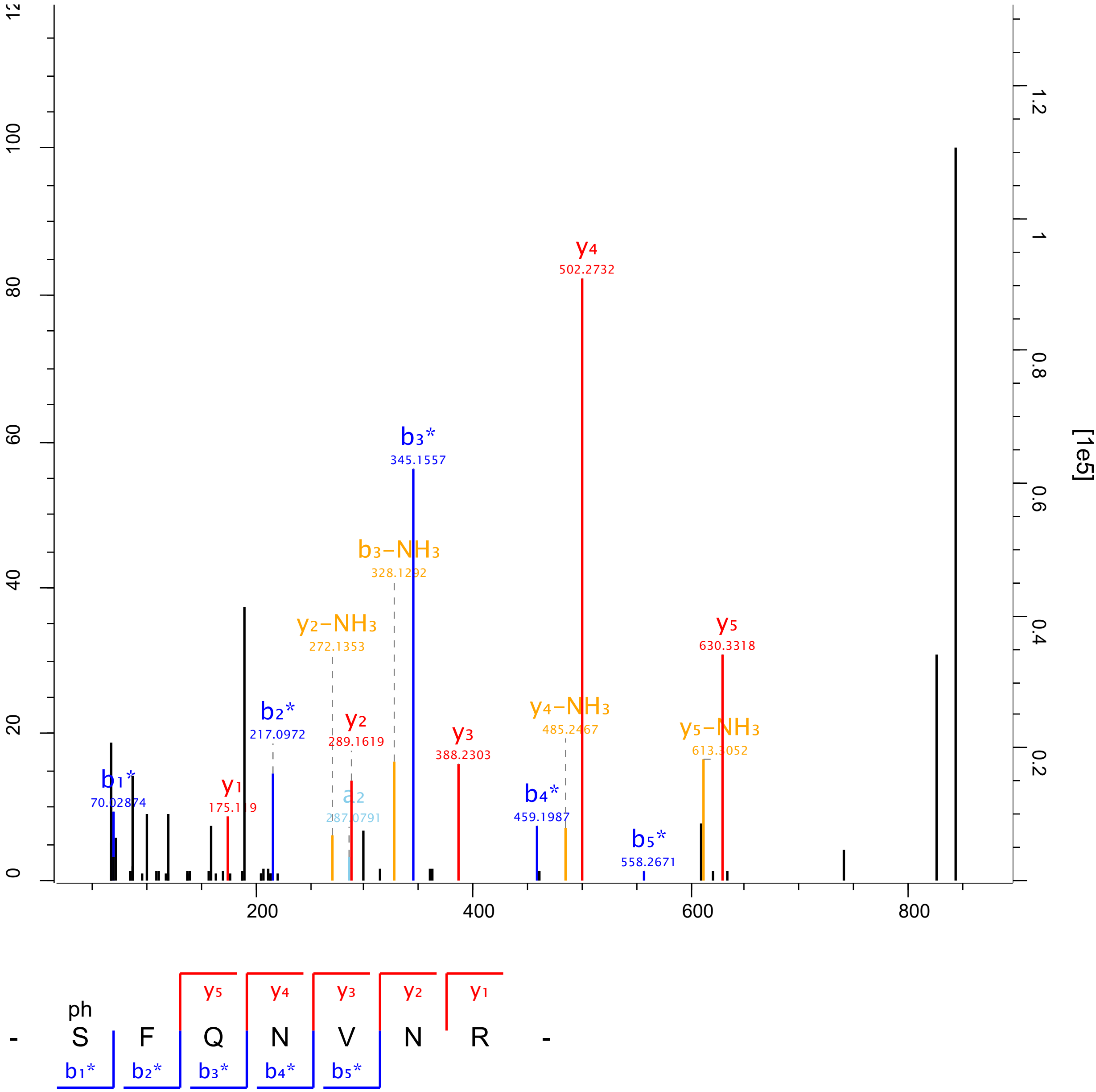

Raw file	Scan	Method	Score	m/z
sys_15_1	5137	FTMS; HCD	64.1	451.69

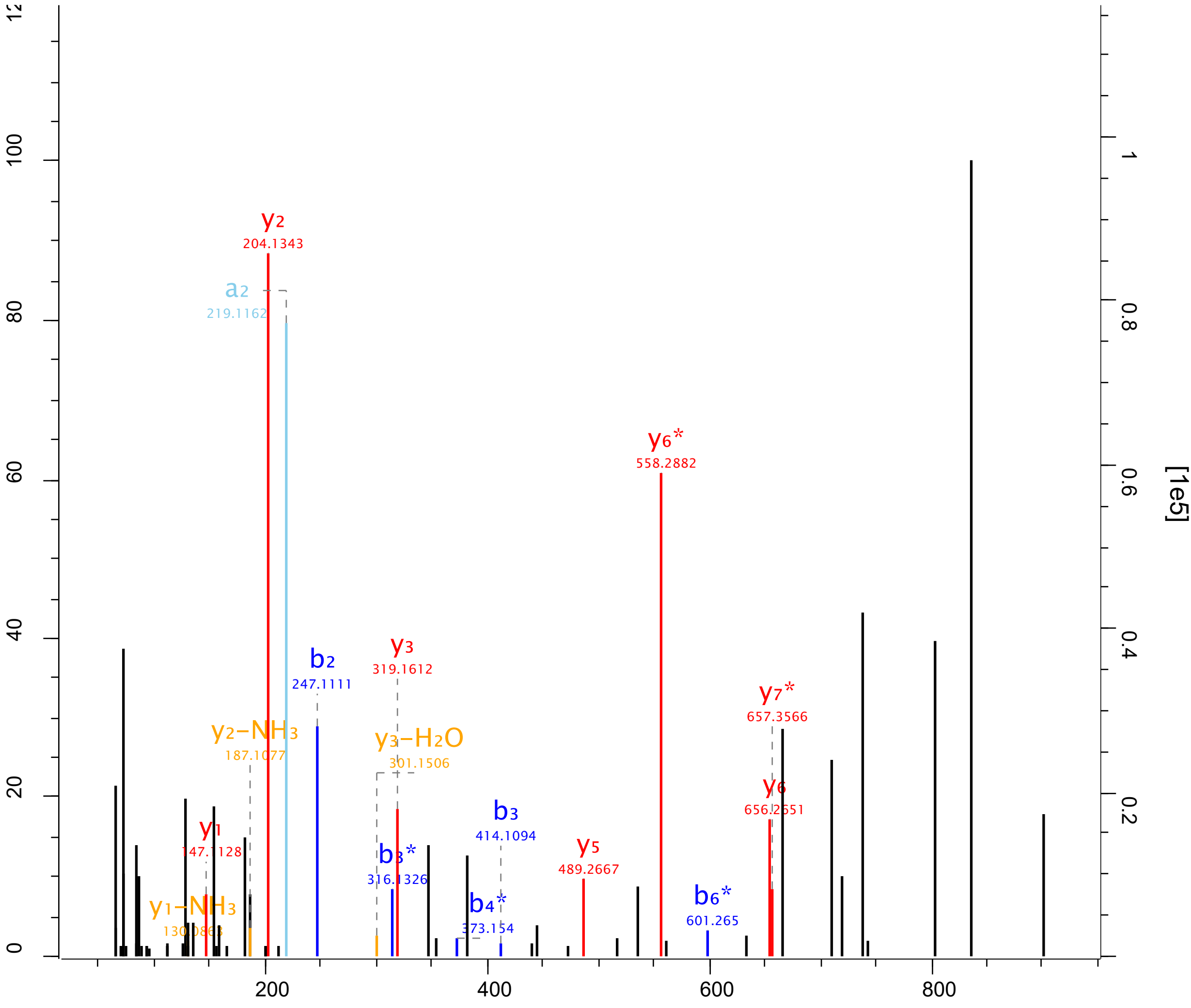

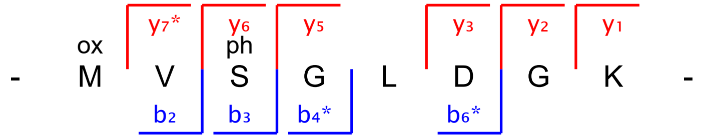

Raw file	Scan	Method	Score	m/z
sys_15_1	5155	FTMS; HCD	83.05	532.26

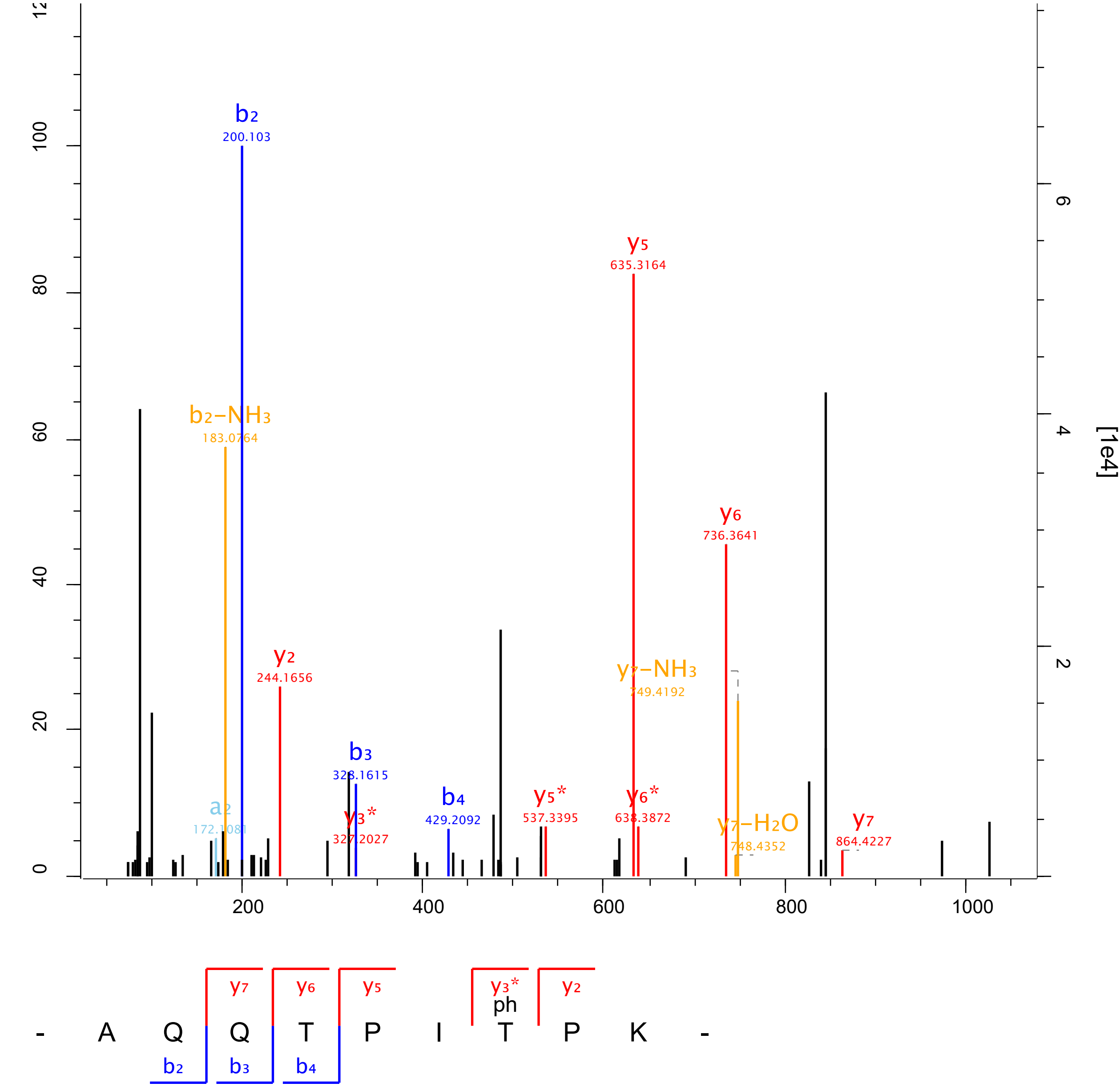

Raw file	Scan	Method	Score	m/z
sys_15_1	5170	FTMS; HCD	93.48	656.25

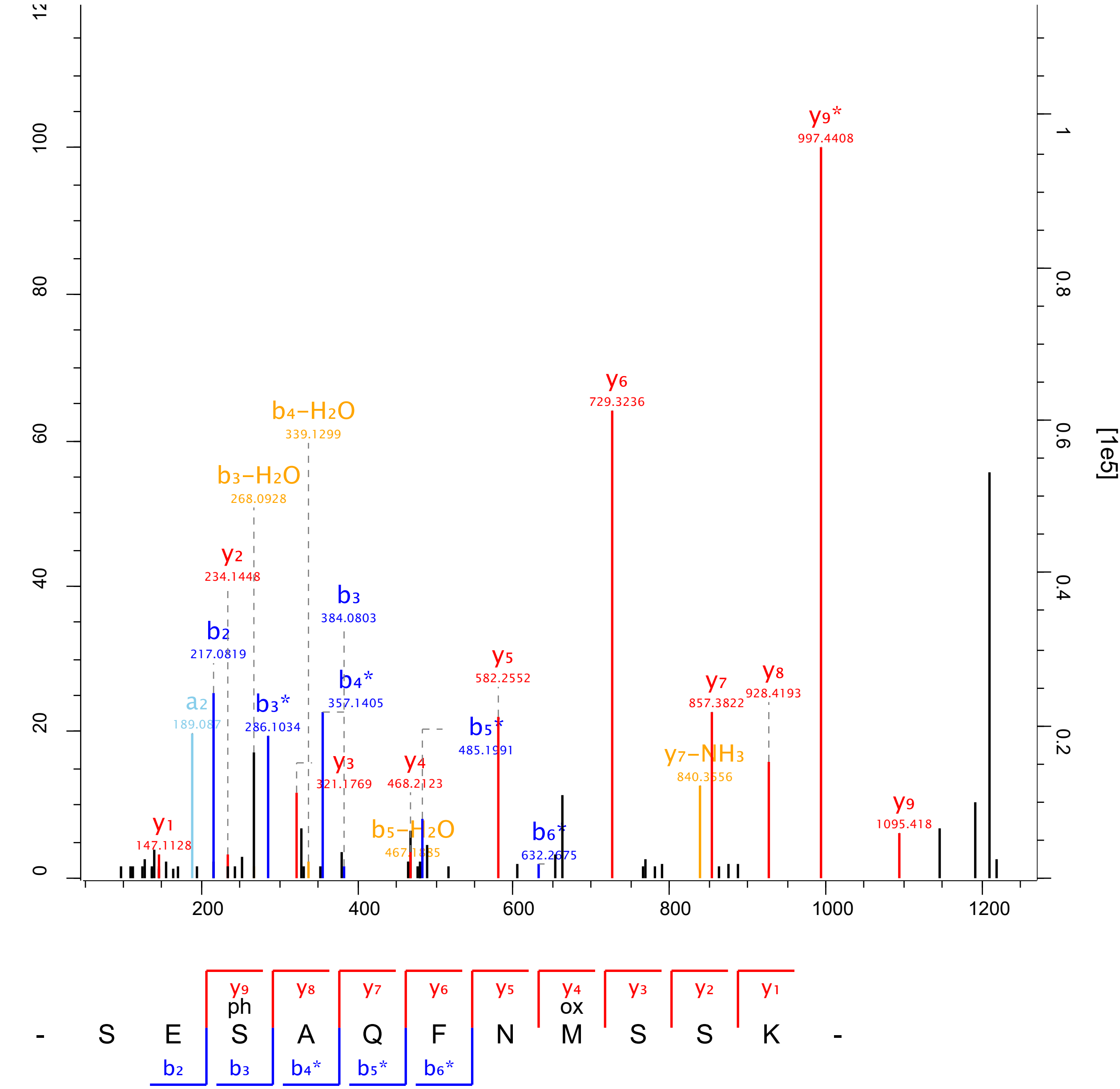

Raw file	Scan	Method	Score	m/z
sys_15_1	5203	FTMS; HCD	86.77	545.23

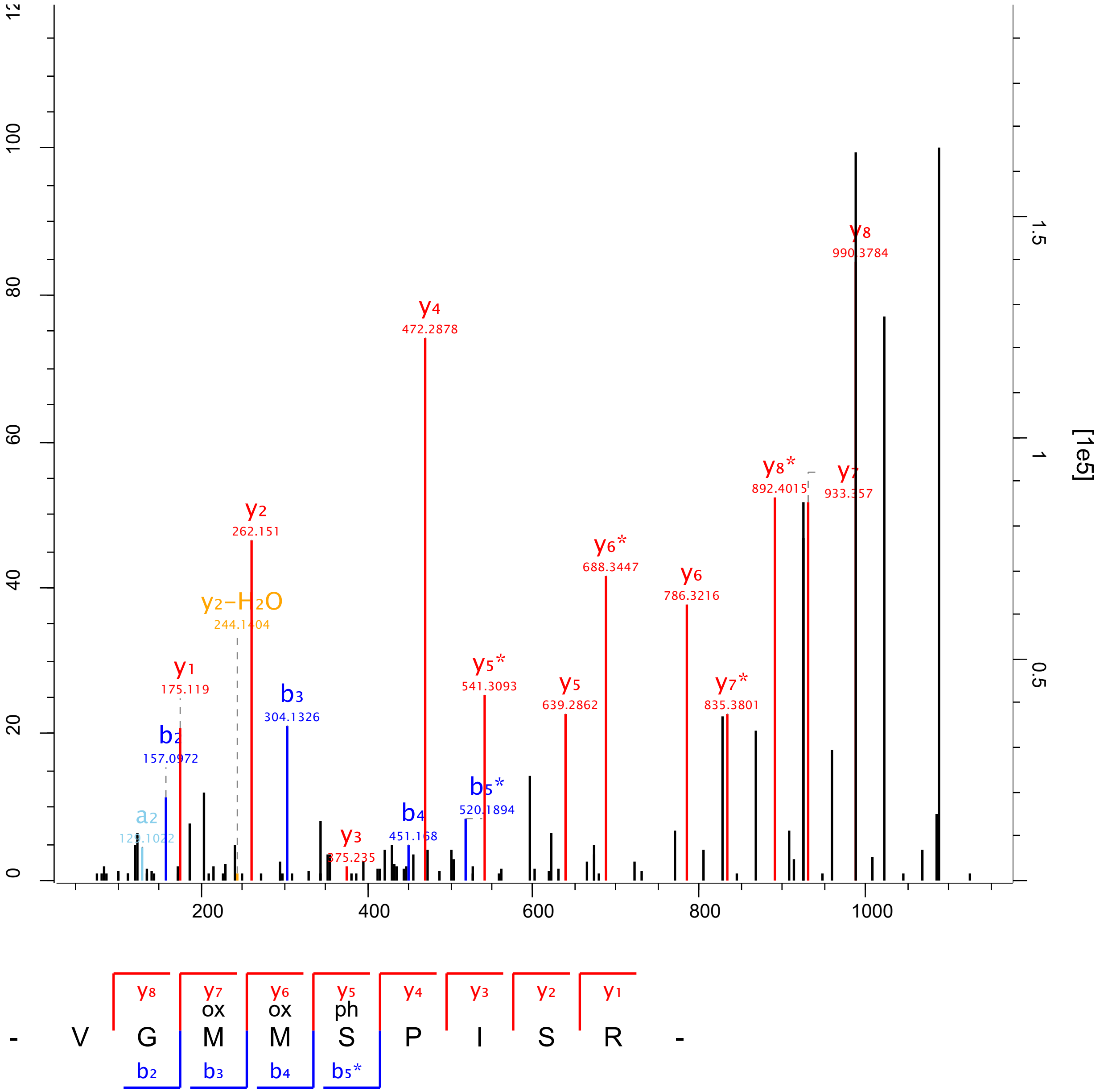

Raw file	Scan	Method	Score	m/z
sys_15_1	5233	FTMS; HCD	102.52	659.25

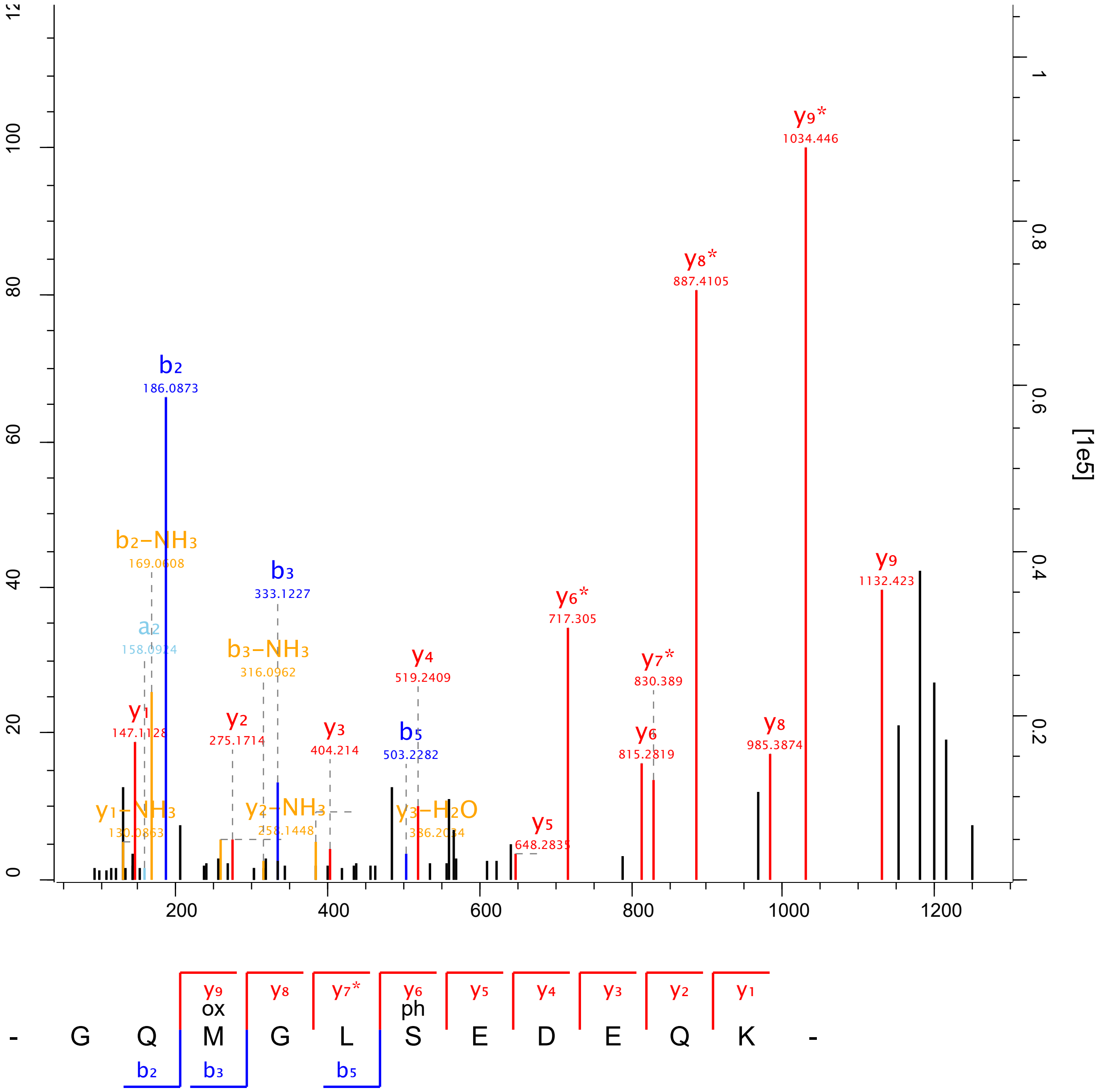

Raw file	Scan	Method	Score	m/z
sys_15_1	5280	FTMS; HCD	63.3	847.83

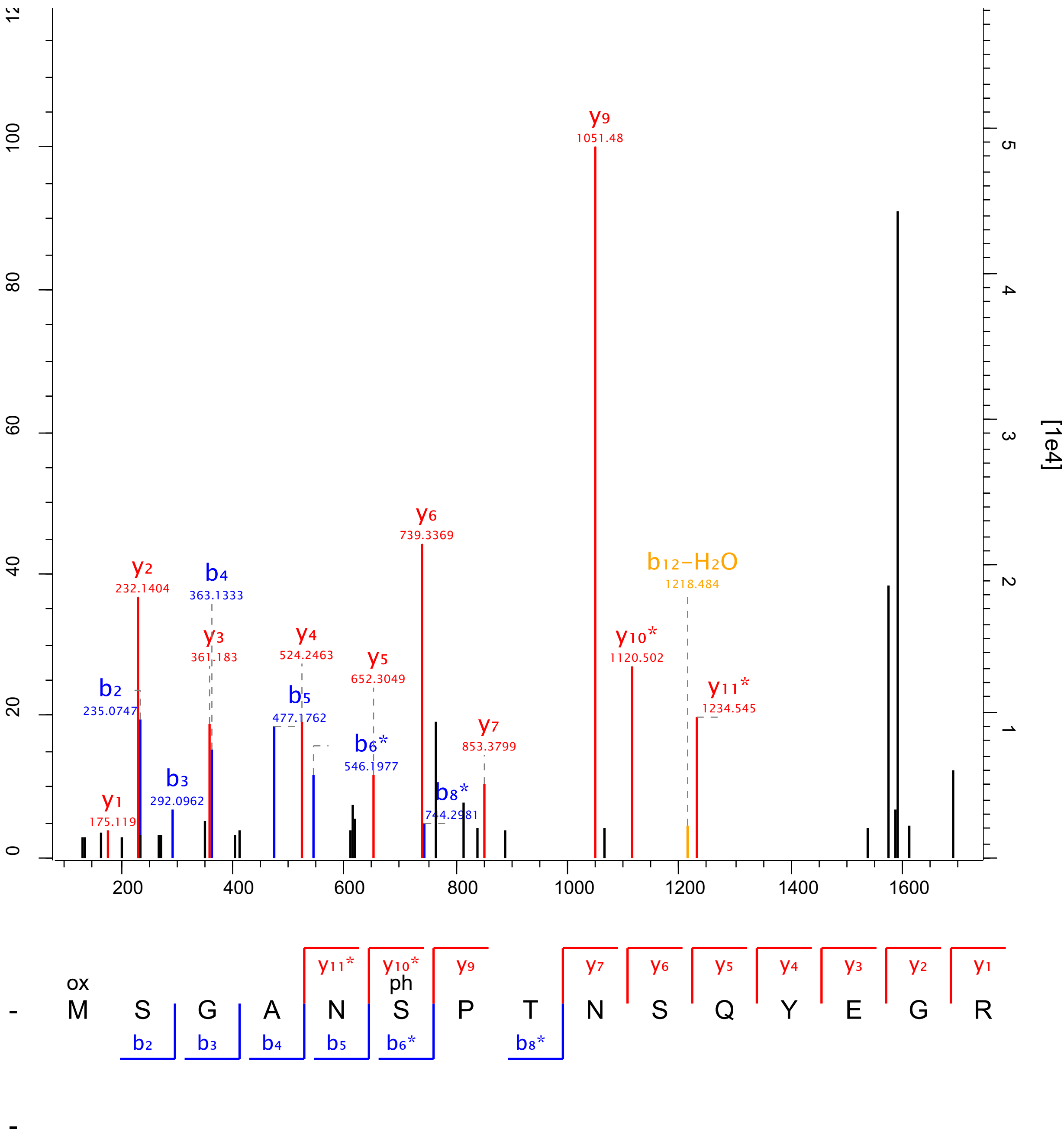

Raw file	Scan	Method	Score	m/z
sys_15_1	5382	FTMS; HCD	65.72	489.7

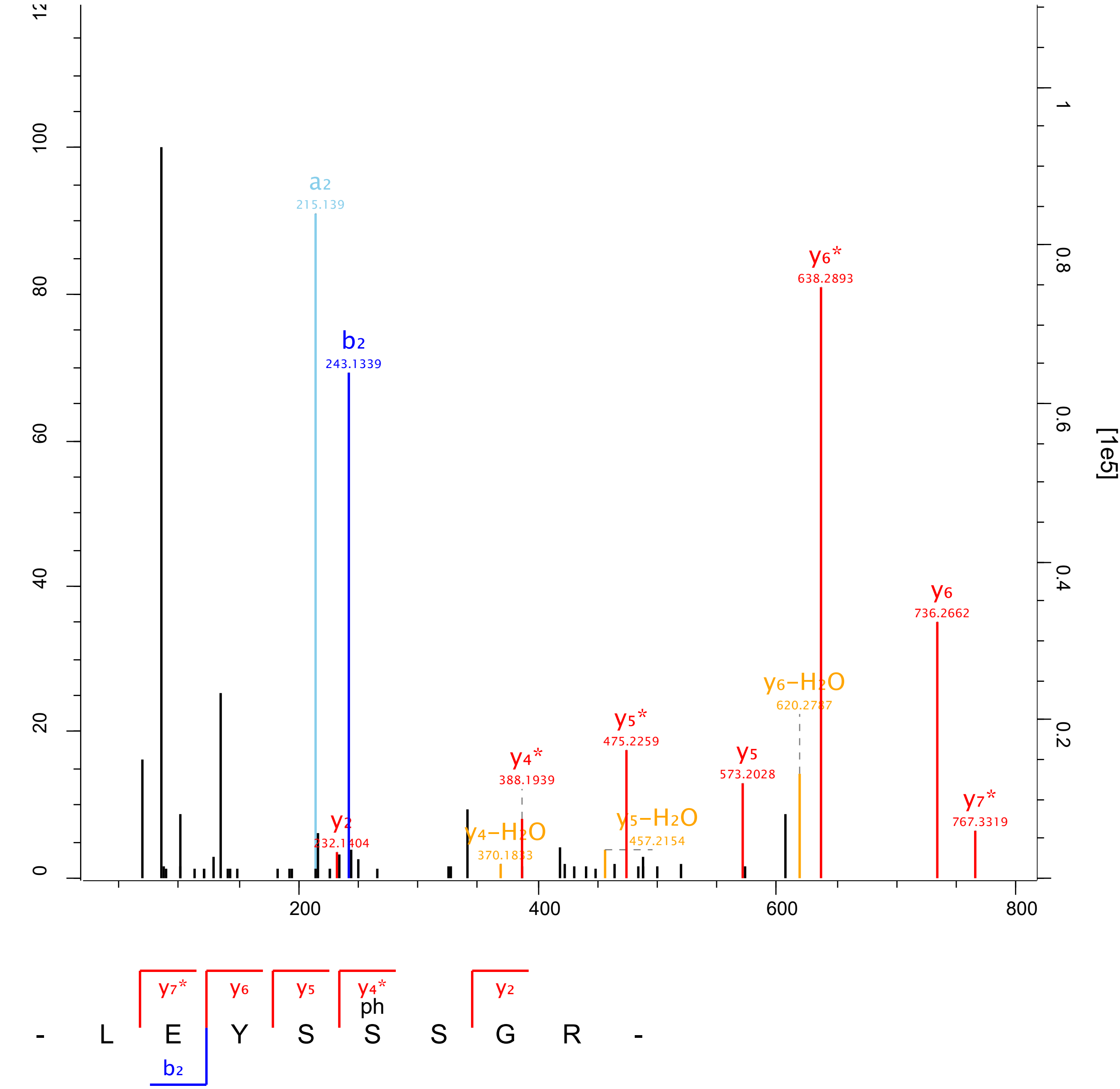

Raw file	Scan	Method	Score	m/z
sys_15_1	5390	FTMS; HCD	61.44	606.72

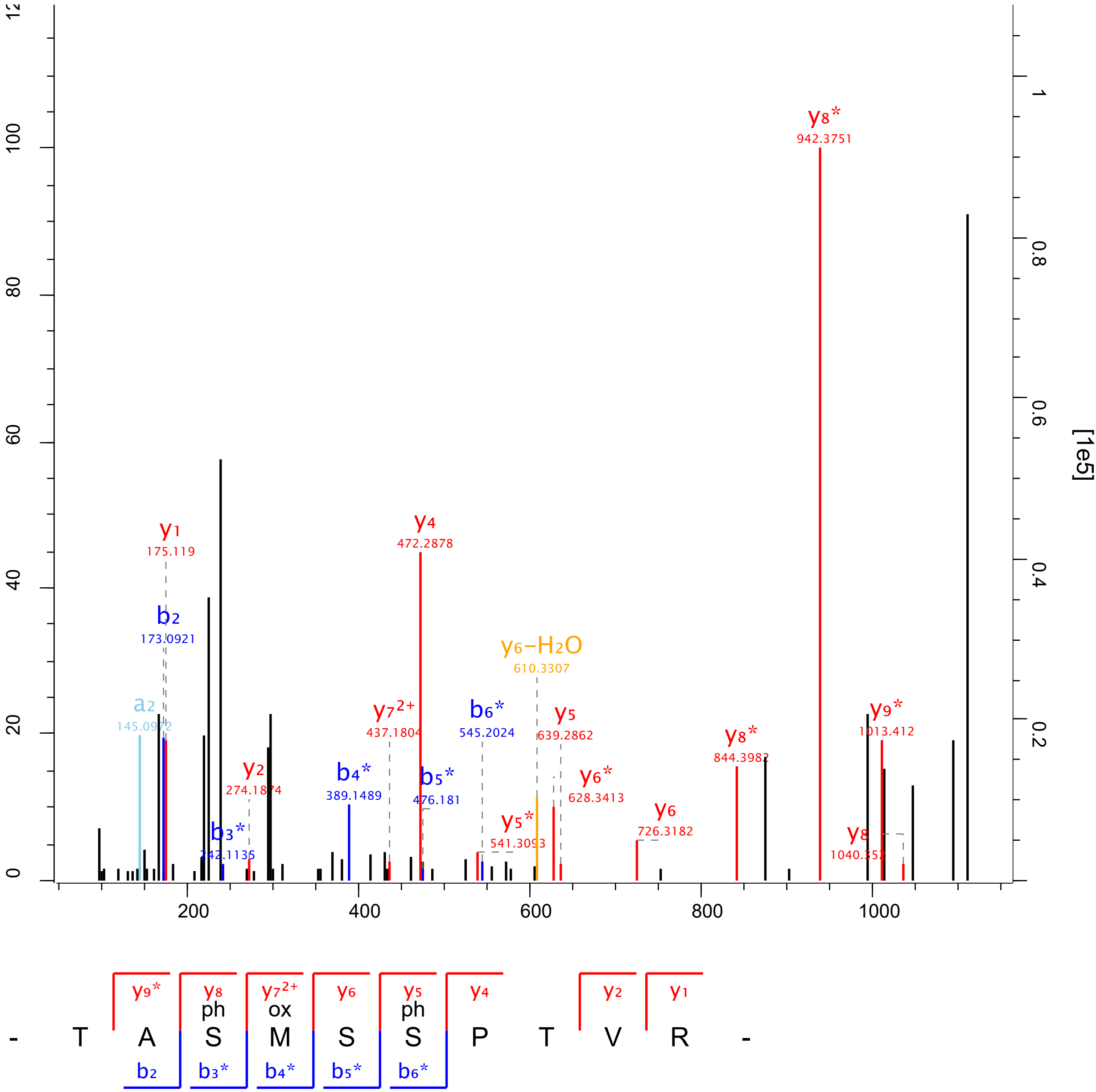

Raw file	Scan	Method	Score	m/z
sys_15_1	5415	FTMS; HCD	71.56	529.23

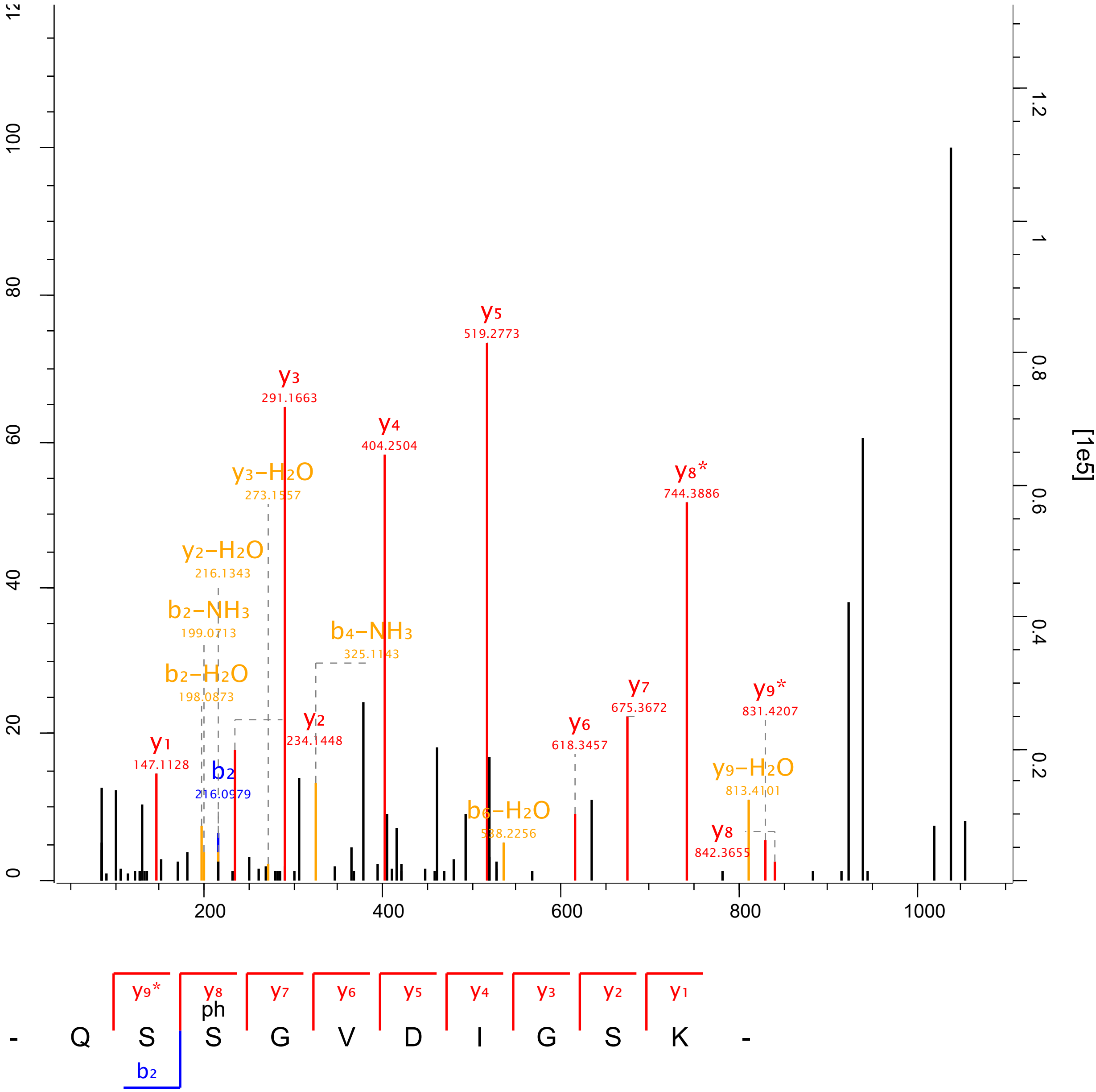

Raw file	Scan	Method	Score	m/z
sys_15_1	5445	FTMS; HCD	107.06	457.71

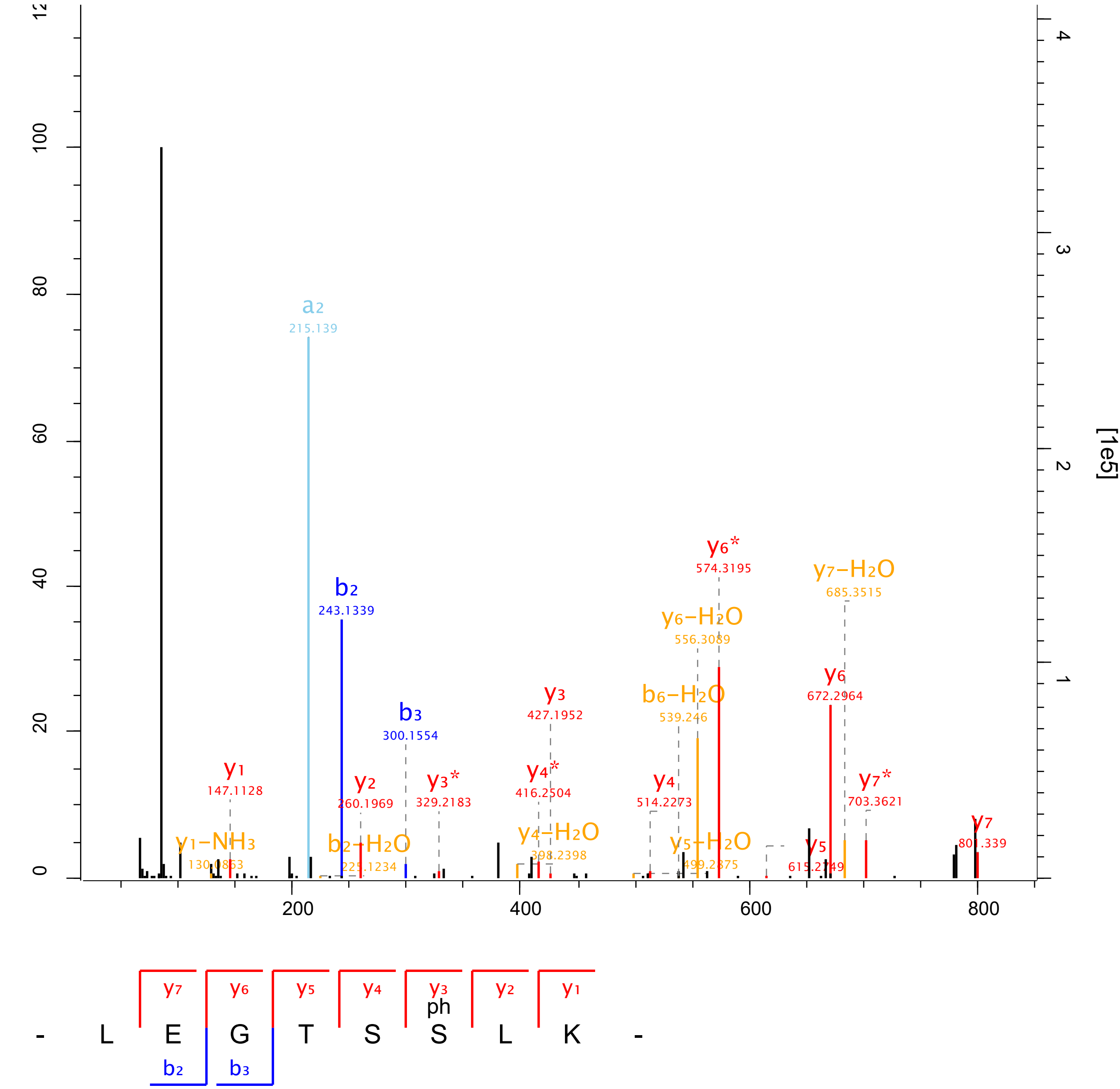

Raw file	Scan	Method	Score	m/z
sys_15_1	5463	FTMS; HCD	103.67	541.24

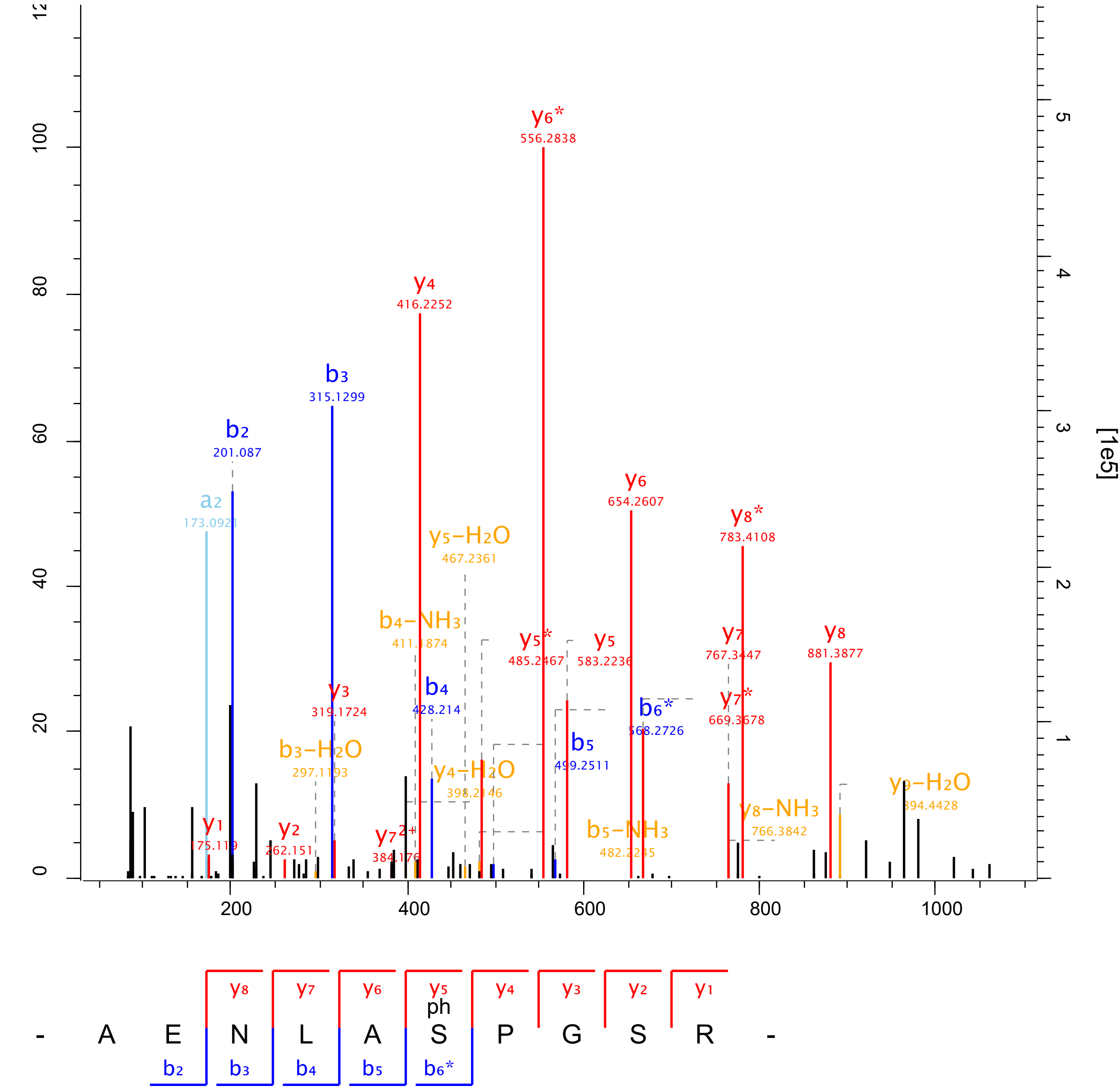

Raw file	Scan	Method	Score	m/z
sys_15_1	5653	FTMS; HCD	150.22	578.24

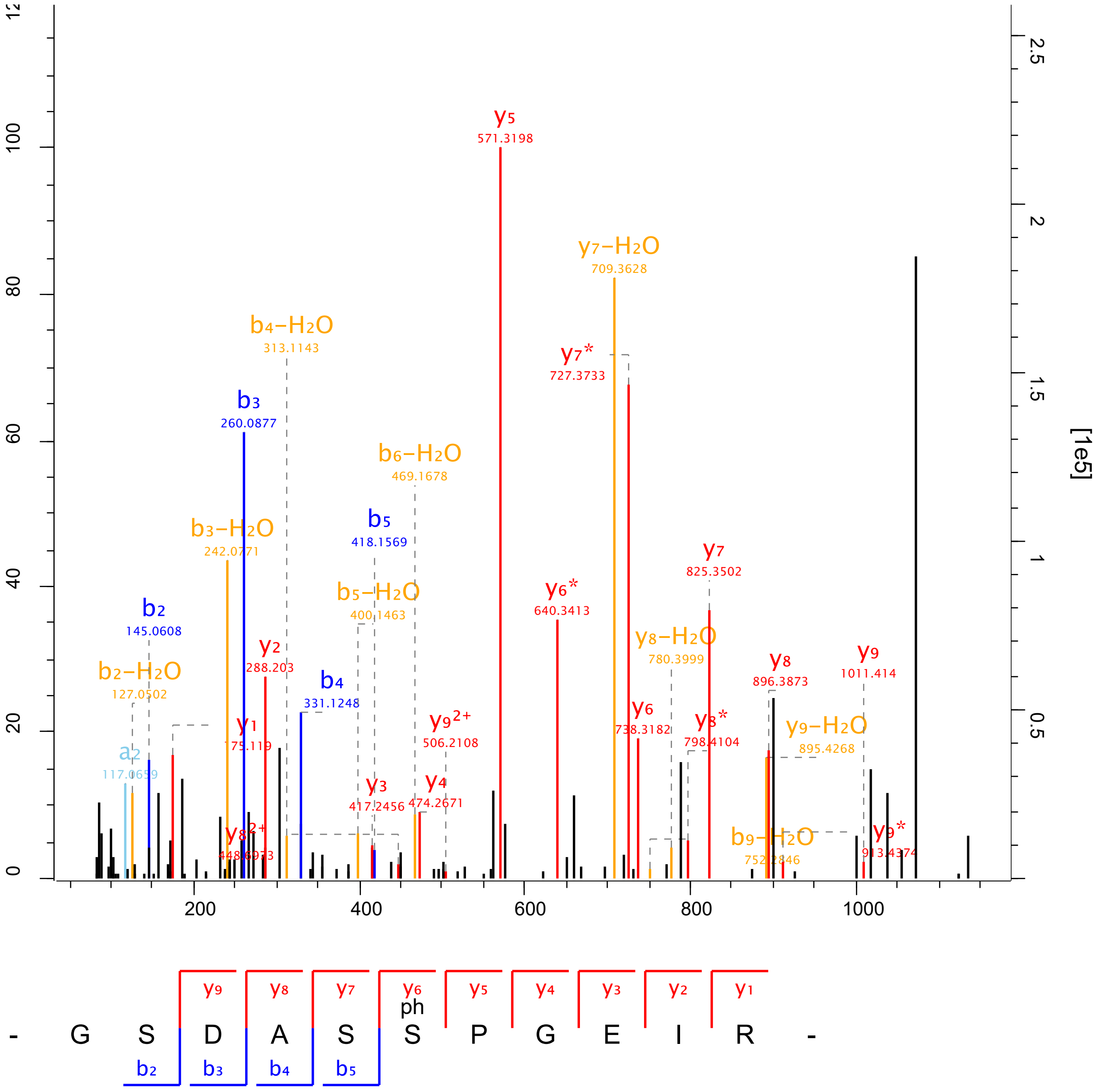

Raw file	Scan	Method	Score	m/z
sys_15_1	5754	FTMS; HCD	92.87	462.7

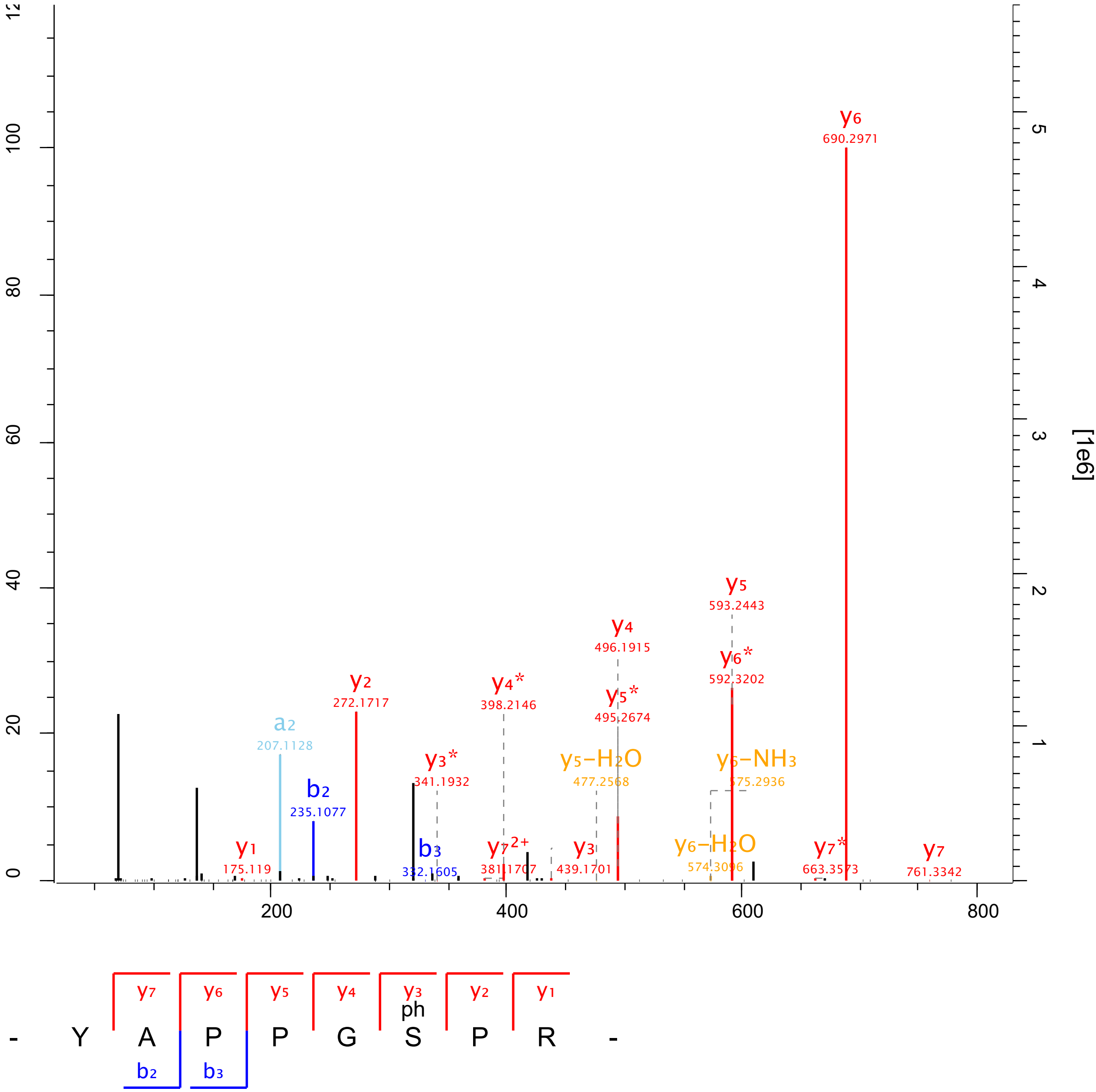

Raw file	Scan	Method	Score	m/z
sys_15_1	5758	FTMS; HCD	112.36	496.69

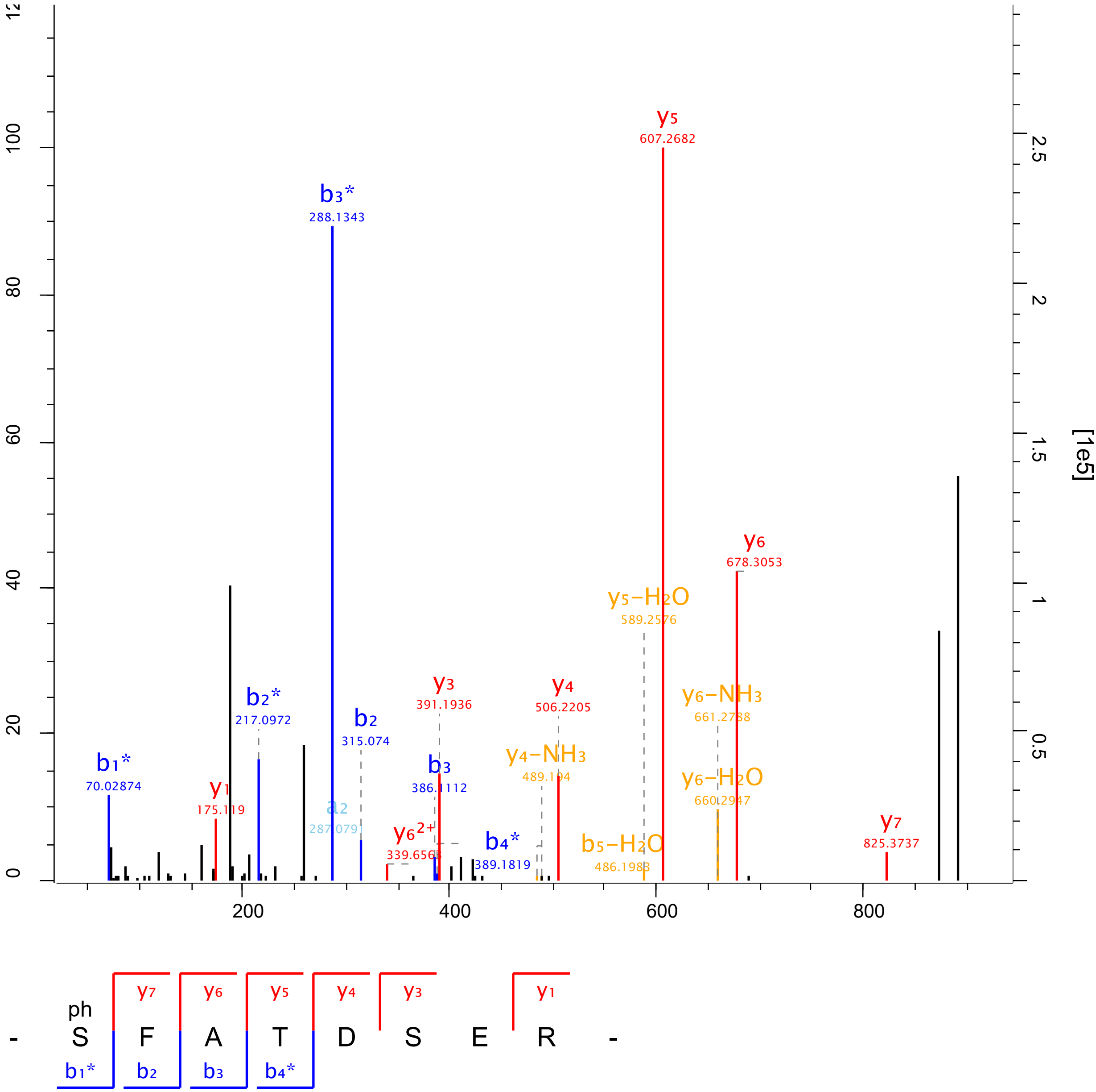

Raw file	Scan	Method	Score	m/z
sys_15_1	5771	FTMS; HCD	143.25	525.26

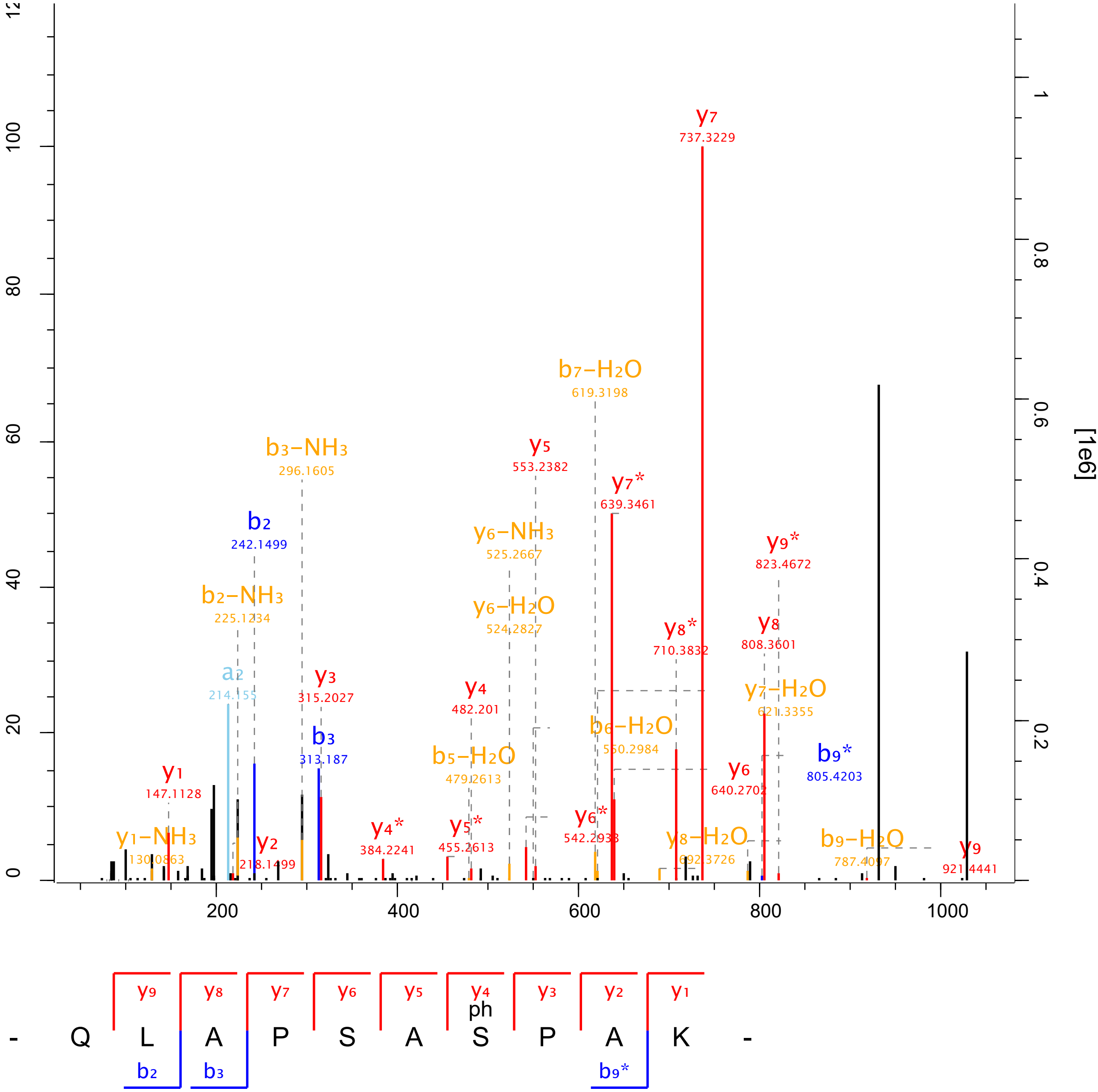

Raw file	Scan	Method	Score	m/z
sys_15_1	5791	FTMS; HCD	216.01	635.76

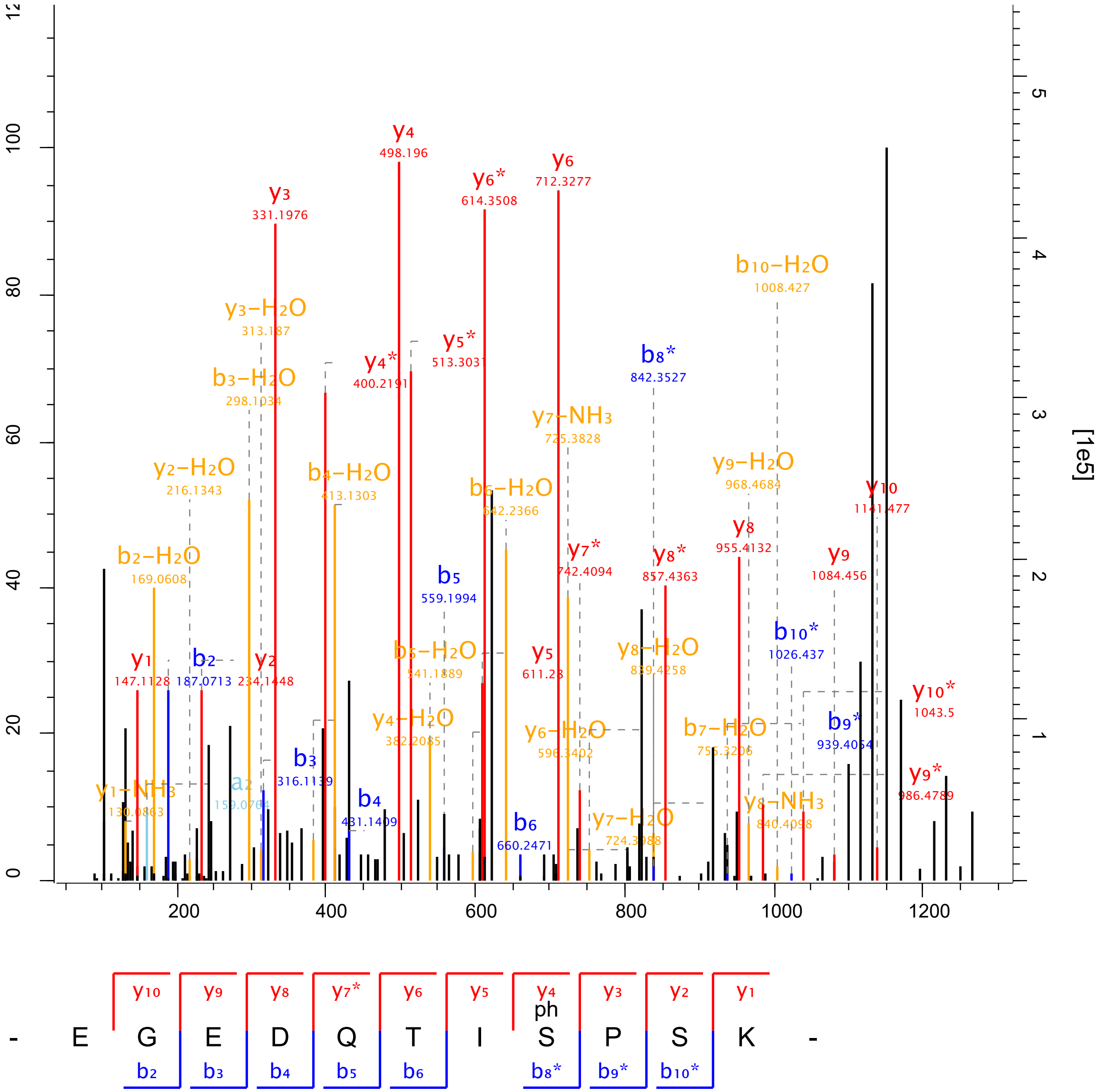

Raw file	Scan	Method	Score	m/z
sys_15_1	5796	FTMS; HCD	158.47	580.24

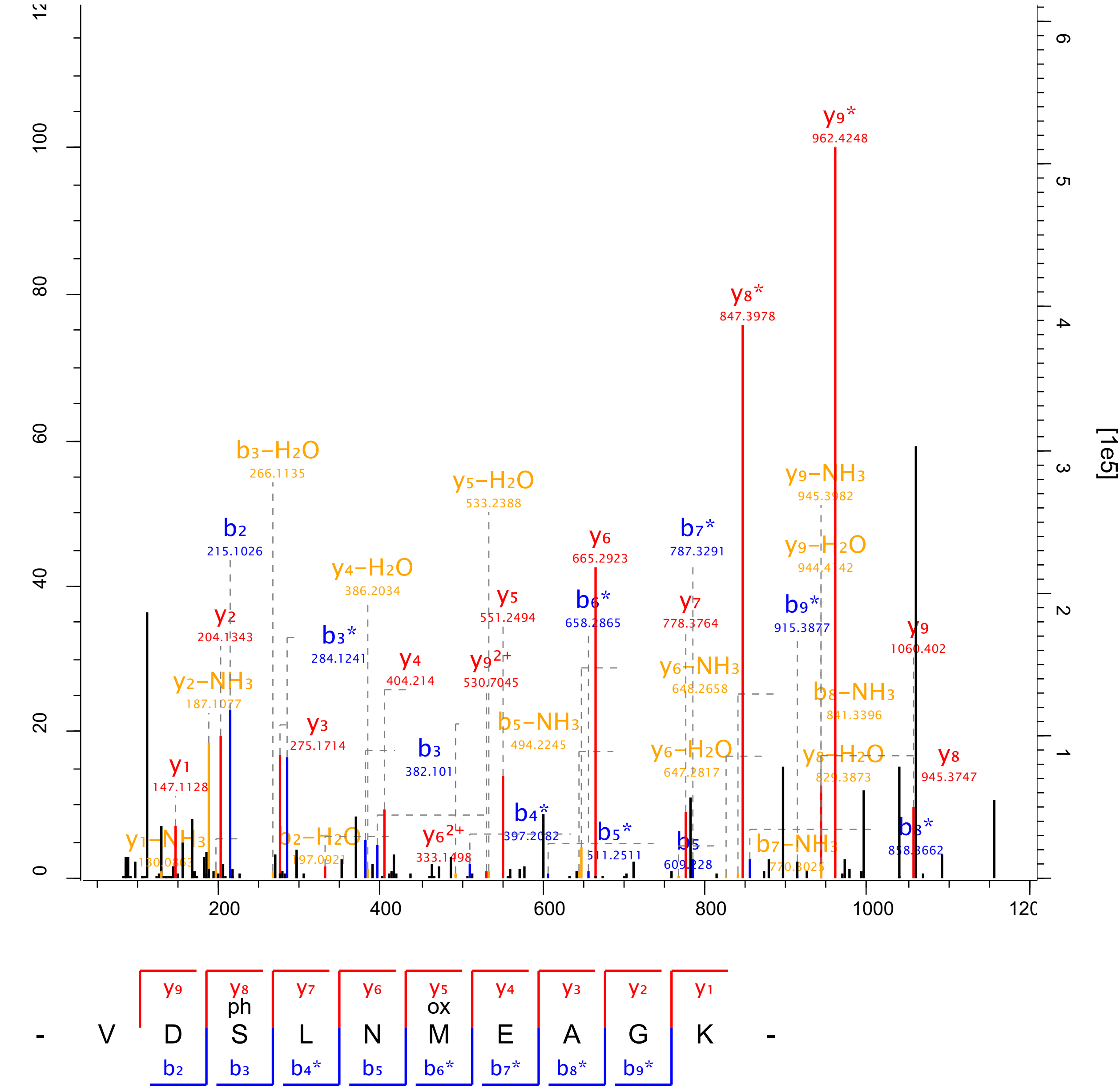

Raw file	Scan	Method	Score	m/z
sys_15_1	5819	FTMS; HCD	45.83	473.19

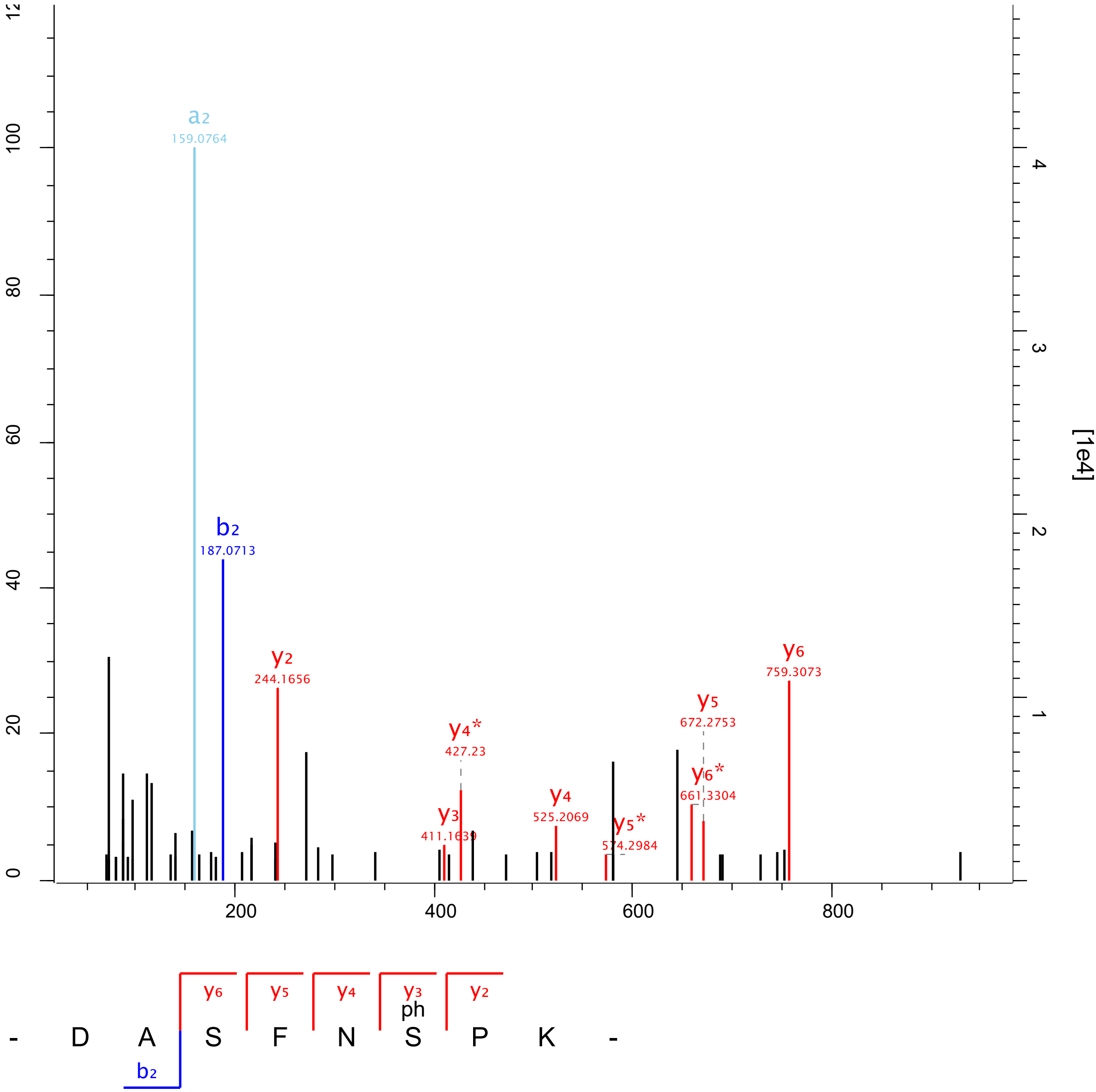

Raw file	Scan	Method	Score	m/z
sys_15_1	5841	FTMS; HCD	96.17	459.7

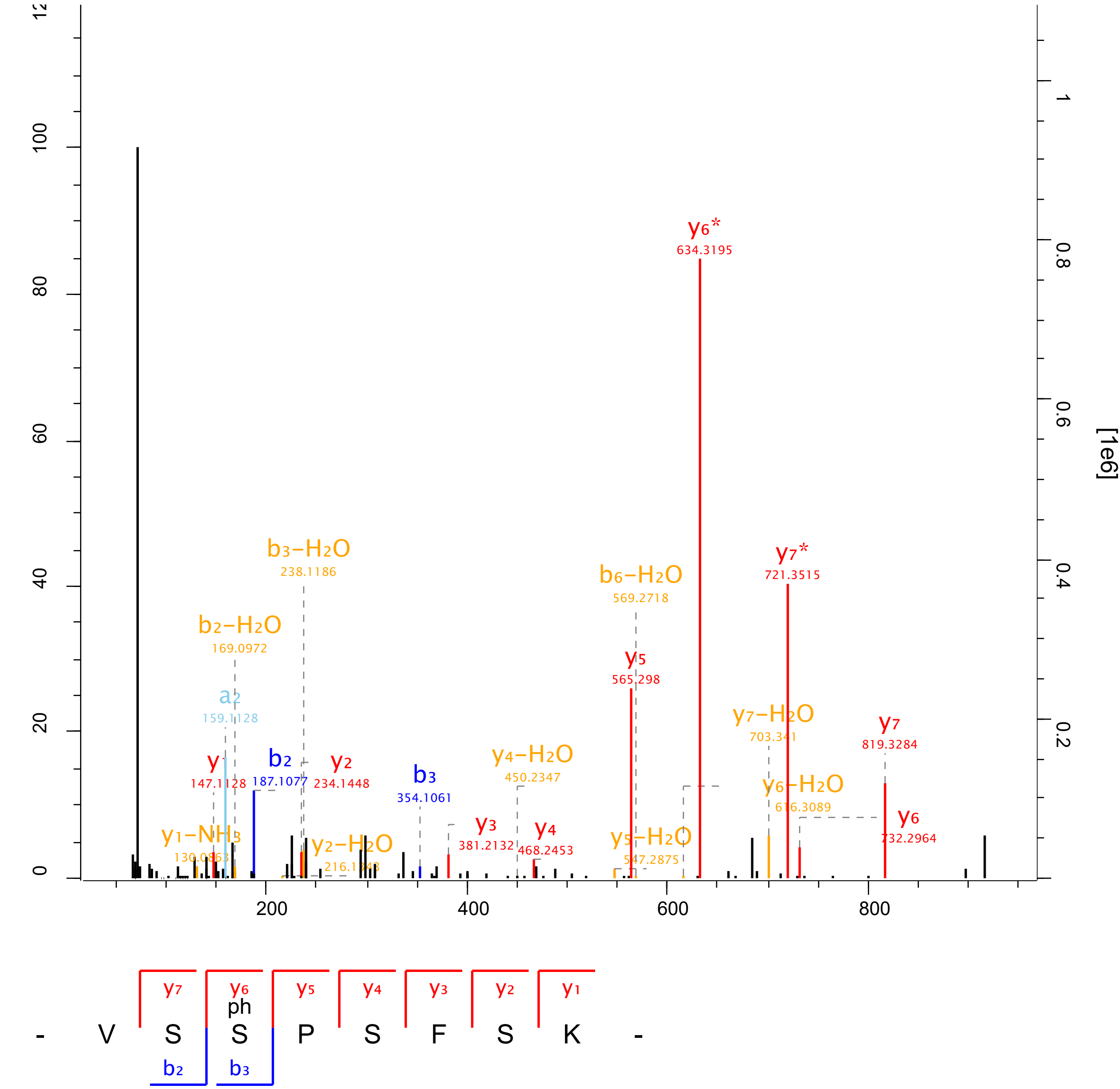

Raw file	Scan	Method	Score	m/z
sys_15_1	5895	FTMS; HCD	50.9	657.76

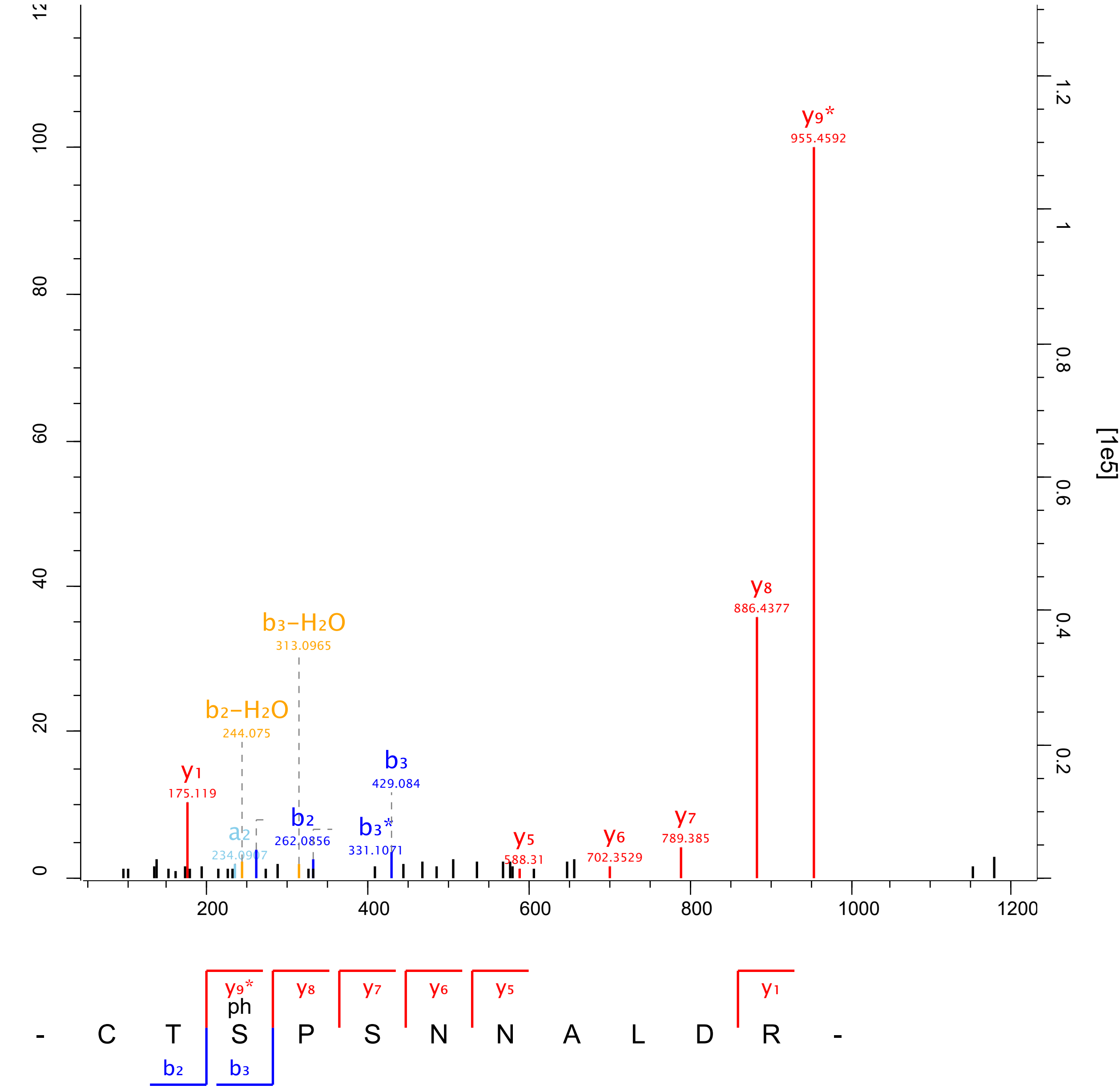

Raw file	Scan	Method	Score	m/z
sys_15_1	5980	FTMS; HCD	51.45	508.73

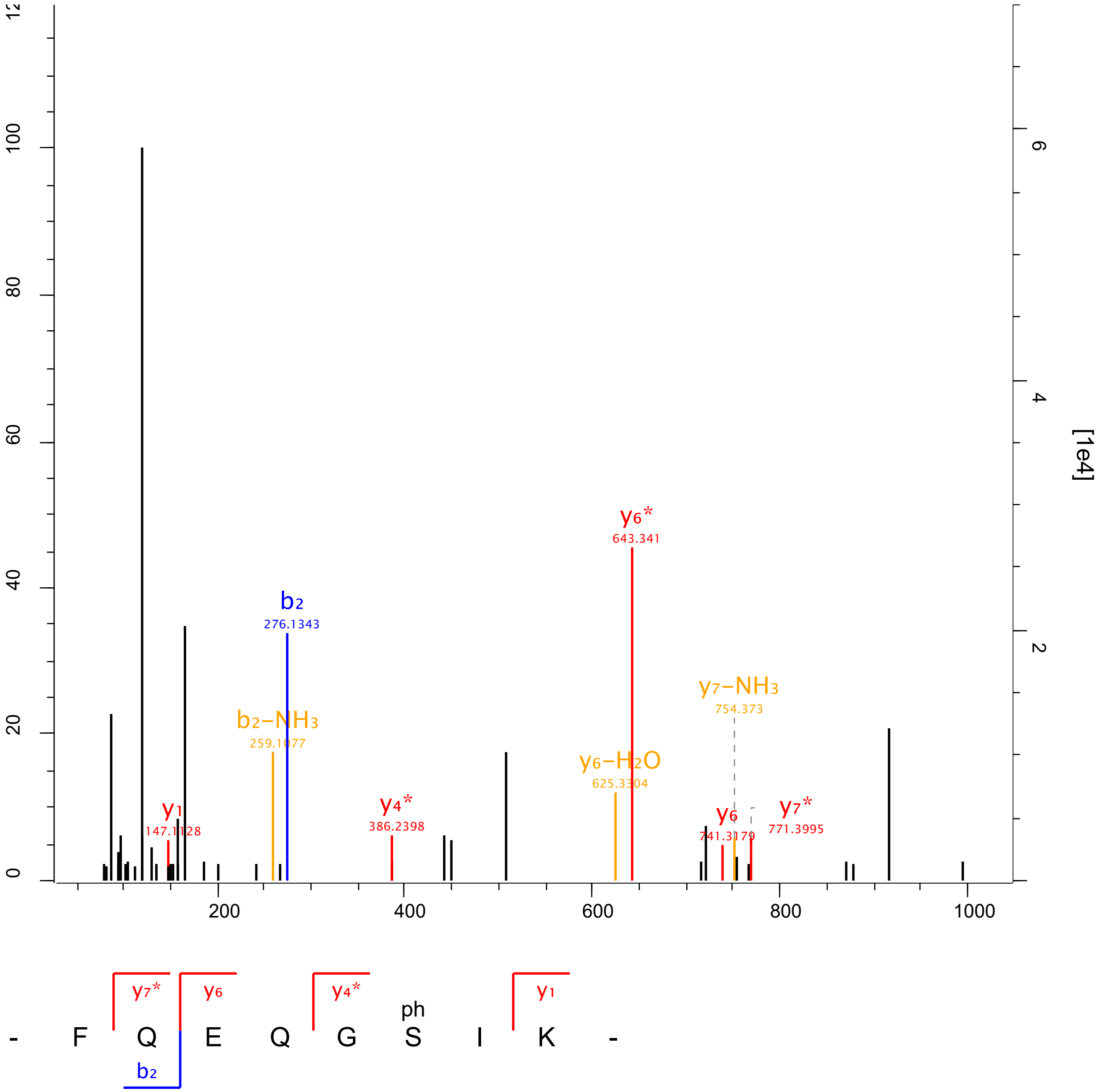

Raw file	Scan	Method	Score	m/z
sys_15_1	6010	FTMS; HCD	123.26	532.21

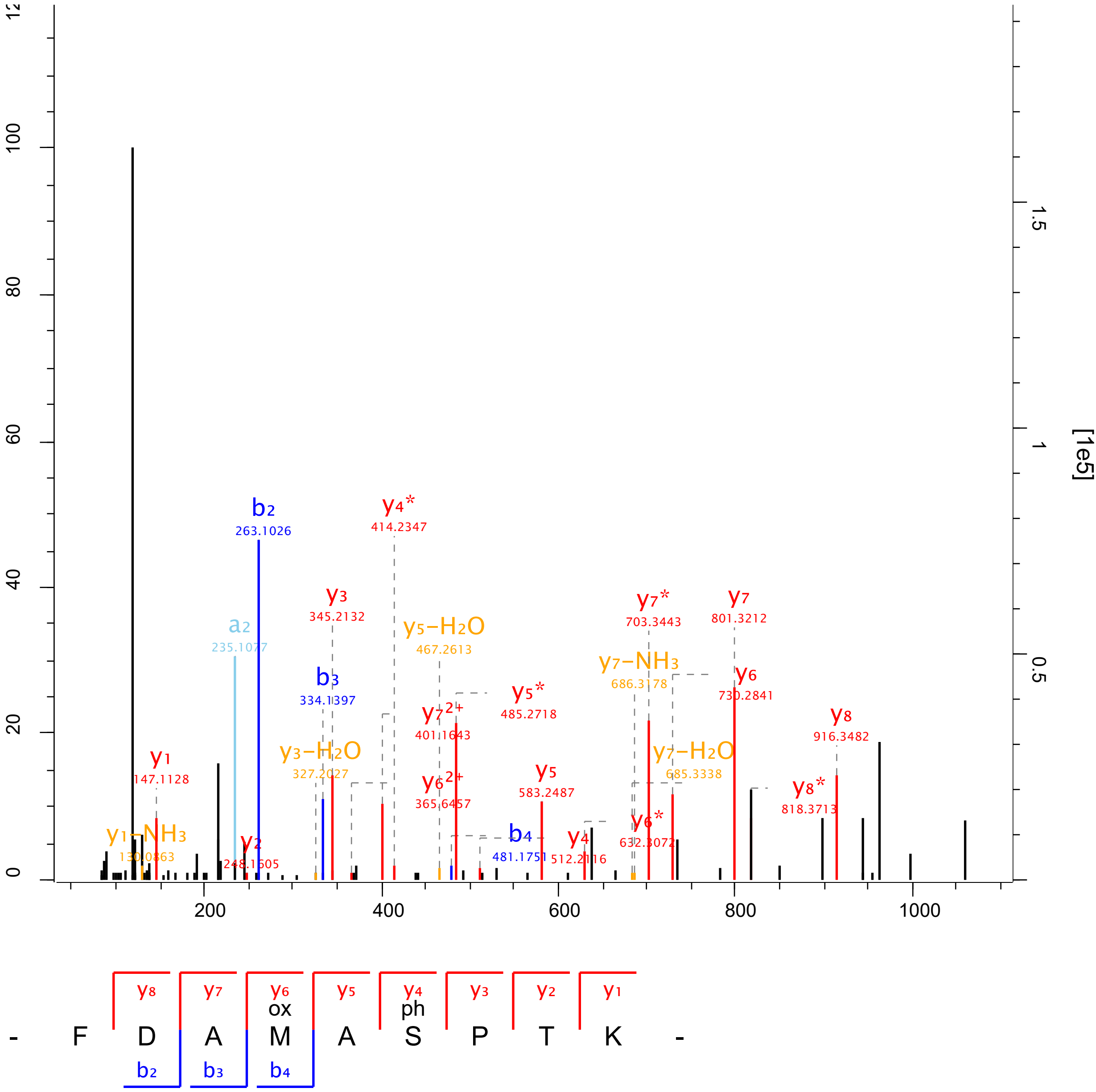

Raw file	Scan	Method	Score	m/z
sys_15_1	6053	FTMS; HCD	49.45	545.71

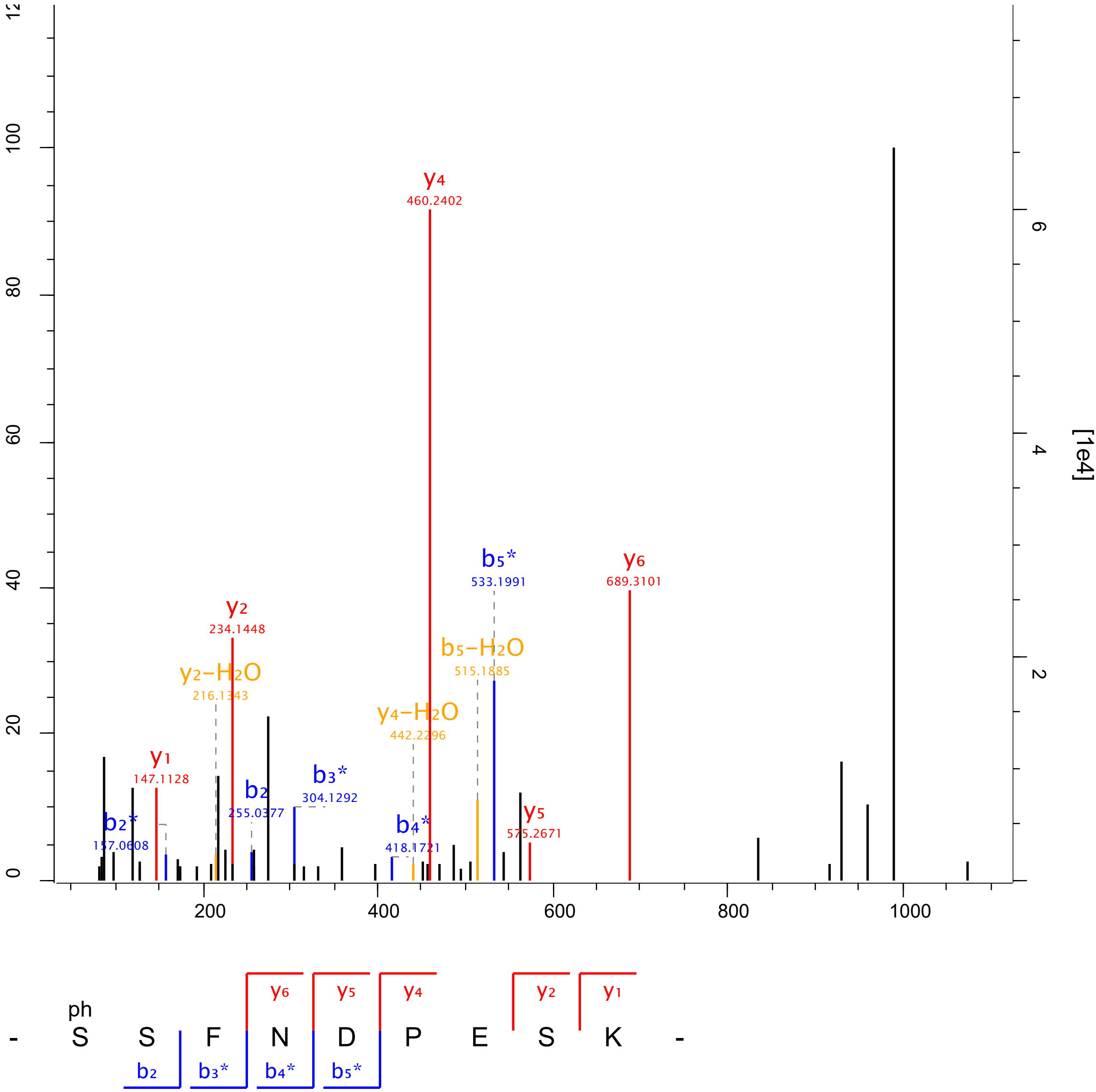

Raw file	Scan	Method	Score	m/z
sys_15_1	6138	FTMS; HCD	92.25	576.21

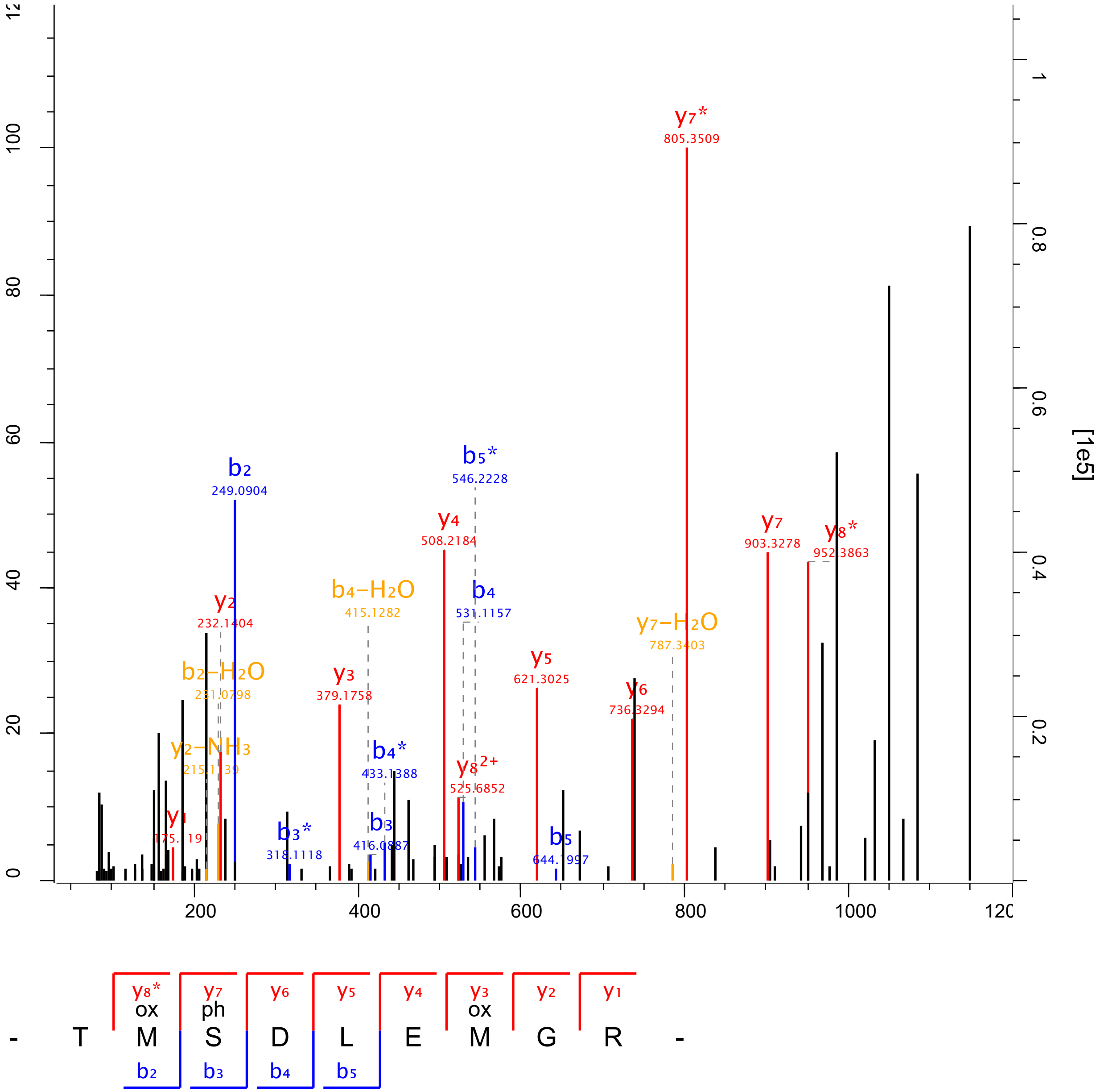

Raw file	Scan	Method	Score	m/z
sys_15_1	6191	FTMS; HCD	71.35	584.21

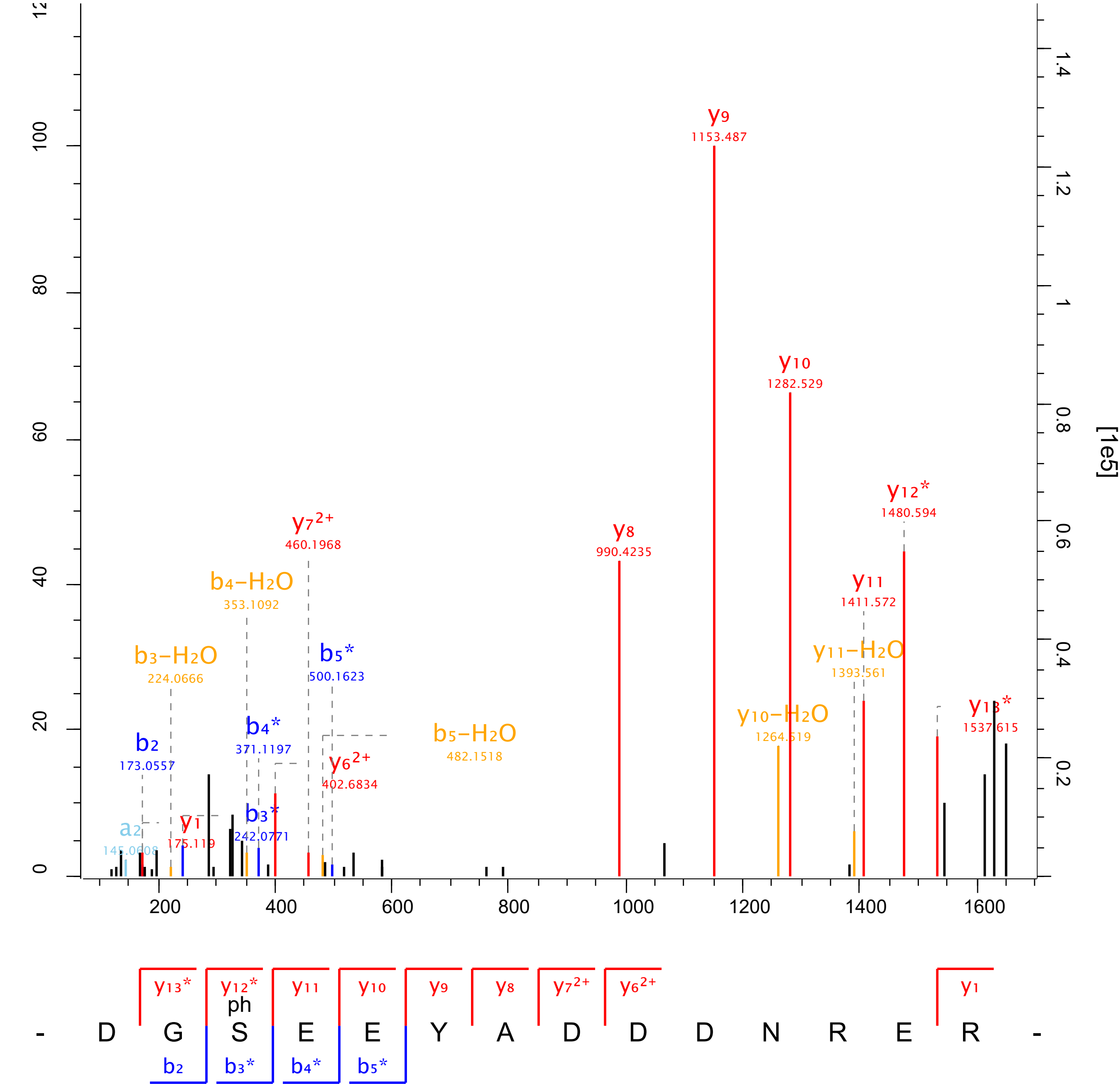

Raw file	Scan	Method	Score	m/z
sys_15_1	6215	FTMS; HCD	110.12	644.79

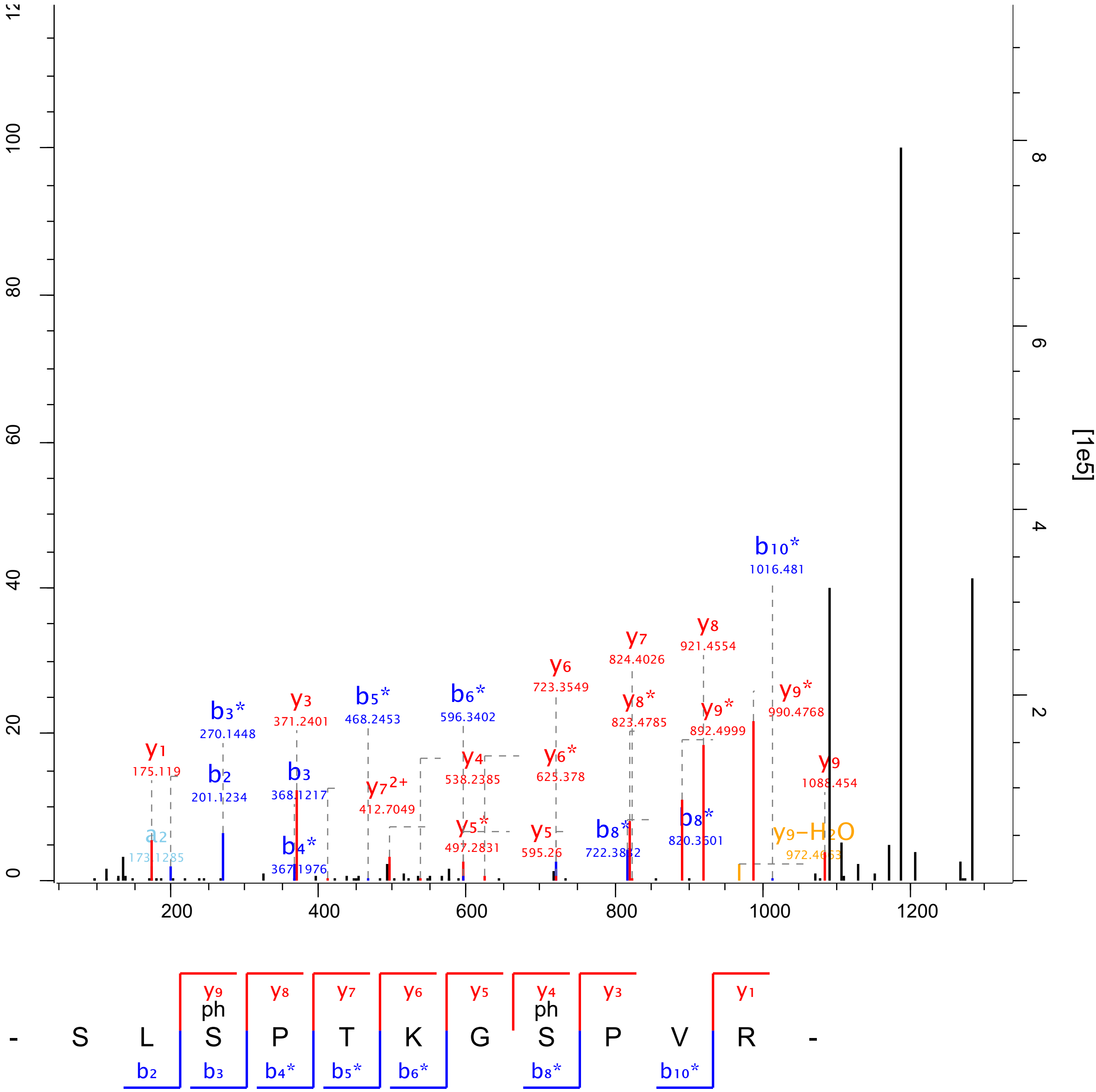

Raw file	Scan	Method	Score	m/z
sys_15_1	6484	FTMS; HCD	217.67	644.29

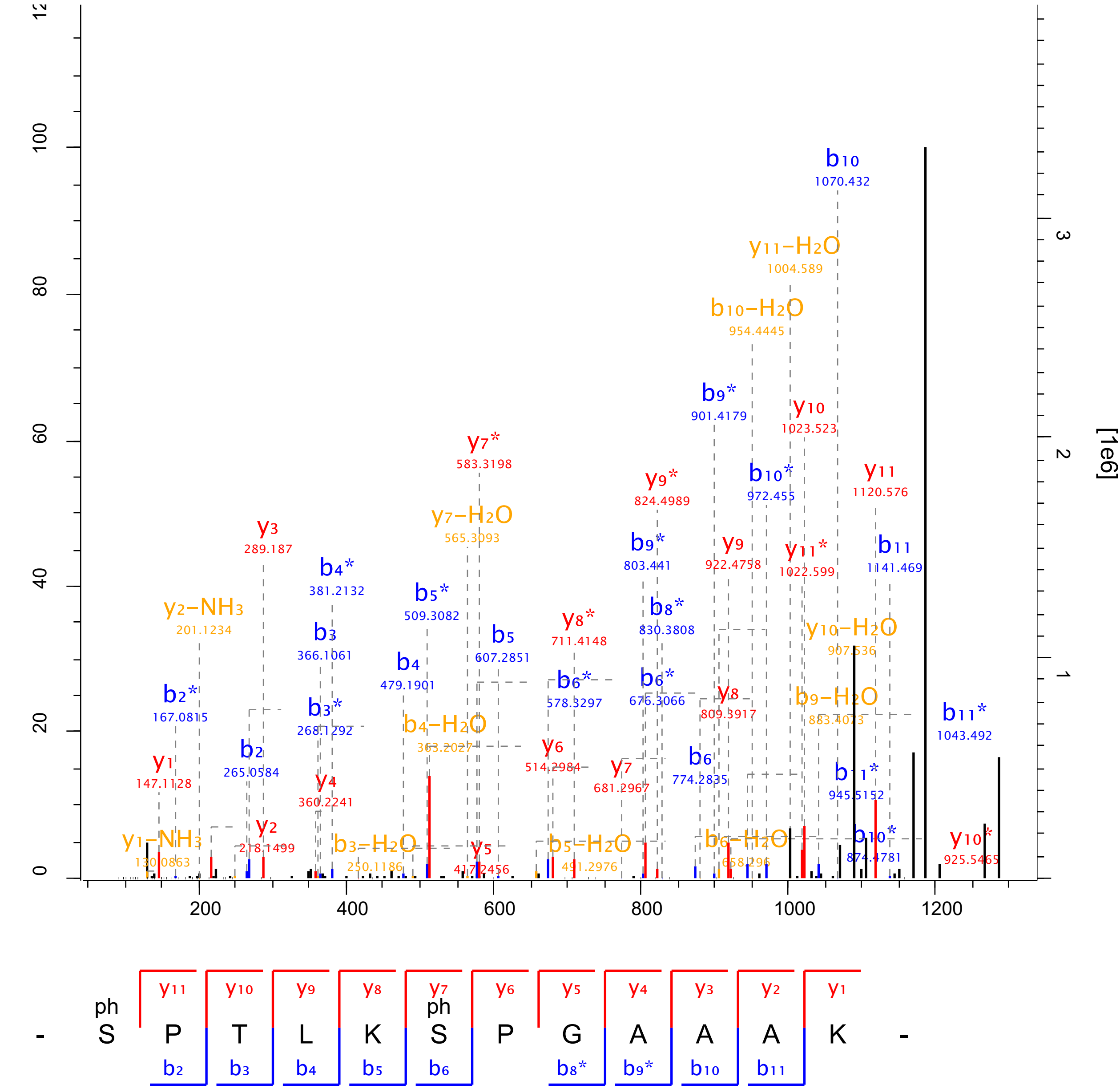

Raw file	Scan	Method	Score	m/z
sys_15_1	6488	FTMS; HCD	121.03	695.75

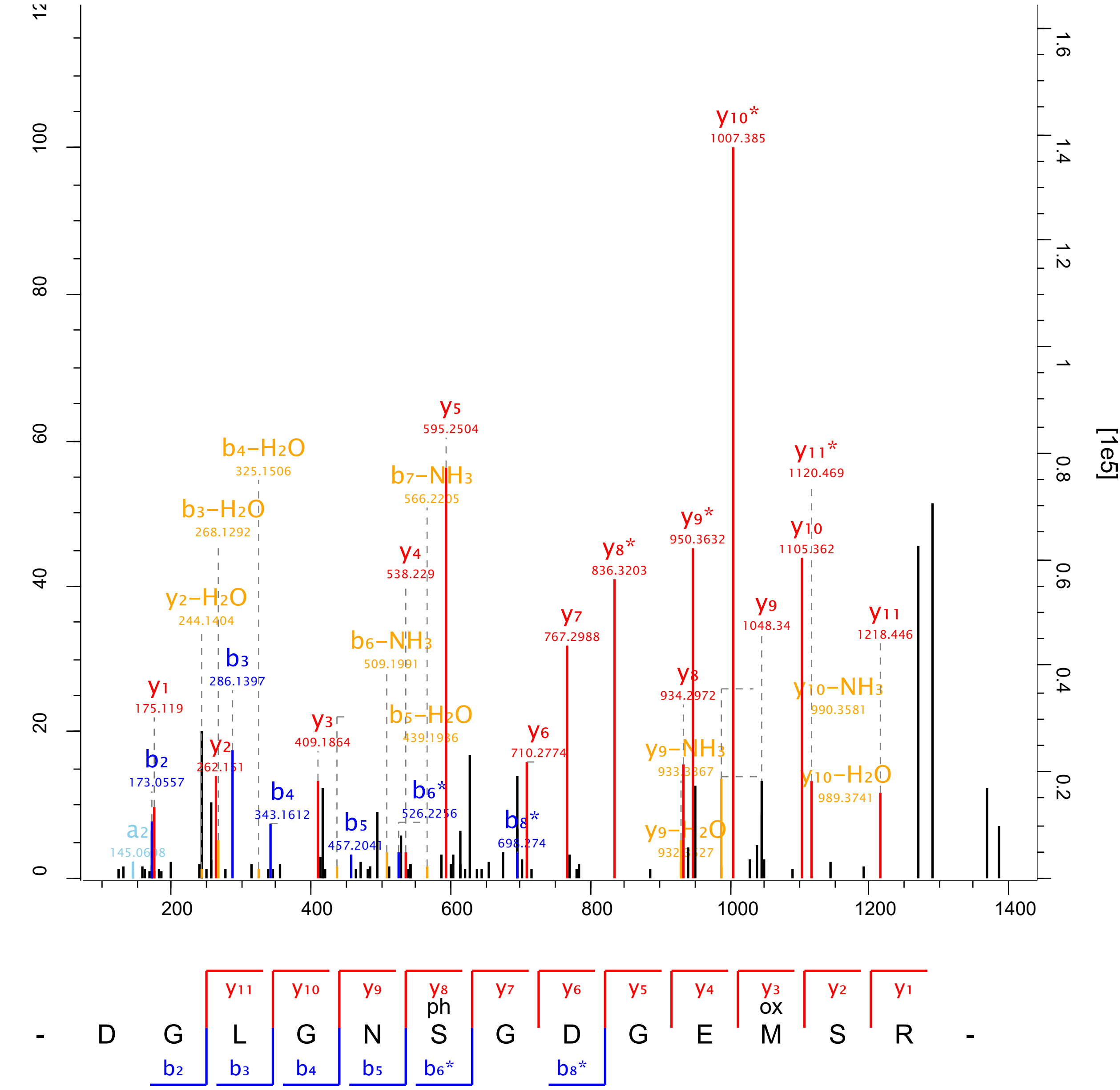

Raw file	Scan	Method	Score	m/z
sys_15_1	6498	FTMS; HCD	191.93	777.32

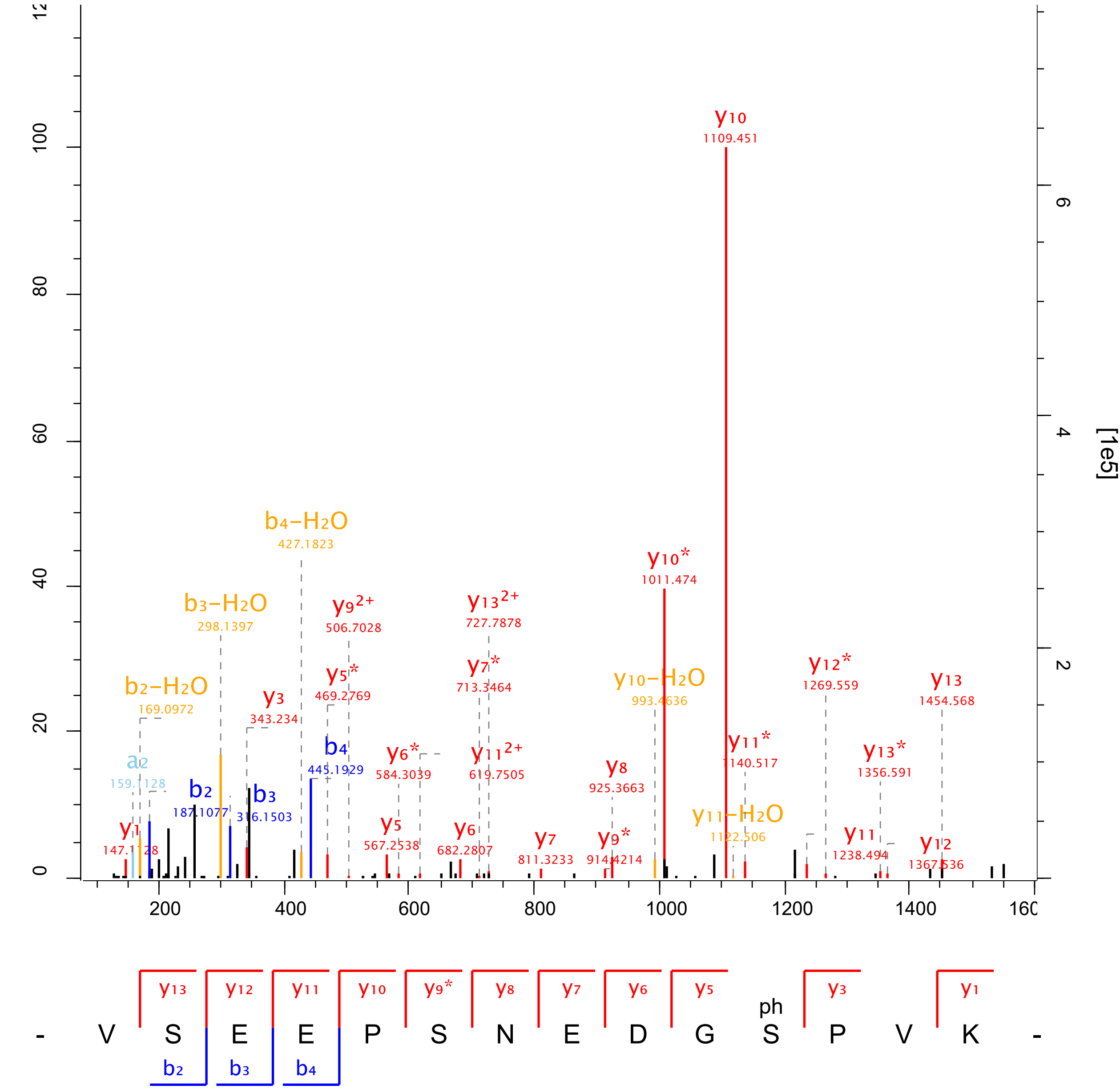

Raw file	Scan	Method	Score	m/z
sys_15_1	6565	FTMS; HCD	65.54	445.2

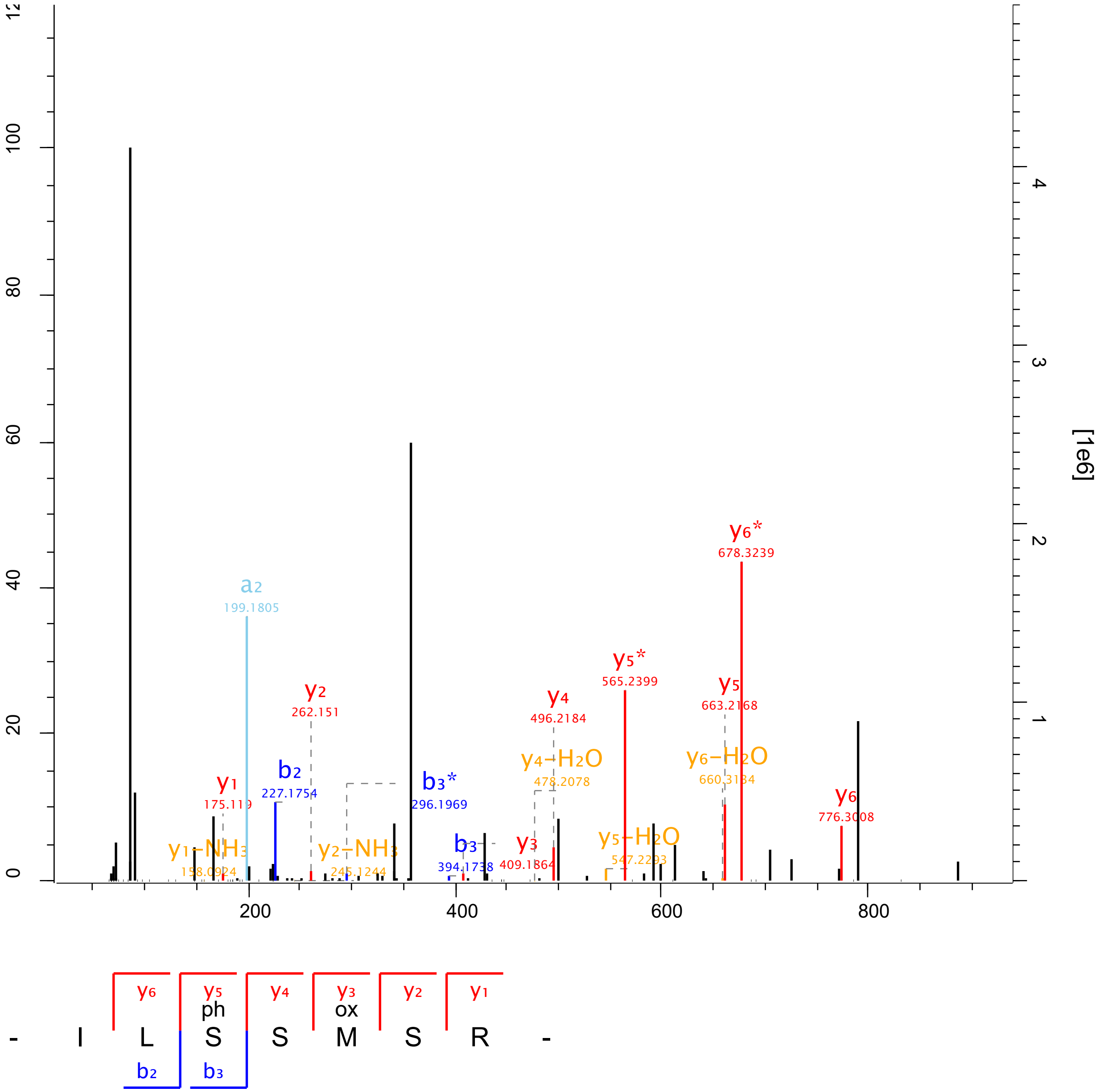

Raw file	Scan	Method	Score	m/z
sys_15_1	6585	FTMS; HCD	57.16	600.74

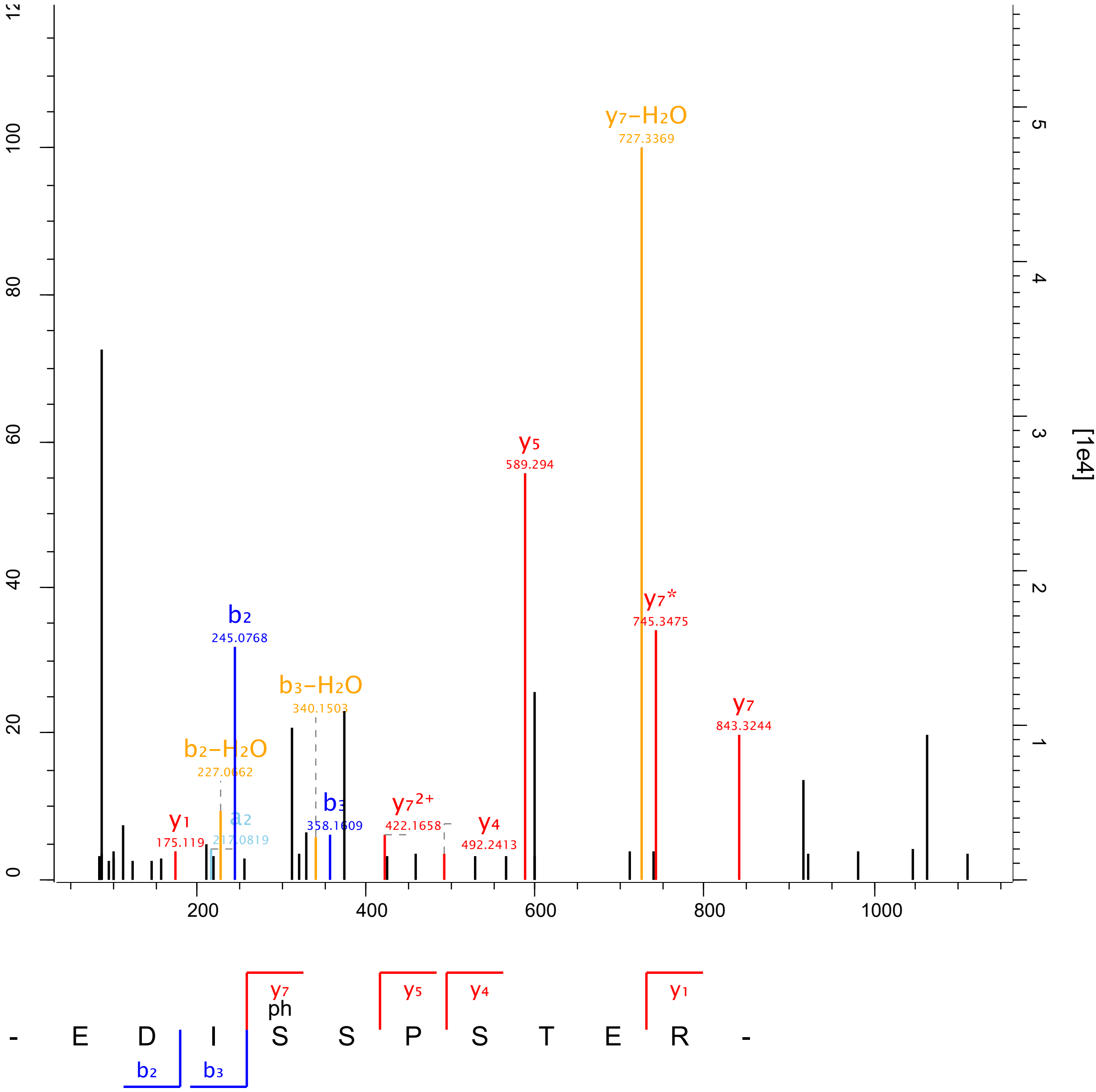

Raw file	Scan	Method	Score	m/z
sys_15_1	6603	FTMS; HCD	49.3	610.26

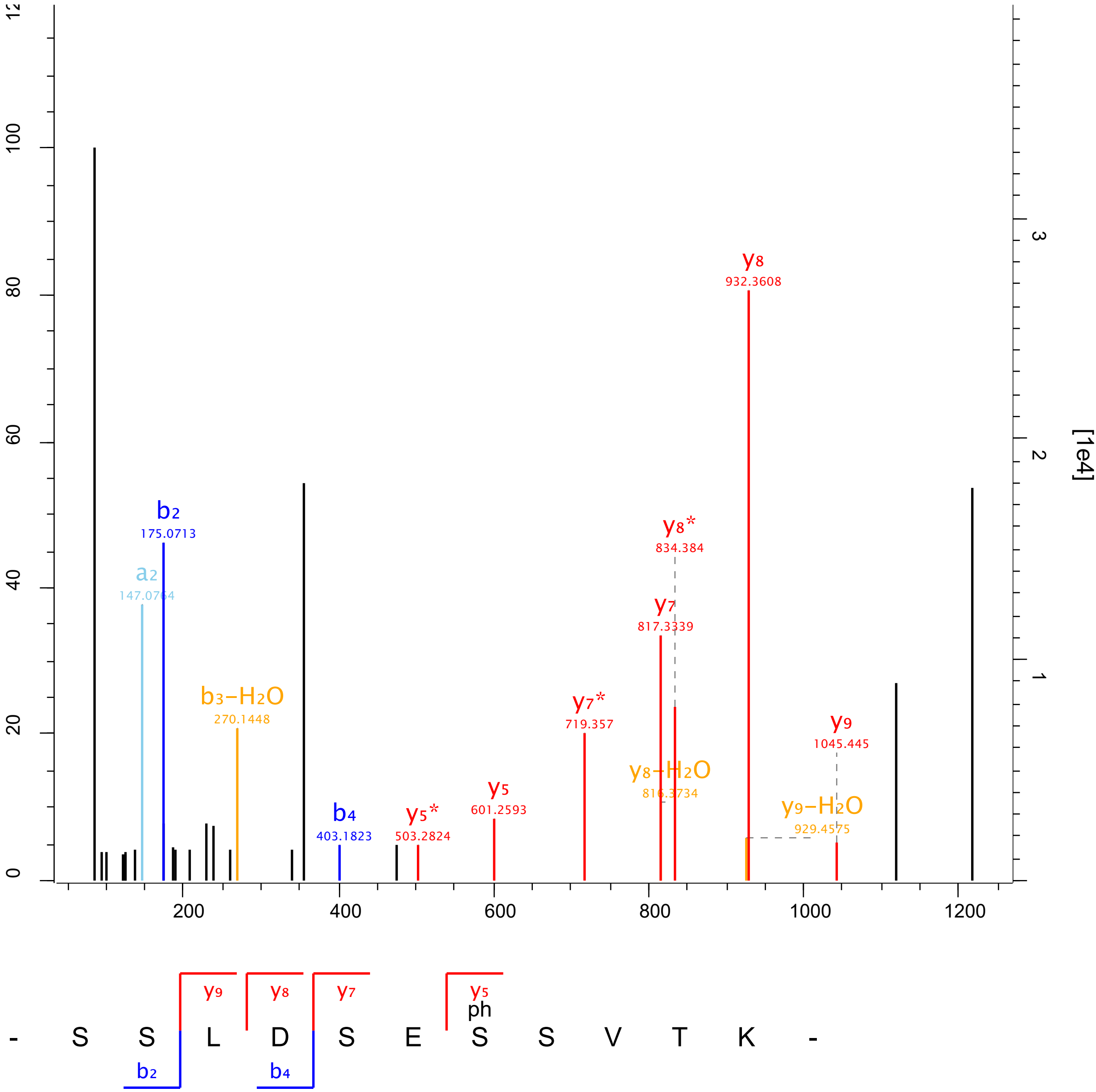

Raw file	Scan	Method	Score	m/z
sys_15_1	6715	FTMS; HCD	169.54	571.23

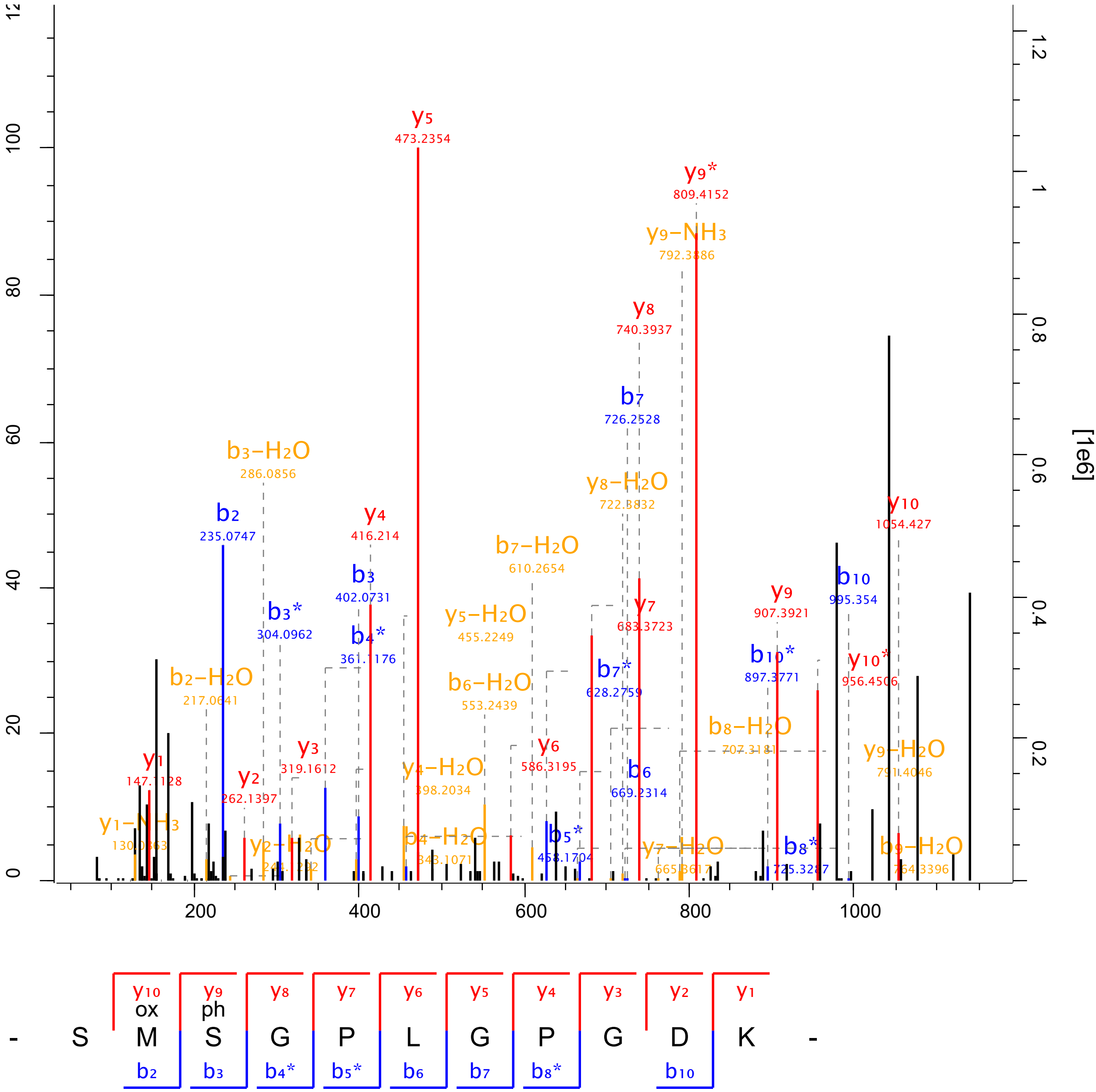

Raw file	Scan	Method	Score	m/z
sys_15_1	6718	FTMS; HCD	182.02	675.76

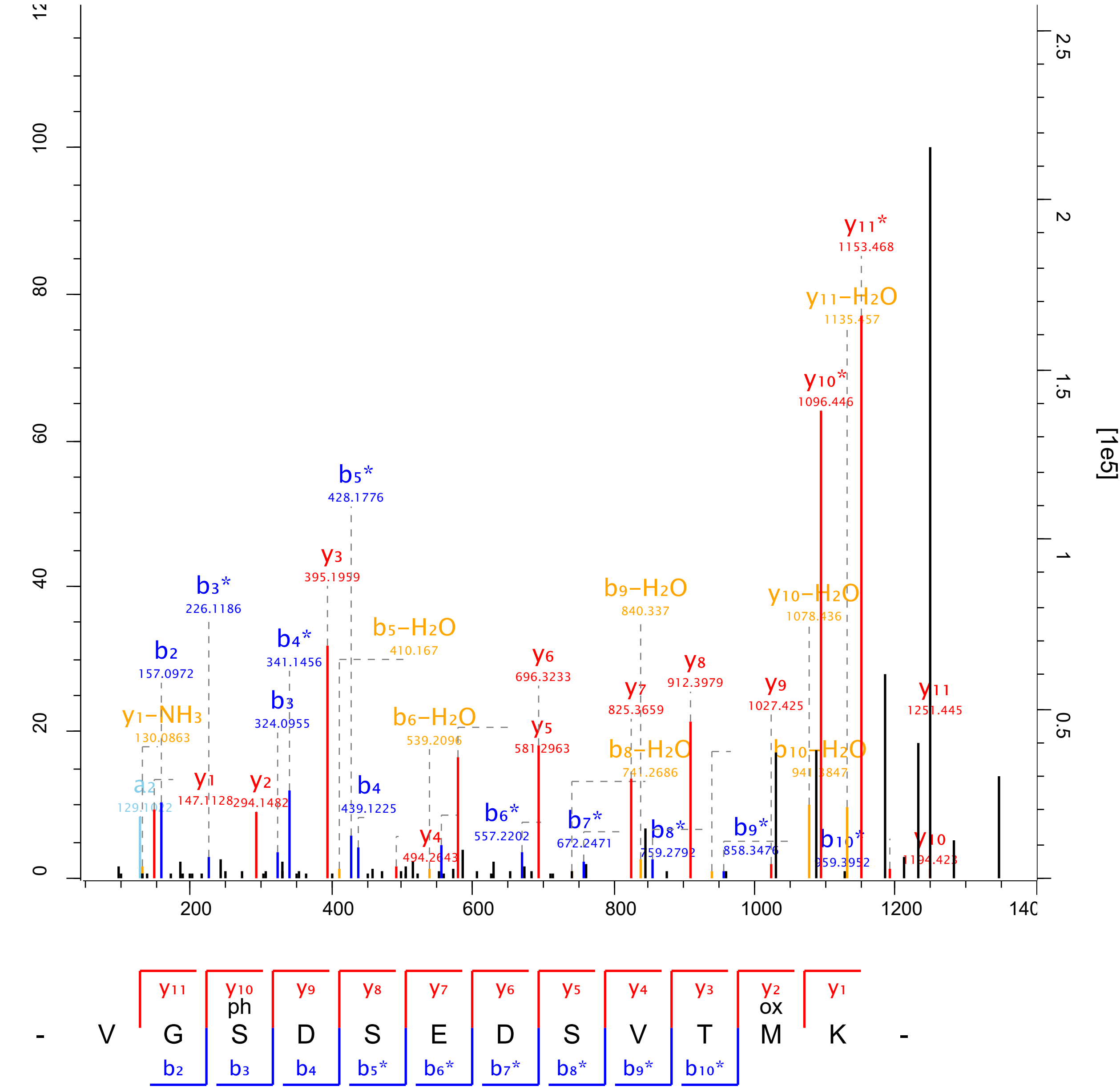

Raw file	Scan	Method	Score	m/z
sys_15_1	6732	FTMS; HCD	210.98	762.35

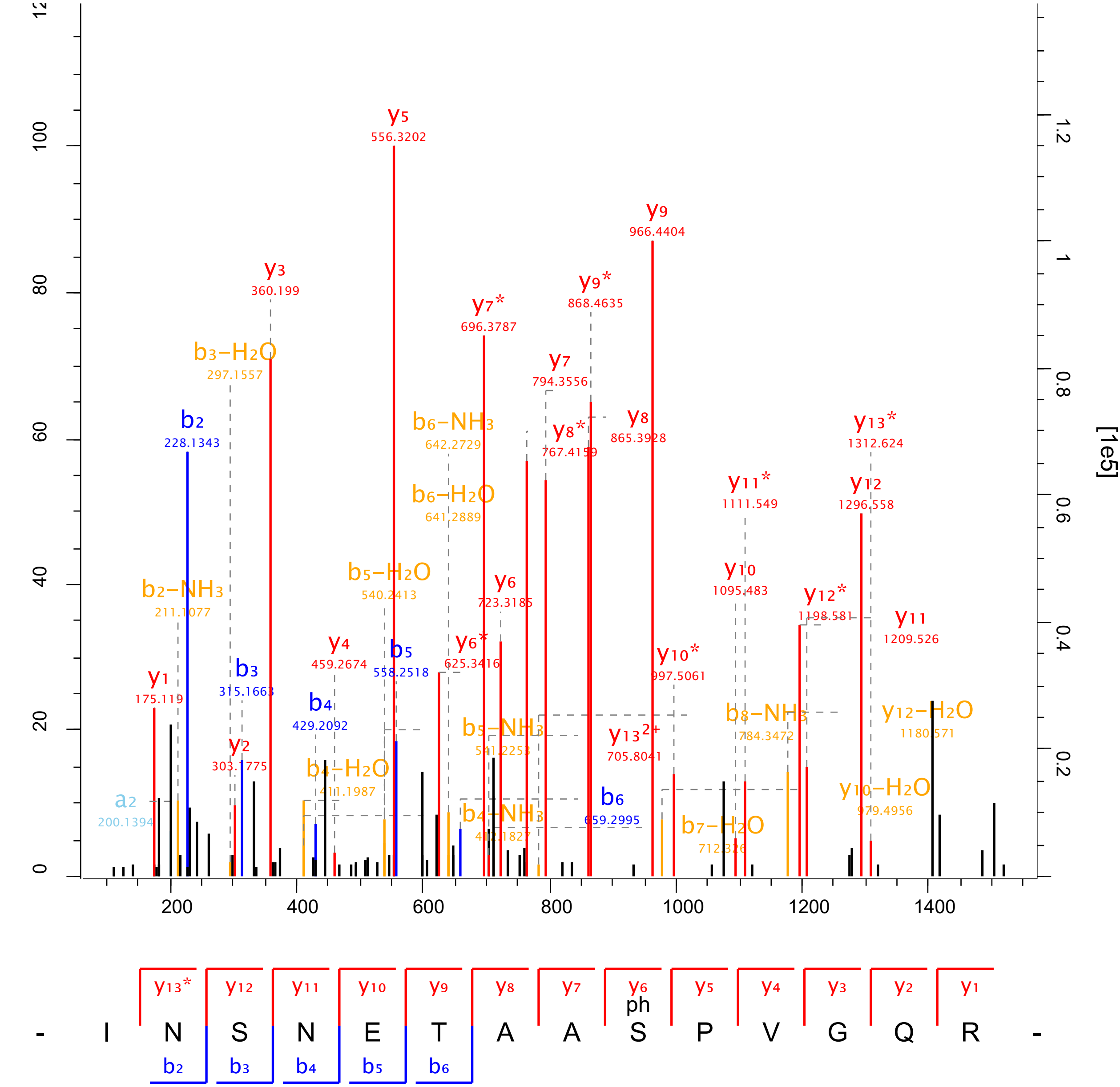

Raw file	Scan	Method	Score	m/z
sys_15_1	6849	FTMS; HCD	69.03	638.72

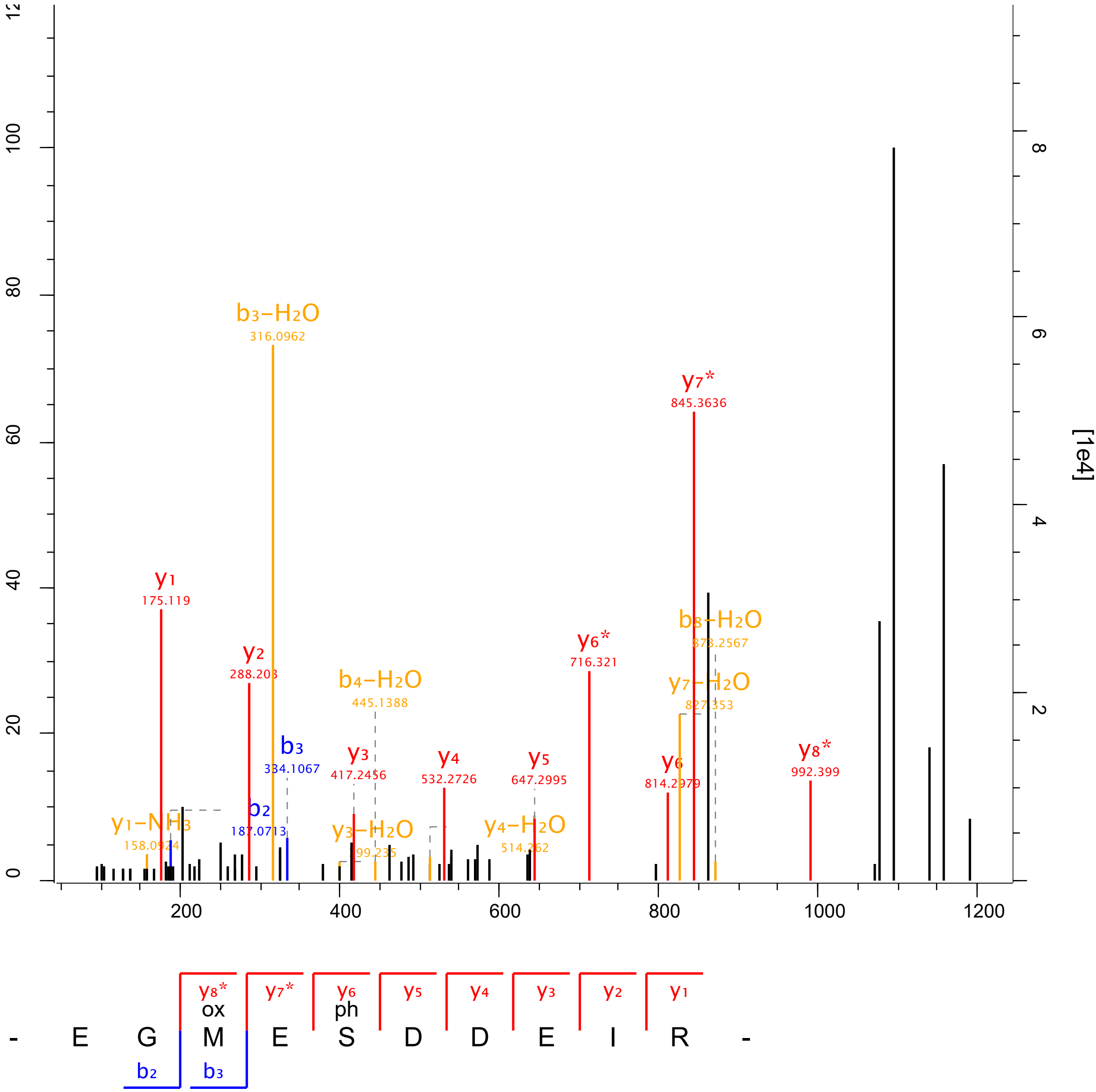

Raw file	Scan	Method	Score	m/z
sys_15_1	6870	FTMS; HCD	41.62	623.25

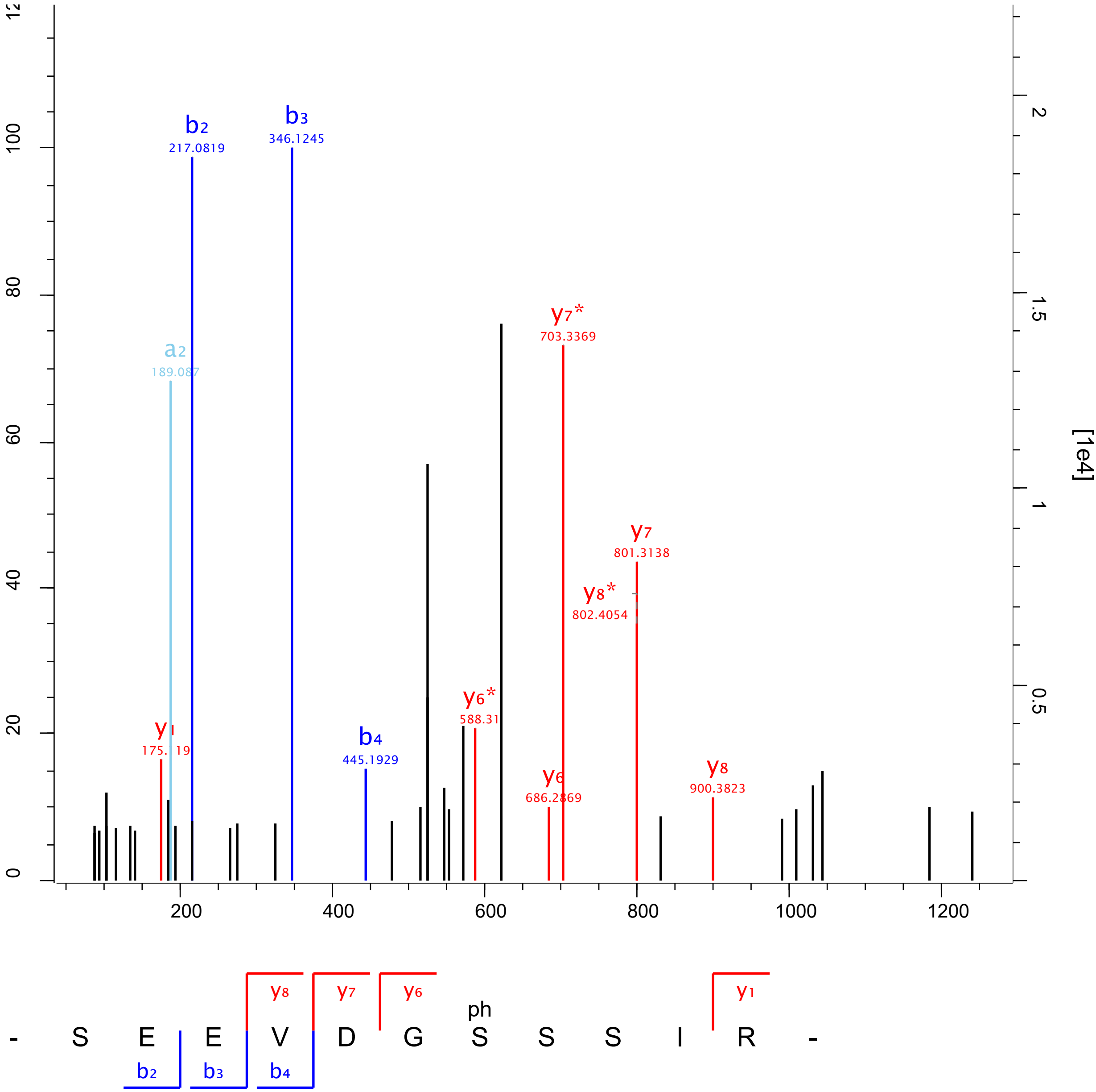

Raw file	Scan	Method	Score	m/z
sys_15_1	7019	FTMS; HCD	73.63	525.2

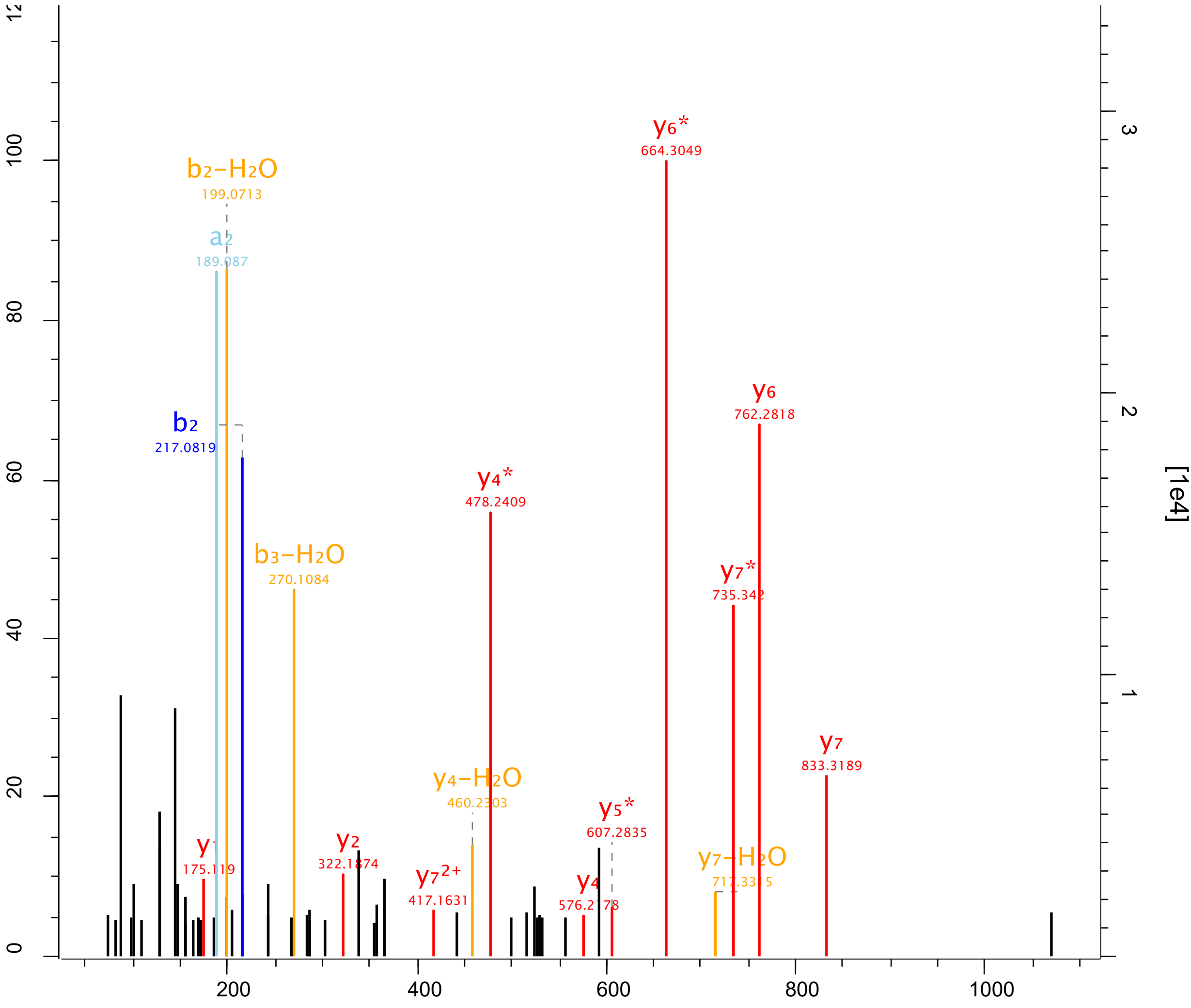

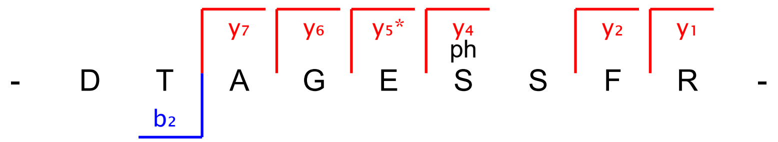

Raw file	Scan	Method	Score	m/z
sys_15_1	7060	FTMS; HCD	41.62	793.86

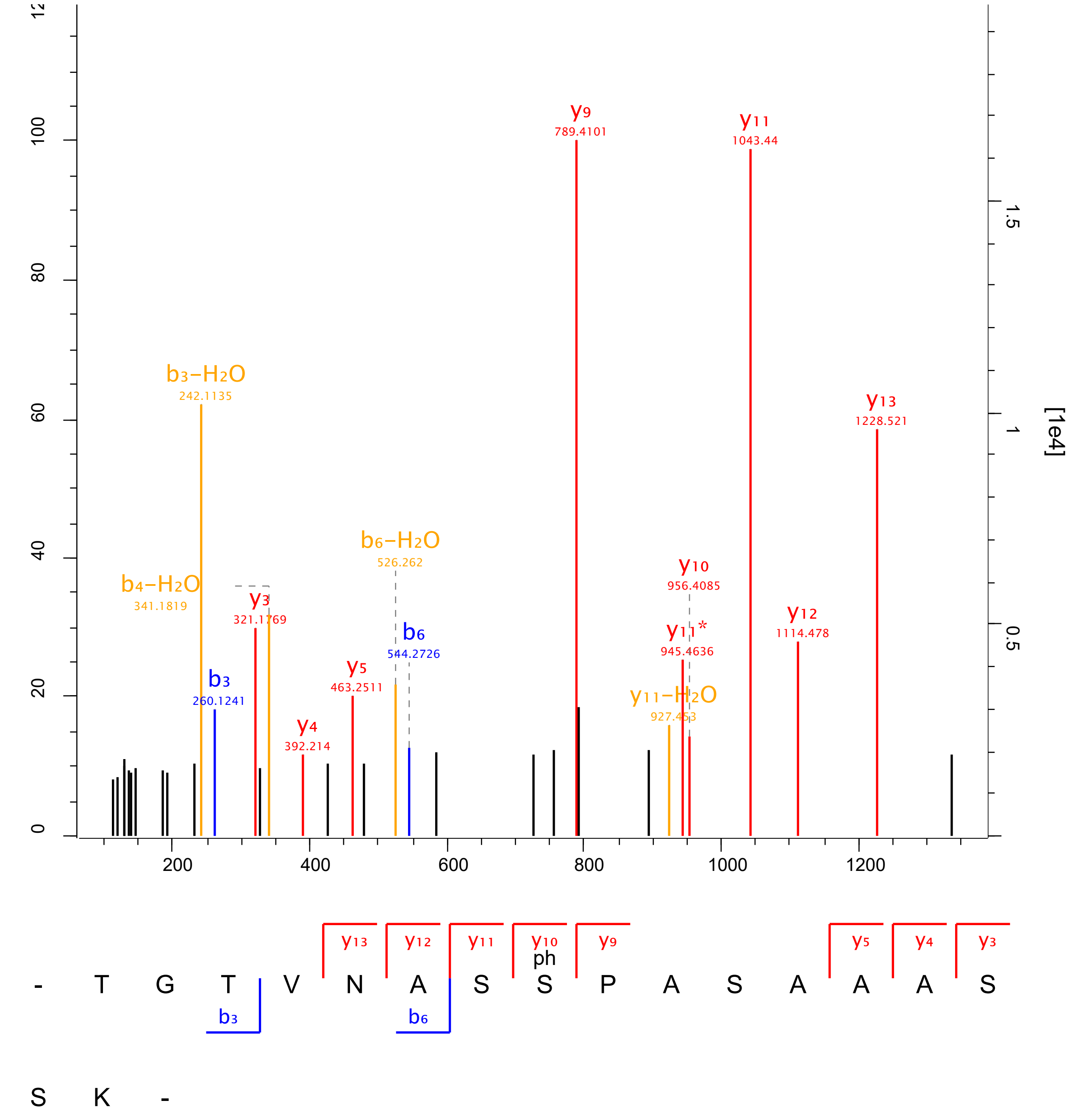

Raw file	Scan	Method	Score	m/z
sys_15_1	7062	FTMS; HCD	43.76	688.27

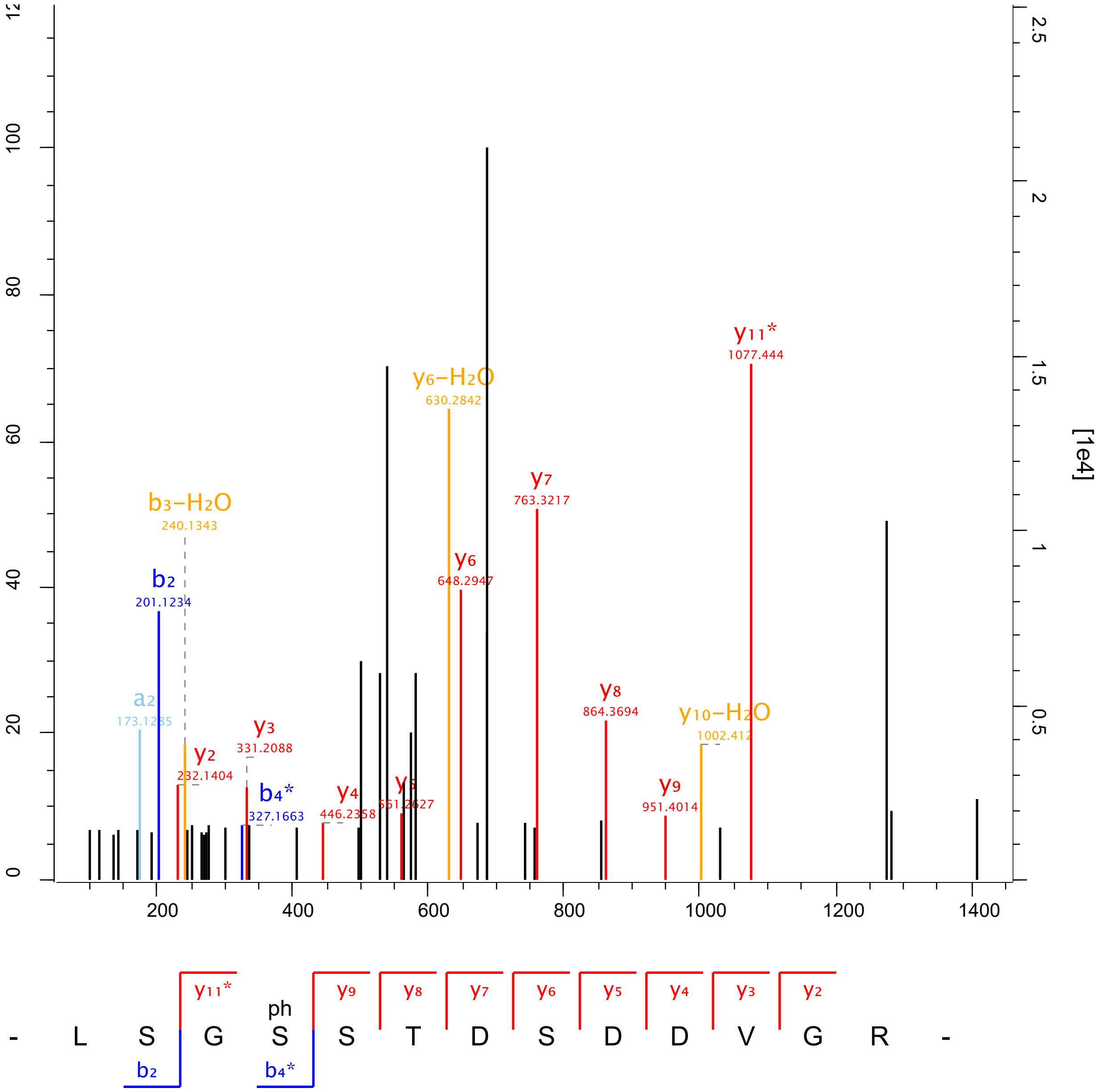

Raw file	Scan	Method	Score	m/z
sys_15_1	7068	FTMS; HCD	162.31	709.25

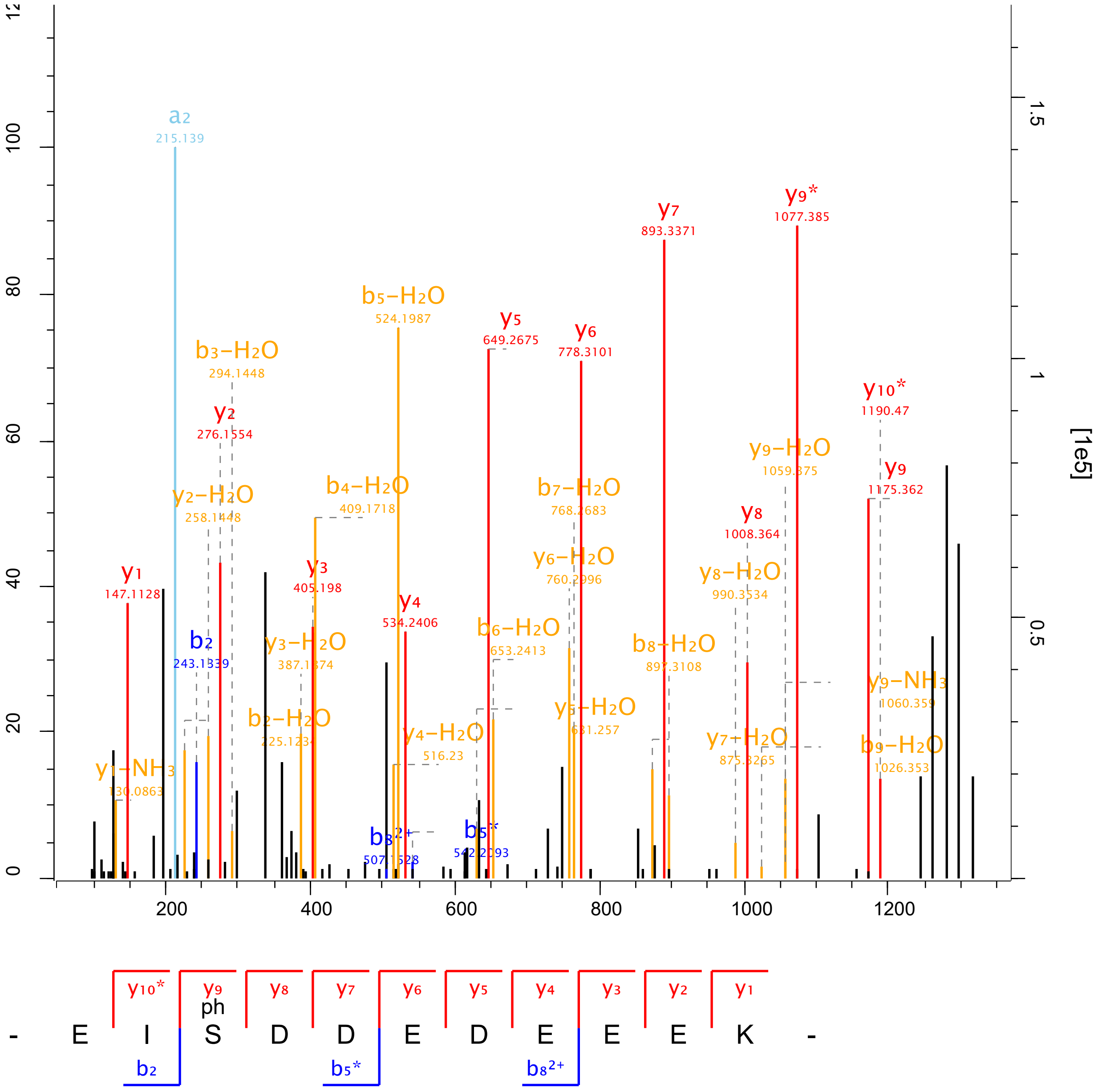

Raw file	Scan	Method	Score	m/z
sys_15_1	7132	FTMS; HCD	87.18	499.22

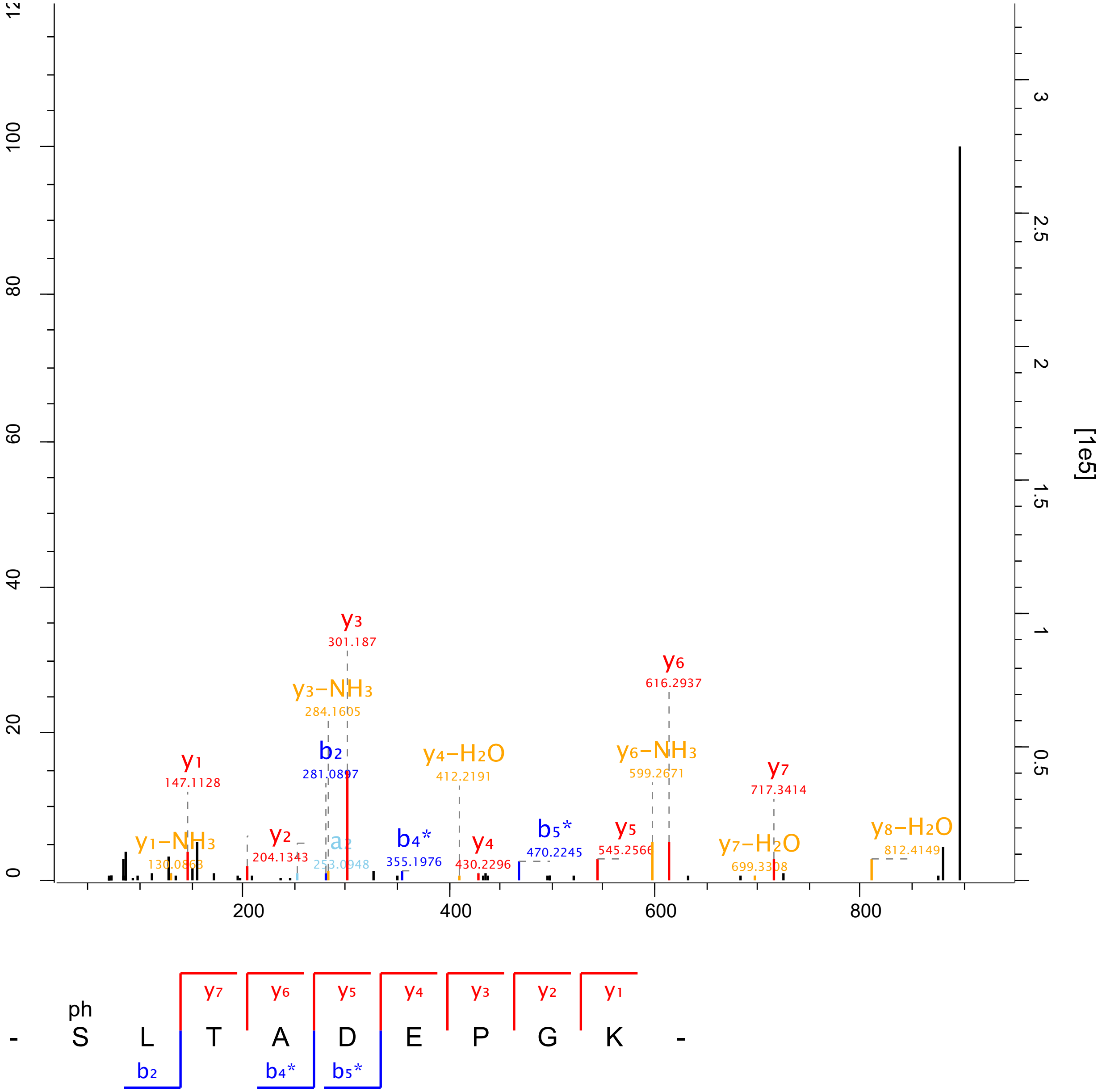

Raw file	Scan	Method	Score	m/z
sys_15_1	7194	FTMS; HCD	72.29	550.25

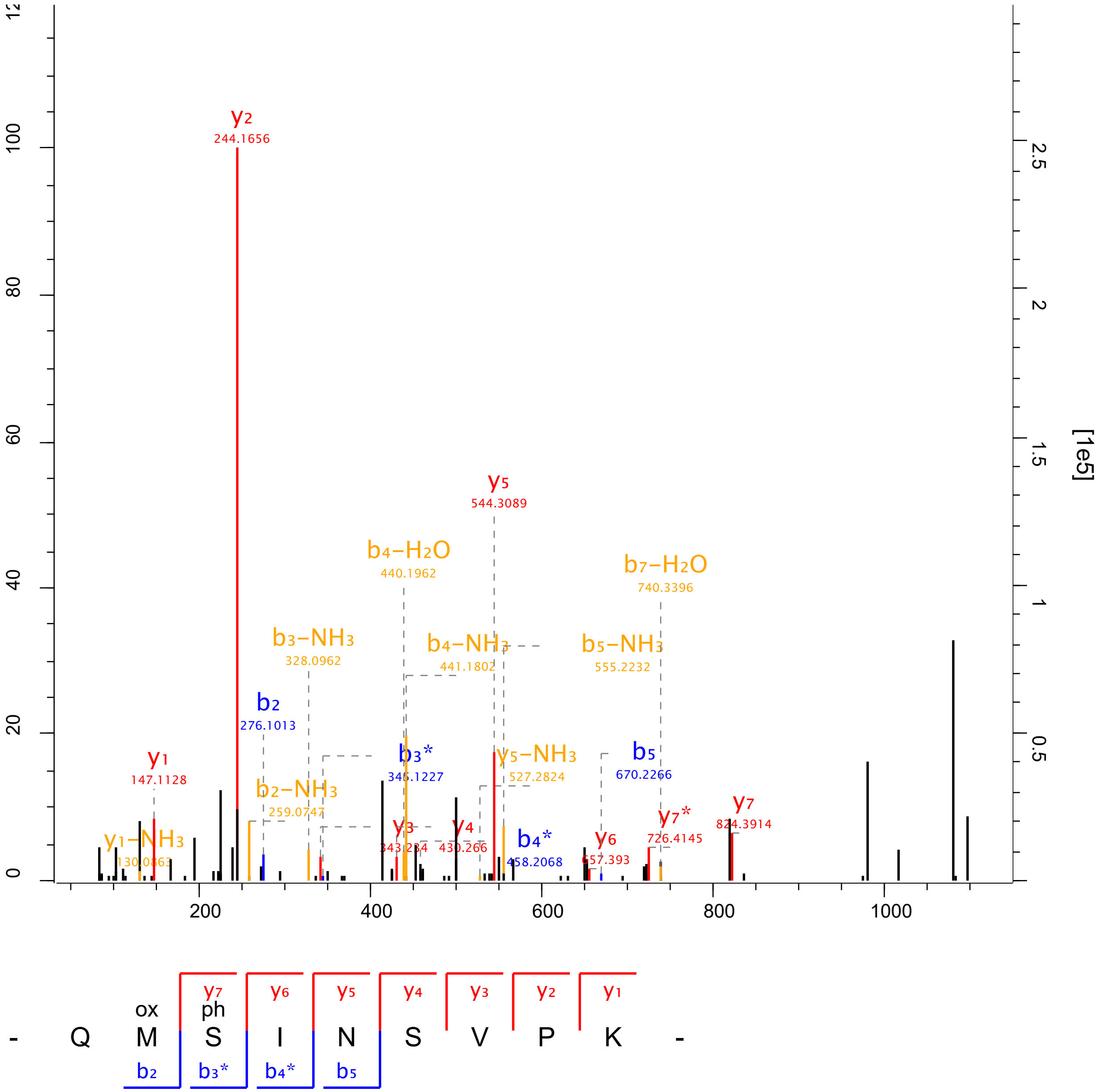

Raw file	Scan	Method	Score	m/z
sys_15_1	7232	FTMS; HCD	72.89	591.71

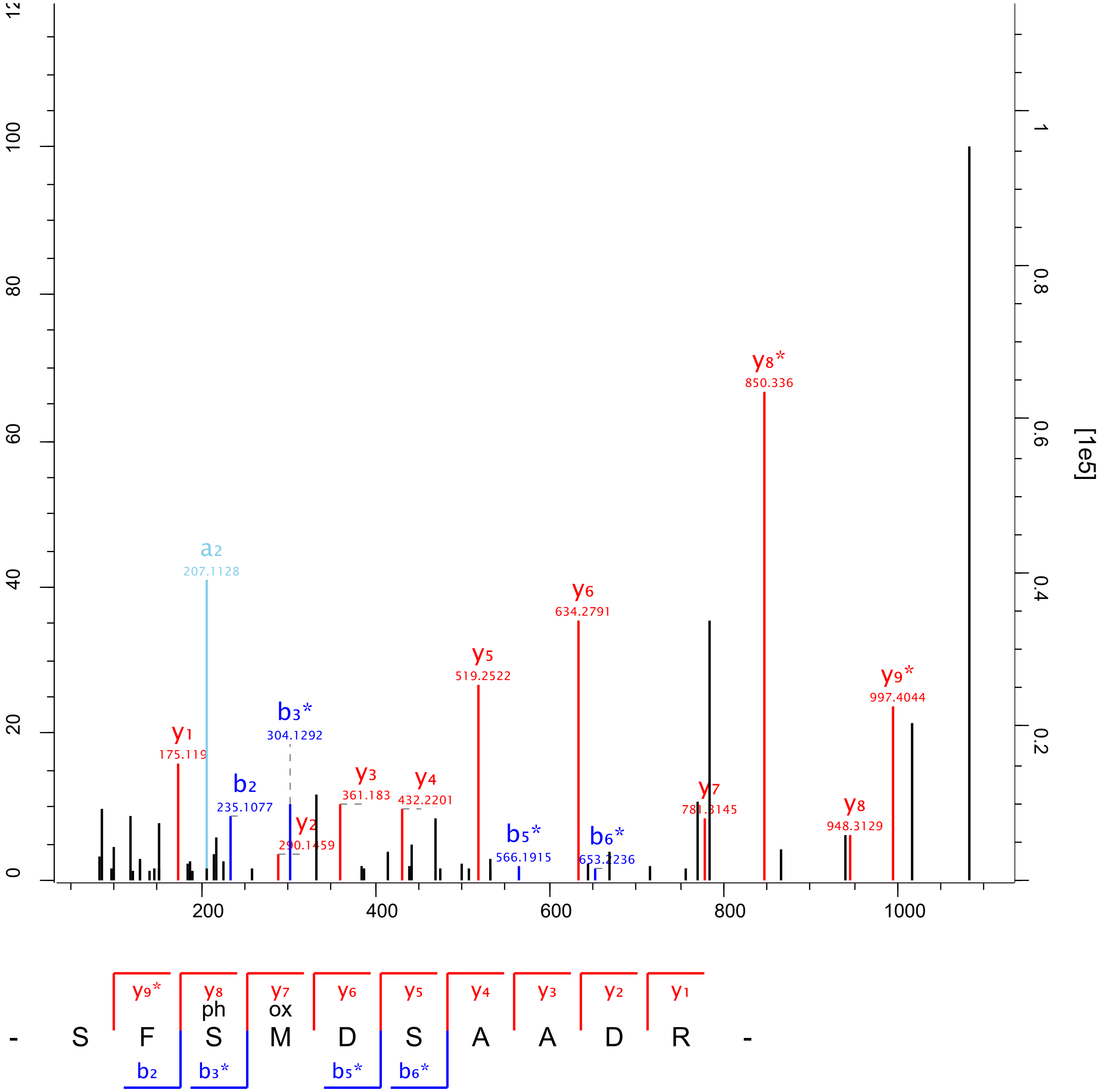

Raw file	Scan	Method	Score	m/z
sys_15_1	7368	FTMS; HCD	85.91	643.74

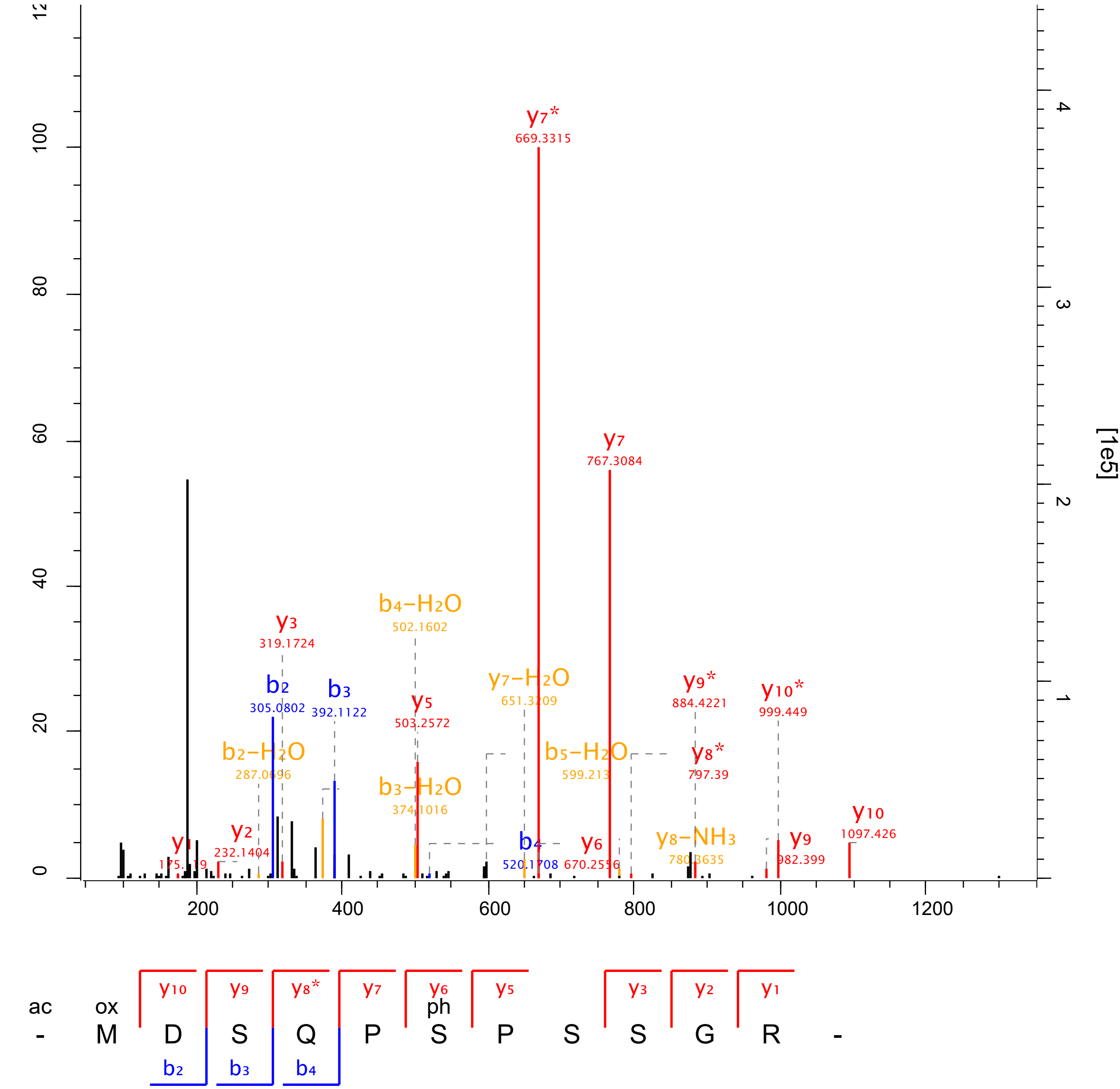

Raw file	Scan	Method	Score	m/z
sys_15_1	7398	FTMS; HCD	101.75	690.28

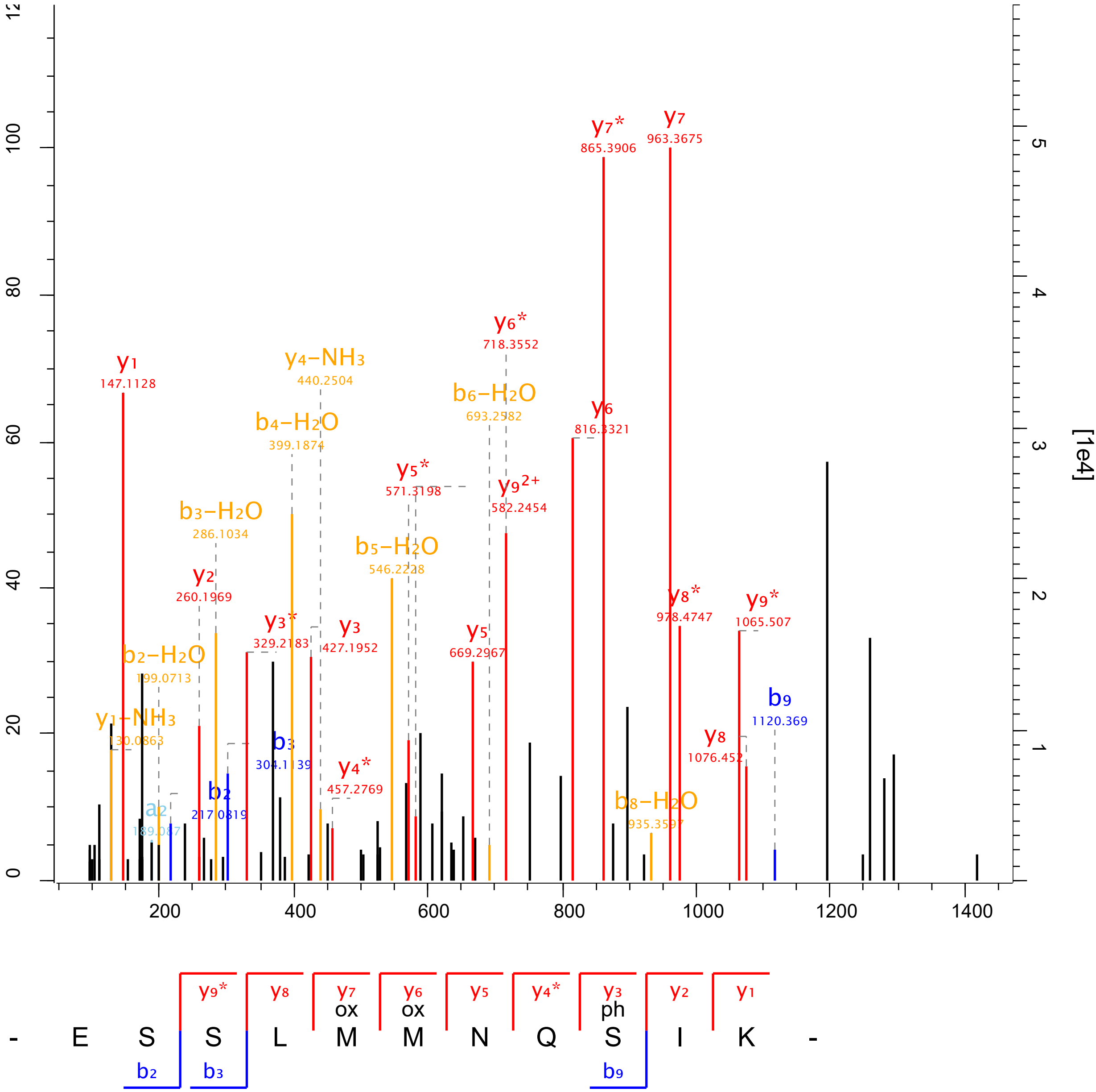

Raw file	Scan	Method	Score	m/z
sys_15_1	7409	FTMS; HCD	68.48	756.32

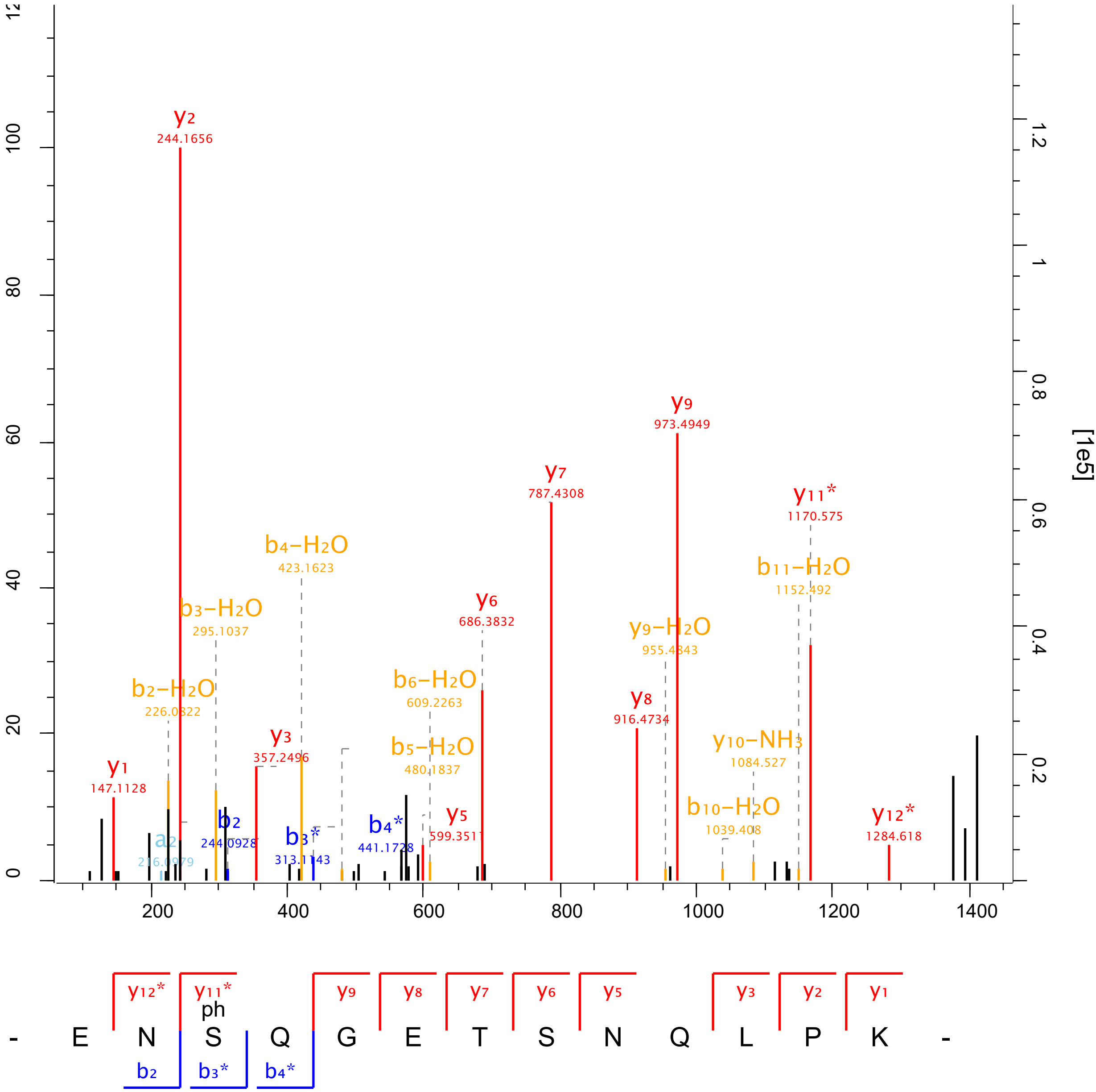

Raw file	Scan	Method	Score	m/z
sys_15_1	7423	FTMS; HCD	99.06	658.29

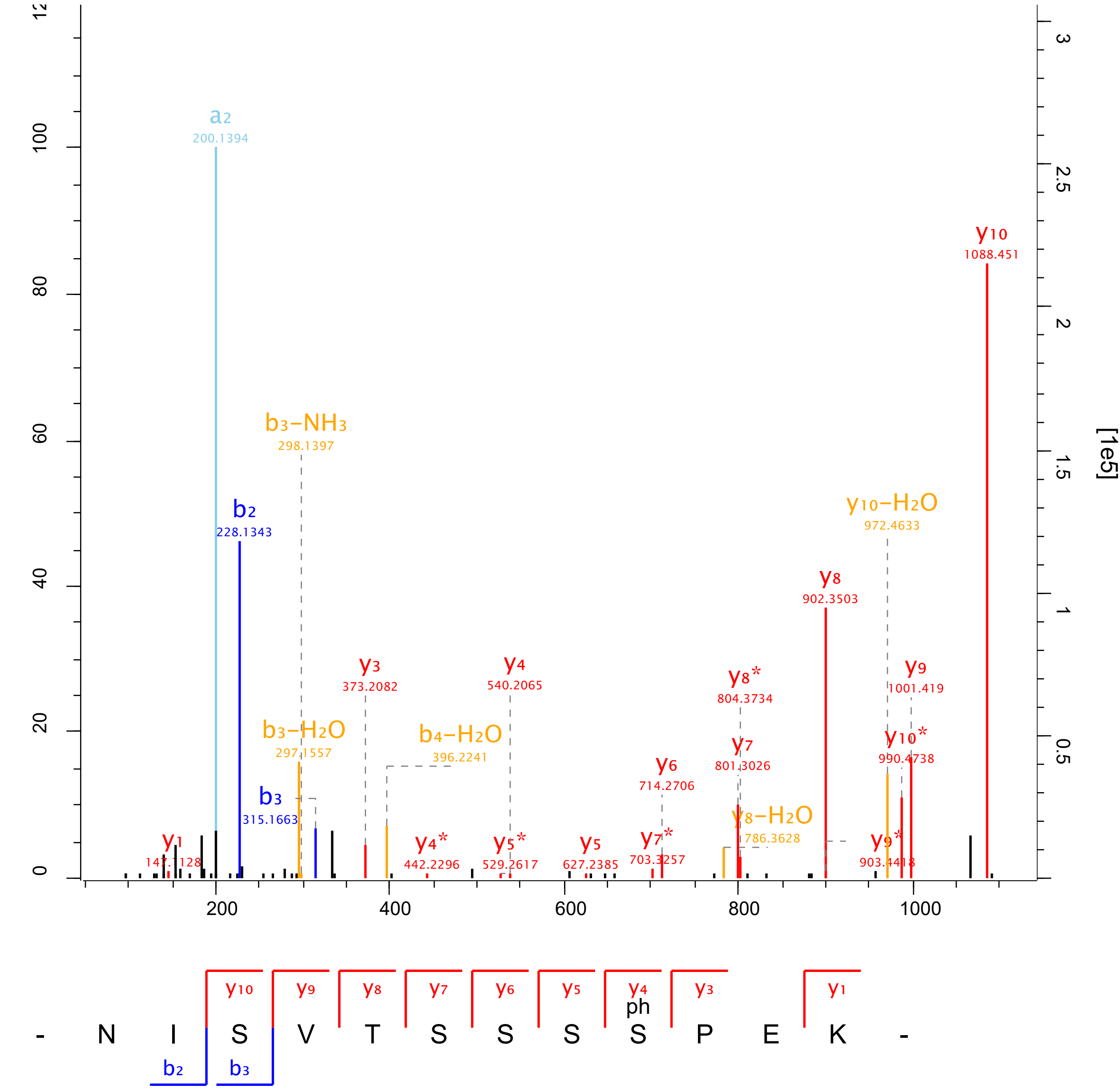

Raw file	Scan	Method	Score	m/z
sys_15_1	7559	FTMS; HCD	61.59	452.2

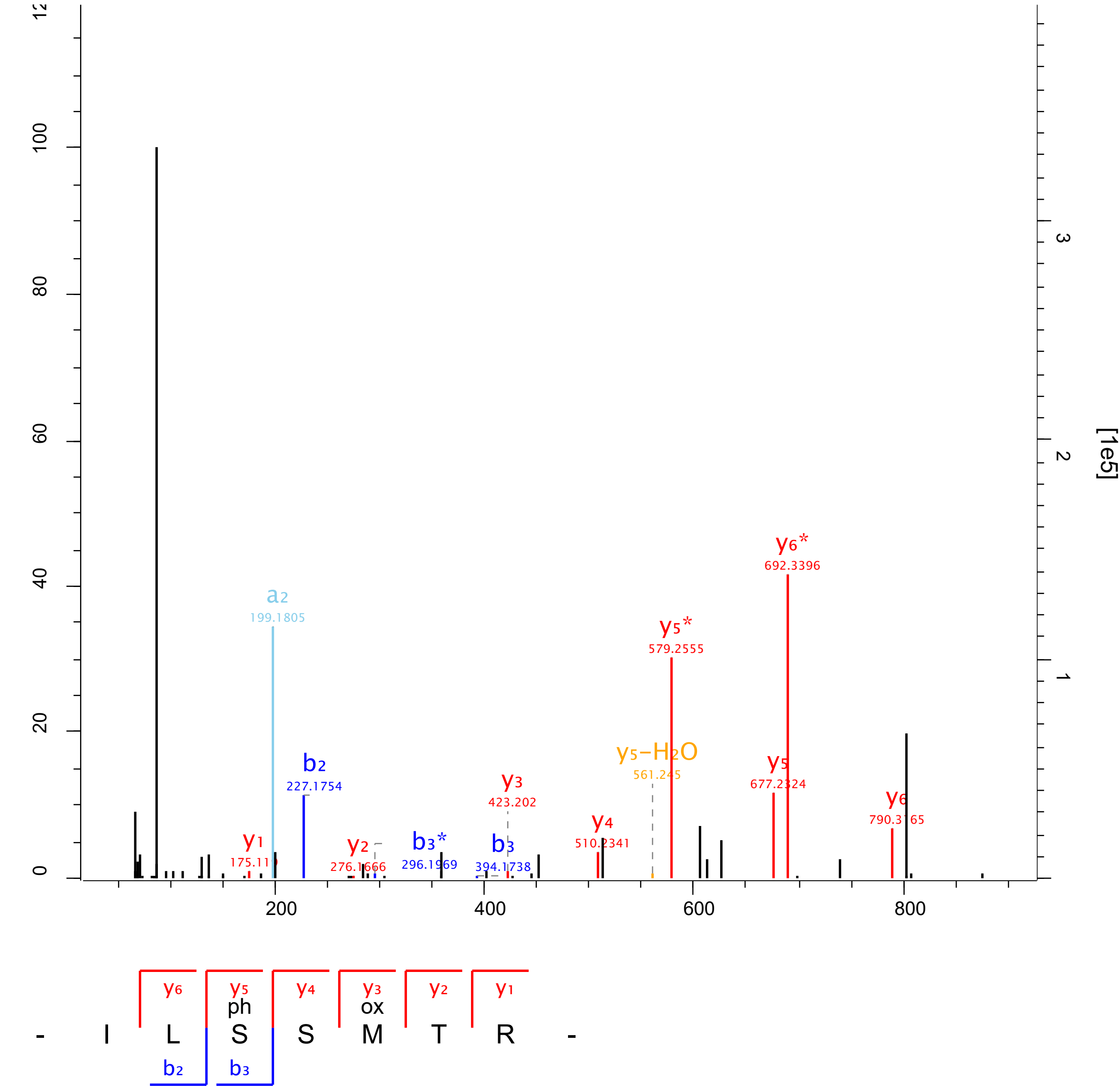

Raw file	Scan	Method	Score	m/z
sys_15_1	7591	FTMS; HCD	51.76	768.77

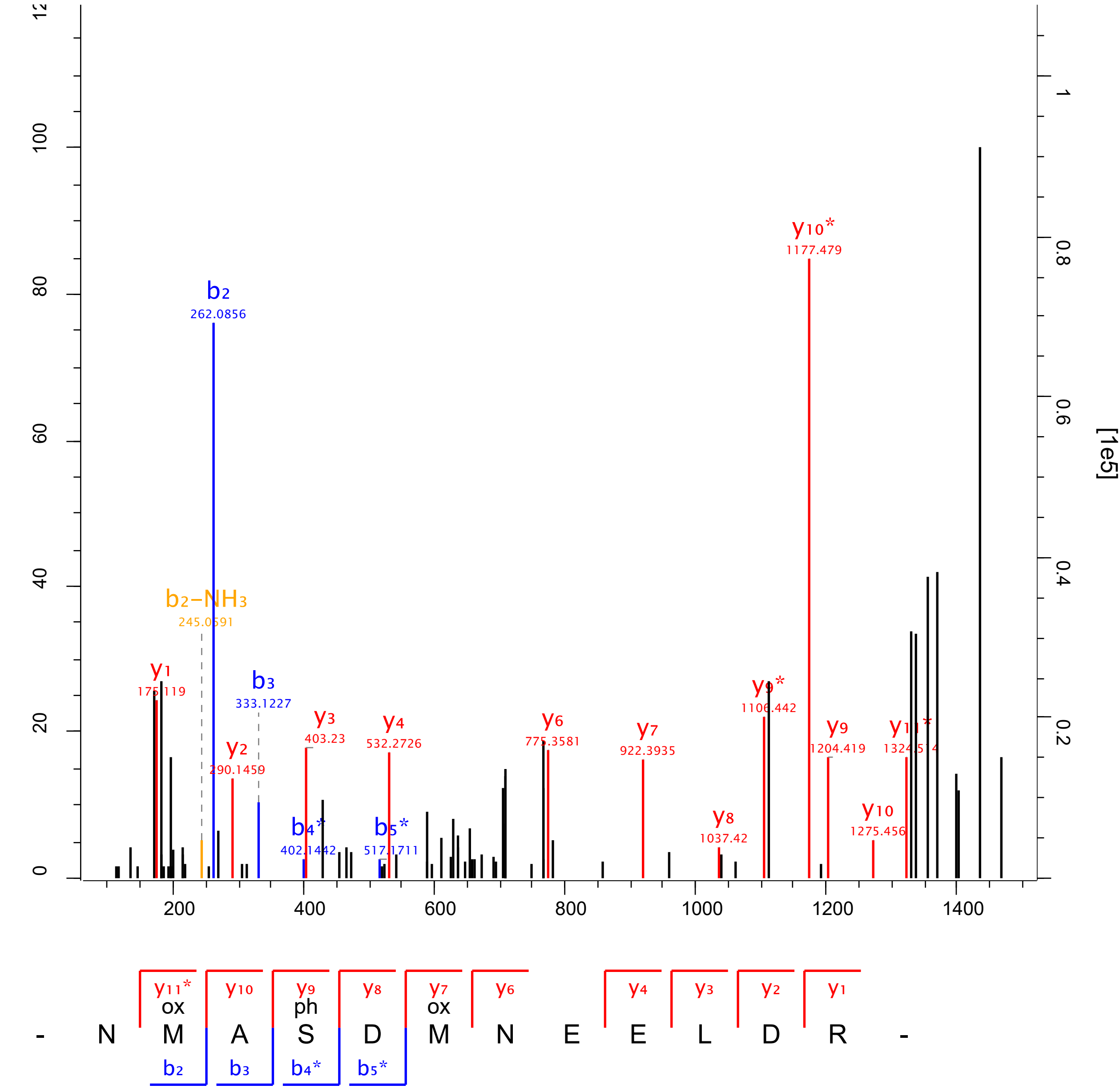

Raw file	Scan	Method	Score	m/z
sys_15_1	7627	FTMS; HCD	67.14	673.74

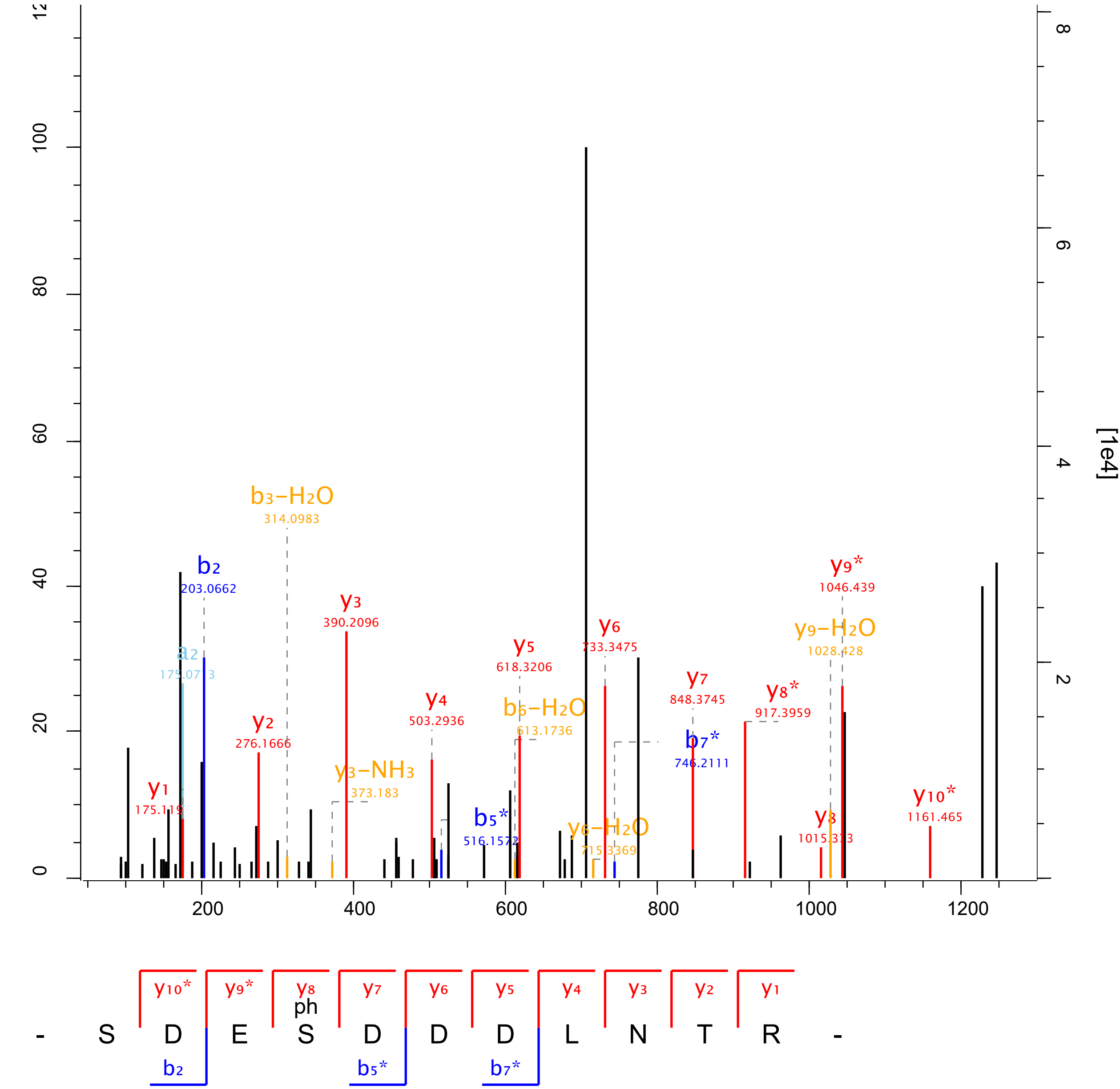

Raw file	Scan	Method	Score	m/z
sys_15_1	7720	FTMS; HCD	139.86	597.26

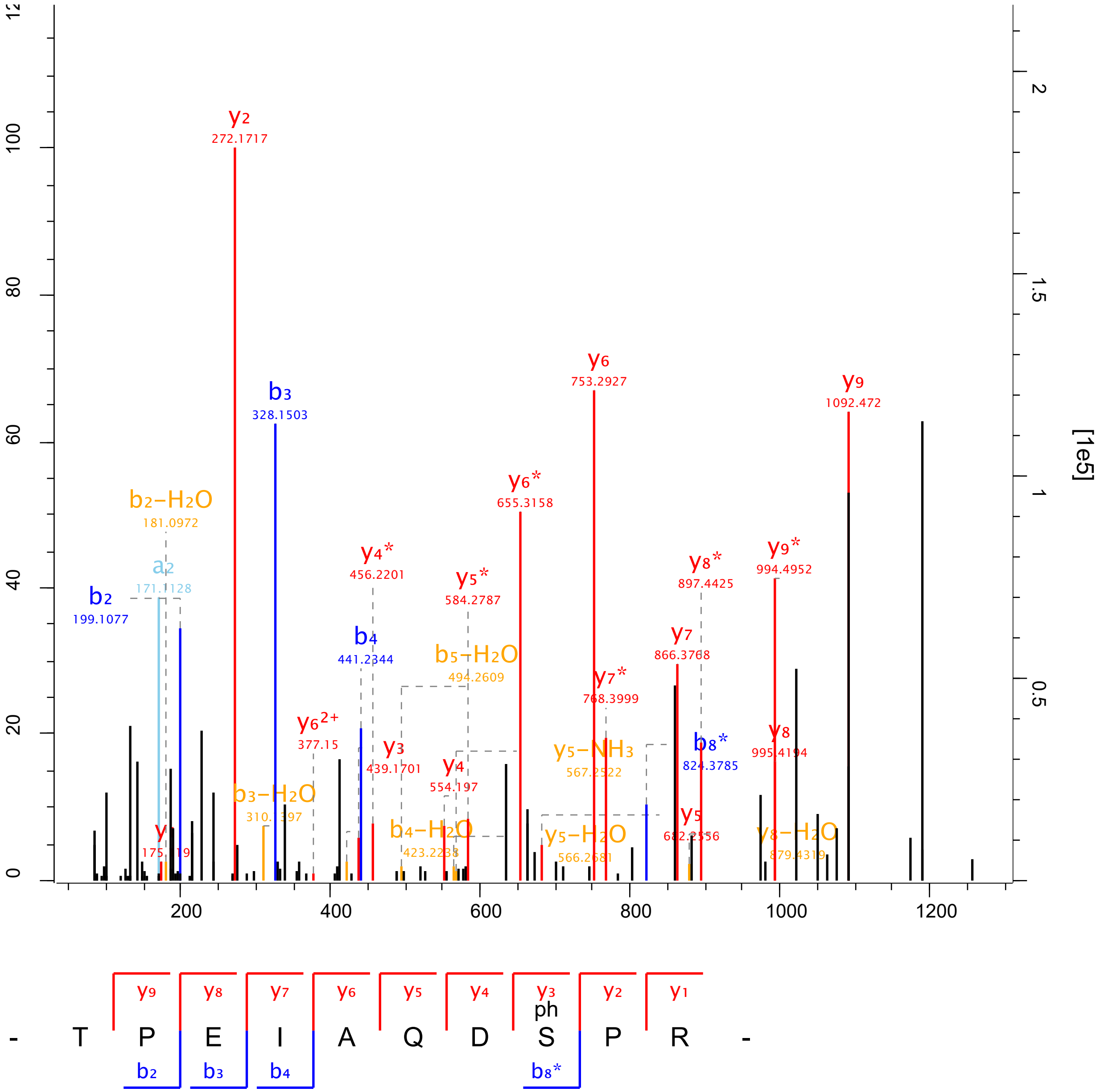

Raw file	Scan	Method	Score	m/z
sys_15_1	7756	FTMS; HCD	109.72	678.8

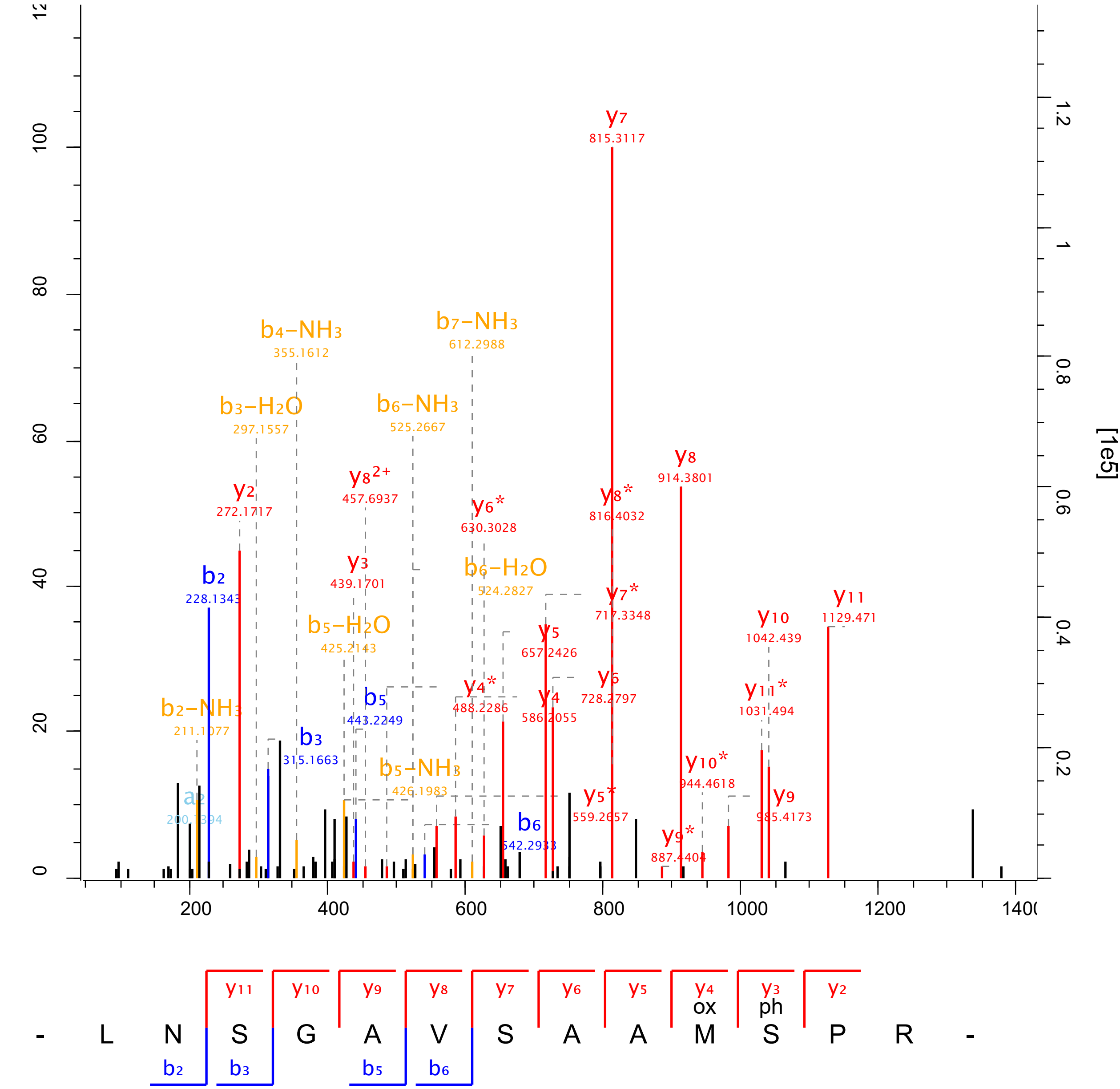

Raw file	Scan	Method	Score	m/z
sys_15_1	7768	FTMS; HCD	118.31	530.74

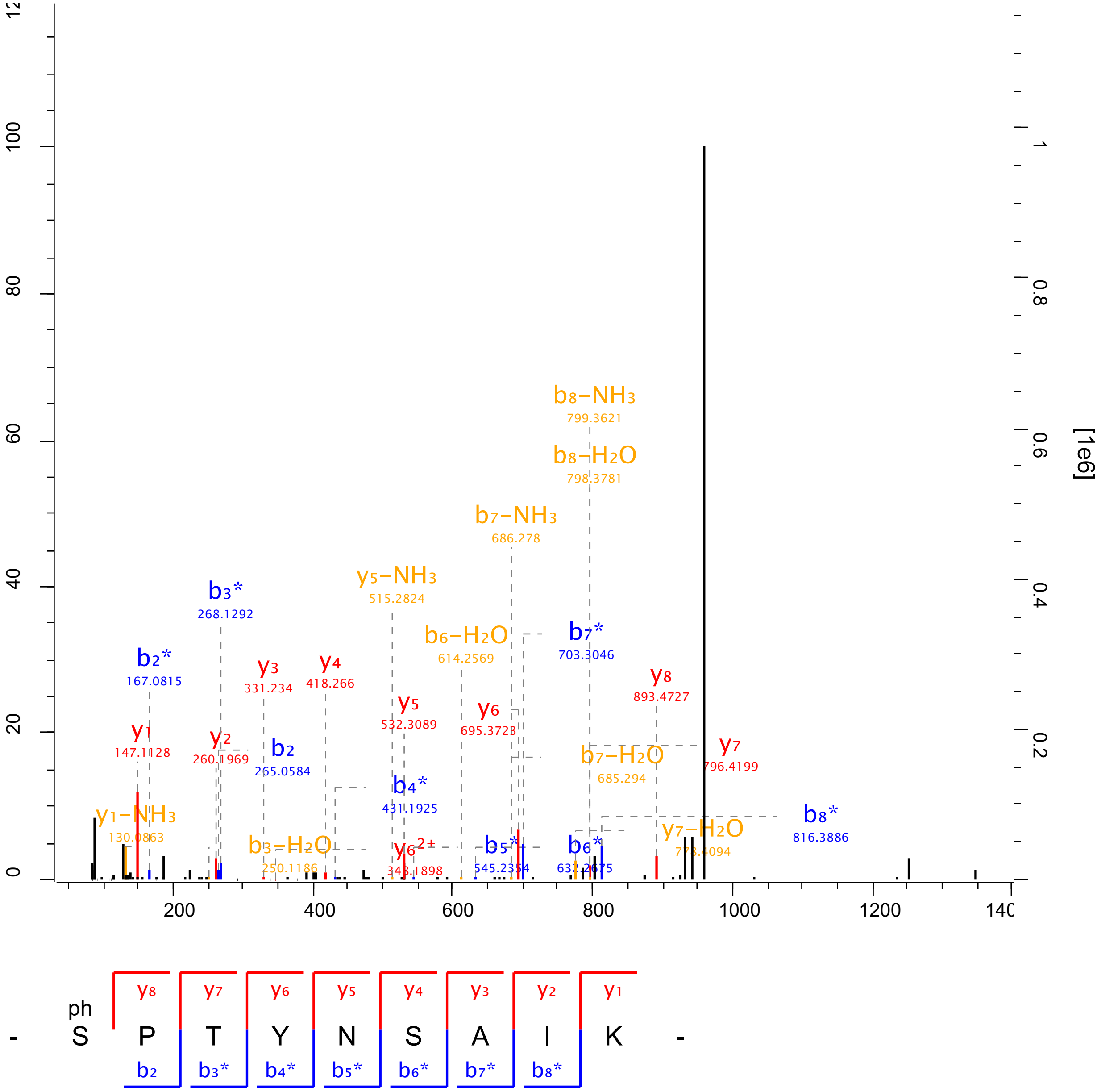

Raw file	Scan	Method	Score	m/z
sys_15_1	7846	FTMS; HCD	67.14	680.76

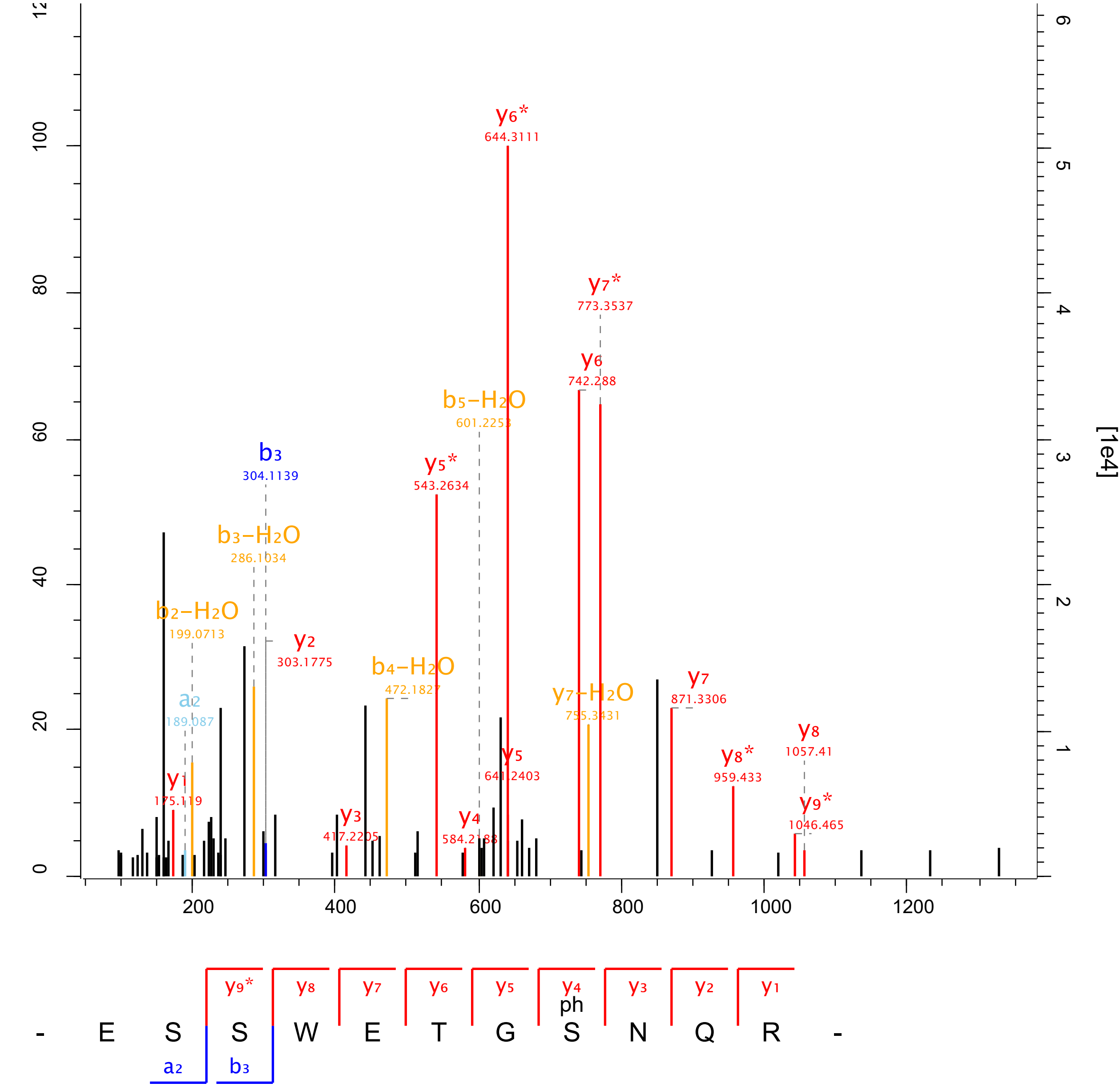

Raw file	Scan	Method	Score	m/z
sys_15_1	7861	FTMS; HCD	47.6	528.23

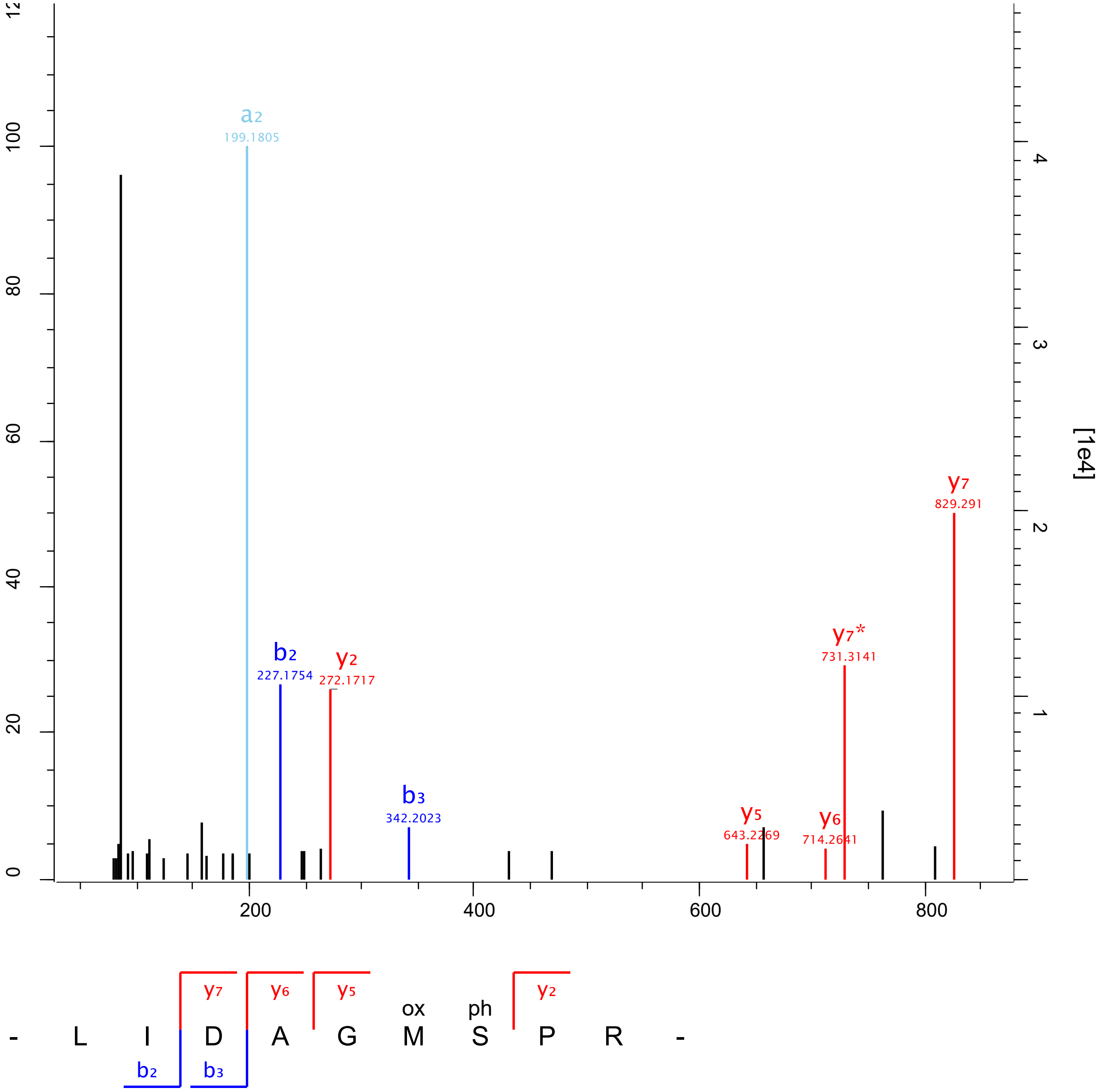

Raw file	Scan	Method	Score	m/z
sys_15_1	7873	FTMS; HCD	70.89	686.77

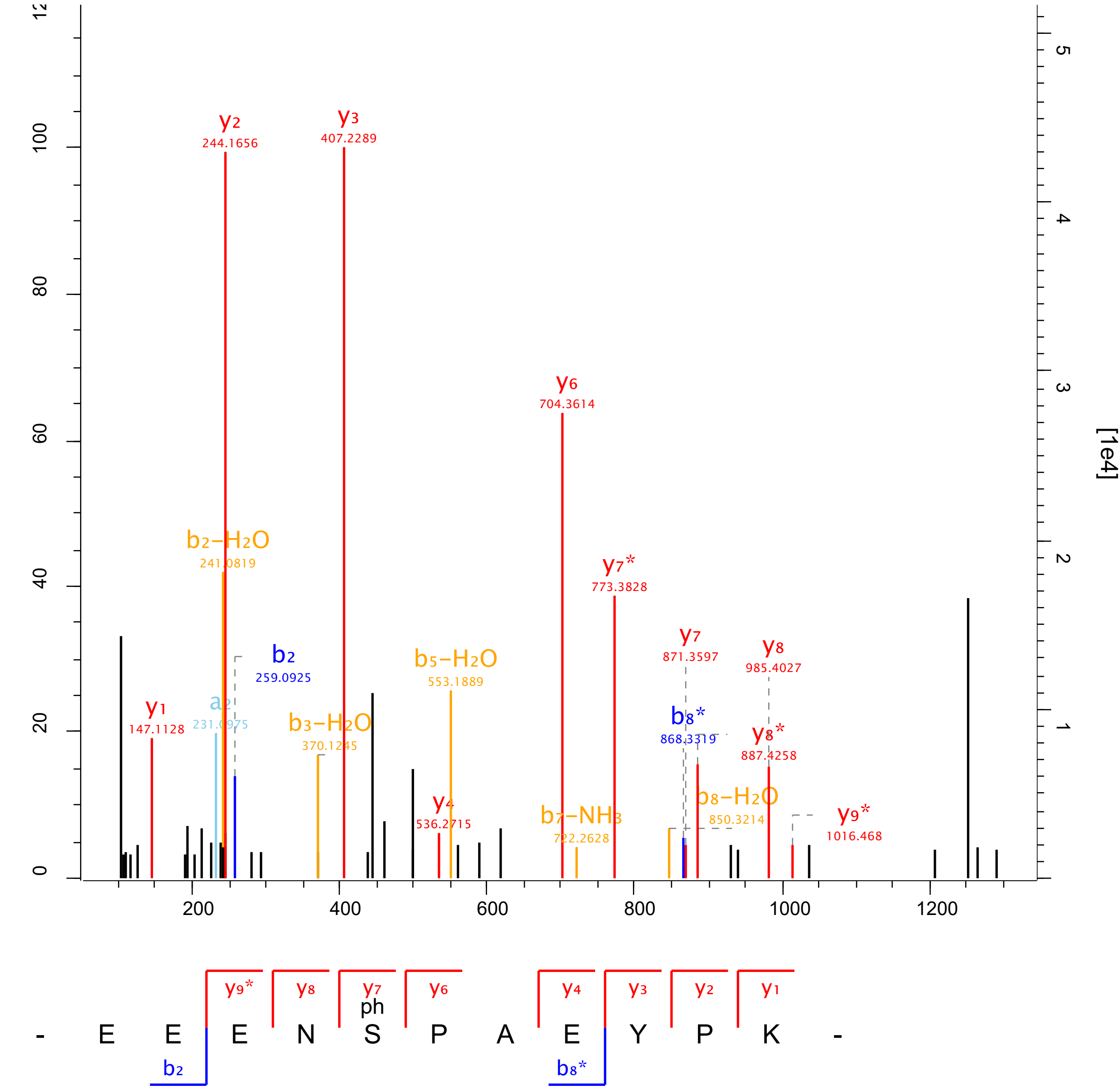

Raw file	Scan	Method	Score	m/z
sys_15_1	7904	FTMS; HCD	88.02	603.78

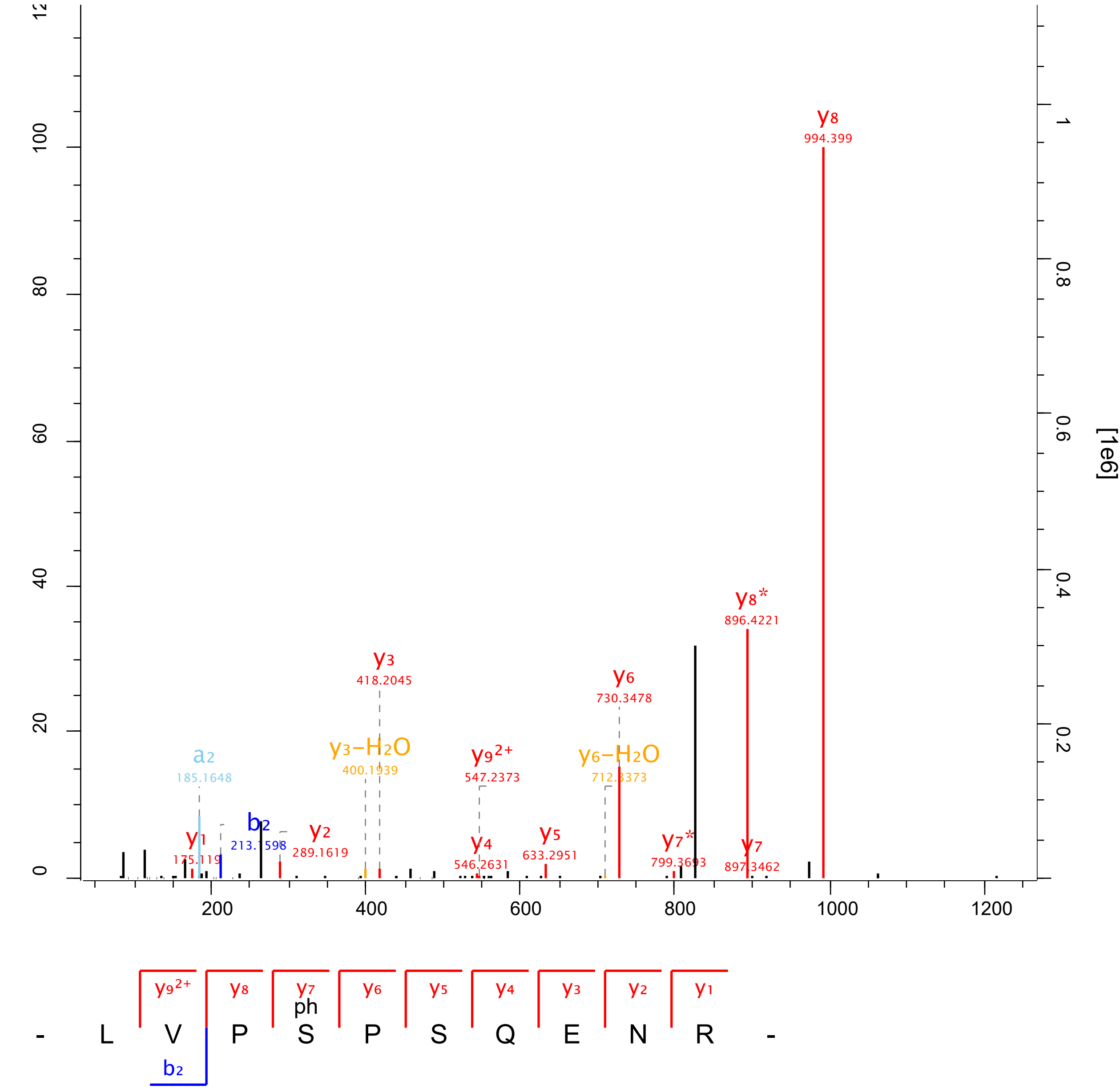

Raw file	Scan	Method	Score	m/z
sys_15_1	7980	FTMS; HCD	179.14	783.79

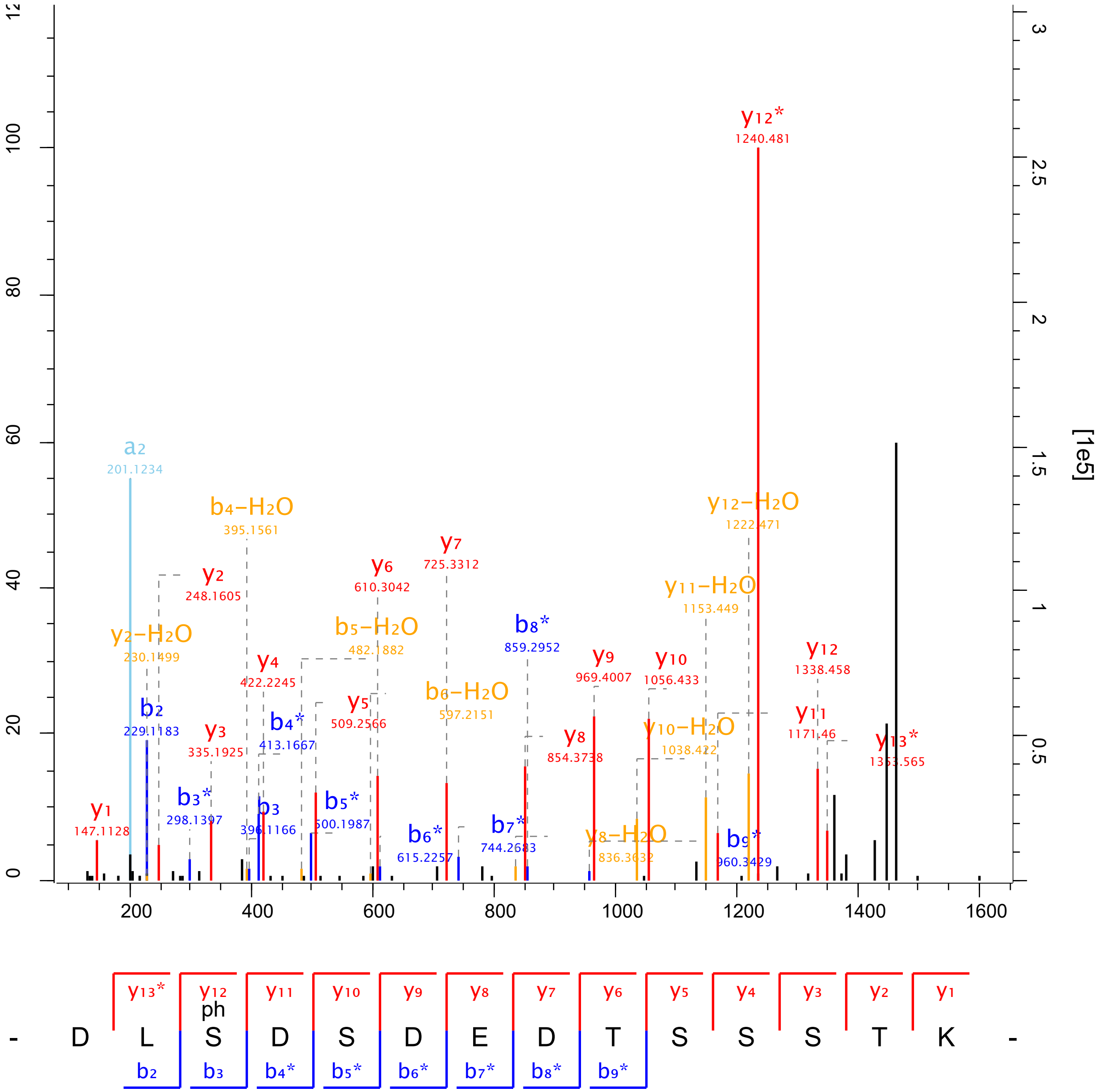

Raw file	Scan	Method	Score	m/z
sys_15_1	8139	FTMS; HCD	84.76	636.26

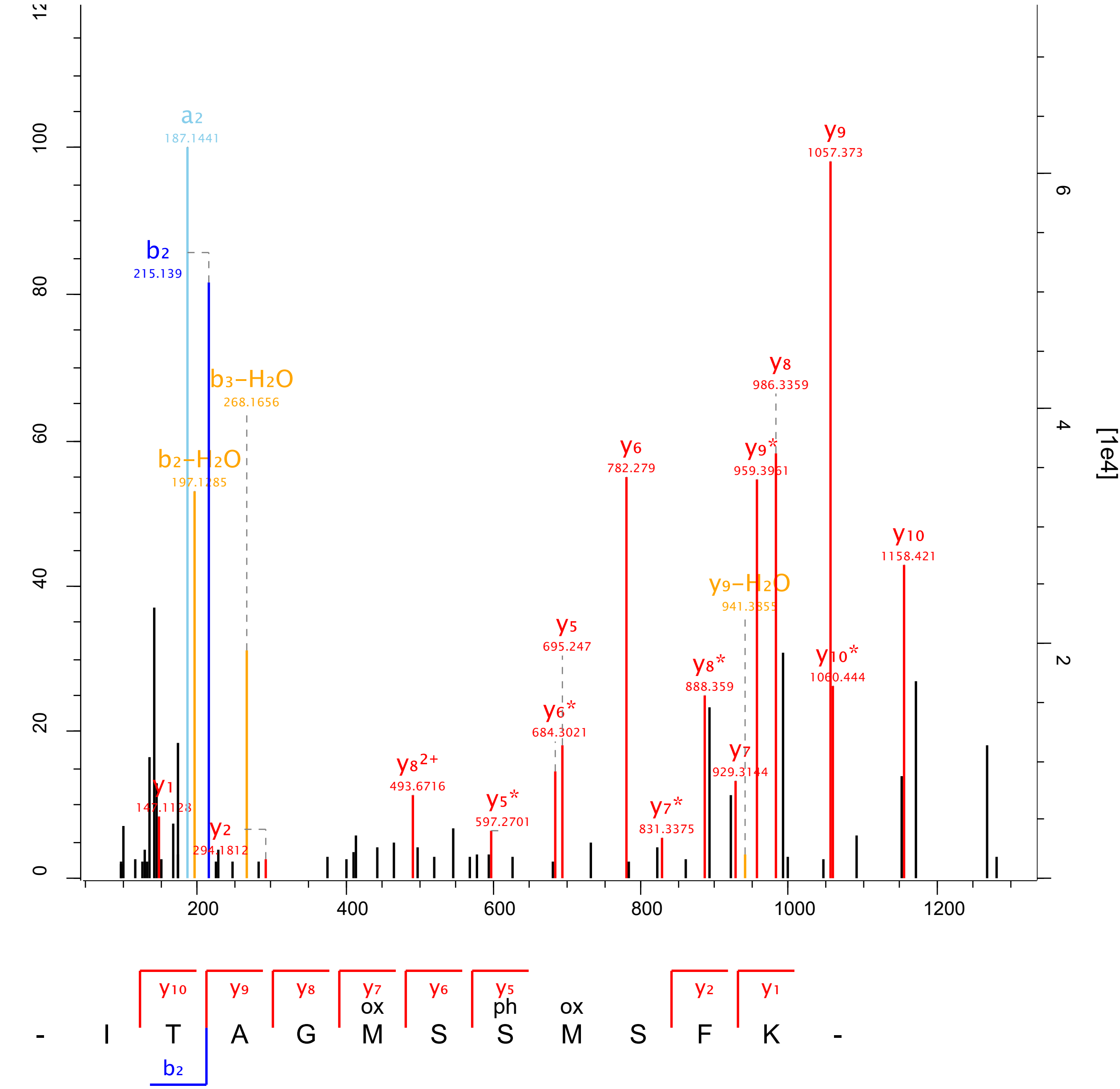

Raw file	Scan	Method	Score	m/z
sys_15_1	8143	FTMS; HCD	65.04	659.75

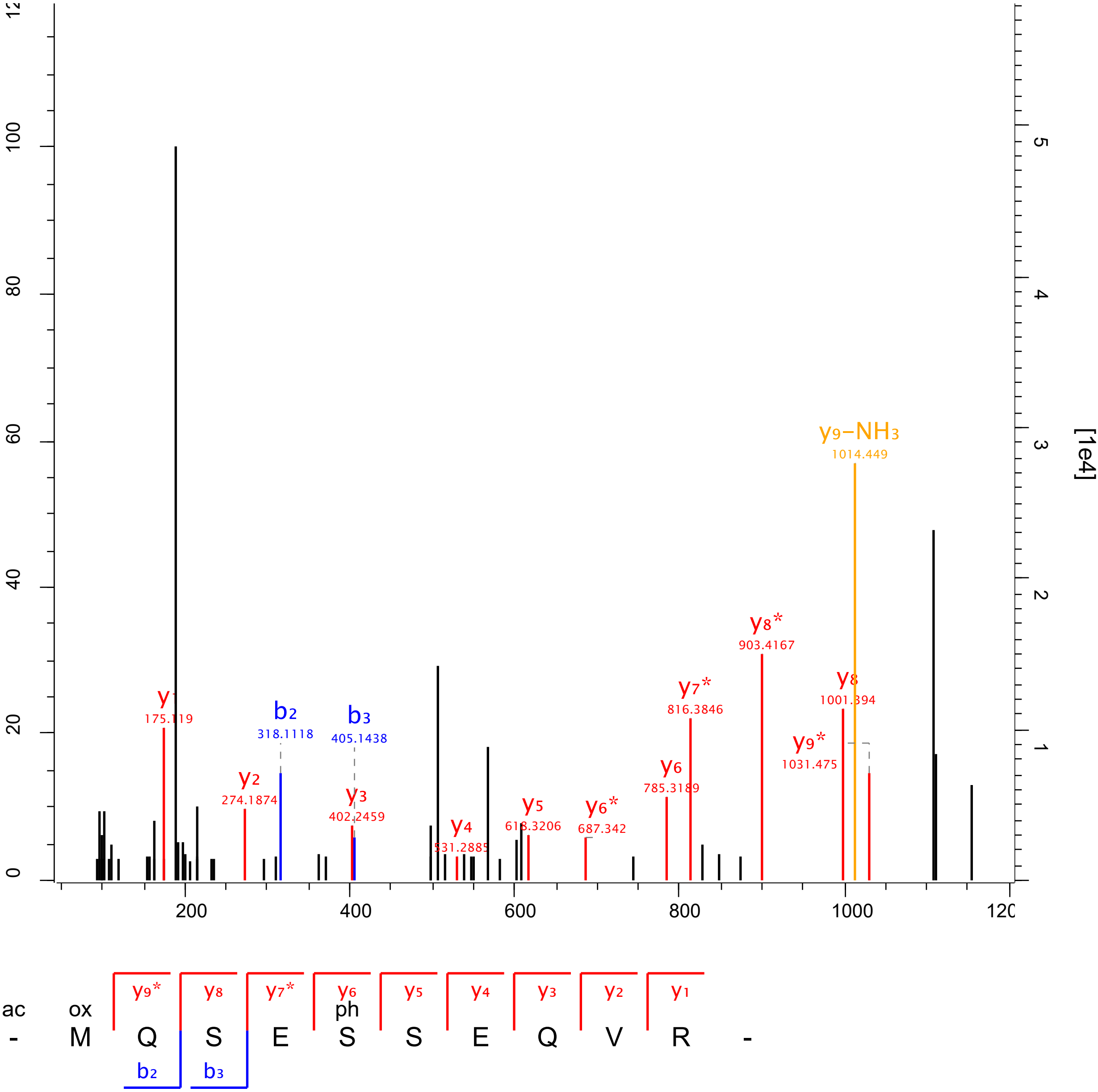

Raw file	Scan	Method	Score	m/z
sys_15_1	8169	FTMS; HCD	198.04	735.8

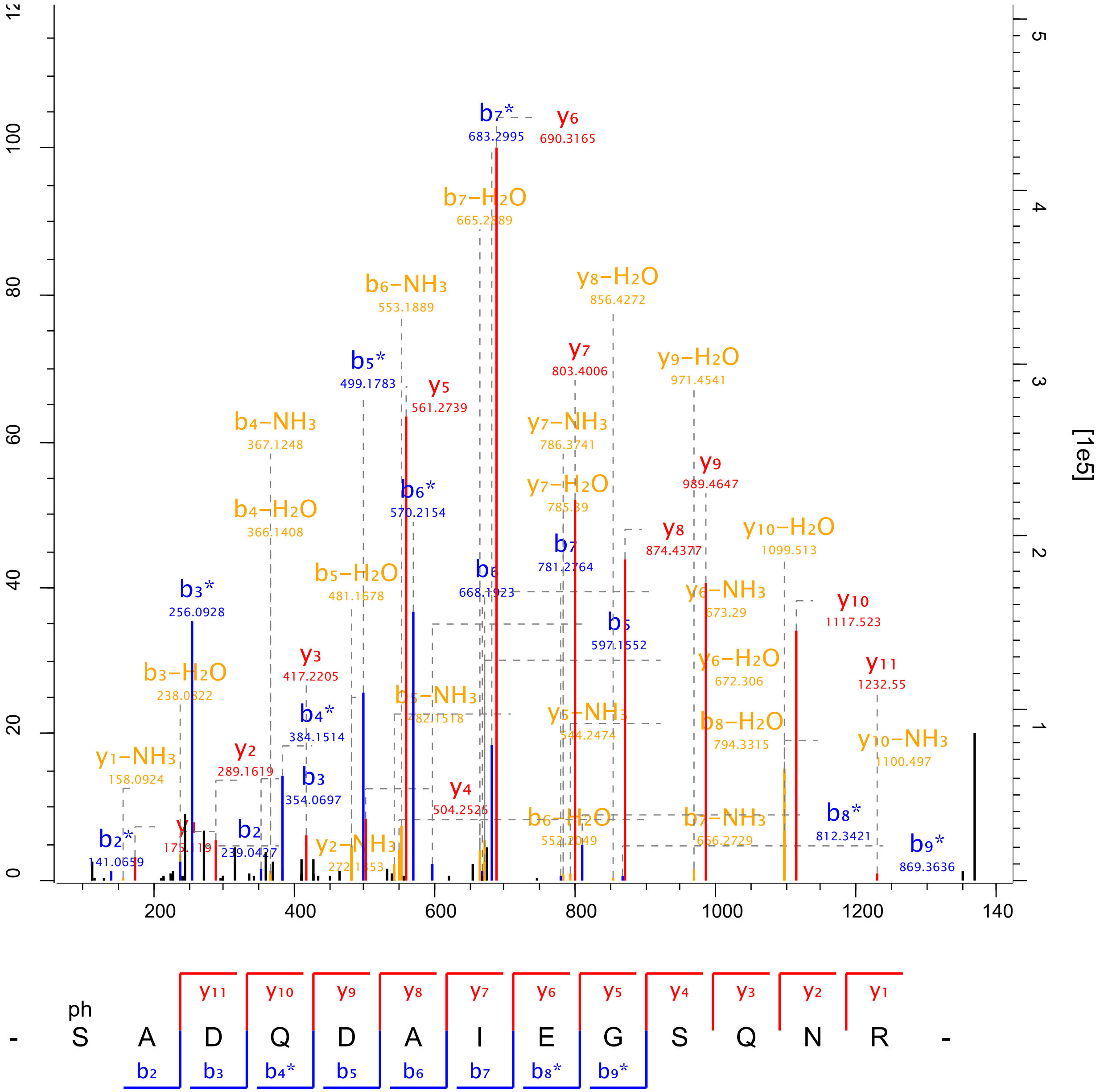

Raw file	Scan	Method	Score	m/z
sys_15_1	8189	FTMS; HCD	51.29	541.68

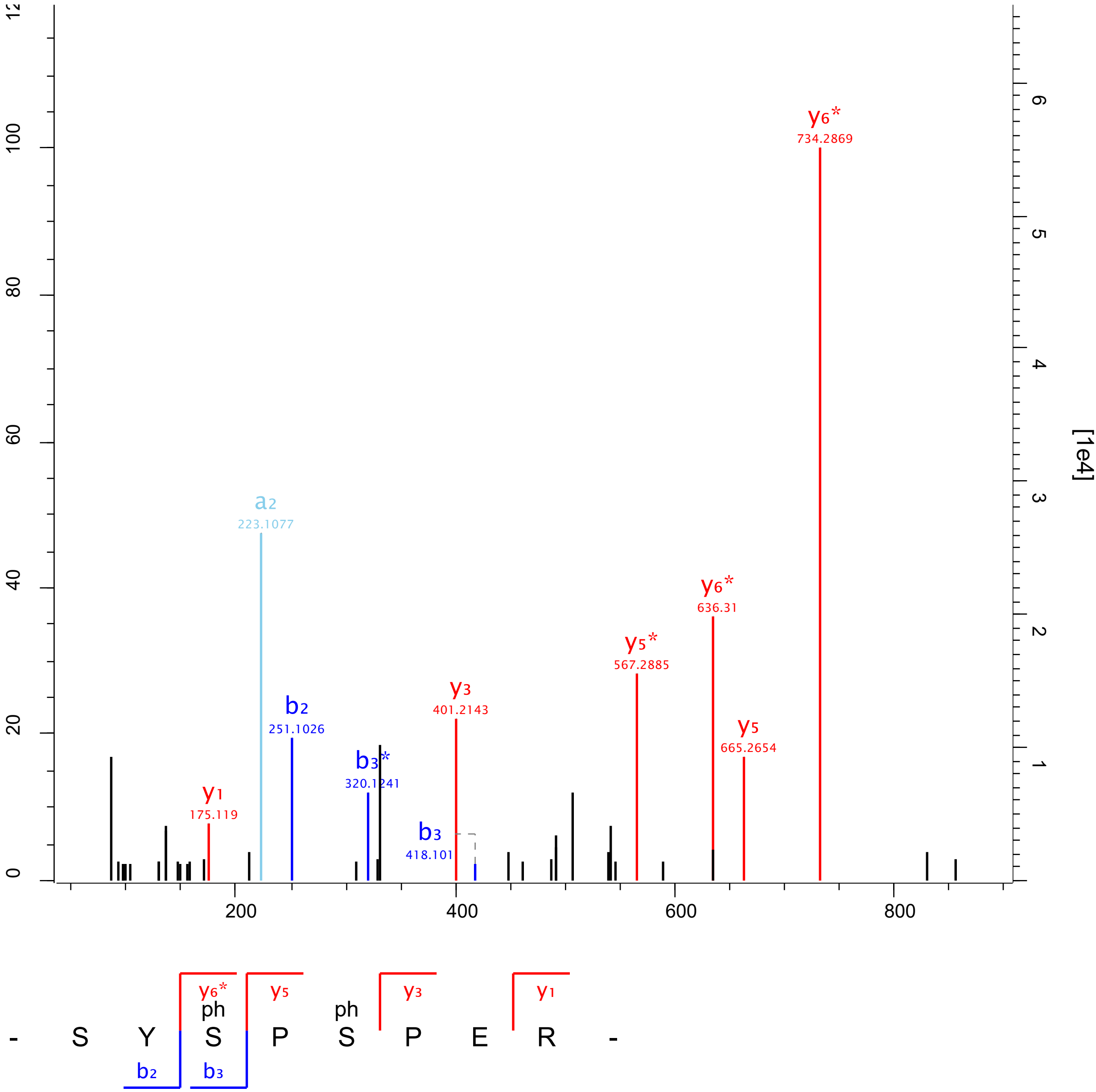

Raw file	Scan	Method	Score	m/z
sys_15_1	8202	FTMS; HCD	125.82	587.75

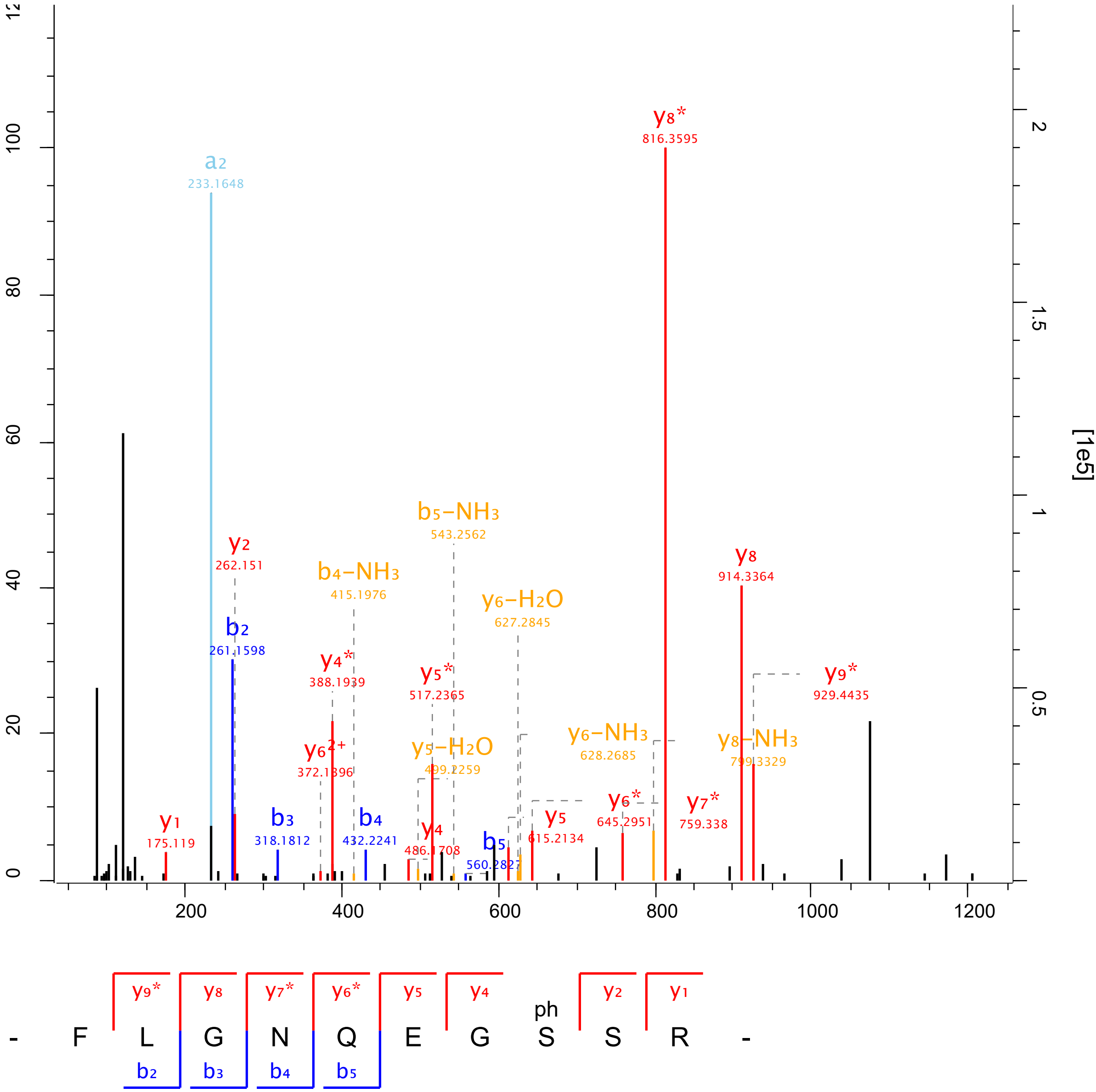

Raw file	Scan	Method	Score	m/z
sys_15_1	8239	FTMS; HCD	54.09	643.58

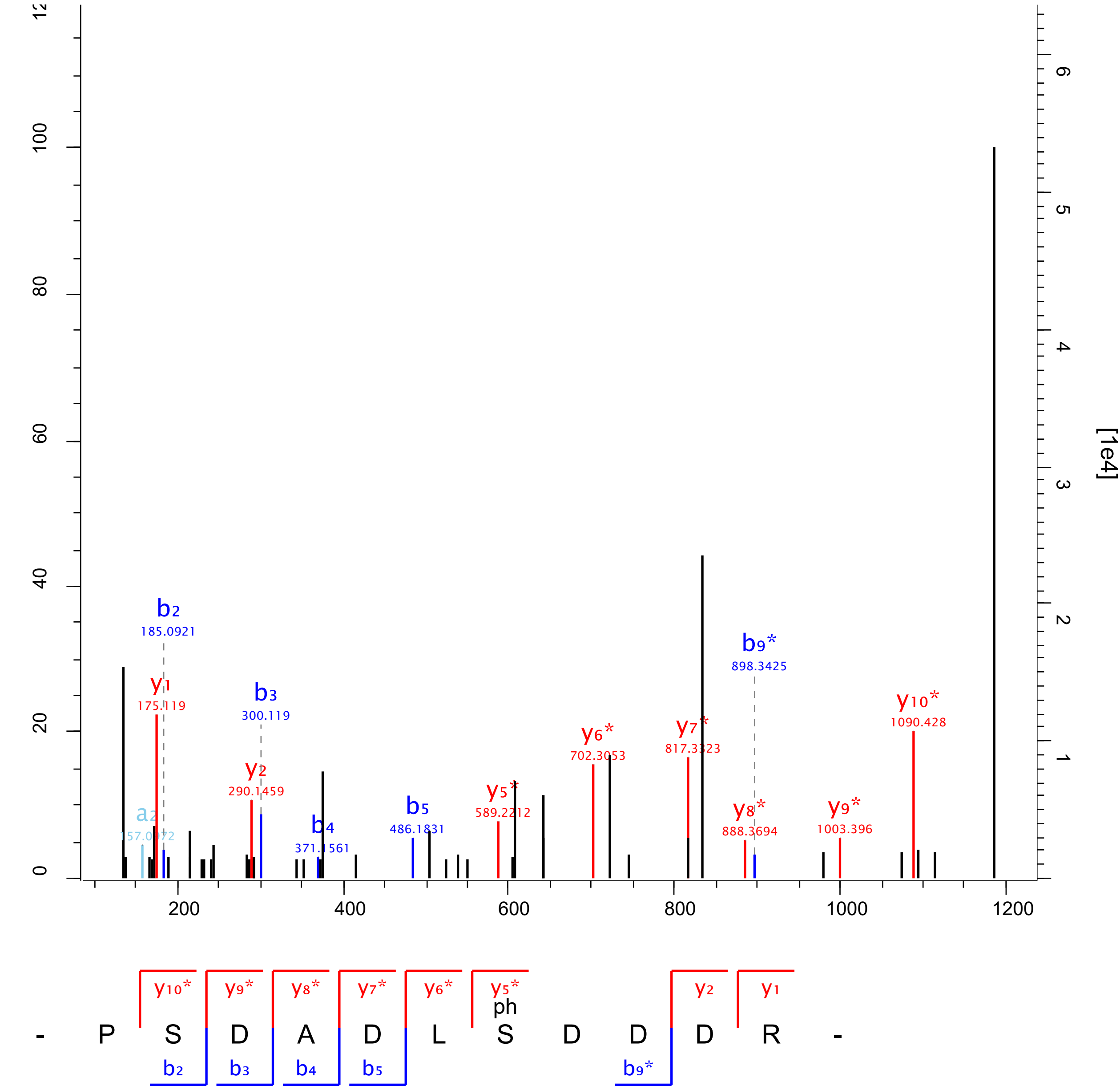

Raw file	Scan	Method	Score	m/z
sys_15_1	8287	FTMS; HCD	161.2	869.35

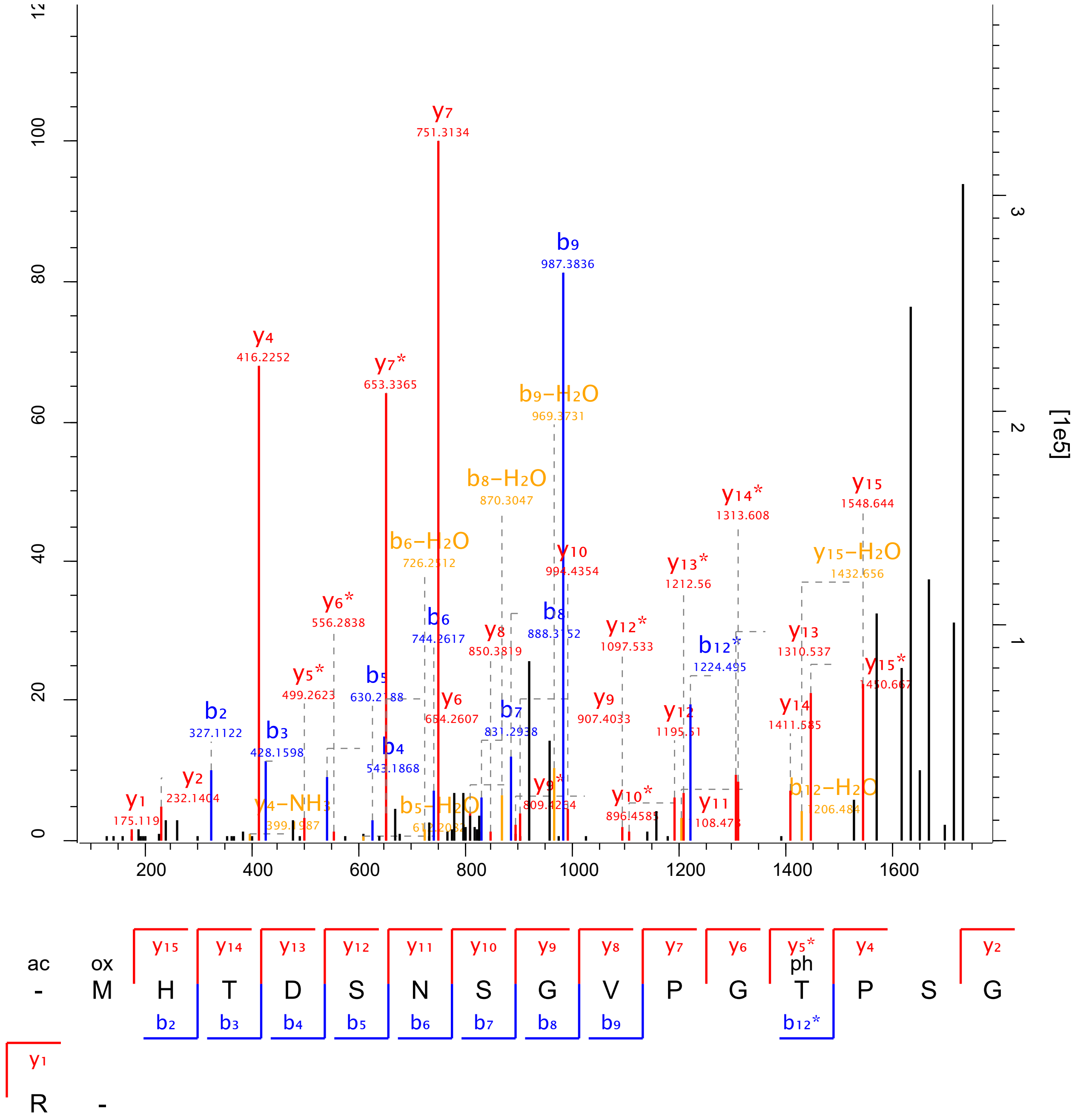

Raw file	Scan	Method	Score	m/z
sys_15_1	8369	FTMS; HCD	191.47	655.77

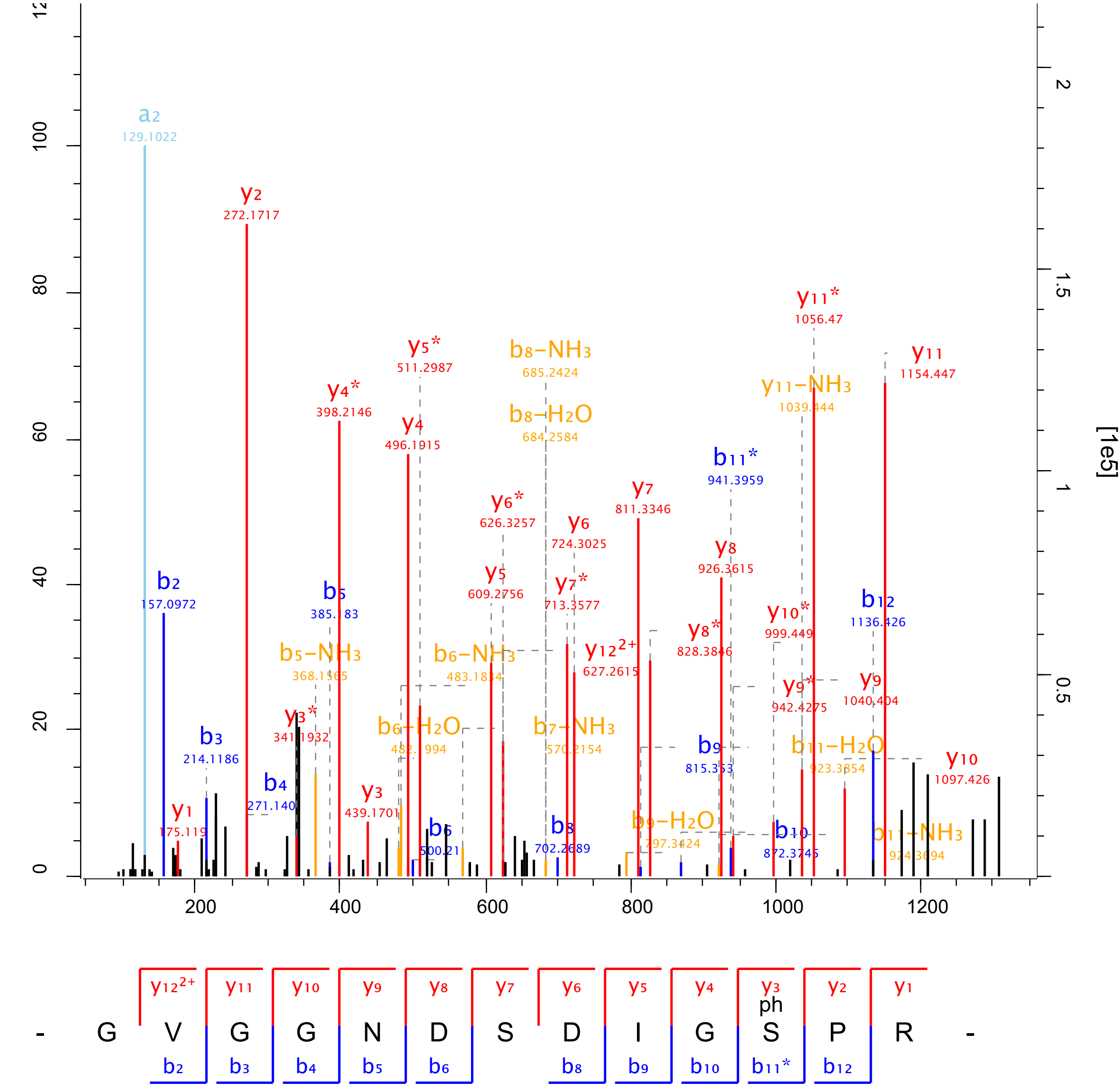

Raw file	Scan	Method	Score	m/z
sys_15_1	8523	FTMS; HCD	44.85	559.23

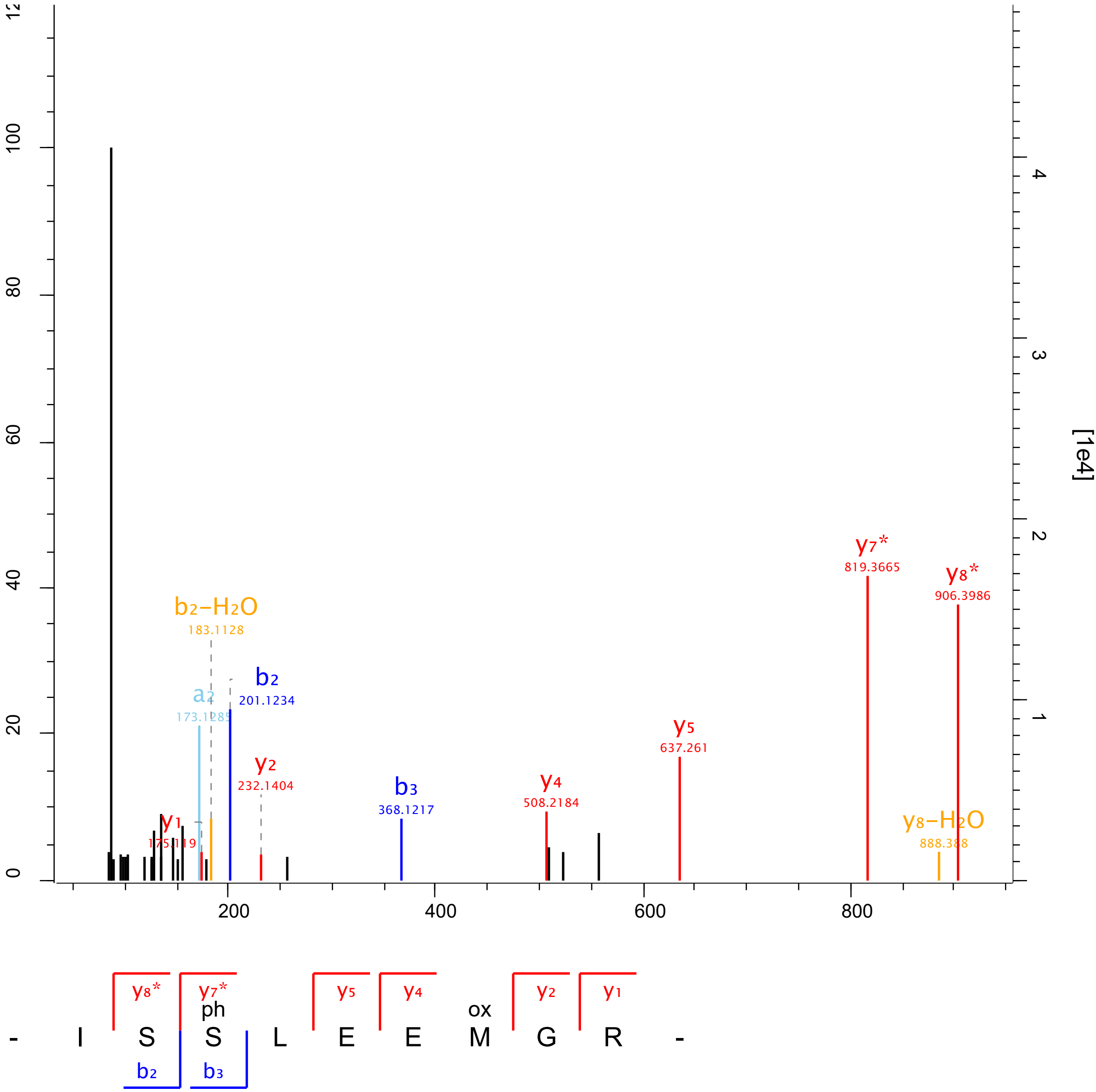

Raw file	Scan	Method	Score	m/z
sys_15_1	8632	FTMS; HCD	210.91	622.28

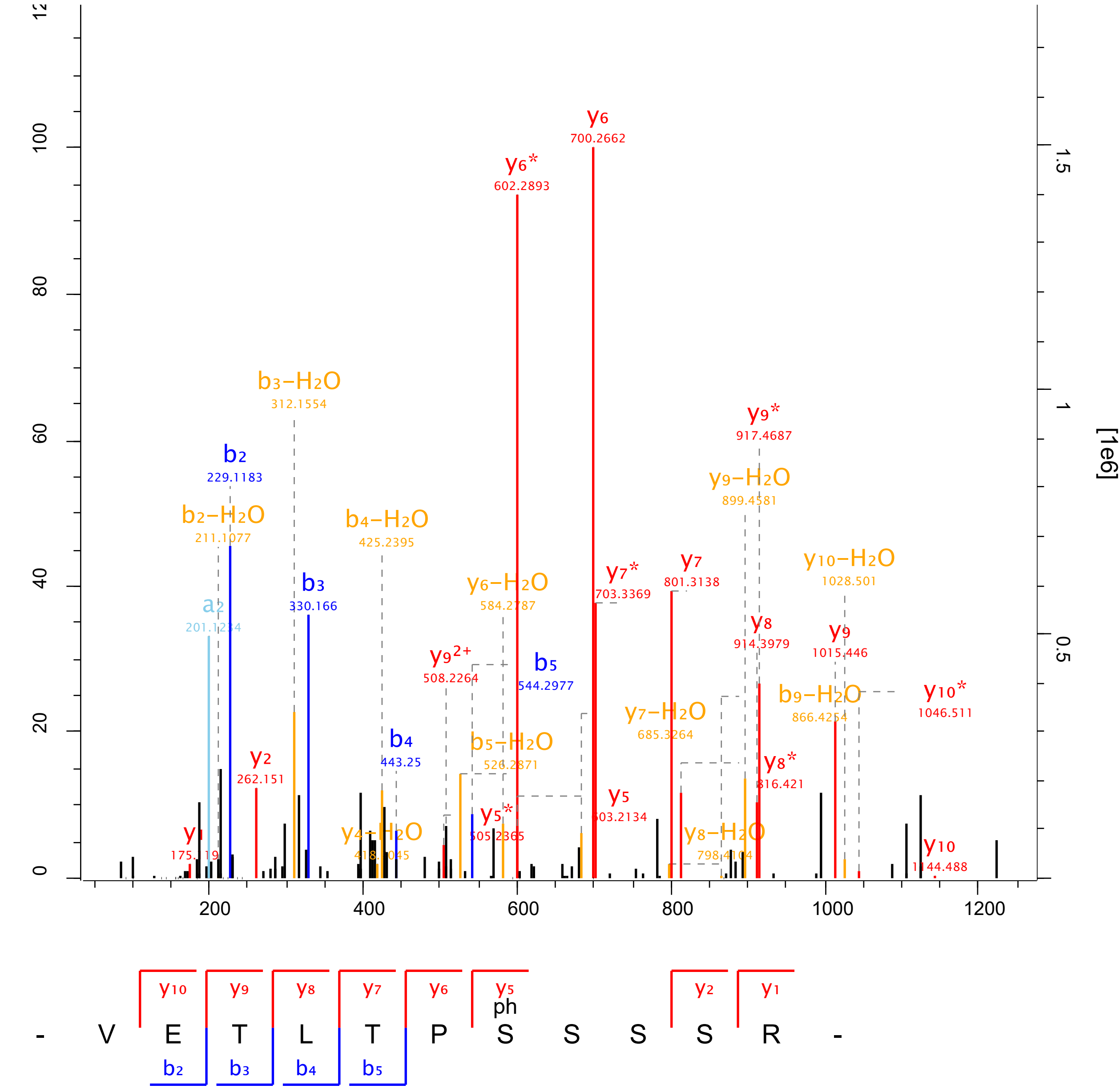

Raw file	Scan	Method	Score	m/z
sys_15_1	8737	FTMS; HCD	63.68	649.31

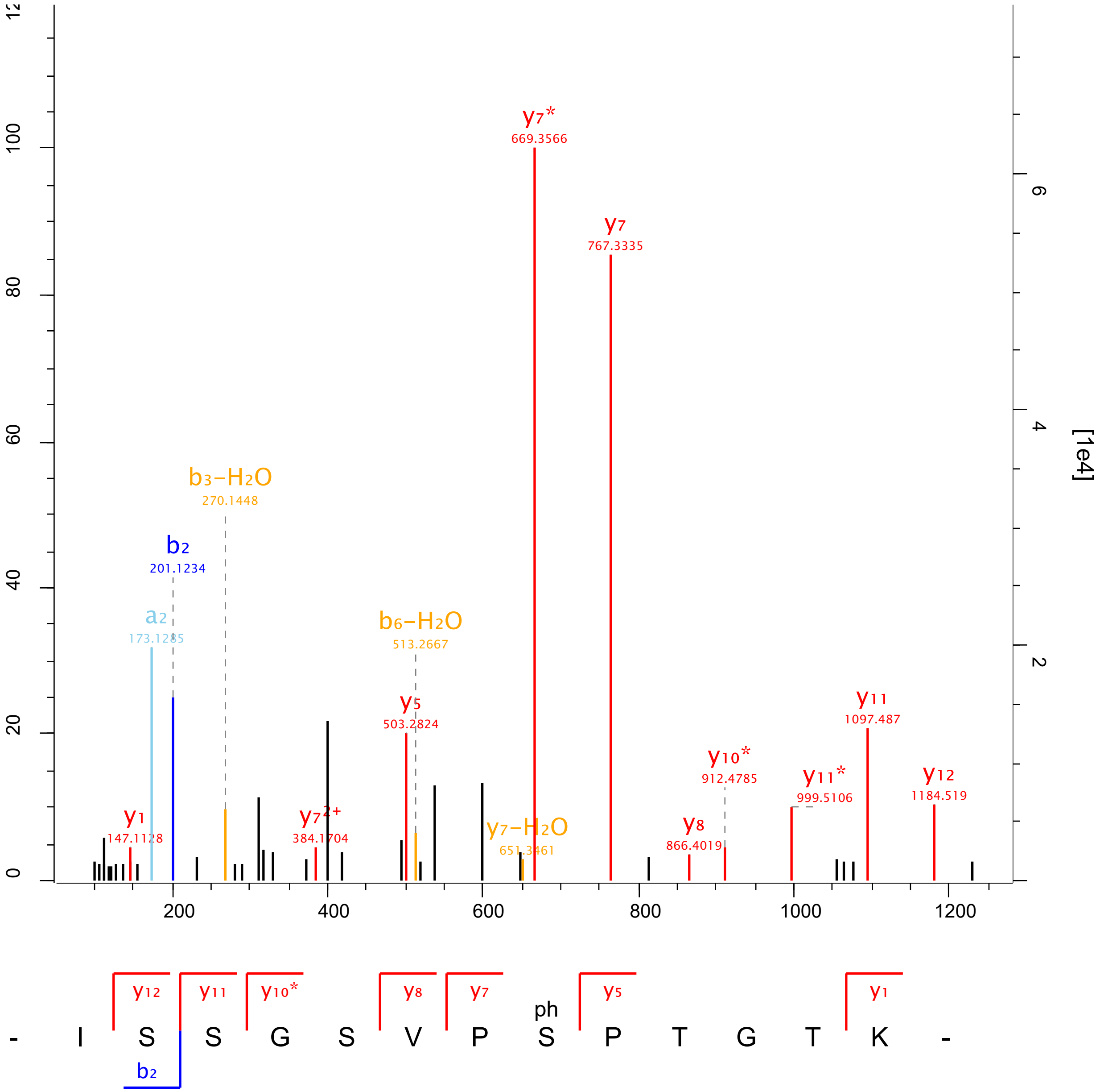

Raw file	Scan	Method	Score	m/z
sys_15_1	8780	FTMS; HCD	103.9	880.86

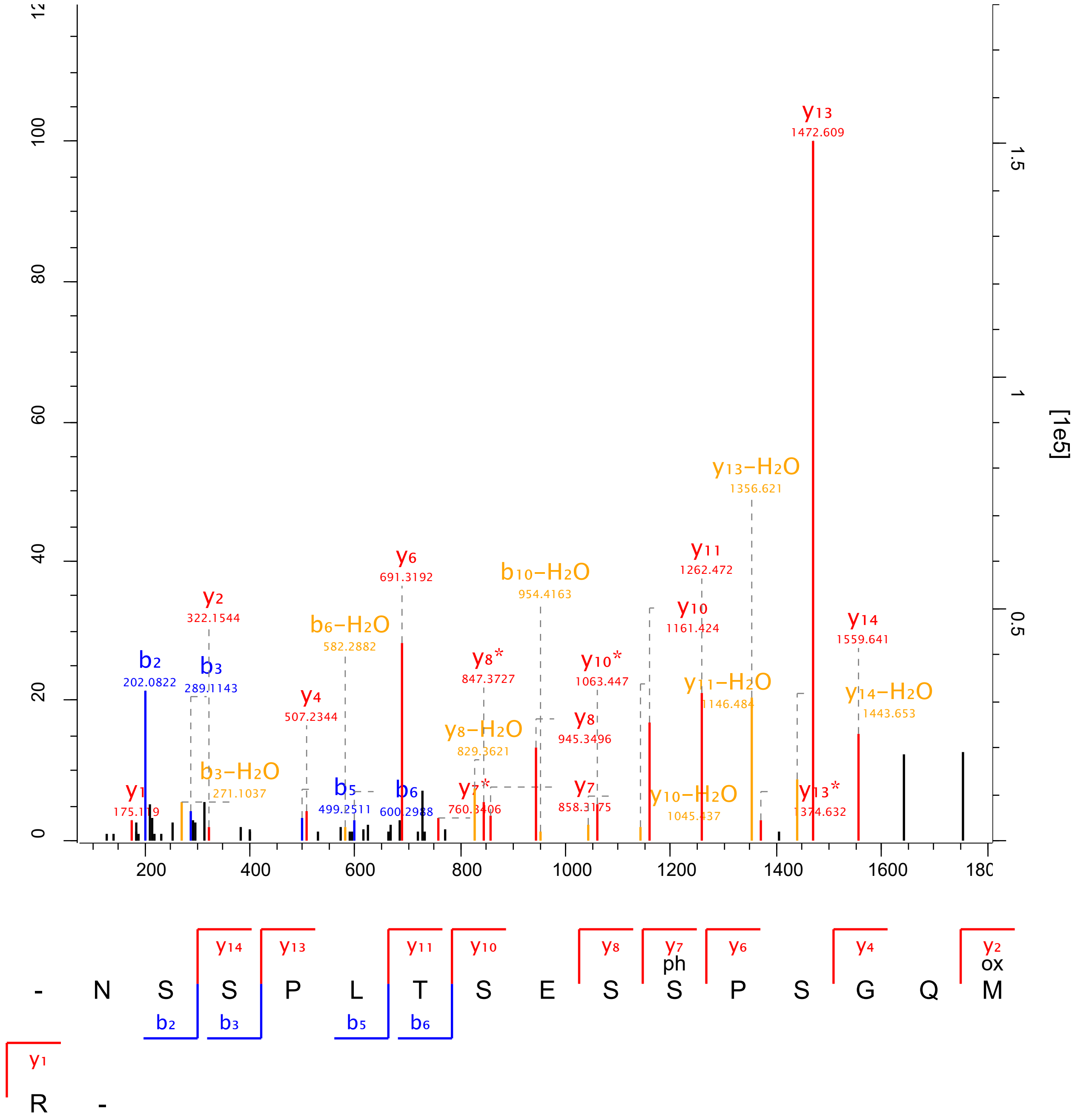

Raw file	Scan	Method	Score	m/z
sys_15_1	8826	FTMS; HCD	43.76	574.24

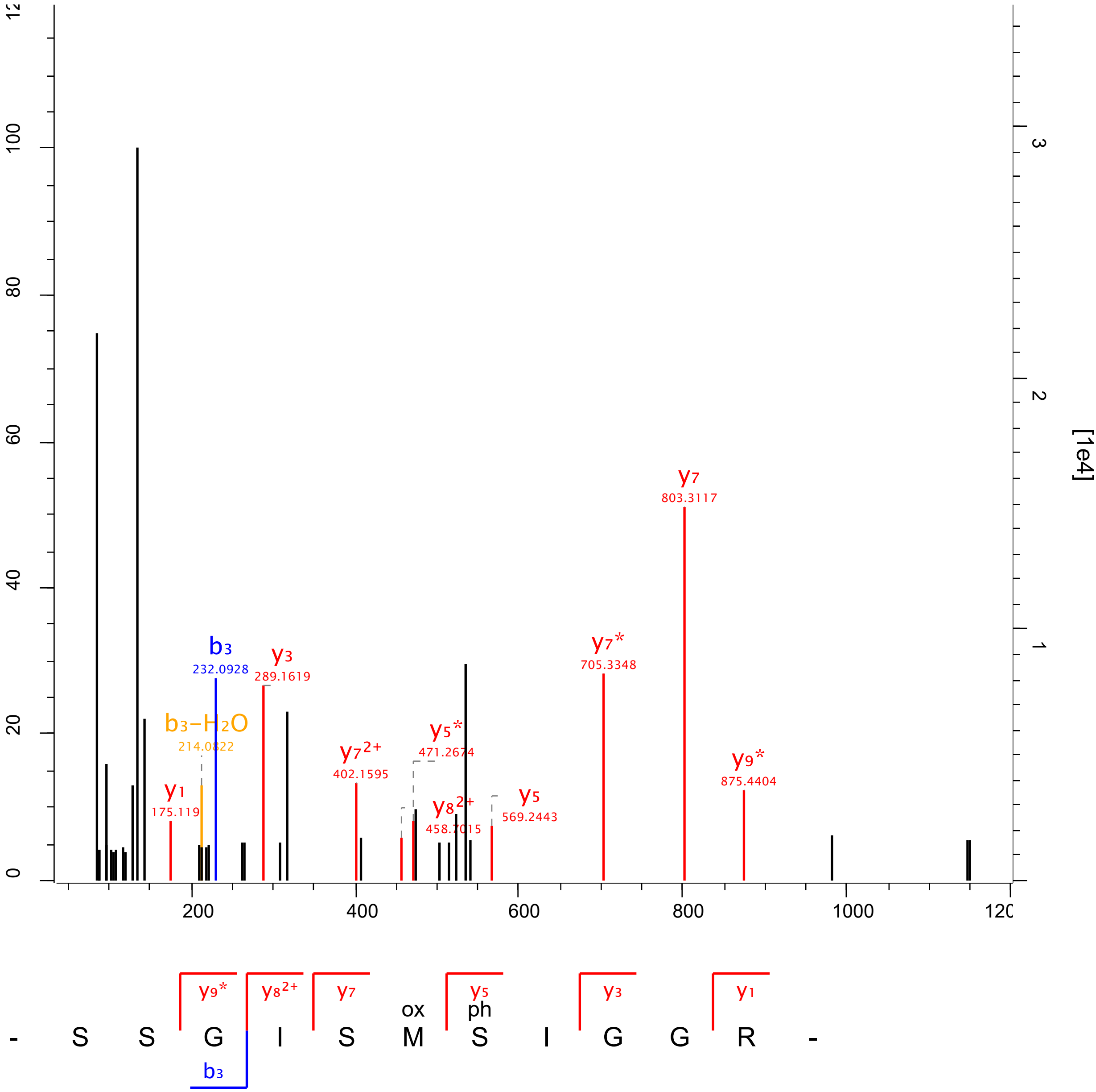

Raw file	Scan	Method	Score	m/z
sys_15_1	8840	FTMS; HCD	93.92	638.59

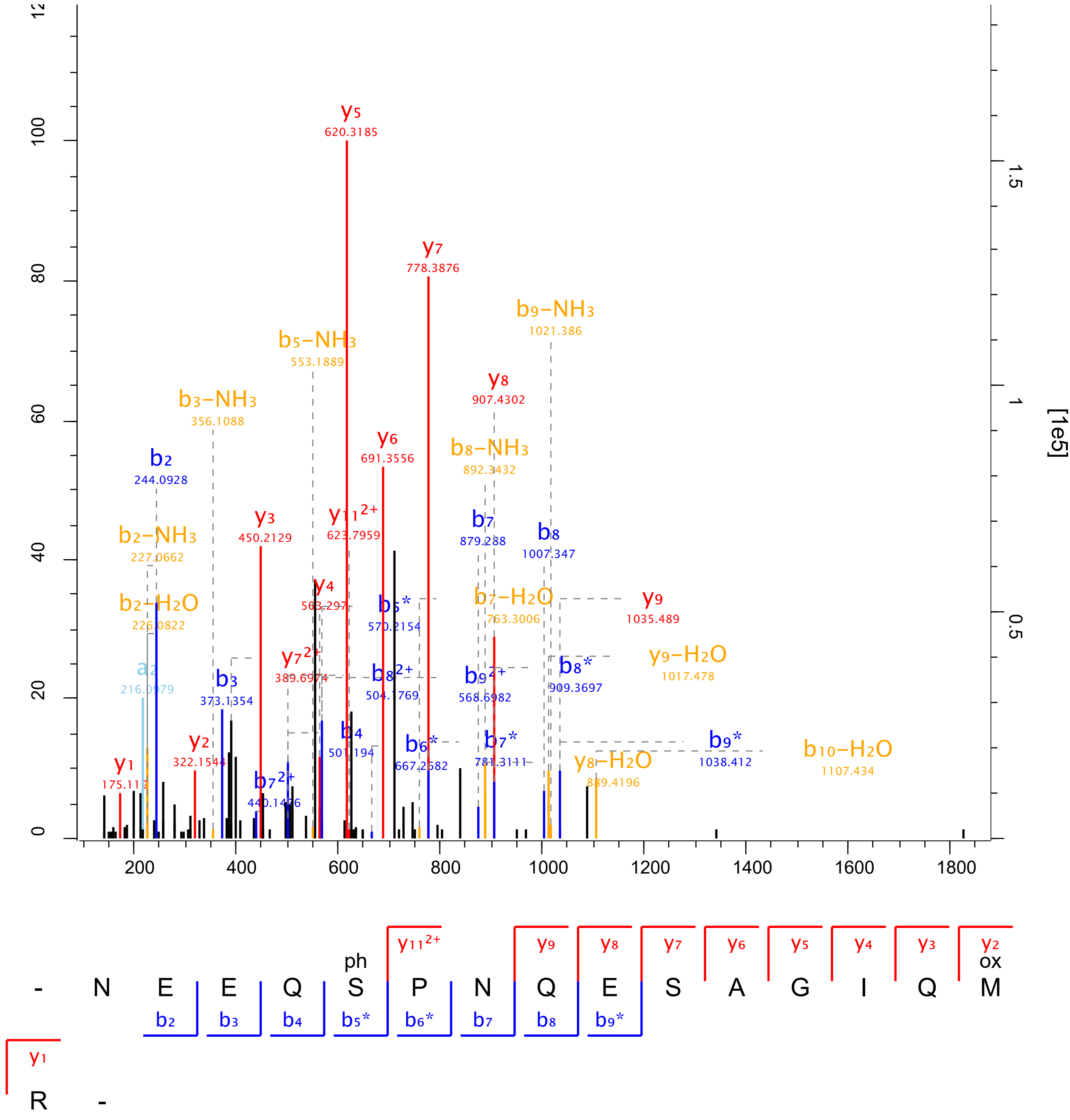

Raw file	Scan	Method	Score	m/z
sys_15_1	8973	FTMS; HCD	61.26	758.82

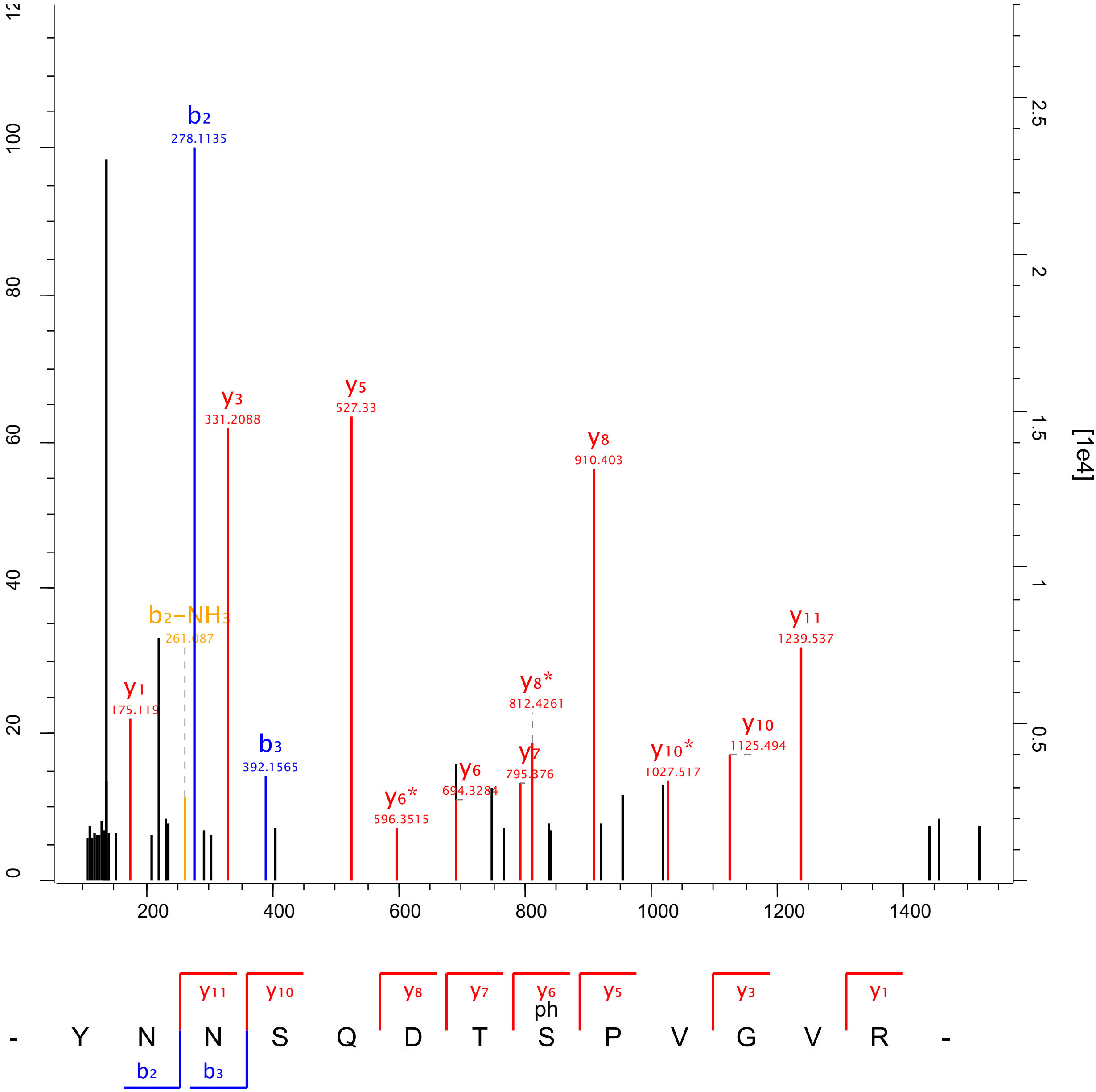

Raw file	Scan	Method	Score	m/z
sys_15_1	9007	FTMS; HCD	74.46	454.19

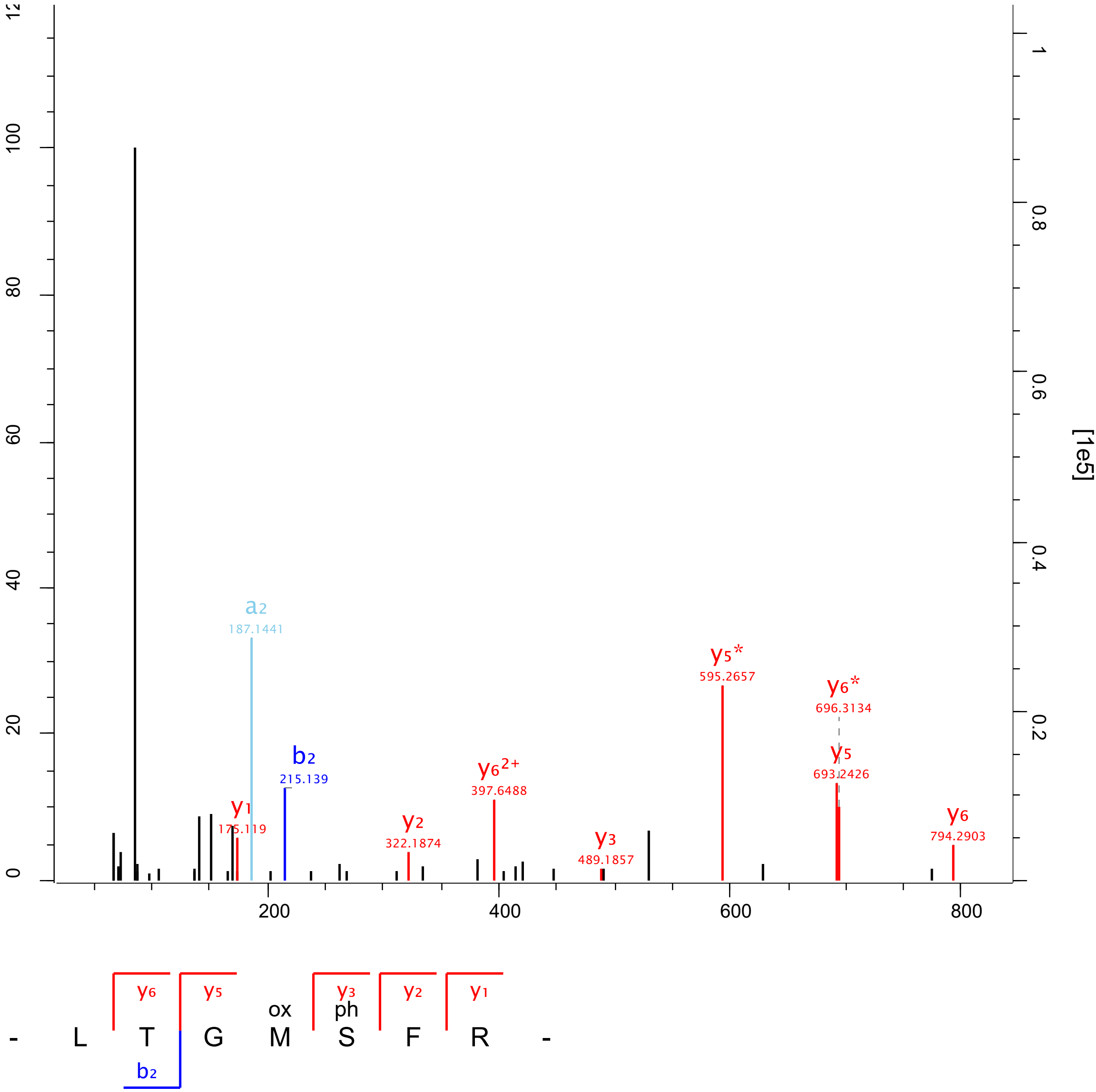

Raw file	Scan	Method	Score	m/z
sys_15_1	9103	FTMS; HCD	81.68	524.23

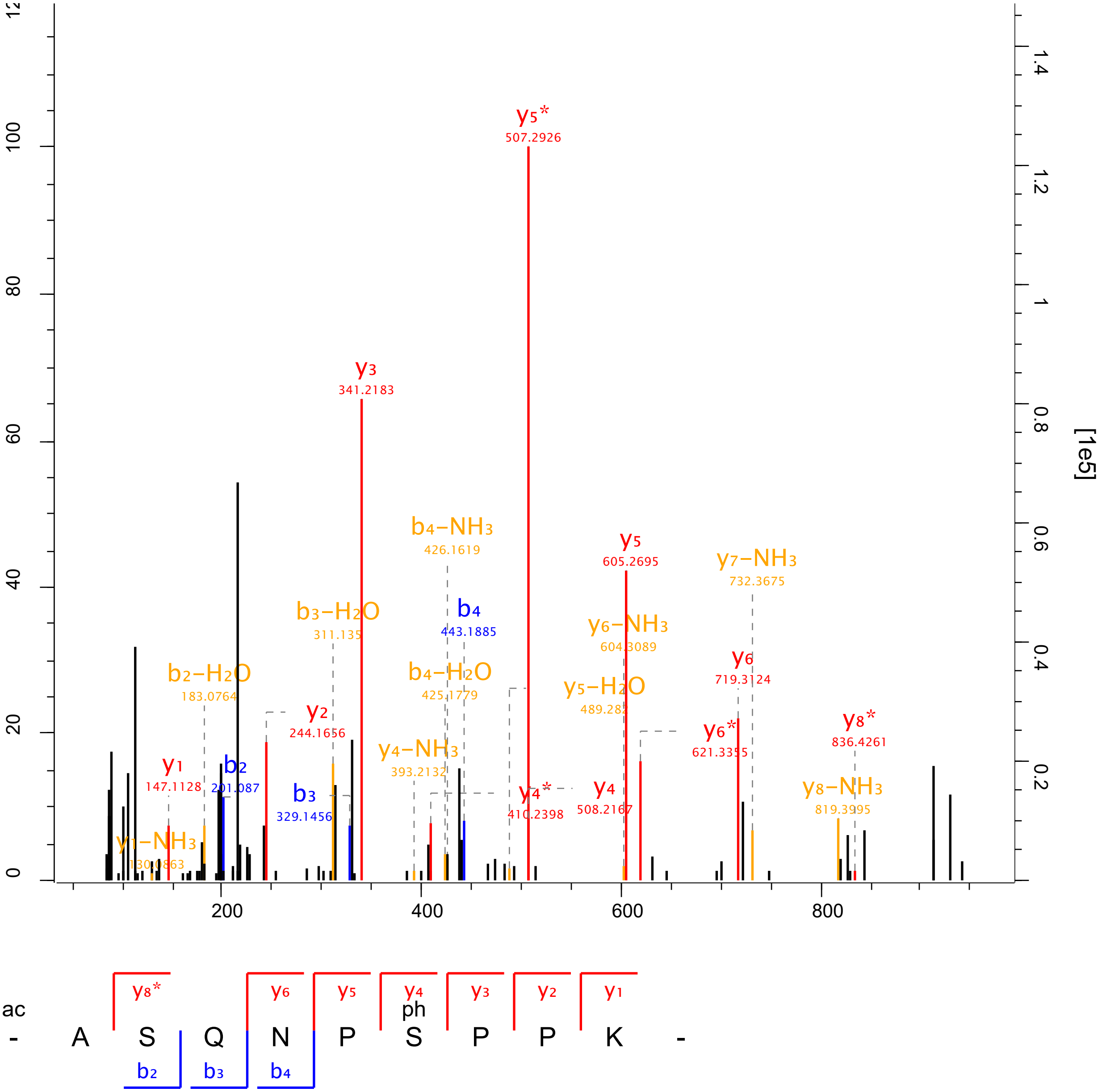

Raw file	Scan	Method	Score	m/z
sys_15_1	9115	FTMS; HCD	197.41	888.84

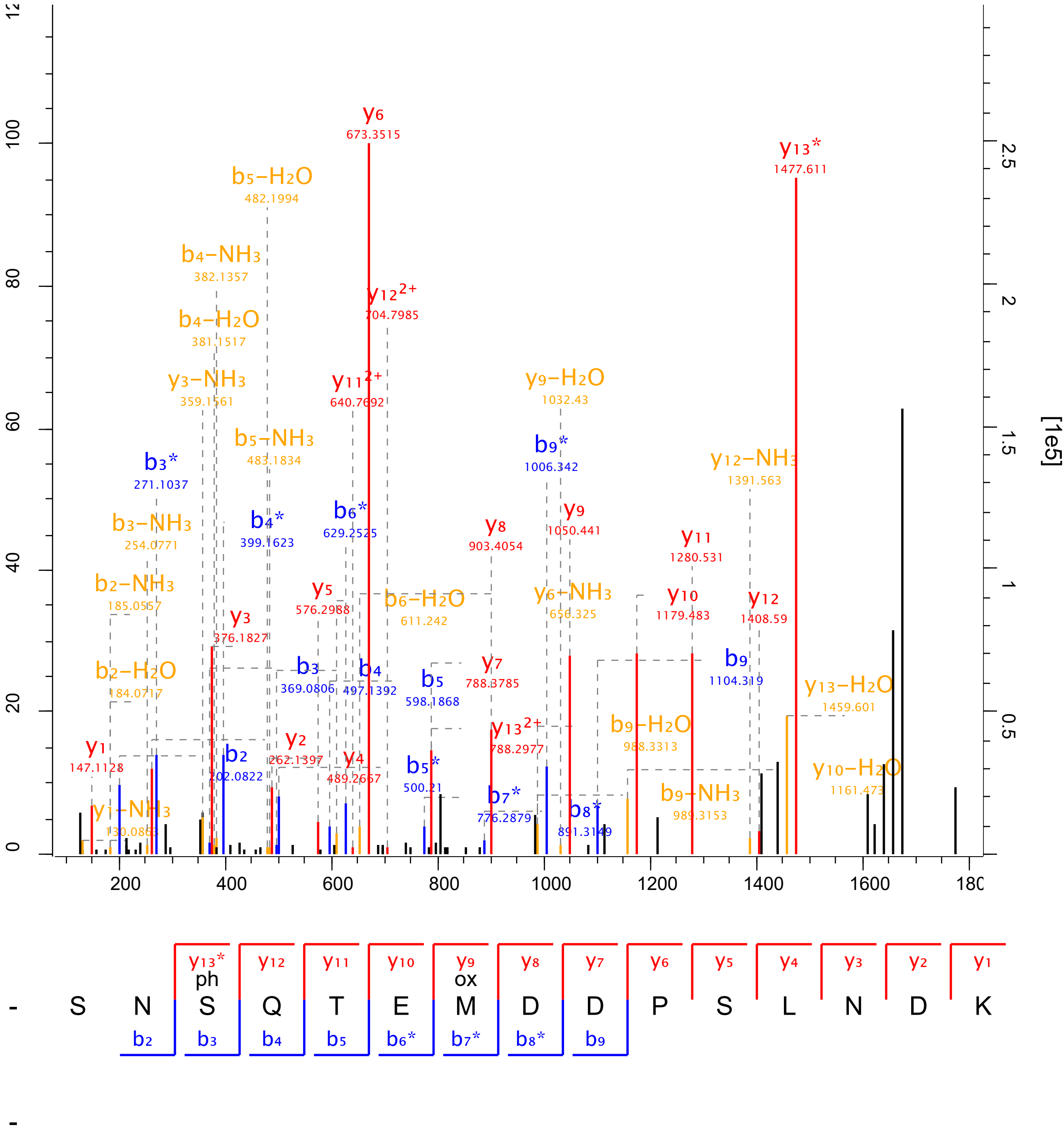

Raw file	Scan	Method	Score	m/z
sys_15_1	9140	FTMS; HCD	42.34	452.72

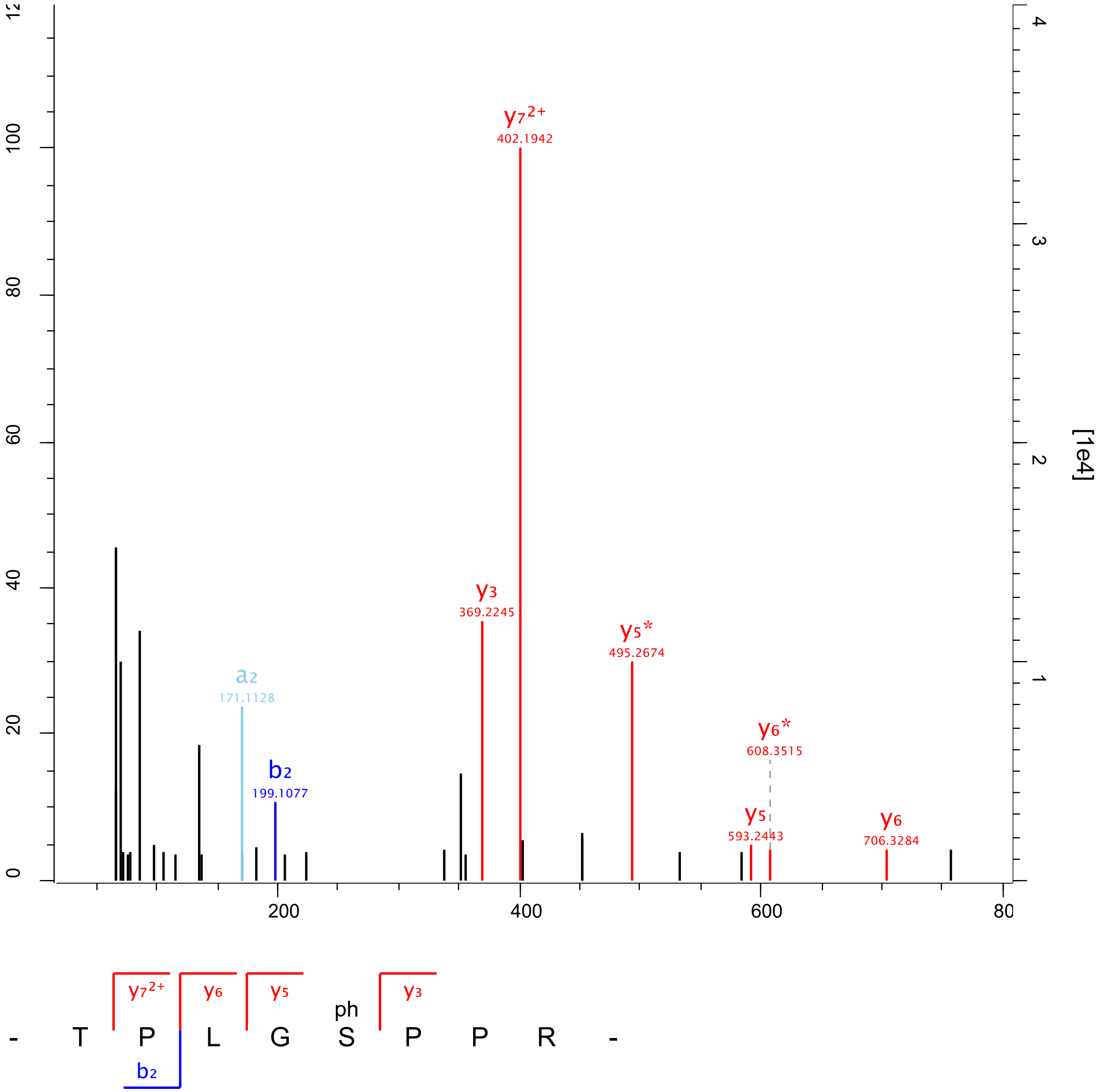

Raw file	Scan	Method	Score	m/z
sys_15_1	9177	FTMS; HCD	174.59	734.78

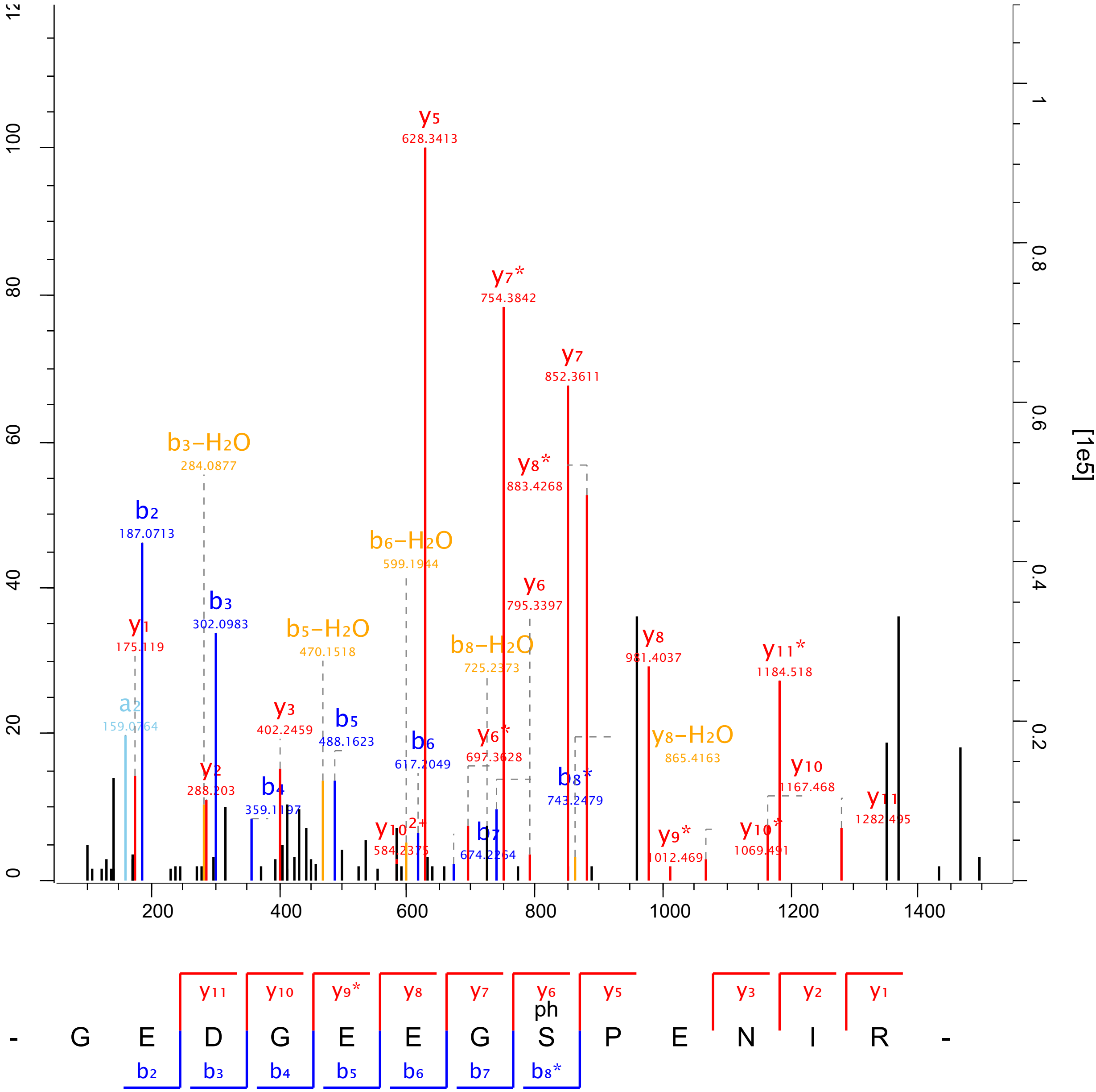

Raw file	Scan	Method	Score	m/z
sys_15_1	9212	FTMS; HCD	155.57	663.79

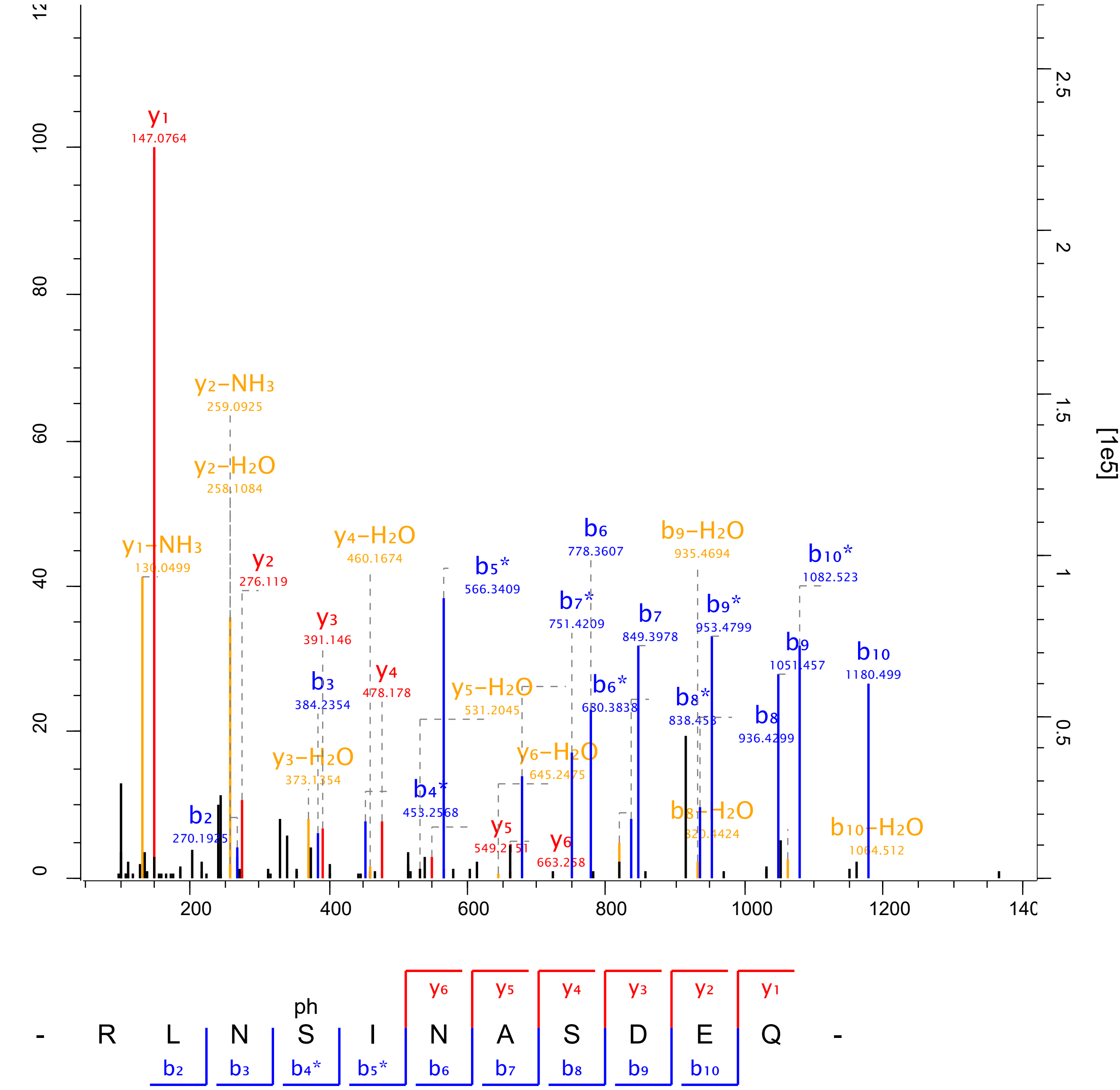

Raw file	Scan	Method	Score	m/z
sys_15_1	9379	FTMS; HCD	160.22	563.23

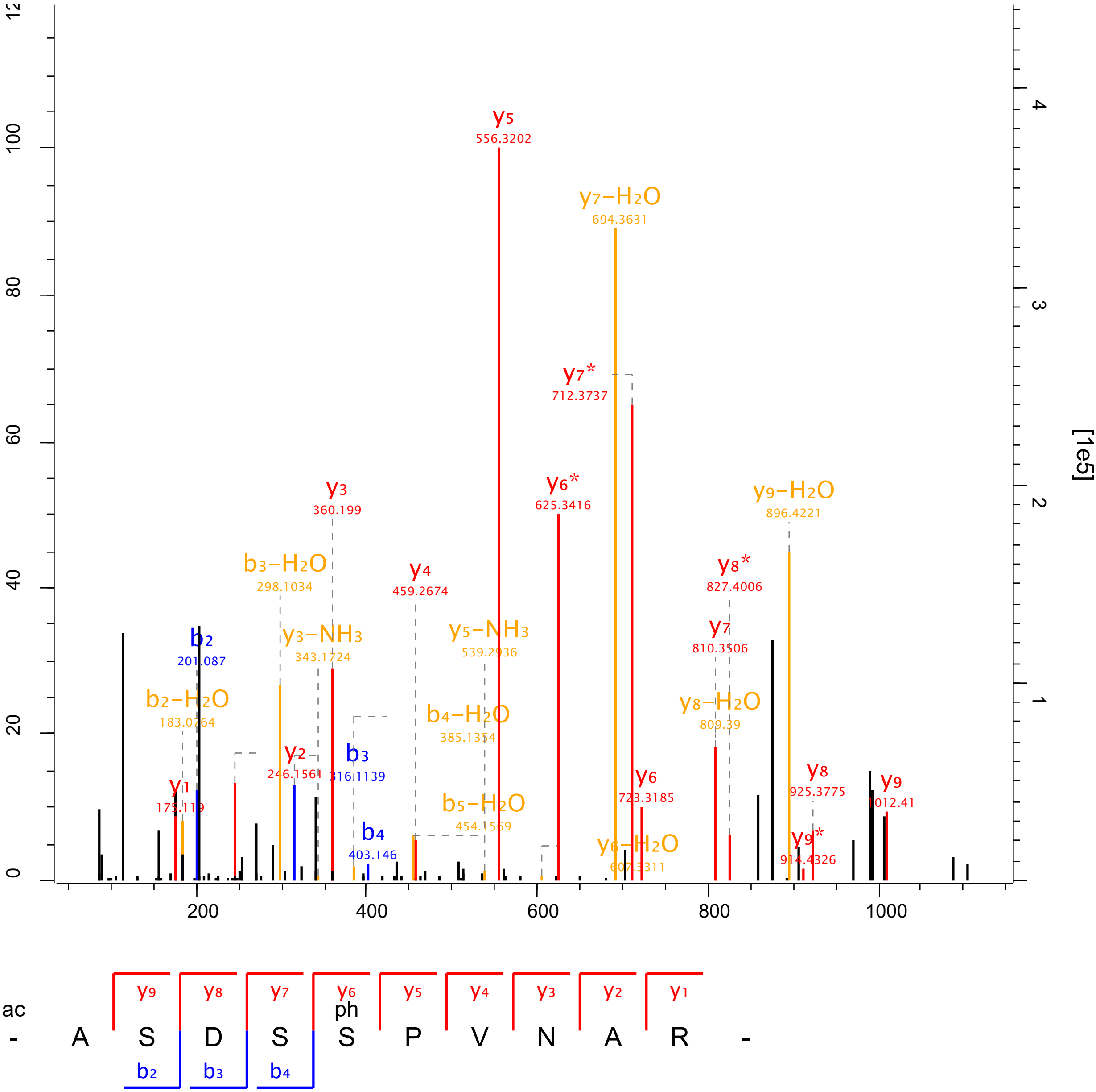

Raw file	Scan	Method	Score	m/z
sys_15_1	9389	FTMS; HCD	148.52	630.77

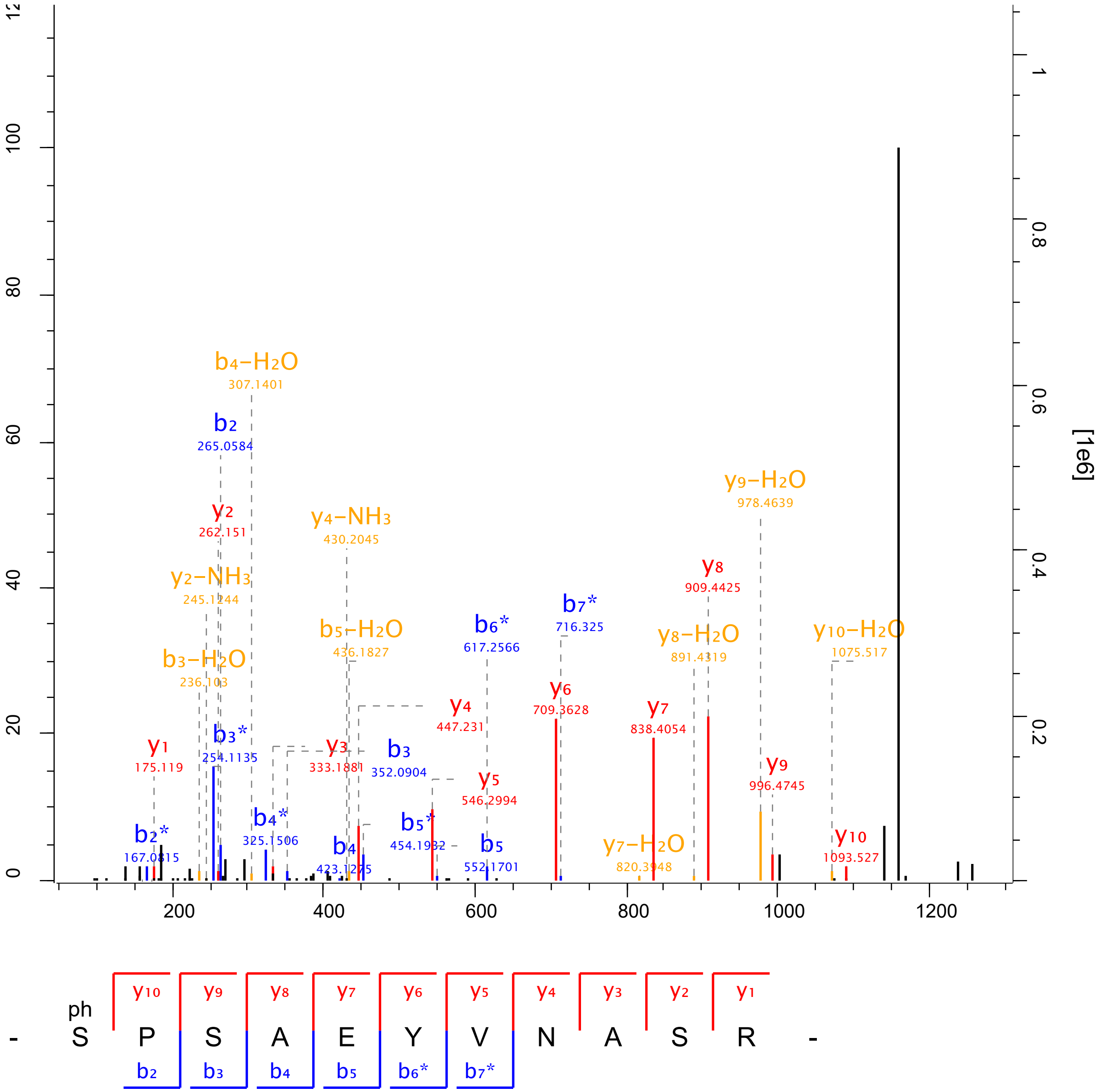

Raw file	Scan	Method	Score	m/z
sys_15_1	9410	FTMS; HCD	125.97	733.24

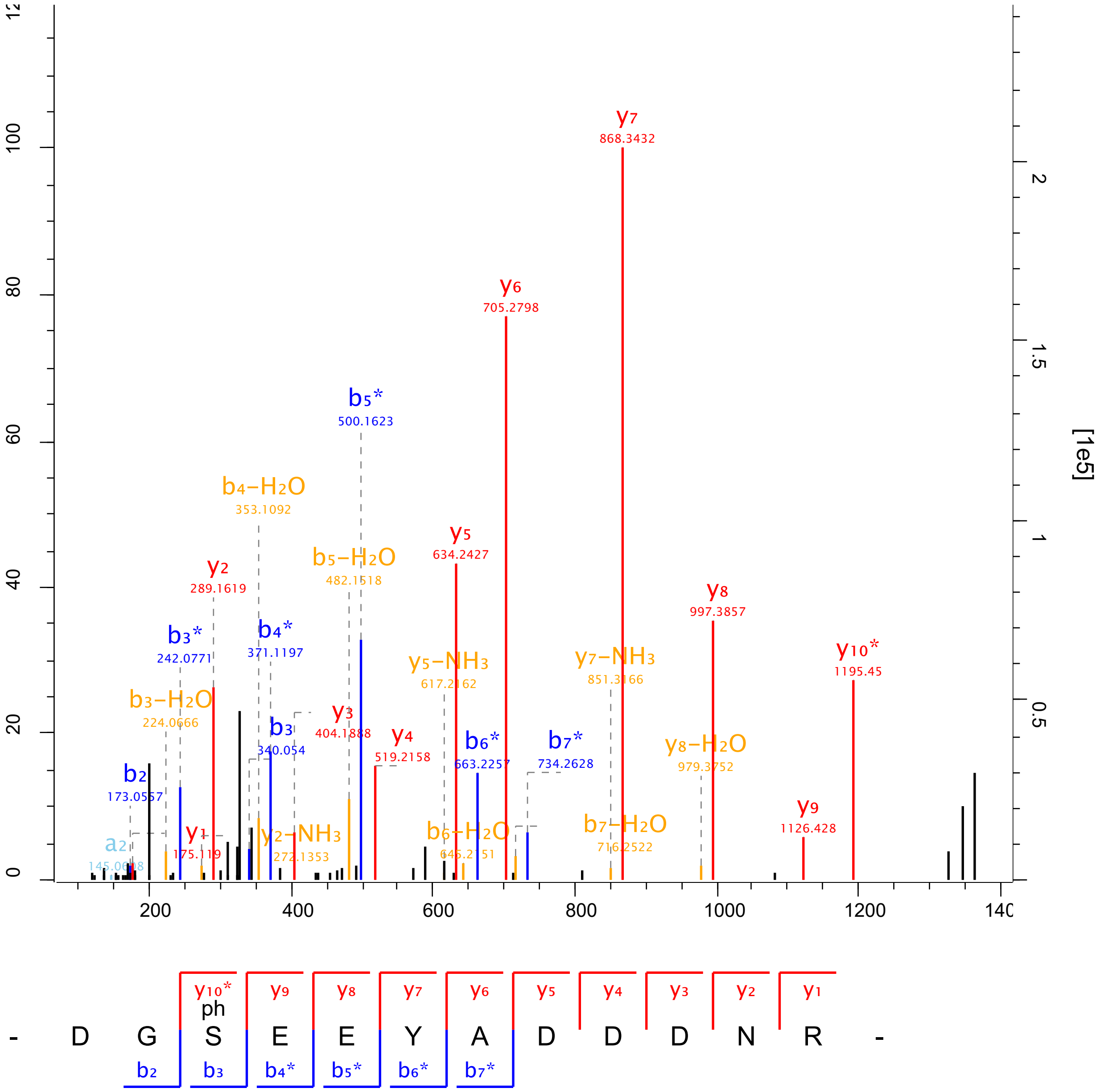

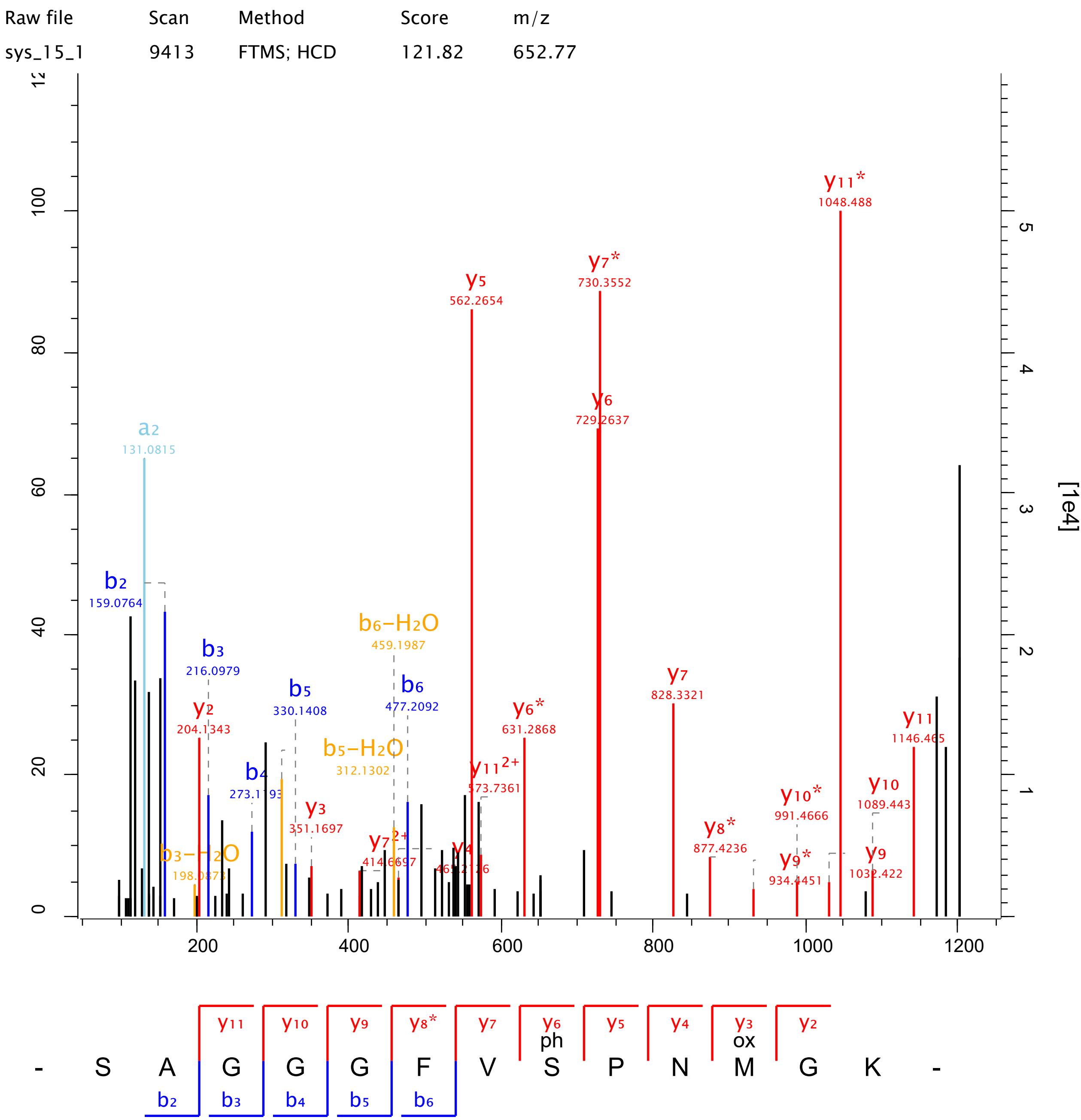

Raw file Scan Method Score m/z
sys_15_1 9413 FTMS; HCD 121.82 652.77
a2 131.0815
b2 159.0764
b3 216.0979
b4 273.1193
b5 330.1408
b6 477.2092
b3−H2O 198.0873
b5−H2O 312.1302
b6−H2O 459.1987
y2 204.1343
y3 351.1697
y4 465.2126
y5 562.2654
y6 729.2637
y6* 631.2868
y7 828.3321
y7* 730.3552
y7 2+ 414.6697
y8* 877.4236
y9 1032.422
y9* 934.4451
y10 1089.443
y10* 991.4666
y11 1146.465
y11* 1048.488
y11 2+ 573.7361
[1e4]
- S A G G G F V S P N M G K -
ph
ox

Raw file	Scan	Method	Score	m/z
sys_15_1	9477	FTMS; HCD	174.18	777.79

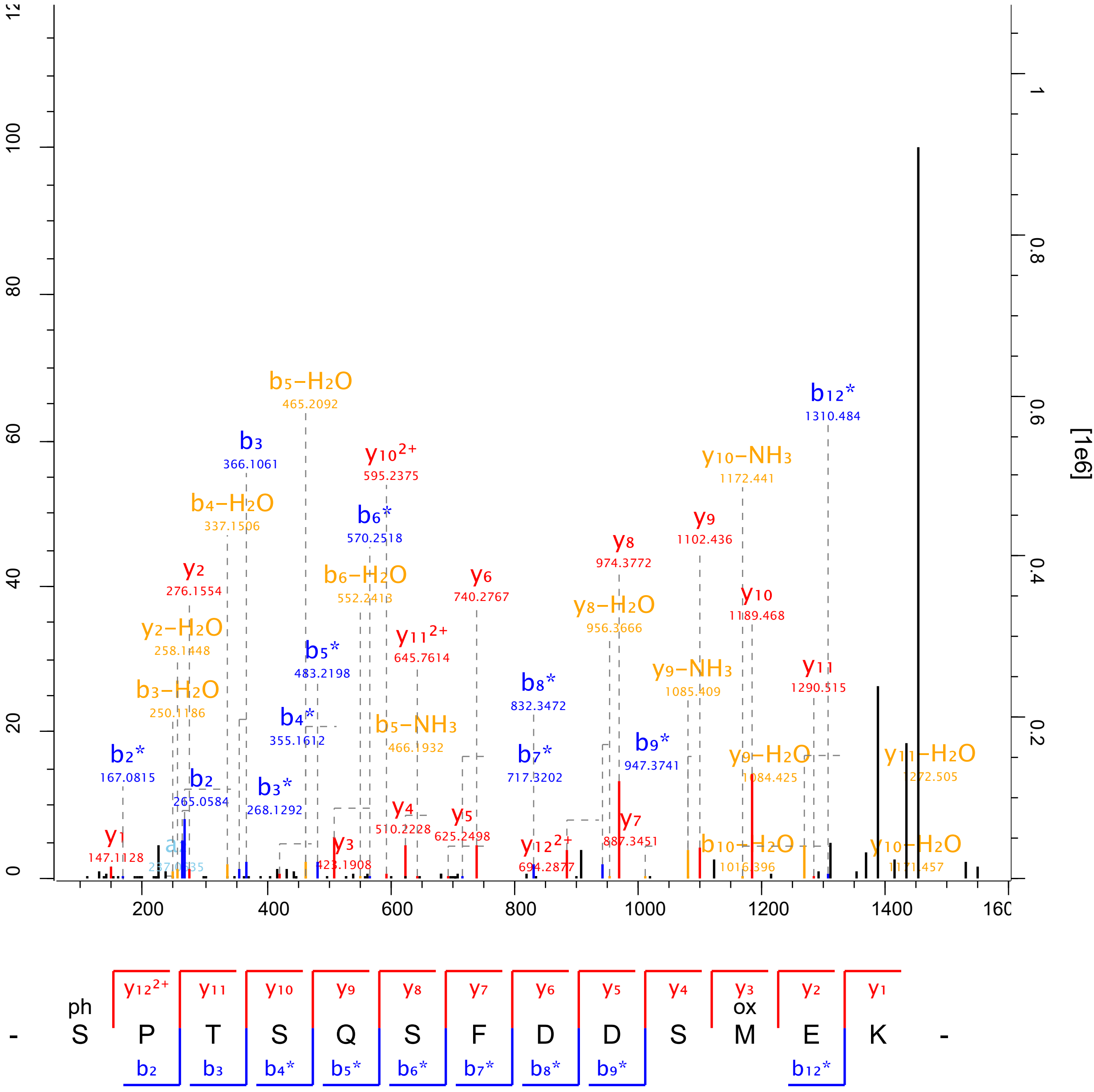

Raw file	Scan	Method	Score	m/z
sys_15_1	9494	FTMS; HCD	253.73	763.85

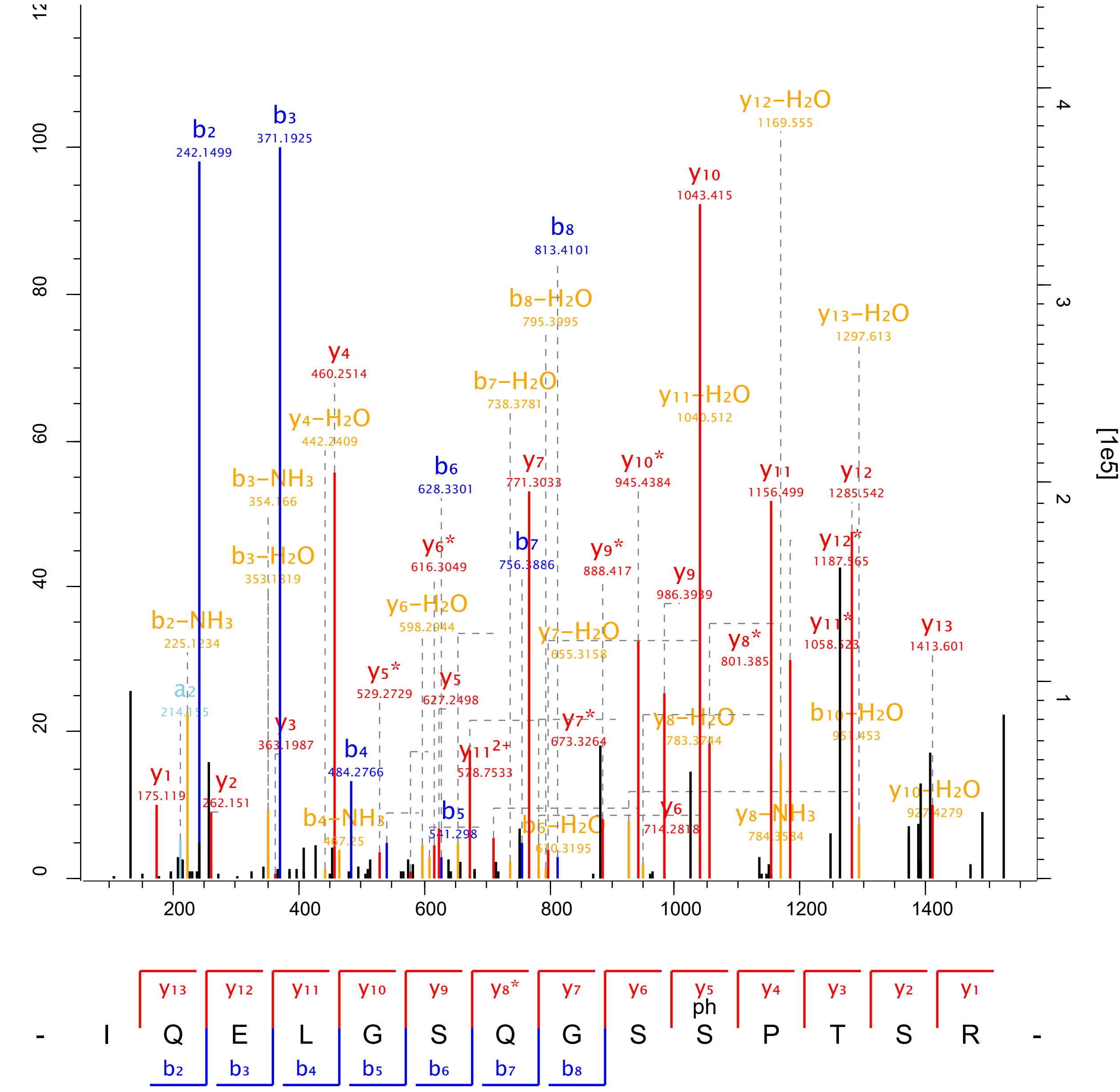

Raw file	Scan	Method	Score	m/z
sys_15_1	9500	FTMS; HCD	81.63	602.78

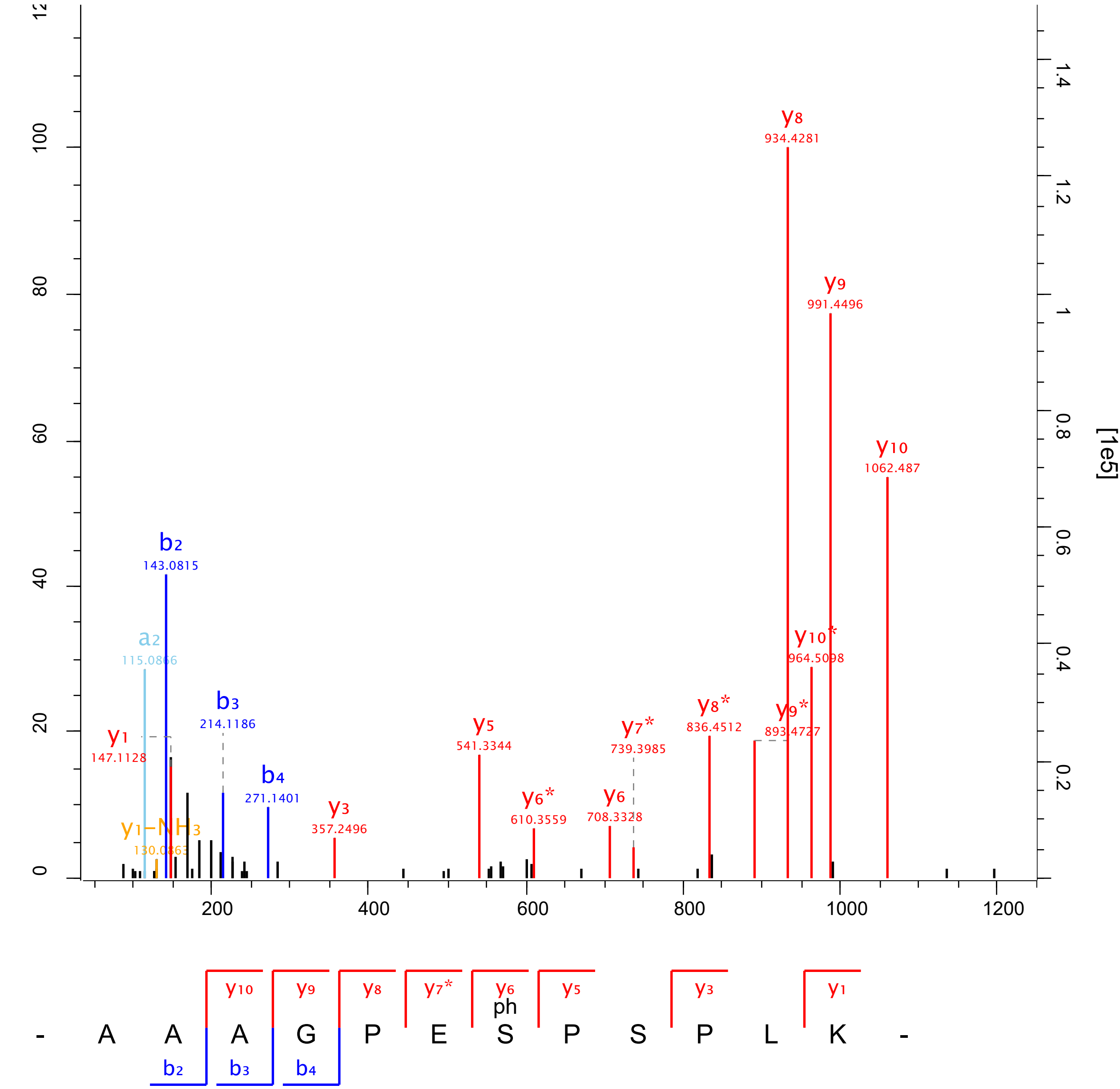

Raw file	Scan	Method	Score	m/z
sys_15_1	9506	FTMS; HCD	60.94	818.3

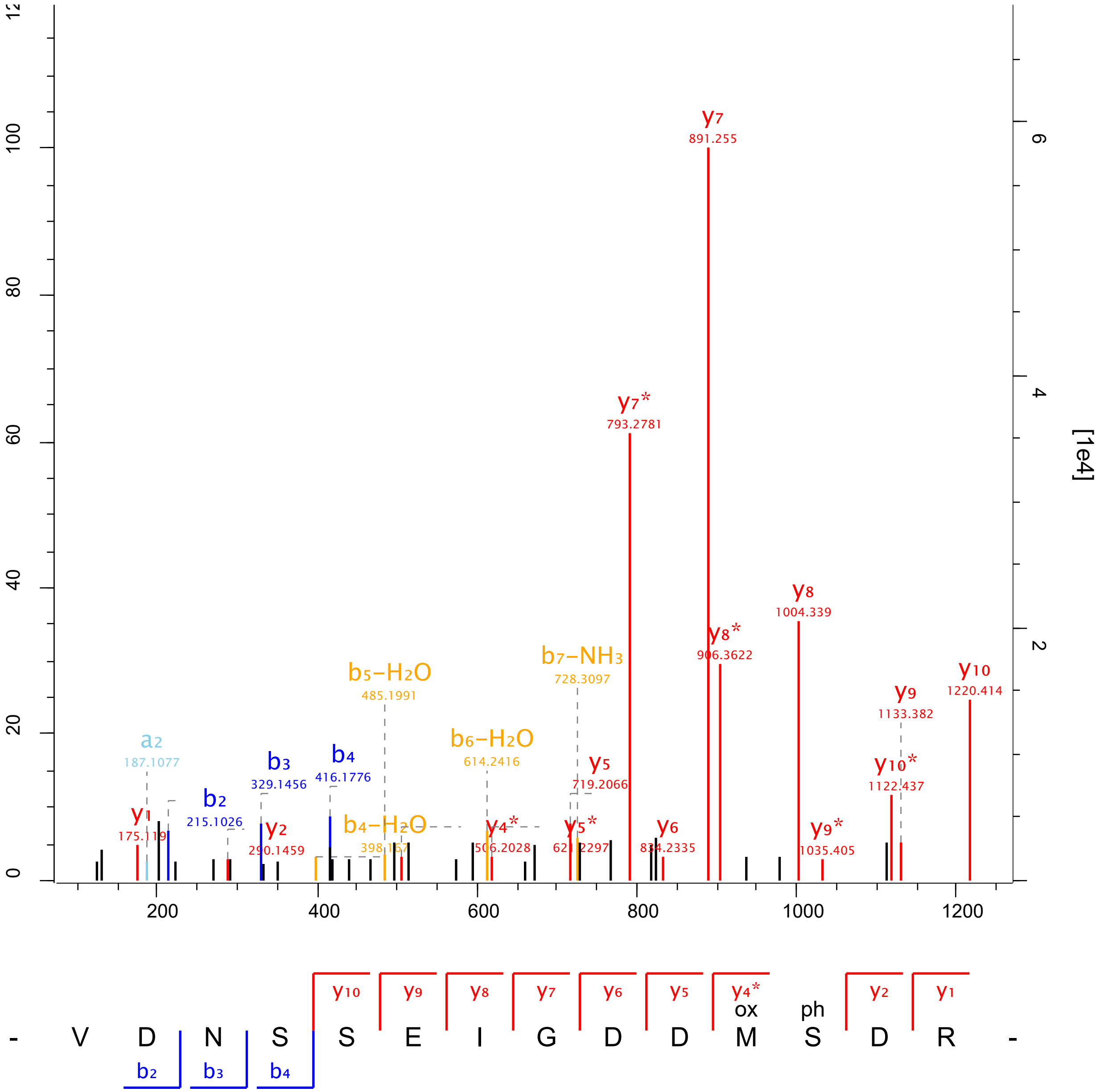

Raw file	Scan	Method	Score	m/z
sys_15_1	9541	FTMS; HCD	66.25	695.3

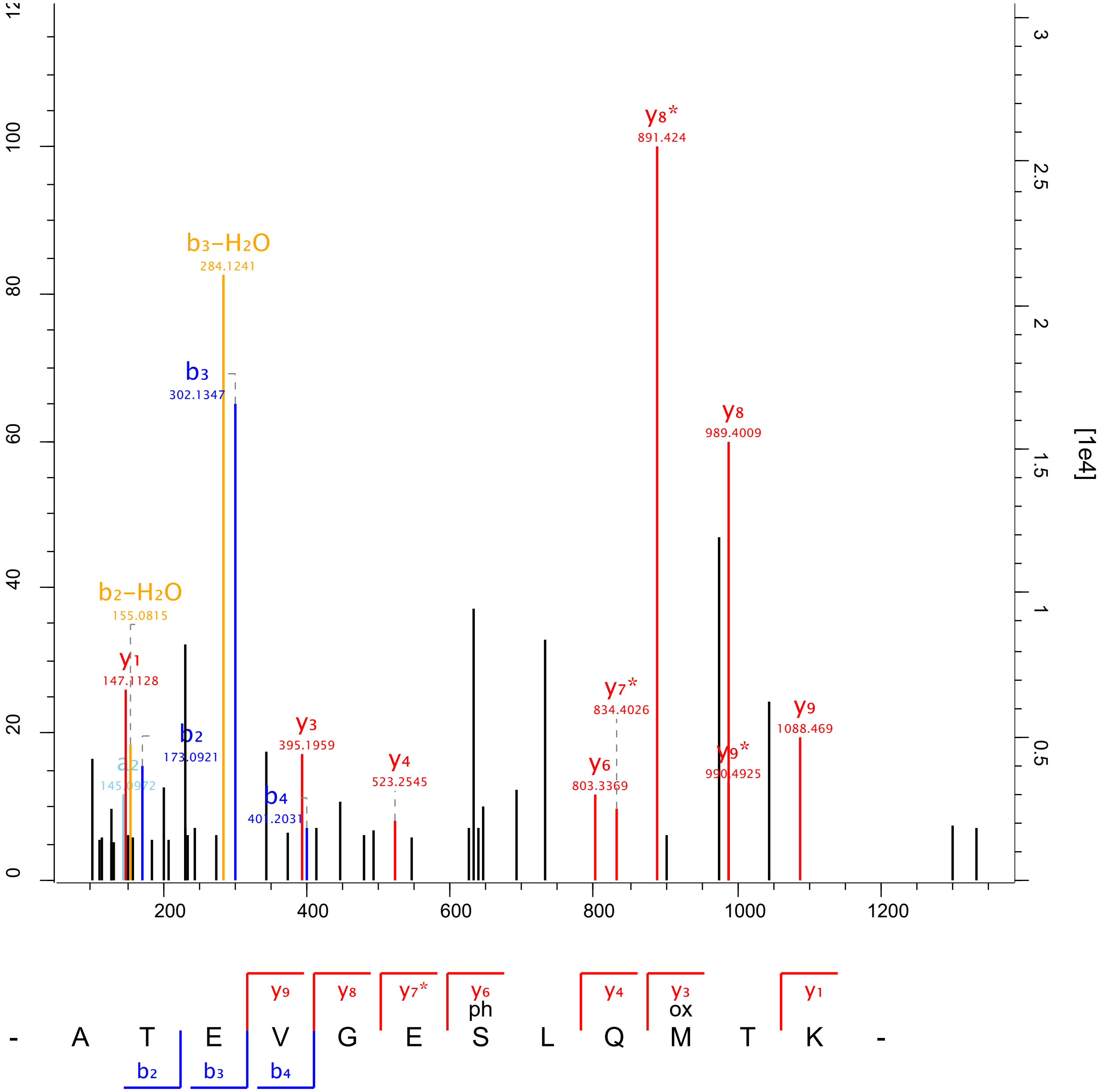

Raw file	Scan	Method	Score	m/z
sys_15_1	9560	FTMS; HCD	94.2	716.31

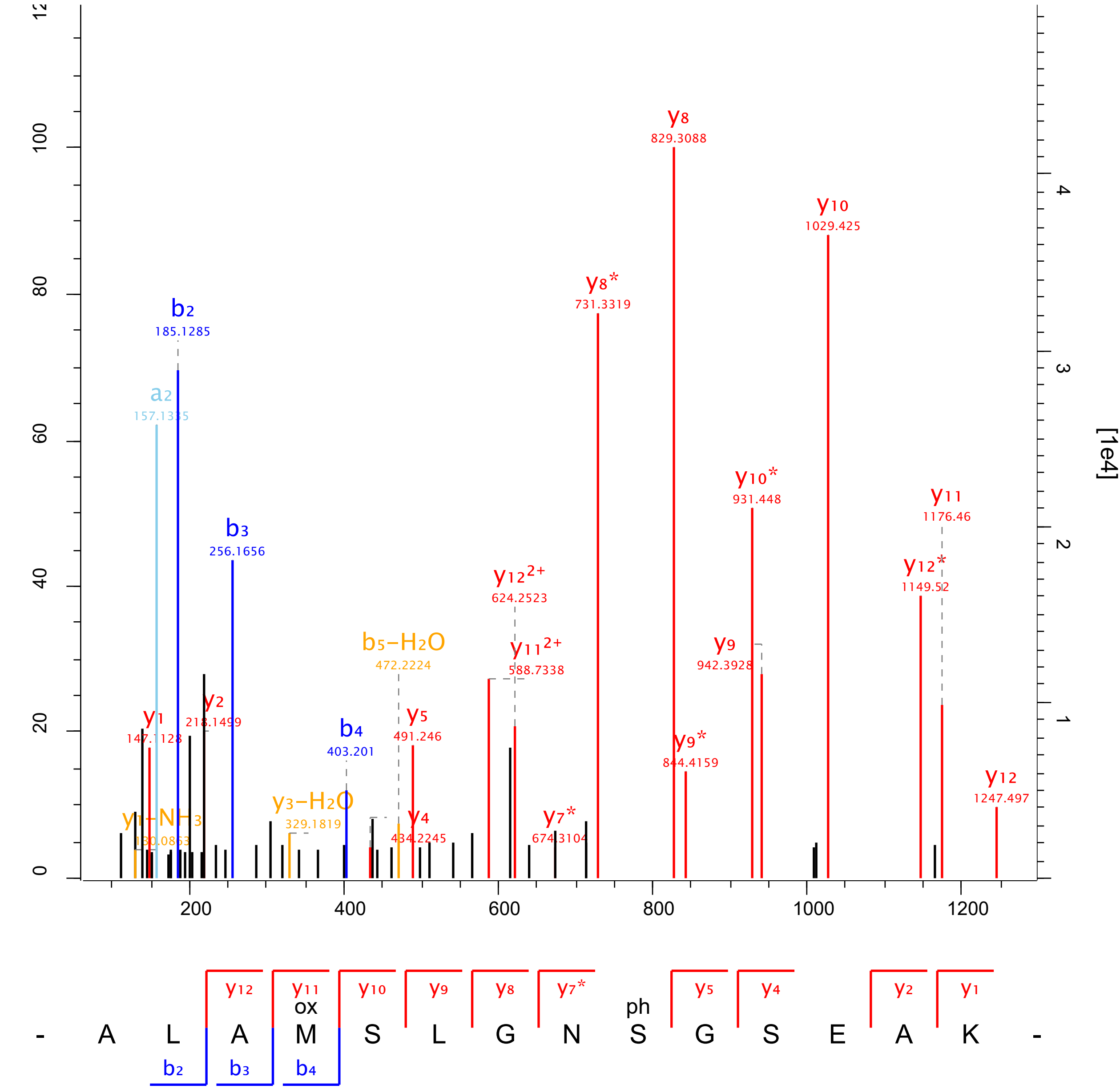

Raw file	Scan	Method	Score	m/z
sys_15_1	9581	FTMS; HCD	222.54	692.94

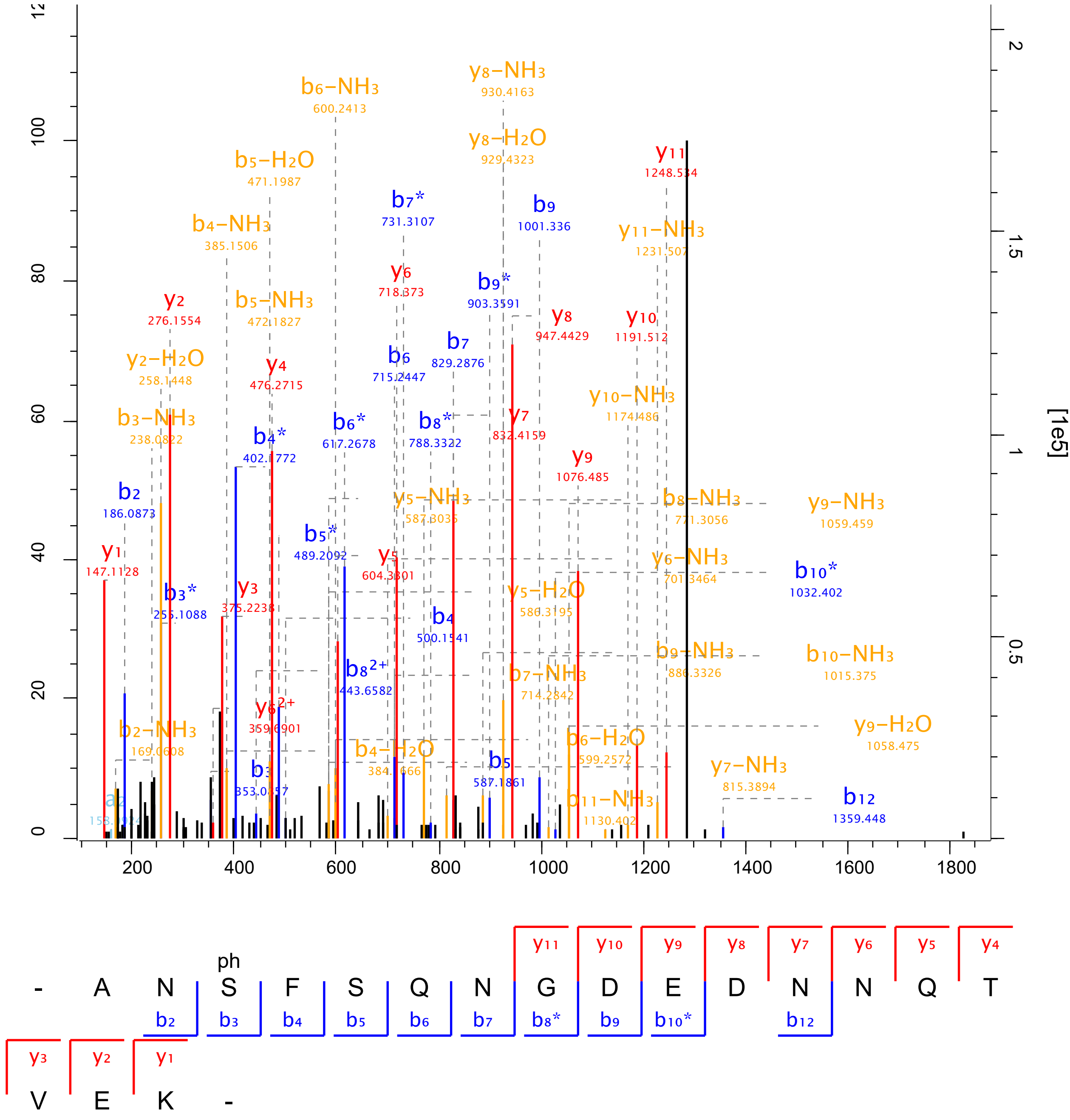

Raw file	Scan	Method	Score	m/z
sys_15_1	9626	FTMS; HCD	248.23	824.83

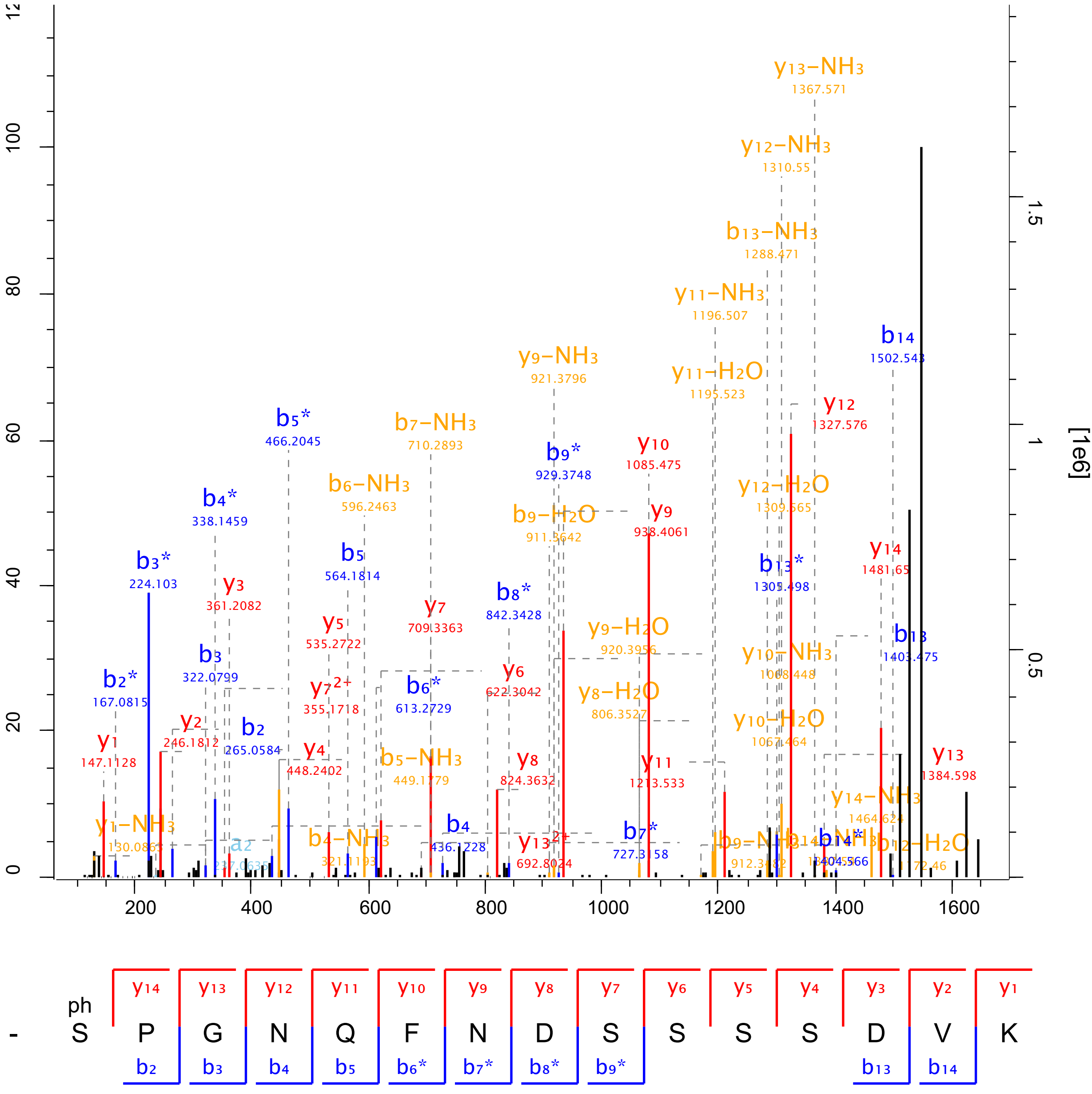

Raw file	Scan	Method	Score	m/z
sys_15_1	9633	FTMS; HCD	70.09	514.72

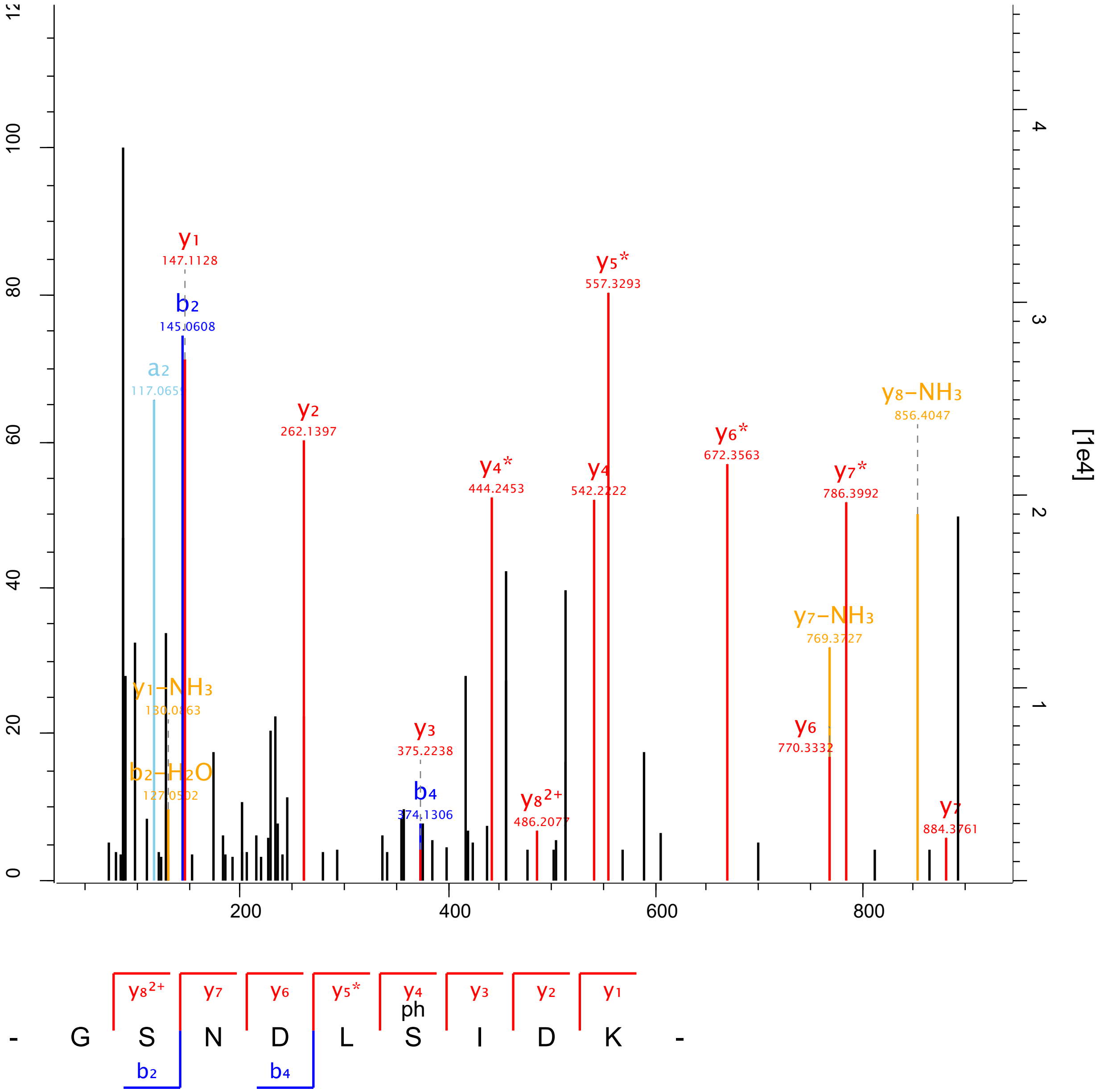

Raw file	Scan	Method	Score	m/z
sys_15_1	9646	FTMS; HCD	138.55	621.28

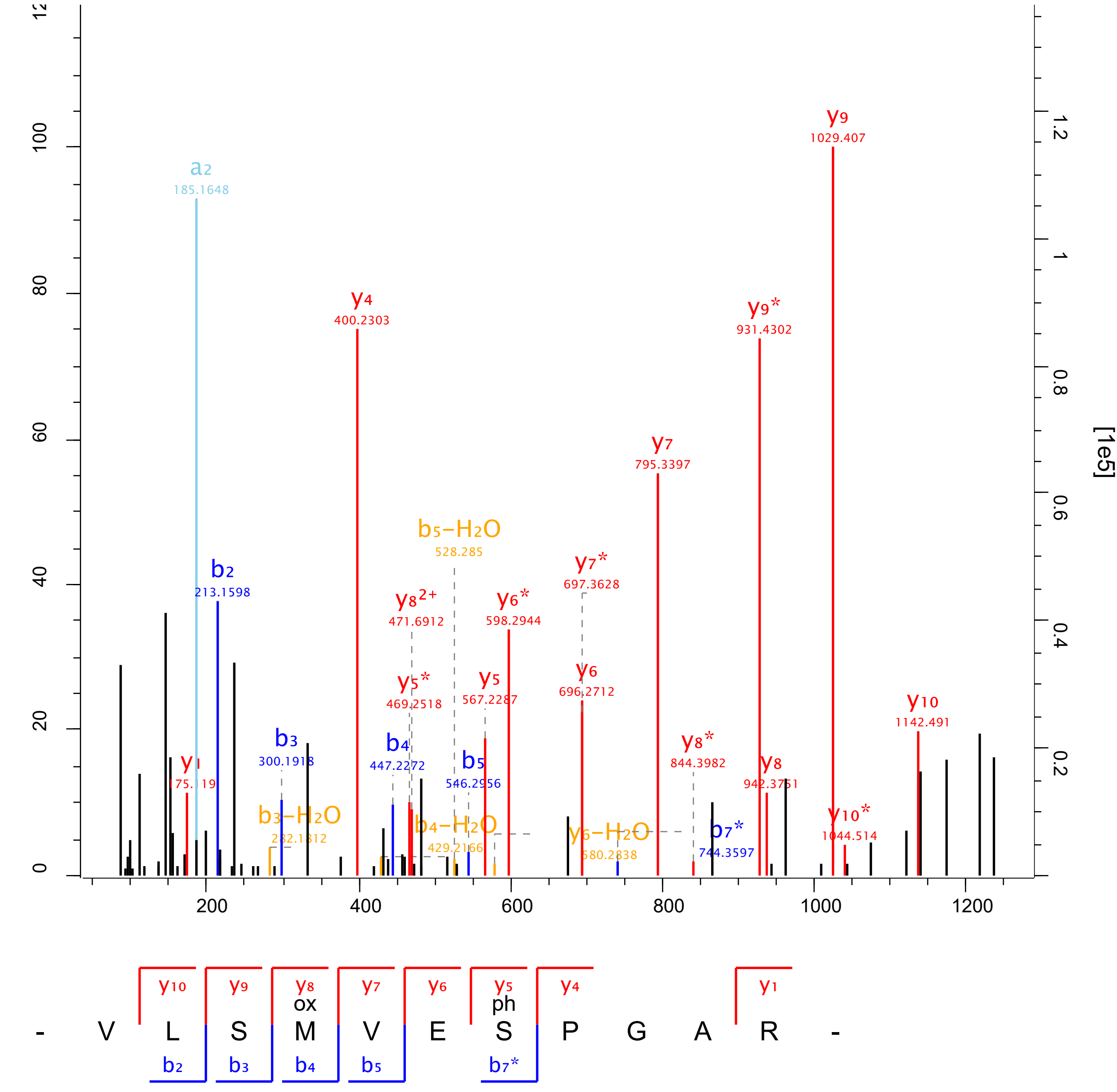

Raw file	Scan	Method	Score	m/z
sys_15_1	9647	FTMS; HCD	73.93	586.75

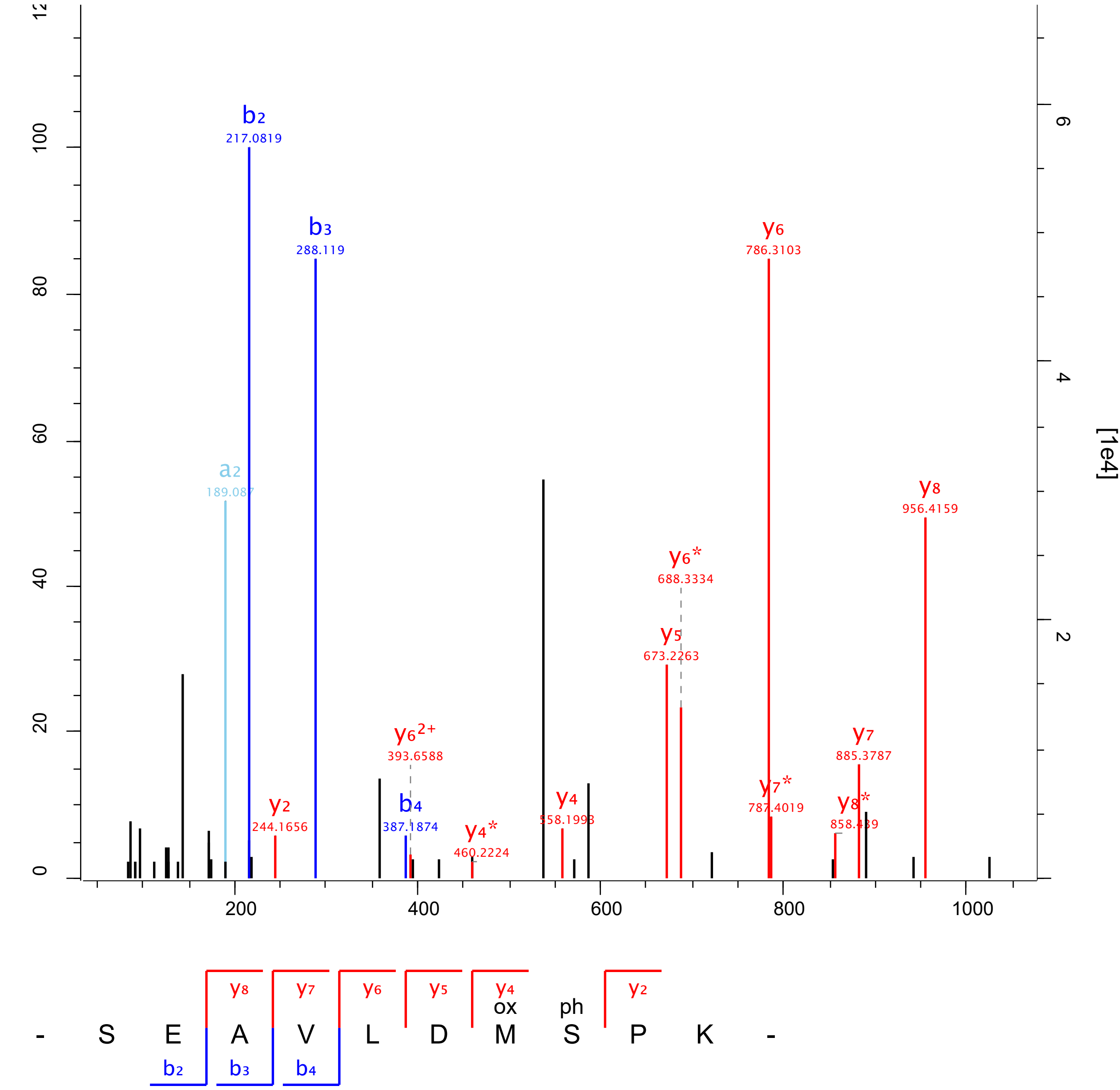

Raw file	Scan	Method	Score	m/z
sys_15_1	9650	FTMS; HCD	63.69	690.26

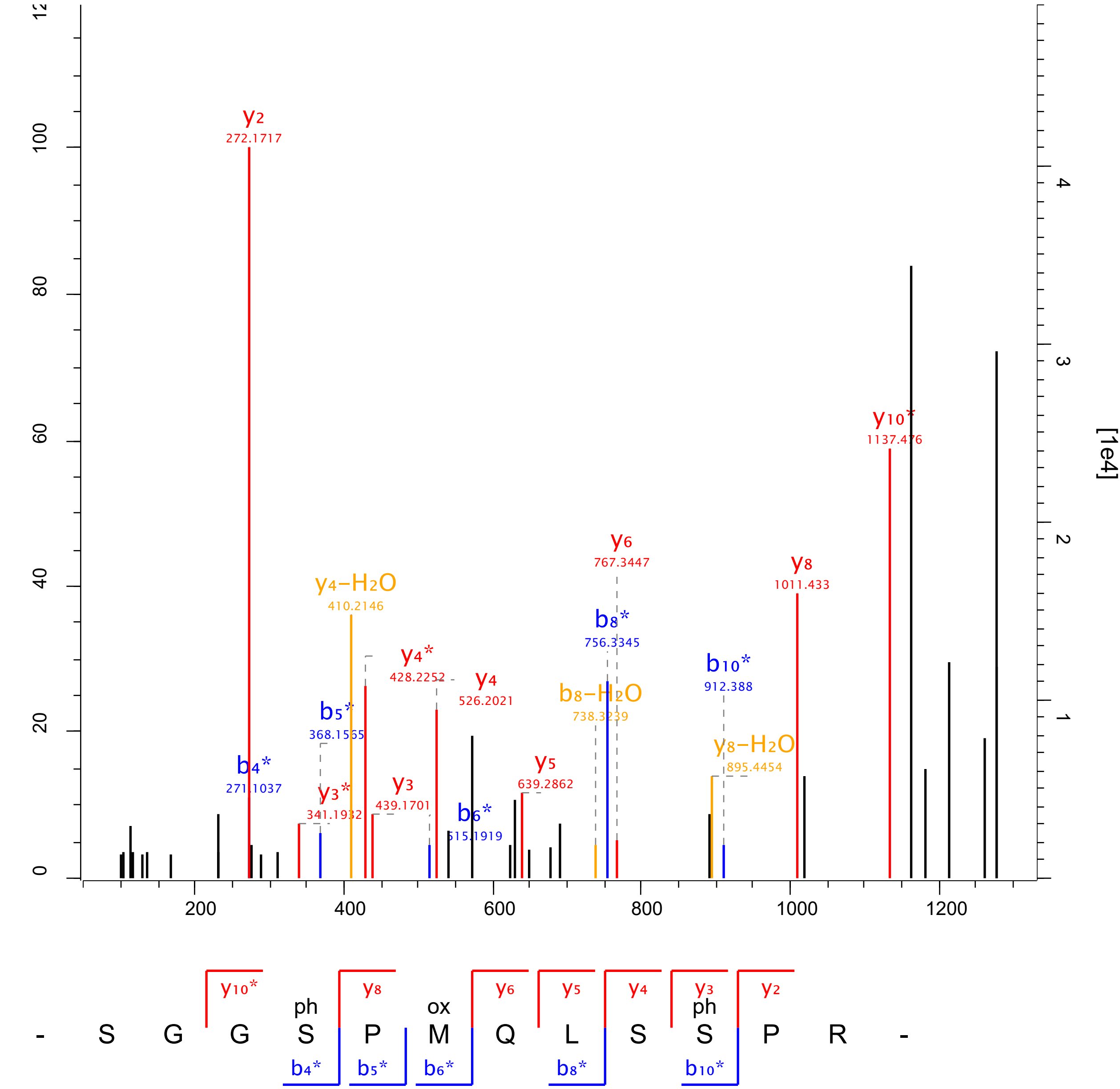

Raw file	Scan	Method	Score	m/z
sys_15_1	9755	FTMS; HCD	43.03	594.25

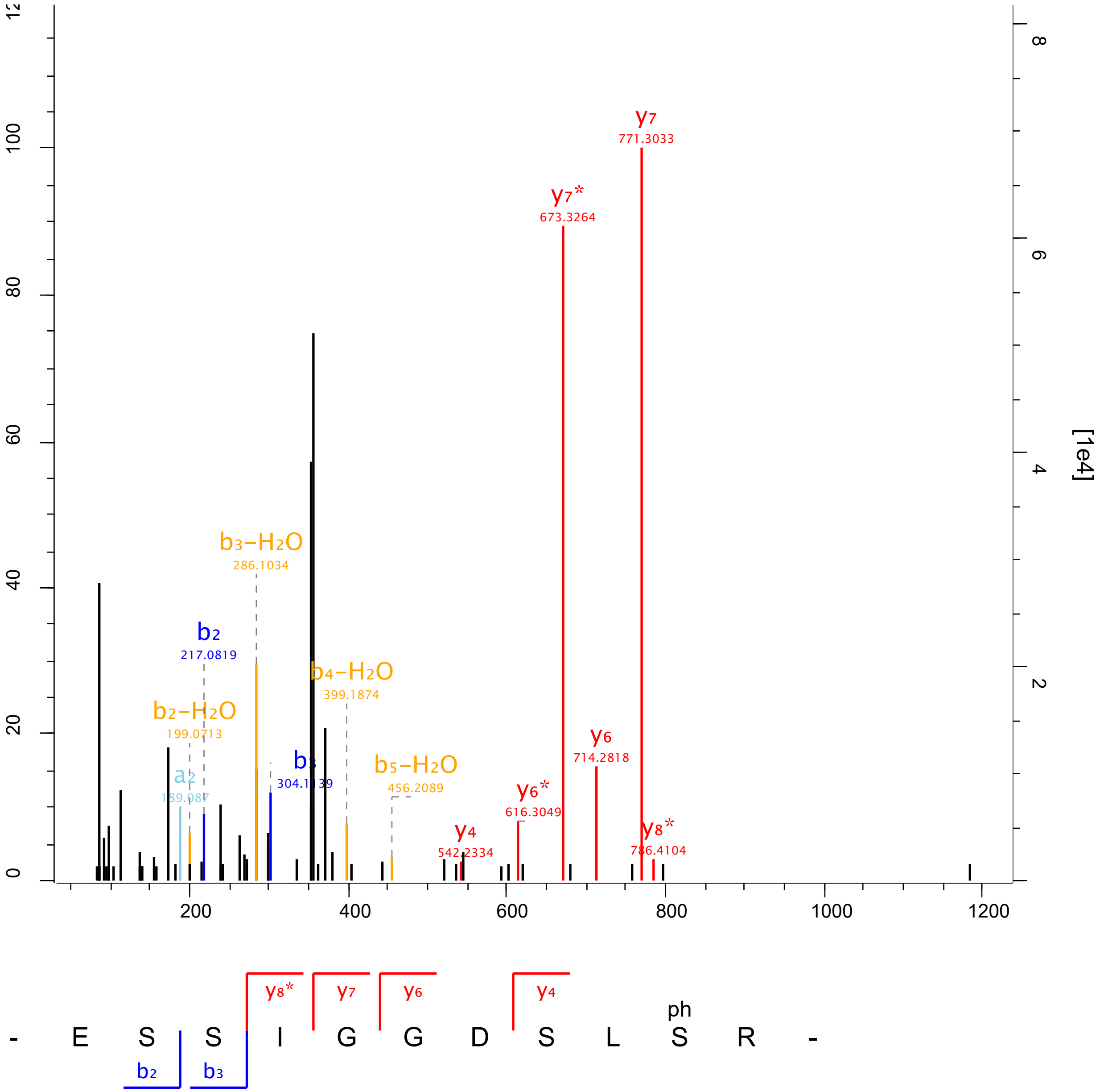

Raw file	Scan	Method	Score	m/z
sys_15_1	9763	FTMS; HCD	92.38	484.87

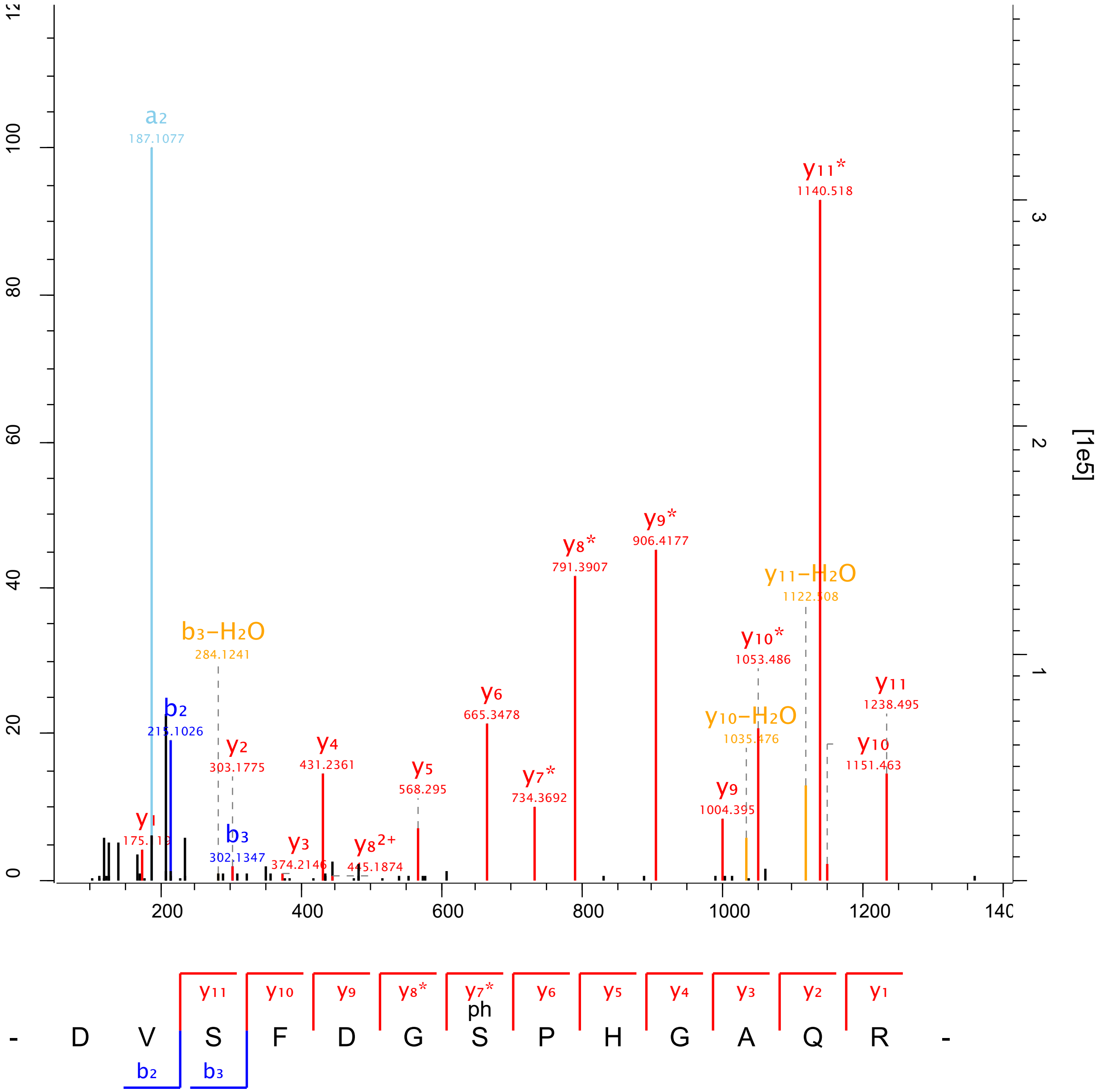

Raw file	Scan	Method	Score	m/z
sys_15_1	9783	FTMS; HCD	52.17	693.3

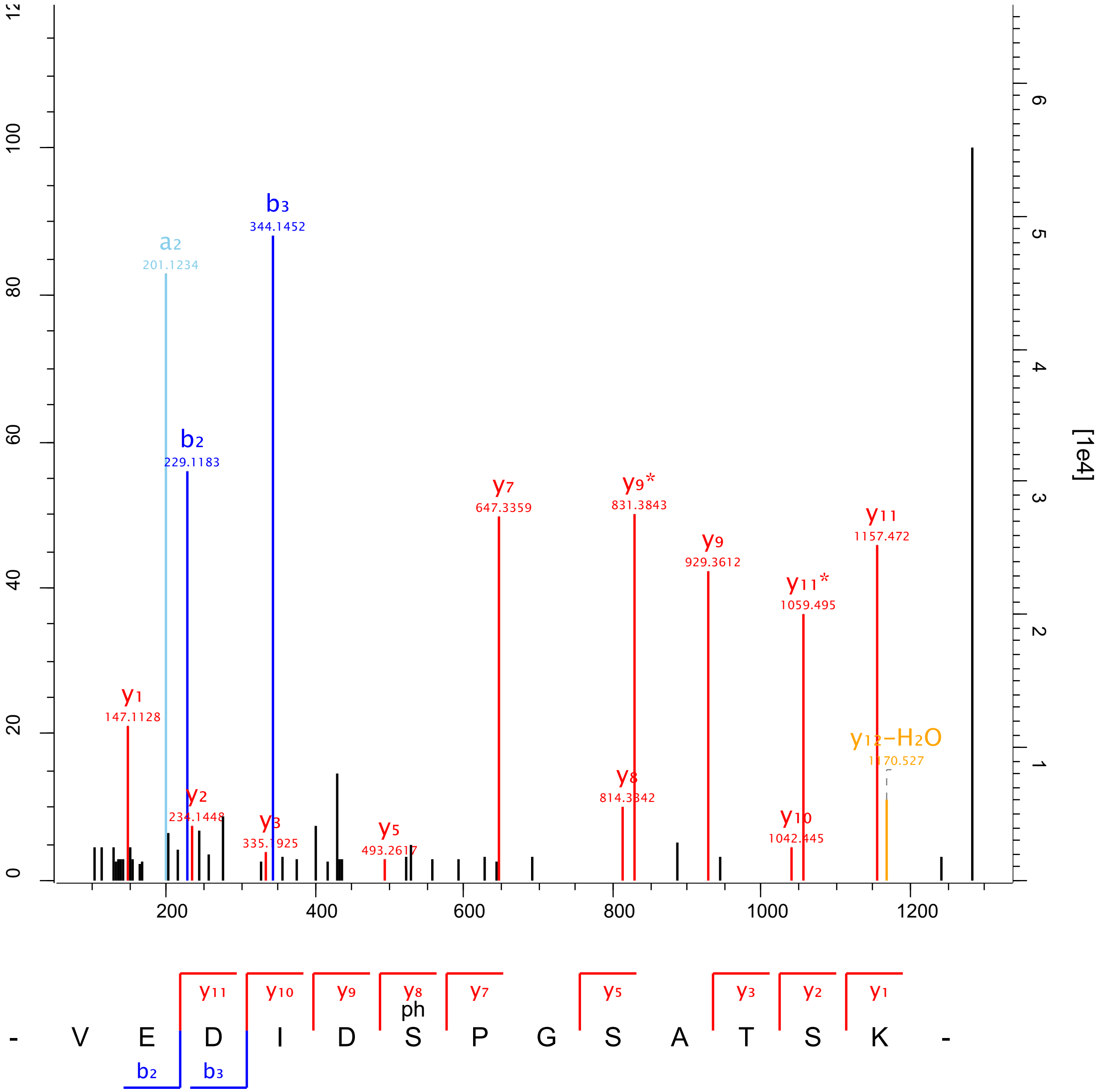

Raw file	Scan	Method	Score	m/z
sys_15_1	9788	FTMS; HCD	86.21	624.6

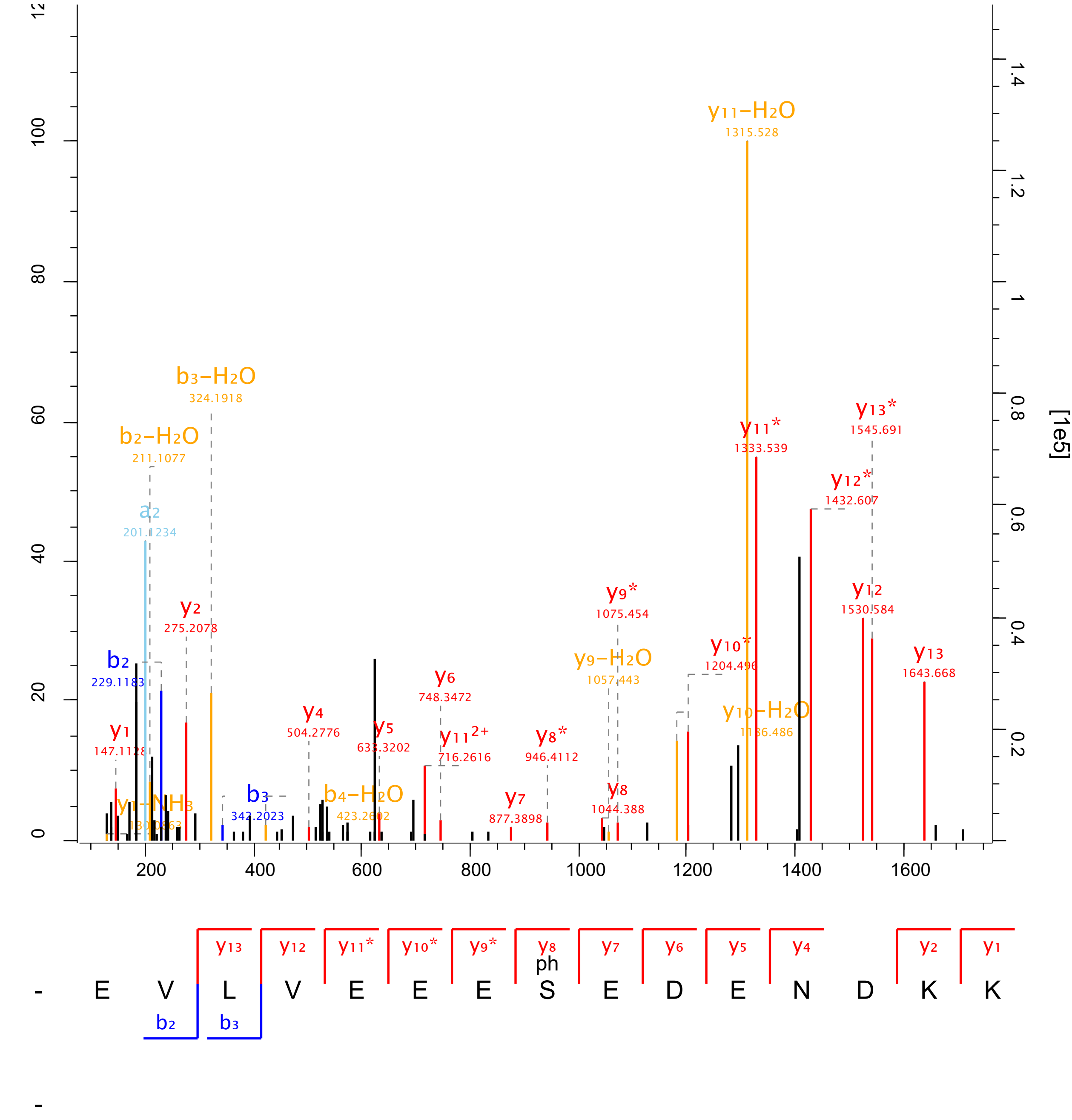

Raw file	Scan	Method	Score	m/z
sys_15_1	9832	FTMS; HCD	50.04	644.79

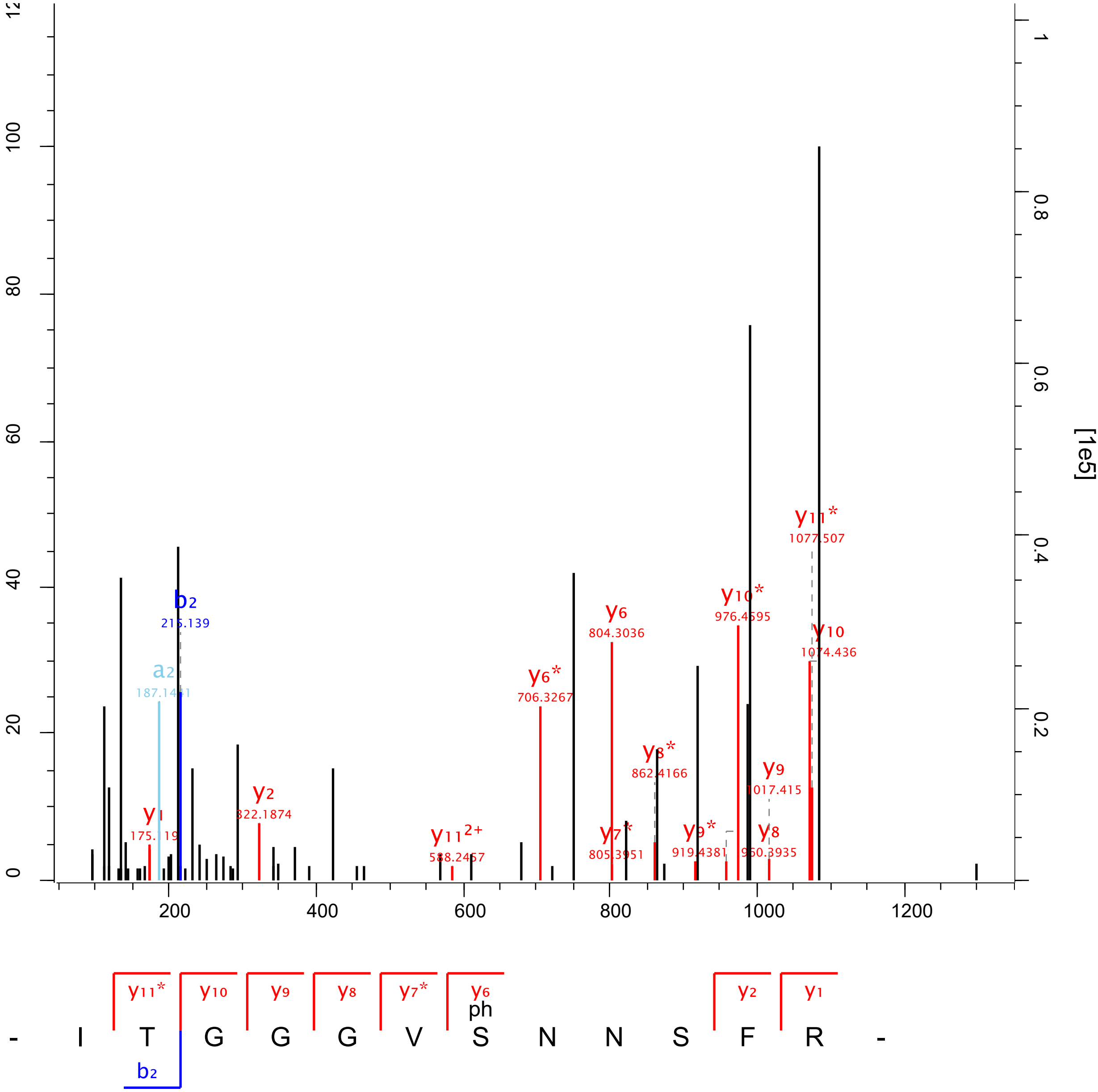

Raw file	Scan	Method	Score	m/z
sys_15_1	9901	FTMS; HCD	80.23	418.22

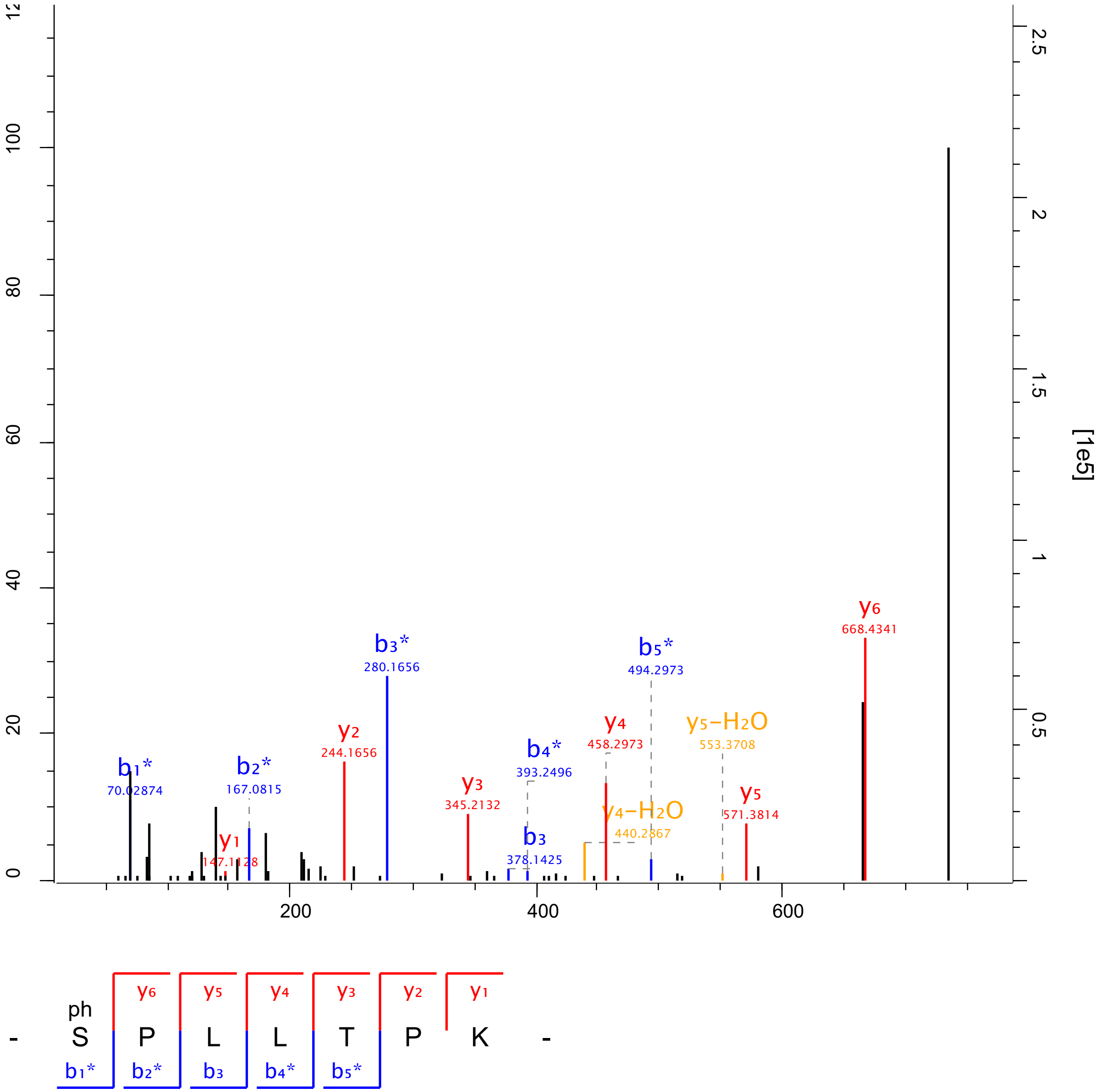

Raw file	Scan	Method	Score	m/z
sys_15_1	9961	FTMS; HCD	96.49	635.26

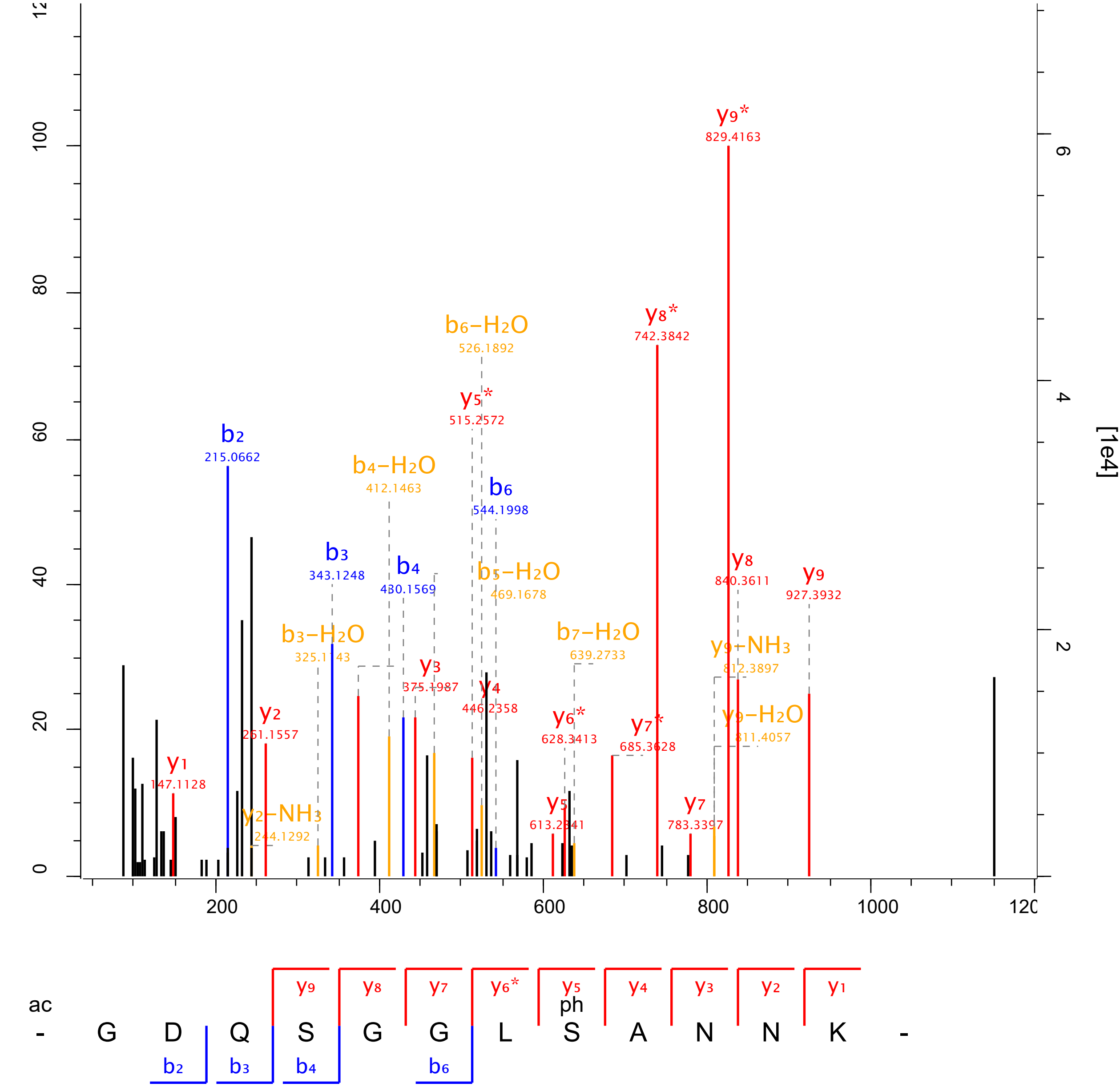

Raw file	Scan	Method	Score	m/z
sys_15_1	9998	FTMS; HCD	79.92	707.82

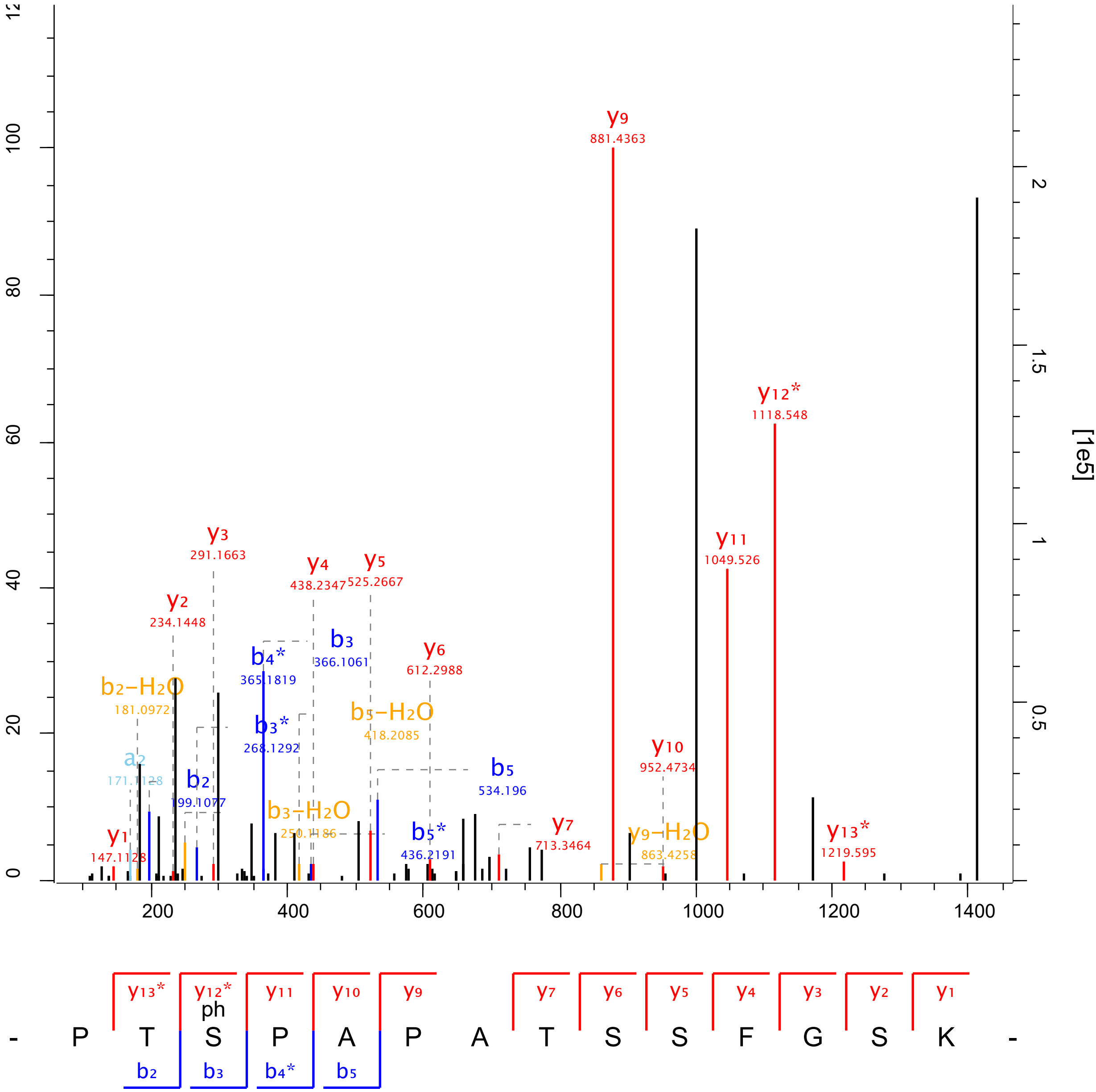

Raw file	Scan	Method	Score	m/z
sys_15_2	30107	FTMS; HCD	185.25	798.87

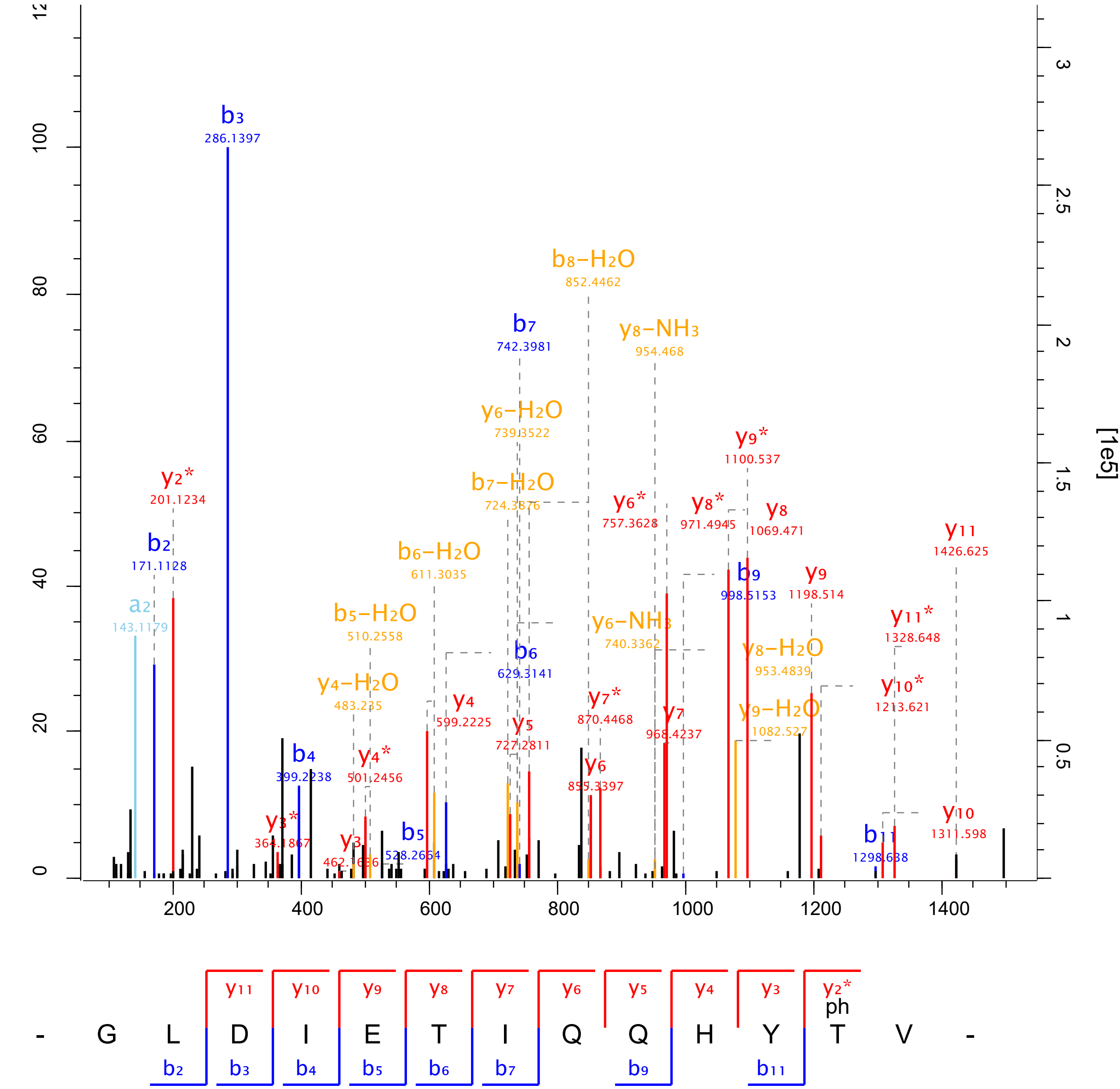

Raw file	Scan	Method	Score	m/z
sys_15_2	30707	FTMS; HCD	109.53	832.7

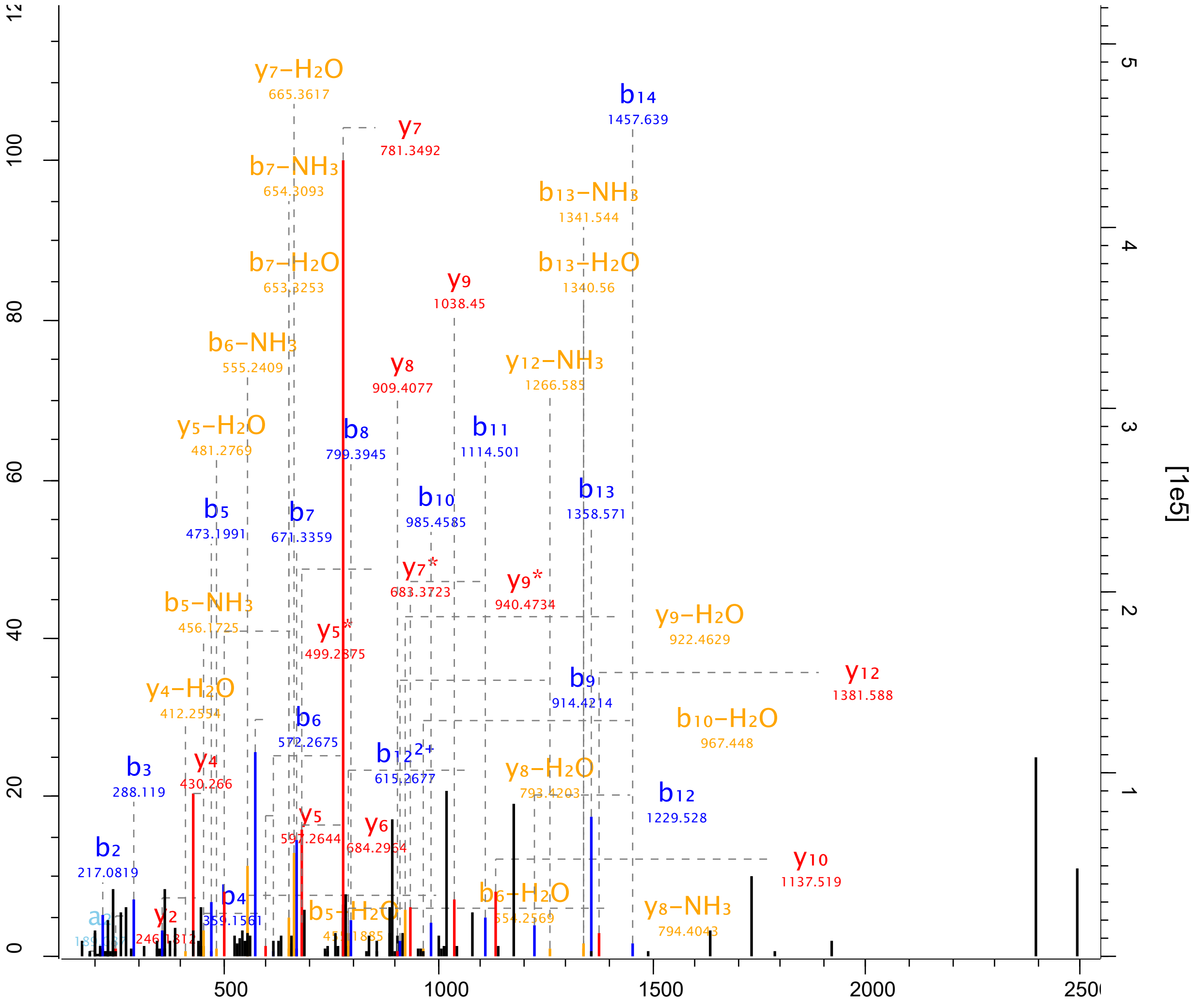

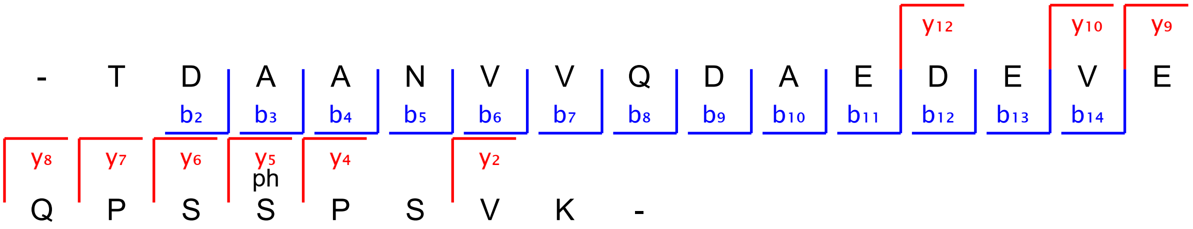

Raw file	Scan	Method	Score	m/z
sys_15_2	33237	FTMS; HCD	54.47	1184.18

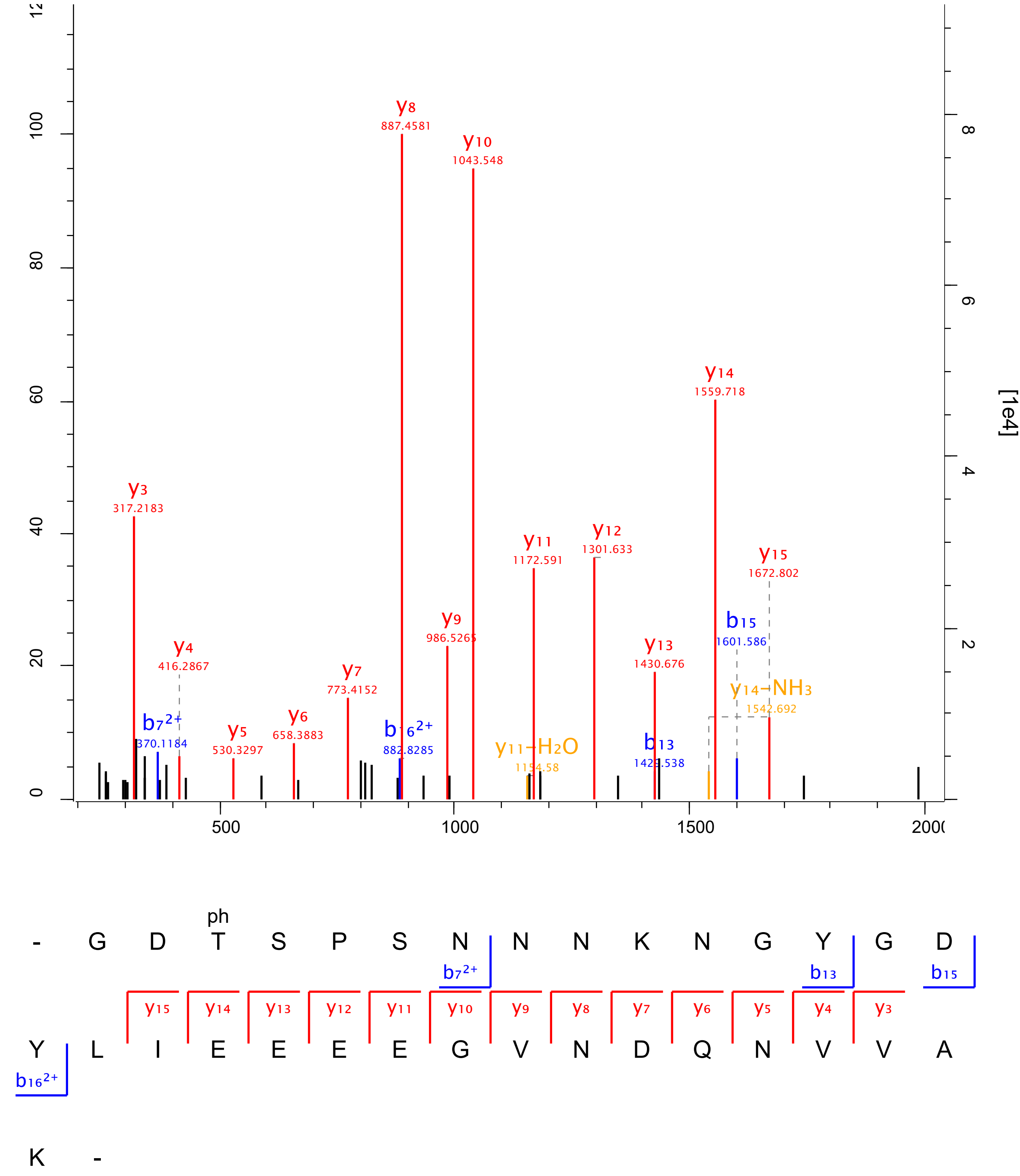

Raw file	Scan	Method	Score	m/z
sys_15_3	10504	FTMS; HCD	174.59	692.3

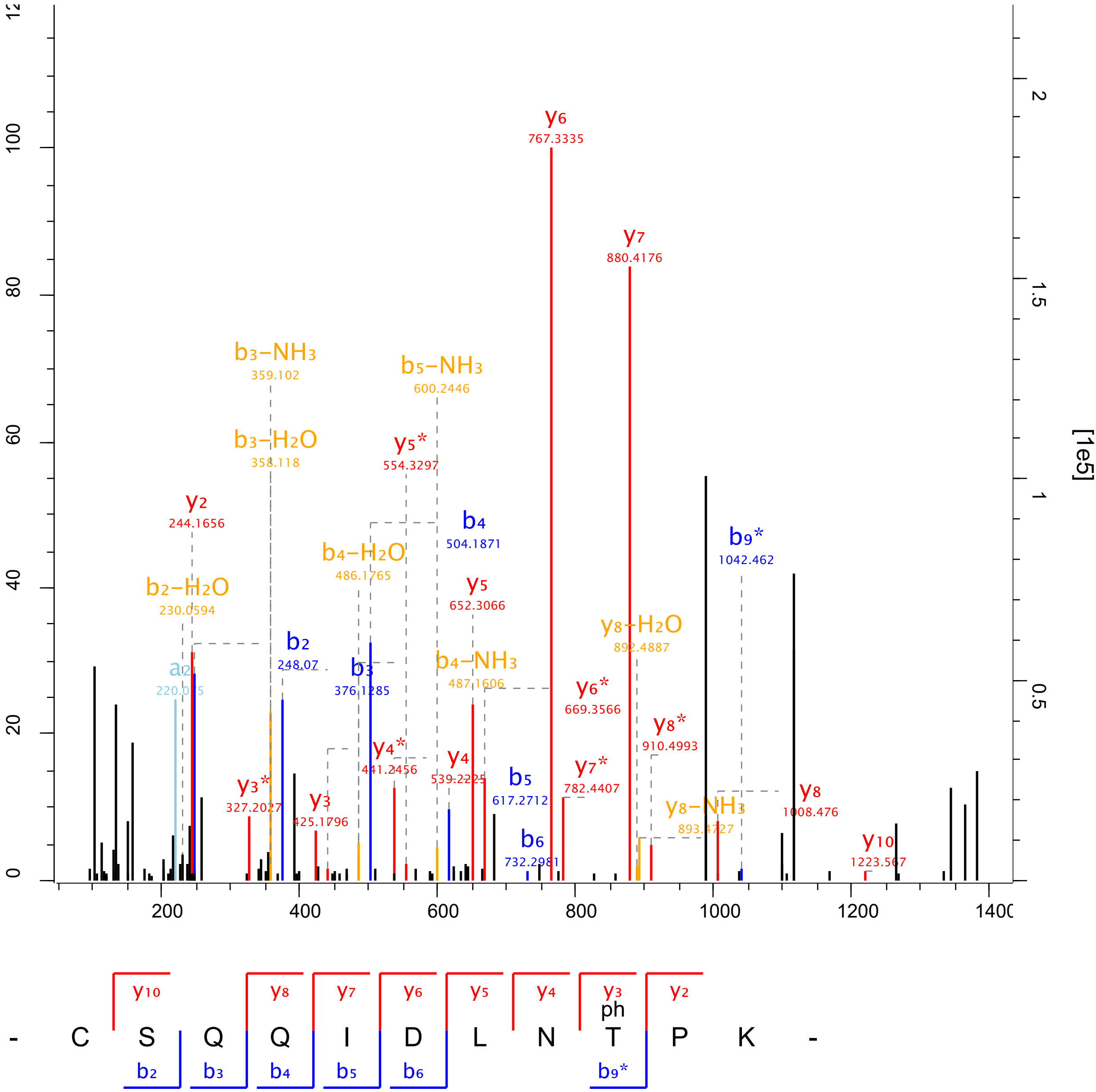

Raw file	Scan	Method	Score	m/z
sys_15_3	10529	FTMS; HCD	100.02	450.2

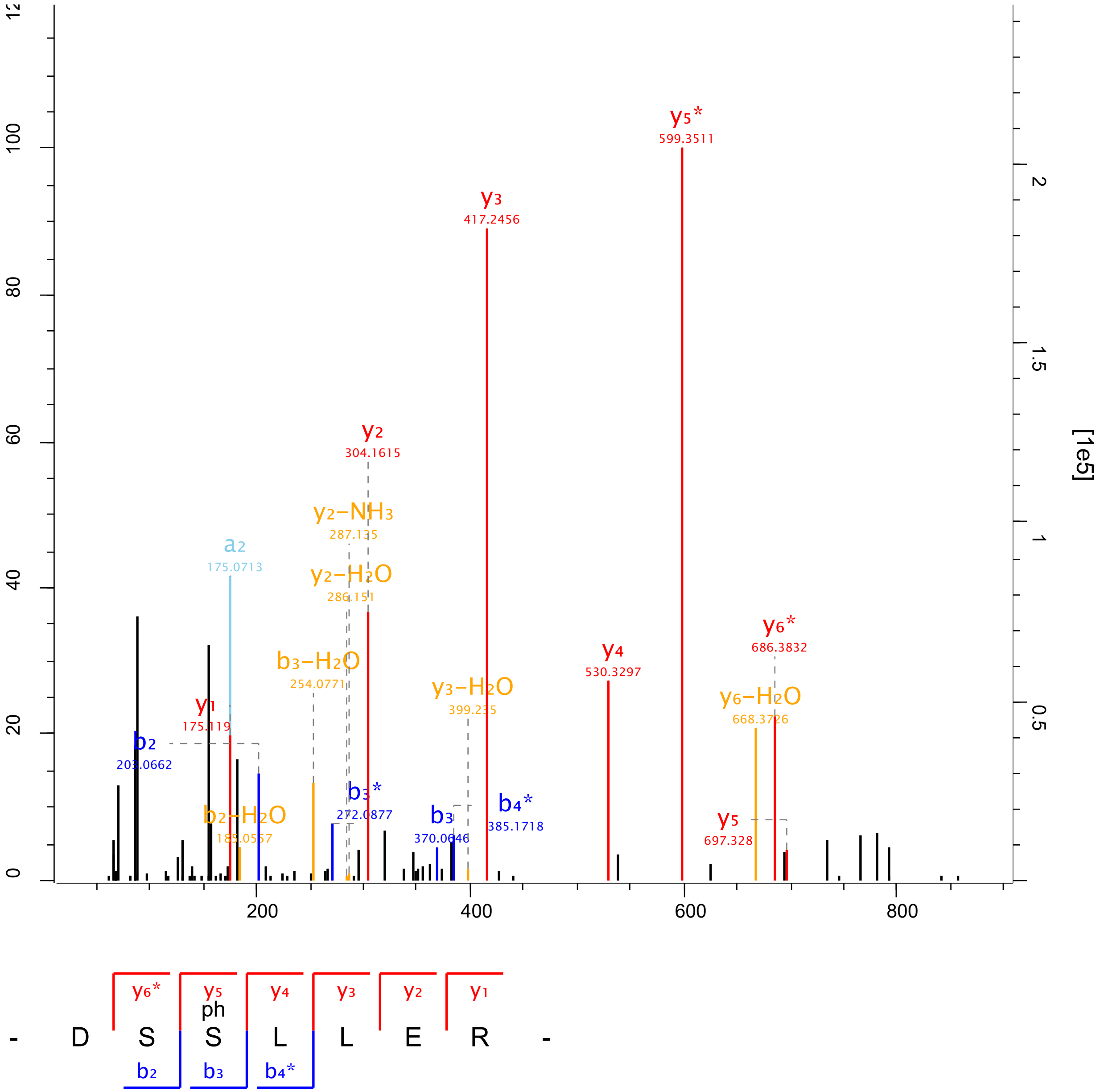

Raw file	Scan	Method	Score	m/z
sys_15_3	10634	FTMS; HCD	119.74	492.24

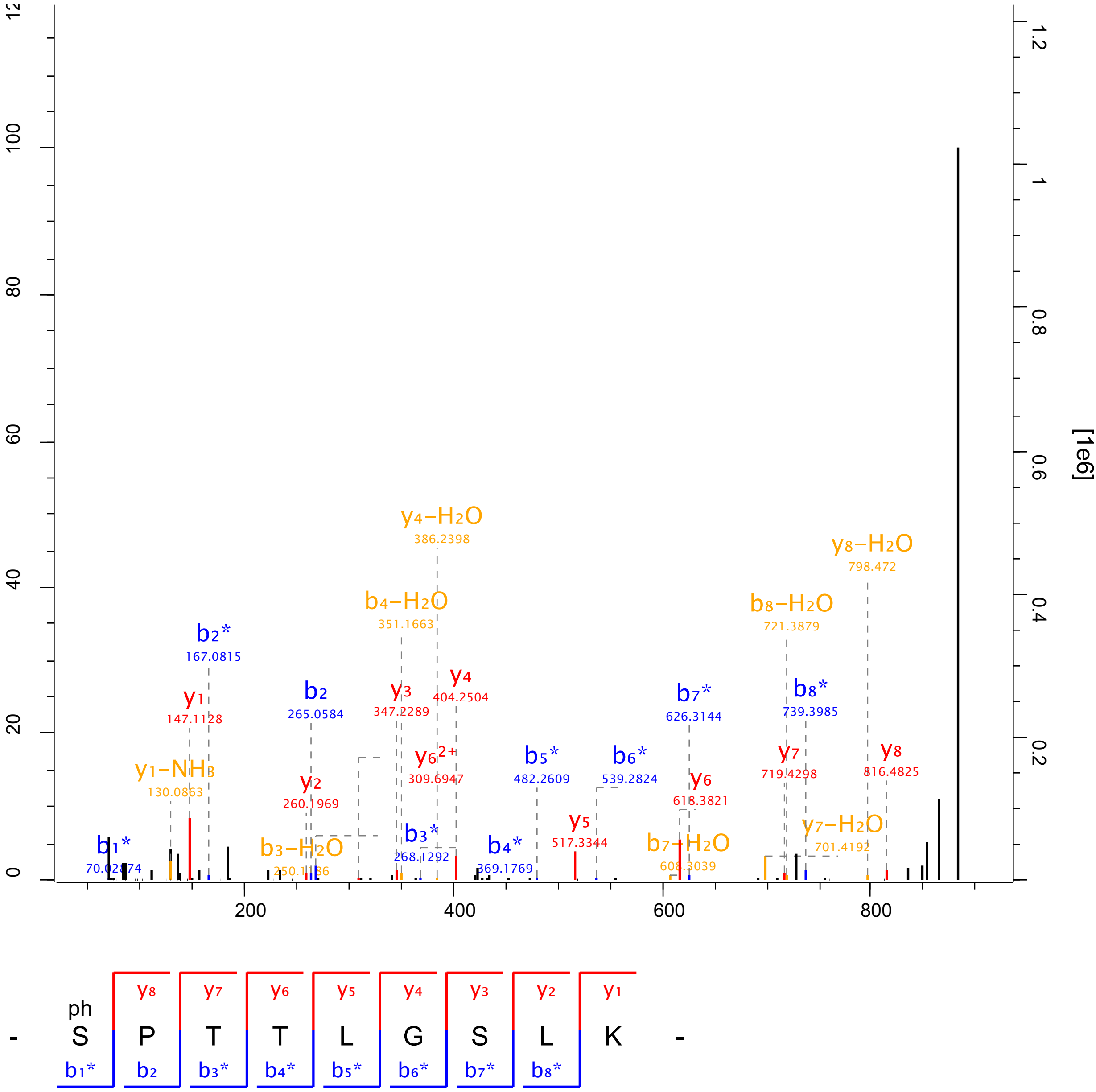

Raw file	Scan	Method	Score	m/z
sys_15_3	10648	FTMS; HCD	302.34	763.82

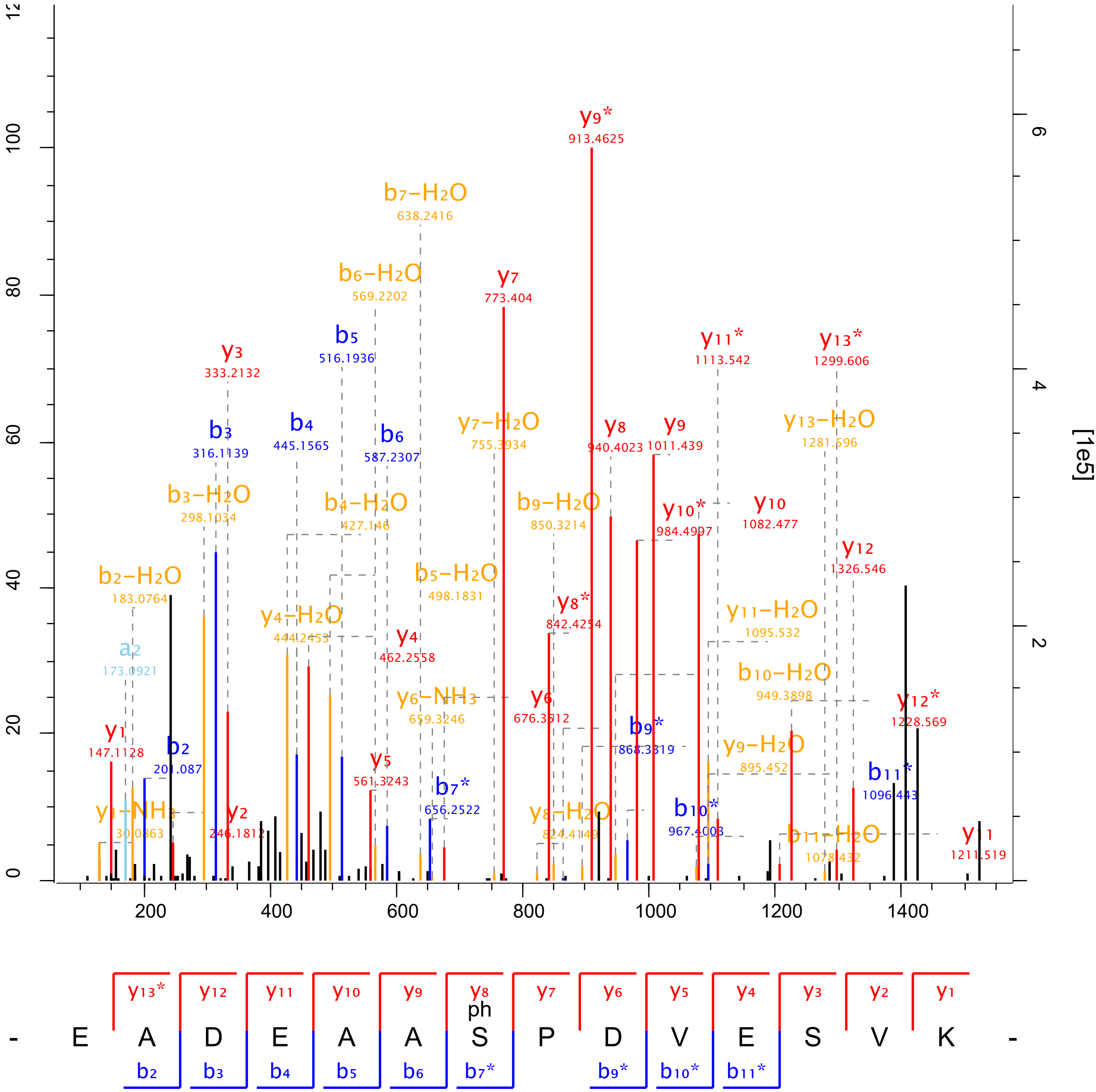

Raw file	Scan	Method	Score	m/z
sys_15_3	11470	FTMS; HCD	60.55	570.75

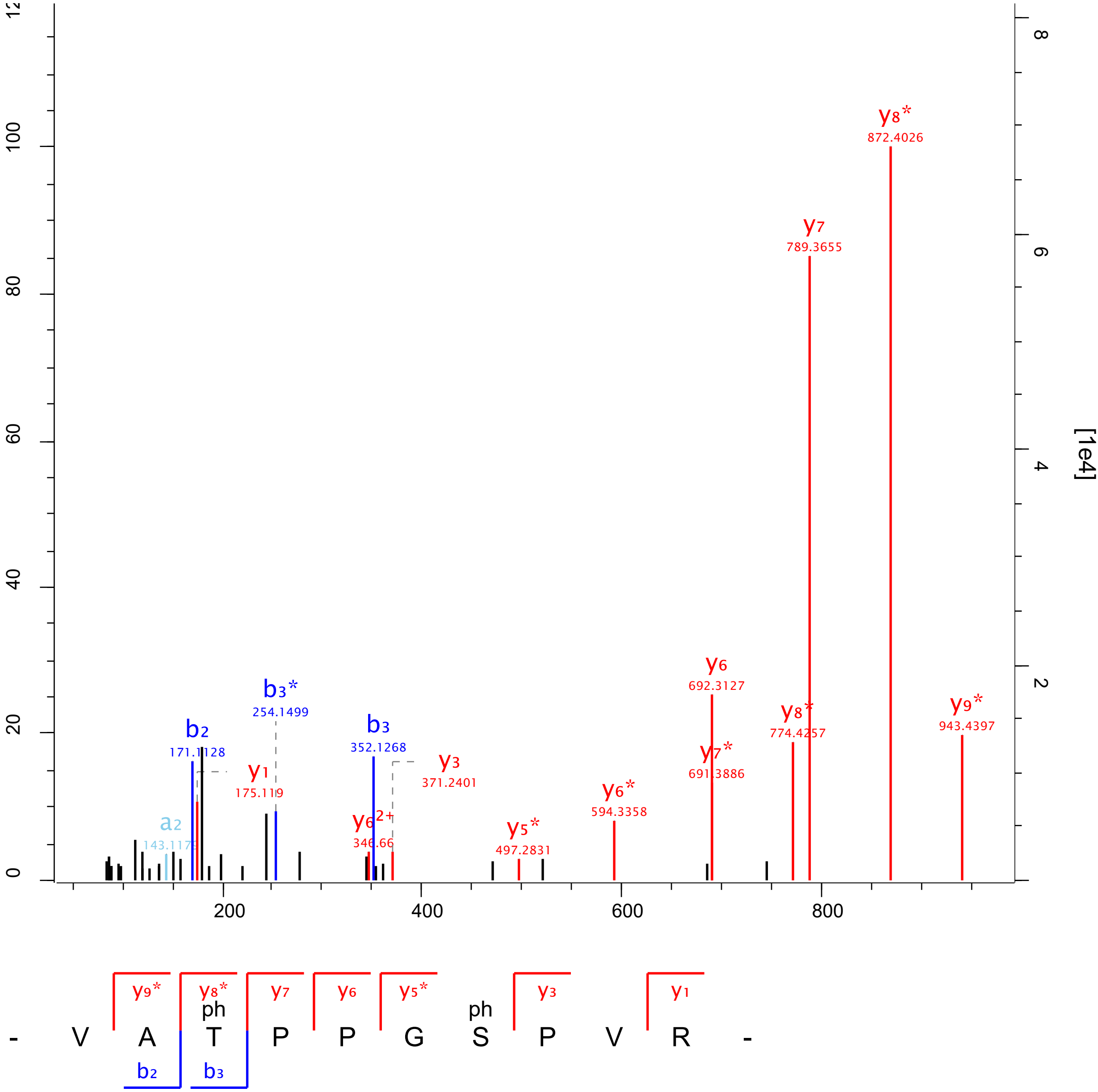

Raw file	Scan	Method	Score	m/z
sys_15_3	11940	FTMS; HCD	48.4	598.8

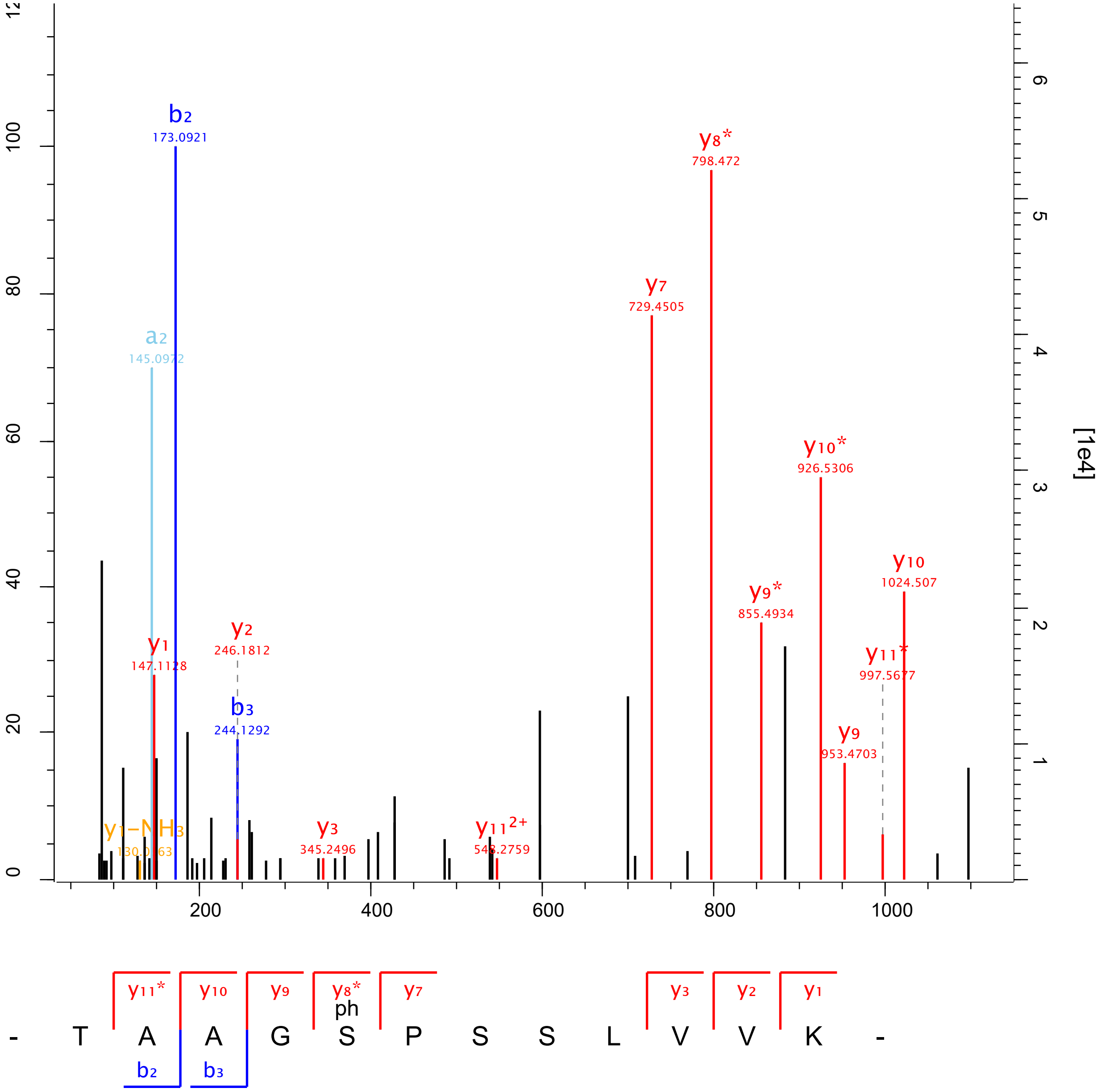

Raw file	Scan	Method	Score	m/z
sys_15_3	12055	FTMS; HCD	45.28	583.27

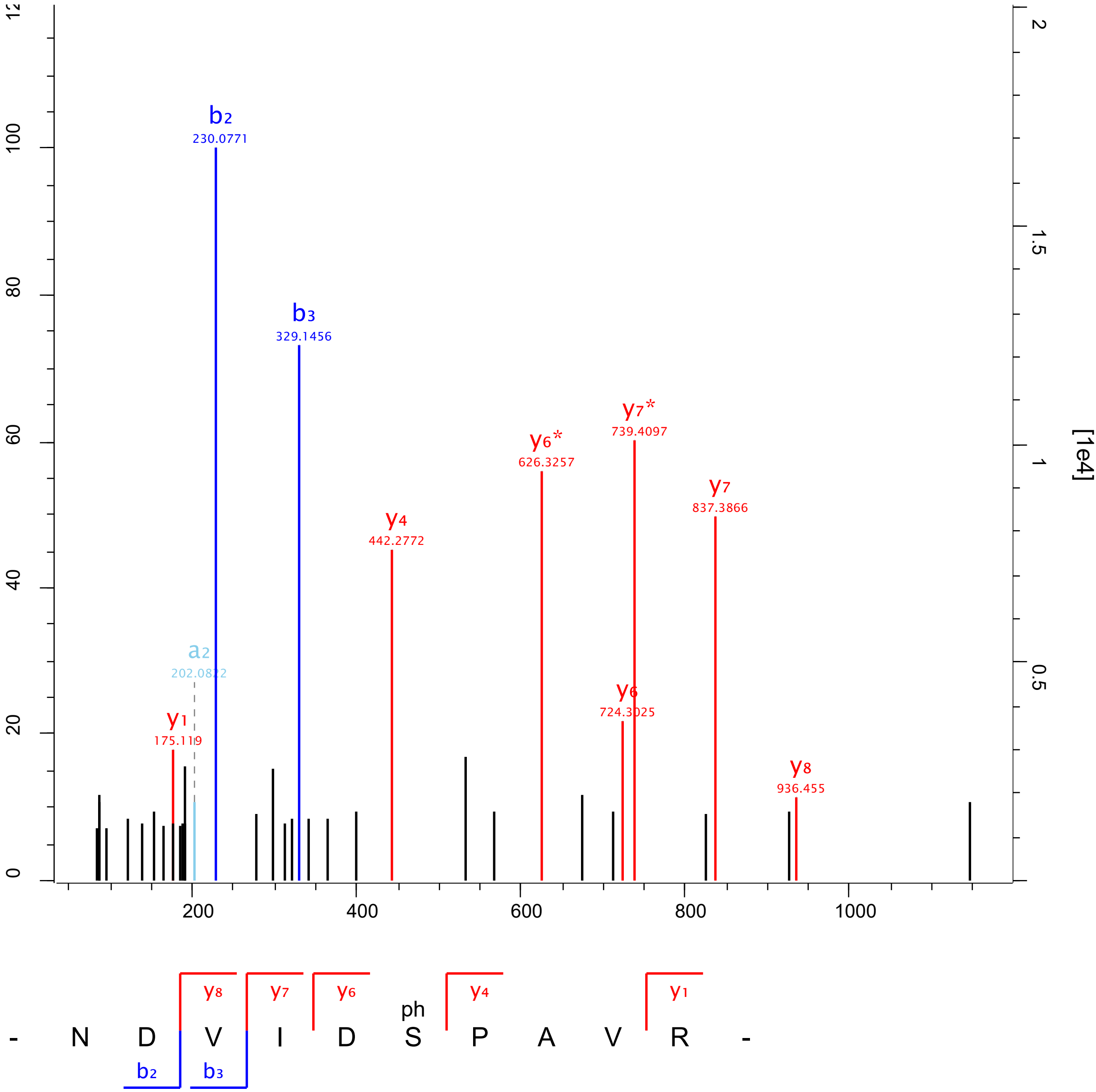

Raw file	Scan	Method	Score	m/z
sys_15_3	12963	FTMS; HCD	48.95	568.21

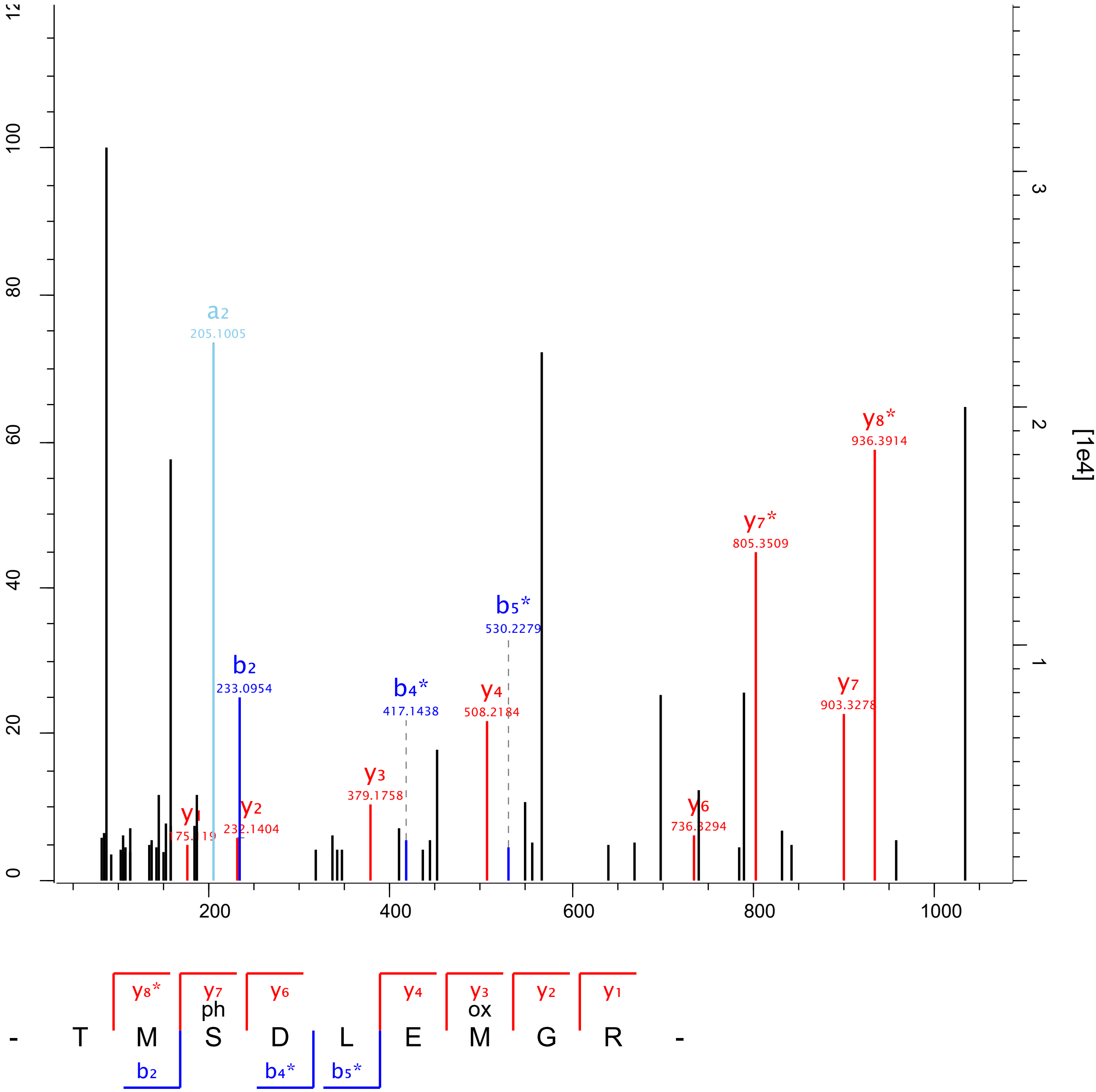

Raw file	Scan	Method	Score	m/z
sys_15_3	14088	FTMS; HCD	71.18	551.75

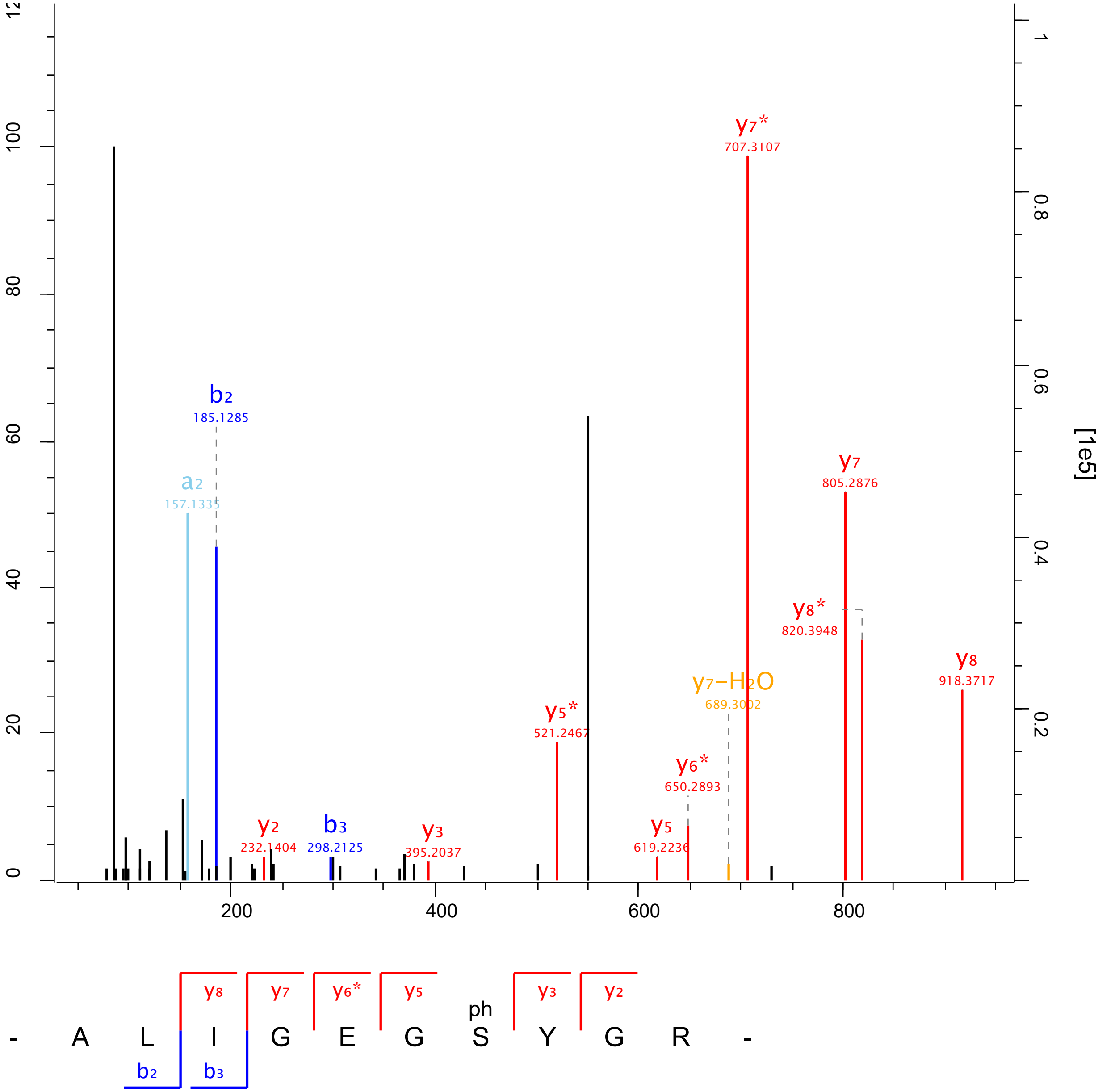

Raw file	Scan	Method	Score	m/z
sys_15_3	14270	FTMS; HCD	42.98	521.22

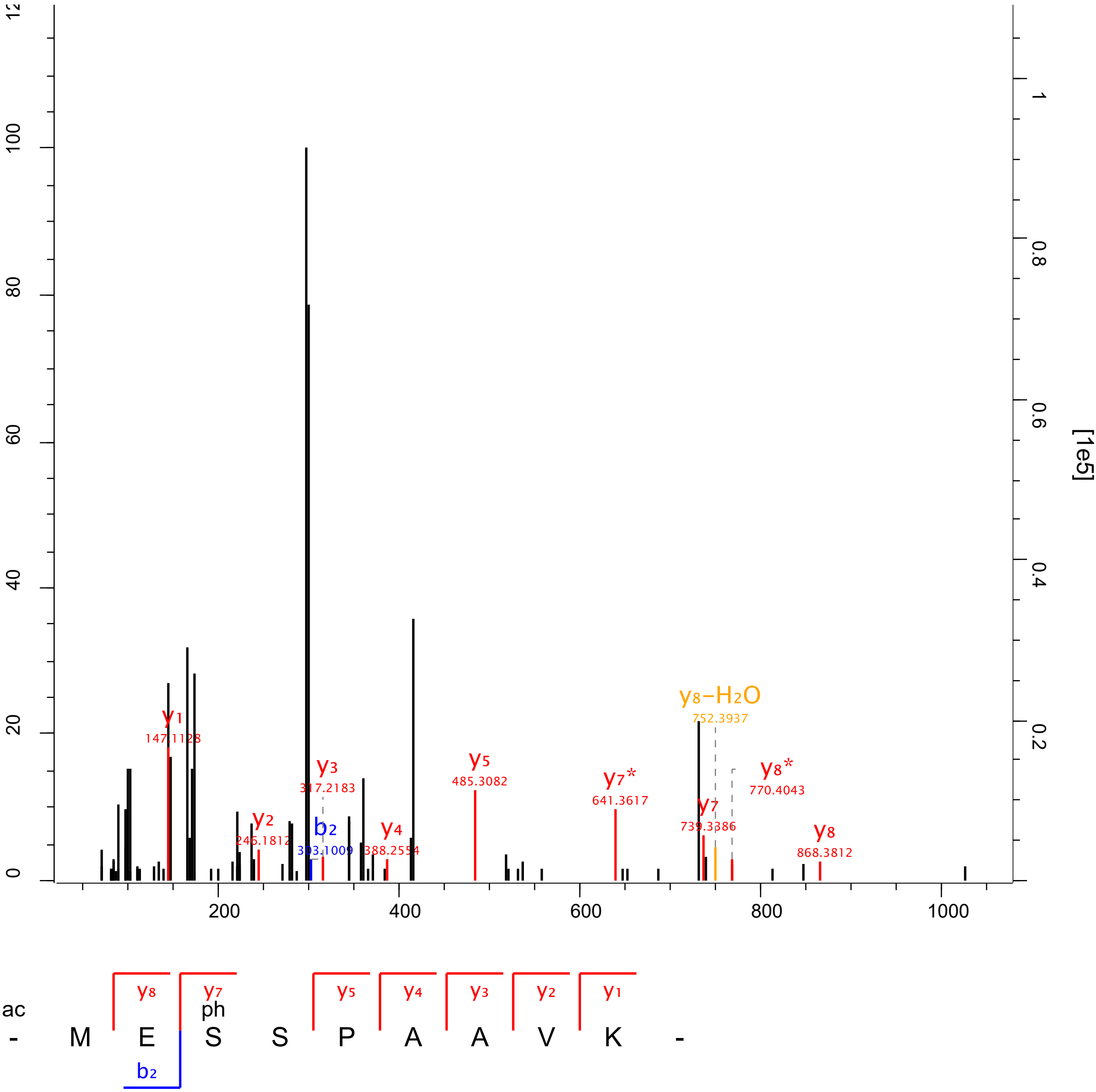

Raw file	Scan	Method	Score	m/z
sys_15_3	14303	FTMS; HCD	119.62	437.2

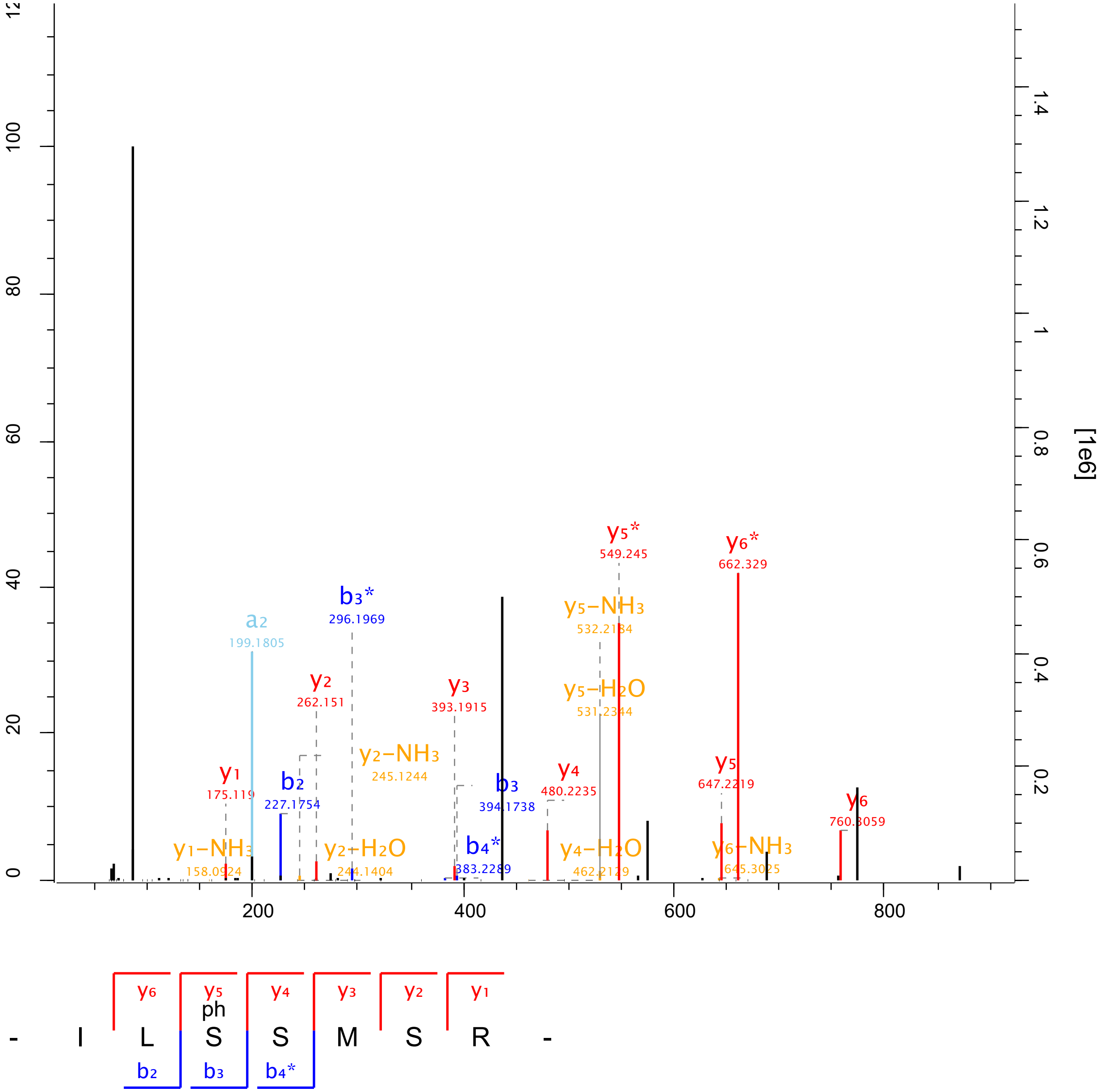

Raw file	Scan	Method	Score	m/z
sys_15_3	15358	FTMS; HCD	69.72	542.78

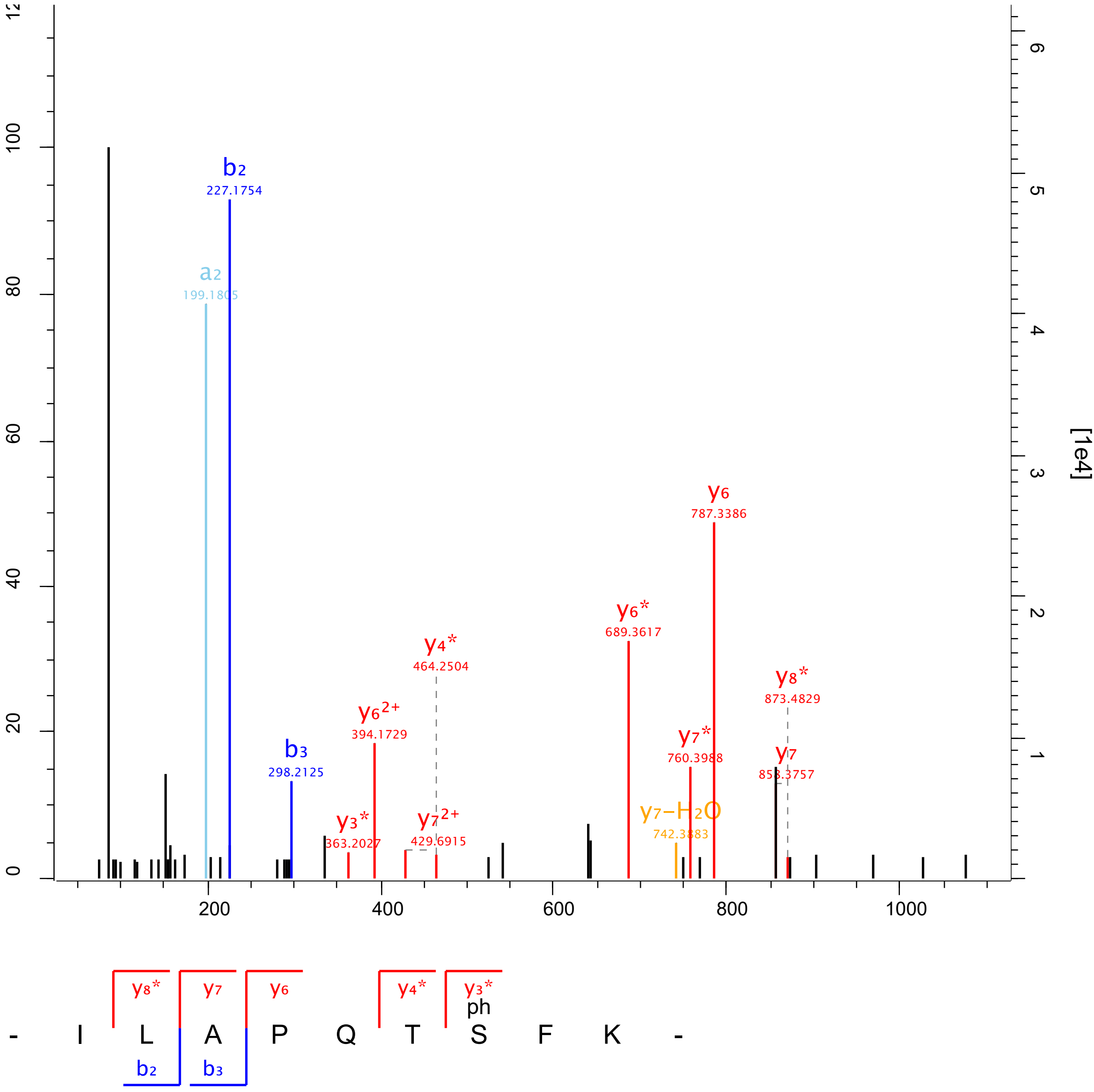

Raw file	Scan	Method	Score	m/z
sys_15_3	17757	FTMS; HCD	201.15	938.36

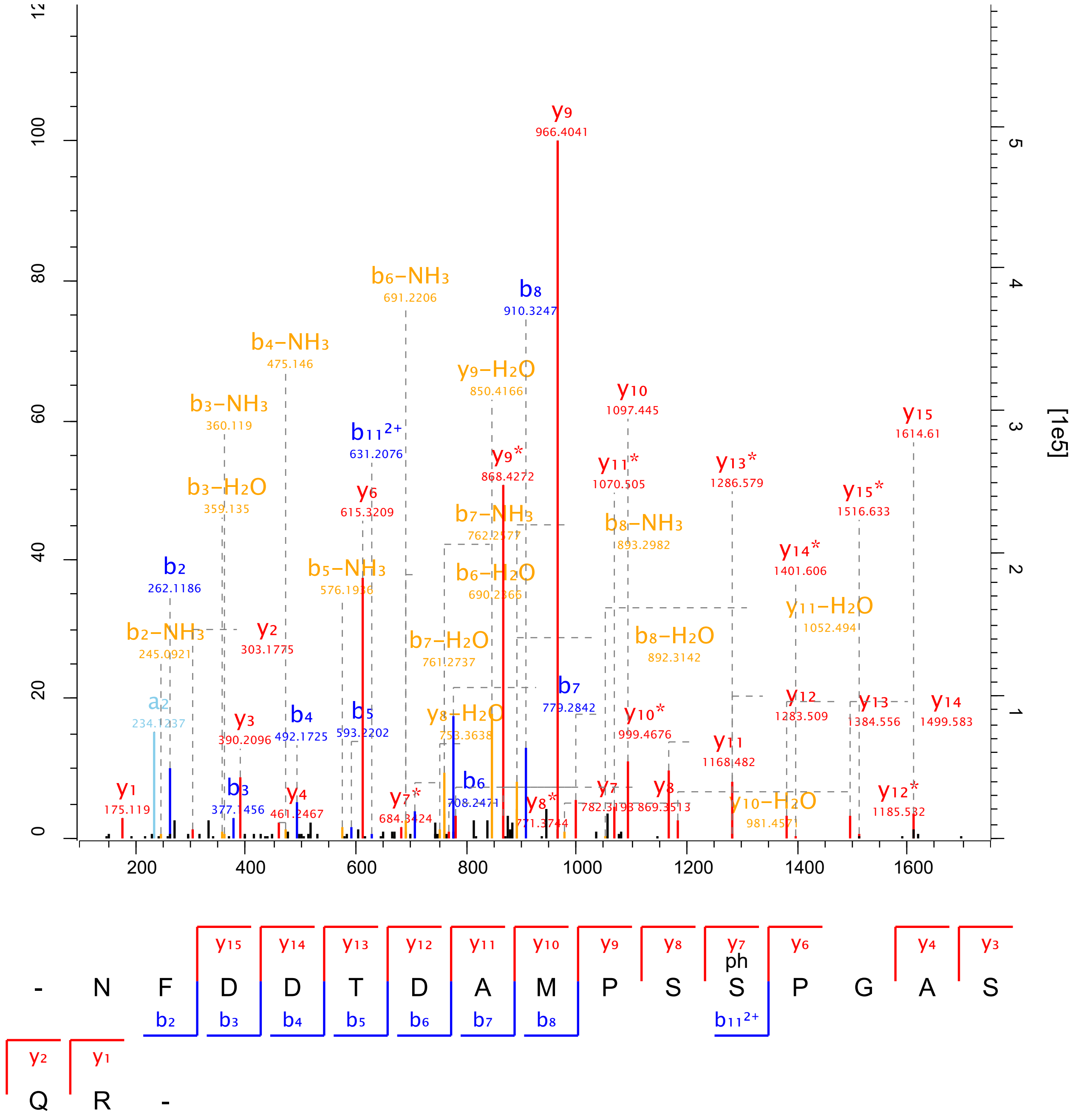

Raw file	Scan	Method	Score	m/z
sys_15_3	17903	FTMS; HCD	45.61	569.27

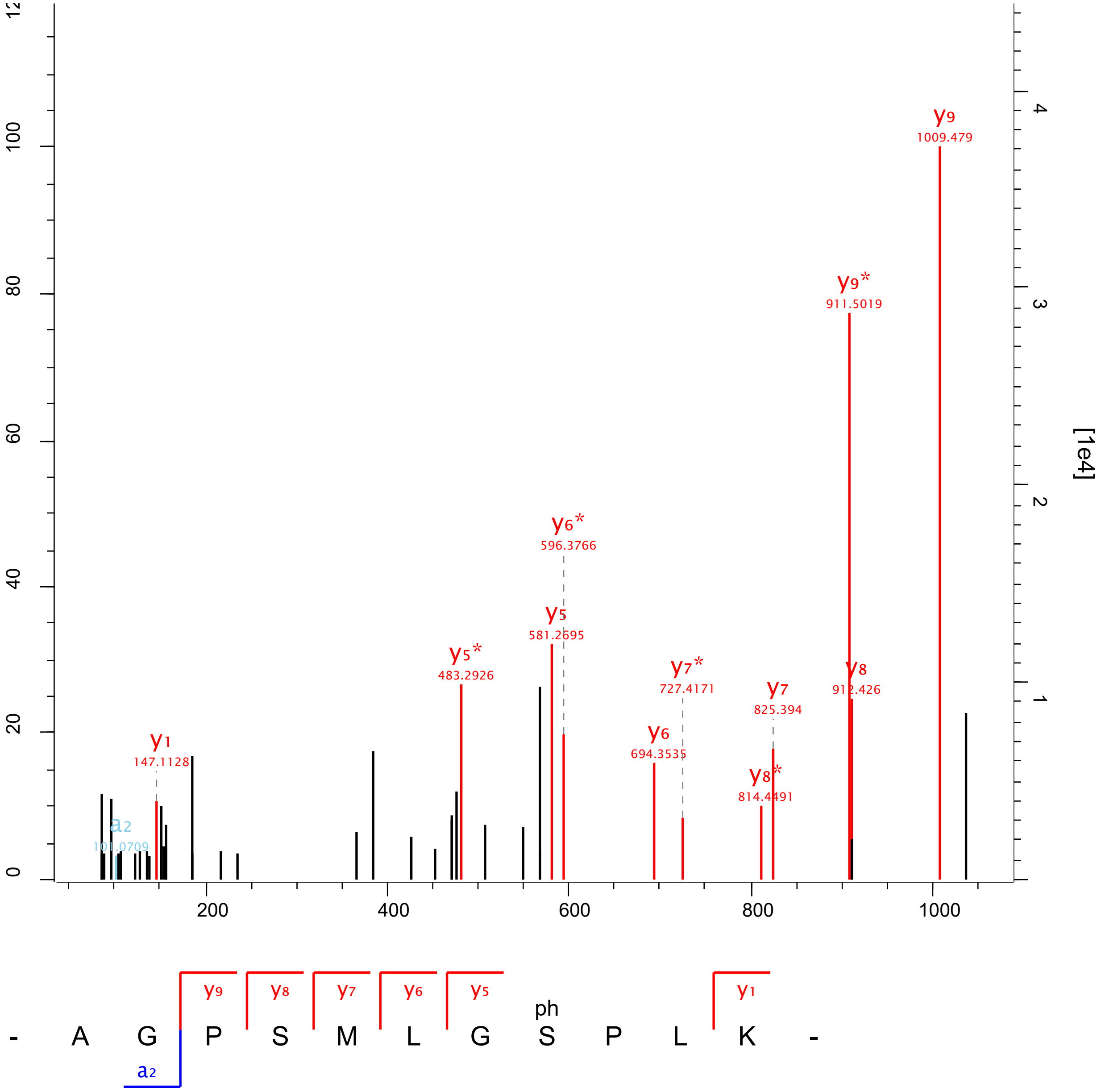

Raw file	Scan	Method	Score	m/z
sys_15_3	19207	FTMS; HCD	87.43	712.83

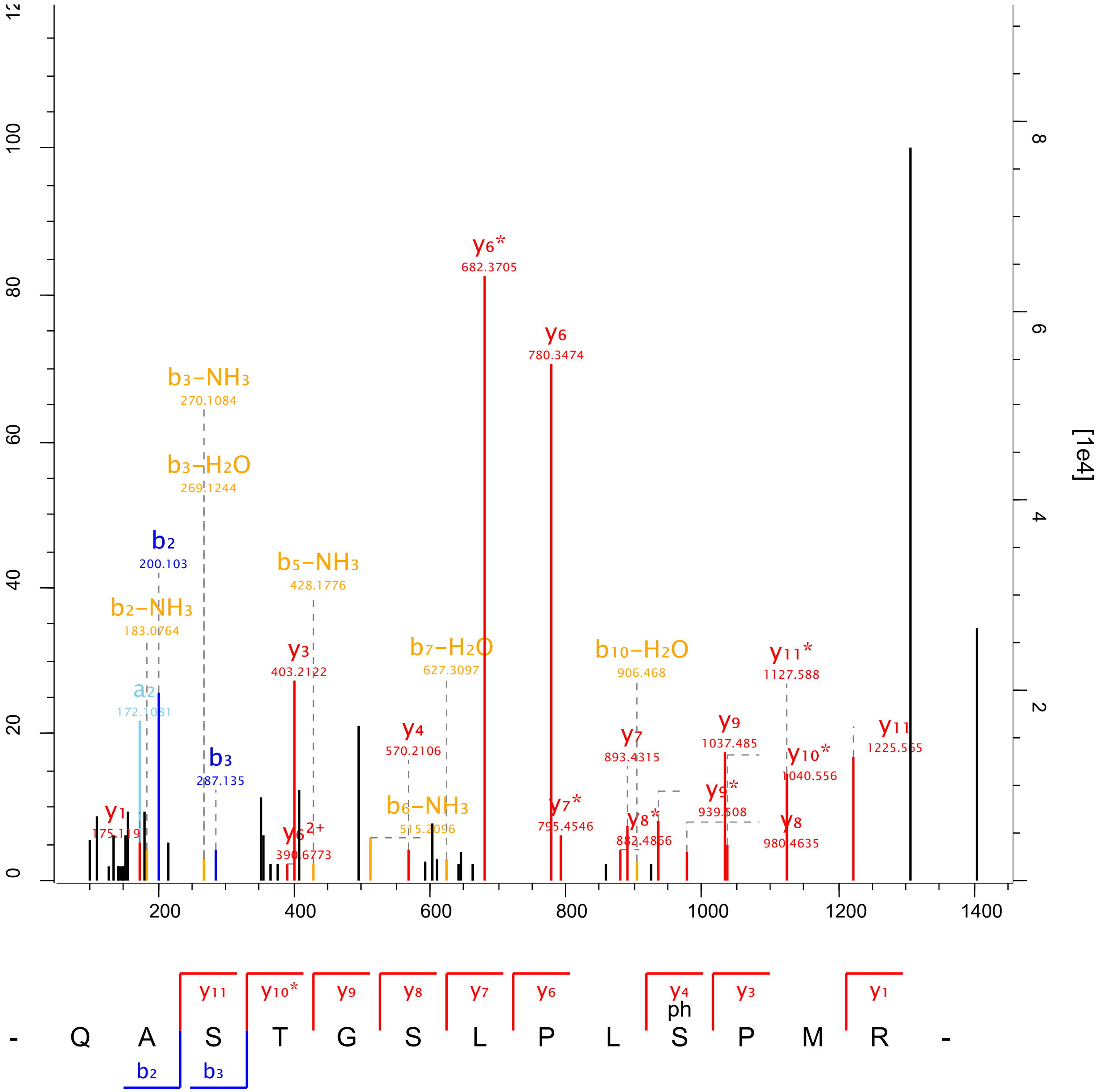

Raw file	Scan	Method	Score	m/z
sys_15_3	21149	FTMS; HCD	75.59	671.27

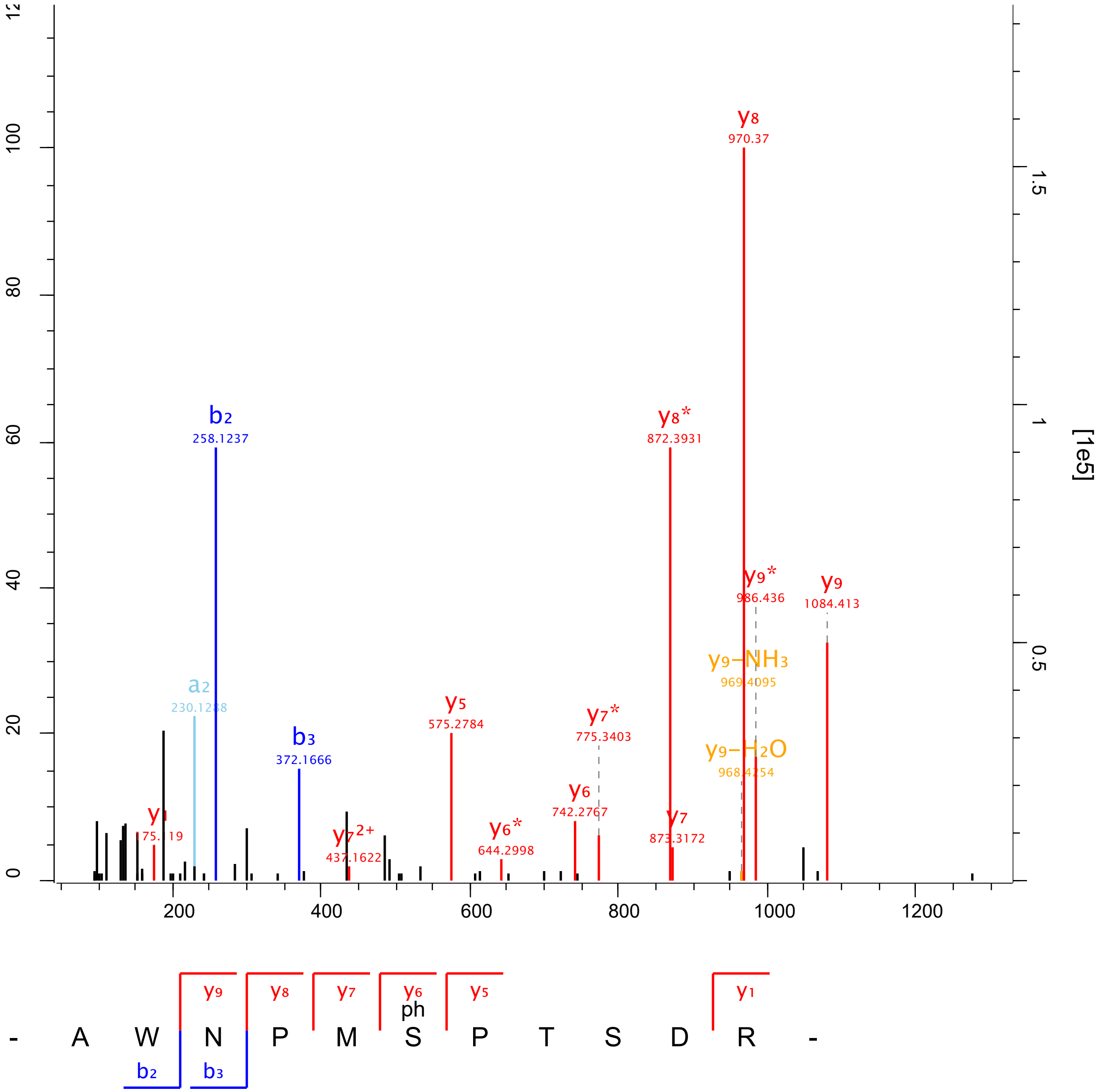

Raw file	Scan	Method	Score	m/z
sys_15_3	35303	FTMS; HCD	81.81	969.39

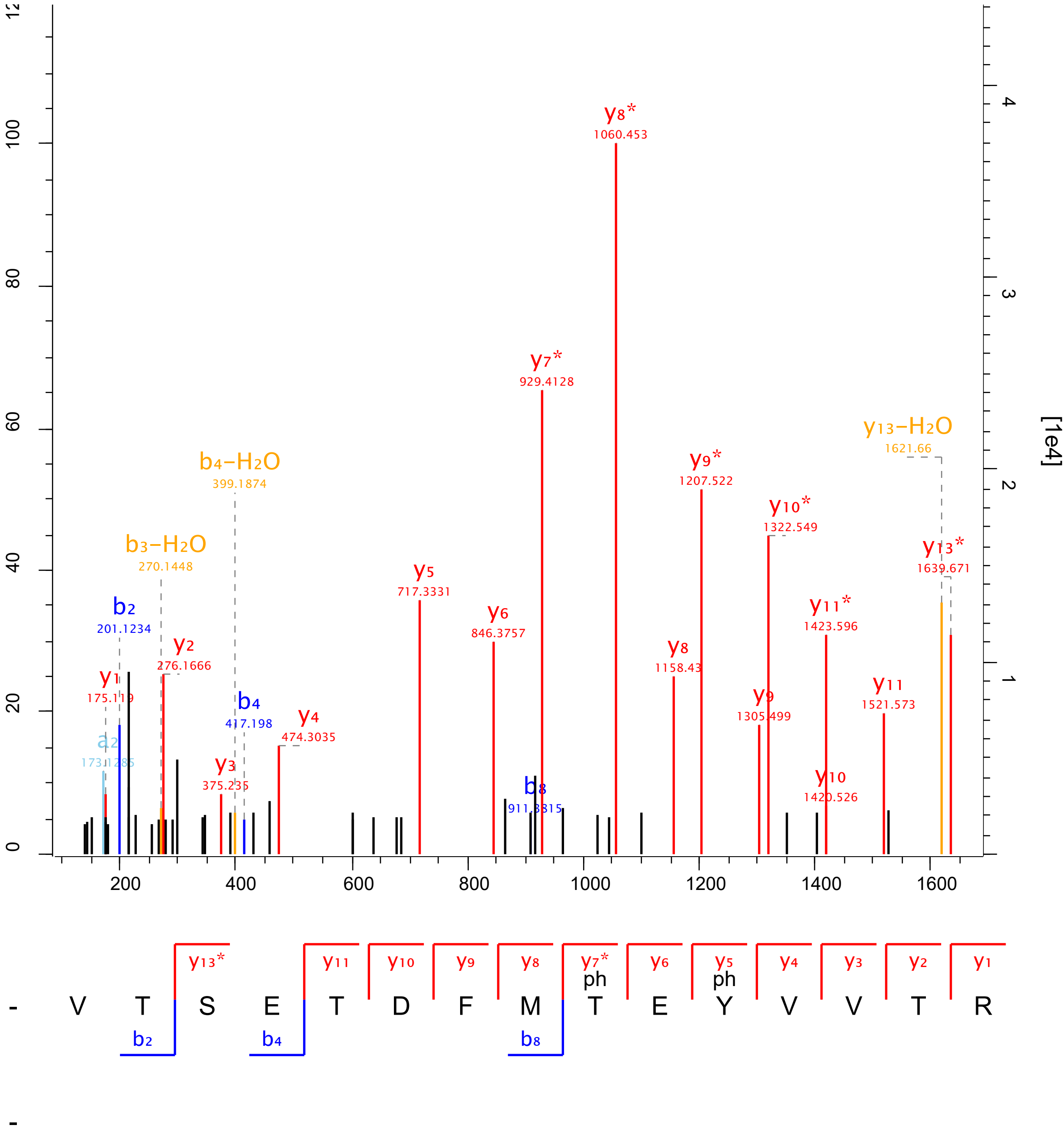

Raw file	Scan	Method	Score	m/z
sys_15_3	5952	FTMS; HCD	62.34	529.74

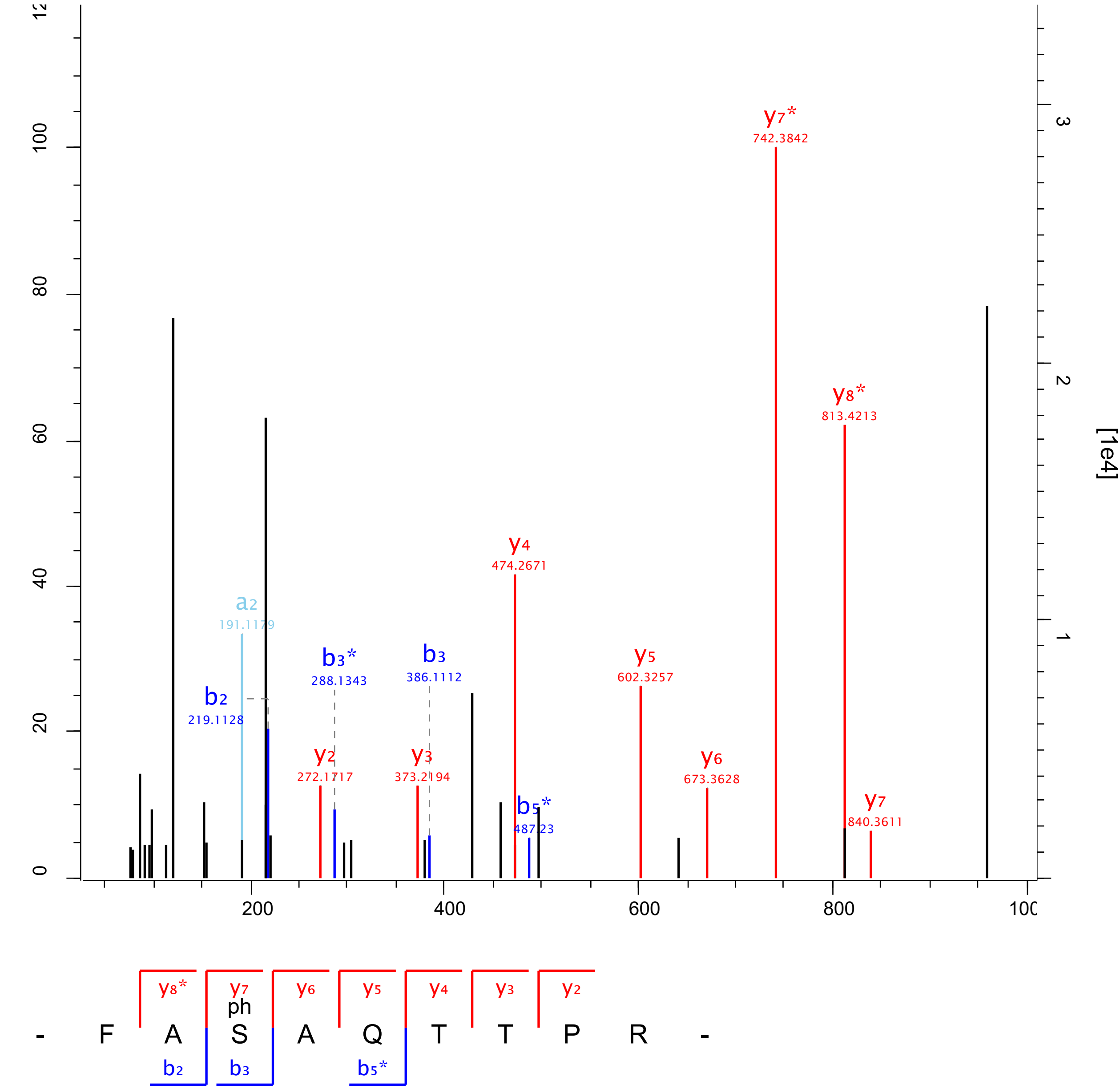

Raw file	Scan	Method	Score	m/z
sys_15_3	7075	FTMS; HCD	63.69	515.23

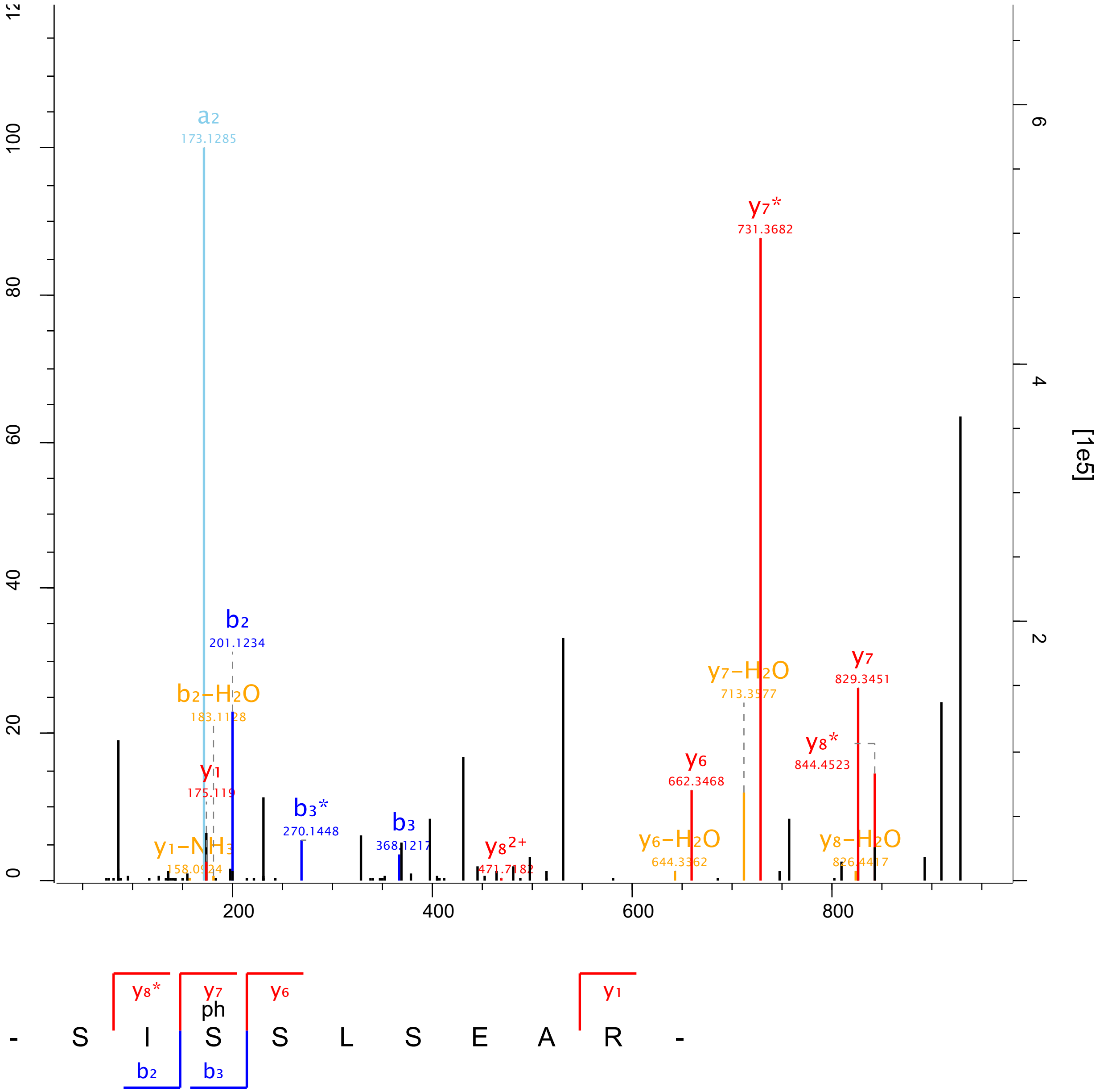

Raw file	Scan	Method	Score	m/z
sys_15_3	8180	FTMS; HCD	93.1	525.21

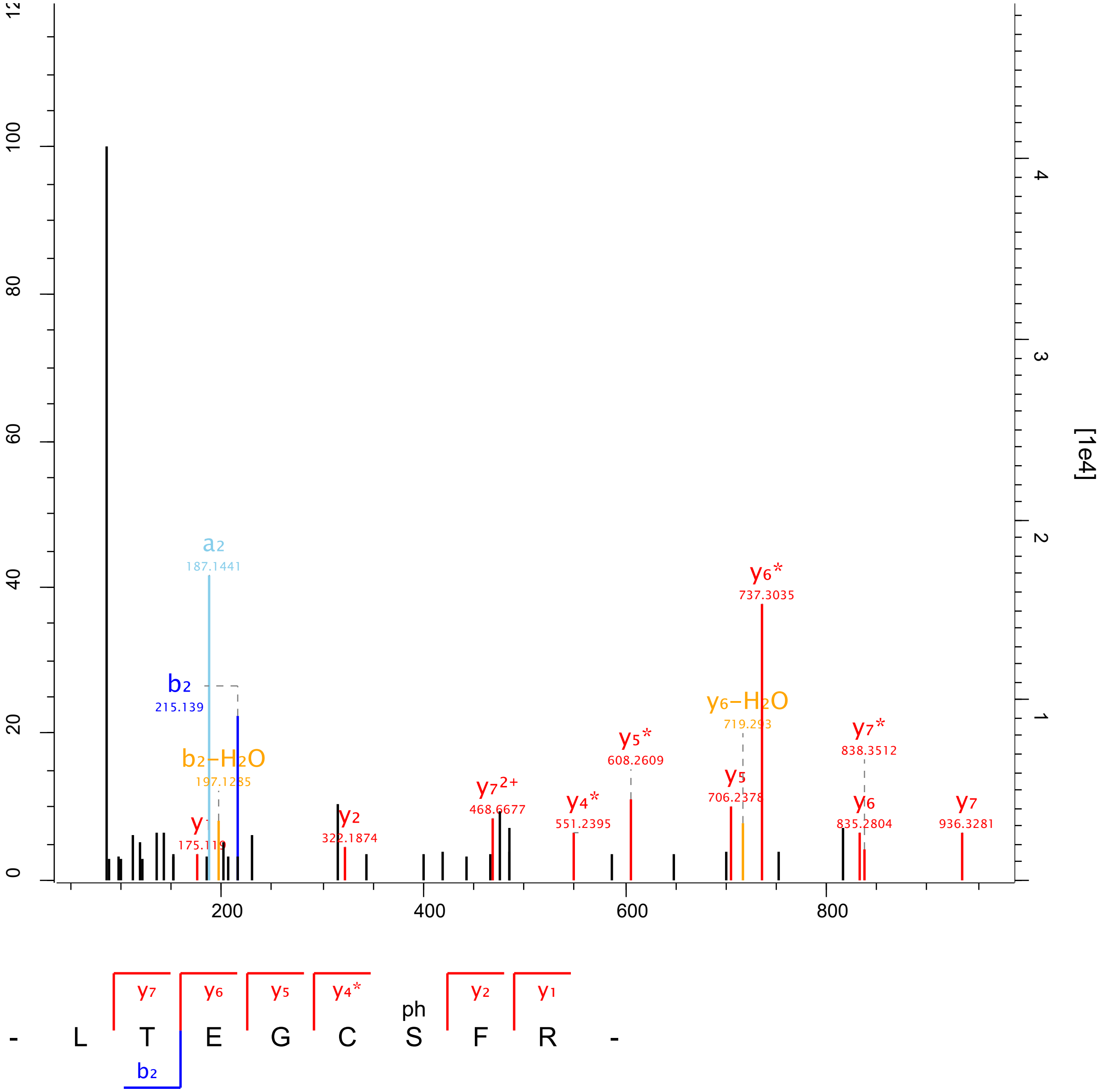

Raw file	Scan	Method	Score	m/z
sys_15_3	8618	FTMS; HCD	99.82	486.23

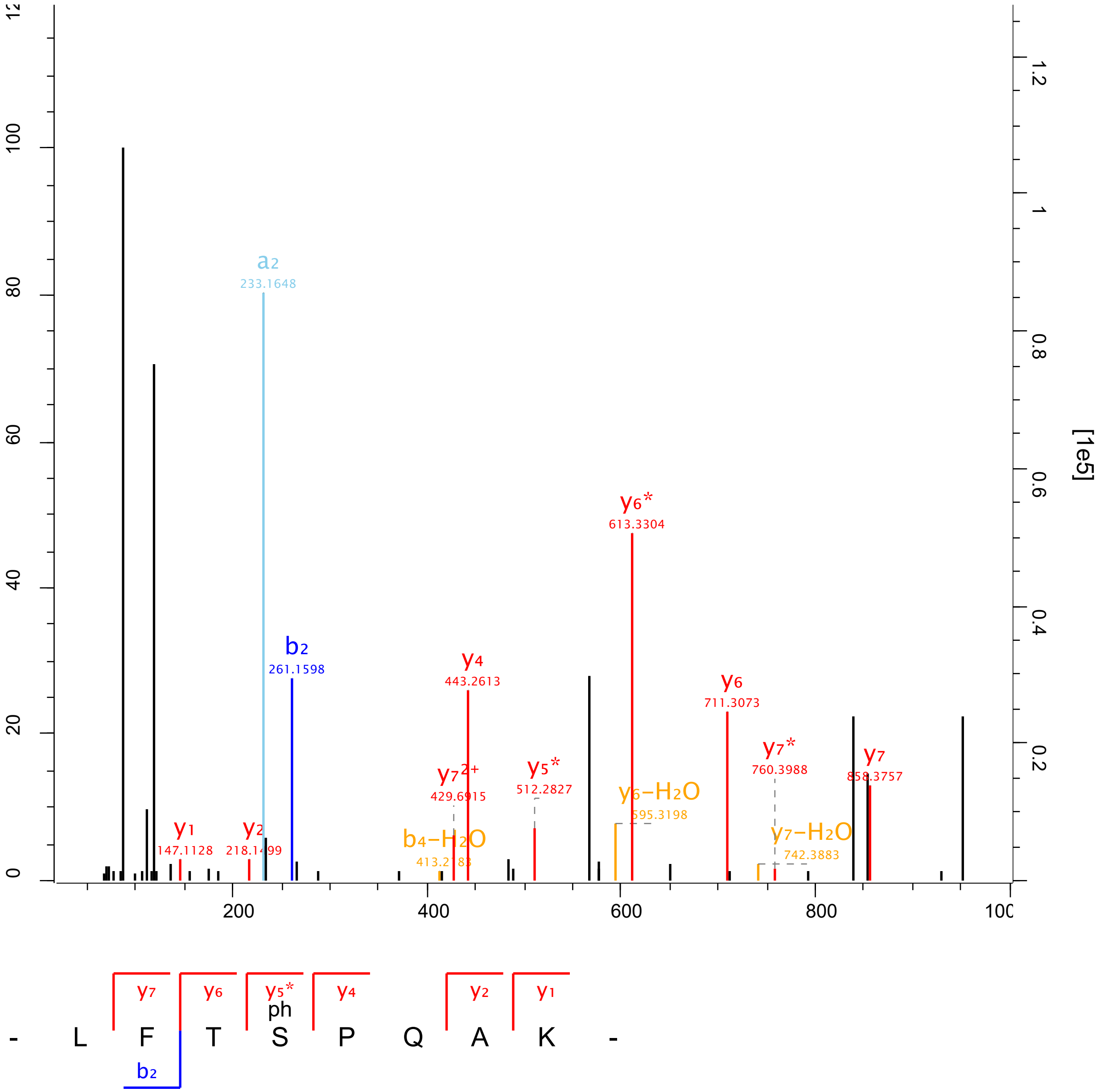

Raw file	Scan	Method	Score	m/z
sys_15_3	9628	FTMS; HCD	160.63	548.28

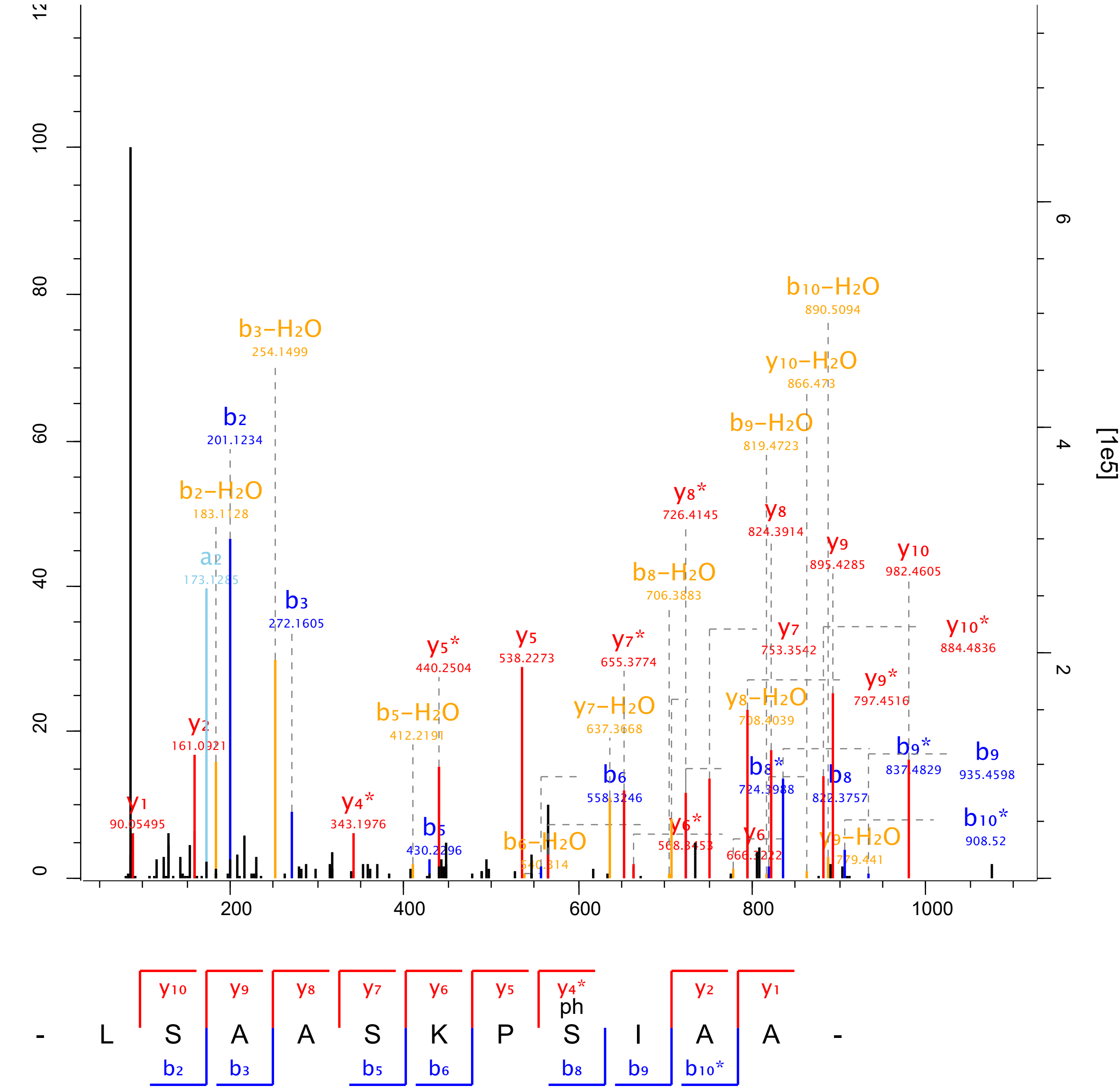

Raw file	Scan	Method	Score	m/z
sys_15_3	9648	FTMS; HCD	69.03	524.21

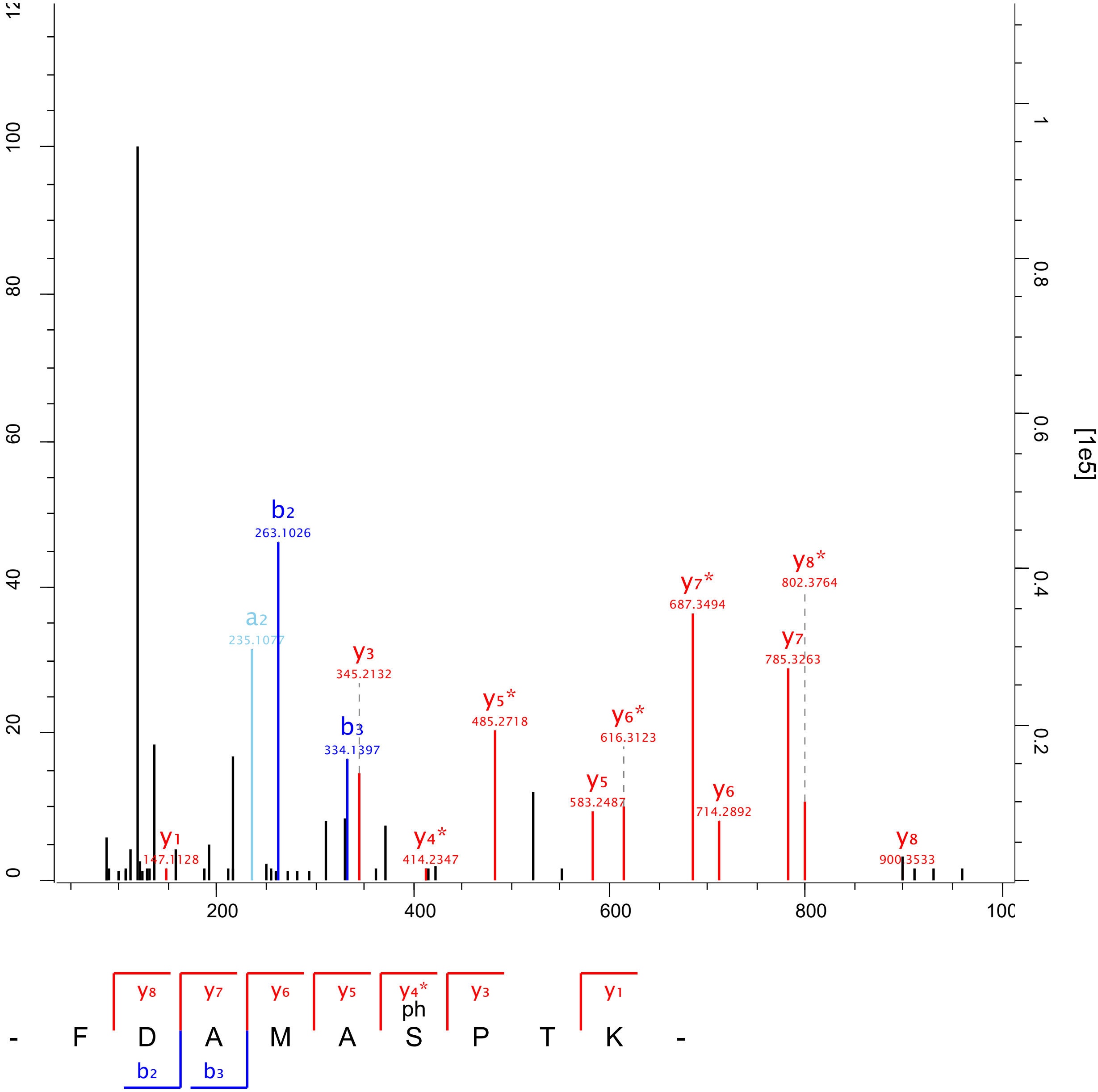

Raw file	Scan	Method	Score	m/z
sys_30_1	14749	FTMS; HCD	120.63	785.82

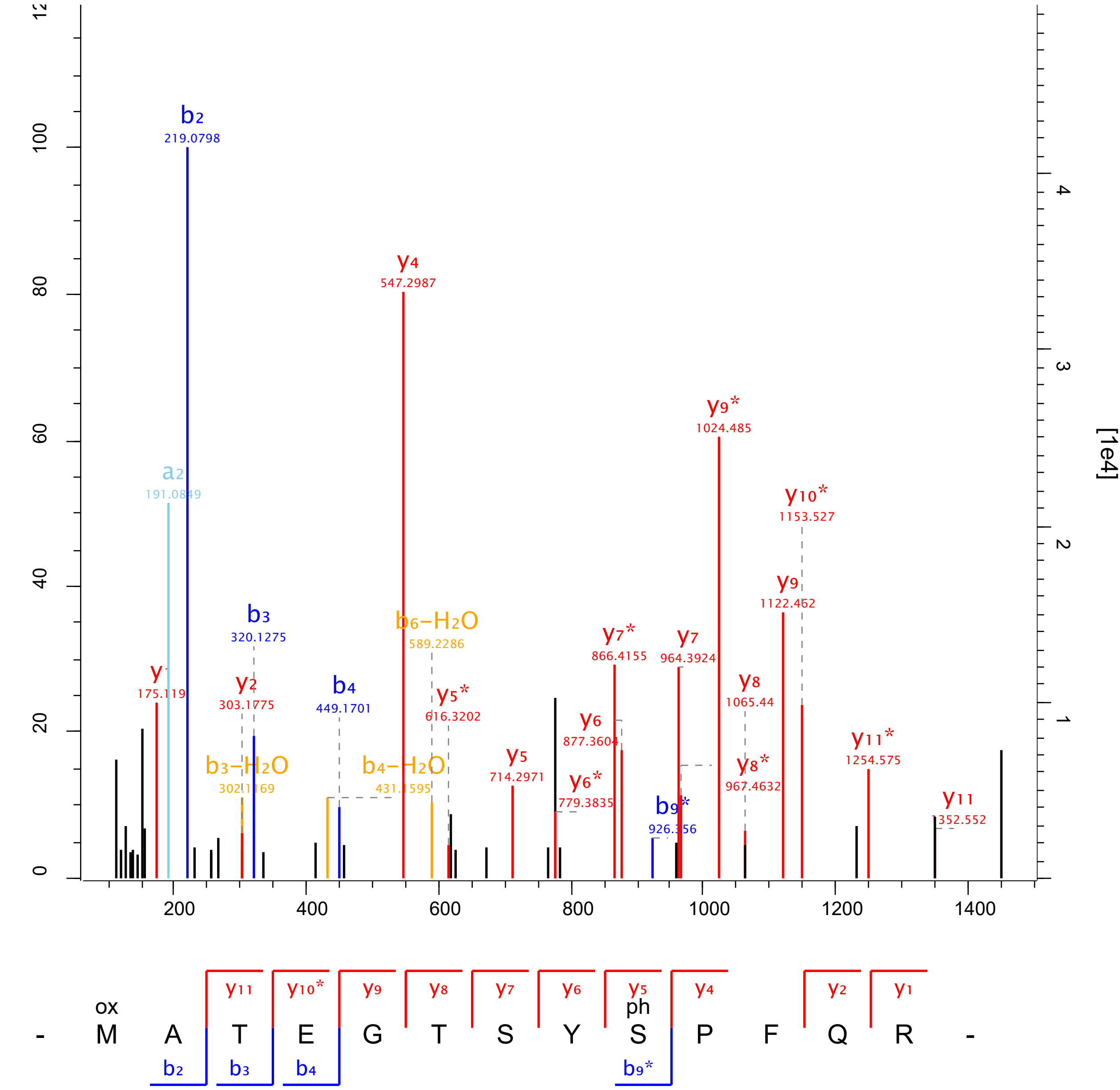

Raw file	Scan	Method	Score	m/z
sys_30_1	16440	FTMS; HCD	139.5	749.81

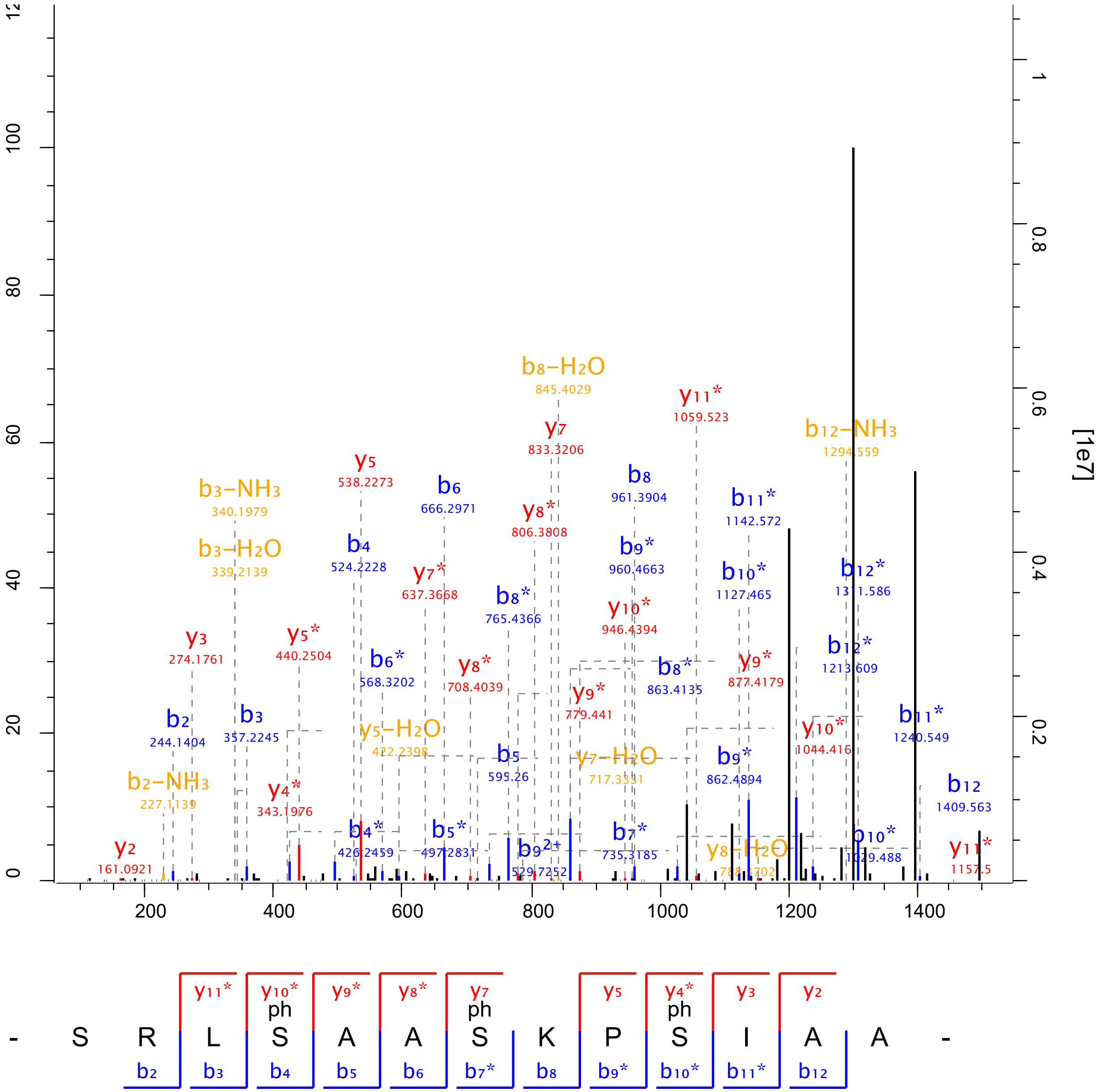

Raw file	Scan	Method	Score	m/z
sys_30_1	26002	FTMS; HCD	126.25	725.8

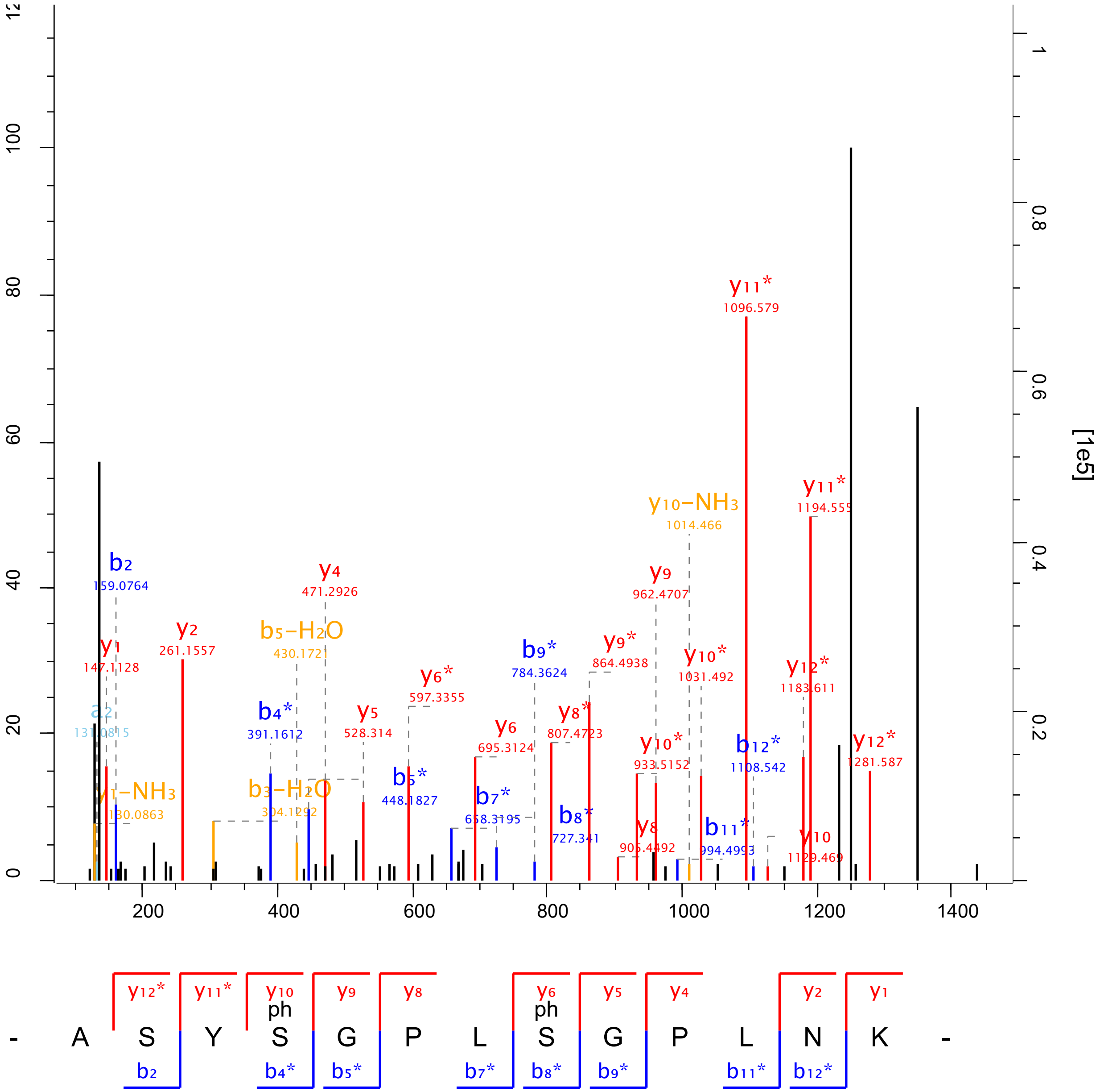

Raw file	Scan	Method	Score	m/z
sys_30_1	36281	FTMS; HCD	141.13	736.32

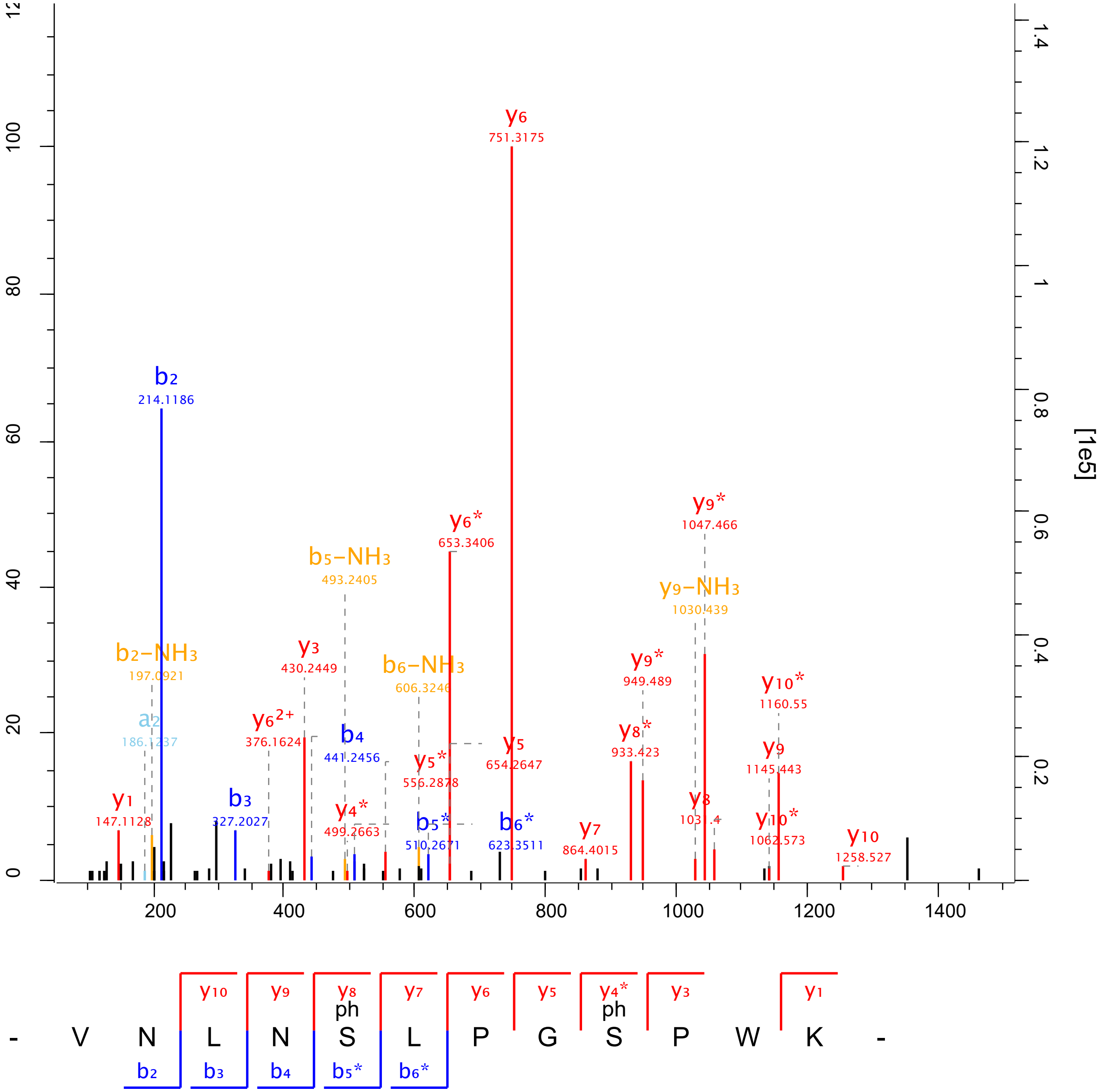

Raw file	Scan	Method	Score	m/z
sys_30_1	8283	FTMS; HCD	114.97	450.7

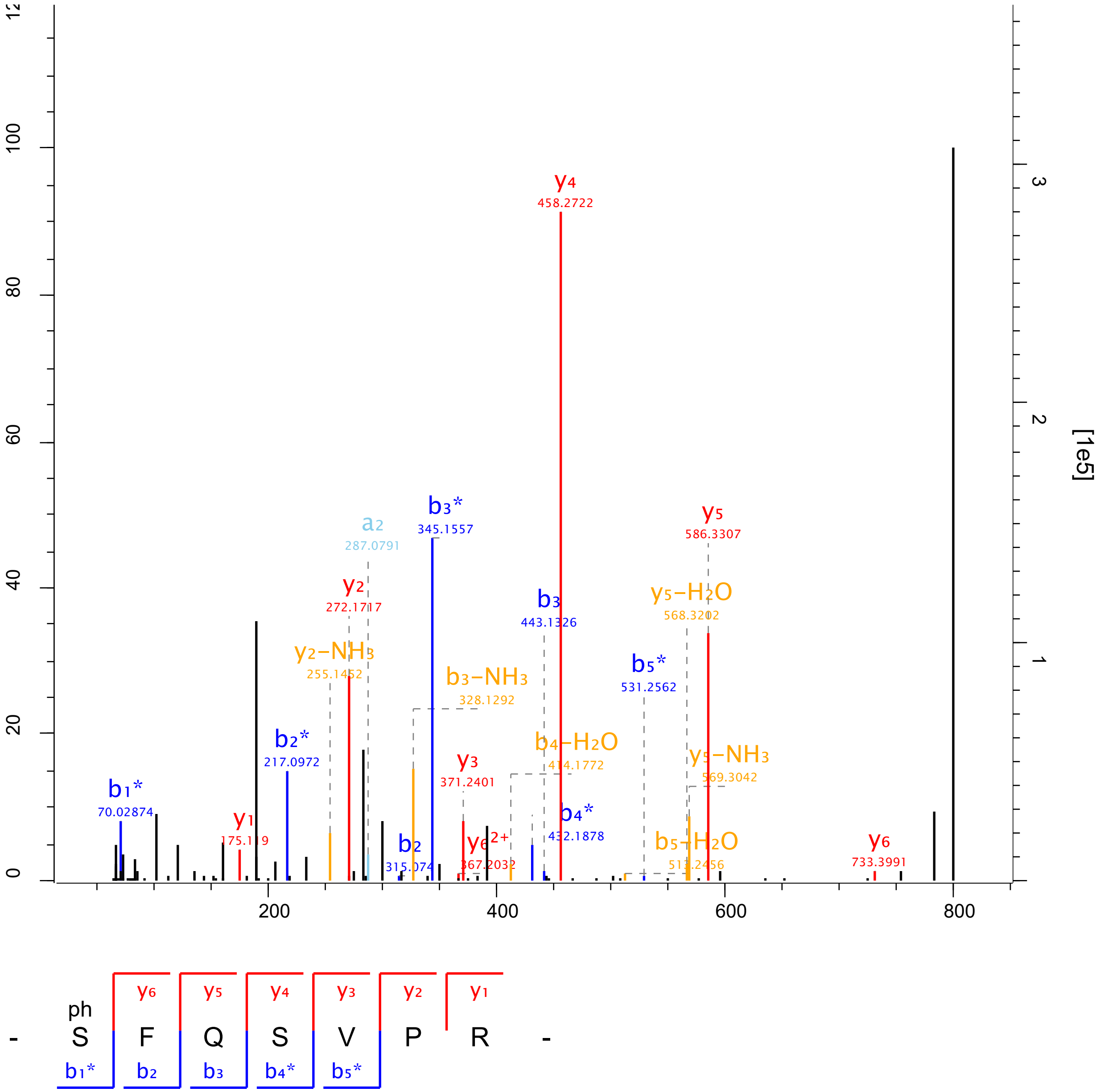

Raw file	Scan	Method	Score	m/z
sys_30_1	8449	FTMS; HCD	47.6	427.22

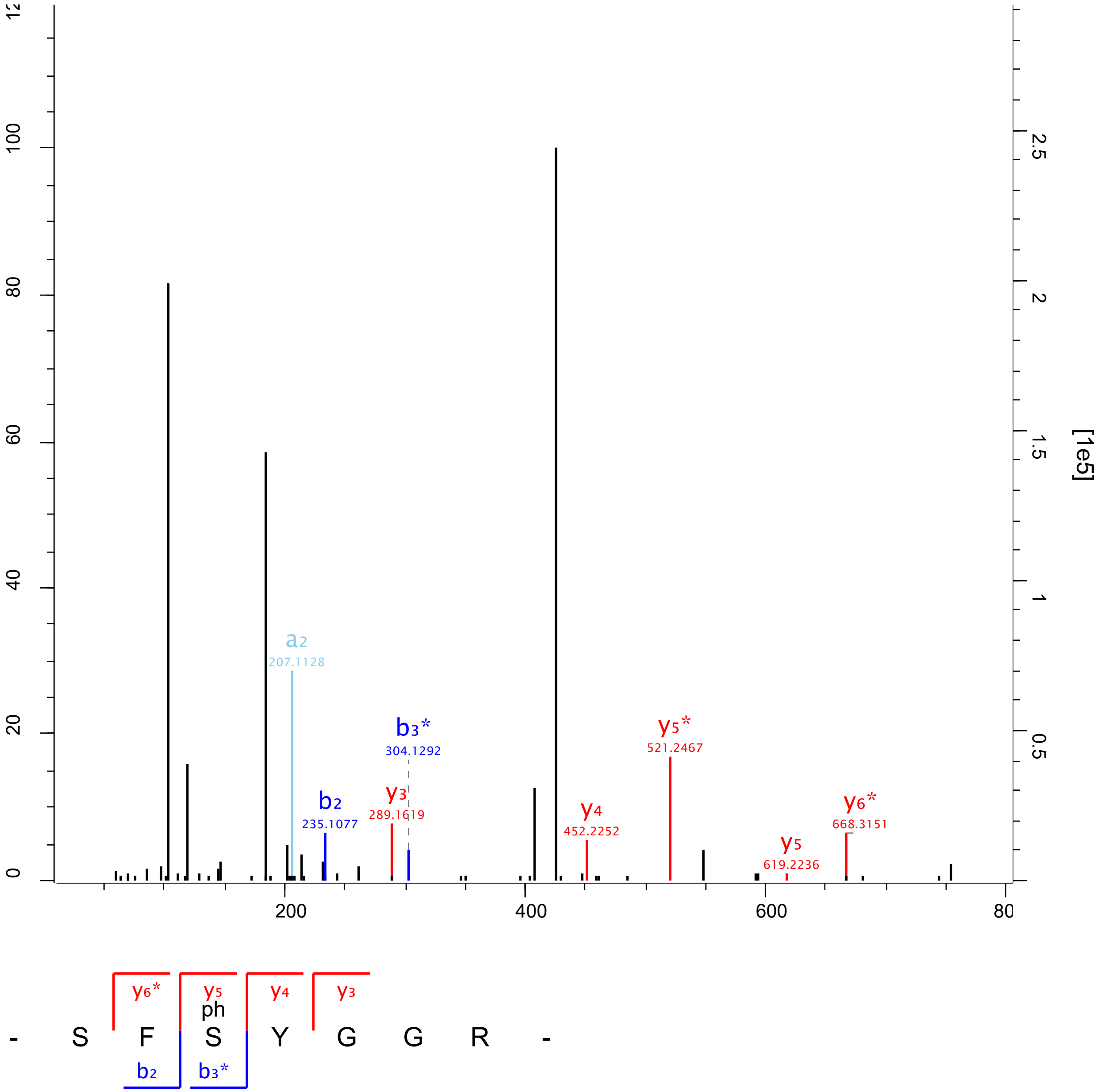

Raw file	Scan	Method	Score	m/z
sys_30_1	8770	FTMS; HCD	153.24	441.71

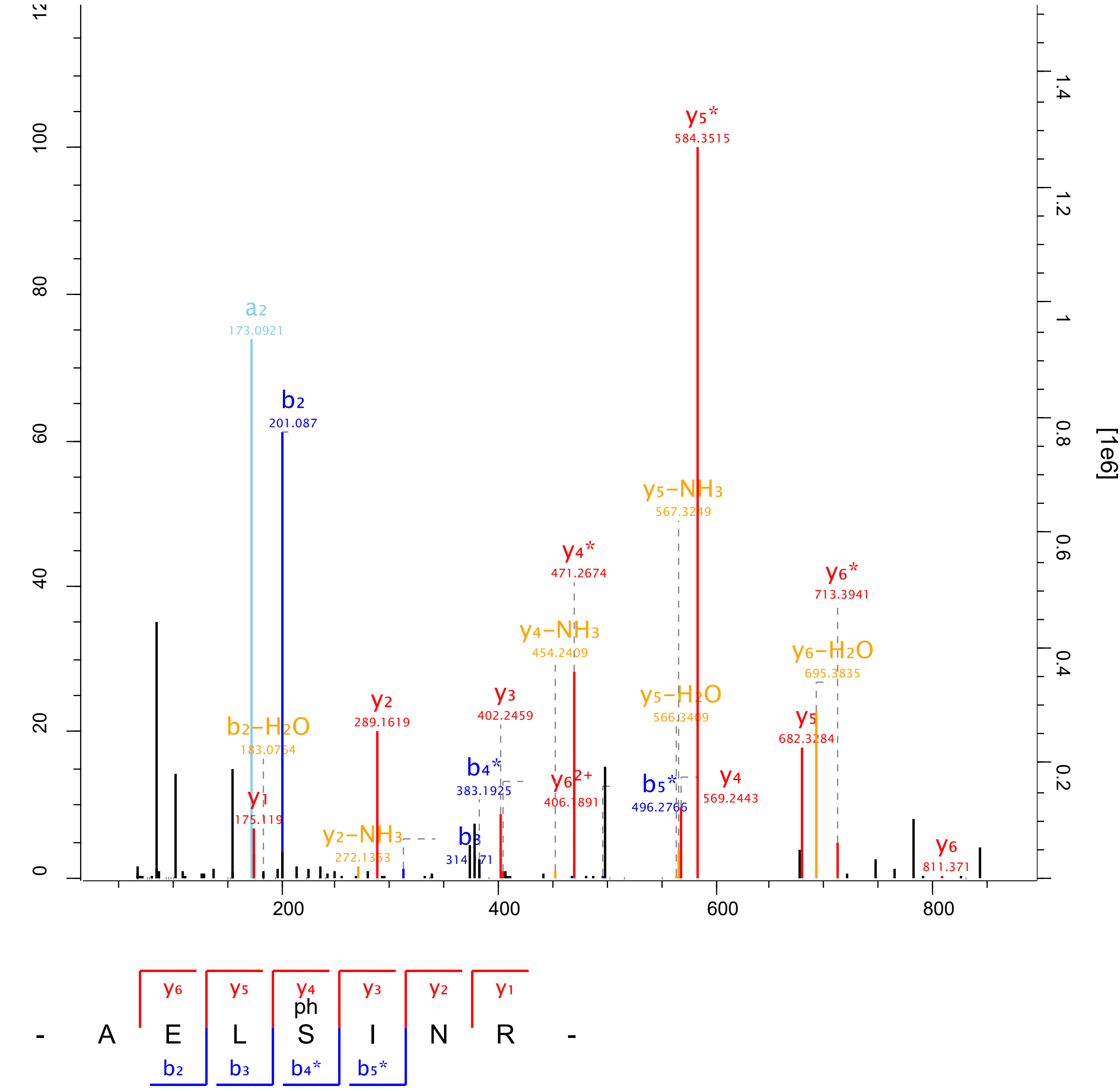

Raw file	Scan	Method	Score	m/z
sys_30_1	8861	FTMS; HCD	75.82	525.7

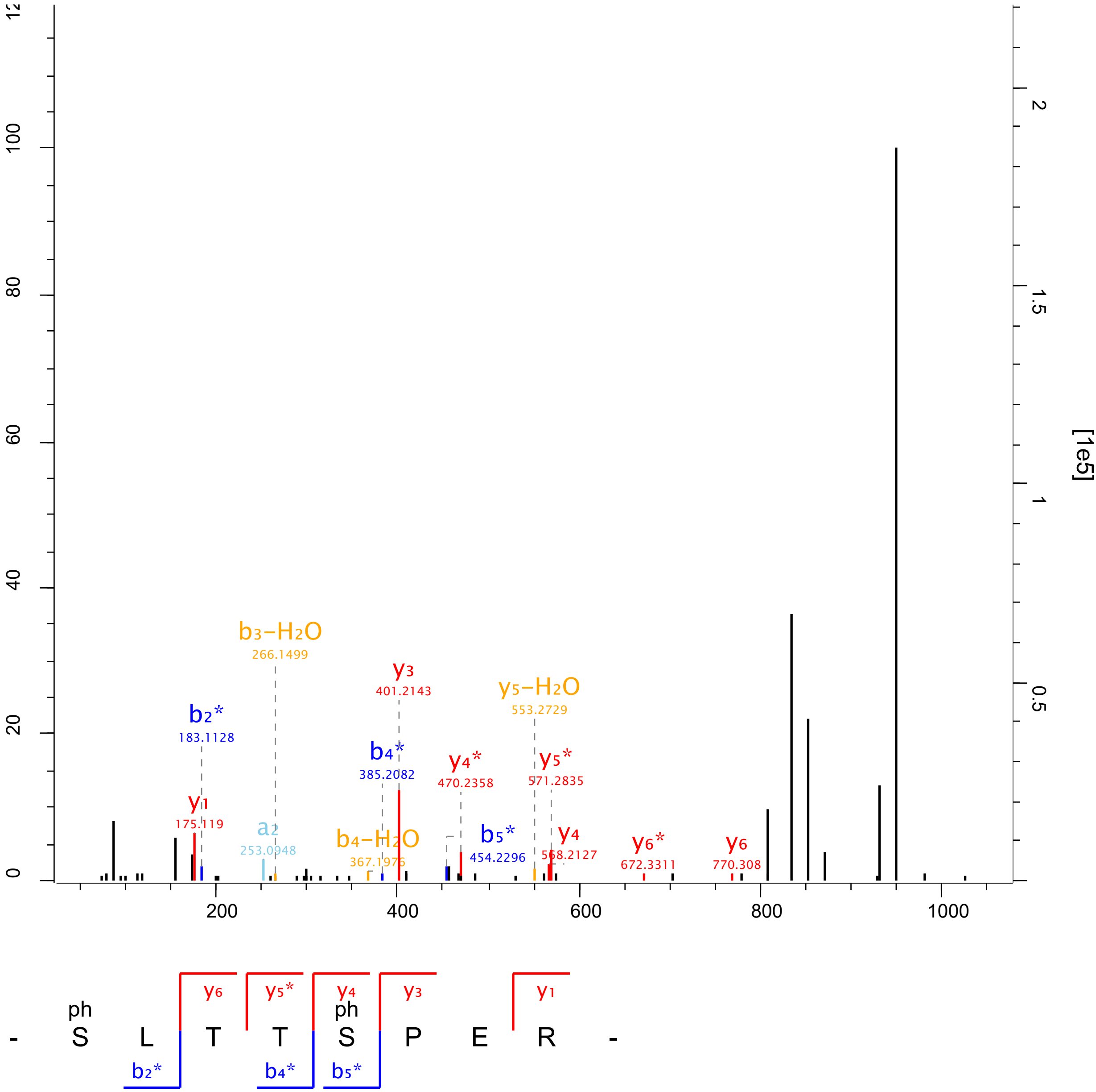

Raw file	Scan	Method	Score	m/z
sys_30_1	9198	FTMS; HCD	153.54	534.74

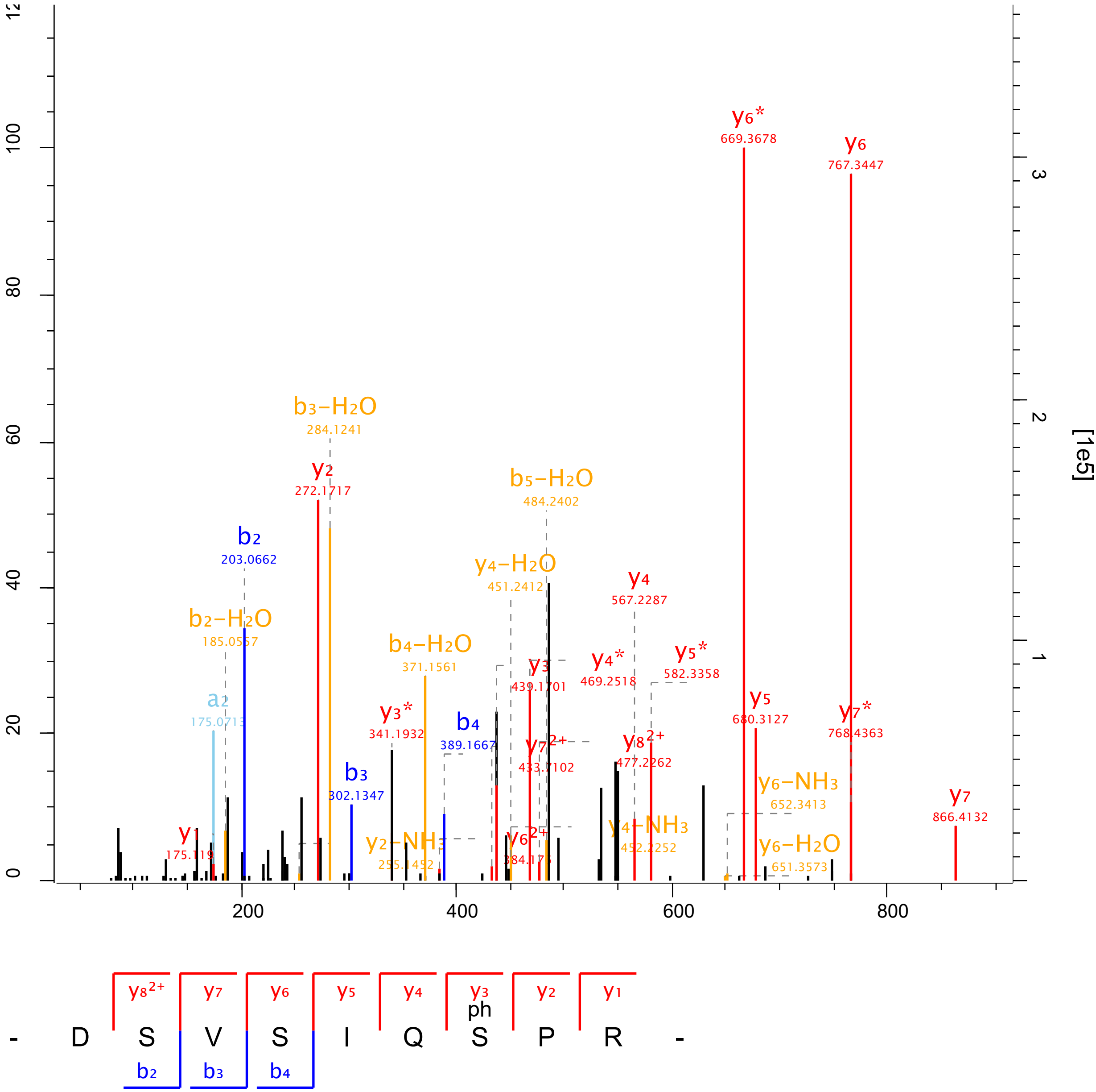

Supplement: Supplementary Figure S2-8 [file 143488_2_supp_337930_ps5hgk.pdf]
