# Supplementary material for: The Systemin Signaling Cascade As Derived from Time Course Analyses of the Systemin-responsive Phosphoproteome
Source: Mol Cell Proteomics. 2019 May 28;18(8):1526–42. doi: 10.1074/mcp.RA119.001367 (PMC6683004; doi:10.1074/mcp.RA119.001367)

Supplementary Figure 2: Representative annotated spectra of identified phosphopeptides under systemin,A17 and water treatment as exported from MaxQuant.

Raw file	Scan	Method	Score	m/z
sys_30_2	10272	FTMS; HCD	218.01	602.27

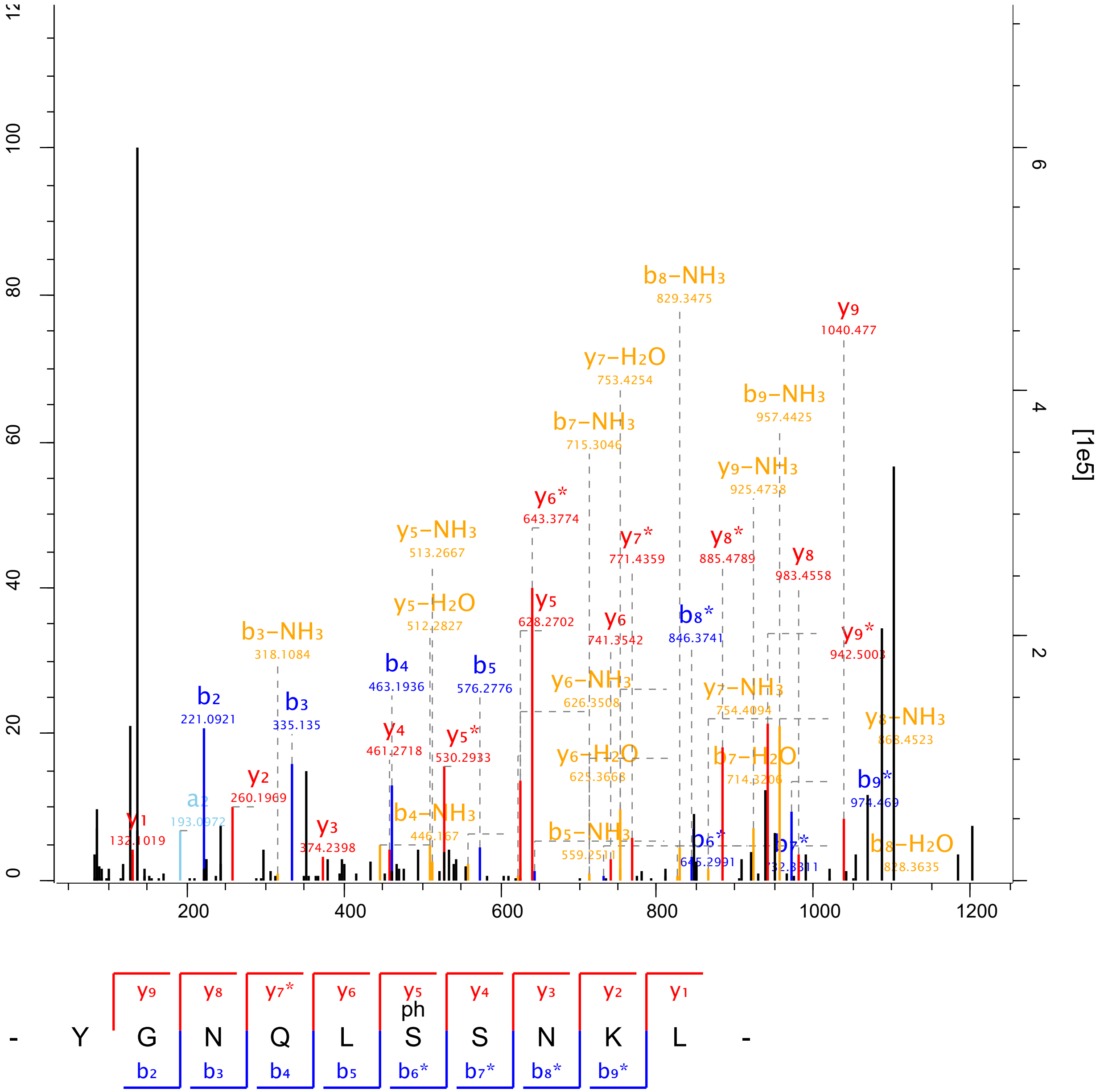

Raw file	Scan	Method	Score	m/z
sys_30_2	20145	FTMS; HCD	102.55	459.19

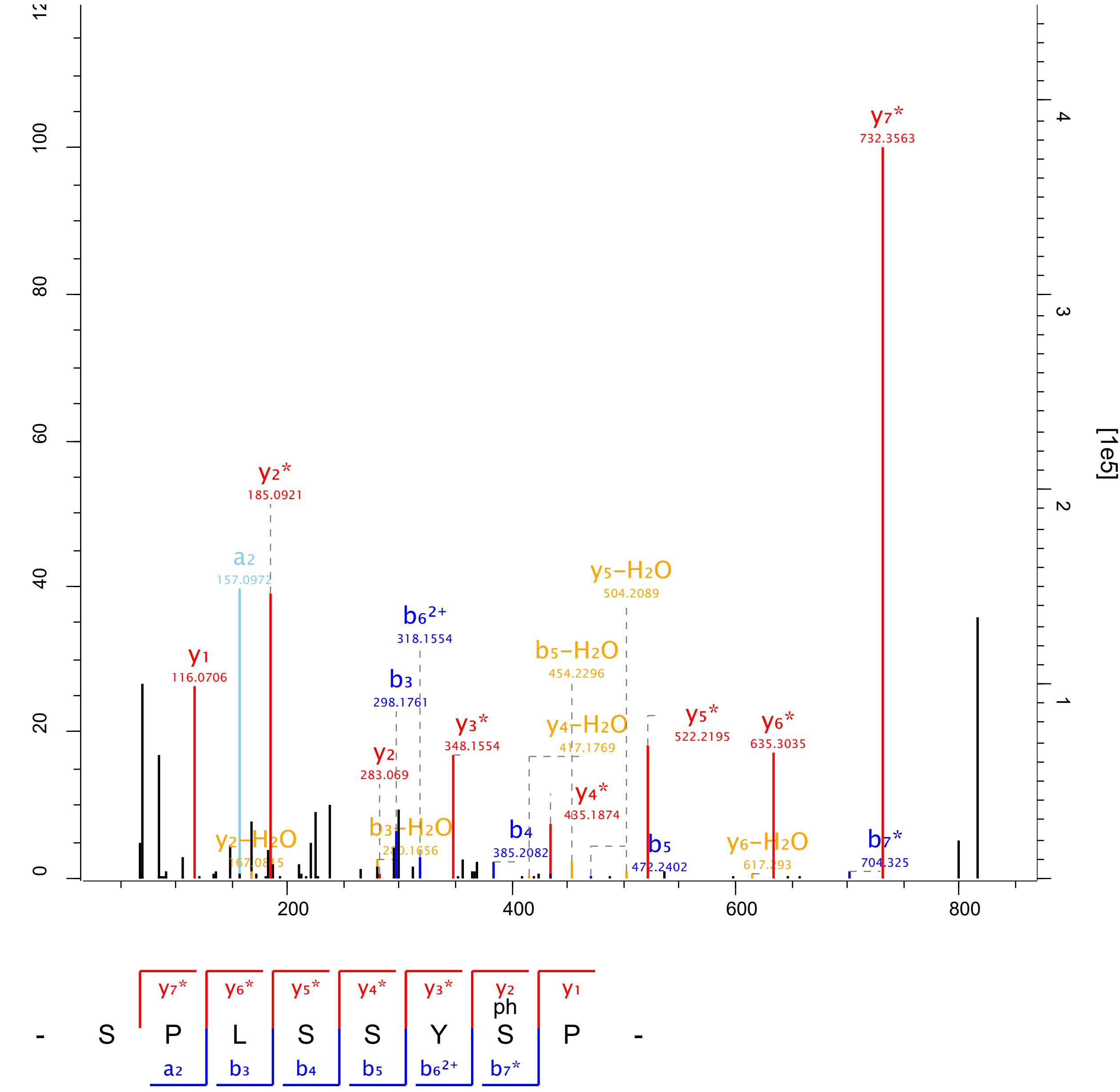

Raw file	Scan	Method	Score	m/z
sys_30_2	3964	FTMS; HCD	81.92	512.74

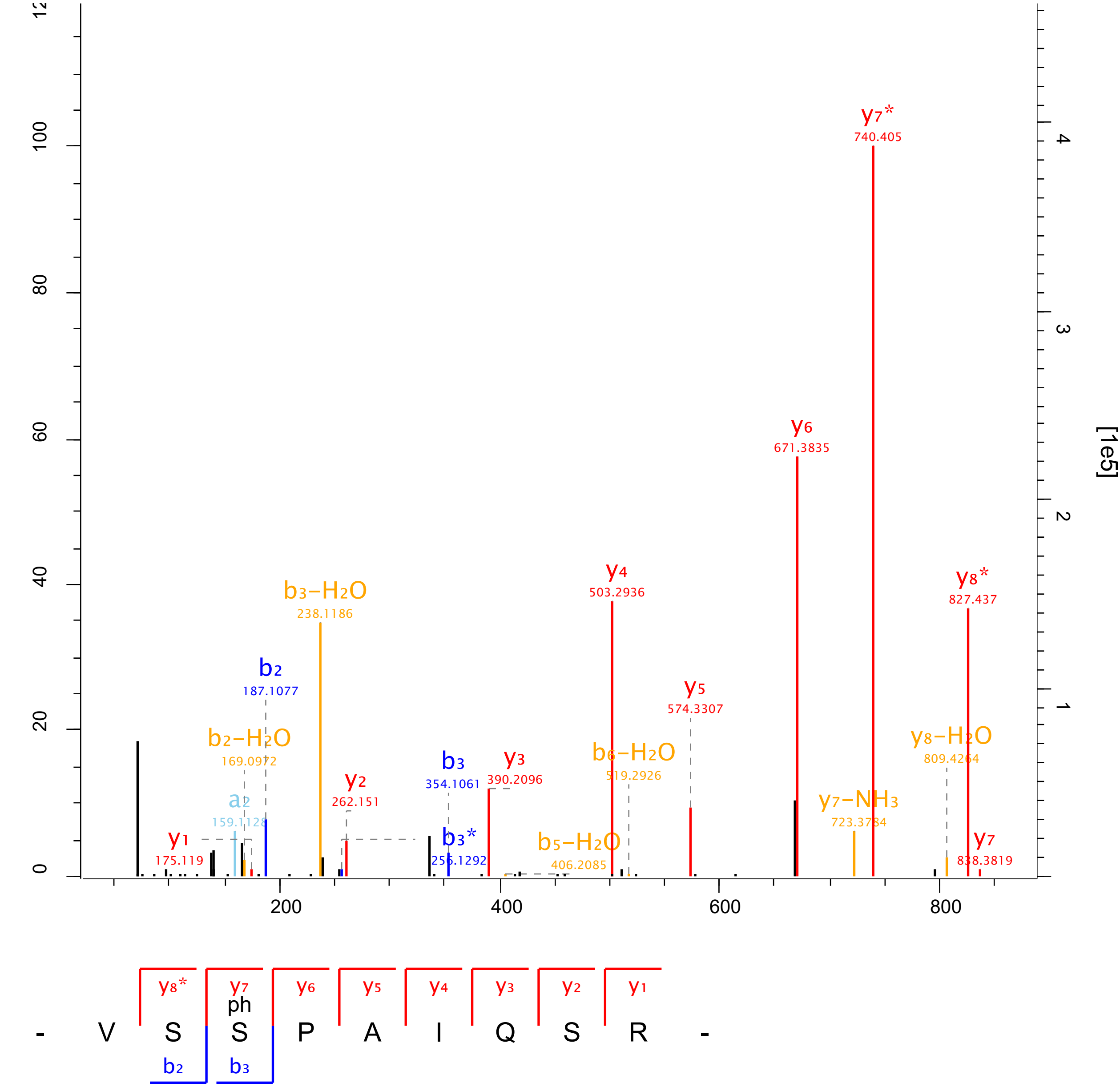

Raw file	Scan	Method	Score	m/z
sys_30_2	5526	FTMS; HCD	66.27	407.2

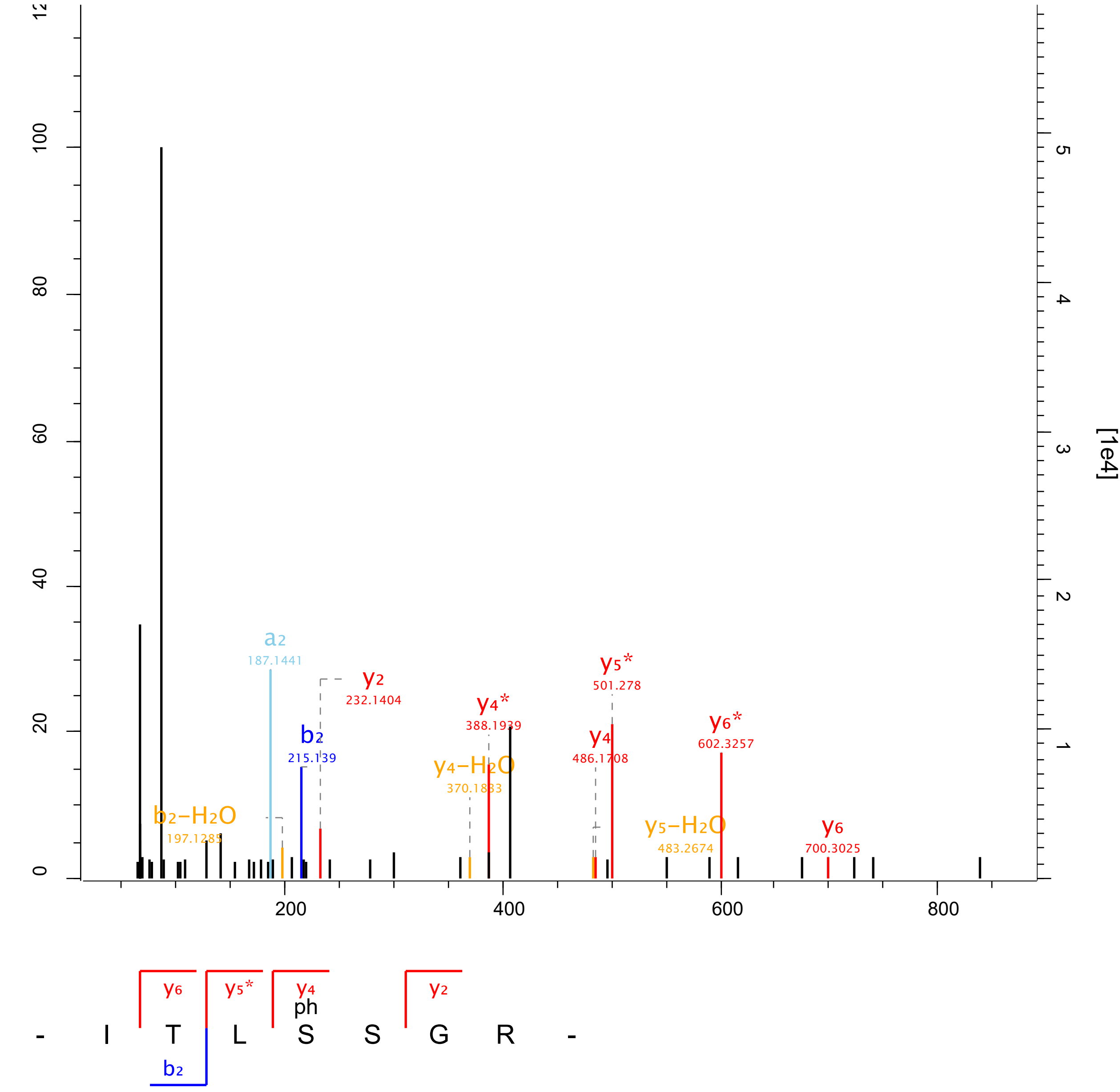

Raw file	Scan	Method	Score	m/z
sys_30_2	6657	FTMS; HCD	119.75	441.22

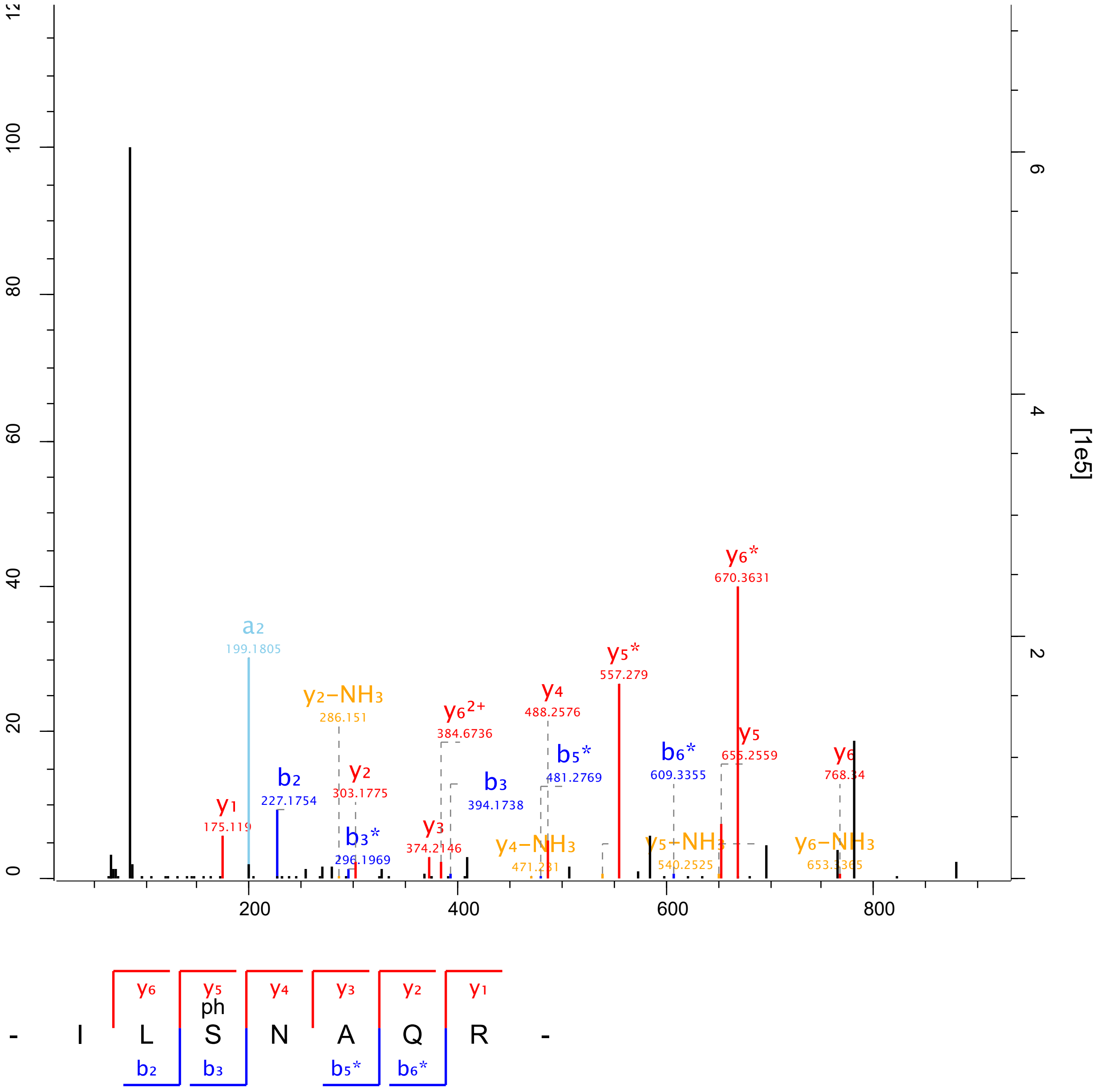

Raw file	Scan	Method	Score	m/z
sys_30_2	8708	FTMS; HCD	152.23	501.72

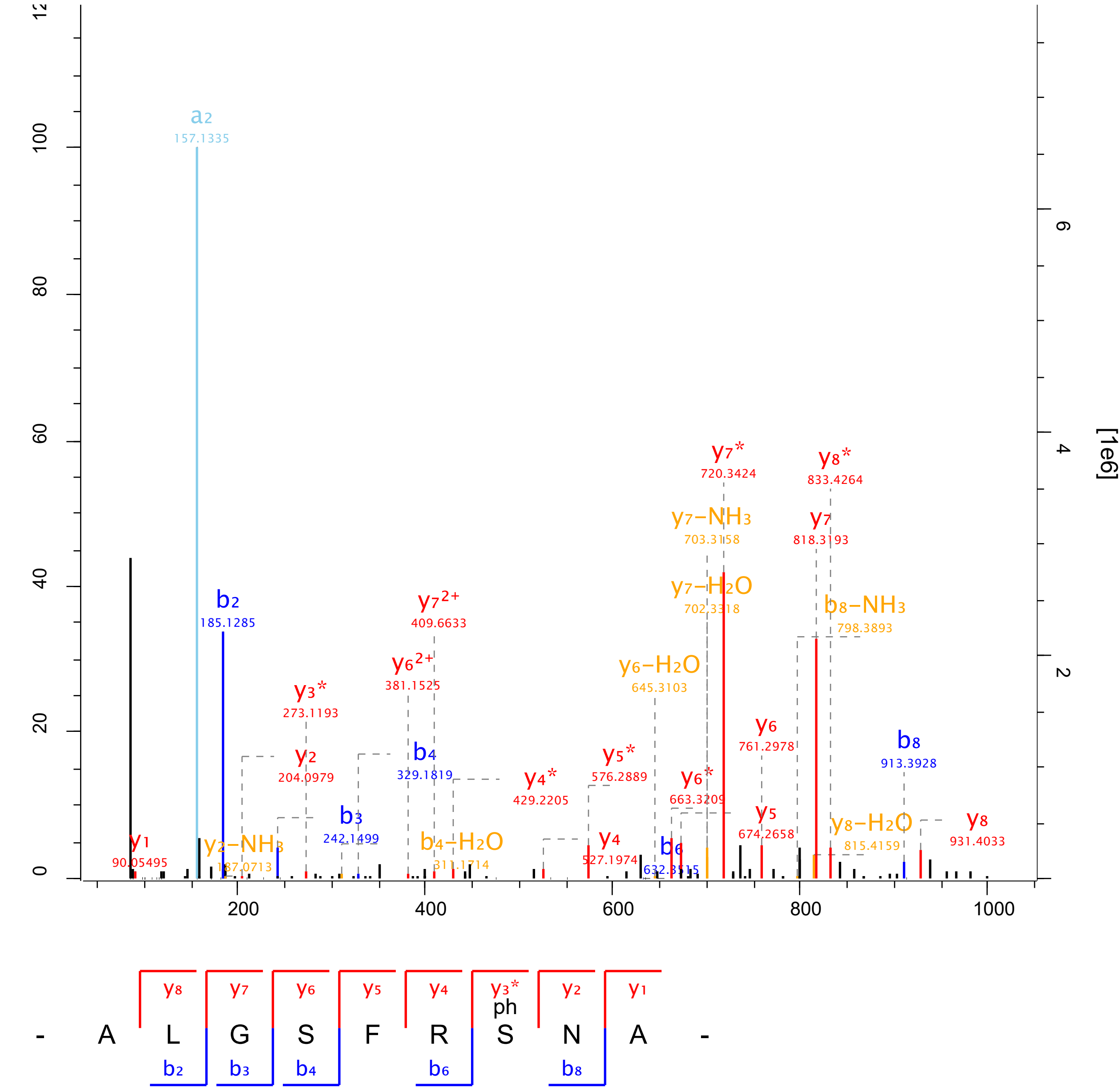

Raw file	Scan	Method	Score	m/z
sys_30_3	10802	FTMS; HCD	150.13	709.83

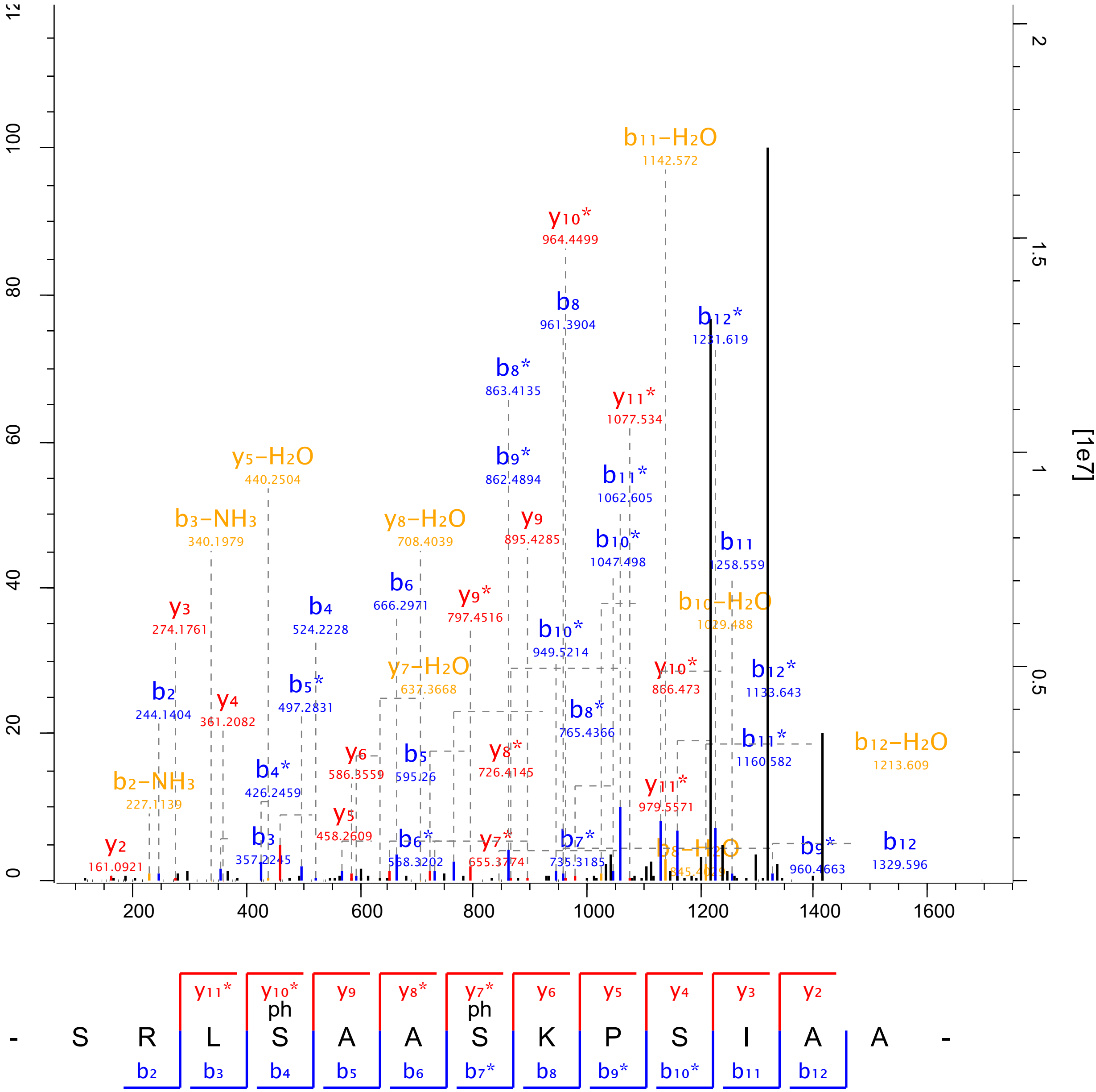

Raw file	Scan	Method	Score	m/z
sys_30_3	13930	FTMS; HCD	57.53	598.77

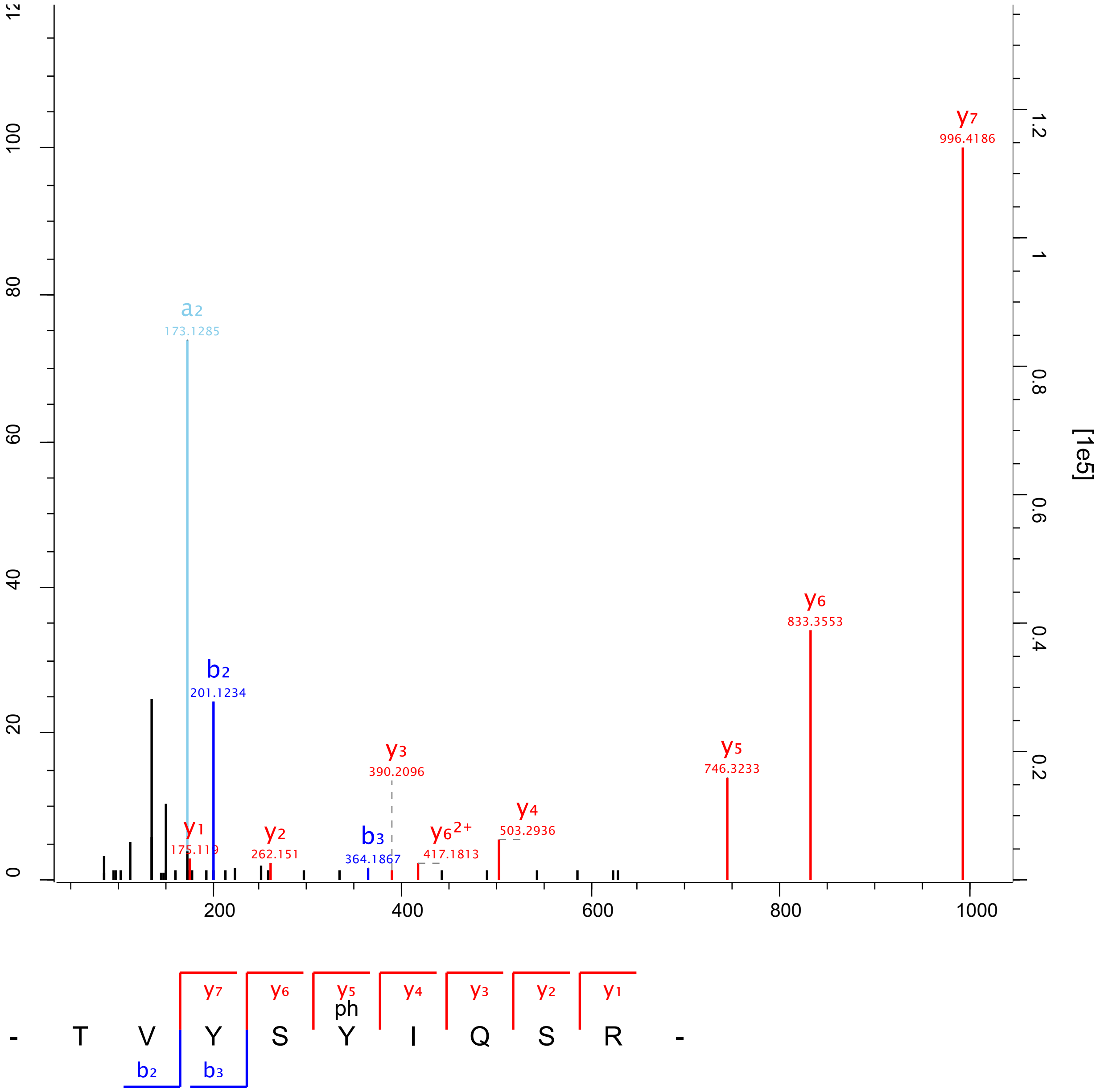

Raw file	Scan	Method	Score	m/z
sys_30_3	19654	FTMS; HCD	47.55	722.82

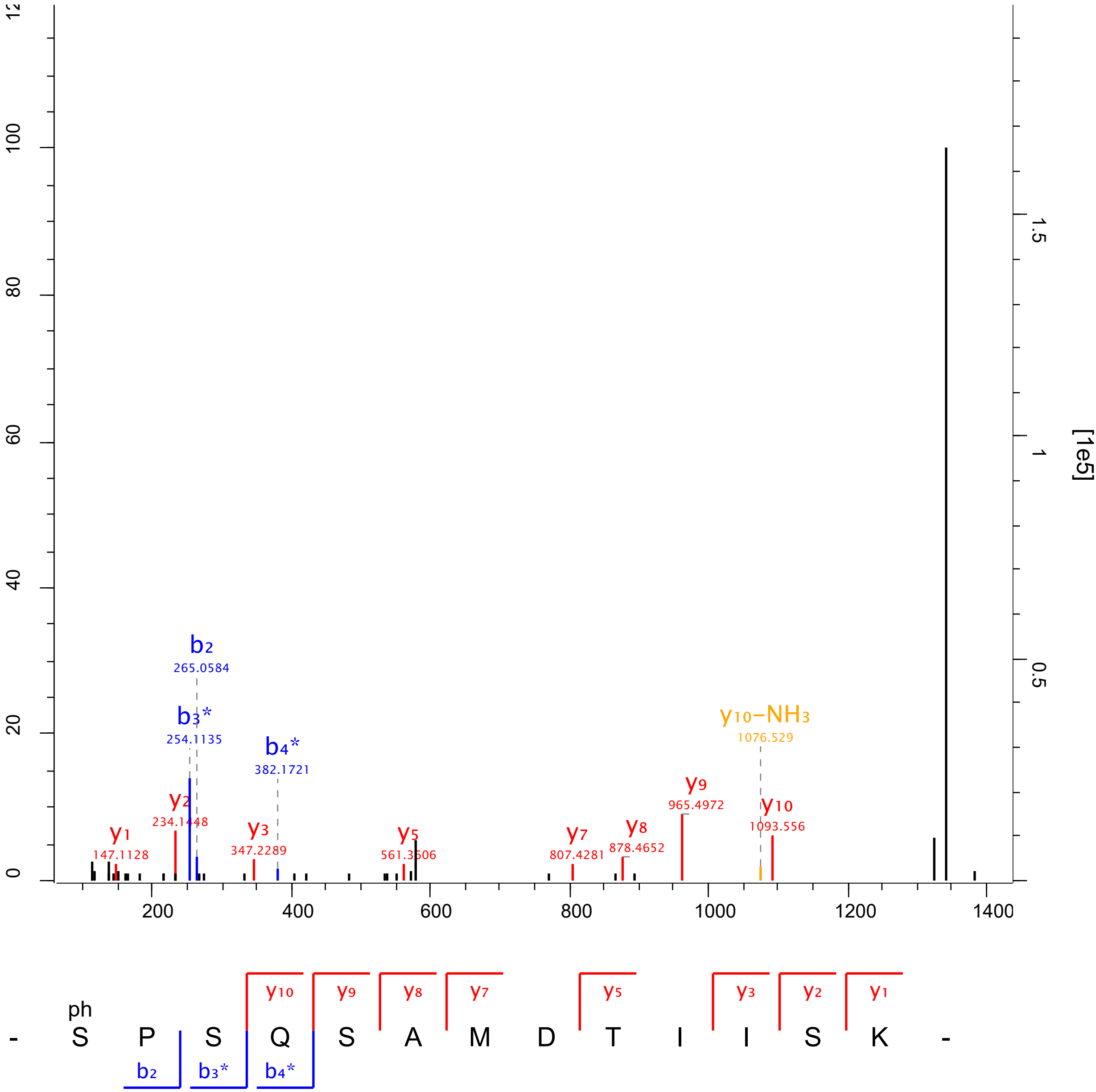

Raw file	Scan	Method	Score	m/z
sys_30_3	25568	FTMS; HCD	106.31	628.24

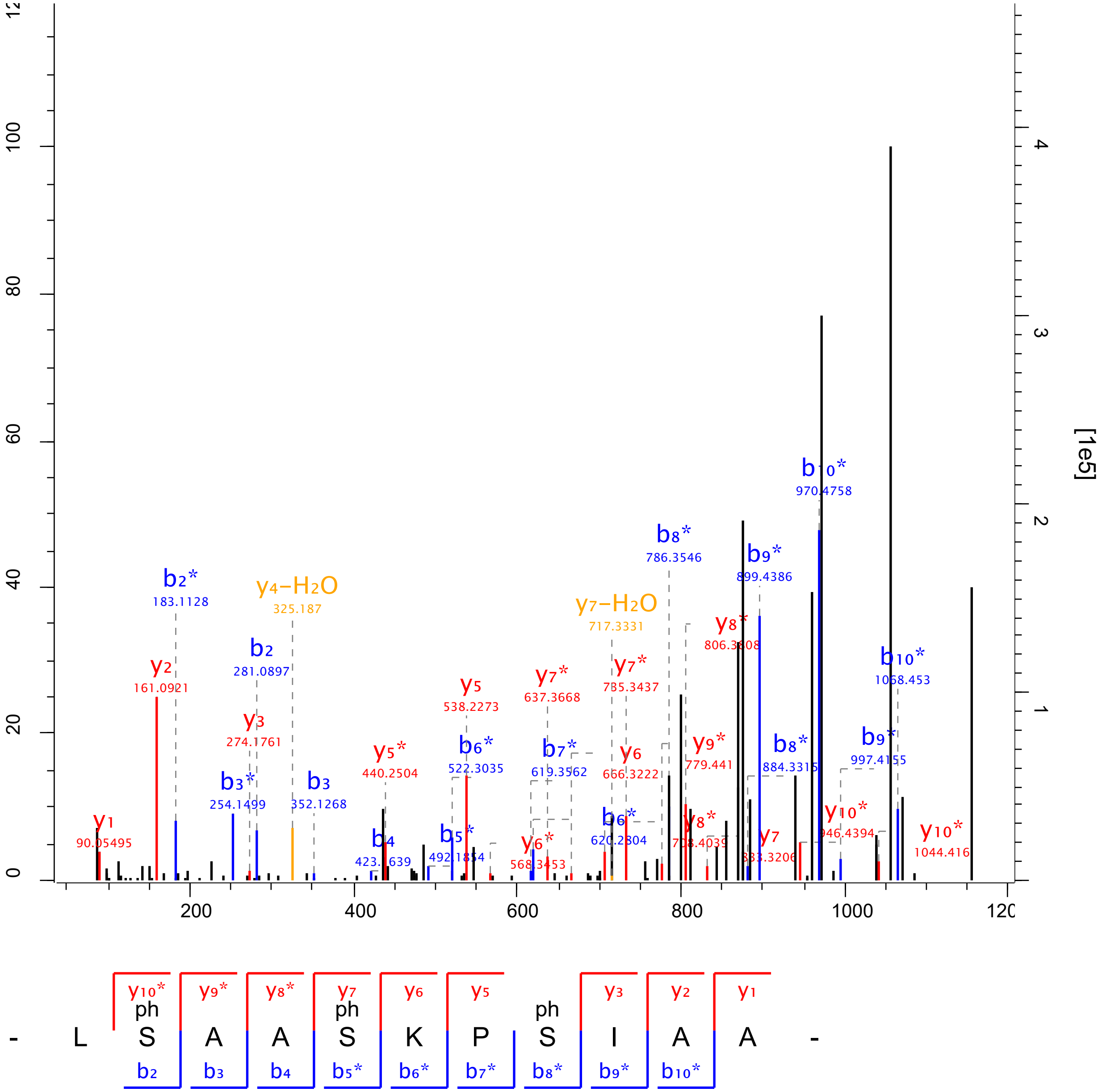

Raw file	Scan	Method	Score	m/z
sys_30_3	3325	FTMS; HCD	66.57	422.17

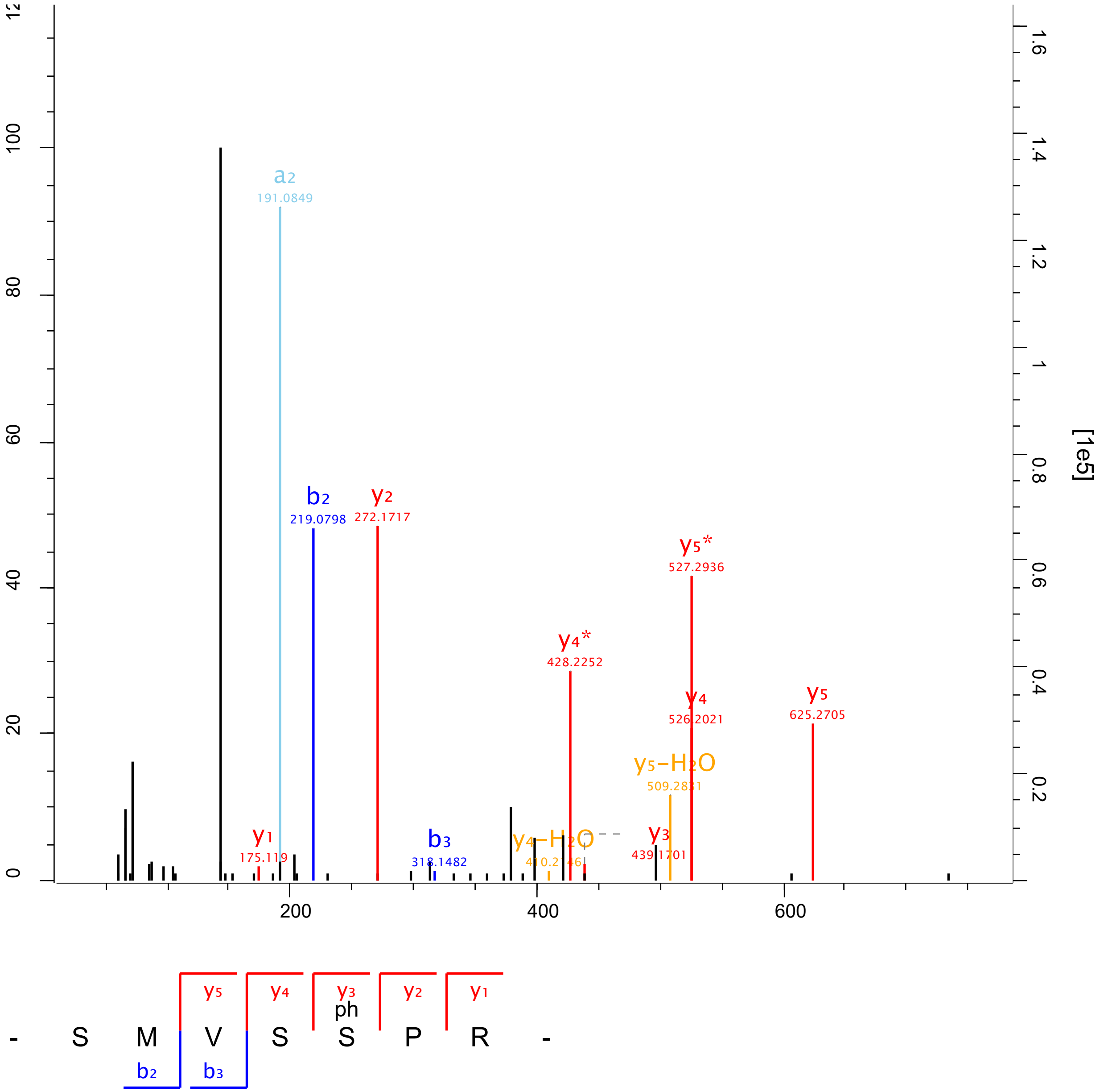

Raw file	Scan	Method	Score	m/z
sys_30_3	5822	FTMS; HCD	78.81	570.75

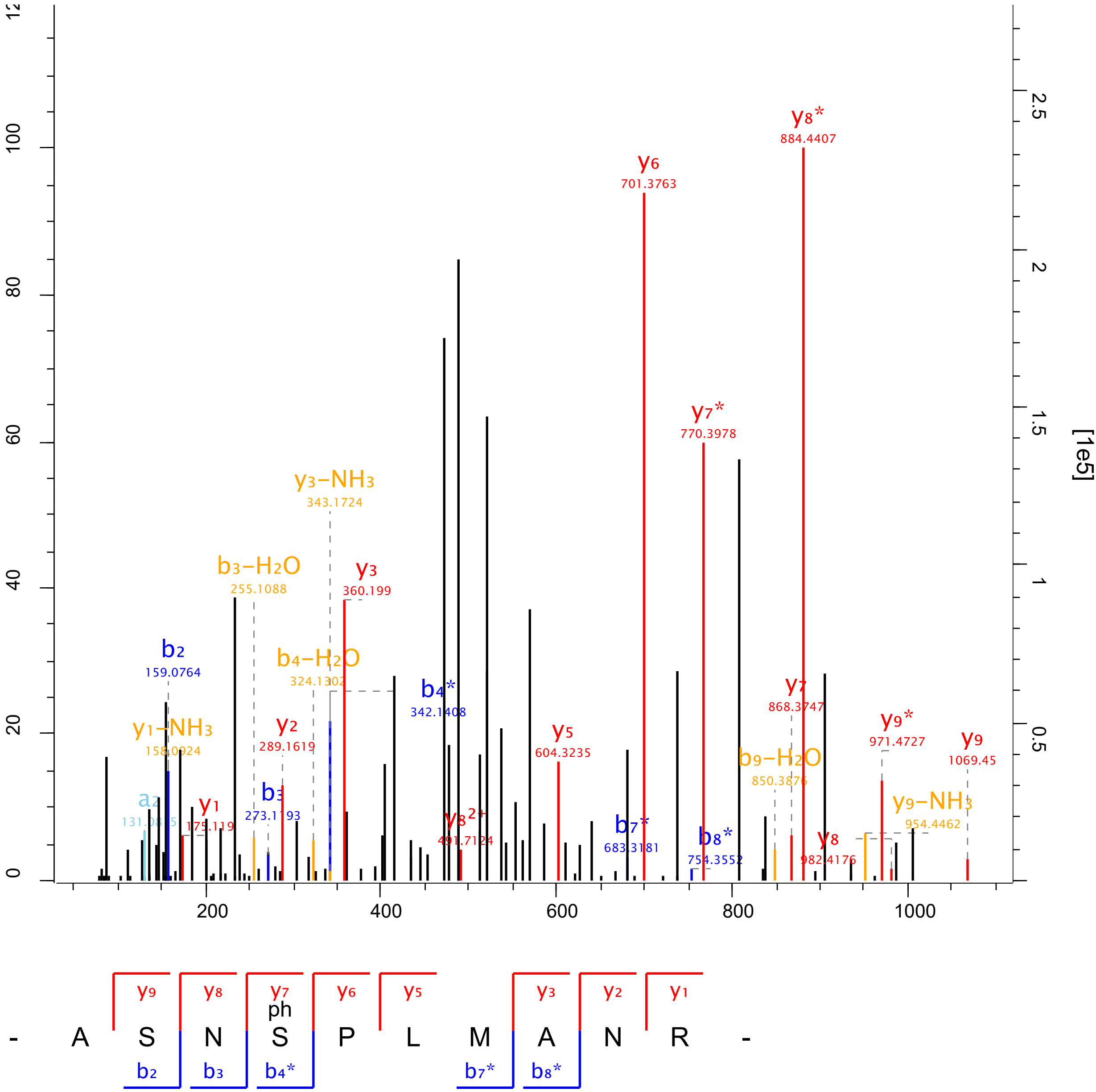

Raw file	Scan	Method	Score	m/z
sys_30_3	8493	FTMS; HCD	139.86	480.22

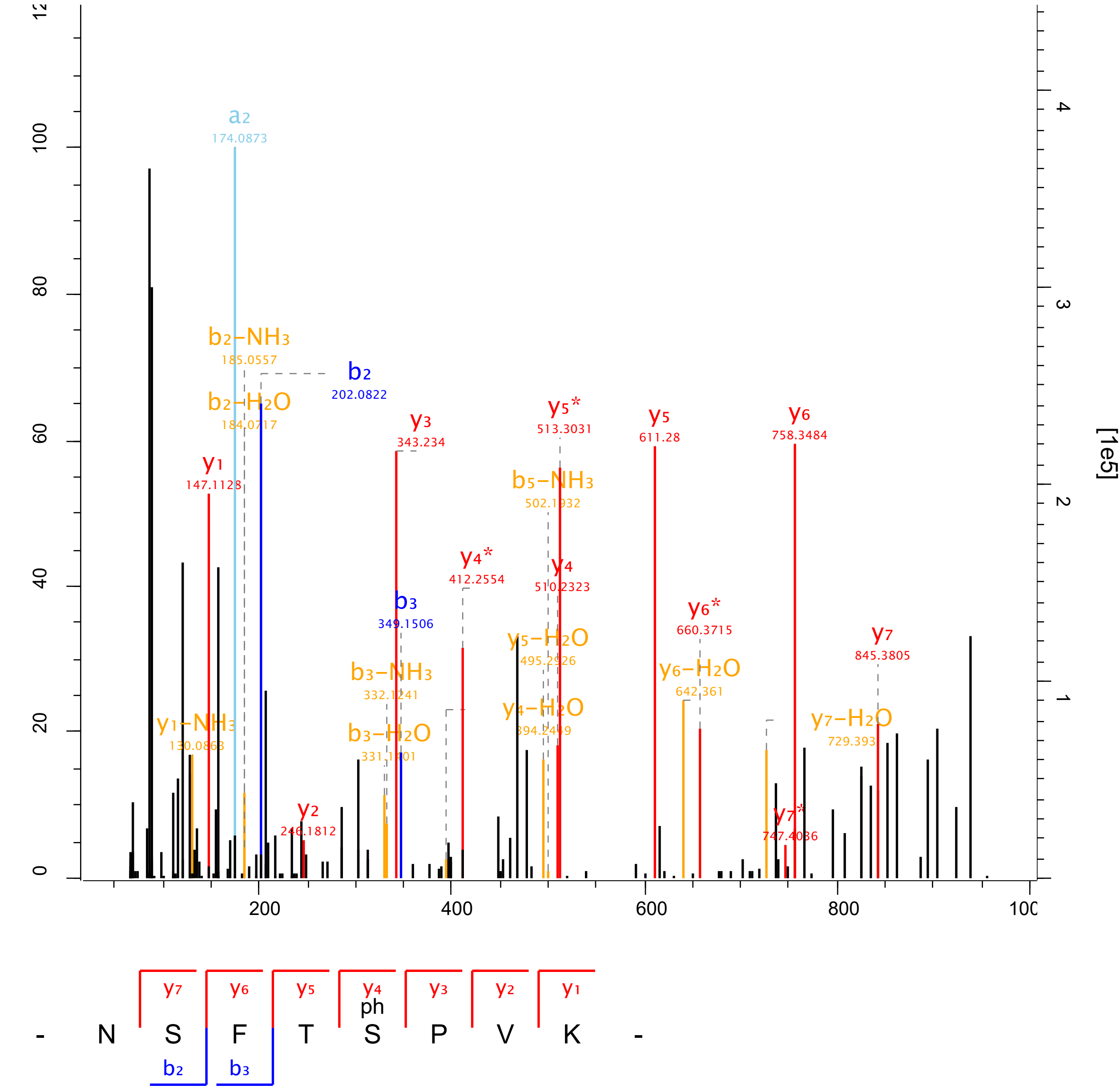

Raw file	Scan	Method	Score	m/z
sys_30_3	9507	FTMS; HCD	140.31	621.28

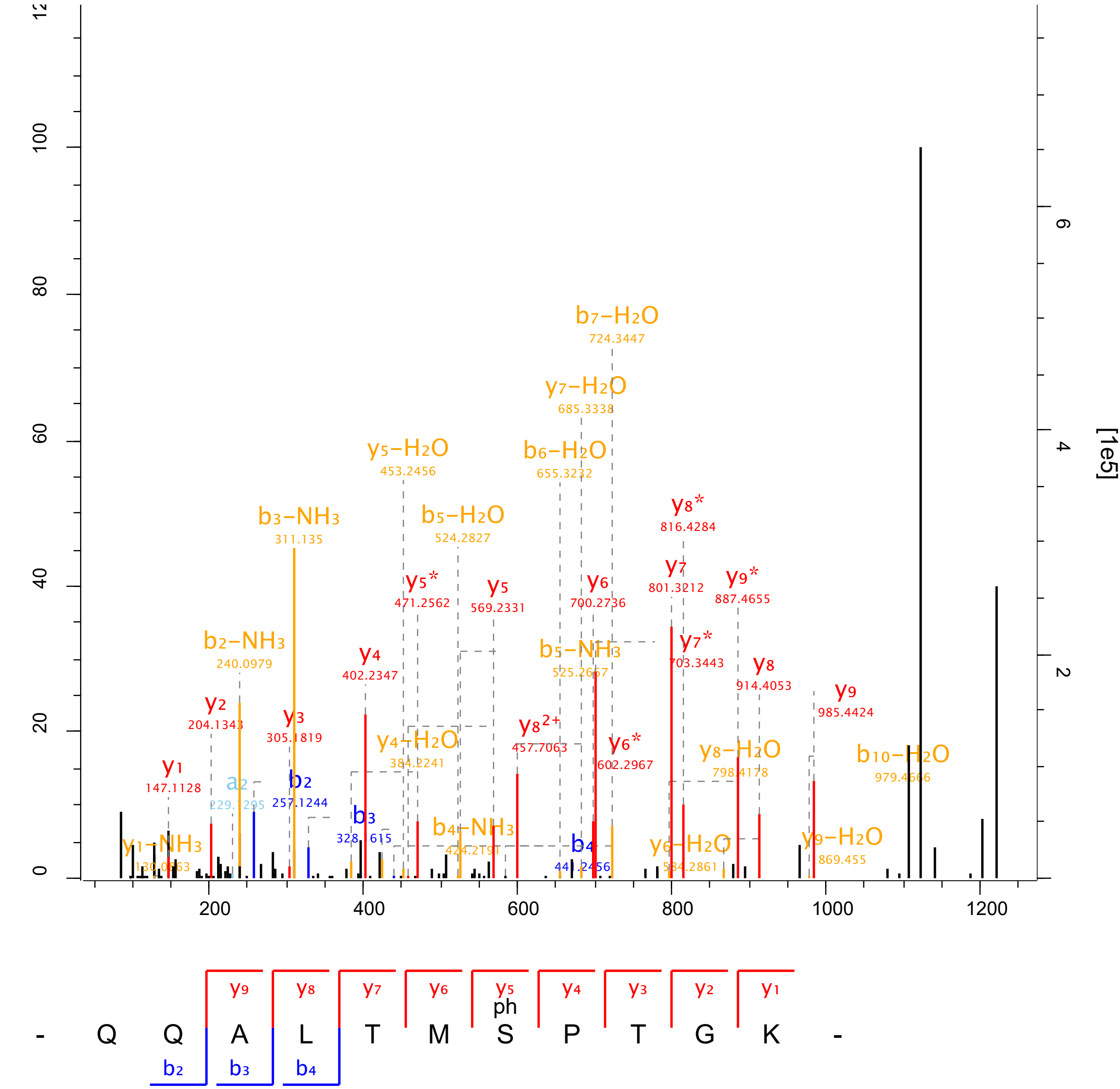

Supplement: Supplementary Figure S2-9 [file 143488_2_supp_337931_ps5hgk.pdf]
